# Chirality Matters: Fine-Tuning of Novel Monoamine Reuptake Inhibitors Selectivity through Manipulation of Stereochemistry

**DOI:** 10.3390/biom13091415

**Published:** 2023-09-19

**Authors:** Predrag Kalaba, Katharina Pacher, Philip John Neill, Vladimir Dragacevic, Martin Zehl, Judith Wackerlig, Michael Kirchhofer, Simone B. Sartori, Hubert Gstach, Shima Kouhnavardi, Anna Fabisikova, Matthias Pillwein, Francisco Monje-Quiroga, Karl Ebner, Alexander Prado-Roller, Nicolas Singewald, Ernst Urban, Thierry Langer, Christian Pifl, Jana Lubec, Johann Jakob Leban, Gert Lubec

**Affiliations:** 1 Department of Pharmaceutical Sciences, Faculty of Life Sciences, University of Vienna, 1090 Vienna, Austria; predrag.kalaba@univie.ac.at (P.K.); katharina.pacher@gmx.net (K.P.); phneill@gmx.at (P.J.N.); dragacevicvladimir@gmail.com (V.D.); judith.wackerlig@univie.ac.at (J.W.); mikey.k.mb@gmail.com (M.K.); hubert.gstach@univie.ac.at (H.G.); shima.kouhnavardi@gmail.com (S.K.); matthias.pillwein@icloud.com (M.P.); ernst.urban@univie.ac.at (E.U.); thierry.langer@univie.ac.at (T.L.); 2 Mass Spectrometry Centre, Faculty of Chemistry, University of Vienna, Währinger Straße 38, 1090 Vienna, Austria; martin.zehl@univie.ac.at (M.Z.); anna.fabisikova@univie.ac.at (A.F.); 3 Department of Analytical Chemistry, Faculty of Chemistry, University of Vienna, Währinger Straße 38, 1090 Vienna, Austria; 4 Department of Pharmacology and Toxicology, Institute of Pharmacy and Center for Molecular Biosciences Innsbruck (CMBI), Leopold Franzens University Innsbruck, 6020 Innsbruck, Austria; simone.sartori@uibk.ac.at (S.B.S.); karl.ebner@uibk.ac.at (K.E.); nicolas.singewald@uibk.ac.at (N.S.); 5 Center for Physiology and Pharmacology, Department of Neurophysiology and Neuropharmacology, Medical University of Vienna, 1090 Vienna, Austria; francisco.monjequiroga@meduniwien.ac.at; 6 X-ray Structure Analysis Centre, Faculty of Chemistry, University of Vienna, 1090 Vienna, Austria; alexander.roller@univie.ac.at; 7 Center for Brain Research, Medical University of Vienna, 1090 Vienna, Austria; christian.pifl@meduniwien.ac.at; 8 Programme for Proteomics, Paracelsus Medical University, 5020 Salzburg, Austria; jana.aradska@gmail.com (J.L.); j-e-leban@outlook.com (J.J.L.)

**Keywords:** selective dopamine reuptake inhibition, chirality, modafinil analogues, dopamine transporter

## Abstract

The high structural similarity, especially in transmembrane regions, of dopamine, norepinephrine, and serotonin transporters, as well as the lack of all crystal structures of human isoforms, make the specific targeting of individual transporters rather challenging. Ligand design itself is also rather limited, as many chemists, fully aware of the synthetic and analytical challenges, tend to modify lead compounds in a way that reduces the number of chiral centers and hence limits the potential chemical space of synthetic ligands. We have previously shown that increasing molecular complexity by introducing additional chiral centers ultimately leads to more selective and potent dopamine reuptake inhibitors. Herein, we significantly extend our structure-activity relationship of dopamine transporter-selective ligands and further demonstrate how stereoisomers of defined absolute configuration may fine-tune and direct the activity towards distinct targets. From the pool of active compounds, using the examples of stereoisomers **7h** and **8h**, we further showcase how in vitro activity significantly differs in in vivo drug efficacy experiments, calling for proper validation of individual stereoisomers in animal studies. Furthermore, by generating a large library of compounds with defined absolute configurations, we lay the groundwork for computational chemists to further optimize and rationally design specific monoamine transporter reuptake inhibitors.

## 1. Introduction

The dopamine transporter (DAT) belongs to the same family of solute carrier (SLC) neurotransmitter transporters as norepinephrine and serotonin transporters (NET and SERT). 

These three transmembrane proteins show a high sequence similarity (67% between hDAT and hNET, 50% between hDAT and hSERT, and 53% between hNET and hSERT), which also results in overlapping substrate specificities, especially between DAT and NET, both of which are able to carry out dopamine and norepinephrine uptake [1,2,3,4,5,6]. Combined with the existence of only a single resolved 3D structure, namely the one of hSERT, it hampers the elucidation of the molecular basis of dopamine transporter inhibition [2,7].

Over the last decade, our lab has successfully pursued modafinil-derived, atypical, high affinity, and selective DAT/dopamine reuptake inhibitors able to improve distinct forms of memory [8,9,10,11,12,13,14,15], along with several other research groups with the primary focus on treating addiction and psychostimulant abuse [16,17,18,19,20,21]. From our pool of derivatives, *S*-CE-123 was distinguished with an atypical dopamine reuptake inhibitor profile and was successfully tested in several rodent memory tasks, albeit with in vitro potency towards DAT in the low micromolar range [8,12,13,14,15]. In our efforts to further optimize the selectivity and potency of dopamine reuptake inhibitors, we have turned towards generating diastereomeric compounds, keeping in mind that such an approach could potentially turn out to be a double-edged sword. While introducing an additional chiral center adds to the molecular complexity of the ligands, therefore generating potential for better binding properties [22], the separation methods and (enantioselective) chemistry necessary to develop these compounds are often rather challenging [23]. The rationale behind this approach was the fact that drug targets (DNA, RNA, and proteins) are chiral structures and therefore recognize their ligands in a three-dimensional (3D) manner—the higher the degree of 3D complexity of the molecule, the higher the potential to interact with the biological target with increased affinity and higher specificity [22]. Additionally, individual stereoisomers of the same drug are known to exhibit marked differences in pharmacodynamic, pharmacokinetic, and toxicological properties [24].

Previously, we have generated a series of novel modafinil derivatives by replacing the carboxamide group of modafinil with a series of substituted and unsubstituted thiazoles and, in some cases, by performing additional modifications (substitutions on the *para*-position of single phenyl rings), which led to the introduction of an additional chiral center and the generation of diastereomeric compounds [25]. Obtained results for seven quadruples of stereoisomers indicated a correlation of the absolute configuration of isolated diastereomers with the activity and selectivity of hDAT, hNET, and hSERT [25]. Strongly encouraged by the observed significant differences in compound potency and selectivity towards DAT with respect to individual stereoisomers, we have continued with further modifications, which ultimately led to the discovery of compound *S*,*S*-CE-158, a novel atypical selective DAT inhibitor with increased potency as compared to both R-Modafinil and *S*-CE-123 [26,27,28].

The objective of the current study was to further extend knowledge on the structure-activity relationship (SAR) of a series of modafinil-derived analogues, i.e., by establishing a well-defined stereochemistry to ultimately obtain new high-affinity and selective dopamine reuptake inhibitors. By generating higher structural complexity (the presence of two chiral centers that are fully resolved), we aim to gain a better understanding of defined stereochemistry in interactions with DAT (but also NET and SERT) and further expand our portfolio of attributed diastereomeric compounds so that the described SAR may also serve as a useful tool for computational chemists to rationally design the next generation of dopamine reuptake inhibitors, a daunting task especially in the absence of the human 3D structure. Furthermore, we selected the two most potent stereoisomers, a pair of enantiomers, and evaluated them in vitro in a series of pharmacological and toxicological assays, thereby demonstrating via pharmacokinetics that this class of compounds is able to successfully penetrate the blood–brain barrier. Furthermore, we compared the drug efficacy of these two selected stereoisomers in vivo and demonstrated the effect of this drug class on synaptic plasticity and basal synaptic transmission. 

## 2. Materials and Methods

### 2.1. Synthetic Procedures

General procedure A-reaction of substituted diphenylmethanol with thiophen-2-ylmethanethiol

An amount of 3.0–10.0 mmol of substituted diphenylmethanol and 3.0–10.0 mmol of thiophen-2-ylmethanethiol (molar ratio 1:1) were dissolved in glacial acetic acid. Then, 1.1 equivalents of 48% BF_3_·Et_2_O were added, and the reaction mixture was left under stirring overnight at room temperature. The reaction mixture was then poured over ice, a small amount of water was added, and the acetic acid was neutralized with the addition of solid NaHCO_3_. The product was then extracted (3×) with 50 mL of EtOAc. Organic layers were collected, combined, dried over anhydrous Na_2_SO_4_, filtered, and concentrated under reduced pressure.

General procedure B-oxydation to sulfoxide with 30% H_2_O_2_

In total, 0.96–5.1 mmol of unoxidized products were dissolved in 5–12 mL of glacial acetic acid and 0.15–0.77 mL (0.15 mL per 1 mmol of unoxidized material) of 30% H_2_O_2_ and left to stir overnight (12–14 h) at room tem sperature. The reaction mixture was then neutralized with a cold 5% sodium bicarbonate solution. The reaction product was extracted (3 × 50 mL) with EtOAc and dried over anhydrous Na_2_SO_4._ The organic solution was concentrated under reduced pressure, and the residue was purified by flash column chromatography on silica gel (2.5 or 5% MeOH in DCM). The product was dried in a high vacuum.

1. Synthesis of 2-((((2-fluorophenyl)(phenyl)methyl)sulfinyl)methyl)thiophene (**5a**–**8a**)

Following general procedure A, 1.0 g (4.9 mmol) of (2-fluorophenyl)(phenyl)methanol, 0.64 g (4.9 mmol) of thiophen-2-ylmethanethiol, and (0.65 mL, 5.4 mmol) of 48% BF_3_·Et_2_O were reacted, affording 1.4 g of **3a** as a yellow oily product (yield 91%).

Following general procedure B, 1.4 g (4.5 mmol) of **3a** dissolved in 12 mL of glacial acetic acid was reacted with 30% H_2_O_2_ (0.54 mL) to give 1.17 g of **4a** as a yellow solid (yield 79%).

Racemic **4a** was further separated into two individual pairs of enantiomers by means of an MPLC system equipped with a glass column packed with silica gel. Toluol/EtOAc = 80/20 was used as the mobile phase, and the separation was carried out with a flow rate of 5 mL/min. Individual pairs of enantiomers were further separated into the individual enantiomers **5a**–**8a** by means of semipreparative chiral HPLC. A semipreparative HPLC instrument was equipped with a CHIRALPAK IA column (10 mm Φ × 25 cm L) (Daicel Inc., Tokyo, Japan), and Hexane/EtOAc = 50/50 was used as the mobile phase in the cases of **5a** and **6a** and 100% EtOAc in the cases of **7a** and **8a**.

**5a**. (R,R)-2-((((2-fluorophenyl)(phenyl)methyl)sulfinyl)methyl)thiophene. HRESIMS *m*/*z* 331.0622 [M+H]^+^ (calcd for C_18_H_16_FOS_2_^+^, 331.0621, Δ = −0.3 ppm) (Figure S1). HPLC R_t_ 24.25 min, purity > 99% (Figure S1a). Chiral HPLC R_t_ 11.93 min, 95.4% ee (Figure S1b). ^1^H NMR (500 MHz, CDCl_3_, 23 °C): δ = 7.67 (td, 1H, CH-6, aromatic), 7.47 (m, 2H, CH-2,6, phenyl), 7.39 (m, ov, 2H, CH-3,5, phenyl), 7.37 (ov, 1H, CH-4, phenyl), 7.34 (ov, 1H, CH-4, aromatic), 7.32 (dd, ov, 1H, CH-5, thiophene), 7.22 (td, 1H, CH-5, aromatic), 7.11 (m, 1H, CH-3, aromatic), 7.03 (dd, 1H, CH-4, thiophene), 6.96 (dd, 1H, CH-3, thiophene), 5.10 (s, 1H, CH), 4.12/3.97 (AB, 2H, CH_2_). ^13^C NMR (125.75 MHz, CDCl_3_, 23 °C): δ = 159.91 (C_q_-2, aromatic), 133.15 (C_q_-1, phenyl), 130.3 (C_q_, thiophene-2), 129.99 (CH-4, aromatic), 129.86 (CH-2,6, phenyl), 129.64 (CH-6, aromatic), 128.91 (CH-3, thiophene), 128.73 (CH-3,5, phenyl), 128.56 (CH-4, phenyl), 127.39 (CH-4, thiophene), 126.89 (CH-5, thiophene), 124.79 (CH-5, aromatic), 123.78 (C_q_-1, aromatic), 116.1 (CH-3, aromatic), 61.39 (CH), 50.84 (CH_2_) (Figure S1c).

**6a**. (S,S)-2-((((2-fluorophenyl)(phenyl)methyl)sulfinyl)methyl)thiophene. HRESIMS *m*/*z* 331.0621 [M+H]^+^ (calcd for C_18_H_16_FOS_2_^+^, 331.0621, Δ = 0.1 ppm) (Figure S2). HPLC R_t_ 24.23 min, purity > 99% (Figure S2a). Chiral HPLC R_t_ 12.76 min, 95.4% ee (Figure S2b). ^1^H NMR (500 MHz, CDCl_3_, 23 °C): δ = 7.67 (CH-6, aromatic), 7.47 (CH-2,6, phenyl), 7.39 (CH-3,5, phenyl), 7.37 (CH-4, phenyl), 7.34 (CH-4, aromatic), 7.32 (s, 1H, CH-5, thiophene), 7.22 (CH-5, aromatic), 7.11 (CH-3, aromatic), 7.03 (s, 1H, CH-4, thiophene), 6.96 (s, 1H, CH-3, thiophene), 5.10 (s, 1H, CH), 4.12/3.97 (AB, 2H, CH_2_). ^13^C NMR (125.75 MHz, CDCl_3_, 23 °C): δ = 159.91 (C_q_-2, aromatic), 133.15 (C_q_-1, phenyl), 130.30 (C_q_, thiophene-2), 129.99 (CH-4, aromatic), 129.87 (CH-2,6, phenyl), 129.64 (CH-6, aromatic), 128.92 (CH-3, thiophene), 128.74 (CH-3,5, phenyl), 128.56 (CH-4, phenyl), 127.39 (CH-4, thiophene), 126.89 (CH-5, thiophene), 124.79 (CH-5, aromatic), 123.78 (C_q_-1, aromatic), 116.10 (CH-3, aromatic), 61.39 (CH), 50.84 (CH_2_) (Figure S2c).

**7a**. (R,S)-2-((((2-fluorophenyl)(phenyl)methyl)sulfinyl)methyl)thiophene. HRESIMS *m*/*z* 331.0623 [M+H]^+^ (calcd for C_18_H_16_FOS_2_^+^, 331.0621, Δ = −0.6 ppm) (Figure S3). HPLC R_t_ 24.48 min, purity > 99% (Figure S3a). Chiral HPLC R_t_ 6.96 min, >99% ee (Figure S3b). ^1^H NMR (500 MHz, CDCl_3_, 23 °C): δ = 7.73 (CH-6, aromatic), 7.47 (CH-2,6, phenyl), 7.39 (CH-3,5, phenyl), 7.34 (CH-4, phenyl), 7.33 (CH-4, aromatic), 7.32 (s, 1H, CH-5, thiophene), 7.21 (CH-5, aromatic), 7.12 (CH-3, aromatic), 7.03 (s, 1H, CH-4, thiophene), 6.99 (s, 1H, CH-3, thiophene), 5.28 (s, 1H, CH), 4.16/3.91 (AB, 2H, CH_2_). ^13^C NMR (125.75 MHz, CDCl_3_, 23 °C): δ = 160.84 (C_q_-2, aromatic), 135.47 (C_q_-1, phenyl), 130.49 (CH-6, aromatic), 130.40 (C_q_, thiophene-2), 129.90 (CH-4, aromatic), 129.24 (CH-3,5, phenyl), 129.06 (CH-3, thiophene), 128.81 (CH-2,6, phenyl), 128.46 (CH-4, phenyl), 127.4 (CH-4, thiophene), 126.91 (CH-5, thiophene), 124.55 (CH-5, aromatic), 121.93 (C_q_-1, aromatic), 115.68 (CH-3, aromatic), 61.31 (CH), 50.82 (CH_2_) (Figure S3c).

**8a**. (S,R)-2-((((2-fluorophenyl)(phenyl)methyl)sulfinyl)methyl)thiophene. HRESIMS *m*/*z* 331.0622 [M+H]^+^ (calcd for C_18_H_16_FOS_2_^+^, 331.0621, Δ = −0.2 ppm) (Figure S4). HPLC R_t_ 24.48 min, purity 98.6% (Figure S4a). Chiral HPLC R_t_ 10.94 min, >99% ee (Figure S4b). ^1^H NMR (500 MHz, CDCl_3_, 23 °C): δ = 7.73 (CH-6, aromatic), 7.47 (CH-2,6, phenyl), 7.39 (CH-3,5, phenyl), 7.34 (CH-4, phenyl), 7.33 (CH-4, aromatic), 7.32 (s, 1H, CH-5, thiophene), 7.21 (CH-5, aromatic), 7.12 (CH-3, aromatic), 7.03 (s, 1H, CH-4, thiophene), 6.99 (s, 1H, CH-3, thiophene), 5.28 (s, 1H, CH), 4.16/3.91 (AB, 2H, CH_2_). ^13^C NMR (125.75 MHz, CDCl_3_, 23 °C): δ = 160.84 (C_q_-2, aromatic), 135.47 (C_q_-1, phenyl), 130.48 (CH-6, aromatic), 130.39 (C_q_, thiophene-2) 129.89 (CH-4, aromatic), 129.23 (CH-3,5, phenyl), 129.06 (CH-3, thiophene), 128.80 (CH-2,6, phenyl), 128.45 (CH-4, phenyl), 127.40 (CH-4, thiophene), 126.91 (CH-5, thiophene), 124.55 (CH-5, aromatic), 121.93 (C_q_-1, aromatic), 115.67 (CH-3, aromatic), 61.30 (CH), 50.82 (CH_2_) (Figure S4c).

2. Synthesis of 2-((((3-fluorophenyl)(phenyl)methyl)sulfinyl)methyl)thiophene (**5b–8b**)

Following general procedure A, 1.0 g (4.9 mmol) of (3-fluorophenyl)(phenyl)methanol, 0.64 g (4.9 mmol) of thiophen-2-ylmethanethiol, and (0.65 mL, 5.4 mmol) of 48% BF_3_·Et_2_O were reacted, affording 0.8 g of **3b** as a yellow oily product (yield 53%).

Following general procedure B, 0.8 g (5.2 mmol) of **3b** dissolved in 12 mL of glacial acetic acid was reacted with 30% H_2_O_2_ (0.54 mL) to give 0.7 g of **4b** as a yellow solid (yield 84%).

Racemic **4b** was further separated into two individual pairs of enantiomers by means of an MPLC system equipped with a glass column packed with silica gel. Toluol/EtOAc = 90/10 was used as the mobile phase, and the separation was carried out with a flow rate of 5 mL/min. Individual pairs of enantiomers were further separated into the individual enantiomers **5b**–**8b** by means of semipreparative chiral HPLC. A semipreparative HPLC instrument was equipped with a CHIRALPAK IA column (10 mm Φ × 25 cm L) (Daicel Inc., Osaka, Japan), and EtOAc was used as the mobile phase.

**5b.** HRESIMS *m*/*z* 331.0613 [M+H]^+^ (calcd for C_18_H_16_FOS_2_^+^, 331.0621, Δ = 2.6 ppm) (Figure S5). HPLC R_t_ 24.52 min, purity 96.0% (Figure S5a). Chiral HPLC R_t_ 6.24 min, >99% ee (but contains 2.4% 7b) (Figure S5b). ^1^H NMR (500 MHz, CDCl_3_, 23 °C): δ = 7.44 (C*H*-3,5, phenyl), 7.40 (C*H*-2,6, phenyl), 7.40 (C*H*-4, phenyl), 7.34 (C*H*-5, thiophene), 7.33 (C*H*, aromatic-5), 7.20 (C*H*, aromatic-6), 7.15 (C*H*, aromatic-2), 7.06 (C*H*-4, thiophene), 7.03 (C*H*, aromatic-4), 6.96 (C*H*-3, thiophene), 4.73 (s, 1H, C*H*), 4.16/3.89 (AB, 2H, C*H*_2_). ^13^C NMR (125.75 MHz, CDCl_3_, 23 °C): δ = 162.71 (*C*-3, aromatic), 136.70 (*C*-1, aromatic), 134.91 (*C*-1, phenyl), 130.08 (*C*-5, aromatic), 129.85 (*C*-2, thiophene), 129.44 (*C*H-3,5, phenyl), 129.16 (*C*-3, thiophene), 128.77 (*C*H-2,6, phenyl), 128.71 (*C*H-4, phenyl), 127.33 (*C*-4, thiophene), 129.96 (*C*-5, thiophene), 125.45 (*C*-6, aromatic), 116.56 (*C*-2, aromatic), 115.39 (*C*-4, aromatic), 68.71 (*C*H), 50.09 (*C*H_2_) (Figure S5c).

**6b.** HRESIMS *m*/*z* 331.0616 [M+H]^+^ (calcd for C_18_H_16_FOS_2_^+^, 331.0621, Δ = 1.6 ppm) (Figure S6). HPLC R_t_ 24.48 min, purity > 99% (Figure S6a). Chiral HPLC R_t_ 9.21 min, >99% ee (Figure S6b). ^1^H NMR (500 MHz, CDCl_3_, 23 °C): δ = 7.44 (C*H*-3,5, phenyl), 7.40 (C*H*-2,6, phenyl), 7.40 (C*H*-4, phenyl), 7.34 (C*H*-5, thiophene), 7.33 (C*H*, aromatic-5), 7.20 (C*H*, aromatic-6), 7.15 (C*H*, aromatic-2), 7.06 (C*H*-4, thiophene), 7.03 (C*H*, aromatic-4), 6.96 (C*H*-3, thiophene), 4.73 (s, 1H, C*H*), 4.11/3.89 (AB, 2H, C*H*_2_). ^13^C NMR (125.75 MHz, CDCl_3_, 23 °C): δ = 162.70 (*C*-3, aromatic), 136.70 (*C*-1, aromatic), 134.91 (*C*-1, phenyl), 130.08 (*C*-5, aromatic), 129.85 (*C*-2, thiophene), 129.44 (*C*H-3,5, phenyl), 129.16 (*C*-3, thiophene), 128.77 (*C*H-2,6, phenyl), 128.70 (*C*H-4, phenyl), 127.32 (*C*-4, thiophene), 129.95 (*C*-5, thiophene), 125.45 (*C*-6, aromatic), 116.56 (*C*-2, aromatic), 115.39 (*C*-4, aromatic), 68.71 (*C*H), 50.09 (*C*H_2_) (Figure S6c).

**7b.** HRESIMS *m*/*z* 331.0614 [M+H]^+^ (calcd for C_18_H_16_FOS_2_^+^, 331.0621, Δ = 2.3 ppm) (Figure S7). HPLC R_t_ 24.27 min, purity 98.2% (Figure S7a). Chiral HPLC R_t_ 6.72 min, >99% ee (but contains 2.9% 5b) (Figure S7b). ^1^H NMR (500 MHz, CDCl_3_, 23 °C): δ = 7.43 (C*H*-2,6, phenyl), 7.40 (C*H*-3,5, phenyl), 7.39 (C*H*-4, phenyl), 7.39 (C*H*, aromatic-5), 7.34 (C*H*-5, thiophene), 7.23 (C*H*, aromatic-6), 7.14 (C*H*, aromatic-2), 7.05 (C*H*-4, thiophene), 7.05 (C*H*, aromatic-4), 6.98 (C*H*-3, thiophene), 4.72 (s, 1H, C*H*), 4.09/3.90 (AB, 2H, C*H*_2_). ^13^C NMR (125.75 MHz, CDCl_3_, 23 °C): δ = 162.97 (*C*-3, aromatic), 138.12 (*C*-1, aromatic), 133.33 (*C*-1, phenyl), 130.79 (*C*-5, aromatic), 130.09 (*C*-2, thiophene), 129.72 (*C*H-2,6, phenyl), 129.02 (*C*-3, thiophene), 128.81 (*C*H-3,5, phenyl), 128.67 (*C*H-4, phenyl), 127.42 (*C*-4, thiophene), 126.89 (*C*-5, thiophene), 124.48 (*C*-6, aromatic), 115.95 (*C*-2, aromatic), 115.42 (*C*-4, aromatic), 68.19 (*C*H), 50.28 (*C*H_2_) (Figure S7c).

**8b.** HRESIMS *m*/*z* 331.0615 [M+H]^+^ (calcd for C_18_H_16_FOS_2_^+^, 331.0621, Δ = 1.9 ppm) (Figure S8). HPLC R_t_ 24.23 min, purity > 99% (Figure S8a). Chiral HPLC R_t_ 10.60 min, >99% ee (Figure S8b). ^1^H NMR (500 MHz, CDCl_3_, 23 °C): δ = 7.43 (C*H*-2,6, phenyl), 7.40 (C*H*-3,5, phenyl), 7.39 (C*H*-4, phenyl), 7.39 (C*H*, aromatic-5), 7.34 (C*H*-5, thiophene), 7.23 (C*H*, aromatic-6), 7.14 (C*H*, aromatic-2), 7.05 (C*H*-4, thiophene), 7.05 (C*H*, aromatic-4), 6.98 (C*H*-3, thiophene), 4.72 (s, 1H, C*H*), 4.09/3.90 (AB, 2H, C*H*_2_). ^13^C NMR (125.75 MHz, CDCl_3_, 23 °C): δ = 162.97 (*C*-3, aromatic), 138.12 (*C*-1, aromatic), 133.33 (*C*-1, phenyl), 130.79 (*C*-5, aromatic), 130.08 (*C*-2, thiophene), 129.71 (*C*H-2,6, phenyl), 129.02 (*C*-3, thiophene), 128.81 (*C*H-3,5, phenyl), 128.67 (*C*H-4, phenyl), 127.42 (*C*-4, thiophene), 126.89 (*C*-5, thiophene), 124.48 (*C*-6, aromatic), 115.94 (*C*-2, aromatic), 115.41 (*C*-4, aromatic), 68.18 (*C*H), 50.28 (*C*H_2_) (Figure S8c).

3. Synthesis of 2-((((4-fluorophenyl)(phenyl)methyl)sulfinyl)methyl)thiophene (**5c**–**8c**)

Following general procedure A, 0.6 g (3.0 mmol) of (4-fluorophenyl)(phenyl)methanol, 0.4 g (3.0 mmol) of thiophen-2-ylmethanethiol, and (0.41 mL, 3.3 mmol) of 48% BF_3_·Et_2_O were reacted, affording 0.8 g of **3c** as a yellow oily product (yield 92%).

Following general procedure B, 0.8 g (2.7 mmol) of **3c** dissolved in 10 mL of glacial acetic acid was reacted with 30% H_2_O_2_ (0.31 mL) to give 0.4 g of **4c** as orange oil (yield 45%).

Racemic **4c** was further separated into two individual pairs of enantiomers by means of an MPLC system equipped with a glass column packed with silica gel. Toluol/EtOAc = 80/20 was used as the mobile phase, and the separation was carried out with a flow rate of 5 mL/min. Individual pairs of enantiomers were further separated into the individual enantiomers **5c**–**8c** by means of semipreparative chiral HPLC. A semipreparative HPLC instrument was equipped with a CHIRALPAK IA column (10 mm Φ × 25 cm L) (Daicel Inc.), and EtOAc was used as the mobile phase.

**5c.** HRESIMS *m*/*z* 353.0443 [M+Na]^+^ (calcd for C_18_H_15_FOS_2_Na^+^, 353.0441, Δ = −0.7 ppm) (Figure S9). HPLC R_t_ 23.03 min, purity 97.2% (Figure S9a). Chiral HPLC R_t_ 6.52 min, >99% ee (but contains 1.1% 7c) (Figure S9b). ^1^H NMR (500 MHz, CDCl_3_, 23 °C): δ = 7.44 (C*H*-3,5, phenyl), 7.39 (C*H*-2,6, phenyl), 7.39 (C*H*-4, phenyl), 7.39 (C*H*-2,6, aromatic), 7.34 (C*H*-5, thiophene), 7.06 (C*H*-3,5, aromatic), 7.05 (C*H*-4, thiophene), 6.96 (C*H*-3, thiophene), 4.73 (s, 1H, C*H*), 4.09/3.88 (AB, 2H, C*H*_2_). ^13^C NMR (125.75 MHz, CDCl_3_, 23 °C): δ = 162.82 (*C*-4, aromatic), 135.29 (*C*-1, phenyl), 131.38 (*C*H-2,6, aromatic), 129.97 (*C*-2, thiophene), 129.92 (*C*-1, aromatic), 129.40 (*C*H-3,5, phenyl), 129.08 (*C*-3, thiophene), 128.77 (*C*H-2,6, phenyl), 128.58 (*C*-4, phenyl), 127.32 (*C*-4, thiophene), 126.89 (*C*-5, thiophene), 115.62 (*C*H-3,5, aromatic), 68.20 (*C*H), 50.01 (*C*H_2_) (Figure S9c).

**6c.** HRESIMS *m*/*z* 353.0442 [M+Na]^+^ (calcd for C_18_H_15_FOS_2_Na^+^, 353.0441, Δ = −0.5 ppm) (Figure S10). HPLC R_t_ 23.07 min, purity 98.3% (Figure S10a). Chiral HPLC R_t_ 10.18 min, >99% ee (Figure S10b). ^1^H NMR (500 MHz, CDCl_3_, 23 °C): δ = 7.44 (C*H*-3,5, phenyl), 7.39 (C*H*-2,6, phenyl), 7.39 (C*H*-4, phenyl), 7.39 (C*H*-2,6, aromatic), 7.34 (C*H*-5, thiophene), 7.06 (C*H*-3,5, aromatic), 7.05 (C*H*-4, thiophene), 6.96 (C*H*-3, thiophene), 4.73 (s, 1H, C*H*), 4.09/3.88 (AB, 2H, C*H*_2_). ^13^C NMR (125.75 MHz, CDCl_3_, 23 °C): δ = 162.82 (*C*-4, aromatic), 135.29 (*C*-1, phenyl), 131.38 (*C*H-2,6, aromatic), 129.97 (*C*-2, thiophene), 129.92 (*C*-1, aromatic), 129.40 (*C*H-3,5, phenyl), 129.08 (*C*-3, thiophene), 128.77 (*C*H-2,6, phenyl), 128.58 (*C*-4, phenyl), 127.32 (*C*-4, thiophene), 126.89 (*C*-5, thiophene), 115.62 (*C*H-3,5, aromatic), 68.20 (*C*H), 50.01 (*C*H_2_) (Figure S10c).

**7c.** HRESIMS *m*/*z* 353.0443 [M+Na]^+^ (calcd for C_18_H_15_FOS_2_Na^+^, 353.0441, Δ = −0.6 ppm) (Figure S11). HPLC R_t_ 22.93 min, purity > 99% (Figure S11a). Chiral HPLC R_t_ 7.50 min, >99% ee (Figure S11b). ^1^H NMR (500 MHz, CDCl_3_, 23 °C): δ = 7.42 (C*H*-2,6, phenyl), 7.41 (C*H*-2,6, aromatic), 7.39 (C*H*-3,5, phenyl), 7.37 (C*H*-4, phenyl), 7.34 (C*H*-5, thiophene), 7.10 (C*H*-3,5, aromatic), 7.05 (C*H*-4, thiophene), 6.96 (C*H*-3, thiophene), 4.73 (s, 1H, C*H*), 4.08/3.89 (AB, 2H, C*H*_2_). ^13^C NMR (125.75 MHz, CDCl_3_, 23 °C): δ = 162.53 (*C*-4, aromatic), 135.79 (*C*-1, phenyl), 131.52 (*C*-1, aromatic), 130.55 (*C*H-2,6, aromatic), 130.17 (*C*-2, thiophene), 129.63 (*C*H-2,6, phenyl), 128.98 (*C*-3, thiophene), 128.76 (*C*H-3,5, phenyl), 128.55 (*C*-4, phenyl), 127.39 (*C*-4, thiophene), 126.84 (*C*-5, thiophene), 116.24 (*C*H-3,5, aromatic), 67.87 (*C*H), 50.20 (*C*H_2_) (Figure S11c).

**8c.** HRESIMS *m*/*z* 353.0441 [M+Na]^+^ (calcd for C_18_H_15_FOS_2_Na^+^, 353.0441, Δ = 0.1 ppm)% (Figure S12). HPLC R_t_ 21.80 min, purity > 99% (Figure S12a). Chiral HPLC R_t_ 11.61 min, >99% ee (Figure S12b). ^1^H NMR (500 MHz, CDCl_3_, 23 °C): δ = 7.42 (C*H*-2,6, phenyl), 7.41 (C*H*-2,6, aromatic), 7.39 (C*H*-3,5, phenyl), 7.37 (C*H*-4, phenyl), 7.34 (C*H*-5, thiophene), 7.10 (C*H*-3,5, aromatic), 7.05 (C*H*-4, thiophene), 6.96 (C*H*-3, thiophene), 4.73 (s, 1H, C*H*), 4.08/3.89 (AB, 2H, C*H*_2_). ^13^C NMR (125.75 MHz, CDCl_3_, 23 °C): δ = 162.53 (*C*-4, aromatic), 135.79 (*C*-1, phenyl), 131.52 (*C*-1, aromatic), 130.55 (*C*H-2,6, aromatic), 130.17 (*C*-2, thiophene), 129.63 (*C*H-2,6, phenyl), 128.98 (*C*-3, thiophene), 128.76 (*C*H-3,5, phenyl), 128.55 (*C*-4, phenyl), 127.39 (*C*-4, thiophene), 126.84 (*C*-5, thiophene), 116.24 (*C*H-3,5, aromatic), 67.87 (*C*H), 50.20 (*C*H_2_) (Figure S12c).

4. Synthesis of 2-((((2-chlorophenyl)(phenyl)methyl)sulfinyl)methyl)thiophene (**5d**–**8d**)

Following general procedure A, 1.0 g (4.5 mmol) of (2-chlorophenyl)(phenyl)methanol, 0.59 g (4.5 mmol) of thiophen-2-ylmethanethiol, and (0.61 mL, 4.9 mmol) of 48% BF_3_·Et_2_O were reacted, affording 1.3 g of **3d** as a yellow oily product (yield 88%).

Following general procedure B, 1.3 g (3.9 mmol) of **3d** dissolved in 12 mL of glacial acetic acid was reacted with 30% H_2_O_2_ (0.5 mL) to give 1.0 g of **4d** as a yellow solid (yield 78%).

Racemic **4d** was further separated into the individual enantiomers **5d**–**8d** by means of a semipreparative chiral HPLC method. A semipreparative HPLC instrument was equipped with a CHIRALPAK IA column (10 mm Φ × 25 cm L) (Daicel Inc.), and EtOAc was used as the mobile phase. During this process, **5d** and **8d** were fully separated, whereas the unresolved pair of **6d** and **7d** was further separated using Hexane/THF = 80/20 (*v*/*v*) as the mobile phase.

**5d**. (*R*,*R*)-*2-((((2-chlorophenyl)(phenyl)methyl)sulfinyl)methyl)thiophene.* HRESIMS *m*/*z* 347.0323 [M+H]^+^ (calcd for C_18_H_16_ClOS_2_^+^, 347.0326, Δ = 0.7 ppm) (Figure S13). HPLC R_t_ 25.60 min, purity 98.8% (Figure S13a). Chiral HPLC R_t_ 4.98 min, 96.5% ee (Figure S13b). ^1^H NMR (500 MHz, CDCl_3_, 23 °C): δ = 7.77 (C*H*-6, aromatic), 7.48 (C*H*-2,6, phenyl), 7.42 (C*H*-3, aromatic), 7.39 (C*H*-3,5, phenyl), 7.36 (C*H*-4, phenyl), 7.35 (C*H*-5, aromatic), 7.32 (s, 1H, C*H*-5, thiophene), 7.28 (C*H*-4, aromatic), 7.02 (s, 1H, C*H*-4, thiophene), 6.95 (s, 1H, C*H*-3, thiophene), 5.32 (s, 1H, C*H*), 4.13/4.02 (AB, 2H, C*H*_2_). ^13^C NMR (125.75 MHz, CDCl_3_, 23 °C): δ = 134.39 (*C*_q_-1, aromatic), 134.04 (*C*_q_-2, aromatic), 132.78 (*C*_q_-1, phenyl), 130.47 (*C*_q_, thiophene-2), 130.33 (*C*H-3, aromatic), 130.09 (*C*H-2,6, phenyl), 129.53 (*C*H-6, aromatic), 129.39 (*C*H-4, aromatic), 128.92 (*C*H-3, thiophene), 128.68 (*C*H-3,5, phenyl), 128.56 (*C*H-4, phenyl), 127.48 (*C*H-5, aromatic), 127.45 (*C*H-4, thiophene), 126.88 (*C*H-5, thiophene), 64.77 (*C*H), 50.99 (*C*H_2_) (Figure S13b).

**6d**. (*S*,*S*)-*2-((((2-chlorophenyl)(phenyl)methyl)sulfinyl)methyl)thiophene.* HRESIMS *m*/*z* 347.0323 [M+H]^+^ (calcd for C_18_H_16_ClOS_2_^+^, 347.0326, Δ = 0.7 ppm) (Figure S14). HPLC R_t_ 25.60 min, purity > 99% (Figure S14a). Chiral HPLC R_t_ 21.95 min, >99% ee (Figure S14b). ^1^H NMR (500 MHz, CDCl_3_, 23 °C): δ = 7.77 (C*H*-6, aromatic), 7.48 (C*H*-2,6, phenyl), 7.42 (C*H*-3, aromatic), 7.39 (C*H*-3,5, phenyl), 7.36 (C*H*-4, phenyl), 7.35 (C*H*-5, aromatic), 7.32 (s, 1H, C*H*-5, thiophene), 7.28 (C*H*-4, aromatic), 7.02 (s, 1H, C*H*-4, thiophene), 6.95 (s, 1H, C*H*-3, thiophene), 5.32 (s, 1H, C*H*), 4.13/4.02 (AB, 2H, C*H*_2_). ^13^C NMR (125.75 MHz, CDCl_3_, 23 °C): δ = 134.39 (*C*_q_-1, aromatic), 134.04 (*C*_q_-2, aromatic) 132.78 (*C*_q_-1, phenyl), 130.47 (*C*_q_, thiophene-2), 130.33 (*C*H-3, aromatic), 130.09 (*C*H-2,6, phenyl), 129.53 (*C*H-6, aromatic), 129.39 (*C*H-4, aromatic), 128.92 (*C*H-3, thiophene), 128.68 (*C*H-3,5, phenyl), 128.56 (*C*H-4, phenyl), 127.48 (*C*H-5, aromatic), 127.45 (*C*H-4, thiophene), 126.88 (*C*H-5, thiophene), 64.77 (*C*H), 50.99 (*C*H_2_) (Figure S14c).

**7d**. (*R*,*S*)-*2-((((2-chlorophenyl)(phenyl)methyl)sulfinyl)methyl)thiophene.* HRESIMS *m*/*z* 347.0324 [M+H]^+^ (calcd for C_18_H_16_ClOS_2_^+^, 347.0326, Δ = 0.4 ppm) (Figure S15). HPLC R_t_ 25.82 min, purity 95.9% (Figure S15a). Chiral HPLC R_t_ 22.37 min, >99% ee (Figure S15b). ^1^H NMR (500 MHz, CDCl_3_, 23 °C): δ = 7.95 (C*H*-6, aromatic), 7.47 (C*H*-2,6, phenyl), 7.43 (C*H*-3, aromatic), 7.39 (C*H*-5, aromatic), 7.39 (C*H*-3,5, phenyl), 7.35 (C*H*-4, phenyl), 7.32 (s, 1H, C*H*-5, thiophene), 7.28 (C*H*-4, aromatic), 7.03 (s, 1H, C*H*-4, thiophene), 6.97 (s, 1H, C*H*-3, thiophene), 5.53 (s, 1H, C*H*), 4.19/3.92 (AB, 2H, C*H*_2_). ^13^C NMR (125.75 MHz, CDCl_3_, 23 °C): δ = 135.21 (*C*_q_-1, phenyl), 134.91 (*C*_q_-2, aromatic), 132.96 (*C*_q_-1, aromatic), 130.43 (*C*_q_, thiophene-2), 130.15 (*C*H-6, aromatic), 130.00 (*C*H-3, aromatic), 129.28 (*C*H-4, aromatic), 129.21 (*C*H-3,5, phenyl), 129.13 (*C*H-3, thiophene), 128.91 (*C*H-2,6, phenyl), 128.47 (*C*H-4, phenyl), 127.38 (*C*H-4, thiophene), 127.32 (*C*H-5, aromatic), 126.90 (*C*H-5, thiophene), 64.75 (*C*H), 50.54 (*C*H_2_) (Figure S15c).

**8d**. (*S*,*R*)-*2-((((2-chlorophenyl)(phenyl)methyl)sulfinyl)methyl)thiophene.* HRESIMS *m*/*z* 347.0325 [M+H]^+^ (calcd for C_18_H_16_ClOS_2_^+^, 347.0326, Δ = 0.3 ppm) (Figure S16). HPLC R_t_ 25.82 min, purity 98.5% (Figure S16a). Chiral HPLC R_t_ 8.32 min, >99% ee (Figure S16b). ^1^H NMR (500 MHz, CDCl_3_, 23 °C): δ = 7.95 (C*H*-6, aromatic), 7.47 (C*H*-2,6, phenyl), 7.43 (C*H*-3, aromatic), 7.39 (C*H*-5, aromatic), 7.39 (C*H*-3,5, phenyl), 7.35 (C*H*-4, phenyl), 7.32 (s, 1H, C*H*-5, thiophene), 7.28 (C*H*-4, aromatic), 7.03 (s, 1H, C*H*-4, thiophene), 6.97 (s, 1H, C*H*-3, thiophene), 5.53 (s, 1H, C*H*), 4.19/3.92 (AB, 2H, C*H*_2_). ^13^C NMR (125.75 MHz, CDCl_3_, 23 °C): δ = 135.21 (*C*_q_-1, phenyl), 134.91 (*C*_q_-2, aromatic), 132.96 (*C*_q_-1, aromatic), 130.43 (*C*_q_, thiophene-2), 130.15 (*C*H-6, aromatic), 130.00 (*C*H-3, aromatic), 129.28 (*C*H-4, aromatic), 129.21 (*C*H-3,5, phenyl), 129.13 (*C*H-3, thiophene), 128.91 (*C*H-2,6, phenyl), 128.47 (*C*H-4, phenyl), 127.38 (*C*H-4, thiophene), 127.32 (*C*H-5, aromatic), 126.90 (*C*H-5, thiophene), 64.75 (*C*H), 50.54 (*C*H_2_) (Figure S16c).

5. Synthesis of 2-((((3-chlorophenyl)(phenyl)methyl)sulfinyl)methyl)thiophene (**5e**–**8e**)

Following general procedure A, 2.2 g (10.0 mmol) of (3-chlorophenyl)(phenyl)methanol, 1.3 g (10.0 mmol) of thiophen-2-ylmethanethiol, and (1.4 mL, 11 mmol) of 48% BF_3_·Et_2_O were reacted, affording 1.6 g of **3e** as a yellow oily product (yield 50%).

Following general procedure B, 1.6 g (4.8 mmol) of **3e** dissolved in 20 mL of glacial acetic acid was reacted with 30% H_2_O_2_ (0.5 mL) to give 0.5 g of **4e** as a yellow-brown solid (yield 9%).

Racemic **4e** was further separated into two individual pairs of enantiomers by means of an MPLC system equipped with a glass column packed with silica gel. Toluol/EtOAc = 80/20 was used as the mobile phase, and the separation was carried out with a flow rate of 5 mL/min. Individual pairs of enantiomers were further separated into the individual enantiomers **5e**–**8e** by means of semipreparative chiral HPLC. A semipreparative HPLC instrument was equipped with a CHIRALPAK IA column (10 mm *Φ* × 25 cm L) (Daicel Inc.), and EtOAc was used as the mobile phase.

**5e**. (*S*,*R*)-*2-((((3-chlorophenyl)(phenyl)methyl)sulfinyl)methyl)thiophene.* HRESIMS *m*/*z* 347.0322 [M+H]^+^ (calcd for C_18_H_16_ClOS_2_^+^, 347.0326, Δ = 1.1 ppm) (Figure S17). HPLC R_t_ 24.65 min, purity 95.3% (Figure S17a). Chiral HPLC R_t_ 6.68 min, >99% ee (but contains 4.7% 7e) (Figure S17b). ^1^H NMR (500 MHz, CDCl_3_, 23 °C): δ = 7.44 (C*H*-3,5, phenyl), 7.40 (C*H*-2, aromatic), 7.40 (C*H*-2,6, phenyl), 7.40 (C*H*-4, phenyl), 7.34 (C*H*-5, thiophene), 7.32 (C*H*-6, aromatic), 7.31 (C*H*-4, aromatic), 7.31 (C*H*-5, aromatic), 7.06 (C*H*-4, thiophene), 6.96 (C*H*-3, thiophene), 4.71 (s, 1H, C*H*), 4.10/3.89 (AB, 2H, C*H*_2_). ^13^C NMR (125.75 MHz, CDCl_3_, 23 °C): δ = 136.33 (*C*-1, aromatic), 134.53 (*C*-3, aromatic), 134.53 (*C*-4, phenyl), 134.53 (*C*-2, thiophene), 129.85 (*C*-5, aromatic), 129.55 (*C*-2, aromatic), 129.48 (*C*H-3,5, phenyl), 129.19 (*C*-3, thiophene), 128.76 (*C*H-2,6, phenyl), 128.73 (*C*-4, phenyl), 128.59 (*C*-4, aromatic), 127.89 (*C*-6, aromatic), 127.34 (*C*-4, thiophene), 126.98 (*C*-4, thiophene), 68.72 (*C*H), 50.08 (*C*H_2_) (Figure S17c).

**6e**. (*R*,*S*)-*2-((((3-chlorophenyl)(phenyl)methyl)sulfinyl)methyl)thiophene.* HRESIMS *m*/*z* 347.0322 [M+H]^+^ (calcd for C_18_H_16_ClOS_2_^+^, 347.0326, Δ = 1.0 ppm) (Figure S18). HPLC R_t_ 26.03 min, purity 98.5% (Figure S18a). Chiral HPLC R_t_ 9.04 min, >99% ee (Figure S18b). ^1^H NMR (500 MHz, CDCl_3_, 23 °C): δ = 7.44 (C*H*-3,5, phenyl), 7.40 (C*H*-2, aromatic), 7.40 (C*H*-2,6, phenyl), 7.40 (C*H*-4, phenyl), 7.34 (C*H*-5, thiophene), 7.32 (C*H*-6, aromatic), 7.31 (C*H*-4, aromatic), 7.31 (C*H*-5, aromatic), 7.06 (C*H*-4, thiophene), 6.96 (C*H*-3, thiophene), 4.71 (s, 1H, C*H*), 4.10/3.89 (AB, 2H, C*H*_2_). ^13^C NMR (125.75 MHz, CDCl_3_, 23 °C): δ = 136.33 (*C*-1, aromatic), 134.53 (*C*-3, aromatic), 134.53 (*C*-4, phenyl), 134.53 (*C*-2, thiophene), 129.85 (*C*-5, aromatic), 129.55 (*C*-2, aromatic), 129.48 (*C*H-3,5, phenyl), 129.19 (*C*-3, thiophene), 128.76 (*C*H-2,6, phenyl), 128.73 (*C*-4, phenyl), 128.59 (*C*-4, aromatic), 127.89 (*C*-6, aromatic), 127.34 (*C*-4, thiophene), 126.98 (*C*-4, thiophene), 68.72 (*C*H), 50.08 (*C*H_2_) (Figure S18c).

**7e**. (*S*,*S*)-*2-((((3-chlorophenyl)(phenyl)methyl)sulfinyl)methyl)thiophene.* HRESIMS *m*/*z* 347.0324 [M+H]^+^ (calcd for C_18_H_16_ClOS_2_^+^, 347.0326, Δ = 0.3 ppm) (Figure S19). HPLC R_t_ 25.87 min, purity > 99% (Figure S19a). Chiral HPLC R_t_ 6.95 min, >99% ee (but contains 5.0% 5e) (Figure S19b). ^1^H NMR (500 MHz, CDCl_3_, 23 °C): δ = 7.43 (C*H*-2,6, phenyl), 7.43 (C*H*-2, aromatic), 7.40 (C*H*-6, aromatic), 7.40 (C*H*-3,5, phenyl), 7.39 (C*H*-4, phenyl), 7.34 (C*H*-5, thiophene), 7.34 (C*H*-4, aromatic), 7.34 (C*H*-5, aromatic), 7.06 (C*H*-4, thiophene), 6.97 (C*H*-3, thiophene), 4.70 (s, 1H, C*H*), 4.10/3.90 (AB, 2H, C*H*_2_). ^13^C NMR (125.75 MHz, CDCl_3_, 23 °C): δ = 137.77 (*C*-1, aromatic), 135.06 (*C*-3, aromatic), 133.23 (*C*-1, phenyl), 130.43 (*C*-5, aromatic), 130.05 (*C*-2, thiophene), 129.71 (*C*-2, aromatic), 129.71 (*C*H-2,6, phenyl), 129.01 (*C*-3, thiophene), 128.92 (*C*-6, aromatic), 128.82 (*C*H-3,5, phenyl), 128.68 (*C*-4, phenyl), 127.43 (*C*-4, phenyl), 126.90 (*C*-5, phenyl), 68.08 (*C*H), 50.31 (*C*H_2_) (Figure S19c).

**8e**. (*R*,*R*)-*2-((((3-chlorophenyl)(phenyl)methyl)sulfinyl)methyl)thiophene.* HRESIMS *m*/*z* 347.0321 [M+H]^+^ (calcd for C_18_H_16_ClOS_2_^+^, 347.0326, Δ = 1.3 ppm) (Figure S20). HPLC R_t_ 25.87 min, purity > 99% (Figure S20a). Chiral HPLC R_t_ 9.29 min, >99% ee (Figure S20b). ^1^H NMR (500 MHz, CDCl_3_, 23 °C): δ = 7.43 (C*H*-2,6, phenyl), 7.43 (C*H*-2, aromatic), 7.40 (C*H*-6, aromatic), 7.40 (C*H*-3,5, phenyl), 7.39 (C*H*-4, phenyl), 7.34 (C*H*-5, thiophene), 7.34 (C*H*-4, aromatic), 7.34 (C*H*-5, aromatic), 7.06 (C*H*-4, thiophene), 6.97 (C*H*-3, thiophene), 4.70 (s, 1H, C*H*), 4.10/3.90 (AB, 2H, C*H*_2_). ^13^C NMR (125.75 MHz, CDCl_3_, 23 °C): δ = 137.77 (*C*-1, aromatic), 135.06 (*C*-3, aromatic), 133.23 (*C*-1, phenyl), 130.43 (*C*-5, aromatic), 130.05 (*C*-2, thiophene), 129.71 (*C*-2, aromatic), 129.71 (*C*H-2,6, phenyl), 129.01 (*C*-3, thiophene), 128.92 (*C*-6, aromatic), 128.82 (*C*H-3,5, phenyl), 128.68 (*C*-4, phenyl), 127.43 (*C*-4, phenyl), 126.90 (*C*-5, phenyl), 68.08 (*C*H), 50.31 (*C*H_2_) (Figure S20c).

6. Synthesis of 2-((((4-chlorophenyl)(phenyl)methyl)sulfinyl)methyl)thiophene (**5f**–**8f**)

Following general procedure A, 1.68 g (7.7 mmol) of (4-chlorophenyl)(phenyl)methanol, 1.0 g (7.7 mmol) of thiophen-2-ylmethanethiol, and (1.0 mL, 8.5 mmol) of 48% BF_3_·Et_2_O were reacted, affording 1.7 g of **3f** as a yellow oily product (yield 67%).

Following general procedure B, 1.7 g (5.1 mmol) of **3f** dissolved in 15 mL of glacial acetic acid was reacted with 30% H_2_O_2_ (0.6 mL) to give 0.22 g of **4f** as a pale-yellow solid (yield 11%).

Racemic **4f** was further separated into two individual pairs of enantiomers by means of an MPLC system equipped with a glass column packed with silica gel. Toluol/EtOAc = 80/20 was used as the mobile phase, and the separation was carried out with a flow rate of 5 mL/min. Individual pairs of enantiomers were further separated into the individual enantiomers **5f**–**8f** by means of semipreparative chiral HPLC. A semipreparative HPLC instrument was equipped with a CHIRALPAK IA column (10 mm Φ × 25 cm L) (Daicel Inc.), and EtOAc was used as the mobile phase.

**5f**. (*S*,*R*)-*2-((((4-chlorophenyl)(phenyl)methyl)sulfinyl)methyl)thiophene.* HRESIMS *m*/*z* 369.0143 [M+Na]^+^ (calcd for C_18_H_15_ClNaOS_2_^+^, 369.0145, Δ = 0.6 ppm) (Figure S21). HPLC Rt 24.67 min, purity > 99% (Figure S21a). Chiral HPLC R_t_ 7.10 min, >99% ee (but contains 1.8% 7f) (Figure S21b). ^1^H NMR (500 MHz, CDCl_3_, 23 °C): δ = 7.43 (C*H*-2,6, phenyl), 7.39 (C*H*-3,5, phenyl), 7.39 (C*H*-4, phenyl), 7.35 (C*H*-3,5, aromatic), 7.34 (C*H*-2,6, aromatic), 7.34 (s, 1H, C*H*-5, thiophene), 7.05 (s, 1H, C*H*-4, thiophene), 6.95 (s, 1H, C*H*-3, thiophene), 4.71 (s, 1H, C*H*), 4.09/3.88 (AB, 2H, C*H*_2_). ^13^C NMR (125.75 MHz, CDCl_3_, 23 °C): δ = 135.01 (*C*_q_-1, phenyl), 134.56 (*C*_q_-4, aromatic), 132.65 (*C*_q_-1, aromatic), 130.96 (*C*H-2,6, aromatic), 130.08 (*C*_q_, thiophene-2), 129.64 (*C*H-2,6, phenyl), 129.00 (*C*H-3, thiophene), 128.81 (*C*H-3,5, aromatic), 128.79 (*C*H-3,5, phenyl), 128.61 (*C*H-4, phenyl), 127.40 (*C*H-4, thiophene), 126.87 (*C*H-5, thiophene), 68.27 (*C*H), 50.05 (*C*H_2_) (Figure S21c).

**6f**. (*R*,*S*)-*2-((((4-chlorophenyl)(phenyl)methyl)sulfinyl)methyl)thiophene.* HRESIMS *m*/*z* 369.0145 [M+Na]^+^ (calcd for C_18_H_15_ClNaOS_2_^+^, 369.0145, Δ = −0.1 ppm) (Figure S22). HPLC R_t_ 24.67 min, purity 97.9% (Figure S22a). Chiral HPLC R_t_ 12.94 min, >99% ee (Figure S22b). ^1^H NMR (500 MHz, CDCl_3_, 23 °C): δ = 7.43 (C*H*-2,6, phenyl), 7.39 (C*H*-3,5, phenyl), 7.39 (C*H*-4, phenyl), 7.35 (C*H*-3,5, aromatic), 7.34 (C*H*-2,6, aromatic), 7.34 (s, 1H, C*H*-5, thiophene), 7.05 (s, 1H, C*H*-4, thiophene), 6.95 (s, 1H, C*H*-3, thiophene), 4.71 (s, 1H, C*H*), 4.09/3.88 (AB, 2H, C*H*_2_). ^13^C NMR (125.75 MHz, CDCl_3_, 23 °C): δ = 135.01 (*C*_q_-1, phenyl), 134.56 (*C*_q_-4, aromatic), 132.65 (*C*_q_-1, aromatic), 130.96 (*C*H-2,6, aromatic), 130.08 (*C*_q_, thiophene-2), 129.64 (*C*H-2,6, phenyl), 129.00 (*C*H-3, thiophene), 128.81 (*C*H-3,5, aromatic), 128.79 (*C*H-3,5, phenyl), 128.61 (*C*H-4, phenyl), 127.40 (*C*H-4, thiophene), 126.87 (*C*H-5, thiophene), 68.27 (*C*H), 50.05 (*C*H_2_) (Figure S22c).

**7f**. (*S*,*S*)-*2-((((4-chlorophenyl)(phenyl)methyl)sulfinyl)methyl)thiophene.* HRESIMS *m*/*z* 369.0144 [M+Na]^+^ (calcd for C_18_H_15_ClNaOS_2_^+^, 369.0145, Δ = 0.3 ppm) (Figure S23). HPLC R_t_ 24.70 min, purity > 99% (Figure S23a). Chiral HPLC R_t_ 7.63 min, >99% ee (but contains 0.9% 5f) (Figure S23b). ^1^H NMR (500 MHz, CDCl_3_, 23 °C): δ = 7.42 (C*H*-2,6, aromatic), 7.42 (C*H*-2,6, phenyl), 7.39 (C*H*-3,5, phenyl), 7.38 (C*H*-3,5, aromatic), 7.37 (C*H*-4, phenyl), 7.34 (s, 1H, C*H*-5, thiophene), 7.05 (s, 1H, C*H*-4, thiophene), 6.96 (s, 1H, C*H*-3, thiophene), 4.71 (s, 1H, C*H*), 4.09/3.89 (AB, 2H, C*H*_2_). ^13^C NMR (125.75 MHz, CDCl_3_, 23 °C): δ = 134.40 (*C*_q_-4, aromatic), 134.22 (*C*_q_-1, aromatic), 133.50 (*C*_q_-1, phenyl), 131.14 (*C*H-2,6, aromatic), 129.88 (*C*_q_, thiophene-2), 129.64 (*C*H-2,6, phenyl), 129.40 (*C*H-3,5, aromatic), 129.11 (*C*H-3, thiophene), 128.79 (*C*H-3,5, phenyl), 128.64 (*C*H-4, phenyl), 127.34 (*C*H-4, thiophene), 126.92 (*C*H-5, thiophene), 67.94 (*C*H), 50.23 (*C*H_2_) (Figure S23c).

**8f**. (*R*,*R*)-*2-((((4-chlorophenyl)(phenyl)methyl)sulfinyl)methyl)thiophene.* HRESIMS *m*/*z* 369.0145 [M+Na]^+^ (calcd for C_18_H_15_ClNaOS_2_^+^, 369.0145, Δ = 0.1 ppm) (Figure S24). HPLC R_t_ 24.72 min, purity > 99% (Figure S24a). Chiral HPLC R_t_ 11.39 min, >99% ee (but contains 0.8% 6f) (Figure S24b). ^1^H NMR (500 MHz, CDCl_3_, 23 °C): δ = 7.42 (C*H*-2,6, aromatic), 7.42 (C*H*-2,6, phenyl), 7.39 (C*H*-3,5, phenyl), 7.38 (C*H*-3,5, aromatic), 7.37 (C*H*-4, phenyl), 7.34 (s, 1H, C*H*-5, thiophene), 7.05 (s, 1H, C*H*-4, thiophene), 6.96 (s, 1H, C*H*-3, thiophene), 4.71 (s, 1H, C*H*), 4.09/3.89 (AB, 2H, C*H*_2_). ^13^C NMR (125.75 MHz, CDCl_3_, 23 °C): δ = 134.40 (*C*_q_-4, aromatic), 134.22 (*C*_q_-1, aromatic), 133.50 (*C*_q_-1, phenyl), 131.14 (*C*H-2,6, aromatic), 129.88 (*C*_q_, thiophene-2), 129.64 (*C*H-2,6, phenyl), 129.40 (*C*H-3,5, aromatic), 129.11 (*C*H-3, thiophene), 128.79 (*C*H-3,5, phenyl), 128.64 (*C*H-4, phenyl), 127.34 (*C*H-4, thiophene), 126.92 (*C*H-5, thiophene), 67.94 (*C*H), 50.23 (*C*H_2_) (Figure S24c).

7. Synthesis of 2-((((2-bromophenyl)(phenyl)methyl)sulfinyl)methyl)thiophene (**5g**–**8g**)

Following general procedure A, 1.0 g (3.8 mmol) of (2-bromophenyl)(phenyl)methanol, 0.49 g (3.8 mmol) of thiophen-2-ylmethanethiol, and (0.5 mL, 4.2 mmol) of 48% BF_3_·Et_2_O were reacted, affording 1.34 g of **3g** as a white oily product (yield 94%).

Following general procedure B, 1.34 g (3.5 mmol) of **3g** dissolved in 12 mL of glacial acetic acid was reacted with 30% H_2_O_2_ (0.37 mL) to give 0.6 g of **4g** as a white solid (yield 43%).

Racemic **4g** was further separated into two individual pairs of enantiomers by means of flash column chromatography on silica gel with Toluol/EtOAc = 80/20 as the mobile phase. Individual pairs of enantiomers were further separated into the individual enantiomers **5g**–**8g** by means of semipreparative chiral HPLC. A semipreparative HPLC instrument was equipped with a CHIRALPAK IA column (10 mm Φ × 25 cm L) (Daicel Inc.), and Heptane/EtOAc = 70/30 was used as the mobile phase in the case of **5g/6g** and 100% EtOAc in the case of **7g/8g**.

**5g.** HRESIMS *m*/*z* 390.9820 [M+H]^+^ (calcd for C_18_H_16_BrOS_2_^+^, 390.9820, Δ = 0.1 ppm) (Figure S25). HPLC R_t_ 26.00 min, purity > 99% (Figure S25a). Chiral HPLC R_t_ 4.99 min, >99% ee (Figure S25b). ^1^H NMR (500 MHz, CDCl_3_, 23 °C): δ = 7.77 (C*H*-6, aromatic), 7.61 (C*H*-3, aromatic), 7.49 (C*H*-2,6, phenyl), 7.40 (C*H*-5, aromatic), 7.40 (C*H*-3,5, phenyl), 7.38 (C*H*-4, phenyl), 7.32 (s, 1H, C*H*-5, thiophene), 7.20 (C*H*-4, aromatic), 7.02 (s, 1H, C*H*-4, thiophene), 6.96 (s, 1H, C*H*-3, thiophene), 5.33 (s, 1H, C*H*), 4.14/4.04 (AB, 2H, C*H*_2_). ^13^C NMR (125.75 MHz, CDCl_3_, 23 °C): δ = 136.02 (*C*_q_-1, aromatic), 133.69 (*C*H-3, aromatic), 132.76 (*C*_q_-1, phenyl), 130.53 (*C*_q_, thiophene-2), 130.13 (*C*H-2,6, phenyl), 129.71 (*C*H-6, aromatic), 129.65 (*C*H-4, aromatic), 128.94 (*C*H-3, thiophene), 128.68 (*C*H-3,5, phenyl), 128.57 (*C*H-4, phenyl), 128.11 (*C*H-5, aromatic), 127.48 (*C*H-4, thiophene), 126.89 (*C*H-5, thiophene), 124.90 (*C*_q_-2, aromatic) 67.28 (*C*H), 51.04 (*C*H_2_) (Figure S25c).

**6g.** HRESIMS *m*/*z* 390.9817 [M+H]^+^ (calcd for C_18_H_16_BrOS_2_^+^, 390.9820, Δ = 0.9 ppm) (Figure S26). HPLC R_t_ 25.98 min, purity > 99% (Figure S26a). Chiral HPLC R_t_ 25.78 min, 97.0% ee (Figure S26b). ^1^H NMR (500 MHz, CDCl_3_, 23 °C): δ = 7.77 (C*H*-6, aromatic), 7.61 (C*H*-3, aromatic), 7.49 (C*H*-2,6, phenyl), 7.39 (C*H*-5, aromatic), 7.39 (C*H*-3,5, phenyl), 7.37 (C*H*-4, phenyl), 7.32 (s, 1H, C*H*-5, thiophene), 7.20 (C*H*-4, aromatic), 7.02 (s, 1H, C*H*-4, thiophene), 6.96 (s, 1H, C*H*-3, thiophene), 5.33 (s, 1H, C*H*), 4.13/4.04 (AB, 2H, C*H*_2_). ^13^C NMR (125.75 MHz, CDCl_3_, 23 °C): δ = 136.02 (*C*_q_-1, aromatic), 133.68 (*C*H-3, aromatic), 132.75 (*C*_q_-1, phenyl), 130.53 (*C*_q_, thiophene-2), 130.13 (*C*H-2,6, phenyl), 129.71 (*C*H-6, aromatic), 129.65 (*C*H-4, aromatic), 128.93 (*C*H-3, thiophene), 128.67 (*C*H-3,5, phenyl), 128.56 (*C*H-4, phenyl), 128.10 (*C*H-5, aromatic), 127.47 (*C*H-4, thiophene), 126.87 (*C*H-5, thiophene), 124.90 (*C*_q_-2, aromatic), 67.27 (*C*H), 51.04 (*C*H_2_) (Figure S26c).

**7g.** HRESIMS *m*/*z* 390.9819 [M+H]^+^ (calcd for C_18_H_16_BrOS_2_^+^, 390.9820, Δ = 0.3 ppm) (Figure S27). HPLC R_t_ 26.20 min, purity 97.6% (Figure S27a). Chiral HPLC R_t_ 27.22 min, 91.2% ee (Figure S27b). ^1^H NMR (500 MHz, CDCl_3_, 23 °C): δ = 7.96 (C*H*-6, aromatic), 7.61 (C*H*-3, aromatic), 7.48 (C*H*-2,6, phenyl), 7.42 (C*H*-5, aromatic), 7.39 (C*H*-3,5, phenyl), 7.34 (C*H*-4, phenyl), 7.32 (s, 1H, C*H*-5, thiophene), 7.20 (C*H*-4, aromatic), 7.03 (s, 1H, C*H*-4, thiophene), 6.97 (s, 1H, C*H*-3, thiophene), 5.53 (s, 1H, C*H*), 4.21/3.92 (AB, 2H, C*H*_2_). ^13^C NMR (125.75 MHz, CDCl_3_, 23 °C): δ = 135.18 (*C*_q_-1, phenyl), 134.67 (*C*_q_-1, aromatic), 133.36 (*C*H-3, aromatic), 130.40 (*C*_q_, thiophene-2), 130.27 (*C*H-6, aromatic), 129.56 (*C*H-4, aromatic), 129.20 (*C*H-3,5, phenyl), 129.16 (*C*H-3, thiophene), 128.91 (*C*H-2,6, phenyl), 128.47 (*C*H-4, phenyl), 127.96 (*C*H-5, aromatic), 127.39 (*C*H-4, thiophene), 126.91 (*C*H-5, thiophene), 125.92 (*C*_q_-2, aromatic), 67.22 (*C*H), 50.47 (*C*H_2_) (Figure S27c).

**8g.** HRESIMS *m*/*z* 390.9819 [M+H]^+^ (calcd for C_18_H_16_BrOS_2_^+^, 390.9820, Δ = 0.3 ppm) (Figure S28). HPLC R_t_ 26.20 min, purity 97.2% (Figure S28a). Chiral HPLC R_t_ 8.51 min, >99% ee (Figure S28b). ^1^H NMR (500 MHz, CDCl_3_, 23 °C): δ = 7.96 (C*H*-6, aromatic), 7.61 (C*H*-3, aromatic), 7.48 (C*H*-2,6, phenyl), 7.42 (C*H*-5, aromatic), 7.39 (C*H*-3,5, phenyl), 7.34 (C*H*-4, phenyl), 7.32 (s, 1H, C*H*-5, thiophene), 7.19 (C*H*-4, aromatic), 7.03 (s, 1H, C*H*-4, thiophene), 6.97 (s, 1H, C*H*-3, thiophene), 5.53 (s, 1H, C*H*), 4.20/3.92 (AB, 2H, C*H*_2_). ^13^C NMR (125.75 MHz, CDCl_3_, 23 °C): δ = 135.18 (*C*_q_-1, phenyl), 134.67 (*C*_q_-1, aromatic), 133.37 (*C*H-3, aromatic), 130.40 (*C*_q_, thiophene-2), 130.27 (*C*H-6, aromatic), 129.56 (*C*H-4, aromatic), 129.21 (*C*H-3,5, phenyl), 129.17 (*C*H-3, thiophene), 128.92 (*C*H-2,6, phenyl), 128.48 (*C*H-4, phenyl), 127.96 (*C*H-5, aromatic), 127.40 (*C*H-4, thiophene), 126.91 (*C*H-5, thiophene), 125.92 (*C*_q_-2, aromatic) 67.24 (*C*H), 50.47 (*C*H_2_) (Figure S28c).

8. Synthesis of 2-((((3-bromophenyl)(phenyl)methyl)sulfinyl)methyl)thiophene (**5h**–**8h**)

Following general procedure A, 1.0 g (3.8 mmol) of (3-bromophenyl)(phenyl)methanol, 0.49 g (3.8 mmol) of thiophen-2-ylmethanethiol, and (0.5 mL, 4.18 mmol) of 48% BF_3_·Et_2_O were reacted, affording 1.13 g of **3h** as a yellow oily product (yield 79%).

Following general procedure B, 1.13 g (3.0 mmol) of **3h** dissolved in 12 mL of glacial acetic acid was reacted with 30% H_2_O_2_ (0.3 mL) to give 0.71 g of **4h** as a pale-yellow solid (yield 60%).

Racemic **4h** was further separated into two individual pairs of enantiomers by means of an MPLC system equipped with a glass column packed with silica gel. Toluol/EtOAc = 80/20 was used as the mobile phase, and the separation was carried out with a flow rate of 5 mL/min. Individual pairs of enantiomers were further separated into the individual enantiomers **5h**–**8h** by means of semipreparative chiral HPLC. A semipreparative HPLC instrument was equipped with a CHIRALPAK IA column (10 mm Φ × 25 cm L) (Daicel Inc.), and EtOAc was used as the mobile phase.

**5h**. (*S*,*R*)-*2-((((3-bromophenyl)(phenyl)methyl)sulfinyl)methyl)thiophene.* HRESIMS *m*/*z* 390.9811 [M+H]^+^ (calcd for C_18_H_16_BrOS_2_^+^, 390.9820, Δ = 2.3 ppm) (Figure S29). HPLC R_t_ 28.23 min, purity 98.4% (Figure S29a). Chiral HPLC R_t_ 6.75 min, >99% ee (Figure S29b). ^1^H NMR (500 MHz, CDCl_3_, 23 °C): δ = 7.54 (C*H*-2, aromatic), 7.46 (C*H*-4, aromatic), 7.44 (C*H*-3,5, phenyl), 7.39 (C*H*-6, aromatic), 7.39 (C*H*-2,6, aromatic), 7.39 (C*H*-4, phenyl), 7.34 (C*H*-5, thiophene), 7.24 (C*H*-5, aromatic), 7.06 (C*H*-4, thiophene), 6.95 (C*H*-3, thiophene), 4.69 (s, 1H, C*H*), 4.10/3.89 (AB, 2H, C*H*_2_). ^13^C NMR (125.75 MHz, CDCl_3_, 23 °C): δ = 136.60 (*C*-1, aromatic), 134.79 (*C*-1, phenyl), 132.40 (*C*-2, aromatic), 131.50 (*C*-4, aromatic), 130.12 (*C*-5, aromatic), 129.78 (*C*-2, thiophene), 129.48 (*C*H-3,5, phenyl), 129.18 (*C*-3, thiophene), 128.74 (*C*H-2,6, phenyl), 128.74 (*C*-4, phenyl), 128.33 (*C*-6, aromatic), 126.98 (*C*-5, thiophene), 127.33 (*C*-4, thiophene), 122.70 (*C*-3, aromatic), 68.67 (*C*H), 50.09 (*C*H_2_) (Figure S29c).

**6h**. (*R*,*S*)-*2-((((3-bromophenyl)(phenyl)methyl)sulfinyl)methyl)thiophene.* HRESIMS *m*/*z* 390.9810 [M+H]^+^ (calcd for C_18_H_16_BrOS_2_^+^, 390.9820, Δ = 2.7 ppm) (Figure S30). HPLC R_t_ 26.50 min, purity 96.9% (Figure S30a). Chiral HPLC R_t_ 9.10 min, >99% ee (Figure S30b). ^1^H NMR (500 MHz, CDCl_3_, 23 °C): δ = 7.54 (C*H*-2, aromatic), 7.46 (C*H*-4, aromatic), 7.44 (C*H*-3,5, phenyl), 7.39 (C*H*-6, aromatic), 7.39 (C*H*-2,6, aromatic), 7.39 (C*H*-4, phenyl), 7.34 (C*H*-5, thiophene), 7.24 (C*H*-5, aromatic), 7.06 (C*H*-4, thiophene), 6.95 (C*H*-3, thiophene), 4.69 (s, 1H, C*H*), 4.10/3.89 (AB, 2H, C*H*_2_). ^13^C NMR (125.75 MHz, CDCl_3_, 23 °C): δ = 136.60 (*C*-1, aromatic), 134.79 (*C*-1, phenyl), 132.40 (*C*-2, aromatic), 131.50 (*C*-4, aromatic), 130.12 (*C*-5, aromatic), 129.78 (*C*-2, thiophene), 129.48 (*C*H-3,5, phenyl), 129.18 (*C*-3, thiophene), 128.74 (*C*H-2,6, phenyl), 128.74 (*C*-4, phenyl), 128.33 (*C*-6, aromatic), 126.98 (*C*-5, thiophene), 127.33 (*C*-4, thiophene), 122.70 (*C*-3, aromatic), 68.67 (*C*H), 50.09 (*C*H_2_) (Figure S30c).

**7h**. (*S*,*S*)-*2-((((3-bromophenyl)(phenyl)methyl)sulfinyl)methyl)thiophene.* HRESIMS *m*/*z* 390.9812 [M+H]^+^ (calcd for C_18_H_16_BrOS_2_^+^, 390.9820, Δ = 2.2 ppm) (Figure S31). HPLC R_t_ 26.36 min, purity 96.8% (Figure S31a). Chiral HPLC Rt 7.15 min, >99% ee (but contains 0.8% 5m) (Figure S31b). ^1^H NMR (500 MHz, CDCl_3_, 23 °C): δ = 7.55 (C*H*-2, aromatic), 7.49 (C*H*-4, aromatic), 7.42 (C*H*-2,6, aromatic), 7.41 (C*H*-3,5, phenyl), 7.39 (C*H*-6, aromatic), 7.38 (C*H*-4, phenyl), 7.34 (C*H*-5, thiophene), 7.28 (C*H*-5, aromatic), 7.06 (C*H*-4, thiophene), 6.97 (C*H*-3, thiophene), 4.69 (s, 1H, C*H*), 4.10/3.90 (AB, 2H, C*H*_2_). ^13^C NMR (125.75 MHz, CDCl_3_, 23 °C): δ = 138.04 (*C*-1, aromatic), 133.22 (*C*-1, phenyl), 131.79 (*C*-2, aromatic), 131.50 (*C*-4, aromatic), 130.69 (*C*-5, aromatic), 130.04 (*C*-2, thiophene), 129.71 (*C*H-2,6, phenyl), 129.02 (*C*-3, thiophene), 128.83 (*C*H-3,5, phenyl), 128.69 (*C*-4, phenyl), 127.43 (*C*-4, thiophene), 127.35 (*C*-6, aromatic), 126.90 (*C*-5, thiophene), 123.21 (*C*-3, aromatic), 68.03 (*C*H), 50.33 (*C*H_2_) (Figure S31c).

**8h**. (*R*,*R*)-*2-((((3-bromophenyl)(phenyl)methyl)sulfinyl)methyl)thiophene.* HRESIMS *m*/*z* 390.9810 [M+H]^+^ (calcd for C_18_H_16_BrOS_2_^+^, 390.9820, Δ = 2.6 ppm) (Figure S32). HPLC R_t_ 26.36 min, purity 96.4% (Figure S32a). Chiral HPLC R_t_ 10.28 min, >99% ee (Figure S32b). ^1^H NMR (500 MHz, CDCl_3_, 23 °C): δ = 7.55 (C*H*-2, aromatic), 7.49 (C*H*-4, aromatic), 7.42 (C*H*-2,6, aromatic), 7.41 (C*H*-3,5, phenyl), 7.39 (C*H*-6, aromatic), 7.38 (C*H*-4, phenyl), 7.34 (C*H*-5, thiophene), 7.28 (C*H*-5, aromatic), 7.06 (C*H*-4, thiophene), 6.97 (C*H*-3, thiophene), 4.69 (s, 1H, C*H*), 4.10/3.90 (AB, 2H, C*H*_2_). ^13^C NMR (125.75 MHz, CDCl_3_, 23 °C): δ = 138.04 (*C*-1, aromatic), 133.22 (*C*-1, phenyl), 131.79 (*C*-2, aromatic), 131.50 (*C*-4, aromatic), 130.69 (*C*-5, aromatic), 130.04 (*C*-2, thiophene), 129.71 (*C*H-2,6, phenyl), 129.02 (*C*-3, thiophene), 128.83 (*C*H-3,5, phenyl), 128.69 (*C*-4, phenyl), 127.43 (*C*-4, thiophene), 127.35 (*C*-6, aromatic), 126.90 (*C*-5, thiophene), 123.21 (*C*-3, aromatic), 68.03 (*C*H), 50.33 (*C*H_2_) (Figure S32c).

9. Synthesis of 2-((((2-iodophenyl)(phenyl)methyl)sulfinyl)methyl)thiophene (**5i**–**8i**)

Following general procedure A, 0.5 g (1.61 mmol) of (2-iodophenyl)(phenyl)methanol, 0.21 g (1.61 mmol) of thiophen-2-ylmethanethiol, and 0.22 mL (1.8 mmol) of 48% BF_3_·Et_2_O were reacted, affording 0.49 g of **3i** as a white oily product (yield 72%).

Following general procedure B, 0.49 g (1.17 mmol) of **3i** dissolved in 6 mL of glacial acetic acid was reacted with 30% H_2_O_2_ (0.12 mL) to give 1.5 g of **4i** as white oil (yield >95%).

Racemic **4i** was further separated into the four individual diastereomers **5i**–**8i** by means of semipreparative chiral HPLC. A semipreparative HPLC instrument was equipped with a CHIRALPAK IA column (10 mm Φ × 25 cm L) (Daicel Inc.), and EtOAc was used as the mobile phase.

**5i**. HRESIMS *m*/*z* 438.9682 [M+H]^+^ (calcd for C_18_H_16_IOS_2_^+^, 438.9682, Δ = −0.1 ppm) (Figure S33). HPLC R_t_ 26.52 min, purity 95.9% (Figure S33a). Chiral HPLC R_t_ 5.06 min, 93.9% ee (Figure S33b). ^1^H NMR (500 MHz, CDCl_3_, 23 °C): δ = 7.90 (C*H*-3, aromatic), 7.73 (C*H*-6, aromatic), 7.51 (C*H*-2,6, phenyl), 7.44 (C*H*-5, aromatic), 7.40 (C*H*-3,5, phenyl), 7.37 (C*H*-4, phenyl), 7.32 (C*H*-5, thiophene), 7.03 (C*H*-4, thiophene), 7.03 (C*H*-4, aromatic), 6.96 (C*H*-3, thiophene), 5.22 (s, 1H, C*H*), 4.14/4.06 (AB, 2H, C*H*_2_). ^13^C NMR (125.75 MHz, CDCl_3_, 23 °C): δ = 140.46 (*C*H-3, aromatic), 139.01 (*C*_q_-1, aromatic), 132.76 (*C*_q_-1, phenyl), 130.57 (*C*_q_, thiophene-2), 130.22 (*C*H-2,6, phenyl), 129.83 (*C*H-4, aromatic), 129.24 (*C*H-6, aromatic), 128.99 (*C*H-5, aromatic), 128.93 (*C*H-3, thiophene), 128.66 (*C*H-3,5, phenyl), 128.56 (*C*H-4, phenyl), 127.55 (*C*H-4, thiophene), 126.90 (*C*H-5, thiophene), 101.75 (*C*_q_-2, aromatic), 71.95 (*C*H), 51.02 (*C*H_2_) (Figure S33c).

**6i**. HRESIMS *m*/*z* 438.9678 [M+H]^+^ (calcd for C_18_H_16_IOS_2_^+^, 438.9682, Δ = 0.8 ppm) (Figure S34). HPLC R_t_ 26.52 min, purity 98.6% (Figure S34a). Chiral HPLC R_t_ 6.27 min, >99% ee (Figure S34b). ^1^H NMR (500 MHz, CDCl_3_, 23 °C): δ = 7.90 (C*H*-3, aromatic), 7.73 (C*H*-6, aromatic), 7.51 (C*H*-2,6, phenyl), 7.44 (C*H*-5, aromatic), 7.40 (C*H*-3,5, phenyl), 7.37 (C*H*-4, phenyl), 7.32 (C*H*-5, thiophene), 7.03 (C*H*-4, thiophene), 7.03 (C*H*-4, aromatic), 6.96 (C*H*-3, thiophene), 5.22 (s, 1H, C*H*), 4.14/4.05 (AB, 2H, C*H*_2_). ^13^C NMR (125.75 MHz, CDCl_3_, 23 °C): δ = 140.46 (*C*H-3, aromatic), 139.02 (*C*_q_-1, aromatic), 132.76 (*C*_q_-1, phenyl), 130.57 (*C*_q_, thiophene-2), 130.22 (*C*H-2,6, phenyl), 129.83 (*C*H-4, aromatic), 129.24 (*C*H-6, aromatic), 128.99 (*C*H-5, aromatic), 128.93 (*C*H-3, thiophene), 128.66 (*C*H-3,5, phenyl), 128.56 (*C*H-4, phenyl), 127.55 (*C*H-4, thiophene), 126.90 (*C*H-5, thiophene), 101.76 (*C*_q_-2, aromatic), 71.95 (*C*H), 51.02 (*C*H_2_) (Figure S34c).

**7i**. HRESIMS *m*/*z* 438.9679 [M+H]^+^ (calcd for C_18_H_16_IOS_2_^+^, 438.9682, Δ = 2.0 ppm) (Figure S35). HPLC R_t_ 26.67 min, purity 97.0% (Figure S35a). Chiral HPLC R_t_ 6.82 min, 93.0% ee (Figure S35b). ^1^H NMR (500 MHz, CDCl_3_, 23 °C): δ = 7.95 (C*H*-6, aromatic), 7.89 (C*H*-3, aromatic), 7.50 (C*H*-2,6, phenyl), 7.45 (C*H*-5, aromatic), 7.39 (C*H*-3,5, phenyl), 7.34 (C*H*-4, phenyl), 7.33 (C*H*-5, thiophene), 7.04 (C*H*-4, thiophene), 7.02 (C*H*-4, aromatic), 6.97 (C*H*-3, thiophene), 5.41 (s, 1H, C*H*), 4.21/3.92 (AB, 2H, C*H*_2_). ^13^C NMR (125.75 MHz, CDCl_3_, 23 °C): δ = 140.16 (*C*H-3, aromatic), 137.89 (*C*_q_-1, aromatic), 135.12 (*C*_q_-1, phenyl), 130.26 (*C*_q_, thiophene-2), 129.77 (*C*H-4, aromatic), 129.67 (*C*H-6, aromatic), 129.23 (*C*H-3, thiophene), 129.20 (*C*H-3,5, phenyl), 128.94 (*C*H-2,6, phenyl), 128.82 (*C*H-5, aromatic), 128.50 (*C*H-4, phenyl), 127.43 (*C*H-4, thiophene), 126.96 (*C*H-5, thiophene), 103.01 (*C*_q_-2, aromatic), 71.83 (*C*H), 50.27 (*C*H_2_) (Figure S35c).

**8i**. HRESIMS *m*/*z* 438.9679 [M+H]^+^ (calcd for C_18_H_16_IOS_2_^+^, 438.9682, Δ = 0.5 ppm) (Figure S36). HPLC R_t_ 26.67 min, purity 97.0% (Figure S36a). Chiral HPLC R_t_ 8.37 min, 97.9% ee (Figure S36b). ^1^H NMR (500 MHz, CDCl_3_, 23 °C): δ = 7.95 (C*H*-6, aromatic), 7.89 (C*H*-3, aromatic), 7.50 (C*H*-2,6, phenyl), 7.45 (C*H*-5, aromatic), 7.39 (C*H*-3,5, phenyl), 7.35 (C*H*-4, phenyl), 7.33 (C*H*-5, thiophene), 7.04 (C*H*-4, thiophene), 7.02 (C*H*-4, aromatic), 6.97 (C*H*-3, thiophene), 5.41 (s, 1H, C*H*), 4.21/3.92 (AB, 2H, C*H*_2_). ^13^C NMR (125.75 MHz, CDCl_3_, 23 °C): δ = 140.17 (*C*H-3, aromatic), 137.94 (*C*_q_-1, aromatic), 135.19 (*C*_q_-1, phenyl), 130.35 (*C*_q_, thiophene-2), 129.77 (*C*H-4, aromatic), 129.71 (*C*H-6, aromatic), 129.21 (*C*H-3,5, phenyl), 129.21 (*C*H-3, thiophene), 128.96 (*C*H-2,6, phenyl), 128.82 (*C*H-5, aromatic), 128.49 (*C*H-4, phenyl), 127.44 (*C*H-4, thiophene), 126.94 (*C*H-5, thiophene), 103.02 (*C*_q_-2, aromatic), 71.89 (*C*H), 50.36 (*C*H_2_) (Figure S36c).

10. Synthesis of 2-((((3-iodophenyl)(phenyl)methyl)sulfinyl)methyl)thiophene (**5j**–**8j**)

Following general procedure A, 1.5 g (1.61 mmol) of (3-iodophenyl)(phenyl)methanol, 0.21 g (1.61 mmol) of thiophen-2-ylmethanethiol and 0.22 mL (1.8 mmol) of 48% BF_3_·Et_2_O were reacted, affording 0.58 g of **3j** as an orange oily product (yield 86%).

Following general procedure B, 0.58 g (1.39 mmol) of **3j** dissolved in 6 mL of glacial acetic acid was reacted with 30% H_2_O_2_ (0.14 mL) to give 0.38 g of **4j** as a yellow solid (yield 63%).

Racemic **4j** was further separated into two individual pairs of enantiomers by means of an MPLC system equipped with a glass column packed with silica gel. Toluol/EtOAc = 80/20 was used as the mobile phase, and the separation was carried out with a flow rate of 5 mL/min. Individual pairs of enantiomers were further separated into the individual enantiomers **5j**–**8j** by means of semipreparative chiral HPLC. A semipreparative HPLC instrument was equipped with a CHIRALPAK IA column (10 mm Φ × 25 cm L) (Daicel Inc.), and EtOAc was used as the mobile phase.

**5j**. HRESIMS *m*/*z* 438.9681 [M+H]^+^ (calcd for C_18_H_16_IOS_2_^+^, 438.9682, Δ = 0.3 ppm) (Figure S37). HPLC R_t_ 27.10 min, purity > 99% (Figure S37a). Chiral HPLC R_t_ 6.59 min, >99% ee (Figure S37b). ^1^H NMR (500 MHz, CDCl_3_, 23 °C): δ = 7.72 (C*H*-2, aromatic), 7.66 (C*H*-4, aromatic), 7.44 (C*H*-3,5, phenyl), 7.43 (C*H*-6, aromatic), 7.38 (C*H*-2,6, phenyl), 7.38 (C*H*-4, phenyl), 7.34 (s, 1H, C*H*-5, thiophene), 7.11 (C*H*-5, aromatic), 7.06 (s, 1H, C*H*-4, thiophene), 6.95 (s, 1H, C*H*_-_3, thiophene), 4.66 (s, 1H, C*H*), 4.09/3.89 (AB, 2H, C*H*_2_). ^13^C NMR (125.75 MHz, CDCl_3_, 23 °C): δ = 138.24 (*C*H-2, aromatic), 137.44 (*C*H-4, aromatic), 136.65 (*C*_q_-1, aromatic), 134.84 (*C*_q_-1, phenyl), 130.28 (*C*H-5, aromatic), 129.83 (*C*_q_, thiophene-2), 129.46 (*C*H-3,5, phenyl), 129.15 (*C*H-3, thiophene), 128.88 (*C*H-6, aromatic), 128.72 (*C*H-4, phenyl), 128.72 (*C*H-2,6, phenyl), 127.34 (*C*H-4, thiophene), 126.96 (*C*H-5, thiophene), 94.51 (*C*_q_-3, aromatic), 68.59 (*C*H), 50.12 (*C*H_2_) (Figure S37c).

**6j**. HRESIMS *m*/*z* 438.9683 [M+H]^+^ (calcd for C_18_H_16_IOS_2_^+^, 438.9682, Δ = −0.3 ppm) (Figure S38). HPLC R_t_ 27.10 min, purity > 99% (Figure S38a). Chiral HPLC R_t_ 8.69 min, 98.6% ee (Figure S38b). ^1^H NMR (500 MHz, CDCl_3_, 23 °C): δ = 7.72 (C*H*-2, aromatic), 7.66 (C*H*-4, aromatic), 7.44 (C*H*-3,5, phenyl), 7.43 (C*H*-6, aromatic), 7.38 (C*H*-2,6, phenyl), 7.38 (C*H*-4, phenyl), 7.34 (s, 1H, C*H*-5, thiophene), 7.11 (C*H*-5, aromatic), 7.06 (s, 1H, C*H*-4, thiophene), 6.95 (s, 1H, C*H*-3, thiophene), 4.66 (s, 1H, C*H*), 4.09/3.89 (AB, 2H, C*H*_2_). ^13^C NMR (125.75 MHz, CDCl_3_, 23 °C): δ = 138.24 (*C*H-2, aromatic), 137.44 (*C*H-4, aromatic), 136.65 (*C*_q_-1, aromatic), 134.84 (*C*_q_-1, phenyl), 130.28 (*C*H-5, aromatic), 129.83 (*C*_q_, thiophene-2), 129.46 (*C*H-3,5, phenyl), 129.15 (*C*H-3, thiophene), 128.88 (*C*H-6, aromatic), 128.72 (*C*H-4, phenyl), 128.72 (*C*H-2,6, phenyl), 127.34 (*C*H-4, thiophene), 126.96 (*C*H-5, thiophene), 94.51 (*C*_q_-3, aromatic), 68.59 (*C*H), 50.12 (*C*H_2_) (Figure S38c).

**7j**. HRESIMS *m*/*z* 438.9681 [M+H]^+^ (calcd for C_18_H_16_IOS_2_^+^, 438.9682, Δ = 0.1 ppm) (Figure S39). HPLC R_t_ 26.97 min, purity 98.9% (Figure S39a). Chiral HPLC R_t_ 7.04 min, >99% ee (but contains 1.4% 5j) (Figure S39b). ^1^H NMR (500 MHz, CDCl_3_, 23 °C): δ = 7.73 (C*H*-2, aromatic), 7.69 (C*H*-4, aromatic), 7.44 (C*H*-6, aromatic), 7.42 (C*H*-2,6, phenyl), 7.41 (C*H*-3,5, phenyl), 7.38 (C*H*-4, phenyl), 7.34 (s, 1H, C*H*-5, thiophene), 7.14 (C*H*-5, aromatic), 7.06 (s, 1H, C*H*-4, thiophene), 6.97 (s, 1H, C*H*-3, thiophene), 4.65 (s, 1H, C*H*), 4.10/3.90 (AB, 2H, C*H*_2_). ^13^C NMR (125.75 MHz, CDCl_3_, 23 °C): δ = 138.08 (*C*_q_-1, aromatic), 137.63 (*C*H-2, aromatic), 137.44 (*C*H-4, aromatic), 133.25 (*C*q-1, phenyl), 130.03 (*C*H-5, aromatic), 130.03 (*C*_q_, thiophene-2), 129.68 (*C*H-2,6, phenyl), 129.02 (*C*H-3, thiophene), 128.82 (*C*H-3,5, phenyl), 128.67 (*C*H-4, phenyl), 127.92 (*C*H-6, aromatic), 127.42 (*C*H-4, thiophene), 126.91 (*C*H-5, thiophene), 95.00 (*C*_q_-3, aromatic), 67.95 (*C*H), 50.34 (*C*H_2_) (Figure S39c).

**8j**. HRESIMS *m*/*z* 438.9683 [M+H]^+^ (calcd for C_18_H_16_IOS_2_^+^, 438.9682, Δ = −0.3 ppm) (Figure S40). HPLC R_t_ 26.98 min, purity > 99% (Figure S40a). Chiral HPLC R_t_ 9.33 min, >99% ee (but contains 1.2% 6j) (Figure S40b). ^1^H NMR (500 MHz, CDCl_3_, 23 °C): δ = 7.72 (C*H*-2, aromatic), 7.69 (C*H*-4, aromatic), 7.44 (C*H*-6, aromatic), 7.42 (C*H*-2,6, phenyl), 7.41 (C*H*-3,5, phenyl), 7.38 (C*H*-4, phenyl), 7.34 (s, 1H, C*H*-5, thiophene), 7.14 (C*H*-5, aromatic), 7.06 (s, 1H, C*H*-4, thiophene), 6.97 (s, 1H, C*H*-3, thiophene), 4.65 (s, 1H, C*H*), 4.09/3.90 (AB, 2H, C*H*_2_). ^13^C NMR (125.75 MHz, CDCl_3_, 23 °C): δ = 138.08 (*C*_q_-1, aromatic), 137.63 (*C*H-2, aromatic) 137.44 (*C*H-4, aromatic), 133.25 (*C*_q_-1, phenyl), 130.79 (*C*H-5, aromatic), 130.03 (*C*_q_, thiophene-2), 129.68 (*C*H-2,6, phenyl), 129.02 (*C*H-3, thiophene), 128.82 (*C*H-3,5, phenyl), 128.67 (*C*H-4, phenyl), 127.92 (*C*H-6, aromatic), 127.42 (*C*H-4, thiophene), 126.91 (*C*H-5, thiophene), 95.00 (*C*_q_-3, aromatic), 67.95 (*C*H), 50.34 (*C*H_2_) (Figure S40c).

11. Synthesis of 2-((((4-iodophenyl)(phenyl)methyl)sulfinyl)methyl)thiophene (**5k**–**8k**)

Following general procedure A, 0.3 g (0.97 mmol) of (4-iodophenyl)(phenyl)methanol, 0.13 g (0.97 mmol) of thiophen-2-ylmethanethiol, and (0.13 mL, 1.1 mmol) of 48% BF_3_·Et_2_O were reacted, affording 0.4 g of **3k** as a yellow oily product (yield >95%).

Following general procedure B, 0.4 g (0.96 mmol) of **3k** dissolved in 6 mL of glacial acetic acid was reacted with 30% H_2_O_2_ (0.54 mL) to give 0.37 g of **4k** as white oil (yield 88%).

Racemic **4k** was further separated into two individual pairs of enantiomers by means of an MPLC system equipped with a glass column packed with silica gel. Toluol/EtOAc = 80/20 was used as the mobile phase, and the separation was carried out with a flow rate of 5 mL/min. Individual pairs of enantiomers were further separated into the individual enantiomers **5k**–**8k** by means of semipreparative chiral HPLC. A semipreparative HPLC instrument was equipped with a CHIRALPAK IA column (10 mm Φ × 25 cm L) (Daicel Inc), and EtOAc was used as the mobile phase.

**5k**. (*S*,*R*)-*2-((((4-iodophenyl)(phenyl)methyl)sulfinyl)methyl)thiophene.* HRESIMS *m*/*z* 438.9678 [M+H]^+^ (calcd for C_18_H_16_IOS_2_^+^, 438.9682, Δ = 0.9 ppm) (Figure S41). HPLC R_t_ 27.28 min, purity 98.8% (Figure S41a). Chiral HPLC R_t_ 7.38 min, >99% ee (Figure S41b). ^1^H NMR (500 MHz, CDCl_3_, 23 °C): δ = 7.69 (C*H*-3,5, aromatic), 7.43 (C*H*-3,5, phenyl), 7.38 (C*H*-2,6, phenyl), 7.38 (C*H*-4, phenyl), 7.34 (s, 1H, C*H*-5, thiophene), 7.15 (C*H*-2,6, aromatic), 7.05 (s, 1H, C*H*-4, thiophene), 6.95 (s, 1H, C*H*-3, thiophene), 4.68 (s, 1H, C*H*), 4.09/3.88 (AB, 2H, C*H*_2_). ^13^C NMR (125.75 MHz, CDCl_3_, 23 °C): δ = 137.72 (*C*H-3,5, aromatic), 134.92 (*C*_q_-1, phenyl), 133.85 (*C*_q_-1, aromatic), 131.48 (*C*H-2,6, aromatic), 129.87 (*C*_q_, thiophene-2), 129.40 (*C*H-3,5, phenyl), 129.12 (*C*H-3, thiophene), 128.74 (*C*H-2,6, phenyl), 128.64 (*C*H-4, phenyl), 127.34 (*C*H-4, thiophene), 126.93 (*C*H-5, thiophene), 94.62 (*C*_q_-4, aromatic), 68.45 (*C*H), 50.07 (*C*H_2_) (Figure S41c).

**6k**. (*R*,*S*)-*2-((((4-iodophenyl)(phenyl)methyl)sulfinyl)methyl)thiophene.* HRESIMS *m*/*z* 438.9679 [M+H]^+^ (calcd for C_18_H_16_IOS_2_^+^, 438.9682, Δ = 0.7 ppm) (Figure S42). HPLC R_t_ 27.28 min, purity 98.9% (Figure S42a). Chiral HPLC R_t_ 13.96 min, 98.8% ee (Figure S42b). ^1^H NMR (500 MHz, CDCl_3_, 23 °C): δ = 7.69 (C*H*-3,5, aromatic), 7.43 (C*H*-3,5, phenyl), 7.38 (C*H*-4, phenyl), 7.38 (C*H*-2,6, phenyl), 7.34 (s, 1H, C*H*-5, thiophene), 7.15 (C*H*-2,6, aromatic), 7.05 (s, 1H, C*H*-4, thiophene), 6.95 (s, 1H, C*H*-3, thiophene), 4.68 (s, 1H, C*H*), 4.09/3.88 (AB, 2H, C*H*_2_). ^13^C NMR (125.75 MHz, CDCl_3_, 23 °C): δ = 137.72 (*C*H-3,5, aromatic), 134.92 (*C*_q_-1, phenyl), 133.85 (*C*_q_-1, aromatic), 131.48 (*C*H-2,6, aromatic), 129.87 (*C*_q_, thiophene-2), 129.40 (*C*H-3,5, phenyl), 129.11 (*C*H-3, thiophene), 128.74 (*C*H-2,6, phenyl), 128.64 (*C*H-4, phenyl), 127.34 (*C*H-4, thiophene), 126.93 (*C*H-5, thiophene), 94.62 (*C*_q_-4, aromatic), 68.45 (*C*H), 50.07 (*C*H_2_) (Figure S42c).

**7k**. (*S*,*S*)-*2-((((4-iodophenyl)(phenyl)methyl)sulfinyl)methyl)thiophene.* HRESIMS *m*/*z* 438.9677 [M+H]^+^ (calcd for C_18_H_16_IOS_2_^+^, 438.9682, Δ = 1.2 ppm) (Figure S43). HPLC R_t_ 27.33 min, purity 98.4% (Figure S43a). Chiral HPLC R_t_ 7.56 min, >99% ee (Figure S43b). ^1^H NMR (500 MHz, CDCl_3_, 23 °C): δ = 7.74 (C*H*-3,5, aromatic), 7.40 (C*H*-3,5, phenyl), 7.40 (C*H*-2,6, phenyl), 7.37 (C*H*-4, phenyl), 7.34 (s, 1H, C*H*-5, thiophene), 7.17 (C*H*-2,6, aromatic), 7.05 (s, 1H, C*H*-4, thiophene), 6.96 (s, 1H, C*H*-3, thiophene), 4.67 (s, 1H, C*H*), 4.09/3.89 (AB, 2H, C*H*_2_). ^13^C NMR (125.75 MHz, CDCl_3_, 23 °C): δ = 138.30 (*C*H-3,5, aromatic), 135.39 (*C*_q_-1, aromatic), 133.41 (*C*_q_-1, phenyl), 130.62 (*C*H-2,6, aromatic), 130.04 (*C*_q_, thiophene-2), 129.62 (*C*H-2,6, phenyl), 129.01 (*C*H-3, thiophene), 128.79 (*C*H-3,5, phenyl), 128.61 (*C*H-4, phenyl), 127.39 (*C*H-4, thiophene), 126.87 (*C*H-5, thiophene), 94.19 (*C*_q_-4, aromatic), 68.15 (*C*H), 50.25 (*C*H_2_) (Figure S43c).

**8k**. (*R*,*R*)-*2-((((4-iodophenyl)(phenyl)methyl)sulfinyl)methyl)thiophene.* HRESIMS *m*/*z* 438.9676 [M+H]^+^ (calcd for C_18_H_16_IOS_2_^+^, 438.9682, Δ = 1.2 ppm) (Figure S44). HPLC R_t_ 27.35 min, purity > 99% (Figure S44a). Chiral HPLC R_t_ 10.83 min, >99% ee (Figure S44b). ^1^H NMR (500 MHz, CDCl_3_, 23 °C): δ = 7.74 (C*H*-3,5, aromatic), 7.40 (C*H*-3,5, phenyl), 7.40 (C*H*-2,6, phenyl), 7.37 (C*H*-4, phenyl), 7.34 (s, 1H, C*H*-5, thiophene), 7.17 (C*H*-2,6, aromatic), 7.05 (s, 1H, C*H*-4, thiophene), 6.96 (s, 1H, C*H*-3, thiophene), 4.67 (s, 1H, C*H*), 4.09/3.89 (AB, 2H, C*H*_2_). ^13^C NMR (125.75 MHz, CDCl_3_, 23 °C): δ = 138.30 (*C*H-3,5, aromatic), 135.39 (*C*_q_-1, aromatic), 133.41 (*C*_q_-1, phenyl), 130.62 (*C*H-2,6, aromatic), 130.04 (*C*_q_, thiophene-2), 129.62 (*C*H-2,6, phenyl), 129.02 (*C*H-3, thiophene), 128.79 (*C*H-3,5, phenyl), 128.62 (*C*H-4, phenyl), 127.39 (*C*H-4, thiophene), 126.87 (*C*H-5, thiophene), 94.19 (*C*_q_-4, aromatic), 68.15 (*C*H), 50.25 (*C*H_2_) (Figure S44c).

12. Synthesis of 2-(((phenyl(o-tolyl)methyl)sulfinyl)methyl)thiophene (**5l**–**8l**)

Following general procedure A, 1.0 g (5.0 mmol) of phenyl(*o*-tolyl)methanol, 0.65 g (5.0 mmol) of thiophen-2-ylmethanethiol, and (0.7 mL, 5.5 mmol) of 48% BF_3_·Et_2_O were reacted, affording 1.53 g of **3l** as a yellow oily product (yield > 95%).

Following general procedure B, 1.53 g (4.92 mmol) of **3l** dissolved in 12 mL of glacial acetic acid was reacted with 30% H_2_O_2_ (0.54 mL) to give 1.3 g of **4l** as pale-yellow solid (yield 81%).

Racemic **4l** was further separated into two individual pairs of enantiomers by means of an MPLC system equipped with a glass column packed with silica gel. Toluol/EtOAc = 80/20 was used as the mobile phase, and the separation was carried out with a flow rate of 5 mL/min. Individual pairs of enantiomers were further separated into the individual enantiomers **5l**–**8l** by means of semipreparative chiral HPLC. A semipreparative HPLC instrument was equipped with a CHIRALPAK IA column (10 mm Φ × 25 cm L) (Daicel Inc.), and Hexane/EtOAc = 50/50 was used as the mobile phase in the case of **5l/6l** and 100% EtOAc in the case of **7l/8l**.

**5l**. HRESIMS *m*/*z* 349.0688 [M+Na]^+^ (calcd for C_19_H_18_NaOS_2_^+^, 349.0691, Δ = 1.1 ppm) (Figure S45). HPLC R_t_ 25.45 min, purity > 99% (Figure S45a). Chiral HPLC R_t_ 12.77 min, 97.6% ee (Figure S45b). ^1^H NMR (500 MHz, CDCl_3_, 23 °C): δ = 7.59 (C*H*-6, aromatic), 7.37 (C*H*-2,6, phenyl), 7.34 (C*H*-3,5, phenyl), 7.33 (s, 1H, C*H*-5, thiophene), 7.32 (C*H*-5, aromatic), 7.32 (C*H*-4, phenyl), 7.26 (C*H*-4, aromatic), 7.21 (C*H*-3, aromatic), 7.03 (s, 1H, C*H*-4, thiophene), 6.85 (s, 1H, C*H*-3, thiophene), 4.82 (s, 1H, C*H*), 4.18/4.01 (AB, 2H, C*H*_2_), 2.10 (C*H*_3_). ^13^C NMR (125.75 MHz, CDCl_3_, 23 °C): δ = 136.50 (*C*_q_-2, aromatic), 134.81 (*C*_q_-1, aromatic), 133.57 (*C*_q_-1, phenyl), 131.34 (*C*H-3, aromatic), 130.33 (*C*_q_, thiophene-2), 130.03 (*C*H-2,6, phenyl), 128.84 (*C*H-3, thiophene), 128.58 (*C*H-3,5, phenyl), 128.36 (*C*H-4, phenyl), 128.12 (*C*H-4, aromatic), 127.59 (*C*H-6, aromatic), 127.22 (*C*H-4, thiophene), 126.85 (*C*H-5, thiophene), 126.61 (*C*H-5, aromatic), 65.63 (*C*H), 50.26 (*C*H_2_), 19.61 (*C*H_3_) (Figure S45c).

**6l.** HRESIMS *m*/*z* 349.0689 [M+Na]^+^ (calcd for C_19_H_18_NaOS_2_^+^, 349.0691, Δ = 0.6 ppm) (Figure S46). HPLC R_t_ 25.43 min, purity > 99% (Figure S46a). Chiral HPLC R_t_ 13.82 min, 96.5% ee (Figure S46b). ^1^H NMR (500 MHz, CDCl_3_, 23 °C): δ = 7.59 (C*H*-6, aromatic), 7.37 (C*H*-2,6, phenyl), 7.34 (C*H*-3,5, phenyl), 7.33 (s, 1H, C*H*-5, thiophene), 7.32 (C*H*-5, aromatic), 7.32 (C*H*-4, phenyl), 7.26 (C*H*-4, aromatic), 7.21 (C*H*-3, aromatic), 7.03 (s, 1H, C*H*-4, thiophene), 6.85 (s, 1H, C*H*-3, thiophene), 4.82 (s, 1H, C*H*), 4.18/4.01 (AB, 2H, C*H*_2_), 2.10 (C*H*_3_). ^13^C NMR (125.75 MHz, CDCl_3_, 23 °C): δ = 136.50 (*C*_q_-2, aromatic), 134.81 (*C*_q_-1, aromatic), 133.57 (*C*_q_-1, phenyl), 131.34 (*C*H-3, aromatic), 130.33 (*C*_q_, thiophene-2), 130.03 (*C*H-2,6, phenyl), 128.84 (*C*H-3, thiophene), 128.58 (*C*H-3,5, phenyl), 128.36 (*C*H-4, phenyl), 128.12 (*C*H-4, aromatic), 127.59 (*C*H-6, aromatic), 127.22 (*C*H-4, thiophene), 126.85 (*C*H-5, thiophene), 126.61 (*C*H-5, aromatic), 65.63 (*C*H), 50.26 (*C*H_2_), 19.61 (*C*H_3_) (Figure S46c).

**7l.** HRESIMS *m*/*z* 349.0690 [M+Na]^+^ (calcd for C_19_H_18_NaOS_2_^+^, 349.0691, Δ = 0.3 ppm) (Figure S47). HPLC R_t_ 25.22 min, purity 95.32% (Figure S47a). Chiral HPLC R_t_ 6.66 min, >99% ee (Figure S47b). ^1^H NMR (500 MHz, CDCl_3_, 23 °C): δ = 7.84 (C*H*-6, aromatic), 7.40 (C*H*-2,6, phenyl), 7.40 (C*H*-3,5, phenyl), 7.33 (s, 1H, C*H*-5, thiophene), 7.33 (C*H*-4, phenyl), 7.28 (C*H*-5, aromatic), 7.20 (C*H*-4, aromatic), 7.16 (C*H*-3, aromatic), 7.04 (s, 1H, C*H*-4, thiophene), 6.92 (s, 1H, C*H*-3, thiophene), 5.11 (s, 1H, C*H*), 4.18/3.96 (AB, 2H, C*H*_2_), 2.16 (C*H*_3_). ^13^C NMR (125.75 MHz, CDCl_3_, 23 °C): δ = 137.60 (*C*_q_-2, aromatic), 135.23 (*C*_q_-1, phenyl), 133.95 (*C*_q_-1, aromatic), 130.99 (*C*H-3, aromatic), 130.16 (*C*_q_, thiophene-2), 129.24 (*C*H-3,5, phenyl), 129.06 (*C*H-3, thiophene), 129.04 (*C*H-2,6, phenyl), 128.35 (*C*H-4, phenyl), 127.95 (*C*H-4, aromatic), 127.93 (*C*H-6, aromatic), 127.22 (*C*H-4, thiophene), 126.78 (*C*H-5, thiophene), 126.52 (*C*H-5, aromatic), 65.20 (*C*H), 49.69 (*C*H_2_), 19.87 (*C*H_3_) (Figure S47c).

**8l.** HRESIMS *m*/*z* 349.0690 [M+Na]^+^ (calcd for C_19_H_18_NaOS_2_^+^, 349.0691, Δ = 0.4 ppm) (Figure S48). HPLC R_t_ 25.22 min, purity 95.68% (Figure S48a). Chiral HPLC R_t_ 11.48 min, >96.1% ee (Figure S48b). ^1^H NMR (500 MHz, CDCl_3_, 23 °C): δ = 7.84 (C*H*-6, aromatic), 7.40 (C*H*-2,6, phenyl), 7.40 (C*H*-3,5, phenyl), 7.34 (s, 1H, C*H*-5, thiophene), 7.33 (C*H*-4, phenyl), 7.28 (C*H*-5, aromatic), 7.20 (C*H*-4, aromatic), 7.16 (C*H*-3, aromatic), 7.04 (s, 1H, C*H*-4, thiophene), 6.92 (s, 1H, C*H*-3, thiophene), 5.11 (s, 1H, C*H*), 4.18/3.96 (AB, 2H, C*H*_2_), 2.16 (C*H*_3_). ^13^C NMR (125.75 MHz, CDCl_3_, 23 °C): δ = 137.60 (*C*_q_-2, aromatic), 135.23 (*C*_q_-1, phenyl), 133.95 (*C*_q_-1, aromatic), 130.99 (*C*H-3, aromatic), 130.18 (*C*_q_, thiophene-2), 129.24 (*C*H-3,5, phenyl), 129.06 (*C*H-3, thiophene), 129.04 (*C*H-2,6, phenyl), 128.35 (*C*H-4, phenyl), 127.95 (*C*H-4, aromatic), 127.93 (*C*H-6, aromatic), 127.22 (*C*H-4, thiophene), 126.79 (*C*H-5, thiophene), 126.52 (*C*H-5, aromatic), 65.20 (*C*H), 49.69 (*C*H_2_), 19.87 (*C*H_3_) (Figure S48c).

13. Synthesis of 2-(((phenyl(m-tolyl)methyl)sulfinyl)methyl)thiophene (**5m**–**8m**)

Following general procedure A, 1.0 g (5.0 mmol) of phenyl(*m*-tolyl)methanol, 0.65 g (5.0 mmol) of thiophen-2-ylmethanethiol, and (0.7 mL, 5.5 mmol) of 48% BF_3_·Et_2_O were reacted, affording 1.49 g of **3m** as a yellow oily product (yield 95%).

Following general procedure B, 1.49 g (4.8 mmol) of **3m** dissolved in 10 mL of glacial acetic acid was reacted with 30% H_2_O_2_ (0.5 mL) to give 1.4 g of **4m** as pale-yellow solid (yield 89%).

Racemic **4m** was further separated into two individual pairs of enantiomers by means of an MPLC system equipped with a glass column packed with silica gel. Toluol/EtOAc = 80/20 was used as the mobile phase, and the separation was carried out with a flow rate of 5 mL/min. Individual pairs of enantiomers were further separated into the individual enantiomers **5m**–**8m** by means of semipreparative chiral HPLC. A semipreparative HPLC instrument was equipped with a CHIRALPAK IA column (10 mm Φ × 25 cm L) (Daicel Inc.), and EtOAc was used as the mobile phase.

**5m**. (*S*,*R*)-*2-(((phenyl(m-tolyl)methyl)sulfinyl)methyl)thiophene.* HRESIMS *m*/*z* 349.0685 [M+Na]^+^ (calcd for C_19_H_18_NaOS_2_^+^, 349.0691, Δ = 1.7 ppm) (Figure S49). HPLC R_t_ 23.93 min, purity 98.4% (Figure S49a). Chiral HPLC R_t_ 8.13 min, >99% ee (but contains 1.0% 7m) (Figure S49b). ^1^H NMR (500 MHz, CDCl_3_, 23 °C): δ = 7.43 (C*H*-2,6, phenyl), 7.42 (C*H*-3,5, phenyl), 7.36 (C*H*-4, phenyl), 7.32 (C*H*-5, thiophene), 7.25 (C*H*-5, aromatic), 7.25 (C*H*-6, aromatic), 7.24 (C*H*-2, aromatic), 7.14 (C*H*-4, aromatic), 7.04 (C*H*-4, thiophene), 6.96 (C*H*-3, thiophene), 4.72 (s, 1H, C*H*), 4.09/3.88 (AB, 2H, C*H*_2_), 2.35 (s, 3H, C*H_3_*). ^13^C NMR (125.75 MHz, CDCl_3_, 23 °C): δ = 138.38 (*C*-3, aromatic), 135.78 (*C*-1, phenyl), 134.22 (*C*-1, aromatic), 130.38 (*C*-2, thiophene), 130.27 (*C*-2, aromatic), 129.25 (*C*H-3,5, phenyl), 129.20 (*C*-4, aromatic), 128.99 (*C*-3, thiophene), 128.74 (*C*H-2,6, phenyl), 128.59 (*C*-5, aromatic), 128.35 (*C*-4, phenyl), 127.27 (*C*-4, thiophene), 126.74 (*C*-5, thiophene), 126.59 (*C*-6, aromatic), 64.59 (*C*H), 50.17 (*C*H_2_), 21.51 (*C*H_3_) (Figure S49c).

**6m**. (*R*,*S*)-*2-(((phenyl(m-tolyl)methyl)sulfinyl)methyl)thiophene.* HRESIMS *m*/*z* 349.0694 [M+Na]^+^ (calcd for C_19_H_18_NaOS_2_^+^, 349.0691, Δ = −0.7 ppm) (Figure S50). HPLC R_t_ 23.95 min, purity 99.0% (Figure S50a). Chiral HPLC R_t_ 14.67 min, >99% ee (Figure S50b). ^1^H NMR (500 MHz, CDCl_3_, 23 °C): δ = 7.43 (C*H*-2,6, phenyl), 7.42 (C*H*-3,5, phenyl), 7.36 (C*H*-4, phenyl), 7.32 (C*H*-5, thiophene), 7.25 (C*H*-5, aromatic), 7.25 (C*H*-6, aromatic), 7.24 (C*H*-2, aromatic), 7.14 (C*H*-4, aromatic), 7.04 (C*H*-4, thiophene), 6.96 (C*H*-3, thiophene), 4.72 (s, 1H, C*H*), 4.09/3.88 (AB, 2H, C*H*_2_), 2.35 (s, 3H, C*H_3_*). ^13^C NMR (125.75 MHz, CDCl_3_, 23 °C): δ = 138.38 (*C*-3, aromatic), 135.78 (*C*-1, phenyl), 134.22 (*C*-1, aromatic), 130.38 (*C*-2, thiophene), 130.27 (*C*-2, aromatic), 129.25 (*C*H-3,5, phenyl), 129.20 (*C*-4, aromatic), 128.99 (*C*-3, thiophene), 128.74 (*C*H-2,6, phenyl), 128.59 (*C*-5, aromatic), 128.35 (*C*-4, phenyl), 127.27 (*C*-4, thiophene), 126.74 (*C*-5, thiophene), 126.59 (*C*-6, aromatic), 64.59 (*C*H), 50.17 (*C*H_2_), 21.51 (*C*H_3_) (Figure S50c).

**7m**. (*S*,*S*)-*2-(((phenyl(m-tolyl)methyl)sulfinyl)methyl)thiophene.* HRESIMS *m*/*z* 349.0690 [M+Na]^+^ (calcd for C_19_H_18_NaOS_2_^+^, 349.0691, Δ = −0.4 ppm) (Figure S51). HPLC R_t_ 24.02 min, purity > 99% (Figure S51a). Chiral HPLC R_t_ 7.54 min, >99% ee (but contains 15.5% 5m) (Figure S51b). ^1^H NMR (500 MHz, CDCl_3_, 23 °C): δ = 7.43 (C*H*-2,6, phenyl), 7.37 (C*H*-3,5, phenyl), 7.33 (C*H*-4, phenyl), 7.33 (C*H*-5, thiophene), 7.30 (C*H*-5, aromatic), 7.22 (C*H*-2, aromatic), 7.22 (C*H*-6, aromatic), 7.17 (C*H*-4, aromatic), 7.04 (C*H*-4, thiophene), 6.96 (C*H*-3, thiophene), 4.72 (s, 1H, C*H*), 4.09/3.88 (AB, 2H, C*H*_2_), 2.37 (s, 3H, C*H_3_*). ^13^C NMR (125.75 MHz, CDCl_3_, 23 °C): δ = 139.09 (*C*-3, aromatic), 135.47 (*C*-1, aromatic), 134.34 (*C*-1, phenyl), 130.40 (*C*-2, thiophene), 129.61 (*C*H-2,6, phenyl), 129.42 (*C*-2, aromatic), 129.18 (*C*-4, aromatic), 128.99 (*C*-3, thiophene), 128.99 (*C*-5, aromatic), 128.65 (*C*H-3,5, phenyl), 128.32 (*C*-4, phenyl), 127.27 (*C*-4, thiophene), 126.74 (*C*-5, thiophene), 125.80 (*C*-6, aromatic), 64.43 (*C*H), 50.20 (*C*H_2_), 21.50 (*C*H_3_) (Figure S51c).

**8m**. (*R*,*R*)-*2-(((phenyl(m-tolyl)methyl)sulfinyl)methyl)thiophene.* HRESIMS *m*/*z* 349.0691 [M+Na]^+^ (calcd for C_19_H_18_NaOS_2_^+^, 349.0691, Δ = 0.1 ppm) (Figure S52). HPLC R_t_ 24.02 min, purity > 99% (Figure S52a). Chiral HPLC R_t_ 11.26 min, >99% ee (Figure S52b). ^1^H NMR (500 MHz, CDCl_3_, 23 °C): δ = 7.43 (C*H*-2,6, phenyl), 7.37 (C*H*-3,5, phenyl), 7.33 (C*H*-4, phenyl), 7.33 (C*H*-5, thiophene), 7.30 (C*H*-5, aromatic), 7.22 (C*H*-2, aromatic), 7.22 (C*H*-6, aromatic), 7.17 (C*H*-4, aromatic), 7.04 (C*H*-4, thiophene), 6.96 (C*H*-3, thiophene), 4.72 (s, 1H, C*H*), 4.09/3.88 (AB, 2H, C*H*_2_), 2.37 (s, 3H, C*H_3_*). ^13^C NMR (125.75 MHz, CDCl_3_, 23 °C): δ = 139.09 (*C*-3, aromatic), 135.47 (*C*-1, aromatic), 134.34 (*C*-1, phenyl), 130.40 (*C*-2, thiophene), 129.61 (*C*H-2,6, phenyl), 129.42 (*C*-2, aromatic), 129.18 (*C*-4, aromatic), 128.99 (*C*-3, thiophene), 128.99 (*C*-5, aromatic), 128.65 (*C*H-3,5, phenyl), 128.32 (*C*-4, phenyl), 127.27 (*C*-4, thiophene), 126.74 (*C*-5, thiophene), 125.80 (*C*-6, aromatic), 64.43 (*C*H), 50.20 (*C*H_2_), 21.50 (*C*H_3_) (Figure S52c).

14. Synthesis of 2-(((phenyl(p-tolyl)methyl)sulfinyl)methyl)thiophene (**5n**–**8n**)

Following general procedure A, 1.0 g (5.0 mmol) of phenyl(*p*-tolyl)methanol, 0.65 g (5.0 mmol) of thiophen-2-ylmethanethiol, and (0.7 mL, 5.5 mmol) of 48% BF_3_·Et_2_O were reacted, affording 1.5 g of **3n** as a yellow oily product (yield >95%).

Following general procedure B, 1.5 g (4.8 mmol) of **3n** dissolved in 12 mL of glacial acetic acid was reacted with 30% H_2_O_2_ (0.5 mL) to give 1.47 g of **4n** as a pale-yellow solid (yield 94%).

Racemic **4n** was further separated into two individual pairs of enantiomers by means of an MPLC system equipped with a glass column packed with silica gel. Toluol/EtOAc = 80/20 was used as the mobile phase, and the separation was carried out with a flow rate of 5 mL/min. Individual pairs of enantiomers were further separated into the individual enantiomers **5n**–**8n** by means of semipreparative chiral HPLC. A semipreparative HPLC instrument was equipped with a CHIRALPAK IA column (10 mm Φ × 25 cm L) (Daicel Inc.), and EtOAc was used as the mobile phase.

**5n.** HRESIMS *m*/*z* 349.0694 [M+H]^+^ (calcd for C_19_H_18_NaOS_2_^+^, 349.0691, Δ = −0.6 ppm) (Figure S53). HPLC R_t_ 24.08 min, purity > 99% (Figure S53a). Chiral HPLC R_t_ 7.94 min, >99% ee (Figure S53b). ^1^H NMR (500 MHz, CDCl_3_, 23 °C): δ = 7.42 (C*H*-2,6, phenyl), 7.41 (C*H*-3,5, phenyl), 7.35 (C*H*-4, phenyl), 7.32 (C*H*-5, thiophene), 7.32 (C*H*-2,6, aromatic), 7.18 (C*H*-3,5, phenyl), 7.04 (C*H*-4, thiophene), 6.96 (C*H*-3, thiophene), 4.72 (s, 1H, C*H*), 4.08/3.87 (AB, 2H, C*H*_2_), and 2.33 (s, 3H, C*H_3_*). ^13^C NMR (125.75 MHz, CDCl_3_, 23 °C): δ = 138.26 (*C*-4, aromatic), 135.84 (*C*-1, phenyl), 131.04 (*C*-1, aromatic), 130.44 (*C*-2, thiophene), 129.54 (*C*H-2,6, aromatic), 129.40 (*C*H-3,5, aromatic), 129.21 (*C*H-3,5, phenyl), 128.94 (*C*-3, thiophene), 128.76 (*C*H-2,6, phenyl), 128.30 (*C*-4, phenyl), 127.28 (*C*-4, thiophene), 126.72 (*C*-5, thiophene), 69.00 (*C*H), 50.16 (*C*H_2_), 21.14 (*C*H_3_) (Figure S53c).

**6n.** HRESIMS *m*/*z* 349.0689 [M+Na]^+^ (calcd for C_19_H_18_NaOS_2_^+^, 349.0691, Δ = 0.5 ppm) (Figure S54). HPLC R_t_ 24.08 min, purity > 99% (Figure S54a). Chiral HPLC R_t_ 14.87 min, >99% ee (Figure S54b). ^1^H NMR (500 MHz, CDCl_3_, 23 °C): δ = 7.42 (C*H*-2,6, phenyl), 7.41 (C*H*-3,5, phenyl), 7.35 (C*H*-4, phenyl), 7.32 (C*H*-5, thiophene), 7.32 (C*H*-2,6, aromatic), 7.18 (C*H*-3,5, phenyl), 7.04 (C*H*-4, thiophene), 6.96 (C*H*-3, thiophene), 4.72 (s, 1H, C*H*), 4.08/3.87 (AB, 2H, C*H*_2_), and 2.33 (s, 3H, C*H_3_*). ^13^C NMR (125.75 MHz, CDCl_3_, 23 °C): δ = 138.26 (*C*-4, aromatic), 135.85 (*C*-1, phenyl), 131.04 (*C*-1, aromatic), 130.44 (*C*-2, thiophene), 129.54 (*C*H-2,6, aromatic), 129.40 (*C*H-3,5, aromatic), 129.21 (*C*H-3,5, phenyl), 128.95 (*C*-3, thiophene), 128.77 (*C*H-2,6, phenyl), 128.30 (*C*-4, phenyl), 127.29 (*C*-4, thiophene), 126.72 (*C*-5, thiophene), 69.00 (*C*H), 50.16 (*C*H_2_), 21.14 (*C*H_3_) (Figure S54c).

7n. (*S*,*S*)-*2-(((phenyl(p-tolyl)methyl)sulfinyl)methyl)thiophene.* HRESIMS *m*/*z* 349.0690 [M+Na]^+^ (calcd for C_19_H_18_NaOS_2_^+^, 349.0691, Δ = 0.5 ppm) (Figure S55). HPLC R_t_ 24.18 min, purity > 99% (Figure S55a). Chiral HPLC R_t_ 7.85 min, >99% ee (Figure S55b). ^1^H NMR (500 MHz, CDCl_3_, 23 °C): δ = 7.42 (C*H*-2,6, phenyl), 7.36 (C*H*-3,5, phenyl), 7.33 (C*H*-4, phenyl), 7.33 (C*H*-5, thiophene), 7.31 (C*H*-2,6, aromatic), 7.22 (C*H*-3,5, phenyl), 7.04 (C*H*-4, thiophene), 6.97 (C*H*-3, thiophene), 4.73 (s, 1H, C*H*), 4.09/3.87 (AB, 2H, C*H*_2_), 2.37 (s, 3H, C*H_3_*). ^13^C NMR (125.75 MHz, CDCl_3_, 23 °C): δ = 138.33 (*C*-4, aromatic), 134.50 (*C*-1, phenyl), 132.43 (*C*-1, aromatic), 130.35 (*C*-2, thiophene), 129.96 (*C*H-3,5, aromatic), 129.56 (*C*H-2,6, phenyl), 129.02 (*C*-3, thiophene), 128.68 (*C*H-2,6, aromatic), 128.64 (*C*H-3,5, phenyl), 128.27 (*C*-4, phenyl), 127.24 (*C*-4, thiophene), 126.75 (*C*-5, thiophene), 69.11 (*C*H), 50.05 (*C*H_2_), 21.15 (*C*H_3_) (Figure S55c).

8n. (*R*,*R*)-*2-(((phenyl(p-tolyl)methyl)sulfinyl)methyl)thiophene.* HRESIMS *m*/*z* 349.0692 [M+Na]^+^ (calcd for C_19_H_18_NaOS_2_^+^, 349.0691, Δ = −0.3 ppm) (Figure S56). HPLC R_t_ 24.20 min, purity > 99% (Figure S56a). Chiral HPLC R_t_ 14.42 min, 9 8.8% (Figure S56b). ^1^H NMR (500 MHz, CDCl_3_, 23 °C): δ = 7.42 (C*H*-2,6, phenyl), 7.36 (C*H*-3,5, phenyl), 7.33 (C*H*-4, phenyl), 7.33 (C*H*-5, thiophene), 7.31 (C*H*-2,6, aromatic), 7.22 (C*H*-3,5, phenyl), 7.05 (C*H*-4, thiophene), 6.97 (C*H*-3, thiophene), 4.73 (s, 1H, C*H*), 4.09/3.87 (AB, 2H, C*H*_2_), 2.37 (s, 3H, C*H_3_*). ^13^C NMR (125.75 MHz, CDCl_3_, 23 °C): δ = 138.32 (*C*-4, aromatic), 134.49 (*C*-1, phenyl), 132.42 (*C*-1, aromatic), 130.35 (*C*-2, thiophene), 129.96 (*C*H-3,5, aromatic), 129.56 (*C*H-2,6, phenyl), 129.01 (*C*-3, thiophene), 128.67 (*C*H-2,6, aromatic), 128.64 (*C*H-3,5, phenyl), 128.27 (*C*-4, phenyl), 127.24 (*C*-4, thiophene), 126.74 (*C*-5, thiophene), 69.11 (*C*H), 50.04 (*C*H_2_), 21.15 (*C*H_3_) (Figure S56c).

15. Synthesis of 2-(((phenyl(2-(trifluoromethyl)phenyl)methyl)sulfinyl)methyl)thiophene (**5o**–**8o**)

Following general procedure A, 1.0 g (4.0 mmol) of phenyl(2-(trifluoromethyl)phenyl)methanol, 0.52 g (4.0 mmol) of thiophen-2-ylmethanethiol, and (0.54 mL, 4.4 mmol) of 48% BF_3_·Et_2_O were reacted, affording 1.37 g of **3o** as a yellow oily product (yield 95%).

Following general procedure B, 1.37 g (4.4 mmol) of **3o** dissolved in 12 mL of glacial acetic acid was reacted with 30% H_2_O_2_ (0.4 mL) to give 1.2 g of **4o** as a white solid (yield 81%).

Racemic **4o** was further separated into two individual pairs of enantiomers by means of a flash column chromatography system filled with silica gel and using Hexane/EtOAc = 2/1 as the mobile phase. Individual pairs of enantiomers were further separated into the individual enantiomers **5o**–**8o** by means of semipreparative chiral HPLC. A semipreparative HPLC instrument was equipped with a CHIRALPAK IA column (10 mm Φ × 25 cm L) (Daicel Inc.), and EtOAc was used as the mobile phase.

**5o.** HRESIMS *m*/*z* 381.0592 [M+H]^+^ (calcd for C_19_H_16_F_3_OS_2_^+^, 381.0589, Δ = −0.6 ppm) (Figure S57). HPLC R_t_ 26.31 min, purity 98.7% (Figure S57a). Chiral HPLC R_t_ 4.09 min, >99% ee (Figure S57b). ^1^H NMR (500 MHz, CDCl_3_, 23 °C): δ = 8.00 (C*H*-6, aromatic), 7.71 (C*H*-3, aromatic), 7.65 (C*H*-5, aromatic), 7.54 (C*H*-2,6, phenyl), 7.46 (C*H*-4, aromatic), 7.41 (C*H*-3,5, phenyl), 7.38 (C*H*-4, phenyl), 7.29 (s, 1H, C*H*-5, thiophene), 6.99 (s, 1H, C*H*-4, thiophene), 6.95 (s, 1H, C*H*-3, thiophene), 5.22 (s, 1H, C*H*), 4.04 (AB, 2H, C*H*_2_). ^13^C NMR (125.75 MHz, CDCl_3_, 23 °C): δ = 135.06 (*C*_q_-1, aromatic), 133.21 (*C*_q_-1, phenyl), 132.52 (*C*H-5, aromatic), 131.15 (*C*_q_, thiophene-2), 130.35 (*C*H-6, aromatic), 129.73 (*C*H-2,6, phenyl), 128.72 (*C*H-3,5, phenyl), 128.67 (*C*H-4, phenyl), 128.67 (*C*H-3, thiophene), 128.41 (*C*_q_-2, aromatic), 128.25 (*C*H-4, aromatic), 127.52 (*C*H-4, thiophene), 126.79 (*C*H-3, aromatic), 126.79 (*C*H-5, thiophene), 123.98 (*C*F_3_), 64.58 (*C*H), 52.02 (*C*H_2_) (Figure S57c).

**6o.** HRESIMS *m*/*z* 381.0586 [M+H]^+^ (calcd for C_19_H_16_F_3_OS_2_^+^, 381.0589, Δ = 0.8 ppm) (Figure S58). HPLC R_t_ 26.31 min, purity > 99% (Figure S58a). Chiral HPLC R_t_ 5.70 min, >99% ee (Figure S58b). ^1^H NMR (500 MHz, CDCl_3_, 23 °C): δ = 8.00 (C*H*-6, aromatic), 7.71 (C*H*-3, aromatic), 7.65 (C*H*-5, aromatic), 7.54 (C*H*-2,6, phenyl), 7.46 (C*H*-4, aromatic), 7.41 (C*H*-3,5, phenyl), 7.38 (C*H*-4, phenyl), 7.29 (s, 1H, C*H*-5, thiophene), 6.99 (s, 1H, C*H*-4, thiophene), 6.95 (s, 1H, C*H*-3, thiophene), 5.22 (s, 1H, C*H*), 4.04 (AB, 2H, C*H*_2_). ^13^C NMR (125.75 MHz, CDCl_3_, 23 °C): δ = 135.06 (*C*_q_-1, aromatic), 133.21 (*C*_q_-1, phenyl), 132.52 (*C*H-5, aromatic), 131.15 (*C*_q_, thiophene-2), 130.35 (*C*H-6, aromatic), 129.73 (*C*H-2,6, phenyl), 128.72 (*C*H-3,5, phenyl), 128.67 (*C*H-4, phenyl), 128.67 (*C*H-3, thiophene), 128.41 (*C*_q_-2, aromatic), 128.25 (*C*H-4, aromatic), 127.52 (*C*H-4, thiophene), 126.79 (*C*H-3, aromatic), 126.79 (*C*H-5, thiophene), 123.98 (*C*F_3_), 64.58 (*C*H), 52.02 (*C*H_2_) (Figure S58c).

**7o.** HRESIMS *m*/*z* 381.0580 [M+H]^+^ (calcd for C_19_H_16_F_3_OS_2_^+^, 381.0589, Δ = 2.4 ppm) (Figure S59). HPLC R_t_ 26.28 min, purity > 99% (Figure S59a). Chiral HPLC R_t_ 4.80 min, >99% ee (Figure S59b). ^1^H NMR (500 MHz, CDCl_3_, 23 °C): δ = 8.16 (C*H*-6, aromatic), 7.70 (C*H*-3, aromatic), 7.67 (C*H*-5, aromatic), 7.49 (C*H*-2,6, phenyl), 7.44 (C*H*-4, aromatic), 7.40 (C*H*-3,5, phenyl), 7.34 (C*H*-4, phenyl), 7.32 (s, 1H, C*H*-5, thiophene), 7.03 (s, 1H, C*H*-4, thiophene), 6.98 (s, 1H, C*H*-3, thiophene), 5.35 (s, 1H, C*H*), 4.16/3.83 (AB, 2H, C*H*_2_). ^13^C NMR (125.75 MHz, CDCl_3_, 23 °C): δ = 135.27 (*C*_q_-1, phenyl), 134.25 (*C*_q_-1, aromatic), 132.37 (*C*H-5, aromatic), 130.47 (*C*H-6, aromatic), 130.12 (*C*_q_, thiophene-2), 129.62 (*C*_q_-2, aromatic), 129.30 (*C*H-3,5, phenyl), 129.19 (*C*H-3, thiophene), 128.63 (*C*H-2,6, phenyl), 128.63 (*C*H-4, phenyl), 128.05 (*C*H-4, aromatic), 127.26 (*C*H-4, thiophene), 126.94 (*C*H-5, thiophene), 126.58 (*C*H-3, aromatic), 124.08 (*C*F_3_), 64.85 (*C*H), 50.43 (*C*H_2_) (Figure S59c).

**8o.** HRESIMS *m*/*z* 381.0585 [M+H]^+^ (calcd for C_19_H_16_F_3_OS_2_^+^, 381.0589, Δ = 1.0 ppm) (Figure S60). HPLC R_t_ 26.28 min, purity > 99% (Figure S60a). Chiral HPLC R_t_ 6.48 min, >99% ee (Figure S60b). ^1^H NMR (500 MHz, CDCl_3_, 23 °C): δ = 8.16 (C*H*-6, aromatic), 7.70 (C*H*-3, aromatic), 7.67 (C*H*-5, aromatic), 7.49 (C*H*-2,6, phenyl), 7.44 (C*H*-4, aromatic), 7.40 (C*H*-3,5, phenyl), 7.34 (C*H*-4, phenyl), 7.32 (s, 1H, C*H*-5, thiophene), 7.03 (s, 1H, C*H*-4, thiophene), 6.98 (s, 1H, C*H*-3, thiophene), 5.35 (s, 1H, C*H*), 4.16/3.83 (AB, 2H, C*H*_2_). ^13^C NMR (125.75 MHz, CDCl_3_, 23 °C): δ = 135.27 (*C*_q_-1, phenyl), 134.25 (*C*_q_-1, aromatic), 132.37 (*C*H-5, aromatic), 130.47 (*C*H-6, aromatic), 130.12 (*C*_q_, thiophene-2), 129.62 (*C*_q_-2, aromatic), 129.30 (*C*H-3,5, phenyl), 129.19 (*C*H-3, thiophene), 128.63 (*C*H-2,6, phenyl), 128.63 (*C*H-4, phenyl), 128.05 (*C*H-4, aromatic), 127.26 (*C*H-4, thiophene), 126.94 (*C*H-5, thiophene), 126.58 (*C*H-3, aromatic), 124.08 (*C*F_3_), 64.85 (*C*H), 50.43 (*C*H_2_) (Figure S60c).

16. Synthesis of 2-(((phenyl(3-(trifluoromethyl)phenyl)methyl)sulfinyl)methyl)thiophene (**5p**–**8p**)

Following general procedure A, 1.0 g (4.0 mmol) of phenyl(3-(trifluoromethyl)phenyl)methanol, 0.52 g (4.0 mmol) of thiophen-2-ylmethanethiol, and (0.54 mL, 4.4 mmol) of 48% BF_3_·Et_2_O were reacted, affording 1.3 g of **3p** as a yellow oily product (yield 87%).

Following general procedure B, 1.3 g (3.6 mmol) of **3p** dissolved in 12 mL of glacial acetic acid was reacted with 30% H_2_O_2_ (0.36 mL) to give 0.85 g of **4p** as a pale-yellow solid (yield 63%).

Racemic **4p** was further separated into two individual pairs of enantiomers by means of an MPLC system equipped with a glass column packed with silica gel. Toluol/EtOAc = 80/20 was used as the mobile phase, and the separation was carried out with a flow rate of 5 mL/min. Individual pairs of enantiomers were further separated into the individual enantiomers **5p**–**8p** by means of semipreparative chiral HPLC. A semipreparative HPLC instrument was equipped with a CHIRALPAK IA column (10 mm Φ × 25 cm L) (Daicel Inc.), and EtOAc was used as the mobile phase.

**5p.** HRESIMS *m*/*z* 381.0588 [M+H]^+^ (calcd for C_19_H_16_F_3_OS_2_^+^, 381.0589, Δ = 0.2 ppm) (Figure S61). HPLC R_t_ 26.42 min, purity > 99% (Figure S61a). Chiral HPLC R_t_ 4.81 min, >99% ee (Figure S61b). ^1^H NMR (500 MHz, CDCl_3_, 23 °C): δ = 7.66 (C*H*-6, aromatic), 7.63 (C*H*-2, aromatic), 7.60 (C*H*-4, aromatic), 7.50 (C*H*-5, aromatic), 7.46 (C*H*-3,5, phenyl), 7.46 (C*H*-4, phenyl), 7.41 (C*H*-2,6, phenyl), 7.35 (C*H*-5, thiophene), 7.06 (C*H*-4, thiophene), 6.96 (C*H*-3, thiophene), 4.80 (s, 1H, C*H*), 4.09/3.91 (AB, 2H, C*H*_2_). ^13^C NMR (125.75 MHz, CDCl_3_, 23 °C): δ = 135.35 (*C*-1, aromatic), 134.56 (*C*-1, phenyl), 133.07 (*C*-6, aromatic), 130.93 (*C*-3, aromatic), 129.68 (*C*-2, thiophene), 129.55 (*C*H-3,5, phenyl), 129.20 (*C*-3, thiophene), 129.10 (*C*-5, aromatic), 128.84 (*C*-4, phenyl), 128.76 (*C*H-2,6, phenyl), 127.37 (*C*-4, thiophene), 127.04 (*C*-5, thiophene), 126.25 (*C*-2, aromatic), 125.23 (*C*-4, aromatic), 123.85 (*C*F_3_), 68.72 (*C*H), 52.12 (*C*H_2_) (Figure S61c).

**6p.** HRESIMS *m*/*z* 381.0587 [M+H]^+^ (calcd for C_19_H_16_F_3_OS_2_^+^, 381.0589, Δ = 0.5 ppm) (Figure S62). HPLC R_t_ 26.43 min, purity 98.3% (Figure S62a). Chiral HPLC R_t_ 6.12 min, >99% ee (but contains 7.6% **7p**) (Figure S62b). ^1^H NMR (500 MHz, CDCl_3_, 23 °C): δ = 7.66 (C*H*-6, aromatic), 7.63 (C*H*-2, aromatic), 7.60 (C*H*-4, aromatic), 7.50 (C*H*-5, aromatic), 7.46 (C*H*-3,5, phenyl), 7.46 (C*H*-4, phenyl), 7.41 (C*H*-2,6, phenyl), 7.35 (C*H*-5, thiophene), 7.06 (C*H*-4, thiophene), 6.96 (C*H*-3, thiophene), 4.80 (s, 1H, C*H*), 4.09/3.91 (AB, 2H, C*H*_2_). ^13^C NMR (125.75 MHz, CDCl_3_, 23 °C): δ = 135.35 (*C*-1, aromatic), 134.56 (*C*-1, phenyl), 133.07 (*C*-6, aromatic), 130.93 (*C*-3, aromatic), 129.68 (*C*-2, thiophene), 129.55 (*C*H-3,5, phenyl), 129.20 (*C*-3, thiophene), 129.10 (*C*-5, aromatic), 128.84 (*C*-4, phenyl), 128.76 (*C*H-2,6, phenyl), 127.37 (*C*-4, thiophene), 127.04 (*C*-5, thiophene), 126.25 (*C*-2, aromatic), 125.23 (*C*-4, aromatic), 123.85 (*C*F_3_), 68.72 (*C*H), 52.12 (*C*H_2_) (Figure S62c).

**7p.** HRESIMS *m*/*z* 381.0588 [M+H]^+^ (calcd for C_19_H_16_F_3_OS_2_^+^, 381.0589, Δ = 0.3 ppm) (Figure S63). HPLC R_t_ 26.02 min, purity 97.7% (Figure S63a). Chiral HPLC R_t_ 5.50 min, >99% ee (Figure S63b). ^1^H NMR (500 MHz, CDCl_3_, 23 °C): δ = 7.68 (C*H*-6, aromatic), 7.64 (C*H*-2, aromatic), 7.62 (C*H*-4, aromatic), 7.54 (C*H*-5, aromatic), 7.44 (C*H*-2,6, phenyl), 7.43 (C*H*-3,5, phenyl), 7.40 (C*H*-4, phenyl), 7.35 (C*H*-5, thiophene), 7.05 (C*H*-4, thiophene), 6.96 (C*H*-3, thiophene), 4.77 (s, 1H, C*H*), 4.09/3.90 (AB, 2H, C*H*_2_). ^13^C NMR (125.75 MHz, CDCl_3_, 23 °C): δ = 136.98 (*C*-1, aromatic), 132.89 (*C*-1, phenyl), 132.08 (*C*-6, aromatic), 131.50 (*C*-3, aromatic), 129.95 (*C*H-2,6, phenyl), 129.94 (*C*-2, thiophene), 129.70 (*C*-5, aromatic), 129.00 (*C*-3, thiophene), 128.90 (*C*H-3,5, phenyl), 128.82 (*C*-4, phenyl), 127.48 (*C*-4, thiophene), 126.95 (*C*-5, thiophene), 125.68 (*C*-2, aromatic), 125.17 (*C*-4, aromatic), 123.71 (*C*F_3_), 67.85 (*C*H), 50.41 (*C*H_2_) (Figure S63c).

**8p.** HRESIMS *m*/*z* 381.0585 [M+H]^+^ (calcd for C_19_H_16_F_3_OS_2_^+^, 381.0589, Δ = 1.2 ppm) (Figure S64). HPLC R_t_ 26.03 min, purity > 99% (Figure S64a). Chiral HPLC R_t_ 7.90 min, >99% ee (Figure S64b). ^1^H NMR (500 MHz, CDCl_3_, 23 °C): δ = 7.68 (C*H*-6, aromatic), 7.64 (C*H*-2, aromatic), 7.62 (C*H*-4, aromatic), 7.54 (C*H*-5, aromatic), 7.44 (C*H*-2,6, phenyl), 7.43 (C*H*-3,5, phenyl), 7.40 (C*H*-4, phenyl), 7.35 (C*H*-5, thiophene), 7.05 (C*H*-4, thiophene), 6.96 (C*H*-3, thiophene), 4.77 (s, 1H, C*H*), 4.09/3.90 (AB, 2H, C*H*_2_). ^13^C NMR (125.75 MHz, CDCl_3_, 23 °C): δ = 136.98 (*C*-1, aromatic), 132.89 (*C*-1, phenyl), 132.08 (*C*-6, aromatic), 131.50 (*C*-3, aromatic), 129.95 (*C*H-2,6, phenyl), 129.94 (*C*-2, thiophene), 129.70 (*C*-5, aromatic), 129.00 (*C*-3, thiophene), 128.90 (*C*H-3,5, phenyl), 128.82 (*C*-4, phenyl), 127.48 (*C*-4, thiophene), 126.95 (*C*-5, thiophene), 125.68 (*C*-2, aromatic), 125.17 (*C*-4, aromatic), 123.71 (*C*F_3_), 67.85 (*C*H), 50.41 (*C*H_2_) (Figure S64c).

17. Synthesis of 2-(((phenyl(4-(trifluoromethyl)phenyl)methyl)sulfinyl)methyl)thiophene (**5q**–**8q**)

Following general procedure A, 1.0 g (4.0 mmol) of phenyl(4-(trifluoromethyl)phenyl)methanol, 0.52 g (4.0 mmol) of thiophen-2-ylmethanethiol, and (0.54 mL, 4.4 mmol) of 48% BF_3_·Et_2_O were reacted, affording 0.95 g of **3q** as a yellow oily product (yield 67%).

Following general procedure B, 1.95 g (2.6 mmol) of **3q** dissolved in 10 mL of glacial acetic acid was reacted with 30% H_2_O_2_ (0.36 mL) to give 0.8 g of **4q** as a yellow solid (yield 80%).

Racemic **4q** was further separated into two individual pairs of enantiomers by means of an MPLC system equipped with a glass column packed with silica gel. Toluol/EtOAc = 80/20 was used as the mobile phase, and the separation was carried out with a flow rate of 5 mL/min. Individual pairs of enantiomers were further separated into the individual enantiomers **5q**–**8q** by means of semipreparative chiral HPLC. A semipreparative HPLC instrument was equipped with a CHIRALPAK IA column (10 mm Φ × 25 cm L) (Daicel Inc.), and EtOAc was used as the mobile phase.

**5q.** HRESIMS *m*/*z* 381.0585 [M+H]^+^ (calcd for C_19_H_16_F_3_OS_2_^+^, 381.0589, Δ = 1.2 ppm) (Figure S65). HPLC R_t_ 26.65 min, purity > 99% (Figure S65a). Chiral HPLC R_t_ 5.51 min, >99% ee (Figure S65b). ^1^H NMR (500 MHz, CDCl_3_, 23 °C): δ = 7.62 (C*H*-3,5, aromatic), 7.53 (C*H*-2,6, aromatic), 7.45 (C*H*-3,5, phenyl), 7.41 (C*H*-2,6, phenyl), 7.39 (C*H*-4, phenyl), 7.36 (C*H*-5, thiophene), 7.07 (C*H*-4, thiophene), 6.97 (C*H*-3, thiophene), 4.80 (s, 1H, C*H*), 4.12/3.91 (AB, 2H, C*H*_2_). ^13^C NMR (125.75 MHz, CDCl_3_, 23 °C): δ = 138.28 (*C*-1, aromatic), 134.56 (*C*-1, phenyl), 130.46 (*C*-4, aromatic), 129.99 (*C*H-2,6, aromatic), 129.60 (*C*H-3,5, phenyl), 129.60 (*C*-2, thiophene), 129.27 (*C*-3, thiophene), 128.91 (*C*-4, phenyl), 128.79 (*C*H-2,6, phenyl), 127.37 (*C*-4, thiophene), 127.06 (*C*-5, thiophene), 125.54 (*C*H-3,5, aromatic), 123.94 (*C*F_3_), 68.59 (*C*H), 50.05 (*C*H_2_) (Figure S65a).

**6q.** HRESIMS *m*/*z* 381.0583 [M+H]^+^ (calcd for C_19_H_16_F_3_OS_2_^+^, 381.0589, Δ = 1.7 ppm) (Figure S66). HPLC R_t_ 26.63 min, purity > 99% (Figure S66a). Chiral HPLC R_t_ 10.55 min, >99% ee (Figure S66b). ^1^H NMR (500 MHz, CDCl_3_, 23 °C): δ = 7.62 (C*H*-3,5, aromatic), 7.53 (C*H*-2,6, aromatic), 7.45 (C*H*-3,5, phenyl), 7.41 (C*H*-2,6, phenyl), 7.39 (C*H*-4, phenyl), 7.36 (C*H*-5, thiophene), 7.07 (C*H*-4, thiophene), 6.97 (C*H*-3, thiophene), 4.80 (s, 1H, C*H*), 4.12/3.91 (AB, 2H, C*H*_2_). ^13^C NMR (125.75 MHz, CDCl_3_, 23 °C): δ = 138.28 (*C*-1, aromatic), 134.56 (*C*-1, phenyl), 130.46 (*C*-4, aromatic), 129.99 (*C*H-2,6, aromatic), 129.60 (*C*H-3,5, phenyl), 129.60 (*C*-2, thiophene), 129.27 (*C*-3, thiophene), 128.91 (*C*-4, phenyl), 128.79 (*C*H-2,6, phenyl), 127.37 (*C*-4, thiophene), 127.06 (*C*-5, thiophene), 125.54 (*C*H-3,5, aromatic), 123.94 (*C*F_3_), 68.59 (*C*H), 50.05 (*C*H_2_) (Figure S66c).

**7q.** HRESIMS *m*/*z* 381.0583 [M+H]^+^ (calcd for C_19_H_16_F_3_OS_2_^+^, 381.0589, Δ = 1.6 ppm) (Figure S67). HPLC R_t_ 26.40 min, purity > 99% (Figure S67a). Chiral HPLC R_t_ 6.58 min, >99% ee (Figure S67b). ^1^H NMR (500 MHz, CDCl_3_, 23 °C): δ = 7.66 (C*H*-3,5, aromatic), 7.57 (C*H*-2,6, aromatic), 7.44 (C*H*-2,6, phenyl), 7.43 (C*H*-3,5, phenyl), 7.40 (C*H*-4, phenyl), 7.35 (C*H*-5, thiophene), 7.06 (C*H*-4, thiophene), 6.97 (C*H*-3, thiophene), 4.78 (s, 1H, C*H*), 4.10/3.92 (AB, 2H, C*H*_2_). ^13^C NMR (125.75 MHz, CDCl_3_, 23 °C): δ = 139.95 (*C*-1, aromatic), 132.93 (*C*-1, phenyl), 130.49 (*C*-4, aromatic), 129.22 (*C*H-2,6, aromatic), 129.99 (*C*-2, thiophene), 129.77 (*C*H-2,6, phenyl), 129.00 (*C*-3, thiophene), 128.89 (*C*H-3,5, phenyl), 128.81 (*C*-4, phenyl), 127.49 (*C*-4, thiophene), 127.05 (*C*-5, thiophene), 126.09 (*C*H-3,5, aromatic), 123.79 (*C*F_3_), 67.95 (*C*H), 50.41 (*C*H_2_) (Figure S67c).

**8q.** HRESIMS *m*/*z* 381.0586 [M+H]^+^ (calcd for C_19_H_16_F_3_OS_2_^+^, 381.0589, Δ = 0.7 ppm) (Figure S68). HPLC R_t_ 26.38 min, purity 95.7% (Figure S68a). Chiral HPLC R_t_ 9.28 min, >99% ee (Figure S68b). ^1^H NMR (500 MHz, CDCl_3_, 23 °C): δ = 7.66 (C*H*-3,5, aromatic), 7.57 (C*H*-2,6, aromatic), 7.44 (C*H*-2,6, phenyl), 7.43 (C*H*-3,5, phenyl), 7.40 (C*H*-4, phenyl), 7.35 (C*H*-5, thiophene), 7.06 (C*H*-4, thiophene), 6.97 (C*H*-3, thiophene), 4.78 (s, 1H, C*H*), 4.10/3.92 (AB, 2H, C*H*_2_). ^13^C NMR (125.75 MHz, CDCl_3_, 23 °C): δ = 139.95 (*C*-1, aromatic), 132.93 (*C*-1, phenyl), 130.49 (*C*-4, aromatic), 129.22 (*C*H-2,6, aromatic), 129.99 (*C*-2, thiophene), 129.77 (*C*H-2,6, phenyl), 129.00 (*C*-3, thiophene), 128.89 (*C*H-3,5, phenyl), 128.81 (*C*-4, phenyl), 127.49 (*C*-4, thiophene), 127.05 (*C*-5, thiophene), 126.09 (*C*H-3,5, aromatic), 123.79 (*C*F_3_), 67.95 (*C*H), 50.41 (*C*H_2_) (Figure S68c).

18. Synthesis of 2-((((2-methoxyphenyl)(phenyl)methyl)sulfinyl)methyl)thiophene (**5r**–**8r**)

Following general procedure A, 0.4 g (1.8 mmol) of (2-methoxyphenyl)(phenyl)methanol, 0.24 g (1.8 mmol) of thiophen-2-ylmethanethiol, and (0.24 mL, 1.98 mmol) of 48% BF_3_·Et_2_O were reacted, affording 0.44 g of **3r** as a yellow oily product (yield 75%).

Following general procedure B, 0.44 g (1.3 mmol) of **3r** dissolved in 12 mL of glacial acetic acid was reacted with 30% H_2_O_2_ (0.15 mL) to give 0.21 g of **4r** as a yellow solid (yield 46%).

Racemic **4r** was further separated into two individual pairs of enantiomers by means of an MPLC system equipped with a glass column packed with silica gel. Toluol/EtOAc = 80/20 was used as the mobile phase, and the separation was carried out with a flow rate of 5 mL/min. Individual pairs of enantiomers were further separated into the individual enantiomers **5r**–**8r** by means of semipreparative chiral HPLC. A semipreparative HPLC instrument was equipped with a CHIRALPAK IA column (10 mm Φ × 25 cm L) (Daicel Inc.), and EtOAc was used as the mobile phase in the case of **7r/8r** and i-PrOH in the case of **5r/6r**.

**5r**. (*R*,*R*)-*2-((((2-methoxyphenyl)(phenyl)methyl)sulfinyl)methyl)thiophene.* HRESIMS *m*/*z* 365.0643 [M+Na]^+^ (calcd for C_19_H_18_NaO_2_S_2_^+^, 365.0640, Δ = −0.8 ppm) (Figure S69). HPLC R_t_ 24.02 min, purity 96.6% (Figure S69a). Chiral HPLC R_t_ 13.84 min, >90% ee (Figure S69b). ^1^H NMR (500 MHz, CDCl_3_, 23 °C): δ = 7.54 (C*H*-6, aromatic), 7.49 (C*H*-2,6, phenyl), 7.36 (C*H*-3,5, phenyl), 7.32 (C*H*-4, phenyl), 7.32 (C*H*-4, aromatic), 7.30 (C*H*-5, thiophene), 7.02 (C*H*-5, aromatic), 7.04 (C*H*-4, thiophene), 6.91 (C*H*-3, thiophene), 5.34 (s, 1H, C*H*), 4.09/3.95 (AB, 2H, C*H*_2_), 3.80 (s, 3H, OC*H_3_*). ^13^C NMR (125.75 MHz, CDCl_3_, 23 °C): δ = 156.47 (*C*-2, aromatic), 134.39 (*C*-1, phenyl), 131.25 (*C*-2, thiophene), 129.92 (*C*H-2,6, phenyl), 129.40 (*C*-4, aromatic), 129.27 (*C*-6, aromatic), 128.72 (*C*-3, thiophene), 128.50 (*C*H-3,5, phenyl), 128.11 (*C*-4, phenyl), 127.17 (*C*-4, thiophene), 126.56 (*C*-5, thiophene), 124.52 (*C*-1, aromatic), 120.97 (*C*-5, aromatic), 111.12 (*C*-3, aromatic), 62.95 (*C*H), 55.49 (O*C*H_3_), 50.80 (*C*H_2_) (Figure S69c).

**6r**. (*S*,*S*)-*2-((((2-methoxyphenyl)(phenyl)methyl)sulfinyl)methyl)thiophene.* HRESIMS *m*/*z* 365.0641 [M+Na]^+^ (calcd for C_19_H_18_NaO_2_S_2_^+^, 365.0640, Δ = −0.1 ppm) (Figure S70). HPLC R_t_ 24.00 min, purity 96.9% (Figure S70a). Chiral HPLC R_t_ 14.36 min, ~75% ee (Figure S70b). ^1^H NMR (500 MHz, CDCl_3_, 23 °C): δ = 7.54 (C*H*-6, aromatic), 7.49 (C*H*-2,6, phenyl), 7.36 (C*H*-3,5, phenyl), 7.32 (C*H*-4, phenyl), 7.32 (C*H*-4, aromatic), 7.30 (C*H*-5, thiophene), 7.02 (C*H*-5, aromatic), 7.04 (C*H*-4, thiophene), 6.92 (C*H*-3, aromatic), 6.91 (C*H*-3, thiophene), 5.34 (s, 1H, C*H*), 4.09/3.95 (AB, 2H, C*H*_2_), 3.80 (s, 3H, OC*H_3_*). ^13^C NMR (125.75 MHz, CDCl_3_, 23 °C): δ = 156.47 (*C*-2, aromatic), 134.39 (*C*-1, phenyl), 131.25 (*C*-2, thiophene), 129.92 (*C*H-2,6, phenyl), 129.40 (*C*-4, aromatic), 129.27 (*C*-6, aromatic), 128.72 (*C*-3, thiophene), 128.50 (*C*H-3,5, phenyl), 128.11 (*C*-4, phenyl), 127.17 (*C*-4, thiophene), 126.56 (*C*-5, thiophene), 124.52 (*C*-1, aromatic), 120.97 (*C*-5, aromatic), 111.12 (*C*-3, aromatic), 62.95 (*C*H), 55.49 (O*C*H_3_), 50.80 (*C*H_2_) (Figure S70c).

**7r**. (*S*,*R*)-*2-((((2-methoxyphenyl)(phenyl)methyl)sulfinyl)methyl)thiophene.* HRESIMS *m*/*z* 365.0641 [M+Na]^+^ (calcd for C_19_H_18_NaO_2_S_2_^+^, 365.0640, Δ = −0.2 ppm) (Figure S71). HPLC R_t_ 23.15 min, purity 97.9% (Figure S71a). Chiral HPLC R_t_ 12.70 min, >99% ee (Figure S71b). ^1^H NMR (500 MHz, CDCl_3_, 23 °C): δ = 7.54 (C*H*-6, aromatic), 7.49 (C*H*-2,6, phenyl), 7.36 (C*H*-3,5, phenyl), 7.32 (C*H*-4, aromatic), 7.30 (C*H*-5, thiophene), 7.29 (C*H*-4, phenyl), 7.04 (C*H*-5, aromatic), 7.01 (C*H*-4, thiophene), 6.97 (C*H*-3, thiophene), 6.94 (C*H*-3, aromatic), 5.53 (s, 1H, C*H*), 4.14/3.84 (AB, 2H, C*H*_2_), 3.84 (s, 3H, OC*H_3_*). ^13^C NMR (125.75 MHz, CDCl_3_, 23 °C): δ = 157.19 (*C*-2, aromatic), 136.67 (*C*-1, phenyl), 131.42 (*C*-2, thiophene), 130.25 (*C*-6, aromatic), 129.28 (*C*-4, aromatic), 128.94 (*C*H-3,5, phenyl), 128.88 (*C*H-2,6, phenyl), 128.78 (*C*-3, thiophene), 127.95 (*C*-4, phenyl), 127.27 (*C*-4, thiophene), 126.52 (*C*-5, thiophene), 122.91 (*C*-1, aromatic), 120.95 (*C*-5, aromatic), 110.88 (*C*-3, aromatic), 62.96 (*C*H), 55.60 (O*C*H_3_), 50.85 (*C*H_2_) (Figure S71c).

**8r**. (*S*,*S*)-*2-((((2-methoxyphenyl)(phenyl)methyl)sulfinyl)methyl)thiophene.* HRESIMS *m*/*z* 365.0642 [M+Na]^+^ (calcd for C_19_H_18_NaO_2_S_2_^+^, 365.0640, Δ = −0.5 ppm) (Figure S72). HPLC R_t_ 23.15 min, purity 97.0% (Figure S72a). Chiral HPLC R_t_ 15.24 min, >99% ee (Figure S72b). ^1^H NMR (500 MHz, CDCl_3_, 23 °C): δ = 7.54 (C*H*-6, aromatic), 7.49 (C*H*-2,6, phenyl), 7.36 (C*H*-3,5, phenyl), 7.32 (C*H*-4, aromatic), 7.30 (C*H*-5, thiophene), 7.29 (C*H*-4, phenyl), 7.04 (C*H*-5, aromatic), 7.01 (C*H*-4, thiophene), 6.97 (C*H*-3, thiophene), 6.94 (C*H*-3, aromatic), 5.53 (s, 1H, C*H*), 4.14/3.84 (AB, 2H, C*H*_2_), 3.84 (s, 3H, OC*H_3_*). ^13^C NMR (125.75 MHz, CDCl_3_, 23 °C): δ = 157.19 (*C*-2, aromatic), 136.67 (*C*-1, phenyl), 131.42 (*C*-2, thiophene), 130.25 (*C*-6, aromatic), 129.28 (*C*-4, aromatic), 128.94 (*C*H-3,5, phenyl), 128.88 (*C*H-2,6, phenyl), 128.78 (*C*-3, thiophene), 127.95 (*C*-4, phenyl), 127.27 (*C*-4, thiophene), 126.52 (*C*-5, thiophene), 122.91 (*C*-1, aromatic), 120.95 (*C*-5, aromatic), 110.88 (*C*-3, aromatic), 62.96 (*C*H), 55.60 (O*C*H_3_), 50.85 (*C*H_2_) (Figure S72c).

19. Synthesis of 2-((((3-methoxyphenyl)(phenyl)methyl)sulfinyl)methyl)thiophene (**5s**–**8s**)

Following general procedure A, 1.0 g (4.6 mmol) of (3-methoxyphenyl)(phenyl)methanol, 0.6 g (4.6 mmol) of thiophen-2-ylmethanethiol, and 0.62 mL (5.1 mmol) of 48% BF_3_·Et_2_O were reacted, affording 1.1 g of **3s** as a red-yellow oily product (yield 73%).

Following general procedure B, 1.1 g (3.4 mmol) of **3s** dissolved in 12 mL of glacial acetic acid was reacted with 30% H_2_O_2_ (0.35 mL) to give 1.1 g of **4s** as a yellow solid (yield 94%).

Racemic **4s** was further separated into two individual pairs of enantiomers by means of an MPLC system equipped with a glass column packed with silica gel. Toluol/EtOAc = 80/20 was used as the mobile phase, and the separation was carried out with a flow rate of 5 mL/min. Individual pairs of enantiomers were further separated into the individual enantiomers **5s**–**8s** by means of semipreparative chiral HPLC. A semipreparative HPLC instrument was equipped with a CHIRALPAK IA column (10 mm Φ × 25 cm L) (Daicel Inc.), and EtOAc was used as the mobile phase.

**5s.** HRESIMS *m*/*z* 365.0652 [M+Na]^+^ (calcd for C_19_H_18_NaO_2_S_2_^+^, 365.0640, Δ = −3.2 ppm) (Figure S73). HPLC R_t_ 23.83 min, purity 98.2% (Figure S73a). Chiral HPLC R_t_ 7.05 min, >99% ee (Figure S73b). ^1^H NMR (500 MHz, CDCl_3_, 23 °C): δ = 7.43 (C*H*-2,6, phenyl), 7.37 (C*H*-3,5, phenyl), 7.34 (C*H*-4, phenyl), 7.34 (C*H*-5, aromatic), 7.33 (C*H*-5, thiophene), 7.05 (C*H*-4, thiophene), 7.02 (C*H*-6, aromatic), 6.98 (C*H*-3, thiophene), 6.95 (C*H*-2, aromatic), 6.89 (C*H*-4, aromatic), 4.72 (s, 1H, C*H*), 4.11/3.89 (AB, 2H, C*H*_2_), 3.82 (s, 3H, OC*H_3_*). ^13^C NMR (125.75 MHz, CDCl_3_, 23 °C): δ = 160.09 (*C*-3, aromatic), 136.92 (*C*-1, aromatic), 134.16 (*C*-1, phenyl), 130.34 (*C*-5, aromatic), 130.27 (*C*-2, thiophene), 129.60 (*C*H-2,6, phenyl), 129.08 (*C*-3, thiophene), 128.68 (*C*H-3,5, phenyl), 128.39 (*C*-4, phenyl), 127.28 (*C*-4, thiophene), 126.79 (*C*-5, thiophene), 120.97 (*C*-6, aromatic), 114.72 (*C*-2, aromatic), 113.57 (*C*-4, aromatic), 69.36 (*C*H), 55.31 (O*C*H_3_), 50.14 (*C*H_2_) (Figure S73a).

**6s.** HRESIMS *m*/*z* 365.0645 [M+Na]^+^ (calcd for C_19_H_18_NaO_2_S_2_^+^, 365.0640, Δ = −1.3 ppm) (Figure S74). HPLC R_t_ 22.57 min, purity > 99% (Figure S74a). Chiral HPLC R_t_ 10.55 min, 98.7% ee (Figure S74b). ^1^H NMR (500 MHz, CDCl_3_, 23 °C): δ = 7.43 (C*H*-2,6, phenyl), 7.37 (C*H*-3,5, phenyl), 7.34 (C*H*-4, phenyl), 7.34 (C*H*-5, aromatic), 7.33 (C*H*-5, thiophene), 7.05 (C*H*-4, thiophene), 7.02 (C*H*-6, aromatic), 6.98 (C*H*-3, thiophene), 6.95 (C*H*-2, aromatic), 6.89 (C*H*-4, aromatic), 4.72 (s, 1H, C*H*), 4.11/3.89 (AB, 2H, C*H*_2_), 3.82 (s, 3H, OC*H_3_*). ^13^C NMR (125.75 MHz, CDCl_3_, 23 °C): δ = 160.09 (*C*-3, aromatic), 136.92 (*C*-1, aromatic), 134.16 (*C*-1, phenyl), 130.34 (*C*-5, aromatic), 130.27 (*C*-2, thiophene), 129.60 (*C*H-2,6, phenyl), 129.08 (*C*-3, thiophene), 128.68 (*C*H-3,5, phenyl), 128.39 (*C*-4, phenyl), 127.28 (*C*-4, thiophene), 126.79 (*C*-5, thiophene), 120.97 (*C*-6, aromatic), 114.72 (*C*-2, aromatic), 113.57 (*C*-4, aromatic), 69.36 (*C*H), 55.31 (O*C*H_3_), 50.14 (*C*H_2_) (Figure S74c).

**7s.** HRESIMS *m*/*z* 365.0644 [M+Na]^+^ (calcd for C_19_H_18_NaO_2_S_2_^+^, 365.0640, Δ = −0.8 ppm) (Figure S75). HPLC R_t_ 24.05 min, purity 96.7% (Figure S75a). Chiral HPLC R_t_ 7.04 min, >99% ee (Figure S75b). ^1^H NMR (500 MHz, CDCl_3_, 23 °C): δ = 7.43 (C*H*-2,6, phenyl), 7.41 (C*H*-3,5, phenyl), 7.36 (C*H*-4, phenyl), 7.32 (C*H*-5, thiophene), 7.29 (C*H*-5, aromatic), 7.04 (C*H*-4, thiophene), 7.03 (C*H*-6, aromatic), 7.00 (C*H*-2, aromatic), 6.96 (C*H*-3, thiophene), 6.87 (C*H*-4, aromatic), 4.72 (s, 1H, C*H*), 4.09/3.89 (AB, 2H, C*H*_2_), 3.79 (s, 3H, OC*H_3_*). ^13^C NMR (125.75 MHz, CDCl_3_, 23 °C): δ = 159.66 (*C*-3, aromatic), 135.75 (*C*-1, aromatic), 133.57 (*C*-1, phenyl), 130.37 (*C*-2, thiophene), 129.66 (*C*-5, aromatic), 129.27 (*C*H-3,5, phenyl), 129.01 (*C*-3, thiophene), 128.75 (*C*H-2,6, phenyl), 128.43 (*C*-4, phenyl), 127.30 (*C*-4, thiophene), 126.77 (*C*-5, thiophene), 121.98 (*C*-6, aromatic), 115.42 (*C*-2, aromatic), 113.69 (*C*-4, aromatic), 69.37 (*C*H), 55.25 (O*C*H_3_), 50.25 (*C*H_2_) (Figure S75c).

**8s.** HRESIMS *m*/*z* 365.0642 [M+Na]^+^ (calcd for C_19_H_18_NaO_2_S_2_^+^, 365.0640, Δ = −0.4 ppm) (Figure S76). HPLC R_t_ 22.80 min, purity > 99% (Figure S76a). Chiral HPLC R_t_ 13.05 min, >99% ee (Figure S76b). ^1^H NMR (500 MHz, CDCl_3_, 23 °C): δ = 7.43 (C*H*-2,6, phenyl), 7.41 (C*H*-3,5, phenyl), 7.36 (C*H*-4, phenyl), 7.32 (C*H*-5, thiophene), 7.29 (C*H*-5, aromatic), 7.04 (C*H*-4, thiophene), 7.03 (C*H*-6, aromatic), 7.00 (C*H*-2, aromatic), 6.96 (C*H*-3, thiophene), 6.87 (C*H*-4, aromatic), 4.72 (s, 1H, C*H*), 4.09/3.89 (AB, 2H, C*H*_2_), 3.79 (s, 3H, OC*H_3_*). ^13^C NMR (125.75 MHz, CDCl_3_, 23 °C): δ = 159.66 (*C*-3, aromatic), 135.75 (*C*-1, aromatic), 133.57 (*C*-1, phenyl), 130.37 (*C*-2, thiophene), 129.66 (*C*-5, aromatic), 129.27 (*C*H-3,5, phenyl), 129.01 (*C*-3, thiophene), 128.75 (*C*H-2,6, phenyl), 128.43 (*C*-4, phenyl), 127.30 (*C*-4, thiophene), 126.77 (*C*-5, thiophene), 121.98 (*C*-6, aromatic), 115.42 (*C*-2, aromatic), 113.69 (*C*-4, aromatic), 69.37 (*C*H), 55.25 (O*C*H_3_), 50.25 (*C*H_2_) (Figure S76c).

20. Synthesis of 2-((((4-methoxyphenyl)(phenyl)methyl)sulfinyl)methyl)thiophene (**5t**–**8t**)

Following general procedure A, 1.64 g (7.7 mmol) of (2-fluorophenyl)(phenyl)methanol, 1.0 g (7.7 mmol) of thiophen-2-ylmethanethiol, and 1.0 mL (8.5 mmol) of 48% BF_3_·Et_2_O were reacted, affording 0.8 g of **3t** as a yellow oily product (yield 30%).

Following general procedure B, 0.8 g (2.3 mmol) of **3t** dissolved in 12 mL of glacial acetic acid was reacted with 30% H_2_O_2_ (0.27 mL) to give 0.4 g of **4t** as dark orange-brown oil (yield 44%).

Racemic **4t** was further separated into two individual pairs of enantiomers by means of an MPLC system equipped with a glass column packed with silica gel. Toluol/EtOAc = 80/20 was used as the mobile phase, and the separation was carried out with a flow rate of 5 mL/min. Individual pairs of enantiomers were further separated into the individual enantiomers **5t**–**8t** by means of semipreparative chiral HPLC. A semipreparative HPLC instrument was equipped with a CHIRALPAK IA column (10 mm Φ × 25 cm L) (Daicel Inc.), and EtOAc was used as the mobile phase.

**5t**. (*S*,*S*)-*2-((((4-methoxyphenyl)(phenyl)methyl)sulfinyl)methyl)thiophene.* HRESIMS *m*/*z* 365.0639 [M+Na]^+^ (calcd for C_19_H_18_NaO_2_S_2_^+^, 365.0640, Δ = 0.4 ppm) (Figure S77). HPLC R_t_ 22.37 min, purity 91.7% (Figure S77a). Chiral HPLC R_t_ 8.72 min, >99% ee (but contains 2.8% **7t**) (Figure S77b). ^1^H NMR (500 MHz, CDCl_3_, 23 °C): δ = 7.42 (C*H*-2,6, phenyl), 7.37 (C*H*-3,5, phenyl), 7.34 (C*H*-2,6, aromatic), 7.33 (C*H*-4, phenyl), 7.32 (C*H*-5, thiophene), 7.04 (C*H*-4, thiophene), 6.97 (C*H*-3, thiophene), 6.94 (C*H*-3,5, aromatic), 4.72 (s, 1H, C*H*), 4.09/3.87 (AB, 2H, C*H*_2_), 3.82 (s, 3H, OC*H_3_*). ^13^C NMR (125.75 MHz, CDCl_3_, 23 °C): δ = 159.55 (*C*-4, aromatic), 134.60 (*C*-1, phenyl), 130.37 (*C*-2, thiophene), 130.02 (*C*H-2,6, aromatic), 129.52 (*C*H-2,6, phenyl), 128.99 (*C*-3, thiophene), 128.64 (*C*H-3,5, phenyl), 128.26 (*C*-4, phenyl), 127.32 (*C*-1, aromatic), 127.26 (*C*-4, thiophene), 126.74 (*C*-5, thiophene), 114.64 (*C*H-3,5, aromatic), 68.61 (*C*H), 55.32 (O*C*H_3_), 50.02 (*C*H_2_) (Figure S77c).

**6t**. (*R*,*R*)-*2-((((4-methoxyphenyl)(phenyl)methyl)sulfinyl)methyl)thiophene.* HRESIMS *m*/*z* 365.0639 [M+Na]^+^ (calcd for C_19_H_18_NaO_2_S_2_^+^, 365.0640, Δ = 0.3 ppm) (Figure S78). HPLC R_t_ 23.63 min, purity > 99% (Figure S78a). Chiral HPLC R_t_ 14.51 min, >99% ee (Figure S78b). ^1^H NMR (500 MHz, CDCl_3_, 23 °C): δ = 7.42 (C*H*-2,6, phenyl), 7.37 (C*H*-3,5, phenyl), 7.34 (C*H*-2,6, aromatic), 7.33 (C*H*-4, phenyl), 7.32 (C*H*-5, thiophene), 7.04 (C*H*-4, thiophene), 6.97 (C*H*-3, thiophene), 6.94 (C*H*-3,5, aromatic), 4.72 (s, 1H, C*H*), 4.09/3.87 (AB, 2H, C*H*_2_), 3.82 (s, 3H, OC*H_3_*). ^13^C NMR (125.75 MHz, CDCl_3_, 23 °C): δ = 159.55 (*C*-4, aromatic), 134.60 (*C*-1, phenyl), 130.37 (*C*-2, thiophene), 130.02 (*C*H-2,6, aromatic), 129.52 (*C*H-2,6, phenyl), 128.99 (*C*-3, thiophene), 128.64 (*C*H-3,5, phenyl), 128.26 (*C*-4, phenyl), 127.32 (*C*-1, aromatic), 127.26 (*C*-4, thiophene), 126.74 (*C*-5, thiophene), 114.64 (*C*H-3,5, aromatic), 68.61 (*C*H), 55.32 (O*C*H_3_), 50.02 (*C*H_2_) (Figure S78c).

**7t**. (*S*,*R*)-*2-((((4-methoxyphenyl)(phenyl)methyl)sulfinyl)methyl)thiophene.* HRESIMS *m*/*z* 365.0639 [M+Na]^+^ (calcd for C_19_H_18_NaO_2_S_2_^+^, 365.0640, Δ = 0.4 ppm) (Figure S79). HPLC R_t_ 23.88 min, purity > 99% (Figure S79a). Chiral HPLC R_t_ 8.13 min, >99% ee (but contains 7.6% **5t**) (Figure S79b). ^1^H NMR (500 MHz, CDCl_3_, 23 °C): δ = 7.41 (C*H*-2,6, phenyl), 7.41 (C*H*-3,5, phenyl), 7.35 (C*H*-2,6, aromatic), 7.35 (C*H*-4, phenyl), 7.32 (C*H*-5, thiophene), 7.04 (C*H*-4, thiophene), 6.96 (C*H*-3, thiophene), 6.91 (C*H*-3,5, aromatic), 4.71 (s, 1H, C*H*), 4.07/3.87 (AB, 2H, C*H*_2_), 3.79 (s, 3H, OC*H_3_*). ^13^C NMR (125.75 MHz, CDCl_3_, 23 °C): δ = 159.69 (*C*-4, aromatic), 135.89 (*C*-1, phenyl), 130.89 (*C*H-2,6, aromatic), 130.42 (*C*-2, thiophene), 129.23 (*C*H-3,5, phenyl), 128.93 (*C*-3, thiophene), 128.76 (*C*H-2,6, phenyl), 128.30 (*C*-4, phenyl), 127.29 (*C*-4, thiophene), 126.73 (*C*-5, thiophene), 125.91 (*C*-1, aromatic), 114.09 (*C*H-3,5, aromatic), 68.52 (*C*H), 55.32 (O*C*H_3_), 50.10 (*C*H_2_) (Figure S79c).

**8t**. (*R*,*S*)-*2-((((4-methoxyphenyl)(phenyl)methyl)sulfinyl)methyl)thiophene.* HRESIMS *m*/*z* 365.0641 [M+Na]^+^ (calcd for C_19_H_18_NaO_2_S_2_^+^, 365.0640, Δ = −0.2 ppm) (Figure S80). HPLC R_t_ 22.65 min, purity 98.4% (Figure S80a). Chiral HPLC R_t_ 14.38 min, >99% ee (Figure S80b). ^1^H NMR (500 MHz, CDCl_3_, 23 °C): δ = 7.41 (C*H*-2,6, phenyl), 7.41 (C*H*-3,5, phenyl), 7.35 (C*H*-2,6, aromatic), 7.35 (C*H*-4, phenyl), 7.32 (C*H*-5, thiophene), 7.04 (C*H*-4, thiophene), 6.96 (C*H*-3, thiophene), 6.91 (C*H*-3,5, aromatic), 4.71 (s, 1H, C*H*), 4.07/3.87 (AB, 2H, C*H*_2_), 3.79 (s, 3H, OC*H_3_*). ^13^C NMR (125.75 MHz, CDCl_3_, 23 °C): δ = 159.69 (*C*-4, aromatic), 135.89 (*C*-1, phenyl), 130.89 (*C*H-2,6, aromatic), 130.42 (*C*-2, thiophene), 129.23 (*C*H-3,5, phenyl), 128.93 (*C*-3, thiophene), 128.76 (*C*H-2,6, phenyl), 128.30 (*C*-4, phenyl), 127.29 (*C*-4, thiophene), 126.73 (*C*-5, thiophene), 125.91 (*C*-1, aromatic), 114.09 (*C*H-3,5, aromatic), 68.52 (*C*H), 55.32 (O*C*H_3_), 50.10 (*C*H_2_) (Figure S80c).

21. Synthesis of 2-((((4-chlorophenyl)(4-fluorophenyl)methyl)sulfinyl)methyl)thiophene (**5u**–**8u**)

Following general procedure A, 1.0 g (3.9 mmol) of (4-chlorophenyl)(4-fluorophenyl)methanol, 0.52 g (3.9 mmol) of thiophen-2-ylmethanethiol, and 0.54 mL (4.3 mmol) of 48% BF_3_·Et_2_O were reacted, affording 0.6 g of **3u** as a yellow oily product (yield 44%).

Following general procedure B, 0.6 g (1.72 mmol) of **3u** dissolved in 12 mL of glacial acetic acid was reacted with 30% H_2_O_2_ (0.18 mL) to give 0.52 g of **4u** as a yellow solid (yield 83%).

Racemic **4u** was further separated into two individual pairs of enantiomers by means of an MPLC system equipped with a glass column packed with silica gel. Toluol/EtOAc = 80/20 was used as the mobile phase, and the separation was carried out with a flow rate of 5 mL/min. Individual pairs of enantiomers were further separated into the individual enantiomers **5u**–**8u** by means of semipreparative chiral HPLC. A semipreparative HPLC instrument was equipped with a CHIRALPAK IA column (10 mm Φ × 25 cm L) (Daicel Inc.), and EtOAc was used as the mobile phase.

**5u.** HRESIMS *m*/*z* 387.0052 [M+Na]^+^ (calcd for C_18_H_14_ClFNaOS_2_^+^, 387.0051, Δ = −0.2 ppm) (Figure S81). HPLC R_t_ 26.13 min, purity > 99% (Figure S81a). Chiral HPLC R_t_ 7.22 min, >99% ee (but contains 15.5% **5m**) (Figure S81b). ^1^H NMR (500 MHz, CDCl_3_, 23 °C): δ = 7.37 (C*H*-2’,6’, phenyl), 7.35 (C*H*-3,5, phenyl), 7.34 (C*H*-2,6, phenyl), 7.34 (C*H*-5, thiophene), 7.12 (C*H*-3’,5’, phenyl), 7.06 (C*H*-4, thiophene), 6.96 (C*H*-3, thiophene), 4.69 (s, 1H, C*H*), 4.08/3.89 (AB, 2H, C*H*_2_). ^13^C NMR (125.75 MHz, CDCl_3_, 23 °C): δ = 162.62 (*C*-4, aromatic), 134.75 (*C*-4, phenyl), 132.26 (*C*-1, phenyl), 130.97 (*C*-1, aromatic), 130.91 (*C*H-2,6, phenyl), 130.51 (*C*H-2,6, aromatic), 129.77 (*C*-2, thiophene), 129.97 (*C*-3, thiophene), 128.92 (*C*H-3,5, phenyl), 127.44 (*C*-4, thiophene), 126.97 (*C*-5, thiophene), 116.41 (*C*H-3,5, aromatic), 66.93 (*C*H), 50.11 (*C*H_2_) (Figure S81c).

**6u.** HRESIMS *m*/*z* 387.0053 [M+Na]^+^ (calcd for C_18_H_14_ClFNaOS_2_^+^, 387.0051, Δ = −0.5 ppm) (Figure S82). HPLC R_t_ 26.17 min, purity > 99% (Figure S82a). Chiral HPLC R_t_ 12.64 min, >99% ee (Figure S82b). ^1^H NMR (500 MHz, CDCl_3_, 23 °C): δ = 7.37 (C*H*-2’,6’, phenyl), 7.35 (C*H*-3,5, phenyl), 7.34 (C*H*-2,6, phenyl), 7.34 (C*H*-5, thiophene), 7.12 (C*H*-3’,5’, phenyl), 7.06 (C*H*-4, thiophene), 6.96 (C*H*-3, thiophene), 4.69 (s, 1H, C*H*), 4.08/3.89 (AB, 2H, C*H*_2_). ^13^C NMR (125.75 MHz, CDCl_3_, 23 °C): δ = 162.62 (*C*-4, aromatic), 134.75 (*C*-4, phenyl), 132.26 (*C*-1, phenyl), 130.97 (*C*-1, aromatic), 130.93 (*C*H-2,6, phenyl), 130.51 (*C*H-2,6, aromatic), 129.76 (*C*-2, thiophene), 129.07 (*C*-3, thiophene), 128.92 (*C*H-3,5, phenyl), 127.44 (*C*-4, thiophene), 126.97 (*C*-5, thiophene), 116.41 (*C*H-3,5, aromatic), 66.92 (*C*H), 50.11 (*C*H_2_) (Figure S82c).

**7u.** HRESIMS *m*/*z* 387.0052 [M+Na]^+^ (calcd for C_18_H_14_ClFNaOS_2_^+^, 387.0051, Δ = −0.3 ppm) (Figure S83). HPLC R_t_ 26.27 min, purity 97.4% (Figure S83a). Chiral HPLC R_t_ 6.38 min, >99% ee (Figure S83b). ^1^H NMR (500 MHz, CDCl_3_, 23 °C): δ = 7.40 (C*H*-2,6, phenyl), 7.37 (C*H*-2’,6’, phenyl), 7.35 (C*H*-5, thiophene), 7.33 (C*H*-3,5, phenyl), 7.08 (C*H*-3’,5’, phenyl), 7.06 (C*H*-4, thiophene), 6.96 (C*H*-3, thiophene), 4.68 (s, 1H, C*H*), 4.08/3.89 (AB, 2H, C*H*_2_). ^13^C NMR (125.75 MHz, CDCl_3_, 23 °C): δ = 162.62 (*C*-4, aromatic), 134.59 (*C*-4, phenyl), 133.94 (*C*-1, phenyl), 131.38 (*C*H-2,6, aromatic), 130.08 (*C*H-2,6, phenyl), 129.77 (*C*-2, thiophene), 129.53 (*C*H-3,5, phenyl), 129.26 (*C*-1, aromatic), 129.06 (*C*-3, thiophene), 127.44 (*C*-4, thiophene), 126.96 (*C*-5, thiophene), 115.77 (*C*H-3,5, aromatic), 66.93 (*C*H), 50.11 (*C*H_2_) (Figure S83c).

**8u.** HRESIMS *m*/*z* 387.0050 [M+Na]^+^ (calcd for C_18_H_14_ClFNaOS_2_^+^, 387.0051, Δ = 0.1 ppm) (Figure S84). HPLC R_t_ 26.27 min, purity > 99% (Figure S84a). Chiral HPLC R_t_ 10.81 min, >99% ee (Figure S84b). ^1^H NMR (500 MHz, CDCl_3_, 23 °C): δ = 7.40 (C*H*-3,5, phenyl), 7.37 (C*H*-2’,6’, phenyl), 7.35 (C*H*-5, thiophene), 7.33 (C*H*-2,6, phenyl), 7.08 (C*H*-3’,5’, phenyl), 7.06 (C*H*-4, thiophene), 6.96 (C*H*-3, thiophene), 4.68 (s, 1H, C*H*), 4.08/3.89 (AB, 2H, C*H*_2_). ^13^C NMR (125.75 MHz, CDCl_3_, 23 °C): δ = 162.62 (*C*-4, aromatic), 134.59 (*C*-4, phenyl), 133.93 (*C*-1, phenyl), 131.38 (*C*H-2,6, aromatic), 130.08 (*C*H-2,6, phenyl), 129.77 (*C*-2, thiophene), 129.53 (*C*H-3,5, phenyl), 129.26 (*C*-1, aromatic), 129.06 (*C*-3, thiophene), 127.44 (*C*-4, thiophene), 126.96 (*C*-5, thiophene), 115.77 (*C*H-3,5, aromatic), 66.93 (*C*H), 50.11 (*C*H_2_) (Figure S84c).

### 2.2. Compound Analytics

^1^H and ^13^C NMR spectra were recorded on a Bruker Avance 500 NMR spectrometer (UltraShield) using a 5-mm switchable probe (PA BBO 500SB BBF-H-D-05-Z, 1H, BB = ^19^F and ^31^P-^15^N) with z axis gradients and an automatic tuning and matching accessory (Bruker BioSpin, Billerica, MA, USA). The resonance frequency for ^1^H NMR was 500.13 MHz and for ^13^C NMR, 125.75 MHz. All measurements were performed for a solution in fully deuterated chloroform or DMSO at 298 K. Standard 1D and gradient-enhanced (ge) 2D experiments, such as double quantum filtered (DQF) COSY, NOESY, HSQC, and HMBC, were used as supplied by the manufacturer. Chemical shifts are referenced internally to the residual, non-deuterated solvent signal for chloroform ^1^H (*δ* = 7.26 ppm) or DMSO ^1^H (*δ* = 2.50 ppm) and to the carbon signal of the solvent for chloroform ^13^C (*δ* = 77.00 ppm) or DMSO ^13^C (*δ* = 39.57 ppm).

HRESIMS spectra were obtained on a maXis UHR ESI-Qq-TOF mass spectrometer (Bruker Daltonics, Bremen, Germany) in the positive-ion mode. Samples were dissolved in ACN/MeOH/H_2_O 99:99:2 (*v*/*v*/*v*) and directly infused into the ESI source with a syringe pump. The ESI ion source was operated as follows: capillary voltage: 4.2 kV, nebulizer: 0.4 bar (N_2_), dry gas flow: 4 L/min (N_2_), and dry temperature: 150 or 180 °C. The sum formulas of the detected ions were determined using Bruker Compass DataAnalysis 5.1 based on mass accuracy (Δ*m*/*z* ≤ 5 ppm) and isotopic pattern matching (SmartFormula algorithm).

The overall purity of the compounds was determined by HPLC on an UltiMate 3000 series system equipped with a VWD detector (Dionex/Thermo Fisher Scientific, Germering, Germany). Separation was carried out on an Acclaim 120 C18, 2.1 × 150 mm, 3 µm HPLC column (Thermo Fisher Scientific) using LC-MS-grade water and acetonitrile as mobile phases A and B, respectively. The sample components were separated and eluted with a linear gradient from 10% to 90% B in 25 min, followed by an isocratic column cleaning and re-equilibration step. The flow rate was 0.2 mL/min, and the column oven temperature was set to 25 °C. The purity was determined from the UV chromatogram (254 nm) as the ratio of the peak area of the compound to the total peak area (i.e., the sum of the areas of all peaks that were not present in the solvent blank). Based on the HPLC data, all final compounds are ≥95% pure.

Enantio-purity of the compounds was determined by HPLC on a LC- 2010A HT liquid chromatograph device (Shimadzu Corporation, Tokyo, Japan), equipped with a Chiralpak IA column (4.6 mm ⌀ × 250 mm L) (Daicel Inc., Tokyo, Japan), using the same mobile phase as for separation. Except for isopropanol, which allowed a maximum flowrate of only 0.4 mL/min due to its high viscosity, the general flowrate was set to 1mL/min and the temperature was kept at 25 °C. The purity was determined from the UV chromatogram (254 nm) as the ratio of the peak area of the compound to the total peak area, including other stereoisomers.

### 2.3. Pharmacological Characterization

Cell culture. Human embryonic kidney (HEK) 293 or SK-N-MC (human neuroblastoma) cells were grown in minimum essential medium with Earle’s salts and L-glutamine, 10% heat-inactivated fetal bovine serum, and 50 mg/L gentamicin. Cells were grown in 100- or 60-mm diameter tissue culture dishes (polystyrene, Falcon) at 37 °C under an atmosphere of 5% CO_2_/95% air. The human dopamine transporter/SLC6A3 (DAT), noradrenaline transporter/SLC6A2 (NET), or human serotonin transporter/SLC6A4 (SERT) cDNA was stably expressed using methods as described previously [28].

Cellular uptake experiments. The transporter-expressing HEK293 cells were seeded in poly-D-lysine-coated 24-well plates (1 × 10^5^ cells/well) and, one day later, each well was washed with 0.5 mL uptake buffer and incubated with 0.5 mL buffer containing various concentrations of the drugs [29]. Uptake was started by the addition of [^3^H]-dopamine, [^3^H]-noradrenaline, or [^3^H]-serotonin at a final concentration of 1 µM (specific activity 0.14 Ci/mmol) after 5 min of preincubation. After incubation for 2.5 min (SERT-cells: 5 min) at 25 °C, it was stopped by aspirating the uptake buffer and washing each well twice with 1 mL ice-cold buffer. Nonspecific uptake was determined in the presence of 10 µM mazindol (DAT- and NET-cells) or 10 µM clomipramine (SERT-cells). The radioactivity remaining in each well was determined by incubating with 0.4 mL 1% sodium dodecyl sulphate and transferring this solution into scintillation vials containing 3 mL of scintillation cocktail (Ultima Gold MV, Packard, Dovners Grove, IL, USA). The uptake buffer consisted of (mmol/L): 4 Tris–HCl; 6.25 4-(2-hydroxyethyl)-1-piperazineethanesulfonic acid (HEPES); 120 NaCl; 5 KCl; 1.2 CaCl_2_; 1.2 MgSO_4_; 5.6 D-glucose; 0.5 ascorbic acid; pH 7.1.

Superfusion experiments. Superfusion experiments were performed as described recently with minor modifications [30]. DAT-expressing SK-N-MC cells were seeded onto poly-D-lysine-coated 5-mm-diameter glass cover slips in 96-well tissue culture plates (5 × 10^4^ cells/well). On the next day, cells were loaded with 6 µM [^3^H] MPP^+^ (0.2 Ci/mmol) in uptake buffer at 37 °C for 20 min. After loading, coverslips were transferred to small chambers and superfused (25 °C, 1.0 mL/min) with the uptake buffer mentioned above. After a washout period of 45 min to establish a stable efflux of radioactivity, the experiment was started with the collection of 4-min fractions. For the determination of a releasing effect, substances were added for three fractions to the superfusion buffer after three fractions of baseline and then removed for another three fractions. For substance/d-amphetamine interaction experiments, substances were added to the superfusion buffer one fraction before 3 µM amphetamine after two fractions of baseline. At the end of the experiment, cells were lysed by superfusion with 4 mL of 1% SDS. The radioactivity in the superfusate fractions and the SDS-lysates was determined by liquid scintillation counting. The release of tritium was expressed as a fractional rate, i.e., the radioactivity released during a fraction was expressed as a percentage of the total radioactivity present in the cells at the beginning of that fraction.

Binding experiments. Binding affinity to the human DAT, SERT, and NET stable proteins expressed in CHO cells was evaluated in in vitro radioligand binding assays. Non-specific binding was determined using [^3^H]BTCP, [^3^H]imipramine, and ^[3^H] nisoxetine for hDAT, hSERT, and hNET, respectively. The IC50 values were calculated by non-linear regression analysis of the competition curves generated from the mean replicate values. The Ki values were calculated using the Cheng–Prusoff equation (Ki = IC_50_/(1 + (L + KD))). Binding assays were carried out by Eurofins (Cerep SA, Celle l’Evescault, France), Study No. 100053935. For an in-depth description, please refer to the report in the supporting information file.

Neurite outgrowth assay. Binding assays were carried out by Cyprotex (an Evotec Company, Toulouse, France), Study No. CYP2052-R1. For an in-depth description, please refer to the report in the supporting information files. Cryo-preserved rat cortical neurons (QBM Cell Science, R-Cx-500) were quickly thawed and slowly diluted with neurobasal medium supplemented with L-glutamine (Invitrogen, Cat. No. 25030-081), penicillin-streptomycin (Invitrogen, Cat. No. 15140-122), and B27 (NB/B27 medium, Invitrogen, Cat. No. 17504-044). After a gentle centrifugation step, the neurons were resuspended in NB/B27 medium at a density of 4.0 × 10^6^ cells/mL. One hour prior to treatment, the neurons were plated in 384-well plates (Corning 354663) pre-coated with a laminin solution (Sigma L2020 1 mg/mL) at a density of 10,000 cells per well. After treatment with test articles and control compounds, the neurons were maintained in a humidified environment at 37 °C with 5% CO_2_ for 72 h. At the end of the treatment period, the neurons were fixed for 1 h with a 3.7% formaldehyde solution (Acros Organics, 41073-0010). The neurons were washed with phosphate buffered saline (PBS) (SH30264.02) and permeabilized for 10 min with a 0.5% solution of Triton X-100 (Millipore Sigma, X100). After another wash step, the neurons were stained for one hour with Hoechst 33342 (ThermoFisher Scientific, H3570) and the anti-beta III tubulin antibody, Alexa Fluor^®^ 488 conjugate (Millipore Sigma AB15708A4), diluted in a blocking buffer solution (Thermo Scientific, 37525). Upon completion of the staining step, the neurons were washed again, and the plates were sealed in preparation for the imaging process. The neurons were evaluated for neurite outgrowth and cell health using the high-content imaging platform, ArrayScan VTi (Thermo Scientific ArrayScan VTi), with an optimized neuronal profiling bio-application.

This assay assessed cell health and neurite outgrowth utilizing a neuronal profiling bio-application applied to images obtained on an ArrayScan VTi. Valid neuron count, mean neurite average length, and neurite total length per neuron were determined and reported. Raw data were normalized to the vehicle control and reported as a normalized% response. Dose response graphs were generated with GraphPad Prism using a sigmoidal dose-response (variable slope) algorithm.

Compound brain levels. For an in-depth description of the brain and blood at **7h**, we refer to the protocol of Evotec (France, Study No. DMPK-2020-494) in the supplementary information file.

### 2.4. In Vivo Micro-Dialysis in Freely Moving Mice

Male, adult C57BL76J mice (Janvier-Labs, Saint Berthevin, France) were group-housed in individually ventilated cages under standard laboratory conditions (temperature 22 ± 2 °C, humidity 45–65%, with a 12:12 h light-dark cycle lights on at 07:00 h). Animals had free access to food and water. All experimental procedures were approved by the Austrian Animal Experimentation Ethical Committee (Bundesministerium für Wissenschaft, Forschung und Wirtschaft, Kommission für Tierversuchsangelegenheiten) in compliance with international laws and policies.

Under isoflurane anesthesia (at 5% and 1.5% for induction and maintenance, respectively), a commercially available guide cannula (MAB 4.15 with OD 0.48 mm and ID 0.35 mm, Microbiotech, Stockholm, Sweden) was implanted above the right hippocampus (AP = −2.1 mm, ML = +1.5 mm, DV = −0.7 mm from Bregma) and closed with a dummy probe as described previously [12,25]. After surgery, animals received buprenorphine (0.5 mg/kg s.c.) and meloxicam (1.0 mg/kg p.o. via the drinking water) for analgesic care. They were allowed to recover for 5–7 days while being single-housed and habituated to the experimenter and experimental procedures. On the day before the experiment, the dummy was replaced by a microdialysis probe (MAB 4.15.1 PES, Microbiotech, Sweden) that extended the guide cannula by 1 mm, thus reaching into the dorsal hippocampus. Microdialysis probes were connected to a microinfusion pump (CMA, Stockholm, Sweden) and a swivel-tether system via polyethylene tubing. Probes were constantly superfused with artificial cerebrospinal fluid (aCSF); consisting of in mM: 140 NaCl, 3.0 KCl, 1.2 CaCl_2_, 1.0 MgCl_2_, 1.0 Na_2_HPO4, pH 7.4, at a flow rate of 0.5 µL/min overnight and 1.0 µL/min during sample collection, respectively. Microdialysis fractions were collected every 20 min in pre-cooled microtubes containing antioxidative protection solution (in mM: 0.27 Na_2_EDTA, 100 acetic acid, 0.0125 ascorbic acid), vortexed, and immediately frozen at −80 °C until further analysis. At any time, the dead volumes generated by the inlet and outlet systems were considered to cause a temporal delay in fraction collection.

On experimental day 1, vehicle (30% Kolliphor EL) or one of the two enantiomers -**7h** and **8h** (1 mg/kg) was administered i.p. after six baseline samples, and another 12 samples were collected. On experimental day 2, the collection of dialysates was resumed. Again, 6 baseline samples and 12 samples after the administration of either vehicle (30% Kolliphor EL) or **7h**/**8h** (10 mg/kg) were collected. At the end of the experiment, microdialysis probes were superfused with high (100 mM) depolarizing concentrations of potassium (K^+^) containing aCSF in order to elicit local hyperpolarization for testing the functionality of the microdialysis system. Thereafter, animals were euthanized with an overdose of thiopental, decapitated, and their brains removed. The localization of the microdialysis probe was verified by observing cresyl violet-stained coronal sections (40 µm) under a light microscope. Three animals were removed from the analysis due to misplaced probes.

Quantification of dopamine in microdialysates. The quantification of dopamine in microdialysates was performed using a high-performance liquid chromatography (HPLC) system for electrochemical detection, as previously described [12]. The HPLC system consisted of a Shimadzu (Kyoto, Japan) system controller (CBM-20A), a degassing unit (DGU-20A3R), and a micro HPLC pump (LC-20AD) operating at a flow rate of 55 µL/min. Microdialysis samples (5 µL) were injected via a SIL-20ACHT autosampler (Shimadzu, Japan) and separated on a C18 reversed-phase column (Inertsil ODS-3; 50 mm × 1.0 mm ID; 3 µm particle sizes, GL Sciences Inc., Tokyo, Japan) placed in a DECADE II electrochemical detector equipped with an amperometric flow cell (Antec, Zoeterwoude, The Netherlands). Detection was carried out at 35 °C with an applied potential of +460 mV vs. the Ag/AgCl reference electrode. The mobile phase consisted of 93% *v*/*v* buffered aqueous solution (50 mM phosphoric acid, 50 mM citric acid, 2.36 mM octane–sulfonic acid, 0.1 mM Na_2_EDTA, pH adjusted to 5.6) and 7% (*v*/*v*) methanol. Instrument control and data acquisition were carried out by LabSolution chromatography software (version 5; Shimadzu, Japan). Thus, the area under the curve (AUC) was determined and converted into concentration units using a standard calibration equation. Dopamine concentrations for each microdialysate were expressed as relative values to the mean baseline concentrations constituting four samples immediately before treatment. The detection limit for dopamine was 0.15 fmol per sample.

Statistical analysis. Data are presented as mean ± SEM. A 2-way ANOVA with repeated measures was conducted for statistical analysis using Statistica Software v13 (StatSoft GmbH, Hamburg, Germany). A *p*-value *p* < 0.05 was considered a statistically significant effect.

### 2.5. Ex Vivo Electrophysiology

Animals. Male Sprague–Dawley rats (22–23 months old) were bred and kept in the Core Unit of Biomedical Research, Division of Laboratory Animal Science and Genetics, Medical University of Vienna. Rats were housed in groups of two in standard Makrolon cages filled with autoclaved woodchips (temperature: 22 ± 2 °C; humidity: 55 ± 5%; 12 h artificial light/12 h dark cycle: light on at 7:00 am). Tap water and food were provided ad libitum. The study was carried out according to the guidelines of the ethics committee at the Medical University of Vienna and was approved by the Federal Ministry of Education, Science, and Culture, Austria (BMBWF-66.009/235/V/3b/2018).

Hippocampal Slices Preparation. Acutely dissected hippocampal slices obtained from rat singles administered with vehicle or 10 mg/kg for **7h** (1 h prior to brain extraction) were prepared as previously described [26], with slight modifications. Briefly, rats were anesthetized by 50 s isoflurane inhalation exposure [31], and (upon the absence of corneal reflexes), animals were decapitated using a high-precision, hardened, sharp-blade rodent guillotine (DCAP-M, World Precision Instruments, Inc., Sarasota, FL, USA). The brains were removed gently and immediately submerged in an ice-cold artificial cerebrospinal fluid (aCSF) solution of the following composition (in mM): 125 NaCl, 2.5 KCl, 20 NaHCO_3_, 2.5 CaCl_2_, 1 MgCl_2_, 25 D-glucose, and 1 NaH_2_PO_4_ (pH 7.40). Hippocampi were carefully extracted and sectioned using a tissue chopper (McIlwain TC752, Campden Instruments Ltd., Loughborough, UK). Hippocampal slices were immediately transferred to a custom-made recovery chamber filled with carbogenated aCSF at 22 ± 2 °C and allowed to recover for a minimum of 1 h before electrophysiological recording.

Extracellular recording. Hippocampal slices were transferred to a customized low-volume (2 mL) submersion-type recording chamber perfused with carbogenated aCSF solution at 30 ± 2 °C at a constant flow rate of 4 mL/min. Evoked field excitatory postsynaptic potentials (fEPSPs) were acquired using borosilicate glass micropipettes made in a horizontal puller (Sutter Instrument, Novato, CA, USA). Micropipette tips (3 ± 1 MΩ) were filled with aCSF. Teflon-coated tungsten wire bipolar stimulating electrodes (~50 μm diameter) connected to an ISO-STIM 01D isolator stimulator (NPI Electronics, Tamm, Germany) were used to evoke fEPSPs. An AxoClamp-2B amplifier, Digidata-1440 interface (Axon Instruments, Molecular Devices, Berkshire, UK), and pClamp-11 software (Molecular Devices, Berkshire, UK) were used for data acquisition and analysis. fEPSPs were recorded at 0.033 Hz.

As in the previously described protocol for CA1 [32,33], a stimulus was delivered at the CA3-Schaffer collateral axons and a recording electrode was placed at the dendritic region of the CA1 pyramidal neurons.

Basal synaptic transmission was accessed by administering square pulses of increasing stimulus intensity (200 μs pulse length, 5 s inter-pulse interval, 0–9 V, 1 V increments) and measuring the corresponding changes in fEPSP slopes (input/output (I/O) curves). The stimulus intensity eliciting 40–50% of the maximum response as estimated from I/O curves was used for baseline and post-high-frequency-induced electrical stimulation (HFS) recording.

Hippocampal synaptic potentiation was induced by delivering 4 trains of 20 pulses at 100 Hz (200 μs/pulse) with a 500 ms inter-train interval, a variant of the different forms of HFS protocol known to induce both post-tetanic potentiation (PTP) and long-term potentiation (LTP) in hippocampal CA3-CA1 hippocampal synapses in young rodents. [32,33,34] Synaptic plasticity was assessed by quantifying the time course of changes in fEPSP slope (decaying phase) after applying the plasticity-inducing protocol, normalized to the average of all fEPSP slopes recorded during the preceding baseline recording period. A two-way RM-ANOVA with a Sidak post hoc test was used to identify differences between experimental groups.

## 3. Results and Discussion

### 3.1. Compound Design and Synthesis

**Scheme 1:**
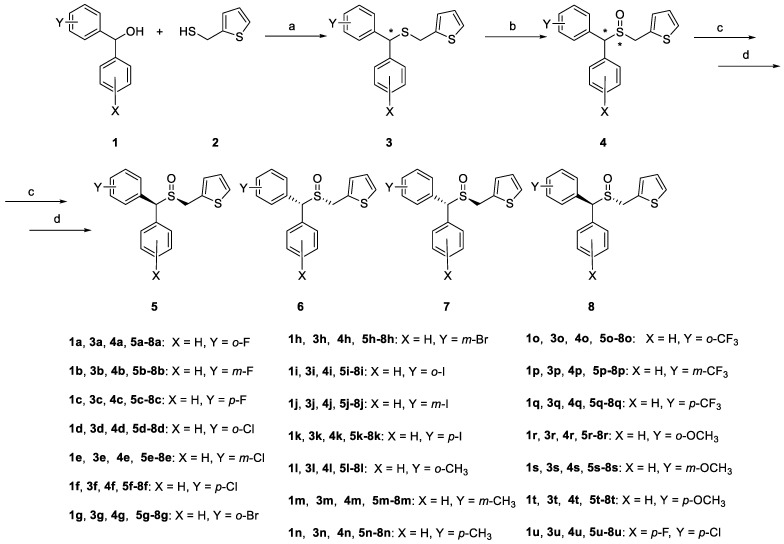
Synthetic routes of compounds **5a**–**u**, **6a**–**u**, **7a**–**u**, and **8a**–**u**. Reagents and conditions: (a) BF_3_•Et_2_O, CH_3_COOH, r.t. 12–14h; (b) CH_3_COOH, H_2_O_2_, r.t. 12h; (c) separation of diastereomers via column chromatography on silica gel (for conditions see Supporting Information); (d) chiral-phase separation of enantiomers (for conditions see Supporting Information); note that the drawn stereochemistry of compounds is only for representation purposes and does not represent the actual chirality of the compound (see Table 1); * indicates a chiral center.

The synthetic pathway of novel modafinil-derived thiophen-containing analogues with mono-substitution on one or both phenyl rings (**5a**–**8a**–**5u**–**8u**) is depicted in Scheme 1. Commercially purchased substituted benzhydrols (**1a**–**1u**) were condensed with thiophen-2-ylmethanethiol (**2**) to yield unoxidized intermediates **3a**–**3u** (yield 30% to >95%) in a single step. In the second and final synthetic step, oxidation of the sulfanyl containing intermediates in acetic acid with hydrogen peroxide gave the desired products **4a**–**4u** (yield 9% to >95%). At this stage, all compounds **4a**–**4u** possess two chiral centers and, as such, were obtained as racemic mixtures with four stereoisomers each. Hence, the further strategy focused on the chiral resolution of racemic compound mixtures into individual stereoisomers. Screening by means of thin-layer chromatography revealed that separation of diastereomeric fractions (each consisting of a pair of enantiomers) could be readily achieved by column chromatography with silica gel as the stationary phase and with a mobile phase consisting of toluene and ethyl acetate in different ratios (see supporting information). Fractions containing pairs of enantiomers were further subjected to chiral phase separations by means of HPLC equipped with a CHIRALPAK IA column (10 mm diameter × 20 cm length) (Daicel Inc., Tokyo, Japan) (for experimental details, refer to supporting information). This separation strategy yielded four individual stereoisomers from each racemic mixture (**5a**–**8a**–**5u**–**8u**), which were all tested for bioactivity. However, only for 32 of them was the absolute configuration established by X-ray crystallography and VCD (see supporting information).

### 3.2. In Vitro Pharmacology

#### SARs at Monoamine Transporters

All 84 stereoisomers were profiled for inhibition of monoamine reuptake (dopamine, norepinephrine, and serotonin) in order to establish a clear structure-activity relationship. Screening was initially performed with a cut-off value of 50 µM. Analogues that showed more than 50% radioligand displacement at this concentration were tested in the full concentration range, and the IC_50_ value was determined. The results are summarized in Table 1. The parent scaffold (Scheme 1) was first modified through the introduction of a single strong electron-withdrawing group on one of the phenyl rings. The ‘‘fluorine walk’’ was performed on *ortho*- (**5a**–**8a**), *meta*- (**5b**–**8b**), and *para*-positions (**5c**–**8c**), yielding several compounds with low micromolar activity on DAT, such as **8a** (IC_50_ = 1.09 ± 0.07 µM), **5b** (IC_50_ = 3.2 ± 0.4 µM), **7b** (IC_50_ = 2.3 ± 0.2 µM), and **7c** (IC_50_ = 2.1 ± 0.6 µM) and no activity on NET or SERT at 50 µM concentration. Within the *ortho*-fluorinated isomers, the most pronounced difference in DAT activity was observed between the (*S*,*R*)-stereoisomer (**8a**, IC_50_ = 1.09 ± 0.07 µM) and the (*R*,*R*)-stereoisomer (**5a**, IC_50_ = 32.8 ± 0.2 µM). The ‘‘chlorine walk’’ on *ortho*- (**5d**–**8d**), *meta*-(**5e**–**8e**), and *para*-positions (**5f**–**8f**) also yielded a couple of compounds with low micromolar activity on DAT, such as **8d** (IC_50_ = 2.55 ± 0.04 µM), **5e** (IC_50_ = 3.0 ± 0.1 µM), **7e** (IC_50_ = 0.63 ± 0.17 µM), and **8e** (IC_50_ = 0.83 ± 0.16 µM). Herein, the chirality of the compounds has influenced not only the activity on the DAT but also the selectivity towards NET and SERT. Within the *ortho*-chlorinated isomers, the (*S*,*R*)-stereoisomer (**8d**, IC_50_ = 2.55 ± 0.04 µM) had the strongest potency on DAT, followed by the (*S*,*S*)-stereoisomer (**6d**, IC_50_ = 14.4 ± 2.4 µM). The (*R*,*R*)-stereoisomer (**5d**) and the (*R*,*S*)-stereoisomer (**7d**) did not achieve ≥50% of radioligand displacement at 50 µM on any of the transporters. Within the *meta*-chlorinated isomers, the (*S*,*S*)-stereoisomer (**7e**) showed the strongest potency on DAT (IC_50_ = 0.63 ± 0.17 µM), but also exerted weak activity on NET (IC_50_ = 29 ± 4 µM). Interestingly, the (R,*R*)-stereoisomer (**8e**) showed strong potency on DAT (IC_50_ = 0.83 ± 0.16 µM) with weak activity on both NET (IC_50_ = 48 ± 6 µM) and SERT (IC_50_ = 19 ± 1 µM). A 10-fold difference in activity on DAT was observed between *para*-chlorinated (*S*,*S*)- and (*R*,*S*)-stereoisomers **7f** (IC_50_ = 4.0 ± 1 µM) and **6f** (IC_50_ = 46 ± 1 µM), respectively. The ‘‘bromine walk’’ on *ortho*- (**5g**–**8g**) and *meta*-positions (**5h**–**8h**) further emphasized the influence of stereochemistry on activity and selectivity towards DAT. Within the *ortho*-brominated isomers, whose crystal structure was not measured, **8g** showed the highest potency of DAT inhibition (IC_50_ = 5.06 ± 0.31 µM), followed by **6g** (IC_50_ = 12.1 ± 1.0 µM), with both of the compounds showing no activity on NET and SERT at tested concentrations. The other two isomers, **5g** and **7g**, had no effect on DAT, NET, or SERT at 50 µM. Bromine introduction to the *meta*-position yielded three isomers with low micromolar activity on DAT, namely the (*S*,*R*)-isomer (**5h**, IC_50_ = 1.8 ± 0.4 µM), the (*S*,*S*)-isomer (**7h**, IC_50_ = 0.48 ± 0.11 µM), and the (*R*,*R*)-isomer (**8h**, IC_50_ = 0.30 ± 0.03 µM). Within the same enantiomeric pair, a significant difference was observed in selectivity towards DAT against NET or SERT. The **7h** exerted moderate activity on NET (IC_50_ = 16.4 ± 0.6 µM) with no activity on SERT at 50 µM concentration, while **8h** exerted moderate activity on SERT (IC_50_ = 14 ± 3 µM) and no activity on NET. The ‘‘iodine walk’’ on *ortho*- (**5i**–**8i**), *meta*- (**5j**–**8j**) and *para*-positions (**5k**–**8k**) provided further activity dependency on not only substitution pattern but also on stereochemistry. *Ortho*-substitution (**5i**–**8i**) was generally less favored compared to *meta-*substitution (**5j**–**8j**). One stereoisomer (**5i**) was found to be inactive on DAT (IC_50_ larger than the 50 µM cut-off value), two (**6i**, **7i**) were rather weak (IC_50_ = 20.3 ± 0.6 µM, 28.4 ± 2.8 µM, respectively), while the fourth (**8i**) was at least five times more potent (IC_50_ = 9.6 ± 1.2 µM) as compared to **5i** and more than two times as compared to **6i** and **7i**. Interestingly, the most active iodine-containing derivative was substituted in *para*-position, namely the (R,S)-isomer (**6k**, IC50 DAT = 1.1 ± 0.3 µM), which also showed a strong inhibition of SERT (IC50 = 3.8 ± 0.5 µM). None of the other iodine derivatives exerted any activity on NET or SERT at the tested concentration range. Many analogues obtained through ‘‘methyl’’ walking on *ortho*- (**5l**–**8l**), *meta*- (**5m**–**8m**), and *para*- (**5n**–**8n**) positions had low micromolar activity on DA reuptake (IC_50_ = 3.8 ± 0.5 µM for **7l**, IC_50_ = 2.2 ± 0.4 µM for **5m**, IC_50_ = 1.2 ± 0.1 µM for **7m**, IC_50_ = 1.56 ± 0.25 µM for **8m**, IC_50_ = 1.63 ± 0.1 µM for **7n**) positions with no activity on SERT or NET in the tested concentration range. As was already observed for the m-Cl and m-Br derivatives, both (*S*,*S*) and (*R*,*R*) isomers of m-CH3 (**7m** and **8m**, respectively) showed good activity on DAT, while in the case of the methyl-substitution, the (R,R) configuration did not trigger inhibitory activity on SERT. Instead, the (*R*,*R*) configuration of the *para*-substituted analogue (**8n**) had a dual effect on dopamine and serotonin reuptake inhibition (IC_50_ DAT = 4.0 ± 0.4 µM, IC_50_ SERT = 25 ± 4 µM). Moving on with the ‘‘trifluoromethyl’’ walk, generally lower activity on DAT was observed compared to the previous substituents. An *ortho*-substituted stereoisomer **5o** exerted moderate NET activity (IC_50_ = 25 ± 1 µM), while the stereoisomer **8o** exerted low micromolar DAT activity (IC_50_ = 8.9 ± 0.7 µM), along with the *meta*-substituted stereoisomers **5p** and **7p** (IC_50_ = 5 ± 1 µM and 10 ± 1 µM), respectively. Compound **8p** exerted dual activity on DAT and SERT (IC_50_ DAT = 6.2 ± 0.6 µM, IC_50_ SERT = 31 ± 5 µM). Such dual activity was also observed in one of the *para*-substituted stereoisomers, where the analogue **8q** exerted a stronger effect on SERT (IC_50_ = 4.7 ± 0.5 µM) compared to DAT (IC_50_ = 21 ± 2 µM). Nevertheless, no clear relation with regards to absolute configuration could be established as single crystal X-ray diffraction was not performed. Introduction of an electron-donating group, i.e., the ‘‘methoxy’’ walk, unexpectedly yielded analogues substituted on *ortho*- and *meta*-positions with low micromolar activity on DAT (**7r**, IC_50_ = 1.7 ± 0.1 µM, and **7s**, IC_50_ = 1.56 ± 0.24 µM, respectively). From the *para*-substituted analogues, the stereoisomer with (*R*,*R*) configuration (**6t**) exerted moderate yet dual activity on DAT (IC_50_ = 17 ± 3 µM) and SERT (IC_50_ = 11 ± 3 µM). Lastly, a combination of *para*-fluoro substitution on one and *para*-chloro substitution on the other phenyl ring also yielded a series of stereoisomers with activity on DAT in the low micromolar range (**5u**, IC_50_ = 4.5 ± 1.8 µM; **7u**, IC_50_ = 6.4 ± 0.4 µM; **8u**, IC_50_ = 5.4 ± 1.0 µM). Based on the reuptake inhibition data, the two most potent analogues (**7h**, **8h**), which are enantiomers of each other, were selected for further pharmacological and biological evaluation.

### 3.3. Type of Interaction of **7h** and **8h** at Monoamine Transporters 

**Figure 1:**
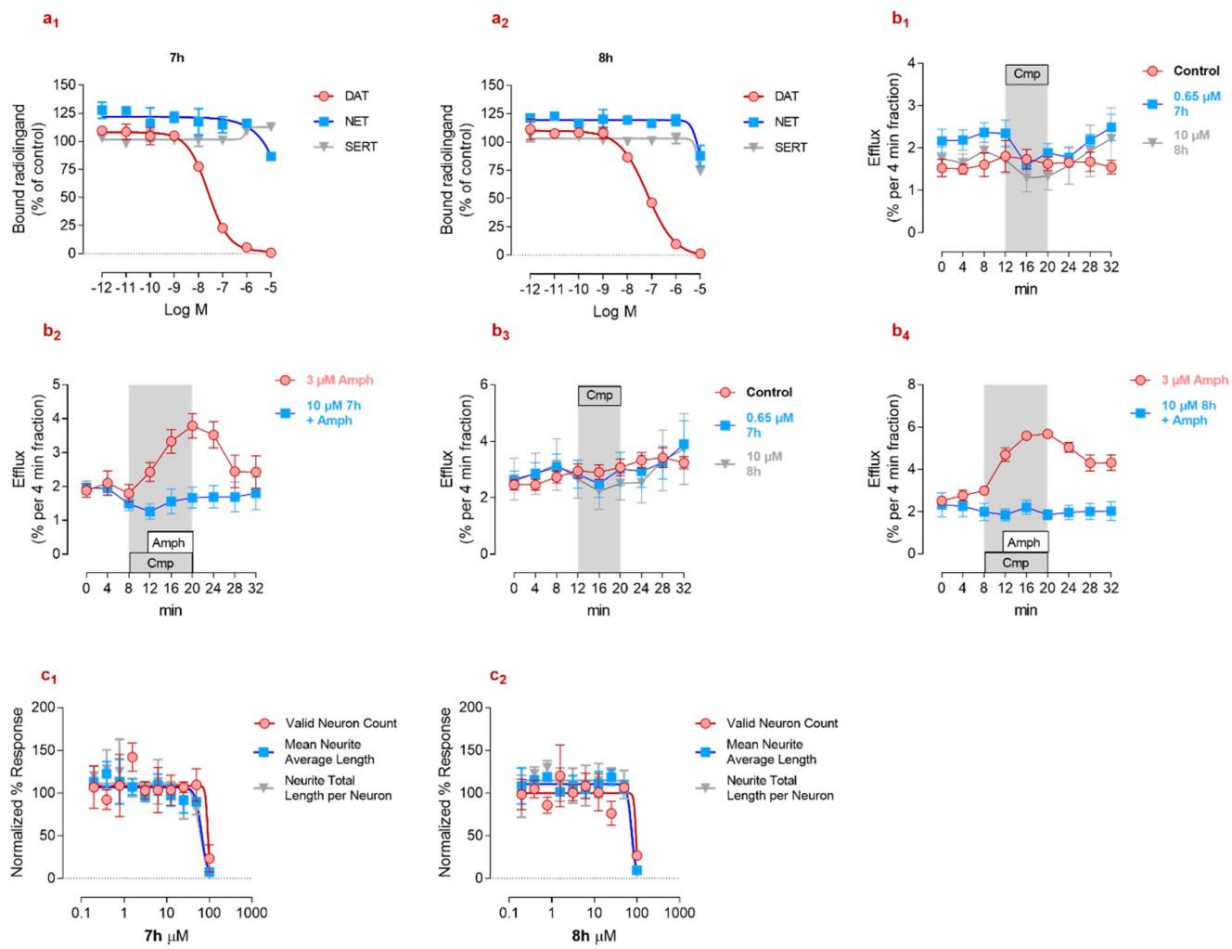
**In vitro pharmacology of 7h and 8h.** Radioligand binding assays on monoamine transporters (DAT, NET, SERT) of **7h** (**a_1_**) and **8h** (**a_2_**). Dopamine release effect of **7h** alone (**b_1_**) and effect on amphetamine-induced dopamine release (**b_2_**). Dopamine release effect of **8h** alone (**b_3_**) and effect on amphetamine-induced dopamine release (**b_4_**). Neurite outgrowth assay as dose-dependent basic neurotoxicity screening of **7h** (**c_1_**) and **8h** (**c_2_**).

Compounds **7h** and **8h** potently displaced the radioligand [^3^H]BTCP, a selective blocker of dopamine transport, from the DAT with K_i_ values of 13 and 35 nM, respectively, whereas they were almost inactive on the binding sites of the NET (against [^3^H]nisoxetine) and SERT (against [^3^H]imipramine) with K_i_ values > 10 µM (Figure 1(a_1_,a_2_)). The absence of an efflux-increasing action on DAT-expressing and substrate-loaded super-fused HEK cells (Figure 1(b_1_,b_3_)) and the potent inhibiting action on the dopamine-releasing effect of 3 µM D-amphetamine (Figure 1(b_2_,b_4_)) characterize compounds **7h** and **8h** as pure uptake-inhibiting drugs without releasing effects. 

### 3.4. In Vitro Neurotoxicity of **7h** and **8h**

Compounds **7h** and **8h** were evaluated in the neurite outgrowth assay using cryopreserved rat cortical neurons by Cyprotex (Framingham, MA, USA, Study Number CYP2052–R1). The positive control, nocodazole, had an IC_50_ of 2.22 μM for valid neuron count, an IC_50_ of 0.097 μM for mean neurite average length, and an IC_50_ of 0.0997 μM for neurite total length per neuron. The negative control, chlorpromazine, had an IC_50_ of 7.99 μM for valid neuron count, an IC_50_ of 9.02 μM for mean neurite average length, and an IC_50_ of 13.8 μM for neurite total length per neuron. Nocodazole was positive for substantially affecting neurite outgrowth while nominally affecting cell viability. Chlorpromazine’s effect on neurite outgrowth was not independent of its effect on cell viability. Only at 100 µM, **7h** and **8h** caused significant decreases in the valid neuron count, mean neurite average length, and neurite total length per neuron. The effects on neurite outgrowth for **7h** and **8h** were not independent of their effect on cell viability (Table 2, Figure 1(c_1_,c_2_)).

Despite the neurite outgrowth effect of **7h** and **8h** at the top concentration tested (100 µM), there is more than 100-fold window compared to the active concentration, which renders **7h** and **8h** suitable for subsequent in vivo pharmacological evaluation.

### 3.5. In Vivo Drug Efficacy

#### Compound Brain Levels 

**Figure 2:**
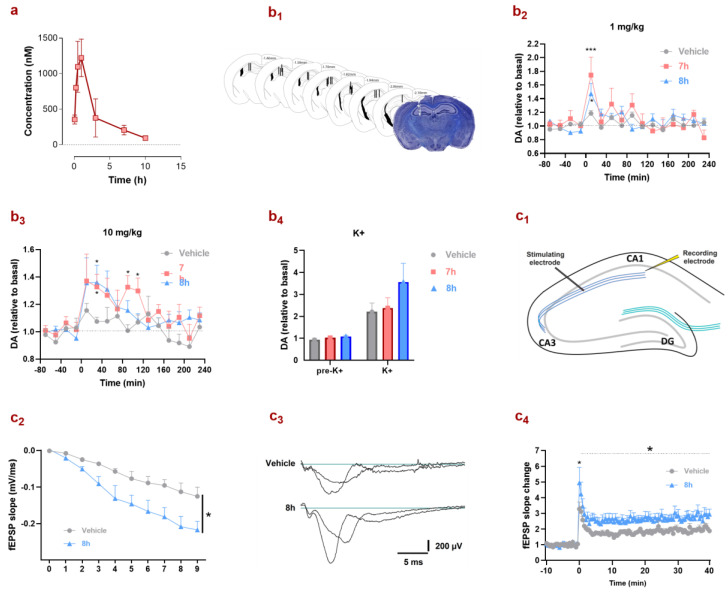
**In vivo pharmacology of 7h and 8h.** Mean total brain concentrations of **7h** in nM (**a**) following IP administration to male Sprague–Dawley rats at 10 mg/kg (each point represents the mean of 3 animals ± SD, except for 0.25 and 1 h time points with only 2 animals w/o SD calculation). Schematic diagrams of coronal mouse brain sections summarizing the placement of the microdialysis probes in the dorsal hippocampus, including a representative image (**b_1_**) Dynamic changes in the extracellular DA levels after administration of **7h** and **8h** enantiomers at a dose of 1 mg/kg on experimental day 1 (**b_2_**) and at a dose of 10 mg/kg on experimental day 2 (**b_3_**). Data represent relative values to the mean baseline DA levels. Analyzed by two-way ANOVA with repeated measures and a post-Fisher‘s LSD test; n = 7–9/group. A final local stimulation with high potassium (100 nM; K^+^) increased DA levels as compared to pre-potassium (pre-K^+^) levels (**b_4_**). Diagram of a hippocampal slice with the positioning of the stimulating electrode in the Schaffer collateral axons from CA3 pyramidal cells and the recording electrode in the dendritic region of the CA1 (**c_1_**). Input-output curve determined in CA1 synapses (22–23 months, n = 5–6/group, two-way RM-ANOVA) (**c_2_**). Representative traces obtained 5 min before and 30 min after high frequency stimulation (HFS) in **8h** and vehicle-treated rats (**c_3_**). Long-term potentiation induced by four trains of HFS with significant enhancement in **8h** group (22–23 months, n = 5–6/group, two-way RM-ANOVA with Sidak post hoc) (**c_4_**). Data are presented as mean ± SEM; *p* = 0.08, * *p* < 0.05, *** *p* < 0.001.

Prior to drug efficacy evaluation, we aimed to determine the bioavailability of this compound class in the brain. For this purpose, **7h** was acutely administered intraperitoneally in Sprague–Dawley rats (for in-detail procedures and parameters, see Supplementary Information) at a dose of 10 mg/kg body weight. Following a single intraperitoneal injection, a C_max_ of 478 ng/g was observed at a median T_max_ of 1 h with a long half-life of 3.0 h in the brain (Figure 2a). The **7h** not only penetrated through the blood–brain barrier and reached necessary brain concentrations above the IC_50_ value (of DAT inhibition) but was also detectable up to 10 h post-administration. Although pharmacokinetic parameters can vary between two enantiomers of the same drug, as showcased in the case of the *R*- (longer lasting) and *S*- (shorter lasting) enantiomers of the marketed drug Modafinil [36], only **7h** was evaluated here as a representative compound of its class.

### 3.6. In Vivo Micro-Dialysis in Freely Moving Mice

Next, we determined whether the two most potent DAT-inhibiting compounds (**7h** and **8h**) are able to increase dopamine (DA) levels in a physiologically relevant system. In the mouse hippocampus, extracellular DA levels were stable 80 min prior to drug injection at an average concentration of 0.20 ± 0.02 fmol/5µL sample on experimental day 1 and of 0.23 ± 0.02 fmol/5µL sample on experimental day 2. After low-dose (1 mg/kg) drug treatment on experimental day 1, extracellular DA levels changed over time [time effect; F(15,270) = 3.991; *p* < 0.001], but no treatment effect was revealed [treatment effect: F(2,18) = 0.495, *p* = 0.618; treatment x time interaction: F(30,270) = 1.28, *p* = 0.160] despite an apparent transient increase in DA levels immediately after drug administration (Figure 2b_1_,b_2_). 

In contrast, when the higher dose of drug (10 mg/kg) was injected on experimental day 2, there was a significant main effect of the drug on extracellular DA levels [drug effect: F(2,20) = 5.66, *p* = 0.011] over time [time effect: F(15,270) = 3.991; *p* < 0.001]. As shown in Figure 2b_3_, the administration of 10 mg/kg for **7h** as well as for **8h** increased extracellular DA levels by approximately 35% within 20 min of administration. While DA levels constantly declined from the peak values elicited by **8h**, they remained elevated throughout a 2 h time period after administration of **7h**. Stimulation with high K^+^ caused a pronounced increase in extracellular DA levels in the hippocampus as compared with baseline, pre-K^+^ conditions [K^+^ effect: F(2,40) = 24.42, *p* < 0.001; treatment × K^+^ interaction: F(2,40) = 1.675, *p* = 0.175; Figure 2b_4_] indicating that the microdialysis systems were functional and that **7h** and **8h** enantiomers did not exhaust dopamine reserves. 

### 3.7. **8h** Enhances Basal Synaptic Transmission and Synaptic Plasticity 

Activity-dependent synaptic plasticity, particularly long-term potentiation (LTP), is considered a possible cellular correlate of learning and memory. Aged rats have previously been used as plausible models to test the learning- and memory-enhancing effects of DAT inhibitors [26,37]. Therefore, we used aged (22–23 months) rats to examine the effect of **8h** on basal synaptic transmission and synaptic plasticity at Schaffer collateral (SC)-CA1 synapses (Figure 2c_1_). 

We generated I/O curves by incrementally increasing stimulus intensity (0–9 V, 1 V increments) applied to SC fibers. As shown in Figure 2c_2_, the fEPSP slopes were significantly enhanced in slices from the **8h**-treated aged rats compared to vehicle-treated rats (F(1,9) = 6.6639, *p* = 0.0299, n = 5–6, two-way RM-ANOVA), suggesting that acute treatment with 10 mg/kg **8h** effectively enhances basal synaptic transmission. A similar effect on basal synaptic transmission was also observed for methamphetamine; however, unlike **8h** (see below), systemic exposure to methamphetamine resulted in deficient LTP [38]. 

A high-frequency stimulation (HFS) protocol induced robust synaptic strengthening responses in both treatment groups. Acute treatment of aged rats with **8h** significantly increased and maintained LTP for at least 40 min after HFS (F(1,9) = 8.983, *p* = 0.015, n = 5–6/group, RM-ANOVA), as compared to the vehicle group. Sidak post hoc analysis revealed a statistically significant (*p* < 0.0001) enhancement of the early post-tetanic potentiation (PTP; the response that occurs right after the HFS) responses in slices obtained from **8h**-treated rats (Figure 2(c_3_,c_4_)). Our findings indicate that acute systemic treatment for **8h** is sufficient to enhance hippocampal synaptic plasticity. Several other DAT inhibitors, including cocaine, GBR12935, (*S*,*S*)-CE-158, and MK-26, have been shown to augment hippocampal plasticity-related responses [26,37,38,39]. It has been demonstrated that exogenously applied dopamine receptor agonists and antagonists can modulate the magnitude and/or persistence of LTP in the hippocampus CA1 region [38]. Since DAT inhibition profoundly increases the concentration of extracellular dopamine, it is feasible that enhanced LTP is mediated by dopamine receptor activation. 

## 4. Conclusions

Herein, we have significantly extended our portfolio of modafinil-derived selective dopamine reuptake inhibitors, first by generating diastereomeric mixtures and later by separating individual diastereomers. This strategy led to the identification not only of dopamine-selective reuptake inhibitors but also of compounds with dual activity on DAT and NET (**7e**, **7h**, and **7m**). Additionally, compounds with dual activity on DAT and SERT (**8c**, **8e**, **8h**, **6k**, **8n**, **8p**, **8q**, **6s**, and **6t**) were found. The systematic substitution of the *ortho*-, *meta*-, and *para*-positions on one phenyl ring of the parent structure and the determination of the absolute configuration of many derivatives enabled the establishment of a SAR study. For the majority of substituents (Cl, Br, CH_3_, CF_3_, and OCH_3_), modification in *meta*-position gave the most active compounds, with both (*S*,*S*)- and (*R*,*R*)-configurations preferred where absolute configuration was successfully attributed. Also, the three derivatives with dual activity on DAT and NET were substituted in *meta*-position but had all (*S*,*S*)-configuration. Dual activity on DAT and SERT was observed for *meta*- and *para*-substituted structures, and in four out of five cases (**8e**, **8h**, **8n**, **6t**) with assigned absolute configuration, their activity seems to be governed by (*R*,*R*)-configuration. The two most potent DAT inhibitors (**7h** and **8h**) were further evaluated in a series of in vitro assays. The observed high binding affinity towards DAT (13 and 35 nM), a lack of amphetamine-like effects, i.e., release of dopamine, and the absence of cell-toxic effects up to a concentration of 50 µM, recommended them for further in vivo evaluations. Accordingly, to demonstrate that this compound class can successfully penetrate the blood–brain barrier, a pharmacokinetic study was performed with a single intraperitoneal injection of **7h**, where a C_max_ of 478 ng/g was observed at a median T_max_ of 1 h with a long half-life of 3.0 h in the brain. In vivo drug efficacy was demonstrated by micro-dialysis in freely moving animals, where both **7h** and **8h** induced an increase in extracellular dopamine levels in the hippocampus. Furthermore, acute systemic treatment for **8h** was sufficient to enhance hippocampal synaptic plasticity, a phenomenon thought to underlie learning and memory. 

## Figures and Tables

**Table 1:** Reuptake inhibition of compounds on cells stably expressing DAT, NET and SERT.

Cmpd. No.	Cmpd. ID	Chirality		Reuptake Inhibition (IC_50_ ± SE, µM)		
		S Atom	C Atom	DAT	NET	SERT
**1**	**5a**	*R*	*R*	32.8 ± 0.2	>50	>50
**2**	**6a**	*(S)*	*(S)*	13.3 ± 1.6	>50	>50
**3**	**7a**	*R*	*S*	8.25 ± 0.16	>50	>50
**4**	**8a**	*S*	*R*	1.09 ± 0.07	>50	>50
**5**	**5b**	-	-	3.2 ± 0.4	>50	>50
**6**	**6b**	-	-	28 ± 7	>50	>50
**7**	**7b**	-	-	2.3 ± 0.2	>50	>50
**8**	**8b**	-	-	9 ± 3	>50	>50
**9**	**5c**	-	-	7.8 ± 0.7	>50	>50
**10**	**6c**	-	-	10.1 ± 0.3	>50	>50
**11**	**7c**	-	-	2.1 ± 0.6	>50	>50
**12**	**8c**	-	-	5.2 ± 0.1	>50	24 ± 6
**13**	**5d**	*R*	*R*	>50	>50	>50
**14**	**6d**	*S*	*S*	14.4 ± 2.4	>50	>50
**15**	**7d**	*(R)*	*(S)*	>50	>50	>50
**16**	**8d**	*S*	*R*	2.55 ± 0.04	>50	>50
**17**	**5e**	*S*	*R*	3.0 ± 0.1	>50	>50
**18**	**6e**	*(R)*	*(S)*	14.1 ± 0.5	>50	>50
**19**	**7e**	*S*	*S*	0.63 ± 0.17	29 ± 4	>50
**20**	**8e**	*R*	*R*	0.83 ± 0.16	48 ± 6	19 ± 1
**21**	**5f**	*S*	*R*	12 ± 1	>50	>50
**22**	**6f**	*R*	*S*	46 ± 1	>50	>50
**23**	**7f**	*S*	*S*	4.0 ± 1	>50	>50
**24**	**8f**	*R*	*R*	4.9 ± 0.6	>50	>50
**25**	**5g**	-	-	>50	>50	>50
**26**	**6g**	-	-	12.1 ± 1.0	>50	>50
**27**	**7g**	-	-	>50	>50	>50
**28**	**8g**	-	-	5.06 ± 0.31	>50	>50
**29**	**5h**	*S*	*R*	1.8 ± 0.4	>50	>50
**30**	**6h**	*R*	*S*	15 ± 1	>50	>50
**31**	**7h**	*S*	*S*	0.48 ± 0.11	16.4 ± 0.6	>50
**32**	**8h**	*R*	*R*	0.30 ± 0.03	>50	14 ± 3
**33**	**5i**	-	-	>50	>50	>50
**34**	**6i**	-	-	20.3 ± 0.6	>50	>50
**35**	**7i**	-	-	28.4 ± 2.8	>50	>50
**36**	**8i**	-	-	9.6 ± 1.2	>50	>50
**37**	**5j**	-	-	2.09 ± 0.21	>50	>50
**38**	**6j**	-	-	17.7 ± 2.2	>50	>50
**39**	**7j**	-	-	1.46 ± 0.06	>50	>50
**40**	**8j**	-	-	1.50 ± 0.17	>50	>50
**41**	**5k**	*S*	*R*	5.1 ± 0.6	>50	>50
**42**	**6k**	*R*	*S*	1.1 ± 0.3	>50	3.8 ± 0.5
**43**	**7k**	*S*	*S*	>50	>50	>50
**44**	**8k**	*R*	*R*	2.6 ± 0.1	>50	>50
**45**	**5l**	-	-	15.6 ± 1.0	>50	>50
**46**	**6l**	-	-	>50	>50	>50
**47**	**7l**	-	-	3.8 ± 0.5	>50	>50
**48**	**8l**	-	-	9.5 ± 0.9	>50	>50
**49**	**5m**	*(S)*	*(R)*	2.20 ± 0.40	>50	>50
**50**	**6m**	*R*	*S*	>50	>50	>50
**51**	**7m**	*S*	*S*	1.20 ± 0.09	49 ± 3	>50
**52**	**8m**	*R*	*R*	1.56 ± 0.25	>50	>50
**53**	**5n**	-	-	3.97 ± 0.21	>50	>50
**54**	**6n**	-	-	>50	>50	>50
**55**	**7n**	*S*	*S*	1.63 ± 0.10	>50	>50
**56**	**8n**	*R*	*R*	4.01 ± 0.41	>50	25 ± 4
**57**	**5o**	-	-	>50	25 ± 1	>50
**58**	**6o**	-	-	>50	>50	>50
**59**	**7o**	-	-	>50	>50	>50
**60**	**8o**	-	-	8.9 ± 0.7	>50	>50
**61**	**5p**	-	-	5 ± 1	>50	>50
**62**	**6p**	-	-	22 ± 1	>50	>50
**63**	**7p**	-	-	10 ± 1	>50	>50
**64**	**8p**	-	-	6.2 ± 0.6	>50	31 ± 5
**65**	**5q**	-	-	17 ± 2	>50	>50
**66**	**6q**	-	-	40 ± 9	>50	>50
**67**	**7q**	-	-	24 ± 1	>50	>50
**68**	**8q**	-	-	21 ± 2	>50	4.7 ± 0.5
**69**	**5r**	*(R)*	*(R)*	31 ± 8	>50	>50
**70**	**6r**	*S*	*S*	>50	>50	>50
**71**	**7r**	*S*	*R*	1.7 ± 0.1	>50	>50
**72**	**8r**	*R*	*S*	>50	>50	>50
**73**	**5s**	-	-	2.89 ± 0.47	>50	>50
**74**	**6s**	-	-	5.41 ± 0.99	>50	35 ± 3
**75**	**7s**	-	-	1.56 ± 0.24	>50	>50
**76**	**8s**	-	-	>50	>50	>50
**77**	**5t**	*S*	*S*	16 ± 6	>50	>50
**78**	**6t**	*(R)*	*(R)*	17 ± 3	>50	11 ± 3
**79**	**7t**	*S*	*R*	15 ± 2	>50	>50
**80**	**8t**	*R*	*S*	>50	>50	>50
**81**	**5u**	-	-	4.5 ± 1.8	>50	>50
**82**	**6u**	-	-	12 ± 2	>50	>50
**83**	**7u**	-	-	6.4 ± 0.4	>50	>50
**84**	**8u**	-	-	5.4 ± 1.0	>50	>50
R-Modafinil *		*(R)*	-	6.6 ± 0.7	169 ± 90	232 ± 196

* [35].

**Table 2:** Results for valid neuron count, mean neurite average length, and neurite total length per neuron.

Compound ID	Top Conc. Tested (μM)	Valid Neuron Count IC_50_ (μM)	Mean Neurite Avg. Length IC_50_ (μM)	Neurite Total Length per Neuron IC_50_ (μM)	Comments
Nocodazole	1	2.22	0.097	0.0997	Control
Chlorpromazine	100	7.99	9.022	13.8	Control
7h	100	92.16	65.82	59.76	Test compound
8h	100	94.6	75.22	72.15	Test compound

## Data Availability

No new data were created.

## Supplementary Materials

The following supporting information can be downloaded at: https://www.mdpi.com/article/10.3390/biom13091415/s1.
