# Supplementary material for: Chirality Matters: Fine-Tuning of Novel Monoamine Reuptake Inhibitors Selectivity through Manipulation of Stereochemistry
Source: Biomolecules. 2023 Sep 19;13(9):1415. doi: 10.3390/biom13091415 (PMC10527105; doi:10.3390/biom13091415)
Supplement: Supplementary file 1 [file biomolecules-2521332-supplementary.pdf › biomolecules-2521332-supplementary.pdf]

Supporting Information

Chirality matters: Fine-tuning of novel monoamine reuptake inhibitors selectivity through manipulation of stereochemistry

Predrag Kalaba[1], Katharina Pacher[1], Philip John Neill[1], Vladimir Dragacevic[1], Martin Zehl[2,3], Judith Wackerlig[1], Michael Kirchhofer[1], Simone Sartori[4], Hubert Gstach[1], Shima Kouhnavardi[1], Anna Fabisikova[2], Matthias Pillwein[1], Francisco Monje Quiroga[5], Karl Ebner[4], Alexander Prado-Roller[6], Nicolas Singewald[4], Ernst Urban[1], Thierry Langer[1], Christian Pifl[7], Jana Lubec[8], Johann Jakob Leban[8], Gert Lubec[8, #]

Compound analytics (HRESIMS, ^{1}H, ^{13}C and 2D NMR, RP-HPLC and chiral-phase HPLC)

Compound brain levels study

In vitro assays

- Binding assays on DAT, NET and SERT
- Neurite Outgrowth Assay

Absolute configuration

- VCD method
- single-crystal X-ray diffraction

Figure S1. HRESIMS spectrum of compound **5a**

Generic Display Report

Analysis Info

Analysis Name	D:\Data\Kalaba\78522000001.d	Acquisition Date	26/03/2021 15:41:08
Method	tune_low_MS_Service_03_21.m	Operator	msc
Sample Name	PN-20-peak-1	Instrument	maXis
Comment	Kalaba / Zehl<br>Ergebnis +/- 5 ppm<br>ACN / MeOH + 1% H2O		

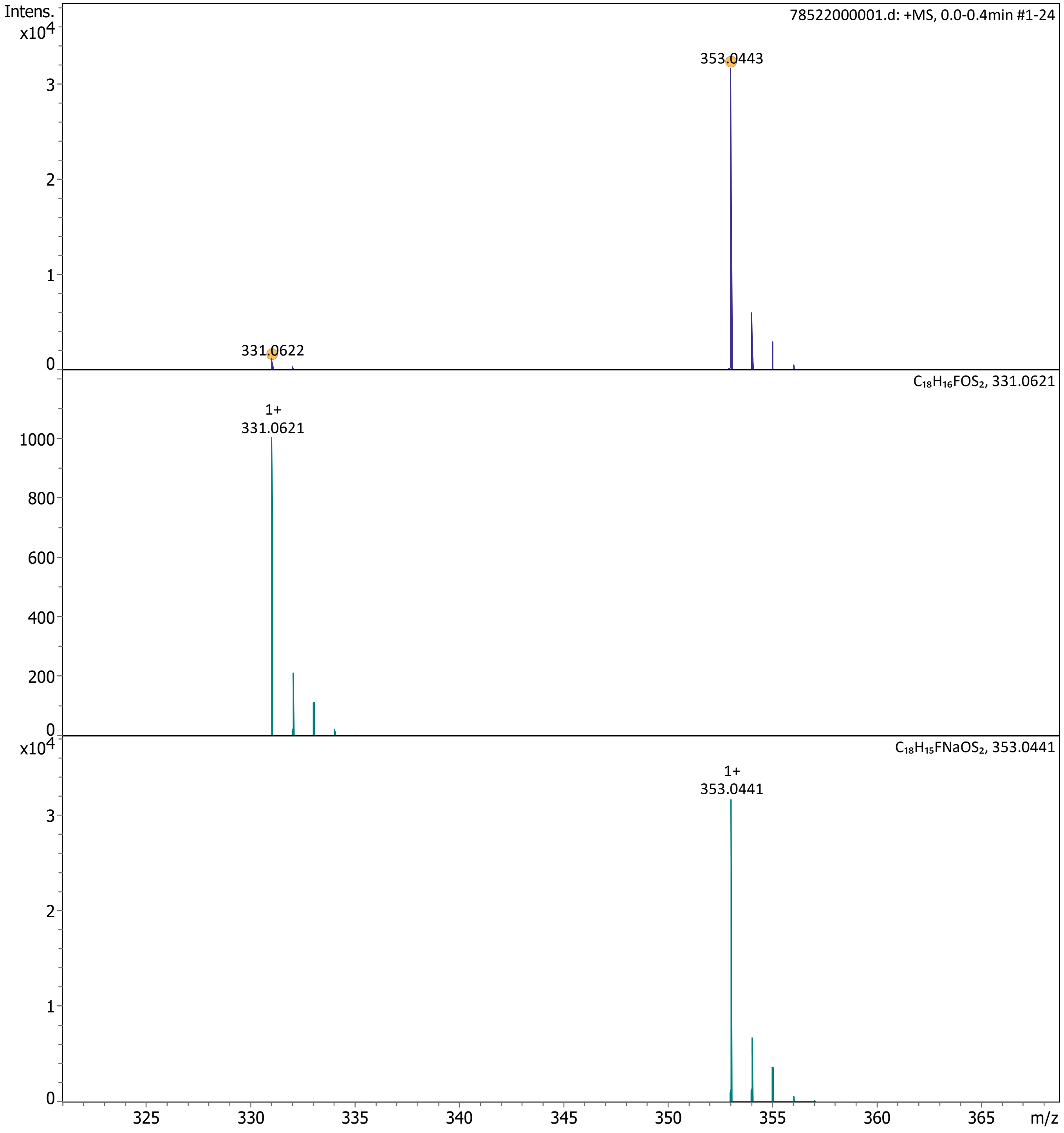

Mass Spectrum SmartFormula Report

Analysis Info

Analysis Name	D:\Data\Kalaba\78522000001.d	Acquisition Date	26/03/2021 15:41:08
Method	tune_low_MS_Service_03_21.m	Operator	msc
Sample Name	PN-20-peak-1	Instrument	maXis 255552.00016
Comment	Kalaba / Zehl<br>Ergebnis +/- 5 ppm<br>ACN / MeOH + 1% H2O		

Acquisition Parameter

Source Type	ESI	Ion Polarity	Positive	Set Nebulizer	0.4 Bar
Focus	Not active	Set Capillary	4200 V	Set Dry Heater	180 Â°C
Scan Begin	80 m/z	Set End Plate Offset	-500 V	Set Dry Gas	4.0 l/min
Scan End	1900 m/z	Set Charging Voltage	0 V	Set Divert Valve	Source
		Set Corona	0 nA	Set APCI Heater	0 Â°C

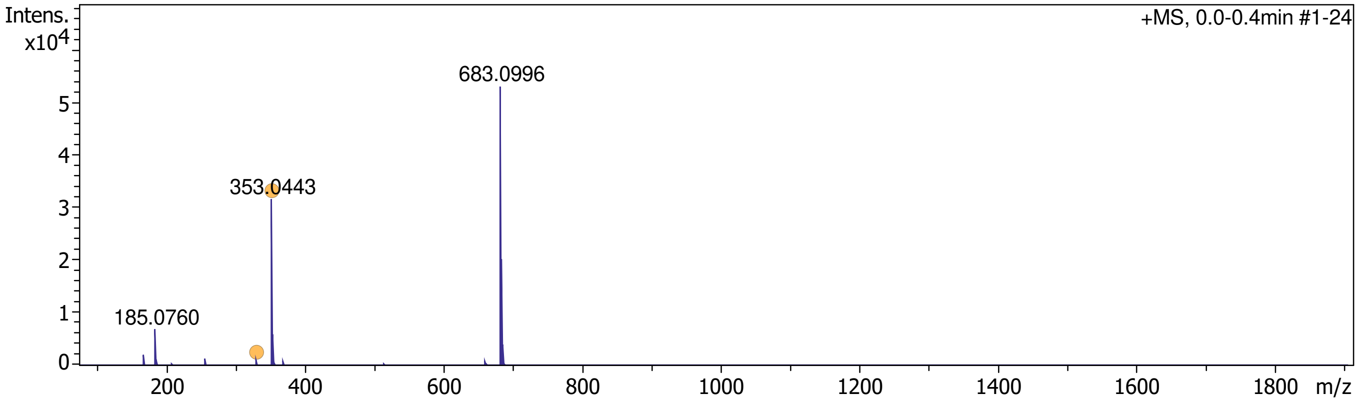

Meas. m/z	#	Ion Formula	Score	m/z	err [mDa]	err [ppm]	mSigma	rdb	eÂ¯ Conf	N-Rule
331.0622	1	C18H16FOS2	100.00	331.0621	-0.1	-0.3	15.1	15.0	even	ok
	2	C15H17F2O2S2	49.47	331.0633	1.1	3.2	25.7	11.0	even	ok
	3	C15H14F3O3S	39.72	331.0610	-1.2	-3.6	31.8	10.0	even	ok
	4	C13H16F5S2	31.65	331.0608	-1.4	-4.2	35.4	8.0	even	ok
	5	C11H13F2N6S2	26.94	331.0606	-1.6	-4.9	35.5	12.0	even	ok
	6	C13H11F4N4S	31.95	331.0635	1.3	3.9	37.2	11.0	even	ok
	7	C11H8FN10S	36.94	331.0633	1.1	3.2	37.2	15.0	even	ok
	8	C18H8FN4O2	34.76	331.0626	0.4	1.1	42.0	17.0	even	ok
	9	C17H12FO6	23.59	331.0612	-1.0	-2.9	45.2	12.0	even	ok
	10	C10H20FN2O3S3	43.31	331.0615	-0.7	-2.2	45.9	8.0	even	ok
	11	C12H15F4O4S	47.07	331.0622	-0.0	-0.1	46.7	6.0	even	ok
	12	C10H12FN6O4S	42.49	331.0619	-0.3	-0.8	46.7	10.0	even	ok
	13	C10H17F6OS2	37.77	331.0620	-0.2	-0.8	50.9	4.0	even	ok
	14	C15H9F2N4O3	13.82	331.0637	1.5	4.6	51.5	13.0	even	ok
	15	C14H13F2O7	23.16	331.0624	0.2	0.6	58.1	8.0	even	ok
	16	C11H5F2N10O	11.91	331.0610	-1.2	-3.5	62.9	14.0	even	ok
	17	C13H8F5N4O	13.69	331.0613	-0.9	-2.8	62.9	10.0	even	ok
	18	C11H14F3O8	7.41	331.0635	1.3	4.0	73.2	4.0	even	ok
	19	C10H9F6N4O2	11.49	331.0624	0.2	0.7	77.0	6.0	even	ok
353.0443	1	C15H16F2NaO2S2	84.24	353.0452	0.9	2.5	8.6	11.0	even	ok
	2	C18H15FNaOS2	100.00	353.0441	-0.3	-0.8	15.0	15.0	even	ok
	3	C13H15F5NaS2	47.24	353.0428	-1.6	-4.5	16.1	8.0	even	ok
	4	C15H13F3NaO3S	53.15	353.0430	-1.4	-3.9	17.3	10.0	even	ok
	5	C11H7FN10NaS	62.90	353.0452	0.9	2.5	22.8	15.0	even	ok
	6	C13H10F4N4NaS	54.82	353.0455	1.1	3.2	22.8	11.0	even	ok
	7	C12H14F4NaO4S	74.46	353.0441	-0.2	-0.6	29.5	6.0	even	ok
	8	C10H11FN6NaO4S	66.75	353.0439	-0.5	-1.3	29.5	10.0	even	ok
	9	C10H16F6NaOS2	63.75	353.0439	-0.4	-1.2	31.9	4.0	even	ok
	10	C10H19FN2NaO3S3	60.67	353.0434	-0.9	-2.6	32.7	8.0	even	ok

Mass Spectrum SmartFormula Report

Meas. m/z	#	Ion Formula	Score	m/z	err [mDa]	err [ppm]	mSigma	rdb	eÂ¯ Conf	N-Rule
	11	C10H19F5NaS3	34.62	353.0461	1.8	5.1	32.9	5.0	even	ok
	12	C17H11FNaO6	28.21	353.0432	-1.1	-3.2	37.7	12.0	even	ok
	13	C18H7FN4NaO2	41.20	353.0445	0.2	0.5	41.6	17.0	even	ok
	14	C15H8F2N4NaO3	21.97	353.0457	1.3	3.8	42.5	13.0	even	ok
	15	C14H12F2NaO7	41.60	353.0443	-0.0	-0.0	44.1	8.0	even	ok
	16	C11H4F2N10NaO	17.34	353.0430	-1.3	-3.8	50.3	14.0	even	ok
	17	C13H7F5N4NaO	20.09	353.0432	-1.1	-3.1	50.3	10.0	even	ok
	18	C11H13F3NaO8	16.57	353.0455	1.1	3.2	55.8	4.0	even	ok
	19	C10H8F6N4NaO2	24.37	353.0444	0.0	0.1	60.8	6.0	even	ok

Figure S2. HRESIMS spectrum of compound **6a**

Generic Display Report

Analysis Info

Analysis Name	D:\Data\Kalaba\78523000001.d	Acquisition Date	26/03/2021 15:50:02
Method	tune_low_MS_Service_03_21.m	Operator	msc
Sample Name	PN-20-peak-2	Instrument	maXis
Comment	Kalaba / Zehl<br>Ergebnis +/- 5 ppm<br>ACN / MeOH + 1% H2O		

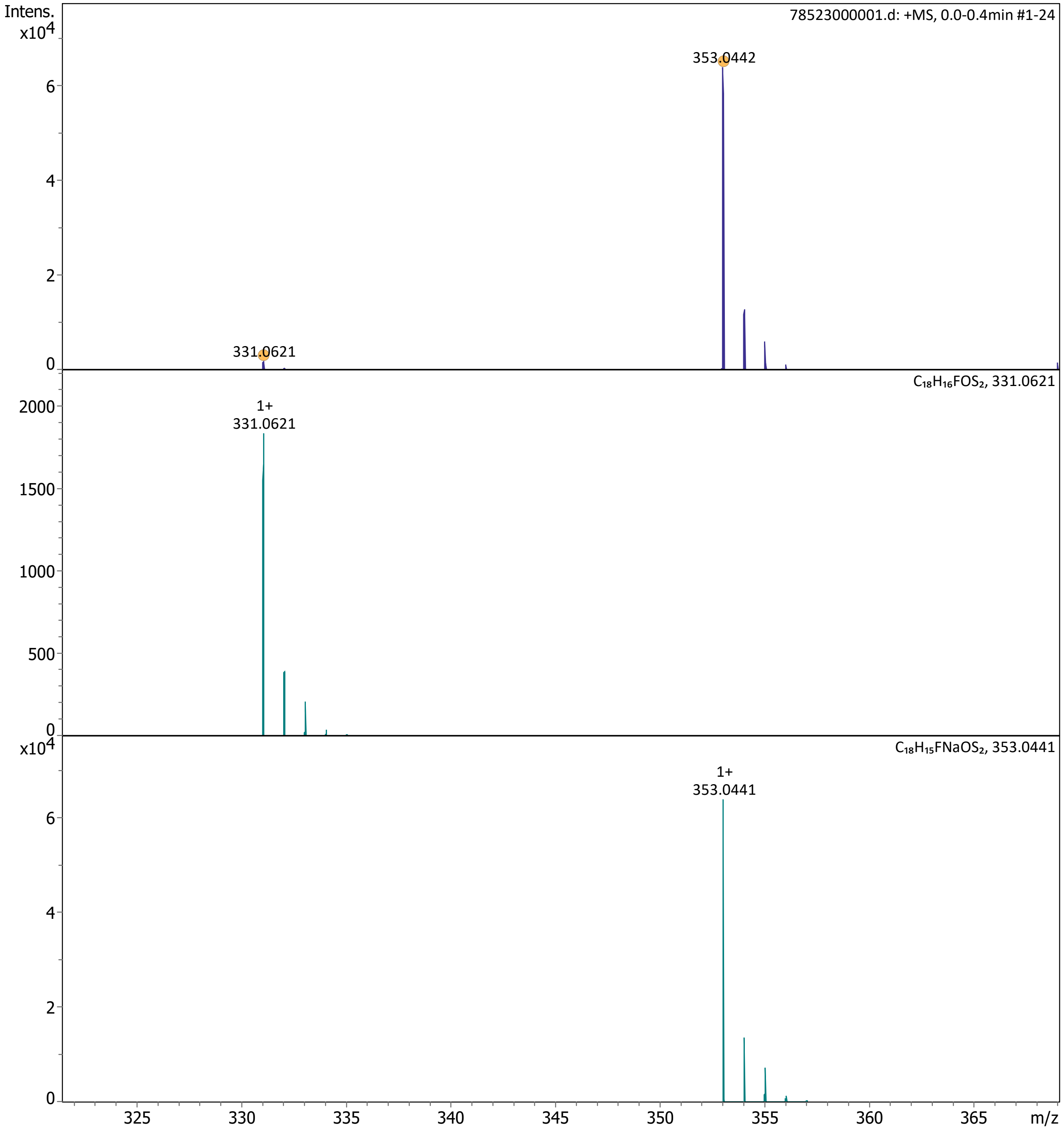

Mass Spectrum SmartFormula Report

Analysis Info

Analysis Name	D:\Data\Kalaba\78523000001.d	Acquisition Date	26/03/2021 15:50:02
Method	tune_low_MS_Service_03_21.m	Operator	msc
Sample Name	PN-20-peak-2	Instrument	maXis 255552.00016
Comment	Kalaba / Zehl<br>Ergebnis +/- 5 ppm<br>ACN / MeOH + 1% H2O		

Acquisition Parameter

Source Type	ESI	Ion Polarity	Positive	Set Nebulizer	0.4 Bar
Focus	Not active	Set Capillary	4200 V	Set Dry Heater	180 Â°C
Scan Begin	80 m/z	Set End Plate Offset	-500 V	Set Dry Gas	4.0 l/min
Scan End	1900 m/z	Set Charging Voltage	0 V	Set Divert Valve	Source
		Set Corona	0 nA	Set APCI Heater	0 Â°C

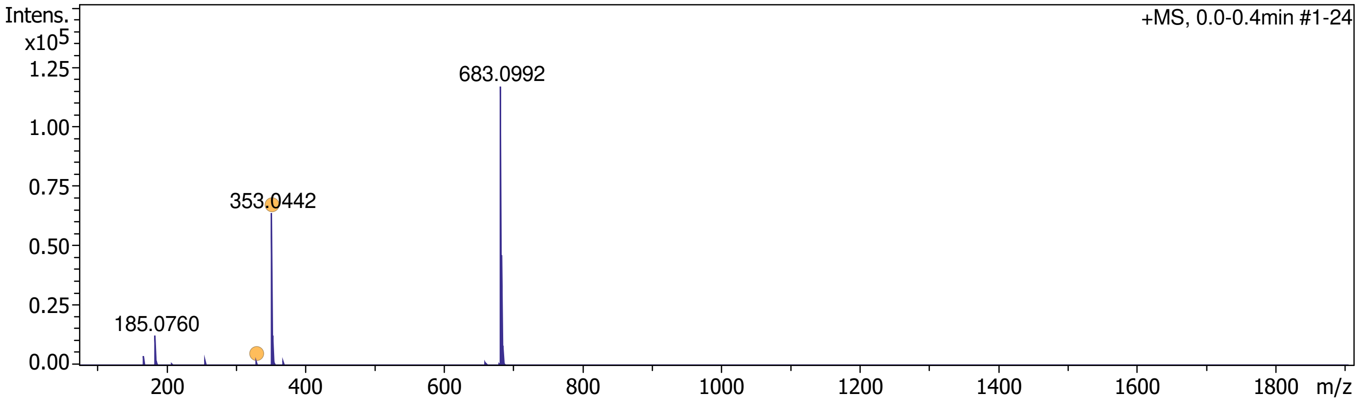

Meas. m/z	#	Ion Formula	Score	m/z	err [mDa]	err [ppm]	mSigma	rdb	eÂ¯ Conf	N-Rule
331.0621	1	C15H17F2O2S2	60.17	331.0633	1.2	3.5	12.5	11.0	even	ok
	2	C18H16FOS2	100.00	331.0621	0.0	0.1	15.8	15.0	even	ok
	3	C13H16F5S2	49.04	331.0608	-1.3	-3.8	19.6	8.0	even	ok
	4	C11H13F2N6S2	41.98	331.0606	-1.5	-4.6	19.6	12.0	even	ok
	5	C15H14F3O3S	55.59	331.0610	-1.1	-3.2	19.8	10.0	even	ok
	6	C11H8FN10S	45.89	331.0633	1.2	3.6	25.1	15.0	even	ok
	7	C13H11F4N4S	39.46	331.0635	1.4	4.3	25.1	11.0	even	ok
	8	C12H15F4O4S	67.56	331.0622	0.1	0.3	32.2	6.0	even	ok
	9	C10H12FN6O4S	65.66	331.0619	-0.2	-0.5	32.2	10.0	even	ok
	10	C10H20FN2O3S3	62.24	331.0615	-0.6	-1.9	34.5	8.0	even	ok
	11	C10H17F6OS2	62.34	331.0620	-0.1	-0.4	34.6	4.0	even	ok
	12	C17H12FO6	29.29	331.0612	-0.8	-2.5	39.4	12.0	even	ok
	13	C18H8FN4O2	32.16	331.0626	0.5	1.5	42.4	17.0	even	ok
	14	C15H9F2N4O3	15.52	331.0637	1.6	5.0	44.5	13.0	even	ok
	15	C14H13F2O7	30.95	331.0624	0.3	0.9	46.8	8.0	even	ok
	16	C11H5F2N10O	17.62	331.0610	-1.0	-3.2	52.9	14.0	even	ok
	17	C13H8F5N4O	20.13	331.0613	-0.8	-2.4	52.9	10.0	even	ok
	18	C11H14F3O8	11.31	331.0635	1.4	4.4	58.9	4.0	even	ok
	19	C10H9F6N4O2	17.62	331.0624	0.3	1.0	63.8	6.0	even	ok
353.0442	1	C15H16F2NaO2S2	67.65	353.0452	1.0	2.7	11.4	11.0	even	ok
	2	C18H15FNaOS2	100.00	353.0441	-0.2	-0.5	11.7	15.0	even	ok
	3	C15H13F3NaO3S	47.96	353.0430	-1.3	-3.6	20.1	10.0	even	ok
	4	C13H15F5NaS2	41.38	353.0428	-1.5	-4.2	20.5	8.0	even	ok
	5	C11H12F2N6NaS2	35.08	353.0425	-1.7	-4.9	20.5	12.0	even	ok
	6	C11H7FN10NaS	49.81	353.0452	1.0	2.8	25.7	15.0	even	ok
	7	C13H10F4N4NaS	43.22	353.0455	1.2	3.5	25.7	11.0	even	ok
	8	C12H14F4NaO4S	63.23	353.0441	-0.1	-0.3	33.5	6.0	even	ok
	9	C10H11FN6NaO4S	56.91	353.0439	-0.4	-1.0	33.5	10.0	even	ok
	10	C10H19FN2NaO3S3	53.82	353.0434	-0.8	-2.3	35.3	8.0	even	ok

Mass Spectrum SmartFormula Report

Meas. m/z	#	Ion Formula	Score	m/z	err [mDa]	err [ppm]	mSigma	rdb	eÂ¯ Conf	N-Rule
	11	C10H16F6NaOS2	53.41	353.0439	-0.3	-0.9	36.4	4.0	even	ok
	12	C17H11FNaO6	26.26	353.0432	-1.0	-3.0	38.5	12.0	even	ok
	13	C18H7FN4NaO2	36.23	353.0445	0.3	0.8	40.7	17.0	even	ok
	14	C15H8F2N4NaO3	17.74	353.0457	1.4	4.1	43.8	13.0	even	ok
	15	C14H12F2NaO7	32.96	353.0443	0.1	0.3	46.9	8.0	even	ok
	16	C11H4F2N10NaO	15.34	353.0430	-1.2	-3.5	52.8	14.0	even	ok
	17	C13H7F5N4NaO	17.68	353.0432	-1.0	-2.9	52.8	10.0	even	ok
	18	C11H13F3NaO8	12.26	353.0455	1.2	3.5	59.7	4.0	even	ok
	19	C10H8F6N4NaO2	18.47	353.0444	0.1	0.4	64.4	6.0	even	ok

Figure S3. HRESIMS spectrum of compound **7a**

Generic Display Report

Analysis Info

Analysis Name	D:\Data\Kalaba\71703000001.d	Acquisition Date	16/07/2020 12:53:24
Method	tune_low_MS_Service_07_20.m	Operator	msc
Sample Name	PN-20-3	Instrument	maXis
Comment	Kalaba/Zehl<br>Ergebnis +/- 5ppm<br>ACN/MeOH + 1 % H2O		

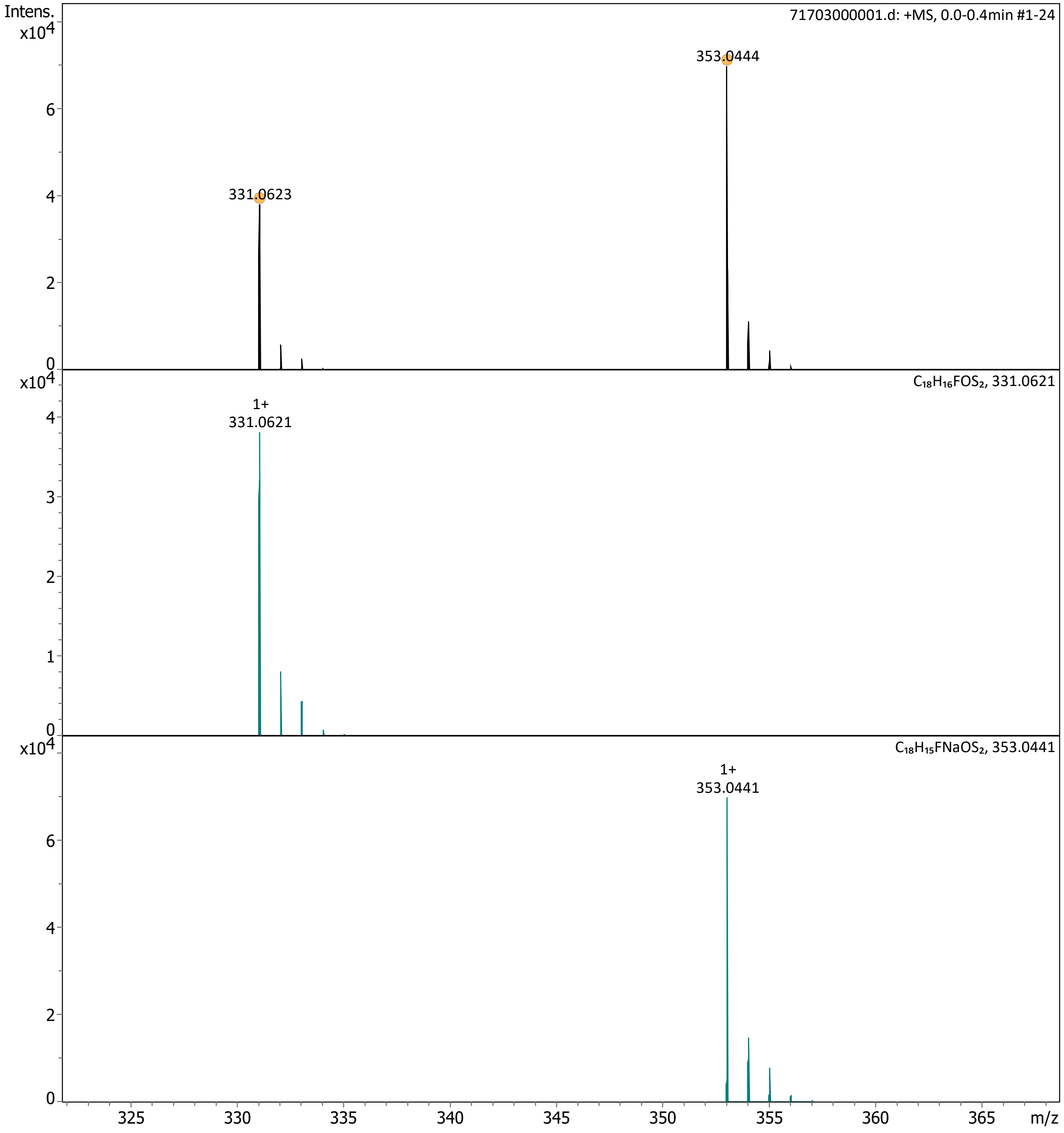

Mass Spectrum SmartFormula Report

Analysis Info

Analysis Name	D:\Data\Kalaba\71703000001.d	Acquisition Date	16/07/2020 12:53:24
Method	tune_low_MS_Service_07_20.m	Operator	msc
Sample Name	PN-20-3	Instrument	maXis 255552.00016
Comment	Kalaba/Zehl<br>Ergebnis +/- 5ppm<br>ACN/MeOH + 1 % H2O		

Acquisition Parameter

Source Type	ESI	Ion Polarity	Positive	Set Nebulizer	0.4 Bar
Focus	Not active	Set Capillary	4200 V	Set Dry Heater	180 Â°C
Scan Begin	50 m/z	Set End Plate Offset	-500 V	Set Dry Gas	4.0 l/min
Scan End	1900 m/z	Set Charging Voltage	0 V	Set Divert Valve	Source
		Set Corona	0 nA	Set APCI Heater	0 Â°C

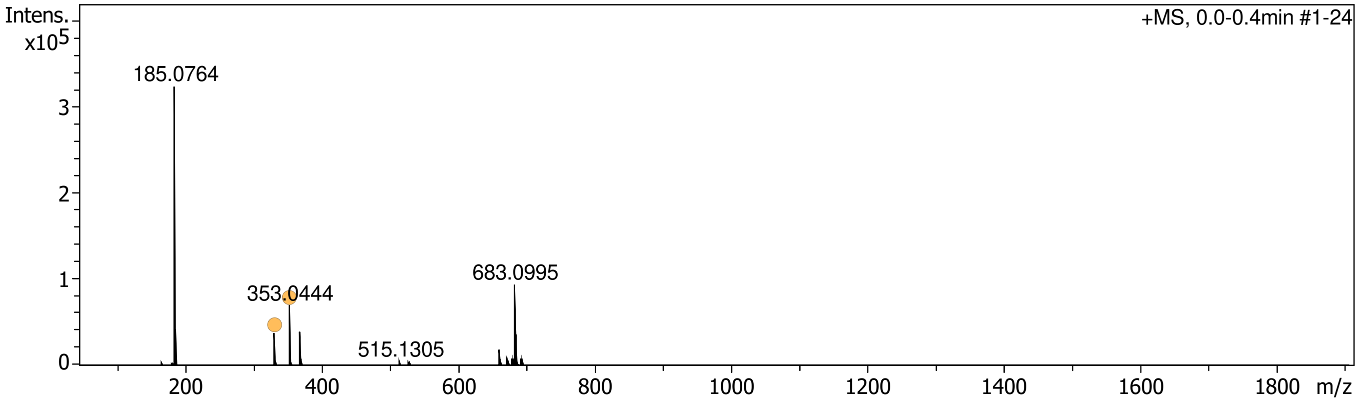

Meas. m/z	#	Ion Formula	m/z	err [ppm]	mSigma	# mSigma	Score	rdb	eÂ¯ Conf	N-Rule
331.0623	1	C12H15F4O4S	331.0622	-0.5	6.4	1	100.00	6.0	even	ok
	2	C13H11F4N4S	331.0635	3.6	8.2	2	57.14	11.0	even	ok
	3	C13H11N6O3S	331.0608	-4.6	10.6	3	43.67	14.0	even	ok
	4	C15H14F3O3S	331.0610	-3.9	10.6	4	51.01	10.0	even	ok
	5	C13H16F5S2	331.0608	-4.6	17.3	5	38.87	8.0	even	ok
	6	C17H15O5S	331.0635	3.5	21.9	6	44.33	13.0	even	ok
	7	C14H13F2O7	331.0624	0.2	24.1	7	54.62	8.0	even	ok
	8	C15H17F2O2S2	331.0633	2.8	25.5	8	46.35	11.0	even	ok
	9	C17H12FO6	331.0612	-3.3	30.2	9	28.62	12.0	even	ok
	10	C15H9F2N4O3	331.0637	4.2	30.8	10	23.15	13.0	even	ok
	11	C13H8F5N4O	331.0613	-3.2	31.1	11	28.56	10.0	even	ok
	12	C18H16FOS2	331.0621	-0.6	38.3	12	48.22	15.0	even	ok
	13	C18H8FN4O2	331.0626	0.8	41.8	13	32.17	17.0	even	ok
	14	C21H15S2	331.0610	-4.1	53.2	14	16.99	19.0	even	ok
	15	C21H7N4O	331.0614	-2.7	56.7	15	15.04	21.0	even	ok
353.0444	1	C13H10F4N4NaS	353.0455	2.9	4.0	1	75.85	11.0	even	ok
	2	C13H10N6NaO3S	353.0427	-4.8	6.7	2	47.67	14.0	even	ok
	3	C15H13F3NaO3S	353.0430	-4.1	6.8	3	56.08	10.0	even	ok
	4	C12H14F4NaO4S	353.0441	-0.9	9.5	4	100.00	6.0	even	ok
	5	C17H14NaO5S	353.0454	2.8	18.6	5	58.71	13.0	even	ok
	6	C13H15F5NaS2	353.0428	-4.7	19.2	6	38.11	8.0	even	ok
	7	C14H12F2NaO7	353.0443	-0.2	21.7	7	64.38	8.0	even	ok
	8	C17H11FNaO6	353.0432	-3.5	25.5	8	33.01	12.0	even	ok
	9	C15H16F2NaO2S2	353.0452	2.2	25.5	9	56.84	11.0	even	ok
	10	C15H8F2N4NaO3	353.0457	3.5	26.6	10	31.78	13.0	even	ok
	11	C13H7F5N4NaO	353.0432	-3.4	28.6	11	31.31	10.0	even	ok
	12	C18H15FNaOS2	353.0441	-1.0	36.8	12	53.00	15.0	even	ok
	13	C18H7FN4NaO2	353.0445	0.3	36.8	13	44.36	17.0	even	ok
	14	C21H14NaS2	353.0429	-4.3	51.1	14	18.59	19.0	even	ok

Mass Spectrum SmartFormula Report

Meas. m/z	#	Ion Formula	m/z	err [ppm]	mSigma	# mSigma	Score	rdb	eÂ¯ Conf	N-Rule
	15	C21H6N4NaO	353.0434	-2.9	51.7	15	18.28	21.0	even	ok

Figure S4. HRESIMS spectrum of compound **8a**

Generic Display Report

Analysis Info

Analysis Name	D:\Data\Kalaba\71704000001.d	Acquisition Date	16/07/2020 12:55:37
Method	tune_low_MS_Service_07_20.m	Operator	msc
Sample Name	PN-20-4	Instrument	maXis
Comment	Kalaba/Zehl<br>Ergebnis +/- 5ppm<br>ACN/MeOH + 1 % H2O		

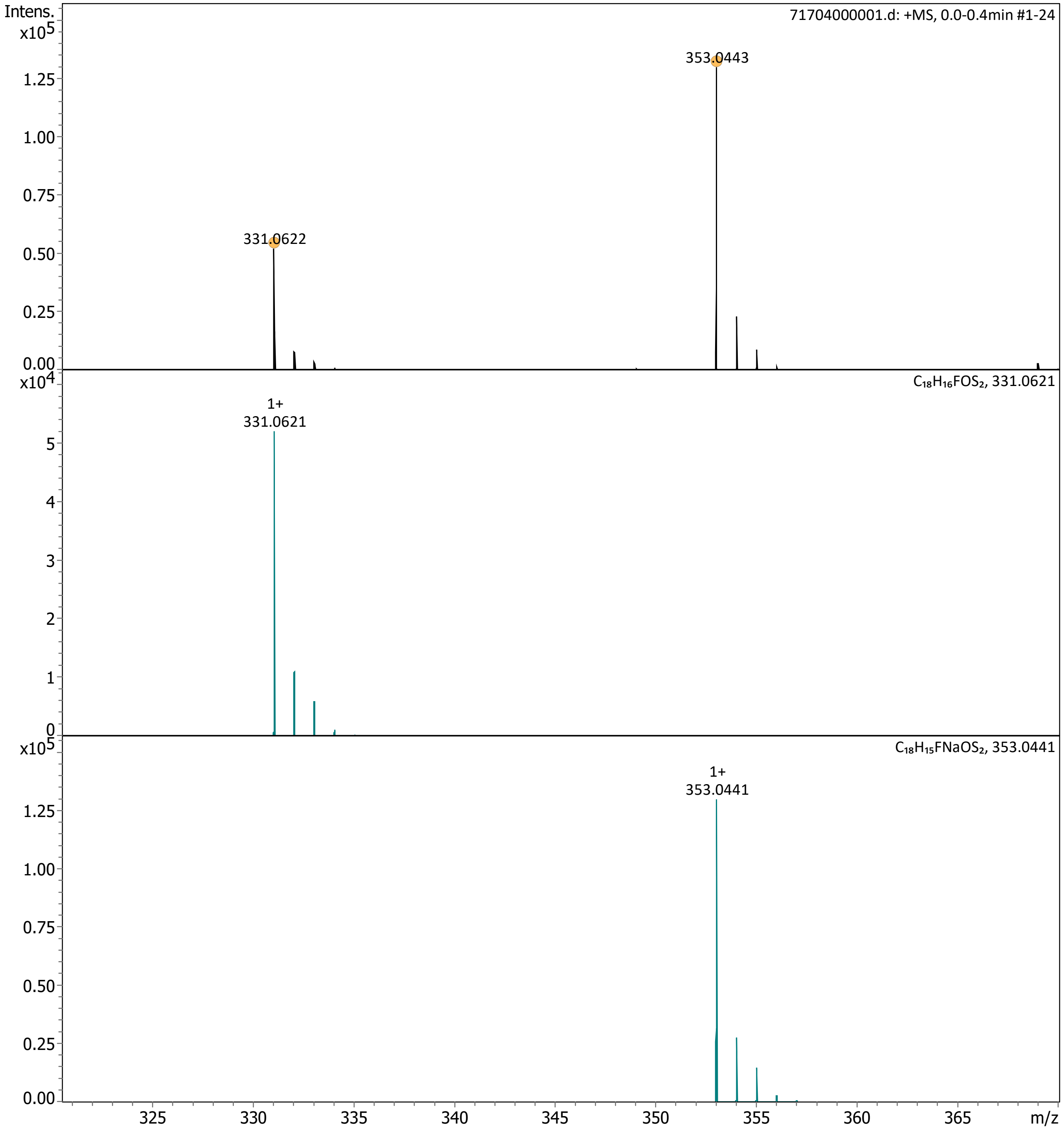

Mass Spectrum SmartFormula Report

Analysis Info

Analysis Name	D:\Data\Kalaba\71704000001.d	Acquisition Date	16/07/2020 12:55:37
Method	tune_low_MS_Service_07_20.m	Operator	msc
Sample Name	PN-20-4	Instrument	maXis 255552.00016
Comment	Kalaba/Zehl<br>Ergebnis +/- 5ppm<br>ACN/MeOH + 1 % H2O		

Acquisition Parameter

Source Type	ESI	Ion Polarity	Positive	Set Nebulizer	0.4 Bar
Focus	Not active	Set Capillary	4200 V	Set Dry Heater	180 Â°C
Scan Begin	50 m/z	Set End Plate Offset	-500 V	Set Dry Gas	4.0 l/min
Scan End	1900 m/z	Set Charging Voltage	0 V	Set Divert Valve	Source
		Set Corona	0 nA	Set APCI Heater	0 Â°C

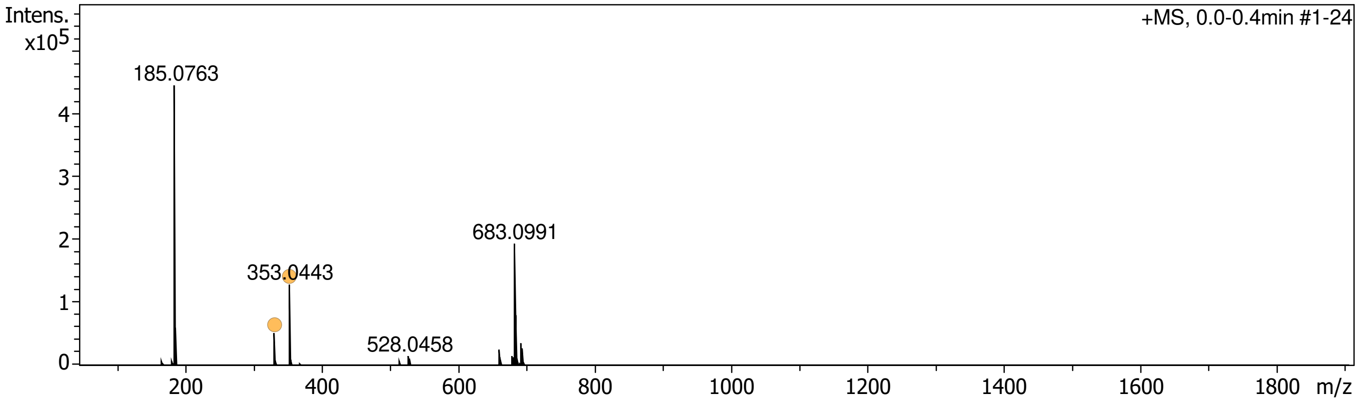

Meas. m/z	#	Ion Formula	m/z	err [ppm]	mSigma	# mSigma	Score	rdb	eÂ¯ Conf	N-Rule
331.0622	1	C12H15F4O4S	331.0622	-0.1	7.2	1	100.00	6.0	even	ok
	2	C13H11F4N4S	331.0635	4.0	8.0	2	50.91	11.0	even	ok
	3	C13H11N6O3S	331.0608	-4.3	10.0	3	46.18	14.0	even	ok
	4	C15H14F3O3S	331.0610	-3.5	10.0	4	53.63	10.0	even	ok
	5	C13H16F5S2	331.0608	-4.2	16.7	5	41.15	8.0	even	ok
	6	C17H15O5S	331.0635	3.9	21.2	6	40.04	13.0	even	ok
	7	C14H13F2O7	331.0624	0.6	24.6	7	49.14	8.0	even	ok
	8	C15H17F2O2S2	331.0633	3.2	24.7	8	42.17	11.0	even	ok
	9	C17H12FO6	331.0612	-2.9	30.1	9	29.65	12.0	even	ok
	10	C15H9F2N4O3	331.0637	4.6	30.9	10	20.38	13.0	even	ok
	11	C13H8F5N4O	331.0613	-2.8	31.7	11	29.12	10.0	even	ok
	12	C18H16FOS2	331.0621	-0.2	37.5	12	50.02	15.0	even	ok
	13	C18H8FN4O2	331.0626	1.2	41.5	13	29.40	17.0	even	ok
	14	C21H15S2	331.0610	-3.7	52.4	14	18.15	19.0	even	ok
	15	C21H7N4O	331.0614	-2.3	56.2	15	15.72	21.0	even	ok
353.0443	1	C15H13F3NaO3S	353.0430	-3.7	2.8	1	73.75	10.0	even	ok
	2	C13H10N6NaO3S	353.0427	-4.4	2.8	2	63.06	14.0	even	ok
	3	C13H10F4N4NaS	353.0455	3.3	8.4	3	72.72	11.0	even	ok
	4	C17H14NaO5S	353.0454	3.2	9.4	4	72.95	13.0	even	ok
	5	C12H14F4NaO4S	353.0441	-0.5	18.5	5	100.00	6.0	even	ok
	6	C13H15F5NaS2	353.0428	-4.3	19.4	6	46.87	8.0	even	ok
	7	C15H16F2NaO2S2	353.0452	2.6	21.0	7	65.56	11.0	even	ok
	8	C17H11FNaO6	353.0432	-3.1	23.0	8	42.60	12.0	even	ok
	9	C15H8F2N4NaO3	353.0457	3.9	26.7	9	32.65	13.0	even	ok
	10	C14H12F2NaO7	353.0443	0.1	27.2	10	64.81	8.0	even	ok
	11	C18H15FNaOS2	353.0441	-0.6	29.1	11	76.87	15.0	even	ok
	12	C18H7FN4NaO2	353.0445	0.7	31.2	12	54.31	17.0	even	ok
	13	C13H7F5N4NaO	353.0432	-3.0	33.4	13	33.99	10.0	even	ok
	14	C21H14NaS2	353.0429	-3.9	42.5	14	29.45	19.0	even	ok

Mass Spectrum SmartFormula Report

Meas. m/z	#	Ion Formula	m/z	err [ppm]	mSigma	# mSigma	Score	rdb	eÂ¯ Conf	N-Rule
	15	C21H6N4NaO	353.0434	-2.5	43.5	15	28.35	21.0	even	ok

Figure S5. HRESIMS spectrum of compound **5b**

Generic Display Report

Analysis Info

Analysis Name	D:\MZ\maXis_data\temp\60653000001.d	Acquisition Date	11/8/2018 1:01:54 PM
Method	tune_low_MS_Service_11_18.m	Operator	msc
Sample Name	CE-148-spot1-peak1	Instrument	maXis
Comment	Kalaba/Zehl<br>Ergebnis: +/- 5ppm<br>ACN/MeOH + 1%H2O		

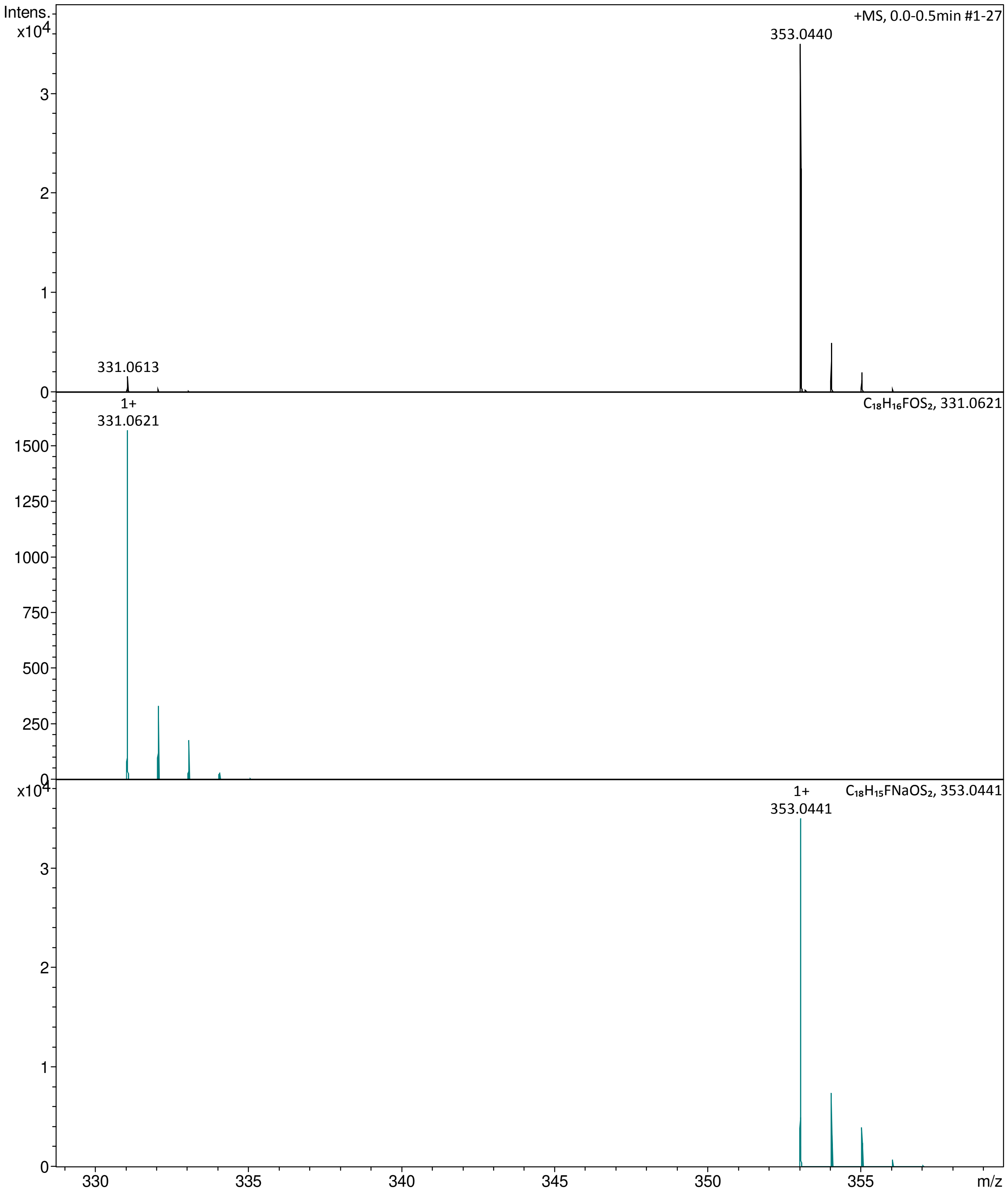

Mass Spectrum SmartFormula Report

Analysis Info

		Acquisition Date	11/8/2018 1:01:54 PM
Analysis Name	D:\MZ\maXis_data\temp\60653000001.d		
Method	tune_low_MS_Service_11_18.m	Operator	msc
Sample Name	CE-148-spot1-peak1	Instrument	maXis 255552.00016
Comment	Kalaba/Zehl<br>Ergebnis: +/- 5ppm<br>ACN/MeOH + 1%H2O		

Acquisition Parameter

Source Type	ESI	Ion Polarity	Positive	Set Nebulizer	0.4 Bar
Focus	Not active	Set Capillary	4500 V	Set Dry Heater	150 °C
Scan Begin	50 m/z	Set End Plate Offset	-500 V	Set Dry Gas	4.0 l/min
Scan End	1800 m/z	Set Charging Voltage	0 V	Set Divert Valve	Source
		Set Corona	0 nA	Set APCI Heater	0 °C

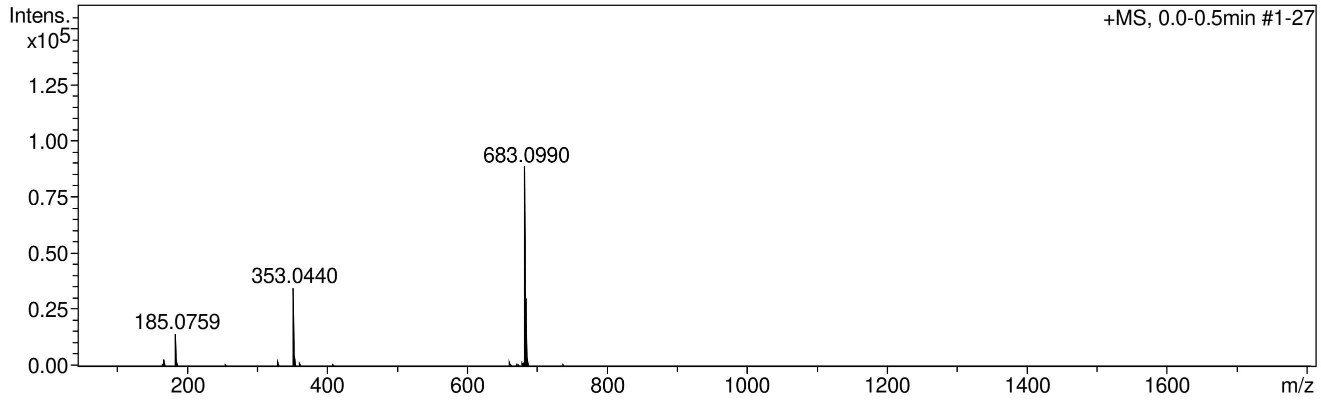

Meas. m/z	#	Ion Formula	Score	m/z	err [mDa]	err [ppm]	mSigma	rdb	e^- Conf	N-Rule
167.9670	1	C5H5NaOS2	100.00	167.9674	-0.4	-2.6	7.0	3.0	odd	ok
	2	C4HNaO6	61.02	167.9665	-0.4	-2.6	11.7	4.0	odd	ok
	3	C2H3F2NaO3S	60.44	167.9663	0.7	3.9	26.3	0.0	odd	ok
	4	CH2FNaO7	37.21	167.9677	-0.7	-4.2	28.6	0.0	odd	ok
185.0759	1	C13H10F	100.00	185.0761	0.2	1.3	14.7	8.5	even	ok
	2	C5H14FN2O2S	51.15	185.0755	-0.4	-2.2	39.8	-0.5	even	ok
331.0613	1	C13H11N6O3S	89.58	331.0608	-0.5	-1.4	11.9	11.5	even	ok
	2	C15H14F3O3S	100.00	331.0610	0.2	0.7	11.9	7.5	even	ok
	3	C18H13F2O2S	52.51	331.0599	1.4	4.2	13.5	11.5	even	ok
	4	C13H16F5S2	81.11	331.0608	0.5	1.4	17.5	3.5	even	ok
	5	C11H13F2N6S2	71.95	331.0606	-0.7	-2.1	17.5	7.5	even	ok
	6	C18H16FOS2	62.54	331.0621	0.8	2.6	20.4	10.5	even	ok
	7	C20H11O5	32.09	331.0601	1.2	3.5	24.0	15.5	even	ok
	8	C12H15F4O4S	55.04	331.0622	-0.9	-2.7	24.7	3.5	even	ok
	9	C17H12FO6	71.38	331.0612	-0.0	-0.1	31.5	11.5	even	ok
	10	C21H15S2	61.70	331.0610	-0.3	-0.9	32.7	14.5	even	ok
	11	C13H19N2O2S3	43.71	331.0603	0.9	2.9	33.3	5.5	even	ok
	12	C18H8FN4O2	32.13	331.0626	1.3	4.0	36.8	16.5	even	ok
	13	C14H13F2O7	35.59	331.0624	1.1	3.4	37.4	7.5	even	ok
	14	C21H7N4O	38.02	331.0614	0.2	0.5	37.8	20.5	even	ok
	15	C14H4FN10	29.68	331.0599	1.4	4.1	38.5	17.5	even	ok
	16	C16H7F4N4	34.39	331.0601	1.1	3.4	38.5	13.5	even	ok
	17	C11H5F2N10O	47.56	331.0610	0.2	0.7	43.5	13.5	even	ok
	18	C13H8F5N4O	51.95	331.0613	-0.0	-0.0	43.6	9.5	even	ok
	19	C12H12F5O5	23.32	331.0599	1.3	4.0	48.0	4.5	even	ok
353.0440	1	C12H14F4NaO4S	100.00	353.0441	-0.1	-0.2	2.5	3.5	even	ok
	2	C10H11FN6NaO4S	97.08	353.0439	-0.2	-0.4	2.6	7.5	even	ok
	3	C11H7FN10NaS	49.11	353.0452	1.2	3.3	11.3	12.5	even	ok
	4	C13H10F4N4NaS	42.21	353.0455	-1.4	-4.0	11.3	8.5	even	ok
	5	C9H15FN2NaO8S	38.91	353.0425	1.5	4.2	13.2	2.5	even	ok
	6	C13H10N6NaO3S	41.77	353.0427	1.3	3.7	15.9	11.5	even	ok
	7	C15H13F3NaO3S	48.28	353.0430	-1.1	-3.0	16.0	7.5	even	ok
	8	C9H15F5NaO5S	43.04	353.0453	1.2	3.5	16.6	-0.5	even	ok
	9	C7H12F2N6NaO5S	49.59	353.0450	1.0	2.8	16.6	3.5	even	ok
	10	C14H12F2NaO7	48.92	353.0443	-0.3	-0.9	20.1	7.5	even	ok

Mass Spectrum SmartFormula Report

Meas. m/z	#	Ion Formula	Score	m/z	err [mDa]	err [ppm]	mSigma	rdb	e⁻ Conf	N-Rule
	11	C8H13F3N6NaOS2	60.64	353.0437	0.4	1.1	22.0	3.5	even	ok
	12	C9H10N6NaO8	28.97	353.0452	-1.2	-3.4	22.4	7.5	even	ok
	13	C11H13F3NaO8	24.88	353.0455	1.4	4.1	22.4	3.5	even	ok
	14	C13H15F5NaS2	36.24	353.0428	-1.3	-3.6	23.3	3.5	even	ok
	15	C11H12F2N6NaS2	31.02	353.0425	-1.5	-4.3	23.4	7.5	even	ok
	16	C9H18N2NaO7S2	47.35	353.0448	-0.7	-2.1	25.1	1.5	even	ok
	17	C11H4F2N10NaO	28.75	353.0430	1.0	3.0	26.8	13.5	even	ok
	18	C13H7F5N4NaO	32.84	353.0432	-0.8	-2.3	26.9	9.5	even	ok
	19	C17H14NaO5S	30.37	353.0454	1.4	3.9	27.9	10.5	even	ok
	20	C8H5F3N10NaO2	44.15	353.0441	0.1	0.3	28.6	9.5	even	ok
	21	C15H16F2NaO2S2	33.81	353.0452	-1.2	-3.3	29.0	6.5	even	ok
	22	C8H14N2NaO12	42.89	353.0439	-0.1	-0.4	29.2	2.5	even	ok
	23	C15H8F2N4NaO3	18.27	353.0457	1.6	4.6	30.1	12.5	even	ok
	24	C17H11FNaO6	29.05	353.0432	0.8	2.4	31.2	11.5	even	ok
	25	C7H9F3N6NaO6	21.91	353.0428	1.2	3.5	33.3	4.5	even	ok
	26	C18H15FNaOS2	44.54	353.0441	0.0	0.1	40.5	10.5	even	ok
	27	C10H19FN2NaO3S3	33.02	353.0434	0.6	1.8	41.6	1.5	even	ok
	28	C18H7FN4NaO2	24.56	353.0445	0.5	1.4	44.1	16.5	even	ok
	29	C21H14NaS2	17.60	353.0429	-1.1	-3.2	53.7	14.5	even	ok
	30	C21H6N4NaO	13.67	353.0434	-0.6	-1.8	60.5	20.5	even	ok

Figure S6. HRESIMS spectrum of compound **6b**

Generic Display Report

Analysis Info

Analysis Name	D:\MZ\maXis_data\temp\60654000001.d	Acquisition Date	11/8/2018 1:00:26 PM
Method	tune_low_MS_Service_11_18.m	Operator	msc
Sample Name	CE-148-spot1-peak2	Instrument	maXis
Comment	Kalaba/Zehl<br>Ergebnis: +/- 5ppm<br>ACN/MeOH + 1%H2O		

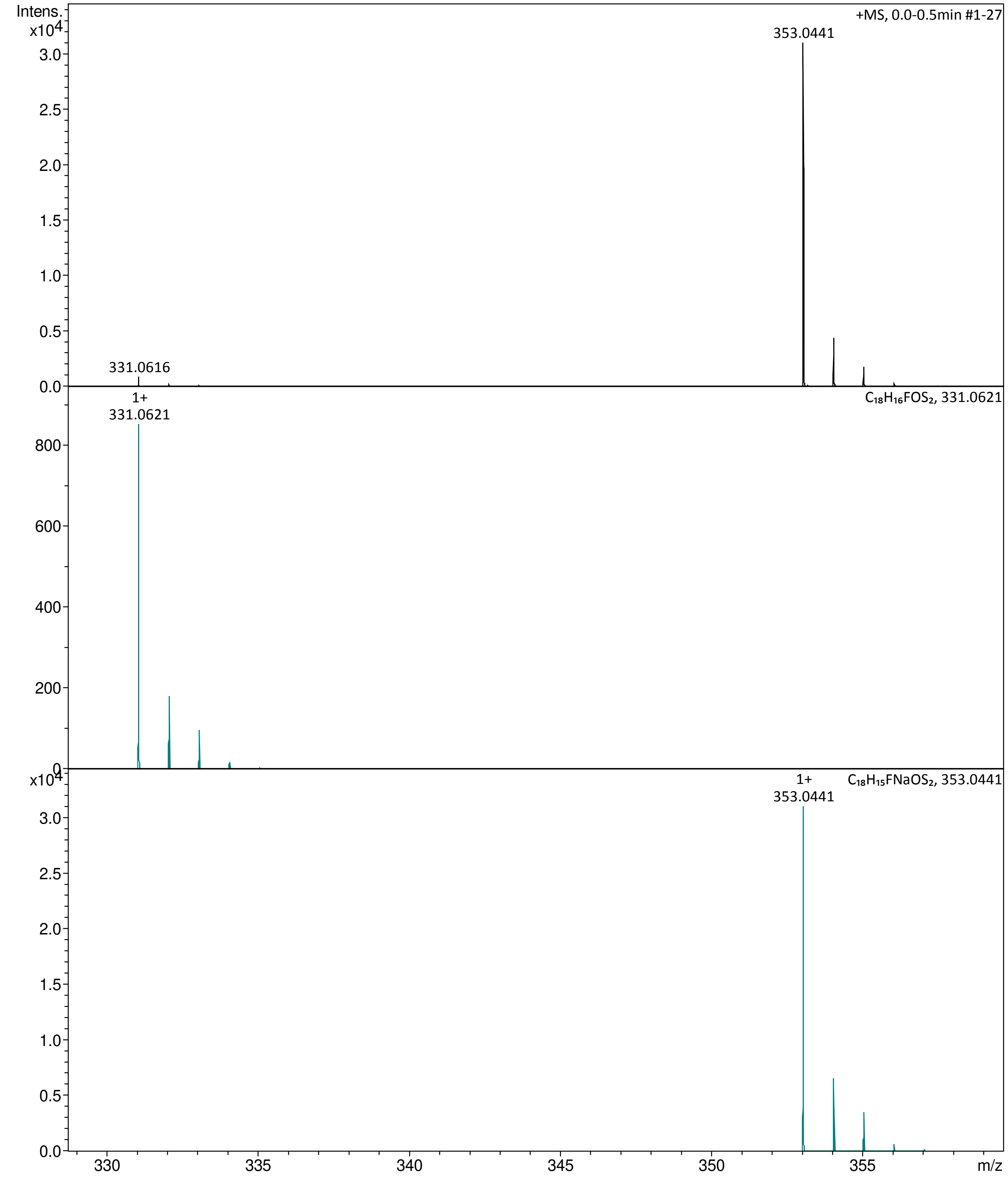

Mass Spectrum SmartFormula Report

Analysis Info

Analysis Name	D:\MZ\maXis_data\temp\60654000001.d	Acquisition Date	11/8/2018 1:00:26 PM
Method	tune_low_MS_Service_11_18.m	Operator	msc
Sample Name	CE-148-spot1-peak2	Instrument	maXis 255552.00016
Comment	Kalaba/Zehl<br>Ergebnis: +/- 5ppm<br>ACN/MeOH + 1%H2O		

Acquisition Parameter

Source Type	ESI	Ion Polarity	Positive	Set Nebulizer	0.4 Bar
Focus	Not active	Set Capillary	4500 V	Set Dry Heater	150 °C
Scan Begin	50 m/z	Set End Plate Offset	-500 V	Set Dry Gas	4.0 l/min
Scan End	1800 m/z	Set Charging Voltage	0 V	Set Divert Valve	Source
		Set Corona	0 nA	Set APCI Heater	0 °C

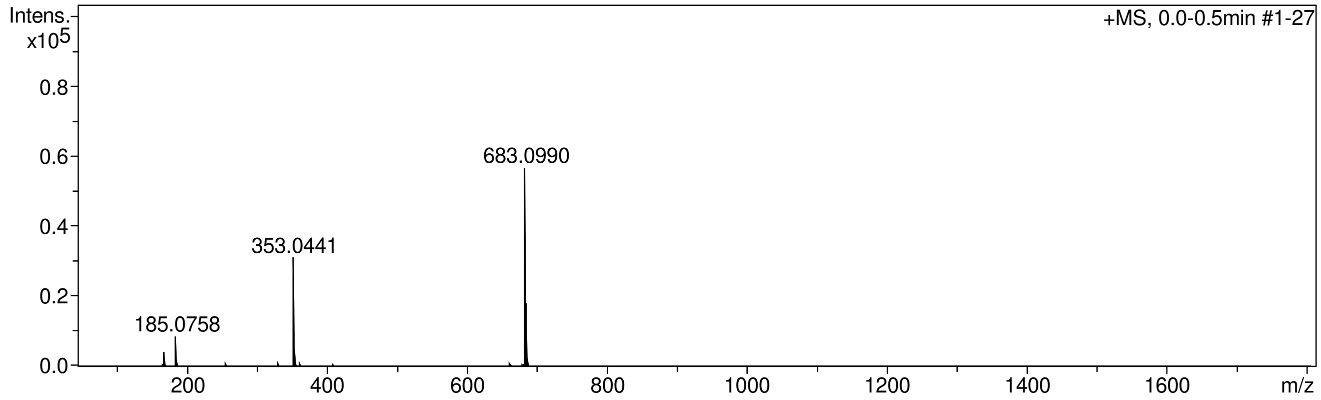

Meas. m/z	#	Ion Formula	Score	m/z	err [mDa]	err [ppm]	mSigma	rdb	e⁻ Conf	N-Rule
167.9671	1	C5H5NaOS2	100.00	167.9674	-0.3	-2.0	6.5	3.0	odd	ok
	2	C4HNaO6	55.46	167.9665	0.5	3.2	11.2	4.0	odd	ok
	3	C2H3F2NaO3S	52.43	167.9663	0.8	4.5	27.8	0.0	odd	ok
	4	CH2FNaO7	37.61	167.9677	0.6	3.6	27.9	0.0	odd	ok
185.0758	1	C13H10F	100.00	185.0761	-0.3	-1.7	10.5	8.5	even	ok
331.0616	1	C18H16FOS2	100.00	331.0621	0.5	1.6	17.0	10.5	even	ok
	2	C15H14F3O3S	93.65	331.0610	0.6	1.7	19.3	7.5	even	ok
	3	C13H11N6O3S	82.74	331.0608	-0.8	-2.4	19.3	11.5	even	ok
	4	C20H11O5	48.17	331.0601	-1.5	-4.5	25.2	15.5	even	ok
	5	C21H15S2	78.52	331.0610	-0.6	-1.9	25.9	14.5	even	ok
	6	C13H16F5S2	70.78	331.0608	-0.8	-2.4	26.9	3.5	even	ok
	7	C17H12FO6	76.70	331.0612	-0.3	-1.0	32.4	11.5	even	ok
	8	C18H8FN4O2	54.65	331.0626	-1.0	-3.0	32.7	16.5	even	ok
	9	C21H7N4O	75.24	331.0614	-0.2	-0.5	36.4	20.5	even	ok
	10	C13H19N2O2S3	41.88	331.0603	-1.3	-3.8	36.6	5.5	even	ok
	11	C16H7F4N4	35.09	331.0601	-1.5	-4.4	38.9	13.5	even	ok
	12	C14H13F2O7	45.36	331.0624	0.8	2.4	43.6	7.5	even	ok
	13	C13H8F5N4O	49.63	331.0613	-0.3	-0.9	48.8	9.5	even	ok
353.0441	1	C12H14F4NaO4S	100.00	353.0441	0.0	0.1	2.7	3.5	even	ok
	2	C10H11FN6NaO4S	92.93	353.0439	-0.2	-0.6	2.8	7.5	even	ok
	3	C11H7FN10NaS	49.43	353.0452	-1.1	-3.2	11.7	12.5	even	ok
	4	C13H10F4N4NaS	42.58	353.0455	1.4	3.9	11.7	8.5	even	ok
	5	C13H10N6NaO3S	39.42	353.0427	-1.4	-3.8	16.3	11.5	even	ok
	6	C15H13F3NaO3S	45.67	353.0430	1.1	3.1	16.3	7.5	even	ok
	7	C14H12F2NaO7	49.08	353.0443	0.2	0.7	20.2	7.5	even	ok
	8	C11H13F3NaO8	25.41	353.0455	-1.4	-3.9	22.2	3.5	even	ok
	9	C13H15F5NaS2	34.34	353.0428	-1.3	-3.8	23.5	3.5	even	ok
	10	C11H12F2N6NaS2	29.32	353.0425	1.6	4.4	23.5	7.5	even	ok
	11	C11H4F2N10NaO	27.35	353.0430	-1.1	-3.1	26.9	13.5	even	ok
	12	C13H7F5N4NaO	31.31	353.0432	0.9	2.4	27.0	9.5	even	ok
	13	C17H14NaO5S	30.61	353.0454	-1.3	-3.8	28.2	10.5	even	ok
	14	C15H16F2NaO2S2	34.11	353.0452	1.1	3.2	29.2	6.5	even	ok
	15	C15H8F2N4NaO3	18.50	353.0457	-1.6	-4.5	30.3	12.5	even	ok
	16	C17H11FNaO6	27.53	353.0432	-0.9	-2.5	31.5	11.5	even	ok
	17	C18H15FNaOS2	43.55	353.0441	0.0	0.1	40.8	10.5	even	ok

Mass Spectrum SmartFormula Report

Meas. m/z	#	Ion Formula	Score	m/z	err [mDa]	err [ppm]	mSigma	rdb	e⁻ Conf	N-Rule
	18	C10H19FN2NaO3S3	31.57	353.0434	-0.7	-1.9	41.6	1.5	even	ok
	19	C18H7FN4NaO2	24.52	353.0445	-0.4	-1.3	44.4	16.5	even	ok
	20	C21H14NaS2	16.63	353.0429	1.2	3.3	54.0	14.5	even	ok
	21	C21H6N4NaO	12.92	353.0434	-0.7	-2.0	60.8	20.5	even	ok

Figure S7. HRESIMS spectrum of compound **7b**

Generic Display Report

Analysis Info

Analysis Name	D:\MZ\maXis_data\temp\60655000001.d
Method	tune_low_MS_Service_11_18.m
Sample Name	CE-148-spot2-peak1
Comment	Kalaba/Zehl<br>Ergebnis: +/- 5ppm<br>ACN/MeOH + 1%H2O
Acquisition Date	11/8/2018 1:11:11 PM
Operator	msc
Instrument	maXis

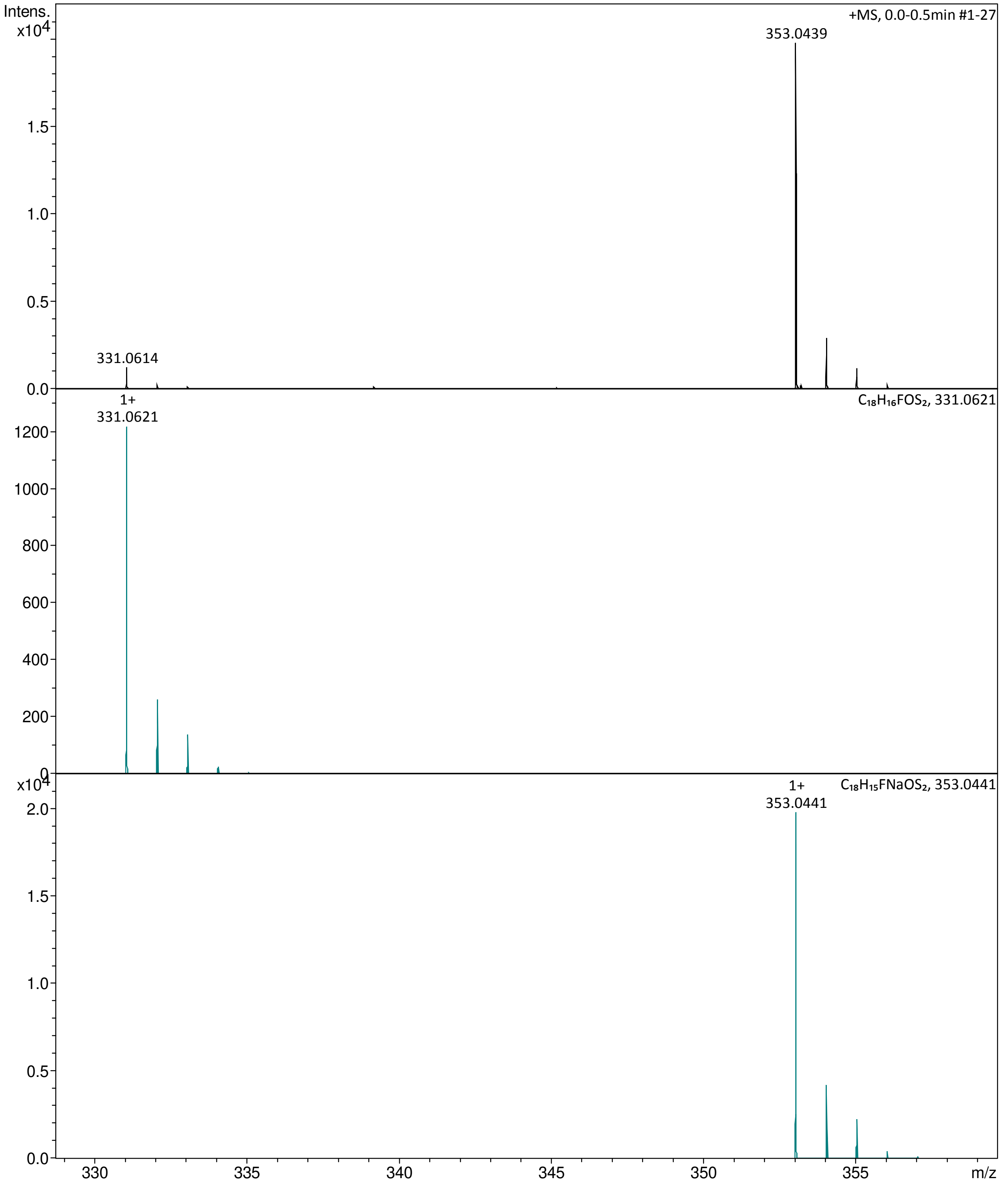

Mass Spectrum SmartFormula Report

Analysis Info

Analysis Name	D:\MZ\maXis_data\temp\60655000001.d	Acquisition Date	11/8/2018 1:11:11 PM
Method	tune_low_MS_Service_11_18.m	Operator	msc
Sample Name	CE-148-spot2-peak1	Instrument	maXis 255552.00016
Comment	Kalaba/Zehl<br>Ergebnis: +/- 5ppm<br>ACN/MeOH + 1%H2O		

Acquisition Parameter

Source Type	ESI	Ion Polarity	Positive	Set Nebulizer	0.4 Bar
Focus	Not active	Set Capillary	4500 V	Set Dry Heater	150 °C
Scan Begin	50 m/z	Set End Plate Offset	-500 V	Set Dry Gas	4.0 l/min
Scan End	1800 m/z	Set Charging Voltage	0 V	Set Divert Valve	Source
		Set Corona	0 nA	Set APCI Heater	0 °C

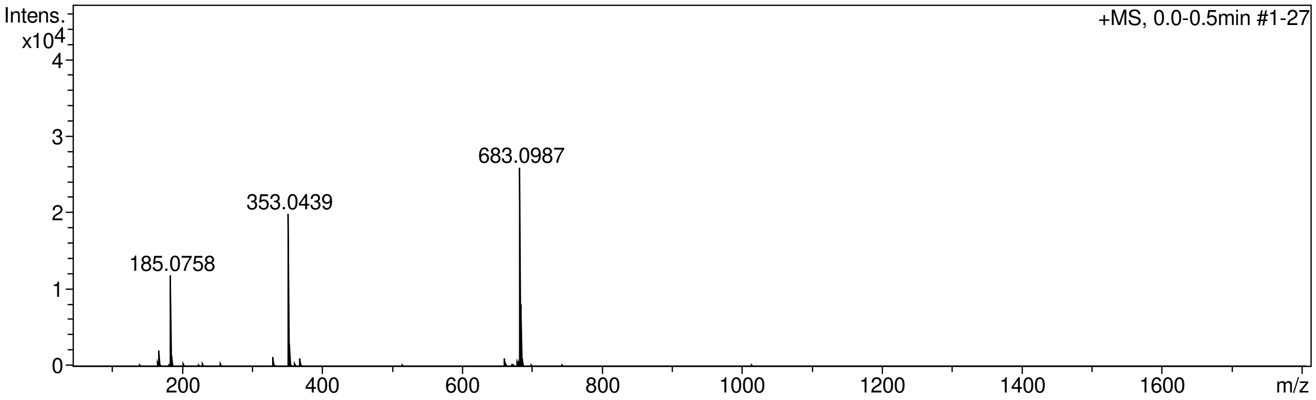

Meas. m/z	#	Ion Formula	Score	m/z	err [mDa]	err [ppm]	mSigma	rdb	e⁻ Conf	N-Rule
167.9669	1	C3H3N3NaS2	92.03	167.9661	0.8	4.8	2.6	3.5	even	ok
	2	C5H5NaOS2	100.00	167.9674	0.5	3.2	5.6	3.0	odd	ok
	3	C4HNaO6	65.73	167.9665	-0.3	-2.0	11.8	4.0	odd	ok
	4	CH2FNaO7	35.97	167.9677	0.8	4.8	28.7	0.0	odd	ok
	5	C2H3F2NaO3S	60.83	167.9663	-0.5	-3.2	29.5	0.0	odd	ok
185.0758	1	C13H10F	100.00	185.0761	-0.3	-1.5	13.9	8.5	even	ok
	2	C5H14FN2O2S	34.95	185.0755	0.4	2.0	39.8	-0.5	even	ok
331.0614	1	C16H7F4N4	48.75	331.0601	1.2	3.7	12.2	13.5	even	ok
	2	C18H13F2O2S	61.31	331.0599	1.5	4.5	13.0	11.5	even	ok
	3	C18H16FOS2	92.57	331.0621	-0.7	-2.3	14.1	10.5	even	ok
	4	C18H8FN4O2	45.90	331.0626	-1.2	-3.7	15.7	16.5	even	ok
	5	C17H12FO6	75.86	331.0612	0.1	0.4	18.5	11.5	even	ok
	6	C20H11O5	41.50	331.0601	-1.3	-3.8	19.1	15.5	even	ok
	7	C13H11N6O3S	89.20	331.0608	-0.6	-1.8	20.1	11.5	even	ok
	8	C15H14F3O3S	100.00	331.0610	0.3	1.0	20.1	7.5	even	ok
	9	C13H16F5S2	84.68	331.0608	-0.6	-1.7	23.0	3.5	even	ok
	10	C21H15S2	87.27	331.0610	0.4	1.2	25.1	14.5	even	ok
	11	C21H7N4O	59.96	331.0614	-0.1	-0.2	29.8	20.5	even	ok
	12	C14H13F2O7	37.28	331.0624	-1.0	-3.1	30.2	7.5	even	ok
	13	C13H19N2O2S3	53.01	331.0603	-1.0	-3.2	31.7	5.5	even	ok
	14	C12H15F4O4S	57.63	331.0622	0.8	2.4	33.8	3.5	even	ok
	15	C12H12F5O5	22.17	331.0599	-1.4	-4.3	40.7	4.5	even	ok
	16	C14H4FN10	29.42	331.0599	-1.5	-4.4	44.0	17.5	even	ok
	17	C13H8F5N4O	48.19	331.0613	-0.1	-0.3	52.1	9.5	even	ok
353.0439	1	C12H14F4NaO4S	93.39	353.0441	0.2	0.6	4.1	3.5	even	ok
	2	C10H11FN6NaO4S	100.00	353.0439	-0.0	-0.1	4.1	7.5	even	ok
	3	C11H7FN10NaS	48.56	353.0452	-1.3	-3.7	8.4	12.5	even	ok
	4	C13H10F4N4NaS	41.51	353.0455	1.5	4.4	8.4	8.5	even	ok
	5	C13H10N6NaO3S	48.34	353.0427	-1.2	-3.3	12.5	11.5	even	ok
	6	C15H13F3NaO3S	55.59	353.0430	0.9	2.7	12.6	7.5	even	ok
	7	C14H12F2NaO7	46.13	353.0443	0.4	1.2	20.7	7.5	even	ok
	8	C13H15F5NaS2	41.36	353.0428	-1.2	-3.3	20.9	3.5	even	ok
	9	C11H12F2N6NaS2	35.59	353.0425	1.4	4.0	20.9	7.5	even	ok
	10	C17H14NaO5S	30.71	353.0454	-1.5	-4.3	24.4	10.5	even	ok
	11	C11H13F3NaO8	21.47	353.0455	-1.6	-4.4	25.8	3.5	even	ok

Mass Spectrum SmartFormula Report

Meas. m/z	#	Ion Formula	Score	m/z	err [mDa]	err [ppm]	mSigma	rdb	e⁻ Conf	N-Rule
	12	C15H16F2NaO2S2	42.37	353.0452	1.3	3.6	26.2	6.5	even	ok
	13	C11H4F2N10NaO	30.37	353.0430	-0.9	-2.6	27.7	13.5	even	ok
	14	C13H7F5N4NaO	34.52	353.0432	0.7	1.9	27.7	9.5	even	ok
	15	C15H8F2N4NaO3	17.42	353.0457	-1.8	-5.0	28.9	12.5	even	ok
	16	C17H11FNaO6	32.67	353.0432	-0.7	-2.1	29.2	11.5	even	ok
	17	C18H15FNaOS2	58.17	353.0441	-0.1	-0.4	37.4	10.5	even	ok
	18	C10H19FN2NaO3S3	45.78	353.0434	-0.5	-1.4	40.2	1.5	even	ok
	19	C18H7FN4NaO2	25.04	353.0445	-0.6	-1.7	41.6	16.5	even	ok
	20	C13H18N2NaO2S3	21.52	353.0423	1.6	4.7	43.3	5.5	even	ok
	21	C21H14NaS2	26.21	353.0429	1.0	2.8	50.5	14.5	even	ok
	22	C21H6N4NaO	16.15	353.0434	-0.5	-1.5	57.5	20.5	even	ok

Figure S8. HRESIMS spectrum of compound **8b**

Generic Display Report

Analysis Info

Analysis Name	D:\MZ\maXis_data\temp\60656000001.d	Acquisition Date	11/8/2018 1:13:17 PM
Method	tune_low_MS_Service_11_18.m	Operator	msc
Sample Name	CE-148-spot2-peak2	Instrument	maXis
Comment	Kalaba/Zehl<br>Ergebnis: +/- 5ppm<br>ACN/MeOH + 1%H2O		

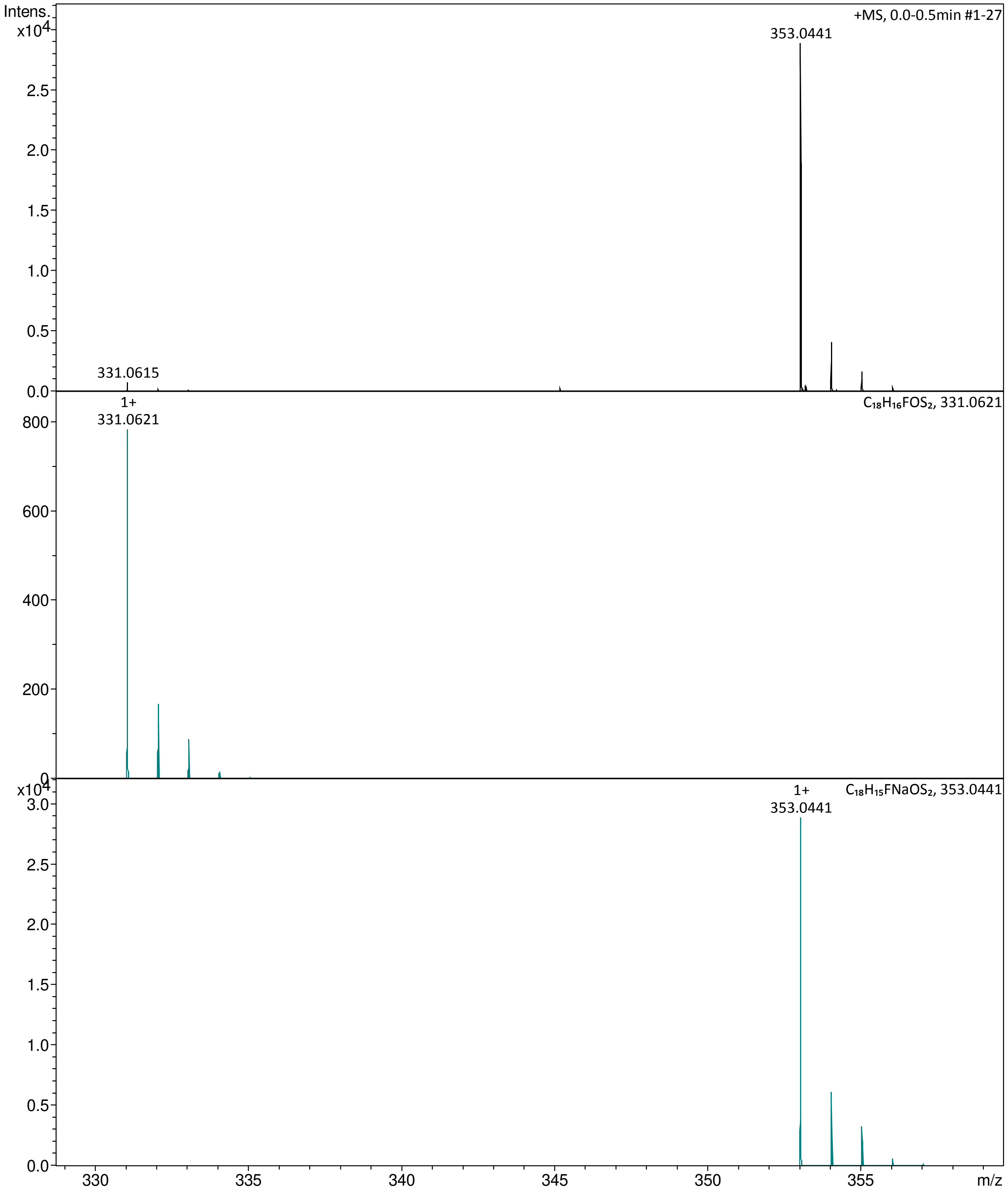

Mass Spectrum SmartFormula Report

Analysis Info

Analysis Name	D:\MZ\maXis_data\temp\60656000001.d	Acquisition Date	11/8/2018 1:13:17 PM
Method	tune_low_MS_Service_11_18.m	Operator	msc
Sample Name	CE-148-spot2-peak2	Instrument	maXis 255552.00016
Comment	Kalaba/Zehl<br>Ergebnis: +/- 5ppm<br>ACN/MeOH + 1%H2O		

Acquisition Parameter

Source Type	ESI	Ion Polarity	Positive	Set Nebulizer	0.4 Bar
Focus	Not active	Set Capillary	4500 V	Set Dry Heater	150 °C
Scan Begin	50 m/z	Set End Plate Offset	-500 V	Set Dry Gas	4.0 l/min
Scan End	1800 m/z	Set Charging Voltage	0 V	Set Divert Valve	Source
		Set Corona	0 nA	Set APCI Heater	0 °C

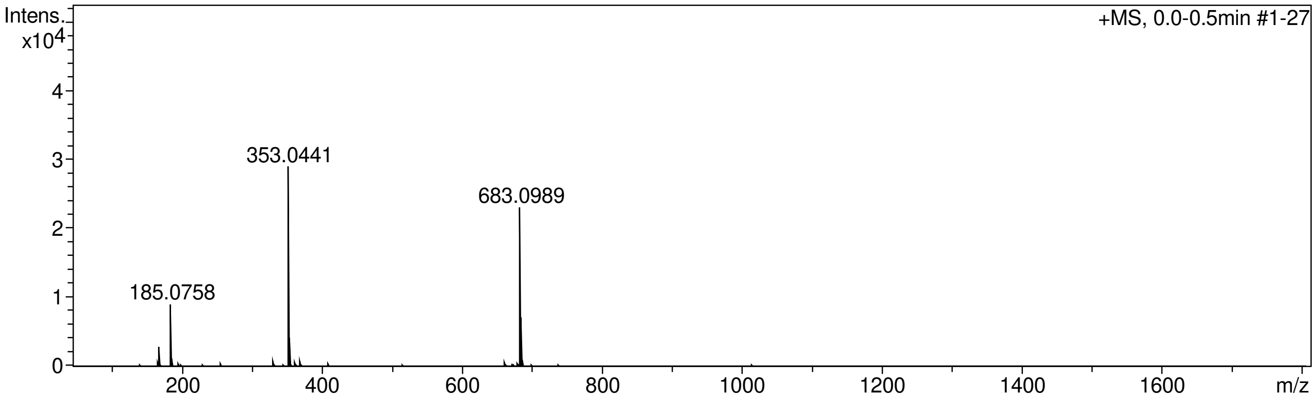

Meas. m/z	#	Ion Formula	Score	m/z	err [mDa]	err [ppm]	mSigma	rdb	e⁻ Conf	N-Rule
167.9670	1	C5H5NaOS2	100.00	167.9674	-0.4	-2.5	7.3	3.0	odd	ok
	2	C4HNaO6	62.70	167.9665	-0.4	-2.6	10.2	4.0	odd	ok
	3	CH2FNaO7	39.18	167.9677	0.7	4.2	26.7	0.0	odd	ok
	4	C2H3F2NaO3S	58.52	167.9663	-0.7	-3.9	27.7	0.0	odd	ok
185.0758	1	C13H10F	100.00	185.0761	-0.3	-1.8	12.1	8.5	even	ok
331.0615	1	C18H13F2O2S	56.57	331.0599	-1.6	-4.8	10.9	11.5	even	ok
	2	C15H14F3O3S	100.00	331.0610	0.5	1.4	15.5	7.5	even	ok
	3	C13H11N6O3S	88.76	331.0608	-0.7	-2.1	15.5	11.5	even	ok
	4	C18H16FOS2	88.20	331.0621	-0.6	-1.9	17.6	10.5	even	ok
	5	C13H16F5S2	79.65	331.0608	-0.7	-2.0	21.3	3.5	even	ok
	6	C20H11O5	44.73	331.0601	1.4	4.2	28.6	15.5	even	ok
	7	C21H15S2	72.52	331.0610	-0.5	-1.6	28.8	14.5	even	ok
	8	C12H15F4O4S	65.59	331.0622	0.7	2.1	29.5	3.5	even	ok
	9	C17H12FO6	75.20	331.0612	0.2	0.7	32.5	11.5	even	ok
	10	C13H19N2O2S3	45.19	331.0603	1.2	3.5	33.7	5.5	even	ok
	11	C18H8FN4O2	44.91	331.0626	1.1	3.3	35.6	16.5	even	ok
	12	C14H4FN10	29.69	331.0599	1.6	4.8	39.4	17.5	even	ok
	13	C16H7F4N4	34.76	331.0601	-1.3	-4.1	39.4	13.5	even	ok
	14	C14H13F2O7	43.42	331.0624	0.9	2.7	41.0	7.5	even	ok
	15	C21H7N4O	63.62	331.0614	-0.0	-0.1	41.9	20.5	even	ok
	16	C13H8F5N4O	51.88	331.0613	0.2	0.6	46.7	9.5	even	ok
	17	C12H12F5O5	20.98	331.0599	1.5	4.7	52.3	4.5	even	ok
353.0441	1	C12H14F4NaO4S	100.00	353.0441	-0.0	-0.1	3.2	3.5	even	ok
	2	C10H11FN6NaO4S	90.30	353.0439	-0.3	-0.7	3.2	7.5	even	ok
	3	C11H7FN10NaS	51.82	353.0452	-1.1	-3.1	10.9	12.5	even	ok
	4	C13H10F4N4NaS	44.75	353.0455	1.3	3.7	10.9	8.5	even	ok
	5	C13H10N6NaO3S	38.76	353.0427	-1.4	-4.0	15.5	11.5	even	ok
	6	C15H13F3NaO3S	45.01	353.0430	1.2	3.3	15.6	7.5	even	ok
	7	C14H12F2NaO7	51.05	353.0443	0.2	0.6	19.5	7.5	even	ok
	8	C11H13F3NaO8	26.22	353.0455	-1.3	-3.8	22.3	3.5	even	ok
	9	C13H15F5NaS2	33.27	353.0428	1.4	3.9	23.5	3.5	even	ok
	10	C11H12F2N6NaS2	28.34	353.0425	1.6	4.6	23.5	7.5	even	ok
	11	C11H4F2N10NaO	26.98	353.0430	-1.1	-3.3	26.2	13.5	even	ok
	12	C13H7F5N4NaO	30.96	353.0432	-0.9	-2.6	26.3	9.5	even	ok
	13	C17H14NaO5S	32.19	353.0454	-1.3	-3.6	27.5	10.5	even	ok

Mass Spectrum SmartFormula Report

Meas. m/z	#	Ion Formula	Score	m/z	err [mDa]	err [ppm]	mSigma	rdb	e⁻ Conf	N-Rule
	14	C15H16F2NaO2S2	44.29	353.0452	1.1	3.0	29.0	6.5	even	ok
	15	C15H8F2N4NaO3	19.63	353.0457	-1.5	-4.3	29.3	12.5	even	ok
	16	C17H11FNaO6	27.48	353.0432	0.9	2.7	30.5	11.5	even	ok
	17	C18H15FNaOS2	54.03	353.0441	0.1	0.2	40.3	10.5	even	ok
	18	C10H19FN2NaO3S3	38.24	353.0434	0.7	2.1	41.8	1.5	even	ok
	19	C18H7FN4NaO2	25.90	353.0445	0.4	1.1	43.4	16.5	even	ok
	20	C21H14NaS2	20.51	353.0429	1.2	3.5	53.4	14.5	even	ok
	21	C21H6N4NaO	8.53	353.0434	-0.8	-2.1	60.4	20.5	even	ok

Figure S9. HRESIMS spectrum of compound **5c**

Generic Display Report

Analysis Info

Analysis Name	E:\Data\MS_MessService\58959000001.d	Acquisition Date	8/14/2018 6:04:02 PM
Method	tune_low_MS_Service_08_18.m	Operator	msc
Sample Name	CE-155-SPOT1-PEAK1	Instrument	maXis
Comment	Kalaba/Zehl<br>Ergebnis: +/- 5ppm<br>ACN/MeOH +0.1% H2O		

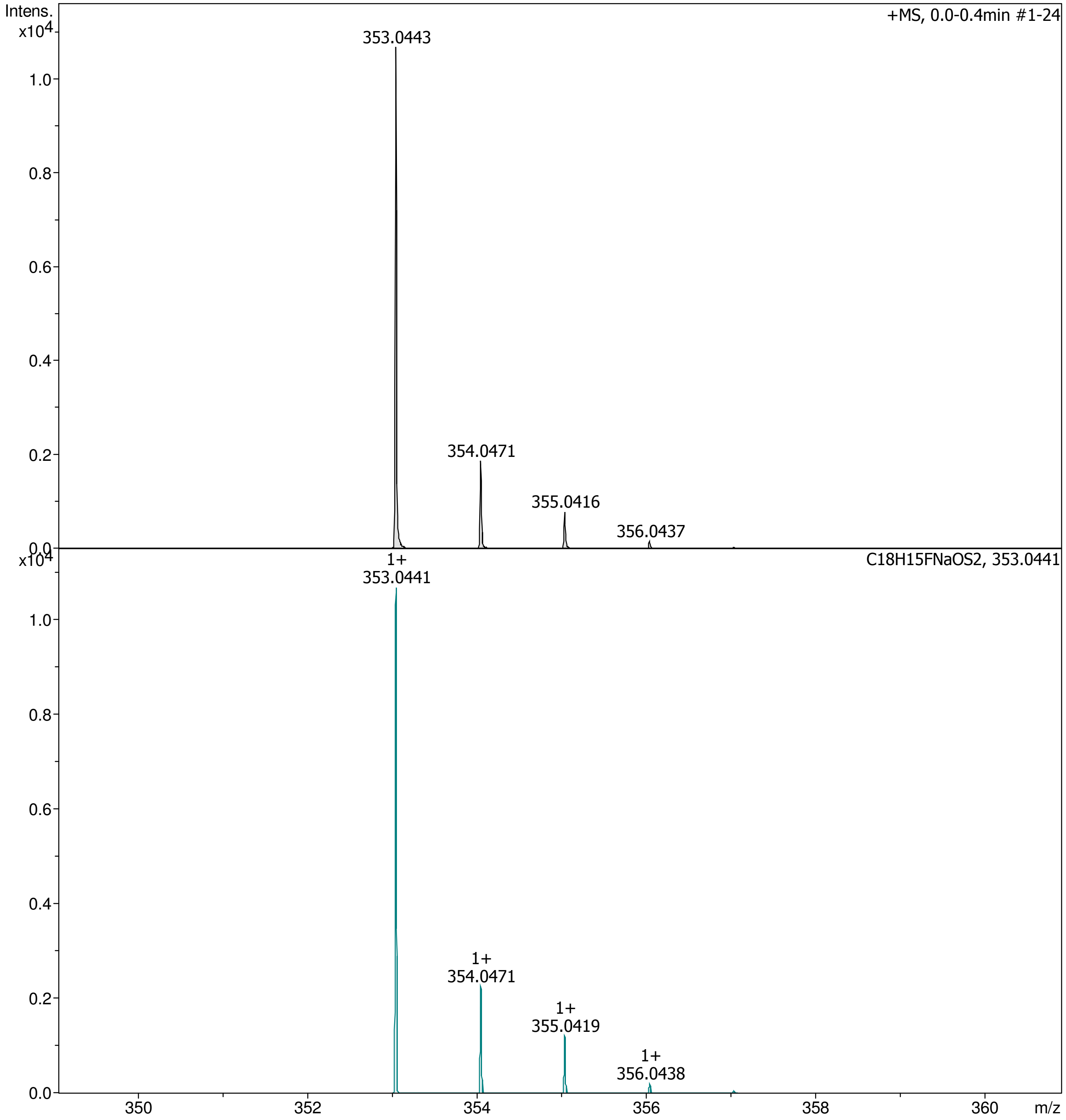

Mass Spectrum SmartFormula Report

Analysis Info

Analysis Name	E:\Data\MS_MessService\58959000001.d	Acquisition Date	8/14/2018 6:04:02 PM
Method	tune_low_MS_Service_08_18.m	Operator	msc
Sample Name	CE-155-SPOT1-PEAK1	Instrument	maXis 255552.00016
Comment	Kalaba/Zehl<br>Ergebnis: +/- 5ppm<br>ACN/MeOH +0.1% H2O		

Acquisition Parameter

Source Type	ESI	Ion Polarity	Positive	Set Nebulizer	0.4 Bar
Focus	Not active	Set Capillary	4500 V	Set Dry Heater	180 °C
Scan Begin	50 m/z	Set End Plate Offset	-500 V	Set Dry Gas	4.0 l/min
Scan End	1900 m/z	Set Charging Voltage	0 V	Set Divert Valve	Source
		Set Corona	0 nA	Set APCI Heater	0 °C

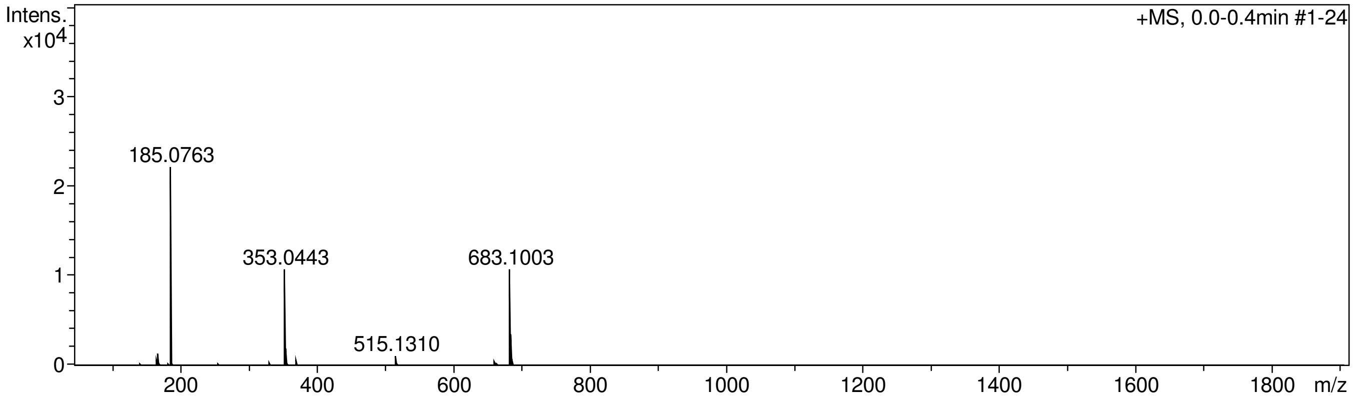

Meas. m/z	#	Ion Formula	Score	m/z	err [mDa]	err [ppm]	mSigma	rdb	e⁻ Conf	N-Rule
353.044287	1	C13H10N6NaO3S	47.99	353.042730	-1.6	-4.4	5.1	11.5	even	ok
	2	C15H13F3NaO3S	56.12	353.042971	1.3	3.7	5.2	7.5	even	ok
	3	C15H12N3NaO4S	100.00	353.044073	-0.2	-0.6	5.5	11.0	odd	ok
	4	C11H7FN10NaS	64.73	353.045210	-0.9	-2.6	10.2	12.5	even	ok
	5	C13H10F4N4NaS	56.30	353.045451	1.2	3.3	10.2	8.5	even	ok
	6	C17H14NaO5S	57.43	353.045415	1.1	3.2	10.3	10.5	even	ok
	7	C12H13FN3NaO5S	61.82	353.045215	-0.9	-2.6	12.5	7.0	odd	ok
	8	C13H14F2N3NaOS2	76.61	353.043856	0.4	1.2	14.6	7.0	odd	ok
	9	C15H16F2NaO2S2	57.81	353.045199	-0.9	-2.6	16.3	6.5	even	ok
	10	C13H15F5NaS2	39.51	353.042754	-1.5	-4.3	16.3	3.5	even	ok
	11	C16H8N7NaS	50.66	353.045410	1.1	3.2	16.9	16.0	odd	ok
	12	C18H11F3NNaS	43.65	353.045651	1.4	3.9	16.9	12.0	odd	ok
	13	C12H14F4NaO4S	79.60	353.044114	-0.2	-0.5	18.3	3.5	even	ok
	14	C10H11FN6NaO4S	71.46	353.043873	-0.4	-1.2	18.4	7.5	even	ok
	15	C16H13FN3NaS2	35.68	353.042713	1.6	4.5	19.9	11.0	odd	ok
	16	C10H15F3N3NaO2S2	54.81	353.044999	-0.7	-2.0	23.8	3.0	odd	ok
	17	C10H12F4N3NaO3S	33.73	353.042771	1.5	4.3	24.2	4.0	odd	ok
	18	C18H15FNaOS2	67.62	353.044056	0.2	0.7	24.7	10.5	even	ok
	19	C17H11FNaO6	31.15	353.043187	-1.1	-3.1	26.6	11.5	even	ok
	20	C10H16F6NaOS2	58.78	353.043897	0.4	1.1	27.6	-0.5	even	ok
	21	C10H12F8NNaS	36.42	353.045492	1.2	3.4	29.5	1.0	odd	ok
	22	C14H12F2NaO7	48.84	353.044330	0.0	0.1	29.7	7.5	even	ok
	23	C15H8F2N4NaO3	24.12	353.045667	-1.4	-3.9	30.1	12.5	even	ok
	24	C13H6F2N7NaO2	45.63	353.044325	0.0	0.1	32.5	13.0	odd	ok
	25	C15H9F5NNaO2	41.16	353.044565	-0.3	-0.8	32.5	9.0	odd	ok
	26	C16H5FN7NaO	26.35	353.043182	-1.1	-3.1	33.3	17.0	odd	ok
	27	C18H8F4NNaO	30.18	353.043422	0.9	2.4	33.4	13.0	odd	ok
	28	C12H10F2N3NaO6	22.72	353.042987	-1.3	-3.7	34.5	8.0	odd	ok
	29	C18H7FN4NaO2	39.83	353.044524	-0.2	-0.7	34.6	16.5	even	ok

Mass Spectrum SmartFormula Report

Meas. m/z	#	Ion Formula	Score	m/z	err [mDa]	err [ppm]	mSigma	rdb	e⁻ Conf	N-Rule
	30	C11H4F2N10NaO	21.74	353.042982	-1.3	-3.7	36.1	13.5	even	ok
	31	C13H7F5N4NaO	25.13	353.043223	1.1	3.0	36.1	9.5	even	ok
	32	C20H9FNNaO3	17.71	353.045867	-1.6	-4.5	37.0	16.0	odd	ok
	33	C21H14NaS2	26.88	353.042913	1.4	3.9	37.3	14.5	even	ok
	34	C10H19FN2NaO3S3	35.80	353.043404	-0.9	-2.5	37.5	1.5	even	ok
	35	C10H7F3N7NaO3	20.75	353.045467	-1.2	-3.3	40.7	9.0	odd	ok
	36	C12H10F6NNaO3	17.84	353.045708	1.4	4.0	40.7	5.0	odd	ok
	37	C11H13F3NaO8	20.31	353.045473	-1.2	-3.4	41.3	3.5	even	ok
	38	C10H8F6N4NaO2	30.98	353.044366	0.1	0.2	46.2	5.5	even	ok
	39	C21H6N4NaO	20.70	353.043382	0.9	2.6	46.3	20.5	even	ok
	40	C23H8NNaO2	23.08	353.044724	0.4	1.2	50.7	20.0	odd	ok
	41	C10H9F9NNaO	16.37	353.043264	1.0	2.9	51.9	2.0	odd	ok
	42	C10H22N2NaO2S4	15.96	353.045633	-1.3	-3.8	55.6	0.5	even	ok

Figure S10. HRESIMS spectrum of compound **6c**

Generic Display Report

Analysis Info

Analysis Name	E:\Data\MS_MessService\58960000001.d	Acquisition Date	8/14/2018 6:07:58 PM
Method	tune_low_MS_Service_08_18.m	Operator	msc
Sample Name	CE-155-SPOT1-PEAK2	Instrument	maXis
Comment	Kalaba/Zehl<br>Ergebnis: +/- 5ppm<br>ACN/MeOH +0.1% H2O		

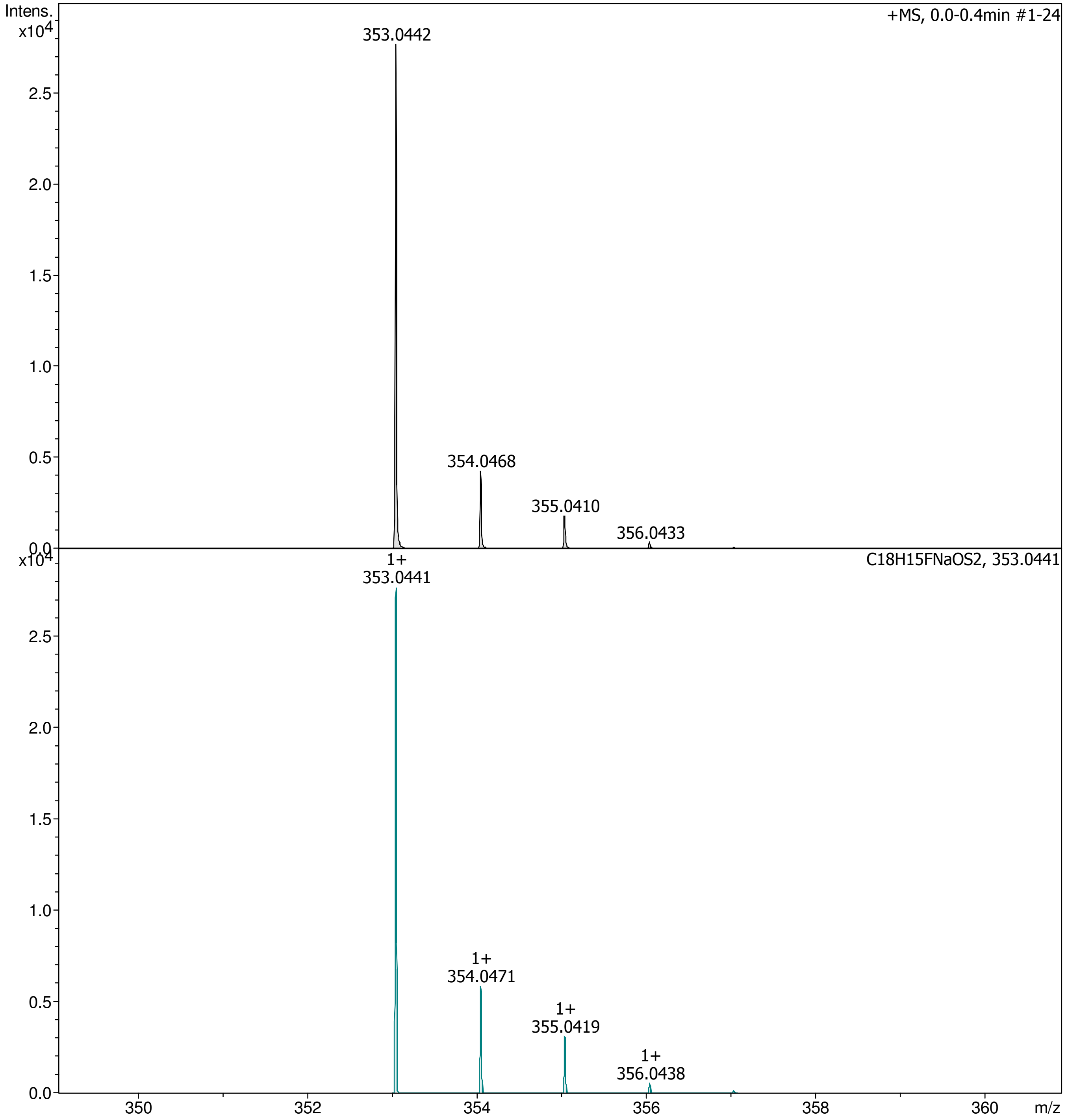

Mass Spectrum SmartFormula Report

Analysis Info

Analysis Name	E:\Data\MS_MessService\58960000001.d	Acquisition Date	8/14/2018 6:07:58 PM
Method	tune_low_MS_Service_08_18.m	Operator	msc
Sample Name	CE-155-SPOT1-PEAK2	Instrument	maXis 255552.00016
Comment	Kalaba/Zehl<br>Ergebnis: +/- 5ppm<br>ACN/MeOH +0.1% H2O		

Acquisition Parameter

Source Type	ESI	Ion Polarity	Positive	Set Nebulizer	0.4 Bar
Focus	Not active	Set Capillary	4500 V	Set Dry Heater	180 °C
Scan Begin	50 m/z	Set End Plate Offset	-500 V	Set Dry Gas	4.0 l/min
Scan End	1900 m/z	Set Charging Voltage	0 V	Set Divert Valve	Source
		Set Corona	0 nA	Set APCI Heater	0 °C

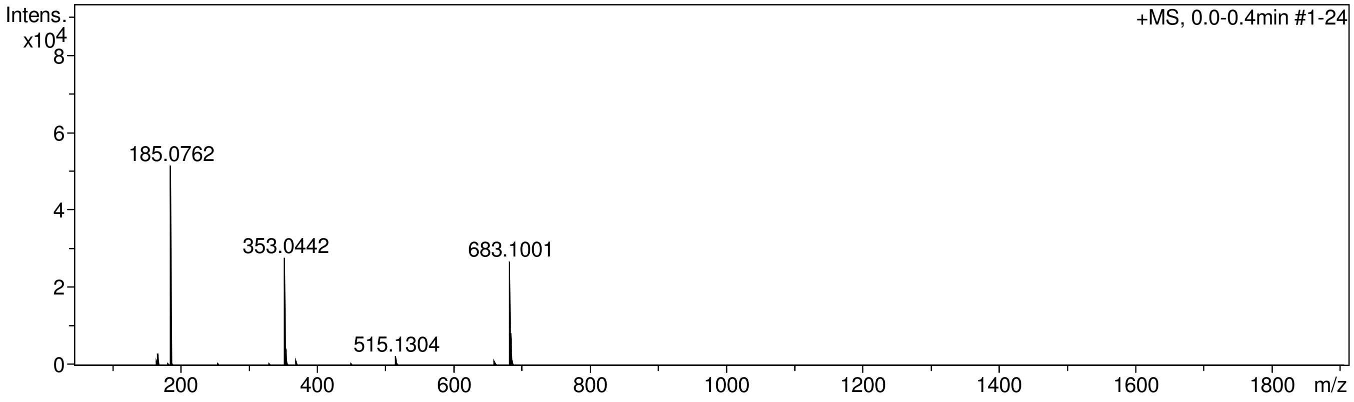

Meas. m/z	#	Ion Formula	Score	m/z	err [mDa]	err [ppm]	mSigma	rdb	e⁻ Conf	N-Rule
353.044219	1	C12H13FN3NaO5S	70.34	353.045215	-1.0	-2.8	1.7	7.0	odd	ok
	2	C12H14F4NaO4S	100.00	353.044114	-0.1	-0.3	6.8	3.5	even	ok
	3	C10H11FN6NaO4S	90.02	353.043873	0.3	1.0	6.8	7.5	even	ok
	4	C11H7FN10NaS	64.56	353.045210	-1.0	-2.8	6.9	12.5	even	ok
	5	C13H10F4N4NaS	55.95	353.045451	1.2	3.5	7.0	8.5	even	ok
	6	C13H10N6NaO3S	45.17	353.042730	-1.5	-4.2	9.8	11.5	even	ok
	7	C15H13F3NaO3S	52.65	353.042971	1.2	3.5	9.8	7.5	even	ok
	8	C10H12F4N3NaO3S	43.95	353.042771	1.4	4.1	12.6	4.0	odd	ok
	9	C15H12N3NaO4S	83.46	353.044073	-0.1	-0.4	15.4	11.0	odd	ok
	10	C10H12F8NNaS	44.28	353.045492	1.3	3.6	17.8	1.0	odd	ok
	11	C13H15F5NaS2	38.88	353.042754	1.5	4.1	18.2	3.5	even	ok
	12	C11H12F2N6NaS2	32.98	353.042514	1.7	4.8	18.2	7.5	even	ok
	13	C13H14F2N3NaOS2	70.02	353.043856	-0.4	-1.0	19.3	7.0	odd	ok
	14	C10H15F3N3NaO2S2	55.89	353.044999	0.8	2.2	20.2	3.0	odd	ok
	15	C17H14NaO5S	43.09	353.045415	-1.2	-3.4	21.3	10.5	even	ok
	16	C10H16F6NaOS2	67.89	353.043897	-0.3	-0.9	21.6	-0.5	even	ok
	17	C14H12F2NaO7	54.33	353.044330	0.1	0.3	22.9	7.5	even	ok
	18	C15H16F2NaO2S2	46.88	353.045199	1.0	2.8	23.3	6.5	even	ok
	19	C12H10F2N3NaO6	28.81	353.042987	-1.2	-3.5	25.5	8.0	odd	ok
	20	C16H8N7NaS	37.99	353.045410	-1.2	-3.4	27.0	16.0	odd	ok
	21	C18H11F3NNaS	32.63	353.045651	1.4	4.1	27.1	12.0	odd	ok
	22	C17H11FNaO6	30.08	353.043187	-1.0	-2.9	28.7	11.5	even	ok
	23	C13H6F2N7NaO2	47.45	353.044325	-0.1	-0.3	28.8	13.0	odd	ok
	24	C15H9F5NNaO2	42.68	353.044565	0.3	1.0	28.9	9.0	odd	ok
	25	C16H13FN3NaS2	29.57	353.042713	1.5	4.3	29.2	11.0	odd	ok
	26	C15H8F2N4NaO3	22.95	353.045667	-1.4	-4.1	29.3	12.5	even	ok
	27	C11H13F3NaO8	25.78	353.045473	-1.3	-3.6	29.6	3.5	even	ok
	28	C11H4F2N10NaO	25.91	353.042982	-1.2	-3.5	29.8	13.5	even	ok
	29	C13H7F5N4NaO	29.85	353.043223	-1.0	-2.8	29.9	9.5	even	ok

Mass Spectrum SmartFormula Report

Meas. m/z	#	Ion Formula	Score	m/z	err [mDa]	err [ppm]	mSigma	rdb	e⁻ Conf	N-Rule
	30	C10H7F3N7NaO3	25.10	353.045467	-1.2	-3.5	30.9	9.0	odd	ok
	31	C12H10F6NNaO3	21.50	353.045708	-1.5	-4.2	30.9	5.0	odd	ok
	32	C18H15FNaOS2	53.91	353.044056	0.2	0.5	34.3	10.5	even	ok
	33	C10H8F6N4NaO2	39.65	353.044366	0.1	0.4	35.3	5.5	even	ok
	34	C16H5FN7NaO	24.40	353.043182	-1.0	-2.9	36.9	17.0	odd	ok
	35	C18H8F4NNaO	27.86	353.043422	0.8	2.3	37.0	13.0	odd	ok
	36	C10H19FN2NaO3S3	35.30	353.043404	0.8	2.3	38.5	1.5	even	ok
	37	C10H9F9NNaO	23.28	353.043264	1.0	2.7	40.4	2.0	odd	ok
	38	C18H7FN4NaO2	32.31	353.044524	0.3	0.9	40.4	16.5	even	ok
	39	C20H9FNNaO3	13.36	353.045867	-1.6	-4.7	44.7	16.0	odd	ok
	40	C21H14NaS2	20.59	353.042913	1.3	3.7	47.5	14.5	even	ok
	41	C21H6N4NaO	15.86	353.043382	-0.8	-2.4	55.4	20.5	even	ok
	42	C10H22N2NaO2S4	13.70	353.045633	-1.4	-4.0	58.2	0.5	even	ok
	43	C23H8NNaO2	15.79	353.044724	-0.5	-1.4	60.7	20.0	odd	ok

Figure S11. HRESIMS spectrum of compound **7c**

Generic Display Report

Analysis Info

Analysis Name	E:\Data\MS_MessService\58961000001.d	Acquisition Date	8/14/2018 6:10:41 PM
Method	tune_low_MS_Service_08_18.m	Operator	msc
Sample Name	CE-155-SPOT2-PEAK1	Instrument	maXis
Comment	Kalaba/Zehl<br>Ergebnis: +/- 5ppm<br>ACN/MeOH +0.1% H2O		

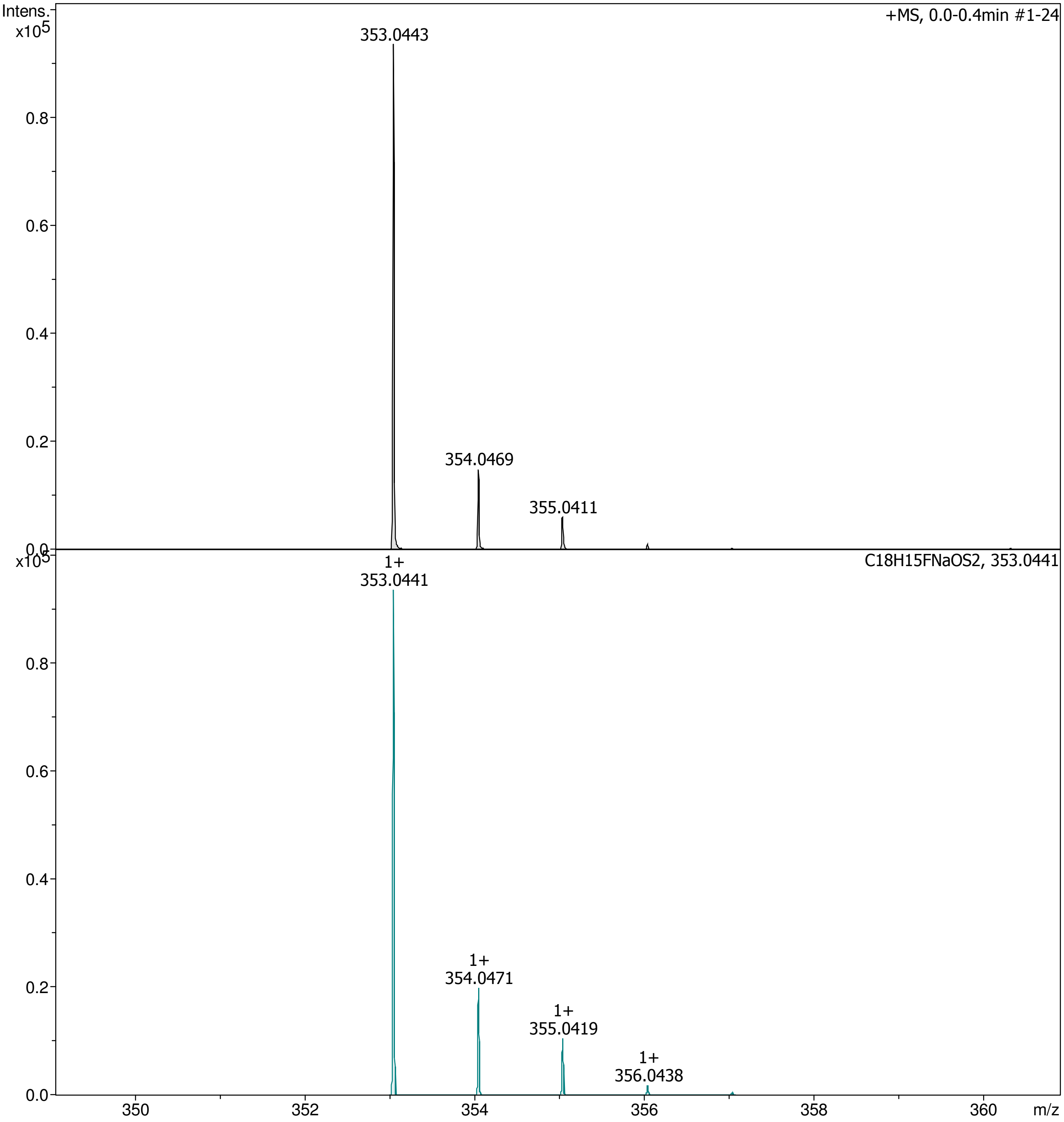

Mass Spectrum SmartFormula Report

Analysis Info

Analysis Name	E:\Data\MS_MessService\58961000001.d	Acquisition Date	8/14/2018 6:10:41 PM
Method	tune_low_MS_Service_08_18.m	Operator	msc
Sample Name	CE-155-SPOT2-PEAK1	Instrument	maXis 255552.00016
Comment	Kalaba/Zehl<br>Ergebnis: +/- 5ppm<br>ACN/MeOH +0.1% H2O		

Acquisition Parameter

Source Type	ESI	Ion Polarity	Positive	Set Nebulizer	0.4 Bar
Focus	Not active	Set Capillary	4500 V	Set Dry Heater	180 °C
Scan Begin	50 m/z	Set End Plate Offset	-500 V	Set Dry Gas	4.0 l/min
Scan End	1900 m/z	Set Charging Voltage	0 V	Set Divert Valve	Source
		Set Corona	0 nA	Set APCI Heater	0 °C

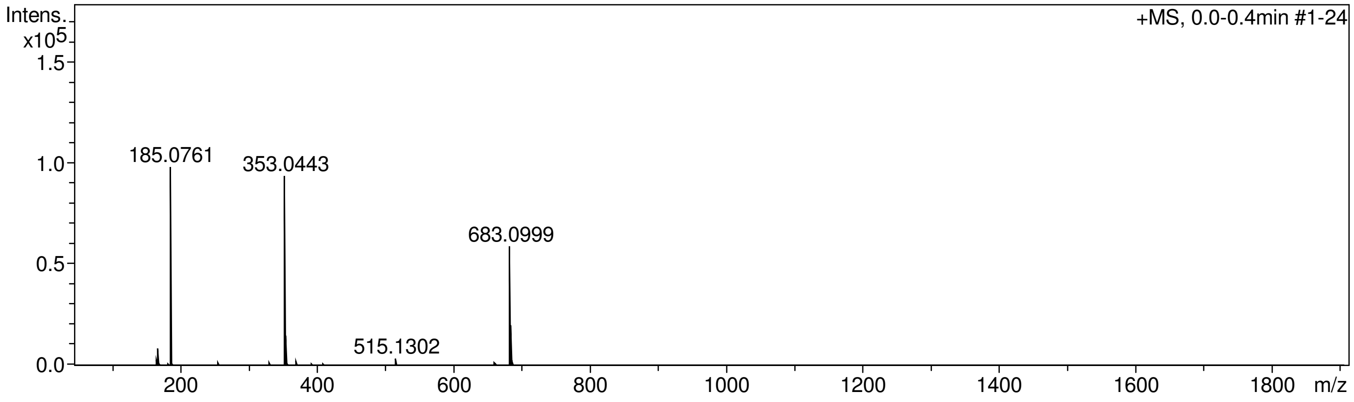

Meas. m/z	#	Ion Formula	Score	m/z	err [mDa]	err [ppm]	mSigma	rdb	e⁻ Conf	N-Rule
353.044273	1	C12H13FN3NaO5S	74.99	353.045215	-0.9	-2.7	3.6	7.0	odd	ok
	2	C11H7FN10NaS	73.90	353.045210	0.9	2.7	4.6	12.5	even	ok
	3	C13H10F4N4NaS	64.20	353.045451	-1.2	-3.3	4.6	8.5	even	ok
	4	C13H10N6NaO3S	48.72	353.042730	-1.5	-4.4	7.1	11.5	even	ok
	5	C15H13F3NaO3S	56.94	353.042971	-1.3	-3.7	7.2	7.5	even	ok
	6	C12H14F4NaO4S	100.00	353.044114	-0.2	-0.5	9.1	3.5	even	ok
	7	C10H11FN6NaO4S	89.81	353.043873	-0.4	-1.1	9.1	7.5	even	ok
	8	C15H12N3NaO4S	91.35	353.044073	0.2	0.6	12.9	11.0	odd	ok
	9	C10H12F4N3NaO3S	43.23	353.042771	-1.5	-4.3	14.9	4.0	odd	ok
	10	C13H15F5NaS2	39.63	353.042754	1.5	4.3	18.6	3.5	even	ok
	11	C11H12F2N6NaS2	33.53	353.042514	-1.8	-5.0	18.7	7.5	even	ok
	12	C17H14NaO5S	49.97	353.045415	-1.1	-3.2	18.9	10.5	even	ok
	13	C13H14F2N3NaOS2	91.50	353.043856	-0.4	-1.2	19.1	7.0	odd	ok
	14	C10H12F8NNaS	46.65	353.045492	1.2	3.5	20.0	1.0	odd	ok
	15	C10H15F3N3NaO2S2	59.10	353.044999	-0.7	-2.1	21.9	3.0	odd	ok
	16	C14H12F2NaO7	60.01	353.044330	-0.1	-0.2	22.2	7.5	even	ok
	17	C15H16F2NaO2S2	65.37	353.045199	-0.9	-2.6	22.6	6.5	even	ok
	18	C10H16F6NaOS2	67.35	353.043897	0.4	1.1	23.8	-0.5	even	ok
	19	C16H8N7NaS	44.41	353.045410	-1.1	-3.2	24.4	16.0	odd	ok
	20	C18H11F3NNaS	38.24	353.045651	-1.4	-3.9	24.5	12.0	odd	ok
	21	C12H10F2N3NaO6	29.59	353.042987	1.3	3.6	25.7	8.0	odd	ok
	22	C17H11FNaO6	32.94	353.043187	1.1	3.1	26.3	11.5	even	ok
	23	C15H8F2N4NaO3	26.55	353.045667	1.4	4.0	27.4	12.5	even	ok
	24	C13H6F2N7NaO2	53.40	353.044325	-0.1	-0.1	27.5	13.0	odd	ok
	25	C15H9F5NNaO2	48.13	353.044565	-0.3	-0.8	27.5	9.0	odd	ok
	26	C16H13FN3NaS2	39.21	353.042713	-1.6	-4.4	27.9	11.0	odd	ok
	27	C11H4F2N10NaO	27.15	353.042982	1.3	3.7	29.2	13.5	even	ok
	28	C13H7F5N4NaO	31.36	353.043223	-1.1	-3.0	29.2	9.5	even	ok
	29	C11H13F3NaO8	27.48	353.045473	1.2	3.4	31.0	3.5	even	ok

Mass Spectrum SmartFormula Report

Meas. m/z	#	Ion Formula	Score	m/z	err [mDa]	err [ppm]	mSigma	rdb	e⁻ Conf	N-Rule
	30	C10H7F3N7NaO3	27.29	353.045467	1.2	3.4	31.4	9.0	odd	ok
	31	C12H10F6NNaO3	23.44	353.045708	-1.4	-4.1	31.4	5.0	odd	ok
	32	C18H15FNaOS2	72.76	353.044056	-0.2	-0.6	32.9	10.5	even	ok
	33	C16H5FN7NaO	26.97	353.043182	1.1	3.1	34.3	17.0	odd	ok
	34	C18H8F4NNaO	30.86	353.043422	-0.9	-2.4	34.4	13.0	odd	ok
	35	C10H8F6N4NaO2	42.15	353.044366	0.1	0.3	36.3	5.5	even	ok
	36	C18H7FN4NaO2	38.07	353.044524	0.3	0.7	37.6	16.5	even	ok
	37	C10H19FN2NaO3S3	44.69	353.043404	-0.9	-2.5	39.3	1.5	even	ok
	38	C10H9F9NNaO	23.23	353.043264	-1.0	-2.9	41.7	2.0	odd	ok
	39	C20H9FNNaO3	16.08	353.045867	-1.6	-4.5	41.7	16.0	odd	ok
	40	C21H14NaS2	27.88	353.042913	1.4	3.9	45.8	14.5	even	ok
	41	C21H6N4NaO	18.08	353.043382	0.9	2.5	52.4	20.5	even	ok
	42	C23H8NNaO2	19.14	353.044724	0.5	1.3	57.6	20.0	odd	ok
	43	C10H22N2NaO2S4	18.55	353.045633	-1.4	-3.9	58.8	0.5	even	ok

Figure S12. HRESIMS spectrum of compound **8c**

Generic Display Report

Analysis Info

Analysis Name	E:\Data\MS_MessService\58962000001.d	Acquisition Date	8/14/2018 6:12:55 PM
Method	tune_low_MS_Service_08_18.m	Operator	msc
Sample Name	CE-155-SPOT2-PEAK2	Instrument	maXis
Comment	Kalaba/Zehl<br>Ergebnis: +/- 5ppm<br>ACN/MeOH +0.1% H2O		

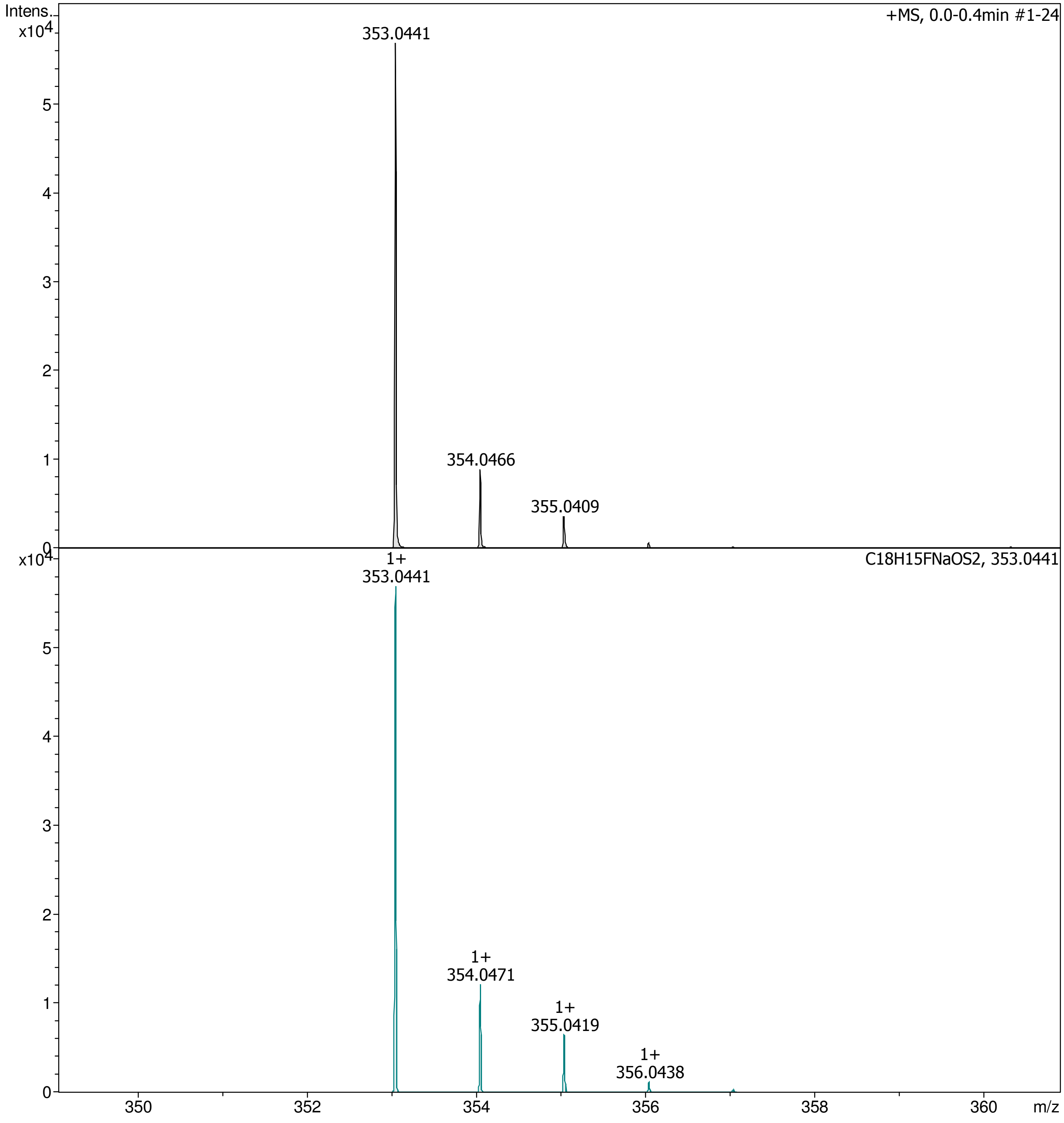

Mass Spectrum SmartFormula Report

Analysis Info

Analysis Name	E:\Data\MS_MessService\58962000001.d	Acquisition Date	8/14/2018 6:12:55 PM
Method	tune_low_MS_Service_08_18.m	Operator	msc
Sample Name	CE-155-SPOT2-PEAK2	Instrument	maXis 255552.00016
Comment	Kalaba/Zehl<br>Ergebnis: +/- 5ppm<br>ACN/MeOH +0.1% H2O		

Acquisition Parameter

Source Type	ESI	Ion Polarity	Positive	Set Nebulizer	0.4 Bar
Focus	Not active	Set Capillary	4500 V	Set Dry Heater	180 °C
Scan Begin	50 m/z	Set End Plate Offset	-500 V	Set Dry Gas	4.0 l/min
Scan End	1900 m/z	Set Charging Voltage	0 V	Set Divert Valve	Source
		Set Corona	0 nA	Set APCI Heater	0 °C

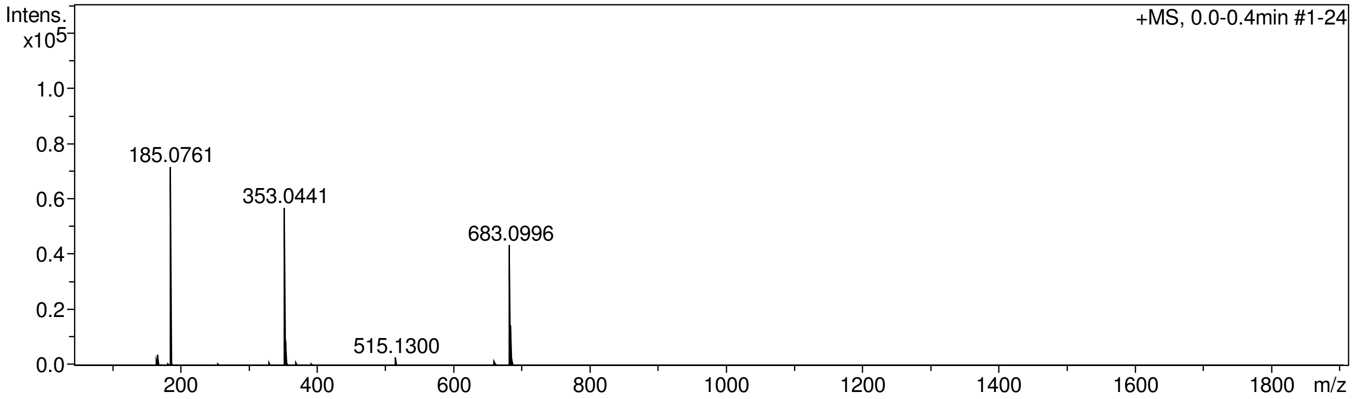

Meas. m/z	#	Ion Formula	Score	m/z	err [mDa]	err [ppm]	mSigma	rdb	e⁻ Conf	N-Rule
353.044084	1	C12H13FN3NaO5S	62.89	353.045215	-1.1	-3.2	2.3	7.0	odd	ok
	2	C11H7FN10NaS	59.69	353.045210	-1.1	-3.2	5.6	12.5	even	ok
	3	C13H10F4N4NaS	51.42	353.045451	-1.4	-3.9	5.6	8.5	even	ok
	4	C12H14F4NaO4S	100.00	353.044114	-0.0	-0.1	7.2	3.5	even	ok
	5	C10H11FN6NaO4S	92.64	353.043873	0.2	0.6	7.2	7.5	even	ok
	6	C13H10N6NaO3S	48.81	353.042730	-1.4	-3.8	9.0	11.5	even	ok
	7	C15H13F3NaO3S	56.56	353.042971	-1.1	-3.2	9.1	7.5	even	ok
	8	C10H12F4N3NaO3S	46.47	353.042771	-1.3	-3.7	13.0	4.0	odd	ok
	9	C15H12N3NaO4S	87.16	353.044073	-0.0	-0.0	14.9	11.0	odd	ok
	10	C10H12F8NNaS	39.54	353.045492	1.4	4.0	18.0	1.0	odd	ok
	11	C13H15F5NaS2	40.47	353.042754	1.3	3.8	19.3	3.5	even	ok
	12	C11H12F2N6NaS2	34.55	353.042514	-1.6	-4.4	19.3	7.5	even	ok
	13	C13H14F2N3NaOS2	89.40	353.043856	0.2	0.6	20.1	7.0	odd	ok
	14	C17H14NaO5S	39.11	353.045415	-1.3	-3.8	20.8	10.5	even	ok
	15	C14H12F2NaO7	51.64	353.044330	-0.2	-0.7	21.5	7.5	even	ok
	16	C10H15F3N3NaO2S2	49.23	353.044999	-0.9	-2.6	21.6	3.0	odd	ok
	17	C10H16F6NaOS2	68.26	353.043897	0.2	0.5	23.0	-0.5	even	ok
	18	C15H16F2NaO2S2	52.28	353.045199	-1.1	-3.2	23.8	6.5	even	ok
	19	C12H10F2N3NaO6	31.32	353.042987	1.1	3.1	24.4	8.0	odd	ok
	20	C16H8N7NaS	34.72	353.045410	-1.3	-3.8	26.3	16.0	odd	ok
	21	C18H11F3NNaS	29.64	353.045651	-1.6	-4.4	26.3	12.0	odd	ok
	22	C17H11FNaO6	32.91	353.043187	-0.9	-2.5	27.1	11.5	even	ok
	23	C13H6F2N7NaO2	45.39	353.044325	-0.2	-0.7	27.3	13.0	odd	ok
	24	C15H9F5NNaO2	40.60	353.044565	0.5	1.4	27.3	9.0	odd	ok
	25	C15H8F2N4NaO3	21.28	353.045667	-1.6	-4.5	27.7	12.5	even	ok
	26	C11H4F2N10NaO	28.39	353.042982	1.1	3.1	28.5	13.5	even	ok
	27	C13H7F5N4NaO	32.51	353.043223	0.9	2.4	28.5	9.5	even	ok
	28	C11H13F3NaO8	23.44	353.045473	1.4	3.9	29.1	3.5	even	ok
	29	C16H13FN3NaS2	39.21	353.042713	-1.4	-3.9	29.4	11.0	odd	ok

Mass Spectrum SmartFormula Report

Meas. m/z	#	Ion Formula	Score	m/z	err [mDa]	err [ppm]	mSigma	rdb	e⁻ Conf	N-Rule
	30	C10H7F3N7NaO3	23.06	353.045467	1.4	3.9	29.9	9.0	odd	ok
	31	C12H10F6NNaO3	19.64	353.045708	-1.6	-4.6	29.9	5.0	odd	ok
	32	C18H15FNaOS2	69.31	353.044056	-0.0	-0.1	34.5	10.5	even	ok
	33	C10H8F6N4NaO2	37.25	353.044366	-0.3	-0.8	34.6	5.5	even	ok
	34	C16H5FN7NaO	26.80	353.043182	0.9	2.6	35.4	17.0	odd	ok
	35	C18H8F4NNaO	30.42	353.043422	-0.7	-1.9	35.4	13.0	odd	ok
	36	C18H7FN4NaO2	30.88	353.044524	0.4	1.2	38.9	16.5	even	ok
	37	C10H19FN2NaO3S3	44.94	353.043404	-0.7	-1.9	39.6	1.5	even	ok
	38	C10H9F9NNaO	24.90	353.043264	-0.8	-2.3	39.8	2.0	odd	ok
	39	C21H14NaS2	27.33	353.042913	1.2	3.3	47.5	14.5	even	ok
	40	C21H6N4NaO	17.34	353.043382	0.7	2.0	54.1	20.5	even	ok
	41	C10H22N2NaO2S4	14.80	353.045633	-1.5	-4.4	59.2	0.5	even	ok
	42	C23H8NNaO2	15.03	353.044724	0.6	1.8	59.5	20.0	odd	ok

Figure S13. HRESIMS spectrum of compound **5d**

Generic Display Report

Analysis Info

Analysis Name	D:\Data\Kalaba\71582000001.d	Acquisition Date	10/07/2020 17:54:47
Method	tune_low_MS_Service_07_20.m	Operator	msc
Sample Name	PN-18-1	Instrument	maXis
Comment	Kalaba/Zehl<br>Ergebnis +/- 5ppm<br>ACN/MeOH + 1 % H2O		

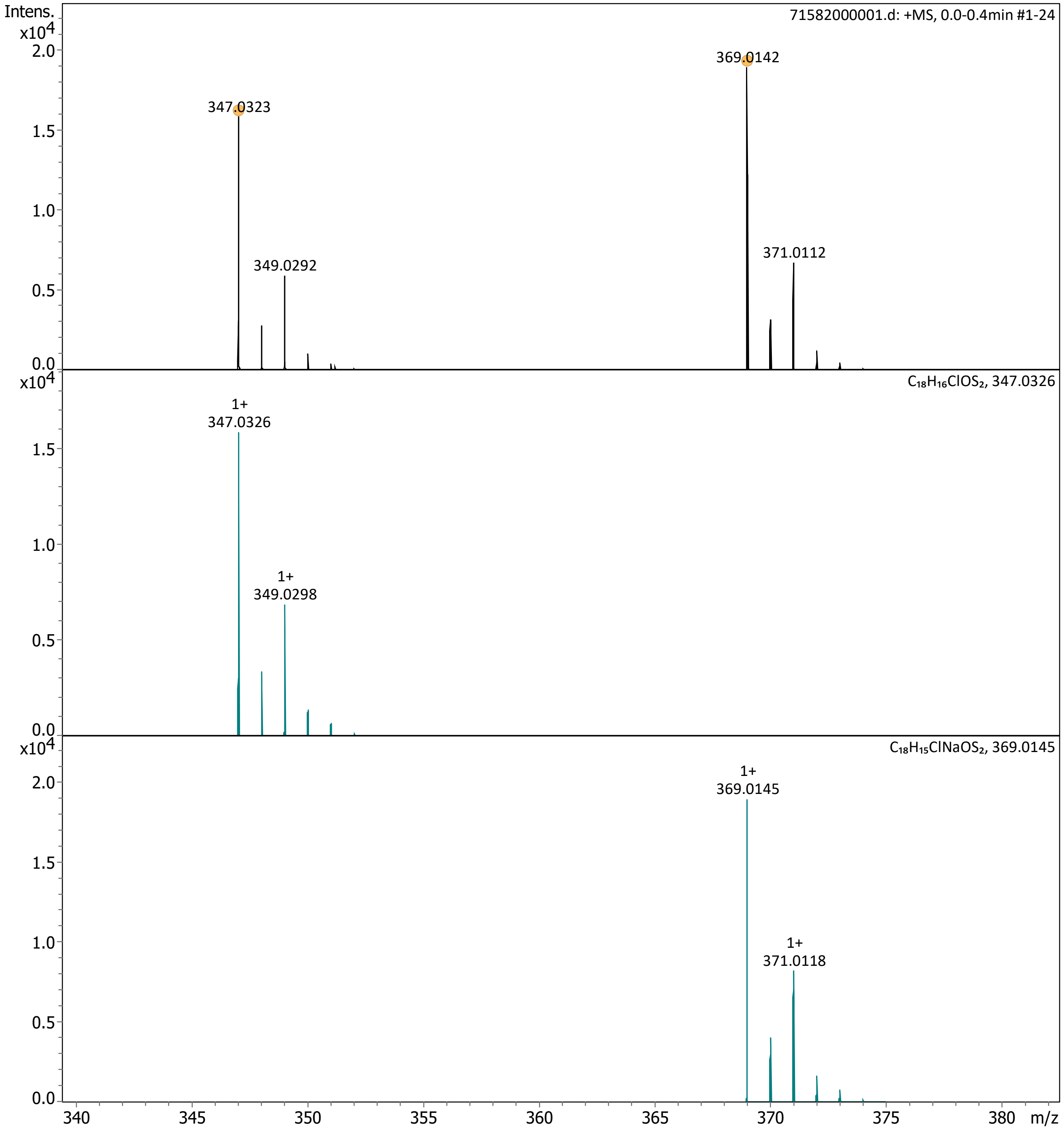

Mass Spectrum SmartFormula Report

Analysis Info

Analysis Name	D:\Data\Kalaba\71582000001.d	Acquisition Date	10/07/2020 17:54:47
Method	tune_low_MS_Service_07_20.m	Operator	msc
Sample Name	PN-18-1	Instrument	maXis 255552.00016
Comment	Kalaba/Zehl<br>Ergebnis +/- 5ppm<br>ACN/MeOH + 1 % H2O		

Acquisition Parameter

Source Type	ESI	Ion Polarity	Positive	Set Nebulizer	0.4 Bar
Focus	Not active	Set Capillary	4200 V	Set Dry Heater	180 Â°C
Scan Begin	50 m/z	Set End Plate Offset	-500 V	Set Dry Gas	4.0 l/min
Scan End	1900 m/z	Set Charging Voltage	0 V	Set Divert Valve	Source
		Set Corona	0 nA	Set APCI Heater	0 Â°C

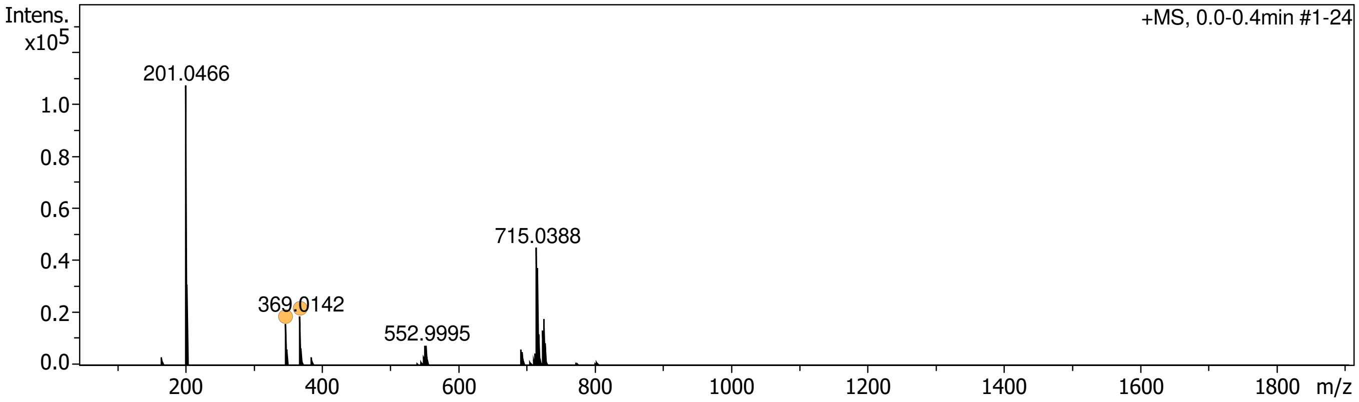

Meas. m/z	#	Ion Formula	m/z	err [ppm]	mSigma	# mSigma	Score	rdb	eÂ¯ Conf	N-Rule
347.0323	1	C11H8ClN10S	347.0337	4.1	6.7	1	60.51	18.0	even	ok
	2	C17H12ClO6	347.0317	-1.8	14.7	2	82.46	15.0	even	ok
	3	C10H12ClN6O4S	347.0324	0.2	17.2	3	100.00	13.0	even	ok
	4	C18H8ClN4O2	347.0330	2.1	22.1	4	66.60	20.0	even	ok
	5	C18H16ClOS2	347.0326	0.7	33.2	5	64.19	18.0	even	ok
	6	C10H20ClN2O3S3	347.0319	-1.1	43.1	6	55.52	11.0	even	ok
	7	C13H9Cl2N8	347.0322	-0.4	120.8	7	2.52	19.0	even	ok
	8	C12H13Cl2N4O4	347.0308	-4.2	122.7	8	1.11	14.0	even	ok
	9	C12H14Cl3N6	347.0340	4.9	254.0	9	0.00	17.0	even	ok
	10	C11H18Cl3N2O4	347.0327	1.0	255.9	10	0.00	12.0	even	ok
369.0142	1	C11H7ClN10NaS	369.0157	4.1	9.8	1	55.87	18.0	even	ok
	2	C17H11ClNaO6	369.0136	-1.4	11.6	2	95.16	15.0	even	ok
	3	C14H3ClN10Na	369.0123	-5.1	14.5	3	31.68	21.0	even	ok
	4	C10H11ClN6NaO4S	369.0143	0.5	17.2	4	100.00	13.0	even	ok
	5	C18H7ClN4NaO2	369.0150	2.2	21.6	5	66.56	20.0	even	ok
	6	C9H15ClN2NaO8S	369.0130	-3.2	27.5	6	47.65	8.0	even	ok
	7	C18H15ClNaOS2	369.0145	0.9	42.2	7	50.33	18.0	even	ok
	8	C10H19ClN2NaO3S3	369.0139	-0.8	49.4	8	50.04	11.0	even	ok
	9	C13H8Cl2N8Na	369.0141	-0.1	128.3	9	1.79	19.0	even	ok
	10	C12H12Cl2N4NaO4	369.0128	-3.7	129.9	10	0.81	14.0	even	ok
	11	C12H20Cl2NaO3S2	369.0123	-5.0	159.3	11	0.11	12.0	even	ok
	12	C9H24Cl2NaO3S3	369.0157	4.1	178.4	12	0.03	9.0	even	ok
	13	C12H13Cl3N6Na	369.0159	4.9	260.6	13	0.00	17.0	even	ok
	14	C11H17Cl3N2NaO4	369.0146	1.2	262.4	14	0.00	12.0	even	ok

Figure S14. HRESIMS spectrum of compound **6d**

Generic Display Report

Analysis Info

Analysis Name	D:\Data\Kalaba\78520000001.d	Acquisition Date	26/03/2021 15:27:38
Method	tune_low_MS_Service_03_21.m	Operator	msc
Sample Name	PN-18-peak-2	Instrument	maXis
Comment	Kalaba / Zehl<br>Ergebnis +/- 5 ppm<br>ACN / MeOH + 1% H2O		

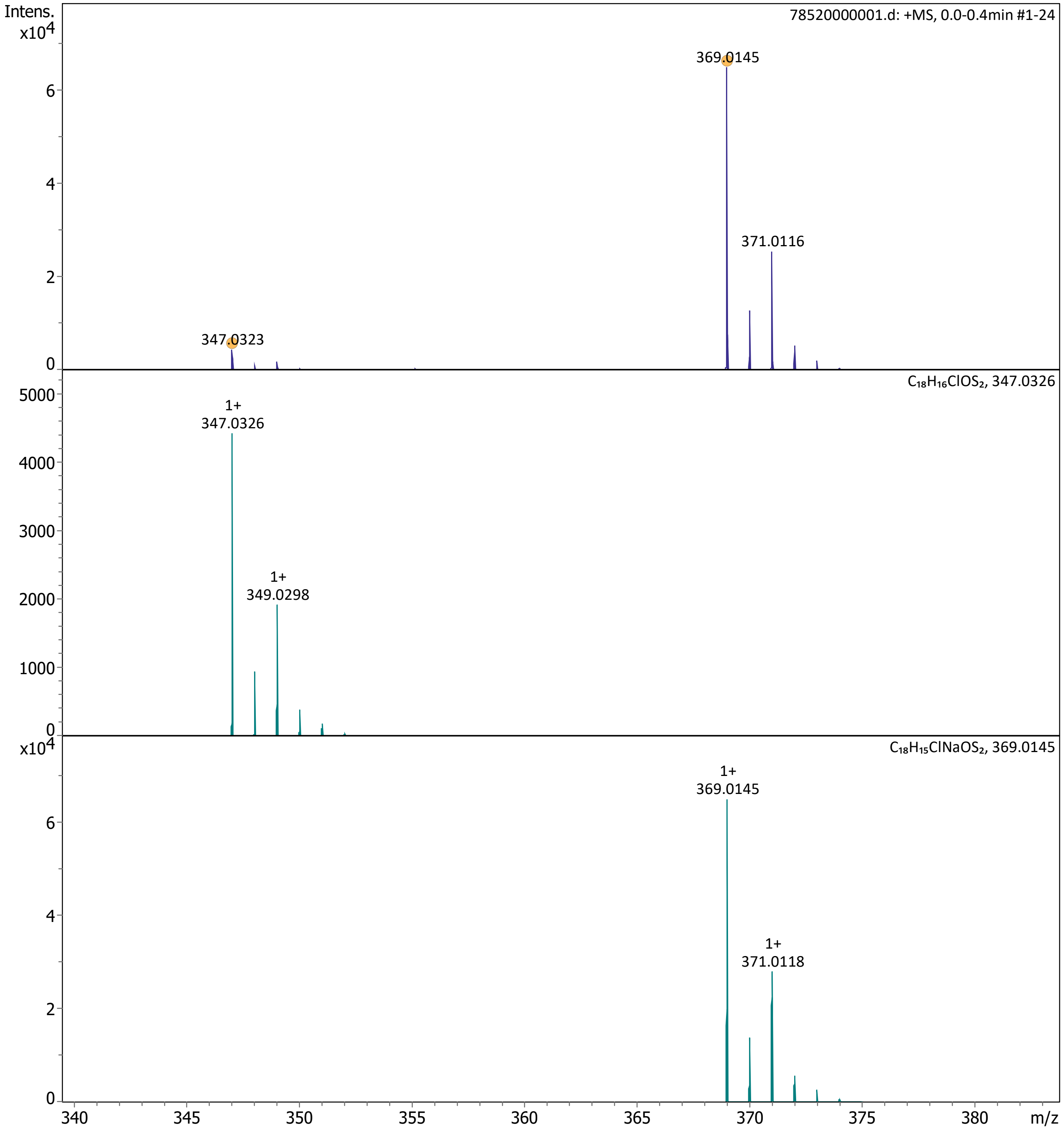

Mass Spectrum SmartFormula Report

Analysis Info

Analysis Name	D:\Data\Kalaba\78520000001.d	Acquisition Date	26/03/2021 15:27:38
Method	tune_low_MS_Service_03_21.m	Operator	msc
Sample Name	PN-18-peak-2	Instrument	maXis 255552.00016
Comment	Kalaba / Zehl<br>Ergebnis +/- 5 ppm<br>ACN / MeOH + 1% H2O		

Acquisition Parameter

Source Type	ESI	Ion Polarity	Positive	Set Nebulizer	0.4 Bar
Focus	Not active	Set Capillary	4200 V	Set Dry Heater	180 Â°C
Scan Begin	80 m/z	Set End Plate Offset	-500 V	Set Dry Gas	4.0 l/min
Scan End	1900 m/z	Set Charging Voltage	0 V	Set Divert Valve	Source
		Set Corona	0 nA	Set APCI Heater	0 Â°C

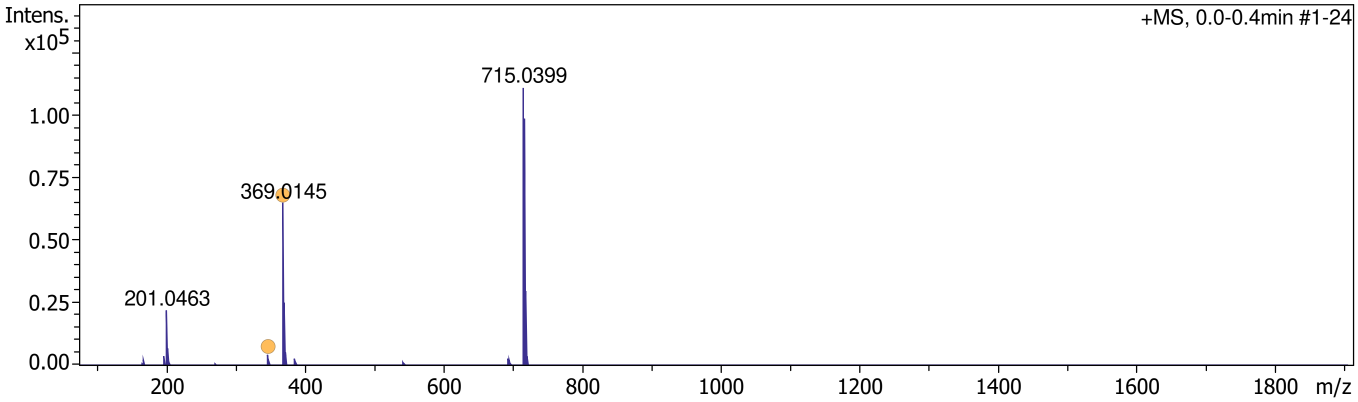

Meas. m/z	#	Ion Formula	Score	m/z	err [mDa]	err [ppm]	mSigma	rdb	eÂ¯ Conf	N-Rule
347.0323	1	C18H16ClOS2	100.00	347.0326	0.2	0.7	7.7	18.0	even	ok
	2	C11H8ClN10S	32.08	347.0337	1.4	4.0	31.9	18.0	even	ok
	3	C10H20ClN2O3S3	58.23	347.0319	-0.4	-1.2	36.8	11.0	even	ok
	4	C18H8ClN4O2	41.69	347.0330	0.7	2.0	37.2	20.0	even	ok
	5	C17H12ClO6	42.71	347.0317	-0.6	-1.8	37.6	15.0	even	ok
	6	C10H12ClN6O4S	52.89	347.0324	0.1	0.1	39.5	13.0	even	ok
	7	C13H9Cl2N8	5.84	347.0322	-0.2	-0.4	102.1	19.0	even	ok
	8	C12H13Cl2N4O4	2.37	347.0308	-1.5	-4.3	105.8	14.0	even	ok
	9	C12H14Cl3N6	0.00	347.0340	1.7	4.8	236.9	17.0	even	ok
	10	C11H18Cl3N2O4	0.00	347.0327	0.3	1.0	239.7	12.0	even	ok
369.0145	1	C11H7ClN10NaS	59.26	369.0157	1.1	3.0	19.3	18.0	even	ok
	2	C18H15ClNaOS2	100.00	369.0145	-0.0	-0.1	20.1	18.0	even	ok
	3	C17H11ClNaO6	61.01	369.0136	-0.9	-2.4	23.7	15.0	even	ok
	4	C18H7ClN4NaO2	75.22	369.0150	0.4	1.2	25.1	20.0	even	ok
	5	C10H11ClN6NaO4S	76.01	369.0143	-0.2	-0.6	28.8	13.0	even	ok
	6	C10H19ClN2NaO3S3	54.36	369.0139	-0.7	-1.9	40.3	11.0	even	ok
	7	C13H8Cl2N8Na	3.58	369.0141	-0.4	-1.1	112.6	19.0	even	ok
	8	C12H12Cl2N4NaO4	1.40	369.0128	-1.8	-4.8	115.6	14.0	even	ok
	9	C12H13Cl3N6Na	0.00	369.0159	1.4	3.8	246.6	17.0	even	ok
	10	C11H17Cl3N2NaO4	0.00	369.0146	0.1	0.2	249.0	12.0	even	ok

Figure S15. HRESIMS spectrum of compound **7d**

Generic Display Report

Analysis Info

Analysis Name	D:\Data\Kalaba\78521000001.d	Acquisition Date	26/03/2021 15:29:31
Method	tune_low_MS_Service_03_21.m	Operator	msc
Sample Name	PN-18-peak-3	Instrument	maXis
Comment	Kalaba / Zehl<br>Ergebnis +/- 5 ppm<br>ACN / MeOH + 1% H2O		

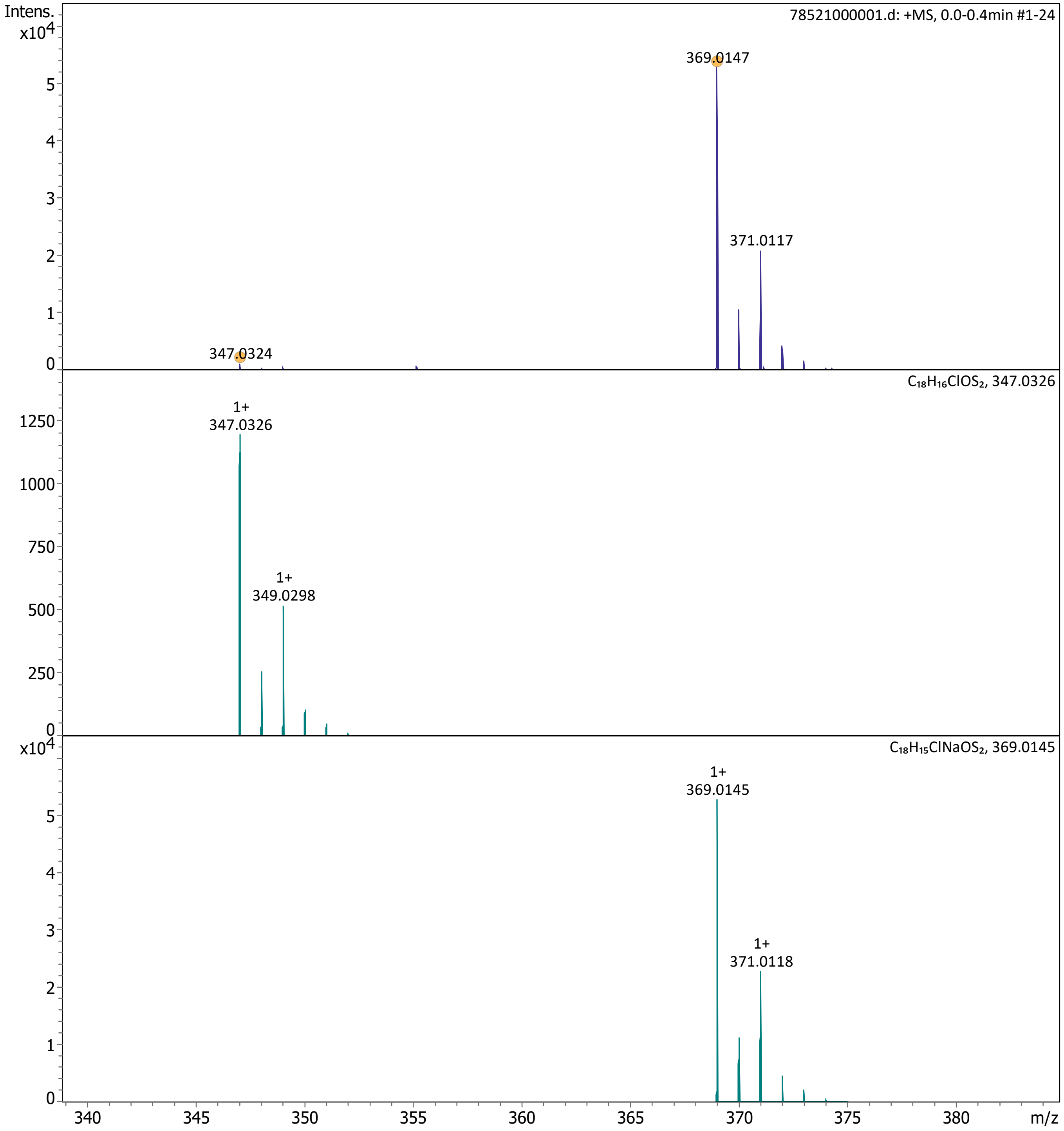

Mass Spectrum SmartFormula Report

Analysis Info

Analysis Name	D:\Data\Kalaba\78521000001.d	Acquisition Date	26/03/2021 15:29:31
Method	tune_low_MS_Service_03_21.m	Operator	msc
Sample Name	PN-18-peak-3	Instrument	maXis 255552.00016
Comment	Kalaba / Zehl<br>Ergebnis +/- 5 ppm<br>ACN / MeOH + 1% H2O		

Acquisition Parameter

Source Type	ESI	Ion Polarity	Positive	Set Nebulizer	0.4 Bar
Focus	Not active	Set Capillary	4200 V	Set Dry Heater	180 Â°C
Scan Begin	80 m/z	Set End Plate Offset	-500 V	Set Dry Gas	4.0 l/min
Scan End	1900 m/z	Set Charging Voltage	0 V	Set Divert Valve	Source
		Set Corona	0 nA	Set APCI Heater	0 Â°C

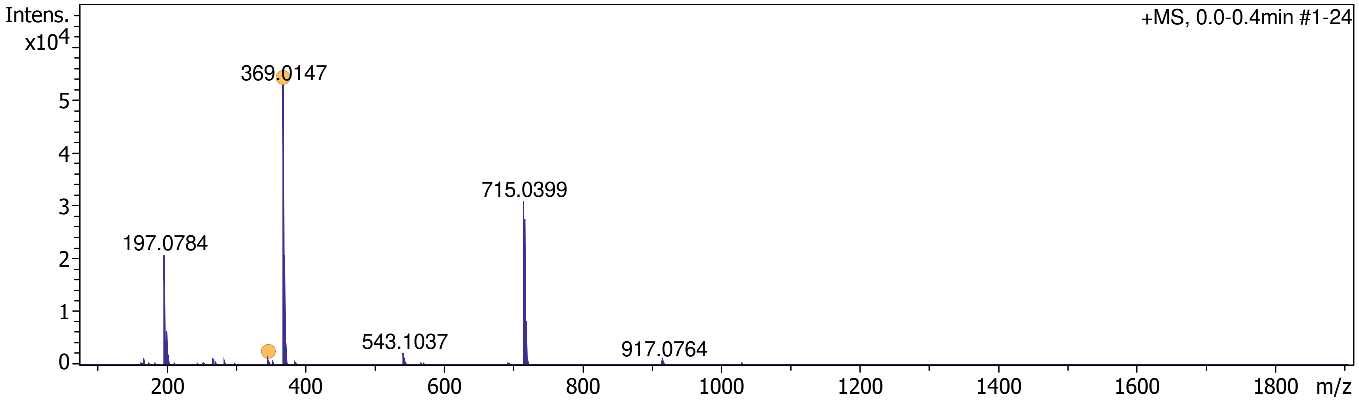

Meas. m/z	#	Ion Formula	Score	m/z	err [mDa]	err [ppm]	mSigma	rdb	eÂ¯ Conf	N-Rule
347.0324	1	C18H16ClOS2	100.00	347.0326	0.1	0.4	19.4	18.0	even	ok
	2	C11H8ClN10S	39.74	347.0337	1.3	3.7	33.4	18.0	even	ok
	3	C18H8ClN4O2	55.88	347.0330	0.6	1.7	35.1	20.0	even	ok
	4	C17H12ClO6	49.92	347.0317	-0.7	-2.1	36.4	15.0	even	ok
	5	C10H12ClN6O4S	59.63	347.0324	-0.1	-0.2	41.6	13.0	even	ok
	6	C10H20ClN2O3S3	54.49	347.0319	-0.5	-1.5	43.9	11.0	even	ok
	7	C13H9Cl2N8	5.03	347.0322	-0.3	-0.8	107.8	19.0	even	ok
	8	C12H13Cl2N4O4	1.96	347.0308	-1.6	-4.6	111.6	14.0	even	ok
	9	C12H14Cl3N6	0.00	347.0340	1.6	4.5	243.6	17.0	even	ok
	10	C11H18Cl3N2O4	0.00	347.0327	0.2	0.7	246.4	12.0	even	ok
369.0147	1	C18H15ClNaOS2	100.00	369.0145	-0.2	-0.4	18.1	18.0	even	ok
	2	C11H7ClN10NaS	62.07	369.0157	1.0	2.7	21.3	18.0	even	ok
	3	C17H11ClNaO6	55.49	369.0136	-1.0	-2.8	25.2	15.0	even	ok
	4	C18H7ClN4NaO2	79.55	369.0150	0.3	0.8	25.8	20.0	even	ok
	5	C10H11ClN6NaO4S	69.33	369.0143	-0.3	-0.9	30.7	13.0	even	ok
	6	C10H19ClN2NaO3S3	51.35	369.0139	-0.8	-2.2	40.4	11.0	even	ok
	7	C13H8Cl2N8Na	3.59	369.0141	-0.6	-1.5	111.6	19.0	even	ok
	8	C12H13Cl3N6Na	0.00	369.0159	1.3	3.5	245.7	17.0	even	ok
	9	C11H17Cl3N2NaO4	0.00	369.0146	-0.1	-0.2	248.2	12.0	even	ok
	10	C10H22Cl4NaO4	0.00	369.0164	1.8	4.8	285.9	10.0	even	ok

Figure S16. HRESIMS spectrum of compound **8d**

Generic Display Report

Analysis Info

Analysis Name	D:\Data\Kalaba\71583000001.d	Acquisition Date	10/07/2020 17:56:30
Method	tune_low_MS_Service_07_20.m	Operator	msc
Sample Name	PN-18-4	Instrument	maXis
Comment	Kalaba/Zehl<br>Ergebnis +/- 5ppm<br>ACN/MeOH + 1 % H2O		

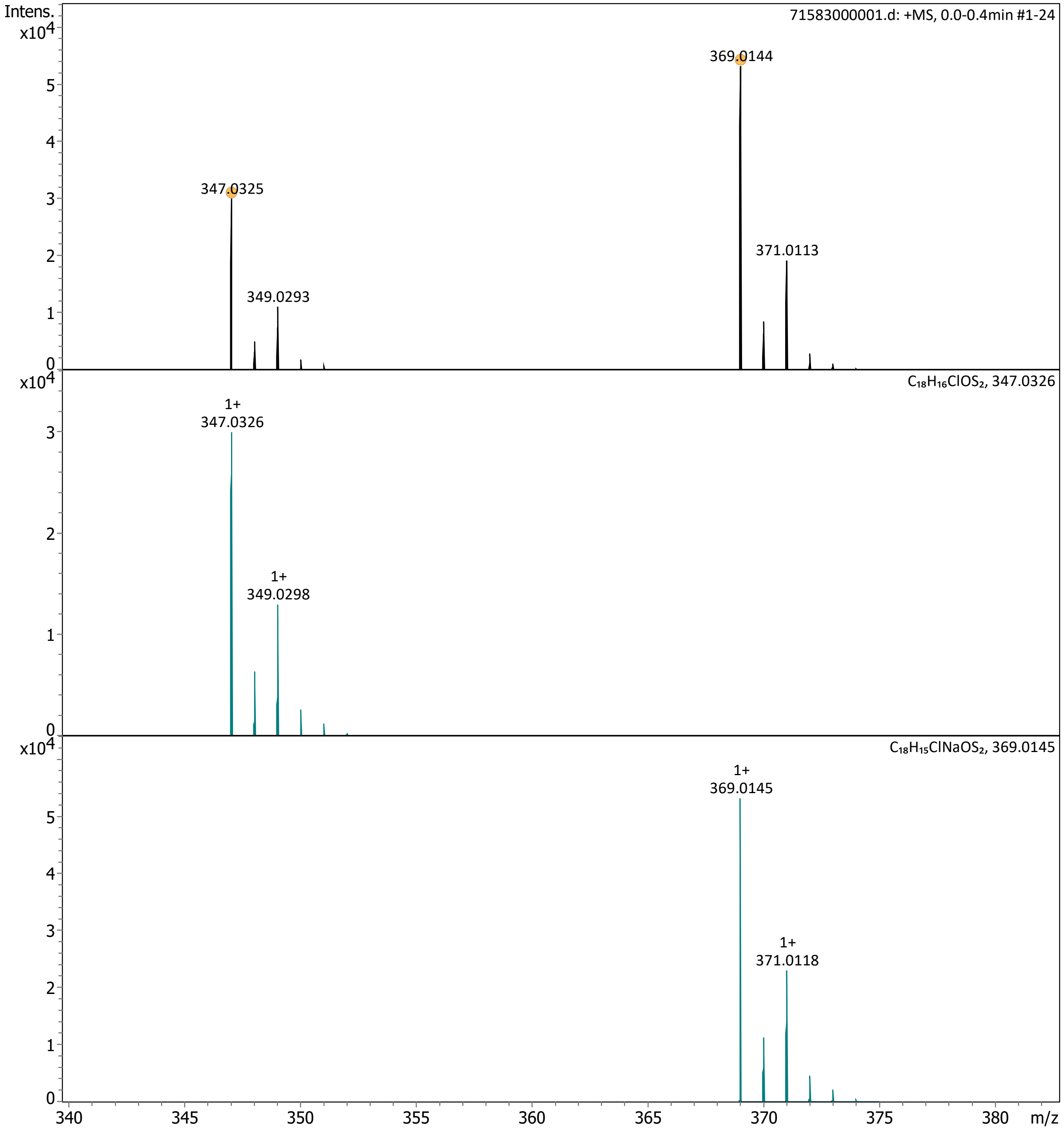

Mass Spectrum SmartFormula Report

Analysis Info

Analysis Name	D:\Data\Kalaba\71583000001.d	Acquisition Date	10/07/2020 17:56:30
Method	tune_low_MS_Service_07_20.m	Operator	msc
Sample Name	PN-18-4	Instrument	maXis 255552.00016
Comment	Kalaba/Zehl<br>Ergebnis +/- 5ppm<br>ACN/MeOH + 1 % H2O		

Acquisition Parameter

Source Type	ESI	Ion Polarity	Positive	Set Nebulizer	0.4 Bar
Focus	Not active	Set Capillary	4200 V	Set Dry Heater	180 Â°C
Scan Begin	50 m/z	Set End Plate Offset	-500 V	Set Dry Gas	4.0 l/min
Scan End	1900 m/z	Set Charging Voltage	0 V	Set Divert Valve	Source
		Set Corona	0 nA	Set APCI Heater	0 Â°C

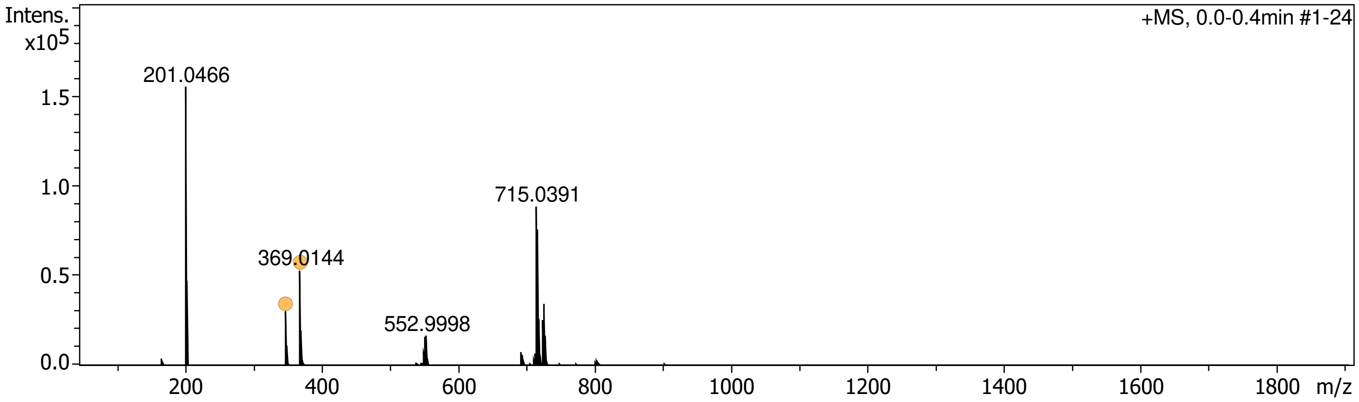

Meas. m/z	#	Ion Formula	m/z	err [ppm]	mSigma	# mSigma	Score	rdb	eÂ¯ Conf	N-Rule
347.0325	1	C11H8ClN10S	347.0337	3.6	4.6	1	66.09	18.0	even	ok
	2	C17H12ClO6	347.0317	-2.2	14.1	2	73.00	15.0	even	ok
	3	C10H12ClN6O4S	347.0324	-0.2	14.5	3	100.00	13.0	even	ok
	4	C18H8ClN4O2	347.0330	1.6	23.1	4	67.16	20.0	even	ok
	5	C18H16ClOS2	347.0326	0.3	36.9	5	59.39	18.0	even	ok
	6	C10H20ClN2O3S3	347.0319	-1.6	44.3	6	47.30	11.0	even	ok
	7	C13H9Cl2N8	347.0322	-0.8	122.9	7	1.99	19.0	even	ok
	8	C12H13Cl2N4O4	347.0308	-4.7	124.5	8	0.85	14.0	even	ok
	9	C12H14Cl3N6	347.0340	4.4	255.9	9	0.00	17.0	even	ok
	10	C11H18Cl3N2O4	347.0327	0.6	257.7	10	0.00	12.0	even	ok
369.0144	1	C11H7ClN10NaS	369.0157	3.4	7.5	1	60.95	18.0	even	ok
	2	C10H11ClN6NaO4S	369.0143	-0.2	13.0	2	100.00	13.0	even	ok
	3	C17H11ClNaO6	369.0136	-2.1	14.2	3	70.77	15.0	even	ok
	4	C9H15ClN2NaO8S	369.0130	-3.9	23.3	4	39.82	8.0	even	ok
	5	C18H7ClN4NaO2	369.0150	1.5	24.8	5	62.77	20.0	even	ok
	6	C18H15ClNaOS2	369.0145	0.3	42.9	6	48.88	18.0	even	ok
	7	C10H19ClN2NaO3S3	369.0139	-1.5	47.4	7	41.97	11.0	even	ok
	8	C13H8Cl2N8Na	369.0141	-0.8	127.1	8	1.53	19.0	even	ok
	9	C12H12Cl2N4NaO4	369.0128	-4.4	128.4	9	0.67	14.0	even	ok
	10	C9H16Cl2N4NaO4S	369.0162	4.7	148.2	10	0.18	11.0	even	ok
	11	C9H24Cl2NaO3S3	369.0157	3.5	177.1	11	0.04	9.0	even	ok
	12	C12H13Cl3N6Na	369.0159	4.2	259.6	12	0.00	17.0	even	ok
	13	C11H17Cl3N2NaO4	369.0146	0.6	261.2	13	0.00	12.0	even	ok

Figure S17. HRESIMS spectrum of compound **5e**

Generic Display Report

Analysis Info

Analysis Name	D:\Data\Kalaba\72415000001.d	Acquisition Date	20/08/2020 16:56:28
Method	tune_low_MS_Service_08_20.m	Operator	msc
Sample Name	CE-167-1-1-b2	Instrument	maXis
Comment	Kalaba/Zehl<br>Ergebnis +/- 5ppm<br>ACN / MeOH + 1% H2O		

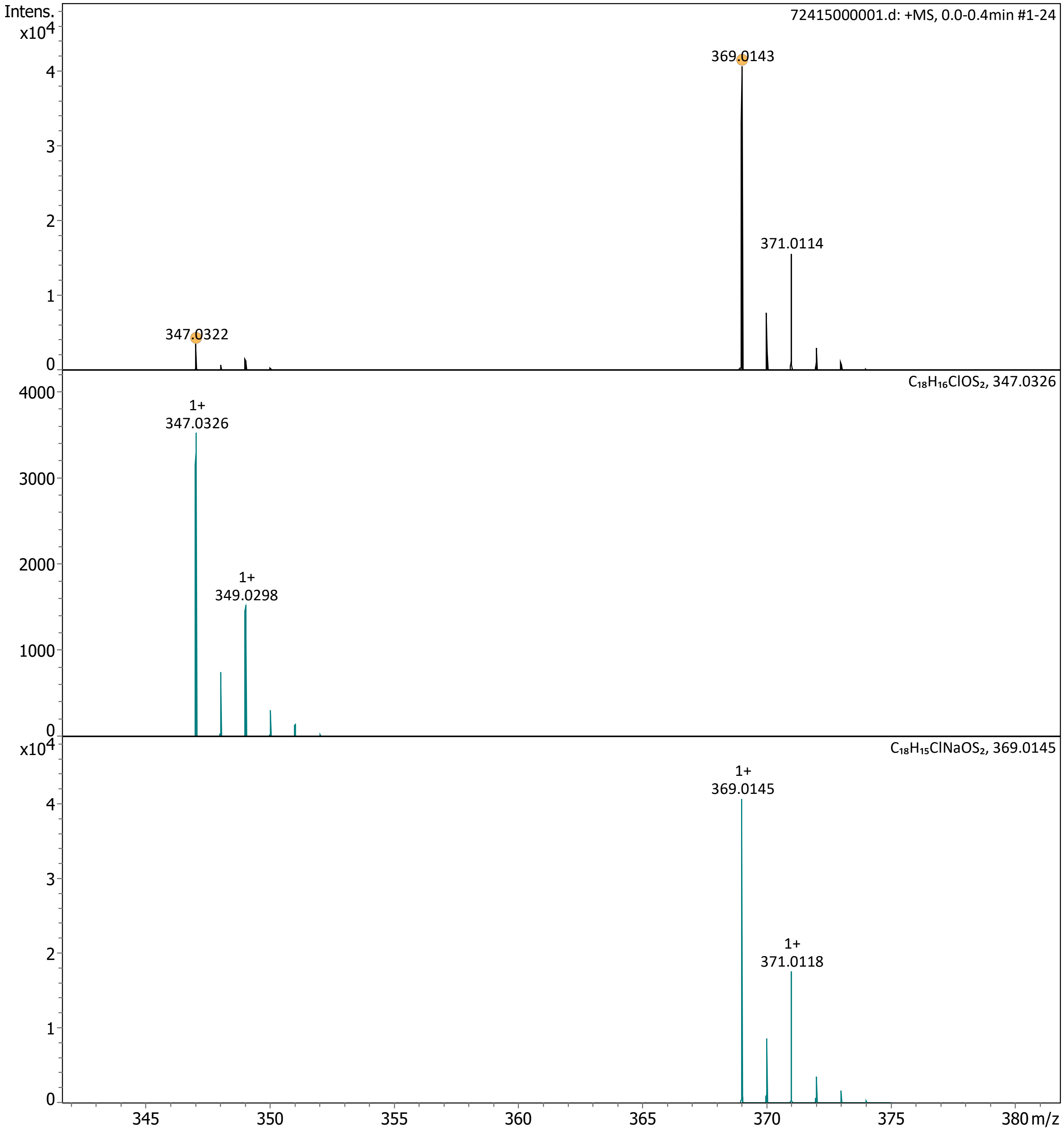

Mass Spectrum SmartFormula Report

Analysis Info

Analysis Name	D:\Data\Kalaba\72415000001.d	Acquisition Date	20/08/2020 16:56:28
Method	tune_low_MS_Service_08_20.m	Operator	msc
Sample Name	CE-167-1-1-b2	Instrument	maXis 255552.00016
Comment	Kalaba/Zehl<br>Ergebnis +/- 5ppm<br>ACN / MeOH + 1% H2O		

Acquisition Parameter

Source Type	ESI	Ion Polarity	Positive	Set Nebulizer	0.4 Bar
Focus	Not active	Set Capillary	4200 V	Set Dry Heater	180 Â°C
Scan Begin	50 m/z	Set End Plate Offset	-500 V	Set Dry Gas	4.0 l/min
Scan End	1900 m/z	Set Charging Voltage	0 V	Set Divert Valve	Source
		Set Corona	0 nA	Set APCI Heater	0 Â°C

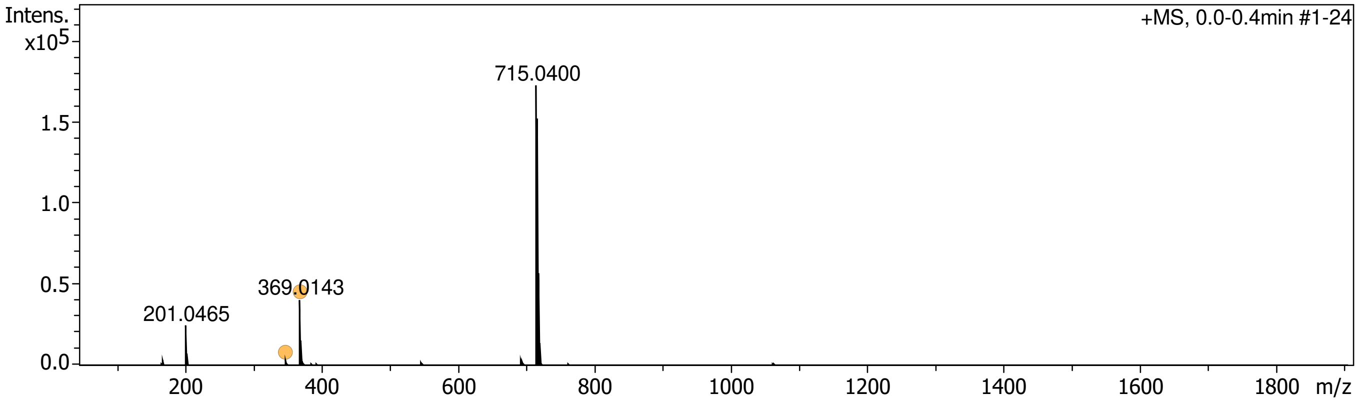

Meas. m/z	#	Ion Formula	m/z	err [ppm]	mSigma	# mSigma	Score	rdb	eÂ¯ Conf	N-Rule
347.0322	1	C18H16ClOS2	347.0326	1.1	19.5	1	100.00	18.0	even	ok
	2	C11H8ClN10S	347.0337	4.4	30.3	2	40.79	18.0	even	ok
	3	C18H8ClN4O2	347.0330	2.4	34.4	3	55.79	20.0	even	ok
	4	C17H12ClO6	347.0317	-1.4	34.6	4	66.71	15.0	even	ok
	5	C10H12ClN6O4S	347.0324	0.6	38.1	5	69.53	13.0	even	ok
	6	C10H20ClN2O3S3	347.0319	-0.8	41.5	6	73.67	11.0	even	ok
369.0143	1	C11H7ClN10NaS	369.0157	3.6	13.5	1	63.81	18.0	even	ok
	2	C17H11ClNaO6	369.0136	-1.9	18.7	2	80.98	15.0	even	ok
	3	C18H7ClN4NaO2	369.0150	1.7	22.3	3	78.21	20.0	even	ok
	4	C10H11ClN6NaO4S	369.0143	-0.1	23.6	4	100.00	13.0	even	ok
	5	C18H15ClNaOS2	369.0145	0.4	25.5	5	90.45	18.0	even	ok
	6	C10H19ClN2NaO3S3	369.0139	-1.3	41.2	6	62.80	11.0	even	ok

Figure S18. HRESIMS spectrum of compound **6e**

Generic Display Report

Analysis Info

Analysis Name	D:\Data\Kalaba\72416000001.d	Acquisition Date	20/08/2020 16:59:35
Method	tune_low_MS_Service_08_20.m	Operator	msc
Sample Name	CE-167-1-2-b2	Instrument	maXis
Comment	Kalaba/Zehl<br>Ergebnis +/- 5ppm<br>ACN / MeOH + 1% H2O		

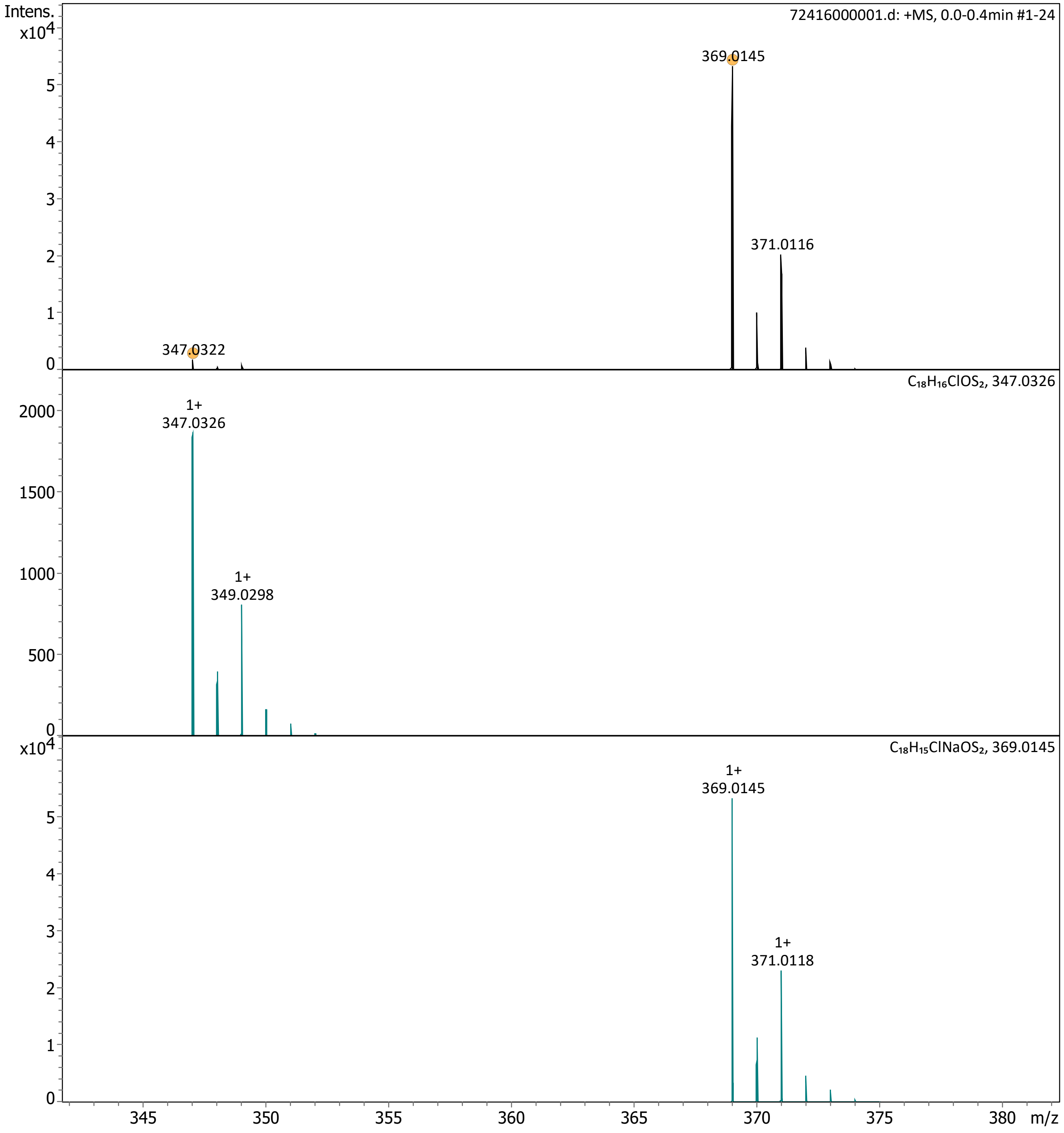

Mass Spectrum SmartFormula Report

Analysis Info

Analysis Name	D:\Data\Kalaba\72416000001.d	Acquisition Date	20/08/2020 16:59:35
Method	tune_low_MS_Service_08_20.m	Operator	msc
Sample Name	CE-167-1-2-b2	Instrument	maXis 255552.00016
Comment	Kalaba/Zehl<br>Ergebnis +/- 5ppm<br>ACN / MeOH + 1% H2O		

Acquisition Parameter

Source Type	ESI	Ion Polarity	Positive	Set Nebulizer	0.4 Bar
Focus	Not active	Set Capillary	4200 V	Set Dry Heater	180 Â°C
Scan Begin	50 m/z	Set End Plate Offset	-500 V	Set Dry Gas	4.0 l/min
Scan End	1900 m/z	Set Charging Voltage	0 V	Set Divert Valve	Source
		Set Corona	0 nA	Set APCI Heater	0 Â°C

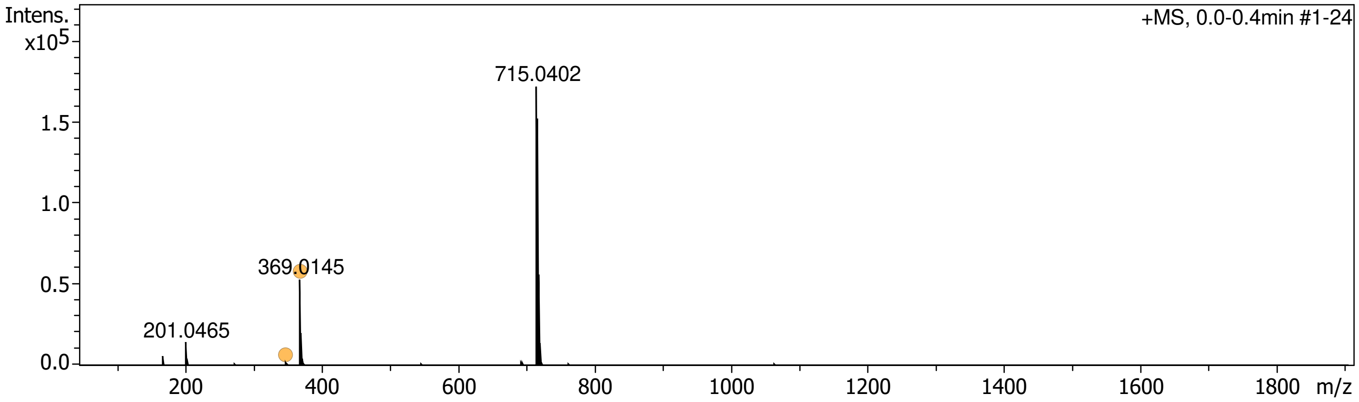

Meas. m/z	#	Ion Formula	m/z	err [ppm]	mSigma	# mSigma	Score	rdb	eÂ¯ Conf	N-Rule
347.0322	1	C18H16ClOS2	347.0326	1.0	43.4	1	93.74	18.0	even	ok
	2	C11H8ClN10S	347.0337	4.3	43.7	2	48.73	18.0	even	ok
	3	C10H12ClN6O4S	347.0324	0.5	47.3	3	90.86	13.0	even	ok
	4	C10H20ClN2O3S3	347.0319	-0.9	48.3	4	100.00	11.0	even	ok
	5	C17H12ClO6	347.0317	-1.5	48.7	5	74.43	15.0	even	ok
	6	C18H8ClN4O2	347.0330	2.4	50.2	6	60.82	20.0	even	ok
369.0145	1	C11H7ClN10NaS	369.0157	3.1	13.3	1	76.94	18.0	even	ok
	2	C17H11ClNaO6	369.0136	-2.4	17.2	2	81.82	15.0	even	ok
	3	C18H7ClN4NaO2	369.0150	1.2	20.8	3	95.14	20.0	even	ok
	4	C10H11ClN6NaO4S	369.0143	-0.6	23.8	4	99.49	13.0	even	ok
	5	C18H15ClNaOS2	369.0145	-0.1	26.9	5	100.00	18.0	even	ok
	6	C10H19ClN2NaO3S3	369.0139	-1.8	42.7	6	59.33	11.0	even	ok

Figure S19. HRESIMS spectrum of compound **7e**

Generic Display Report

Analysis Info

Analysis Name	D:\Data\Kalaba\72417000001.d	Acquisition Date	20/08/2020 17:00:59
Method	tune_low_MS_Service_08_20.m	Operator	msc
Sample Name	CE-167-2-1-b2	Instrument	maXis
Comment	Kalaba/Zehl<br>Ergebnis +/- 5ppm<br>ACN / MeOH + 1% H2O		

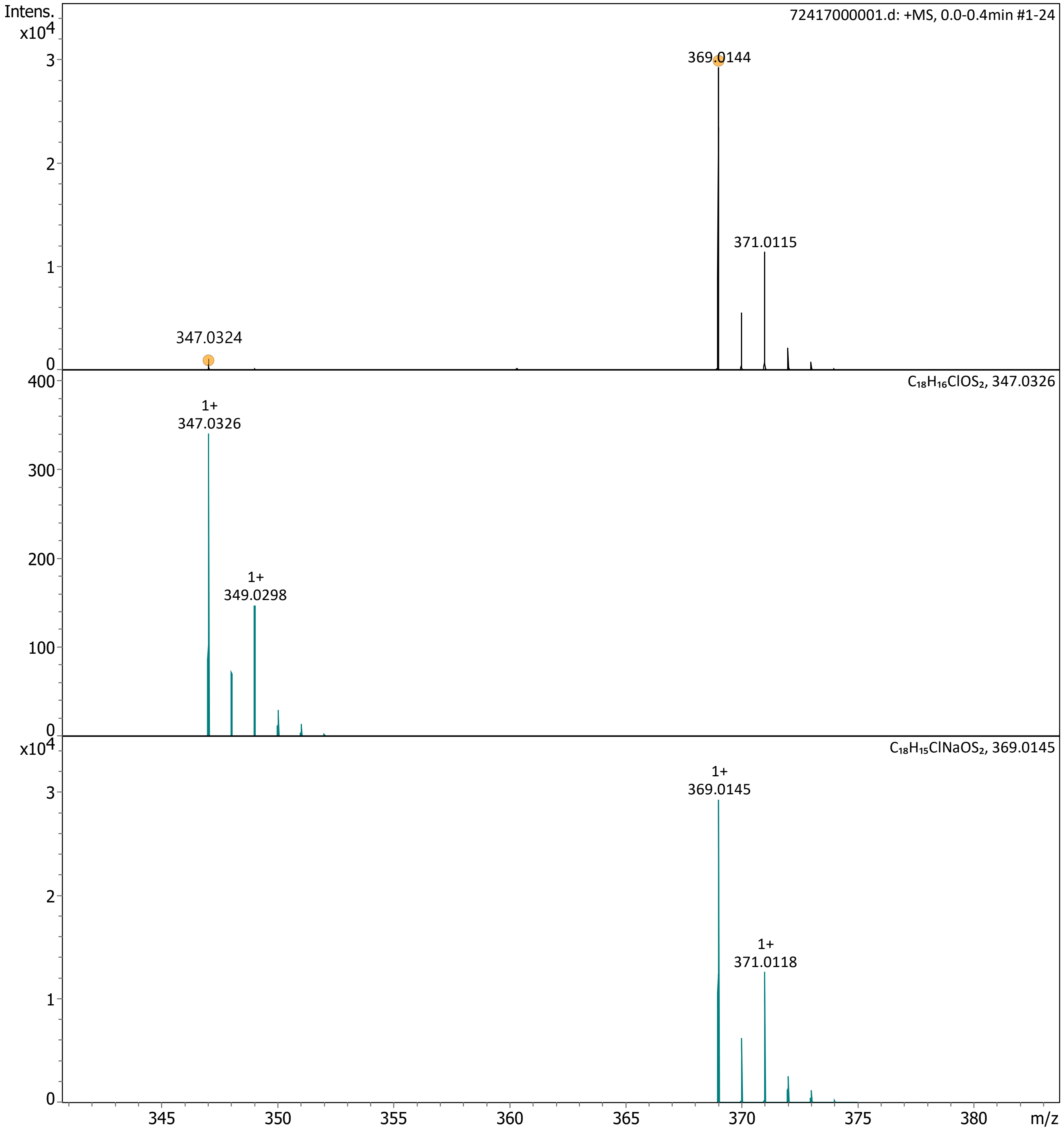

Mass Spectrum SmartFormula Report

Analysis Info

Analysis Name	D:\Data\Kalaba\72417000001.d	Acquisition Date	20/08/2020 17:00:59
Method	tune_low_MS_Service_08_20.m	Operator	msc
Sample Name	CE-167-2-1-b2	Instrument	maXis 255552.00016
Comment	Kalaba/Zehl<br>Ergebnis +/- 5ppm<br>ACN / MeOH + 1% H2O		

Acquisition Parameter

Source Type	ESI	Ion Polarity	Positive	Set Nebulizer	0.4 Bar
Focus	Not active	Set Capillary	4200 V	Set Dry Heater	180 Â°C
Scan Begin	50 m/z	Set End Plate Offset	-500 V	Set Dry Gas	4.0 l/min
Scan End	1900 m/z	Set Charging Voltage	0 V	Set Divert Valve	Source
		Set Corona	0 nA	Set APCI Heater	0 Â°C

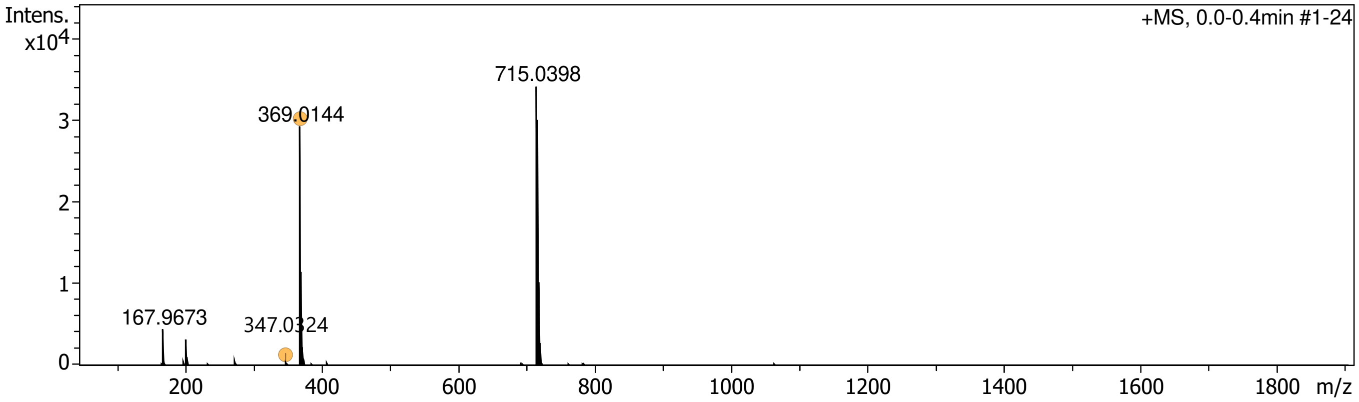

Meas. m/z	#	Ion Formula	m/z	err [ppm]	mSigma	# mSigma	Score	rdb	eÂ¯ Conf	N-Rule
347.0324	1	C10H20ClN2O3S3	347.0319	-1.5	68.1	1	100.00	11.0	even	ok
	2	C10H12ClN6O4S	347.0324	-0.2	79.1	2	67.28	13.0	even	ok
	3	C11H8ClN10S	347.0337	3.7	89.6	3	23.45	18.0	even	ok
	4	C17H12ClO6	347.0317	-2.2	104.6	4	15.69	15.0	even	ok
	5	C18H16ClOS2	347.0326	0.3	106.1	5	19.70	18.0	even	ok
	6	C18H8ClN4O2	347.0330	1.7	114.7	6	10.23	20.0	even	ok
369.0144	1	C11H7ClN10NaS	369.0157	3.5	15.7	1	63.73	18.0	even	ok
	2	C17H11ClNaO6	369.0136	-2.0	21.3	2	76.81	15.0	even	ok
	3	C18H15ClNaOS2	369.0145	0.3	22.6	3	100.00	18.0	even	ok
	4	C18H7ClN4NaO2	369.0150	1.6	24.1	4	78.19	20.0	even	ok
	5	C10H11ClN6NaO4S	369.0143	-0.2	25.3	5	96.70	13.0	even	ok
	6	C10H19ClN2NaO3S3	369.0139	-1.4	39.8	6	65.35	11.0	even	ok

Figure S20. HRESIMS spectrum of compound **8e**

Generic Display Report

Analysis Info

Analysis Name	D:\Data\Kalaba\72418000001.d	Acquisition Date	20/08/2020 17:03:35
Method	tune_low_MS_Service_08_20.m	Operator	msc
Sample Name	CE-167-2-2-b2	Instrument	maXis
Comment	Kalaba/Zehl<br>Ergebnis +/- 5ppm<br>ACN / MeOH + 1% H2O		

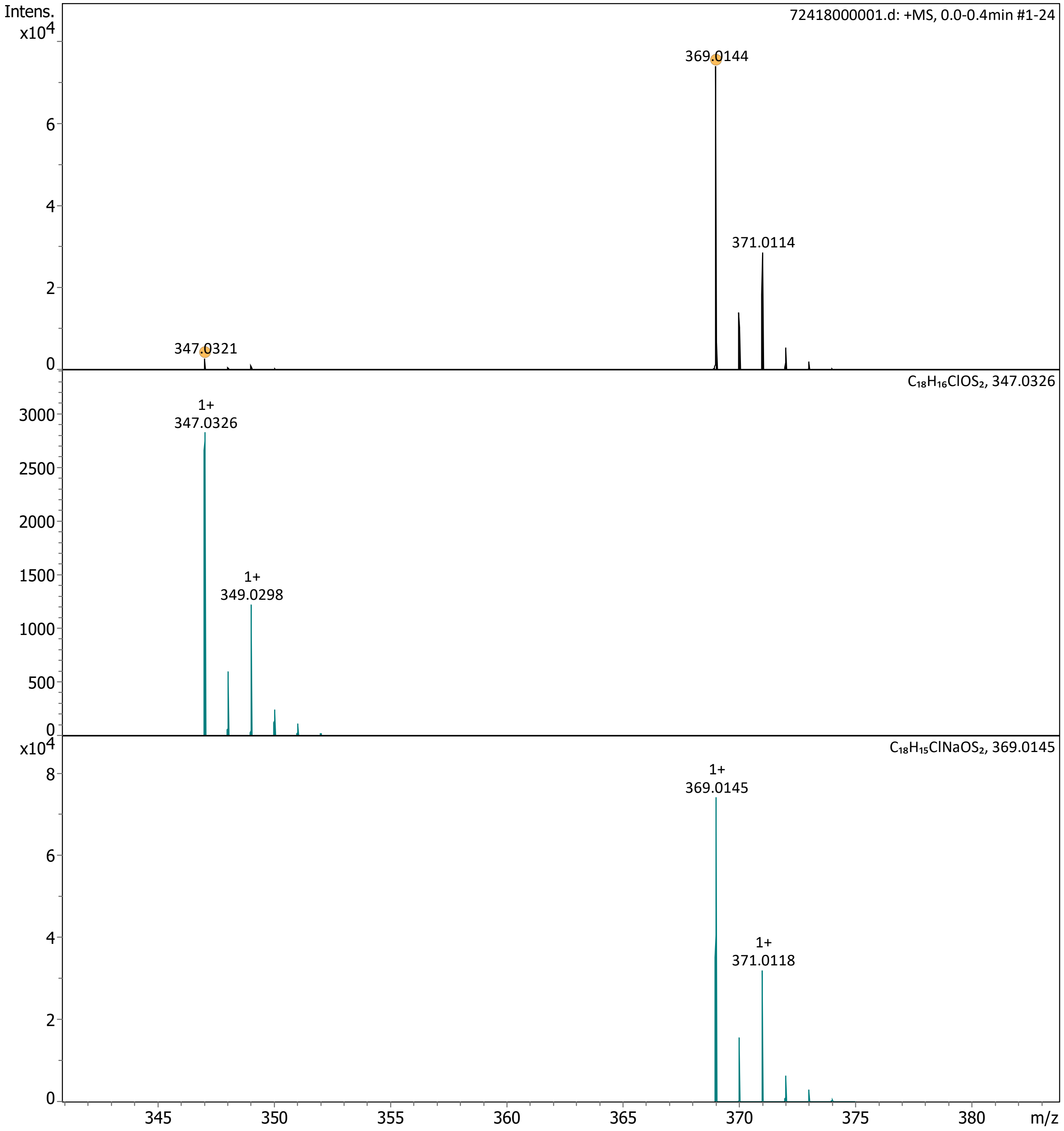

Mass Spectrum SmartFormula Report

Analysis Info

Analysis Name	D:\Data\Kalaba\72418000001.d	Acquisition Date	20/08/2020 17:03:35
Method	tune_low_MS_Service_08_20.m	Operator	msc
Sample Name	CE-167-2-2-b2	Instrument	maXis 255552.00016
Comment	Kalaba/Zehl<br>Ergebnis +/- 5ppm<br>ACN / MeOH + 1% H2O		

Acquisition Parameter

Source Type	ESI	Ion Polarity	Positive	Set Nebulizer	0.4 Bar
Focus	Not active	Set Capillary	4200 V	Set Dry Heater	180 Â°C
Scan Begin	50 m/z	Set End Plate Offset	-500 V	Set Dry Gas	4.0 l/min
Scan End	1900 m/z	Set Charging Voltage	0 V	Set Divert Valve	Source
		Set Corona	0 nA	Set APCI Heater	0 Â°C

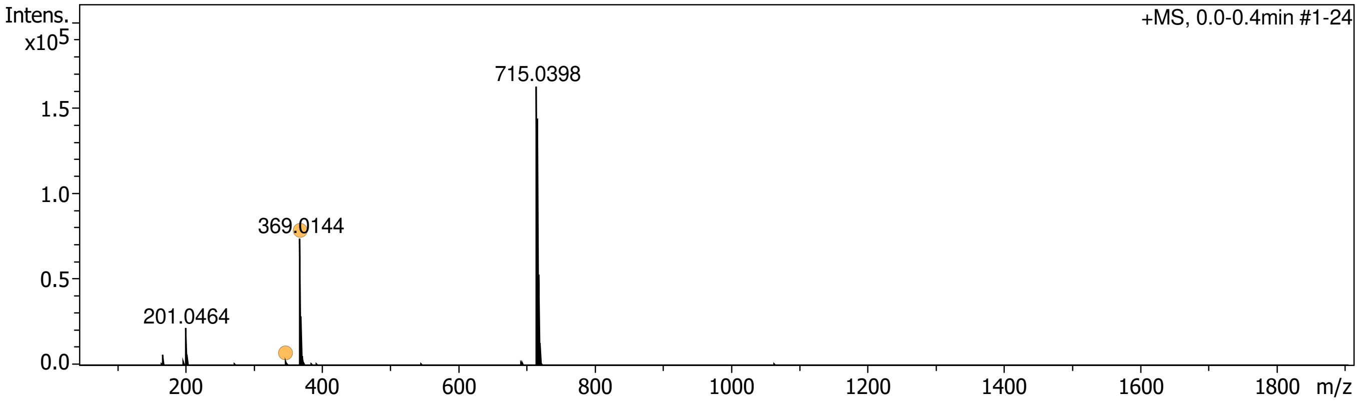

Meas. m/z	#	Ion Formula	m/z	err [ppm]	mSigma	# mSigma	Score	rdb	eÂ¯ Conf	N-Rule
347.0321	1	C18H16ClOS2	347.0326	1.3	19.9	1	100.00	18.0	even	ok
	2	C18H8ClN4O2	347.0330	2.6	33.5	2	57.32	20.0	even	ok
	3	C11H8ClN10S	347.0337	4.6	34.4	3	36.71	18.0	even	ok
	4	C17H12ClO6	347.0317	-1.2	35.9	4	68.62	15.0	even	ok
	5	C10H12ClN6O4S	347.0324	0.7	43.2	5	61.01	13.0	even	ok
	6	C10H20ClN2O3S3	347.0319	-0.6	46.1	6	68.41	11.0	even	ok
369.0144	1	C11H7ClN10NaS	369.0157	3.5	14.3	1	67.89	18.0	even	ok
	2	C17H11ClNaO6	369.0136	-2.0	19.6	2	83.24	15.0	even	ok
	3	C18H7ClN4NaO2	369.0150	1.6	22.7	3	83.69	20.0	even	ok
	4	C18H15ClNaOS2	369.0145	0.3	24.3	4	100.00	18.0	even	ok

Figure S21. HRESIMS spectrum of compound **5f**

Generic Display Report

Analysis Info

Analysis Name	E:\Data\MS_MessService\58951000001.d	Acquisition Date	8/14/2018 5:17:54 PM
Method	tune_low_MS_Service_08_18.m	Operator	msc
Sample Name	CE-140-SPOT1-PEAK1	Instrument	maXis
Comment	Kalaba/Zehl<br>Ergebnis: +/- 5ppm<br>ACN/MeOH +0.1% H2O		

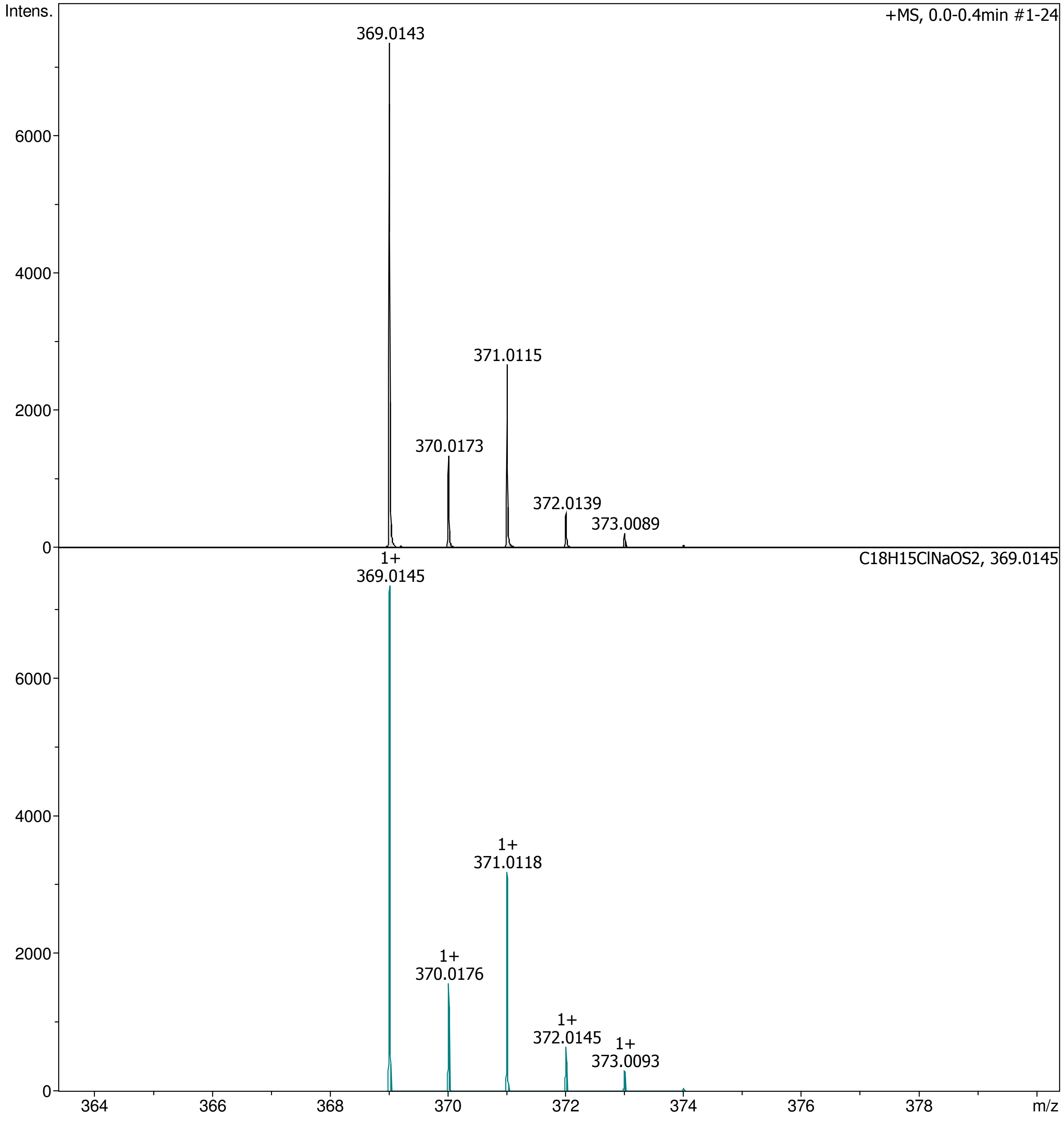

Mass Spectrum SmartFormula Report

Analysis Info

Analysis Name	E:\Data\MS_MessService\58951000001.d	Acquisition Date	8/14/2018 5:17:54 PM
Method	tune_low_MS_Service_08_18.m	Operator	msc
Sample Name	CE-140-SPOT1-PEAK1	Instrument	maXis 255552.00016
Comment	Kalaba/Zehl<br>Ergebnis: +/- 5ppm<br>ACN/MeOH +0.1% H2O		

Acquisition Parameter

Source Type	ESI	Ion Polarity	Positive	Set Nebulizer	0.4 Bar
Focus	Not active	Set Capillary	4500 V	Set Dry Heater	180 °C
Scan Begin	50 m/z	Set End Plate Offset	-500 V	Set Dry Gas	4.0 l/min
Scan End	1900 m/z	Set Charging Voltage	0 V	Set Divert Valve	Source
		Set Corona	0 nA	Set APCI Heater	0 °C

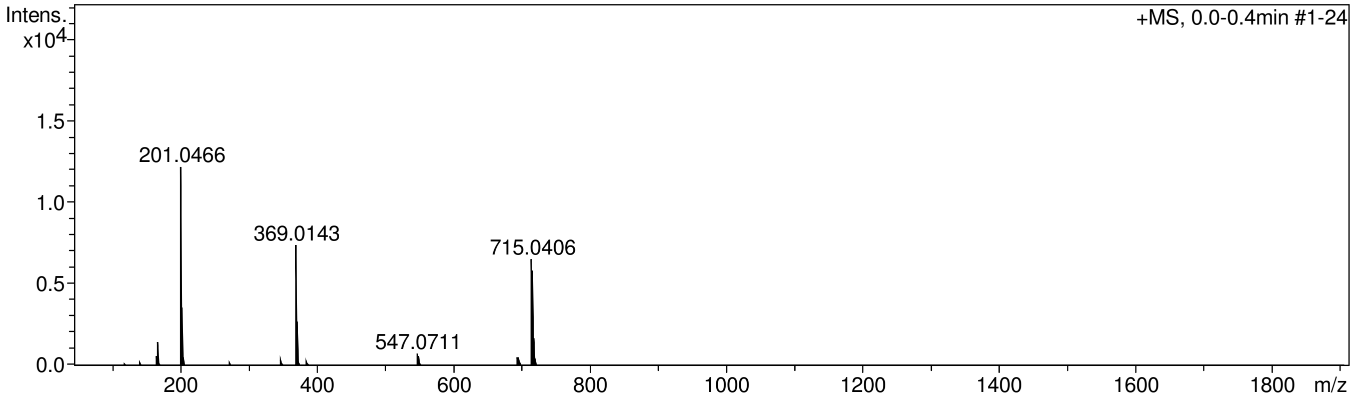

Meas. m/z	#	Ion Formula	Score	m/z	err [mDa]	err [ppm]	mSigma	rdb	e⁻ Conf	N-Rule
369.014277	1	C17H11ClNaO6	100.00	369.013636	-0.6	-1.7	10.8	11.5	even	ok
	2	C11H7ClN10NaS	64.20	369.015660	-1.4	-3.7	11.7	12.5	even	ok
	3	C16H5ClN7NaO	90.72	369.013631	0.6	1.8	15.6	17.0	odd	ok
	4	C18H7ClN4NaO2	85.42	369.014974	0.7	1.9	17.3	16.5	even	ok
	5	C12H13ClN3NaO5S	56.56	369.015665	-1.4	-3.8	17.9	7.0	odd	ok
	6	C16H13ClN3NaS2	52.29	369.013163	-1.1	-3.0	29.0	11.0	odd	ok
	7	C18H15ClNaOS2	75.20	369.014505	0.2	0.6	32.7	10.5	even	ok

Figure S22. HRESIMS spectrum of compound **6f**

Generic Display Report

Analysis Info

Analysis Name	E:\Data\MS_MessService\58952000001.d	Acquisition Date	8/14/2018 5:19:55 PM
Method	tune_low_MS_Service_08_18.m	Operator	msc
Sample Name	CE-140-SPOT1-PEAK2	Instrument	maXis
Comment	Kalaba/Zehl<br>Ergebnis: +/- 5ppm<br>ACN/MeOH +0.1% H2O		

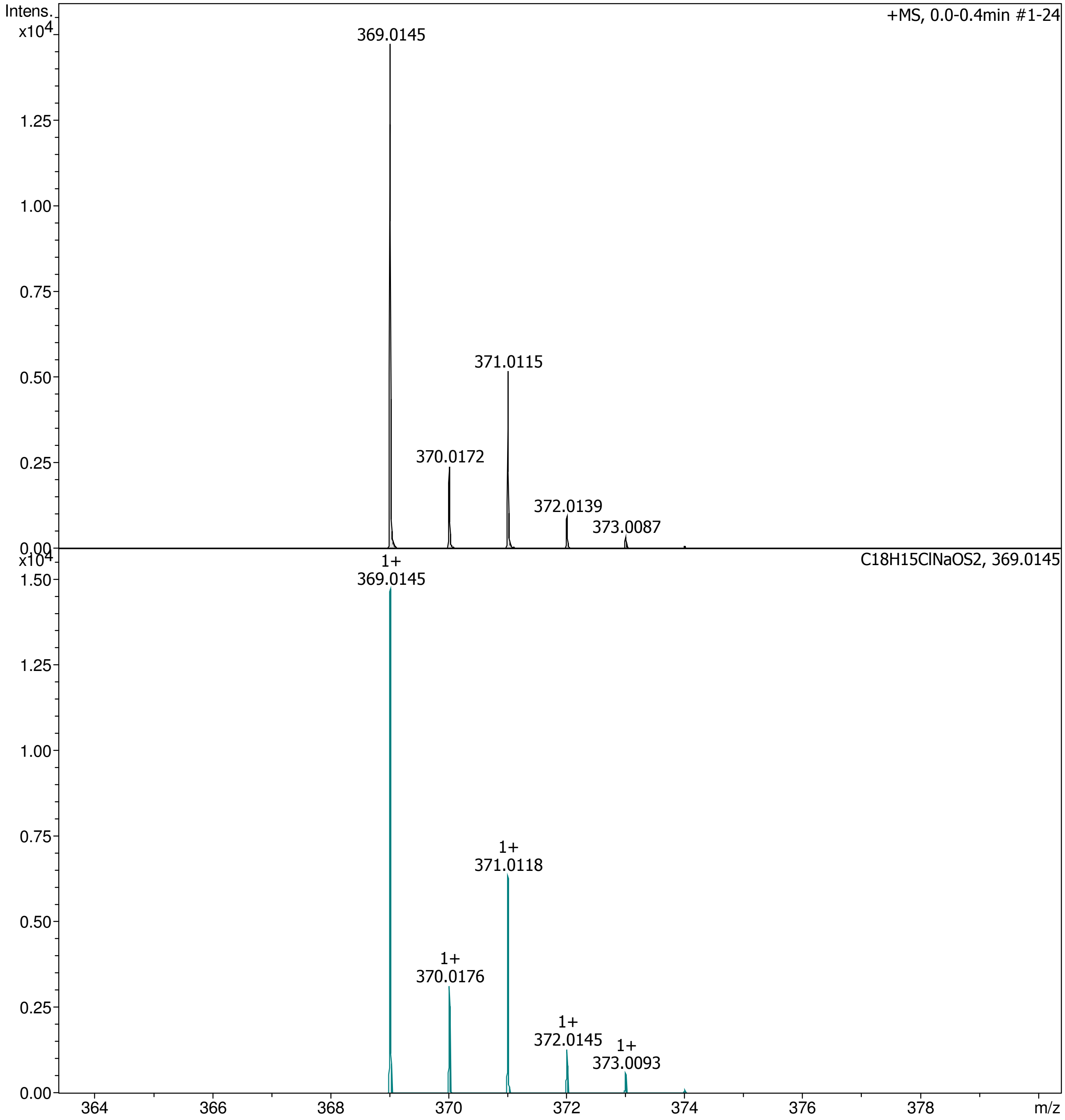

Mass Spectrum SmartFormula Report

Analysis Info

Analysis Name	E:\Data\MS_MessService\58952000001.d	Acquisition Date	8/14/2018 5:19:55 PM
Method	tune_low_MS_Service_08_18.m	Operator	msc
Sample Name	CE-140-SPOT1-PEAK2	Instrument	maXis 255552.00016
Comment	Kalaba/Zehl<br>Ergebnis: +/- 5ppm<br>ACN/MeOH +0.1% H2O		

Acquisition Parameter

Source Type	ESI	Ion Polarity	Positive	Set Nebulizer	0.4 Bar
Focus	Not active	Set Capillary	4500 V	Set Dry Heater	180 °C
Scan Begin	50 m/z	Set End Plate Offset	-500 V	Set Dry Gas	4.0 l/min
Scan End	1900 m/z	Set Charging Voltage	0 V	Set Divert Valve	Source
		Set Corona	0 nA	Set APCI Heater	0 °C

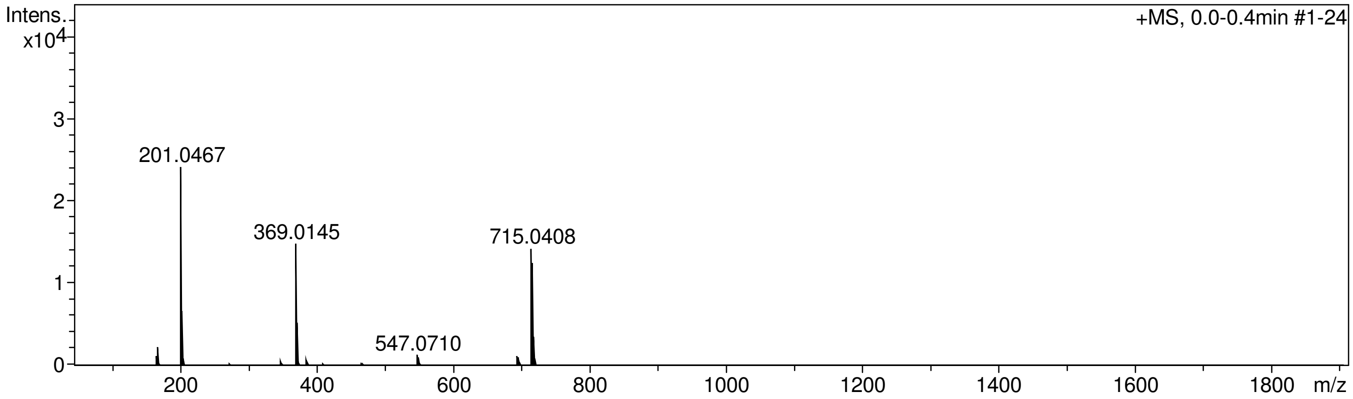

Meas. m/z	#	Ion Formula	Score	m/z	err [mDa]	err [ppm]	mSigma	rdb	e⁻ Conf	N-Rule
369.014535	1	C11H7ClN10NaS	87.10	369.015660	-1.1	-3.0	11.8	12.5	even	ok
	2	C17H11ClNaO6	97.55	369.013636	0.9	2.4	12.7	11.5	even	ok
	3	C12H13ClN3NaO5S	79.47	369.015665	-1.1	-3.1	16.3	7.0	odd	ok
	4	C16H5ClN7NaO	86.24	369.013631	0.9	2.4	18.7	17.0	odd	ok
	5	C18H7ClN4NaO2	100.00	369.014974	-0.4	-1.2	22.8	16.5	even	ok
	6	C20H9ClNNaO3	40.52	369.016317	-1.8	-4.8	27.5	16.0	odd	ok
	7	C16H13ClN3NaS2	42.85	369.013163	1.4	3.7	36.5	11.0	odd	ok
	8	C18H15ClNaOS2	76.70	369.014505	-0.0	-0.1	40.7	10.5	even	ok

Figure S23. HRESIMS spectrum of compound **7f**

Generic Display Report

Analysis Info

Analysis Name	E:\Data\MS_MessService\58953000001.d	Acquisition Date	8/14/2018 5:21:38 PM
Method	tune_low_MS_Service_08_18.m	Operator	msc
Sample Name	CE-140-SPOT2-PEAK1	Instrument	maXis
Comment	Kalaba/Zehl<br>Ergebnis: +/- 5ppm<br>ACN/MeOH +0.1% H2O		

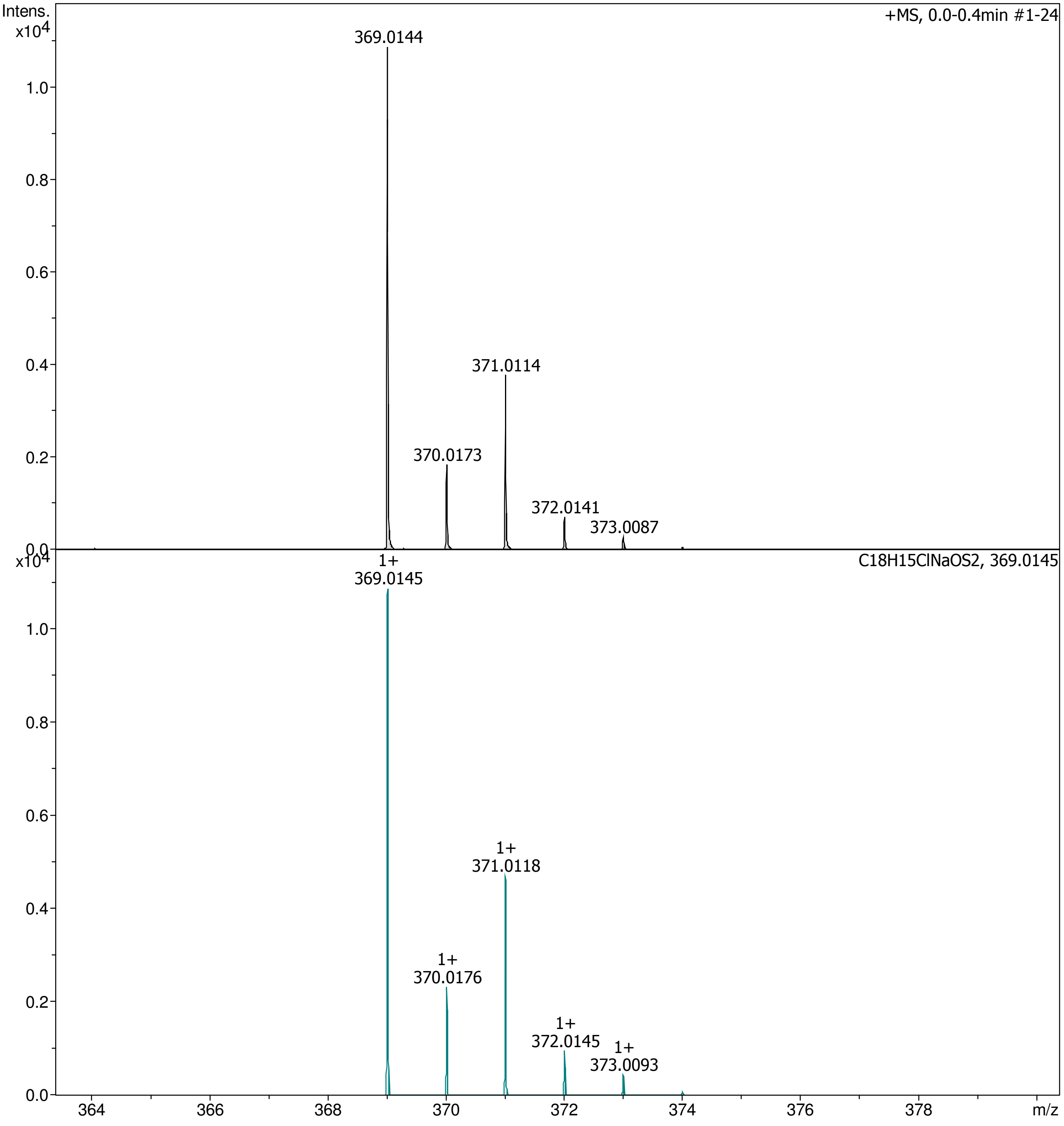

Mass Spectrum SmartFormula Report

Analysis Info

Acquisition Date 8/14/2018 5:21:38 PM

Analysis Name	E:\Data\MS_MessService\58953000001.d		
Method	tune_low_MS_Service_08_18.m	Operator	msc
Sample Name	CE-140-SPOT2-PEAK1	Instrument	maXis 255552.00016
Comment	Kalaba/Zehl<br>Ergebnis: +/- 5ppm<br>ACN/MeOH +0.1% H2O		

Acquisition Parameter

Source Type	ESI	Ion Polarity	Positive	Set Nebulizer	0.4 Bar
Focus	Not active	Set Capillary	4500 V	Set Dry Heater	180 °C
Scan Begin	50 m/z	Set End Plate Offset	-500 V	Set Dry Gas	4.0 l/min
Scan End	1900 m/z	Set Charging Voltage	0 V	Set Divert Valve	Source
		Set Corona	0 nA	Set APCI Heater	0 °C

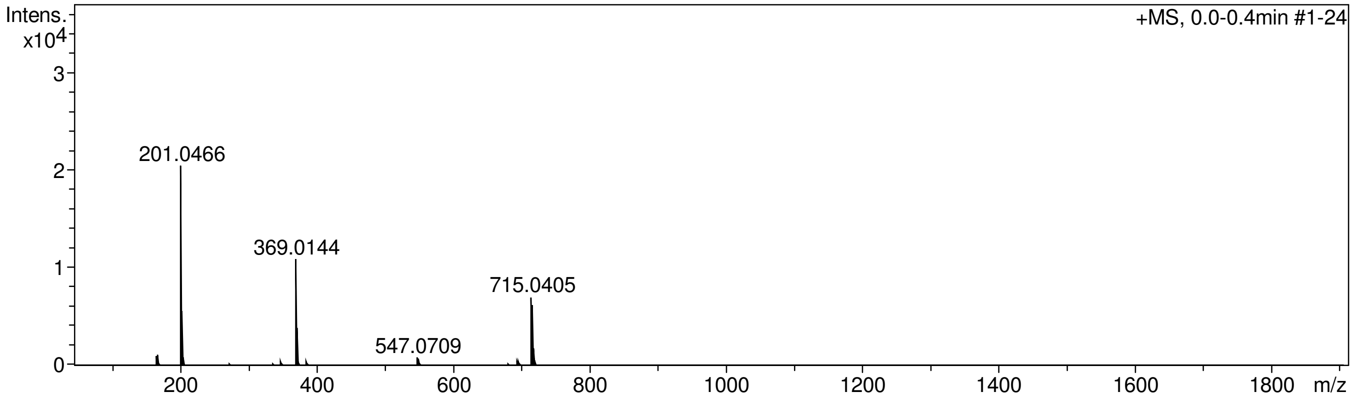

Meas. m/z	#	Ion Formula	Score	m/z	err [mDa]	err [ppm]	mSigma	rdb	e⁻ Conf	N-Rule
369.014397	1	C17H11ClNaO6	100.00	369.013636	-0.8	-2.1	11.1	11.5	even	ok
	2	C11H7ClN10NaS	70.58	369.015660	1.3	3.4	14.3	12.5	even	ok
	3	C16H5ClN7NaO	89.82	369.013631	-0.8	-2.1	16.4	17.0	odd	ok
	4	C12H13ClN3NaO5S	63.52	369.015665	1.3	3.4	19.2	7.0	odd	ok
	5	C18H7ClN4NaO2	90.63	369.014974	-0.6	-1.6	20.6	16.5	even	ok
	6	C16H13ClN3NaS2	42.21	369.013163	-1.2	-3.3	37.3	11.0	odd	ok
	7	C18H15ClNaOS2	67.50	369.014505	0.1	0.3	41.3	10.5	even	ok

Figure S24. HRESIMS spectrum of compound **8f**

Generic Display Report

Analysis Info

Analysis Name	E:\Data\MS_MessService\58954000001.d	Acquisition Date	8/14/2018 5:23:13 PM
Method	tune_low_MS_Service_08_18.m	Operator	msc
Sample Name	CE-140-SPOT2-PEAK2	Instrument	maXis
Comment	Kalaba/Zehl<br>Ergebnis: +/- 5ppm<br>ACN/MeOH +0.1% H2O		

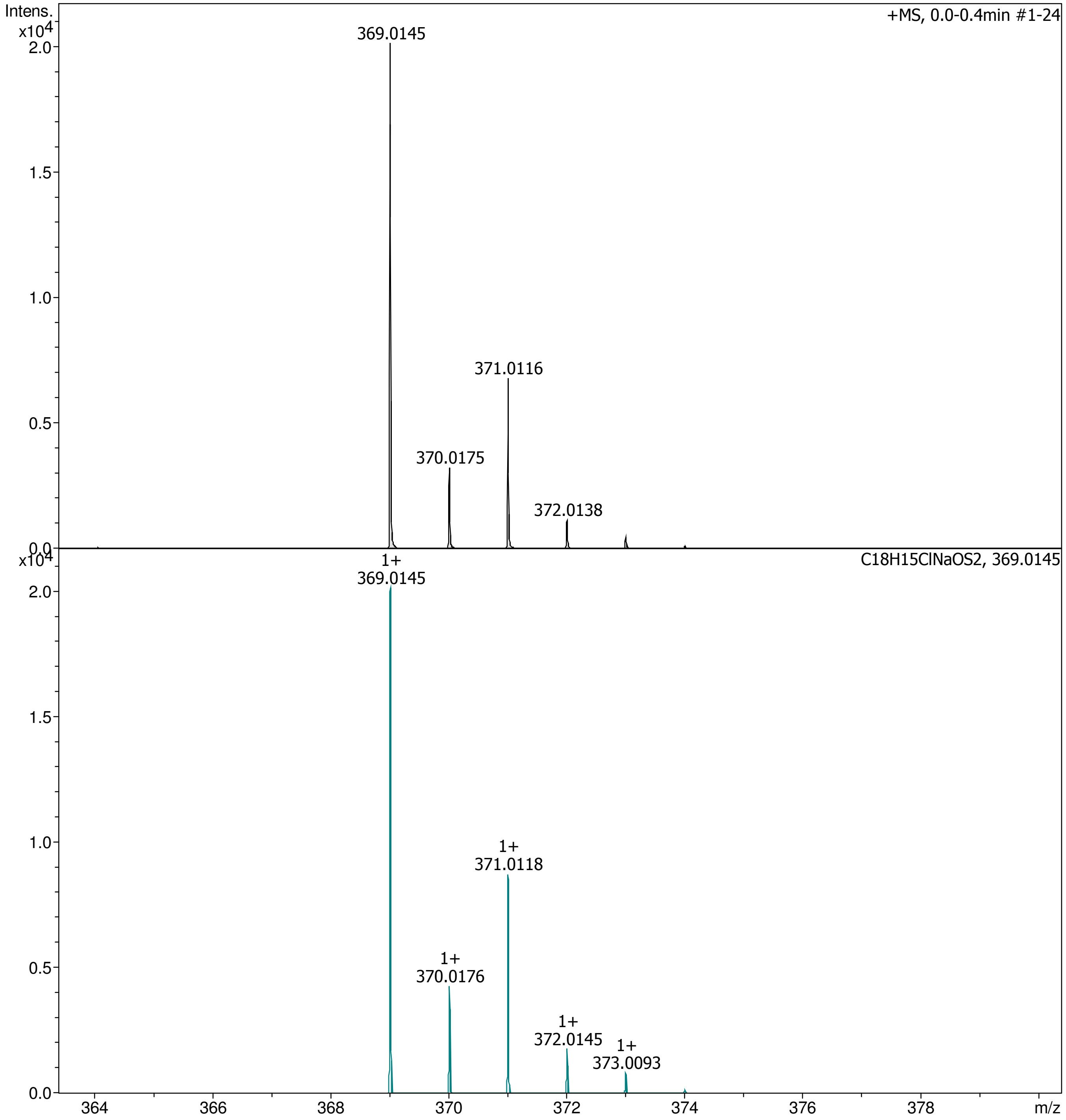

Mass Spectrum SmartFormula Report

Analysis Info

Analysis Name	E:\Data\MS_MessService\58954000001.d	Acquisition Date	8/14/2018 5:23:13 PM
Method	tune_low_MS_Service_08_18.m	Operator	msc
Sample Name	CE-140-SPOT2-PEAK2	Instrument	maXis 255552.00016
Comment	Kalaba/Zehl<br>Ergebnis: +/- 5ppm<br>ACN/MeOH +0.1% H2O		

Acquisition Parameter

Source Type	ESI	Ion Polarity	Positive	Set Nebulizer	0.4 Bar
Focus	Not active	Set Capillary	4500 V	Set Dry Heater	180 °C
Scan Begin	50 m/z	Set End Plate Offset	-500 V	Set Dry Gas	4.0 l/min
Scan End	1900 m/z	Set Charging Voltage	0 V	Set Divert Valve	Source
		Set Corona	0 nA	Set APCI Heater	0 °C

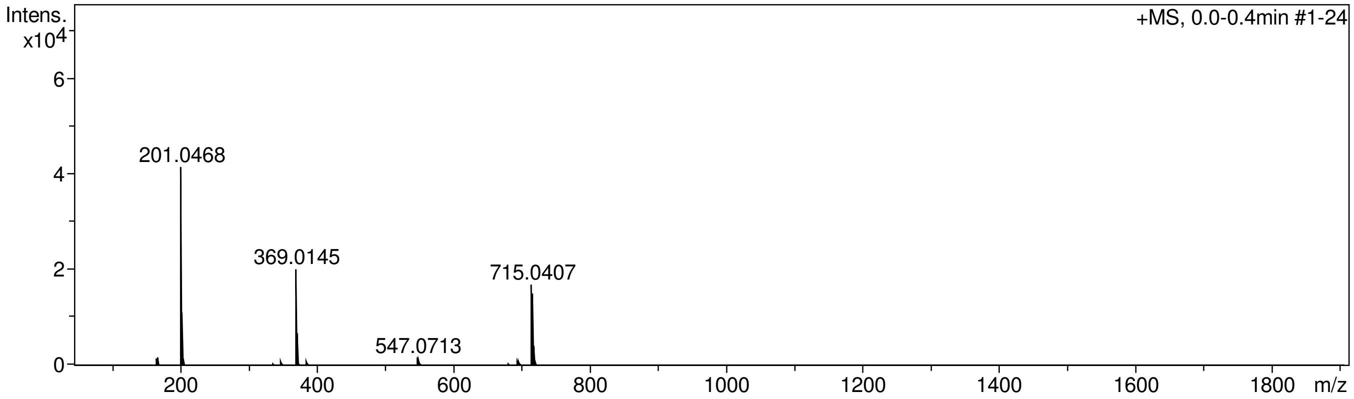

Meas. m/z	#	Ion Formula	Score	m/z	err [mDa]	err [ppm]	mSigma	rdb	e⁻ Conf	N-Rule
369.014529	1	C17H11ClNaO6	97.86	369.013636	-0.9	-2.4	14.6	11.5	even	ok
	2	C11H7ClN10NaS	79.69	369.015660	-1.1	-3.1	18.0	12.5	even	ok
	3	C16H5ClN7NaO	88.56	369.013631	-0.9	-2.4	19.3	17.0	odd	ok
	4	C12H13ClN3NaO5S	73.18	369.015665	-1.1	-3.1	21.9	7.0	odd	ok
	5	C18H7ClN4NaO2	100.00	369.014974	-0.4	-1.2	24.4	16.5	even	ok
	6	C20H9ClNNaO3	39.80	369.016317	-1.8	-4.8	29.7	16.0	odd	ok
	7	C16H13ClN3NaS2	37.71	369.013163	1.4	3.7	42.7	11.0	odd	ok
	8	C18H15ClNaOS2	67.06	369.014505	0.0	0.1	46.8	10.5	even	ok

Figure S25. HRESIMS spectrum of compound **5g**

Generic Display Report

Analysis Info

Analysis Name	D:\Data\Kalaba\71580000001.d	Acquisition Date	10/07/2020 17:47:41
Method	tune_low_MS_Service_07_20.m	Operator	msc
Sample Name	PN-14-1	Instrument	maXis
Comment	Kalaba/Zehl<br>Ergebnis +/- 5ppm<br>ACN/MeOH + 1 % H2O		

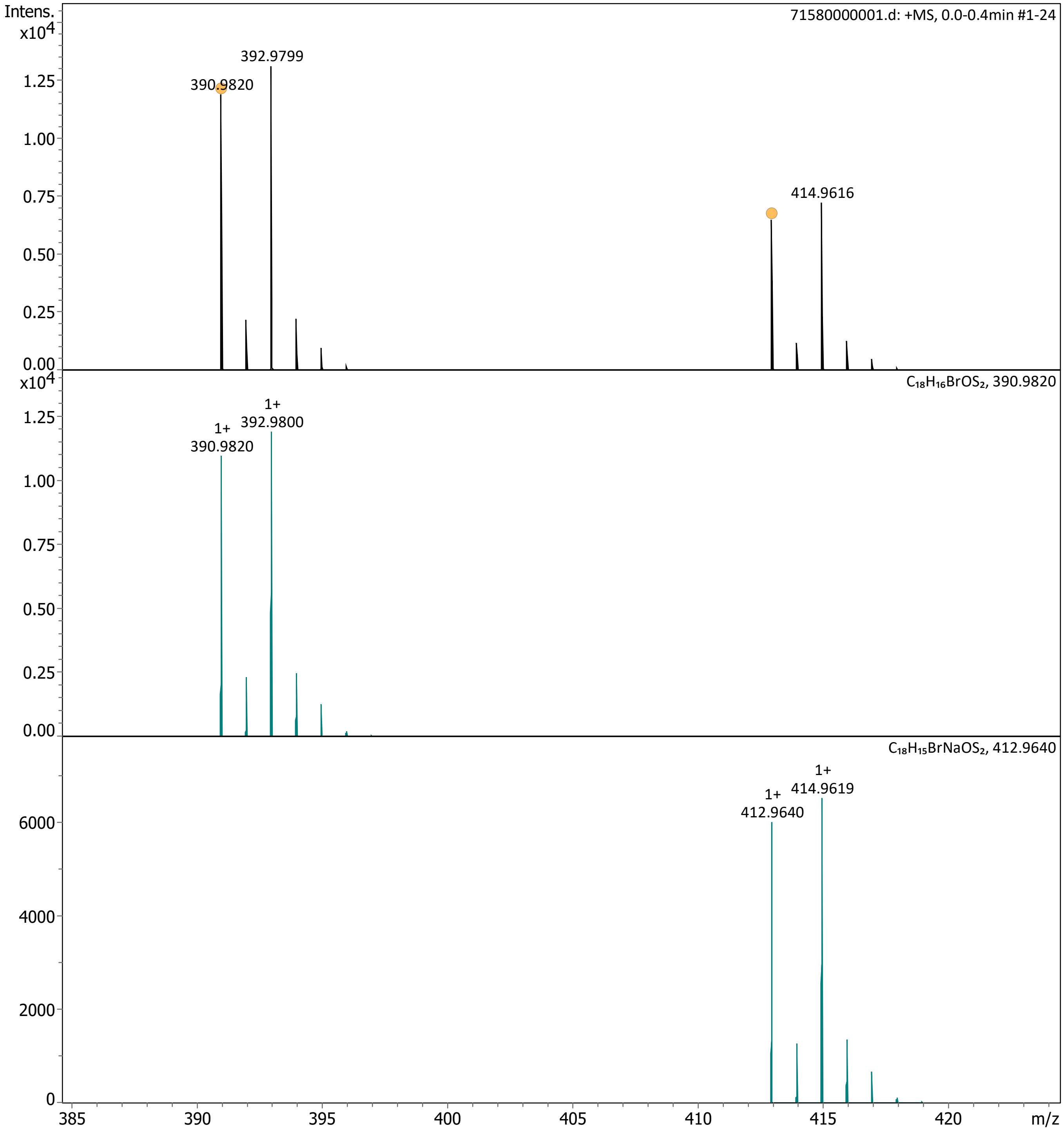

Mass Spectrum SmartFormula Report

Analysis Info

Analysis Name	D:\Data\Kalaba\71580000001.d	Acquisition Date	10/07/2020 17:47:41
Method	tune_low_MS_Service_07_20.m	Operator	msc
Sample Name	PN-14-1	Instrument	maXis 255552.00016
Comment	Kalaba/Zehl<br>Ergebnis +/- 5ppm<br>ACN/MeOH + 1 % H2O		

Acquisition Parameter

Source Type	ESI	Ion Polarity	Positive	Set Nebulizer	0.4 Bar
Focus	Not active	Set Capillary	4200 V	Set Dry Heater	180 Â°C
Scan Begin	50 m/z	Set End Plate Offset	-500 V	Set Dry Gas	4.0 l/min
Scan End	1900 m/z	Set Charging Voltage	0 V	Set Divert Valve	Source
		Set Corona	0 nA	Set APCI Heater	0 Â°C

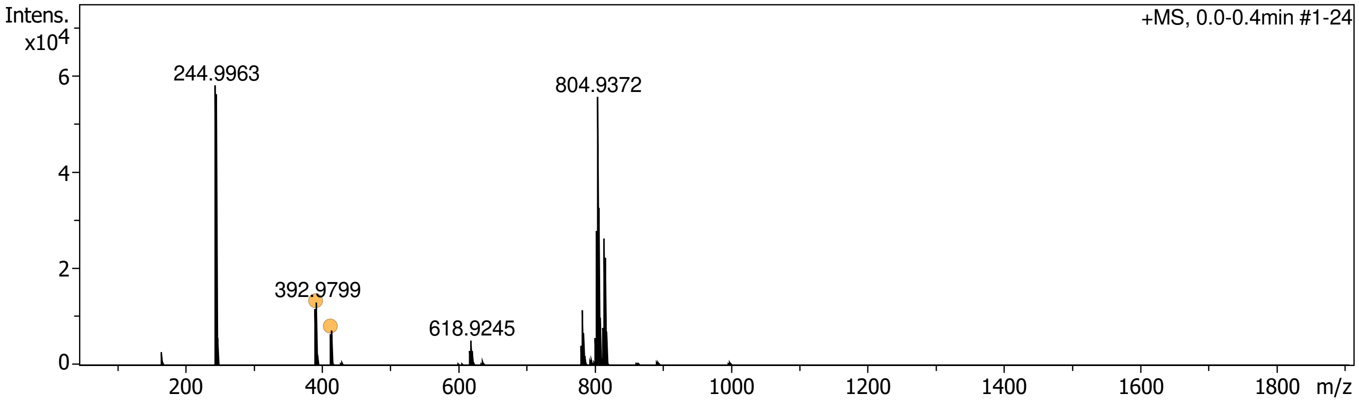

Meas. m/z	#	Ion Formula	m/z	err [ppm]	mSigma	# mSigma	Score	rdb	eÂ¯ Conf	N-Rule
390.9820	1	C18H16BrOS2	390.9820	0.1	24.2	1	100.00	17.0	even	ok
	2	C11H8BrN10S	390.9832	3.1	30.3	2	40.23	17.0	even	ok
	3	C17H12BrO6	390.9812	-2.1	47.5	3	31.80	14.0	even	ok
	4	C18H8BrN4O2	390.9825	1.3	54.2	4	29.92	19.0	even	ok
412.9635	1	C18H15BrNaOS2	412.9640	1.1	24.0	1	100.00	17.0	even	ok
	2	C11H7BrN10NaS	412.9651	3.9	32.4	2	34.90	17.0	even	ok
	3	C17H11BrNaO6	412.9631	-1.0	48.4	3	45.60	14.0	even	ok
	4	C14H3BrN10Na	412.9618	-4.2	51.7	4	18.75	20.0	even	ok
	5	C18H7BrN4NaO2	412.9645	2.3	54.5	5	28.57	19.0	even	ok

Figure S26. HRESIMS spectrum of compound **6g**

Generic Display Report

Analysis Info

Analysis Name	D:\Data\Kalaba\78518000001.d	Acquisition Date	26/03/2021 13:40:00
Method	tune_low_MS_Service_03_21.m	Operator	msc
Sample Name	PN-14-peak-2	Instrument	maXis
Comment	Kalaba / Zehl<br>Ergebnis +/- 5 ppm<br>ACN / MeOH + 1% H2O		

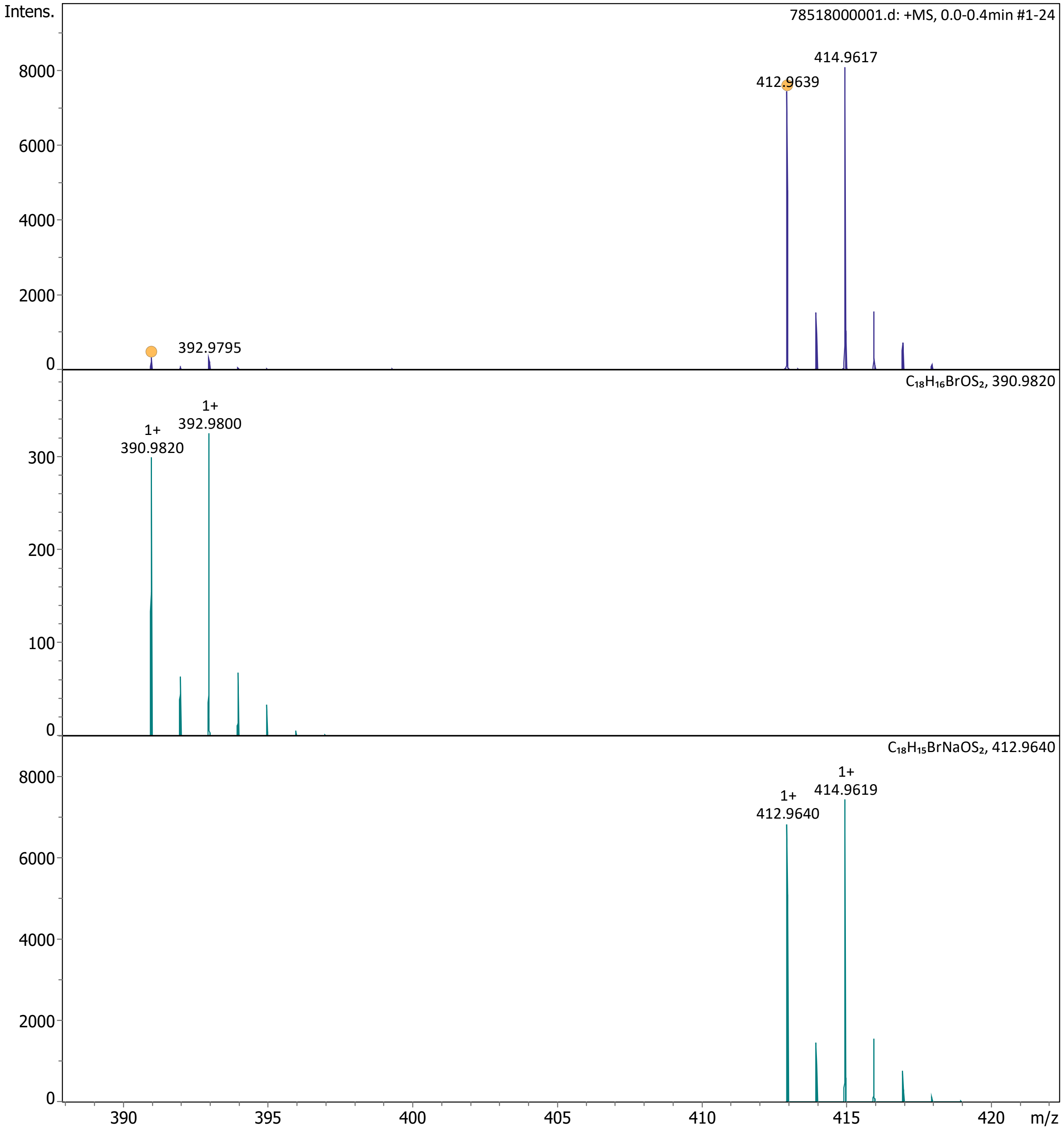

Mass Spectrum SmartFormula Report

Analysis Info

Analysis Name	D:\Data\Kalaba\78518000001.d	Acquisition Date	26/03/2021 13:40:00
Method	tune_low_MS_Service_03_21.m	Operator	msc
Sample Name	PN-14-peak-2	Instrument	maXis 255552.00016
Comment	Kalaba / Zehl<br>Ergebnis +/- 5 ppm<br>ACN / MeOH + 1% H2O		

Acquisition Parameter

Source Type	ESI	Ion Polarity	Positive	Set Nebulizer	0.4 Bar
Focus	Not active	Set Capillary	4200 V	Set Dry Heater	180 Â°C
Scan Begin	80 m/z	Set End Plate Offset	-500 V	Set Dry Gas	4.0 l/min
Scan End	1900 m/z	Set Charging Voltage	0 V	Set Divert Valve	Source
		Set Corona	0 nA	Set APCI Heater	0 Â°C

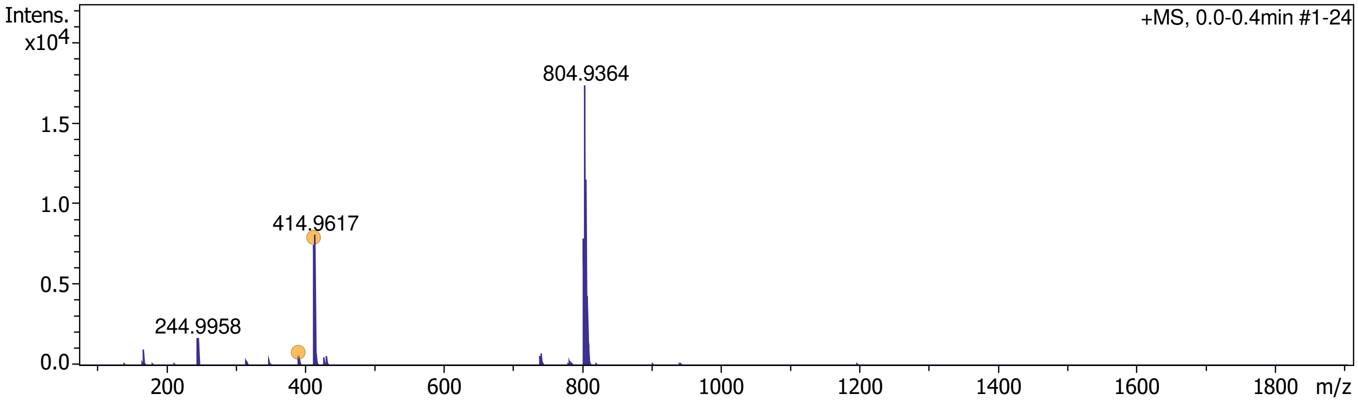

Meas. m/z	#	Ion Formula	Score	m/z	err [mDa]	err [ppm]	mSigma	rdb	eÂ¯ Conf	N-Rule
390.9817	1	C18H16BrOS2	100.00	390.9820	0.4	0.9	24.4	17.0	even	ok
	2	C18H8BrN4O2	56.20	390.9825	0.8	2.1	31.2	19.0	even	ok
	3	C17H12BrO6	57.54	390.9812	-0.5	-1.3	36.5	14.0	even	ok
	4	C14H4BrN10	22.69	390.9798	-1.9	-4.8	40.7	20.0	even	ok
	5	C11H8BrN10S	28.85	390.9832	1.5	3.9	40.9	17.0	even	ok
	6	C10H12BrN6O4S	39.65	390.9819	0.2	0.4	54.7	12.0	even	ok
	7	C10H20BrN2O3S3	32.80	390.9814	-0.3	-0.8	64.1	10.0	even	ok
412.9639	1	C18H15BrNaOS2	100.00	412.9640	0.1	0.3	9.7	17.0	even	ok
	2	C11H7BrN10NaS	27.10	412.9651	1.3	3.1	33.6	17.0	even	ok
	3	C10H19BrN2NaO3S3	42.41	412.9633	-0.5	-1.3	39.8	10.0	even	ok
	4	C10H11BrN6NaO4S	41.20	412.9638	-0.0	-0.1	41.8	12.0	even	ok
	5	C17H11BrNaO6	27.30	412.9631	-0.7	-1.8	45.1	14.0	even	ok
	6	C18H7BrN4NaO2	26.72	412.9645	0.6	1.5	48.1	19.0	even	ok
	7	C14H3BrN10Na	10.05	412.9618	-2.1	-5.0	49.3	20.0	even	ok

Figure S27. HRESIMS spectrum of compound **7g**

Generic Display Report

Analysis Info

Analysis Name	D:\Data\Kalaba\78519000001.d	Acquisition Date	26/03/2021 15:25:17
Method	tune_low_MS_Service_03_21.m	Operator	msc
Sample Name	PN-14-peak-3	Instrument	maXis
Comment	Kalaba / Zehl<br>Ergebnis +/- 5 ppm<br>ACN / MeOH + 1% H2O		

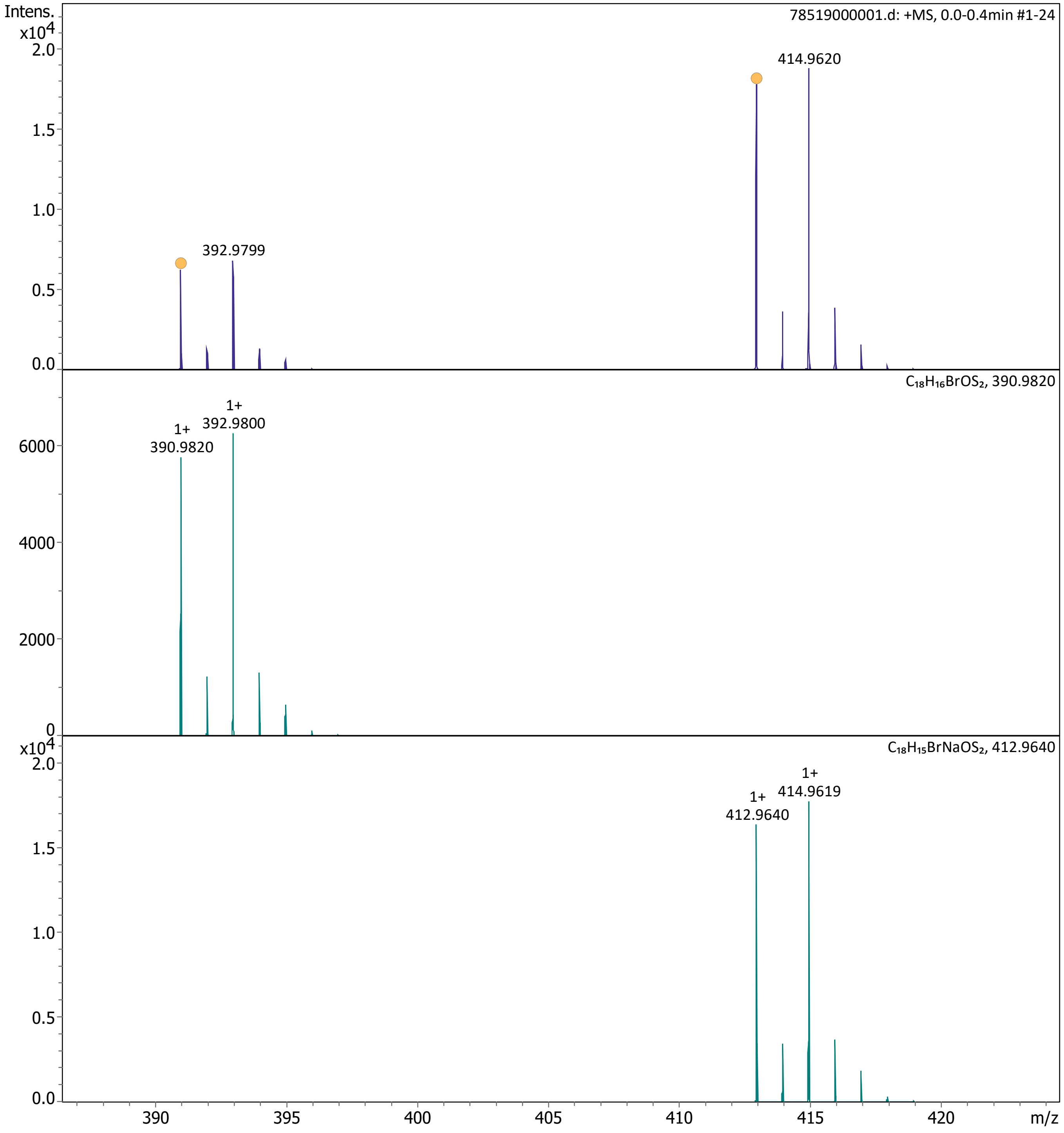

Mass Spectrum SmartFormula Report

Analysis Info

Analysis Name	D:\Data\Kalaba\78519000001.d
Method	tune_low_MS_Service_03_21.m
Sample Name	PN-14-peak-3
Comment	Kalaba / Zehl<br>Ergebnis +/- 5 ppm<br>ACN / MeOH + 1% H2O

Acquisition Date	26/03/2021 15:25:17	
Operator	msc	
Instrument	maXis	255552.00016

Acquisition Parameter

Source Type	ESI	Ion Polarity	Positive	Set Nebulizer	0.4 Bar
Focus	Not active	Set Capillary	4200 V	Set Dry Heater	180 Â°C
Scan Begin	80 m/z	Set End Plate Offset	-500 V	Set Dry Gas	4.0 l/min
Scan End	1900 m/z	Set Charging Voltage	0 V	Set Divert Valve	Source
		Set Corona	0 nA	Set APCI Heater	0 Â°C

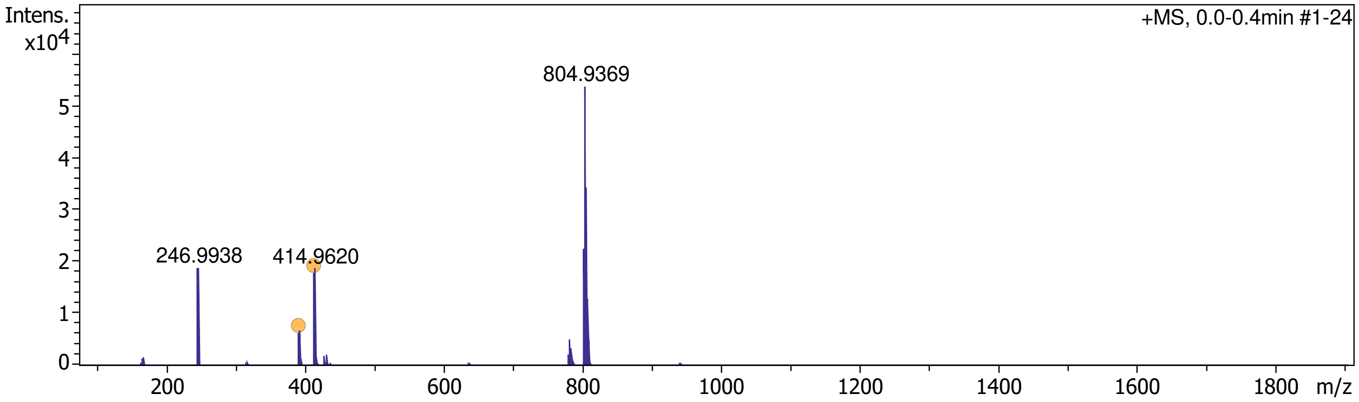

Meas. m/z	#	Ion Formula	Score	m/z	err [mDa]	err [ppm]	mSigma	rdb	eÂ¯ Conf	N-Rule
390.9819	1	C18H16BrOS2	100.00	390.9820	0.1	0.3	6.8	17.0	even	ok
	2	C11H8BrN10S	23.84	390.9832	1.3	3.2	36.8	17.0	even	ok
	3	C10H20BrN2O3S3	36.58	390.9814	-0.5	-1.4	42.3	10.0	even	ok
	4	C10H12BrN6O4S	34.30	390.9819	-0.1	-0.2	45.6	12.0	even	ok
	5	C17H12BrO6	24.23	390.9812	-0.8	-1.9	46.5	14.0	even	ok
	6	C18H8BrN4O2	25.26	390.9825	0.6	1.5	48.3	19.0	even	ok
412.9641	1	C18H15BrNaOS2	100.00	412.9640	-0.1	-0.2	13.5	17.0	even	ok
	2	C11H7BrN10NaS	35.57	412.9651	1.1	2.6	30.3	17.0	even	ok
	3	C17H11BrNaO6	33.08	412.9631	-1.0	-2.3	35.5	14.0	even	ok
	4	C18H7BrN4NaO2	43.42	412.9645	0.4	0.9	36.6	19.0	even	ok
	5	C10H11BrN6NaO4S	39.07	412.9638	-0.3	-0.7	42.2	12.0	even	ok
	6	C10H19BrN2NaO3S3	30.07	412.9633	-0.7	-1.8	49.6	10.0	even	ok

Figure S28. HRESIMS spectrum of compound **8g**

Generic Display Report

Analysis Info

Analysis Name	D:\Data\Kalaba\71581000001.d	Acquisition Date	10/07/2020 17:52:35
Method	tune_low_MS_Service_07_20.m	Operator	msc
Sample Name	PN-14-4	Instrument	maXis
Comment	Kalaba/Zehl<br>Ergebnis +/- 5ppm<br>ACN/MeOH + 1 % H2O		

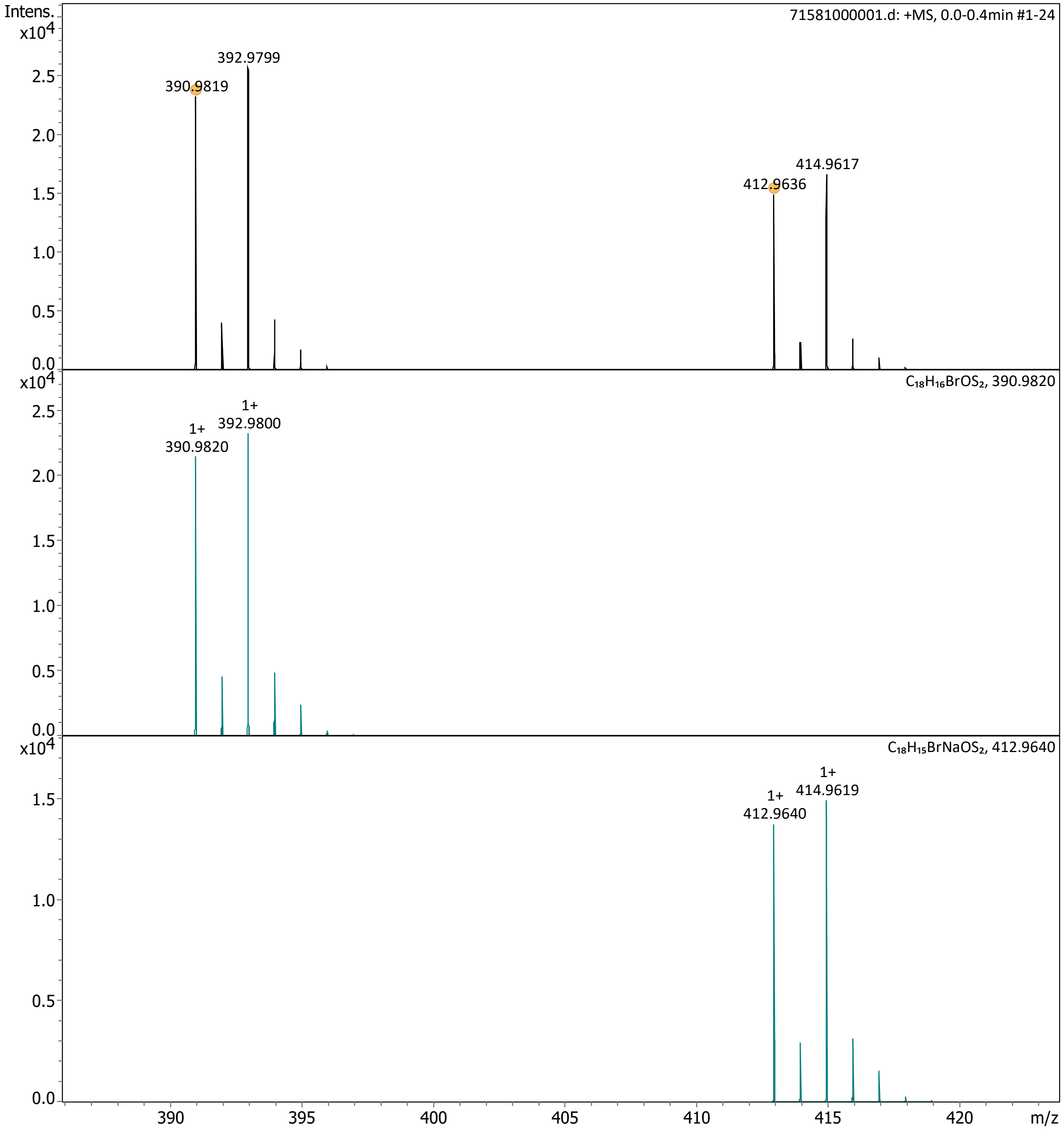

Mass Spectrum SmartFormula Report

Analysis Info

Analysis Name	D:\Data\Kalaba\71581000001.d	Acquisition Date	10/07/2020 17:52:35
Method	tune_low_MS_Service_07_20.m	Operator	msc
Sample Name	PN-14-4	Instrument	maXis 255552.00016
Comment	Kalaba/Zehl<br>Ergebnis +/- 5ppm<br>ACN/MeOH + 1 % H2O		

Acquisition Parameter

Source Type	ESI	Ion Polarity	Positive	Set Nebulizer	0.4 Bar
Focus	Not active	Set Capillary	4200 V	Set Dry Heater	180 Â°C
Scan Begin	50 m/z	Set End Plate Offset	-500 V	Set Dry Gas	4.0 l/min
Scan End	1900 m/z	Set Charging Voltage	0 V	Set Divert Valve	Source
		Set Corona	0 nA	Set APCI Heater	0 Â°C

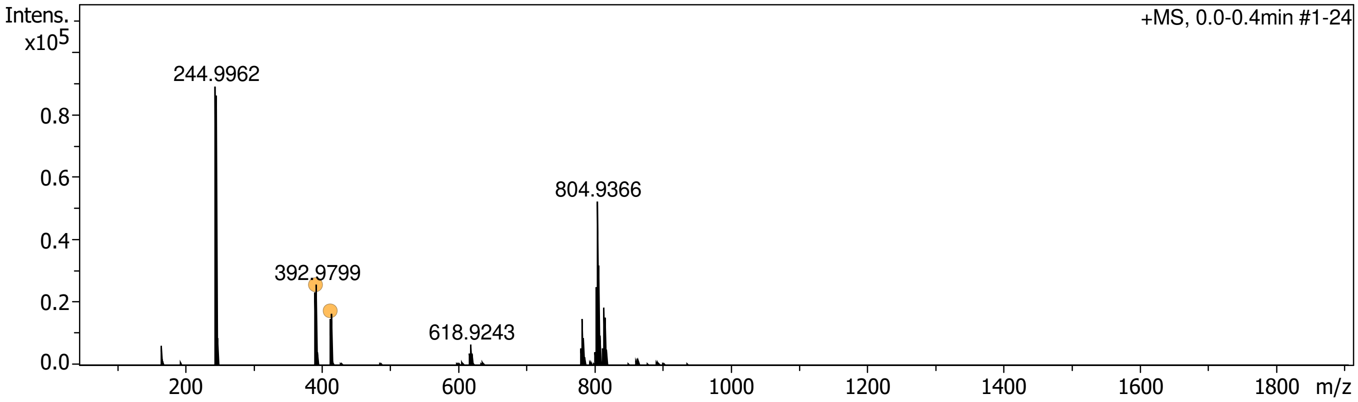

Meas. m/z	#	Ion Formula	m/z	err [ppm]	mSigma	# mSigma	Score	rdb	eÂ¯ Conf	N-Rule
390.9819	1	C18H16BrOS2	390.9820	0.3	29.0	1	100.00	17.0	even	ok
	2	C11H8BrN10S	390.9832	3.2	30.2	2	44.74	17.0	even	ok
	3	C10H12BrN6O4S	390.9819	-0.2	31.4	3	79.95	12.0	even	ok
	4	C10H20BrN2O3S3	390.9814	-1.4	32.4	4	75.75	10.0	even	ok
	5	C17H12BrO6	390.9812	-1.9	48.5	5	36.33	14.0	even	ok
	6	C18H8BrN4O2	390.9825	1.5	56.2	6	31.29	19.0	even	ok
412.9636	1	C10H19BrN2NaO3S3	412.9633	-0.7	31.2	1	100.00	10.0	even	ok
	2	C10H11BrN6NaO4S	412.9638	0.4	32.3	2	85.42	12.0	even	ok
	3	C11H7BrN10NaS	412.9651	3.7	33.9	3	39.58	17.0	even	ok
	4	C18H15BrNaOS2	412.9640	0.9	35.2	4	87.69	17.0	even	ok
	5	C9H15BrN2NaO8S	412.9625	-2.8	36.9	5	45.67	7.0	even	ok
	6	C17H11BrNaO6	412.9631	-1.2	53.3	6	40.48	14.0	even	ok
	7	C14H3BrN10Na	412.9618	-4.5	56.1	7	16.34	20.0	even	ok
	8	C18H7BrN4NaO2	412.9645	2.0	61.9	8	25.99	19.0	even	ok
	9	C9H20Br2N4NaOS	412.9617	-4.7	233.3	9	0.00	7.0	even	ok

Figure S29. HRESIMS spectrum of compound **5h**

Generic Display Report

Analysis Info

Analysis Name	D:\MZ\maXis_data\temp\64021000001.d	Acquisition Date	20/05/2019 15:54:18
Method	tune_low_MS_Service_05_19.m	Operator	msc
Sample Name	CE-196 spot 1 peak 1	Instrument	maXis
Comment	Predrag Kalaba<br>Ergebnis: +/- 5ppm<br>ACN / MeOH + 1% H2O		

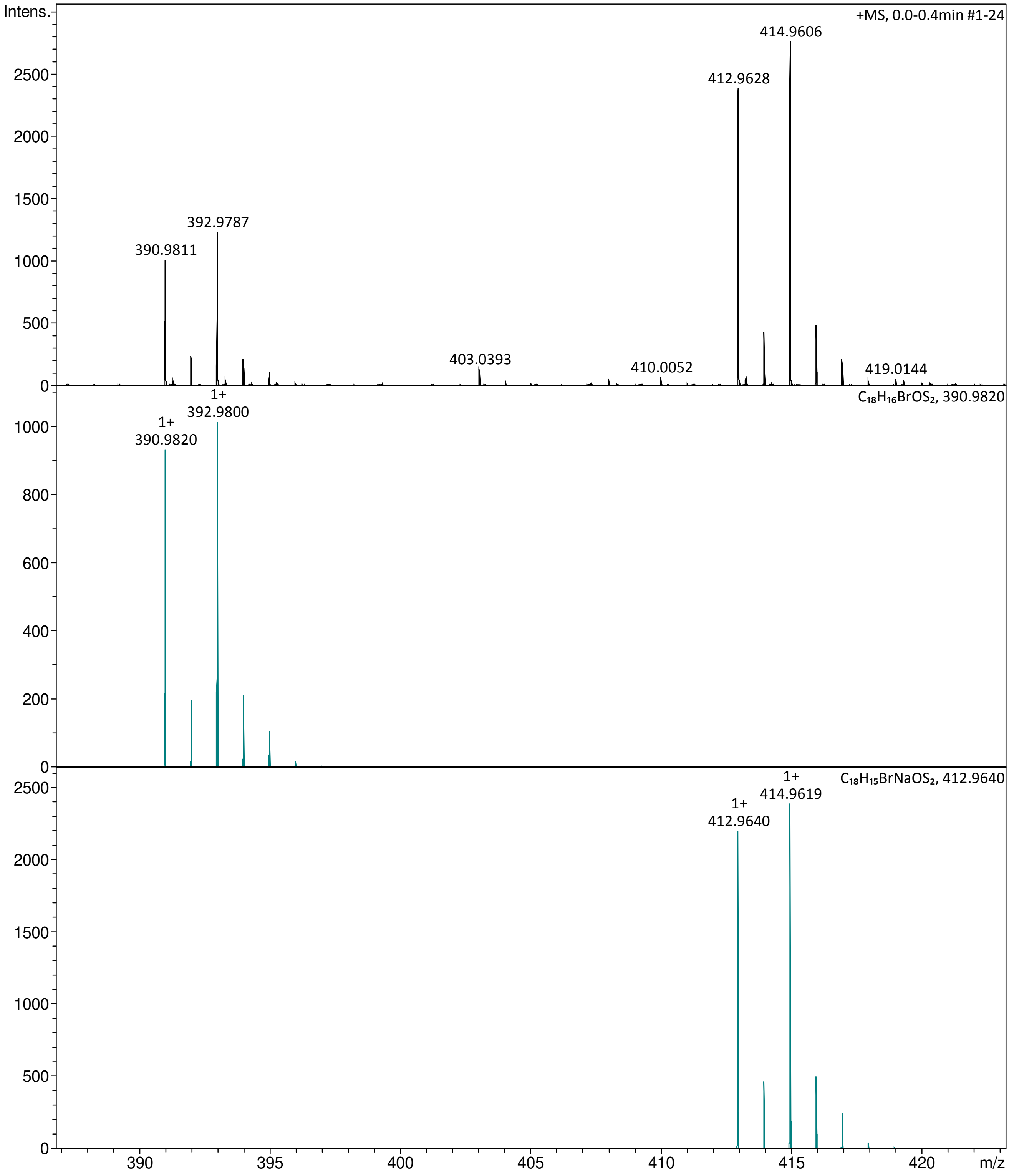

Mass Spectrum SmartFormula Report

Analysis Info

Analysis Name	D:\MZ\maXis_data\temp\64021000001.d	Acquisition Date	20/05/2019 15:54:18
Method	tune_low_MS_Service_05_19.m	Operator	msc
Sample Name	CE-196 spot 1 peak 1	Instrument	maXis 255552.00016
Comment	Predrag Kalaba<br>Ergebnis: +/- 5ppm<br>ACN / MeOH + 1% H2O		

Acquisition Parameter

Source Type	ESI	Ion Polarity	Positive	Set Nebulizer	0.4 Bar
Focus	Not active	Set Capillary	4500 V	Set Dry Heater	150 °C
Scan Begin	50 m/z	Set End Plate Offset	-500 V	Set Dry Gas	4.0 l/min
Scan End	1900 m/z	Set Charging Voltage	0 V	Set Divert Valve	Source
		Set Corona	0 nA	Set APCI Heater	0 °C

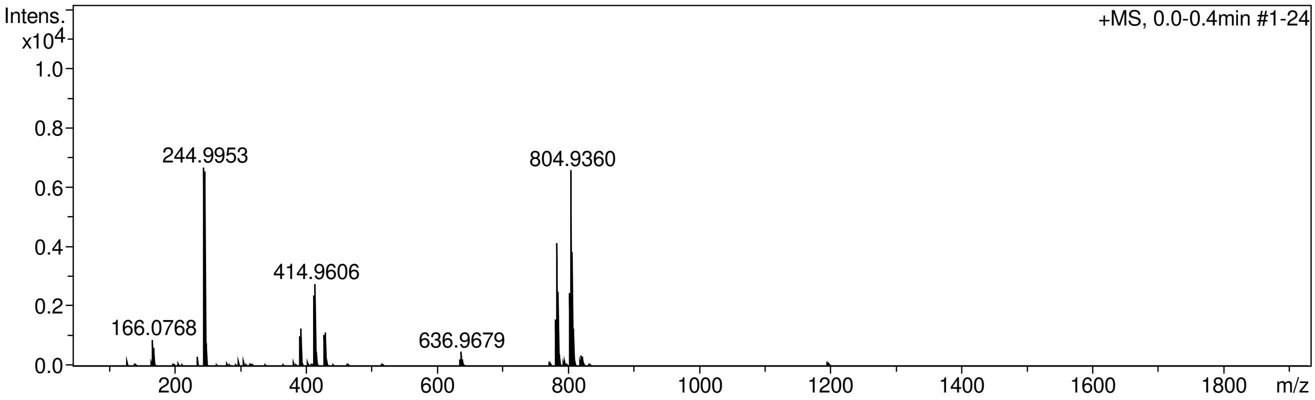

Meas. m/z	#	Ion Formula	Score	m/z	err [mDa]	err [ppm]	mSigma	rdb	e^- Conf	N-Rule
390.9811	1	C18H16BrOS2	78.22	390.9820	0.9	2.3	39.9	10.5	even	ok
	2	C10H20BrN2O3S3	100.00	390.9814	-0.2	-0.6	42.7	1.5	even	ok
	3	C10H12BrN6O4S	37.01	390.9819	0.7	1.8	66.7	7.5	even	ok
	4	C9H16BrN2O8S	32.77	390.9805	0.6	1.6	71.3	2.5	even	ok
	5	C9H21Br2N4OS	0.00	390.9797	-1.4	-3.6	212.4	0.5	even	ok
	6	C15H19S6	0.00	390.9806	0.6	1.5	303.9	6.5	even	ok
	7	C11H19O5S5	0.00	390.9831	-1.9	-4.9	353.4	2.5	even	ok
	8	C14H15O5S4	0.00	390.9797	1.5	3.7	371.0	7.5	even	ok
	9	C15H11N4OS4	0.00	390.9810	-0.1	-0.3	372.4	12.5	even	ok
	10	C10H15O10S3	0.00	390.9822	-1.0	-2.7	391.2	3.5	even	ok
	11	C14H7N4O6S2	0.00	390.9802	-1.0	-2.5	410.3	13.5	even	ok
	12	C15H3N8O2S2	0.00	390.9815	-0.3	-0.9	411.9	18.5	even	ok
	13	C22H3N2O4S	0.00	390.9808	-0.3	-0.9	474.4	22.5	even	ok
	14	C9H11O15S	0.00	390.9813	0.2	0.4	477.2	4.5	even	ok
	15	C10H7N4O11S	0.00	390.9827	1.5	3.9	478.8	9.5	even	ok
412.9628	1	C18H15BrNaOS2	100.00	412.9640	-1.2	-2.8	30.5	10.5	even	ok
	2	C15H18NaS6	0.00	412.9625	0.3	0.8	300.5	6.5	even	ok
	3	C14H14NaO5S4	0.00	412.9616	-1.2	-2.9	367.1	7.5	even	ok
	4	C15H10N4NaOS4	0.00	412.9630	-0.1	-0.3	369.0	12.5	even	ok
	5	C14H6N4NaO6S2	0.00	412.9621	-0.7	-1.8	406.7	13.5	even	ok
	6	C15H2N8NaO2S2	0.00	412.9634	-0.6	-1.5	408.7	18.5	even	ok
	7	C22H2N2NaO4S	0.00	412.9627	0.1	0.2	472.4	22.5	even	ok

Figure S30. HRESIMS spectrum of compound **6h**

Generic Display Report

Analysis Info

Analysis Name	D:\MZ\maXis_data\temp\64022000001.d	Acquisition Date	20/05/2019 15:58:19
Method	tune_low_MS_Service_05_19.m	Operator	msc
Sample Name	CE-196 spot 1 peak 2	Instrument	maXis
Comment	Predrag Kalaba<br>Ergebnis: +/- 5ppm<br>ACN / MeOH + 1% H2O		

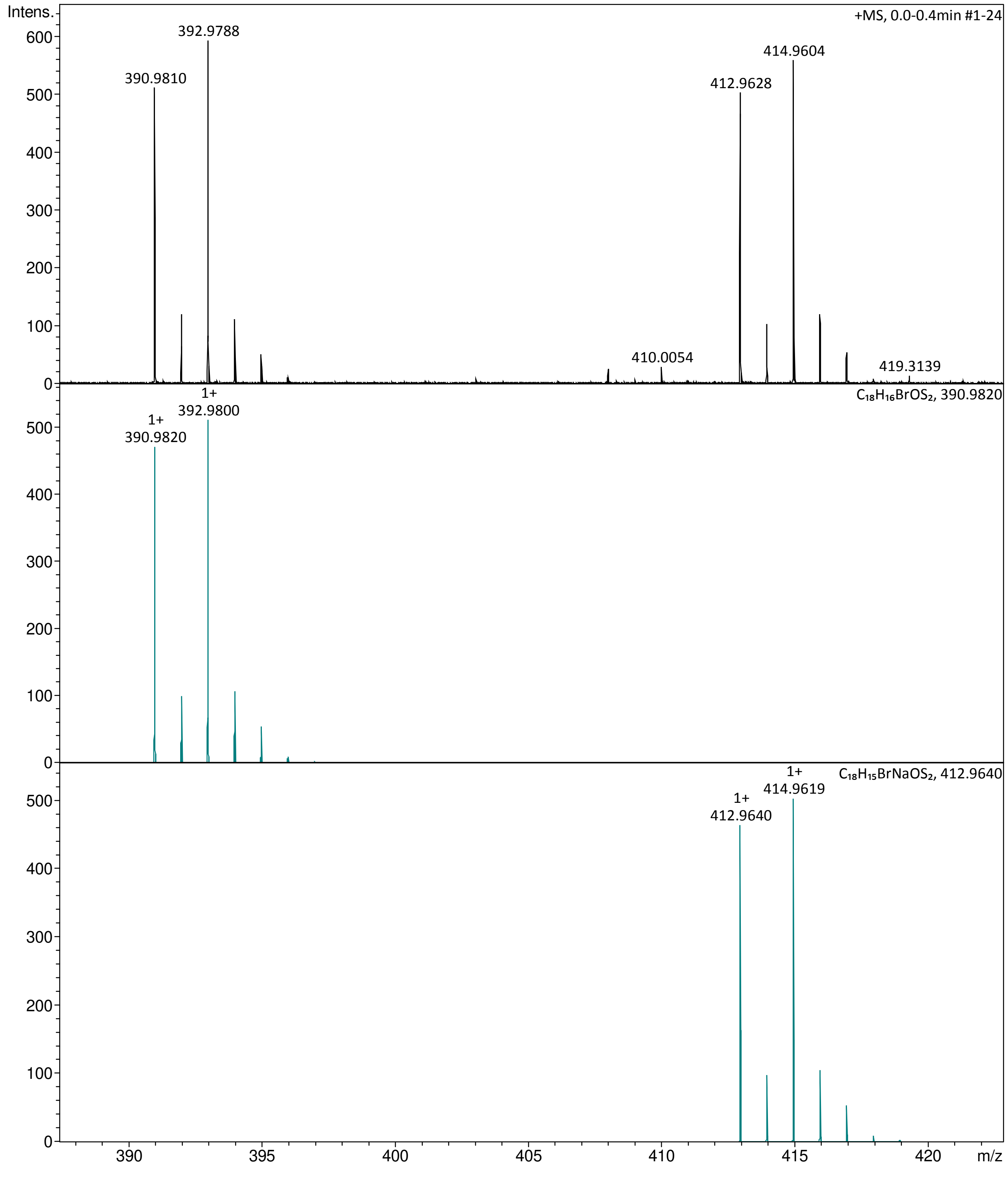

Mass Spectrum SmartFormula Report

Analysis Info

Analysis Name	D:\MZ\maXis_data\temp\64022000001.d	Acquisition Date	20/05/2019 15:58:19
Method	tune_low_MS_Service_05_19.m	Operator	msc
Sample Name	CE-196 spot 1 peak 2	Instrument	maXis 255552.00016
Comment	Predrag Kalaba<br>Ergebnis: +/- 5ppm<br>ACN / MeOH + 1% H2O		

Acquisition Parameter

Source Type	ESI	Ion Polarity	Positive	Set Nebulizer	0.4 Bar
Focus	Not active	Set Capillary	4500 V	Set Dry Heater	150 °C
Scan Begin	50 m/z	Set End Plate Offset	-500 V	Set Dry Gas	4.0 l/min
Scan End	1900 m/z	Set Charging Voltage	0 V	Set Divert Valve	Source
		Set Corona	0 nA	Set APCI Heater	0 °C

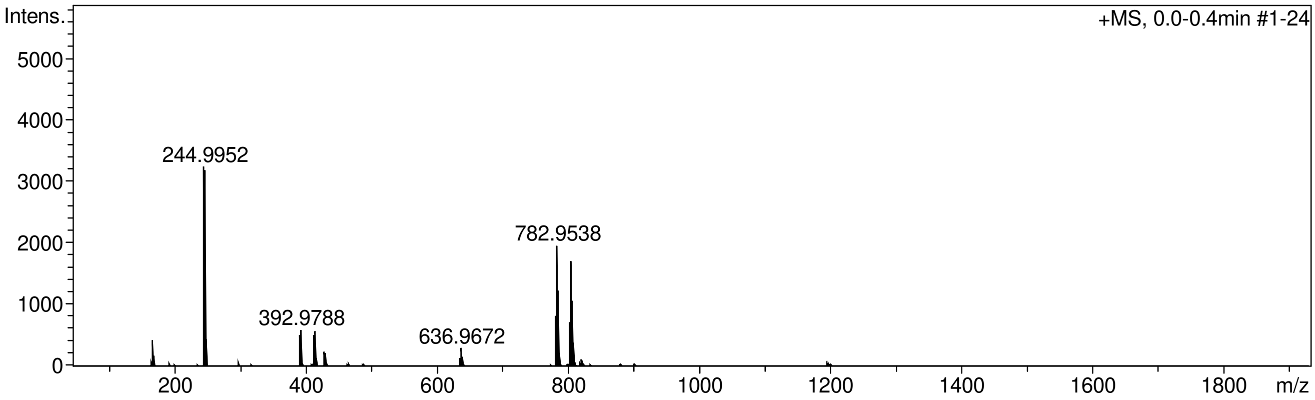

Meas. m/z	#	Ion Formula	Score	m/z	err [mDa]	err [ppm]	mSigma	rdb	e⁻ Conf	N-Rule
390.9810	1	C18H16BrOS2	100.00	390.9820	1.1	2.7	24.9	10.5	even	ok
	2	C10H20BrN2O3S3	95.17	390.9814	0.4	1.0	40.4	1.5	even	ok
	3	C10H12BrN6O4S	47.96	390.9819	-0.9	-2.2	55.4	7.5	even	ok
	4	C9H16BrN2O8S	46.18	390.9805	0.5	1.2	62.7	2.5	even	ok
	5	C9H21Br2N4OS	0.00	390.9797	-1.3	-3.2	224.0	0.5	even	ok
	6	C15H19S6	0.00	390.9806	0.4	1.1	301.0	6.5	even	ok
	7	C14H15O5S4	0.00	390.9797	1.3	3.3	368.4	7.5	even	ok
	8	C15H11N4OS4	0.00	390.9810	-0.0	-0.1	369.7	12.5	even	ok
	9	C10H15O10S3	0.00	390.9822	-1.2	-3.1	389.1	3.5	even	ok
	10	C14H7N4O6S2	0.00	390.9802	-0.8	-2.1	408.1	13.5	even	ok
	11	C15H3N8O2S2	0.00	390.9815	-0.5	-1.3	409.5	18.5	even	ok
	12	C22H3N2O4S	0.00	390.9808	0.2	0.5	471.8	22.5	even	ok
	13	C9H11O15S	0.00	390.9813	-0.3	-0.8	475.3	4.5	even	ok
	14	C10H7N4O11S	0.00	390.9827	1.7	4.3	476.9	9.5	even	ok
412.9628	1	C18H15BrNaOS2	100.00	412.9640	1.2	2.9	12.4	10.5	even	ok
	2	C10H19BrN2NaO3S3	77.14	412.9633	-0.5	-1.3	39.1	1.5	even	ok
	3	C10H11BrN6NaO4S	46.98	412.9638	-1.0	-2.5	47.8	7.5	even	ok
	4	C9H15BrN2NaO8S	50.35	412.9625	-0.3	-0.8	56.9	2.5	even	ok
	5	C9H20Br2N4NaOS	0.00	412.9617	-1.1	-2.7	230.5	0.5	even	ok
	6	C15H18NaS6	0.00	412.9625	-0.3	-0.7	301.1	6.5	even	ok
	7	C14H14NaO5S4	0.00	412.9616	1.2	2.8	368.6	7.5	even	ok
	8	C15H10N4NaOS4	0.00	412.9630	-0.2	-0.4	370.1	12.5	even	ok
	9	C10H14NaO10S3	0.00	412.9641	-1.3	-3.2	389.1	3.5	even	ok
	10	C13H10NaO10S2	0.00	412.9608	2.0	4.9	406.9	8.5	even	ok
	11	C14H6N4NaO6S2	0.00	412.9621	-0.7	-1.7	408.5	13.5	even	ok
	12	C15H2N8NaO2S2	0.00	412.9634	0.6	1.6	410.2	18.5	even	ok
	13	C22H2N2NaO4S	0.00	412.9627	0.0	0.1	472.6	22.5	even	ok
	14	C9H10NaO15S	0.00	412.9633	-0.5	-1.1	474.8	4.5	even	ok
	15	C10H6N4NaO11S	0.00	412.9646	-1.8	-4.4	476.6	9.5	even	ok

Figure S31. HRESIMS spectrum of compound **7h**

Generic Display Report

Analysis Info

Analysis Name	D:\MZ\maXis_data\temp\64023000002.d
Method	tune_low_MS_Service_05_19.m
Sample Name	CE-196 spot 2 peak 1
Comment	Predrag Kalaba<br>Ergebnis: +/- 5ppm<br>ACN / MeOH + 1% H2O
Acquisition Date	20/05/2019 16:04:05
Operator	msc
Instrument	maXis

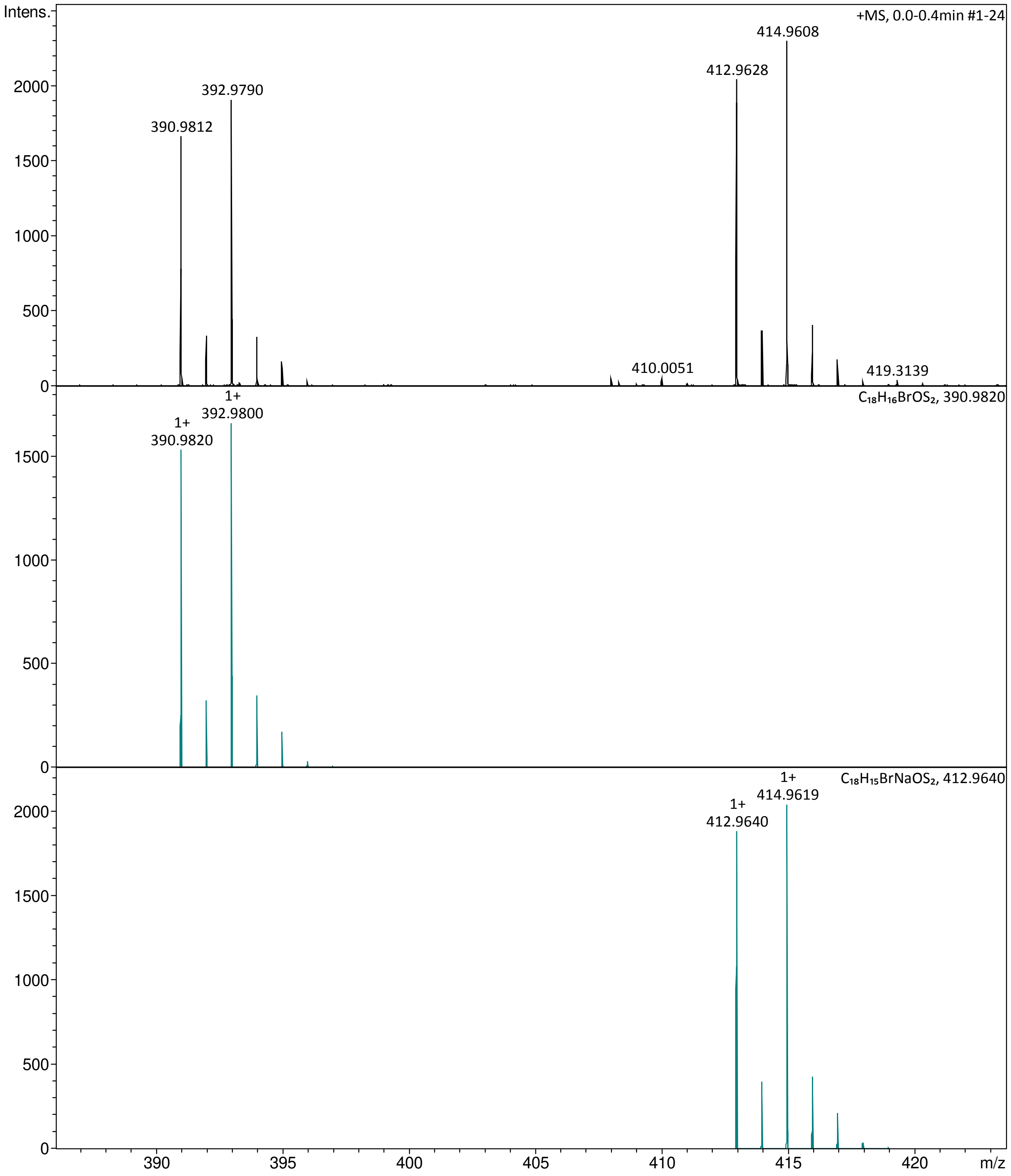

Mass Spectrum SmartFormula Report

Analysis Info

Analysis Name	D:\MZ\maXis_data\temp\64023000002.d	Acquisition Date	20/05/2019 16:04:05
Method	tune_low_MS_Service_05_19.m	Operator	msc
Sample Name	CE-196 spot 2 peak 1	Instrument	maXis 255552.00016
Comment	Predrag Kalaba<br>Ergebnis: +/- 5ppm<br>ACN / MeOH + 1% H2O		

Acquisition Parameter

Source Type	ESI	Ion Polarity	Positive	Set Nebulizer	0.4 Bar
Focus	Not active	Set Capillary	4500 V	Set Dry Heater	150 °C
Scan Begin	50 m/z	Set End Plate Offset	-500 V	Set Dry Gas	4.0 l/min
Scan End	1900 m/z	Set Charging Voltage	0 V	Set Divert Valve	Source
		Set Corona	0 nA	Set APCI Heater	0 °C

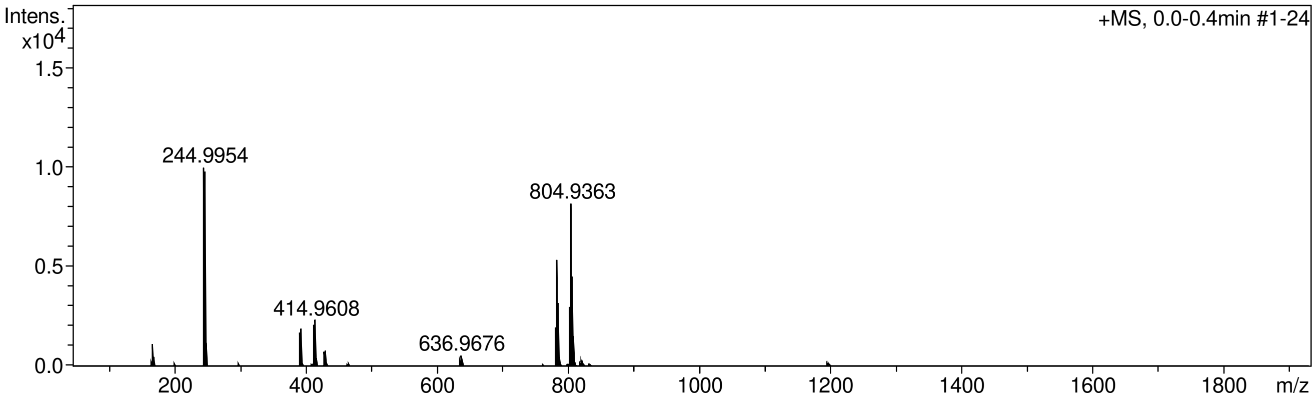

Meas. m/z	#	Ion Formula	Score	m/z	err [mDa]	err [ppm]	mSigma	rdb	e⁻ Conf	N-Rule
390.9812	1	C18H16BrOS2	82.64	390.9820	0.9	2.2	25.3	10.5	even	ok
	2	C10H20BrN2O3S3	100.00	390.9814	0.2	0.5	30.7	1.5	even	ok
	3	C10H12BrN6O4S	53.89	390.9819	0.7	1.7	45.5	7.5	even	ok
	4	C9H16BrN2O8S	45.92	390.9805	0.7	1.7	51.3	2.5	even	ok
	5	C9H21Br2N4OS	0.00	390.9797	-1.4	-3.7	223.9	0.5	even	ok
	6	C15H19S6	0.00	390.9806	0.6	1.6	299.5	6.5	even	ok
	7	C11H19O5S5	0.00	390.9831	1.9	4.8	348.3	2.5	even	ok
	8	C14H15O5S4	0.00	390.9797	-1.5	-3.8	366.4	7.5	even	ok
	9	C15H11N4OS4	0.00	390.9810	0.2	0.4	368.1	12.5	even	ok
	10	C10H15O10S3	0.00	390.9822	1.0	2.6	386.4	3.5	even	ok
	11	C14H7N4O6S2	0.00	390.9802	1.0	2.6	406.1	13.5	even	ok
	12	C15H3N8O2S2	0.00	390.9815	0.3	0.8	407.9	18.5	even	ok
	13	C22H3N2O4S	0.00	390.9808	0.4	1.0	470.7	22.5	even	ok
	14	C9H11O15S	0.00	390.9813	0.1	0.4	472.2	4.5	even	ok
	15	C10H7N4O11S	0.00	390.9827	-1.5	-3.8	474.1	9.5	even	ok
412.9628	1	C18H15BrNaOS2	79.87	412.9640	1.2	2.9	24.0	10.5	even	ok
	2	C10H19BrN2NaO3S3	100.00	412.9633	-0.6	-1.4	29.7	1.5	even	ok
	3	C10H11BrN6NaO4S	63.26	412.9638	-1.0	-2.5	37.9	7.5	even	ok
	4	C9H15BrN2NaO8S	76.60	412.9625	0.3	0.8	44.2	2.5	even	ok
	5	C9H20Br2N4NaOS	0.00	412.9617	-1.1	-2.7	229.1	0.5	even	ok
	6	C15H18NaS6	0.00	412.9625	0.3	0.7	298.9	6.5	even	ok
	7	C14H14NaO5S4	0.00	412.9616	1.2	2.8	365.6	7.5	even	ok
	8	C15H10N4NaOS4	0.00	412.9630	-0.2	-0.5	367.5	12.5	even	ok
	9	C10H14NaO10S3	0.00	412.9641	-1.4	-3.3	385.3	3.5	even	ok
	10	C13H10NaO10S2	0.00	412.9608	-2.0	-4.9	403.5	8.5	even	ok
	11	C14H6N4NaO6S2	0.00	412.9621	-0.7	-1.7	405.4	13.5	even	ok
	12	C15H2N8NaO2S2	0.00	412.9634	-0.7	-1.6	407.3	18.5	even	ok
	13	C22H2N2NaO4S	0.00	412.9627	0.0	0.1	470.8	22.5	even	ok
	14	C9H10NaO15S	0.00	412.9633	0.5	1.2	471.1	4.5	even	ok
	15	C10H6N4NaO11S	0.00	412.9646	1.8	4.4	473.2	9.5	even	ok

Figure S32. HRESIMS spectrum of compound **8h**

Generic Display Report

Analysis Info

Analysis Name	D:\MZ\maXis_data\temp\64024000002.d	Acquisition Date	20/05/2019 16:08:36
Method	tune_low_MS_Service_05_19.m	Operator	msc
Sample Name	CE-196 spot 2 peak 2	Instrument	maXis
Comment	Predrag Kalaba<br>Ergebnis: +/- 5ppm<br>ACN / MeOH + 1% H2O		

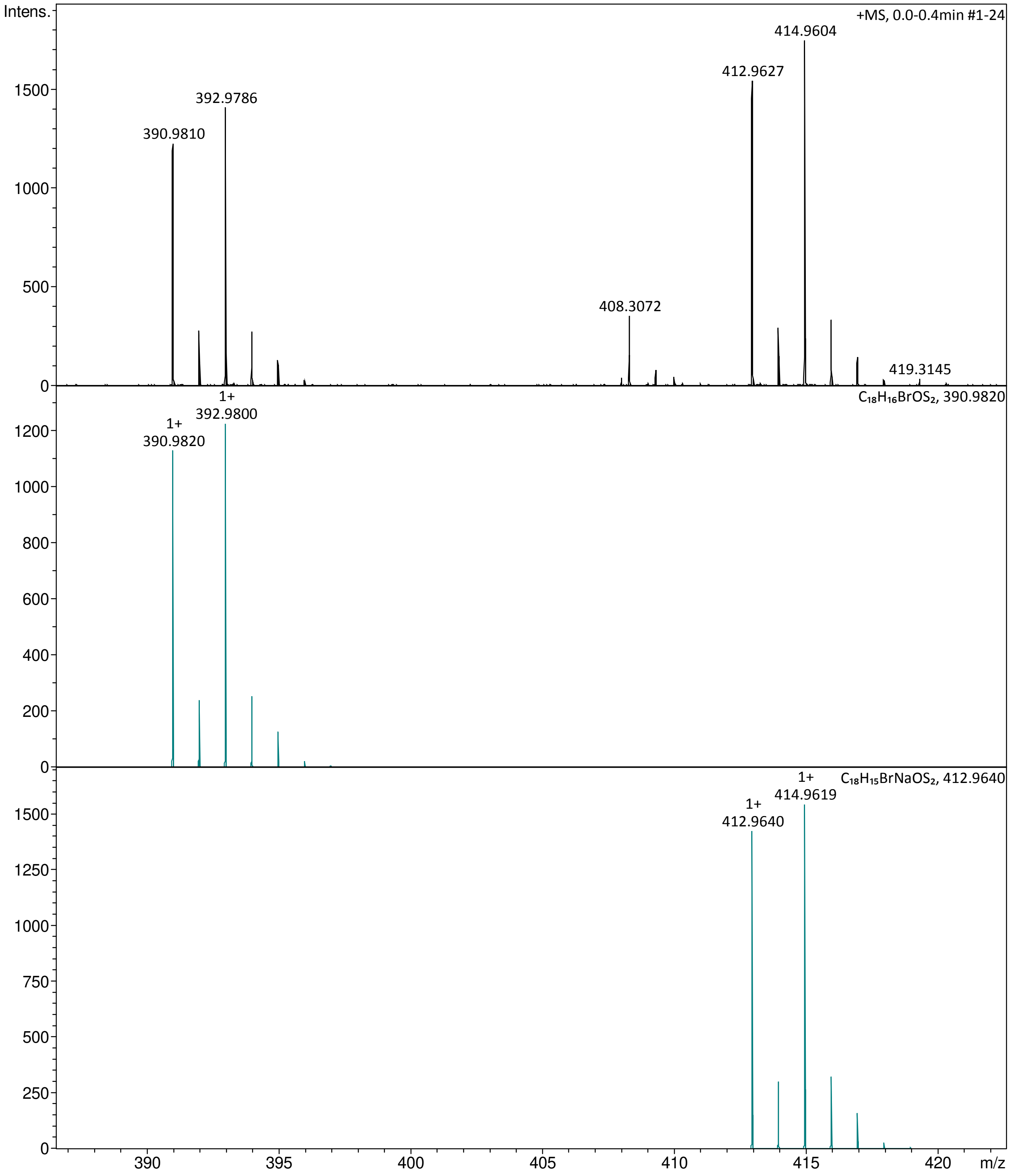

Mass Spectrum SmartFormula Report

Analysis Info

Analysis Name	D:\MZ\maXis_data\temp\64024000002.d	Acquisition Date	20/05/2019 16:08:36
Method	tune_low_MS_Service_05_19.m	Operator	msc
Sample Name	CE-196 spot 2 peak 2	Instrument	maXis 255552.00016
Comment	Predrag Kalaba<br>Ergebnis: +/- 5ppm<br>ACN / MeOH + 1% H2O		

Acquisition Parameter

Source Type	ESI	Ion Polarity	Positive	Set Nebulizer	0.4 Bar
Focus	Not active	Set Capillary	4500 V	Set Dry Heater	150 °C
Scan Begin	50 m/z	Set End Plate Offset	-500 V	Set Dry Gas	4.0 l/min
Scan End	1900 m/z	Set Charging Voltage	0 V	Set Divert Valve	Source
		Set Corona	0 nA	Set APCI Heater	0 °C

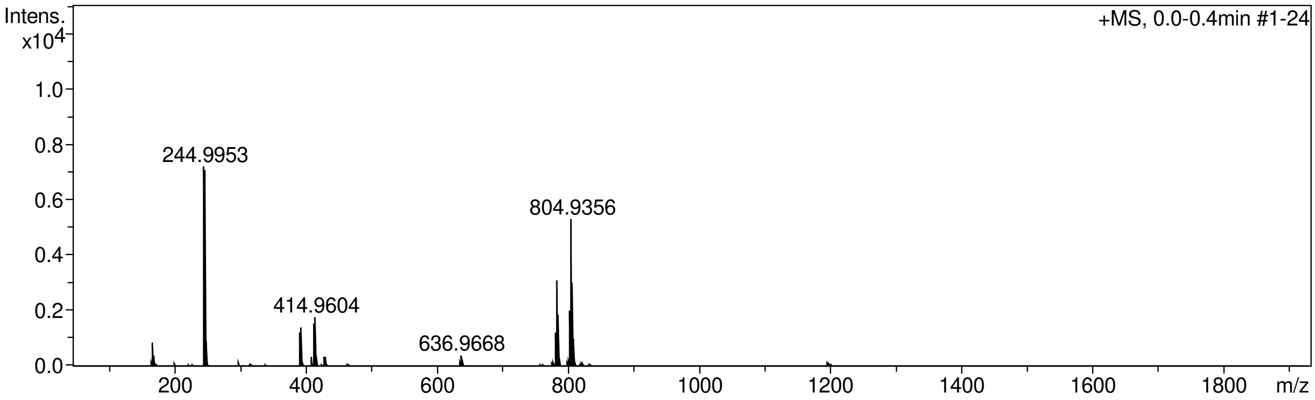

Meas. m/z	#	Ion Formula	Score	m/z	err [mDa]	err [ppm]	mSigma	rdb	e⁻ Conf	N-Rule
390.9810	1	C18H16BrOS2	100.00	390.9820	-1.0	-2.6	20.2	10.5	even	ok
	2	C10H20BrN2O3S3	90.19	390.9814	0.4	1.0	38.4	1.5	even	ok
	3	C10H12BrN6O4S	45.45	390.9819	0.8	2.2	53.8	7.5	even	ok
	4	C9H16BrN2O8S	42.14	390.9805	0.5	1.3	61.5	2.5	even	ok
	5	C9H21Br2N4OS	0.00	390.9797	1.3	3.3	223.7	0.5	even	ok
	6	C15H19S6	0.00	390.9806	0.5	1.2	301.1	6.5	even	ok
	7	C14H15O5S4	0.00	390.9797	-1.3	-3.4	368.6	7.5	even	ok
	8	C15H11N4OS4	0.00	390.9810	-0.0	-0.0	369.9	12.5	even	ok
	9	C10H15O10S3	0.00	390.9822	1.2	3.0	389.2	3.5	even	ok
	10	C14H7N4O6S2	0.00	390.9802	0.9	2.2	408.3	13.5	even	ok
	11	C15H3N8O2S2	0.00	390.9815	-0.5	-1.2	409.8	18.5	even	ok
	12	C22H3N2O4S	0.00	390.9808	0.2	0.6	471.9	22.5	even	ok
	13	C9H11O15S	0.00	390.9813	-0.3	-0.8	475.1	4.5	even	ok
	14	C10H7N4O11S	0.00	390.9827	-1.6	-4.2	476.7	9.5	even	ok
412.9627	1	C18H15BrNaOS2	90.97	412.9640	1.3	3.2	20.5	10.5	even	ok
	2	C10H19BrN2NaO3S3	100.00	412.9633	0.7	1.6	32.4	1.5	even	ok
	3	C21H11BrNaOS	32.31	412.9606	-2.1	-5.0	41.5	15.5	even	ok
	4	C10H11BrN6NaO4S	58.18	412.9638	-1.1	-2.7	43.0	7.5	even	ok
	5	C9H15BrN2NaO8S	75.86	412.9625	0.2	0.5	50.1	2.5	even	ok
	6	C9H20Br2N4NaOS	0.00	412.9617	-1.0	-2.4	227.7	0.5	even	ok
	7	C15H18NaS6	0.00	412.9625	0.2	0.4	300.1	6.5	even	ok
	8	C14H14NaO5S4	0.00	412.9616	1.0	2.5	367.0	7.5	even	ok
	9	C15H10N4NaOS4	0.00	412.9630	-0.3	-0.7	368.7	12.5	even	ok
	10	C10H14NaO10S3	0.00	412.9641	-1.5	-3.5	386.9	3.5	even	ok
	11	C13H10NaO10S2	0.00	412.9608	1.9	4.6	405.0	8.5	even	ok
	12	C14H6N4NaO6S2	0.00	412.9621	0.6	1.4	406.8	13.5	even	ok
	13	C15H2N8NaO2S2	0.00	412.9634	0.8	1.8	408.6	18.5	even	ok
	14	C22H2N2NaO4S	0.00	412.9627	-0.1	-0.2	471.8	22.5	even	ok
	15	C9H10NaO15S	0.00	412.9633	0.6	1.4	472.8	4.5	even	ok
	16	C10H6N4NaO11S	0.00	412.9646	1.9	4.7	474.8	9.5	even	ok

Figure S33. HRESIMS spectrum of compound **5i**

Generic Display Report

Analysis Info

Analysis Name	D:\Data\Kalaba\72419000001.d	Acquisition Date	20/08/2020 17:11:14
Method	tune_low_MS_Service_08_20.m	Operator	msc
Sample Name	MK-31-p1	Instrument	maXis
Comment	Kalaba/Zehl<br>Ergebnis +/- 5ppm<br>ACN / MeOH + 1% H2O		

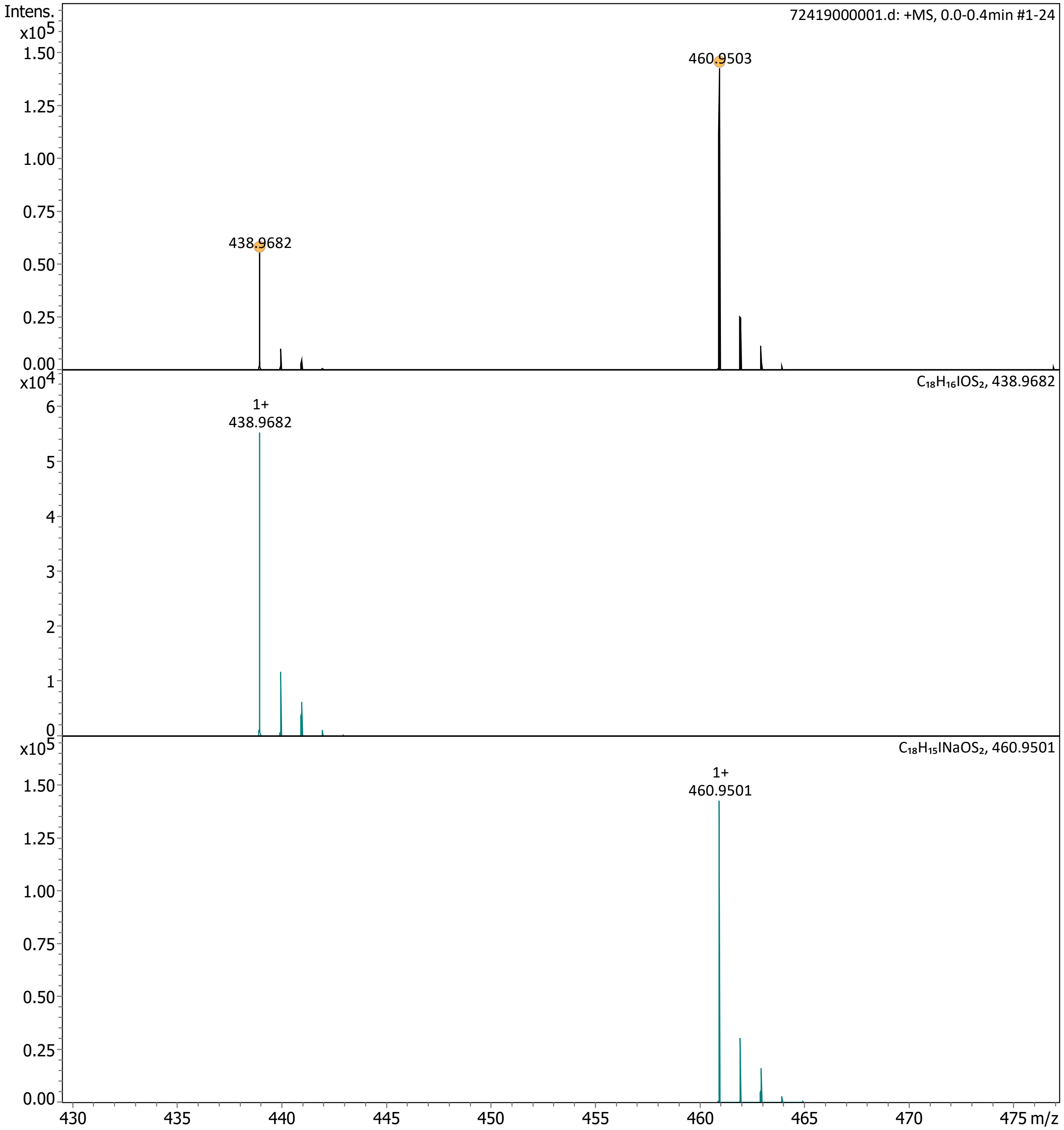

Mass Spectrum SmartFormula Report

Analysis Info

Analysis Name	D:\Data\Kalaba\72419000001.d	Acquisition Date	20/08/2020 17:11:14
Method	tune_low_MS_Service_08_20.m	Operator	msc
Sample Name	MK-31-p1	Instrument	maXis 255552.00016
Comment	Kalaba/Zehl<br>Ergebnis +/- 5ppm<br>ACN / MeOH + 1% H2O		

Acquisition Parameter

Source Type	ESI	Ion Polarity	Positive	Set Nebulizer	0.4 Bar
Focus	Not active	Set Capillary	4200 V	Set Dry Heater	180 Â°C
Scan Begin	50 m/z	Set End Plate Offset	-500 V	Set Dry Gas	4.0 l/min
Scan End	1900 m/z	Set Charging Voltage	0 V	Set Divert Valve	Source
		Set Corona	0 nA	Set APCI Heater	0 Â°C

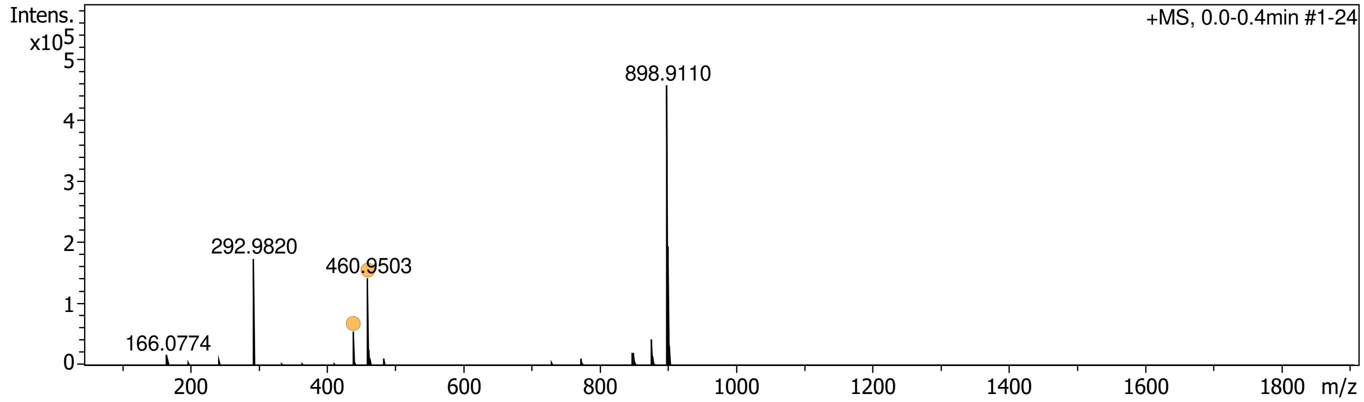

Meas. m/z	#	Ion Formula	m/z	err [ppm]	mSigma	# mSigma	Score	rdb	eÂ¯ Conf	N-Rule
438.9682	1	C11H3N8O10S	438.9687	1.2	11.0	1	100.00	17.0	even	ok
	2	C11H8IN10S	438.9693	2.5	16.9	2	64.64	18.0	even	ok
	3	C18H16IOS2	438.9682	-0.1	21.3	3	99.92	18.0	even	ok
	4	C10H7N4O14S	438.9674	-1.9	21.8	4	68.39	12.0	even	ok
	5	C15H3N8O5S2	438.9662	-4.5	22.9	5	31.38	23.0	even	ok
	6	C10H12IN6O4S	438.9680	-0.5	23.3	6	88.55	13.0	even	ok
	7	C18H3N2O12	438.9680	-0.4	25.2	7	86.72	19.0	even	ok
	8	C19H7N2O7S2	438.9689	1.6	30.8	8	74.40	22.0	even	ok
	9	C10H20IN2O3S3	438.9675	-1.6	33.1	9	70.08	11.0	even	ok
	10	C17H12IO6	438.9673	-2.1	33.5	10	37.05	15.0	even	ok
	11	C12H7N8O5S3	438.9696	3.1	34.0	11	46.13	20.0	even	ok
	12	C11H11N4O9S3	438.9683	0.1	36.6	12	86.59	15.0	even	ok
	13	C18H8IN4O2	438.9686	1.0	39.6	13	40.52	20.0	even	ok
	14	C10H15O13S3	438.9669	-2.9	42.0	14	39.33	10.0	even	ok
	15	C20H3N6O3S2	438.9703	4.6	43.3	15	18.17	27.0	even	ok
	16	C16H7N8S4	438.9671	-2.6	60.3	16	24.85	26.0	even	ok
	17	C27H3O5S	438.9696	3.1	61.6	17	16.59	29.0	even	ok
	18	C23H7N2O2S3	438.9664	-4.1	62.0	18	14.93	28.0	even	ok
	19	C20H11N2O2S4	438.9698	3.6	69.1	19	13.73	25.0	even	ok
	20	C28H7S3	438.9704	5.0	81.2	20	5.31	32.0	even	ok
	21	C31H3S2	438.9671	-2.6	82.4	21	10.61	35.0	even	ok
460.9503	1	C11H2N8NaO10S	460.9507	0.9	9.3	1	100.00	17.0	even	ok
	2	C14H15INaO6S	460.9526	5.1	9.8	2	28.40	12.0	even	ok
	3	C11H7IN10NaS	460.9513	2.2	14.3	3	66.73	18.0	even	ok
	4	C10H6N4NaO14S	460.9493	-2.0	20.7	4	60.24	12.0	even	ok
	5	C10H11IN6NaO4S	460.9499	-0.7	21.3	5	80.65	13.0	even	ok
	6	C18H15INaOS2	460.9501	-0.3	23.5	6	83.16	18.0	even	ok
	7	C18H2N2NaO12	460.9500	-0.6	23.7	7	78.20	19.0	even	ok
	8	C15H2N8NaO5S2	460.9482	-4.6	25.3	8	24.94	23.0	even	ok

Mass Spectrum SmartFormula Report

Meas. m/z	#	Ion Formula	m/z	err [ppm]	mSigma	# mSigma	Score	rdb	eÂ¯ Conf	N-Rule
	9	C17H11INaO6	460.9493	-2.2	31.0	9	33.93	15.0	even	ok
	10	C19H6N2NaO7S2	460.9509	1.3	32.8	10	68.85	22.0	even	ok
	11	C10H19IN2NaO3S3	460.9495	-1.8	34.6	11	58.28	11.0	even	ok
	12	C12H6N8NaO5S3	460.9515	2.7	36.2	12	43.01	20.0	even	ok
	13	C18H7IN4NaO2	460.9506	0.7	37.7	13	41.26	20.0	even	ok
	14	C11H10N4NaO9S3	460.9502	-0.2	38.5	14	74.58	15.0	even	ok
	15	C10H14NaO13S3	460.9489	-3.1	43.6	15	32.06	10.0	even	ok
	16	C20H2N6NaO3S2	460.9522	4.2	45.3	16	17.19	27.0	even	ok
	17	C16H6N8NaS4	460.9490	-2.7	62.6	17	19.68	26.0	even	ok
	18	C27H2NaO5S	460.9515	2.7	62.8	18	15.70	29.0	even	ok
	19	C23H6N2NaO2S3	460.9484	-4.2	64.0	19	11.73	28.0	even	ok
	20	C20H10N2NaO2S4	460.9517	3.1	71.4	20	12.52	25.0	even	ok
	21	C28H6NaS3	460.9524	4.6	83.1	21	4.94	32.0	even	ok
	22	C31H2NaS2	460.9490	-2.8	83.9	22	8.51	35.0	even	ok

Figure S34. HRESIMS spectrum of compound **6i**

Generic Display Report

Analysis Info

Analysis Name	D:\Data\Kalaba\72420000001.d	Acquisition Date	20/08/2020 17:13:58
Method	tune_low_MS_Service_08_20.m	Operator	msc
Sample Name	MK-31-p2	Instrument	maXis
Comment	Kalaba/Zehl<br>Ergebnis +/- 5ppm<br>ACN / MeOH + 1% H2O		

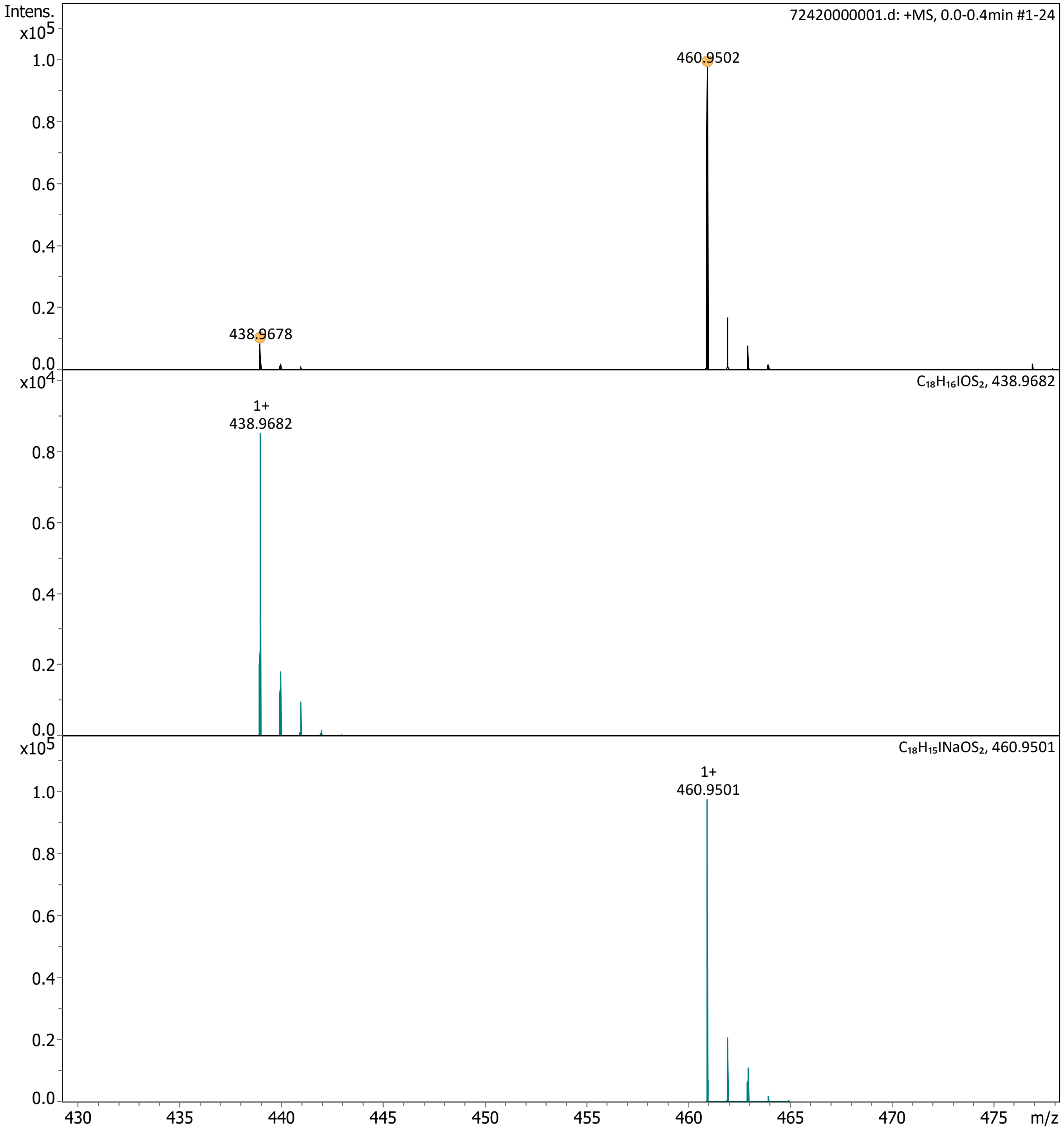

Mass Spectrum SmartFormula Report

Analysis Info

Analysis Name	D:\Data\Kalaba\72420000001.d	Acquisition Date	20/08/2020 17:13:58
Method	tune_low_MS_Service_08_20.m	Operator	msc
Sample Name	MK-31-p2	Instrument	maXis 255552.00016
Comment	Kalaba/Zehl<br>Ergebnis +/- 5ppm<br>ACN / MeOH + 1% H2O		

Acquisition Parameter

Source Type	ESI	Ion Polarity	Positive	Set Nebulizer	0.4 Bar
Focus	Not active	Set Capillary	4200 V	Set Dry Heater	180 Â°C
Scan Begin	50 m/z	Set End Plate Offset	-500 V	Set Dry Gas	4.0 l/min
Scan End	1900 m/z	Set Charging Voltage	0 V	Set Divert Valve	Source
		Set Corona	0 nA	Set APCI Heater	0 Â°C

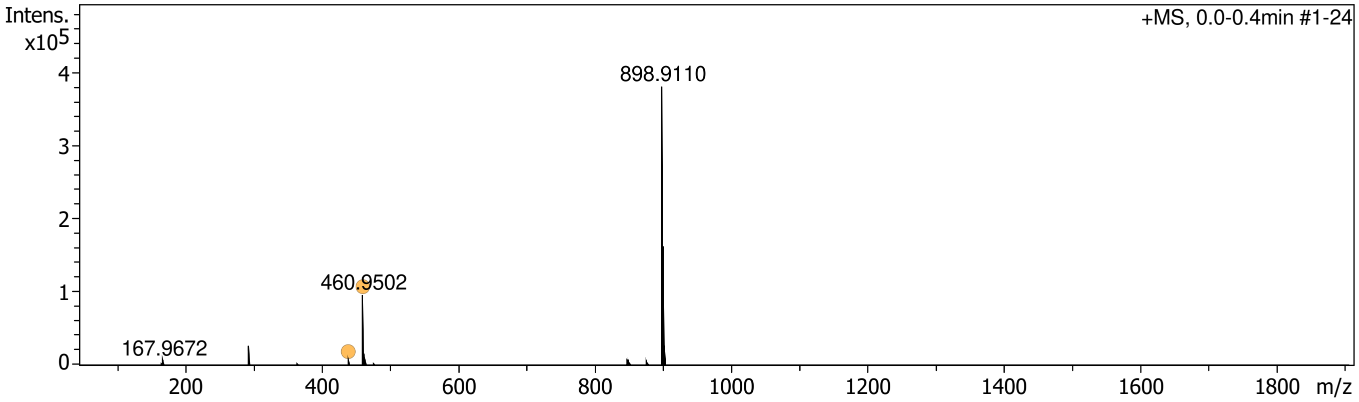

Meas. m/z	#	Ion Formula	m/z	err [ppm]	mSigma	# mSigma	Score	rdb	eÂ¯ Conf	N-Rule
438.9678	1	C18H16IOS2	438.9682	0.8	16.4	1	100.00	18.0	even	ok
	2	C15H3N8O5S2	438.9662	-3.6	18.4	2	47.01	23.0	even	ok
	3	C19H7N2O7S2	438.9689	2.5	25.7	3	69.56	22.0	even	ok
	4	C18H3N2O12	438.9680	0.5	26.0	4	86.05	19.0	even	ok
	5	C12H7N8O5S3	438.9696	4.0	32.3	5	37.96	20.0	even	ok
	6	C17H12IO6	438.9673	-1.2	38.3	6	41.11	15.0	even	ok
	7	C18H8IN4O2	438.9686	1.9	41.4	7	32.64	20.0	even	ok
	8	C14H4IN10	438.9660	-4.3	45.2	8	14.94	21.0	even	ok
	9	C15H11N4O4S4	438.9658	-4.7	54.8	9	15.98	21.0	even	ok
	10	C27H3O5S	438.9696	4.0	54.9	10	16.34	29.0	even	ok
	11	C16H7N8S4	438.9671	-1.7	56.4	11	36.07	26.0	even	ok
	12	C23H7N2O2S3	438.9664	-3.2	56.7	12	23.96	28.0	even	ok
	13	C20H11N2O2S4	438.9698	4.4	64.8	13	12.48	25.0	even	ok
	14	C31H3S2	438.9671	-1.7	76.3	14	17.36	35.0	even	ok
460.9502	1	C11H2N8NaO10S	460.9507	1.0	7.5	1	100.00	17.0	even	ok
	2	C11H7IN10NaS	460.9513	2.3	13.0	2	65.71	18.0	even	ok
	3	C10H6N4NaO14S	460.9493	-1.9	19.0	3	65.80	12.0	even	ok
	4	C10H11IN6NaO4S	460.9499	-0.6	19.4	4	87.57	13.0	even	ok
	5	C18H2N2NaO12	460.9500	-0.5	24.3	5	80.42	19.0	even	ok
	6	C18H15INaOS2	460.9501	-0.2	25.3	6	83.17	18.0	even	ok
	7	C15H2N8NaO5S2	460.9482	-4.4	26.9	7	25.74	23.0	even	ok
	8	C17H11INaO6	460.9493	-2.1	30.7	8	36.03	15.0	even	ok
	9	C19H6N2NaO7S2	460.9509	1.4	34.3	9	63.83	22.0	even	ok
	10	C10H19IN2NaO3S3	460.9495	-1.6	34.5	10	61.46	11.0	even	ok
	11	C12H6N8NaO5S3	460.9515	2.9	37.0	11	40.25	20.0	even	ok
	12	C14H3IN10Na	460.9479	-5.0	37.6	12	11.94	21.0	even	ok
	13	C18H7IN4NaO2	460.9506	0.8	38.2	13	39.49	20.0	even	ok
	14	C11H10N4NaO9S3	460.9502	0.0	38.8	14	76.25	15.0	even	ok
	15	C10H14NaO13S3	460.9489	-2.9	43.5	15	34.05	10.0	even	ok

Mass Spectrum SmartFormula Report

Meas. m/z	#	Ion Formula	m/z	err [ppm]	mSigma	# mSigma	Score	rdb	eÂ¯ Conf	N-Rule
	16	C20H2N6NaO3S2	460.9522	4.3	47.1	16	15.39	27.0	even	ok
	17	C16H6N8NaS4	460.9490	-2.5	63.7	17	19.96	26.0	even	ok
	18	C27H2NaO5S	460.9515	2.8	64.6	18	14.06	29.0	even	ok
	19	C23H6N2NaO2S3	460.9484	-4.0	65.6	19	11.86	28.0	even	ok
	20	C20H10N2NaO2S4	460.9517	3.3	72.7	20	11.35	25.0	even	ok
	21	C28H6NaS3	460.9524	4.7	84.7	21	4.35	32.0	even	ok
	22	C31H2NaS2	460.9490	-2.6	85.6	22	8.39	35.0	even	ok

Figure S35. HRESIMS spectrum of compound **7i**

Generic Display Report

Analysis Info

Analysis Name	D:\Data\Kalaba\72421000001.d	Acquisition Date	20/08/2020 17:15:38
Method	tune_low_MS_Service_08_20.m	Operator	msc
Sample Name	MK-31-p3	Instrument	maXis
Comment	Kalaba/Zehl<br>Ergebnis +/- 5ppm<br>ACN / MeOH + 1% H2O		

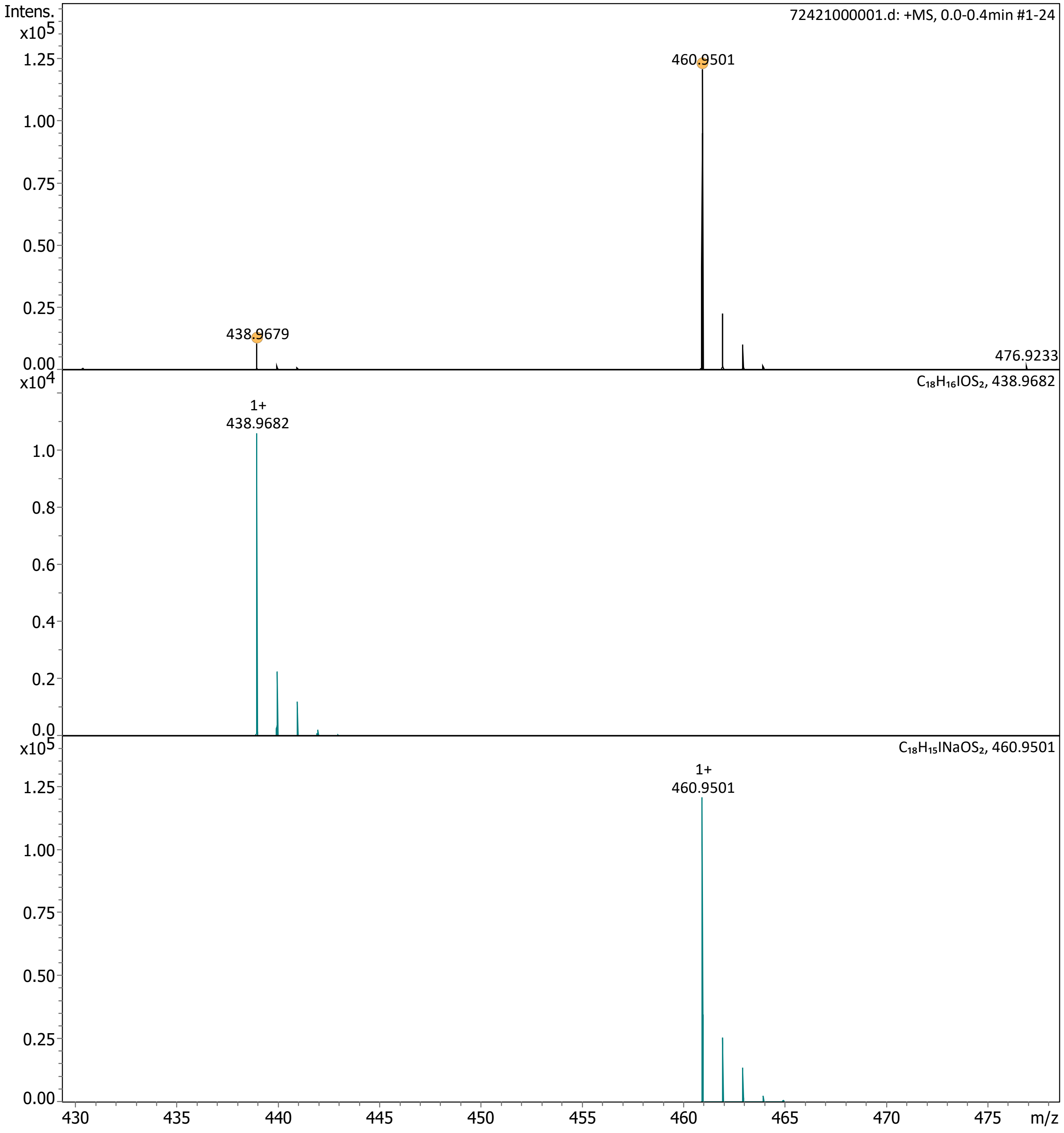

Mass Spectrum SmartFormula Report

Analysis Info

Analysis Name	D:\Data\Kalaba\72421000001.d	Acquisition Date	20/08/2020 17:15:38
Method	tune_low_MS_Service_08_20.m	Operator	msc
Sample Name	MK-31-p3	Instrument	maXis 255552.00016
Comment	Kalaba/Zehl<br>Ergebnis +/- 5ppm<br>ACN / MeOH + 1% H2O		

Acquisition Parameter

Source Type	ESI	Ion Polarity	Positive	Set Nebulizer	0.4 Bar
Focus	Not active	Set Capillary	4200 V	Set Dry Heater	180 Â°C
Scan Begin	50 m/z	Set End Plate Offset	-500 V	Set Dry Gas	4.0 l/min
Scan End	1900 m/z	Set Charging Voltage	0 V	Set Divert Valve	Source
		Set Corona	0 nA	Set APCI Heater	0 Â°C

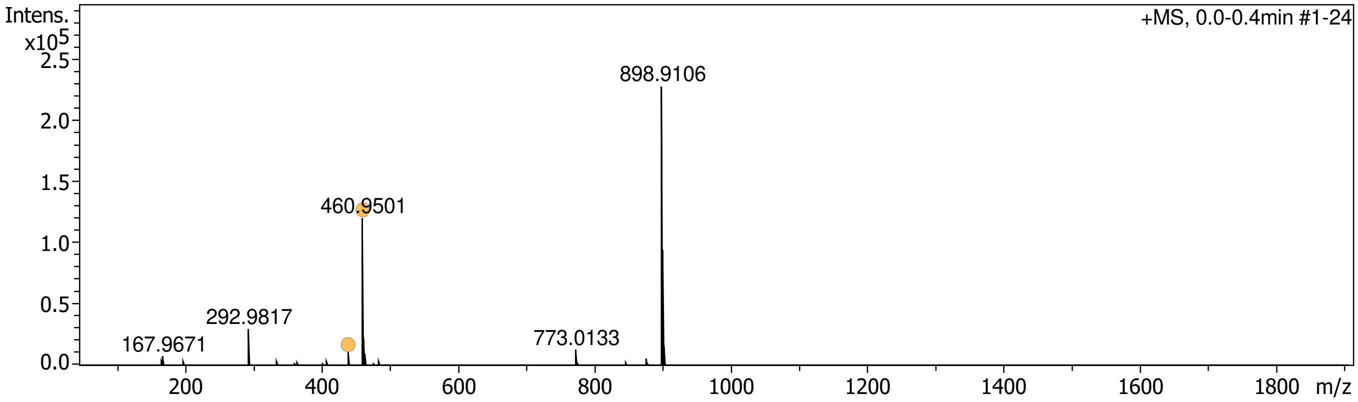

Meas. m/z	#	Ion Formula	m/z	err [ppm]	mSigma	# mSigma	Score	rdb	eÂ¯ Conf	N-Rule
438.9679	1	C11H3N8O10S	438.9687	2.0	16.8	1	79.38	17.0	even	ok
	2	C18H16IOS2	438.9682	0.8	19.0	2	100.00	18.0	even	ok
	3	C15H3N8O5S2	438.9662	-3.7	21.0	3	46.20	23.0	even	ok
	4	C11H8IN10S	438.9693	3.4	21.2	4	50.38	18.0	even	ok
	5	C18H3N2O12	438.9680	0.5	24.9	5	93.13	19.0	even	ok
	6	C19H7N2O7S2	438.9689	2.4	28.3	6	69.26	22.0	even	ok
	7	C12H7N8O5S3	438.9696	4.0	33.7	7	38.90	20.0	even	ok
	8	C17H12IO6	438.9673	-1.2	35.9	8	45.67	15.0	even	ok
	9	C11H11N4O9S3	438.9683	0.9	37.2	9	78.07	15.0	even	ok
	10	C18H8IN4O2	438.9686	1.8	40.0	10	35.83	20.0	even	ok
	11	C14H4IN10	438.9660	-4.3	42.8	11	16.58	21.0	even	ok
	12	C15H11N4O4S4	438.9658	-4.8	56.8	12	15.53	21.0	even	ok
	13	C27H3O5S	438.9696	3.9	57.6	13	15.88	29.0	even	ok
	14	C16H7N8S4	438.9671	-1.7	58.7	14	34.79	26.0	even	ok
	15	C23H7N2O2S3	438.9664	-3.3	59.4	15	22.71	28.0	even	ok
	16	C20H11N2O2S4	438.9698	4.4	67.3	16	12.13	25.0	even	ok
	17	C31H3S2	438.9671	-1.8	79.0	17	16.20	35.0	even	ok
460.9501	1	C11H2N8NaO10S	460.9507	1.3	13.8	1	87.56	17.0	even	ok
	2	C11H7IN10NaS	460.9513	2.6	17.7	2	58.00	18.0	even	ok
	3	C18H15INaOS2	460.9501	0.1	19.6	3	100.00	18.0	even	ok
	4	C15H2N8NaO5S2	460.9482	-4.1	21.8	4	33.67	23.0	even	ok
	5	C18H2N2NaO12	460.9500	-0.2	22.4	5	93.63	19.0	even	ok
	6	C19H6N2NaO7S2	460.9509	1.7	29.2	6	71.20	22.0	even	ok
	7	C17H11INaO6	460.9493	-1.8	31.9	7	39.64	15.0	even	ok
	8	C12H6N8NaO5S3	460.9515	3.2	34.9	8	40.99	20.0	even	ok
	9	C18H7IN4NaO2	460.9506	1.1	36.7	9	40.50	20.0	even	ok
	10	C11H10N4NaO9S3	460.9502	0.3	38.3	10	77.05	15.0	even	ok
	11	C14H3IN10Na	460.9479	-4.7	38.9	11	13.54	21.0	even	ok
	12	C20H2N6NaO3S2	460.9522	4.6	40.9	12	17.40	27.0	even	ok

Mass Spectrum SmartFormula Report

Meas. m/z	#	Ion Formula	m/z	err [ppm]	mSigma	# mSigma	Score	rdb	eÂ¯ Conf	N-Rule
	13	C27H2NaO5S	460.9515	3.1	58.3	13	16.86	29.0	even	ok
	14	C16H6N8NaS4	460.9490	-2.2	60.0	14	25.89	26.0	even	ok
	15	C23H6N2NaO2S3	460.9484	-3.7	60.4	15	16.44	28.0	even	ok
	16	C20H10N2NaO2S4	460.9517	3.6	68.5	16	12.74	25.0	even	ok
	17	C28H6NaS3	460.9524	5.0	79.3	17	5.13	32.0	even	ok
	18	C31H2NaS2	460.9490	-2.3	79.9	18	12.12	35.0	even	ok

Figure S36. HRESIMS spectrum of compound **8i**

Generic Display Report

Analysis Info

Analysis Name	D:\Data\Kalaba\72422000001.d	Acquisition Date	20/08/2020 17:27:15
Method	tune_low_MS_Service_08_20.m	Operator	msc
Sample Name	MK-31-p4	Instrument	maXis
Comment	Kalaba/Zehl<br>Ergebnis +/- 5ppm<br>ACN / MeOH + 1% H2O		

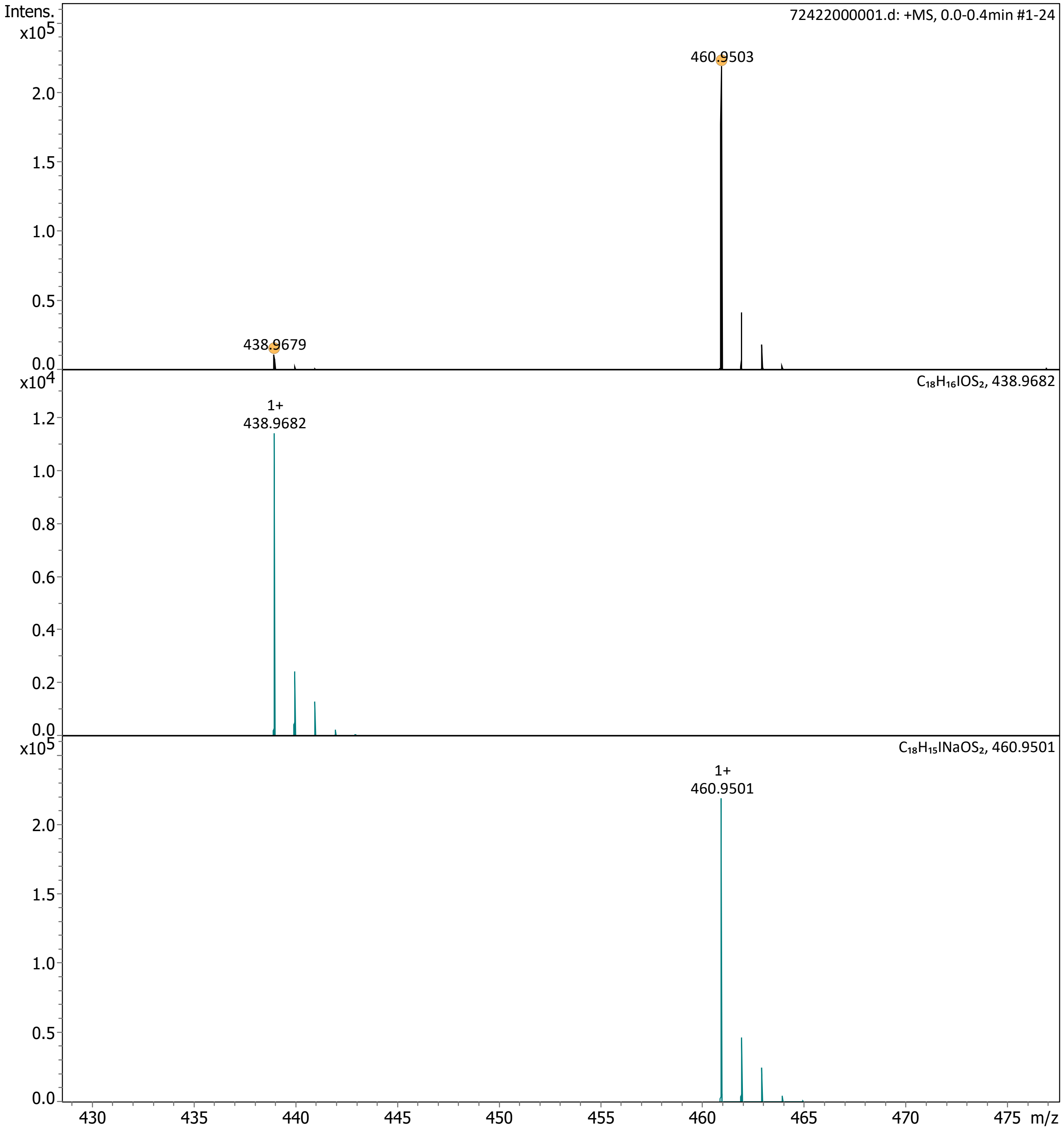

Mass Spectrum SmartFormula Report

Analysis Info

Analysis Name	D:\Data\Kalaba\72422000001.d	Acquisition Date	20/08/2020 17:27:15
Method	tune_low_MS_Service_08_20.m	Operator	msc
Sample Name	MK-31-p4	Instrument	maXis 255552.00016
Comment	Kalaba/Zehl<br>Ergebnis +/- 5ppm<br>ACN / MeOH + 1% H2O		

Acquisition Parameter

Source Type	ESI	Ion Polarity	Positive	Set Nebulizer	0.4 Bar
Focus	Not active	Set Capillary	4200 V	Set Dry Heater	180 Â°C
Scan Begin	50 m/z	Set End Plate Offset	-500 V	Set Dry Gas	4.0 l/min
Scan End	1900 m/z	Set Charging Voltage	0 V	Set Divert Valve	Source
		Set Corona	0 nA	Set APCI Heater	0 Â°C

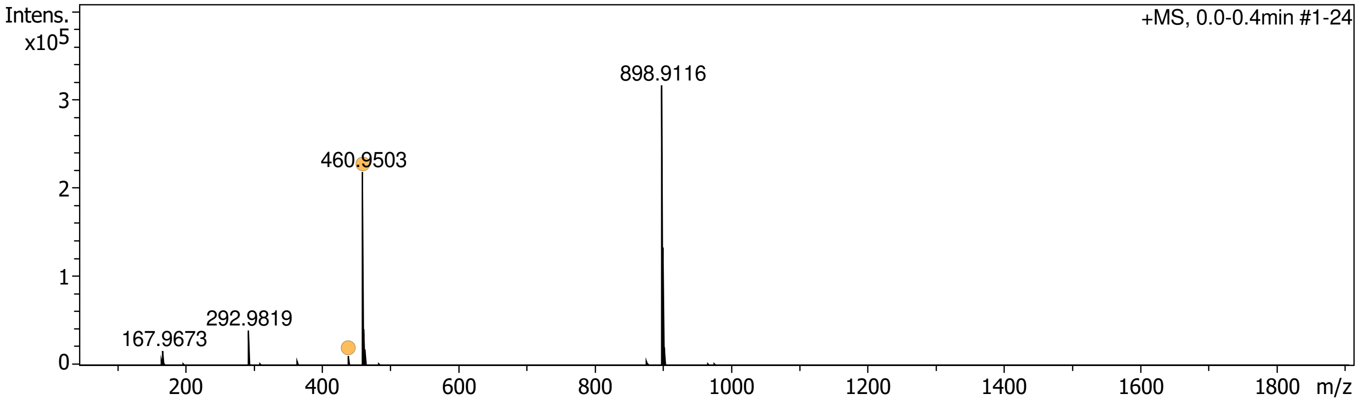

Meas. m/z	#	Ion Formula	m/z	err [ppm]	mSigma	# mSigma	Score	rdb	eÂ¯ Conf	N-Rule
438.9679	1	C18H16IOS2	438.9682	0.5	16.1	1	100.00	18.0	even	ok
	2	C15H3N8O5S2	438.9662	-3.9	18.6	2	41.26	23.0	even	ok
	3	C18H3N2O12	438.9680	0.2	24.4	3	88.52	19.0	even	ok
	4	C19H7N2O7S2	438.9689	2.2	25.3	4	70.78	22.0	even	ok
	5	C12H7N8O5S3	438.9696	3.8	33.5	5	37.70	20.0	even	ok
	6	C17H12IO6	438.9673	-1.4	37.2	6	38.11	15.0	even	ok
	7	C18H8IN4O2	438.9686	1.6	39.6	7	34.42	20.0	even	ok
	8	C14H4IN10	438.9660	-4.5	44.1	8	13.54	21.0	even	ok
	9	C27H3O5S	438.9696	3.7	53.2	9	17.65	29.0	even	ok
	10	C15H11N4O4S4	438.9658	-5.0	55.6	10	13.60	21.0	even	ok
	11	C23H7N2O2S3	438.9664	-3.5	56.3	11	21.47	28.0	even	ok
	12	C16H7N8S4	438.9671	-1.9	56.9	12	31.89	26.0	even	ok
	13	C20H11N2O2S4	438.9698	4.2	65.0	13	12.73	25.0	even	ok
	14	C31H3S2	438.9671	-2.0	75.2	14	16.26	35.0	even	ok
460.9503	1	C11H2N8NaO10S	460.9507	0.7	14.5	1	100.00	17.0	even	ok
	2	C14H15INaO6S	460.9526	4.9	14.6	2	29.24	12.0	even	ok
	3	C11H7IN10NaS	460.9513	2.0	18.3	3	68.41	18.0	even	ok
	4	C18H15INaOS2	460.9501	-0.5	19.1	4	95.68	18.0	even	ok
	5	C15H2N8NaO5S2	460.9482	-4.7	21.4	5	27.81	23.0	even	ok
	6	C18H2N2NaO12	460.9500	-0.8	22.2	6	84.37	19.0	even	ok
	7	C19H6N2NaO7S2	460.9509	1.1	28.6	7	84.20	22.0	even	ok
	8	C17H11INaO6	460.9493	-2.4	32.0	8	34.22	15.0	even	ok
	9	C12H6N8NaO5S3	460.9515	2.6	34.8	9	49.72	20.0	even	ok
	10	C18H7IN4NaO2	460.9506	0.5	36.6	10	46.90	20.0	even	ok
	11	C11H10N4NaO9S3	460.9502	-0.3	38.3	11	78.28	15.0	even	ok
	12	C20H2N6NaO3S2	460.9522	4.0	40.3	12	22.20	27.0	even	ok
	13	C27H2NaO5S	460.9515	2.5	57.6	13	20.83	29.0	even	ok
	14	C16H6N8NaS4	460.9490	-2.8	59.6	14	22.43	26.0	even	ok
	15	C23H6N2NaO2S3	460.9484	-4.3	59.9	15	13.82	28.0	even	ok

Mass Spectrum SmartFormula Report

Meas. m/z	#	Ion Formula	m/z	err [ppm]	mSigma	# mSigma	Score	rdb	eÂ¯ Conf	N-Rule
	16	C20H10N2NaO2S4	460.9517	3.0	68.1	16	15.78	25.0	even	ok
	17	C28H6NaS3	460.9524	4.4	78.7	17	6.65	32.0	even	ok
	18	C31H2NaS2	460.9490	-2.9	79.3	18	10.63	35.0	even	ok

Figure S37. HRESIMS spectrum of compound **5j**

Generic Display Report

Analysis Info

Analysis Name	D:\Data\Kalaba\71415000001.d	Acquisition Date	06/07/2020 18:06:20
Method	tune_low_MS_Service_07_20.m	Operator	msc
Sample Name	MK-30-1-1	Instrument	maXis
Comment	Kalaba/Zehl<br>Ergebnis +/- 5 ppm<br>ACN/MeOH + 1 % H2O		

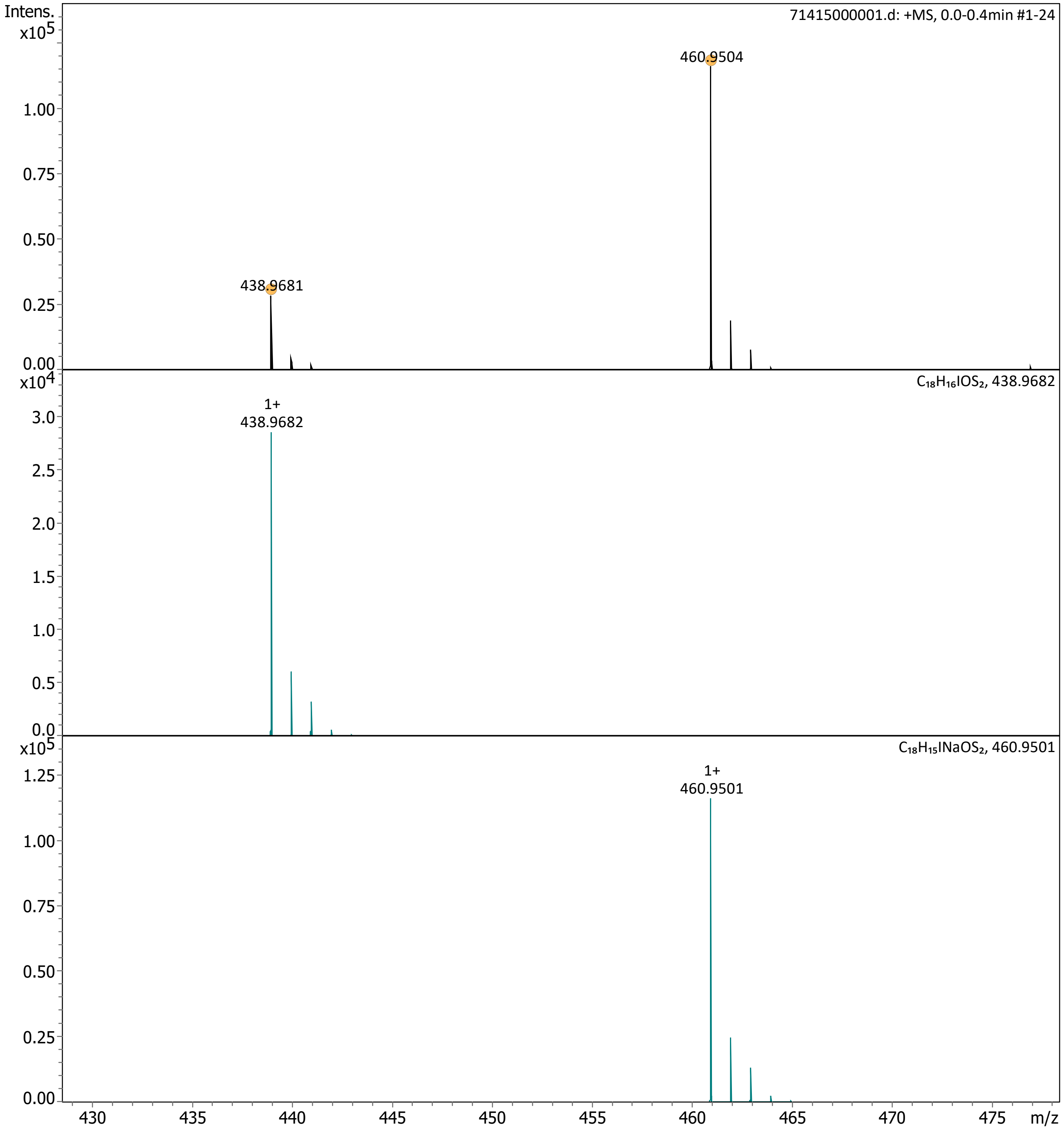

Mass Spectrum SmartFormula Report

Analysis Info

Analysis Name	D:\Data\Kalaba\71415000001.d	Acquisition Date	06/07/2020 18:06:20
Method	tune_low_MS_Service_07_20.m	Operator	msc
Sample Name	MK-30-1-1	Instrument	maXis 255552.00016
Comment	Kalaba/Zehl<br>Ergebnis +/- 5 ppm<br>ACN/MeOH + 1 % H2O		

Acquisition Parameter

Source Type	ESI	Ion Polarity	Positive	Set Nebulizer	0.4 Bar
Focus	Not active	Set Capillary	4200 V	Set Dry Heater	180 Â°C
Scan Begin	50 m/z	Set End Plate Offset	-500 V	Set Dry Gas	4.0 l/min
Scan End	1900 m/z	Set Charging Voltage	0 V	Set Divert Valve	Source
		Set Corona	0 nA	Set APCI Heater	0 Â°C

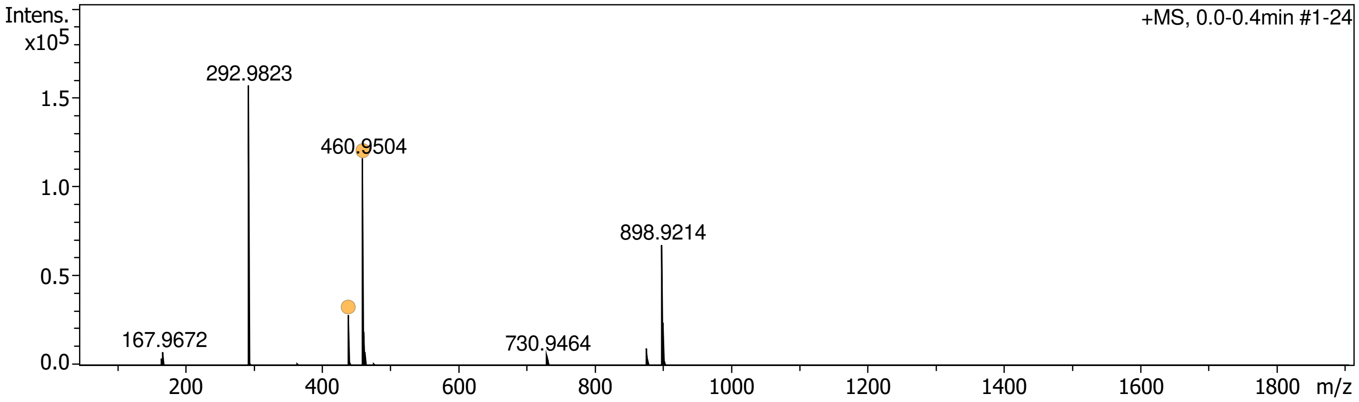

Meas. m/z	#	Ion Formula	m/z	err [ppm]	mSigma	# mSigma	Score	rdb	eÂ¯ Conf	N-Rule
438.9681	1	C11H8IN10S	438.9693	2.9	5.6	1	59.62	18.0	even	ok
	2	C10H12IN6O4S	438.9680	-0.2	10.9	2	100.00	13.0	even	ok
	3	C9H16IN2O8S	438.9667	-3.2	22.2	3	39.34	8.0	even	ok
	4	C17H12IO6	438.9673	-1.7	26.9	4	38.89	15.0	even	ok
	5	C14H4IN10	438.9660	-4.8	33.1	5	13.87	21.0	even	ok
	6	C18H16IOS2	438.9682	0.3	34.5	6	58.25	18.0	even	ok
	7	C18H8IN4O2	438.9686	1.3	37.5	7	32.59	20.0	even	ok
	8	C10H20IN2O3S3	438.9675	-1.2	38.1	8	54.59	11.0	even	ok
460.9504	1	C14H15INaO6S	460.9526	4.8	1.0	1	39.92	12.0	even	ok
	2	C11H7IN10NaS	460.9513	1.9	5.3	2	91.83	18.0	even	ok
	3	C10H11IN6NaO4S	460.9499	-1.0	11.2	3	100.00	13.0	even	ok
	4	C9H15IN2NaO8S	460.9486	-3.9	22.6	4	35.53	8.0	even	ok
	5	C17H11INaO6	460.9493	-2.5	26.7	5	37.37	15.0	even	ok
	6	C18H15INaOS2	460.9501	-0.6	34.2	6	65.36	18.0	even	ok
	7	C18H7IN4NaO2	460.9506	0.4	37.2	7	47.90	20.0	even	ok
	8	C10H19IN2NaO3S3	460.9495	-2.1	38.1	8	53.10	11.0	even	ok

Figure S38. HRESIMS spectrum of compound **6j**

Generic Display Report

Analysis Info

Analysis Name	D:\Data\Kalaba\71416000001.d	Acquisition Date	06/07/2020 18:30:37
Method	tune_low_MS_Service_07_20.m	Operator	msc
Sample Name	MK-30-1-2	Instrument	maXis
Comment	Kalaba/Zehl<br>Ergebnis +/- 5 ppm<br>ACN/MeOH + 1 % H2O		

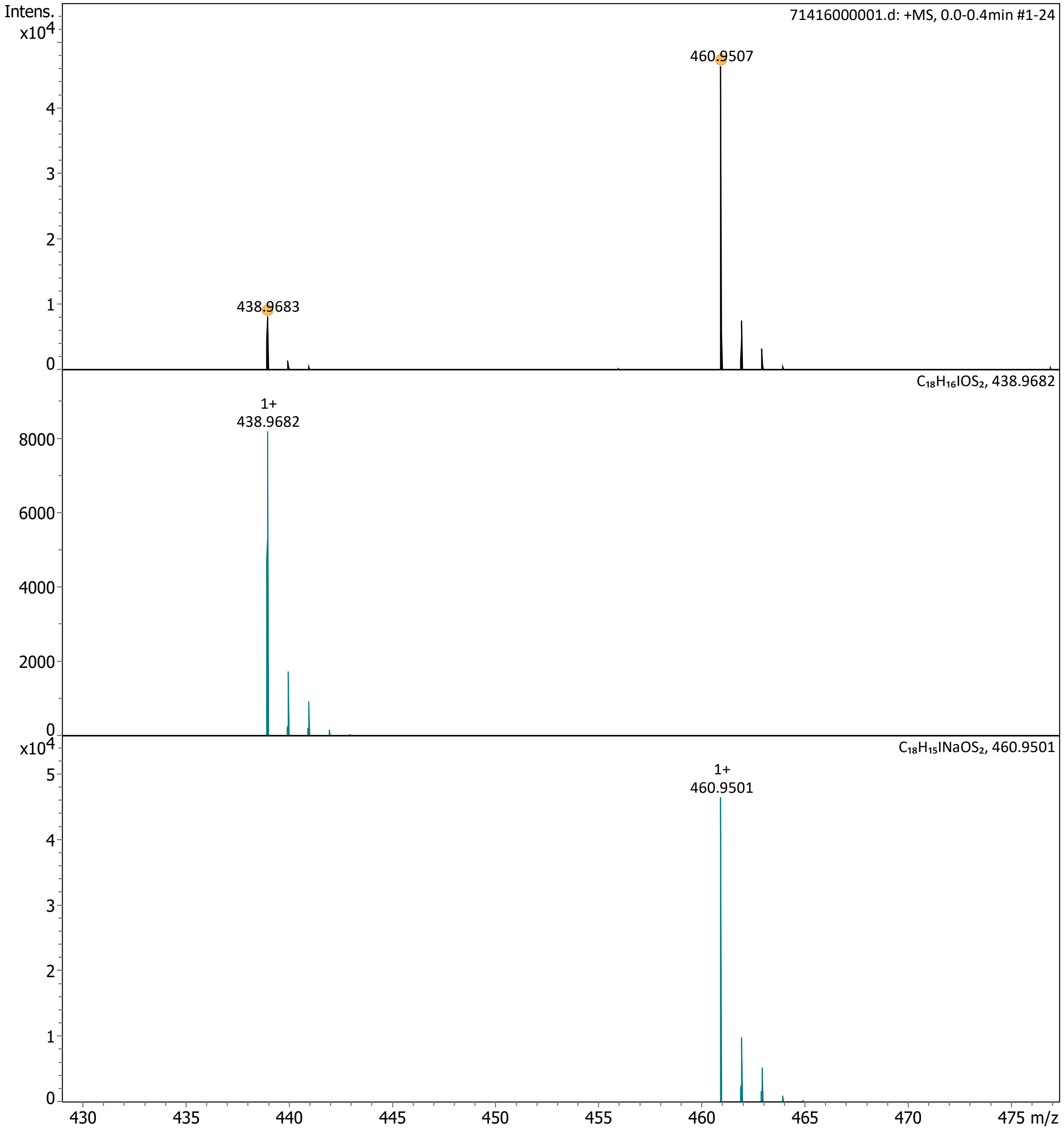

Mass Spectrum SmartFormula Report

Analysis Info

Analysis Name	D:\Data\Kalaba\71416000001.d	Acquisition Date	06/07/2020 18:30:37
Method	tune_low_MS_Service_07_20.m	Operator	msc
Sample Name	MK-30-1-2	Instrument	maXis 255552.00016
Comment	Kalaba/Zehl<br>Ergebnis +/- 5 ppm<br>ACN/MeOH + 1 % H2O		

Acquisition Parameter

Source Type	ESI	Ion Polarity	Positive	Set Nebulizer	0.4 Bar
Focus	Not active	Set Capillary	4200 V	Set Dry Heater	180 Â°C
Scan Begin	50 m/z	Set End Plate Offset	-500 V	Set Dry Gas	4.0 l/min
Scan End	1900 m/z	Set Charging Voltage	0 V	Set Divert Valve	Source
		Set Corona	0 nA	Set APCI Heater	0 Â°C

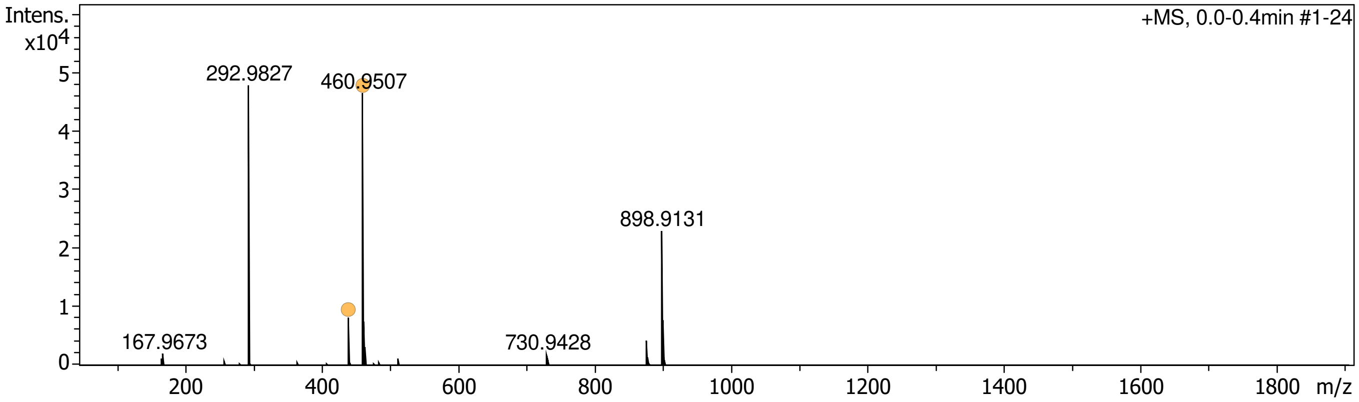

Meas. m/z	#	Ion Formula	m/z	err [ppm]	mSigma	# mSigma	Score	rdb	eÂ¯ Conf	N-Rule
438.9683	1	C11H8IN10S	438.9693	2.3	7.3	1	81.26	18.0	even	ok
	2	C10H12IN6O4S	438.9680	-0.7	15.4	2	100.00	13.0	even	ok
	3	C17H12IO6	438.9673	-2.3	25.7	3	42.31	15.0	even	ok
	4	C18H16IOS2	438.9682	-0.3	30.5	4	77.41	18.0	even	ok
	5	C18H8IN4O2	438.9686	0.8	34.9	5	47.56	20.0	even	ok
	6	C10H20IN2O3S3	438.9675	-1.8	38.1	6	58.42	11.0	even	ok
460.9507	1	C14H15INaO6S	460.9526	4.3	0.8	1	46.99	12.0	even	ok
	2	C11H7IN10NaS	460.9513	1.3	6.2	2	100.00	18.0	even	ok
	3	C10H11IN6NaO4S	460.9499	-1.6	12.3	3	85.08	13.0	even	ok
	4	C9H15IN2NaO8S	460.9486	-4.5	23.4	4	28.52	8.0	even	ok
	5	C17H11INaO6	460.9493	-3.0	27.0	5	31.32	15.0	even	ok
	6	C18H15INaOS2	460.9501	-1.2	33.0	6	58.98	18.0	even	ok
	7	C18H7IN4NaO2	460.9506	-0.1	37.1	7	49.02	20.0	even	ok
	8	C10H19IN2NaO3S3	460.9495	-2.6	37.7	8	45.80	11.0	even	ok

Figure S39. HRESIMS spectrum of compound **7j**

Generic Display Report

Analysis Info

Analysis Name	D:\Data\Kalaba\71417000001.d	Acquisition Date	06/07/2020 18:34:09
Method	tune_low_MS_Service_07_20.m	Operator	msc
Sample Name	MK-30-2-1	Instrument	maXis
Comment	Kalaba/Zehl<br>Ergebnis +/- 5 ppm<br>ACN/MeOH + 1 % H2O		

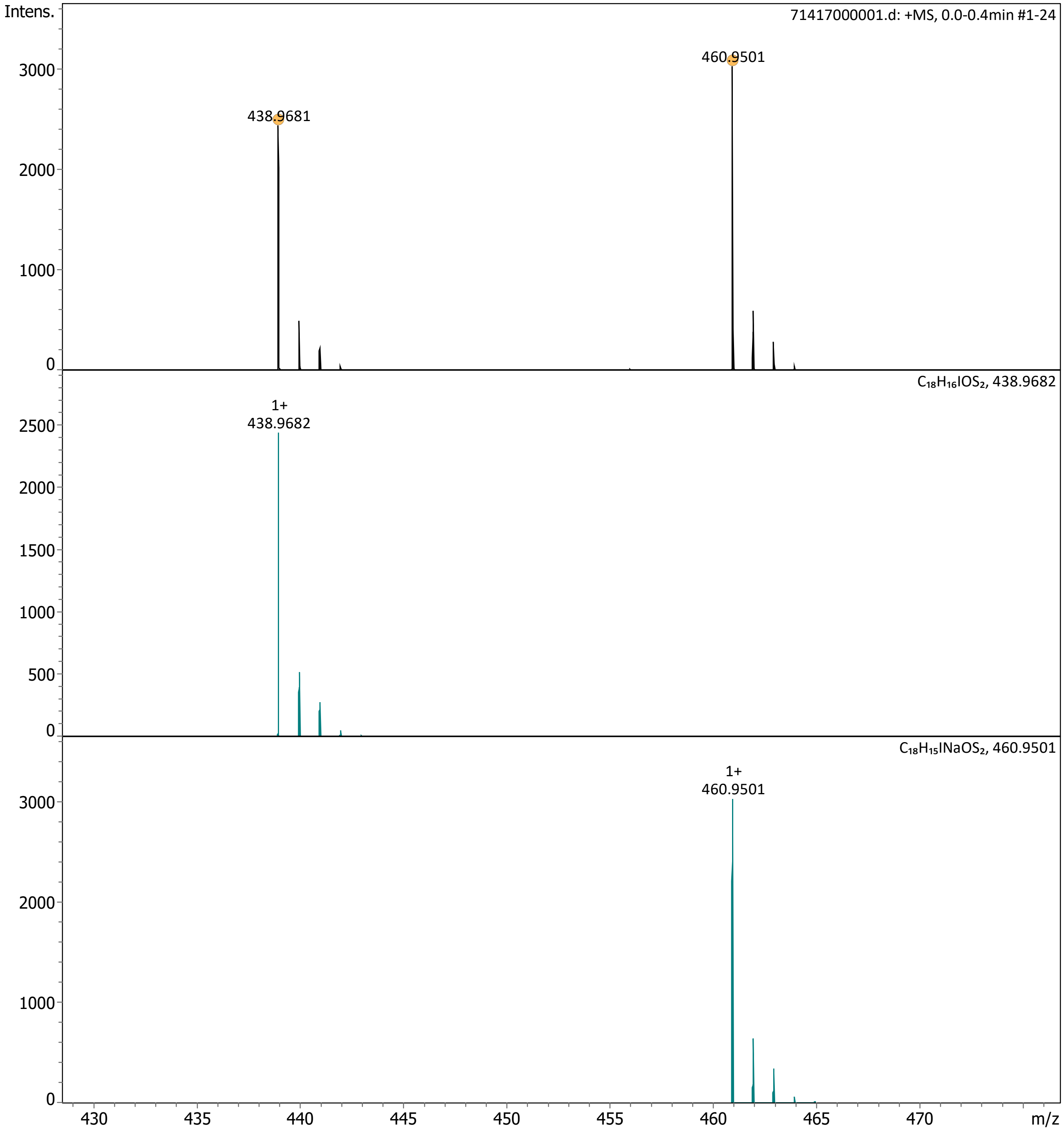

Mass Spectrum SmartFormula Report

Analysis Info

Analysis Name	D:\Data\Kalaba\71417000001.d	Acquisition Date	06/07/2020 18:34:09
Method	tune_low_MS_Service_07_20.m	Operator	msc
Sample Name	MK-30-2-1	Instrument	maXis 255552.00016
Comment	Kalaba/Zehl<br>Ergebnis +/- 5 ppm<br>ACN/MeOH + 1 % H2O		

Acquisition Parameter

Source Type	ESI	Ion Polarity	Positive	Set Nebulizer	0.4 Bar
Focus	Not active	Set Capillary	4200 V	Set Dry Heater	180 Â°C
Scan Begin	50 m/z	Set End Plate Offset	-500 V	Set Dry Gas	4.0 l/min
Scan End	1900 m/z	Set Charging Voltage	0 V	Set Divert Valve	Source
		Set Corona	0 nA	Set APCI Heater	0 Â°C

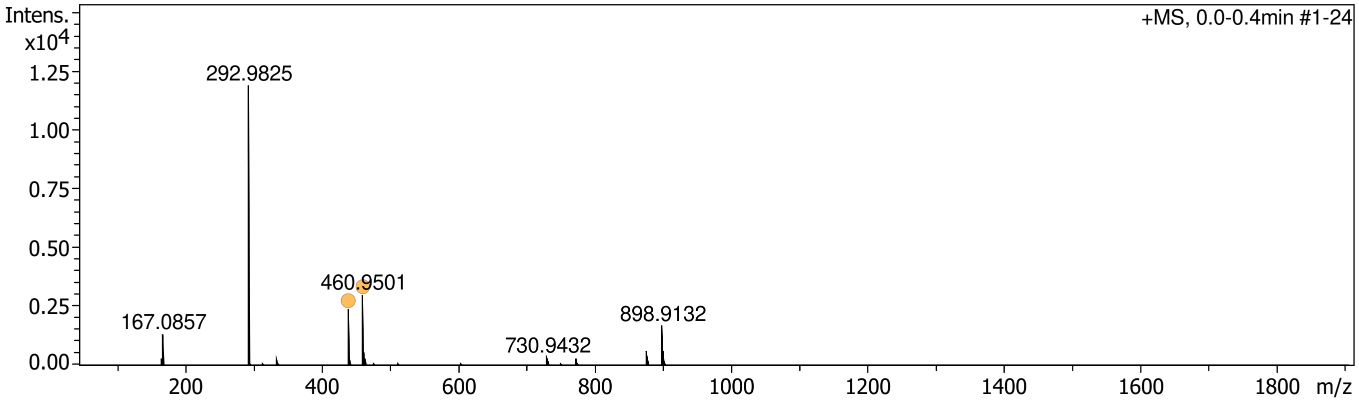

Meas. m/z	#	Ion Formula	Score	m/z	err [mDa]	err [ppm]	mSigma	rdb	eÂ¯ Conf	N-Rule
438.9681	1	C18H16IOS2	100.00	438.9682	0.0	0.1	11.8	18.0	even	ok
	2	C17H12IO6	28.58	438.9673	-0.8	-1.9	37.7	15.0	even	ok
	3	C18H8IN4O2	32.32	438.9686	0.5	1.1	39.3	20.0	even	ok
	4	C14H4IN10	9.65	438.9660	-2.2	-5.0	44.4	21.0	even	ok
460.9501	1	C18H15INaOS2	100.00	460.9501	0.0	0.0	12.4	18.0	even	ok
	2	C17H11INaO6	27.95	460.9493	-0.8	-1.8	38.4	15.0	even	ok
	3	C18H7IN4NaO2	31.36	460.9506	0.5	1.1	40.9	20.0	even	ok
	4	C14H3IN10Na	9.36	460.9479	-2.2	-4.8	45.2	21.0	even	ok

Figure S40. HRESIMS spectrum of compound **8j**

Generic Display Report

Analysis Info

Analysis Name	D:\Data\Kalaba\71418000001.d
Method	tune_low_MS_Service_07_20.m
Sample Name	MK-30-2-2
Comment	Kalaba/Zehl
	Ergebnis +/- 5 ppm
	ACN/MeOH + 1 % H2O

Acquisition Date	06/07/2020 18:36:02
Operator	msc
Instrument	maXis

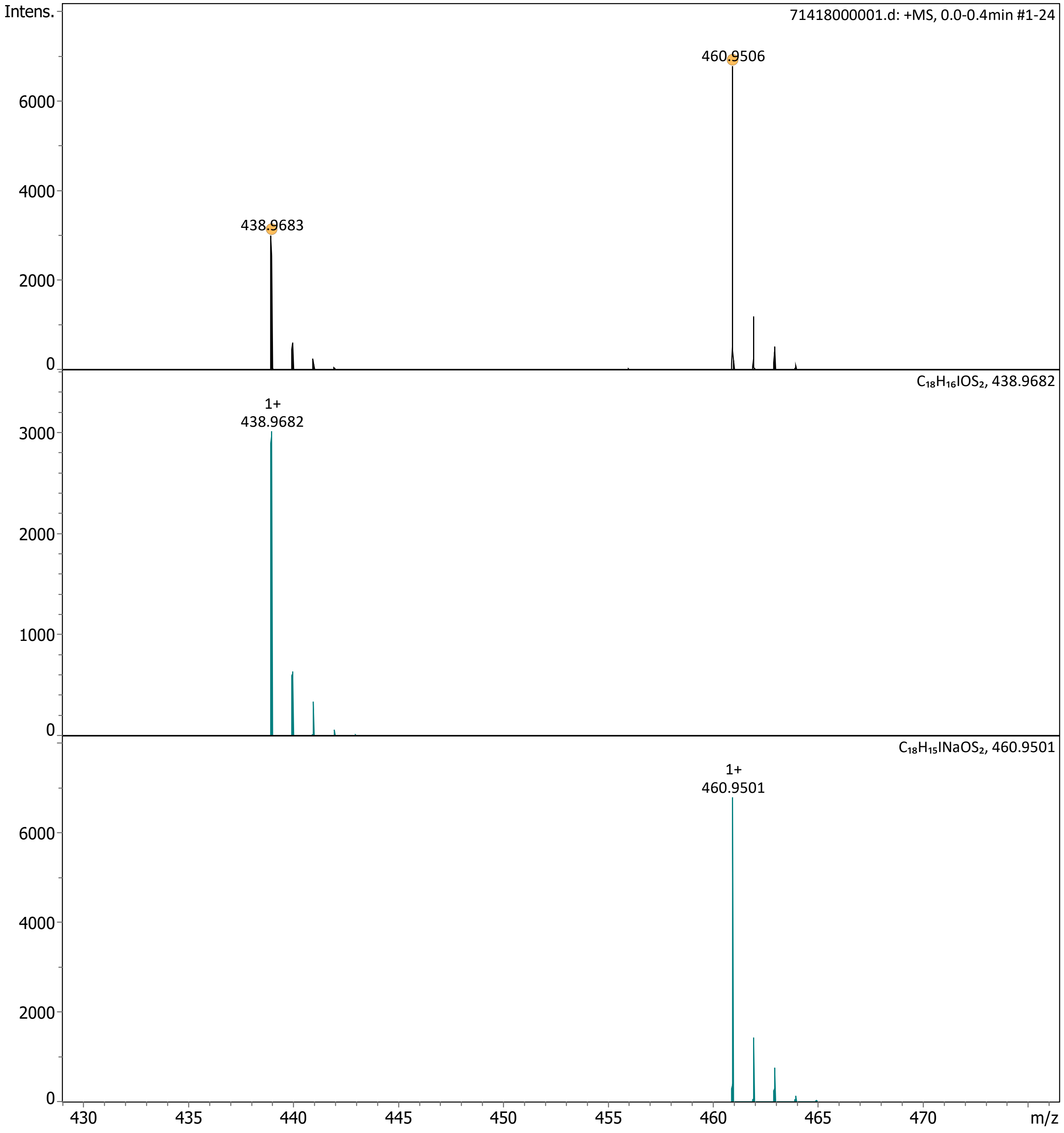

Mass Spectrum SmartFormula Report

Analysis Info

Analysis Name	D:\Data\Kalaba\71418000001.d	Acquisition Date	06/07/2020 18:36:02
Method	tune_low_MS_Service_07_20.m	Operator	msc
Sample Name	MK-30-2-2	Instrument	maXis 255552.00016
Comment	Kalaba/Zehl<br>Ergebnis +/- 5 ppm<br>ACN/MeOH + 1 % H2O		

Acquisition Parameter

Source Type	ESI	Ion Polarity	Positive	Set Nebulizer	0.4 Bar
Focus	Not active	Set Capillary	4200 V	Set Dry Heater	180 Â°C
Scan Begin	50 m/z	Set End Plate Offset	-500 V	Set Dry Gas	4.0 l/min
Scan End	1900 m/z	Set Charging Voltage	0 V	Set Divert Valve	Source
		Set Corona	0 nA	Set APCI Heater	0 Â°C

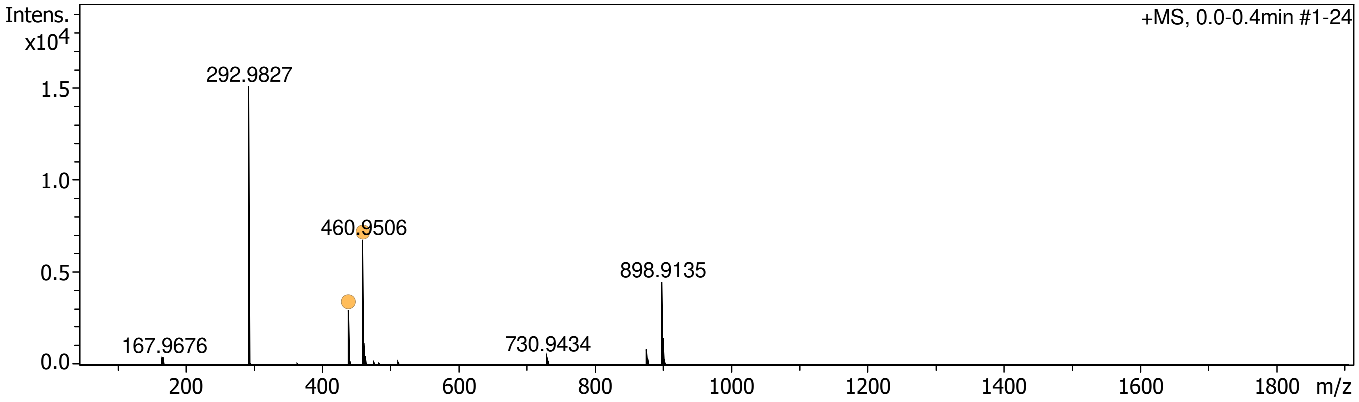

Meas. m/z	#	Ion Formula	Score	m/z	err [mDa]	err [ppm]	mSigma	rdb	eÂ¯ Conf	N-Rule
438.9683	1	C18H16IOS2	100.00	438.9682	-0.1	-0.3	14.7	18.0	even	ok
	2	C17H12IO6	31.67	438.9673	-1.0	-2.3	33.9	15.0	even	ok
	3	C18H8IN4O2	42.72	438.9686	0.3	0.8	35.1	20.0	even	ok
460.9506	1	C14H15INaO6S	46.07	460.9526	2.0	4.4	7.8	12.0	even	ok
	2	C11H7IN10NaS	100.00	460.9513	0.7	1.5	12.4	18.0	even	ok
	3	C10H11IN6NaO4S	88.04	460.9499	-0.7	-1.4	19.3	13.0	even	ok
	4	C18H15INaOS2	83.72	460.9501	-0.5	-1.0	25.7	18.0	even	ok
	5	C17H11INaO6	35.35	460.9493	-1.3	-2.9	29.6	15.0	even	ok
	6	C10H19IN2NaO3S3	58.56	460.9495	-1.1	-2.4	35.2	11.0	even	ok
	7	C18H7IN4NaO2	58.04	460.9506	-0.0	-0.0	37.1	20.0	even	ok

Figure S41. HRESIMS spectrum of compound **5k**

Generic Display Report

Analysis Info

Analysis Name	D:\Data\Kalaba\74278000001.d	Acquisition Date	03/11/2020 14:09:14
Method	tune_low_MS_Service_11_20.m	Operator	msc
Sample Name	SB2-Spot1-P1	Instrument	maXis
Comment	Kalaba/Zehl<br>Ergebnis: +/- 5ppm<br>ACN/MeOH + 1% H2O		

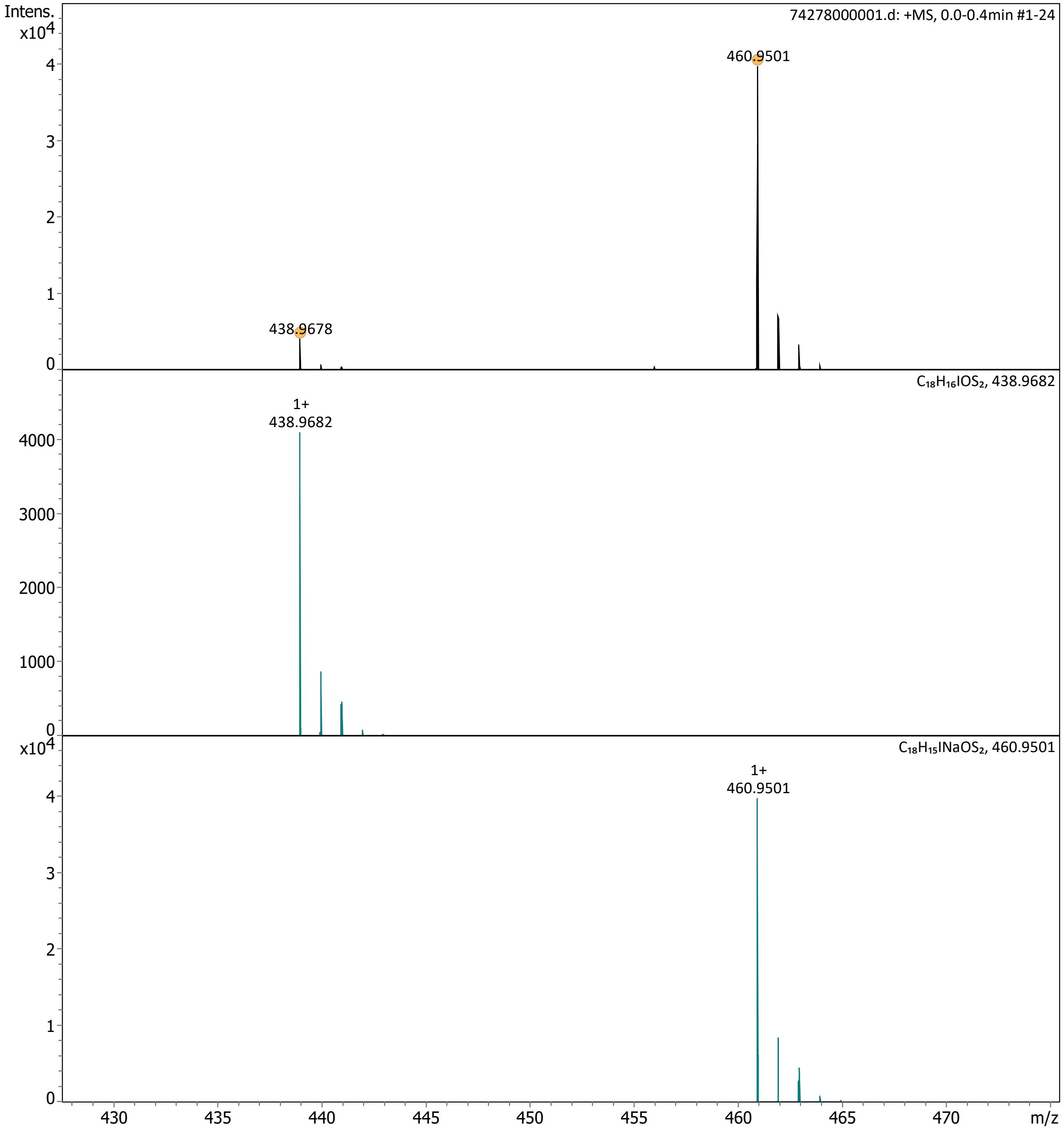

Mass Spectrum SmartFormula Report

Analysis Info

Analysis Name	D:\Data\Kalaba\74278000001.d	Acquisition Date	03/11/2020 14:09:14
Method	tune_low_MS_Service_11_20.m	Operator	msc
Sample Name	SB2-Spot1-P1	Instrument	maXis 255552.00016
Comment	Kalaba/Zehl<br>Ergebnis: +/- 5ppm<br>ACN/MeOH + 1% H2O		

Acquisition Parameter

Source Type	ESI	Ion Polarity	Positive	Set Nebulizer	0.4 Bar
Focus	Not active	Set Capillary	4200 V	Set Dry Heater	180 Â°C
Scan Begin	50 m/z	Set End Plate Offset	-500 V	Set Dry Gas	4.0 l/min
Scan End	1900 m/z	Set Charging Voltage	0 V	Set Divert Valve	Source
		Set Corona	0 nA	Set APCI Heater	0 Â°C

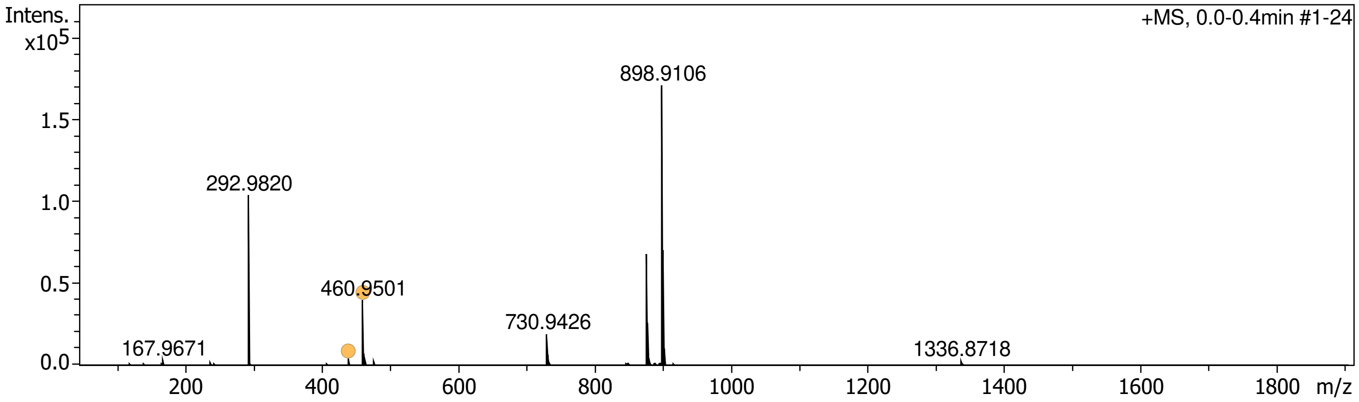

Meas. m/z	#	Ion Formula	Score	m/z	err [mDa]	err [ppm]	mSigma	rdb	eÂ¯ Conf	N-Rule
438.9678	1	C18H16IOS2	100.00	438.9682	0.4	0.9	17.7	18.0	even	ok
	2	C11H3N8O10S	74.74	438.9687	1.0	2.2	18.0	17.0	even	ok
	3	C15H3N8O5S2	50.57	438.9662	-1.5	-3.5	19.6	23.0	even	ok
	4	C11H8IN10S	46.52	438.9693	1.6	3.6	22.8	18.0	even	ok
	5	C18H3N2O12	88.08	438.9680	0.3	0.6	26.2	19.0	even	ok
	6	C19H7N2O7S2	68.75	438.9689	1.1	2.6	27.1	22.0	even	ok
	7	C12H7N8O5S3	38.36	438.9696	1.8	4.2	32.4	20.0	even	ok
	8	C22H3N2O7S	21.65	438.9655	-2.2	-5.1	34.1	25.0	even	ok
	9	C11H11N4O9S3	78.13	438.9683	0.5	1.1	36.1	15.0	even	ok
	10	C17H12IO6	45.39	438.9673	-0.5	-1.1	37.8	15.0	even	ok
	11	C18H8IN4O2	33.17	438.9686	0.9	2.0	41.6	20.0	even	ok
	12	C14H4IN10	16.73	438.9660	-1.8	-4.1	44.7	21.0	even	ok
	13	C15H11N4O4S4	17.40	438.9658	-2.0	-4.6	55.4	21.0	even	ok
	14	C27H3O5S	15.51	438.9696	1.8	4.1	56.9	29.0	even	ok
	15	C16H7N8S4	38.28	438.9671	-0.7	-1.5	57.3	26.0	even	ok
	16	C23H7N2O2S3	25.12	438.9664	-1.4	-3.1	58.1	28.0	even	ok
	17	C20H11N2O2S4	12.16	438.9698	2.0	4.6	65.9	25.0	even	ok
	18	C12H15N4O4S5	15.03	438.9691	1.4	3.1	72.5	18.0	even	ok
	19	C11H19O8S5	27.46	438.9678	0.0	0.1	74.8	13.0	even	ok
	20	C31H3S2	17.68	438.9671	-0.7	-1.6	78.1	35.0	even	ok
	21	C12H23O3S7	6.17	438.9687	0.9	2.0	103.7	16.0	even	ok
460.9501	1	C11H2N8NaO10S	100.00	460.9507	0.6	1.3	10.1	17.0	even	ok
	2	C11H7IN10NaS	64.96	460.9513	1.2	2.6	15.4	18.0	even	ok
	3	C10H6N4NaO14S	72.90	460.9493	-0.8	-1.6	21.2	12.0	even	ok
	4	C10H11IN6NaO4S	94.97	460.9499	-0.2	-0.3	22.1	13.0	even	ok
	5	C18H15INaOS2	99.40	460.9501	0.0	0.1	22.6	18.0	even	ok
	6	C18H2N2NaO12	92.53	460.9500	-0.1	-0.2	24.3	19.0	even	ok
	7	C15H2N8NaO5S2	32.47	460.9482	-1.9	-4.2	24.3	23.0	even	ok
	8	C17H11INaO6	40.71	460.9493	-0.8	-1.8	31.9	15.0	even	ok

Mass Spectrum SmartFormula Report

Meas. m/z	#	Ion Formula	Score	m/z	err [mDa]	err [ppm]	mSigma	rdb	eÂ¯ Conf	N-Rule
	9	C19H6N2NaO7S2	70.86	460.9509	0.8	1.7	31.9	22.0	even	ok
	10	C10H19IN2NaO3S3	71.92	460.9495	-0.6	-1.4	34.1	11.0	even	ok
	11	C12H6N8NaO5S3	43.32	460.9515	1.4	3.1	35.4	20.0	even	ok
	12	C11H10N4NaO9S3	82.86	460.9502	0.1	0.2	37.8	15.0	even	ok
	13	C18H7IN4NaO2	41.25	460.9506	0.5	1.1	38.4	20.0	even	ok
	14	C14H3IN10Na	13.79	460.9479	-2.2	-4.8	38.8	21.0	even	ok
	15	C10H14NaO13S3	40.48	460.9489	-1.2	-2.7	43.0	10.0	even	ok
	16	C20H2N6NaO3S2	16.95	460.9522	2.1	4.6	44.4	27.0	even	ok
	17	C16H6N8NaS4	25.09	460.9490	-1.1	-2.3	61.6	26.0	even	ok
	18	C27H2NaO5S	15.77	460.9515	1.4	3.1	62.2	29.0	even	ok
	19	C23H6N2NaO2S3	15.28	460.9484	-1.7	-3.8	63.2	28.0	even	ok
	20	C20H10N2NaO2S4	12.68	460.9517	1.6	3.5	70.5	25.0	even	ok
	21	C13H10N8NaS5	6.17	460.9524	2.3	5.0	75.7	23.0	even	ok
	22	C12H14N4NaO4S5	15.58	460.9511	1.0	2.1	75.8	18.0	even	ok
	23	C11H18NaO8S5	19.93	460.9497	-0.4	-0.8	77.6	13.0	even	ok
	24	C28H6NaS3	4.89	460.9524	2.3	5.0	82.2	32.0	even	ok
	25	C31H2NaS2	10.82	460.9490	-1.1	-2.4	83.3	35.0	even	ok
	26	C12H22NaO3S7	6.04	460.9506	0.5	1.1	106.8	16.0	even	ok

Figure S42. HRESIMS spectrum of compound **6k**

Generic Display Report

Analysis Info

Analysis Name	D:\Data\Kalaba\74279000001.d
Method	tune_low_MS_Service_11_20.m
Sample Name	SB2-spot1-p2
Comment	Kalaba/Zehl<br>Ergebnis: +/- 5ppm<br>ACN/MeOH + 1% H2O
Acquisition Date	03/11/2020 14:14:26
Operator	msc
Instrument	maXis

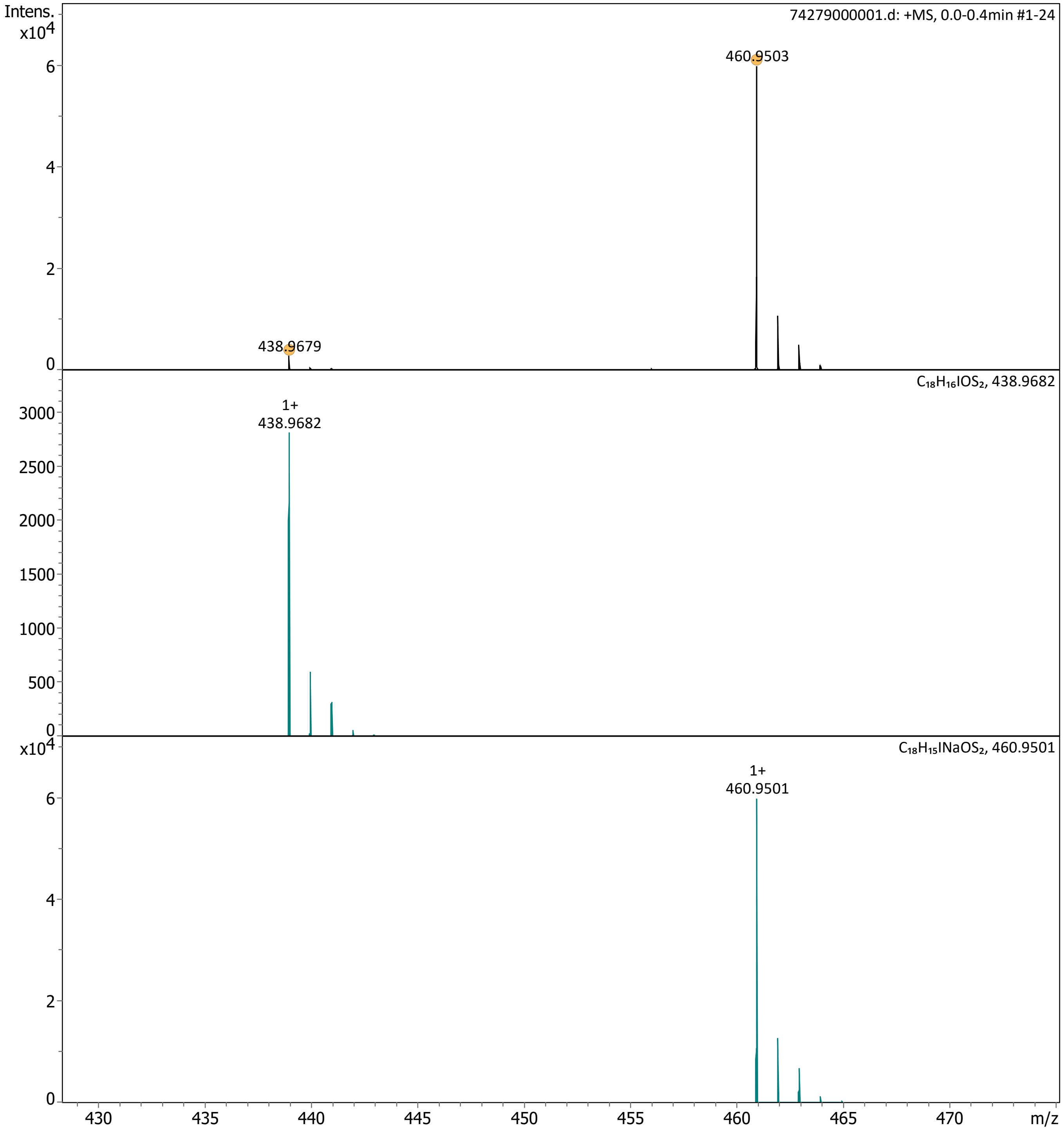

Mass Spectrum SmartFormula Report

Analysis Info

Analysis Name	D:\Data\Kalaba\74279000001.d	Acquisition Date	03/11/2020 14:14:26
Method	tune_low_MS_Service_11_20.m	Operator	msc
Sample Name	SB2-spot1-p2	Instrument	maXis 255552.00016
Comment	Kalaba/Zehl<br>Ergebnis: +/- 5ppm<br>ACN/MeOH + 1% H2O		

Acquisition Parameter

Source Type	ESI	Ion Polarity	Positive	Set Nebulizer	0.4 Bar
Focus	Not active	Set Capillary	4200 V	Set Dry Heater	180 Â°C
Scan Begin	50 m/z	Set End Plate Offset	-500 V	Set Dry Gas	4.0 l/min
Scan End	1900 m/z	Set Charging Voltage	0 V	Set Divert Valve	Source
		Set Corona	0 nA	Set APCI Heater	0 Â°C

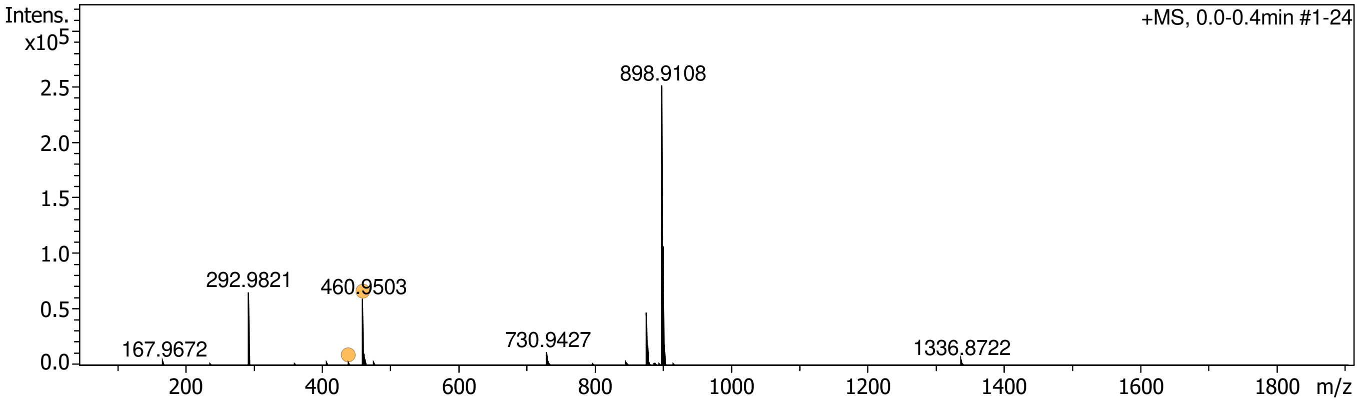

Meas. m/z	#	Ion Formula	Score	m/z	err [mDa]	err [ppm]	mSigma	rdb	eÂ¯ Conf	N-Rule
438.9679	1	C18H16IOS2	100.00	438.9682	0.3	0.7	16.5	18.0	even	ok
	2	C15H3N8O5S2	45.68	438.9662	-1.6	-3.7	18.6	23.0	even	ok
	3	C11H3N8O10S	71.09	438.9687	0.9	2.0	19.6	17.0	even	ok
	4	C11H8IN10S	44.92	438.9693	1.5	3.4	24.1	18.0	even	ok
	5	C19H7N2O7S2	69.75	438.9689	1.1	2.4	25.9	22.0	even	ok
	6	C18H3N2O12	86.19	438.9680	0.2	0.4	26.0	19.0	even	ok
	7	C12H7N8O5S3	38.37	438.9696	1.7	4.0	32.3	20.0	even	ok
	8	C11H11N4O9S3	75.62	438.9683	0.4	0.9	36.4	15.0	even	ok
	9	C17H12IO6	40.32	438.9673	-0.5	-1.3	38.3	15.0	even	ok
	10	C18H8IN4O2	32.77	438.9686	0.8	1.8	41.4	20.0	even	ok
	11	C14H4IN10	14.55	438.9660	-1.9	-4.3	45.2	21.0	even	ok
	12	C15H11N4O4S4	15.51	438.9658	-2.1	-4.8	54.9	21.0	even	ok
	13	C27H3O5S	16.36	438.9696	1.7	3.9	55.2	29.0	even	ok
	14	C16H7N8S4	35.19	438.9671	-0.8	-1.7	56.5	26.0	even	ok
	15	C23H7N2O2S3	23.22	438.9664	-1.4	-3.3	56.9	28.0	even	ok
	16	C20H11N2O2S4	12.56	438.9698	1.9	4.4	64.9	25.0	even	ok
	17	C12H15N4O4S5	15.00	438.9691	1.3	2.9	72.3	18.0	even	ok
	18	C11H19O8S5	25.36	438.9678	-0.1	-0.1	74.8	13.0	even	ok
	19	C31H3S2	16.83	438.9671	-0.8	-1.8	76.5	35.0	even	ok
	20	C12H23O3S7	6.12	438.9687	0.8	1.8	103.5	16.0	even	ok
460.9503	1	C11H2N8NaO10S	100.00	460.9507	0.4	0.8	10.9	17.0	even	ok
	2	C14H15INaO6S	28.54	460.9526	2.3	5.0	11.5	12.0	even	ok
	3	C11H7IN10NaS	66.68	460.9513	1.0	2.1	16.0	18.0	even	ok
	4	C18H15INaOS2	87.29	460.9501	-0.2	-0.4	21.8	18.0	even	ok
	5	C15H2N8NaO5S2	25.98	460.9482	-2.1	-4.6	23.6	23.0	even	ok
	6	C18H2N2NaO12	78.70	460.9500	-0.3	-0.7	23.9	19.0	even	ok
	7	C19H6N2NaO7S2	73.73	460.9509	0.6	1.2	31.2	22.0	even	ok
	8	C17H11INaO6	33.31	460.9493	-1.1	-2.3	32.0	15.0	even	ok
	9	C12H6N8NaO5S3	45.72	460.9515	1.2	2.7	35.1	20.0	even	ok

Mass Spectrum SmartFormula Report

Meas. m/z	#	Ion Formula	Score	m/z	err [mDa]	err [ppm]	mSigma	rdb	eÂ¯ Conf	N-Rule
	10	C11H10N4NaO9S3	76.86	460.9502	-0.1	-0.2	37.8	15.0	even	ok
	11	C18H7IN4NaO2	42.04	460.9506	0.3	0.6	38.1	20.0	even	ok
	12	C20H2N6NaO3S2	18.71	460.9522	1.9	4.1	43.6	27.0	even	ok
	13	C16H6N8NaS4	20.77	460.9490	-1.3	-2.7	61.2	26.0	even	ok
	14	C27H2NaO5S	17.06	460.9515	1.2	2.6	61.3	29.0	even	ok
	15	C23H6N2NaO2S3	12.41	460.9484	-1.9	-4.2	62.5	28.0	even	ok
	16	C20H10N2NaO2S4	13.66	460.9517	1.4	3.1	69.9	25.0	even	ok
	17	C13H10N8NaS5	6.79	460.9524	2.1	4.6	75.4	23.0	even	ok
	18	C12H14N4NaO4S5	16.18	460.9511	0.8	1.7	75.6	18.0	even	ok
	19	C11H18NaO8S5	16.72	460.9497	-0.6	-1.2	77.5	13.0	even	ok
	20	C28H6NaS3	5.46	460.9524	2.1	4.5	81.5	32.0	even	ok
	21	C31H2NaS2	9.10	460.9490	-1.3	-2.8	82.5	35.0	even	ok
	22	C12H22NaO3S7	6.17	460.9506	0.3	0.7	106.6	16.0	even	ok

Figure S43. HRESIMS spectrum of compound **7k**

Generic Display Report

Analysis Info

Analysis Name	D:\Data\Kalaba\74280000001.d	Acquisition Date	03/11/2020 14:17:10
Method	tune_low_MS_Service_11_20.m	Operator	msc
Sample Name	SB2-spot2-p1	Instrument	maXis
Comment	Kalaba/Zehl<br>Ergebnis: +/- 5ppm<br>ACN/MeOH + 1% H2O		

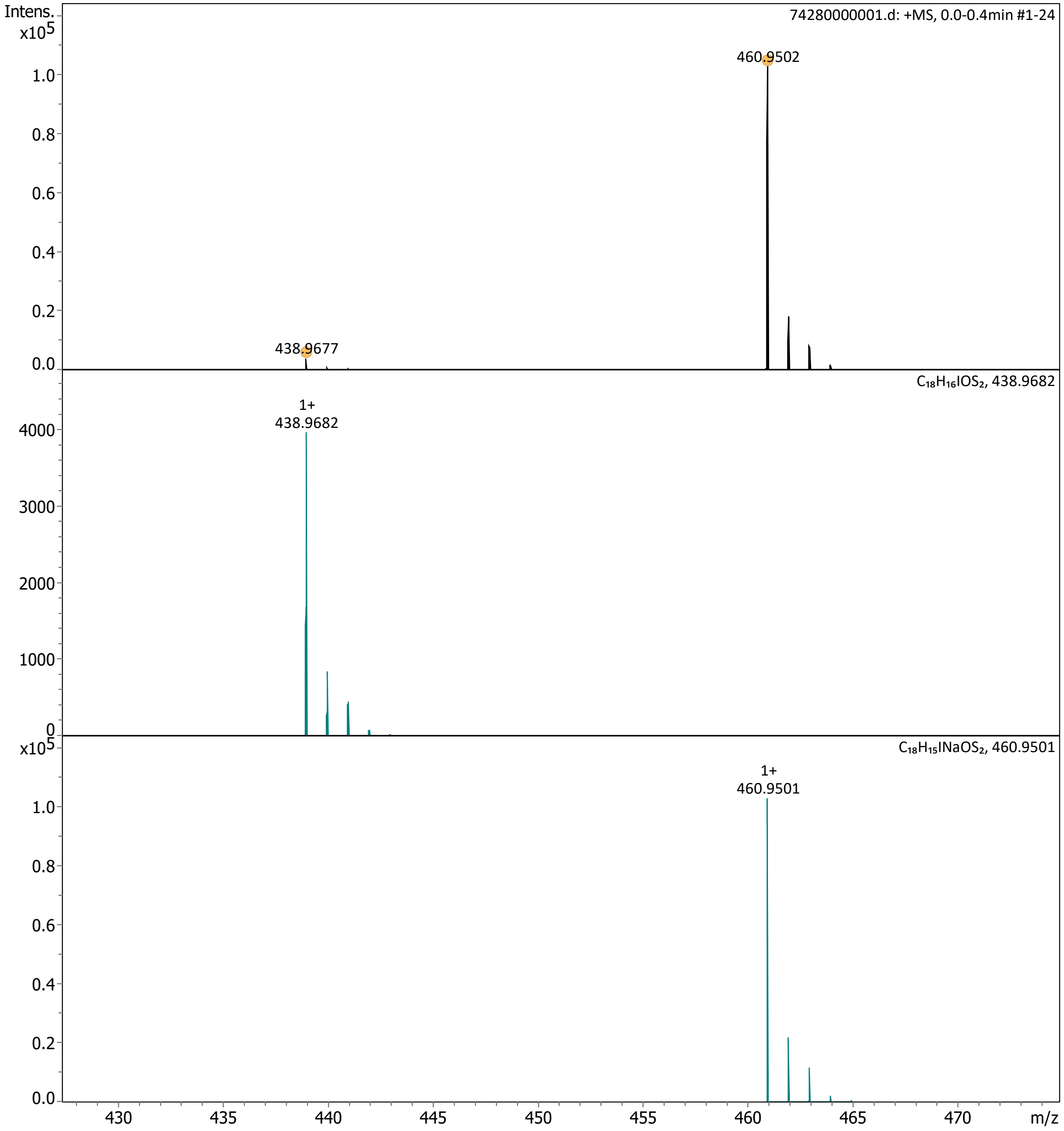

Mass Spectrum SmartFormula Report

Analysis Info

Analysis Name	D:\Data\Kalaba\74280000001.d	Acquisition Date	03/11/2020 14:17:10
Method	tune_low_MS_Service_11_20.m	Operator	msc
Sample Name	SB2-spot2-p1	Instrument	maXis 255552.00016
Comment	Kalaba/Zehl<br>Ergebnis: +/- 5ppm<br>ACN/MeOH + 1% H2O		

Acquisition Parameter

Source Type	ESI	Ion Polarity	Positive	Set Nebulizer	0.4 Bar
Focus	Not active	Set Capillary	4200 V	Set Dry Heater	180 Â°C
Scan Begin	50 m/z	Set End Plate Offset	-500 V	Set Dry Gas	4.0 l/min
Scan End	1900 m/z	Set Charging Voltage	0 V	Set Divert Valve	Source
		Set Corona	0 nA	Set APCI Heater	0 Â°C

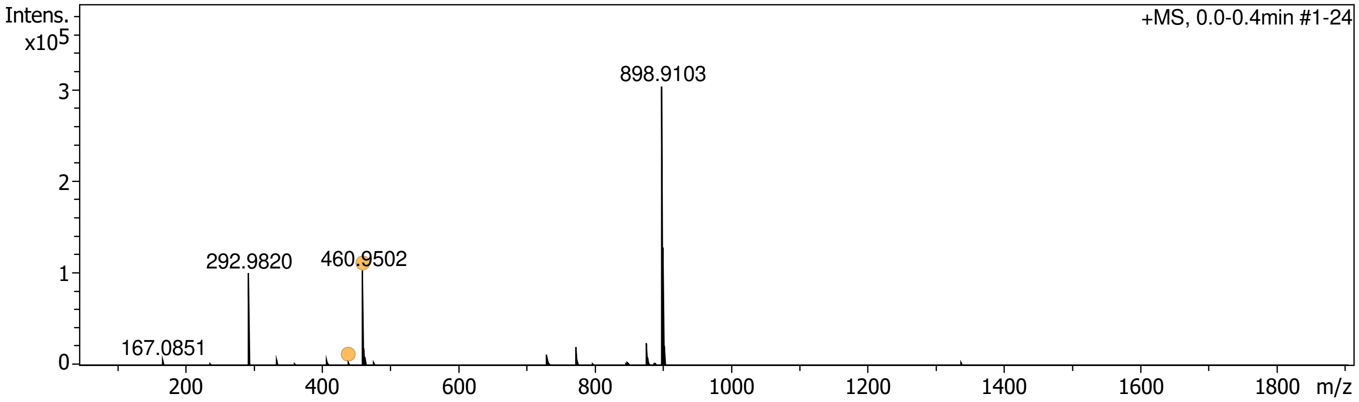

Meas. m/z	#	Ion Formula	Score	m/z	err [mDa]	err [ppm]	mSigma	rdb	eÂ¯ Conf	N-Rule
438.9677	1	C18H16IOS2	100.00	438.9682	0.5	1.2	17.2	18.0	even	ok
	2	C11H3N8O10S	72.08	438.9687	1.1	2.4	18.8	17.0	even	ok
	3	C15H3N8O5S2	56.49	438.9662	-1.4	-3.3	19.2	23.0	even	ok
	4	C11H8IN10S	44.57	438.9693	1.7	3.8	23.3	18.0	even	ok
	5	C18H3N2O12	87.98	438.9680	0.4	0.9	25.9	19.0	even	ok
	6	C19H7N2O7S2	67.94	438.9689	1.2	2.8	26.6	22.0	even	ok
	7	C12H7N8O5S3	36.85	438.9696	1.9	4.4	32.5	20.0	even	ok
	8	C22H3N2O7S	24.87	438.9655	-2.1	-4.8	33.2	25.0	even	ok
	9	C11H11N4O9S3	76.49	438.9683	0.6	1.3	36.4	15.0	even	ok
	10	C17H12IO6	49.32	438.9673	-0.4	-0.8	37.8	15.0	even	ok
	11	C18H8IN4O2	32.86	438.9686	1.0	2.2	41.3	20.0	even	ok
	12	C14H4IN10	18.65	438.9660	-1.7	-3.9	44.7	21.0	even	ok
	13	C15H11N4O4S4	19.51	438.9658	-1.9	-4.4	55.3	21.0	even	ok
	14	C27H3O5S	15.40	438.9696	1.9	4.3	56.0	29.0	even	ok
	15	C16H7N8S4	42.06	438.9671	-0.6	-1.3	57.0	26.0	even	ok
	16	C23H7N2O2S3	28.23	438.9664	-1.3	-2.9	57.6	28.0	even	ok
	17	C20H11N2O2S4	11.80	438.9698	2.1	4.8	65.5	25.0	even	ok
	18	C12H15N4O4S5	14.58	438.9691	1.5	3.3	72.6	18.0	even	ok
	19	C11H19O8S5	27.15	438.9678	0.1	0.3	75.0	13.0	even	ok
	20	C31H3S2	19.86	438.9671	-0.6	-1.4	77.3	35.0	even	ok
	21	C12H23O3S7	6.03	438.9687	1.0	2.3	103.7	16.0	even	ok
460.9502	1	C11H2N8NaO10S	100.00	460.9507	0.5	1.1	9.1	17.0	even	ok
	2	C11H7IN10NaS	65.35	460.9513	1.1	2.4	14.5	18.0	even	ok
	3	C10H6N4NaO14S	68.97	460.9493	-0.8	-1.8	20.4	12.0	even	ok
	4	C10H11IN6NaO4S	90.70	460.9499	-0.2	-0.5	21.1	13.0	even	ok
	5	C18H15INaOS2	92.76	460.9501	-0.0	-0.1	23.6	18.0	even	ok
	6	C18H2N2NaO12	86.58	460.9500	-0.2	-0.4	24.2	19.0	even	ok
	7	C15H2N8NaO5S2	29.18	460.9482	-2.0	-4.3	25.3	23.0	even	ok
	8	C17H11INaO6	38.19	460.9493	-0.9	-2.0	31.4	15.0	even	ok

Mass Spectrum SmartFormula Report

Meas. m/z	#	Ion Formula	Score	m/z	err [mDa]	err [ppm]	mSigma	rdb	eÂ¯ Conf	N-Rule
	9	C19H6N2NaO7S2	68.19	460.9509	0.7	1.5	32.8	22.0	even	ok
	10	C10H19IN2NaO3S3	66.72	460.9495	-0.7	-1.5	34.2	11.0	even	ok
	11	C12H6N8NaO5S3	42.26	460.9515	1.4	3.0	35.9	20.0	even	ok
	12	C11H10N4NaO9S3	80.22	460.9502	0.1	0.1	38.1	15.0	even	ok
	13	C18H7IN4NaO2	40.55	460.9506	0.4	0.9	38.3	20.0	even	ok
	14	C14H3IN10Na	12.77	460.9479	-2.2	-4.9	38.4	21.0	even	ok
	15	C10H14NaO13S3	37.26	460.9489	-1.3	-2.8	43.2	10.0	even	ok
	16	C20H2N6NaO3S2	16.43	460.9522	2.0	4.4	45.4	27.0	even	ok
	17	C16H6N8NaS4	22.63	460.9490	-1.1	-2.4	62.4	26.0	even	ok
	18	C27H2NaO5S	15.16	460.9515	1.4	2.9	63.1	29.0	even	ok
	19	C23H6N2NaO2S3	13.63	460.9484	-1.8	-3.9	64.1	28.0	even	ok
	20	C20H10N2NaO2S4	12.20	460.9517	1.6	3.4	71.3	25.0	even	ok
	21	C12H14N4NaO4S5	15.02	460.9511	0.9	2.0	76.4	18.0	even	ok
	22	C13H10N8NaS5	6.01	460.9524	2.3	4.9	76.4	23.0	even	ok
	23	C11H18NaO8S5	18.25	460.9497	-0.4	-0.9	78.1	13.0	even	ok
	24	C28H6NaS3	4.71	460.9524	2.2	4.8	83.1	32.0	even	ok
	25	C31H2NaS2	9.67	460.9490	-1.1	-2.5	84.1	35.0	even	ok
	26	C12H22NaO3S7	5.78	460.9506	0.5	1.0	107.3	16.0	even	ok

Figure S44. HRESIMS spectrum of compound **8k**

Generic Display Report

Analysis Info

Analysis Name	D:\Data\Kalaba\74281000001.d	Acquisition Date	03/11/2020 14:36:01
Method	tune_low_MS_Service_11_20.m	Operator	msc
Sample Name	SB2-spot2-p2	Instrument	maXis
Comment	Kalaba/Zehl<br>Ergebnis: +/- 5ppm<br>ACN/MeOH + 1% H2O		

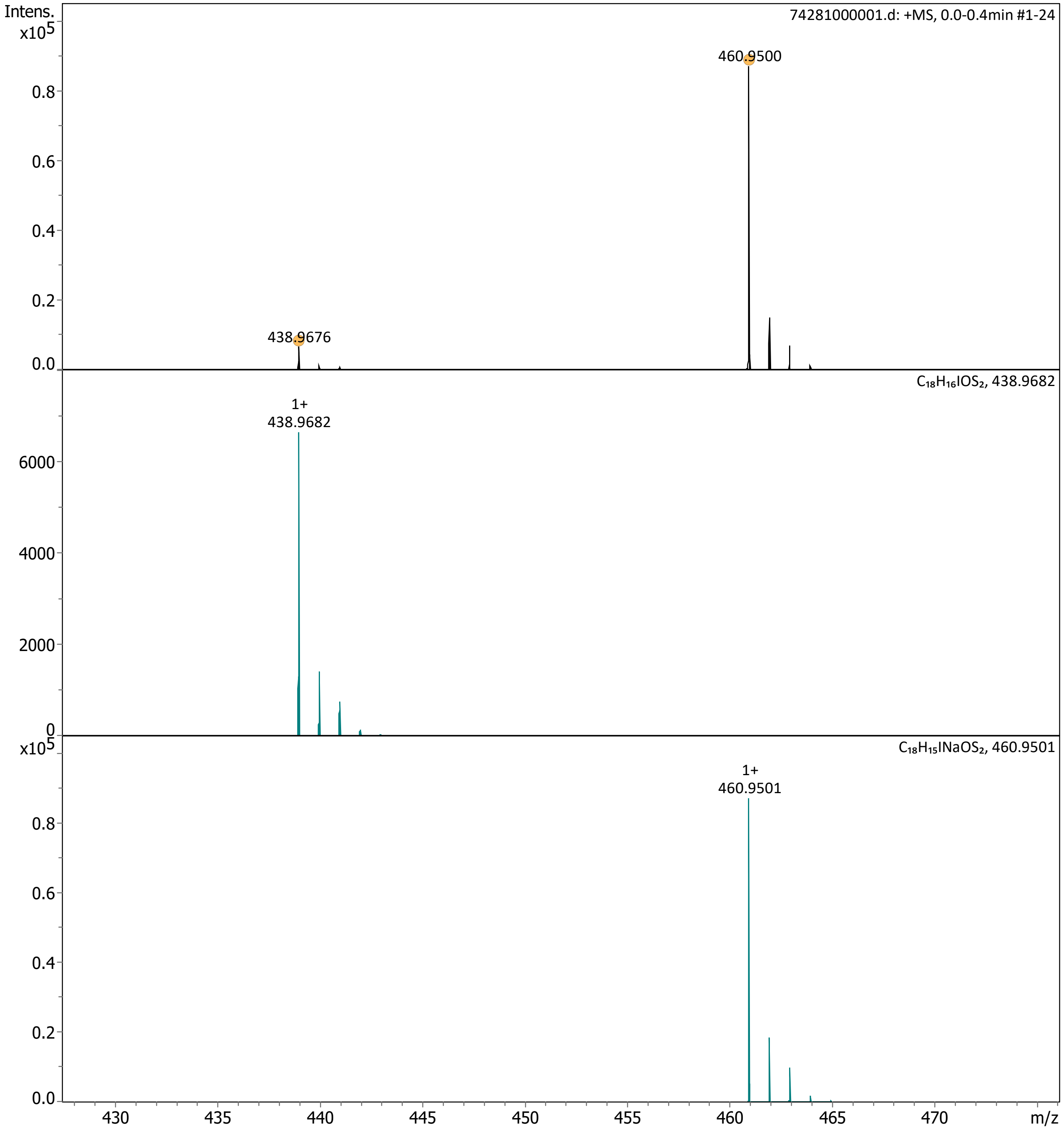

Mass Spectrum SmartFormula Report

Analysis Info

Analysis Name	D:\Data\Kalaba\74281000001.d	Acquisition Date	03/11/2020 14:36:01
Method	tune_low_MS_Service_11_20.m	Operator	msc
Sample Name	SB2-spot2-p2	Instrument	maXis 255552.00016
Comment	Kalaba/Zehl<br>Ergebnis: +/- 5ppm<br>ACN/MeOH + 1% H2O		

Acquisition Parameter

Source Type	ESI	Ion Polarity	Positive	Set Nebulizer	0.4 Bar
Focus	Not active	Set Capillary	4200 V	Set Dry Heater	180 Â°C
Scan Begin	50 m/z	Set End Plate Offset	-500 V	Set Dry Gas	4.0 l/min
Scan End	1900 m/z	Set Charging Voltage	0 V	Set Divert Valve	Source
		Set Corona	0 nA	Set APCI Heater	0 Â°C

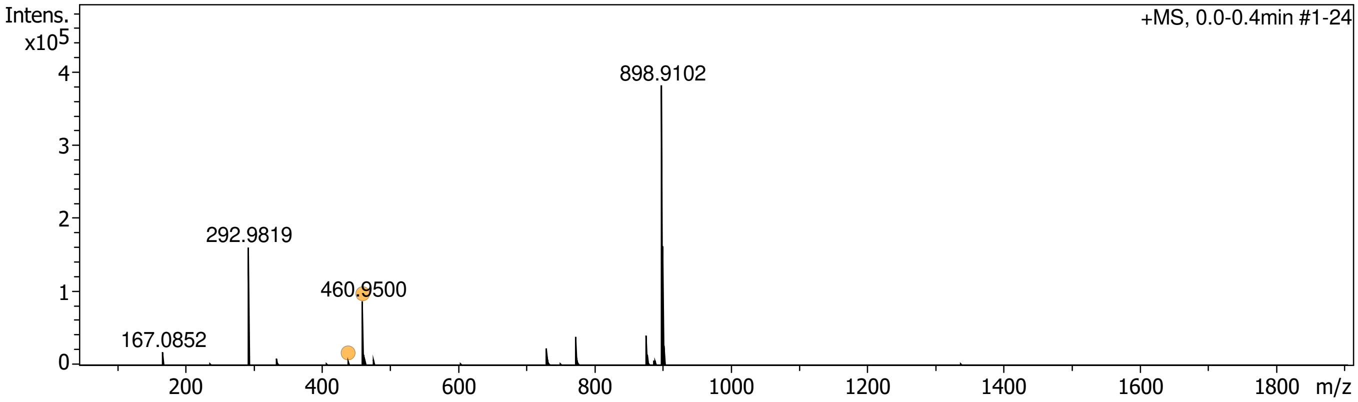

Meas. m/z	#	Ion Formula	Score	m/z	err [mDa]	err [ppm]	mSigma	rdb	eÂ¯ Conf	N-Rule
438.9676	1	C11H3N8O10S	83.40	438.9687	1.1	2.5	15.0	17.0	even	ok
	2	C11H8IN10S	51.51	438.9693	1.7	3.9	19.6	18.0	even	ok
	3	C18H16IOS2	100.00	438.9682	0.5	1.2	20.6	18.0	even	ok
	4	C15H3N8O5S2	58.90	438.9662	-1.4	-3.2	22.4	23.0	even	ok
	5	C18H3N2O12	96.48	438.9680	0.4	0.9	25.0	19.0	even	ok
	6	C19H7N2O7S2	67.19	438.9689	1.3	2.9	29.8	22.0	even	ok
	7	C12H7N8O5S3	37.53	438.9696	2.0	4.5	34.3	20.0	even	ok
	8	C17H12IO6	58.75	438.9673	-0.3	-0.7	35.1	15.0	even	ok
	9	C22H3N2O7S	25.72	438.9655	-2.1	-4.8	36.5	25.0	even	ok
	10	C11H11N4O9S3	80.36	438.9683	0.6	1.4	37.2	15.0	even	ok
	11	C18H8IN4O2	36.41	438.9686	1.0	2.3	40.0	20.0	even	ok
	12	C14H4IN10	22.50	438.9660	-1.7	-3.8	42.1	21.0	even	ok
	13	C15H11N4O4S4	20.24	438.9658	-1.9	-4.3	57.7	21.0	even	ok
	14	C27H3O5S	14.63	438.9696	1.9	4.4	59.5	29.0	even	ok
	15	C16H7N8S4	42.60	438.9671	-0.5	-1.2	59.8	26.0	even	ok
	16	C23H7N2O2S3	28.15	438.9664	-1.2	-2.8	61.0	28.0	even	ok
	17	C20H11N2O2S4	11.24	438.9698	2.1	4.9	68.6	25.0	even	ok
	18	C12H15N4O4S5	14.47	438.9691	1.5	3.4	74.5	18.0	even	ok
	19	C11H19O8S5	27.59	438.9678	0.2	0.4	76.5	13.0	even	ok
	20	C31H3S2	19.25	438.9671	-0.6	-1.3	80.7	35.0	even	ok
	21	C12H23O3S7	5.93	438.9687	1.0	2.3	105.5	16.0	even	ok
460.9500	1	C11H2N8NaO10S	94.62	460.9507	0.7	1.5	7.6	17.0	even	ok
	2	C11H7IN10NaS	60.30	460.9513	1.3	2.8	13.5	18.0	even	ok
	3	C10H6N4NaO14S	77.27	460.9493	-0.7	-1.4	18.9	12.0	even	ok
	4	C10H11IN6NaO4S	100.00	460.9499	-0.1	-0.1	19.7	13.0	even	ok
	5	C18H2N2NaO12	91.18	460.9500	0.0	0.0	24.8	19.0	even	ok
	6	C18H15INaOS2	86.24	460.9501	0.1	0.3	24.9	18.0	even	ok
	7	C15H2N8NaO5S2	31.93	460.9482	-1.8	-3.9	26.5	23.0	even	ok
	8	C17H11INaO6	41.72	460.9493	-0.7	-1.6	31.3	15.0	even	ok

Mass Spectrum SmartFormula Report

Meas. m/z	#	Ion Formula	Score	m/z	err [mDa]	err [ppm]	mSigma	rdb	eÂ¯ Conf	N-Rule
	9	C10H19IN2NaO3S3	72.69	460.9495	-0.5	-1.1	34.0	11.0	even	ok
	10	C19H6N2NaO7S2	60.64	460.9509	0.9	1.9	34.0	22.0	even	ok
	11	C12H6N8NaO5S3	37.45	460.9515	1.6	3.4	36.5	20.0	even	ok
	12	C14H3IN10Na	14.57	460.9479	-2.1	-4.5	38.2	21.0	even	ok
	13	C11H10N4NaO9S3	74.59	460.9502	0.2	0.5	38.3	15.0	even	ok
	14	C18H7IN4NaO2	36.99	460.9506	0.6	1.3	38.8	20.0	even	ok
	15	C10H14NaO13S3	41.26	460.9489	-1.1	-2.4	43.0	10.0	even	ok
	16	C20H2N6NaO3S2	13.86	460.9522	2.2	4.8	46.8	27.0	even	ok
	17	C15H10N4NaO4S4	10.51	460.9477	-2.3	-5.0	60.6	21.0	even	ok
	18	C16H6N8NaS4	24.08	460.9490	-0.9	-2.1	63.3	26.0	even	ok
	19	C27H2NaO5S	12.94	460.9515	1.5	3.3	64.6	29.0	even	ok
	20	C23H6N2NaO2S3	14.64	460.9484	-1.6	-3.5	65.2	28.0	even	ok
	21	C20H10N2NaO2S4	10.51	460.9517	1.7	3.8	72.2	25.0	even	ok
	22	C12H14N4NaO4S5	13.45	460.9511	1.1	2.4	76.8	18.0	even	ok
	23	C11H18NaO8S5	19.41	460.9497	-0.3	-0.5	78.3	13.0	even	ok
	24	C31H2NaS2	10.04	460.9490	-1.0	-2.1	85.4	35.0	even	ok
	25	C12H22NaO3S7	5.23	460.9506	0.6	1.3	107.7	16.0	even	ok

Figure S45. HRESIMS spectrum of compound **5l**

Generic Display Report

Analysis Info

Analysis Name	D:\Data\Kalaba\78516000001.d	Acquisition Date	26/03/2021 13:37:22
Method	tune_low_MS_Service_03_21.m	Operator	msc
Sample Name	PN-6-peak-1	Instrument	maXis
Comment	Kalaba / Zehl<br>Ergebnis +/- 5 ppm<br>ACN / MeOH + 1% H2O		

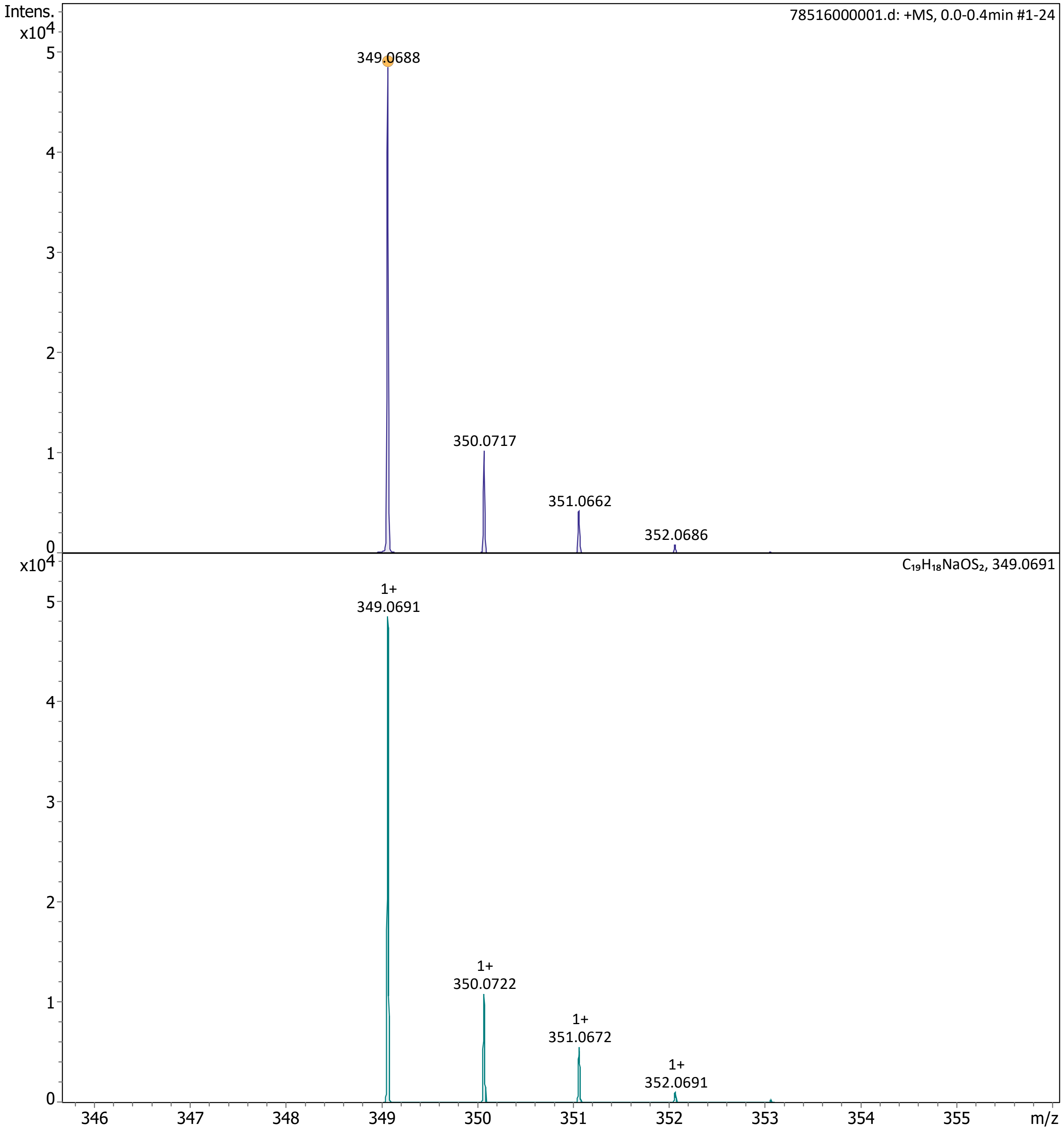

Mass Spectrum SmartFormula Report

Analysis Info

Analysis Name	D:\Data\Kalaba\78516000001.d	Acquisition Date	26/03/2021 13:37:22
Method	tune_low_MS_Service_03_21.m	Operator	msc
Sample Name	PN-6-peak-1	Instrument	maXis 255552.00016
Comment	Kalaba / Zehl<br>Ergebnis +/- 5 ppm<br>ACN / MeOH + 1% H2O		

Acquisition Parameter

Source Type	ESI	Ion Polarity	Positive	Set Nebulizer	0.4 Bar
Focus	Not active	Set Capillary	4200 V	Set Dry Heater	180 Â°C
Scan Begin	80 m/z	Set End Plate Offset	-500 V	Set Dry Gas	4.0 l/min
Scan End	1900 m/z	Set Charging Voltage	0 V	Set Divert Valve	Source
		Set Corona	0 nA	Set APCI Heater	0 Â°C

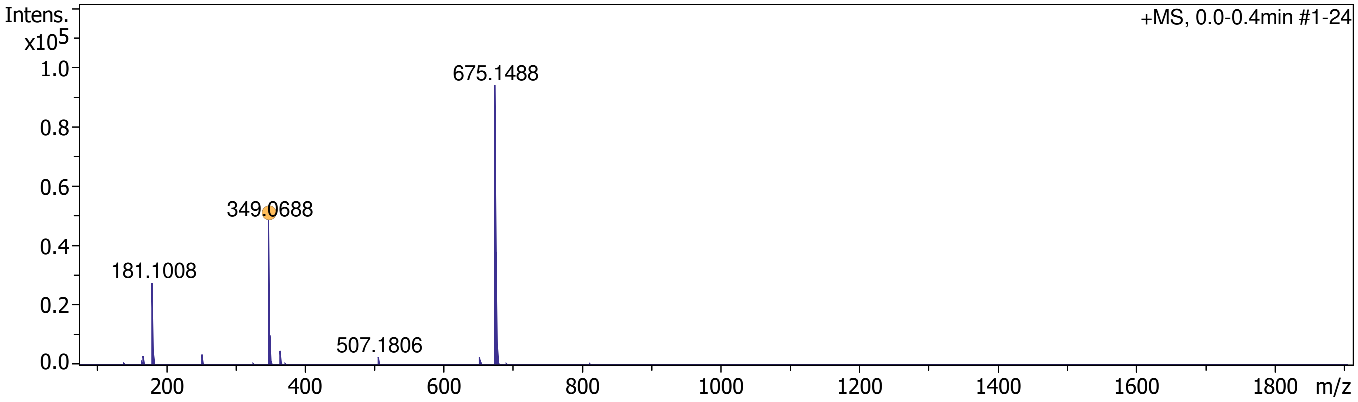

Meas. m/z	#	Ion Formula	Score	m/z	err [mDa]	err [ppm]	mSigma	rdb	eÂ¯ Conf	N-Rule
349.0688	1	C19H18NaOS2	100.00	349.0691	0.4	1.1	15.4	15.0	even	ok
	2	C12H10N10NaS	44.01	349.0703	1.5	4.4	23.4	15.0	even	ok
	3	C11H14N6NaO4S	73.76	349.0689	0.2	0.5	32.5	10.0	even	ok
	4	C18H14NaO6	47.06	349.0683	-0.5	-1.4	33.4	12.0	even	ok
	5	C19H10N4NaO2	37.87	349.0696	0.8	2.4	35.0	17.0	even	ok
	6	C11H22N2NaO3S3	75.34	349.0685	-0.3	-0.8	38.8	8.0	even	ok
	7	C10H18N2NaO8S	34.21	349.0676	-1.1	-3.3	42.9	5.0	even	ok

Figure S46. HRESIMS spectrum of compound **6l**

Generic Display Report

Analysis Info

Analysis Name	D:\Data\Kalaba\78517000001.d	Acquisition Date	26/03/2021 13:38:44
Method	tune_low_MS_Service_03_21.m	Operator	msc
Sample Name	PN-6-peak-2	Instrument	maXis
Comment	Kalaba / Zehl<br>Ergebnis +/- 5 ppm<br>ACN / MeOH + 1% H2O		

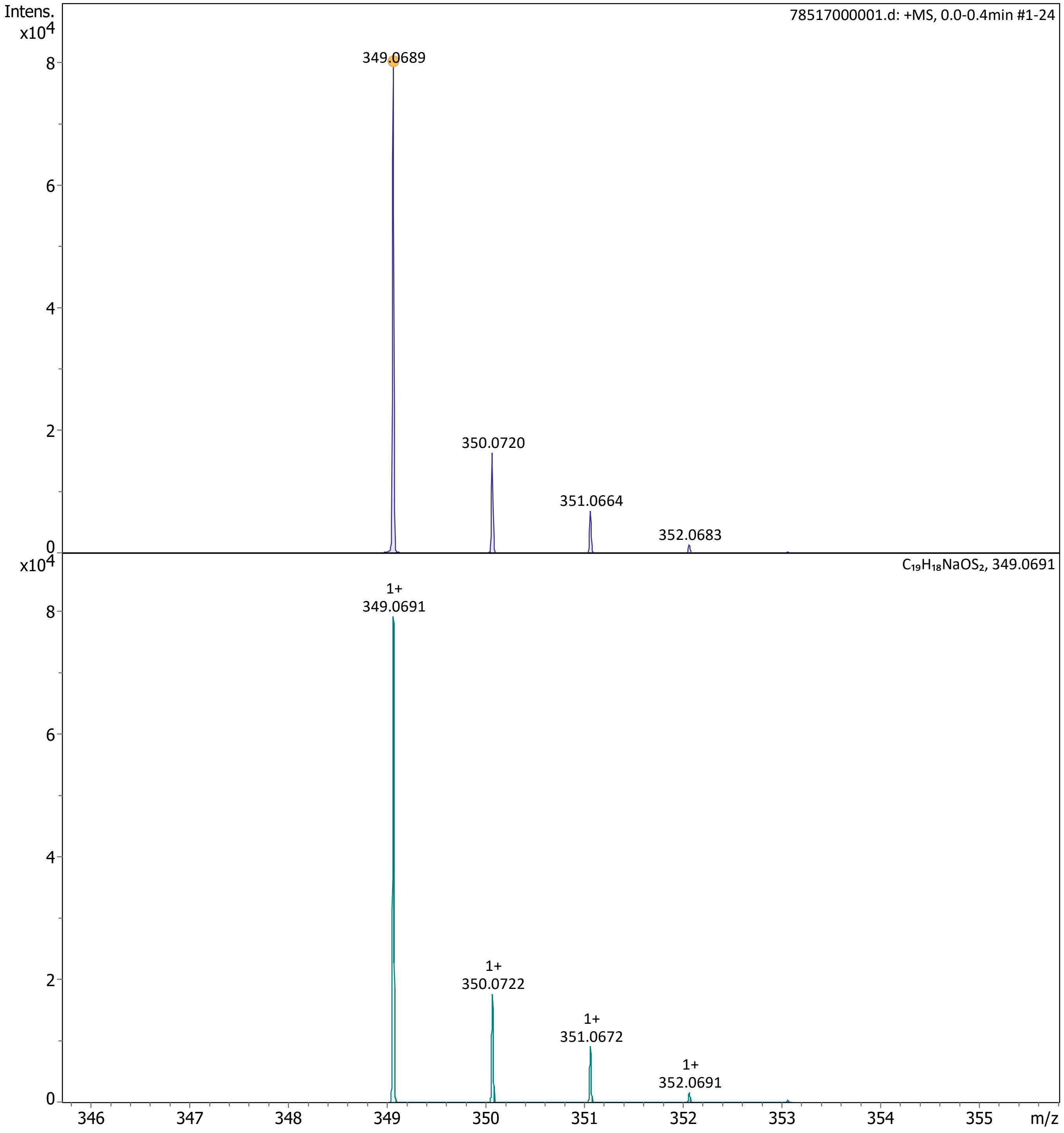

Mass Spectrum SmartFormula Report

Analysis Info

Analysis Name	D:\Data\Kalaba\78517000001.d	Acquisition Date	26/03/2021 13:38:44
Method	tune_low_MS_Service_03_21.m	Operator	msc
Sample Name	PN-6-peak-2	Instrument	maXis 255552.00016
Comment	Kalaba / Zehl<br>Ergebnis +/- 5 ppm<br>ACN / MeOH + 1% H2O		

Acquisition Parameter

Source Type	ESI	Ion Polarity	Positive	Set Nebulizer	0.4 Bar
Focus	Not active	Set Capillary	4200 V	Set Dry Heater	180 Â°C
Scan Begin	80 m/z	Set End Plate Offset	-500 V	Set Dry Gas	4.0 l/min
Scan End	1900 m/z	Set Charging Voltage	0 V	Set Divert Valve	Source
		Set Corona	0 nA	Set APCI Heater	0 Â°C

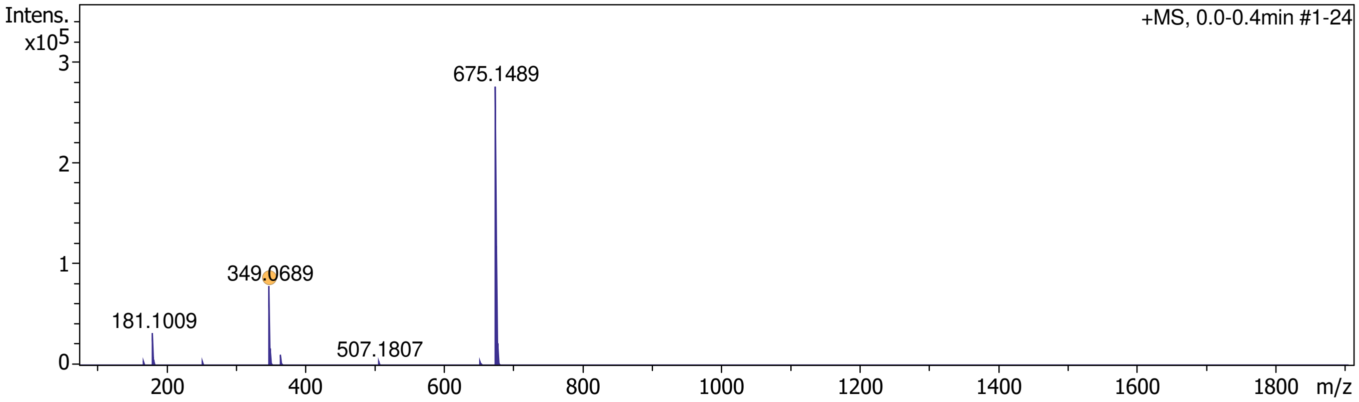

Meas. m/z	#	Ion Formula	Score	m/z	err [mDa]	err [ppm]	mSigma	rdb	eÂ¯ Conf	N-Rule
349.0689	1	C19H18NaOS2	100.00	349.0691	0.2	0.6	16.1	15.0	even	ok
	2	C12H10N10NaS	47.62	349.0703	1.4	3.9	22.1	15.0	even	ok
	3	C11H14N6NaO4S	77.75	349.0689	0.0	0.1	30.7	10.0	even	ok
	4	C18H14NaO6	41.07	349.0683	-0.7	-1.9	33.2	12.0	even	ok
	5	C19H10N4NaO2	38.17	349.0696	0.7	1.9	35.7	17.0	even	ok
	6	C11H22N2NaO3S3	68.07	349.0685	-0.4	-1.3	37.6	8.0	even	ok
	7	C10H18N2NaO8S	30.76	349.0676	-1.3	-3.8	41.0	5.0	even	ok

Figure S47. HRESIMS spectrum of compound **7l**

Generic Display Report

Analysis Info

Analysis Name	W:\MS_MessService\80201000001.d	Acquisition Date	18/05/2021 19:50:55
Method	tune_low_MS_Service_04_21.m	Operator	msc
Sample Name	PN-6-PEAK-3	Instrument	maXis
Comment	Kalaba / Zehl<br>Ergebnis +/- 5 ppm<br>ACN / MeOH + 1% H2O		

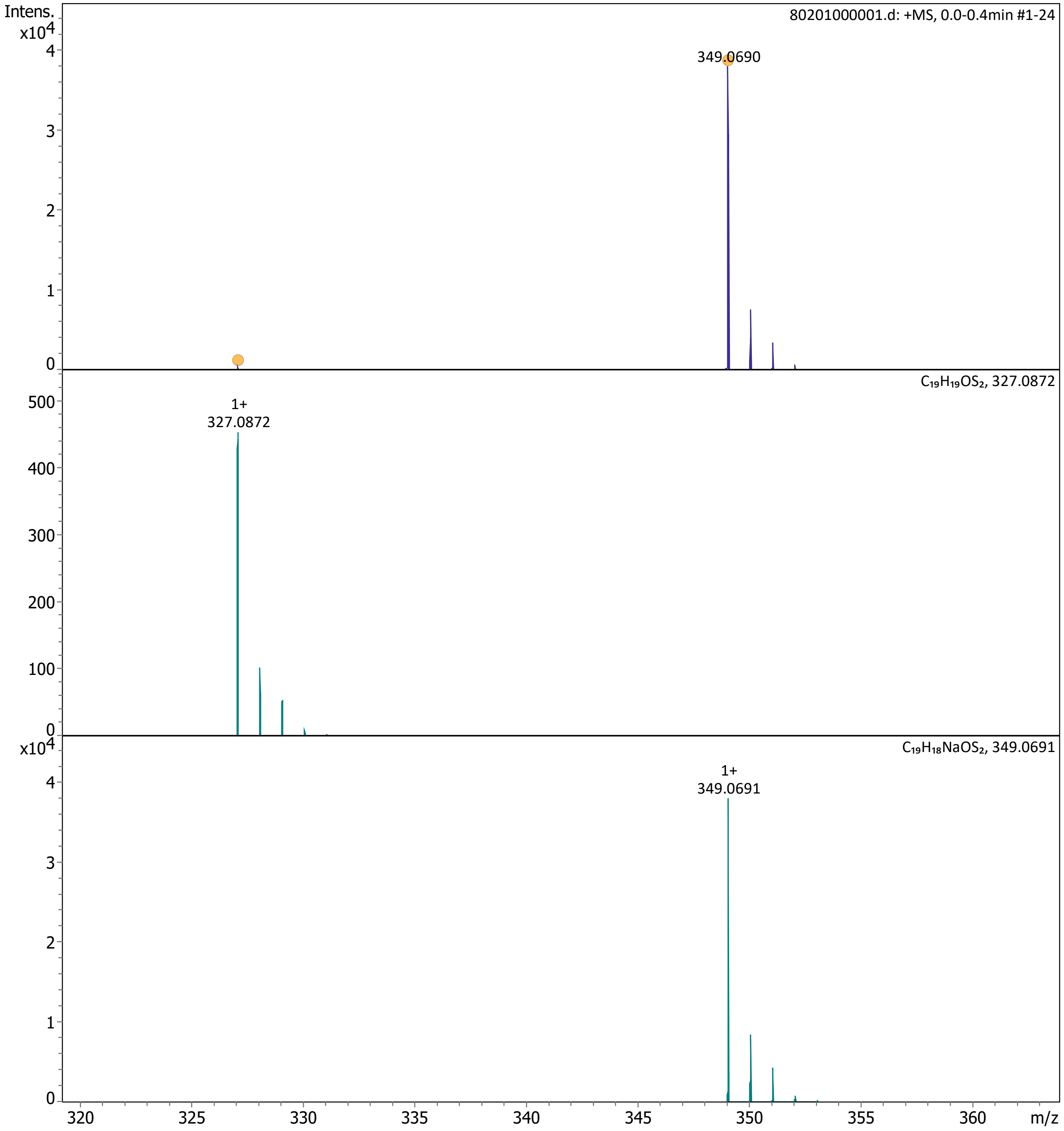

Mass Spectrum SmartFormula Report

Analysis Info

Analysis Name	W:\MS_MessService\80201000001.d	Acquisition Date	18/05/2021 19:50:55
Method	tune_low_MS_Service_04_21.m	Operator	msc
Sample Name	PN-6-PEAK-3	Instrument	maXis 255552.00016
Comment	Kalaba / Zehl<br>Ergebnis +/- 5 ppm<br>ACN / MeOH + 1% H2O		

Acquisition Parameter

Source Type	ESI	Ion Polarity	Positive	Set Nebulizer	0.4 Bar
Focus	Not active	Set Capillary	4200 V	Set Dry Heater	180 Â°C
Scan Begin	50 m/z	Set End Plate Offset	-500 V	Set Dry Gas	4.0 l/min
Scan End	1900 m/z	Set Charging Voltage	0 V	Set Divert Valve	Source
		Set Corona	0 nA	Set APCI Heater	0 Â°C

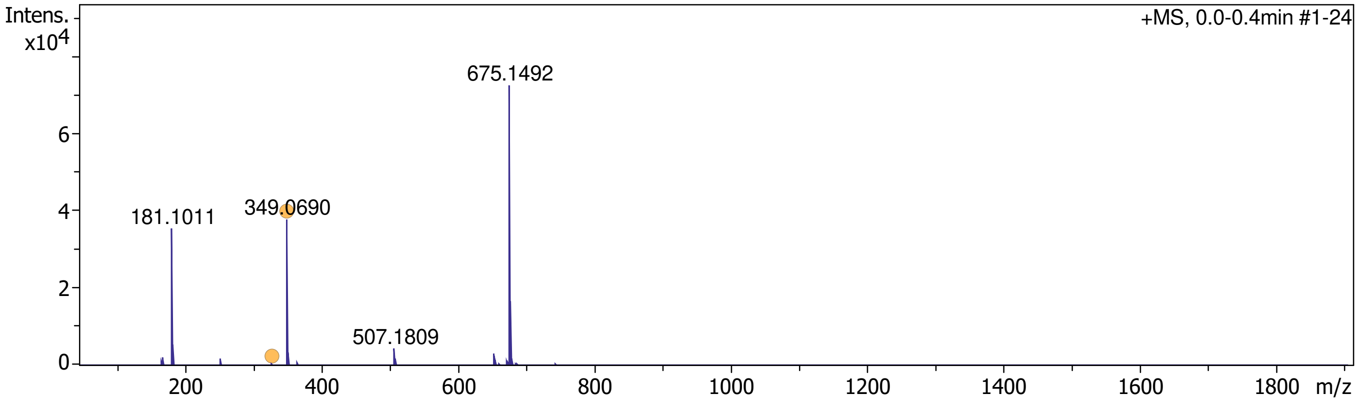

Meas. m/z	#	Ion Formula	Score	m/z	err [mDa]	err [ppm]	mSigma	rdb	eÂ¯ Conf	N-Rule
327.0871	1	C19H11N4O2	100.00	327.0877	0.6	1.7	18.0	17.0	even	ok
	2	C18H15O6	70.75	327.0863	-0.8	-2.4	28.2	12.0	even	ok
	3	C12H11N10S	49.85	327.0883	1.2	3.8	42.7	15.0	even	ok
	4	C11H15N6O4S	65.72	327.0870	-0.1	-0.3	53.1	10.0	even	ok
	5	C19H19OS2	54.43	327.0872	0.1	0.3	59.0	15.0	even	ok
	6	C10H19N2O8S	22.41	327.0857	-1.4	-4.4	64.1	5.0	even	ok
	7	C11H23N2O3S3	27.52	327.0865	-0.6	-1.7	77.8	8.0	even	ok
349.0690	1	C19H18NaOS2	100.00	349.0691	0.1	0.3	17.3	15.0	even	ok
	2	C12H10N10NaS	51.68	349.0703	1.2	3.6	20.6	15.0	even	ok
	3	C18H14NaO6	35.85	349.0683	-0.8	-2.2	35.1	12.0	even	ok
	4	C19H10N4NaO2	35.81	349.0696	0.6	1.6	39.4	17.0	even	ok

Figure S48. HRESIMS spectrum of compound **8l**

Generic Display Report

Analysis Info

Analysis Name	W:\MS_MessService\80202000001.d	Acquisition Date	21/05/2021 16:31:28
Method	tune_low_MS_Service_04_21.m	Operator	msc
Sample Name	PN-6-PEAK-4	Instrument	maXis
Comment	Kalaba / Zehl<br>Ergebnis +/- 5 ppm<br>ACN / MeOH + 1% H2O		

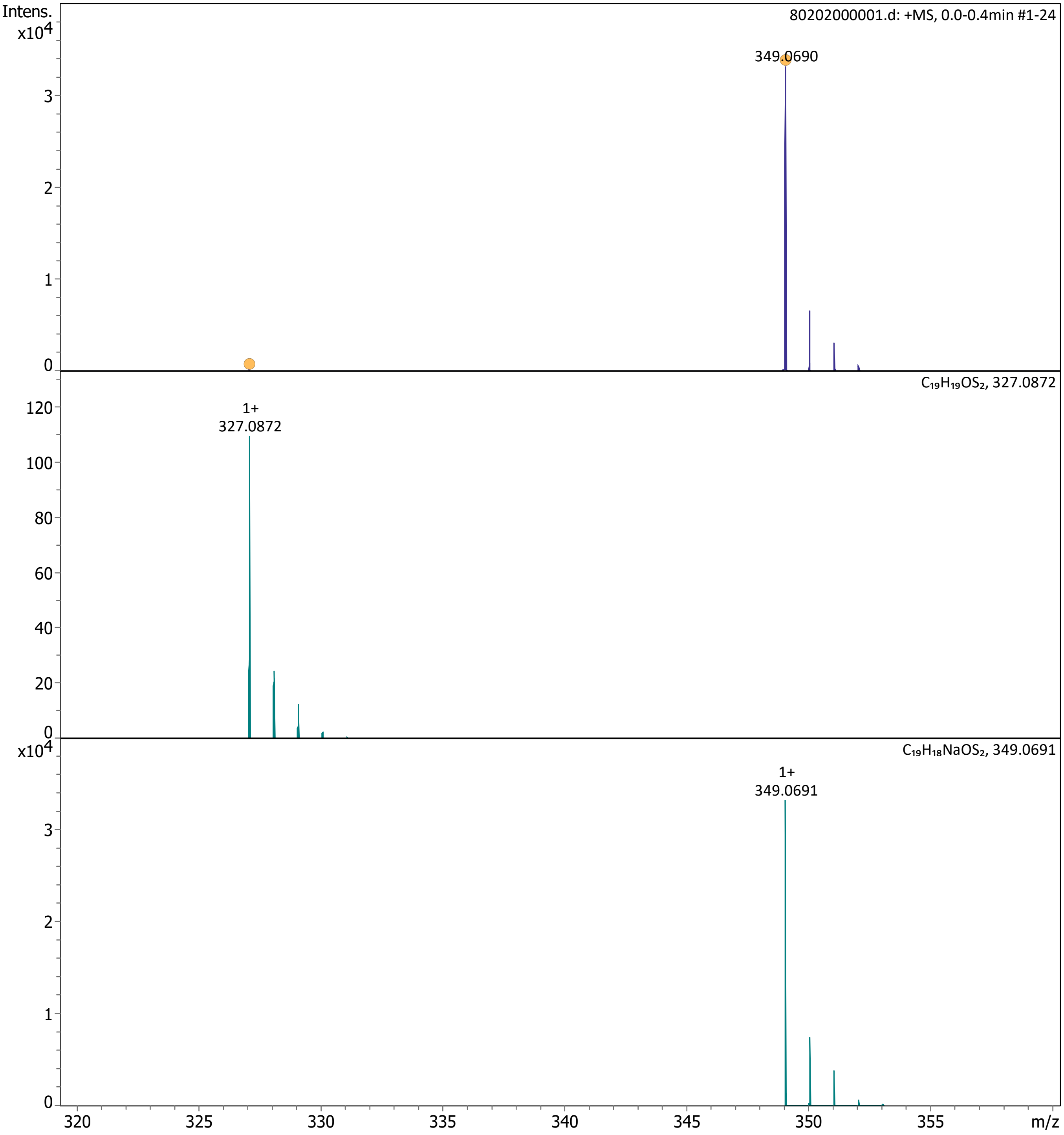

Mass Spectrum SmartFormula Report

Analysis Info

Analysis Name	W:\MS_MessService\80202000001.d	Acquisition Date	21/05/2021 16:31:28
Method	tune_low_MS_Service_04_21.m	Operator	msc
Sample Name	PN-6-PEAK-4	Instrument	maXis 255552.00016
Comment	Kalaba / Zehl<br>Ergebnis +/- 5 ppm<br>ACN / MeOH + 1% H2O		

Acquisition Parameter

Source Type	ESI	Ion Polarity	Positive	Set Nebulizer	0.4 Bar
Focus	Not active	Set Capillary	4200 V	Set Dry Heater	180 Â°C
Scan Begin	50 m/z	Set End Plate Offset	-500 V	Set Dry Gas	4.0 l/min
Scan End	1900 m/z	Set Charging Voltage	0 V	Set Divert Valve	Source
		Set Corona	0 nA	Set APCI Heater	0 Â°C

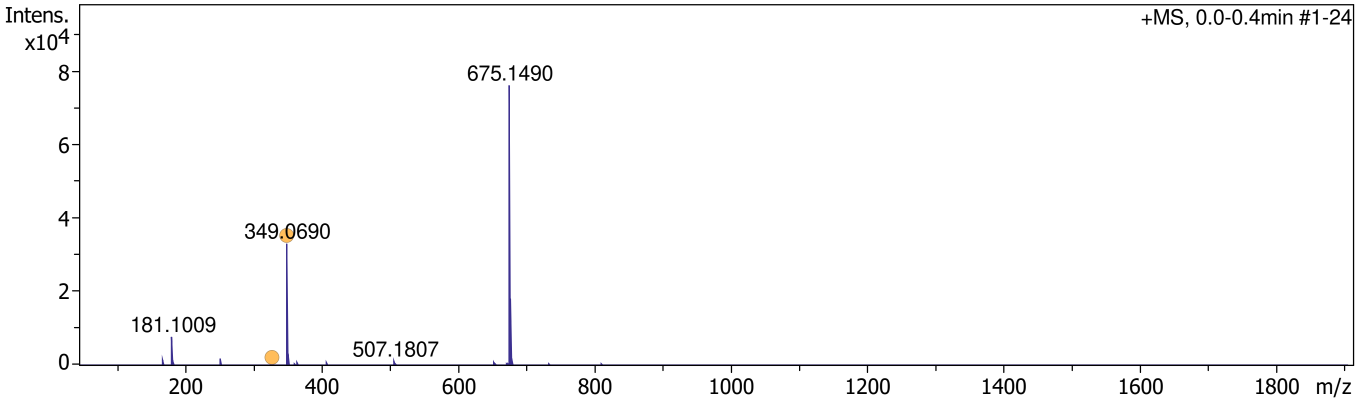

Meas. m/z	#	Ion Formula	Score	m/z	err [mDa]	err [ppm]	mSigma	rdb	eÂ¯ Conf	N-Rule
327.0869	1	C11H15N6O4S	86.28	327.0870	0.1	0.5	n.a.	10.0	even	ok
	2	C11H23N2O3S3	100.00	327.0865	-0.3	-1.0	n.a.	8.0	even	ok
	3	C19H19OS2	79.62	327.0872	0.3	1.0	n.a.	15.0	even	ok
	4	C18H15O6	54.16	327.0863	-0.5	-1.6	n.a.	12.0	even	ok
	5	C19H11N4O2	47.40	327.0877	0.8	2.4	n.a.	17.0	even	ok
	6	C10H19N2O8S	50.59	327.0857	-1.2	-3.6	n.a.	5.0	even	ok
	7	C12H11N10S	41.96	327.0883	1.5	4.5	n.a.	15.0	even	ok
349.0690	1	C19H18NaOS2	100.00	349.0691	0.2	0.4	15.9	15.0	even	ok
	2	C12H10N10NaS	48.04	349.0703	1.3	3.8	22.0	15.0	even	ok
	3	C11H14N6NaO4S	78.89	349.0689	-0.0	-0.1	29.0	10.0	even	ok
	4	C11H22N2NaO3S3	72.37	349.0685	-0.5	-1.4	33.2	8.0	even	ok
	5	C18H14NaO6	35.84	349.0683	-0.7	-2.0	36.4	12.0	even	ok
	6	C10H18N2NaO8S	31.05	349.0676	-1.4	-3.9	38.5	5.0	even	ok
	7	C19H10N4NaO2	33.78	349.0696	0.6	1.8	40.3	17.0	even	ok

Figure S49. HRESIMS spectrum of compound **5m**

Generic Display Report

Analysis Info

Analysis Name	D:\MZ\temp\64677000001.d
Method	tune_low_MS_Service_06_19.m
Sample Name	PN_16_spot_1peak_1
Comment	Kalaba / Zehl<br>Ergebnis: +/- 5ppm<br>ACN / MeOH + 1% H2O
Acquisition Date	01/07/2019 16:05:39
Operator	msc
Instrument	maXis

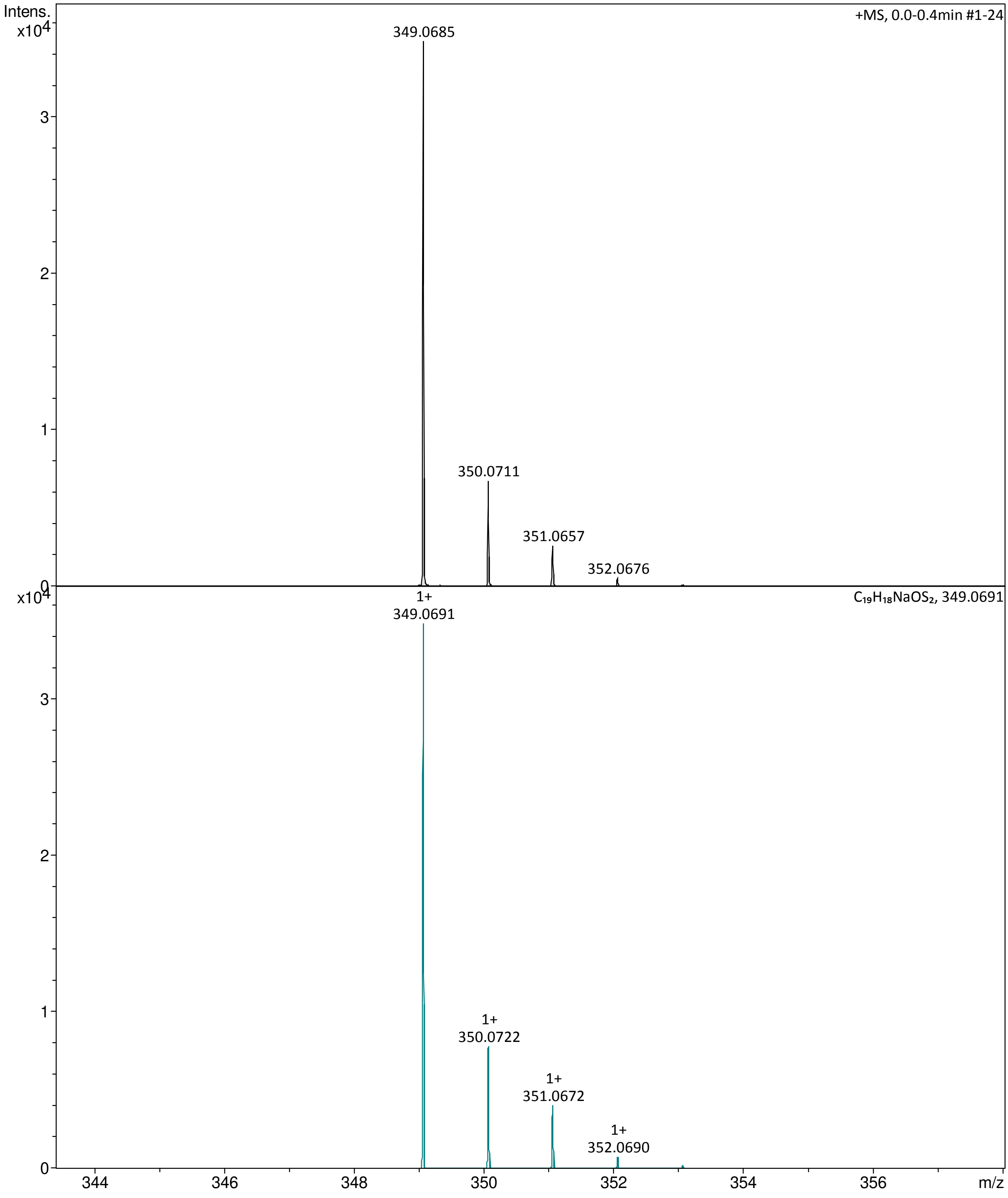

Mass Spectrum SmartFormula Report

Analysis Info

Analysis Name	D:\MZ\temp\64677000001.d	Acquisition Date	01/07/2019 16:05:39
Method	tune_low_MS_Service_06_19.m	Operator	msc
Sample Name	PN_16_spot_1peak_1	Instrument	maXis 255552.00016
Comment	Kalaba / Zehl<br>Ergebnis: +/- 5ppm<br>ACN / MeOH + 1% H2O		

Acquisition Parameter

Source Type	ESI	Ion Polarity	Positive	Set Nebulizer	0.4 Bar
Focus	Not active	Set Capillary	4200 V	Set Dry Heater	150 °C
Scan Begin	50 m/z	Set End Plate Offset	-500 V	Set Dry Gas	4.0 l/min
Scan End	1900 m/z	Set Charging Voltage	0 V	Set Divert Valve	Source
		Set Corona	0 nA	Set APCI Heater	0 °C

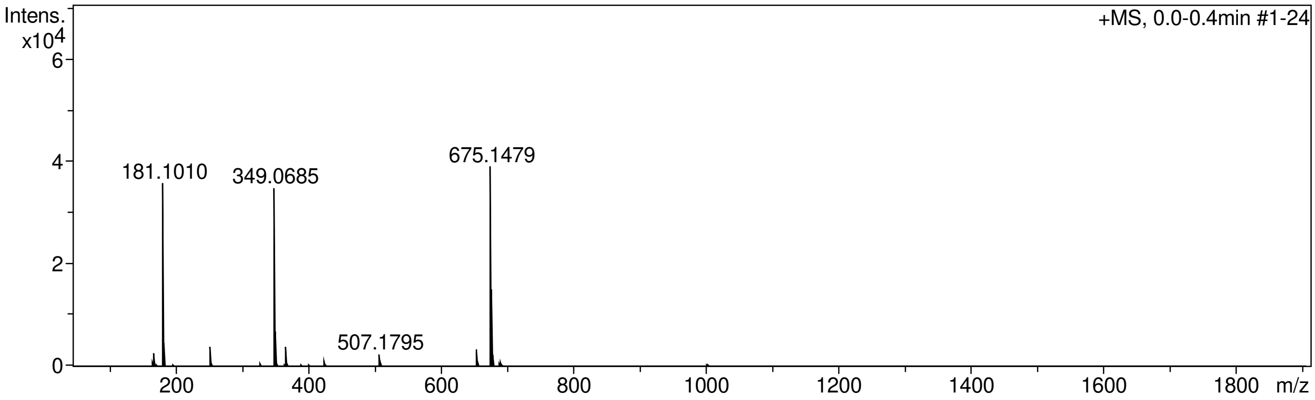

Meas. m/z	#	Ion Formula	m/z	err [ppm]	mSigma	# Sigma	Score	rdb	e⁻ Conf	N-Rule
349.0685	1	C11H14N6NaO4S	349.0689	1.2	21.1	1	100.00	7.5	even	ok
	2	C18H14NaO6	349.0683	0.8	21.8	2	78.87	11.5	even	ok
	3	C19H18NaOS2	349.0691	1.7	23.5	3	86.88	10.5	even	ok
	4	C19H10N4NaO2	349.0696	3.1	31.8	4	41.61	16.5	even	ok
	5	C15H6N10Na	349.0669	4.6	32.1	5	29.09	17.5	even	ok
	6	C10H18N2NaO8S	349.0676	-2.6	32.3	6	59.47	2.5	even	ok
	7	C11H22N2NaO3S3	349.0685	0.2	39.0	7	75.72	1.5	even	ok

Figure S50. HRESIMS spectrum of compound **6m**

Generic Display Report

Analysis Info

Analysis Name	D:\MZ\temp\64678000002.d	Acquisition Date	01/07/2019 16:12:29
Method	tune_low_MS_Service_06_19.m	Operator	msc
Sample Name	PN_16_spot_1peak_2	Instrument	maXis
Comment	Kalaba / Zehl<br>Ergebnis: +/- 5ppm<br>ACN / MeOH + 1% H2O		

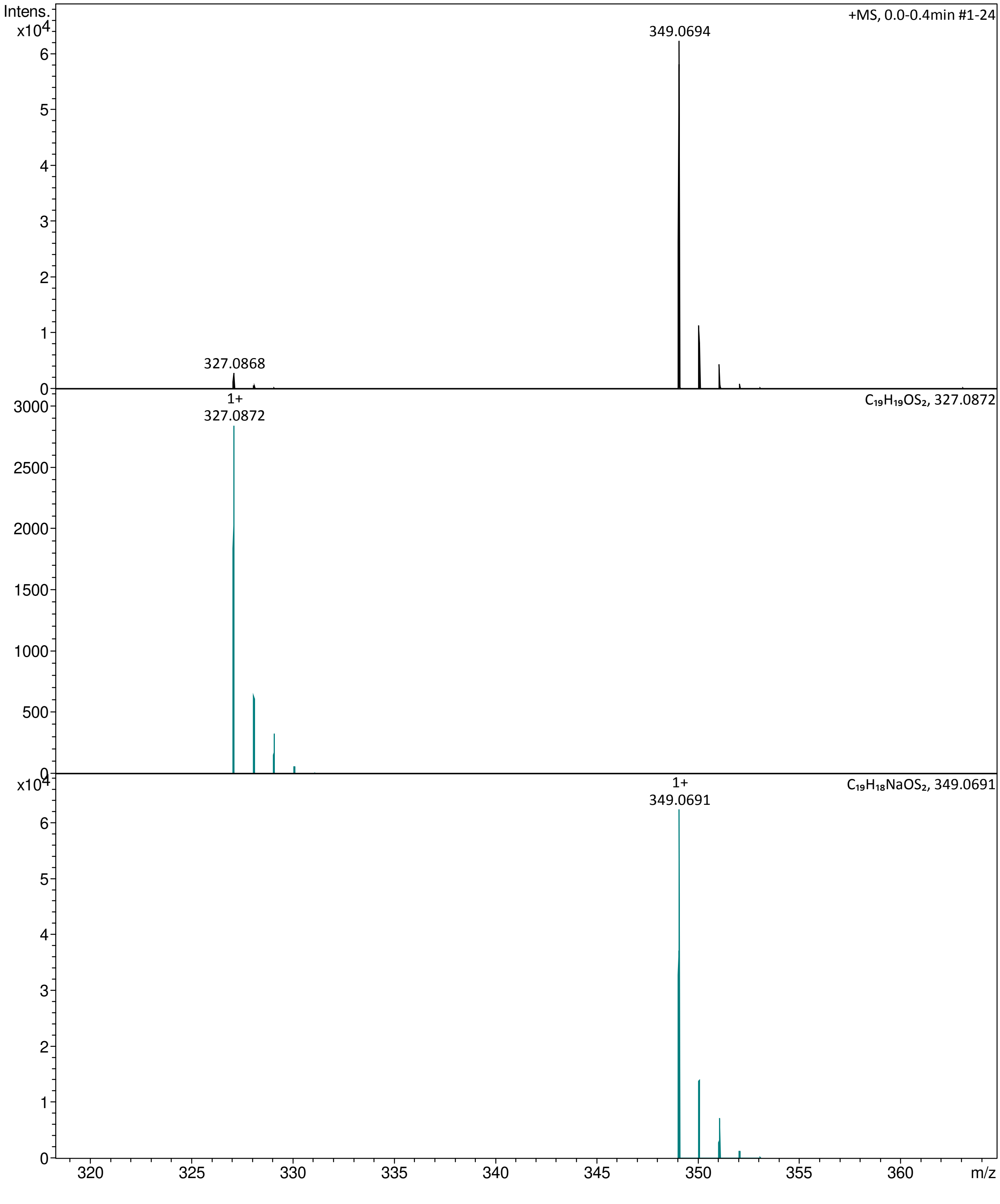

Mass Spectrum SmartFormula Report

Analysis Info

Analysis Name	D:\MZ\temp\64678000002.d	Acquisition Date	01/07/2019 16:12:29
Method	tune_low_MS_Service_06_19.m	Operator	msc
Sample Name	PN_16_spot_1peak_2	Instrument	maXis 255552.00016
Comment	Kalaba / Zehl<br>Ergebnis: +/- 5ppm<br>ACN / MeOH + 1% H2O		

Acquisition Parameter

Source Type	ESI	Ion Polarity	Positive	Set Nebulizer	0.4 Bar
Focus	Not active	Set Capillary	4200 V	Set Dry Heater	150 °C
Scan Begin	50 m/z	Set End Plate Offset	-500 V	Set Dry Gas	4.0 l/min
Scan End	1900 m/z	Set Charging Voltage	0 V	Set Divert Valve	Source
		Set Corona	0 nA	Set APCI Heater	0 °C

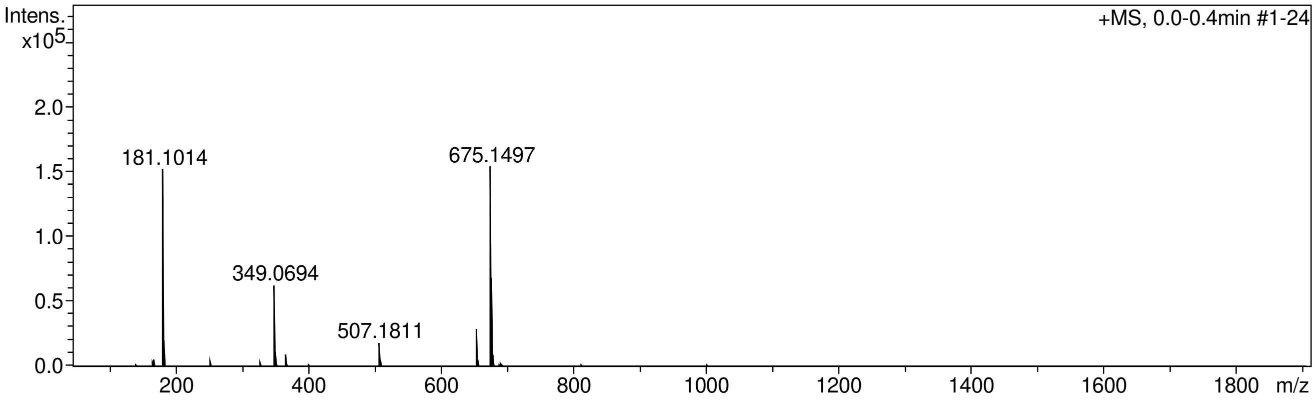

Meas. m/z	#	Ion Formula	m/z	err [ppm]	mSigma	# Sigma	Score	rdb	e⁻ Conf	N-Rule
327.0868	1	C19H19OS2	327.0872	1.1	11.5	1	100.00	10.5	even	ok
	2	C12H11N10S	327.0883	-4.7	30.5	2	34.58	12.5	even	ok
	3	C11H23N2O3S3	327.0865	-0.9	36.3	3	59.68	1.5	even	ok
	4	C18H15O6	327.0863	1.5	37.7	4	51.95	11.5	even	ok
	5	C11H15N6O4S	327.0870	0.6	38.1	5	59.25	7.5	even	ok
	6	C19H11N4O2	327.0877	2.6	44.5	6	36.14	16.5	even	ok
	7	C10H19N2O8S	327.0857	3.5	47.3	7	27.95	2.5	even	ok
349.0694	1	C12H10N10NaS	349.0703	2.6	6.7	1	89.88	12.5	even	ok
	2	C11H14N6NaO4S	349.0689	-1.2	15.6	2	96.38	7.5	even	ok
	3	C18H14NaO6	349.0683	3.2	21.1	3	44.75	11.5	even	ok
	4	C19H18NaOS2	349.0691	-0.7	27.9	4	100.00	10.5	even	ok
	5	C19H10N4NaO2	349.0696	0.6	33.5	5	53.19	16.5	even	ok
	6	C11H22N2NaO3S3	349.0685	-2.6	39.0	6	54.71	1.5	even	ok

Figure S51. HRESIMS spectrum of compound **7m**

Generic Display Report

Analysis Info

Analysis Name	D:\MZ\temp\64679000001.d	Acquisition Date	01/07/2019 16:14:23
Method	tune_low_MS_Service_06_19.m	Operator	msc
Sample Name	PN_16_spot_2_peak_1	Instrument	maXis
Comment	Kalaba / Zehl<br>Ergebnis: +/- 5ppm<br>ACN / MeOH + 1% H2O		

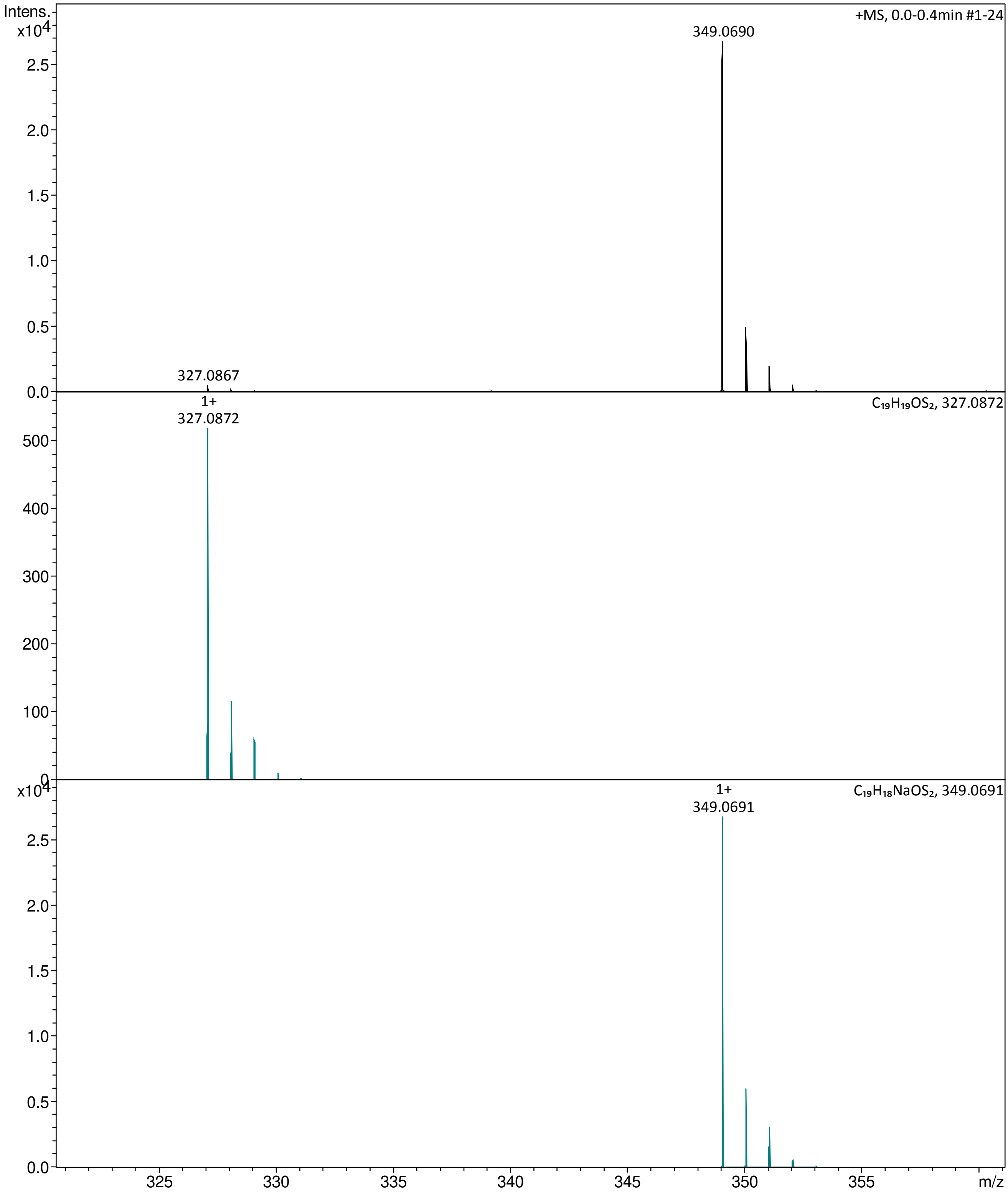

Mass Spectrum SmartFormula Report

Analysis Info

Analysis Name	D:\MZ\temp\64679000001.d	Acquisition Date	01/07/2019 16:14:23
Method	tune_low_MS_Service_06_19.m	Operator	msc
Sample Name	PN_16_spot_2_peak_1	Instrument	maXis 255552.00016
Comment	Kalaba / Zehl<br>Ergebnis: +/- 5ppm<br>ACN / MeOH + 1% H2O		

Acquisition Parameter

Source Type	ESI	Ion Polarity	Positive	Set Nebulizer	0.4 Bar
Focus	Not active	Set Capillary	4200 V	Set Dry Heater	150 °C
Scan Begin	50 m/z	Set End Plate Offset	-500 V	Set Dry Gas	4.0 l/min
Scan End	1900 m/z	Set Charging Voltage	0 V	Set Divert Valve	Source
		Set Corona	0 nA	Set APCI Heater	0 °C

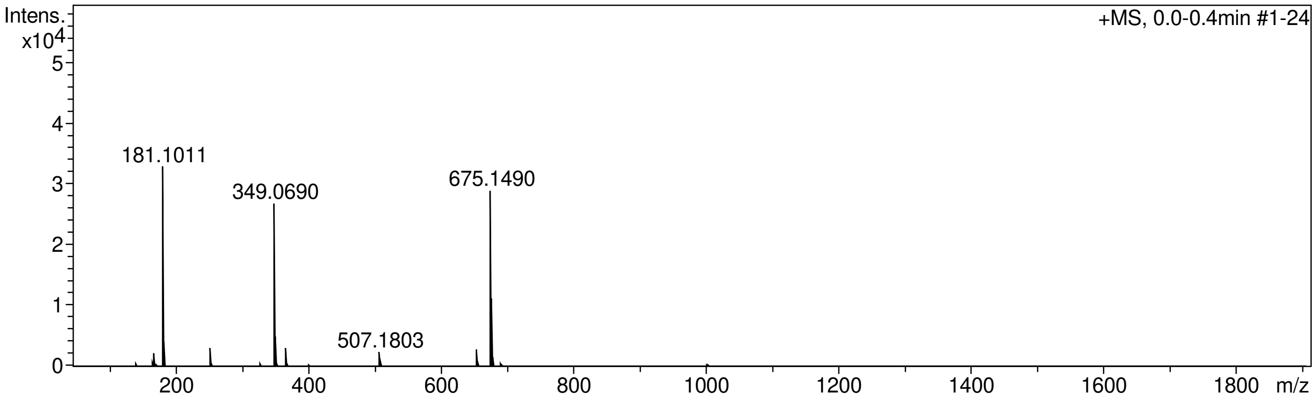

Meas. m/z	#	Ion Formula	m/z	err [ppm]	mSigma	# Sigma	Score	rdb	e⁻ Conf	N-Rule
327.0867	1	C19H11N4O2	327.0877	3.0	29.8	1	88.29	16.5	even	ok
	2	C18H15O6	327.0863	1.1	37.0	2	100.00	11.5	even	ok
	3	C19H19OS2	327.0872	1.5	55.8	3	54.16	10.5	even	ok
	4	C11H15N6O4S	327.0870	-1.0	65.3	4	42.67	7.5	even	ok
	5	C10H19N2O8S	327.0857	-3.1	76.8	5	19.34	2.5	even	ok
	6	C11H23N2O3S3	327.0865	-0.5	84.8	6	21.49	1.5	even	ok
349.0690	1	C12H10N10NaS	349.0703	3.7	9.4	1	62.43	12.5	even	ok
	2	C11H14N6NaO4S	349.0689	-0.1	18.2	2	100.00	7.5	even	ok
	3	C18H14NaO6	349.0683	-2.1	21.9	3	50.46	11.5	even	ok
	4	C19H18NaOS2	349.0691	-0.4	25.5	4	81.66	10.5	even	ok
	5	C10H18N2NaO8S	349.0676	-4.0	29.3	5	38.88	2.5	even	ok
	6	C19H10N4NaO2	349.0696	1.7	33.3	6	40.97	16.5	even	ok
	7	C11H22N2NaO3S3	349.0685	-1.5	38.3	7	50.26	1.5	even	ok

Figure S52. HRESIMS spectrum of compound **8m**

Generic Display Report

Analysis Info

Analysis Name	D:\MZ\temp\64680000001.d	Acquisition Date	01/07/2019 16:16:19
Method	tune_low_MS_Service_06_19.m	Operator	msc
Sample Name	PN_16_spot_2_peak_2	Instrument	maXis
Comment	Kalaba / Zehl		
	Ergebnis: +/- 5ppm		
	ACN / MeOH + 1% H2O		

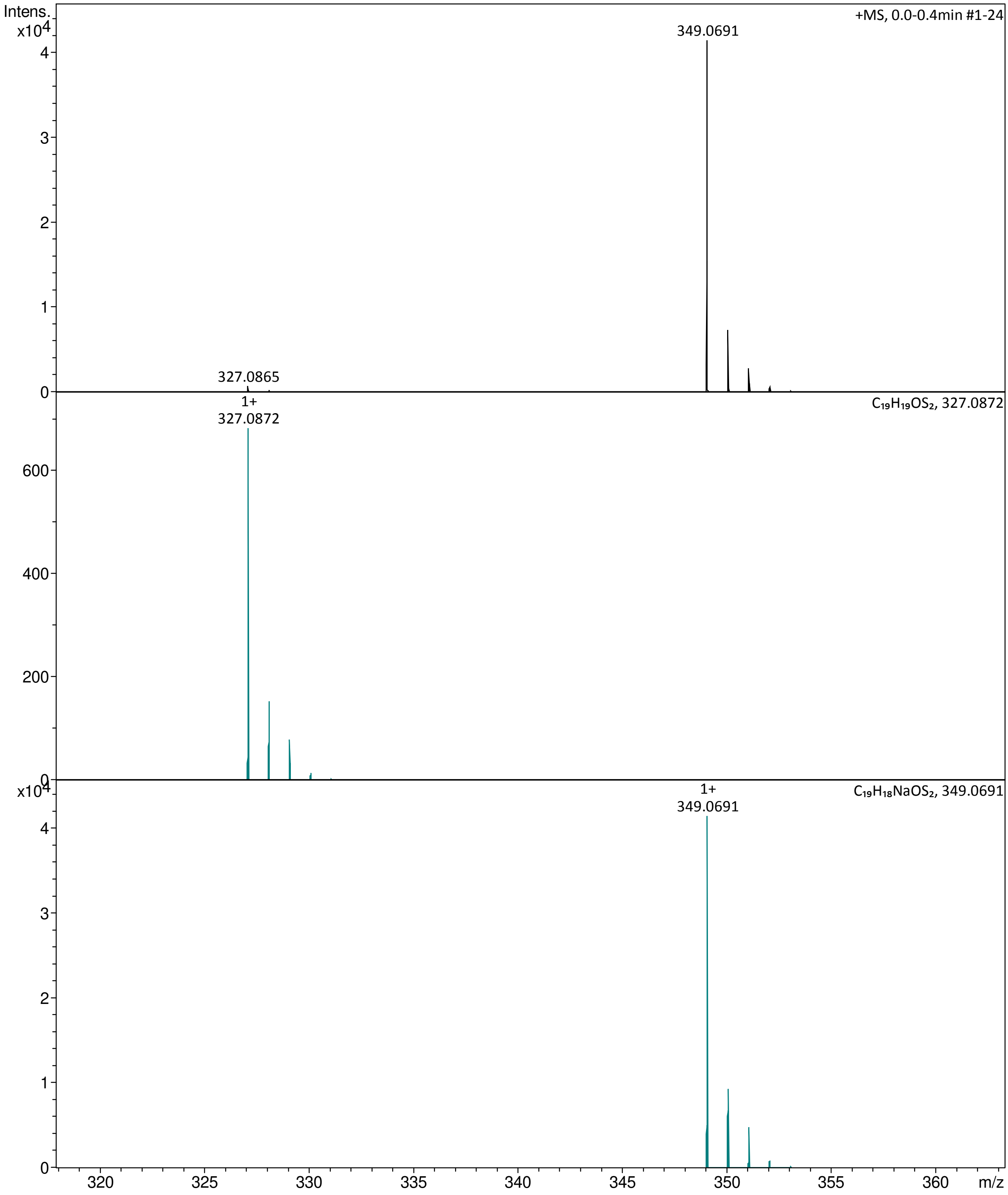

Mass Spectrum SmartFormula Report

Analysis Info

Analysis Name	D:\MZ\temp\64680000001.d	Acquisition Date	01/07/2019 16:16:19
Method	tune_low_MS_Service_06_19.m	Operator	msc
Sample Name	PN_16_spot_2_peak_2	Instrument	maXis 255552.00016
Comment	Kalaba / Zehl<br>Ergebnis: +/- 5ppm<br>ACN / MeOH + 1% H2O		

Acquisition Parameter

Source Type	ESI	Ion Polarity	Positive	Set Nebulizer	0.4 Bar
Focus	Not active	Set Capillary	4200 V	Set Dry Heater	150 °C
Scan Begin	50 m/z	Set End Plate Offset	-500 V	Set Dry Gas	4.0 l/min
Scan End	1900 m/z	Set Charging Voltage	0 V	Set Divert Valve	Source
		Set Corona	0 nA	Set APCI Heater	0 °C

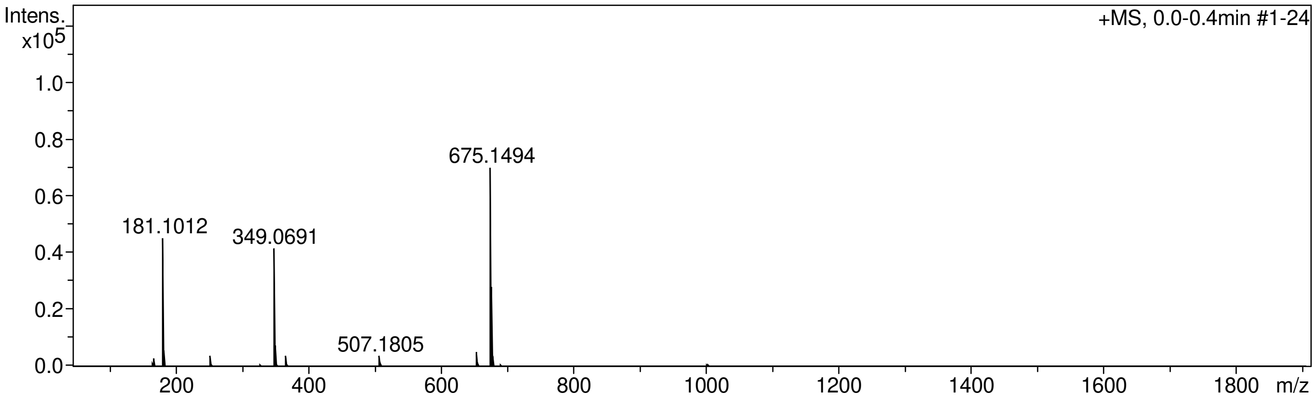

Meas. m/z	#	Ion Formula	m/z	err [ppm]	mSigma	# Sigma	Score	rdb	e⁻ Conf	N-Rule
327.0865	1	C19H11N4O2	327.0877	-3.5	18.2	1	71.90	16.5	even	ok
	2	C15H7N10	327.0850	4.7	24.5	2	48.10	17.5	even	ok
	3	C18H15O6	327.0863	-0.6	24.9	3	100.00	11.5	even	ok
	4	C19H19OS2	327.0872	2.0	52.9	4	37.79	10.5	even	ok
	5	C11H15N6O4S	327.0870	-1.5	53.5	5	40.62	7.5	even	ok
	6	C10H19N2O8S	327.0857	-2.6	64.5	6	23.17	2.5	even	ok
	7	C11H23N2O3S3	327.0865	0.0	78.1	7	20.75	1.5	even	ok
349.0691	1	C12H10N10NaS	349.0703	-3.3	5.1	1	71.52	12.5	even	ok
	2	C11H14N6NaO4S	349.0689	0.6	12.9	2	100.00	7.5	even	ok
	3	C18H14NaO6	349.0683	2.5	21.5	3	44.91	11.5	even	ok
	4	C10H18N2NaO8S	349.0676	4.4	24.4	4	37.76	2.5	even	ok
	5	C19H18NaOS2	349.0691	0.1	30.1	5	74.16	10.5	even	ok
	6	C19H10N4NaO2	349.0696	1.3	35.0	6	40.79	16.5	even	ok
	7	C11H22N2NaO3S3	349.0685	-1.9	39.1	7	43.76	1.5	even	ok

Figure S53. HRESIMS spectrum of compound **5n**

Generic Display Report

Analysis Info

Analysis Name	D:\MZ\temp\64681000001.d	Acquisition Date	01/07/2019 16:18:40
Method	tune_low_MS_Service_06_19.m	Operator	msc
Sample Name	PN_22_spot_1_peak_1	Instrument	maXis
Comment	Kalaba / Zehl<br>Ergebnis: +/- 5ppm<br>ACN / MeOH + 1% H2O		

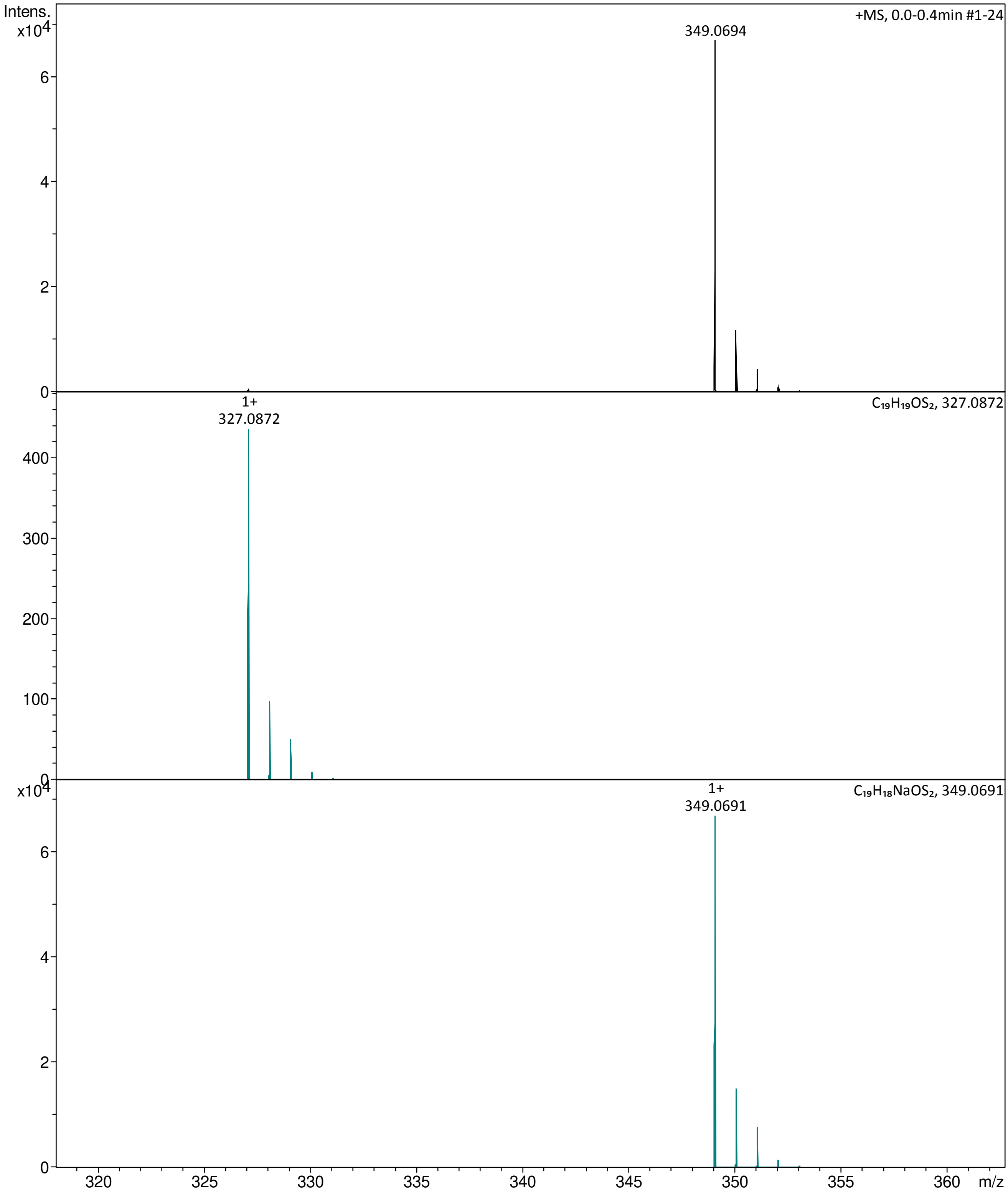

Mass Spectrum SmartFormula Report

Analysis Info

Analysis Name	D:\MZ\temp\64681000001.d	Acquisition Date	01/07/2019 16:18:40
Method	tune_low_MS_Service_06_19.m	Operator	msc
Sample Name	PN_22_spot_1_peak_1	Instrument	maXis 255552.00016
Comment	Kalaba / Zehl<br>Ergebnis: +/- 5ppm<br>ACN / MeOH + 1% H2O		

Acquisition Parameter

Source Type	ESI	Ion Polarity	Positive	Set Nebulizer	0.4 Bar
Focus	Not active	Set Capillary	4200 V	Set Dry Heater	150 °C
Scan Begin	50 m/z	Set End Plate Offset	-500 V	Set Dry Gas	4.0 l/min
Scan End	1900 m/z	Set Charging Voltage	0 V	Set Divert Valve	Source
		Set Corona	0 nA	Set APCI Heater	0 °C

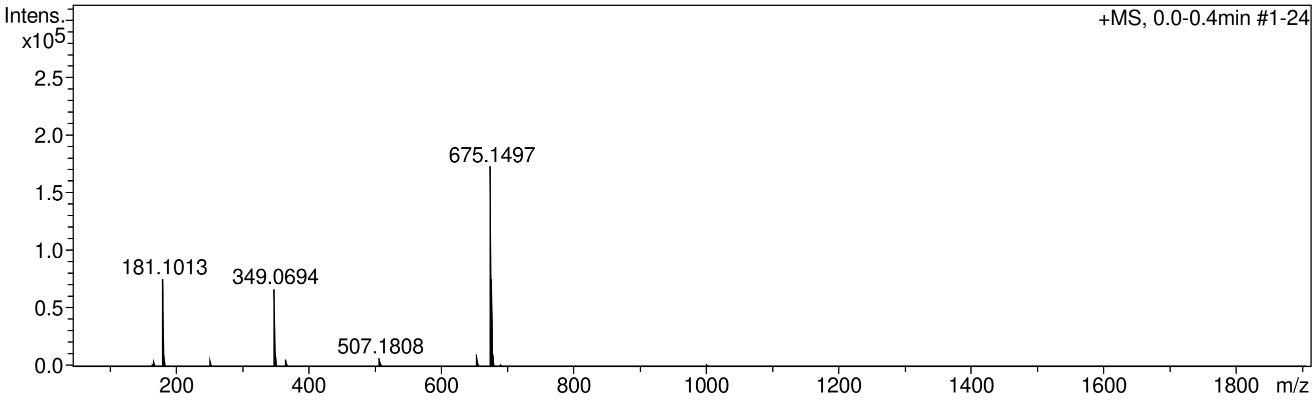

Meas. m/z	#	Ion Formula	m/z	err [ppm]	mSigma	# Sigma	Score	rdb	e⁻ Conf	N-Rule
327.0867	1	C19H11N4O2	327.0877	3.1	718.9	1	-1.#J	16.5	even	ok
	2	C18H15O6	327.0863	-1.0	802.2	2	-1.#J	11.5	even	ok
	3	C11H15N6O4S	327.0870	1.1	803.0	3	-1.#J	7.5	even	ok
	4	C10H19N2O8S	327.0857	-3.0	807.9	4	-1.#J	2.5	even	ok
	5	C19H19OS2	327.0872	1.6	816.5	5	-1.#J	10.5	even	ok
	6	C11H23N2O3S3	327.0865	-0.4	821.9	6	-1.#J	1.5	even	ok
349.0694	1	C12H10N10NaS	349.0703	2.7	3.8	1	90.64	12.5	even	ok
	2	C11H14N6NaO4S	349.0689	-1.2	13.0	2	100.00	7.5	even	ok
	3	C18H14NaO6	349.0683	-3.1	20.3	3	44.98	11.5	even	ok
	4	C19H18NaOS2	349.0691	-0.6	30.7	4	73.60	10.5	even	ok
	5	C19H10N4NaO2	349.0696	-0.7	33.8	5	50.82	16.5	even	ok
	6	C11H22N2NaO3S3	349.0685	2.5	40.2	6	41.76	1.5	even	ok

Figure S54. HRESIMS spectrum of compound **6n**

Generic Display Report

Analysis Info

Analysis Name	D:\MZ\temp\64682000001.d	Acquisition Date	01/07/2019 16:20:33
Method	tune_low_MS_Service_06_19.m	Operator	msc
Sample Name	PN_22_spot_1_peak_2	Instrument	maXis
Comment	Kalaba / Zehl<br>Ergebnis: +/- 5ppm<br>ACN / MeOH + 1% H2O		

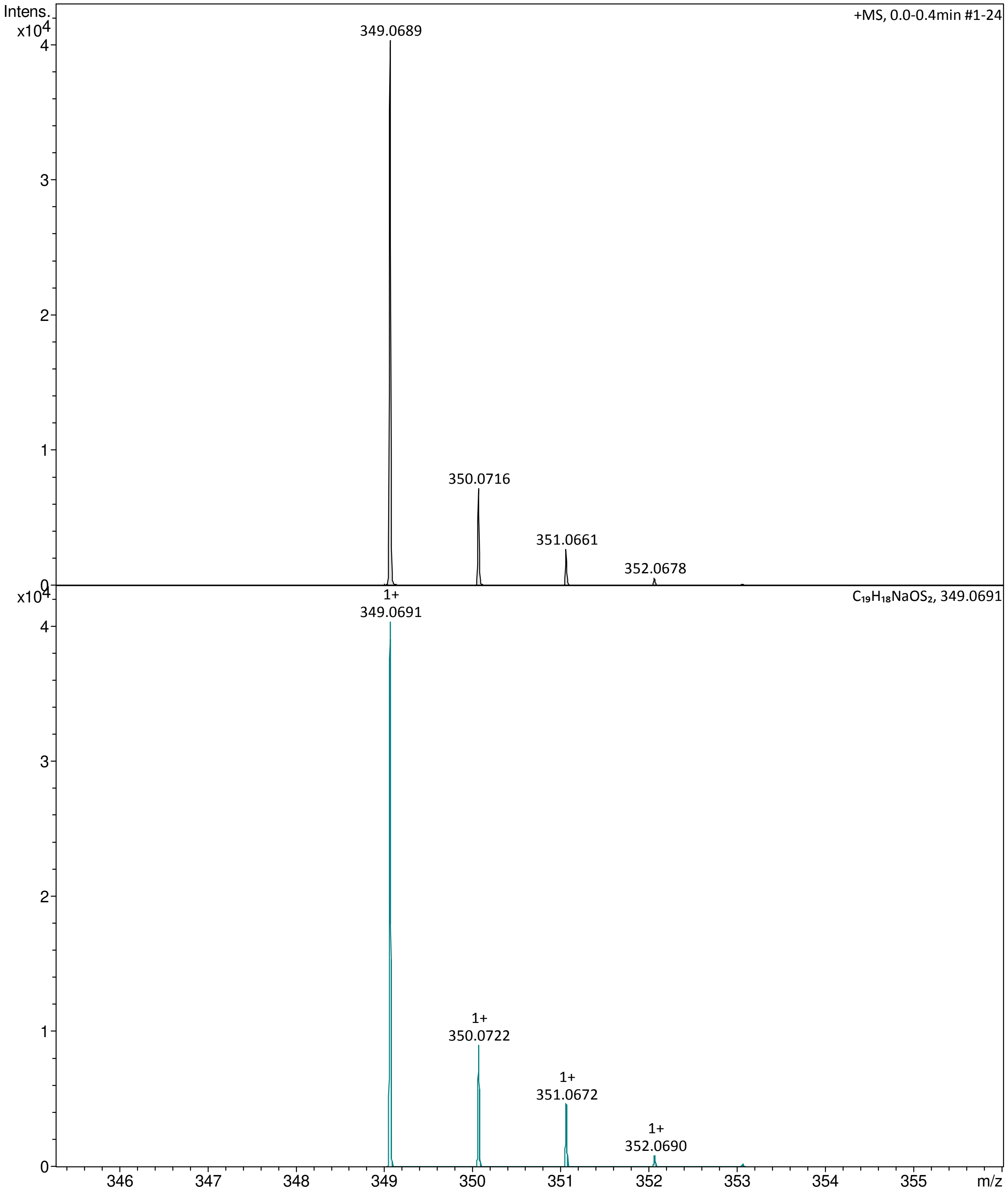

Mass Spectrum SmartFormula Report

Analysis Info

Analysis Name	D:\MZ\temp\64682000001.d	Acquisition Date	01/07/2019 16:20:33
Method	tune_low_MS_Service_06_19.m	Operator	msc
Sample Name	PN_22_spot_1_peak_2	Instrument	maXis 255552.00016
Comment	Kalaba / Zehl<br>Ergebnis: +/- 5ppm<br>ACN / MeOH + 1% H2O		

Acquisition Parameter

Source Type	ESI	Ion Polarity	Positive	Set Nebulizer	0.4 Bar
Focus	Not active	Set Capillary	4200 V	Set Dry Heater	150 °C
Scan Begin	50 m/z	Set End Plate Offset	-500 V	Set Dry Gas	4.0 l/min
Scan End	1900 m/z	Set Charging Voltage	0 V	Set Divert Valve	Source
		Set Corona	0 nA	Set APCI Heater	0 °C

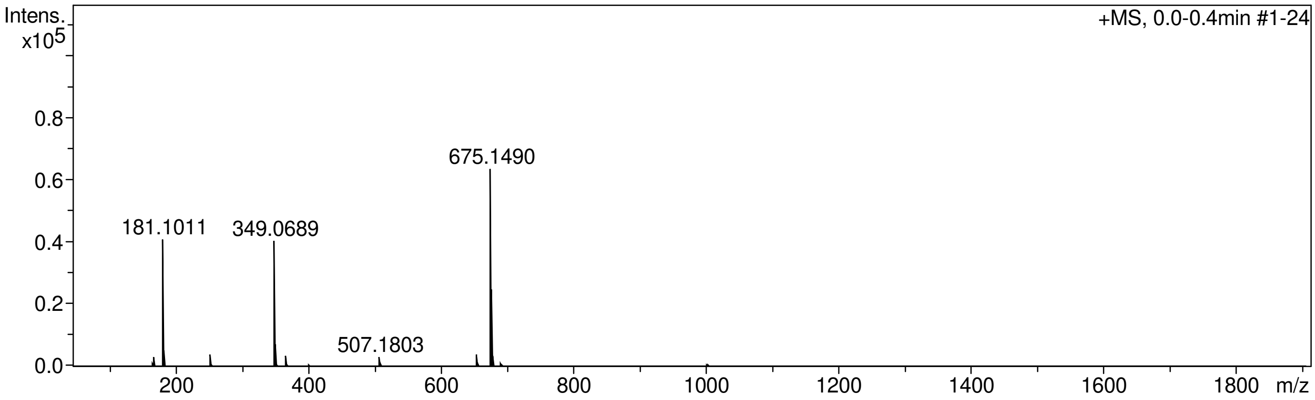

Meas. m/z	#	Ion Formula	m/z	err [ppm]	mSigma	# Sigma	Score	rdb	e⁻ Conf	N-Rule
349.0689	1	C12H10N10NaS	349.0703	-3.8	4.5	1	59.30	12.5	even	ok
	2	C11H14N6NaO4S	349.0689	-0.0	13.4	2	100.00	7.5	even	ok
	3	C18H14NaO6	349.0683	2.0	20.4	3	47.73	11.5	even	ok
	4	C10H18N2NaO8S	349.0676	-3.8	25.0	4	39.58	2.5	even	ok
	5	C19H18NaOS2	349.0691	0.5	30.3	5	64.11	10.5	even	ok
	6	C19H10N4NaO2	349.0696	-1.9	33.7	6	35.41	16.5	even	ok
	7	C11H22N2NaO3S3	349.0685	-1.3	40.0	7	43.99	1.5	even	ok

Figure S55. HRESIMS spectrum of compound **7n**

Generic Display Report

Analysis Info

Analysis Name	D:\MZ\temp\64695000001.d	Acquisition Date	02/07/2019 17:05:50
Method	tune_low_MS_Service_06_19.m	Operator	msc
Sample Name	PN-22_spot-2_peak-1	Instrument	maXis
Comment	Kalaba/Zehl<br>Ergebnis: +/- 5ppm<br>ACN/MeOH + 1% H2O		

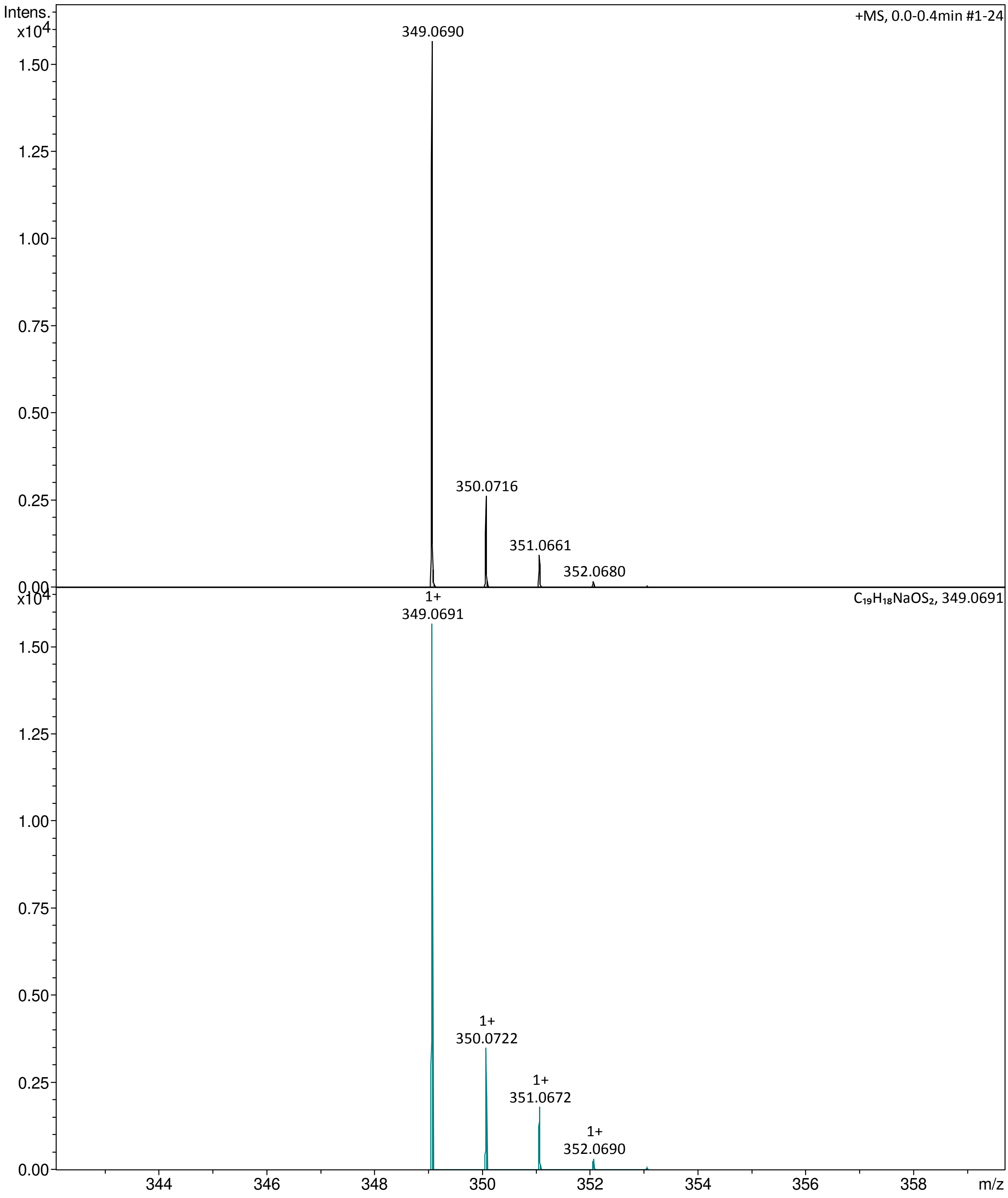

Mass Spectrum SmartFormula Report

Analysis Info

Analysis Name	D:\MZ\temp\64695000001.d	Acquisition Date	02/07/2019 17:05:50
Method	tune_low_MS_Service_06_19.m	Operator	msc
Sample Name	PN-22_spot-2_peak-1	Instrument	maXis 255552.00016
Comment	Kalaba/Zehl<br>Ergebnis: +/- 5ppm<br>ACN/MeOH + 1% H2O		

Acquisition Parameter

Source Type	ESI	Ion Polarity	Positive	Set Nebulizer	0.4 Bar
Focus	Not active	Set Capillary	4200 V	Set Dry Heater	150 °C
Scan Begin	50 m/z	Set End Plate Offset	-500 V	Set Dry Gas	4.0 l/min
Scan End	1900 m/z	Set Charging Voltage	0 V	Set Divert Valve	Source
		Set Corona	0 nA	Set APCI Heater	0 °C

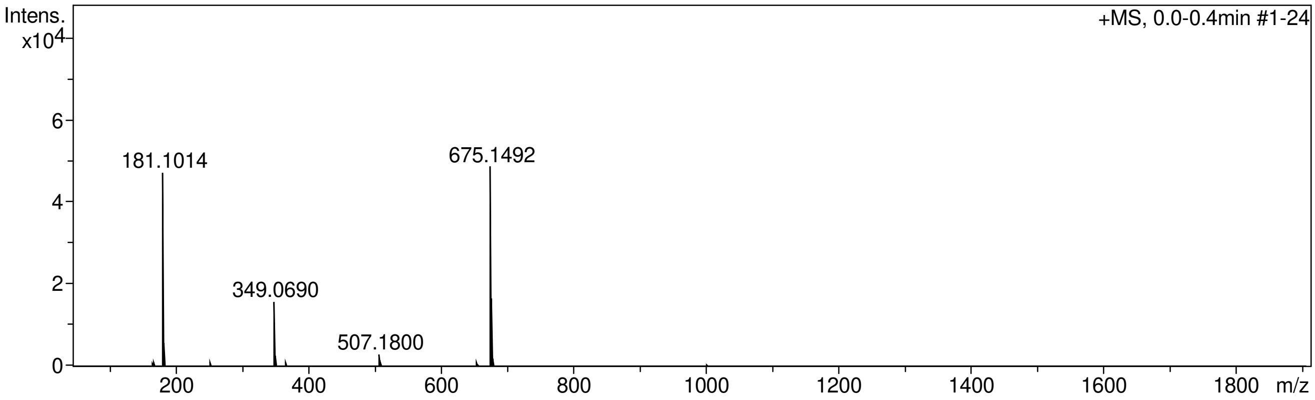

Meas. m/z	#	Ion Formula	m/z	err [ppm]	mSigma	# Sigma	Score	rdb	e⁻ Conf	N-Rule
349.0690	1	C12H10N10NaS	349.0703	3.8	4.3	1	55.41	12.5	even	ok
	2	C11H14N6NaO4S	349.0689	0.1	8.4	2	100.00	7.5	even	ok
	3	C10H18N2NaO8S	349.0676	3.9	20.3	3	39.62	2.5	even	ok
	4	C18H14NaO6	349.0683	-2.0	21.1	4	42.47	11.5	even	ok
	5	C19H18NaOS2	349.0691	0.5	35.7	5	51.80	10.5	even	ok
	6	C19H10N4NaO2	349.0696	-1.8	36.4	6	30.67	16.5	even	ok
	7	C11H22N2NaO3S3	349.0685	-1.4	41.9	7	37.71	1.5	even	ok

Figure S56. HRESIMS spectrum of compound **8n**

Generic Display Report

Analysis Info

Analysis Name	D:\MZ\temp\64696000001.d	Acquisition Date	02/07/2019 17:07:57
Method	tune_low_MS_Service_06_19.m	Operator	msc
Sample Name	PN-22_spot-2_peak-2	Instrument	maXis
Comment	Kalaba/Zehl<br>Ergebnis: +/- 5ppm<br>ACN/MeOH + 1% H2O		

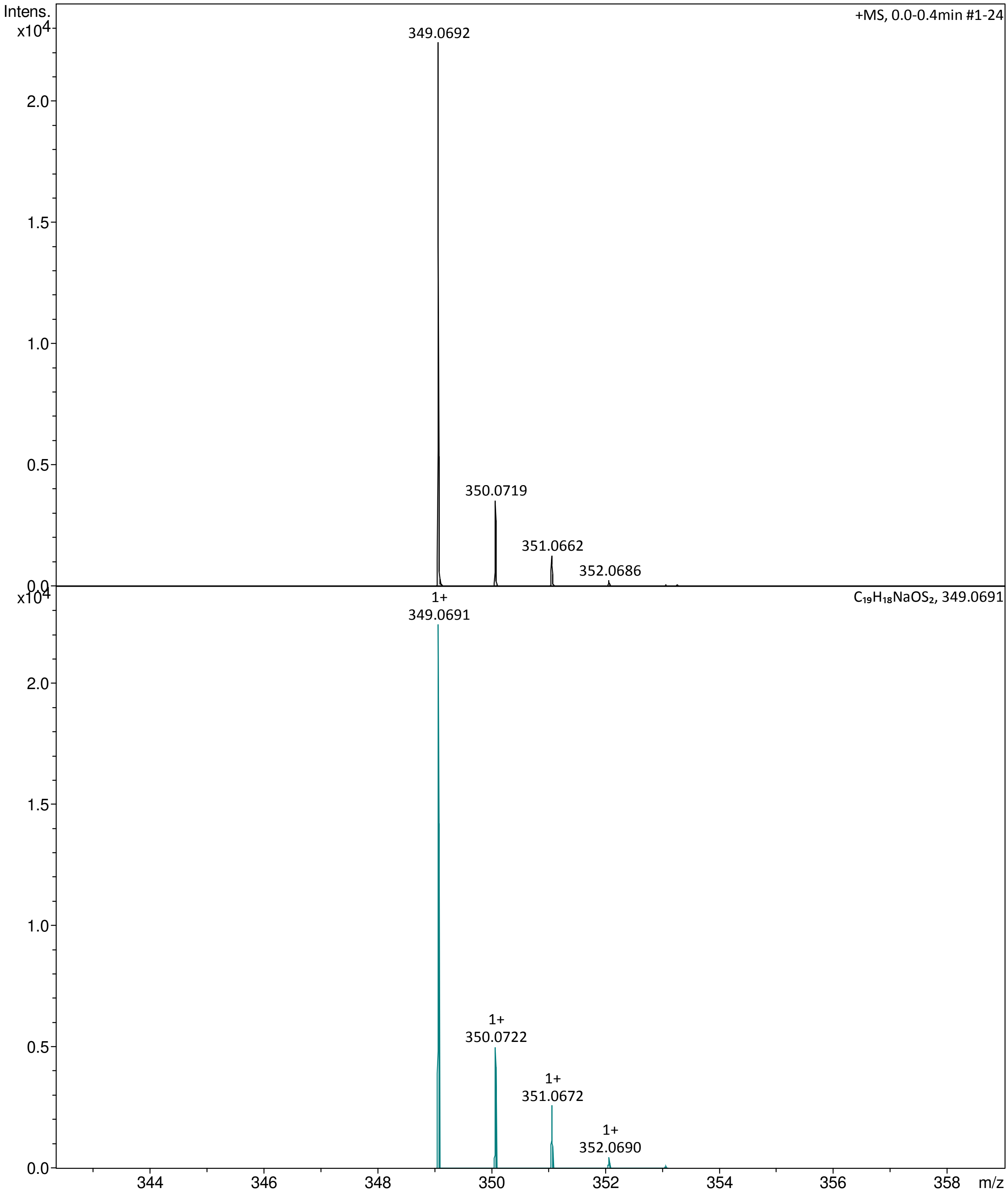

Mass Spectrum SmartFormula Report

Analysis Info

Analysis Name	D:\MZ\temp\64696000001.d	Acquisition Date	02/07/2019 17:07:57
Method	tune_low_MS_Service_06_19.m	Operator	msc
Sample Name	PN-22_spot-2_peak-2	Instrument	maXis 255552.00016
Comment	Kalaba/Zehl<br>Ergebnis: +/- 5ppm<br>ACN/MeOH + 1% H2O		

Acquisition Parameter

Source Type	ESI	Ion Polarity	Positive	Set Nebulizer	0.4 Bar
Focus	Not active	Set Capillary	4200 V	Set Dry Heater	150 °C
Scan Begin	50 m/z	Set End Plate Offset	-500 V	Set Dry Gas	4.0 l/min
Scan End	1900 m/z	Set Charging Voltage	0 V	Set Divert Valve	Source
		Set Corona	0 nA	Set APCI Heater	0 °C

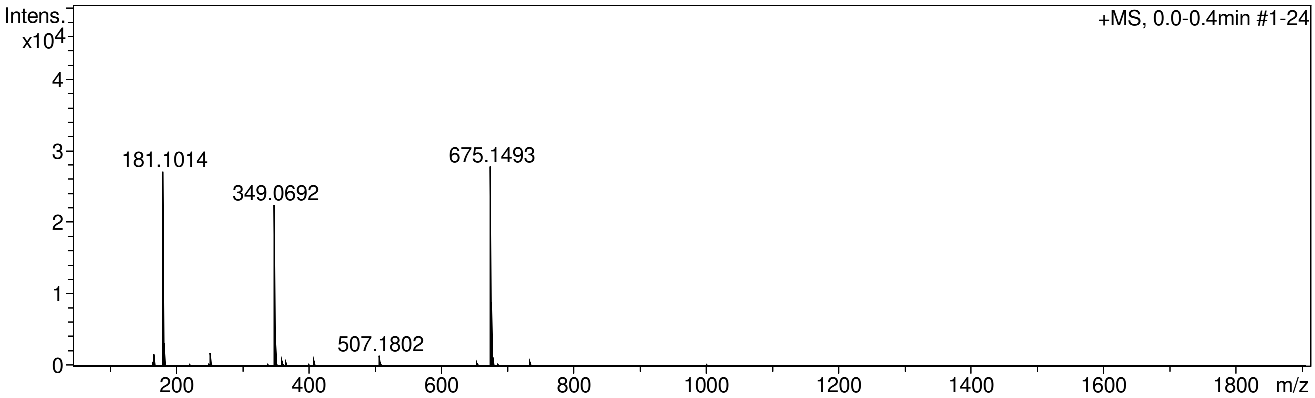

Meas. m/z	#	Ion Formula	m/z	err [ppm]	mSigma	# Sigma	Score	rdb	e^- Conf	N-Rule
349.0692	1	C11H14N6NaO4S	349.0689	-0.8	4.8	1	100.00	7.5	even	ok
	2	C12H10N10NaS	349.0703	3.0	9.0	2	62.66	12.5	even	ok
	3	C10H18N2NaO8S	349.0676	4.6	15.9	3	38.20	2.5	even	ok
	4	C18H14NaO6	349.0683	-2.8	24.2	4	36.08	11.5	even	ok
	5	C19H18NaOS2	349.0691	0.3	39.7	5	50.05	10.5	even	ok
	6	C19H10N4NaO2	349.0696	-1.1	40.7	6	32.46	16.5	even	ok
	7	C11H22N2NaO3S3	349.0685	-2.2	42.7	7	33.92	1.5	even	ok

Figure S57. HRESIMS spectrum of compound **5o**

Generic Display Report

Analysis Info

Analysis Name	D:\MZ\maXis_data\temp\64013000001.d	Acquisition Date	20/05/2019 14:59:37
Method	tune_low_MS_Service_05_19.m	Operator	msc
Sample Name	PN-12 spot 1 peak 1	Instrument	maXis
Comment	Predrag Kalaba<br>Ergebnis: +/- 5ppm<br>ACN / MeOH + 1% H2O		

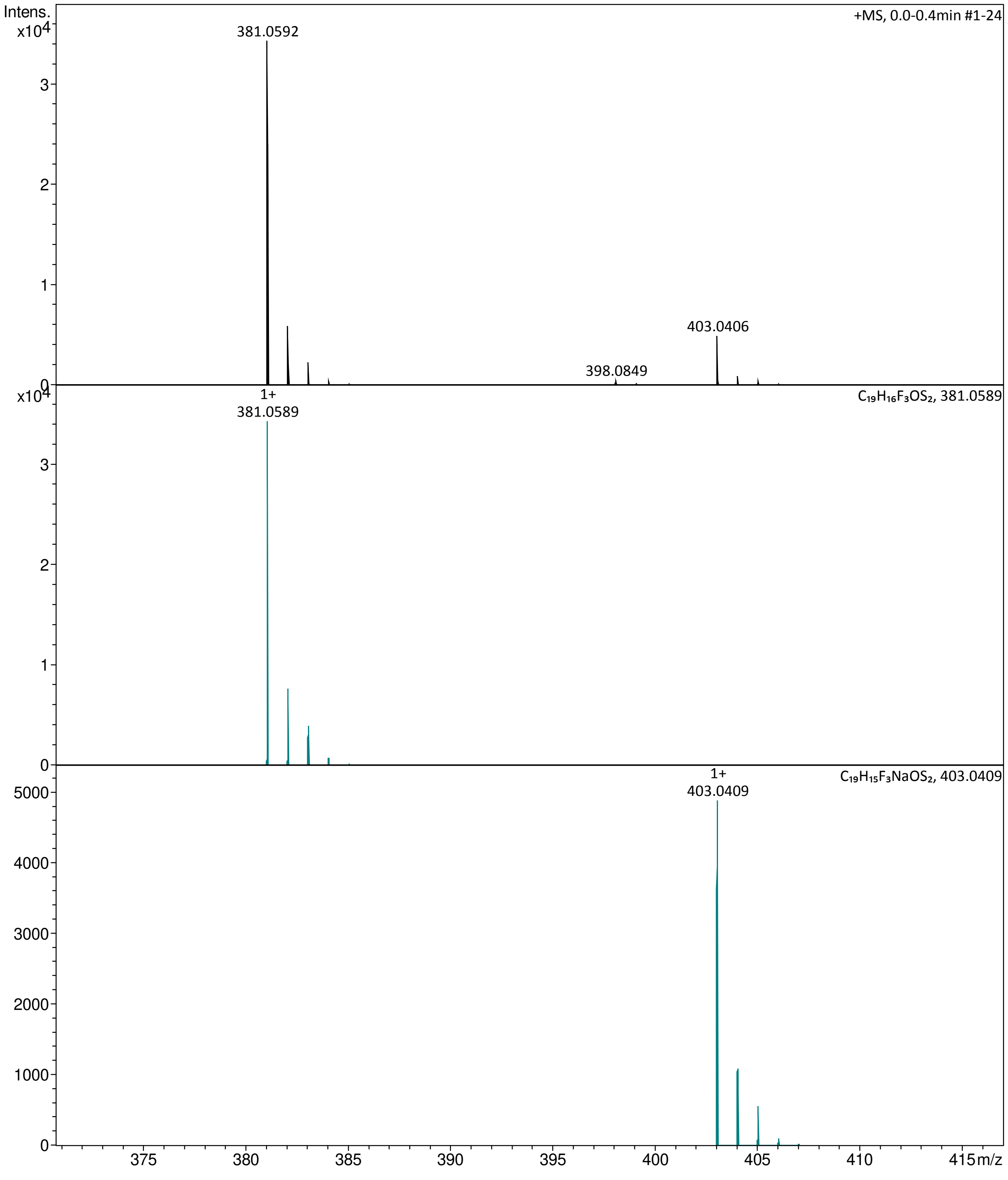

Mass Spectrum SmartFormula Report

Analysis Info

Analysis Name	D:\MZ\maXis_data\temp\64013000001.d	Acquisition Date	20/05/2019 14:59:37
Method	tune_low_MS_Service_05_19.m	Operator	msc
Sample Name	PN-12 spot 1 peak 1	Instrument	maXis 255552.00016
Comment	Predrag Kalaba<br>Ergebnis: +/- 5ppm<br>ACN / MeOH + 1% H2O		

Acquisition Parameter

Source Type	ESI	Ion Polarity	Positive	Set Nebulizer	0.4 Bar
Focus	Not active	Set Capillary	4500 V	Set Dry Heater	150 °C
Scan Begin	50 m/z	Set End Plate Offset	-500 V	Set Dry Gas	4.0 l/min
Scan End	1900 m/z	Set Charging Voltage	0 V	Set Divert Valve	Source
		Set Corona	0 nA	Set APCI Heater	0 °C

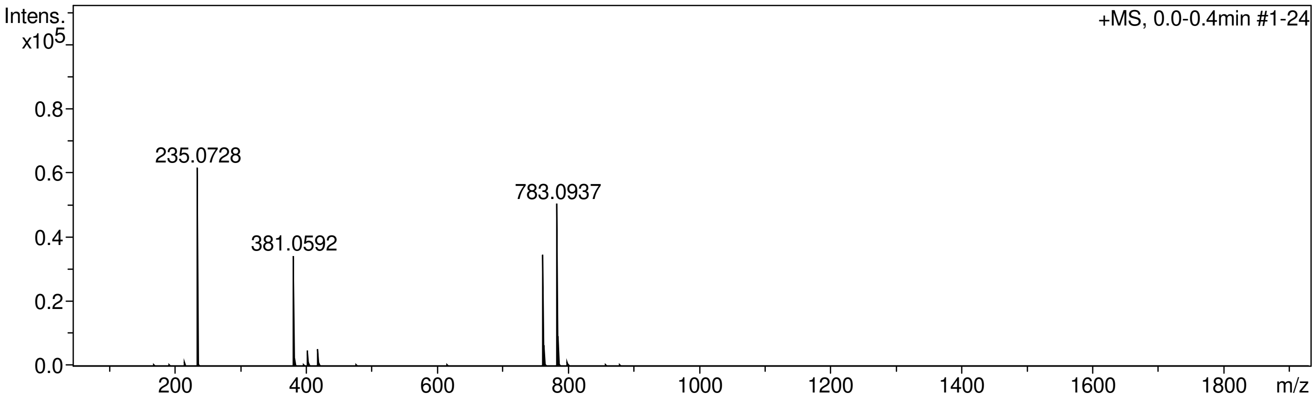

Meas. m/z	#	Ion Formula	Score	m/z	err [mDa]	err [ppm]	mSigma	rdb	e⁻ Conf	N-Rule
381.0592	1	C12H8F3N10S	75.08	381.0601	0.9	2.4	5.1	12.5	even	ok
	2	C14H11F6N4S	65.27	381.0603	1.2	3.1	5.2	8.5	even	ok
	3	C14H11F2N6O3S	49.24	381.0576	1.6	4.1	6.9	11.5	even	ok
	4	C16H14F5O3S	57.58	381.0578	1.3	3.5	6.9	7.5	even	ok
	5	C13H15F6O4S	100.00	381.0590	0.2	0.5	9.6	3.5	even	ok
	6	C11H12F3N6O4S	89.76	381.0587	-0.4	-1.1	9.6	7.5	even	ok
	7	C14H16F7S2	40.49	381.0576	-1.5	-4.0	17.9	3.5	even	ok
	8	C12H13F4N6S2	34.23	381.0574	-1.8	-4.7	17.9	7.5	even	ok
	9	C18H15F2O5S	51.74	381.0603	1.1	3.0	18.5	10.5	even	ok
	10	C11H15F2N6O3S2	40.93	381.0610	-1.8	-4.8	18.7	6.5	even	ok
	11	C10H16F3N2O8S	32.50	381.0574	1.8	4.6	21.1	2.5	even	ok
	12	C13H18FN2O6S2	78.02	381.0585	0.7	1.8	21.6	5.5	even	ok
	13	C16H17F4O2S2	67.97	381.0601	0.9	2.4	21.9	6.5	even	ok
	14	C14H14FN6O2S2	77.25	381.0598	-0.7	-1.8	22.0	10.5	even	ok
	15	C11H17F8OS2	68.63	381.0588	0.4	1.0	23.4	-0.5	even	ok
	16	C10H19F2N2O7S2	78.81	381.0596	-0.5	-1.2	25.3	1.5	even	ok
	17	C10H16F7O5S	48.60	381.0601	-1.0	-2.5	25.5	-0.5	even	ok
	18	C19H16F3OS2	74.39	381.0589	0.2	0.6	32.4	10.5	even	ok
	19	C21H14FO4S	61.59	381.0591	0.0	0.0	34.6	14.5	even	ok
	20	C11H20F3N2O3S3	45.85	381.0583	0.9	2.3	38.7	1.5	even	ok
	21	C22H15F2S2	28.38	381.0578	-1.4	-3.6	45.4	14.5	even	ok
	22	C22H10FN4S	23.05	381.0605	1.3	3.5	46.0	19.5	even	ok
	23	C11H23F2N2O2S4	19.46	381.0605	1.3	3.5	58.2	0.5	even	ok
	24	C14H22FN2OS4	32.63	381.0594	0.2	0.5	60.8	4.5	even	ok
403.0406	1	C14H10F2N6NaO3S	75.87	403.0395	1.0	2.6	6.3	11.5	even	ok
	2	C16H13F5NaO3S	86.71	403.0398	0.8	2.0	6.3	7.5	even	ok
	3	C12H7F3N10NaS	55.96	403.0420	1.4	3.6	10.0	12.5	even	ok
	4	C14H10F6N4NaS	47.56	403.0423	-1.7	-4.2	10.0	8.5	even	ok
	5	C18H14F2NaO5S	46.04	403.0422	1.6	4.1	13.0	10.5	even	ok
	6	C14H15F7NaS2	65.25	403.0396	1.0	2.5	15.0	3.5	even	ok
	7	C12H12F4N6NaS2	56.46	403.0393	-1.3	-3.1	15.1	7.5	even	ok
	8	C13H14F6NaO4S	90.10	403.0409	-0.3	-0.8	16.6	3.5	even	ok
	9	C11H11F3N6NaO4S	100.00	403.0407	-0.1	-0.2	16.6	7.5	even	ok
	10	C16H16F4NaO2S2	49.51	403.0420	-1.4	-3.5	16.7	6.5	even	ok

Mass Spectrum SmartFormula Report

Meas. m/z	#	Ion Formula	Score	m/z	err [mDa]	err [ppm]	mSigma	rdb	e⁻ Conf	N-Rule
	11	C14H13FN6NaO2S2	57.54	403.0418	-1.2	-2.9	16.7	10.5	even	ok
	12	C19H12F4NaO2S	33.35	403.0386	2.0	4.8	17.9	11.5	even	ok
	13	C13H17FN2NaO6S2	93.09	403.0404	-0.2	-0.4	18.8	5.5	even	ok
	14	C11H16F8NaOS2	81.83	403.0407	0.1	0.3	25.4	-0.5	even	ok
	15	C19H15F3NaOS2	74.93	403.0409	0.3	0.7	26.2	10.5	even	ok
	16	C21H13FNaO4S	63.77	403.0411	0.5	1.2	28.8	14.5	even	ok
	17	C14H18F2N2NaO2S3	29.83	403.0391	-1.5	-3.8	35.8	5.5	even	ok
	18	C11H19F3N2NaO3S3	55.88	403.0402	0.4	0.9	36.3	1.5	even	ok
	19	C22H14F2NaS2	40.48	403.0397	-0.9	-2.1	39.2	14.5	even	ok
	20	C22H9FN4NaS	21.14	403.0424	-1.8	-4.5	40.4	19.5	even	ok
	21	C11H22F2N2NaO2S4	13.51	403.0424	1.9	4.6	54.9	0.5	even	ok
	22	C14H21FN2NaOS4	26.36	403.0413	-0.7	-1.8	56.3	4.5	even	ok

Figure S58. HRESIMS spectrum of compound **6o**

Generic Display Report

Analysis Info

Analysis Name	D:\MZ\maXis_data\temp\64014000001.d	Acquisition Date	20/05/2019 15:14:03
Method	tune_low_MS_Service_05_19.m	Operator	msc
Sample Name	PN-12 spot 1 peak 2	Instrument	maXis
Comment	Predrag Kalaba<br>Ergebnis: +/- 5ppm<br>ACN / MeOH + 1% H2O		

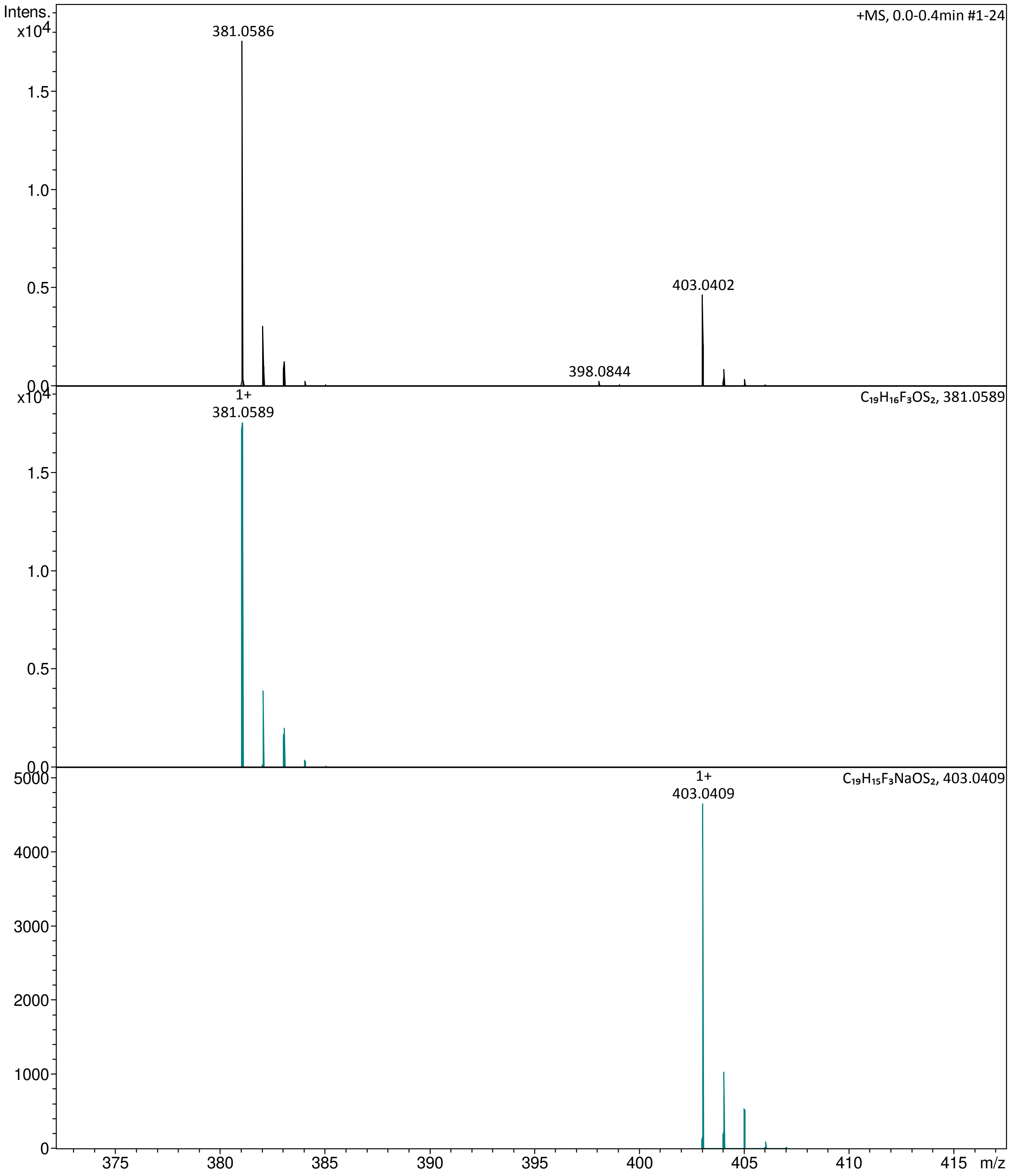

Mass Spectrum SmartFormula Report

Analysis Info

Analysis Name	D:\MZ\maXis_data\temp\64014000001.d	Acquisition Date	20/05/2019 15:14:03
Method	tune_low_MS_Service_05_19.m	Operator	msc
Sample Name	PN-12 spot 1 peak 2	Instrument	maXis 255552.00016
Comment	Predrag Kalaba<br>Ergebnis: +/- 5ppm<br>ACN / MeOH + 1% H2O		

Acquisition Parameter

Source Type	ESI	Ion Polarity	Positive	Set Nebulizer	0.4 Bar
Focus	Not active	Set Capillary	4500 V	Set Dry Heater	150 °C
Scan Begin	50 m/z	Set End Plate Offset	-500 V	Set Dry Gas	4.0 l/min
Scan End	1900 m/z	Set Charging Voltage	0 V	Set Divert Valve	Source
		Set Corona	0 nA	Set APCI Heater	0 °C

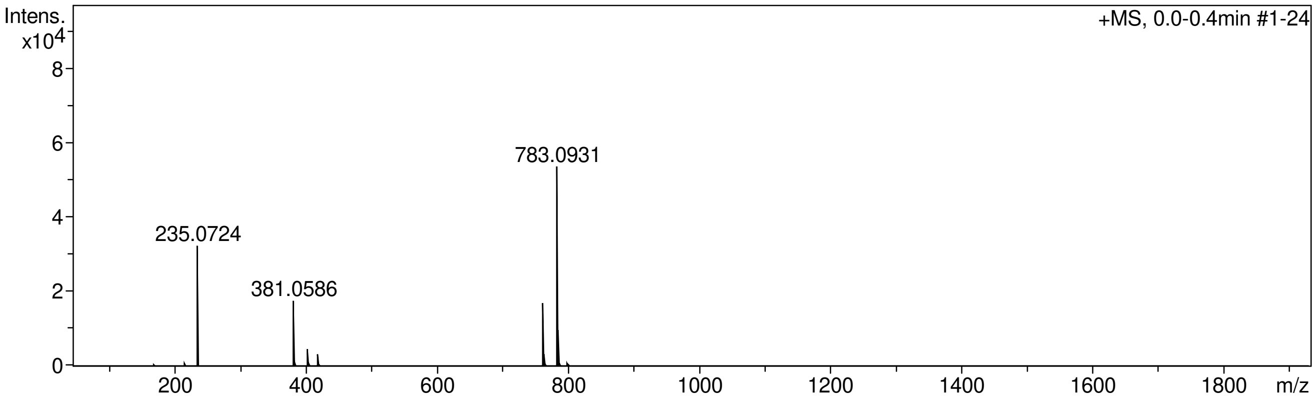

Meas. m/z	#	Ion Formula	Score	m/z	err [mDa]	err [ppm]	mSigma	rdb	e⁻ Conf	N-Rule
381.0586	1	C14H11F2N6O3S	73.93	381.0576	1.0	2.7	5.3	11.5	even	ok
	2	C16H14F5O3S	84.38	381.0578	0.8	2.0	5.3	7.5	even	ok
	3	C12H8F3N10S	54.52	381.0601	1.5	3.8	7.1	12.5	even	ok
	4	C14H11F6N4S	46.25	381.0603	-1.7	-4.5	7.1	8.5	even	ok
	5	C13H15F6O4S	90.00	381.0590	-0.4	-1.0	12.9	3.5	even	ok
	6	C11H12F3N6O4S	100.00	381.0587	-0.1	-0.3	12.9	7.5	even	ok
	7	C18H15F2O5S	40.48	381.0603	1.7	4.4	15.5	10.5	even	ok
	8	C14H16F7S2	61.79	381.0576	1.0	2.6	15.6	3.5	even	ok
	9	C12H13F4N6S2	53.54	381.0574	-1.2	-3.2	15.7	7.5	even	ok
	10	C10H10FN12S2	45.90	381.0571	1.5	3.9	15.7	11.5	even	ok
	11	C16H17F4O2S2	43.65	381.0601	-1.5	-3.8	18.9	6.5	even	ok
	12	C14H14FN6O2S2	50.80	381.0598	-1.2	-3.2	18.9	10.5	even	ok
	13	C13H18FN2O6S2	87.27	381.0585	-0.1	-0.3	19.6	5.5	even	ok
	14	C11H17F8OS2	78.98	381.0588	-0.1	-0.4	23.7	-0.5	even	ok
	15	C10H16F3N2O8S	45.58	381.0574	1.2	3.2	23.8	2.5	even	ok
	16	C10H19F2N2O7S2	49.65	381.0596	1.0	2.7	25.2	1.5	even	ok
	17	C10H16F7O5S	33.83	381.0601	-1.5	-4.0	28.6	-0.5	even	ok
	18	C19H16F3OS2	65.00	381.0589	0.3	0.8	29.1	10.5	even	ok
	19	C21H14FO4S	55.30	381.0591	0.5	1.4	31.6	14.5	even	ok
	20	C11H20F3N2O3S3	52.37	381.0583	0.3	0.9	37.1	1.5	even	ok
	21	C14H19F2N2O2S3	27.30	381.0571	-1.5	-3.9	37.6	5.5	even	ok
	22	C22H15F2S2	35.68	381.0578	-0.8	-2.2	42.2	14.5	even	ok
	23	C22H10FN4S	18.05	381.0605	-1.9	-4.9	43.2	19.5	even	ok
	24	C11H23F2N2O2S4	11.95	381.0605	1.9	4.9	56.2	0.5	even	ok
	25	C14H22FN2OS4	22.91	381.0594	-0.7	-1.9	58.3	4.5	even	ok
403.0402	1	C14H10F2N6NaO3S	89.02	403.0395	-0.6	-1.6	5.6	11.5	even	ok
	2	C16H13F5NaO3S	100.00	403.0398	0.4	1.0	5.6	7.5	even	ok
	3	C12H7F3N10NaS	38.45	403.0420	-1.8	-4.6	10.8	12.5	even	ok
	4	C13H14F2N2NaO7S	32.76	403.0382	-2.0	-4.9	14.2	6.5	even	ok
	5	C17H9FN6NaO2S	37.61	403.0384	1.8	4.4	14.5	15.5	even	ok
	6	C19H12F4NaO2S	44.43	403.0386	-1.5	-3.8	14.5	11.5	even	ok
	7	C16H16F4NaO2S2	34.64	403.0420	1.8	4.6	16.5	6.5	even	ok
	8	C14H13FN6NaO2S2	41.03	403.0418	-1.6	-4.0	16.5	10.5	even	ok
	9	C14H15F7NaS2	72.74	403.0396	-0.6	-1.5	16.9	3.5	even	ok

Mass Spectrum SmartFormula Report

Meas. m/z	#	Ion Formula	Score	m/z	err [mDa]	err [ppm]	mSigma	rdb	e⁻ Conf	N-Rule
	10	C12H12F4N6NaS2	64.09	403.0393	0.9	2.1	17.0	7.5	even	ok
	11	C13H14F6NaO4S	64.63	403.0409	0.7	1.9	19.2	3.5	even	ok
	12	C11H11F3N6NaO4S	72.95	403.0407	0.5	1.3	19.2	7.5	even	ok
	13	C13H17FN2NaO6S2	80.18	403.0404	0.3	0.6	20.2	5.5	even	ok
	14	C19H15F3NaOS2	59.22	403.0409	-0.7	-1.7	24.5	10.5	even	ok
	15	C21H13FNaO4S	51.50	403.0411	-0.9	-2.3	25.6	14.5	even	ok
	16	C11H16F8NaOS2	58.67	403.0407	-0.5	-1.3	28.4	-0.5	even	ok
	17	C11H13F9NaO2S	27.69	403.0385	-1.7	-4.2	30.9	0.5	even	ok
	18	C14H18F2N2NaO2S3	35.47	403.0391	1.1	2.7	36.2	5.5	even	ok
	19	C22H14F2NaS2	49.10	403.0397	0.5	1.1	37.0	14.5	even	ok
	20	C11H19F3N2NaO3S3	57.54	403.0402	-0.0	-0.1	37.8	1.5	even	ok
	21	C14H21FN2NaOS4	19.24	403.0413	1.1	2.8	56.6	4.5	even	ok

Figure S59. HRESIMS spectrum of compound **7o**

Generic Display Report

Analysis Info

Analysis Name	D:\MZ\maXis_data\temp\64015000001.d
Method	tune_low_MS_Service_05_19.m
Sample Name	PN-12 spot 2 peak 1
Comment	Predrag Kalaba<br>Ergebnis: +/- 5ppm<br>ACN / MeOH + 1% H2O
Acquisition Date	20/05/2019 15:19:27
Operator	msc
Instrument	maXis

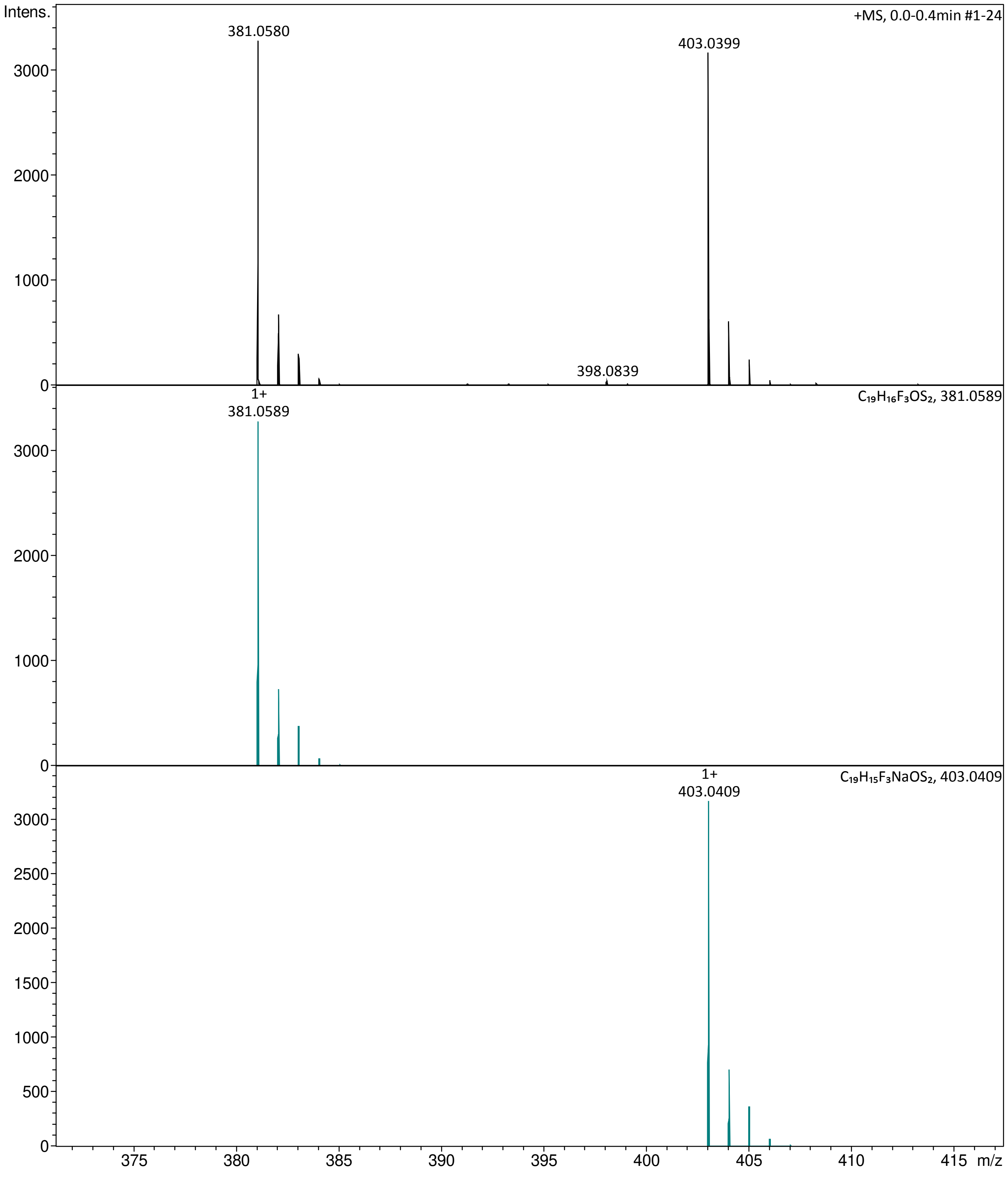

Mass Spectrum SmartFormula Report

Analysis Info

Analysis Name	D:\MZ\maXis_data\temp\64015000001.d	Acquisition Date	20/05/2019 15:19:27
Method	tune_low_MS_Service_05_19.m	Operator	msc
Sample Name	PN-12 spot 2 peak 1	Instrument	maXis 255552.00016
Comment	Predrag Kalaba<br>Ergebnis: +/- 5ppm<br>ACN / MeOH + 1% H2O		

Acquisition Parameter

Source Type	ESI	Ion Polarity	Positive	Set Nebulizer	0.4 Bar
Focus	Not active	Set Capillary	4500 V	Set Dry Heater	150 °C
Scan Begin	50 m/z	Set End Plate Offset	-500 V	Set Dry Gas	4.0 l/min
Scan End	1900 m/z	Set Charging Voltage	0 V	Set Divert Valve	Source
		Set Corona	0 nA	Set APCI Heater	0 °C

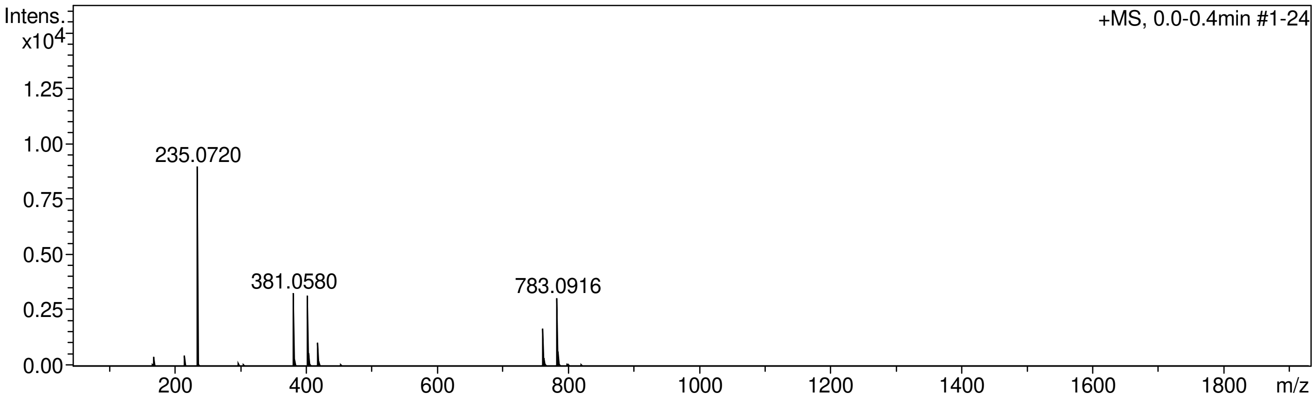

Meas. m/z	#	Ion Formula	Score	m/z	err [mDa]	err [ppm]	mSigma	rdb	e⁻ Conf	N-Rule
381.0580	1	C14H14FN6O2S2	53.36	381.0598	-1.8	-4.8	10.6	10.5	even	ok
	2	C17H10FN6O2S	50.73	381.0564	1.5	4.0	12.1	15.5	even	ok
	3	C19H13F4O2S	59.26	381.0567	1.3	3.4	12.1	11.5	even	ok
	4	C19H16F3OS2	89.22	381.0589	-0.9	-2.4	13.9	10.5	even	ok
	5	C14H11F2N6O3S	88.86	381.0576	0.4	1.0	16.5	11.5	even	ok
	6	C16H14F5O3S	98.87	381.0578	-0.2	-0.4	16.5	7.5	even	ok
	7	C21H14FO4S	58.02	381.0591	1.1	3.0	18.0	14.5	even	ok
	8	C14H16F7S2	85.94	381.0576	-0.4	-1.0	18.6	3.5	even	ok
	9	C12H13F4N6S2	76.49	381.0574	-0.6	-1.6	18.6	7.5	even	ok
	10	C13H18FN2O6S2	100.00	381.0585	0.5	1.3	19.4	5.5	even	ok
	11	C13H15F2N2O7S	34.17	381.0563	-1.7	-4.6	24.5	6.5	even	ok
	12	C22H15F2S2	96.53	381.0578	0.2	0.6	26.7	14.5	even	ok
	13	C14H19F2N2O2S3	65.03	381.0571	-0.9	-2.3	29.9	5.5	even	ok
	14	C13H15F6O4S	48.55	381.0590	1.0	2.6	30.0	3.5	even	ok
	15	C14H22FN2OS4	28.71	381.0594	1.4	3.6	49.2	4.5	even	ok
403.0399	1	C14H10F2N6NaO3S	90.02	403.0395	-0.4	-0.9	8.0	11.5	even	ok
	2	C16H13F5NaO3S	100.00	403.0398	0.1	0.3	8.0	7.5	even	ok
	3	C17H9FN6NaO2S	43.45	403.0384	1.5	3.7	12.8	15.5	even	ok
	4	C19H12F4NaO2S	50.67	403.0386	1.3	3.1	12.8	11.5	even	ok
	5	C14H13FN6NaO2S2	32.49	403.0418	-1.9	-4.6	14.5	10.5	even	ok
	6	C14H15F7NaS2	77.23	403.0396	0.3	0.8	16.6	3.5	even	ok
	7	C12H12F4N6NaS2	68.84	403.0393	0.6	1.4	16.6	7.5	even	ok
	8	C13H14F2N2NaO7S	35.25	403.0382	1.7	4.2	16.6	6.5	even	ok
	9	C13H17FN2NaO6S2	66.25	403.0404	0.5	1.3	19.5	5.5	even	ok
	10	C19H15F3NaOS2	50.11	403.0409	1.0	2.4	21.8	10.5	even	ok
	11	C13H14F6NaO4S	48.49	403.0409	1.0	2.5	21.8	3.5	even	ok
	12	C11H11F3N6NaO4S	55.38	403.0407	0.8	1.9	21.8	7.5	even	ok
	13	C21H13FNaO4S	42.80	403.0411	1.2	2.9	23.2	14.5	even	ok
	14	C11H16F4N2NaO4S2	25.48	403.0380	1.9	4.7	24.4	2.5	even	ok
	15	C11H16F8NaOS2	45.89	403.0407	0.8	2.0	29.4	-0.5	even	ok
	16	C11H13F9NaO2S	28.82	403.0385	-1.4	-3.5	33.6	0.5	even	ok
	17	C22H14F2NaS2	55.05	403.0397	0.2	0.4	34.4	14.5	even	ok
	18	C14H18F2N2NaO2S3	40.09	403.0391	0.8	2.1	34.5	5.5	even	ok
	19	C11H19F3N2NaO3S3	48.39	403.0402	0.3	0.8	37.0	1.5	even	ok

Mass Spectrum SmartFormula Report

Meas. m/z	#	Ion Formula	Score	m/z	err [mDa]	err [ppm]	mSigma	rdb	e⁻ Conf	N-Rule
	20	C17H17FN2NaOS3	17.35	403.0379	2.0	4.9	38.2	9.5	even	ok
	21	C14H21FN2NaOS4	15.96	403.0413	1.4	3.5	54.7	4.5	even	ok

Figure S60. HRESIMS spectrum of compound **8o**

Generic Display Report

Analysis Info

Analysis Name	D:\MZ\maXis_data\temp\64016000001.d	Acquisition Date	20/05/2019 15:22:44
Method	tune_low_MS_Service_05_19.m	Operator	msc
Sample Name	PN-12 spot 2 peak 2	Instrument	maXis
Comment	Predrag Kalaba<br>Ergebnis: +/- 5ppm<br>ACN / MeOH + 1% H2O		

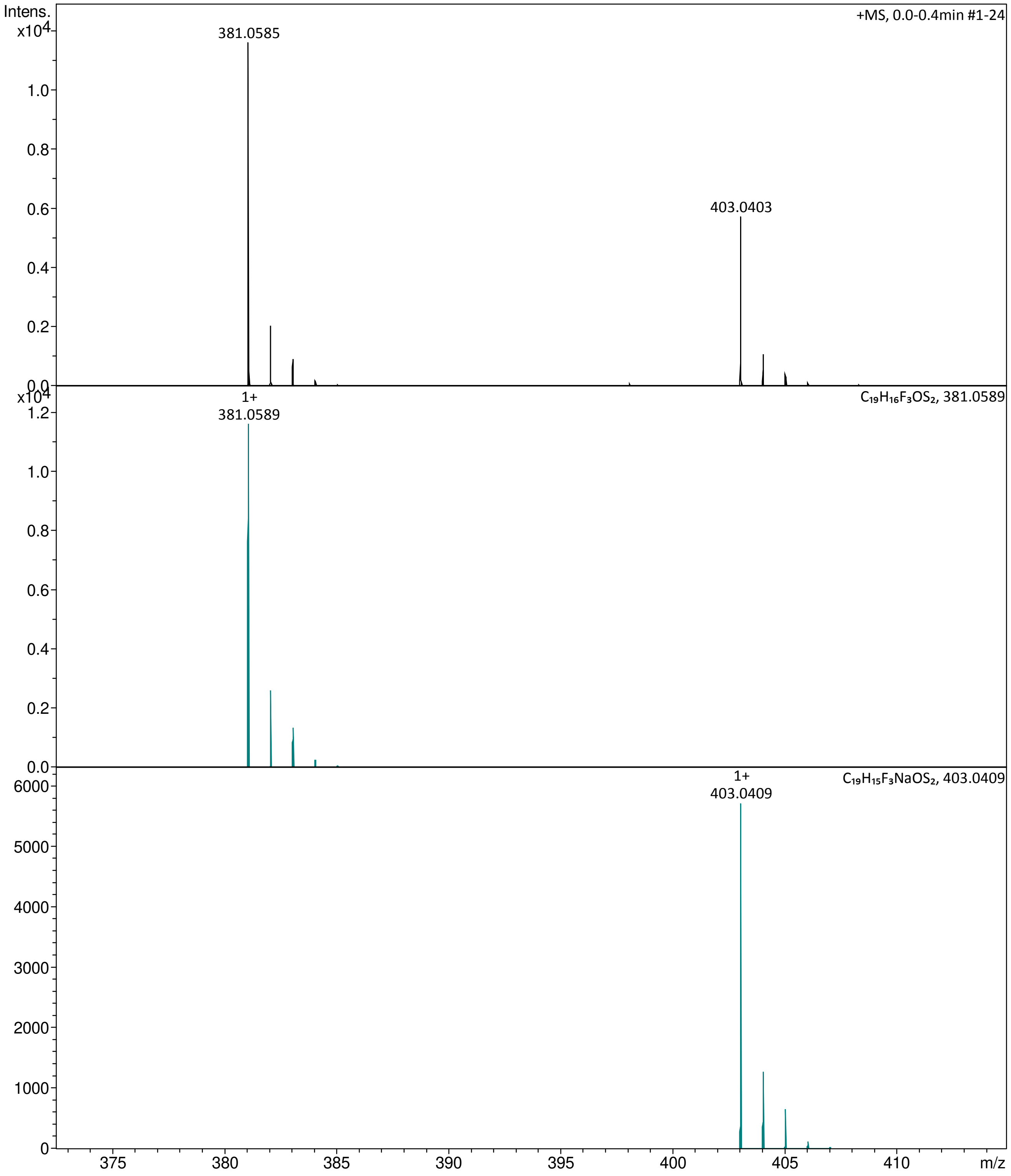

Mass Spectrum SmartFormula Report

Analysis Info

Acquisition Date 20/05/2019 15:22:44

Analysis Name	D:\MZ\maXis_data\temp\64016000001.d
Method	tune_low_MS_Service_05_19.m
Sample Name	PN-12 spot 2 peak 2
Comment	Predrag Kalaba<br>Ergebnis: +/- 5ppm<br>ACN / MeOH + 1% H2O

Operator msc
Instrument maXis 255552.00016

Acquisition Parameter

Source Type	ESI	Ion Polarity	Positive	Set Nebulizer	0.4 Bar
Focus	Not active	Set Capillary	4500 V	Set Dry Heater	150 °C
Scan Begin	50 m/z	Set End Plate Offset	-500 V	Set Dry Gas	4.0 l/min
Scan End	1900 m/z	Set Charging Voltage	0 V	Set Divert Valve	Source
		Set Corona	0 nA	Set APCI Heater	0 °C

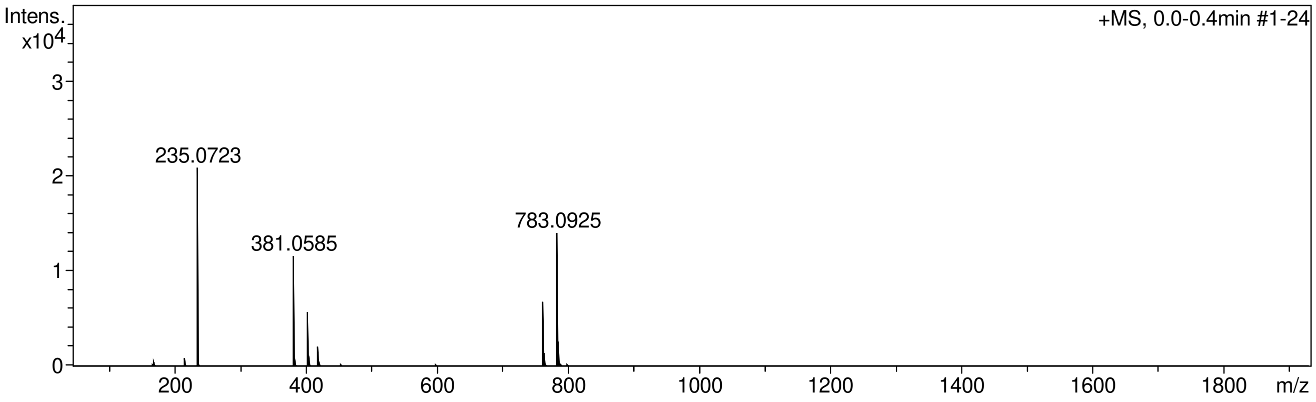

Meas. m/z	#	Ion Formula	Score	m/z	err [mDa]	err [ppm]	mSigma	rdb	e⁻ Conf	N-Rule
381.0585	1	C14H11F2N6O3S	64.20	381.0576	0.9	2.5	6.6	11.5	even	ok
	2	C16H14F5O3S	73.04	381.0578	-0.7	-1.9	6.6	7.5	even	ok
	3	C12H8F3N10S	42.58	381.0601	1.5	4.0	9.4	12.5	even	ok
	4	C14H11F6N4S	36.01	381.0603	1.8	4.7	9.4	8.5	even	ok
	5	C14H16F7S2	57.19	381.0576	-0.9	-2.4	13.5	3.5	even	ok
	6	C12H13F4N6S2	49.72	381.0574	1.2	3.1	13.6	7.5	even	ok
	7	C10H10FN12S2	42.75	381.0571	1.4	3.7	13.6	11.5	even	ok
	8	C13H15F6O4S	72.19	381.0590	0.4	1.1	14.4	3.5	even	ok
	9	C11H12F3N6O4S	80.45	381.0587	0.2	0.5	14.4	7.5	even	ok
	10	C18H15F2O5S	33.25	381.0603	1.7	4.6	14.9	10.5	even	ok
	11	C16H17F4O2S2	46.50	381.0601	-1.5	-4.0	16.7	6.5	even	ok
	12	C14H14FN6O2S2	54.30	381.0598	1.3	3.4	16.8	10.5	even	ok
	13	C13H18FN2O6S2	100.00	381.0585	-0.1	-0.1	17.6	5.5	even	ok
	14	C19H13F4O2S	27.70	381.0567	-1.8	-4.9	19.8	11.5	even	ok
	15	C11H17F8OS2	66.55	381.0588	-0.2	-0.6	23.0	-0.5	even	ok
	16	C10H19F2N2O7S2	52.15	381.0596	1.1	2.9	24.1	1.5	even	ok
	17	C10H16F3N2O8S	39.80	381.0574	1.1	3.0	24.7	2.5	even	ok
	18	C19H16F3OS2	70.09	381.0589	0.4	1.0	27.3	10.5	even	ok
	19	C10H16F7O5S	26.82	381.0601	1.6	4.1	29.7	-0.5	even	ok
	20	C21H14FO4S	46.44	381.0591	0.6	1.6	30.9	14.5	even	ok
	21	C11H20F3N2O3S3	60.67	381.0583	0.3	0.7	35.1	1.5	even	ok
	22	C14H19F2N2O2S3	32.34	381.0571	-1.4	-3.7	35.4	5.5	even	ok
	23	C22H15F2S2	41.24	381.0578	0.8	2.0	40.6	14.5	even	ok
	24	C14H22FN2OS4	25.35	381.0594	0.8	2.1	56.0	4.5	even	ok
403.0403	1	C14H10F2N6NaO3S	88.36	403.0395	-0.8	-2.0	4.4	11.5	even	ok
	2	C16H13F5NaO3S	100.00	403.0398	-0.6	-1.4	4.4	7.5	even	ok
	3	C12H7F3N10NaS	47.94	403.0420	1.7	4.1	9.1	12.5	even	ok
	4	C14H10F6N4NaS	40.30	403.0423	-1.9	-4.7	9.2	8.5	even	ok
	5	C18H14F2NaO5S	40.15	403.0422	-1.9	-4.6	10.8	10.5	even	ok
	6	C17H9FN6NaO2S	34.63	403.0384	-2.0	-4.9	15.3	15.5	even	ok
	7	C19H12F4NaO2S	41.25	403.0386	1.7	4.3	15.3	11.5	even	ok
	8	C14H15F7NaS2	69.46	403.0396	0.8	2.0	17.8	3.5	even	ok
	9	C12H12F4N6NaS2	60.73	403.0393	-1.0	-2.6	17.9	7.5	even	ok
	10	C13H14F6NaO4S	77.43	403.0409	0.6	1.4	17.9	3.5	even	ok

Mass Spectrum SmartFormula Report

Meas. m/z	#	Ion Formula	Score	m/z	err [mDa]	err [ppm]	mSigma	rdb	e⁻ Conf	N-Rule
	11	C11H11F3N6NaO4S	86.75	403.0407	-0.3	-0.8	17.9	7.5	even	ok
	12	C16H16F4NaO2S2	40.64	403.0420	-1.7	-4.1	18.0	6.5	even	ok
	13	C14H13FN6NaO2S2	47.75	403.0418	-1.4	-3.5	18.0	10.5	even	ok
	14	C13H17FN2NaO6S2	90.33	403.0404	-0.1	-0.2	21.2	5.5	even	ok
	15	C19H15F3NaOS2	66.53	403.0409	-0.5	-1.3	26.1	10.5	even	ok
	16	C21H13FNaO4S	58.87	403.0411	0.7	1.8	26.7	14.5	even	ok
	17	C11H16F8NaOS2	68.09	403.0407	0.4	0.9	28.3	-0.5	even	ok
	18	C11H13F9NaO2S	26.80	403.0385	1.9	4.6	29.6	0.5	even	ok
	19	C14H18F2N2NaO2S3	32.74	403.0391	-1.3	-3.2	37.6	5.5	even	ok
	20	C22H14F2NaS2	46.15	403.0397	-0.6	-1.6	38.5	14.5	even	ok
	21	C11H19F3N2NaO3S3	57.42	403.0402	0.1	0.3	38.8	1.5	even	ok
	22	C14H21FN2NaOS4	21.69	403.0413	0.9	2.4	58.0	4.5	even	ok

Figure S61. HRESIMS spectrum of compound **5p**

Generic Display Report

Analysis Info

Analysis Name	D:\Data\Kalaba\72423000001.d	Acquisition Date	20/08/2020 17:45:02
Method	tune_low_MS_Service_08_20.m	Operator	msc
Sample Name	PN-2-1-1-b2	Instrument	maXis
Comment	Kalaba/Zehl<br>Ergebnis +/- 5ppm<br>ACN / MeOH + 1% H2O		

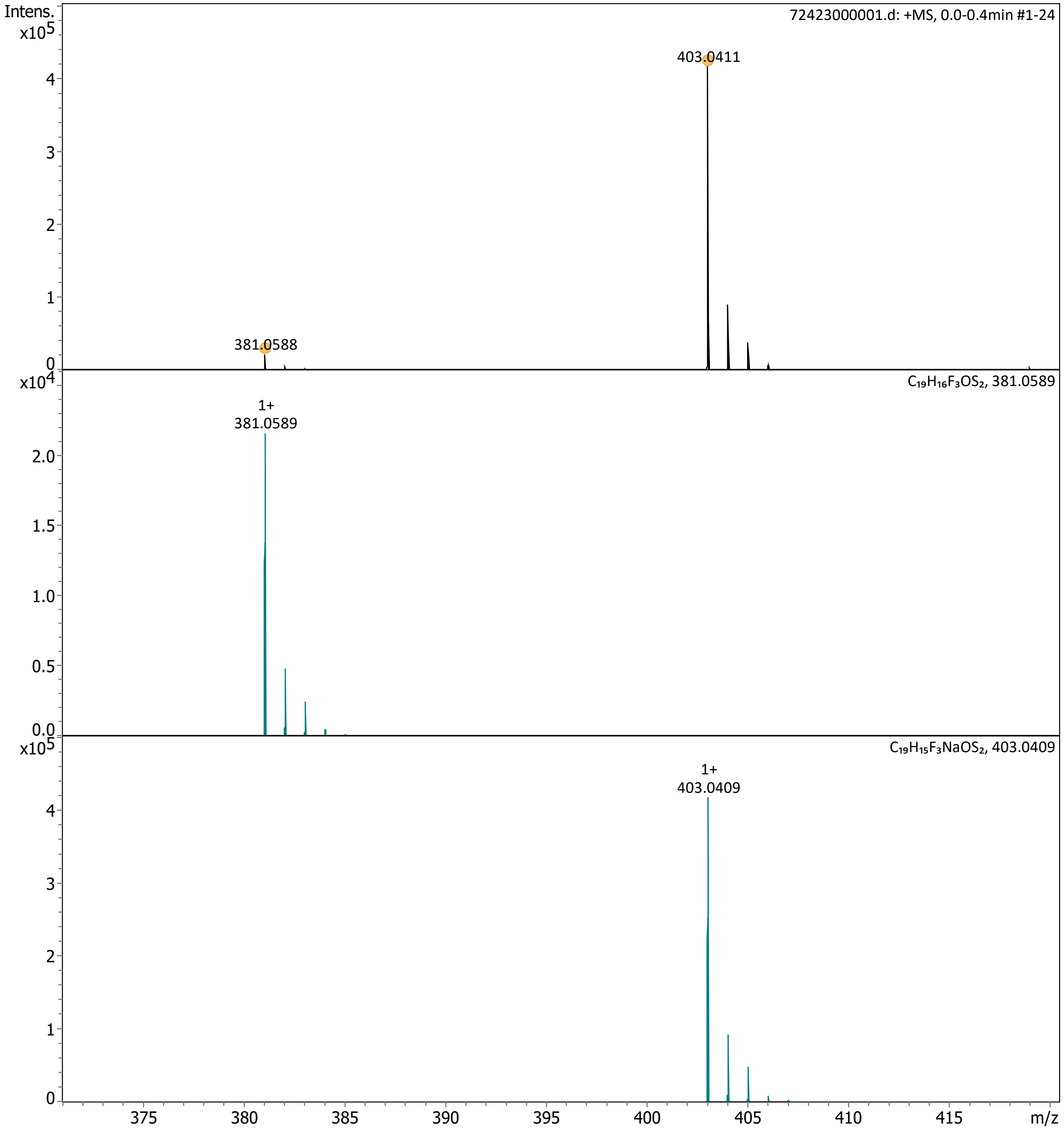

Mass Spectrum SmartFormula Report

Analysis Info

Acquisition Date 20/08/2020 17:45:02

Analysis Name D:\Data\Kalaba\72423000001.d
Method tune_low_MS_Service_08_20.m
Sample Name PN-2-1-1-b2
Comment Kalaba/Zehl
Ergebnis +/- 5ppm
ACN / MeOH + 1% H2O

Operator msc
Instrument maXis 255552.00016

Acquisition Parameter

Source Type	ESI	Ion Polarity	Positive	Set Nebulizer	0.4 Bar
Focus	Not active	Set Capillary	4200 V	Set Dry Heater	180 Â°C
Scan Begin	50 m/z	Set End Plate Offset	-500 V	Set Dry Gas	4.0 l/min
Scan End	1900 m/z	Set Charging Voltage	0 V	Set Divert Valve	Source
		Set Corona	0 nA	Set APCI Heater	0 Â°C

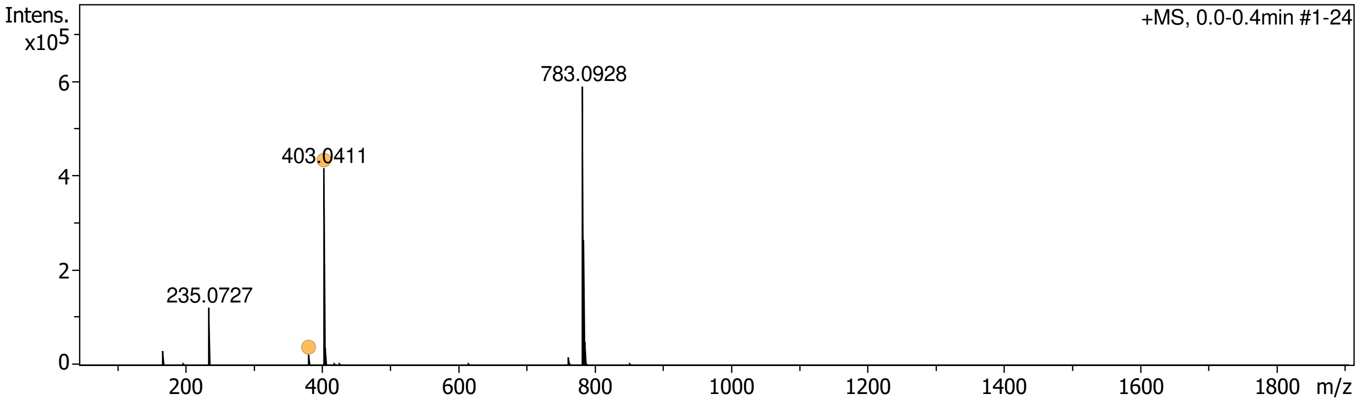

Meas. m/z	#	Ion Formula	m/z	err [ppm]	mSigma	# mSigma	Score	rdb	eÂ¯ Conf	N-Rule
381.0588	1	C18H15F2O5S	381.0603	3.8	12.1	1	52.15	13.0	even	ok
	2	C19H16F3OS2	381.0589	0.2	15.7	2	100.00	15.0	even	ok
	3	C17H13N6OS2	381.0587	-0.4	15.7	3	97.42	19.0	even	ok
	4	C21H14FO4S	381.0591	0.8	16.0	4	90.39	17.0	even	ok
	5	C16H17F4O2S2	381.0601	3.2	16.4	5	54.98	11.0	even	ok
	6	C14H14FN6O2S2	381.0598	2.6	16.4	6	63.33	15.0	even	ok
	7	C16H17N2O5S2	381.0573	-3.9	17.1	7	45.99	14.0	even	ok
	8	C14H11F2N6O3S	381.0576	-3.2	21.0	8	49.74	14.0	even	ok
	9	C13H18FN2O6S2	381.0585	-0.9	26.3	9	71.07	10.0	even	ok
	10	C22H10FN4S	381.0605	4.3	26.5	10	33.71	22.0	even	ok
	11	C22H15F2S2	381.0578	-2.8	27.6	11	47.80	19.0	even	ok
	12	C24H13O3S	381.0580	-2.2	29.8	12	51.18	21.0	even	ok
	13	C14H19F2N2O2S3	381.0571	-4.5	31.9	13	35.43	12.0	even	ok
	14	C20H13O8	381.0605	4.4	32.1	14	21.76	15.0	even	ok
	15	C13H21N2O5S3	381.0607	4.9	35.4	15	28.51	11.0	even	ok
	16	C21H11F2O5	381.0569	-5.0	35.5	16	16.55	16.0	even	ok
	17	C16H9N6O6	381.0578	-2.7	37.8	17	28.25	16.0	even	ok
	18	C18H12F3O6	381.0580	-2.0	37.9	18	32.24	12.0	even	ok
	19	C17H5N10O2	381.0591	0.8	39.1	19	39.18	21.0	even	ok
	20	C19H8F3N4O2	381.0594	1.5	39.1	20	34.94	17.0	even	ok
	21	C22H7F2N4O	381.0582	-1.5	42.6	21	31.27	21.0	even	ok
	22	C14H6FN10O3	381.0603	3.8	43.5	22	18.46	17.0	even	ok
	23	C16H9F4N4O3	381.0605	4.5	43.5	23	15.66	13.0	even	ok
	24	C13H10FN6O7	381.0590	0.3	47.5	24	33.61	12.0	even	ok
	25	C15H13F4O7	381.0592	1.0	47.5	25	30.23	8.0	even	ok
	26	C14H22FN2OS4	381.0594	1.4	50.3	26	42.85	13.0	even	ok
	27	C17H21N2S4	381.0582	-1.6	52.5	27	38.15	17.0	even	ok
	28	C25H6FN4	381.0571	-4.5	52.6	28	11.71	25.0	even	ok
403.0411	1	C18H14F2NaO5S	403.0422	2.7	9.0	1	64.41	13.0	even	ok

Mass Spectrum SmartFormula Report

Meas. m/z	#	Ion Formula	m/z	err [ppm]	mSigma	# mSigma	Score	rdb	eÂ¯ Conf	N-Rule
	2	C21H13FNaO4S	403.0411	-0.1	12.8	2	100.00	17.0	even	ok
	3	C19H15F3NaOS2	403.0409	-0.7	13.5	3	90.06	15.0	even	ok
	4	C17H12N6NaOS2	403.0406	-1.3	13.5	4	80.53	19.0	even	ok
	5	C16H16F4NaO2S2	403.0420	2.2	15.6	5	64.16	11.0	even	ok
	6	C14H13FN6NaO2S2	403.0418	1.6	15.6	6	72.74	15.0	even	ok
	7	C16H16N2NaO5S2	403.0393	-4.6	16.1	7	34.00	14.0	even	ok
	8	C14H10F2N6NaO3S	403.0395	-4.0	19.8	8	37.66	14.0	even	ok
	9	C13H12N6NaO6S	403.0431	4.9	22.6	9	26.65	13.0	even	ok
	10	C22H9FN4NaS	403.0424	3.2	24.3	10	41.87	22.0	even	ok
	11	C22H14F2NaS2	403.0397	-3.5	25.7	11	37.31	19.0	even	ok
	12	C13H17FN2NaO6S2	403.0404	-1.8	26.4	12	55.37	10.0	even	ok
	13	C24H12NaO3S	403.0399	-3.0	27.7	13	40.76	21.0	even	ok
	14	C20H12NaO8	403.0424	3.2	29.5	14	27.42	15.0	even	ok
	15	C19H18F2NaS3	403.0431	4.9	32.8	15	26.84	16.0	even	ok
	16	C16H8N6NaO6	403.0398	-3.4	35.6	16	22.44	16.0	even	ok
	17	C18H11F3NaO6	403.0400	-2.8	35.6	17	26.02	12.0	even	ok
	18	C13H20N2NaO5S3	403.0427	3.8	35.7	18	33.99	11.0	even	ok
	19	C17H4N10NaO2	403.0411	-0.1	36.5	19	43.99	21.0	even	ok
	20	C19H7F3N4NaO2	403.0413	0.5	36.5	20	41.20	17.0	even	ok
	21	C22H6F2N4NaO	403.0402	-2.3	40.1	21	25.86	21.0	even	ok
	22	C14H5FN10NaO3	403.0422	2.7	41.4	22	22.84	17.0	even	ok
	23	C16H8F4N4NaO3	403.0425	3.3	41.4	23	19.70	13.0	even	ok
	24	C13H9FN6NaO7	403.0409	-0.6	45.9	24	31.21	12.0	even	ok
	25	C15H12F4NaO7	403.0411	0.0	45.9	25	34.42	8.0	even	ok
	26	C14H21FN2NaOS4	403.0413	0.4	50.4	26	46.88	13.0	even	ok
	27	C17H20N2NaS4	403.0402	-2.4	52.2	27	29.67	17.0	even	ok

Figure S62. HRESIMS spectrum of compound **6p**

Generic Display Report

Analysis Info

Analysis Name	D:\Data\Kalaba\72424000001.d	Acquisition Date	20/08/2020 17:50:07
Method	tune_low_MS_Service_08_20.m	Operator	msc
Sample Name	PN-2-1-2-b2	Instrument	maXis
Comment	Kalaba/Zehl<br>Ergebnis +/- 5ppm<br>ACN / MeOH + 1% H2O		

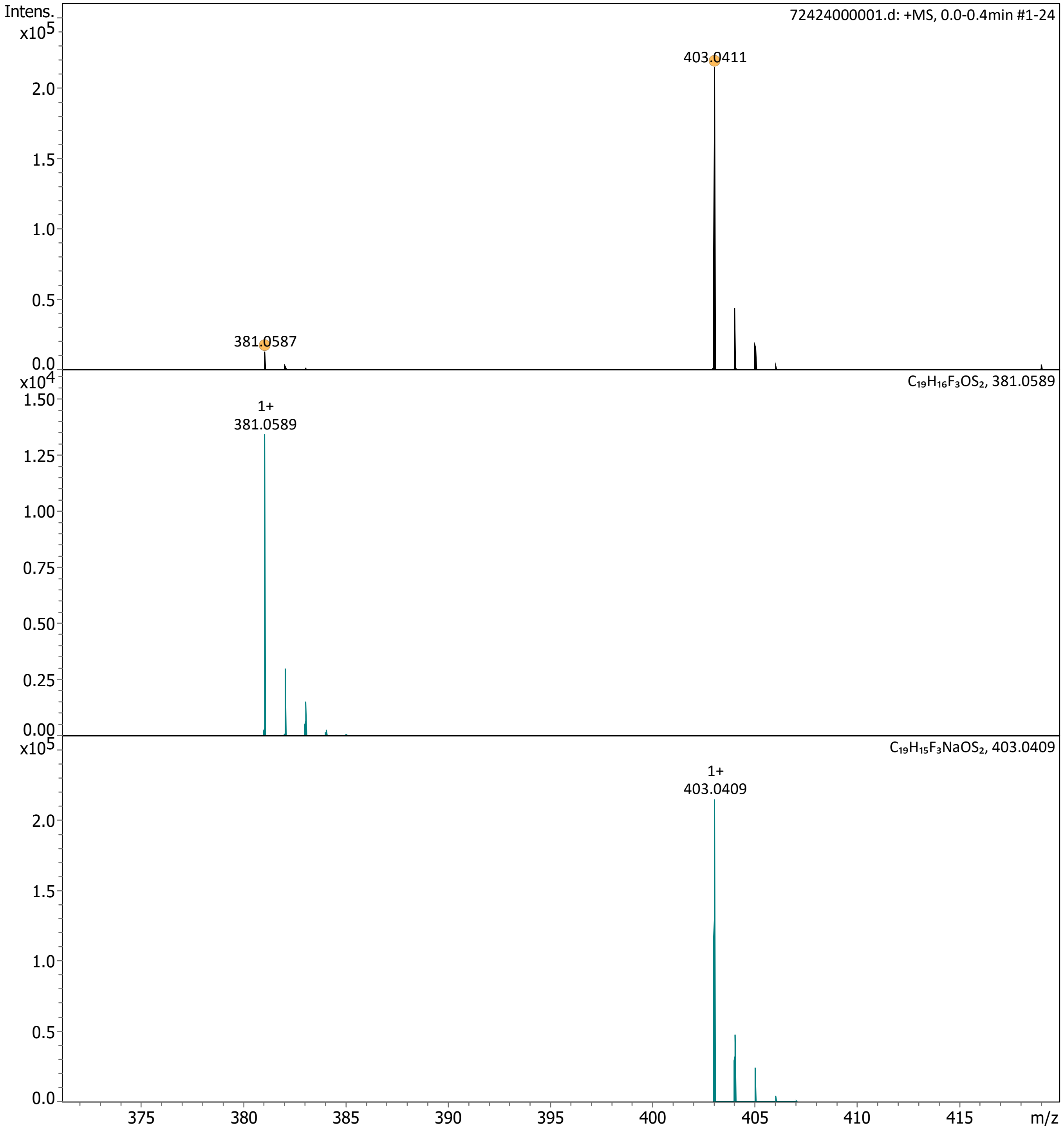

Mass Spectrum SmartFormula Report

Analysis Info

Analysis Name	D:\Data\Kalaba\72424000001.d	Acquisition Date	20/08/2020 17:50:07
Method	tune_low_MS_Service_08_20.m	Operator	msc
Sample Name	PN-2-1-2-b2	Instrument	maXis 255552.00016
Comment	Kalaba/Zehl<br>Ergebnis +/- 5ppm<br>ACN / MeOH + 1% H2O		

Acquisition Parameter

Source Type	ESI	Ion Polarity	Positive	Set Nebulizer	0.4 Bar
Focus	Not active	Set Capillary	4200 V	Set Dry Heater	180 Â°C
Scan Begin	50 m/z	Set End Plate Offset	-500 V	Set Dry Gas	4.0 l/min
Scan End	1900 m/z	Set Charging Voltage	0 V	Set Divert Valve	Source
		Set Corona	0 nA	Set APCI Heater	0 Â°C

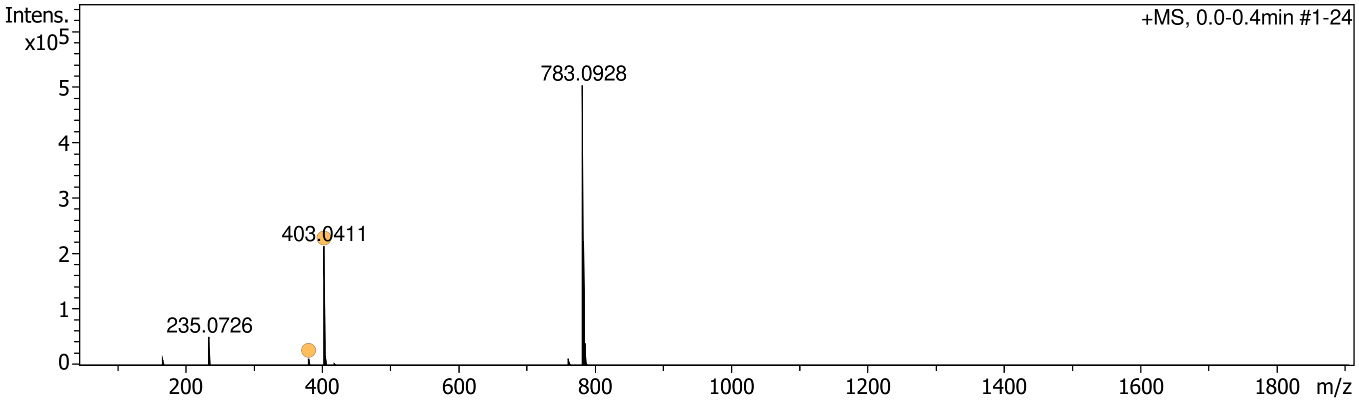

Meas. m/z	#	Ion Formula	m/z	err [ppm]	mSigma	# mSigma	Score	rdb	eÂ¯ Conf	N-Rule
381.0587	1	C21H14FO4S	381.0591	1.1	13.3	1	85.30	17.0	even	ok
	2	C19H16F3OS2	381.0589	0.5	14.0	2	92.88	15.0	even	ok
	3	C17H13N6OS2	381.0587	-0.1	14.0	3	100.00	19.0	even	ok
	4	C18H15F2O5S	381.0603	4.1	15.3	4	42.68	13.0	even	ok
	5	C16H17N2O5S2	381.0573	-3.6	18.9	5	45.23	14.0	even	ok
	6	C16H17F4O2S2	381.0601	3.5	19.7	6	45.01	11.0	even	ok
	7	C14H14FN6O2S2	381.0598	2.9	19.7	7	52.13	15.0	even	ok
	8	C22H10FN4S	381.0605	4.6	22.5	8	32.05	22.0	even	ok
	9	C22H15F2S2	381.0578	-2.5	23.3	9	53.38	19.0	even	ok
	10	C24H13O3S	381.0580	-1.9	25.3	10	57.33	21.0	even	ok
	11	C14H11F2N6O3S	381.0576	-2.9	25.6	11	45.58	14.0	even	ok
	12	C14H19F2N2O2S3	381.0571	-4.2	33.1	12	35.32	12.0	even	ok
	13	C20H13O8	381.0605	4.7	33.3	13	18.32	15.0	even	ok
	14	C21H11F2O5	381.0569	-4.7	35.9	14	16.94	16.0	even	ok
	15	C17H5N10O2	381.0591	1.1	40.3	15	33.92	21.0	even	ok
	16	C19H8F3N4O2	381.0594	1.8	40.3	16	30.10	17.0	even	ok
	17	C16H9N6O6	381.0578	-2.4	41.0	17	26.22	16.0	even	ok
	18	C18H12F3O6	381.0580	-1.7	41.0	18	29.77	12.0	even	ok
	19	C22H7F2N4O	381.0582	-1.2	41.0	19	32.73	21.0	even	ok
	20	C14H6FN10O3	381.0603	4.1	47.0	20	14.54	17.0	even	ok
	21	C16H9F4N4O3	381.0605	4.8	47.0	21	12.27	13.0	even	ok
	22	C25H6FN4	381.0571	-4.2	49.1	22	13.40	25.0	even	ok
	23	C14H22FN2OS4	381.0594	1.7	50.3	23	38.17	13.0	even	ok
	24	C17H21N2S4	381.0582	-1.3	51.2	24	39.83	17.0	even	ok
	25	C15H13F4O7	381.0592	1.3	52.2	25	23.39	8.0	even	ok
403.0411	1	C18H14F2NaO5S	403.0422	2.8	5.1	1	71.32	13.0	even	ok
	2	C14H10F2N6NaO3S	403.0395	-3.9	14.7	2	45.16	14.0	even	ok
	3	C16H16F4NaO2S2	403.0420	2.2	15.1	3	66.94	11.0	even	ok
	4	C14H13FN6NaO2S2	403.0418	1.7	15.2	4	76.02	15.0	even	ok

Meas. m/z	#	Ion Formula	m/z	err [ppm]	mSigma	# mSigma	Score	rdb	eÂ¯ Conf	N-Rule
	5	C21H13FNaO4S	403.0411	-0.0	16.2	5	100.00	17.0	even	ok
	6	C16H16N2NaO5S2	403.0393	-4.5	17.5	6	35.73	14.0	even	ok
	7	C13H12N6NaO6S	403.0431	5.0	17.5	7	30.51	13.0	even	ok
	8	C19H15F3NaOS2	403.0409	-0.6	17.9	8	88.21	15.0	even	ok
	9	C17H12N6NaOS2	403.0406	-1.2	17.9	9	78.99	19.0	even	ok
	10	C13H17FN2NaO6S2	403.0404	-1.7	24.4	10	62.21	10.0	even	ok
	11	C20H12NaO8	403.0424	3.3	27.3	11	29.84	15.0	even	ok
	12	C22H9FN4NaS	403.0424	3.3	27.9	12	39.69	22.0	even	ok
	13	C22H14F2NaS2	403.0397	-3.4	30.9	13	35.57	19.0	even	ok
	14	C16H8N6NaO6	403.0398	-3.3	31.2	14	26.99	16.0	even	ok
	15	C18H11F3NaO6	403.0400	-2.7	31.3	15	31.25	12.0	even	ok
	16	C24H12NaO3S	403.0399	-2.9	32.0	16	39.59	21.0	even	ok
	17	C14H18F2N2NaO2S3	403.0391	-5.0	33.1	17	26.60	12.0	even	ok
	18	C17H4N10NaO2	403.0411	-0.0	34.1	18	50.03	21.0	even	ok
	19	C19H7F3N4NaO2	403.0413	0.6	34.1	19	45.52	17.0	even	ok
	20	C19H18F2NaS3	403.0431	4.9	36.3	20	25.21	16.0	even	ok
	21	C13H20N2NaO5S3	403.0427	3.9	36.4	21	34.41	11.0	even	ok
	22	C14H5FN10NaO3	403.0422	2.8	36.6	22	26.82	17.0	even	ok
	23	C16H8F4N4NaO3	403.0425	3.4	36.6	23	23.10	13.0	even	ok
	24	C13H9FN6NaO7	403.0409	-0.5	40.2	24	39.21	12.0	even	ok
	25	C15H12F4NaO7	403.0411	0.1	40.2	25	42.02	8.0	even	ok
	26	C22H6F2N4NaO	403.0402	-2.3	40.5	26	27.45	21.0	even	ok
	27	C14H21FN2NaOS4	403.0413	0.5	52.4	27	45.84	13.0	even	ok
	28	C25H5FN4Na	403.0390	-5.1	52.8	28	8.97	25.0	even	ok
	29	C17H20N2NaS4	403.0402	-2.3	55.2	29	28.99	17.0	even	ok

Figure S63. HRESIMS spectrum of compound **7p**

Generic Display Report

Analysis Info

Analysis Name	D:\Data\Kalaba\72425000001.d	Acquisition Date	20/08/2020 17:54:56
Method	tune_low_MS_Service_08_20.m	Operator	msc
Sample Name	PN-2-2-1-b2	Instrument	maXis
Comment	Kalaba/Zehl<br>Ergebnis +/- 5ppm<br>ACN / MeOH + 1% H2O		

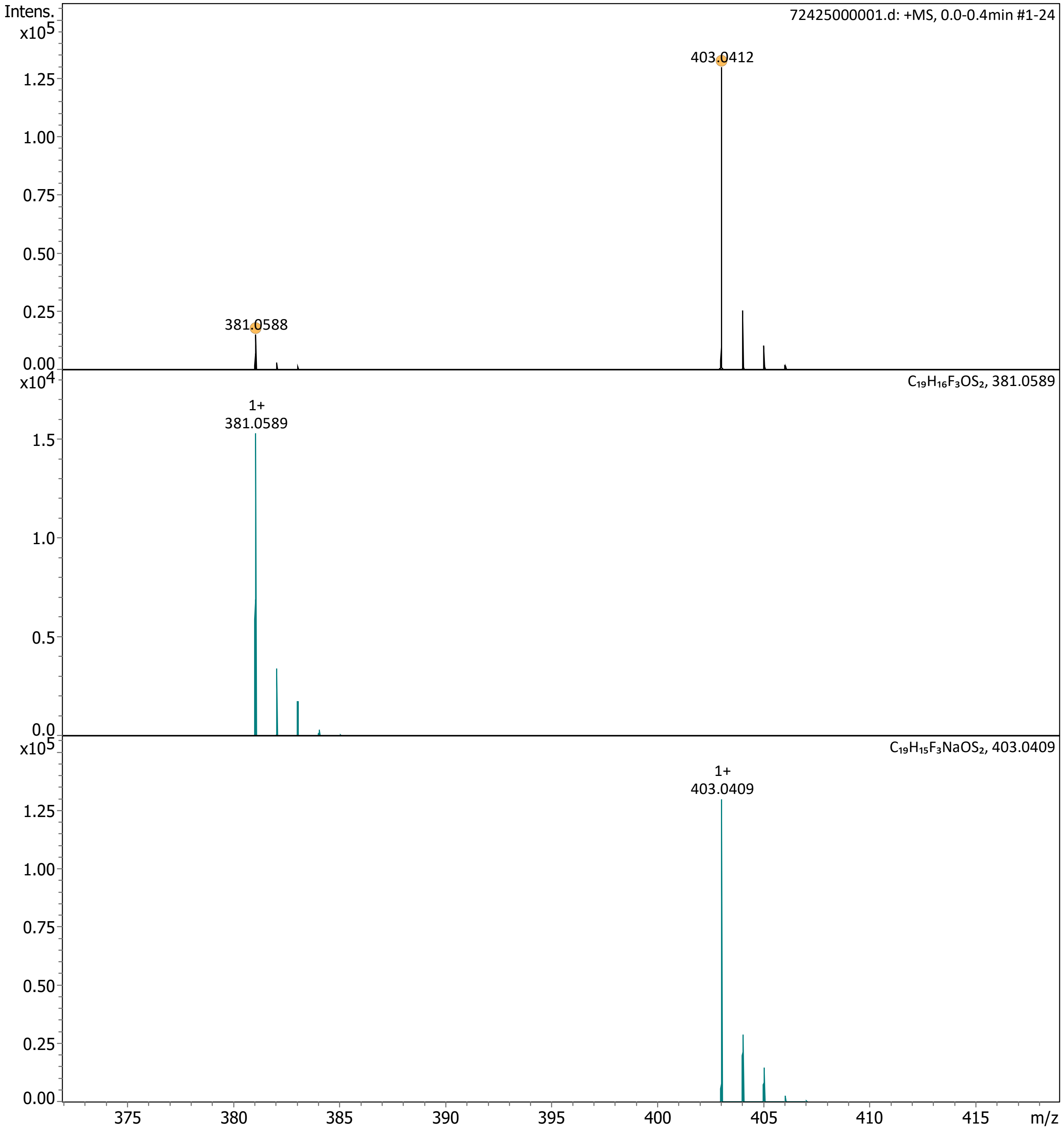

Mass Spectrum SmartFormula Report

Analysis Info

Analysis Name	D:\Data\Kaluba\72425000001.d	Acquisition Date	20/08/2020 17:54:56
Method	tune_low_MS_Service_08_20.m	Operator	msc
Sample Name	PN-2-2-1-b2	Instrument	maXis 255552.00016
Comment	Kalaba/Zehl<br>Ergebnis +/- 5ppm<br>ACN / MeOH + 1% H2O		

Acquisition Parameter

Source Type	ESI	Ion Polarity	Positive	Set Nebulizer	0.4 Bar
Focus	Not active	Set Capillary	4200 V	Set Dry Heater	180 Â°C
Scan Begin	50 m/z	Set End Plate Offset	-500 V	Set Dry Gas	4.0 l/min
Scan End	1900 m/z	Set Charging Voltage	0 V	Set Divert Valve	Source
		Set Corona	0 nA	Set APCI Heater	0 Â°C

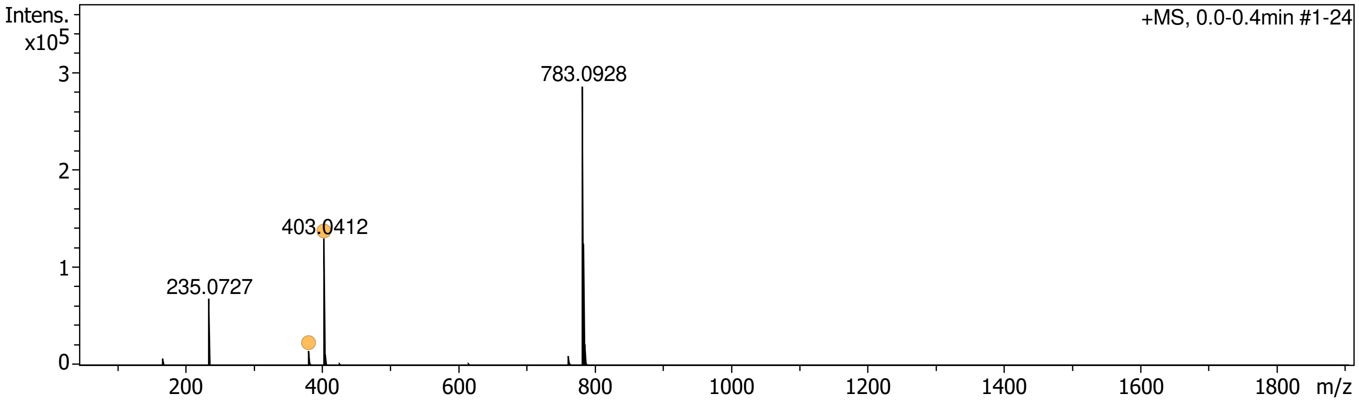

Meas. m/z	#	Ion Formula	m/z	err [ppm]	mSigma	# mSigma	Score	rdb	eÂ¯ Conf	N-Rule
381.0588	1	C19H16F3OS2	381.0589	0.3	13.8	1	100.00	15.0	even	ok
	2	C17H13N6OS2	381.0587	-0.3	13.8	2	99.63	19.0	even	ok
	3	C18H15F2O5S	381.0603	3.9	14.7	3	47.43	13.0	even	ok
	4	C21H14FO4S	381.0591	0.9	14.9	4	88.97	17.0	even	ok
	5	C16H17N2O5S2	381.0573	-3.8	17.4	5	45.36	14.0	even	ok
	6	C16H17F4O2S2	381.0601	3.3	17.8	6	51.16	11.0	even	ok
	7	C14H14FN6O2S2	381.0598	2.7	17.8	7	59.00	15.0	even	ok
	8	C14H11F2N6O3S	381.0576	-3.2	24.4	8	45.72	14.0	even	ok
	9	C22H15F2S2	381.0578	-2.7	24.5	9	50.80	19.0	even	ok
	10	C22H10FN4S	381.0605	4.4	24.5	10	33.72	22.0	even	ok
	11	C24H13O3S	381.0580	-2.1	27.3	11	53.66	21.0	even	ok
	12	C14H19F2N2O2S3	381.0571	-4.4	31.8	12	35.31	12.0	even	ok
	13	C20H13O8	381.0605	4.4	33.9	13	19.86	15.0	even	ok
	14	C21H11F2O5	381.0569	-5.0	36.8	14	15.90	16.0	even	ok
	15	C16H9N6O6	381.0578	-2.6	40.8	15	25.79	16.0	even	ok
	16	C18H12F3O6	381.0580	-2.0	40.8	16	29.40	12.0	even	ok
	17	C17H5N10O2	381.0591	0.9	40.9	17	35.85	21.0	even	ok
	18	C19H8F3N4O2	381.0594	1.5	40.9	18	31.94	17.0	even	ok
	19	C22H7F2N4O	381.0582	-1.5	42.7	19	30.81	21.0	even	ok
	20	C14H6FN10O3	381.0603	3.9	46.6	20	16.12	17.0	even	ok
	21	C16H9F4N4O3	381.0605	4.5	46.7	21	13.66	13.0	even	ok
	22	C14H22FN2OS4	381.0594	1.4	49.4	22	42.39	13.0	even	ok
	23	C17H21N2S4	381.0582	-1.6	50.8	23	39.72	17.0	even	ok
	24	C15H13F4O7	381.0592	1.0	51.2	24	25.97	8.0	even	ok
	25	C25H6FN4	381.0571	-4.5	51.3	25	12.11	25.0	even	ok
403.0412	1	C18H14F2NaO5S	403.0422	2.6	5.6	1	83.68	13.0	even	ok
	2	C14H10F2N6NaO3S	403.0395	-4.1	10.9	2	51.43	14.0	even	ok
	3	C13H12N6NaO6S	403.0431	4.8	13.4	3	39.91	13.0	even	ok
	4	C16H16F4NaO2S2	403.0420	2.0	14.9	4	79.40	11.0	even	ok

Meas. m/z	#	Ion Formula	m/z	err [ppm]	mSigma	# mSigma	Score	rdb	eÂ¯ Conf	N-Rule
	5	C14H13FN6NaO2S2	403.0418	1.4	14.9	5	89.83	15.0	even	ok
	6	C12H7F3N10NaS	403.0420	2.1	16.6	6	76.25	15.0	even	ok
	7	C12H12F4N6NaS2	403.0393	-4.6	18.0	7	38.31	12.0	even	ok
	8	C16H16N2NaO5S2	403.0393	-4.7	18.5	8	36.86	14.0	even	ok
	9	C21H13FNaO4S	403.0411	-0.3	20.1	9	100.00	17.0	even	ok
	10	C19H15F3NaOS2	403.0409	-0.8	21.1	10	88.97	15.0	even	ok
	11	C17H12N6NaOS2	403.0406	-1.4	21.2	11	79.40	19.0	even	ok
	12	C13H17FN2NaO6S2	403.0404	-1.9	22.4	12	70.00	10.0	even	ok
	13	C12H16N2NaO10S	403.0418	1.5	24.1	13	73.21	8.0	even	ok
	14	C20H12NaO8	403.0424	3.1	27.7	14	35.09	15.0	even	ok
	15	C16H8N6NaO6	403.0398	-3.6	29.4	15	30.00	16.0	even	ok
	16	C18H11F3NaO6	403.0400	-3.0	29.4	16	34.87	12.0	even	ok
	17	C22H9FN4NaS	403.0424	3.0	31.8	17	42.91	22.0	even	ok
	18	C17H4N10NaO2	403.0411	-0.2	34.2	18	54.10	21.0	even	ok
	19	C14H5FN10NaO3	403.0422	2.6	34.2	19	33.84	17.0	even	ok
	20	C16H8F4N4NaO3	403.0425	3.2	34.2	20	29.25	13.0	even	ok
	21	C19H7F3N4NaO2	403.0413	0.4	34.2	21	53.01	17.0	even	ok
	22	C22H14F2NaS2	403.0397	-3.6	34.9	22	34.14	19.0	even	ok
	23	C24H12NaO3S	403.0399	-3.1	36.1	23	38.01	21.0	even	ok
	24	C13H20N2NaO5S3	403.0427	3.6	36.3	24	41.15	11.0	even	ok
	25	C13H9FN6NaO7	403.0409	-0.7	36.4	25	46.97	12.0	even	ok
	26	C15H12F4NaO7	403.0411	-0.1	36.4	26	52.02	8.0	even	ok
	27	C19H18F2NaS3	403.0431	4.7	38.6	27	28.49	16.0	even	ok
	28	C12H4F4N10NaO	403.0398	-3.5	42.4	28	21.87	14.0	even	ok
	29	C22H6F2N4NaO	403.0402	-2.5	42.8	29	27.56	21.0	even	ok
	30	C12H13FN2NaO11	403.0396	-4.0	42.9	30	18.48	7.0	even	ok
	31	C14H21FN2NaOS4	403.0413	0.3	53.2	31	52.11	13.0	even	ok
	32	C17H20N2NaS4	403.0402	-2.6	57.0	32	29.28	17.0	even	ok

Figure S64. HRESIMS spectrum of compound **8p**

Generic Display Report

Analysis Info

Analysis Name	D:\Data\Kalaba\72426000001.d	Acquisition Date	20/08/2020 18:02:57
Method	tune_low_MS_Service_08_20.m	Operator	msc
Sample Name	PN-2-2-2-b2	Instrument	maXis
Comment	Kalaba/Zehl<br>Ergebnis +/- 5ppm<br>ACN / MeOH + 1% H2O		

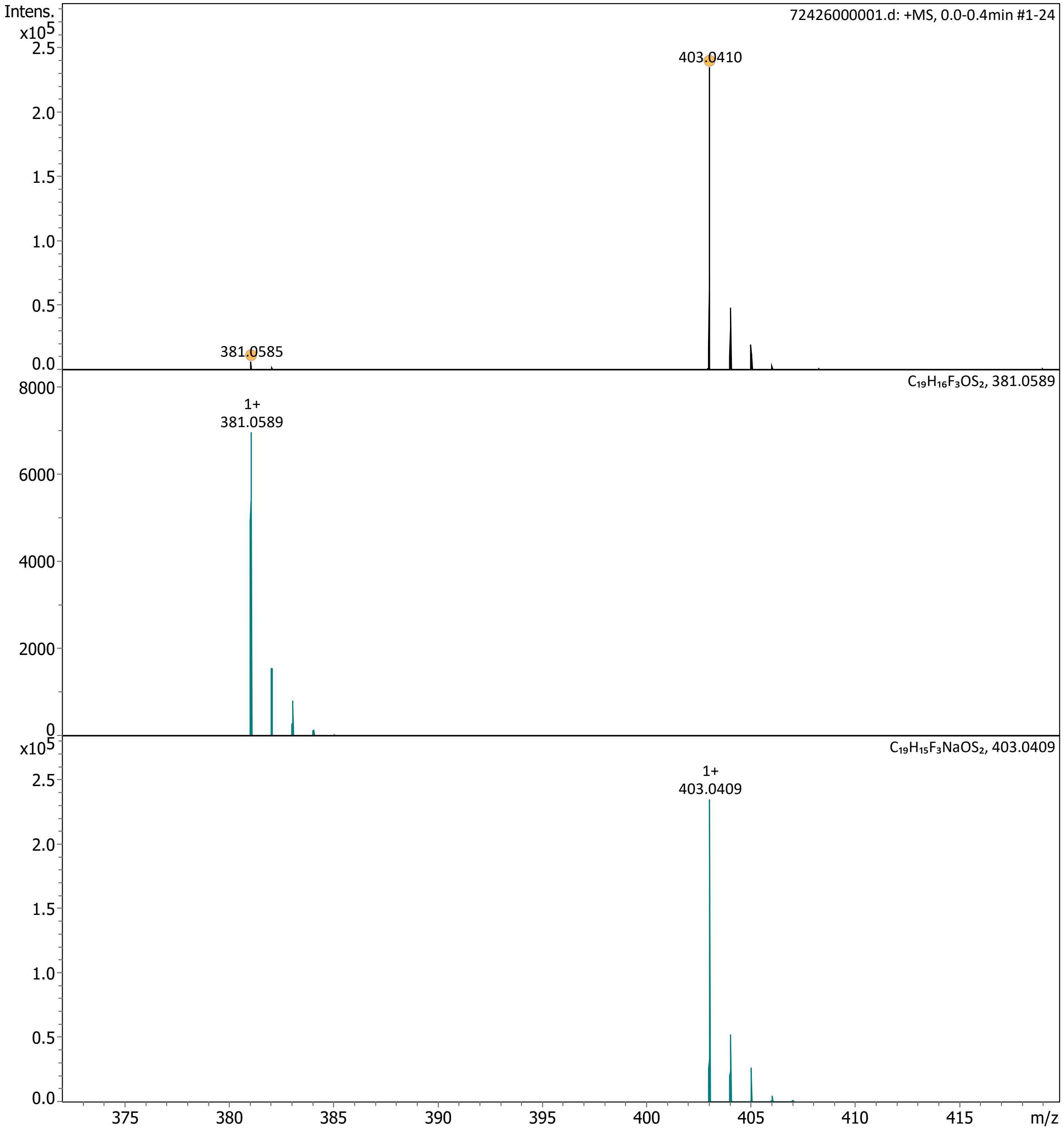

Mass Spectrum SmartFormula Report

Analysis Info

Analysis Name	D:\Data\Kalaba\72426000001.d	Acquisition Date	20/08/2020 18:02:57
Method	tune_low_MS_Service_08_20.m	Operator	msc
Sample Name	PN-2-2-2-b2	Instrument	maXis 255552.00016
Comment	Kalaba/Zehl<br>Ergebnis +/- 5ppm<br>ACN / MeOH + 1% H2O		

Acquisition Parameter

Source Type	ESI	Ion Polarity	Positive	Set Nebulizer	0.4 Bar
Focus	Not active	Set Capillary	4200 V	Set Dry Heater	180 Â°C
Scan Begin	50 m/z	Set End Plate Offset	-500 V	Set Dry Gas	4.0 l/min
Scan End	1900 m/z	Set Charging Voltage	0 V	Set Divert Valve	Source
		Set Corona	0 nA	Set APCI Heater	0 Â°C

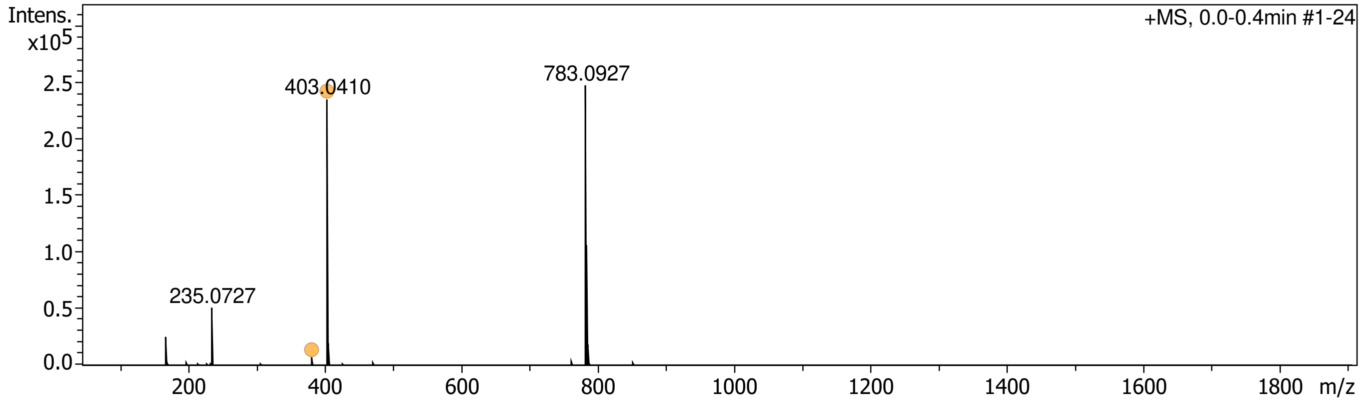

Meas. m/z	#	Ion Formula	m/z	err [ppm]	mSigma	# mSigma	Score	rdb	eÂ¯ Conf	N-Rule
381.0585	1	C19H16F3OS2	381.0589	1.2	13.2	1	89.69	15.0	even	ok
	2	C17H13N6OS2	381.0587	0.6	13.2	2	100.00	19.0	even	ok
	3	C21H14FO4S	381.0591	1.8	13.4	3	80.30	17.0	even	ok
	4	C19H13F4O2S	381.0567	-4.6	17.6	4	37.85	14.0	even	ok
	5	C18H15F2O5S	381.0603	4.8	18.5	5	35.75	13.0	even	ok
	6	C16H17N2O5S2	381.0573	-2.9	20.0	6	55.01	14.0	even	ok
	7	C22H15F2S2	381.0578	-1.8	20.4	7	69.45	19.0	even	ok
	8	C16H17F4O2S2	381.0601	4.2	21.7	8	38.92	11.0	even	ok
	9	C24H13O3S	381.0580	-1.2	23.1	9	72.81	21.0	even	ok
	10	C21H11F2O5	381.0569	-4.1	37.8	10	20.45	16.0	even	ok
	11	C22H7F2N4O	381.0582	-0.6	41.7	11	38.39	21.0	even	ok
	12	C17H5N10O2	381.0591	1.8	42.7	12	29.88	21.0	even	ok
	13	C19H8F3N4O2	381.0594	2.4	42.7	13	26.24	17.0	even	ok
	14	C16H9N6O6	381.0578	-1.7	44.3	14	29.09	16.0	even	ok
	15	C18H12F3O6	381.0580	-1.1	44.3	15	32.68	12.0	even	ok
	16	C25H6FN4	381.0571	-3.6	48.2	16	17.34	25.0	even	ok
	17	C17H21N2S4	381.0582	-0.6	49.7	17	49.96	17.0	even	ok
	18	C15H4F3N10	381.0567	-4.6	50.6	18	12.30	18.0	even	ok
	19	C15H13F4O7	381.0592	1.9	56.1	19	19.42	8.0	even	ok
403.0410	1	C18H14F2NaO5S	403.0422	3.1	5.2	1	67.95	13.0	even	ok
	2	C14H10F2N6NaO3S	403.0395	-3.5	14.9	2	51.12	14.0	even	ok
	3	C16H16F4NaO2S2	403.0420	2.6	15.1	3	64.26	11.0	even	ok
	4	C14H13FN6NaO2S2	403.0418	2.0	15.2	4	73.40	15.0	even	ok
	5	C21H13FNaO4S	403.0411	0.3	16.0	5	100.00	17.0	even	ok
	6	C16H16N2NaO5S2	403.0393	-4.2	17.4	6	40.95	14.0	even	ok
	7	C19H15F3NaOS2	403.0409	-0.2	17.7	7	97.40	15.0	even	ok
	8	C17H12N6NaOS2	403.0406	-0.8	17.7	8	87.70	19.0	even	ok
	9	C13H17FN2NaO6S2	403.0404	-1.3	24.5	9	69.02	10.0	even	ok
	10	C20H12NaO8	403.0424	3.7	27.3	10	28.29	15.0	even	ok

Mass Spectrum SmartFormula Report

Meas. m/z	#	Ion Formula	m/z	err [ppm]	mSigma	# mSigma	Score	rdb	eÂ¯ Conf	N-Rule
	11	C22H9FN4NaS	403.0424	3.6	27.8	11	37.84	22.0	even	ok
	12	C22H14F2NaS2	403.0397	-3.1	30.7	12	40.44	19.0	even	ok
	13	C16H8N6NaO6	403.0398	-3.0	31.4	13	30.40	16.0	even	ok
	14	C18H11F3NaO6	403.0400	-2.4	31.4	14	34.98	12.0	even	ok
	15	C24H12NaO3S	403.0399	-2.5	31.8	15	44.73	21.0	even	ok
	16	C14H18F2N2NaO2S3	403.0391	-4.7	33.0	16	30.65	12.0	even	ok
	17	C17H4N10NaO2	403.0411	0.3	34.1	17	49.34	21.0	even	ok
	18	C19H7F3N4NaO2	403.0413	0.9	34.2	18	44.34	17.0	even	ok
	19	C13H20N2NaO5S3	403.0427	4.2	36.3	19	32.49	11.0	even	ok
	20	C14H5FN10NaO3	403.0422	3.2	36.8	20	25.48	17.0	even	ok
	21	C16H8F4N4NaO3	403.0425	3.8	36.8	21	21.81	13.0	even	ok
	22	C13H9FN6NaO7	403.0409	-0.2	40.4	22	42.85	12.0	even	ok
	23	C15H12F4NaO7	403.0411	0.4	40.4	23	40.93	8.0	even	ok
	24	C22H6F2N4NaO	403.0402	-1.9	40.5	24	30.72	21.0	even	ok
	25	C14H21FN2NaOS4	403.0413	0.8	52.3	25	44.84	13.0	even	ok
	26	C25H5FN4Na	403.0390	-4.8	52.7	26	10.37	25.0	even	ok
	27	C17H20N2NaS4	403.0402	-2.0	55.1	27	32.55	17.0	even	ok

Figure S65. HRESIMS spectrum of compound **5q**

Generic Display Report

Analysis Info

Analysis Name	D:\MZ\maXis_data\temp\64017000001.d	Acquisition Date	20/05/2019 14:29:36
Method	tune_low_MS_Service_05_19.m	Operator	msc
Sample Name	PN-10 spot 1 peak 1	Instrument	maXis
Comment	Predrag Kalaba<br>Ergebnis: +/- 5ppm<br>ACN / MeOH + 1% H2O		

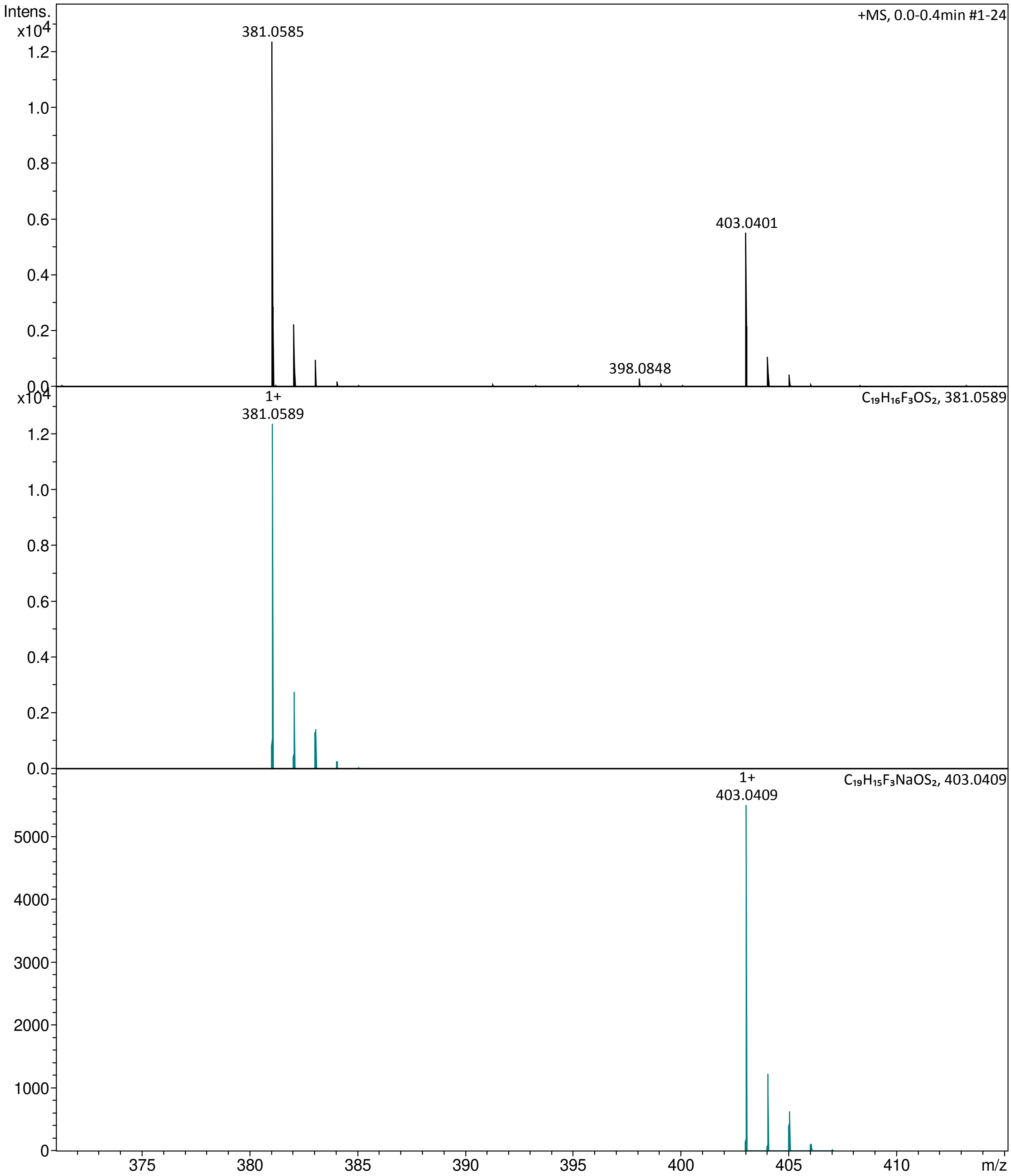

Mass Spectrum SmartFormula Report

Analysis Info

Analysis Name	D:\MZ\maXis_data\temp\64017000001.d	Acquisition Date	20/05/2019 14:29:36
Method	tune_low_MS_Service_05_19.m	Operator	msc
Sample Name	PN-10 spot 1 peak 1	Instrument	maXis 255552.00016
Comment	Predrag Kalaba<br>Ergebnis: +/- 5ppm<br>ACN / MeOH + 1% H2O		

Acquisition Parameter

Source Type	ESI	Ion Polarity	Positive	Set Nebulizer	0.4 Bar
Focus	Not active	Set Capillary	4500 V	Set Dry Heater	150 °C
Scan Begin	50 m/z	Set End Plate Offset	-500 V	Set Dry Gas	4.0 l/min
Scan End	1900 m/z	Set Charging Voltage	0 V	Set Divert Valve	Source
		Set Corona	0 nA	Set APCI Heater	0 °C

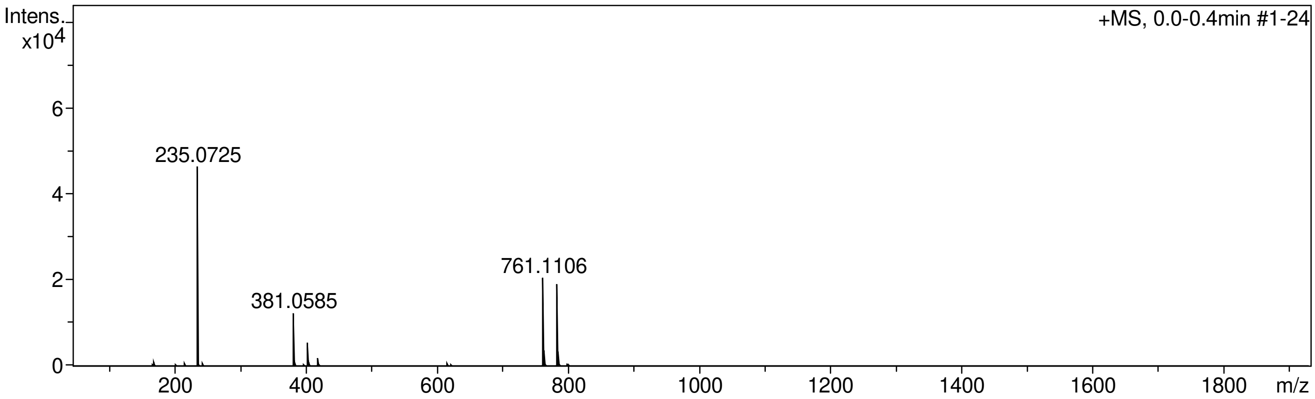

Meas. m/z	#	Ion Formula	Score	m/z	err [mDa]	err [ppm]	mSigma	rdb	e⁻ Conf	N-Rule
381.0585	1	C14H11F2N6O3S	84.18	381.0576	-0.9	-2.3	5.7	11.5	even	ok
	2	C16H14F5O3S	95.52	381.0578	0.6	1.7	5.7	7.5	even	ok
	3	C12H8F3N10S	50.14	381.0601	-1.6	-4.2	10.0	12.5	even	ok
	4	C14H11F6N4S	42.28	381.0603	1.8	4.8	10.0	8.5	even	ok
	5	C18H15F2O5S	41.49	381.0603	-1.8	-4.7	12.4	10.5	even	ok
	6	C14H16F7S2	72.49	381.0576	0.9	2.3	14.4	3.5	even	ok
	7	C12H13F4N6S2	63.19	381.0574	1.1	2.9	14.5	7.5	even	ok
	8	C16H17F4O2S2	44.93	381.0601	1.6	4.2	16.0	6.5	even	ok
	9	C14H14FN6O2S2	52.62	381.0598	1.3	3.5	16.0	10.5	even	ok
	10	C13H15F6O4S	83.13	381.0590	0.5	1.3	16.7	3.5	even	ok
	11	C11H12F3N6O4S	92.88	381.0587	0.3	0.7	16.7	7.5	even	ok
	12	C19H13F4O2S	38.11	381.0567	-1.8	-4.7	17.3	11.5	even	ok
	13	C13H18FN2O6S2	100.00	381.0585	-0.0	-0.0	18.3	5.5	even	ok
	14	C11H17F8OS2	76.49	381.0588	-0.3	-0.7	25.2	-0.5	even	ok
	15	C19H16F3OS2	70.35	381.0589	0.4	1.2	25.6	10.5	even	ok
	16	C21H14FO4S	59.47	381.0591	0.7	1.7	28.3	14.5	even	ok
	17	C14H19F2N2O2S3	33.65	381.0571	1.4	3.5	35.3	5.5	even	ok
	18	C11H20F3N2O3S3	60.83	381.0583	0.2	0.5	35.9	1.5	even	ok
	19	C22H15F2S2	44.79	381.0578	-0.7	-1.8	38.7	14.5	even	ok
	20	C14H22FN2OS4	24.47	381.0594	-0.9	-2.3	55.9	4.5	even	ok
403.0401	1	C14H10F2N6NaO3S	89.23	403.0395	-0.6	-1.4	7.4	11.5	even	ok
	2	C16H13F5NaO3S	100.00	403.0398	-0.3	-0.8	7.4	7.5	even	ok
	3	C17H9FN6NaO2S	41.02	403.0384	1.7	4.3	12.4	15.5	even	ok
	4	C19H12F4NaO2S	48.31	403.0386	1.5	3.7	12.5	11.5	even	ok
	5	C12H7F3N10NaS	35.40	403.0420	1.9	4.7	12.9	12.5	even	ok
	6	C16H16F4NaO2S2	33.80	403.0420	-1.9	-4.7	15.6	6.5	even	ok
	7	C14H13FN6NaO2S2	40.16	403.0418	-1.7	-4.1	15.6	10.5	even	ok
	8	C13H14F2N2NaO7S	32.78	403.0382	-1.9	-4.7	16.6	6.5	even	ok
	9	C14H15F7NaS2	74.11	403.0396	0.5	1.4	17.6	3.5	even	ok
	10	C12H12F4N6NaS2	65.47	403.0393	-0.8	-2.0	17.7	7.5	even	ok
	11	C13H17FN2NaO6S2	77.72	403.0404	0.3	0.8	20.5	5.5	even	ok
	12	C13H14F6NaO4S	59.51	403.0409	0.8	2.0	21.6	3.5	even	ok
	13	C11H11F3N6NaO4S	67.34	403.0407	-0.6	-1.4	21.6	7.5	even	ok
	14	C19H15F3NaOS2	60.08	403.0409	-0.8	-1.9	22.6	10.5	even	ok

Mass Spectrum SmartFormula Report

Meas. m/z	#	Ion Formula	Score	m/z	err [mDa]	err [ppm]	mSigma	rdb	e^- Conf	N-Rule
	15	C21H13FNaO4S	52.68	403.0411	1.0	2.4	23.2	14.5	even	ok
	16	C11H16F8NaOS2	54.74	403.0407	0.6	1.5	30.1	-0.5	even	ok
	17	C11H13F9NaO2S	27.24	403.0385	1.6	4.1	33.4	0.5	even	ok
	18	C22H14F2NaS2	53.60	403.0397	-0.4	-1.0	34.9	14.5	even	ok
	19	C14H18F2N2NaO2S3	37.52	403.0391	-1.0	-2.6	35.6	5.5	even	ok
	20	C11H19F3N2NaO3S3	56.00	403.0402	-0.1	-0.3	38.0	1.5	even	ok
	21	C14H21FN2NaOS4	19.12	403.0413	1.2	2.9	55.7	4.5	even	ok

Figure S66. HRESIMS spectrum of compound **6q**

Generic Display Report

Analysis Info

Analysis Name	D:\MZ\maXis_data\temp\64018000001.d
Method	tune_low_MS_Service_05_19.m
Sample Name	PN-10 spot 1 peak 2
Comment	Predrag Kalaba<br>Ergebnis: +/- 5ppm<br>ACN / MeOH + 1% H2O
Acquisition Date	20/05/2019 14:33:22
Operator	msc
Instrument	maXis

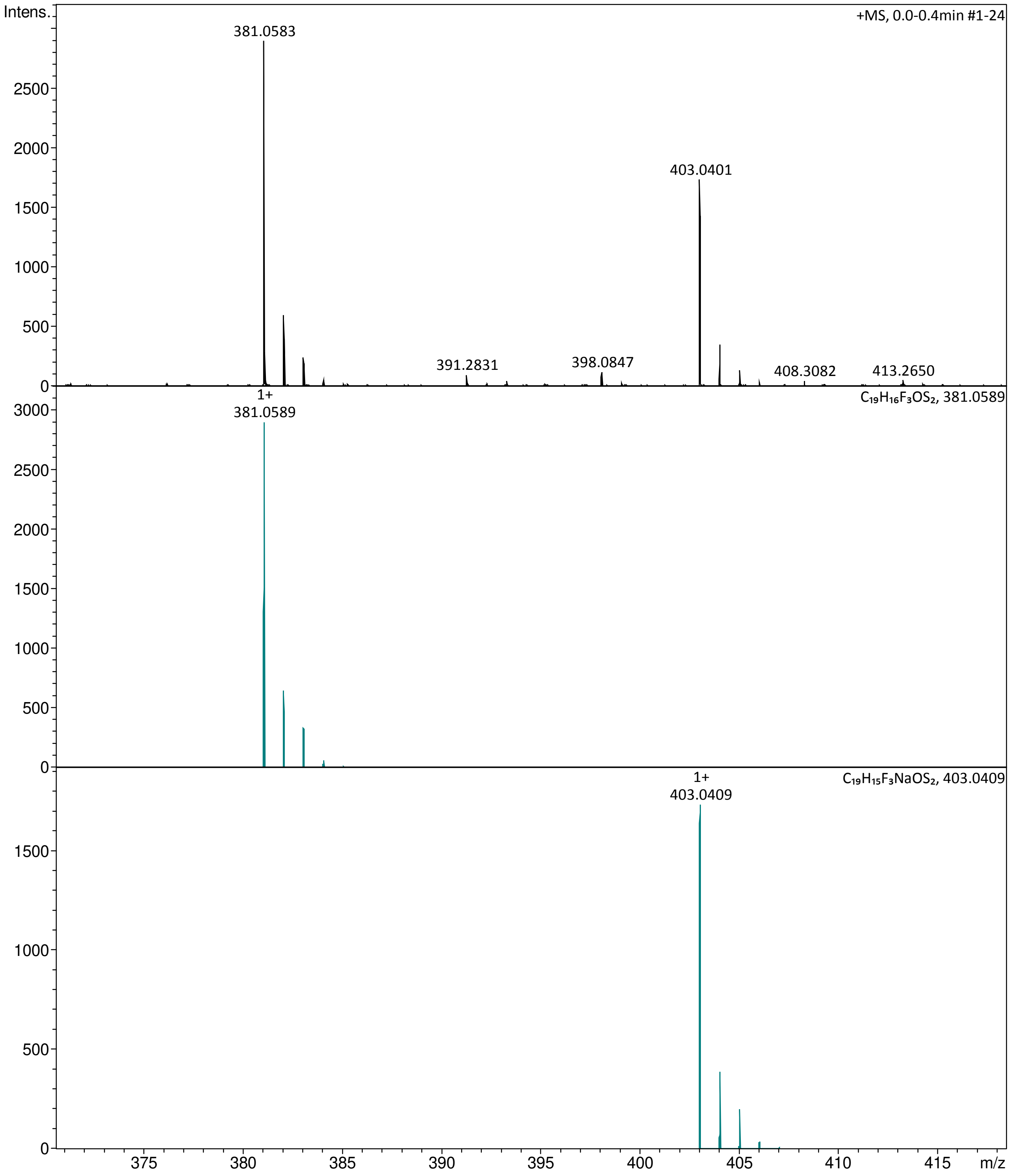

Mass Spectrum SmartFormula Report

Analysis Info

Acquisition Date 20/05/2019 14:33:22

Analysis Name D:\MZ\maXis_data\temp\64018000001.d
Method tune_low_MS_Service_05_19.m
Sample Name PN-10 spot 1 peak 2
Comment Predrag Kalaba
Ergebnis: +/- 5ppm
ACN / MeOH + 1% H2O

Operator msc
Instrument maXis 255552.00016

Acquisition Parameter

Source Type	ESI	Ion Polarity	Positive	Set Nebulizer	0.4 Bar
Focus	Not active	Set Capillary	4500 V	Set Dry Heater	150 °C
Scan Begin	50 m/z	Set End Plate Offset	-500 V	Set Dry Gas	4.0 l/min
Scan End	1900 m/z	Set Charging Voltage	0 V	Set Divert Valve	Source
		Set Corona	0 nA	Set APCI Heater	0 °C

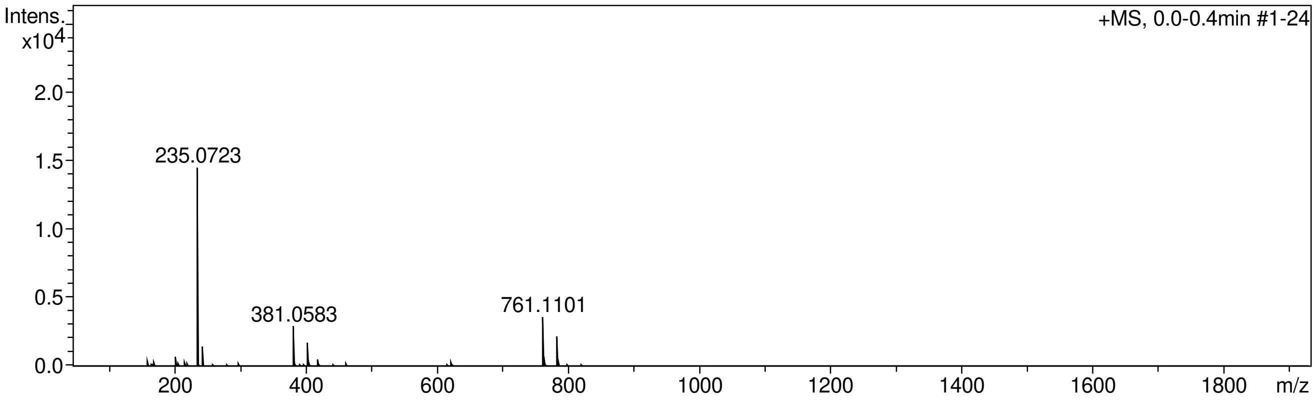

Meas. m/z	#	Ion Formula	Score	m/z	err [mDa]	err [ppm]	mSigma	rdb	e⁻ Conf	N-Rule
381.0583	1	C17H10FN6O2S	39.57	381.0564	-1.8	-4.8	8.5	15.5	even	ok
	2	C19H13F4O2S	46.86	381.0567	-1.6	-4.2	8.5	11.5	even	ok
	3	C16H17F4O2S2	46.28	381.0601	1.8	4.7	13.9	6.5	even	ok
	4	C14H14FN6O2S2	54.68	381.0598	1.5	4.0	13.9	10.5	even	ok
	5	C16H14F5O3S	80.12	381.0578	0.4	1.2	14.9	7.5	even	ok
	6	C14H11F2N6O3S	71.14	381.0576	-0.7	-1.8	14.9	11.5	even	ok
	7	C16H17N2O5S2	76.02	381.0573	0.9	2.5	15.9	9.5	even	ok
	8	C21H14FO4S	63.63	381.0591	0.9	2.2	16.0	14.5	even	ok
	9	C19H16F3OS2	89.01	381.0589	-0.6	-1.7	16.1	10.5	even	ok
	10	C17H13N6OS2	100.00	381.0587	-0.4	-1.0	16.1	14.5	even	ok
	11	C14H16F7S2	63.24	381.0576	0.7	1.7	21.1	3.5	even	ok
	12	C13H18FN2O6S2	95.85	381.0585	0.2	0.5	22.2	5.5	even	ok
	13	C22H15F2S2	73.95	381.0578	-0.5	-1.3	27.5	14.5	even	ok
	14	C13H15F6O4S	51.15	381.0590	0.7	1.8	29.6	3.5	even	ok
	15	C24H13O3S	59.03	381.0580	-0.3	-0.8	31.8	18.5	even	ok
	16	C14H19F2N2O2S3	45.25	381.0571	1.2	3.0	33.2	5.5	even	ok
	17	C14H22FN2OS4	27.63	381.0594	-1.1	-2.8	52.5	4.5	even	ok
	18	C17H21N2S4	41.62	381.0582	-0.1	-0.2	55.2	8.5	even	ok
403.0401	1	C17H9FN6NaO2S	46.37	403.0384	1.8	4.3	9.2	15.5	even	ok
	2	C19H12F4NaO2S	54.71	403.0386	-1.5	-3.8	9.3	11.5	even	ok
	3	C16H13F5NaO3S	100.00	403.0398	-0.4	-0.9	11.3	7.5	even	ok
	4	C14H10F2N6NaO3S	89.08	403.0395	-0.6	-1.5	11.3	11.5	even	ok
	5	C16H16F4NaO2S2	37.96	403.0420	1.9	4.6	15.6	6.5	even	ok
	6	C14H13FN6NaO2S2	45.01	403.0418	-1.6	-4.0	15.6	10.5	even	ok
	7	C12H7F3N10NaS	36.73	403.0420	-1.9	-4.6	16.8	12.5	even	ok
	8	C16H16N2NaO5S2	67.58	403.0393	-0.9	-2.1	18.5	9.5	even	ok
	9	C21H13FNaO4S	64.27	403.0411	-0.9	-2.3	19.0	14.5	even	ok
	10	C19H15F3NaOS2	70.99	403.0409	-0.7	-1.8	19.8	10.5	even	ok
	11	C17H12N6NaOS2	80.03	403.0406	0.5	1.2	19.9	14.5	even	ok
	12	C14H15F7NaS2	75.04	403.0396	0.6	1.5	20.4	3.5	even	ok
	13	C12H12F4N6NaS2	66.19	403.0393	0.8	2.0	20.4	7.5	even	ok
	14	C13H14F2N2NaO7S	31.68	403.0382	1.9	4.8	21.1	6.5	even	ok
	15	C13H17FN2NaO6S2	82.93	403.0404	-0.3	-0.7	22.3	5.5	even	ok
	16	C12H16N2NaO10S	35.86	403.0418	-1.6	-4.1	25.5	5.5	even	ok

Mass Spectrum SmartFormula Report

Meas. m/z	#	Ion Formula	Score	m/z	err [mDa]	err [ppm]	mSigma	rdb	e¯ Conf	N-Rule
	17	C13H14F6NaO4S	59.92	403.0409	0.8	1.9	26.1	3.5	even	ok
	18	C22H14F2NaS2	62.91	403.0397	-0.4	-1.1	31.3	14.5	even	ok
	19	C24H12NaO3S	63.17	403.0399	0.2	0.5	35.0	18.5	even	ok
	20	C14H18F2N2NaO2S3	40.29	403.0391	1.1	2.7	35.4	5.5	even	ok
	21	C14H21FN2NaOS4	21.79	403.0413	1.2	2.9	55.1	4.5	even	ok
	22	C17H20N2NaS4	34.51	403.0402	0.0	0.0	58.4	8.5	even	ok

Figure S67. HRESIMS spectrum of compound **7q**

Generic Display Report

Analysis Info

Analysis Name	D:\MZ\maXis_data\temp\64019000001.d	Acquisition Date	20/05/2019 14:38:15
Method	tune_low_MS_Service_05_19.m	Operator	msc
Sample Name	PN-10 spot 2 peak 1	Instrument	maXis
Comment	Predrag Kalaba<br>Ergebnis: +/- 5ppm<br>ACN / MeOH + 1% H2O		

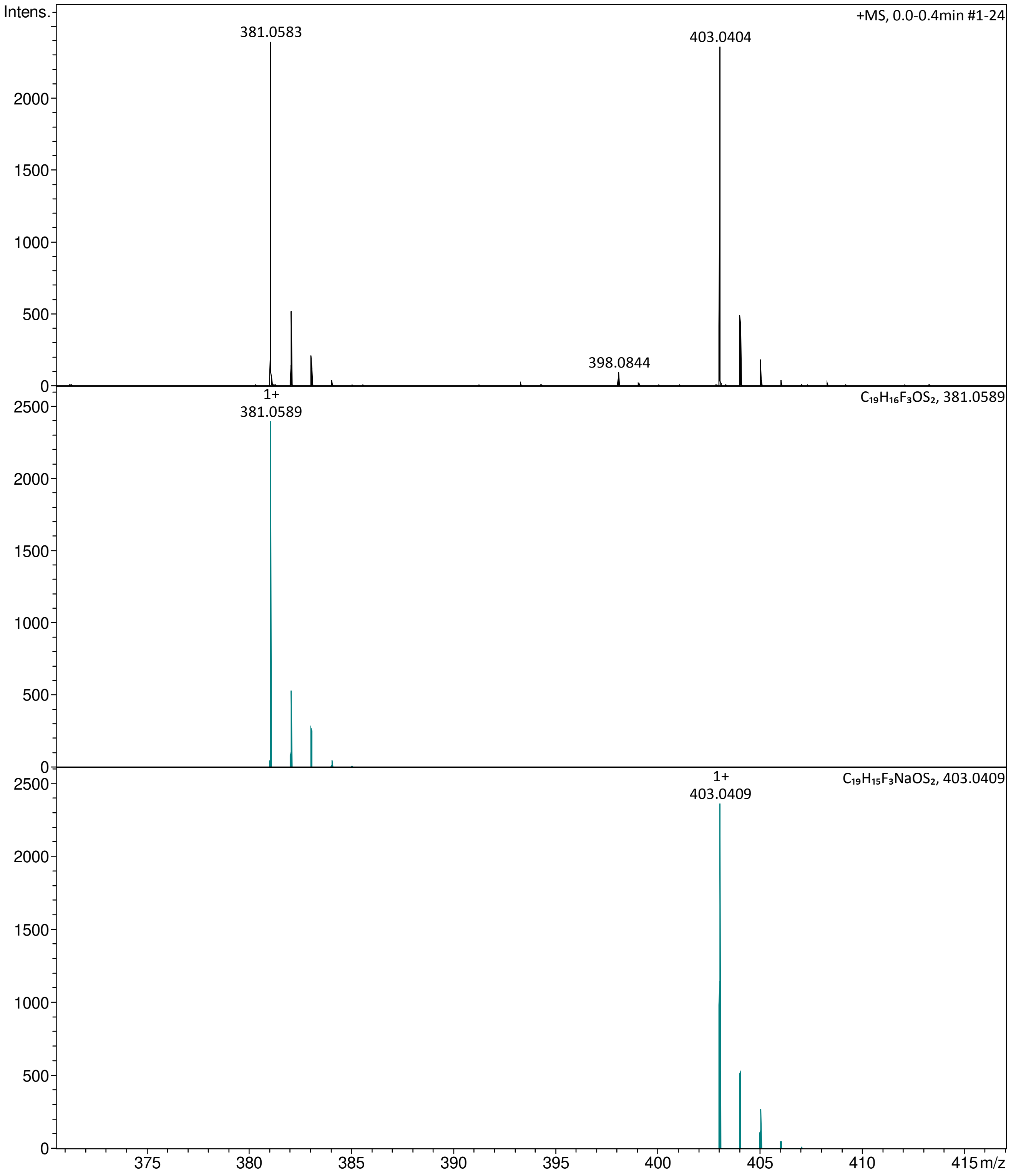

Mass Spectrum SmartFormula Report

Analysis Info

Analysis Name	D:\MZ\maXis_data\temp\64019000001.d	Acquisition Date	20/05/2019 14:38:15
Method	tune_low_MS_Service_05_19.m	Operator	msc
Sample Name	PN-10 spot 2 peak 1	Instrument	maXis 255552.00016
Comment	Predrag Kalaba<br>Ergebnis: +/- 5ppm<br>ACN / MeOH + 1% H2O		

Acquisition Parameter

Source Type	ESI	Ion Polarity	Positive	Set Nebulizer	0.4 Bar
Focus	Not active	Set Capillary	4500 V	Set Dry Heater	150 °C
Scan Begin	50 m/z	Set End Plate Offset	-500 V	Set Dry Gas	4.0 l/min
Scan End	1900 m/z	Set Charging Voltage	0 V	Set Divert Valve	Source
		Set Corona	0 nA	Set APCI Heater	0 °C

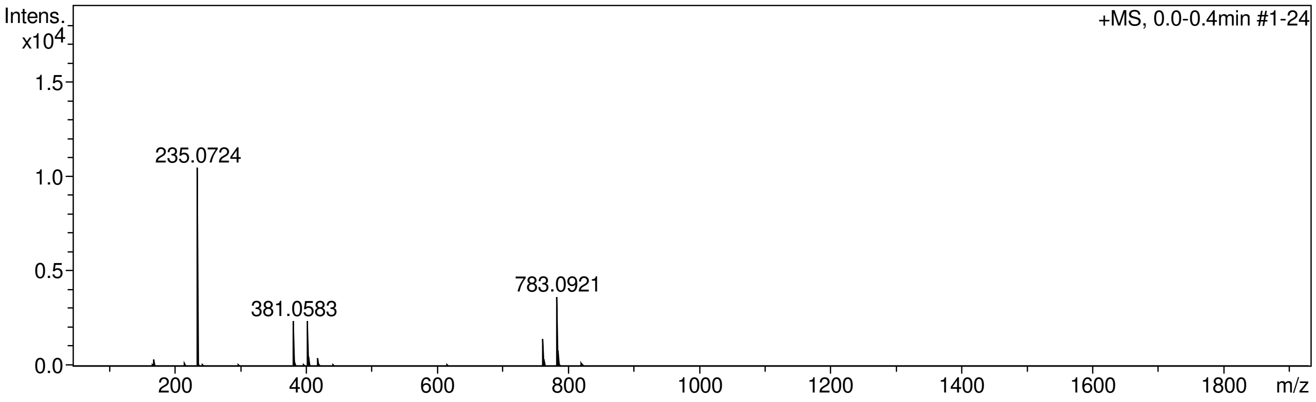

Meas. m/z	#	Ion Formula	Score	m/z	err [mDa]	err [ppm]	mSigma	rdb	e^- Conf	N-Rule
381.0583	1	C17H10FN6O2S	43.29	381.0564	1.9	4.9	9.4	15.5	even	ok
	2	C19H13F4O2S	51.37	381.0567	1.6	4.3	9.4	11.5	even	ok
	3	C21H14FO4S	81.26	381.0591	0.8	2.1	11.7	14.5	even	ok
	4	C19H16F3OS2	89.17	381.0589	0.6	1.6	12.6	10.5	even	ok
	5	C17H13N6OS2	100.00	381.0587	0.4	0.9	12.7	14.5	even	ok
	6	C16H17F4O2S2	42.54	381.0601	1.7	4.6	15.3	6.5	even	ok
	7	C14H14FN6O2S2	50.16	381.0598	-1.5	-3.9	15.3	10.5	even	ok
	8	C16H17N2O5S2	68.41	381.0573	-1.0	-2.6	15.7	9.5	even	ok
	9	C16H14F5O3S	80.57	381.0578	-0.5	-1.3	20.2	7.5	even	ok
	10	C14H11F2N6O3S	71.41	381.0576	0.7	1.9	20.2	11.5	even	ok
	11	C22H15F2S2	74.05	381.0578	0.5	1.4	22.8	14.5	even	ok
	12	C14H16F7S2	65.06	381.0576	0.7	1.8	25.0	3.5	even	ok
	13	C24H13O3S	75.07	381.0580	0.3	0.9	26.7	18.5	even	ok
	14	C14H19F2N2O2S3	40.55	381.0571	1.2	3.1	33.2	5.5	even	ok
	15	C14H22FN2OS4	26.78	381.0594	1.0	2.7	51.5	4.5	even	ok
	16	C17H21N2S4	40.30	381.0582	-0.1	-0.3	52.9	8.5	even	ok
403.0404	1	C18H14F2NaO5S	50.35	403.0422	-1.8	-4.5	4.6	10.5	even	ok
	2	C17H9FN6NaO2S	42.94	403.0384	-2.0	-5.0	6.4	15.5	even	ok
	3	C19H12F4NaO2S	51.26	403.0386	-1.8	-4.4	6.4	11.5	even	ok
	4	C21H13FNaO4S	85.52	403.0411	-0.7	-1.7	14.3	14.5	even	ok
	5	C16H13F5NaO3S	86.51	403.0398	0.6	1.5	15.4	7.5	even	ok
	6	C14H10F2N6NaO3S	76.26	403.0395	0.9	2.1	15.4	11.5	even	ok
	7	C16H16F4NaO2S2	47.02	403.0420	1.6	4.0	16.4	6.5	even	ok
	8	C14H13FN6NaO2S2	55.11	403.0418	-1.4	-3.4	16.5	10.5	even	ok
	9	C19H15F3NaOS2	89.64	403.0409	-0.5	-1.2	17.2	10.5	even	ok
	10	C17H12N6NaOS2	100.00	403.0406	0.2	0.6	17.3	14.5	even	ok
	11	C16H16N2NaO5S2	62.38	403.0393	-1.1	-2.8	18.3	9.5	even	ok
	12	C14H10F6N4NaS	35.74	403.0423	1.9	4.6	21.0	8.5	even	ok
	13	C14H15F7NaS2	64.78	403.0396	-0.8	-2.1	23.7	3.5	even	ok
	14	C13H17FN2NaO6S2	92.26	403.0404	0.0	0.1	24.7	5.5	even	ok
	15	C22H14F2NaS2	64.42	403.0397	-0.7	-1.7	27.6	14.5	even	ok
	16	C24H12NaO3S	67.33	403.0399	-0.5	-1.1	30.2	18.5	even	ok
	17	C13H14F6NaO4S	64.28	403.0409	-0.5	-1.3	30.8	3.5	even	ok
	18	C14H18F2N2NaO2S3	36.34	403.0391	1.3	3.3	35.7	5.5	even	ok

Mass Spectrum SmartFormula Report

Meas. m/z	#	Ion Formula	Score	m/z	err [mDa]	err [ppm]	mSigma	rdb	e⁻ Conf	N-Rule
	19	C14H21FN2NaOS4	26.88	403.0413	-0.9	-2.2	54.7	4.5	even	ok
	20	C17H20N2NaS4	34.66	403.0402	0.2	0.6	57.0	8.5	even	ok

Figure S68. HRESIMS spectrum of compound **8q**

Generic Display Report

Analysis Info

Analysis Name	D:\MZ\maXis_data\temp\64020000001.d	Acquisition Date	20/05/2019 14:41:57
Method	tune_low_MS_Service_05_19.m	Operator	msc
Sample Name	PN-10 spot 2 peak 2	Instrument	maXis
Comment	Predrag Kalaba<br>Ergebnis: +/- 5ppm<br>ACN / MeOH + 1% H2O		

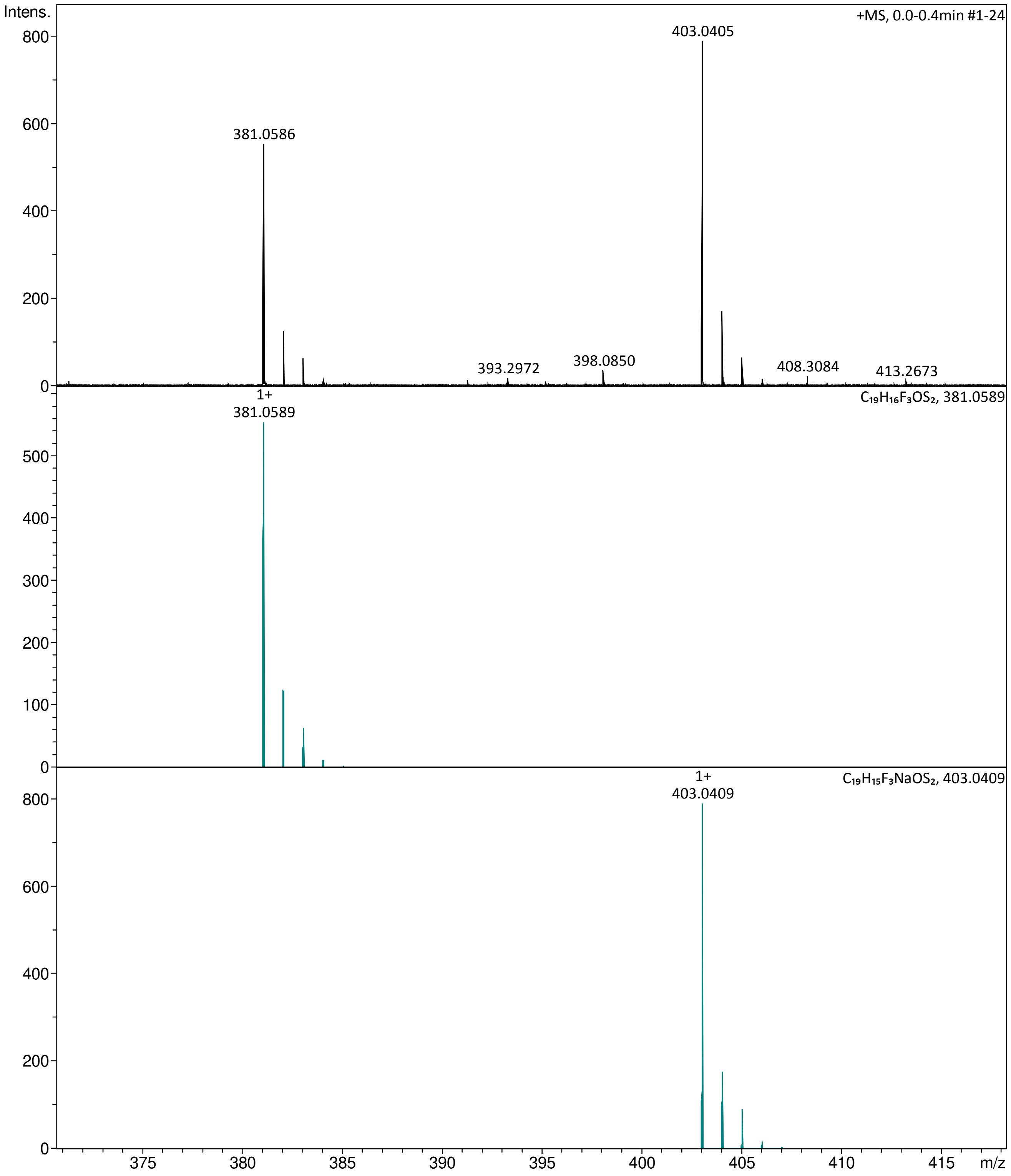

Mass Spectrum SmartFormula Report

Analysis Info

Analysis Name	D:\MZ\maXis_data\temp\64020000001.d	Acquisition Date	20/05/2019 14:41:57
Method	tune_low_MS_Service_05_19.m	Operator	msc
Sample Name	PN-10 spot 2 peak 2	Instrument	maXis 255552.00016
Comment	Predrag Kalaba<br>Ergebnis: +/- 5ppm<br>ACN / MeOH + 1% H2O		

Acquisition Parameter

Source Type	ESI	Ion Polarity	Positive	Set Nebulizer	0.4 Bar
Focus	Not active	Set Capillary	4500 V	Set Dry Heater	150 °C
Scan Begin	50 m/z	Set End Plate Offset	-500 V	Set Dry Gas	4.0 l/min
Scan End	1900 m/z	Set Charging Voltage	0 V	Set Divert Valve	Source
		Set Corona	0 nA	Set APCI Heater	0 °C

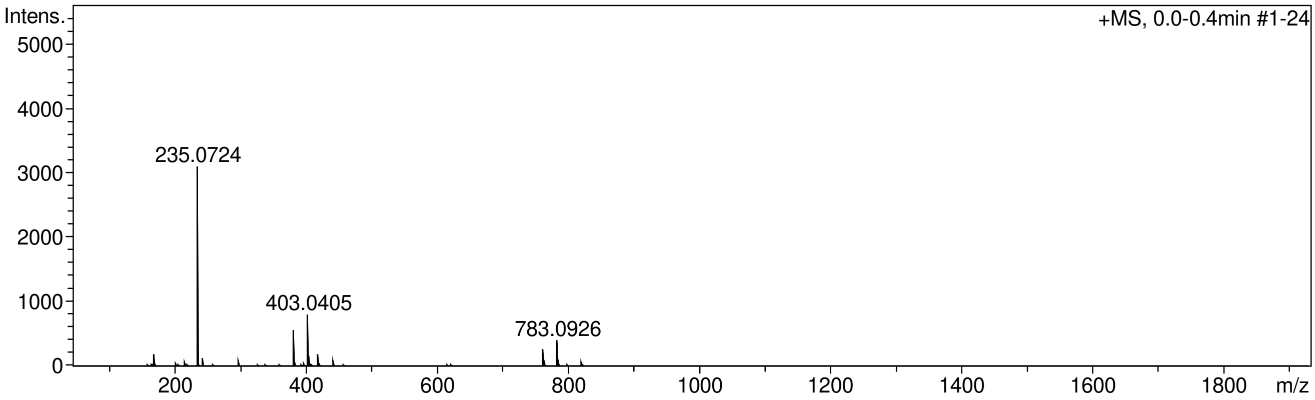

Meas. m/z	#	Ion Formula	Score	m/z	err [mDa]	err [ppm]	mSigma	rdb	e⁻ Conf	N-Rule
381.0586	1	C19H16F3OS2	90.36	381.0589	0.3	0.7	2.9	10.5	even	ok
	2	C17H13N6OS2	100.00	381.0587	-0.0	-0.1	2.9	14.5	even	ok
	3	C16H17N2O5S2	43.49	381.0573	-1.3	-3.4	12.7	9.5	even	ok
	4	C22H15F2S2	55.59	381.0578	-0.9	-2.3	13.3	14.5	even	ok
	5	C16H17F4O2S2	37.95	381.0601	1.4	3.7	16.3	6.5	even	ok
	6	C14H14FN6O2S2	44.09	381.0598	-1.2	-3.1	16.3	10.5	even	ok
	7	C21H14FO4S	61.88	381.0591	-0.5	-1.3	17.8	14.5	even	ok
	8	C18H15F2O5S	28.91	381.0603	-1.6	-4.3	22.3	10.5	even	ok
	9	C22H10FN4S	23.73	381.0605	1.8	4.8	24.9	19.5	even	ok
	10	C24H13O3S	48.14	381.0580	0.7	1.7	25.4	18.5	even	ok
	11	C13H18FN2O6S2	58.77	381.0585	0.2	0.4	26.7	5.5	even	ok
	12	C14H19F2N2O2S3	28.04	381.0571	1.5	4.0	26.7	5.5	even	ok
	13	C14H16F7S2	35.41	381.0576	-1.0	-2.7	29.8	3.5	even	ok
	14	C12H13F4N6S2	30.64	381.0574	1.3	3.3	29.8	7.5	even	ok
	15	C14H11F2N6O3S	32.74	381.0576	-1.1	-2.8	32.4	11.5	even	ok
	16	C16H14F5O3S	37.45	381.0578	0.8	2.1	32.4	7.5	even	ok
	17	C11H20F3N2O3S3	40.89	381.0583	0.4	1.0	37.4	1.5	even	ok
	18	C12H8F3N10S	22.51	381.0601	-1.4	-3.7	38.2	12.5	even	ok
	19	C14H11F6N4S	19.14	381.0603	1.7	4.4	38.2	8.5	even	ok
	20	C17H21N2S4	35.34	381.0582	-0.4	-1.2	41.8	8.5	even	ok
	21	C14H22FN2OS4	30.69	381.0594	-0.7	-1.8	42.2	4.5	even	ok
	22	C12H17N2O10S	22.74	381.0598	-1.2	-3.1	43.1	5.5	even	ok
	23	C13H15F6O4S	33.26	381.0590	0.3	0.9	45.7	3.5	even	ok
	24	C11H12F3N6O4S	36.90	381.0587	-0.1	-0.2	45.8	7.5	even	ok
	25	C11H17F8OS2	36.40	381.0588	0.1	0.3	45.9	-0.5	even	ok
	26	C11H23F2N2O2S4	12.84	381.0605	-1.8	-4.8	47.7	0.5	even	ok
403.0405	1	C19H12F4NaO2S	41.65	403.0386	-1.9	-4.7	7.4	11.5	even	ok
	2	C18H14F2NaO5S	46.92	403.0422	-1.7	-4.2	7.8	10.5	even	ok
	3	C21H13FNaO4S	87.57	403.0411	-0.6	-1.4	10.9	14.5	even	ok
	4	C19H15F3NaOS2	90.08	403.0409	0.3	0.9	14.8	10.5	even	ok
	5	C17H12N6NaOS2	100.00	403.0406	-0.1	-0.3	14.8	14.5	even	ok
	6	C16H16F4NaO2S2	45.57	403.0420	1.5	3.7	17.0	6.5	even	ok
	7	C14H13FN6NaO2S2	53.11	403.0418	-1.2	-3.1	17.0	10.5	even	ok
	8	C16H16N2NaO5S2	52.90	403.0393	-1.2	-3.1	17.8	9.5	even	ok

Mass Spectrum SmartFormula Report

Meas. m/z	#	Ion Formula	Score	m/z	err [mDa]	err [ppm]	mSigma	rdb	e⁻ Conf	N-Rule
	9	C16H13F5NaO3S	67.75	403.0398	0.7	1.8	19.3	7.5	even	ok
	10	C14H10F2N6NaO3S	59.41	403.0395	-1.0	-2.4	19.3	11.5	even	ok
	11	C22H9FN4NaS	30.34	403.0424	-1.9	-4.7	22.5	19.5	even	ok
	12	C22H14F2NaS2	59.15	403.0397	-0.8	-2.0	24.1	14.5	even	ok
	13	C14H10F6N4NaS	32.30	403.0423	1.7	4.3	24.9	8.5	even	ok
	14	C12H7F3N10NaS	38.11	403.0420	-1.5	-3.7	24.9	12.5	even	ok
	15	C14H15F7NaS2	51.82	403.0396	-1.0	-2.4	26.1	3.5	even	ok
	16	C12H12F4N6NaS2	44.98	403.0393	1.2	3.0	26.2	7.5	even	ok
	17	C13H17FN2NaO6S2	78.62	403.0404	-0.1	-0.2	26.3	5.5	even	ok
	18	C24H12NaO3S	62.61	403.0399	-0.6	-1.4	26.5	18.5	even	ok
	19	C12H16N2NaO10S	35.66	403.0418	1.3	3.2	33.8	5.5	even	ok
	20	C13H14F6NaO4S	55.77	403.0409	0.4	1.0	34.7	3.5	even	ok
	21	C11H11F3N6NaO4S	62.06	403.0407	0.2	0.4	34.7	7.5	even	ok
	22	C14H18F2N2NaO2S3	30.70	403.0391	1.4	3.6	35.3	5.5	even	ok
	23	C11H16F8NaOS2	51.70	403.0407	0.2	0.5	41.1	-0.5	even	ok
	24	C11H19F3N2NaO3S3	48.05	403.0402	-0.3	-0.8	41.9	1.5	even	ok
	25	C14H21FN2NaOS4	26.86	403.0413	-0.8	-1.9	53.6	4.5	even	ok
	26	C17H20N2NaS4	31.58	403.0402	-0.4	-0.9	55.0	8.5	even	ok
	27	C11H22F2N2NaO2S4	11.80	403.0424	-1.9	-4.8	56.6	0.5	even	ok

Figure S69. HRESIMS spectrum of compound **5r**

Generic Display Report

Analysis Info

Analysis Name	D:\MZ\maXis_data\temp\63398000001.d	Acquisition Date	4/11/2019 6:26:30 PM
Method	tune_low_MS_Service_04_19.m	Operator	msc
Sample Name	CE-166 SPOT1 PEAK1	Instrument	maXis
Comment	Kalaba/Zehl<br>Ergebnis: +/- 5ppm<br>ACN/MeOH + 1% H2O		

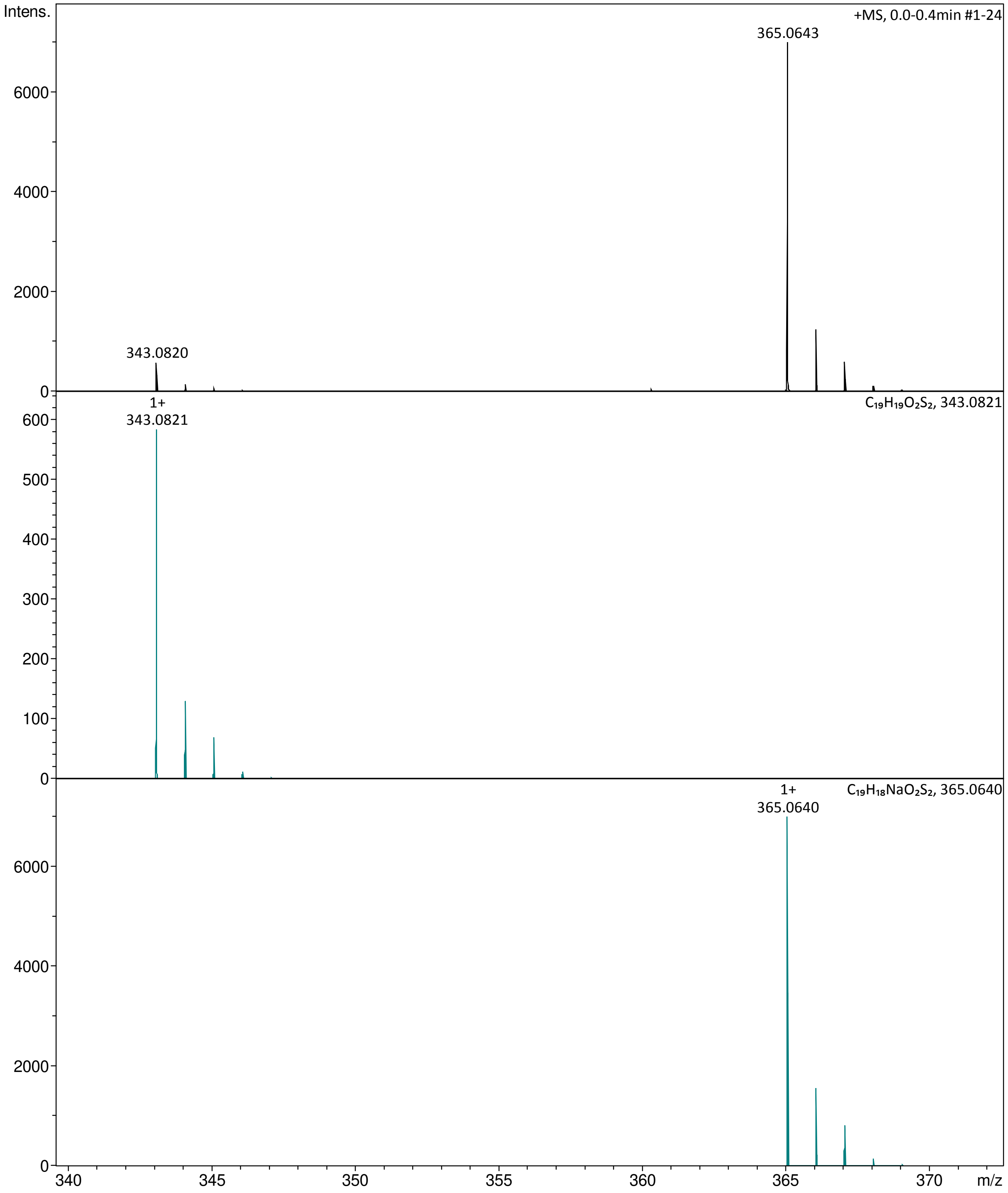

Mass Spectrum SmartFormula Report

Analysis Info

Analysis Name	D:\MZ\maXis_data\temp\63398000001.d	Acquisition Date	4/11/2019 6:26:30 PM
Method	tune_low_MS_Service_04_19.m	Operator	msc
Sample Name	CE-166 SPOT1 PEAK1	Instrument	maXis 255552.00016
Comment	Kalaba/Zehl<br>Ergebnis: +/- 5ppm<br>ACN/MeOH + 1% H2O		

Acquisition Parameter

Source Type	ESI	Ion Polarity	Positive	Set Nebulizer	0.4 Bar
Focus	Not active	Set Capillary	4500 V	Set Dry Heater	150 °C
Scan Begin	50 m/z	Set End Plate Offset	-500 V	Set Dry Gas	4.0 l/min
Scan End	1900 m/z	Set Charging Voltage	0 V	Set Divert Valve	Source
		Set Corona	0 nA	Set APCI Heater	0 °C

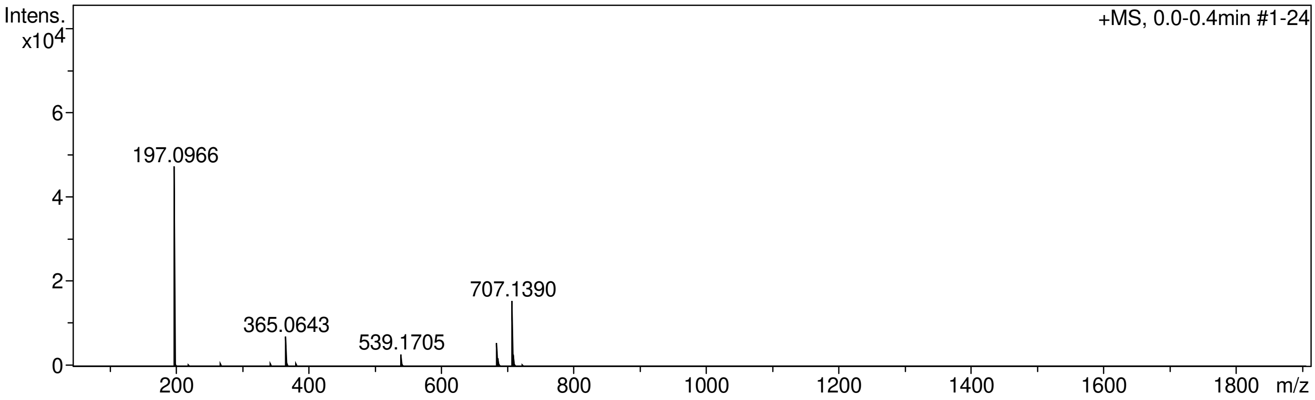

Meas. m/z	#	Ion Formula	Score	m/z	err [mDa]	err [ppm]	mSigma	rdb	e⁻ Conf	N-Rule
197.0966	1	C14H13O	100.00	197.0961	-0.5	-2.8	22.4	8.5	even	ok
343.0820	1	C19H19O2S2	100.00	343.0821	-0.1	-0.4	12.5	10.5	even	ok
	2	C18H15O7	37.40	343.0812	-0.7	-2.1	42.3	11.5	even	ok
	3	C19H11N4O3	35.76	343.0826	-0.6	-1.8	46.2	16.5	even	ok
365.0643	1	C12H10N10NaOS	86.81	365.0652	-0.9	-2.4	13.0	12.5	even	ok
	2	C11H14N6NaO5S	100.00	365.0639	-0.5	-1.3	16.4	7.5	even	ok
	3	C19H18NaO2S2	89.43	365.0640	-0.3	-0.8	25.5	10.5	even	ok
	4	C10H18N2NaO9S	36.96	365.0625	1.8	4.9	25.6	2.5	even	ok
	5	C12H18N6NaS3	78.60	365.0647	-0.4	-1.1	28.5	6.5	even	ok
	6	C18H14NaO7	51.96	365.0632	1.1	3.1	29.4	11.5	even	ok
	7	C11H22N2NaO4S3	55.08	365.0634	-0.9	-2.5	32.1	1.5	even	ok
	8	C19H10N4NaO3	45.99	365.0645	-0.2	-0.5	41.9	16.5	even	ok

Figure S70. HRESIMS spectrum of compound **6r**

Generic Display Report

Analysis Info

Analysis Name	D:\MZ\maXis_data\temp\63399000001.d
Method	tune_low_MS_Service_04_19.m
Sample Name	CE-166 SPOT1 PEAK2
Comment	Kalaba/Zehl<br>Ergebnis: +/- 5ppm<br>ACN/MeOH + 1% H2O
Acquisition Date	4/11/2019 6:31:45 PM
Operator	msc
Instrument	maXis

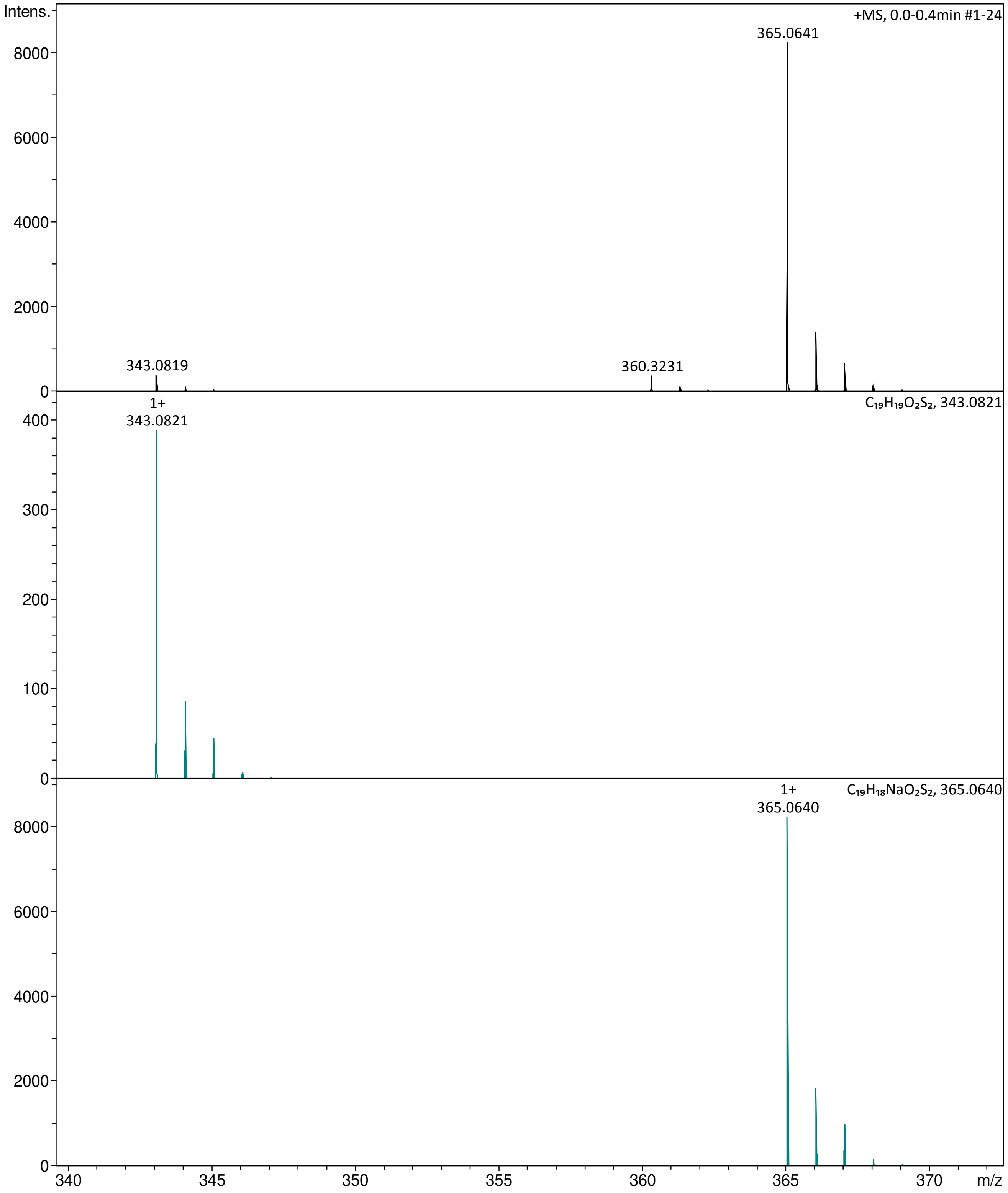

Mass Spectrum SmartFormula Report

Analysis Info

Acquisition Date 4/11/2019 6:31:45 PM

Analysis Name D:\MZ\maXis_data\temp\63399000001.d

Method tune_low_MS_Service_04_19.m

Operator msc

Sample Name CE-166 SPOT1 PEAK2

Instrument maXis 255552.00016

Comment Kalaba/Zehl
Ergebnis: +/- 5ppm
ACN/MeOH + 1% H2O

Acquisition Parameter

Source Type	ESI	Ion Polarity	Positive	Set Nebulizer	0.4 Bar
Focus	Not active	Set Capillary	4500 V	Set Dry Heater	150 °C
Scan Begin	50 m/z	Set End Plate Offset	-500 V	Set Dry Gas	4.0 l/min
Scan End	1900 m/z	Set Charging Voltage	0 V	Set Divert Valve	Source
		Set Corona	0 nA	Set APCI Heater	0 °C

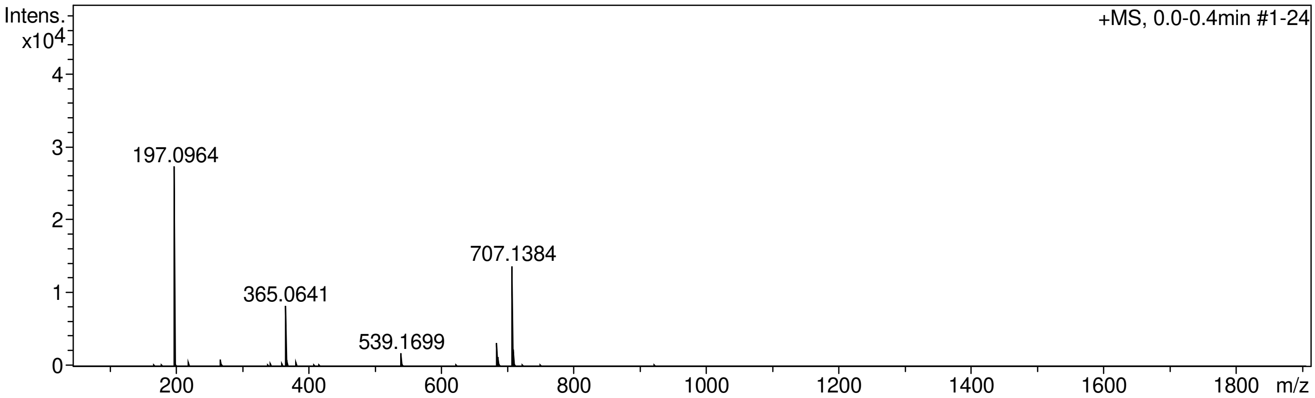

Meas. m/z	#	Ion Formula	Score	m/z	err [mDa]	err [ppm]	mSigma	rdb	e⁻ Conf	N-Rule
197.0964	1	C14H13O	100.00	197.0961	-0.3	-1.5	21.4	8.5	even	ok
343.0819	1	C19H19O2S2	100.00	343.0821	-0.2	-0.5	12.3	10.5	even	ok
	2	C19H11N4O3	40.50	343.0826	-0.6	-1.8	41.6	16.5	even	ok
365.0641	1	C11H14N6NaO5S	100.00	365.0639	-0.2	-0.6	12.0	7.5	even	ok
	2	C19H18NaO2S2	73.93	365.0640	0.1	0.1	29.6	10.5	even	ok
	3	C12H18N6NaS3	54.93	365.0647	0.6	1.7	30.8	6.5	even	ok
	4	C11H22N2NaO4S3	50.02	365.0634	0.7	1.9	33.2	1.5	even	ok
	5	C19H10N4NaO3	33.28	365.0645	-0.4	-1.1	42.9	16.5	even	ok

Figure S71. HRESIMS spectrum of compound **7r**

Generic Display Report

Analysis Info

Analysis Name	D:\MZ\maXis_data\temp\59192000001.d	Acquisition Date	8/24/2018 3:43:18 PM
Method	tune_low_MS_Service_08_18.m	Operator	msc
Sample Name	CE-166-spot2-peak1	Instrument	maXis
Comment	Kalaba/Zehl<br>Ergebnis: +/- 5ppm<br>ACN/MeOH +1%H2O		

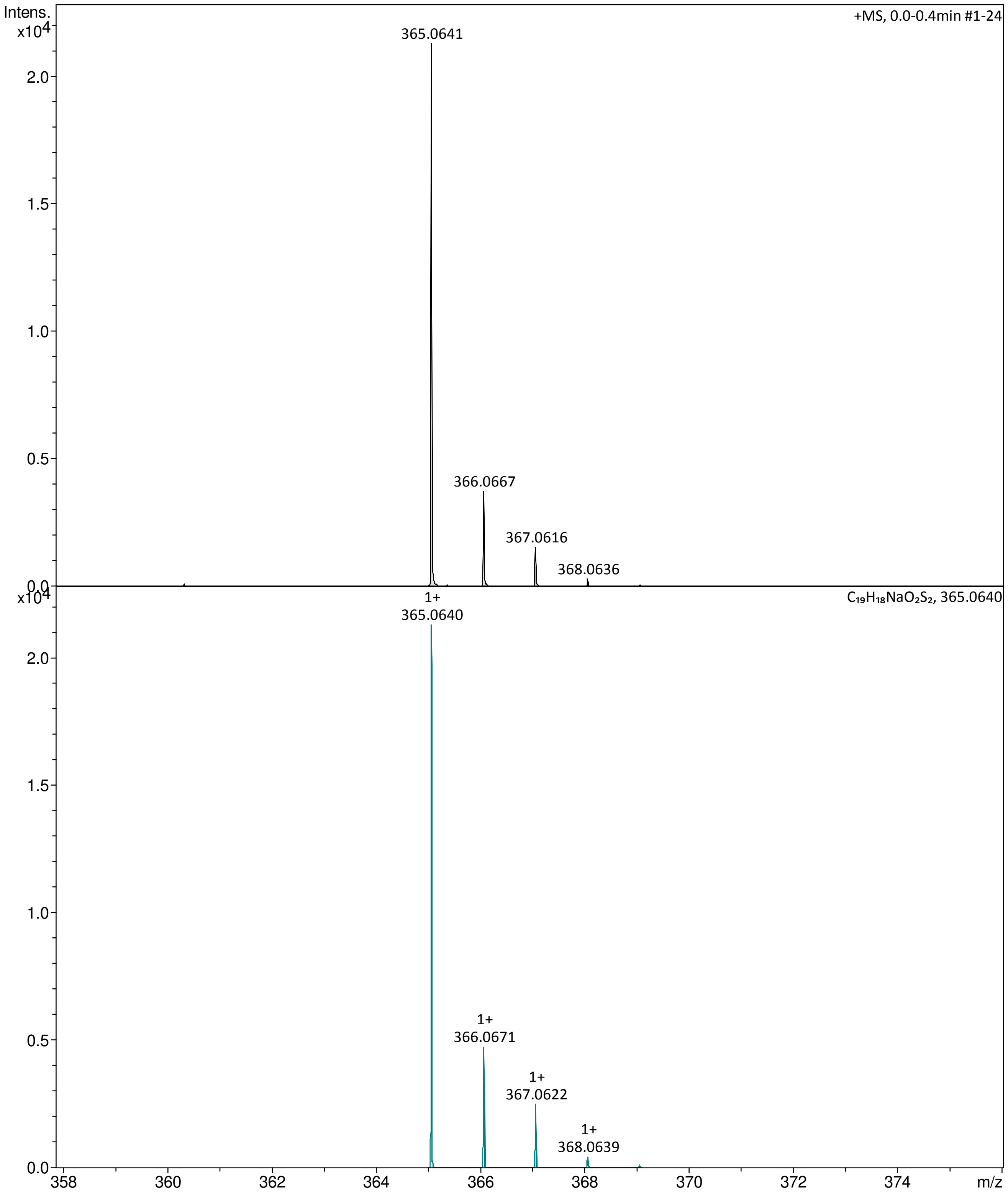

Mass Spectrum SmartFormula Report

Analysis Info

Analysis Name	D:\MZ\maXis_data\temp\59192000001.d	Acquisition Date	8/24/2018 3:43:18 PM
Method	tune_low_MS_Service_08_18.m	Operator	msc
Sample Name	CE-166-spot2-peak1	Instrument	maXis 255552.00016
Comment	Kalaba/Zehl<br>Ergebnis: +/- 5ppm<br>ACN/MeOH +1%H2O		

Acquisition Parameter

Source Type	ESI	Ion Polarity	Positive	Set Nebulizer	0.4 Bar
Focus	Not active	Set Capillary	4500 V	Set Dry Heater	180 °C
Scan Begin	50 m/z	Set End Plate Offset	-500 V	Set Dry Gas	4.0 l/min
Scan End	1900 m/z	Set Charging Voltage	0 V	Set Divert Valve	Source
		Set Corona	0 nA	Set APCI Heater	0 °C

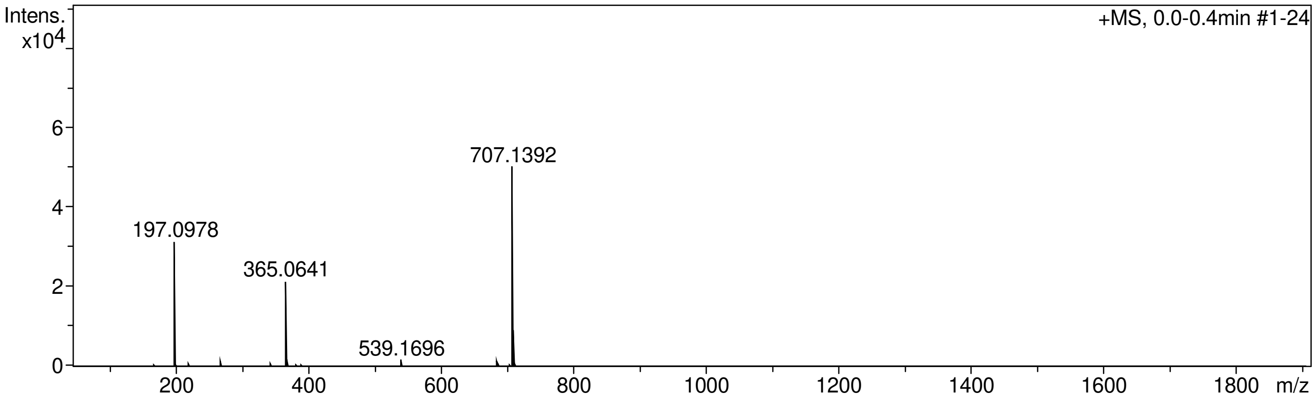

Meas. m/z	#	Ion Formula	Score	m/z	err [mDa]	err [ppm]	mSigma	rdb	e⁻ Conf	N-Rule
365.0641	1	C12H10N10NaOS	72.28	365.0652	1.1	3.0	6.1	12.5	even	ok
	2	C11H14N6NaO5S	100.00	365.0639	0.2	0.7	12.0	7.5	even	ok
	3	C10H18N2NaO9S	37.72	365.0625	1.6	4.3	23.2	2.5	even	ok
	4	C18H14NaO7	55.85	365.0632	0.9	2.6	23.6	11.5	even	ok
	5	C19H18NaO2S2	91.24	365.0640	0.1	0.2	30.1	10.5	even	ok
	6	C12H18N6NaS3	63.12	365.0647	0.6	1.7	34.6	6.5	even	ok
	7	C19H10N4NaO3	39.60	365.0645	-0.4	-1.1	37.0	16.5	even	ok
	8	C11H22N2NaO4S3	55.66	365.0634	0.7	2.0	37.7	1.5	even	ok

Figure S72. HRESIMS spectrum of compound **8r**

Generic Display Report

Analysis Info

Analysis Name	D:\MZ\maXis_data\temp\59193000001.d	Acquisition Date	8/24/2018 3:45:14 PM
Method	tune_low_MS_Service_08_18.m	Operator	msc
Sample Name	CE-166-spot2-peak2	Instrument	maXis
Comment	Kalaba/Zehl<br>Ergebnis: +/- 5ppm<br>ACN/MeOH +1%H2O		

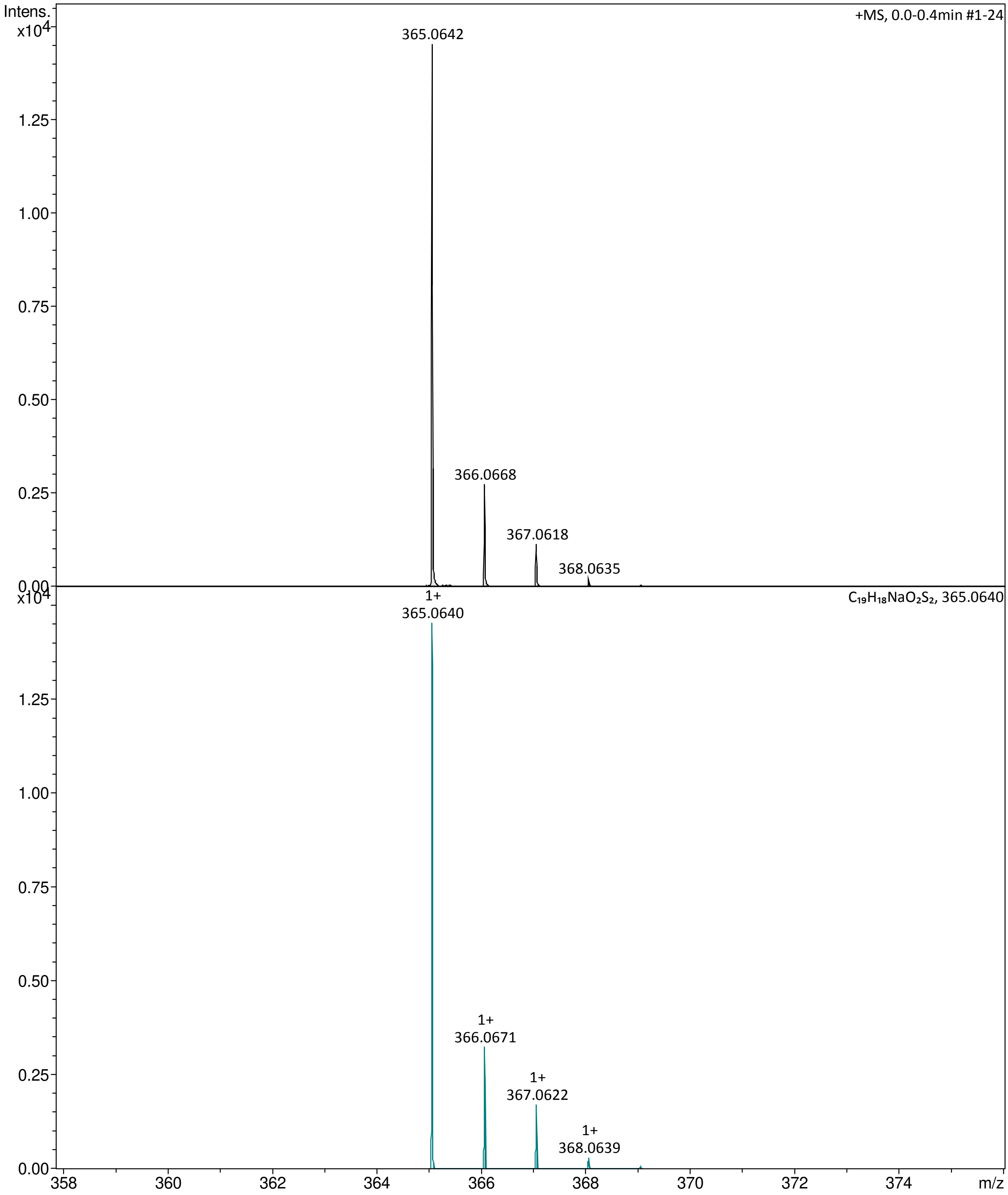

Mass Spectrum SmartFormula Report

Analysis Info

Analysis Name	D:\MZ\maXis_data\temp\59193000001.d	Acquisition Date	8/24/2018 3:45:14 PM
Method	tune_low_MS_Service_08_18.m	Operator	msc
Sample Name	CE-166-spot2-peak2	Instrument	maXis 255552.00016
Comment	Kalaba/Zehl<br>Ergebnis: +/- 5ppm<br>ACN/MeOH +1%H2O		

Acquisition Parameter

Source Type	ESI	Ion Polarity	Positive	Set Nebulizer	0.4 Bar
Focus	Not active	Set Capillary	4500 V	Set Dry Heater	180 °C
Scan Begin	50 m/z	Set End Plate Offset	-500 V	Set Dry Gas	4.0 l/min
Scan End	1900 m/z	Set Charging Voltage	0 V	Set Divert Valve	Source
		Set Corona	0 nA	Set APCI Heater	0 °C

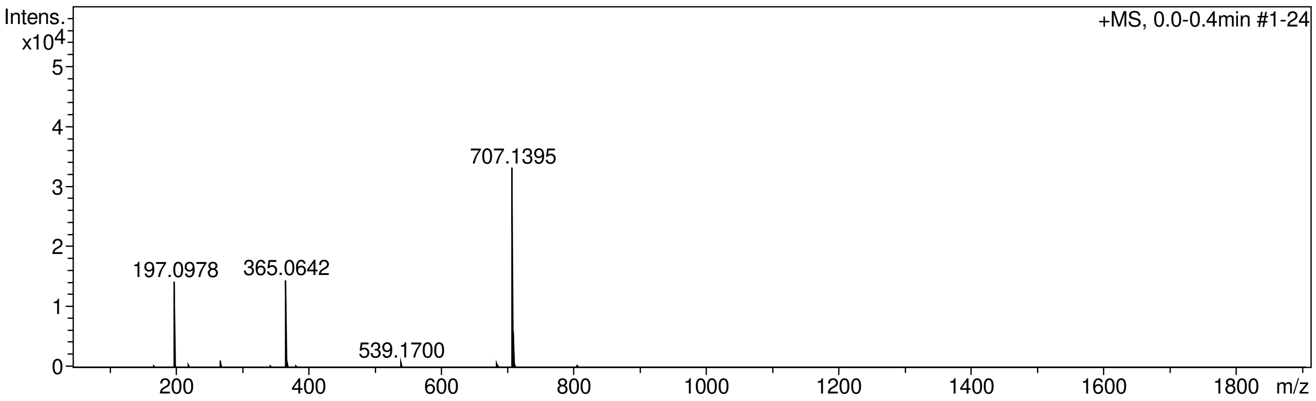

Meas. m/z	#	Ion Formula	Score	m/z	err [mDa]	err [ppm]	mSigma	rdb	e^- Conf	N-Rule
365.064234	1	C12H17N2O9S	96.18	365.064928	0.7	1.9	19.1	5.5	even	ok
	2	C17H13N6S2	100.00	365.063763	0.5	1.3	22.4	14.5	even	ok
	3	C17H5N10O	66.35	365.064231	0.0	0.0	36.0	20.5	even	ok
	1	C11H14N6NaO5S	100.00	365.063859	0.4	1.0	19.2	7.5	even	ok
	2	C19H18NaO2S2	97.26	365.064043	-0.2	-0.5	24.2	10.5	even	ok
	3	C12H18N6NaS3	69.58	365.064728	-0.5	-1.4	32.5	6.5	even	ok
	4	C19H10N4NaO3	55.06	365.064511	-0.3	-0.8	34.3	16.5	even	ok

Figure S73. HRESIMS spectrum of compound **5s**

Generic Display Report

Analysis Info

Analysis Name	D:\MZ\temp\64725000001.d	Acquisition Date	04/07/2019 14:10:01
Method	tune_low_MS_Service_06_19.m	Operator	msc
Sample Name	PN_8_spot_1_peak_1	Instrument	maXis
Comment	Kalaba/Zehl<br>Ergebnis: +/- 5ppm<br>ACN / MeOH + 1% H2O		

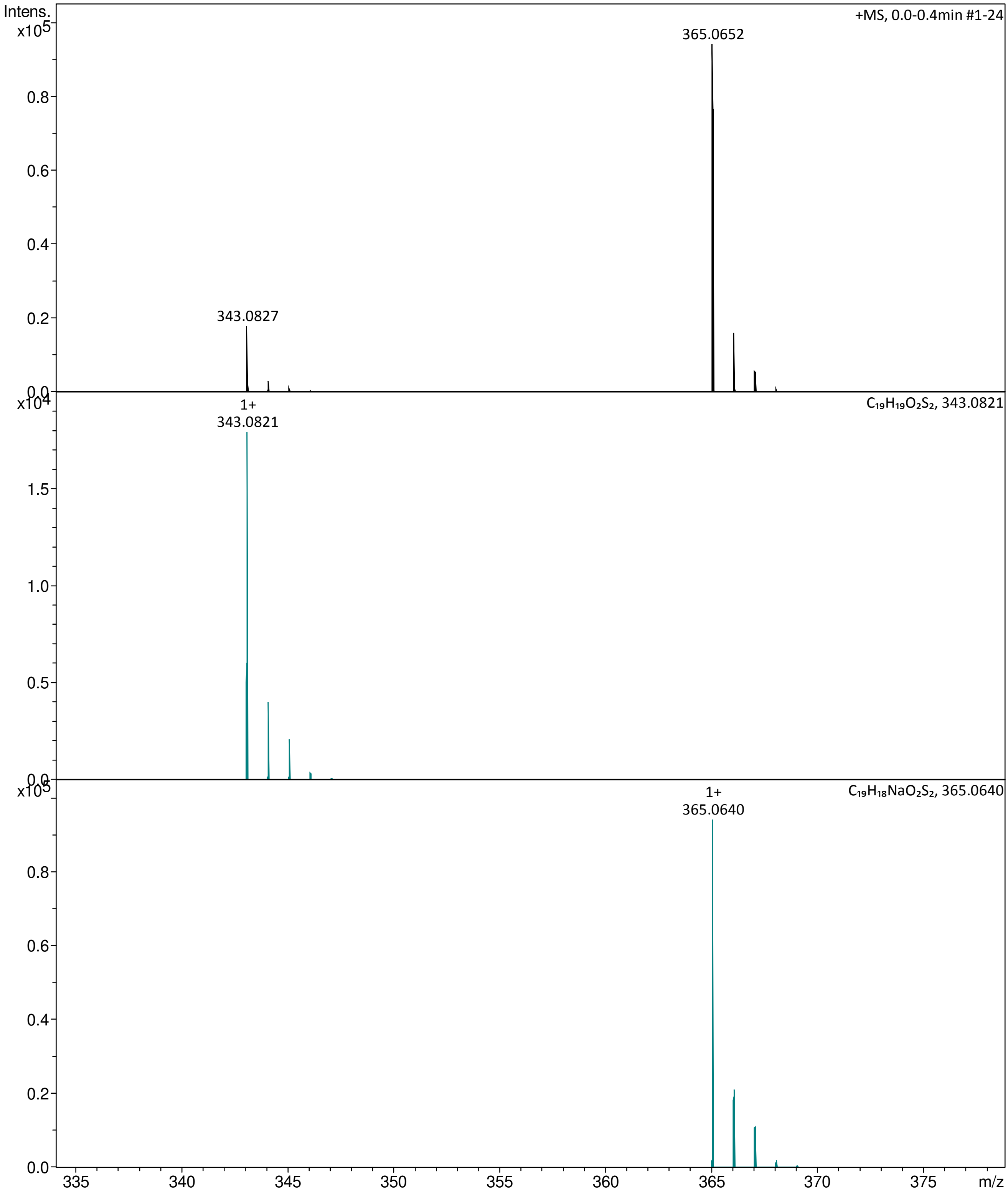

Mass Spectrum SmartFormula Report

Analysis Info

Analysis Name	D:\MZ\temp\64725000001.d	Acquisition Date	04/07/2019 14:10:01
Method	tune_low_MS_Service_06_19.m	Operator	msc
Sample Name	PN_8_spot_1_peak_1	Instrument	maXis 255552.00016
Comment	Kalaba/Zehl<br>Ergebnis: +/- 5ppm<br>ACN / MeOH + 1% H2O		

Acquisition Parameter

Source Type	ESI	Ion Polarity	Positive	Set Nebulizer	0.4 Bar
Focus	Not active	Set Capillary	4200 V	Set Dry Heater	150 °C
Scan Begin	50 m/z	Set End Plate Offset	-500 V	Set Dry Gas	4.0 l/min
Scan End	1900 m/z	Set Charging Voltage	0 V	Set Divert Valve	Source
		Set Corona	0 nA	Set APCI Heater	0 °C

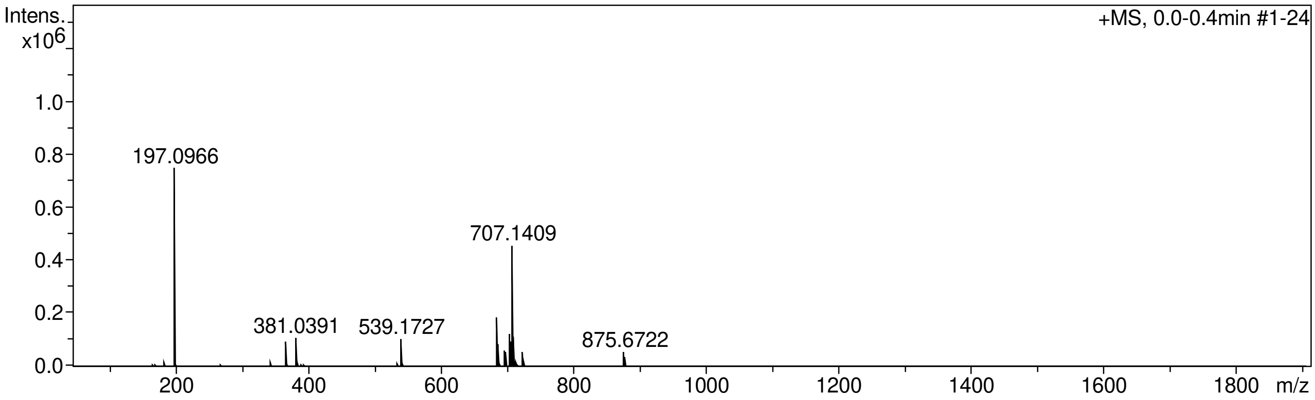

Meas. m/z	#	Ion Formula	Score	m/z	err [mDa]	err [ppm]	mSigma	rdb	e⁻ Conf	N-Rule
343.0827	1	C11H15N6O5S	90.13	343.0819	0.8	2.3	7.9	7.5	even	ok
	2	C12H11N10OS	100.00	343.0833	-0.6	-1.6	8.2	12.5	even	ok
	3	C18H15O7	42.14	343.0812	-1.5	-4.3	25.2	11.5	even	ok
	4	C19H19O2S2	54.31	343.0821	0.6	1.7	35.4	10.5	even	ok
	5	C12H19N6S3	64.49	343.0828	-0.1	-0.3	37.5	6.5	even	ok
	6	C11H23N2O4S3	34.32	343.0814	-1.2	-3.6	39.0	1.5	even	ok
	7	C19H11N4O3	58.55	343.0826	0.1	0.4	40.6	16.5	even	ok
365.0652	1	C12H10N10NaOS	100.00	365.0652	0.0	0.0	3.6	12.5	even	ok
	2	C15H18NaO7S	46.92	365.0665	1.3	3.7	7.6	6.5	even	ok
	3	C11H14N6NaO5S	45.73	365.0639	1.3	3.7	9.0	7.5	even	ok
	4	C19H10N4NaO3	27.94	365.0645	0.7	1.9	35.3	16.5	even	ok
	5	C19H18NaO2S2	27.97	365.0640	-1.2	-3.2	36.3	10.5	even	ok
	6	C5H2N20Na	19.98	365.0664	-1.1	-3.1	38.4	14.5	even	ok
	7	C12H18N6NaS3	35.70	365.0647	0.5	1.3	40.9	6.5	even	ok
	8	C7H14N6NaO10	17.54	365.0664	-1.2	-3.2	42.9	3.5	even	ok
	9	C11H22N2NaO4S3	15.08	365.0634	-1.8	-5.0	43.1	1.5	even	ok

Figure S74. HRESIMS spectrum of compound **6s**

Generic Display Report

Analysis Info

Analysis Name	D:\MZ\temp\64726000001.d	Acquisition Date	04/07/2019 14:13:06
Method	tune_low_MS_Service_06_19.m	Operator	msc
Sample Name	PN_8_spot_1_peak_2	Instrument	maXis
Comment	Kalaba/Zehl<br>Ergebnis: +/- 5ppm<br>ACN / MeOH + 1% H2O		

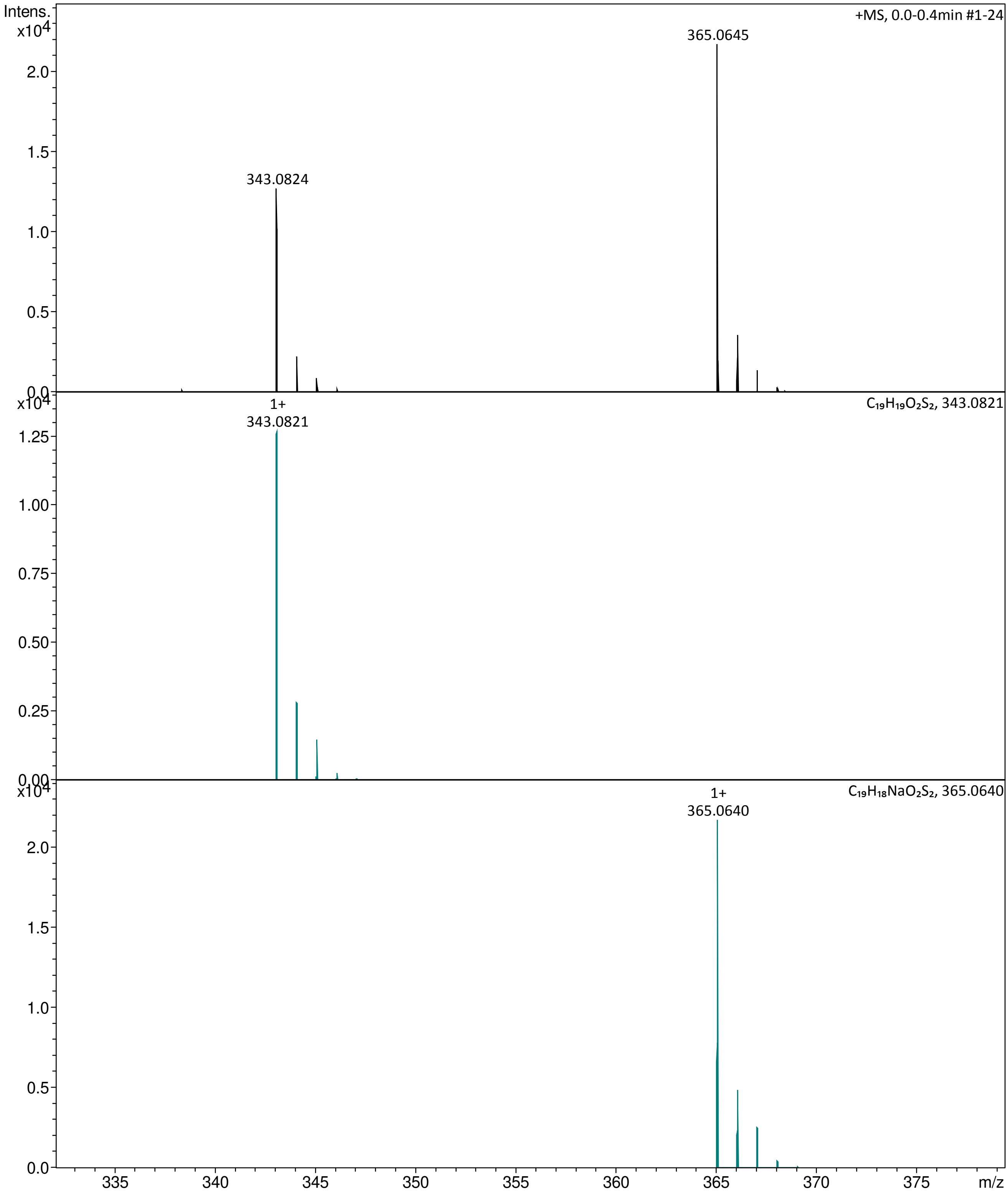

Mass Spectrum SmartFormula Report

Analysis Info

Analysis Name	D:\MZ\temp\64726000001.d	Acquisition Date	04/07/2019 14:13:06
Method	tune_low_MS_Service_06_19.m	Operator	msc
Sample Name	PN_8_spot_1_peak_2	Instrument	maXis 255552.00016
Comment	Kalaba/Zehl<br>Ergebnis: +/- 5ppm<br>ACN / MeOH + 1% H2O		

Acquisition Parameter

Source Type	ESI	Ion Polarity	Positive	Set Nebulizer	0.4 Bar
Focus	Not active	Set Capillary	4200 V	Set Dry Heater	150 °C
Scan Begin	50 m/z	Set End Plate Offset	-500 V	Set Dry Gas	4.0 l/min
Scan End	1900 m/z	Set Charging Voltage	0 V	Set Divert Valve	Source
		Set Corona	0 nA	Set APCI Heater	0 °C

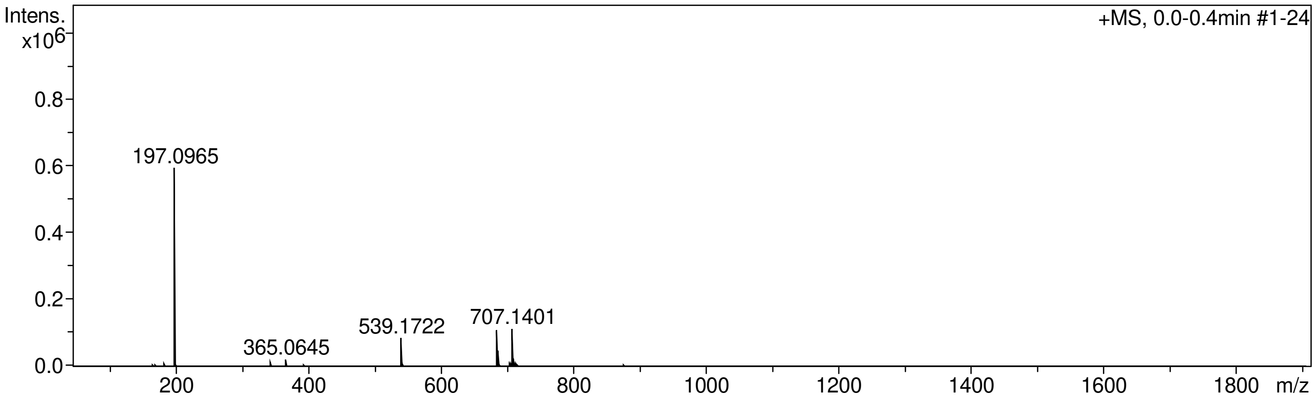

Meas. m/z	#	Ion Formula	Score	m/z	err [mDa]	err [ppm]	mSigma	rdb	e⁻ Conf	N-Rule
343.0824	1	C12H11N10OS	90.55	343.0833	0.9	2.5	6.3	12.5	even	ok
	2	C11H15N6O5S	100.00	343.0819	-0.5	-1.4	12.1	7.5	even	ok
	3	C18H15O7	55.93	343.0812	-1.2	-3.4	22.4	11.5	even	ok
	4	C19H19O2S2	69.92	343.0821	-0.3	-0.8	32.2	10.5	even	ok
	5	C19H11N4O3	65.71	343.0826	-0.2	-0.5	36.3	16.5	even	ok
	6	C12H19N6S3	58.70	343.0828	-0.4	-1.2	36.9	6.5	even	ok
	7	C11H23N2O4S3	41.54	343.0814	0.9	2.7	39.6	1.5	even	ok
365.0645	1	C11H14N6NaO5S	100.00	365.0639	-0.6	-1.8	7.1	7.5	even	ok
	2	C12H10N10NaOS	96.98	365.0652	-0.7	-1.9	7.6	12.5	even	ok
	3	C18H14NaO7	48.79	365.0632	1.3	3.7	23.4	11.5	even	ok
	4	C19H18NaO2S2	55.49	365.0640	-0.5	-1.3	37.8	10.5	even	ok
	5	C19H10N4NaO3	65.31	365.0645	-0.0	-0.0	39.2	16.5	even	ok
	6	C12H18N6NaS3	57.63	365.0647	0.2	0.6	40.5	6.5	even	ok
	7	C11H22N2NaO4S3	35.06	365.0634	1.1	3.1	41.9	1.5	even	ok

Figure S75. HRESIMS spectrum of compound **7s**

Generic Display Report

Analysis Info

Analysis Name	D:\MZ\temp\64727000001.d	Acquisition Date	04/07/2019 14:16:07
Method	tune_low_MS_Service_06_19.m	Operator	msc
Sample Name	PN_8_spot_2_peak_1	Instrument	maXis
Comment	Kalaba/ Zehl<br>Ergebnis: +/- 5ppm<br>ACN / MeOH + 1% H2O		

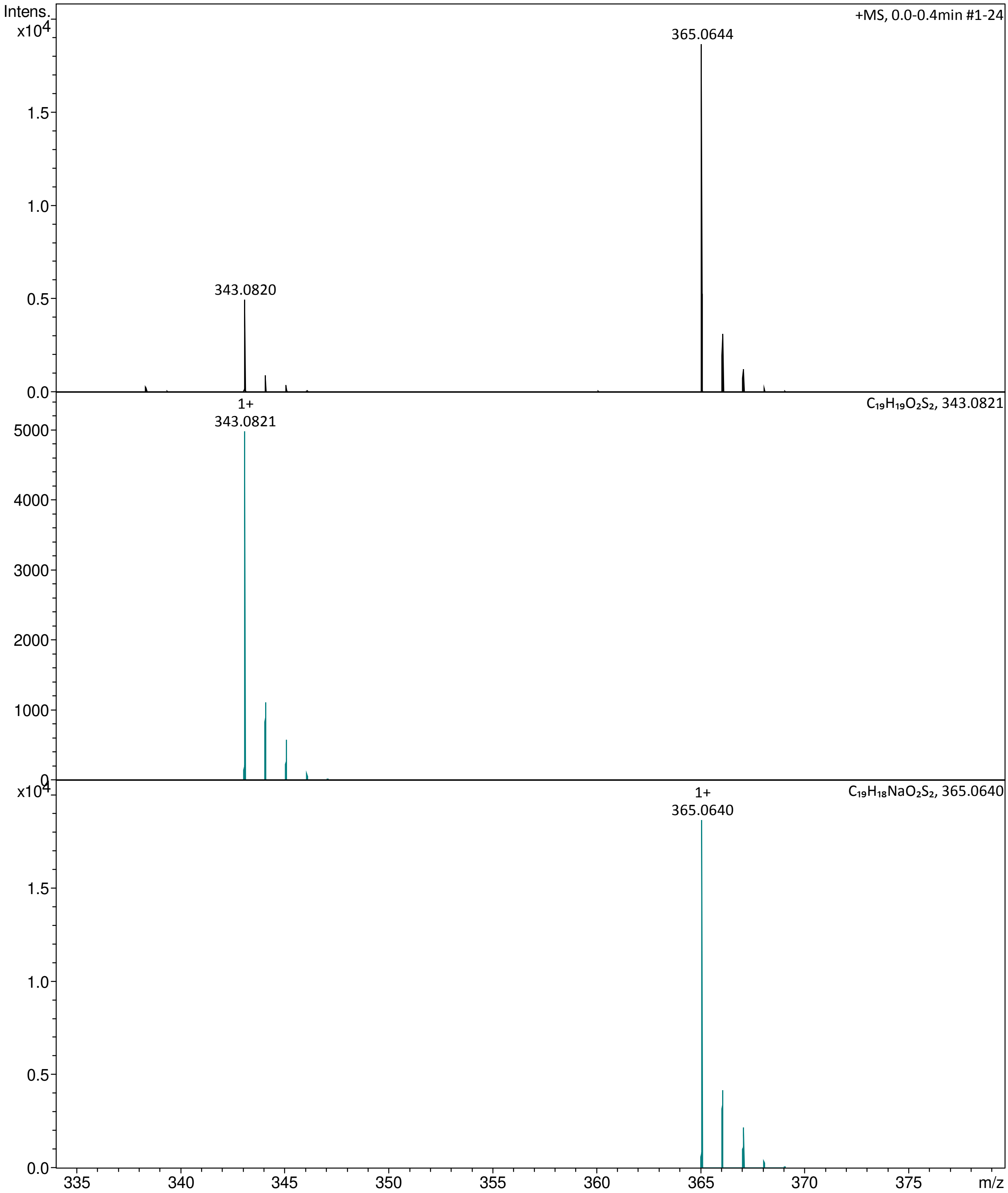

Mass Spectrum SmartFormula Report

Analysis Info

Acquisition Date 04/07/2019 14:16:07

Analysis Name D:\MZ\temp\64727000001.d
Method tune_low_MS_Service_06_19.m
Sample Name PN_8_spot_2_peak_1
Comment Kaliba/ Zehl
Ergebnis: +/- 5ppm
ACN / MeOH + 1% H2O

Operator msc
Instrument maXis 255552.00016

Acquisition Parameter

Source Type	ESI	Ion Polarity	Positive	Set Nebulizer	0.4 Bar
Focus	Not active	Set Capillary	4200 V	Set Dry Heater	150 °C
Scan Begin	50 m/z	Set End Plate Offset	-500 V	Set Dry Gas	4.0 l/min
Scan End	1900 m/z	Set Charging Voltage	0 V	Set Divert Valve	Source
		Set Corona	0 nA	Set APCI Heater	0 °C

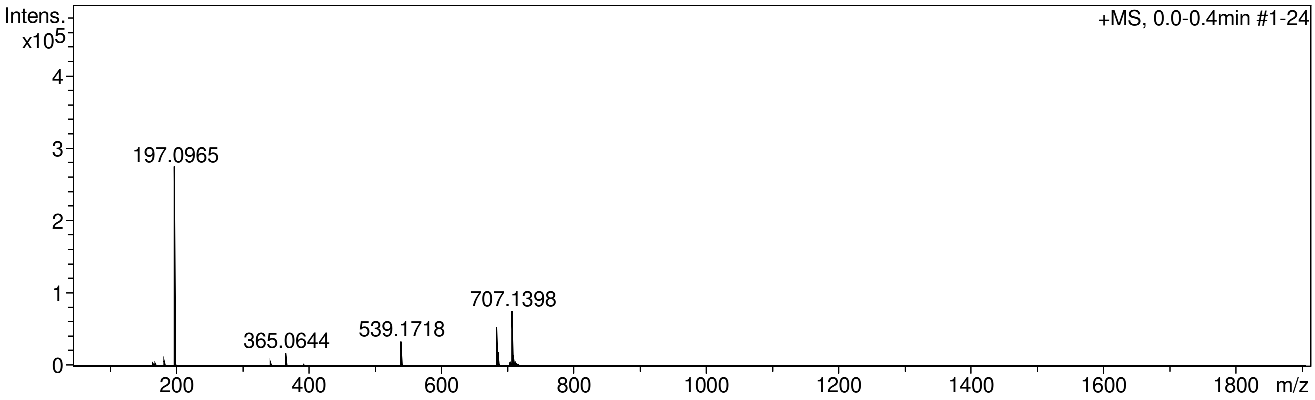

Meas. m/z	#	Ion Formula	Score	m/z	err [mDa]	err [ppm]	mSigma	rdb	e⁻ Conf	N-Rule
343.0820	1	C12H11N10OS	62.76	343.0833	-1.2	-3.6	11.6	12.5	even	ok
	2	C11H15N6O5S	100.00	343.0819	0.1	0.3	17.8	7.5	even	ok
	3	C18H15O7	60.92	343.0812	0.8	2.3	25.7	11.5	even	ok
	4	C19H19O2S2	83.00	343.0821	0.1	0.3	26.4	10.5	even	ok
	5	C10H19N2O9S	39.36	343.0806	-1.4	-4.2	27.9	2.5	even	ok
	6	C12H19N6S3	52.18	343.0828	0.8	2.3	32.2	6.5	even	ok
	7	C11H23N2O4S3	52.62	343.0814	0.6	1.6	36.1	1.5	even	ok
	8	C19H11N4O3	50.69	343.0826	0.6	1.6	37.7	16.5	even	ok
365.0644	1	C12H10N10NaOS	87.79	365.0652	0.8	2.3	6.1	12.5	even	ok
	2	C11H14N6NaO5S	100.00	365.0639	0.5	1.3	8.8	7.5	even	ok
	3	C18H14NaO7	52.84	365.0632	1.2	3.2	22.1	11.5	even	ok
	4	C19H18NaO2S2	59.52	365.0640	0.3	0.8	36.1	10.5	even	ok
	5	C19H10N4NaO3	61.62	365.0645	0.2	0.4	37.3	16.5	even	ok
	6	C12H18N6NaS3	52.30	365.0647	0.4	1.0	39.8	6.5	even	ok
	7	C11H22N2NaO4S3	36.80	365.0634	-1.0	-2.6	41.7	1.5	even	ok

Figure S76. HRESIMS spectrum of compound **8s**

Generic Display Report

Analysis Info

Analysis Name	D:\MZ\temp\64728000001.d	Acquisition Date	04/07/2019 14:18:28
Method	tune_low_MS_Service_06_19.m	Operator	msc
Sample Name	PN_8_spot_2_peak_2	Instrument	maXis
Comment	Kalaba/ Zehl<br>Ergebnis: +/- 5ppm<br>ACN / MeOH + 1% H2O		

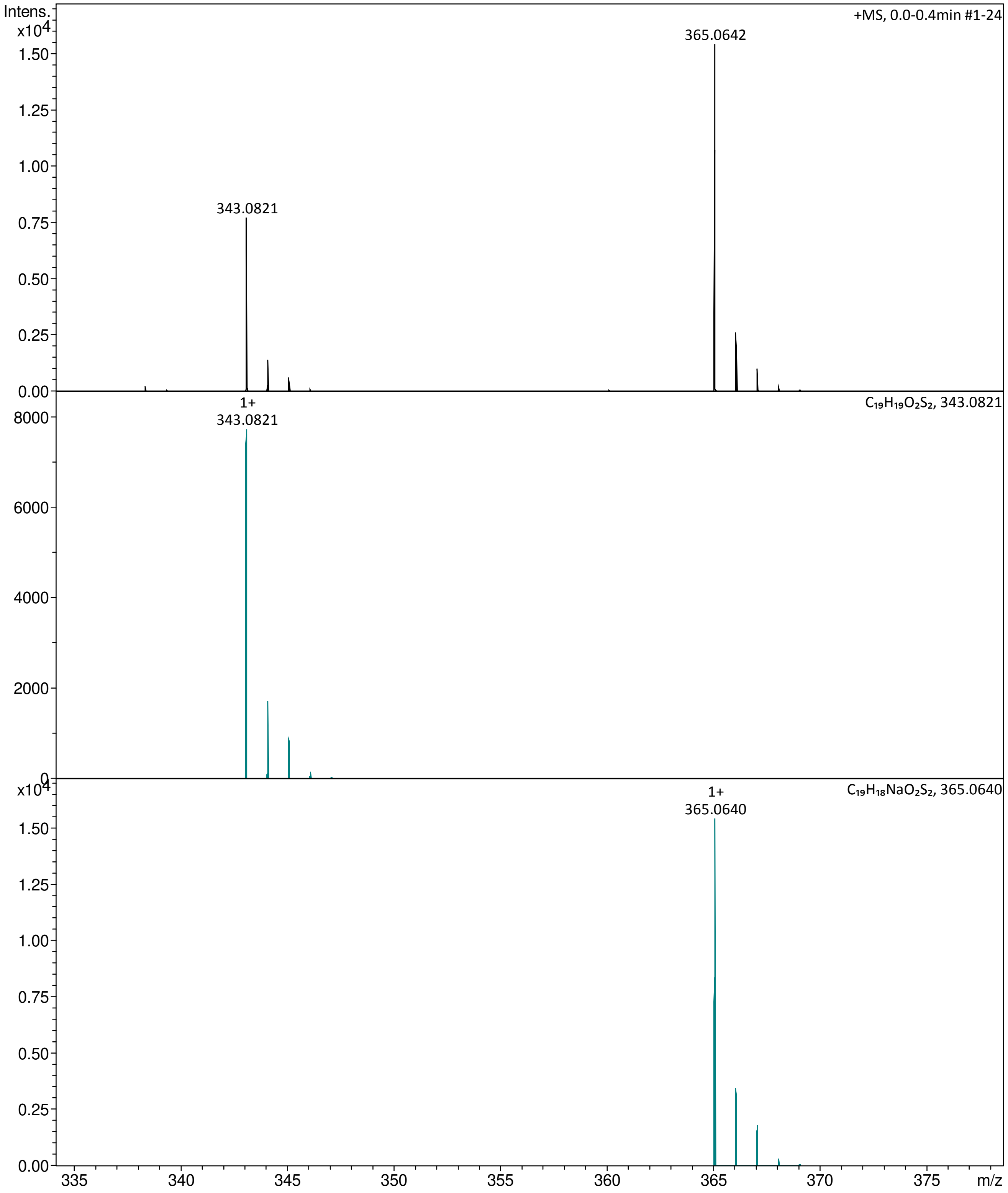

Mass Spectrum SmartFormula Report

Analysis Info

Analysis Name	D:\MZ\temp\64728000001.d	Acquisition Date	04/07/2019 14:18:28
Method	tune_low_MS_Service_06_19.m	Operator	msc
Sample Name	PN_8_spot_2_peak_2	Instrument	maXis 255552.00016
Comment	Kalaba/ Zehl<br>Ergebnis: +/- 5ppm<br>ACN / MeOH + 1% H2O		

Acquisition Parameter

Source Type	ESI	Ion Polarity	Positive	Set Nebulizer	0.4 Bar
Focus	Not active	Set Capillary	4200 V	Set Dry Heater	150 °C
Scan Begin	50 m/z	Set End Plate Offset	-500 V	Set Dry Gas	4.0 l/min
Scan End	1900 m/z	Set Charging Voltage	0 V	Set Divert Valve	Source
		Set Corona	0 nA	Set APCI Heater	0 °C

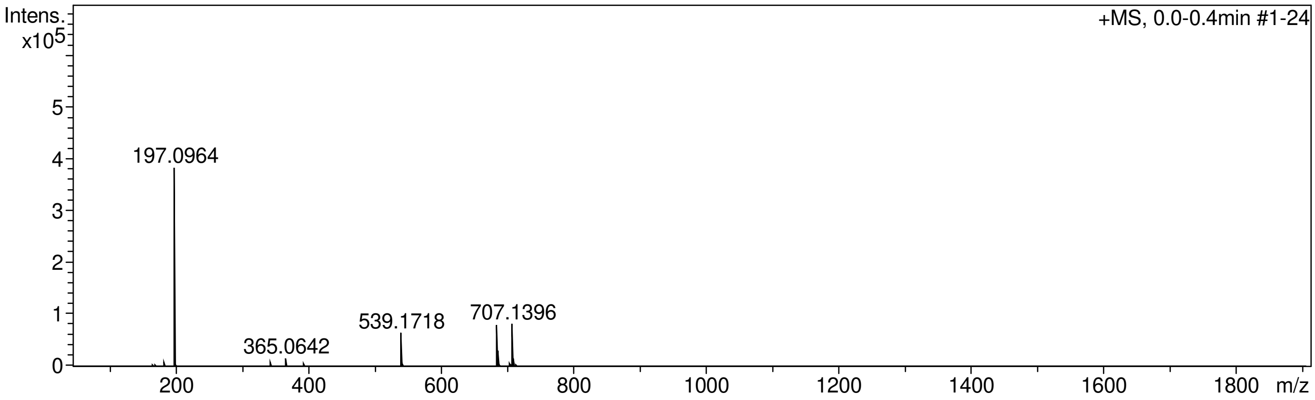

Meas. m/z	#	Ion Formula	Score	m/z	err [mDa]	err [ppm]	mSigma	rdb	e⁻ Conf	N-Rule
343.0821	1	C12H11N10OS	69.38	343.0833	1.2	3.4	10.3	12.5	even	ok
	2	C11H15N6O5S	100.00	343.0819	-0.2	-0.5	17.8	7.5	even	ok
	3	C18H15O7	63.55	343.0812	-0.9	-2.5	23.5	11.5	even	ok
	4	C19H19O2S2	87.01	343.0821	0.0	0.0	26.9	10.5	even	ok
	5	C10H19N2O9S	38.18	343.0806	-1.5	-4.4	28.5	2.5	even	ok
	6	C12H19N6S3	53.48	343.0828	-0.7	-2.0	34.0	6.5	even	ok
	7	C19H11N4O3	57.84	343.0826	0.5	1.4	35.2	16.5	even	ok
	8	C11H23N2O4S3	49.80	343.0814	-0.6	-1.9	38.0	1.5	even	ok
365.0642	1	C12H10N10NaOS	75.99	365.0652	1.0	2.8	5.5	12.5	even	ok
	2	C11H14N6NaO5S	100.00	365.0639	-0.3	-0.9	10.1	7.5	even	ok
	3	C10H18N2NaO9S	37.14	365.0625	1.7	4.5	21.4	2.5	even	ok
	4	C18H14NaO7	55.51	365.0632	1.0	2.8	21.8	11.5	even	ok
	5	C19H18NaO2S2	62.80	365.0640	-0.1	-0.4	34.7	10.5	even	ok
	6	C19H10N4NaO3	55.06	365.0645	-0.3	-0.9	36.5	16.5	even	ok
	7	C12H18N6NaS3	46.63	365.0647	0.5	1.5	38.9	6.5	even	ok
	8	C11H22N2NaO4S3	38.78	365.0634	0.8	2.2	41.2	1.5	even	ok

Figure S77. HRESIMS spectrum of compound **5t**

Generic Display Report

Analysis Info

Analysis Name	E:\Data\MS_MessService\58955000001.d	Acquisition Date	8/14/2018 5:33:13 PM
Method	tune_low_MS_Service_08_18.m	Operator	msc
Sample Name	CE-149-SPOT1-PEAK1	Instrument	maXis
Comment	Kalaba/Zehl<br>Ergebnis: +/- 5ppm<br>ACN/MeOH +0.1% H2O		

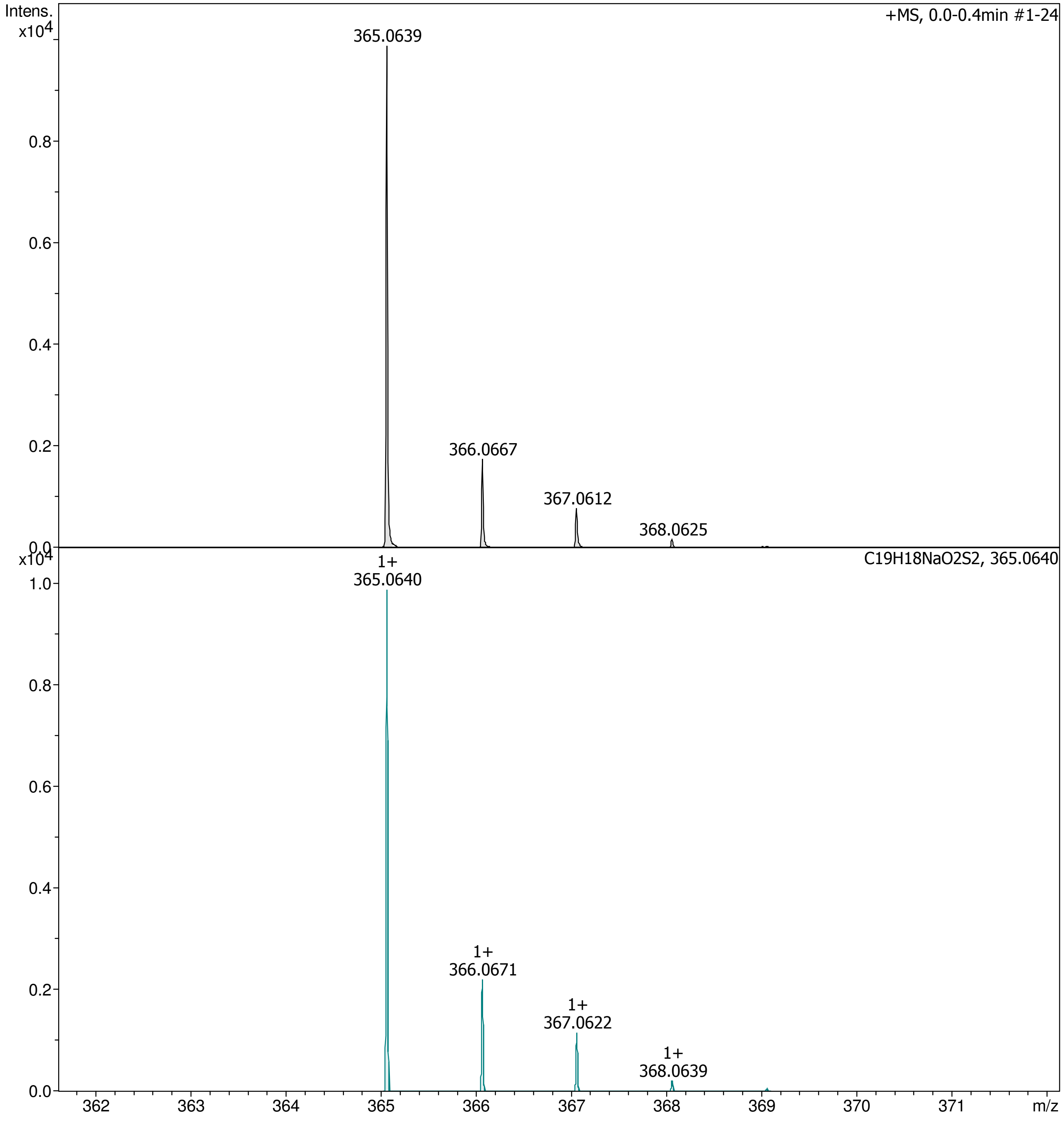

Mass Spectrum SmartFormula Report

Analysis Info

Analysis Name	E:\Data\MS_MessService\58955000001.d	Acquisition Date	8/14/2018 5:33:13 PM
Method	tune_low_MS_Service_08_18.m	Operator	msc
Sample Name	CE-149-SPOT1-PEAK1	Instrument	maXis 255552.00016
Comment	Kalaba/Zehl<br>Ergebnis: +/- 5ppm<br>ACN/MeOH +0.1% H2O		

Acquisition Parameter

Source Type	ESI	Ion Polarity	Positive	Set Nebulizer	0.4 Bar
Focus	Not active	Set Capillary	4500 V	Set Dry Heater	180 °C
Scan Begin	50 m/z	Set End Plate Offset	-500 V	Set Dry Gas	4.0 l/min
Scan End	1900 m/z	Set Charging Voltage	0 V	Set Divert Valve	Source
		Set Corona	0 nA	Set APCI Heater	0 °C

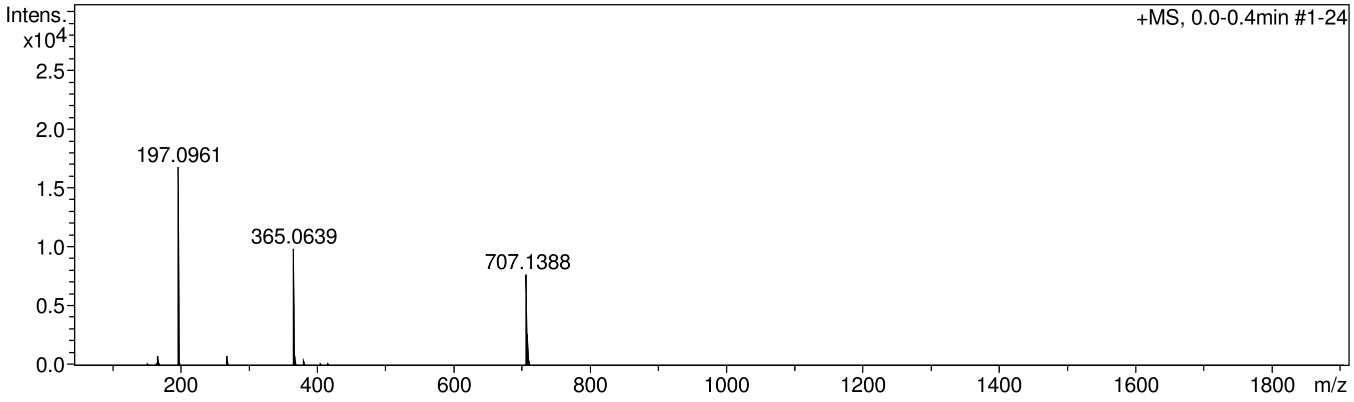

Meas. m/z	#	Ion Formula	Score	m/z	err [mDa]	err [ppm]	mSigma	rdb	e⁻ Conf	N-Rule
365.063889	1	C13H16N3NaO6S	56.16	365.065202	1.3	3.6	8.3	7.0	odd	ok
	2	C12H10N10NaOS	55.50	365.065197	1.3	3.6	9.1	12.5	even	ok
	3	C10H8N13NaS	100.00	365.063854	-0.0	-0.1	12.5	13.0	odd	ok
	4	C11H14N6NaO5S	97.51	365.063859	0.0	0.1	13.9	7.5	even	ok
	5	C17H16N3NaOS2	45.20	365.062700	-1.2	-3.3	22.9	11.0	odd	ok
	6	C10H18N2NaO9S	39.20	365.062522	1.4	3.7	24.3	2.5	even	ok
	7	C18H14NaO7	41.99	365.063173	-0.7	-2.0	25.2	11.5	even	ok
	8	C19H18NaO2S2	68.31	365.064043	0.2	0.4	28.0	10.5	even	ok
	9	C12H18N6NaS3	44.30	365.064728	-0.8	-2.3	32.2	6.5	even	ok
	10	C11H22N2NaO4S3	48.47	365.063391	-0.5	-1.4	35.6	1.5	even	ok
	11	C17H8N7NaO2	31.78	365.063168	-0.7	-2.0	36.4	17.0	odd	ok
	12	C19H10N4NaO3	31.61	365.064511	-0.6	-1.7	38.5	16.5	even	ok

Figure S78. HRESIMS spectrum of compound **6t**

Generic Display Report

Analysis Info

Analysis Name	E:\Data\MS_MessService\58956000001.d	Acquisition Date	8/14/2018 5:38:55 PM
Method	tune_low_MS_Service_08_18.m	Operator	msc
Sample Name	CE-149-SPOT1-PEAK2	Instrument	maXis
Comment	Kalaba/Zehl<br>Ergebnis: +/- 5ppm<br>ACN/MeOH +0.1% H2O		

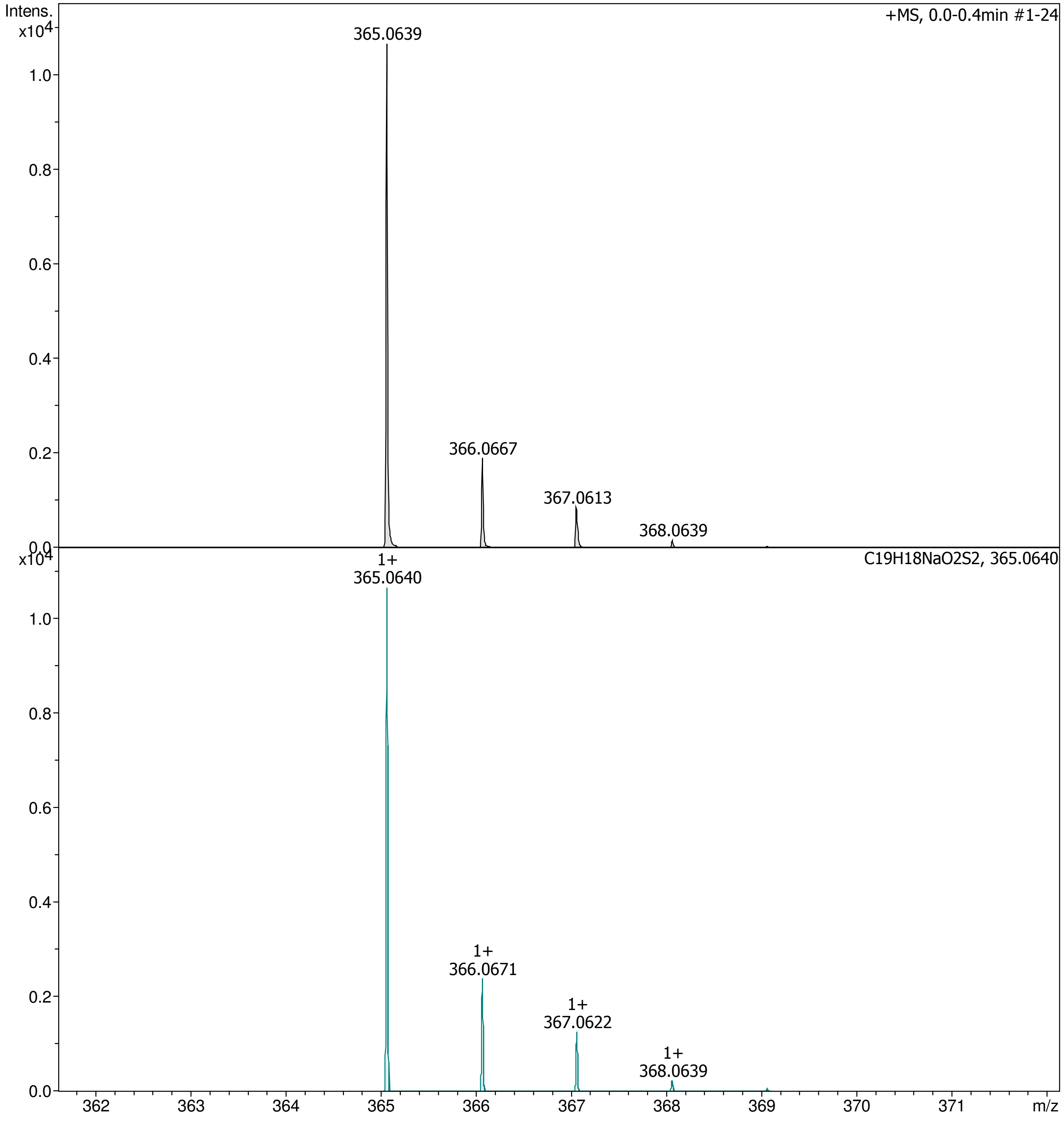

Mass Spectrum SmartFormula Report

Analysis Info

Analysis Name	E:\Data\MS_MessService\58956000001.d	Acquisition Date	8/14/2018 5:38:55 PM
Method	tune_low_MS_Service_08_18.m	Operator	msc
Sample Name	CE-149-SPOT1-PEAK2	Instrument	maXis 255552.00016
Comment	Kalaba/Zehl<br>Ergebnis: +/- 5ppm<br>ACN/MeOH +0.1% H2O		

Acquisition Parameter

Source Type	ESI	Ion Polarity	Positive	Set Nebulizer	0.4 Bar
Focus	Not active	Set Capillary	4500 V	Set Dry Heater	180 °C
Scan Begin	50 m/z	Set End Plate Offset	-500 V	Set Dry Gas	4.0 l/min
Scan End	1900 m/z	Set Charging Voltage	0 V	Set Divert Valve	Source
		Set Corona	0 nA	Set APCI Heater	0 °C

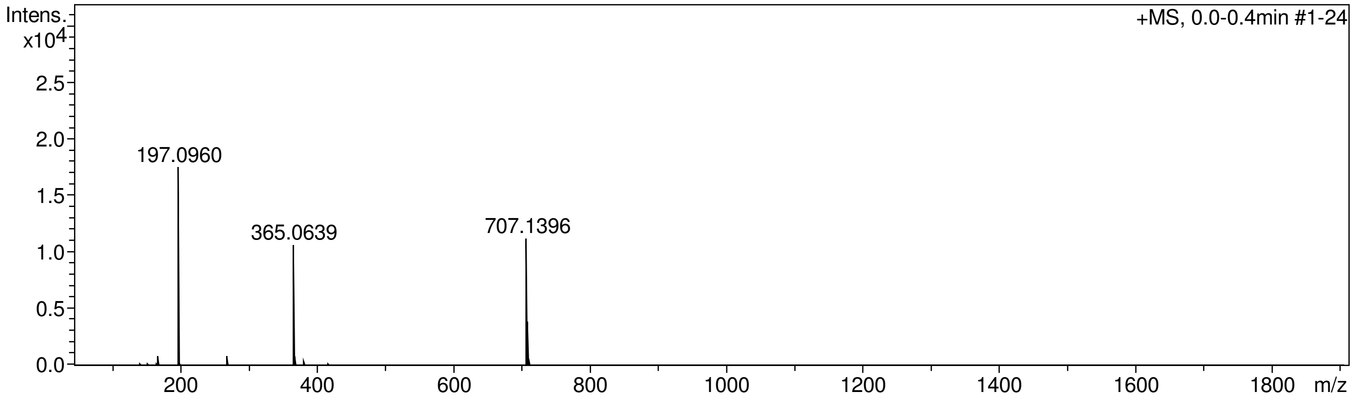

Meas. m/z	#	Ion Formula	Score	m/z	err [mDa]	err [ppm]	mSigma	rdb	e^- Conf	N-Rule
365.063943	1	C13H16N3NaO6S	59.55	365.065202	-1.3	-3.4	8.8	7.0	odd	ok
	2	C12H10N10NaOS	58.89	365.065197	-1.3	-3.4	9.6	12.5	even	ok
	3	C10H8N13NaS	100.00	365.063854	0.1	0.2	13.1	13.0	odd	ok
	4	C11H14N6NaO5S	97.59	365.063859	0.1	0.2	14.5	7.5	even	ok
	5	C17H16N3NaOS2	45.90	365.062700	1.2	3.4	22.2	11.0	odd	ok
	6	C10H18N2NaO9S	38.75	365.062522	-1.4	-3.9	24.7	2.5	even	ok
	7	C18H14NaO7	55.03	365.063173	-0.8	-2.1	26.2	11.5	even	ok
	8	C19H18NaO2S2	73.38	365.064043	0.1	0.3	27.4	10.5	even	ok
	9	C12H18N6NaS3	47.83	365.064728	-0.8	-2.2	31.7	6.5	even	ok
	10	C11H22N2NaO4S3	49.38	365.063391	-0.6	-1.5	35.1	1.5	even	ok
	11	C17H8N7NaO2	31.62	365.063168	-0.8	-2.1	36.8	17.0	odd	ok
	12	C19H10N4NaO3	33.38	365.064511	-0.6	-1.6	38.8	16.5	even	ok

Figure S79. HRESIMS spectrum of compound **7t**

Generic Display Report

Analysis Info

Analysis Name	E:\Data\MS_MessService\58957000001.d
Method	tune_low_MS_Service_08_18.m
Sample Name	CE-149-SPOT2-PEAK1
Comment	Kalaba/Zehl<br>Ergebnis: +/- 5ppm<br>ACN/MeOH +0.1% H2O
Acquisition Date	8/14/2018 5:41:18 PM
Operator	msc
Instrument	maXis

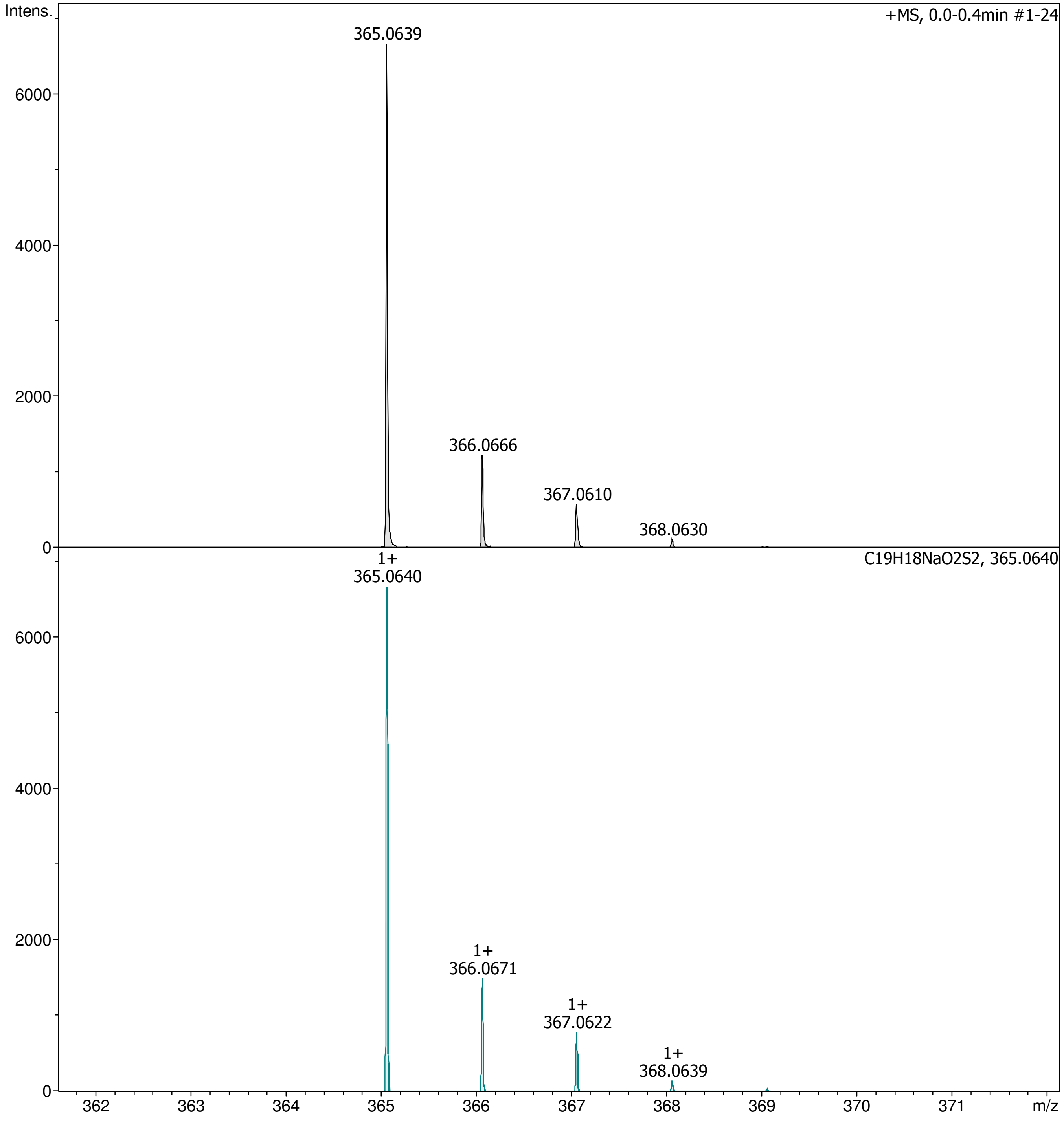

Mass Spectrum SmartFormula Report

Analysis Info

Analysis Name	E:\Data\MS_MessService\58957000001.d	Acquisition Date	8/14/2018 5:41:18 PM
Method	tune_low_MS_Service_08_18.m	Operator	msc
Sample Name	CE-149-SPOT2-PEAK1	Instrument	maXis 255552.00016
Comment	Kalaba/Zehl<br>Ergebnis: +/- 5ppm<br>ACN/MeOH +0.1% H2O		

Acquisition Parameter

Source Type	ESI	Ion Polarity	Positive	Set Nebulizer	0.4 Bar
Focus	Not active	Set Capillary	4500 V	Set Dry Heater	180 °C
Scan Begin	50 m/z	Set End Plate Offset	-500 V	Set Dry Gas	4.0 l/min
Scan End	1900 m/z	Set Charging Voltage	0 V	Set Divert Valve	Source
		Set Corona	0 nA	Set APCI Heater	0 °C

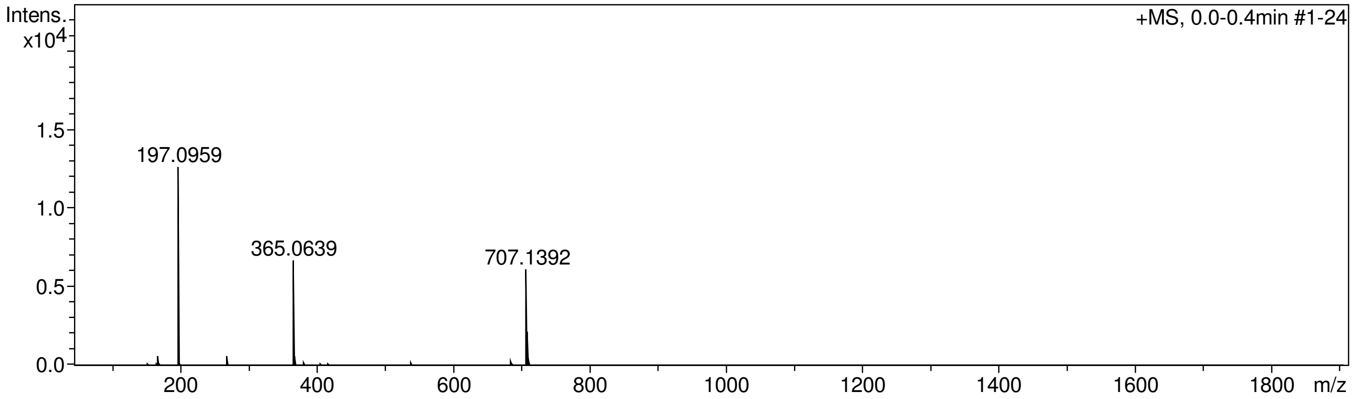

Meas. m/z	#	Ion Formula	Score	m/z	err [mDa]	err [ppm]	mSigma	rdb	e⁻ Conf	N-Rule
365.063898	1	C13H16N3NaO6S	57.17	365.065202	-1.3	-3.6	13.8	7.0	odd	ok
	2	C12H10N10NaOS	57.32	365.065197	-1.3	-3.6	13.9	12.5	even	ok
	3	C17H16N3NaOS2	56.58	365.062700	1.2	3.3	17.6	11.0	odd	ok
	4	C10H8N13NaS	100.00	365.063854	-0.0	-0.1	18.2	13.0	odd	ok
	5	C11H14N6NaO5S	97.45	365.063859	-0.0	-0.1	19.5	7.5	even	ok
	6	C19H18NaO2S2	86.95	365.064043	0.1	0.4	22.7	10.5	even	ok
	7	C18H14NaO7	44.13	365.063173	0.7	2.0	27.9	11.5	even	ok
	8	C12H18N6NaS3	54.80	365.064728	0.8	2.3	28.5	6.5	even	ok
	9	C10H18N2NaO9S	39.10	365.062522	-1.4	-3.8	29.1	2.5	even	ok
	10	C11H22N2NaO4S3	57.66	365.063391	0.5	1.4	33.1	1.5	even	ok
	11	C17H8N7NaO2	33.48	365.063168	0.7	2.0	38.7	17.0	odd	ok
	12	C19H10N4NaO3	34.71	365.064511	0.6	1.7	39.6	16.5	even	ok

Figure S80. HRESIMS spectrum of compound **8t**

Generic Display Report

Analysis Info

Analysis Name	E:\Data\MS_MessService\58958000001.d	Acquisition Date	8/14/2018 5:46:28 PM
Method	tune_low_MS_Service_08_18.m	Operator	msc
Sample Name	CE-149-SPOT2-PEAK2	Instrument	maXis
Comment	Kalaba/Zehl<br>Ergebnis: +/- 5ppm<br>ACN/MeOH +0.1% H2O		

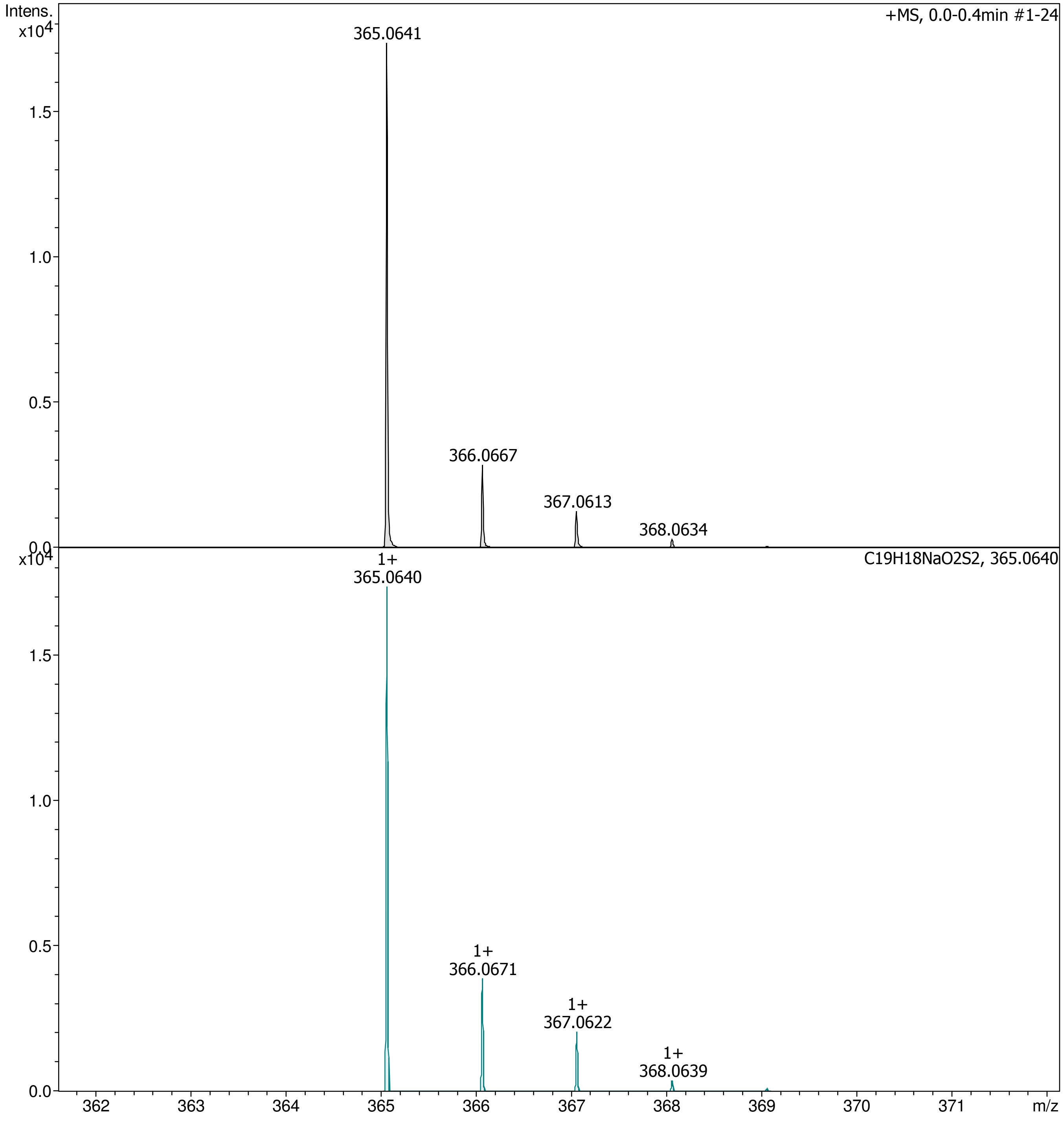

Mass Spectrum SmartFormula Report

Analysis Info

Analysis Name	E:\Data\MS_MessService\58958000001.d	Acquisition Date	8/14/2018 5:46:28 PM
Method	tune_low_MS_Service_08_18.m	Operator	msc
Sample Name	CE-149-SPOT2-PEAK2	Instrument	maXis 255552.00016
Comment	Kalaba/Zehl<br>Ergebnis: +/- 5ppm<br>ACN/MeOH +0.1% H2O		

Acquisition Parameter

Source Type	ESI	Ion Polarity	Positive	Set Nebulizer	0.4 Bar
Focus	Not active	Set Capillary	4500 V	Set Dry Heater	180 °C
Scan Begin	50 m/z	Set End Plate Offset	-500 V	Set Dry Gas	4.0 l/min
Scan End	1900 m/z	Set Charging Voltage	0 V	Set Divert Valve	Source
		Set Corona	0 nA	Set APCI Heater	0 °C

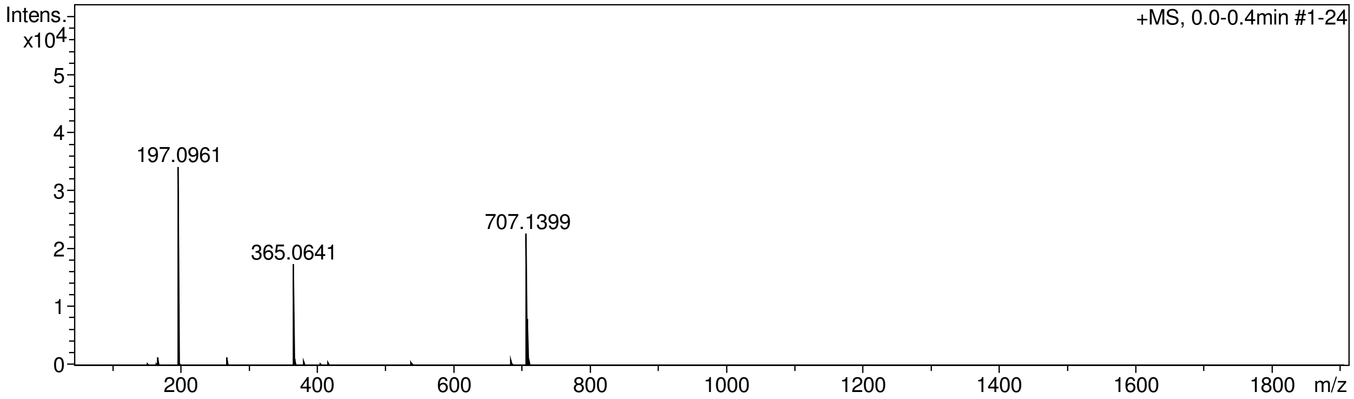

Meas. m/z	#	Ion Formula	Score	m/z	err [mDa]	err [ppm]	mSigma	rdb	e^- Conf	N-Rule
365.064114	1	C13H16N3NaO6S	69.70	365.065202	1.1	3.0	3.1	7.0	odd	ok
	2	C11H14N6NaO5S	100.00	365.063859	0.3	0.7	6.9	7.5	even	ok
	3	C10H8N13NaS	97.87	365.063854	0.3	0.7	8.0	13.0	odd	ok
	4	C12H10N10NaOS	63.26	365.065197	1.1	3.0	8.8	12.5	even	ok
	5	C10H18N2NaO9S	38.74	365.062522	1.6	4.4	17.4	2.5	even	ok
	6	C18H14NaO7	35.78	365.063173	0.9	2.6	26.3	11.5	even	ok
	7	C17H16N3NaOS2	33.44	365.062700	-1.4	-3.9	29.3	11.0	odd	ok
	8	C19H18NaO2S2	59.50	365.064043	0.1	0.2	34.5	10.5	even	ok
	9	C12H18N6NaS3	44.97	365.064728	0.6	1.7	35.8	6.5	even	ok
	10	C11H22N2NaO4S3	40.86	365.063391	0.7	2.0	37.3	1.5	even	ok
	11	C17H8N7NaO2	26.33	365.063168	0.9	2.6	38.5	17.0	odd	ok
	12	C21H12NNaO4	15.27	365.065854	1.7	4.8	40.0	16.0	odd	ok
	13	C19H10N4NaO3	31.74	365.064511	-0.4	-1.1	41.9	16.5	even	ok

Figure S81. HRESIMS spectrum of compound **5u**

Generic Display Report

Analysis Info

Analysis Name	D:\MZ\maXis_data\temp\60657000001.d	Acquisition Date	11/8/2018 1:20:02 PM
Method	tune_low_MS_Service_11_18.m	Operator	msc
Sample Name	CE-156-spot1-peak1	Instrument	maXis
Comment	Kalaba/Zehl<br>Ergebnis: +/- 5ppm<br>ACN/MeOH + 1%H2O		

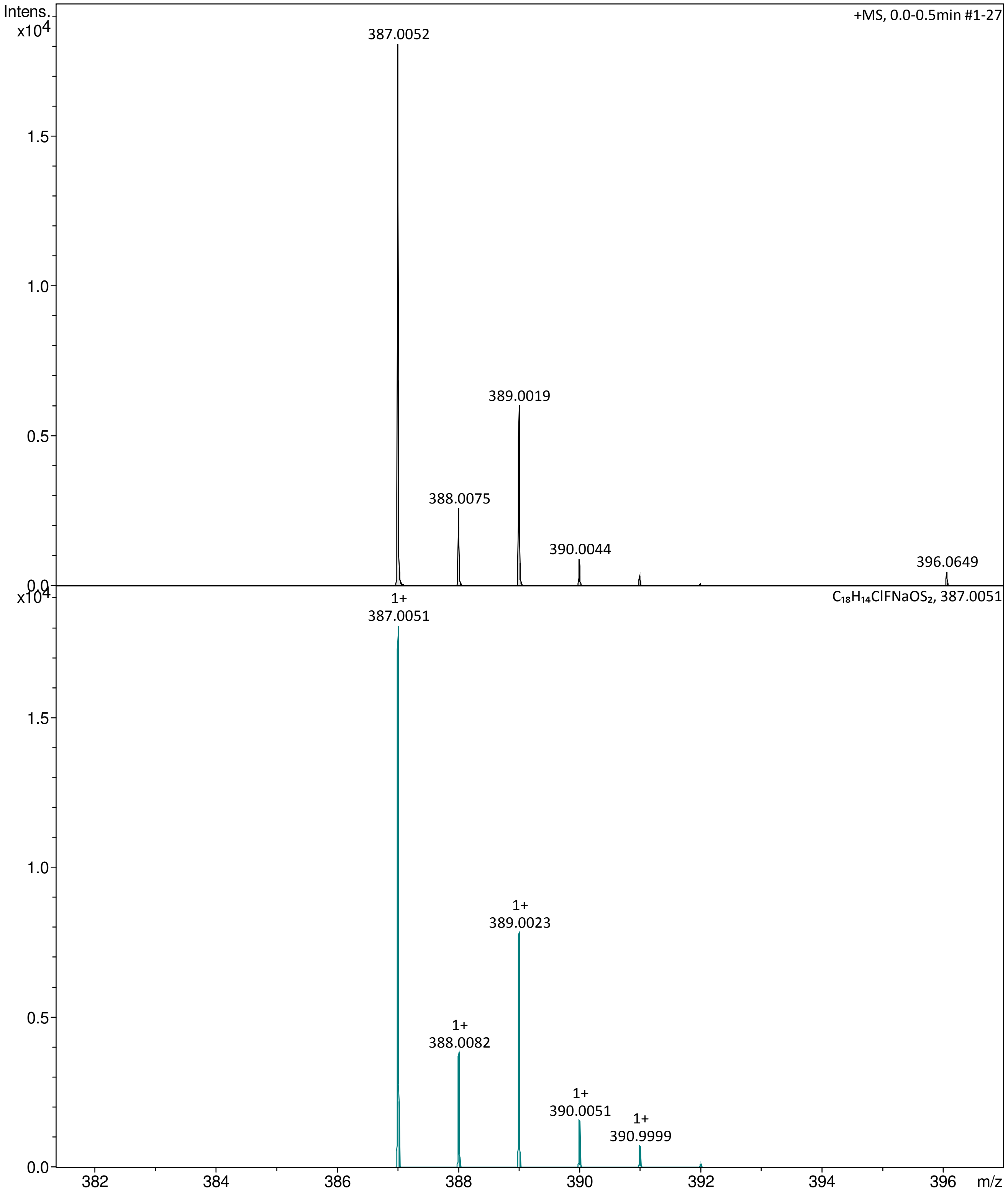

Mass Spectrum SmartFormula Report

Analysis Info

Analysis Name	D:\MZ\maXis_data\temp\60657000001.d	Acquisition Date	11/8/2018 1:20:02 PM
Method	tune_low_MS_Service_11_18.m	Operator	msc
Sample Name	CE-156-spot1-peak1	Instrument	maXis 255552.00016
Comment	Kalaba/Zehl<br>Ergebnis: +/- 5ppm<br>ACN/MeOH + 1%H2O		

Acquisition Parameter

Source Type	ESI	Ion Polarity	Positive	Set Nebulizer	0.4 Bar
Focus	Not active	Set Capillary	4500 V	Set Dry Heater	150 °C
Scan Begin	50 m/z	Set End Plate Offset	-500 V	Set Dry Gas	4.0 l/min
Scan End	1800 m/z	Set Charging Voltage	0 V	Set Divert Valve	Source
		Set Corona	0 nA	Set APCI Heater	0 °C

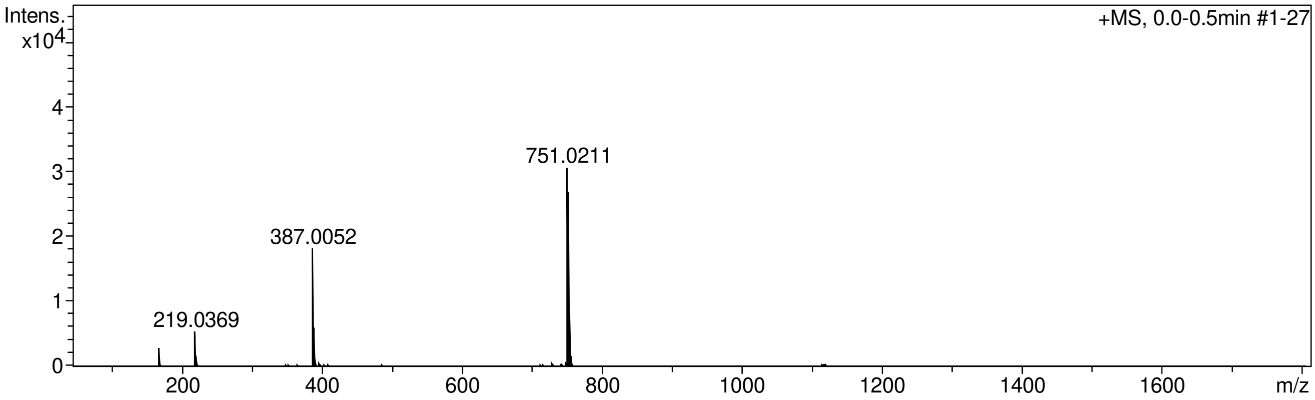

Meas. m/z	#	Ion Formula	Score	m/z	err [mDa]	err [ppm]	mSigma	rdb	e⁻ Conf	N-Rule
167.9670	1	C5H5NaOS2	100.00	167.9674	0.4	2.2	8.0	3.0	odd	ok
	2	C4HNaO6	58.81	167.9665	-0.5	-3.0	11.2	4.0	odd	ok
	3	C2H3F2NaO3S	59.33	167.9663	0.7	4.3	25.0	0.0	odd	ok
	4	CH2FNaO7	38.55	167.9677	0.6	3.8	28.0	0.0	odd	ok
219.0369	1	C13H9ClF	100.00	219.0371	-0.2	-1.0	17.8	8.5	even	ok
387.0052	1	C11H3ClF2N10NaO	59.81	387.0040	1.2	3.0	8.5	13.5	even	ok
	2	C13H6ClF5N4NaO	68.73	387.0043	-0.9	-2.4	8.6	9.5	even	ok
	3	C14H11ClF2NaO7	98.41	387.0054	0.2	0.5	9.0	7.5	even	ok
	4	C8H4ClF3N10NaO2	100.00	387.0052	-0.0	-0.1	11.6	9.5	even	ok
	5	C11H12ClF3NaO8	51.24	387.0065	1.3	3.4	11.7	3.5	even	ok
	6	C9H9ClN6NaO8	59.28	387.0063	-1.1	-2.8	11.7	7.5	even	ok
	7	C15H7ClF2N4NaO3	40.21	387.0067	1.5	3.9	17.5	12.5	even	ok
	8	C7H8ClF3N6NaO6	41.17	387.0038	1.4	3.5	21.2	4.5	even	ok
	9	C12H13ClF4NaO4S	80.50	387.0051	0.0	0.1	22.0	3.5	even	ok
	10	C10H10ClFN6NaO4S	72.63	387.0049	-0.3	-0.7	22.0	7.5	even	ok
	11	C17H10ClFNaO6	51.39	387.0042	1.0	2.5	22.1	11.5	even	ok
	12	C8H13ClN2NaO12	73.09	387.0049	0.3	0.7	22.2	2.5	even	ok
	13	C13H9ClF4N4NaS	41.76	387.0065	-1.3	-3.4	22.3	8.5	even	ok
	14	C11H6ClFN10NaS	48.28	387.0062	1.1	2.7	22.3	12.5	even	ok
	15	C9H14ClF5NaO5S	42.75	387.0063	1.1	2.9	26.5	-0.5	even	ok
	16	C7H11ClF2N6NaO5S	48.97	387.0060	0.9	2.2	26.6	3.5	even	ok
	17	C9H14ClFN2NaO8S	30.49	387.0036	1.6	4.2	27.1	2.5	even	ok
	18	C15H12ClF3NaO3S	40.11	387.0040	-1.2	-3.0	27.4	7.5	even	ok
	19	C13H9ClN6NaO3S	34.49	387.0038	1.4	3.7	27.4	11.5	even	ok
	20	C18H6ClFN4NaO2	54.73	387.0056	0.4	1.0	32.2	16.5	even	ok
	21	C17H13ClNaO5S	38.96	387.0064	1.3	3.3	33.9	10.5	even	ok
	22	C8H12ClF3N6NaOS2	55.89	387.0047	0.5	1.3	36.3	3.5	even	ok
	23	C13H14ClF5NaS2	32.62	387.0038	-1.4	-3.6	37.5	3.5	even	ok
	24	C11H11ClF2N6NaS2	27.76	387.0035	-1.6	-4.2	37.6	7.5	even	ok
	25	C9H17ClN2NaO7S2	45.66	387.0058	-0.6	-1.6	41.6	1.5	even	ok
	26	C15H15ClF2NaO2S2	33.78	387.0062	-1.0	-2.7	43.9	6.5	even	ok
	27	C21H5ClN4NaO	29.90	387.0044	-0.8	-2.0	47.2	20.5	even	ok
	28	C18H14ClFNaOS2	41.66	387.0051	-0.1	-0.2	52.7	10.5	even	ok
	29	C8H15ClF2N6NaS3	15.54	387.0069	1.7	4.5	55.0	2.5	even	ok
	30	C10H18ClFN2NaO3S3	25.72	387.0044	0.7	1.9	58.2	1.5	even	ok

Mass Spectrum SmartFormula Report

Meas. m/z	#	Ion Formula	Score	m/z	err [mDa]	err [ppm]	mSigma	rdb	e⁻ Conf	N-Rule
	31	C13H17ClN2NaO2S3	11.13	387.0033	-1.9	-4.9	61.7	5.5	even	ok
	32	C21H13ClNaS2	16.05	387.0039	-1.2	-3.2	63.9	14.5	even	ok
	33	C10H21ClN2NaO2S4	10.04	387.0067	1.5	3.8	72.4	0.5	even	ok

Figure S82. HRESIMS spectrum of compound **6u**

Generic Display Report

Analysis Info

Analysis Name	D:\MZ\maXis_data\temp\60658000001.d	Acquisition Date	11/8/2018 1:24:10 PM
Method	tune_low_MS_Service_11_18.m	Operator	msc
Sample Name	CE-156-spot1-peak2	Instrument	maXis
Comment	Kalaba/Zehl<br>Ergebnis: +/- 5ppm<br>ACN/MeOH + 1%H2O		

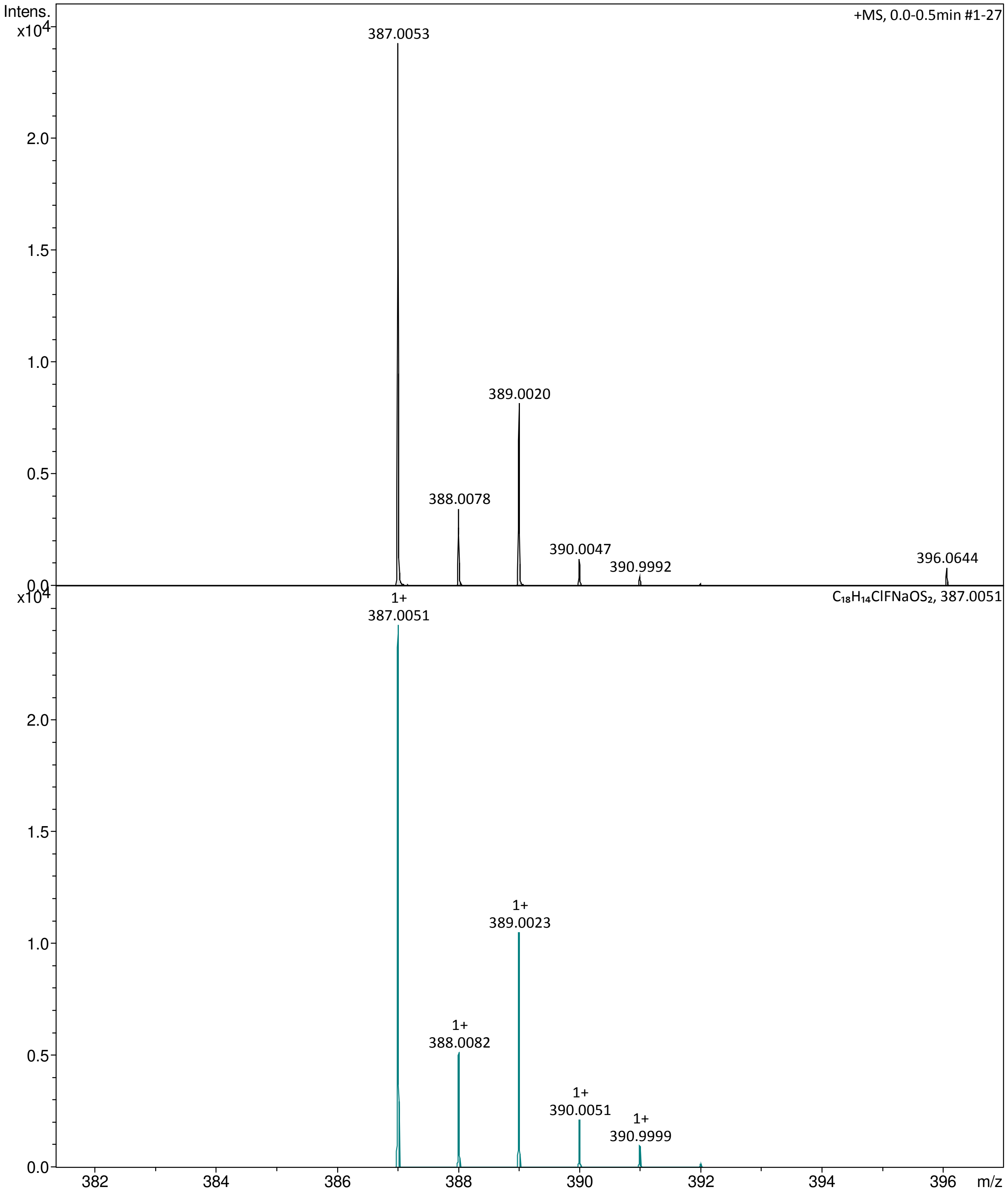

Mass Spectrum SmartFormula Report

Analysis Info

Acquisition Date 11/8/2018 1:24:10 PM

Analysis Name D:\MZ\maXis_data\temp\60658000001.d

Method tune_low_MS_Service_11_18.m

Operator msc

Sample Name CE-156-spot1-peak2

Instrument maXis 255552.00016

Comment Kalaba/Zehl
Ergebnis: +/- 5ppm
ACN/MeOH + 1%H2O

Acquisition Parameter

Source Type	ESI	Ion Polarity	Positive	Set Nebulizer	0.4 Bar
Focus	Not active	Set Capillary	4500 V	Set Dry Heater	150 °C
Scan Begin	50 m/z	Set End Plate Offset	-500 V	Set Dry Gas	4.0 l/min
Scan End	1800 m/z	Set Charging Voltage	0 V	Set Divert Valve	Source
		Set Corona	0 nA	Set APCI Heater	0 °C

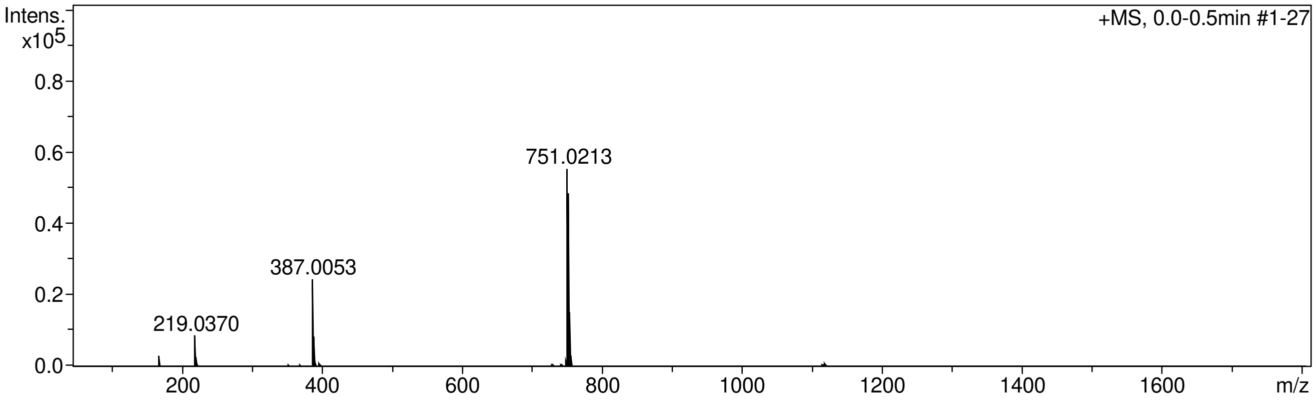

Meas. m/z	#	Ion Formula	Score	m/z	err [mDa]	err [ppm]	mSigma	rdb	e⁻ Conf	N-Rule
167.9670	1	C5H5NaOS2	100.00	167.9674	-0.4	-2.3	9.7	3.0	odd	ok
	2	C4HNaO6	62.02	167.9665	-0.5	-2.9	11.1	4.0	odd	ok
	3	C2H3F2NaO3S	65.42	167.9663	-0.7	-4.2	23.0	0.0	odd	ok
	4	CH2FNaO7	39.92	167.9677	0.7	3.9	27.8	0.0	odd	ok
219.0370	1	C13H9ClF	100.00	219.0371	-0.2	-0.8	24.4	8.5	even	ok
	2	C5H13ClFN2O2S	52.04	219.0365	0.5	2.2	44.3	-0.5	even	ok
387.0053	1	C14H11ClF2NaO7	100.00	387.0054	-0.1	-0.3	8.3	7.5	even	ok
	2	C11H3ClF2N10NaO	55.26	387.0040	1.2	3.2	8.5	13.5	even	ok
	3	C13H6ClF5N4NaO	63.72	387.0043	1.0	2.6	8.6	9.5	even	ok
	4	C11H12ClF3NaO8	53.42	387.0065	1.2	3.2	10.6	3.5	even	ok
	5	C9H9ClN6NaO8	61.58	387.0063	-1.0	-2.6	10.6	7.5	even	ok
	6	C8H4ClF3N10NaO2	94.97	387.0052	-0.1	-0.3	11.0	9.5	even	ok
	7	C15H7ClF2N4NaO3	41.06	387.0067	1.4	3.7	17.7	12.5	even	ok
	8	C7H8ClF3N6NaO6	38.52	387.0038	-1.4	-3.7	20.5	4.5	even	ok
	9	C12H13ClF4NaO4S	77.58	387.0051	0.1	0.3	20.8	3.5	even	ok
	10	C10H10ClFN6NaO4S	69.77	387.0049	-0.4	-0.9	20.8	7.5	even	ok
	11	C8H13ClN2NaO12	69.87	387.0049	-0.3	-0.9	21.2	2.5	even	ok
	12	C13H9ClF4N4NaS	43.37	387.0065	-1.2	-3.2	21.5	8.5	even	ok
	13	C11H6ClFN10NaS	49.97	387.0062	1.0	2.5	21.5	12.5	even	ok
	14	C17H10ClFNaO6	47.59	387.0042	1.0	2.7	22.1	11.5	even	ok
	15	C9H14ClF5NaO5S	44.78	387.0063	1.0	2.7	25.2	-0.5	even	ok
	16	C7H11ClF2N6NaO5S	51.11	387.0060	0.8	2.0	25.3	3.5	even	ok
	17	C9H14ClFN2NaO8S	28.82	387.0036	1.7	4.4	25.9	2.5	even	ok
	18	C15H12ClF3NaO3S	37.67	387.0040	-1.3	-3.3	26.7	7.5	even	ok
	19	C13H9ClN6NaO3S	32.27	387.0038	1.5	3.9	26.7	11.5	even	ok
	20	C18H6ClFN4NaO2	54.75	387.0056	-0.3	-0.8	32.4	16.5	even	ok
	21	C17H13ClNaO5S	40.15	387.0064	1.2	3.1	33.4	10.5	even	ok
	22	C8H12ClF3N6NaOS2	53.60	387.0047	0.6	1.5	35.3	3.5	even	ok
	23	C13H14ClF5NaS2	30.73	387.0038	-1.5	-3.8	36.7	3.5	even	ok
	24	C11H11ClF2N6NaS2	26.05	387.0035	-1.7	-4.4	36.7	7.5	even	ok
	25	C10H13ClN6NaO3S2	22.46	387.0071	-1.9	-4.8	38.1	6.5	even	ok
	26	C9H17ClN2NaO7S2	47.45	387.0058	0.5	1.4	40.6	1.5	even	ok
	27	C15H15ClF2NaO2S2	35.02	387.0062	1.0	2.5	43.3	6.5	even	ok
	28	C21H5ClN4NaO	27.55	387.0044	-0.8	-2.2	47.5	20.5	even	ok
	29	C18H14ClFNaOS2	39.66	387.0051	0.2	0.5	52.3	10.5	even	ok

Mass Spectrum SmartFormula Report

Meas. m/z	#	Ion Formula	Score	m/z	err [mDa]	err [ppm]	mSigma	rdb	e⁻ Conf	N-Rule
	30	C8H15ClF2N6NaS3	16.46	387.0069	1.7	4.3	54.0	2.5	even	ok
	31	C10H18ClFN2NaO3S3	24.64	387.0044	0.8	2.1	57.3	1.5	even	ok
	32	C21H13ClNaS2	14.97	387.0039	-1.3	-3.4	63.5	14.5	even	ok
	33	C10H21ClN2NaO2S4	10.58	387.0067	1.4	3.6	71.6	0.5	even	ok

Figure S83. HRESIMS spectrum of compound **7u**

Generic Display Report

Analysis Info

Analysis Name	D:\MZ\maXis_data\temp\60659000001.d	Acquisition Date	11/8/2018 1:27:05 PM
Method	tune_low_MS_Service_11_18.m	Operator	msc
Sample Name	CE-156-spot2-peak1	Instrument	maXis
Comment	Kalaba/Zehl<br>Ergebnis: +/- 5ppm<br>ACN/MeOH + 1%H2O		

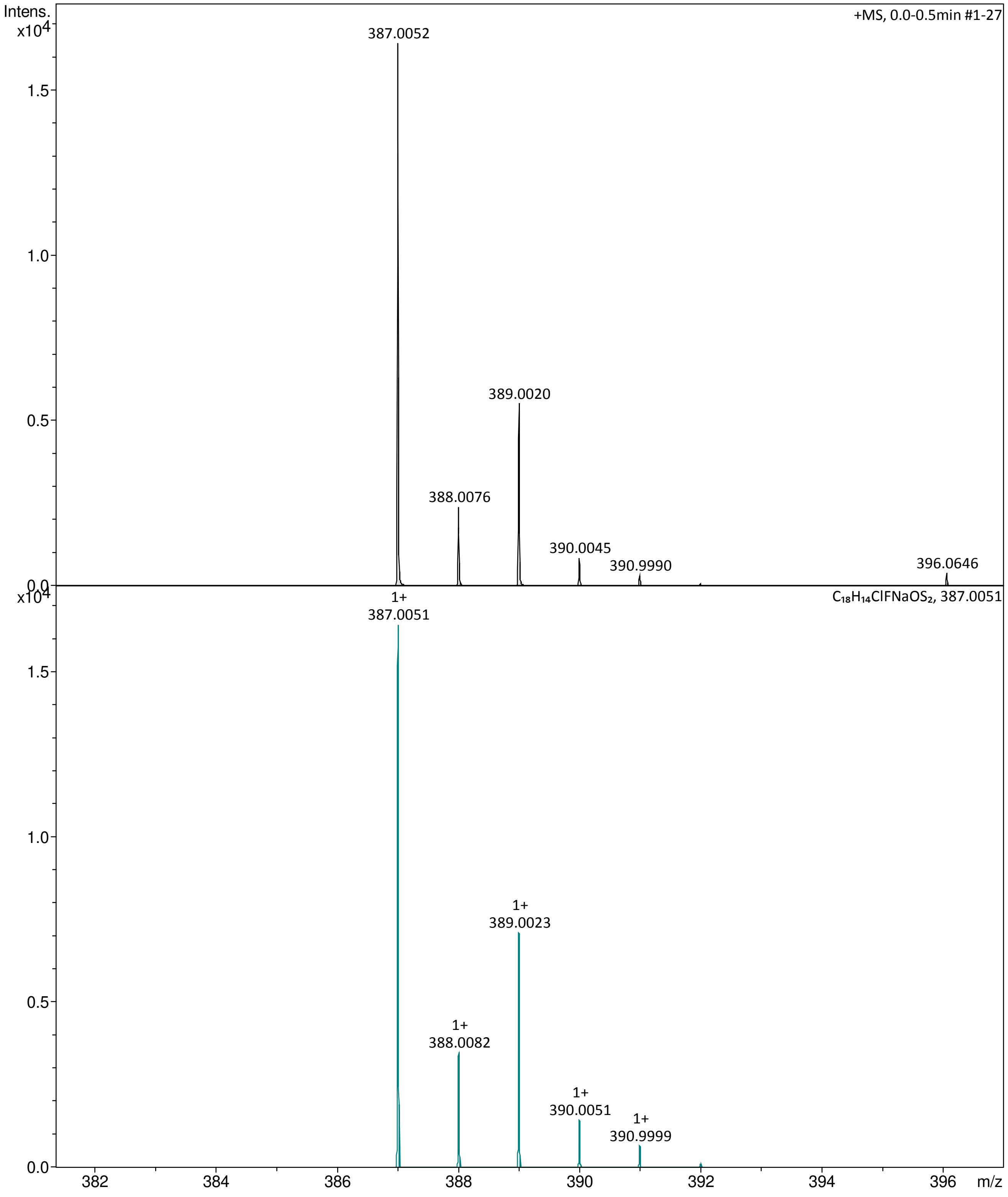

Mass Spectrum SmartFormula Report

Analysis Info

Analysis Name	D:\MZ\maXis_data\temp\60659000001.d	Acquisition Date	11/8/2018 1:27:05 PM
Method	tune_low_MS_Service_11_18.m	Operator	msc
Sample Name	CE-156-spot2-peak1	Instrument	maXis 255552.00016
Comment	Kalaba/Zehl<br>Ergebnis: +/- 5ppm<br>ACN/MeOH + 1%H2O		

Acquisition Parameter

Source Type	ESI	Ion Polarity	Positive	Set Nebulizer	0.4 Bar
Focus	Not active	Set Capillary	4500 V	Set Dry Heater	150 °C
Scan Begin	50 m/z	Set End Plate Offset	-500 V	Set Dry Gas	4.0 l/min
Scan End	1800 m/z	Set Charging Voltage	0 V	Set Divert Valve	Source
		Set Corona	0 nA	Set APCI Heater	0 °C

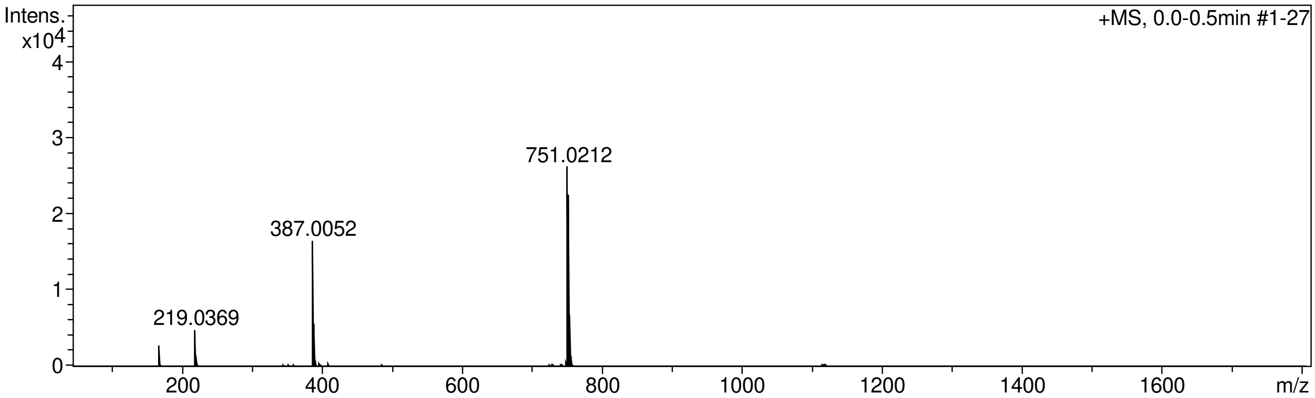

Meas. m/z	#	Ion Formula	Score	m/z	err [mDa]	err [ppm]	mSigma	rdb	e⁻ Conf	N-Rule
167.9670	1	C5H5NaOS2	100.00	167.9674	-0.4	-2.3	5.8	3.0	odd	ok
	2	C4HNaO6	57.13	167.9665	-0.5	-2.9	11.9	4.0	odd	ok
	3	C2H3F2NaO3S	54.17	167.9663	-0.7	-4.1	28.3	0.0	odd	ok
	4	CH2FNaO7	36.26	167.9677	0.7	3.9	28.9	0.0	odd	ok
219.0369	1	C13H9ClF	100.00	219.0371	0.2	1.0	18.3	8.5	even	ok
387.0052	1	C14H11ClF2NaO7	100.00	387.0054	0.2	0.4	7.7	7.5	even	ok
	2	C11H3ClF2N10NaO	59.22	387.0040	-1.2	-3.0	8.2	13.5	even	ok
	3	C13H6ClF5N4NaO	68.07	387.0043	-0.9	-2.4	8.2	9.5	even	ok
	4	C11H12ClF3NaO8	50.54	387.0065	-1.3	-3.4	12.1	3.5	even	ok
	5	C9H9ClN6NaO8	58.46	387.0063	1.1	2.8	12.2	7.5	even	ok
	6	C8H4ClF3N10NaO2	96.58	387.0052	-0.0	-0.1	12.7	9.5	even	ok
	7	C15H7ClF2N4NaO3	40.89	387.0067	-1.5	-3.9	16.5	12.5	even	ok
	8	C12H13ClF4NaO4S	81.70	387.0051	-0.0	-0.1	20.6	3.5	even	ok
	9	C13H9ClF4N4NaS	43.08	387.0065	1.3	3.3	20.6	8.5	even	ok
	10	C11H6ClFN10NaS	49.79	387.0062	-1.1	-2.7	20.7	12.5	even	ok
	11	C10H10ClFN6NaO4S	73.69	387.0049	-0.3	-0.7	20.7	7.5	even	ok
	12	C17H10ClFNaO6	52.00	387.0042	1.0	2.5	20.8	11.5	even	ok
	13	C8H13ClN2NaO12	71.18	387.0049	0.3	0.7	22.7	2.5	even	ok
	14	C15H12ClF3NaO3S	41.06	387.0040	1.2	3.1	25.7	7.5	even	ok
	15	C13H9ClN6NaO3S	35.29	387.0038	-1.4	-3.7	25.7	11.5	even	ok
	16	C9H14ClF5NaO5S	42.99	387.0063	-1.1	-2.8	26.0	-0.5	even	ok
	17	C9H14ClFN2NaO8S	30.54	387.0036	-1.6	-4.2	26.3	2.5	even	ok
	18	C18H6ClFN4NaO2	55.86	387.0056	0.4	0.9	31.1	16.5	even	ok
	19	C17H13ClNaO5S	40.27	387.0064	-1.3	-3.2	32.3	10.5	even	ok
	20	C8H12ClF3N6NaOS2	56.63	387.0047	0.5	1.3	35.2	3.5	even	ok
	21	C13H14ClF5NaS2	33.31	387.0038	1.4	3.6	36.1	3.5	even	ok
	22	C11H11ClF2N6NaS2	28.34	387.0035	1.6	4.2	36.1	7.5	even	ok
	23	C9H17ClN2NaO7S2	46.76	387.0058	-0.6	-1.6	40.5	1.5	even	ok
	24	C15H15ClF2NaO2S2	35.06	387.0062	1.0	2.7	42.4	6.5	even	ok
	25	C21H5ClN4NaO	30.43	387.0044	-0.8	-2.0	46.1	20.5	even	ok
	26	C18H14ClFNaOS2	43.08	387.0051	-0.1	-0.3	51.2	10.5	even	ok
	27	C8H15ClF2N6NaS3	16.08	387.0069	-1.7	-4.5	53.8	2.5	even	ok
	28	C10H18ClFN2NaO3S3	26.41	387.0044	0.8	1.9	56.9	1.5	even	ok
	29	C13H17ClN2NaO2S3	11.49	387.0033	1.9	4.9	60.3	5.5	even	ok
	30	C21H13ClNaS2	16.64	387.0039	1.2	3.2	62.3	14.5	even	ok

Mass Spectrum SmartFormula Report

Meas. m/z	#	Ion Formula	Score	m/z	err [mDa]	err [ppm]	mSigma	rdb	e⁻ Conf	N-Rule
	31	C10H21ClN2NaO2S4	10.45	387.0067	-1.5	-3.8	71.2	0.5	even	ok

Figure S84. HRESIMS spectrum of compound **8u**

Generic Display Report

Analysis Info

Analysis Name	D:\MZ\maXis_data\temp\60660000001.d	Acquisition Date	11/8/2018 1:31:08 PM
Method	tune_low_MS_Service_11_18.m	Operator	msc
Sample Name	CE-156-spot2-peak2	Instrument	maXis
Comment	Kalaba/Zehl<br>Ergebnis: +/- 5ppm<br>ACN/MeOH + 1%H2O		

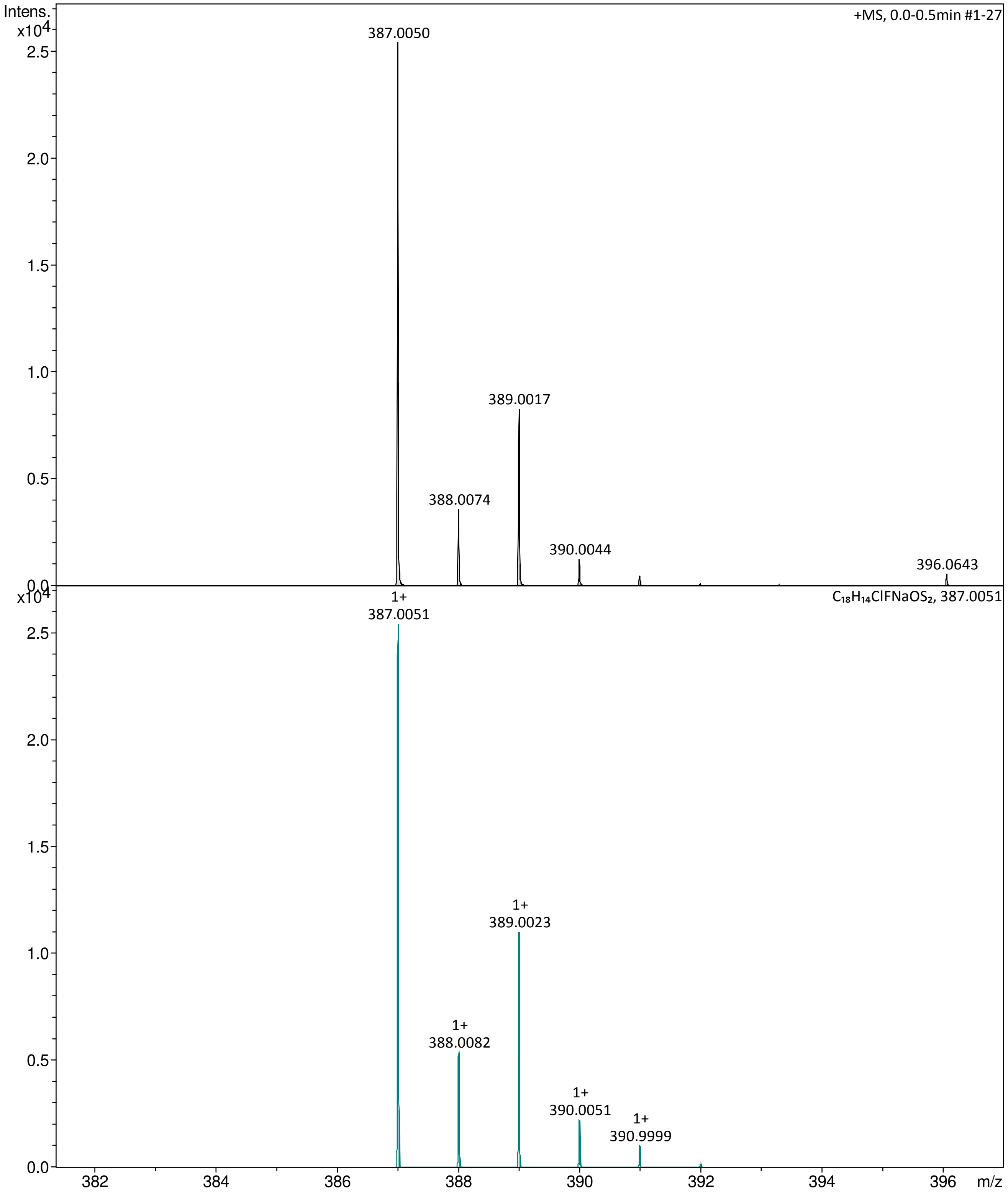

Mass Spectrum SmartFormula Report

Analysis Info

Analysis Name	D:\MZ\maXis_data\temp\60660000001.d	Acquisition Date	11/8/2018 1:31:08 PM
Method	tune_low_MS_Service_11_18.m	Operator	msc
Sample Name	CE-156-spot2-peak2	Instrument	maXis 255552.00016
Comment	Kalaba/Zehl<br>Ergebnis: +/- 5ppm<br>ACN/MeOH + 1%H2O		

Acquisition Parameter

Source Type	ESI	Ion Polarity	Positive	Set Nebulizer	0.4 Bar
Focus	Not active	Set Capillary	4500 V	Set Dry Heater	150 °C
Scan Begin	50 m/z	Set End Plate Offset	-500 V	Set Dry Gas	4.0 l/min
Scan End	1800 m/z	Set Charging Voltage	0 V	Set Divert Valve	Source
		Set Corona	0 nA	Set APCI Heater	0 °C

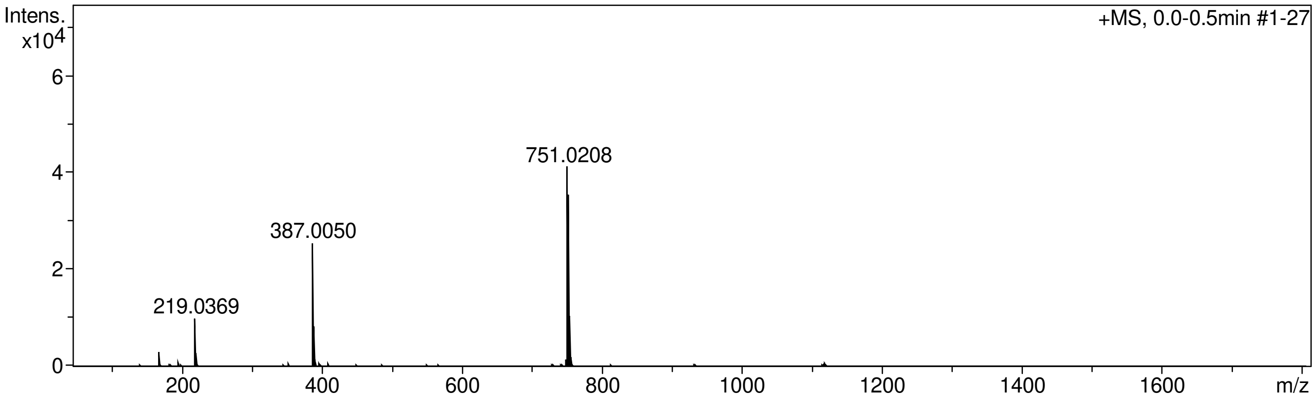

Meas. m/z	#	Ion Formula	Score	m/z	err [mDa]	err [ppm]	mSigma	rdb	e^- Conf	N-Rule
167.9669	1	C5H5NaOS2	100.00	167.9674	0.5	2.9	7.5	3.0	odd	ok
	2	C4HNaO6	64.65	167.9665	0.4	2.3	11.8	4.0	odd	ok
	3	C2H3F2NaO3S	65.31	167.9663	0.6	3.6	25.7	0.0	odd	ok
	4	CH2FNaO7	37.30	167.9677	0.8	4.5	28.7	0.0	odd	ok
219.0369	1	C13H9ClF	100.00	219.0371	0.2	1.0	23.3	8.5	even	ok
387.0050	1	C11H3ClF2N10NaO	65.56	387.0040	1.0	2.6	9.7	13.5	even	ok
	2	C13H6ClF5N4NaO	74.87	387.0043	-0.8	-2.0	9.8	9.5	even	ok
	3	C8H4ClF3N10NaO2	100.00	387.0052	-0.1	-0.3	10.9	9.5	even	ok
	4	C14H11ClF2NaO7	89.80	387.0054	-0.3	-0.8	11.8	7.5	even	ok
	5	C11H12ClF3NaO8	47.06	387.0065	1.5	3.8	12.5	3.5	even	ok
	6	C9H9ClN6NaO8	54.82	387.0063	-1.2	-3.2	12.6	7.5	even	ok
	7	C15H7ClF2N4NaO3	35.90	387.0067	1.7	4.3	19.4	12.5	even	ok
	8	C7H8ClF3N6NaO6	46.92	387.0038	1.2	3.1	20.7	4.5	even	ok
	9	C8H13ClN2NaO12	79.42	387.0049	-0.1	-0.3	22.5	2.5	even	ok
	10	C16H5ClF4N4Na	27.62	387.0031	-1.9	-5.0	23.0	13.5	even	ok
	11	C17H10ClFNaO6	54.56	387.0042	0.8	2.1	24.2	11.5	even	ok
	12	C12H13ClF4NaO4S	74.33	387.0051	-0.1	-0.3	25.3	3.5	even	ok
	13	C10H10ClFN6NaO4S	73.79	387.0049	-0.1	-0.3	25.4	7.5	even	ok
	14	C13H9ClF4N4NaS	36.07	387.0065	-1.4	-3.7	25.8	8.5	even	ok
	15	C11H6ClFN10NaS	41.97	387.0062	1.2	3.1	25.8	12.5	even	ok
	16	C9H14ClF5NaO5S	38.13	387.0063	1.3	3.2	28.7	-0.5	even	ok
	17	C7H11ClF2N6NaO5S	43.97	387.0060	1.0	2.6	28.7	3.5	even	ok
	18	C9H14ClFN2NaO8S	32.48	387.0036	1.5	3.8	29.8	2.5	even	ok
	19	C15H12ClF3NaO3S	41.42	387.0040	-1.0	-2.7	30.8	7.5	even	ok
	20	C13H9ClN6NaO3S	35.85	387.0038	1.3	3.3	30.8	11.5	even	ok
	21	C18H6ClFN4NaO2	50.19	387.0056	0.5	1.4	33.9	16.5	even	ok
	22	C17H13ClNaO5S	33.76	387.0064	1.4	3.7	36.8	10.5	even	ok
	23	C8H12ClF3N6NaOS2	57.04	387.0047	0.3	0.9	39.2	3.5	even	ok
	24	C13H14ClF5NaS2	33.76	387.0038	-1.2	-3.2	40.7	3.5	even	ok
	25	C11H11ClF2N6NaS2	28.93	387.0035	-1.5	-3.8	40.7	7.5	even	ok
	26	C9H17ClN2NaO7S2	40.07	387.0058	-0.8	-2.0	44.5	1.5	even	ok
	27	C15H15ClF2NaO2S2	28.98	387.0062	-1.2	-3.1	47.1	6.5	even	ok
	28	C21H5ClN4NaO	31.54	387.0044	-0.6	-1.6	48.9	20.5	even	ok
	29	C18H14ClFNaOS2	39.50	387.0051	-0.1	-0.1	55.7	10.5	even	ok
	30	C8H15ClF2N6NaS3	13.01	387.0069	1.9	4.9	58.0	2.5	even	ok

Mass Spectrum SmartFormula Report

Meas. m/z	#	Ion Formula	Score	m/z	err [mDa]	err [ppm]	mSigma	rdb	e⁻ Conf	N-Rule
	31	C10H18ClFN2NaO3S3	25.73	387.0044	0.6	1.5	61.3	1.5	even	ok
	32	C13H17ClN2NaO2S3	11.41	387.0033	-1.7	-4.5	64.8	5.5	even	ok
	33	C21H13ClNaS2	16.31	387.0039	-1.1	-2.8	66.7	14.5	even	ok
	34	C10H21ClN2NaO2S4	8.37	387.0067	1.6	4.2	75.2	0.5	even	ok

Figure S1a. RP-Chromatogram of analogue **5a**.

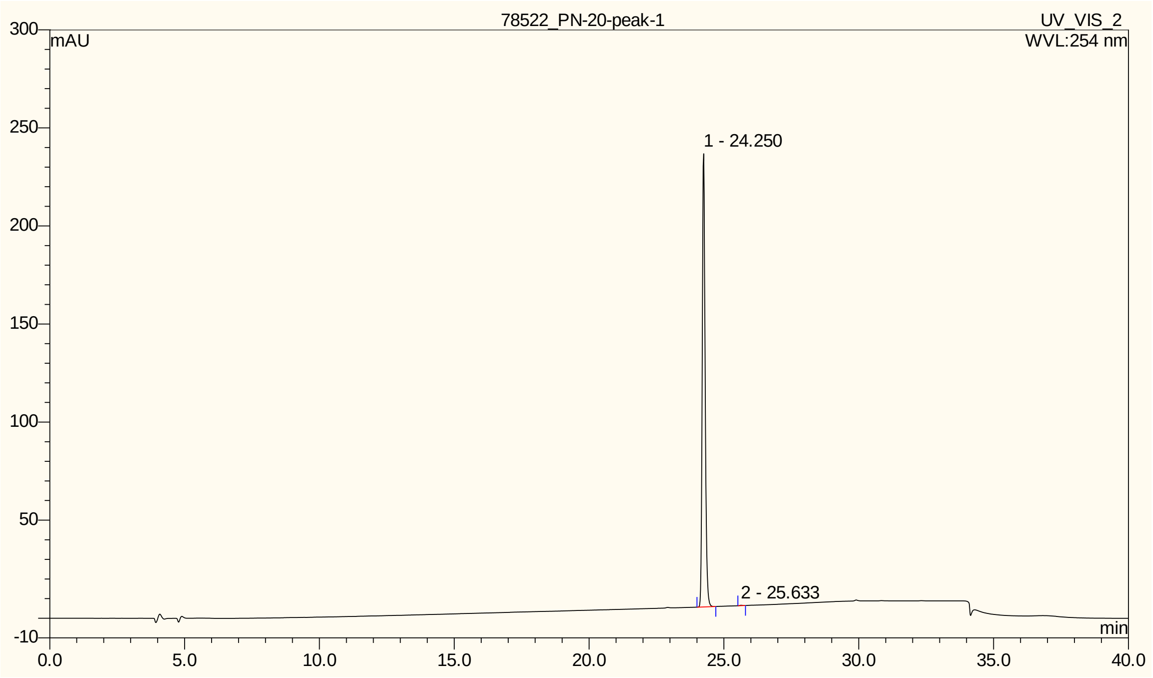

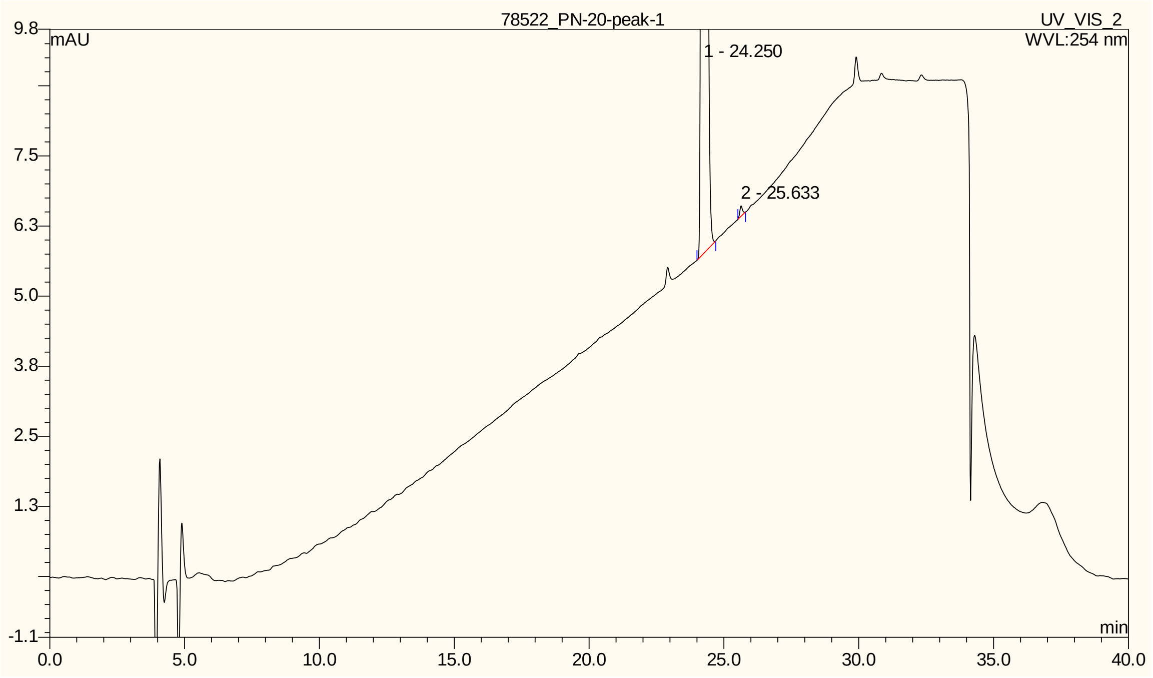

Retention Time: **24.25 min**

Relative Peak Area: **99.93 %**

Figure S2a. RP-Chromatogram of analogue **6a**.

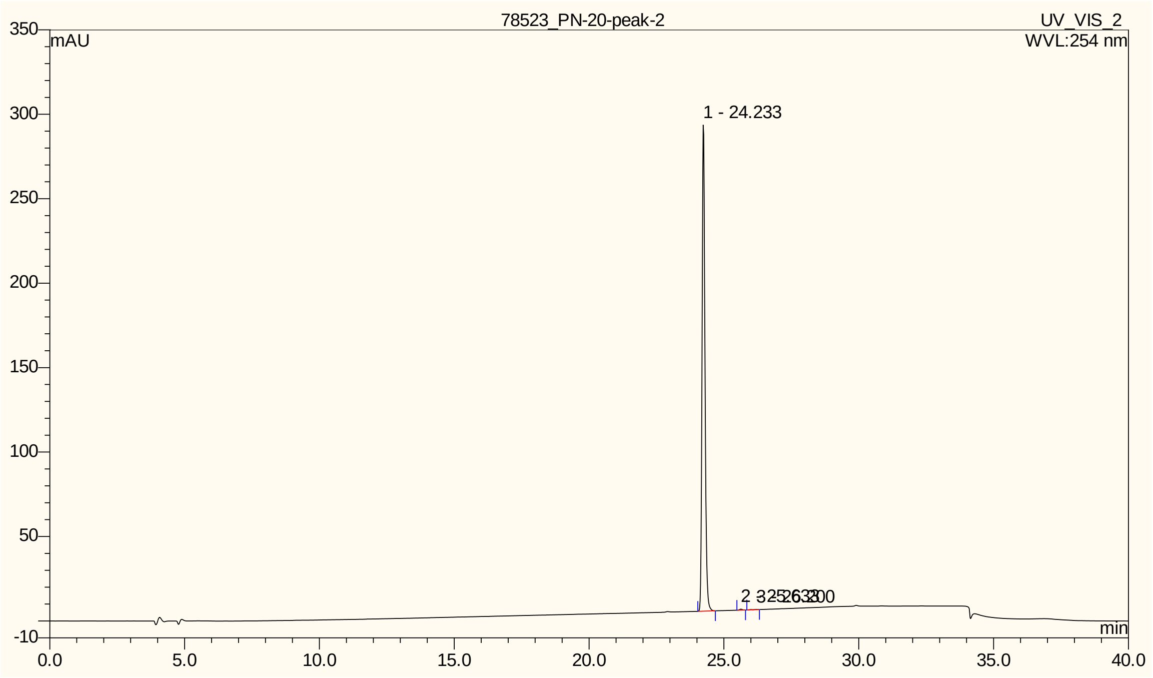

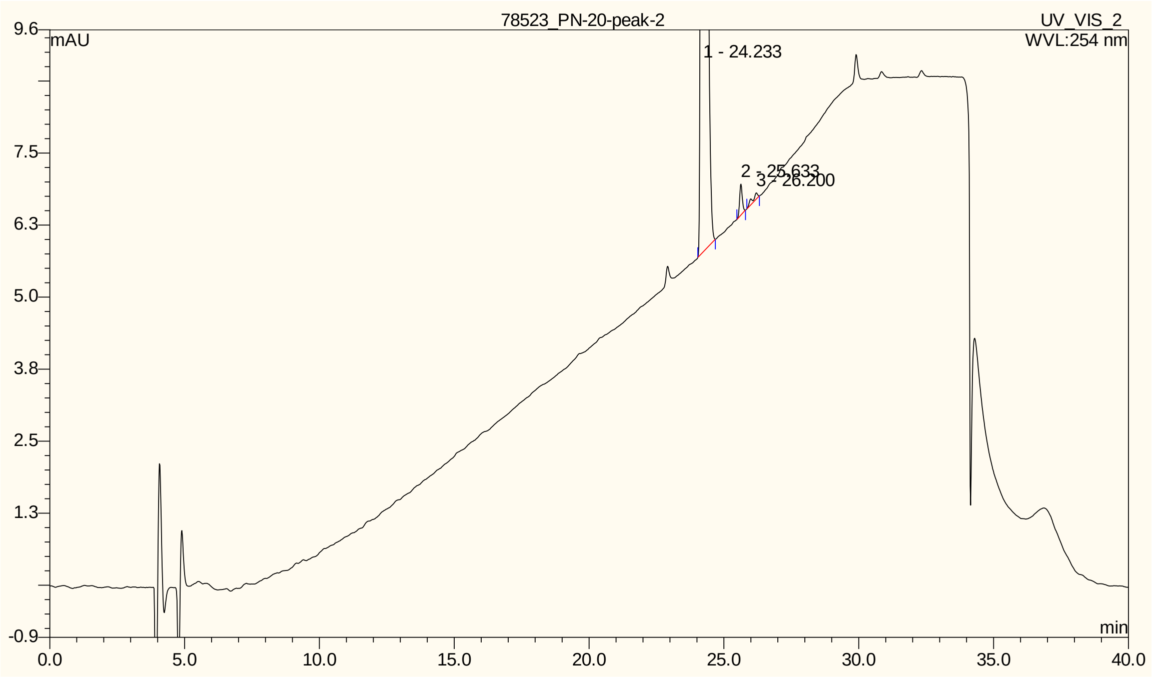

Retention Time: **24.23 min**

Relative Peak Area: **99.76 %**

Figure S3a. RP-Chromatogram of analogue **7a**.

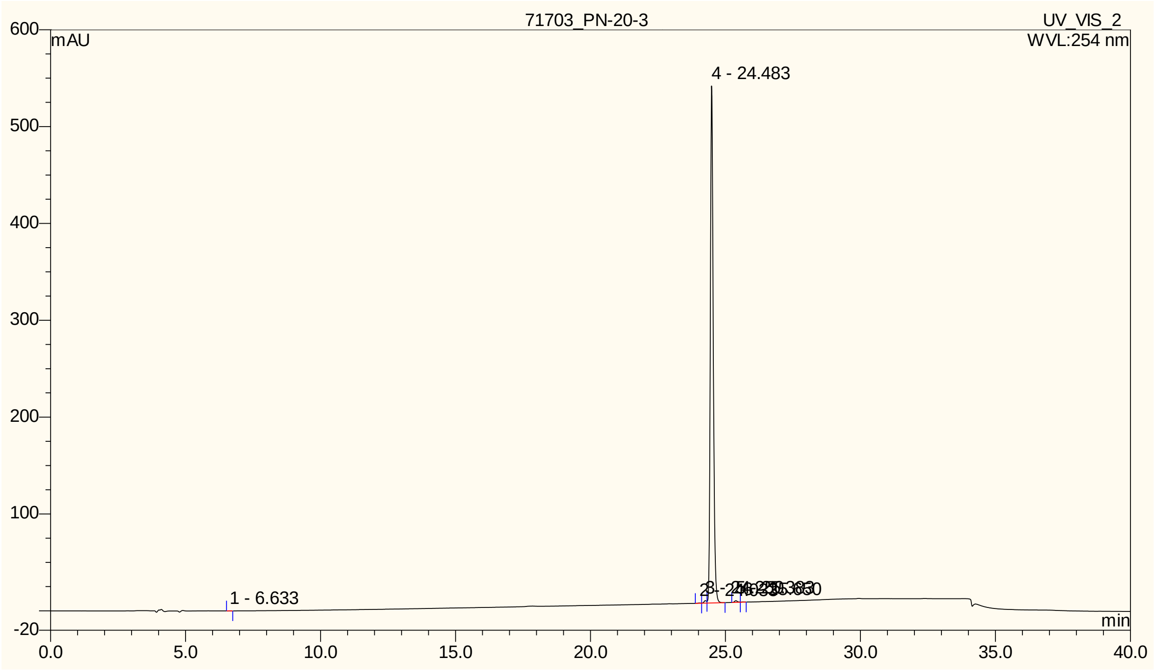

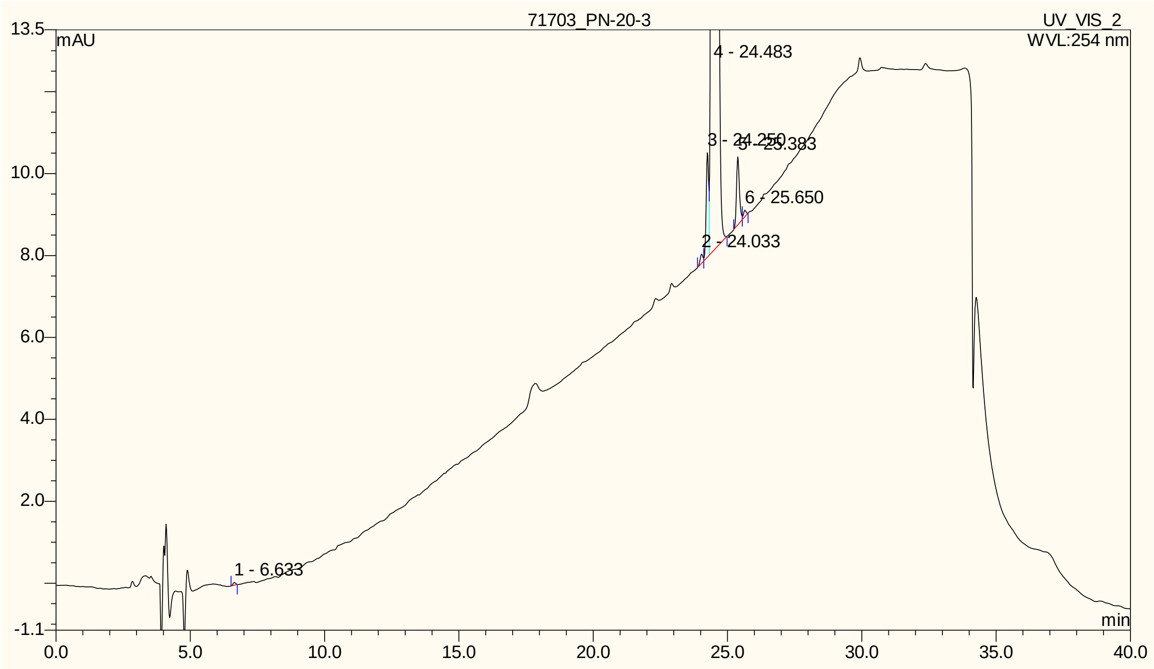

Retention Time: **24.48 min**

Relative Peak Area: **99.22 %**

Figure S4a. RP-Chromatogram of analogue **8a**.

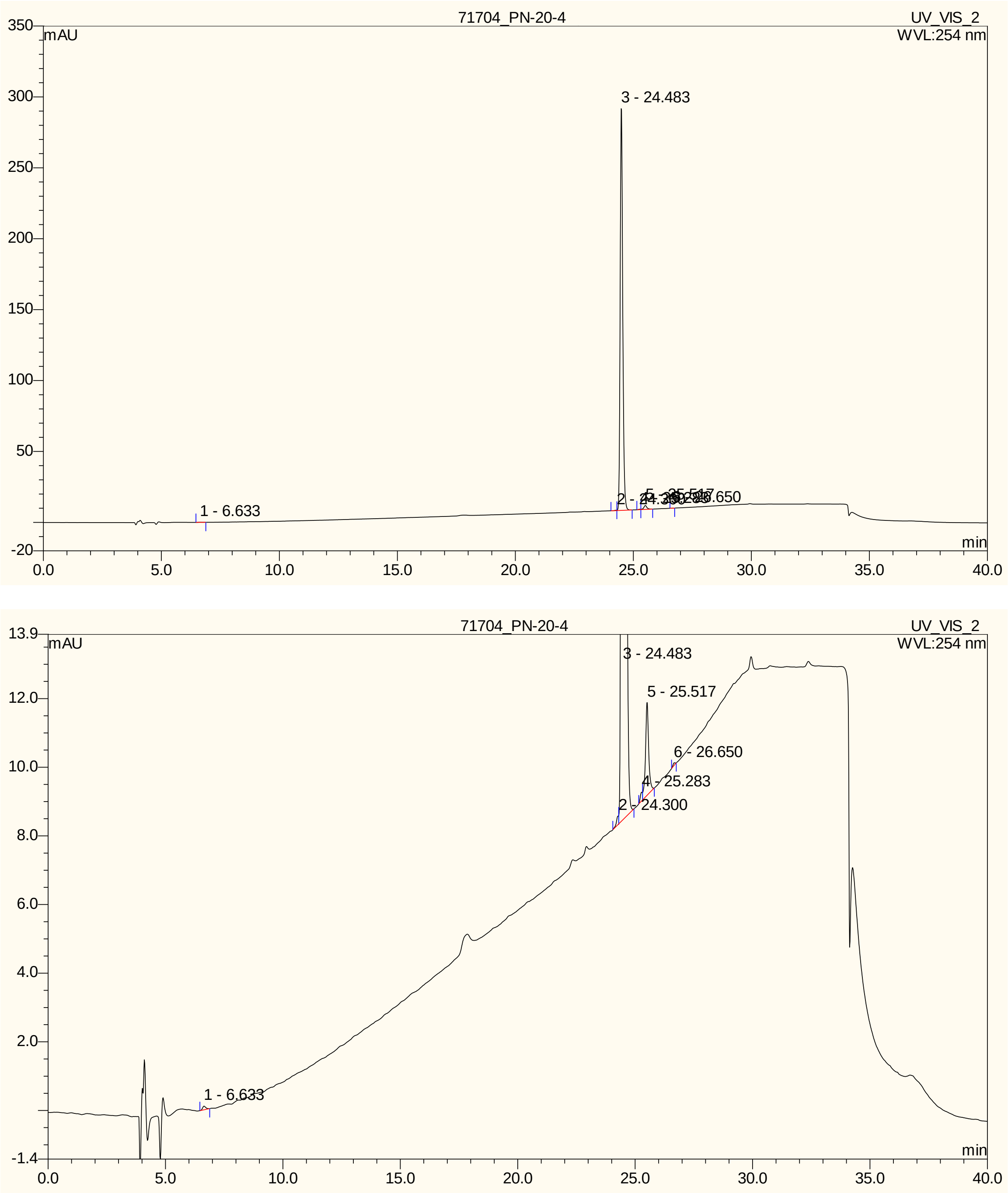

Retention Time: **24.48 min**

Relative Peak Area: **98.62 %**

Figure S5a. RP-Chromatogram of analogue **5b**.

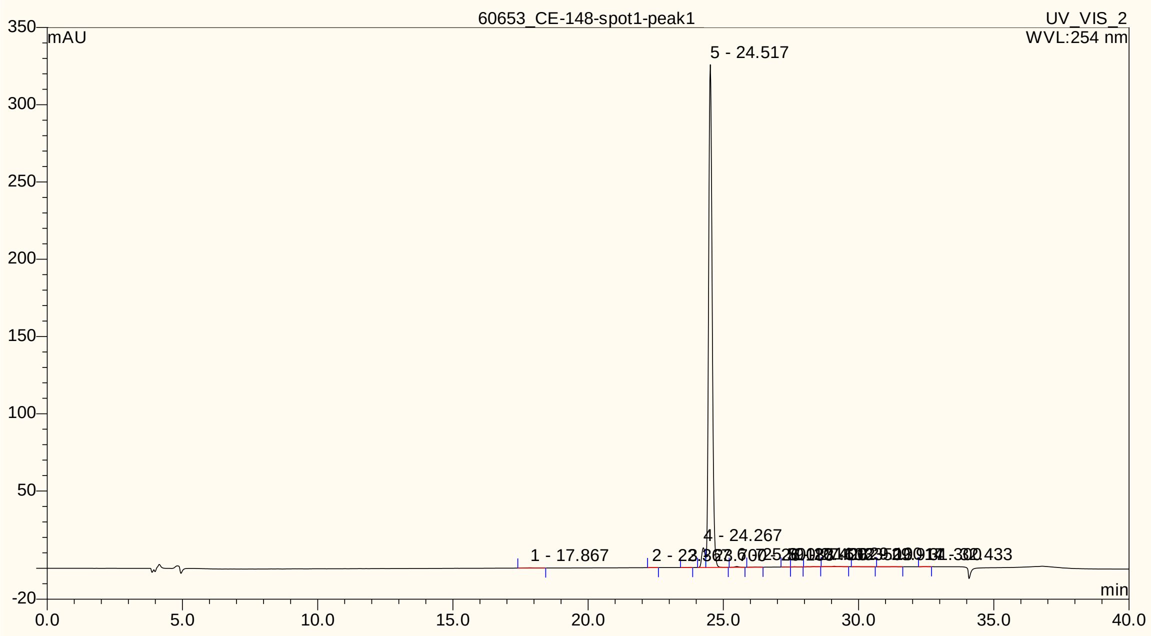

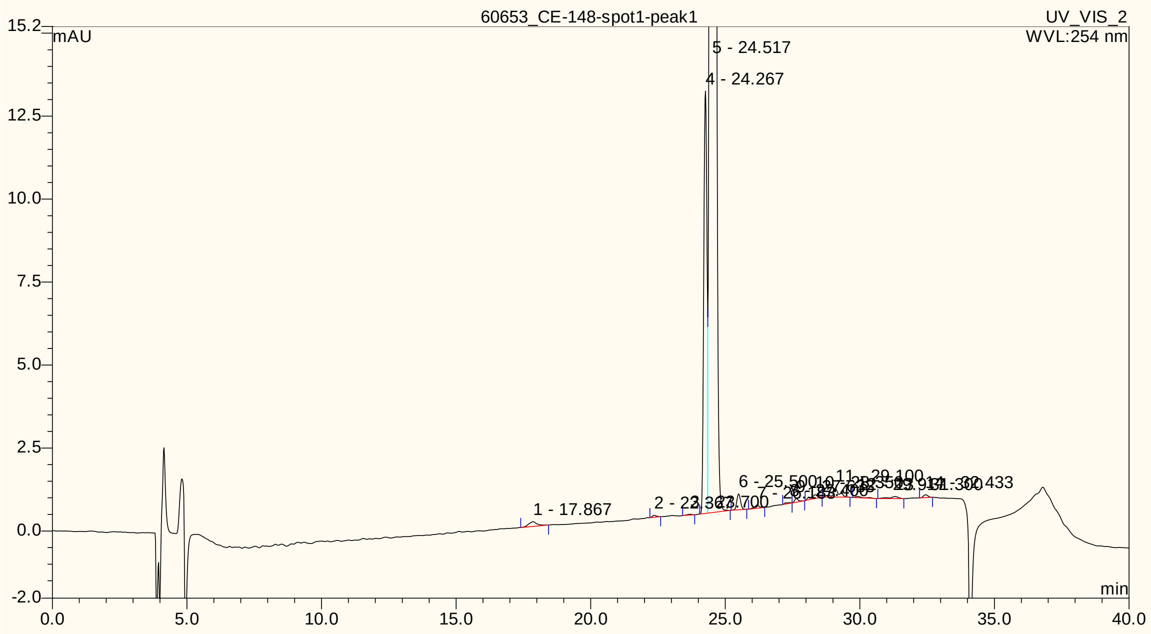

Retention Time: **24.52 min**

Relative Peak Area: **95.97 %**

Figure S6a. RP-Chromatogram of analogue **6b**.

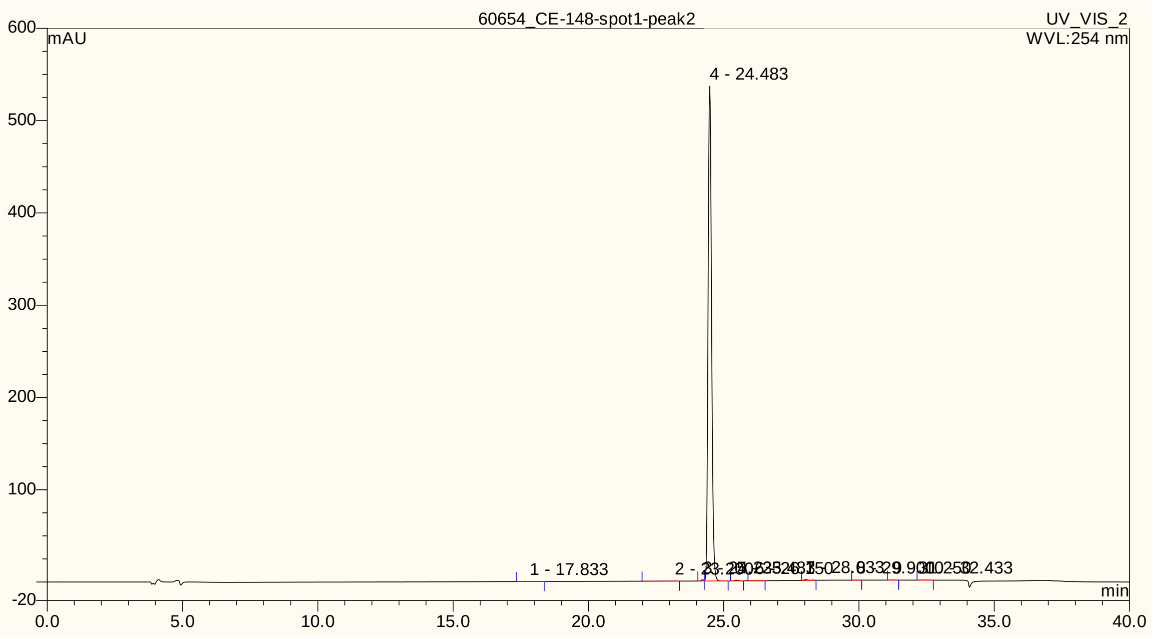

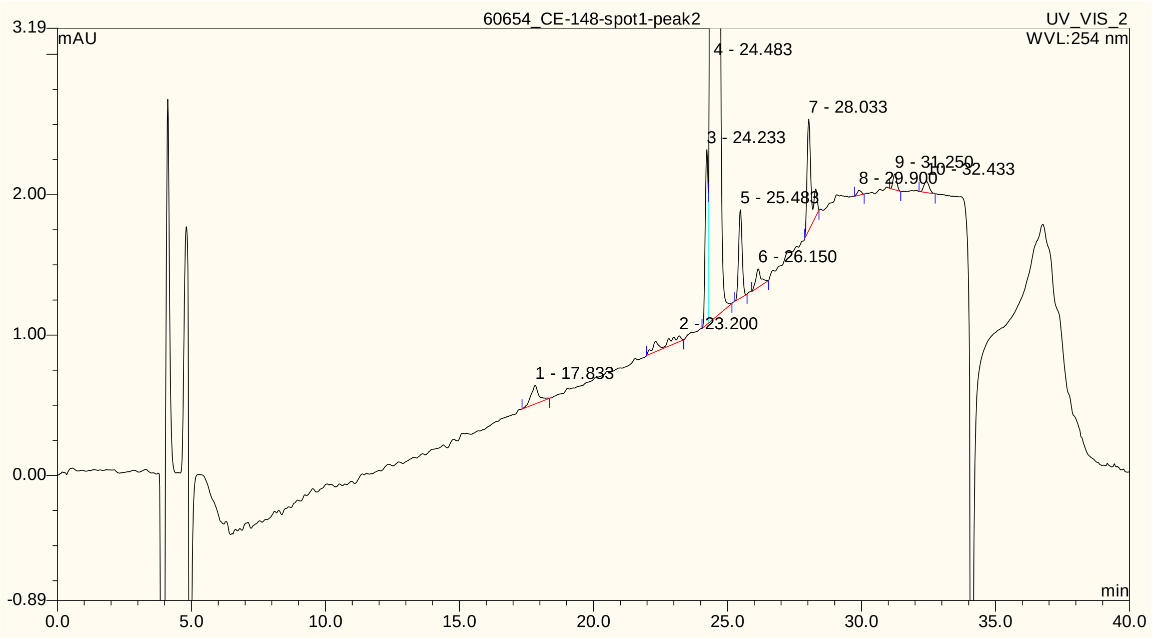

Retention Time: **24.48 min**

Relative Peak Area: **99.32 %**

Figure S7a. RP-Chromatogram of analogue **7b**.

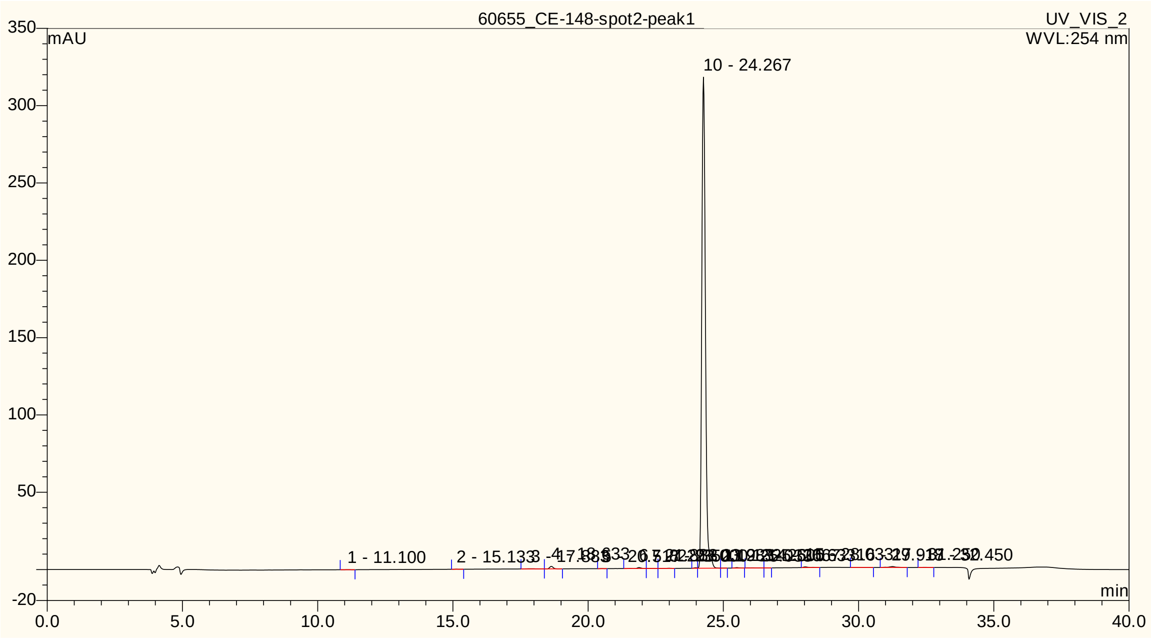

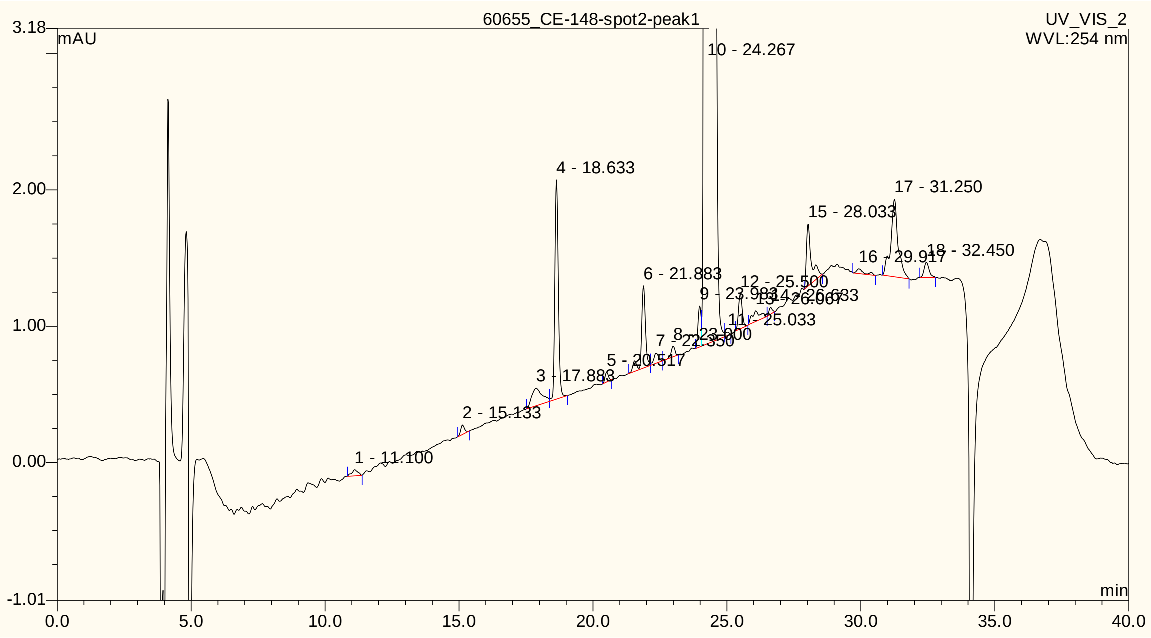

Retention Time: **24.27 min**

Relative Peak Area: **98.20 %**

Figure S8a. RP-Chromatogram of analogue **8b**.

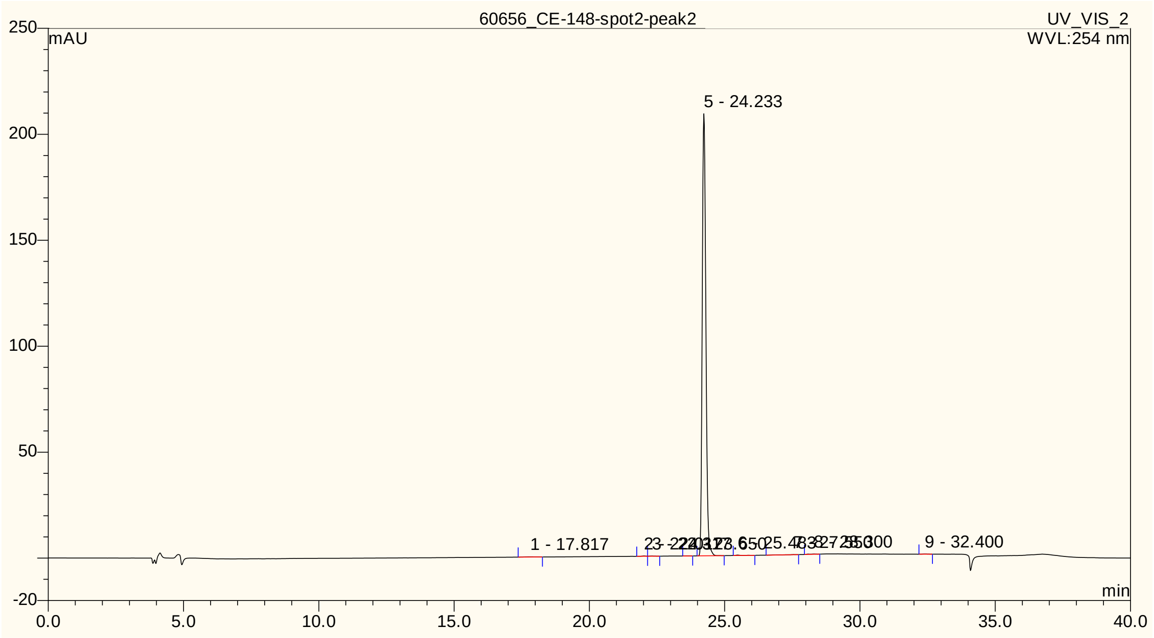

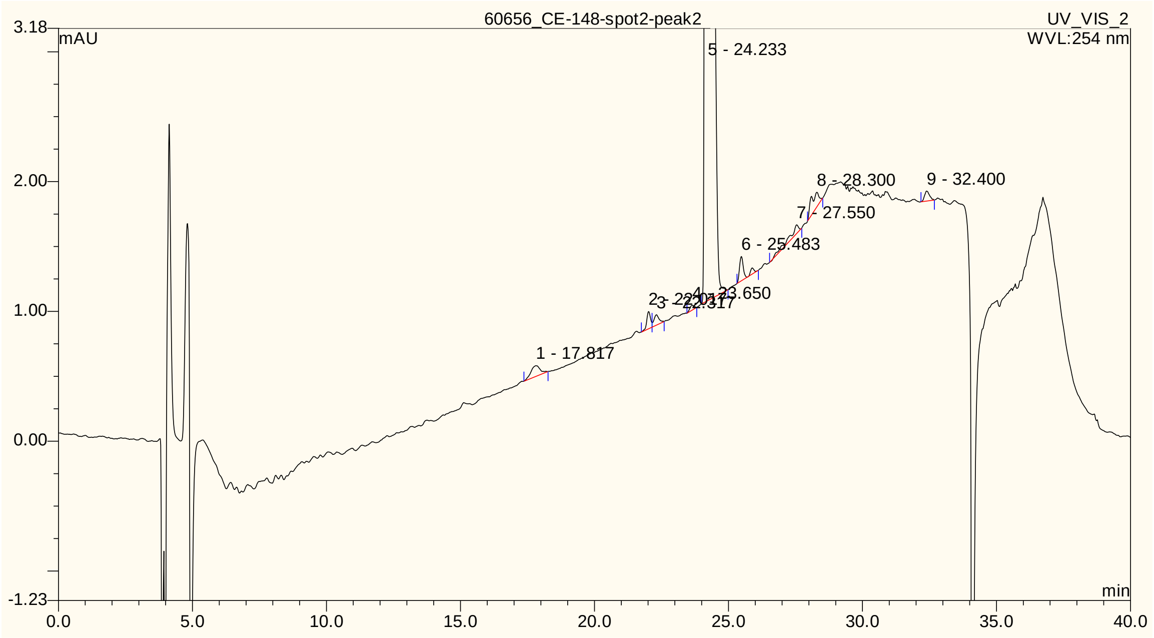

Retention Time: **24.23 min**

Relative Peak Area: **99.37 %**

Figure S9a. RP-Chromatogram of analogue **5c**.

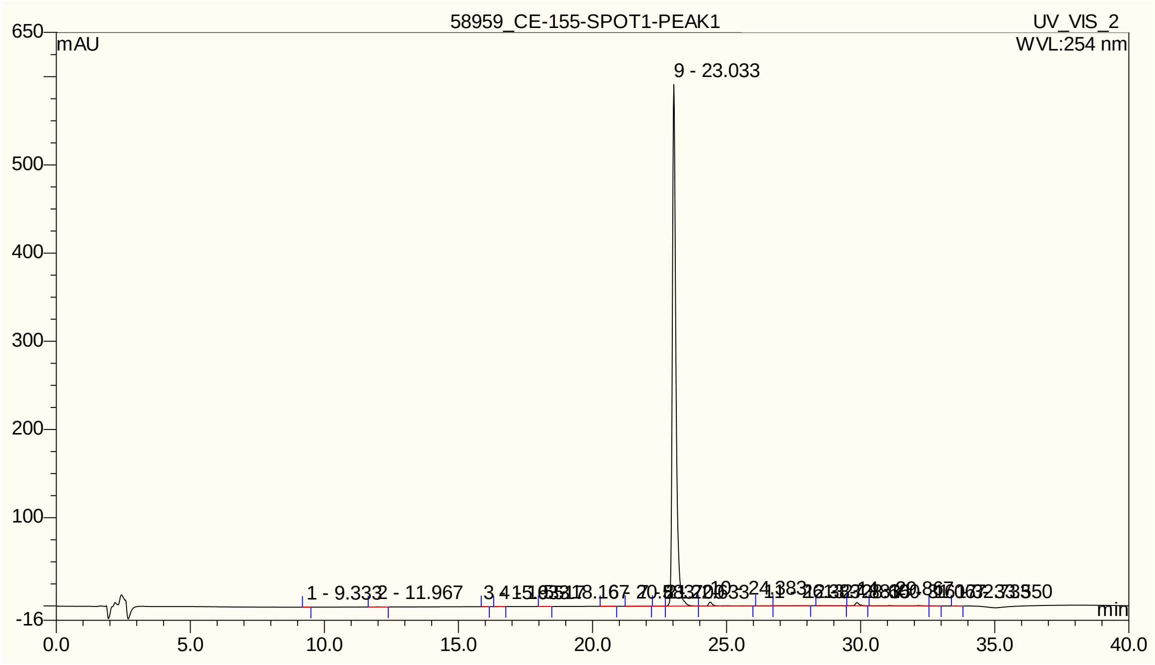

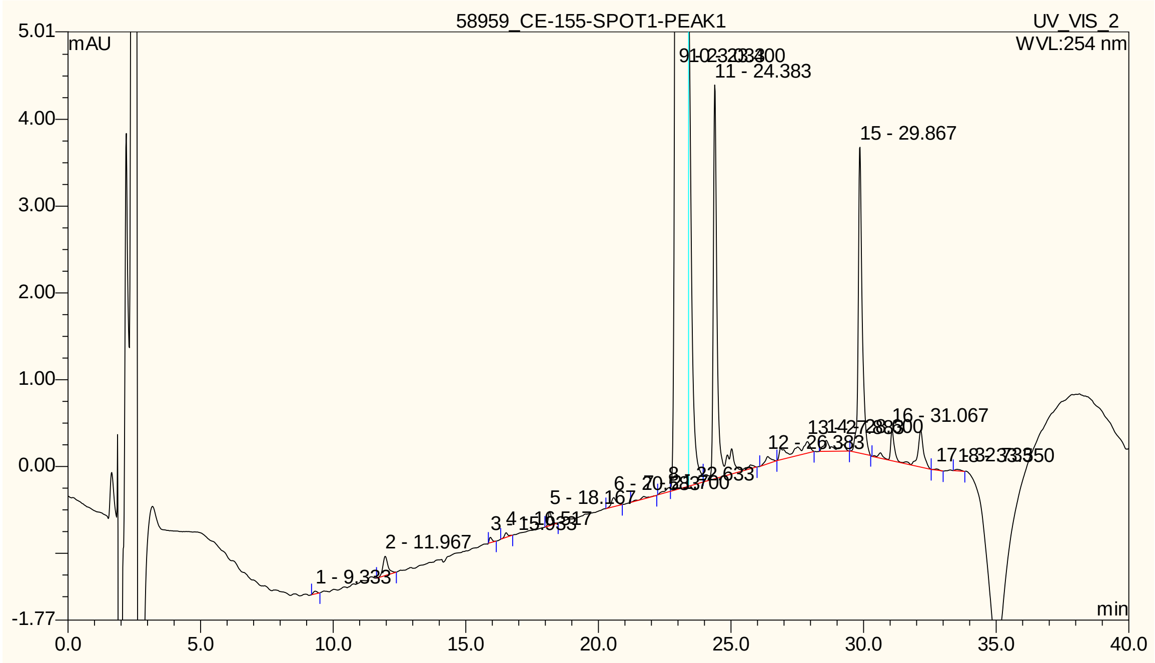

Retention Time: **23.03 min**

Relative Peak Area: **97.17 %**

Figure S10a. RP-Chromatogram of analogue **6c**.

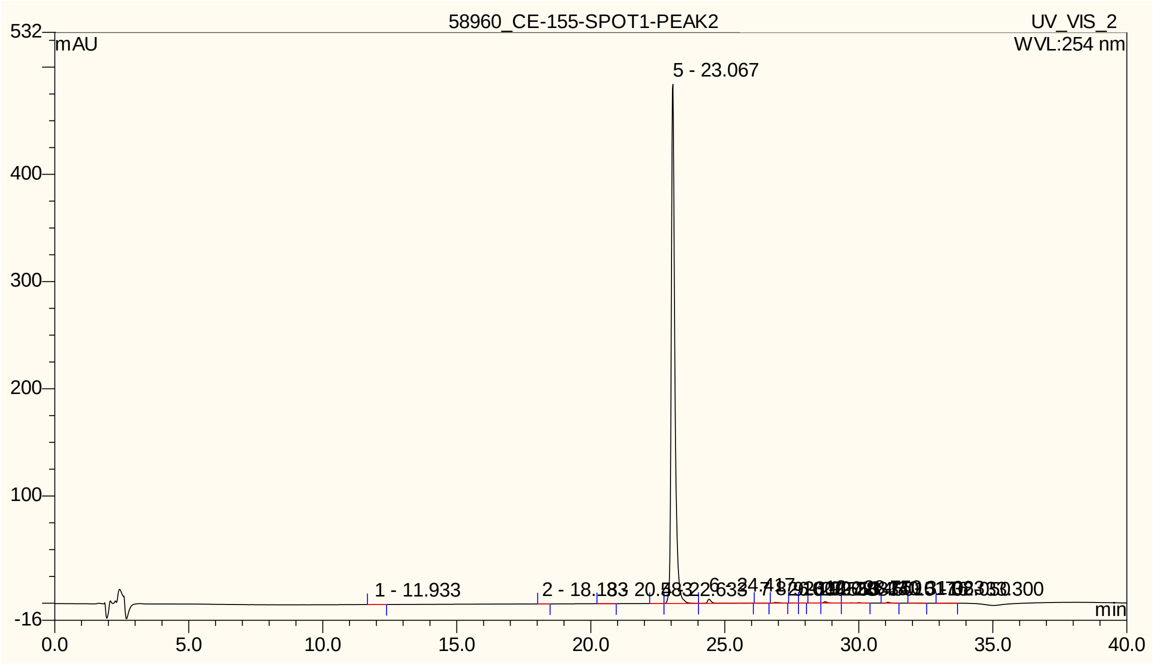

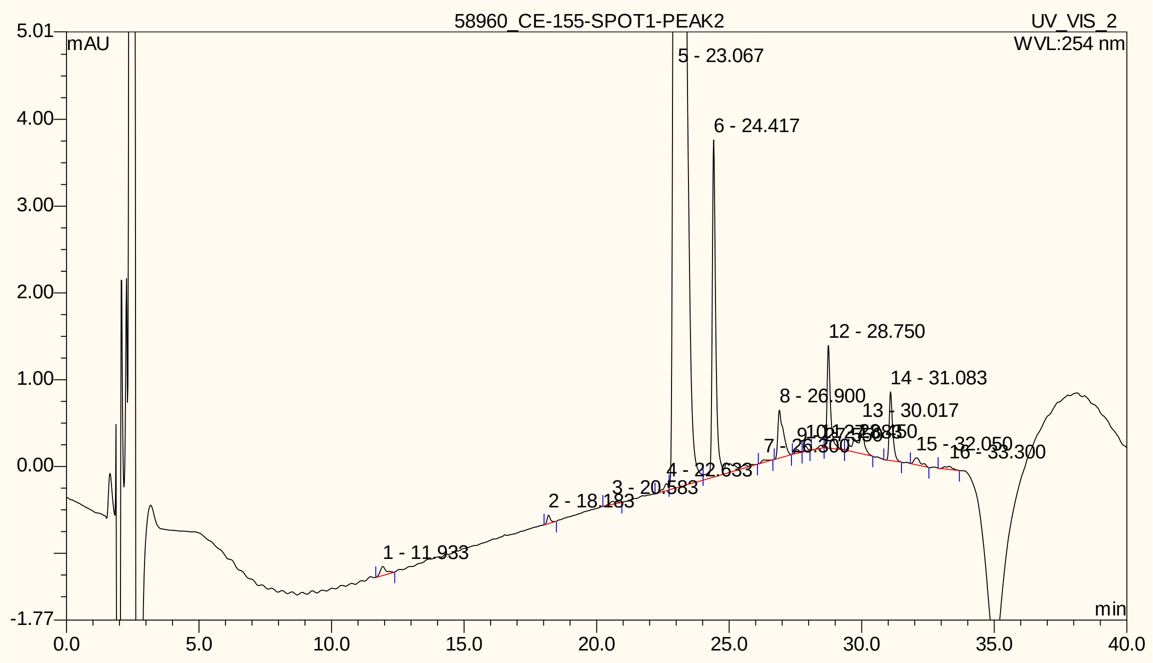

Retention Time: **23.07 min**

Relative Peak Area: **98.29 %**

Figure S11a. RP-Chromatogram of analogue **7c**.

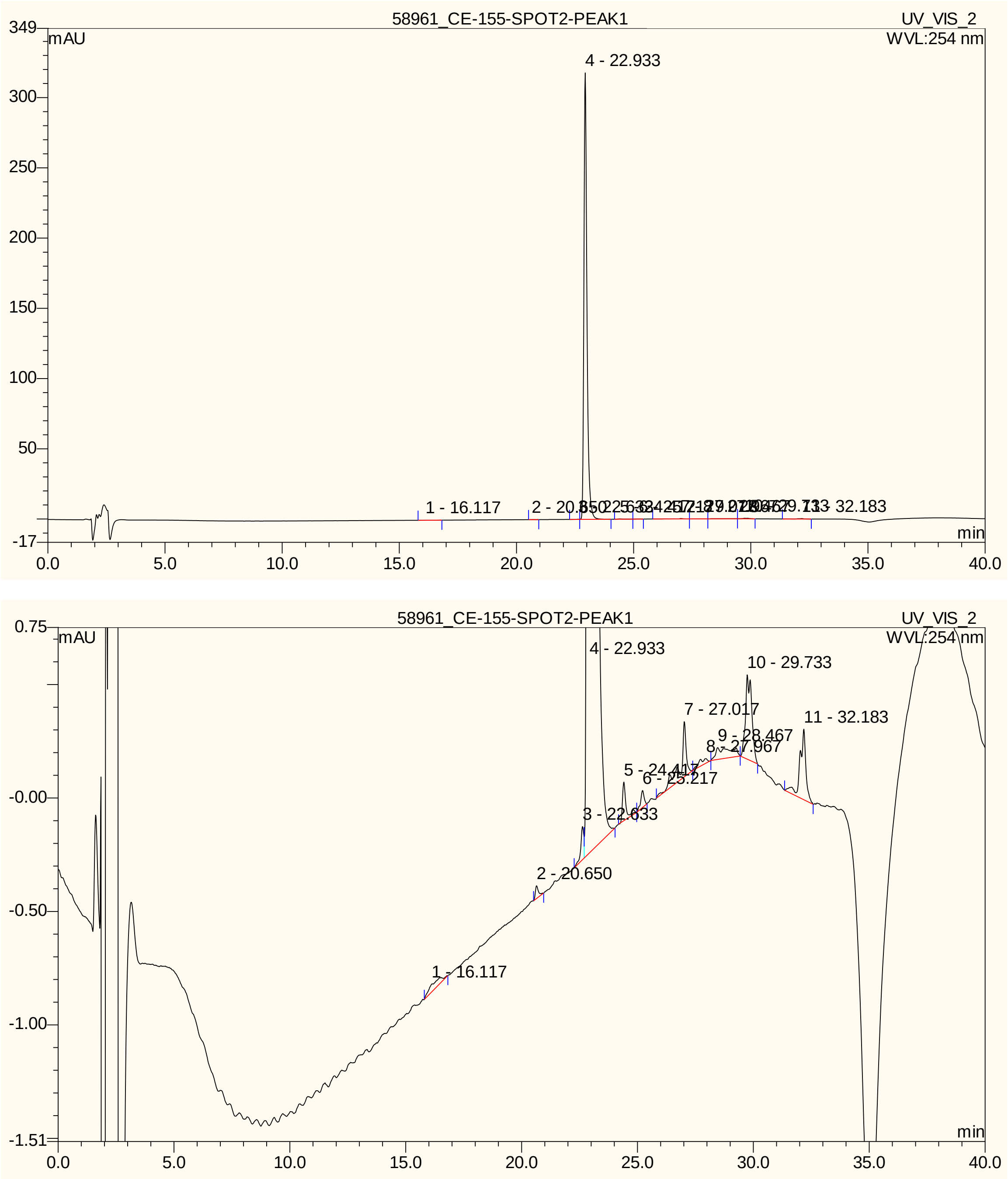

Retention Time: **22.93 min**

Relative Peak Area: **99.15 %**

Figure S12a. RP-Chromatogram of analogue **8c**.

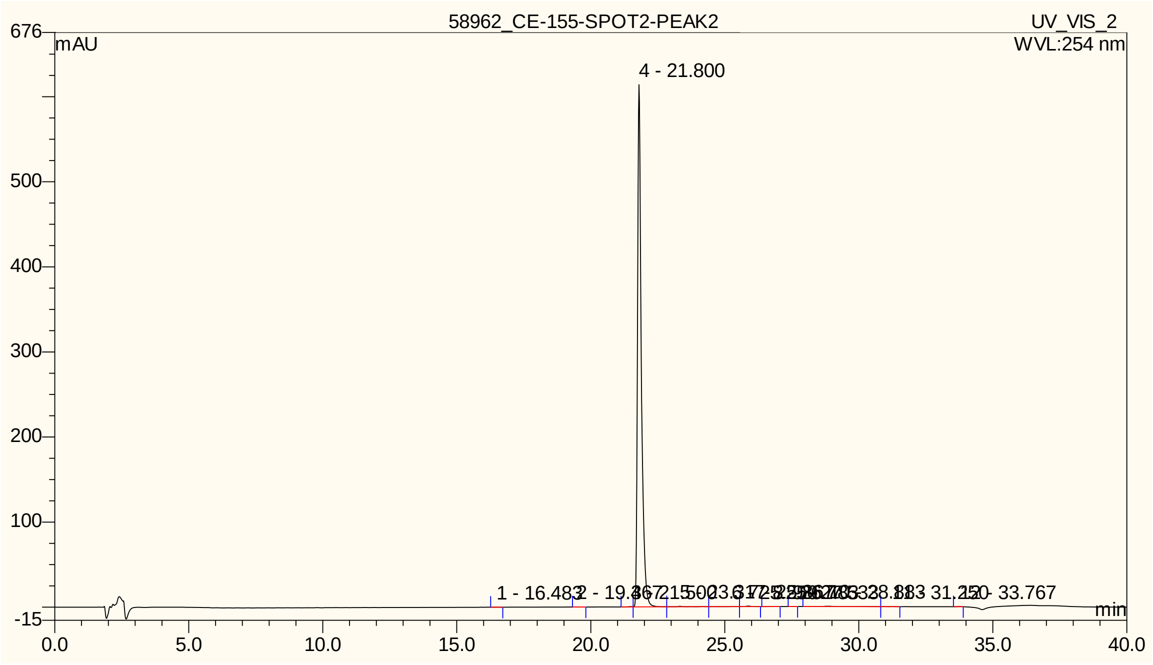

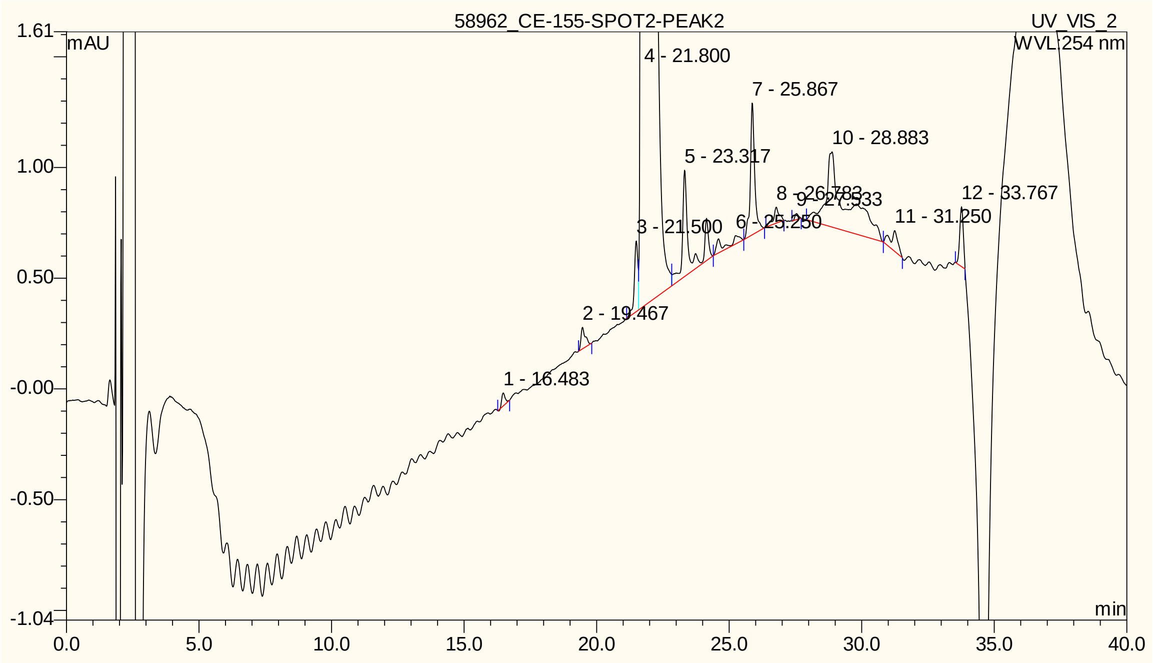

Retention Time: **21.80 min**

Relative Peak Area: **99.27 %**

Figure S13a. RP-Chromatogram of analogue **5d**.

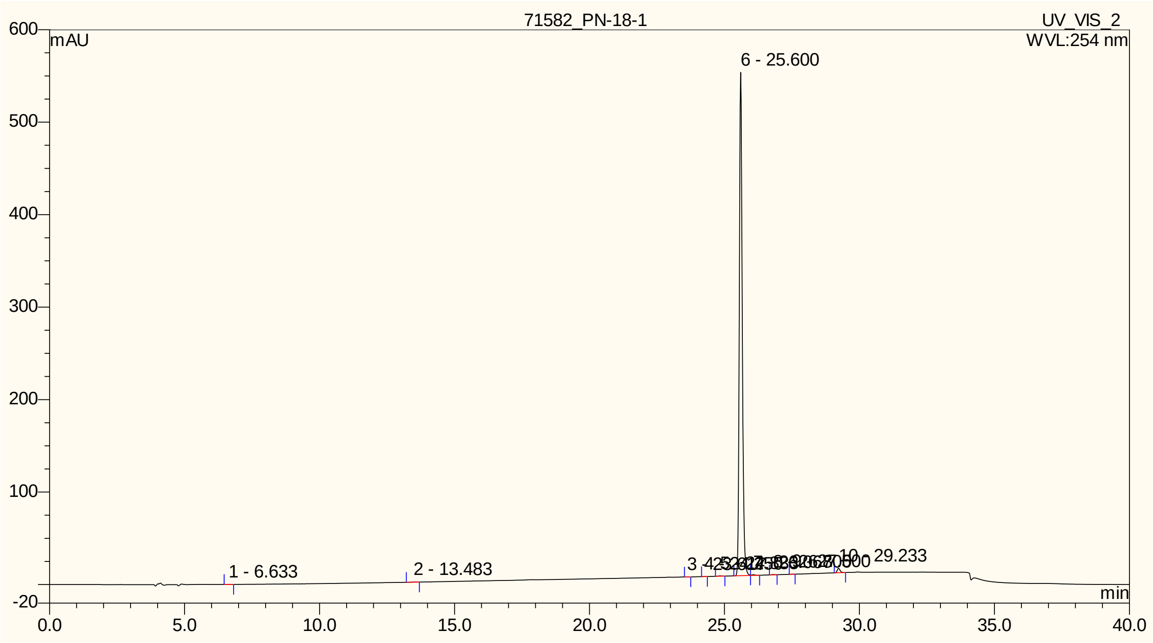

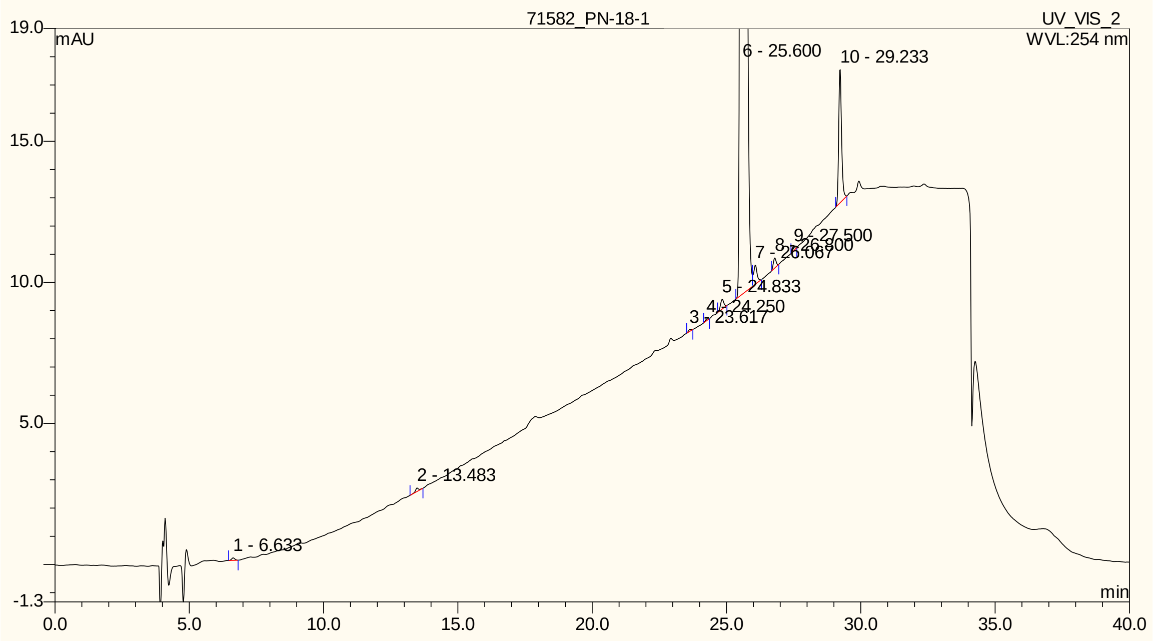

Retention Time: **25.60 min**

Relative Peak Area: **98.81 %**

Figure S14a. RP-Chromatogram of analogue **6d**.

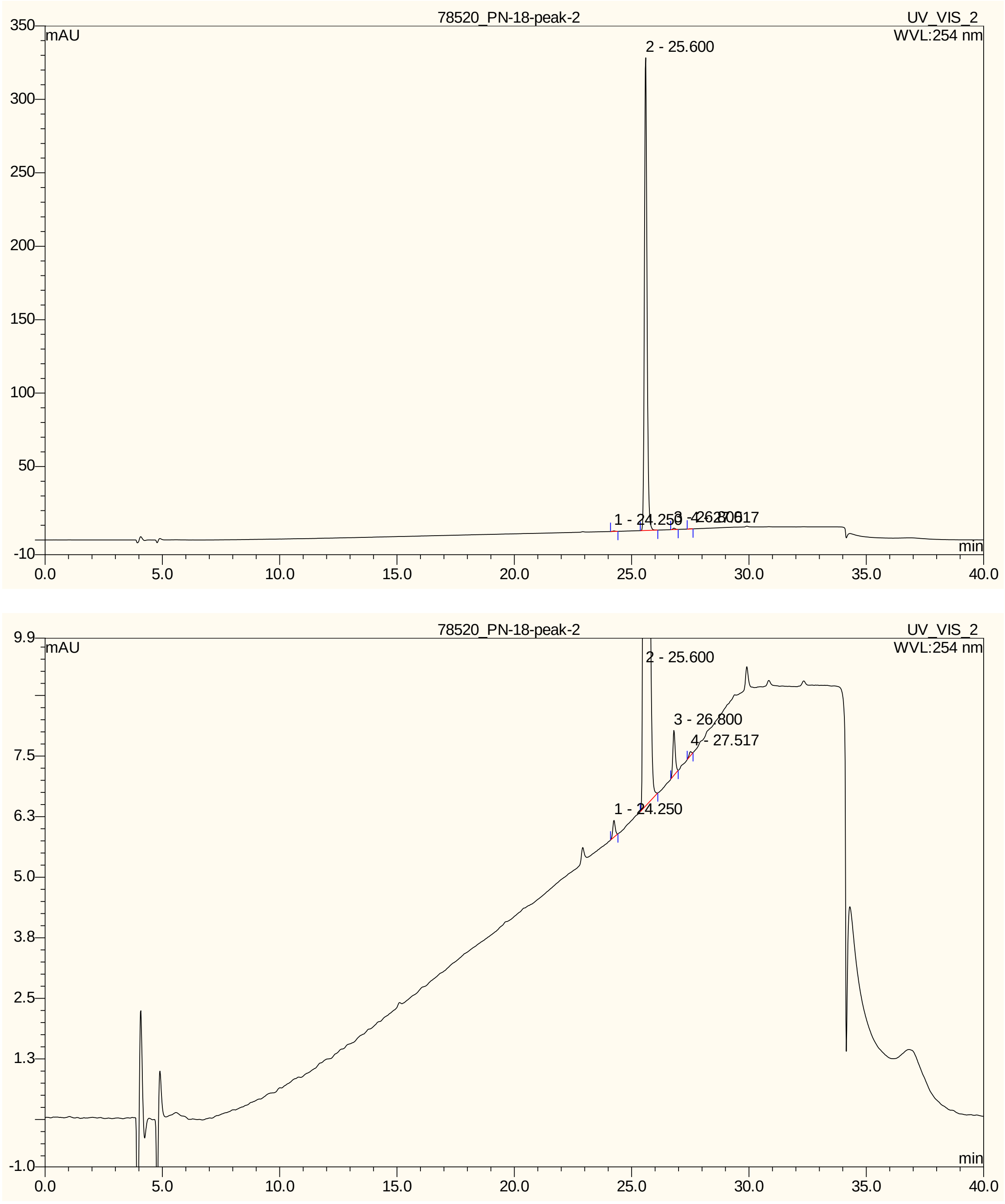

Retention Time: **25.60 min**

Relative Peak Area: **99.61 %**

Figure S15a. RP-Chromatogram of analogue **7d**.

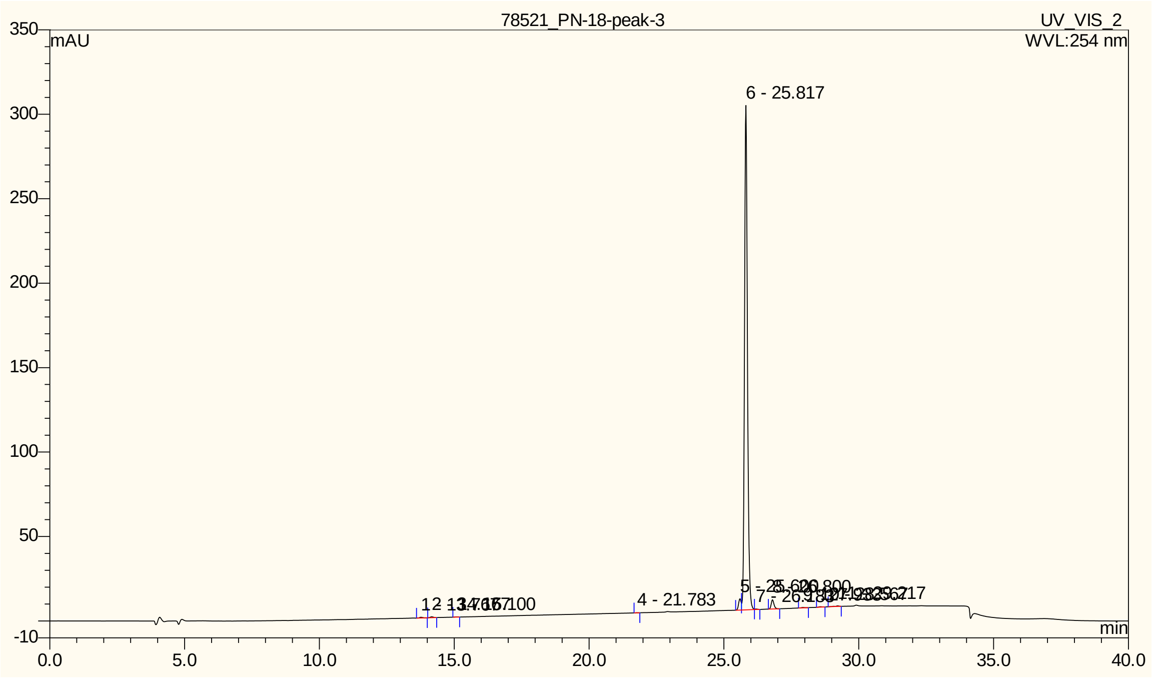

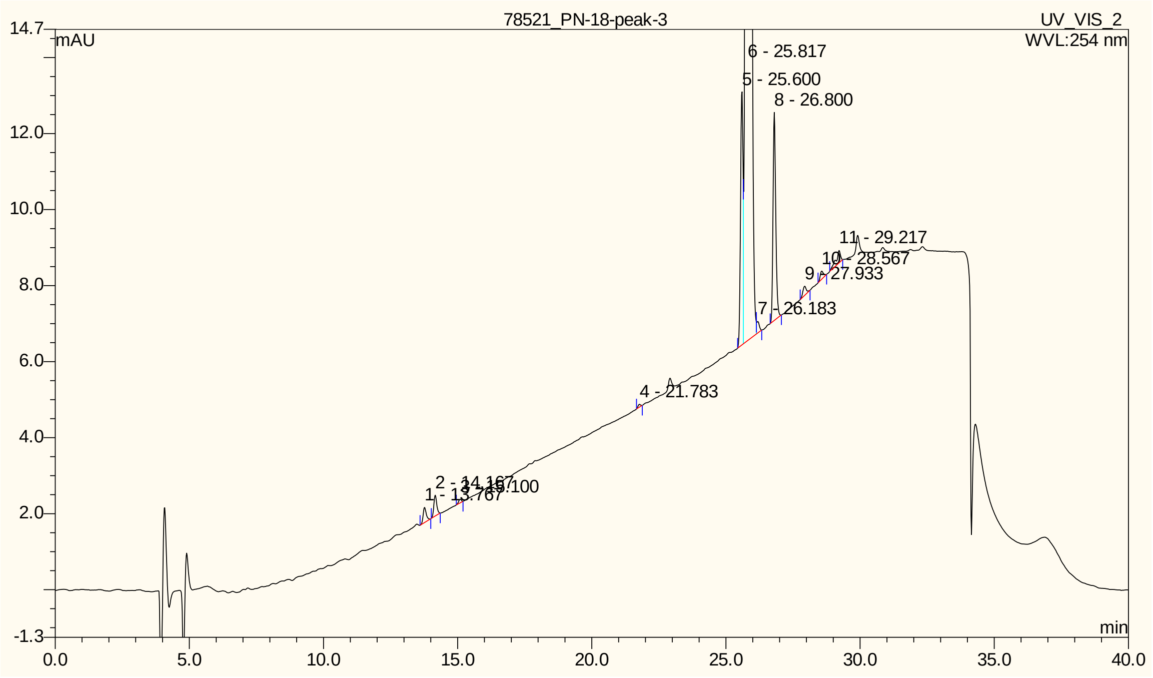

Retention Time: **25.82 min**

Relative Peak Area: **95.86 %**

Figure S16a. RP-Chromatogram of analogue **8d**.

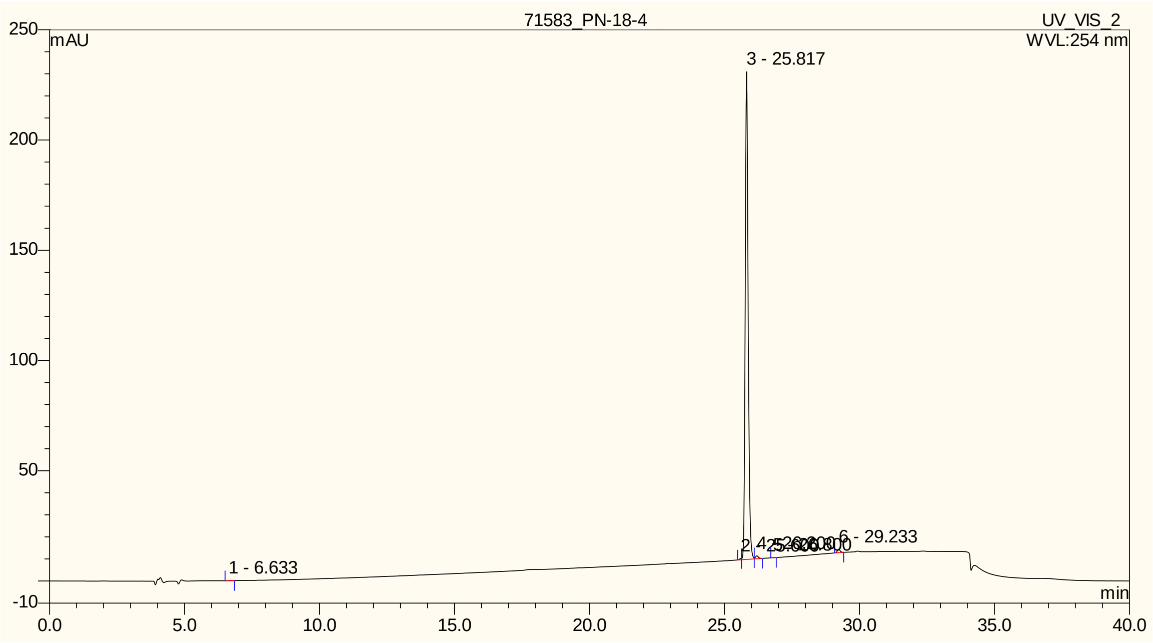

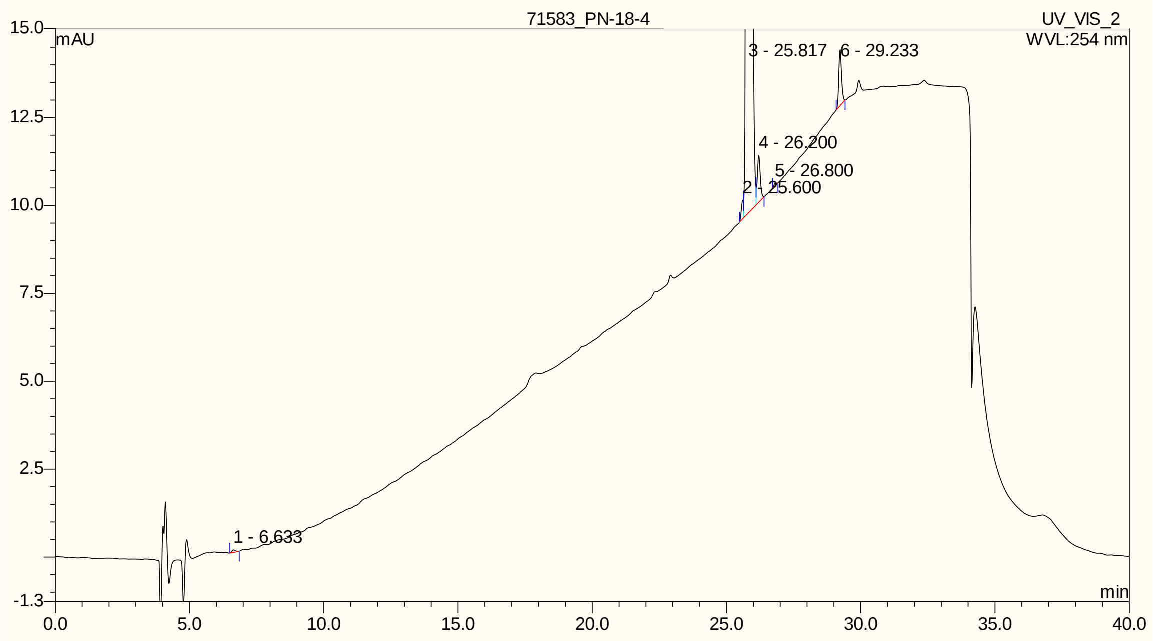

Retention Time: **25.82 min**

Relative Peak Area: **98.49 %**

Figure S17a. RP-Chromatogram of analogue **5e**.

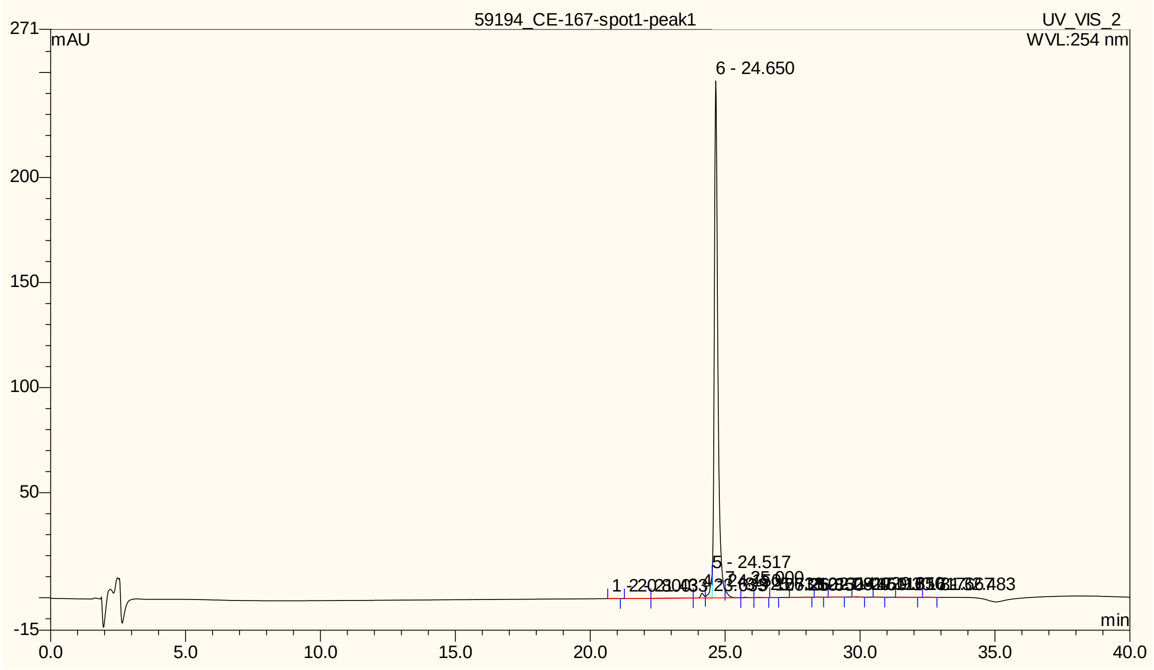

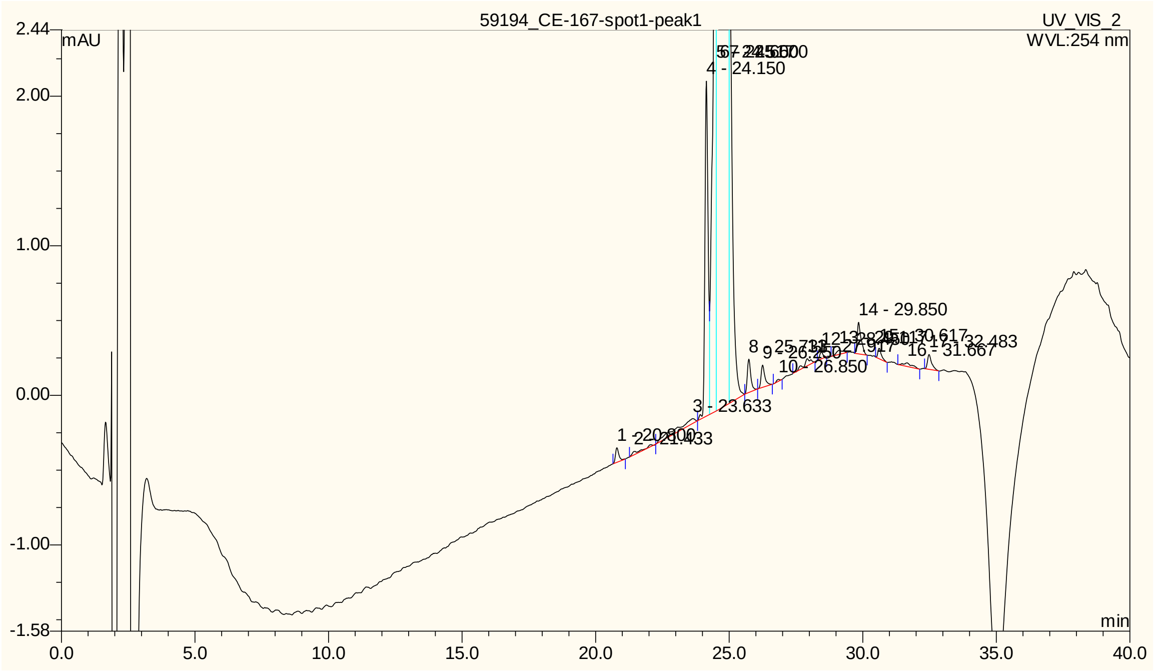

Retention Time: **24.65 min**

Relative Peak Area: **95.31 %**

Figure S18a. RP-Chromatogram of analogue **6e**.

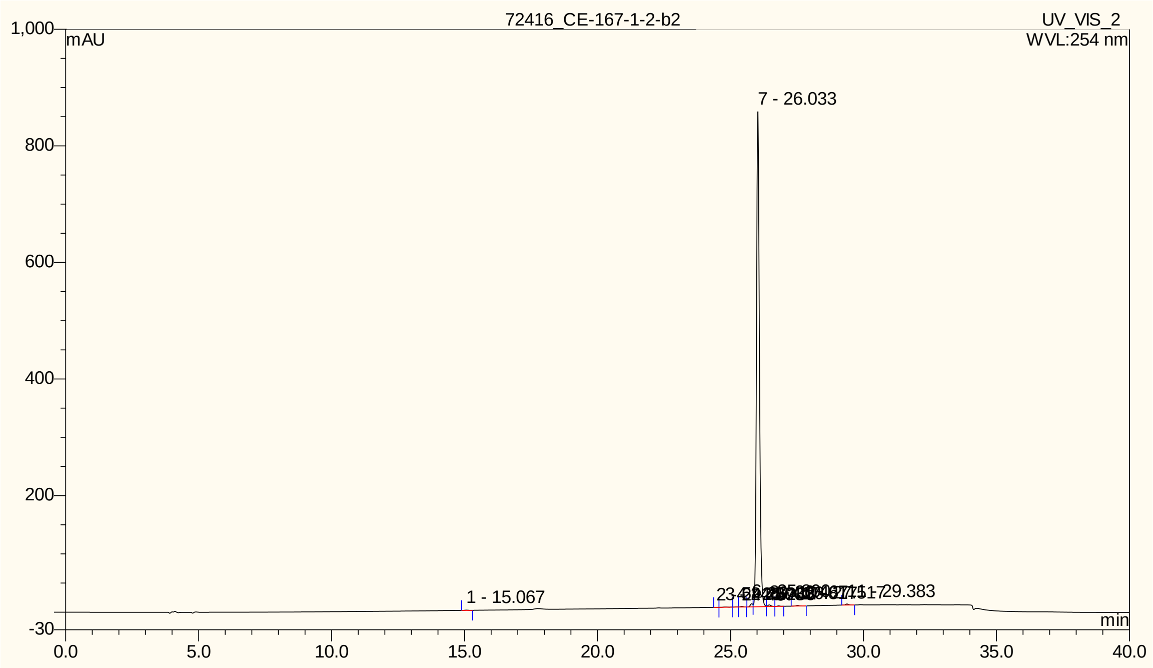

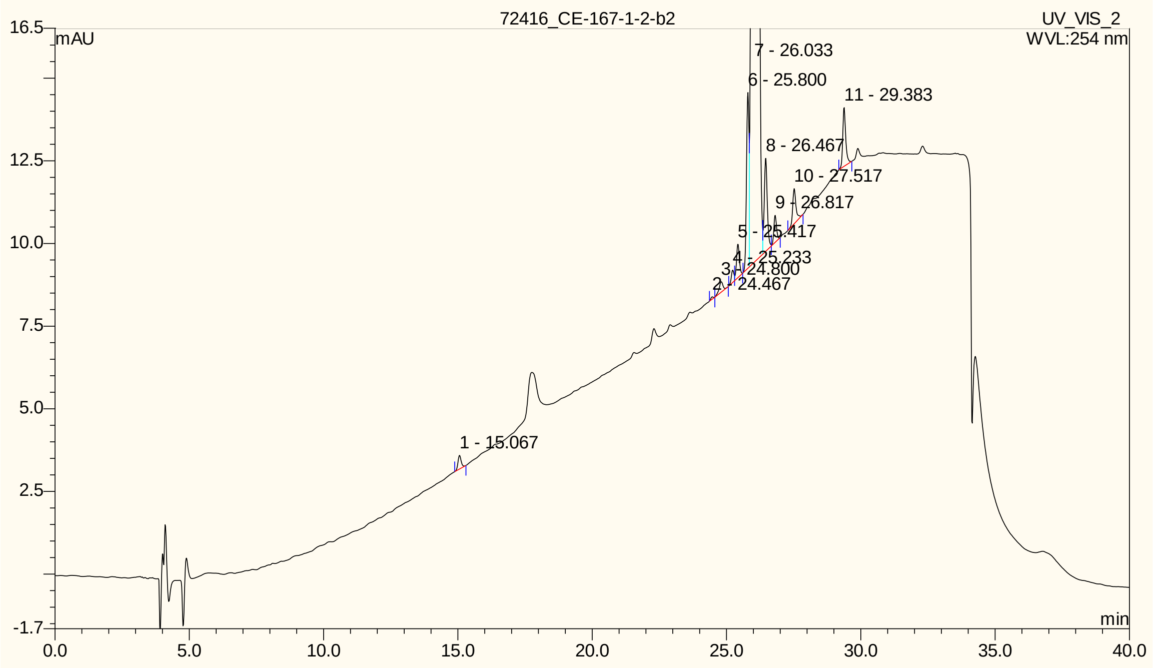

Retention Time: **26.03 min**

Relative Peak Area: **98.46 %**

Figure S19a. RP-Chromatogram of analogue **7e**.

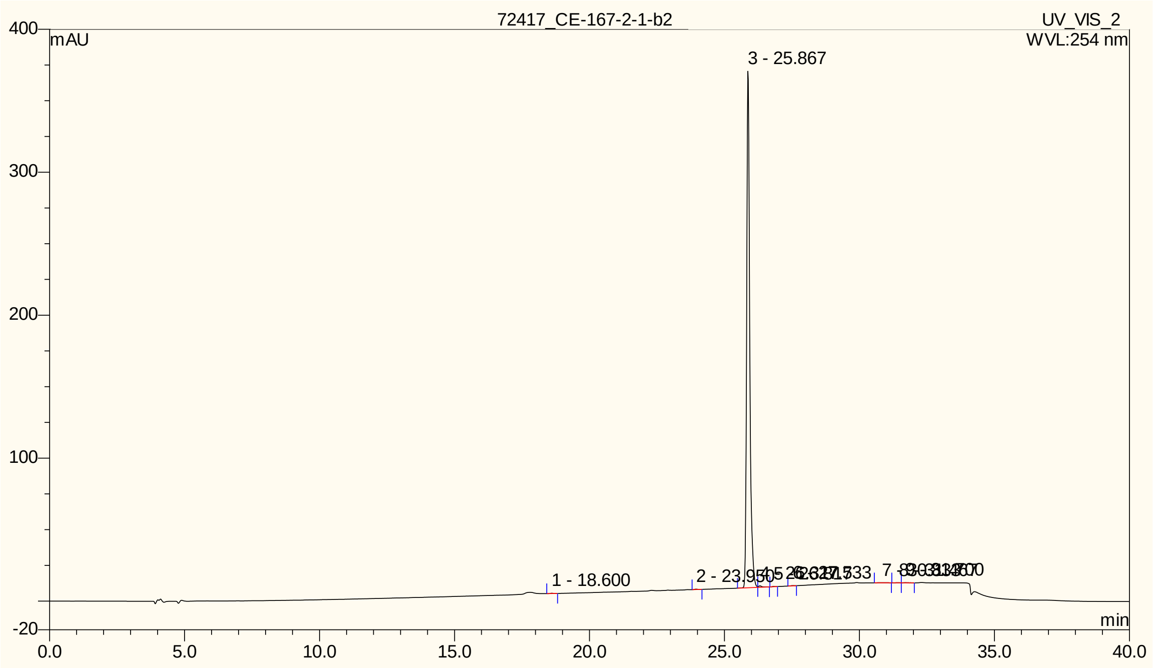

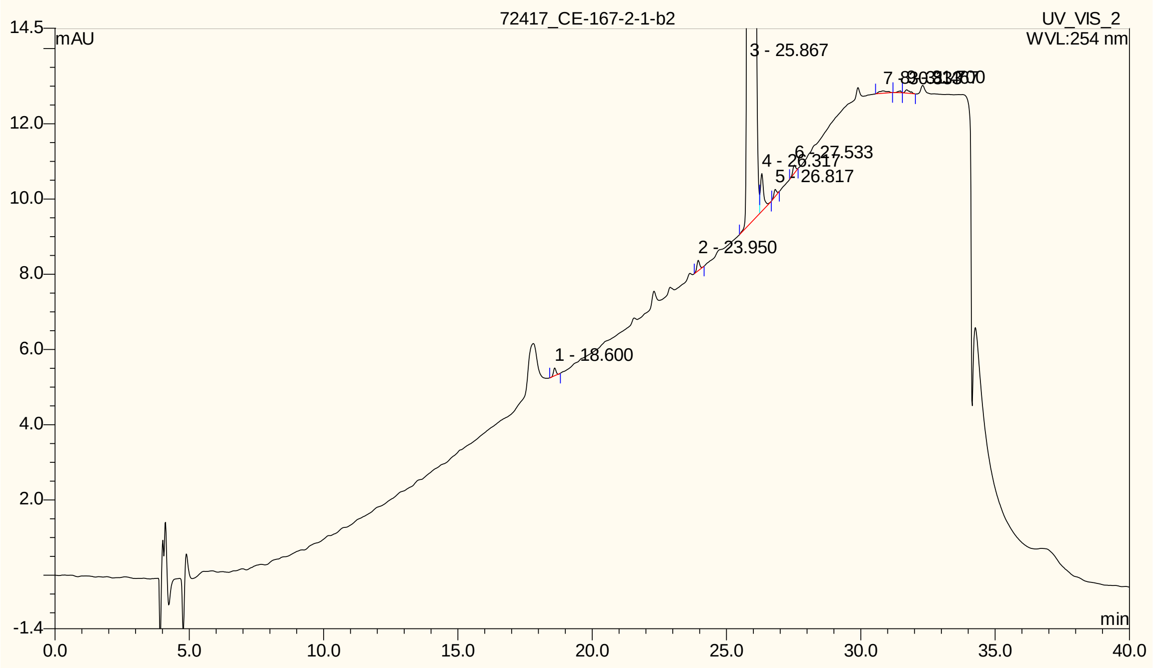

Retention Time: **25.87 min**

Relative Peak Area: **99.41 %**

Figure S20a. RP-Chromatogram of analogue **8e**.

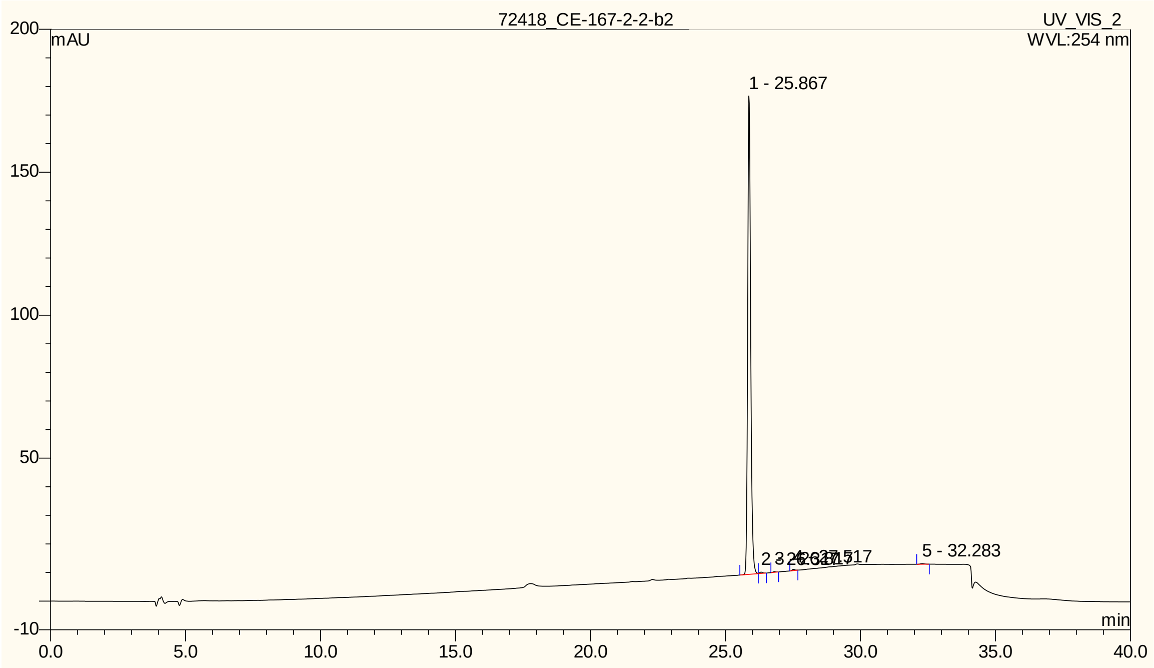

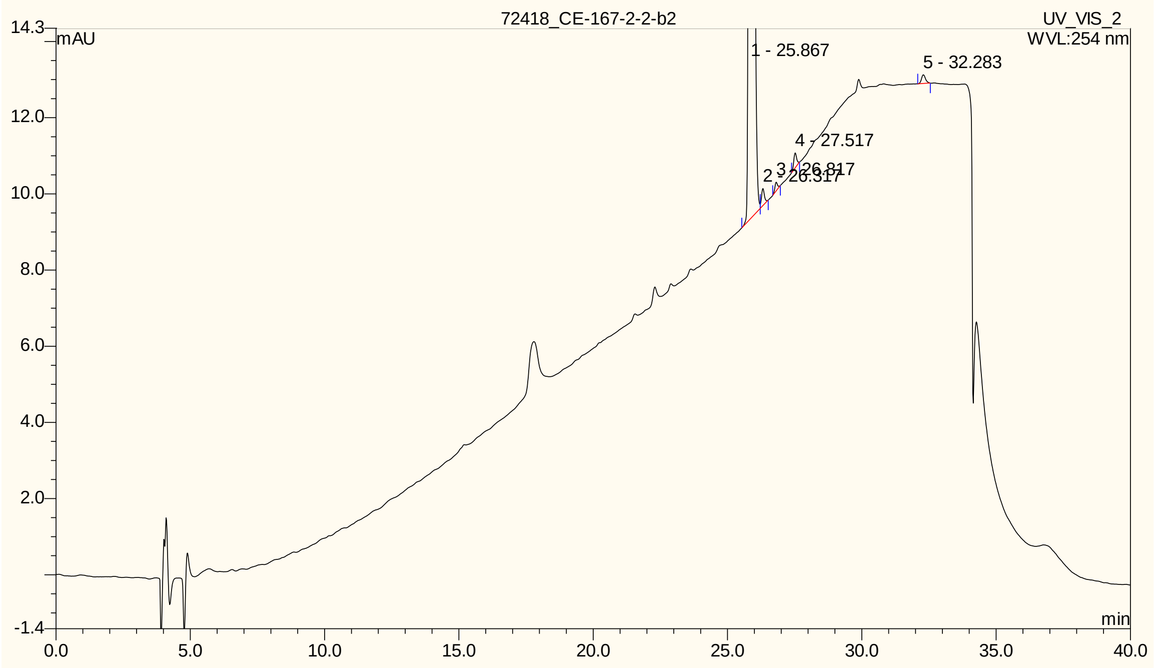

Retention Time: **25.87 min**

Relative Peak Area: **99.21 %**

Figure S21a. RP-Chromatogram of analogue **5f.**

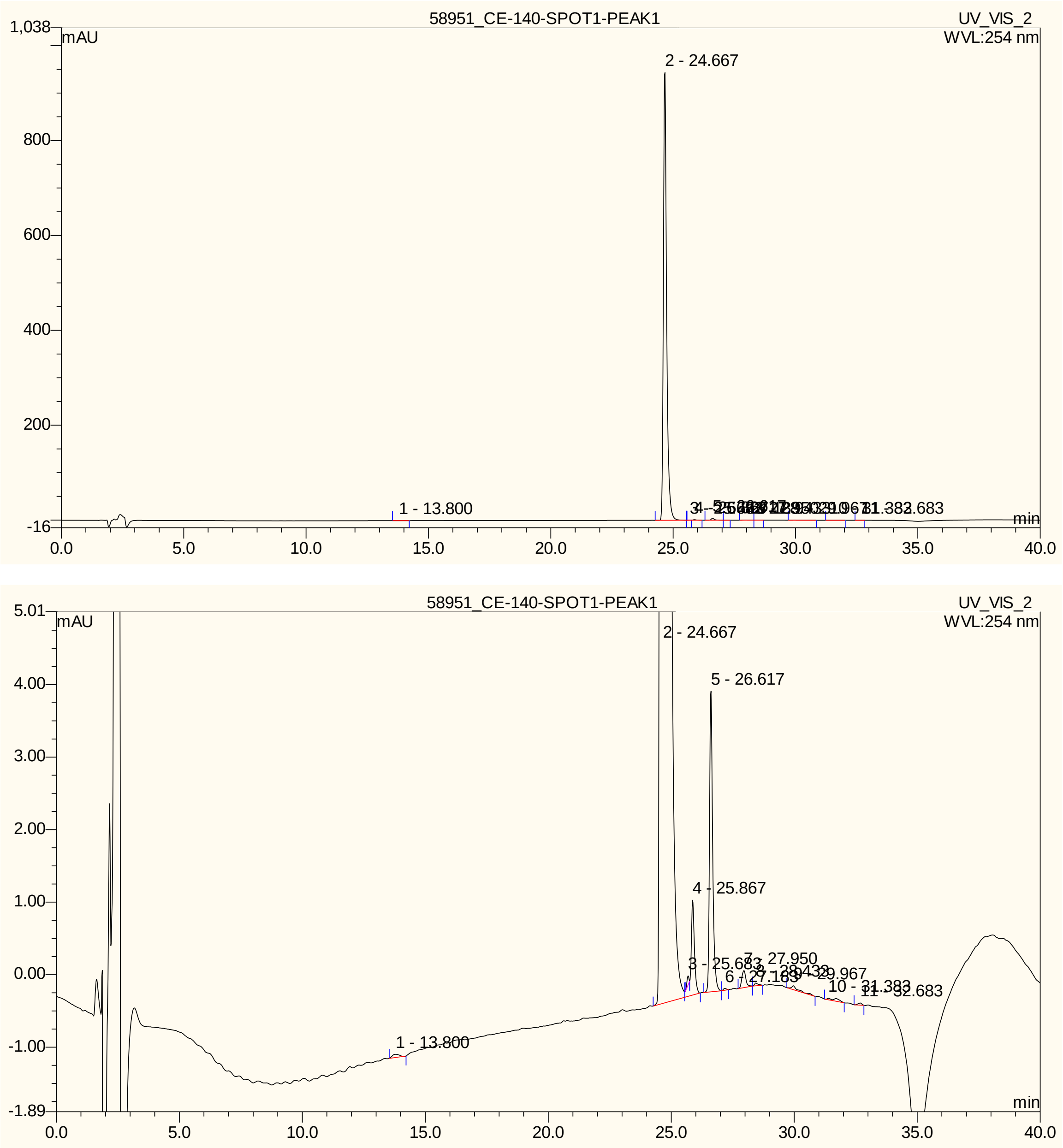

Retention Time: **24.67 min**

Relative Peak Area: **99.34 %**

Figure S22a. RP-Chromatogram of analogue **6f**.

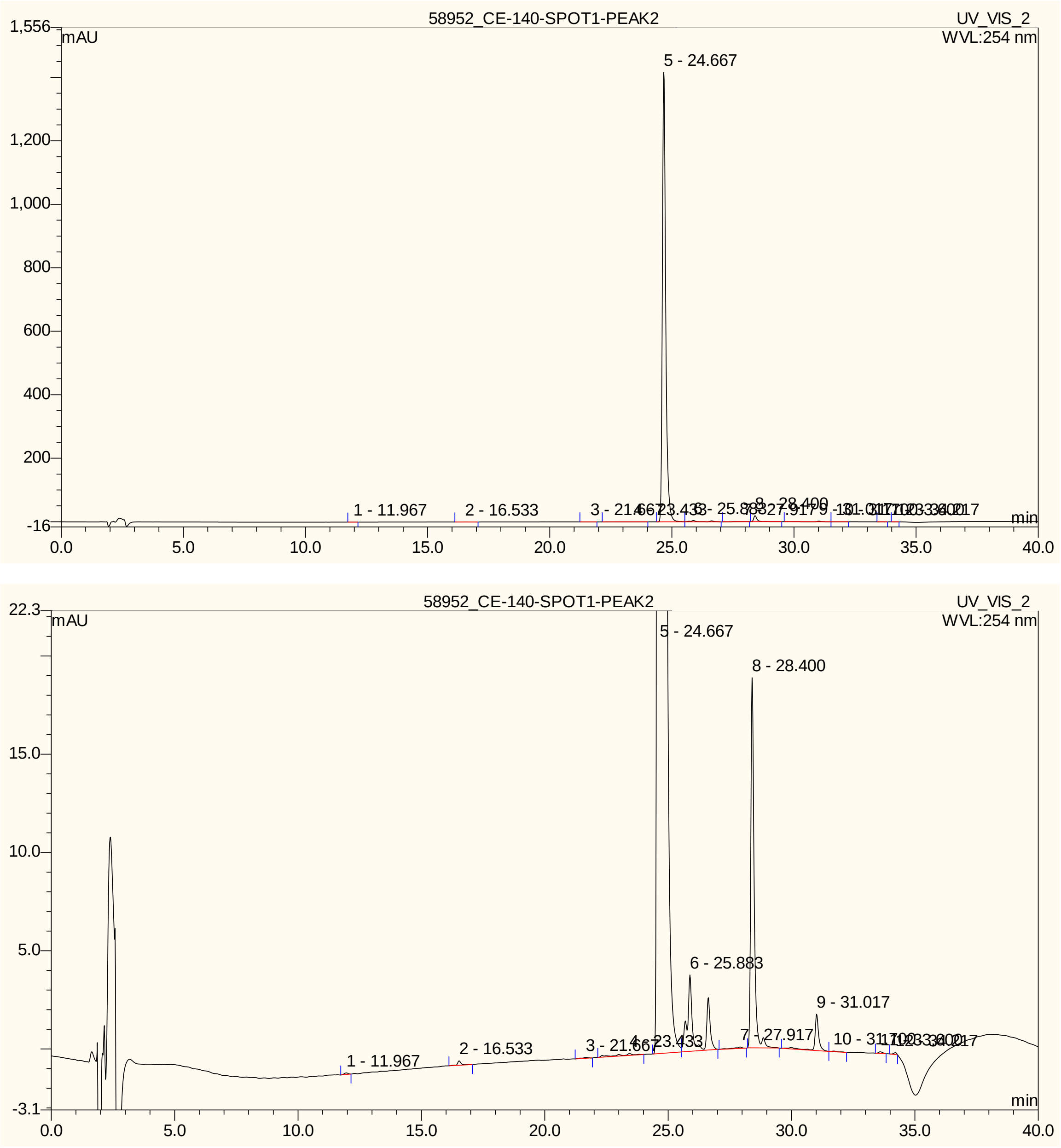

Retention Time: **24.67 min**

Relative Peak Area: **97.90 %**

Figure S23a. RP-Chromatogram of analogue **7f**.

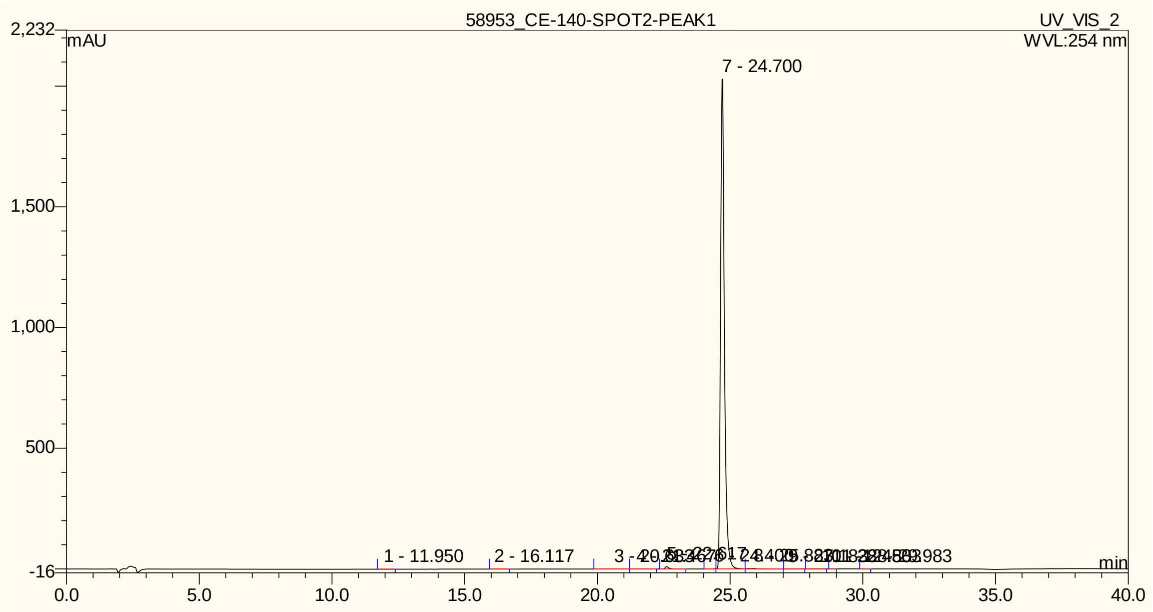

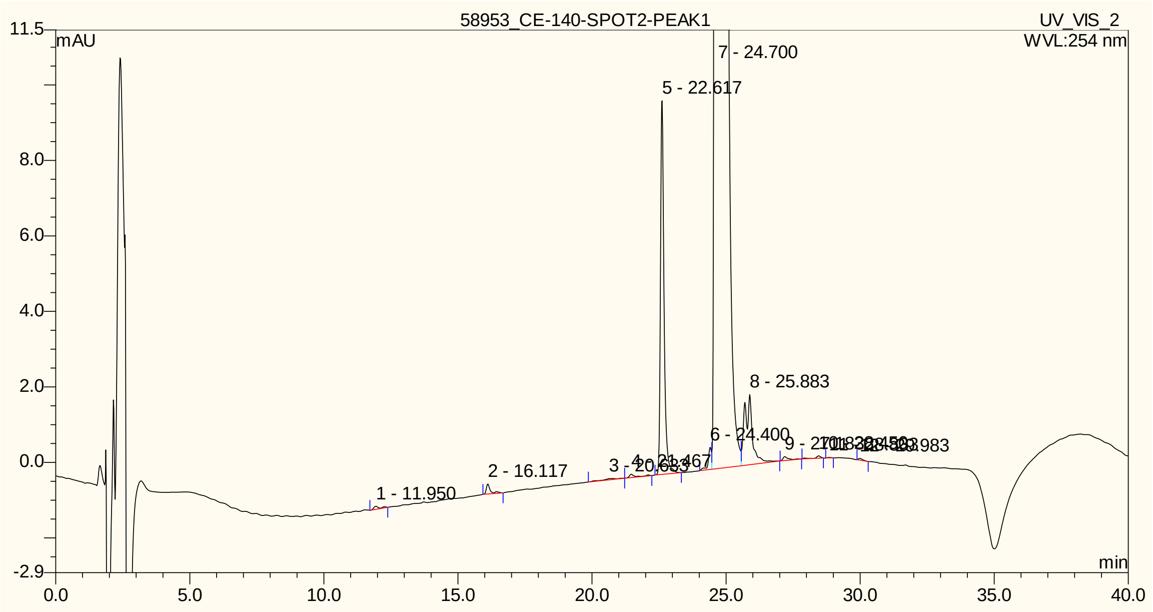

Retention Time: **24.70 min**

Relative Peak Area: **99.31 %**

Figure S24a. RP-Chromatogram of analogue **8f**.

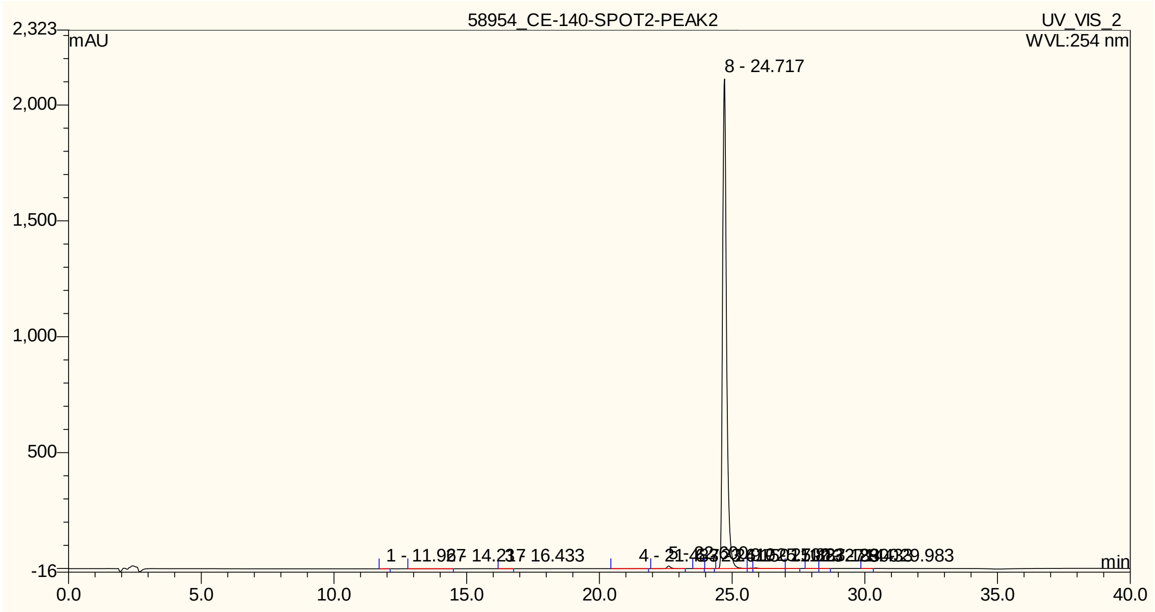

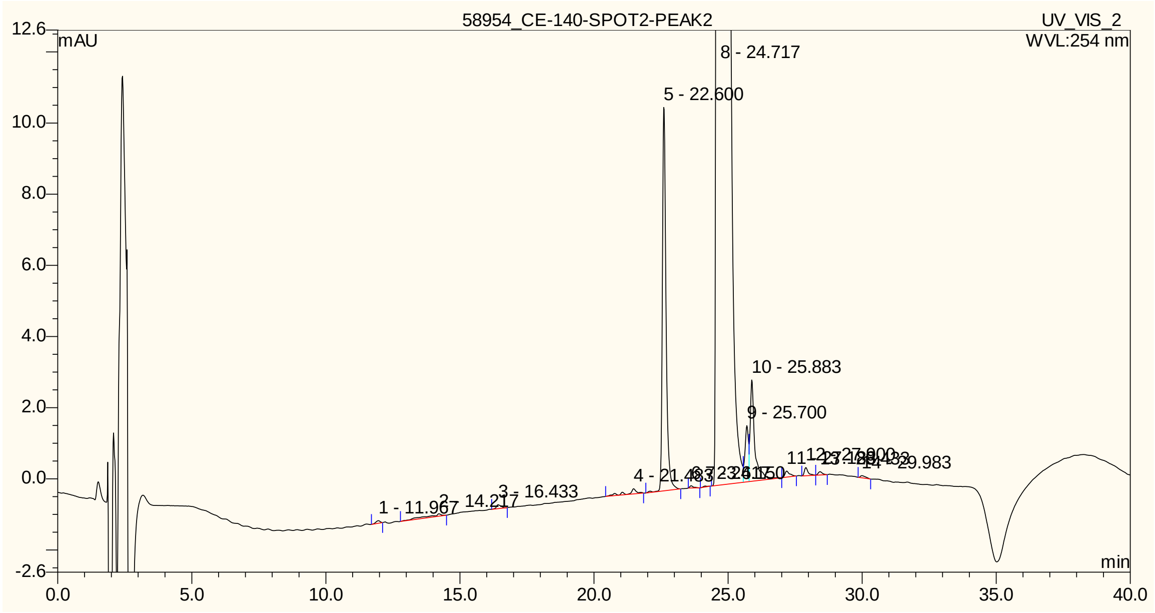

Retention Time: **24.72 min**

Relative Peak Area: **99.26 %**

Figure S25a. RP-Chromatogram of analogue **5g**.

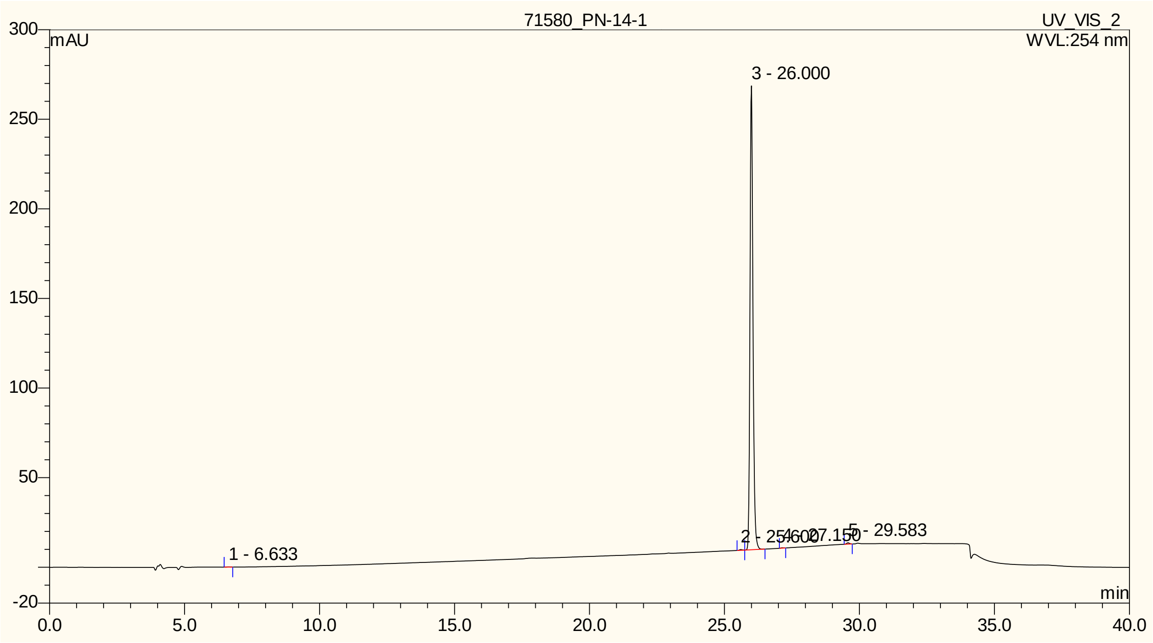

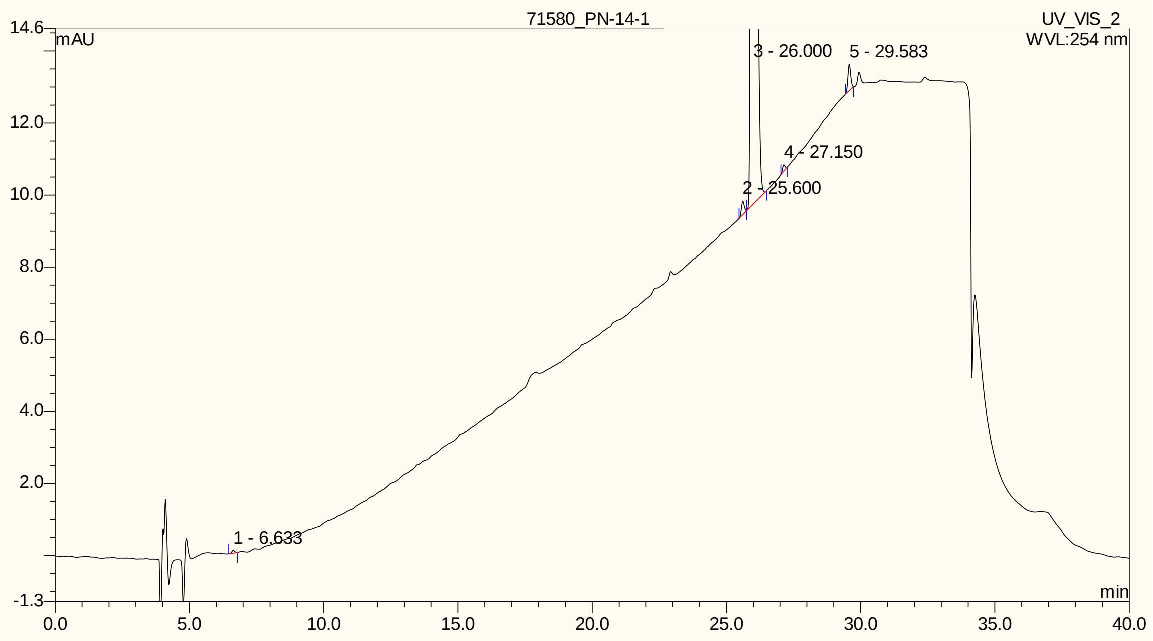

Retention Time: **26.00 min**

Relative Peak Area: **99.51 %**

Figure S26a. RP-Chromatogram of analogue **6g**.

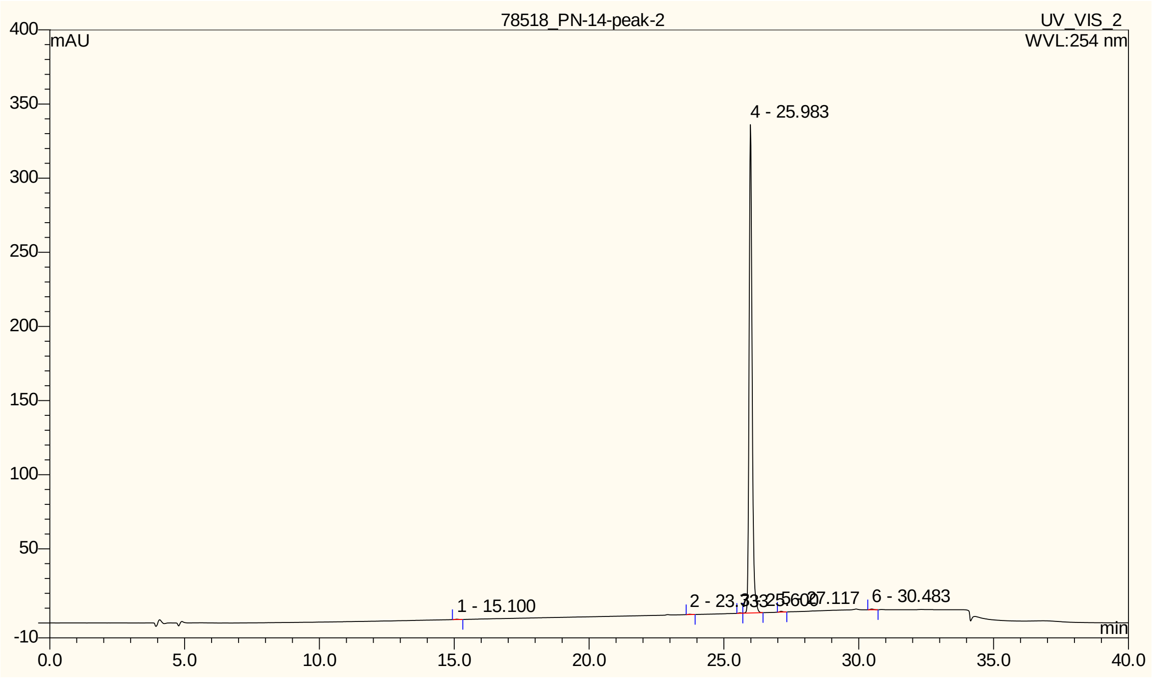

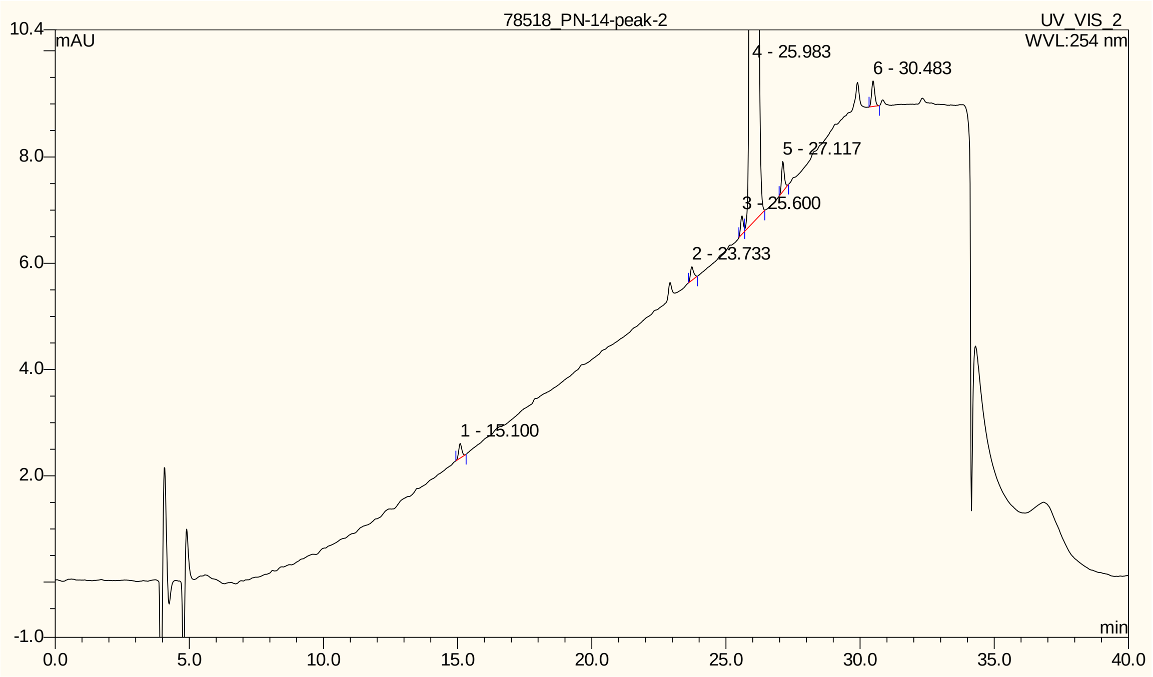

Retention Time: **25.98 min**

Relative Peak Area: **99.44 %**

Figure S27a. RP-Chromatogram of analogue **7g**.

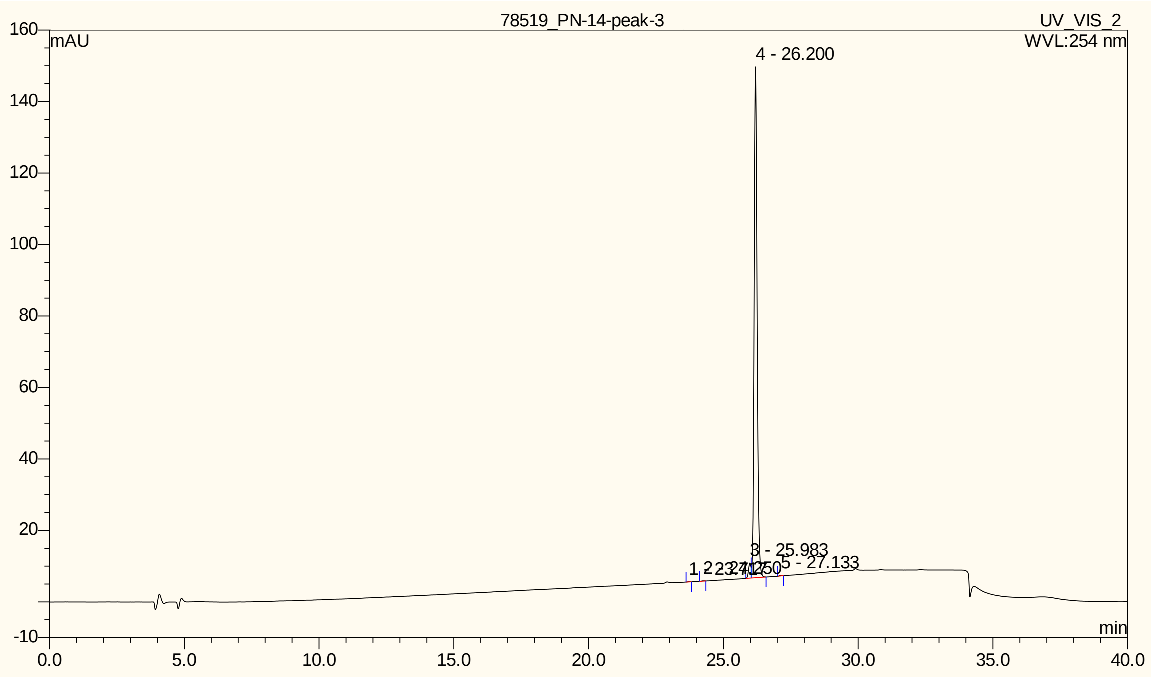

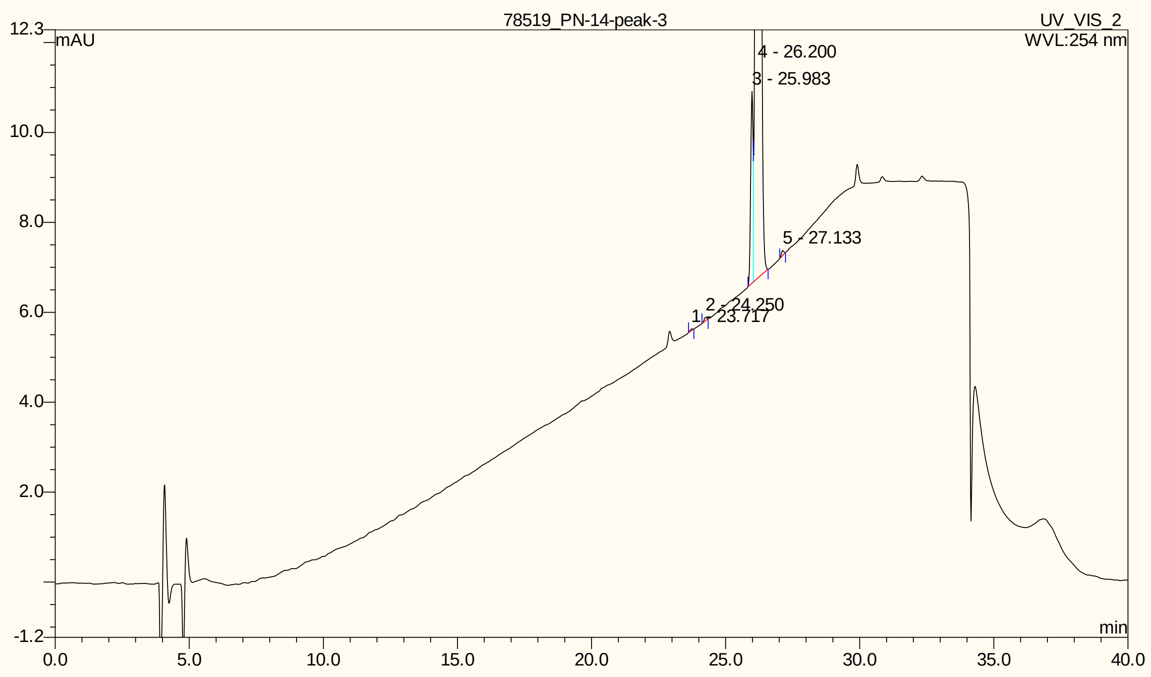

Retention Time: **26.20 min**

Relative Peak Area: **97.61 %**

Figure S28a. RP-Chromatogram of analogue **8g**.

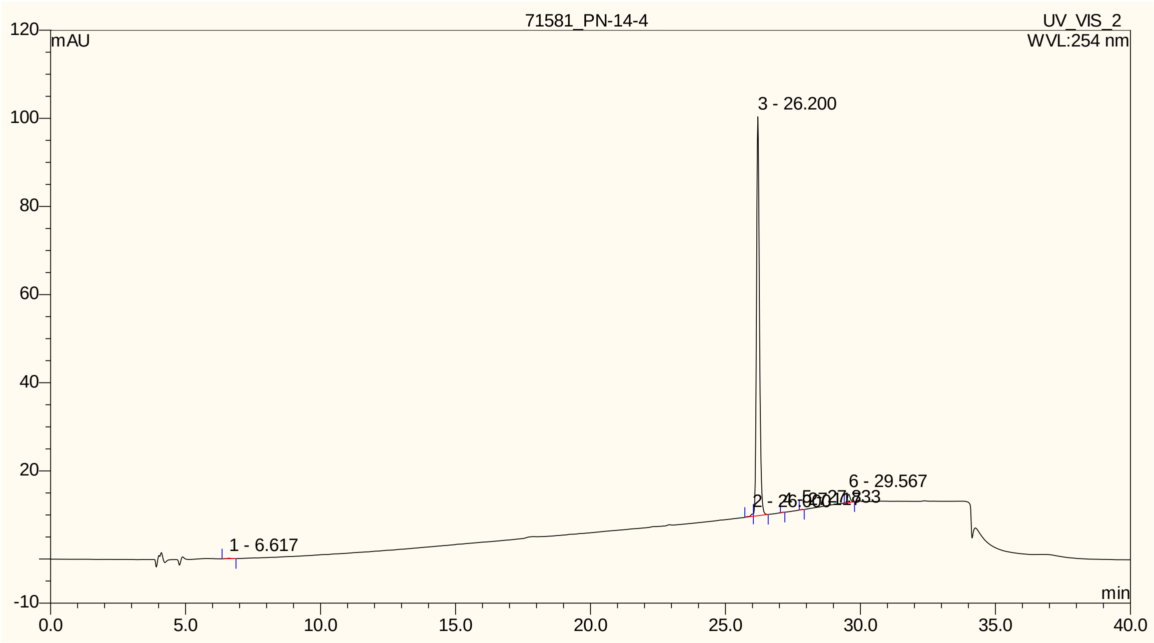

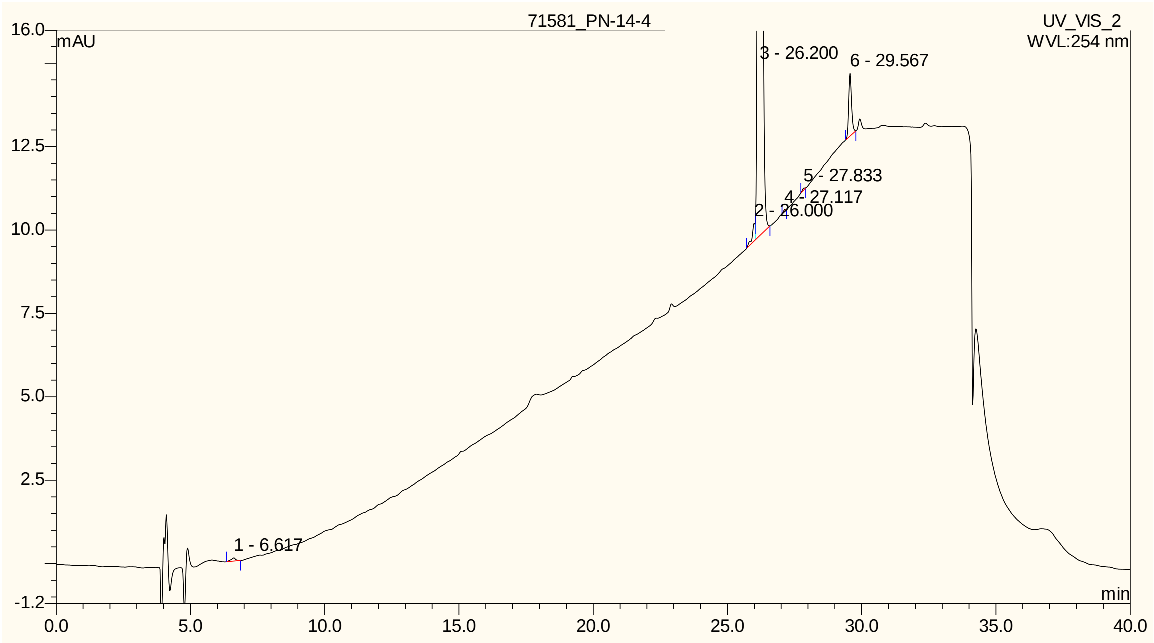

Retention Time: **26.20 min**

Relative Peak Area: **97.24 %**

Figure S29a. RP-Chromatogram of analogue **5h**.

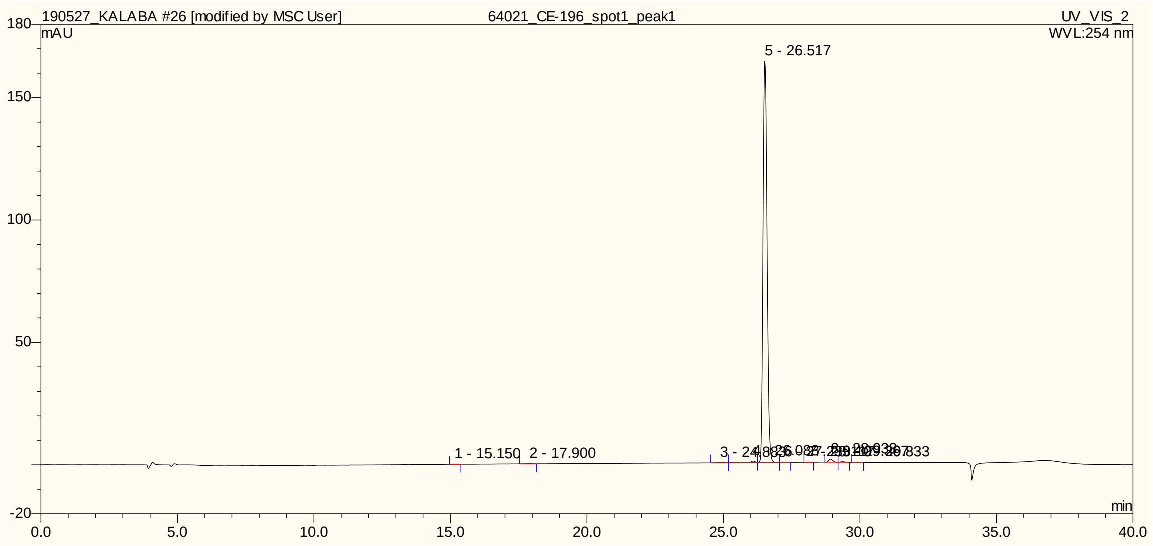

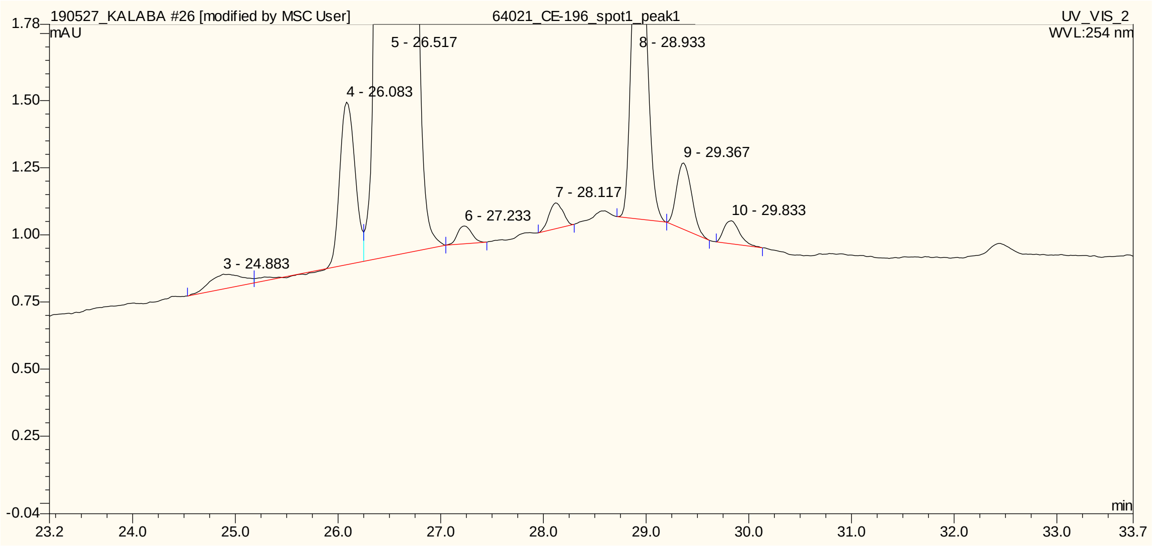

Retention Time: **28.23 min**

Relative Peak Area: **98.42 %**

Figure S30a. RP-Chromatogram of analogue **6h**.

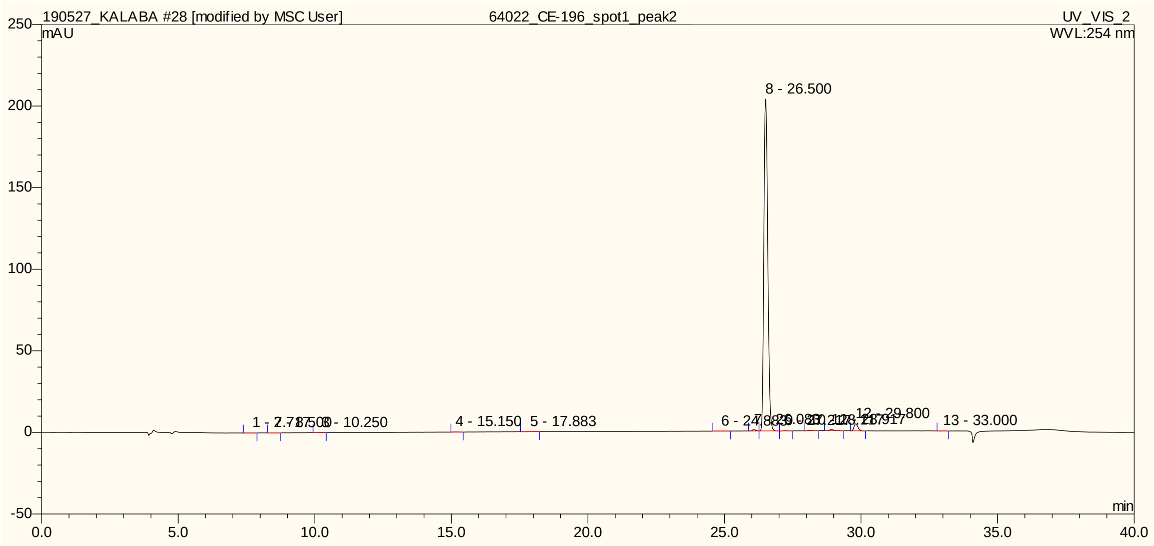

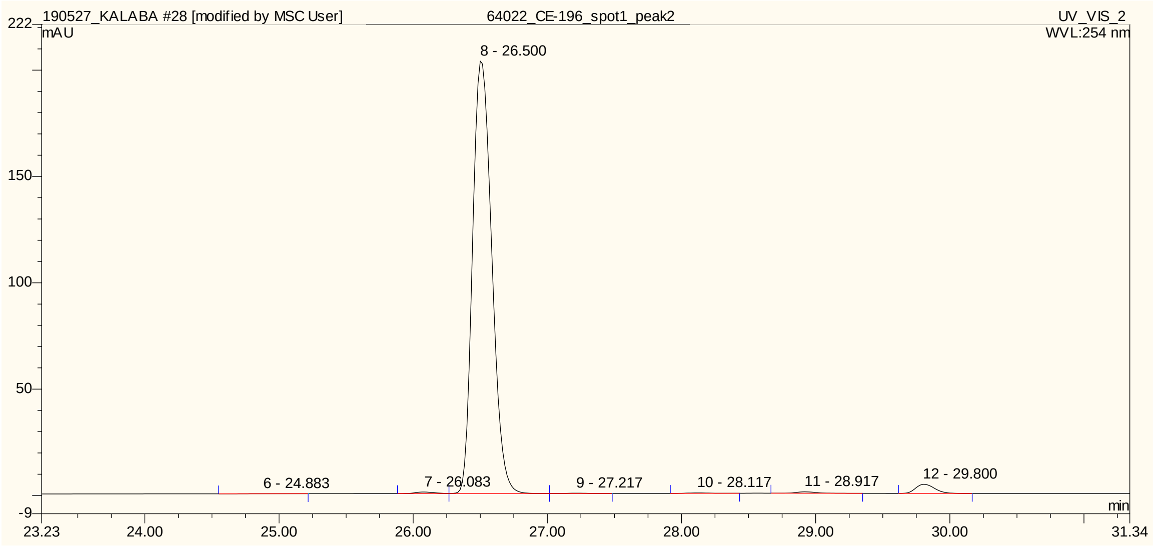

Retention Time: **26.5 min**

Relative Peak Area: **96.86 %**

Figure S31a. RP-Chromatogram of analogue **7h**.

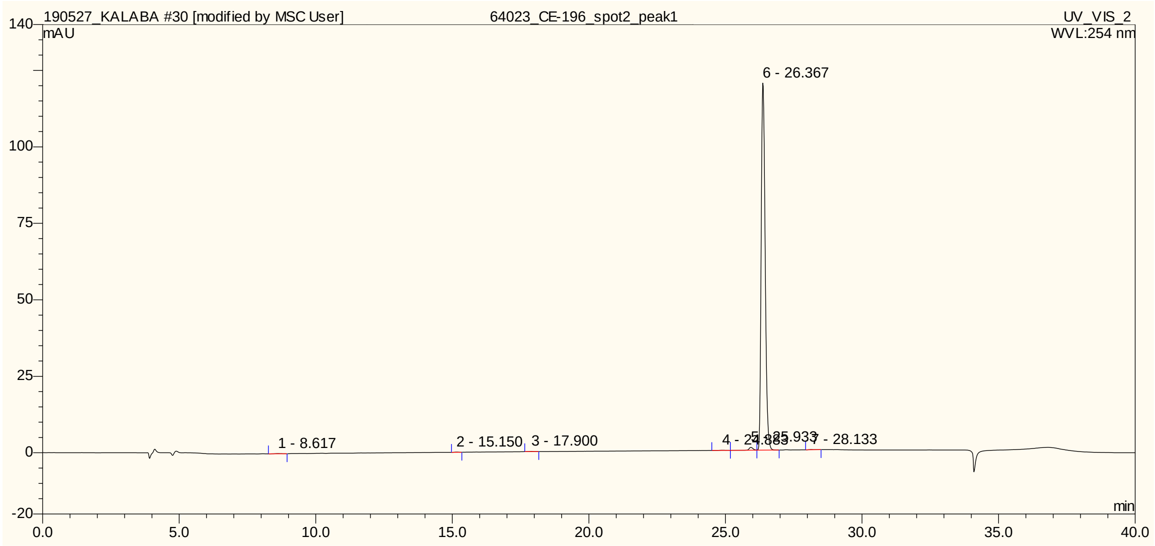

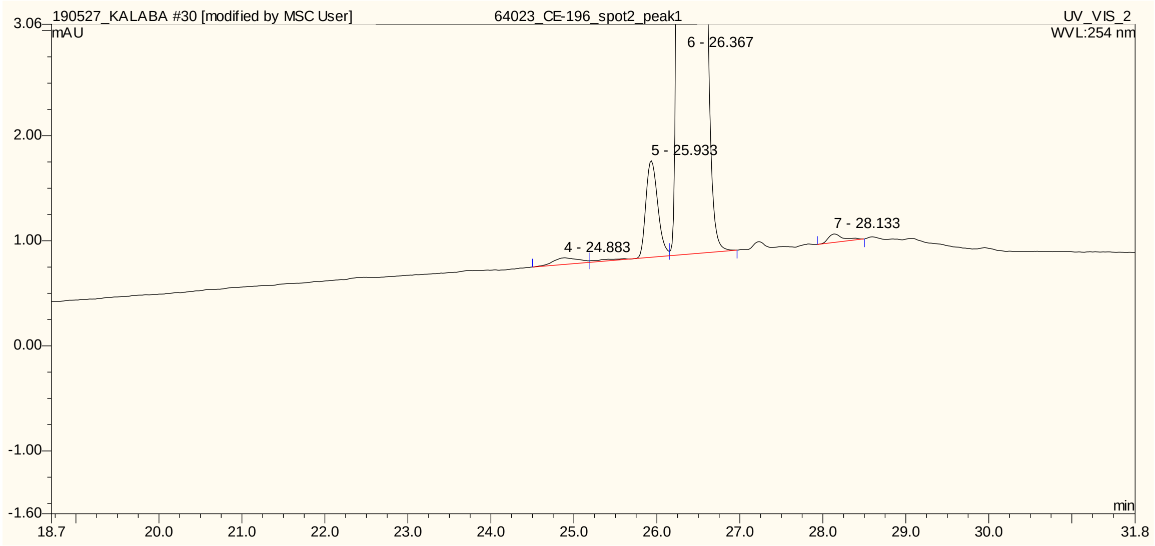

Retention Time: **26.36 min**

Relative Peak Area: **96.83 %**

Figure S32a. RP-Chromatogram of analogue **8h**.

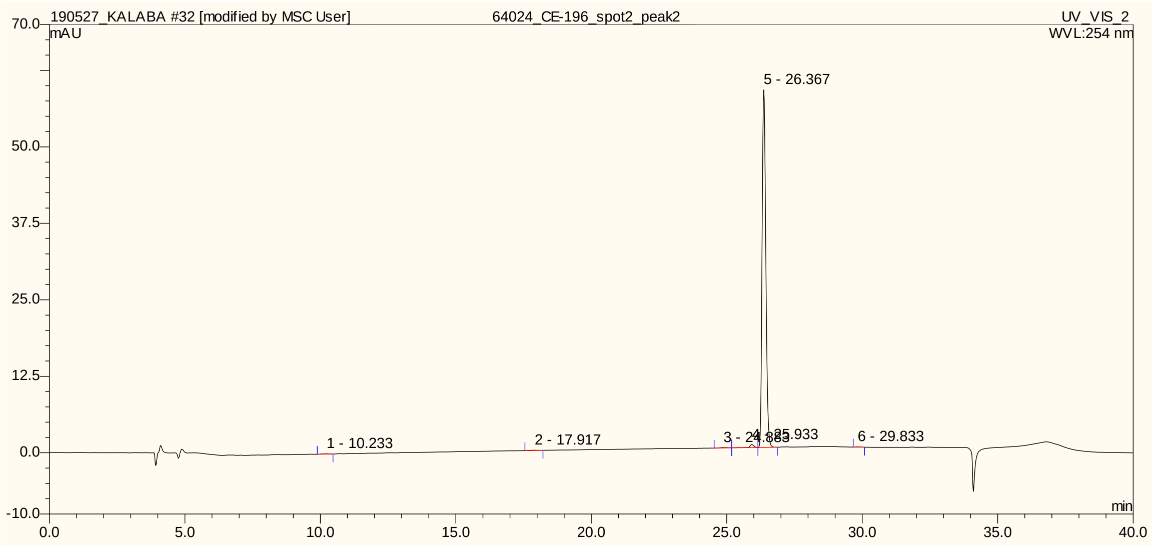

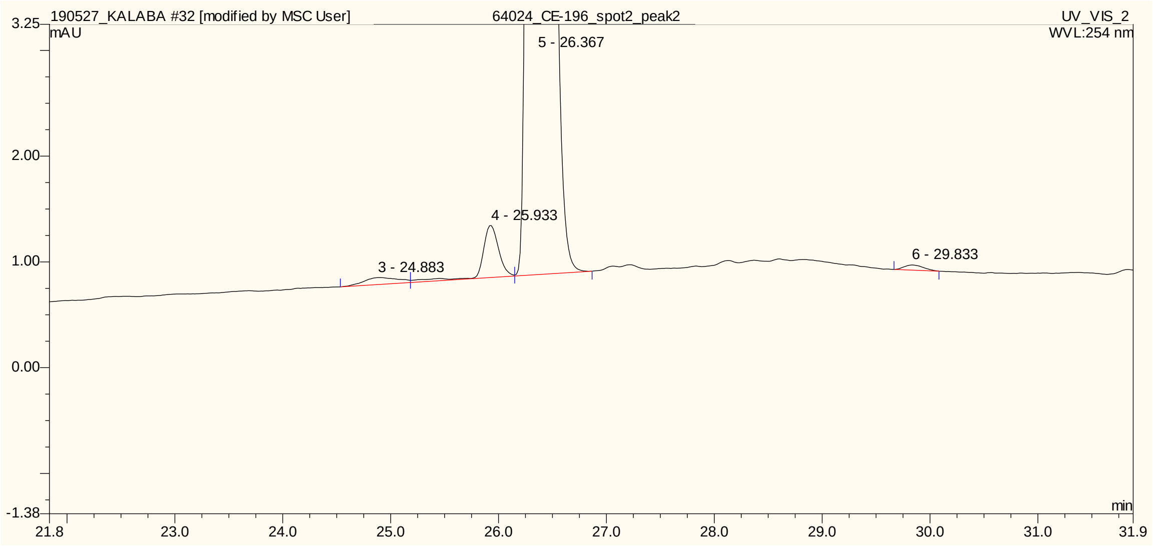

Retention Time: **26.36 min**

Relative Peak Area: **96.4 %**

Figure S33a. RP-Chromatogram of analogue **5i**.

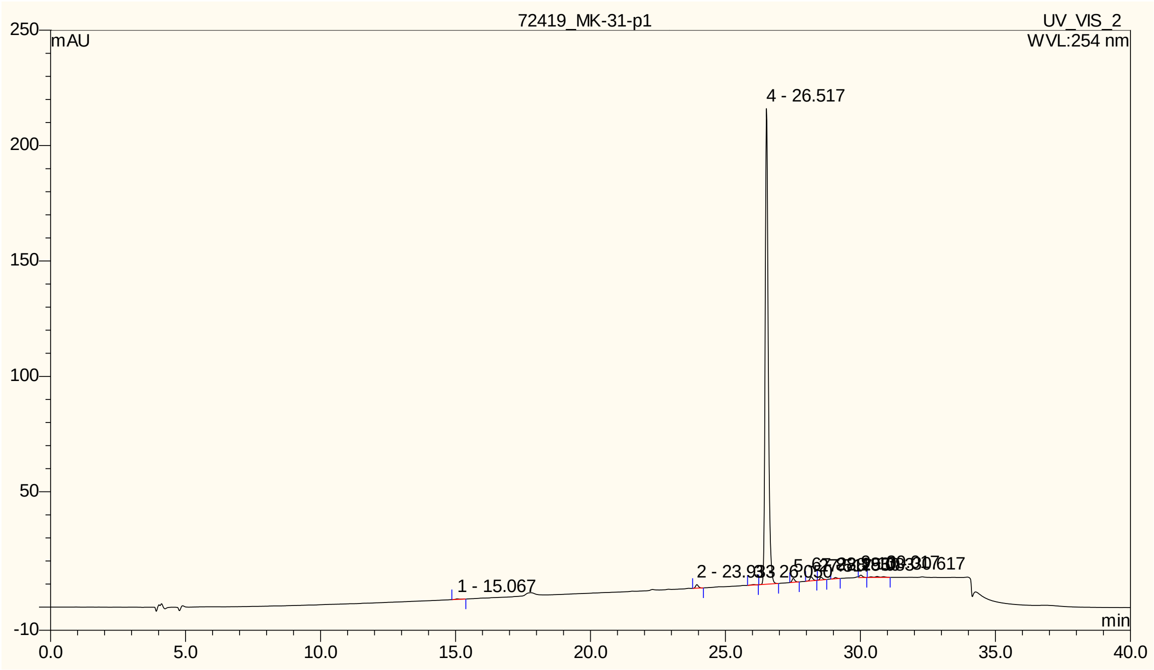

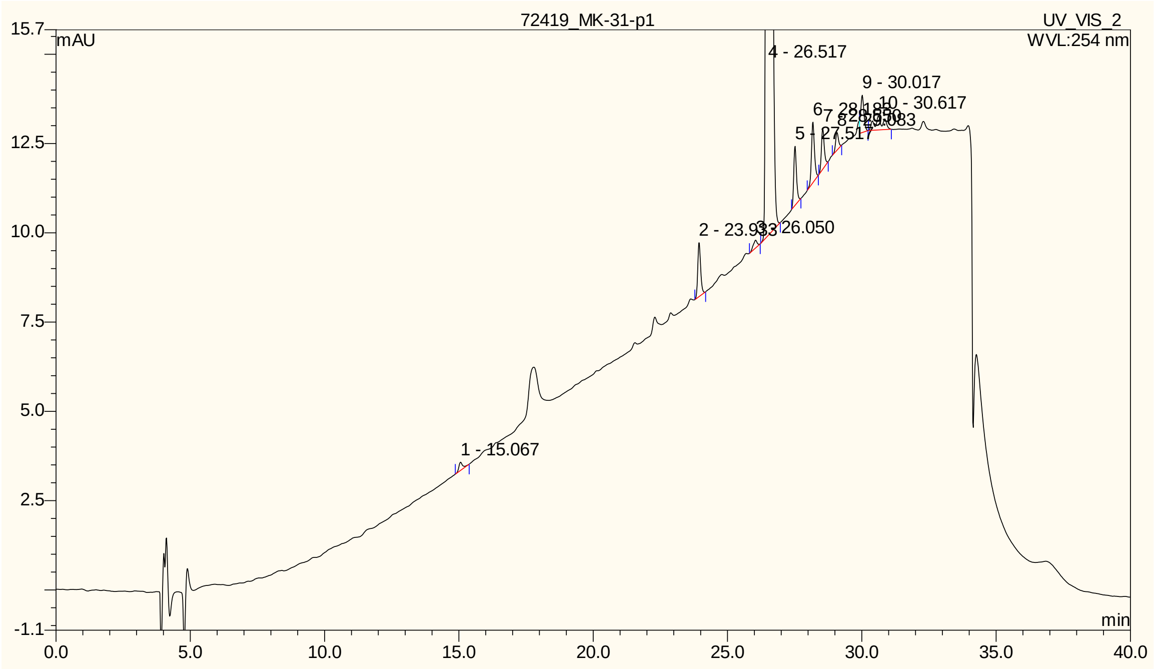

Retention Time: **26.52 min**

Relative Peak Area: **95.88 %**

Figure S34a. RP-Chromatogram of analogue **6i**.

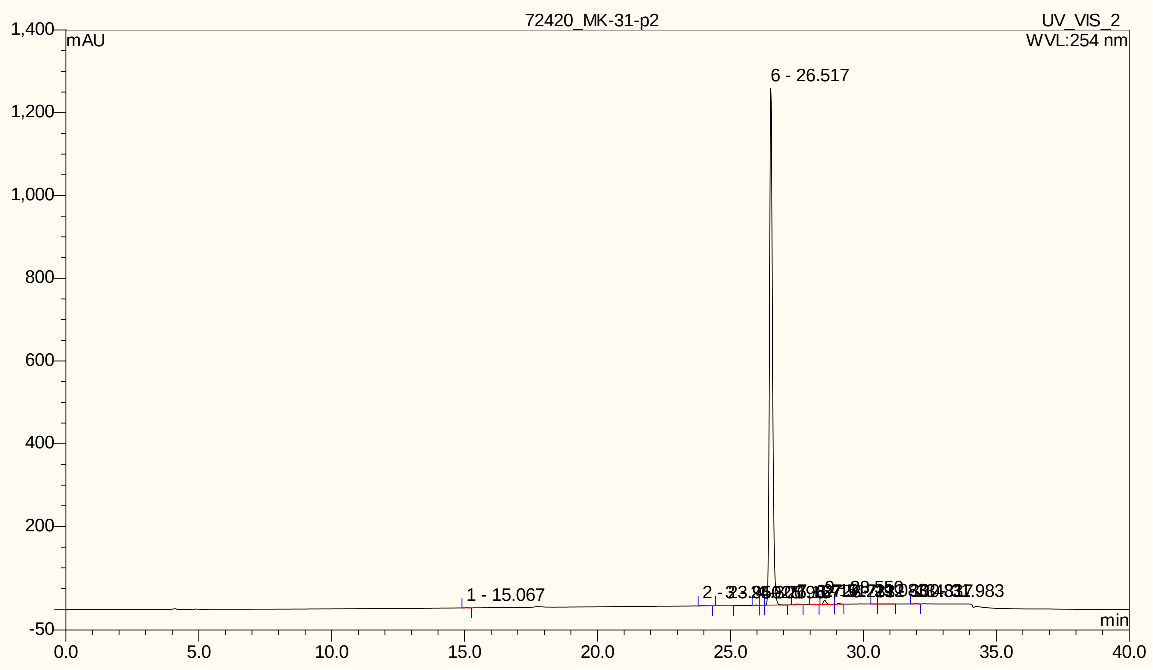

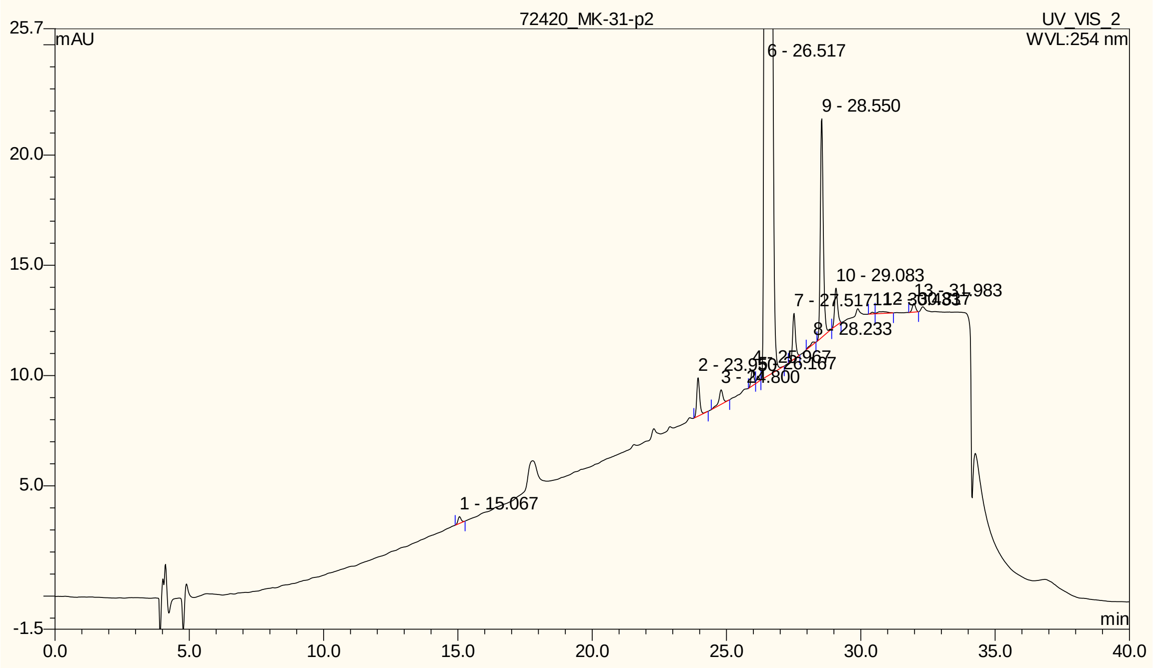

Retention Time: **26.52 min**

Relative Peak Area: **98.58 %**

Figure S35a. RP-Chromatogram of analogue **7i**.

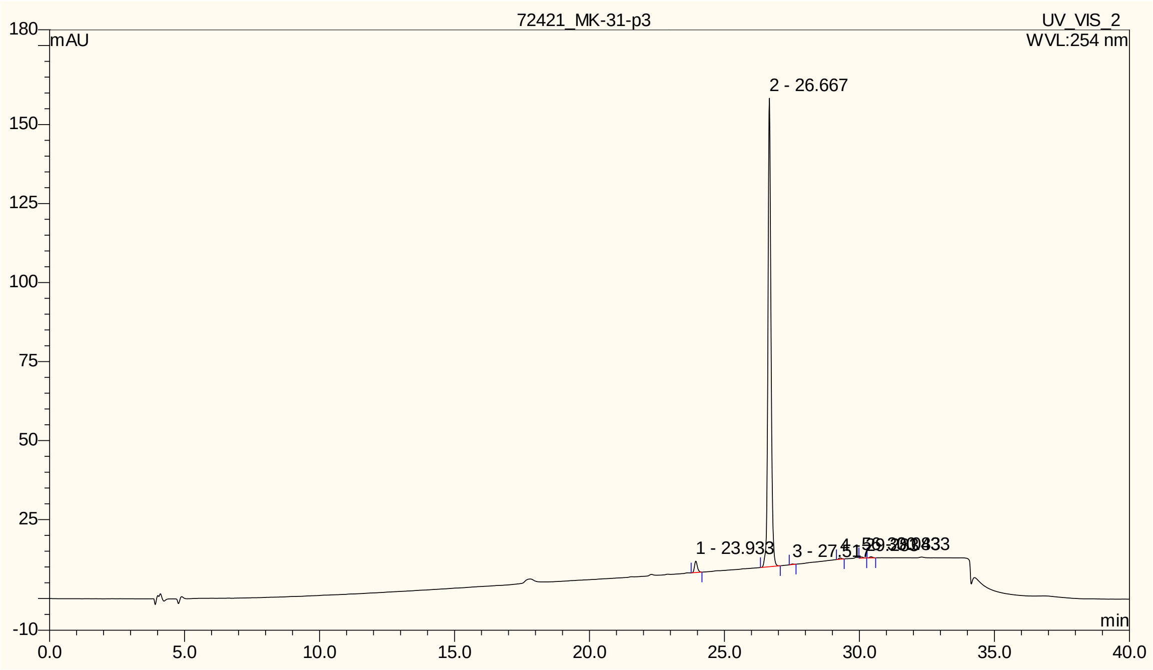

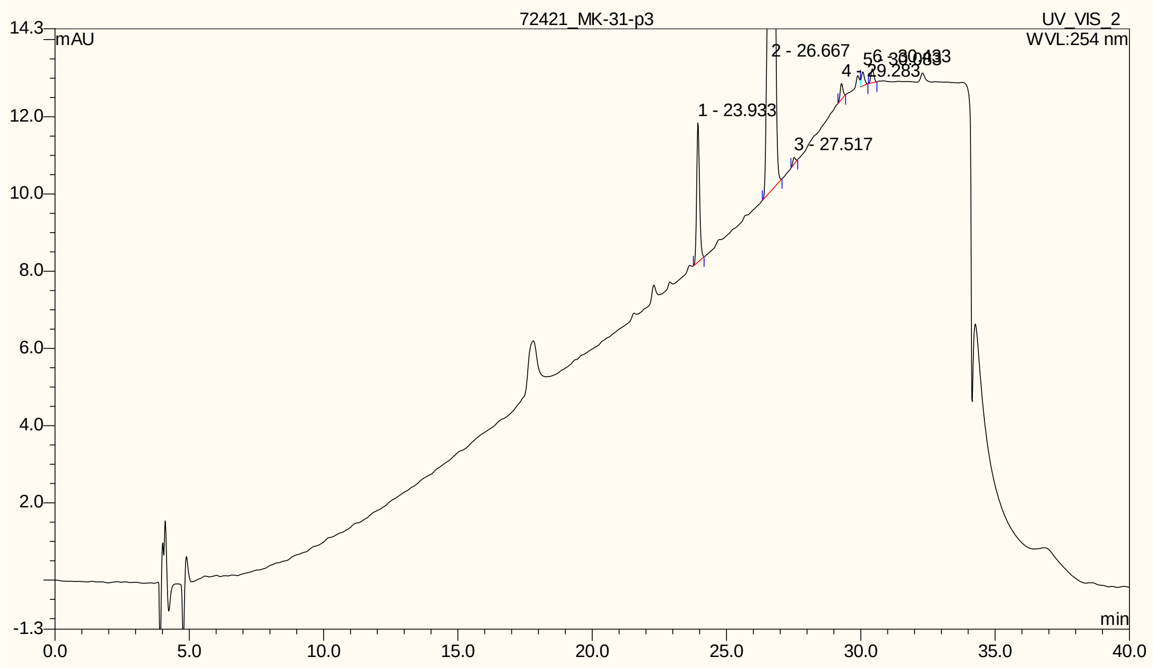

Retention Time: **26.67 min**

Relative Peak Area: **96.96 %**

Figure S36a. RP-Chromatogram of analogue **8i**.

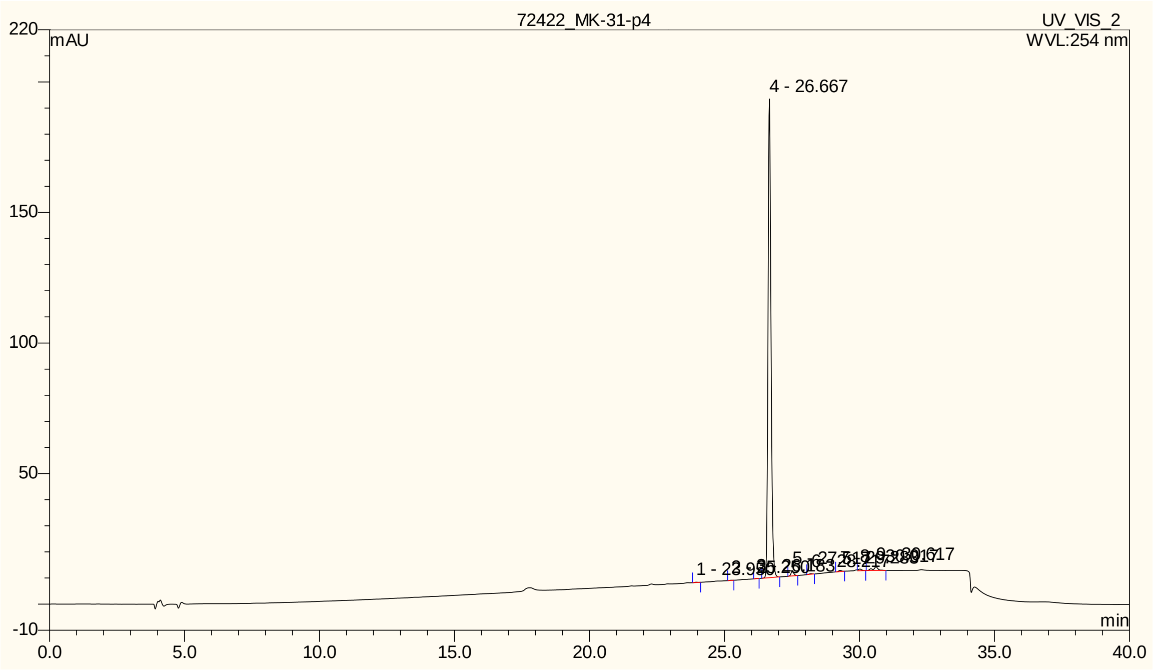

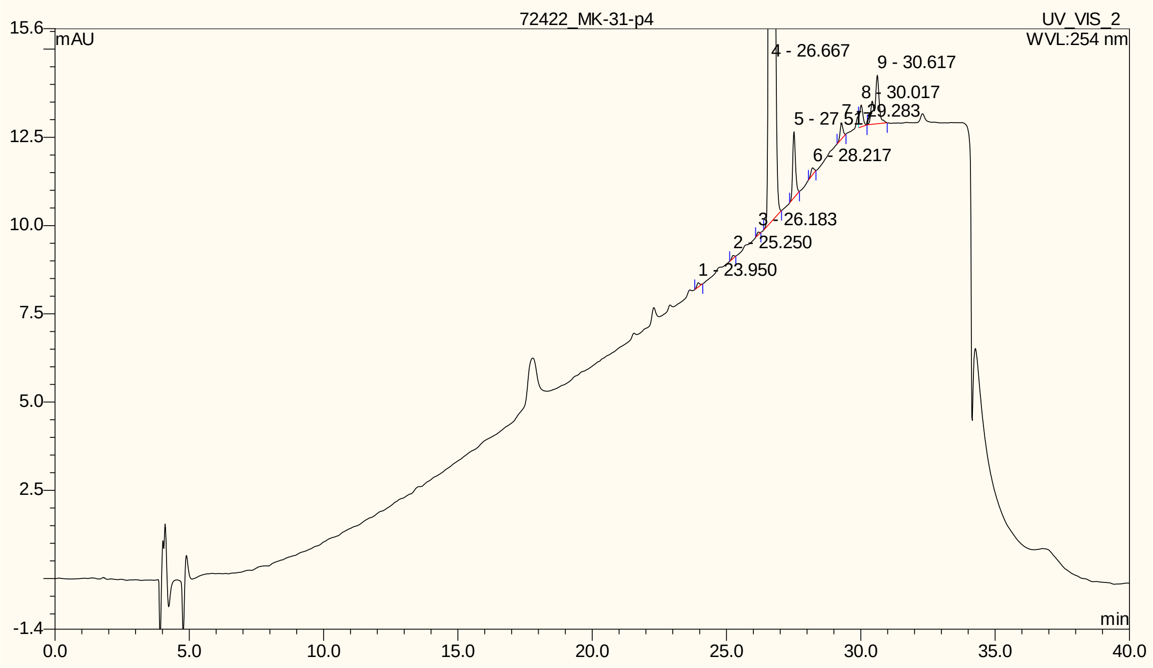

Retention Time: **26.67 min**

Relative Peak Area: **97.04 %**

Figure S37a. RP-Chromatogram of analogue **5j.**

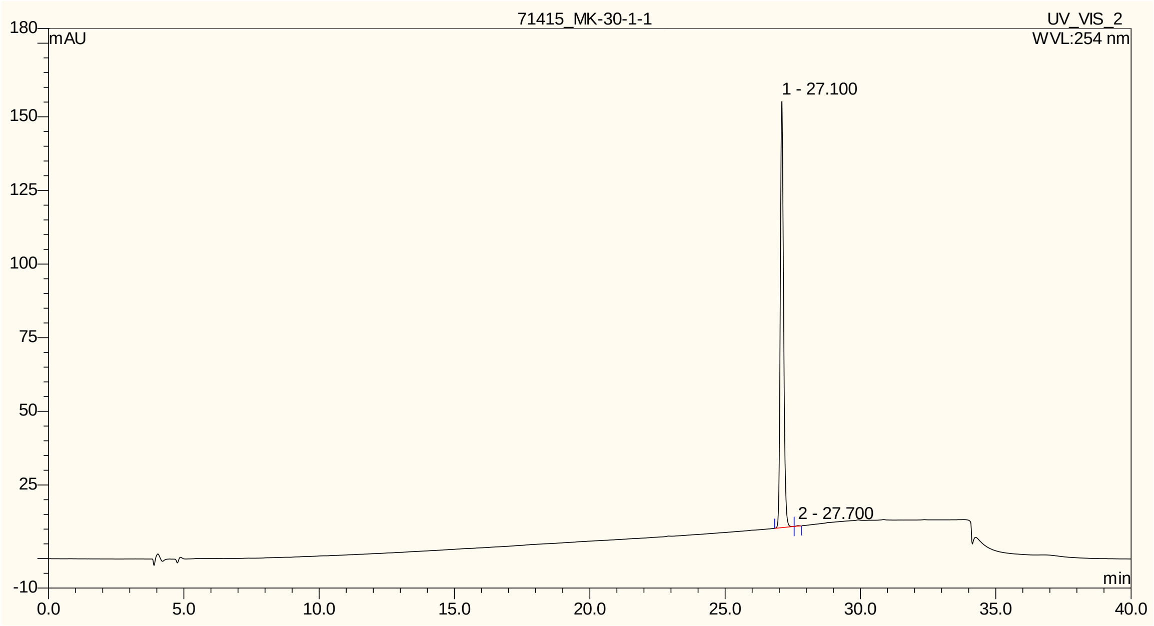

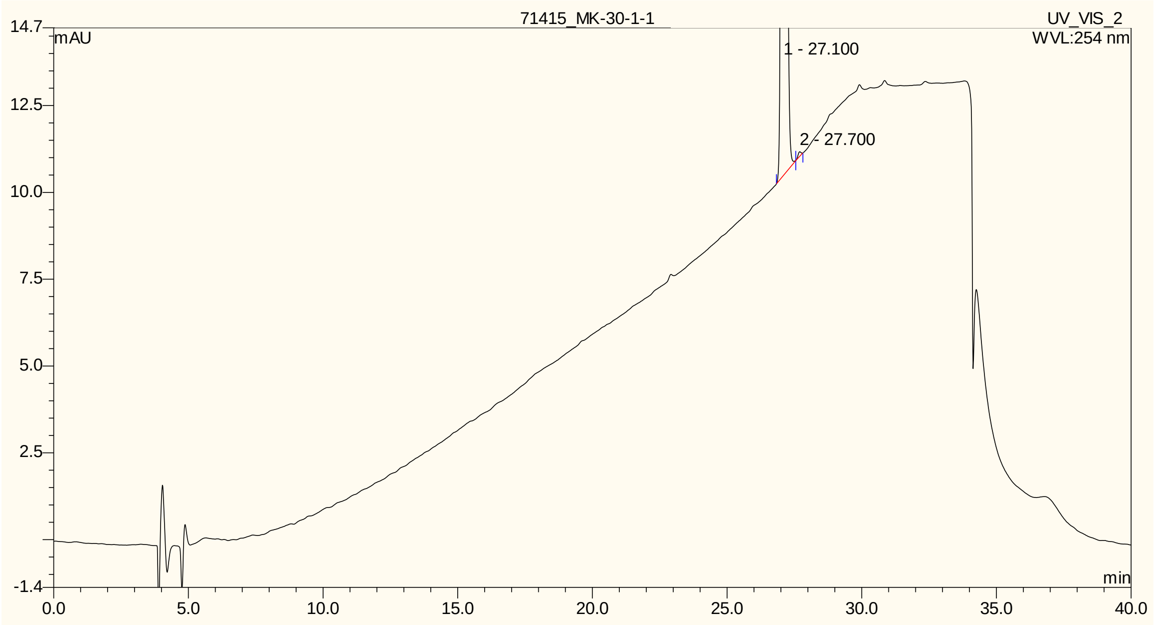

Retention Time: **27.10 min**

Relative Peak Area: **99.92 %**

Figure S38a. RP-Chromatogram of analogue **6j**.

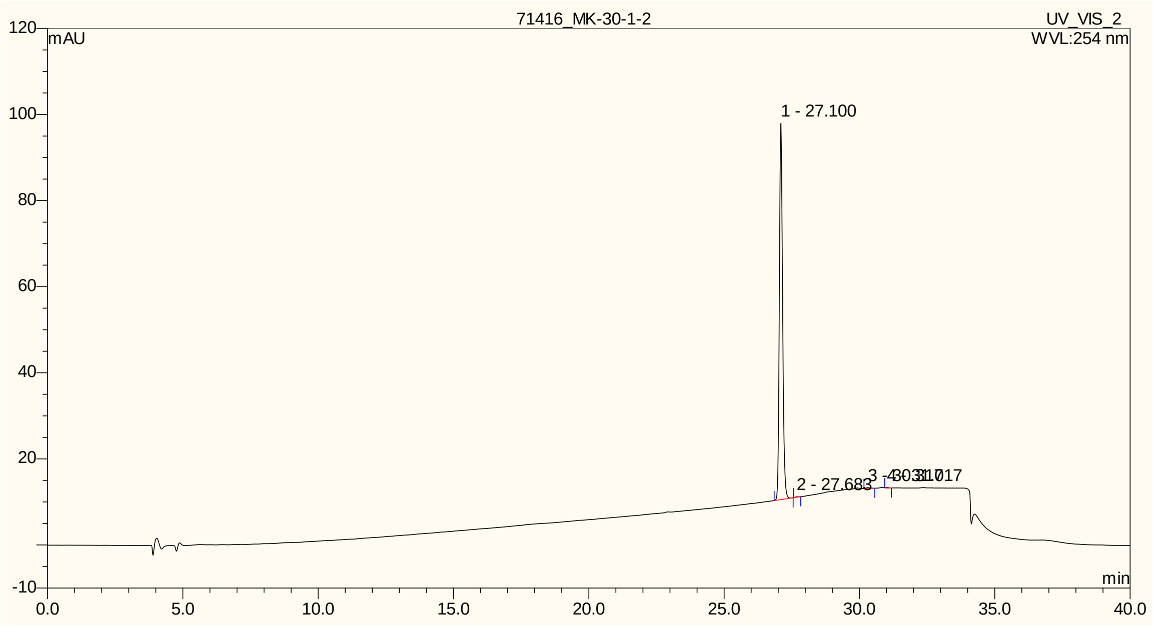

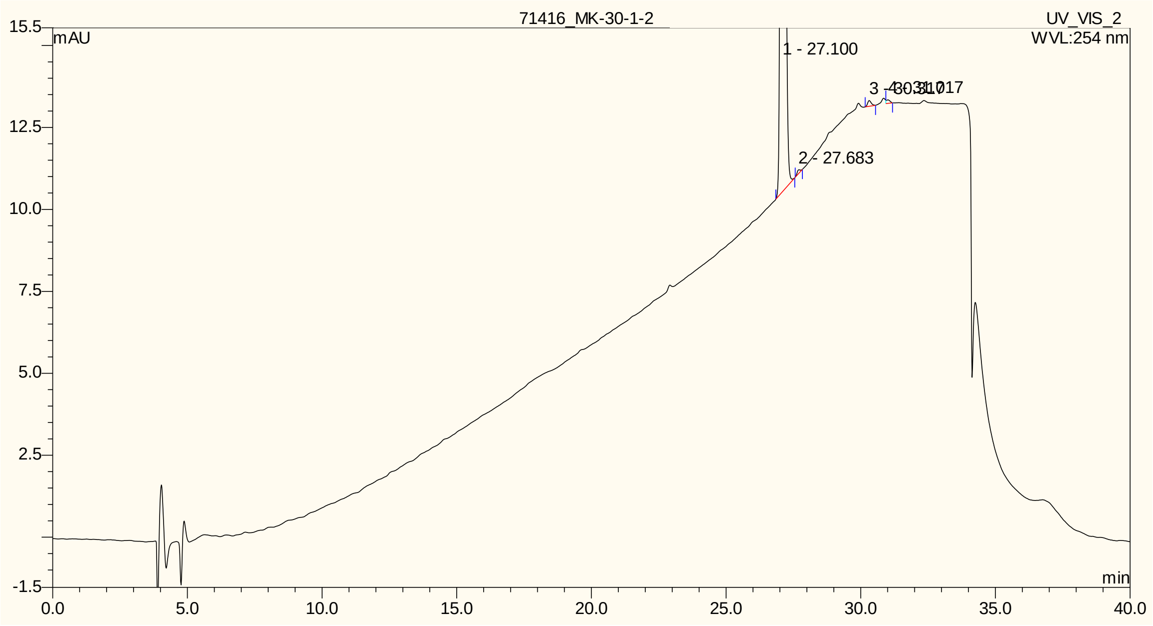

Retention Time: **27.10 min**

Relative Peak Area: **99.52 %**

Figure S39a. RP-Chromatogram of analogue **7j**.

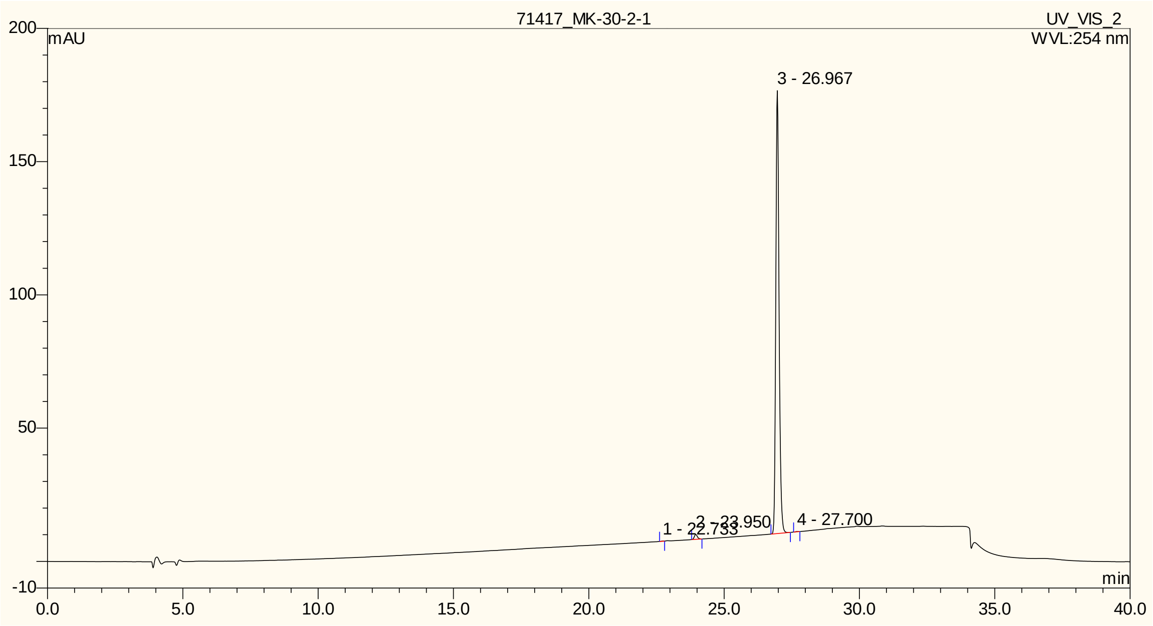

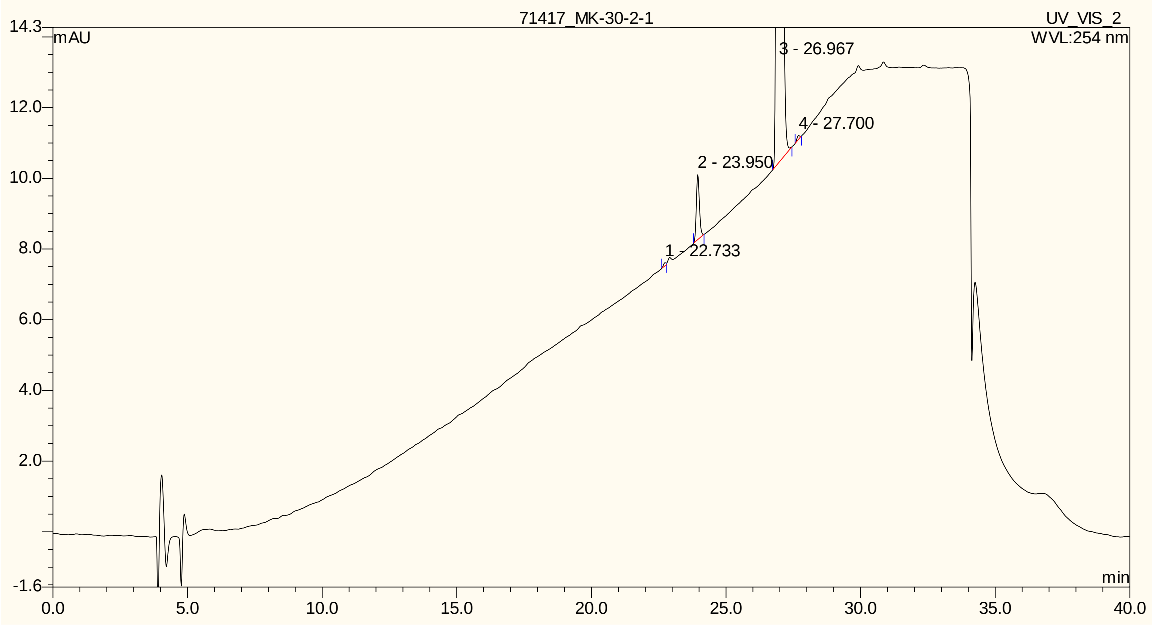

Retention Time: **26.97 min**

Relative Peak Area: **98.87 %**

Figure S40a. RP-Chromatogram of analogue **8j**.

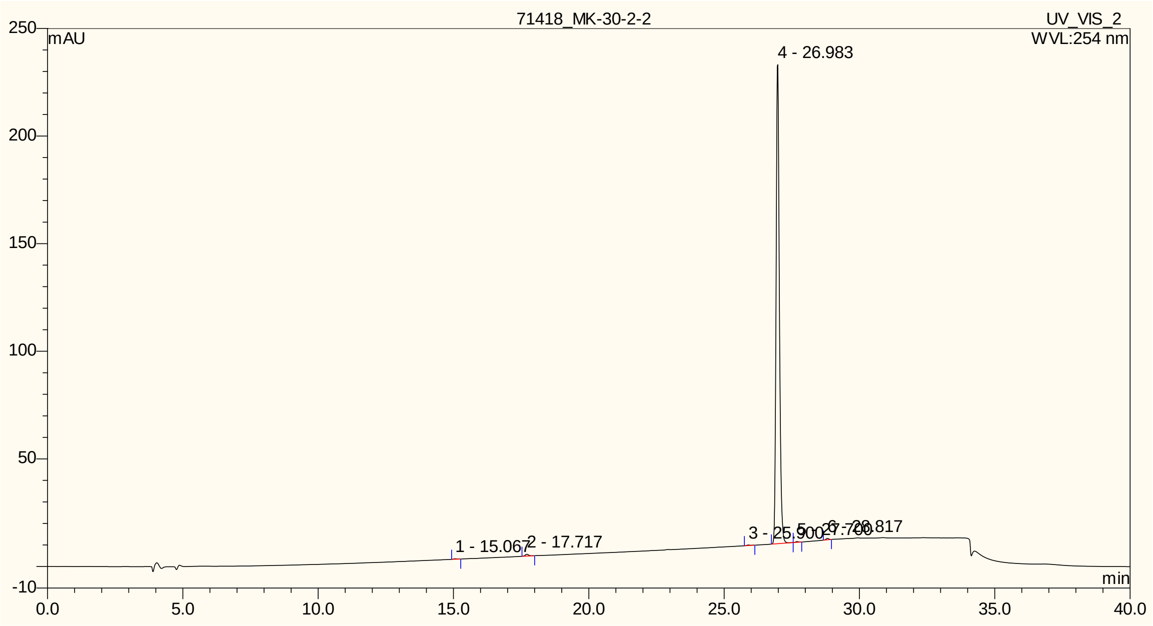

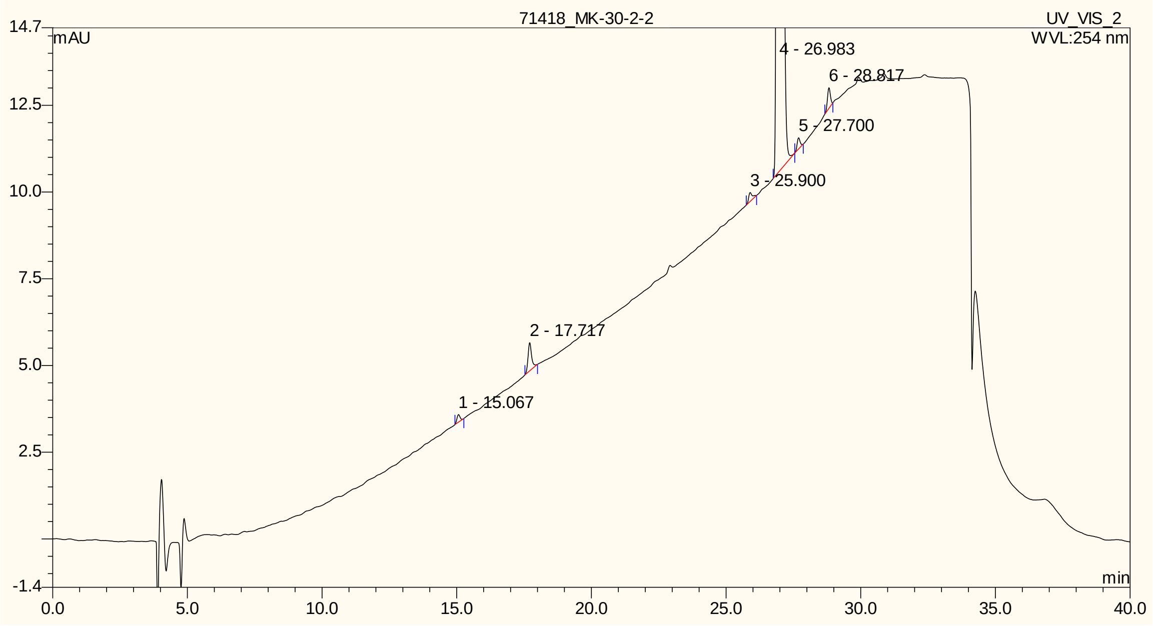

Retention Time: **26.98 min**

Relative Peak Area: **99.03 %**

Figure S41a. RP-Chromatogram of analogue **5k**.

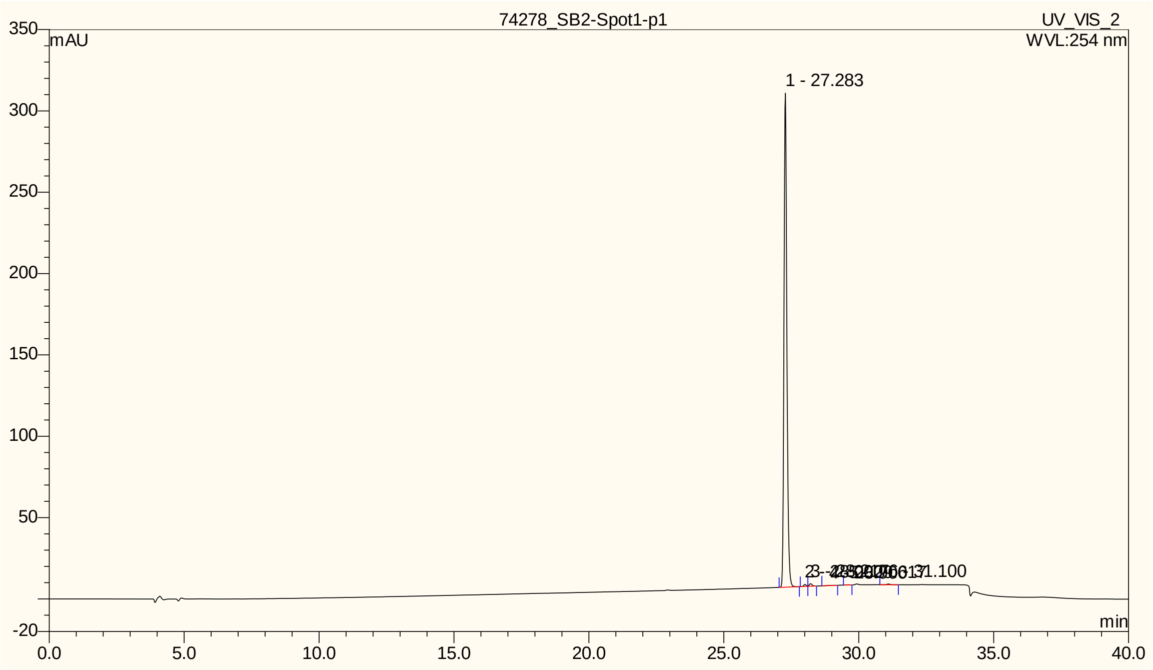

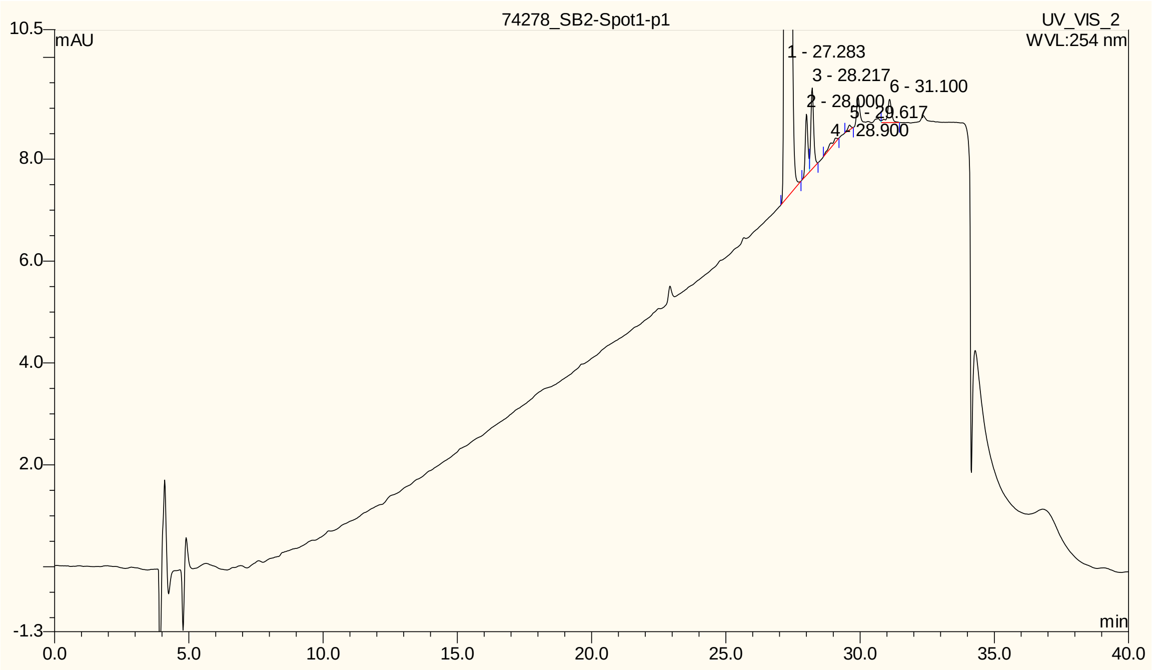

Retention Time: **27.28 min**

Relative Peak Area: **98.82 %**

Figure S42a. RP-Chromatogram of analogue **6k**.

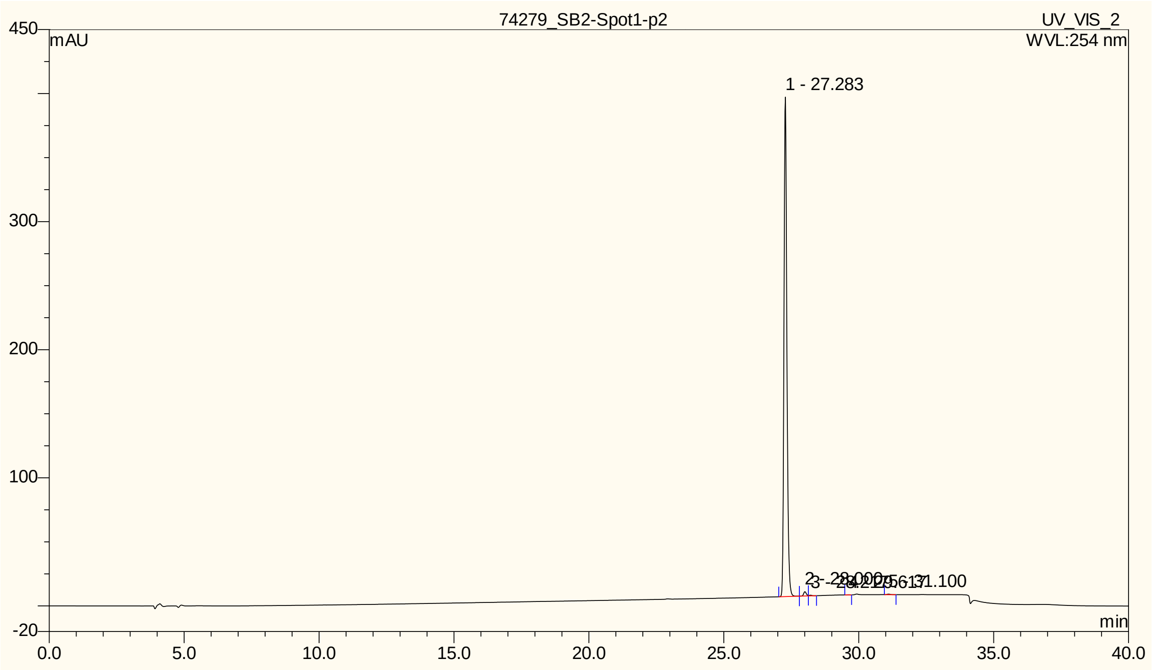

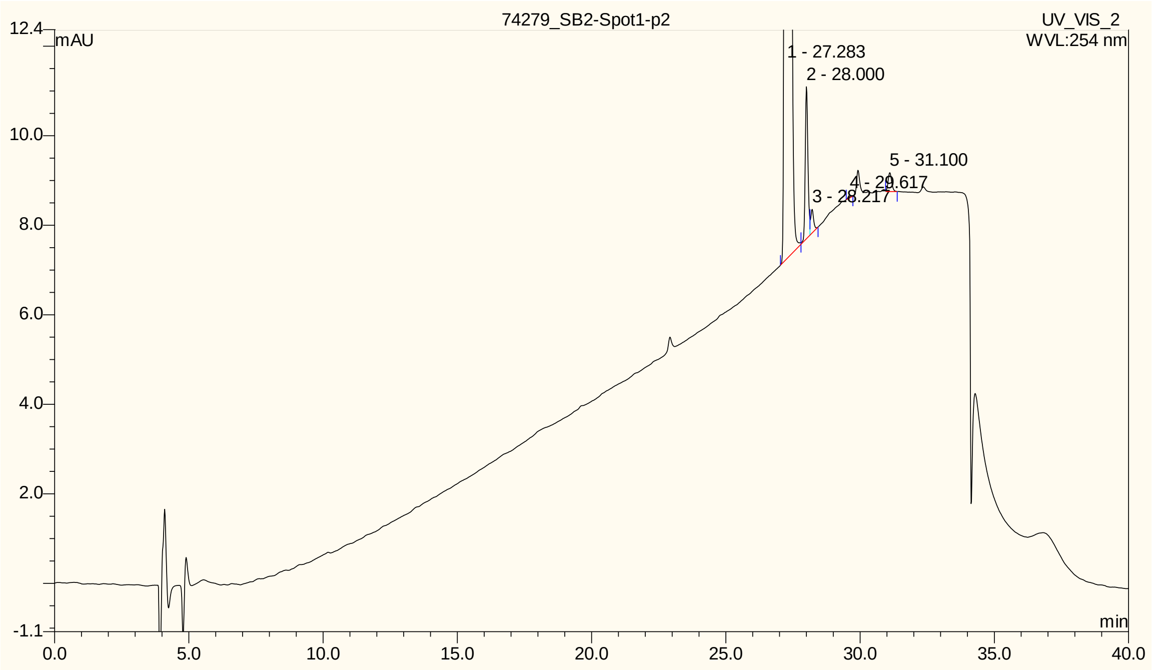

Retention Time: **27.28 min**

Relative Peak Area: **98.92 %**

Figure S43a. RP-Chromatogram of analogue **7k**.

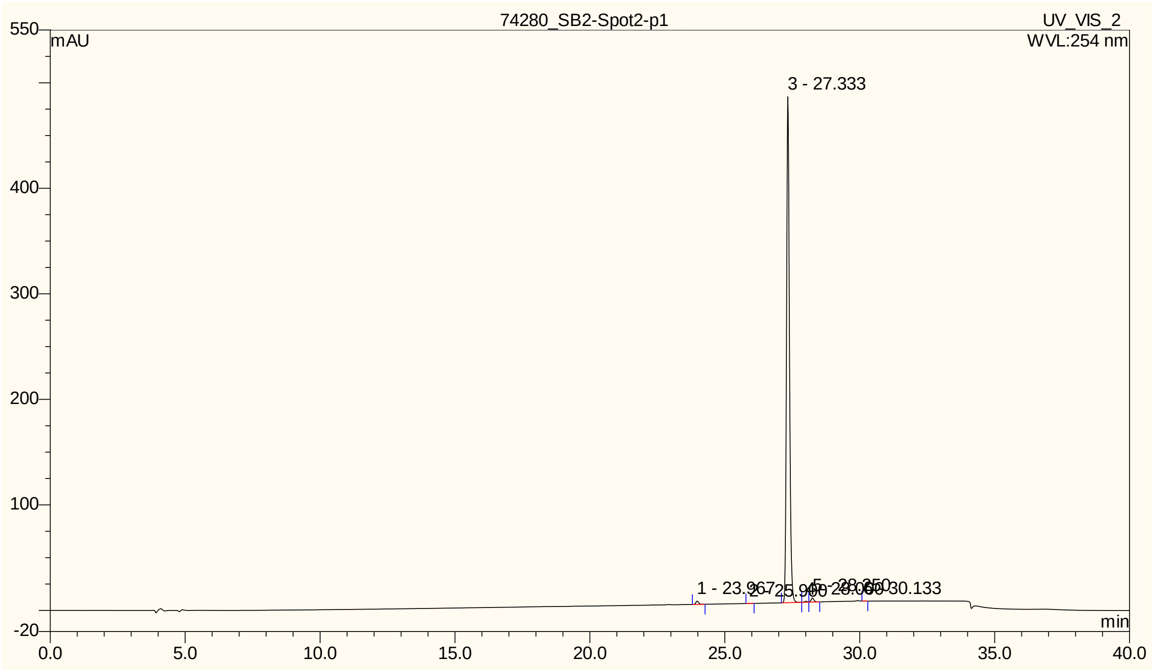

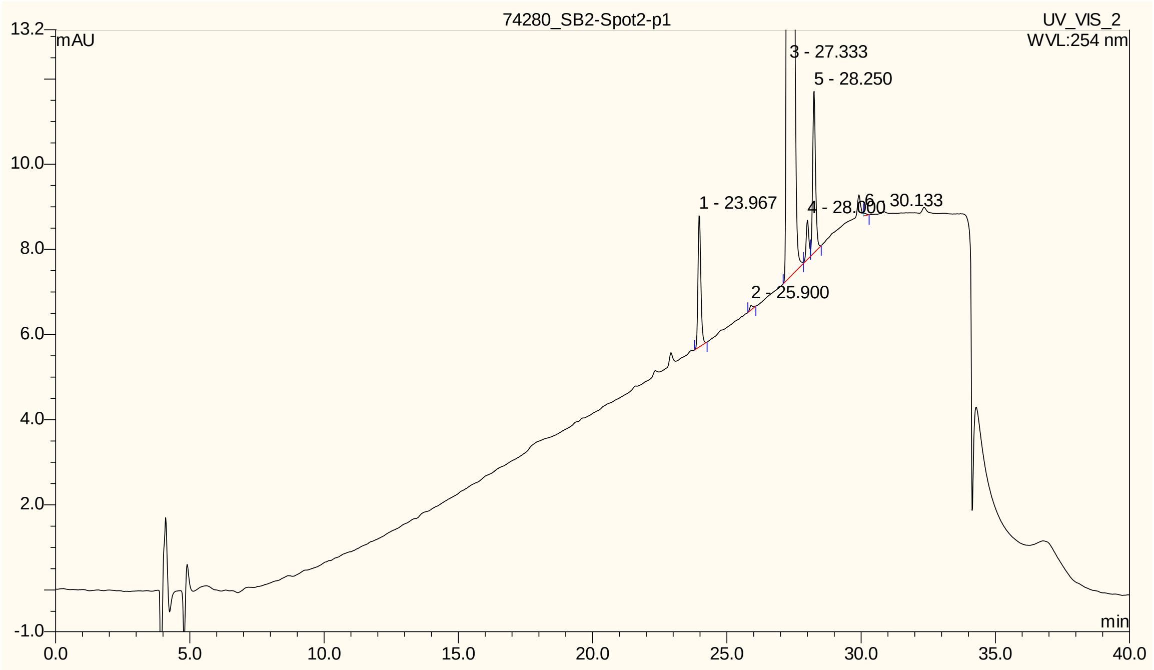

Retention Time: **27.33 min**

Relative Peak Area: **98.41 %**

Figure S44a. RP-Chromatogram of analogue **8k**.

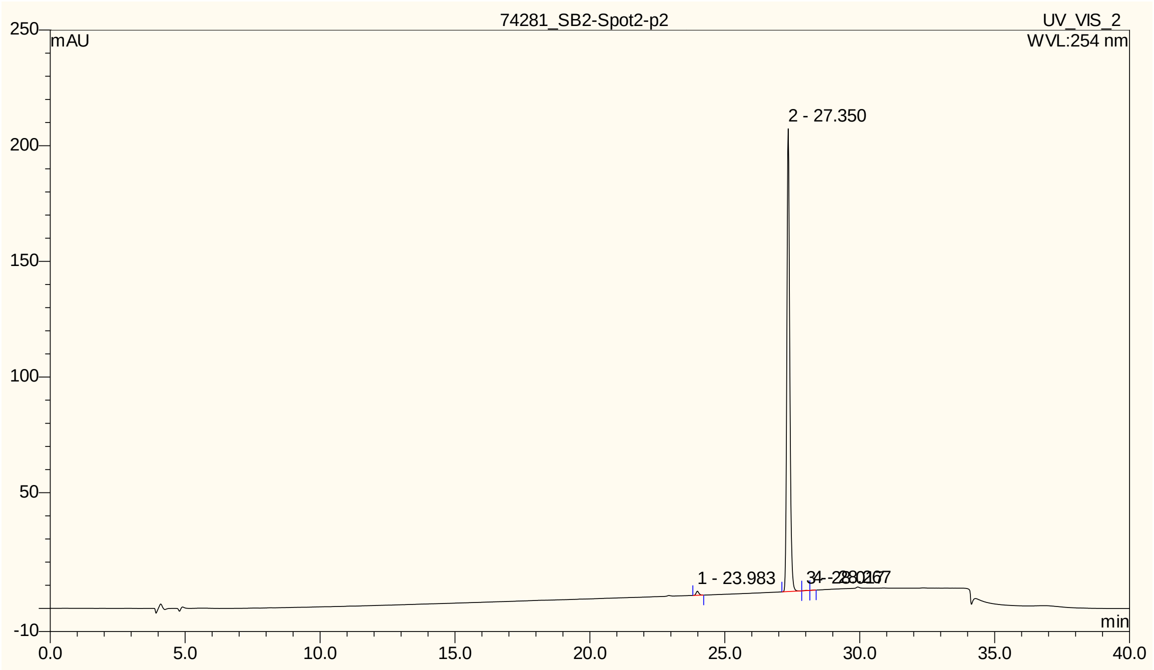

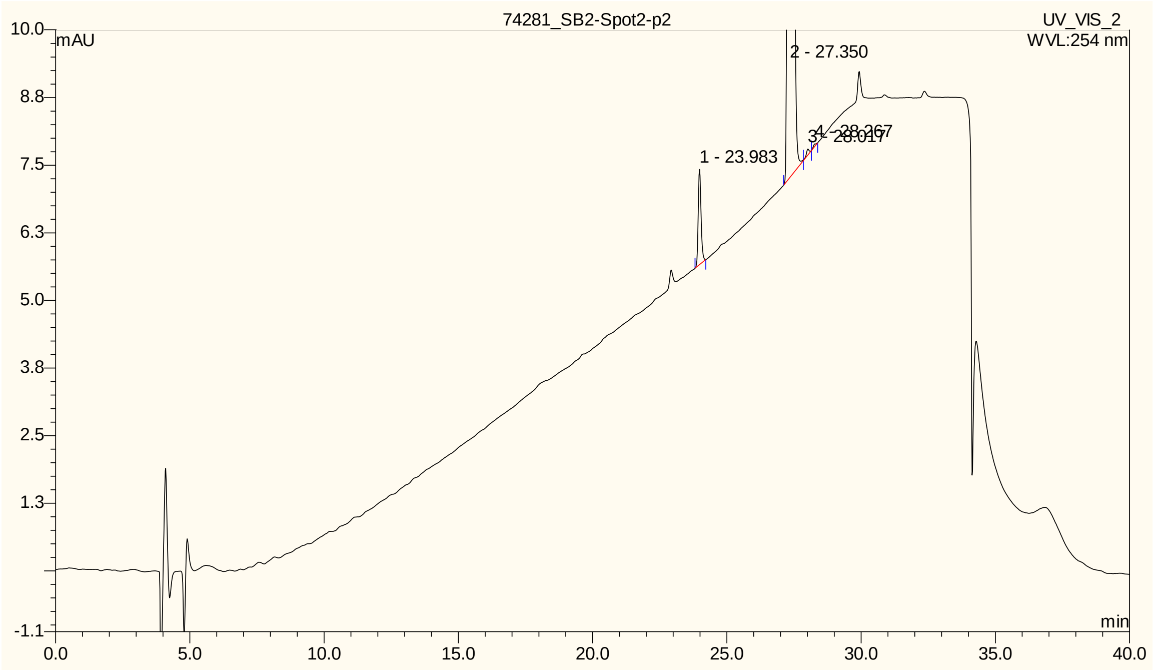

Retention Time: **27.35 min**

Relative Peak Area: **99.08 %**

Figure S45a. RP-Chromatogram of analogue **5l**.

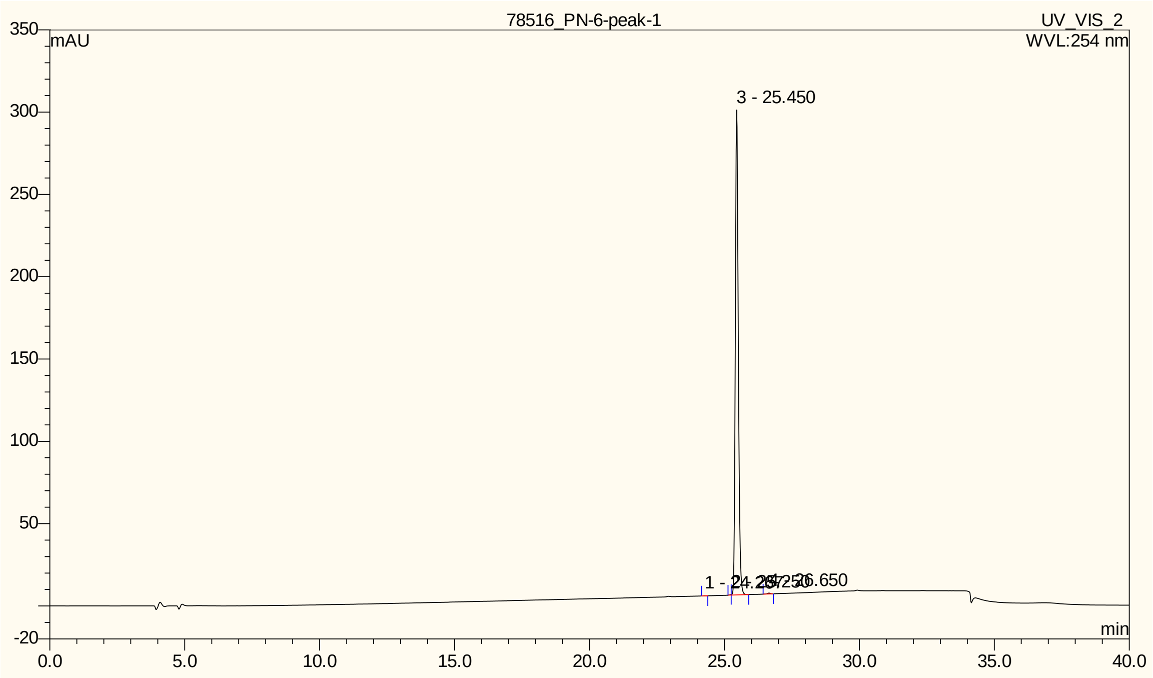

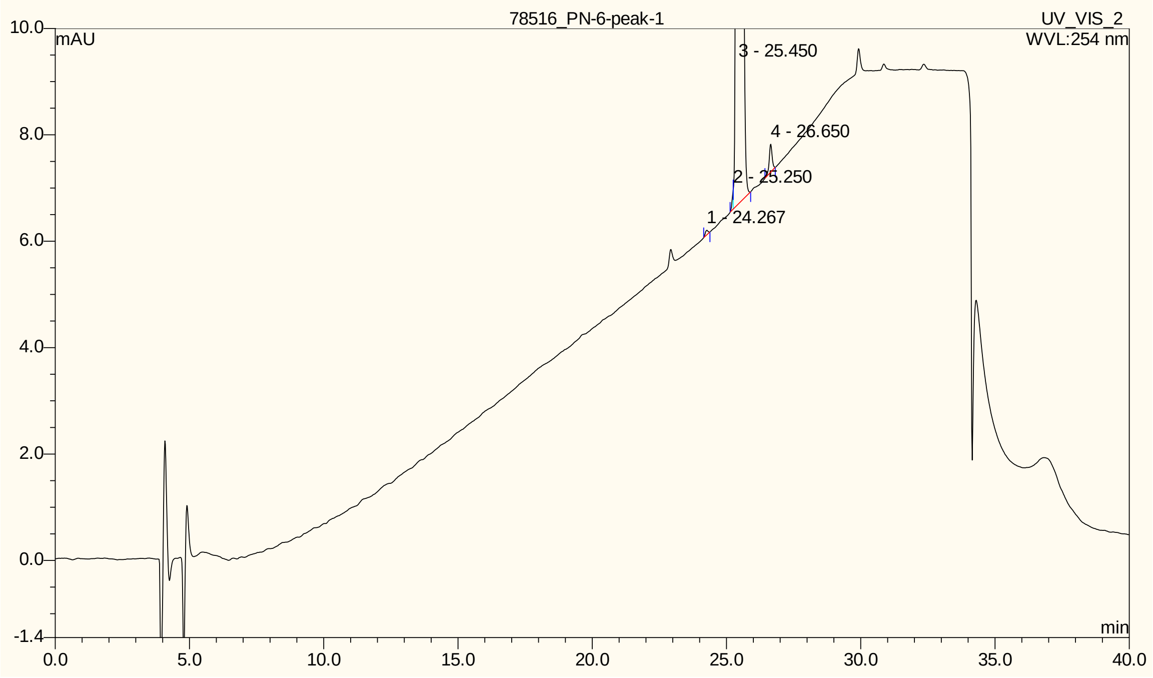

Retention Time: **25.45 min**

Relative Peak Area: **99.75 %**

Figure S46a. RP-Chromatogram of analogue **6l**.

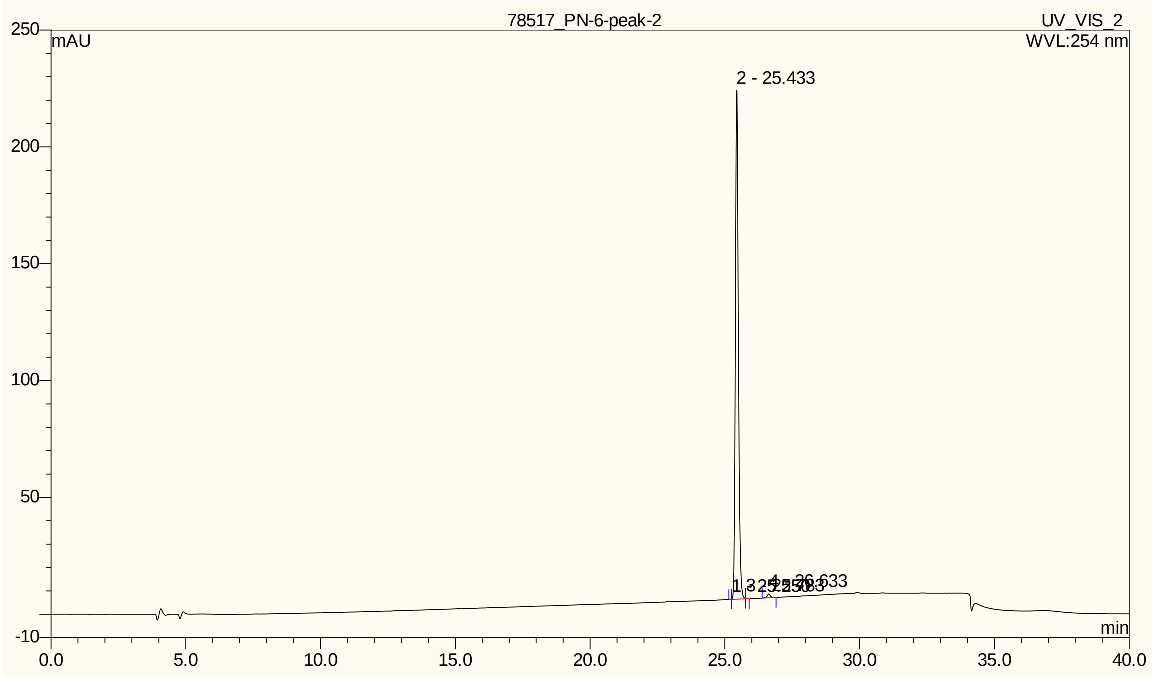

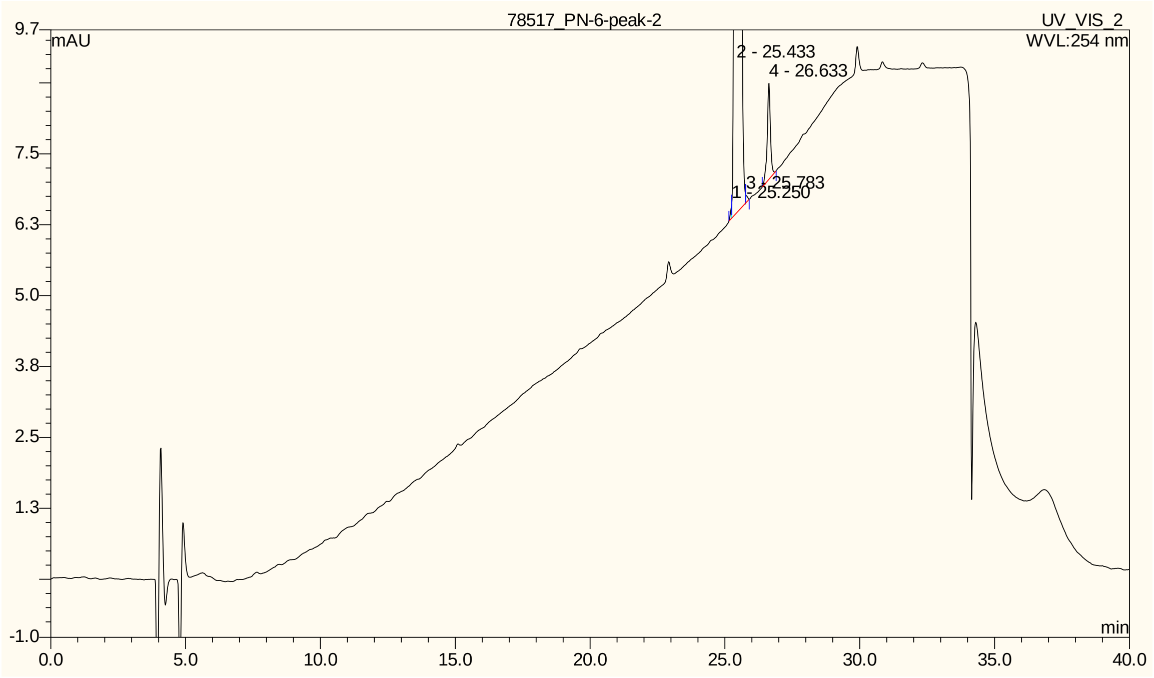

Retention Time: **25.43 min**

Relative Peak Area: **99.12 %**

Figure S47a. RP-Chromatogram of analogue **7l**.

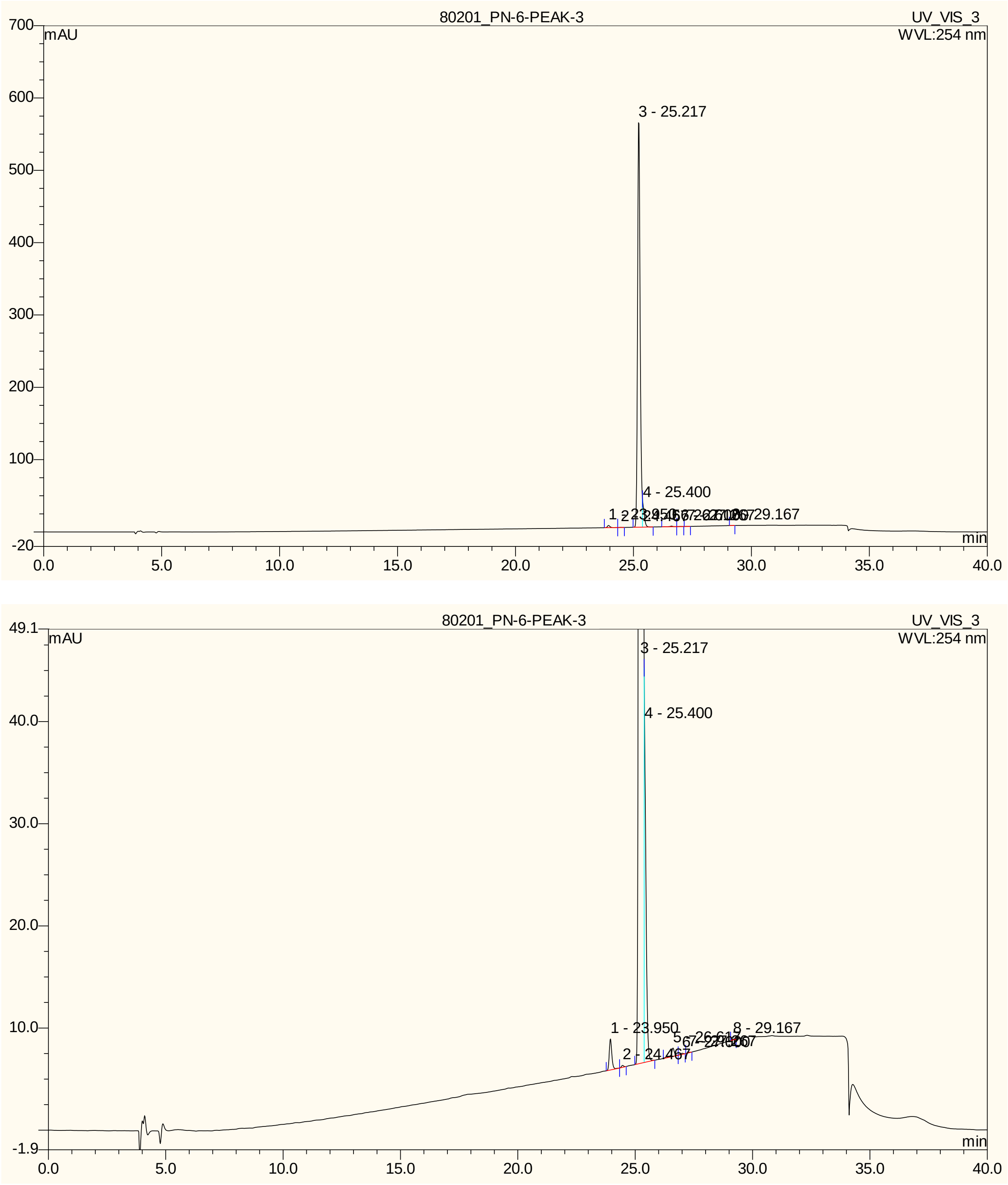

Retention Time: **25.22 min**

Relative Peak Area: **95.32 %**

Figure S48a. RP-Chromatogram of analogue **8l**.

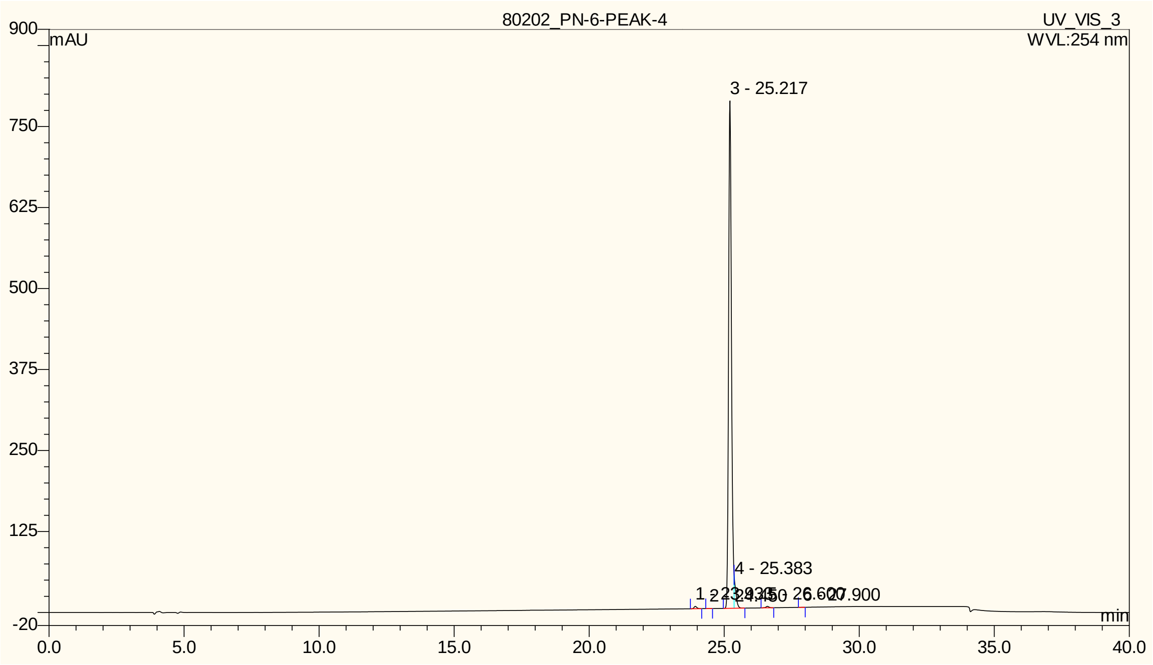

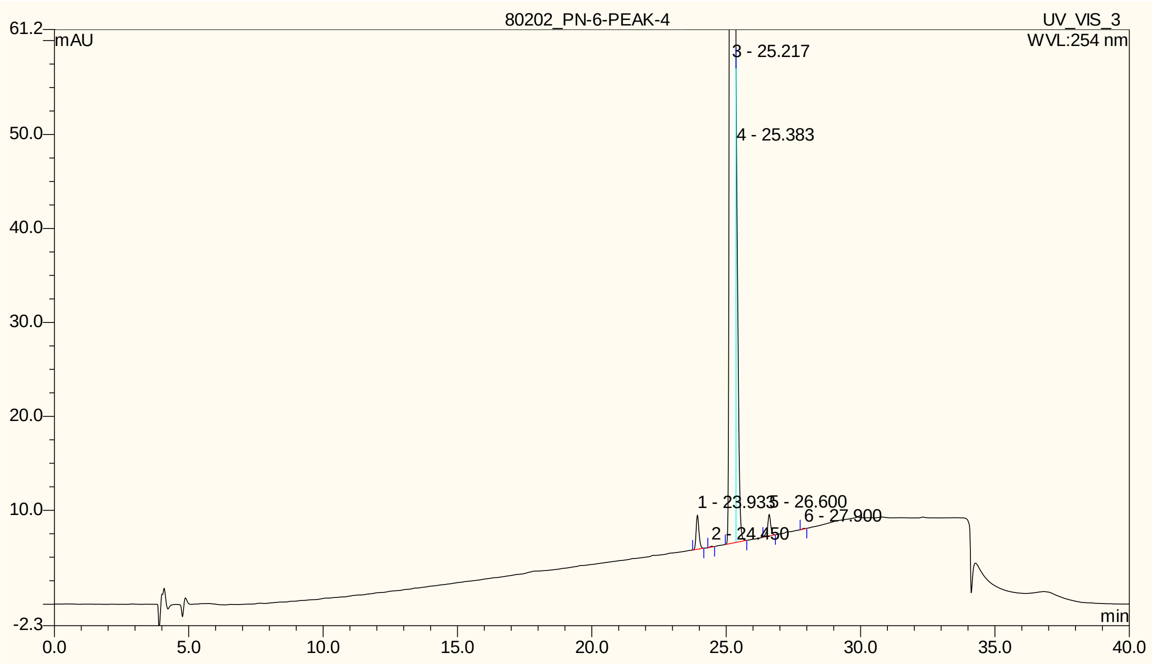

Retention Time: **25.22 min**

Relative Peak Area: **95.68 %**

Figure S49a. RP-Chromatogram of analogue **5m**.

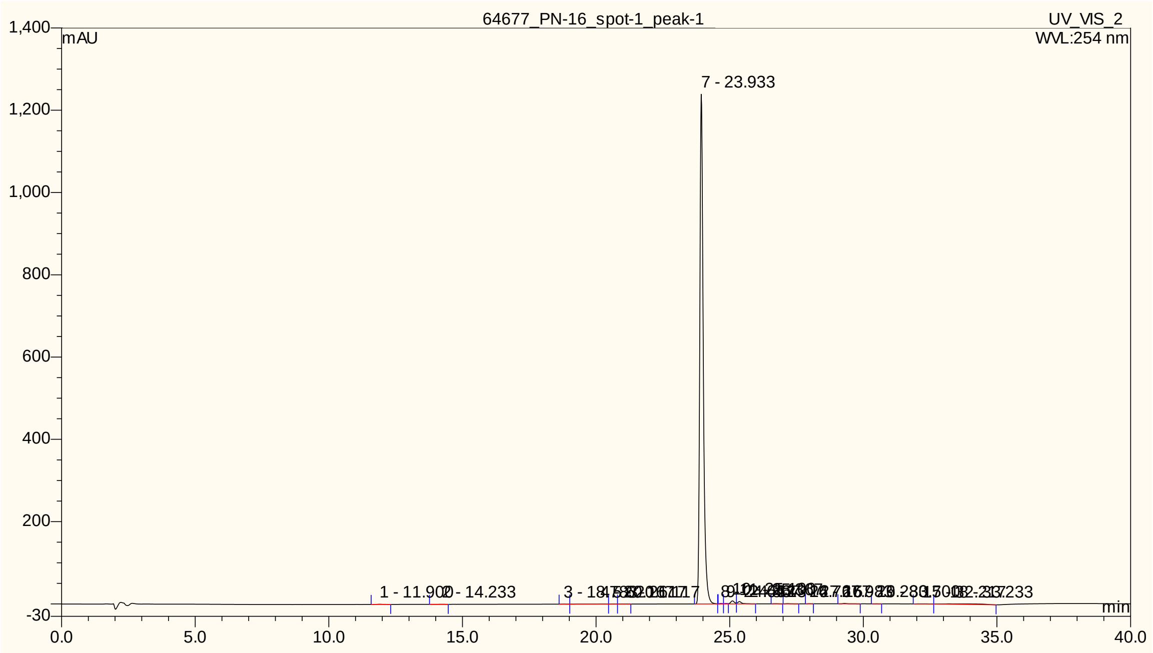

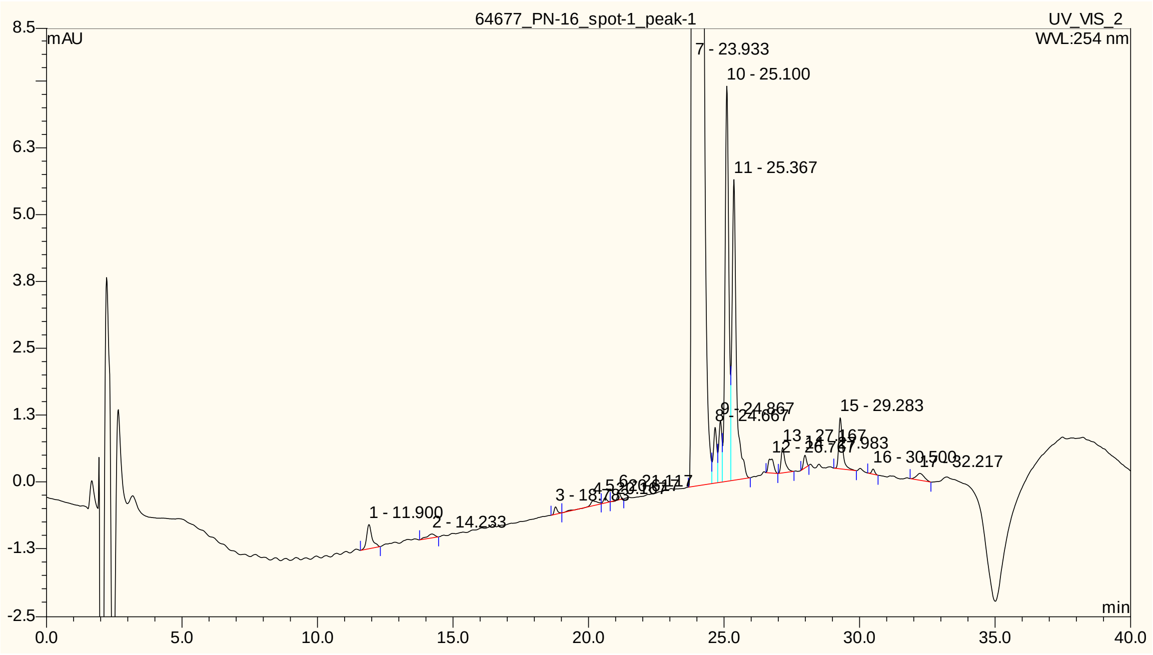

Retention Time: **23.93 min**

Relative Peak Area: **98.36 %**

Figure S50a. RP-Chromatogram of analogue **6m**.

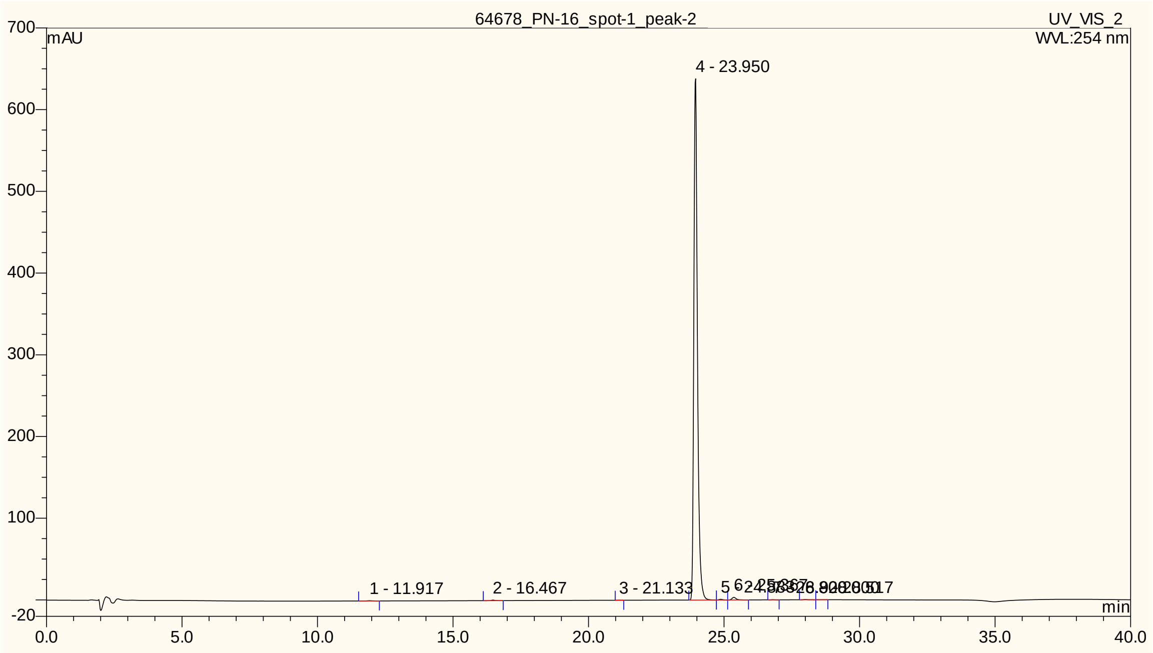

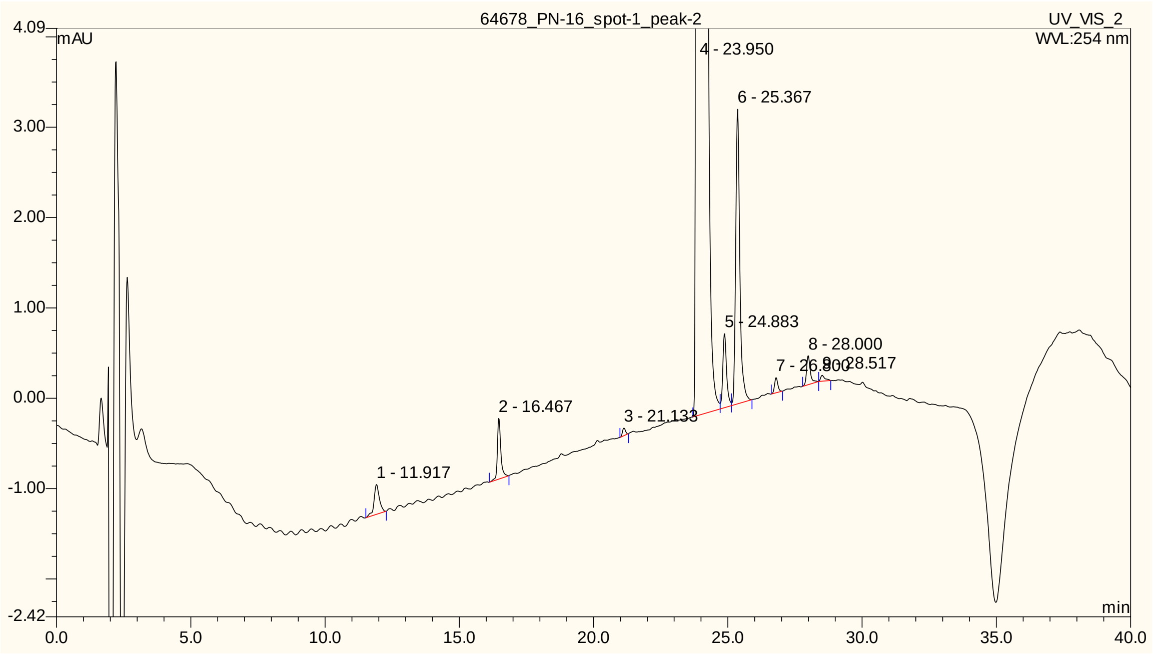

Retention Time: **23.95 min**

Relative Peak Area: **99.00 %**

Figure S51a. RP-Chromatogram of analogue **7m**.

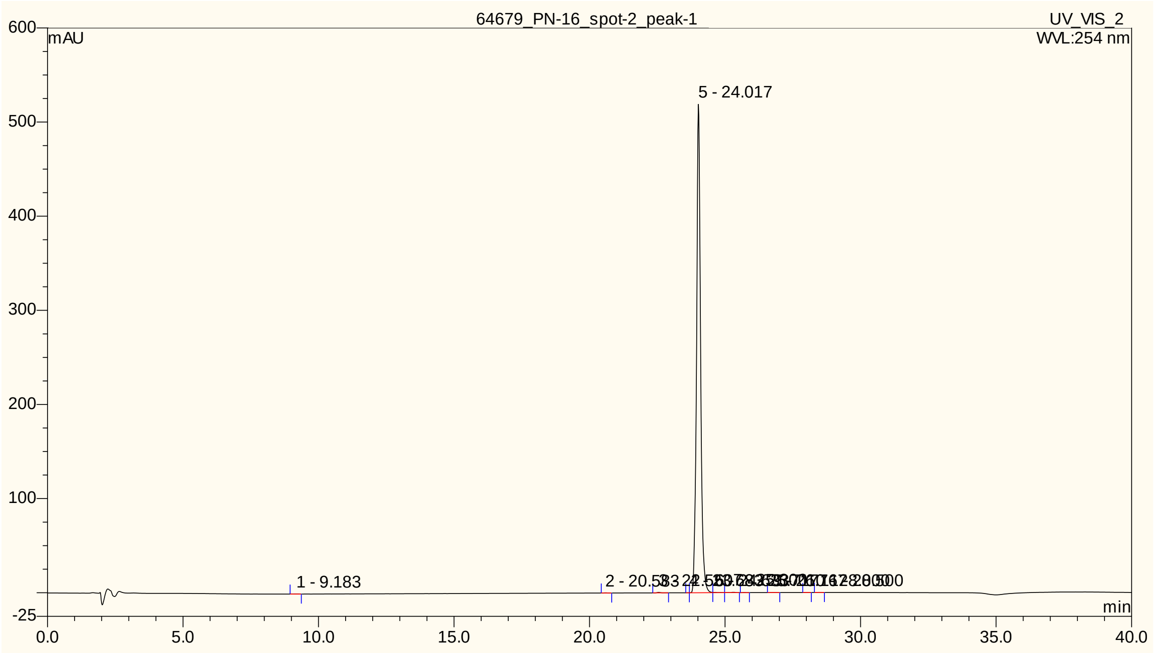

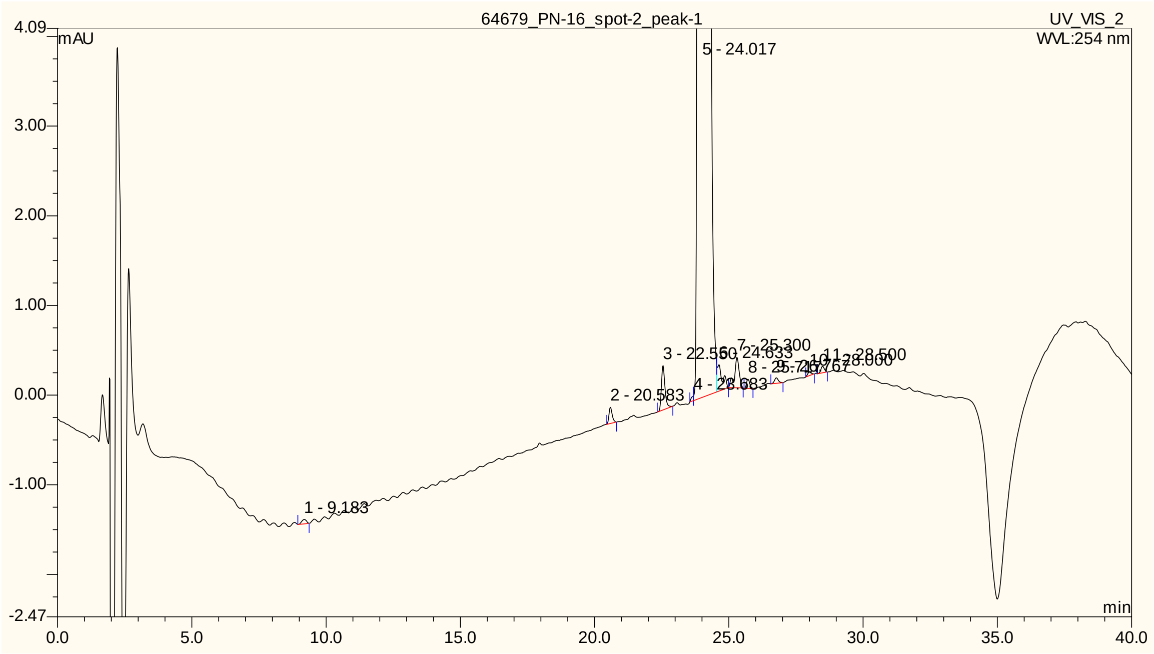

Retention Time: **24.02 min**

Relative Peak Area: **99.67 %**

Figure S52a. RP-Chromatogram of analogue **8m**.

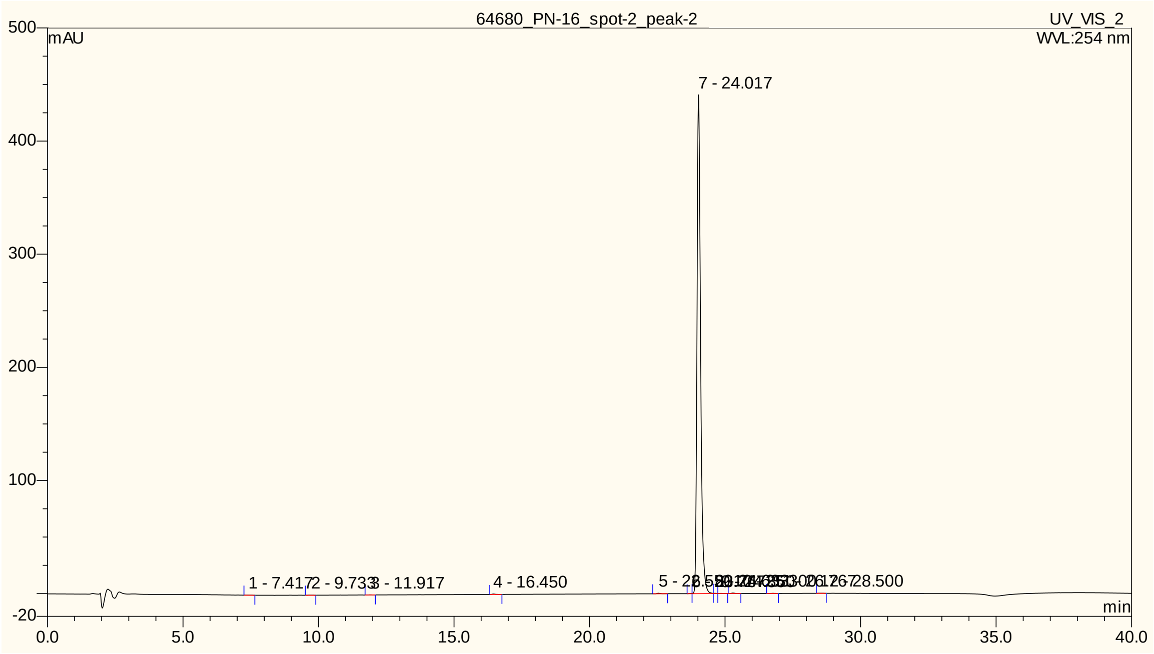

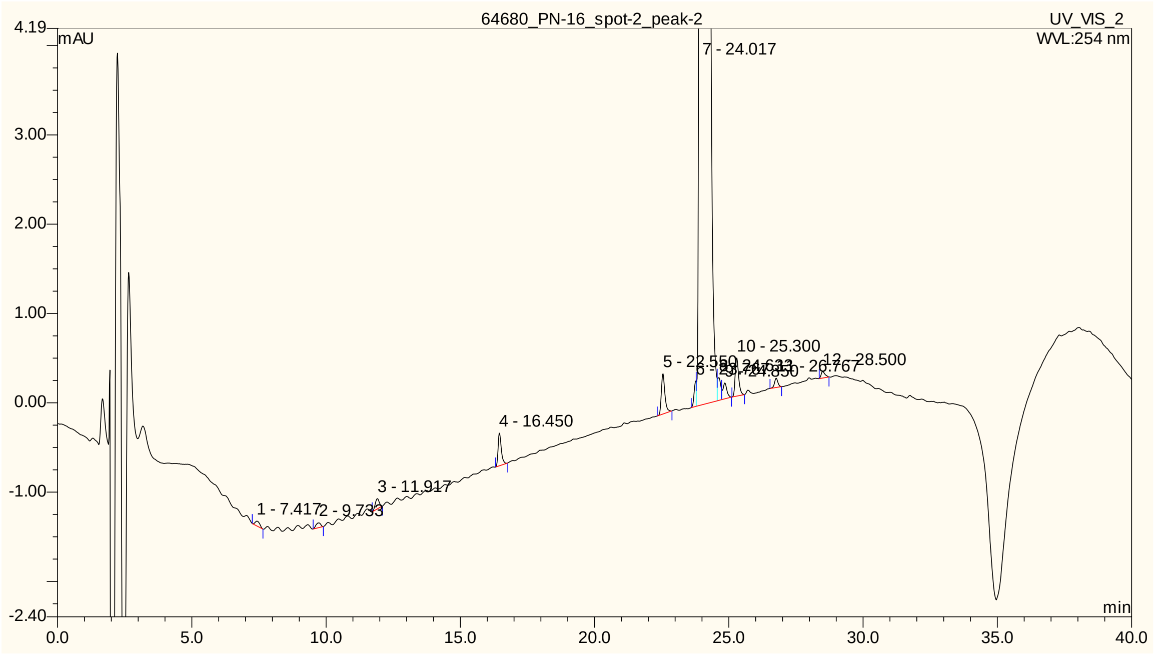

Retention Time: **24.02 min**

Relative Peak Area: **99.50 %**

Figure S53a. RP-Chromatogram of analogue **5n**.

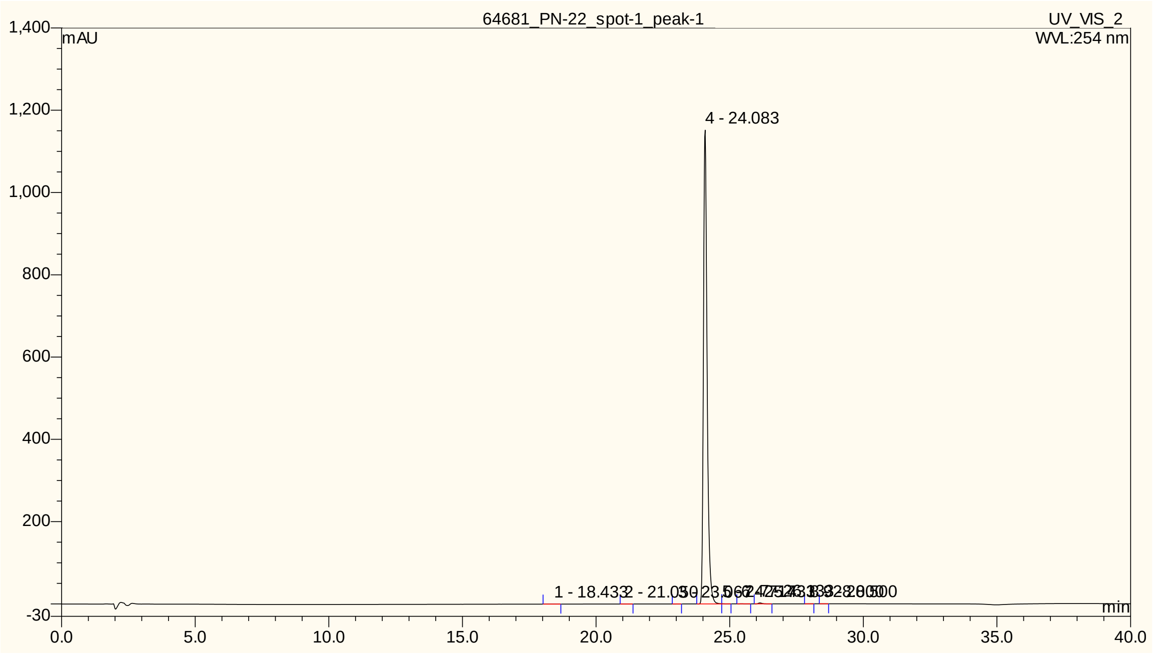

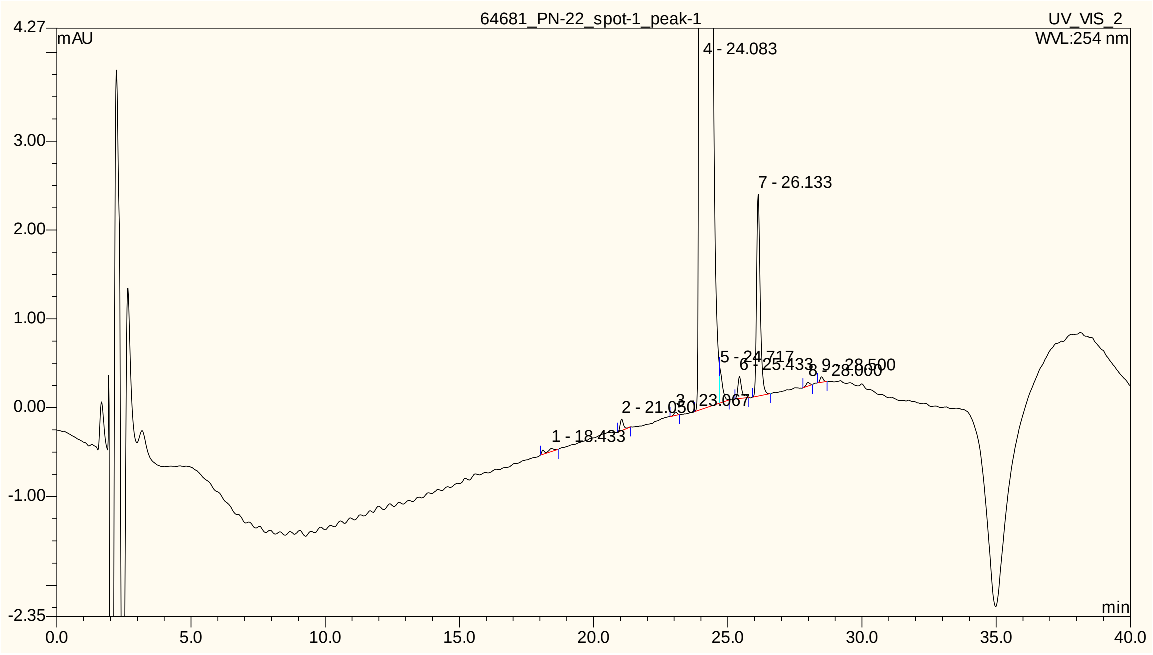

Retention Time: **24.08 min**

Relative Peak Area: **99.73 %**

Figure S54a. RP-Chromatogram of analogue **6n**.

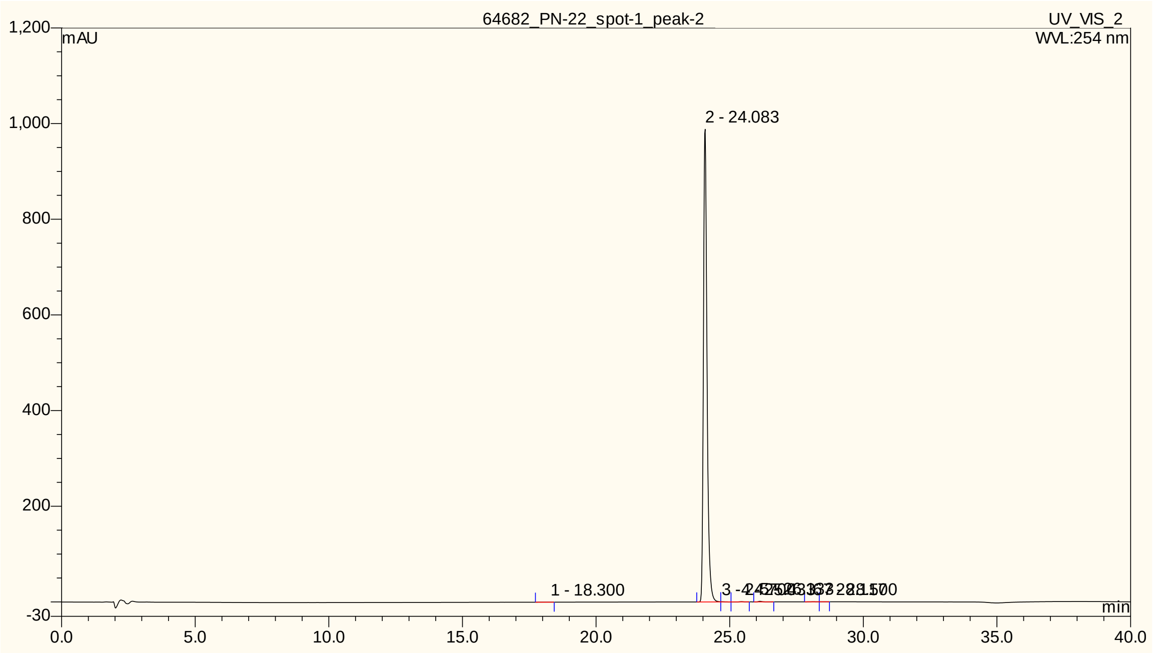

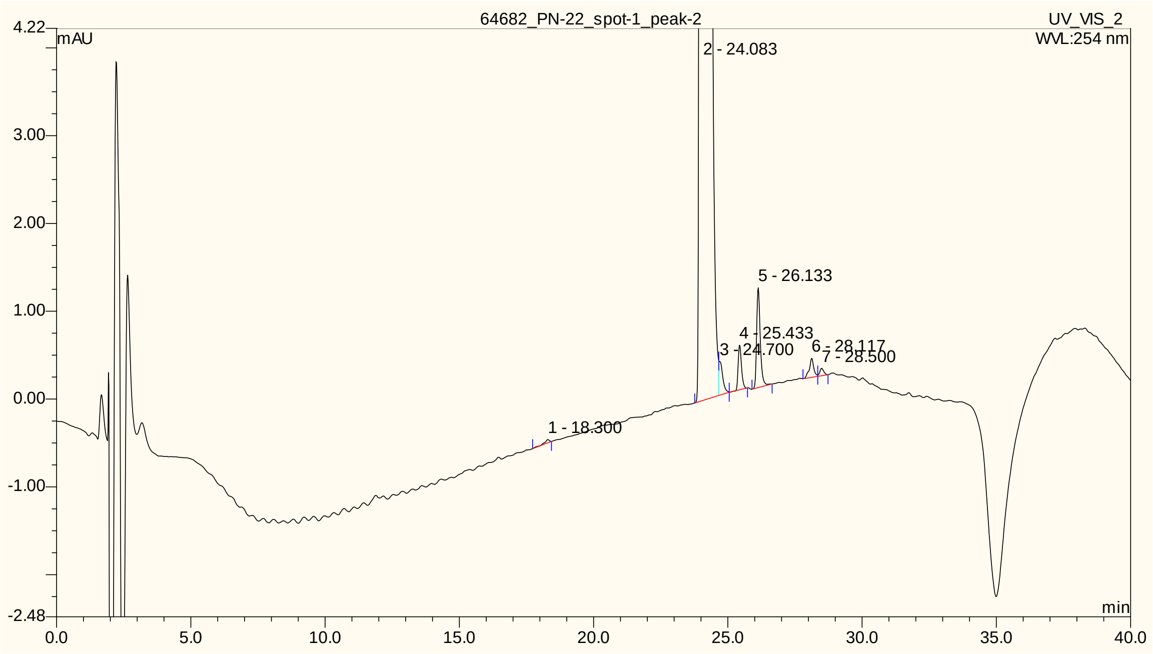

Retention Time: **24.08 min**

Relative Peak Area: **99.76 %**

Figure S55a. RP-Chromatogram of analogue **7n**.

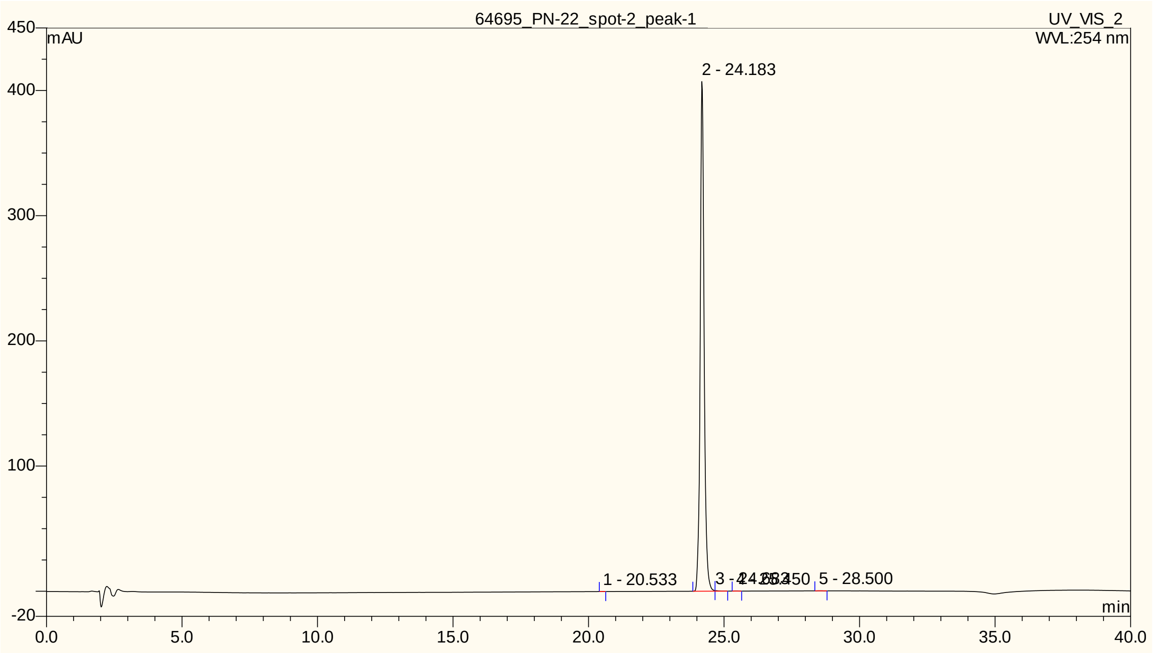

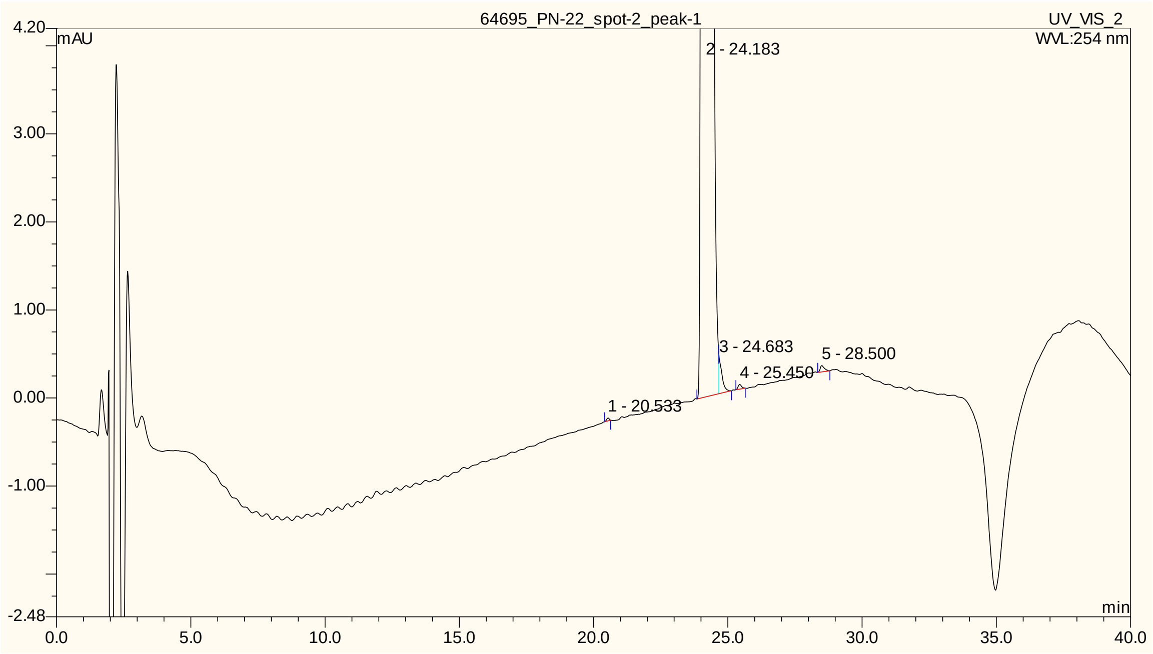

Retention Time: **24.18 min**

Relative Peak Area: **99.89 %**

Figure S56a. RP-Chromatogram of analogue **8n**.

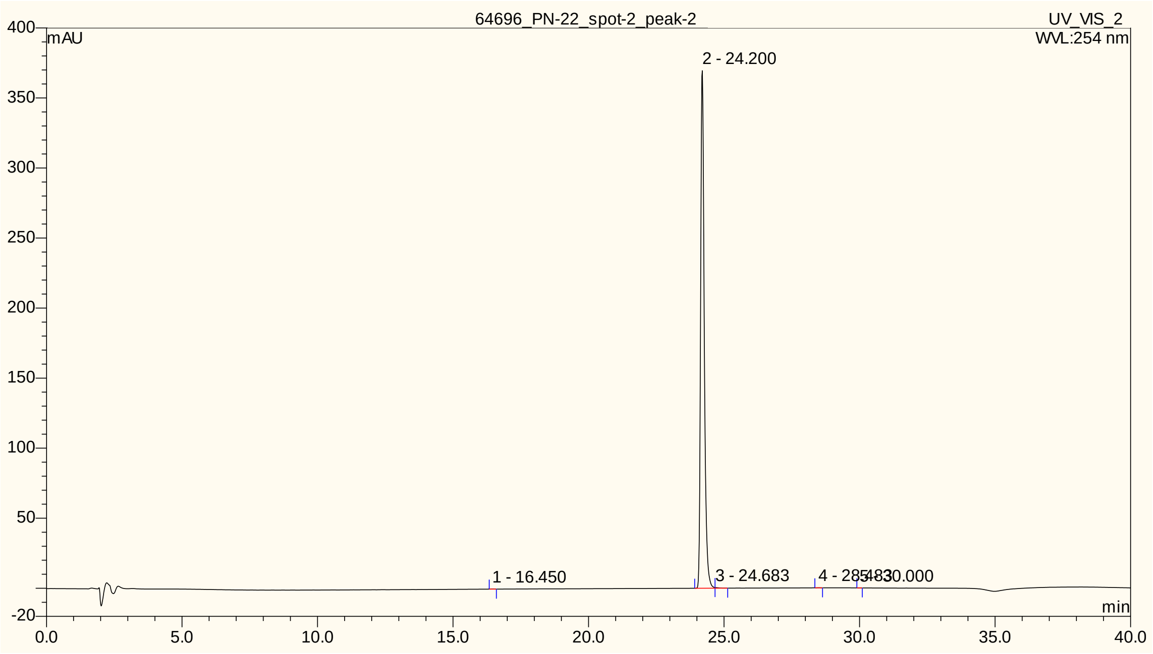

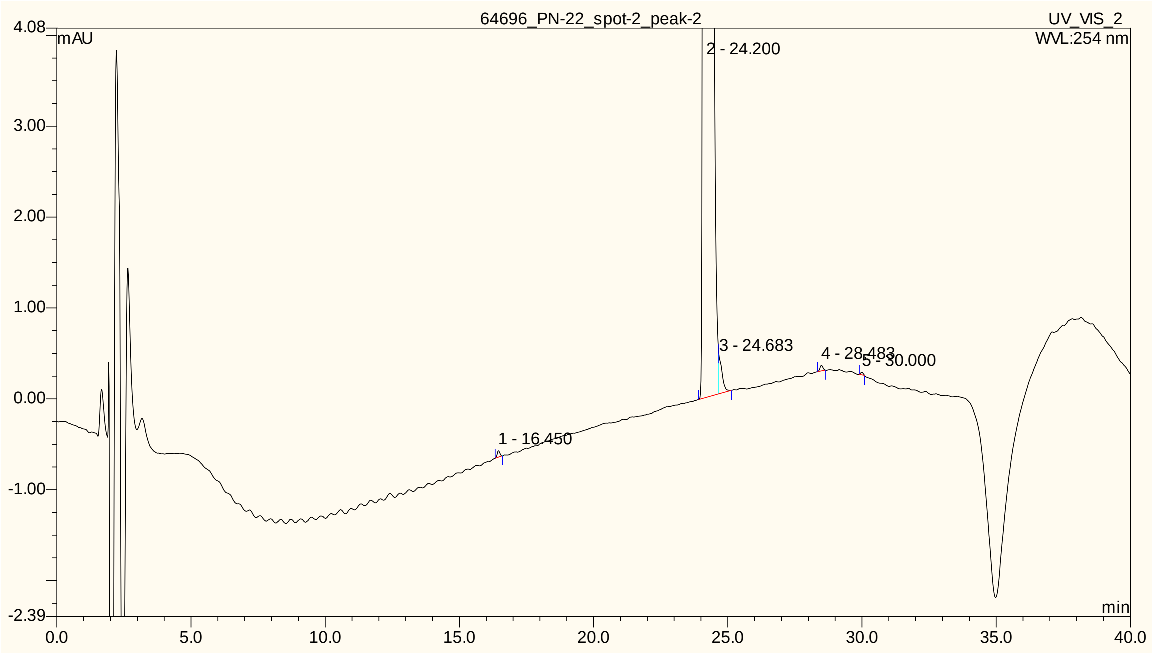

Retention Time: **24.20 min**

Relative Peak Area: **99.87 %**

Figure S57a. RP-Chromatogram of analogue **5o**.

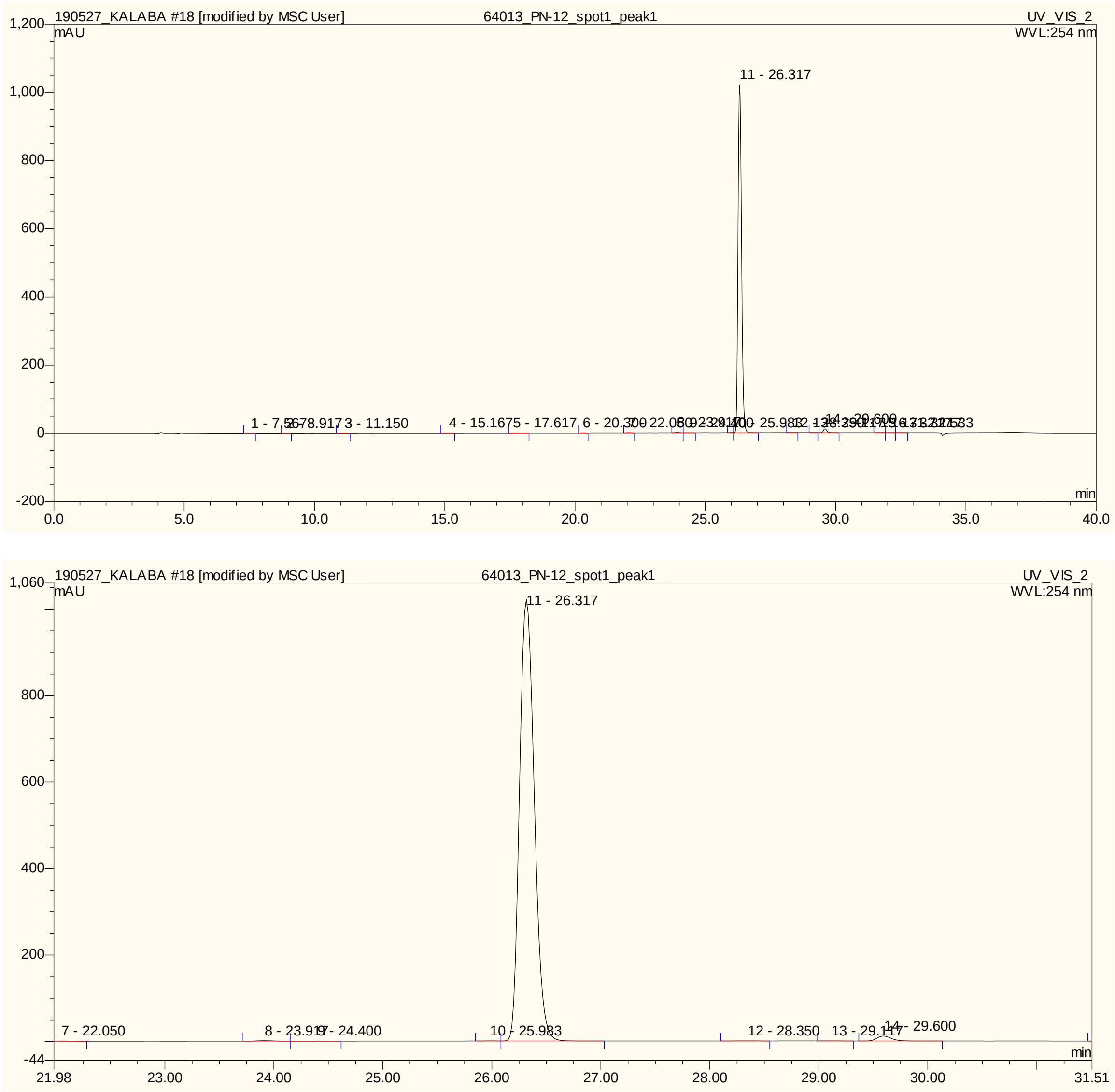

Retention Time: **26.31 min**

Relative Peak Area: **98.66 %**

Figure S58a. RP-Chromatogram of analogue **6o**.

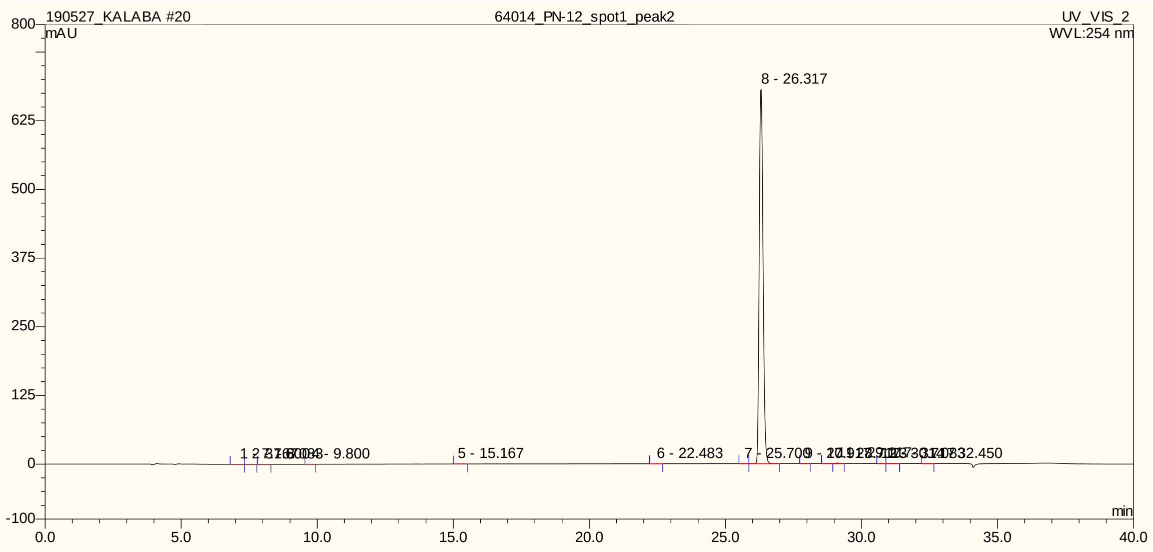

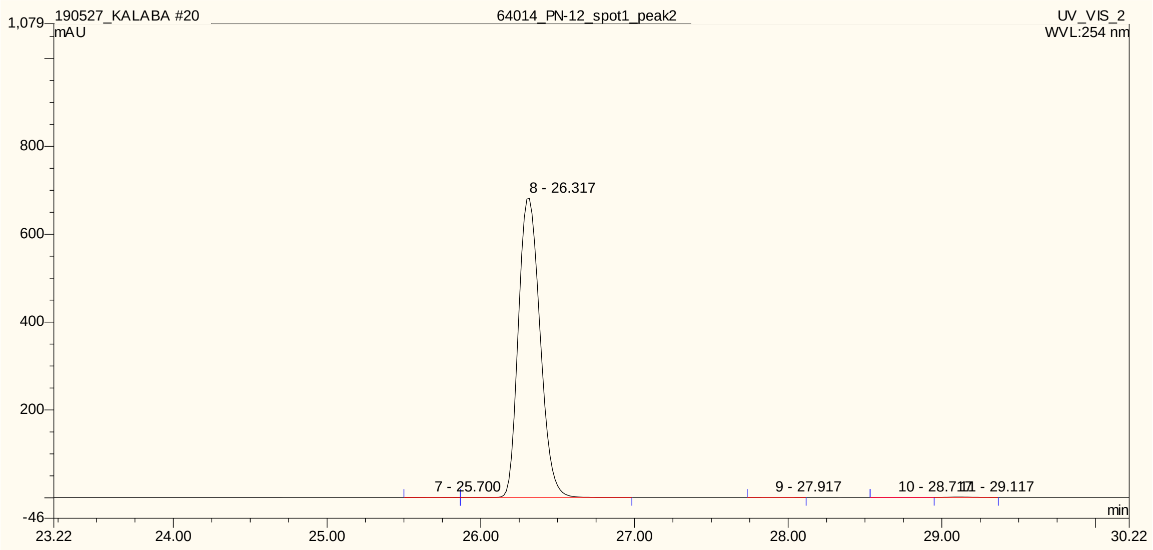

Retention Time: **26.31 min**

Relative Peak Area: **99.66 %**

Figure S59a. RP-Chromatogram of analogue **7o**.

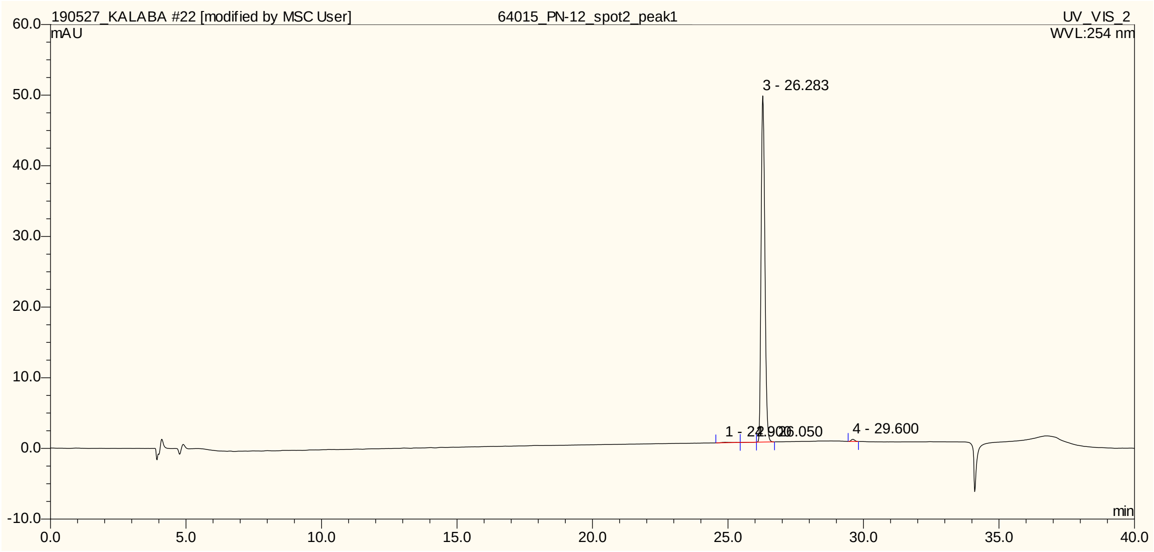

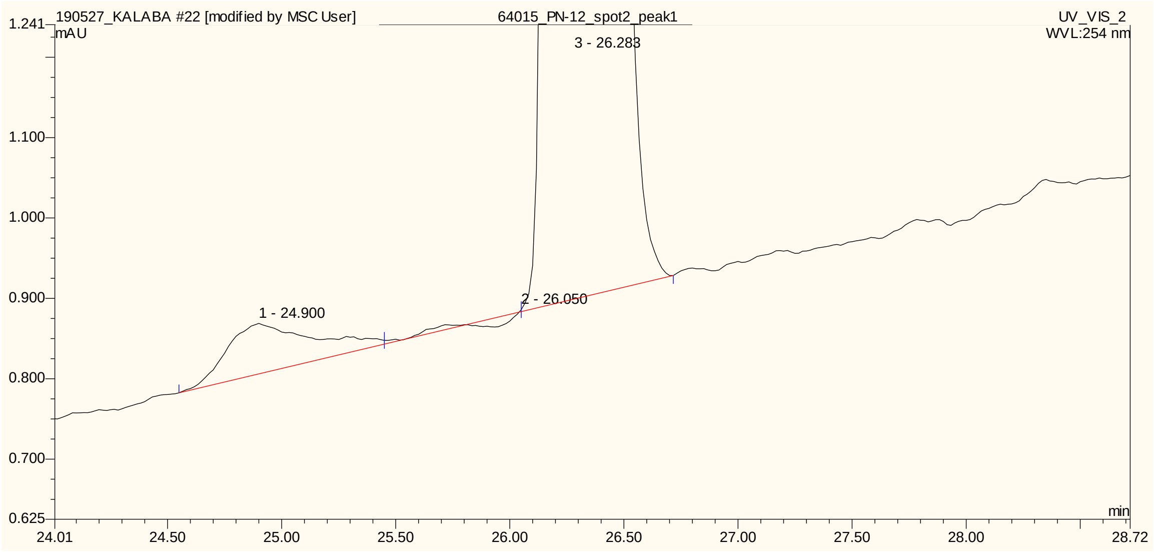

Retention Time: **26.28 min**

Relative Peak Area: **99.09 %**

Figure S60a. RP-Chromatogram of analogue **8o.**

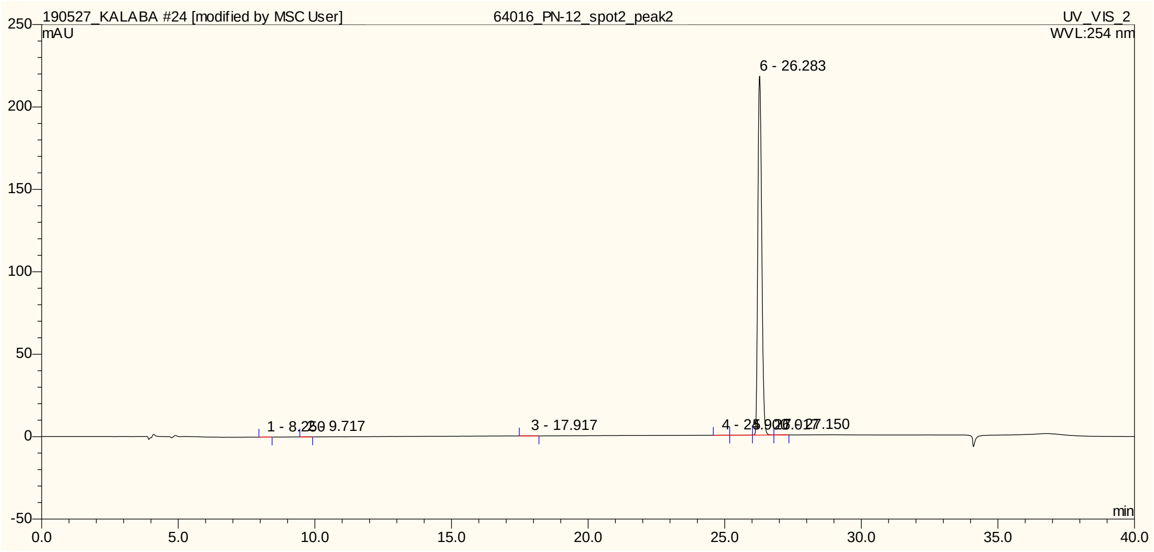

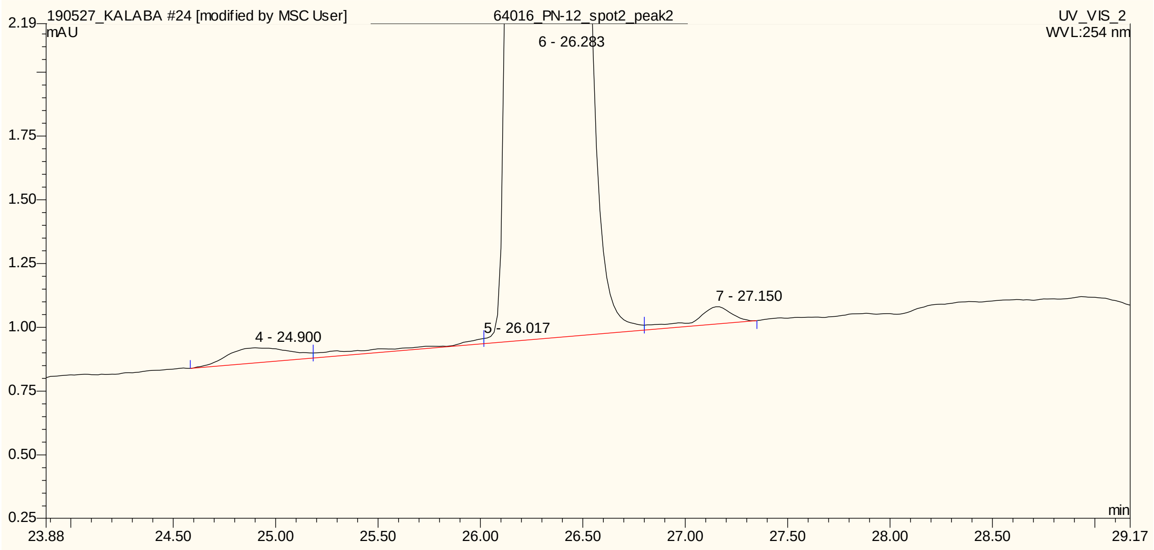

Retention Time: **26.28 min**

Relative Peak Area: **99.76 %**

Figure S61a. RP-Chromatogram of analogue **5p**.

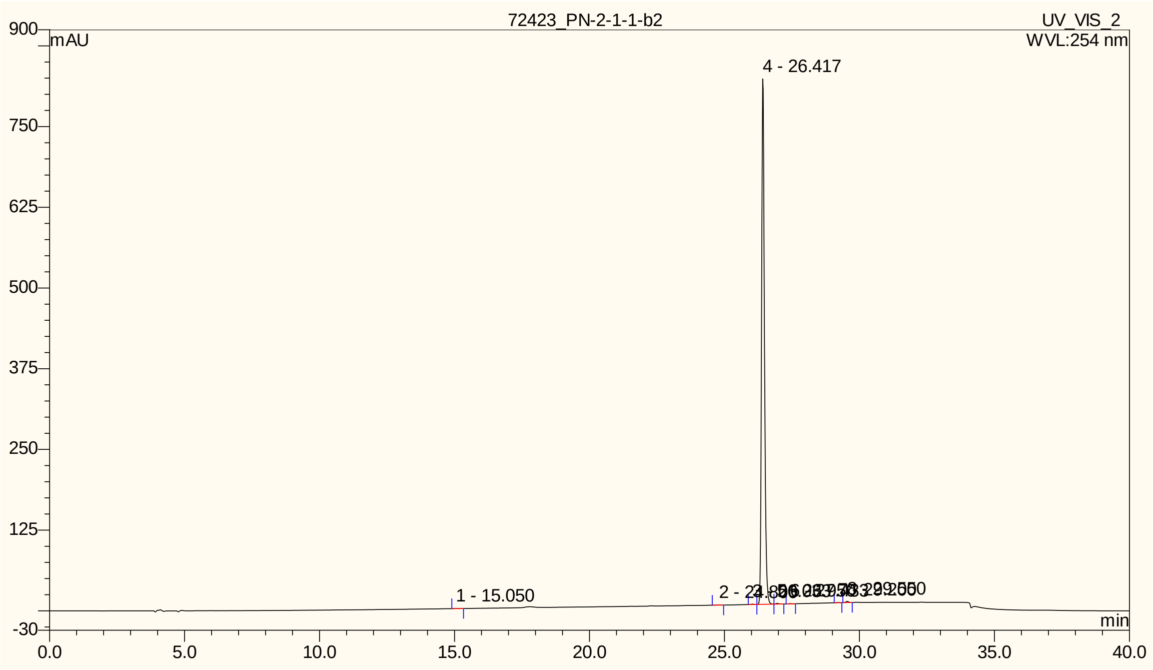

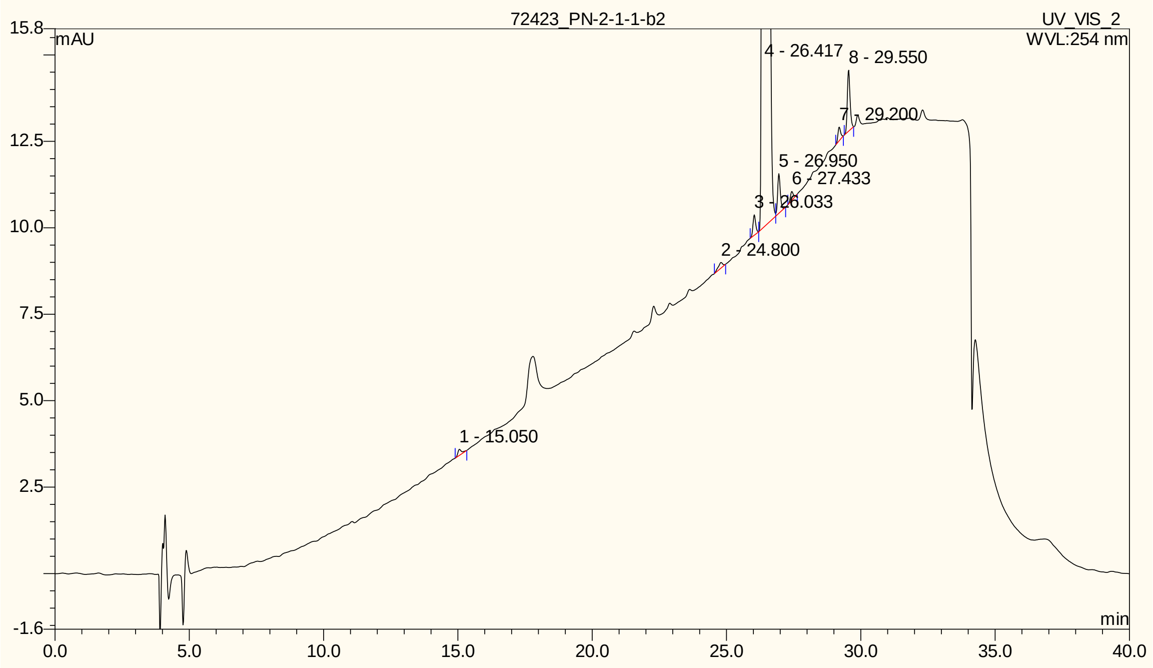

Retention Time: **26.42 min**

Relative Peak Area: **99.46 %**

Figure S62a. RP-Chromatogram of analogue **6p**.

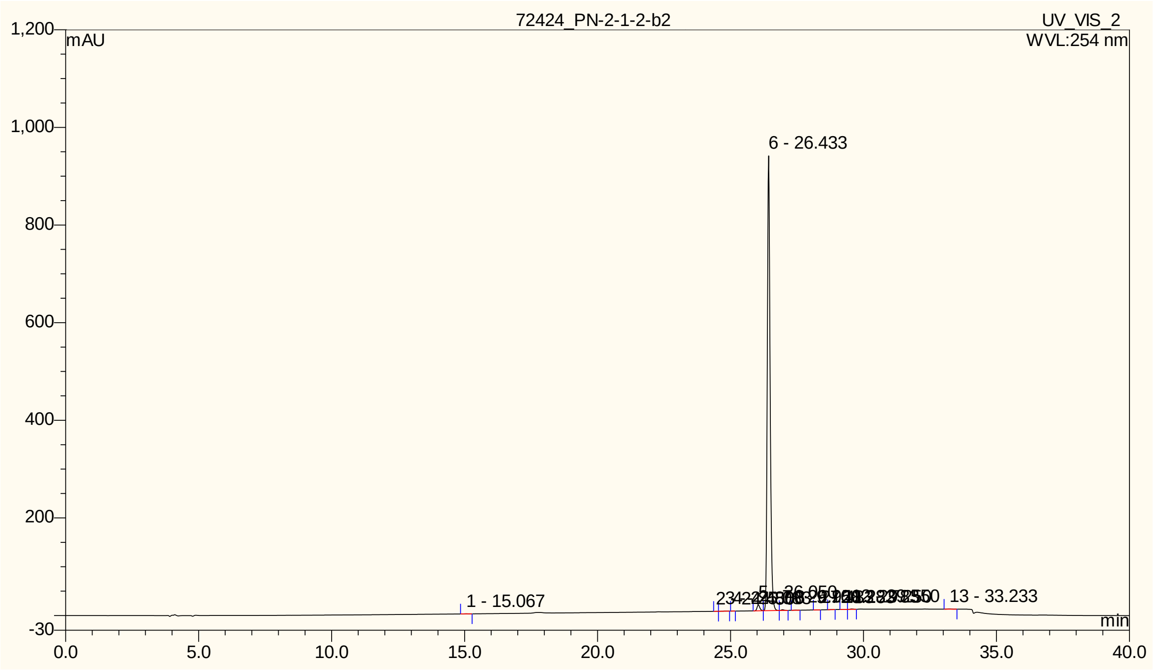

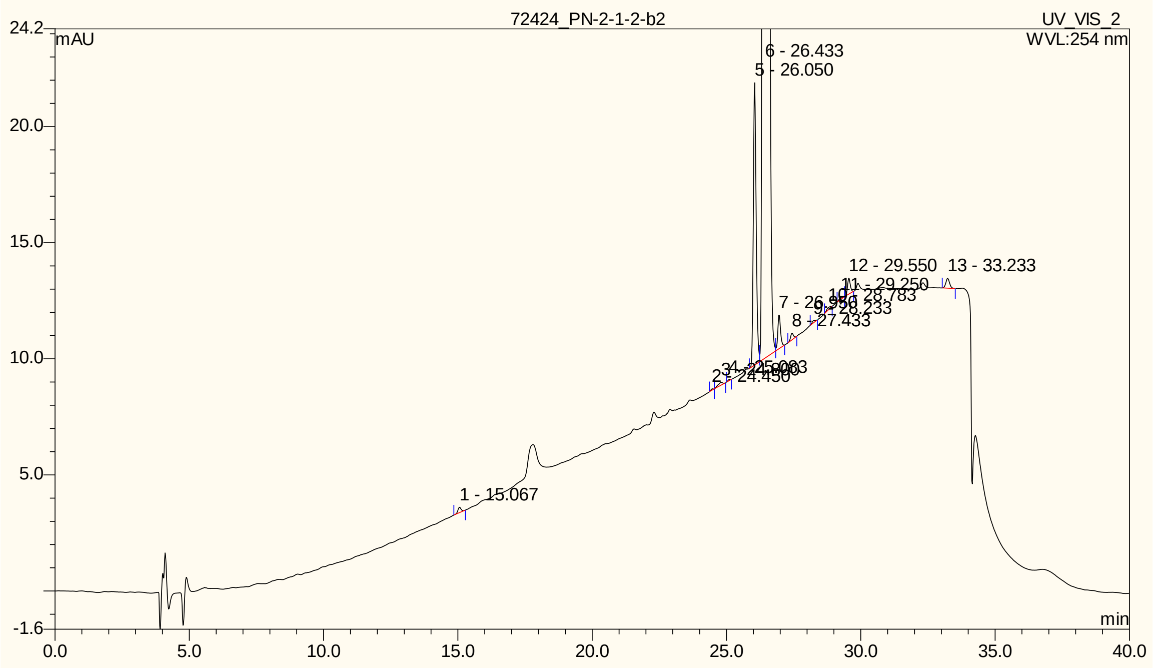

Retention Time: **26.43 min**

Relative Peak Area: **98.34 %**

Figure S63a. RP-Chromatogram of analogue **7p**.

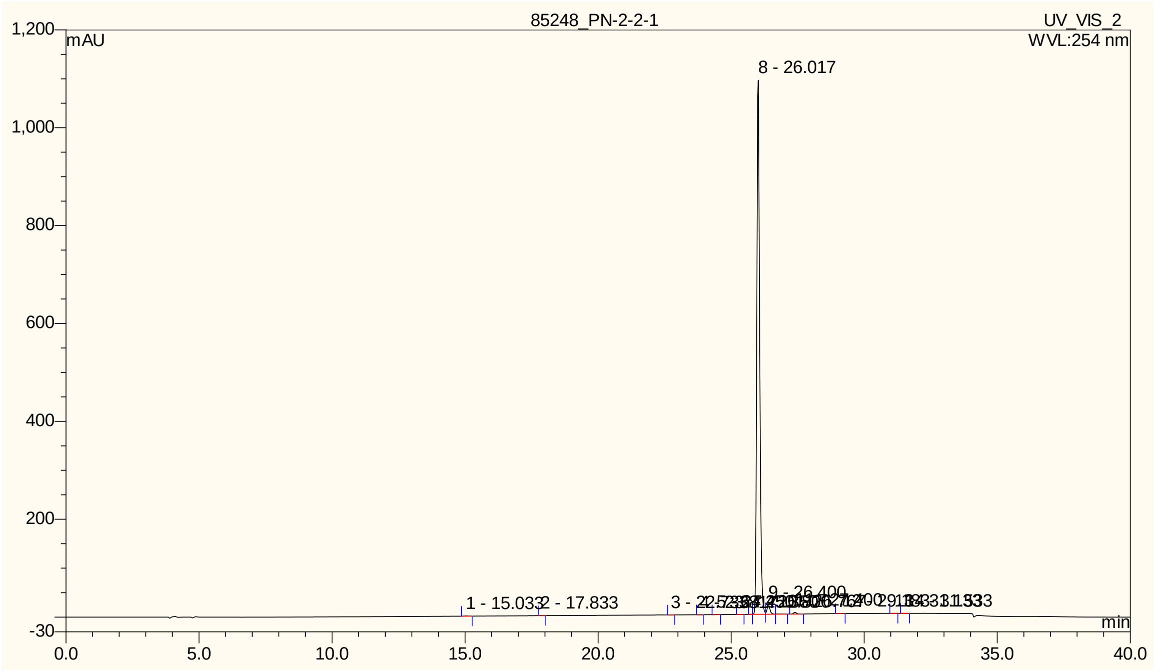

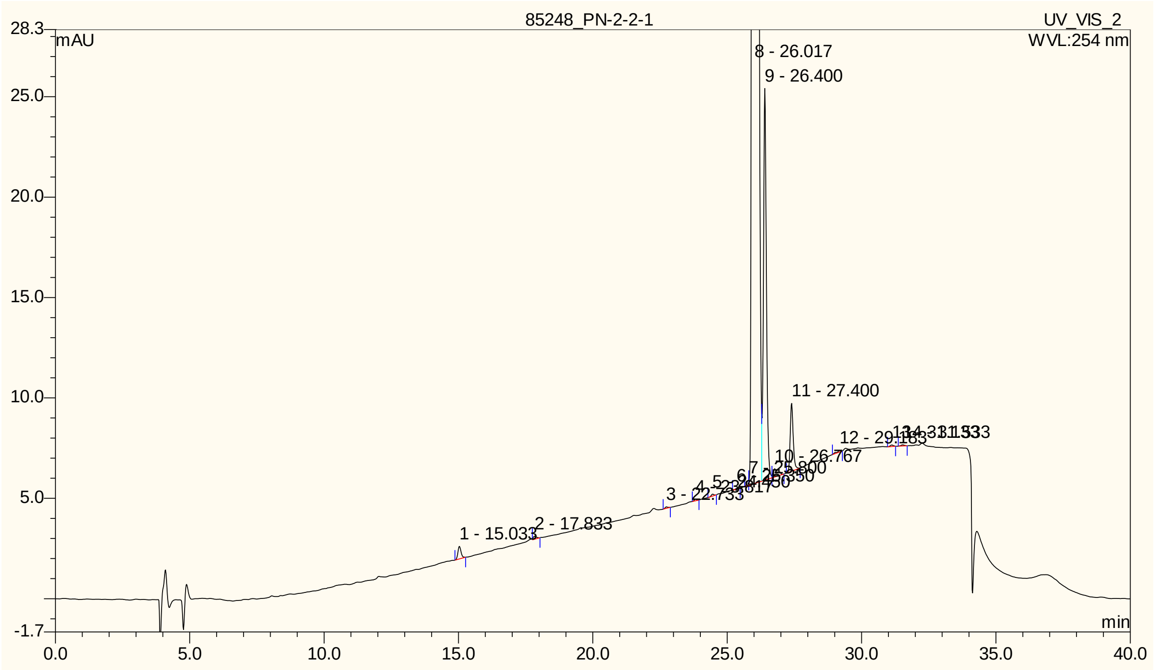

Retention Time: **26.02 min**

Relative Peak Area: **97.72 %**

Figure S64a. RP-Chromatogram of analogue **8p**.

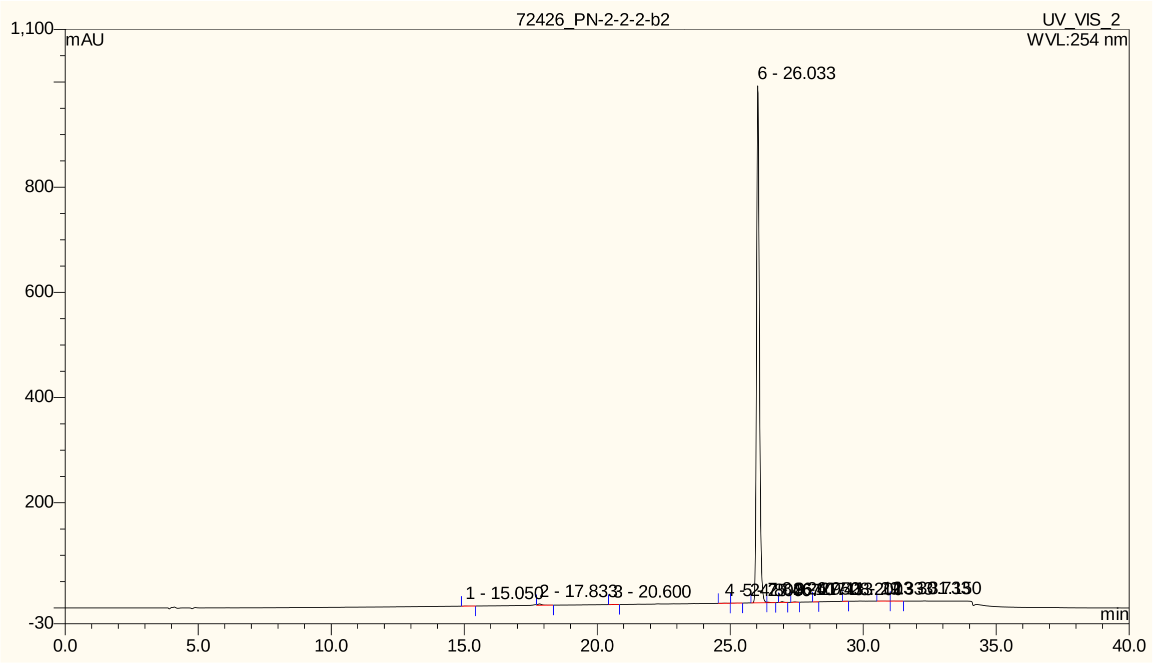

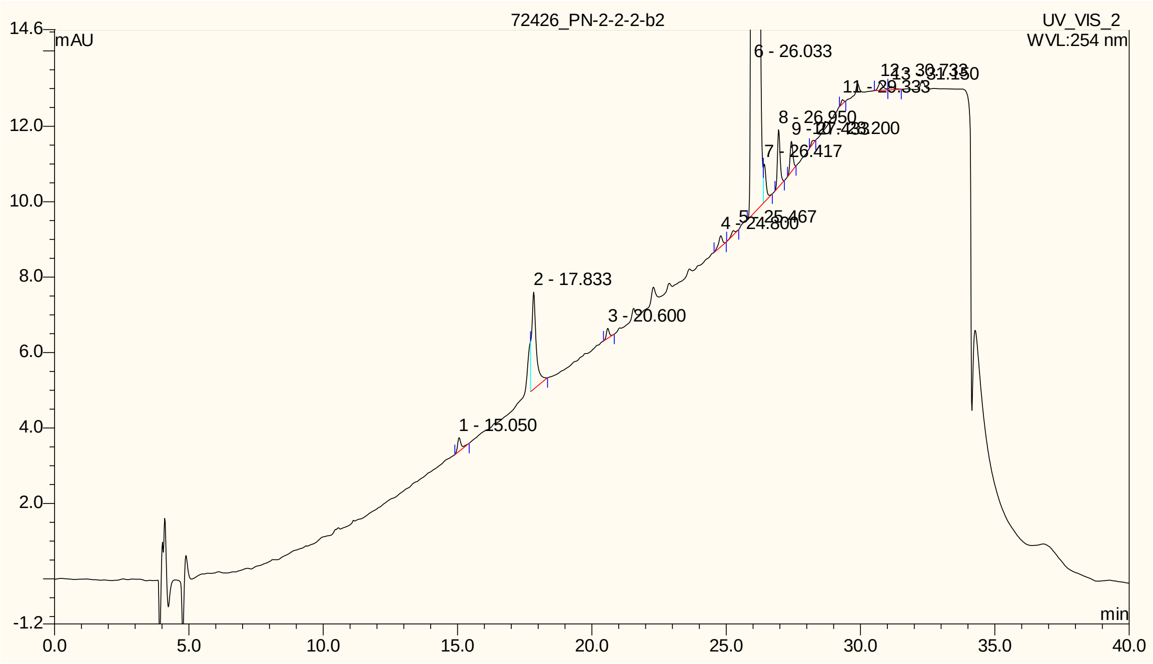

Retention Time: **26.03 min**

Relative Peak Area: **99.09 %**

Figure S65a. RP-Chromatogram of analogue **5q**.

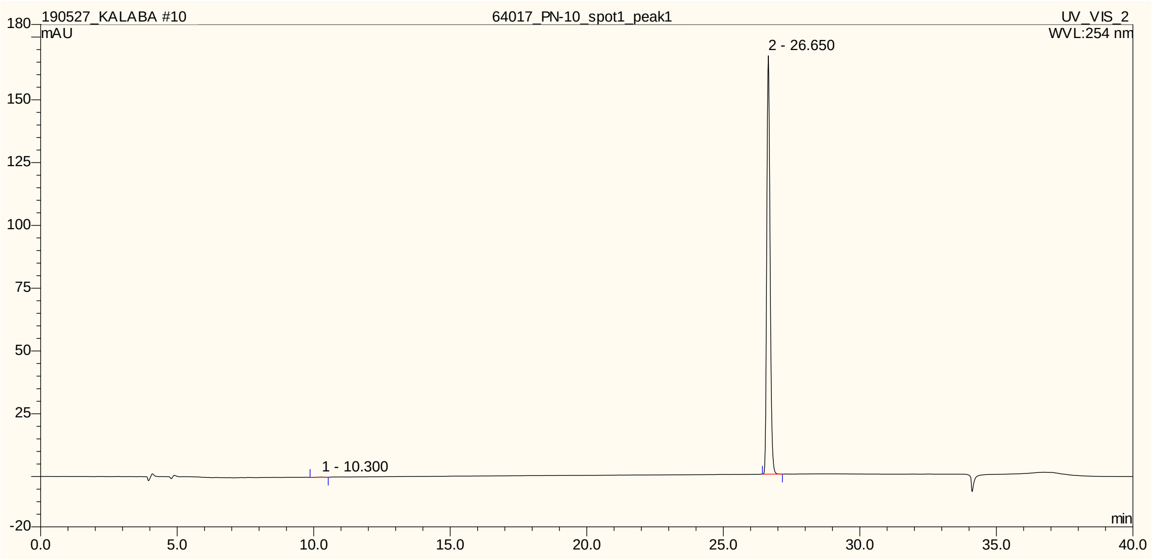

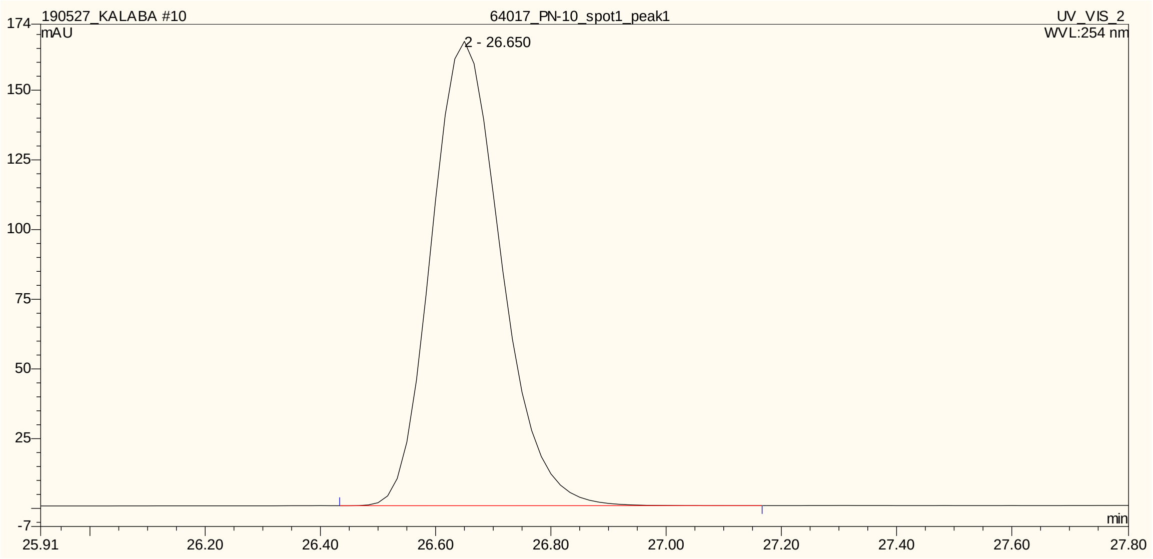

Retention Time: **26.65 min**

Relative Peak Area: **99.89 %**

Figure S66a. RP-Chromatogram of analogue **6q**.

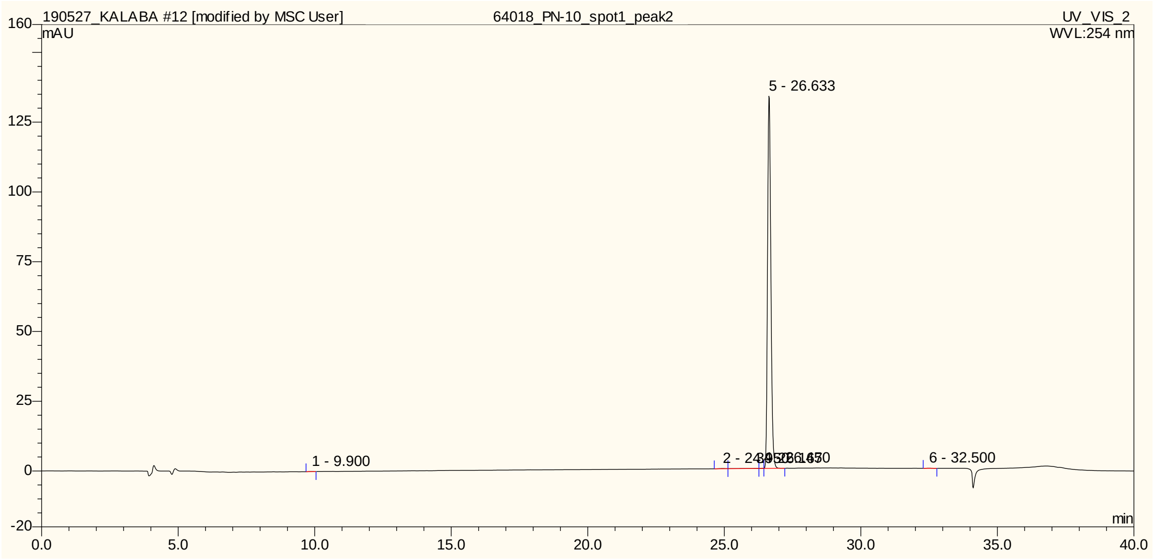

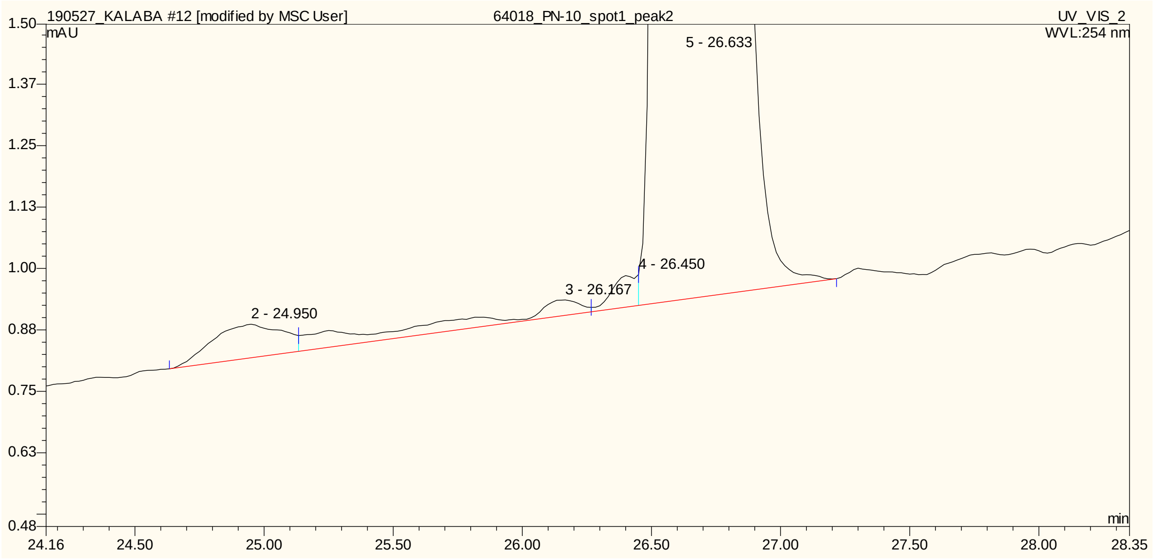

Retention Time: **26.63 min**

Relative Peak Area: **99.61 %**

Figure S67a. RP-Chromatogram of analogue **7q.**

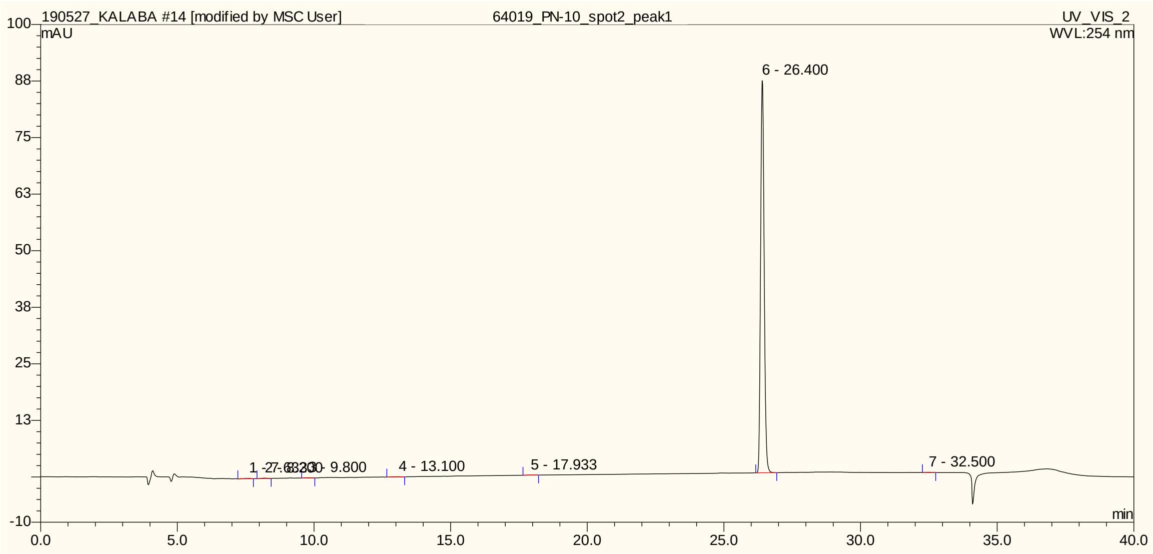

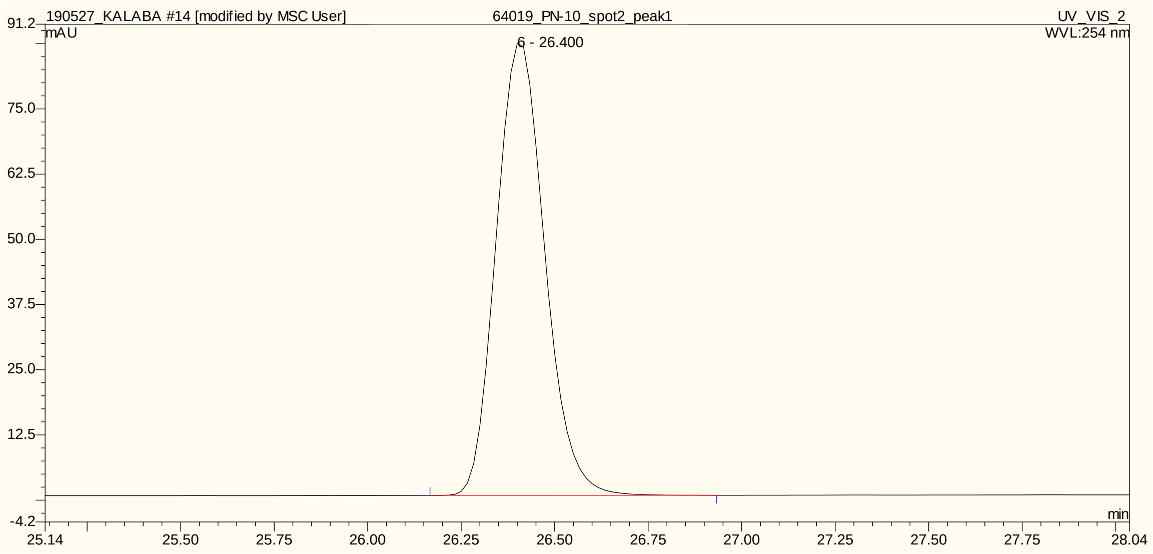

Retention Time: **26.4 min**

Relative Peak Area: **99.29%**

Figure S68a. RP-Chromatogram of analogue **8q**.

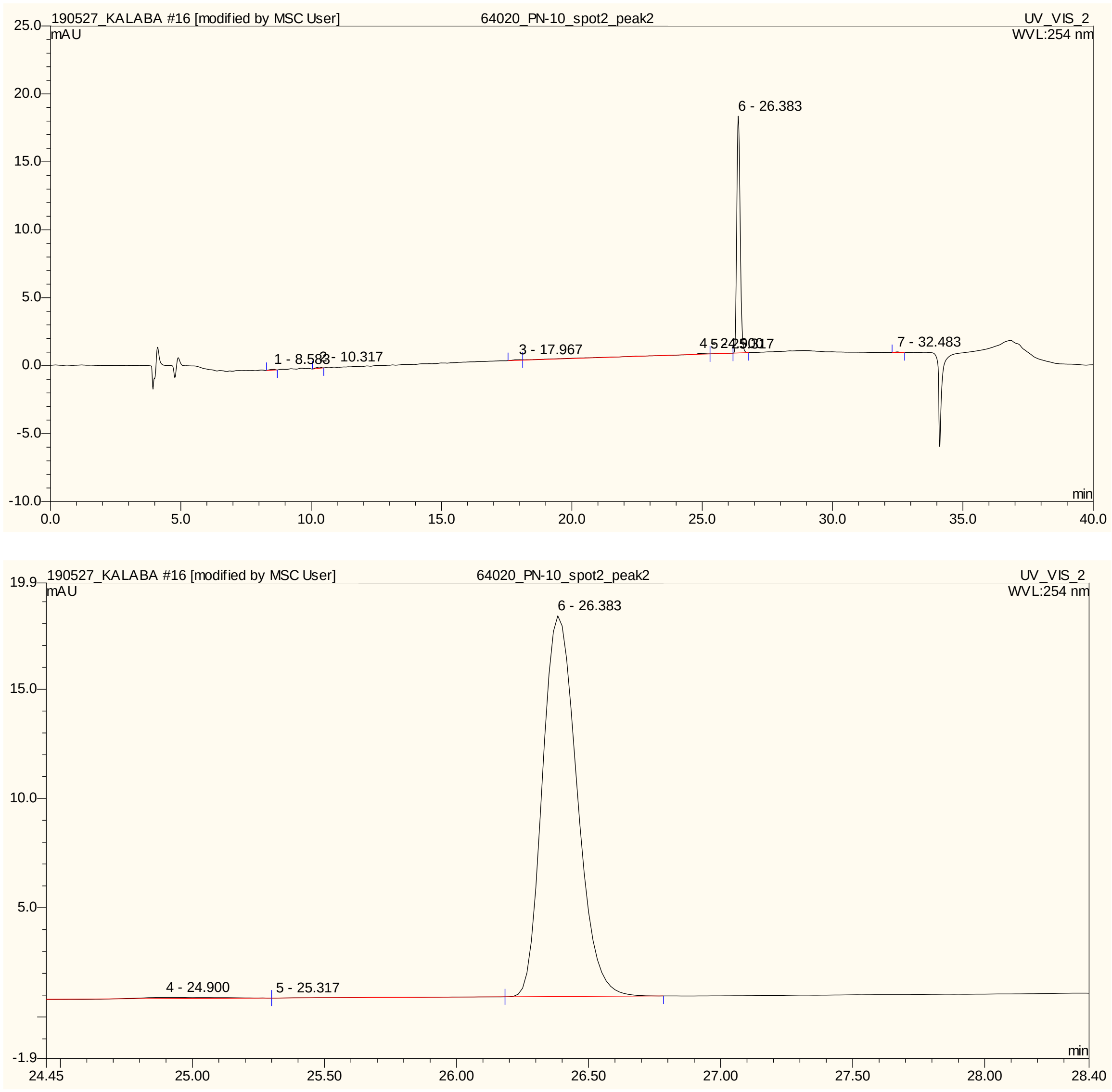

Retention Time: **26.38 min**

Relative Peak Area: **95.74 %**

Figure S69a. RP-Chromatogram of analogue **5r**.

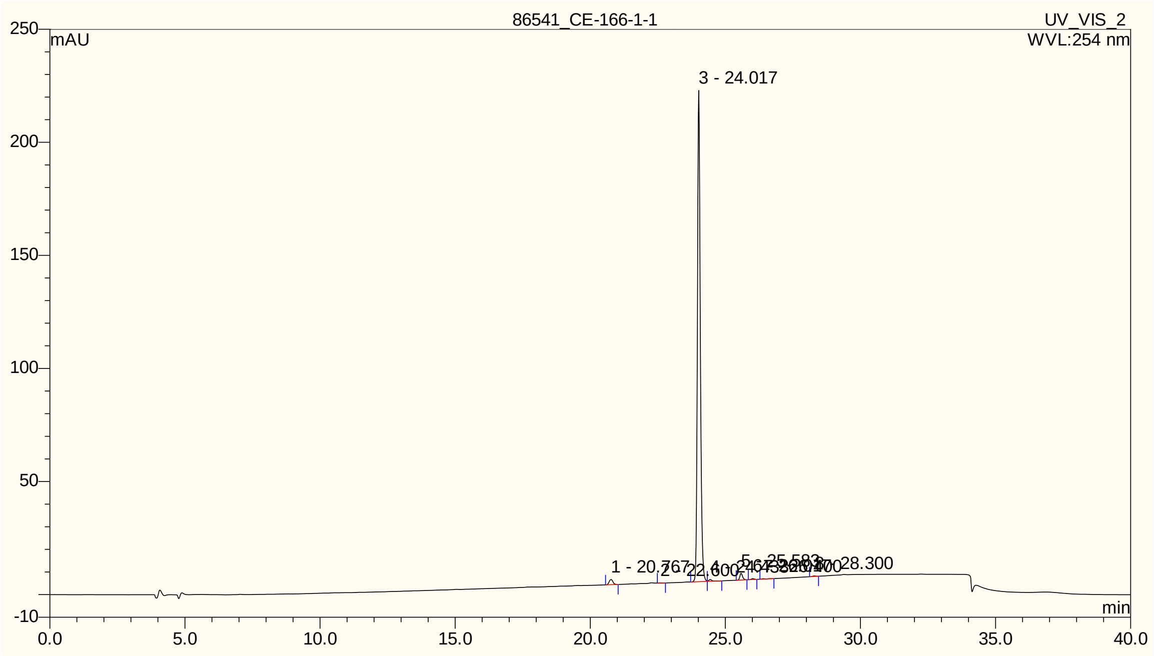

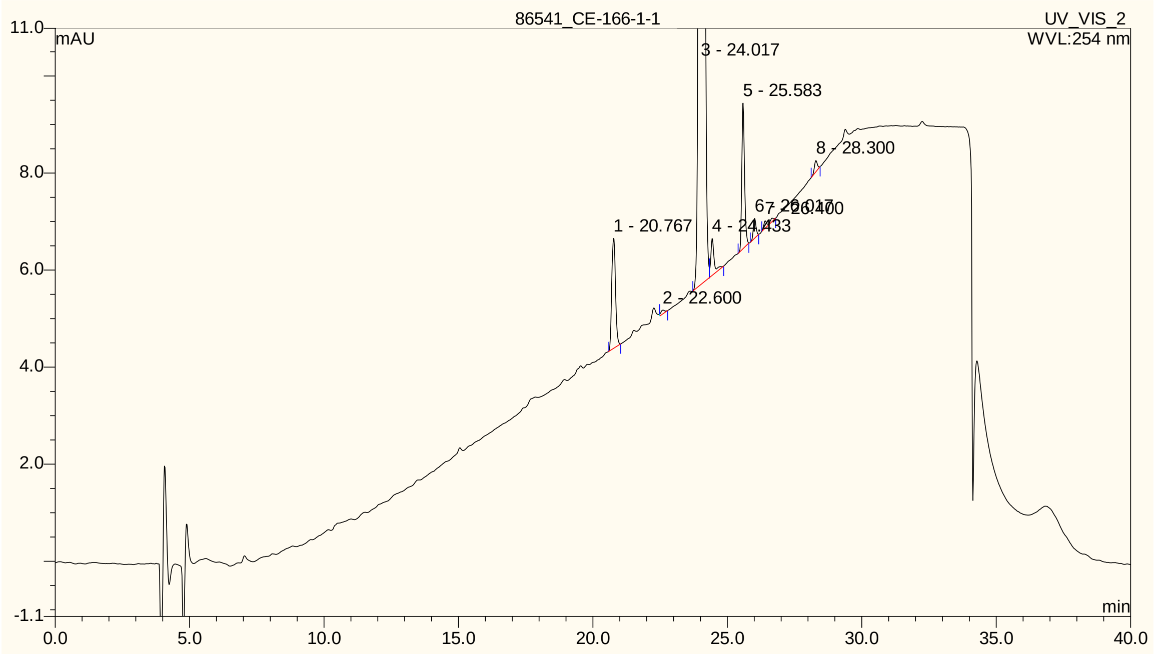

Retention Time: **24.02 min**

Relative Peak Area: **96.62 %**

Figure S70a. RP-Chromatogram of analogue **6r**.

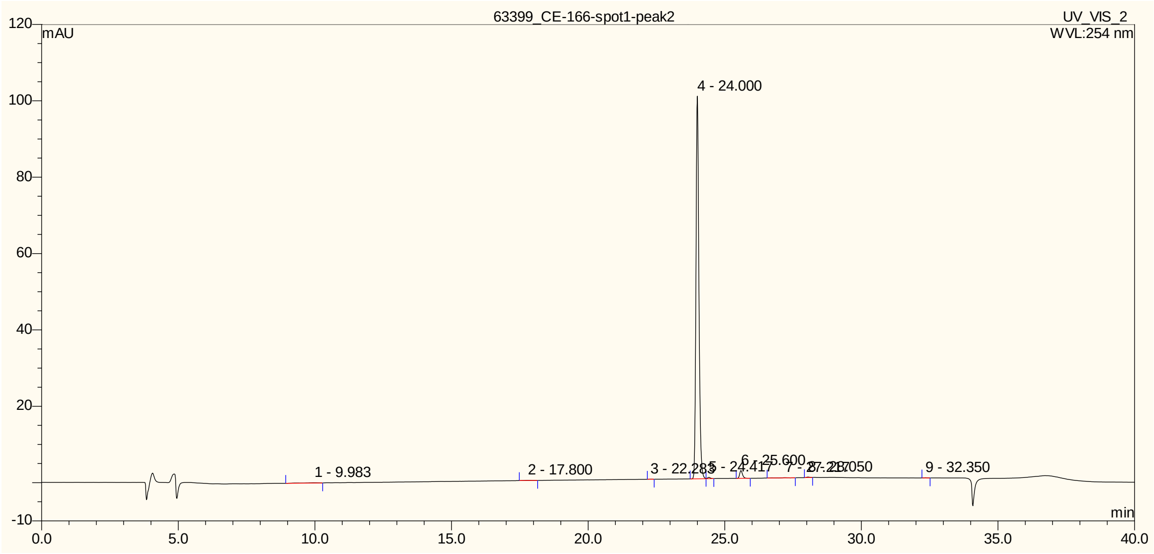

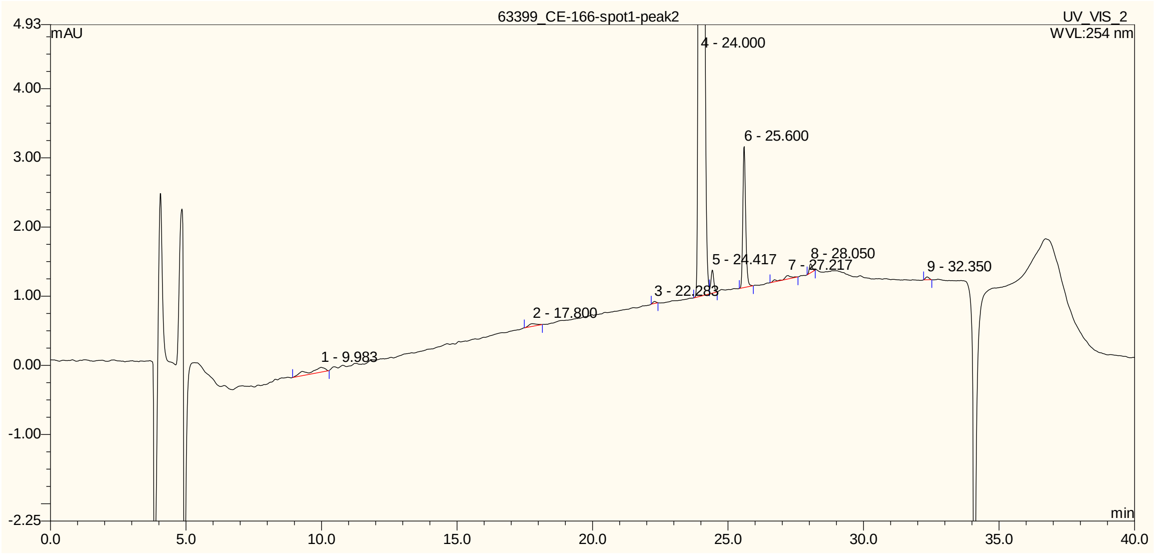

Retention Time: **24.00 min**

Relative Peak Area: **96.94 %**

Figure S71a. RP-Chromatogram of analogue **7r**.

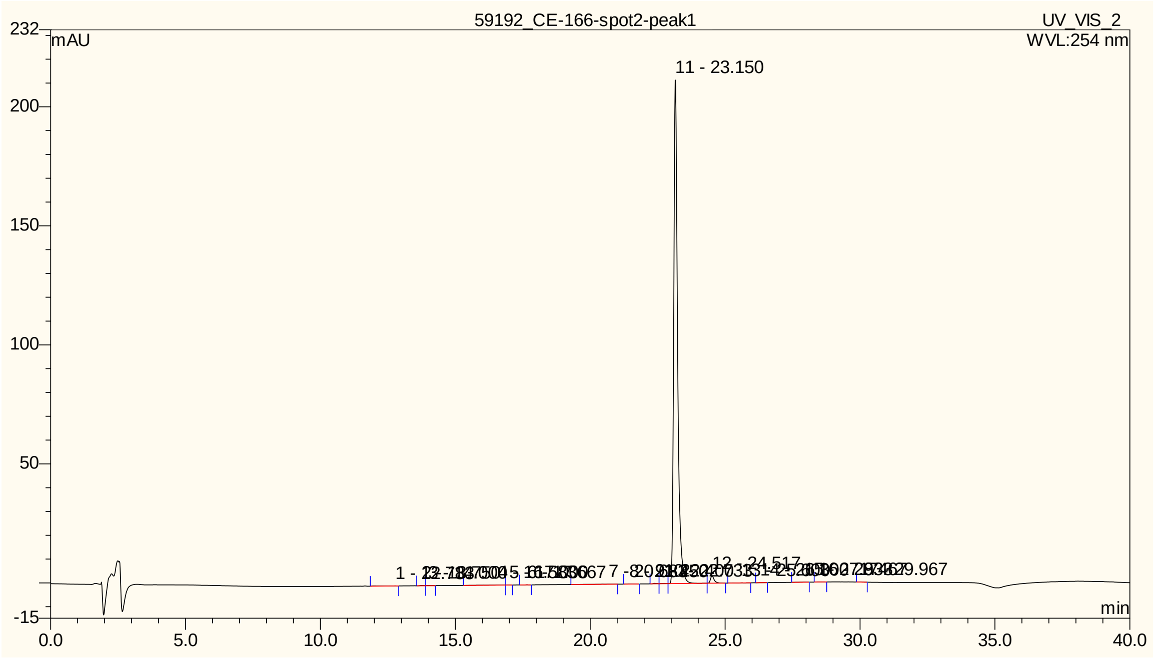

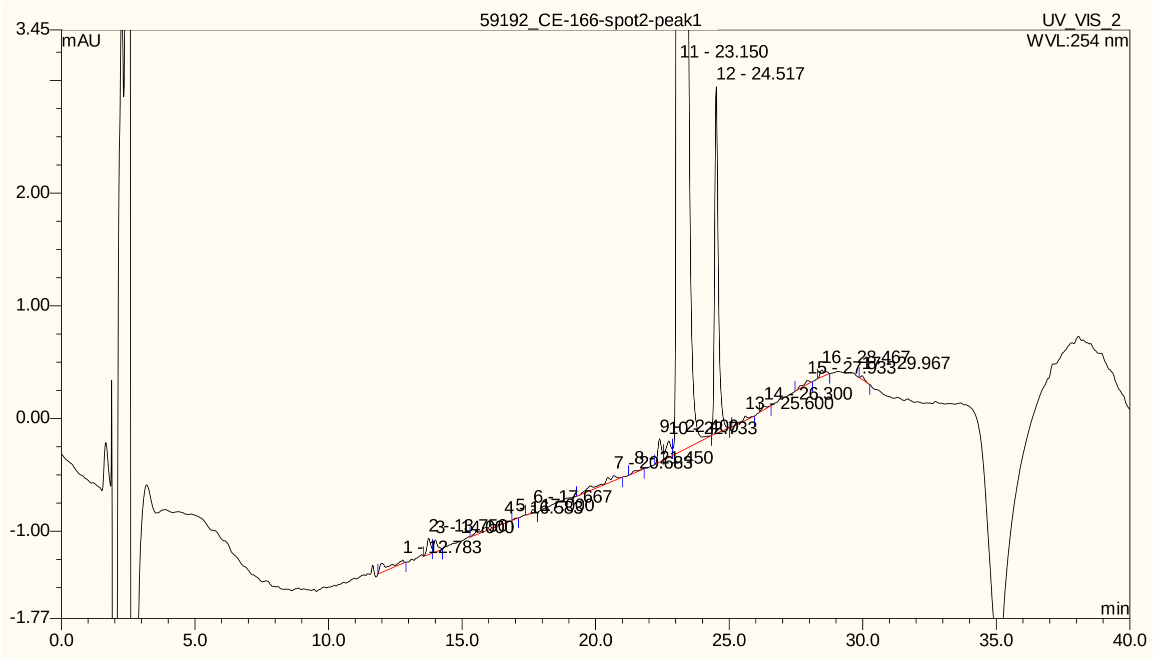

Retention Time: **23.15 min**

Relative Peak Area: **97.94 %**

Figure S72a. RP-Chromatogram of analogue **8r**.

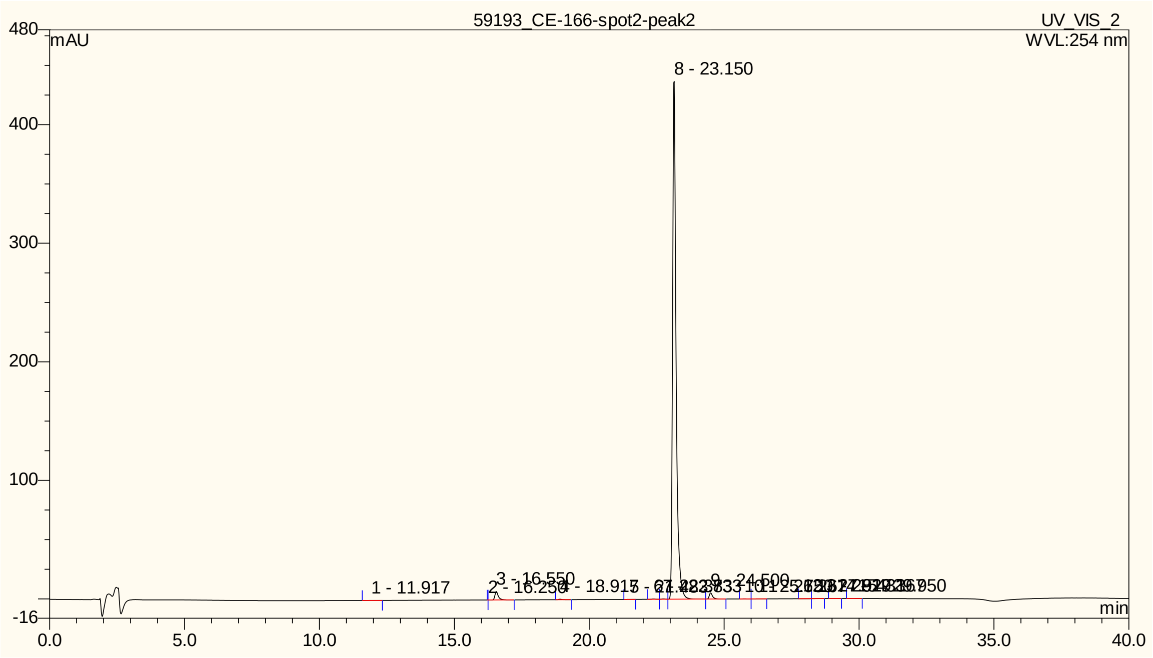

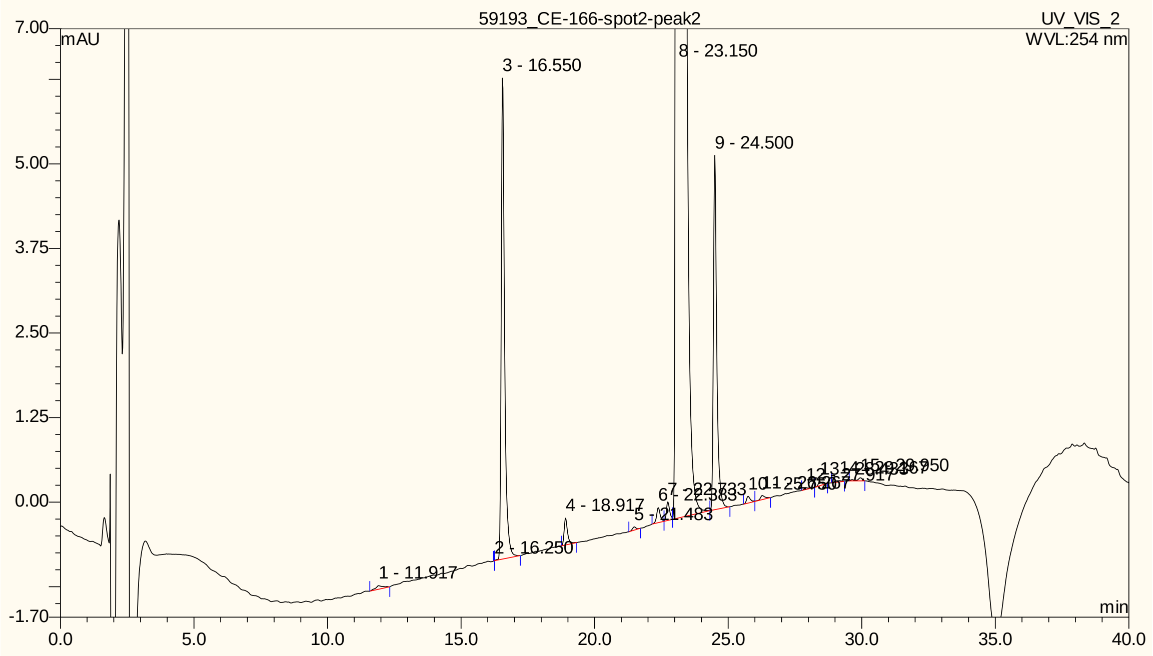

Retention Time: **23.15 min**

Relative Peak Area: **97.04 %**

Figure S73a. RP-Chromatogram of analogue **5s**.

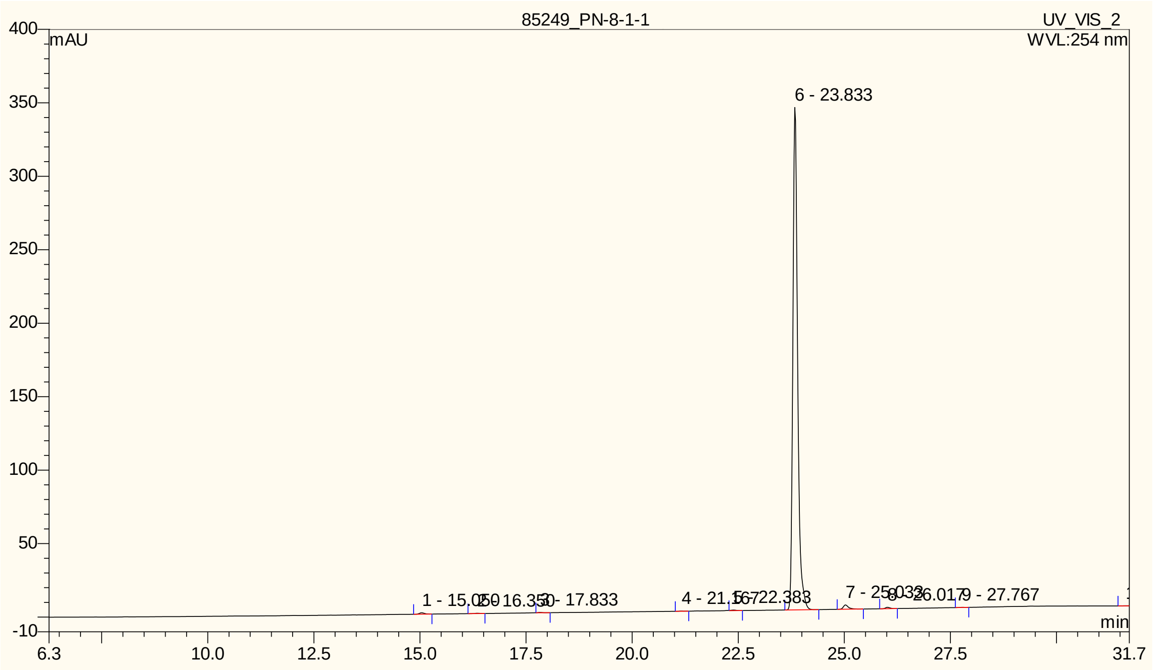

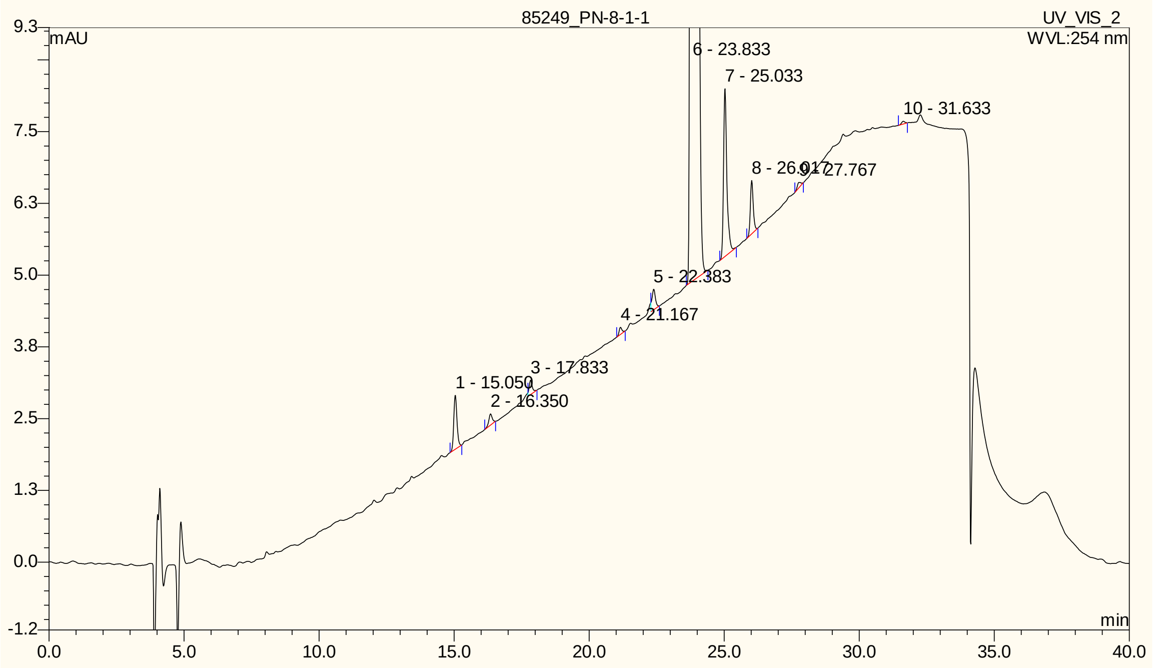

Retention Time: **23.83 min**

Relative Peak Area: **98.20 %**

Figure S74a. RP-Chromatogram of analogue **6s**.

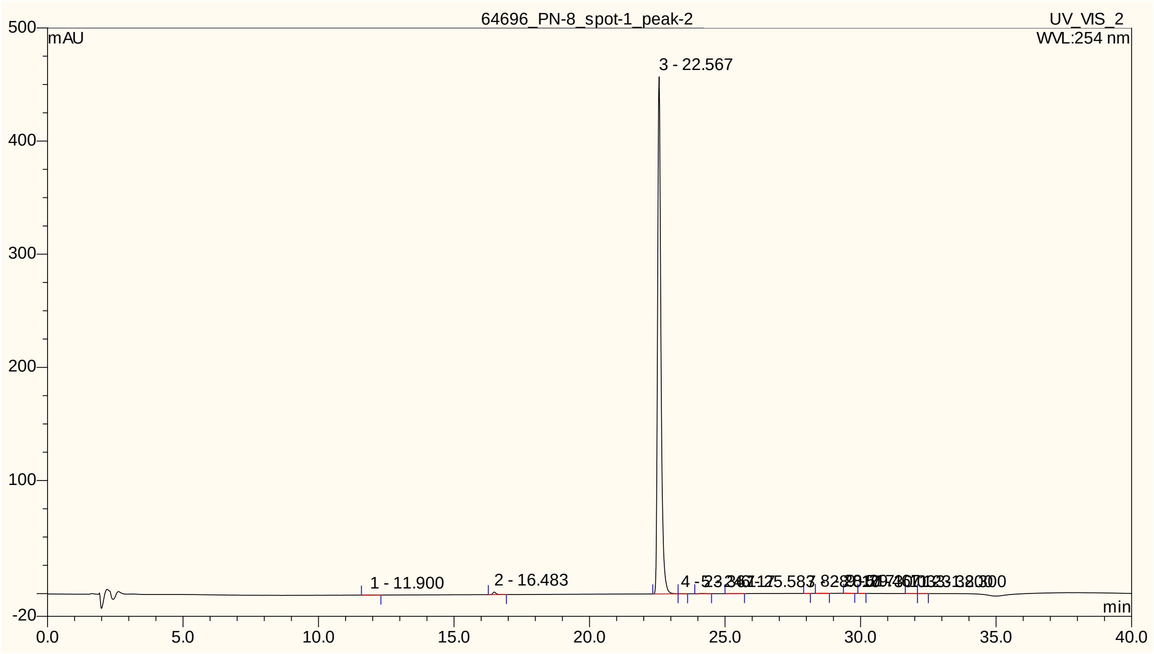

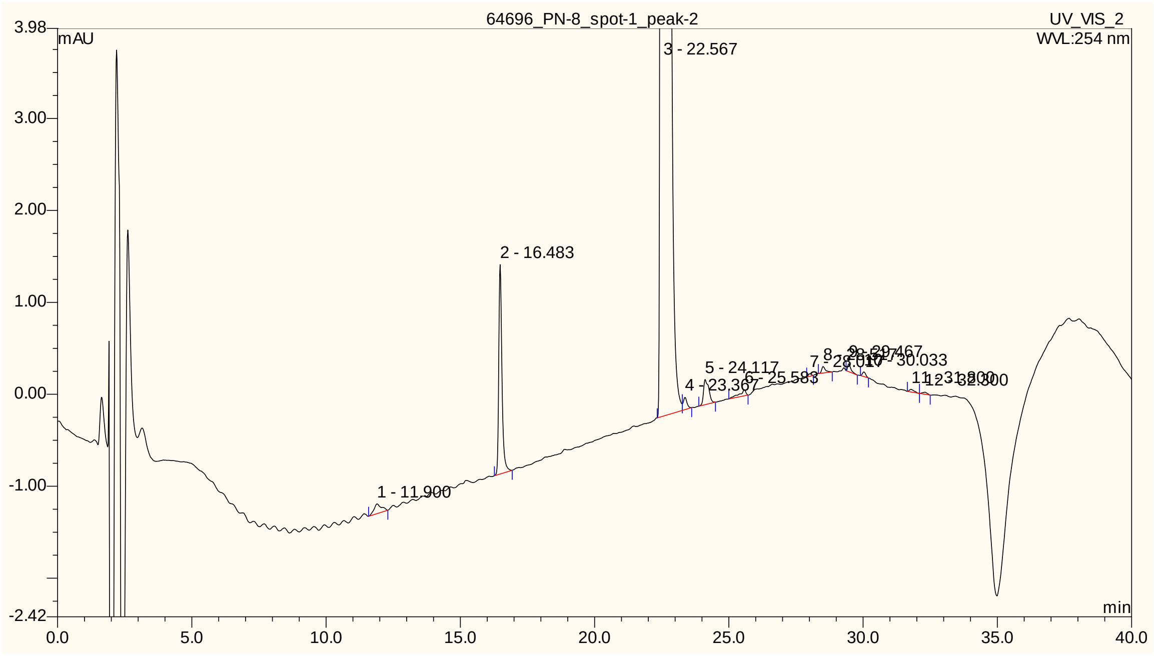

Retention Time: **22.57 min**

Relative Peak Area: **99.26 %**

Figure S75a. RP-Chromatogram of analogue **7s**.

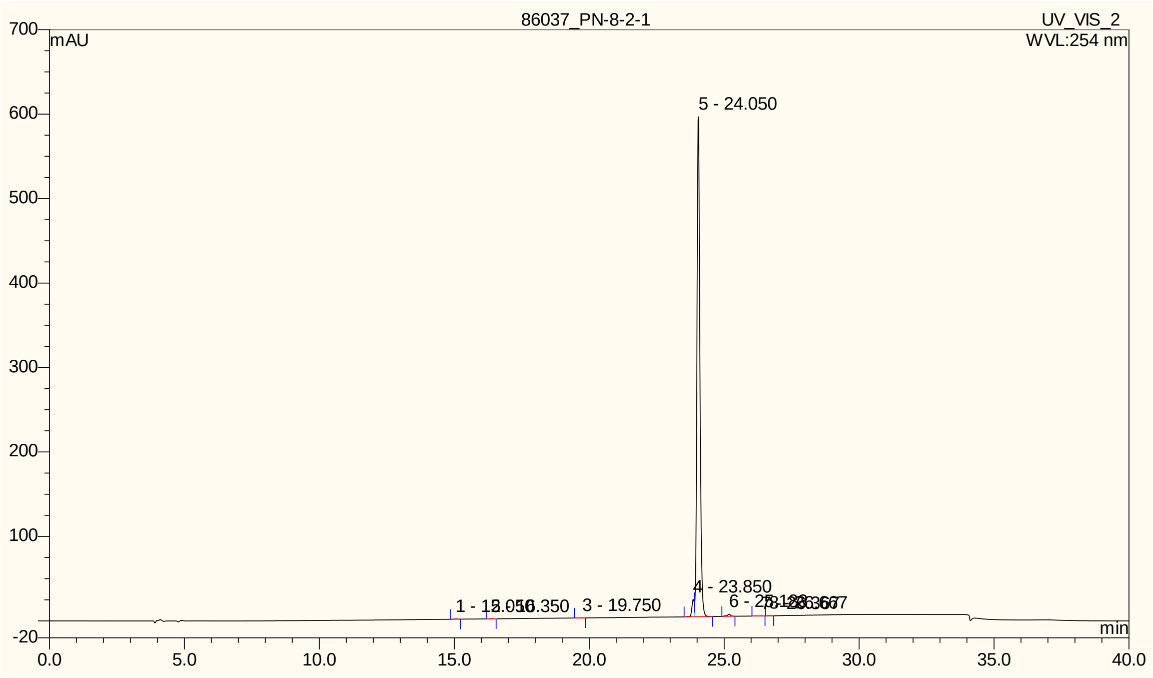

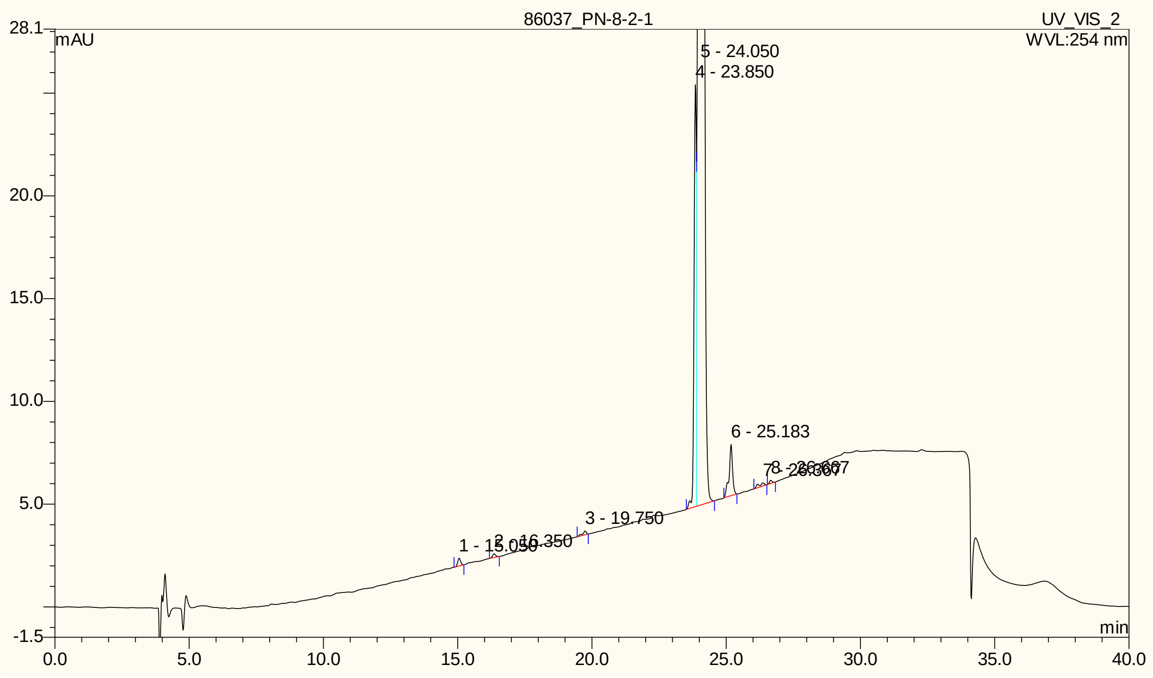

Retention Time: **24.05 min**

Relative Peak Area: **96.70 %**

Figure S76a. RP-Chromatogram of analogue **8s**.

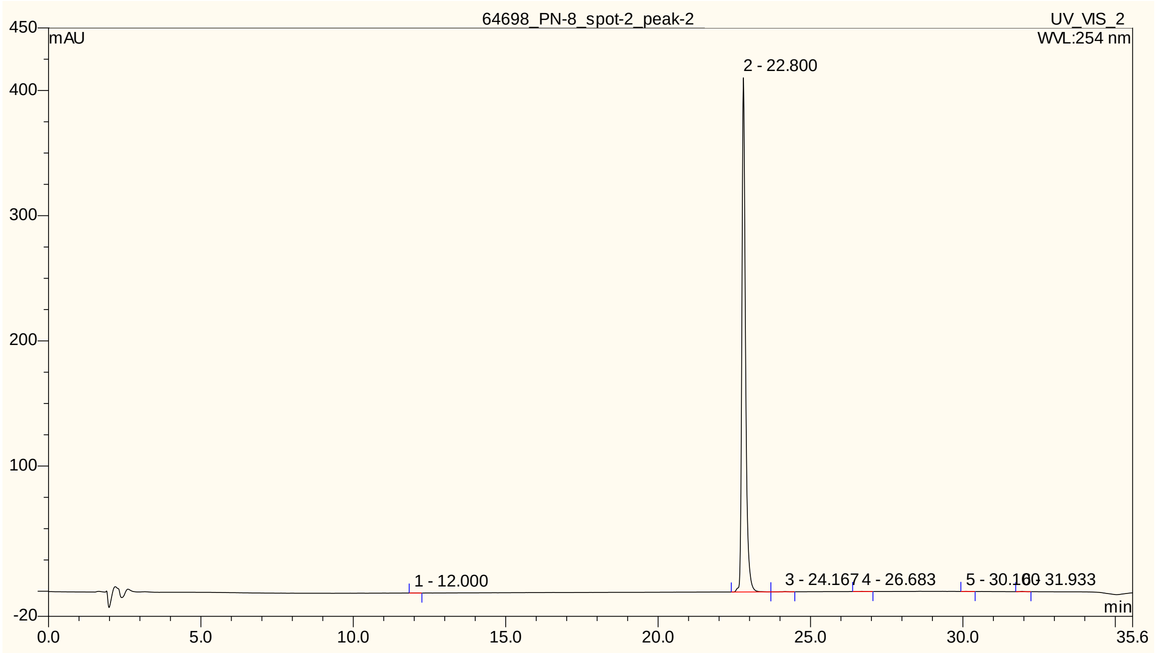

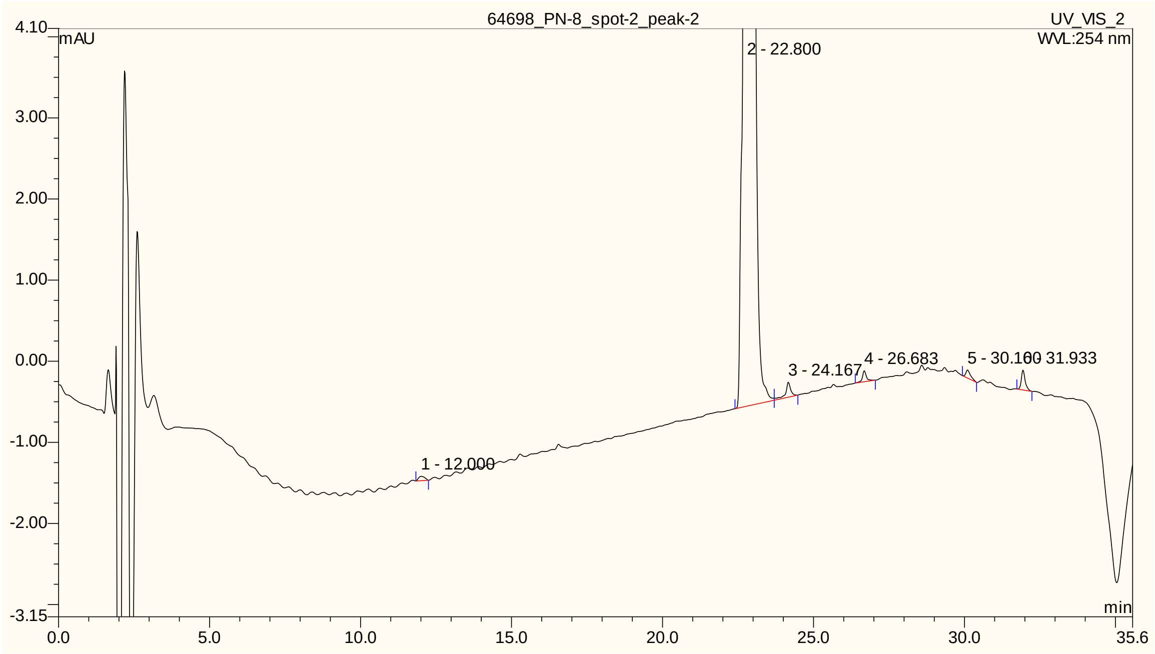

Retention Time: **22.80 min**

Relative Peak Area: **99.78 %**

Figure S77a. RP-Chromatogram of analogue **5t**.

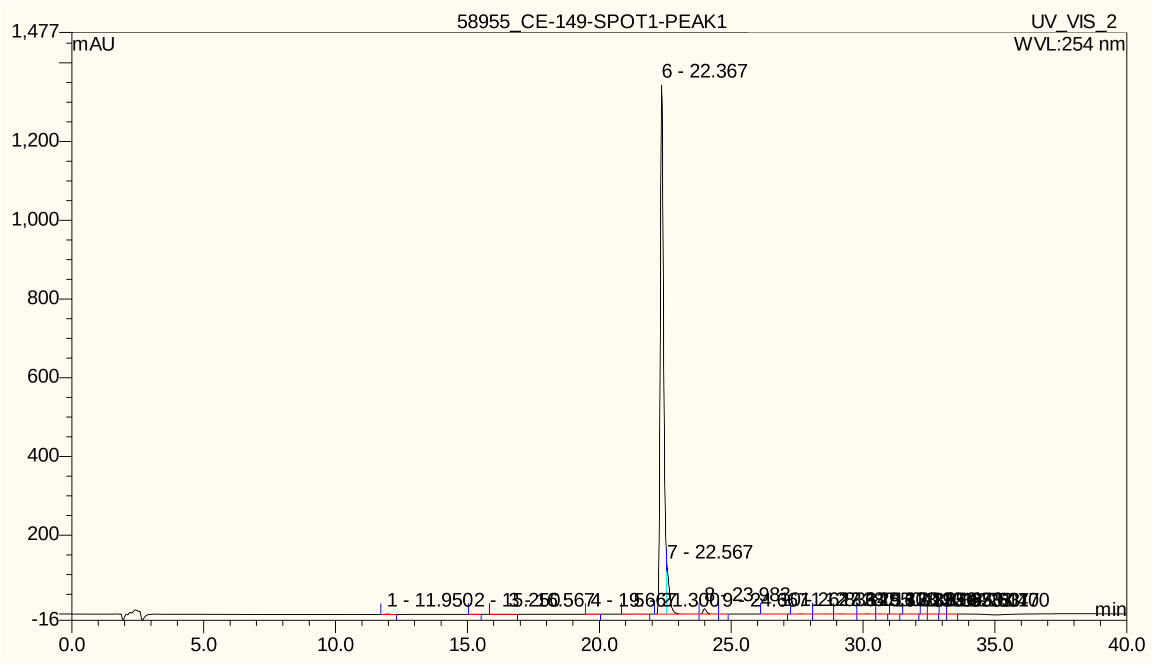

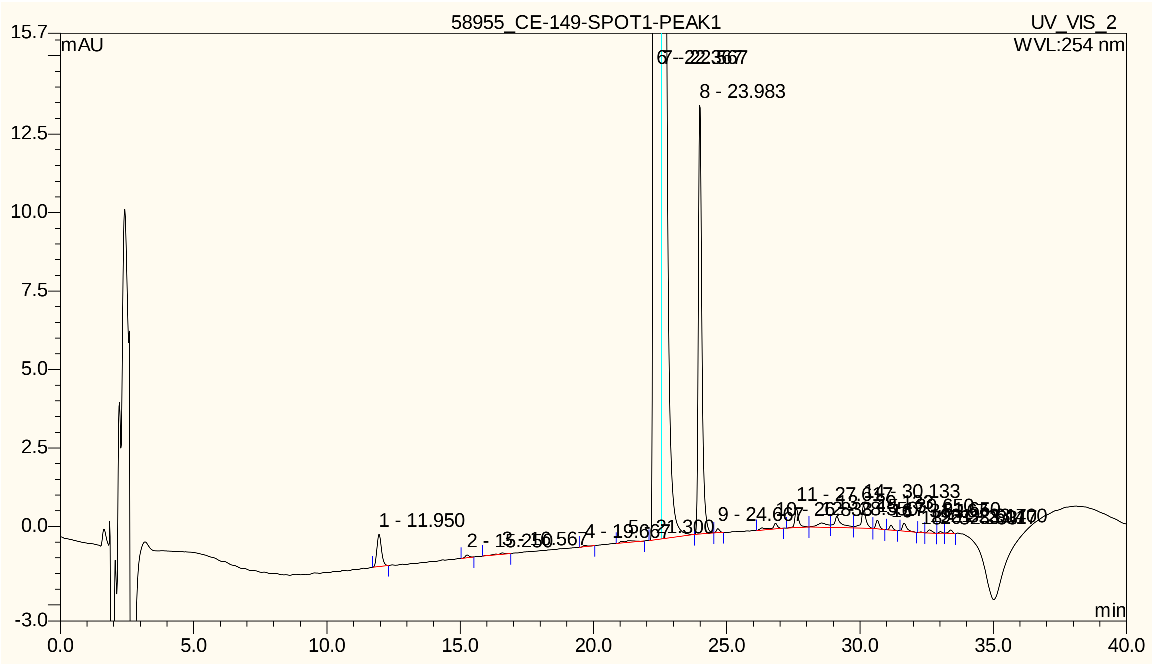

Retention Time:	**22.37 min**
Relative Peak Area:	**91.68 %**

Figure S78a. RP-Chromatogram of analogue **6t**.

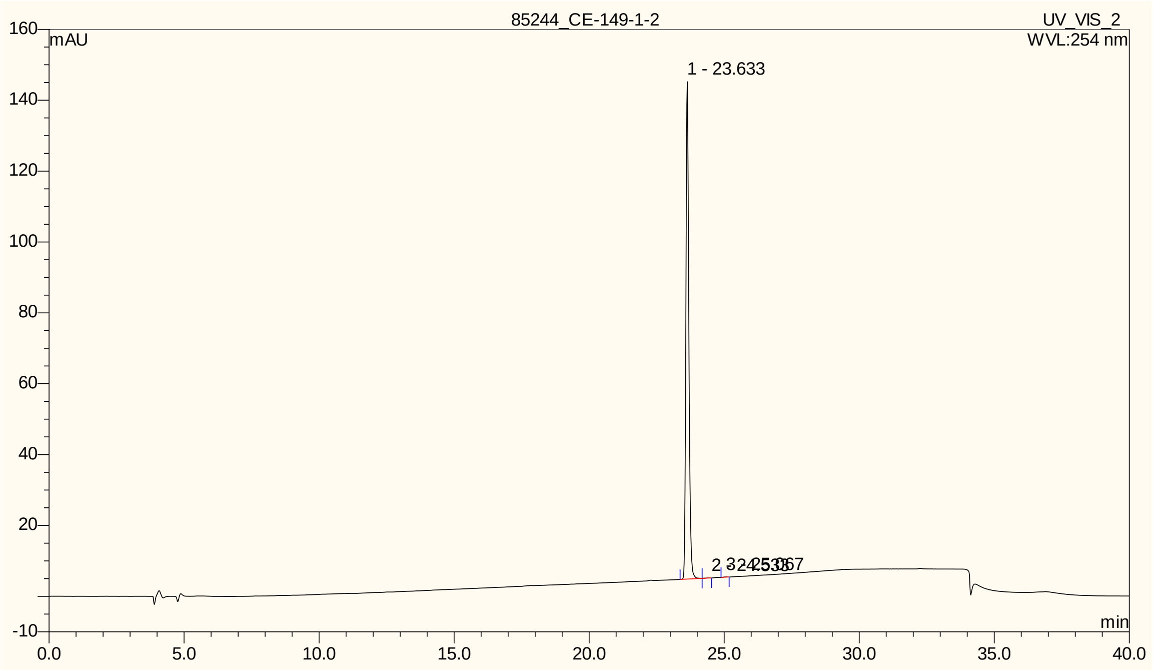

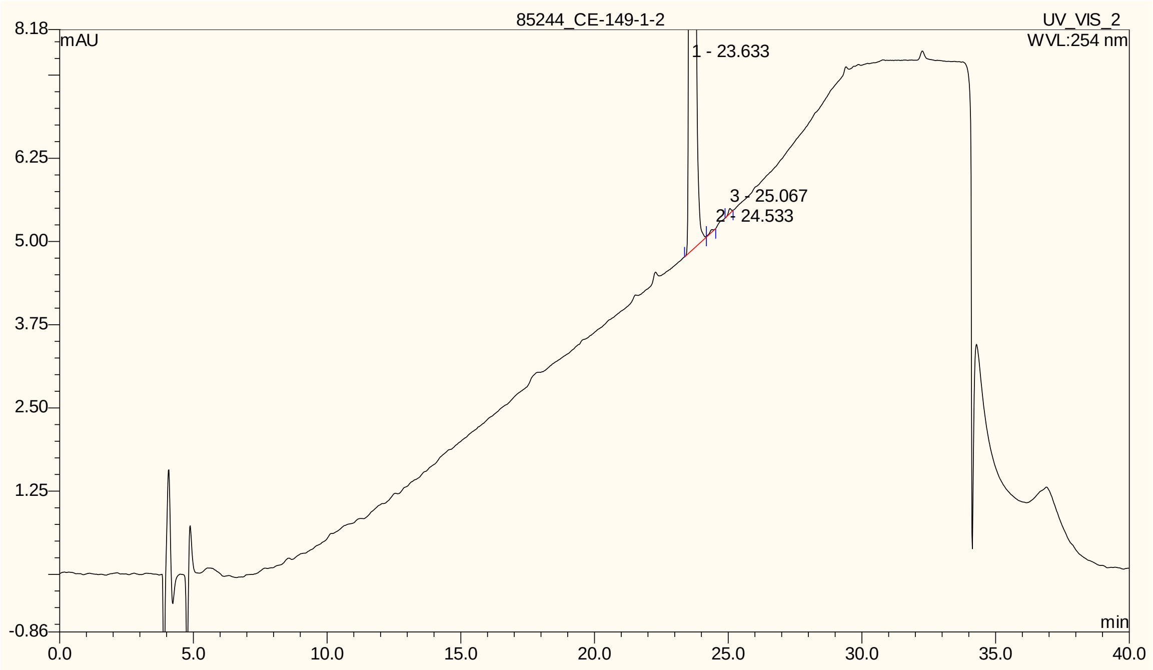

Retention Time: **23.63 min**

Relative Peak Area: **99.92 %**

Figure S79a. RP-Chromatogram of analogue **7t**.

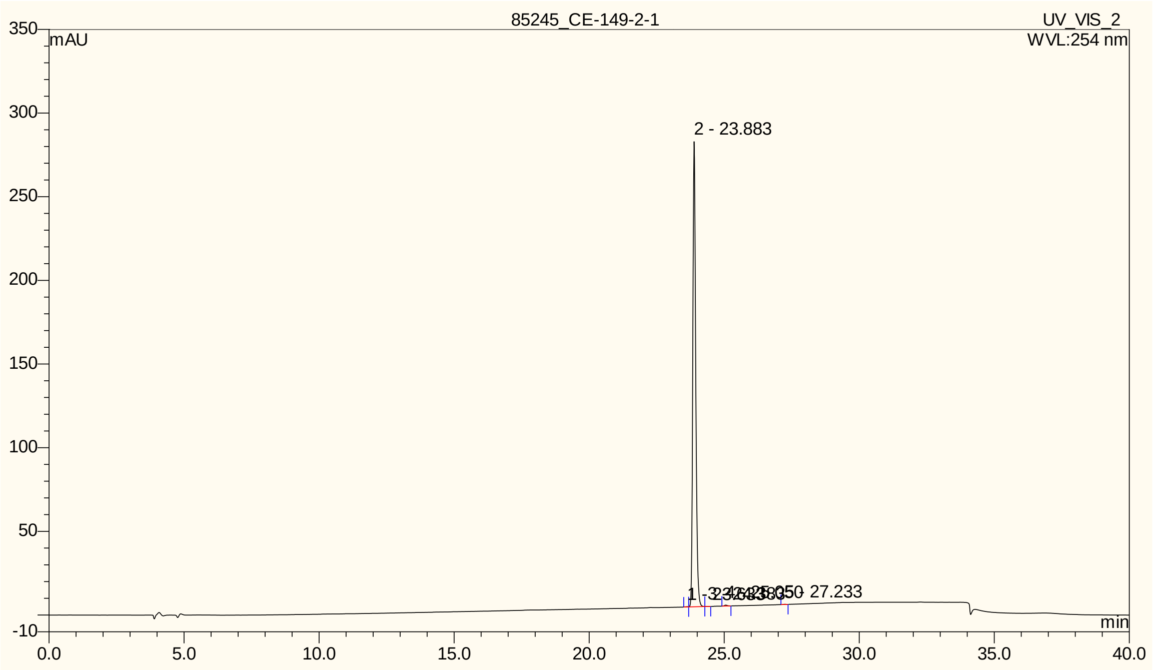

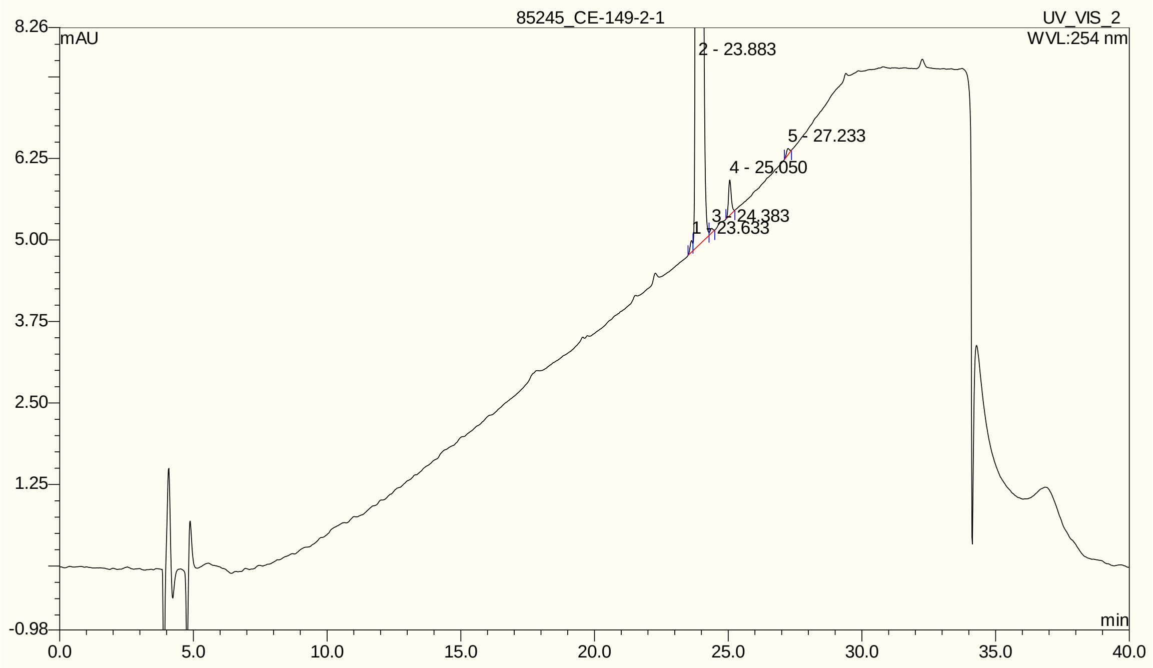

Retention Time: **23.88 min**

Relative Peak Area: **99.70 %**

Figure S80a. RP-Chromatogram of analogue **8t**.

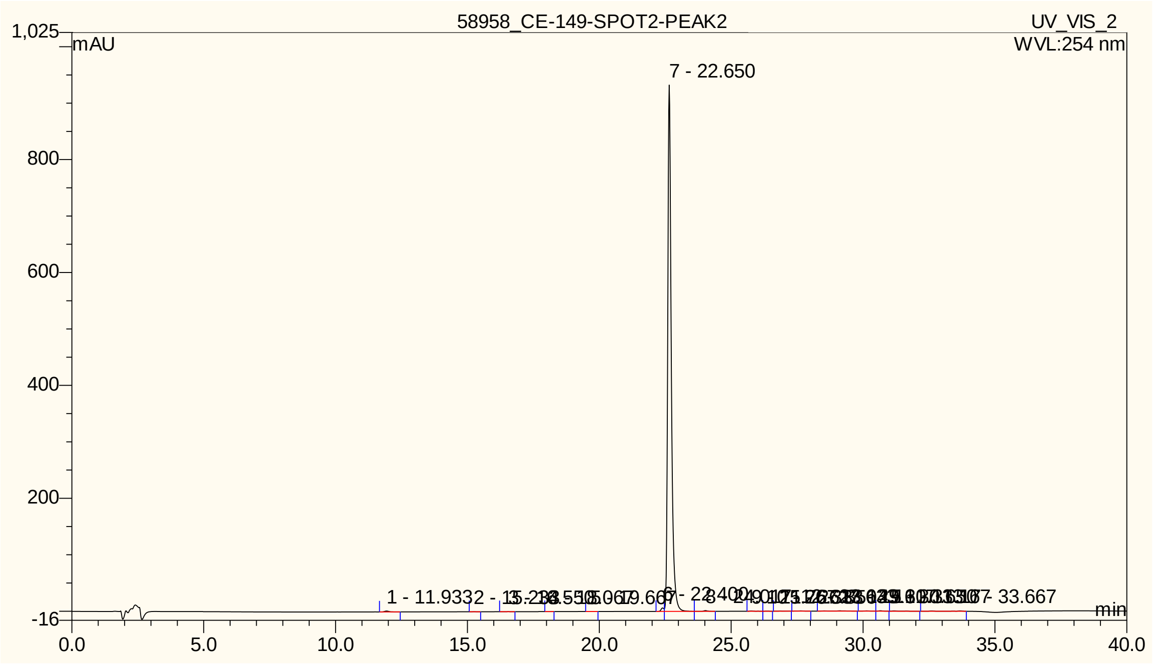

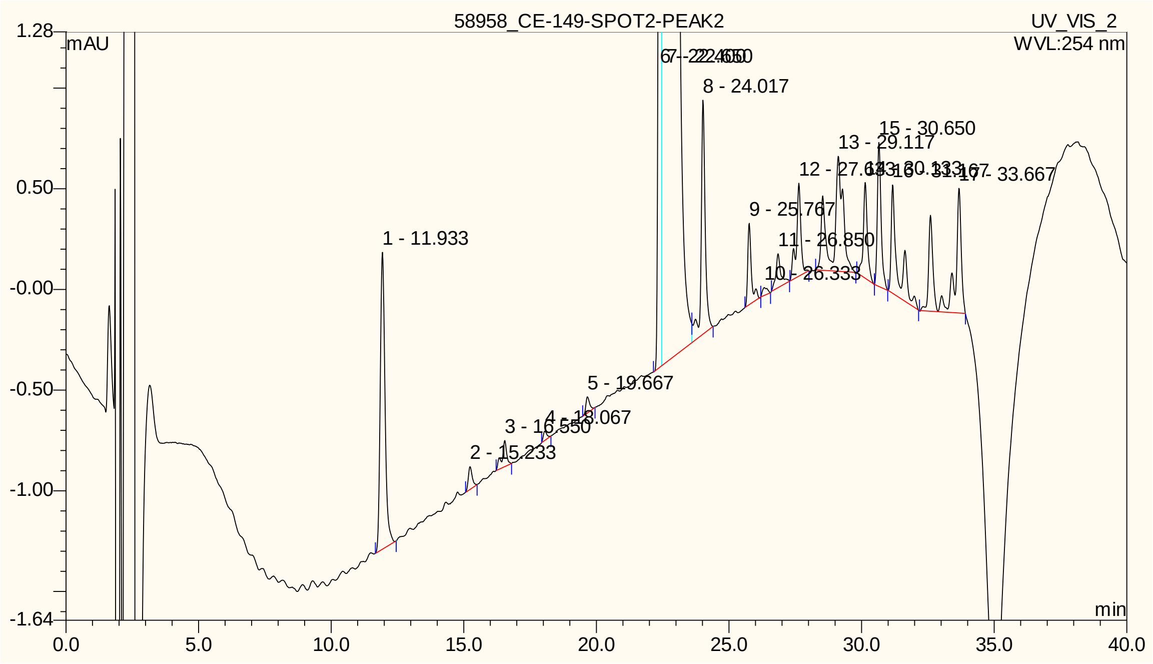

Retention Time: **22.65 min**

Relative Peak Area: **98.42 %**

Figure S81a. RP-Chromatogram of analogue **5u**.

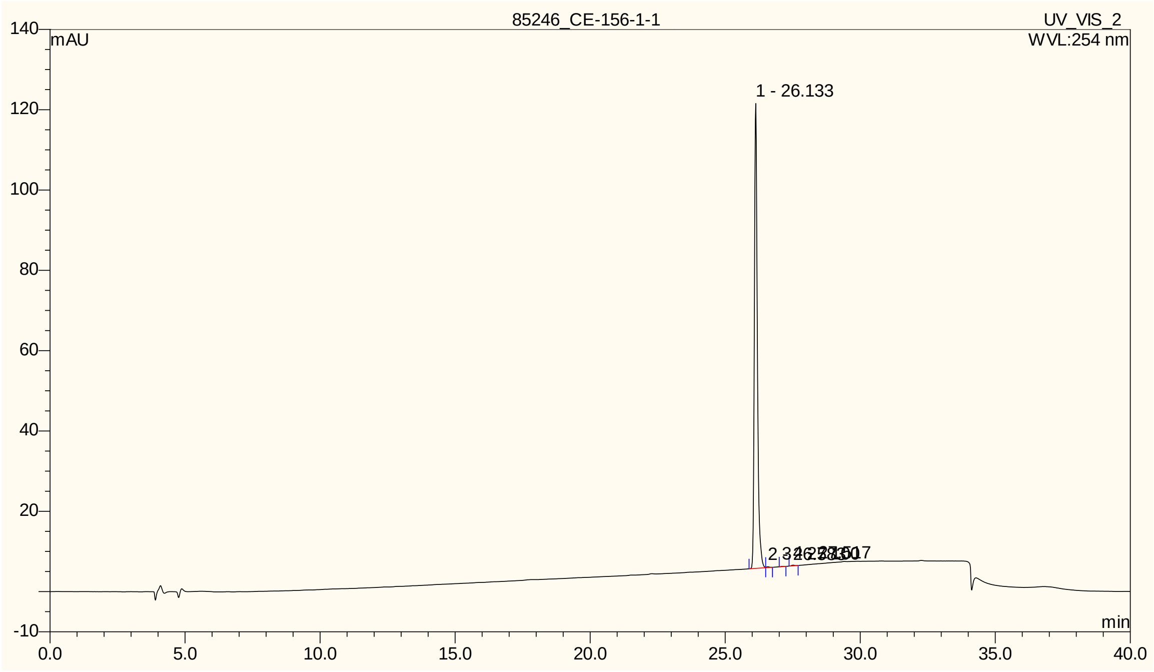

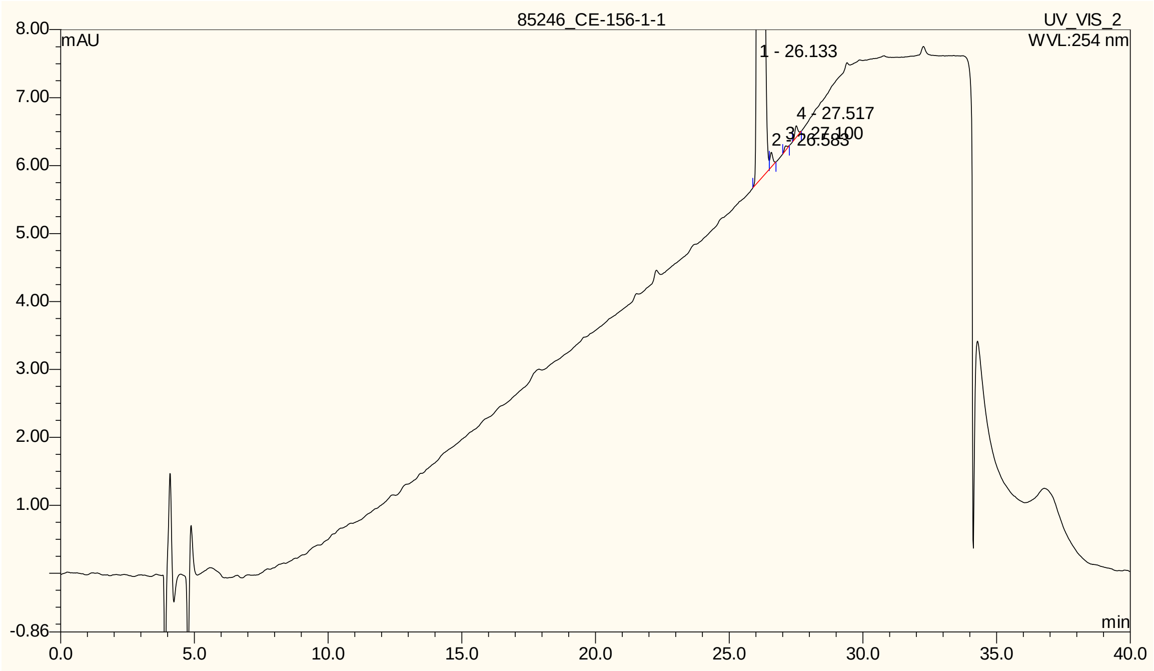

Retention Time: **26.13 min**

Relative Peak Area: **99.62 %**

Figure S82a. RP-Chromatogram of analogue **6u**.

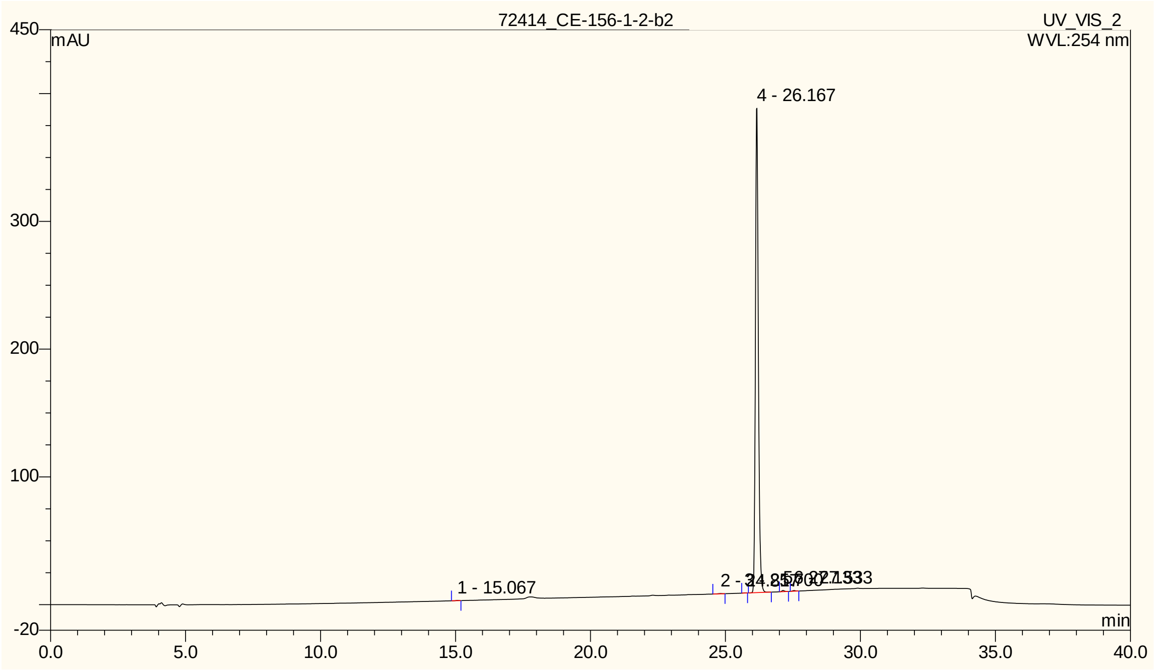

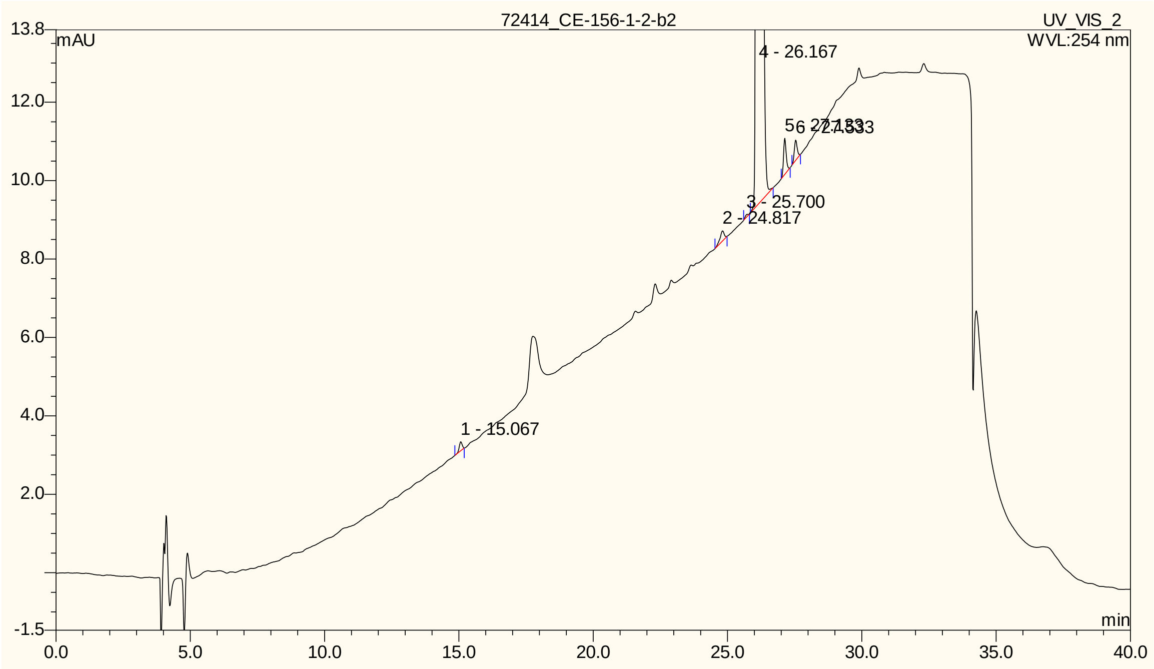

Retention Time: **26.17 min**

Relative Peak Area: **99.47 %**

Figure S83a. RP-Chromatogram of analogue **7u**.

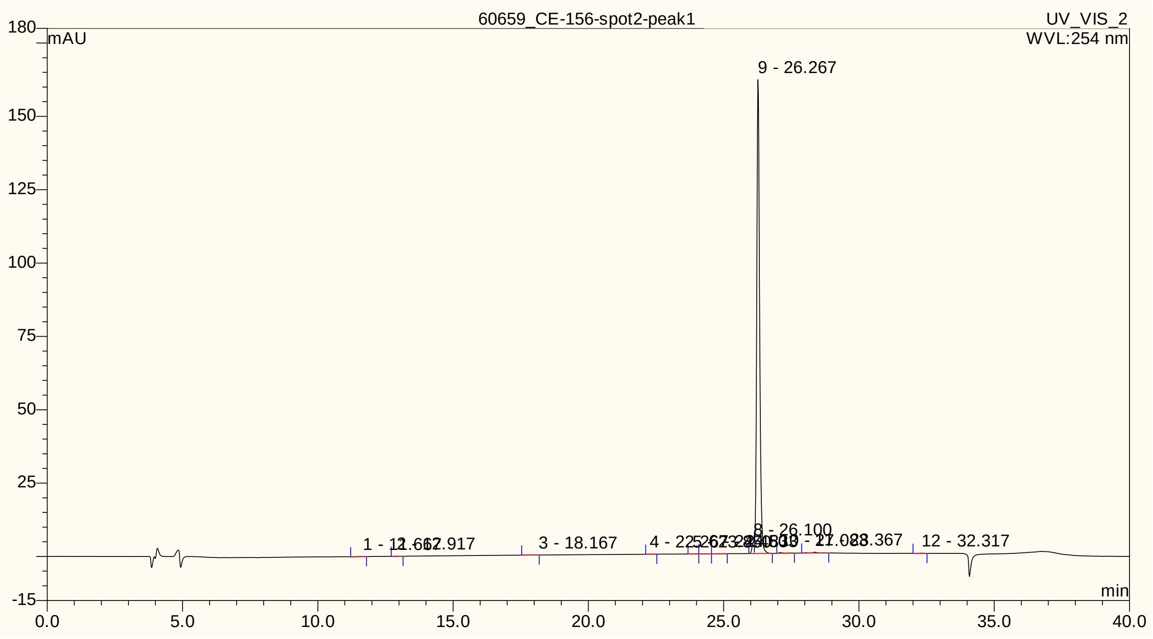

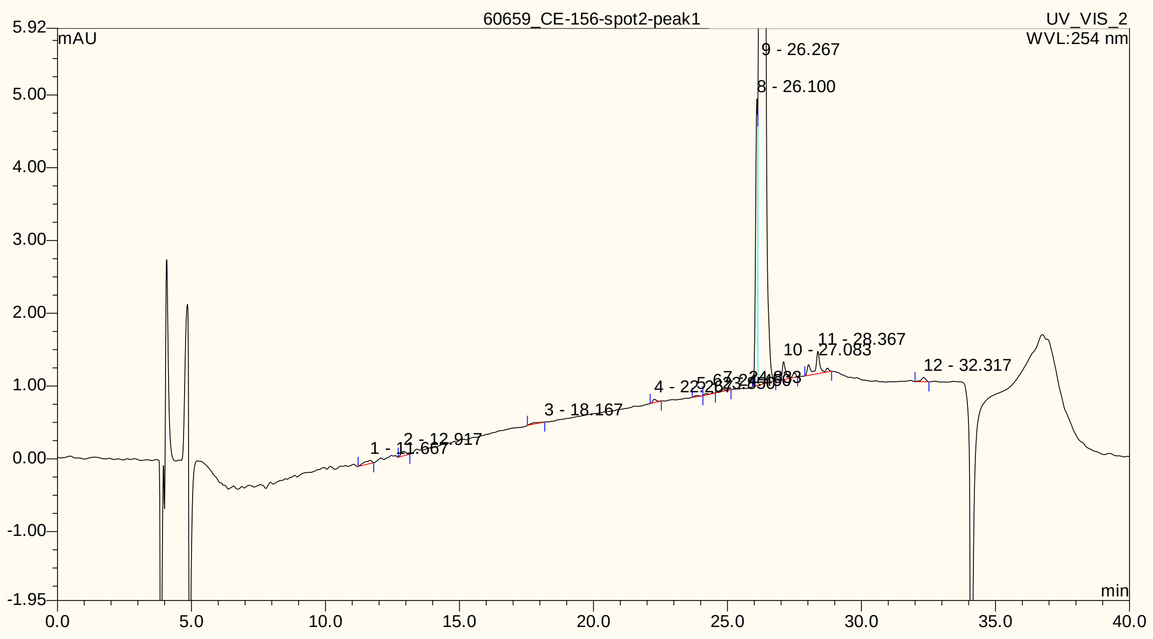

Retention Time: **26.27 min**

Relative Peak Area: **97.35 %**

Figure S84a. RP-Chromatogram of analogue **8u**.

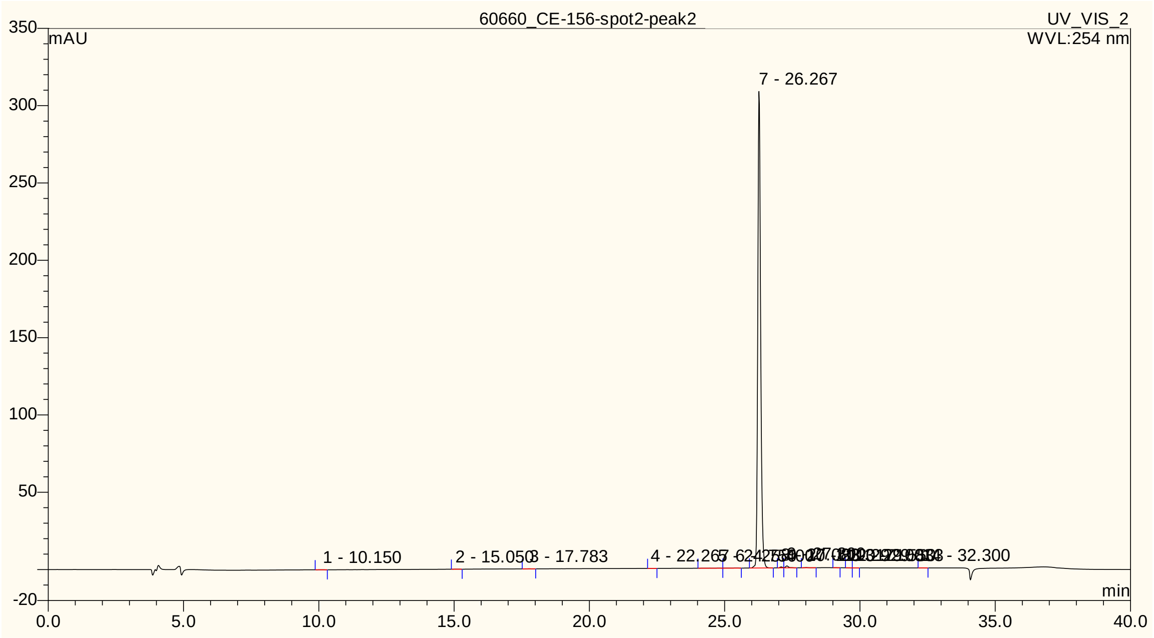

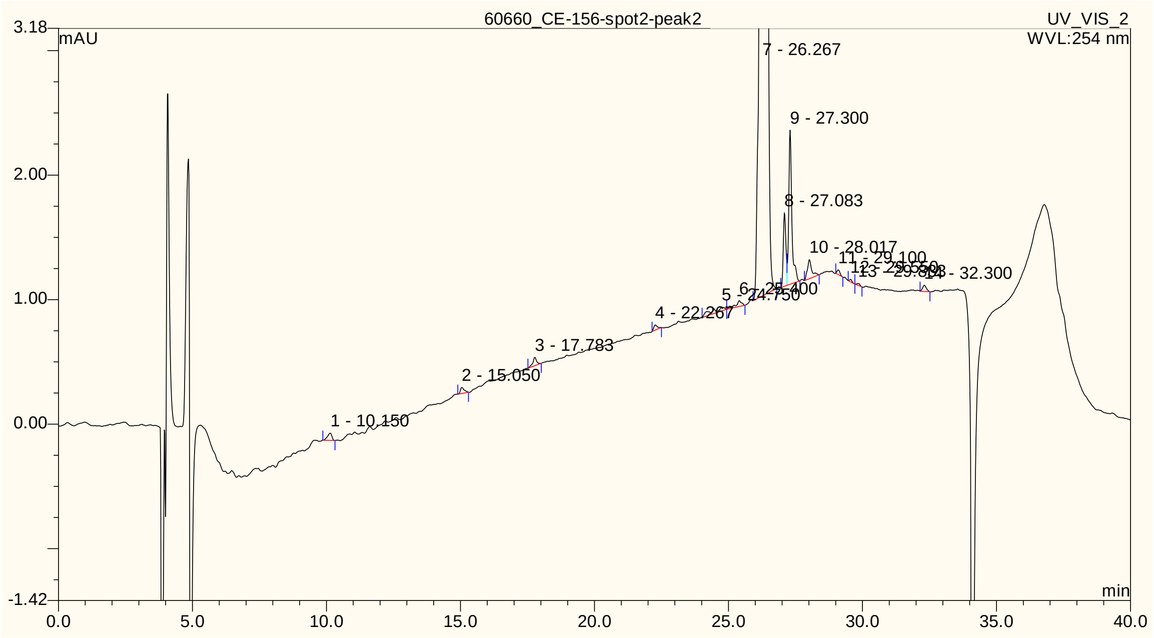

Retention Time: **26.27 min**

Relative Peak Area: **99.09 %**

Figure S1b. Chiral chromatogram of **5a**.

SHIMADZU LabSolutions

Analysis Report

<Sample Information>

Sample Name	: PN-20_Peak_1_		
Sample ID	: PN-20_Peak_1_		
Data Filename	: PN-20_Peak_1_01.lcd		
Method Filename	: Chiralpak_IA_50Hexan_50EtOAc_mind3bar_.lcm		
Batch Filename	:		
Vial #	: 1-1	Sample Type	: Unknown
Injection Volume	: 20 uL		
Date Acquired	: 11.02.2021 11:00:31	Acquired by	: HPLC - Natascha
Date Processed	: 15.02.2021 11:50:45	Processed by	: HPLC - Natascha

<Chromatogram>

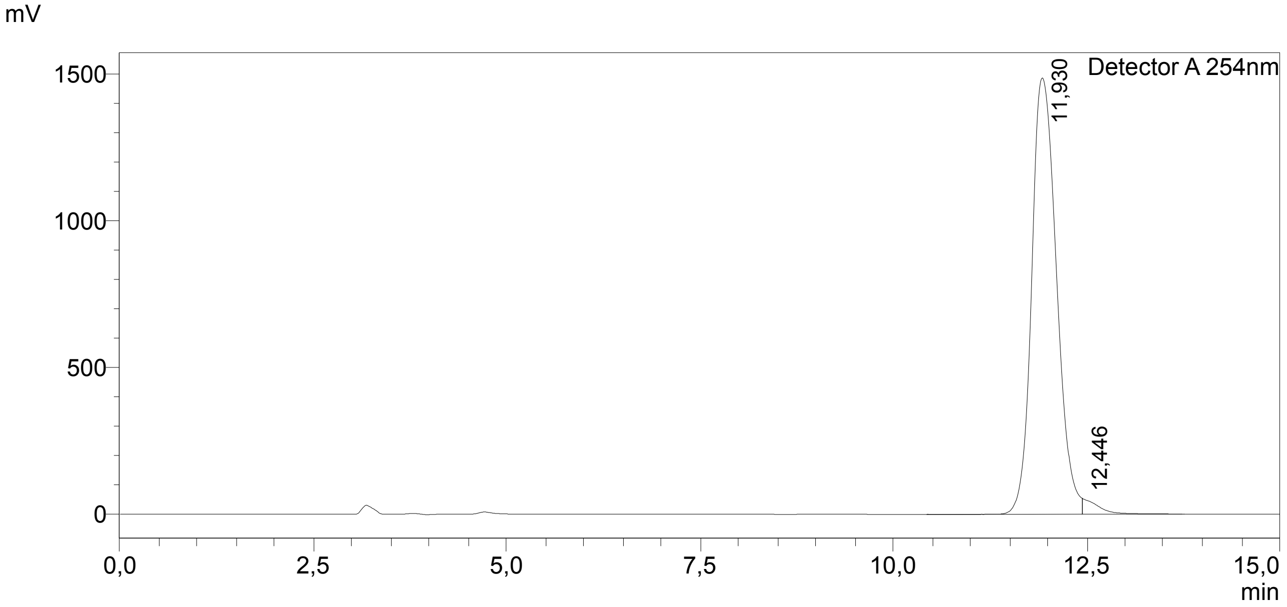

<Peak Table>

Detector A 254nm

Peak#	Ret. Time	Area	Area%
1	11,930	33321625	97,676
2	12,446	792929	2,324
Total		34114554	100,000

Figure S2b. Chiral chromatogram of **6a.**

SHIMADZU LabSolutions

Analysis Report

<Sample Information>

Sample Name	: PN-20_Peak_2_final_
Sample ID	: PN-20_Peak_2_final_
Data Filename	: PN-20_Peak_2_final_02.lcd
Method Filename	: Chiralpak_IA_50Hex_50EtOAc_.lcm
Batch Filename	:
Vial #	: 1-20
Sample Type	: Unknown
Injection Volume	: 20 uL
Date Acquired	: 19.03.2021 14:20:18
Acquired by	: HPLC - Natascha
Date Processed	: 19.03.2021 14:35:19
Processed by	: HPLC - Natascha

<Chromatogram>

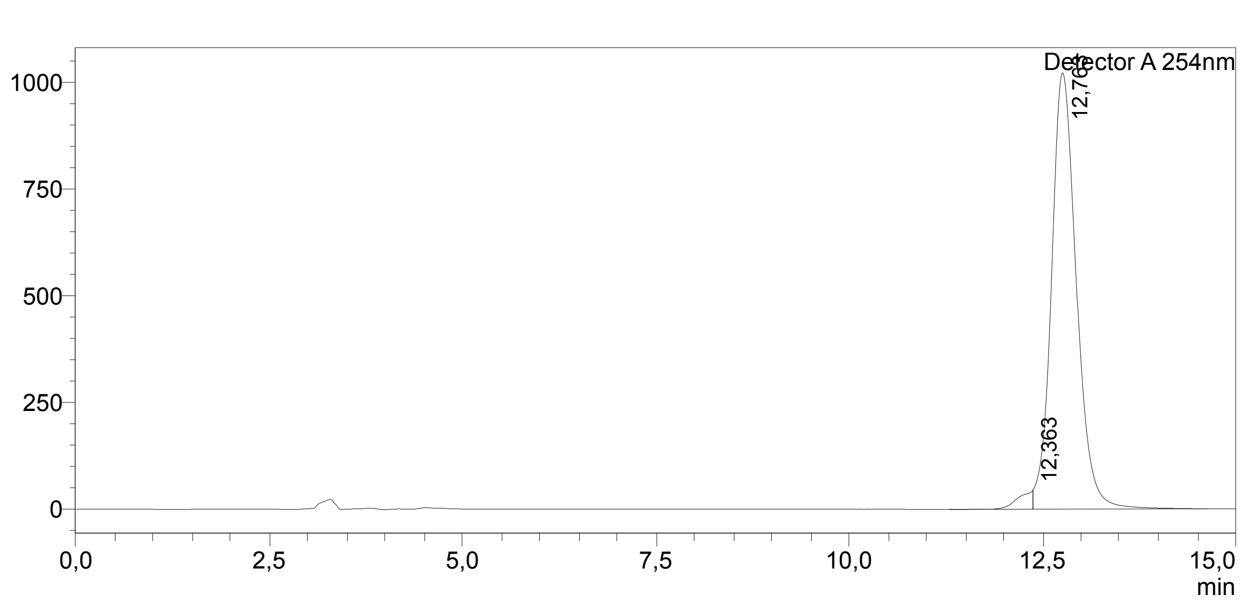

<Peak Table>

Detector A 254nm

Peak#	Ret. Time	Area	Area%
1	12,363	546571	2,322
2	12,763	22989200	97,678
Total		23535771	100,000

Figure S3b. Chiral chromatogram of **7a**.

SHIMADZU LabSolutions

Analysis Report

<Sample Information>

Sample Name	: PN-20-3_		
Sample ID	: PN-20-3_		
Data Filename	: PN-20-3_01.lcd		
Method Filename	: Chiralpak_IA_EtOAc_.lcm		
Batch Filename	:		
Vial #	: 1-2	Sample Type	: Unknown
Injection Volume	: 15 uL		
Date Acquired	: 14.07.2020 13:03:49	Acquired by	: HPLC - Natascha
Date Processed	: 13.10.2020 12:51:17	Processed by	: HPLC - Natascha

<Chromatogram>

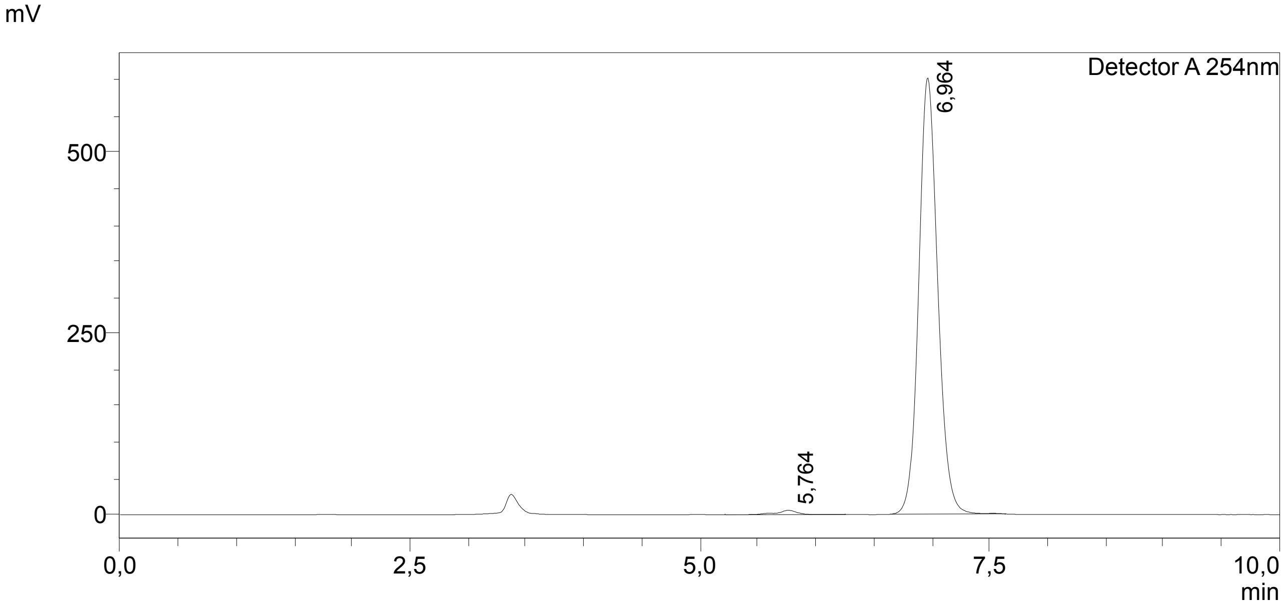

<Peak Table>

Detector A 254nm

Ret. Time	Area	Area%
5,764	72784	1,045
6,964	6889583	98,955
	6962367	100,000

Figure S4b. Chiral chromatogram of **8a**.

Analysis Report

<Sample Information>

Sample Name	: PN-20-4_B_		
Sample ID	: PN-20-4_B_		
Data Filename	: PN-20-4_B_03.lcd		
Method Filename	: Chiralpak_IA_EtOAc_.lcm		
Batch Filename	:		
Vial #	: 1-31	Sample Type	: Unknown
Injection Volume	: 15 uL		
Date Acquired	: 15.07.2020 13:30:38	Acquired by	: HPLC - Natascha
Date Processed	: 01.10.2020 16:58:31	Processed by	: HPLC - Natascha

<Chromatogram>

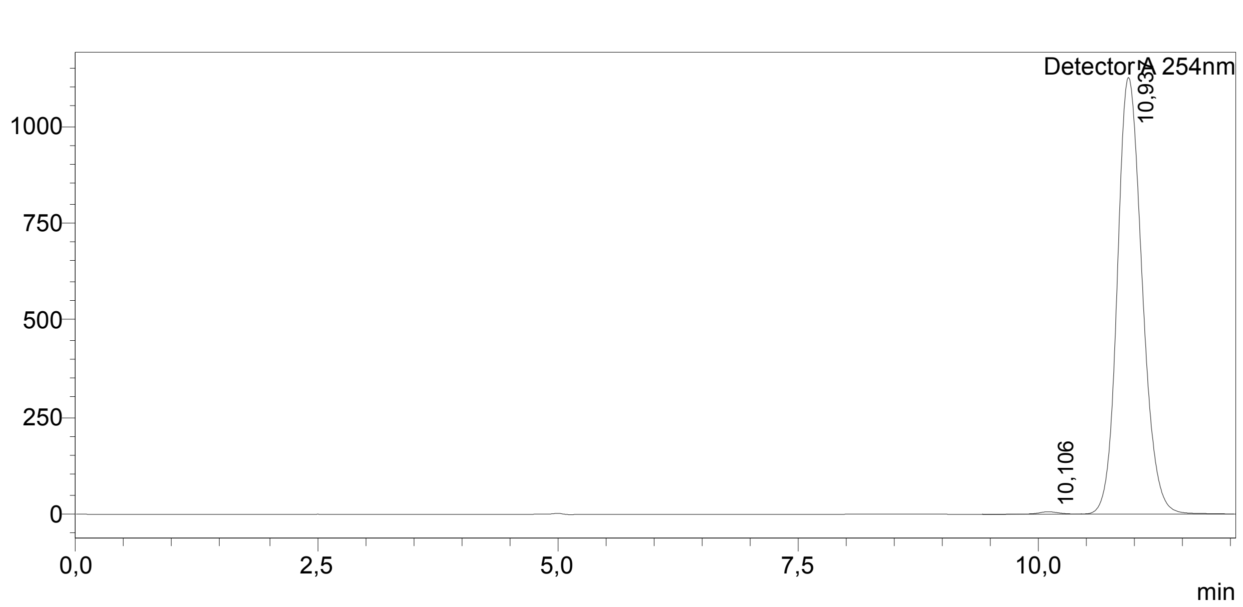

<Peak Table>

Detector A 254nm

Ret. Time	Area	Area%
10,106	96642	0,477
10,937	20183771	99,523
	20280413	100,000

Figure S5b. Chiral chromatogram of **5b**.

SHIMADZU LabSolutions

Analysis Report

<Sample Information>

Sample Name	: CE-148_spot_1_peak_1		
Sample ID	: CE-148_spot_1_peak_1		
Data Filename	: CE-148_spot_1_peak_1_01.lcd		
Method Filename	: Method_12.03.2019.lcm		
Batch Filename	:		
Vial #	: 1-1	Sample Type	: Unknown
Injection Volume	: 10 uL		
Date Acquired	: 12.03.2019 11:08:57	Acquired by	: HPLC - Natascha
Date Processed	: 12.03.2019 15:22:34	Processed by	: HPLC - Natascha

<Chromatogram>

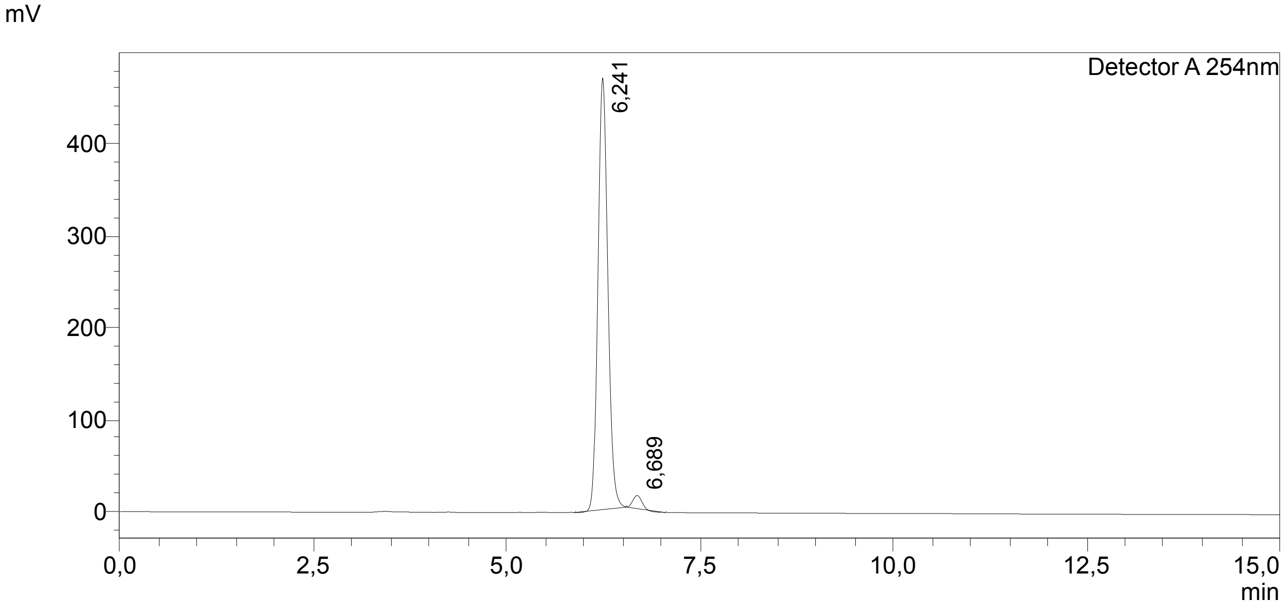

<Peak Table>

Detector A 254nm

Ret. Time	Area	Area%
6,241	4178035	97,568
6,689	104148	2,432
	4282183	100,000

Figure S6b. Chiral chromatogram of **6b**.

SHIMADZU LabSolutions

Analysis Report

<Sample Information>

Sample Name	: CE-148_spot_1_peak_2		
Sample ID	: CE-148_spot_1_peak_2		
Data Filename	: CE-148_spot_1_peak_2_01.lcd		
Method Filename	: Method_12.03.2019.lcm		
Batch Filename	:		
Vial #	: 1-2	Sample Type	: Unknown
Injection Volume	: 10 uL		
Date Acquired	: 12.03.2019 11:24:58	Acquired by	: HPLC - Natascha
Date Processed	: 12.03.2019 11:45:39	Processed by	: HPLC - Natascha

<Chromatogram>

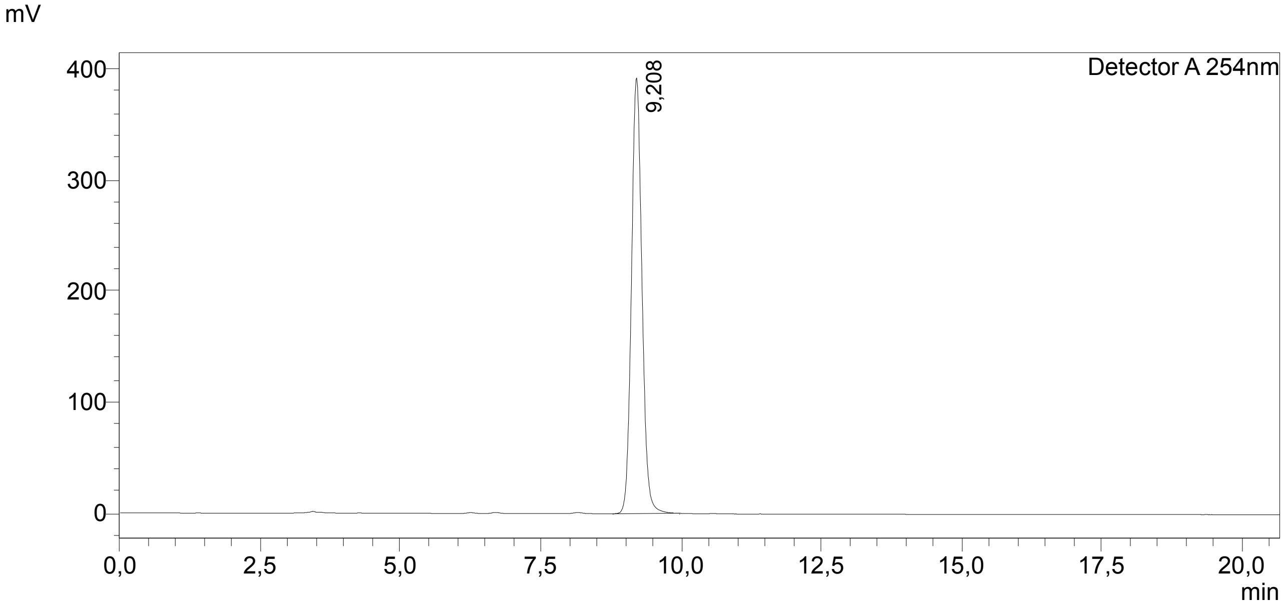

<Peak Table>

Detector A 254nm

Ret. Time	Area	Area%
9,208	5123309	100,000
	5123309	100,000

Figure S7b. Chiral chromatogram of **7b**.

SHIMADZU LabSolutions

Analysis Report

<Sample Information>

Sample Name	: CE-148_spot_2_peak_1		
Sample ID	: CE-148_spot_2_peak_1		
Data Filename	: CE-148_spot_2_peak_1_2nd_meas_01.lcd		
Method Filename	: Method_12.03.2019.lcm		
Batch Filename	:		
Vial #	: 1-3	Sample Type	: Unknown
Injection Volume	: 20 uL		
Date Acquired	: 12.03.2019 18:57:46	Acquired by	: HPLC - Natascha
Date Processed	: 12.03.2019 19:48:36	Processed by	: HPLC - Natascha

<Chromatogram>

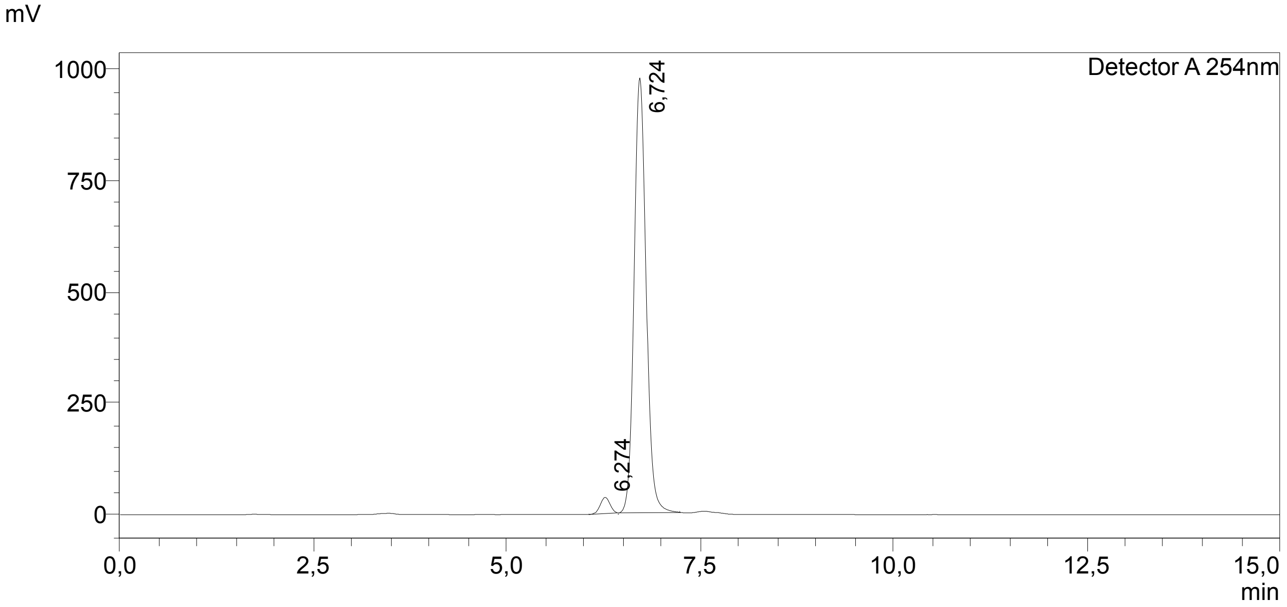

<Peak Table>

Detector A 254nm

Ret. Time	Area	Area%
6,274	318610	2,930
6,724	10555740	97,070
	10874350	100,000

Figure S8b. Chiral chromatogram of **8b**.

SHIMADZU LabSolutions

Analysis Report

<Sample Information>

Sample Name	: CE-148_spot_2_peak_2		
Sample ID	: CE-148_spot_2_peak_2		
Data Filename	: CE-148_spot_2_peak_2_01.lcd		
Method Filename	: Method_12.03.2019.lcm		
Batch Filename	:		
Vial #	: 1-4	Sample Type	: Unknown
Injection Volume	: 10 uL		
Date Acquired	: 12.03.2019 12:04:51	Acquired by	: HPLC - Natascha
Date Processed	: 12.03.2019 15:25:22	Processed by	: HPLC - Natascha

<Chromatogram>

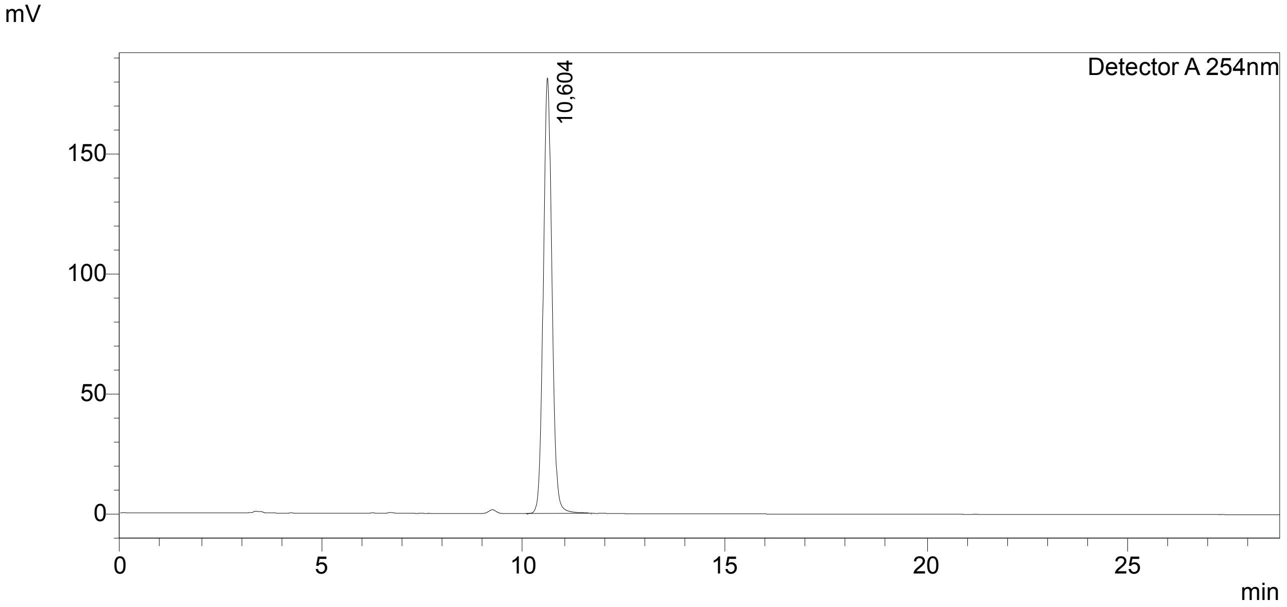

<Peak Table>

Detector A 254nm

Ret. Time	Area	Area%
10,604	2716739	100,000
	2716739	100,000

Figure S9b. Chiral chromatogram of **5c**.

Analysis Report

<Sample Information>

Sample Name : CE_155N_spot_1_peak_1_
Sample ID : CE_155N_spot_1_peak_1_
Data Filename : CE_155N_spot_1_peak_1_.lcd
Method Filename : 100%_EtOAc.lcm
Batch Filename :
Vial # : 1-21
Sample Type : Unknown
Injection Volume : 15 uL
Date Acquired : 09.08.2018 19:49:44
Acquired by : HPLC - Natascha
Date Processed : 09.08.2018 20:04:45
Processed by : HPLC - Natascha

<Chromatogram>

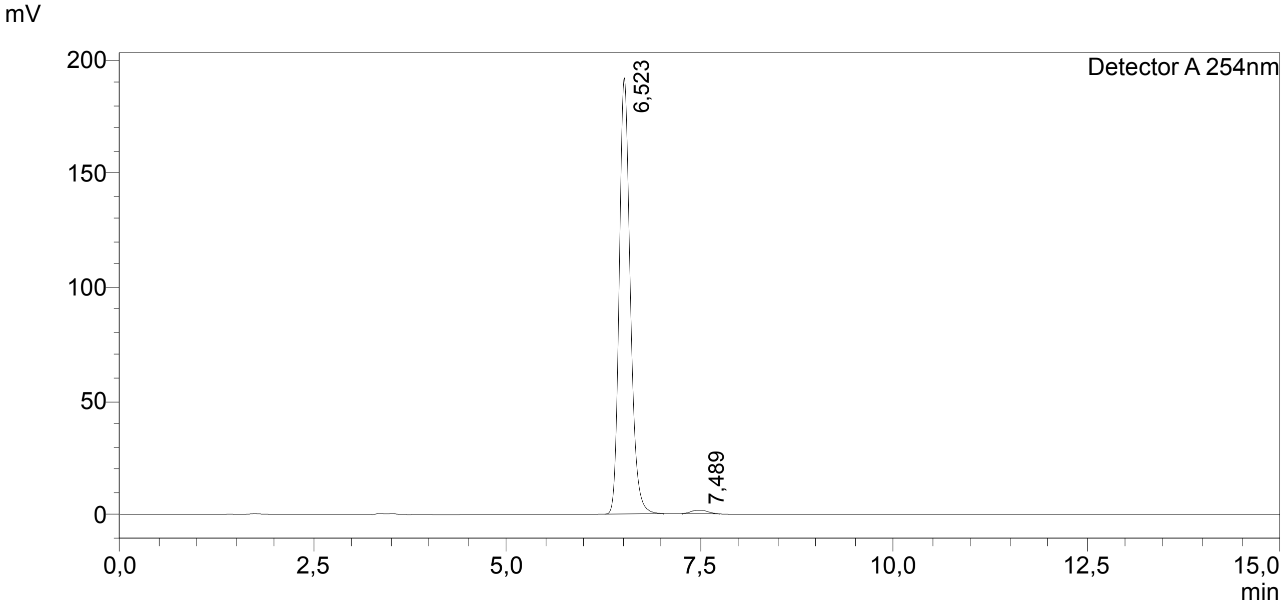

<Peak Table>

Detector A 254nm

Ret. Time	Area	Area%
6,523	1911721	98,866
7,489	21928	1,134
	1933649	100,000

Figure S10b. Chiral chromatogram of **6c**.

SHIMADZU LabSolutions

Analysis Report

<Sample Information>

Sample Name	: CE-155_Spot1_Peak2_		
Sample ID	: CE-155_Spot1_Peak2_		
Data Filename	: CE-155_Spot1_Peak2_01.lcd		
Method Filename	: Chiralpak_IA_100EtOAc_.lcm		
Batch Filename	:		
Vial #	: 1-1	Sample Type	: Unknown
Injection Volume	: 20 uL		
Date Acquired	: 29.04.2022 15:38:54	Acquired by	: HPLC - Natascha
Date Processed	: 29.04.2022 15:53:55	Processed by	: HPLC - Natascha

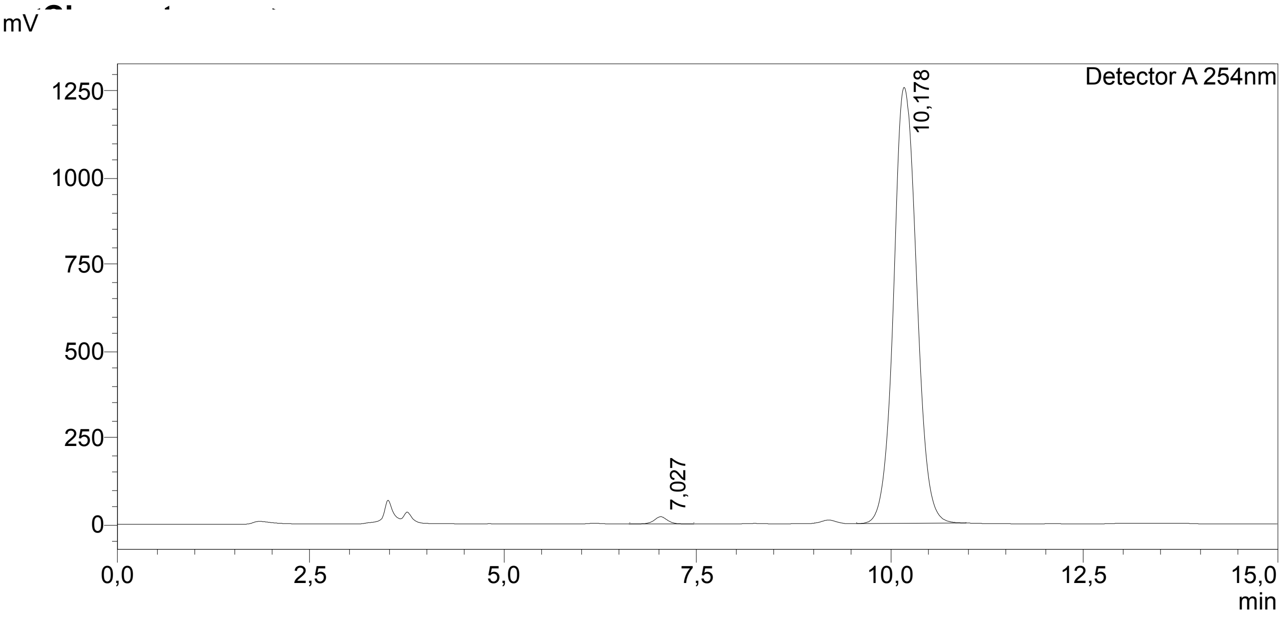

<Peak Table>

Detector A 254nm

Peak#	Ret. Time	Area	Area%
1	7,027	248765	0,947
2	10,178	26020184	99,053
Total		26268949	100,000

Figure S11b. Chiral chromatogram of **7c**.

SHIMADZU LabSolutions

Analysis Report

<Sample Information>

Sample Name	: CE_155_spot_2_peak_1_		
Sample ID	: CE_155_spot_2_peak_1_		
Data Filename	: CE_155_spot_2_peak_1_01.lcd		
Method Filename	: 100%_EtOAc.lcm		
Batch Filename	:		
Vial #	: 1-21	Sample Type	: Unknown
Injection Volume	: 15 uL		
Date Acquired	: 09.08.2018 20:21:30	Acquired by	: HPLC - Natascha
Date Processed	: 09.08.2018 20:36:31	Processed by	: HPLC - Natascha

<Chromatogram>

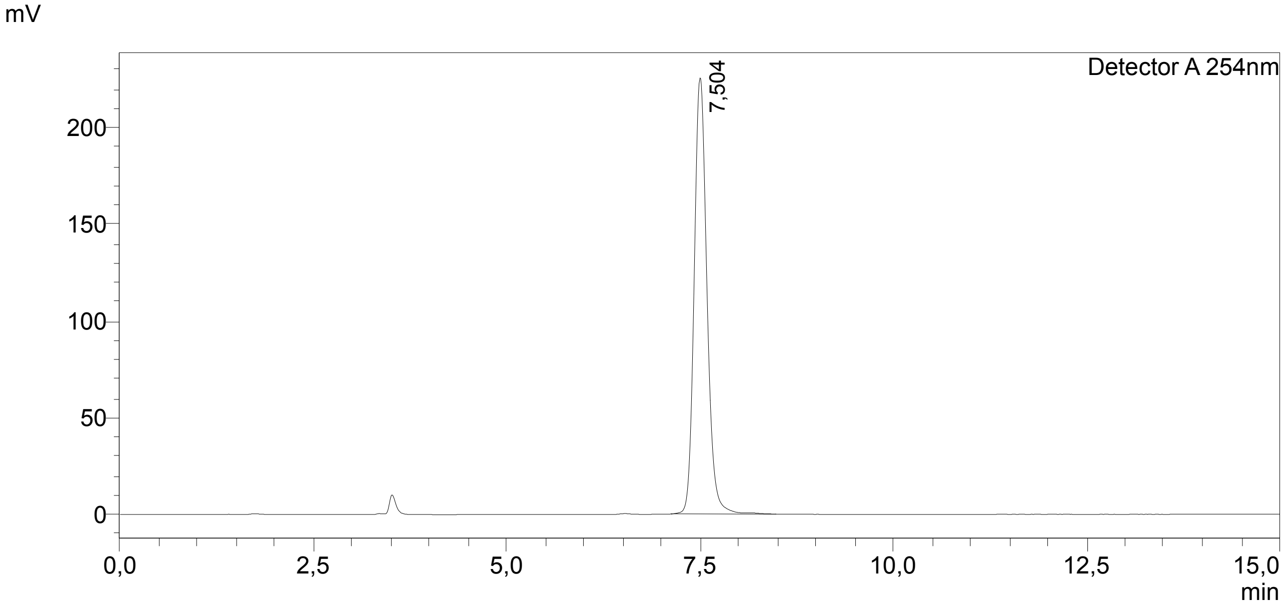

<Peak Table>

Detector A 254nm

Ret. Time	Area	Area%
7,504	2553559	100,000
	2553559	100,000

Figure S12b. Chiral chromatogram of **8c**.

SHIMADZU LabSolutions

Analysis Report

<Sample Information>

Sample Name : CE_155_spot_2_peak_2_
Sample ID : CE_155_spot_2_peak_2_
Data Filename : CE_155_spot_2_peak_2_01.lcd
Method Filename : 100%_EtOAc.lcm
Batch Filename :
Vial # : 1-22
Sample Type : Unknown
Injection Volume : 15 uL
Date Acquired : 09.08.2018 20:37:27
Acquired by : HPLC - Natascha
Date Processed : 09.08.2018 20:52:28
Processed by : HPLC - Natascha

<Chromatogram>

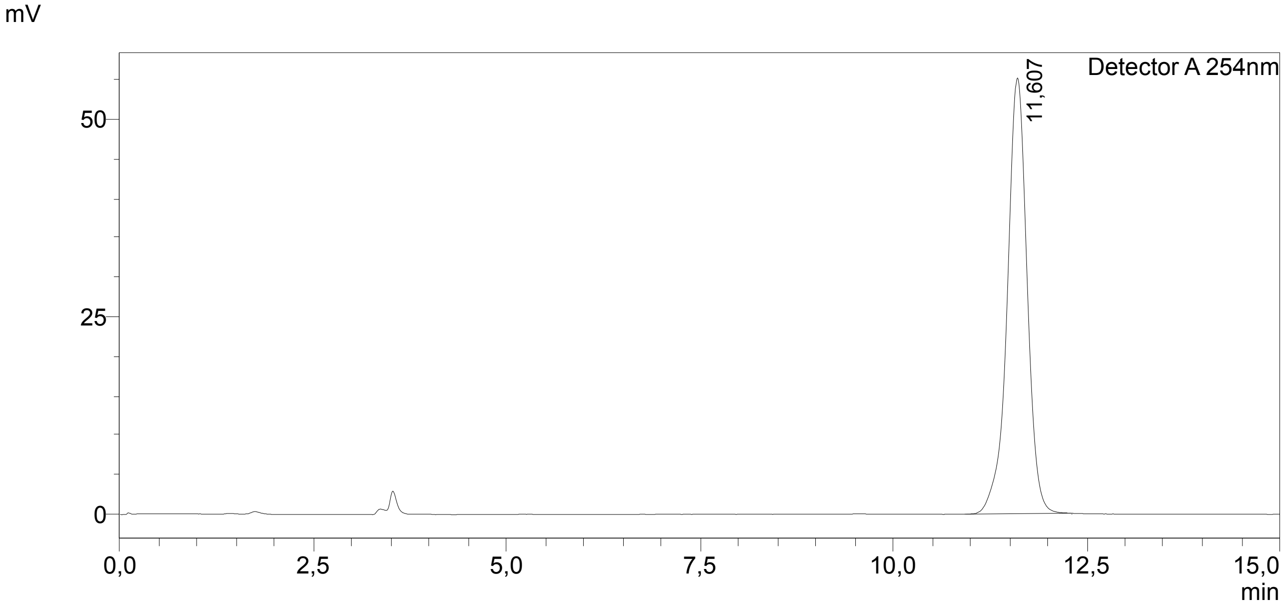

<Peak Table>

Detector A 254nm

Ret. Time	Area	Area%
11,607	977301	100,000
	977301	100,000

Figure S13b. Chiral chromatogram of **5d**.

Analysis Report

<Sample Information>

Sample Name	: PN-18_Spot1_Peak1_		
Sample ID	: PN-18_Spot1_Peak1_		
Data Filename	: PN-18_Spot1_Peak1_01.lcd		
Method Filename	: Chiralpak_IA_100EtOAc_.lcm		
Batch Filename	:		
Vial #	: 1-1	Sample Type	: Unknown
Injection Volume	: 1 uL		
Date Acquired	: 29.04.2022 15:24:36	Acquired by	: HPLC - Natascha
Date Processed	: 29.04.2022 15:37:07	Processed by	: HPLC - Natascha

<Chromatogram>

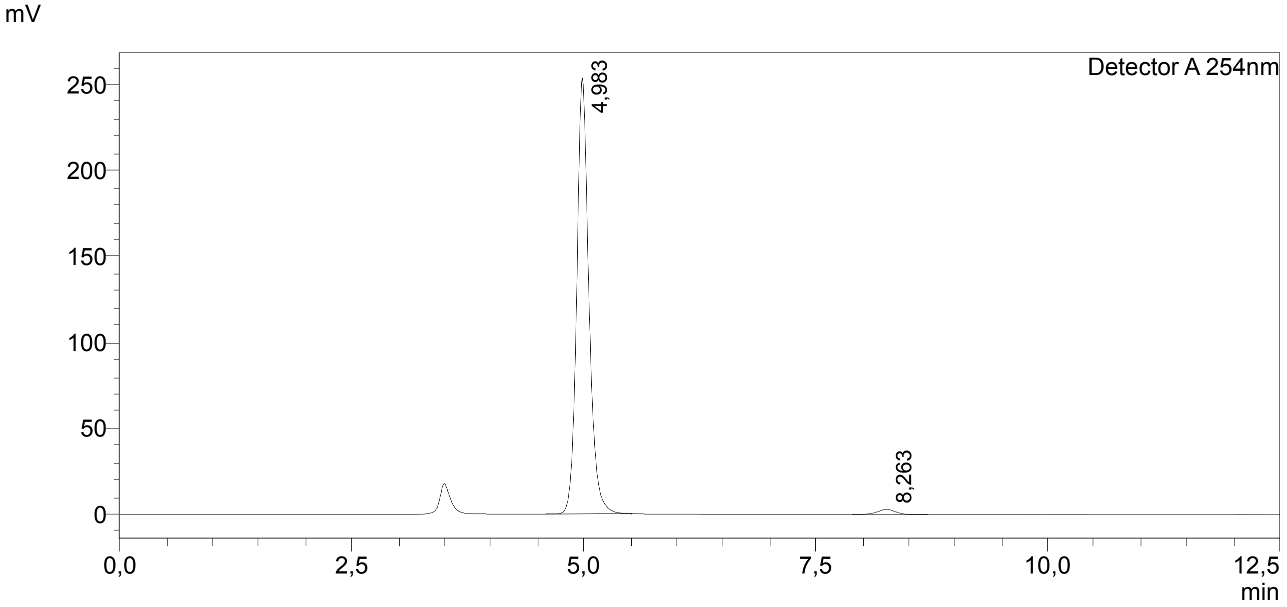

<Peak Table>

Detector A 254nm

Peak#	Ret. Time	Area	Area%
1	4,983	2311423	98,257
2	8,263	41011	1,743
Total		2352434	100,000

Figure S14b. Chiral chromatogram of **6d**.

SHIMADZU LabSolutions

Analysis Report

<Sample Information>

Sample Name	: PN-18_Peak_2_		
Sample ID	: PN-18_Peak_2_		
Data Filename	: PN-18_Peak_2_01.lcd		
Method Filename	: Chiralpak_IA_80HexinC_20THFinD_.lcm		
Batch Filename	:		
Vial #	: 1-18	Sample Type	: Unknown
Injection Volume	: 10 uL		
Date Acquired	: 16.03.2021 11:18:26	Acquired by	: HPLC - Natascha
Date Processed	: 16.03.2021 11:48:26	Processed by	: HPLC - Natascha

<Chromatogram>

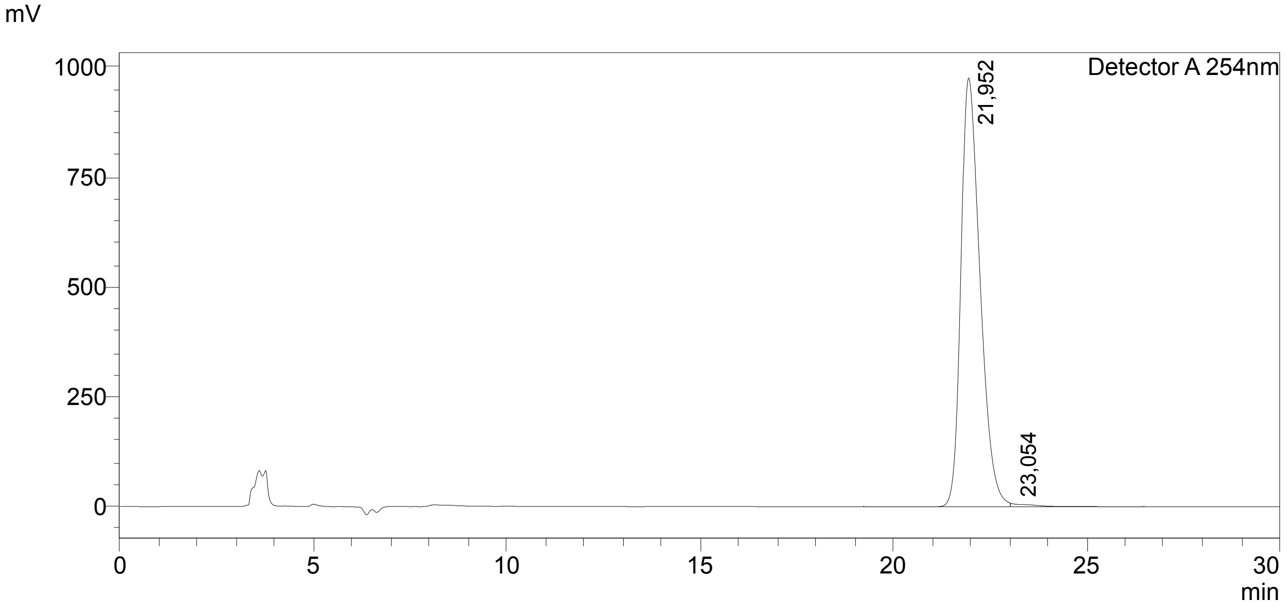

<Peak Table>

Detector A 254nm

Peak#	Ret. Time	Area	Area%
1	21,952	33537115	99,089
2	23,054	308425	0,911
Total		33845540	100,000

Figure S15b. Chiral chromatogram of **7d**.

SHIMADZU LabSolutions

Analysis Report

<Sample Information>

Sample Name : PN-18_Peak_3_final_
Sample ID : PN-18_Peak_3_final_
Data Filename : PN-18_Peak_3_final_01.lcd
Method Filename : Chiralpak_IA_80HexinC_20THFinD_.lcm
Batch Filename :
Vial # : 1-21
Sample Type : Unknown
Injection Volume : 20 uL
Date Acquired : 19.03.2021 09:41:02
Acquired by : HPLC - Natascha
Date Processed : 19.03.2021 10:11:04
Processed by : HPLC - Natascha

<Chromatogram>

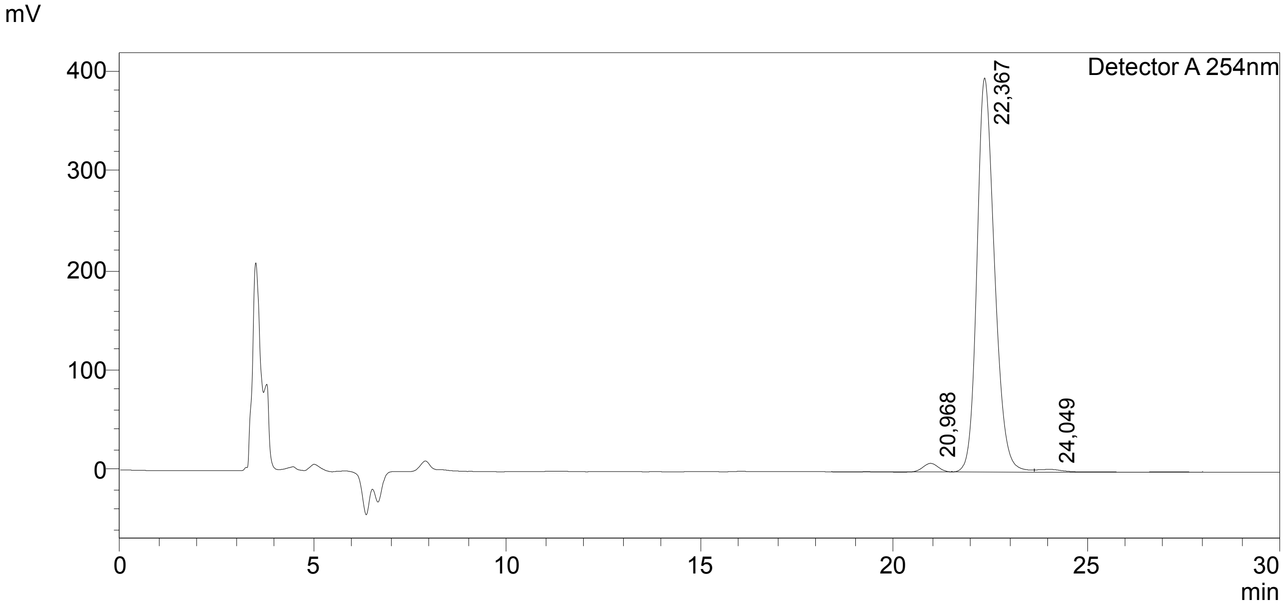

<Peak Table>

Detector A 254nm

Peak#	Ret. Time	Area	Area%
1	20,968	240528	1,860
2	22,367	12537365	96,957
3	24,049	152921	1,183
Total		12930815	100,000

Figure S16b. Chiral chromatogram of **8d**.

SHIMADZU LabSolutions

Analysis Report

<Sample Information>

Sample Name	: PN-18-4_		
Sample ID	: PN-18-4_		
Data Filename	: PN-18-4_01.lcd		
Method Filename	: Chiralpak_IA_EtOAc_.lcm		
Batch Filename	:		
Vial #	: 1-33	Sample Type	: Unknown
Injection Volume	: 15 uL		
Date Acquired	: 09.07.2020 12:48:49	Acquired by	: HPLC - Natascha
Date Processed	: 01.10.2020 16:49:10	Processed by	: HPLC - Natascha

<Chromatogram>

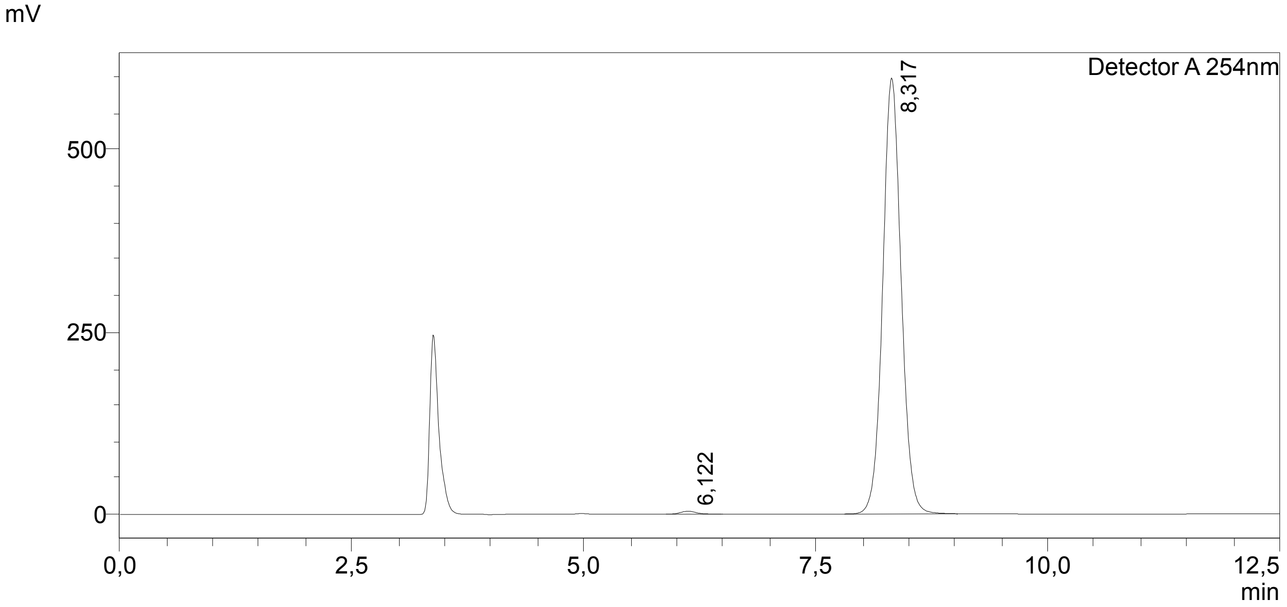

<Peak Table>

Detector A 254nm

Ret. Time	Area	Area%
6,122	46300	0,556
8,317	8274233	99,444
	8320533	100,000

Figure S17b. Chiral chromatogram of **5e**.

Analysis Report

<Sample Information>

Sample Name	: CE_167_spot_1_peak_1_final		
Sample ID	: CE_167_spot_1_peak_1_final		
Data Filename	: 21.08.2020.lcd		
Method Filename	: 100%_EtOAc.lcm		
Batch Filename	:		
Vial #	: 1-21	Sample Type	: Unknown
Injection Volume	: 15 uL		
Date Acquired	: 21.08.2018 16:06:14	Acquired by	: HPLC - Natascha
Date Processed	: 21.08.2018 16:39:24	Processed by	: HPLC - Natascha

<Chromatogram>

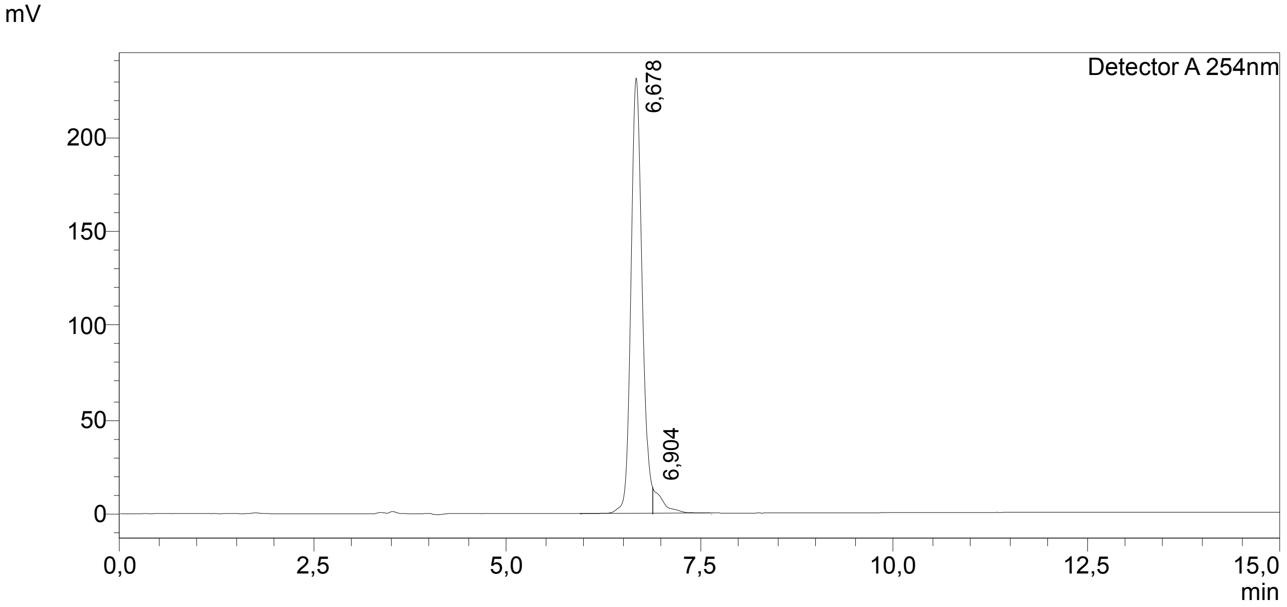

<Peak Table>

Detector A 254nm

Ret. Time	Area	Area%
6,678	2341299	95,319
6,904	114971	4,681
	2456269	100,000

Figure S18b. Chiral chromatogram of **6e**.

SHIMADZU LabSolutions

Analysis Report

<Sample Information>

Sample Name	: CE_167_spot_1_peak_2_final		
Sample ID	: CE_167_spot_1_peak_2_final		
Data Filename	: CE_167_spot_1_peak_2_final01.lcd		
Method Filename	: 100%_EtOAc.lcm		
Batch Filename	:		
Vial #	: 1-22	Sample Type	: Unknown
Injection Volume	: 15 uL		
Date Acquired	: 21.08.2018 16:23:42	Acquired by	: HPLC - Natascha
Date Processed	: 21.08.2018 16:38:43	Processed by	: HPLC - Natascha

<Chromatogram>

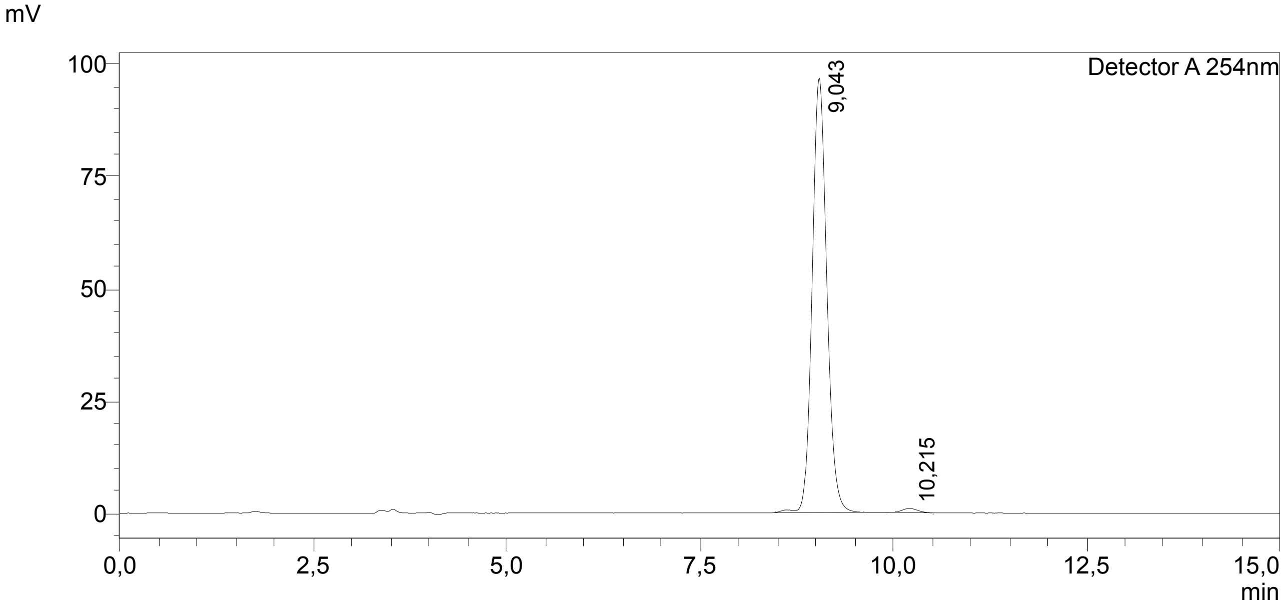

<Peak Table>

Detector A 254nm

Ret. Time	Area	Area%
9,043	1278506	99,124
10,215	11300	0,876
	1289806	100,000

Figure S19b. Chiral chromatogram of **7e**.

SHIMADZU LabSolutions

Analysis Report

<Sample Information>

Sample Name	: CE_167_spot_2_peak_1_final		
Sample ID	: CE_167_spot_2_peak_1_final		
Data Filename	: CE_167_spot_2_peak_1_final01.lcd		
Method Filename	: 100%_EtOAc.lcm		
Batch Filename	:		
Vial #	: 1-21	Sample Type	: Unknown
Injection Volume	: 15 uL		
Date Acquired	: 21.08.2018 16:47:59	Acquired by	: HPLC - Natascha
Date Processed	: 21.08.2018 17:03:00	Processed by	: HPLC - Natascha

<Chromatogram>

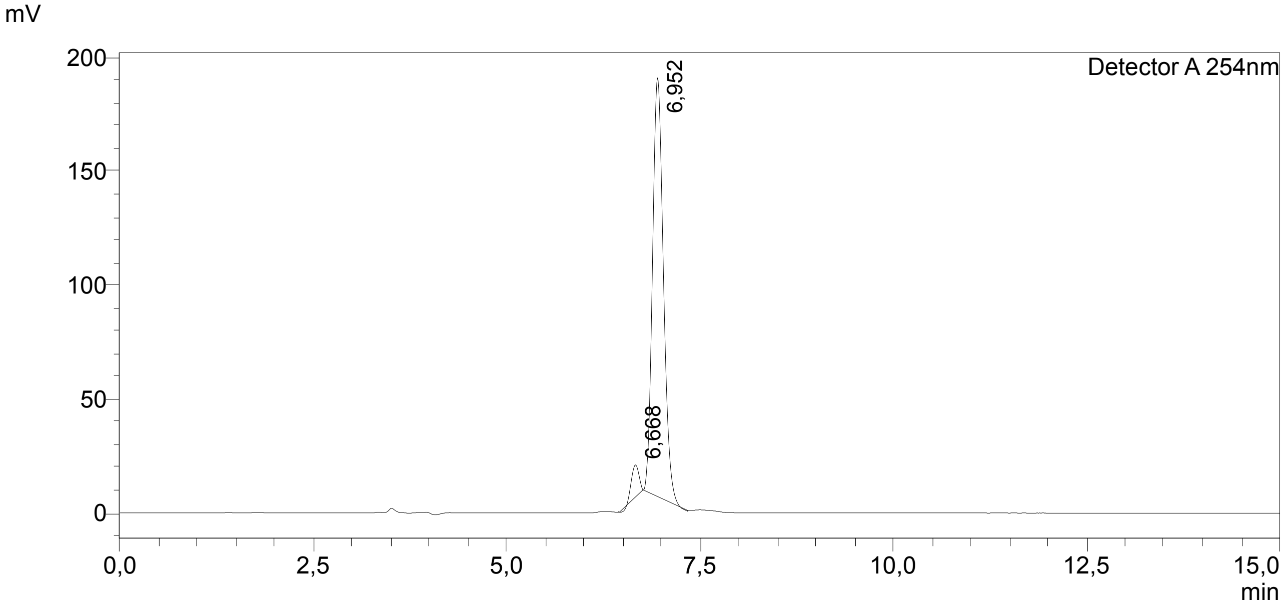

<Peak Table>

Detector A 254nm

Ret. Time	Area	Area%
6,668	95916	5,040
6,952	1807345	94,960
	1903261	100,000

Figure S20b. Chiral chromatogram of **8e**.

SHIMADZU LabSolutions

Analysis Report

<Sample Information>

Sample Name : CE-167_Spot2_Peak2_
Sample ID : CE-167_Spot2_Peak2_
Data Filename : CE-167_Spot2_Peak2_01.lcd
Method Filename : Chiralpak_IA_100EtOAc_.lcm
Batch Filename :
Vial # : 1-1
Sample Type : Unknown
Injection Volume : 17 uL
Date Acquired : 29.04.2022 12:02:31
Acquired by : HPLC - Natascha
Date Processed : 29.04.2022 12:17:32
Processed by : HPLC - Natascha

<Chromatogram>

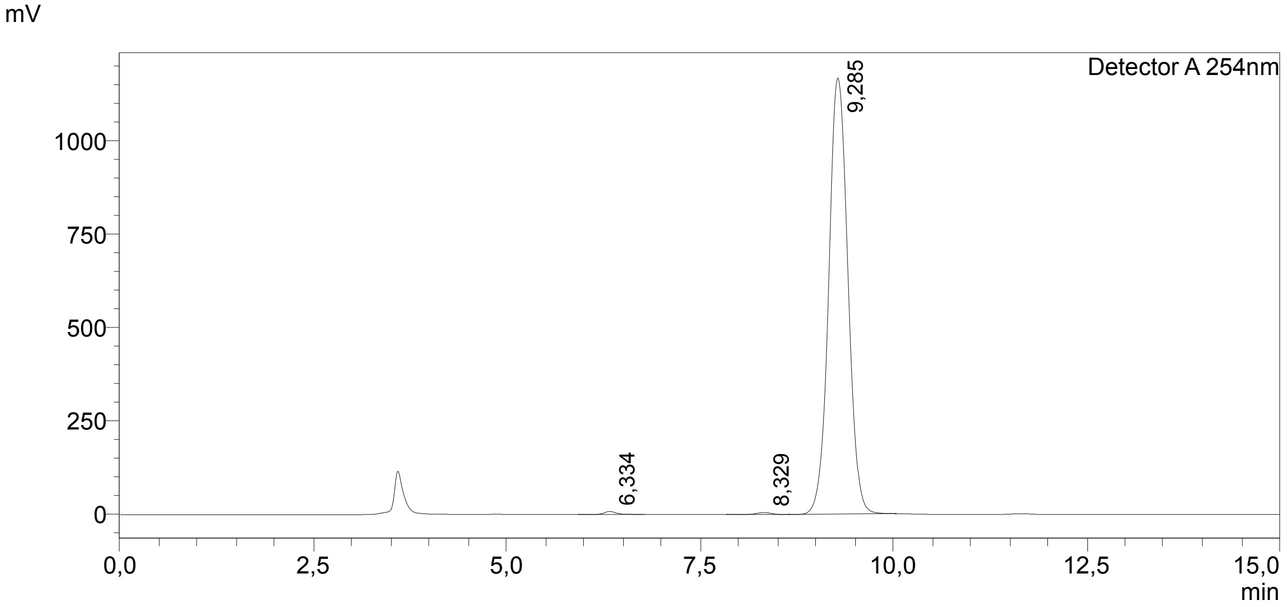

<Peak Table>

Detector A 254nm

Peak#	Ret. Time	Area	Area%
1	6,334	92866	0,454
2	8,329	75040	0,367
3	9,285	20291438	99,179
Total		20459344	100,000

Figure S21b. Chiral chromatogram of **5f**.

SHIMADZU LabSolutions

Analysis Report

<Sample Information>

Sample Name	: CE_140_mix_1_peak_1__final_check		
Sample ID	: CE_140_mix_1_peak_1__final_chec		
Data Filename	: CE_140_mix_1_peak_1__final_check01.lcd		
Method Filename	: 100%_EtOAc.lcm		
Batch Filename	:		
Vial #	: 1-21	Sample Type	: Unknown
Injection Volume	: 15 uL		
Date Acquired	: 25.07.2018 14:52:20	Acquired by	: HPLC - Natascha
Date Processed	: 27.07.2018 17:46:51	Processed by	: HPLC - Natascha

<Chromatogram>

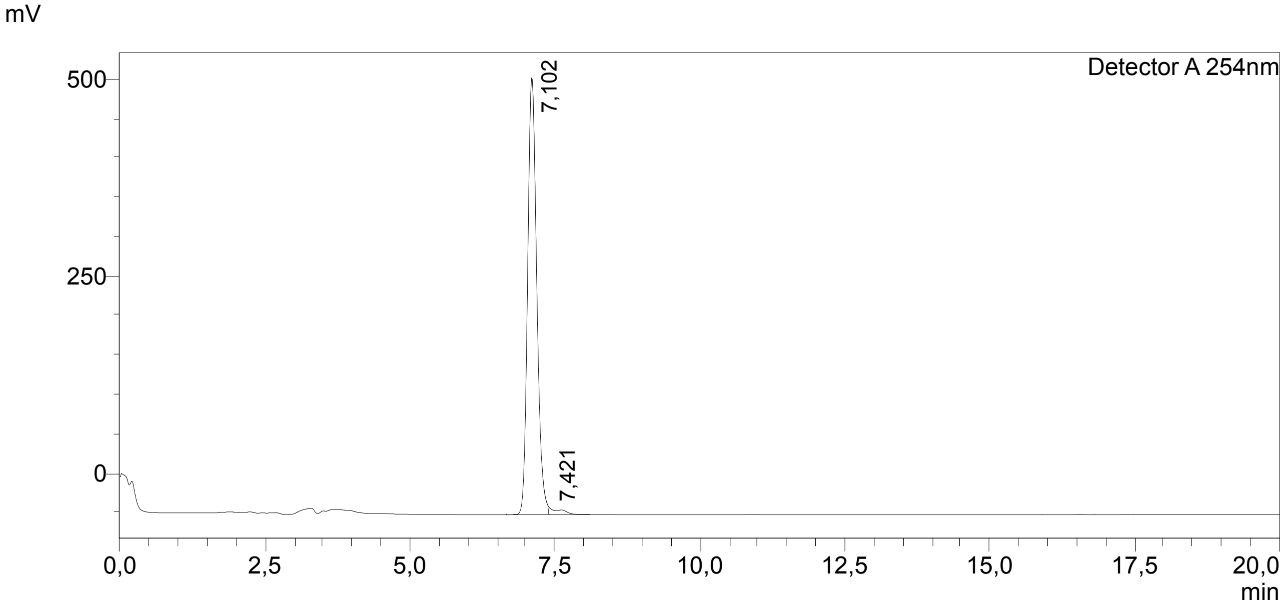

<Peak Table>

Detector A 254nm

Ret. Time	Area	Area%
7,102	6100611	98,222
7,421	110429	1,778
	6211039	100,000

Figure S22b. Chiral chromatogram of **6f**.

SHIMADZU LabSolutions

Analysis Report

<Sample Information>

Sample Name	: CE_140_mix_1_peak_2__final_check		
Sample ID	: CE_140_mix_1_peak_2__final_chec		
Data Filename	: CE_140_mix_1_peak_2__final_check01.lcd		
Method Filename	: 100%_EtOAc.lcm		
Batch Filename	:		
Vial #	: 1-22	Sample Type	: Unknown
Injection Volume	: 15 uL		
Date Acquired	: 25.07.2018 15:18:00	Acquired by	: HPLC - Natascha
Date Processed	: 25.07.2018 15:38:01	Processed by	: HPLC - Natascha

<Chromatogram>

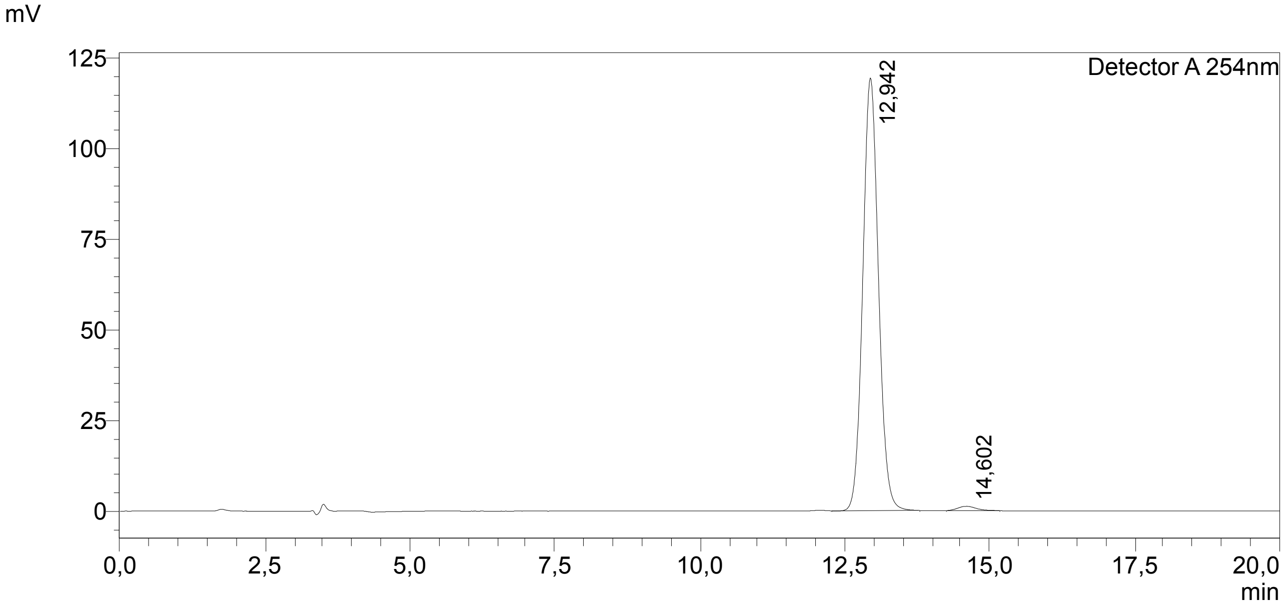

<Peak Table>

Detector A 254nm

Ret. Time	Area	Area%
12,942	2248794	98,859
14,602	25965	1,141
	2274759	100,000

Figure S23b. Chiral chromatogram of **7f.**

SHIMADZU LabSolutions

Analysis Report

<Sample Information>

Sample Name	: CE_140_spot_2_peak_1__final_check		
Sample ID	: CE_140_spot_2_peak_1__final_che		
Data Filename	: CE_140_spot_2_peak_1__final_check01.lcd		
Method Filename	: 100%_EtOAc.lcm		
Batch Filename	:		
Vial #	: 1-21	Sample Type	: Unknown
Injection Volume	: 15 uL		
Date Acquired	: 25.07.2018 17:21:51	Acquired by	: HPLC - Natascha
Date Processed	: 25.07.2018 17:41:52	Processed by	: HPLC - Natascha

<Chromatogram>

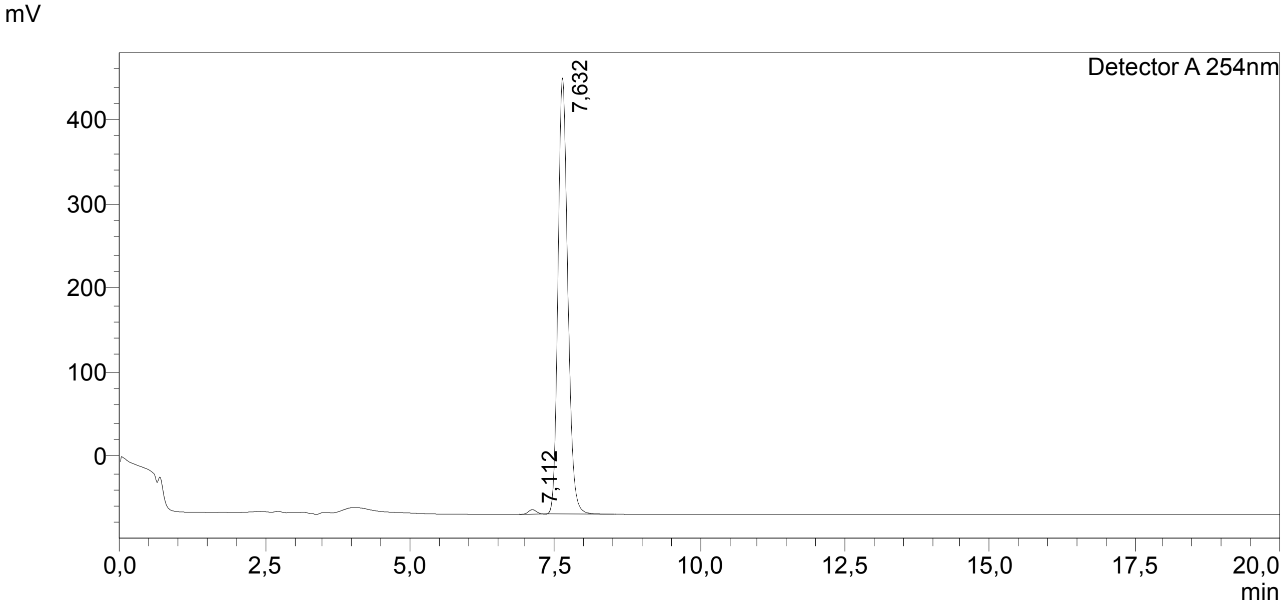

<Peak Table>

Detector A 254nm

Ret. Time	Area	Area%
7,112	53208	0,863
7,632	6110382	99,137
	6163591	100,000

Figure S24b. Chiral chromatogram of **8f**.

SHIMADZU LabSolutions

Analysis Report

<Sample Information>

Sample Name	: CE_140_spot_2_peak_2__final_check		
Sample ID	: CE_140_spot_2_peak_2__final_che		
Data Filename	: CE_140_spot_2_peak_2__final_check01.lcd		
Method Filename	: 100%_EtOAc.lcm		
Batch Filename	:		
Vial #	: 1-22	Sample Type	: Unknown
Injection Volume	: 15 uL		
Date Acquired	: 25.07.2018 17:45:42	Acquired by	: HPLC - Natascha
Date Processed	: 25.07.2018 18:05:43	Processed by	: HPLC - Natascha

<Chromatogram>

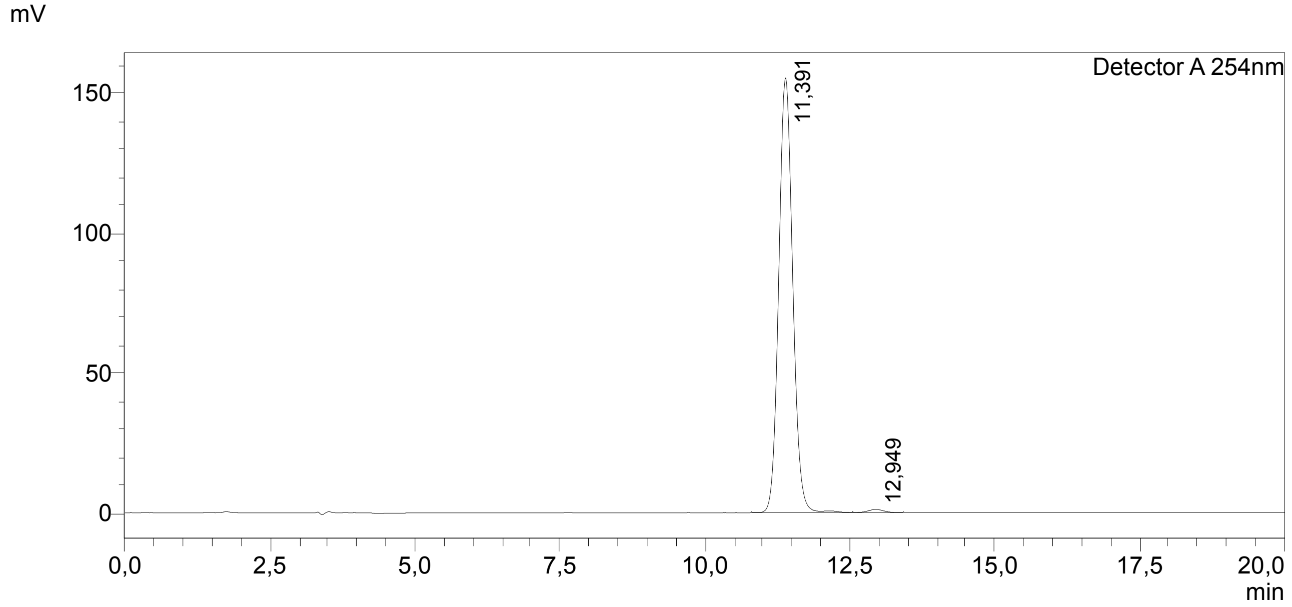

<Peak Table>

Detector A 254nm

Ret. Time	Area	Area%
11,391	2598260	99,224
12,949	20327	0,776
	2618587	100,000

Figure S25b. Chiral chromatogram of **5g**.

SHIMADZU LabSolutions

Analysis Report

<Sample Information>

Sample Name	: PN-14-1_		
Sample ID	: PN-14-1_		
Data Filename	: PN-14-1_01.lcd		
Method Filename	: Chiralpak_IA_EtOAc_.lcm		
Batch Filename	:		
Vial #	: 1-22	Sample Type	: Unknown
Injection Volume	: 15 uL		
Date Acquired	: 07.07.2020 15:33:48	Acquired by	: HPLC - Natascha
Date Processed	: 01.10.2020 16:42:51	Processed by	: HPLC - Natascha

<Chromatogram>

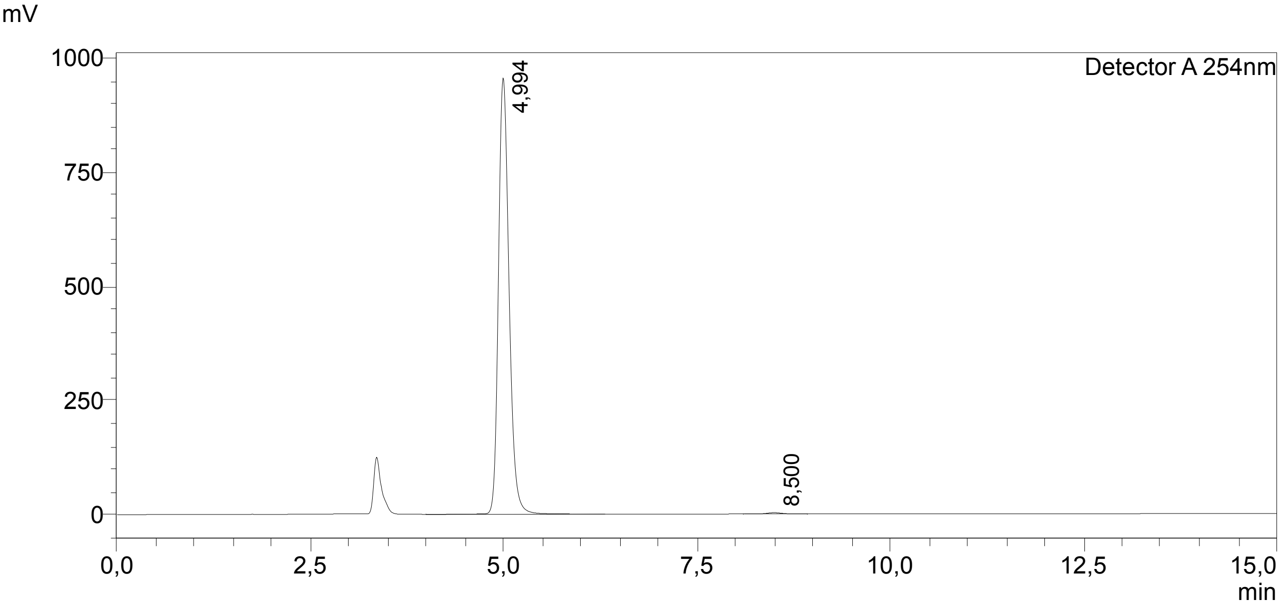

<Peak Table>

Detector A 254nm

Ret. Time	Area	Area%
4,994	9202879	99,593
8,500	37639	0,407
	9240518	100,000

Figure S26b. Chiral chromatogram of **6g**.

Analysis Report

<Sample Information>

Sample Name	: PN-14_Peak_2_		
Sample ID	: PN-14_Peak_2_		
Data Filename	: PN-14_Peak_2_01.lcd		
Method Filename	: Chiralpak_IA_70Heptan_30EtOAc_mind3bar.lcm		
Batch Filename	:		
Vial #	: 1-1	Sample Type	: Unknown
Injection Volume	: 20 uL		
Date Acquired	: 22.01.2021 10:38:55	Acquired by	: HPLC - Natascha
Date Processed	: 22.01.2021 11:08:56	Processed by	: HPLC - Natascha

<Chromatogram>

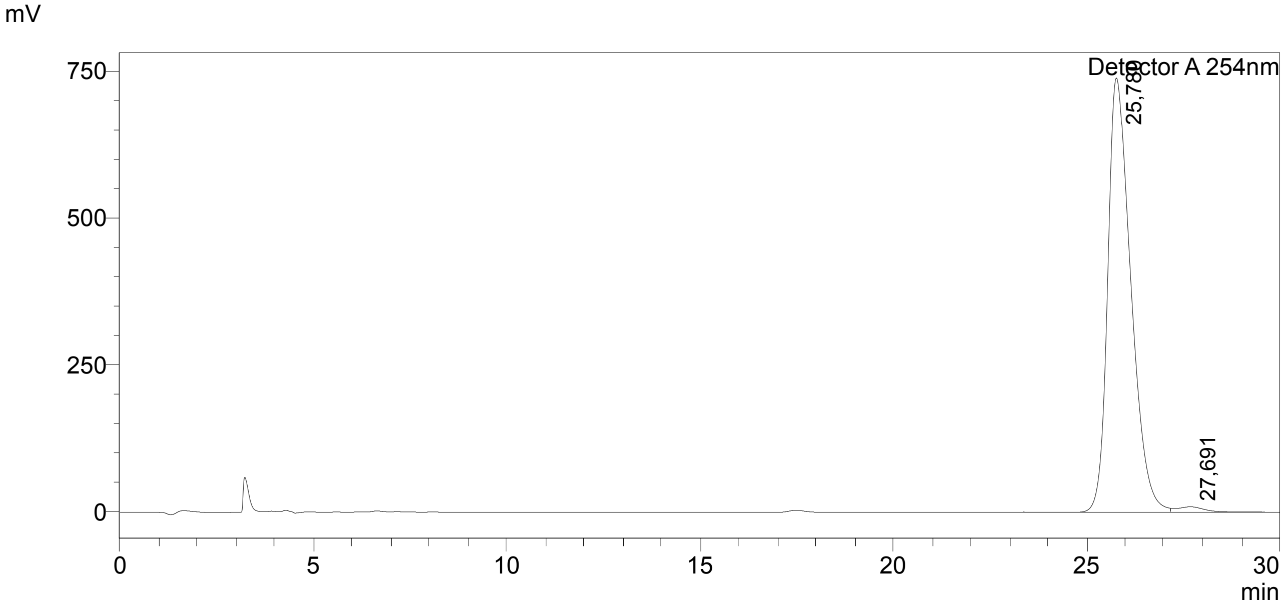

<Peak Table>

Detector A 254nm

Peak#	Ret. Time	Area	Area%
1	25,780	30566879	98,508
2	27,691	462866	1,492
Total		31029746	100,000

Figure S27b. Chiral chromatogram of **7g**.

SHIMADZU LabSolutions

Analysis Report

<Sample Information>

Sample Name	: PN-14_Peak_3_		
Sample ID	: PN-14_Peak_3_		
Data Filename	: PN-14_Peak_3_01.lcd		
Method Filename	: Chiralpak_IA_70Heptan_30EtOAc_mind3bar.lcm		
Batch Filename	:		
Vial #	: 1-2	Sample Type	: Unknown
Injection Volume	: 20 uL		
Date Acquired	: 22.01.2021 11:12:06	Acquired by	: HPLC - Natascha
Date Processed	: 22.01.2021 11:42:07	Processed by	: HPLC - Natascha

<Chromatogram>

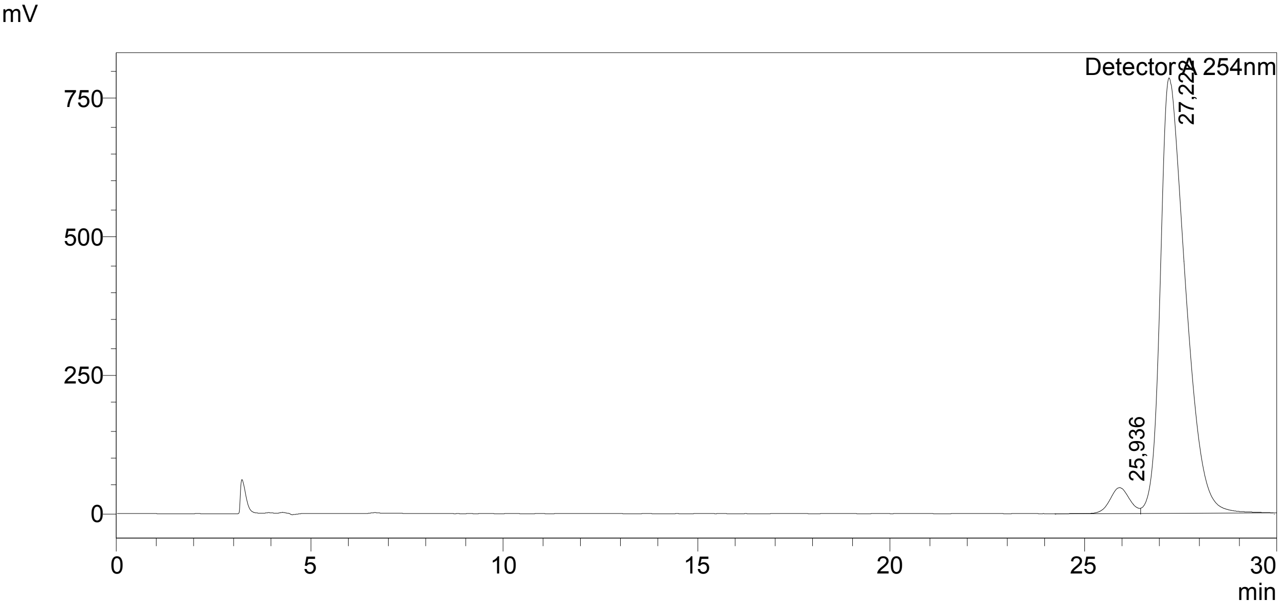

<Peak Table>

Detector A 254nm

Peak#	Ret. Time	Area	Area%
1	25,936	1629543	4,395
2	27,222	35448746	95,605
Total		37078289	100,000

Figure S28b. Chiral chromatogram of **8g**.

SHIMADZU LabSolutions

Analysis Report

<Sample Information>

Sample Name	: PN-14-4_		
Sample ID	: PN-14-4_		
Data Filename	: PN-14-4_01.lcd		
Method Filename	: Chiralpak_IA_EtOAc_.lcm		
Batch Filename	:		
Vial #	: 1-24	Sample Type	: Unknown
Injection Volume	: 10 uL		
Date Acquired	: 07.07.2020 16:08:20	Acquired by	: HPLC - Natascha
Date Processed	: 01.10.2020 16:44:44	Processed by	: HPLC - Natascha

<Chromatogram>

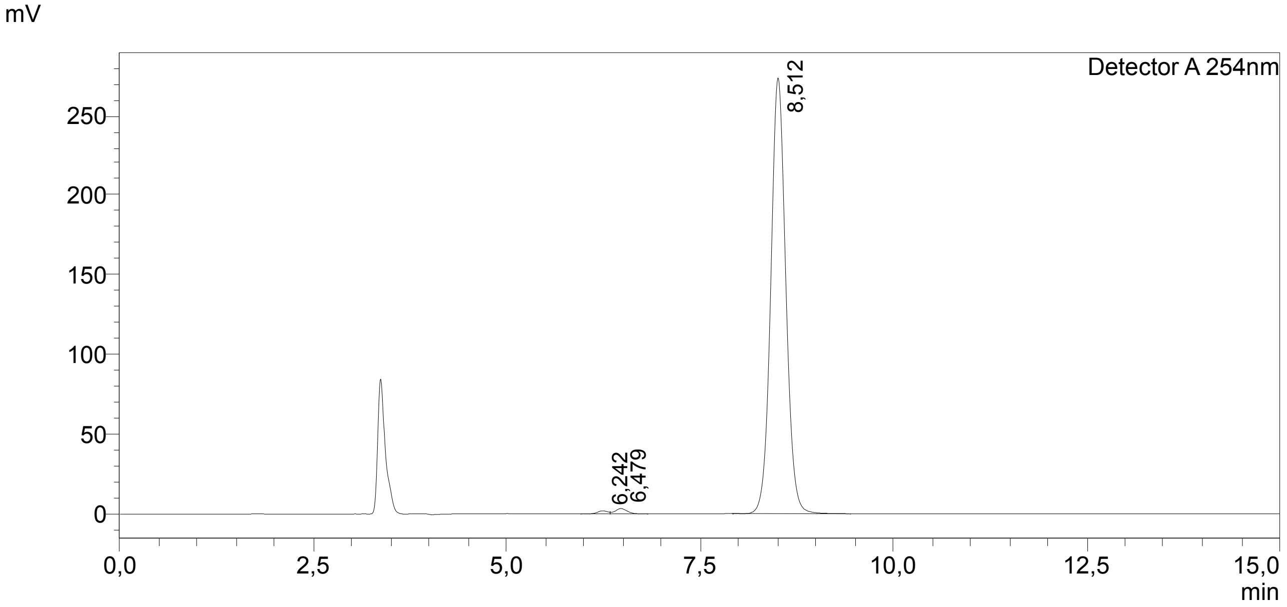

<Peak Table>

Detector A 254nm

Ret. Time	Area	Area%
6,242	18264	0,485
6,479	35811	0,951
8,512	3712718	98,564
	3766792	100,000

Figure S29b. Chiral chromatogram of **5h**.

SHIMADZU LabSolutions

Analysis Report

<Sample Information>

Sample Name : CE-196_spot_1_peak_1
Sample ID : CE-196_spot_1_peak_1
Data Filename : CE-196_spot_1_peak_1_01.lcd
Method Filename : Method_12.03.2019.lcm
Batch Filename :
Vial # : 1-1
Sample Type : Unknown
Injection Volume : 15 uL
Date Acquired : 15.05.2019 18:23:40
Acquired by : HPLC - Natascha
Date Processed : 15.05.2019 18:36:11
Processed by : HPLC - Natascha

<Chromatogram>

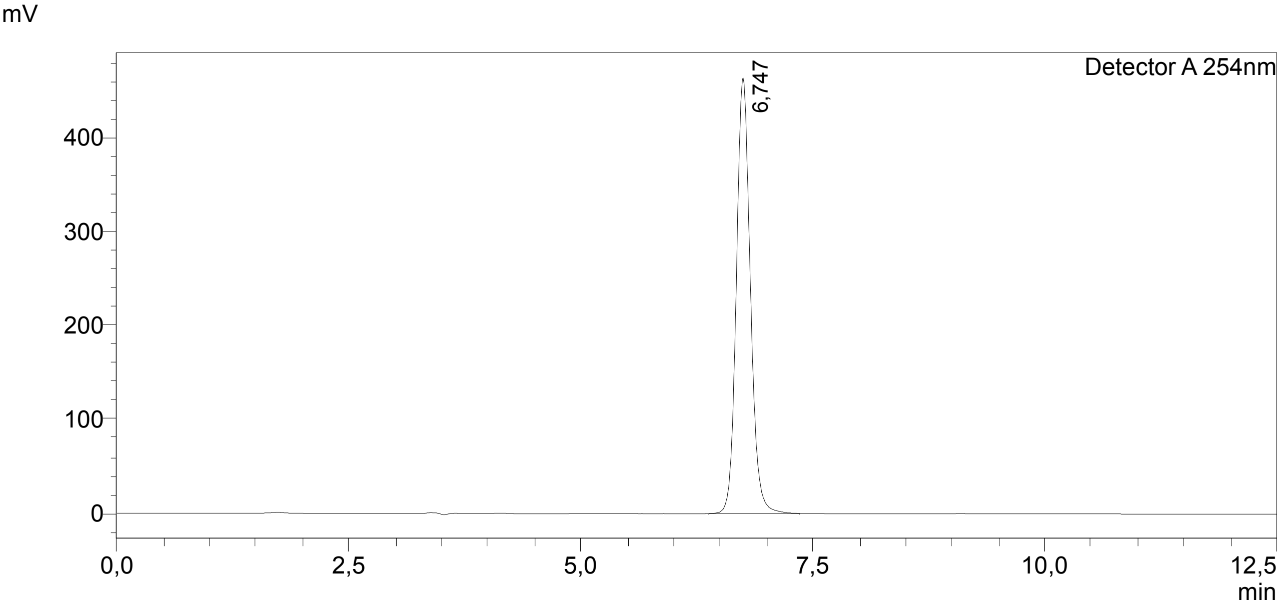

<Peak Table>

Detector A 254nm

Ret. Time	Area	Area%
6,747	4881634	100,000
	4881634	100,000

Figure S30b. Chiral chromatogram of **6h**.

SHIMADZU LabSolutions

Analysis Report

<Sample Information>

Sample Name	: CE-196_spot_1_peak_2		
Sample ID	: CE-196_spot_1_peak_2		
Data Filename	: CE-196_spot_1_peak_2_01.lcd		
Method Filename	: Method_12.03.2019.lcm		
Batch Filename	:		
Vial #	: 1-2	Sample Type	: Unknown
Injection Volume	: 15 uL		
Date Acquired	: 15.05.2019 18:37:21	Acquired by	: HPLC - Natascha
Date Processed	: 15.05.2019 18:49:52	Processed by	: HPLC - Natascha

<Chromatogram>

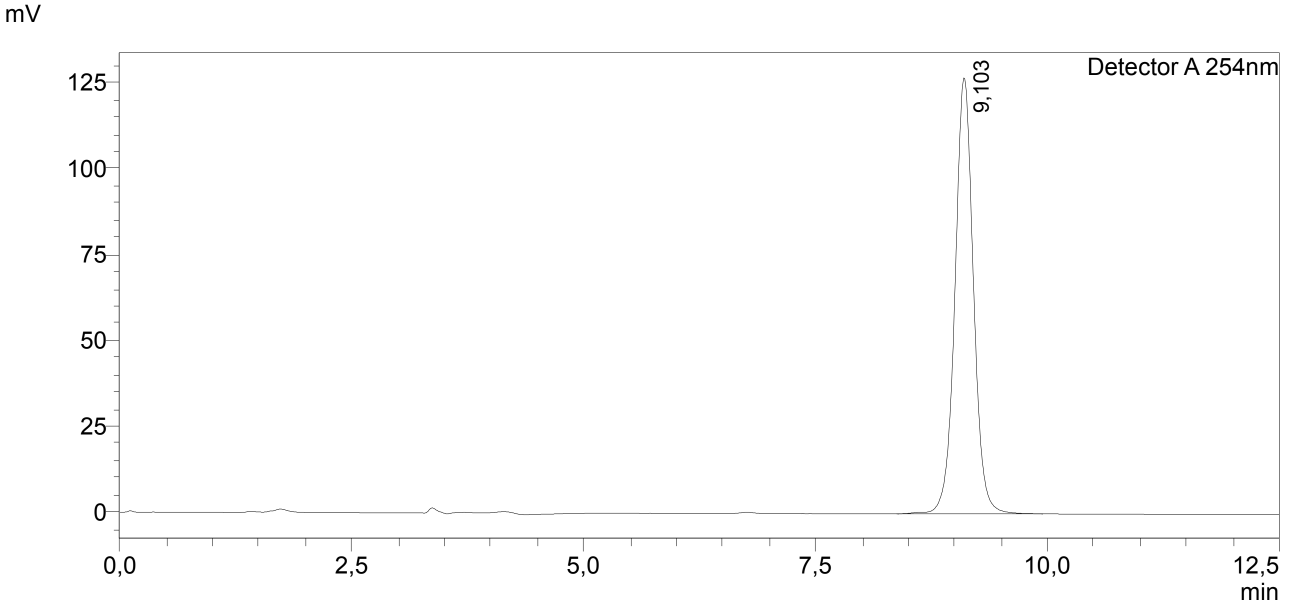

<Peak Table>

Detector A 254nm

Ret. Time	Area	Area%
9,103	1760441	100,000
	1760441	100,000

Figure S31b. Chiral chromatogram of **7h.**

SHIMADZU LabSolutions

Analysis Report

<Sample Information>

Sample Name	: CE-196_spot_2_peak_1		
Sample ID	: CE-196_spot_2_peak_1		
Data Filename	: CE-196_spot_2_peak_1_02.lcd		
Method Filename	: Method_12.03.2019.lcm		
Batch Filename	:		
Vial #	: 1-3	Sample Type	: Unknown
Injection Volume	: 20 uL		
Date Acquired	: 15.05.2019 19:04:23	Acquired by	: HPLC - Natascha
Date Processed	: 15.05.2019 19:16:55	Processed by	: HPLC - Natascha

<Chromatogram>

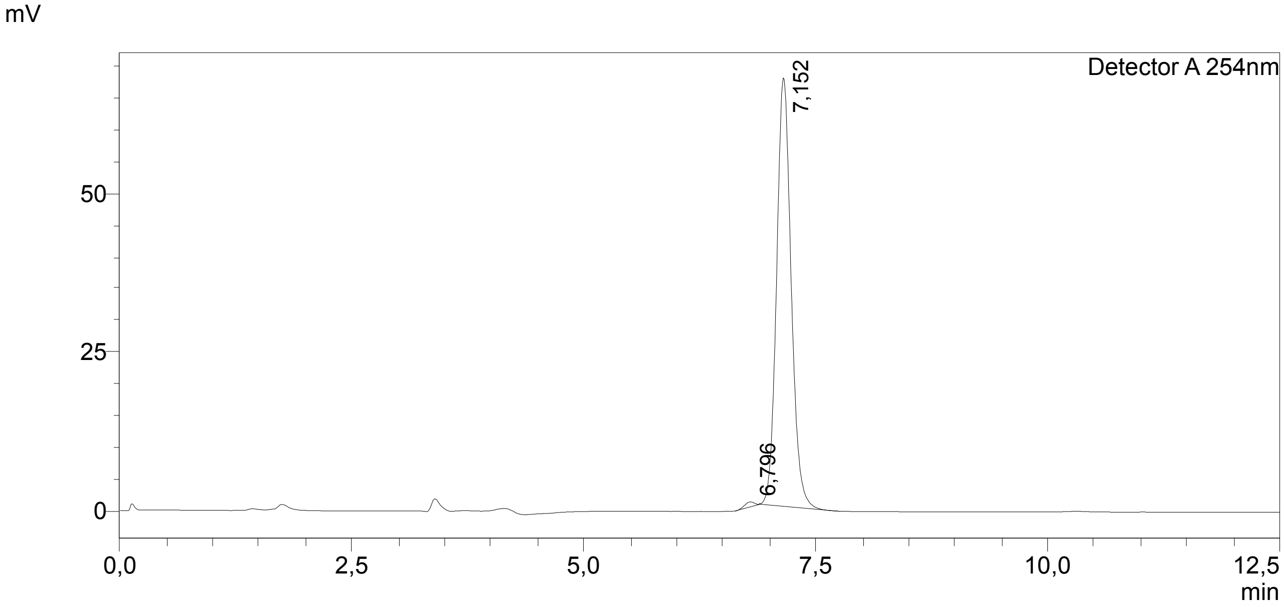

<Peak Table>

Detector A 254nm

Ret. Time	Area	Area%
6,796	5886	0,794
7,152	735162	99,206
	741047	100,000

Figure S32b. Chiral chromatogram of **8h**.

SHIMADZU LabSolutions

Analysis Report

<Sample Information>

Sample Name : CE-196_spot_2_peak_2
Sample ID : CE-196_spot_2_peak_2
Data Filename : CE-196_spot_2_peak_2_01.lcd
Method Filename : Method_12.03.2019.lcm
Batch Filename :
Vial # : 1-4
Sample Type : Unknown
Injection Volume : 15 uL
Date Acquired : 15.05.2019 19:18:22
Acquired by : HPLC - Natascha
Date Processed : 15.05.2019 19:30:53
Processed by : HPLC - Natascha

<Chromatogram>

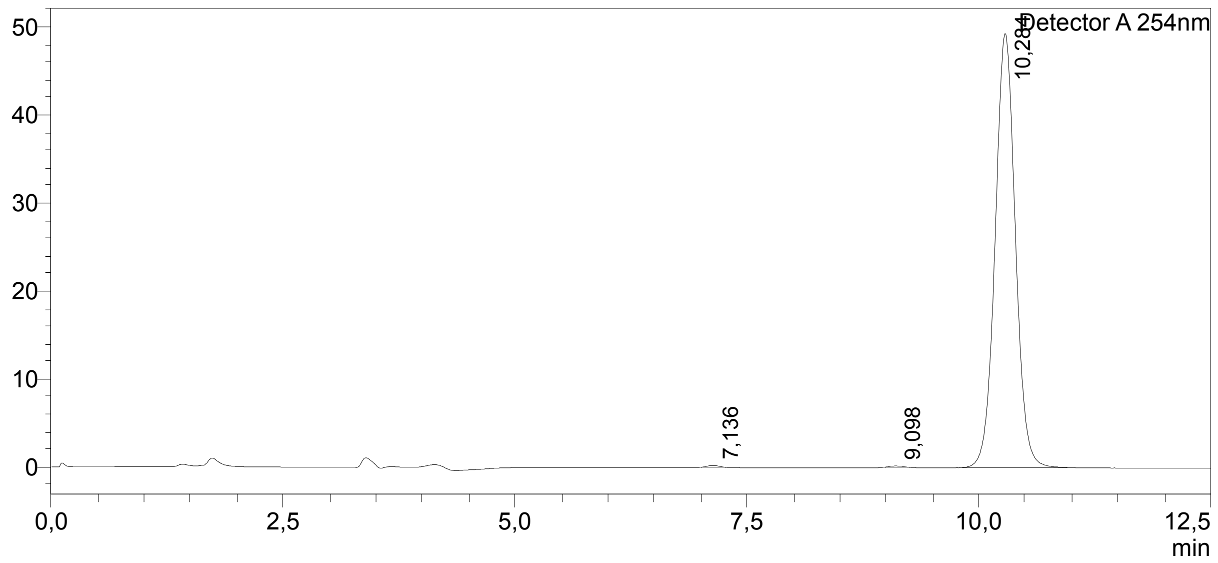

<Peak Table>

Detector A 254nm

Ret. Time	Area	Area%
7,136	1734	0,231
9,098	1187	0,158
10,284	748198	99,611
	751118	100,000

Figure S33b. Chiral chromatogram of **5i**.

SHIMADZU LabSolutions

Analysis Report

<Sample Information>

Sample Name	: MK-31_peak_1_		
Sample ID	: MK-31_peak_1_		
Data Filename	: MK-31_peak_1_01.lcd		
Method Filename	: Chiralpak_IA_EtOAc_.lcm		
Batch Filename	:		
Vial #	: 1-31	Sample Type	: Unknown
Injection Volume	: 20 uL		
Date Acquired	: 14.08.2020 10:11:48	Acquired by	: HPLC - Natascha
Date Processed	: 14.08.2020 10:26:49	Processed by	: HPLC - Natascha

<Chromatogram>

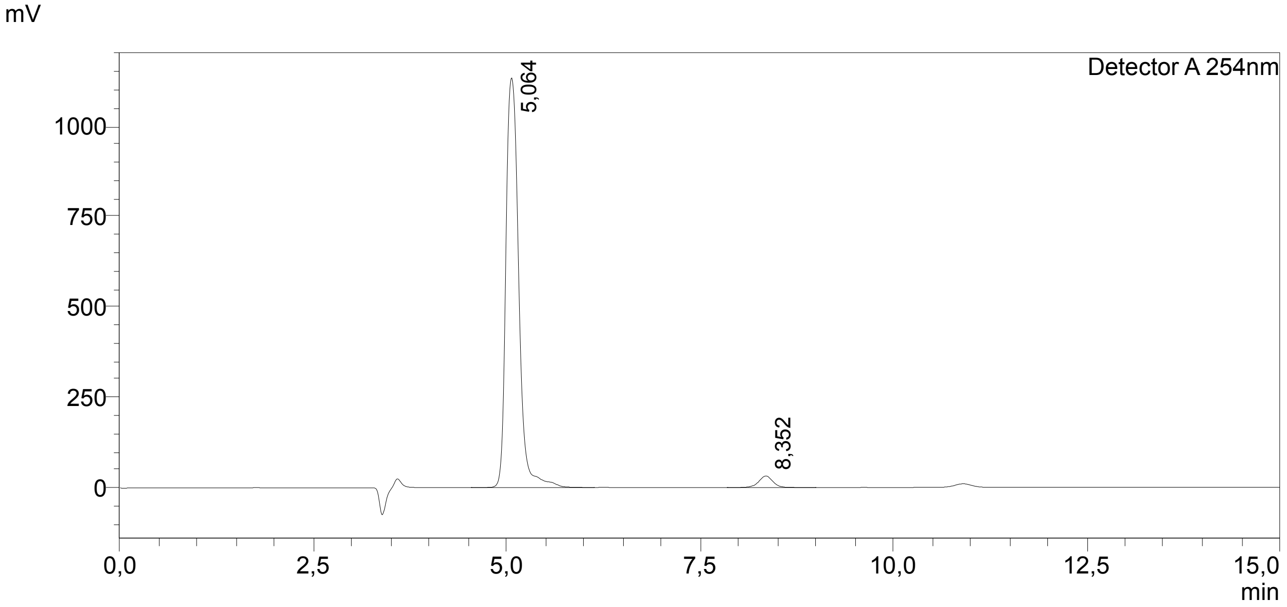

<Peak Table>

Detector A 254nm

Ret. Time	Area	Area%
5,064	13145498	96,972
8,352	410405	3,028
	13555903	100,000

Figure S34b. Chiral chromatogram of **6i**.

SHIMADZU LabSolutions

Analysis Report

<Sample Information>

Sample Name	: MK-31_peak_2_		
Sample ID	: MK-31_peak_2_		
Data Filename	: MK-31_peak_2_01.lcd		
Method Filename	: Chiralpak_IA_EtOAc_.lcm		
Batch Filename	:		
Vial #	: 1-32	Sample Type	: Unknown
Injection Volume	: 20 uL		
Date Acquired	: 14.08.2020 10:28:33	Acquired by	: HPLC - Natascha
Date Processed	: 14.08.2020 10:48:35	Processed by	: HPLC - Natascha

<Chromatogram>

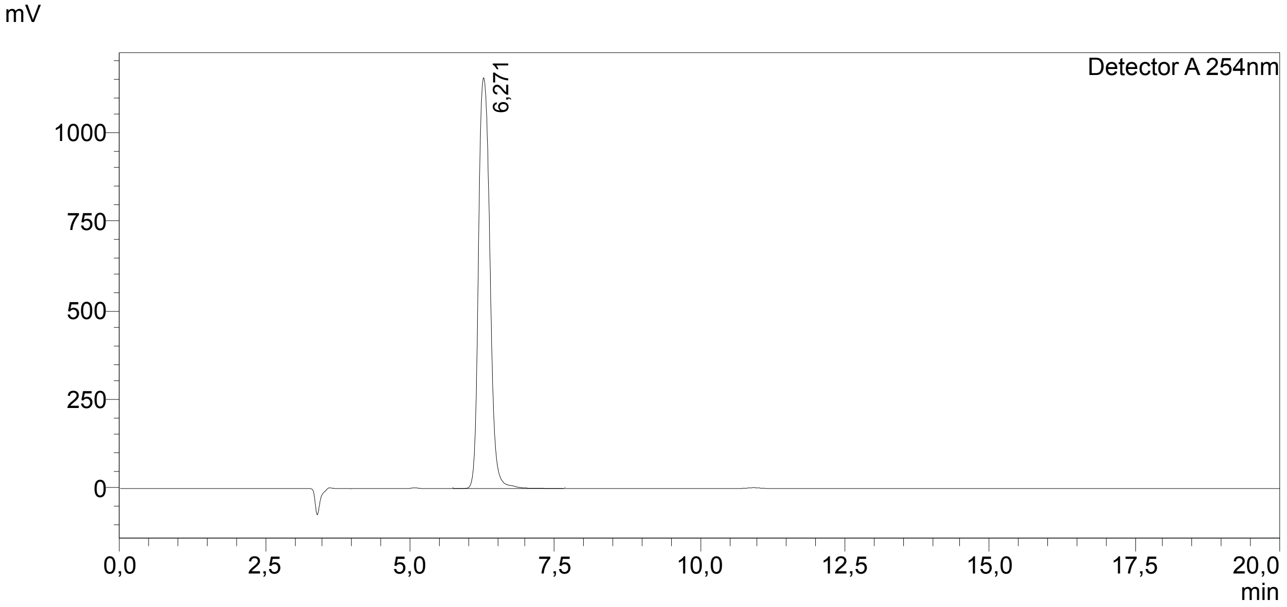

<Peak Table>

Detector A 254nm

Ret. Time	Area	Area%
6,271	15758232	100,000
	15758232	100,000

Figure S35b. Chiral chromatogram of **7i**.

SHIMADZU LabSolutions

Analysis Report

<Sample Information>

Sample Name	: MK-31_Peak_3_		
Sample ID	: MK-31_Peak_3_		
Data Filename	: MK-31_Peak_3_02.lcd		
Method Filename	: Chiralpak_IA_100%_EtOAc_mind3bar.lcm		
Batch Filename	:		
Vial #	: 1-6	Sample Type	: Unknown
Injection Volume	: 25 uL		
Date Acquired	: 29.03.2021 14:43:34	Acquired by	: HPLC - Natascha
Date Processed	: 29.03.2021 14:55:43	Processed by	: HPLC - Natascha

<Chromatogram>

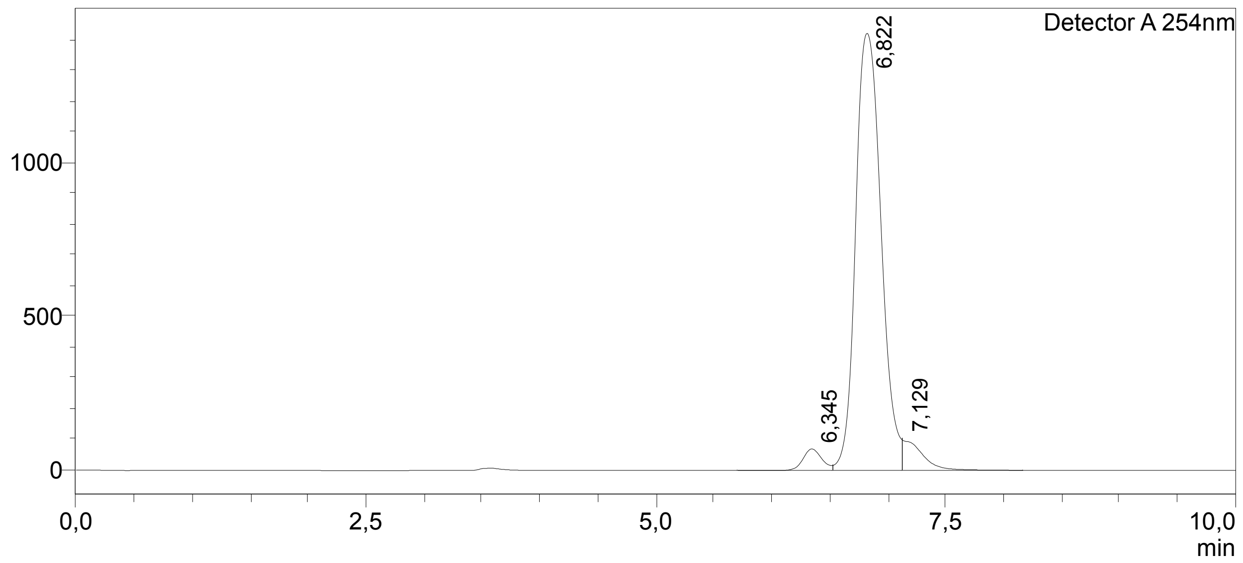

<Peak Table>

Detector A 254nm

Peak#	Ret. Time	Area	Area%
1	6,345	778225	3,301
2	6,822	21586326	91,553
3	7,129	1213302	5,146
Total		23577853	100,000

Figure S36b. Chiral chromatogram of **8i**.

SHIMADZU LabSolutions

Analysis Report

<Sample Information>

Sample Name	: MK-31_peak_4_		
Sample ID	: MK-31_peak_4_		
Data Filename	: MK-31_peak_4_01.lcd		
Method Filename	: Chiralpak_IA_EtOAc_.lcm		
Batch Filename	:		
Vial #	: 1-34	Sample Type	: Unknown
Injection Volume	: 20 uL		
Date Acquired	: 14.08.2020 11:06:40	Acquired by	: HPLC - Natascha
Date Processed	: 14.08.2020 11:21:42	Processed by	: HPLC - Natascha

<Chromatogram>

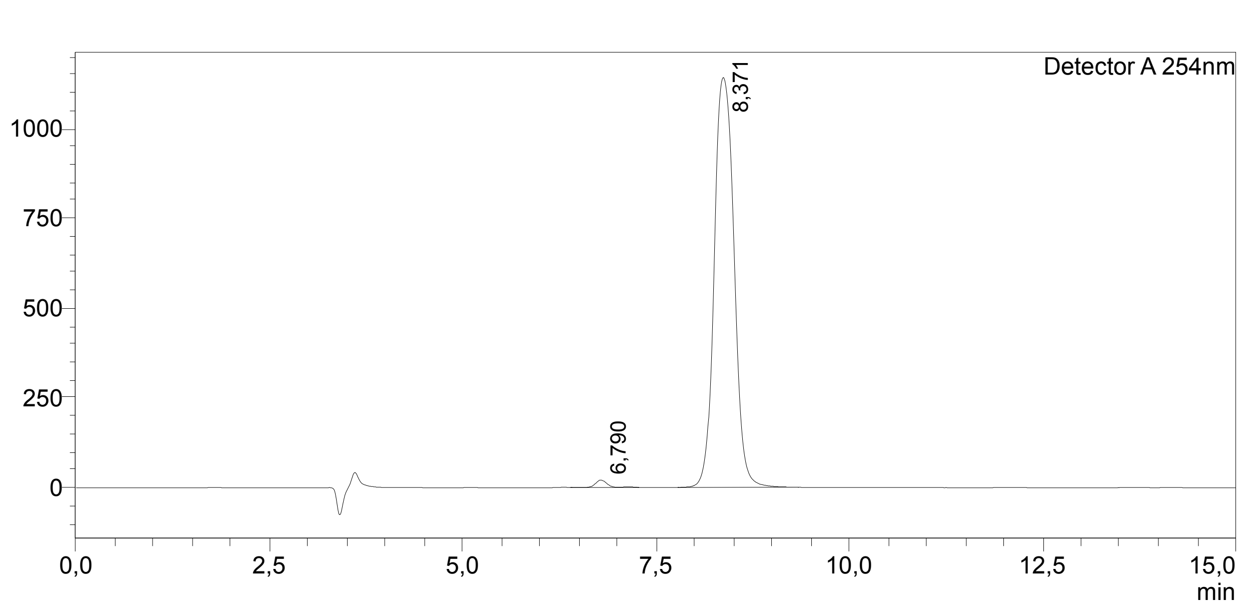

<Peak Table>

Detector A 254nm

Ret. Time	Area	Area%
6,790	216162	1,047
8,371	20422562	98,953
	20638724	100,000

Figure S37b. Chiral chromatogram of **5j**.

SHIMADZU LabSolutions

Analysis Report

<Sample Information>

Sample Name	: MK-30_spot_1_peak_1_		
Sample ID	: MK-30_spot_1_peak_1_		
Data Filename	: MK-30_spot_1_peak_1_01.lcd		
Method Filename	: Chiralpak_IA_EtOAc_.lcm		
Batch Filename	:		
Vial #	: 1-18	Sample Type	: Unknown
Injection Volume	: 15 uL		
Date Acquired	: 26.06.2020 11:17:05	Acquired by	: HPLC - Natascha
Date Processed	: 26.06.2020 11:29:36	Processed by	: HPLC - Natascha

<Chromatogram>

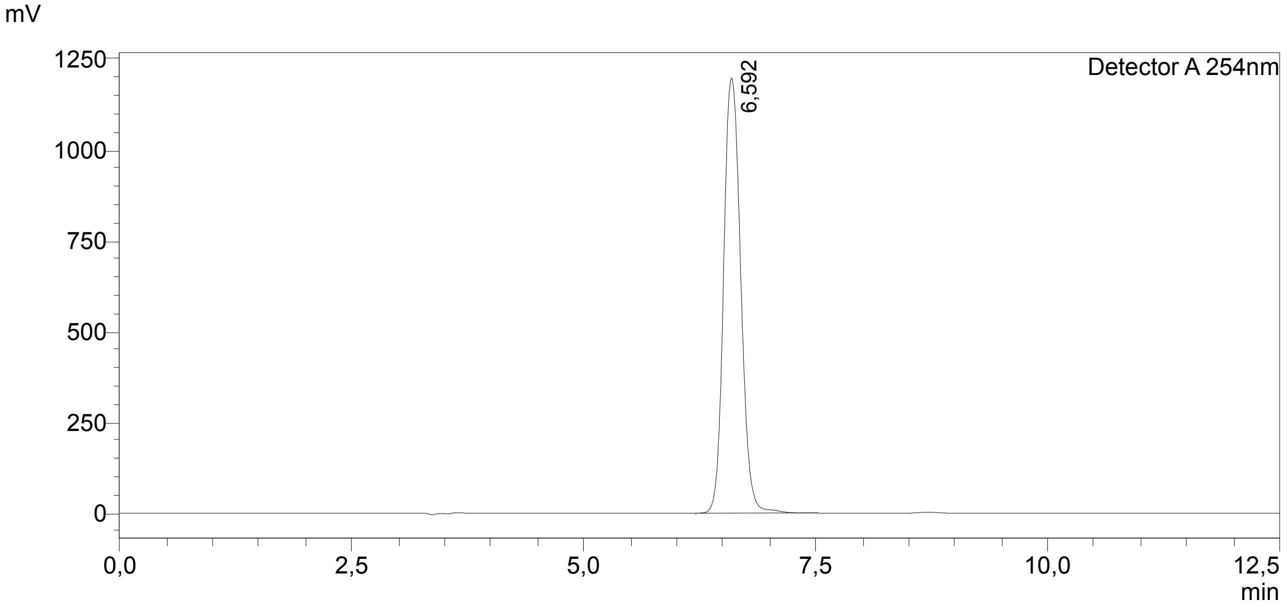

<Peak Table>

Detector A 254nm

Ret. Time	Area	Area%
6,592	15404992	100,000
	15404992	100,000

Figure S38b. Chiral chromatogram of **6j**.

SHIMADZU LabSolutions

Analysis Report

<Sample Information>

Sample Name : MK-30_spot_1_peak_2_
Sample ID : MK-30_spot_1_peak_2_
Data Filename : MK-30_spot_1_peak_2_01.lcd
Method Filename : Chiralpak_IA_EtOAc_.lcm
Batch Filename :
Vial # : 1-19
Sample Type : Unknown
Injection Volume : 15 uL
Date Acquired : 26.06.2020 11:32:20
Acquired by : HPLC - Natascha
Date Processed : 26.06.2020 11:44:51
Processed by : HPLC - Natascha

<Chromatogram>

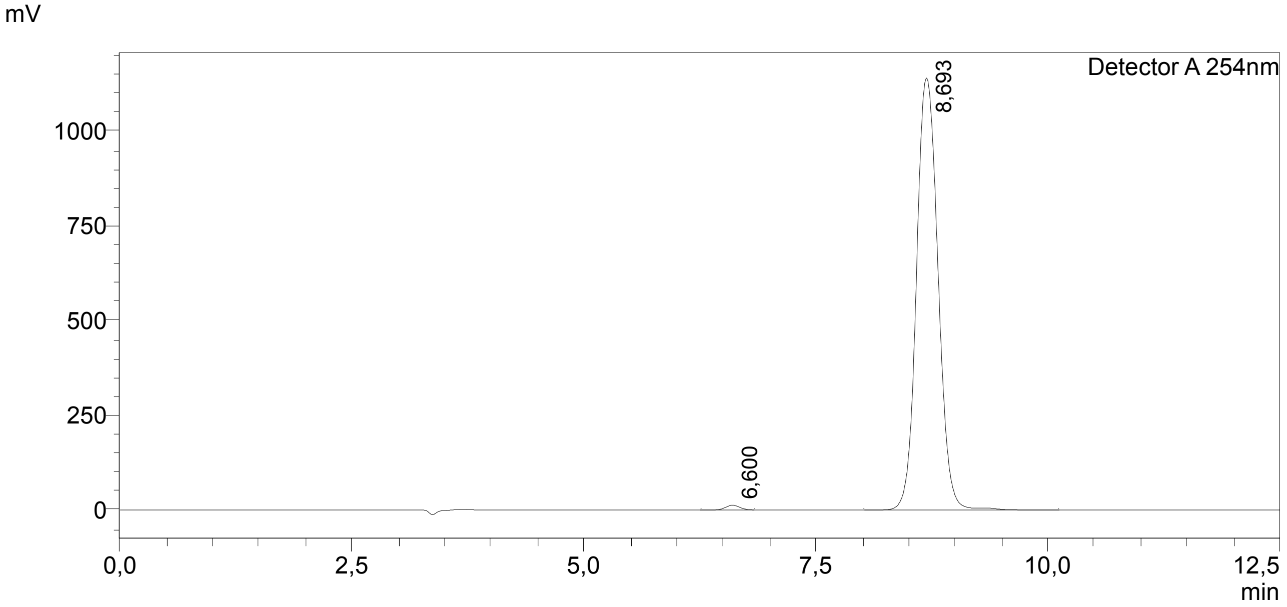

<Peak Table>

Detector A 254nm

Ret. Time	Area	Area%
6,600	131220	0,708
8,693	18414644	99,292
	18545863	100,000

Figure S39b. Chiral chromatogram of **7j**.

SHIMADZU LabSolutions

Analysis Report

<Sample Information>

Sample Name	: MK-30_spot_2_peak_1_final_		
Sample ID	: MK-30_spot_2_peak_1_final_		
Data Filename	: MK-30_spot_2_peak_1_final_01.lcd		
Method Filename	: Chiralpak_IA_EtOAc_.lcm		
Batch Filename	:		
Vial #	: 1-11	Sample Type	: Unknown
Injection Volume	: 20 uL		
Date Acquired	: 25.06.2020 17:54:30	Acquired by	: HPLC - Natascha
Date Processed	: 25.06.2020 18:07:02	Processed by	: HPLC - Natascha

<Chromatogram>

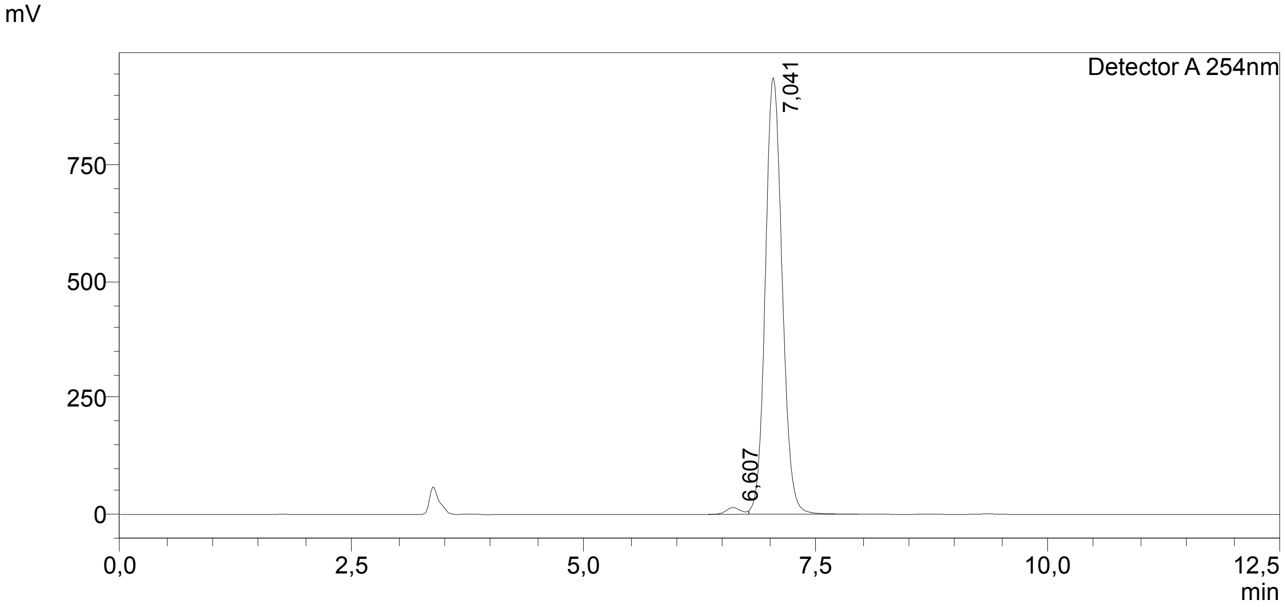

<Peak Table>

Detector A 254nm

Ret. Time	Area	Area%
6,607	165617	1,375
7,041	11882094	98,625
	12047711	100,000

Figure S40b. Chiral chromatogram of **8j**.

SHIMADZU LabSolutions

Analysis Report

<Sample Information>

Sample Name	: MK-30_spot_2_peak_2_final_		
Sample ID	: MK-30_spot_2_peak_2_final_		
Data Filename	: MK-30_spot_2_peak_2_final_01.lcd		
Method Filename	: Chiralpak_IA_EtOAc_.lcm		
Batch Filename	:		
Vial #	: 1-13	Sample Type	: Unknown
Injection Volume	: 20 uL		
Date Acquired	: 25.06.2020 18:07:54	Acquired by	: HPLC - Natascha
Date Processed	: 25.06.2020 18:20:26	Processed by	: HPLC - Natascha

<Chromatogram>

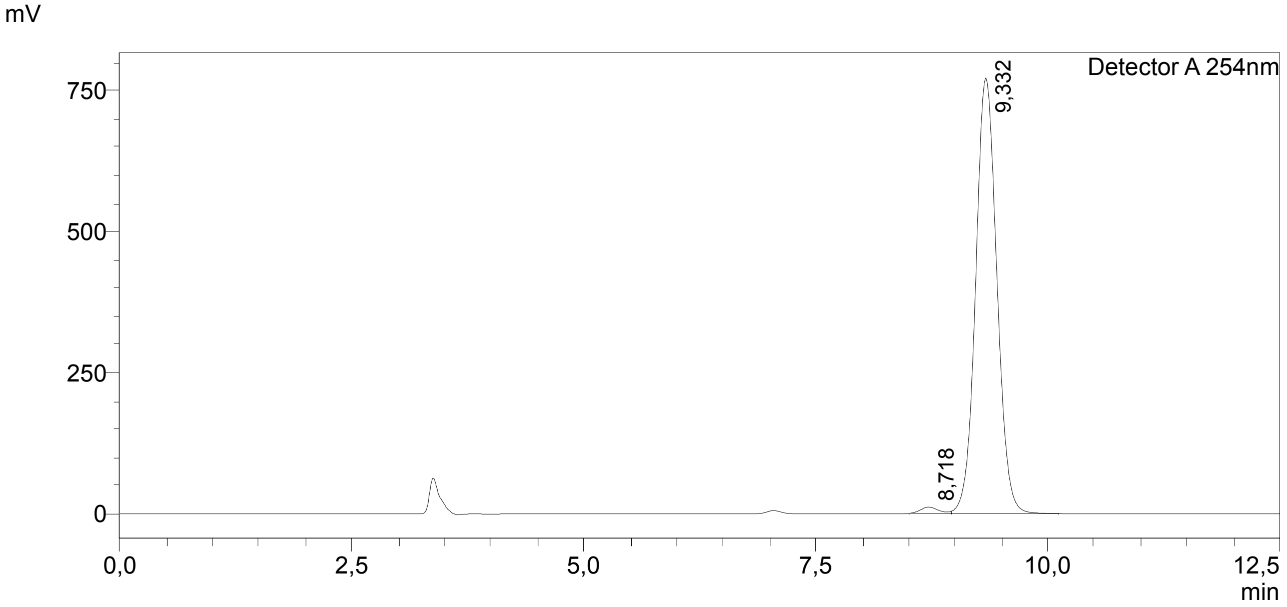

<Peak Table>

Detector A 254nm

Ret. Time	Area	Area%
8,718	147169	1,186
9,332	12262662	98,814
	12409831	100,000

Figure S41b. Chiral chromatogram of **5k**.

SHIMADZU LabSolutions

Analysis Report

<Sample Information>

Sample Name	: SB_1_1_		
Sample ID	: 001		
Data Filename	: SB_alle4Peaks__12.01.2021_001.lcd		
Method Filename	: Chiralpak_IA_100_EtOAc_mind3bar.lcm		
Batch Filename	: SB_alle4Peaks_.lcb		
Vial #	: 1-1	Sample Type	: Unknown
Injection Volume	: 20 uL		
Date Acquired	: 12.01.2021 13:36:41	Acquired by	: HPLC - Natascha
Date Processed	: 12.01.2021 13:56:43	Processed by	: HPLC - Natascha

<Chromatogram>

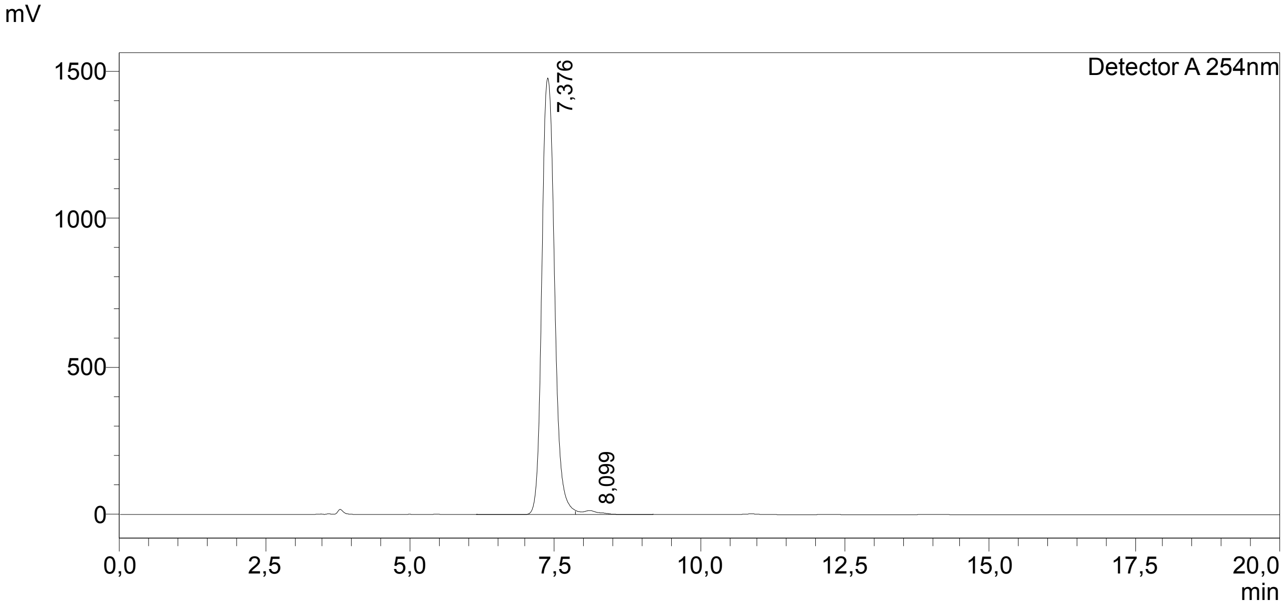

<Peak Table>

Detector A 254nm

Ret. Time	Area	Area%
7,376	22438082	98,591
8,099	320663	1,409
	22758745	100,000

Figure S42b. Chiral chromatogram of **6k**.

SHIMADZU LabSolutions

Analysis Report

<Sample Information>

Sample Name	: SB_1_2_		
Sample ID	: 001		
Data Filename	: SB_alle4Peaks__12.01.2021_002.lcd		
Method Filename	: Chiralpak_IA_100_EtOAc_mind3bar.lcm		
Batch Filename	: SB_alle4Peaks_.lcb		
Vial #	: 1-2	Sample Type	: Unknown
Injection Volume	: 20 uL		
Date Acquired	: 12.01.2021 13:57:06	Acquired by	: HPLC - Natascha
Date Processed	: 12.01.2021 14:17:07	Processed by	: HPLC - Natascha

<Chromatogram>

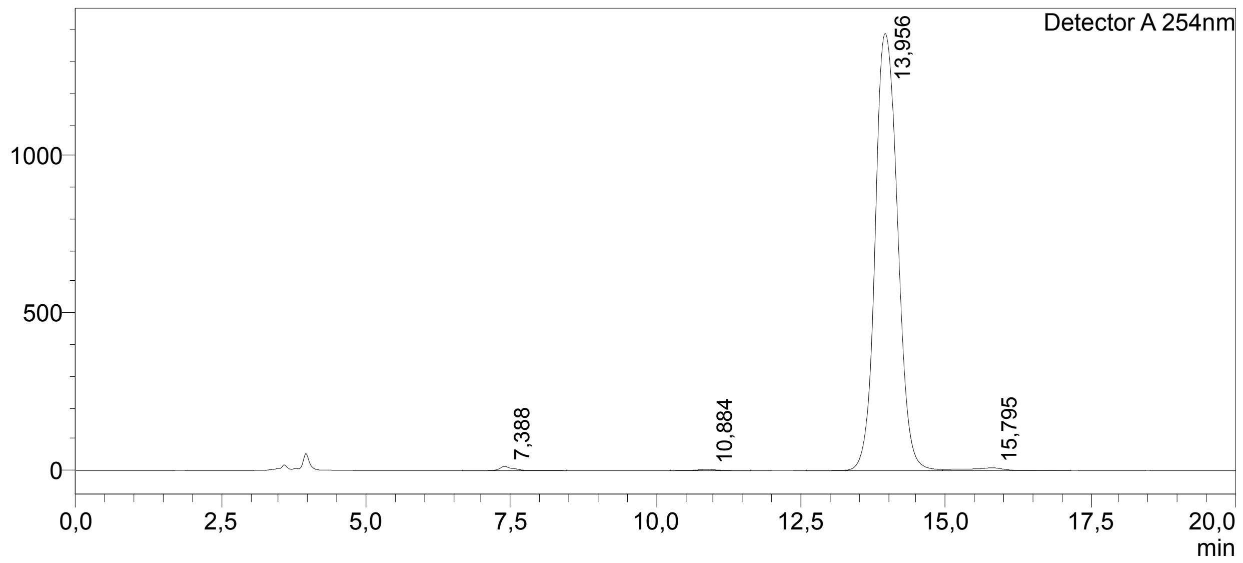

<Peak Table>

Detector A 254nm

Ret. Time	Area	Area%
7,388	219346	0,577
10,884	86961	0,229
13,956	37330799	98,157
15,795	394575	1,037
	38031681	100,000

Figure S43b. Chiral chromatogram of **7k**.

SHIMADZU LabSolutions

Analysis Report

<Sample Information>

Sample Name	: SB_2_1_		
Sample ID	: 001		
Data Filename	: SB_alle4Peaks__12.01.2021_003.lcd		
Method Filename	: Chiralpak_IA_100_EtOAc_mind3bar.lcm		
Batch Filename	: SB_alle4Peaks_.lcb		
Vial #	: 1-3	Sample Type	: Unknown
Injection Volume	: 20 uL		
Date Acquired	: 12.01.2021 14:17:31	Acquired by	: HPLC - Natascha
Date Processed	: 12.01.2021 14:37:32	Processed by	: HPLC - Natascha

<Chromatogram>

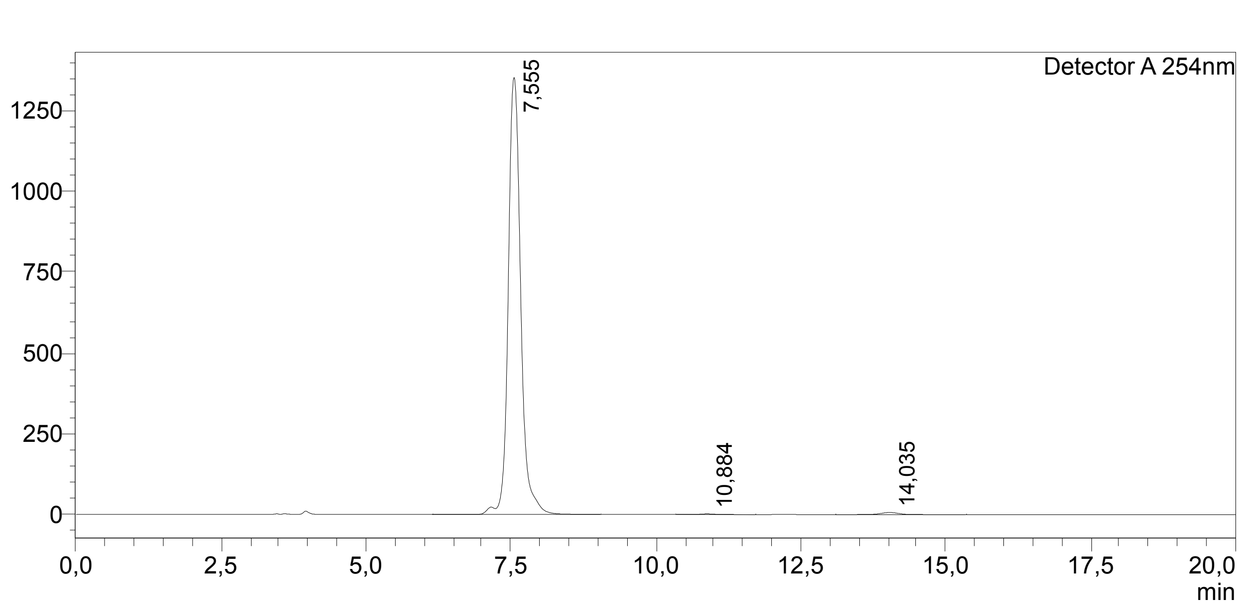

<Peak Table>

Detector A 254nm

Ret. Time	Area	Area%
7,555	20360642	99,124
10,884	36821	0,179
14,035	143022	0,696
	20540485	100,000

Figure S44b. Chiral chromatogram of **8k**.

SHIMADZU LabSolutions

Analysis Report

<Sample Information>

Sample Name	: SB_2_2_		
Sample ID	: 001		
Data Filename	: SB_alle4Peaks__12.01.2021_004.lcd		
Method Filename	: Chiralpak_IA_100_EtOAc_mind3bar.lcm		
Batch Filename	: SB_alle4Peaks_.lcb		
Vial #	: 1-4	Sample Type	: Unknown
Injection Volume	: 20 uL		
Date Acquired	: 12.01.2021 14:37:55	Acquired by	: HPLC - Natascha
Date Processed	: 12.01.2021 14:57:56	Processed by	: HPLC - Natascha

<Chromatogram>

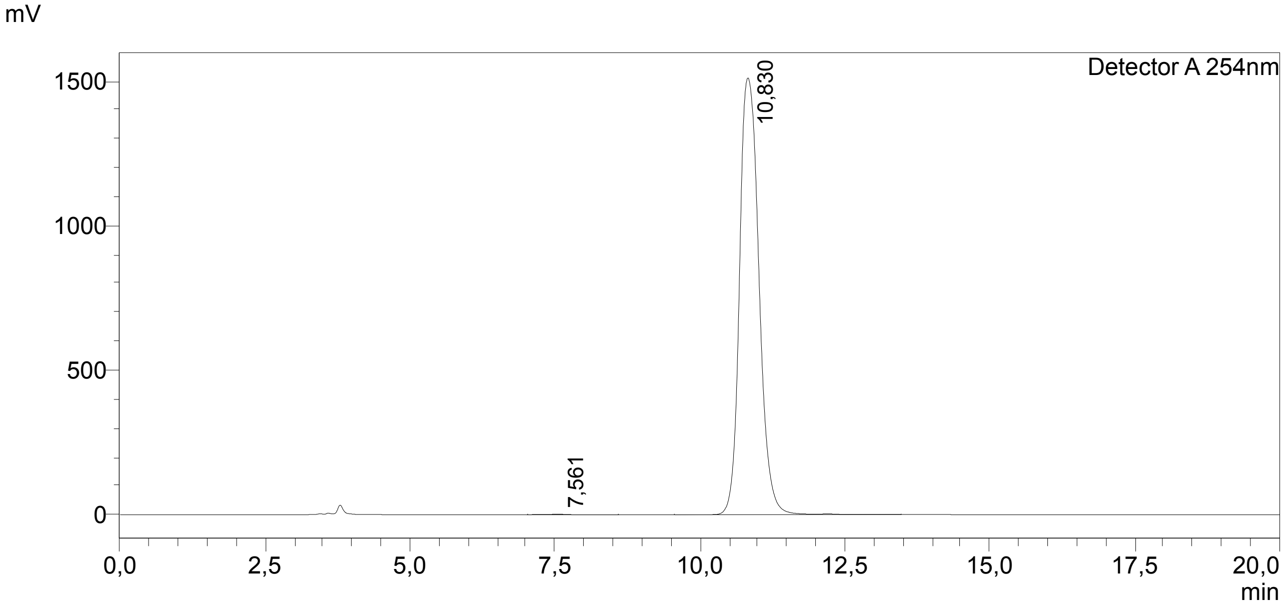

<Peak Table>

Detector A 254nm

Ret. Time	Area	Area%
7,561	38461	0,109
10,830	35181813	99,891
	35220274	100,000

Figure S45b. Chiral chromatogram of **5l.**

SHIMADZU LabSolutions

Analysis Report

<Sample Information>

Sample Name	: PN-6_Peak_1_		
Sample ID	: PN-6_Peak_1_		
Data Filename	: PN-6_Peak_1_01.lcd		
Method Filename	: Chiralpak_IA_50Hex_50EtOAc_.lcm		
Batch Filename	:		
Vial #	: 1-6	Sample Type	: Unknown
Injection Volume	: 20 uL		
Date Acquired	: 19.03.2021 16:12:14	Acquired by	: HPLC - Natascha
Date Processed	: 19.03.2021 16:27:15	Processed by	: HPLC - Natascha

<Chromatogram>

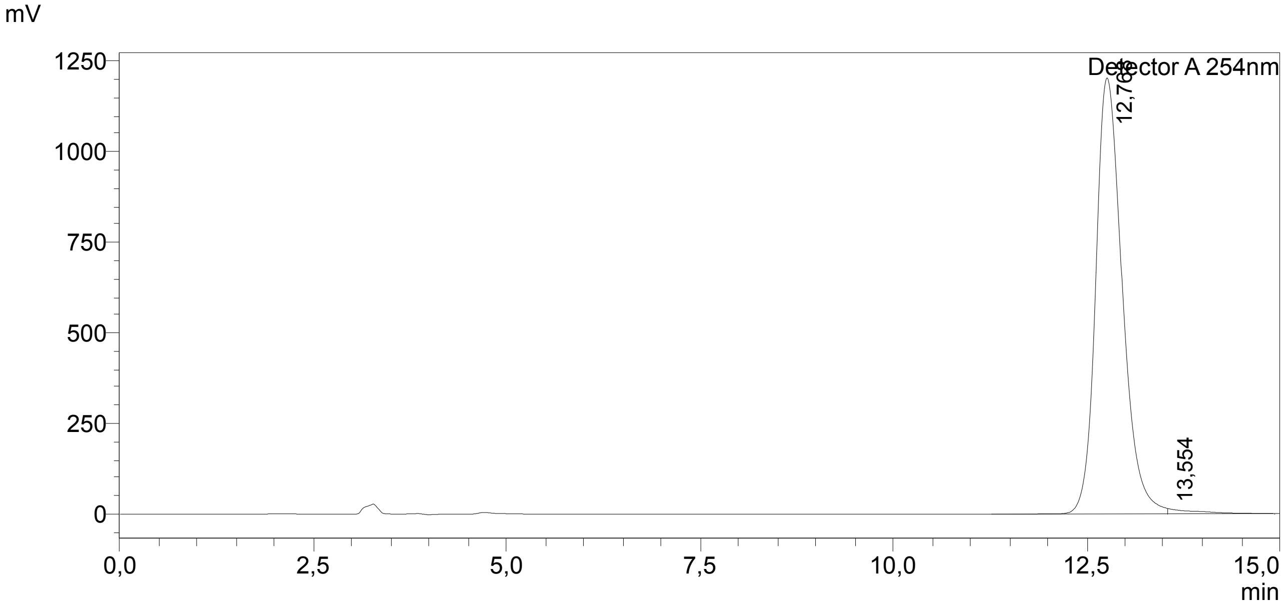

<Peak Table>

Detector A 254nm

Peak#	Ret. Time	Area	Area%
1	12,768	28737649	98,815
2	13,554	344705	1,185
Total		29082354	100,000

Figure S46b. Chiral chromatogram of **6l**.

SHIMADZU LabSolutions

Analysis Report

<Sample Information>

Sample Name	: PN-6_Peak_2_		
Sample ID	: PN-6_Peak_2_		
Data Filename	: PN-6_Peak_2_01.lcd		
Method Filename	: Chiralpak_IA_50Hex_50EtOAc_.lcm		
Batch Filename	:		
Vial #	: 1-6	Sample Type	: Unknown
Injection Volume	: 20 uL		
Date Acquired	: 22.03.2021 14:45:30	Acquired by	: HPLC - Natascha
Date Processed	: 22.03.2021 15:00:31	Processed by	: HPLC - Natascha

<Chromatogram>

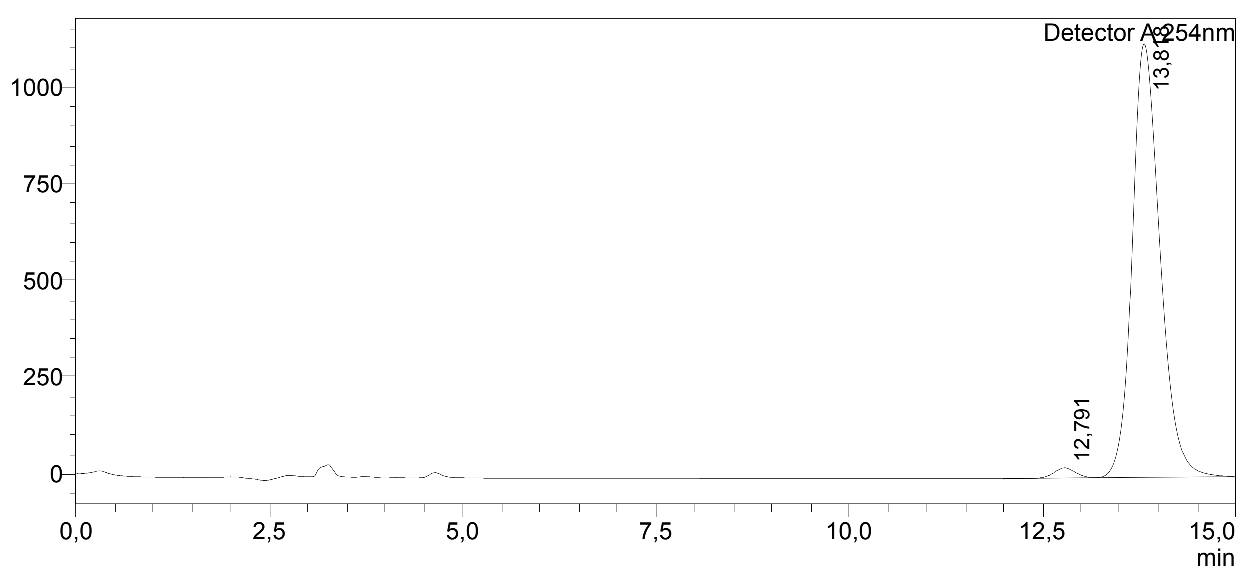

<Peak Table>

Detector A 254nm

Peak#	Ret. Time	Area	Area%
1	12,791	508264	1,771
2	13,818	28185217	98,229
Total		28693481	100,000

Figure S47b. Chiral chromatogram of **7l**.

SHIMADZU LabSolutions

Analysis Report

<Sample Information>

Sample Name : PN-6_Peak_3_
Sample ID : PN-6_Peak_3_
Data Filename : PN-6_Peak_3_01.lcd
Method Filename : Chiralpak_IA_100%_EtOAc_mind3bar.lcm
Batch Filename :
Vial # : 1-1
Sample Type : Unknown
Injection Volume : 20 uL
Date Acquired : 28.05.2021 13:51:17
Acquired by : HPLC - Natascha
Date Processed : 28.05.2021 14:11:19
Processed by : HPLC - Natascha

<Chromatogram>

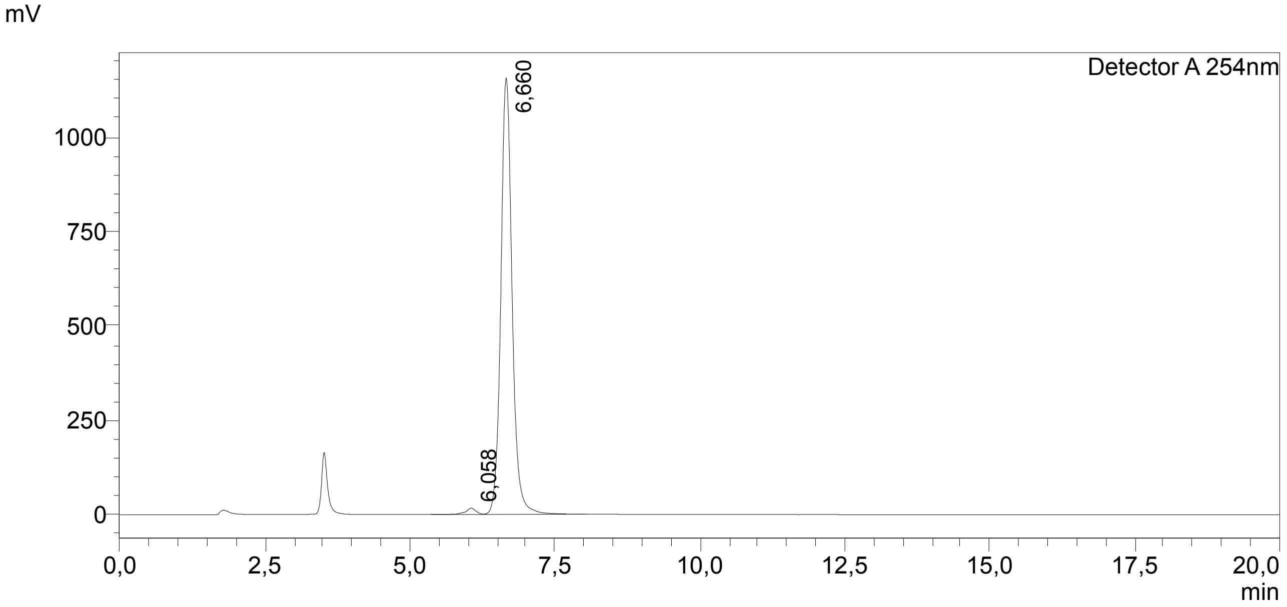

<Peak Table>

Detector A 254nm

Peak#	Ret. Time	Area	Area%
1	6,058	216979	1,366
2	6,660	15667698	98,634
Total		15884678	100,000

Figure S48b. Chiral chromatogram of **8l.**

SHIMADZU LabSolutions

Analysis Report

<Sample Information>

Sample Name	: PN-6_peak_4_		
Sample ID	: PN-6_peak_4_		
Data Filename	: PN-6_peak_4_01.lcd		
Method Filename	: Chiralpak_IA_EtOAc_.lcm		
Batch Filename	:		
Vial #	: 1-8	Sample Type	: Unknown
Injection Volume	: 20 uL		
Date Acquired	: 21.07.2020 12:31:39	Acquired by	: HPLC - Natascha
Date Processed	: 21.07.2020 12:47:26	Processed by	: HPLC - Natascha

<Chromatogram>

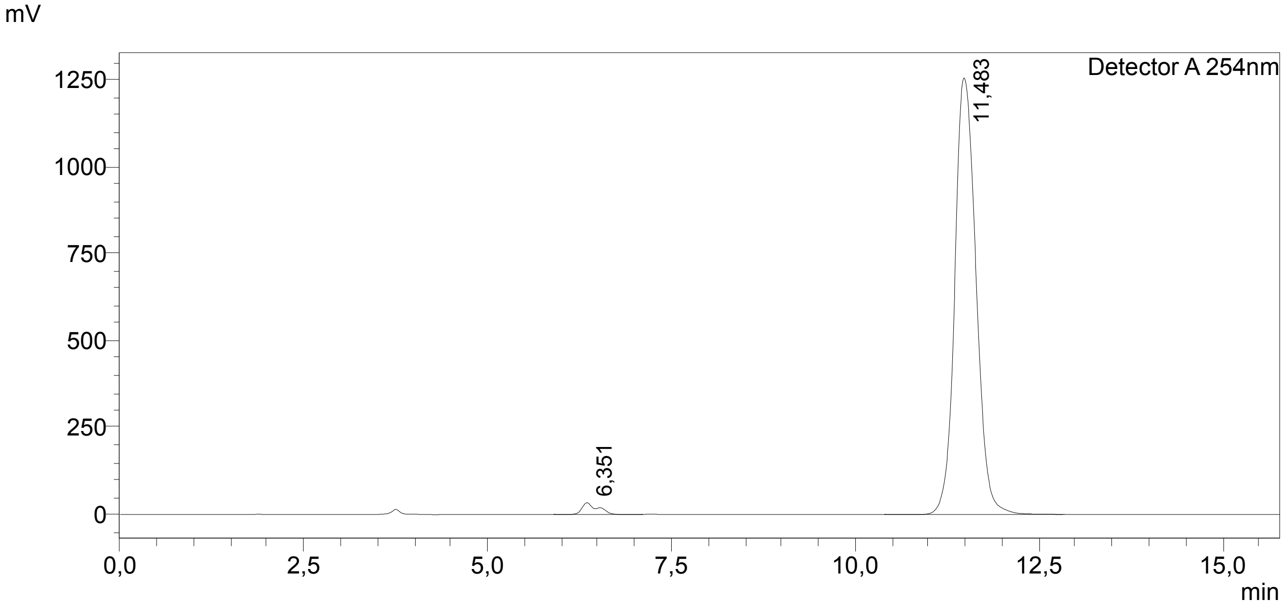

<Peak Table>

Detector A 254nm

Ret. Time	Area	Area%
6,351	502740	1,955
11,483	25216908	98,045
	25719648	100,000

Figure S49b. Chiral chromatogram of **5m**.

SHIMADZU LabSolutions

Analysis Report

<Sample Information>

Sample Name	: PN-16_spot1_peak1_		
Sample ID	: PN-16_spot1_peak1_		
Data Filename	: PN-16_spot1_peak1_01.lcd		
Method Filename	: 100EtOAc_.lcm		
Batch Filename	:		
Vial #	: 1-1	Sample Type	: Unknown
Injection Volume	: 10 uL		
Date Acquired	: 02.07.2019 11:26:21	Acquired by	: HPLC - Natascha
Date Processed	: 02.07.2019 11:46:22	Processed by	: HPLC - Natascha

<Chromatogram>

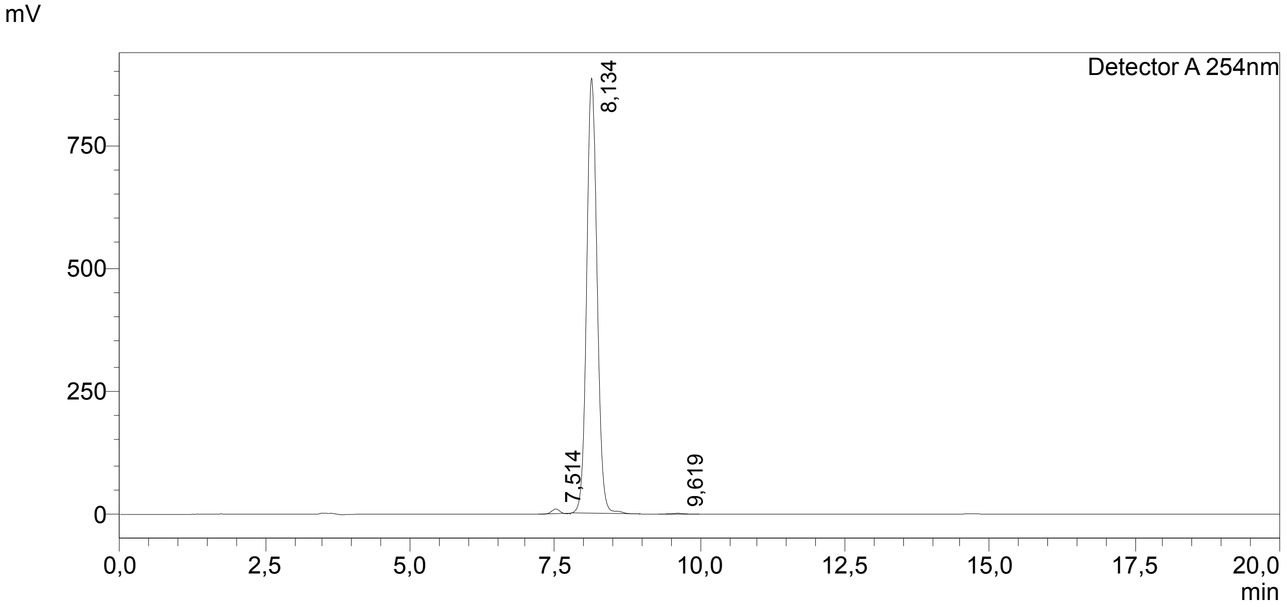

<Peak Table>

Detector A 254nm

Peak#	Ret. Time	Area	Height	Area%	Height%
1	7,514	81294	9265	0,731	1,035
2	8,134	11012292	883931	99,071	98,782
3	9,619	21961	1631	0,198	0,182
Total		11115546	894827	100,000	100,000

Figure S50b. Chiral chromatogram of **6m**.

SHIMADZU LabSolutions

Analysis Report

<Sample Information>

Sample Name	: PN-16_spot1_peak2_		
Sample ID	: PN-16_spot1_peak2_		
Data Filename	: PN-16_spot1_peak2_.lcd		
Method Filename	: 100EtOAc_.lcm		
Batch Filename	: batch_.lcb		
Vial #	: 1-2	Sample Type	: Unknown
Injection Volume	: 10 uL		
Date Acquired	: 02.07.2019 11:57:46	Acquired by	: HPLC - Natascha
Date Processed	: 02.07.2019 12:17:47	Processed by	: HPLC - Natascha

<Chromatogram>

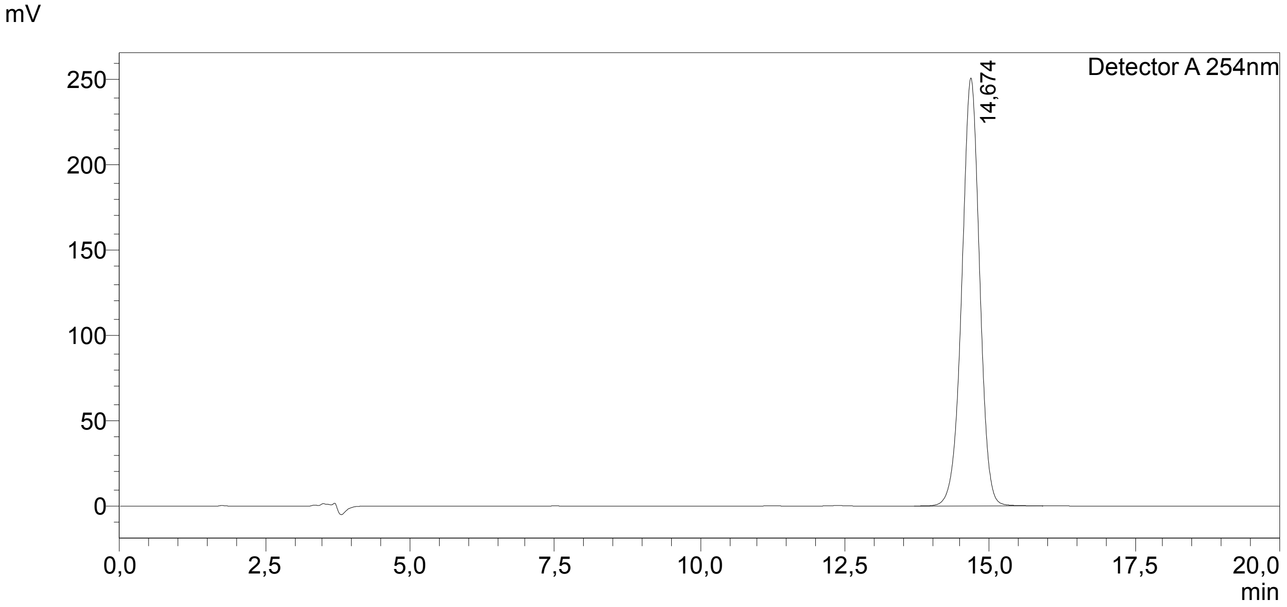

<Peak Table>

Detector A 254nm

Peak#	Ret. Time	Area	Height	Area%	Height%
1	14,674	5358008	250870	100,000	100,000
Total		5358008	250870	100,000	100,000

Figure S51b. Chiral chromatogram of **7m**.

Analysis Report

<Sample Information>

Sample Name : PN-16_spot2_peak1_
Sample ID : PN-16_spot2_peak1_
Data Filename : PN-16_spot2_peak1_.lcd
Method Filename : 100EtOAc_.lcm
Batch Filename : batch_.lcb
Vial # : 1-3
Sample Type : Unknown
Injection Volume : 10 uL
Date Acquired : 02.07.2019 12:18:10
Acquired by : HPLC - Natascha
Date Processed : 02.07.2019 13:50:27
Processed by : HPLC - Natascha

<Chromatogram>

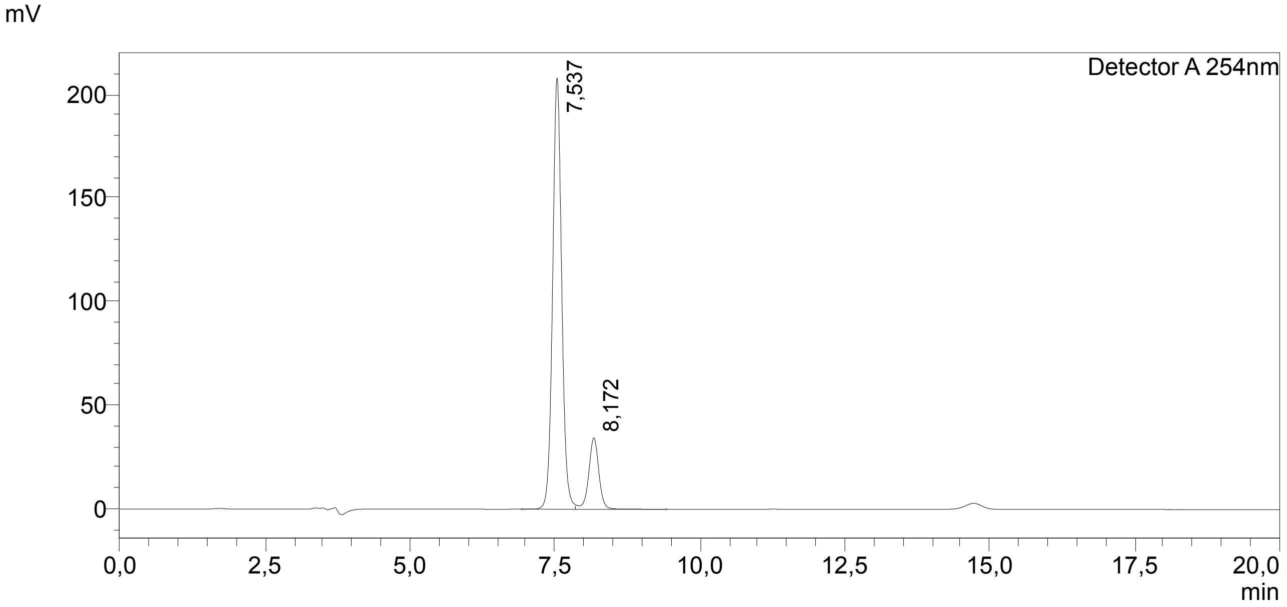

<Peak Table>

Detector A 254nm

Peak#	Ret. Time	Area	Height	Area%	Height%
1	7,537	2275678	208003	84,487	85,828
2	8,172	417833	34346	15,513	14,172
Total		2693511	242349	100,000	100,000

Figure S52b. Chiral chromatogram of **8m**.

SHIMADZU LabSolutions

Analysis Report

<Sample Information>

Sample Name	: PN-16_spot2_peak2_		
Sample ID	: PN-16_spot2_peak2_		
Data Filename	: PN-16_spot2_peak2_.lcd		
Method Filename	: 100EtOAc_.lcm		
Batch Filename	: batch_.lcb		
Vial #	: 1-4	Sample Type	: Unknown
Injection Volume	: 10 uL		
Date Acquired	: 02.07.2019 12:38:33	Acquired by	: HPLC - Natascha
Date Processed	: 02.07.2019 12:58:34	Processed by	: HPLC - Natascha

<Chromatogram>

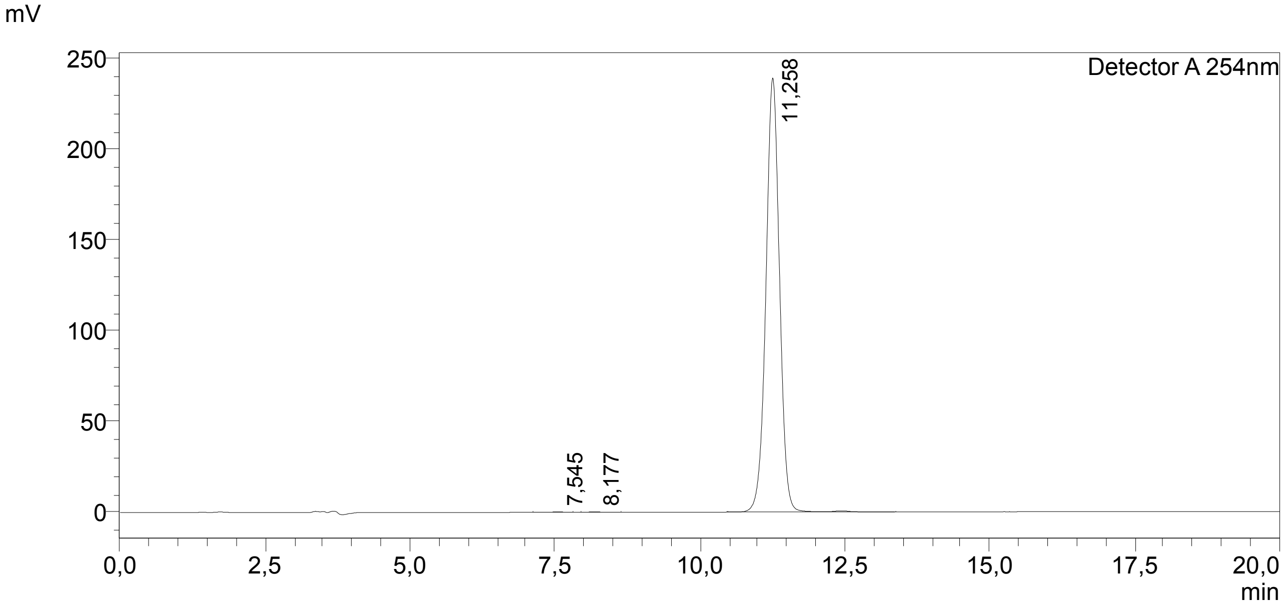

<Peak Table>

Detector A 254nm

Peak#	Ret. Time	Area	Height	Area%	Height%
1	7,545	3188	314	0,081	0,131
2	8,177	3153	275	0,080	0,114
3	11,258	3910182	239325	99,838	99,755
Total		3916523	239913	100,000	100,000

Figure S53b. Chiral chromatogram of **5n**.

Analysis Report

<Sample Information>

Sample Name : PN-22_spot1_peak1_
Sample ID : PN-22_spot1_peak1_
Data Filename : PN-22_spot1_peak1_.lcd
Method Filename : 100EtOAc_.lcm
Batch Filename : batch_.lcb
Vial # : 1-5
Sample Type : Unknown
Injection Volume : 10 uL
Date Acquired : 02.07.2019 12:58:58
Acquired by : HPLC - Natascha
Date Processed : 02.07.2019 13:18:59
Processed by : HPLC - Natascha

<Chromatogram>

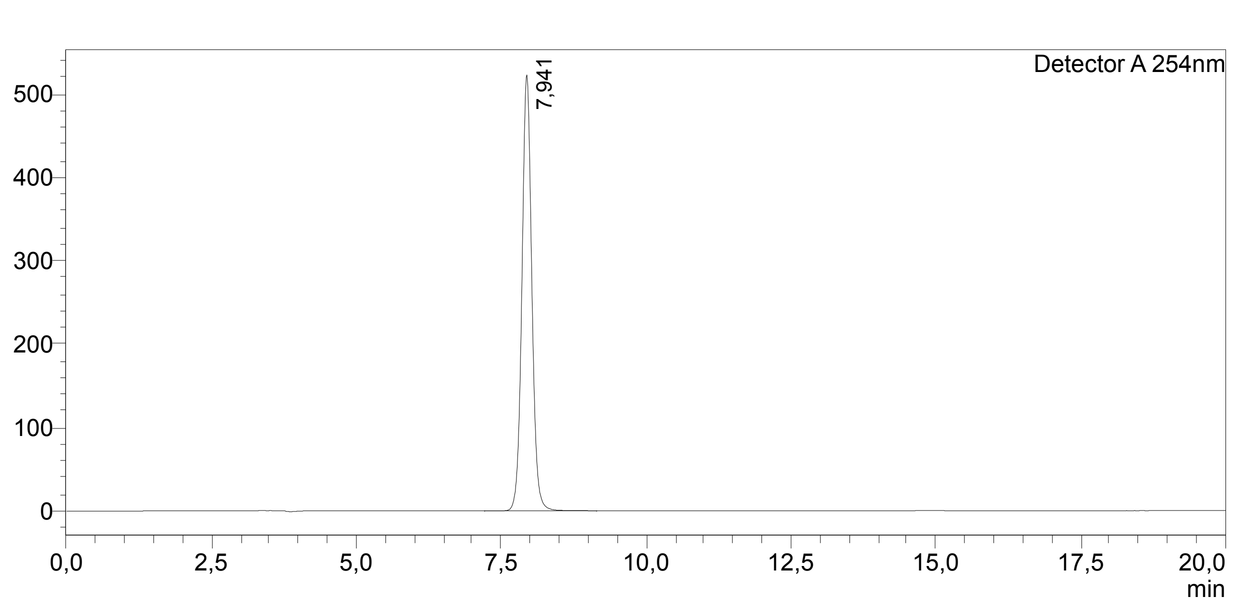

<Peak Table>

Detector A 254nm

Peak#	Ret. Time	Area	Height	Area%	Height%
1	7,941	6236918	522311	100,000	100,000
Total		6236918	522311	100,000	100,000

Figure S54b. Chiral chromatogram of **6n**.

SHIMADZU LabSolutions

Analysis Report

<Sample Information>

Sample Name	: PN-22_spot1_peak2_		
Sample ID	: PN-22_spot1_peak2_		
Data Filename	: PN-22_spot1_peak2_.lcd		
Method Filename	: 100EtOAc_.lcm		
Batch Filename	: batch_.lcb		
Vial #	: 1-6	Sample Type	: Unknown
Injection Volume	: 10 uL		
Date Acquired	: 02.07.2019 13:19:23	Acquired by	: HPLC - Natascha
Date Processed	: 02.07.2019 13:39:25	Processed by	: HPLC - Natascha

<Chromatogram>

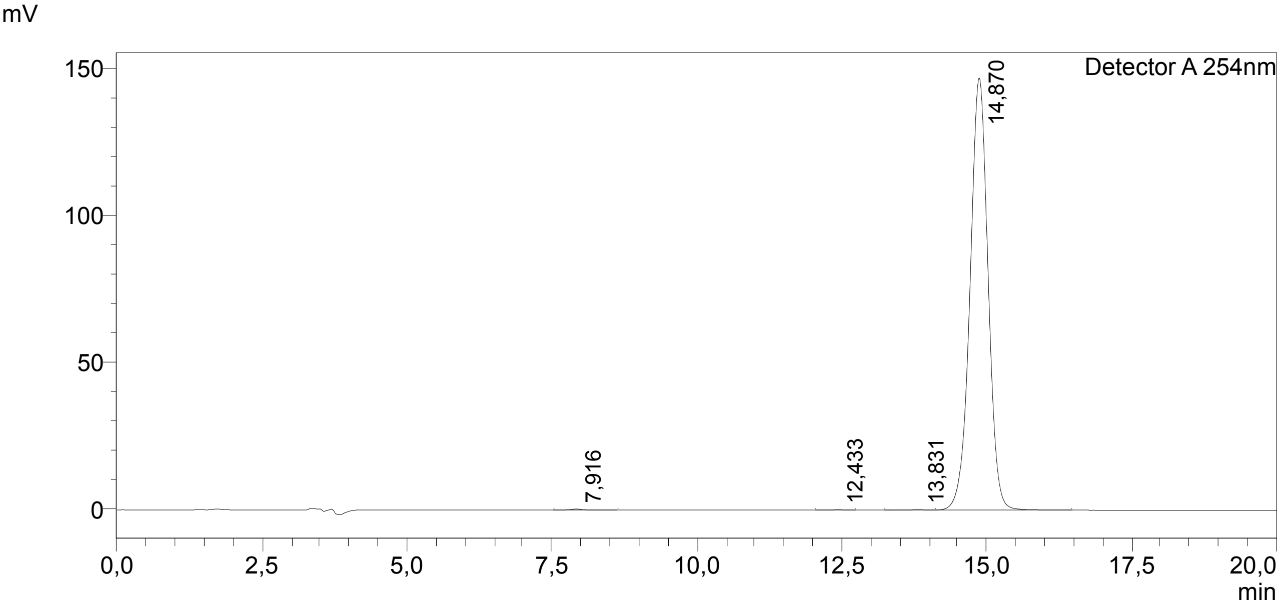

<Peak Table>

Detector A 254nm

Peak#	Ret. Time	Area	Height	Area%	Height%
1	7,916	4104	321	0,129	0,218
2	12,433	1993	109	0,063	0,074
3	13,831	1270	93	0,040	0,063
4	14,870	3174198	146822	99,768	99,645
Total		3181565	147345	100,000	100,000

Figure S55b. Chiral chromatogram of **7n**.

SHIMADZU LabSolutions

Analysis Report

<Sample Information>

Sample Name	: PN-22_spot2_peak1_		
Sample ID	: PN-22_spot2_peak1_		
Data Filename	: PN-22_spot2_peak1_.lcd		
Method Filename	: 100EtOAc_.lcm		
Batch Filename	: batch_.lcb		
Vial #	: 1-7	Sample Type	: Unknown
Injection Volume	: 10 uL		
Date Acquired	: 02.07.2019 13:39:48	Acquired by	: HPLC - Natascha
Date Processed	: 02.07.2019 13:59:49	Processed by	: HPLC - Natascha

<Chromatogram>

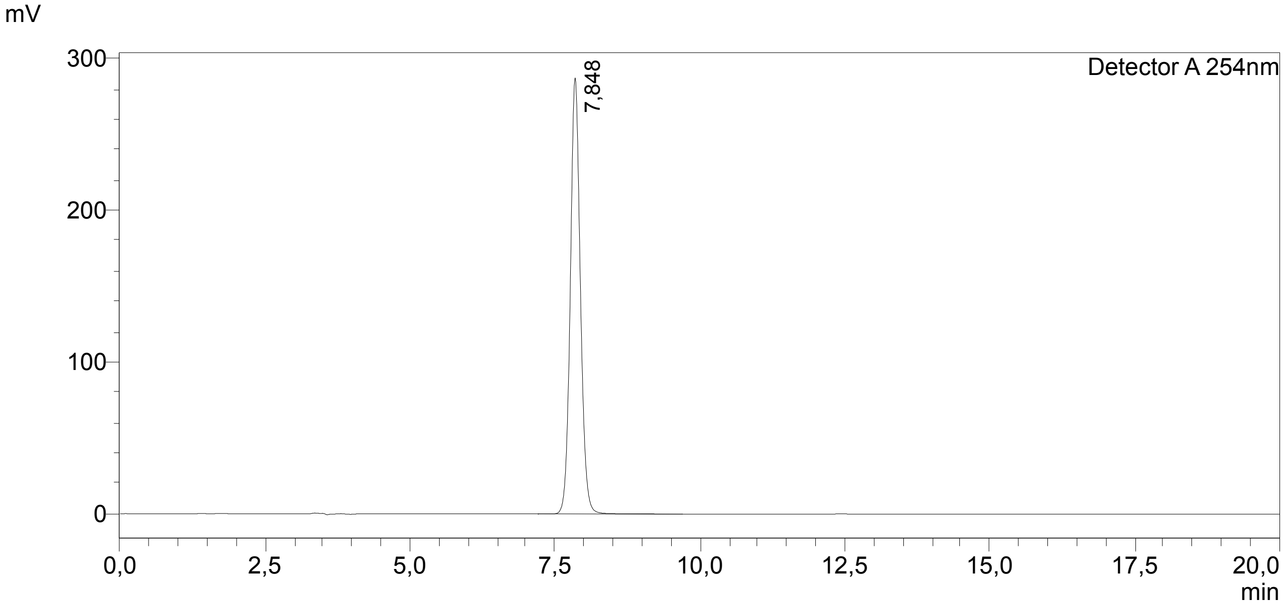

<Peak Table>

Detector A 254nm

Peak#	Ret. Time	Area	Height	Area%	Height%
1	7,848	3473579	287178	100,000	100,000
Total		3473579	287178	100,000	100,000

Figure S56b. Chiral chromatogram of **8n**.

SHIMADZU LabSolutions

Analysis Report

<Sample Information>

Sample Name : PN-22_spot2_peak2_
Sample ID : PN-22_spot2_peak2_
Data Filename : PN-22_spot2_peak2_.lcd
Method Filename : 100EtOAc_.lcm
Batch Filename : batch_.lcb
Vial # : 1-8
Sample Type : Unknown
Injection Volume : 10 uL
Date Acquired : 02.07.2019 14:00:13
Acquired by : HPLC - Natascha
Date Processed : 02.07.2019 14:20:14
Processed by : HPLC - Natascha

<Chromatogram>

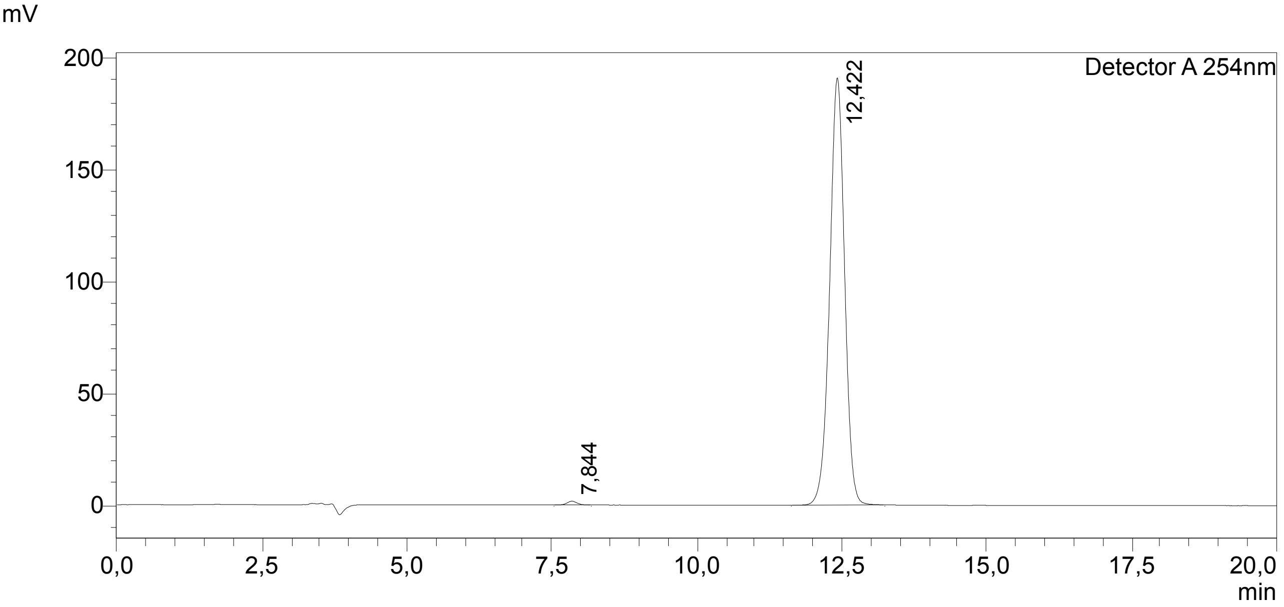

<Peak Table>

Detector A 254nm

Ret. Time	Area	Area%
7,844	20162	0,585
12,422	3427245	99,415
	3447407	100,000

Figure S57b. Chiral chromatogram of **5o**.

SHIMADZU LabSolutions

Analysis Report

<Sample Information>

Sample Name	: PN-12_spot_1_peak_1		
Sample ID	: PN-12_spot_1_peak_1		
Data Filename	: PN-12_spot_1_peak_1_01.lcd		
Method Filename	: Method_12.03.2019.lcm		
Batch Filename	:		
Vial #	: 1-1	Sample Type	: Unknown
Injection Volume	: 20 uL		
Date Acquired	: 16.05.2019 17:24:44	Acquired by	: HPLC - Natascha
Date Processed	: 16.05.2019 17:31:45	Processed by	: HPLC - Natascha

<Chromatogram>

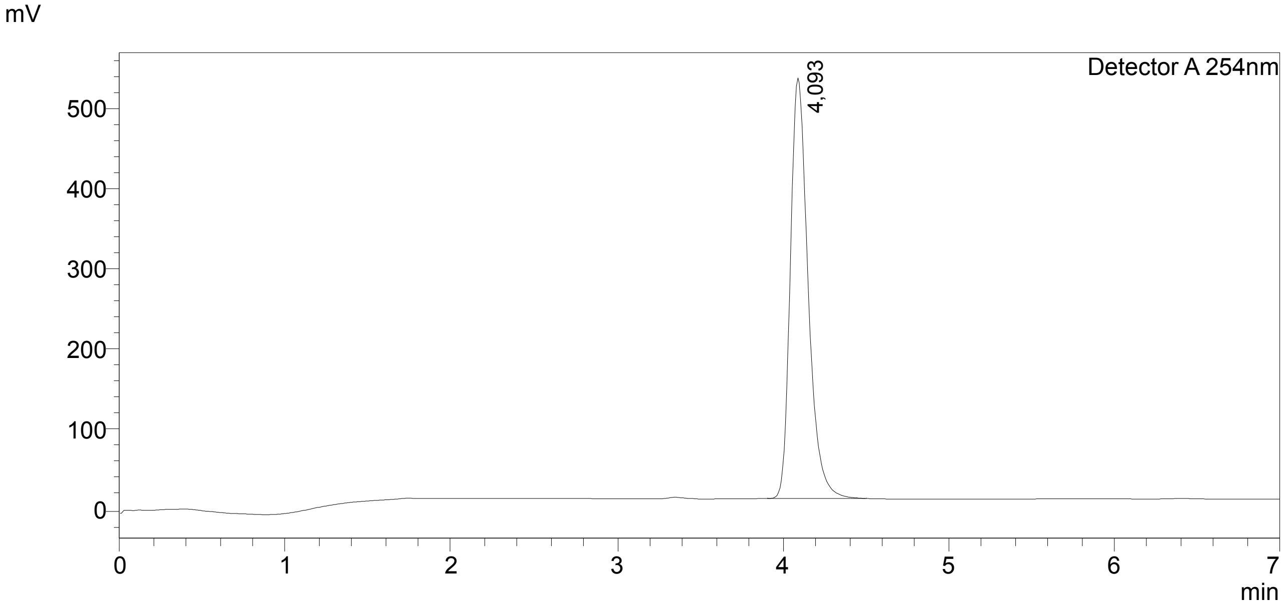

<Peak Table>

Detector A 254nm

Ret. Time	Area	Area%
4,093	3912668	100,000
	3912668	100,000

Figure S58b. Chiral chromatogram of **6o**.

SHIMADZU LabSolutions

Analysis Report

<Sample Information>

Sample Name	: PN-12_spot_1_peak_2		
Sample ID	: PN-12_spot_1_peak_2		
Data Filename	: PN-12_spot_1_peak_2_01.lcd		
Method Filename	: Method_12.03.2019.lcm		
Batch Filename	:		
Vial #	: 1-2	Sample Type	: Unknown
Injection Volume	: 20 uL		
Date Acquired	: 16.05.2019 17:32:47	Acquired by	: HPLC - Natascha
Date Processed	: 16.05.2019 17:39:49	Processed by	: HPLC - Natascha

<Chromatogram>

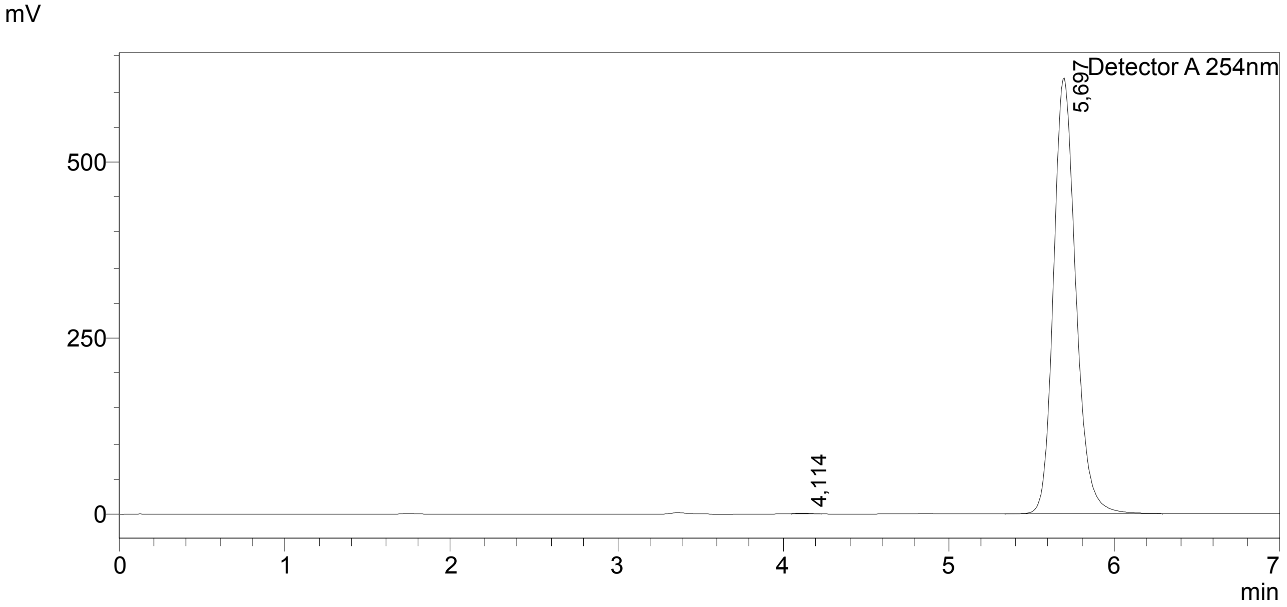

<Peak Table>

Detector A 254nm

Ret. Time	Area	Area%
4,114	5134	0,089
5,697	5783638	99,911
	5788772	100,000

Figure S59b. Chiral chromatogram of **7o**.

SHIMADZU LabSolutions

Analysis Report

<Sample Information>

Sample Name	: PN-12_spot_2_peak_1_2nd_meas		
Sample ID	: PN-12_spot_2_peak_1_2nd_meas		
Data Filename	: PN-12_spot_2_peak_1_2nd_meas02.lcd		
Method Filename	: Method_12.03.2019.lcm		
Batch Filename	:		
Vial #	: 1-3	Sample Type	: Unknown
Injection Volume	: 8 uL		
Date Acquired	: 30.05.2019 14:18:22	Acquired by	: HPLC - Natascha
Date Processed	: 30.05.2019 14:26:23	Processed by	: HPLC - Natascha

<Chromatogram>

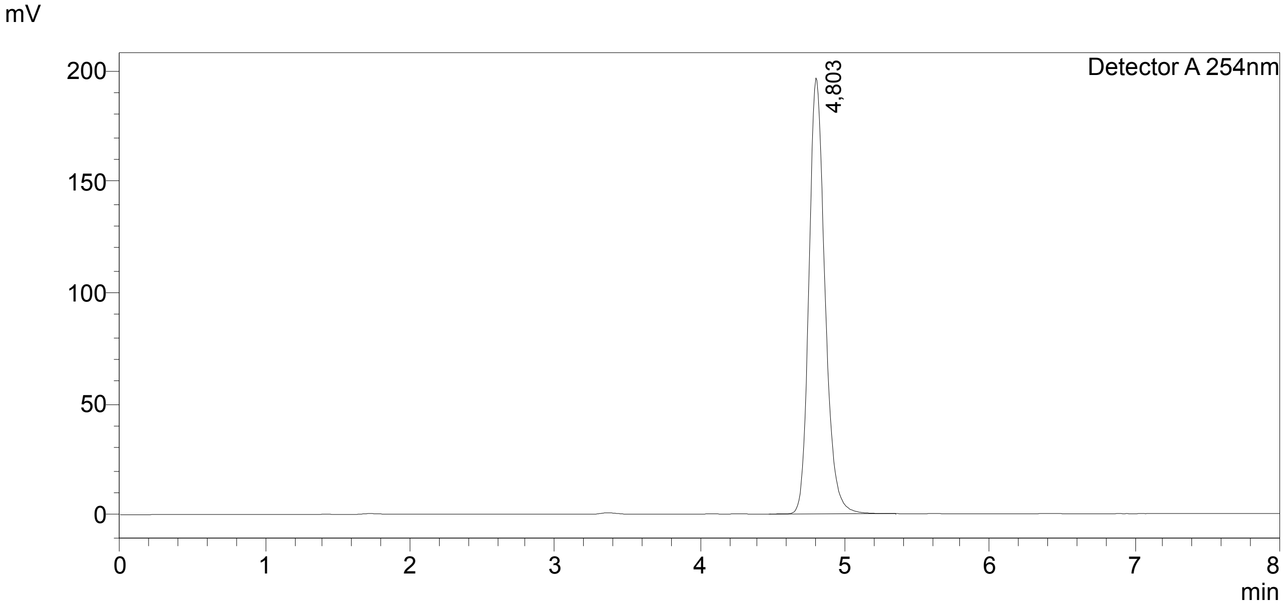

<Peak Table>

Detector A 254nm

Ret. Time	Area	Area%
4,803	1512257	100,000
	1512257	100,000

Figure S60b. Chiral chromatogram of **8o**.

Analysis Report

<Sample Information>

Sample Name	: PN-12_spot_2_peak_2		
Sample ID	: PN-12_spot_2_peak_2		
Data Filename	: PN-12_spot_2_peak_2_01.lcd		
Method Filename	: Method_12.03.2019.lcm		
Batch Filename	:		
Vial #	: 1-4	Sample Type	: Unknown
Injection Volume	: 20 uL		
Date Acquired	: 16.05.2019 17:49:32	Acquired by	: HPLC - Natascha
Date Processed	: 16.05.2019 17:57:03	Processed by	: HPLC - Natascha

<Chromatogram>

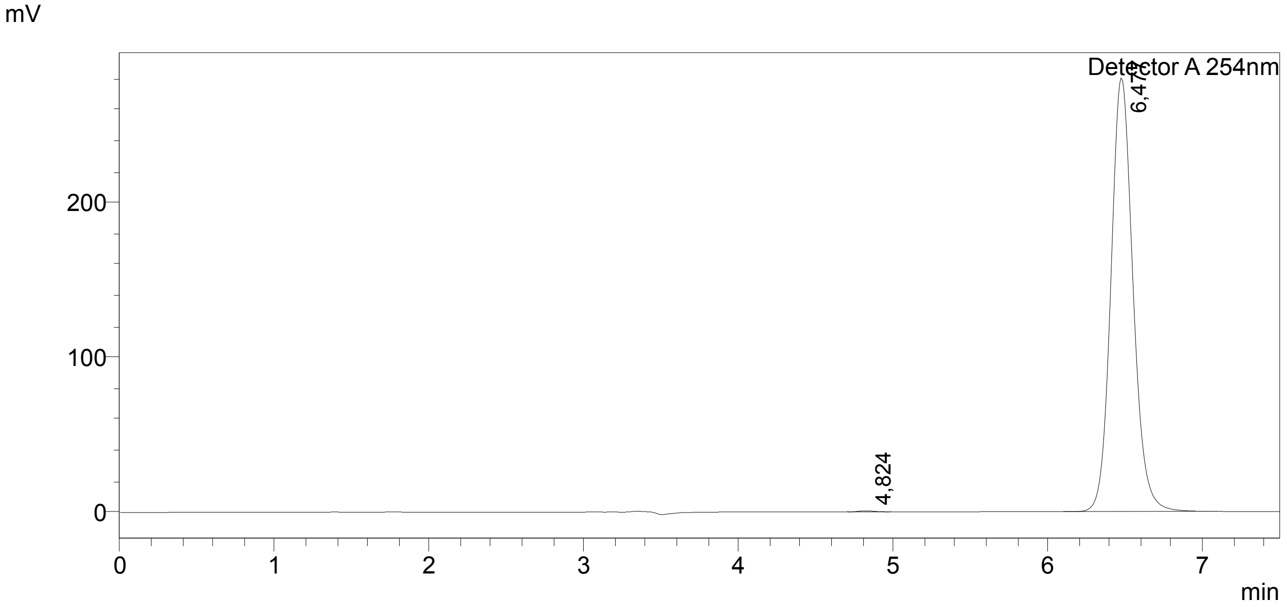

<Peak Table>

Detector A 254nm

Ret. Time	Area	Area%
4,824	4624	0,167
6,477	2761610	99,833
	2766234	100,000

Figure S61b. Chiral chromatogram of **5p**.

SHIMADZU LabSolutions

Analysis Report

<Sample Information>

Sample Name	: PN-2_spot_1_peak_1		
Sample ID	: PN-2_spot_1_peak_1		
Data Filename	: PN-2_spot_1_peak_1_02.lcd		
Method Filename	: Method_12.03.2019.lcm		
Batch Filename	:		
Vial #	: 1-5	Sample Type	: Unknown
Injection Volume	: 20 uL		
Date Acquired	: 15.05.2019 20:05:51	Acquired by	: HPLC - Natascha
Date Processed	: 15.05.2019 20:18:22	Processed by	: HPLC - Natascha

<Chromatogram>

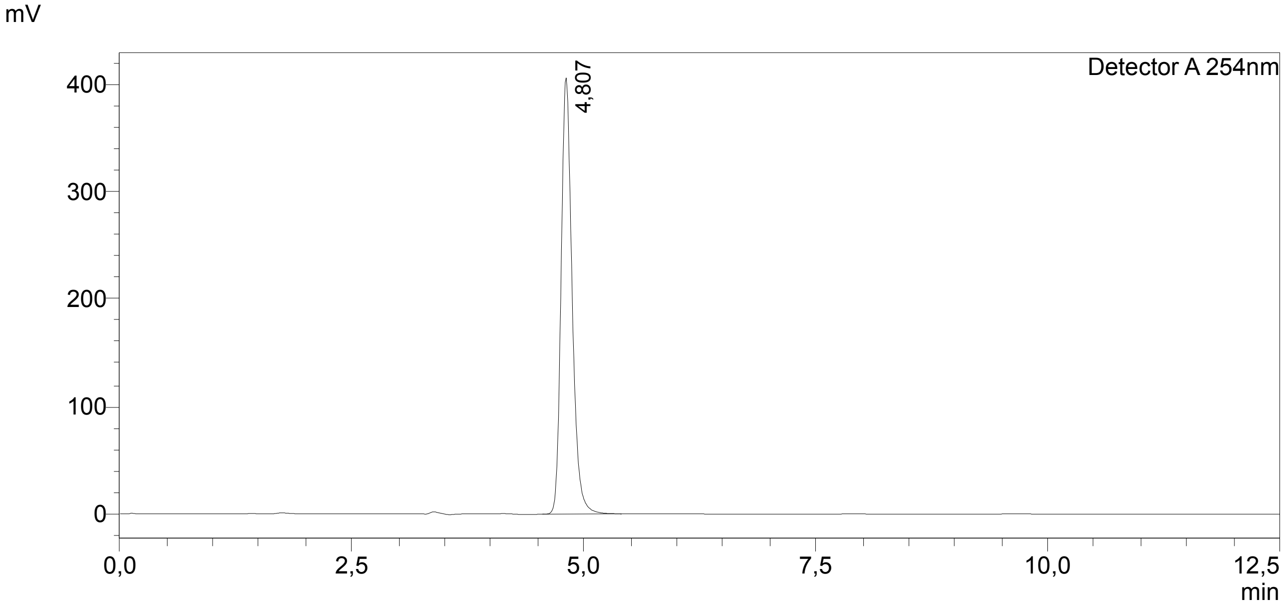

<Peak Table>

Detector A 254nm

Ret. Time	Area	Area%
4,807	3363236	100,000
	3363236	100,000

Figure S62b. Chiral chromatogram of **6p**.

SHIMADZU LabSolutions

Analysis Report

<Sample Information>

Sample Name : PN-2_spot_1_peak_2
Sample ID : PN-2_spot_1_peak_2
Data Filename : PN-2_spot_1_peak_2_01.lcd
Method Filename : Method_12.03.2019.lcm
Batch Filename :
Vial # : 1-6
Sample Type : Unknown
Injection Volume : 20 uL
Date Acquired : 15.05.2019 20:19:29
Acquired by : HPLC - Natascha
Date Processed : 15.05.2019 20:32:01
Processed by : HPLC - Natascha

<Chromatogram>

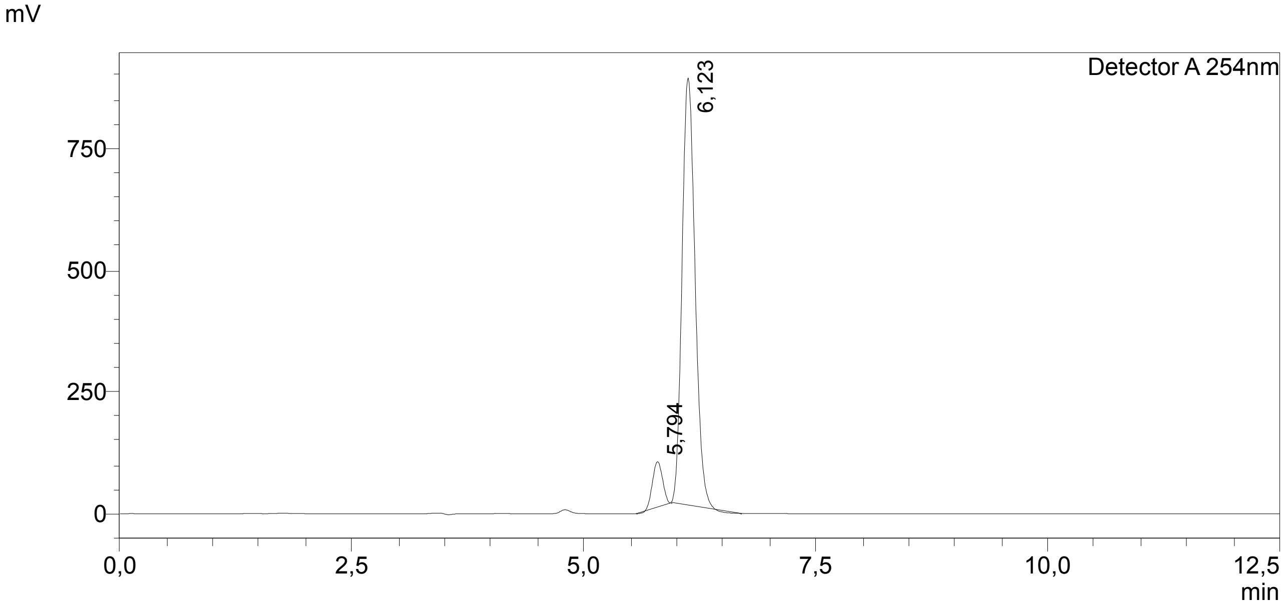

<Peak Table>

Detector A 254nm

Ret. Time	Area	Area%
5,794	700693	7,597
6,123	8522006	92,403
	9222699	100,000

Figure S63b. Chiral chromatogram of **7p**.

SHIMADZU LabSolutions

Analysis Report

<Sample Information>

Sample Name	: PN-2_Spot2_Peak1_		
Sample ID	: PN-2_Spot2_Peak1_		
Data Filename	: PN-2_Spot2_Peak1_04.lcd		
Method Filename	: Chiralpak_IA_100EtOAc_.lcm		
Batch Filename	:		
Vial #	: 1-1	Sample Type	: Unknown
Injection Volume	: 1 uL		
Date Acquired	: 29.04.2022 15:10:41	Acquired by	: HPLC - Natascha
Date Processed	: 29.04.2022 15:23:12	Processed by	: HPLC - Natascha

<Chromatogram>

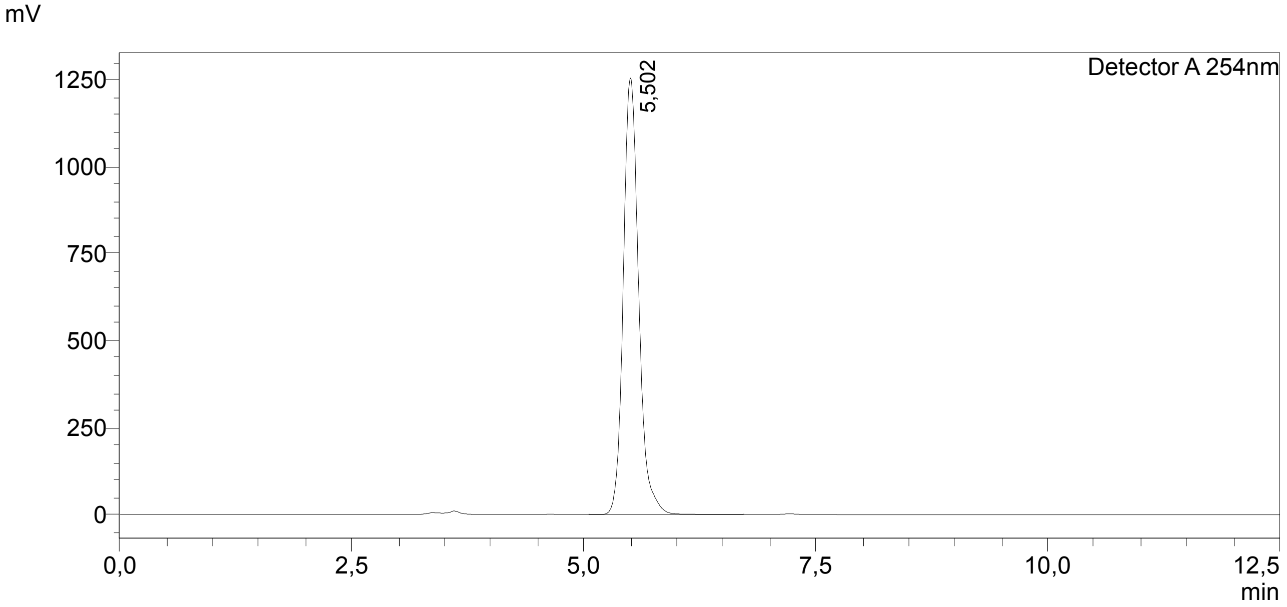

<Peak Table>

Detector A 254nm

Peak#	Ret. Time	Area	Area%
1	5,502	14486571	100,000
Total		14486571	100,000

Figure S64b. Chiral chromatogram of **8p**.

Analysis Report

<Sample Information>

Sample Name	: PN-2_spot_2_peak_2		
Sample ID	: PN-2_spot_2_peak_2		
Data Filename	: PN-2_spot_2_peak_2_01.lcd		
Method Filename	: Method_12.03.2019.lcm		
Batch Filename	:		
Vial #	: 1-8	Sample Type	: Unknown
Injection Volume	: 20 uL		
Date Acquired	: 15.05.2019 20:58:53	Acquired by	: HPLC - Natascha
Date Processed	: 15.05.2019 21:11:24	Processed by	: HPLC - Natascha

<Chromatogram>

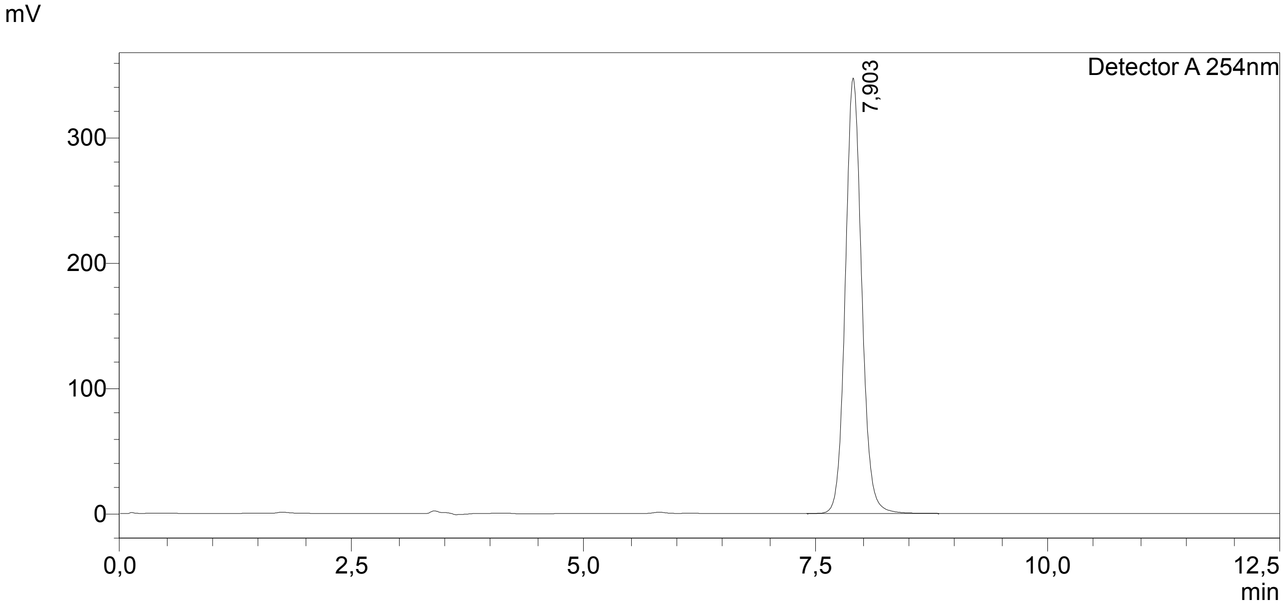

<Peak Table>

Detector A 254nm

Ret. Time	Area	Area%
7,903	4260781	100,000
	4260781	100,000

Figure S65b. Chiral chromatogram of **5q**.

SHIMADZU LabSolutions

Analysis Report

<Sample Information>

Sample Name	: PN-10_spot_1_peak_1		
Sample ID	: PN-10_spot_1_peak_1		
Data Filename	: PN-10_spot_1_peak_1_03.lcd		
Method Filename	: Method_12.03.2019.lcm		
Batch Filename	:		
Vial #	: 1-9	Sample Type	: Unknown
Injection Volume	: 20 uL		
Date Acquired	: 15.05.2019 22:02:47	Acquired by	: HPLC - Natascha
Date Processed	: 15.05.2019 22:15:18	Processed by	: HPLC - Natascha

<Chromatogram>

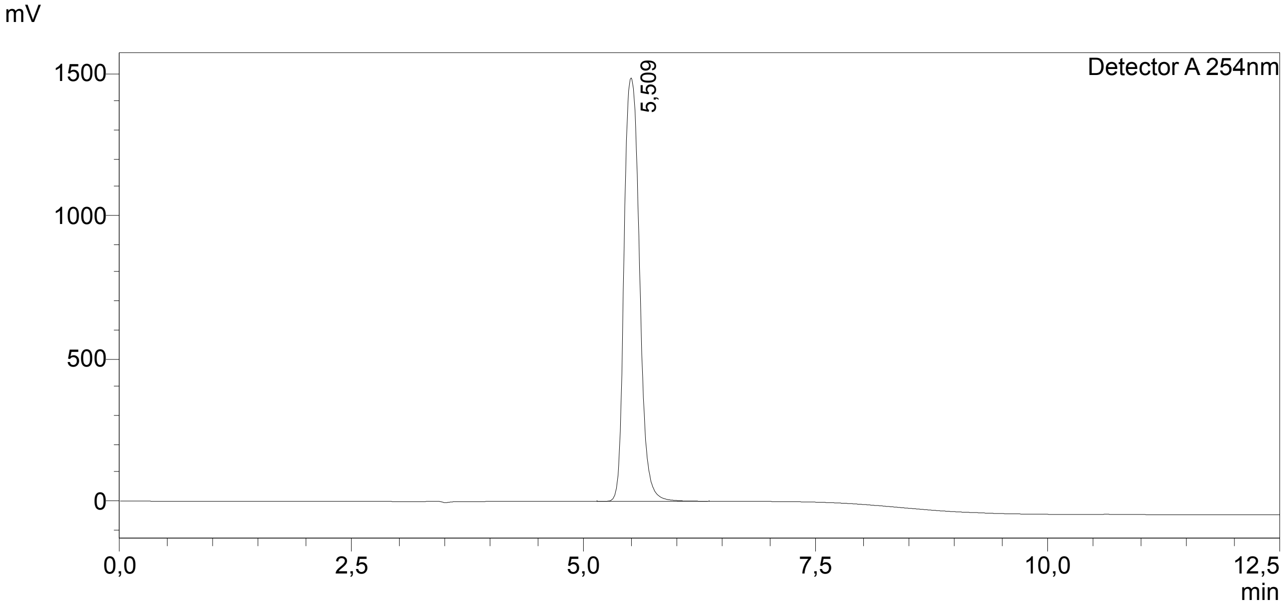

<Peak Table>

Detector A 254nm

Ret. Time	Area	Area%
5,509	17144785	100,000
	17144785	100,000

Figure S66b. Chiral chromatogram of **6q**.

SHIMADZU LabSolutions

Analysis Report

<Sample Information>

Sample Name	: PN-10_spot_1_peak_2		
Sample ID	: PN-10_spot_1_peak_2		
Data Filename	: PN-10_spot_1_peak_2_01.lcd		
Method Filename	: Method_12.03.2019.lcm		
Batch Filename	:		
Vial #	: 1-10	Sample Type	: Unknown
Injection Volume	: 20 uL		
Date Acquired	: 15.05.2019 22:17:24	Acquired by	: HPLC - Natascha
Date Processed	: 15.05.2019 22:29:55	Processed by	: HPLC - Natascha

<Chromatogram>

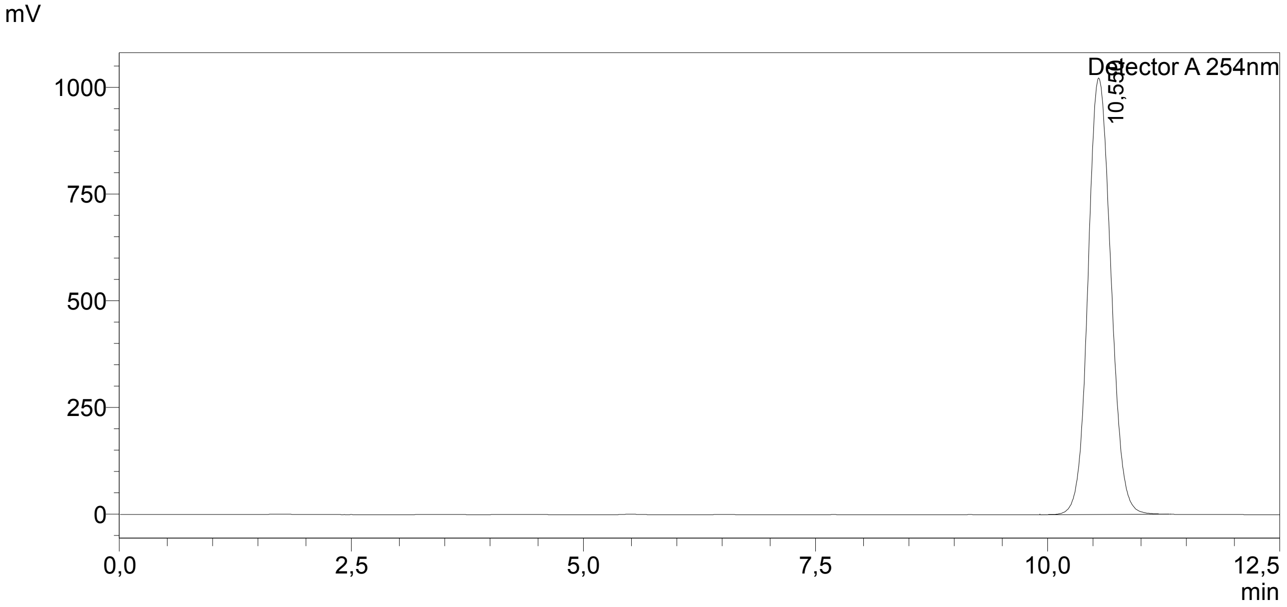

<Peak Table>

Detector A 254nm

Ret. Time	Area	Area%
10,550	17550294	100,000
	17550294	100,000

Figure S67b. Chiral chromatogram of **7q**.

SHIMADZU LabSolutions

Analysis Report

<Sample Information>

Sample Name	: PN-10_spot_2_peak_1		
Sample ID	: PN-10_spot_2_peak_1		
Data Filename	: PN-10_spot_2_peak_1_01.lcd		
Method Filename	: Method_12.03.2019.lcm		
Batch Filename	:		
Vial #	: 1-11	Sample Type	: Unknown
Injection Volume	: 20 uL		
Date Acquired	: 15.05.2019 21:33:17	Acquired by	: HPLC - Natascha
Date Processed	: 15.05.2019 21:45:48	Processed by	: HPLC - Natascha

<Chromatogram>

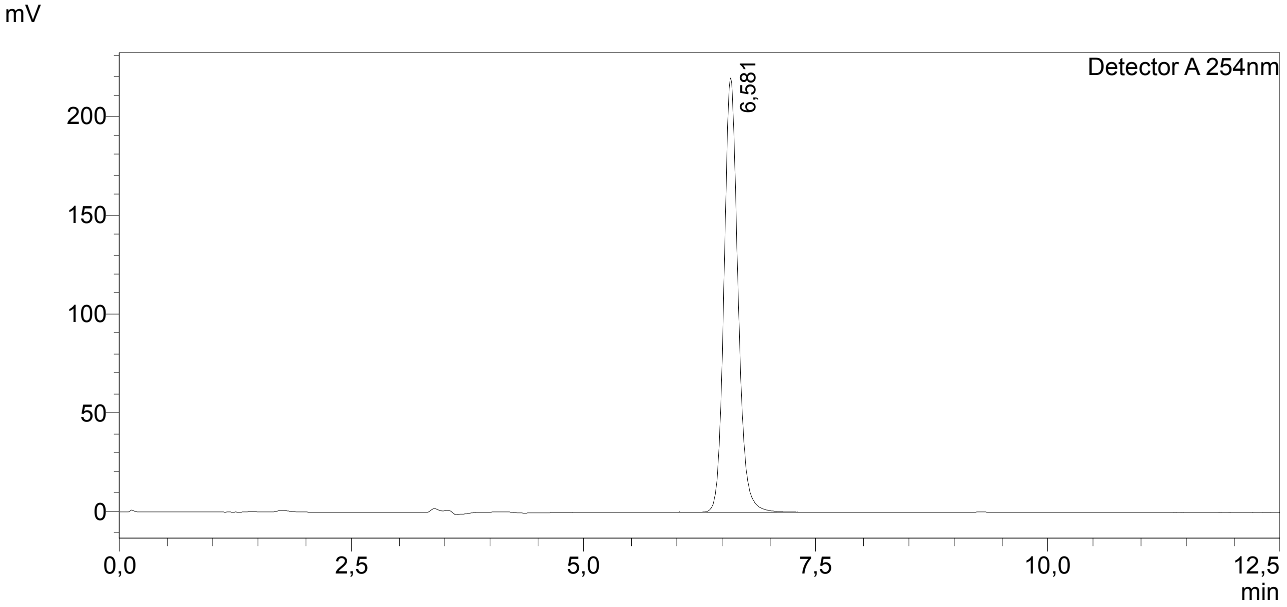

<Peak Table>

Detector A 254nm

Ret. Time	Area	Area%
6,581	2298964	100,000
	2298964	100,000

Figure S68b. Chiral chromatogram of **8q**.

SHIMADZU LabSolutions

Analysis Report

<Sample Information>

Sample Name	: PN-10_spot_2_peak_2		
Sample ID	: PN-10_spot_2_peak_2		
Data Filename	: PN-10_spot_2_peak_2_01.lcd		
Method Filename	: Method_12.03.2019.lcm		
Batch Filename	:		
Vial #	: 1-12	Sample Type	: Unknown
Injection Volume	: 20 uL		
Date Acquired	: 15.05.2019 21:47:05	Acquired by	: HPLC - Natascha
Date Processed	: 15.05.2019 21:59:36	Processed by	: HPLC - Natascha

<Chromatogram>

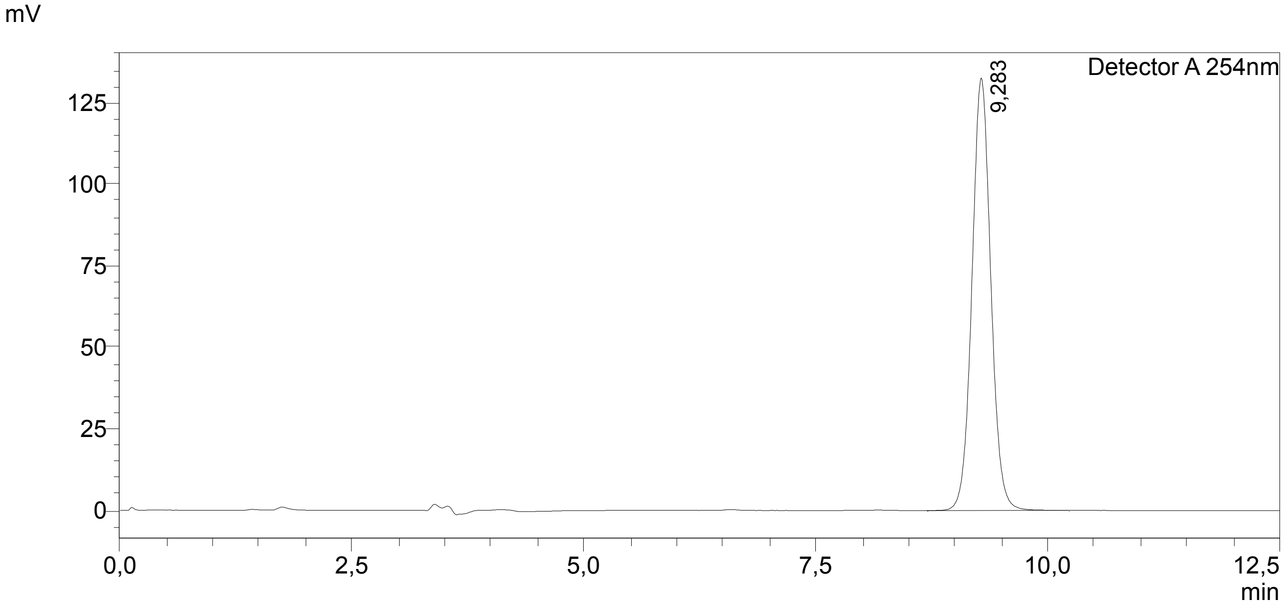

<Peak Table>

Detector A 254nm

Ret. Time	Area	Area%
9,283	1874465	100,000
	1874465	100,000

Figure S69b. Chiral chromatogram of **5r**.

SHIMADZU LabSolutions

Analysis Report

<Sample Information>

Sample Name	: CE-166_spot_1_peak_1		
Sample ID	: CE-166_spot_1_peak_1		
Data Filename	: CE-166_spot_1_peak_1_01.lcd		
Method Filename	: Method_12.03.2019.lcm		
Batch Filename	:		
Vial #	: 1-1	Sample Type	: Unknown
Injection Volume	: 20 uL		
Date Acquired	: 31.05.2019 17:51:24	Acquired by	: HPLC - Natascha
Date Processed	: 20.02.2020 09:46:12	Processed by	: HPLC - Natascha

<Chromatogram>

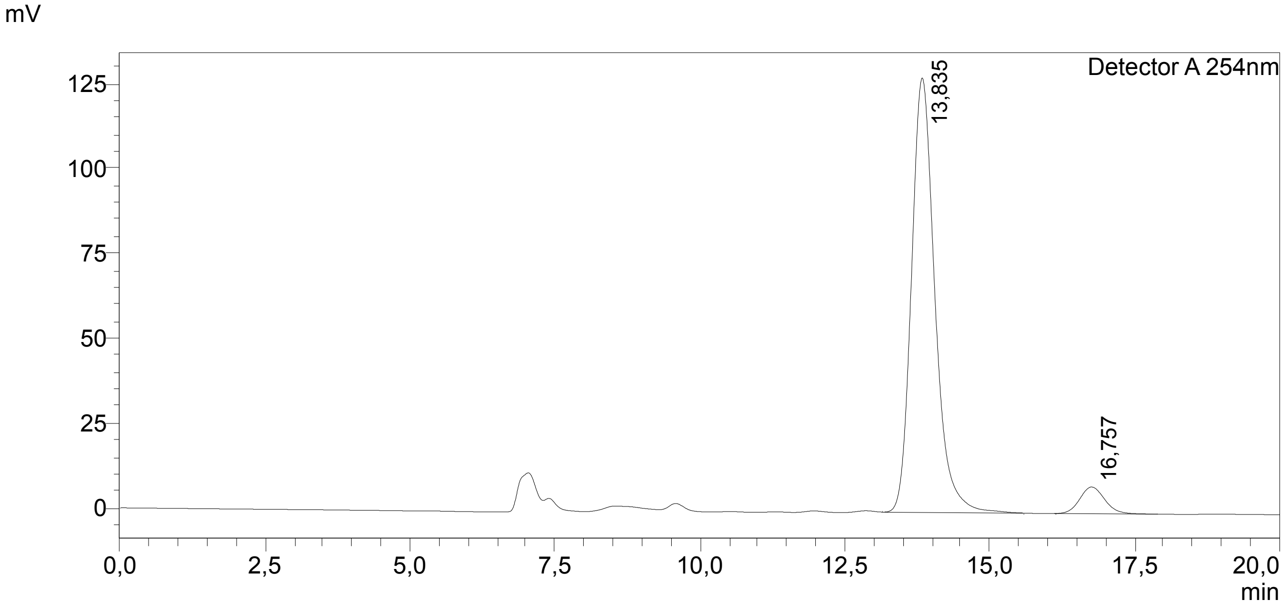

<Peak Table>

Detector A 254nm

Ret. Time	Area	Area%
13,835	3589073	93,810
16,757	236836	6,190
	3825910	100,000

Figure S70b. Chiral chromatogram of **6r**.

SHIMADZU LabSolutions

Analysis Report

<Sample Information>

Sample Name	: CE-166_spot_1_peak_2		
Sample ID	: CE-166_spot_1_peak_2		
Data Filename	: CE-166_spot_1_peak_2_01.lcd		
Method Filename	: Method_12.03.2019.lcm		
Batch Filename	:		
Vial #	: 1-2	Sample Type	: Unknown
Injection Volume	: 20 uL		
Date Acquired	: 31.05.2019 18:17:40	Acquired by	: HPLC - Natascha
Date Processed	: 31.05.2019 18:37:41	Processed by	: HPLC - Natascha

<Chromatogram>

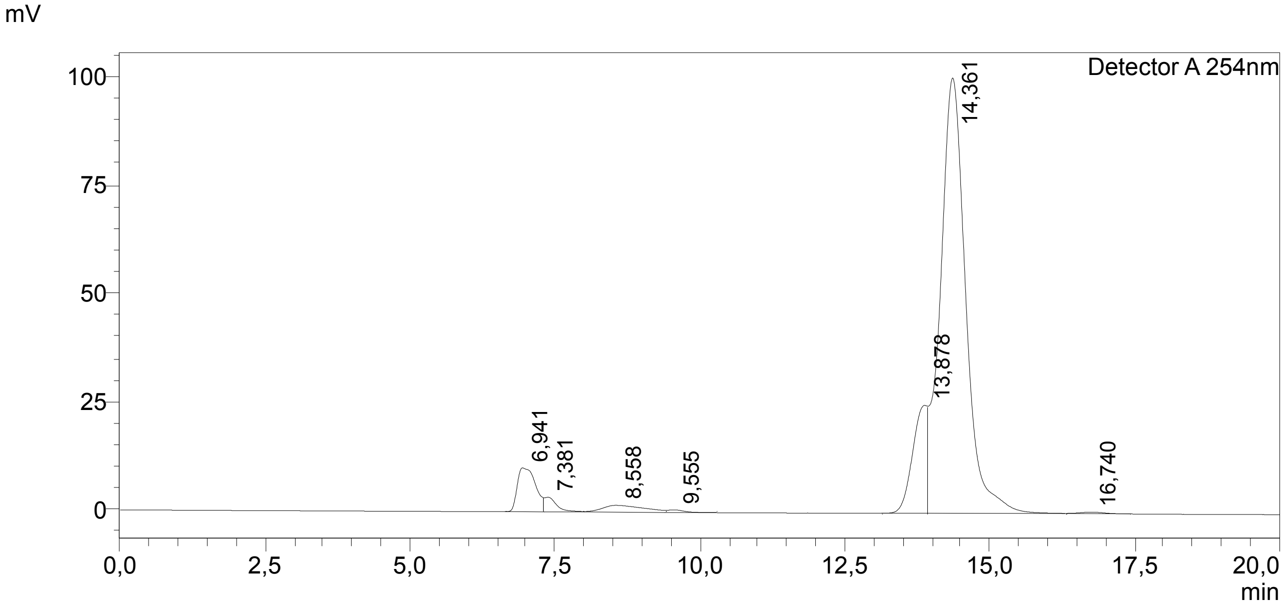

<Peak Table>

Detector A 254nm

Ret. Time	Area	Area%
6,941	225372	5,807
7,381	48400	1,247
8,558	79146	2,039
9,555	12357	0,318
13,878	442291	11,397
14,361	3063880	78,949
16,740	9394	0,242
	3880840	100,000

Figure S71b. Chiral chromatogram of **7r**.

SHIMADZU LabSolutions

Analysis Report

<Sample Information>

Sample Name	: CE_166_spot_2_peak_1_final		
Sample ID	: CE_166_spot_2_peak_1_final		
Data Filename	: CE_166_spot_2_peak_1_final01.lcd		
Method Filename	: 100%_EtOAc.lcm		
Batch Filename	:		
Vial #	: 1-21	Sample Type	: Unknown
Injection Volume	: 15 uL		
Date Acquired	: 22.08.2018 15:28:07	Acquired by	: HPLC - Natascha
Date Processed	: 22.08.2018 15:48:08	Processed by	: HPLC - Natascha

<Chromatogram>

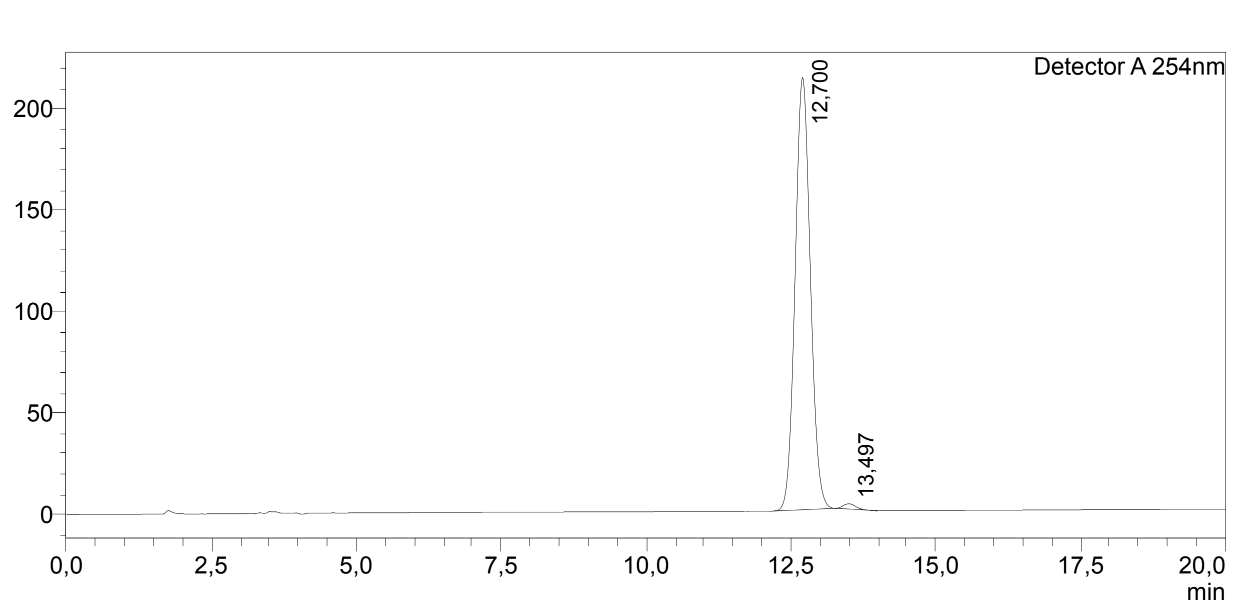

<Peak Table>

Detector A 254nm

Ret. Time	Area	Area%
12,700	3886124	99,026
13,497	38207	0,974
	3924331	100,000

Figure S72b. Chiral chromatogram of **8r**.

SHIMADZU LabSolutions

Analysis Report

<Sample Information>

Sample Name	: CE-166_Spot2_Peak2_		
Sample ID	: CE-166_Spot2_Peak2_		
Data Filename	: CE-166_Spot2_Peak2_01.lcd		
Method Filename	: Chiralpak_IA_100EtOAc_.lcm		
Batch Filename	:		
Vial #	: 1-1	Sample Type	: Unknown
Injection Volume	: 18 uL		
Date Acquired	: 29.04.2022 11:40:10	Acquired by	: HPLC - Natascha
Date Processed	: 29.04.2022 12:00:11	Processed by	: HPLC - Natascha

<Chromatogram>

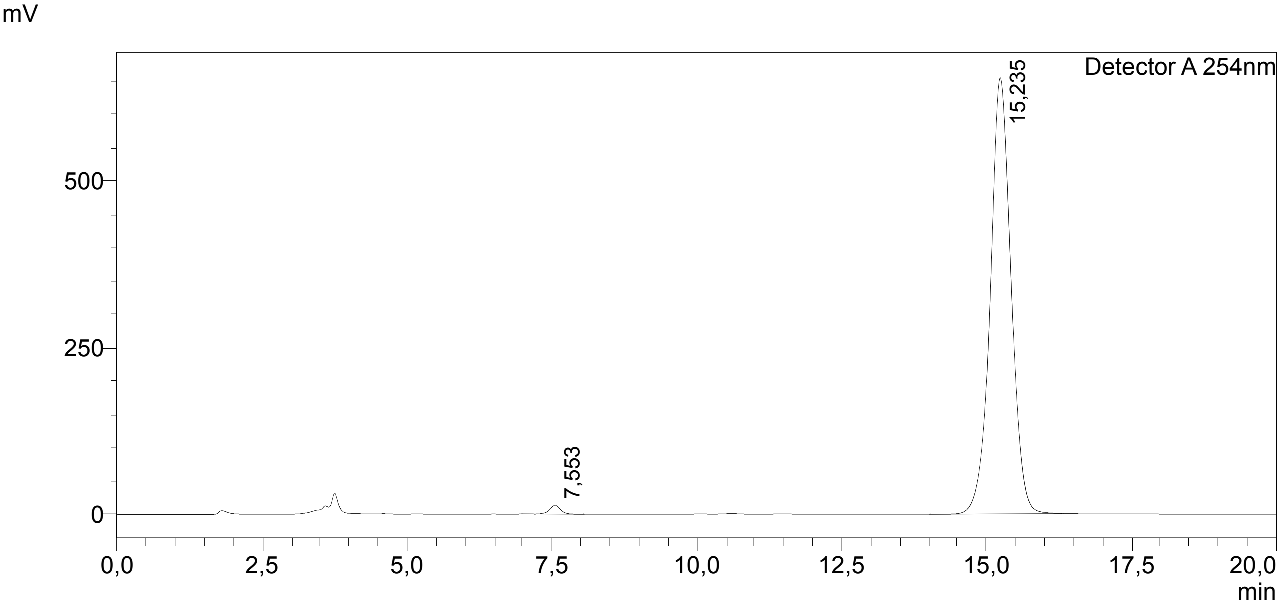

<Peak Table>

Detector A 254nm

Peak#	Ret. Time	Area	Area%
1	7,553	172475	1,046
2	15,235	16315900	98,954
Total		16488375	100,000

Figure S73b. Chiral chromatogram of **5s**.

SHIMADZU LabSolutions

Analysis Report

<Sample Information>

Sample Name	: PN-8_spot1_peak1_		
Sample ID	: PN-8_spot1_peak1_		
Data Filename	: PN-8_spot1_peak1_.lcd		
Method Filename	: 100EtOAc_.lcm		
Batch Filename	: batch_.lcb		
Vial #	: 1-1	Sample Type	: Unknown
Injection Volume	: 10 uL		
Date Acquired	: 03.07.2019 12:42:47	Acquired by	: HPLC - Natascha
Date Processed	: 03.07.2019 13:07:48	Processed by	: HPLC - Natascha

<Chromatogram>

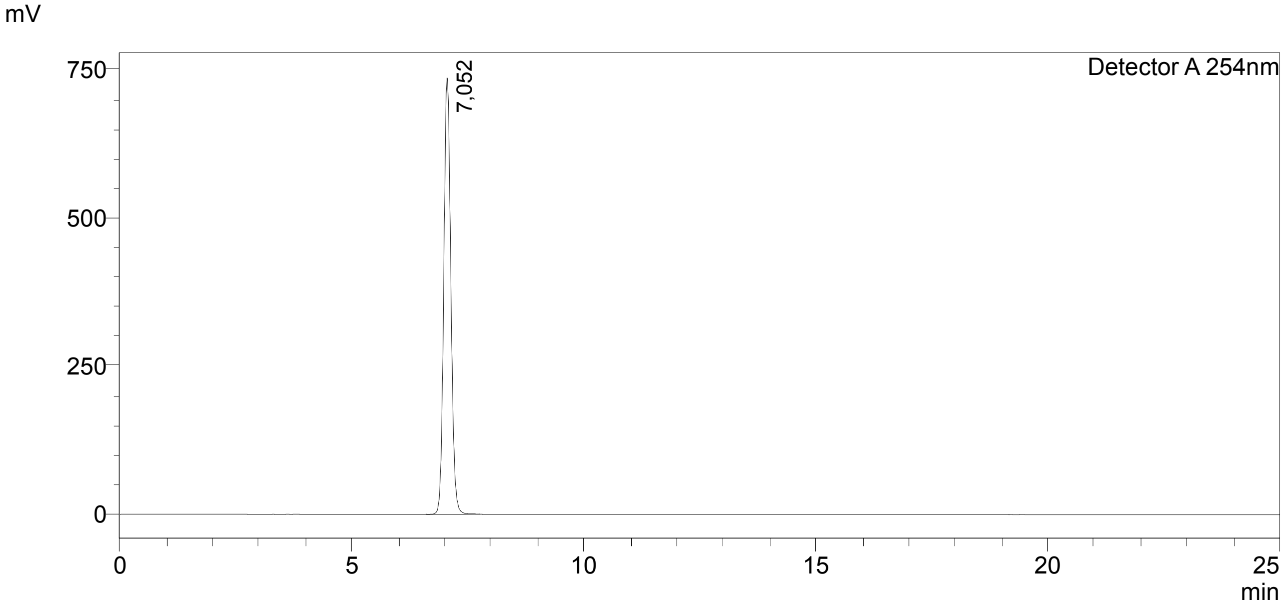

<Peak Table>

Detector A 254nm

Ret. Time	Area	Area%
7,052	7846370	100,000
	7846370	100,000

Figure S74b. Chiral chromatogram of **6s**.

SHIMADZU LabSolutions

Analysis Report

<Sample Information>

Sample Name	: PN-8_spot1_peak2_		
Sample ID	: PN-8_spot1_peak2_		
Data Filename	: PN-8_spot1_peak2_.lcd		
Method Filename	: 100EtOAc_.lcm		
Batch Filename	: batch_.lcb		
Vial #	: 1-2	Sample Type	: Unknown
Injection Volume	: 10 uL		
Date Acquired	: 03.07.2019 13:08:10	Acquired by	: HPLC - Natascha
Date Processed	: 03.07.2019 13:33:11	Processed by	: HPLC - Natascha

<Chromatogram>

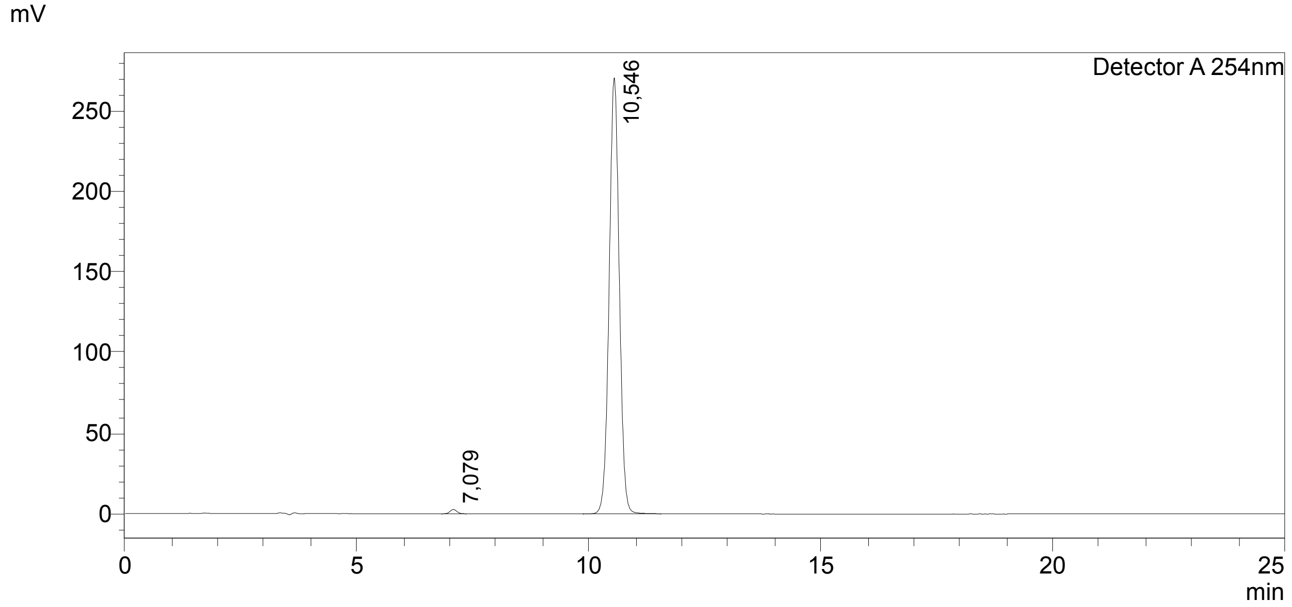

<Peak Table>

Detector A 254nm

Ret. Time	Area	Area%
7,079	26516	0,642
10,546	4103369	99,358
	4129885	100,000

Figure S75b. Chiral chromatogram of **7s**.

SHIMADZU LabSolutions

Analysis Report

<Sample Information>

Sample Name	: PN-8_spot2_peak1_		
Sample ID	: PN-8_spot2_peak1_		
Data Filename	: PN-8_spot2_peak1_.lcd		
Method Filename	: 100EtOAc_.lcm		
Batch Filename	: batch_.lcb		
Vial #	: 1-3	Sample Type	: Unknown
Injection Volume	: 10 uL		
Date Acquired	: 03.07.2019 13:33:33	Acquired by	: HPLC - Natascha
Date Processed	: 03.07.2019 13:58:34	Processed by	: HPLC - Natascha

<Chromatogram>

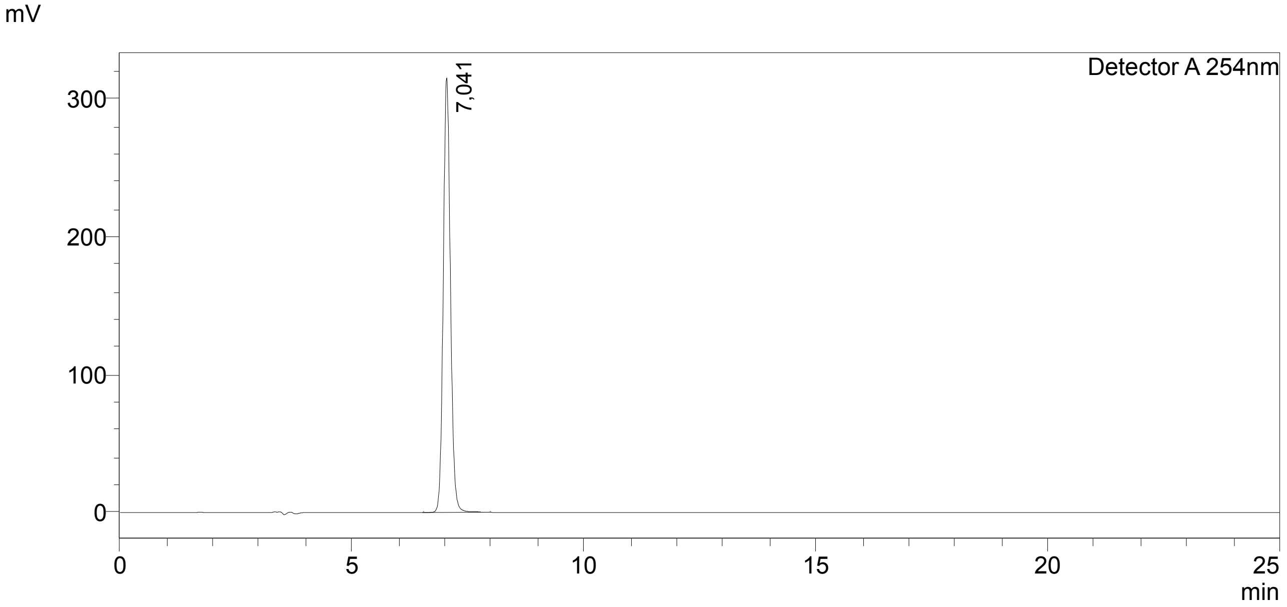

<Peak Table>

Detector A 254nm

Ret. Time	Area	Area%
7,041	3384511	100,000
	3384511	100,000

Figure S76b. Chiral chromatogram of **8s**.

SHIMADZU LabSolutions

Analysis Report

<Sample Information>

Sample Name	: PN-8_spot2_peak2_		
Sample ID	: PN-8_spot2_peak2_		
Data Filename	: PN-8_spot2_peak2_.lcd		
Method Filename	: 100EtOAc_.lcm		
Batch Filename	: batch_.lcb		
Vial #	: 1-4	Sample Type	: Unknown
Injection Volume	: 10 uL		
Date Acquired	: 03.07.2019 13:58:56	Acquired by	: HPLC - Natascha
Date Processed	: 03.07.2019 14:23:57	Processed by	: HPLC - Natascha

<Chromatogram>

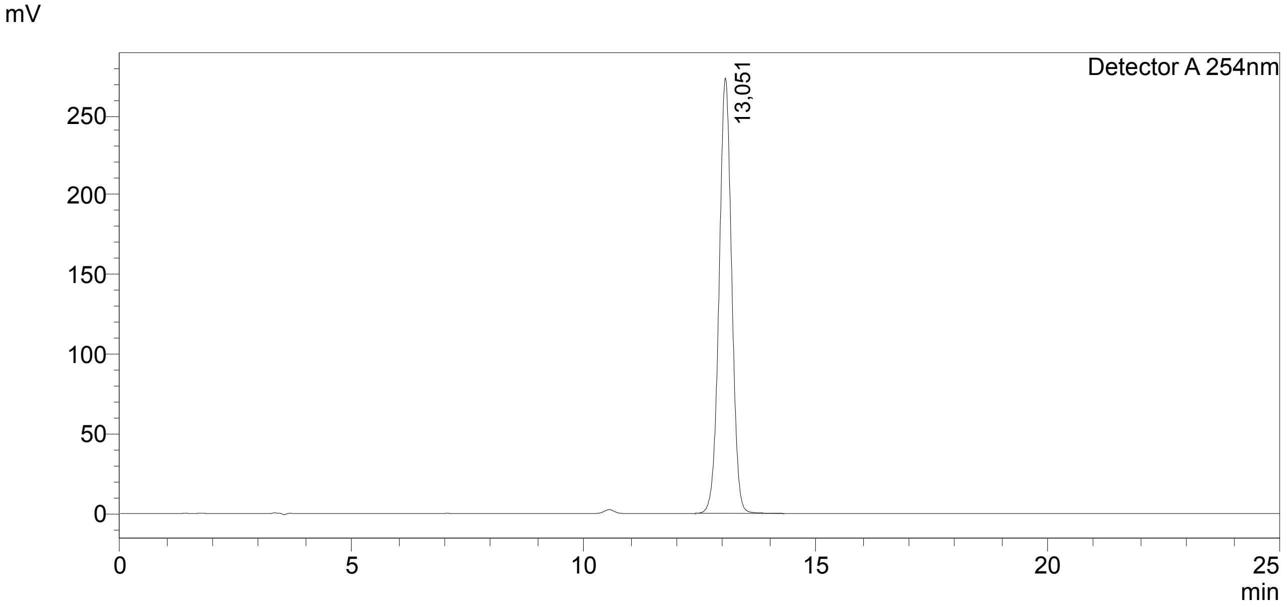

<Peak Table>

Detector A 254nm

Ret. Time	Area	Area%
13,051	5153827	100,000
	5153827	100,000

Figure S77b. Chiral chromatogram of **5t**.

SHIMADZU LabSolutions

Analysis Report

<Sample Information>

Sample Name	: CE_149_spot_1_peak_1__final_check		
Sample ID	: CE_149_spot_1_peak_1__final_che		
Data Filename	: CE_149_spot_1_peak_1__final_check01.lcd		
Method Filename	: 100%_EtOAc.lcm		
Batch Filename	:		
Vial #	: 1-21	Sample Type	: Unknown
Injection Volume	: 15 uL		
Date Acquired	: 25.07.2018 18:49:53	Acquired by	: HPLC - Natascha
Date Processed	: 25.07.2018 19:09:55	Processed by	: HPLC - Natascha

<Chromatogram>

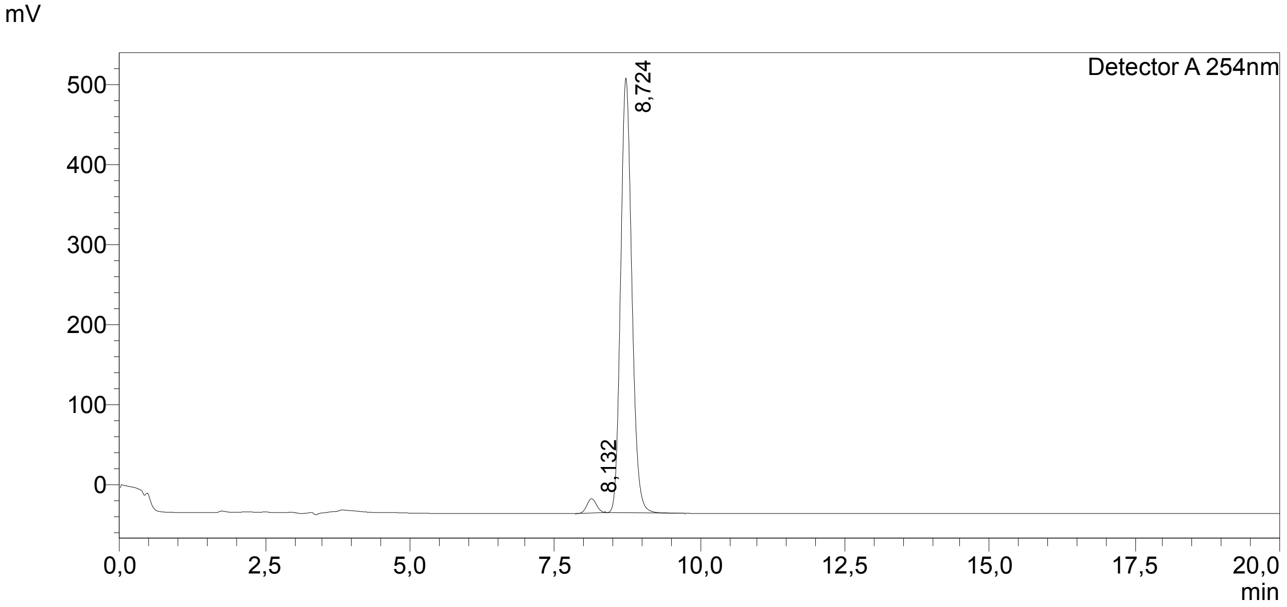

<Peak Table>

Detector A 254nm

Ret. Time	Area	Area%
8,132	206660	2,759
8,724	7283846	97,241
	7490506	100,000

Figure S78b. Chiral chromatogram of **6t**.

SHIMADZU LabSolutions

Analysis Report

<Sample Information>

Sample Name	: CE_149_spot_1_peak_2__final_check		
Sample ID	: CE_149_spot_1_peak_2__final_che		
Data Filename	: CE_149_spot_1_peak_2__final_check02.lcd		
Method Filename	: 100%_EtOAc.lcm		
Batch Filename	:		
Vial #	: 1-22	Sample Type	: Unknown
Injection Volume	: 15 uL		
Date Acquired	: 26.07.2018 17:53:32	Acquired by	: HPLC - Natascha
Date Processed	: 26.07.2018 18:13:34	Processed by	: HPLC - Natascha

<Chromatogram>

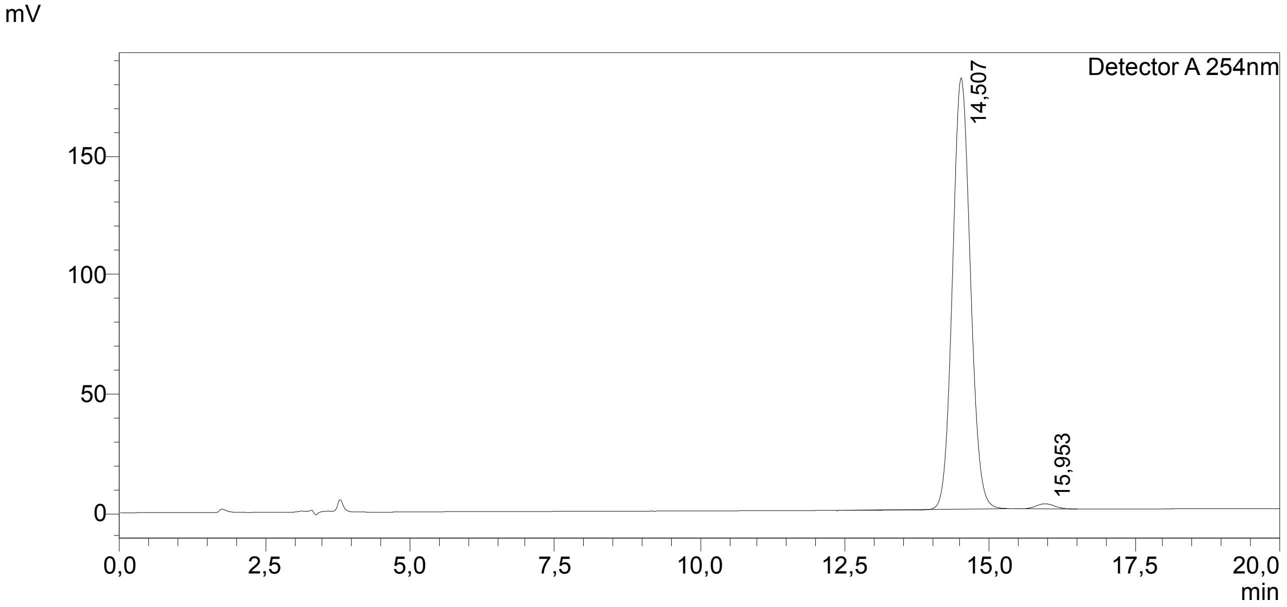

<Peak Table>

Detector A 254nm

Ret. Time	Area	Area%
14,507	3880254	98,778
15,953	47986	1,222
	3928240	100,000

Figure S79b. Chiral chromatogram of **7t**.

SHIMADZU LabSolutions

Analysis Report

<Sample Information>

Sample Name : CE_149_spot_2_peak_1__final_check
Sample ID : CE_149_spot_2_peak_1__final_che
Data Filename : CE_149_spot_2_peak_1__final_check01.lcd
Method Filename : 100%_EtOAc.lcm
Batch Filename :
Vial # : 1-21 Sample Type : Unknown
Injection Volume : 15 uL
Date Acquired : 26.07.2018 18:56:53 Acquired by : HPLC - Natascha
Date Processed : 26.07.2018 19:16:53 Processed by : HPLC - Natascha

<Chromatogram>

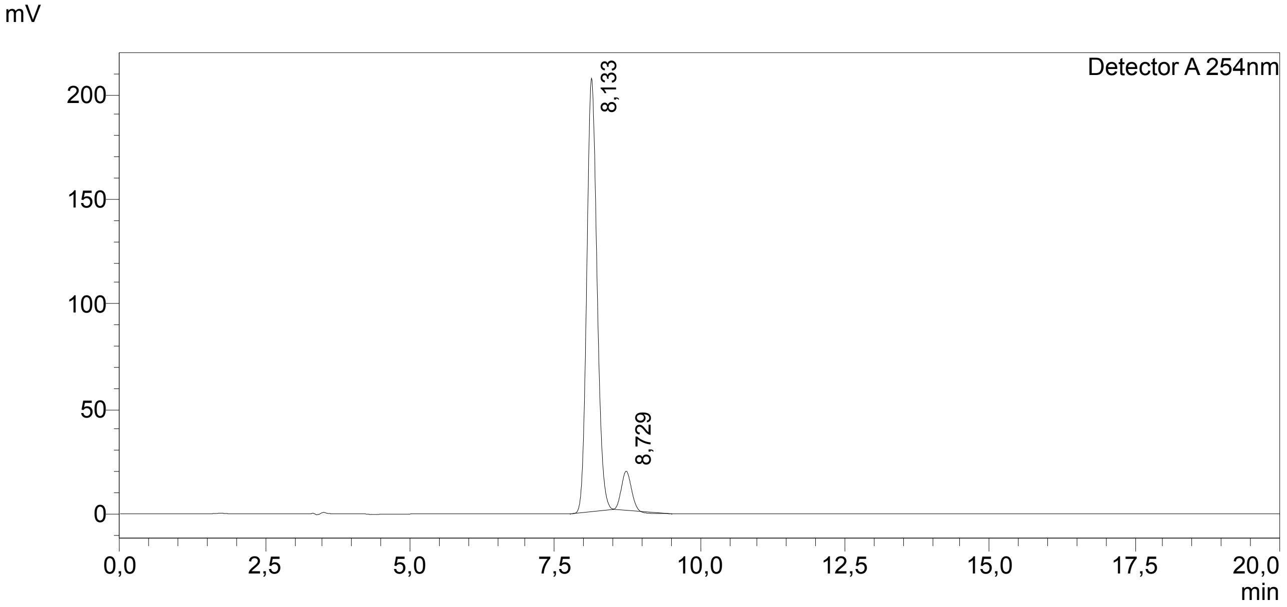

<Peak Table>

Detector A 254nm

Ret. Time	Area	Area%
8,133	2526976	92,357
8,729	209109	7,643
	2736085	100,000

Figure S80b. Chiral chromatogram of **8t**.

SHIMADZU LabSolutions

Analysis Report

<Sample Information>

Sample Name	: CE-149_Spot2_Peak2_		
Sample ID	: CE-149_Spot2_Peak2_		
Data Filename	: CE-149_Spot2_Peak2_01.lcd		
Method Filename	: Chiralpak_IA_100EtOAc_.lcm		
Batch Filename	:		
Vial #	: 1-1	Sample Type	: Unknown
Injection Volume	: 15 uL		
Date Acquired	: 29.04.2022 14:30:08	Acquired by	: HPLC - Natascha
Date Processed	: 29.04.2022 14:52:58	Processed by	: HPLC - Natascha

<Chromatogram>

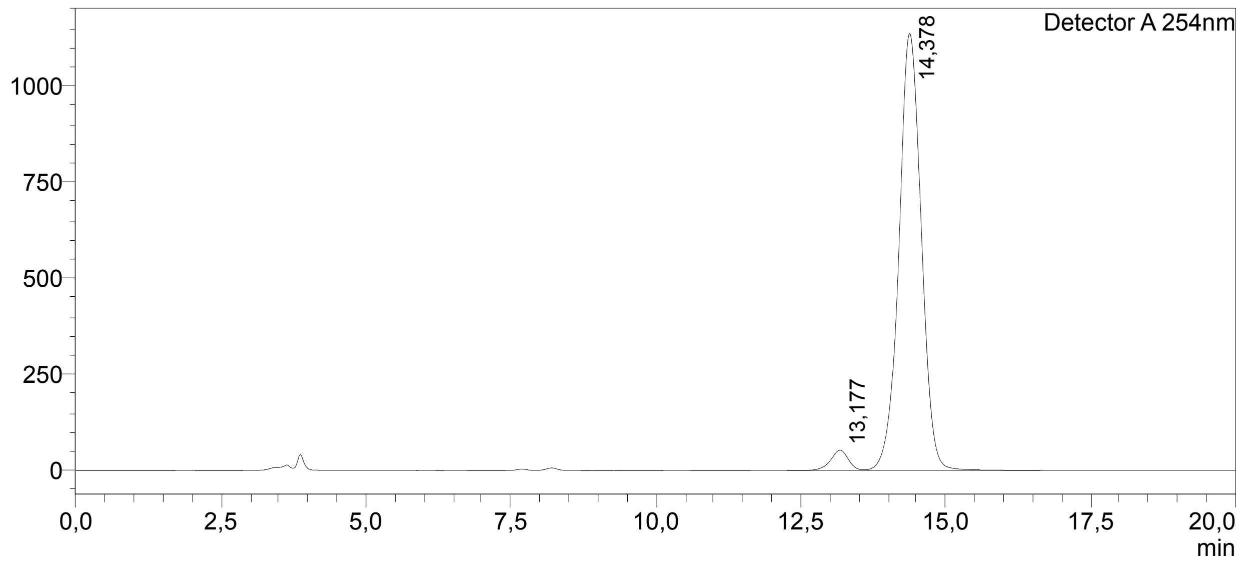

<Peak Table>

Detector A 254nm

Peak#	Ret. Time	Area	Area%
1	13,177	1134420	3,584
2	14,378	30516332	96,416
Total		31650752	100,000

Figure S81b. Chiral chromatogram of **5u**.

Analysis Report

<Sample Information>

Sample Name	: CE-156_spot_1_peak_1		
Sample ID	: CE-156_spot_1_peak_1		
Data Filename	: CE-156_spot_1_peak_1_01.lcd		
Method Filename	: Method_12.03.2019.lcm		
Batch Filename	:		
Vial #	: 1-5	Sample Type	: Unknown
Injection Volume	: 10 uL		
Date Acquired	: 12.03.2019 12:34:52	Acquired by	: HPLC - Natascha
Date Processed	: 28.04.2022 13:25:59	Processed by	: HPLC - Natascha

<Chromatogram>

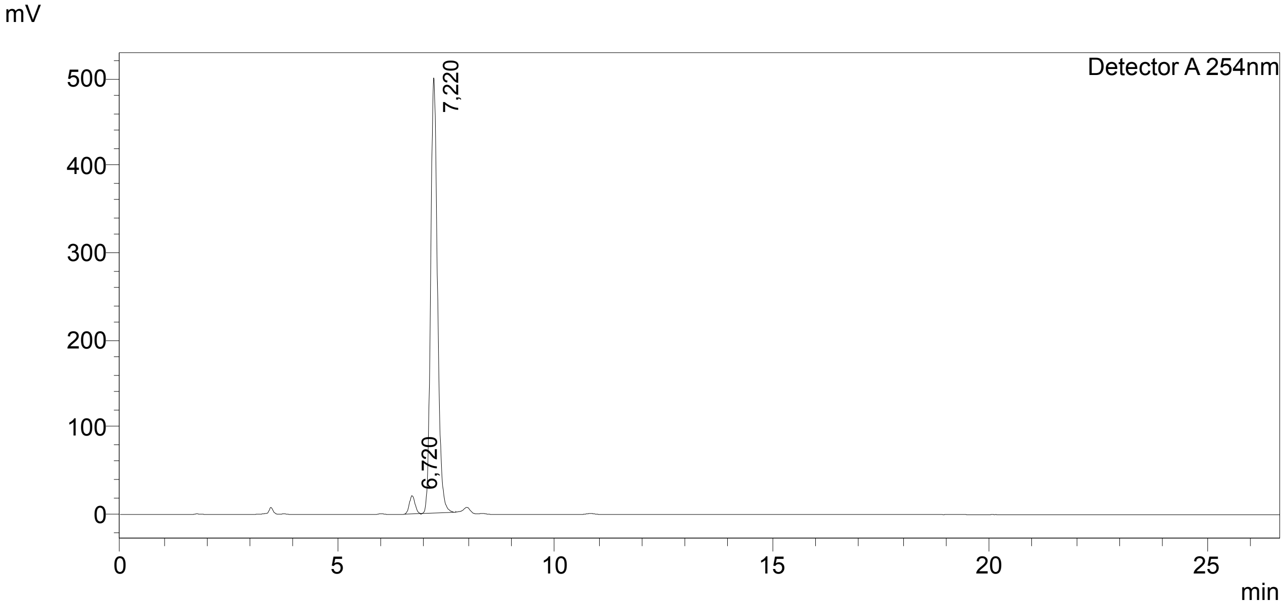

<Peak Table>

Detector A 254nm

Ret. Time	Area	Area%
6,720	189918	3,358
7,220	5465756	96,642
	5655673	100,000

Figure S82b. Chiral chromatogram of **6u**.

SHIMADZU LabSolutions

Analysis Report

<Sample Information>

Sample Name	: CE-156_spot_1_peak_2		
Sample ID	: CE-156_spot_1_peak_2		
Data Filename	: CE-156_spot_1_peak_2_01.lcd		
Method Filename	: Method_12.03.2019.lcm		
Batch Filename	:		
Vial #	: 1-6	Sample Type	: Unknown
Injection Volume	: 10 uL		
Date Acquired	: 12.03.2019 13:02:15	Acquired by	: HPLC - Natascha
Date Processed	: 12.03.2019 13:30:19	Processed by	: HPLC - Natascha

<Chromatogram>

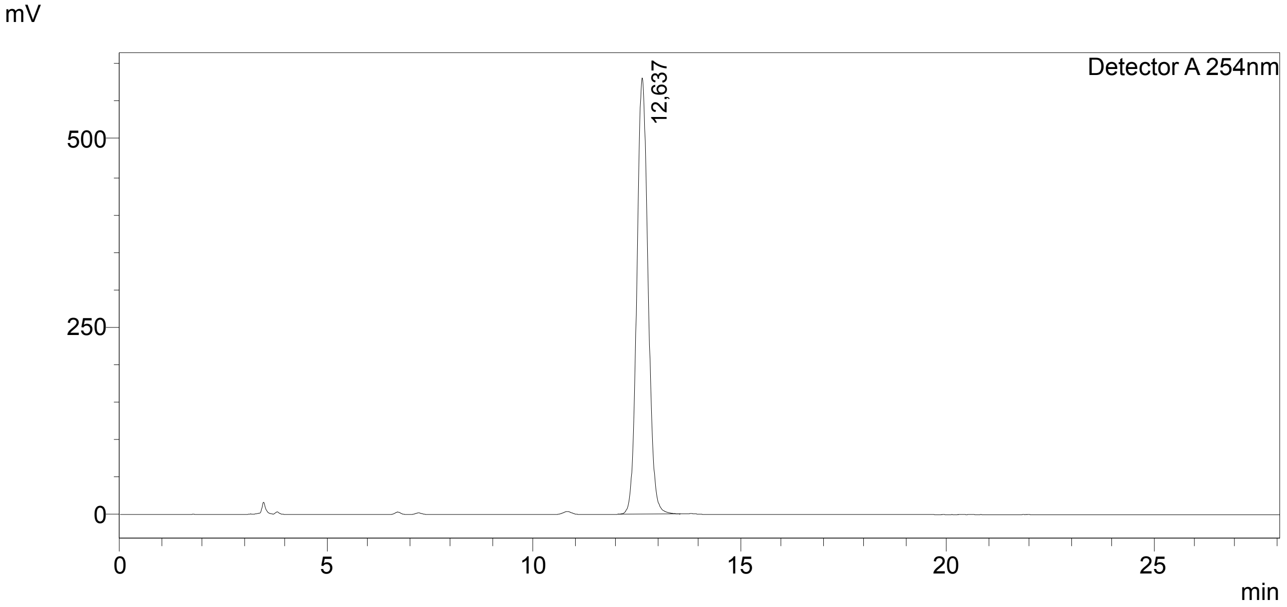

<Peak Table>

Detector A 254nm

Ret. Time	Area	Area%
12,637	10991654	100,000
	10991654	100,000

Figure S83b. Chiral chromatogram of **7u**.

SHIMADZU LabSolutions

Analysis Report

<Sample Information>

Sample Name	: CE-156_Spot2_Peak1_		
Sample ID	: CE-156_Spot2_Peak1_		
Data Filename	: CE-156_Spot2_Peak1_02.lcd		
Method Filename	: Chiralpak_IA_100EtOAc_.lcm		
Batch Filename	:		
Vial #	: 1-1	Sample Type	: Unknown
Injection Volume	: 7 uL		
Date Acquired	: 29.04.2022 11:18:02	Acquired by	: HPLC - Natascha
Date Processed	: 29.04.2022 11:30:04	Processed by	: HPLC - Natascha

<Chromatogram>

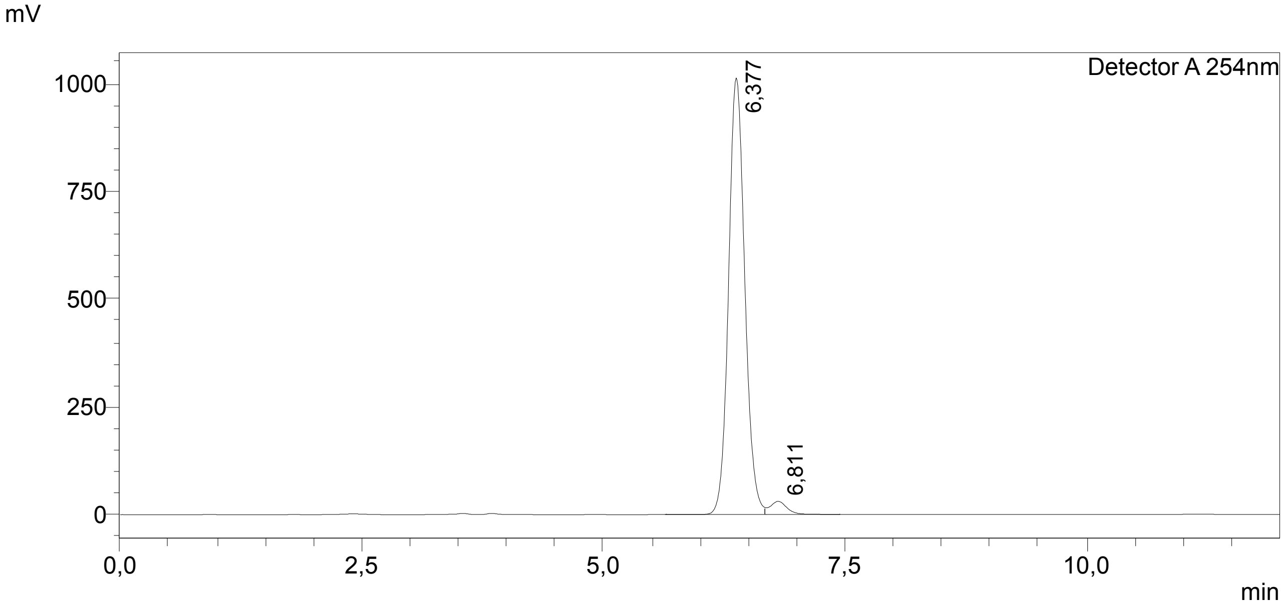

<Peak Table>

Detector A 254nm

Peak#	Ret. Time	Area	Area%
1	6,377	11867874	97,004
2	6,811	366540	2,996
Total		12234415	100,000

Figure S84b. Chiral chromatogram of **8u**.

SHIMADZU LabSolutions

Analysis Report

<Sample Information>

Sample Name	: CE-156_spot_2_peak_2		
Sample ID	: CE-156_spot_2_peak_2		
Data Filename	: CE-156_spot_2_peak_2_01.lcd		
Method Filename	: Method_12.03.2019.lcm		
Batch Filename	:		
Vial #	: 1-8	Sample Type	: Unknown
Injection Volume	: 10 uL		
Date Acquired	: 12.03.2019 14:57:37	Acquired by	: HPLC - Natascha
Date Processed	: 12.03.2019 15:12:38	Processed by	: HPLC - Natascha

<Chromatogram>

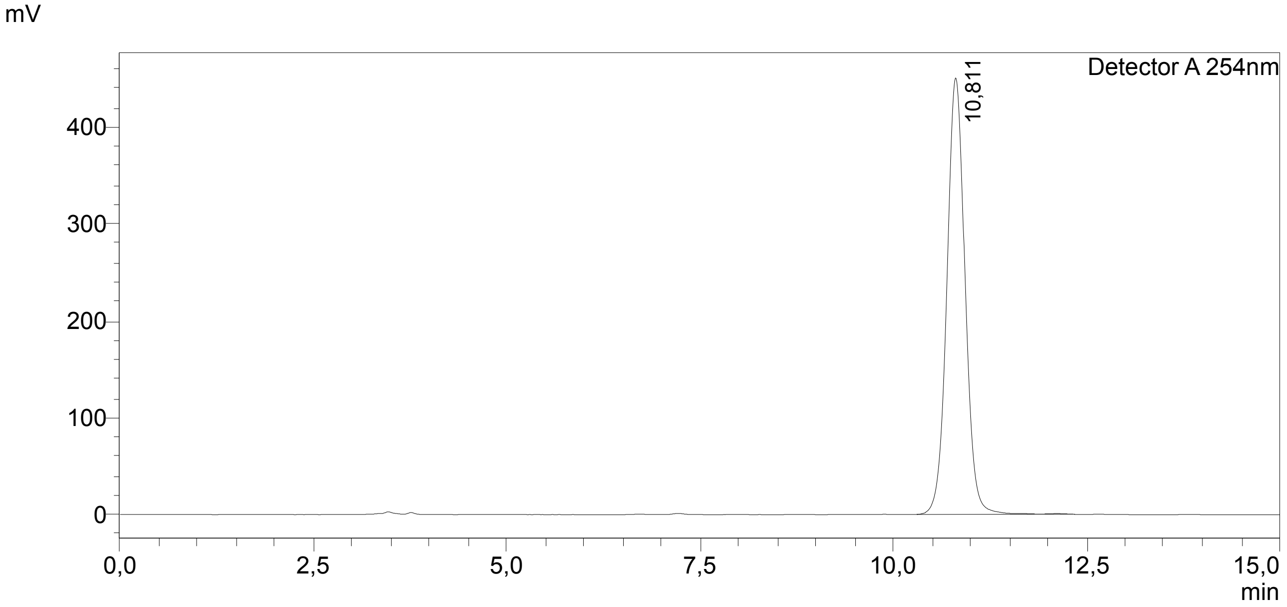

<Peak Table>

Detector A 254nm

Ret. Time	Area	Area%
10,811	7355659	100,000
	7355659	100,000

Figure S1c. NMR spectra of compound **5a**.

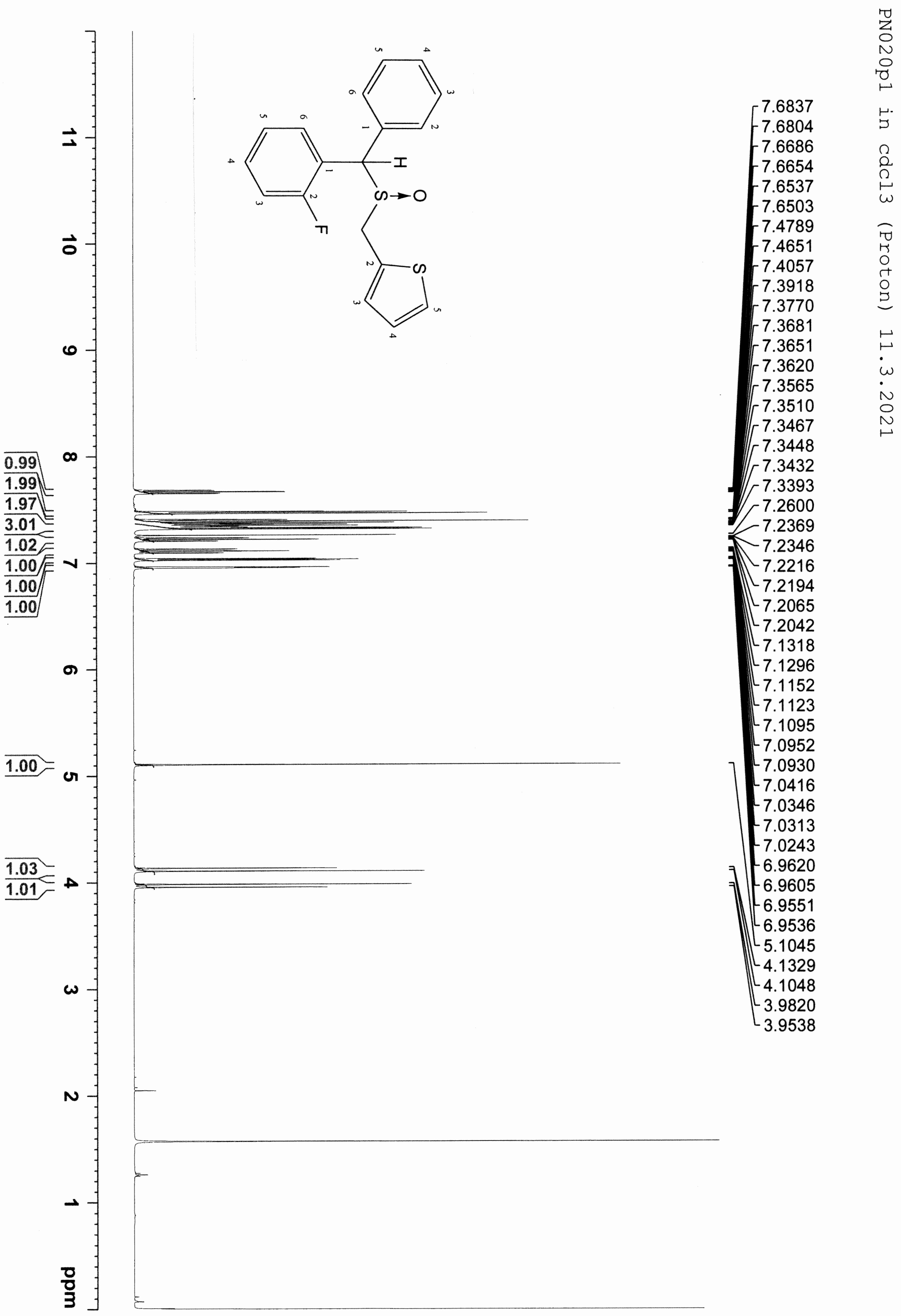

PN020p1 in cdcl3 (Proton) 11.3.2021

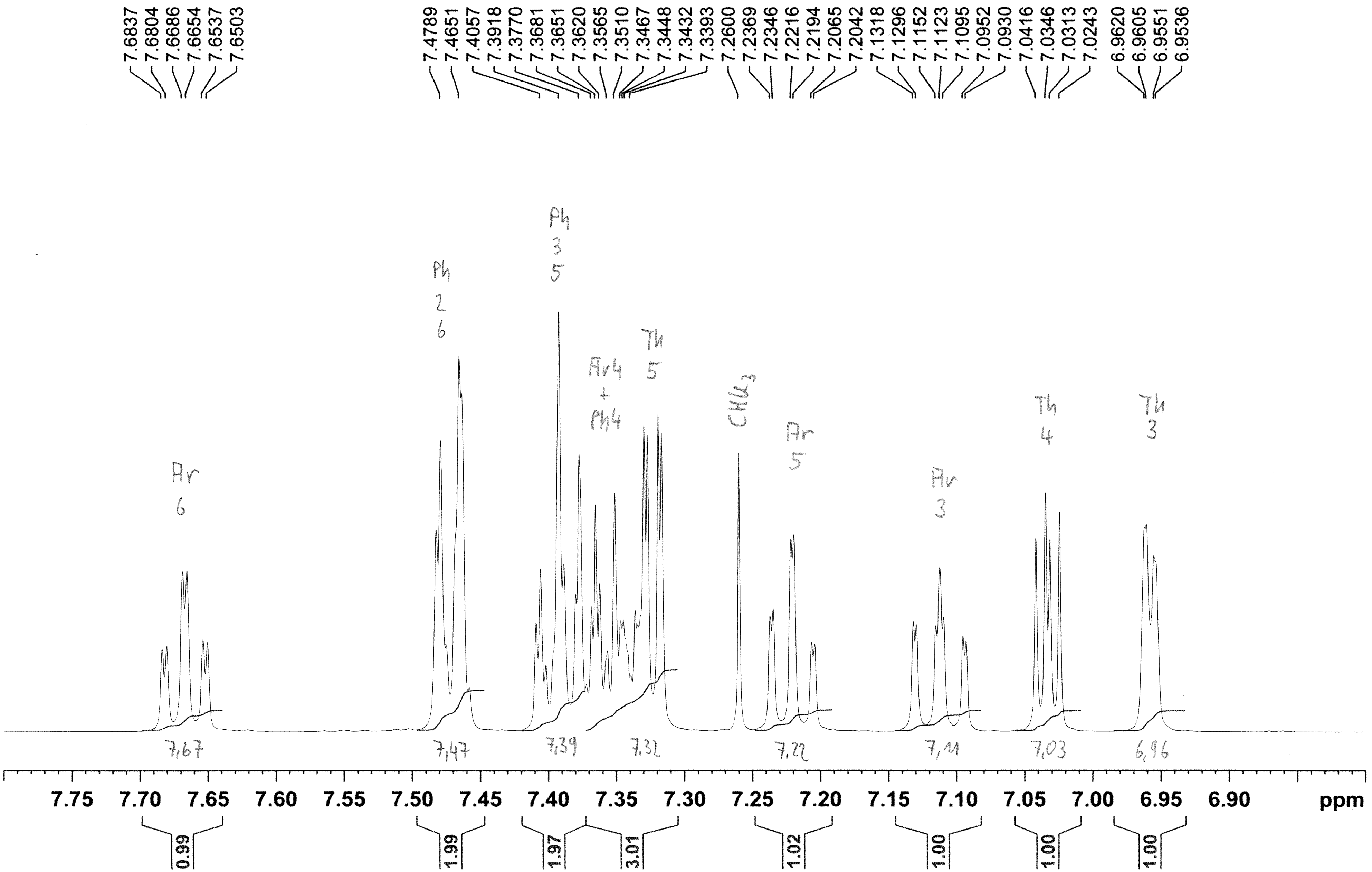

PN020p1 in cdcl3 (Proton) 11.3.2021

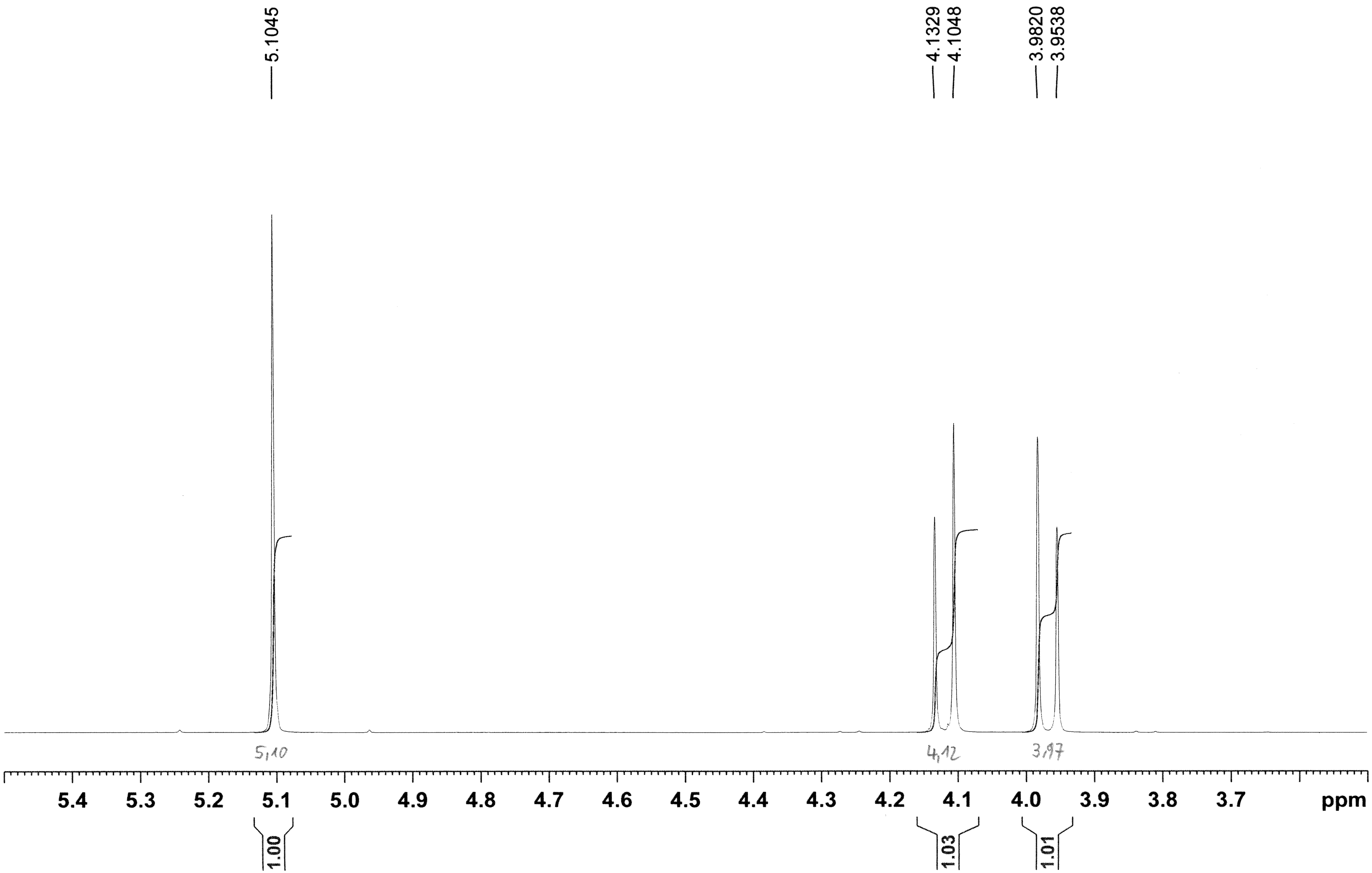

PN020p1 in cdcl3 (APT) 11.3.2021

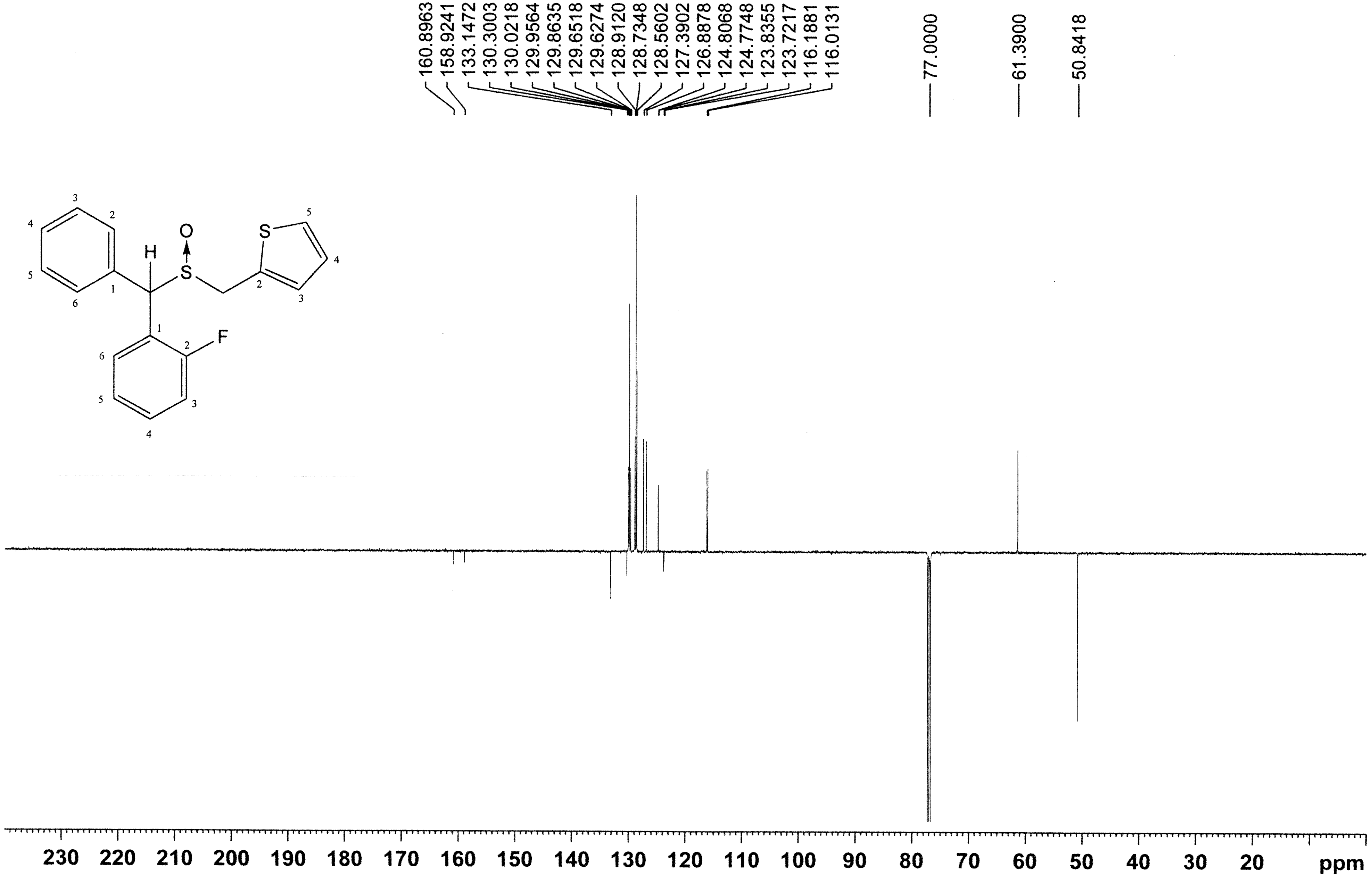

PN020p1 in cdcl3 (APT) 11.3.2021

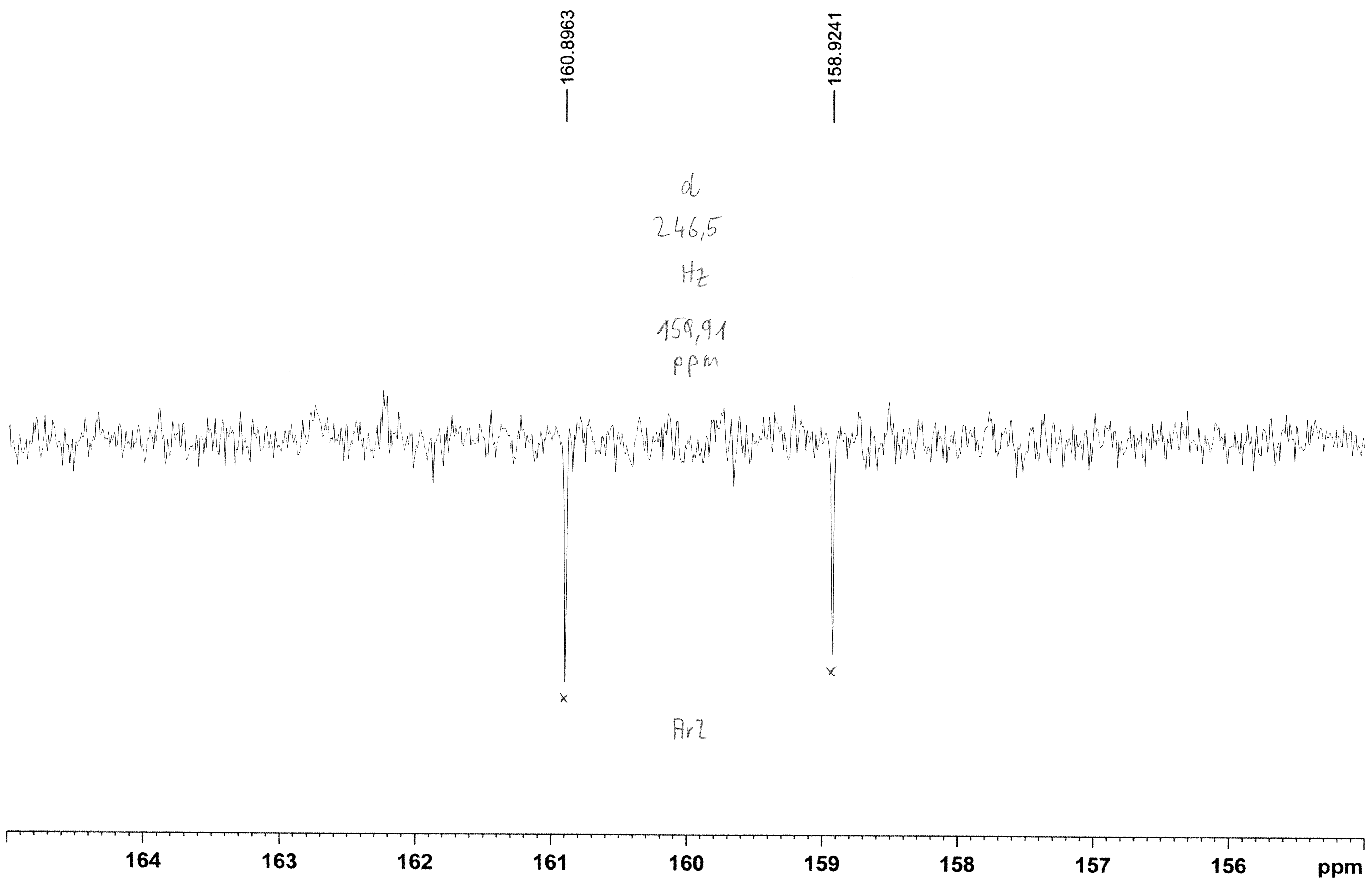

PN020p1 in cdcl3 (APT) 11.3.2021

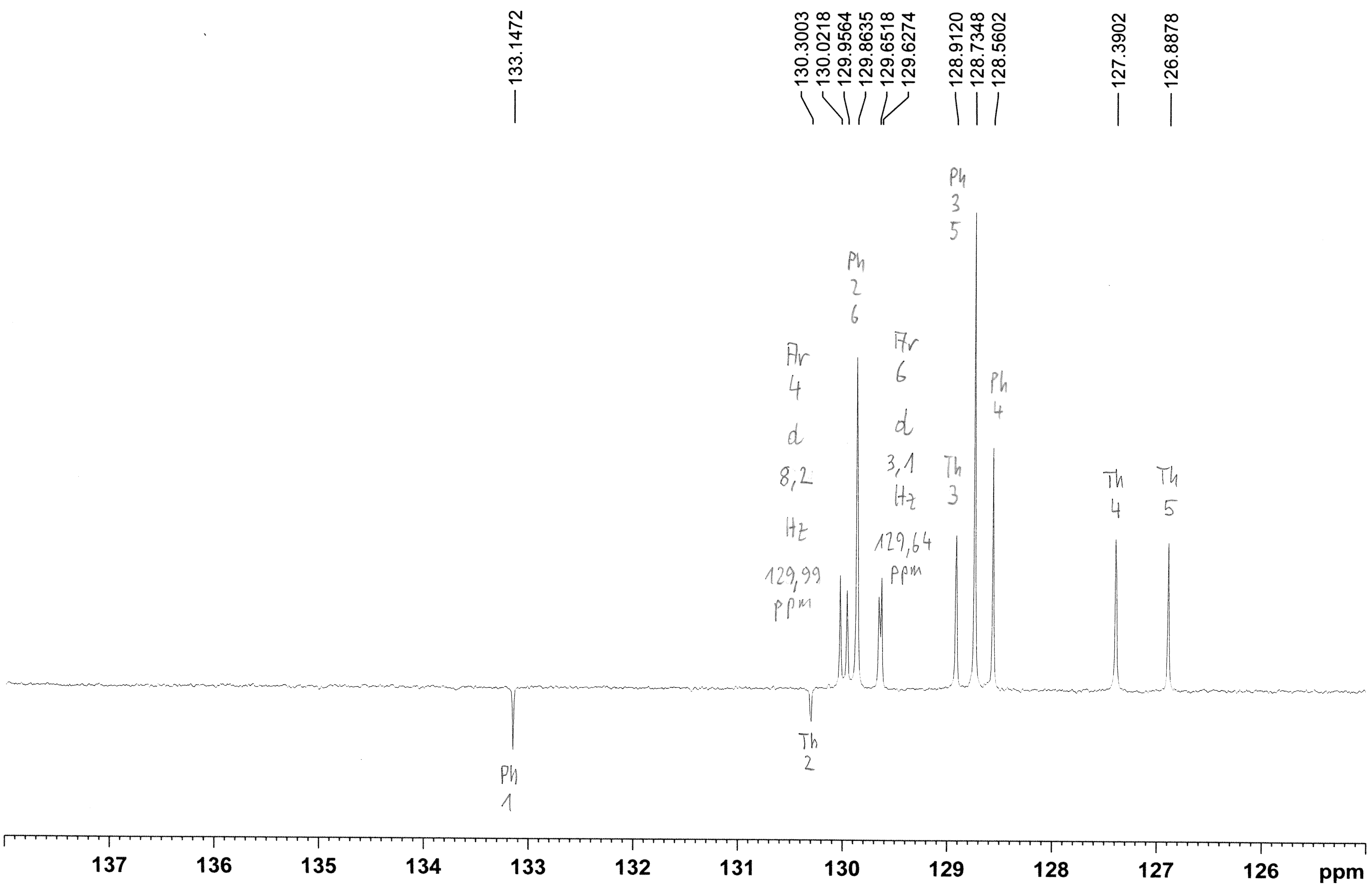

PN020p1 in cdcl3 (APT) 11.3.2021

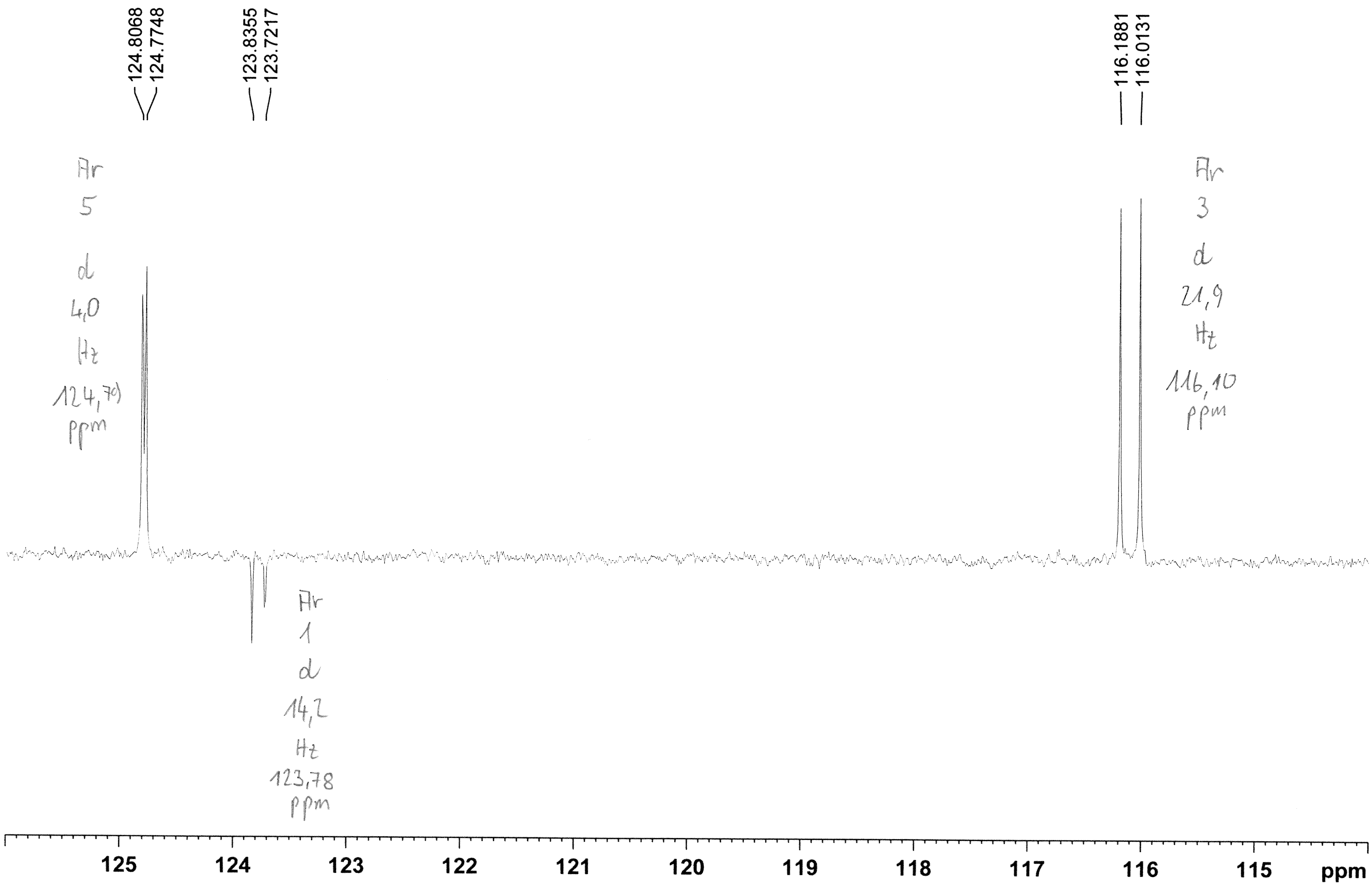

PN020p1 in cdcl3 (APT) 11.3.2021

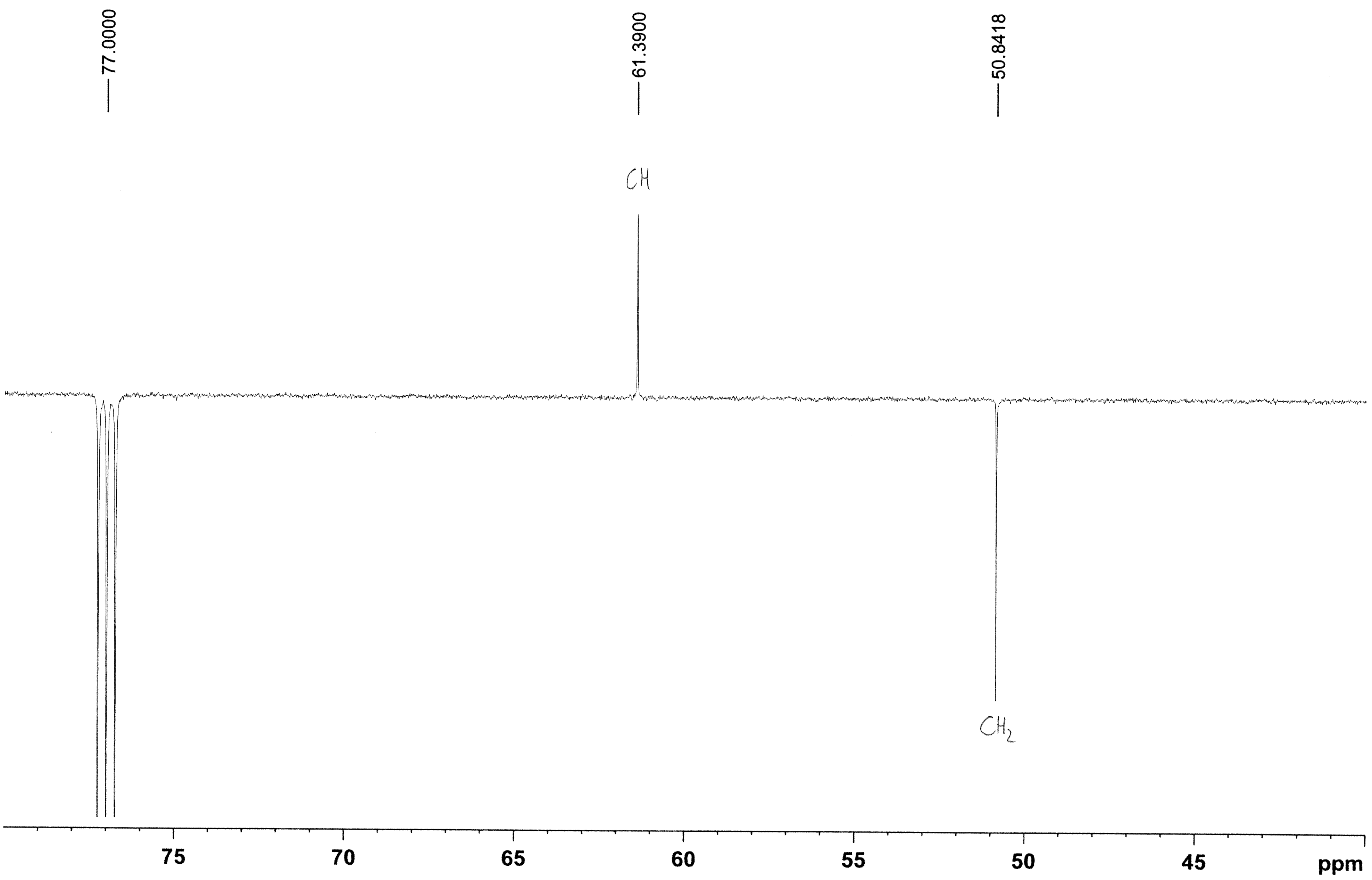

PN020p1 in cdcl3 (COSY) 11.3.2021

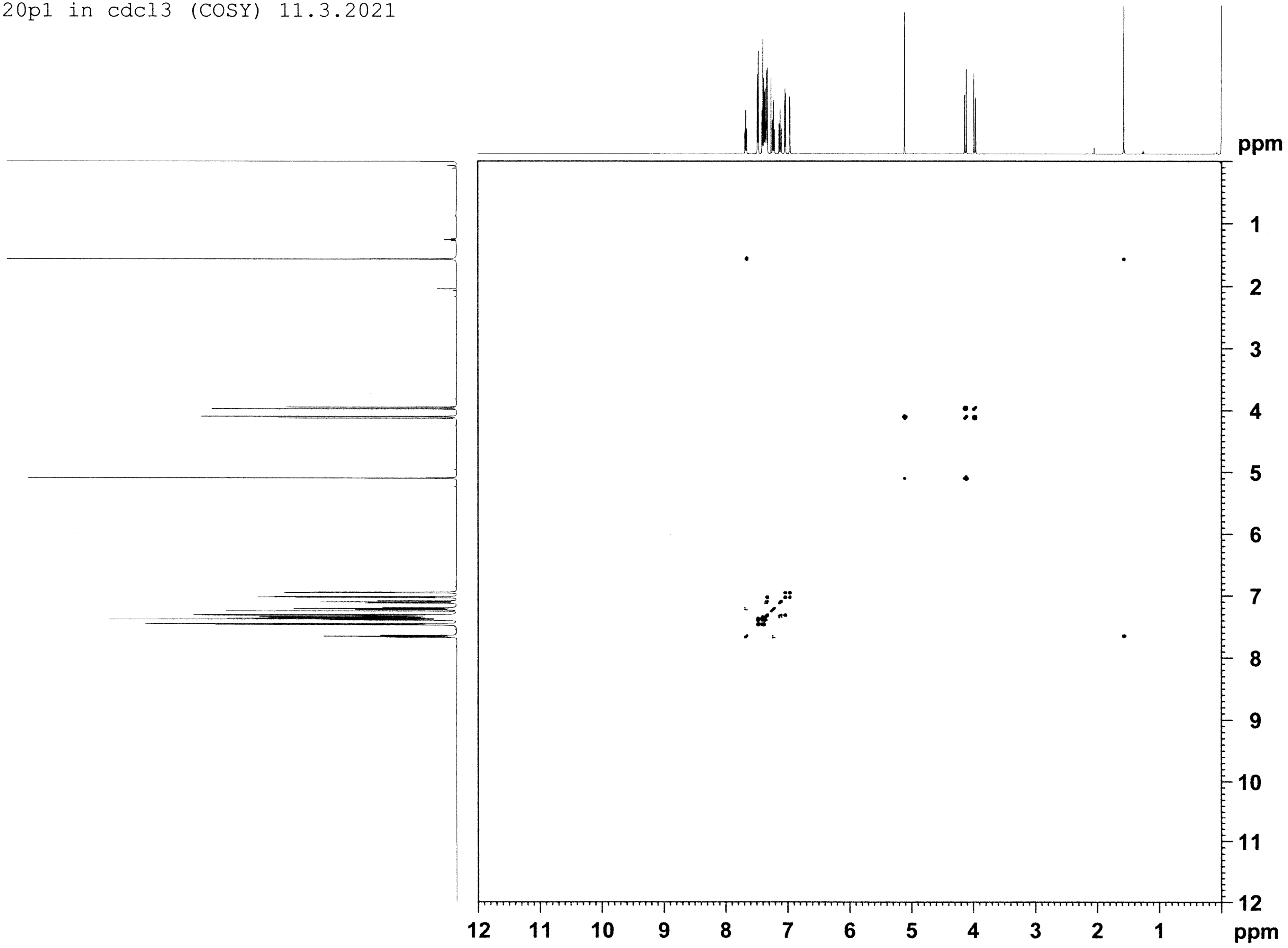

PN020p1 in cdcl3 (COSY) 11.3.2021

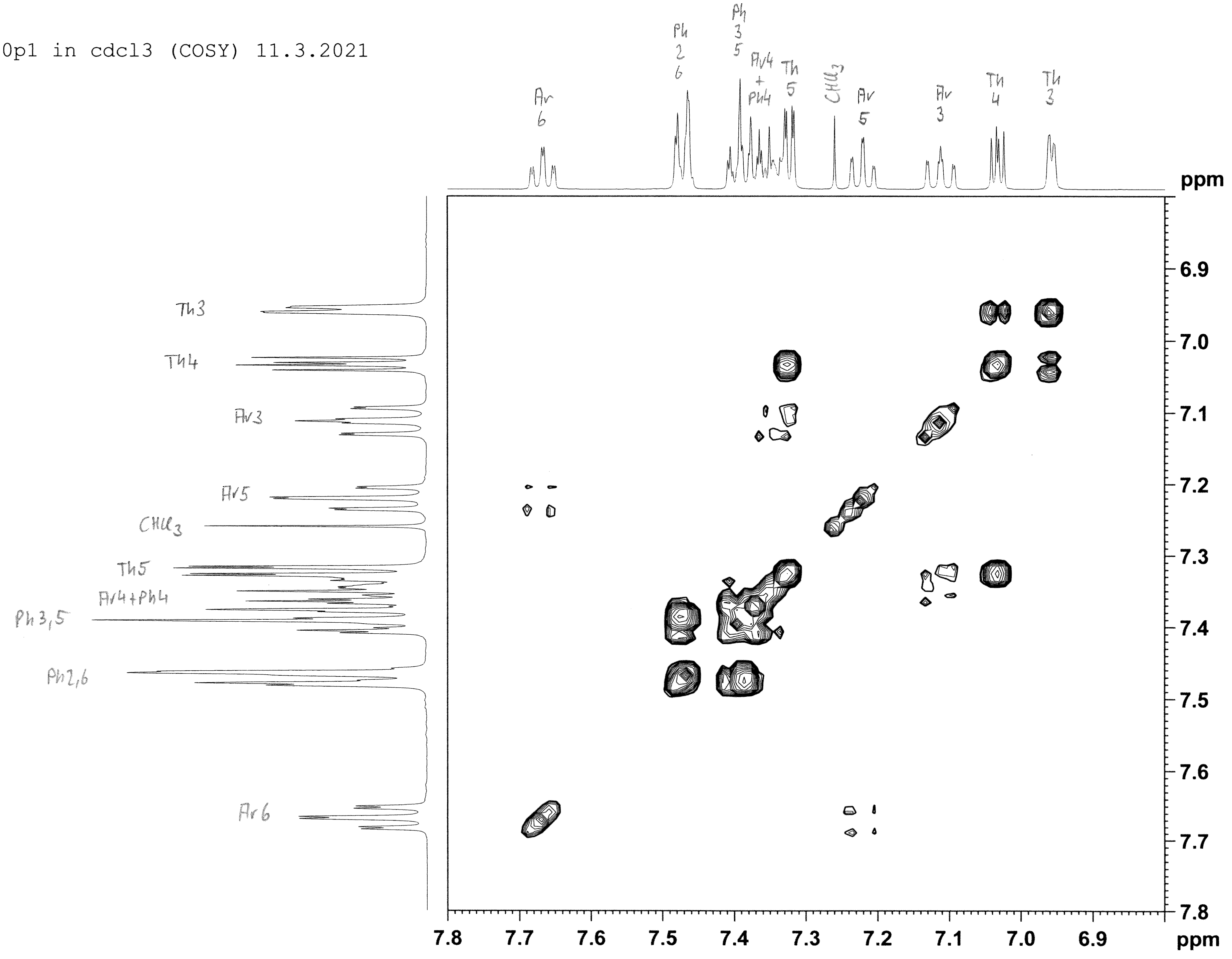

PN020p1 in cdcl3 (COSY) 11.3.2021

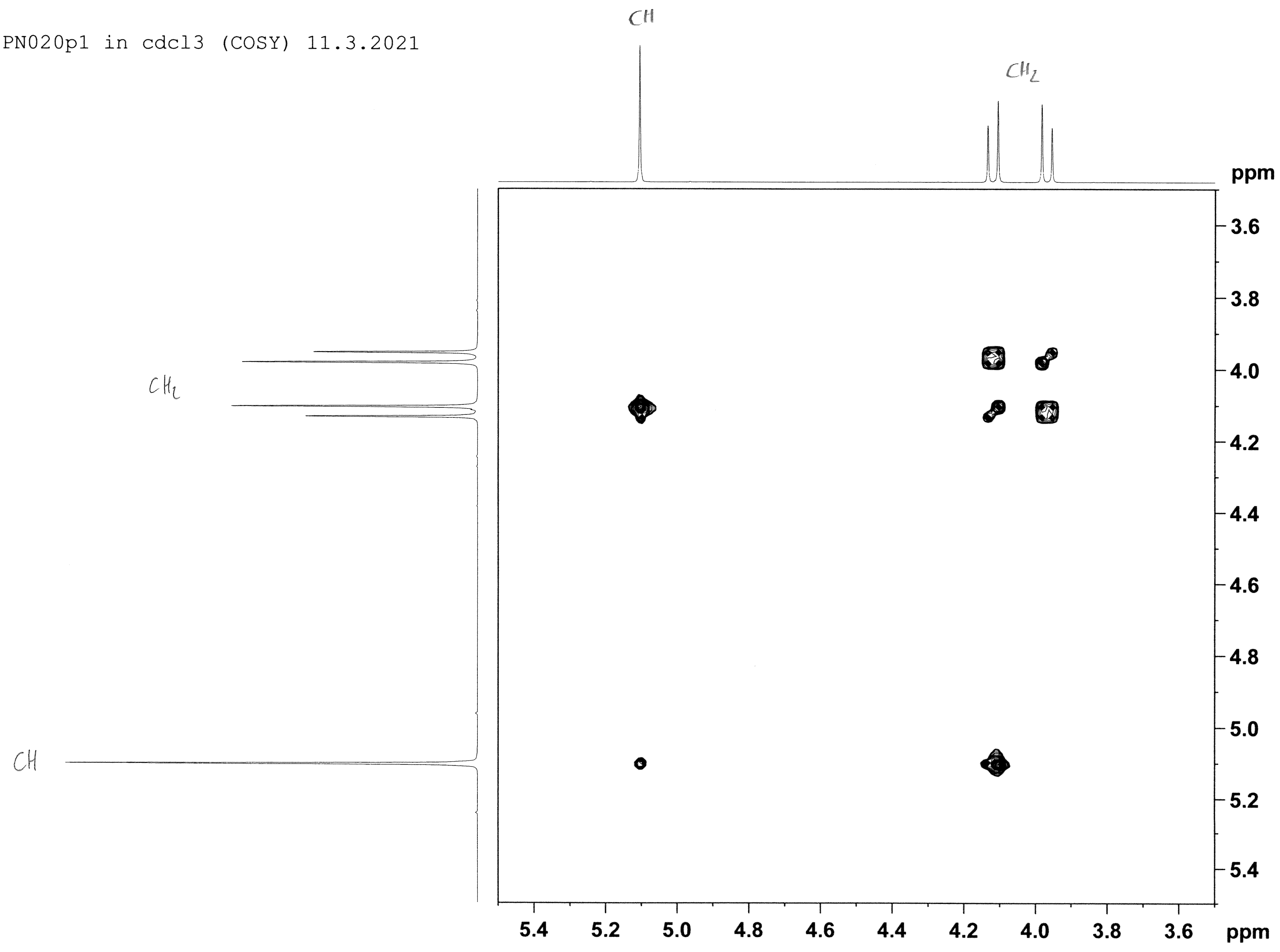

PN020p1 in cdcl3 (HSQC) 11.3.2021

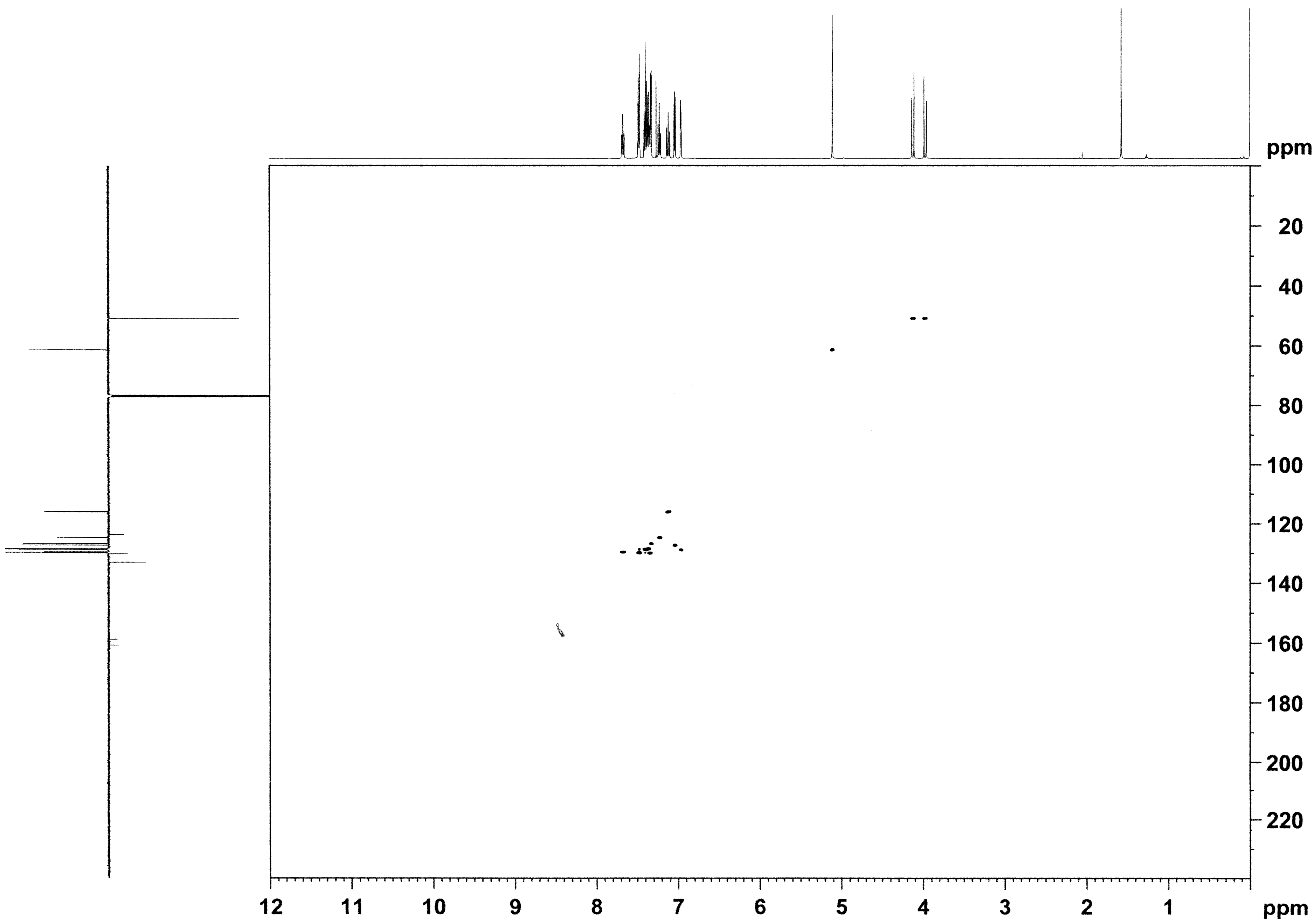

PN020p1 in cdcl3 (HSQC) 11.3.2021

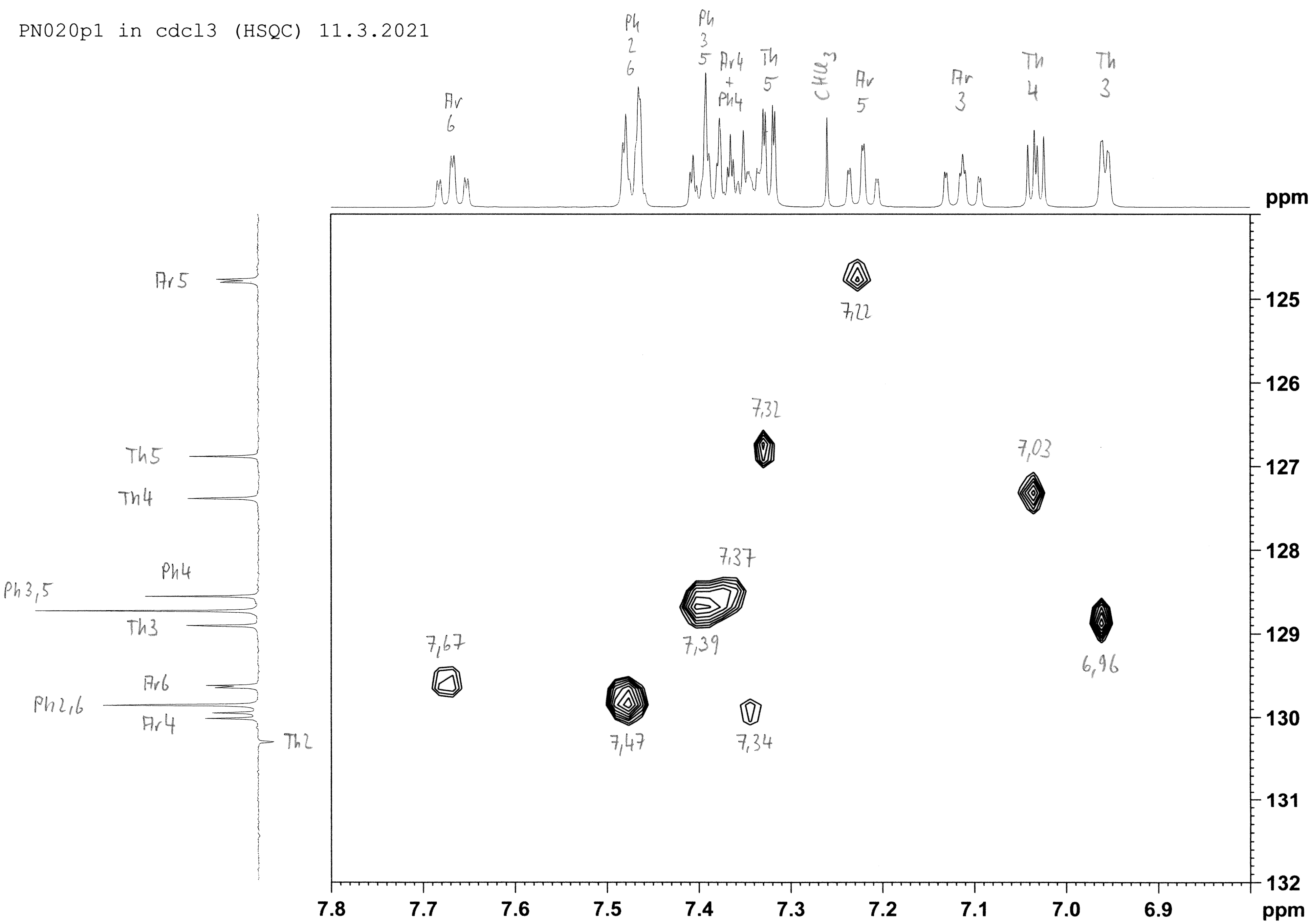

PN020p1 in cdcl3 (HSQC) 11.3.2021

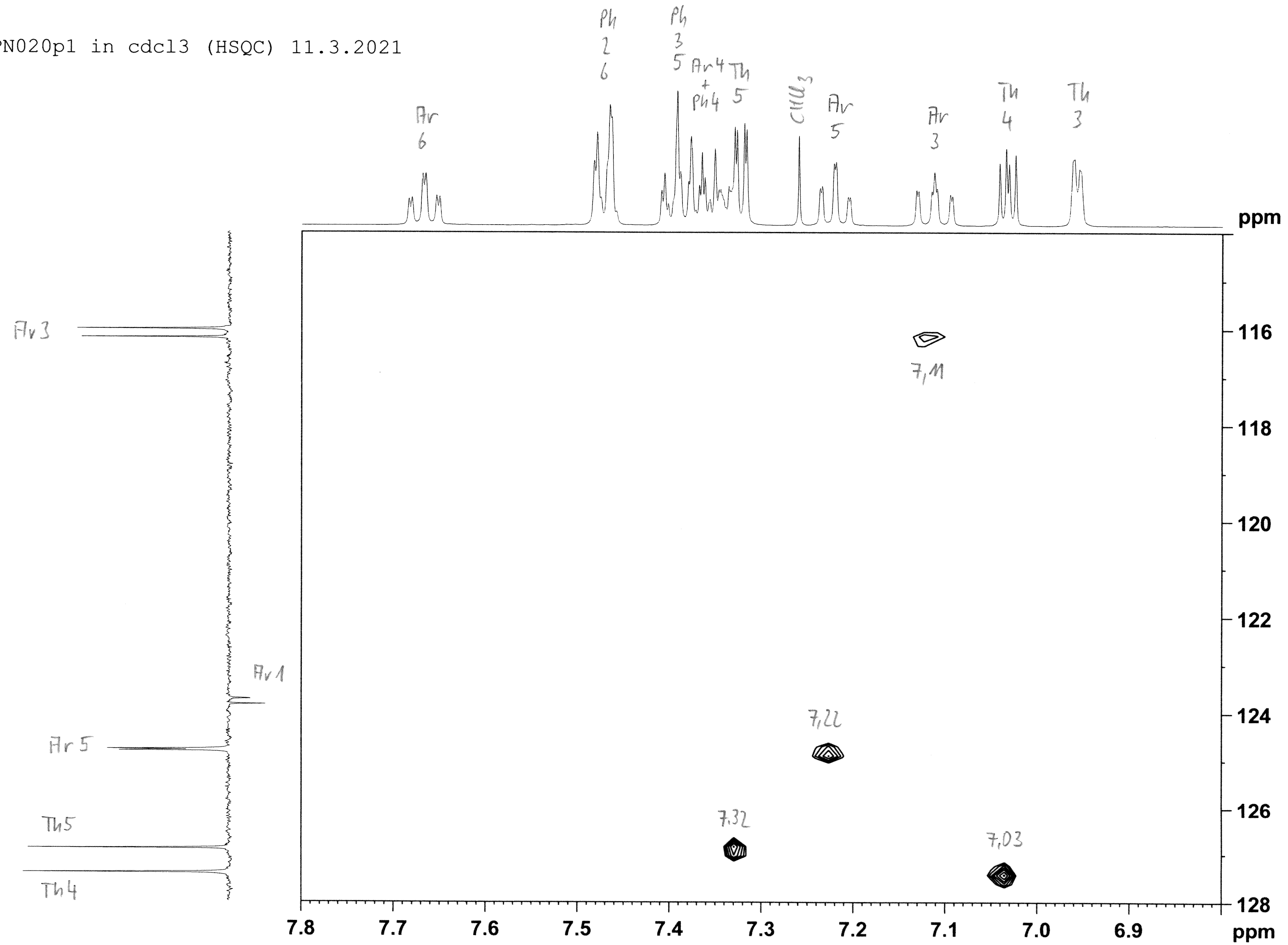

PN020p1 in cdcl3 (HSQC) 11.3.2021

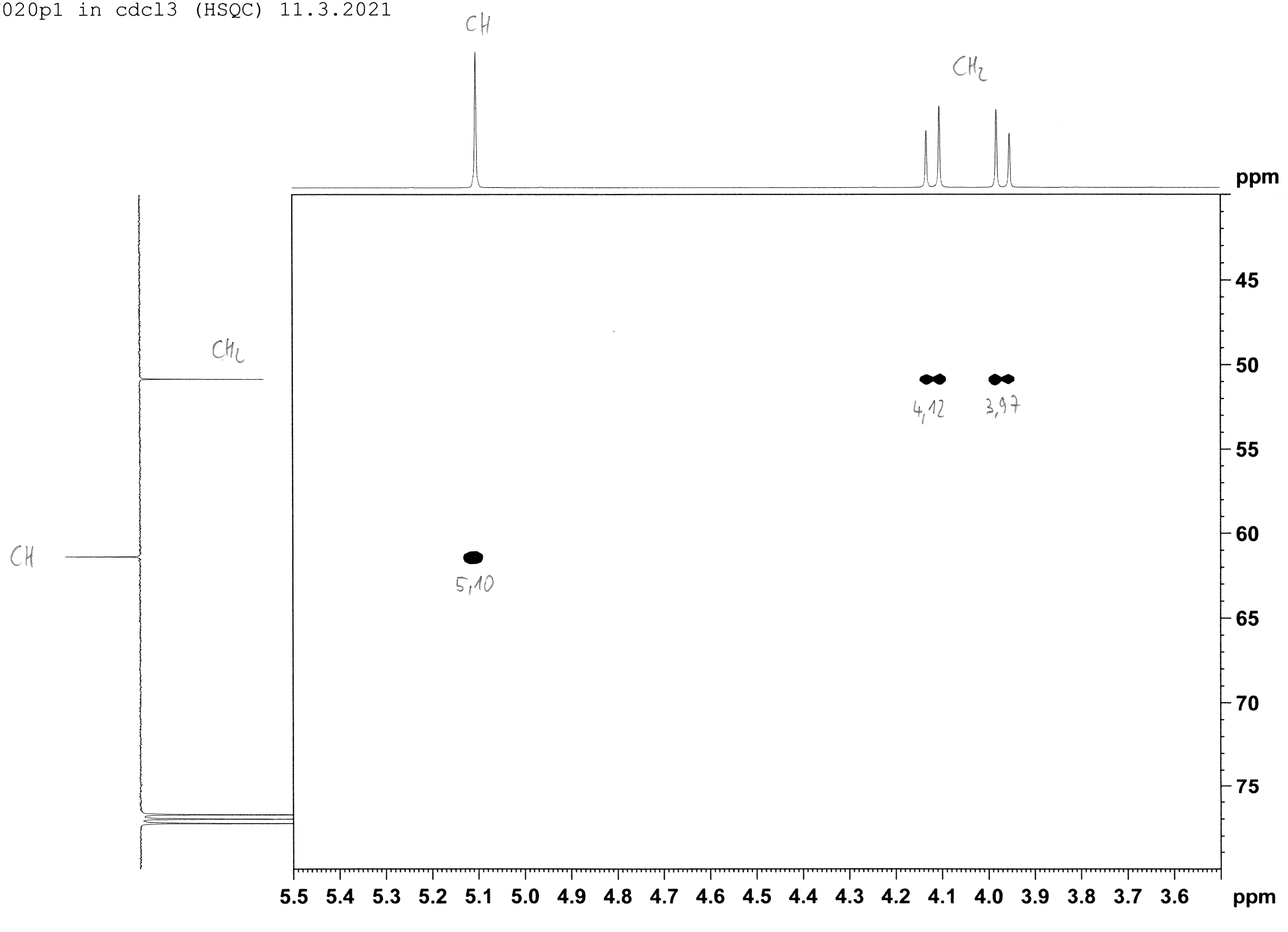

PN020p1 in cdcl3 (HMBC) 11.3.2021

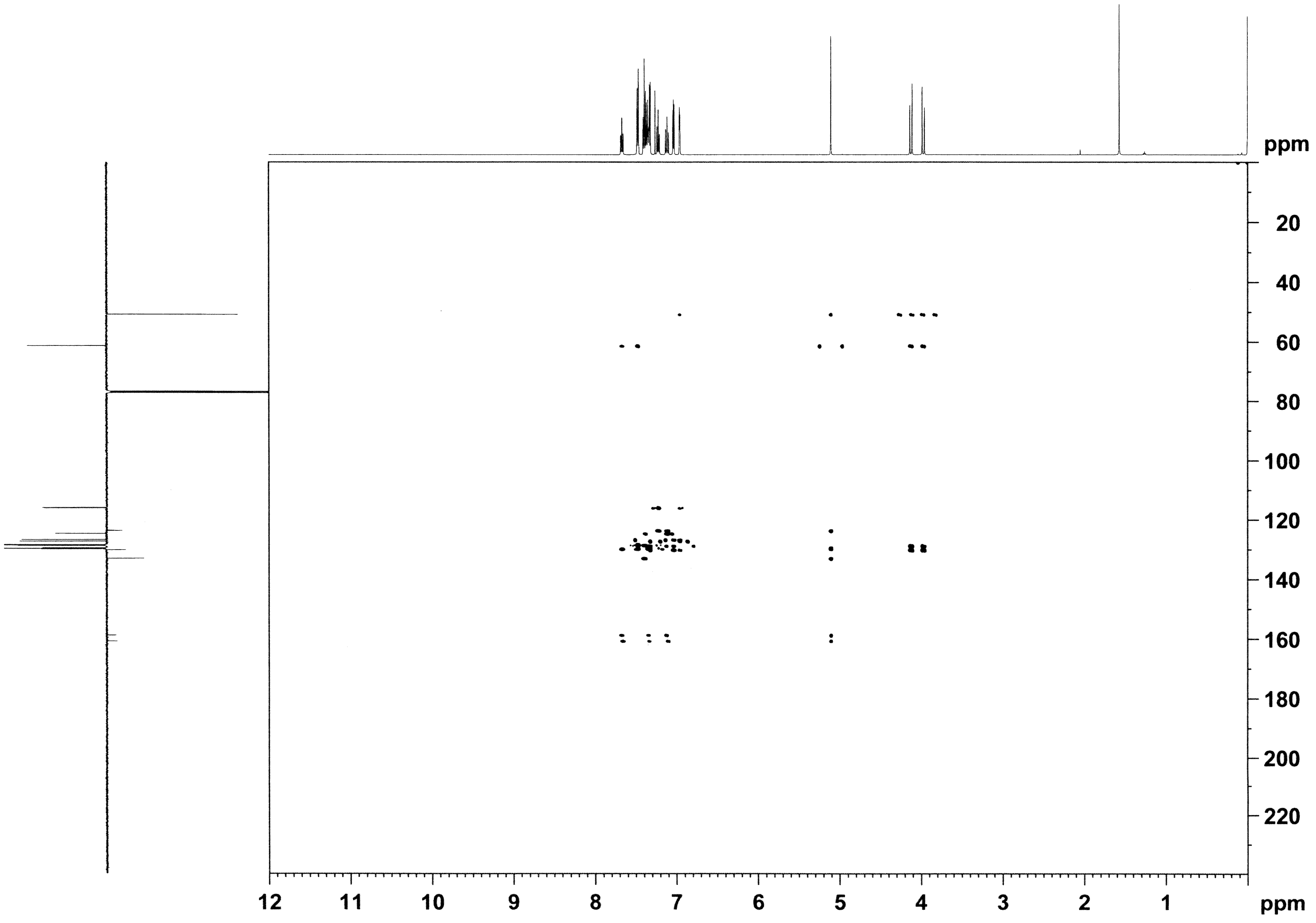

PN020p1 in cdcl3 (HMBC) 11.3.2021

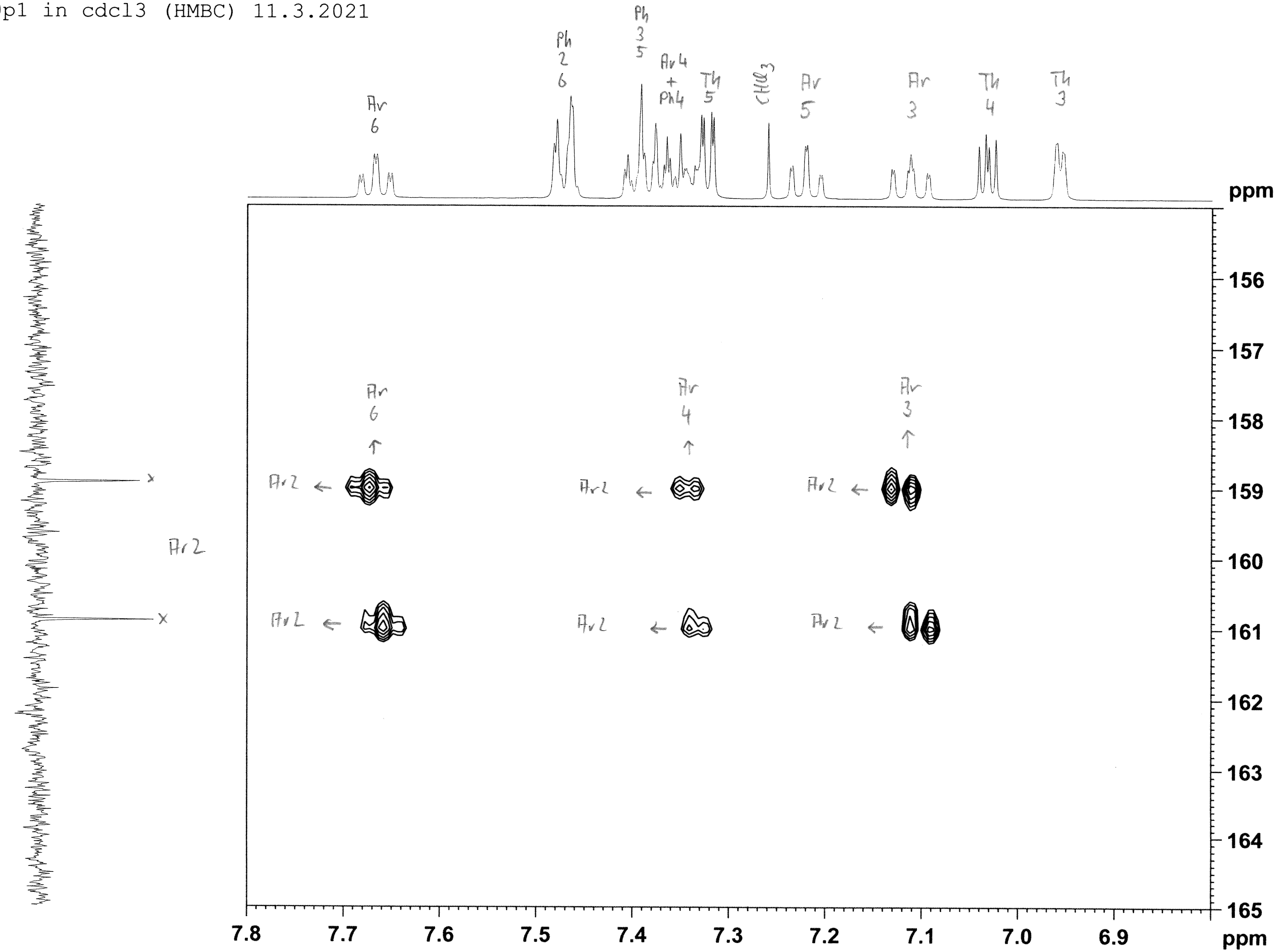

PN020p1 in cdcl3 (HMBC) 11.3.2021

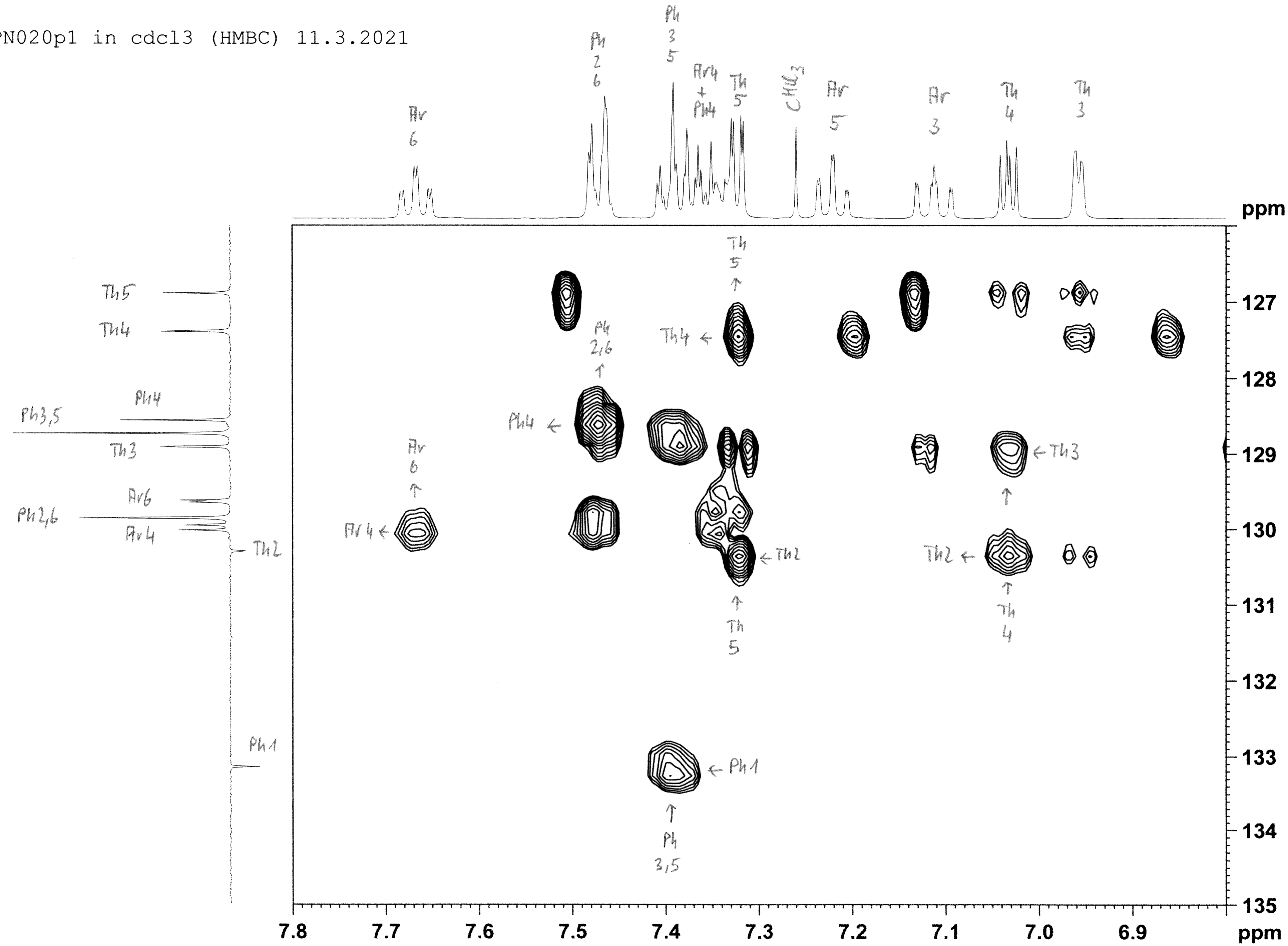

PN020p1 in cdcl3 (HMBC) 11.3.2021

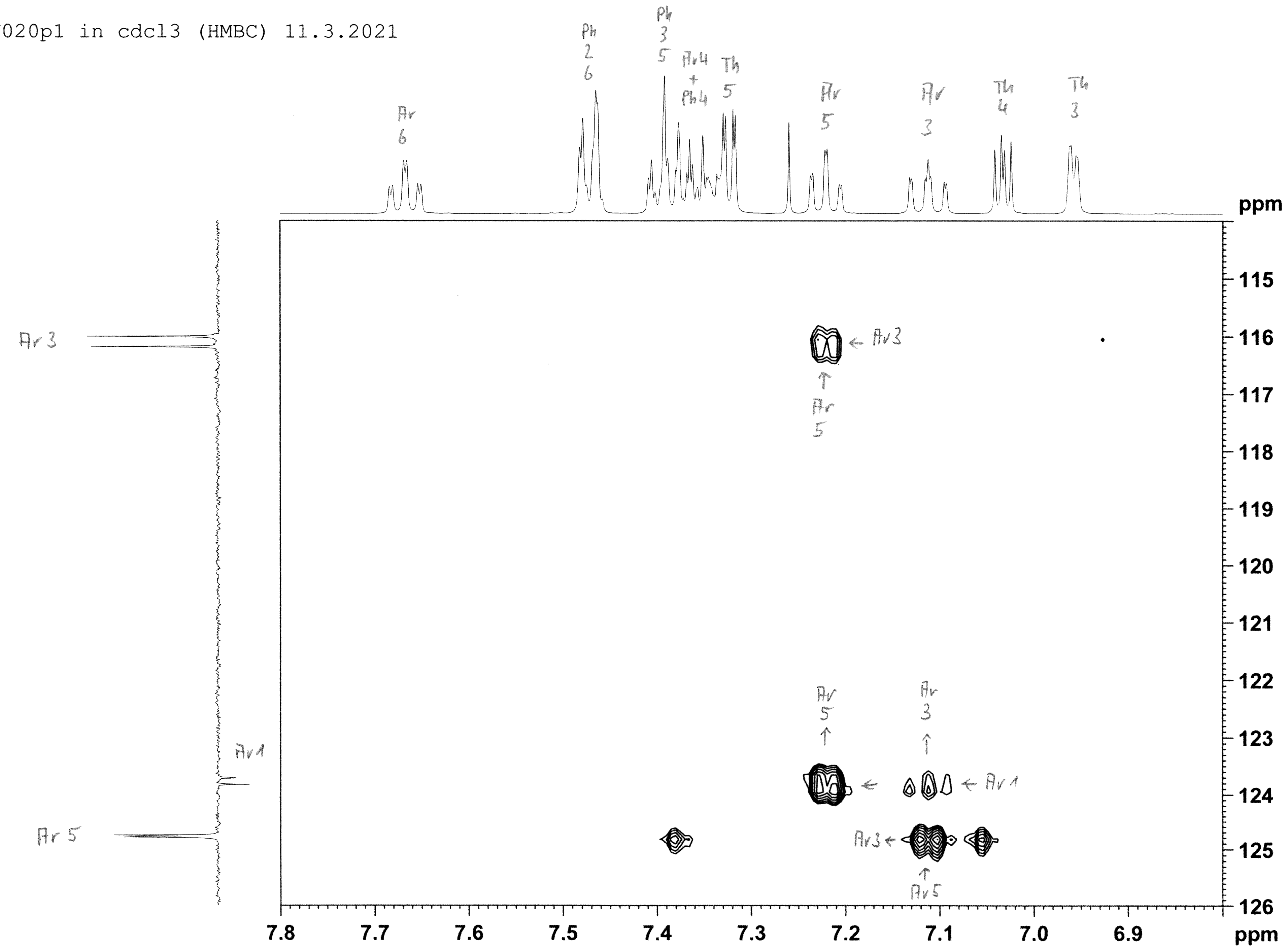

PN020p1 in cdcl3 (HMBC) 11.3.2021

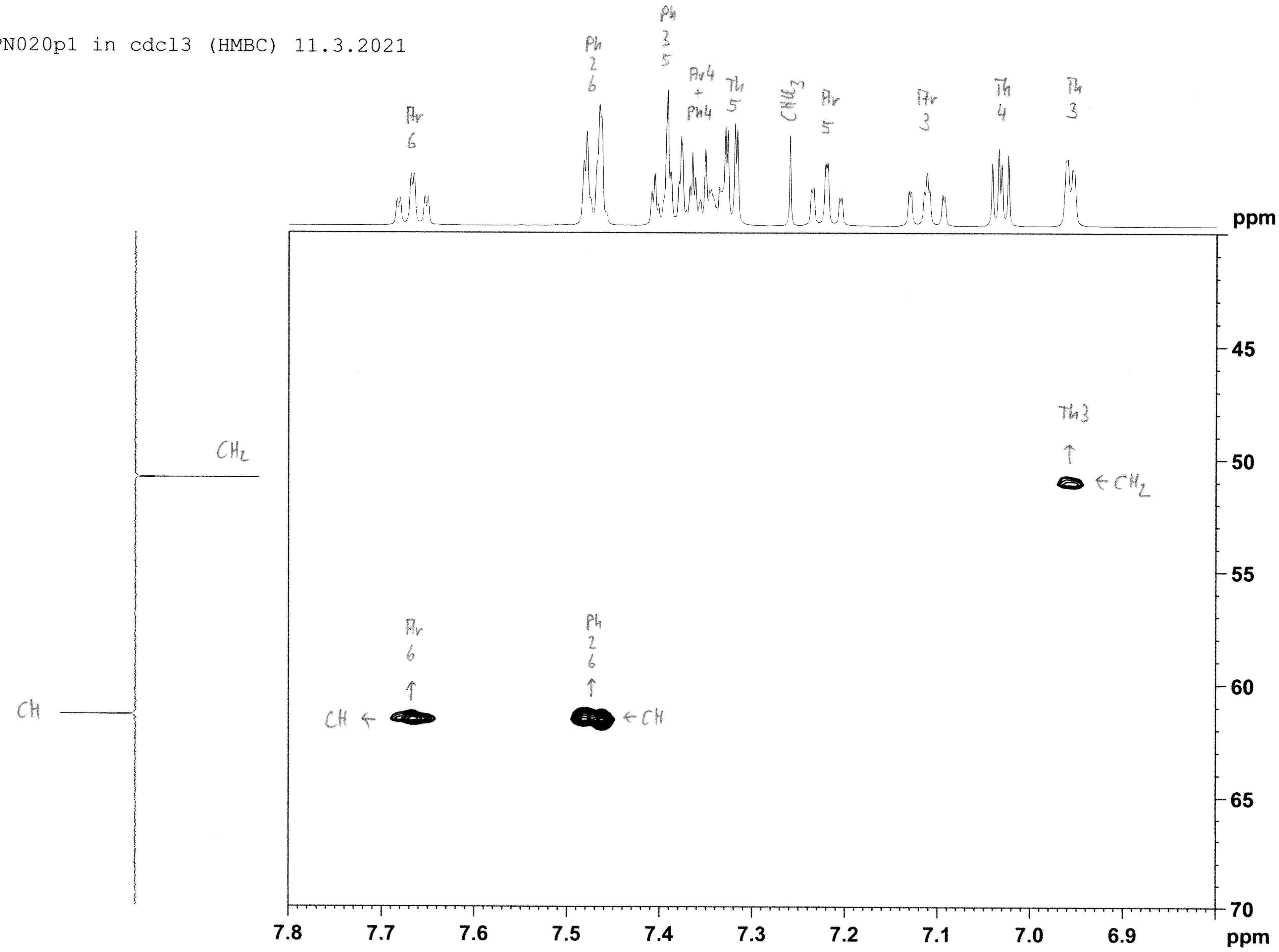

PN020p1 in cdcl3 (HMBC) 11.3.2021

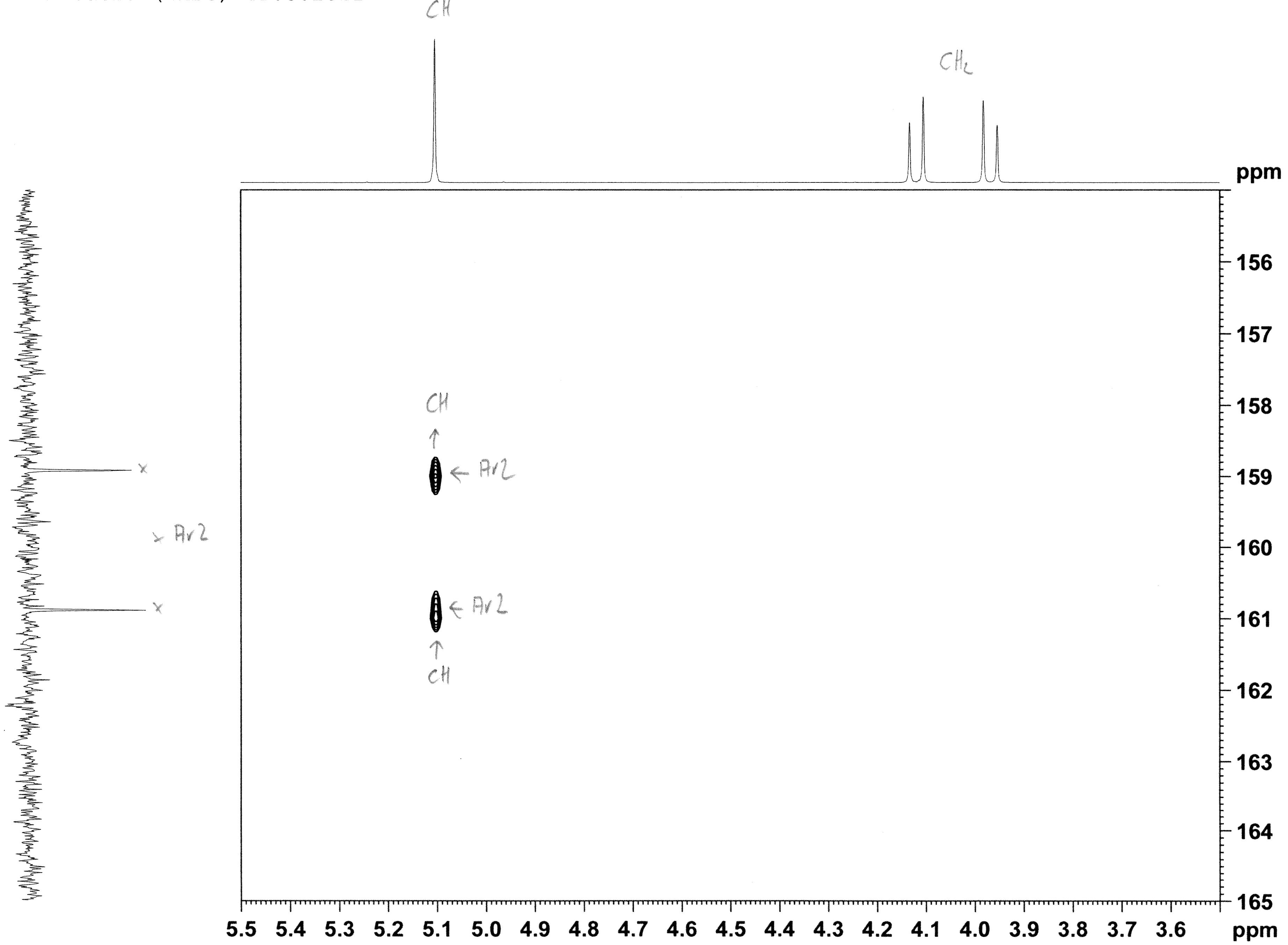

PN020p1 in cdcl3 (HMBC) 11.3.2021

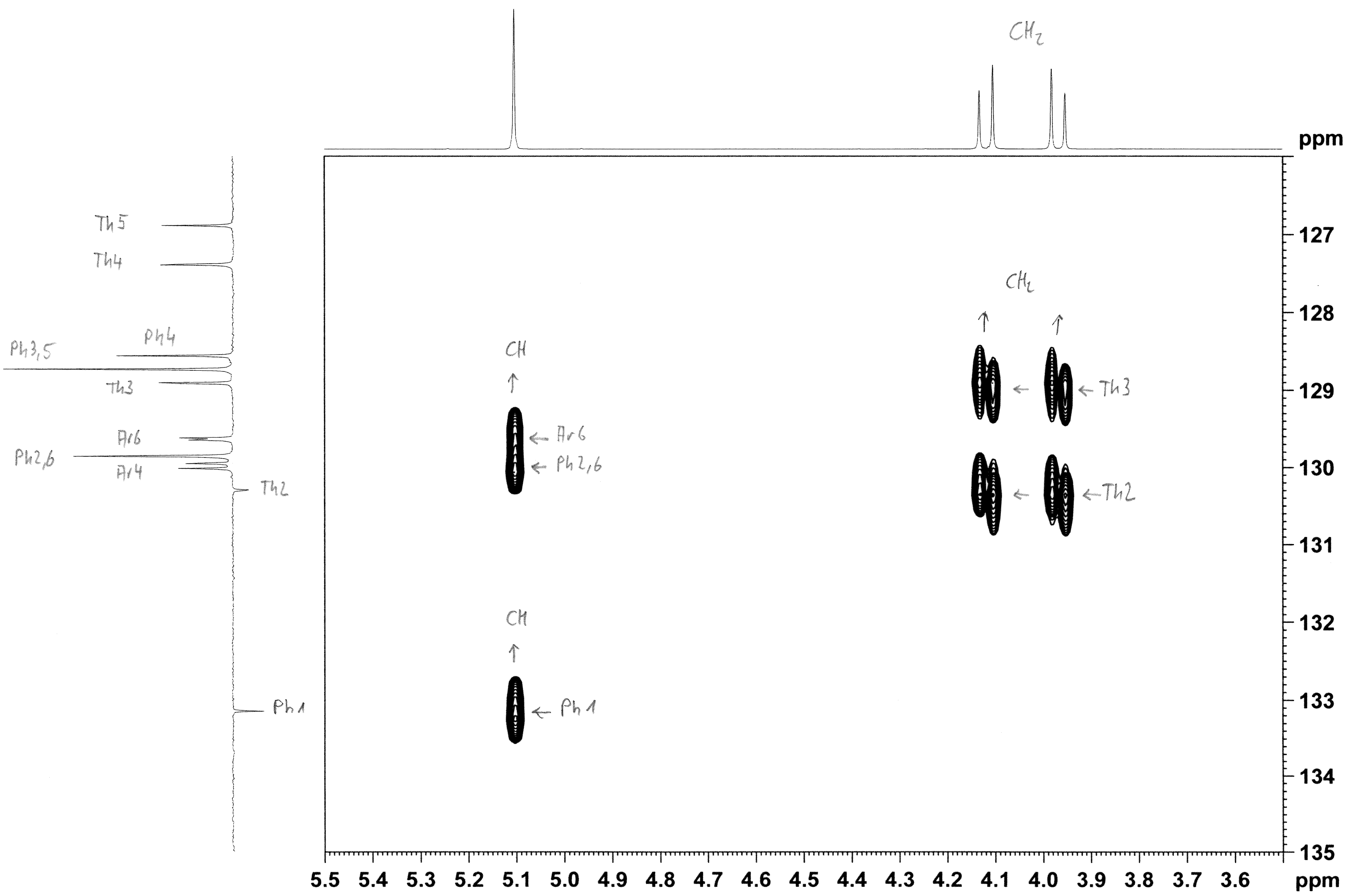

PN020p1 in cdcl3 (HMBC) 11.3.2021

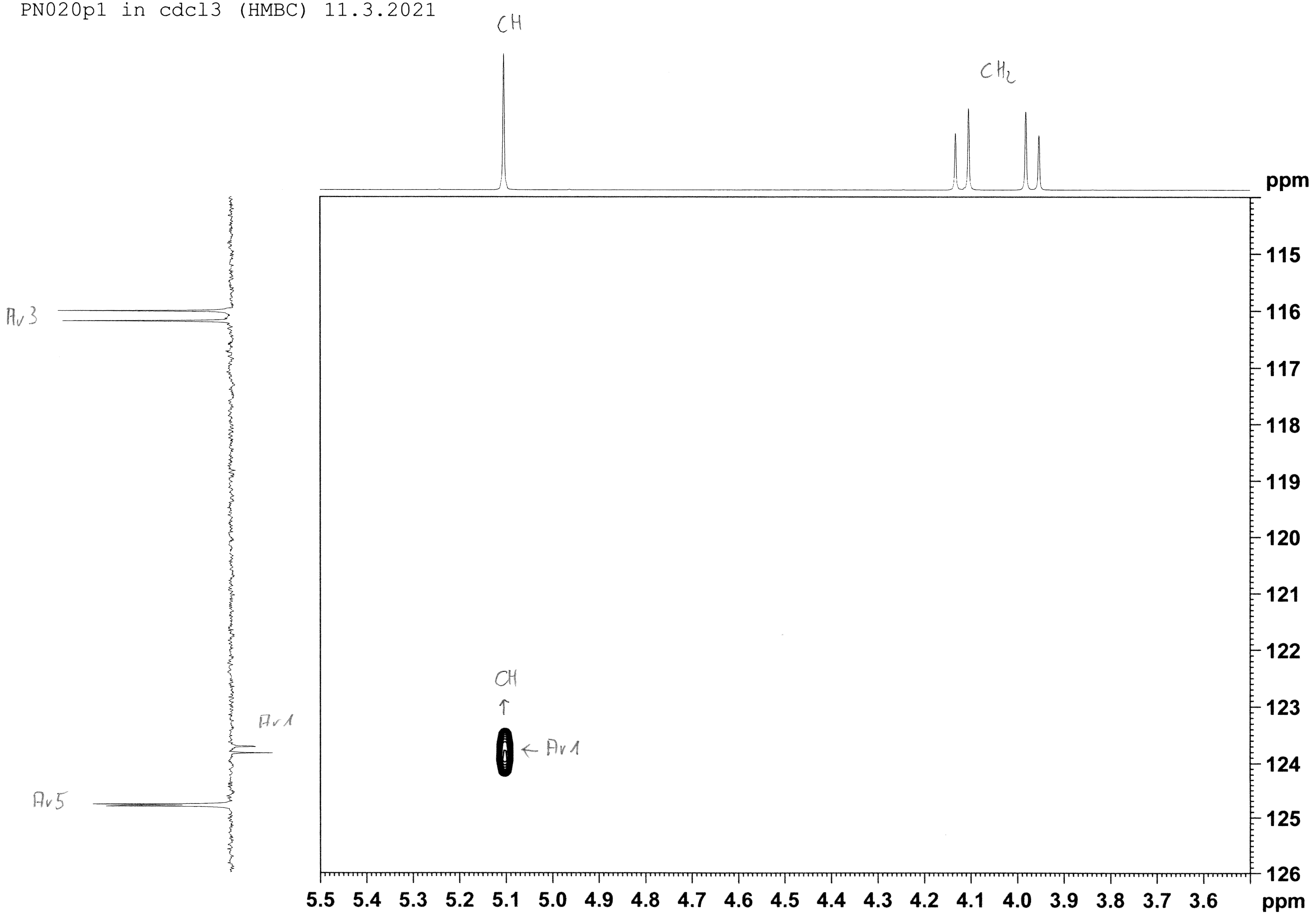

PN020p1 in cdcl3 (HMBC) 11.3.2021

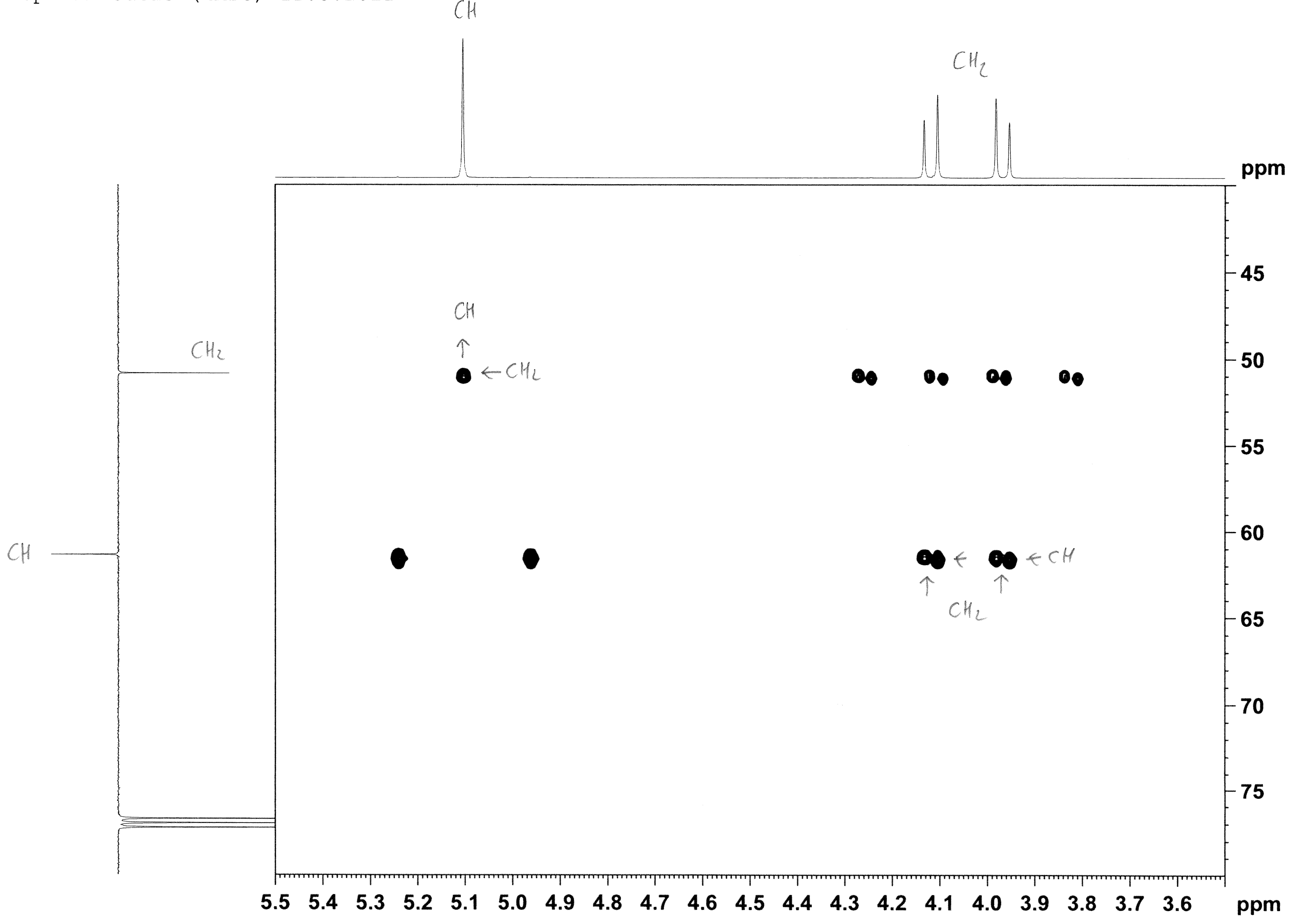

Figure S2c. NMR spectra of compound **6a.**

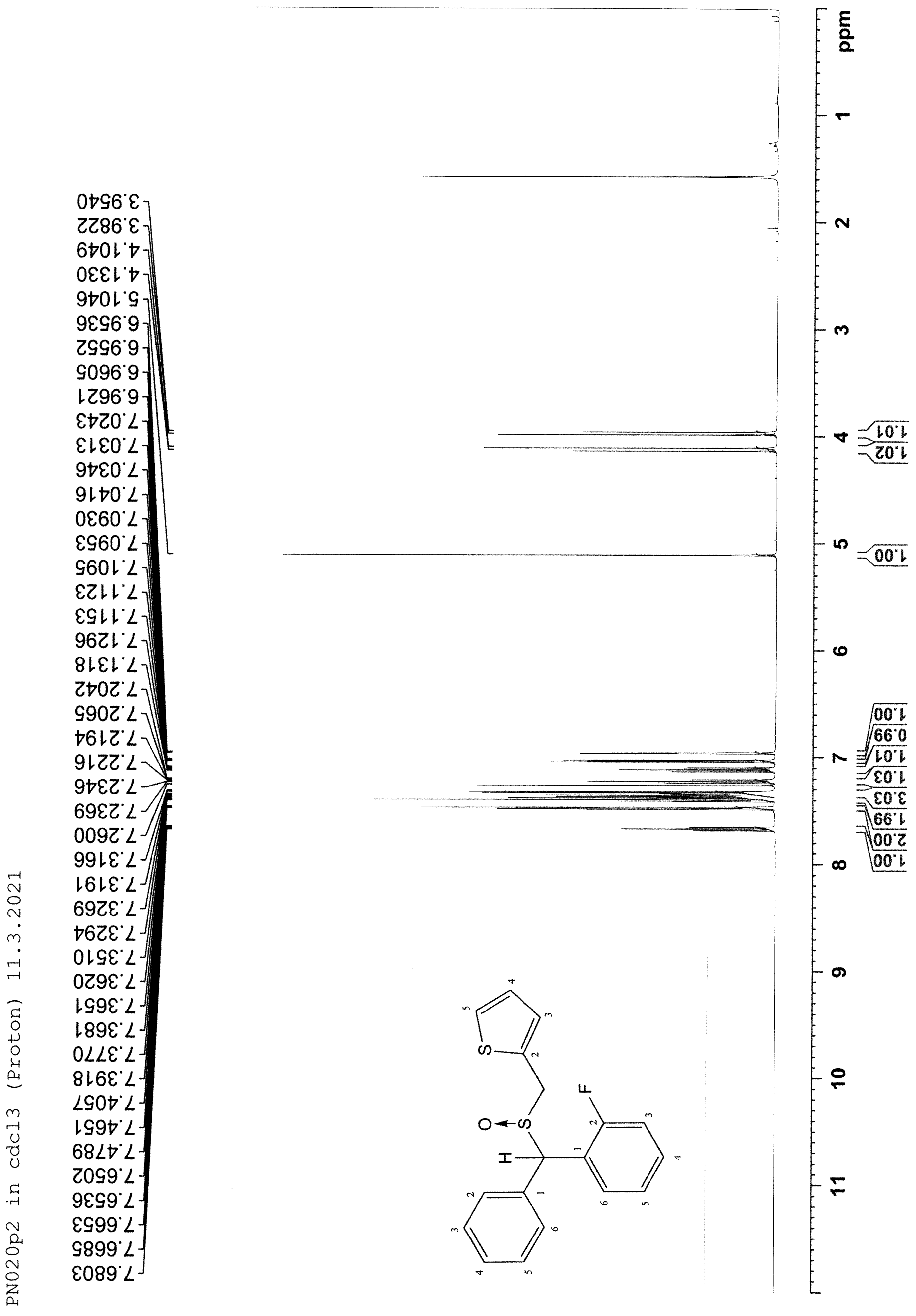

PN020p2 in cdcl3 (Proton) 11.3.2021

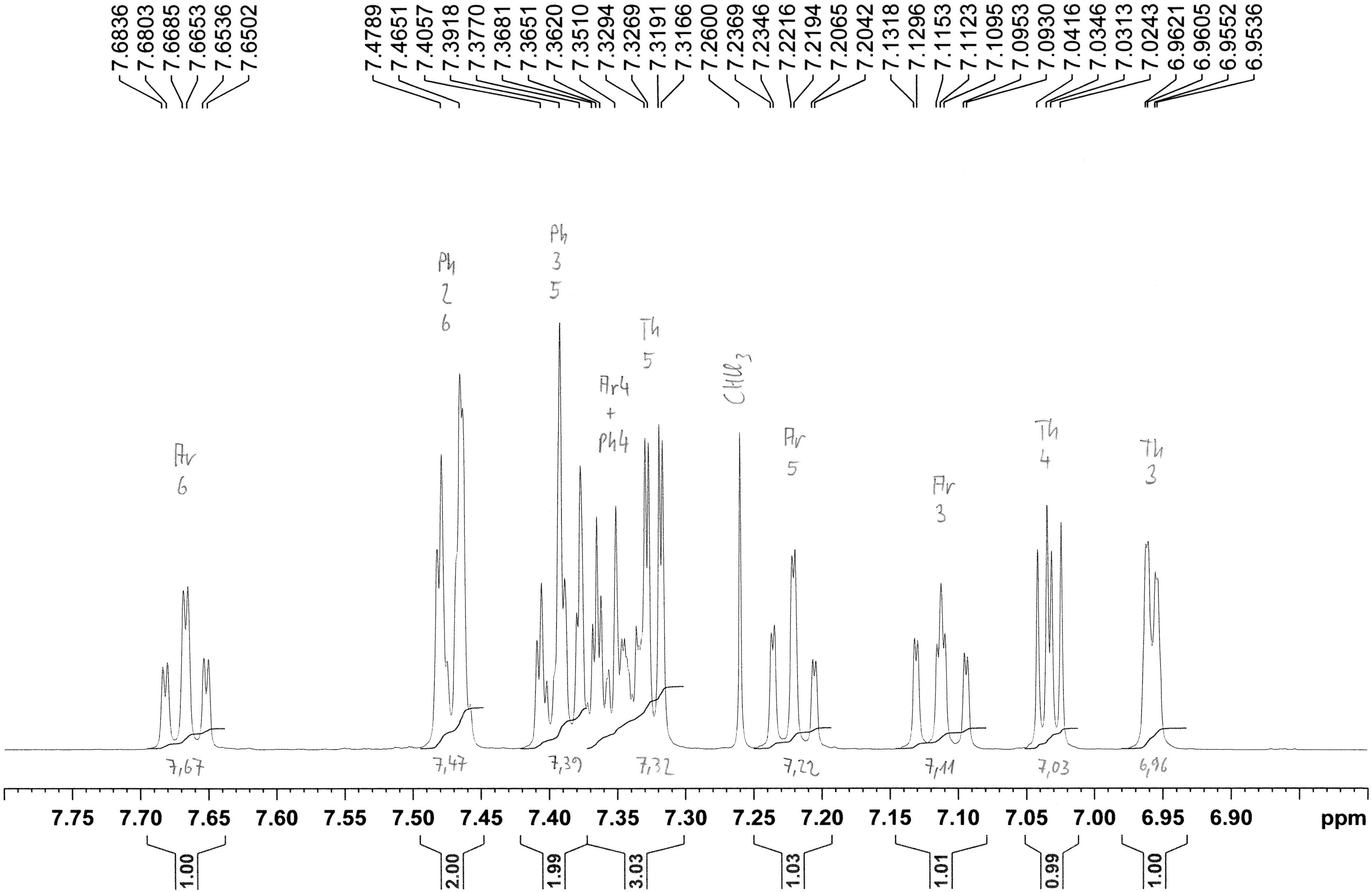

PN020p2 in cdcl3 (Proton) 11.3.2021

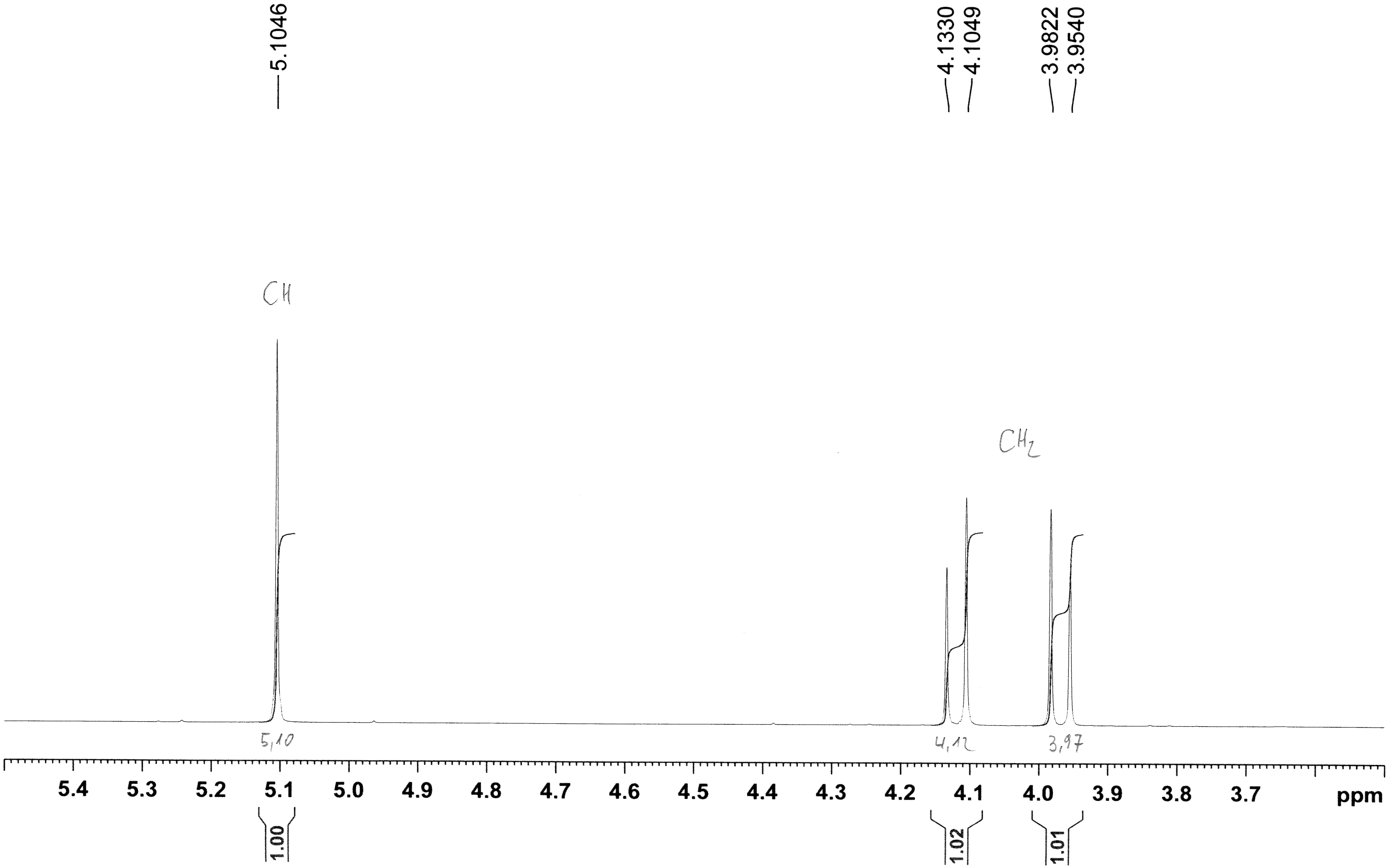

PN020p2 in cdcl3 (APT) 11.3.2021

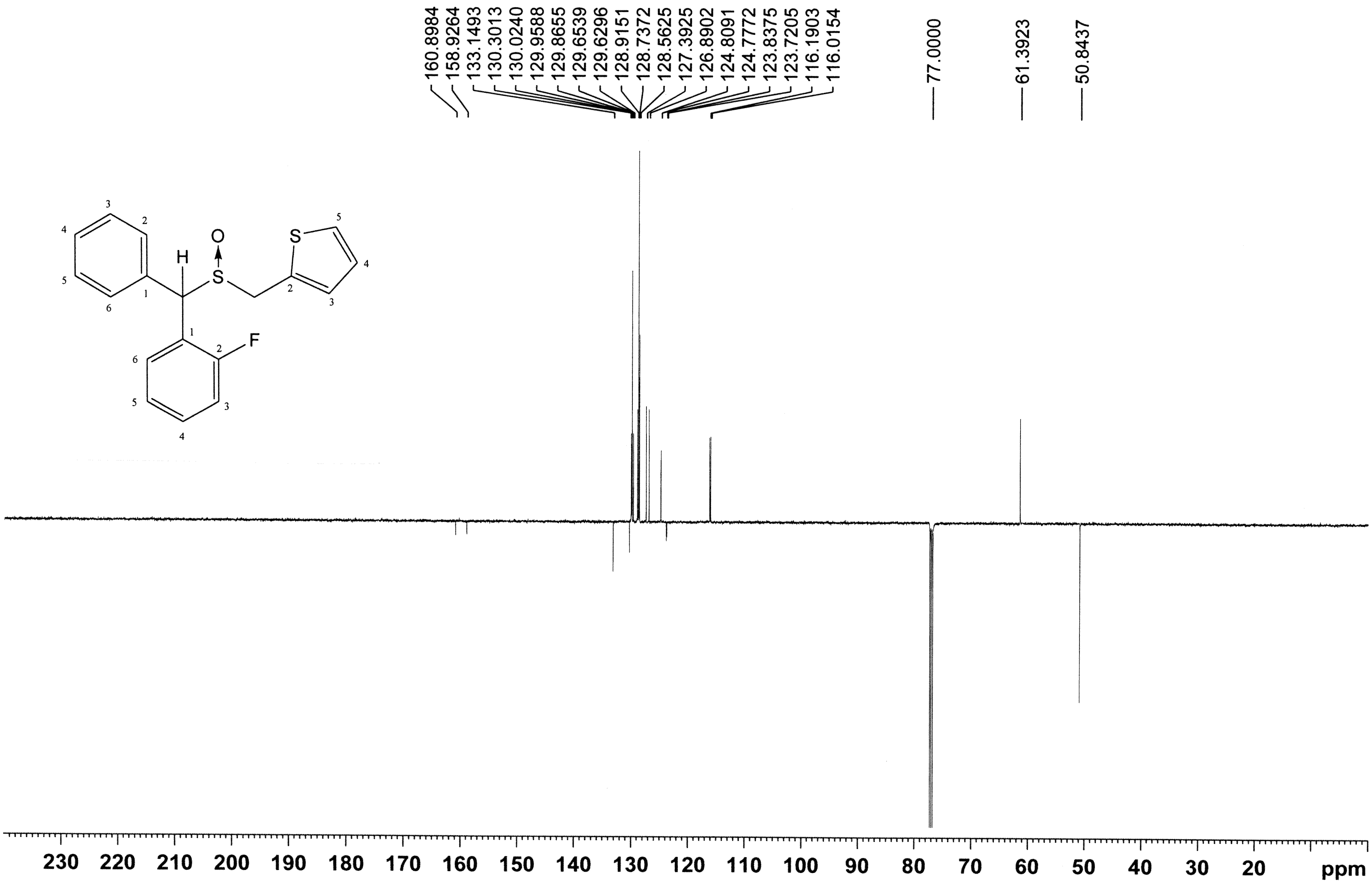

PN020p2 in cdcl3 (APT) 11.3.2021

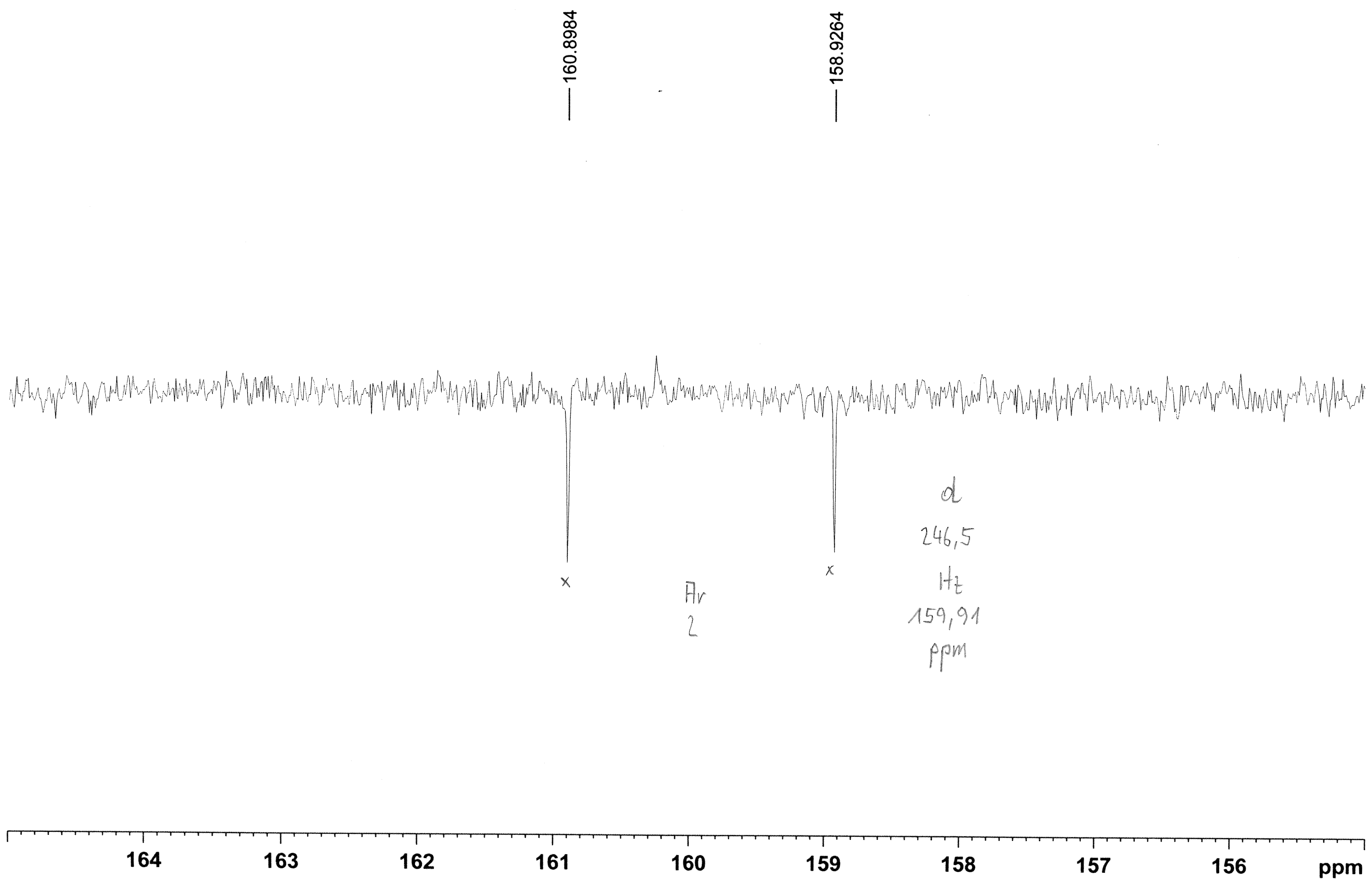

PN020p2 in cdcl3 (APT) 11.3.2021

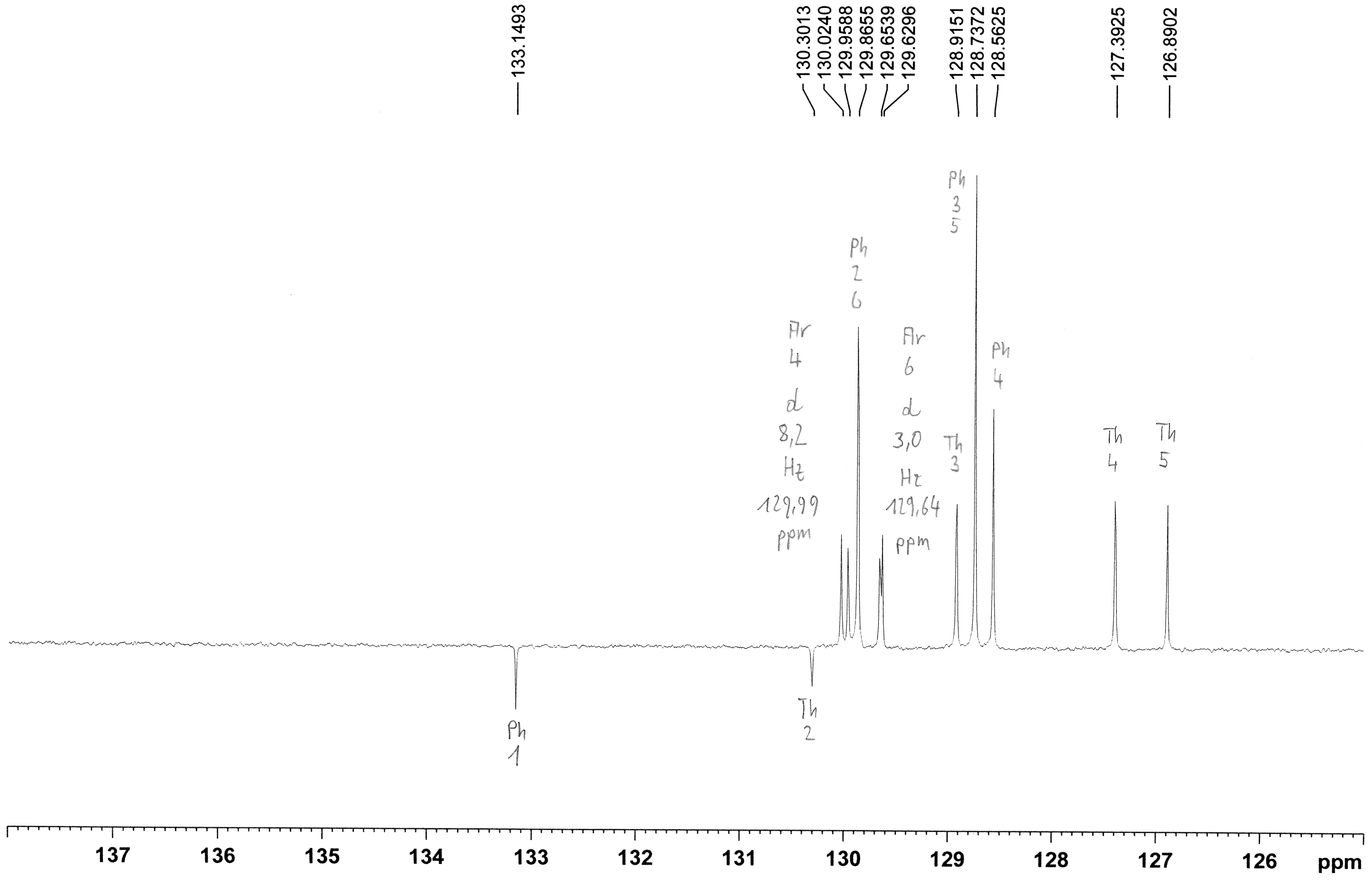

PN020p2 in cdcl3 (APT) 11.3.2021

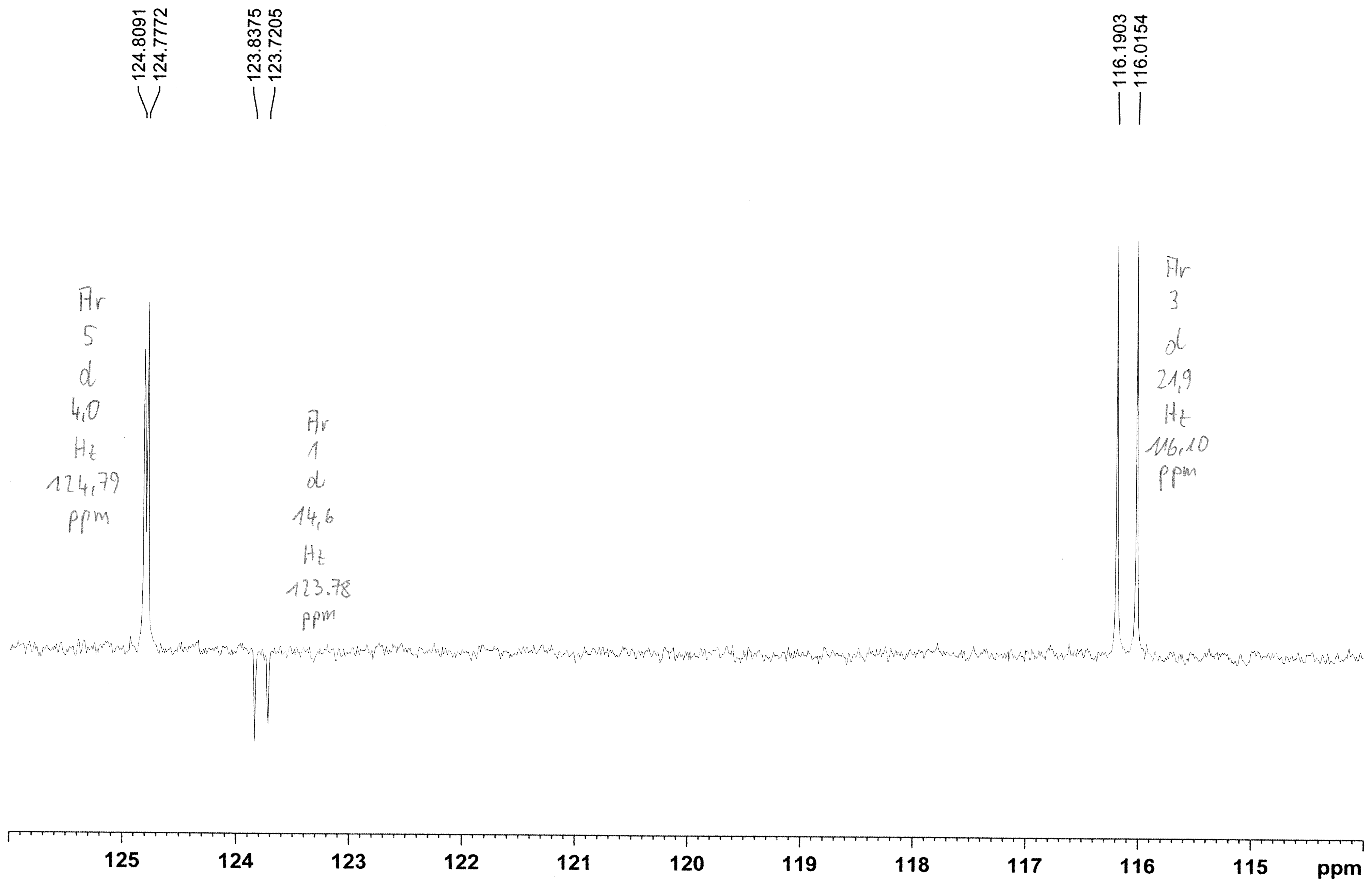

PN020p2 in cdcl3 (APT) 11.3.2021

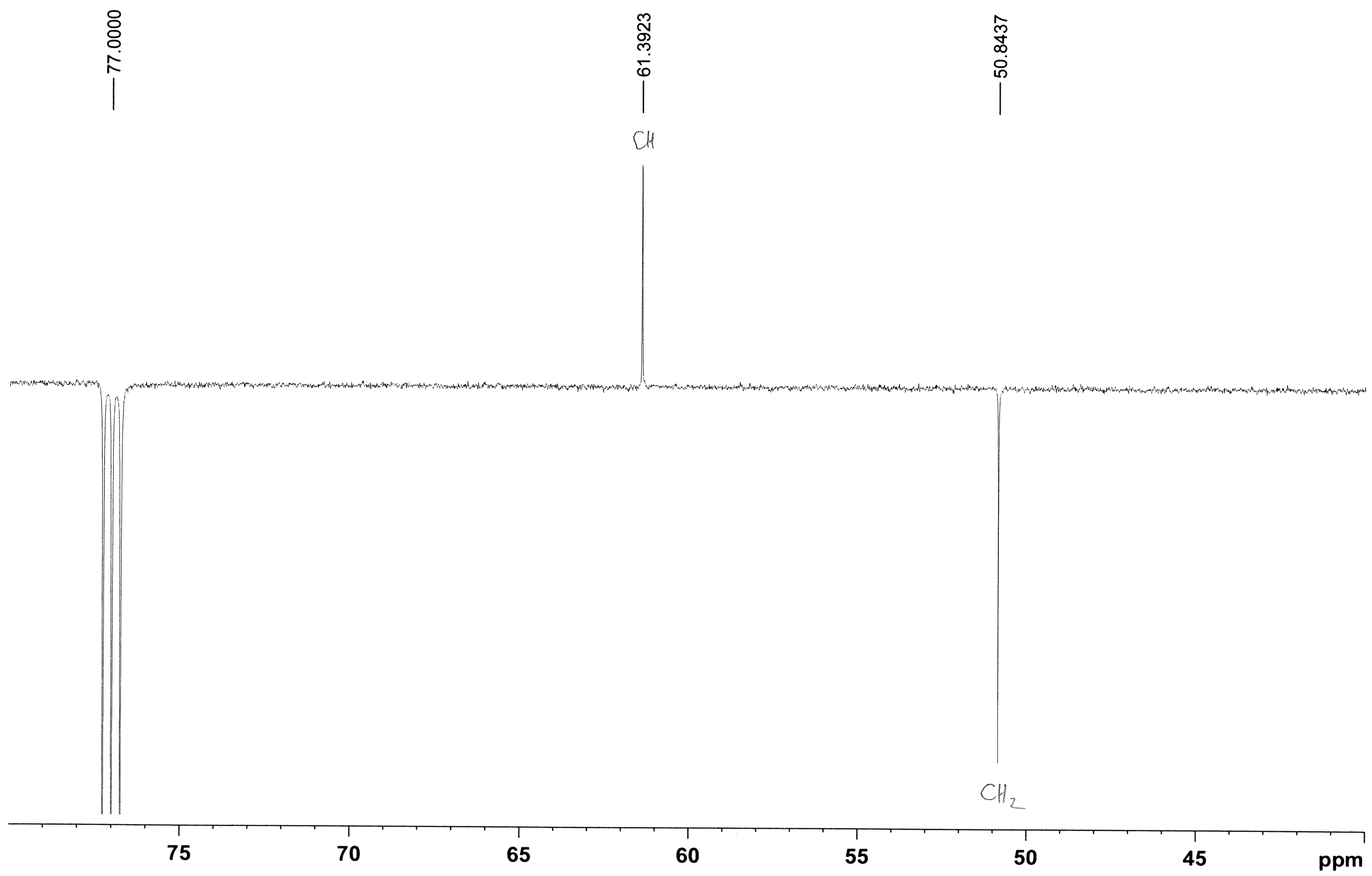

PN020p2 in cdcl3 (COSY) 11.3.2021

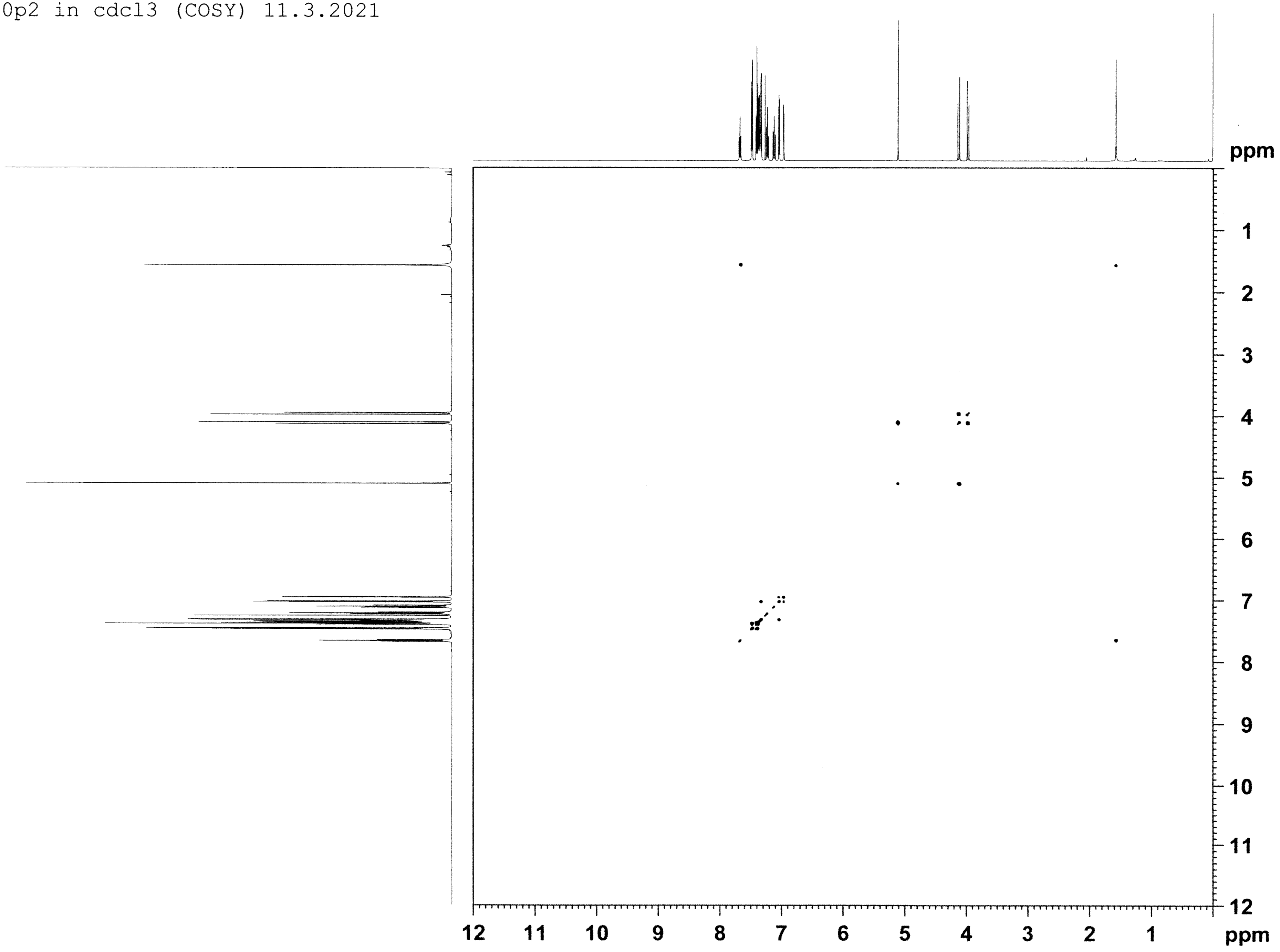

PN020p2 in cdcl3 (COSY) 11.3.2021

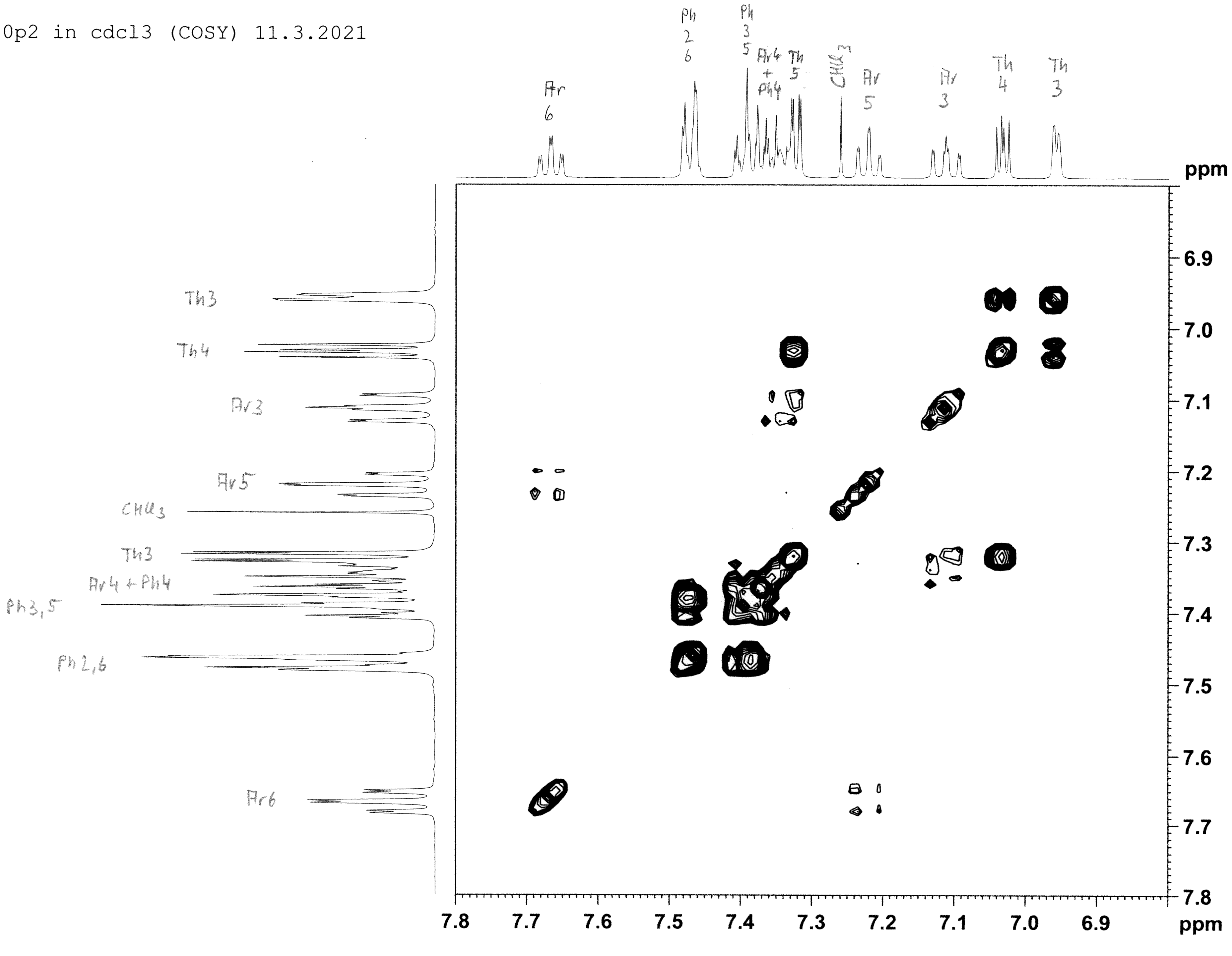

PN020p2 in cdcl3 (COSY) 11.3.2021

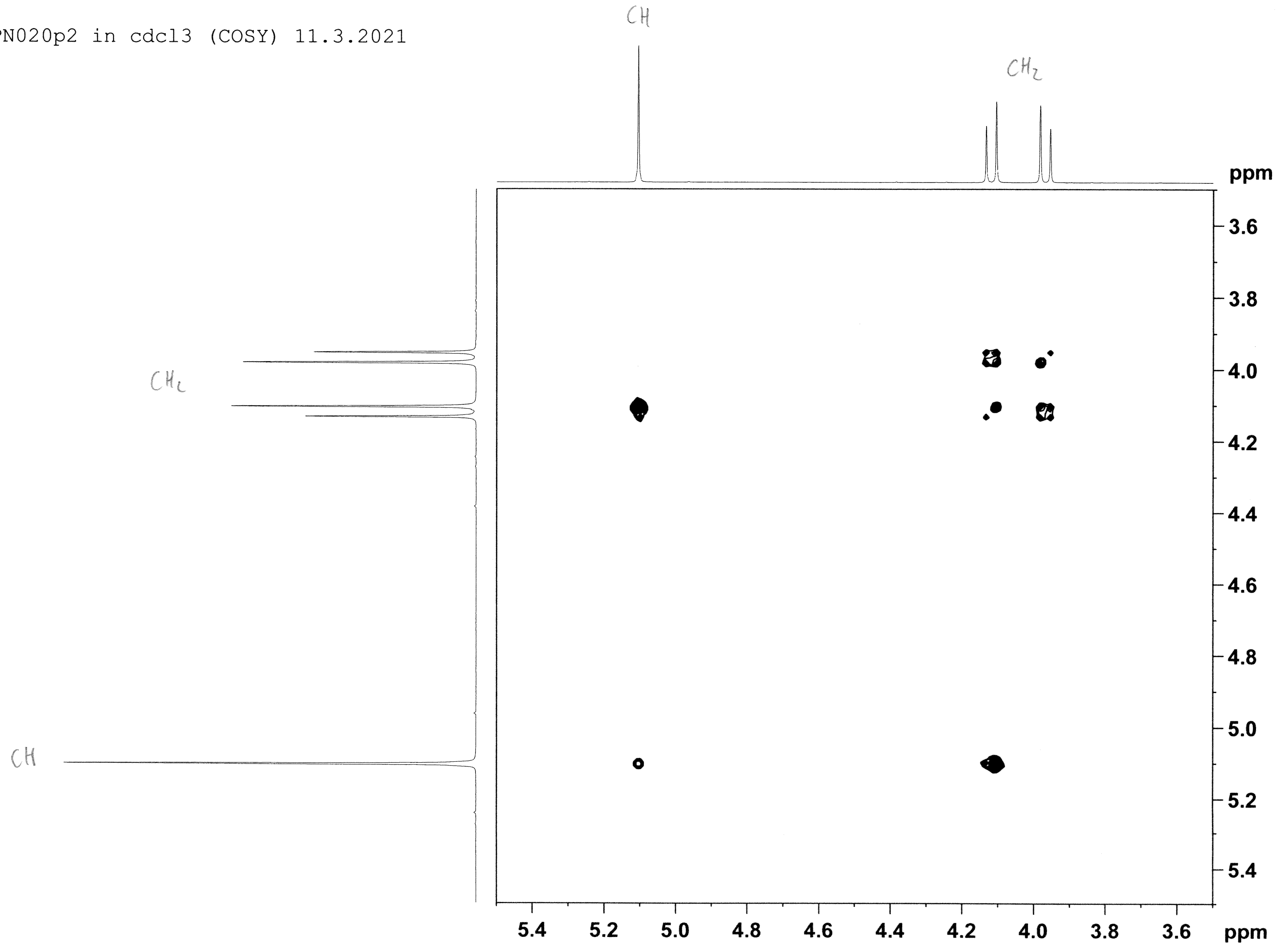

PN020p2 in cdcl3 (HSQC) 11.3.2021

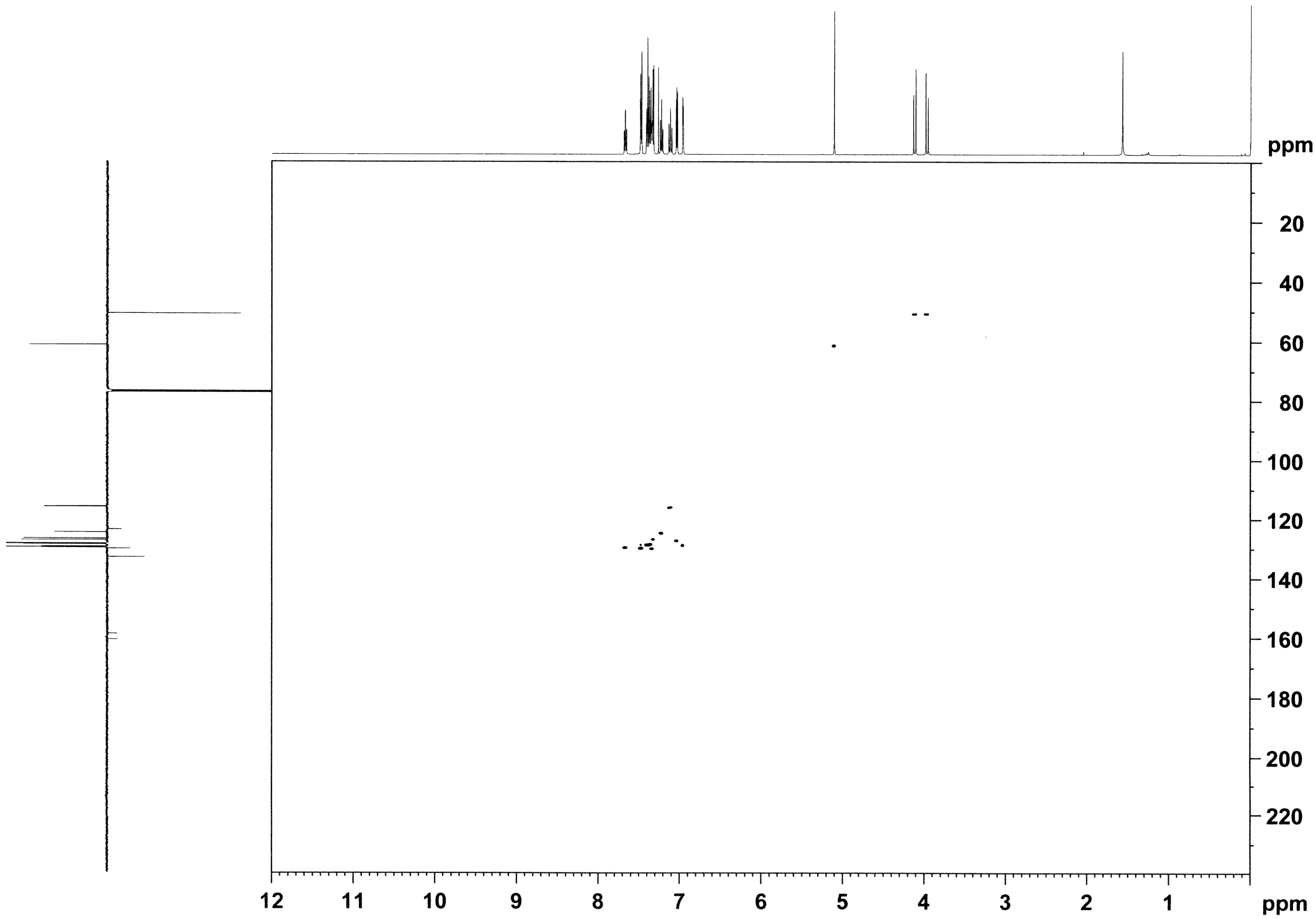

PN020p2 in cdcl3 (HSQC) 11.3.2021

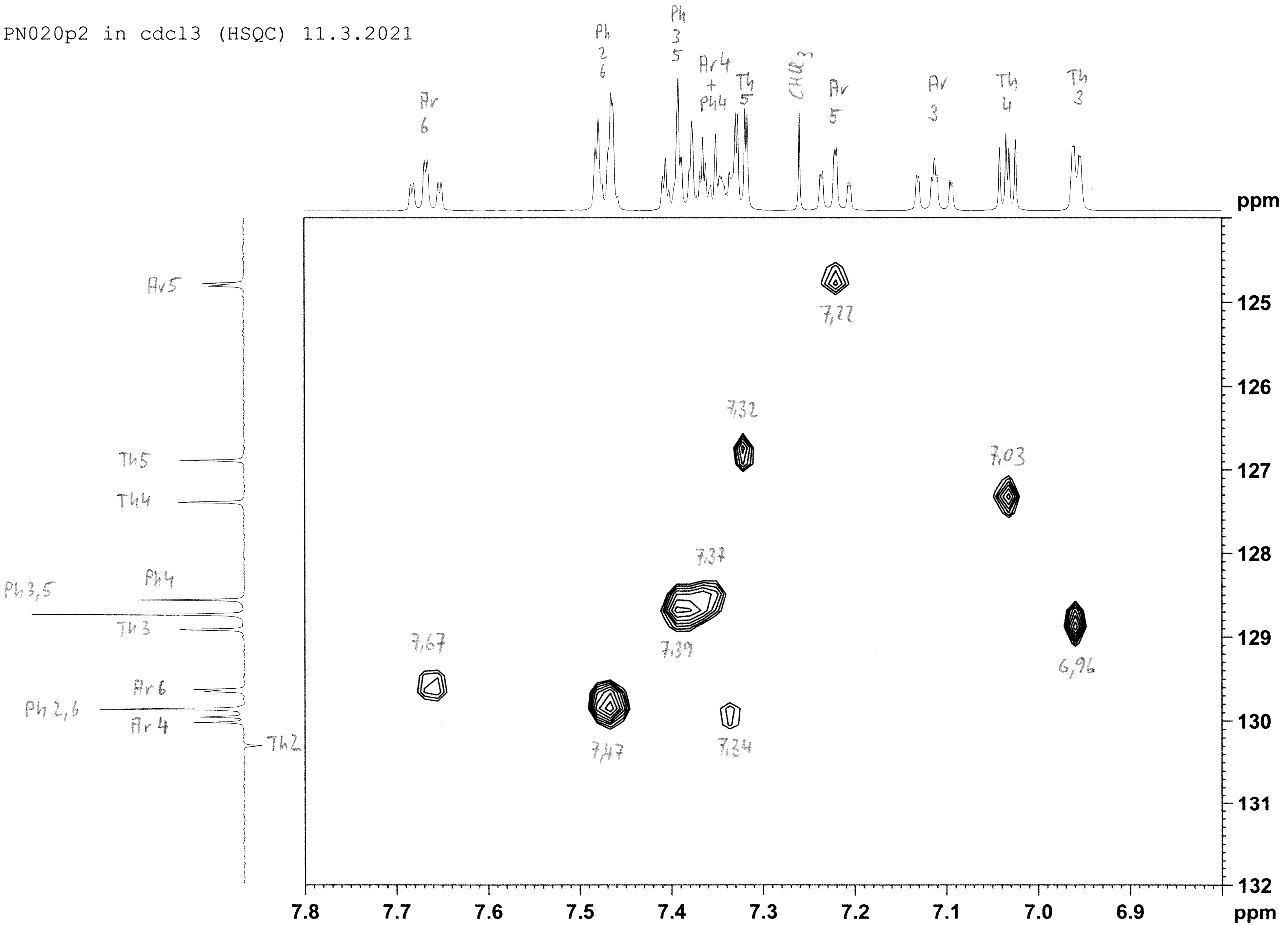

PN020p2 in cdcl3 (HSQC) 11.3.2021

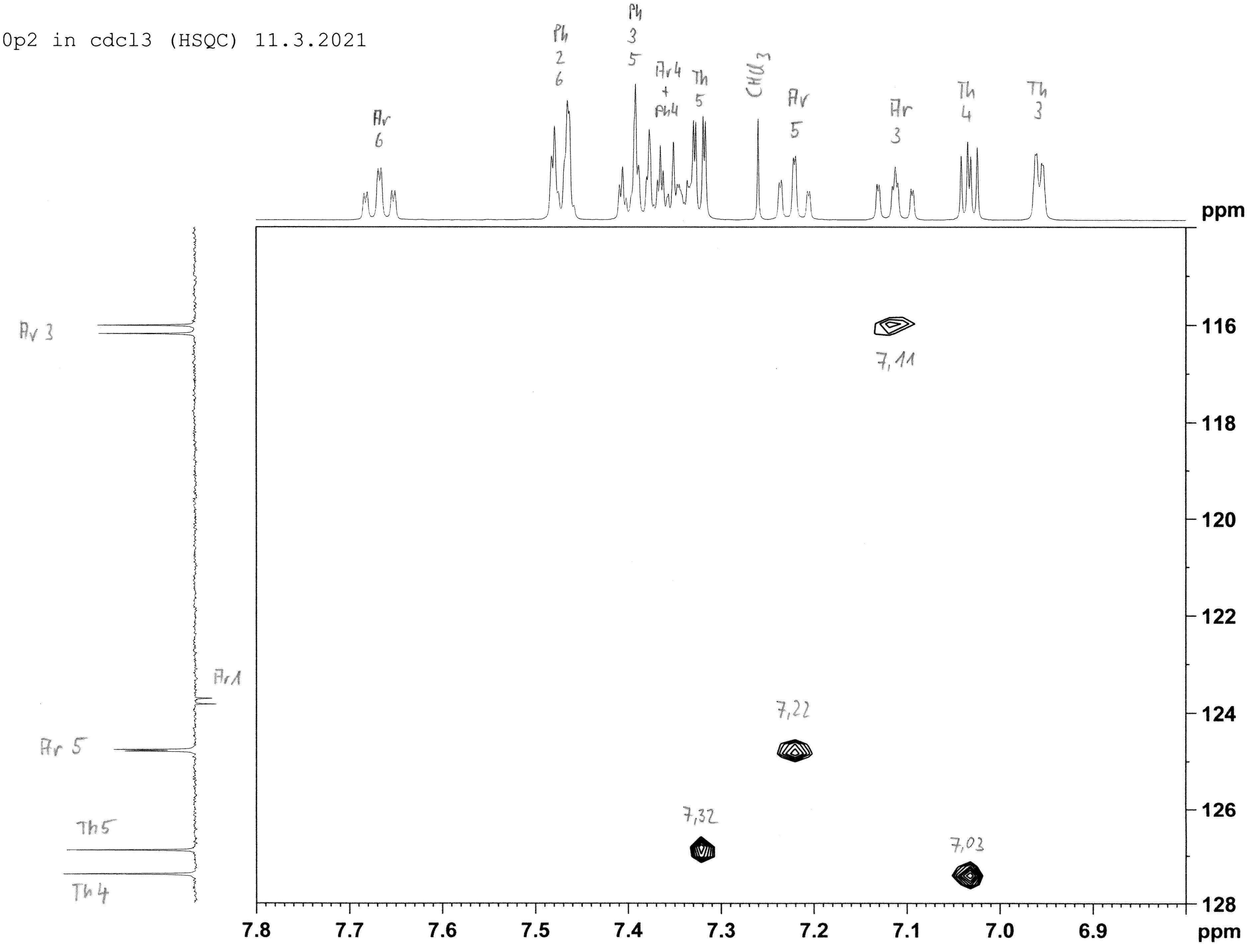

PN020p2 in cdcl3 (HSQC) 11.3.2021

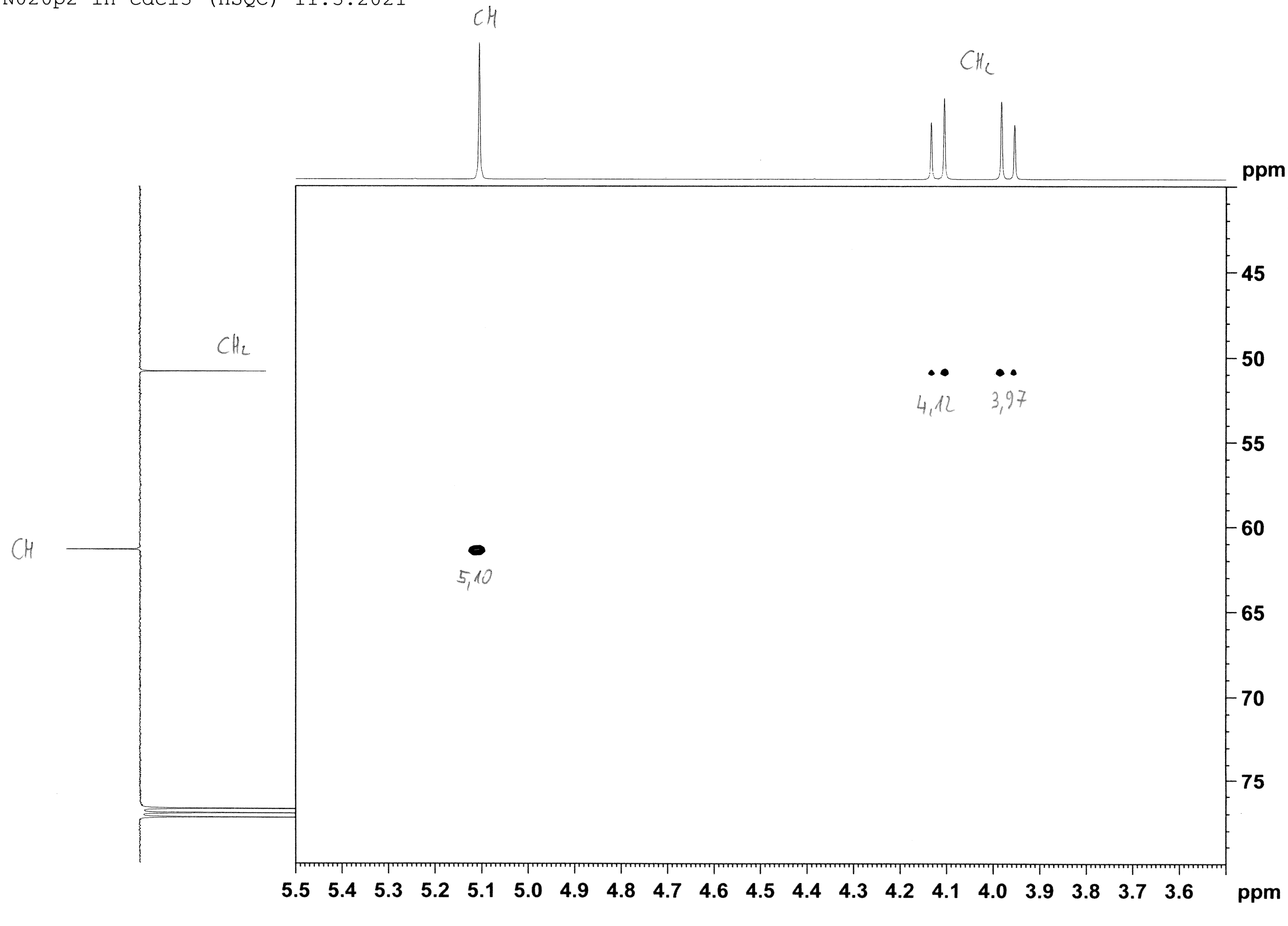

PN020p2 in cdcl3 (HMBC) 11.3.2021

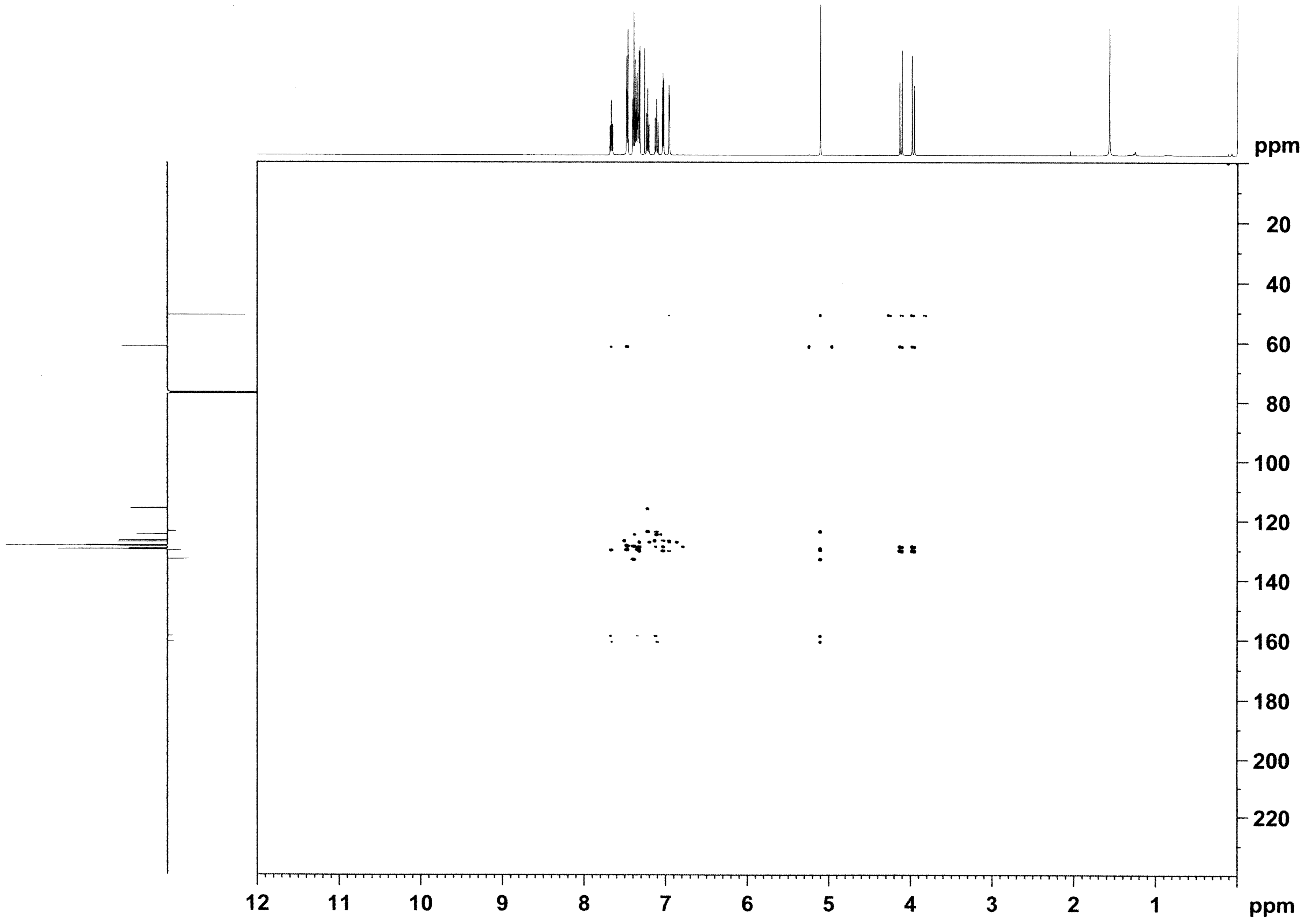

PN020p2 in cdcl3 (HMBC) 11.3.2021

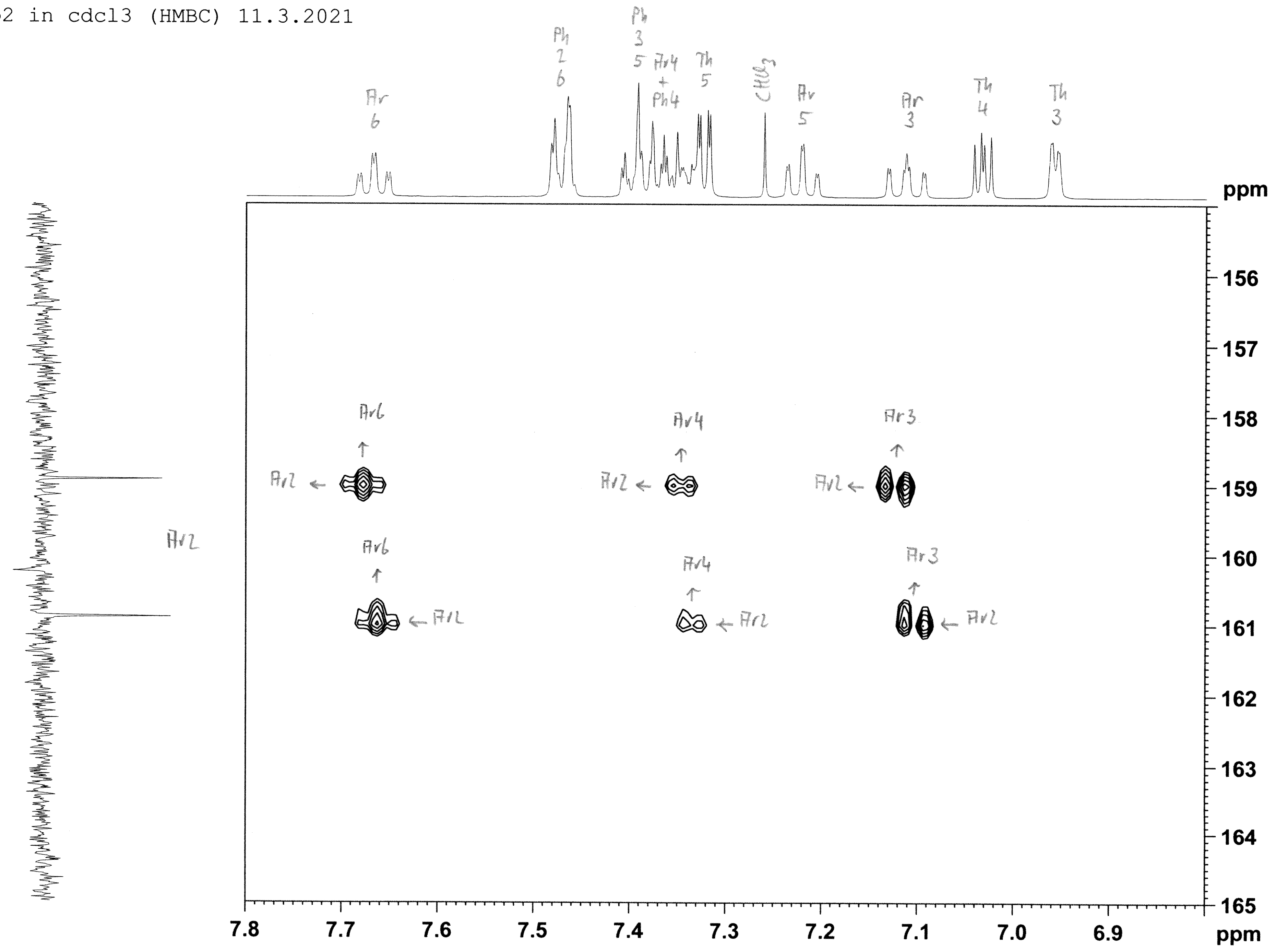

PN020p2 in cdcl3 (HMBC) 11.3.2021

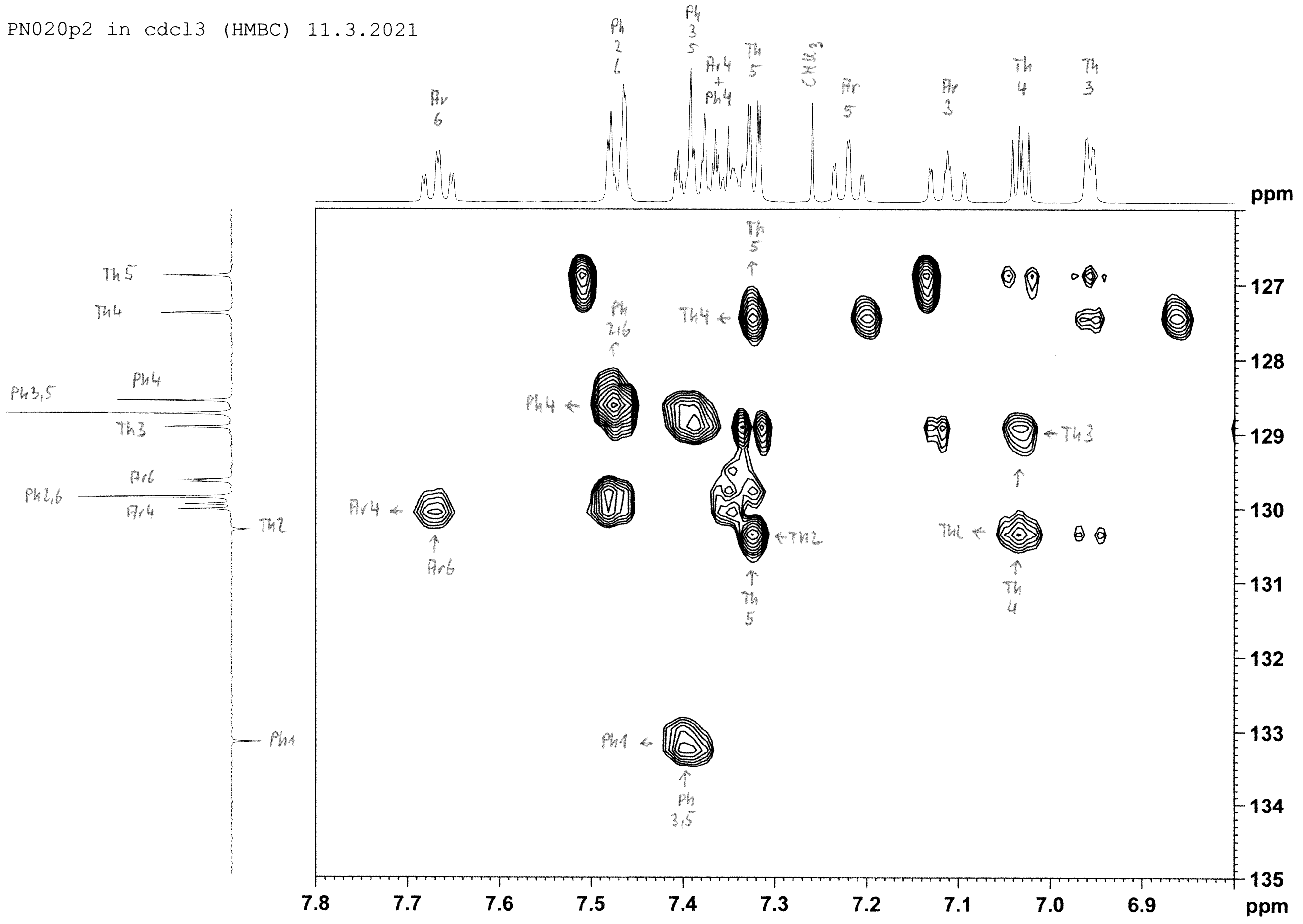

PN020p2 in cdcl3 (HMBC) 11.3.2021

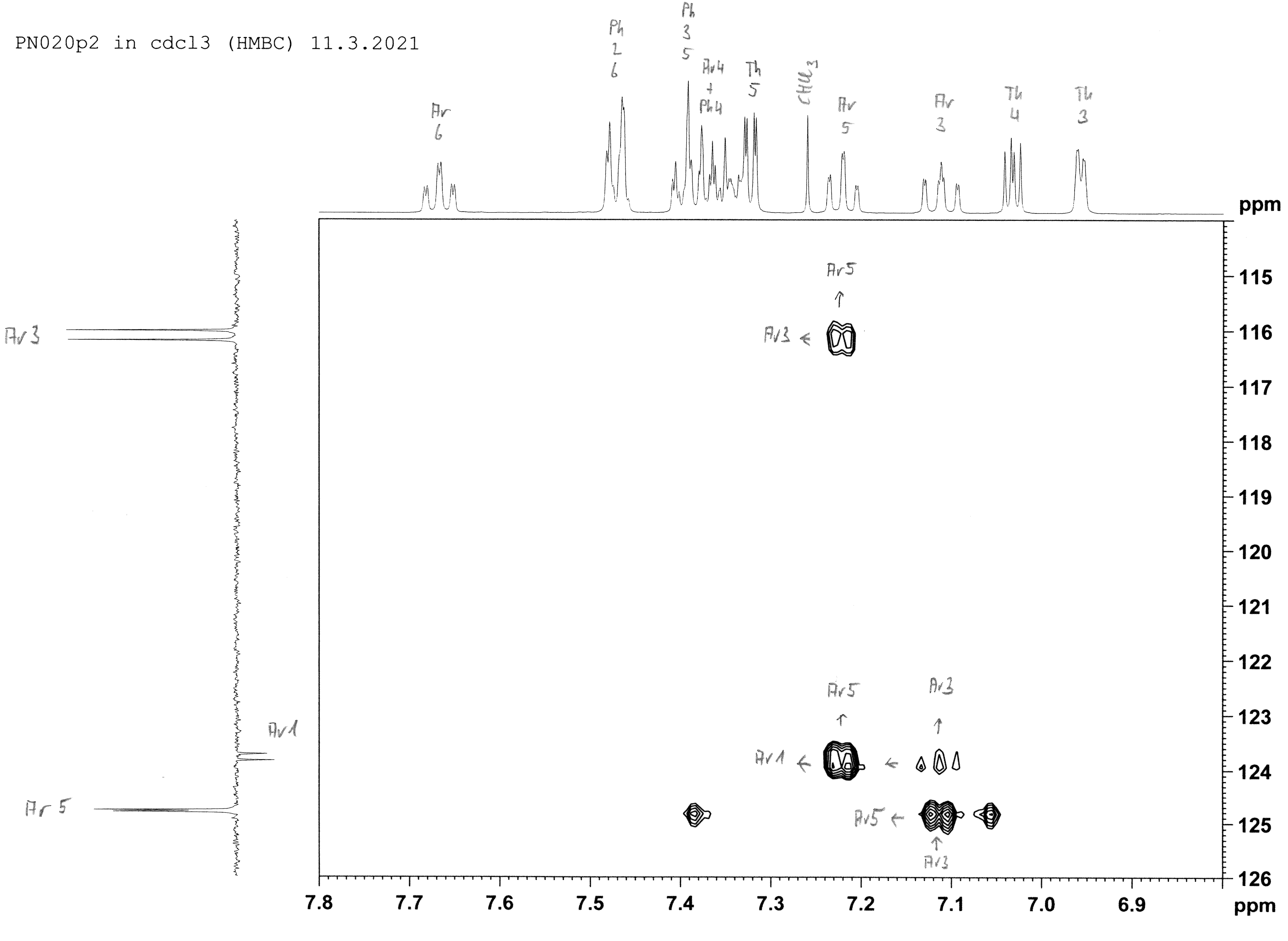

PN020p2 in cdcl3 (HMBC) 11.3.2021

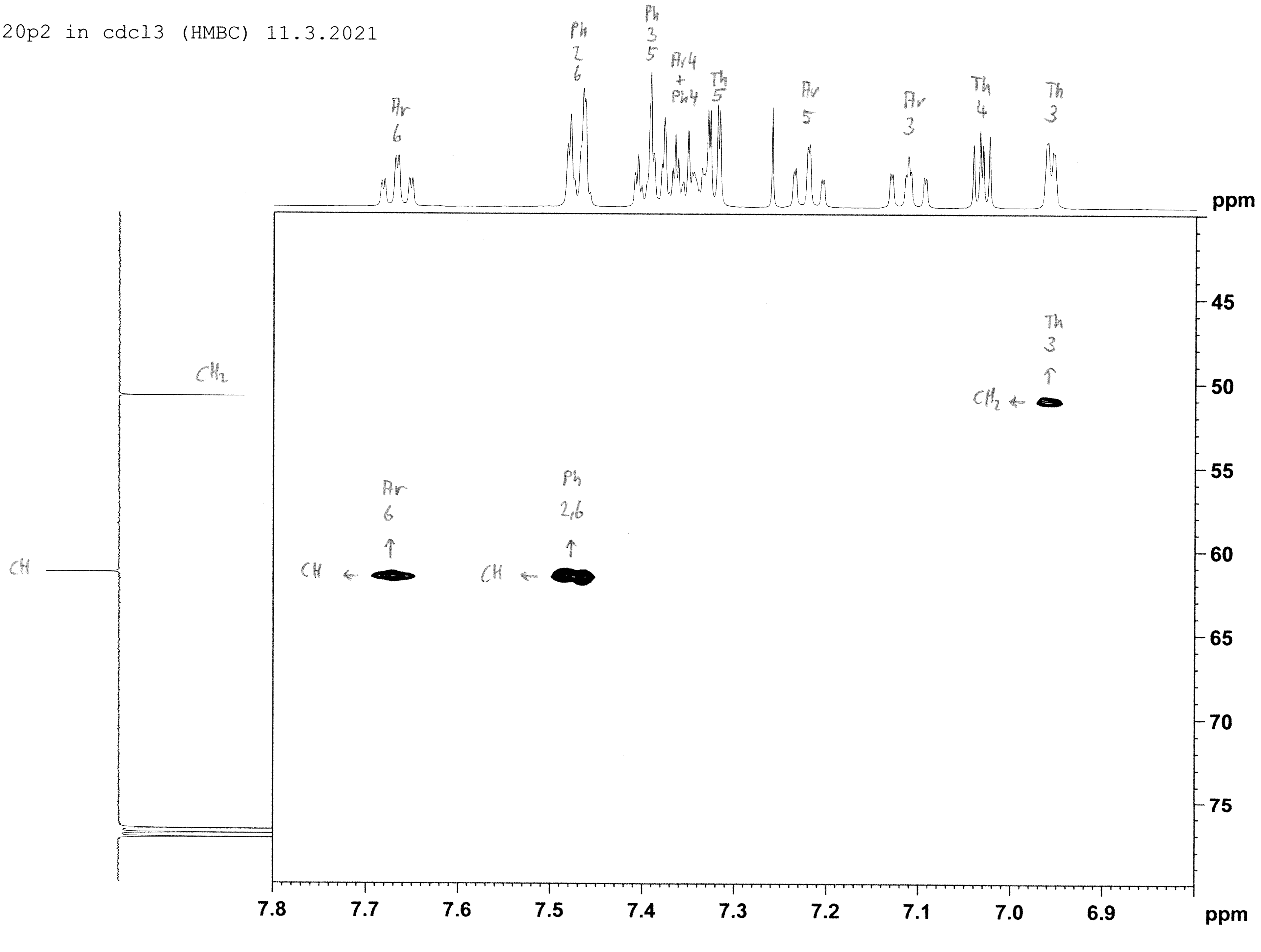

PN020p2 in cdcl3 (HMBC) 11.3.2021

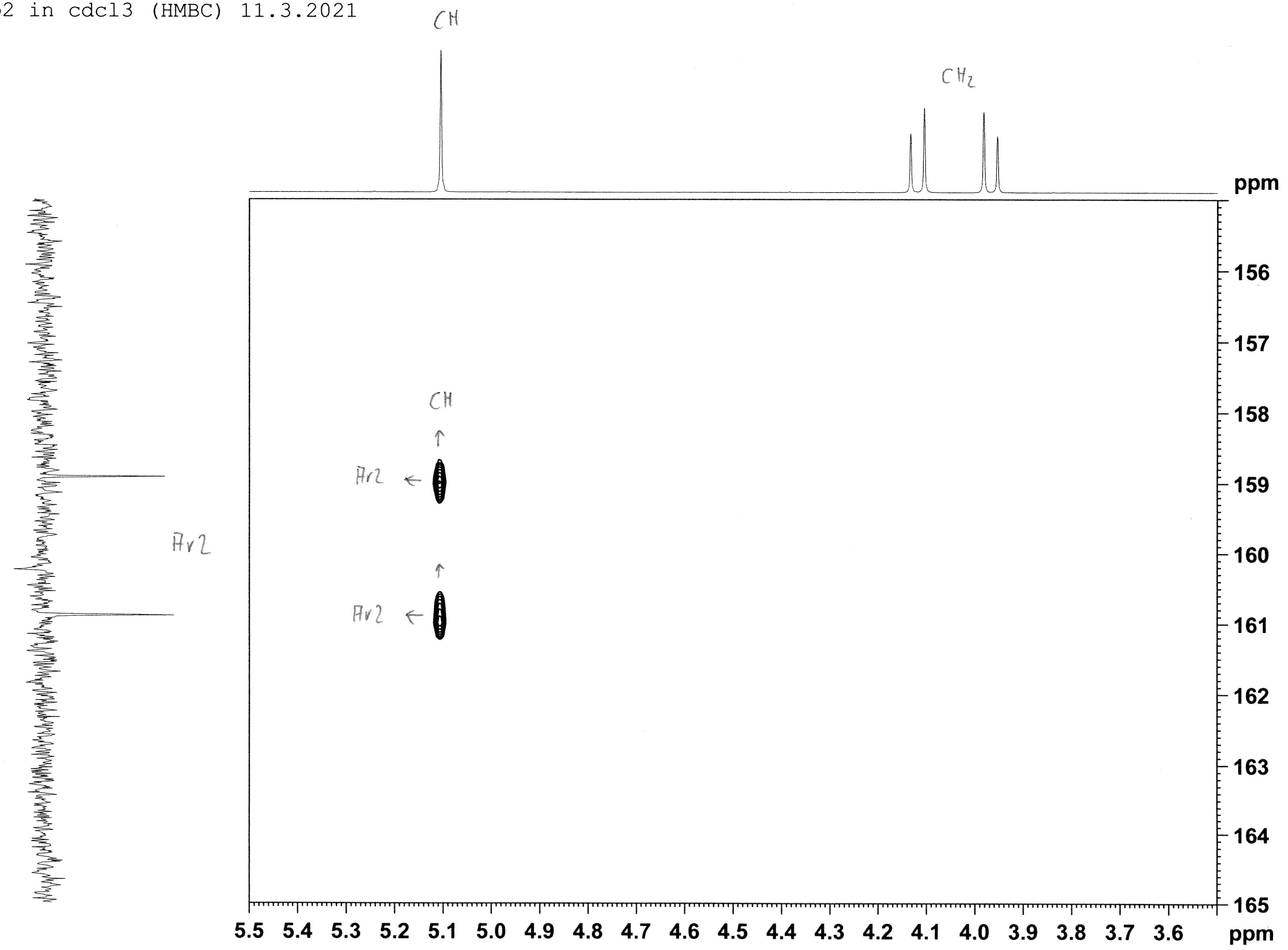

PN020p2 in cdcl3 (HMBC) 11.3.2021

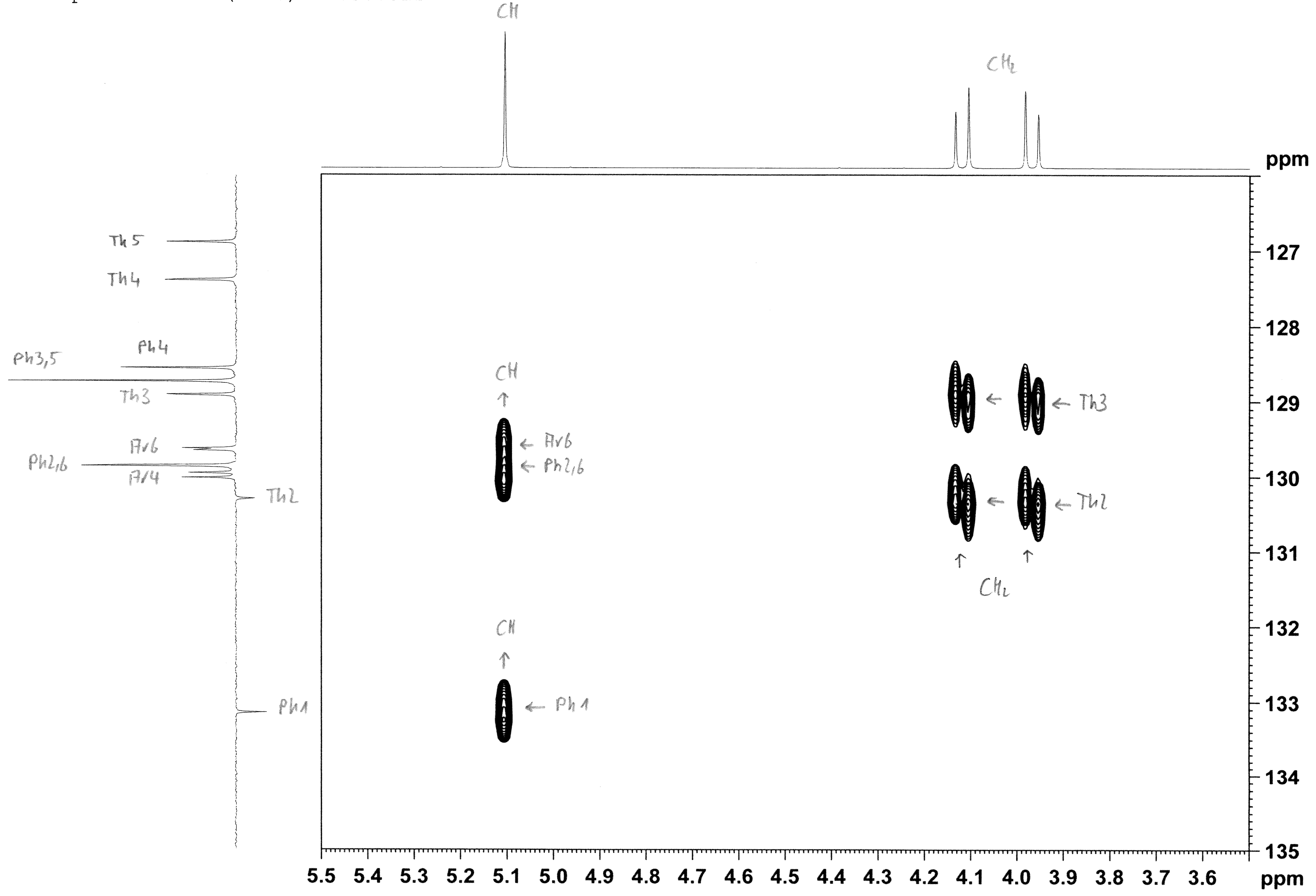

PN020p2 in cdcl3 (HMBC) 11.3.2021

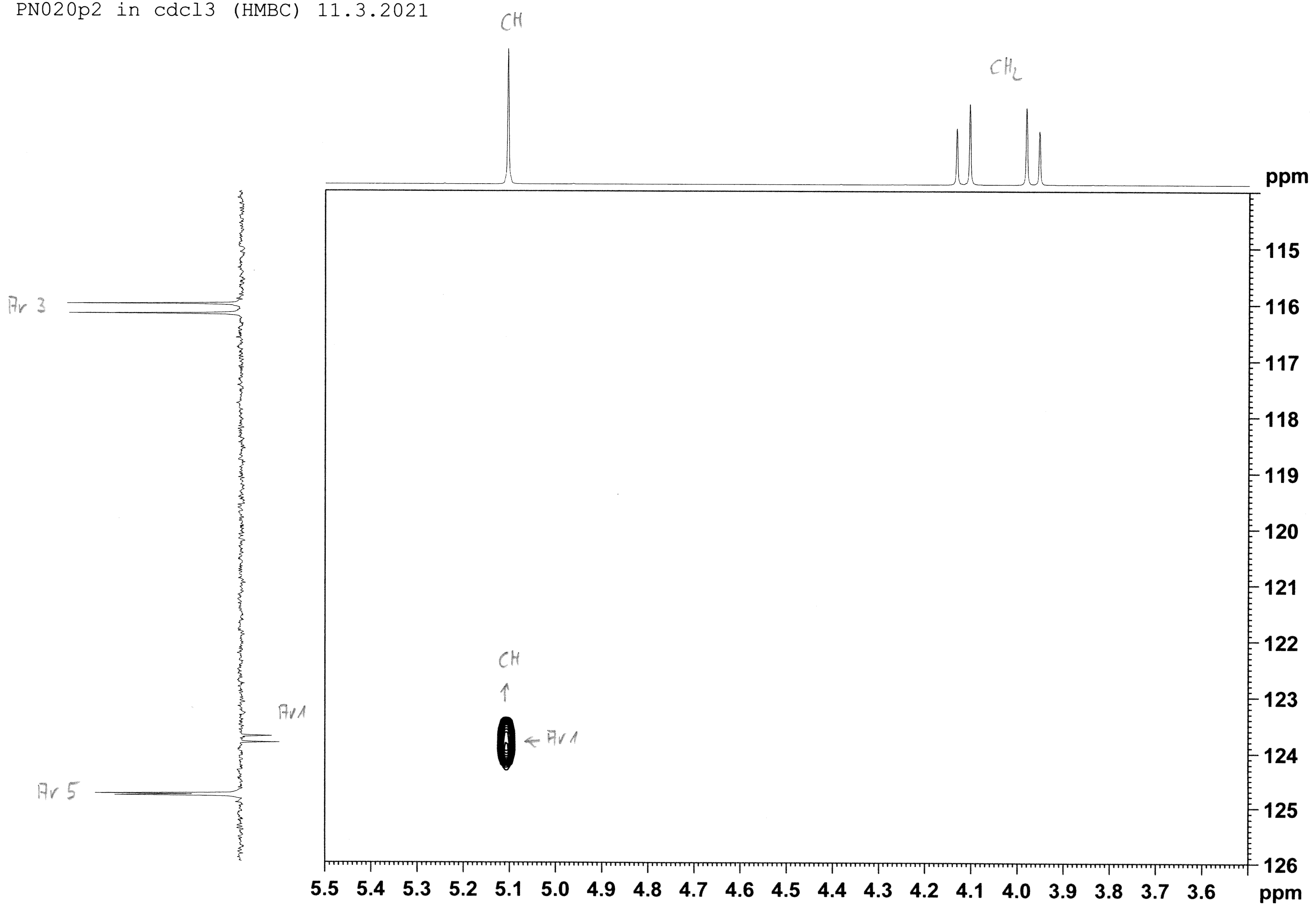

PN020p2 in cdcl3 (HMBC) 11.3.2021

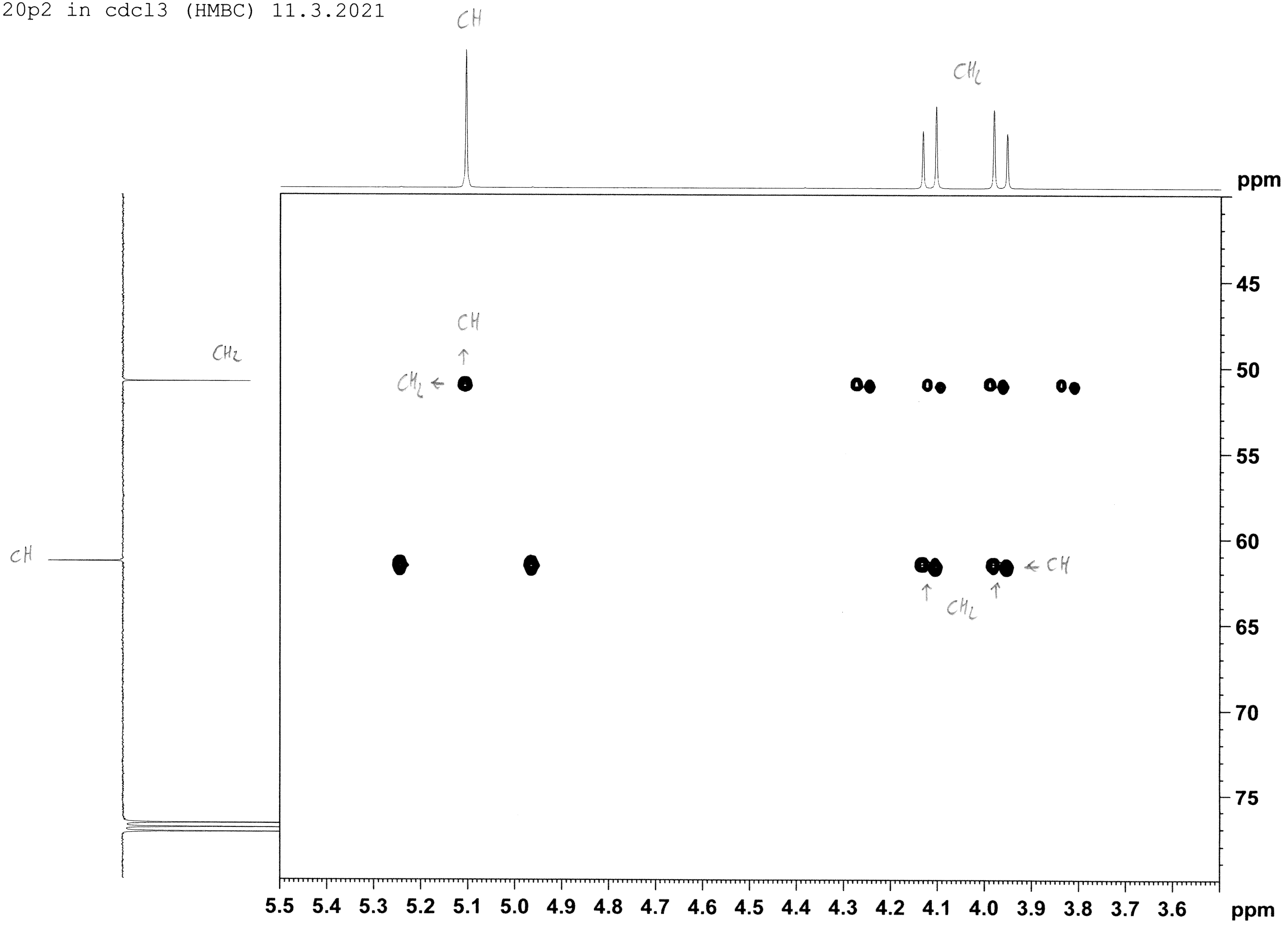

Figure S3c. NMR spectra of compound **7a**.

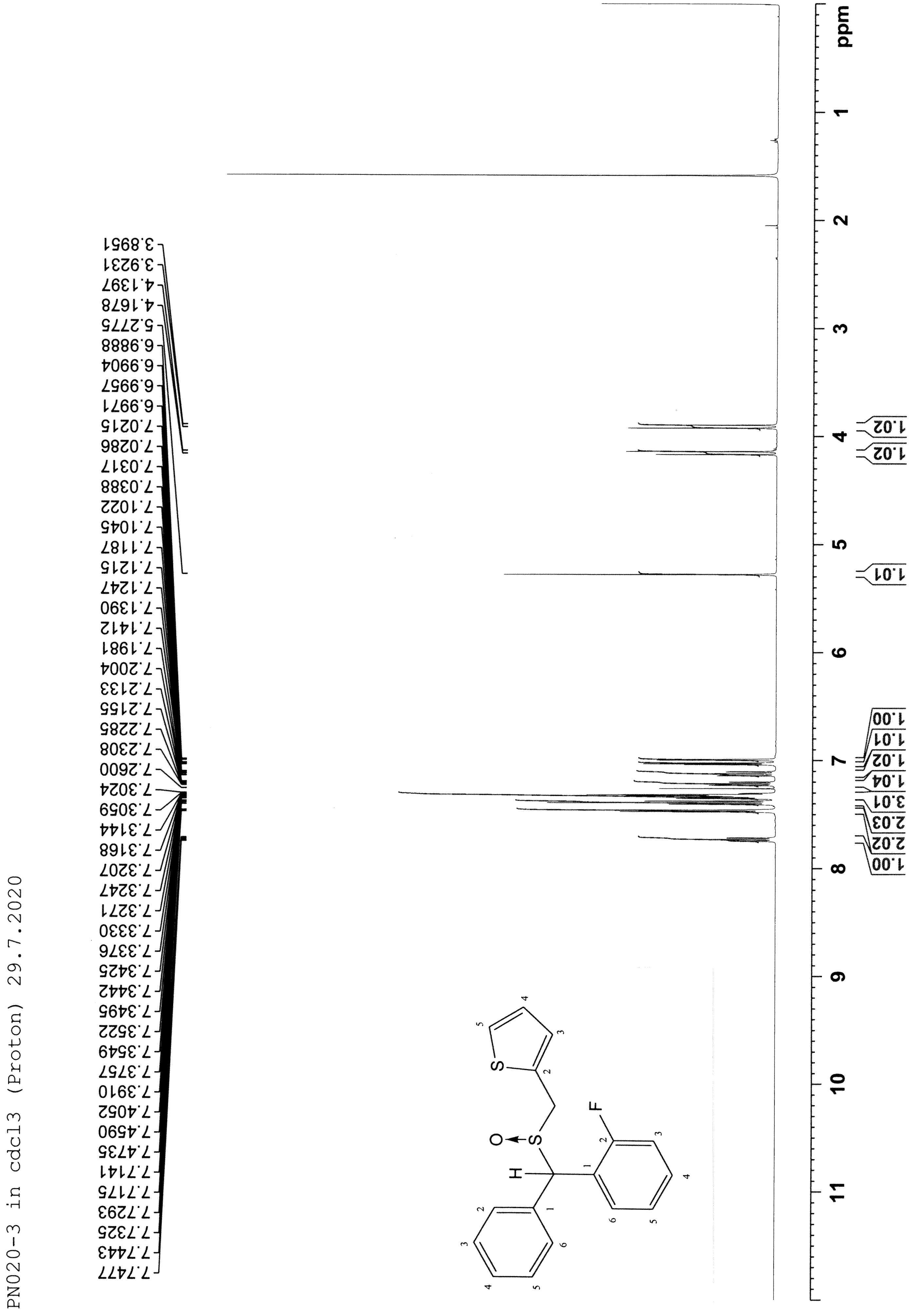

PN020-3 in cdcl3 (Proton) 29.7.2020

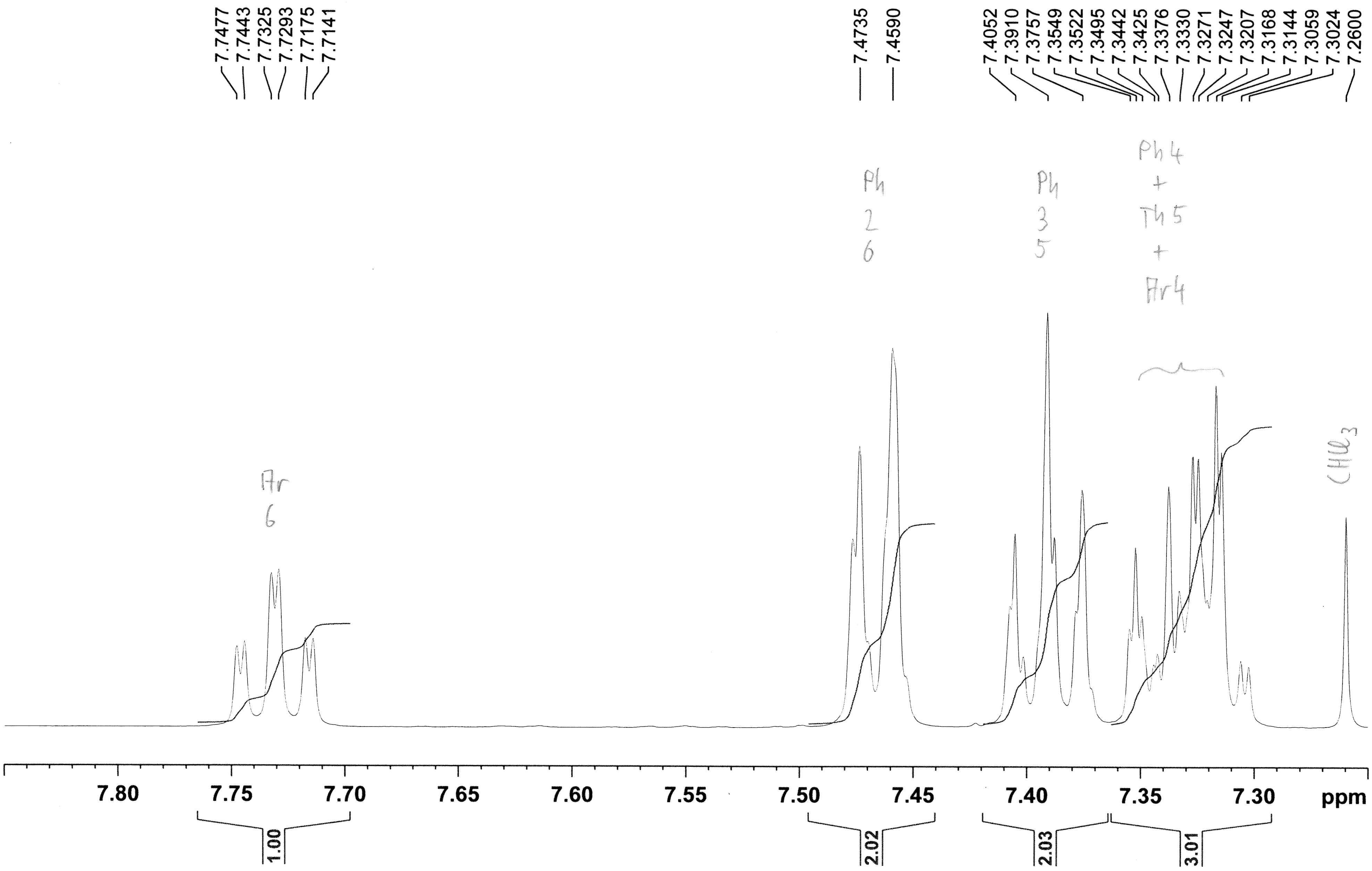

PN020-3 in cdcl3 (Proton) 29.7.2020

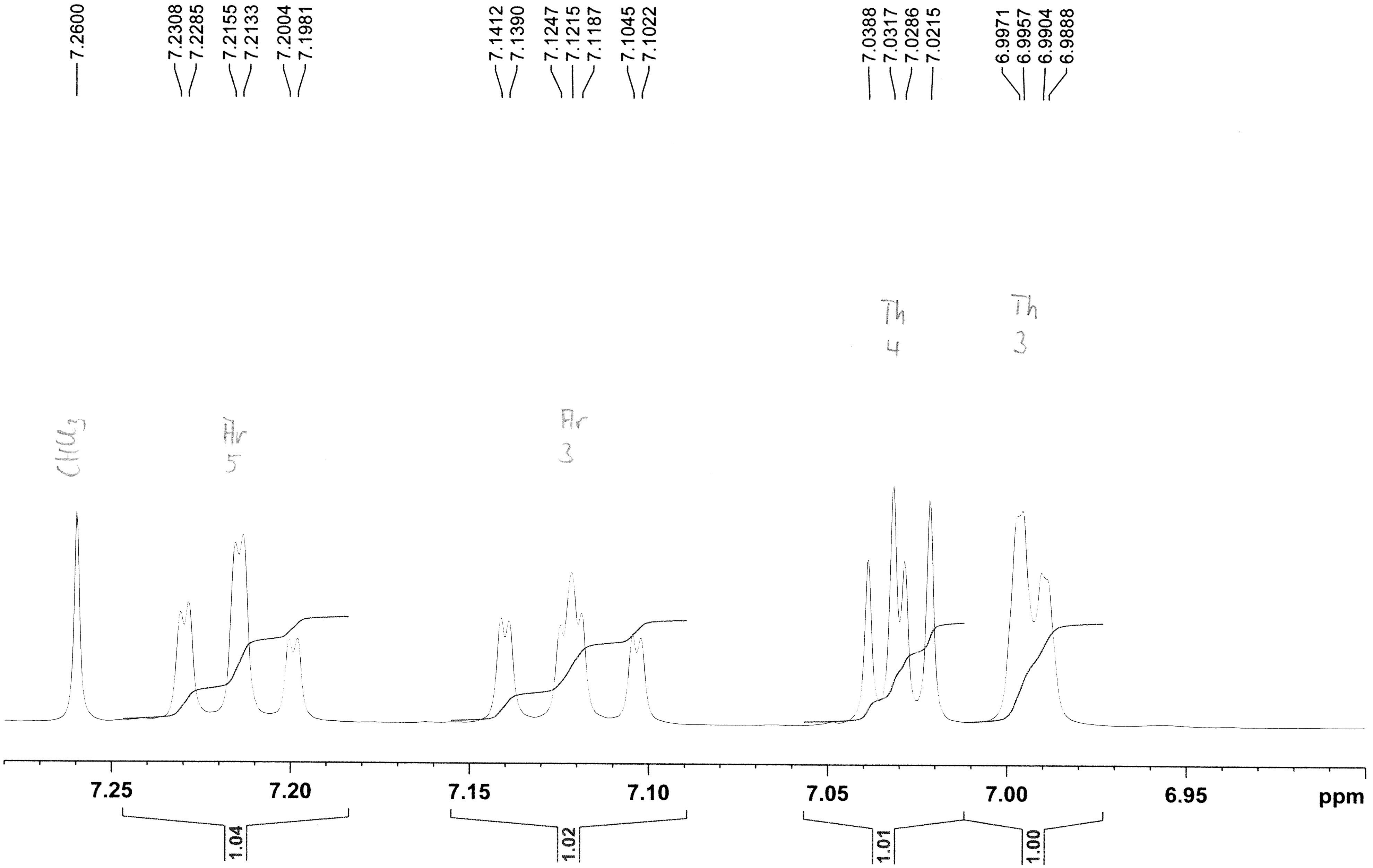

PN020-3 in cdcl3 (Proton) 29.7.2020

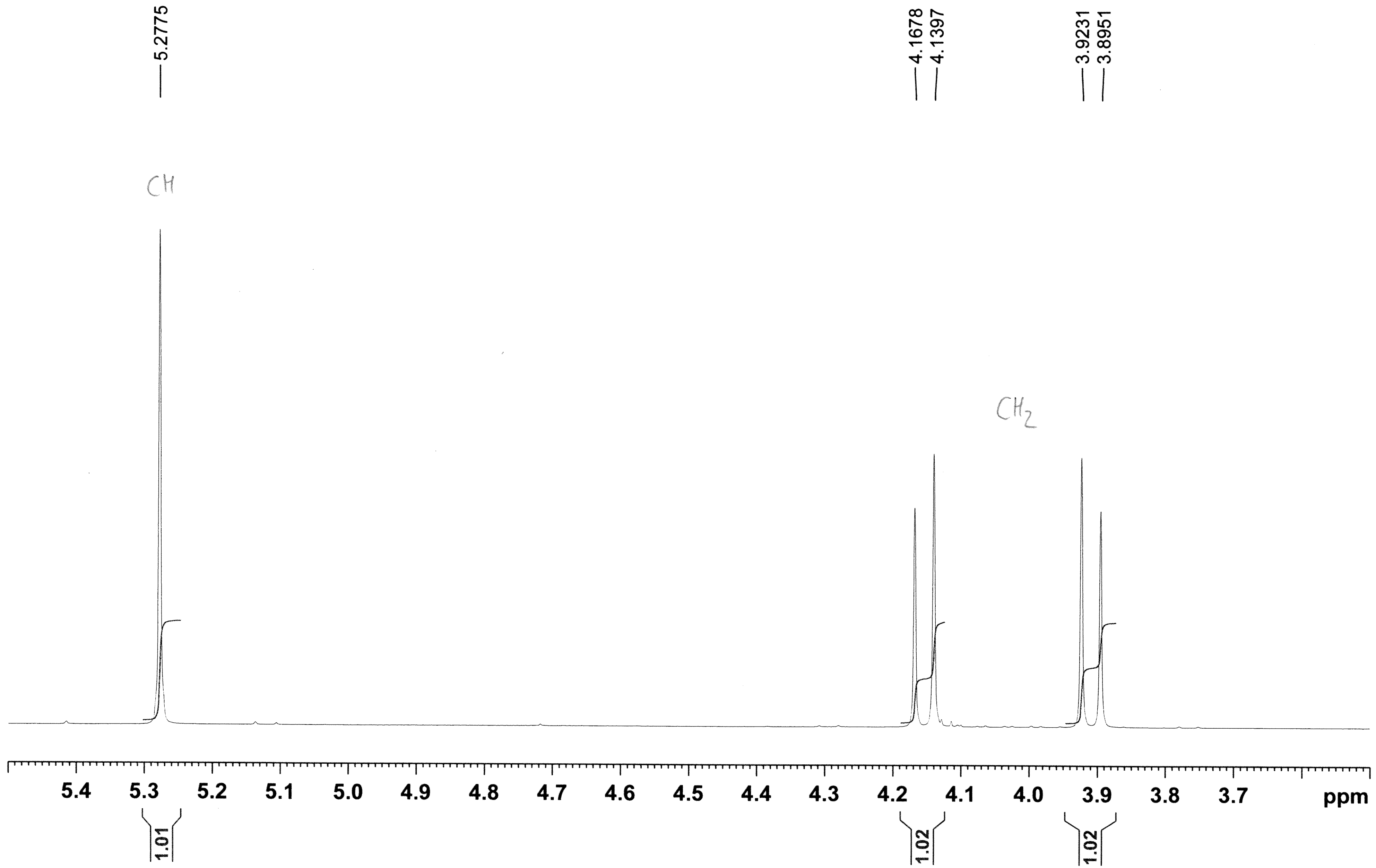

PN020-3 in cdcl3 (APT) 29.7.2020

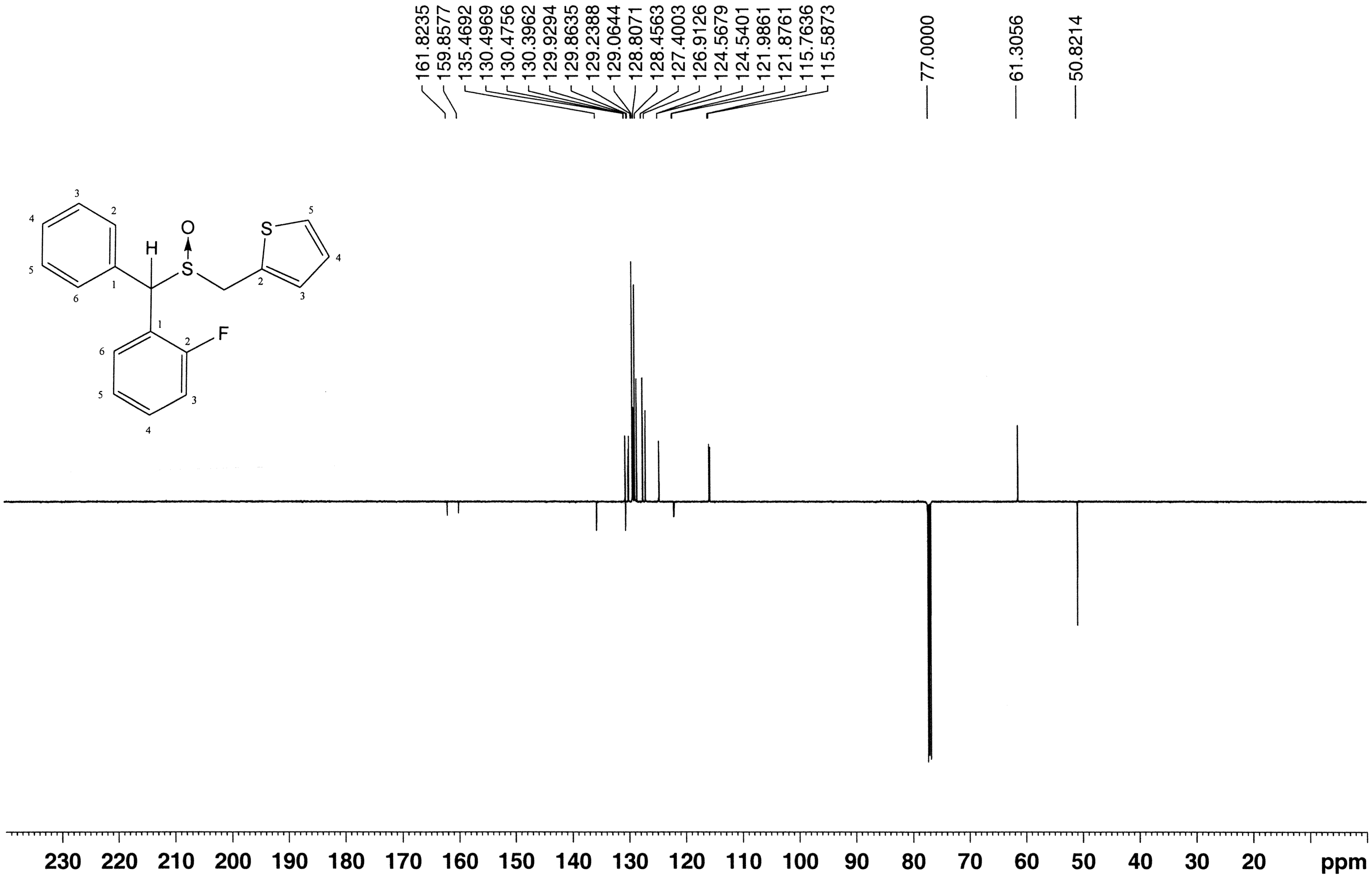

PN020-3 in cdcl3 (APT) 29.7.2020

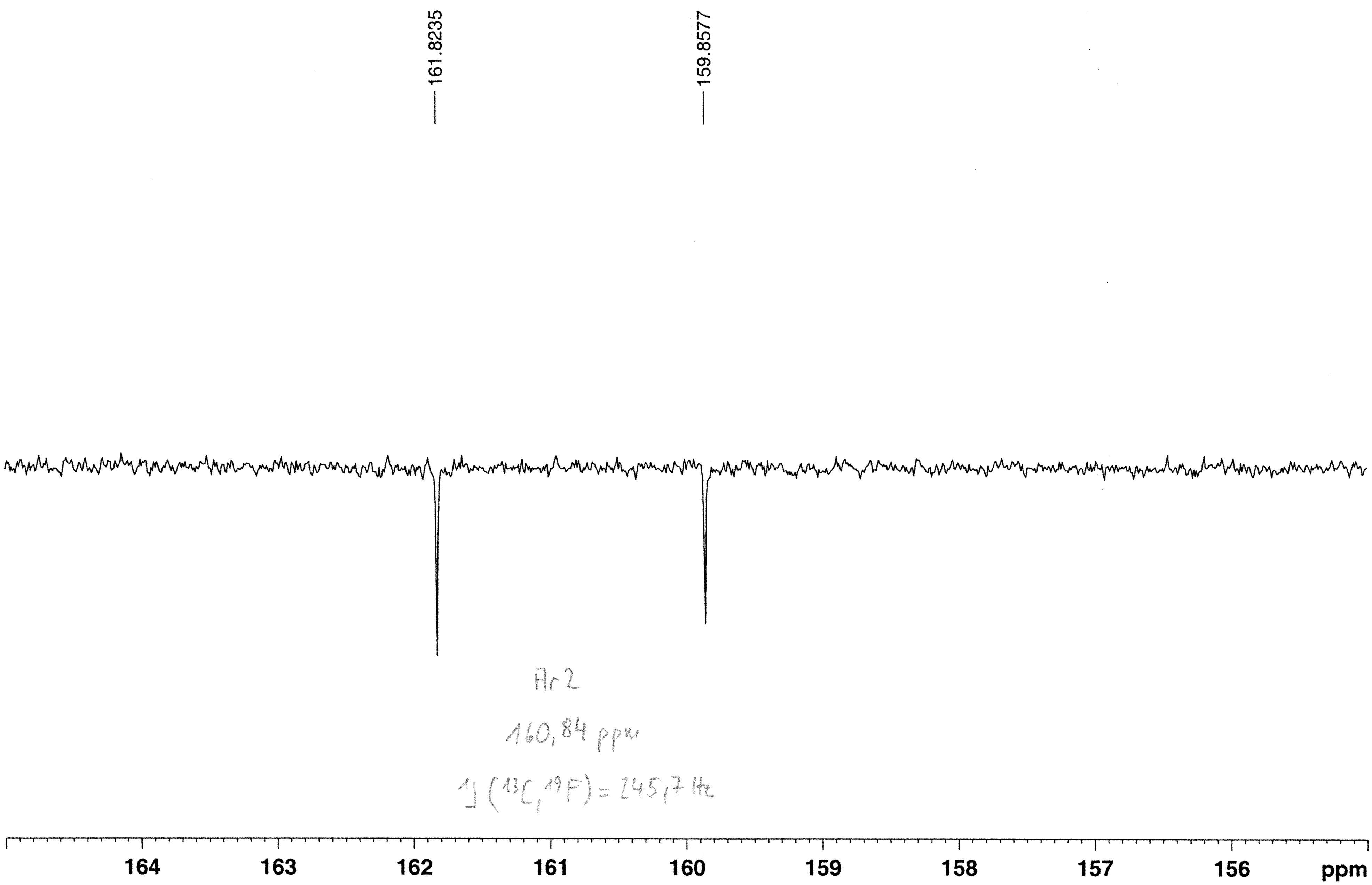

PN020-3 in cdcl3 (APT) 29.7.2020

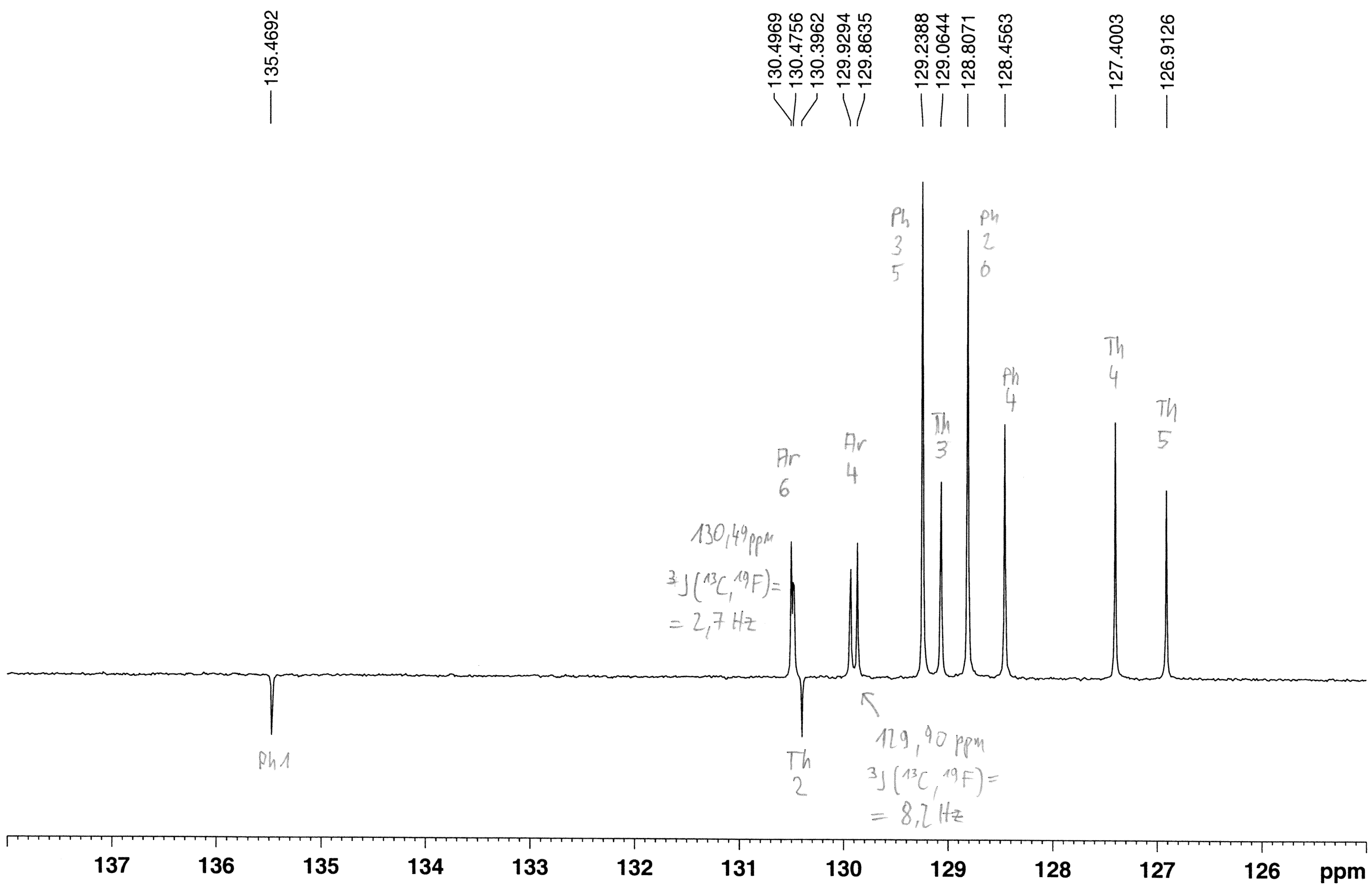

PN020-3 in cdcl3 (APT) 29.7.2020

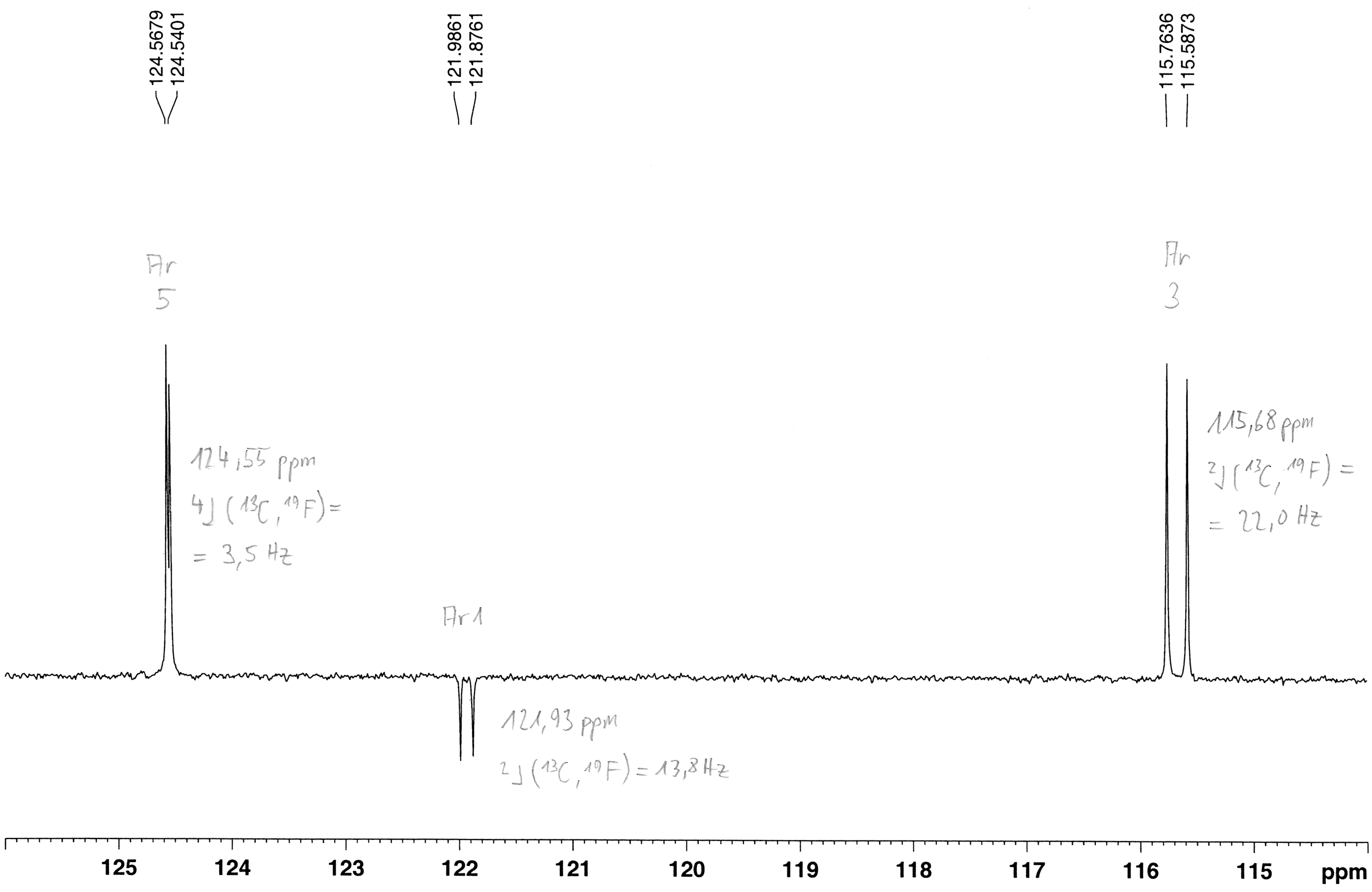

PN020-3 in cdcl3 (APT) 29.7.2020

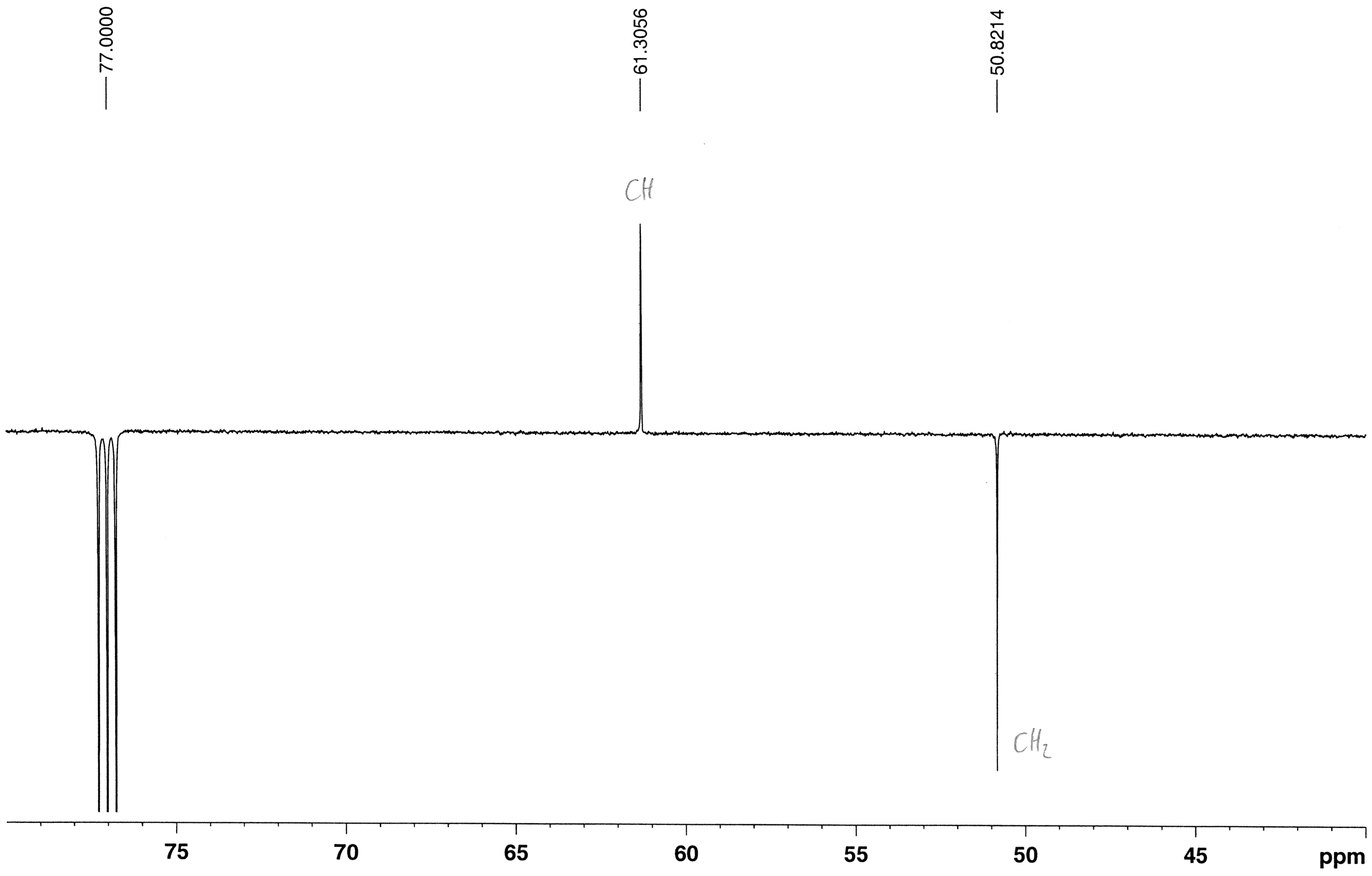

PN020-3 in cdcl3 (COSY) 29.7.2020

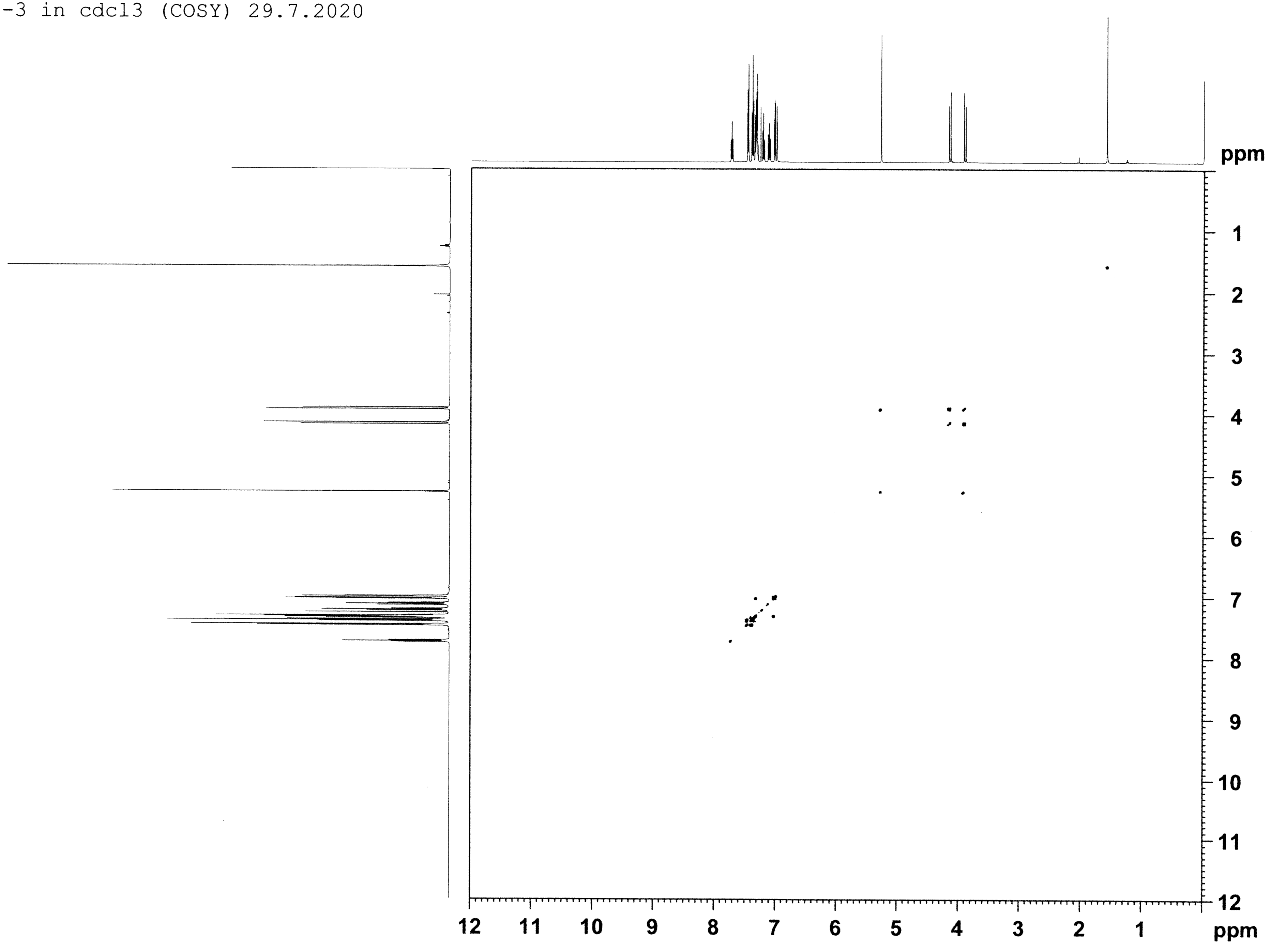

PN020-3 in cdcl3 (COSY) 29.7.2020

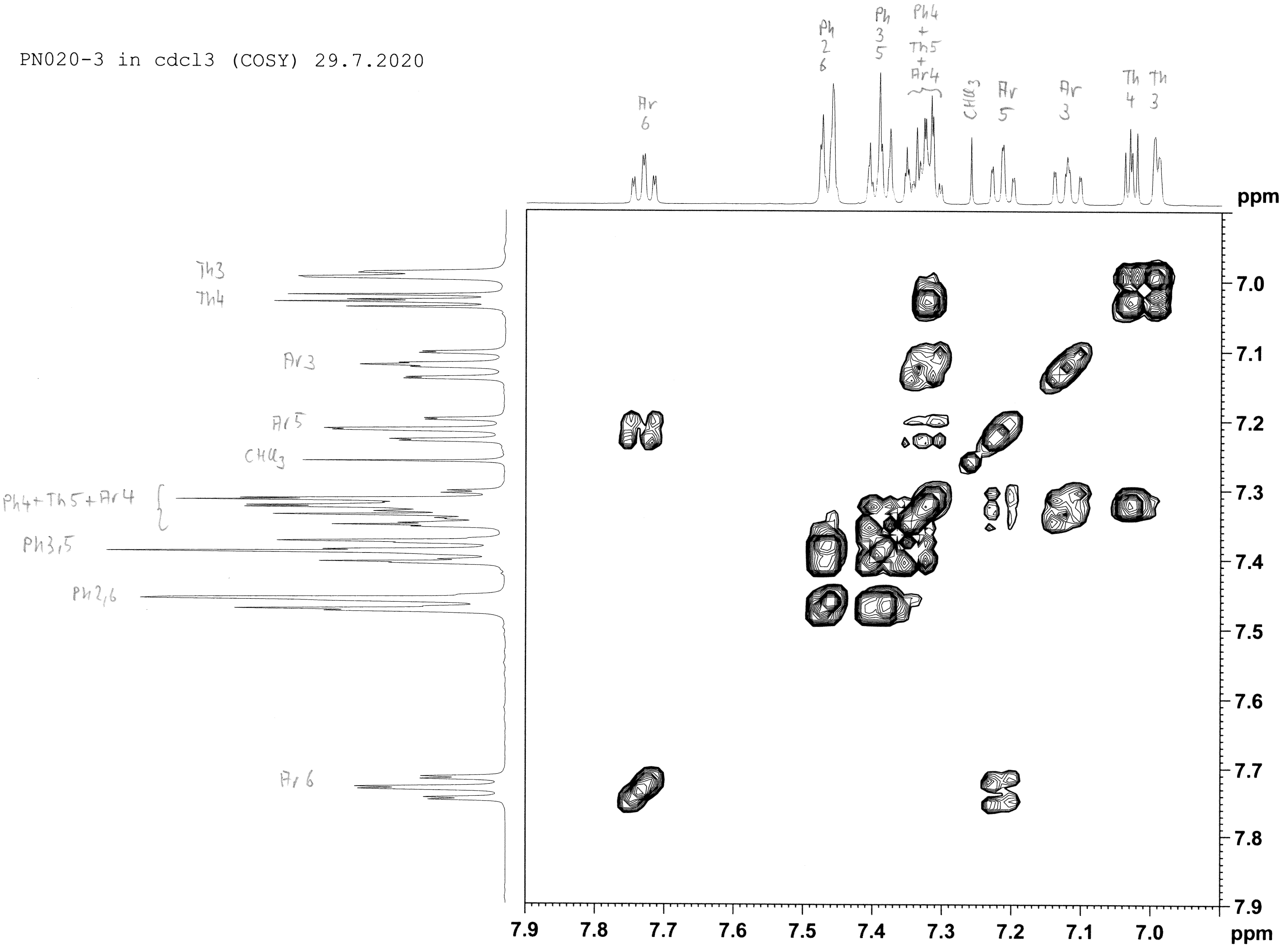

PN020-3 in cdcl3 (COSY) 29.7.2020

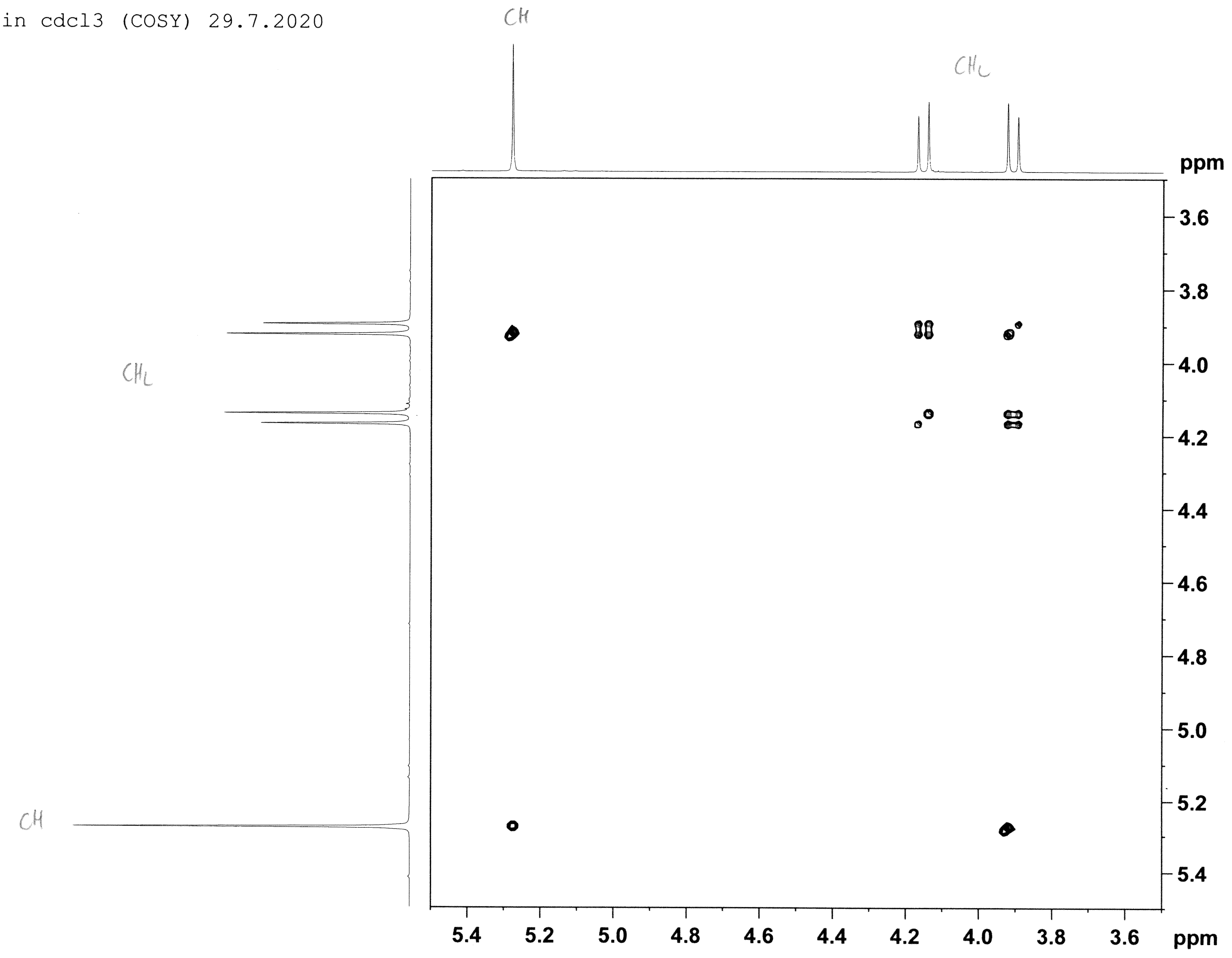

PN020-3 in cdcl3 (HSQC) 29.7.2020

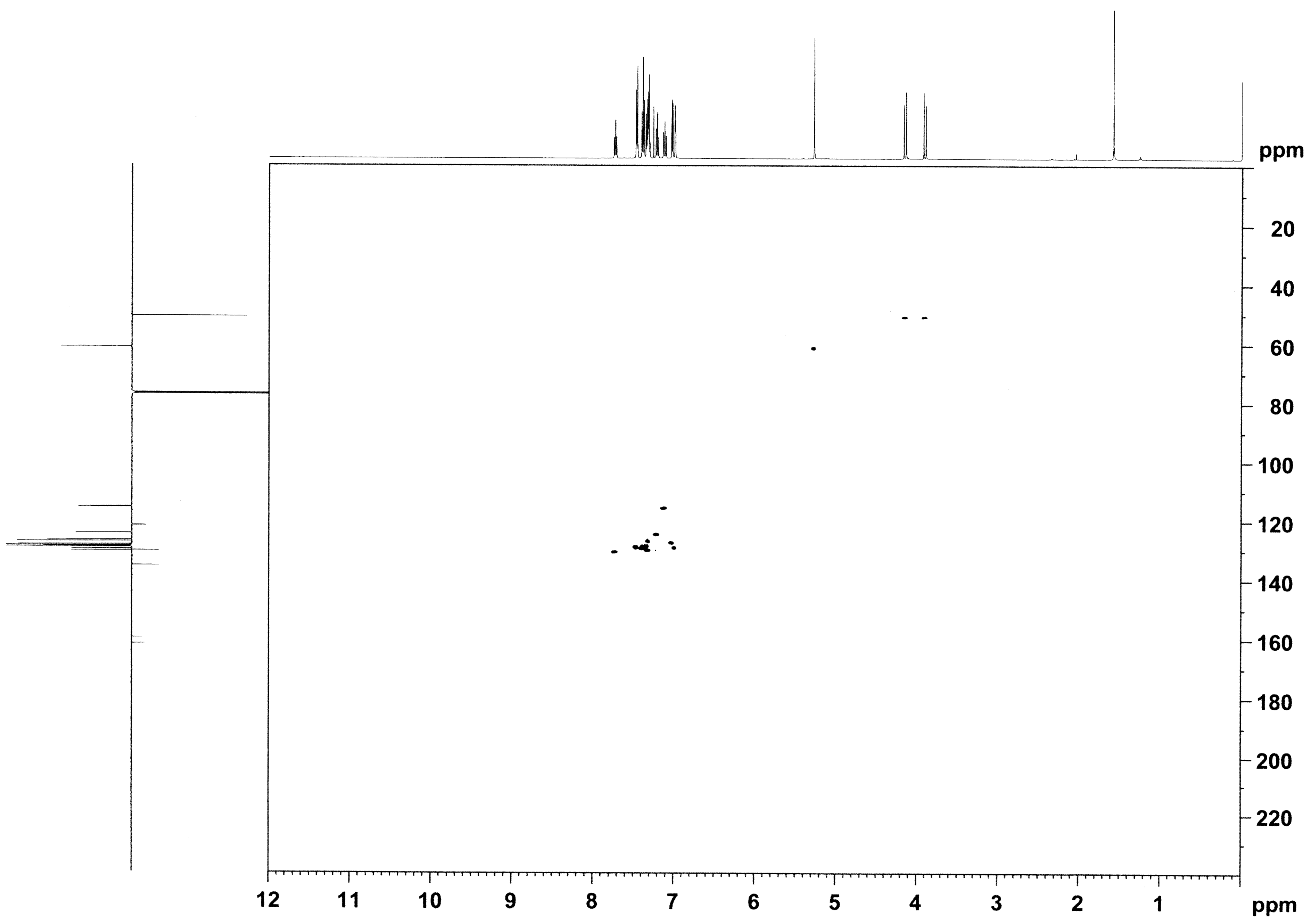

PN020-3 in cdcl3 (HSQC) 29.7.2020

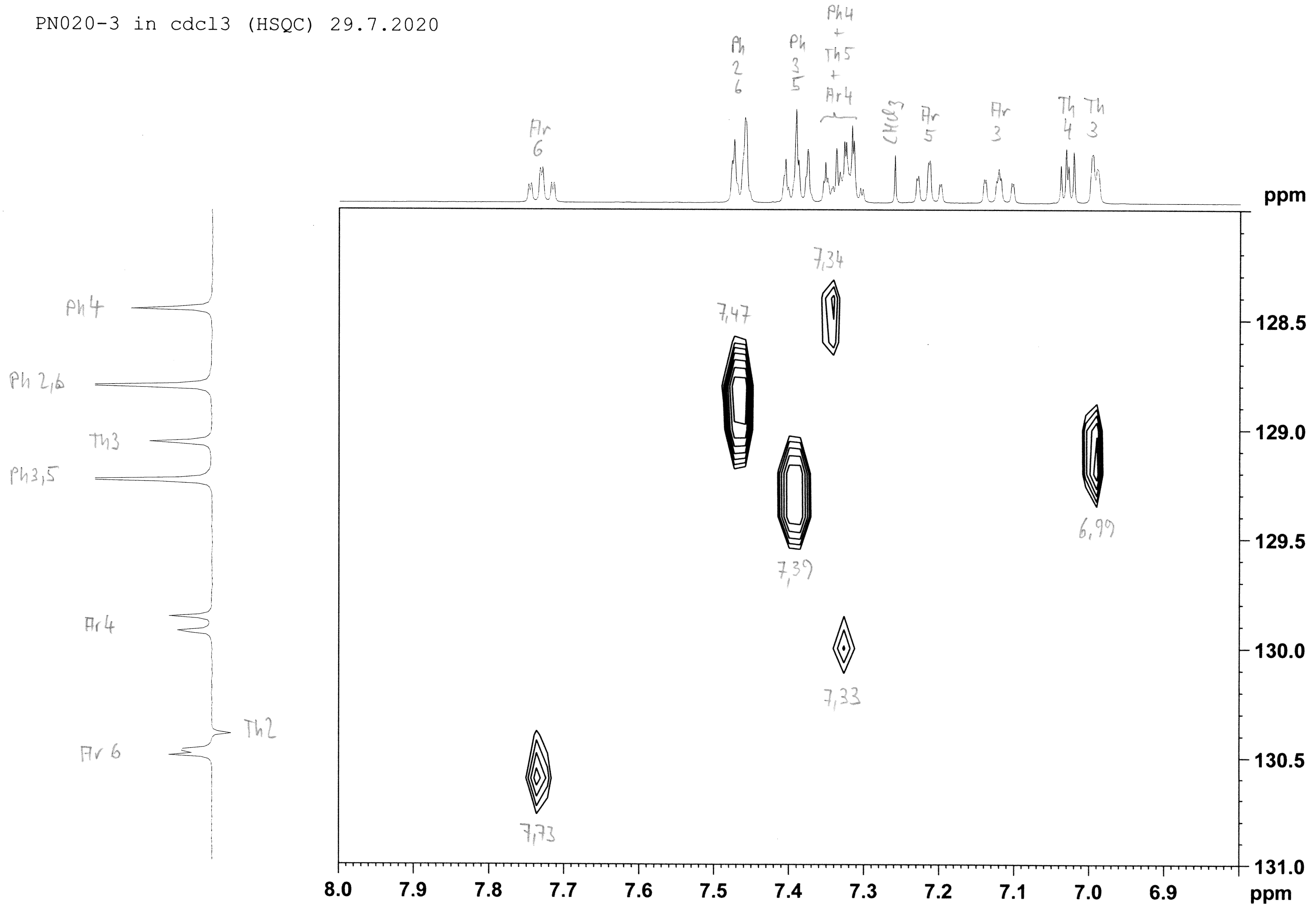

PN020-3 in cdcl3 (HSQC) 29.7.2020

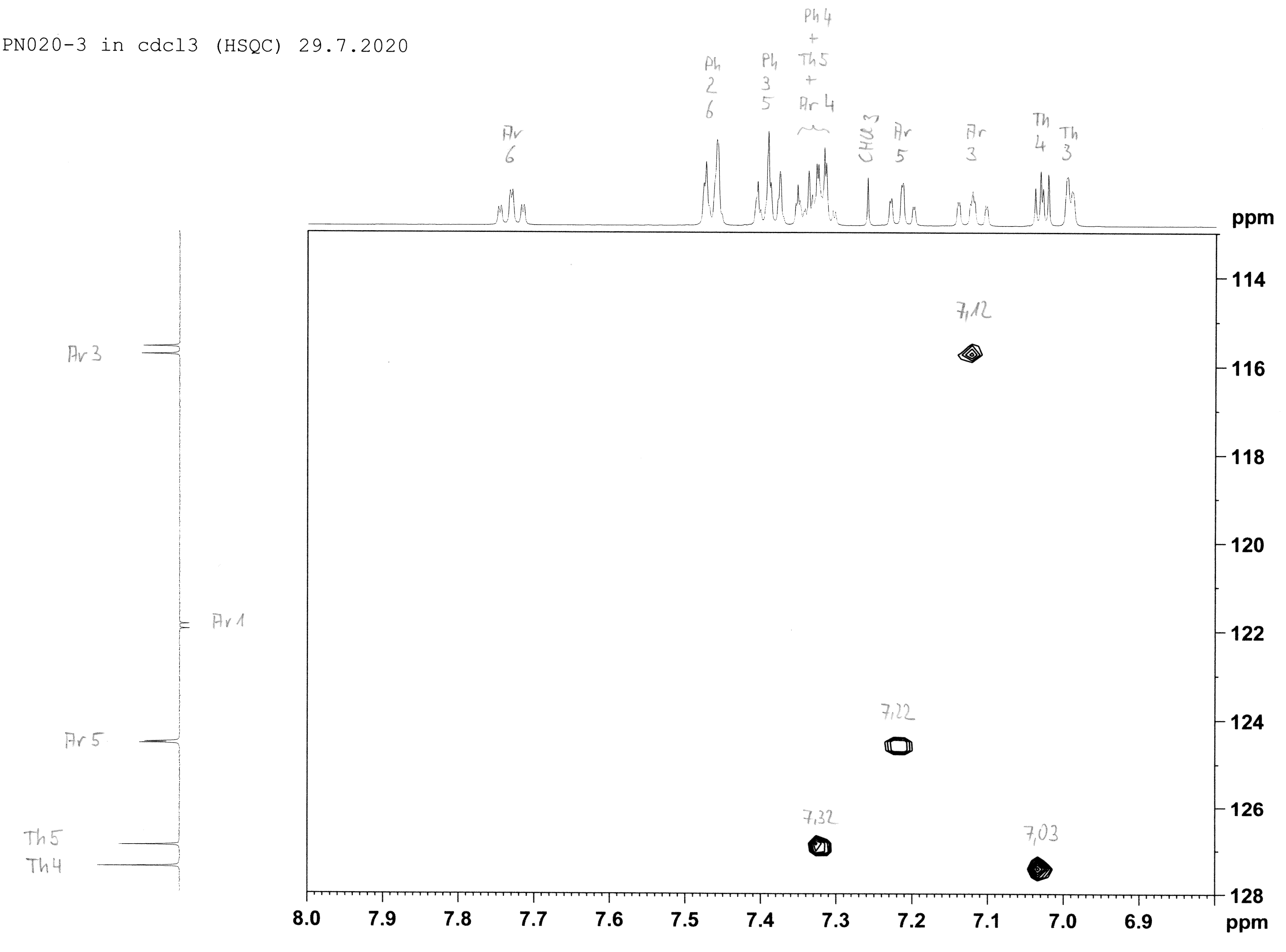

PN020-3 in cdcl3 (HSQC) 29.7.2020

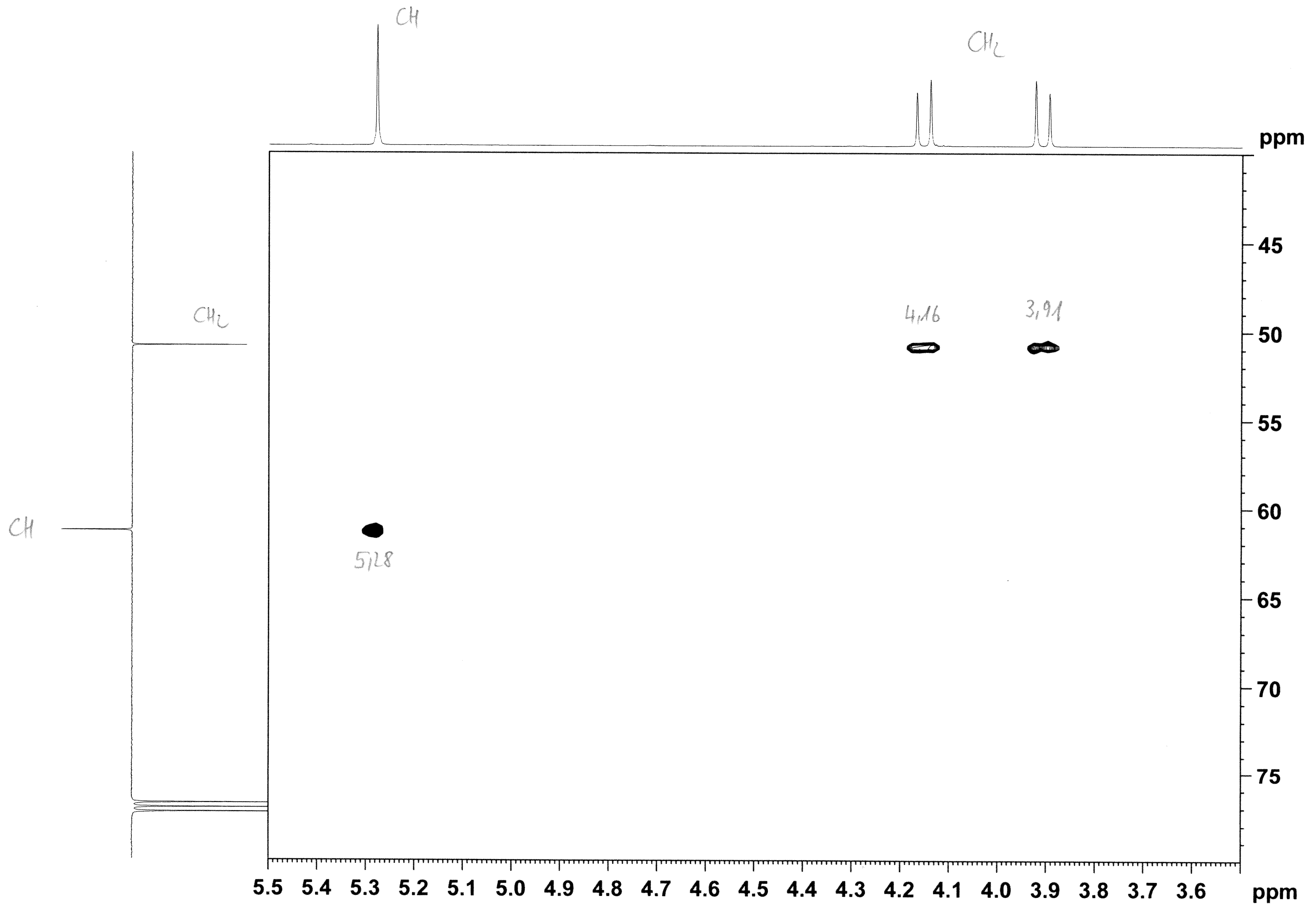

PN020-3 in cdcl3 (HMBC) 29.7.2020

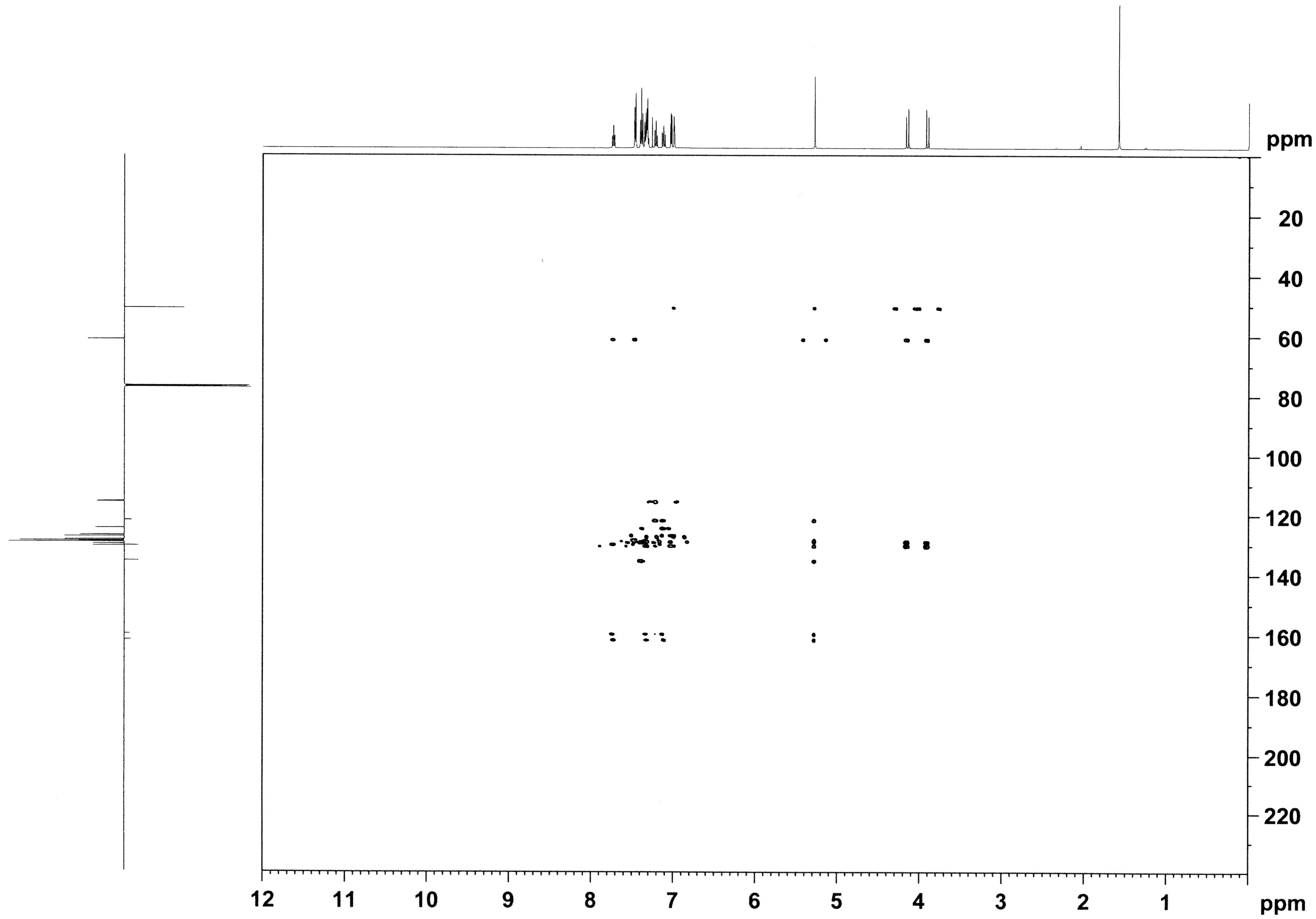

PN020-3 in cdcl3 (HMBC) 29.7.2020

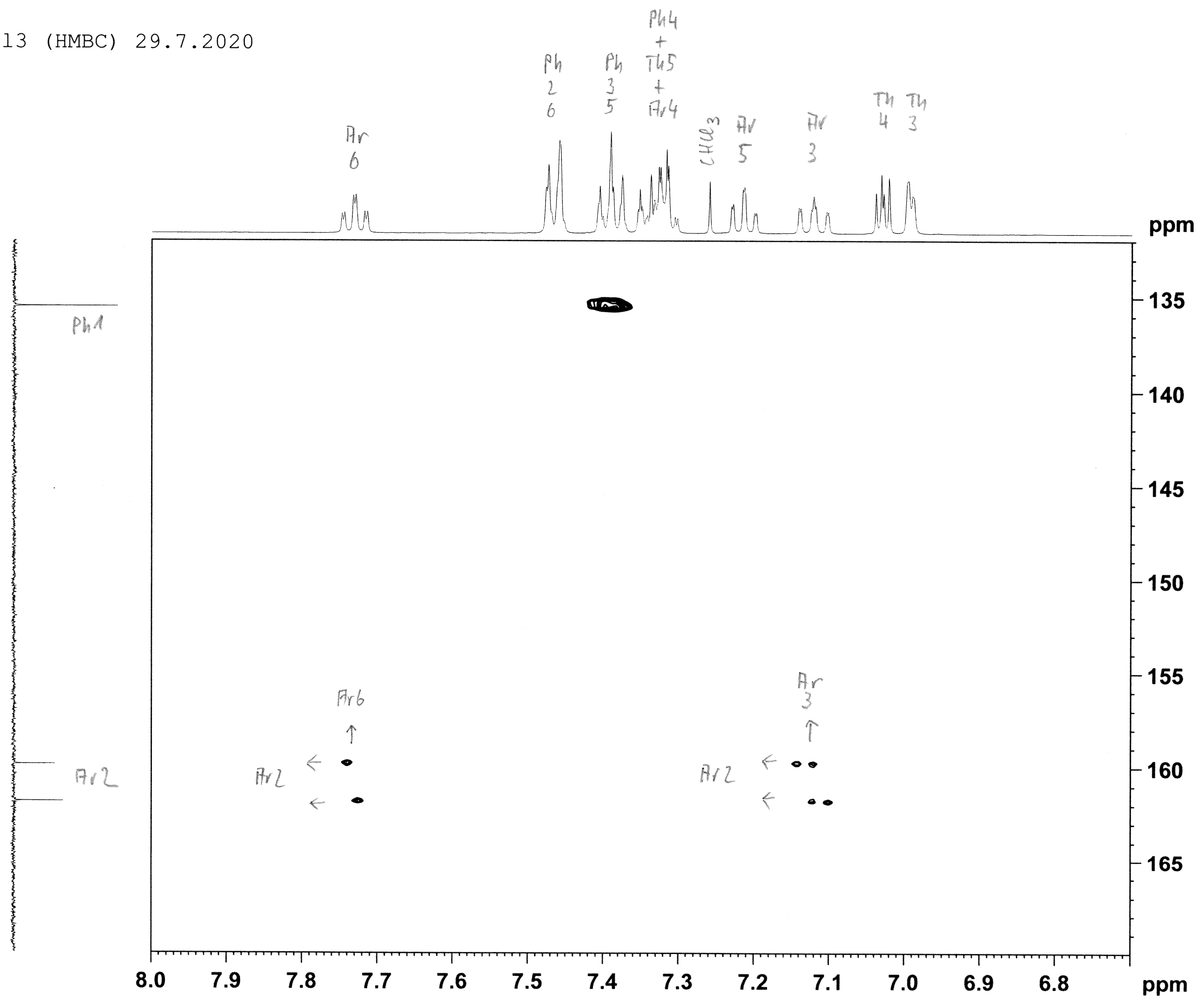

PN020-3 in cdcl3 (HMBC) 29.7.2020

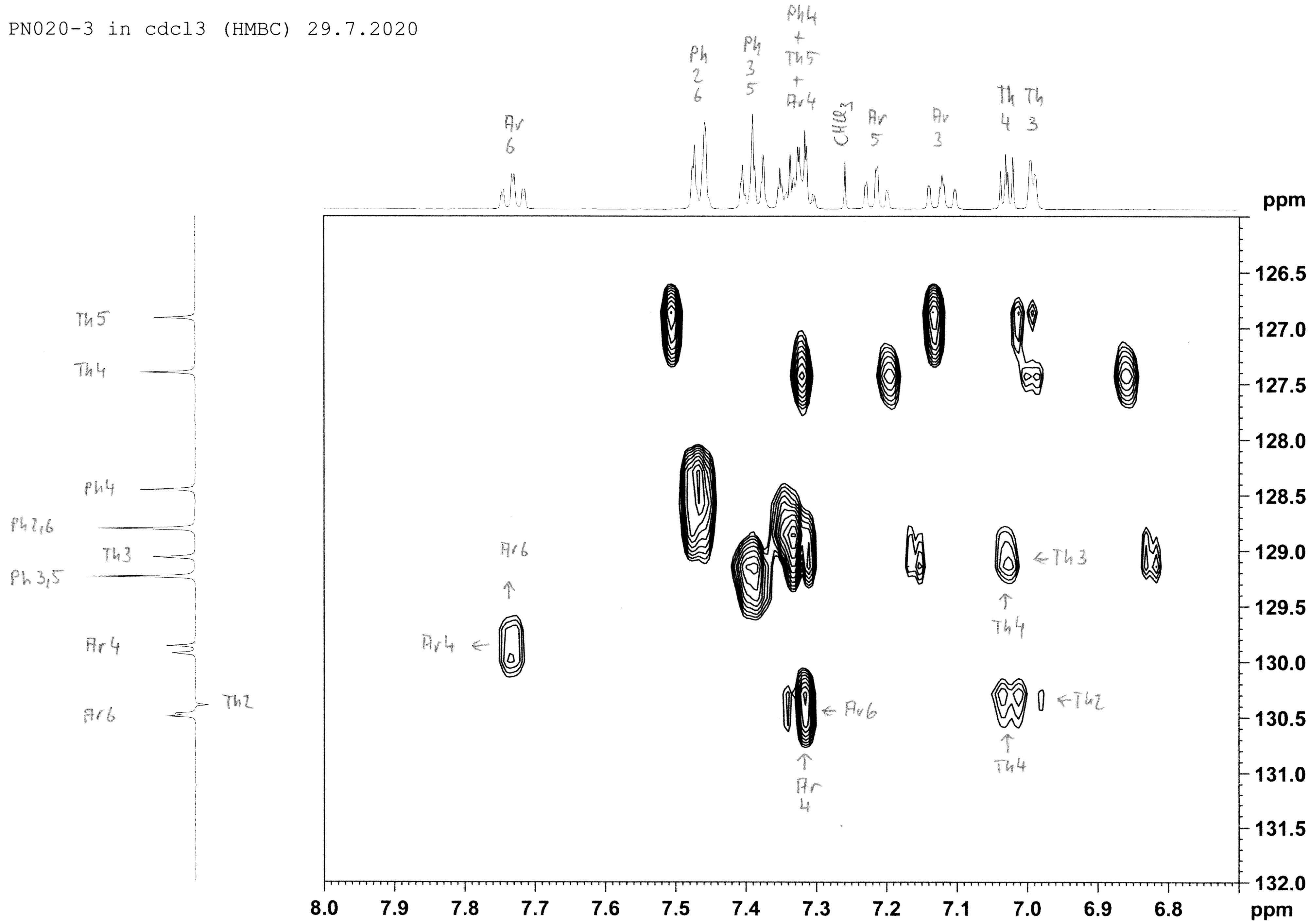

PN020-3 in cdcl3 (HMBC) 29.7.2020

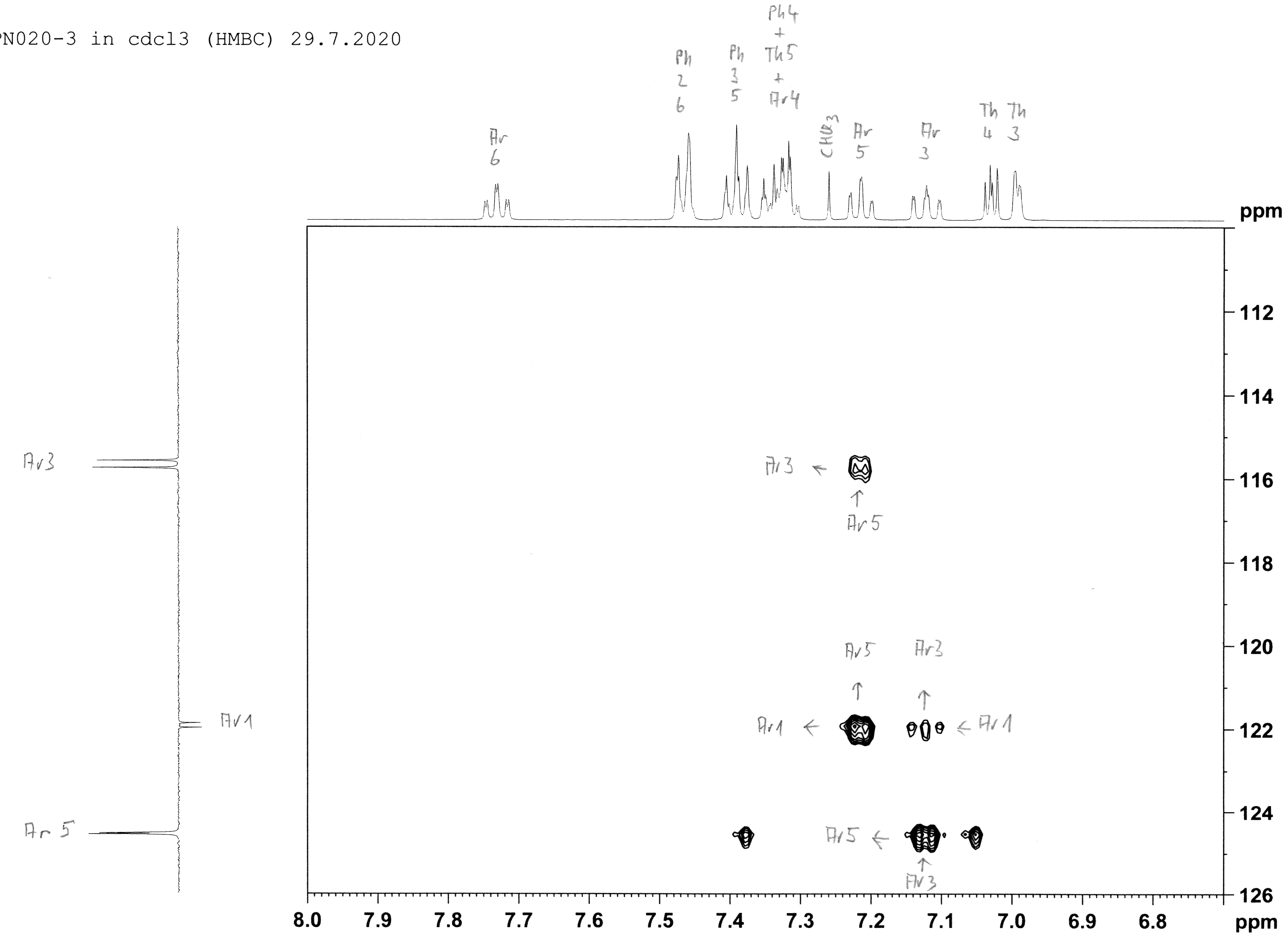

PN020-3 in cdcl3 (HMBC) 29.7.2020

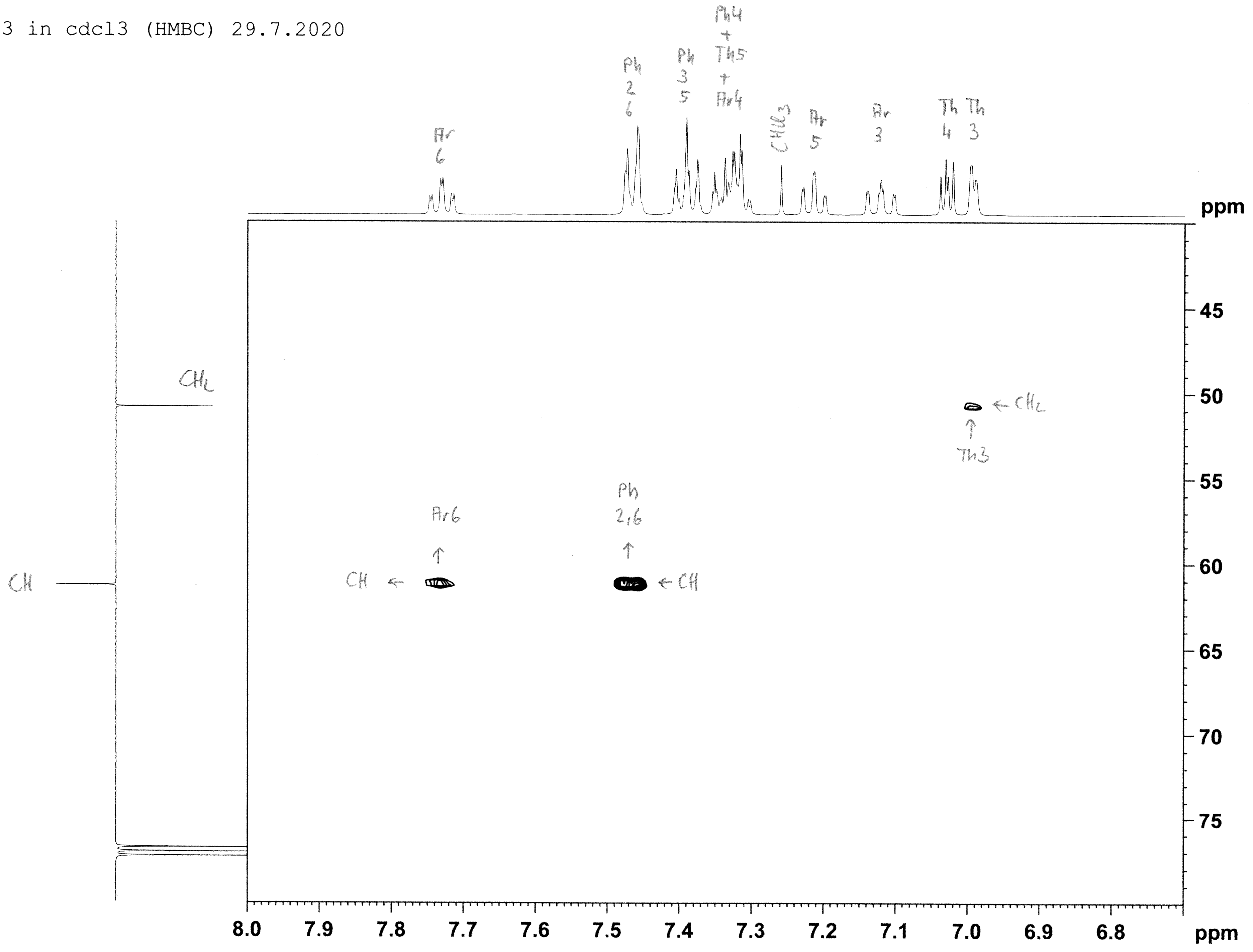

PN020-3 in cdcl3 (HMBC) 29.7.2020

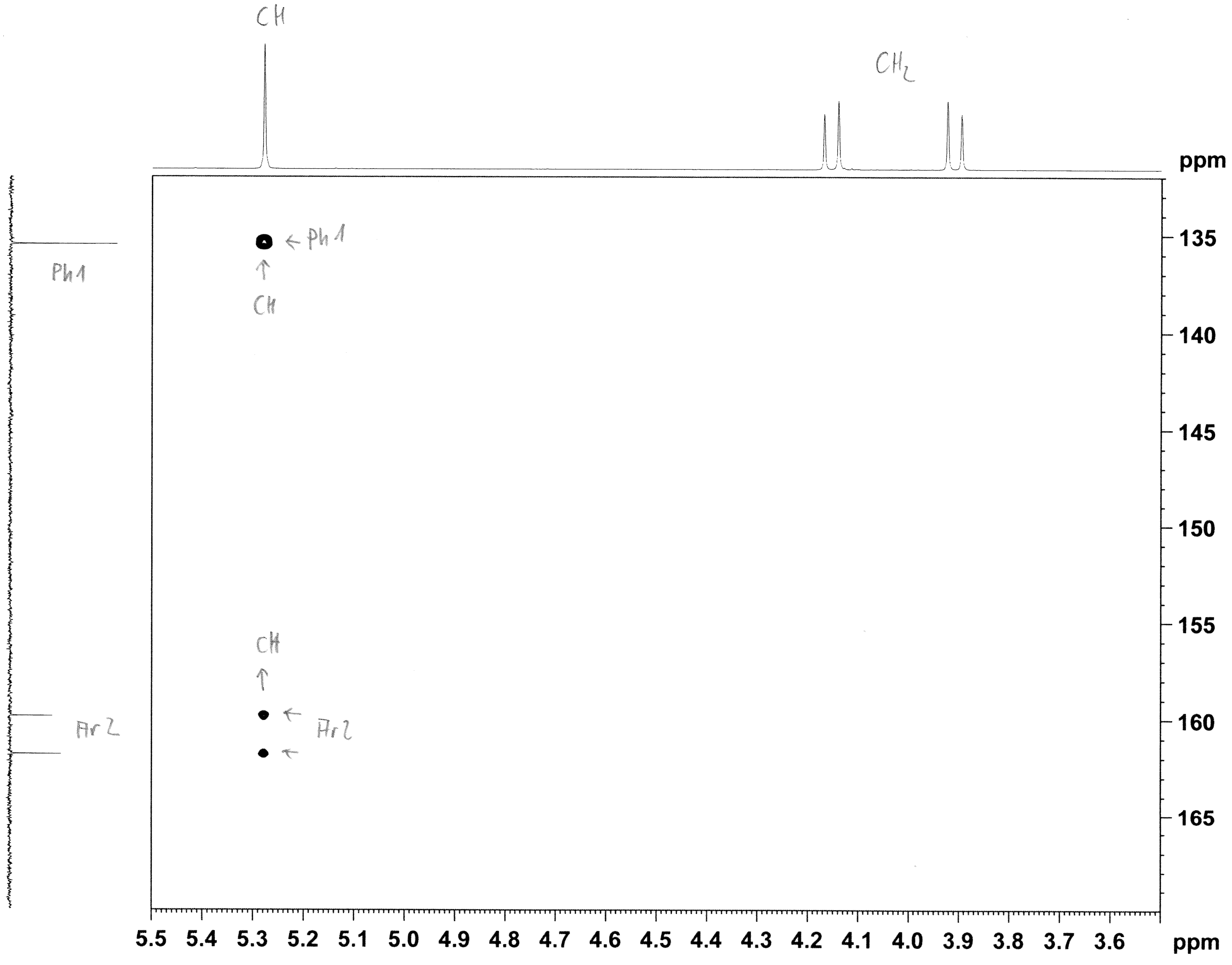

PN020-3 in cdcl3 (HMBC) 29.7.2020

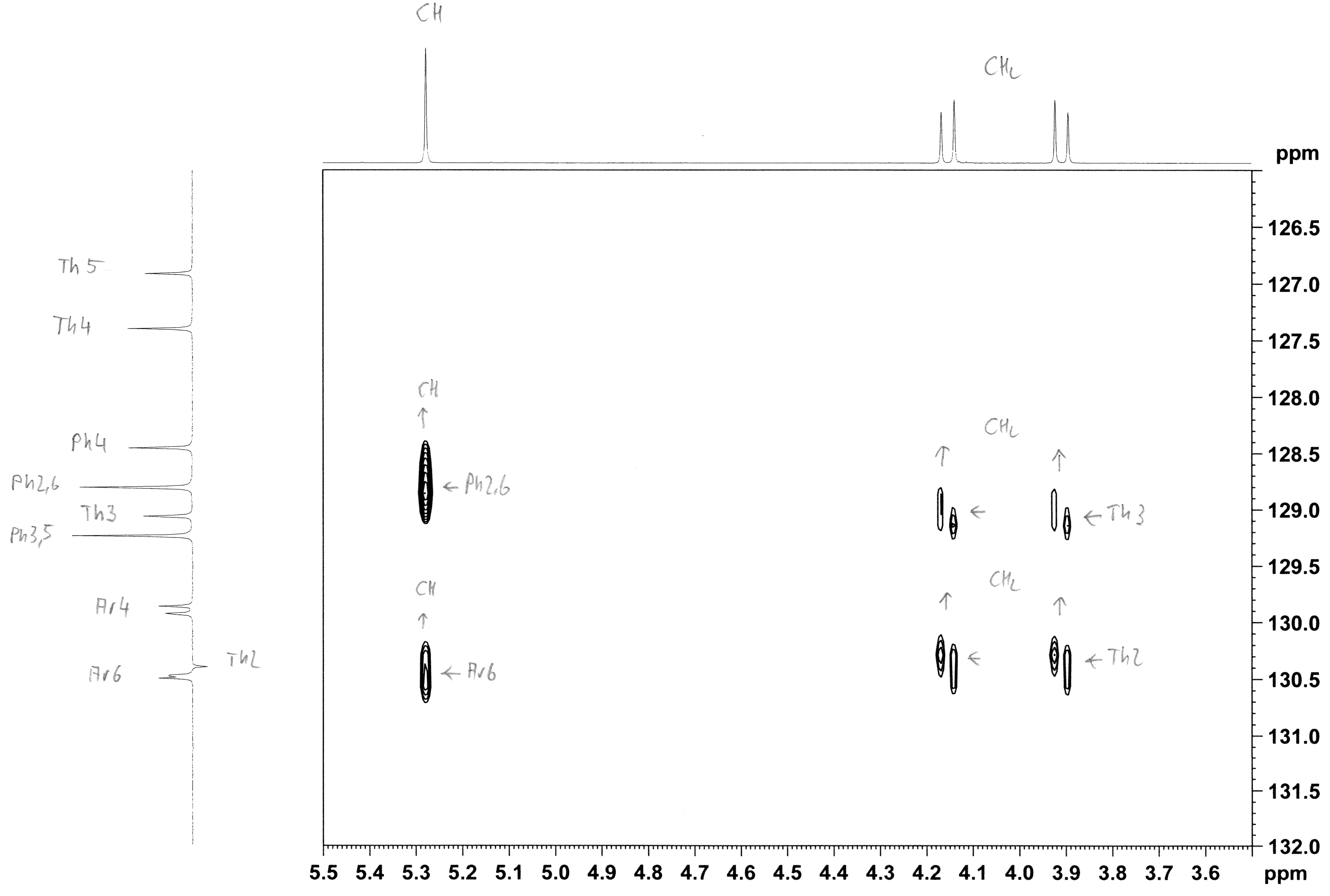

PN020-3 in cdcl3 (HMBC) 29.7.2020

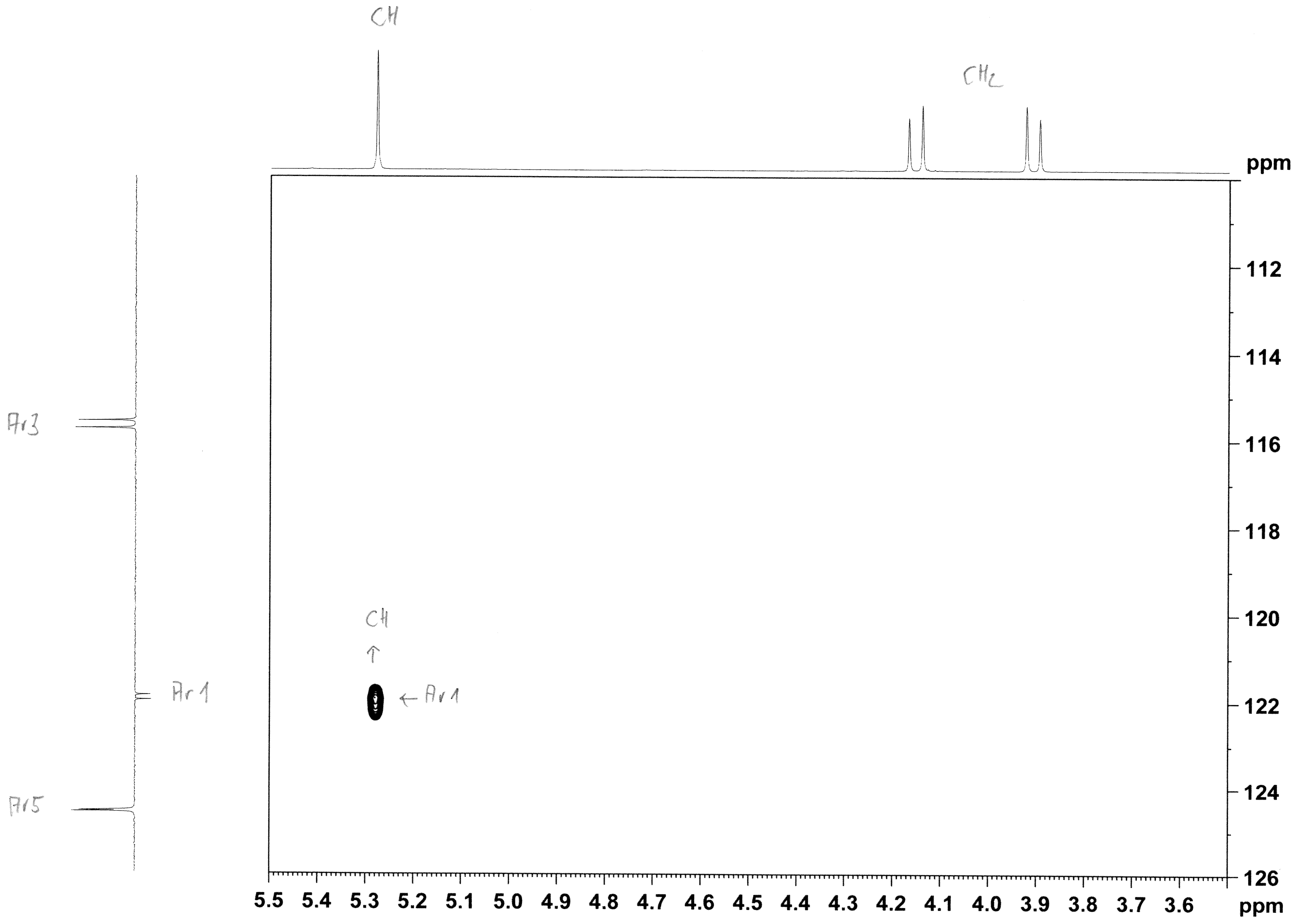

PN020-3 in cdcl3 (HMBC) 29.7.2020

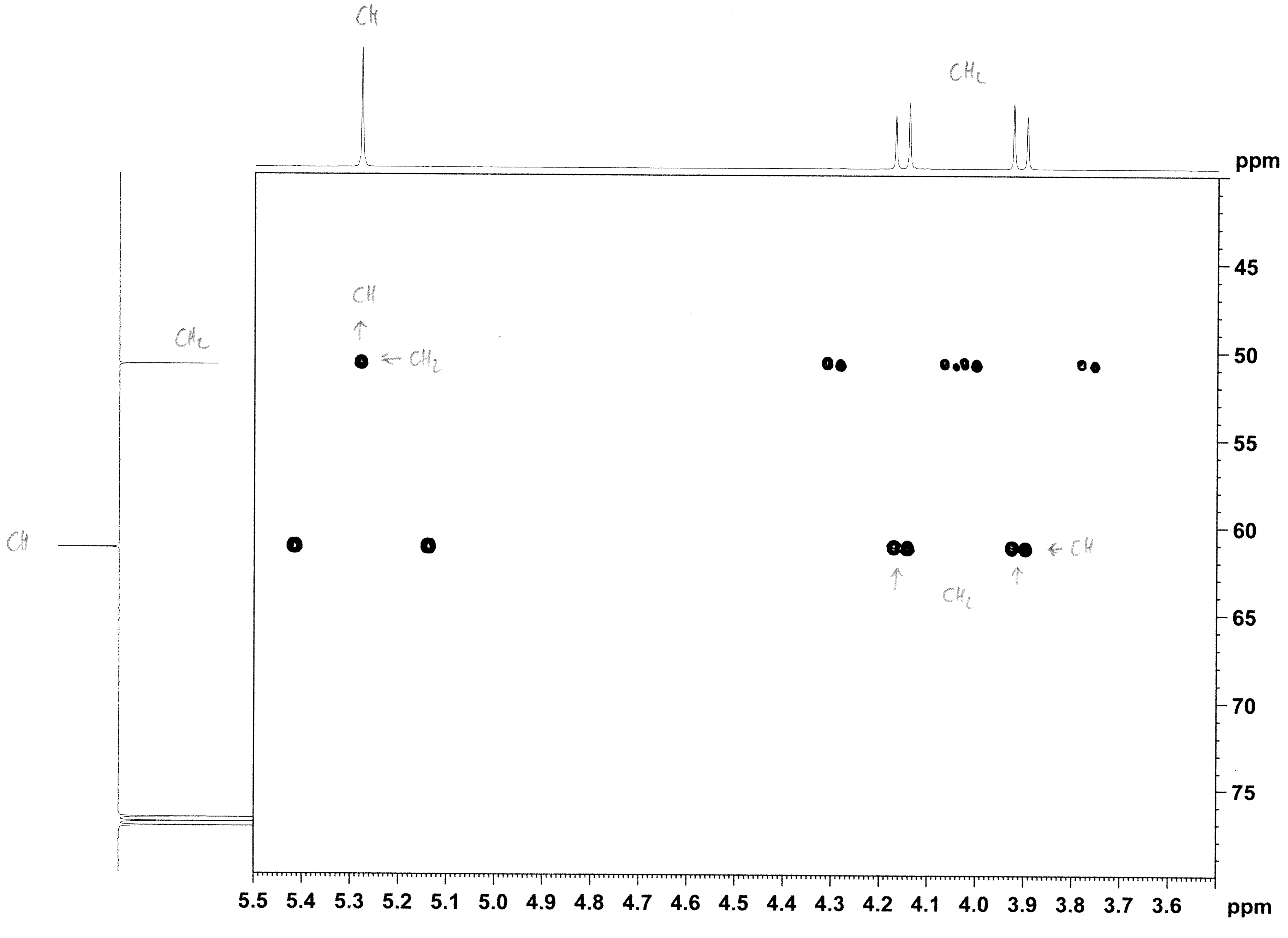

Figure S4c. NMR spectra of compound **8a.**

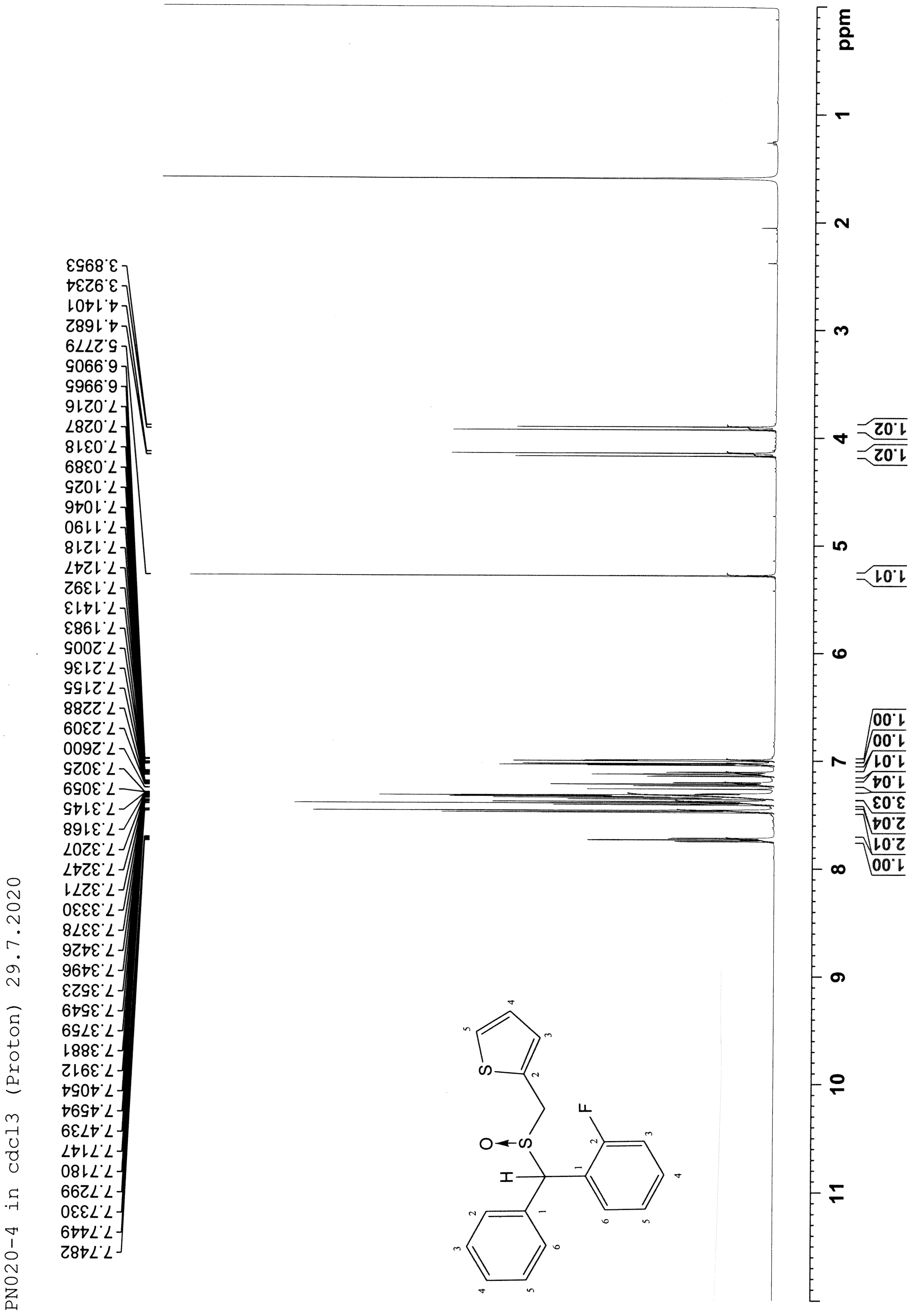

PN020-4 in cdcl3 (Proton) 29.7.2020

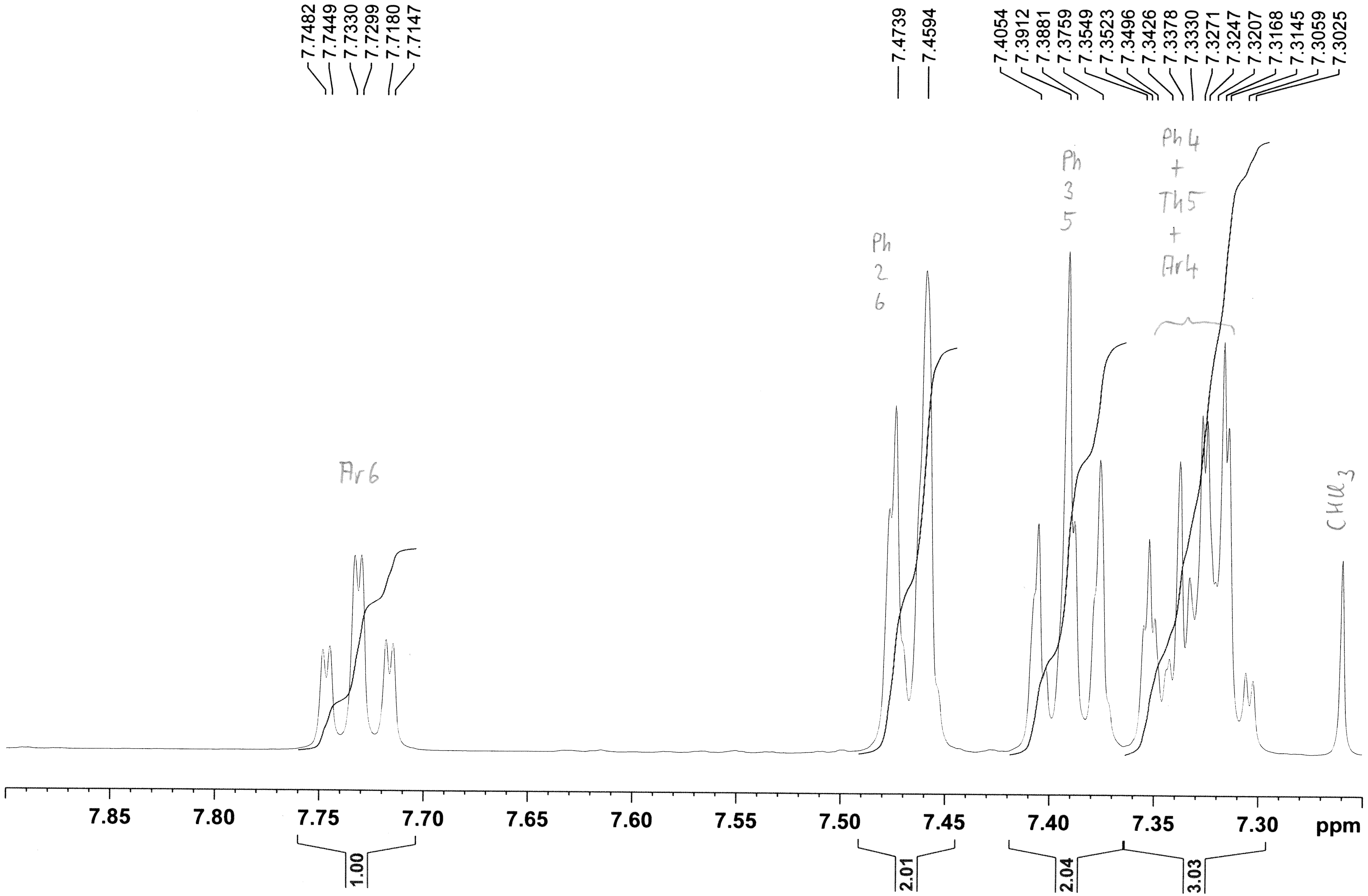

PN020-4 in cdcl3 (Proton) 29.7.2020

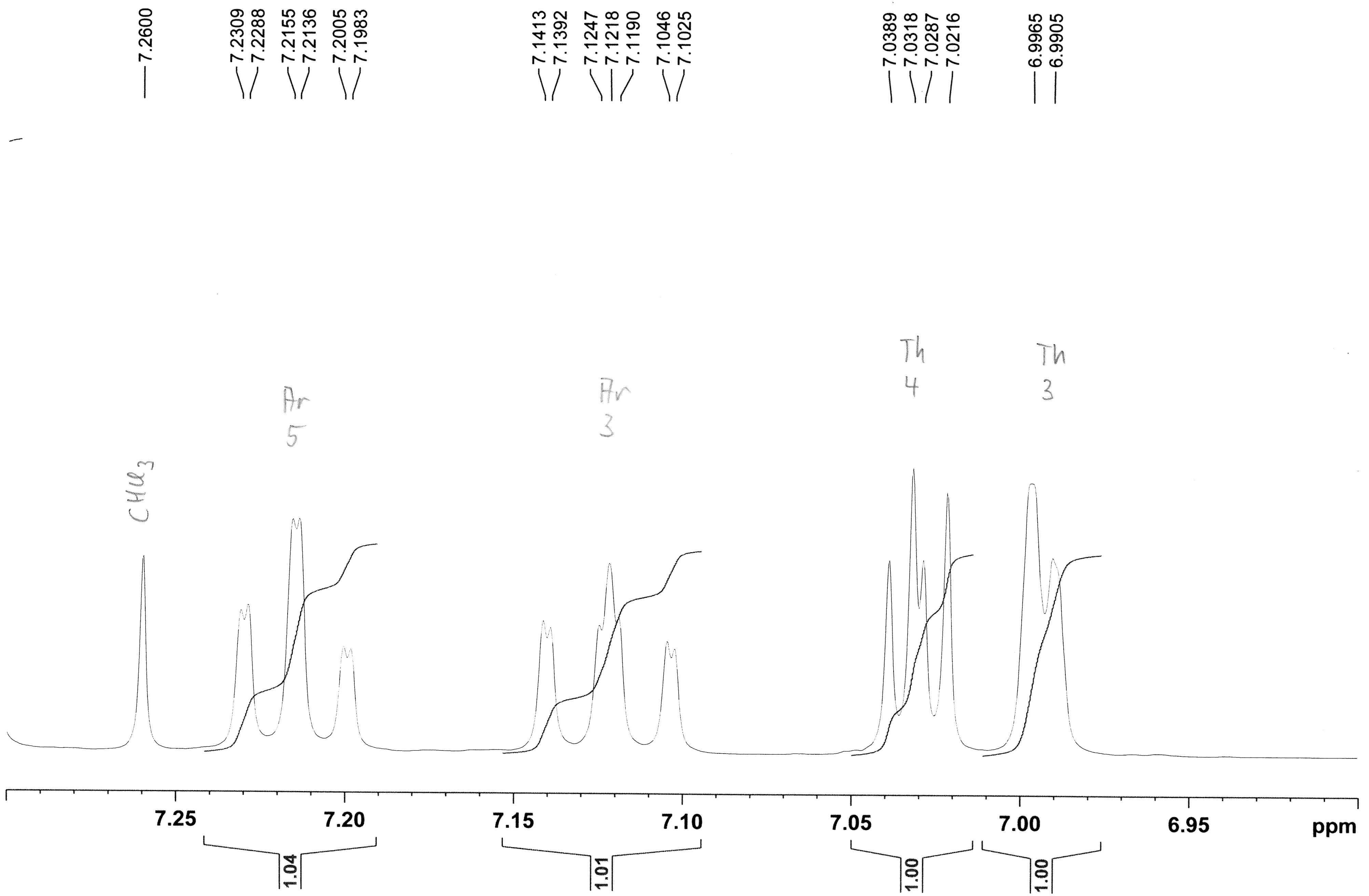

PN020-4 in cdcl3 (Proton) 29.7.2020

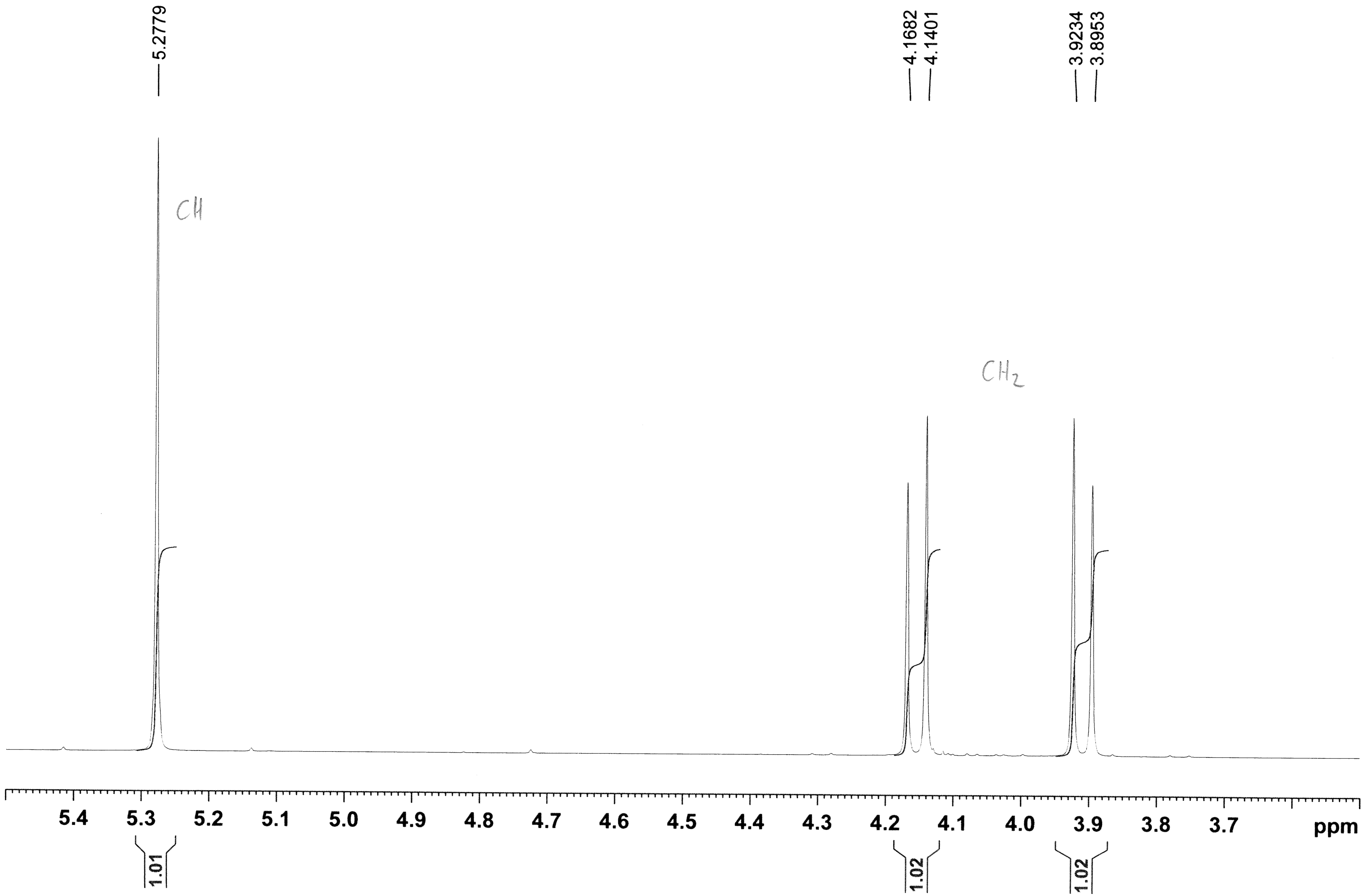

PN020-4 in cdcl3 (APT) 29.7.2020

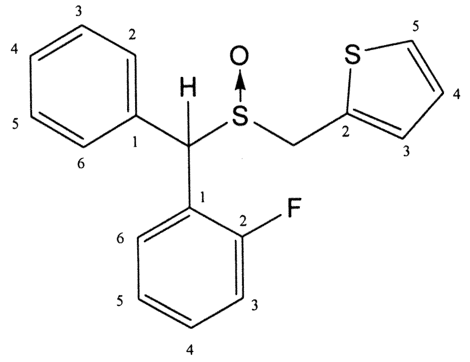

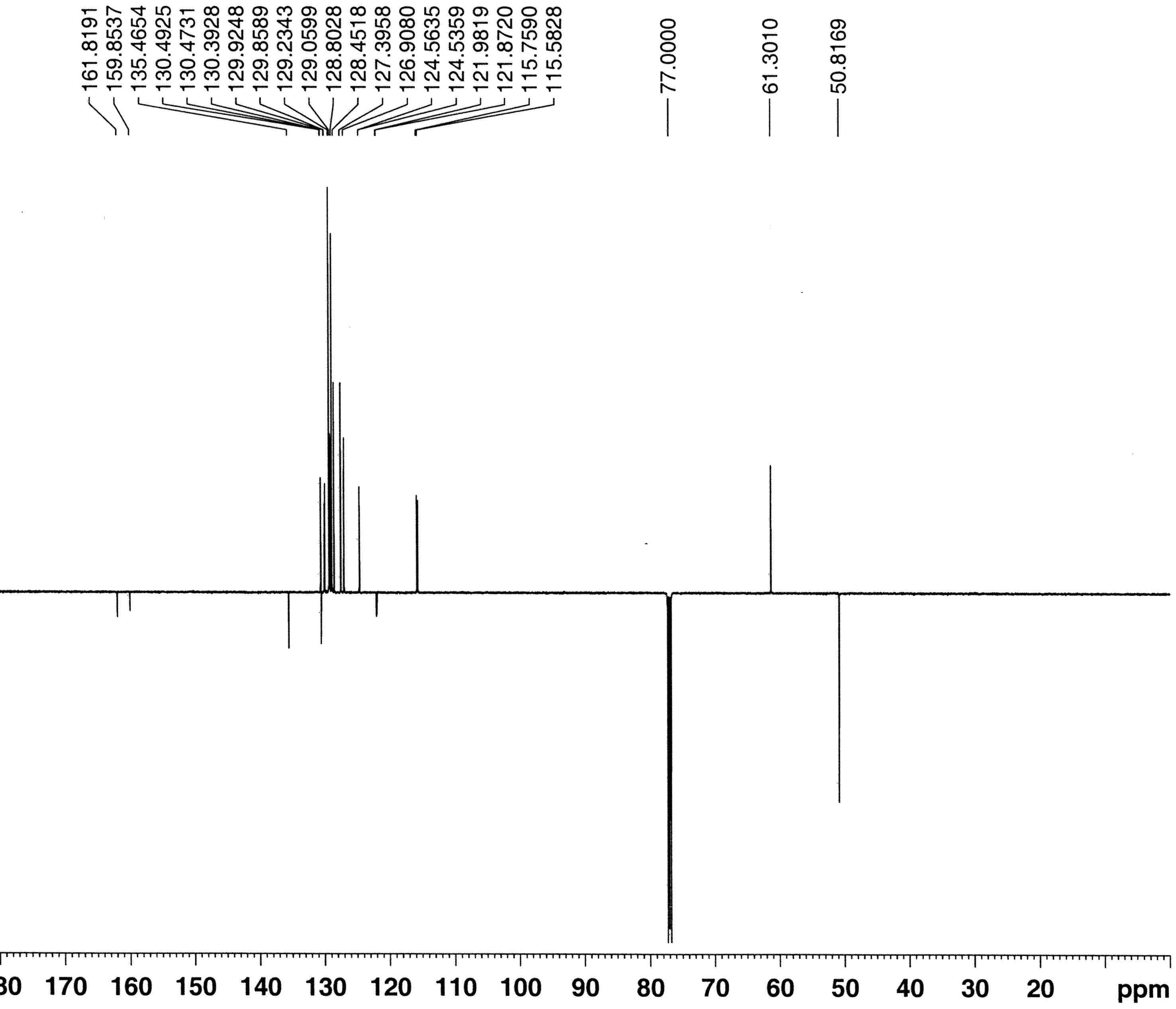

PN020-4 in cdcl3 (APT) 29.7.2020

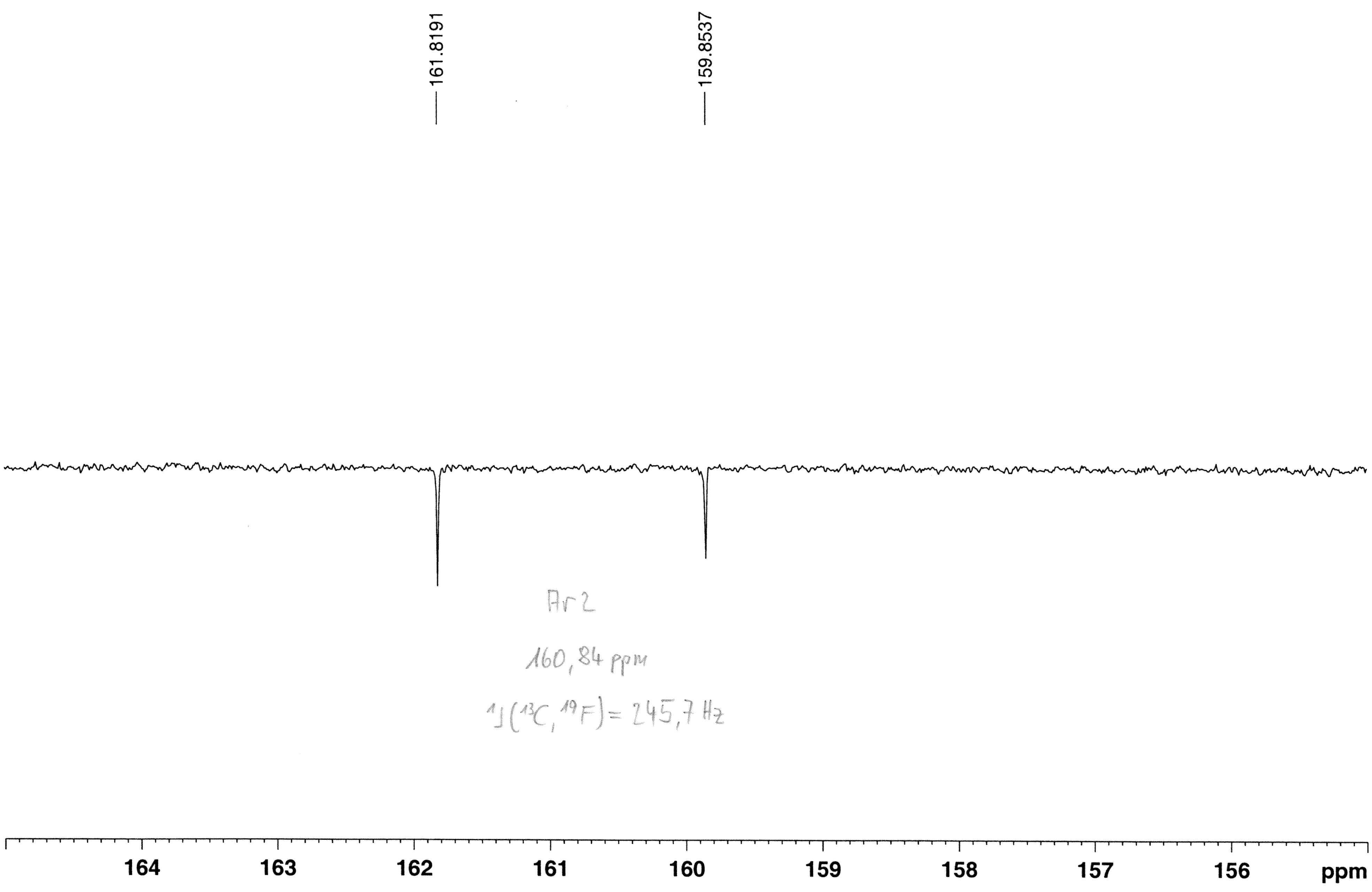

PN020-4 in cdcl3 (APT) 29.7.2020

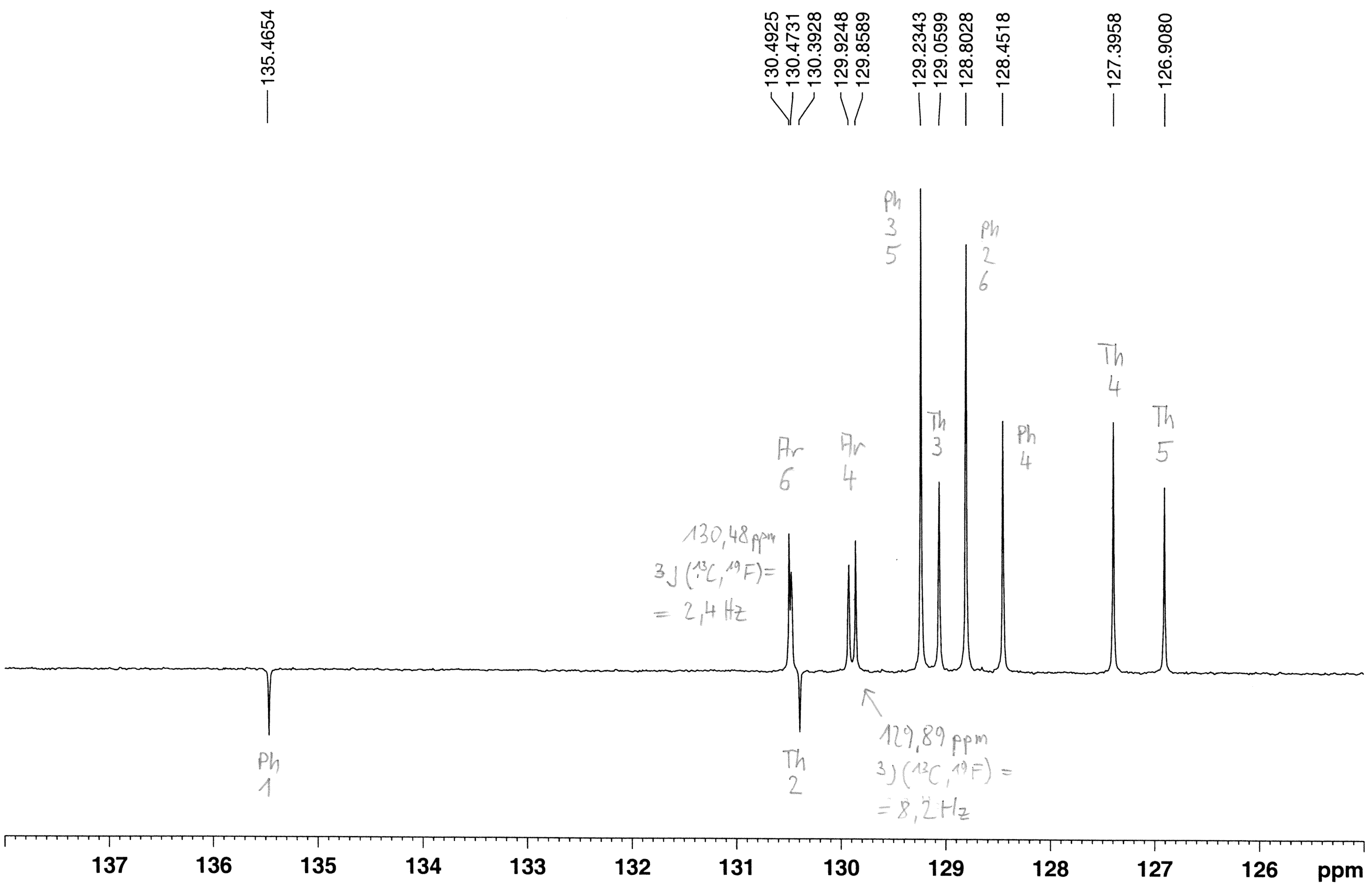

PN020-4 in cdcl3 (APT) 29.7.2020

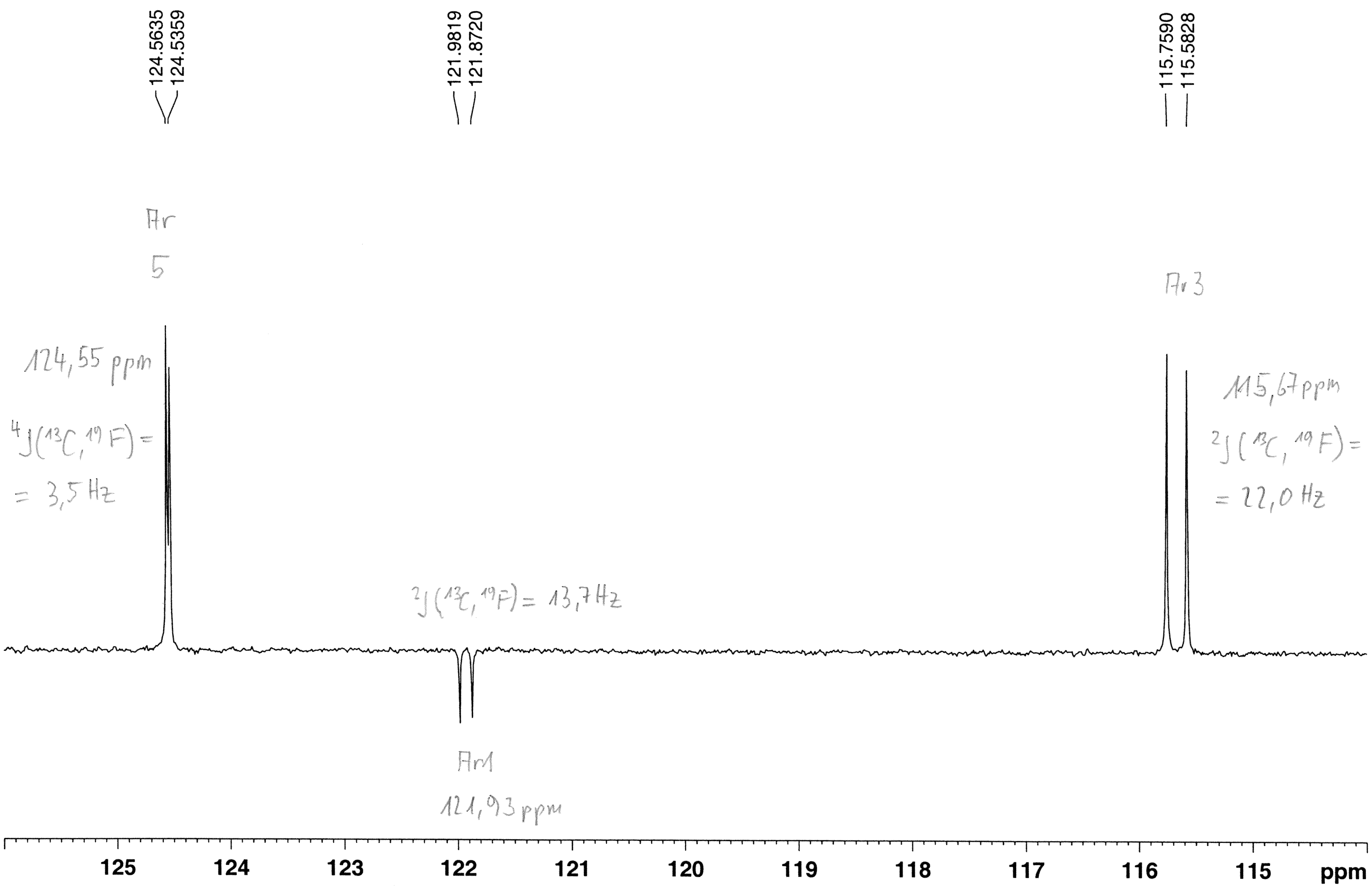

PN020-4 in cdcl3 (APT) 29.7.2020

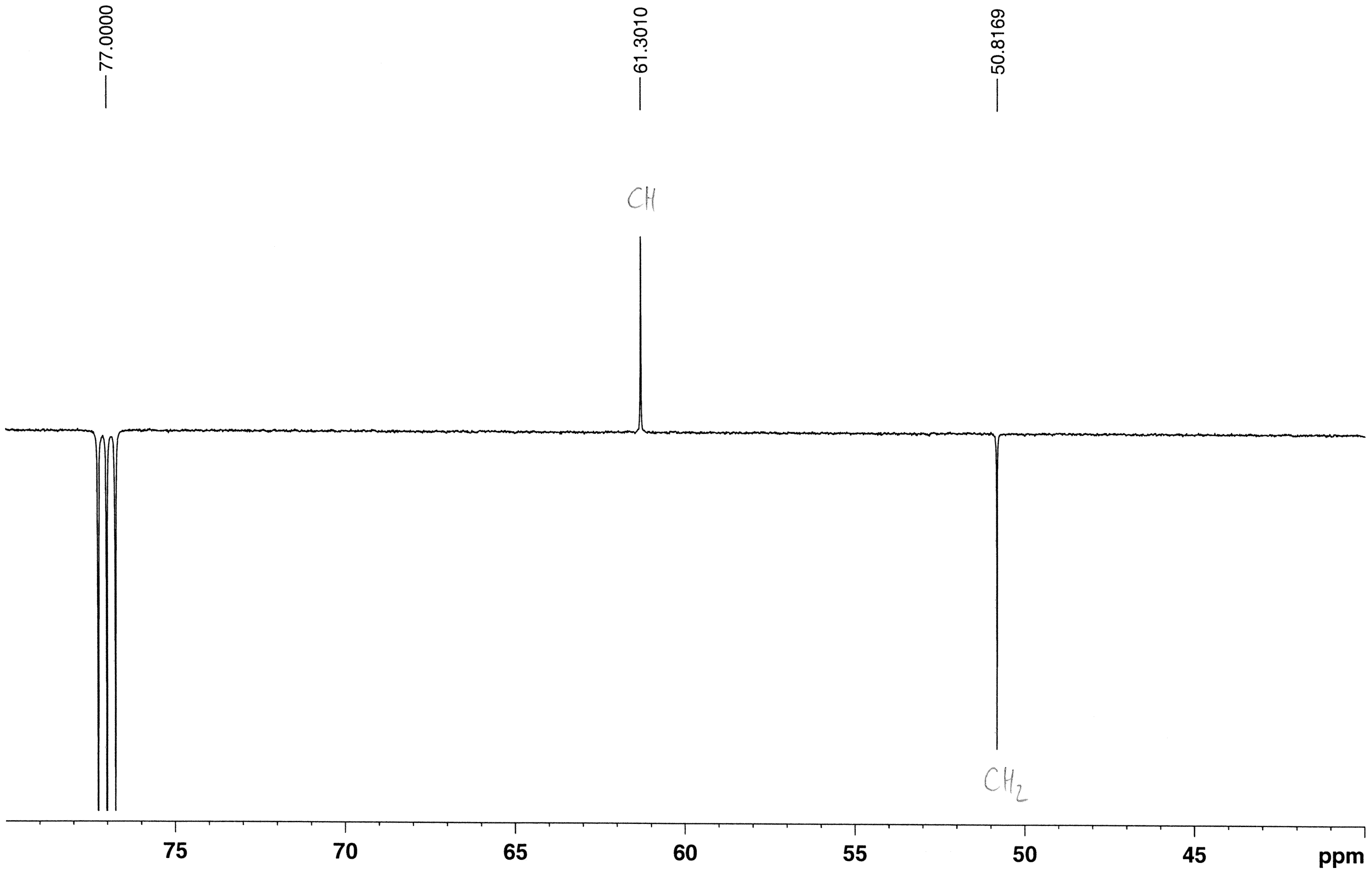

Figure S5c. NMR spectra of compound **5b**.

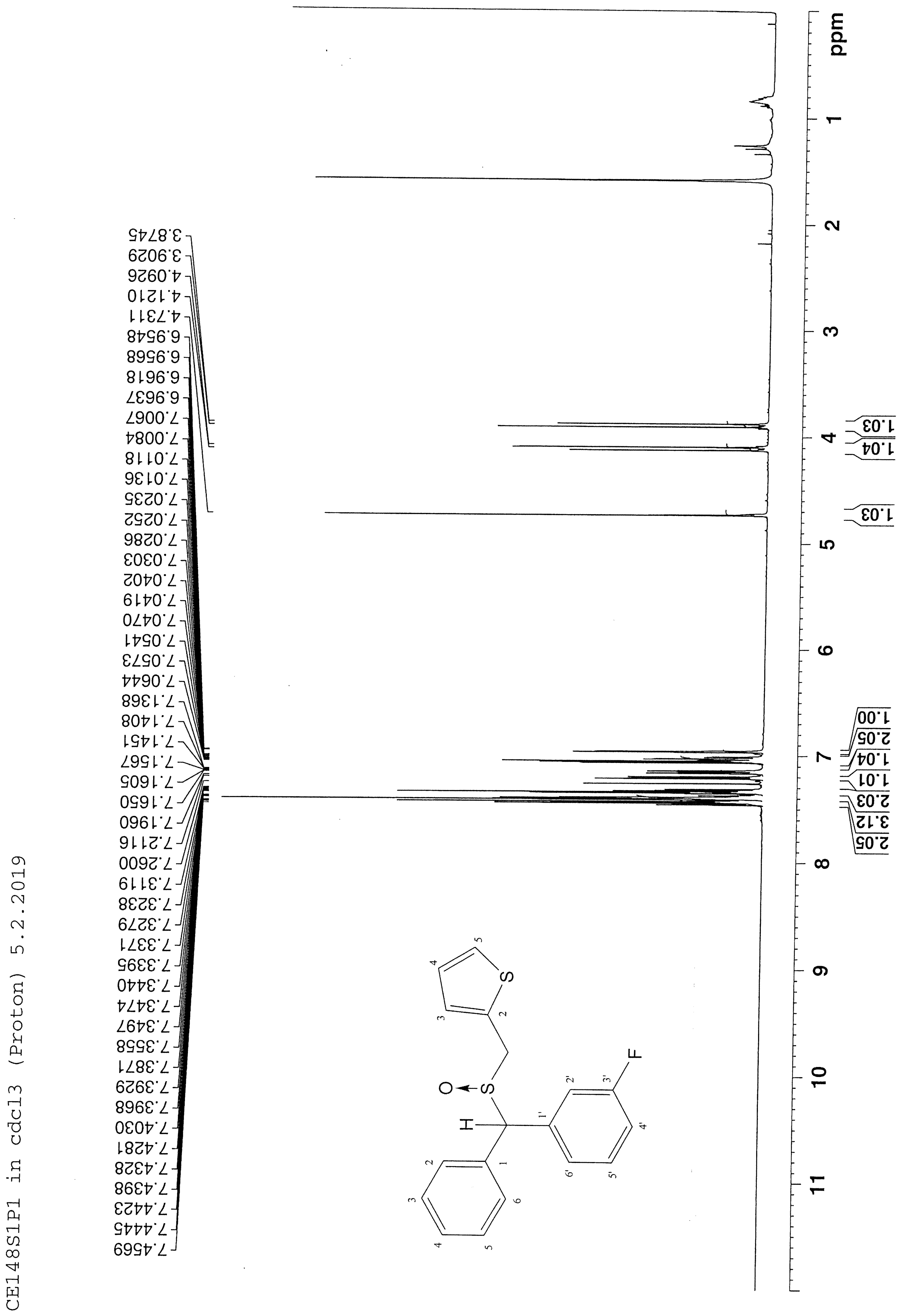

CE148S1P1 in cdcl3 (Proton) 5.2.2019

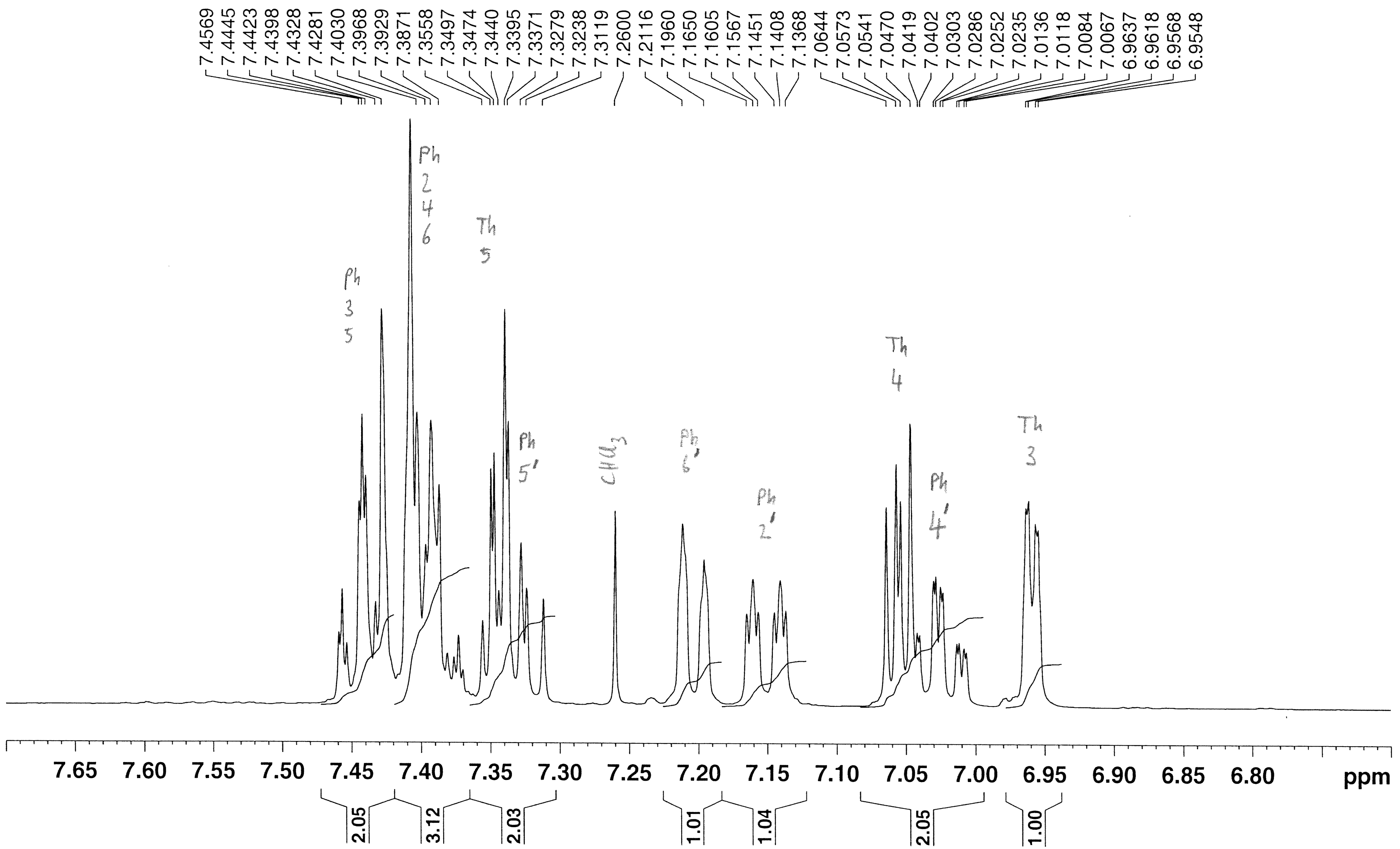

CE148S1P1 in cdcl3 (Proton) 5.2.2019

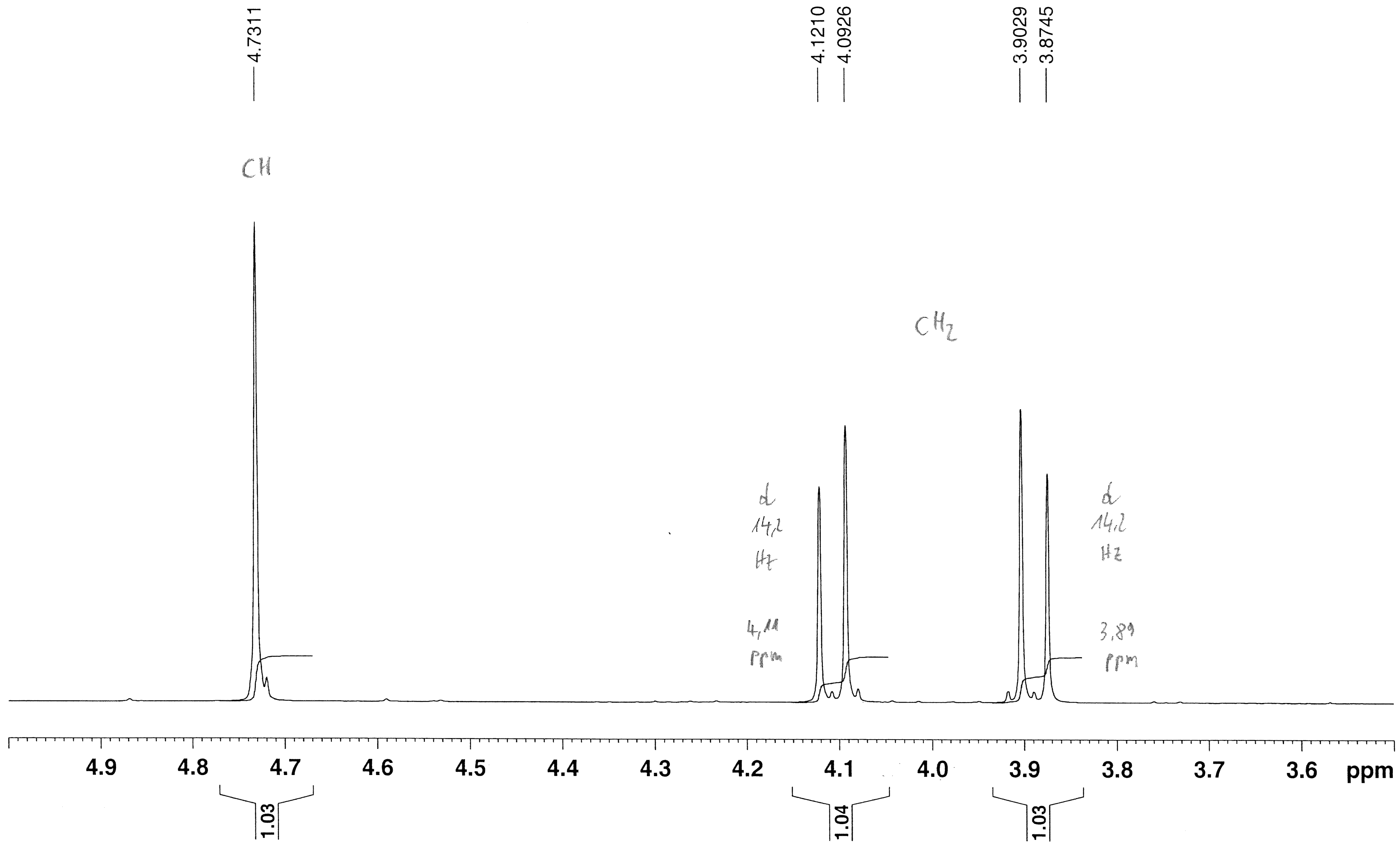

CE148S1P1 in cdcl3 (APT) 5.2.2019

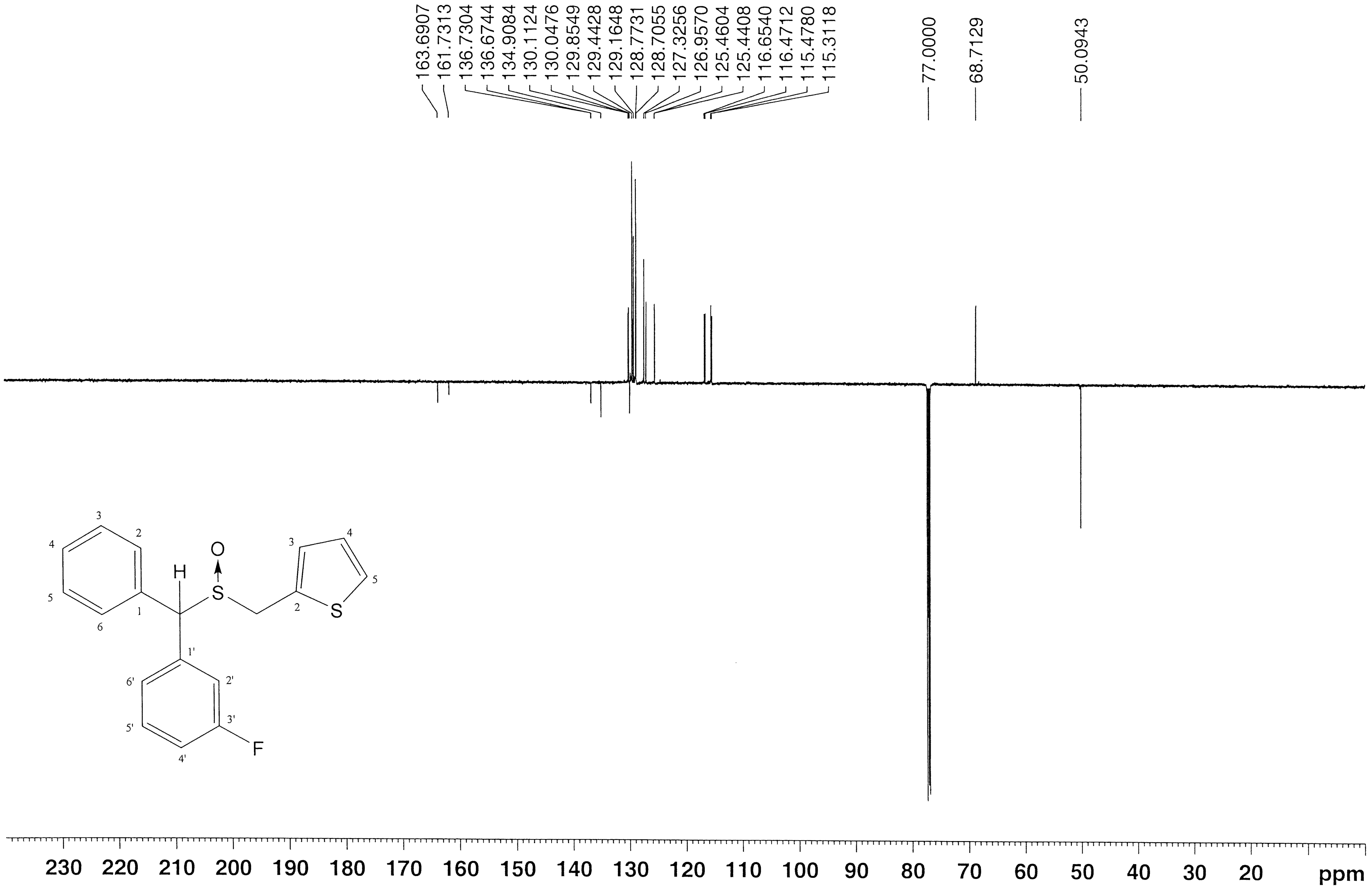

CE148S1P1 in cdcl3 (APT) 5.2.2019

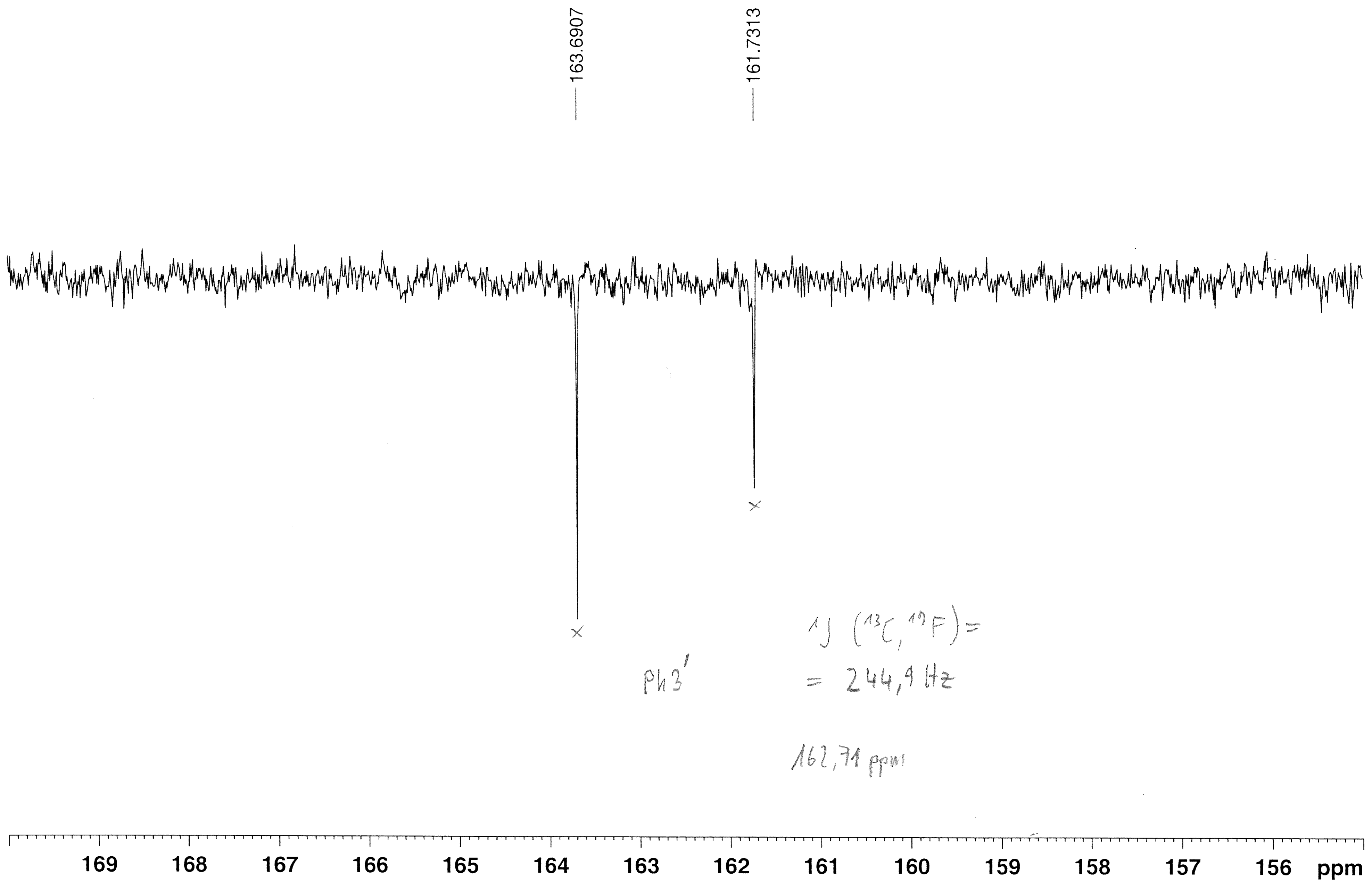

CE148S1P1 in cdcl3 (APT) 5.2.2019

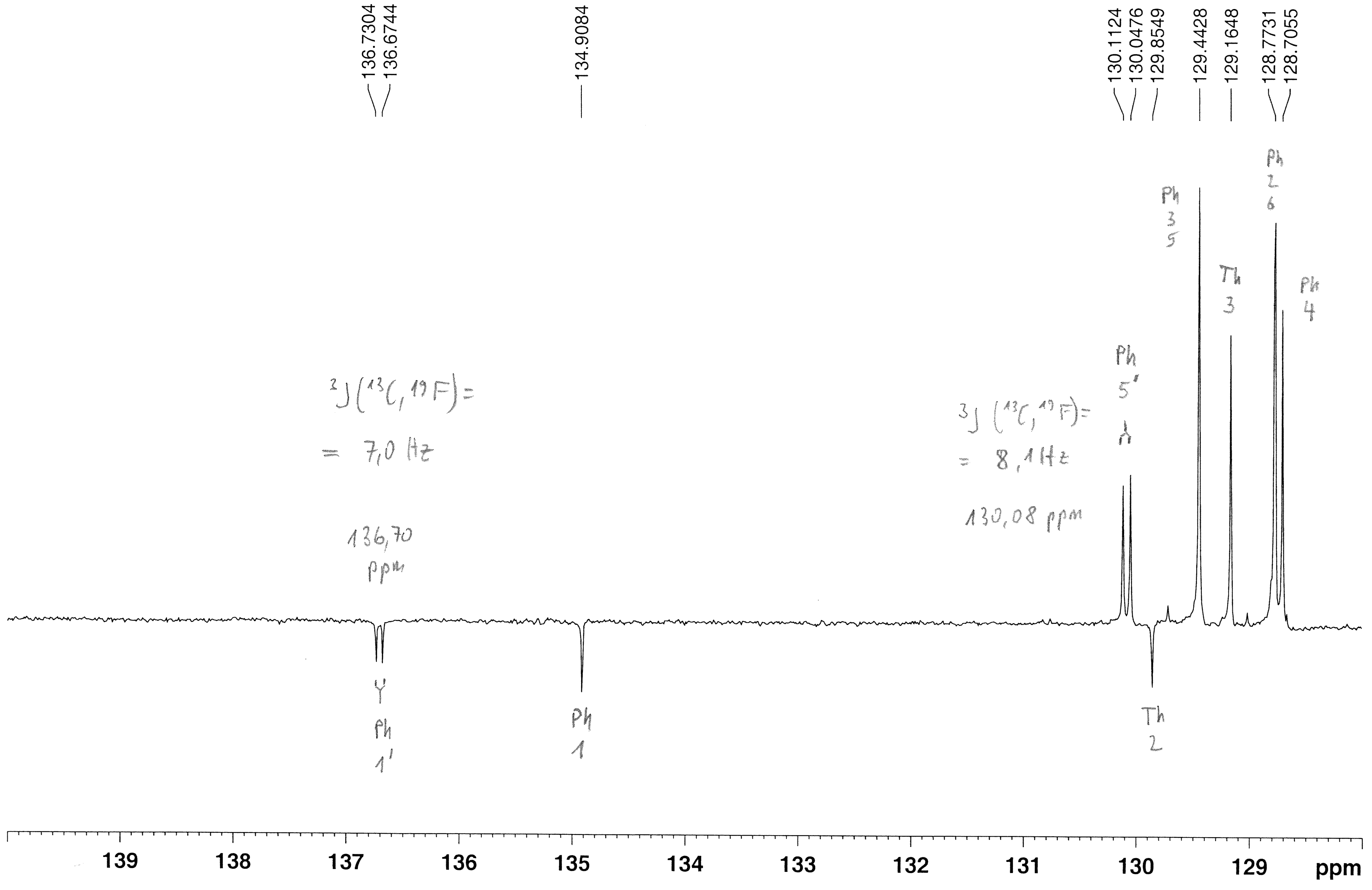

CE148S1P1 in cdcl3 (APT) 5.2.2019

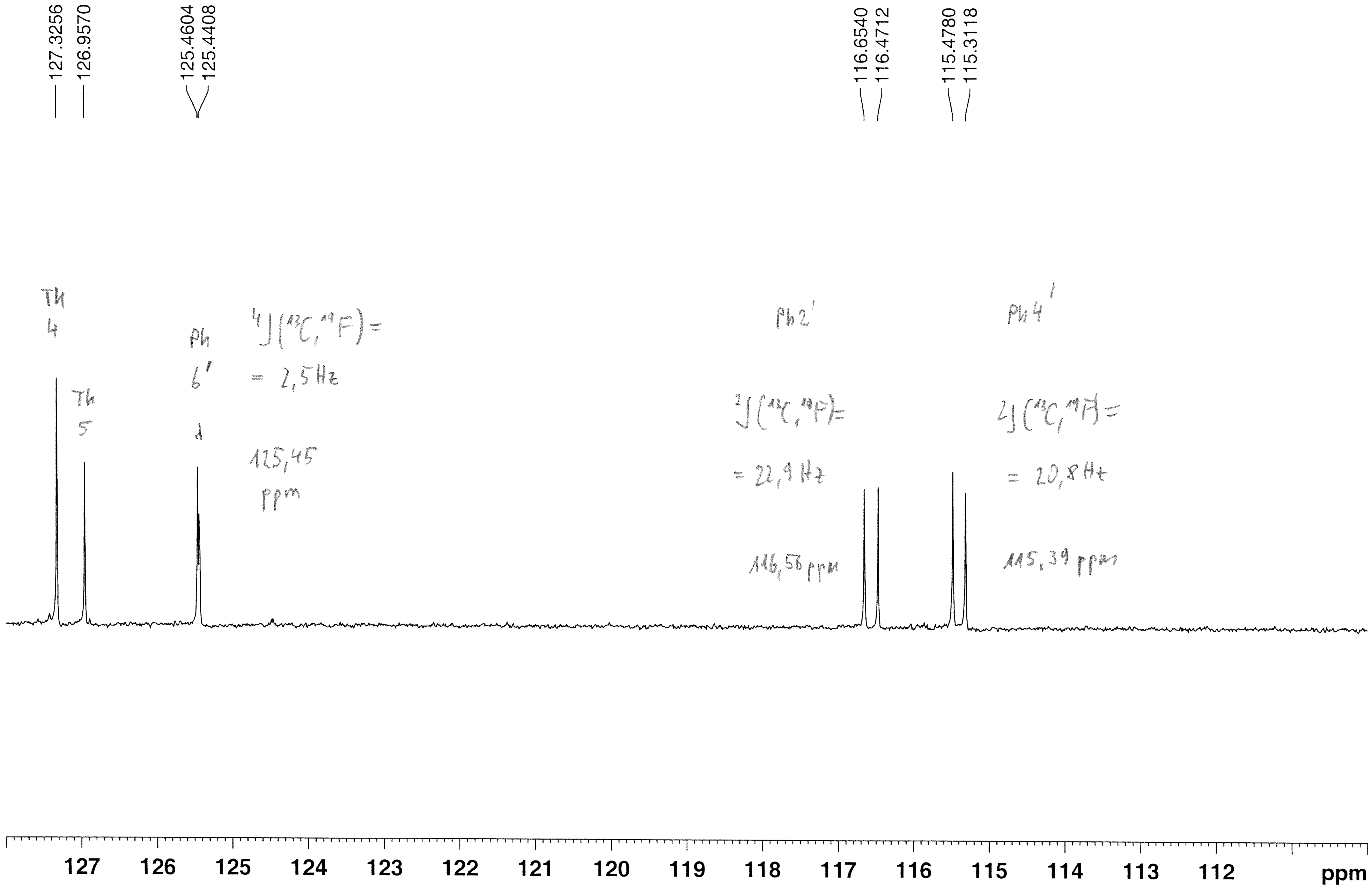

CE148S1P1 in cdcl3 (APT) 5.2.2019

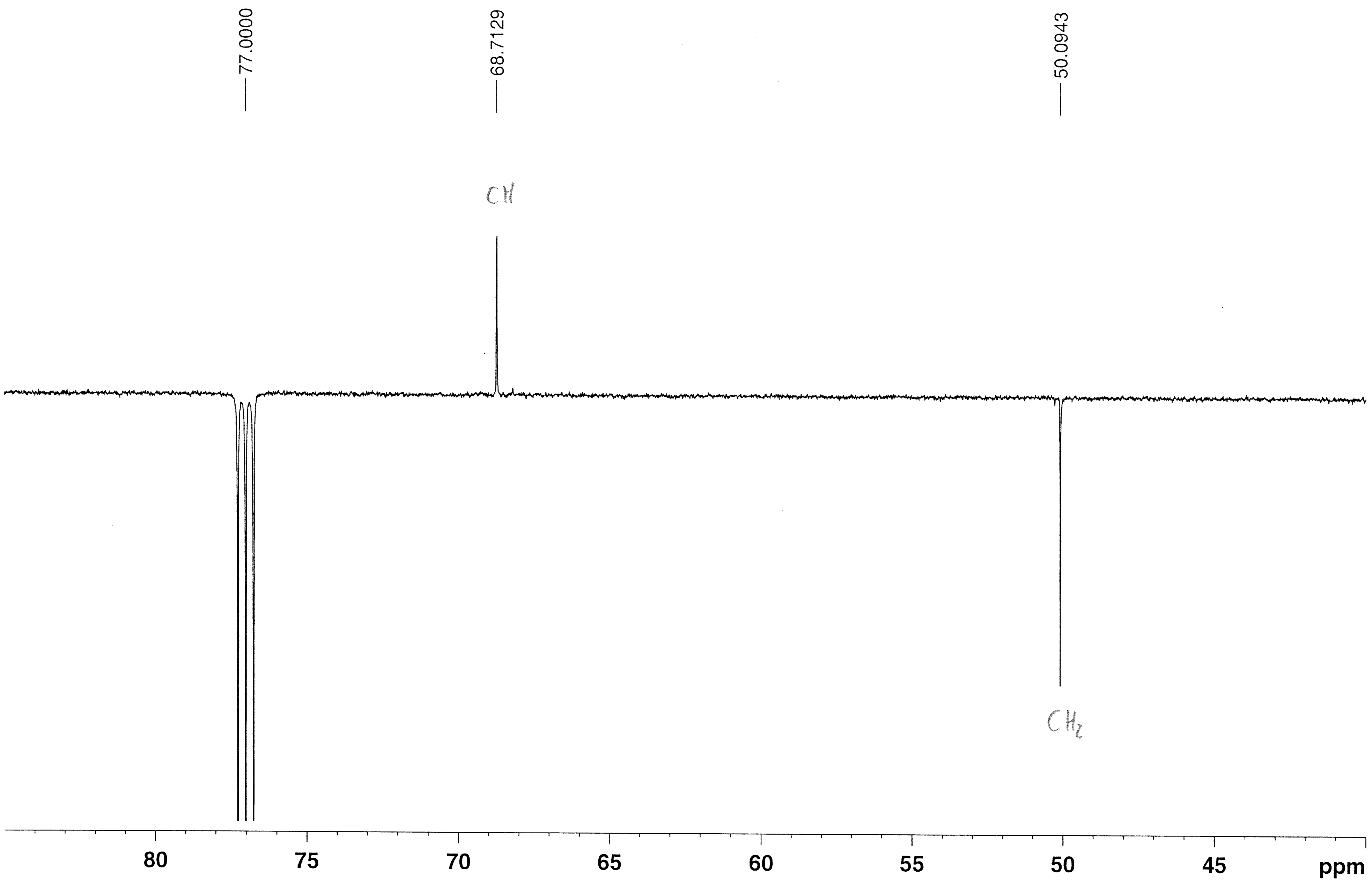

CE148S1P1 in cdcl3 (COSY) 5.2.2019

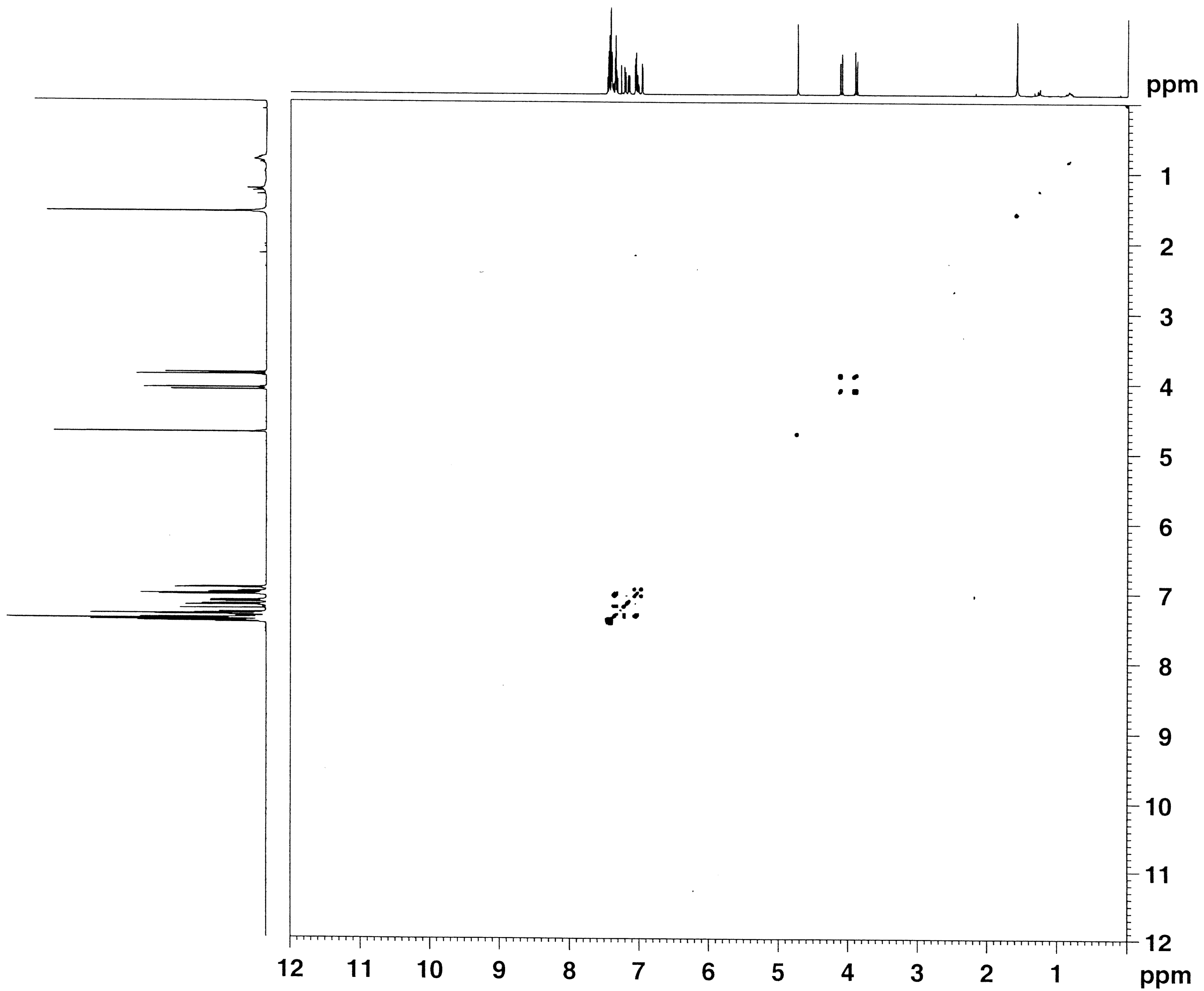

CE148S1P1 in cdcl3 (COSY) 5.2.2019

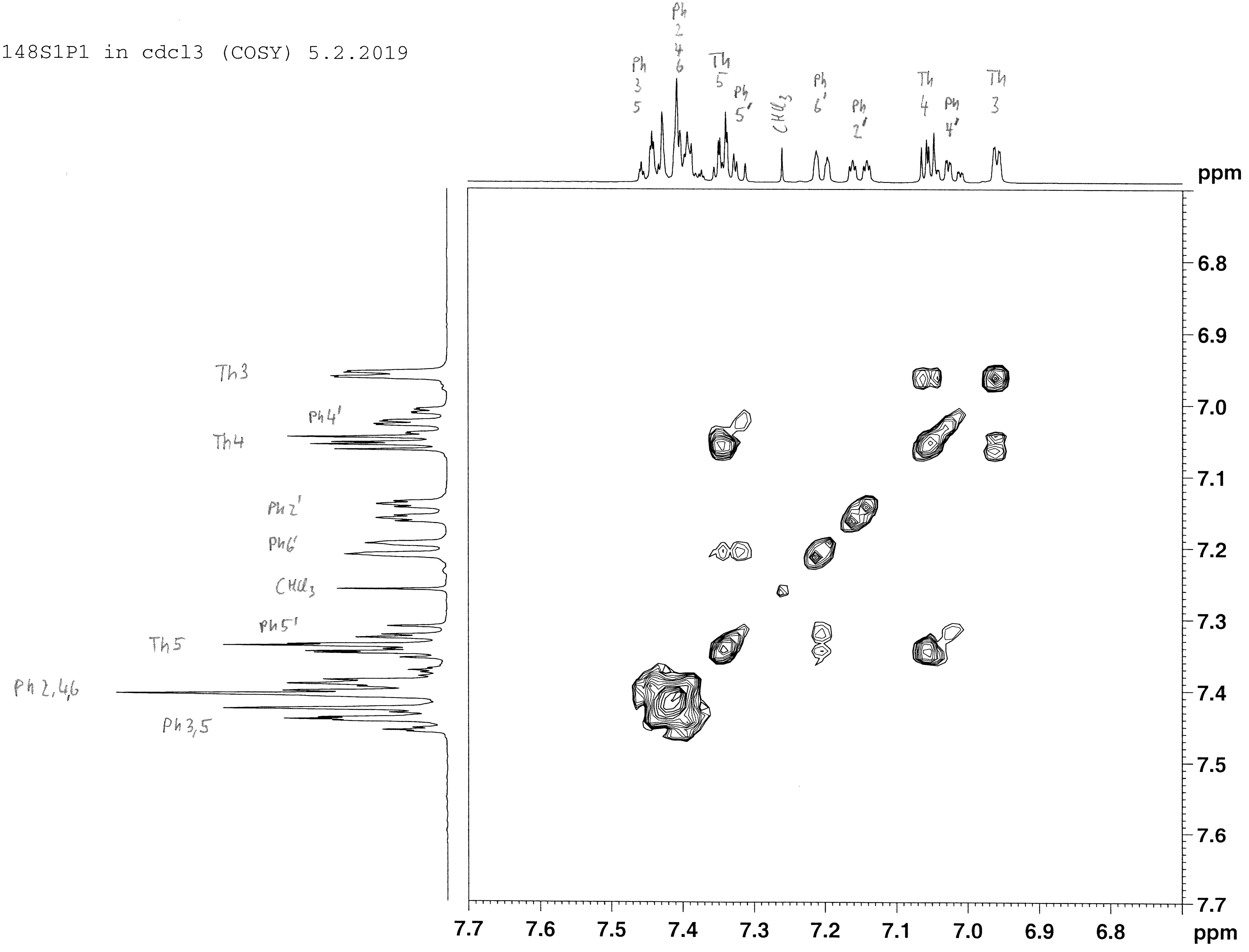

CE148S1P1 in cdcl3 (COSY) 5.2.2019

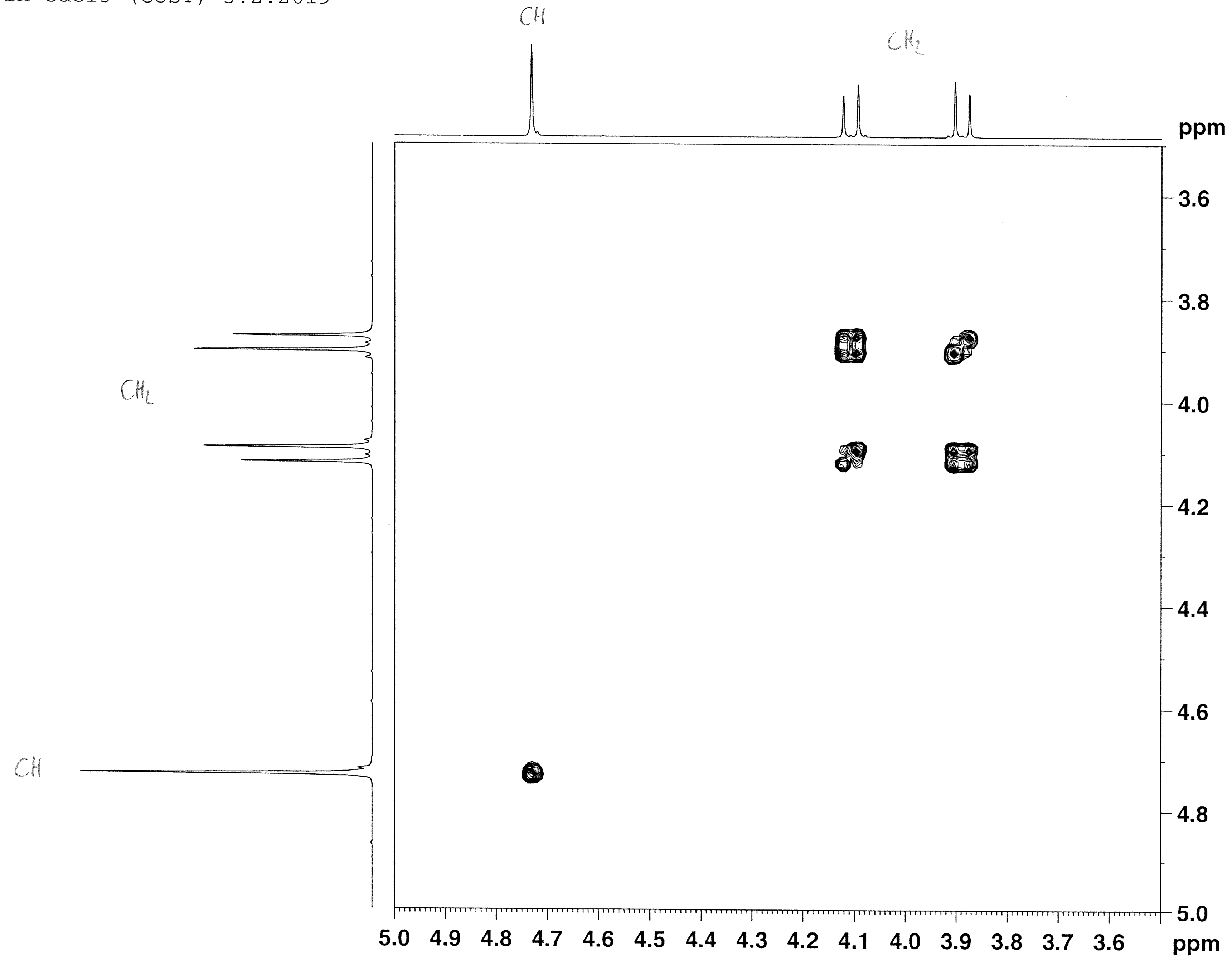

CE148S1P1 in cdcl3 (HSQC) 5.2.2019

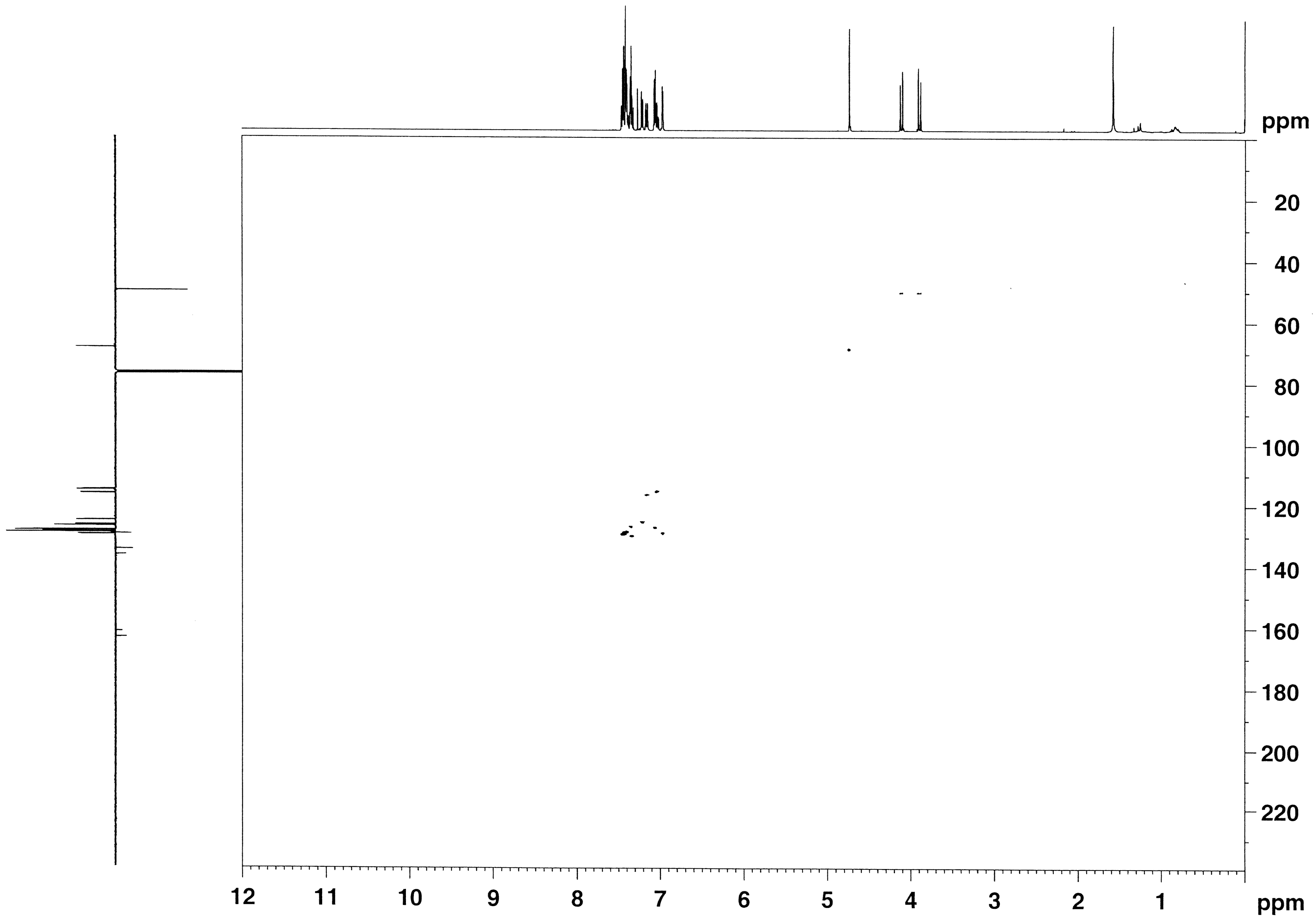

CE148S1P1 in cdcl3 (HSQC) 5.2.2019

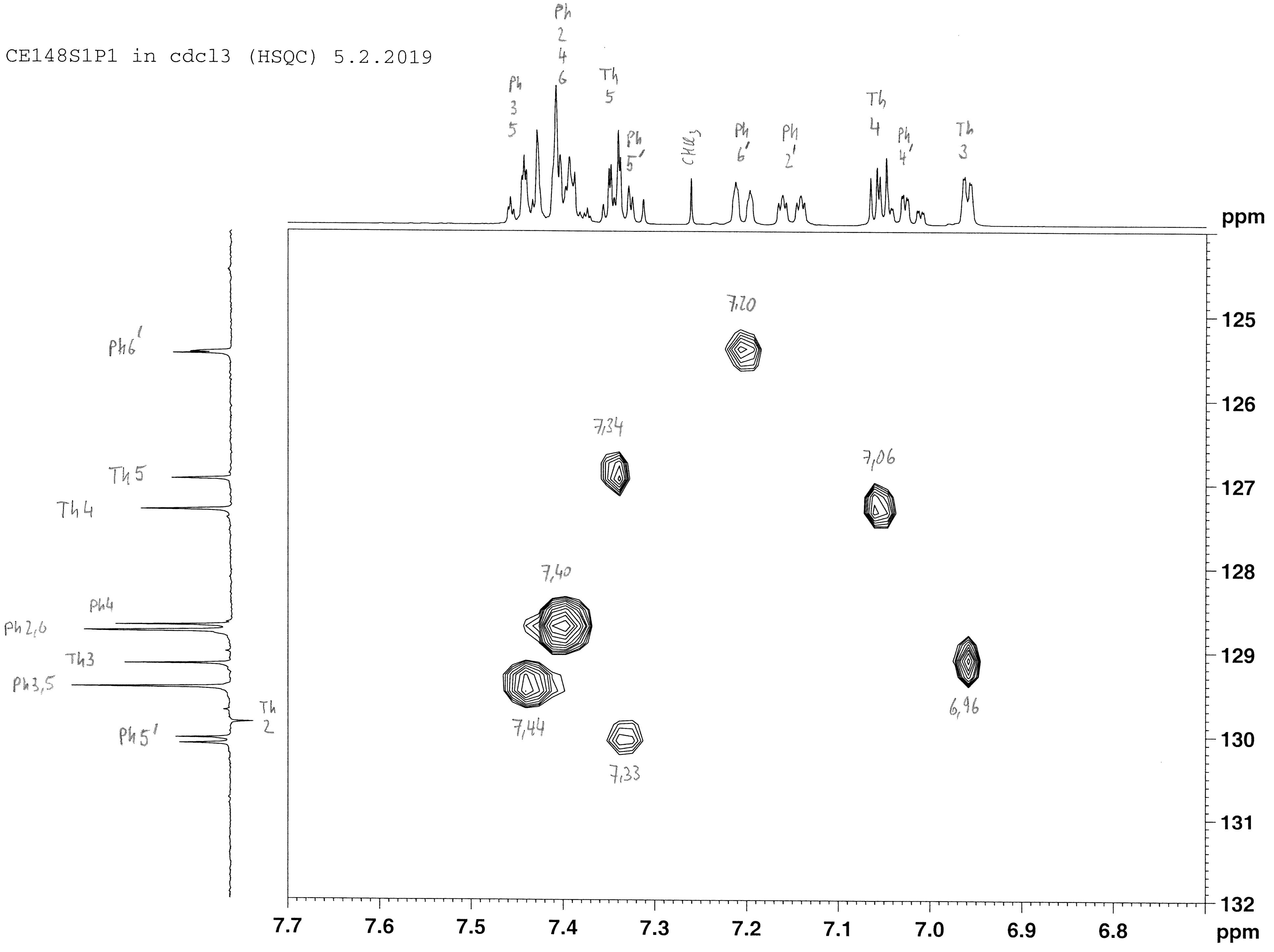

CE148S1P1 in cdcl3 (HSQC) 5.2.2019

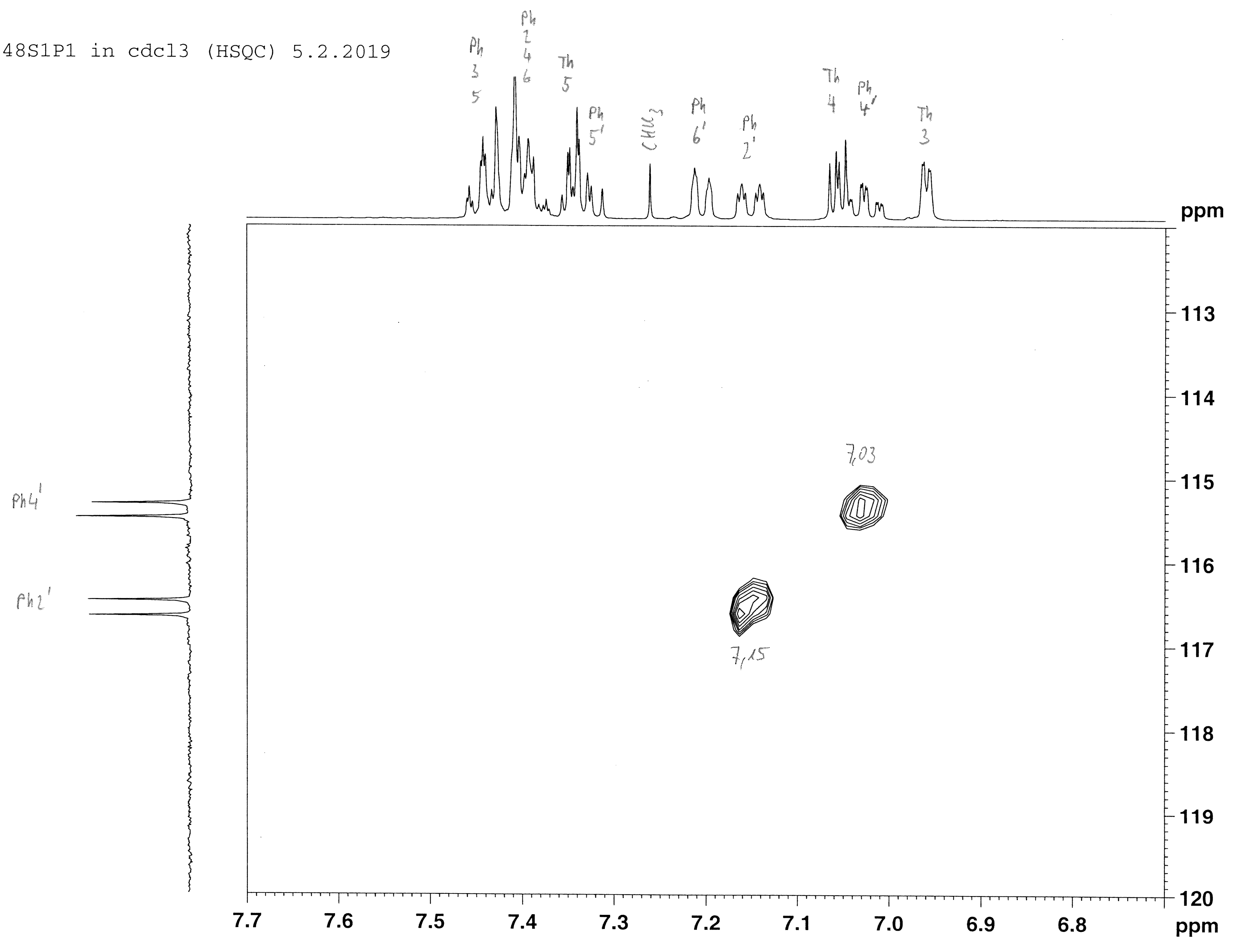

CE148S1P1 in cdcl3 (HSQC) 5.2.2019

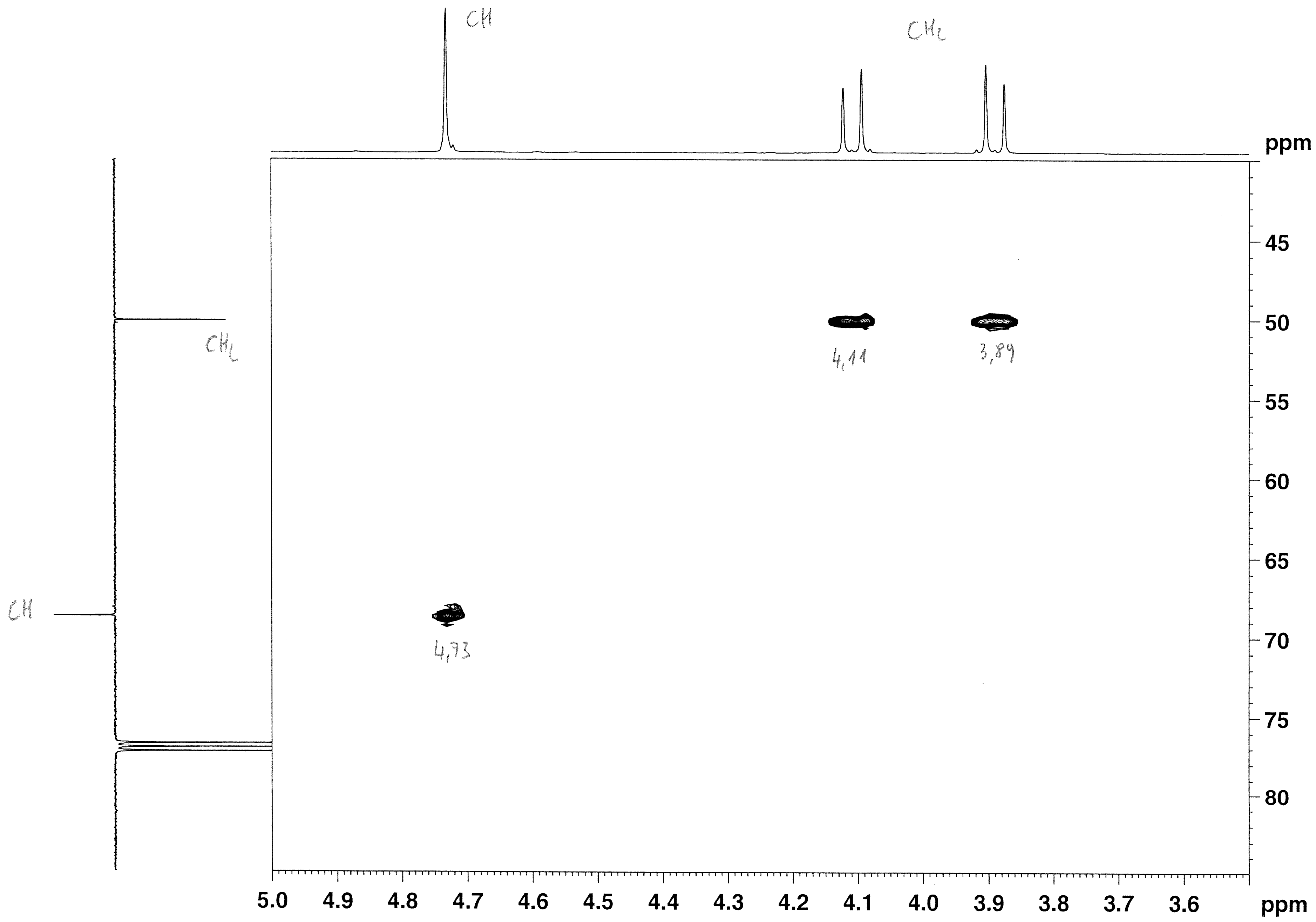

CE148S1P1 in cdcl3 (HMBC) 5.2.2019

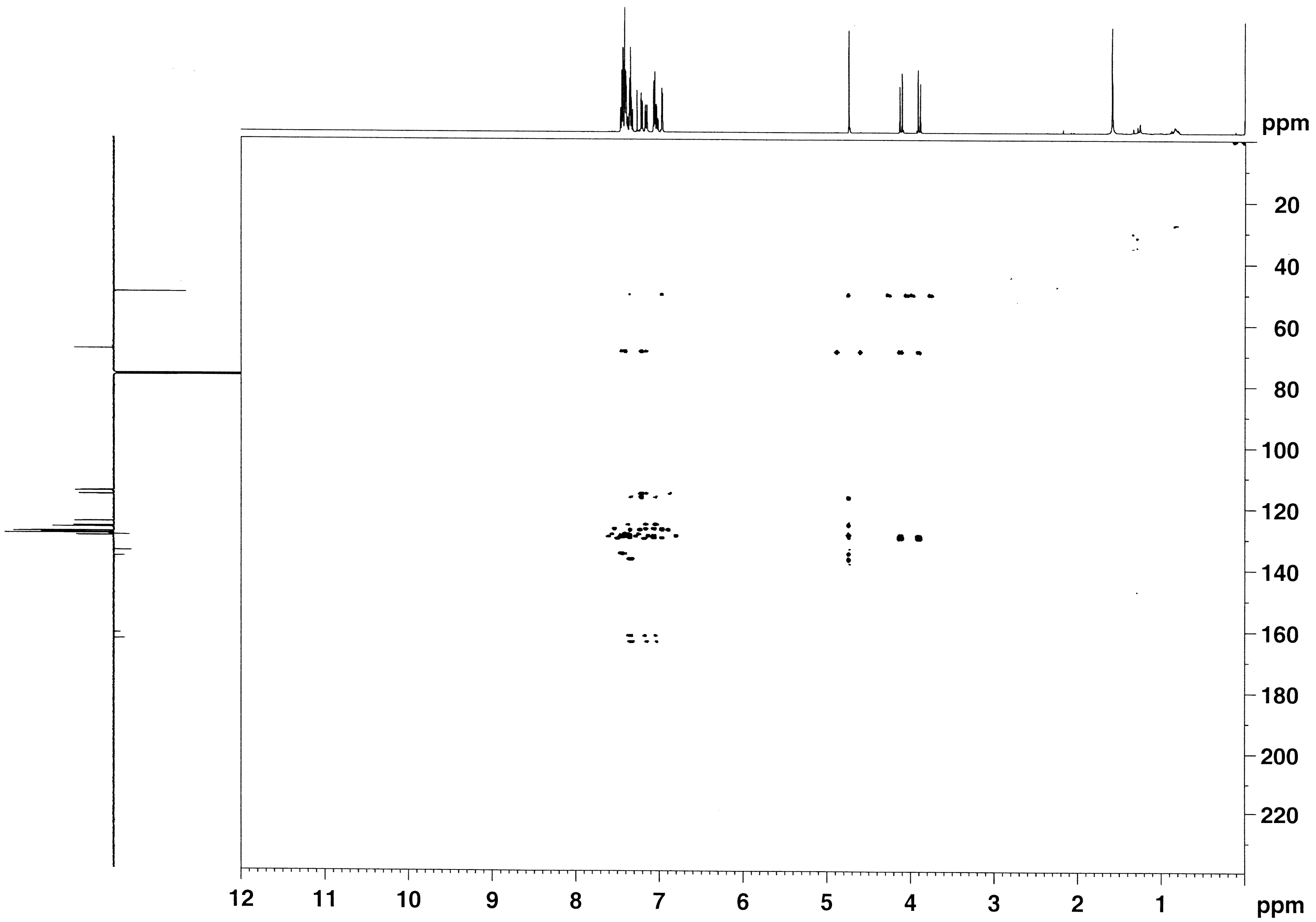

CE148S1P1 in cdcl3 (HMBC) 5.2.2019

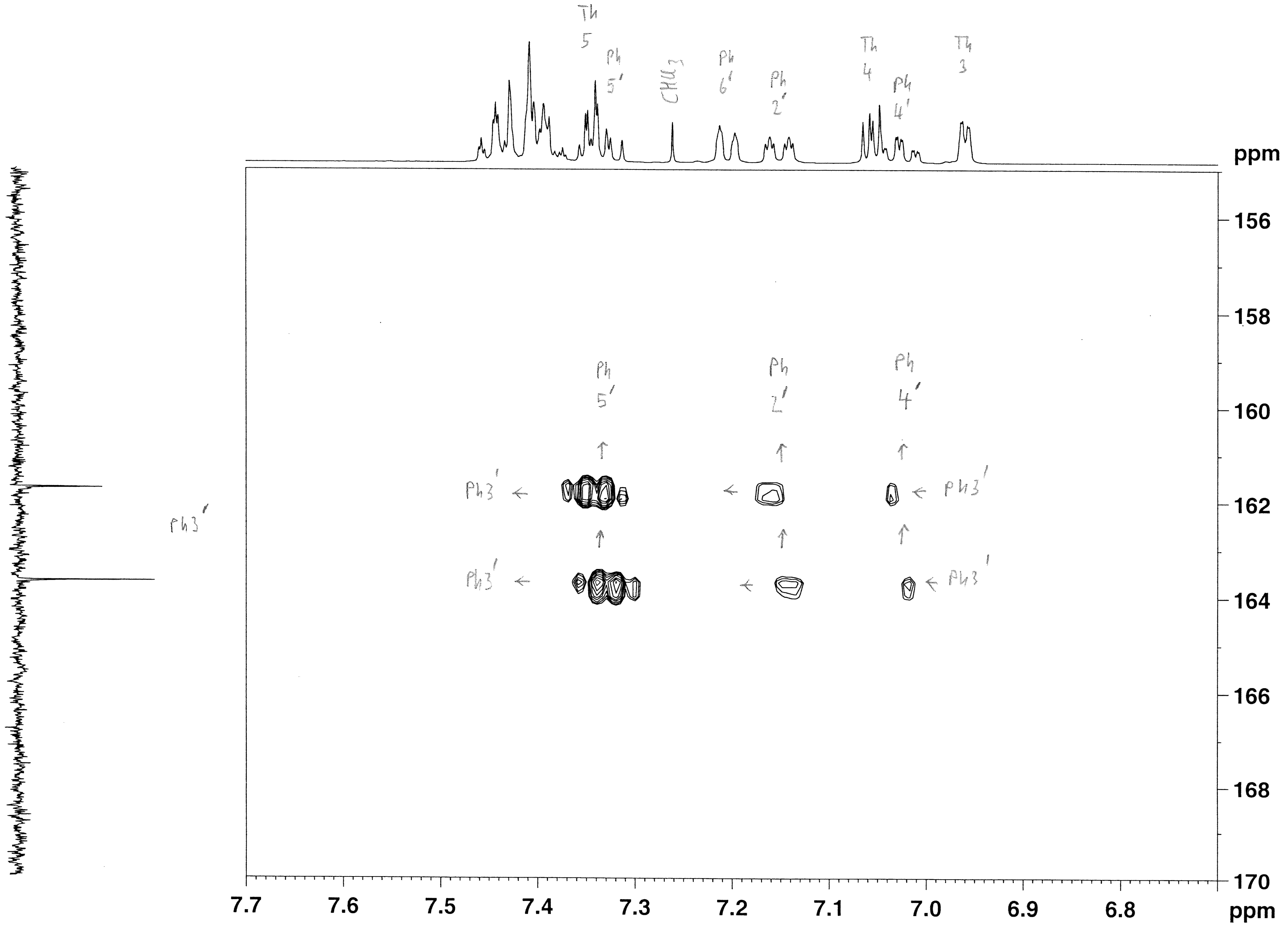

CE148S1P1 in cdcl3 (HMBC) 5.2.2019

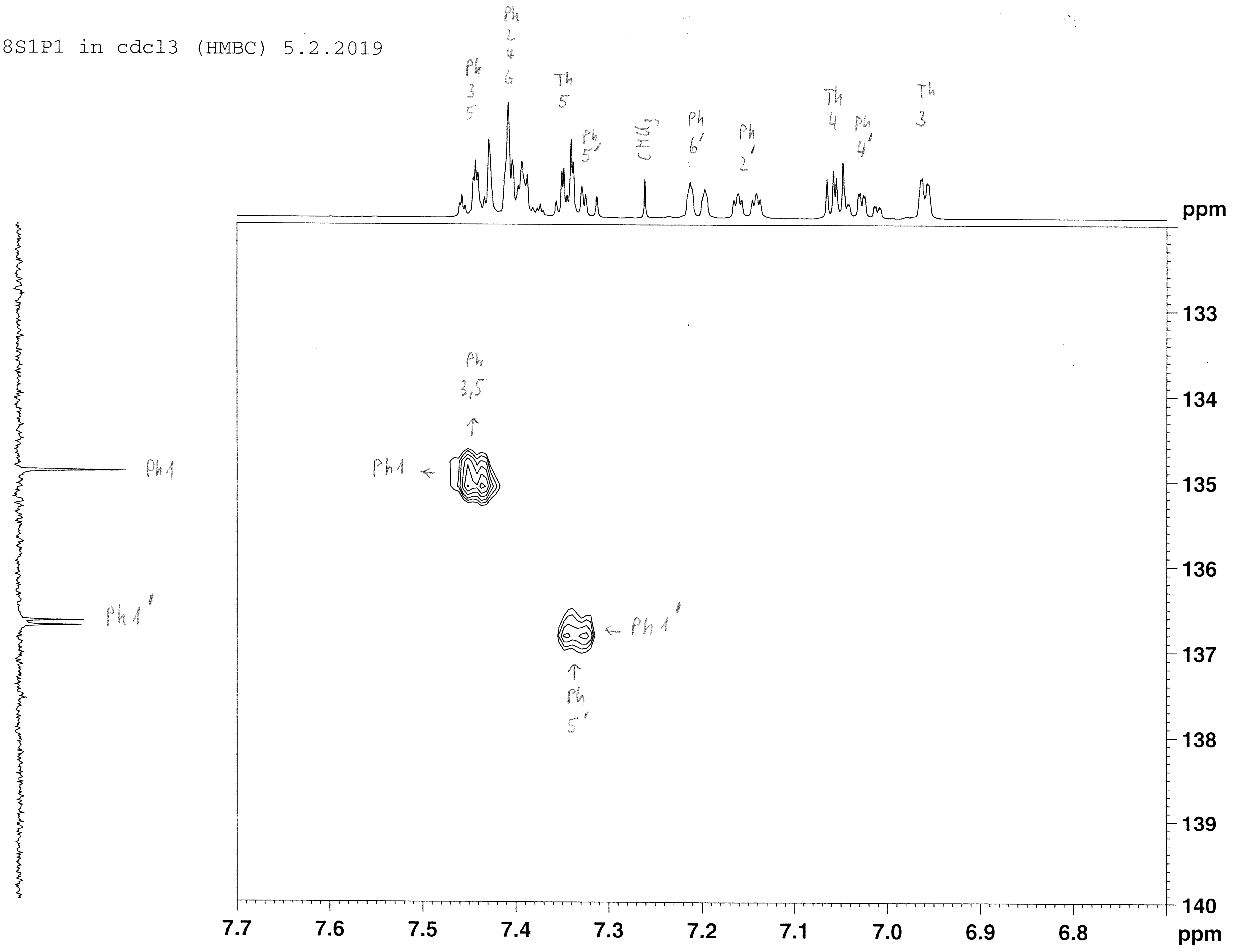

CE148S1P1 in cdcl3 (HMBC) 5.2.2019

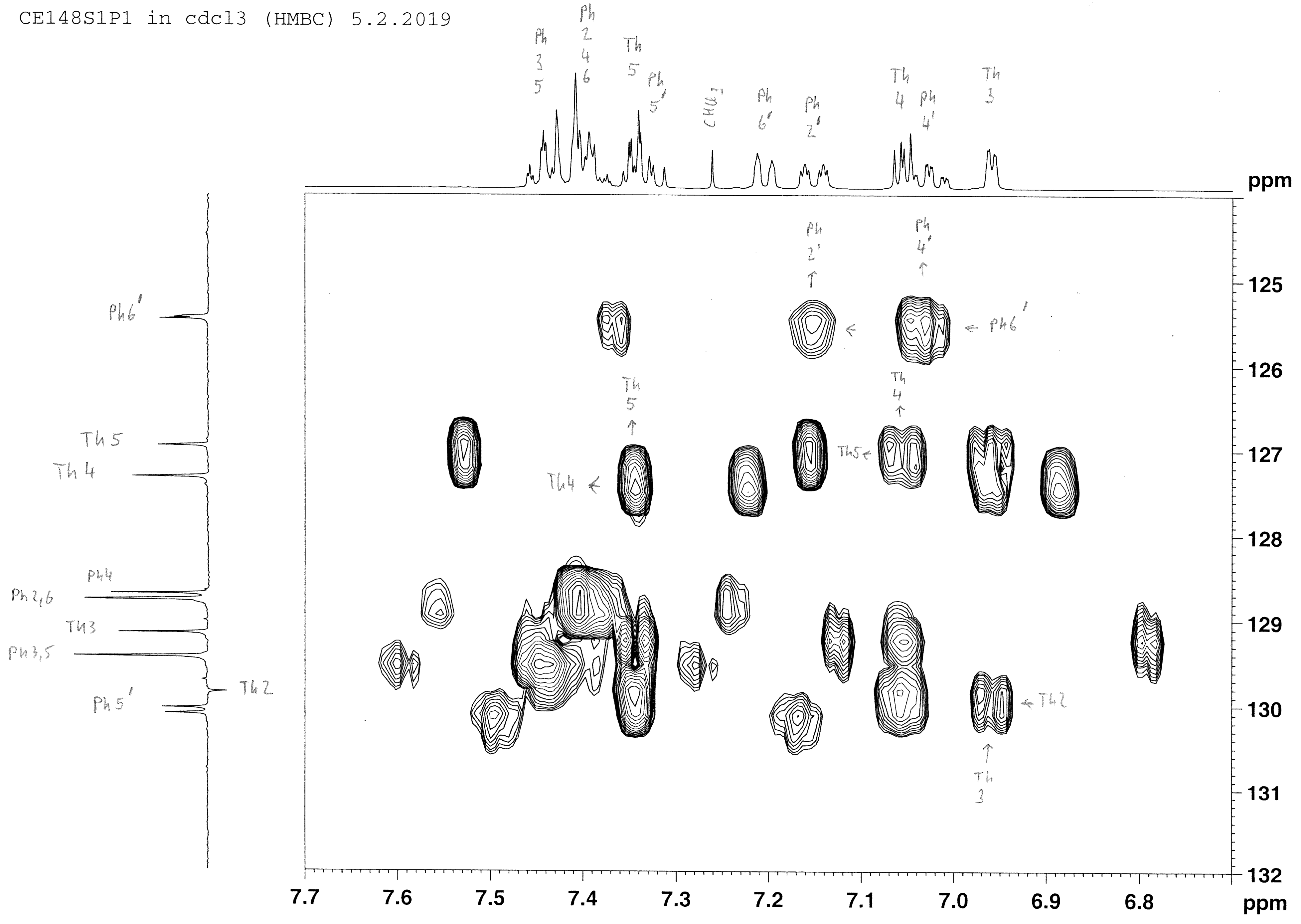

CE148S1P1 in cdcl3 (HMBC) 5.2.2019

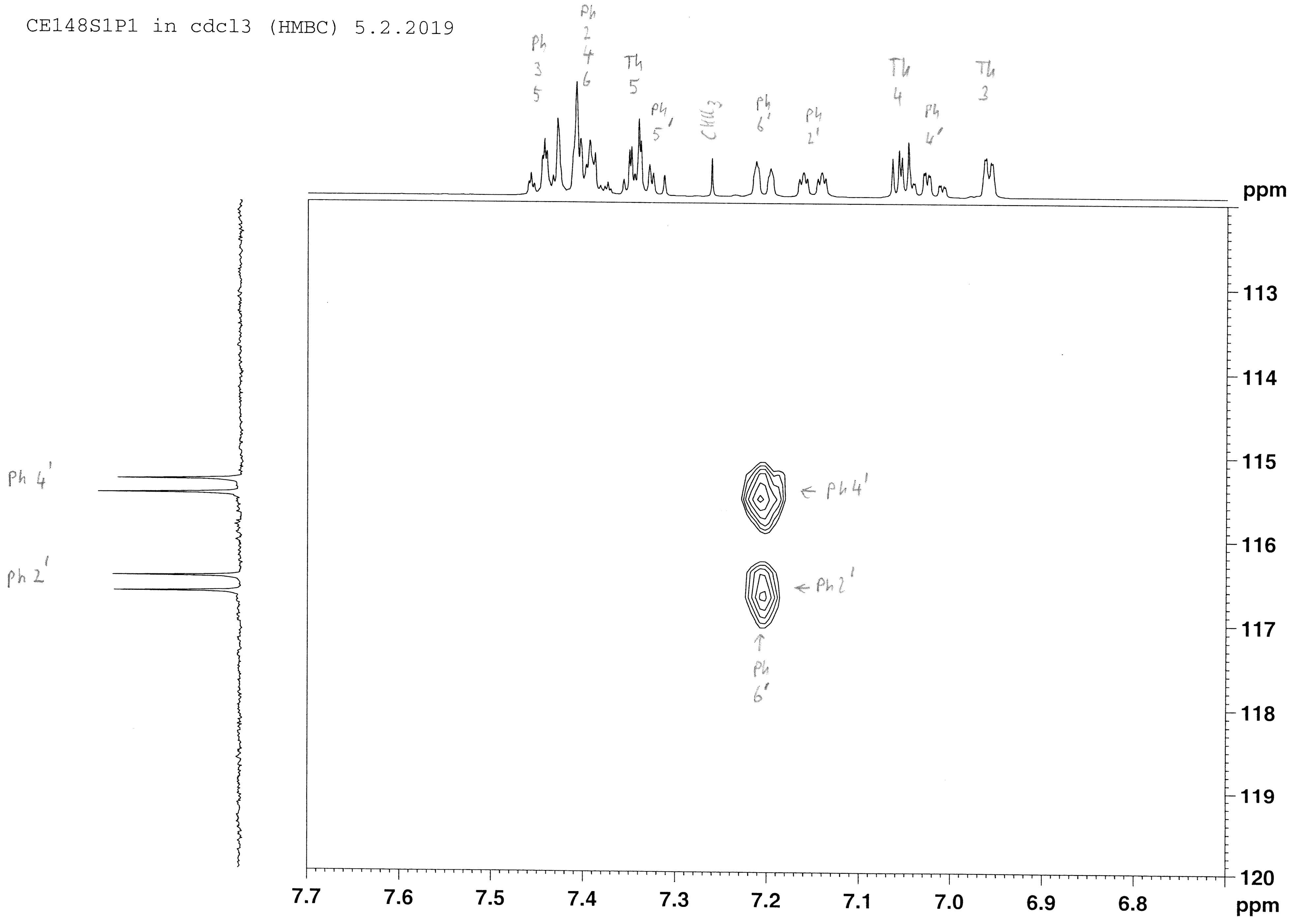

CE148S1P1 in cdcl3 (HMBC) 5.2.2019

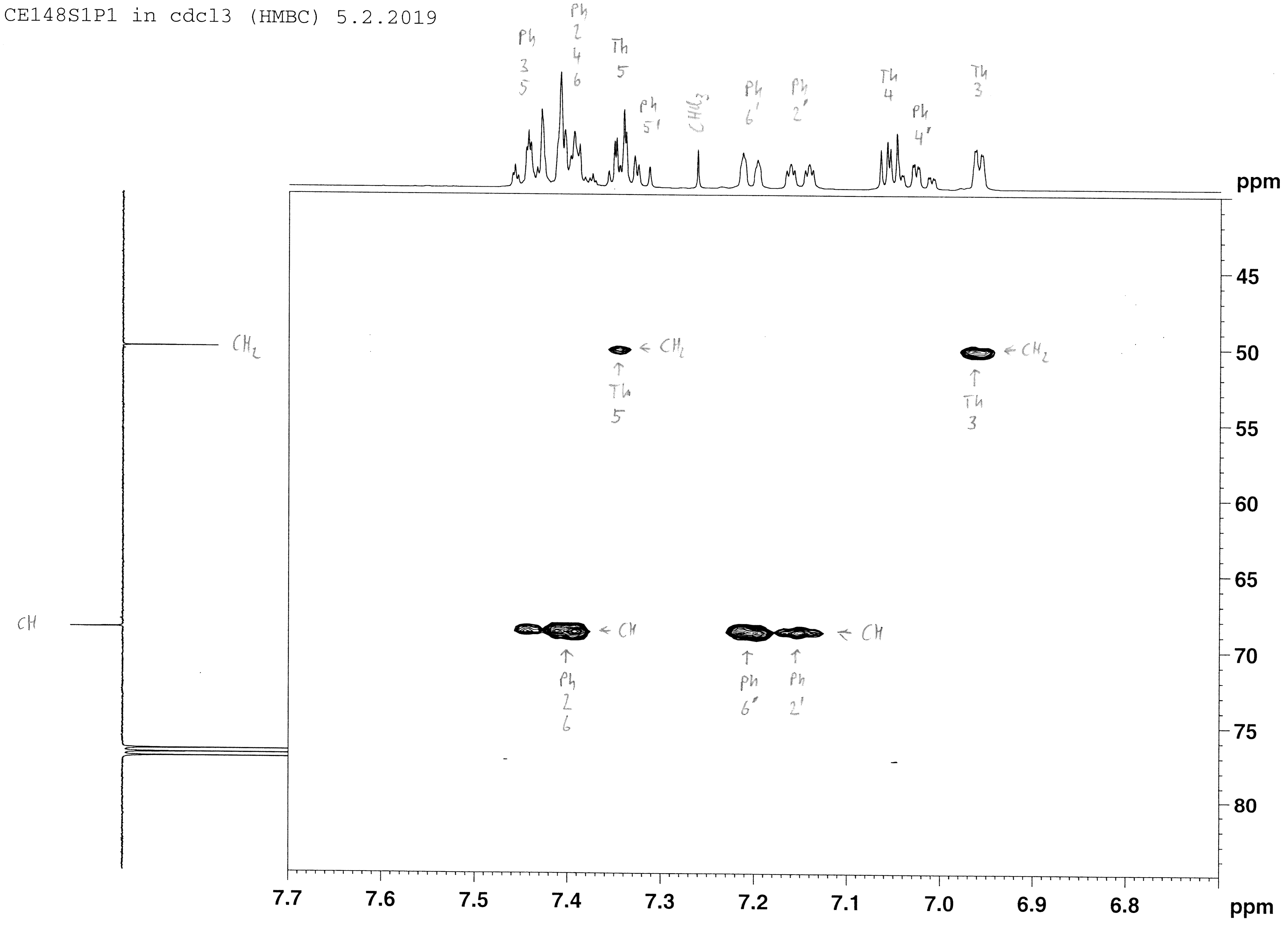

CE148S1P1 in cdcl3 (HMBC) 5.2.2019

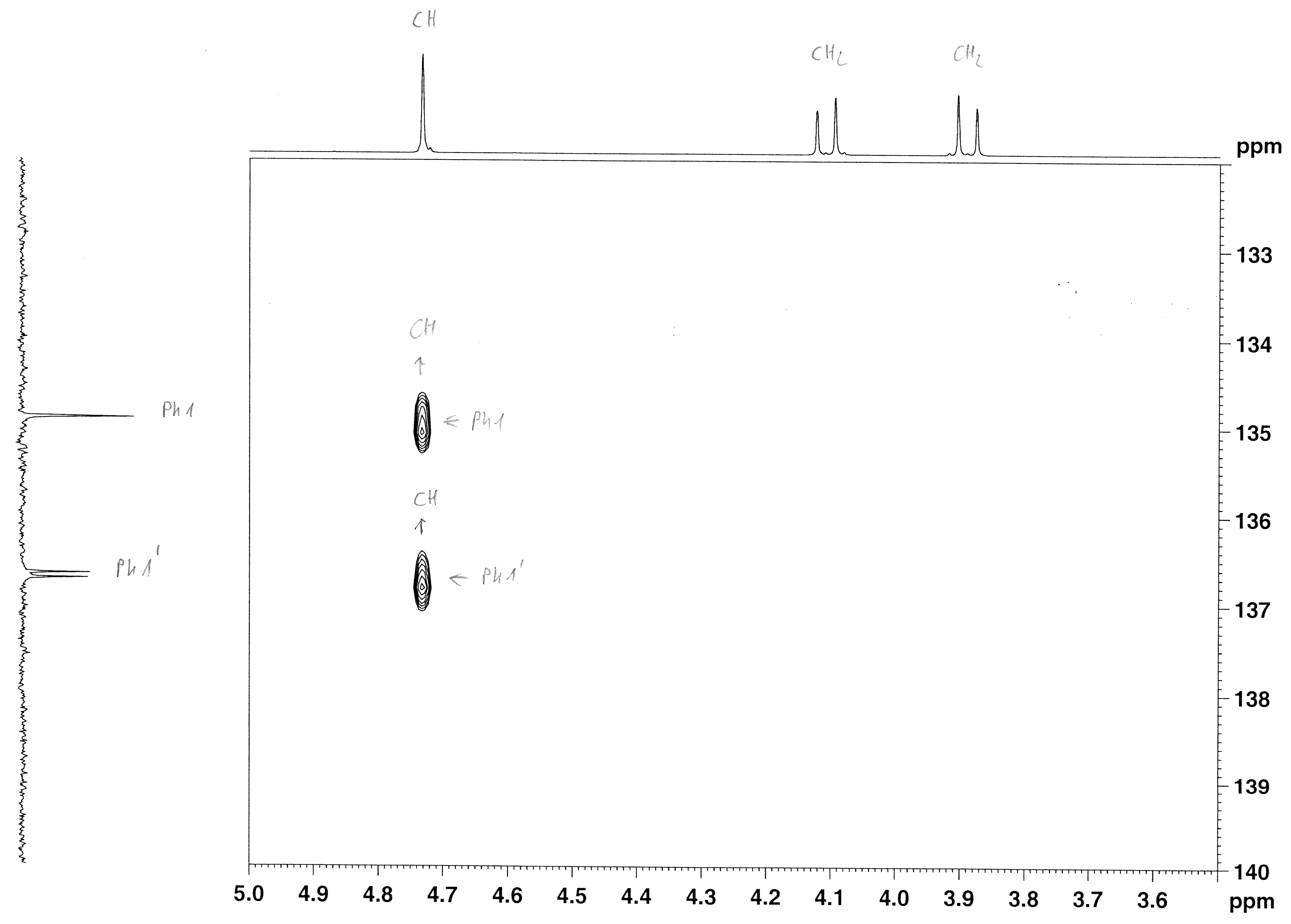

CE148S1P1 in cdcl3 (HMBC) 5.2.2019

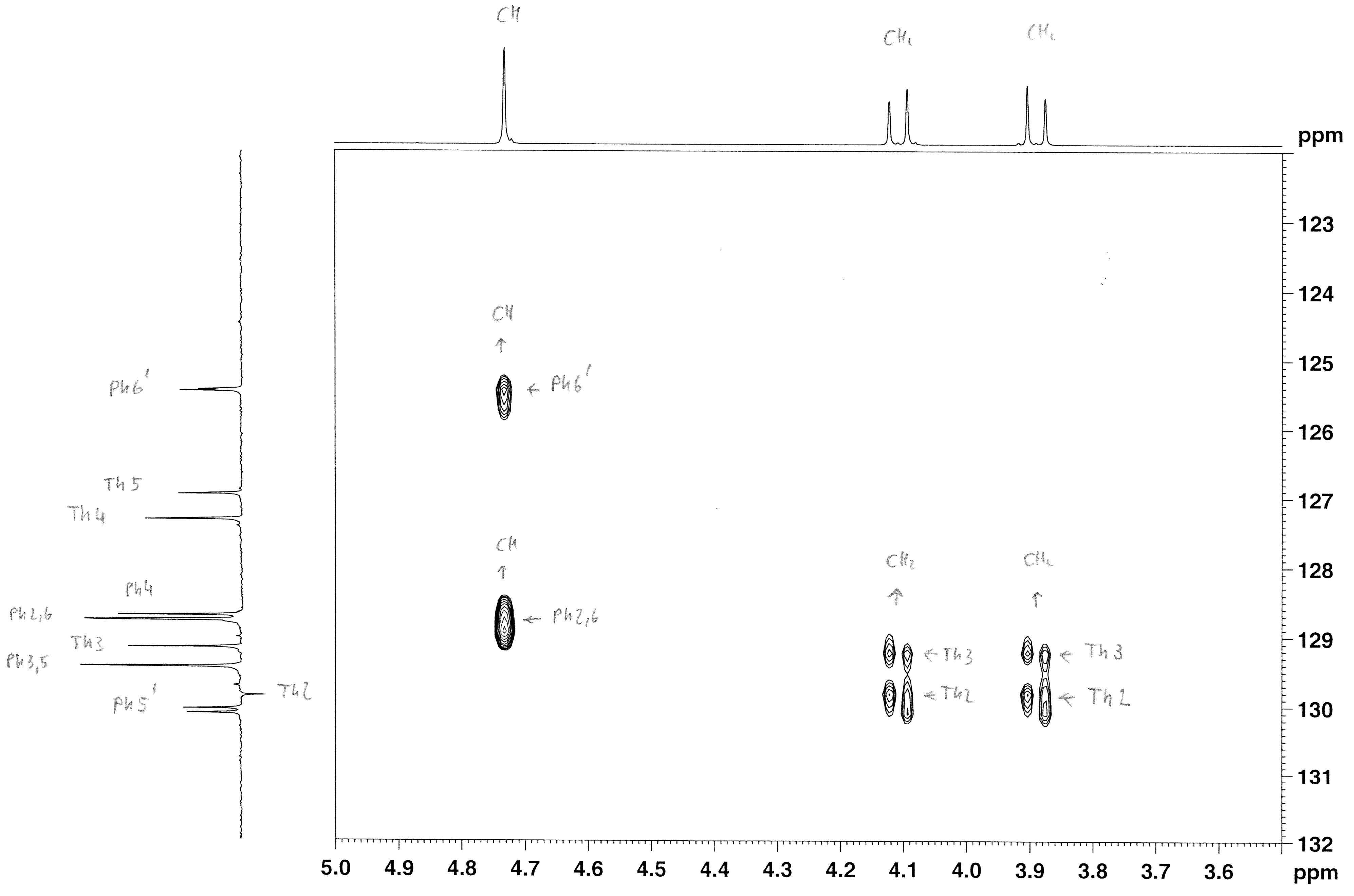

CE148S1P1 in cdcl3 (HMBC) 5.2.2019

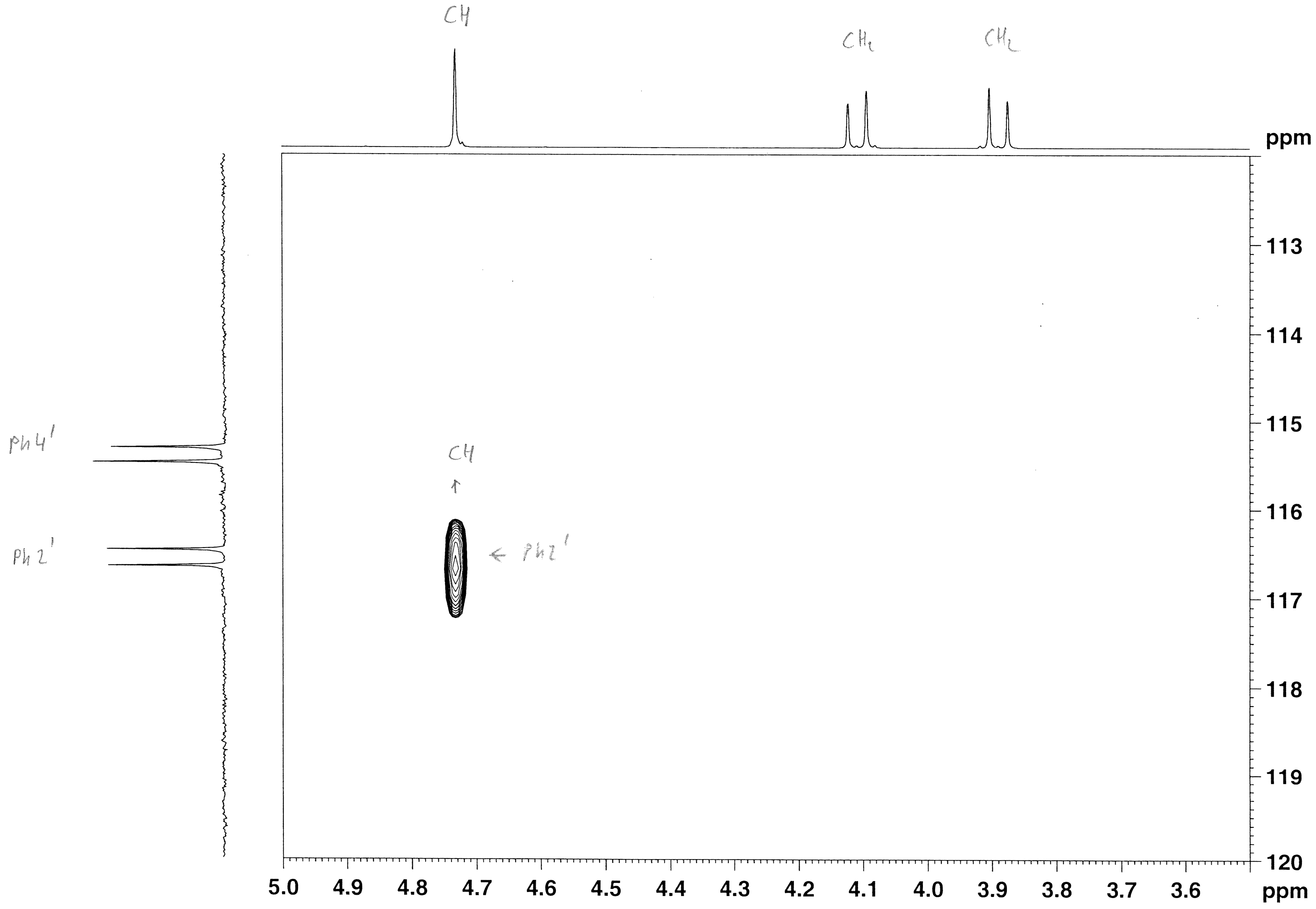

CE148S1P1 in cdcl3 (HMBC) 5.2.2019

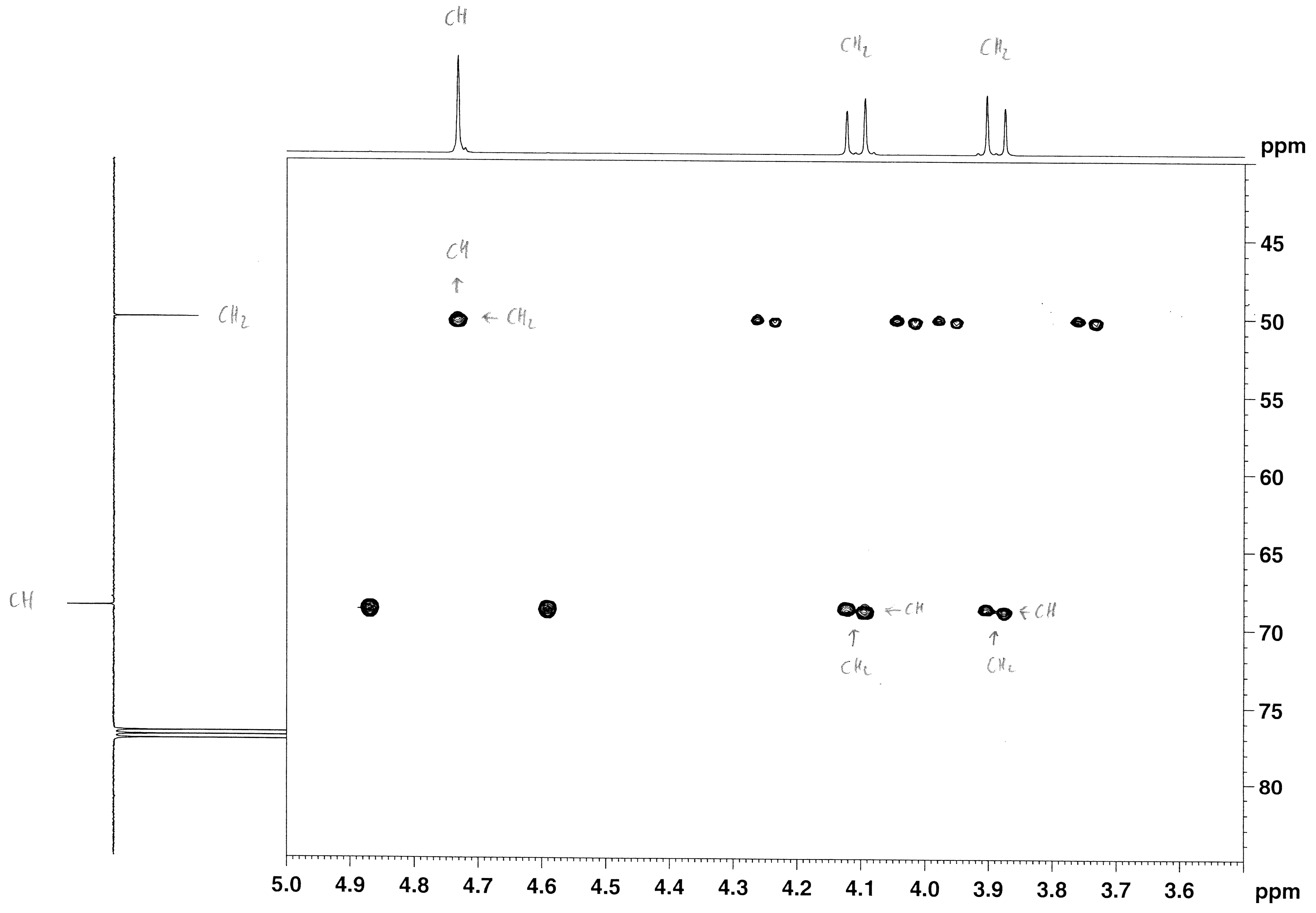

Figure S6c. NMR spectra of compound **6b.**

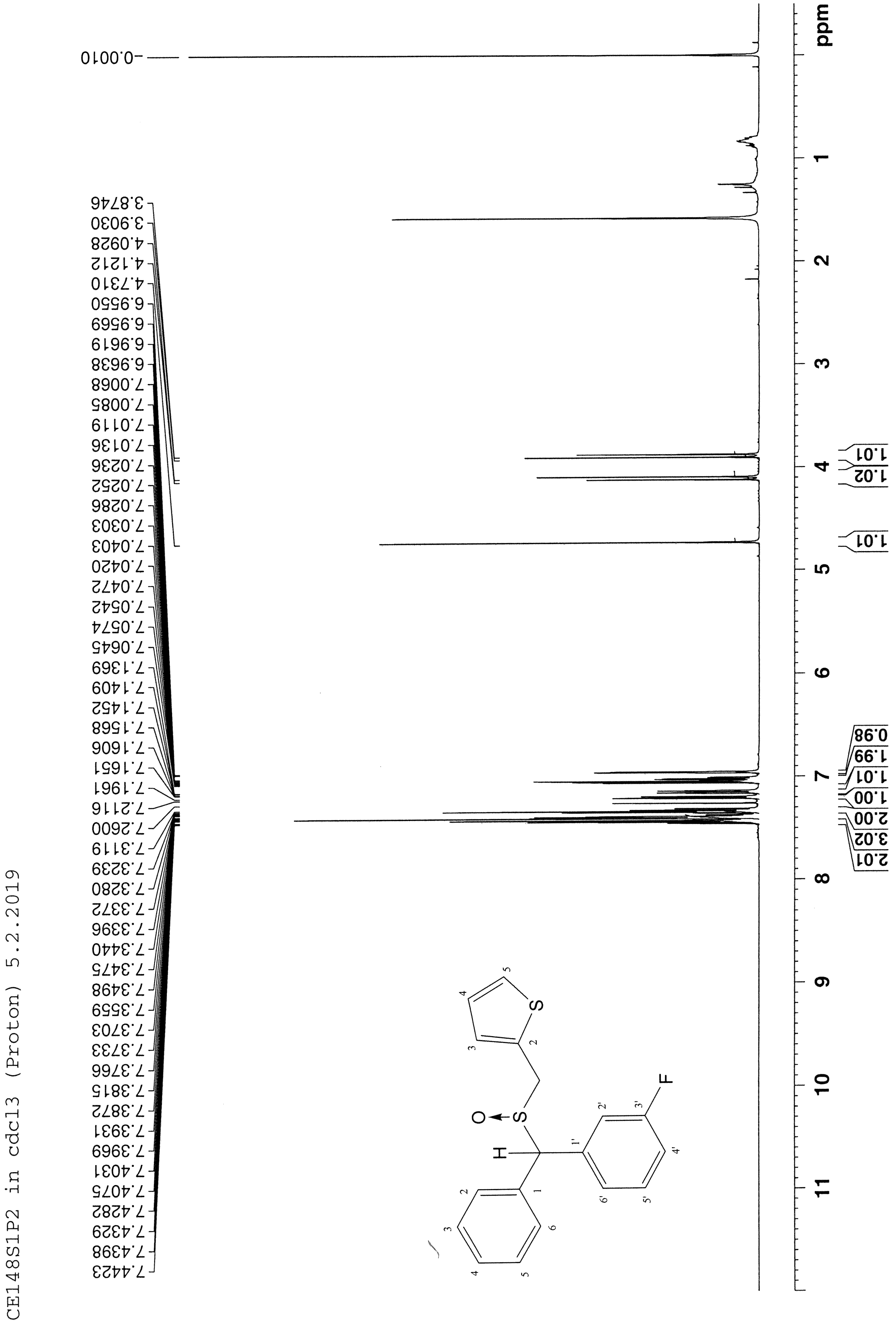

CE148S1P2 in cdcl3 (Proton) 5.2.2019

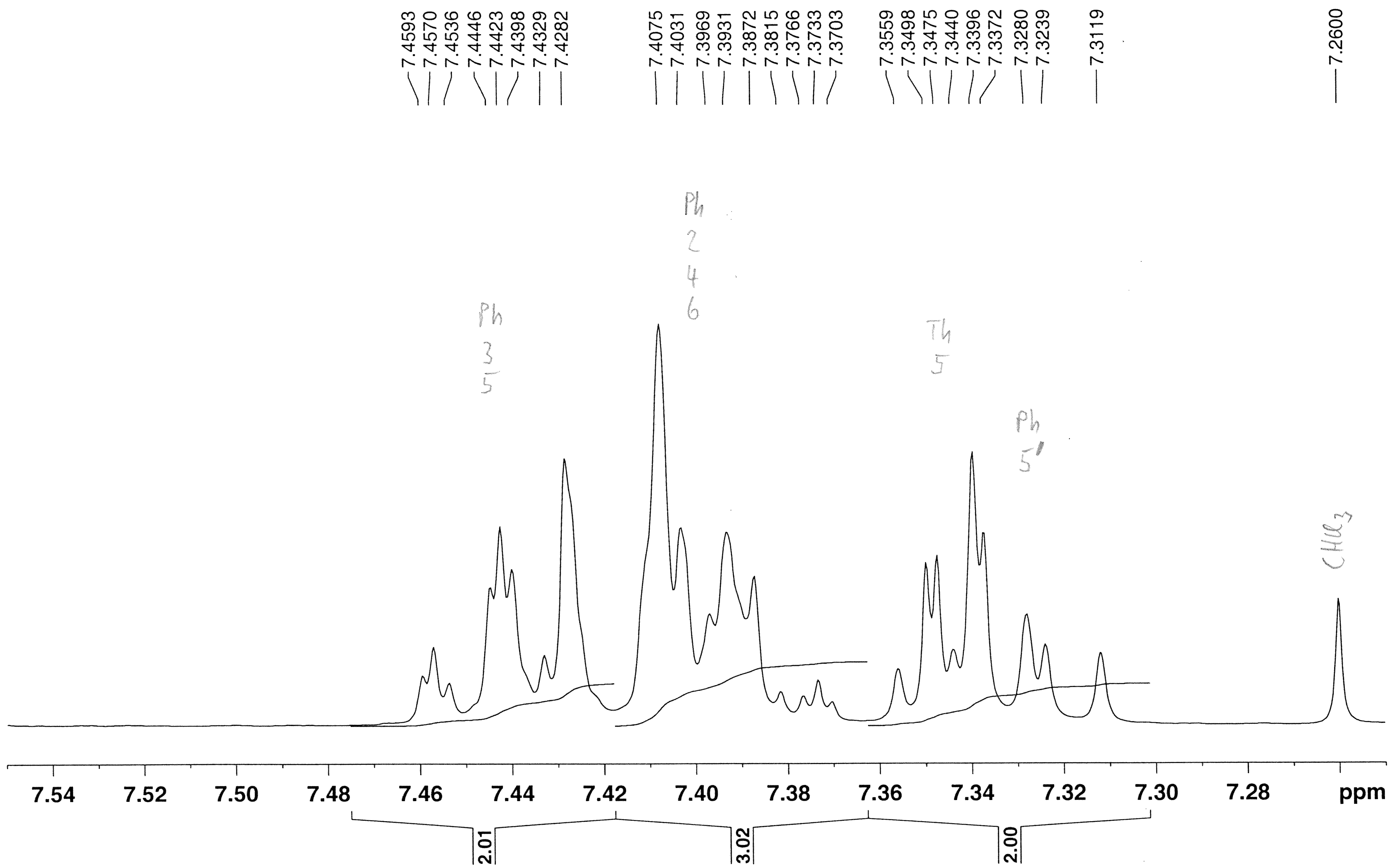

CE148S1P2 in cdcl3 (Proton) 5.2.2019

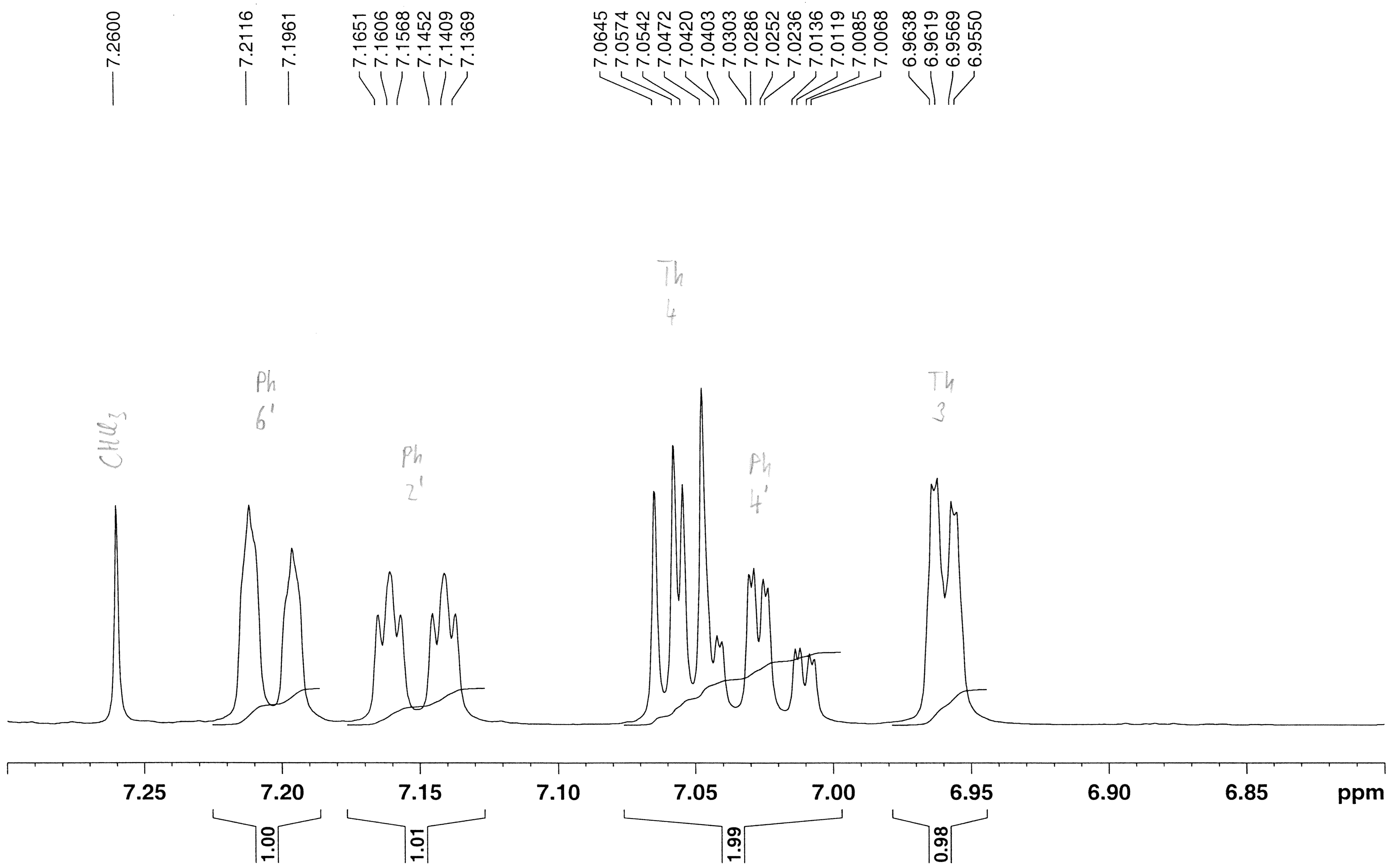

CE148S1P2 in cdcl3 (Proton) 5.2.2019

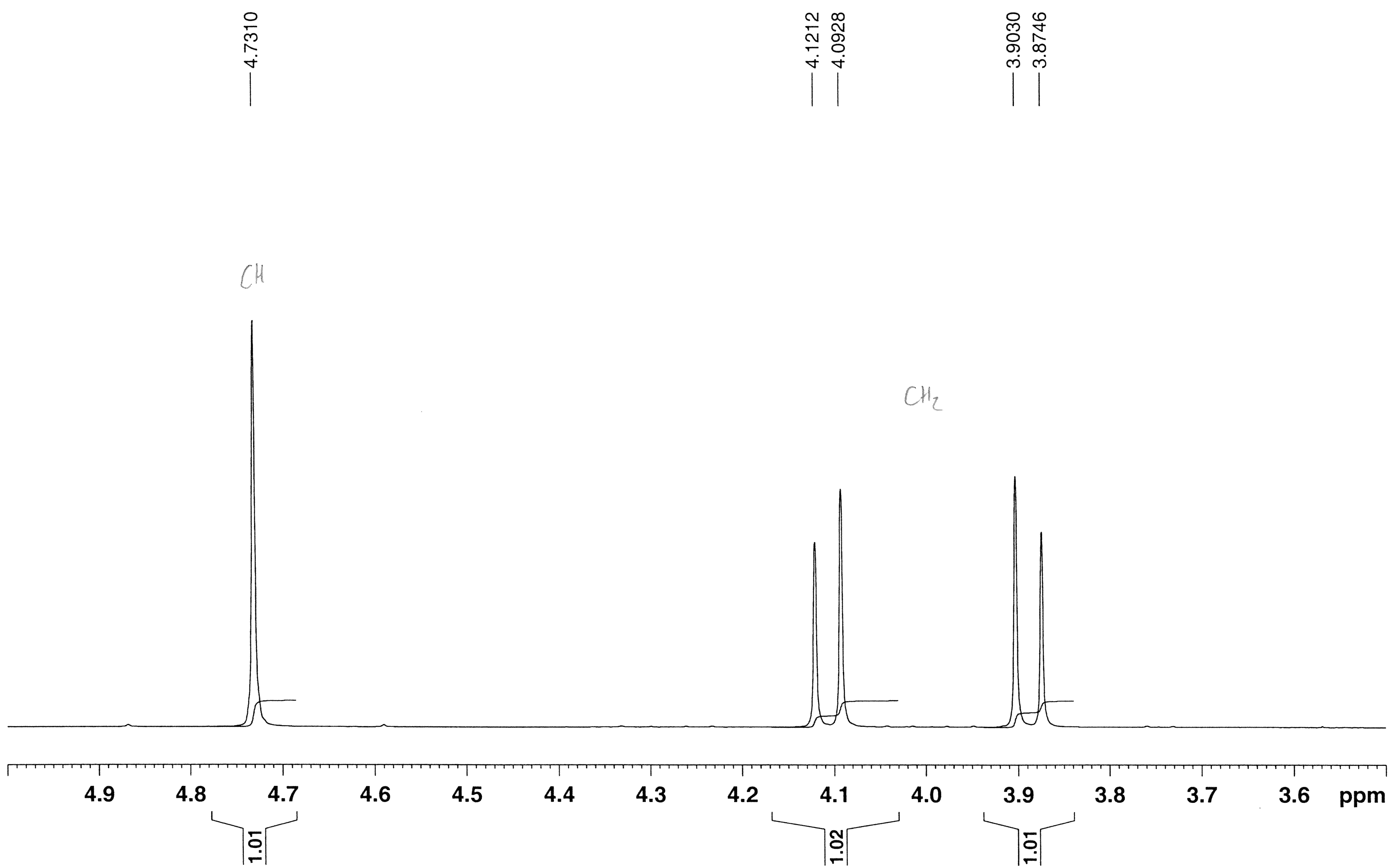

CE148S1P2 in cdcl3 (APT) 5.2.2019

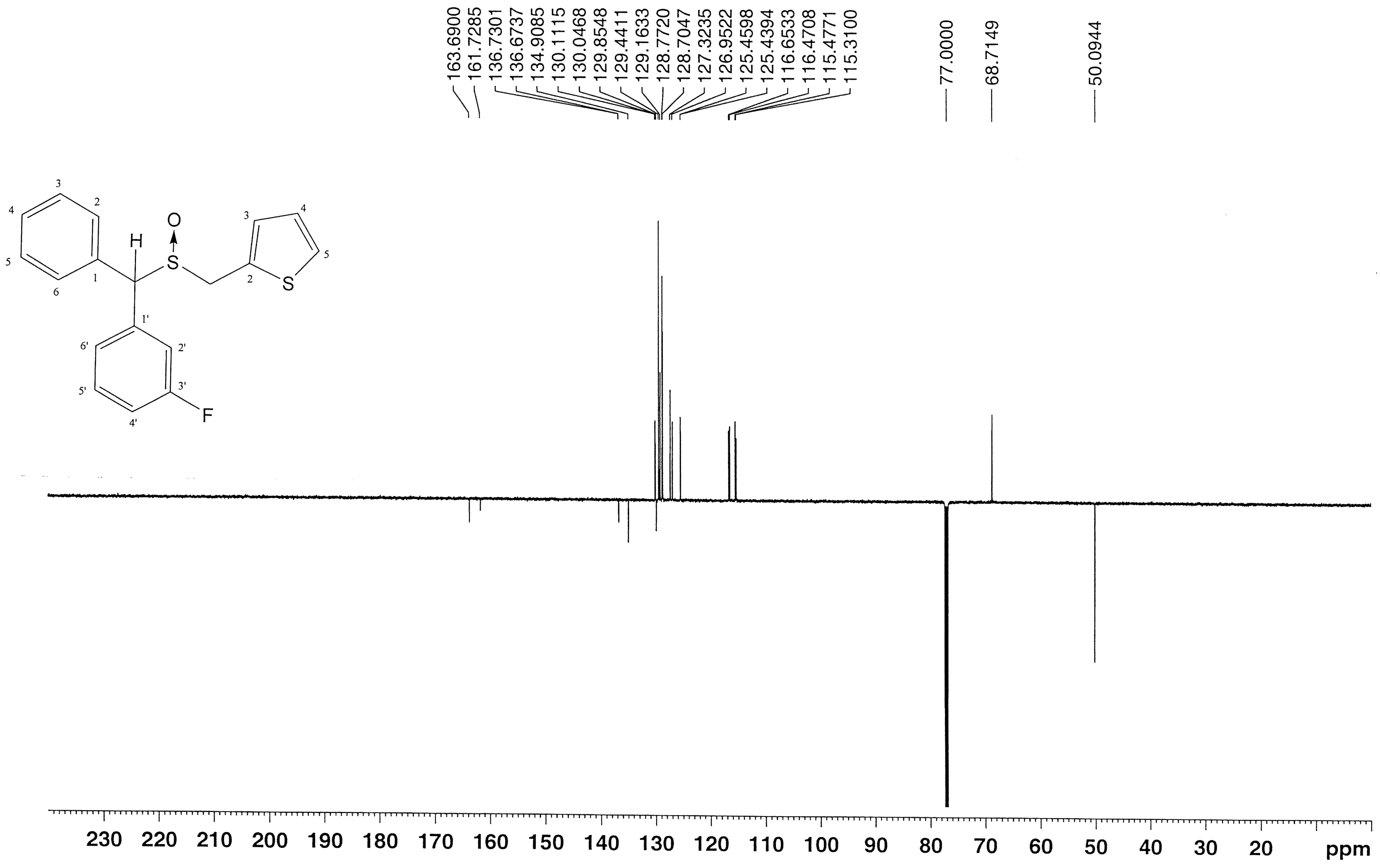

CE148S1P2 in cdcl3 (APT) 5.2.2019

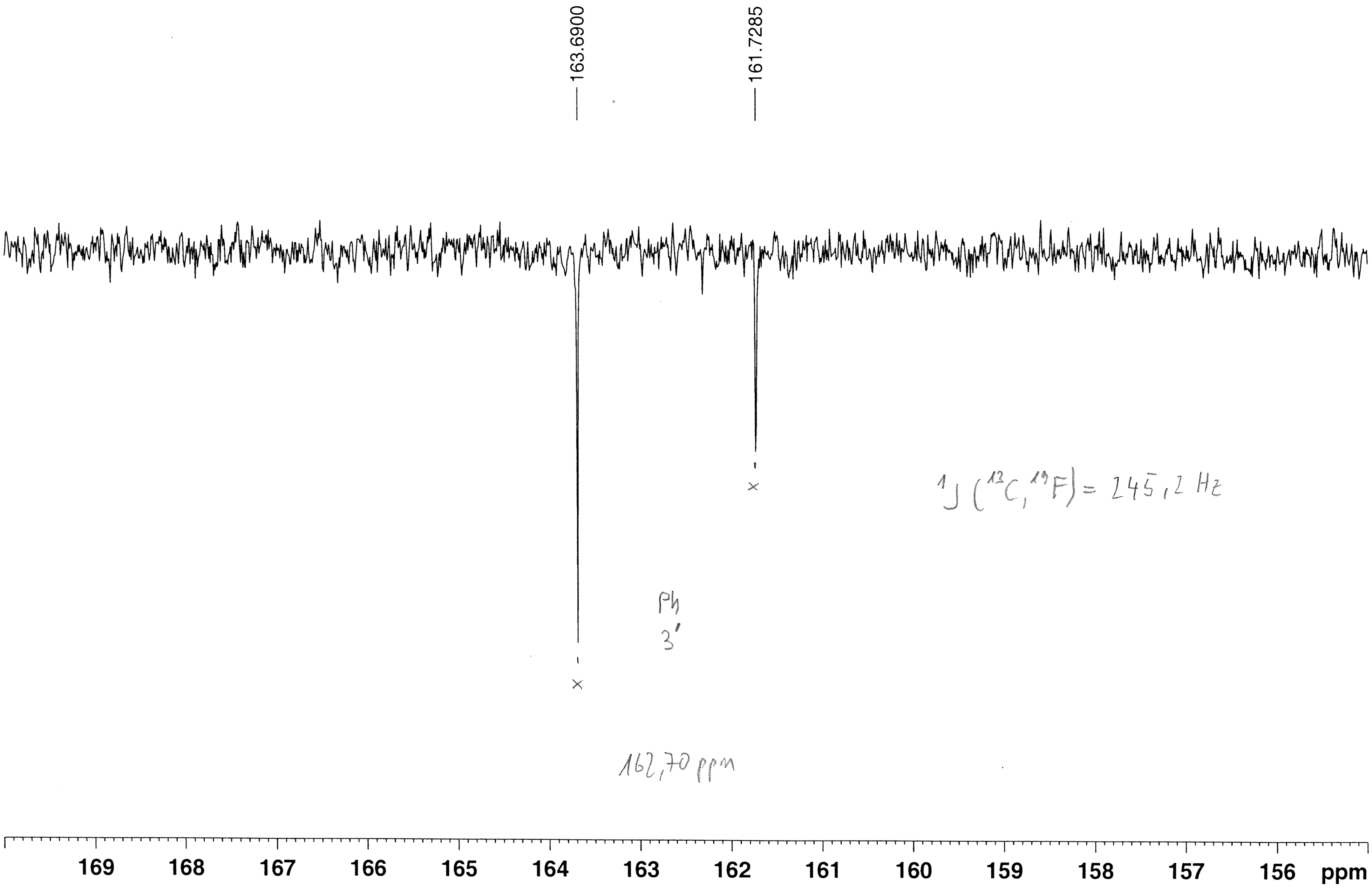

CE148S1P2 in cdcl3 (APT) 5.2.2019

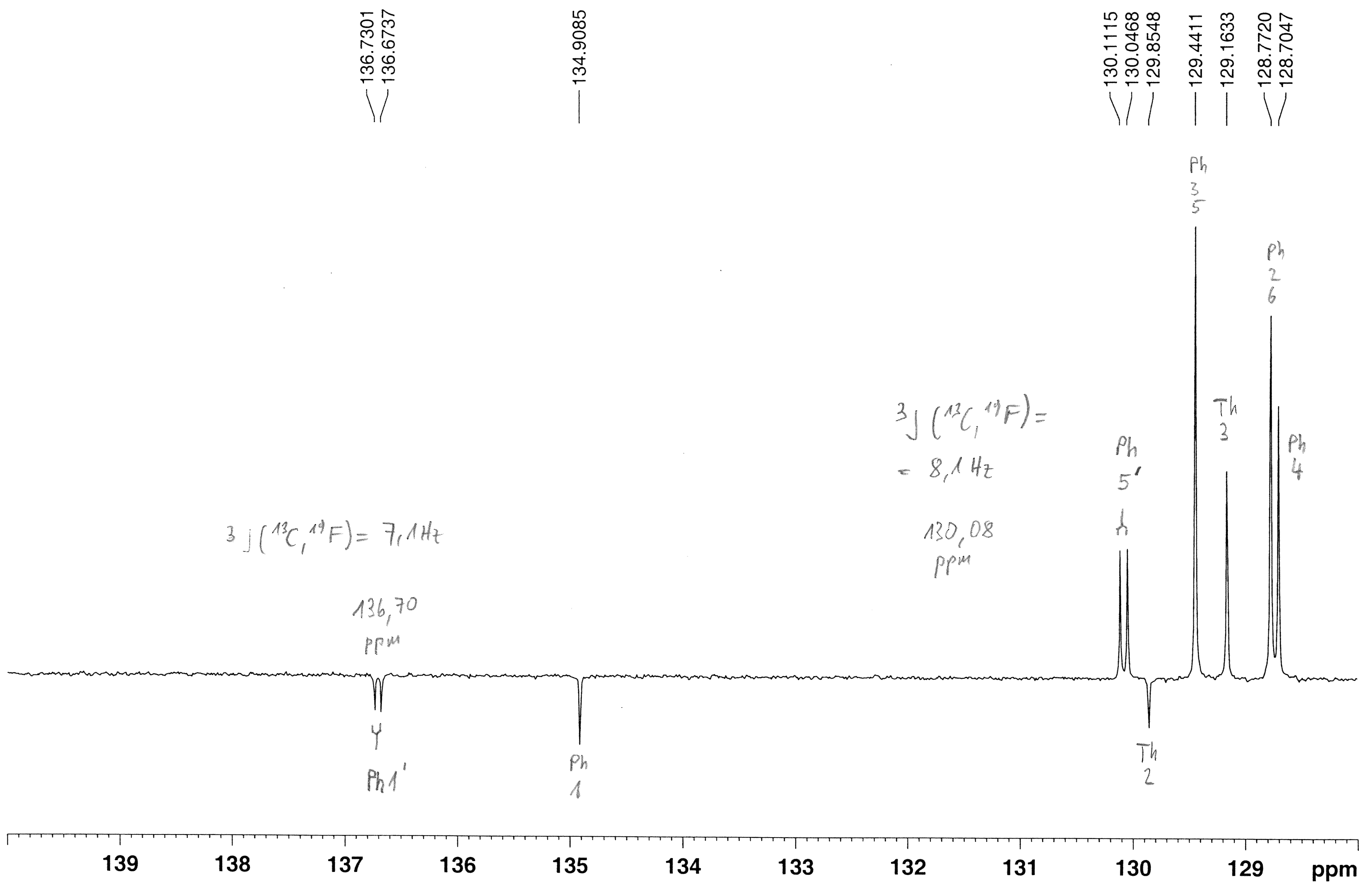

CE148S1P2 in cdcl3 (APT) 5.2.2019

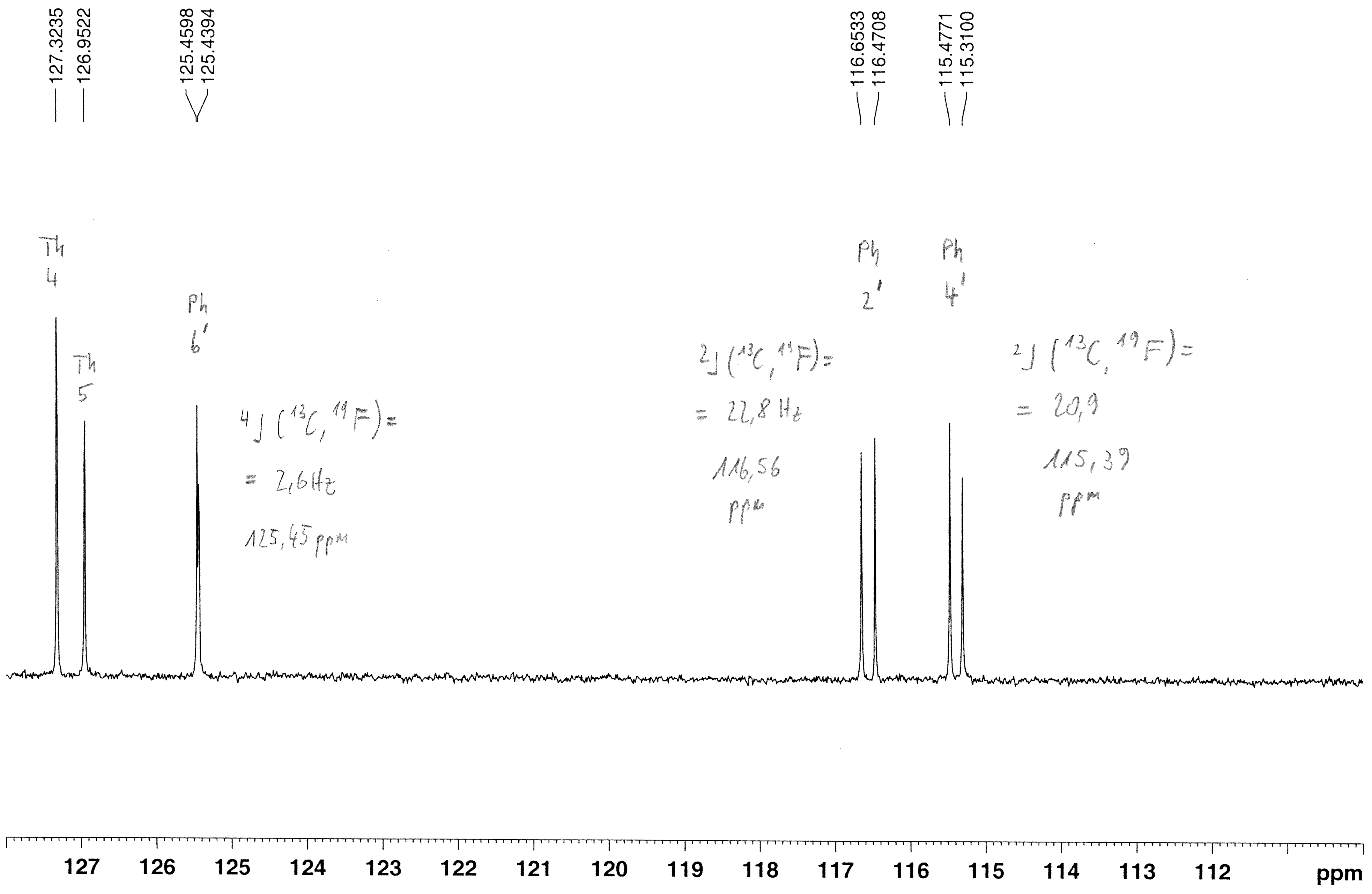

CE148S1P2 in cdcl3 (APT) 5.2.2019

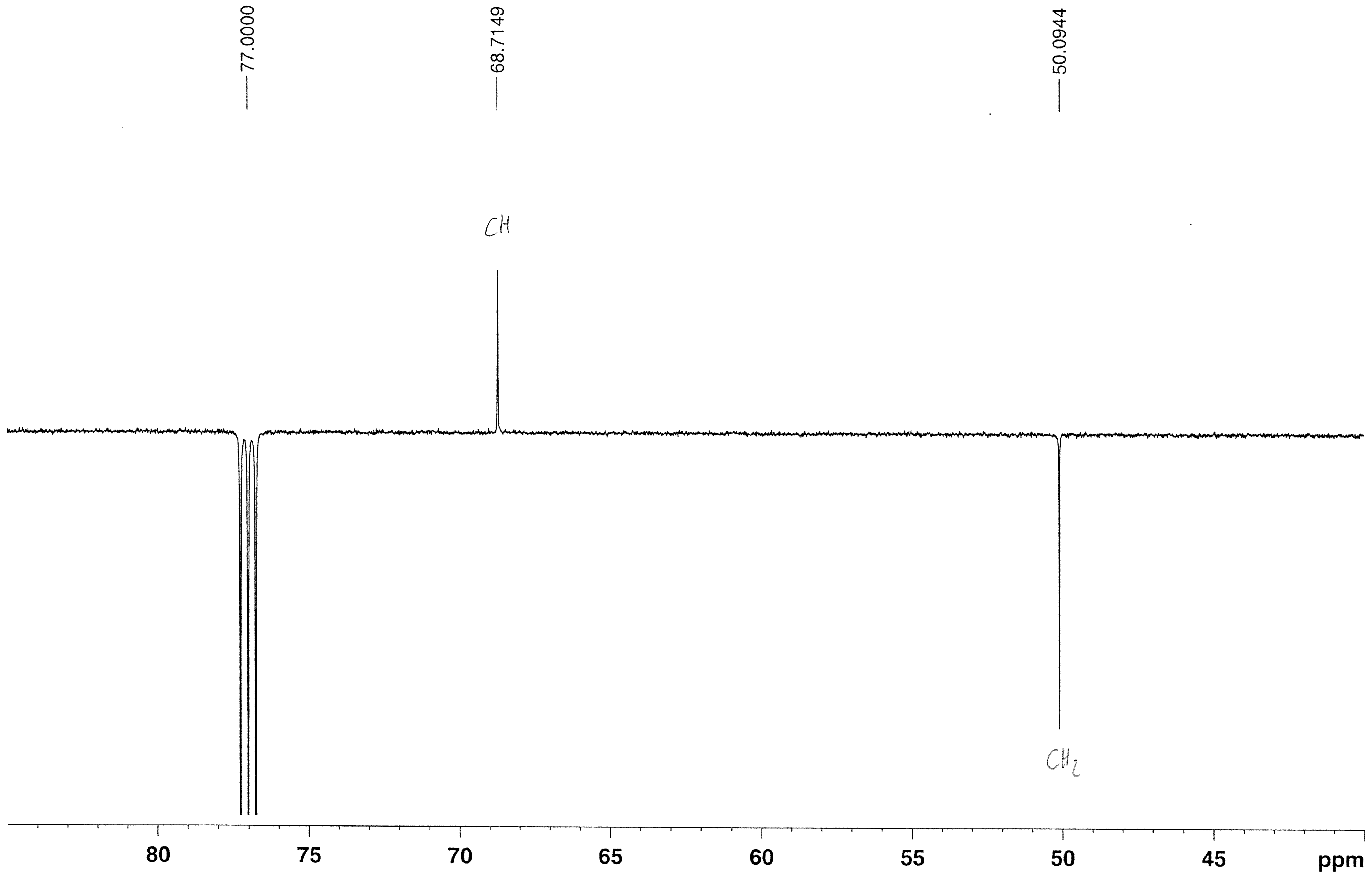

Figure S7c. NMR spectra of compound **7b**.

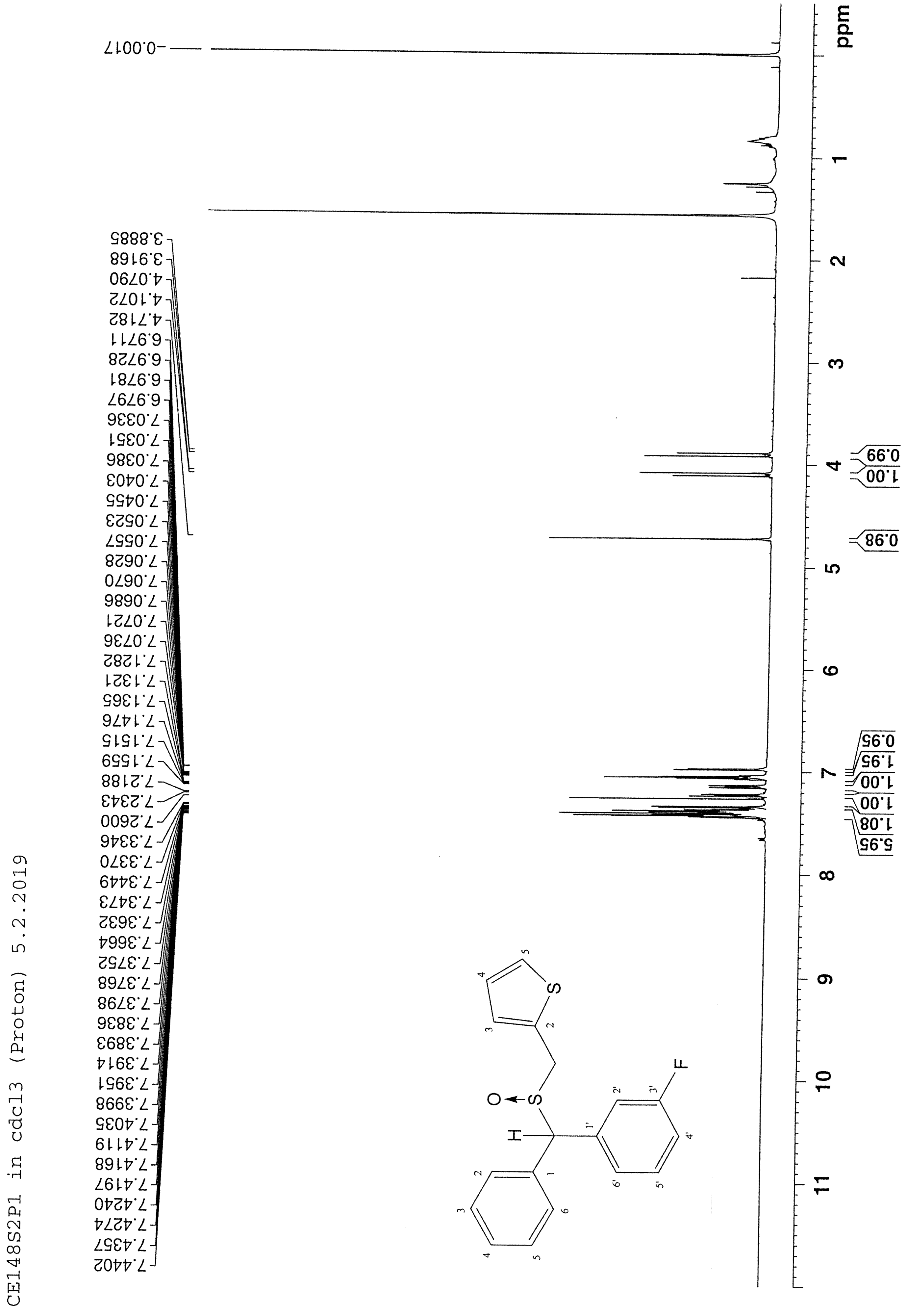

CE148S2P1 in cdcl3 (Proton) 5.2.2019

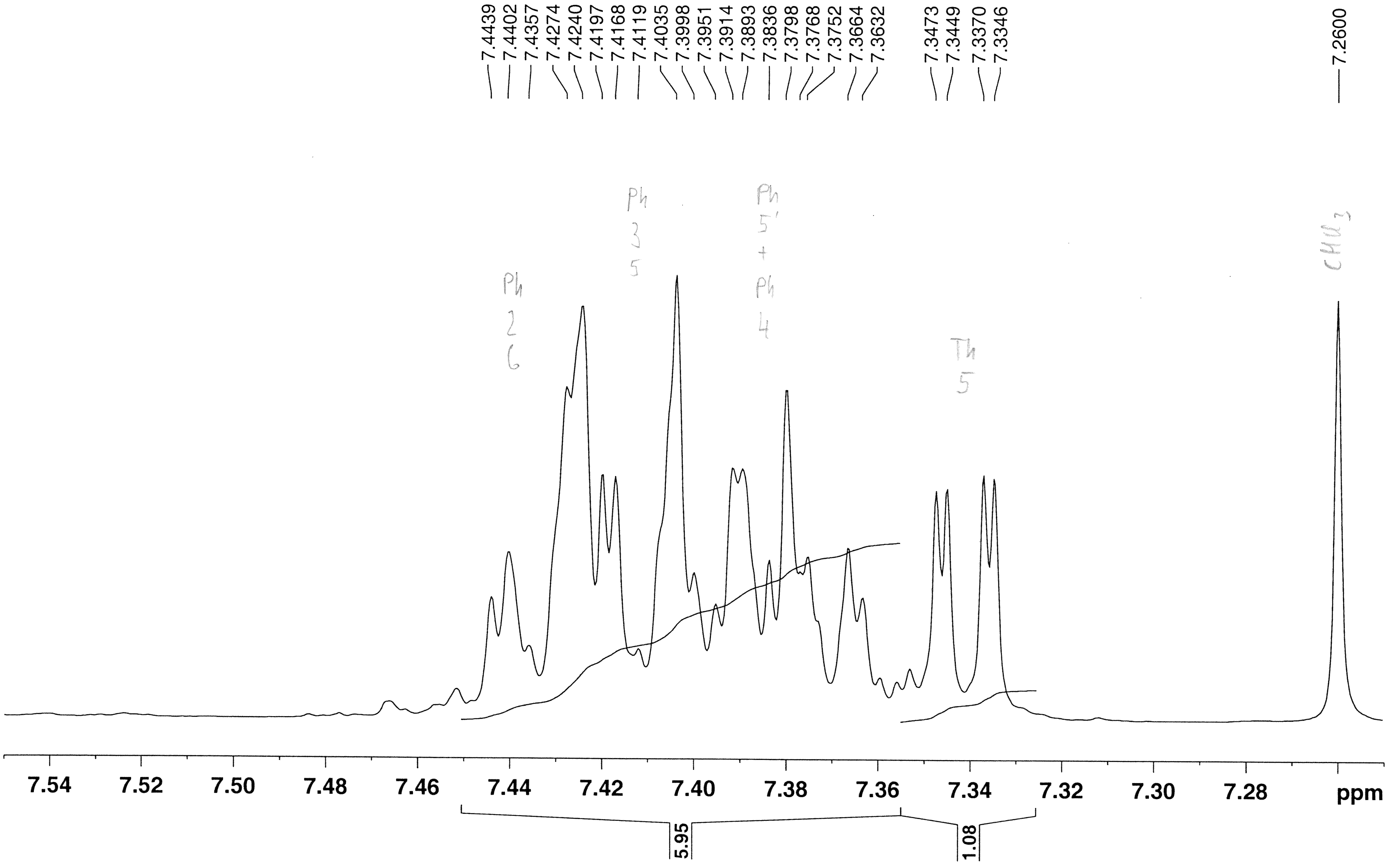

CE148S2P1 in cdcl3 (Proton) 5.2.2019

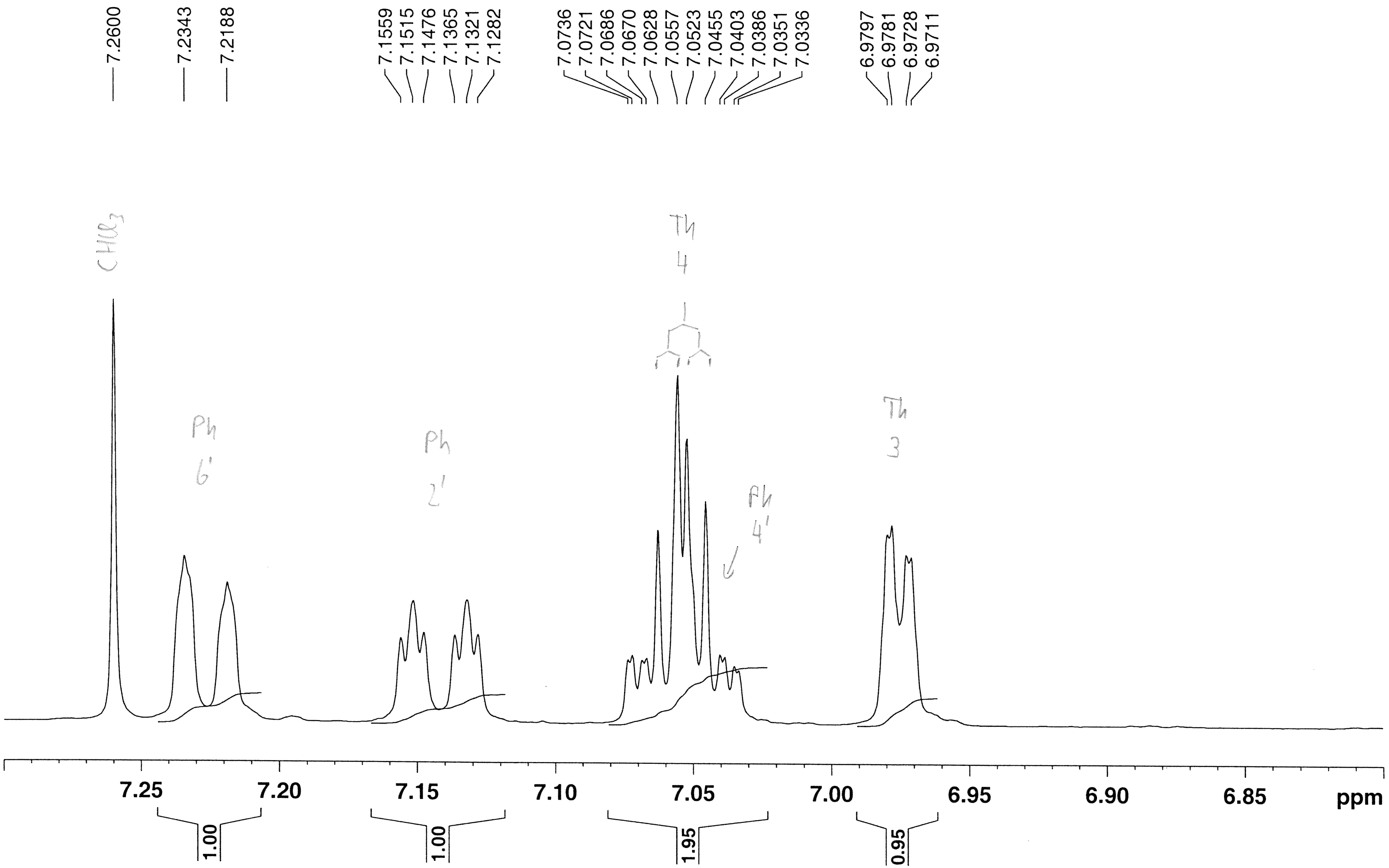

CE148S2P1 in cdcl3 (Proton) 5.2.2019

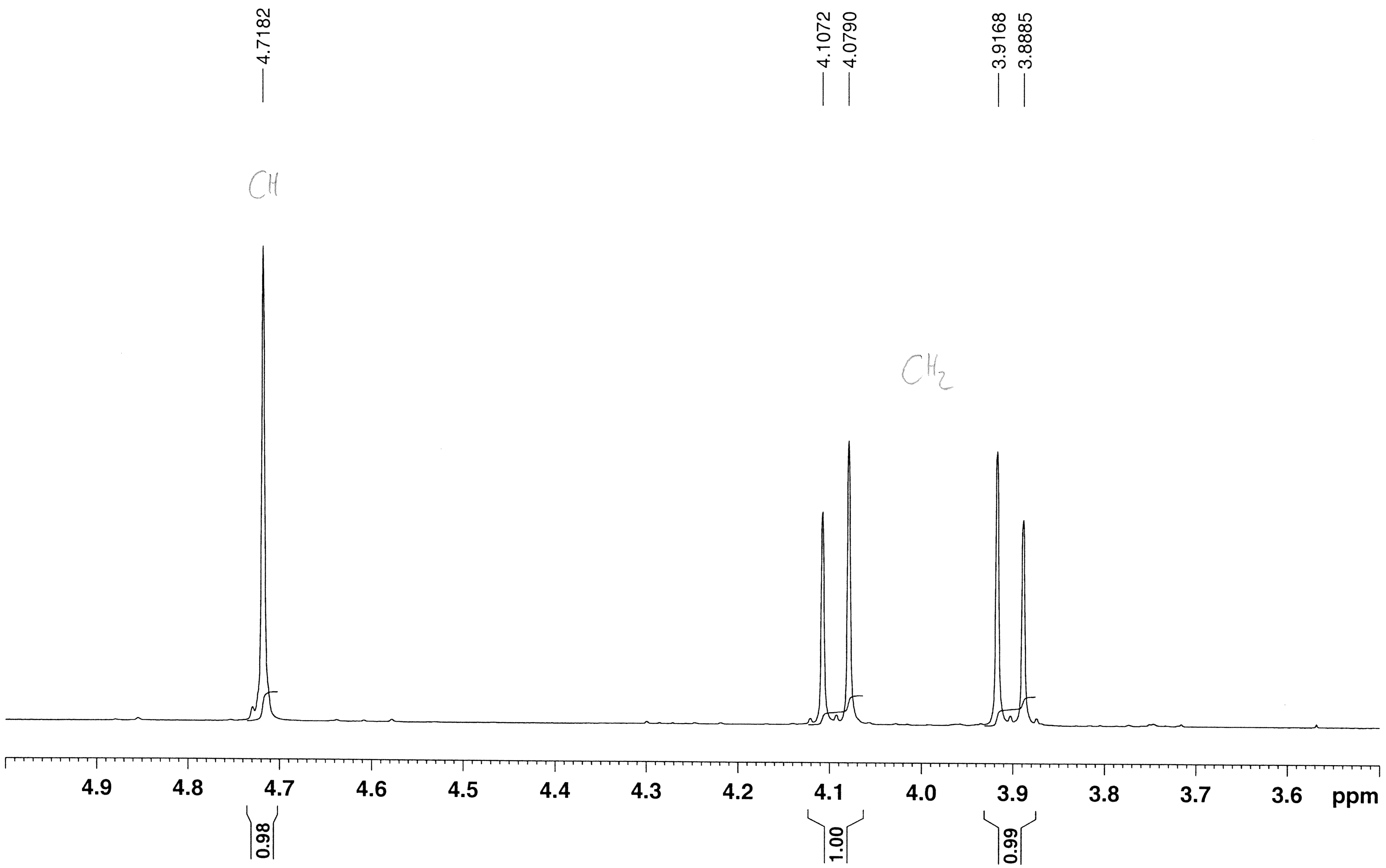

CE148S2P1 in cdcl3 (APT) 5.2.2019

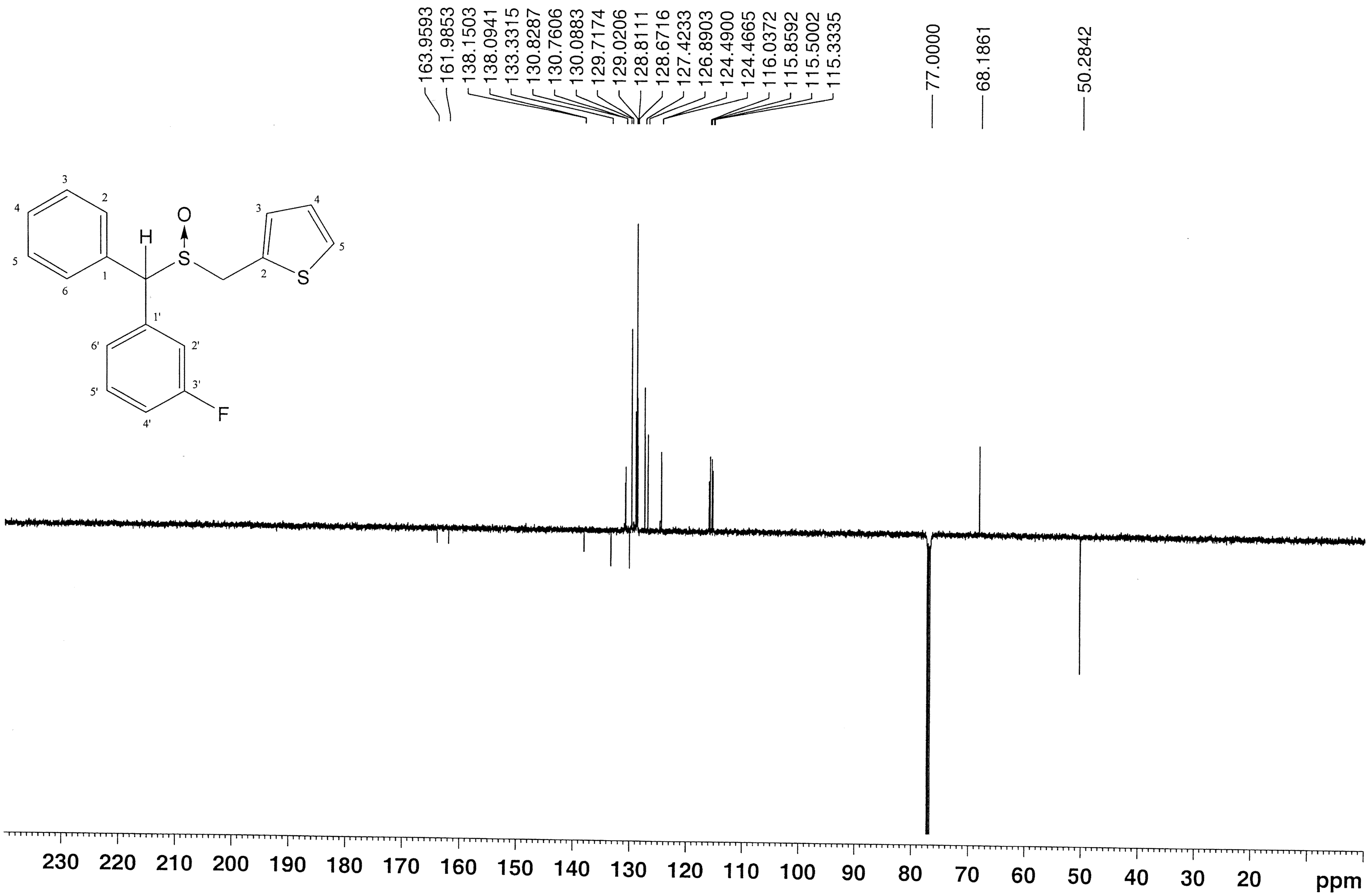

CE148S2P1 in cdcl3 (APT) 5.2.2019

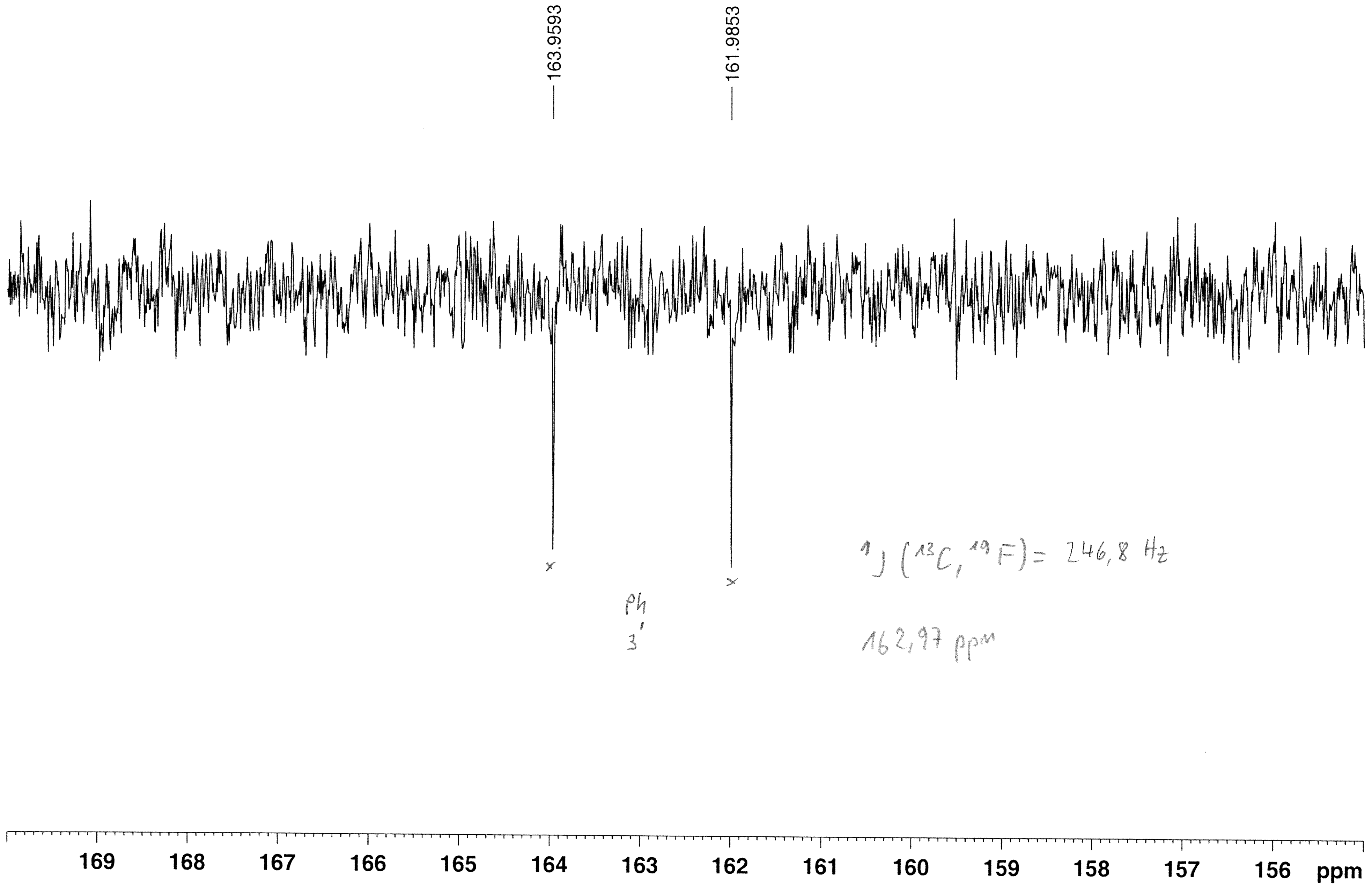

CE148S2P1 in cdcl3 (APT) 5.2.2019

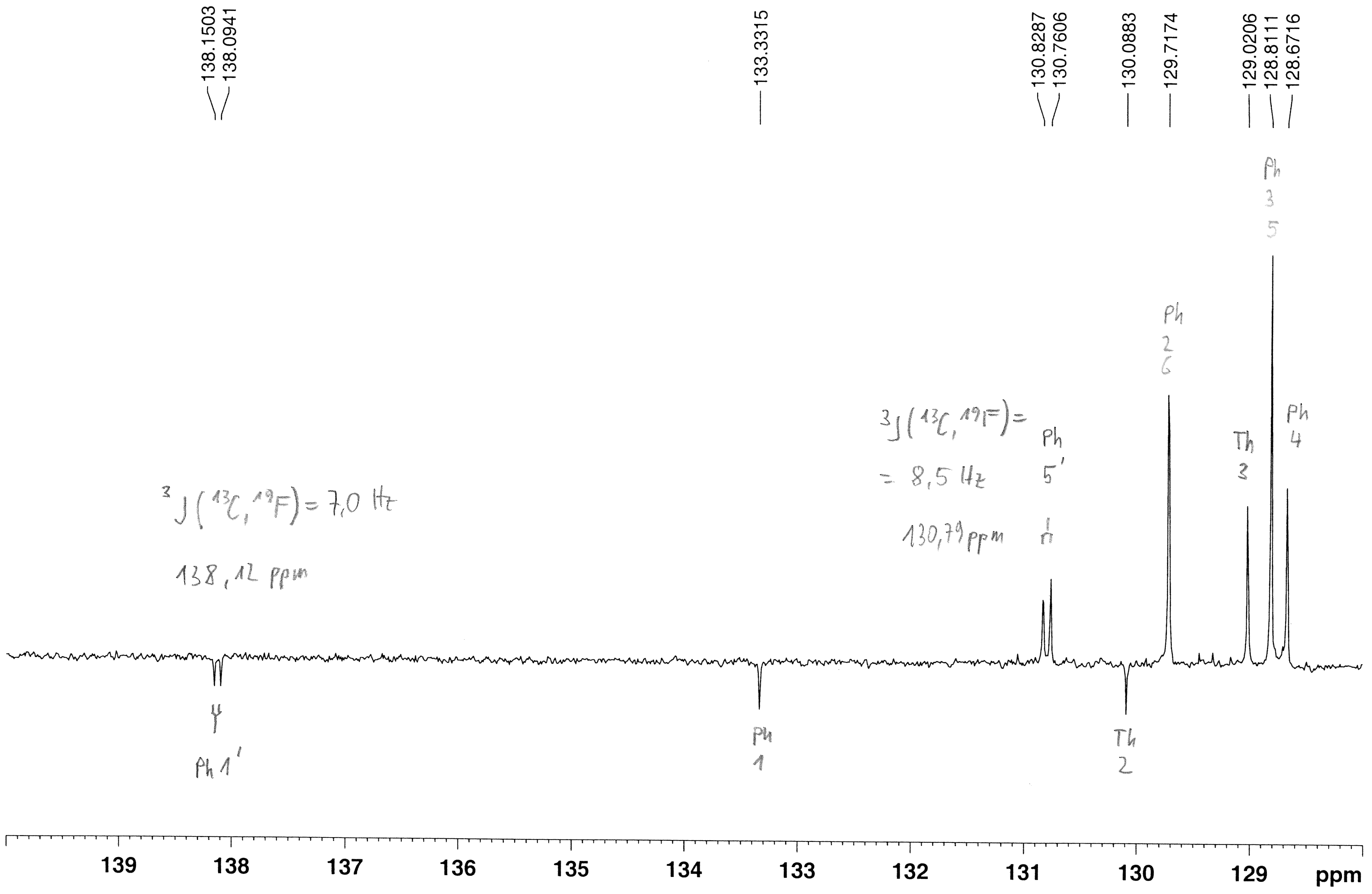

CE148S2P1 in cdcl3 (APT) 5.2.2019

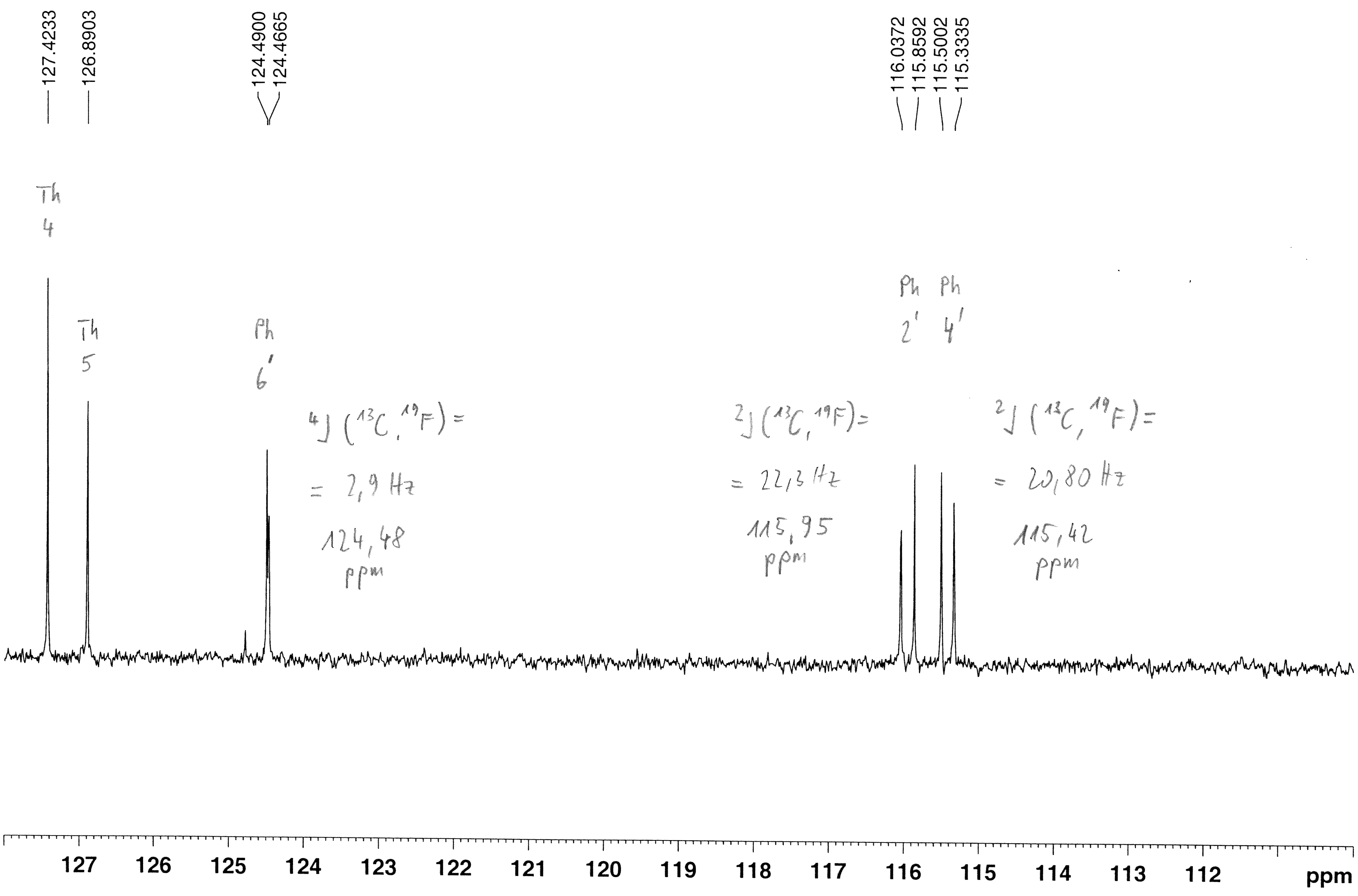

CE148S2P1 in cdcl3 (APT) 5.2.2019

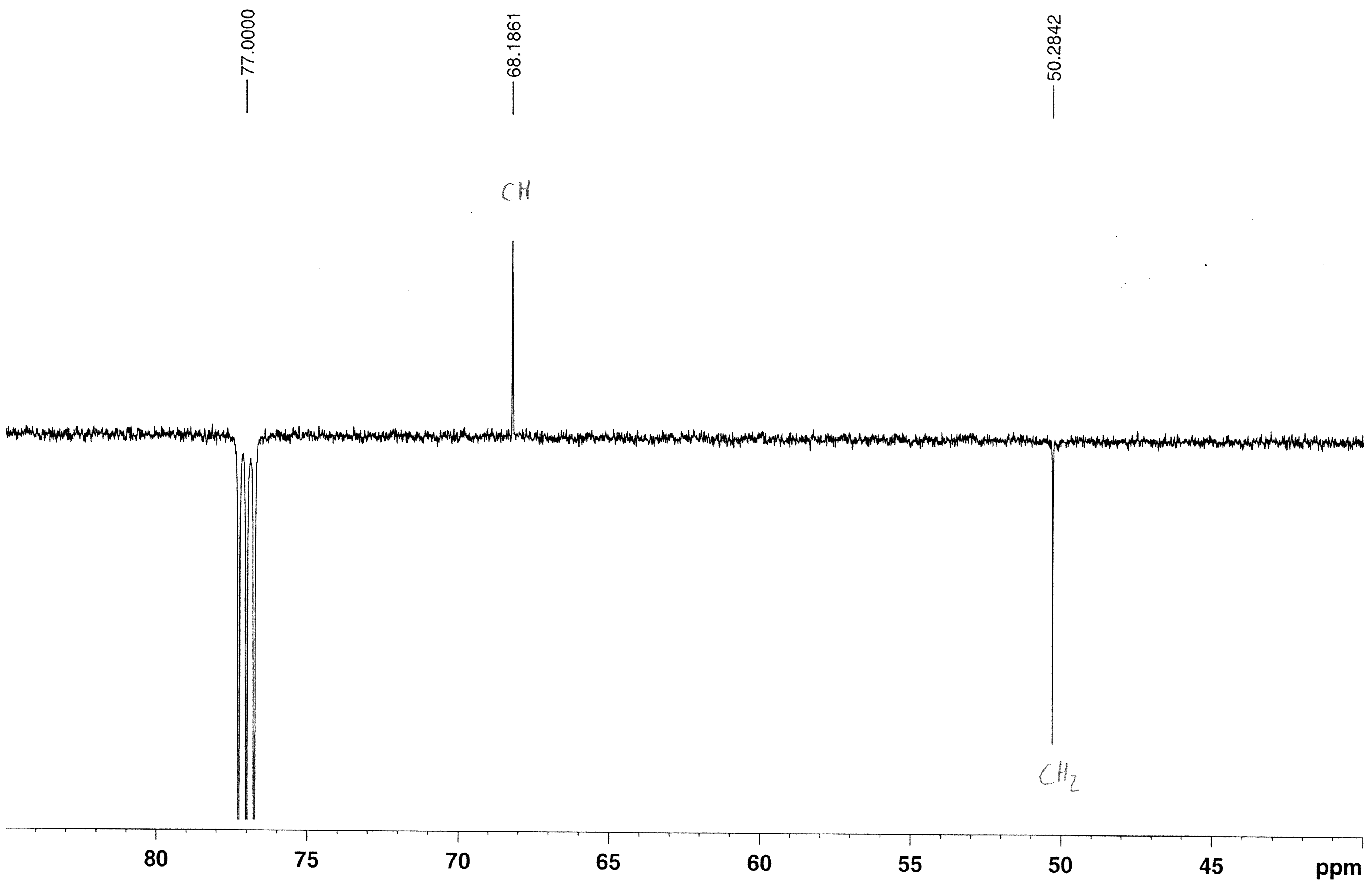

CE148S2P1 in cdcl3 (COSY) 5.2.2019

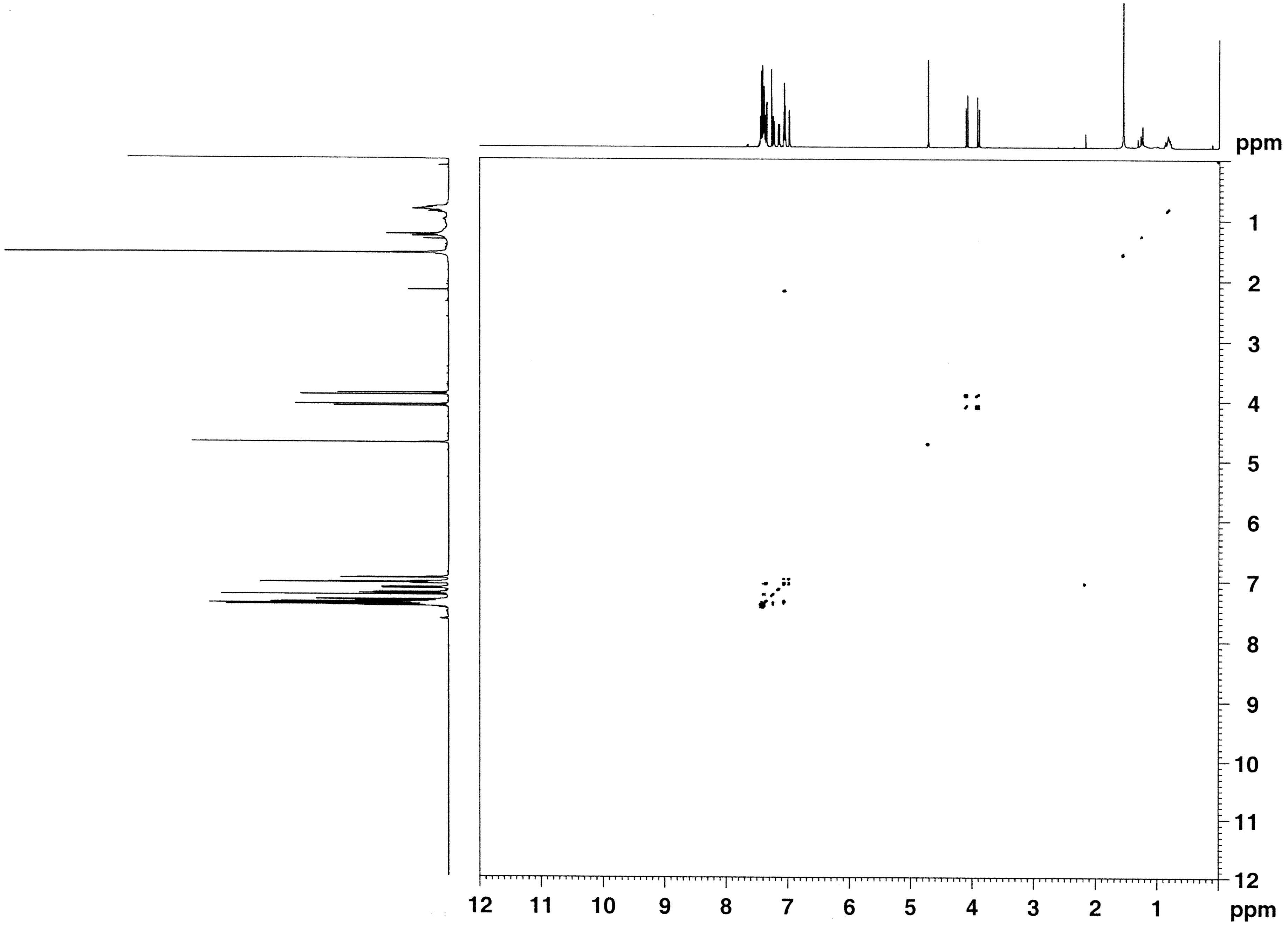

CE148S2P1 in cdcl3 (COSY) 5.2.2019

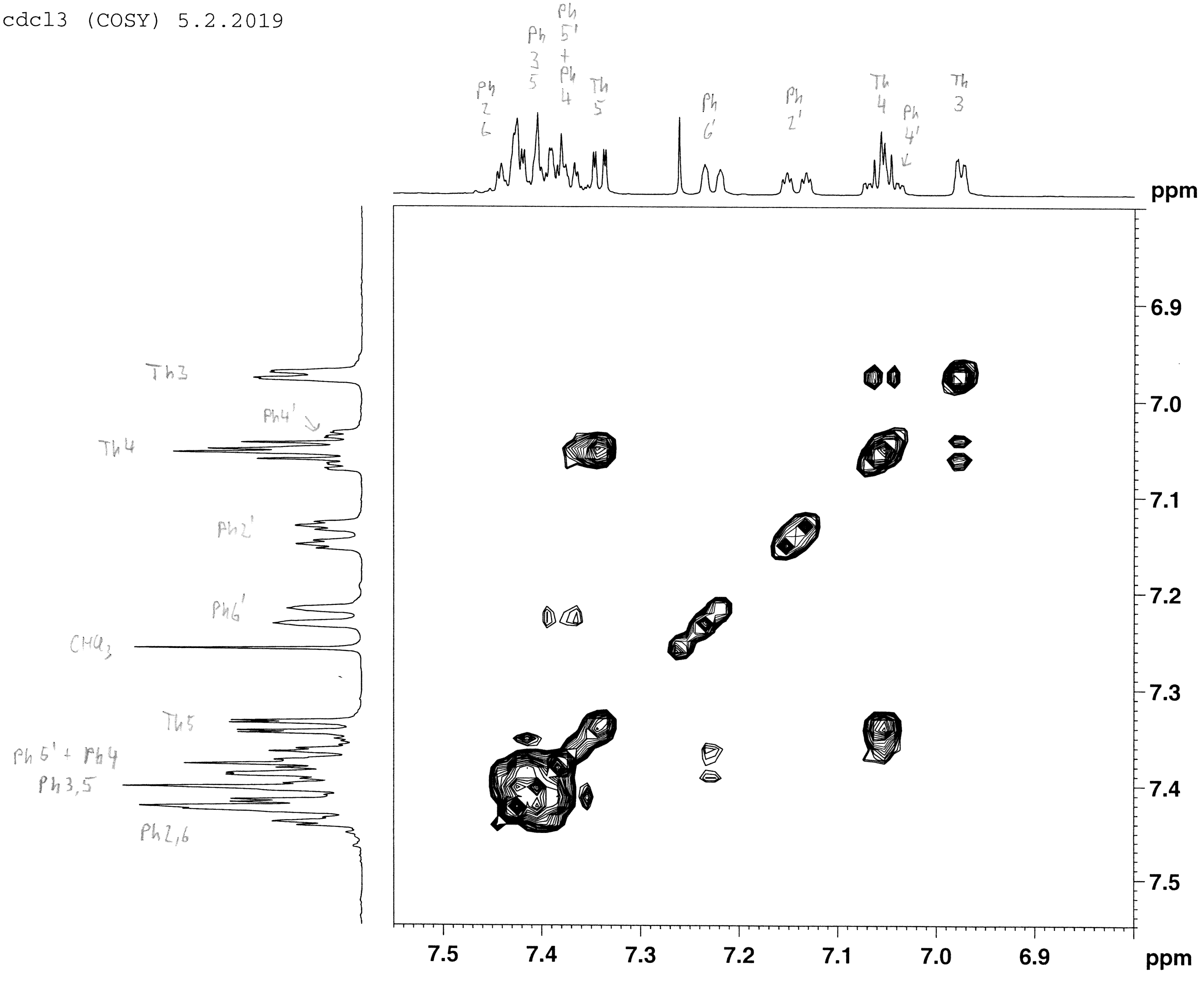

CE148S2P1 in cdcl3 (COSY) 5.2.2019

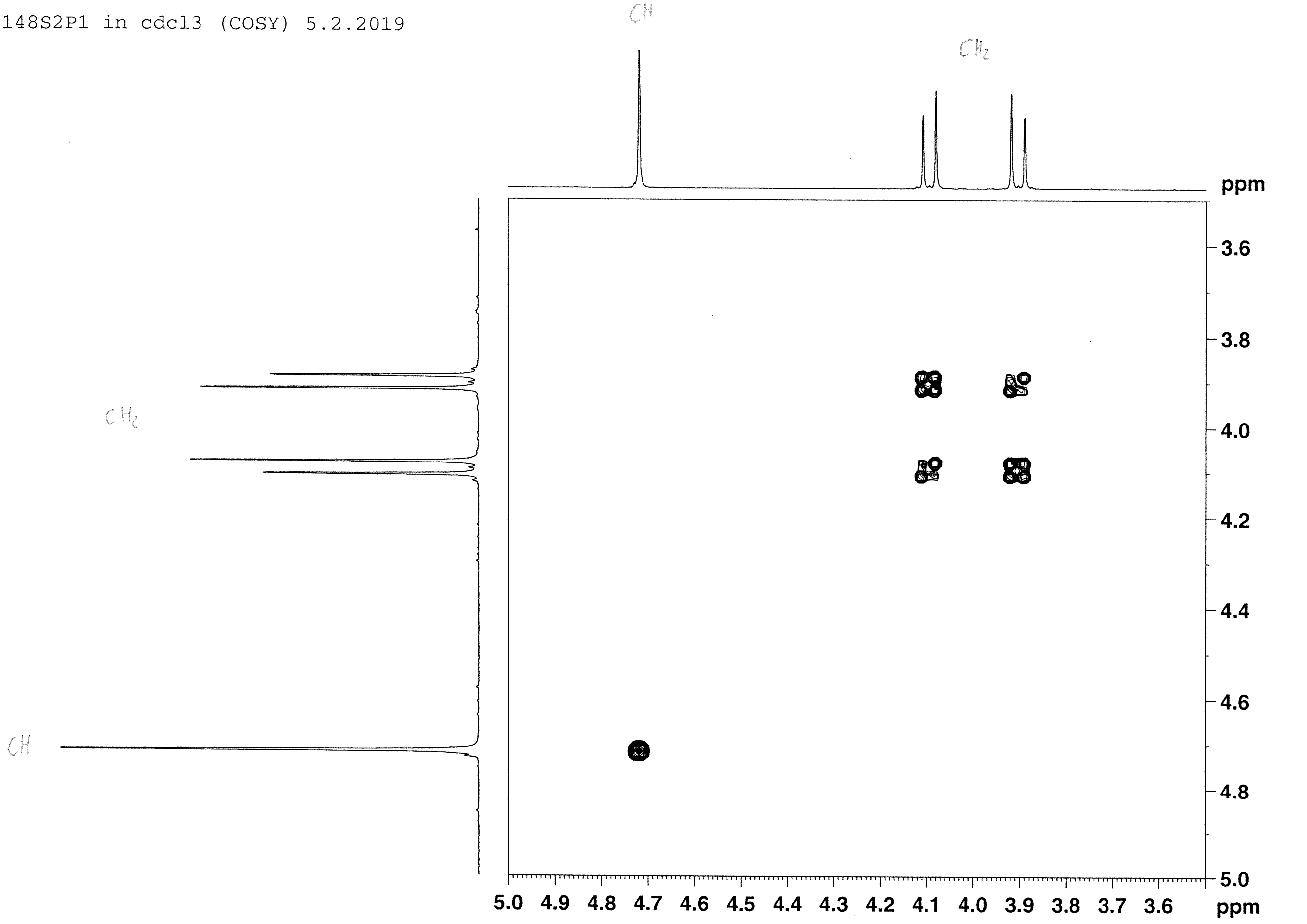

CE148S2P1 in cdcl3 (HSQC) 5.2.2019

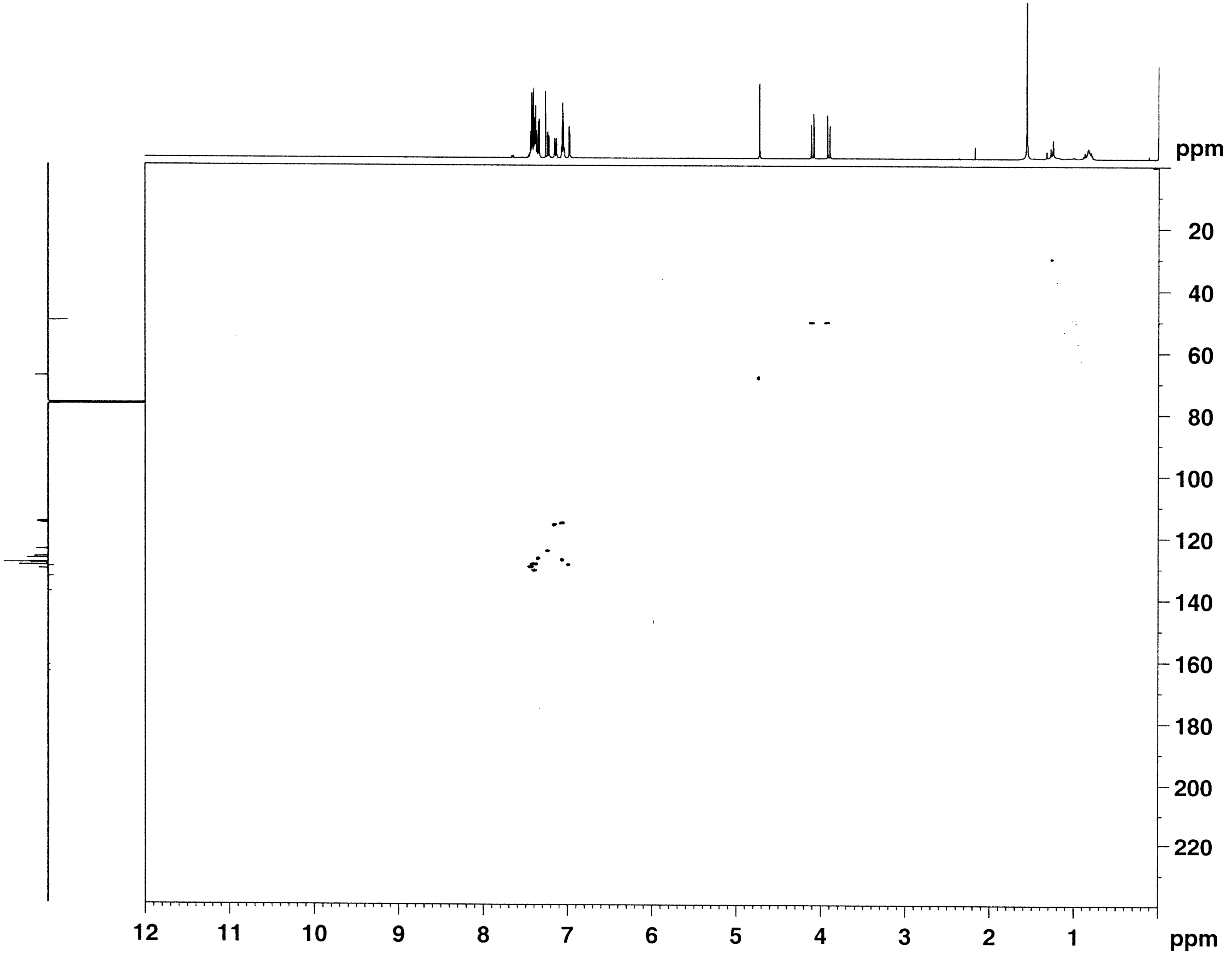

CE148S2P1 in cdcl3 (HSQC) 5.2.2019

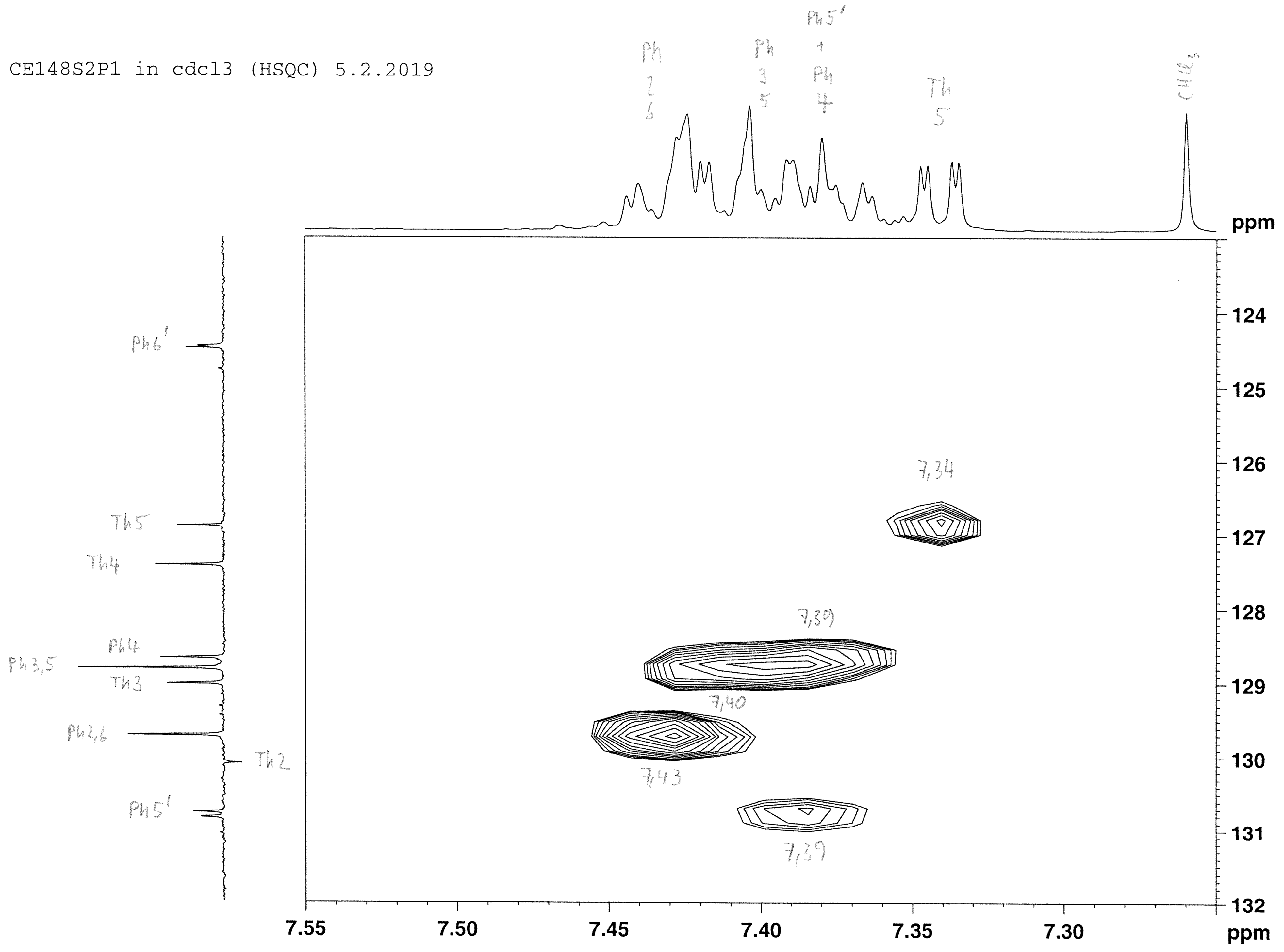

CE148S2P1 in cdcl3 (HSQC) 5.2.2019

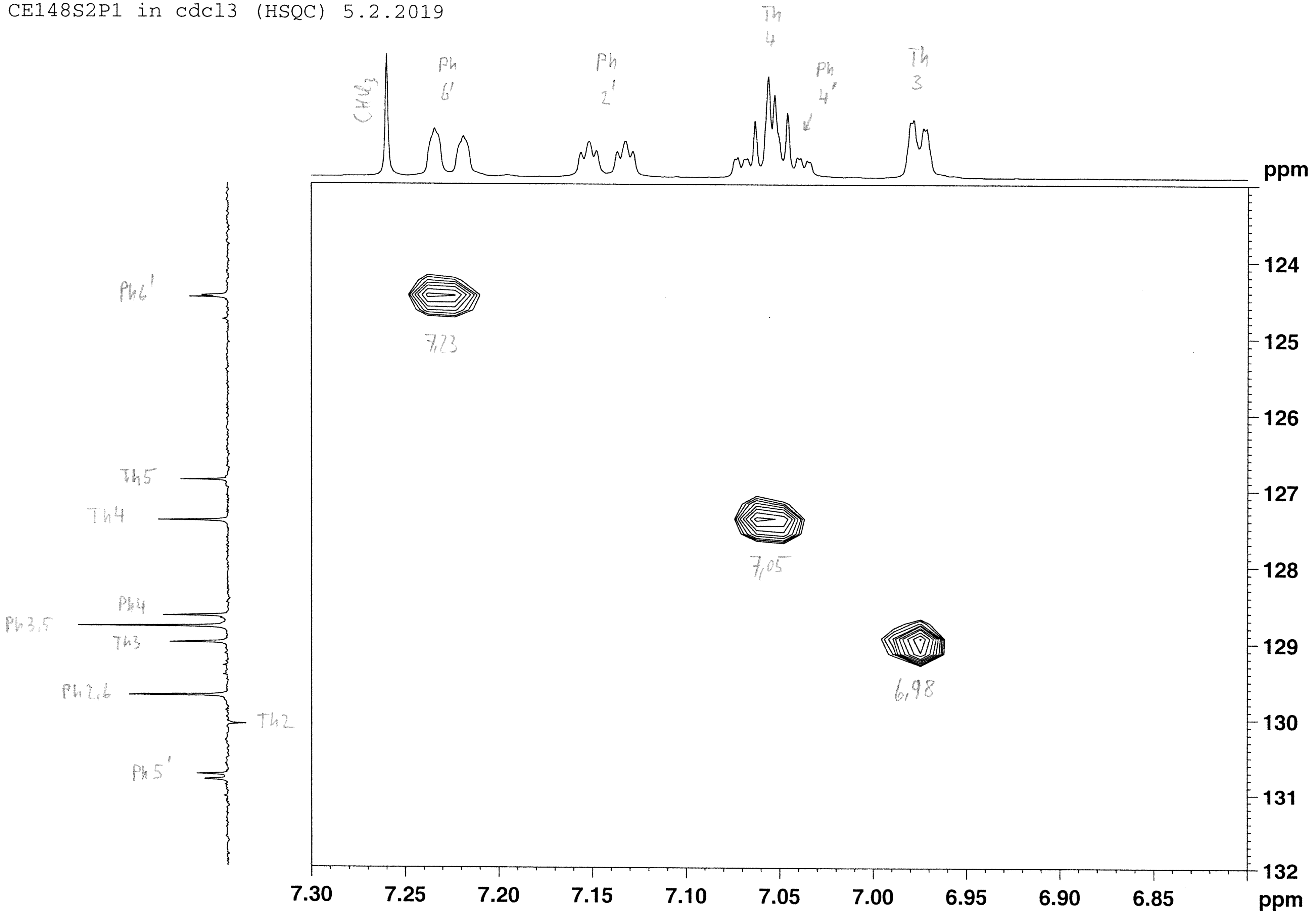

CE148S2P1 in cdcl3 (HSQC) 5.2.2019

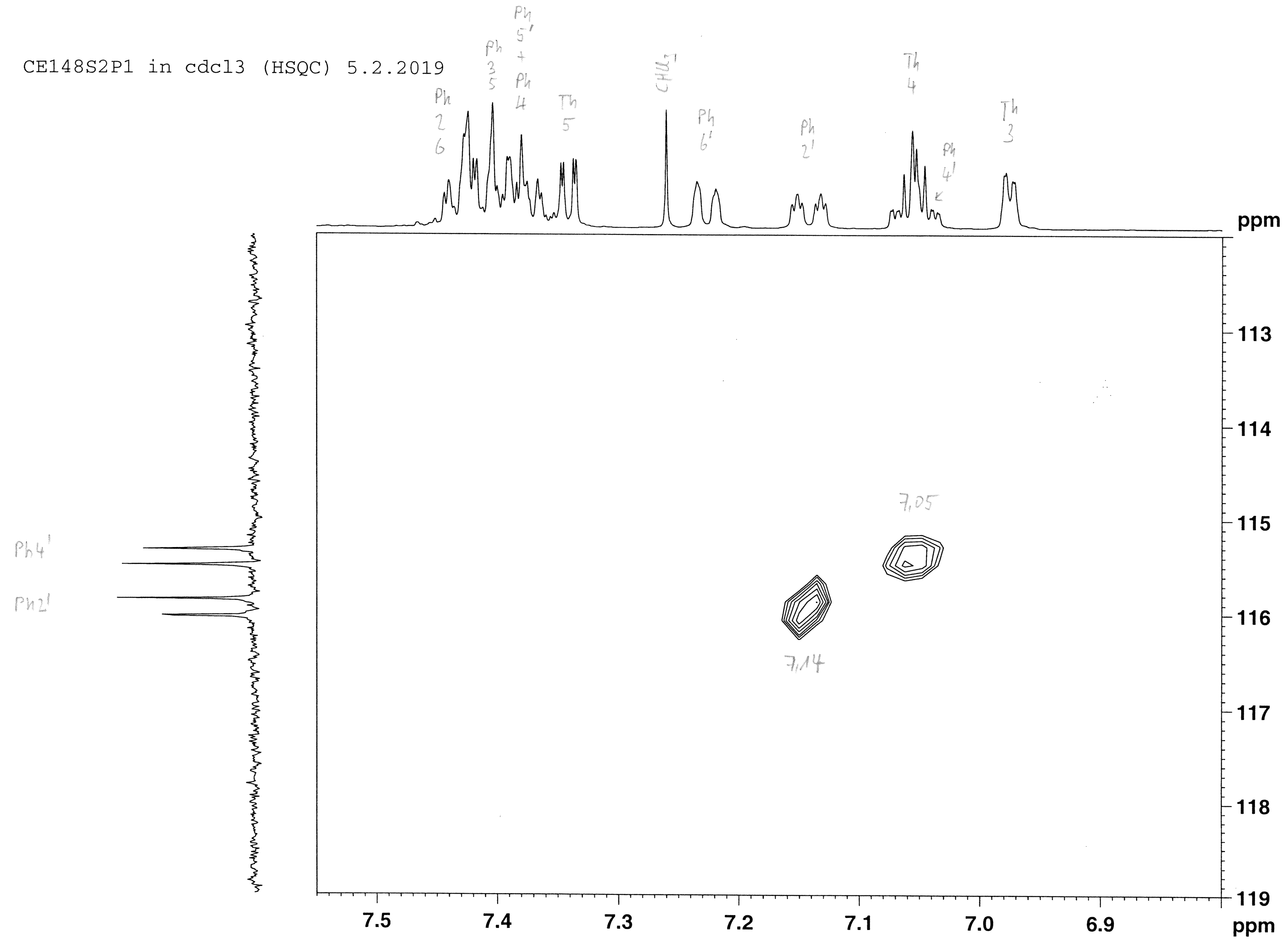

CE148S2P1 in cdcl3 (HSQC) 5.2.2019

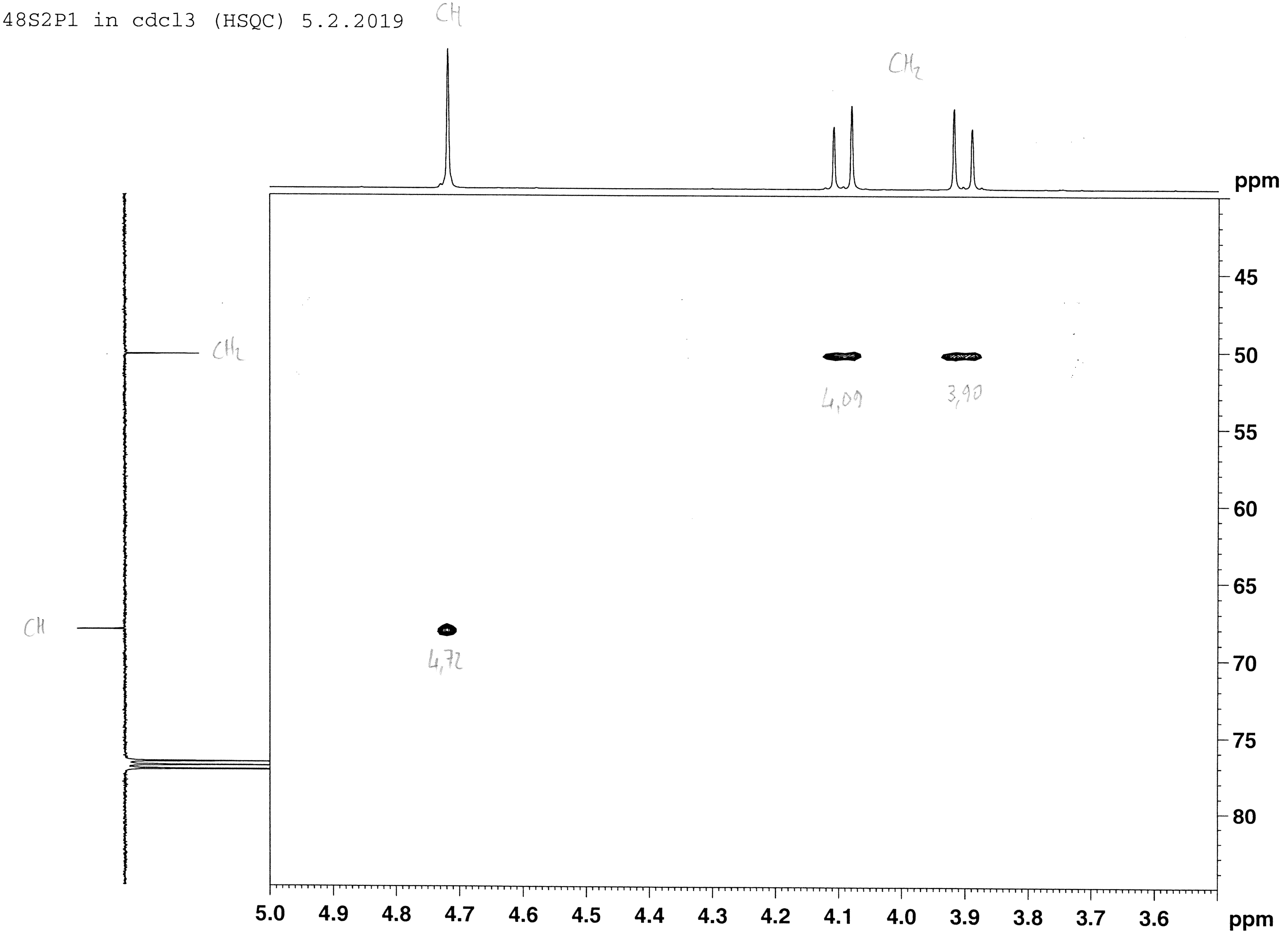

CE148S2P1 in cdcl3 (HMBC) 5.2.2019

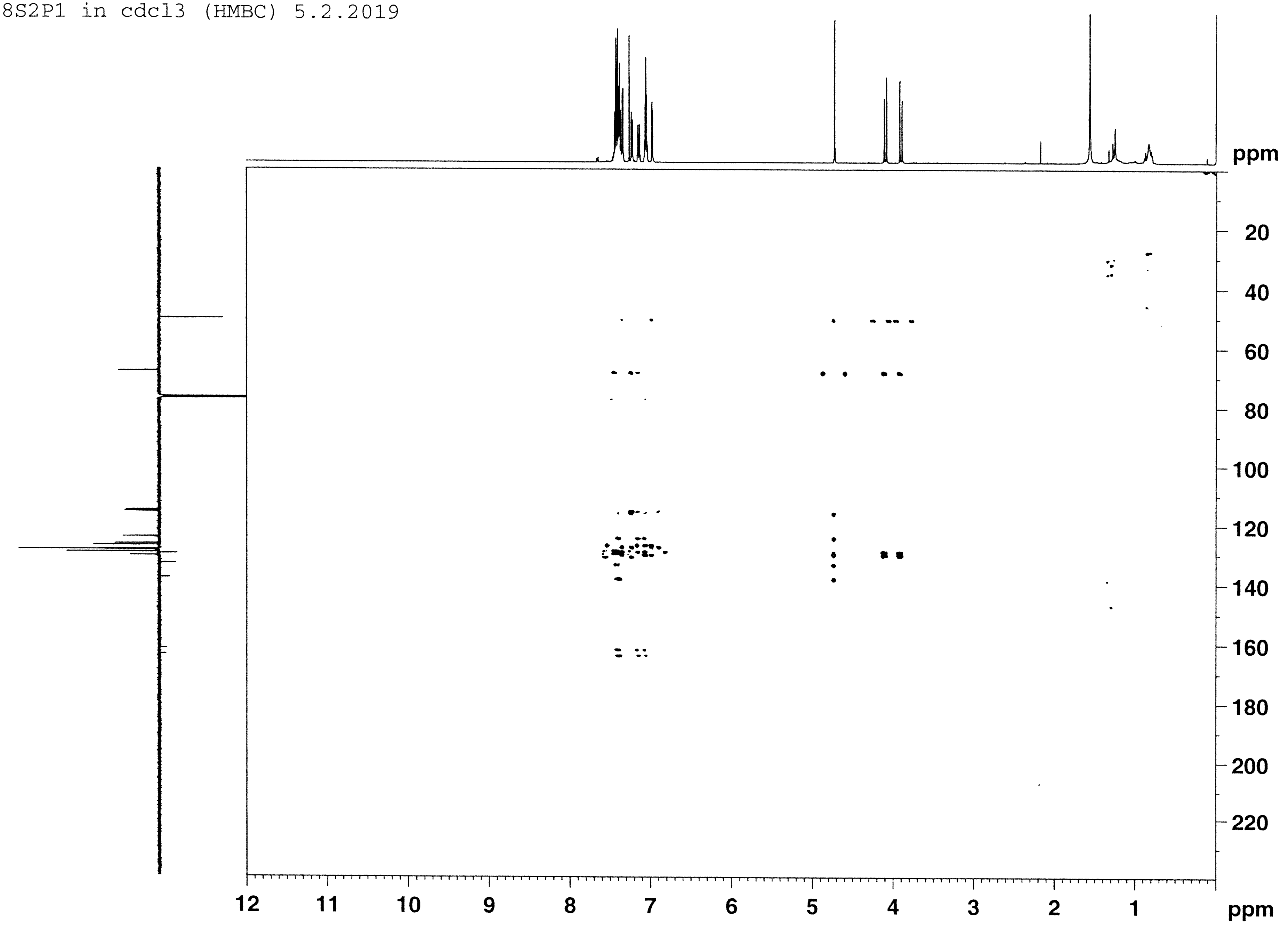

CE148S2P1 in cdcl3 (HMBC) 5.2.2019

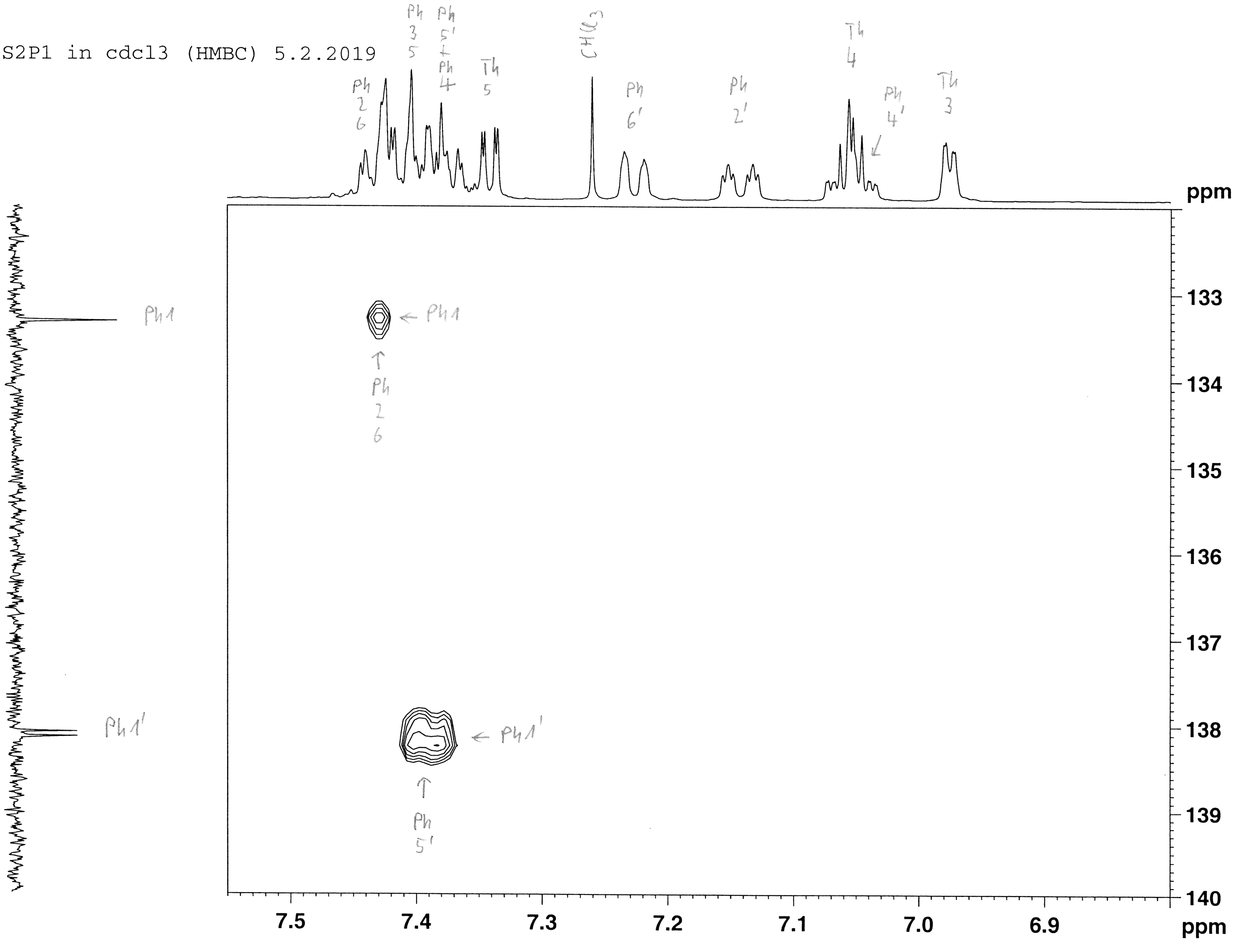

CE148S2P1 in cdcl3 (HMBC) 5.2.2019

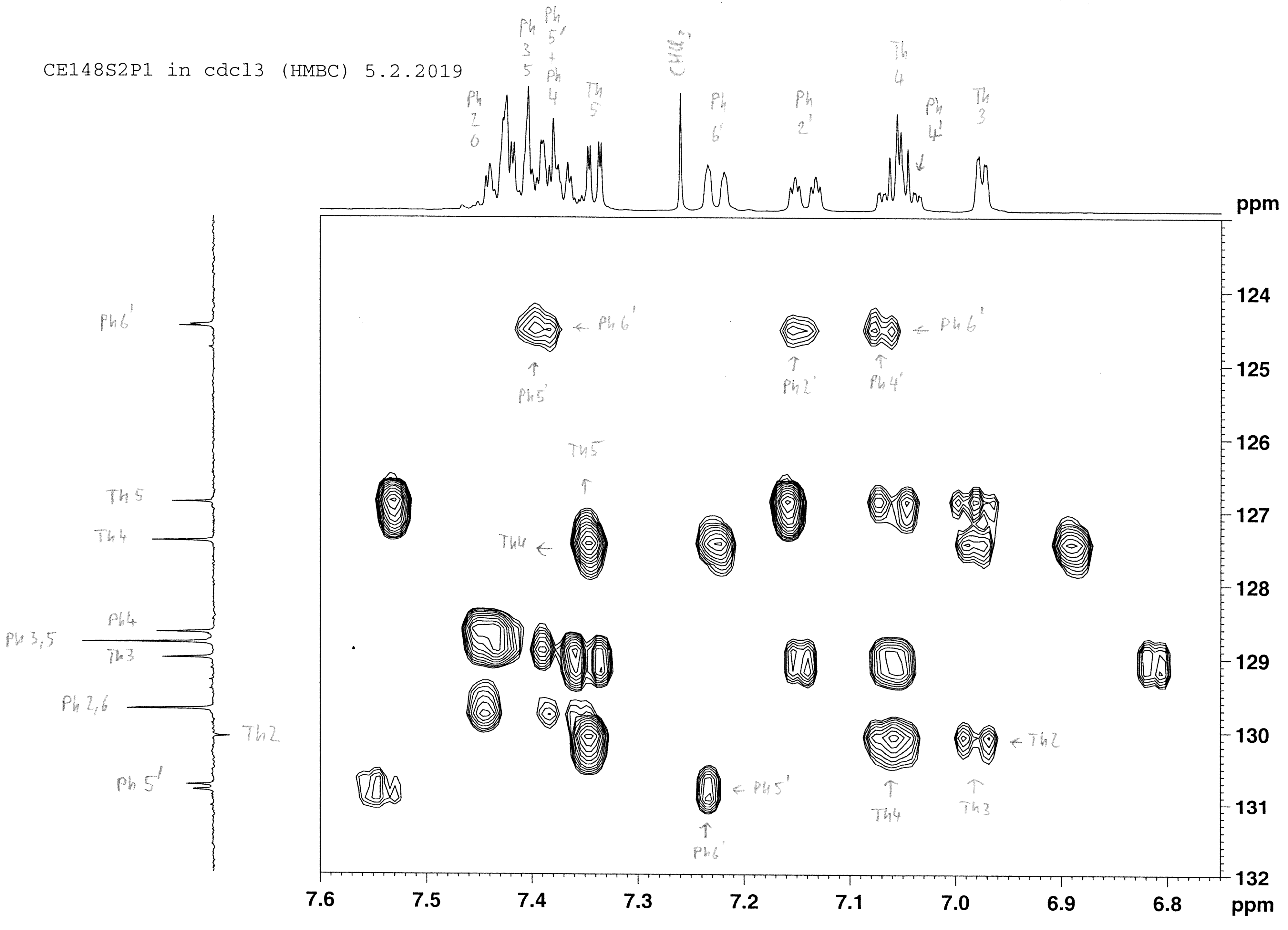

CE148S2P1 in cdcl3 (HMBC) 5.2.2019

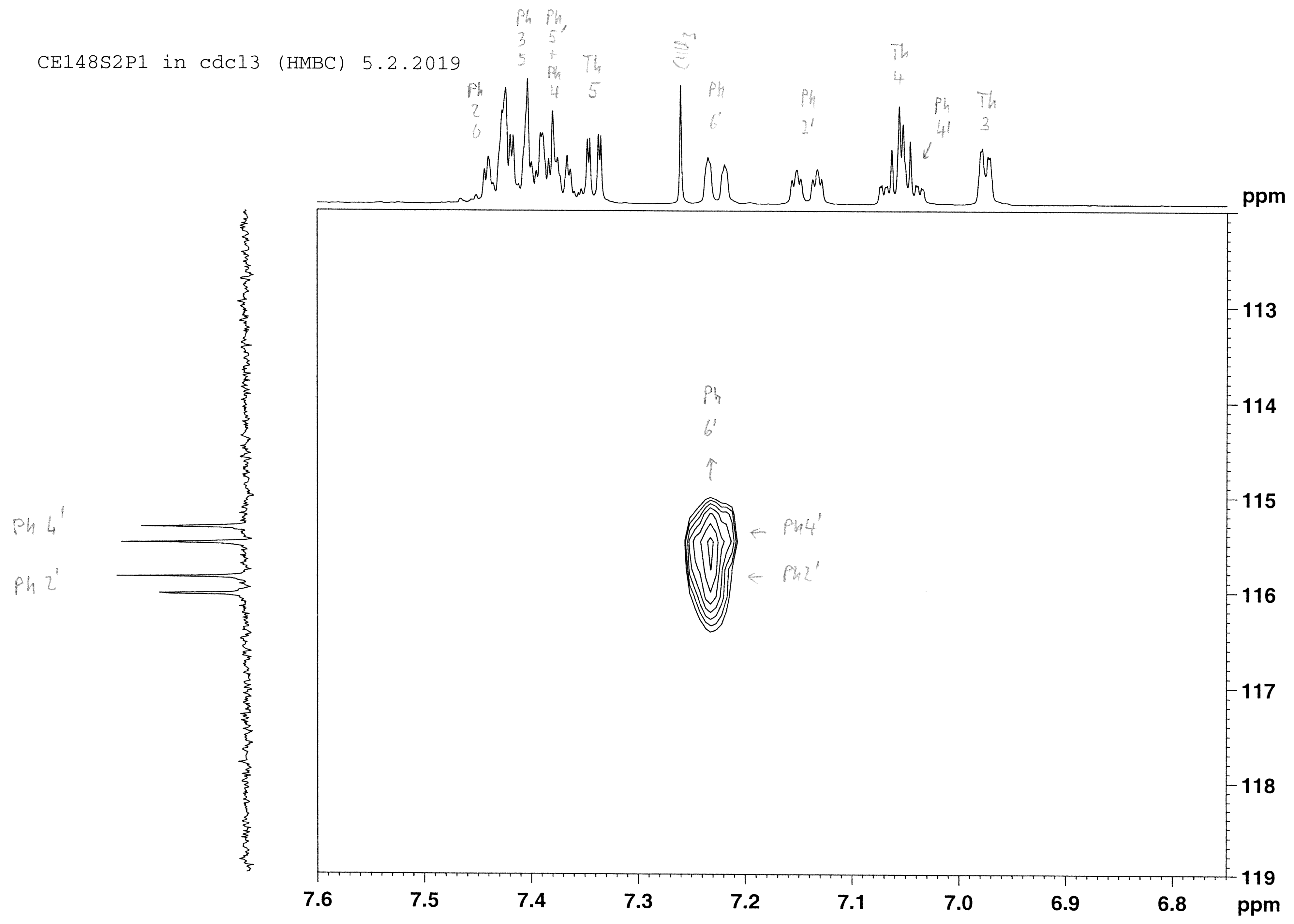

CE148S2P1 in cdcl3 (HMBC) 5.2.2019

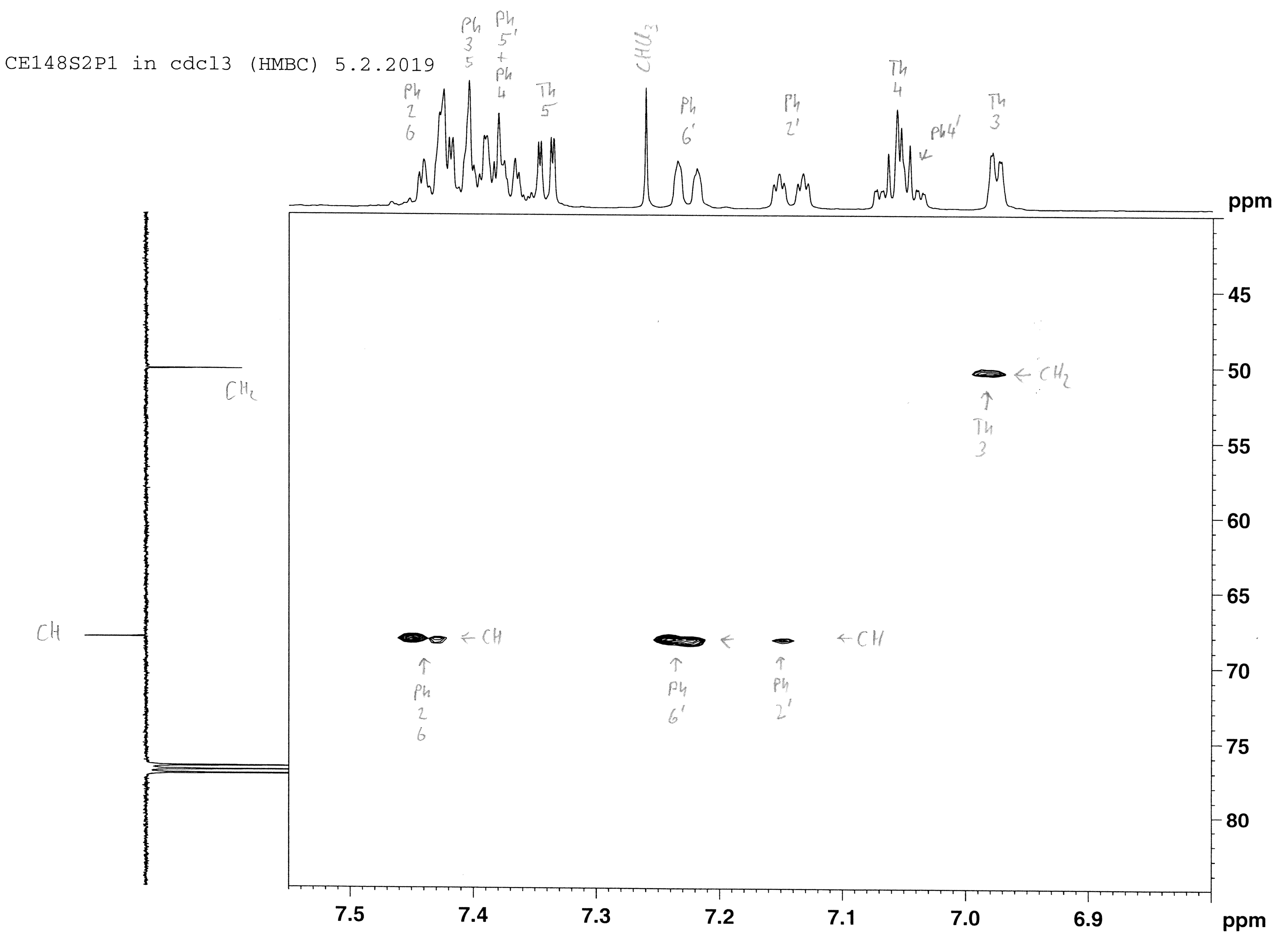

CE148S2P1 in cdcl3 (HMBC) 5.2.2019

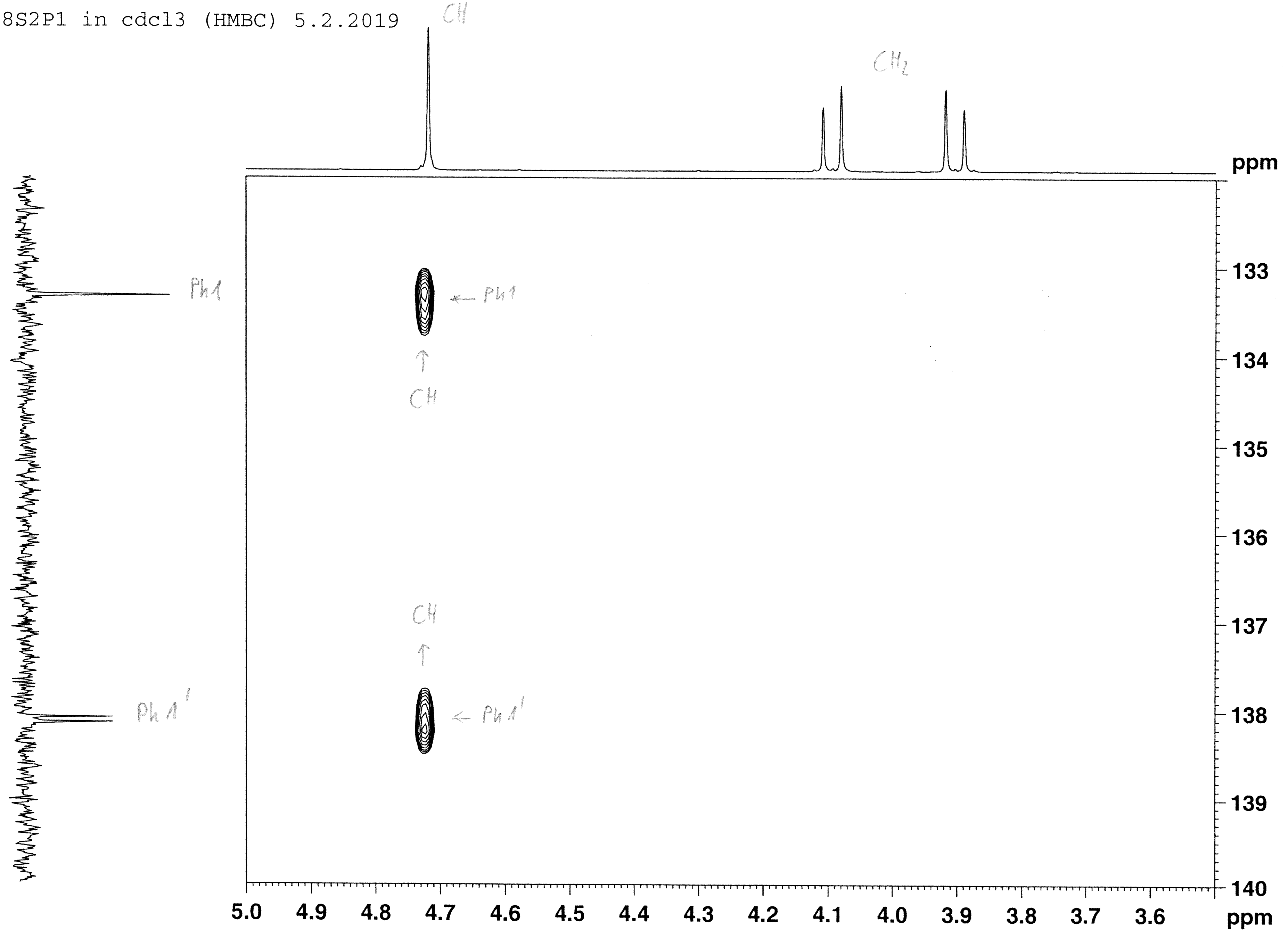

CE148S2P1 in cdcl3 (HMBC) 5.2.2019

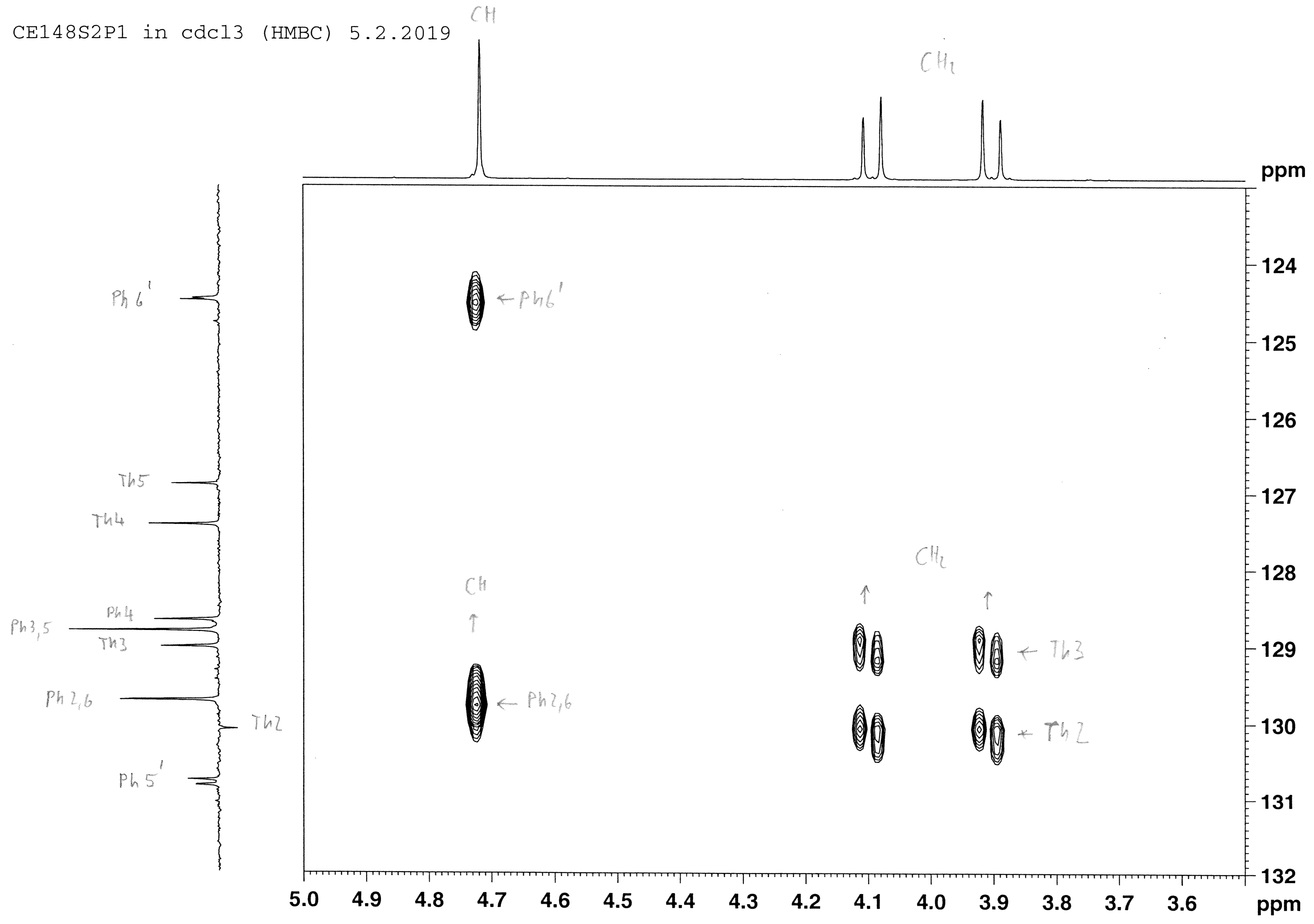

CE148S2P1 in cdcl3 (HMBC) 5.2.2019

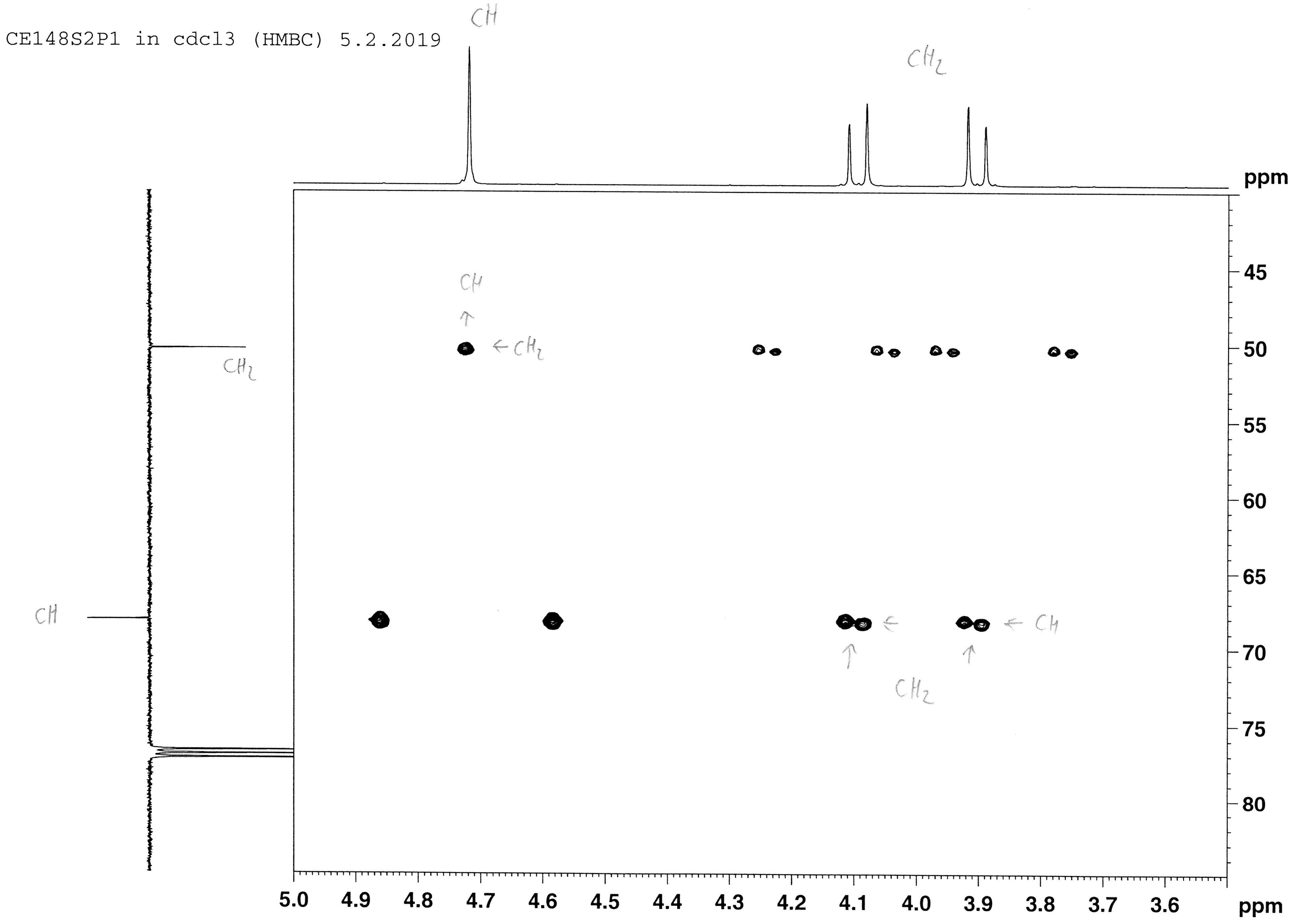

Figure S8c. NMR spectra of compound **8b.**

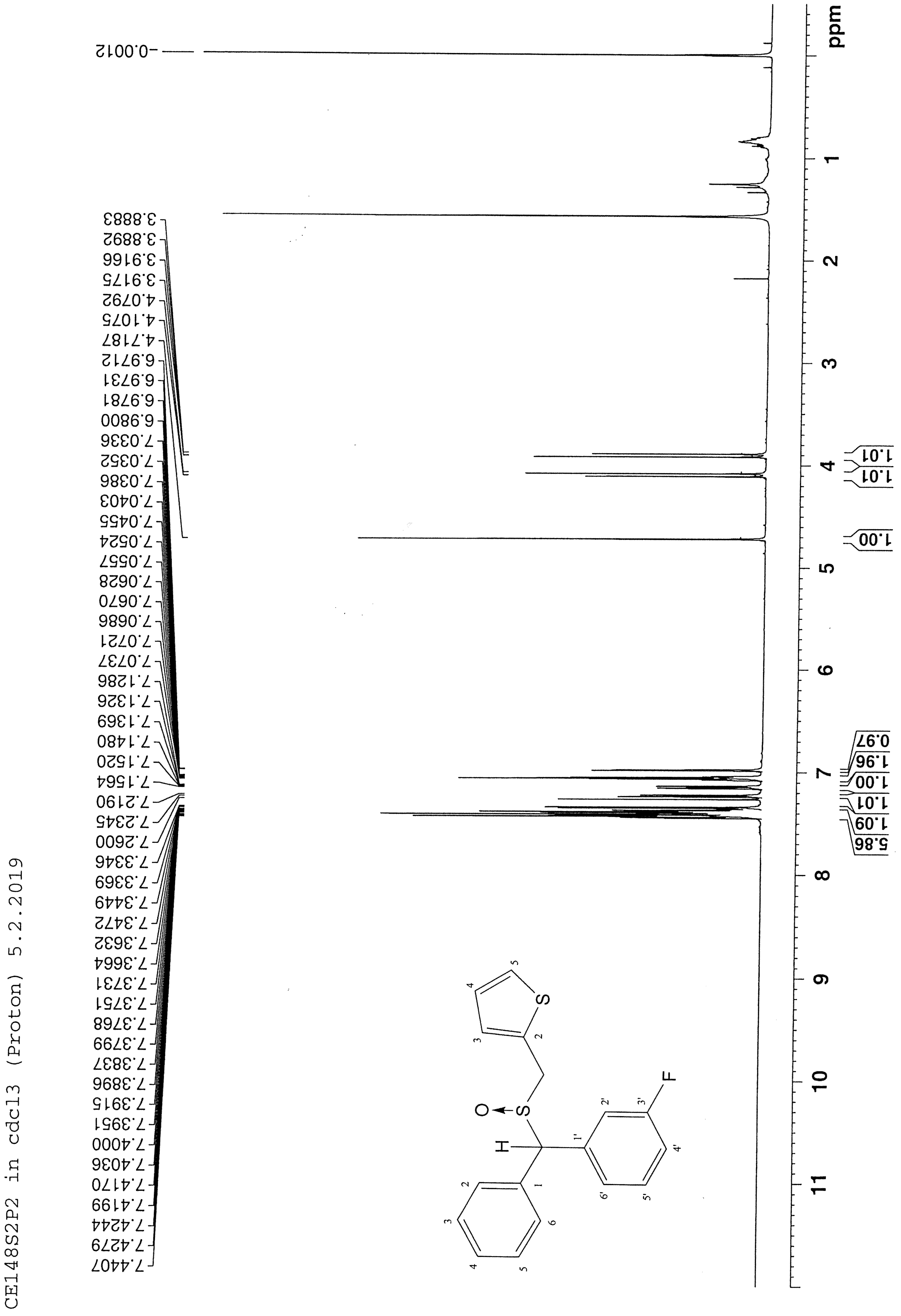

CE148S2P2 in cdcl3 (Proton) 5.2.2019

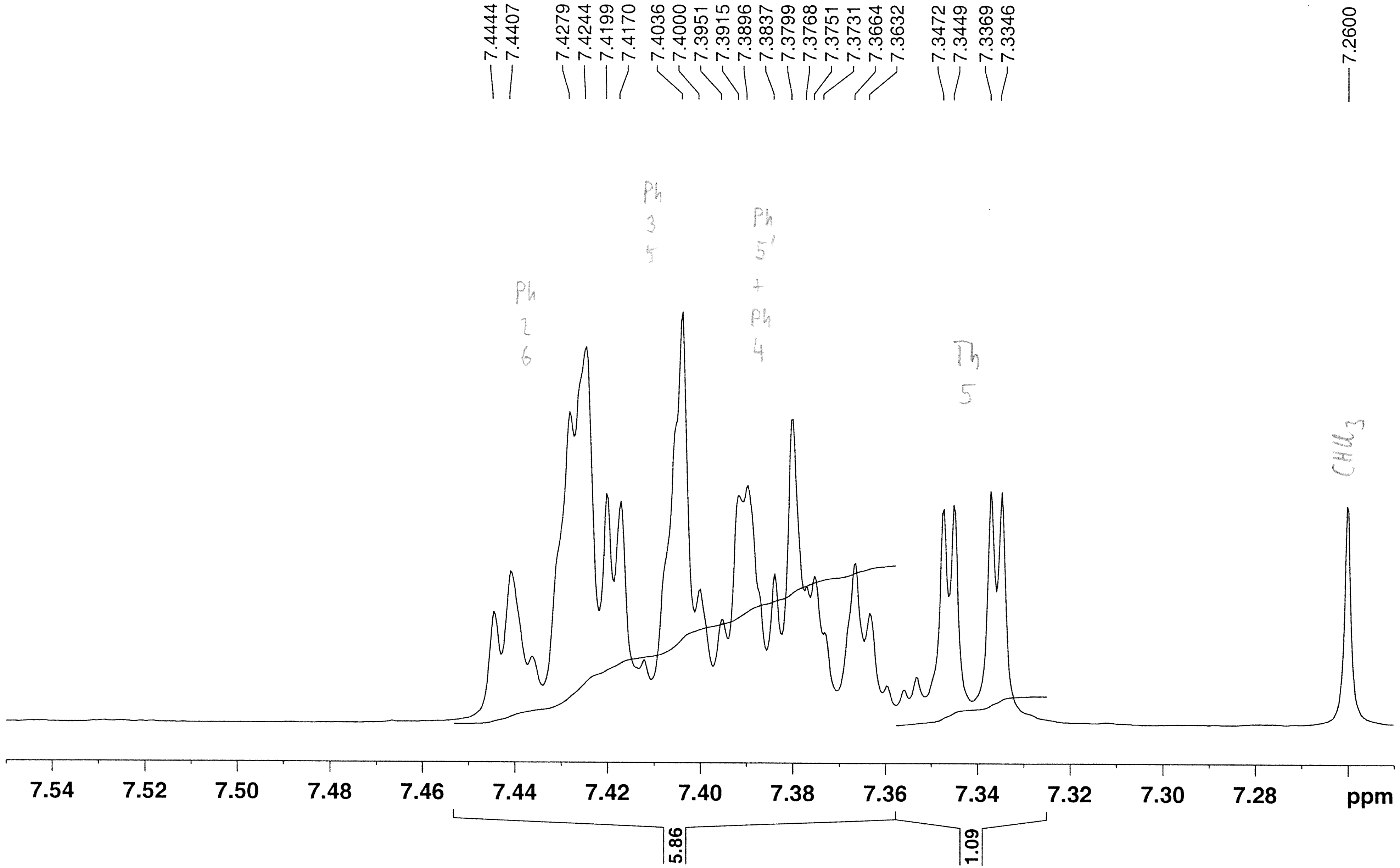

CE148S2P2 in cdcl3 (Proton) 5.2.2019

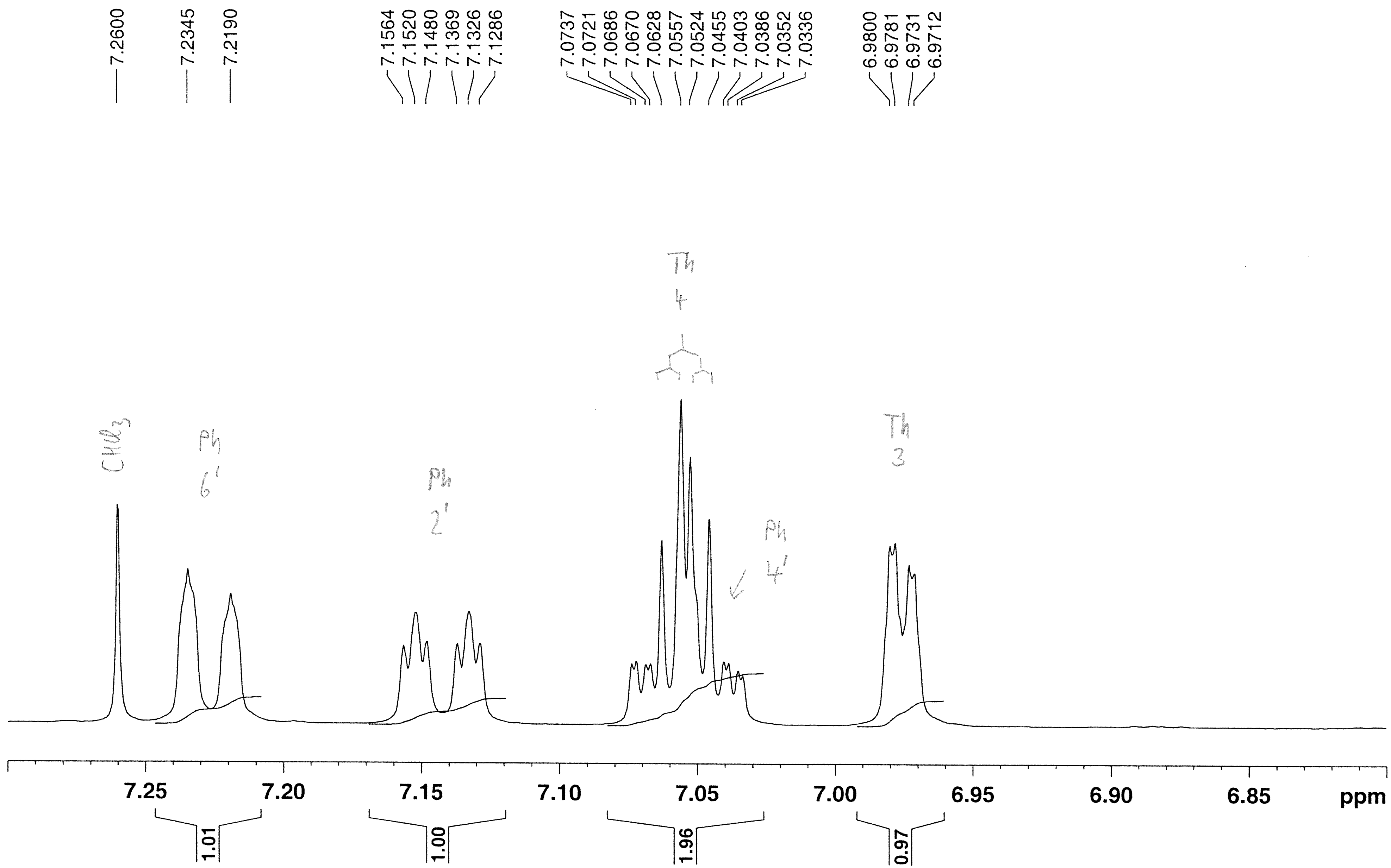

CE148S2P2 in cdcl3 (Proton) 5.2.2019

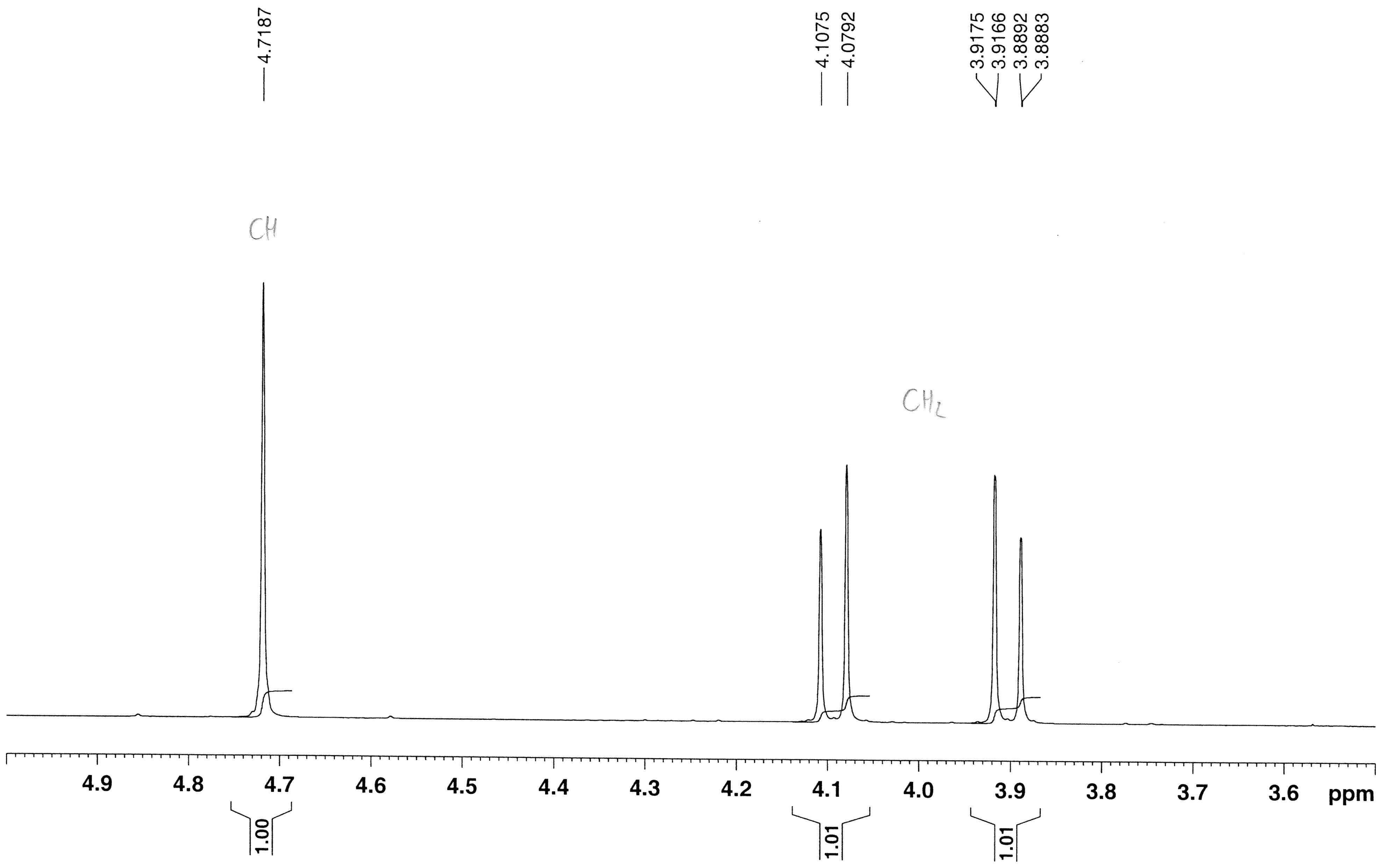

CE148S2P2 in cdcl3 (APT) 5.2.2019

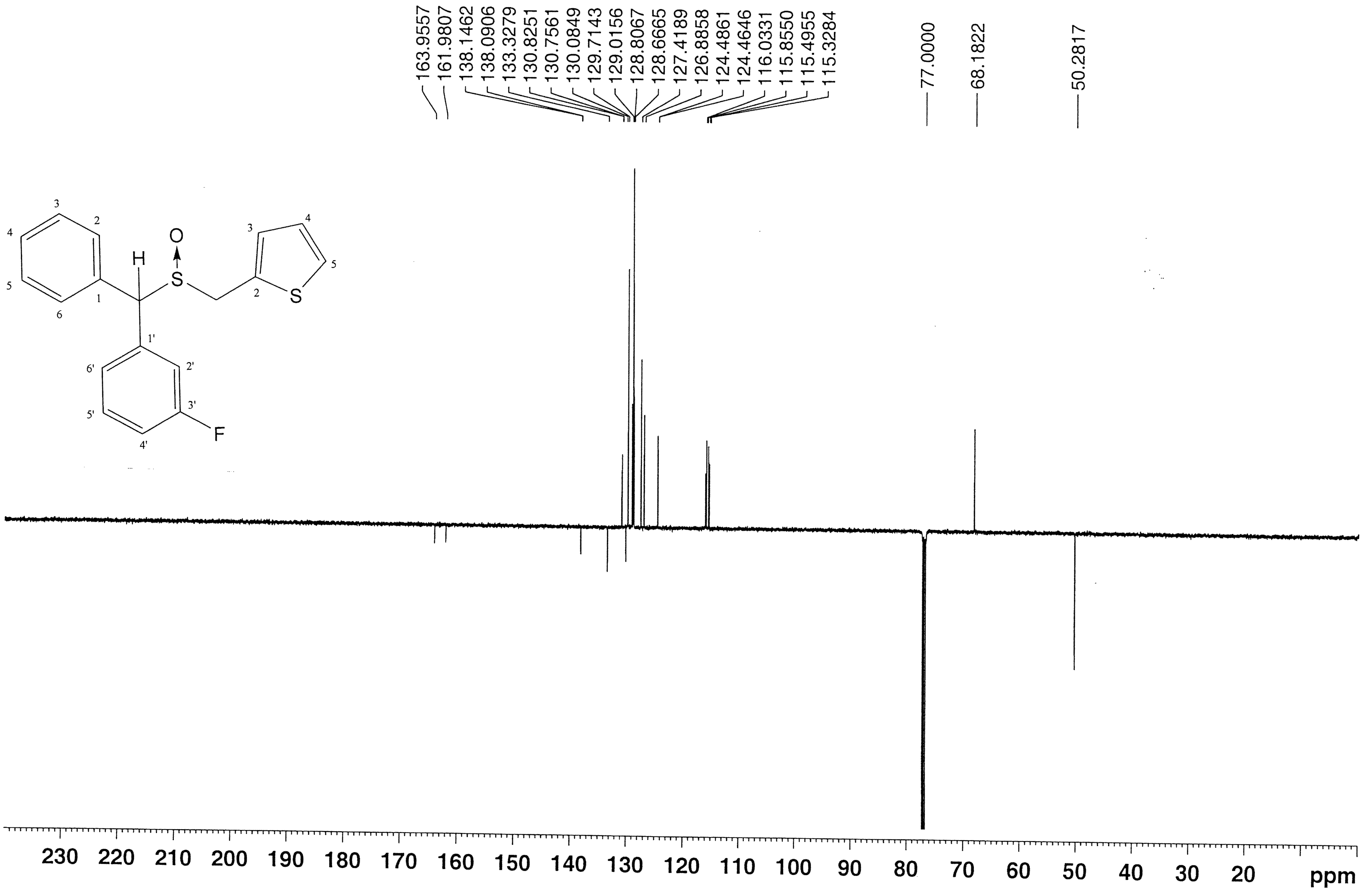

CE148S2P2 in cdcl3 (APT) 5.2.2019

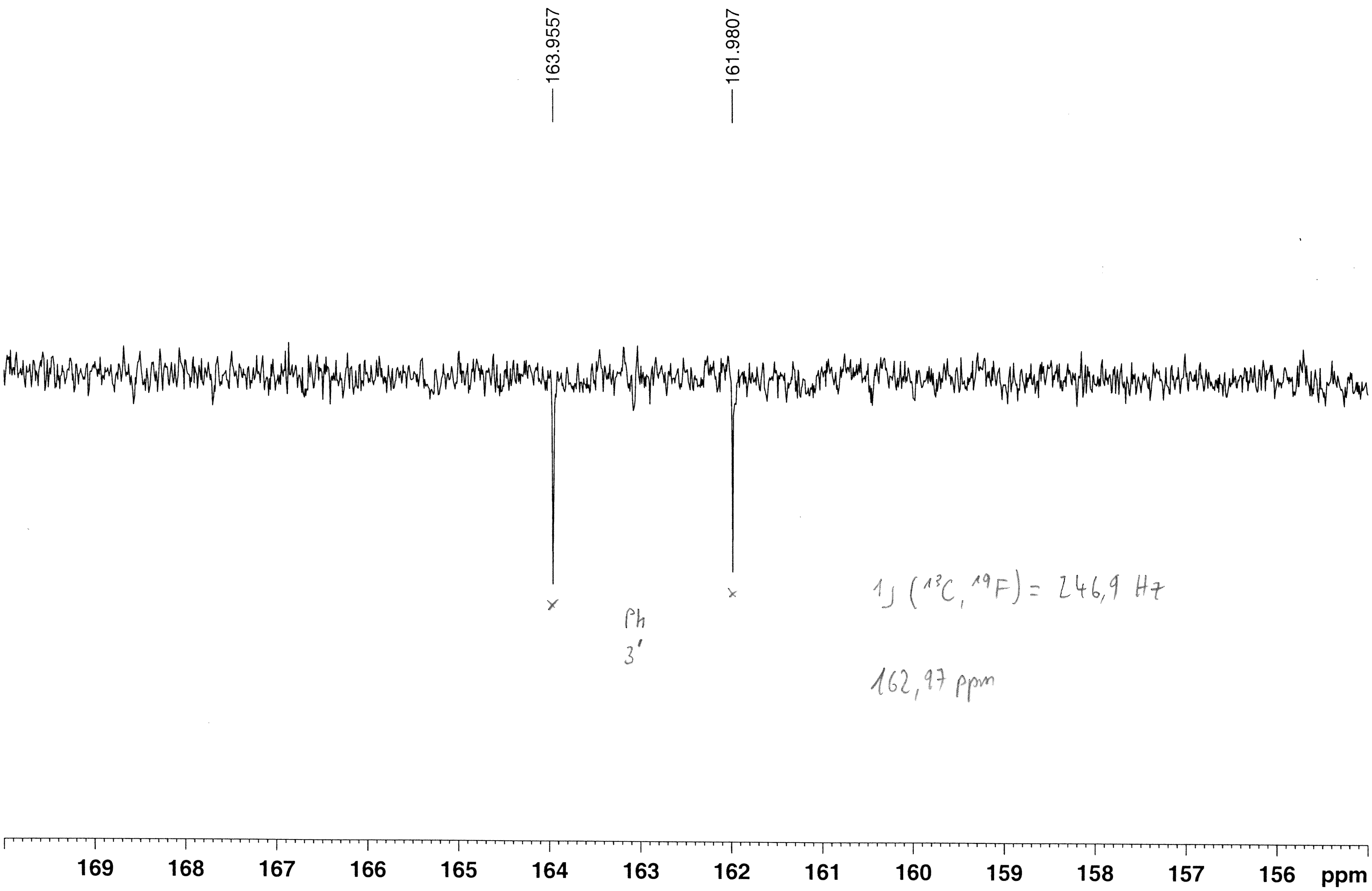

CE148S2P2 in cdcl3 (APT) 5.2.2019

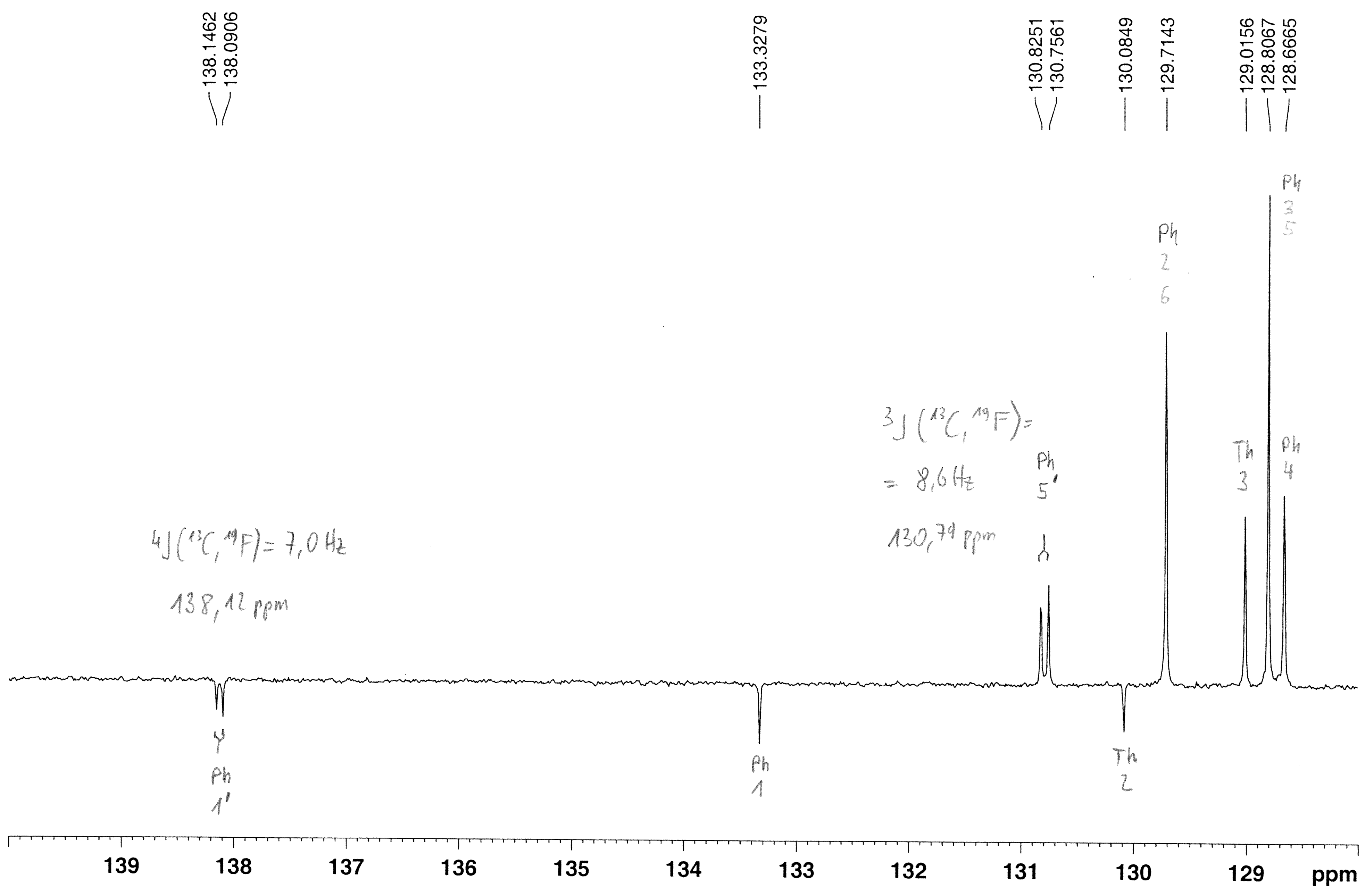

CE148S2P2 in cdcl3 (APT) 5.2.2019

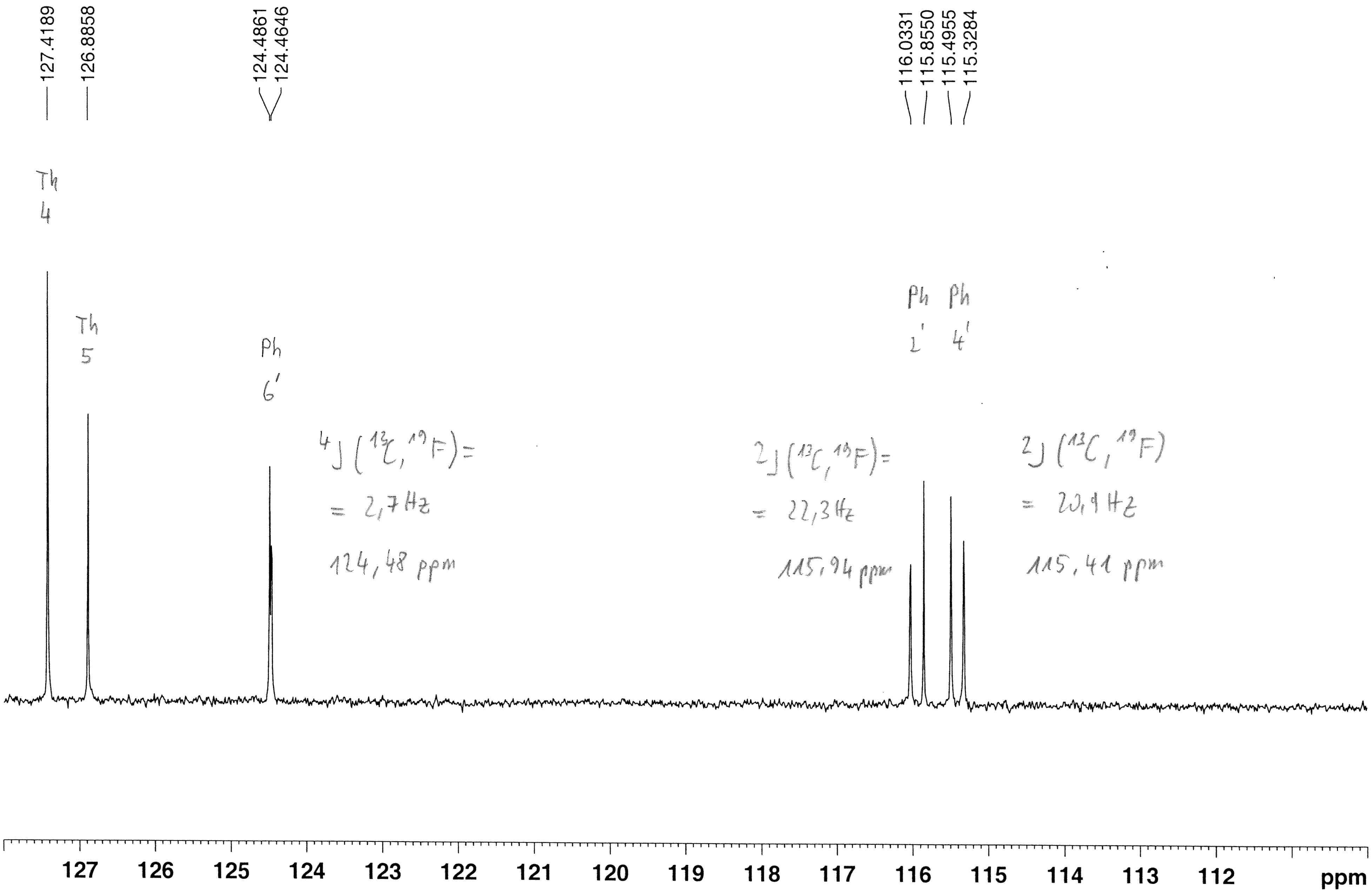

CE148S2P2 in cdcl3 (APT) 5.2.2019

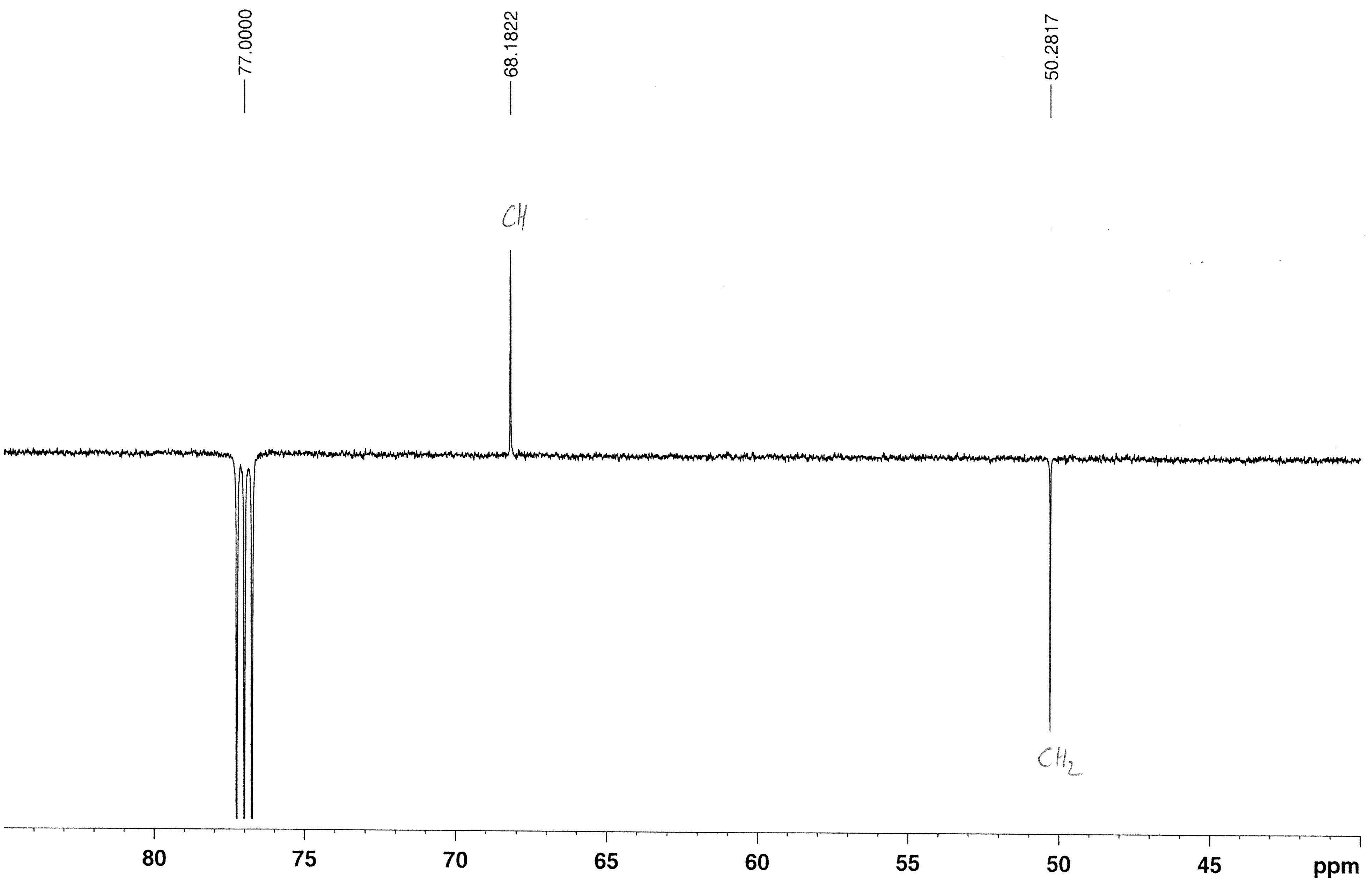

Figure S9c. NMR spectra of compound **5c**.

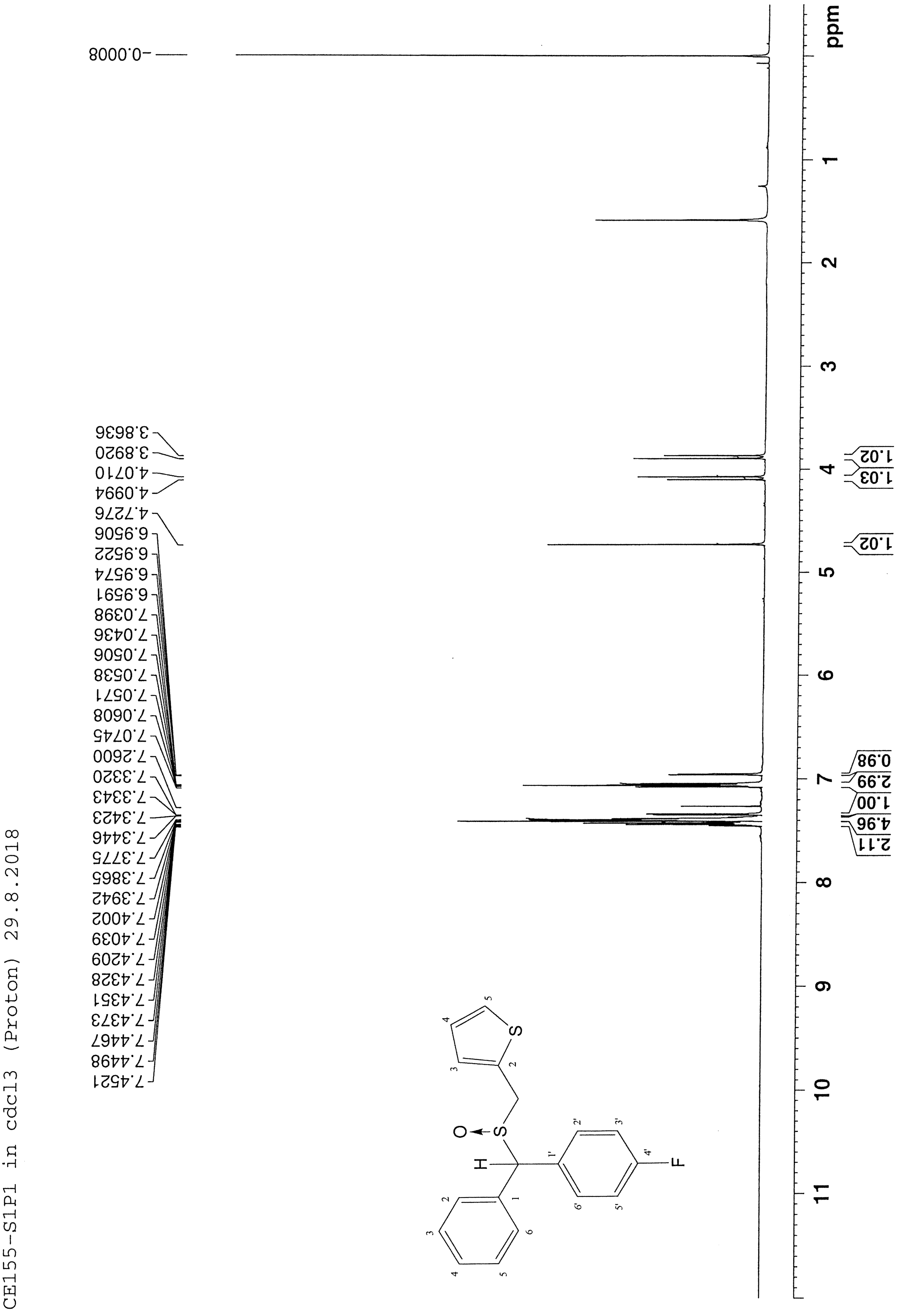

CE155-S1P1 in cdcl3 (Proton) 29.8.2018

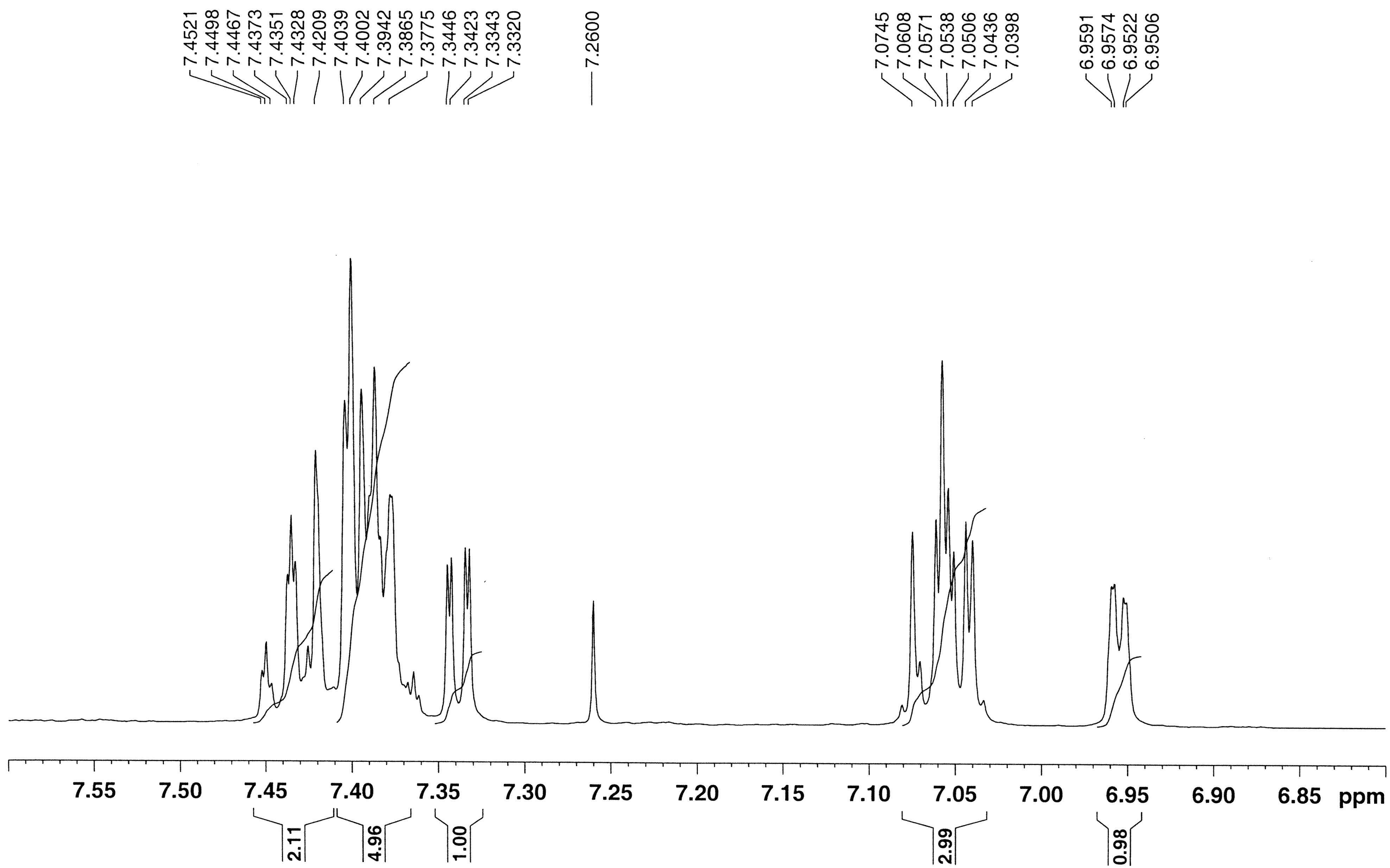

CE155-S1P1 in cdcl3 (Proton) 29.8.2018

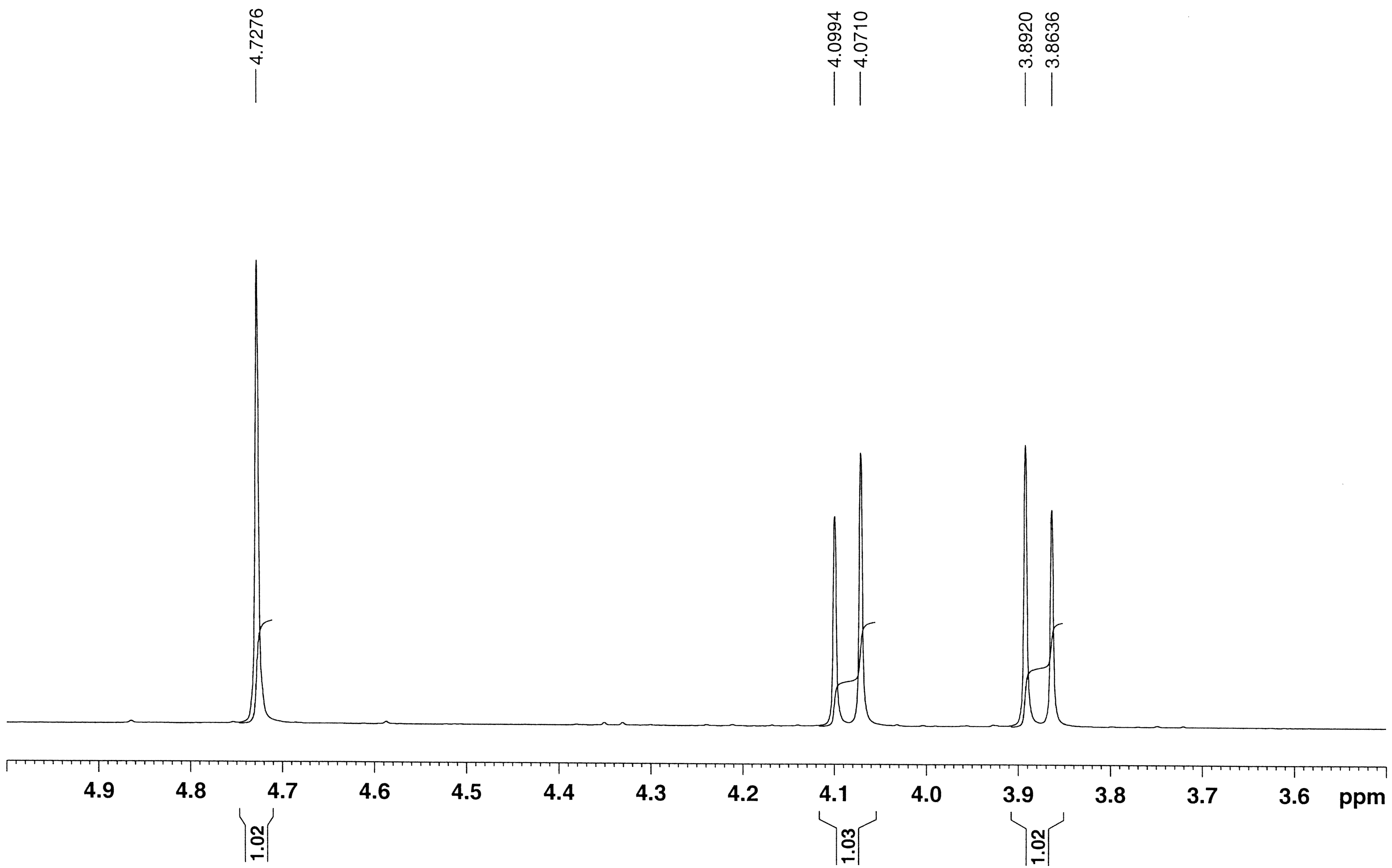

CE155-S1P1 in cdcl3 (APT) 29.8.2018

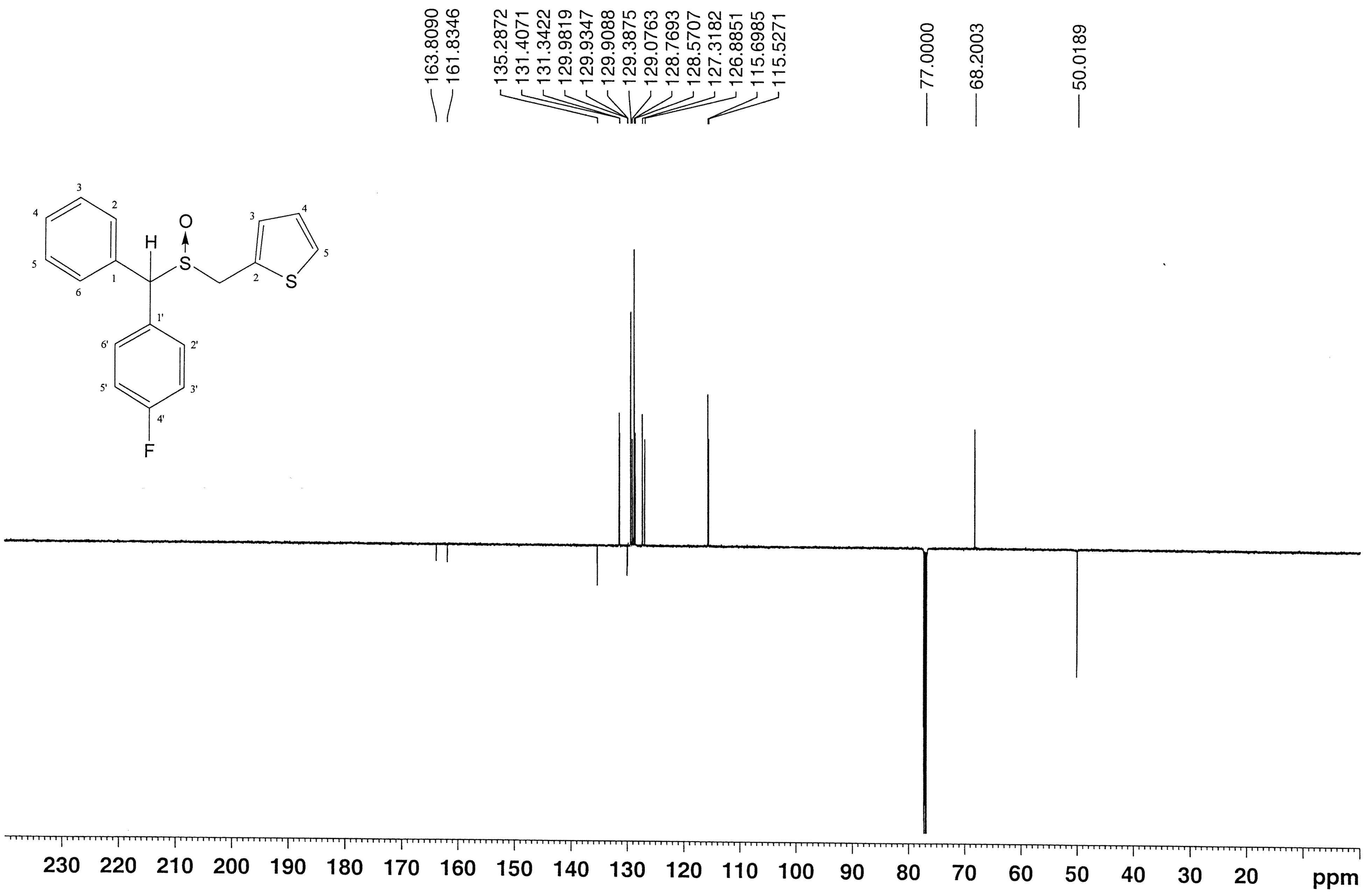

CE155-S1P1 in cdcl3 (APT) 29.8.2018

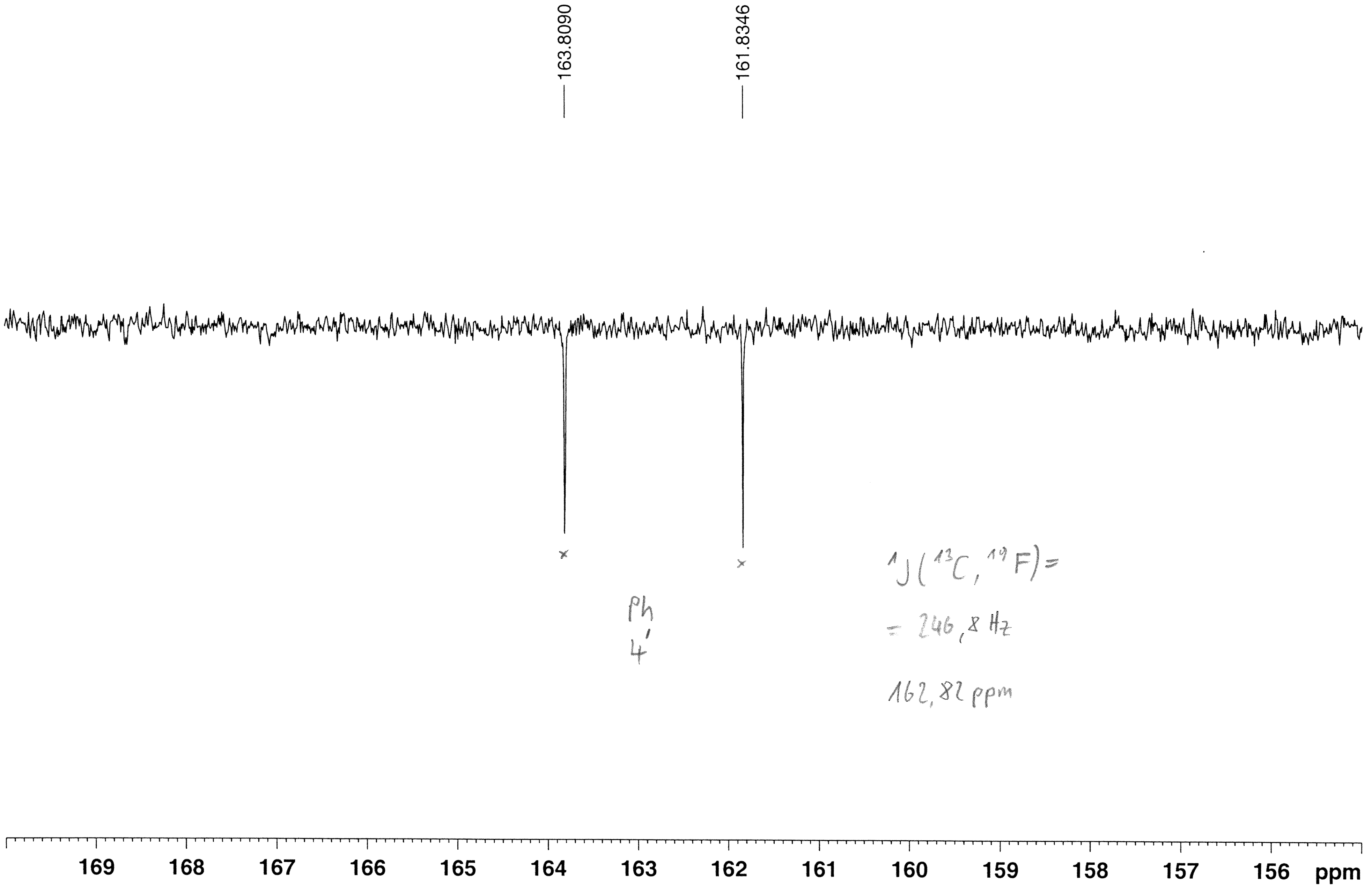

CE155-S1P1 in cdcl3 (APT) 29.8.2018

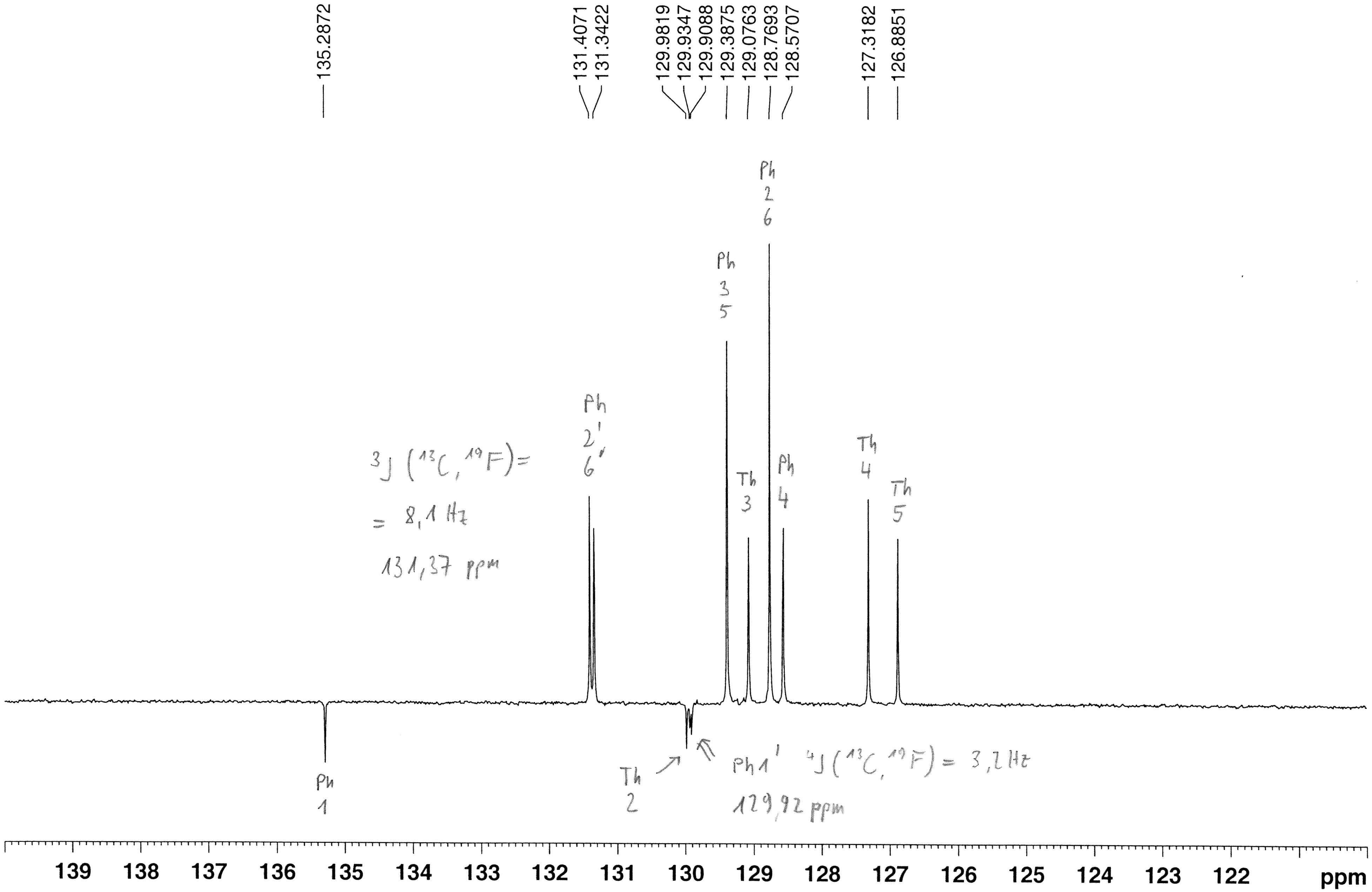

CE155-S1P1 in cdcl3 (APT) 29.8.2018

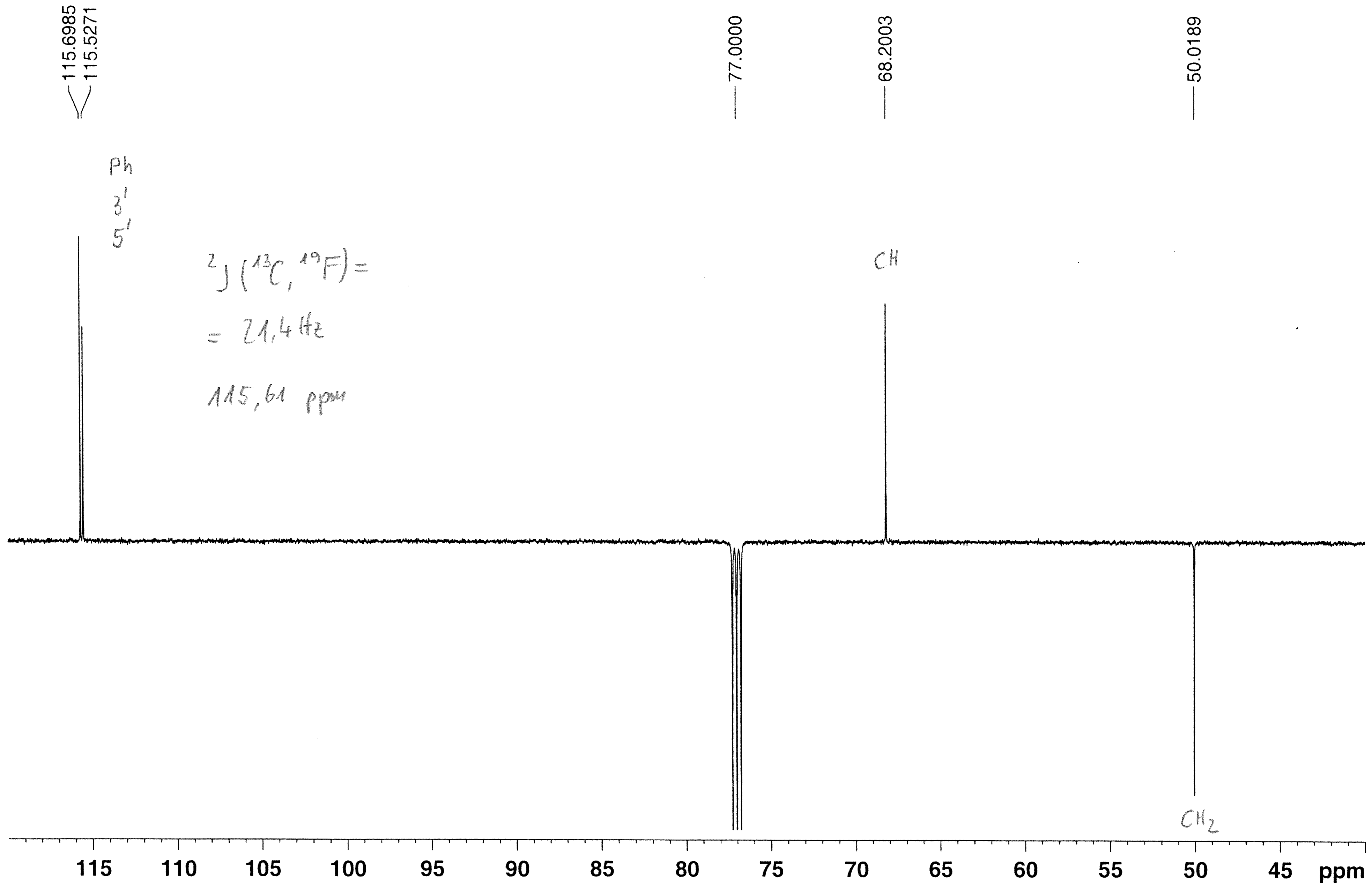

Figure S10c. NMR spectra of compound **6c.**

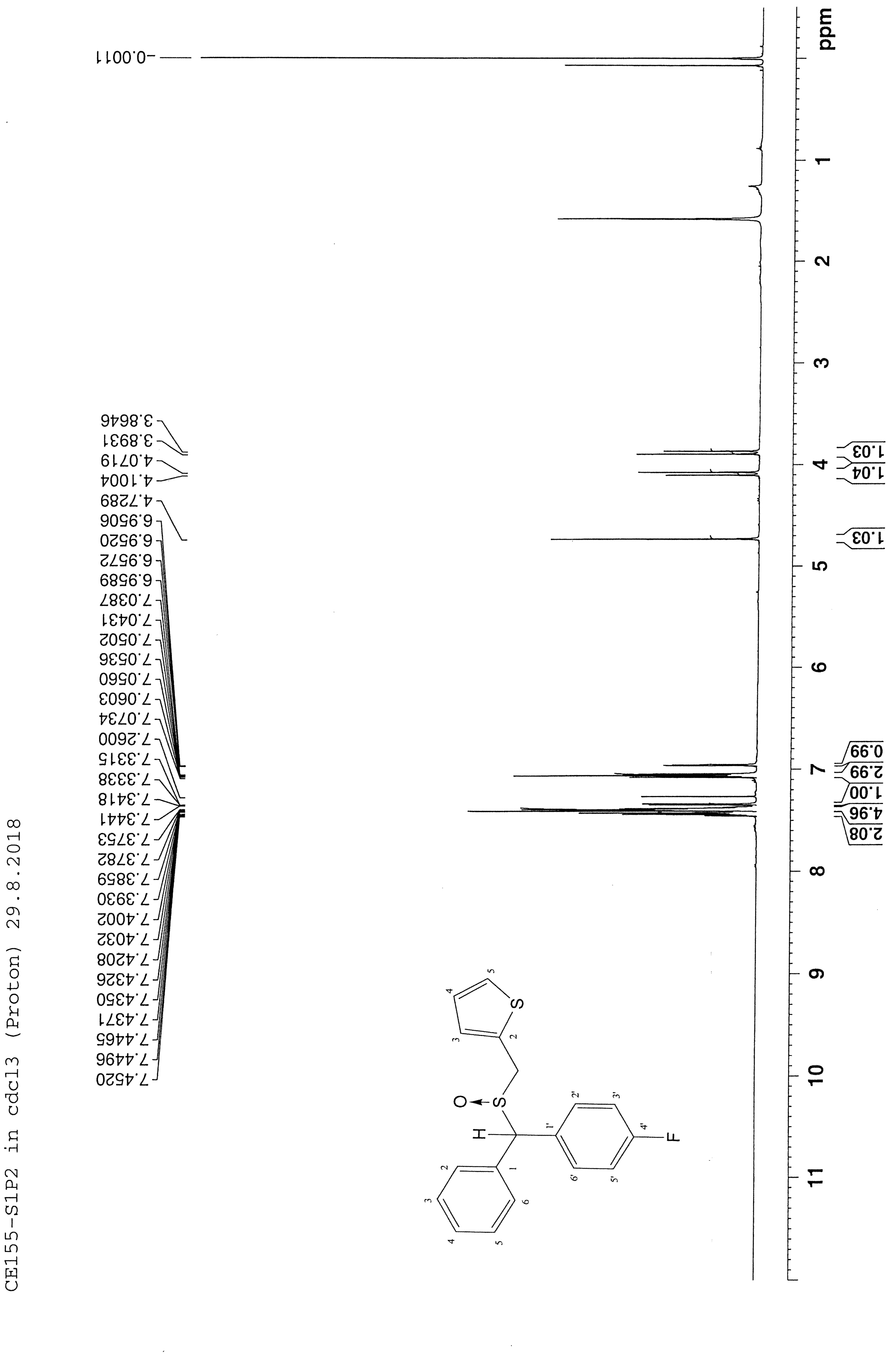

CE155-S1P2 in cdcl3 (Proton) 29.8.2018

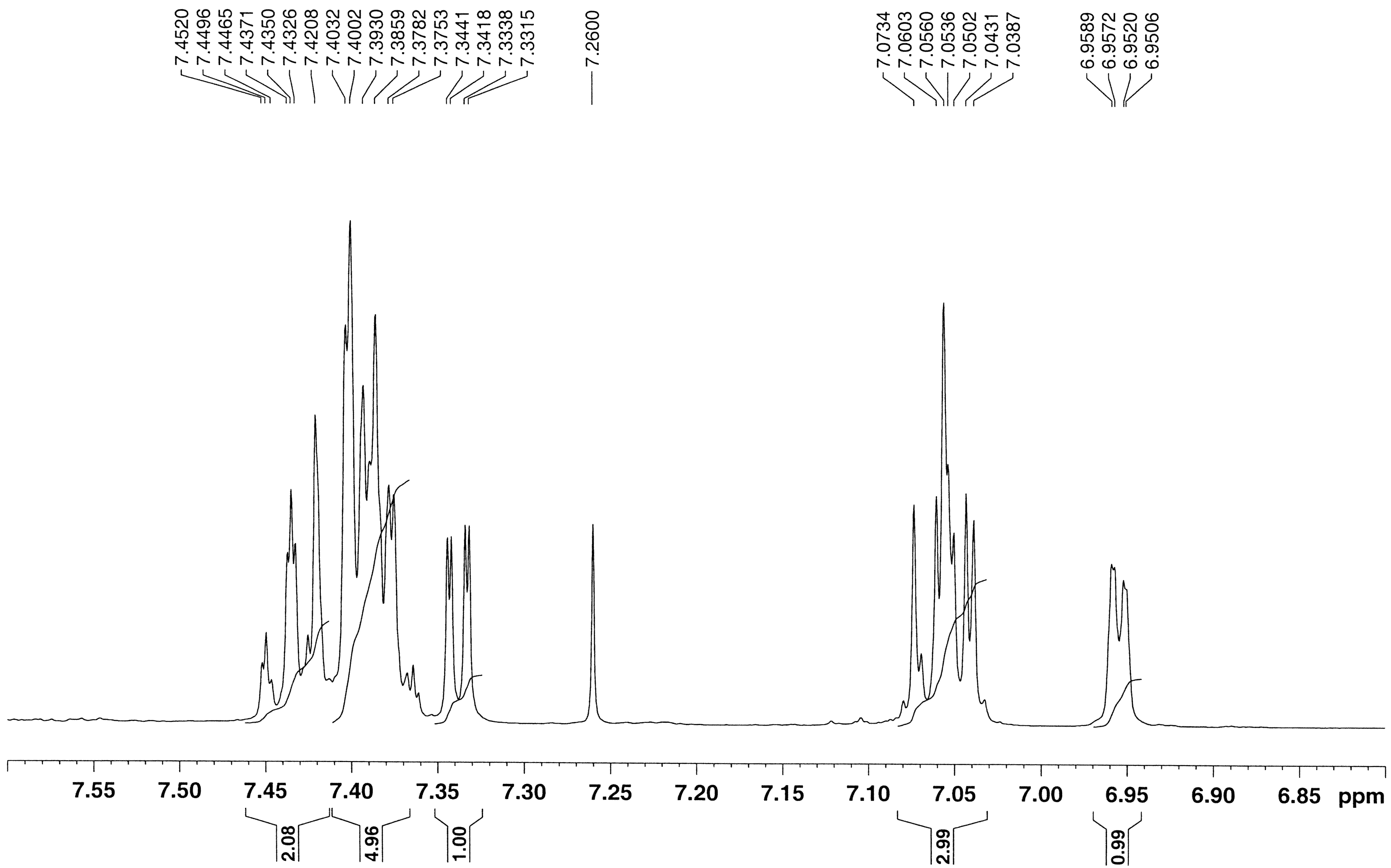

CE155-S1P2 in cdcl3 (Proton) 29.8.2018

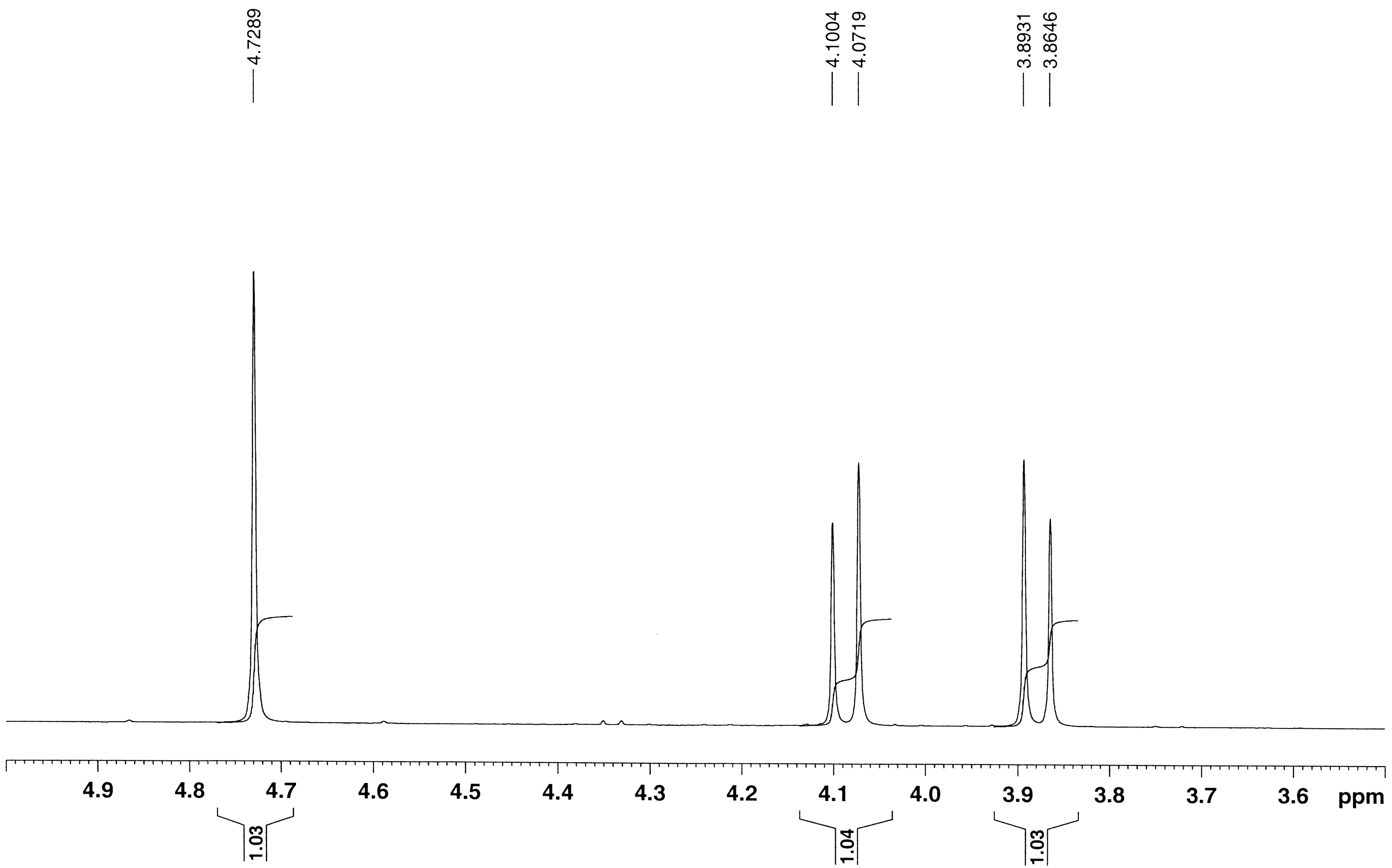

CE155-S1P2 in cdcl3 (APT) 29.8.2018

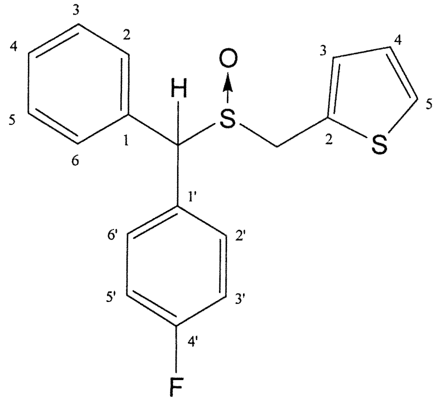

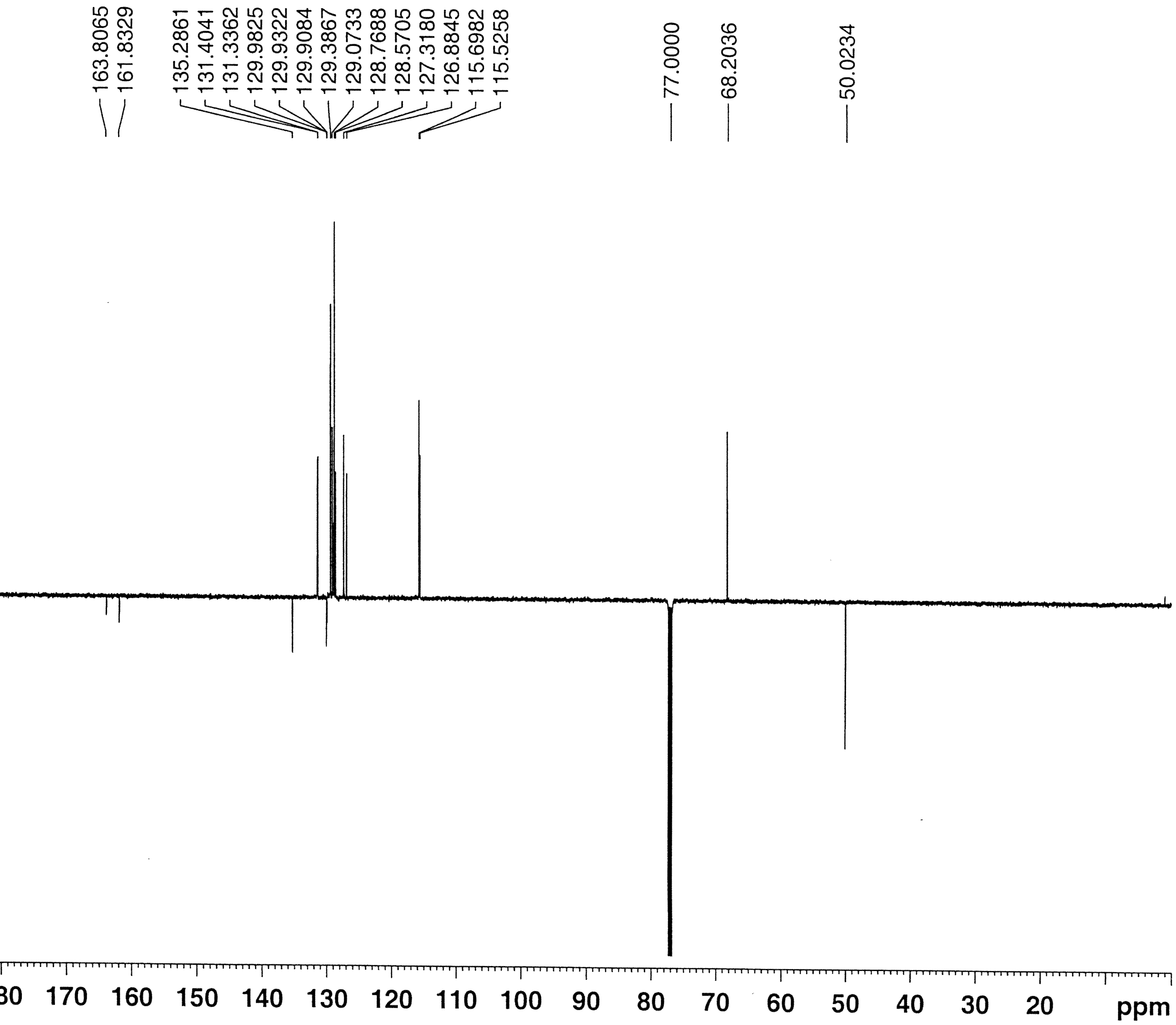

CE155-S1P2 in cdcl3 (APT) 29.8.2018

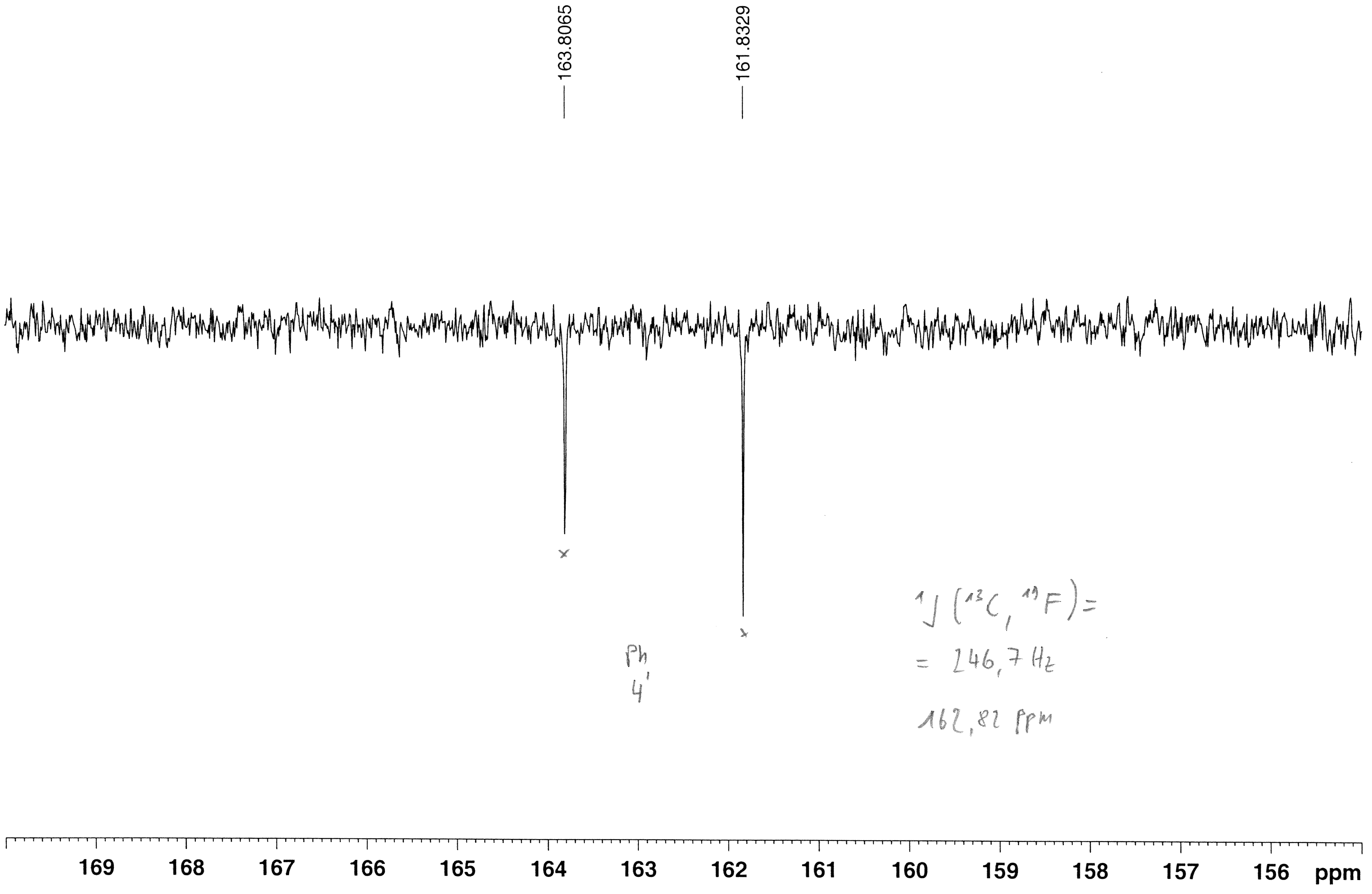

CE155-S1P2 in cdcl3 (APT) 29.8.2018

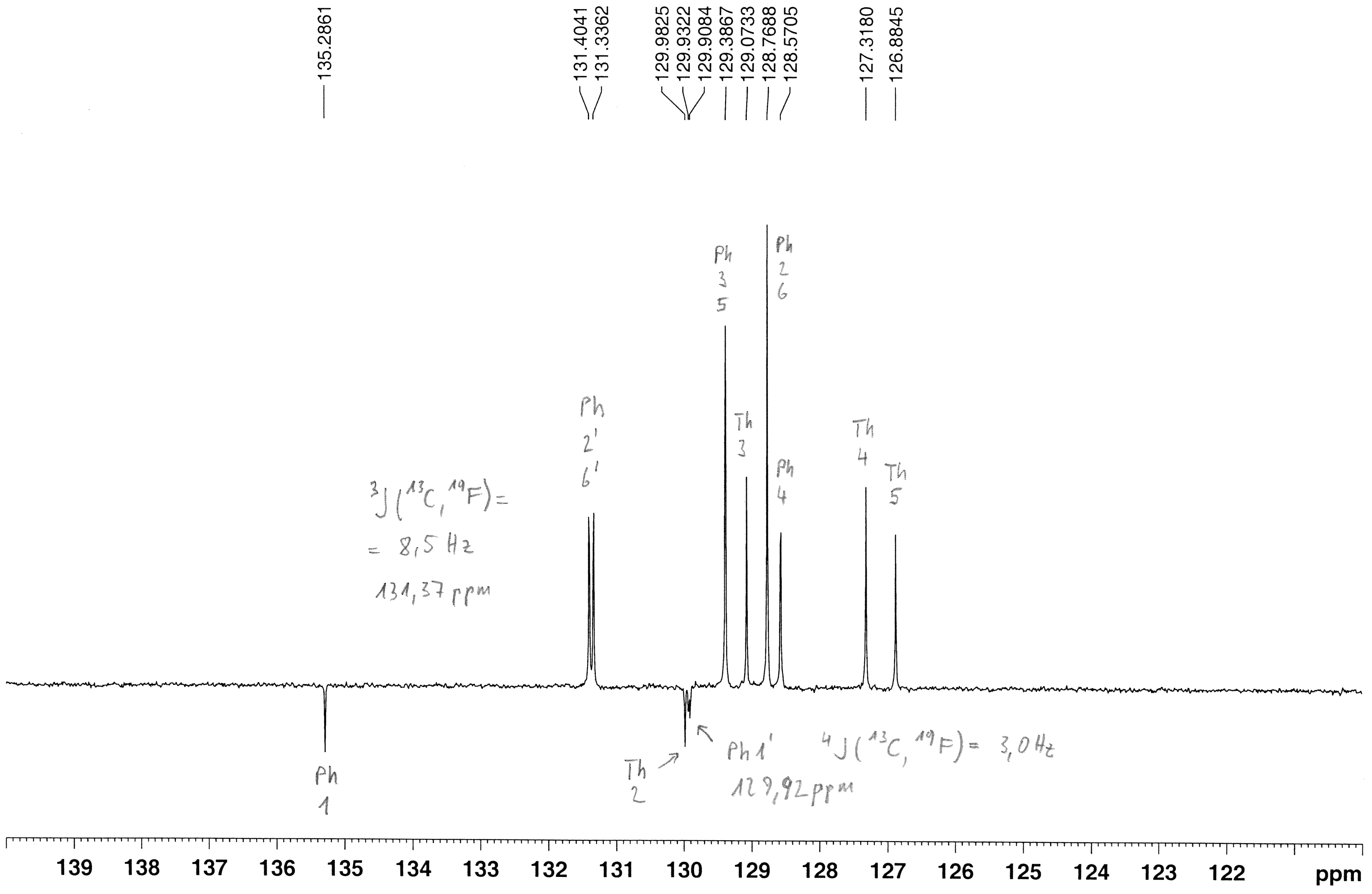

CE155-S1P2 in cdcl3 (APT) 29.8.2018

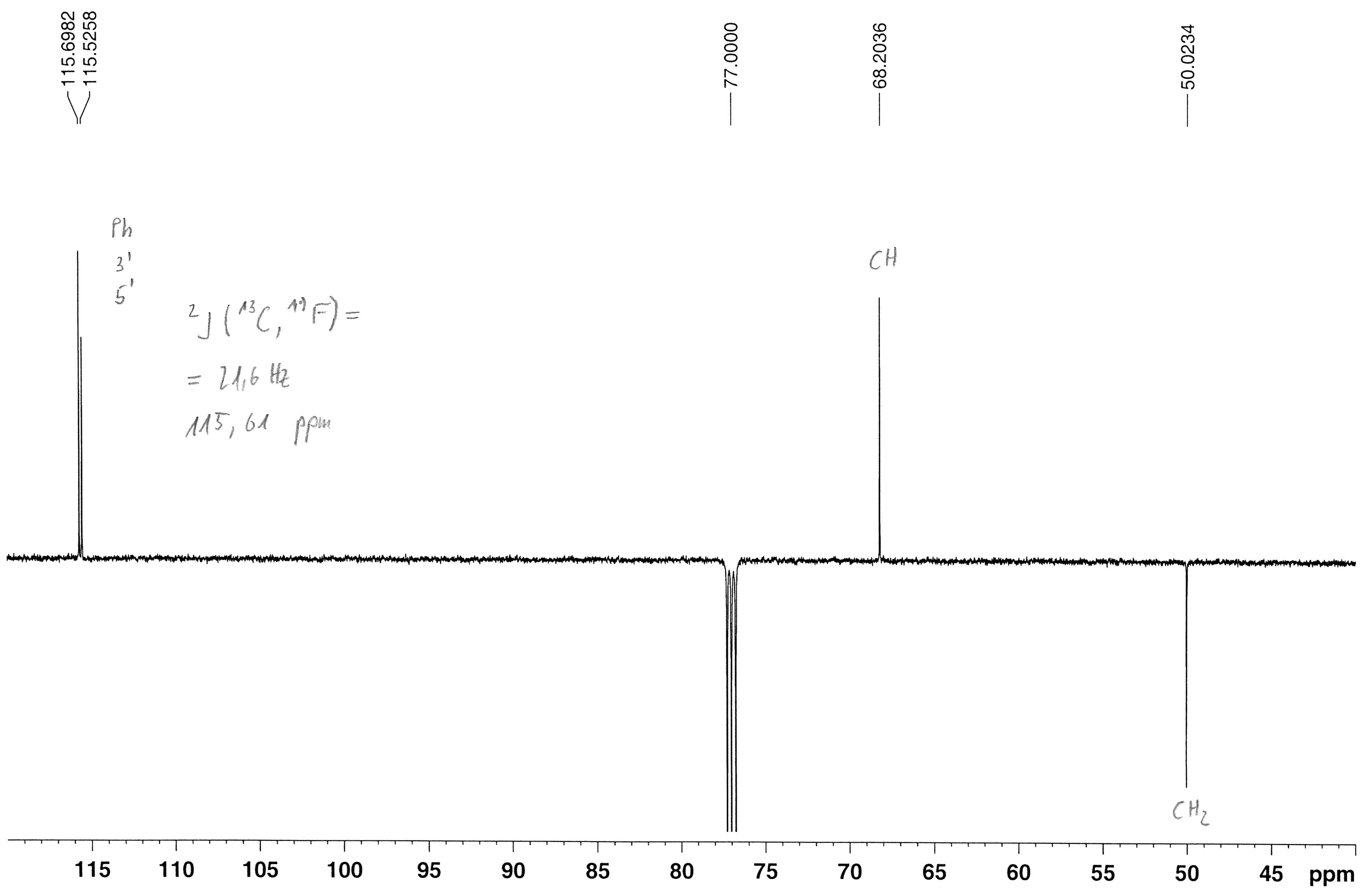

Figure S11c. NMR spectra of compound **7c**.

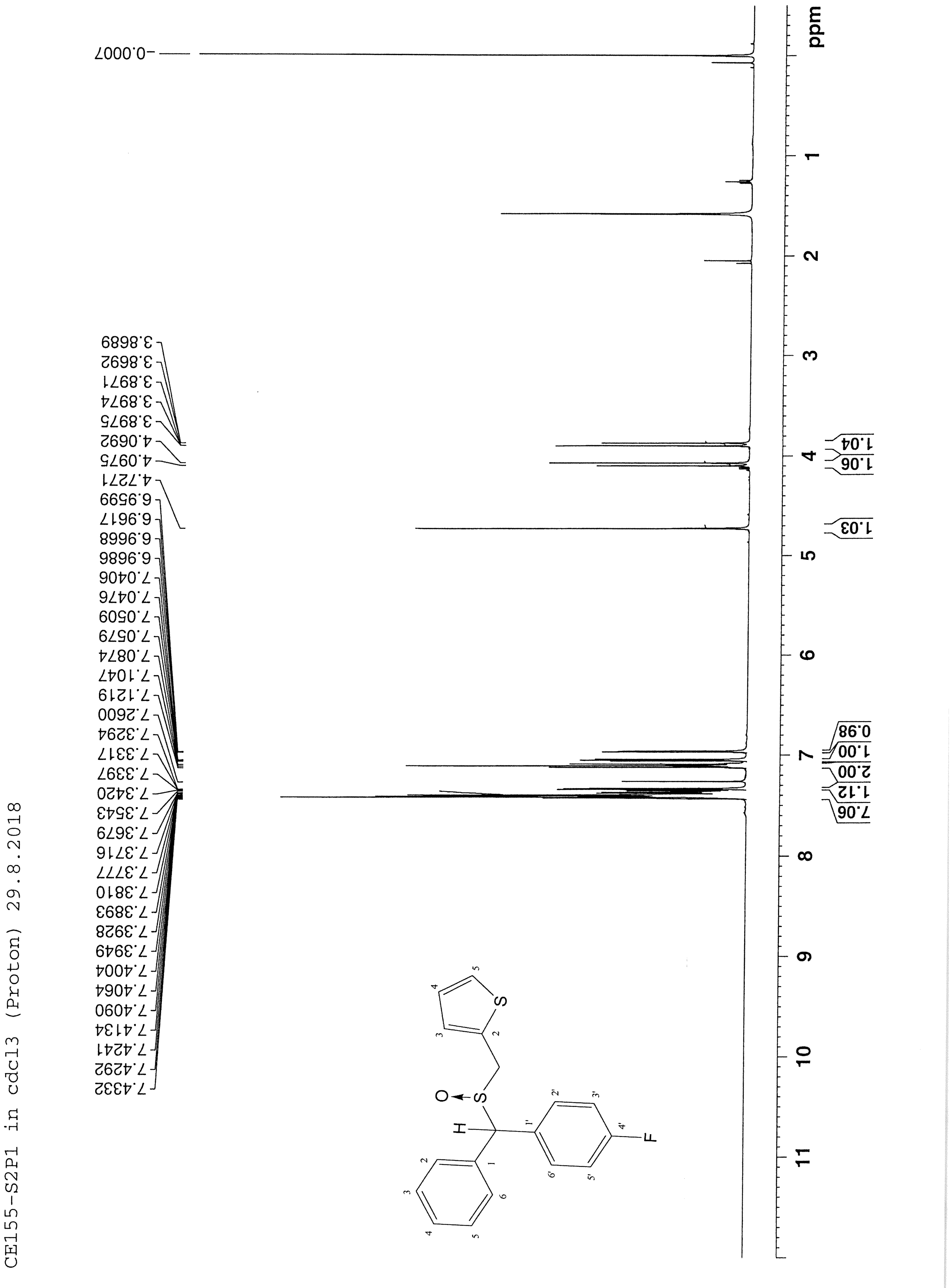

CE155-S2P1 in cdcl3 (Proton) 29.8.2018

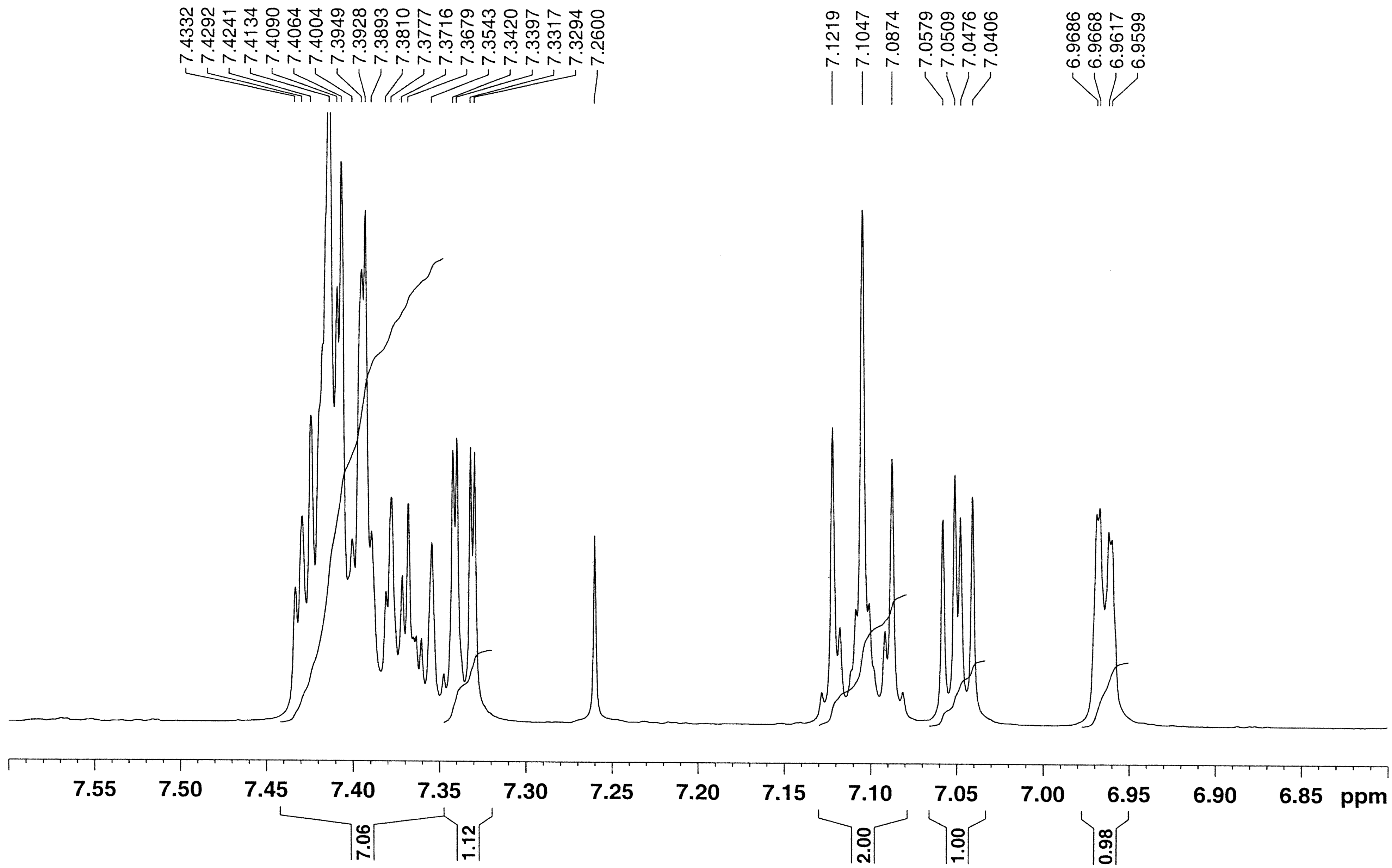

CE155-S2P1 in cdcl3 (Proton) 29.8.2018

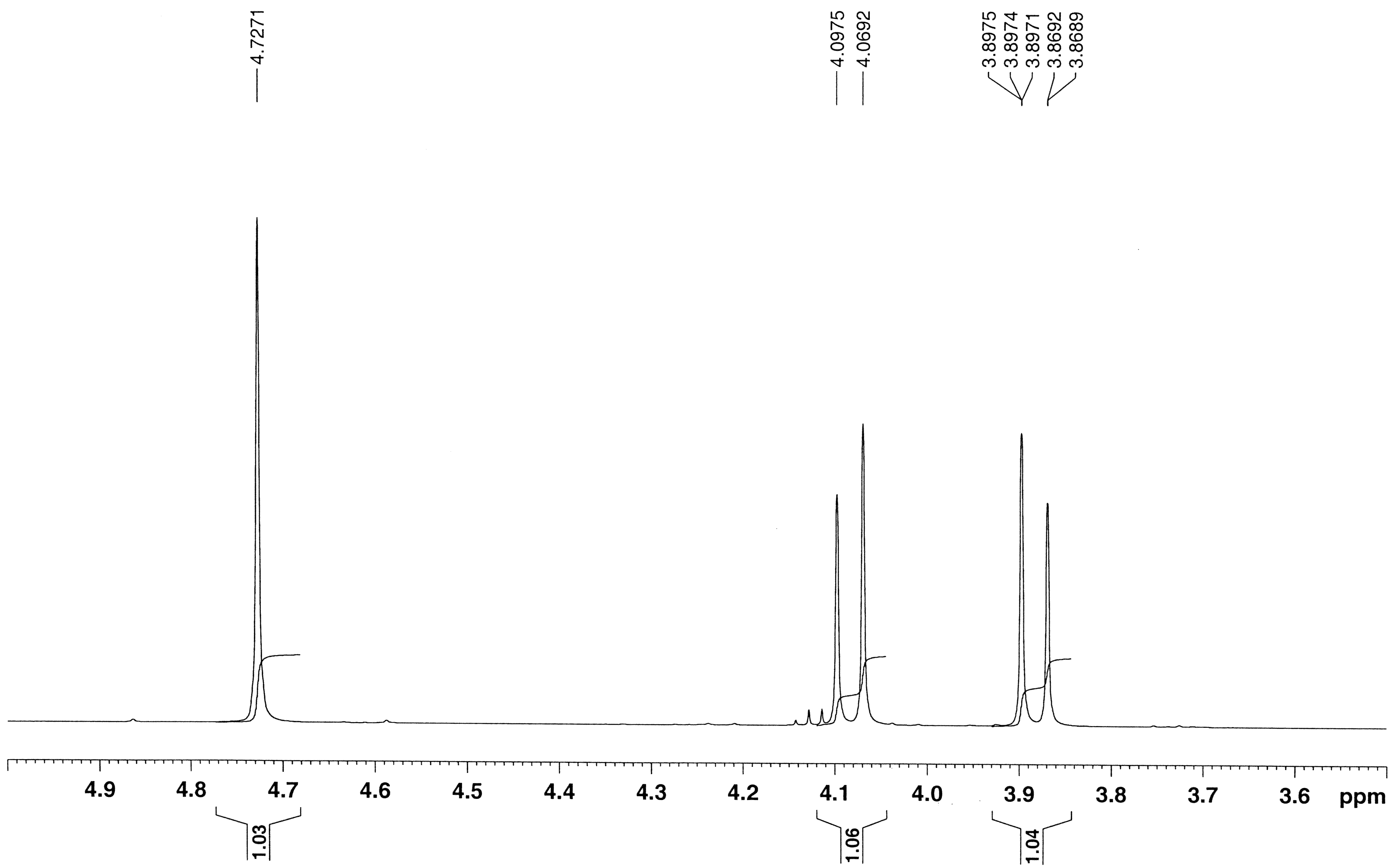

CE155-S2P1 in cdcl3 (APT) 29.8.2018

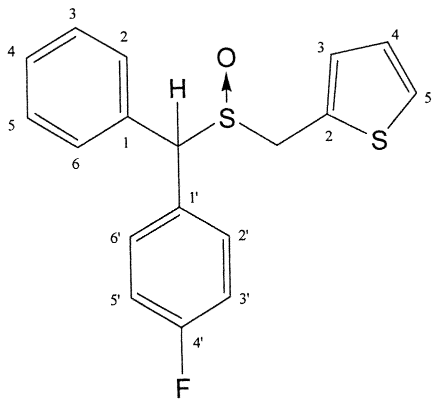

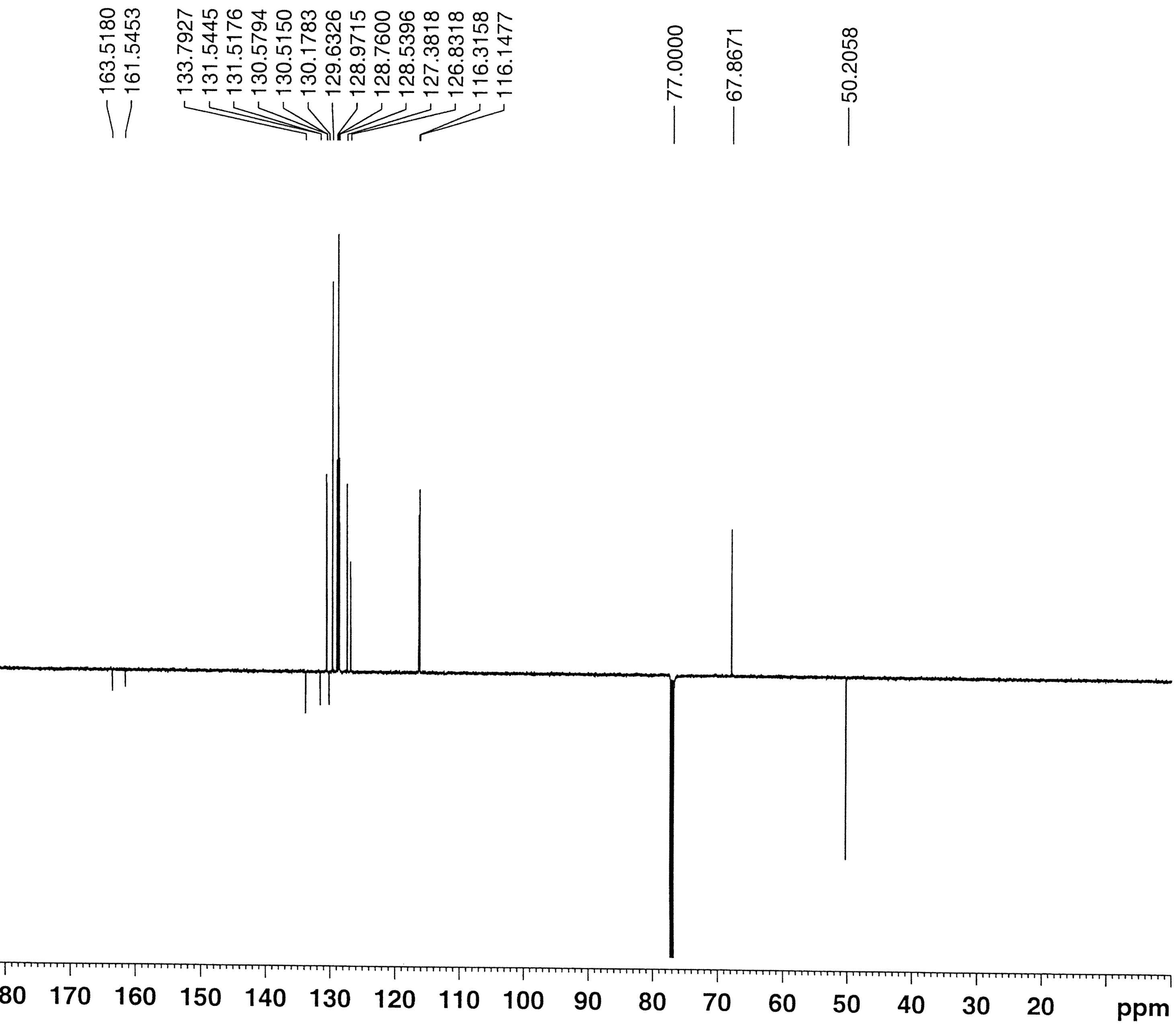

CE155-S2P1 in cdcl3 (APT) 29.8.2018

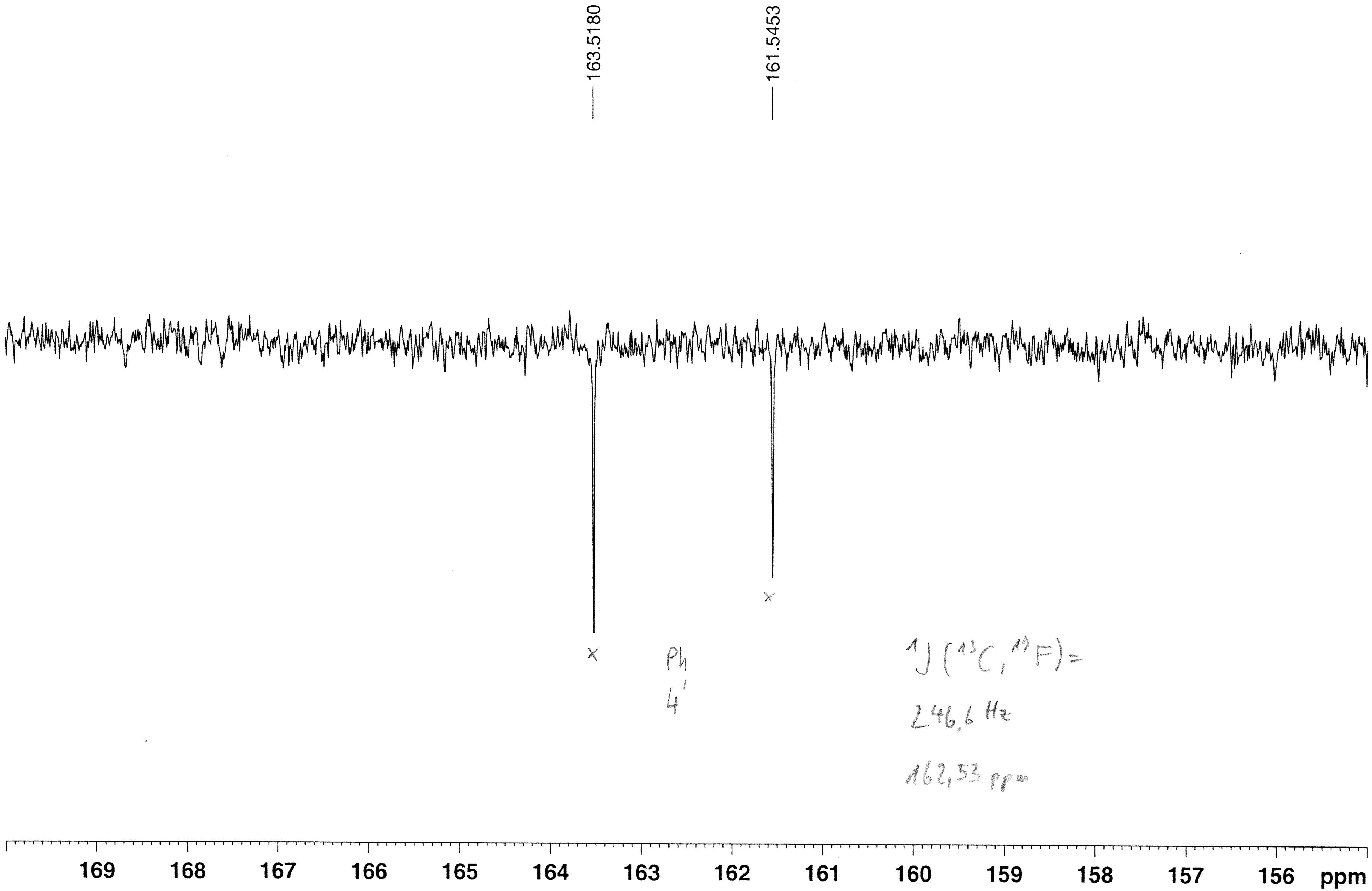

CE155-S2P1 in cdcl3 (APT) 29.8.2018

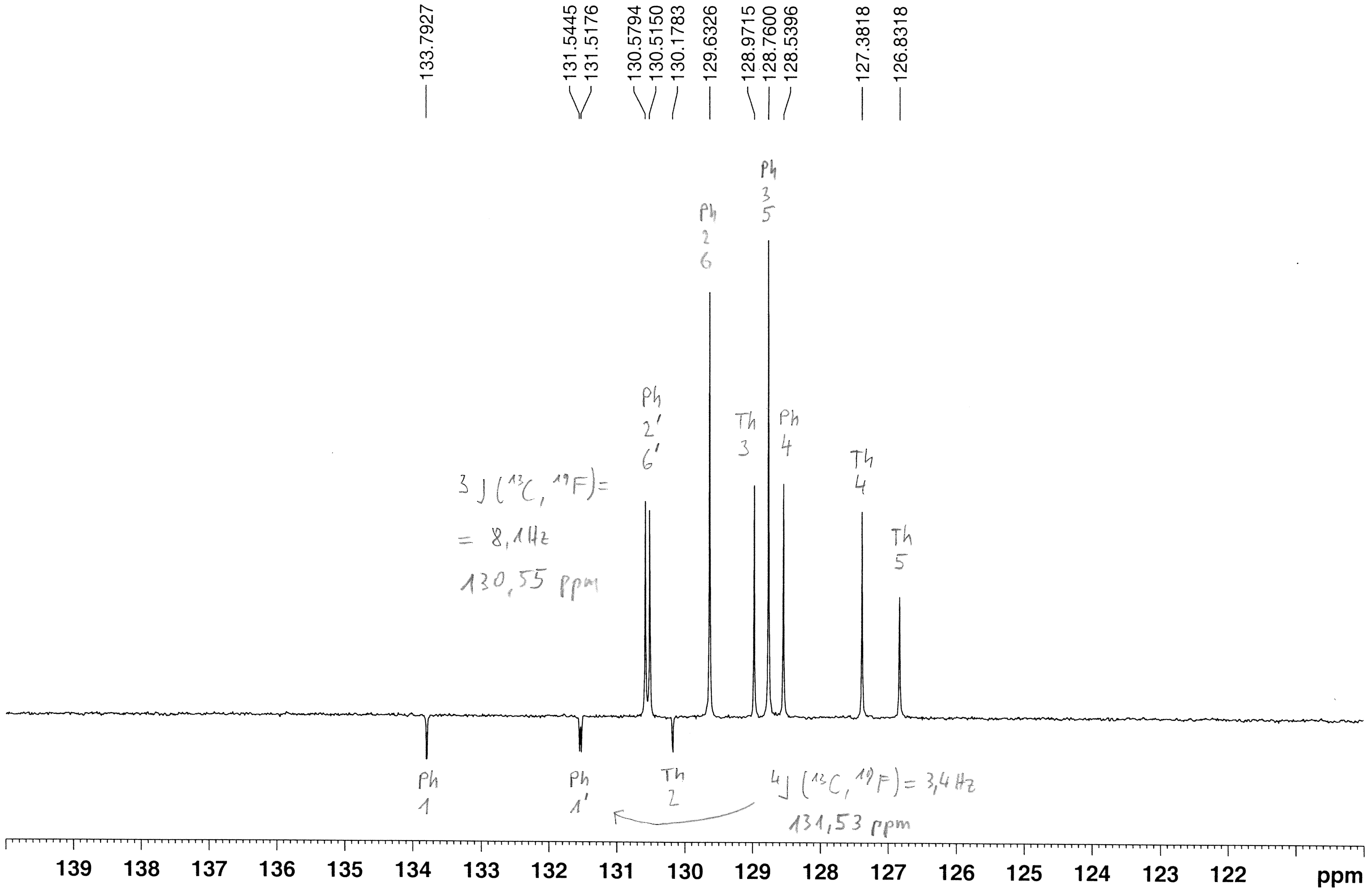

CE155-S2P1 in cdcl3 (APT) 29.8.2018

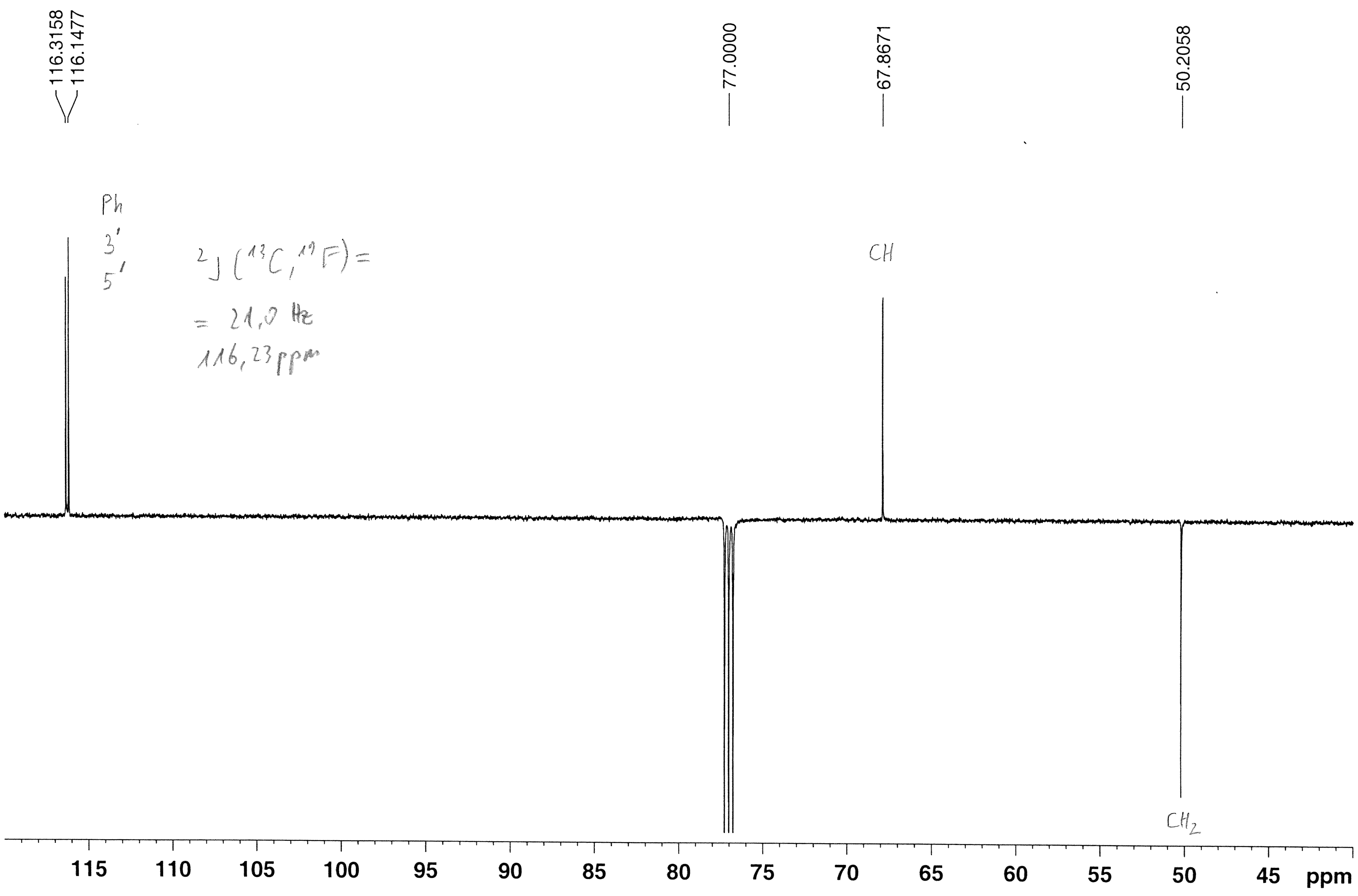

Figure S12c. NMR spectra of compound **8c.**

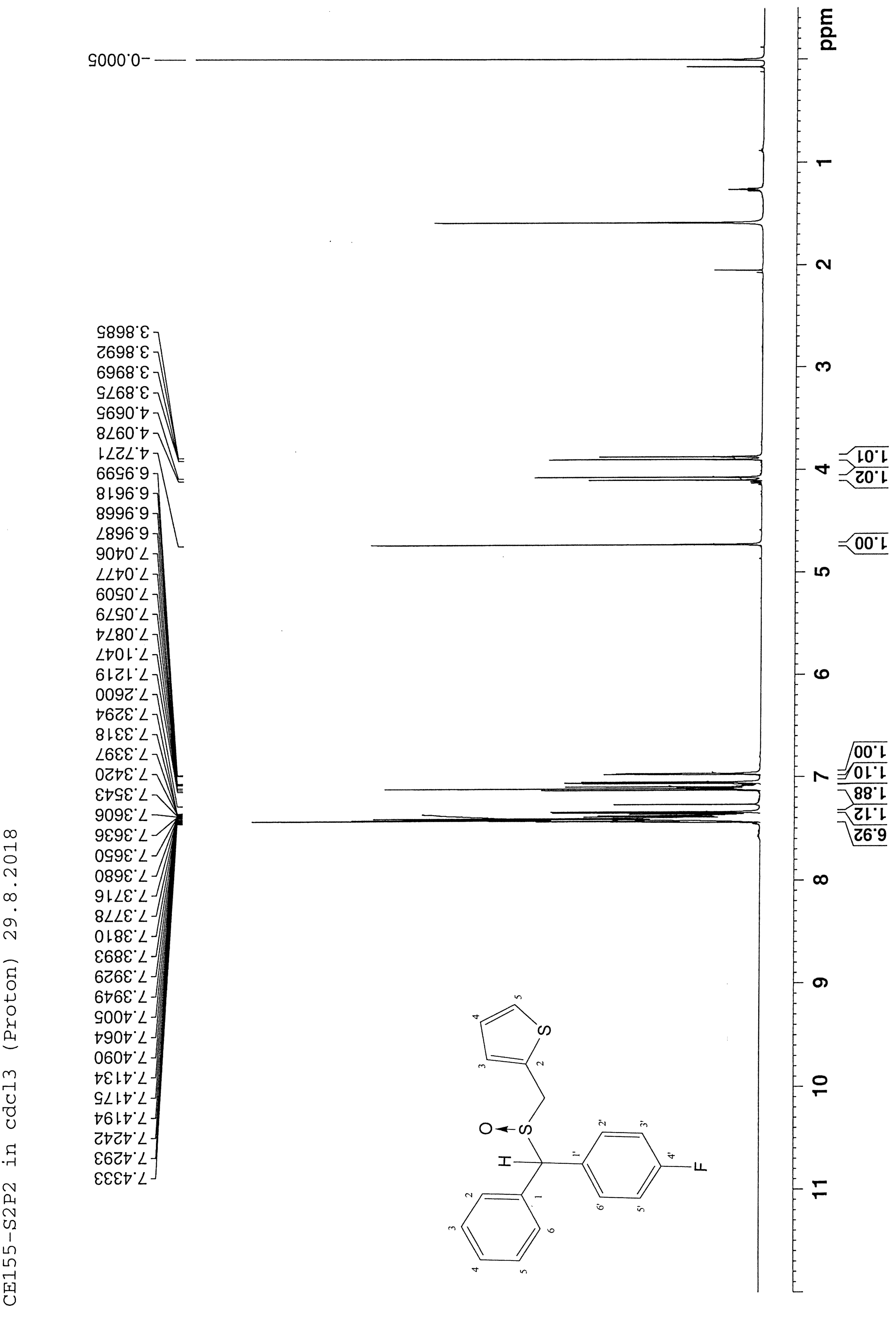

CE155-S2P2 in cdcl3 (Proton) 29.8.2018

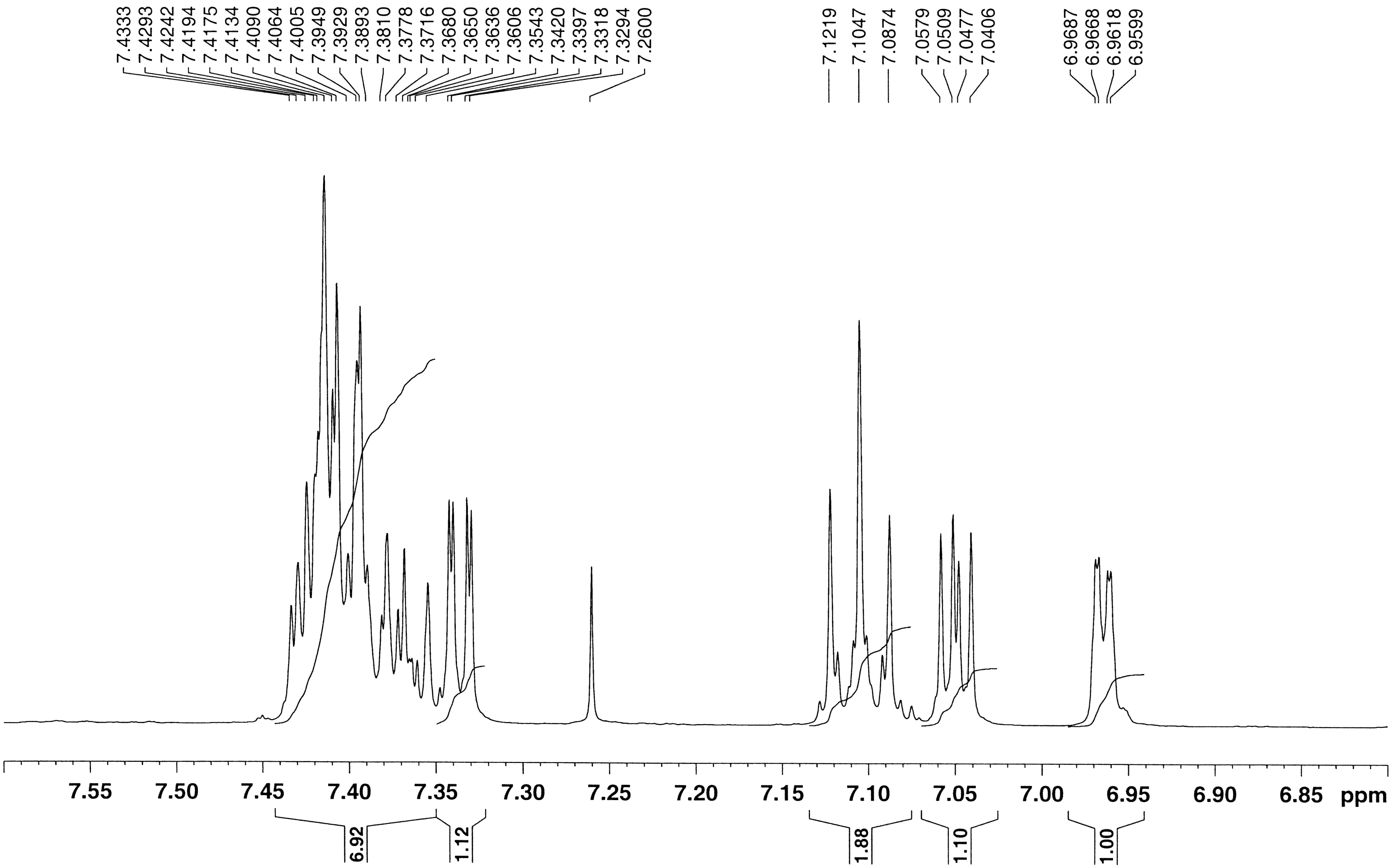

CE155-S2P2 in cdcl3 (Proton) 29.8.2018

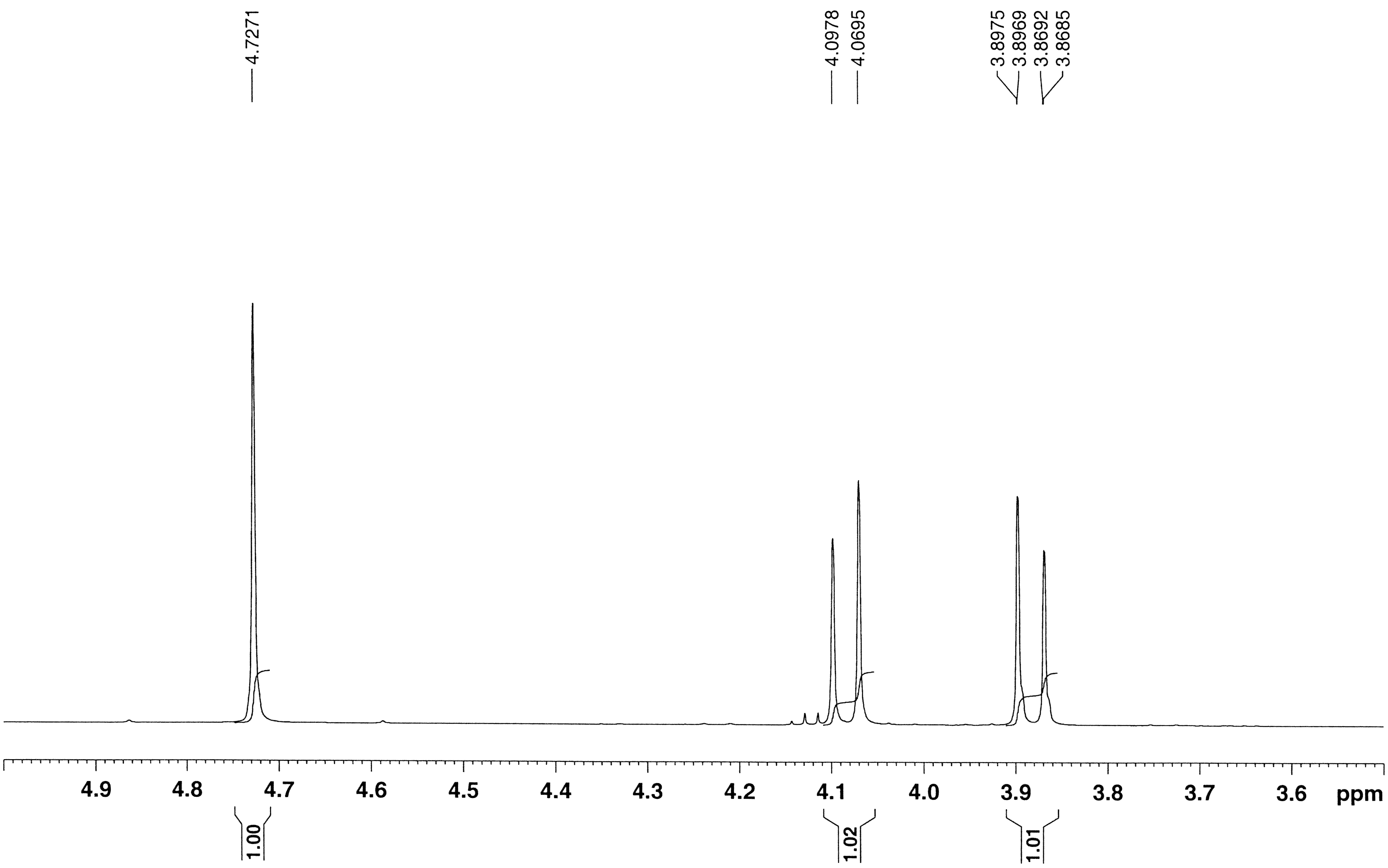

CE155-S2P2 in cdcl3 (APT) 29.8.2018

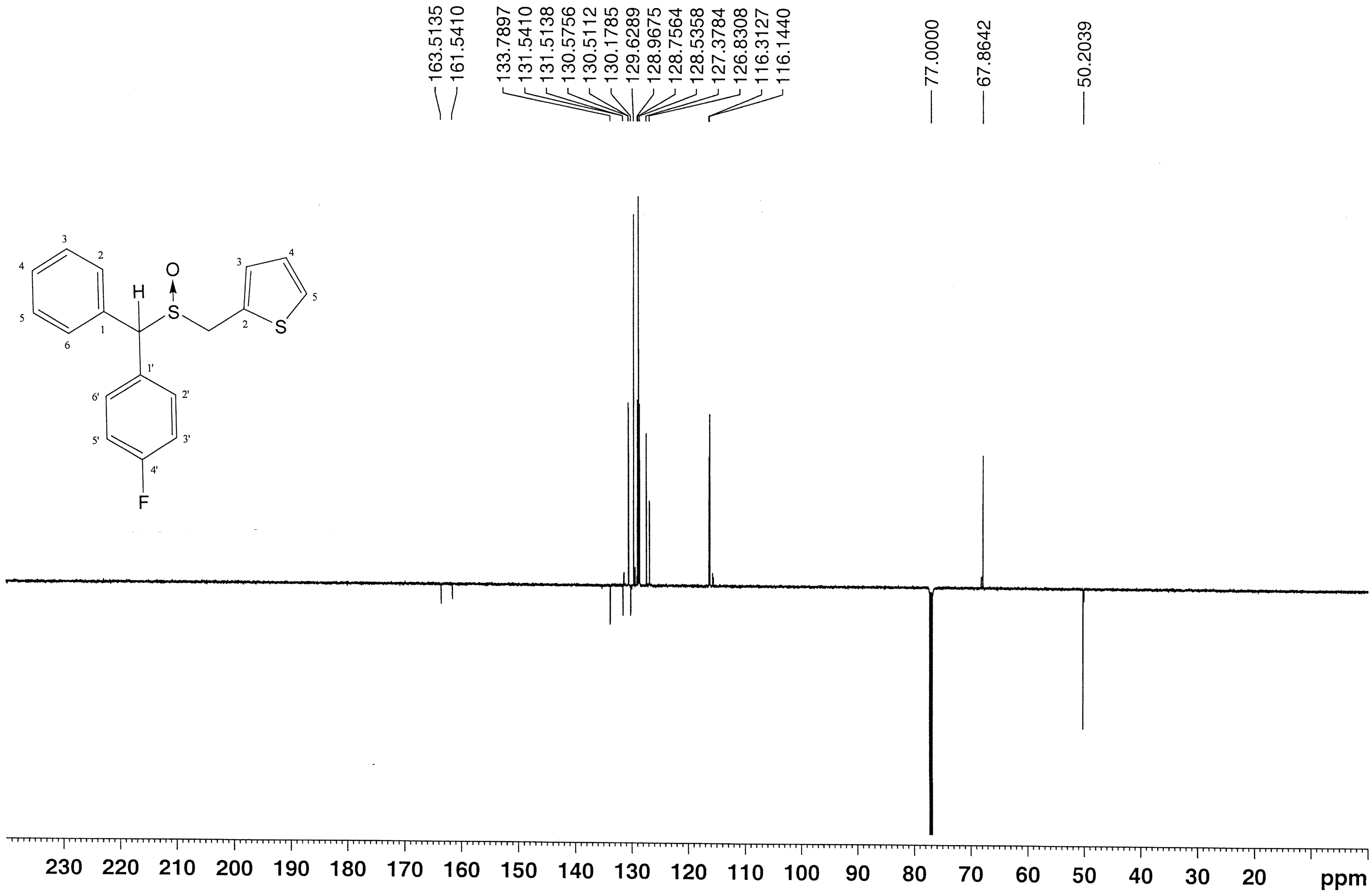

CE155-S2P2 in cdcl3 (APT) 29.8.2018

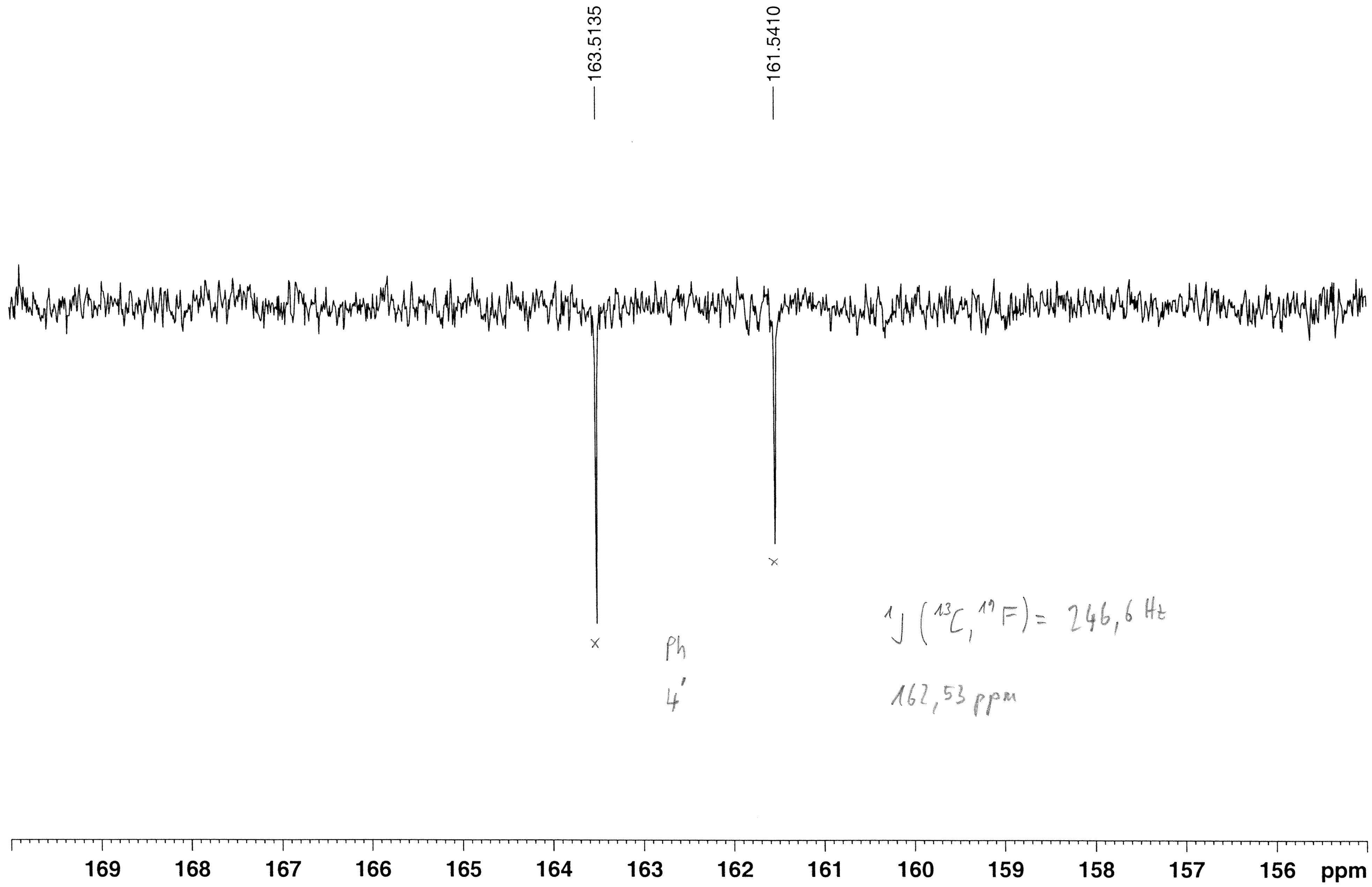

CE155-S2P2 in cdcl3 (APT) 29.8.2018

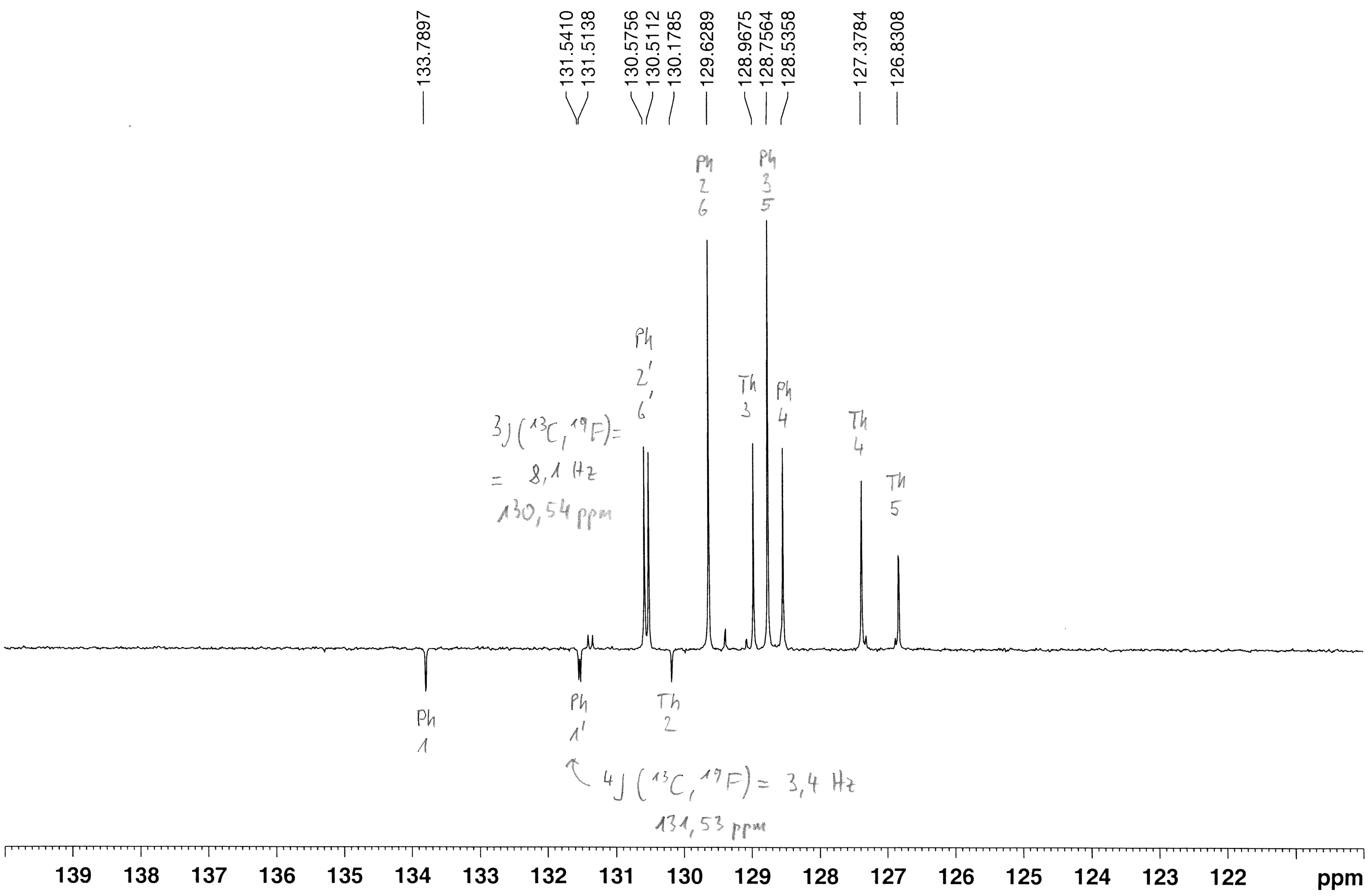

CE155-S2P2 in cdcl3 (APT) 29.8.2018

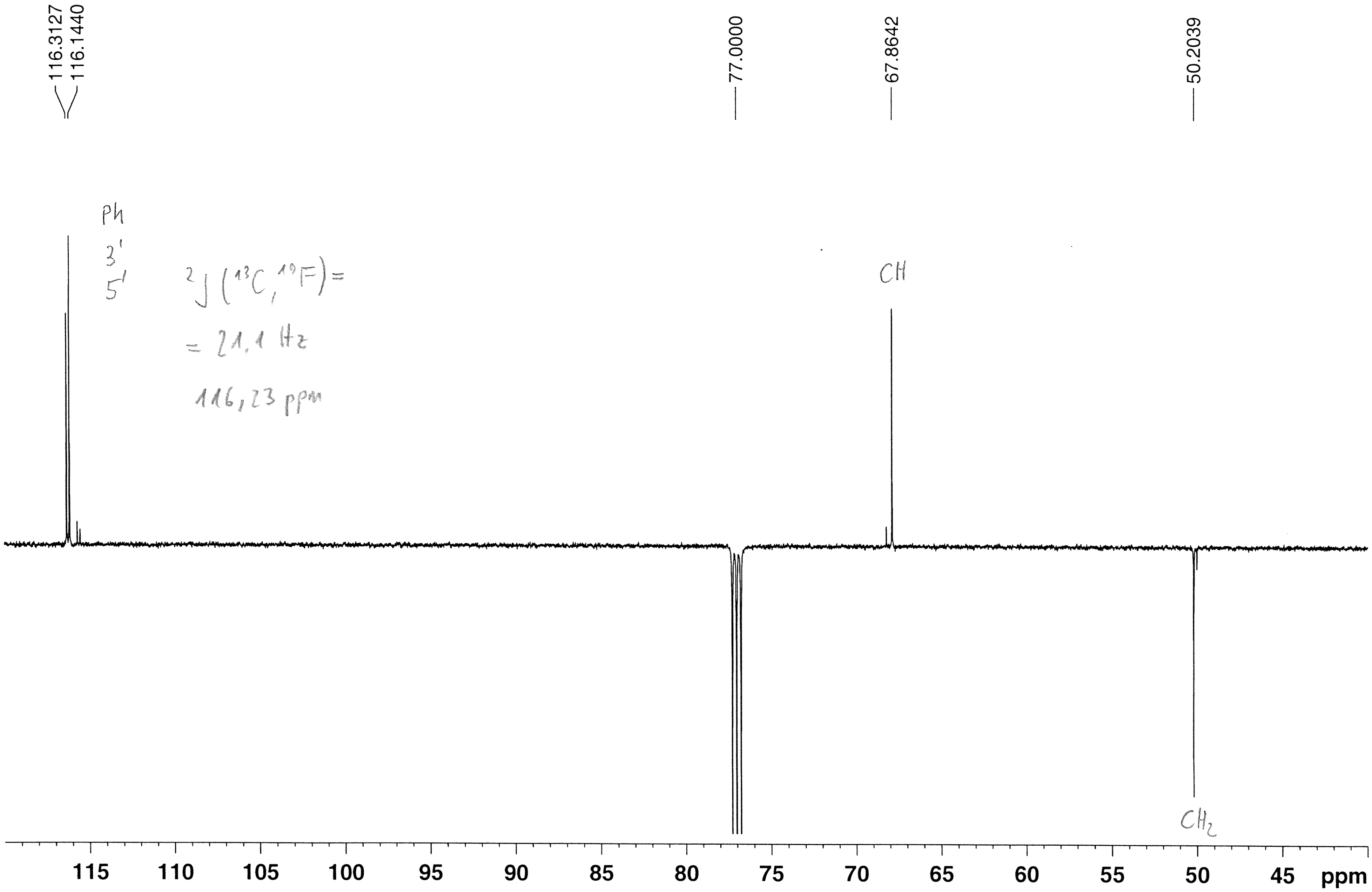

Figure S13c. NMR spectra of compound **5d**.

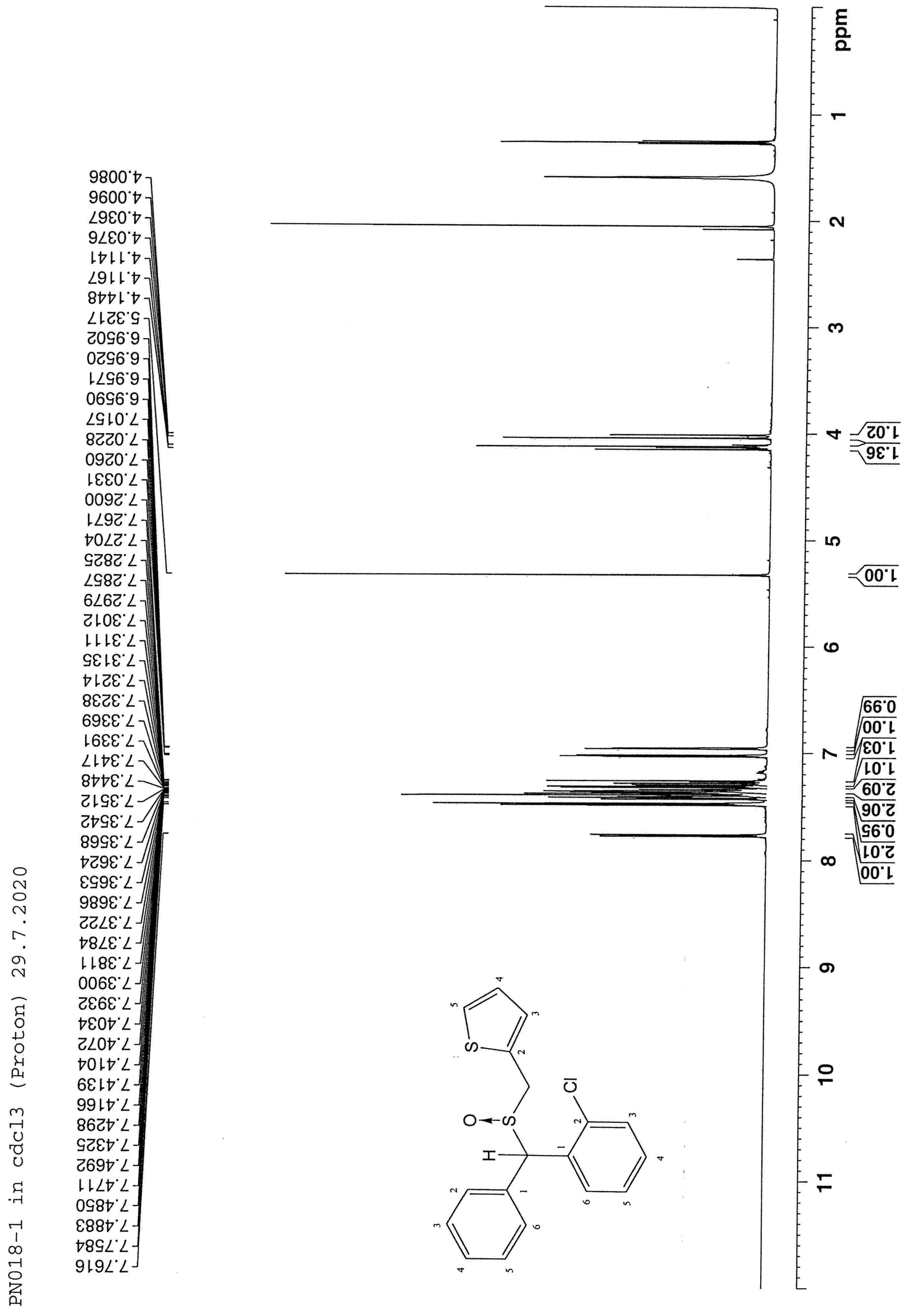

PN018-1 in cdcl3 (Proton) 29.7.2020

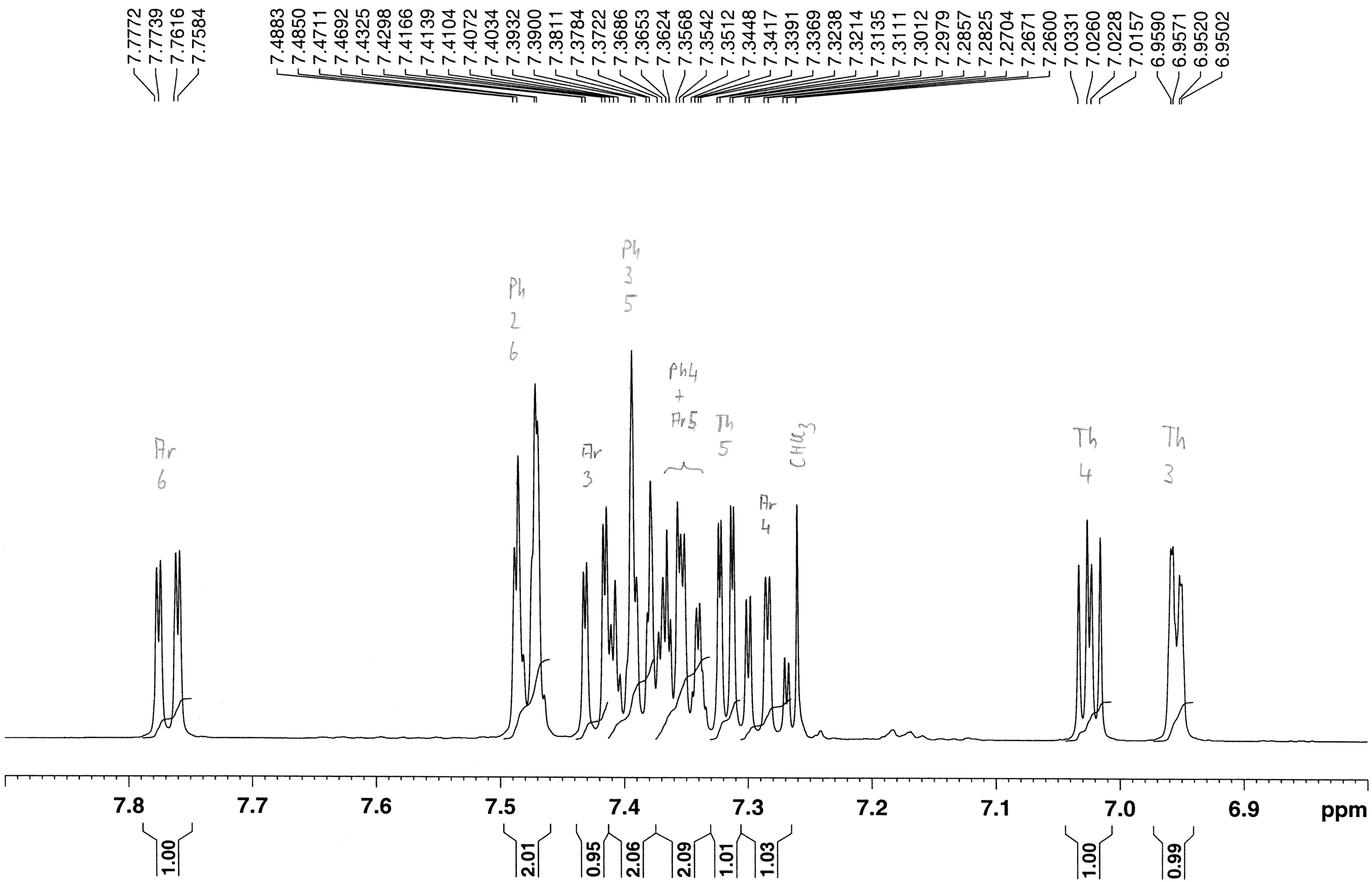

PN018-1 in cdcl3 (Proton) 29.7.2020

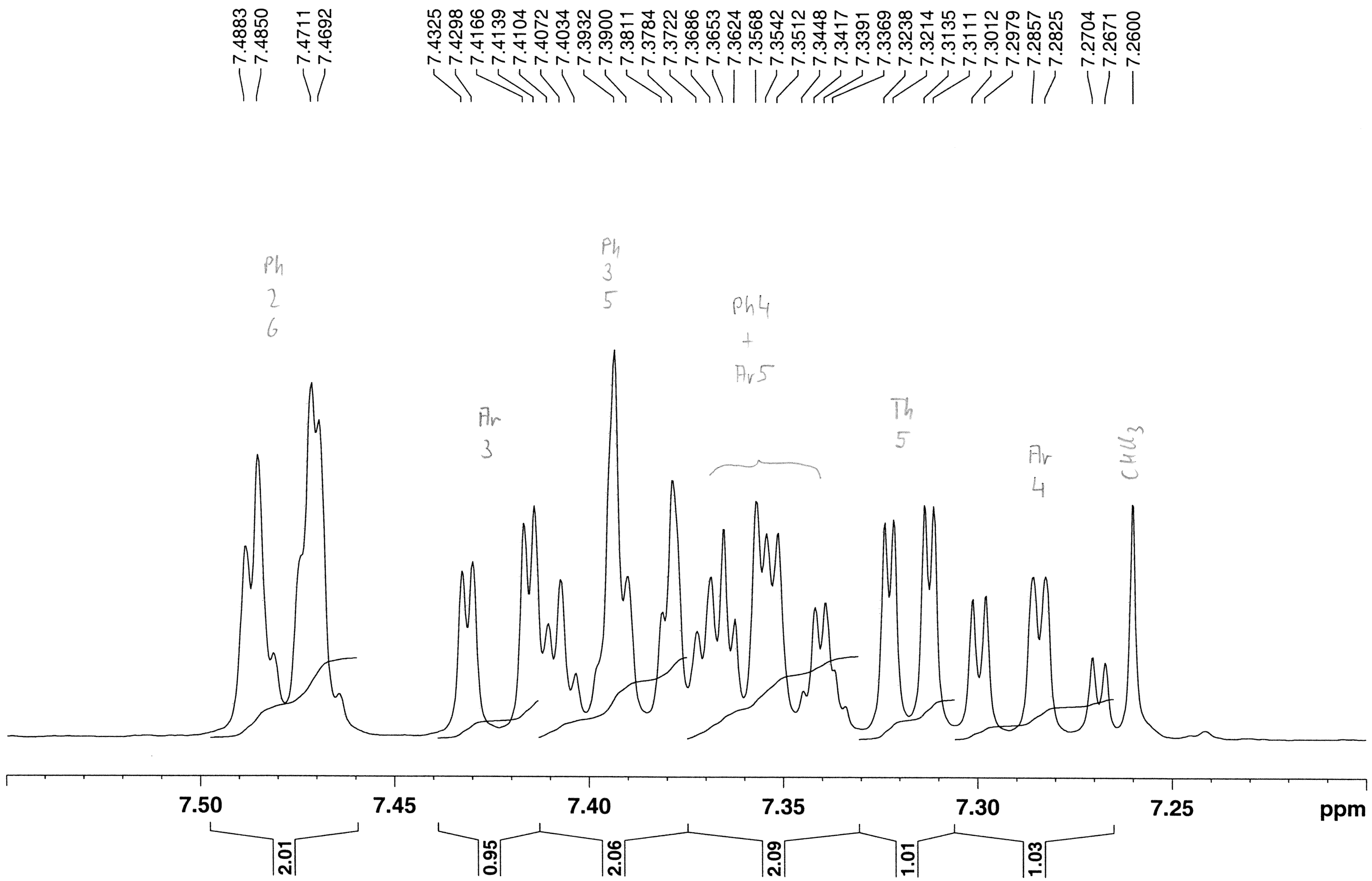

PN018-1 in cdcl3 (Proton) 29.7.2020

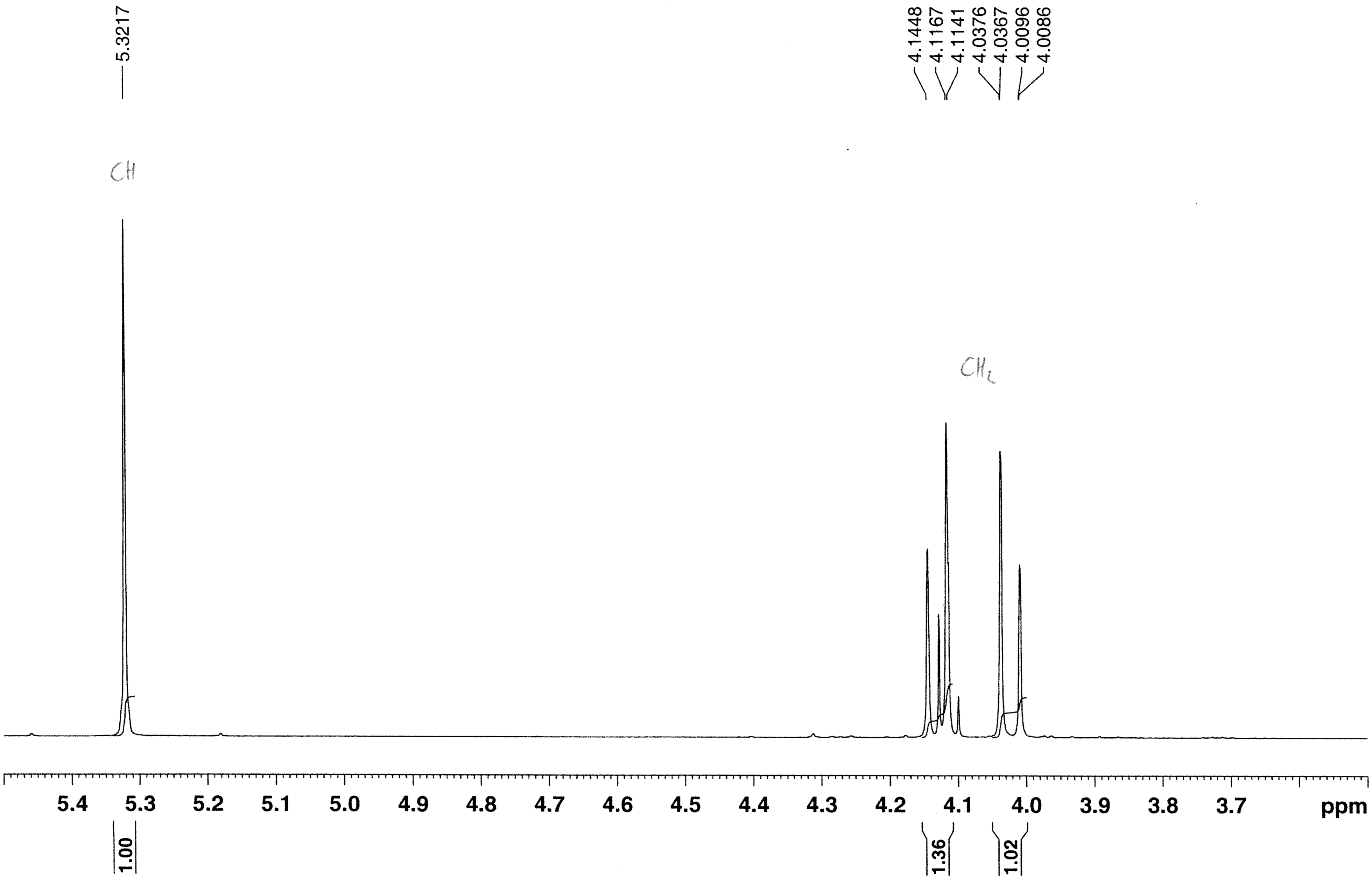

PN018-1 in cdcl3 (APT) 29.7.2020

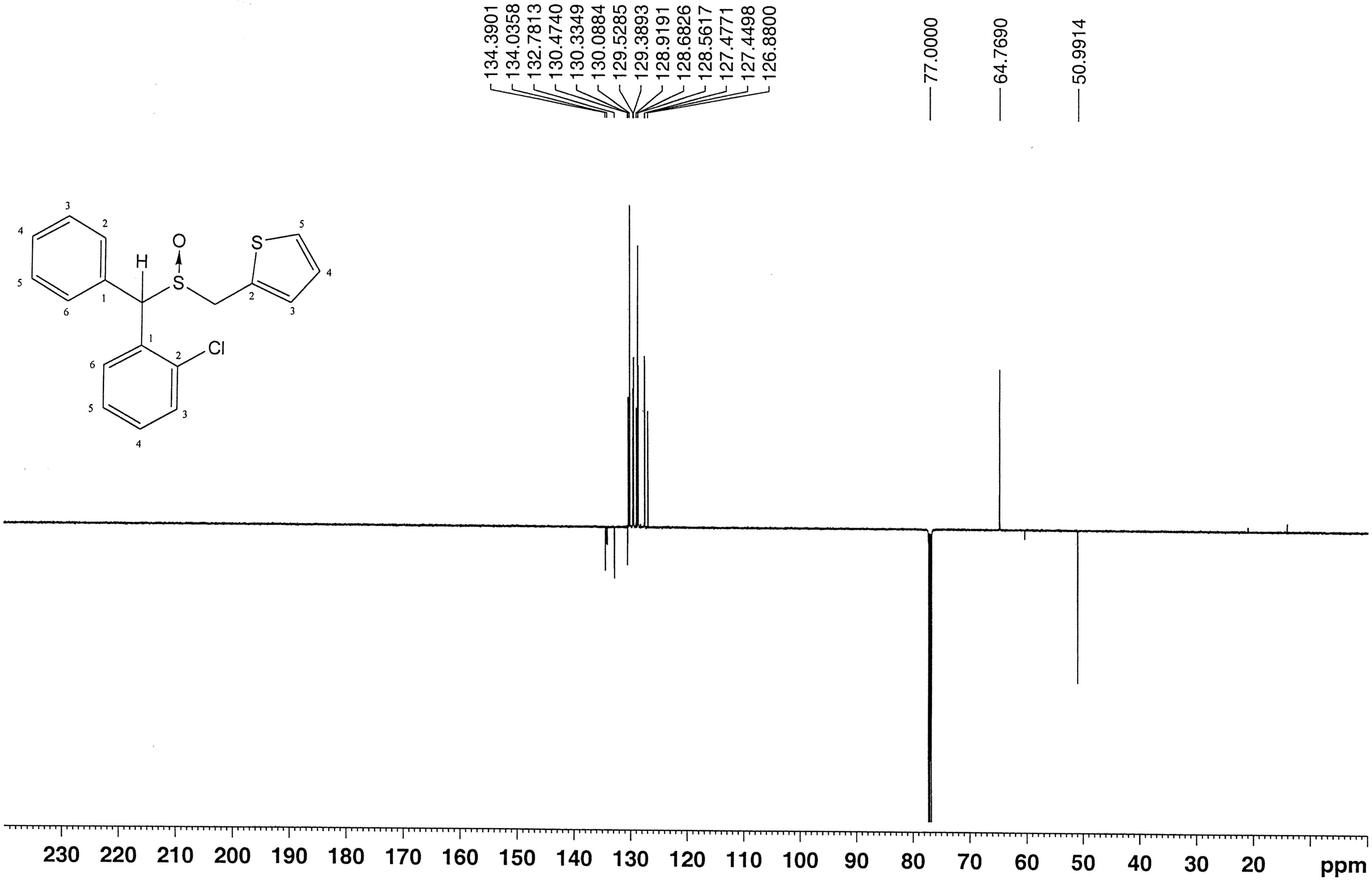

PN018-1 in cdcl3 (APT) 29.7.2020

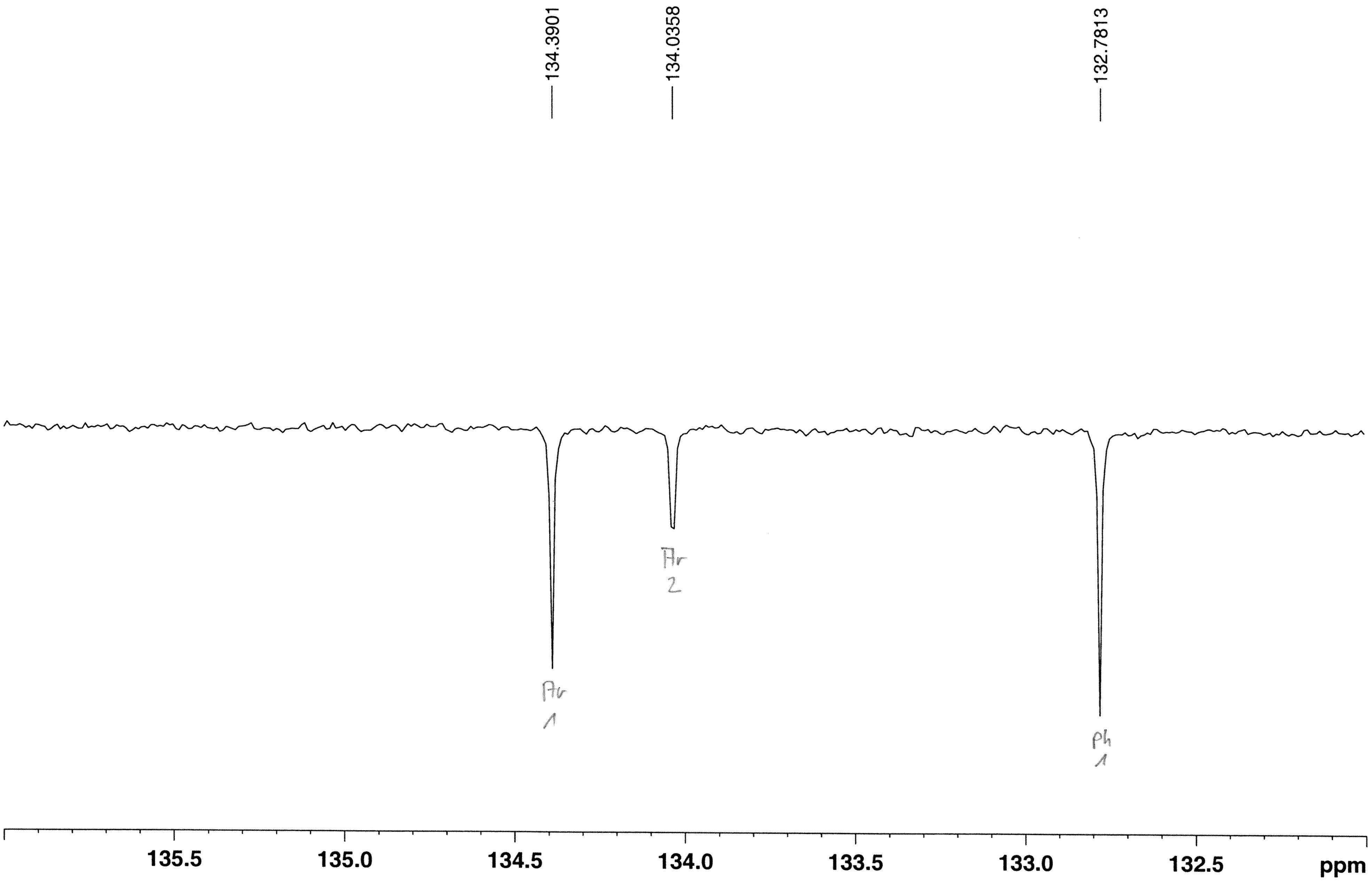

PN018-1 in cdcl3 (APT) 29.7.2020

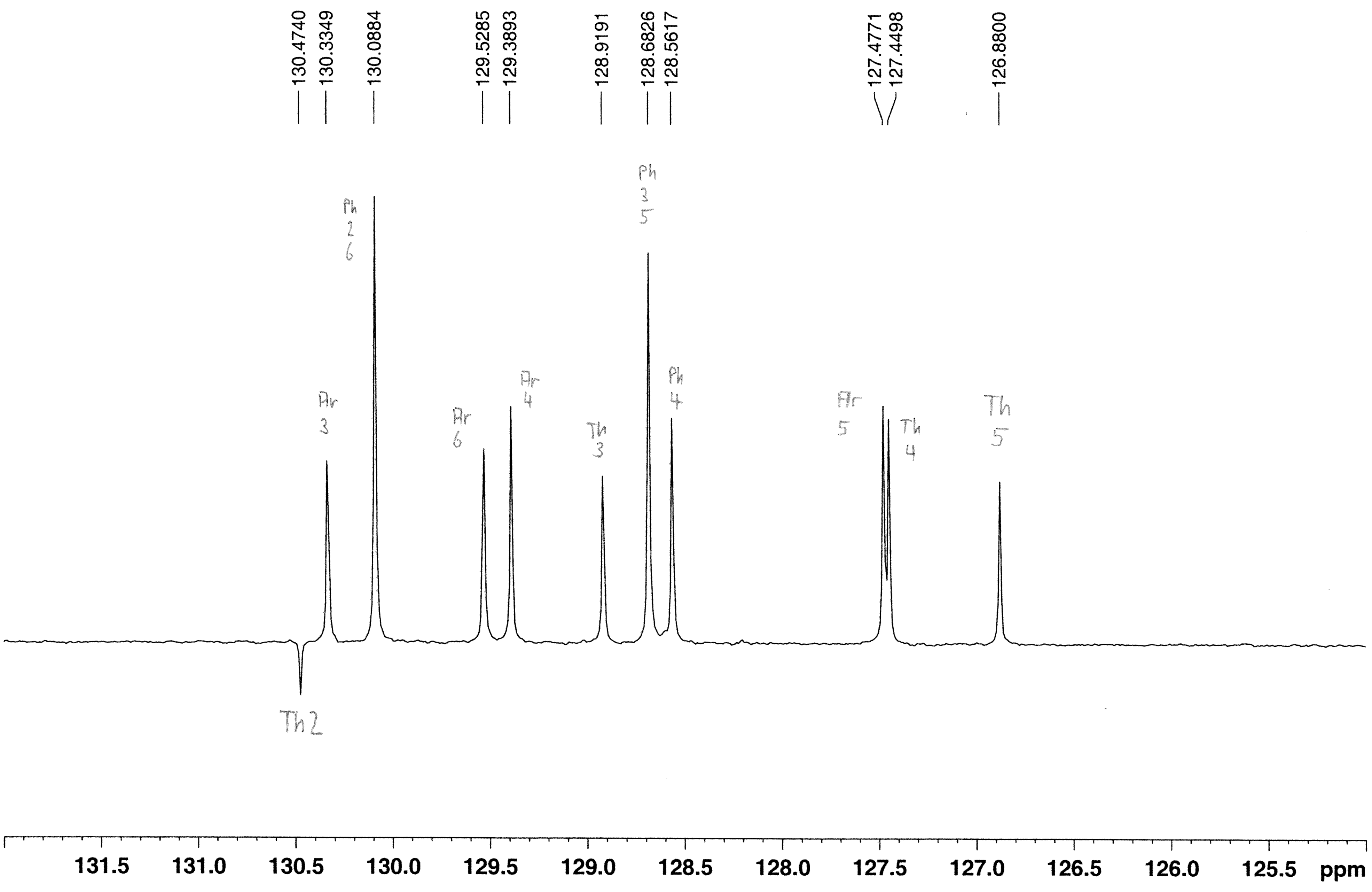

PN018-1 in cdcl3 (APT) 29.7.2020

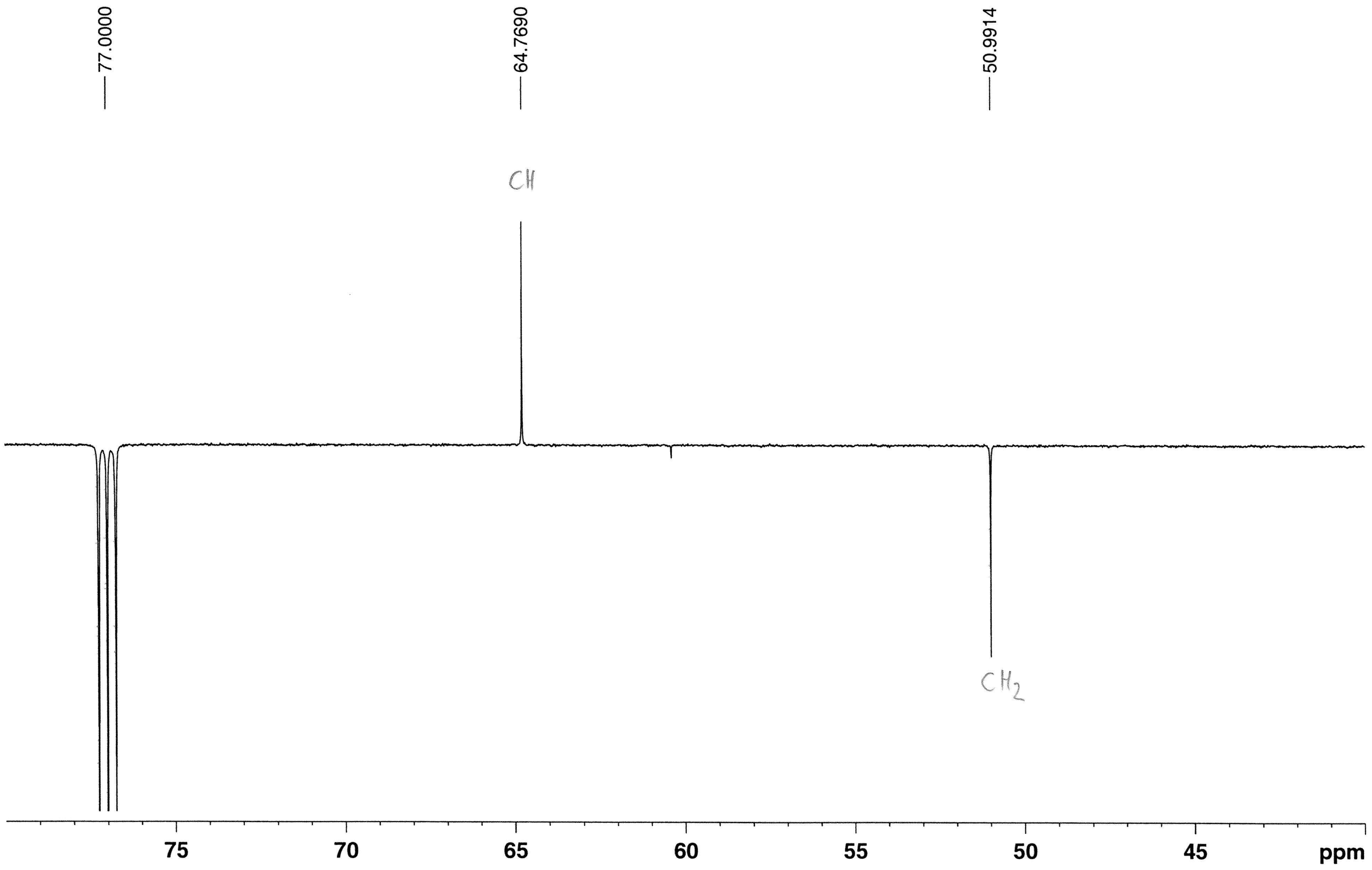

PN018-1 in cdcl3 (COSY) 29.7.2020

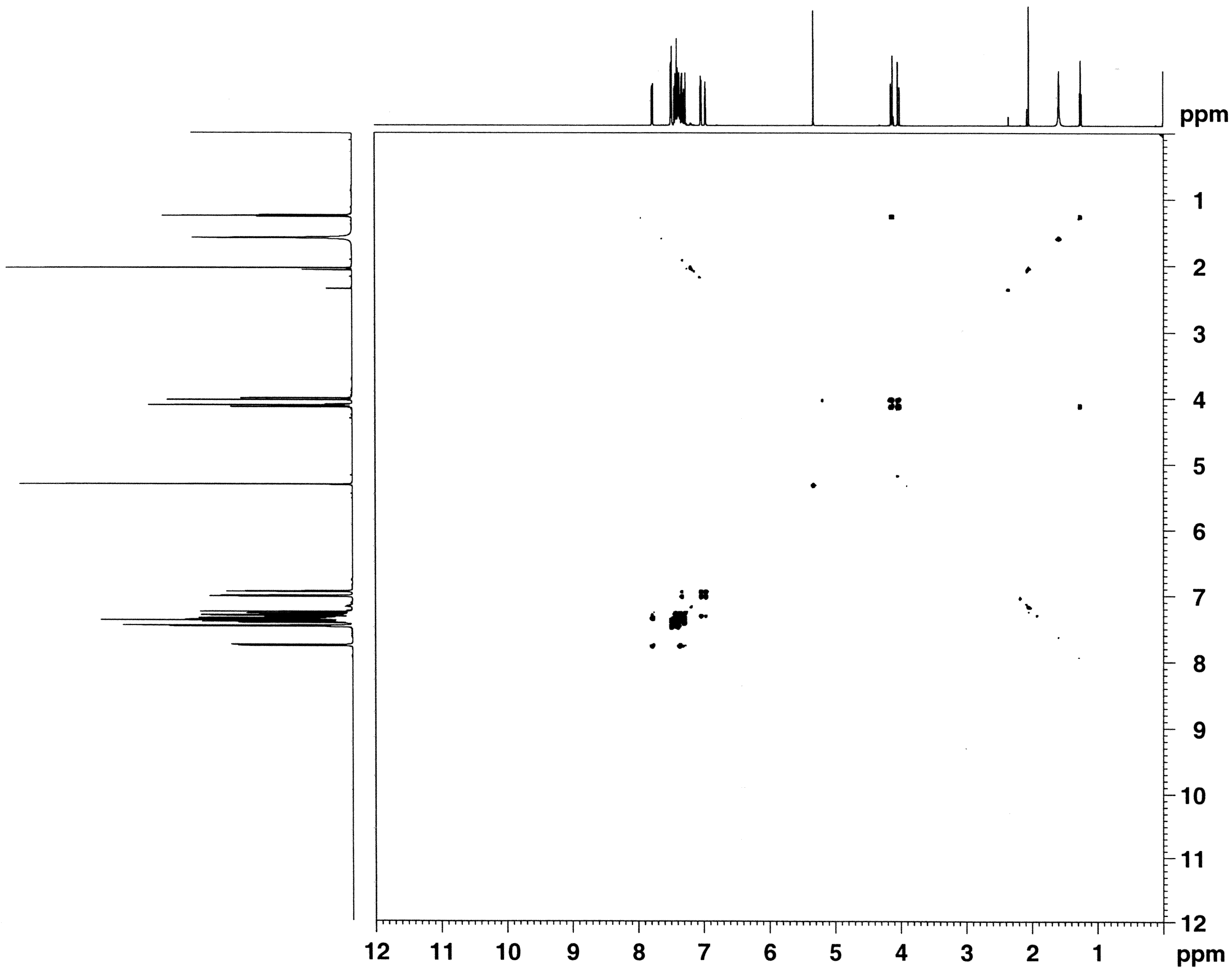

PN018-1 in cdcl3 (COSY) 29.7.2020

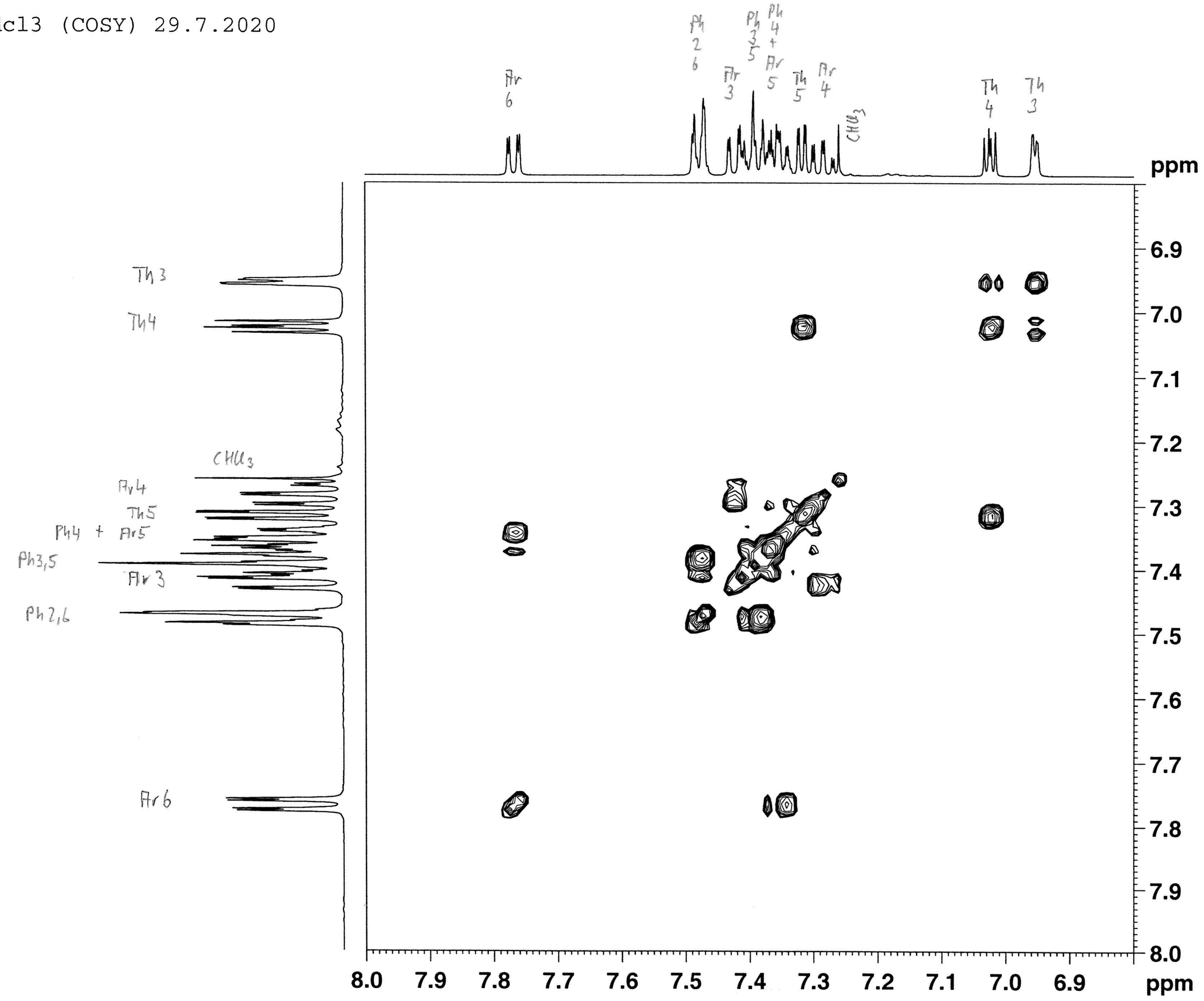

PN018-1 in cdcl3 (COSY) 29.7.2020

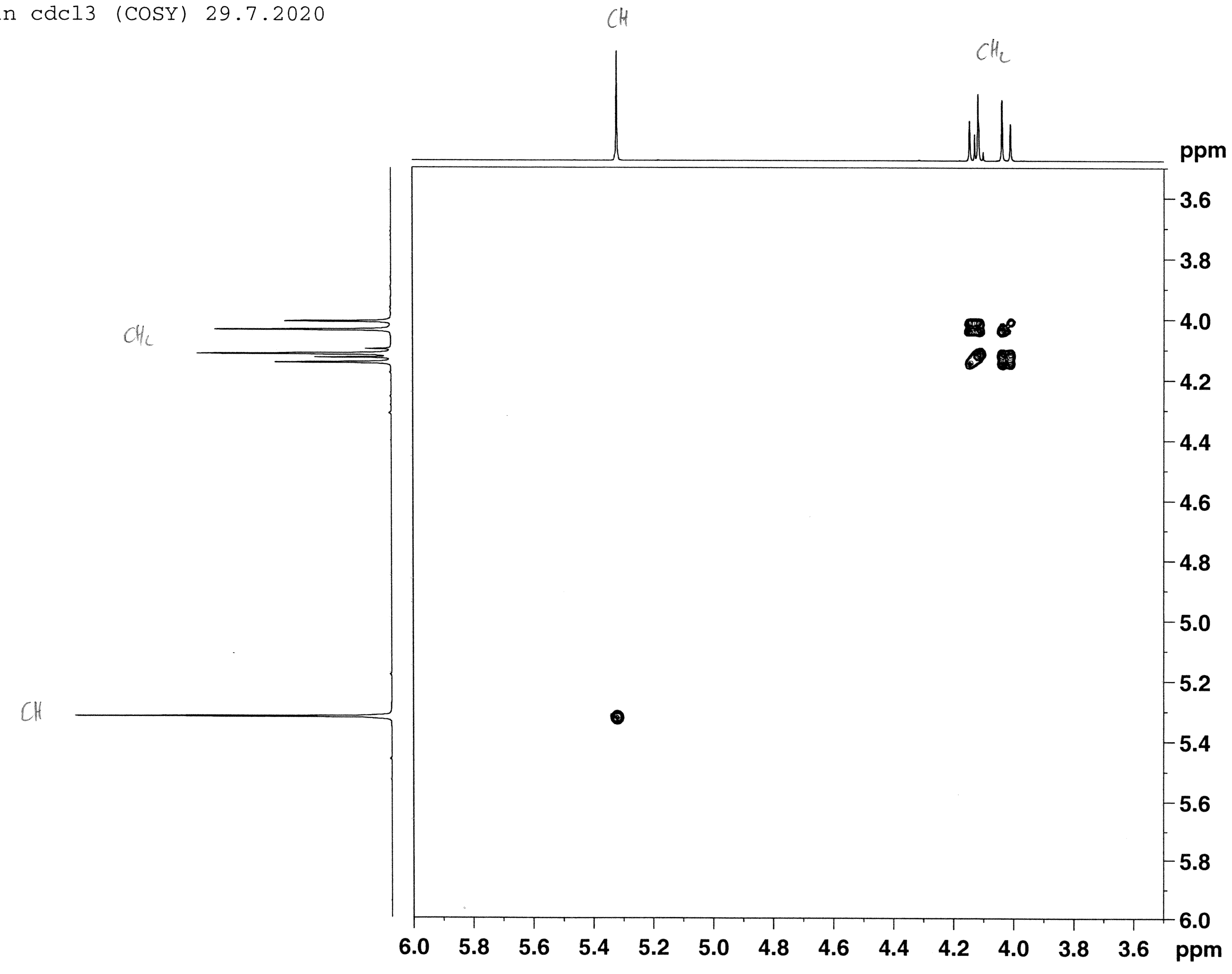

PN018-1 in cdcl3 (HSQC) 29.7.2020

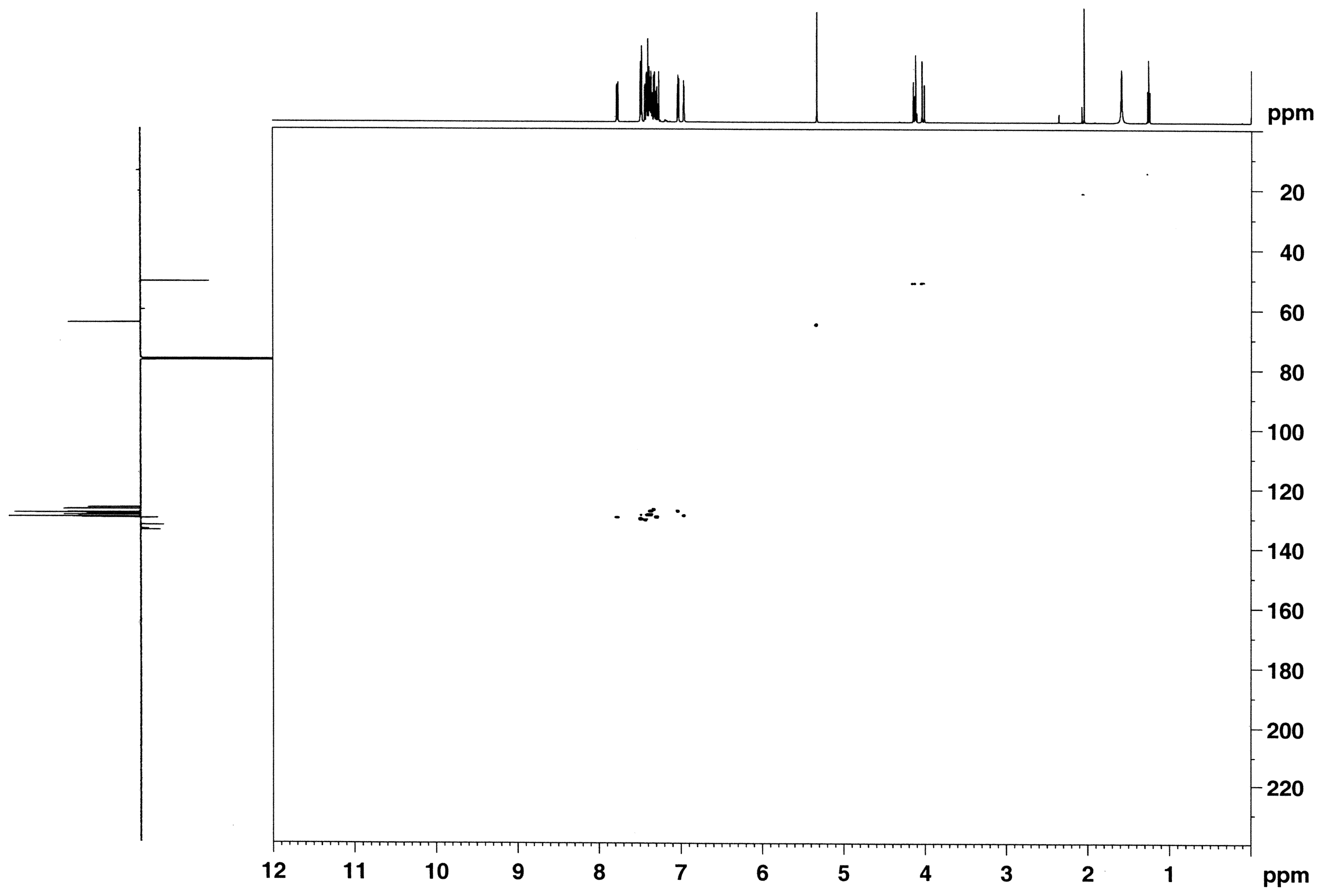

PN018-1 in cdcl3 (HSQC) 29.7.2020

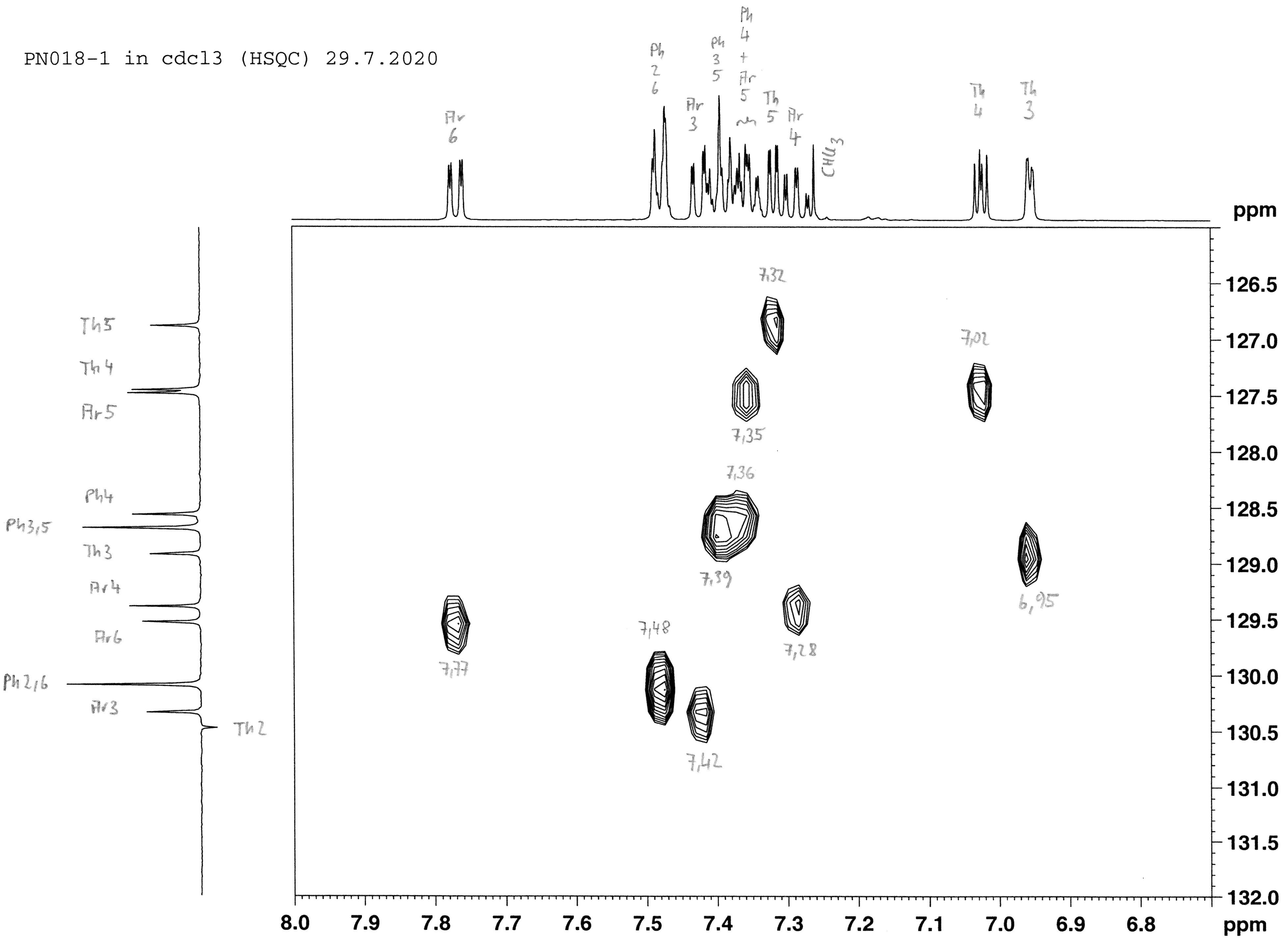

PN018-1 in cdcl3 (HSQC) 29.7.2020

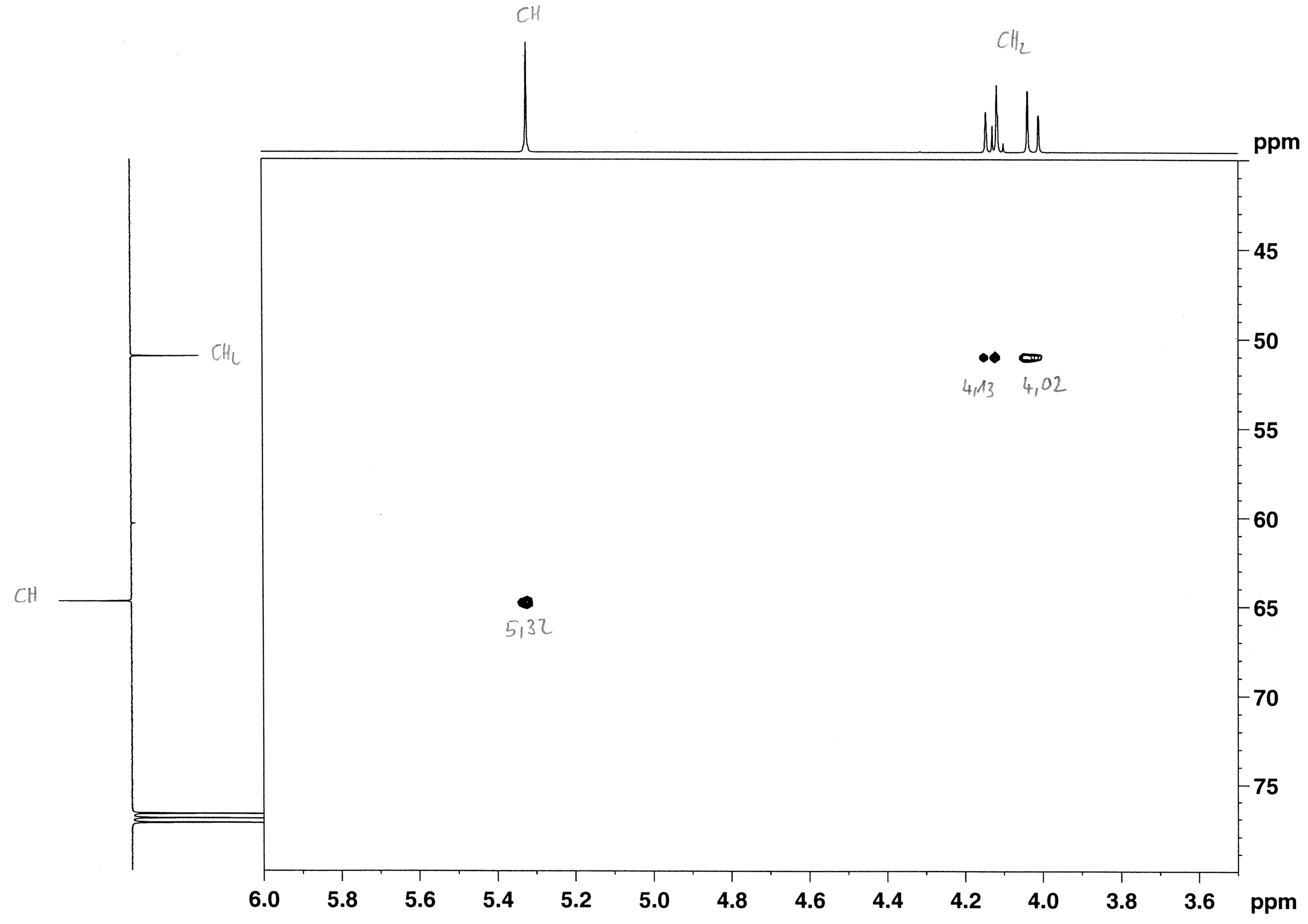

PN018-1 in cdcl3 (HMBC) 29.7.2020

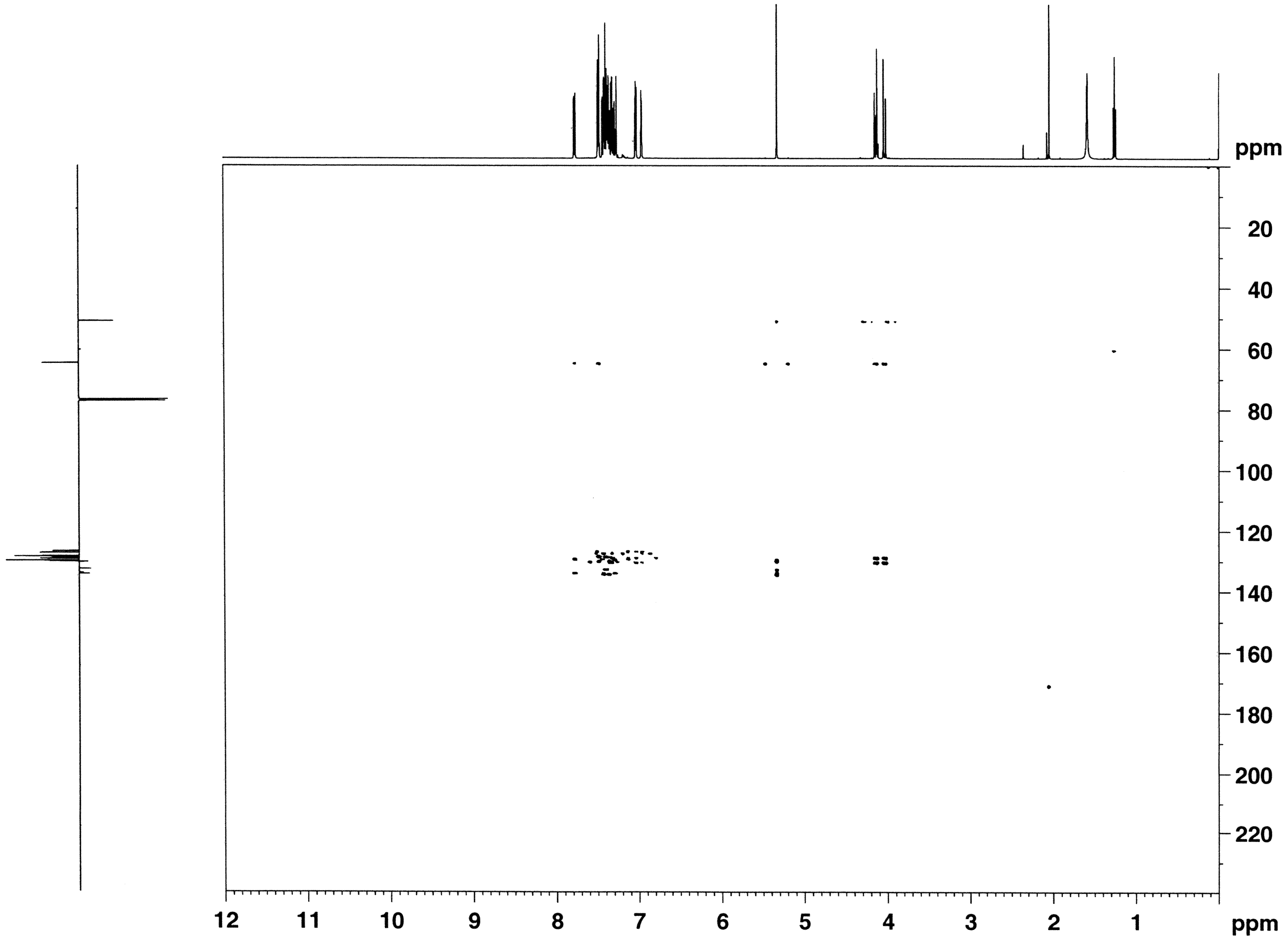

PN018-1 in cdcl3 (HMBC) 29.7.2020

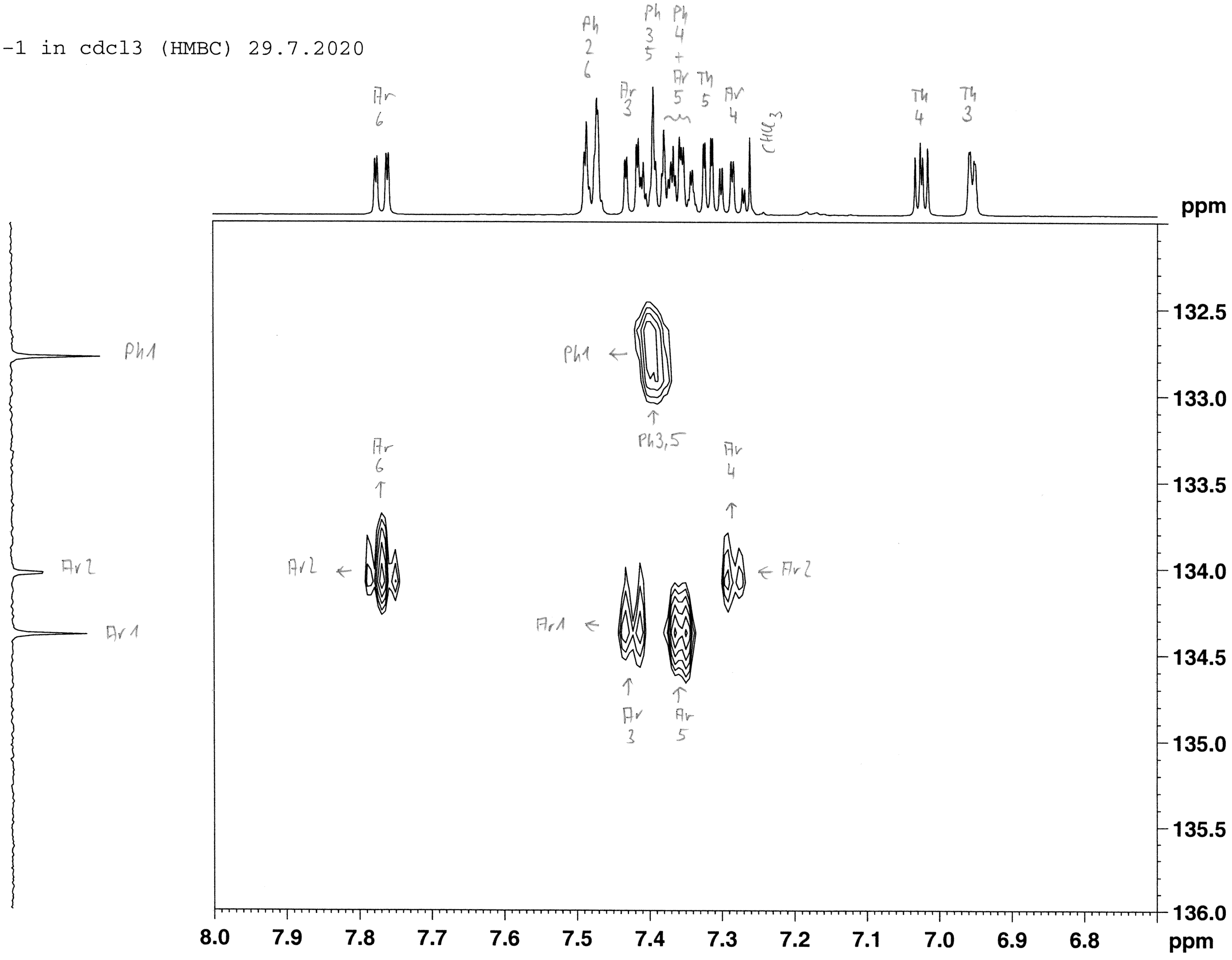

PN018-1 in cdcl3 (HMBC) 29.7.2020

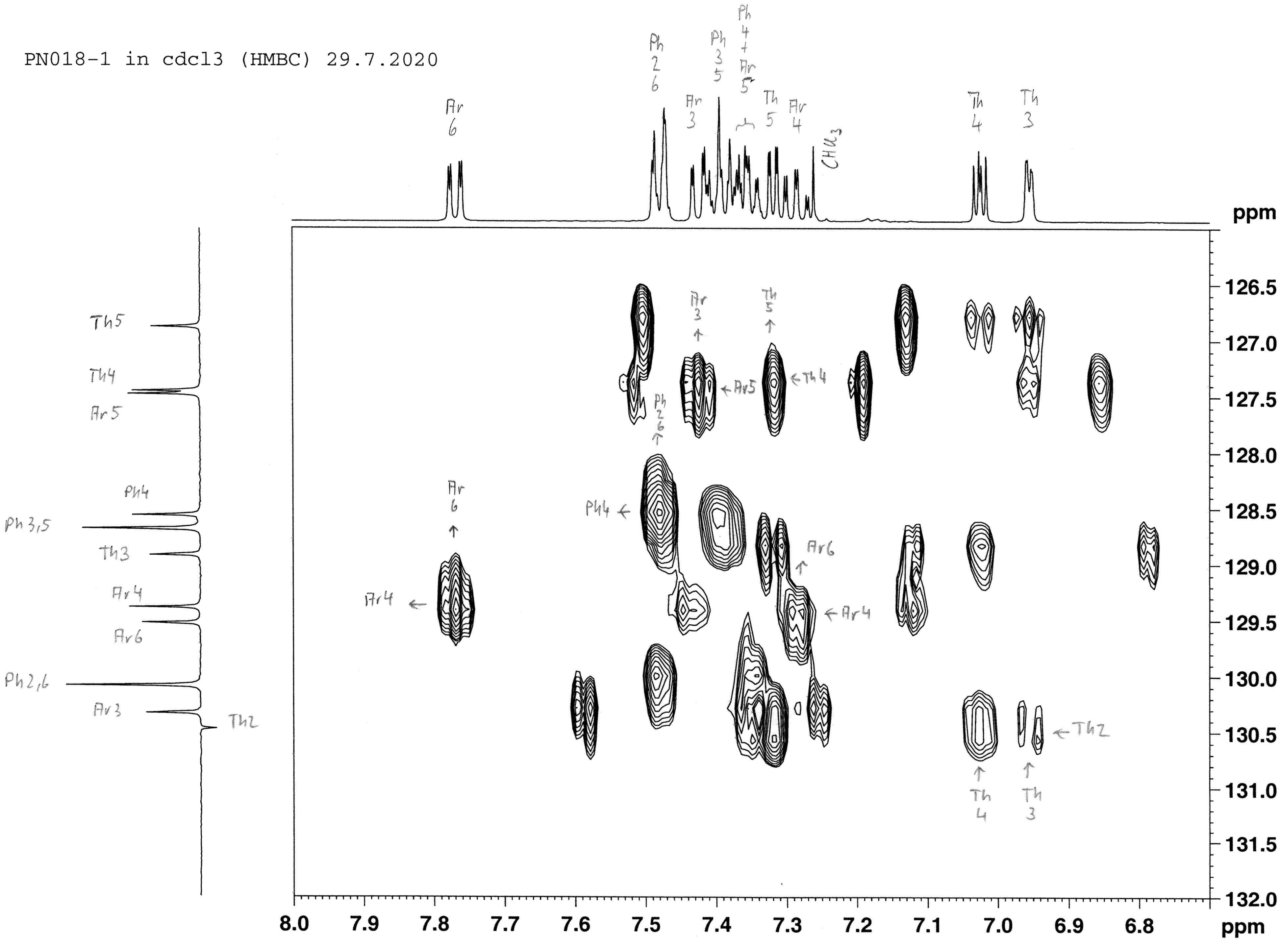

PN018-1 in cdcl3 (HMBC) 29.7.2020

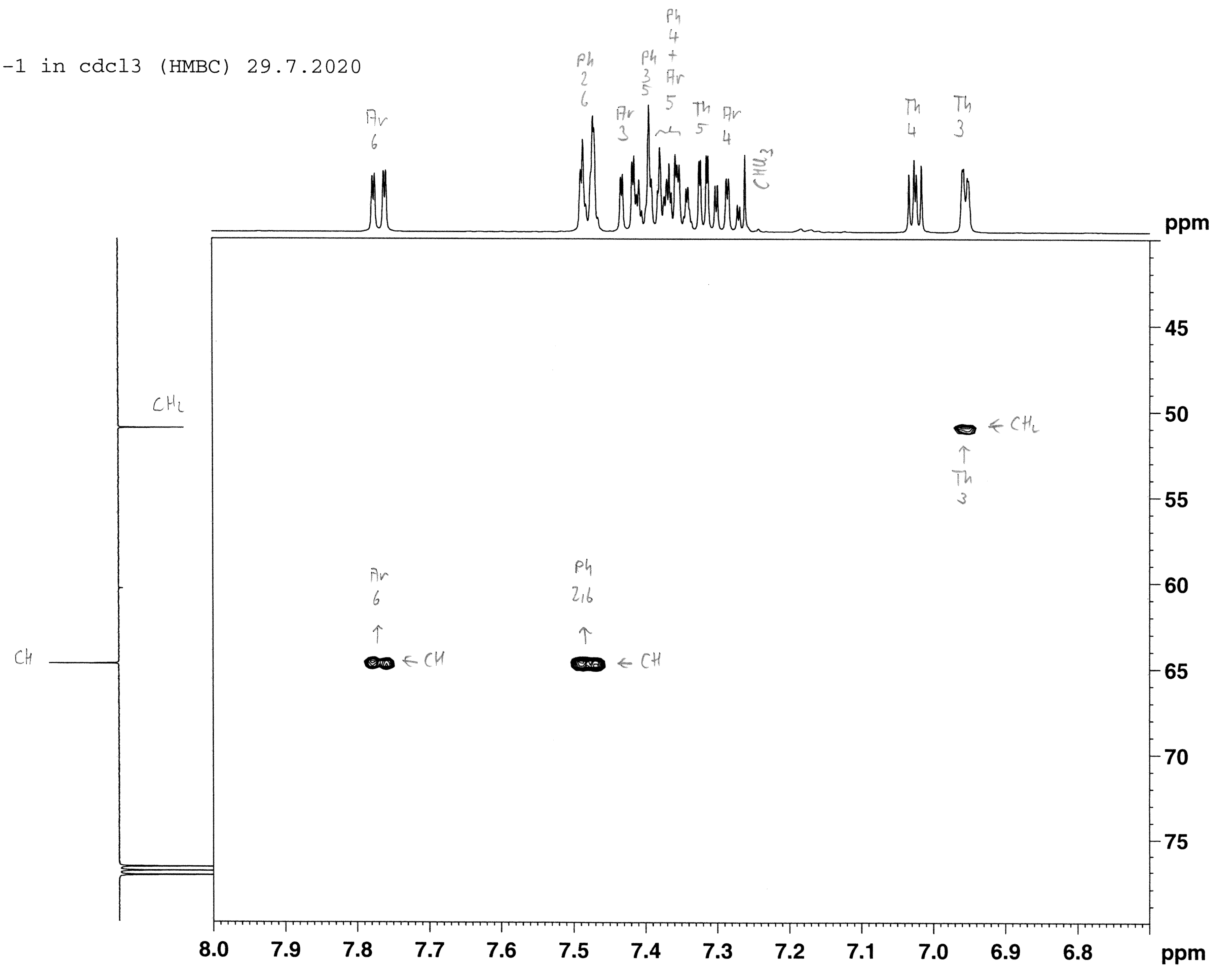

PN018-1 in cdcl3 (HMBC) 29.7.2020

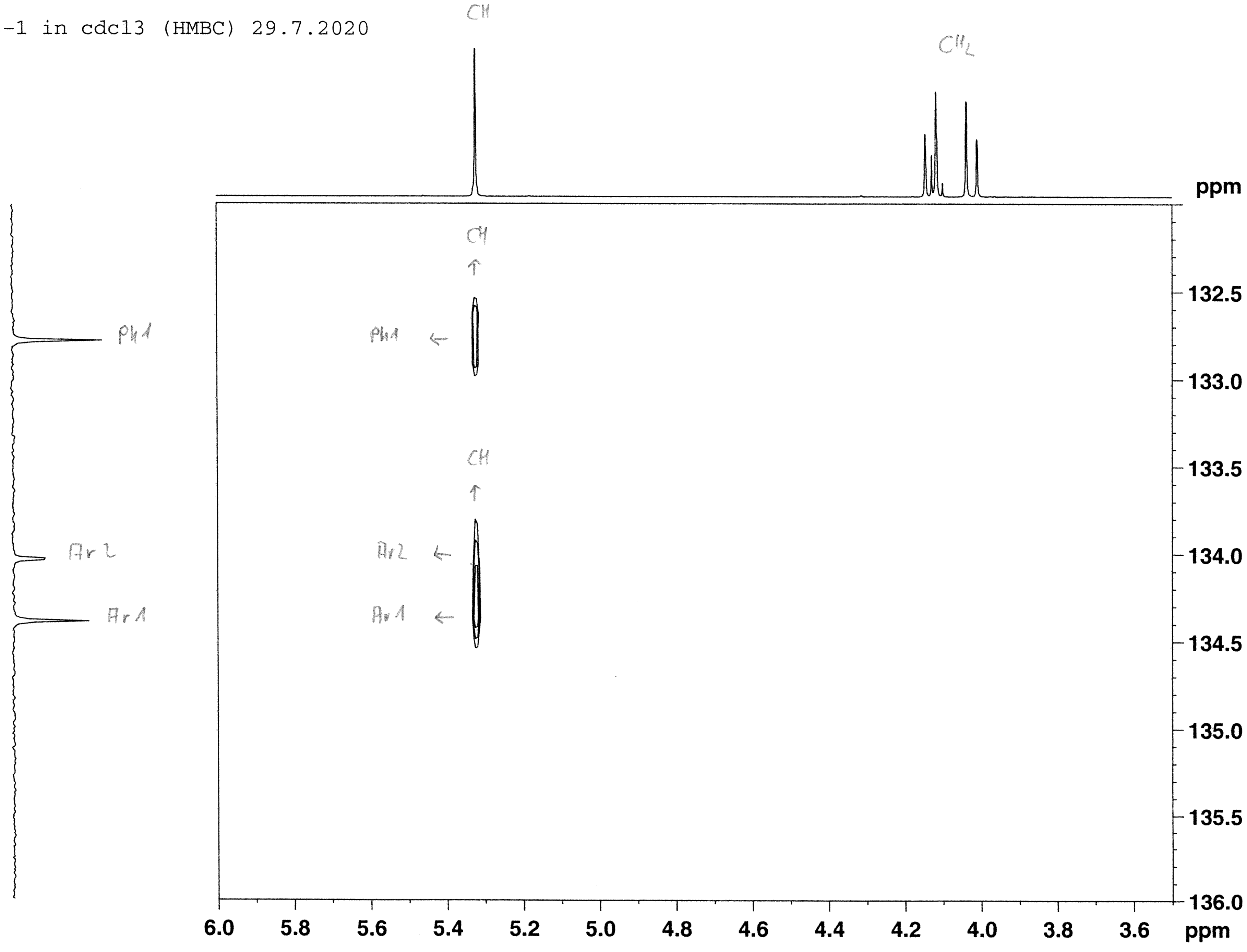

PN018-1 in cdcl3 (HMBC) 29.7.2020

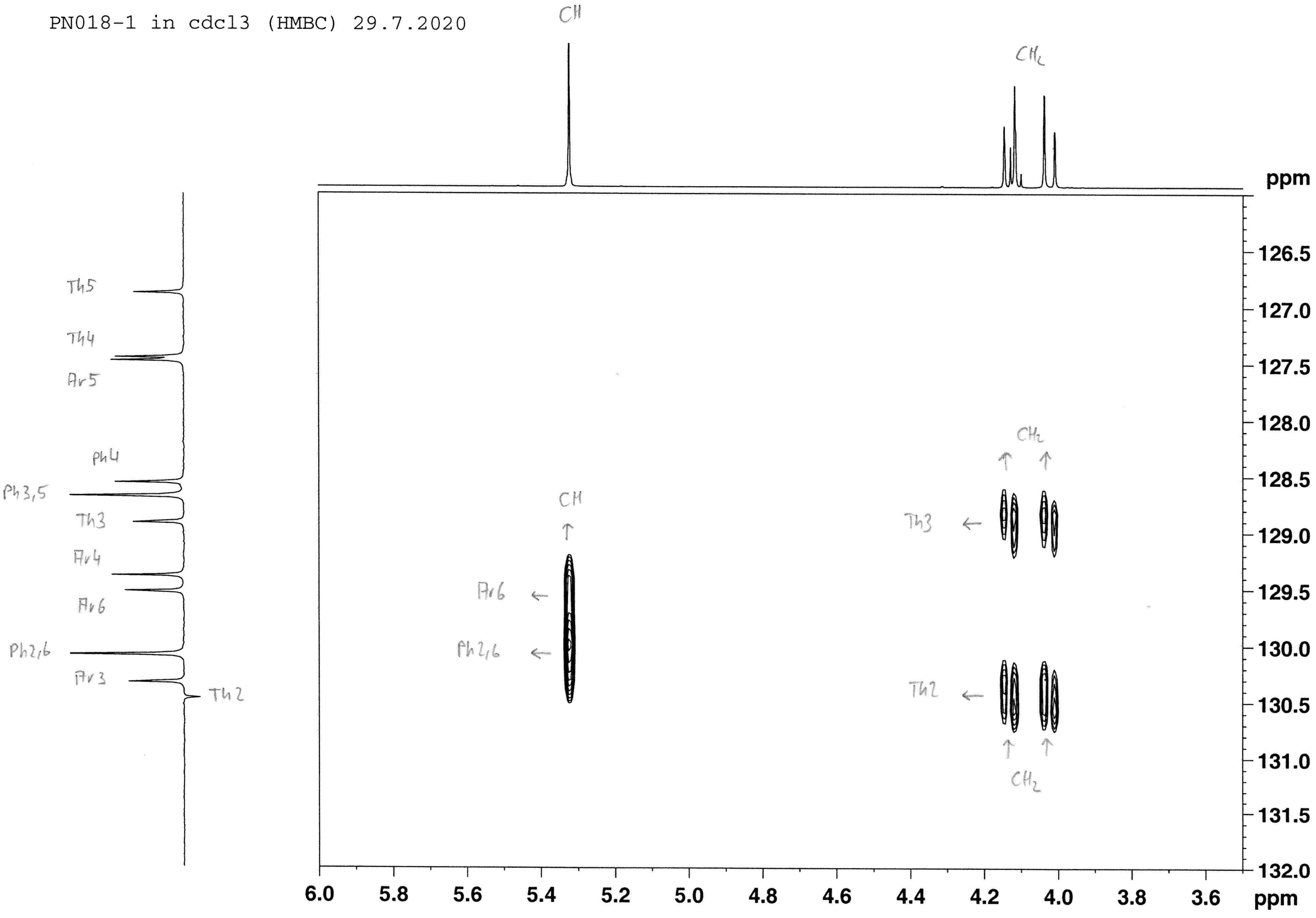

PN018-1 in cdcl3 (HMBC) 29.7.2020

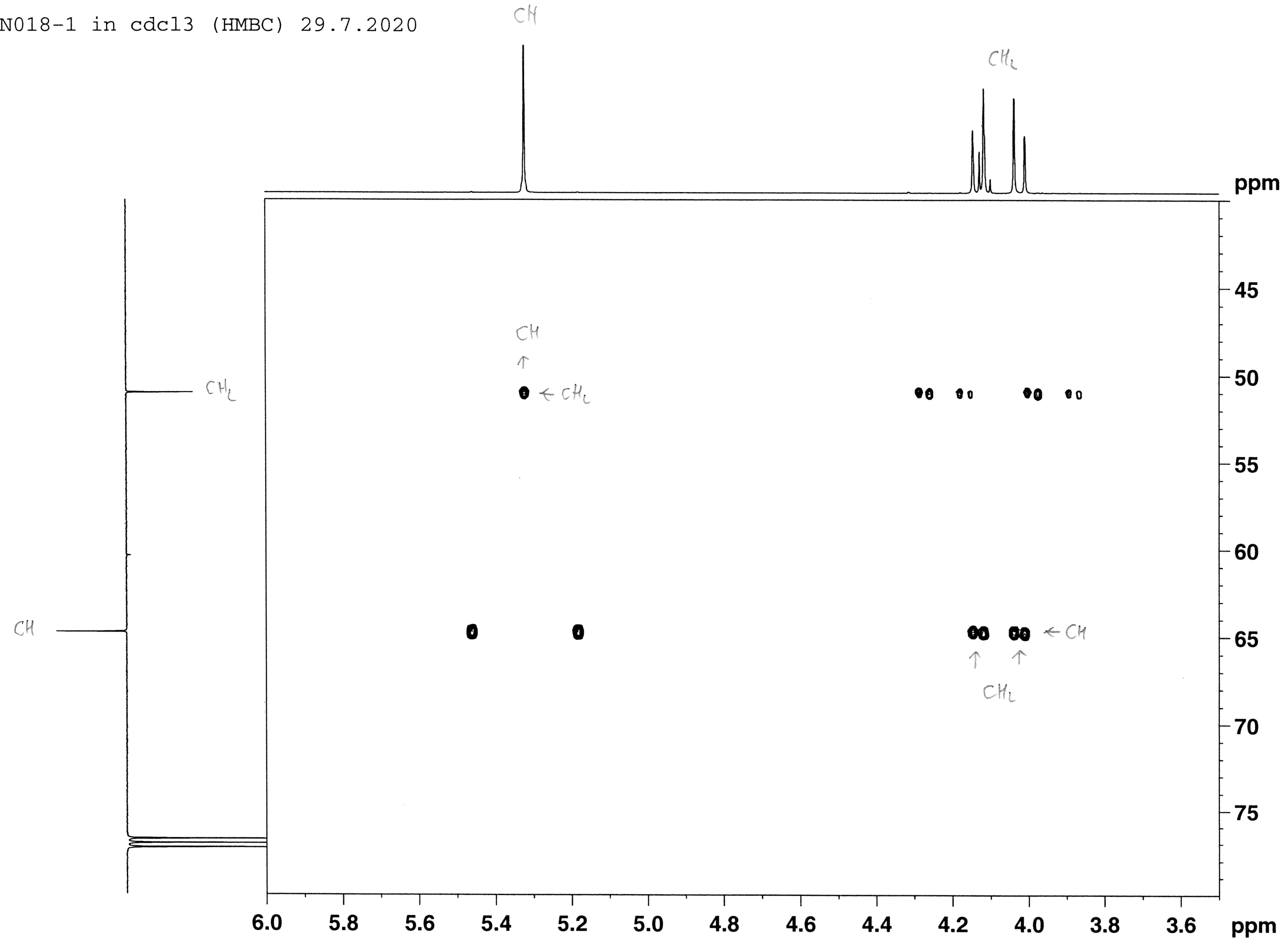

Figure S14c. NMR spectra of compound **6d.**

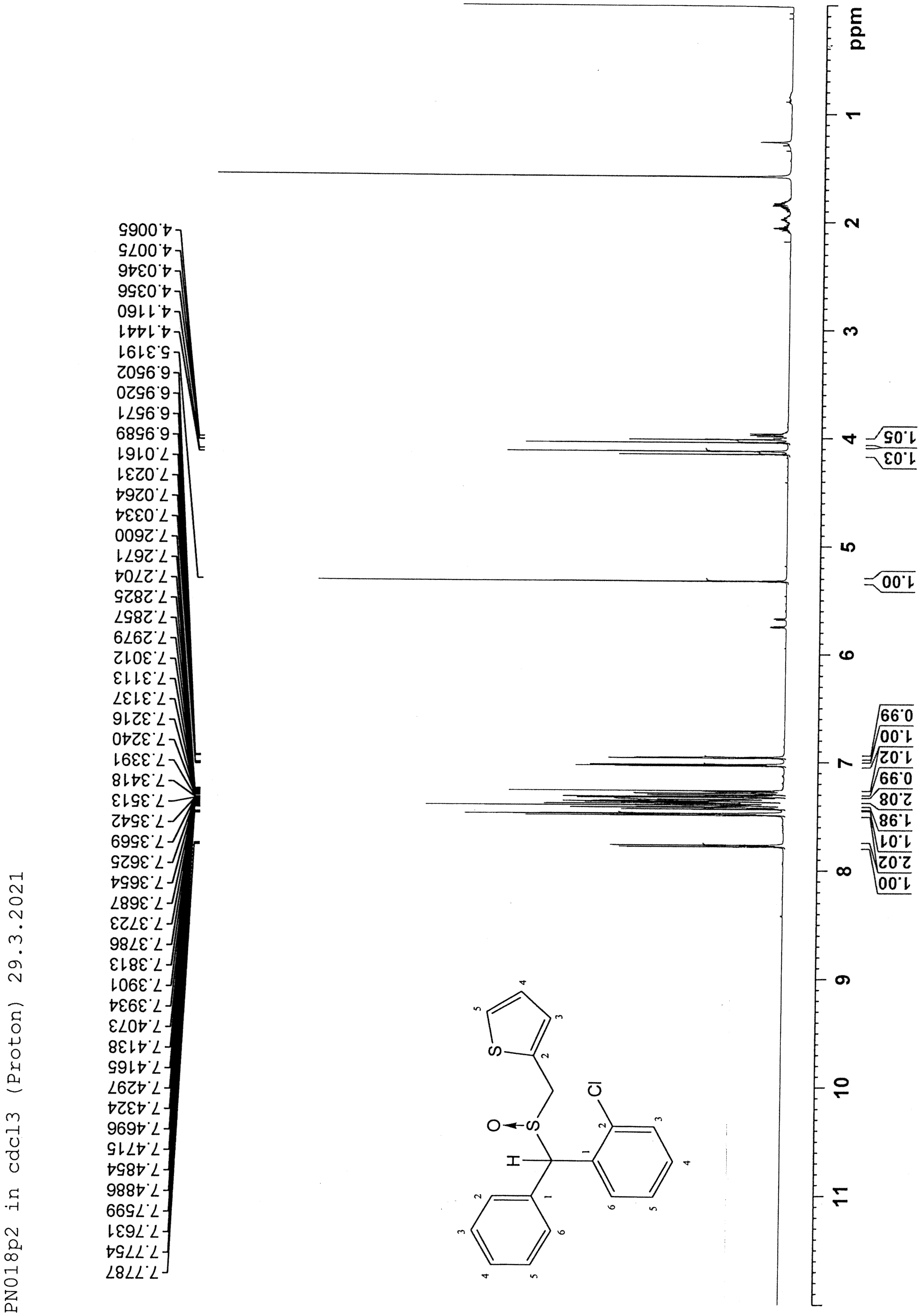

PN018p2 in cdcl3 (Proton) 29.3.2021

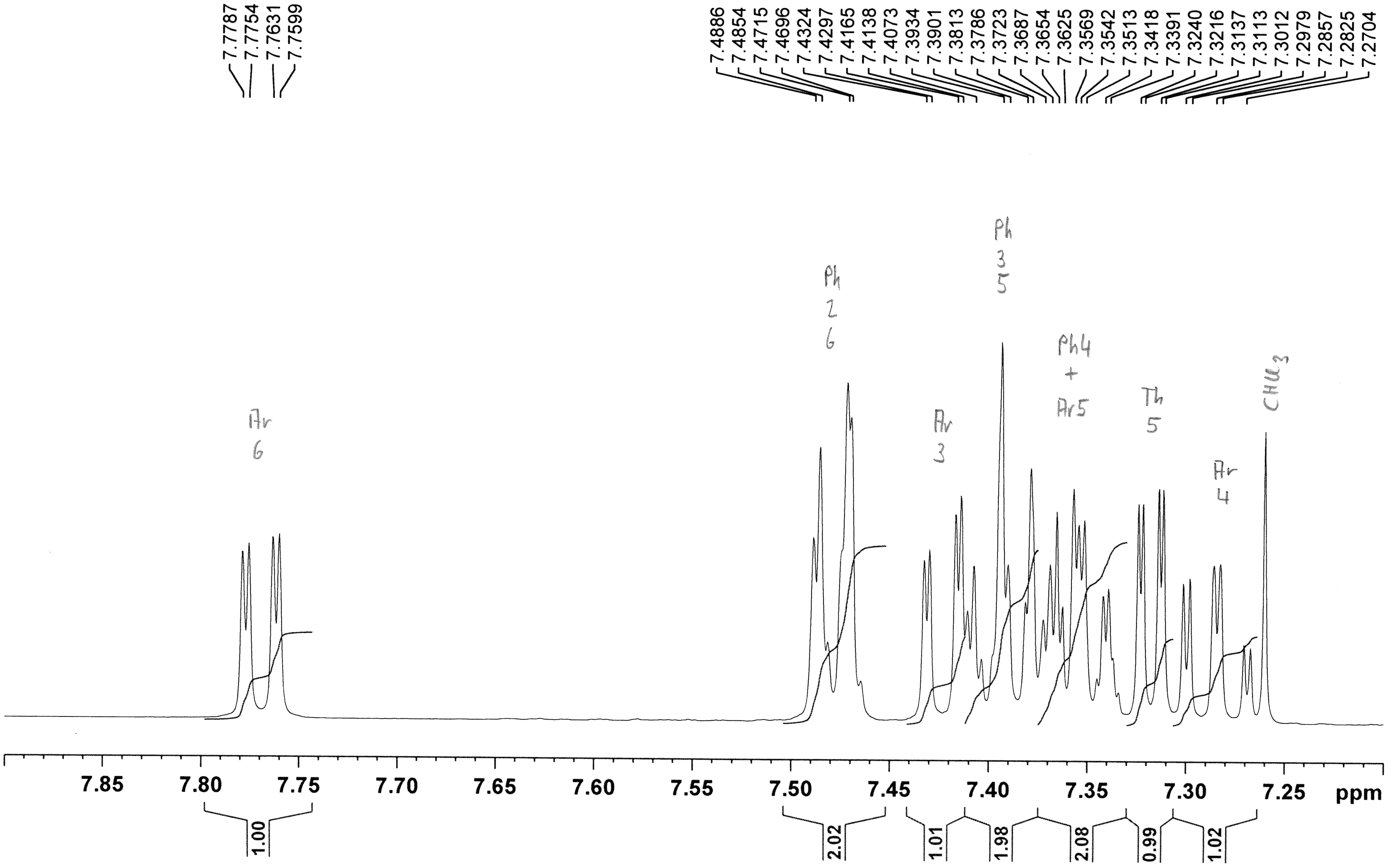

PN018p2 in cdcl3 (Proton) 29.3.2021

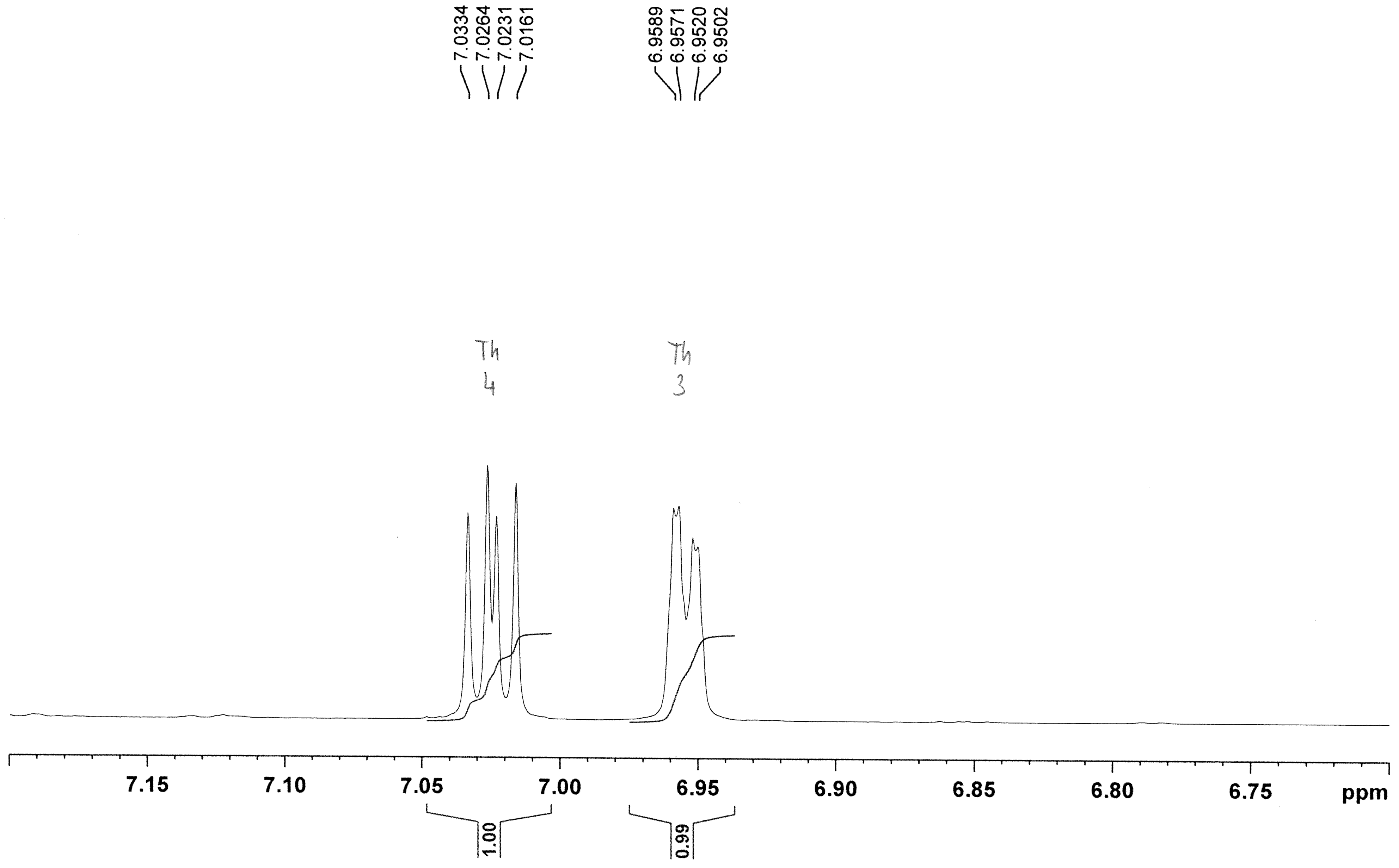

PN018p2 in cdcl3 (Proton) 29.3.2021

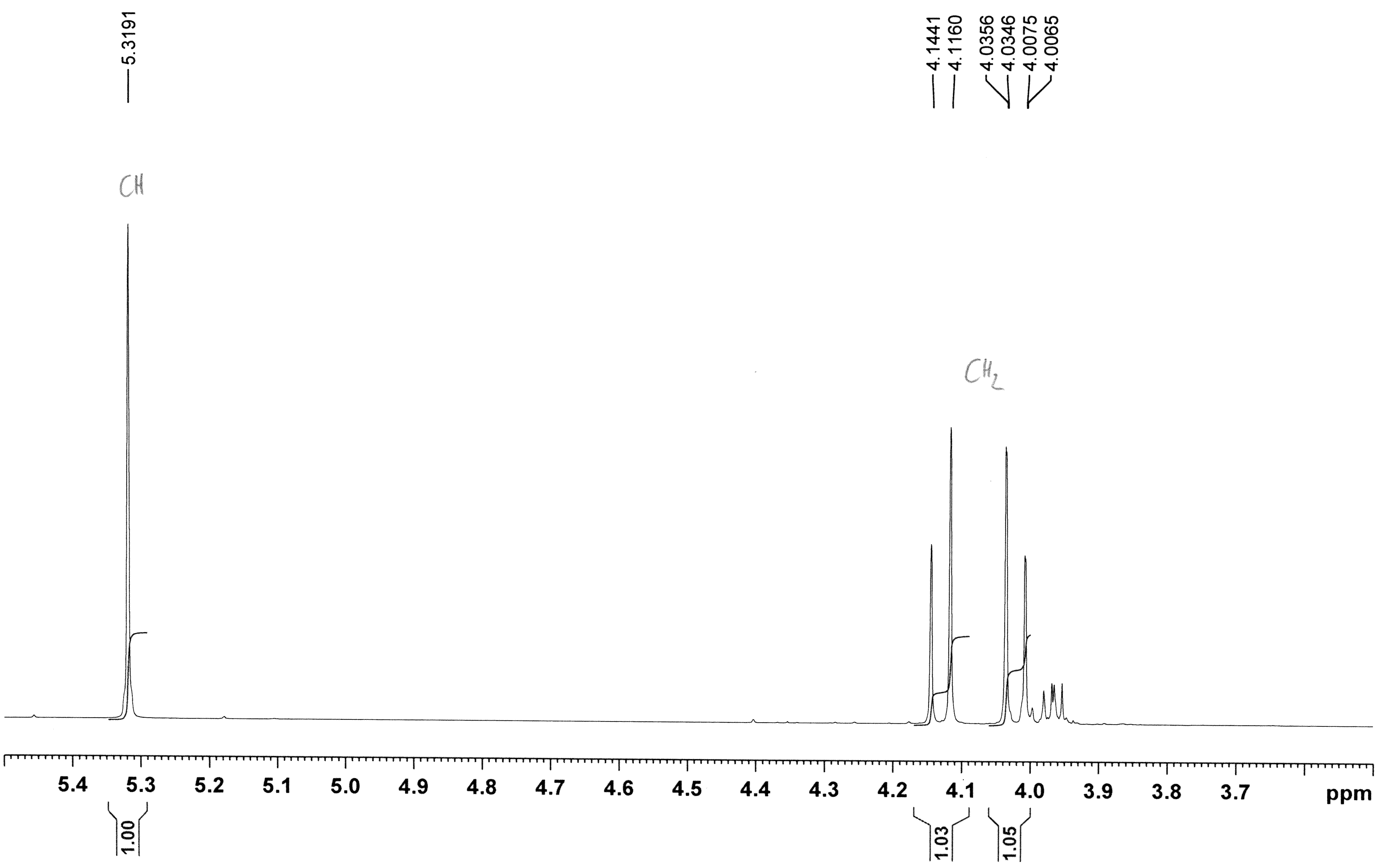

PN018p2 in cdcl3 (APT) 29.3.2021

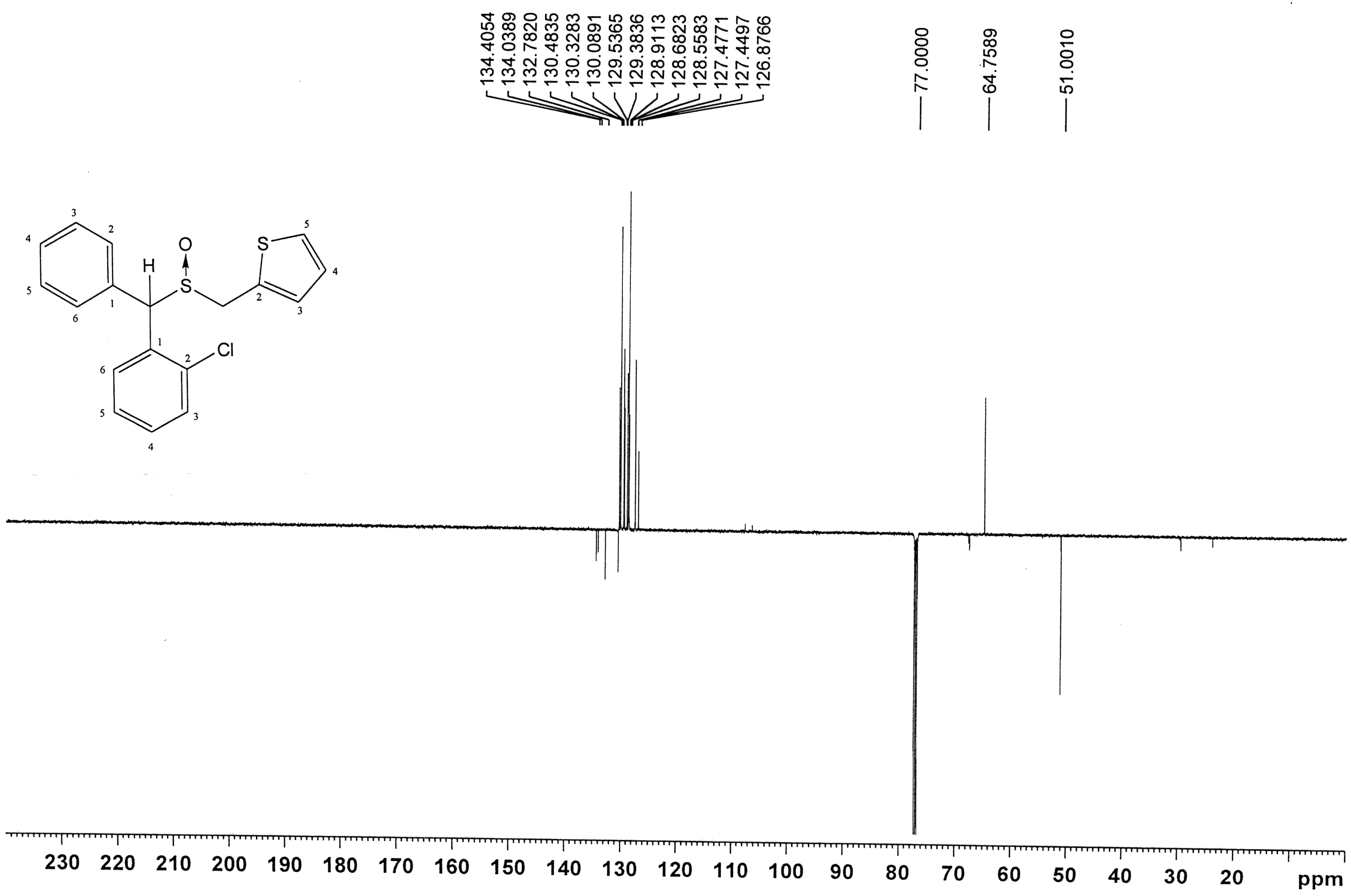

PN018p2 in cdcl3 (APT) 29.3.2021

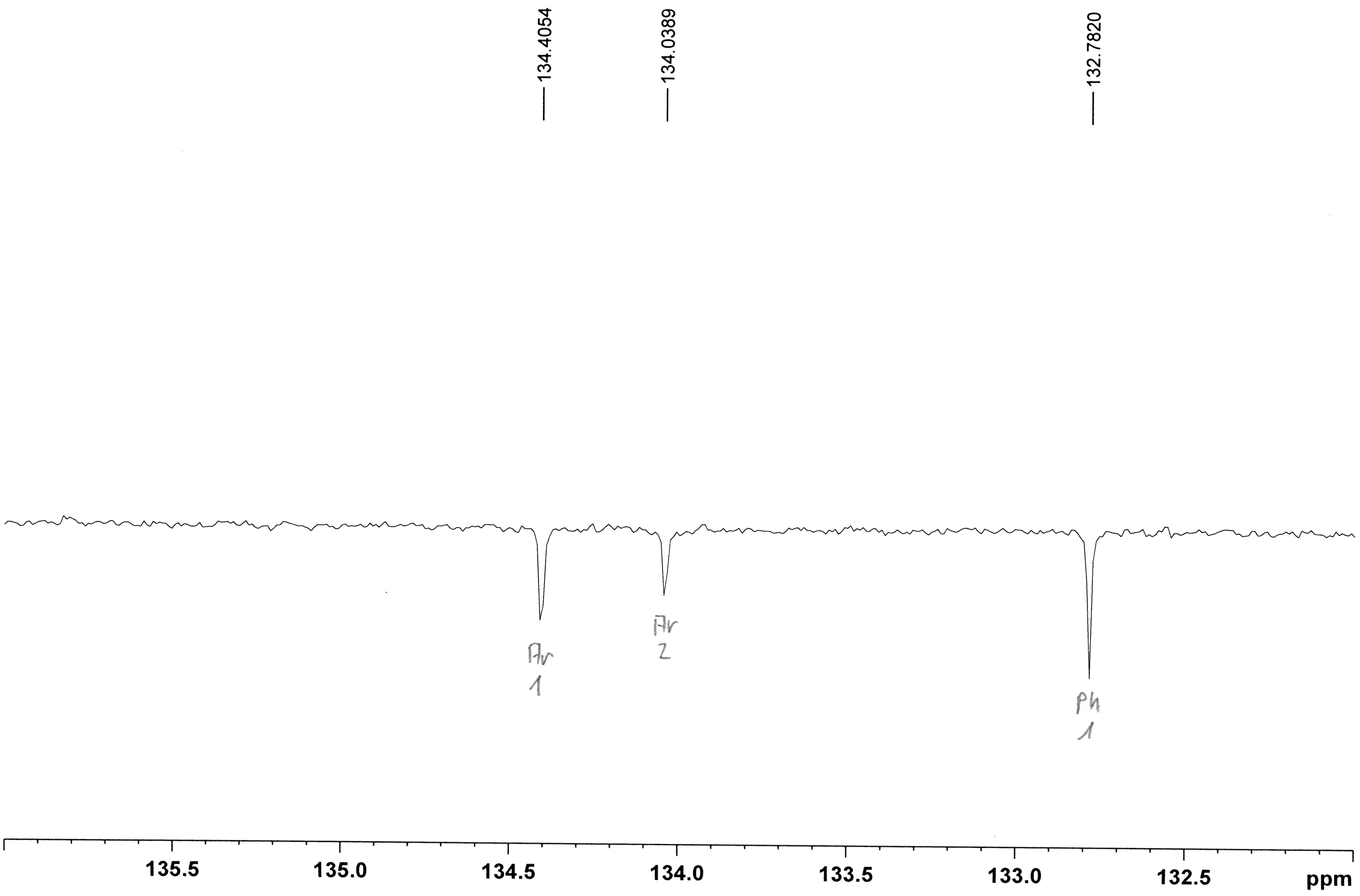

PN018p2 in cdcl3 (APT) 29.3.2021

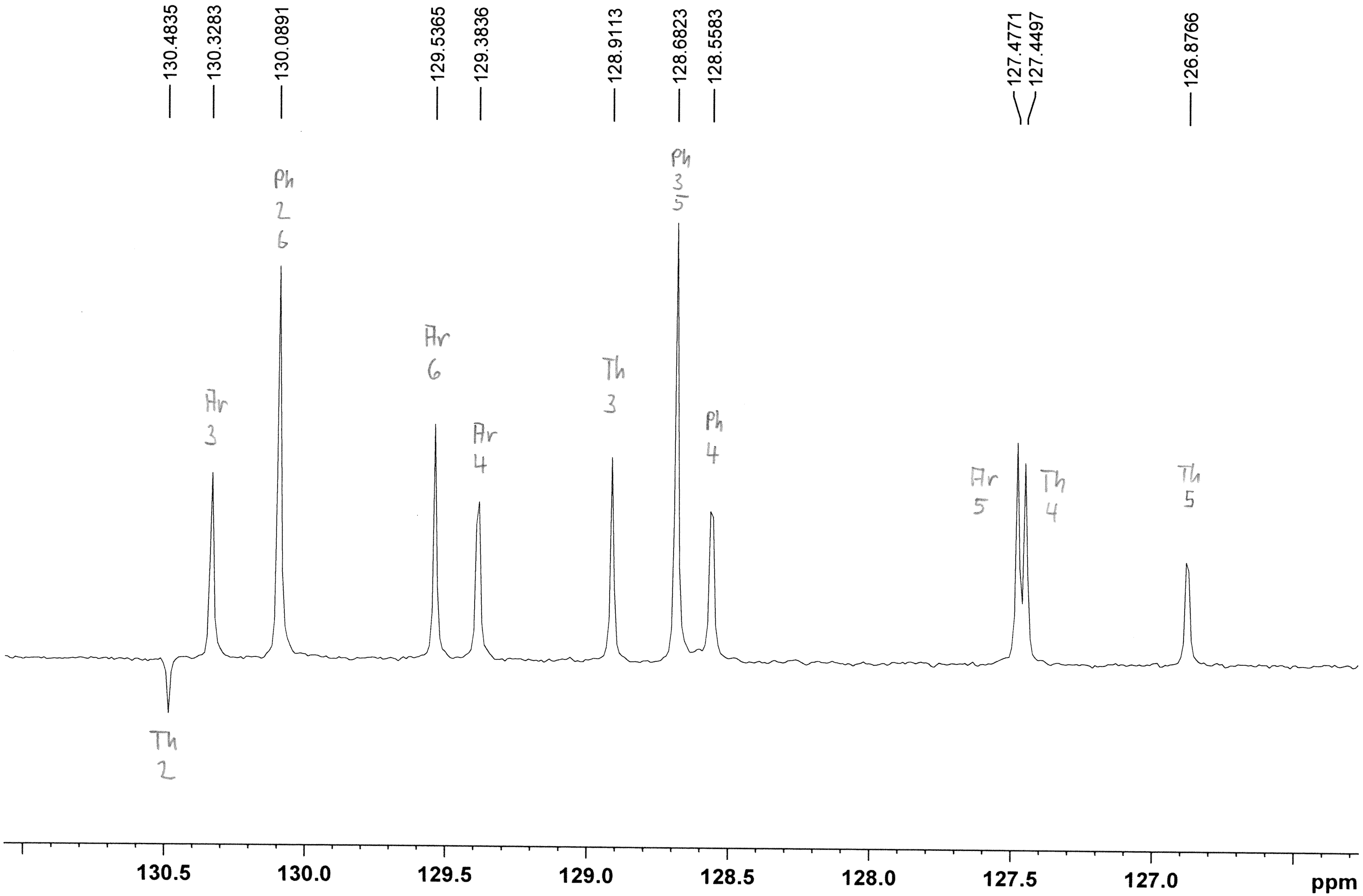

PN018p2 in cdcl3 (APT) 29.3.2021

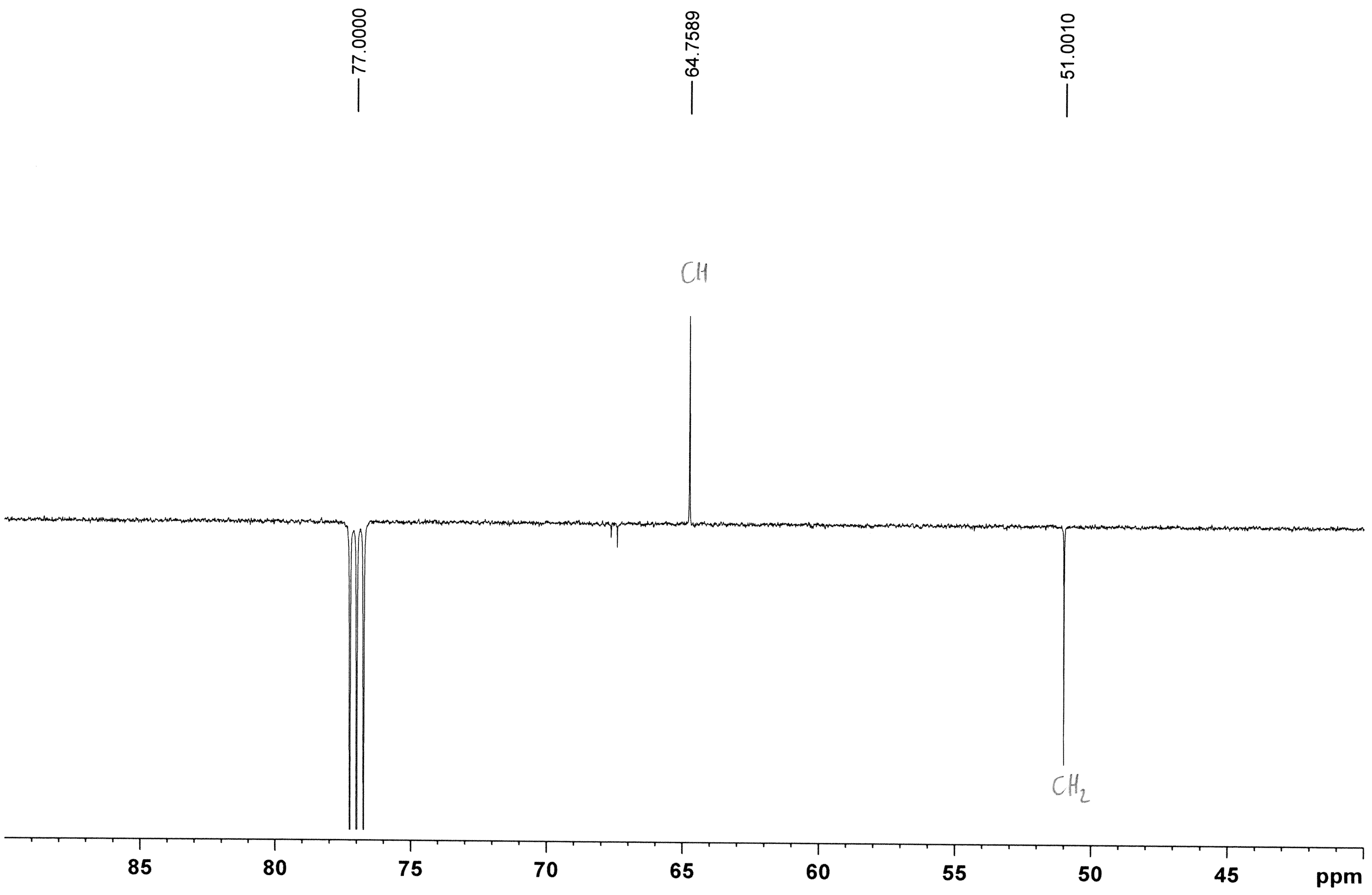

PN018p2 in cdcl3 (COSY) 29.3.2021

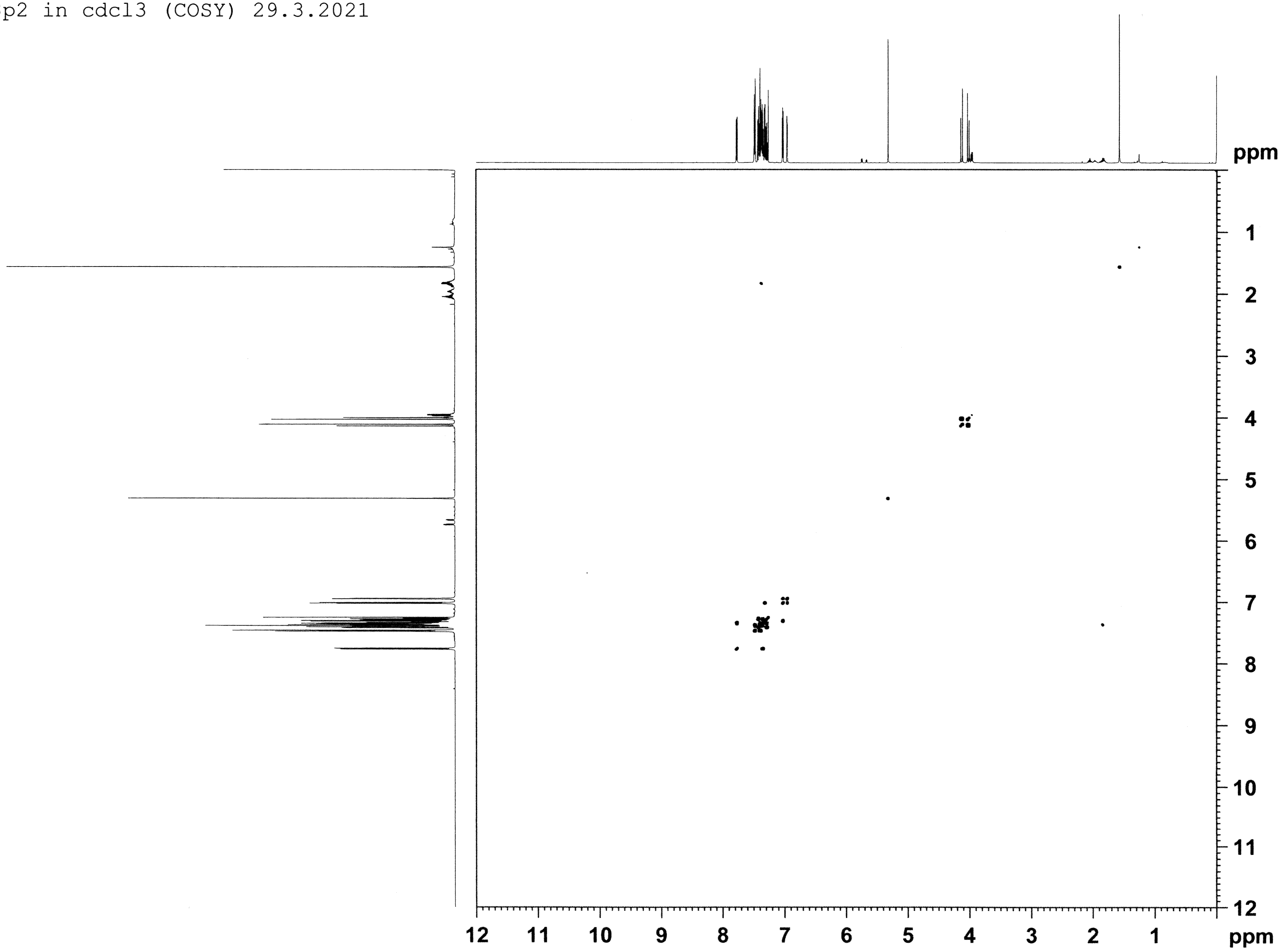

PN018p2 in cdcl3 (COSY) 29.3.2021

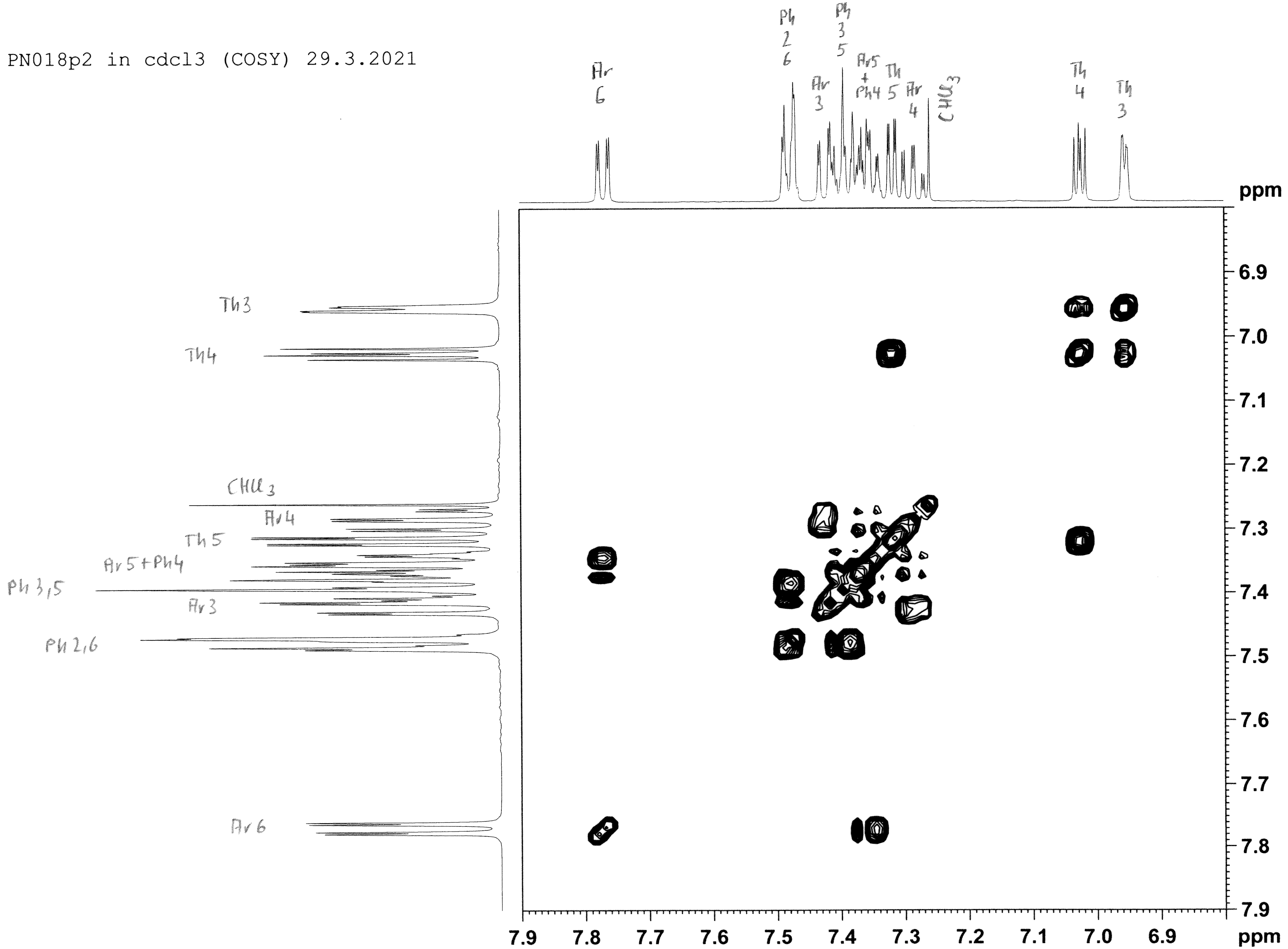

PN018p2 in cdcl3 (COSY) 29.3.2021

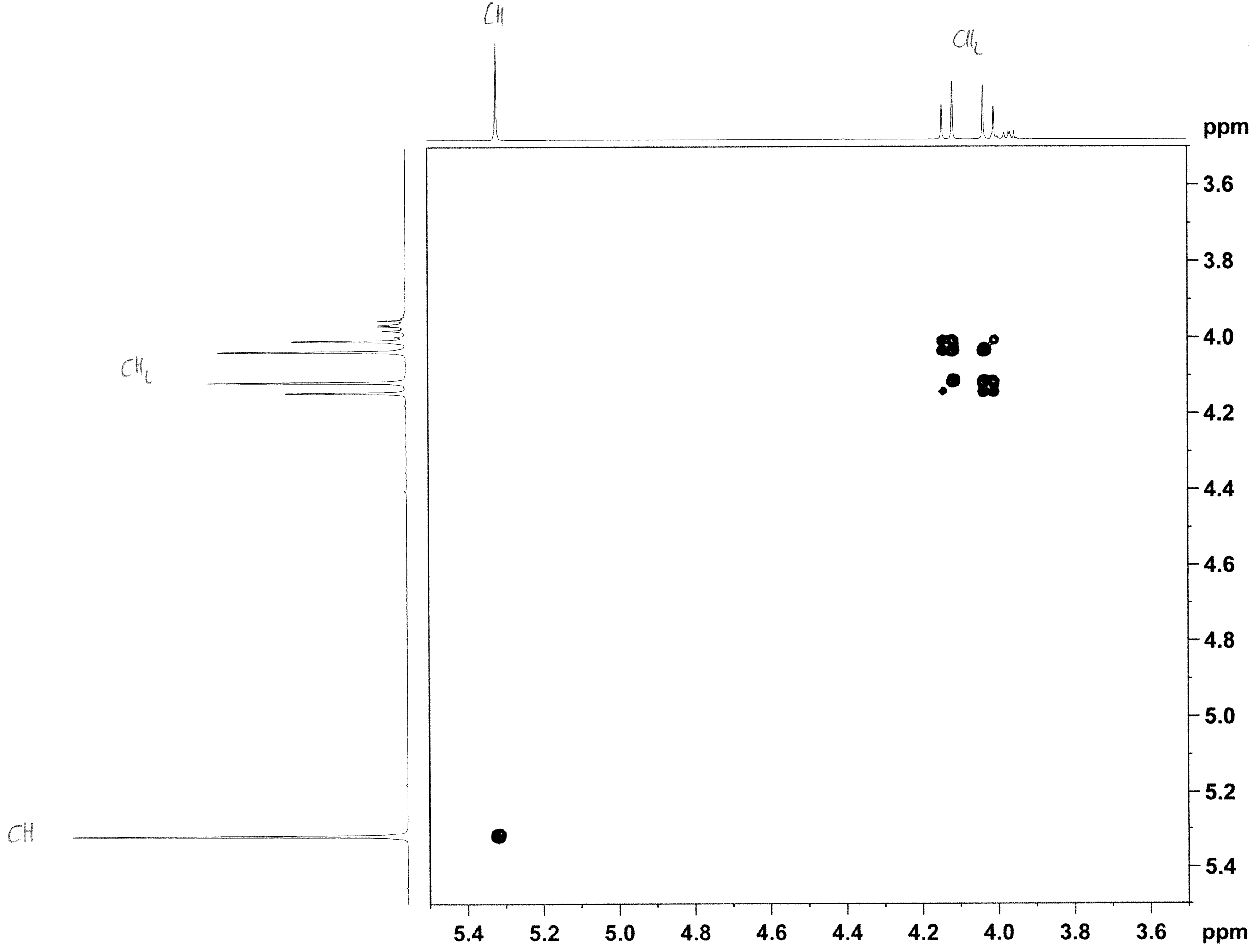

PN018p2 in cdcl3 (HSQC) 29.3.2021

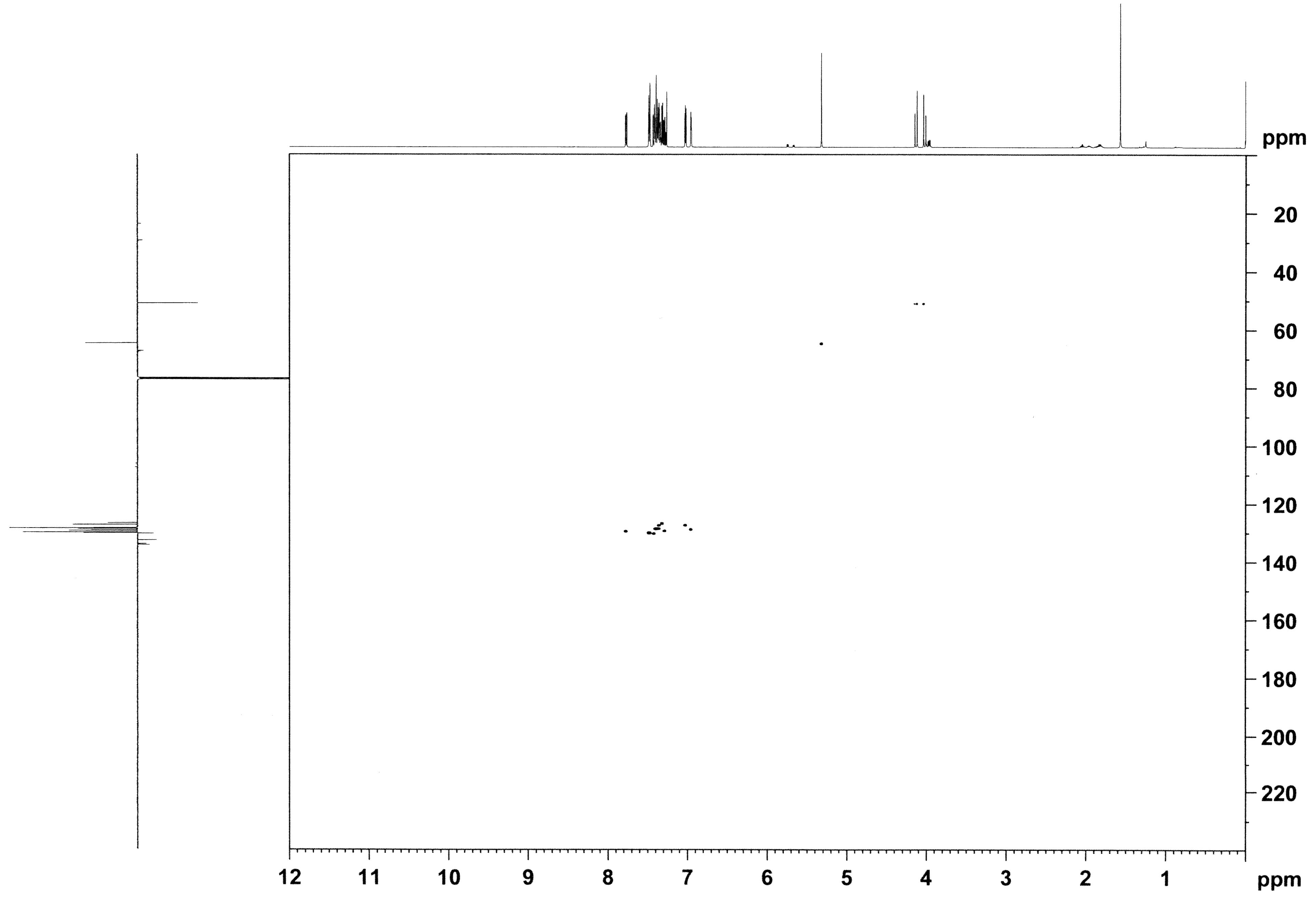

PN018p2 in cdcl3 (HSQC) 29.3.2021

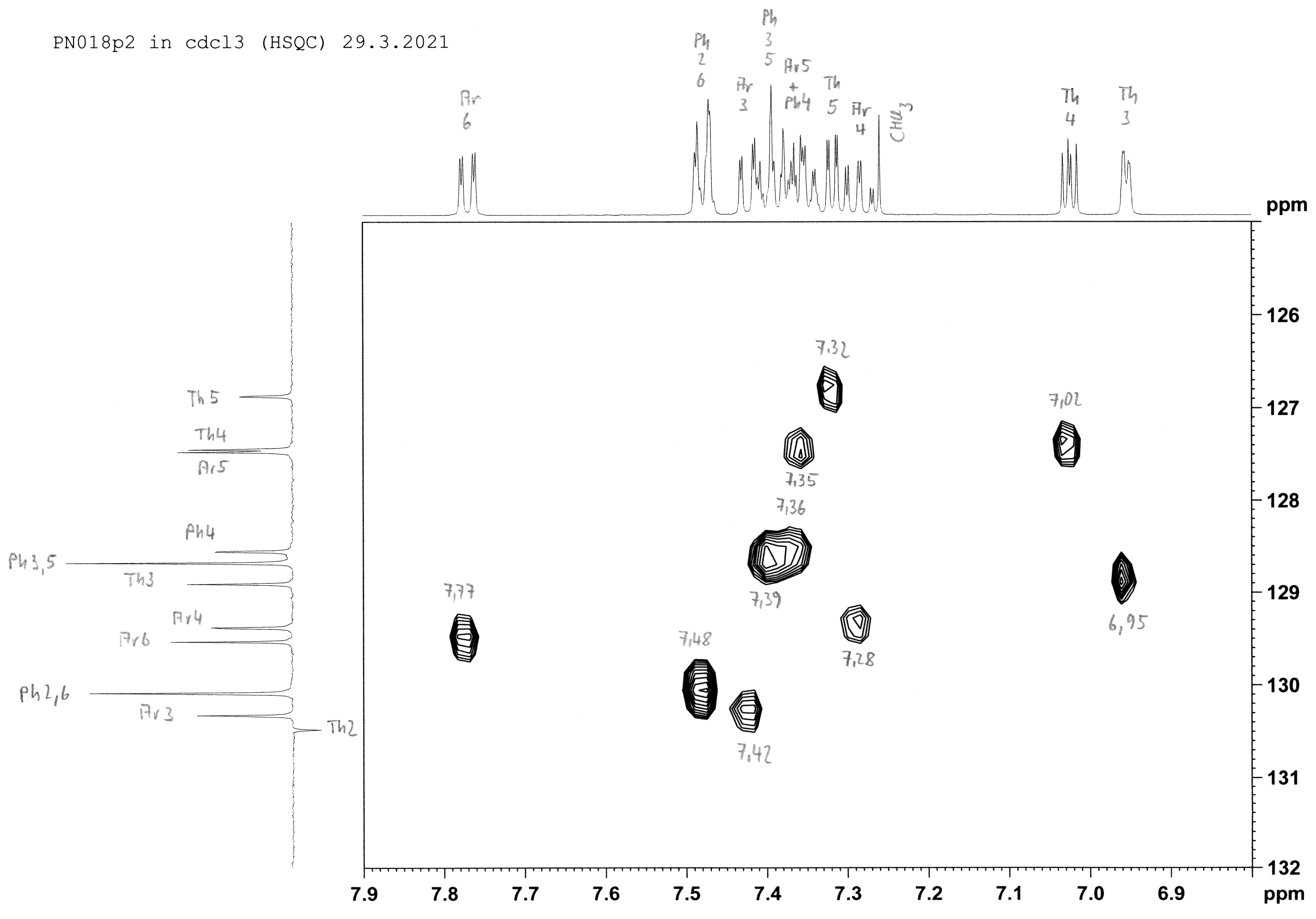

PN018p2 in cdcl3 (HSQC) 29.3.2021

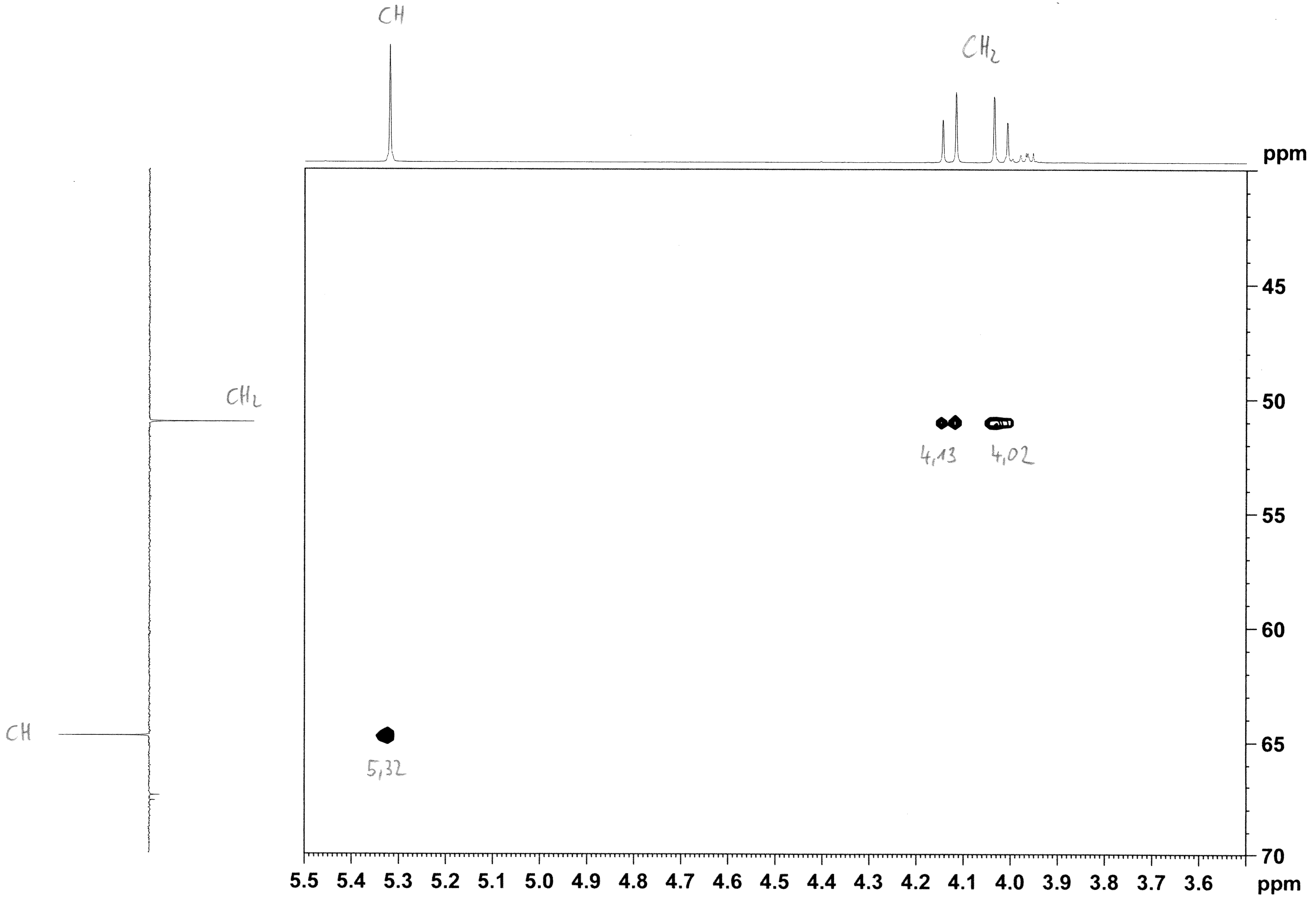

PN018p2 in cdcl3 (HMBC) 29.3.2021

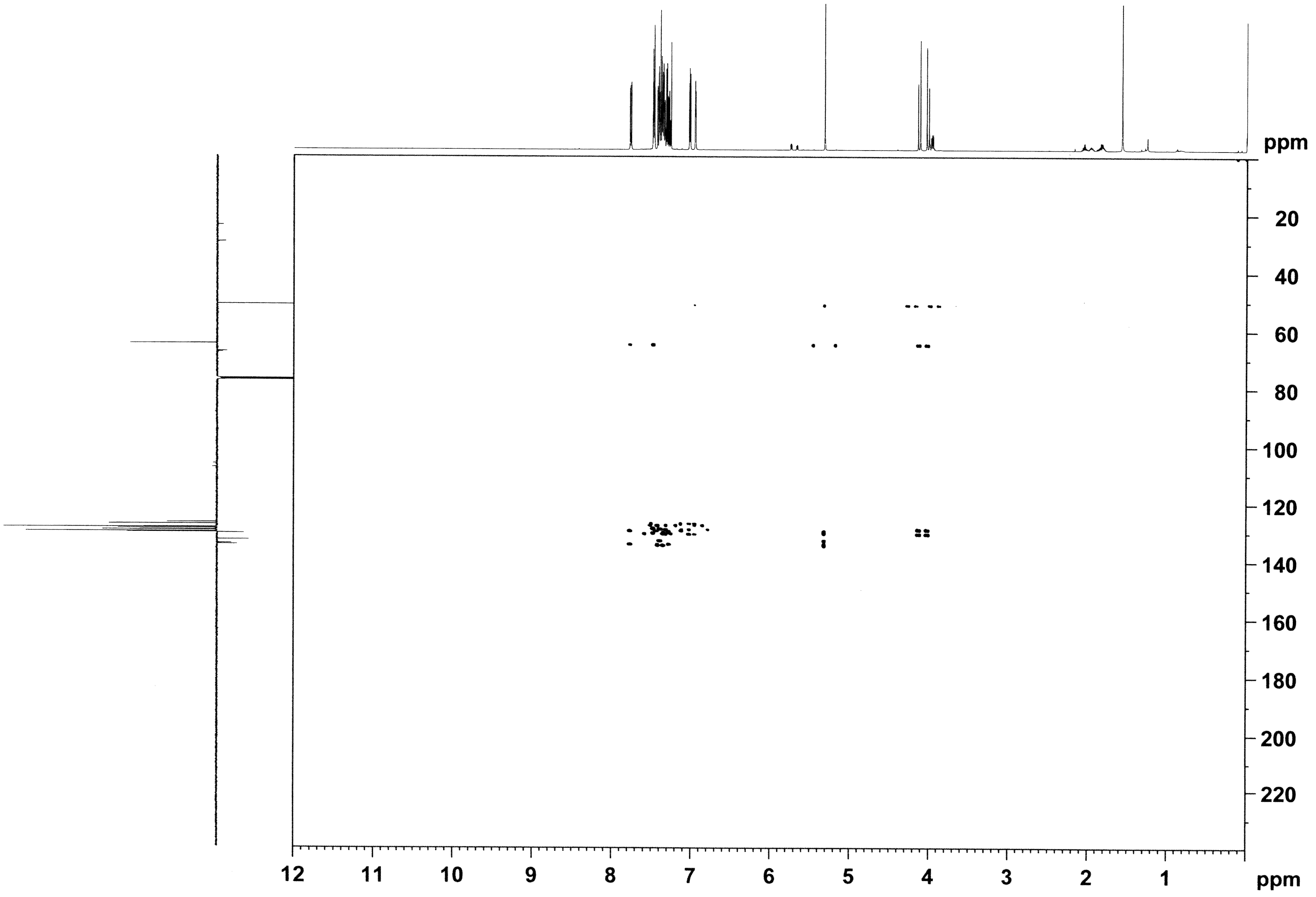

PN018p2 in cdcl3 (HMBC) 29.3.2021

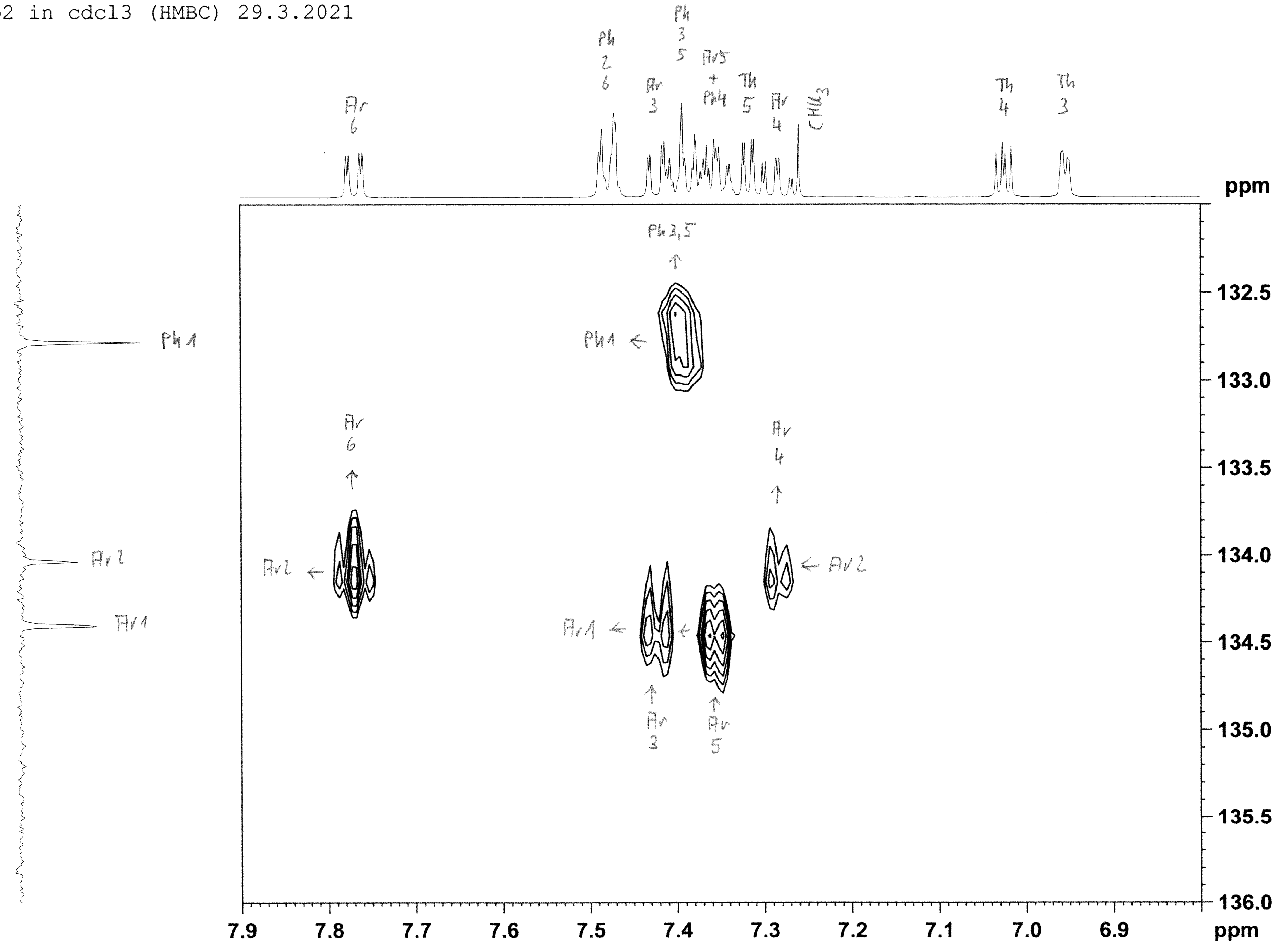

PN018p2 in cdcl3 (HMBC) 29.3.2021

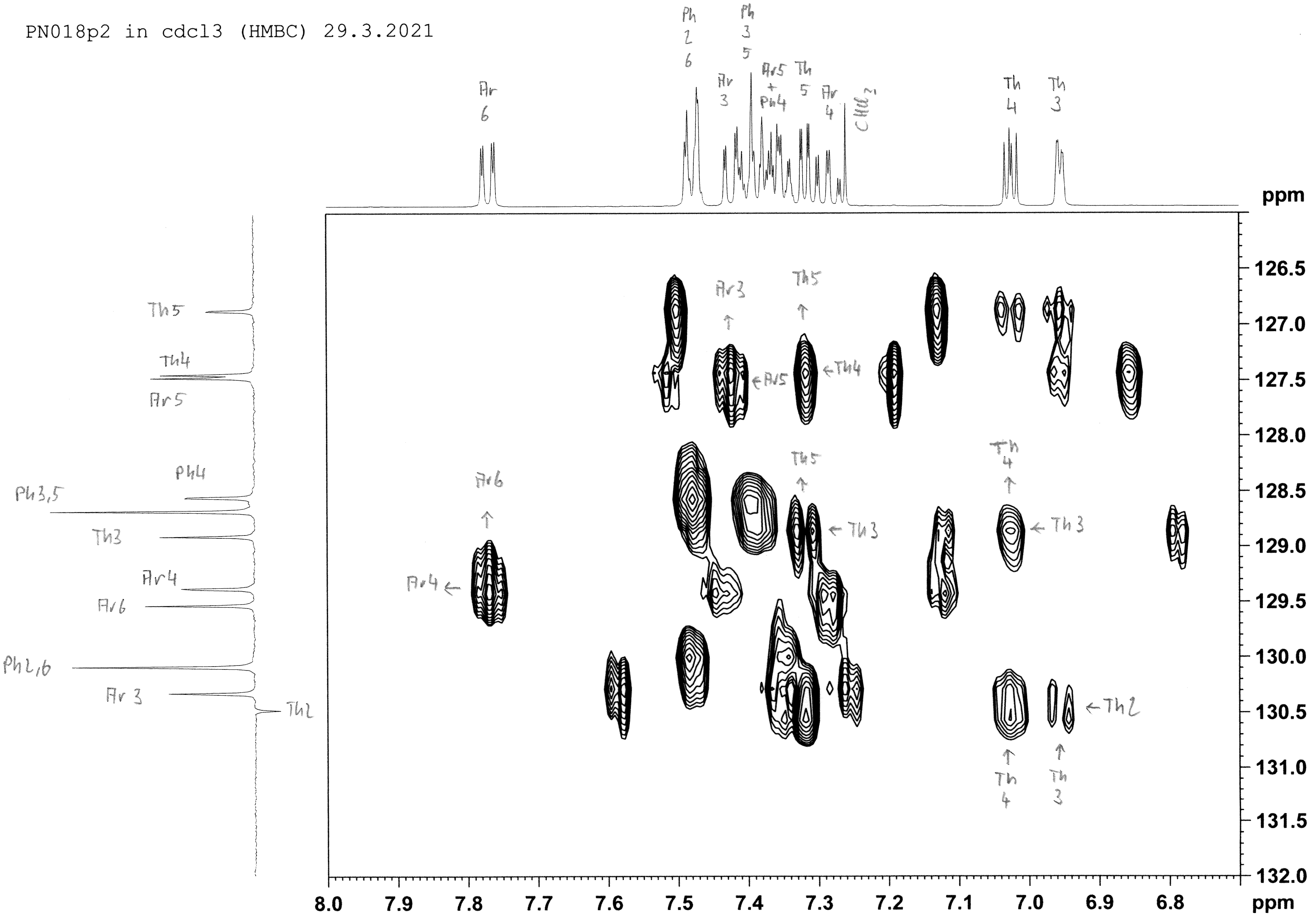

PN018p2 in cdcl3 (HMBC) 29.3.2021

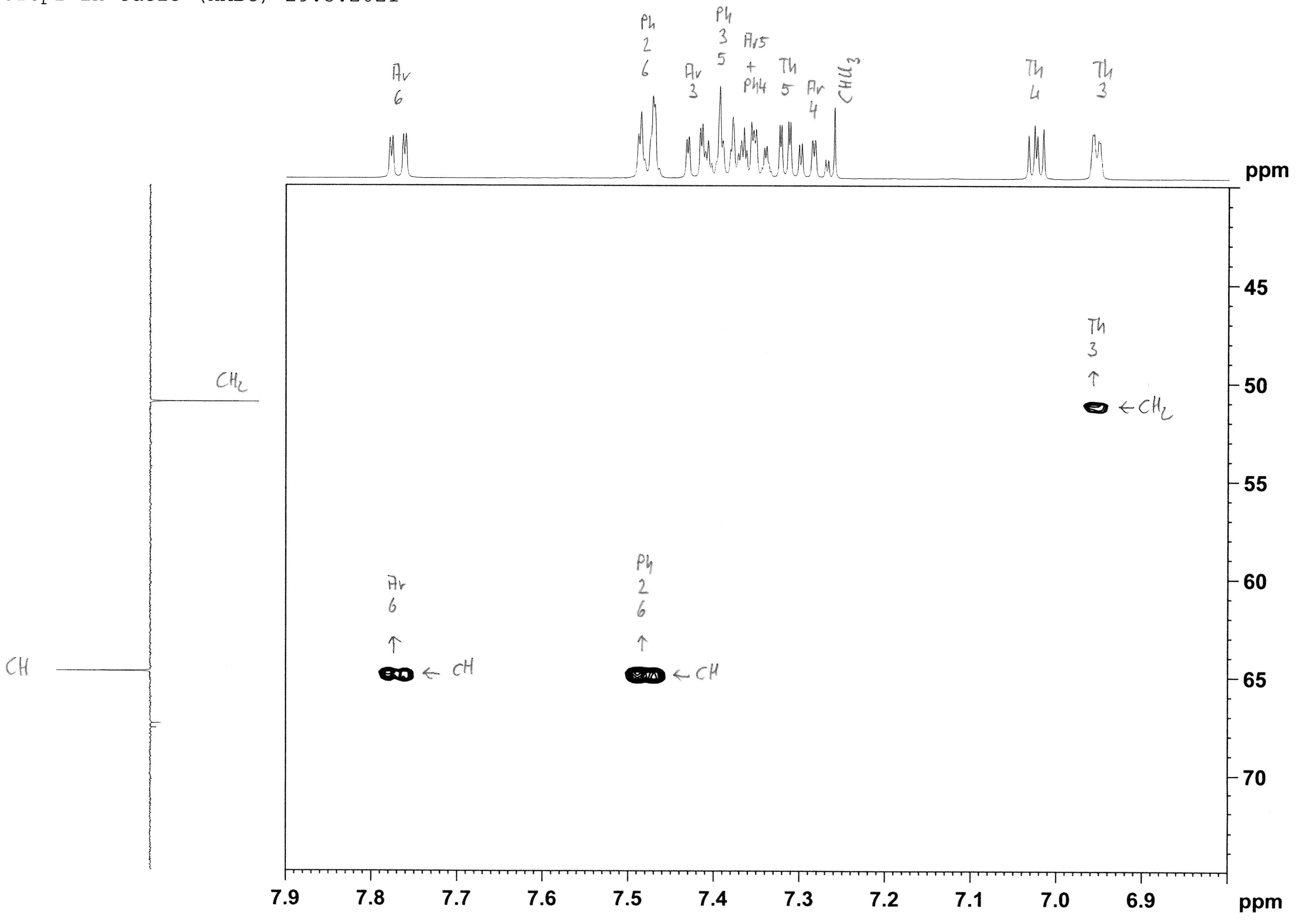

PN018p2 in cdcl3 (HMBC) 29.3.2021

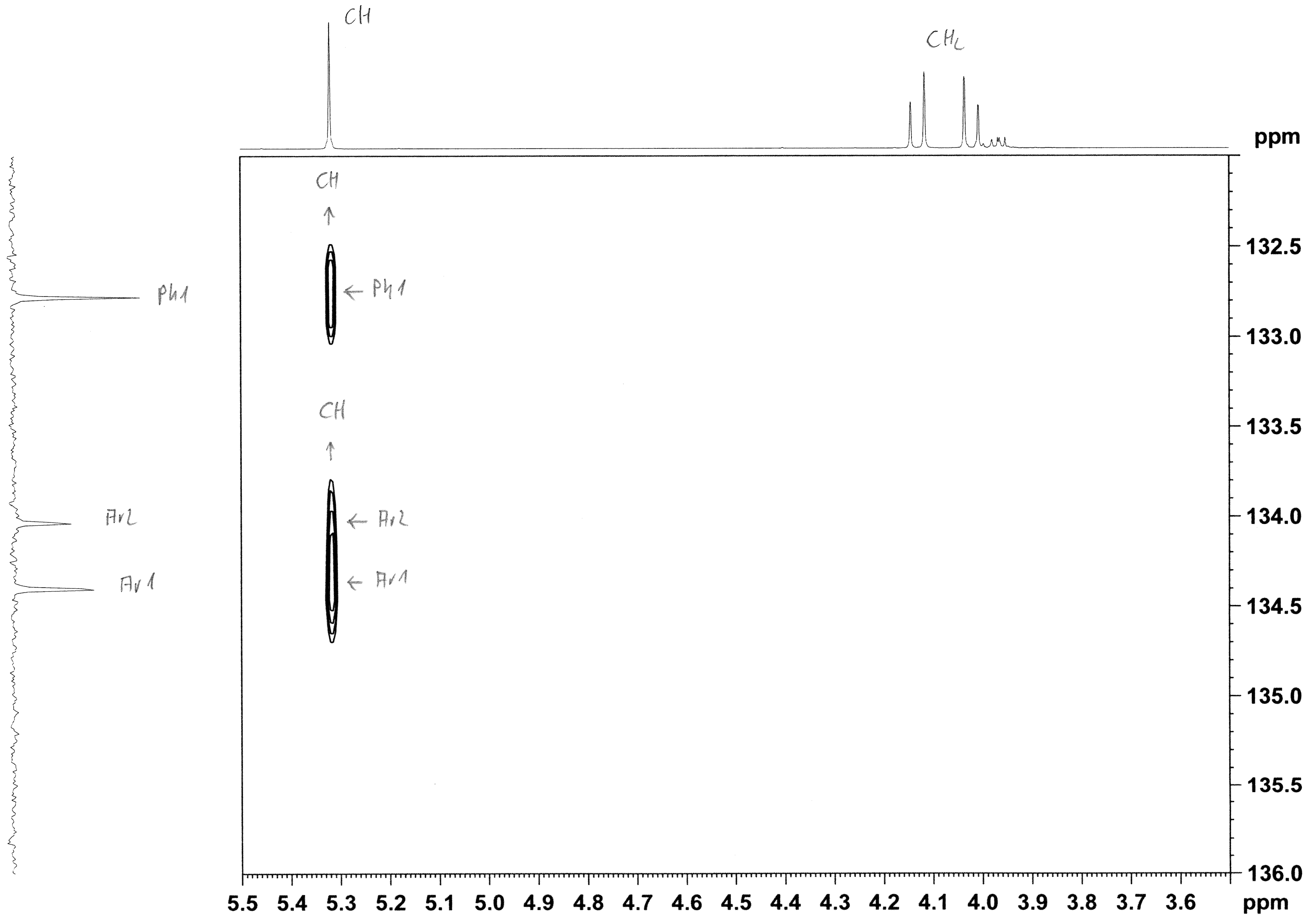

PN018p2 in cdcl3 (HMBC) 29.3.2021

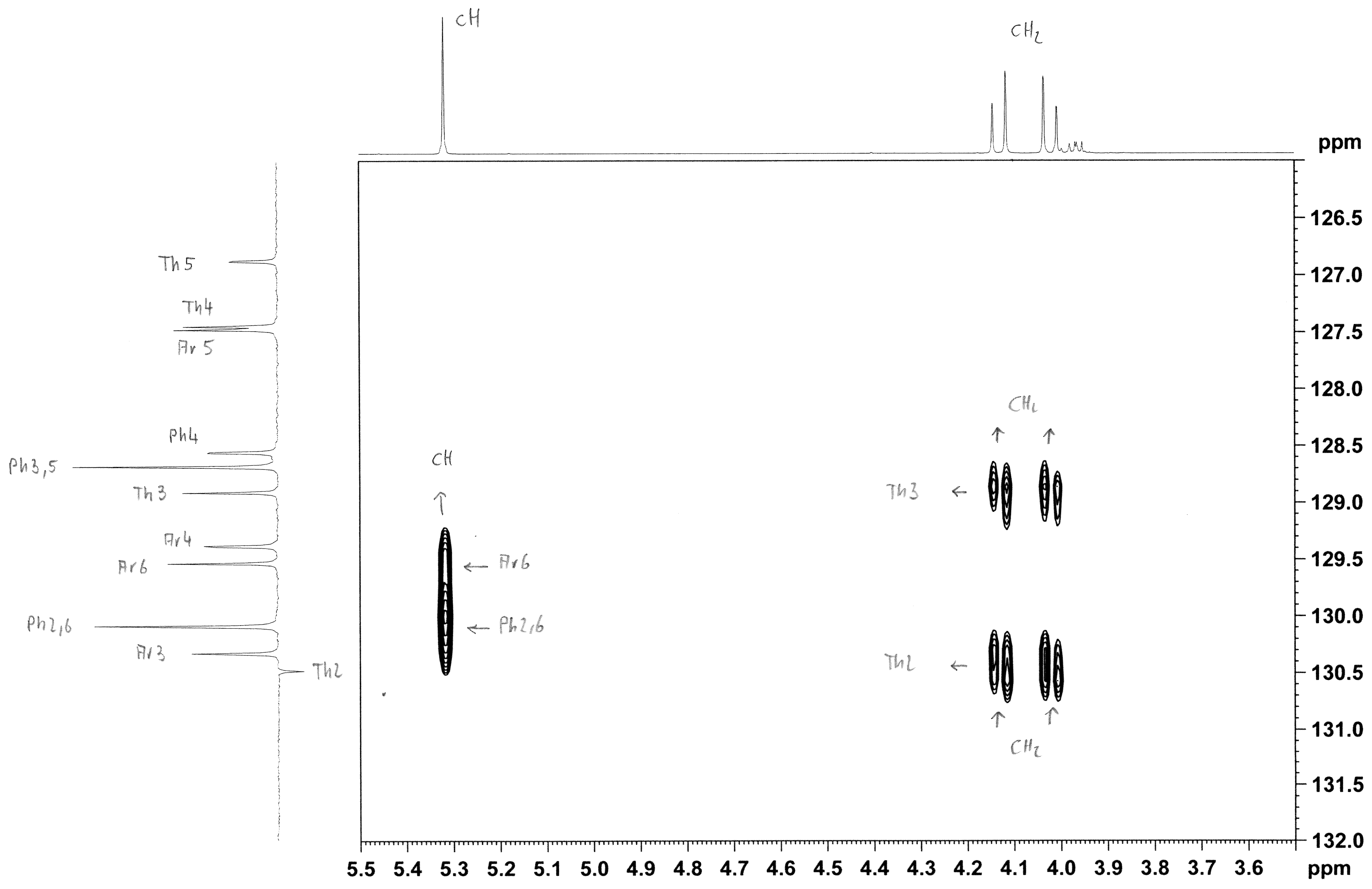

PN018p2 in cdcl3 (HMBC) 29.3.2021

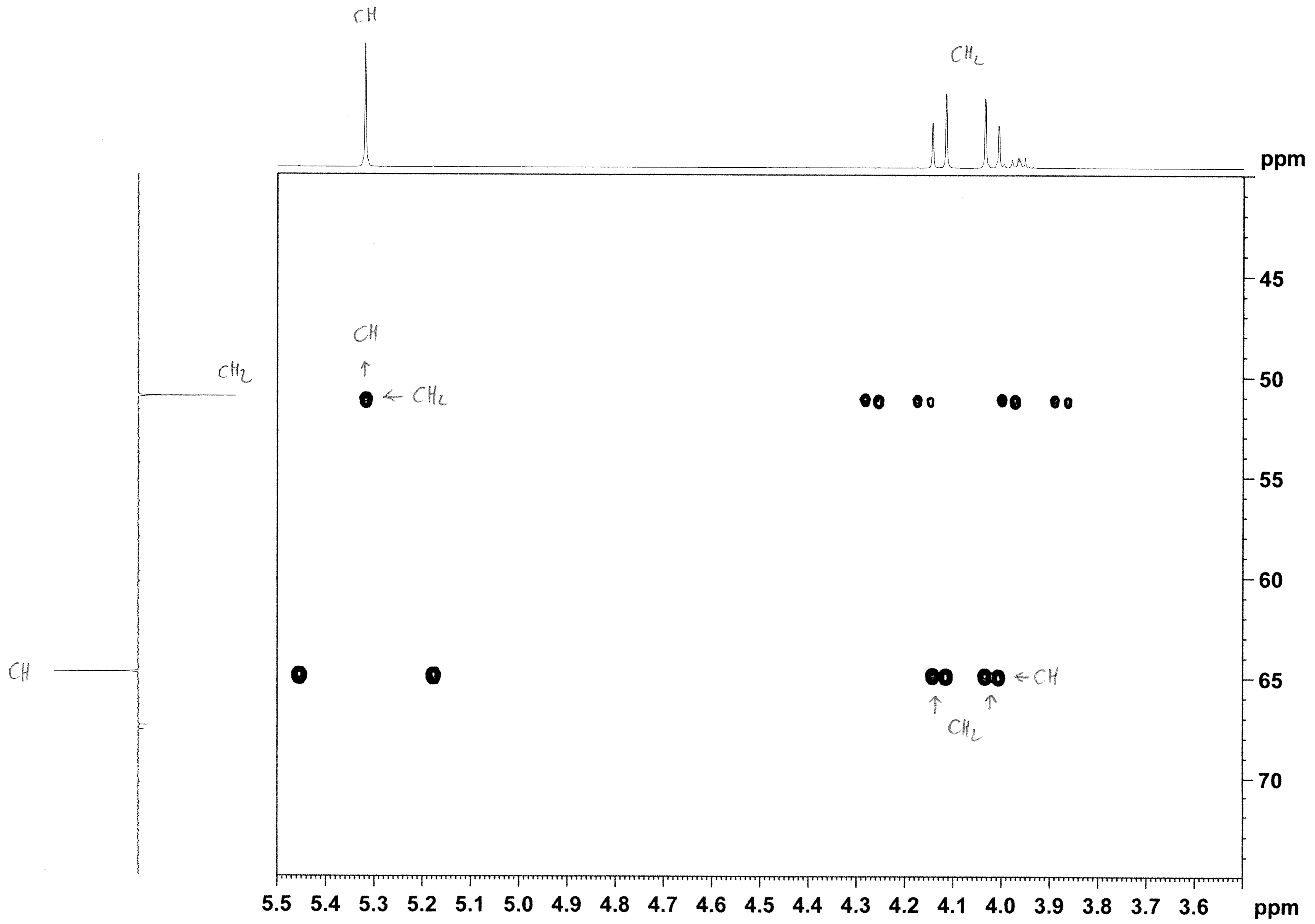

Figure S15c. NMR spectra of compound **7d**.

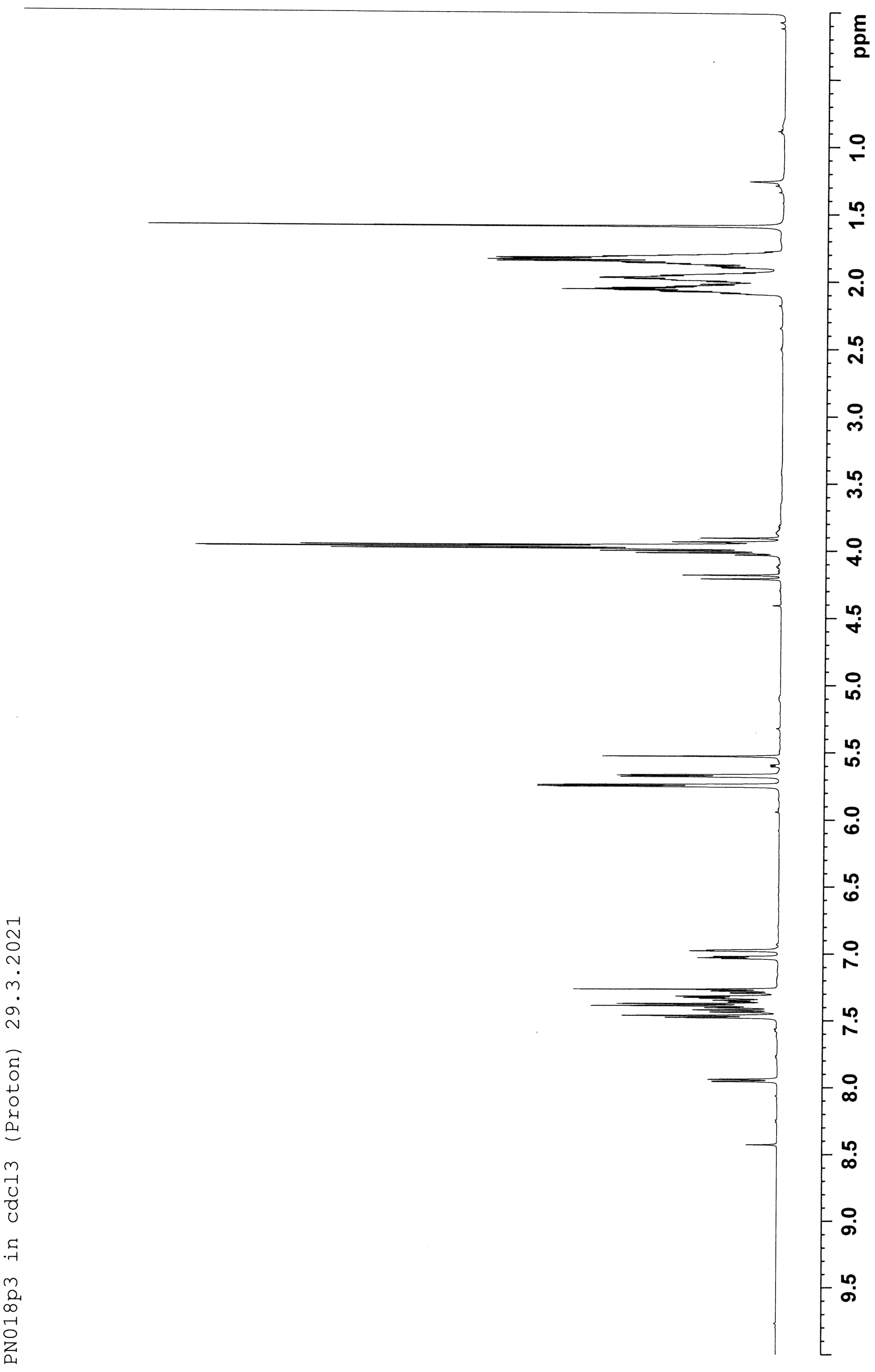

PN018p3 in cdcl3 (APT) 29.3.2021

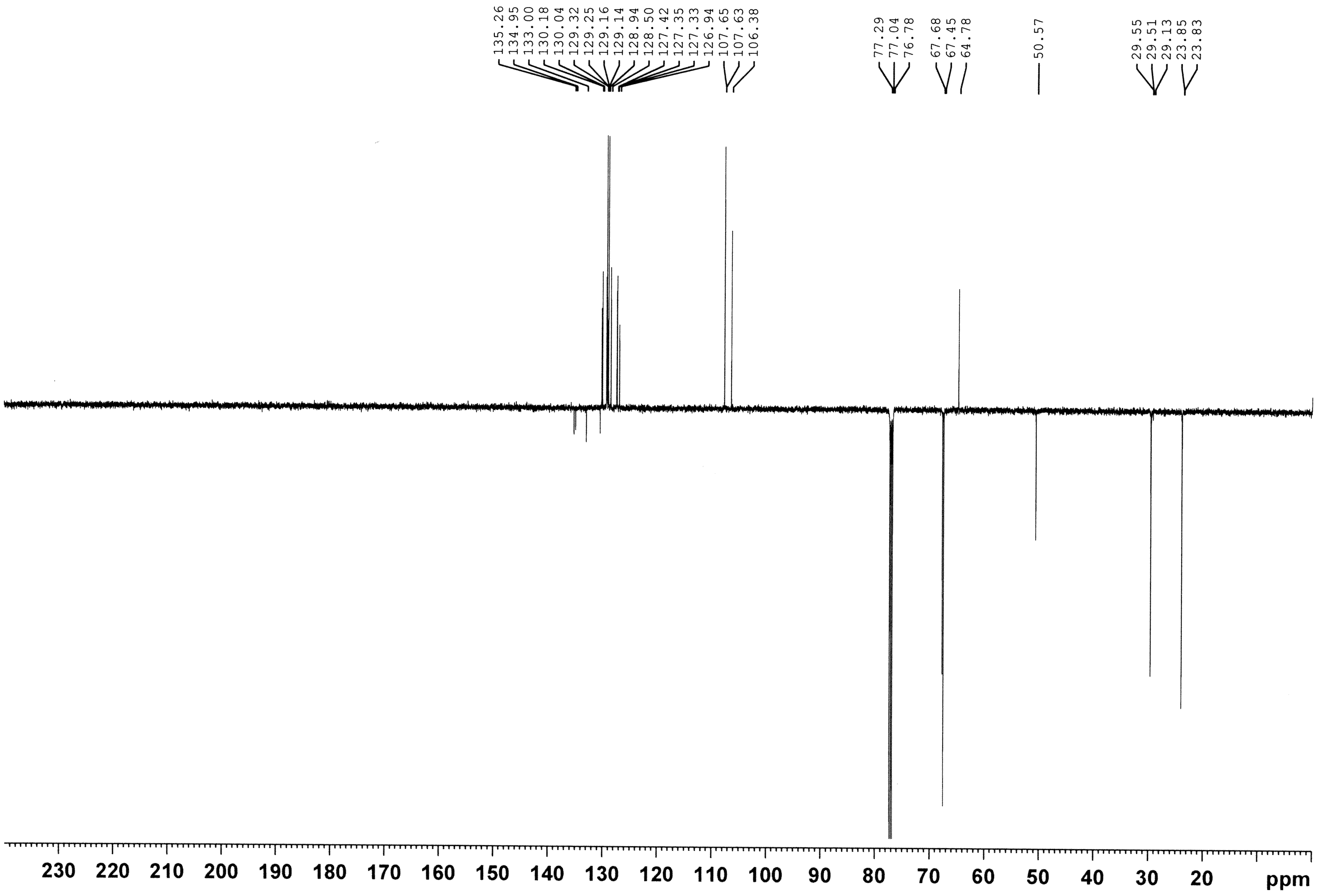

Figure S16c. NMR spectra of compound **8d**.

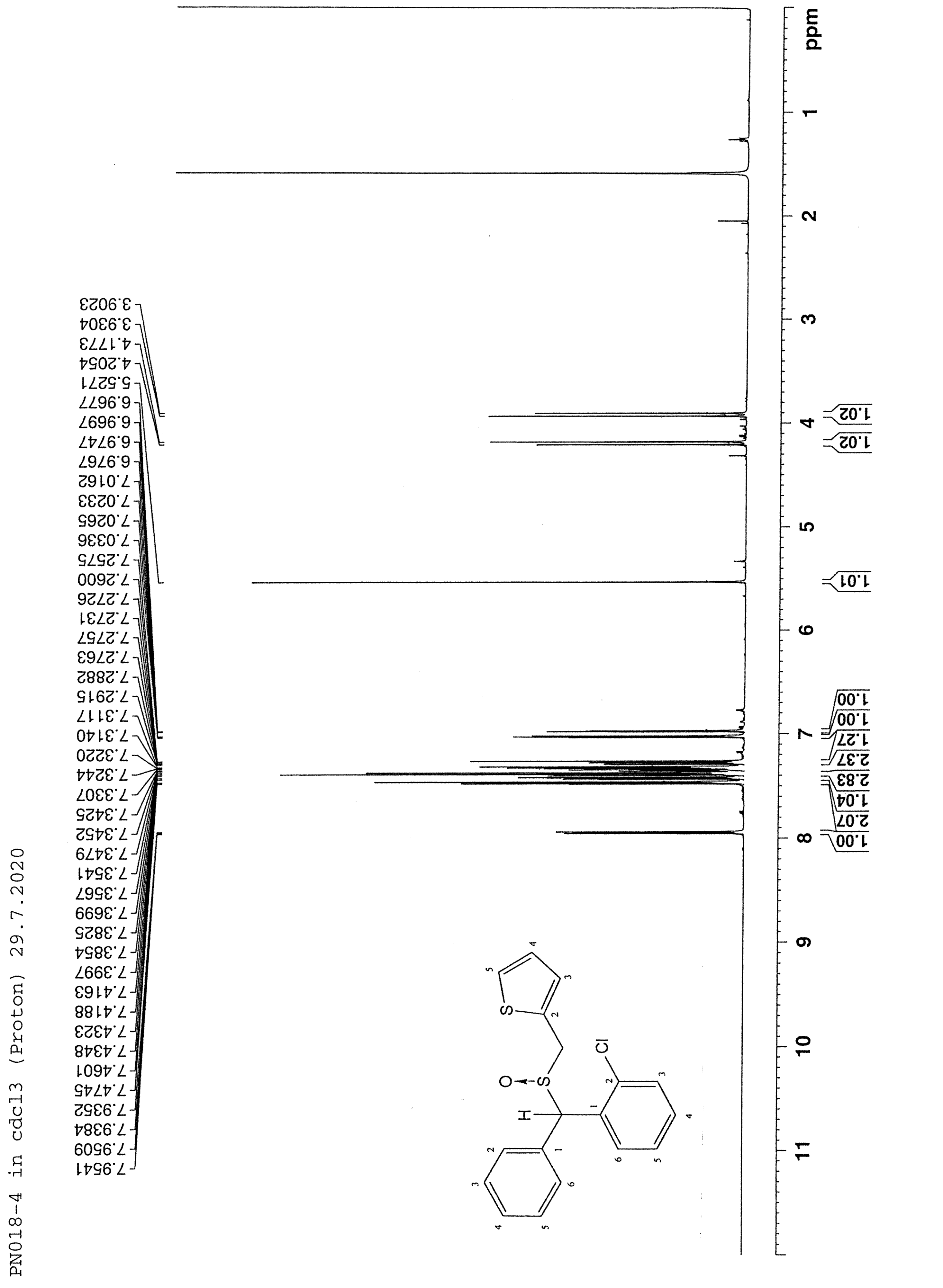

PN018-4 in cdcl3 (Proton) 29.7.2020

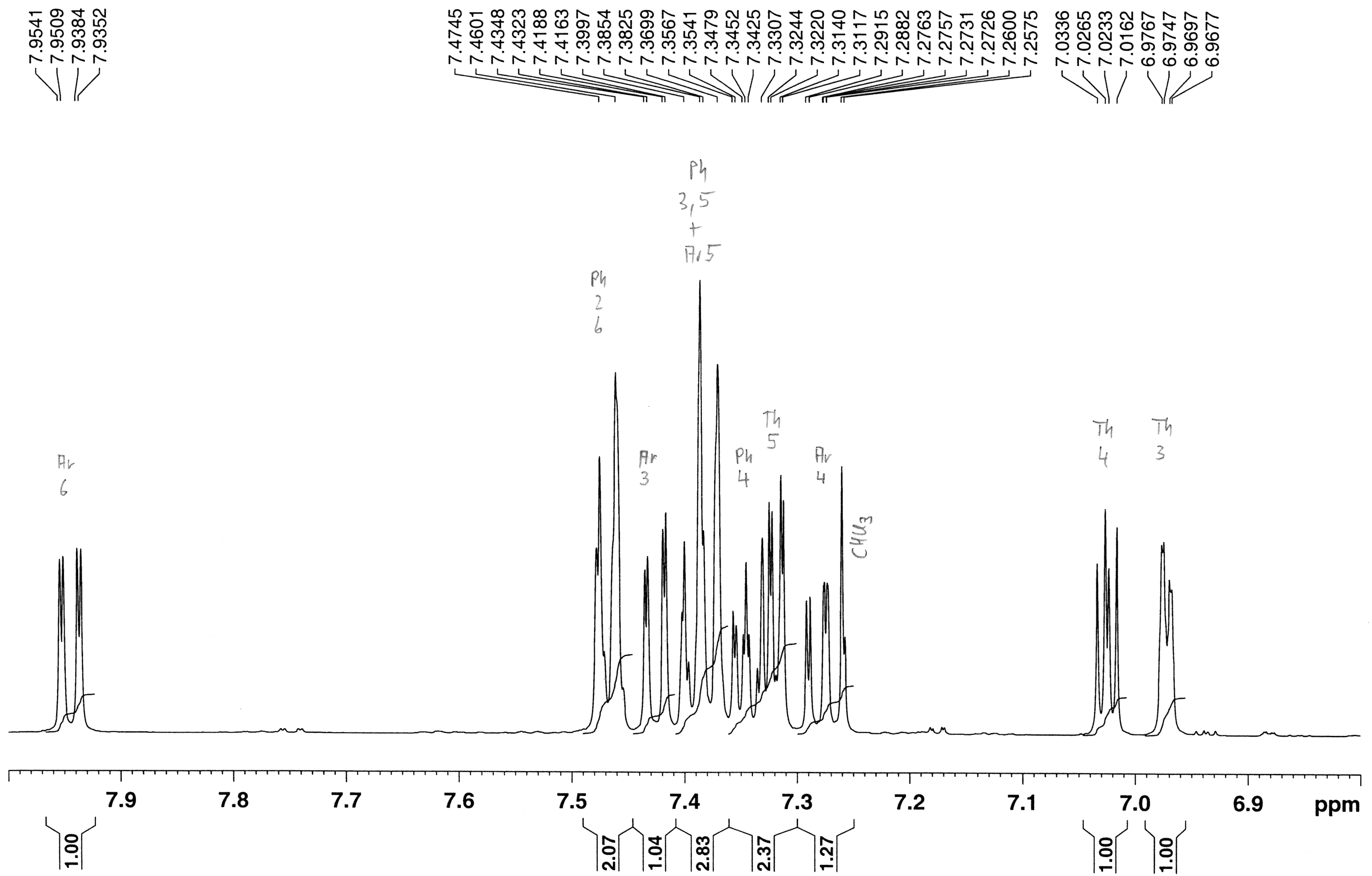

PN018-4 in cdcl3 (Proton) 29.7.2020

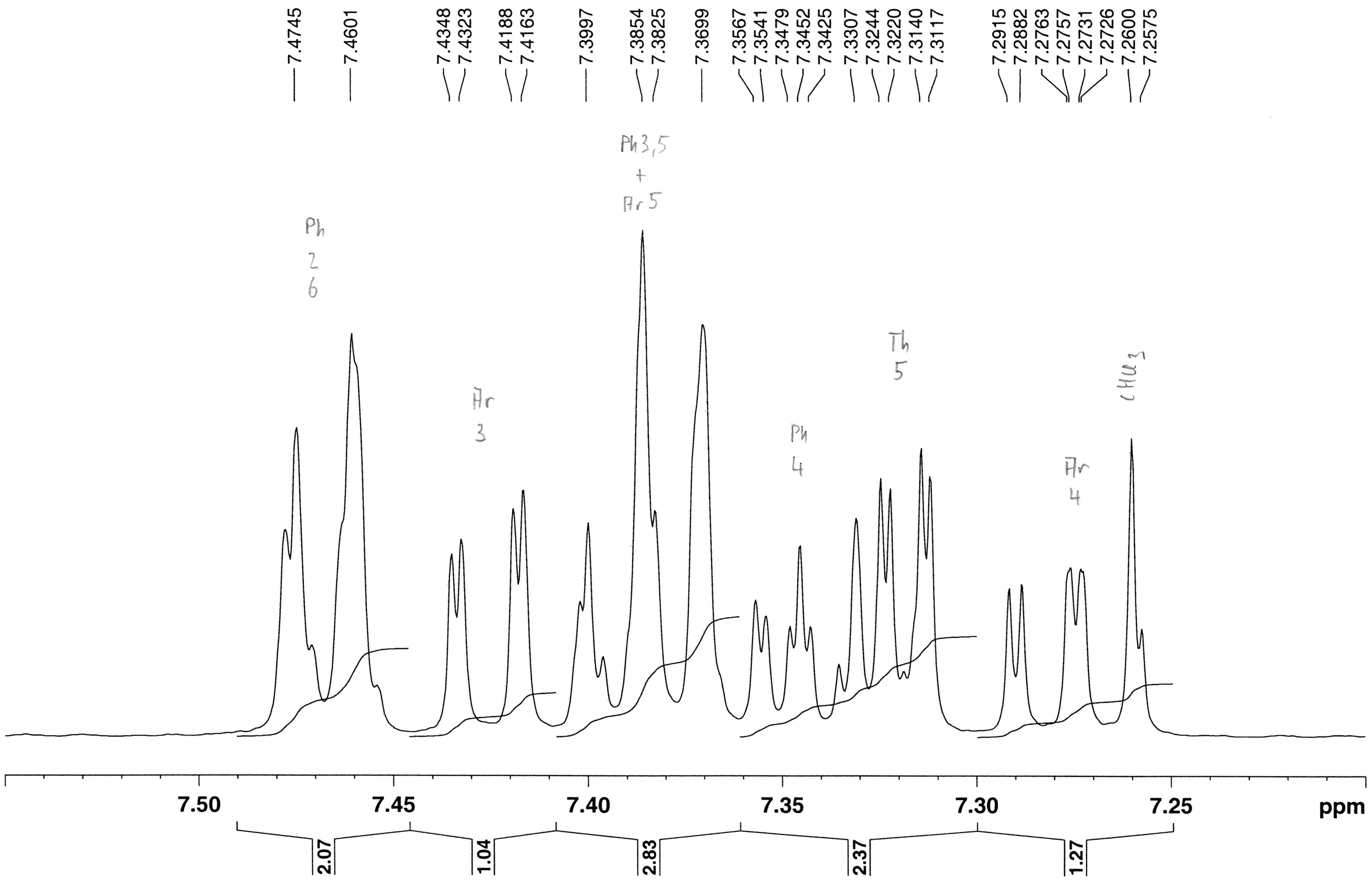

PN018-4 in cdcl3 (Proton) 29.7.2020

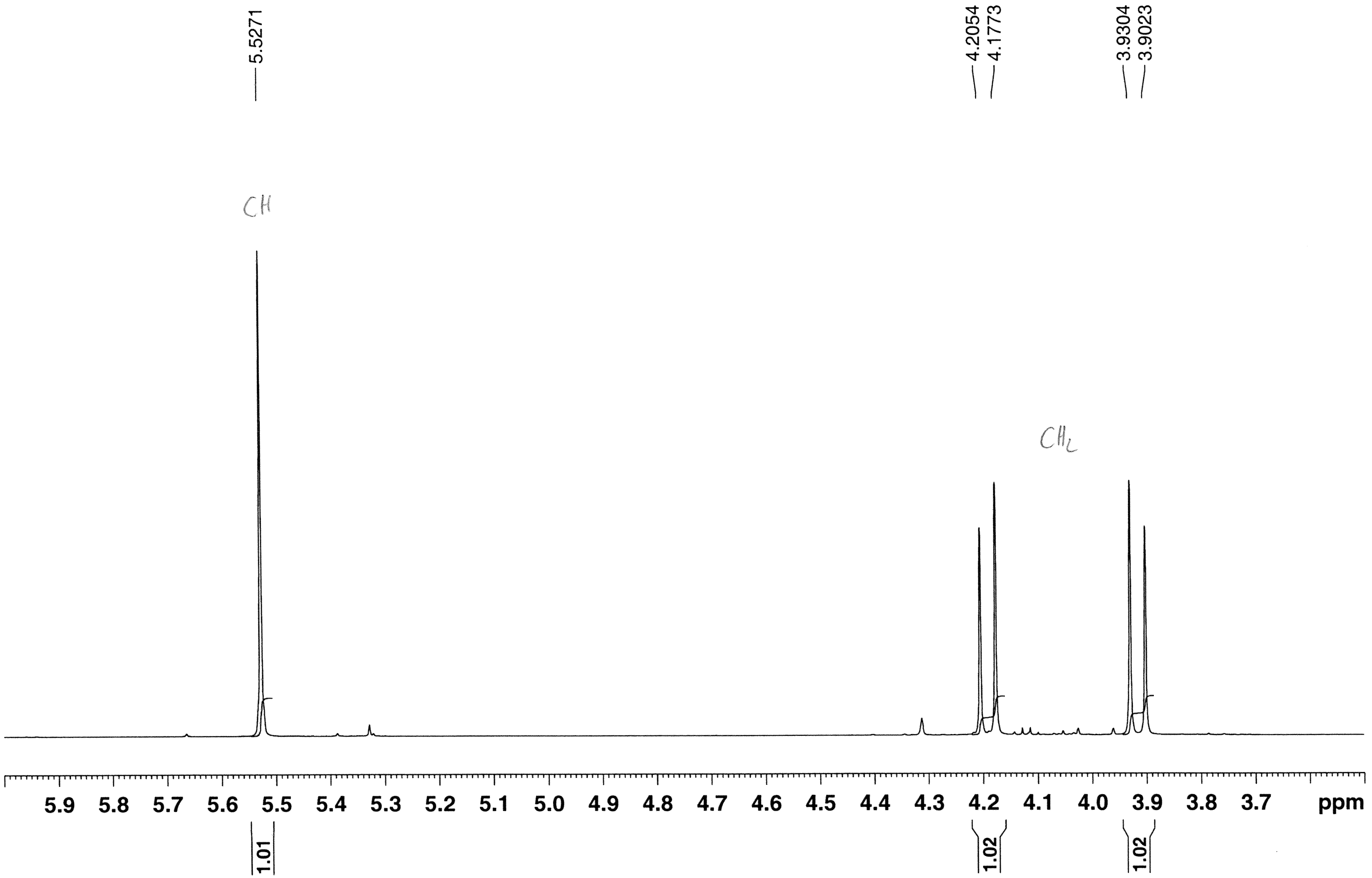

PN018-4 in cdcl3 (APT) 29.7.2020

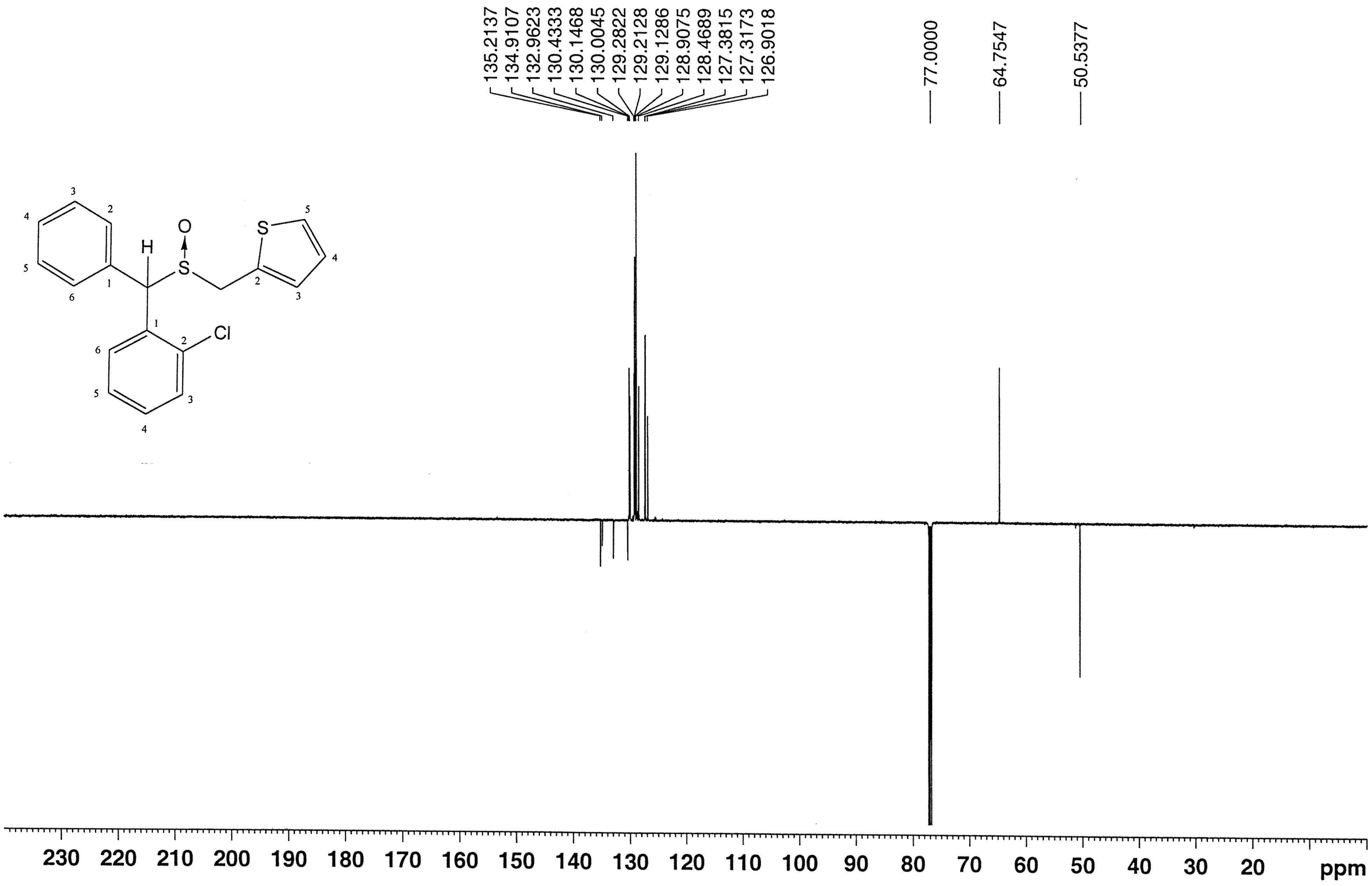

PN018-4 in cdcl3 (APT) 29.7.2020

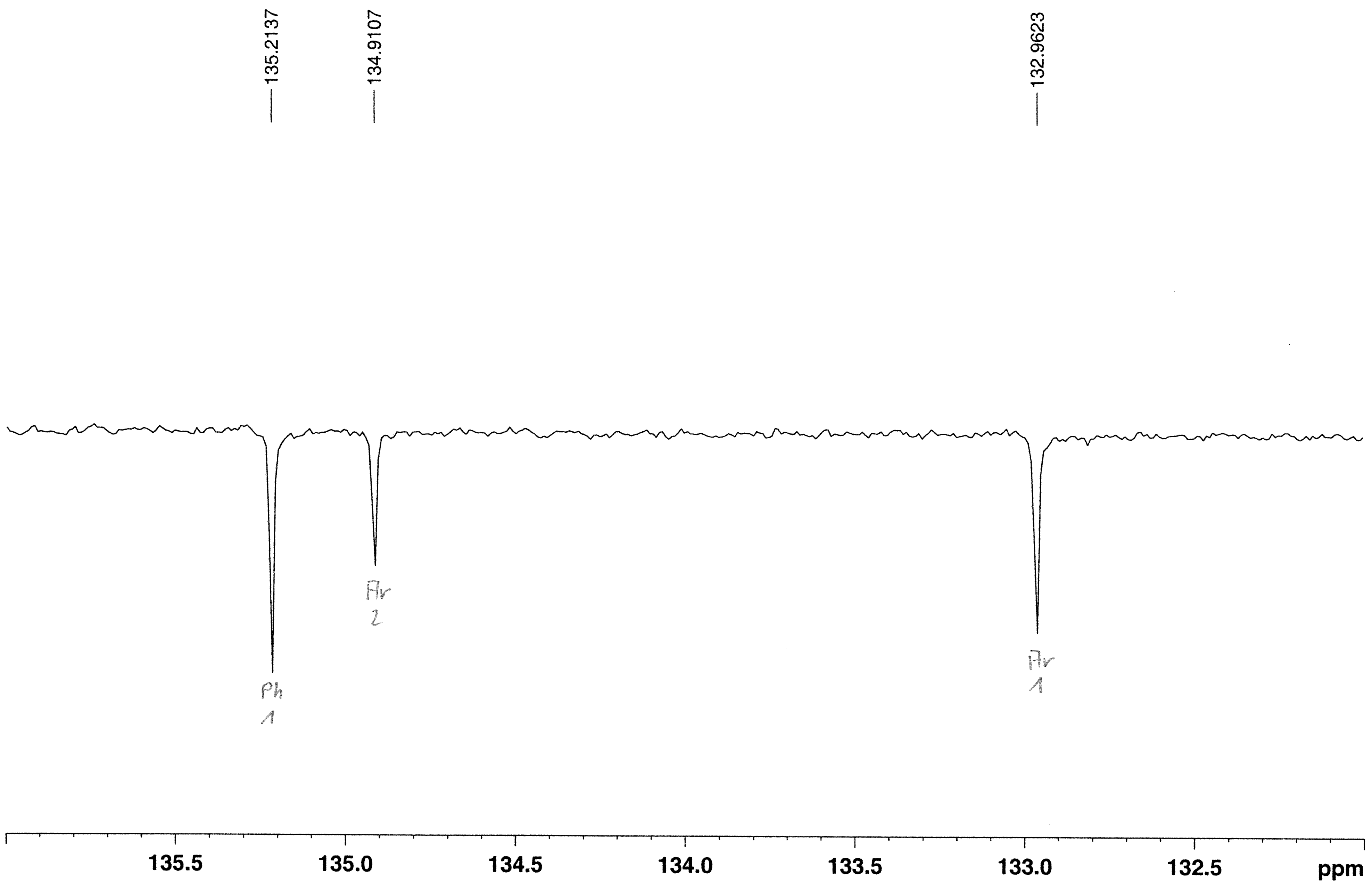

PN018-4 in cdcl3 (APT) 29.7.2020

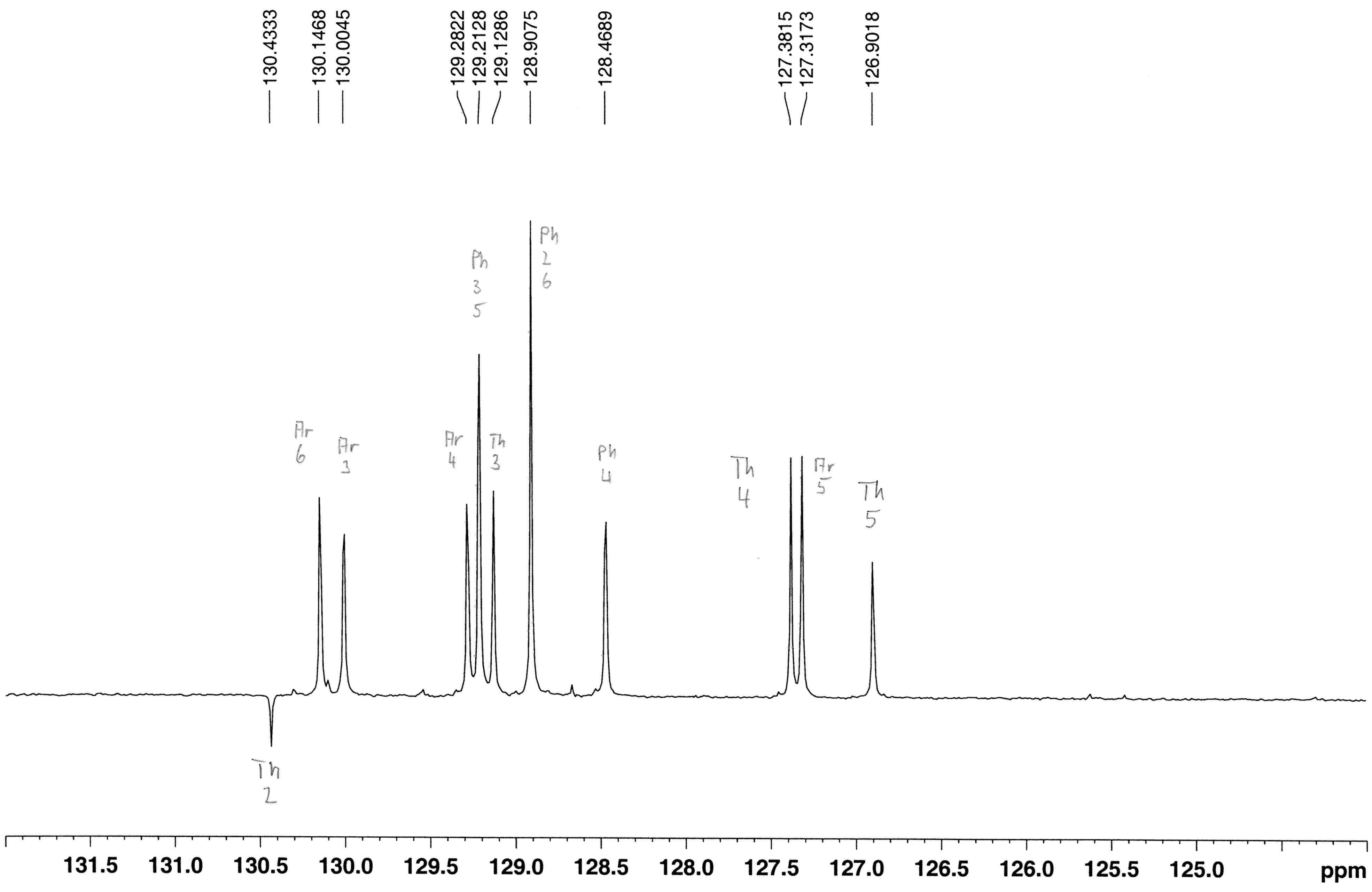

PN018-4 in cdcl3 (APT) 29.7.2020

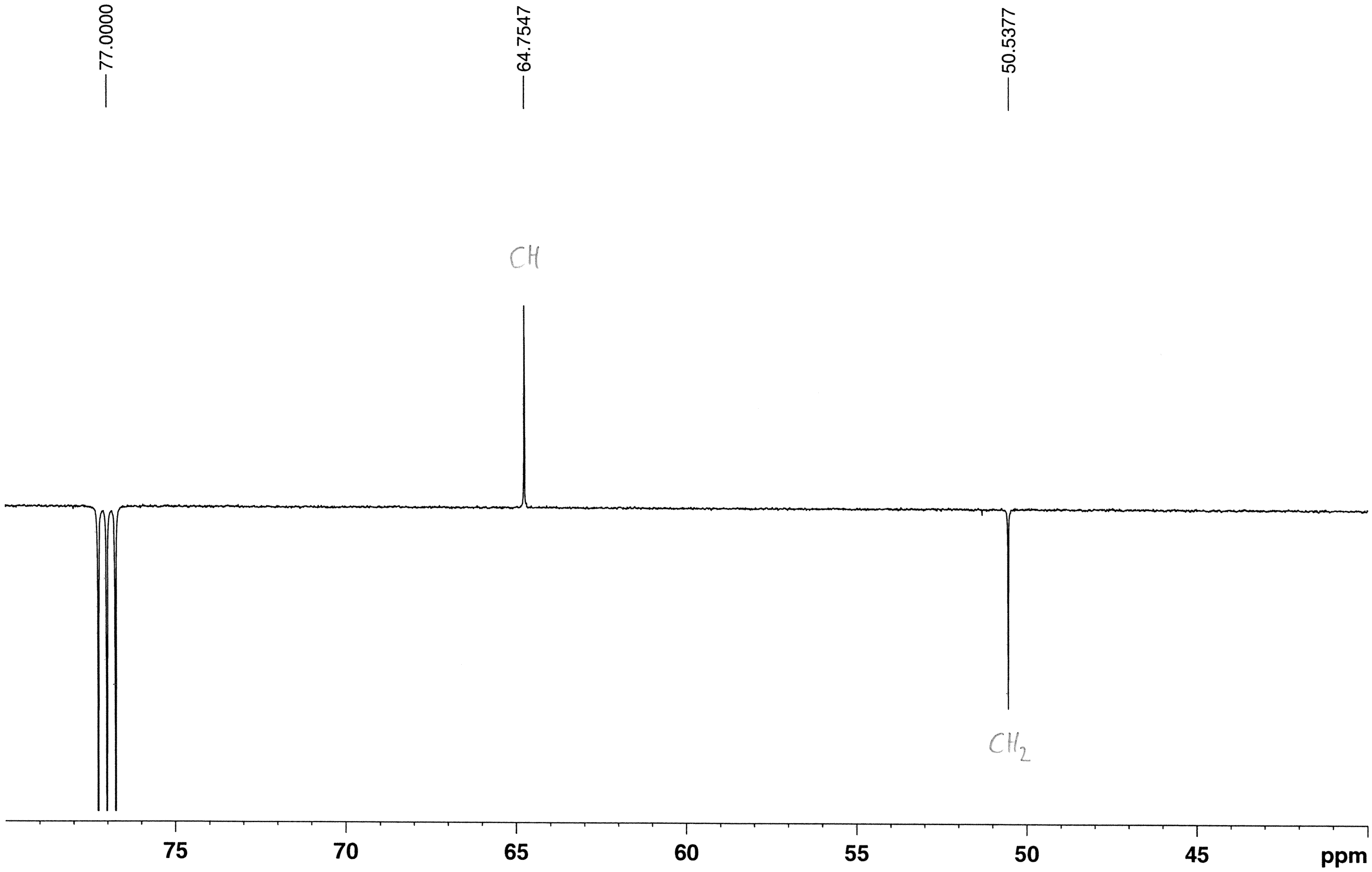

PN018-4 in cdcl3 (COSY) 29.7.2020

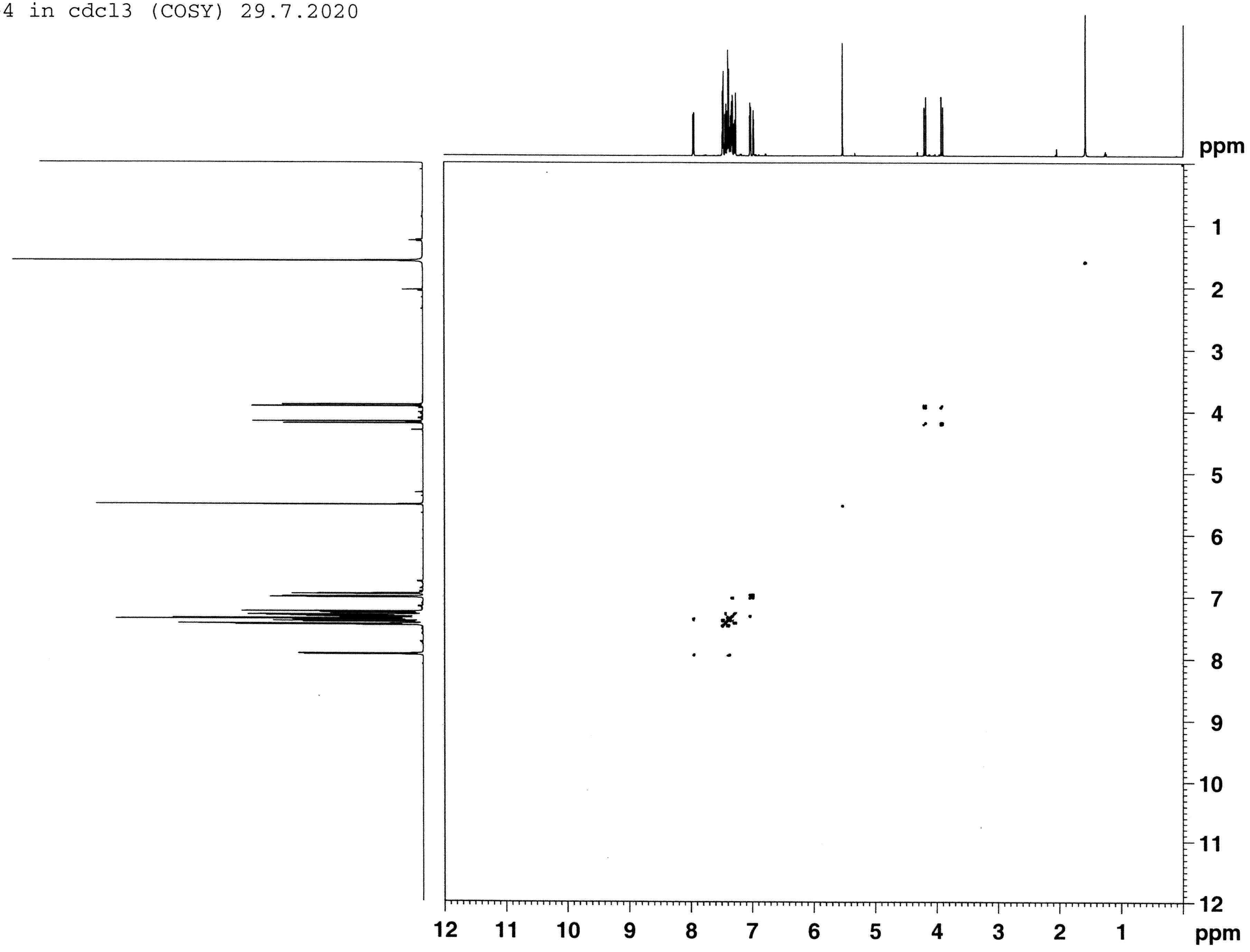

PN018-4 in cdcl3 (COSY) 29.7.2020

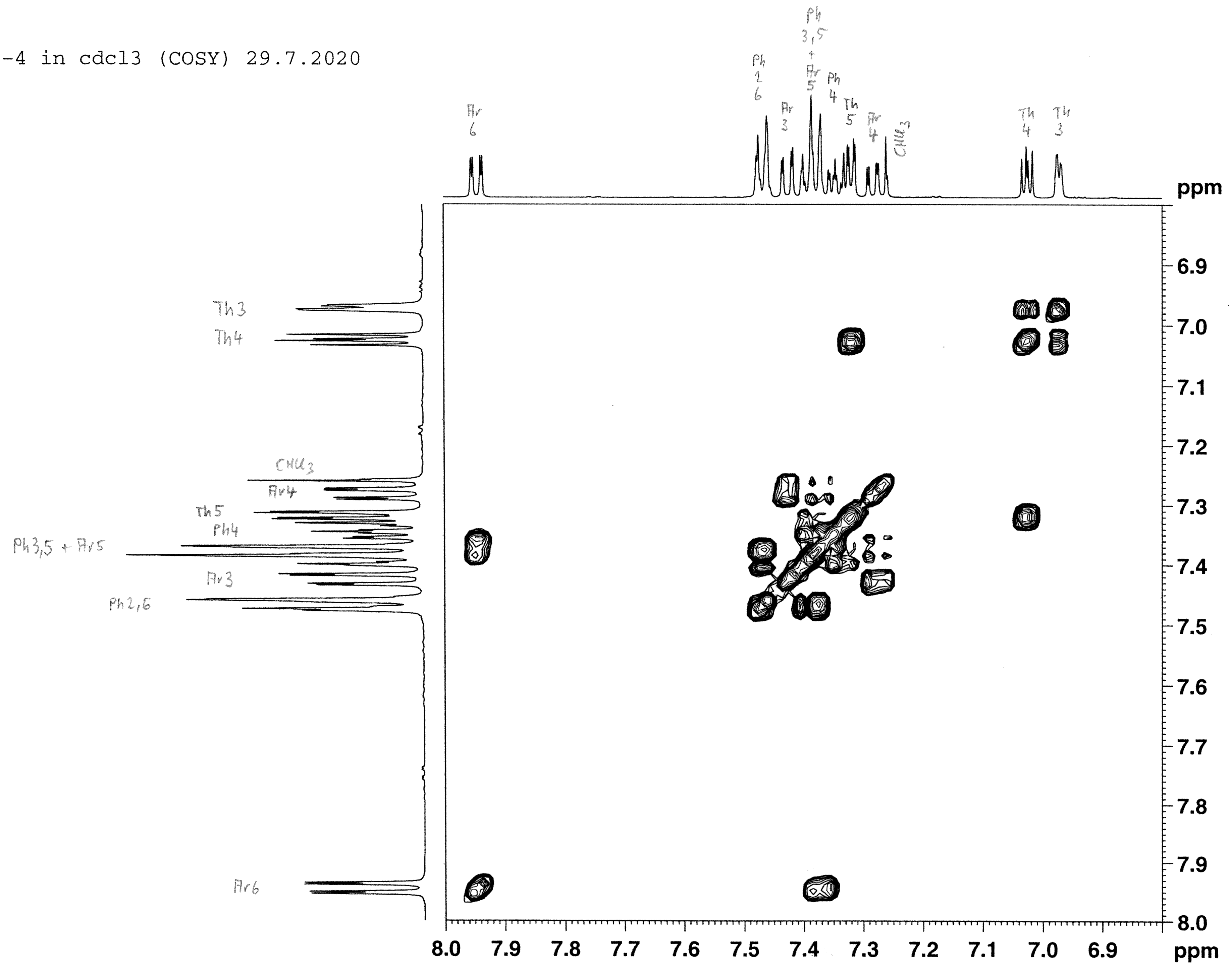

PN018-4 in cdcl3 (COSY) 29.7.2020

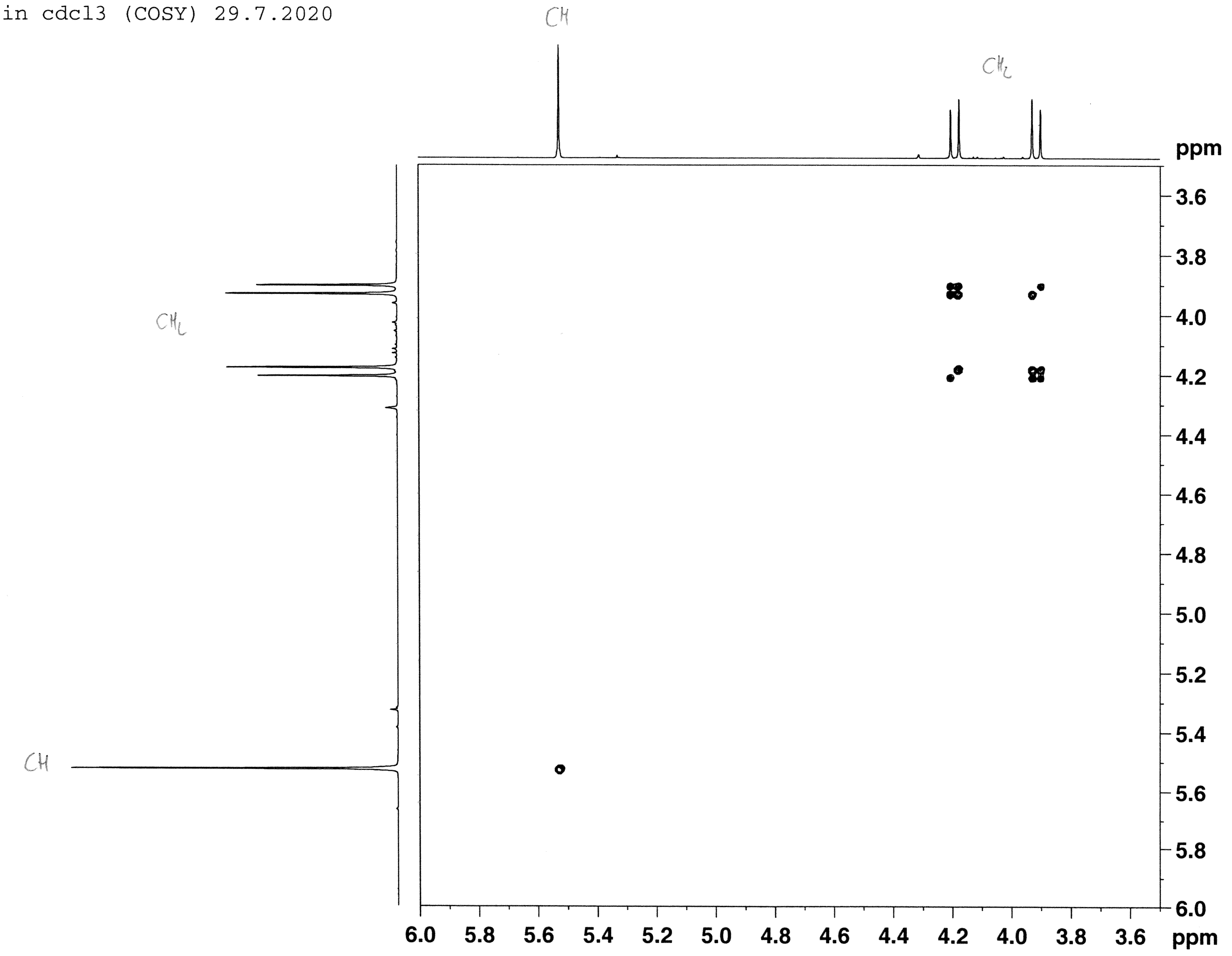

PN018-4 in cdcl3 (HSQC) 29.7.2020

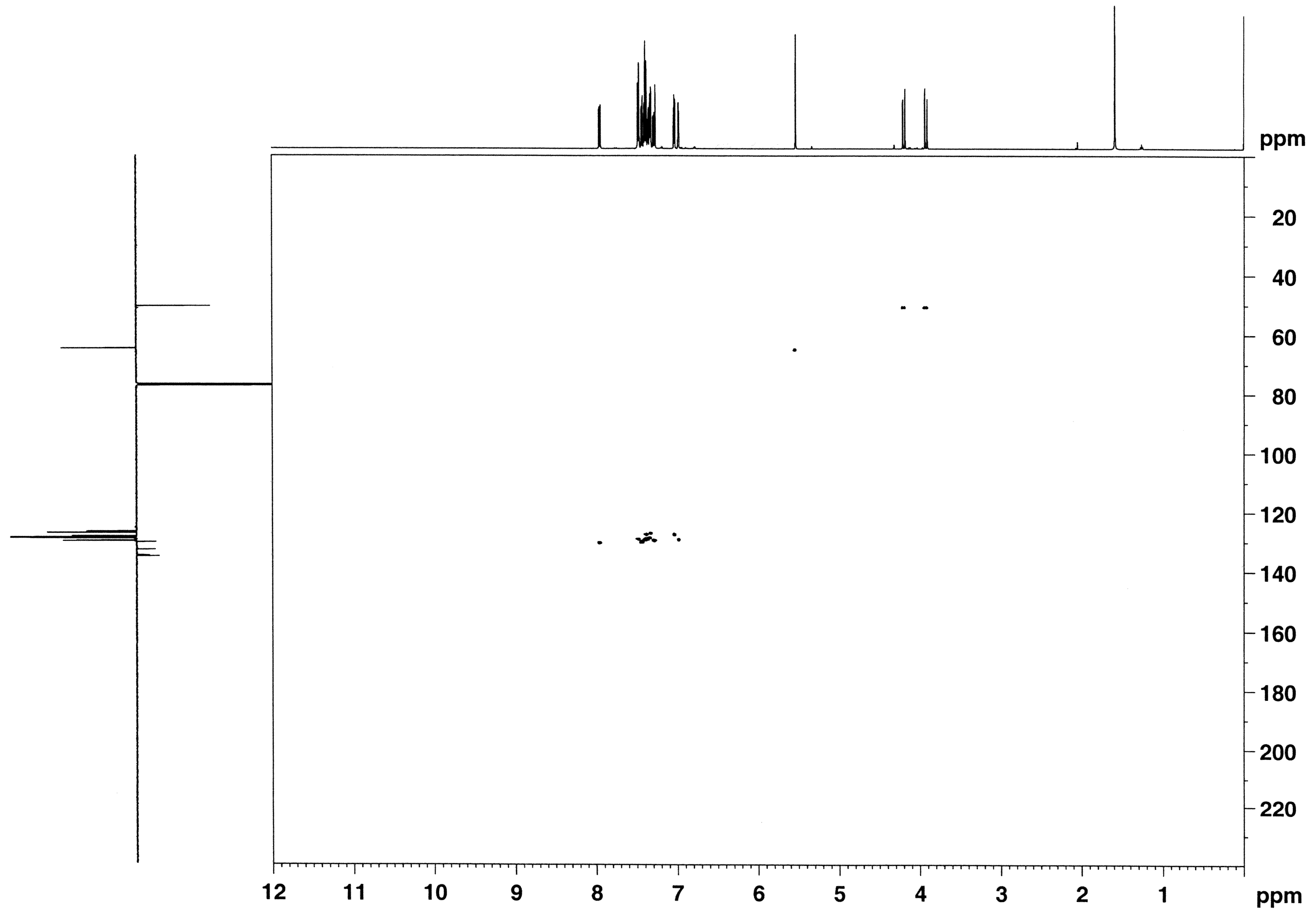

PN018-4 in cdcl3 (HSQC) 29.7.2020

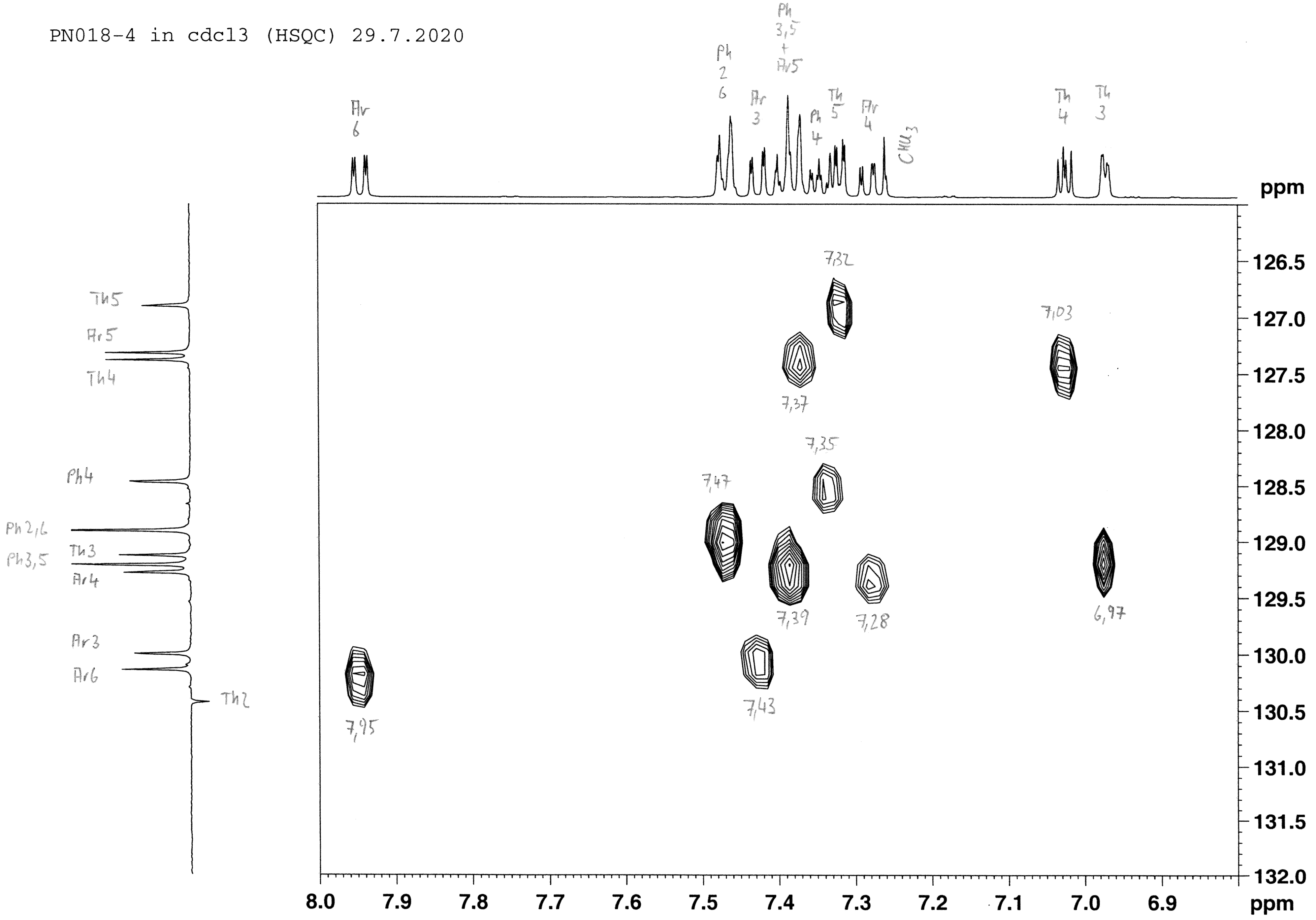

PN018-4 in cdcl3 (HSQC) 29.7.2020

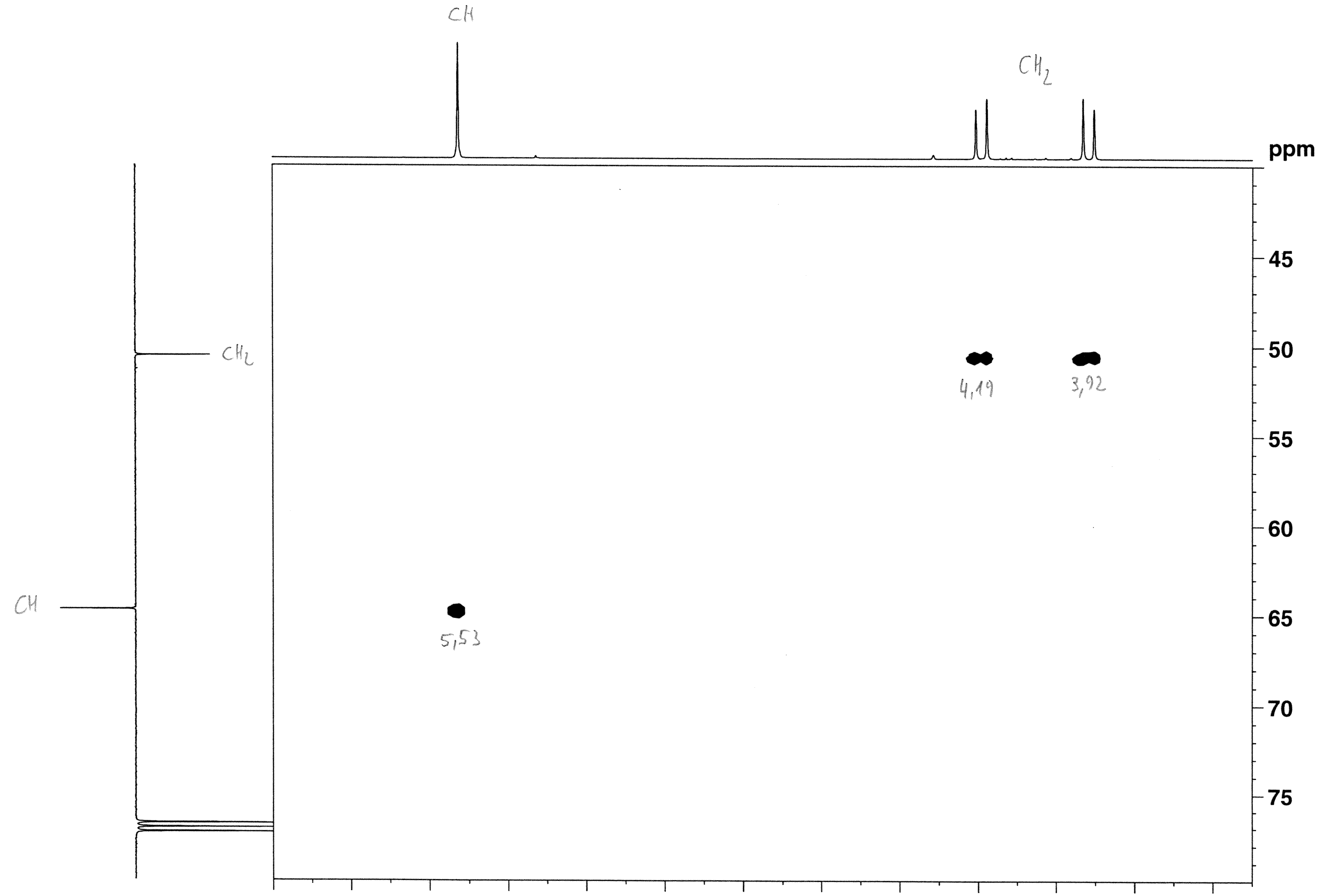

PN018-4 in cdcl3 (HMBC) 29.7.2020

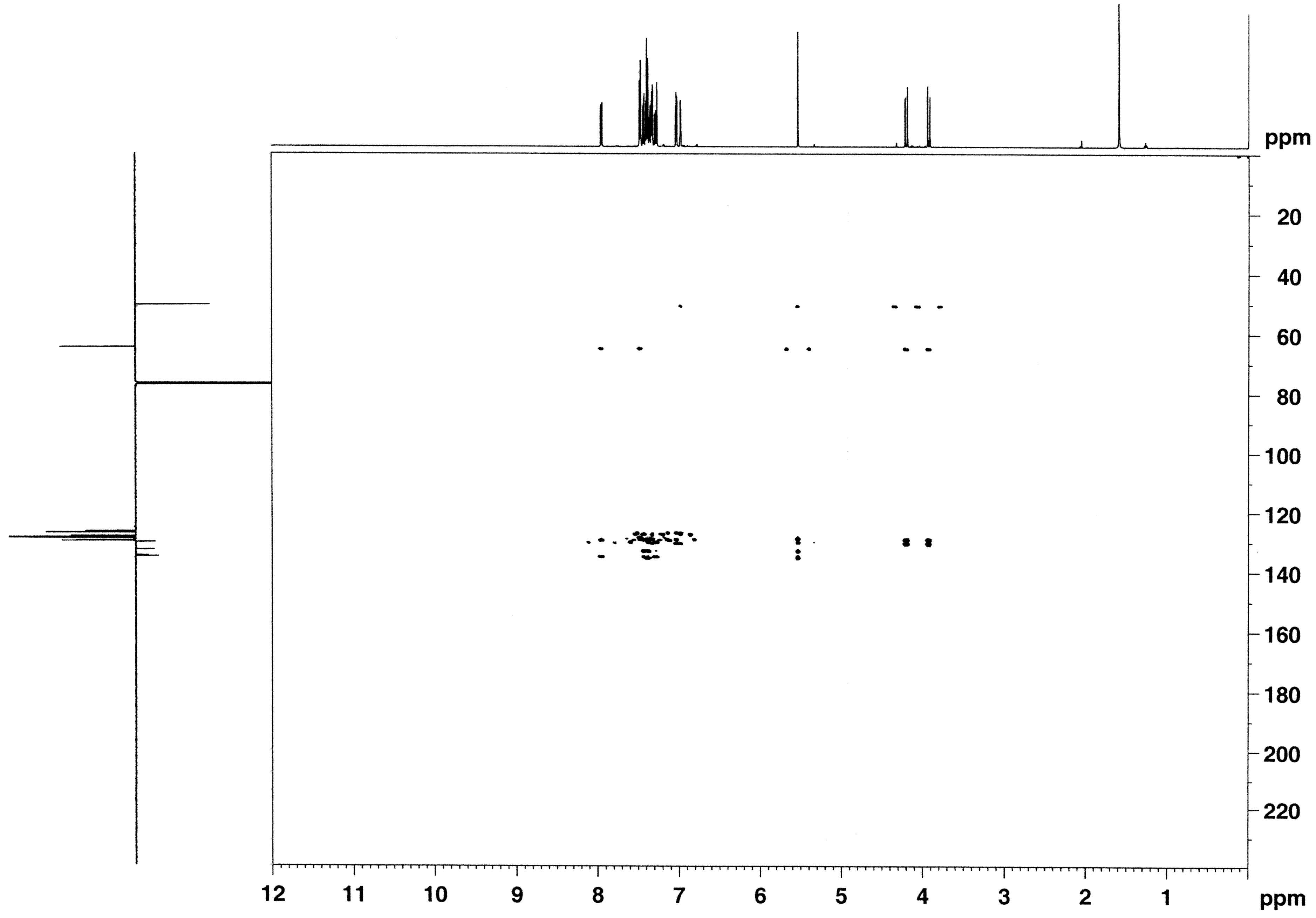

PN018-4 in cdcl3 (HMBC) 29.7.2020

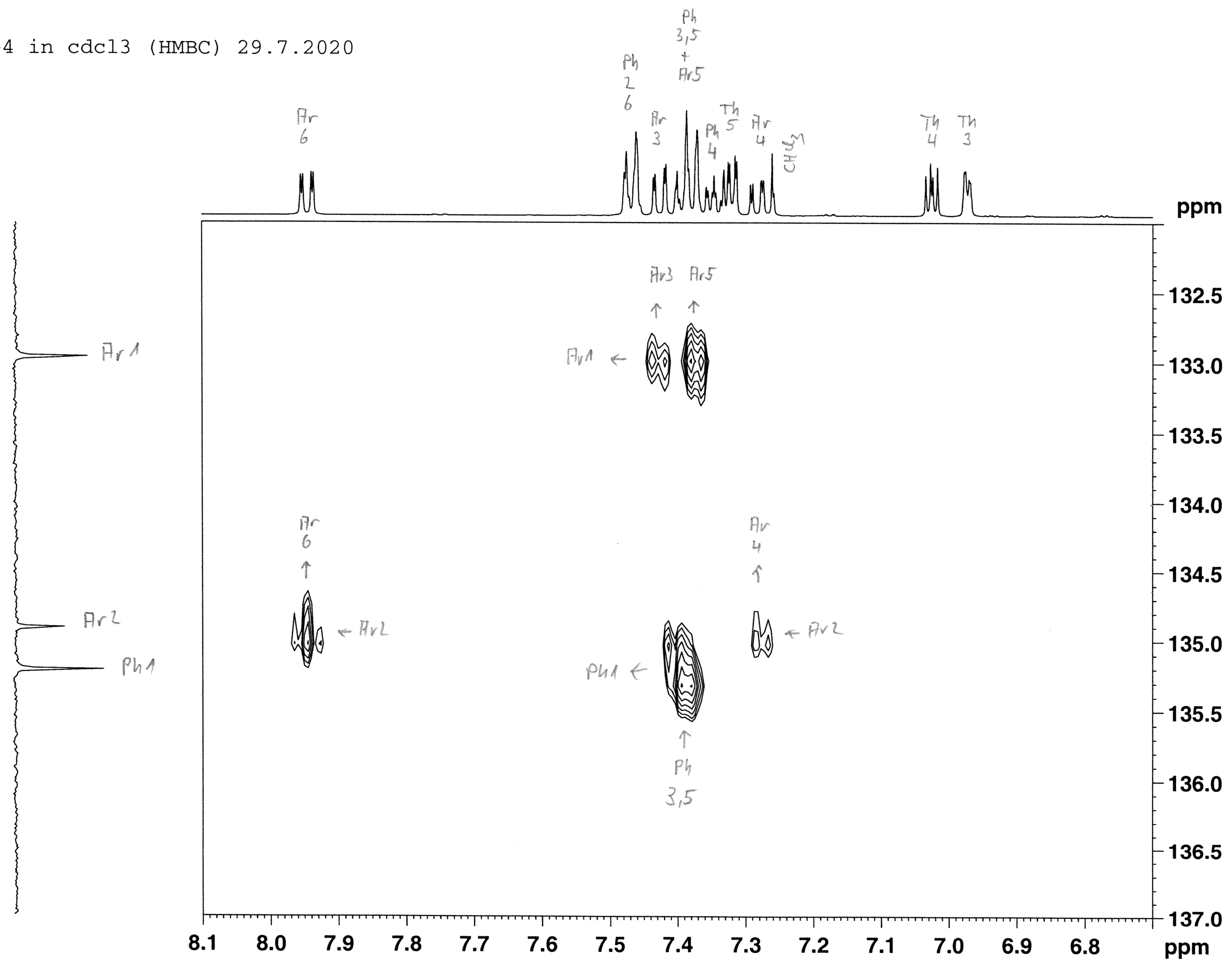

PN018-4 in cdcl3 (HMBC) 29.7.2020

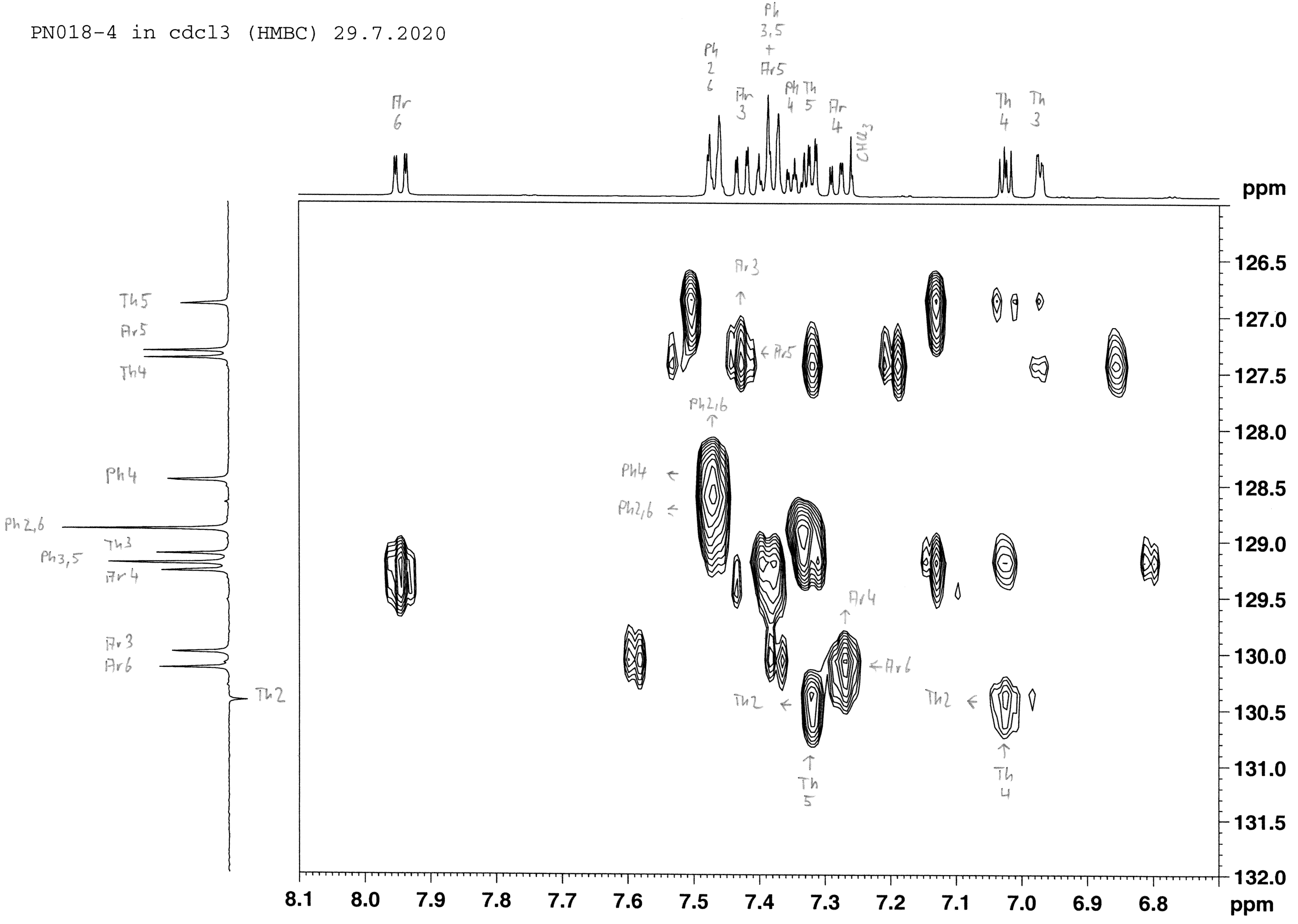

PN018-4 in cdcl3 (HMBC) 29.7.2020

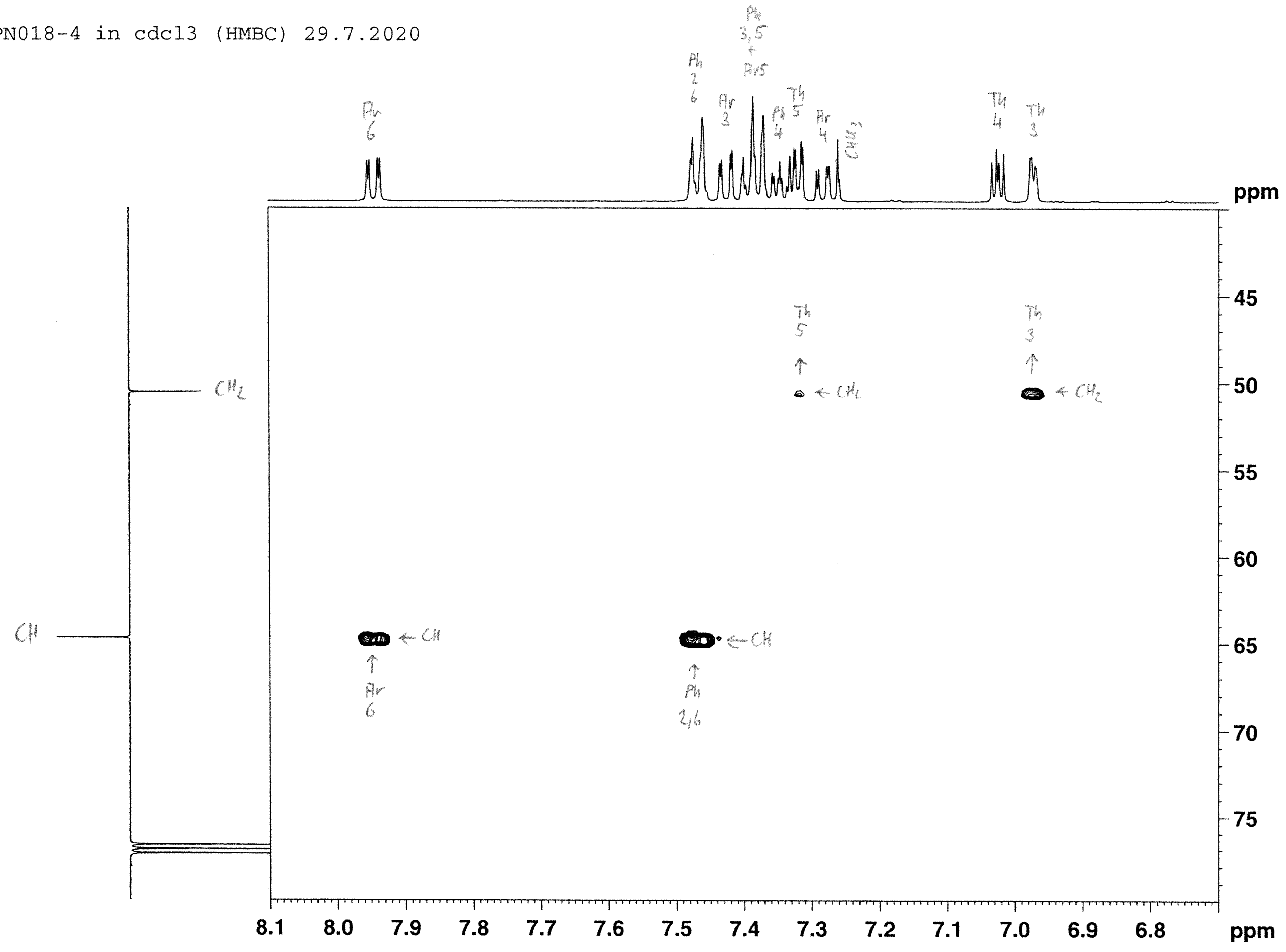

PN018-4 in cdcl3 (HMBC) 29.7.2020

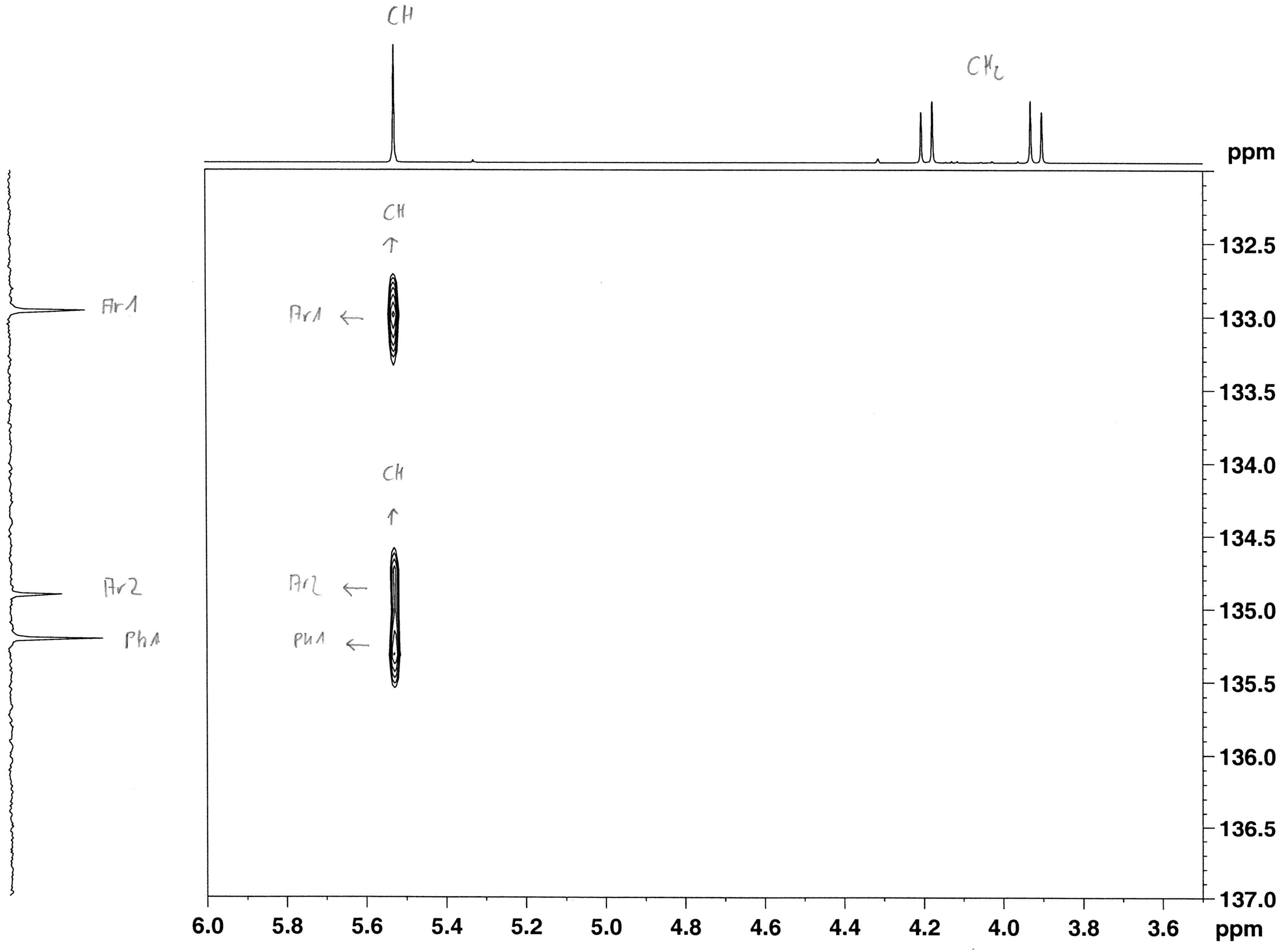

PN018-4 in cdcl3 (HMBC) 29.7.2020

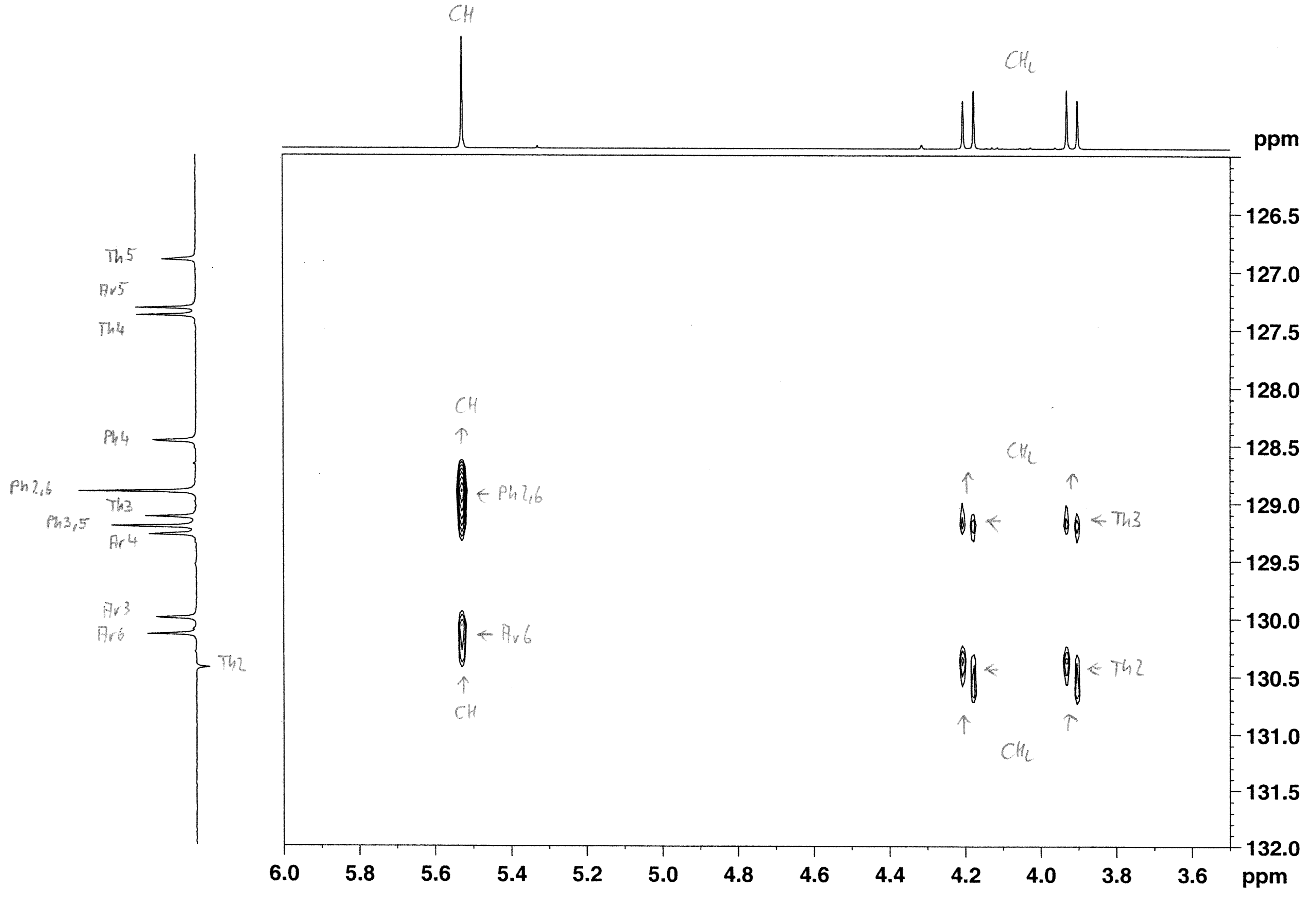

PN018-4 in cdcl3 (HMBC) 29.7.2020

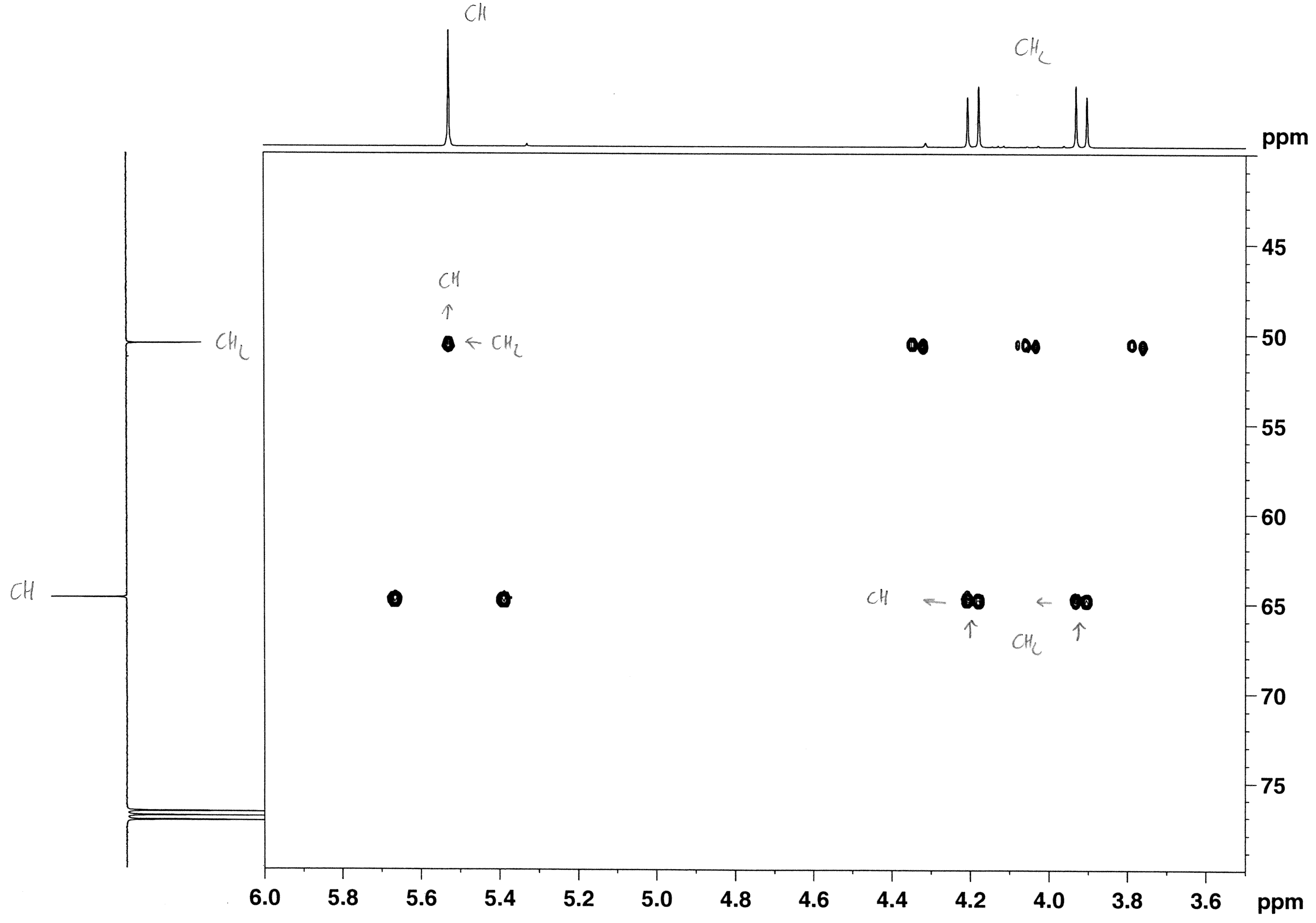

Figure S17c. NMR spectra of compound **5e.**

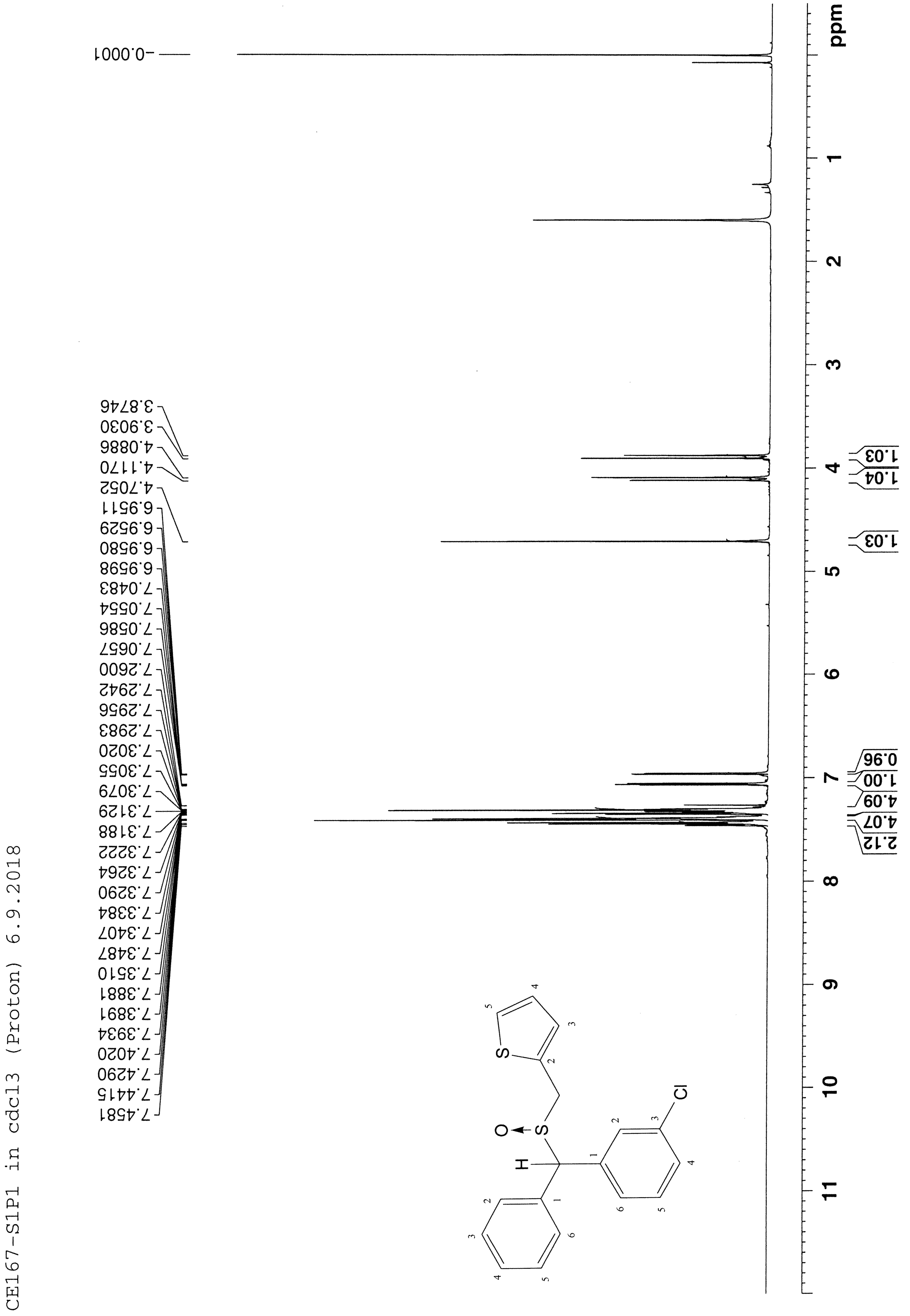

CE167-S1P1 in cdcl3 (Proton) 6.9.2018

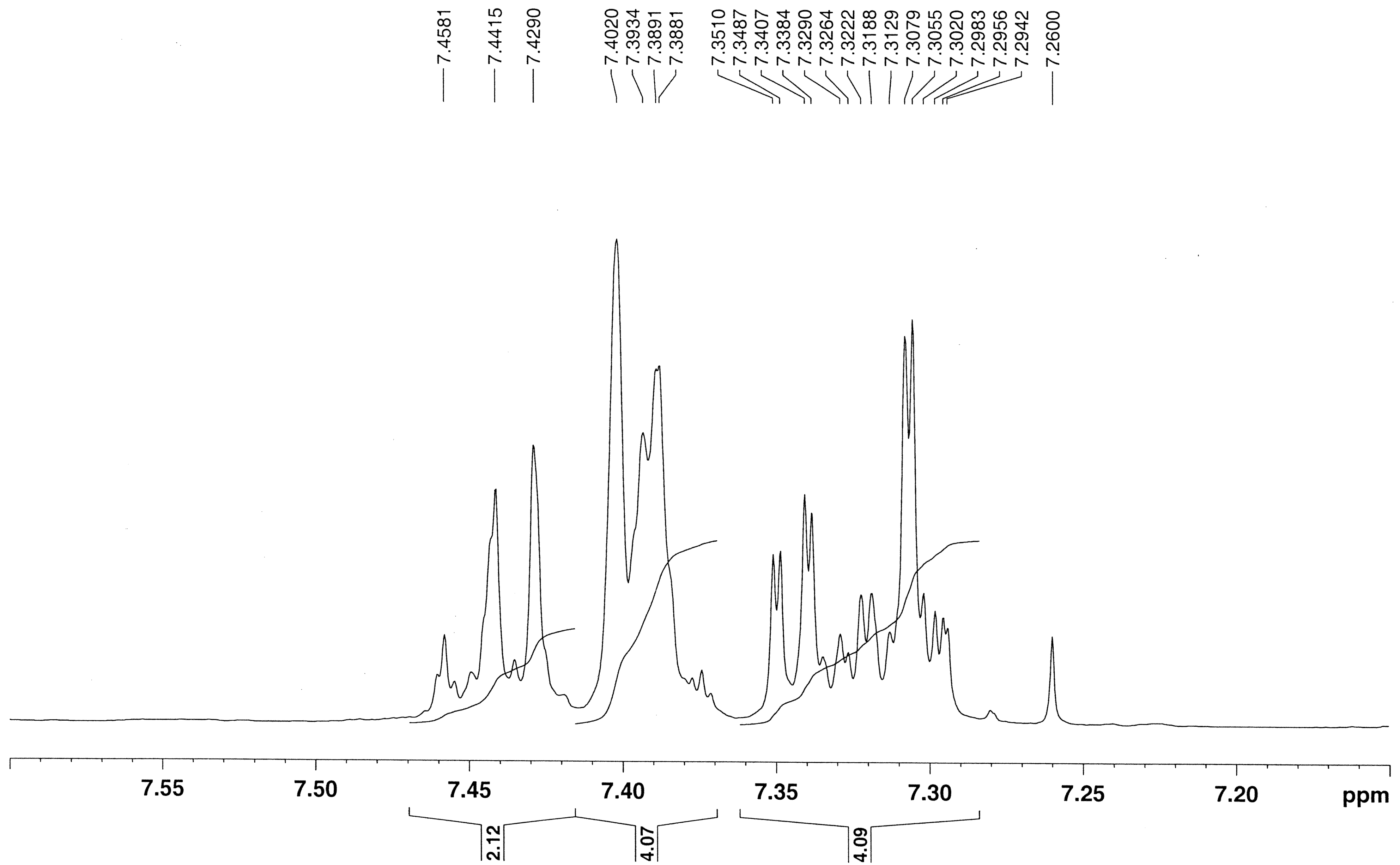

CE167-S1P1 in cdcl3 (Proton) 6.9.2018

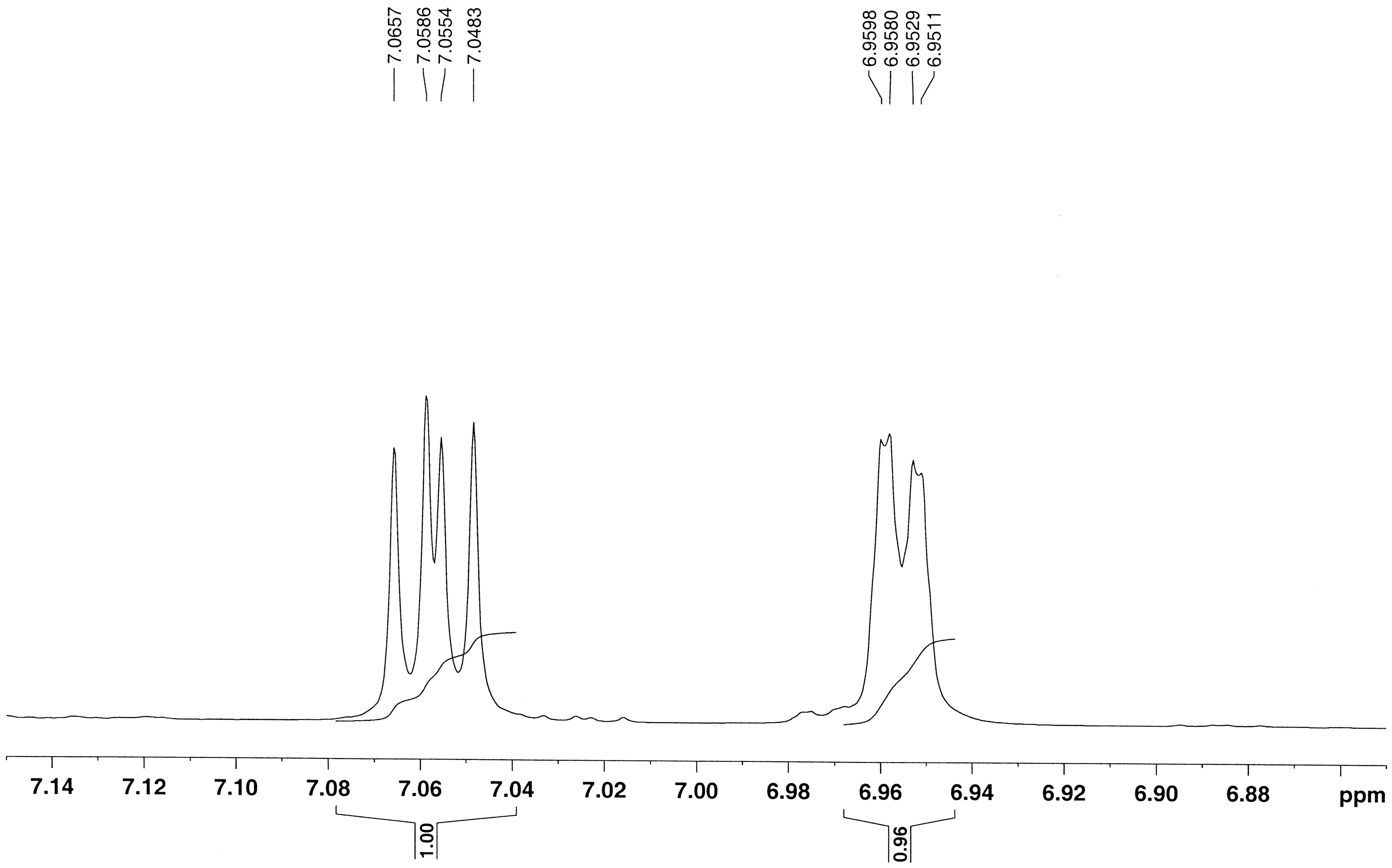

CE167-S1P1 in cdcl3 (Proton) 6.9.2018

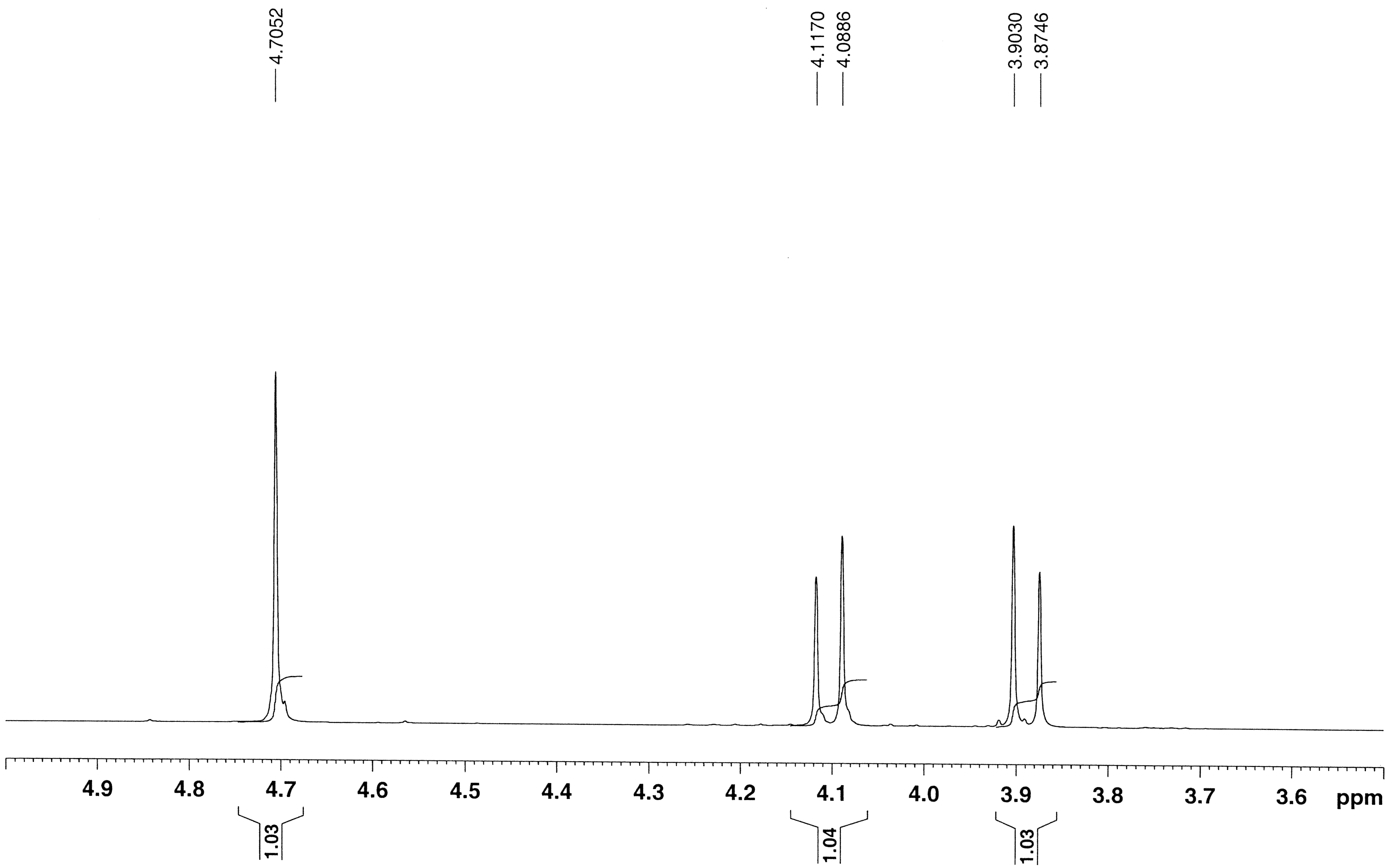

CE167-S1P1 in cdcl3 (APT) 6.9.2018

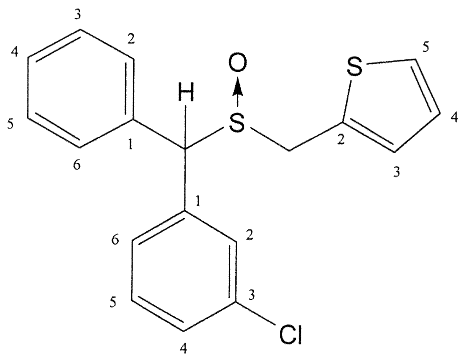

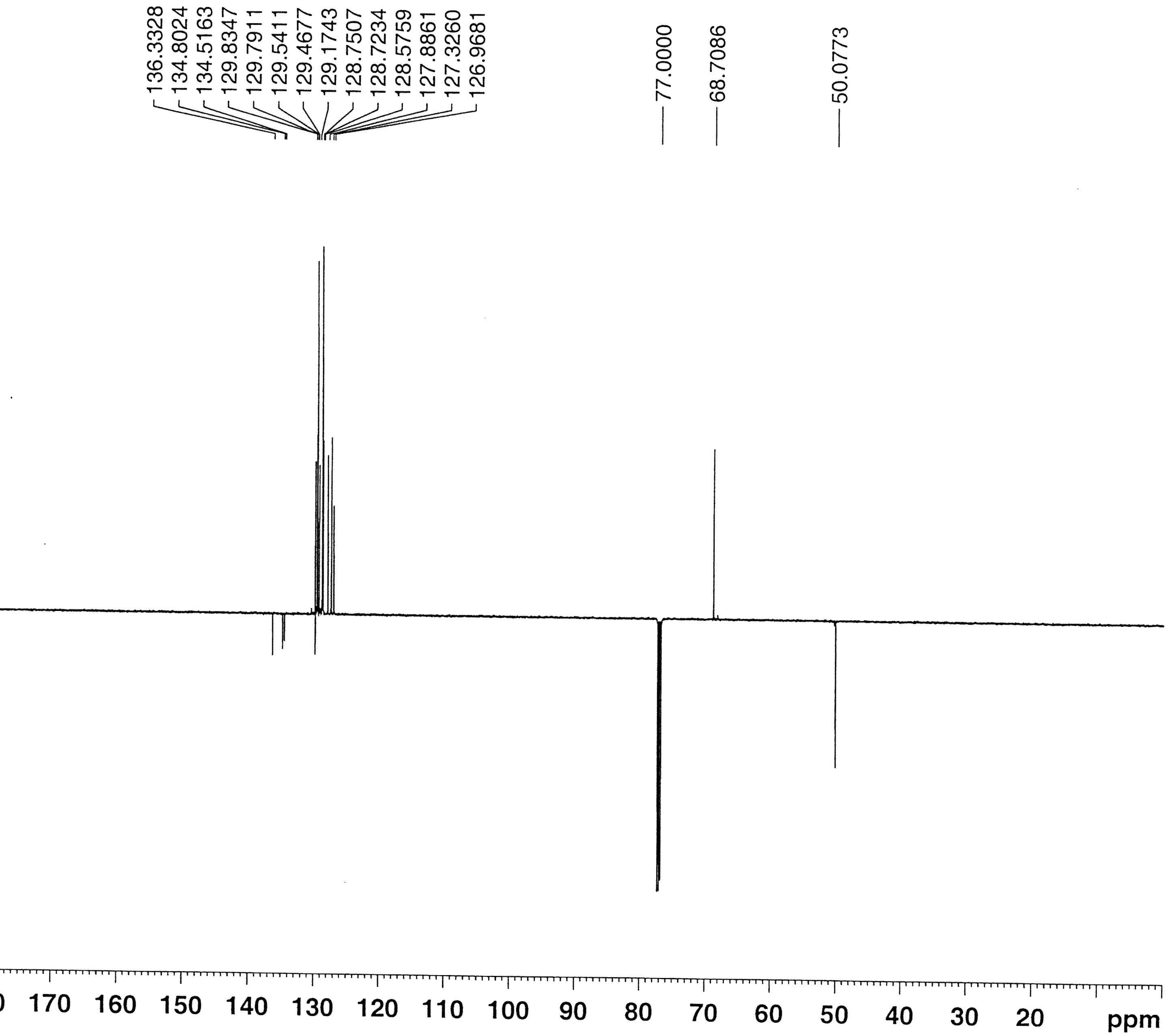

CE167-S1P1 in cdcl3 (APT) 6.9.2018

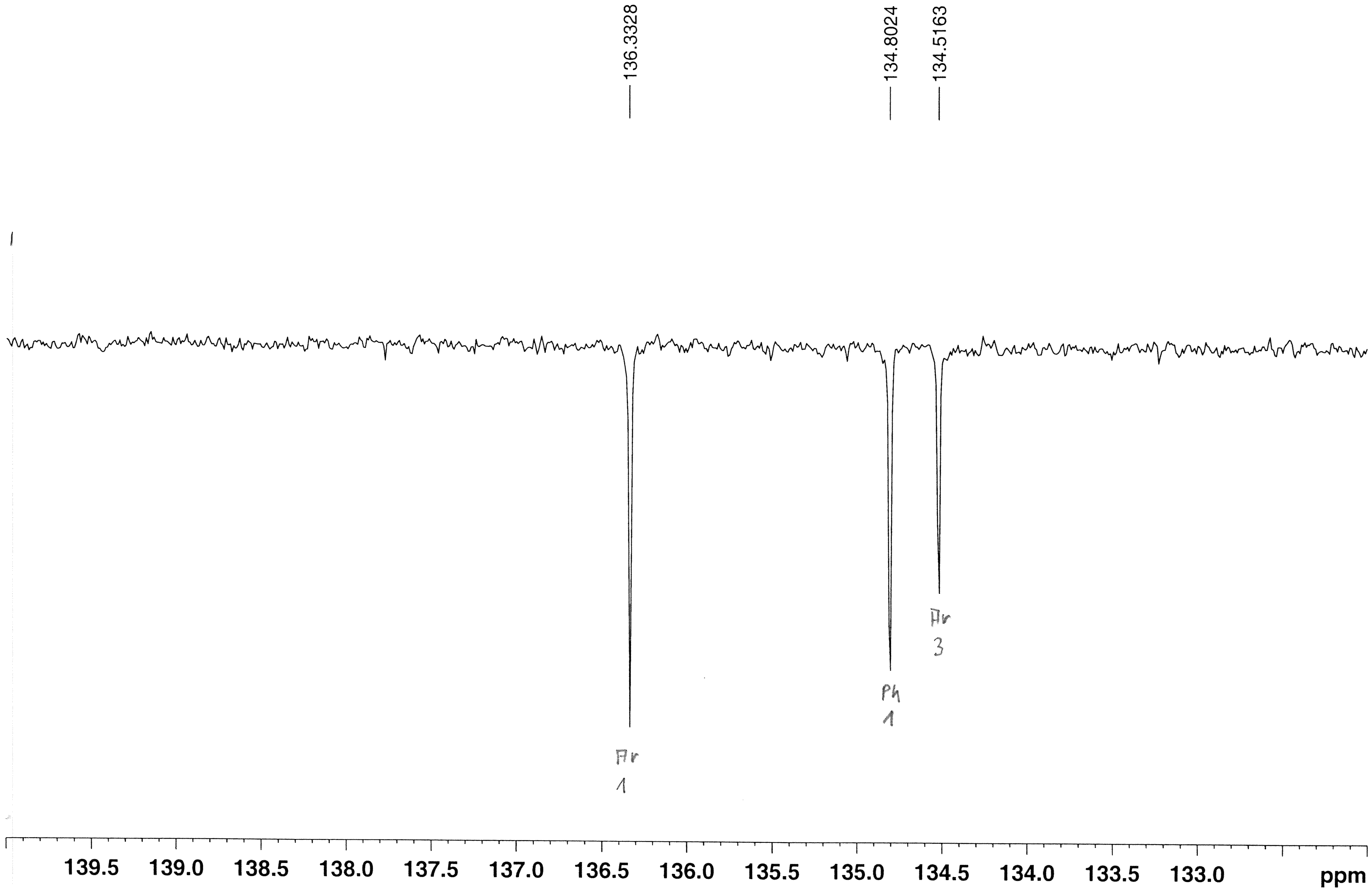

CE167-S1P1 in cdcl3 (APT) 6.9.2018

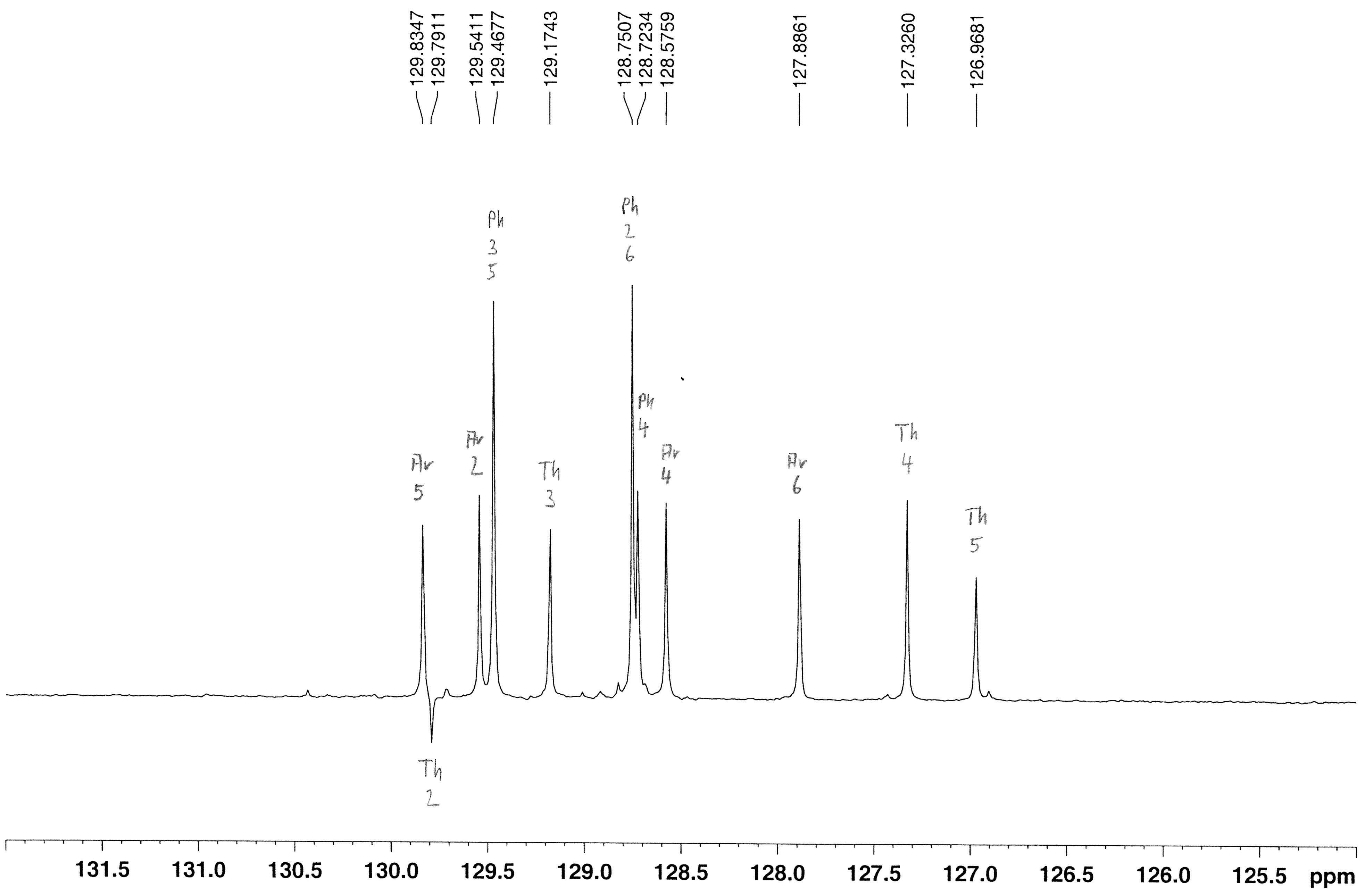

CE167-S1P1 in cdcl3 (APT) 6.9.2018

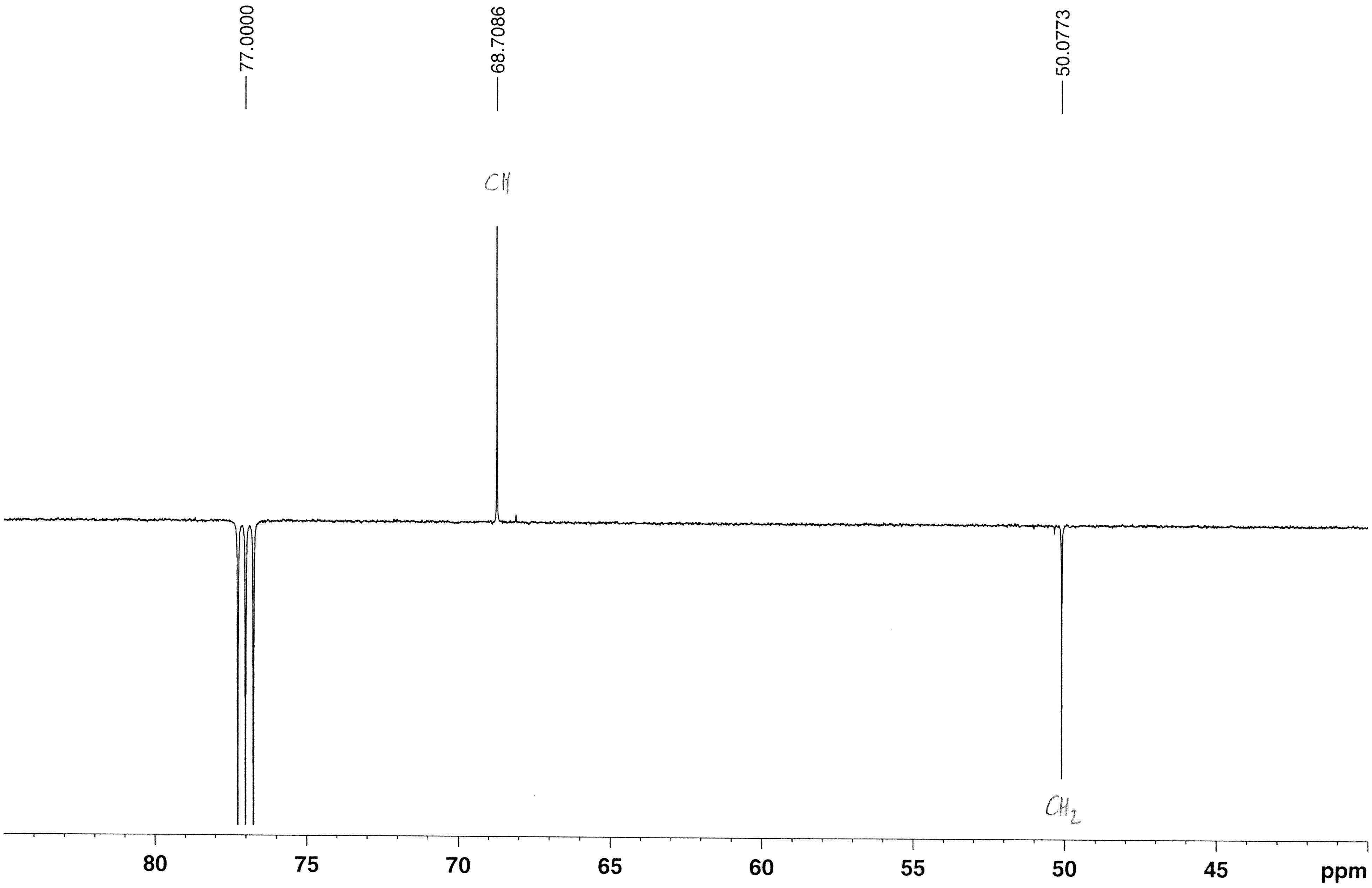

CE167-S1P1 in cdcl3 (HSQC) 6.9.2018

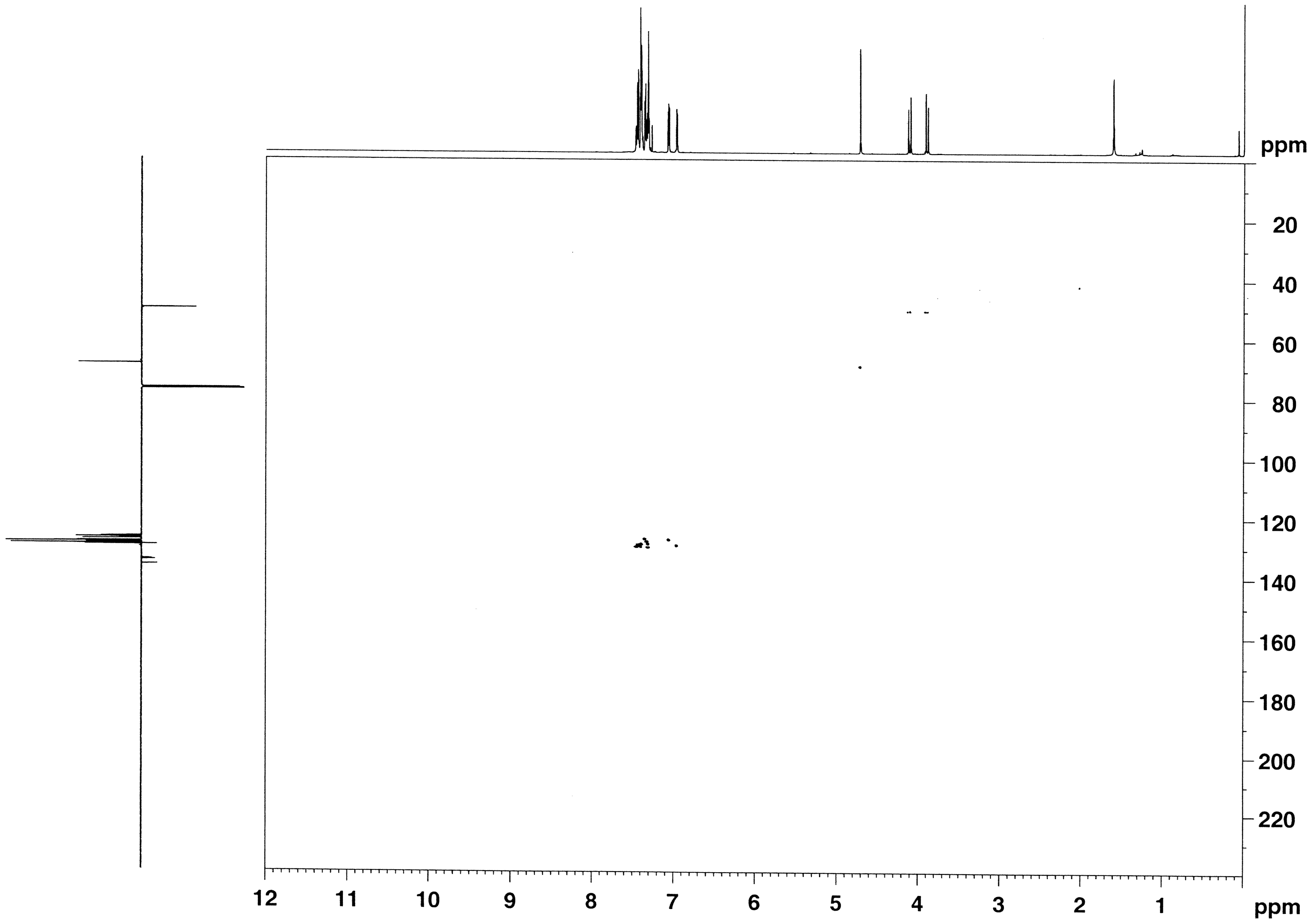

CE167-S1P1 in cdcl3 (HSQC) 6.9.2018

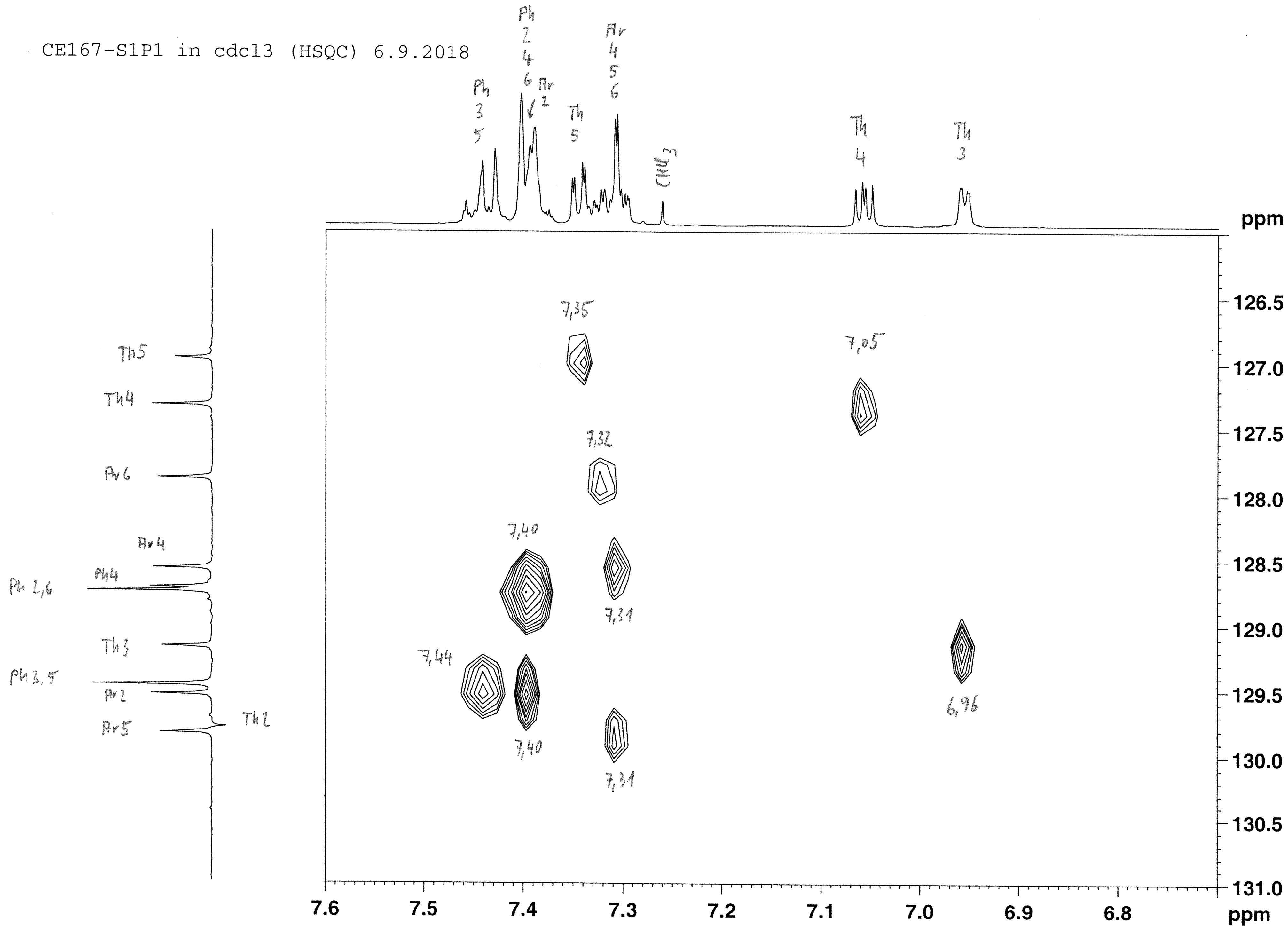

CE167-S1P1 in cdcl3 (HSQC) 6.9.2018

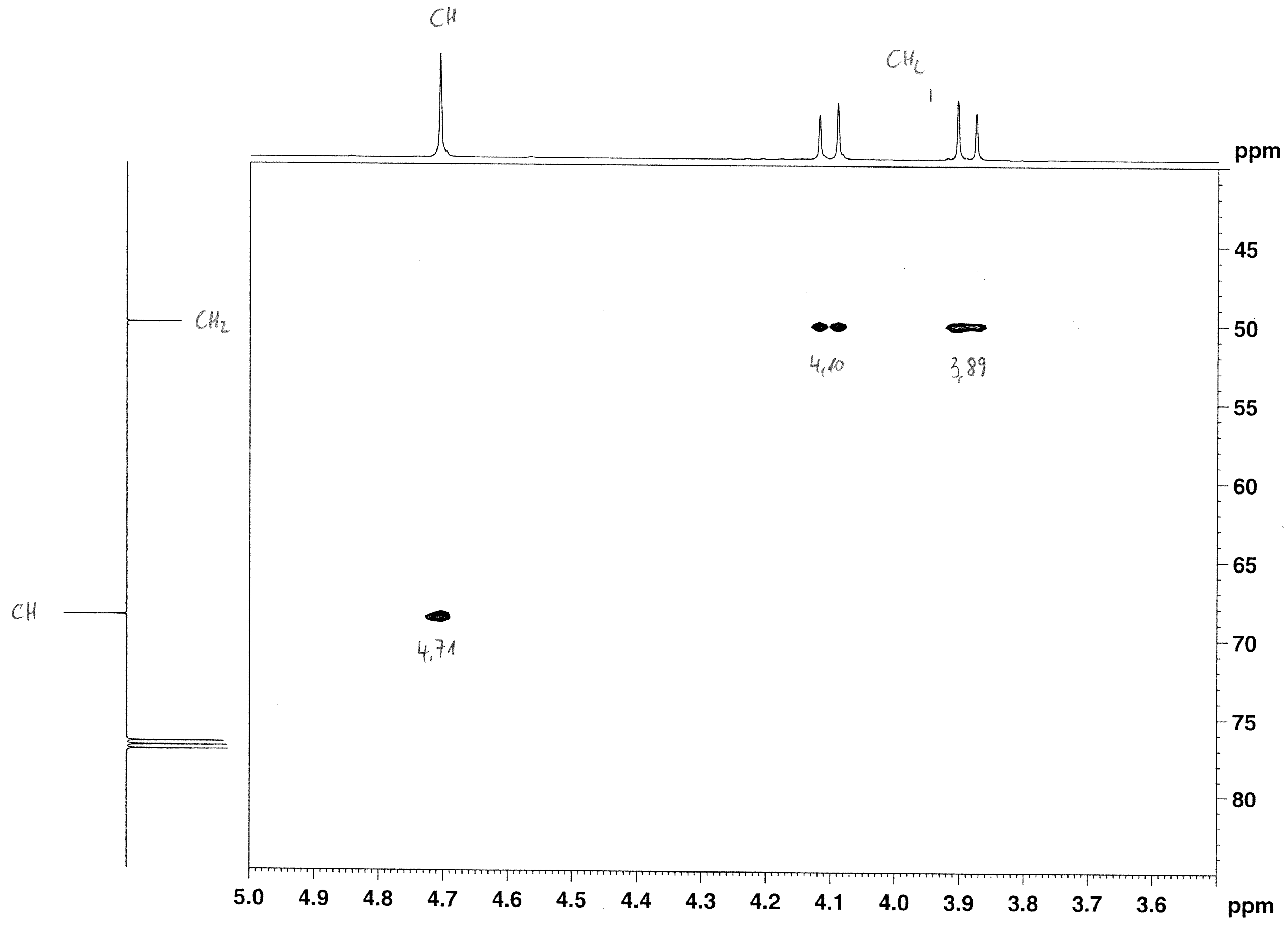

Figure S18c. NMR spectra of compound **6e**.

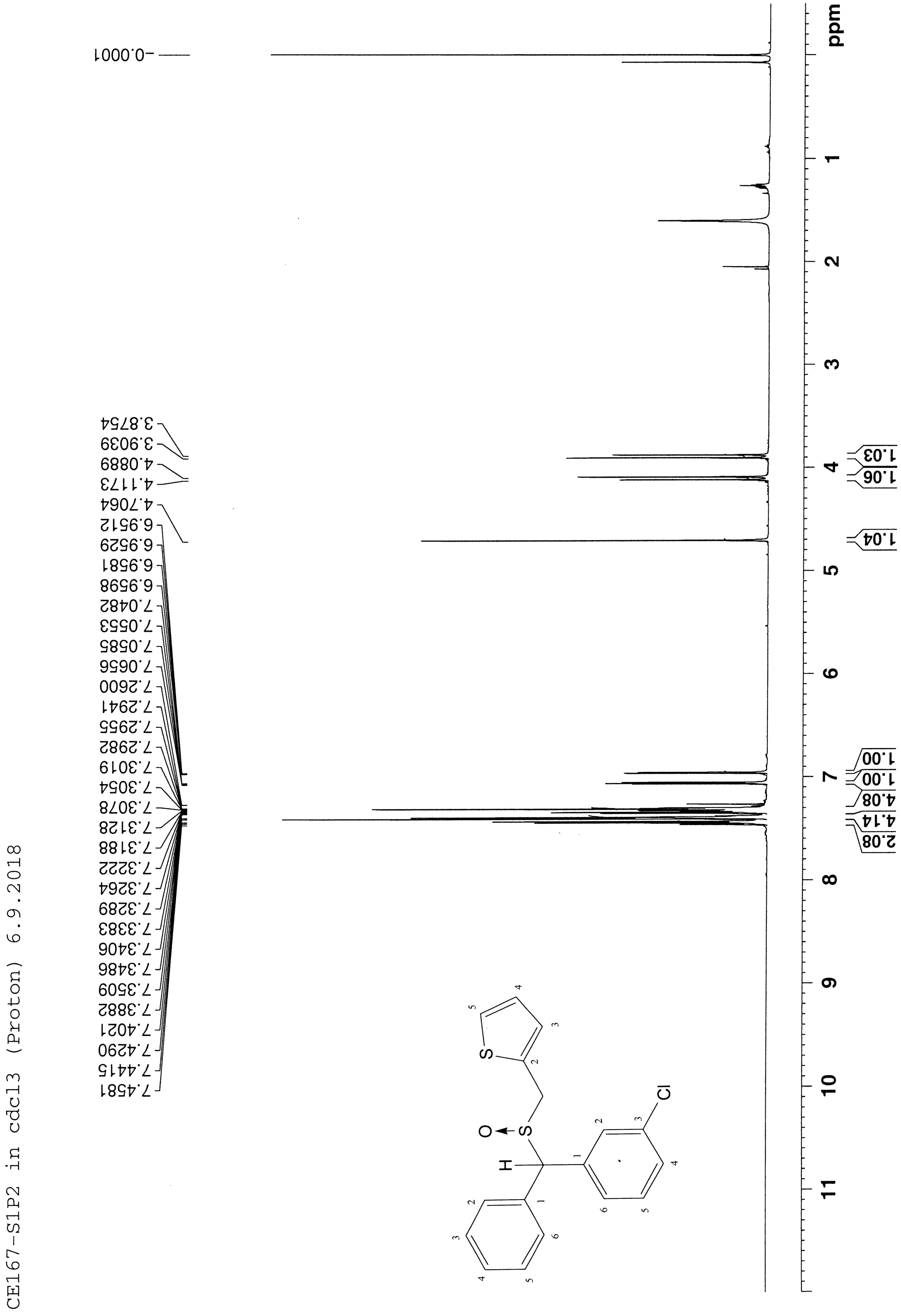

CE167-S1P2 in cdcl3 (Proton) 6.9.2018

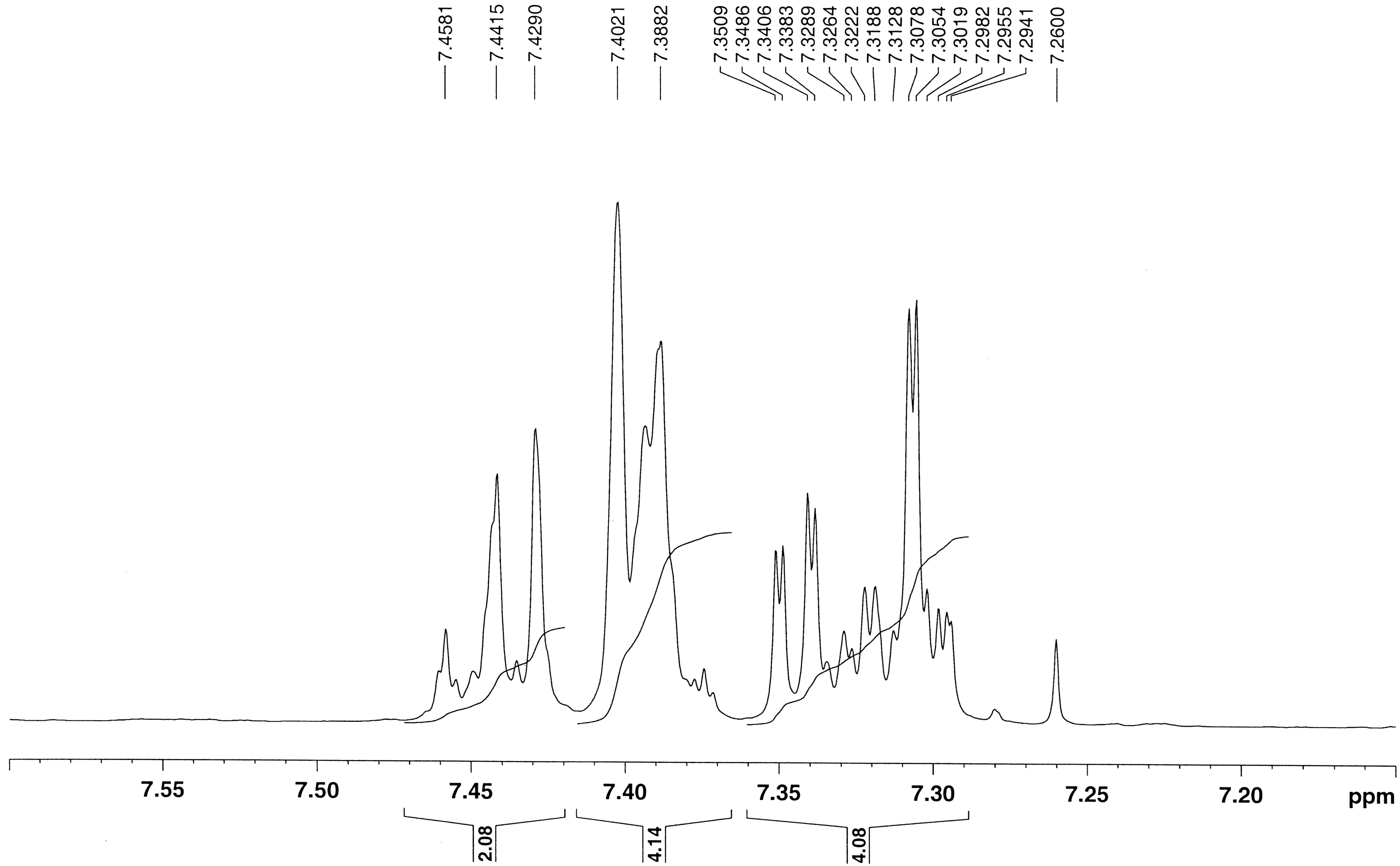

CE167-S1P2 in cdcl3 (Proton) 6.9.2018

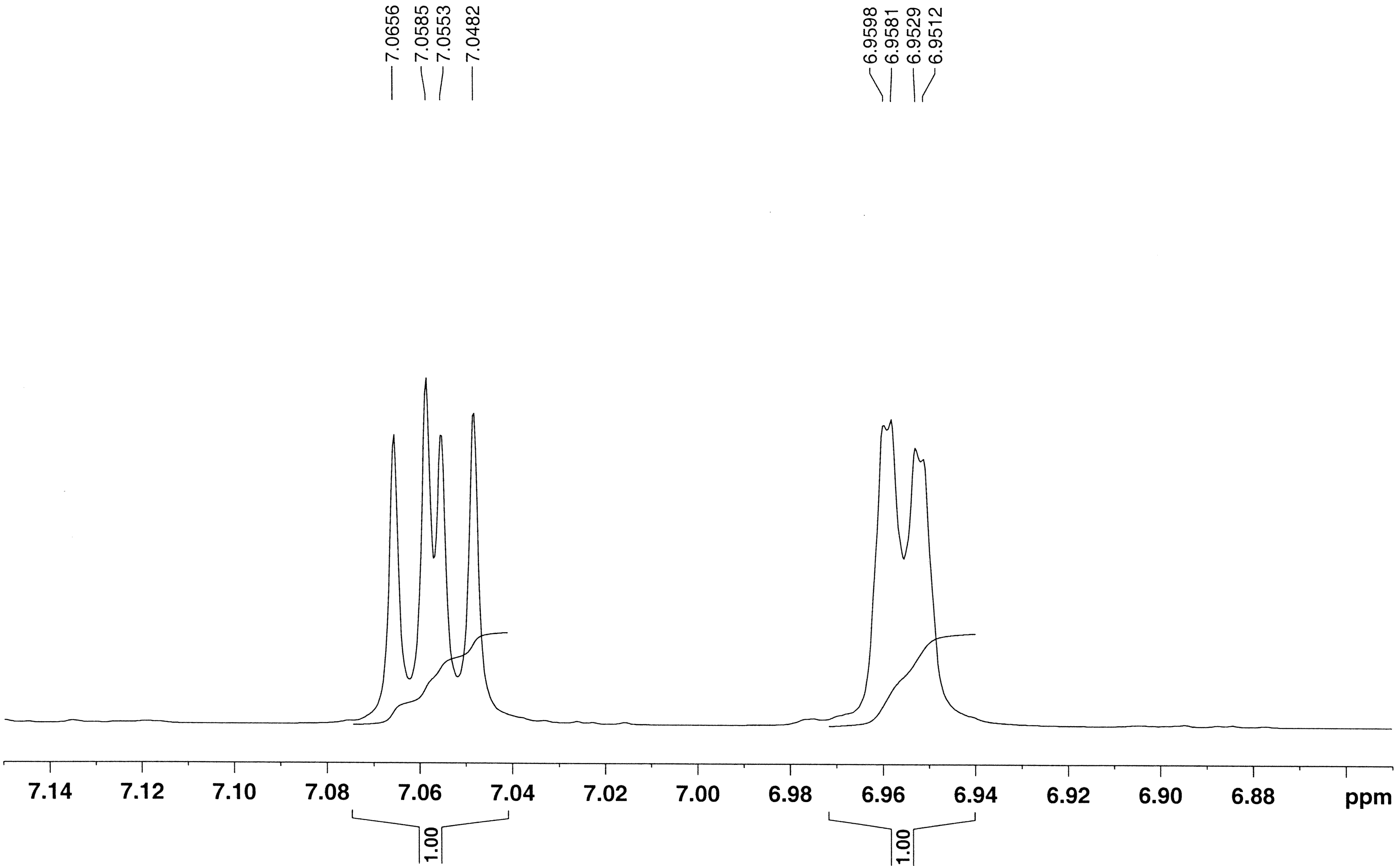

CE167-S1P2 in cdcl3 (Proton) 6.9.2018

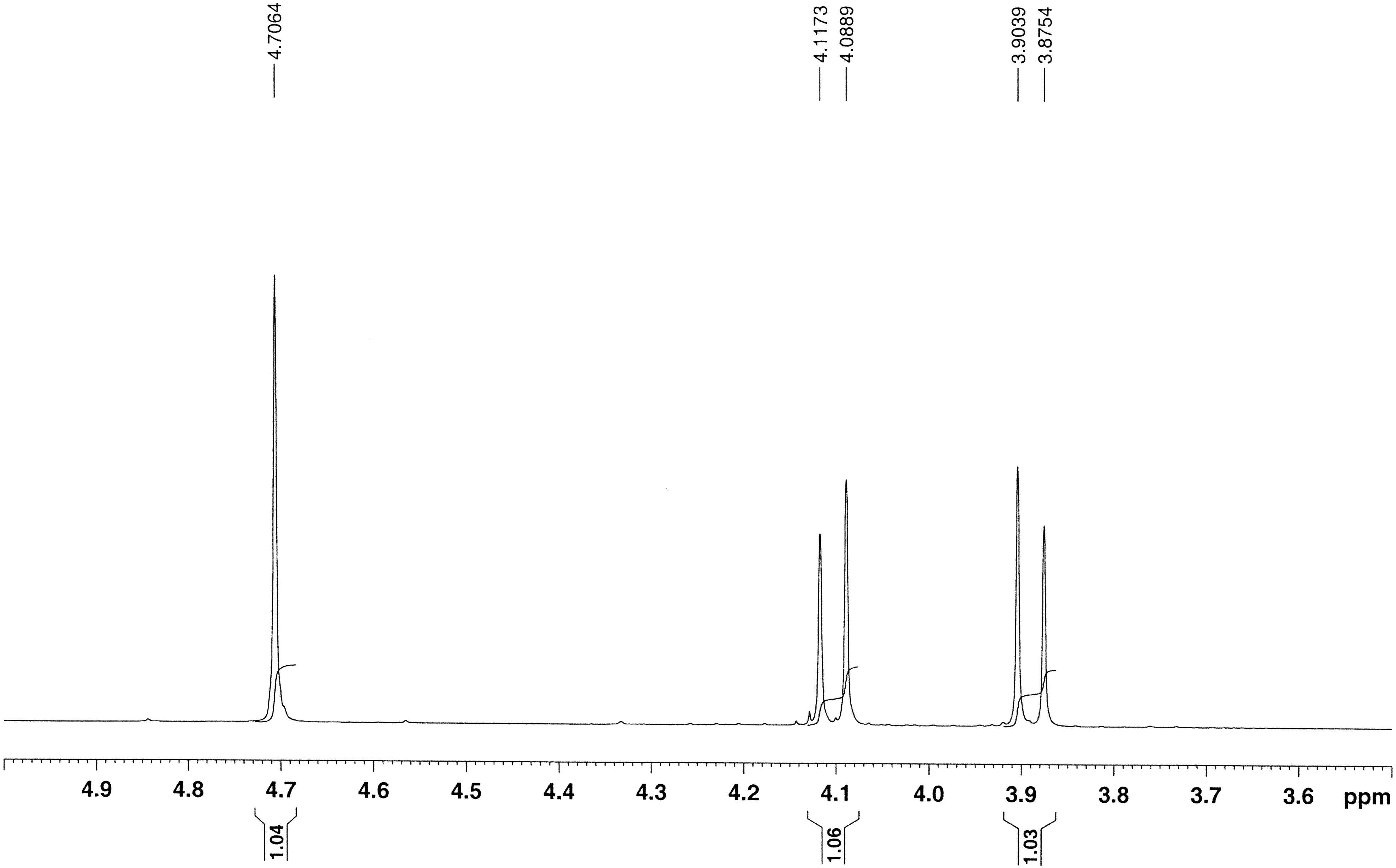

CE167-S1P2 in cdcl3 (APT) 6.9.2018

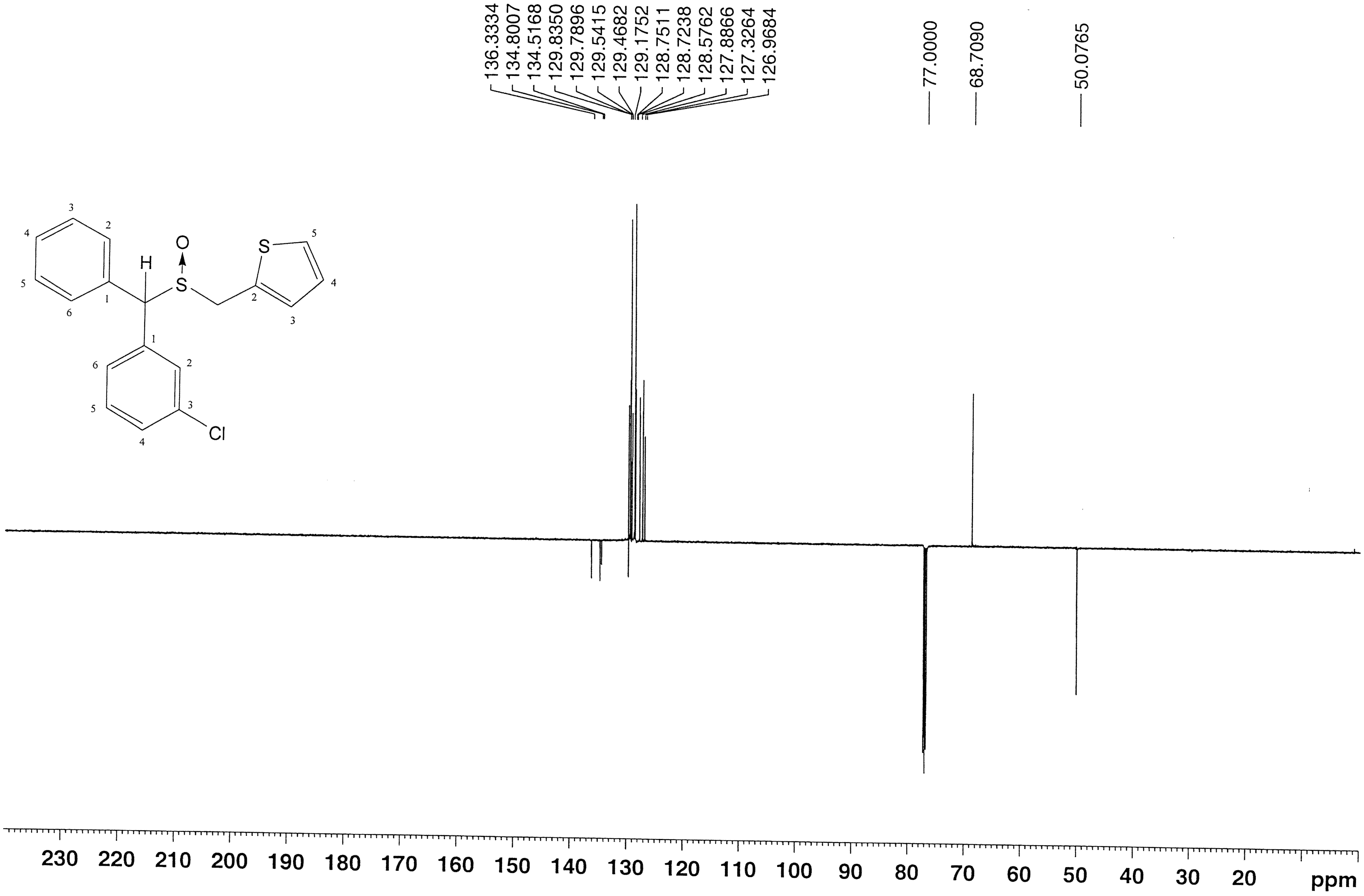

CE167-S1P2 in cdcl3 (APT) 6.9.2018

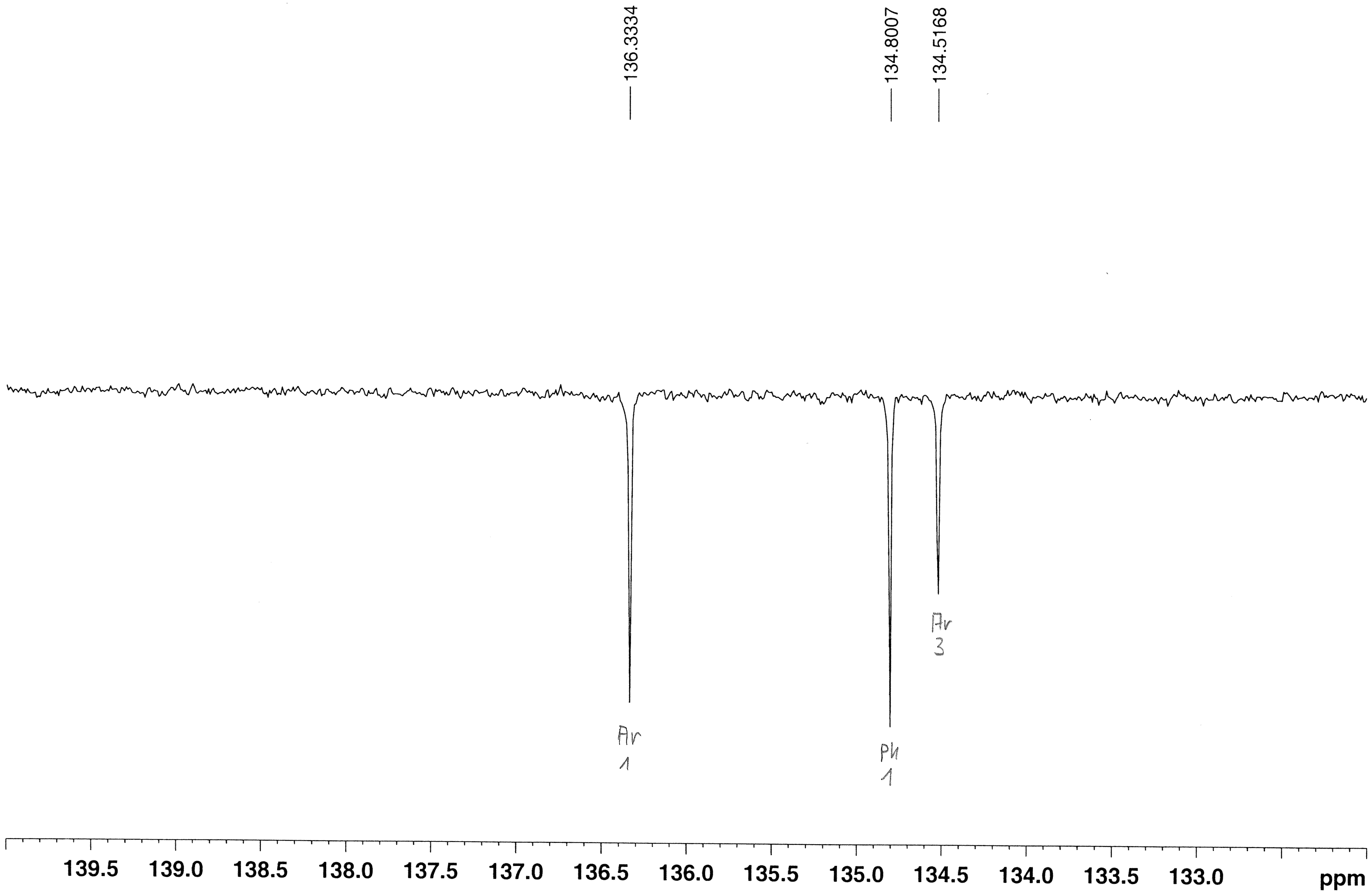

CE167-S1P2 in cdcl3 (APT) 6.9.2018

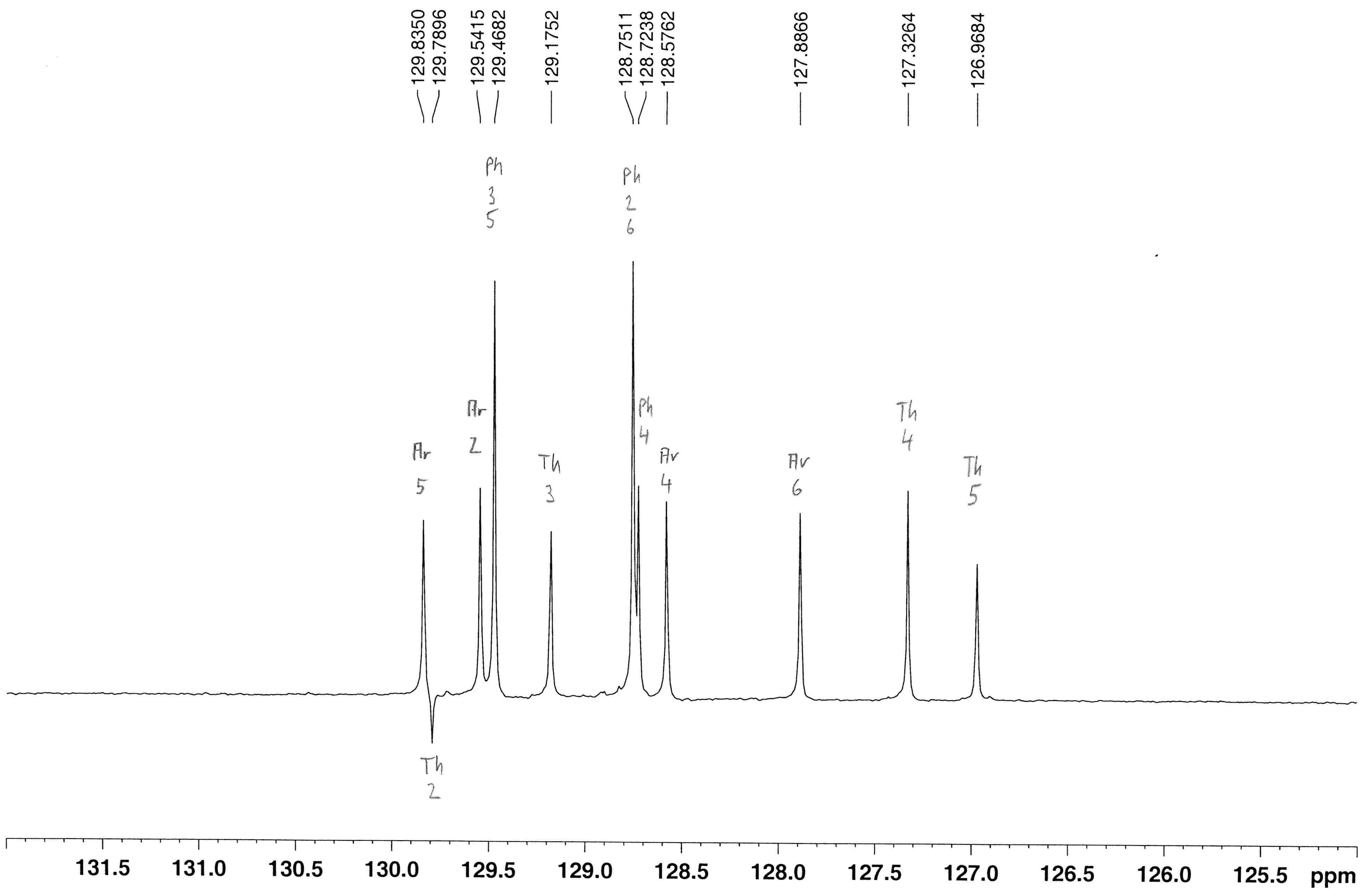

CE167-S1P2 in cdcl3 (APT) 6.9.2018

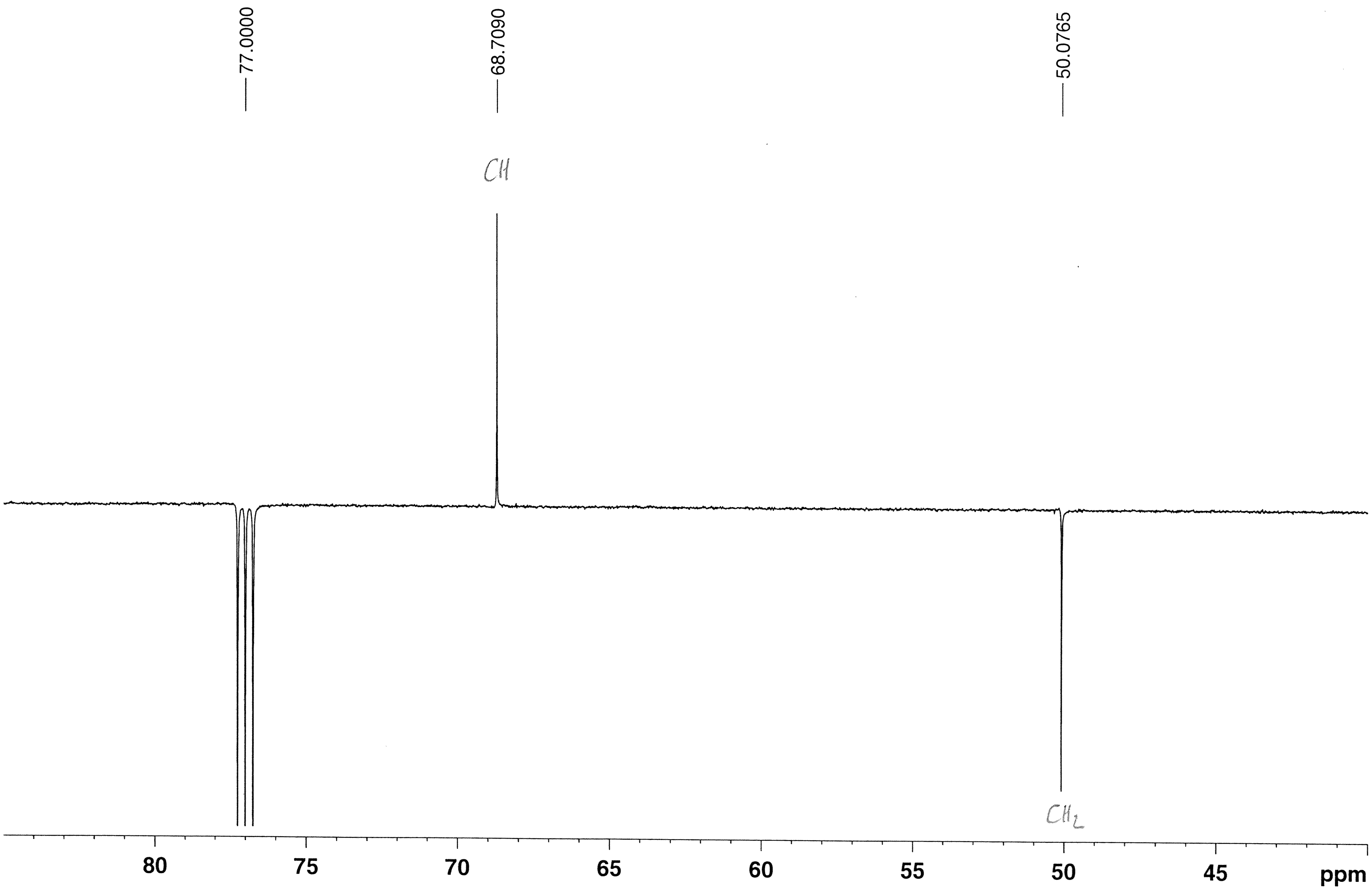

Figure S19c. NMR spectra of compound **7e.**

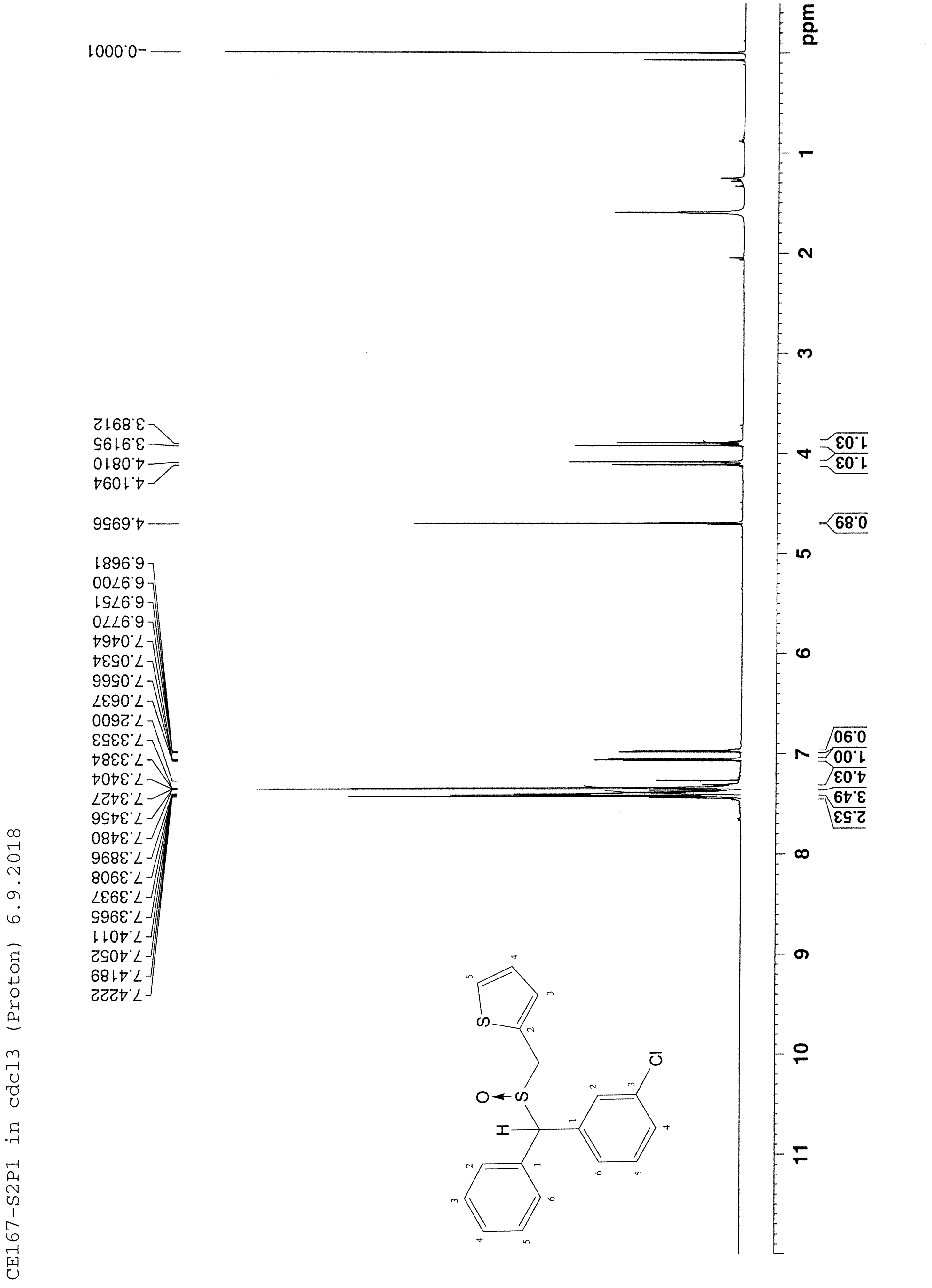

CE167-S2P1 in cdcl3 (Proton) 6.9.2018

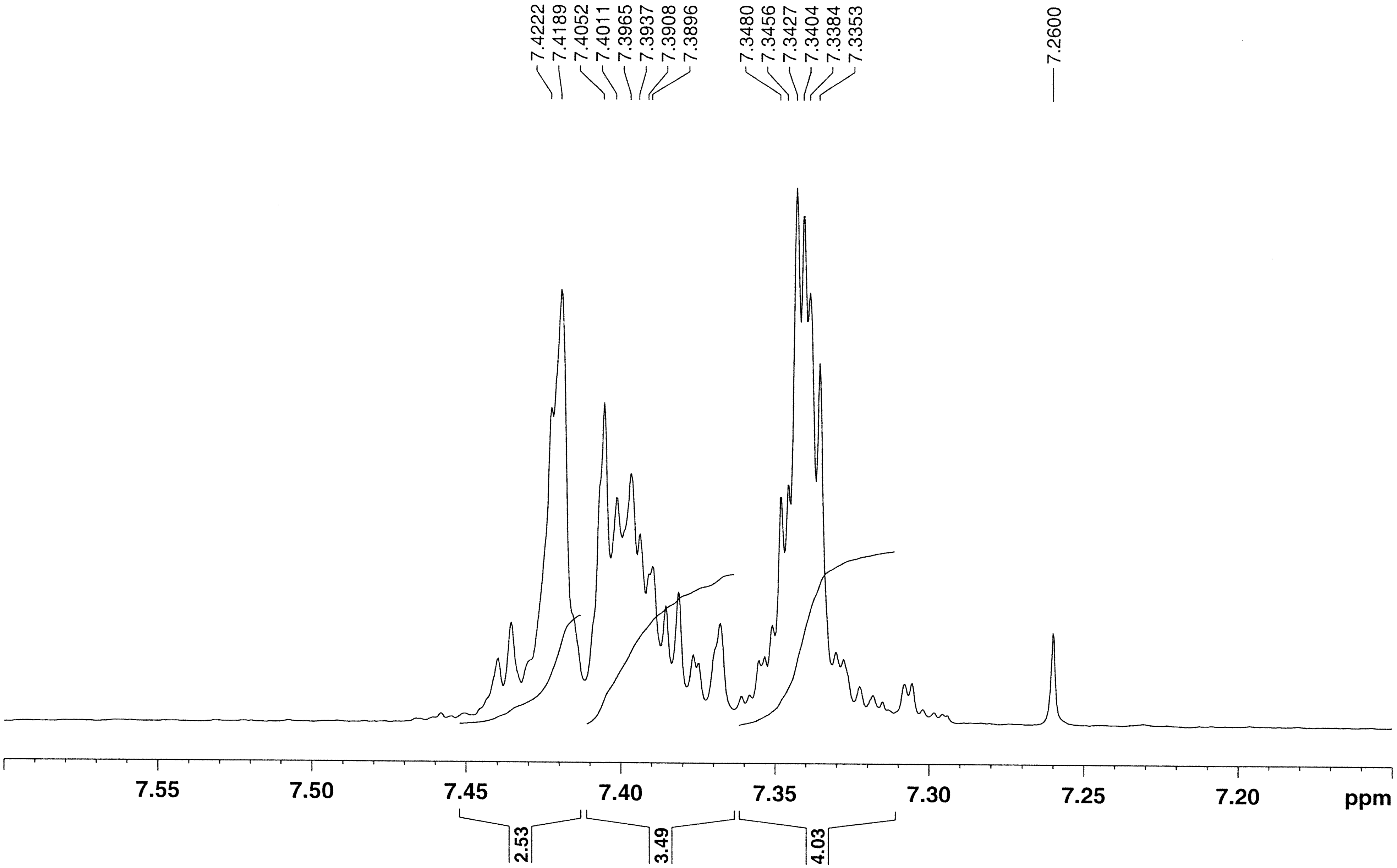

CE167-S2P1 in cdcl3 (Proton) 6.9.2018

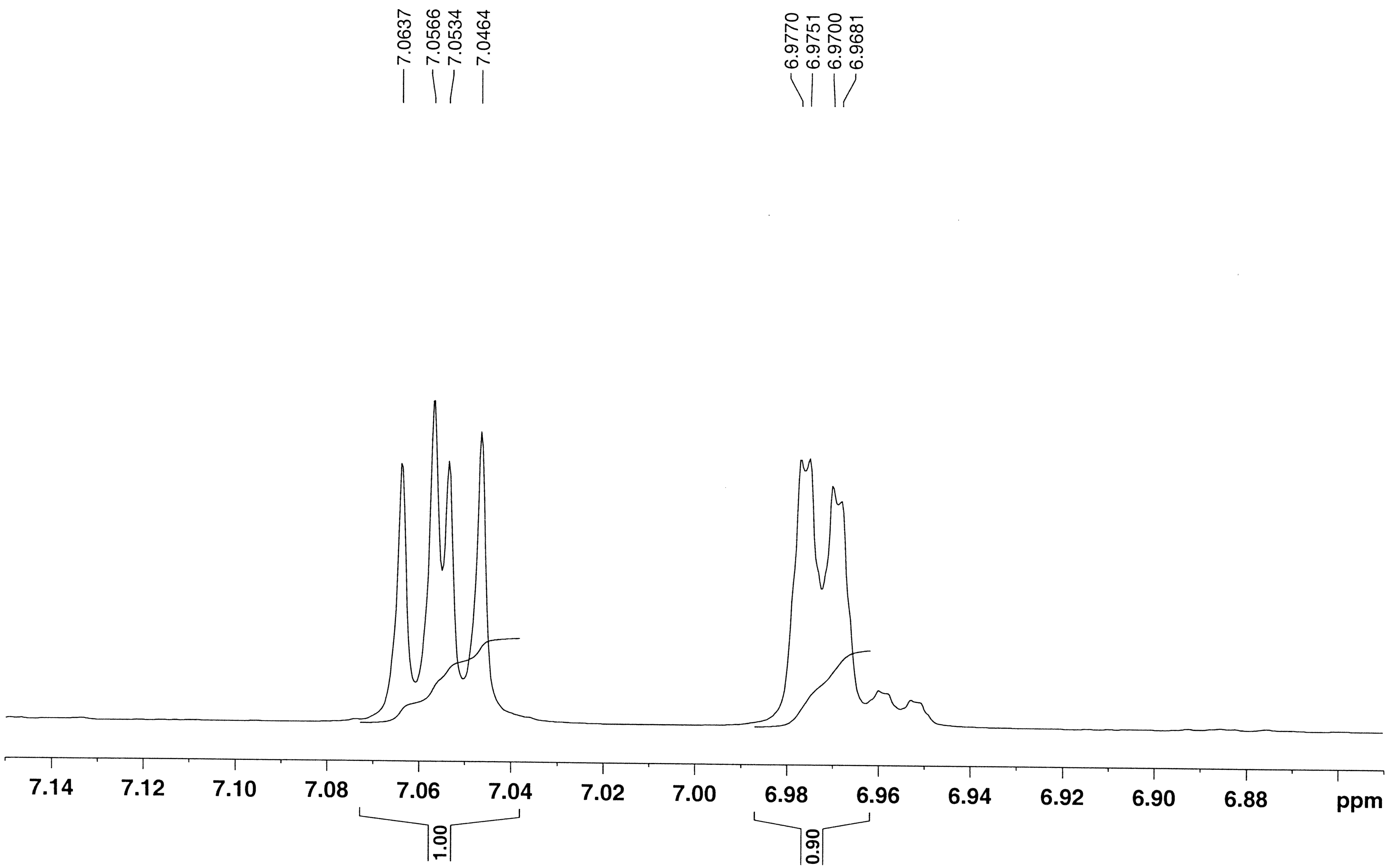

CE167-S2P1 in cdcl3 (Proton) 6.9.2018

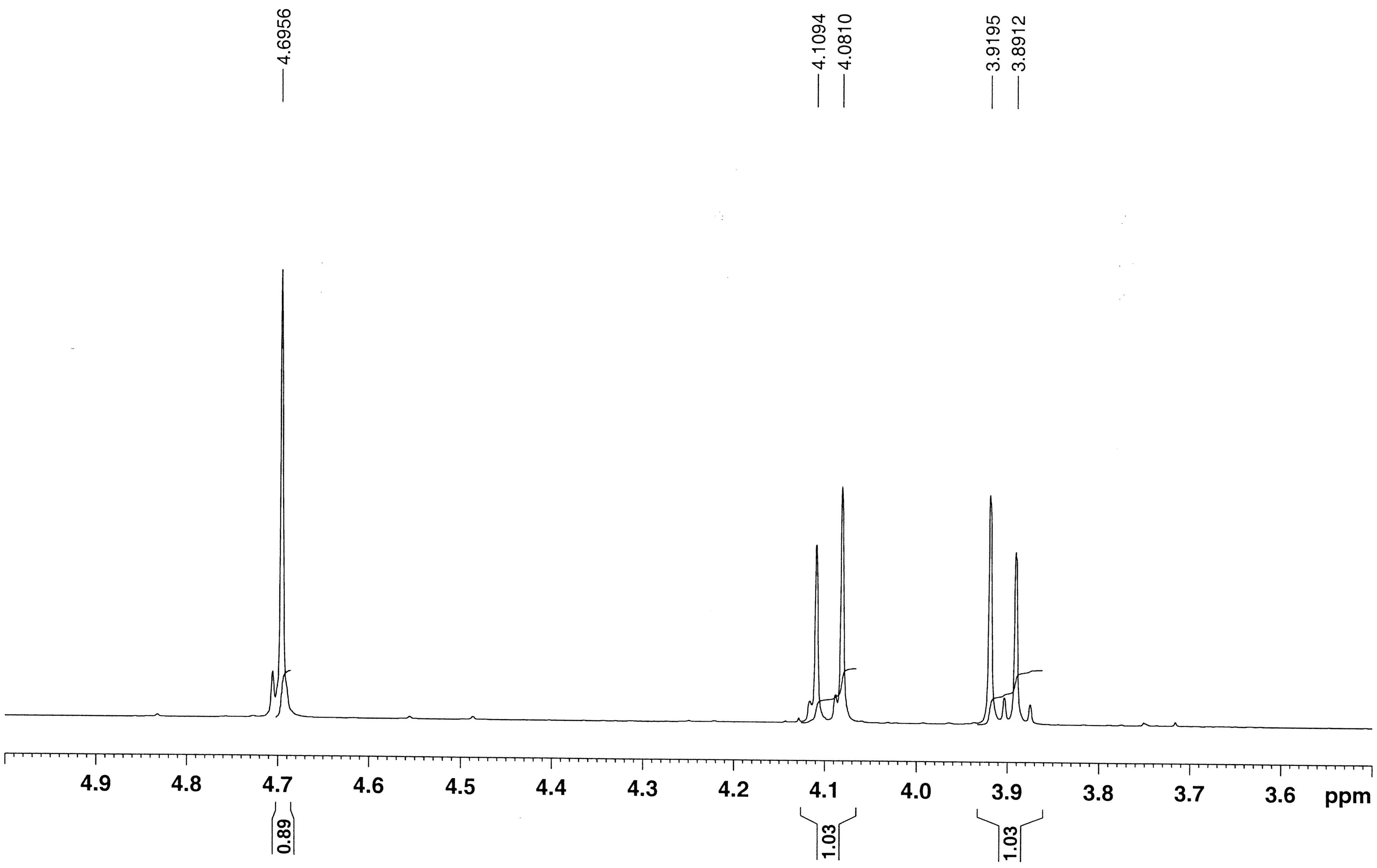

CE167-S2P1 in cdcl3 (APT) 6.9.2018

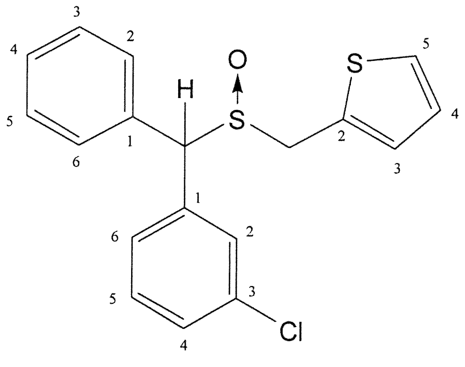

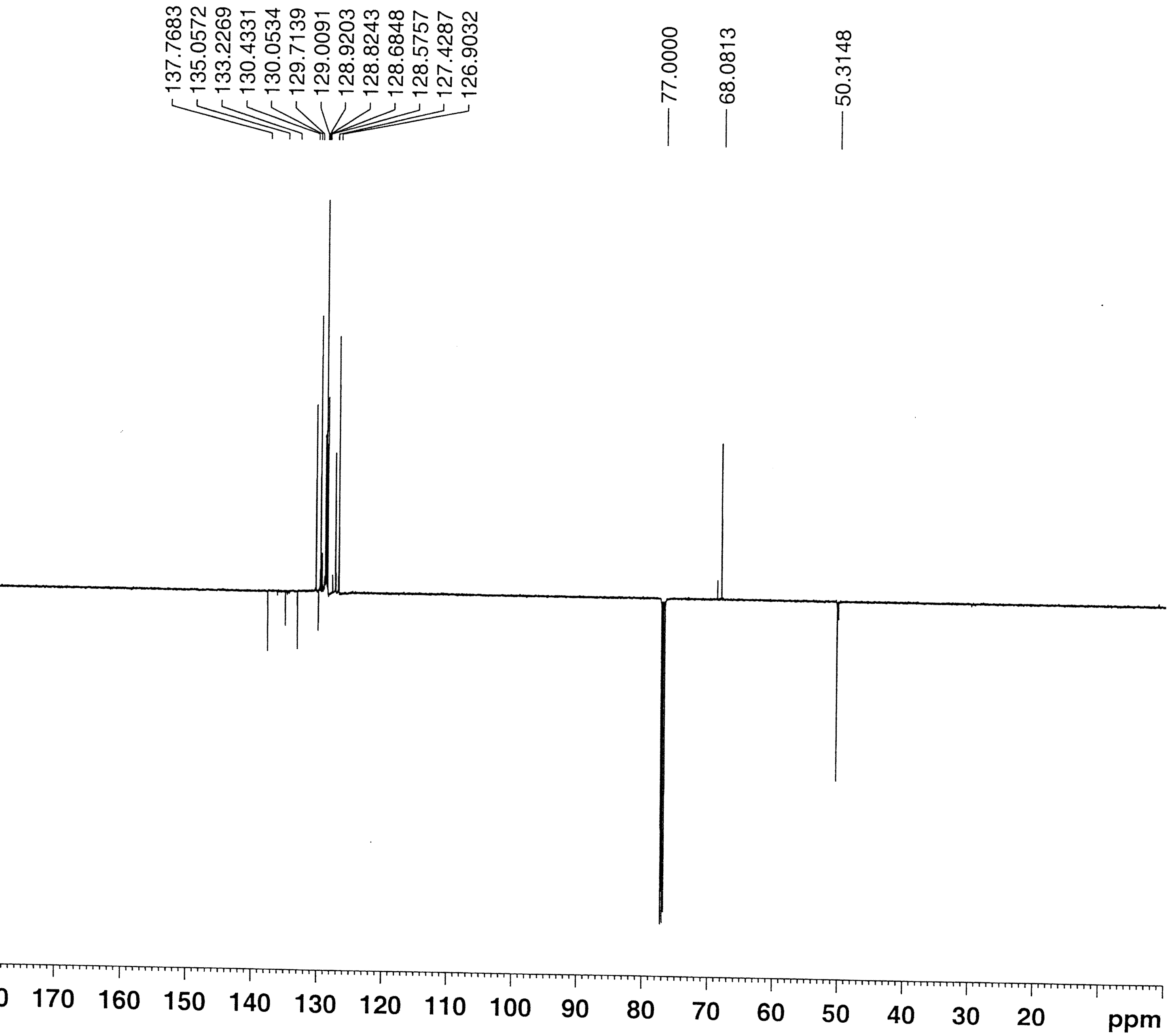

CE167-S2P1 in cdcl3 (APT) 6.9.2018

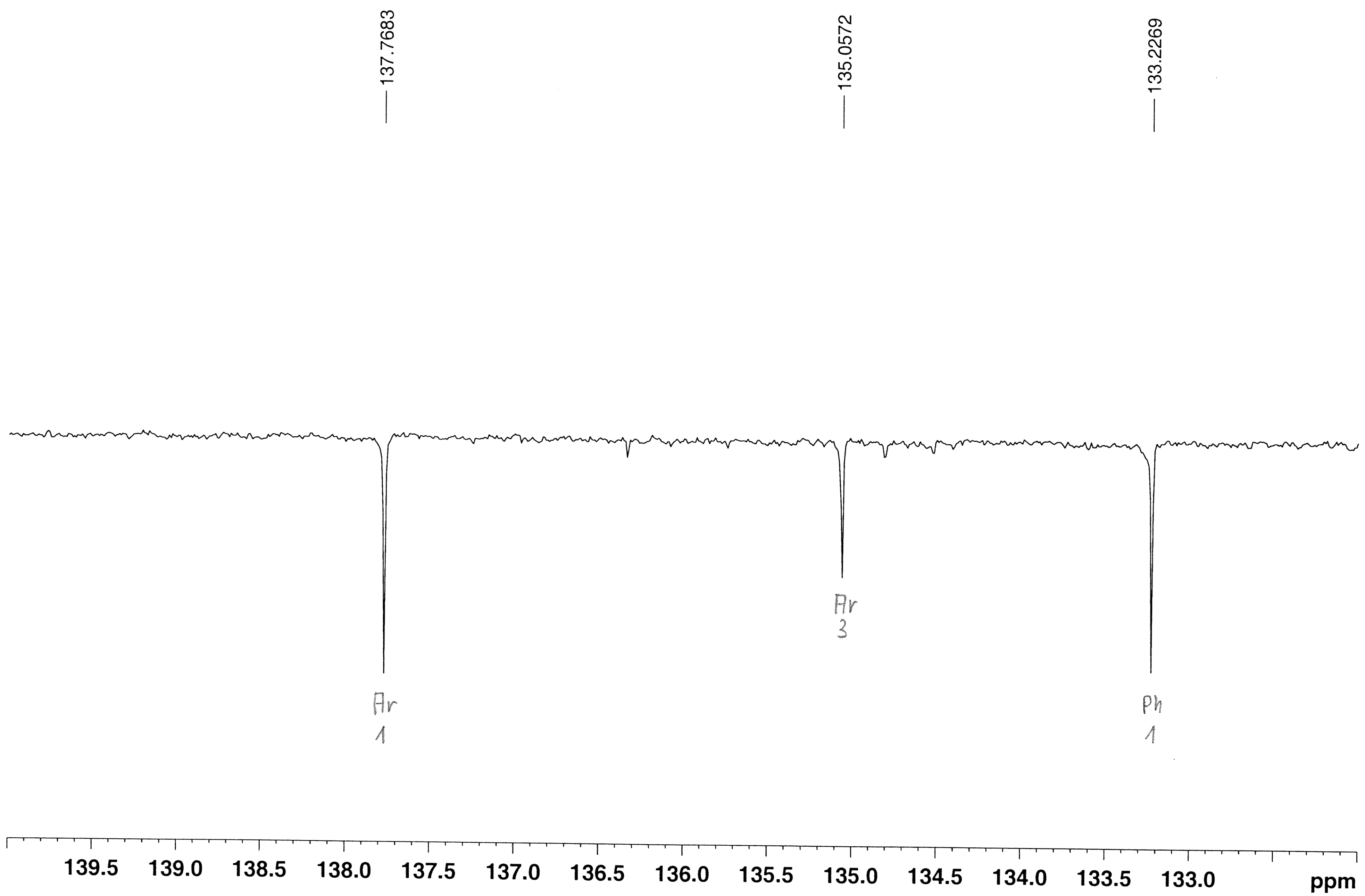

CE167-S2P1 in cdcl3 (APT) 6.9.2018

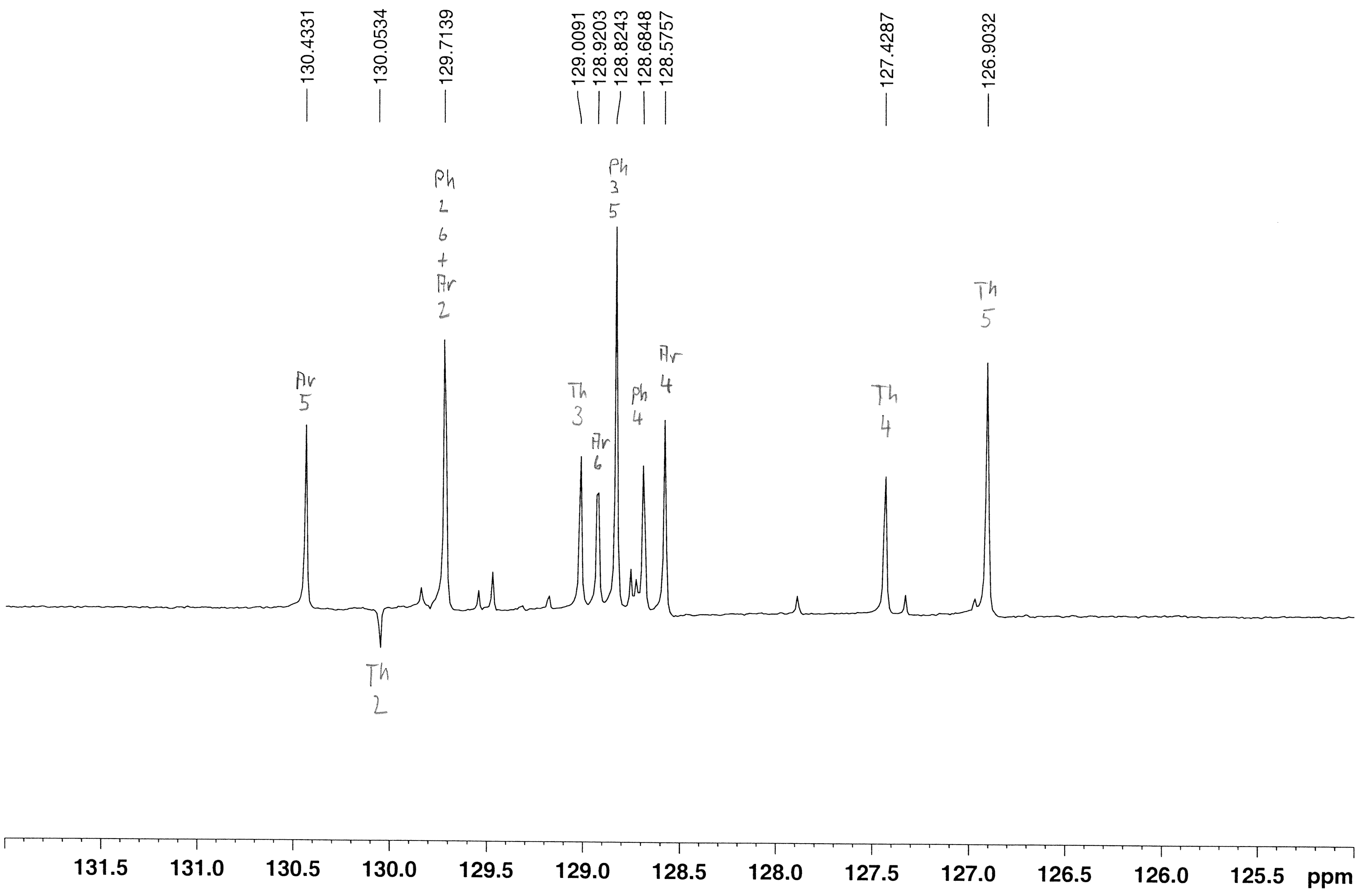

CE167-S2P1 in cdcl3 (APT) 6.9.2018

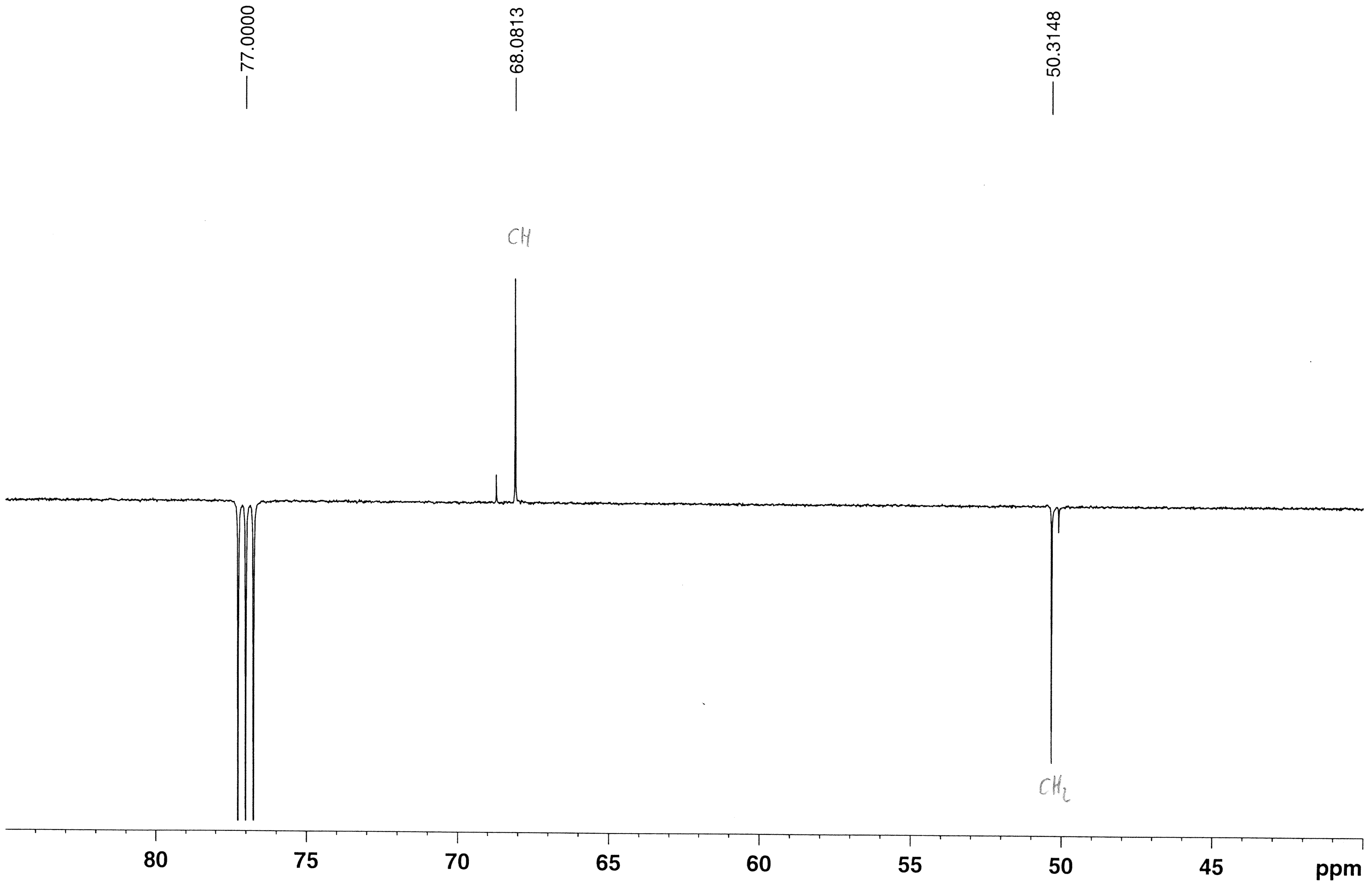

CE167-S2P1 in cdcl3 (HSQC) 6.9.2018

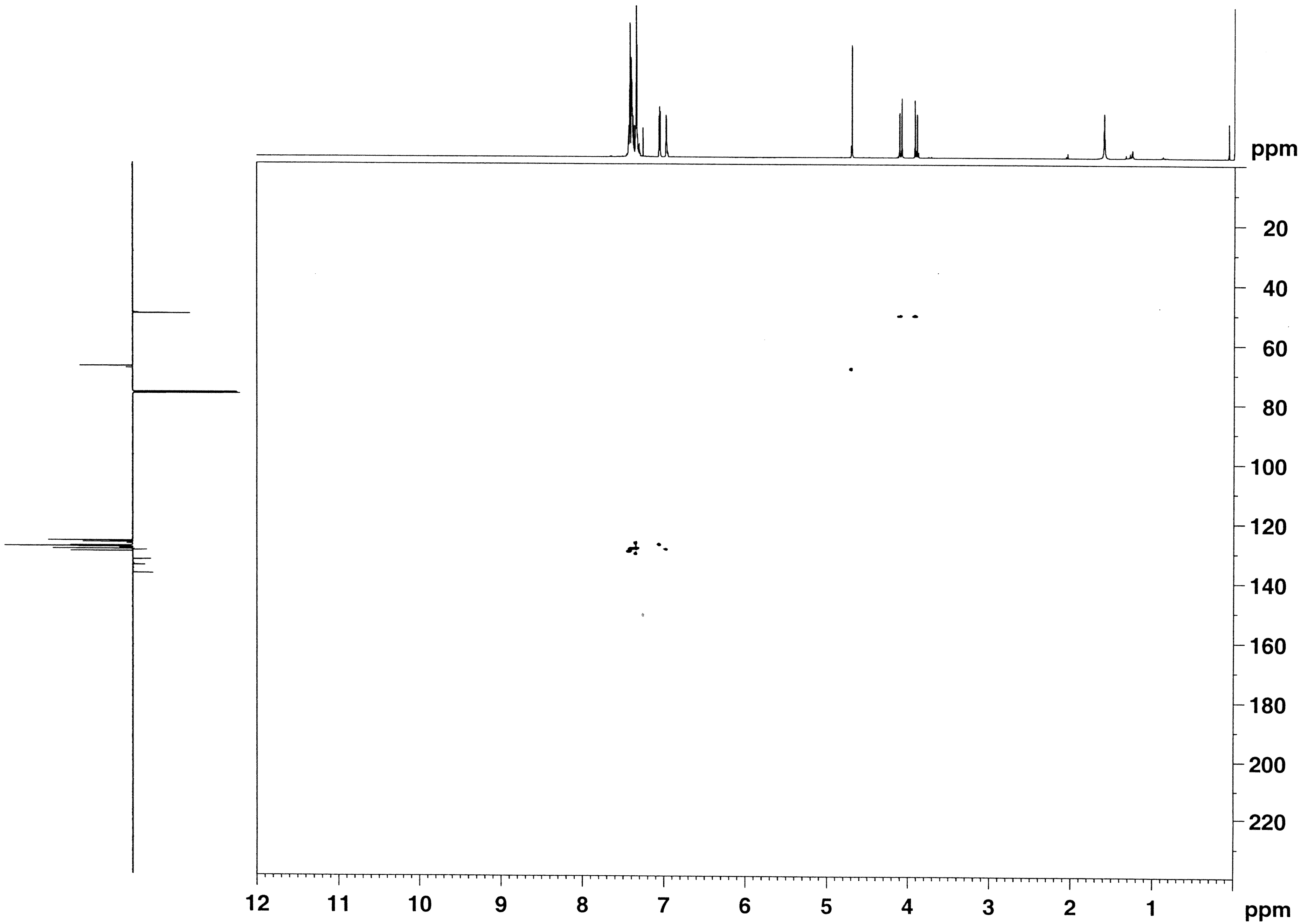

CE167-S2P1 in cdcl3 (HSQC) 6.9.2018

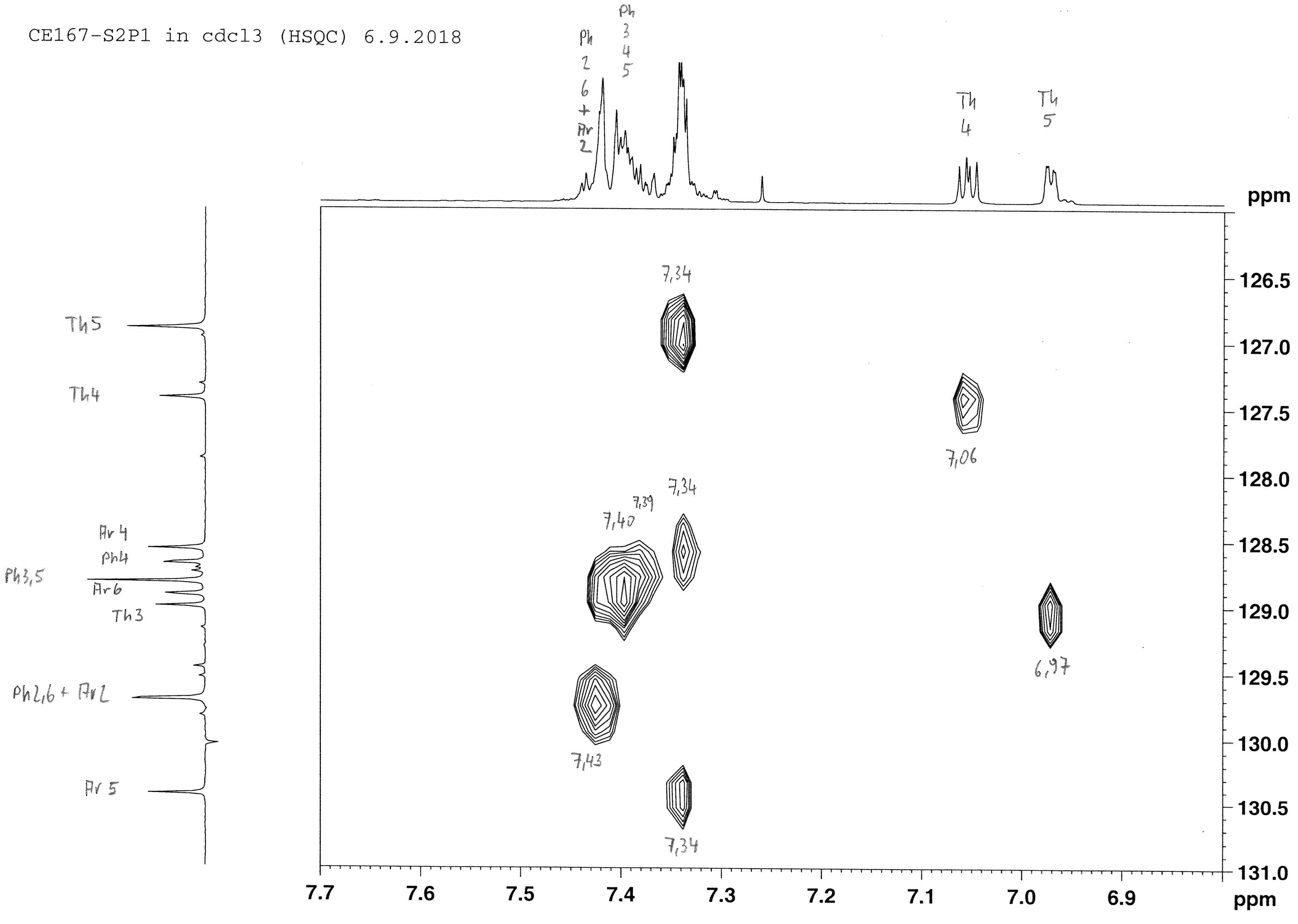

CE167-S2P1 in cdcl3 (HSQC) 6.9.2018

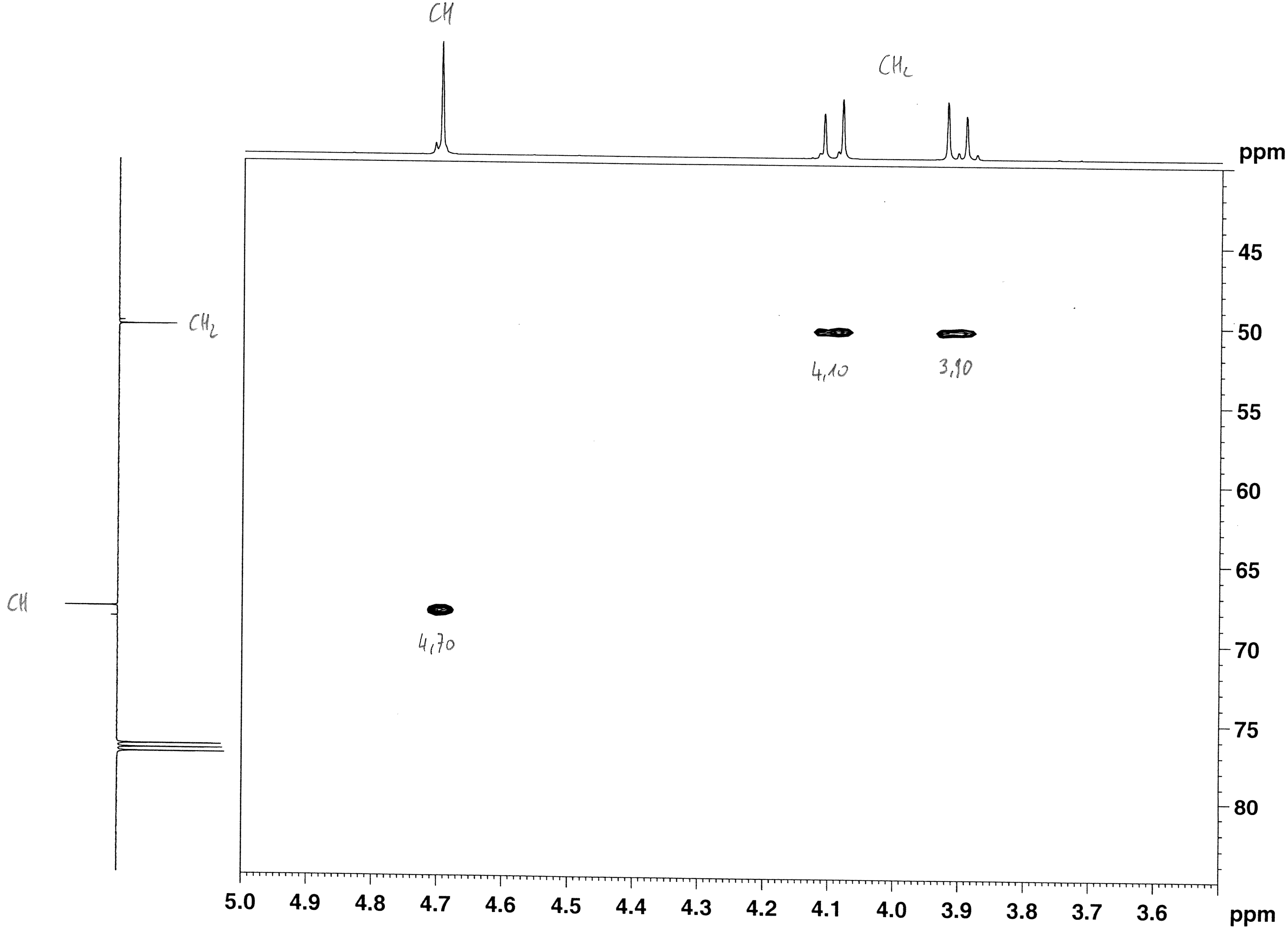

Figure S20c. NMR spectra of compound **8e**.

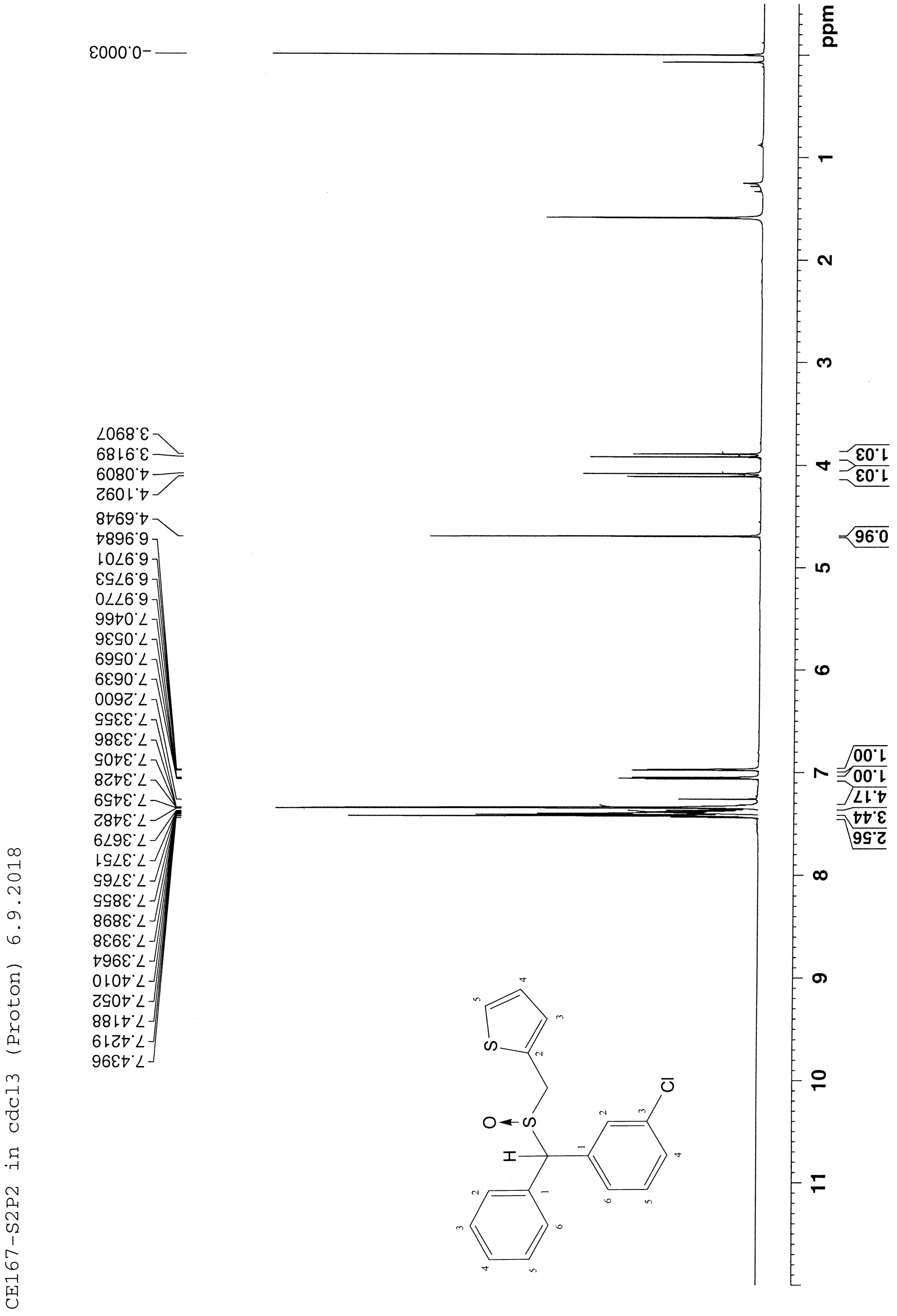

CE167-S2P2 in cdcl3 (Proton) 6.9.2018

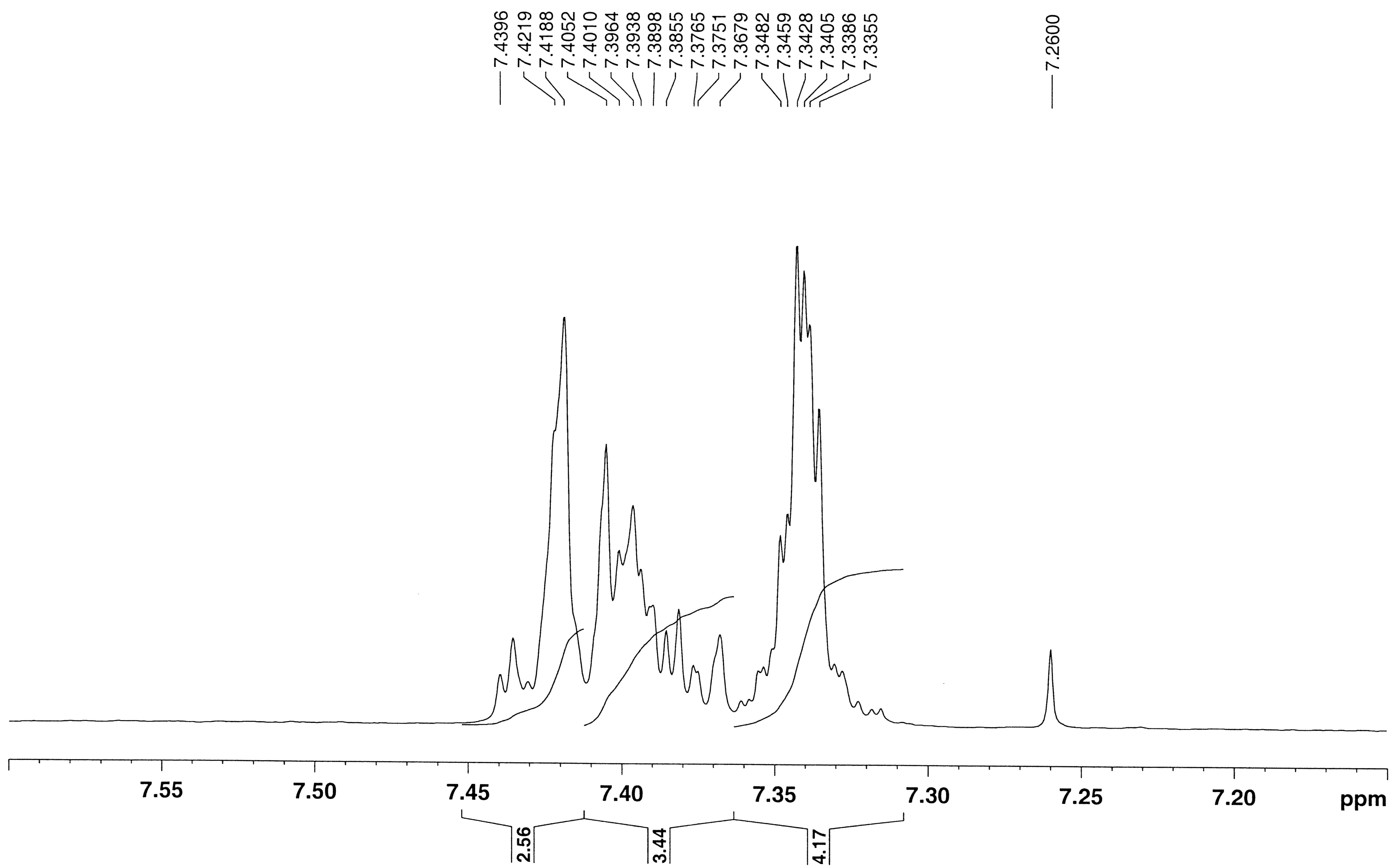

CE167-S2P2 in cdcl3 (Proton) 6.9.2018

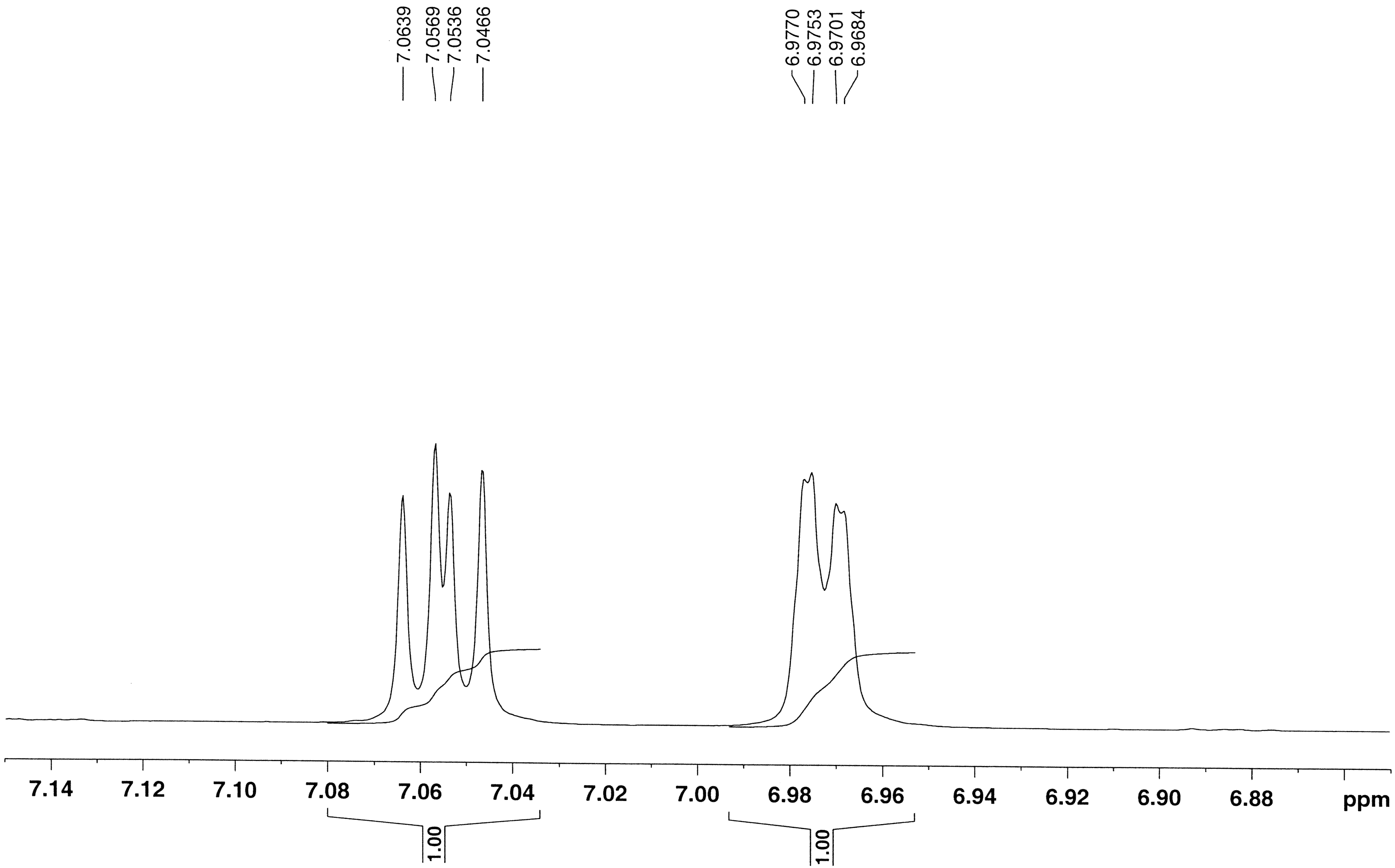

CE167-S2P2 in cdcl3 (Proton) 6.9.2018

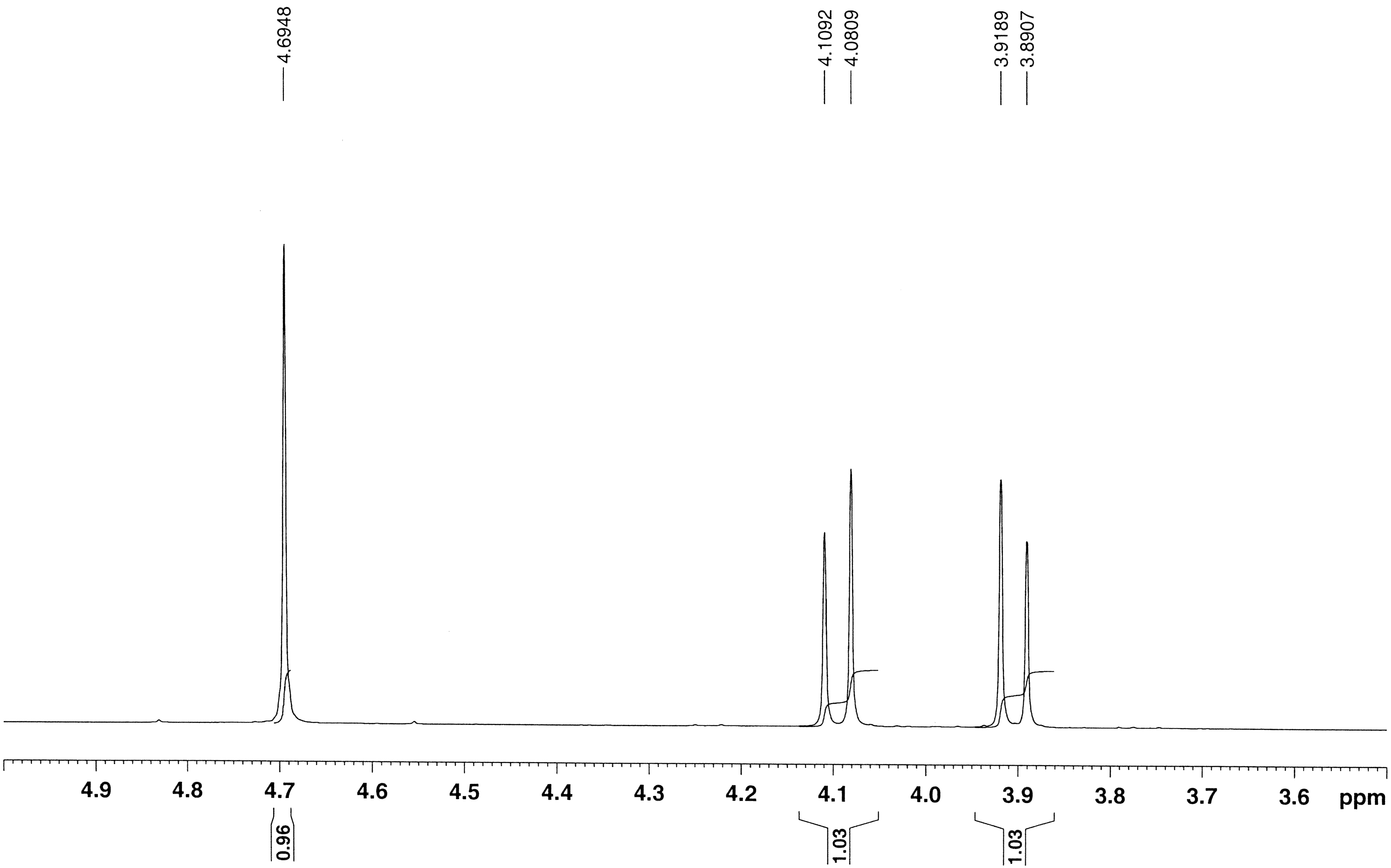

CE167-S2P2 in cdcl3 (APT) 6.9.2018

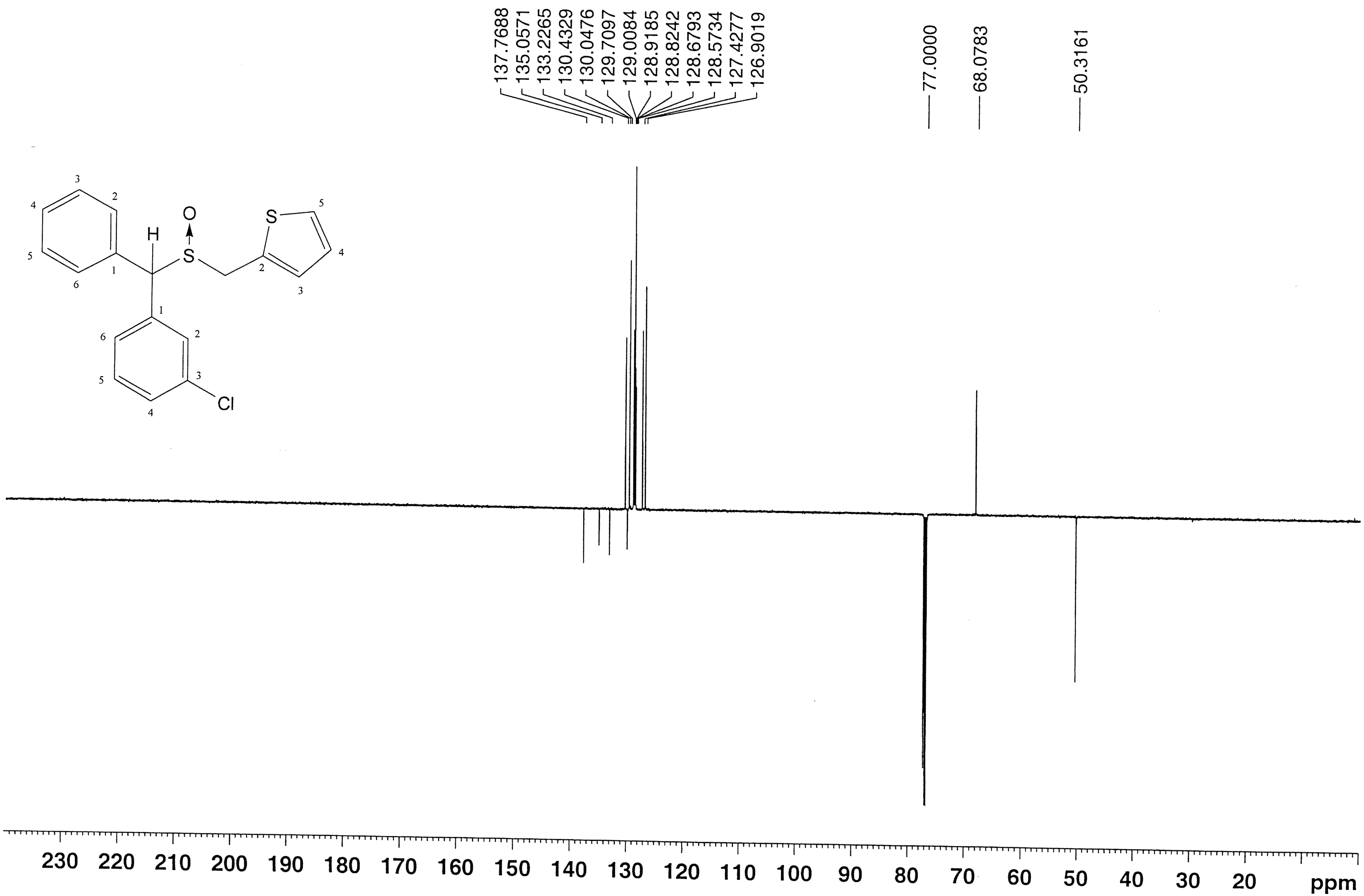

CE167-S2P2 in cdcl3 (APT) 6.9.2018

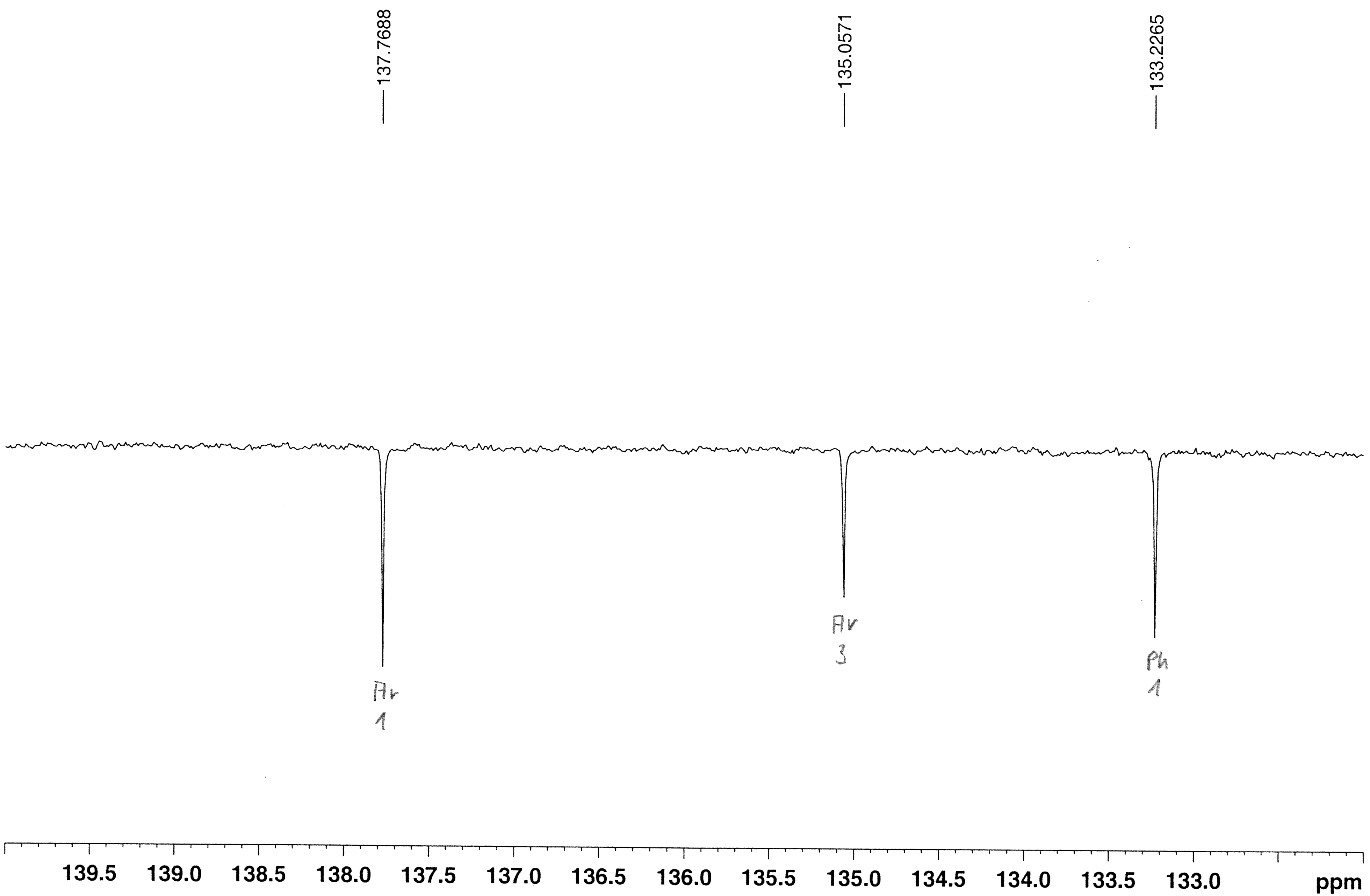

CE167-S2P2 in cdcl3 (APT) 6.9.2018

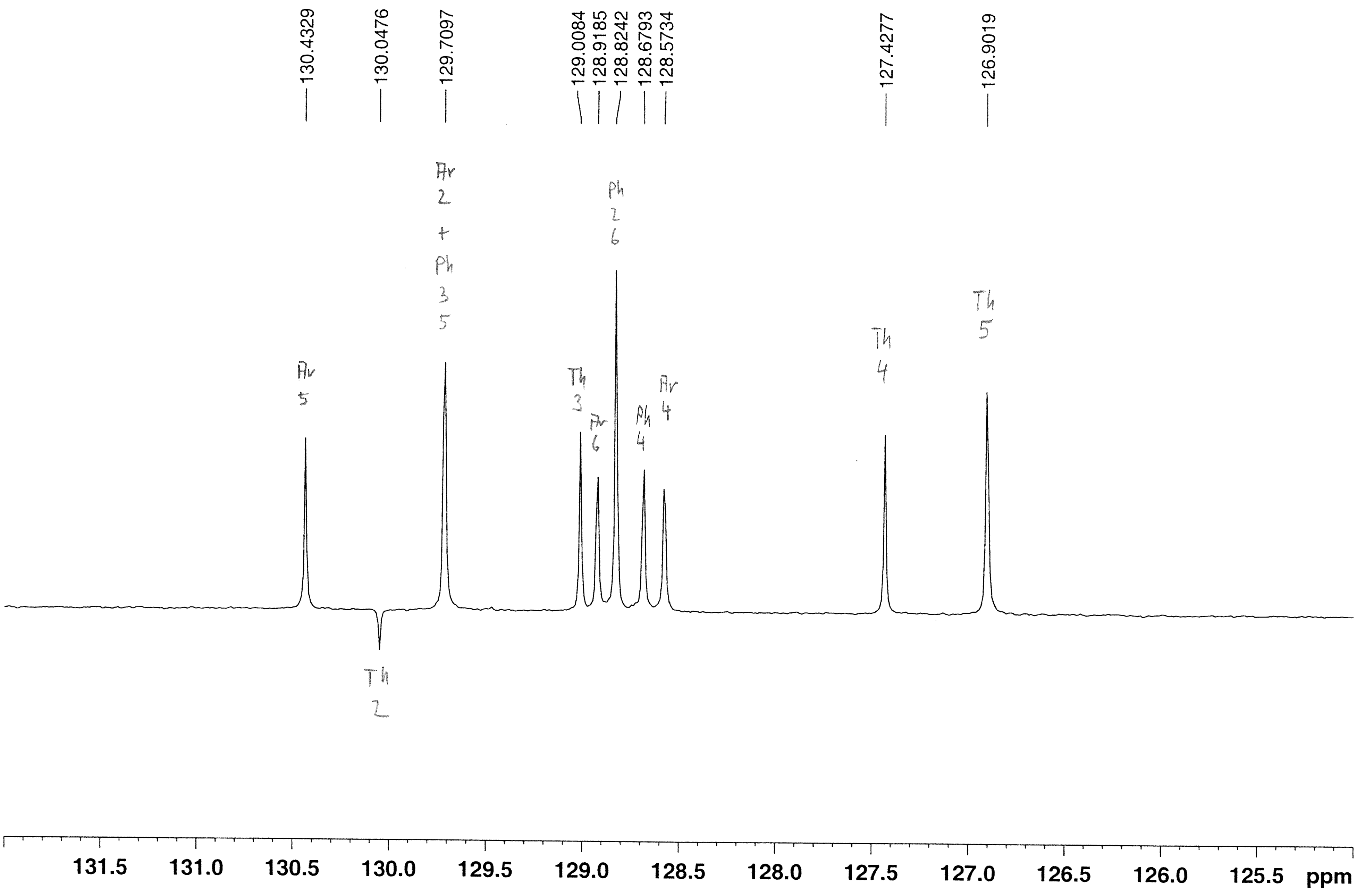

CE167-S2P2 in cdcl3 (APT) 6.9.2018

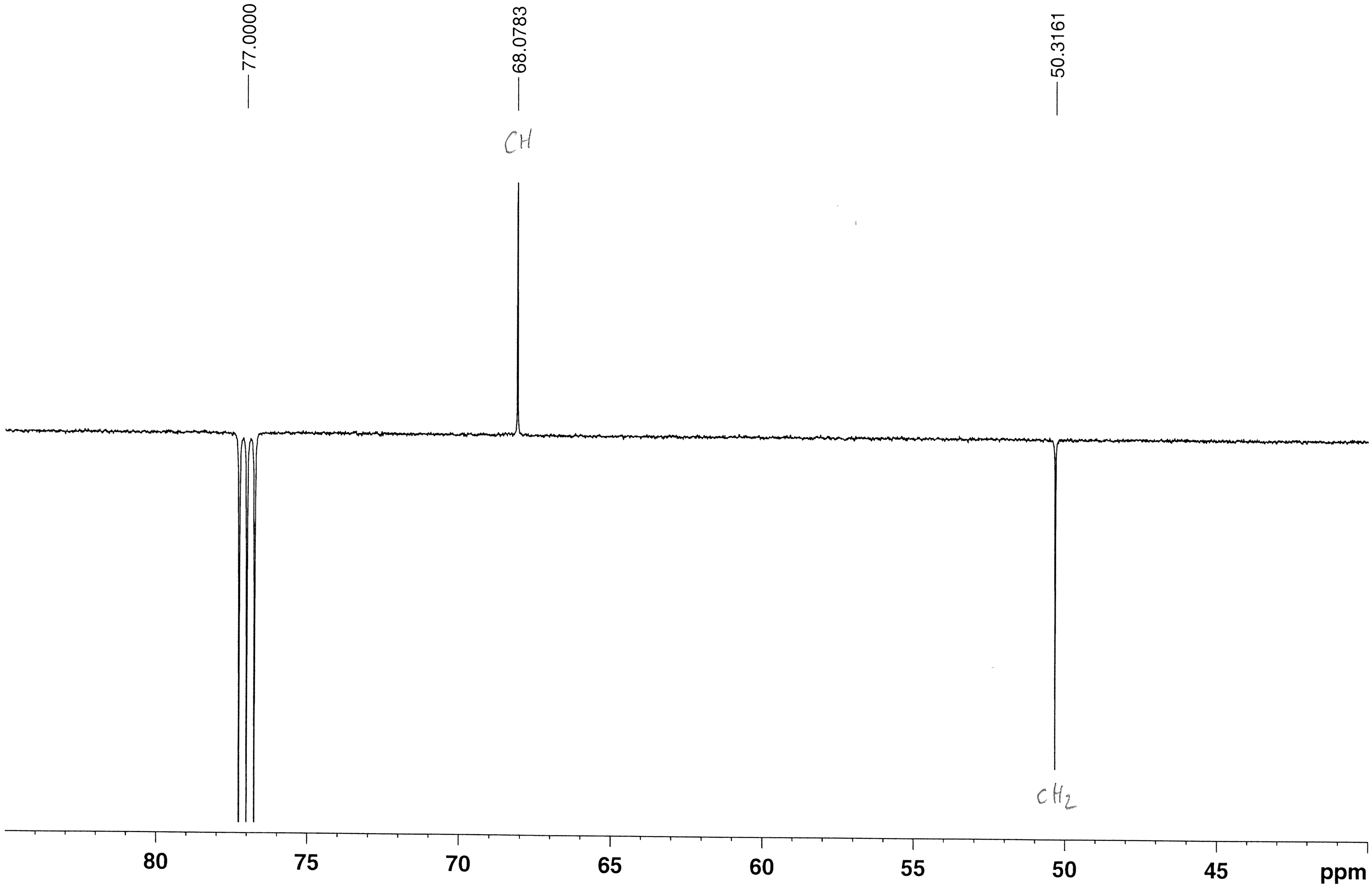

Figure S21c. NMR spectra of compound **5f.**

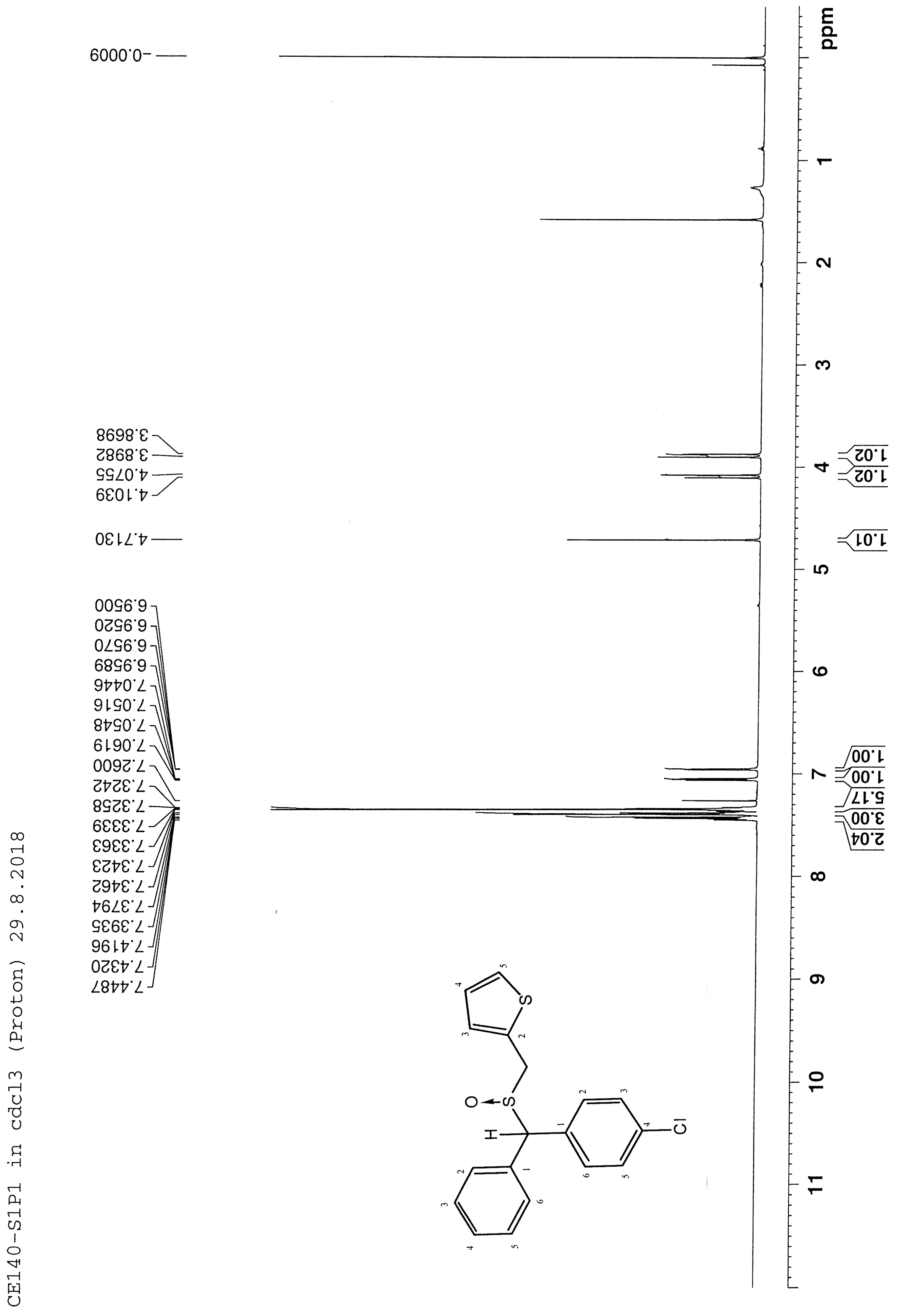

CE140-S1P1 in cdcl3 (Proton) 29.8.2018

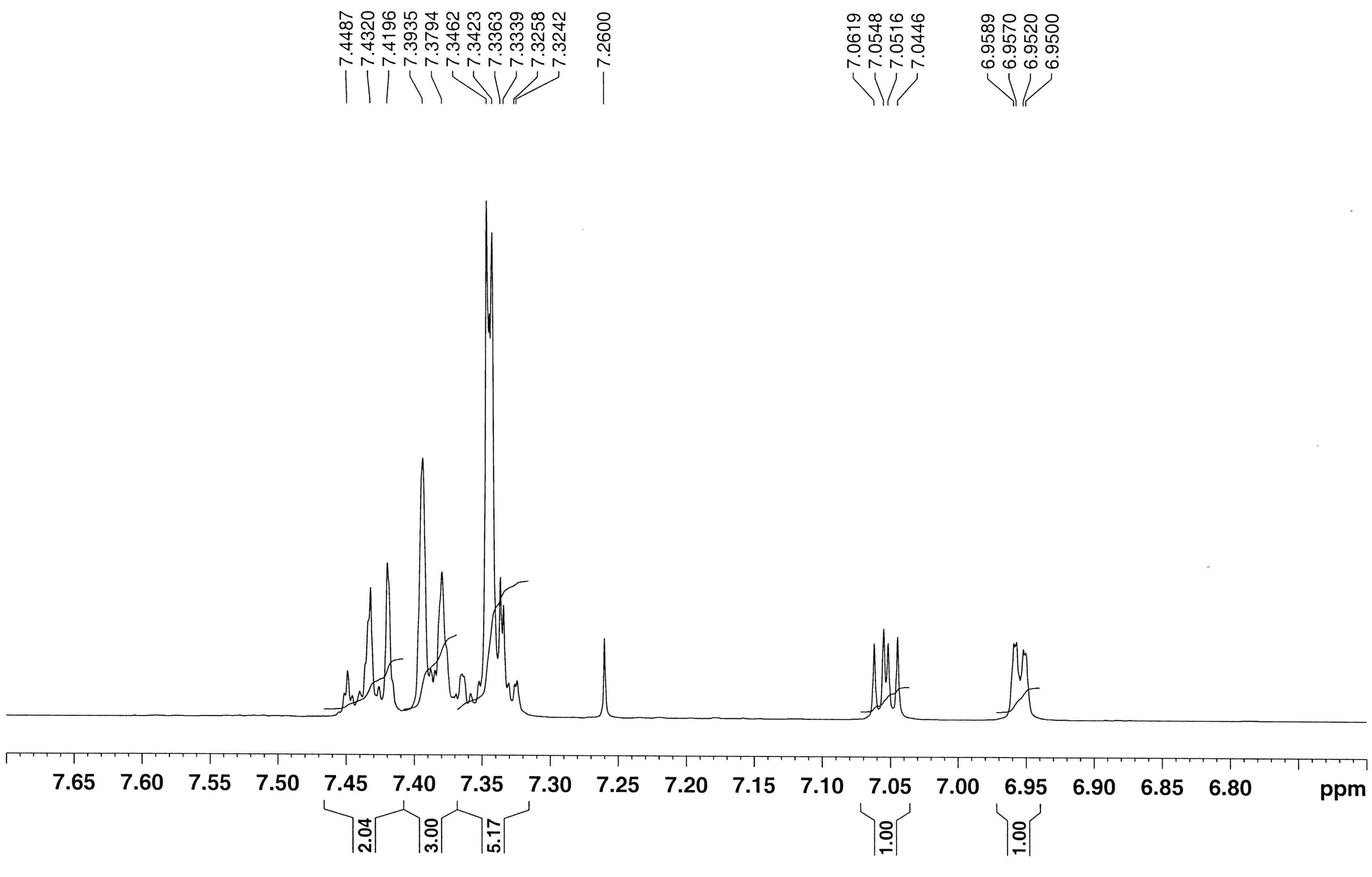

CE140-S1P1 in cdcl3 (Proton) 29.8.2018

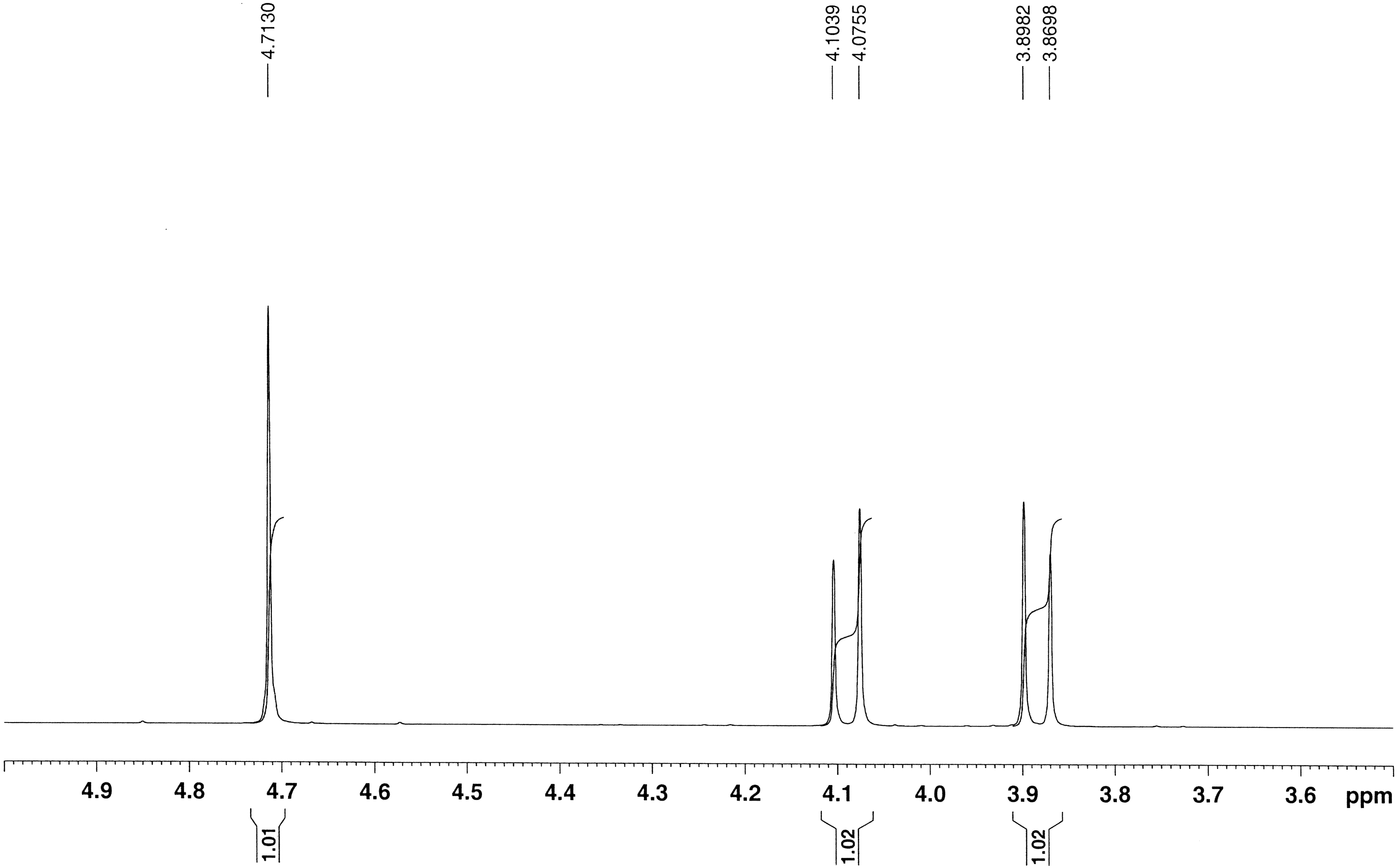

CE140-S1P1 in cdcl3 (APT) 29.8.2018

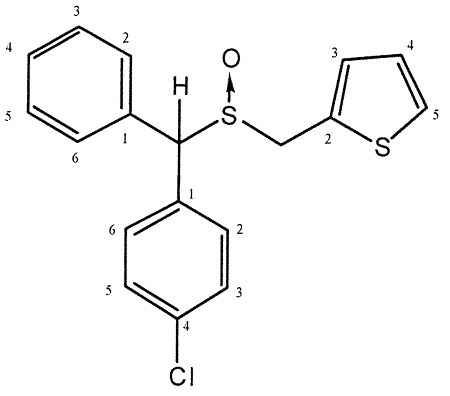

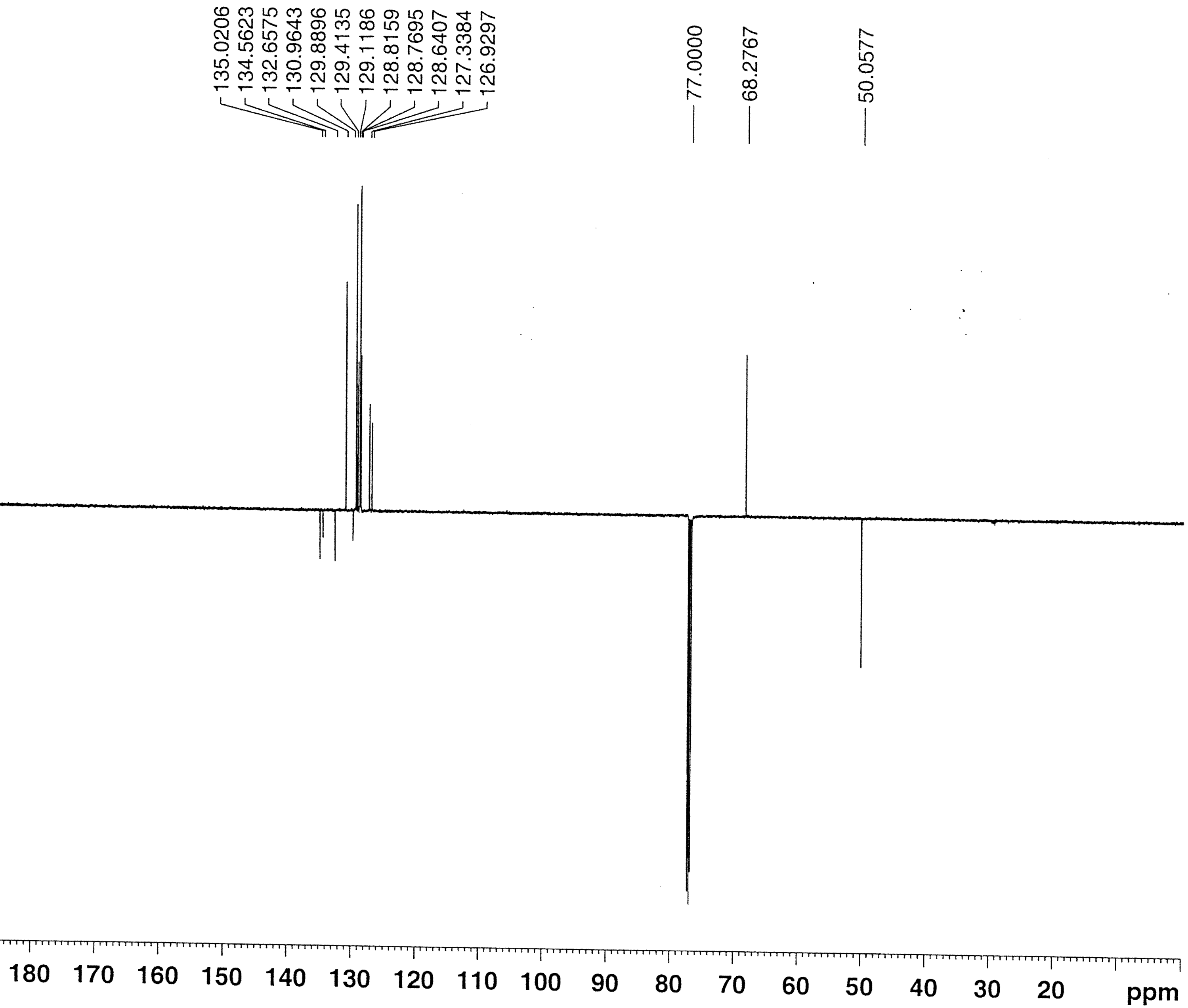

CE140-S1P1 in cdcl3 (APT) 29.8.2018

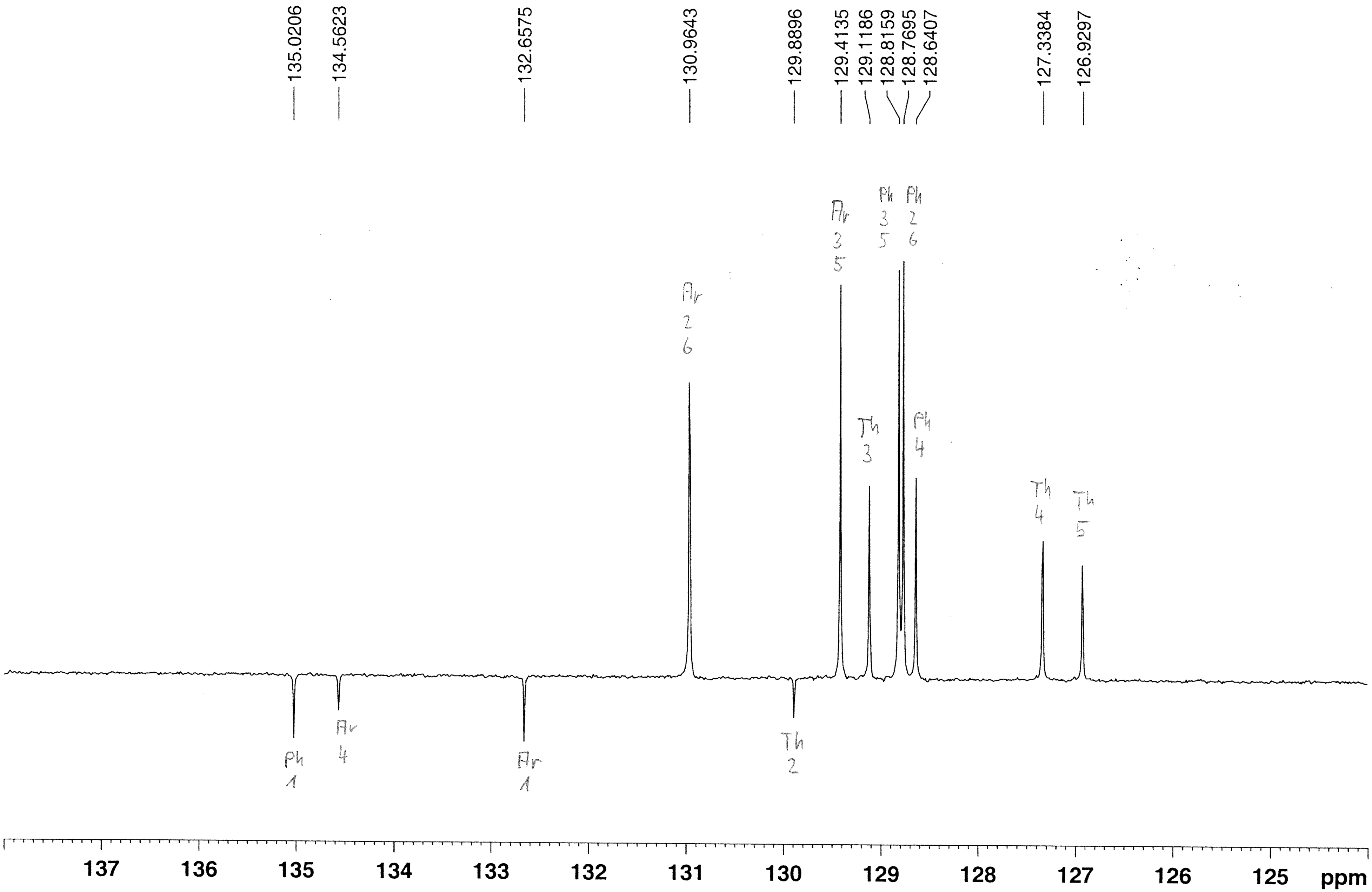

CE140-S1P1 in cdcl3 (APT) 29.8.2018

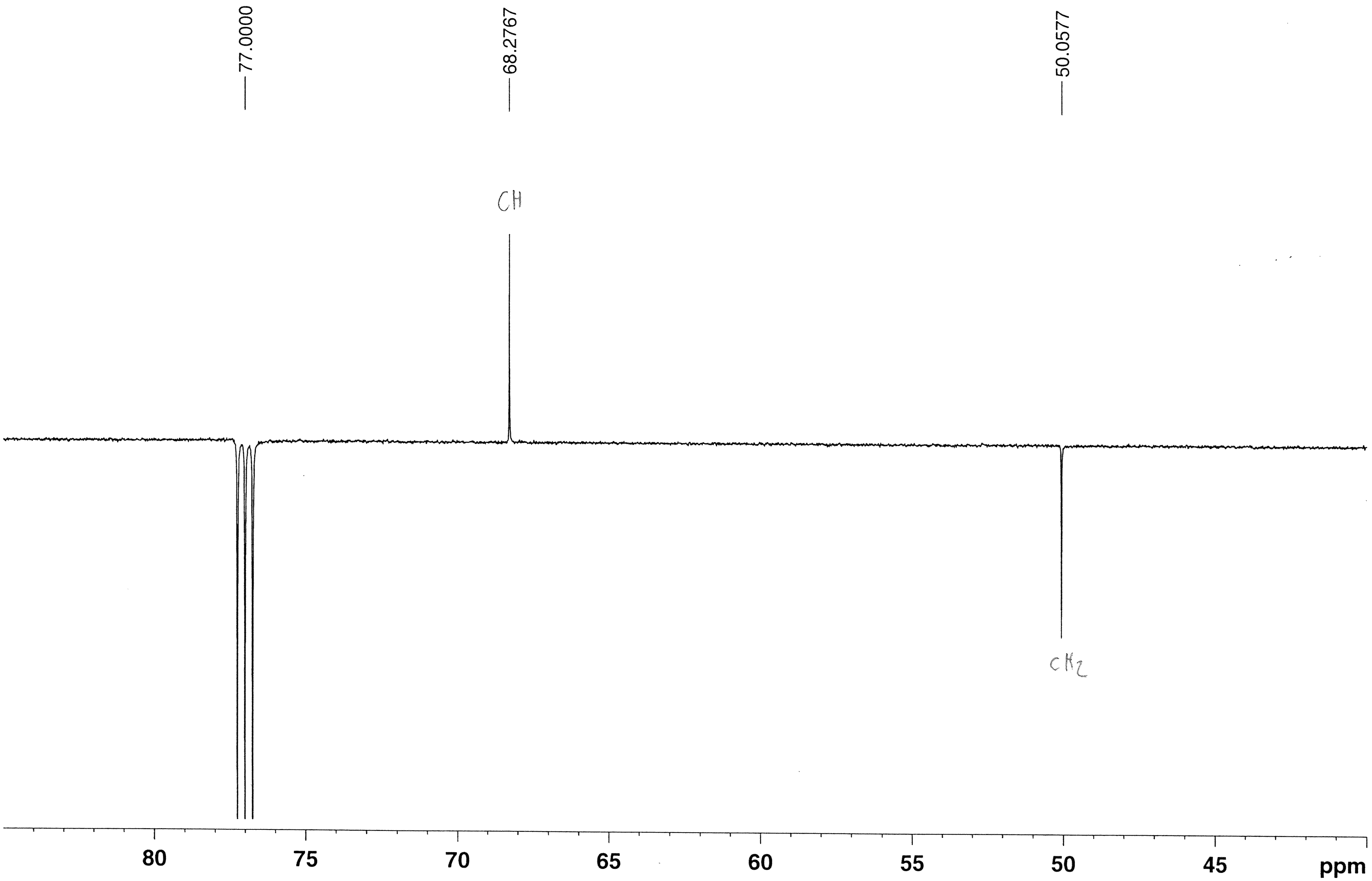

Figure S22c. NMR spectra of compound **6f.**

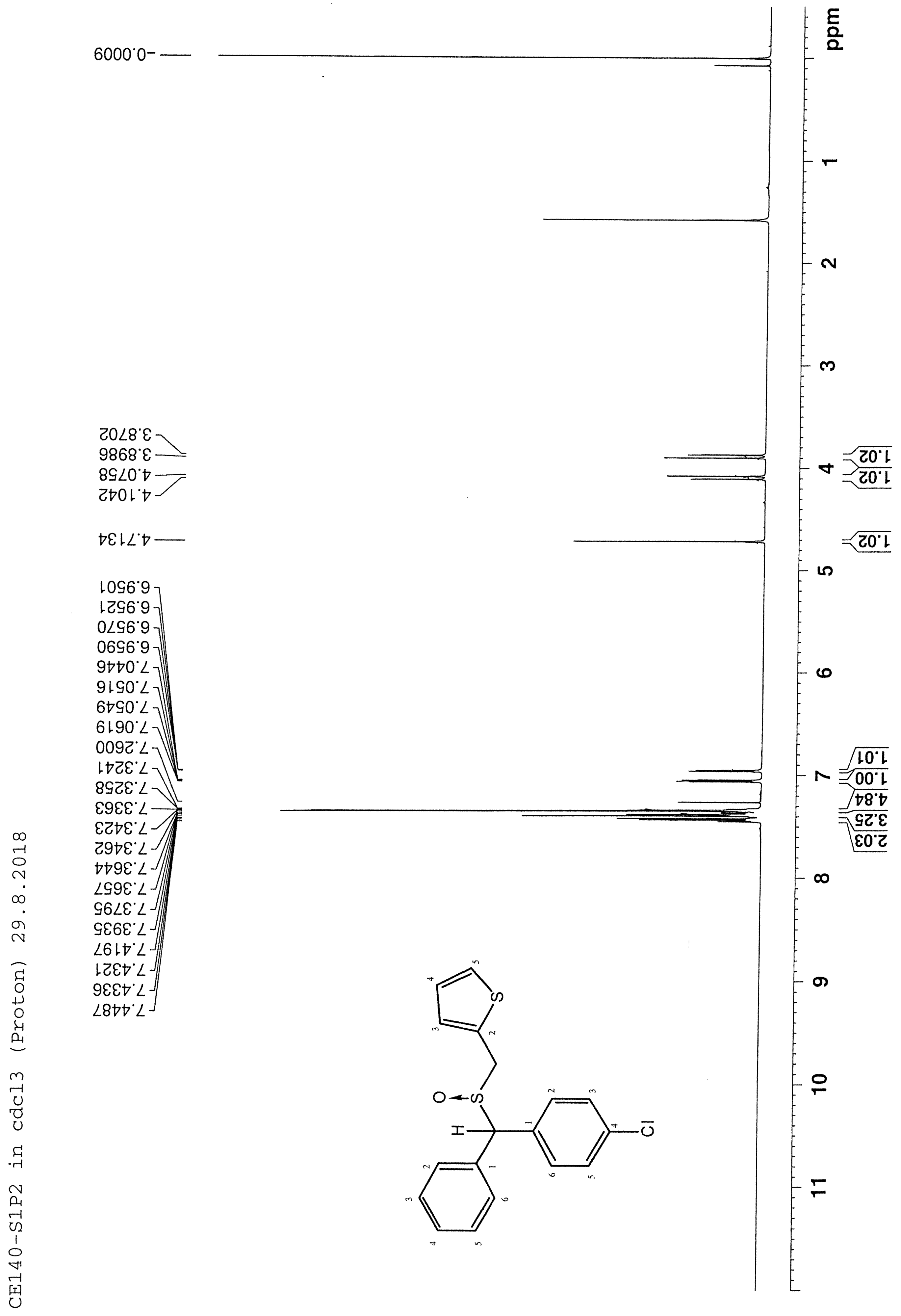

CE140-S1P2 in cdcl3 (Proton) 29.8.2018

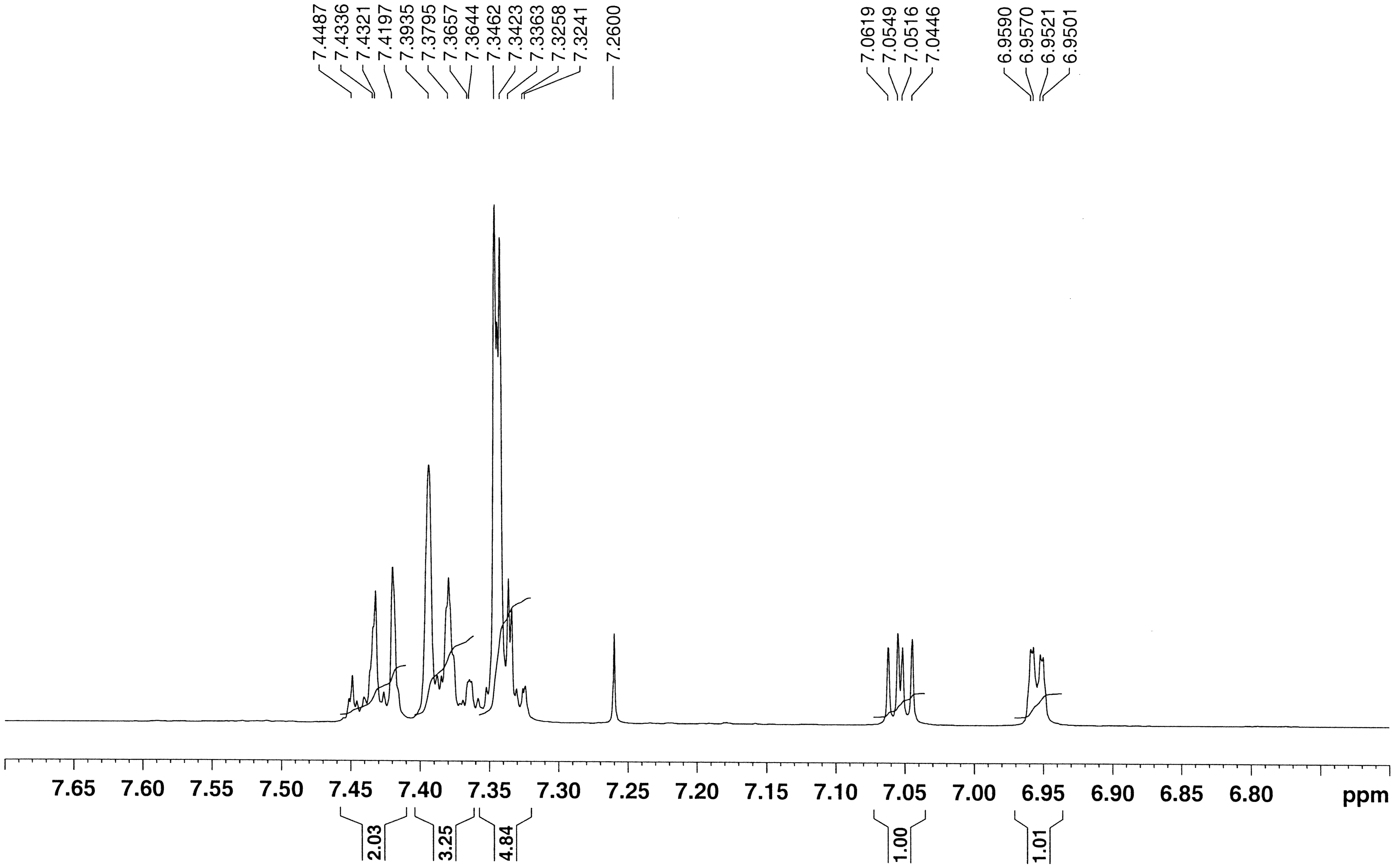

CE140-S1P2 in cdcl3 (Proton) 29.8.2018

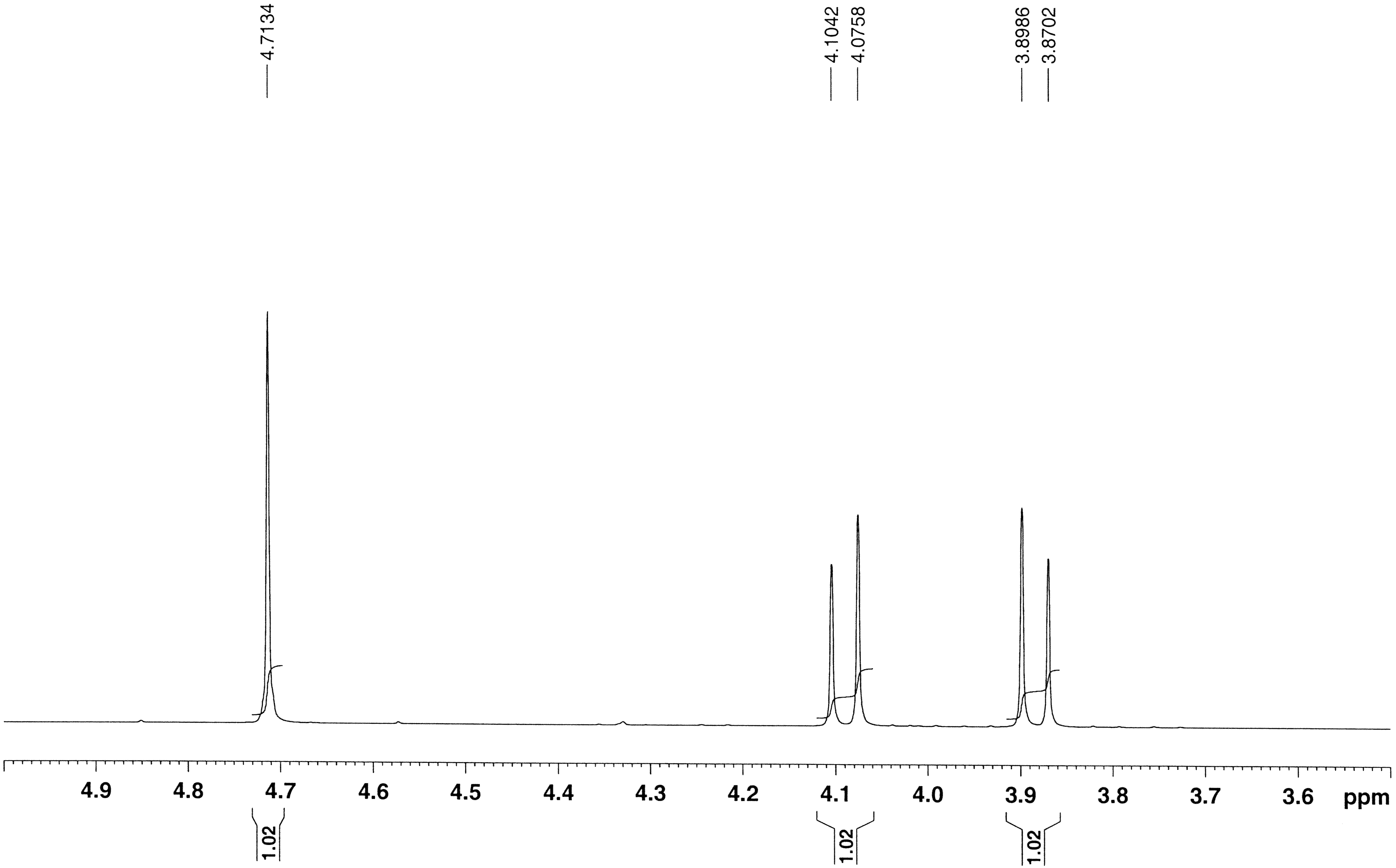

CE140-S1P2 in cdcl3 (APT) 29.8.2018

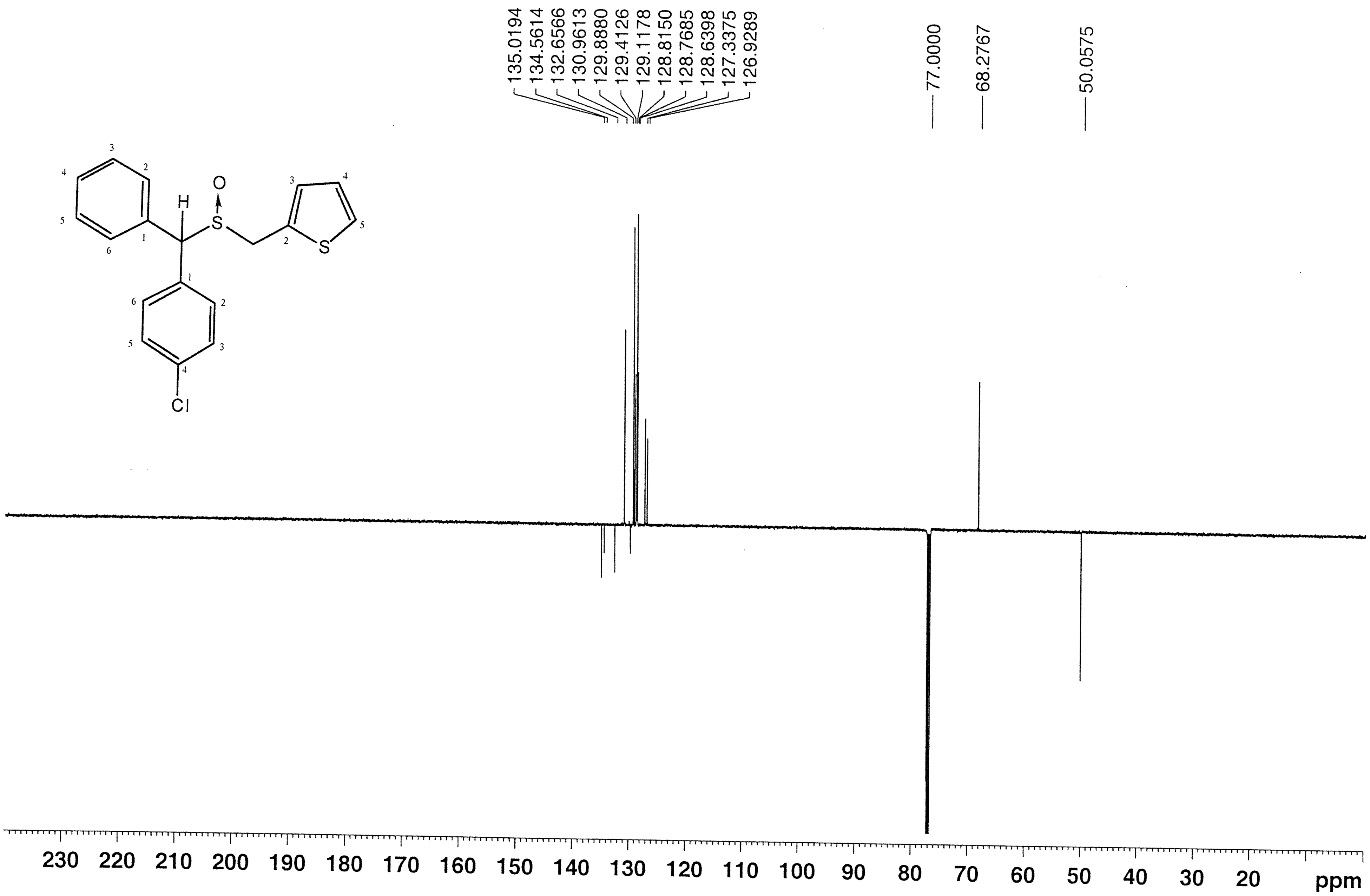

CE140-S1P2 in cdcl3 (APT) 29.8.2018

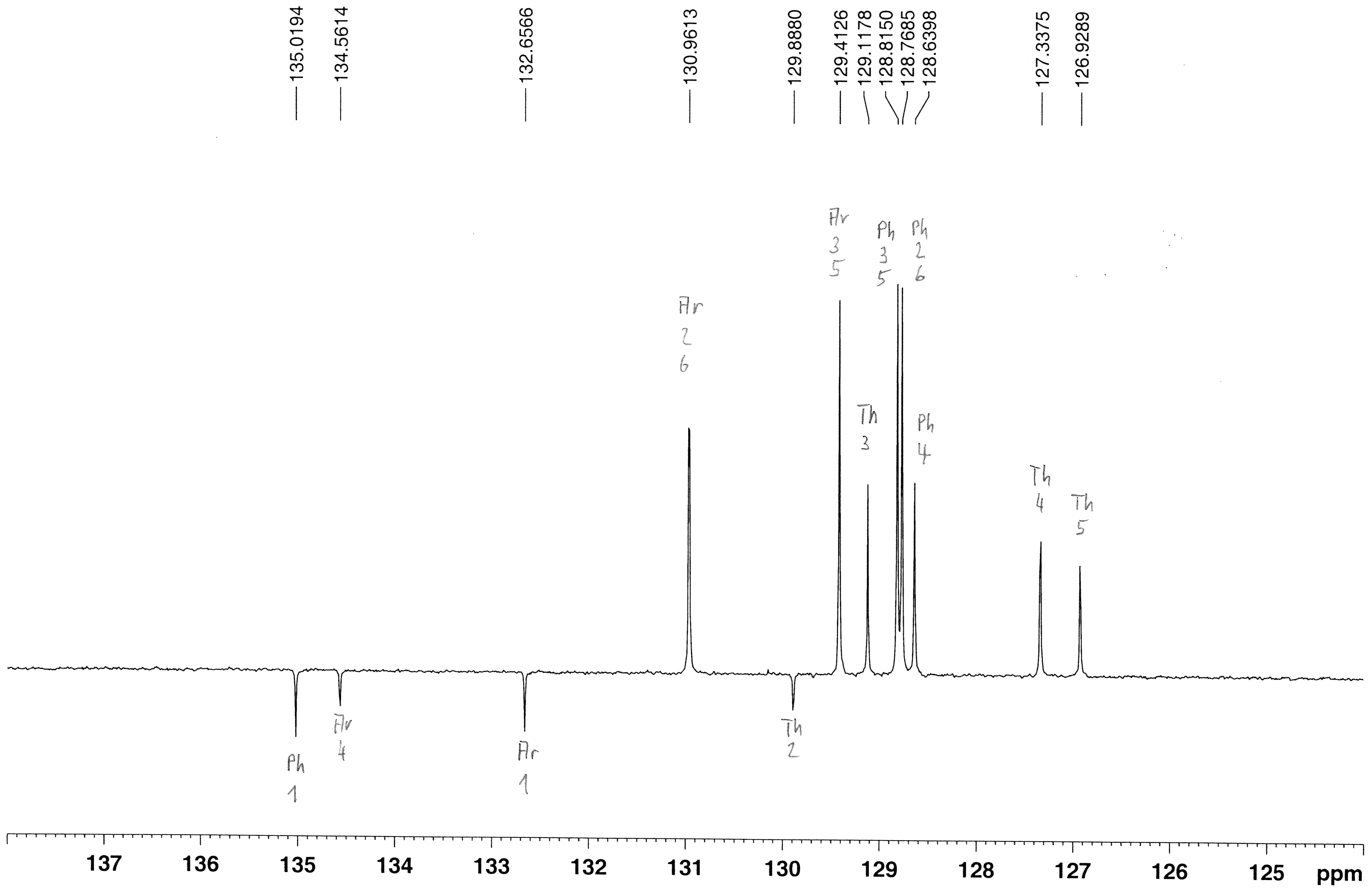

CE140-S1P2 in cdcl3 (APT) 29.8.2018

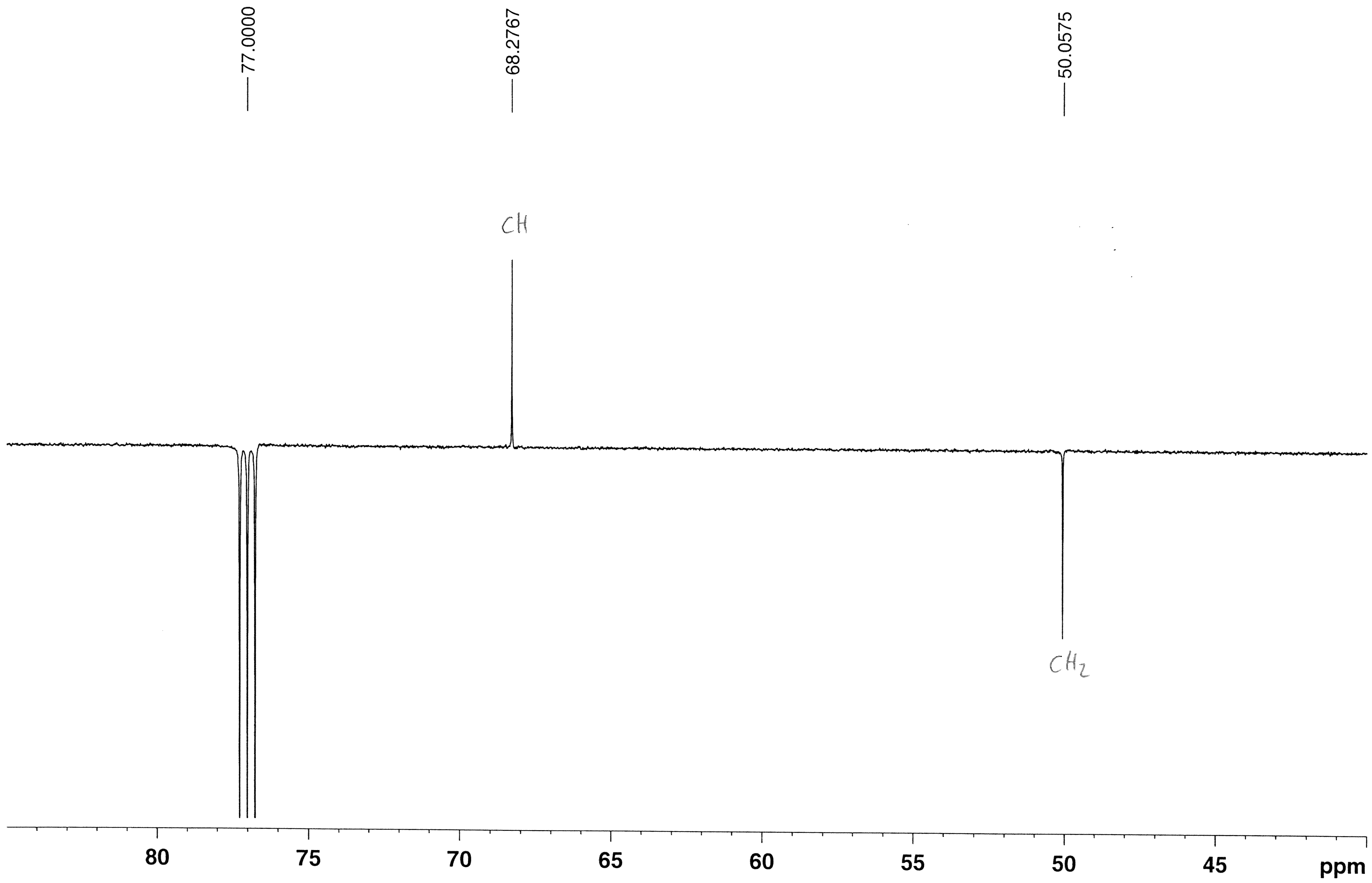

Figure S23c. NMR spectra of compound **7f.**

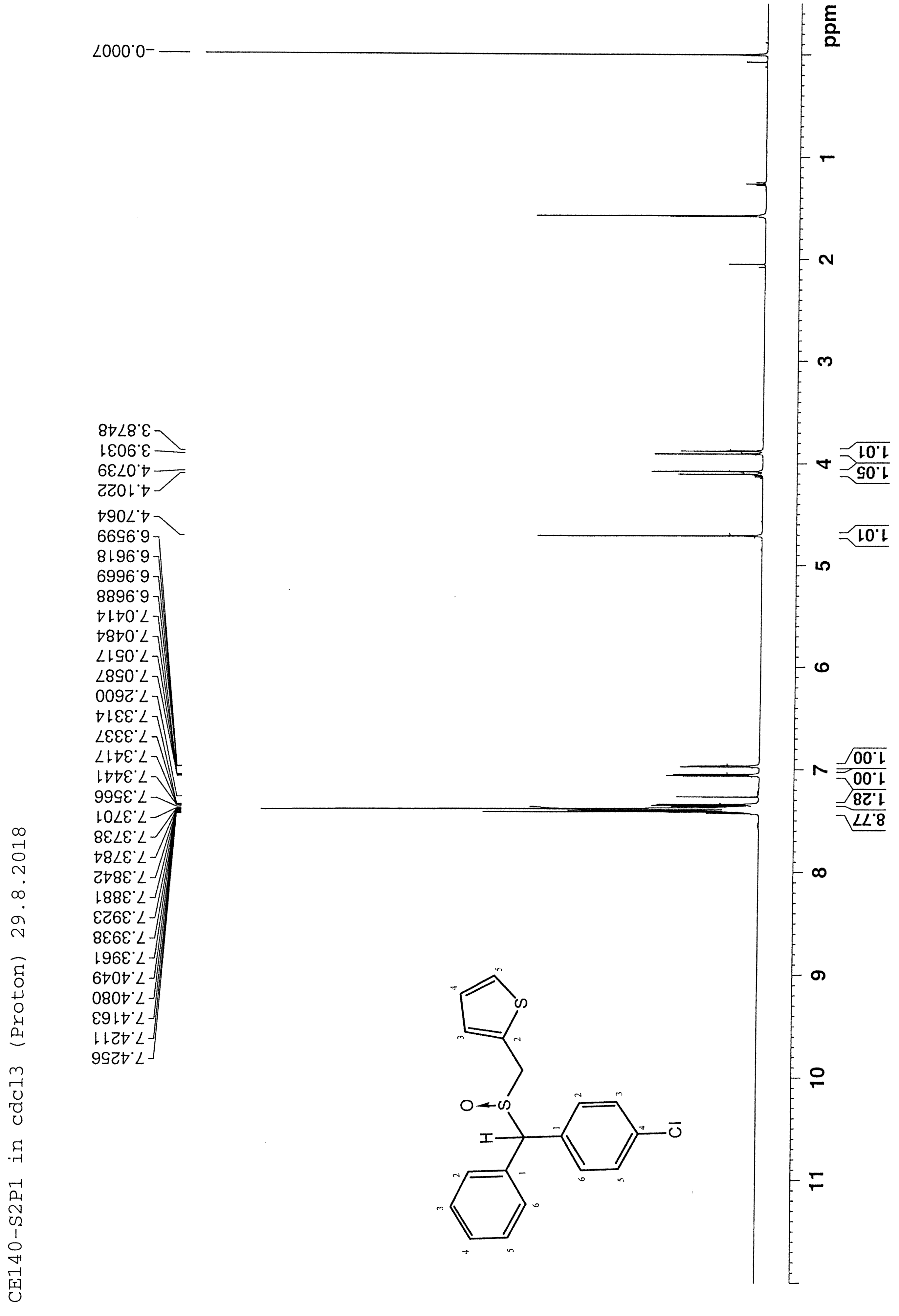

CE140-S2P1 in cdcl3 (Proton) 29.8.2018

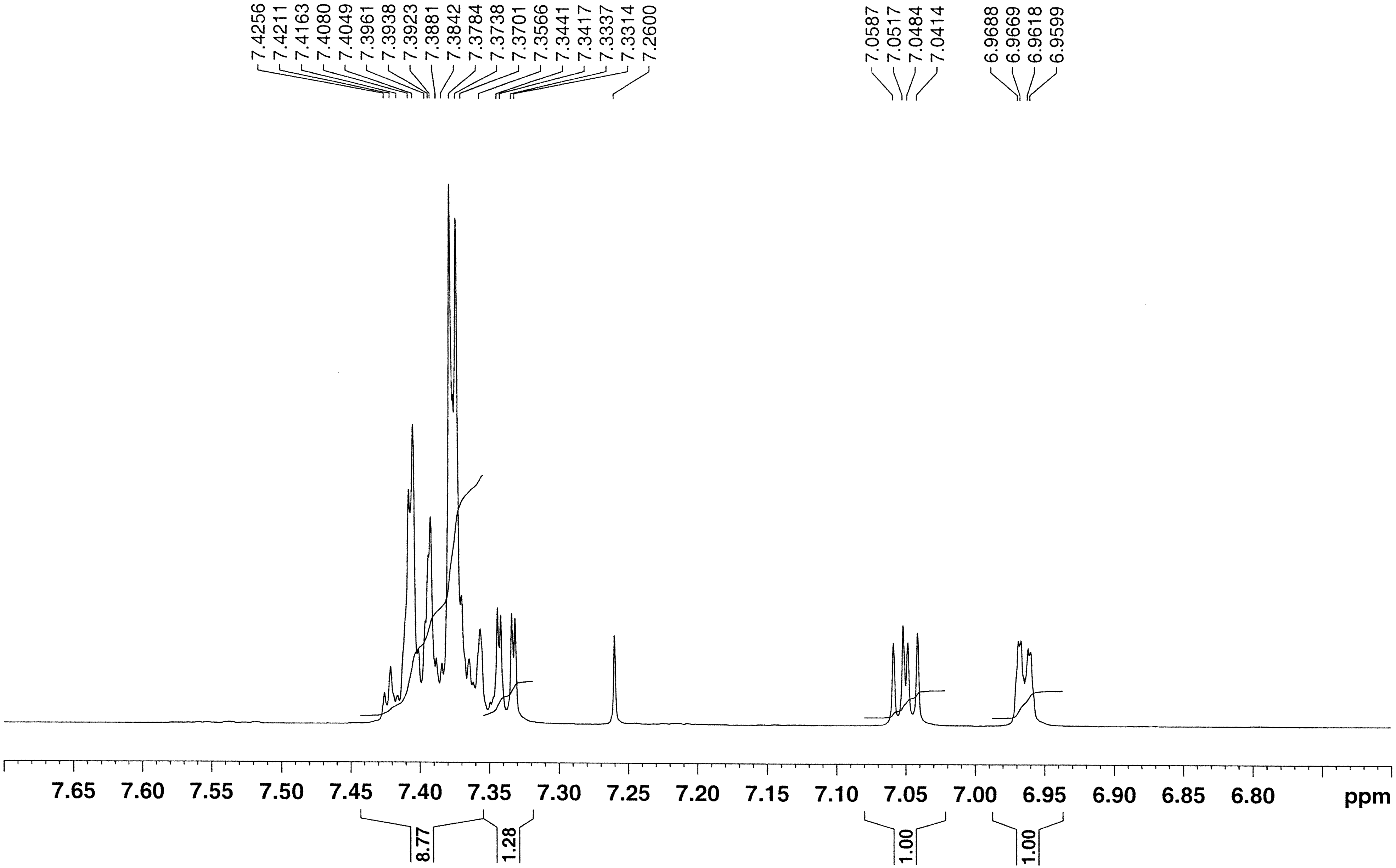

CE140-S2P1 in cdcl3 (Proton) 29.8.2018

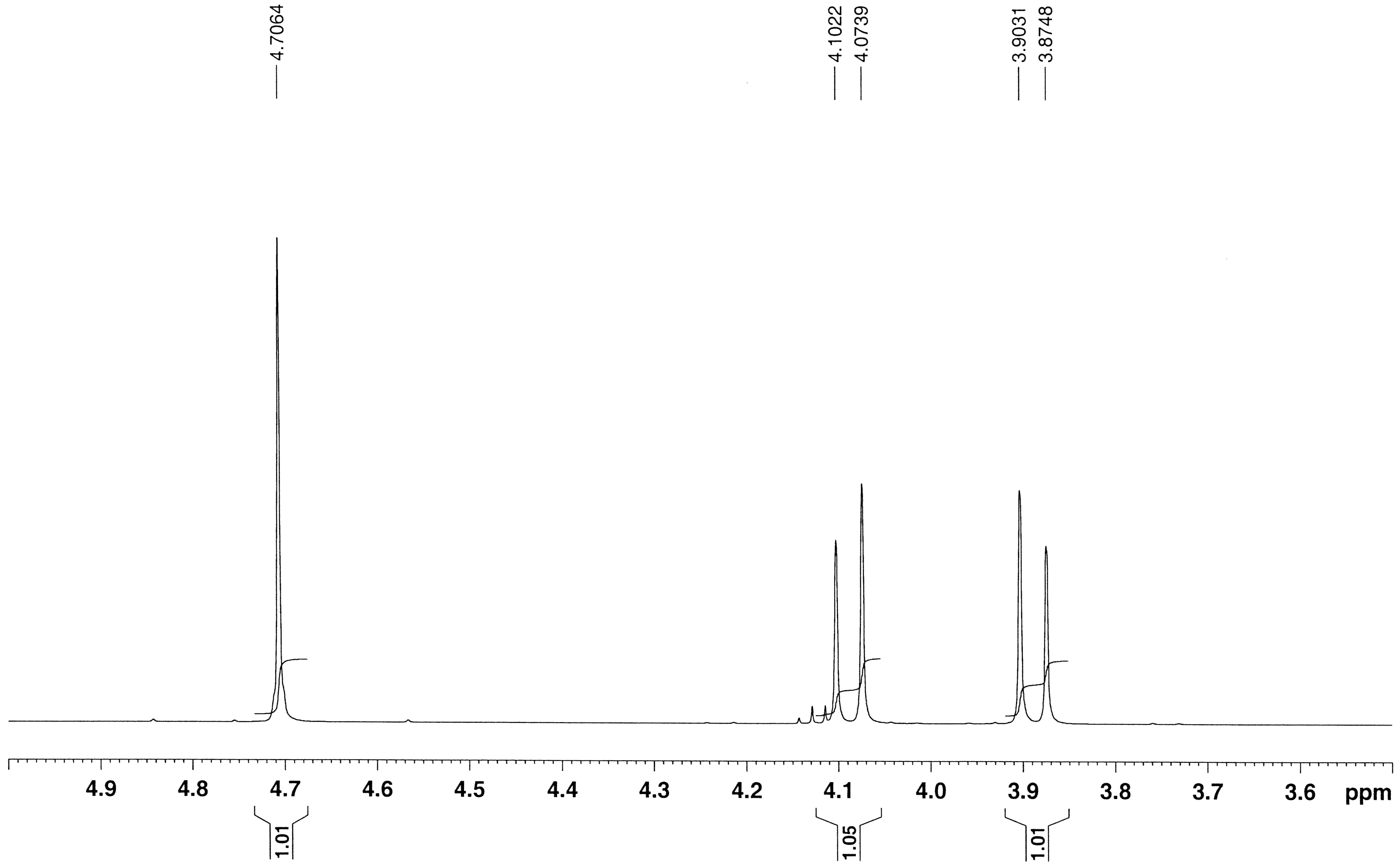

CE140-S2P1 in cdcl3 (APT) 29.8.2018

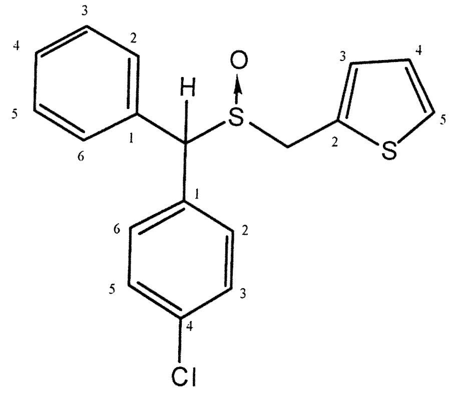

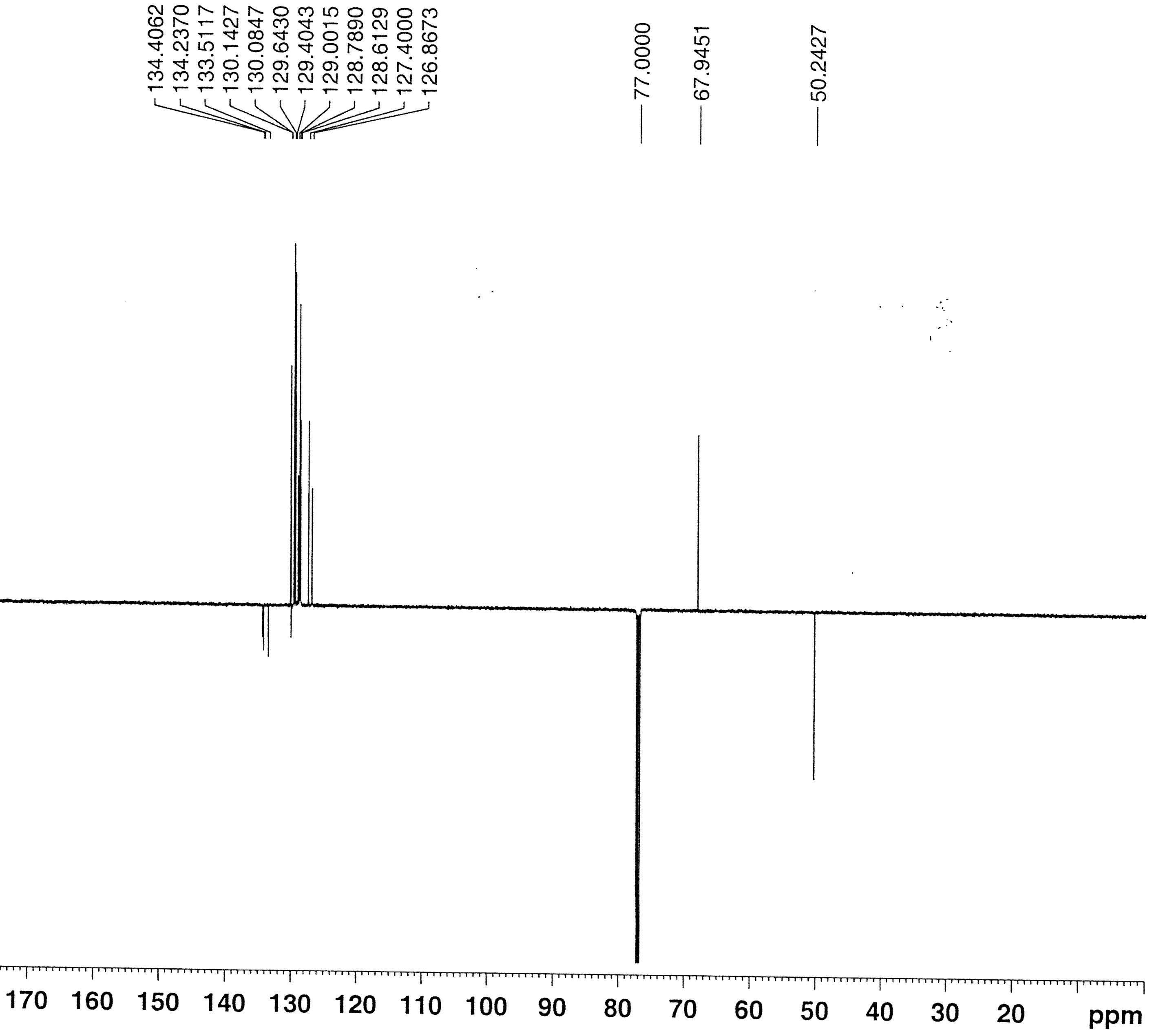

CE140-S2P1 in cdcl3 (APT) 29.8.2018

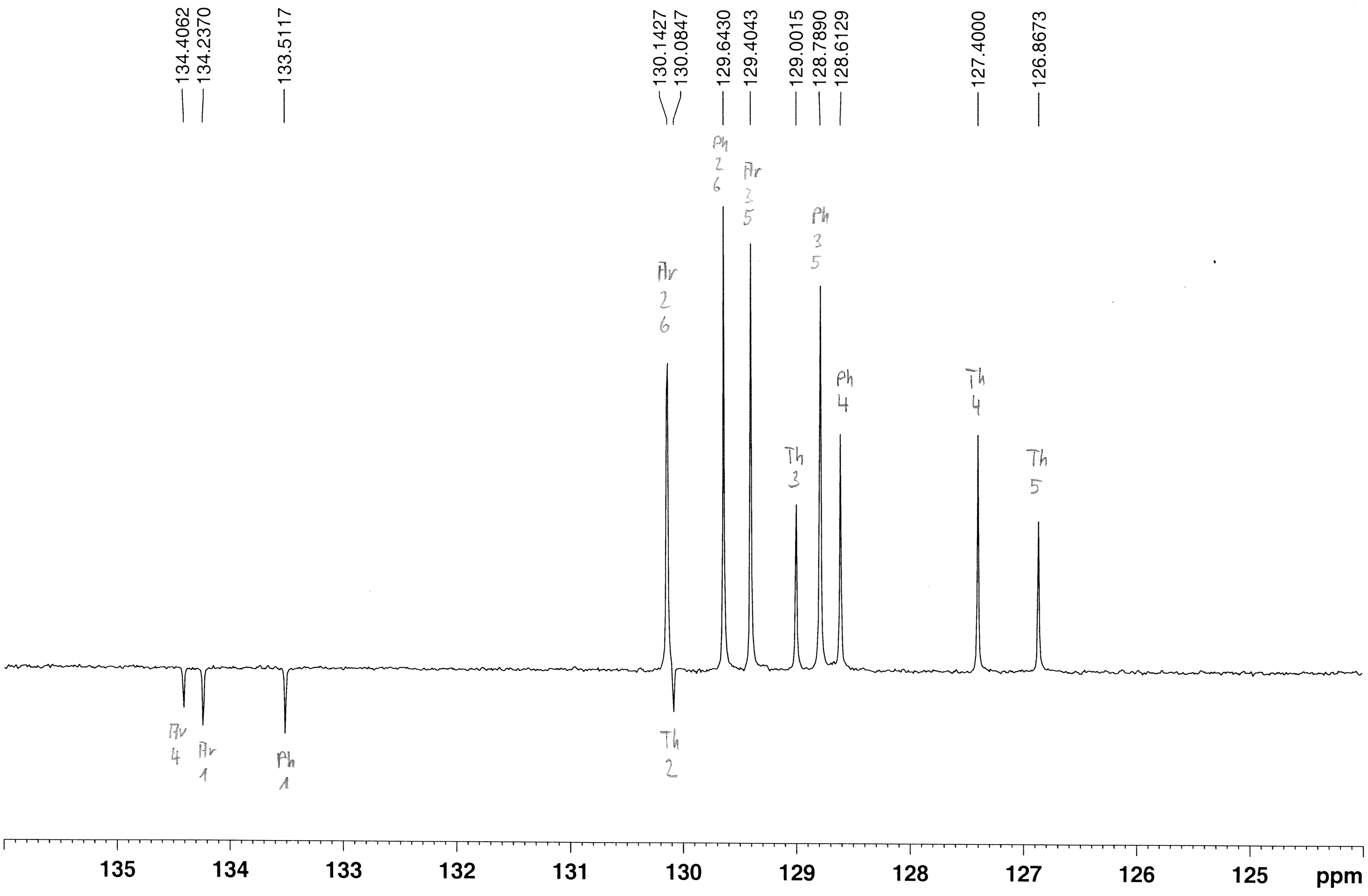

CE140-S2P1 in cdcl3 (APT) 29.8.2018

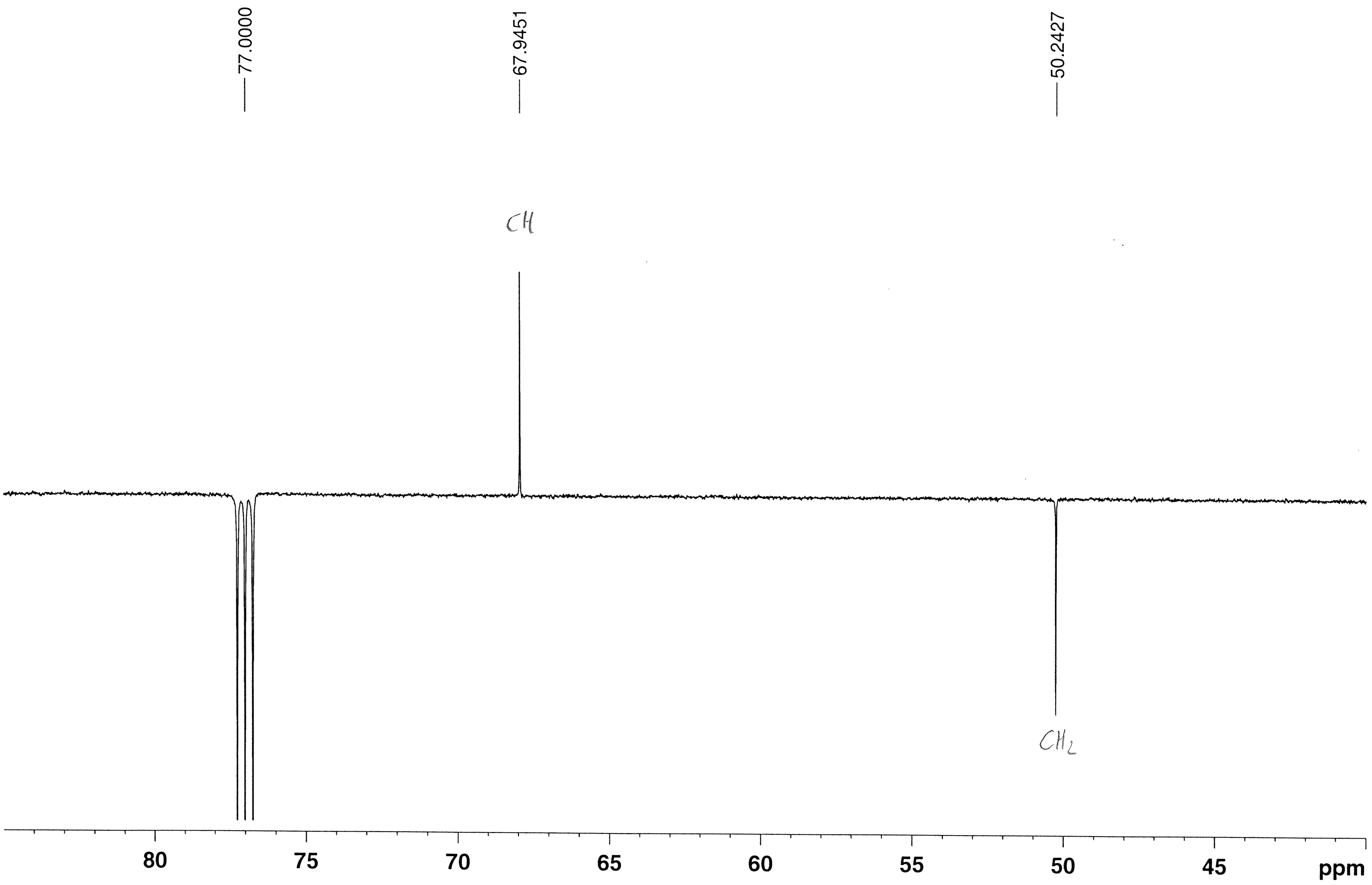

Figure S24c. NMR spectra of compound **8f.**

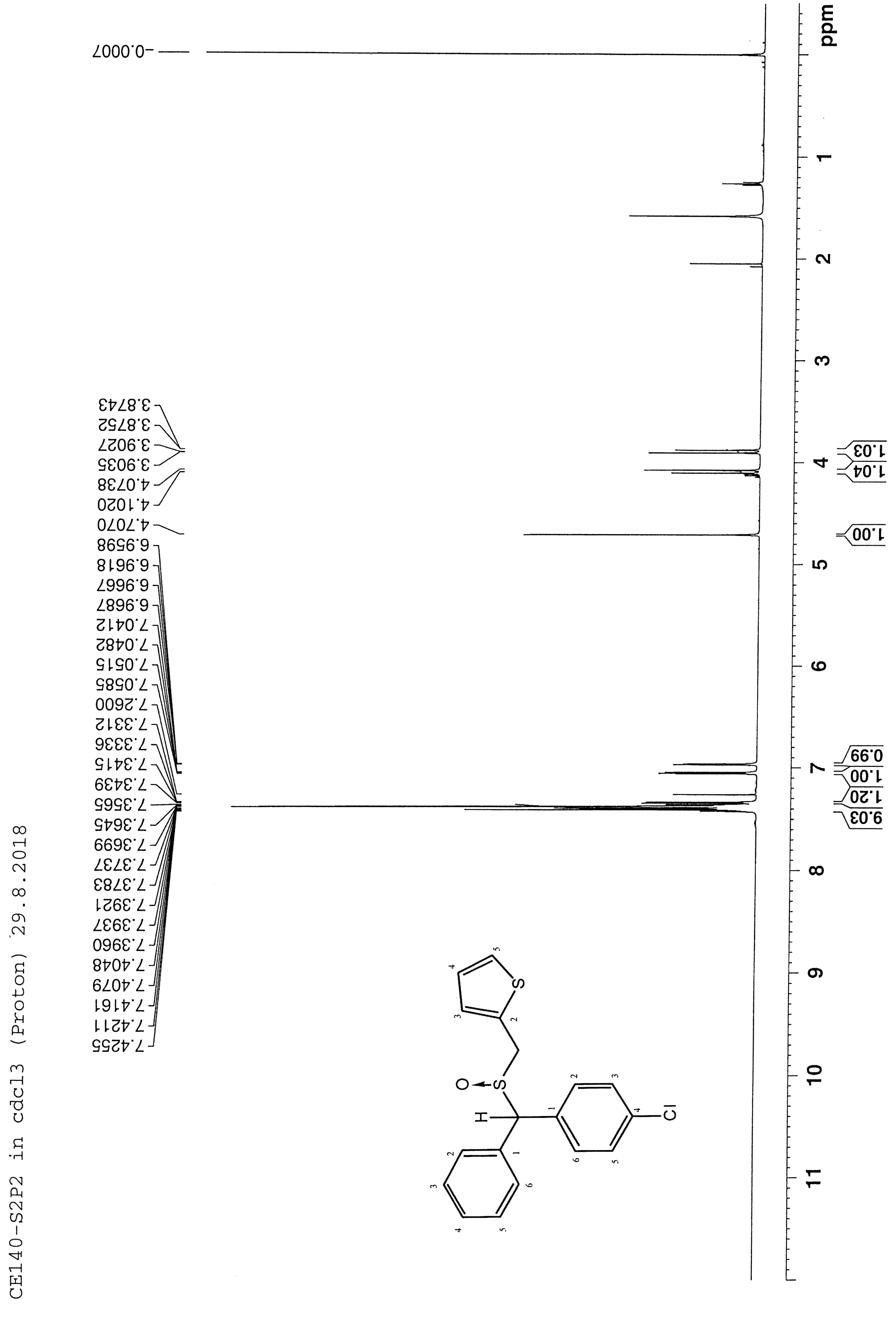

CE140-S2P2 in cdcl3 (Proton) 29.8.2018

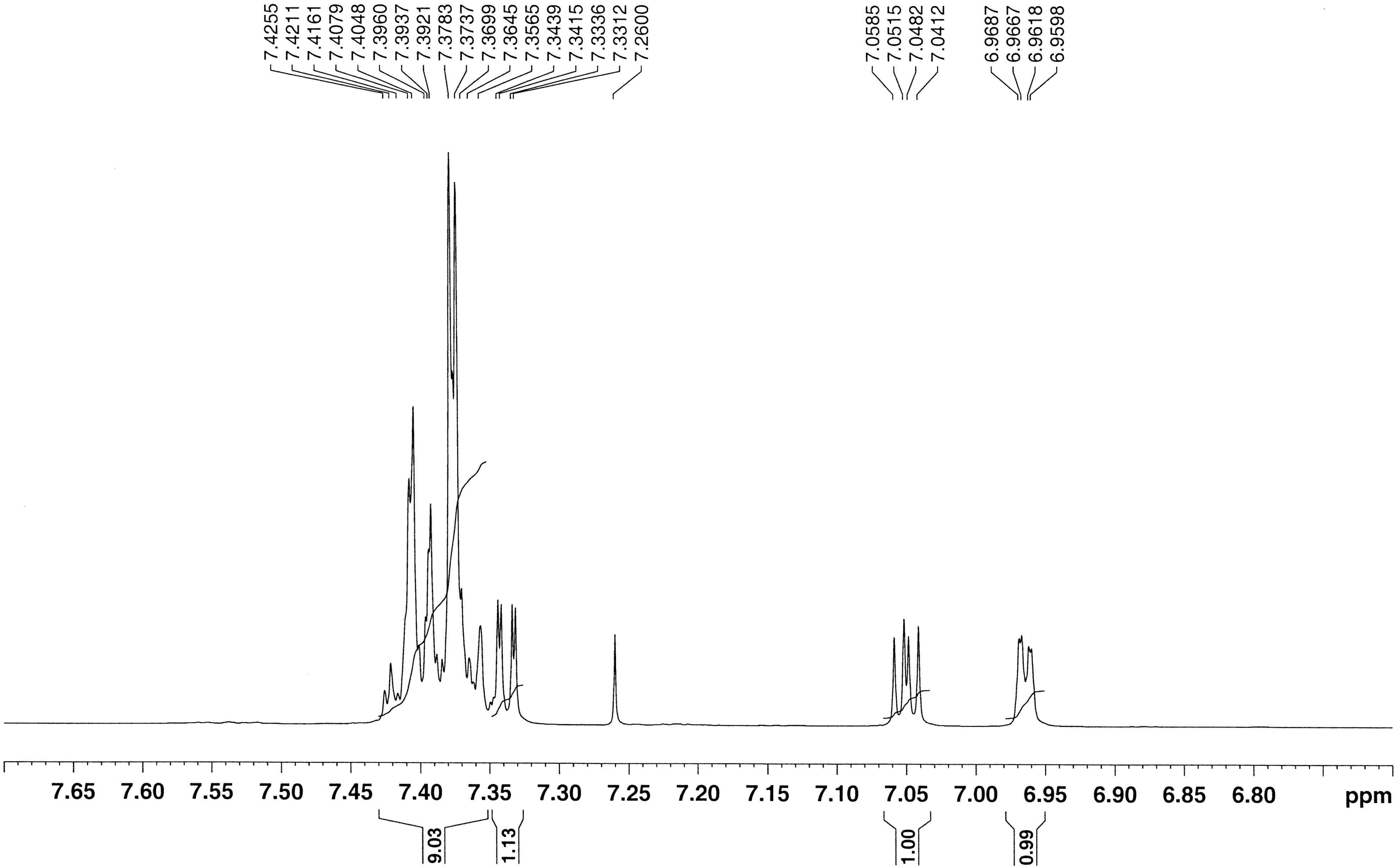

CE140-S2P2 in cdcl3 (Proton) 29.8.2018

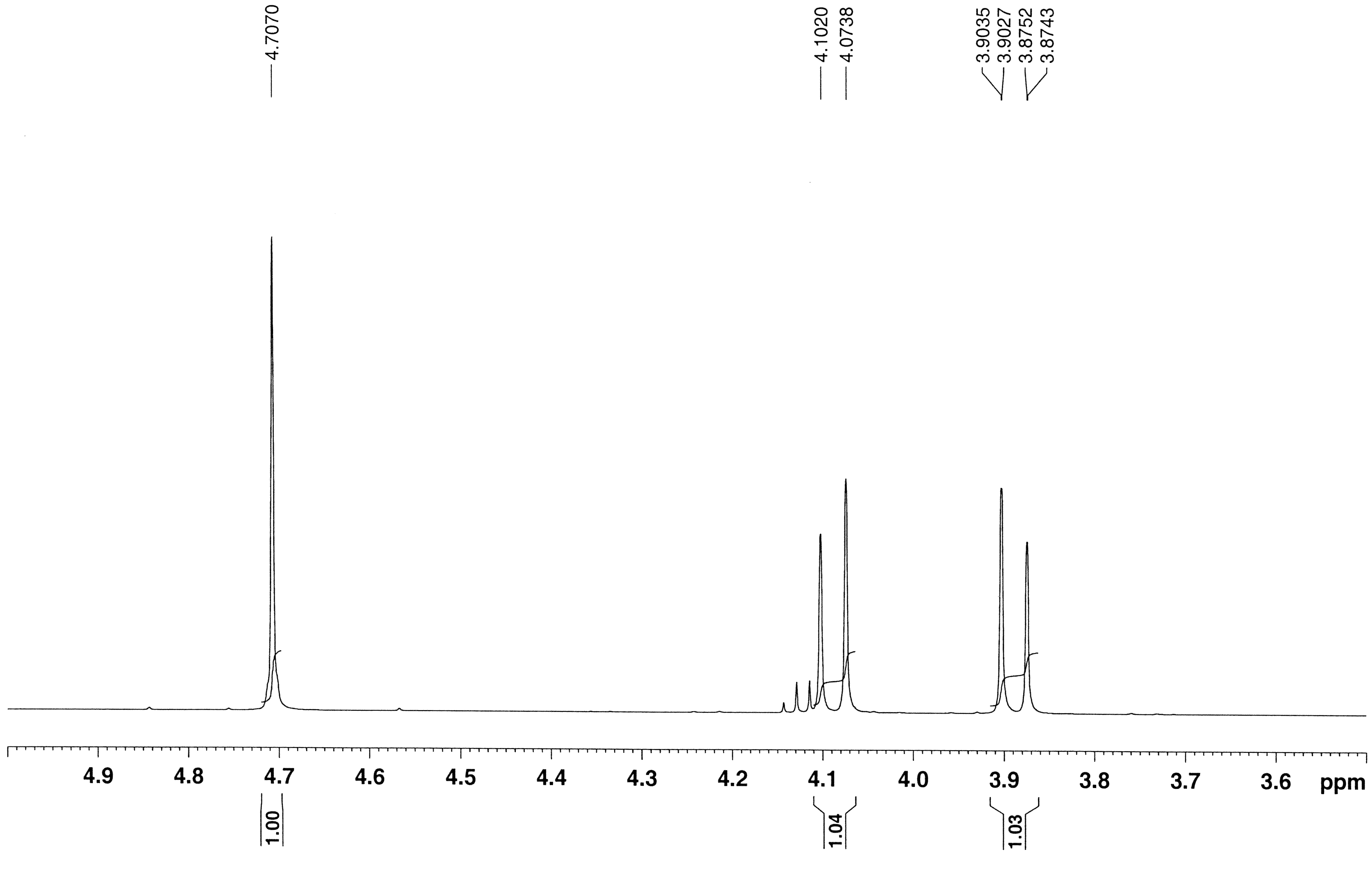

CE140-S2P2 in cdcl3 (APT) 29.8.2018

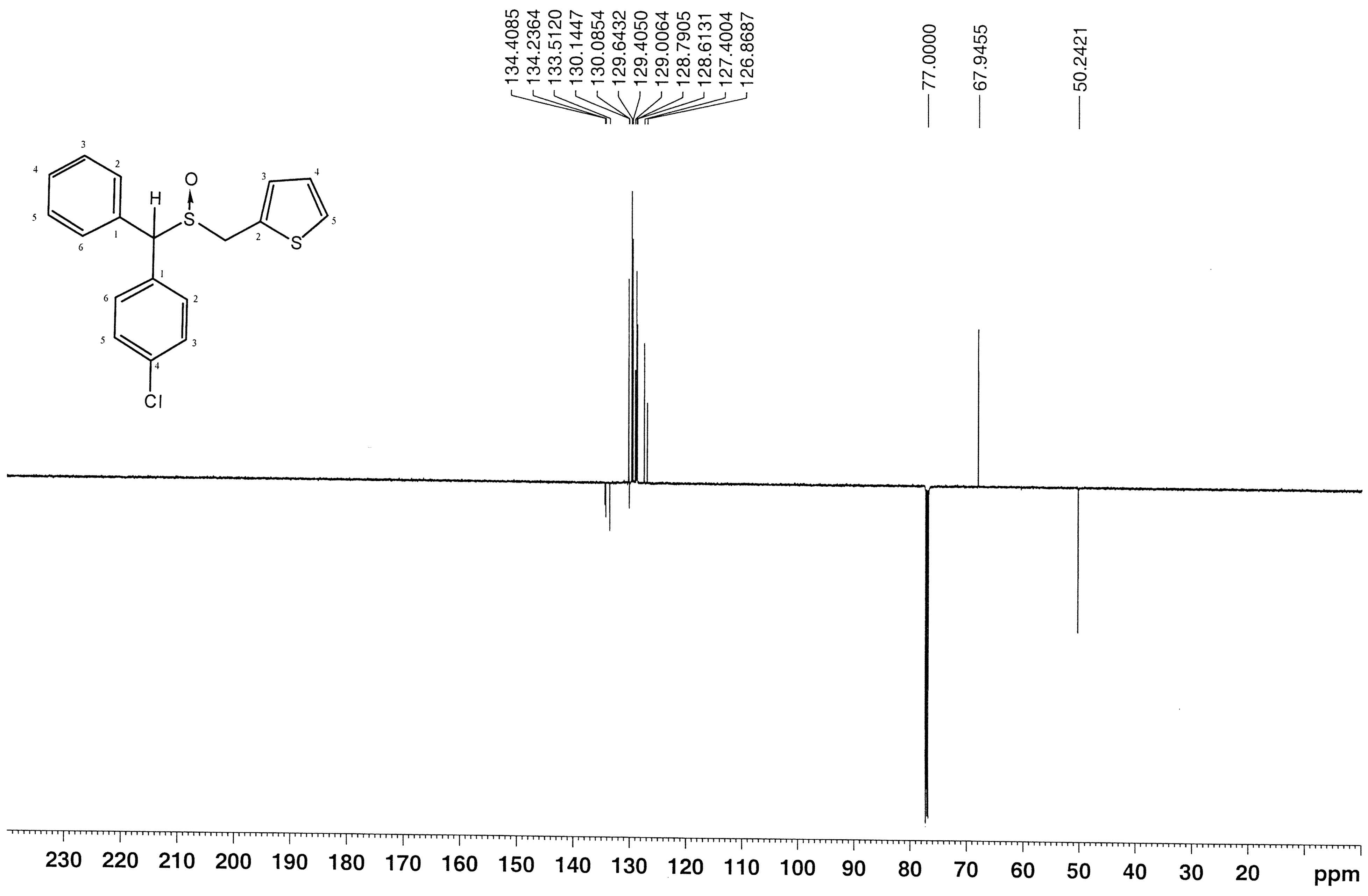

CE140-S2P2 in cdcl3 (APT) 29.8.2018

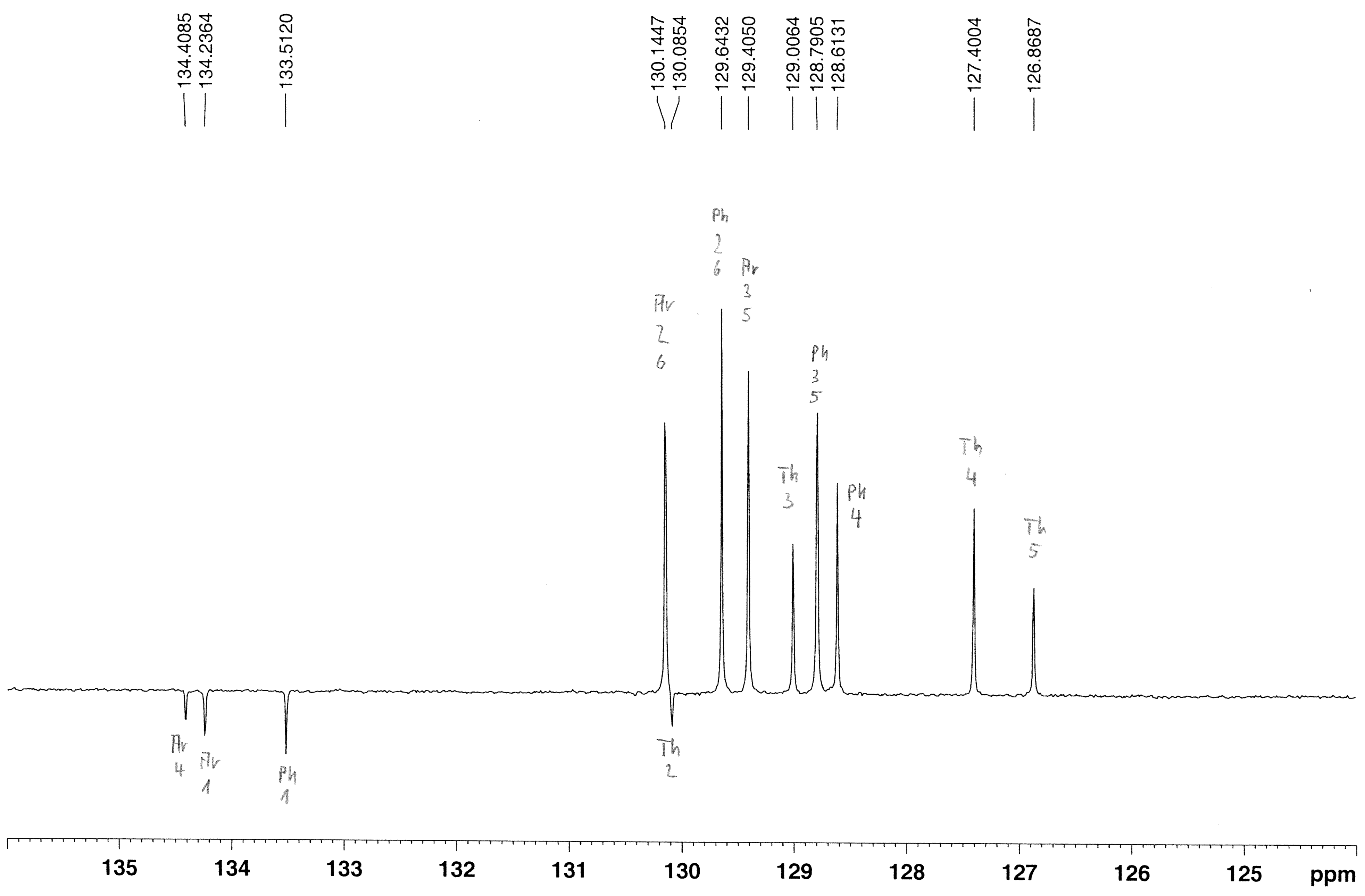

CE140-S2P2 in cdcl3 (APT) 29.8.2018

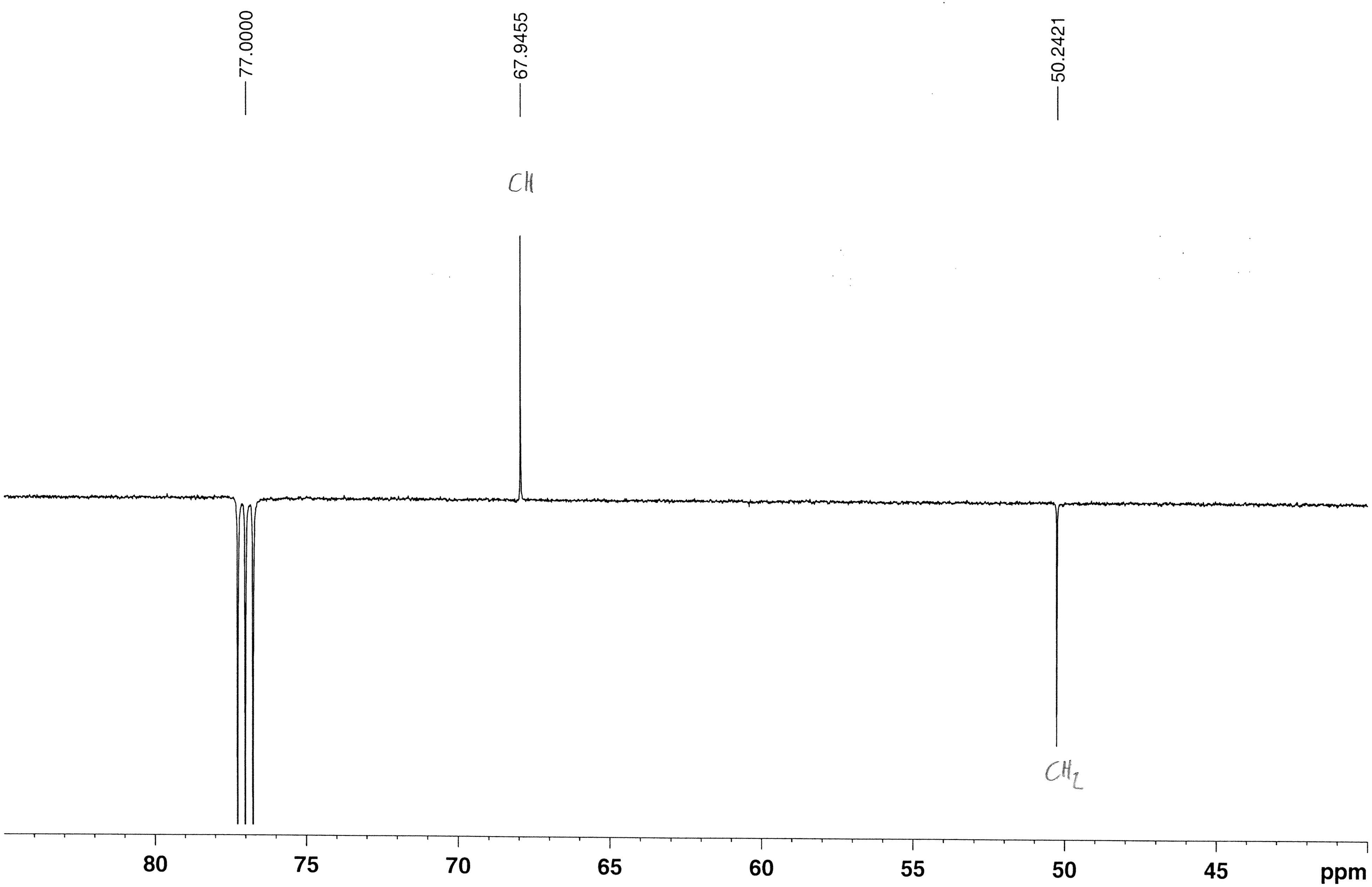

Figure S25c. NMR spectra of compound **5g.**

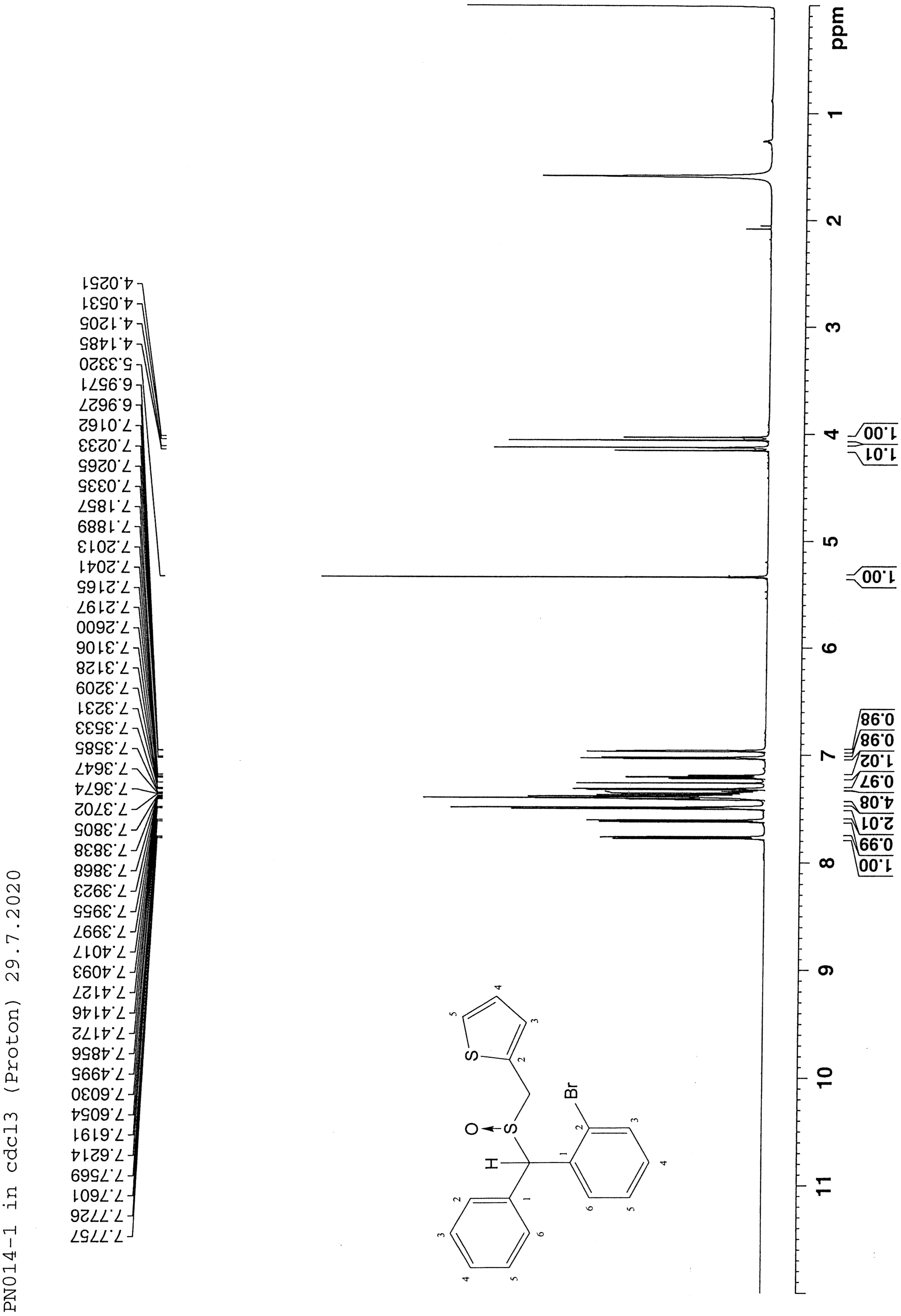

PN014-1 in cdcl3 (Proton) 29.7.2020

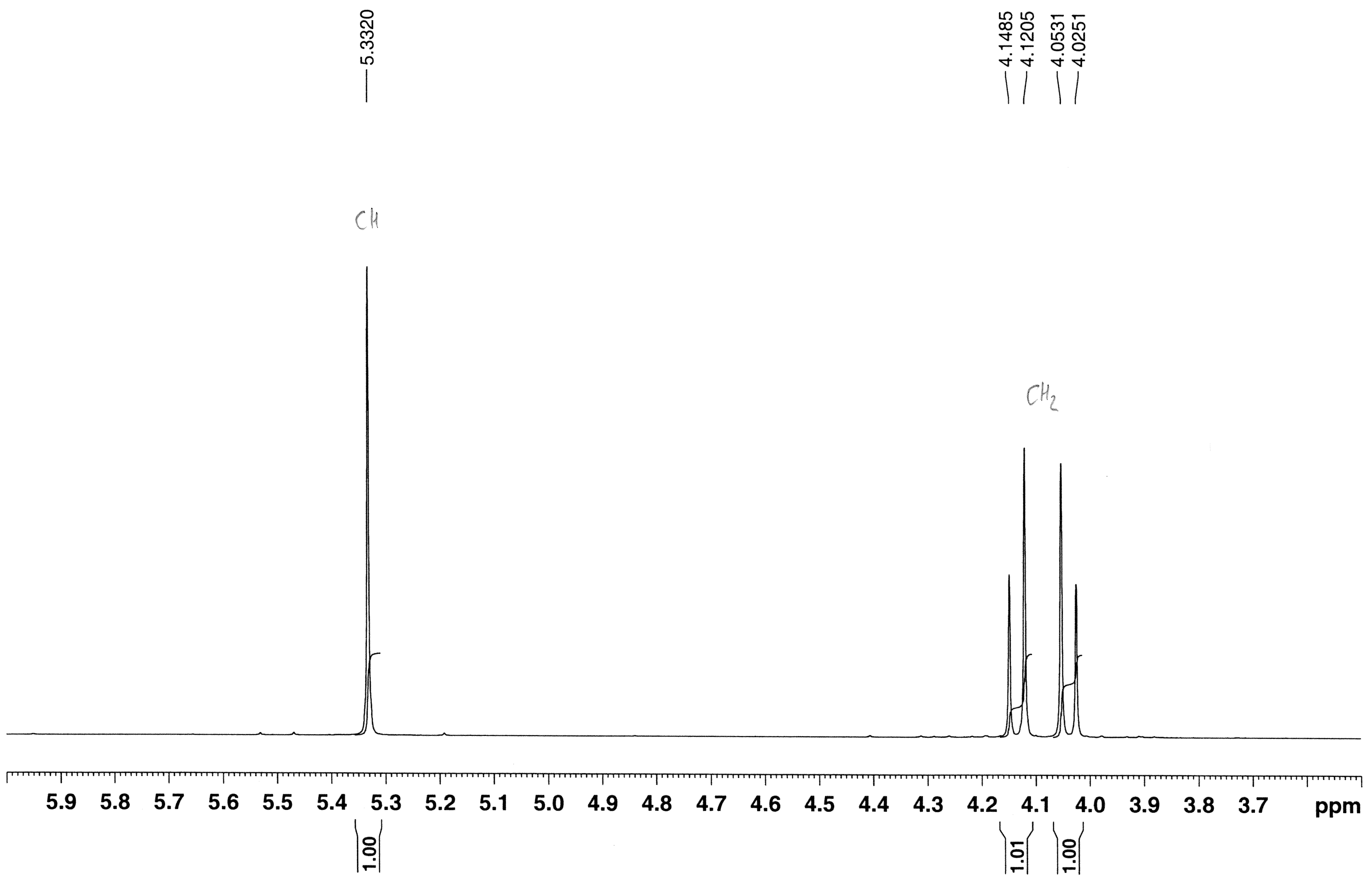

PN014-1 in cdcl3 (Proton) 29.7.2020

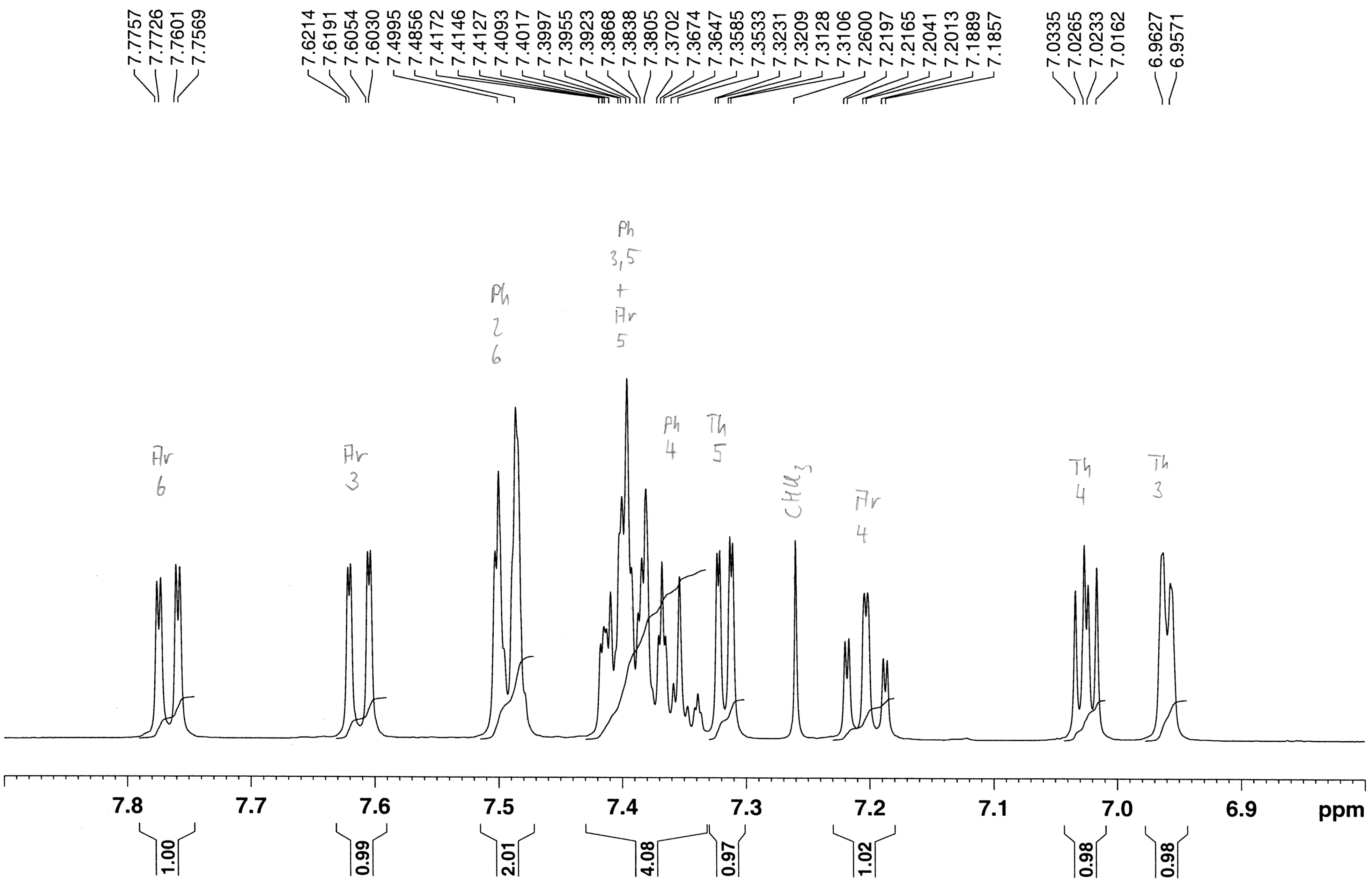

PN014-1 in cdcl3 (APT) 29.7.2020

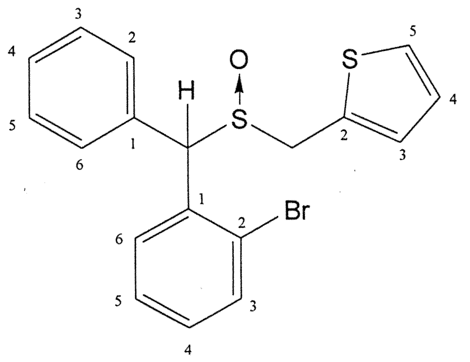

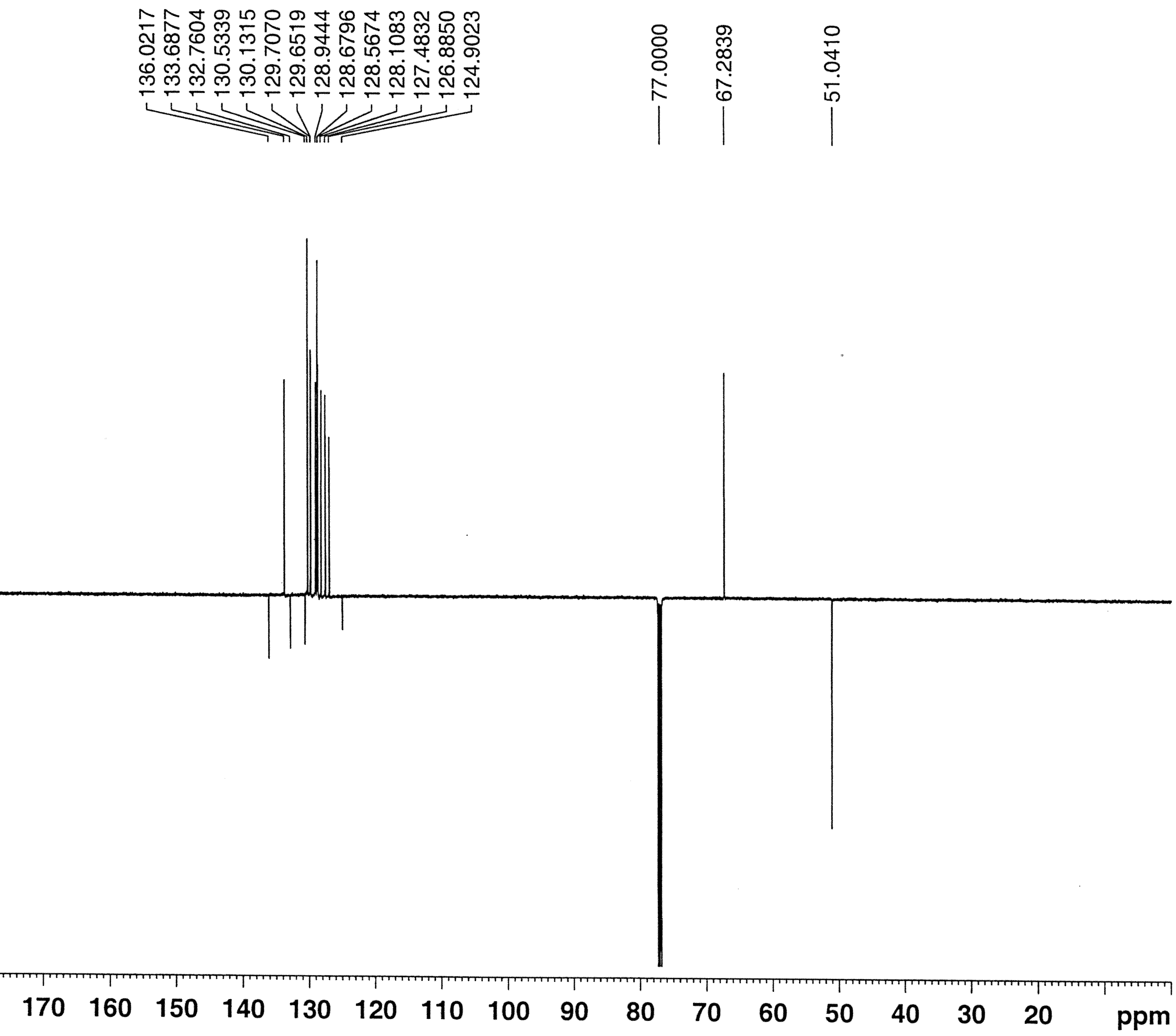

PN014-1 in cdcl3 (APT) 29.7.2020

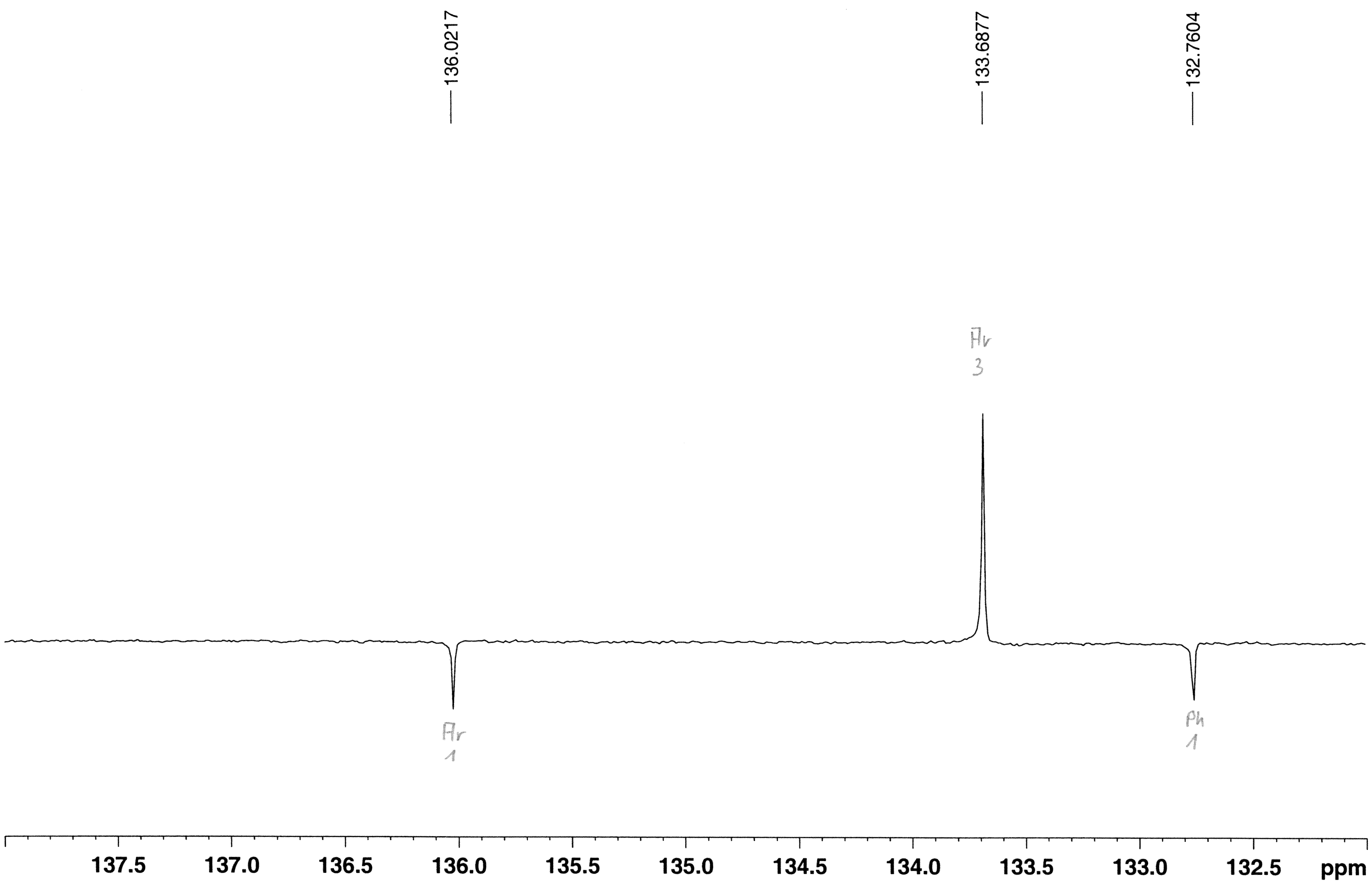

PN014-1 in cdcl3 (APT) 29.7.2020

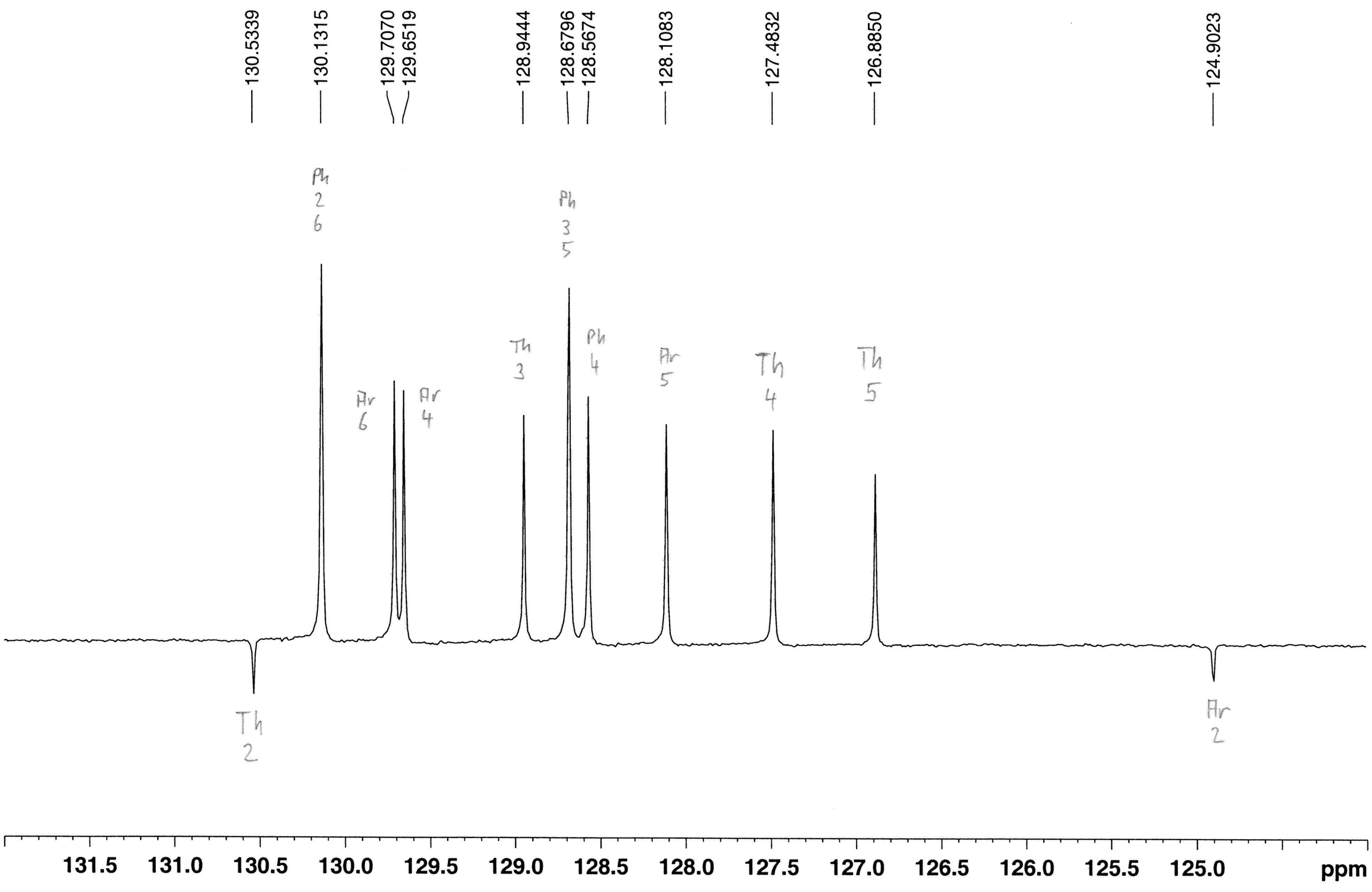

PN014-1 in cdcl3 (APT) 29.7.2020

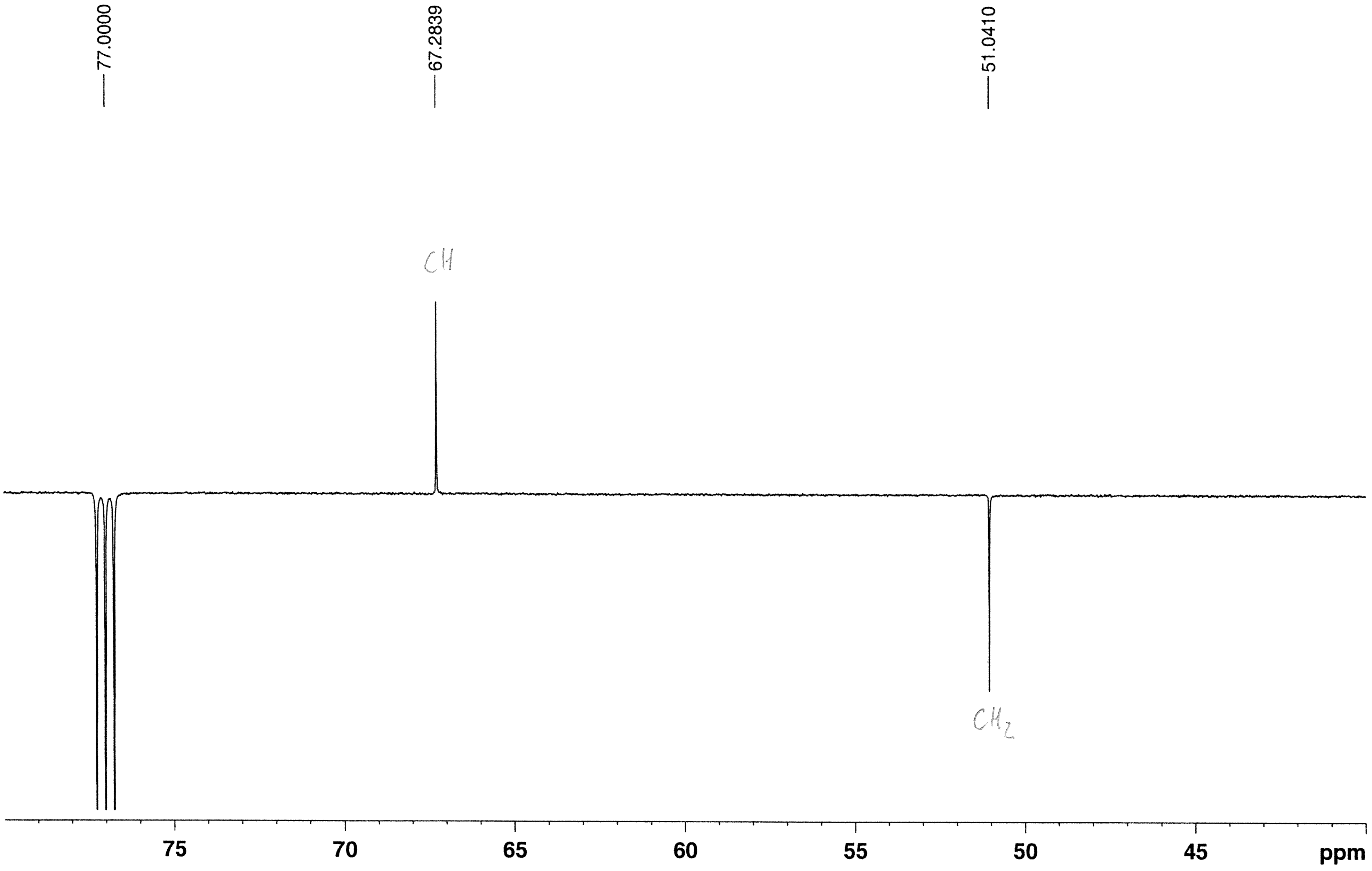

PN014-1 in cdcl3 (COSY) 29.7.2020

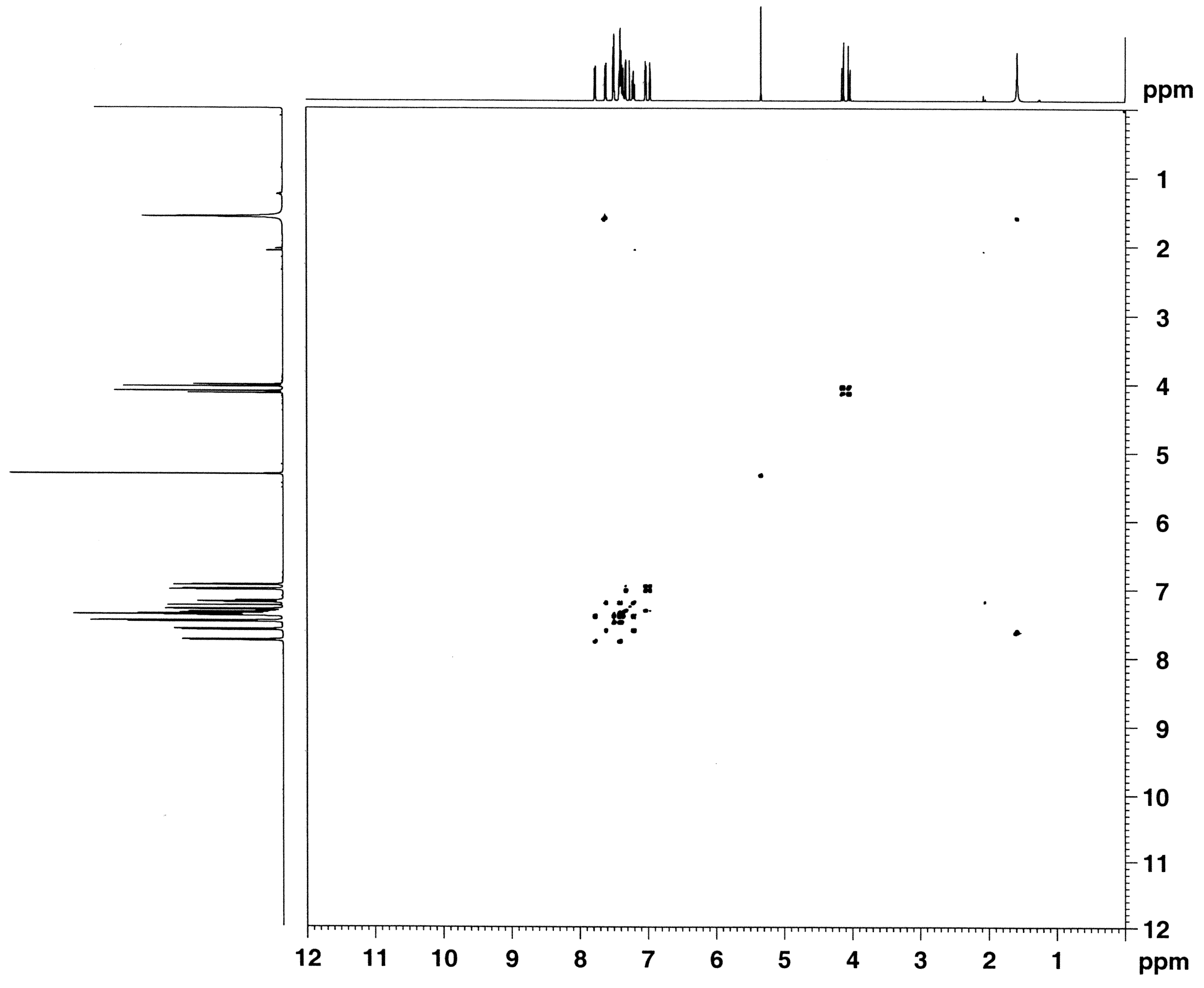

PN014-1 in cdcl3 (COSY) 29.7.2020

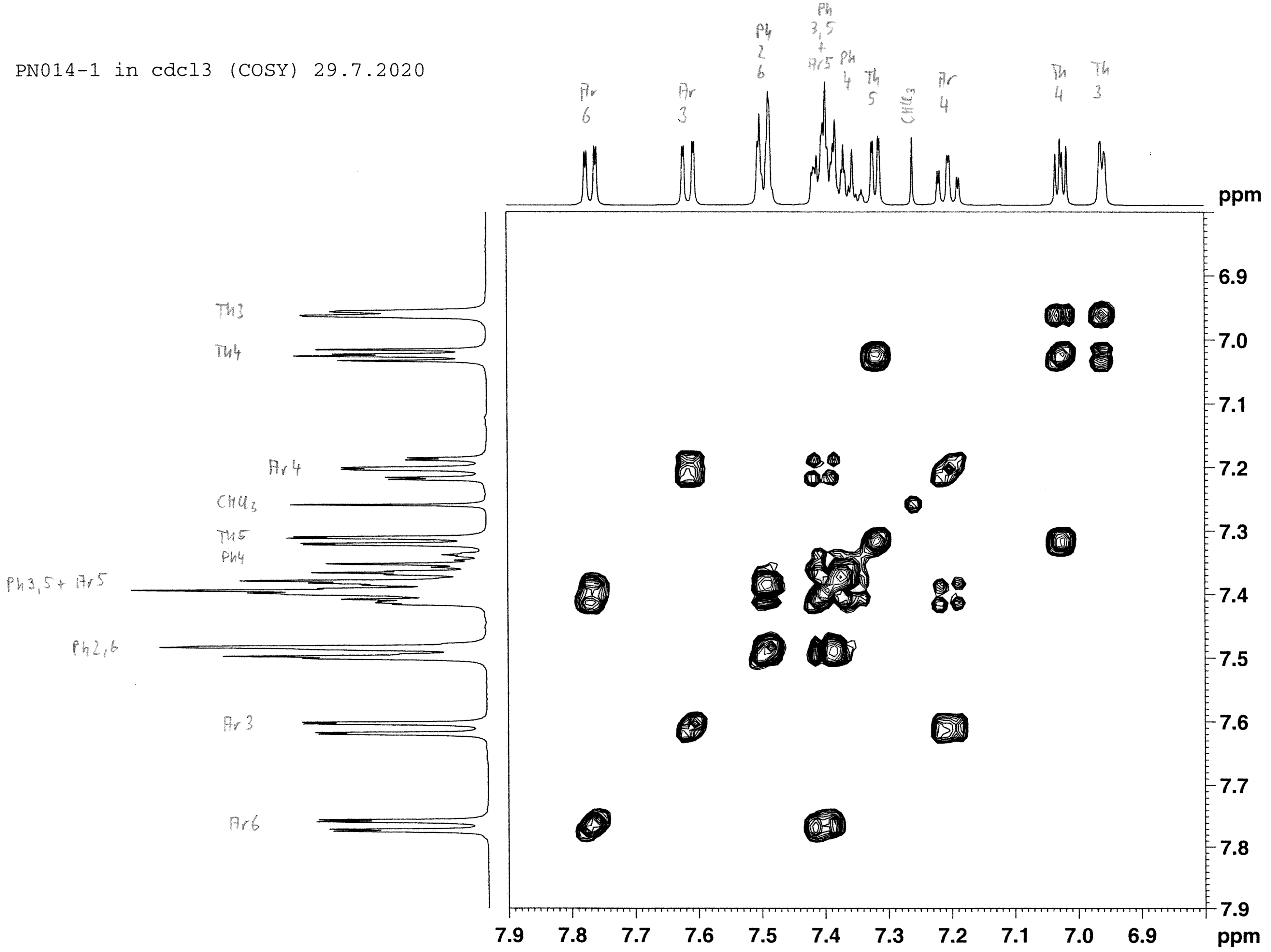

PN014-1 in cdcl3 (COSY) 29.7.2020

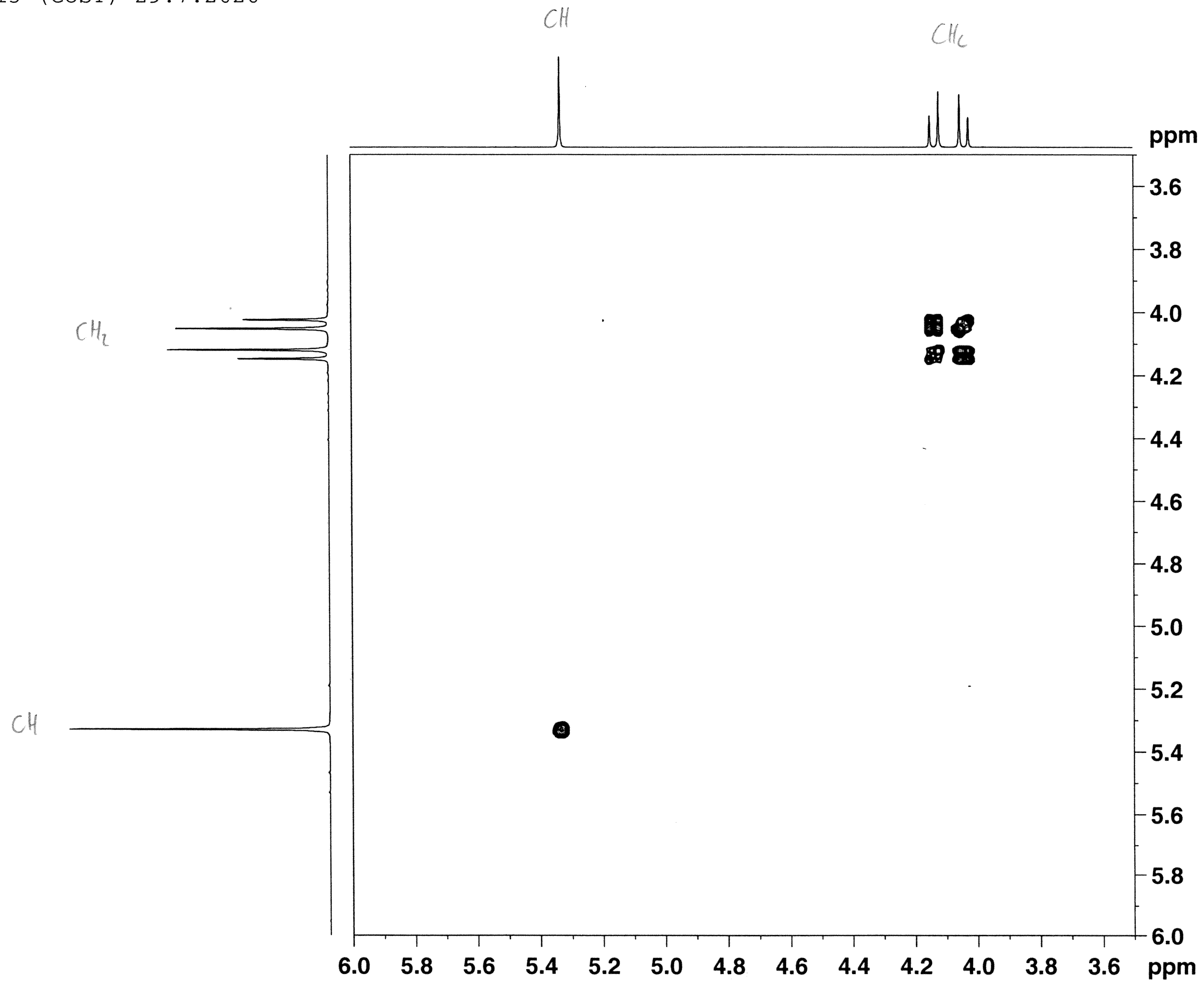

PN014-1 in cdcl3 (HSQC) 29.7.2020

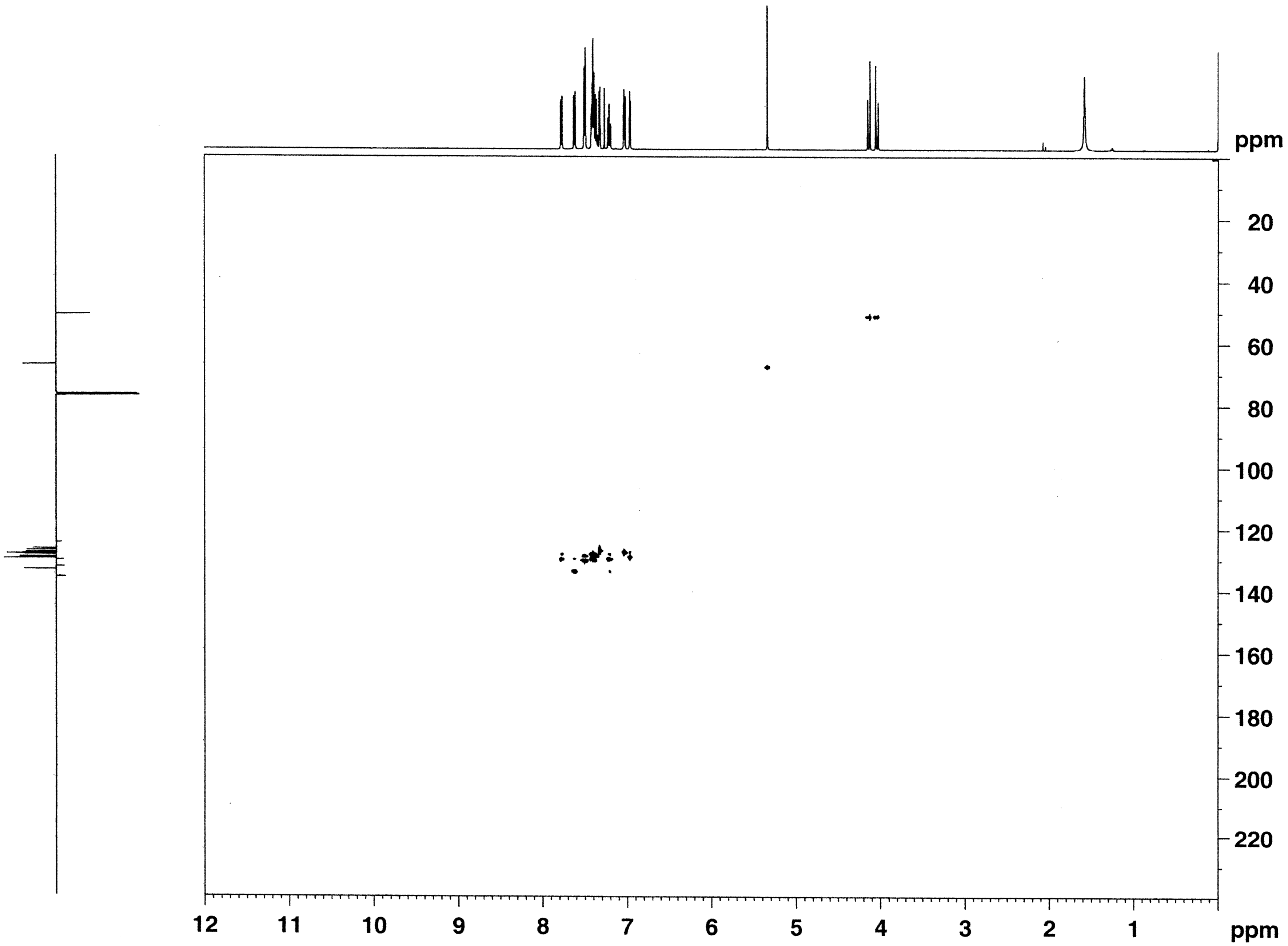

PN014-1 in cdcl3 (HSQC) 29.7.2020

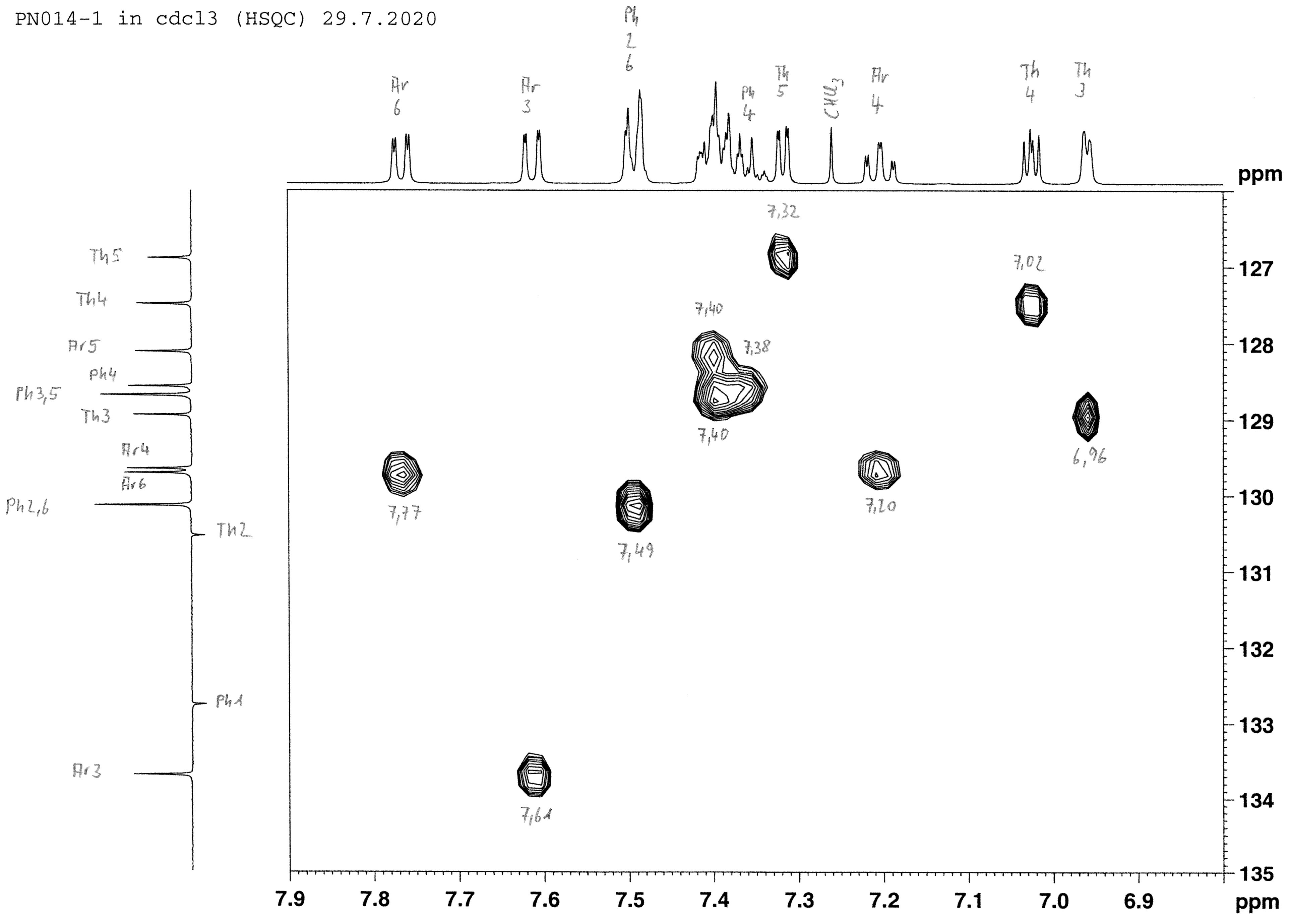

PN014-1 in cdcl3 (HSQC) 29.7.2020

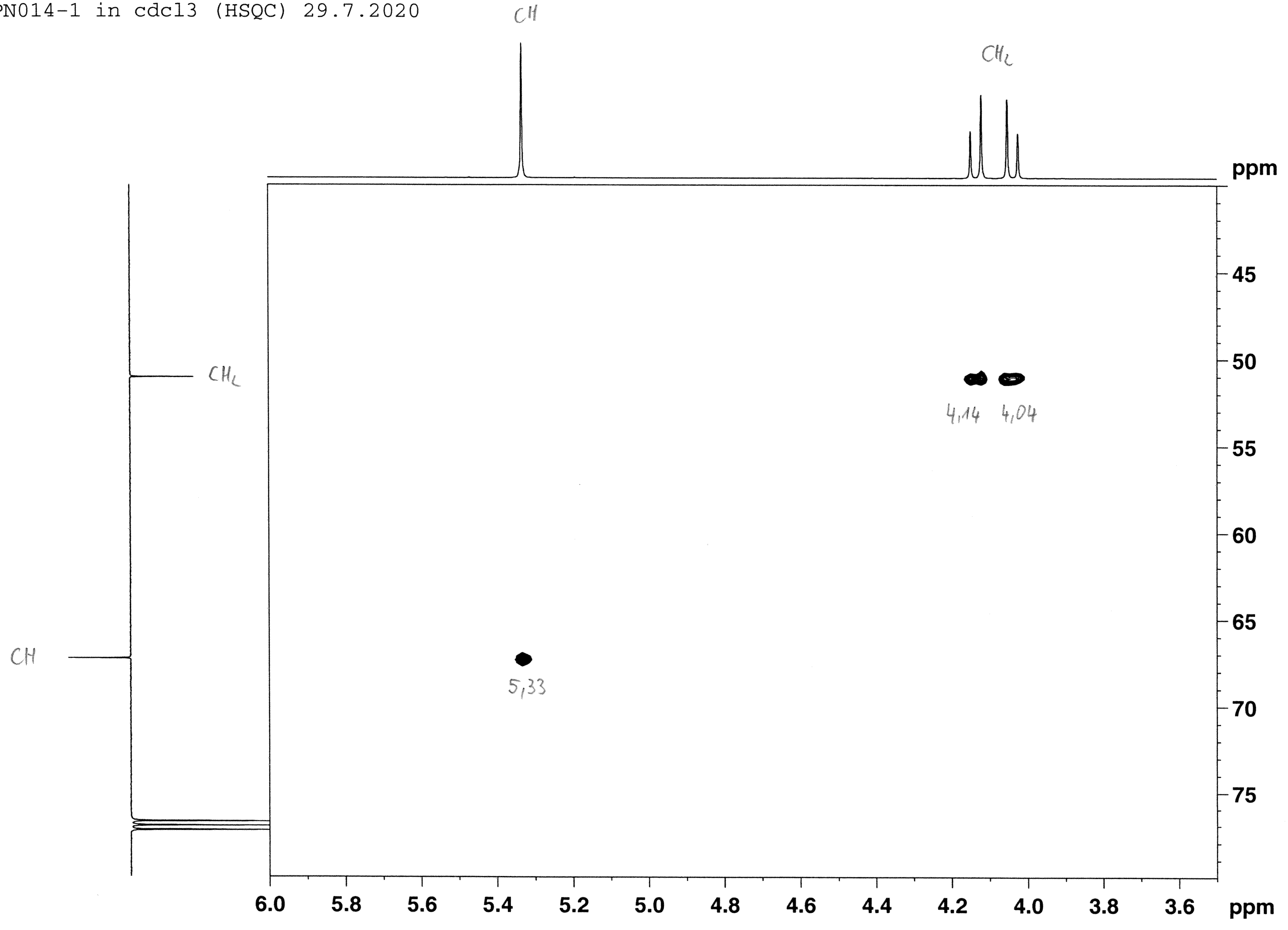

PN014-1 in cdcl3 (HMBC) 29.7.2020

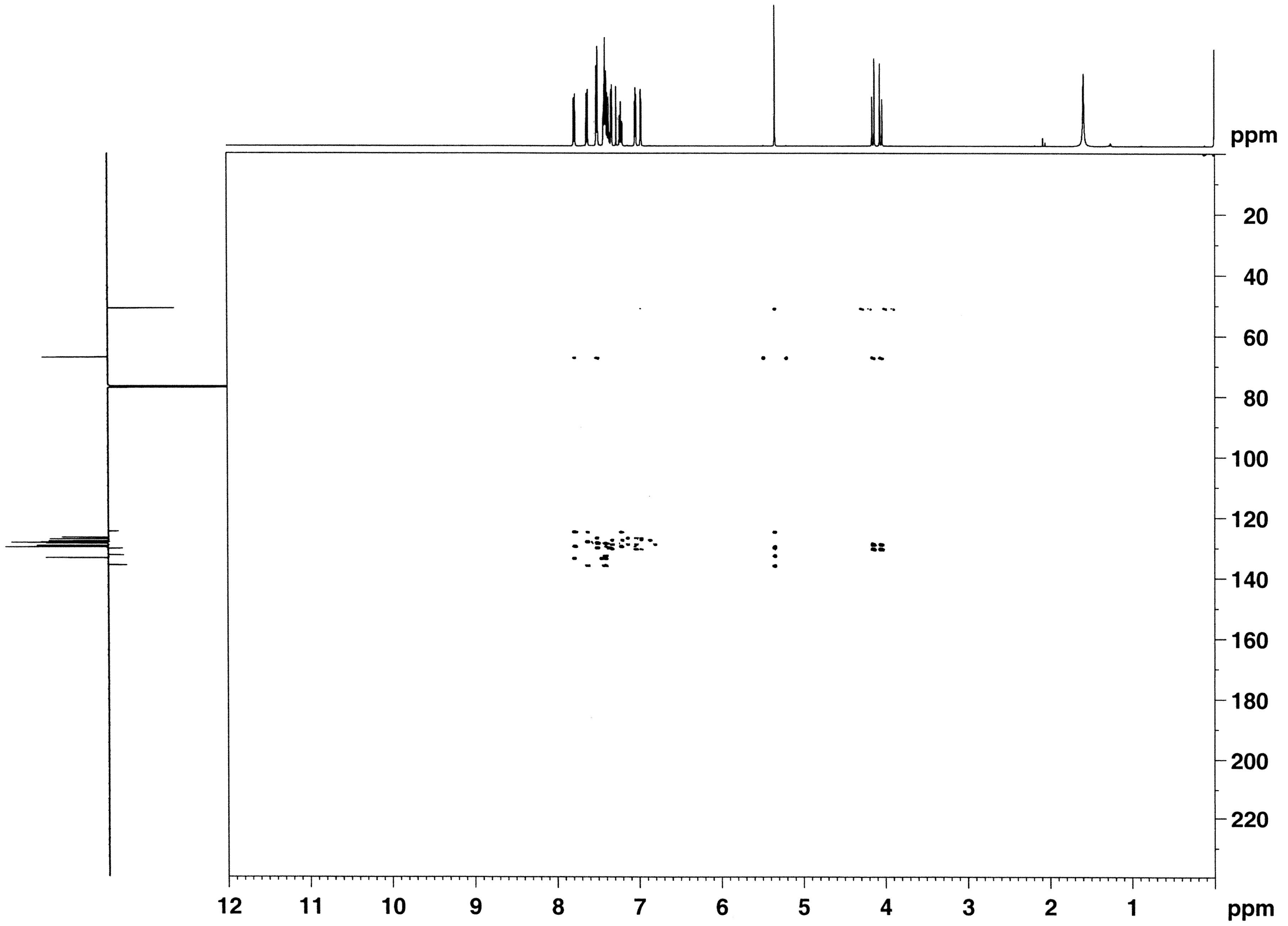

PN014-1 in cdcl3 (HMBC) 29.7.2020

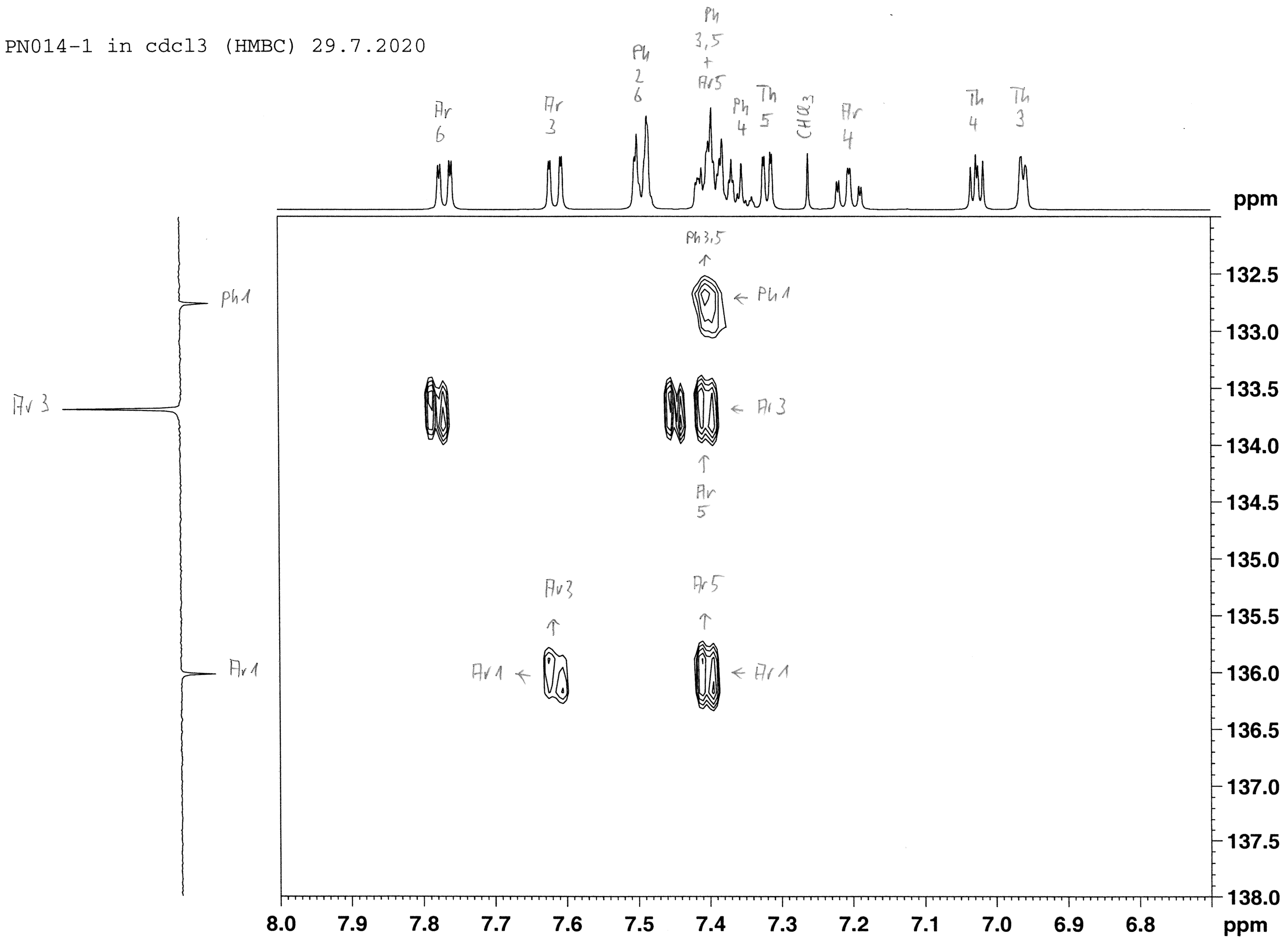

PN014-1 in cdcl3 (HMBC) 29.7.2020

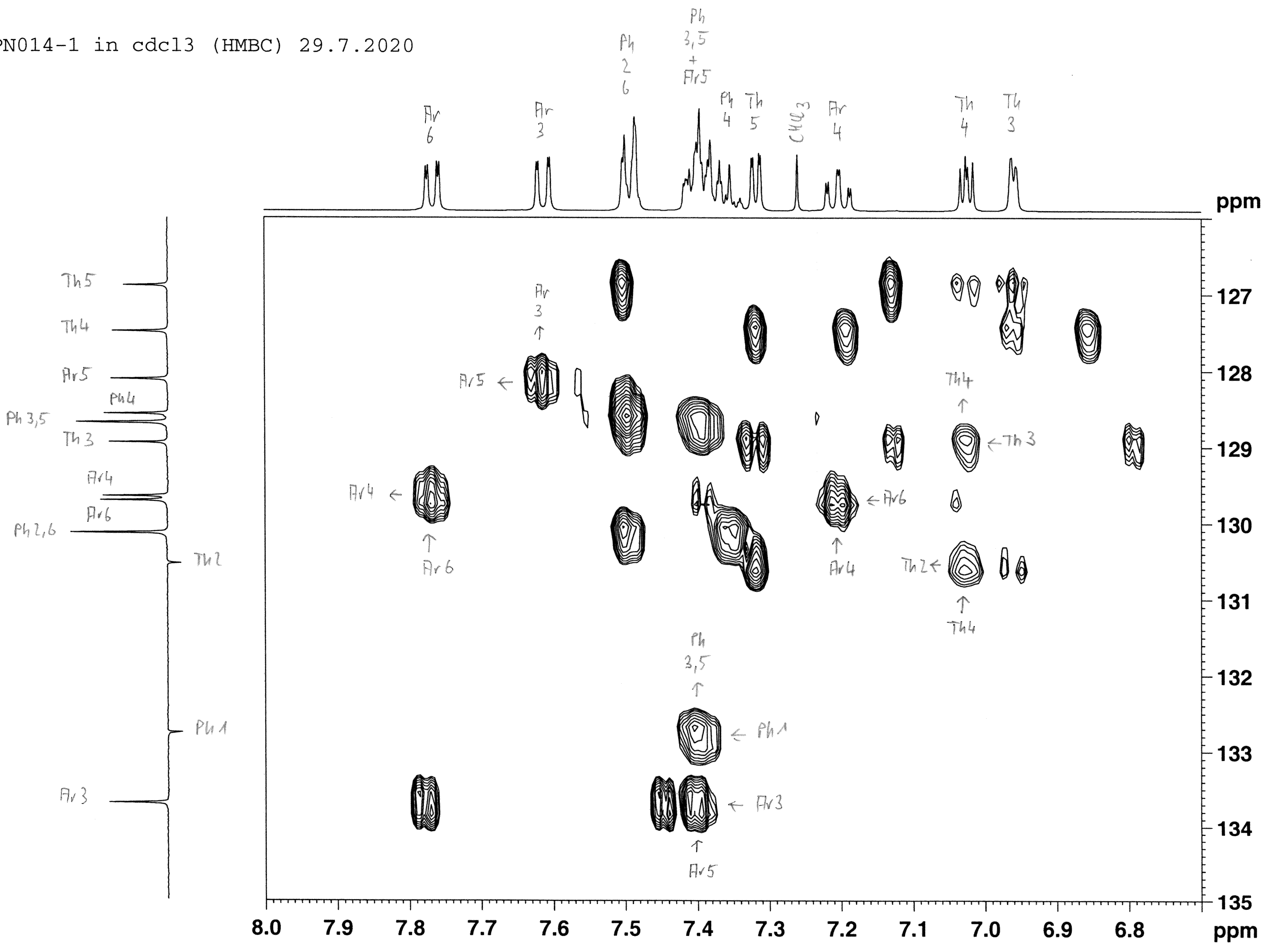

PN014-1 in cdcl3 (HMBC) 29.7.2020

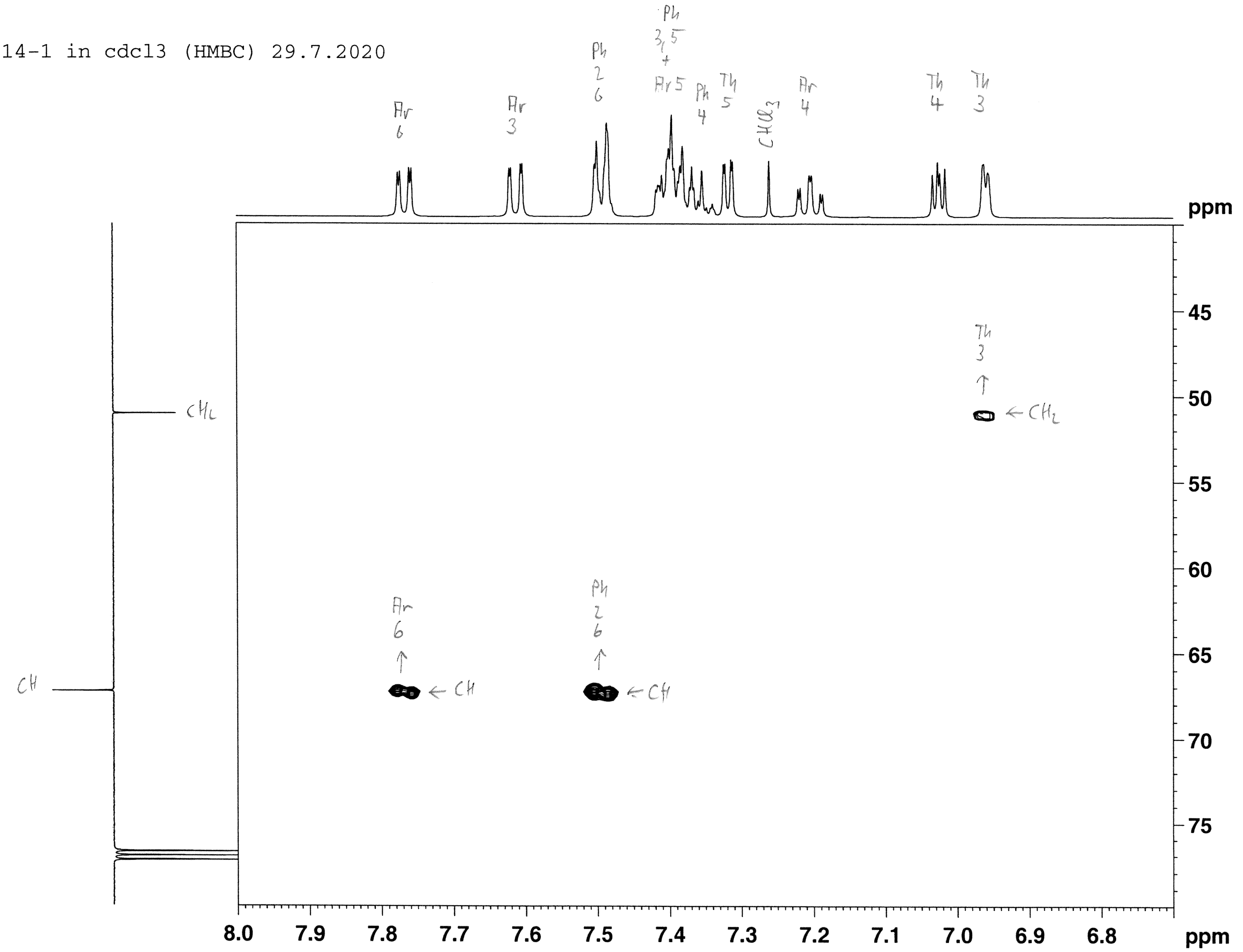

PN014-1 in cdcl3 (HMBC) 29.7.2020

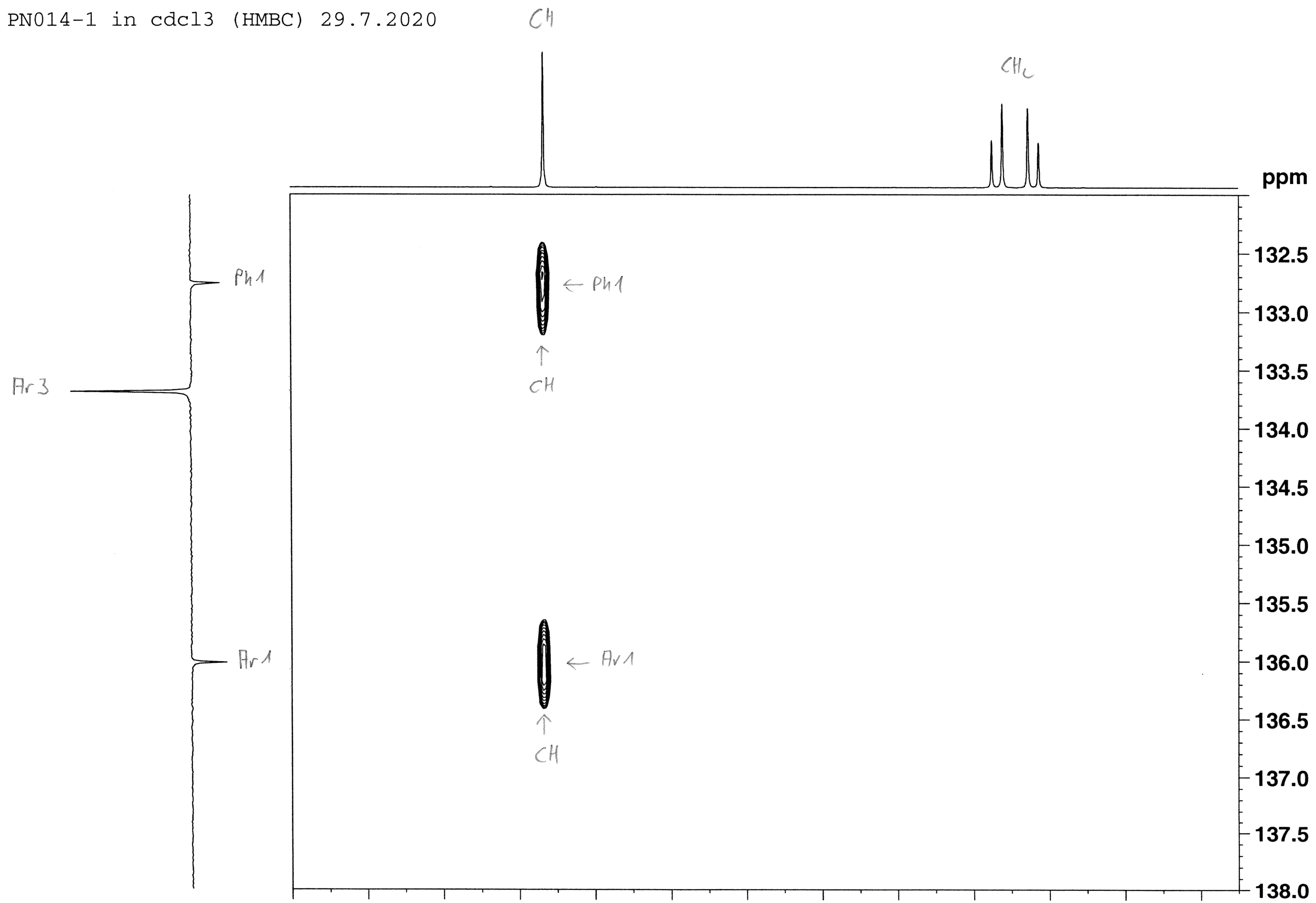

PN014-1 in cdcl3 (HMBC) 29.7.2020

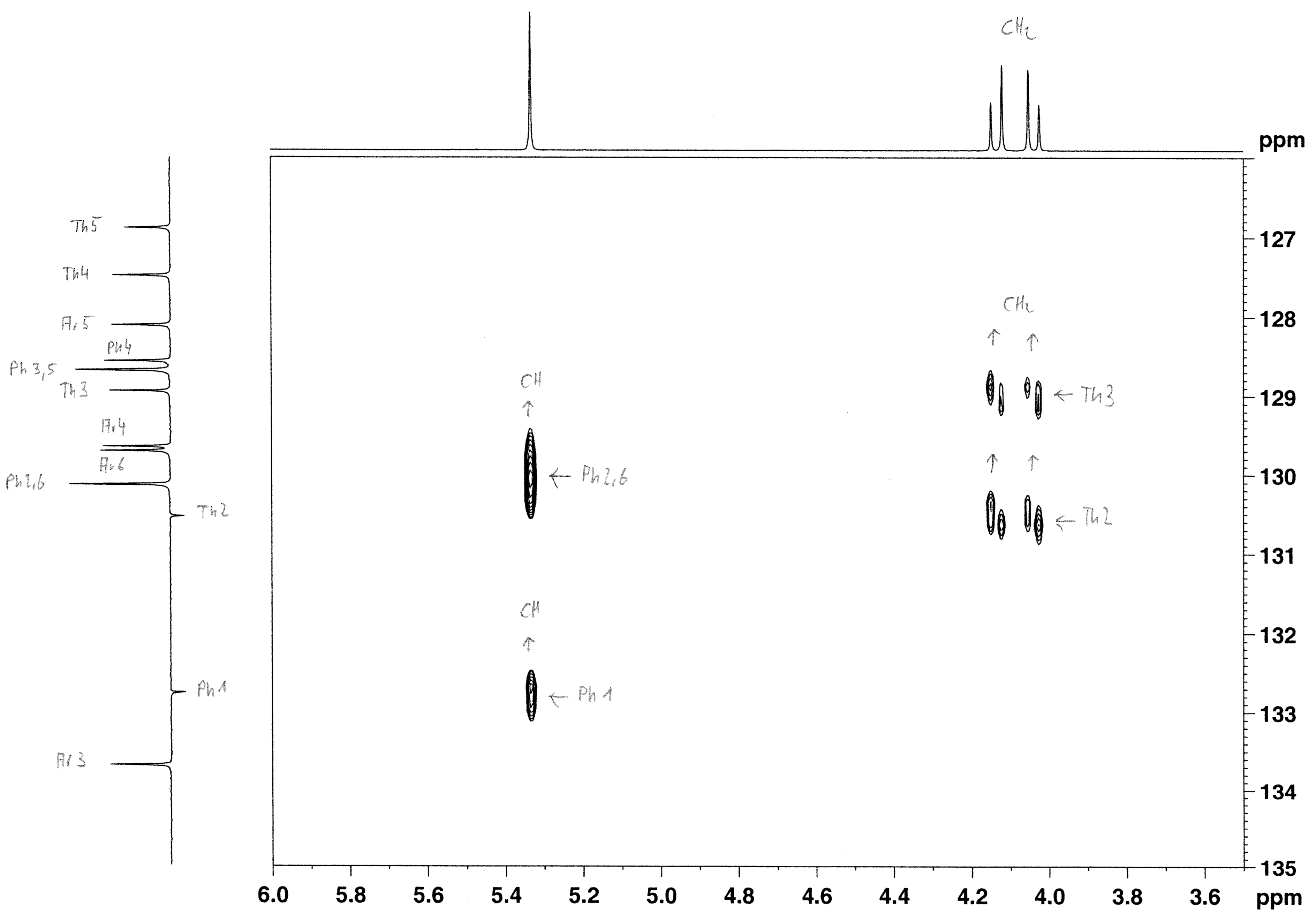

PN014-1 in cdcl3 (HMBC) 29.7.2020

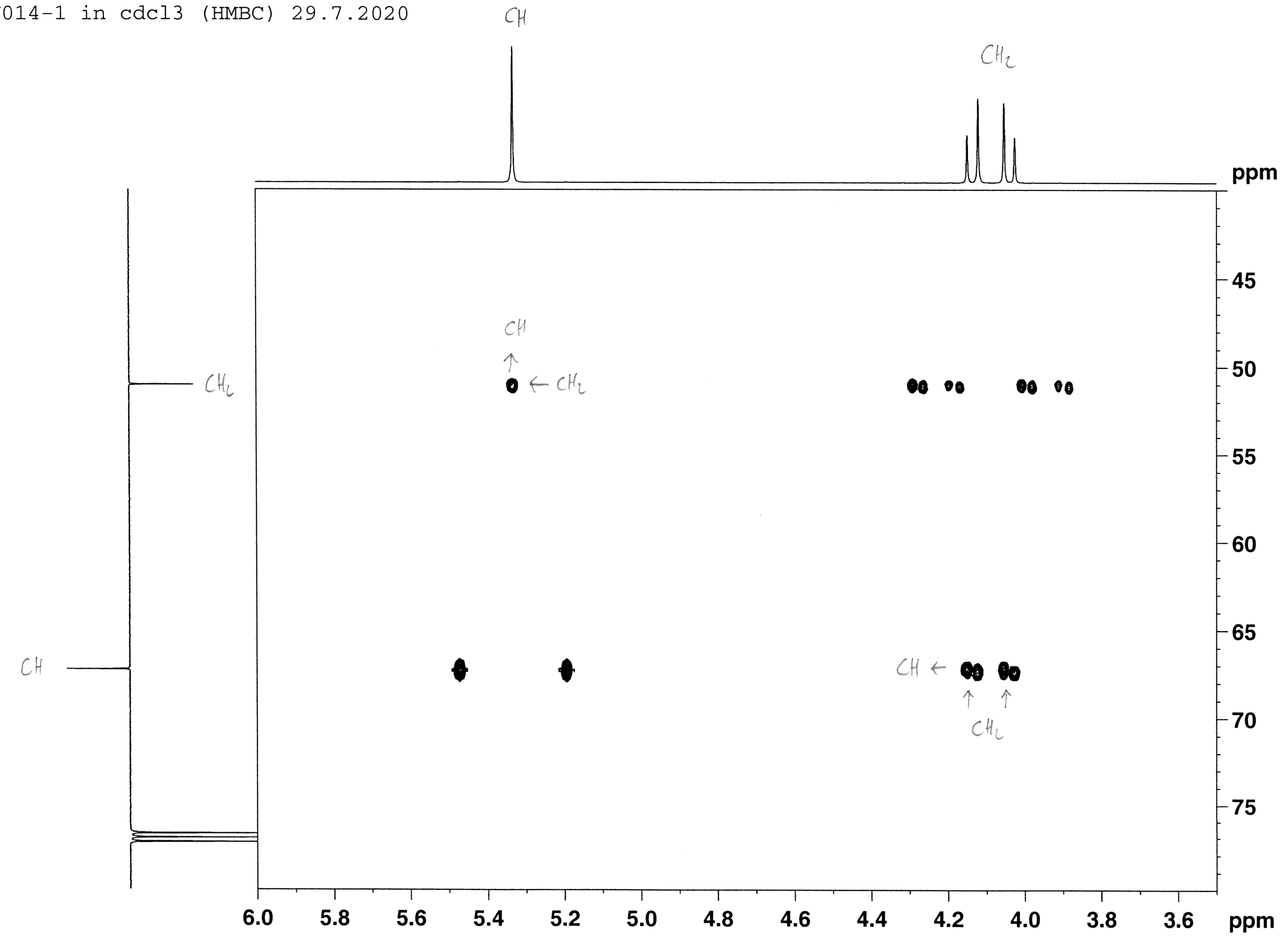

Figure S26c. NMR spectra of compound **6g**.

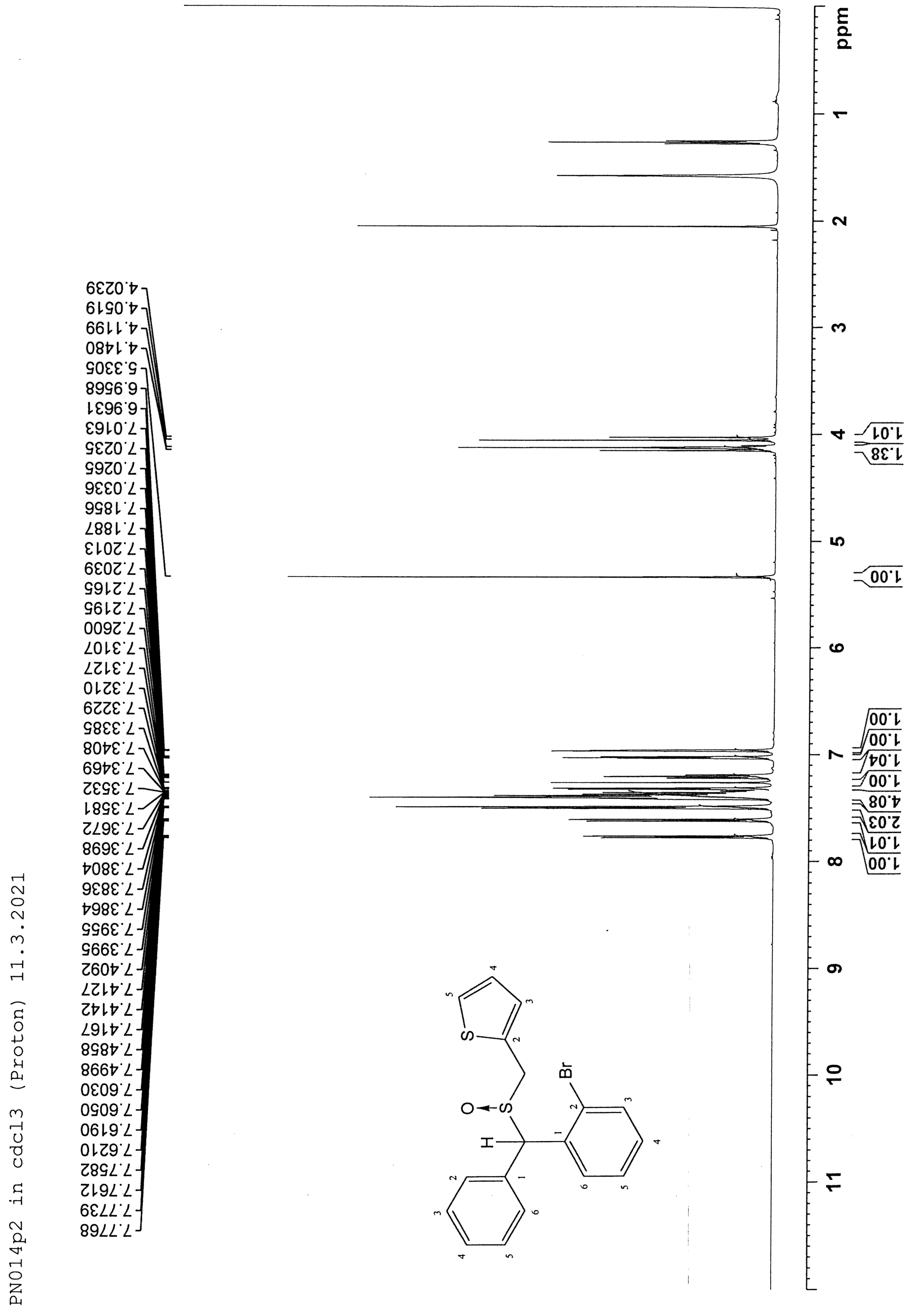

PN014p2 in cdcl3 (Proton) 11.3.2021

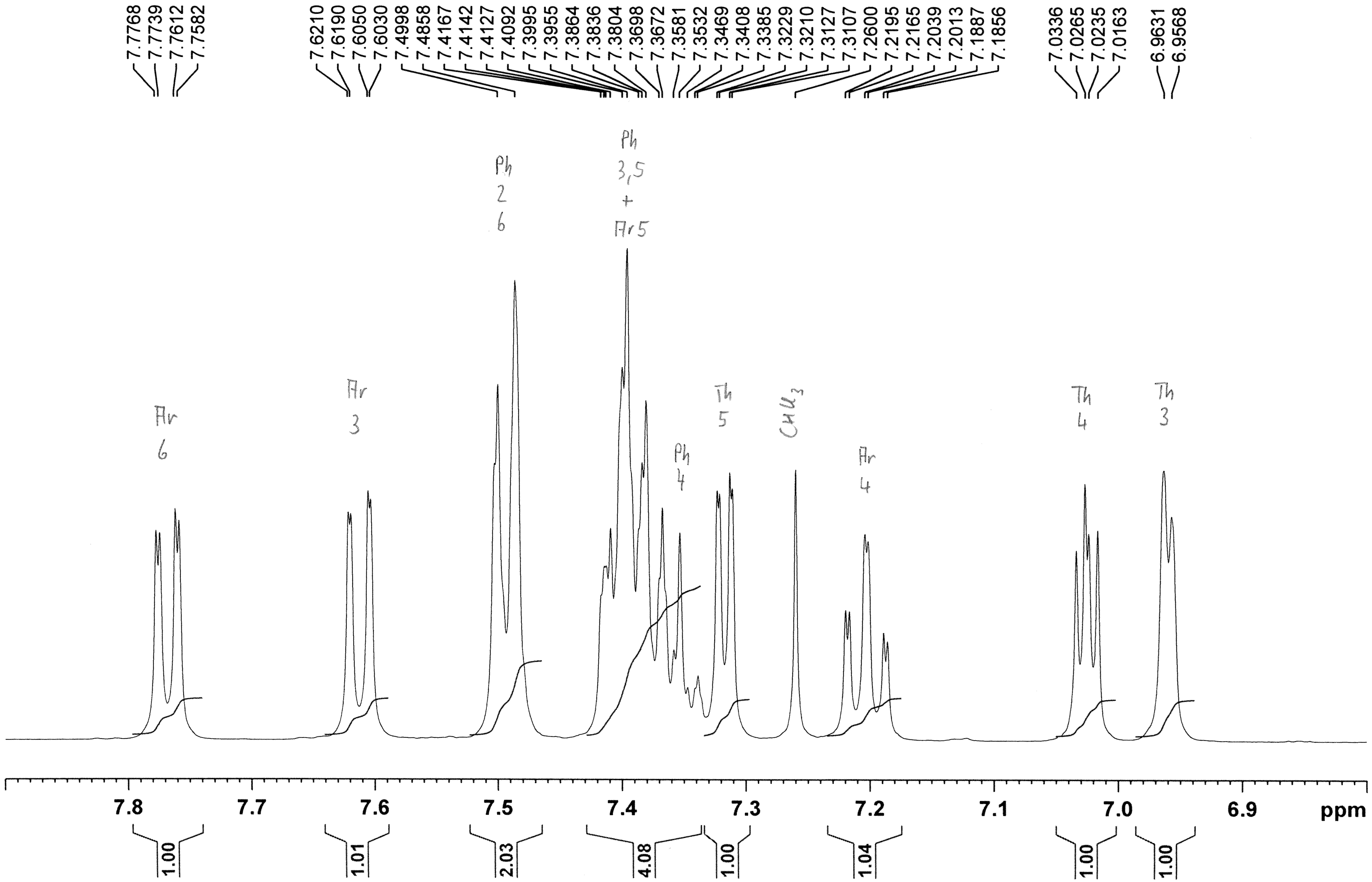

PN014p2 in cdcl3 (Proton) 11.3.2021

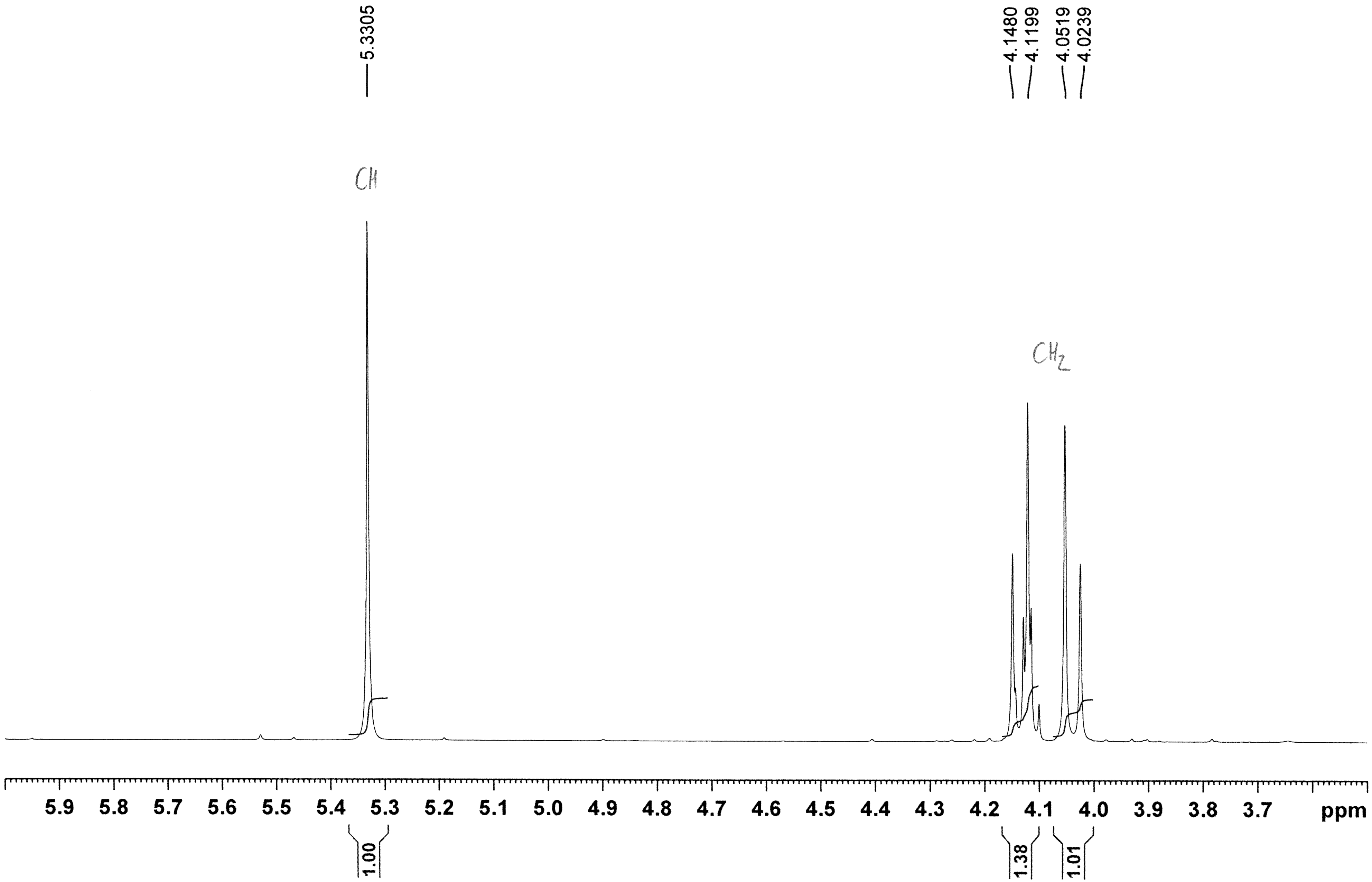

PN014p2 in cdcl3 (APT) 11.3.2021

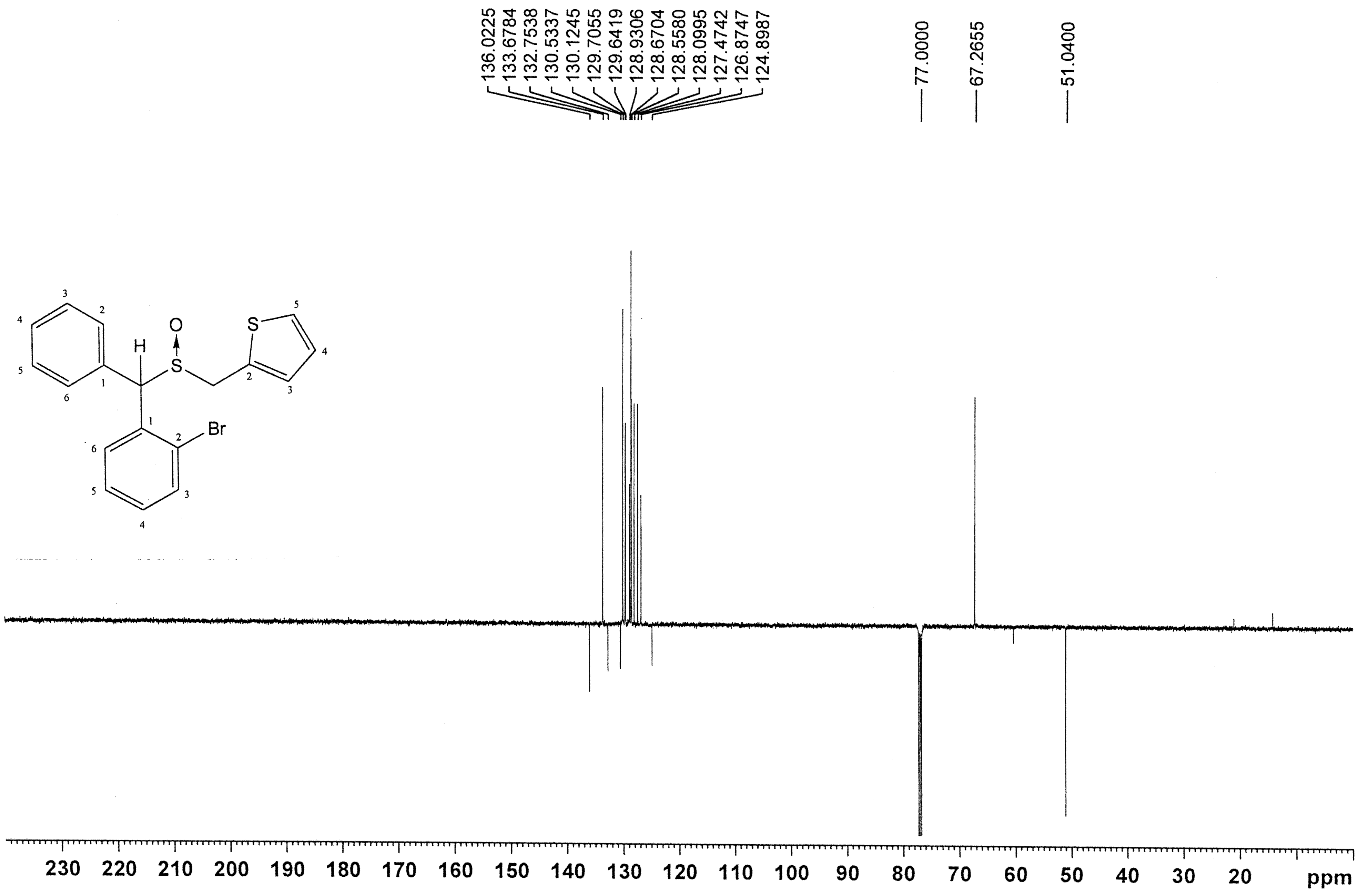

PN014p2 in cdcl3 (APT) 11.3.2021

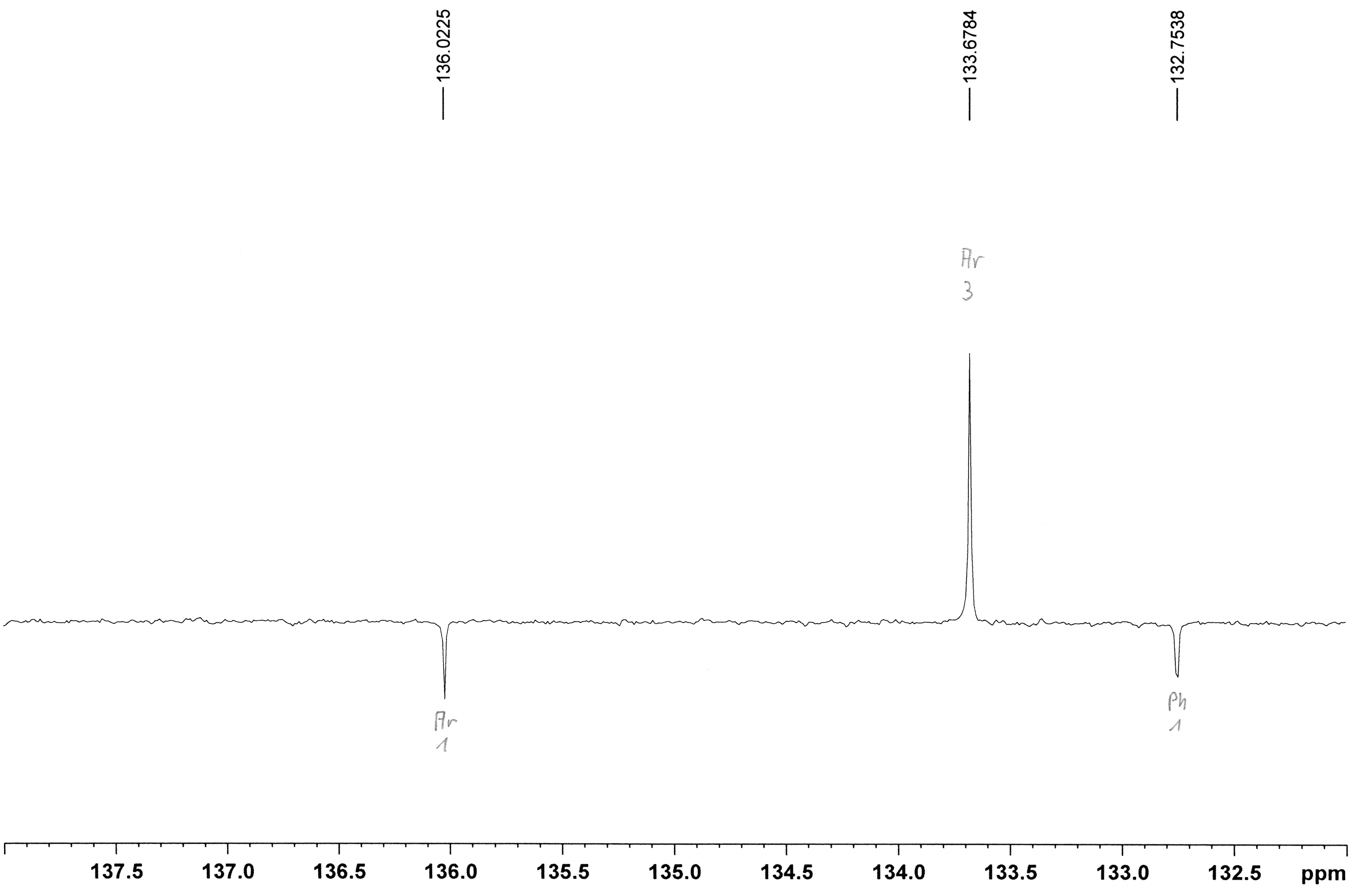

PN014p2 in cdcl3 (APT) 11.3.2021

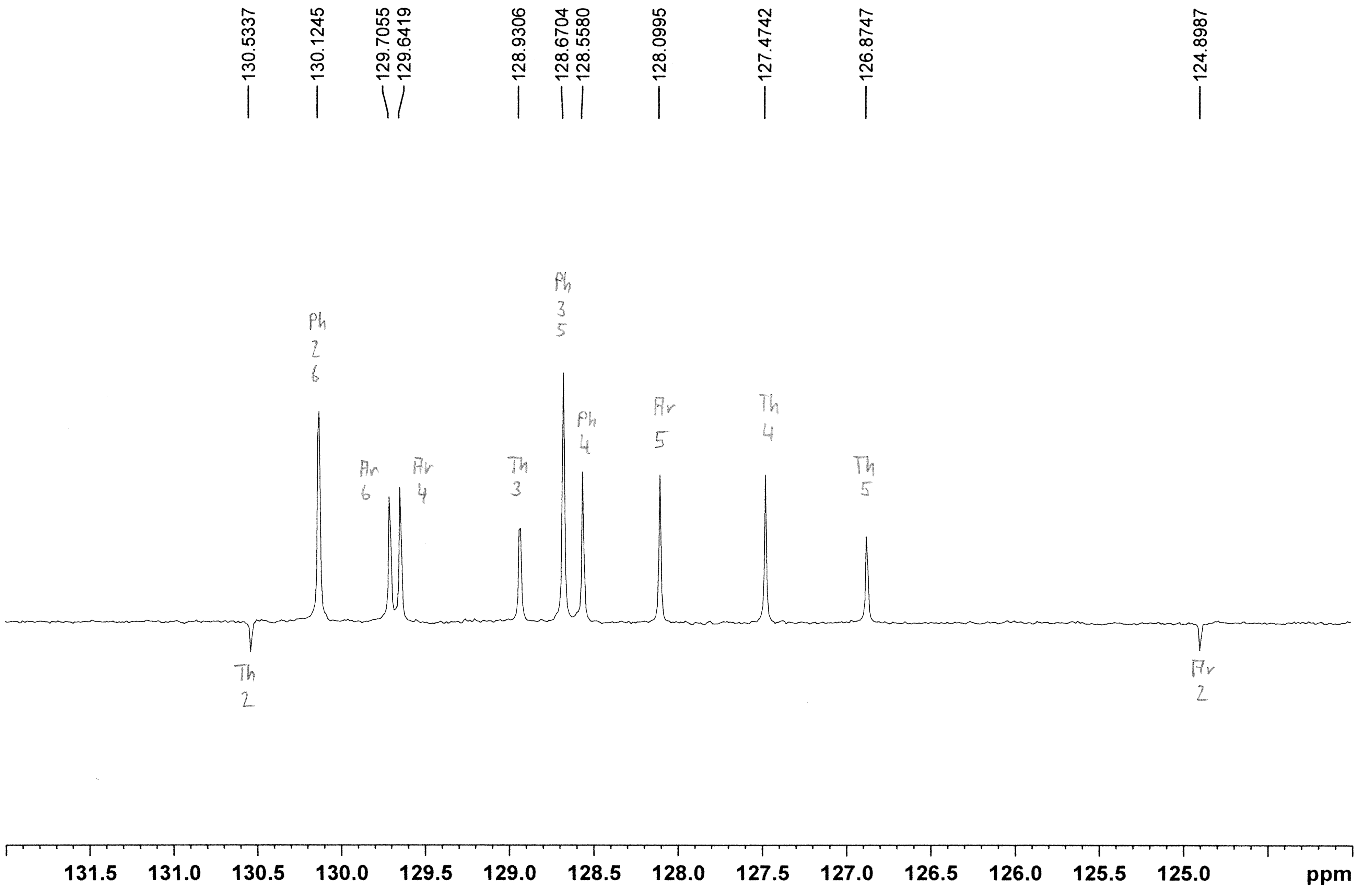

PN014p2 in cdcl3 (APT) 11.3.2021

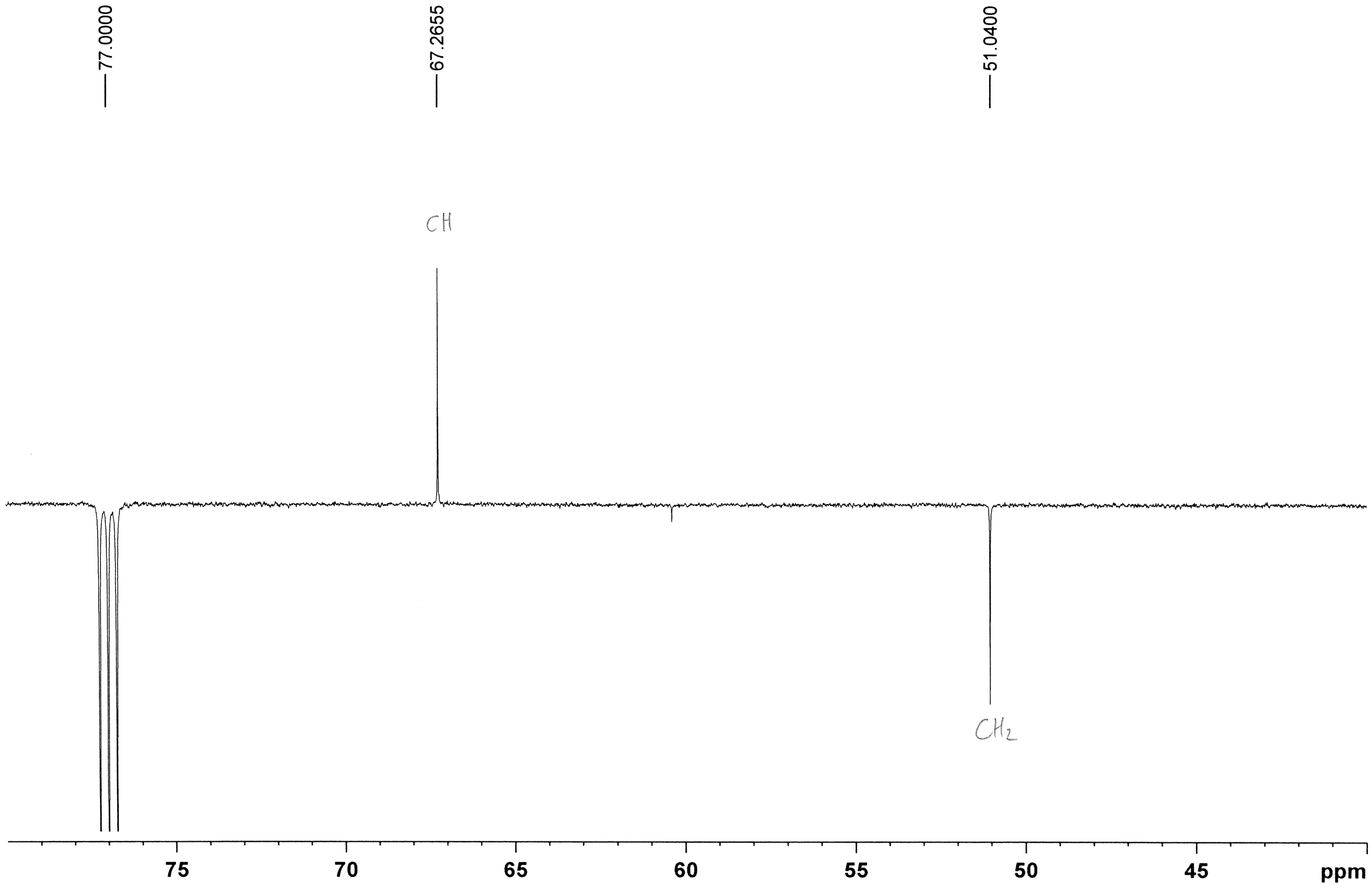

PN014p2 in cdcl3 (COSY) 11.3.2021

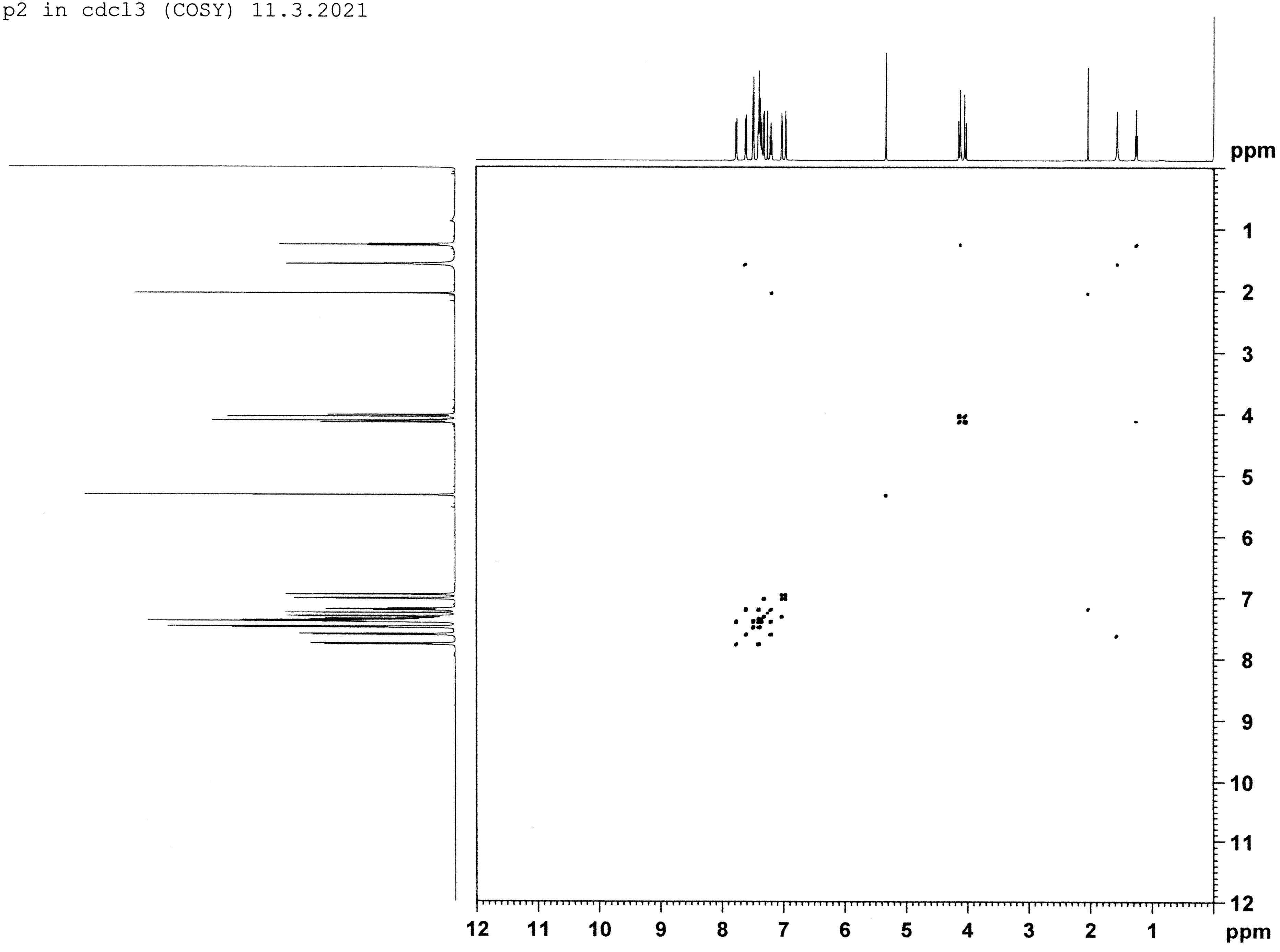

PN014p2 in cdcl3 (COSY) 11.3.2021

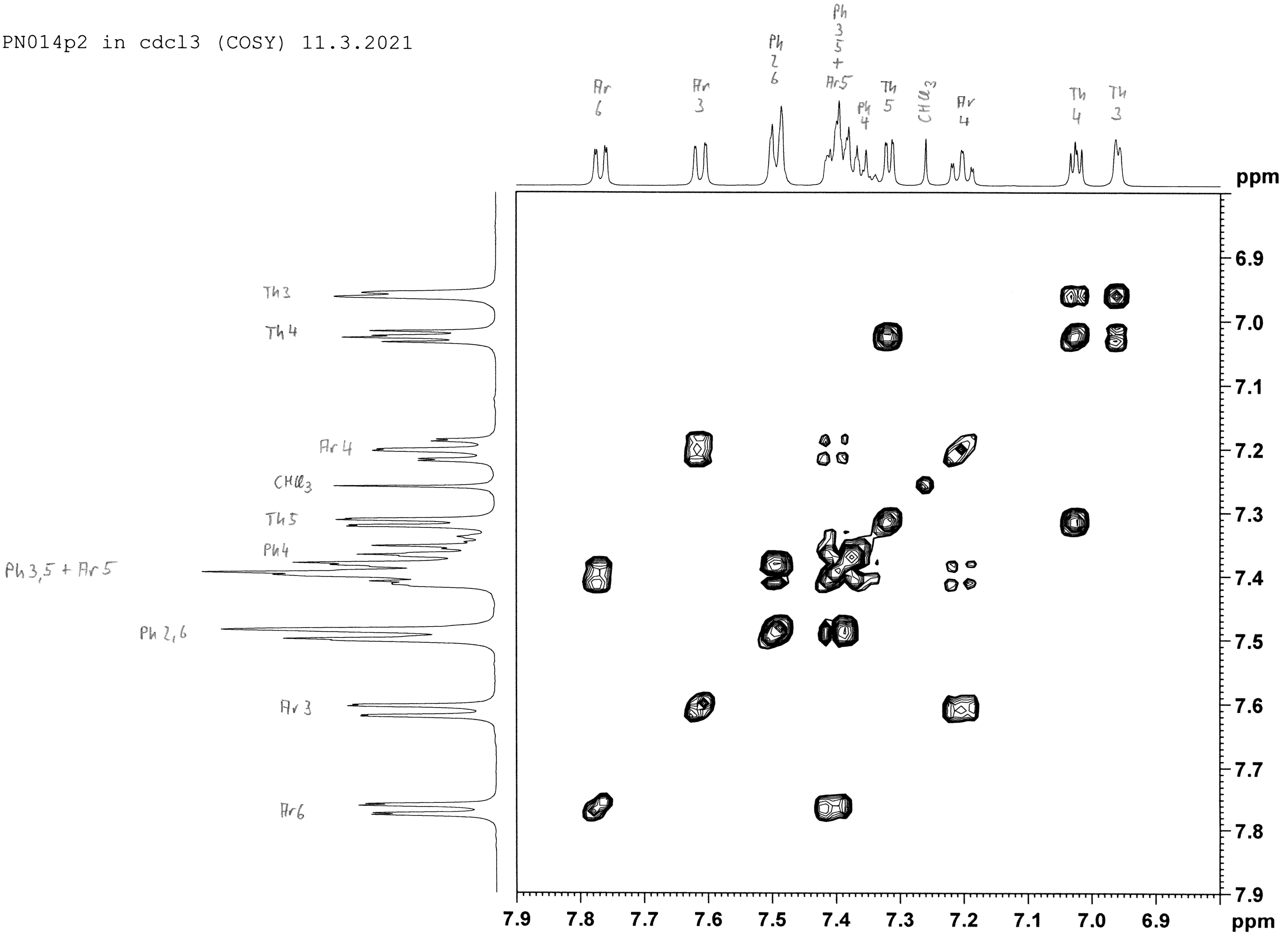

PN014p2 in cdcl3 (COSY) 11.3.2021

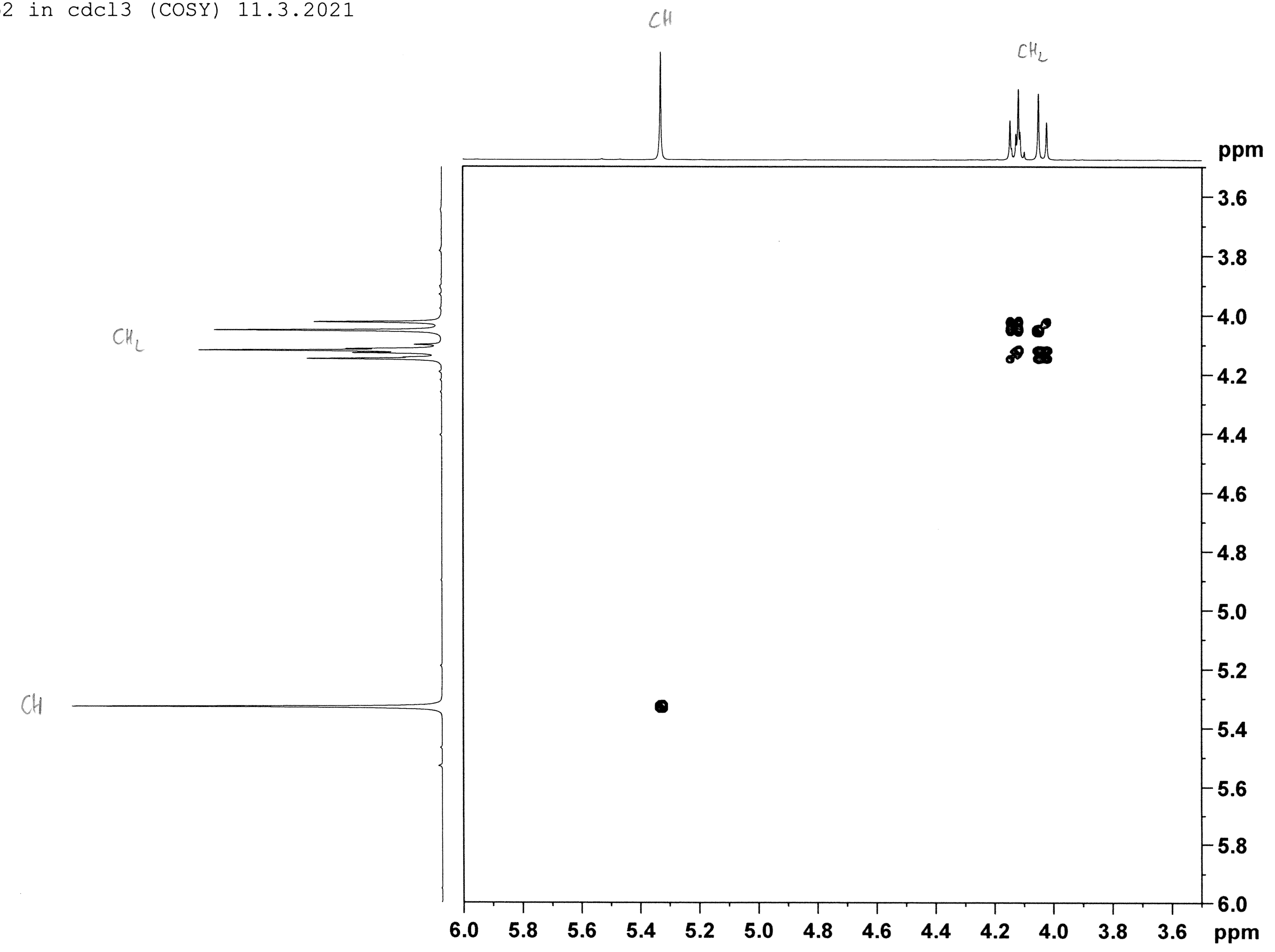

PN014p2 in cdcl3 (HSQC) 11.3.2021

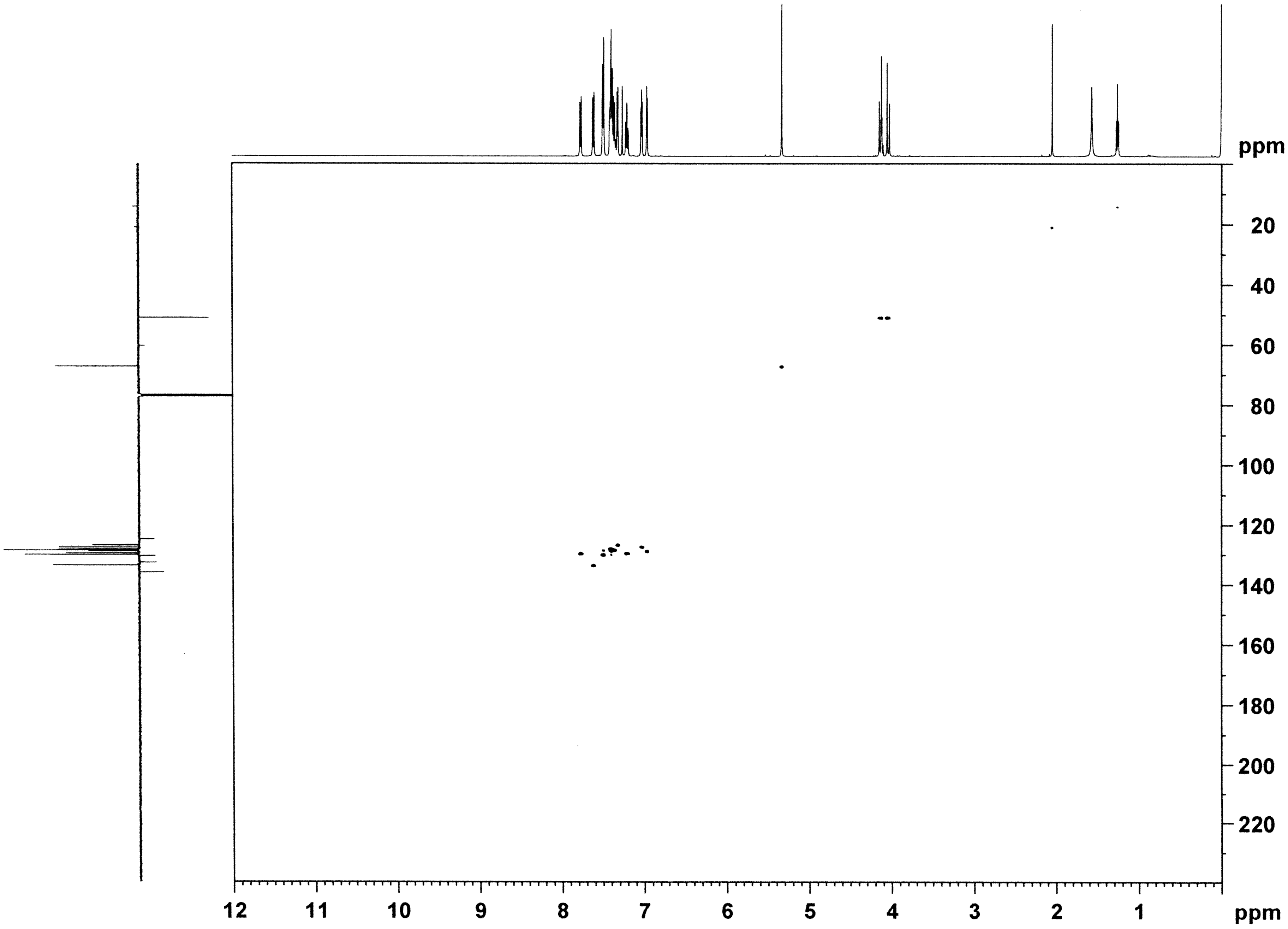

PN014p2 in cdcl3 (HSQC) 11.3.2021

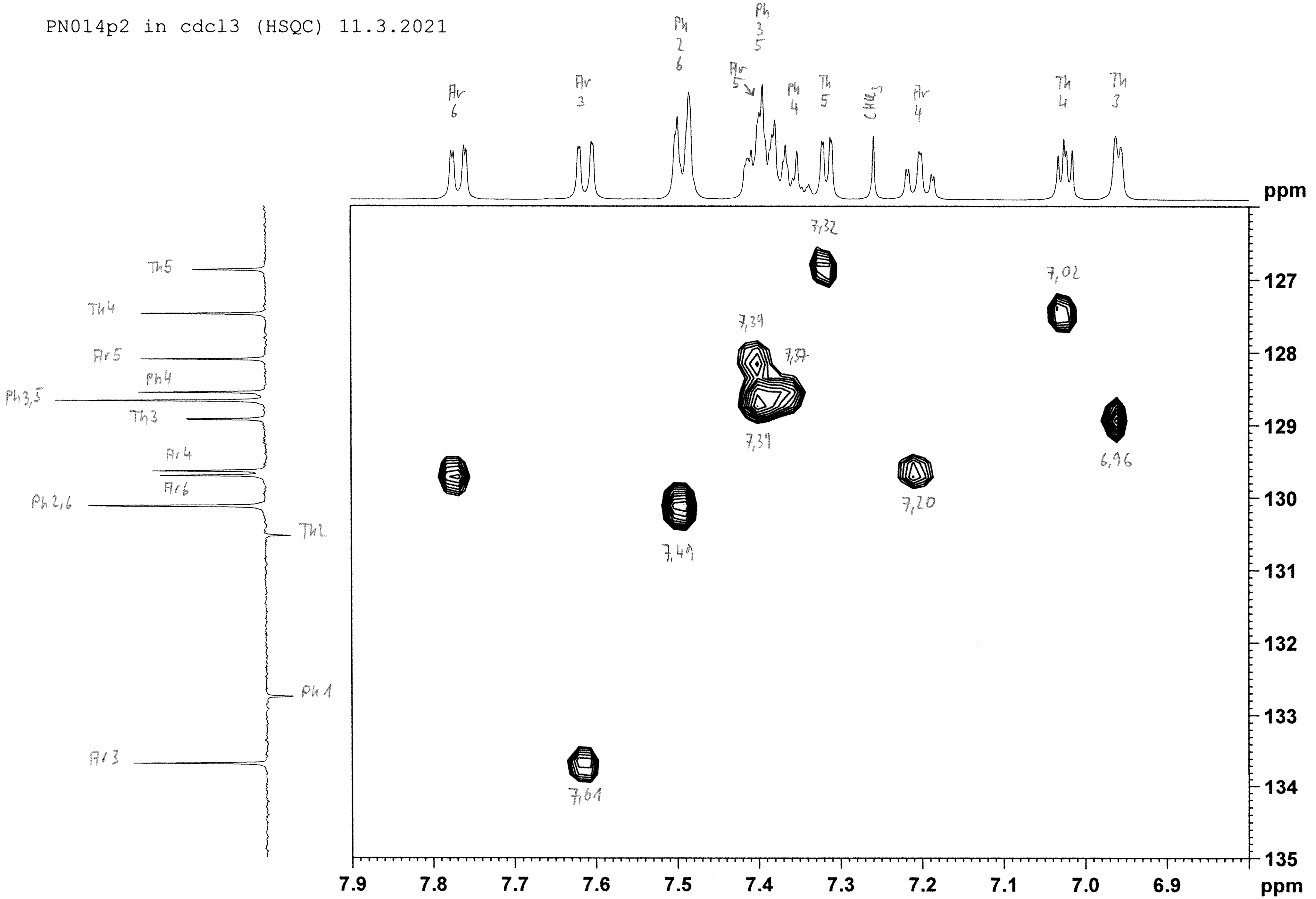

PN014p2 in cdcl3 (HSQC) 11.3.2021

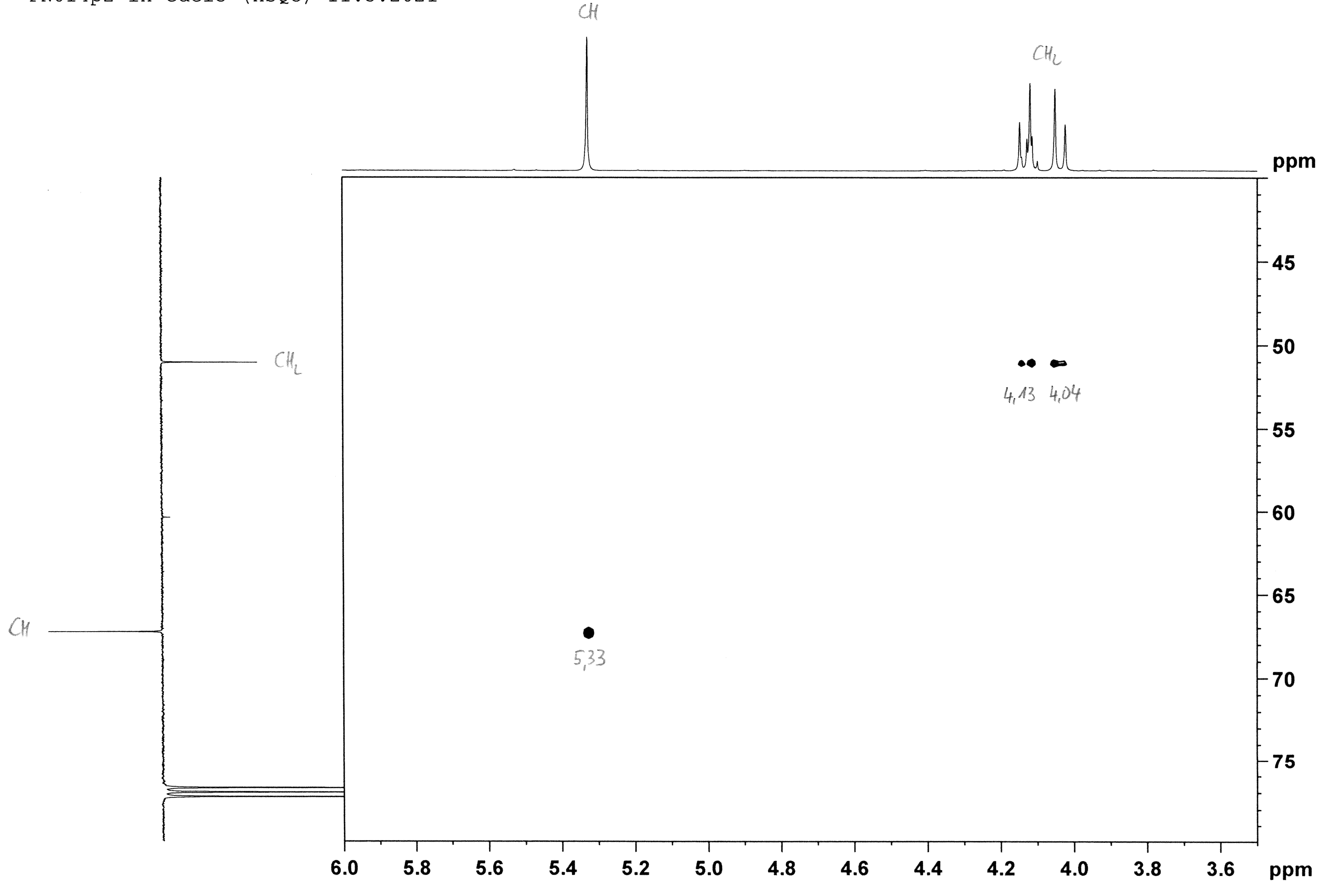

PN014p2 in cdcl3 (HMBC) 11.3.2021

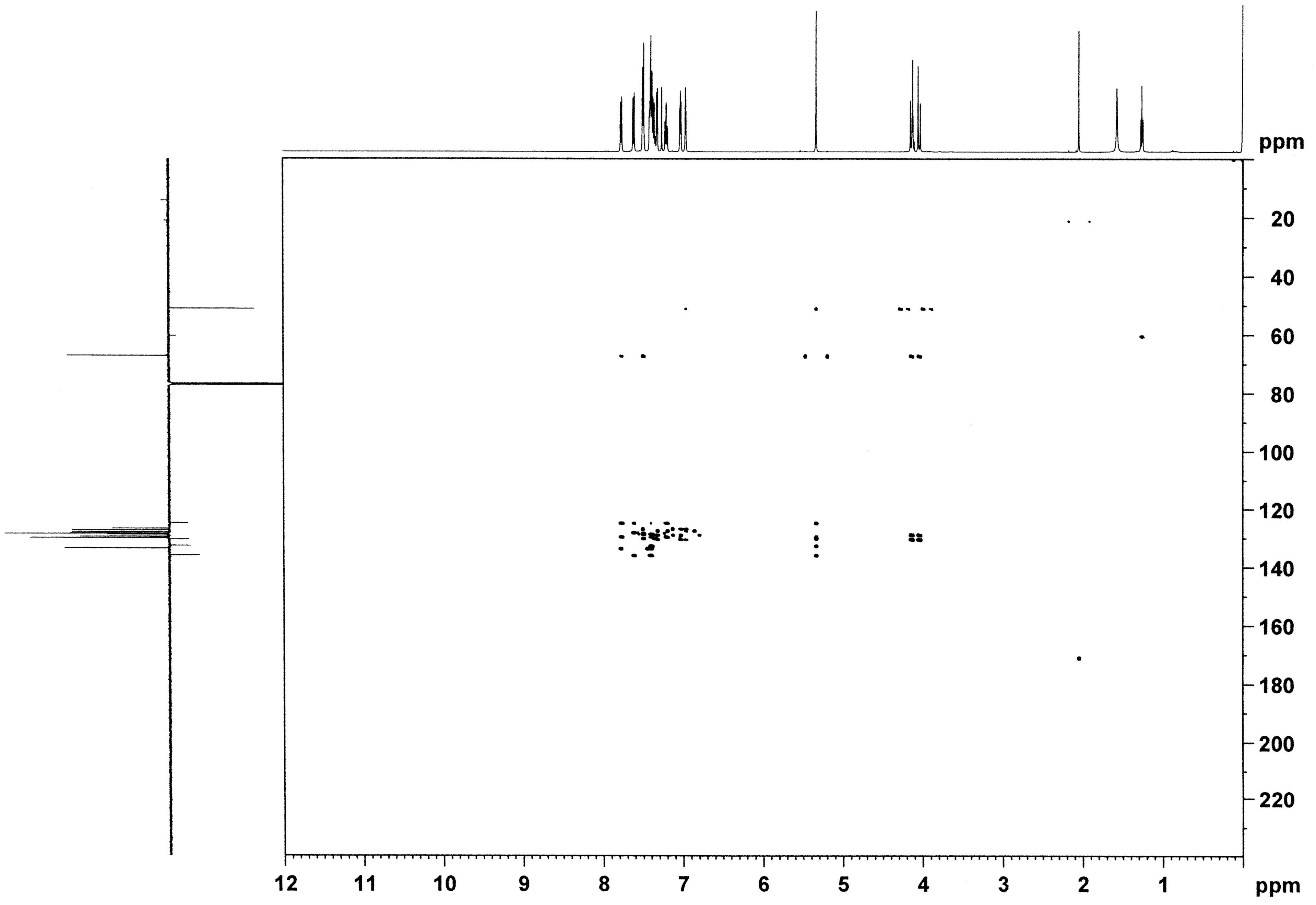

PN014p2 in cdcl3 (HMBC) 11.3.2021

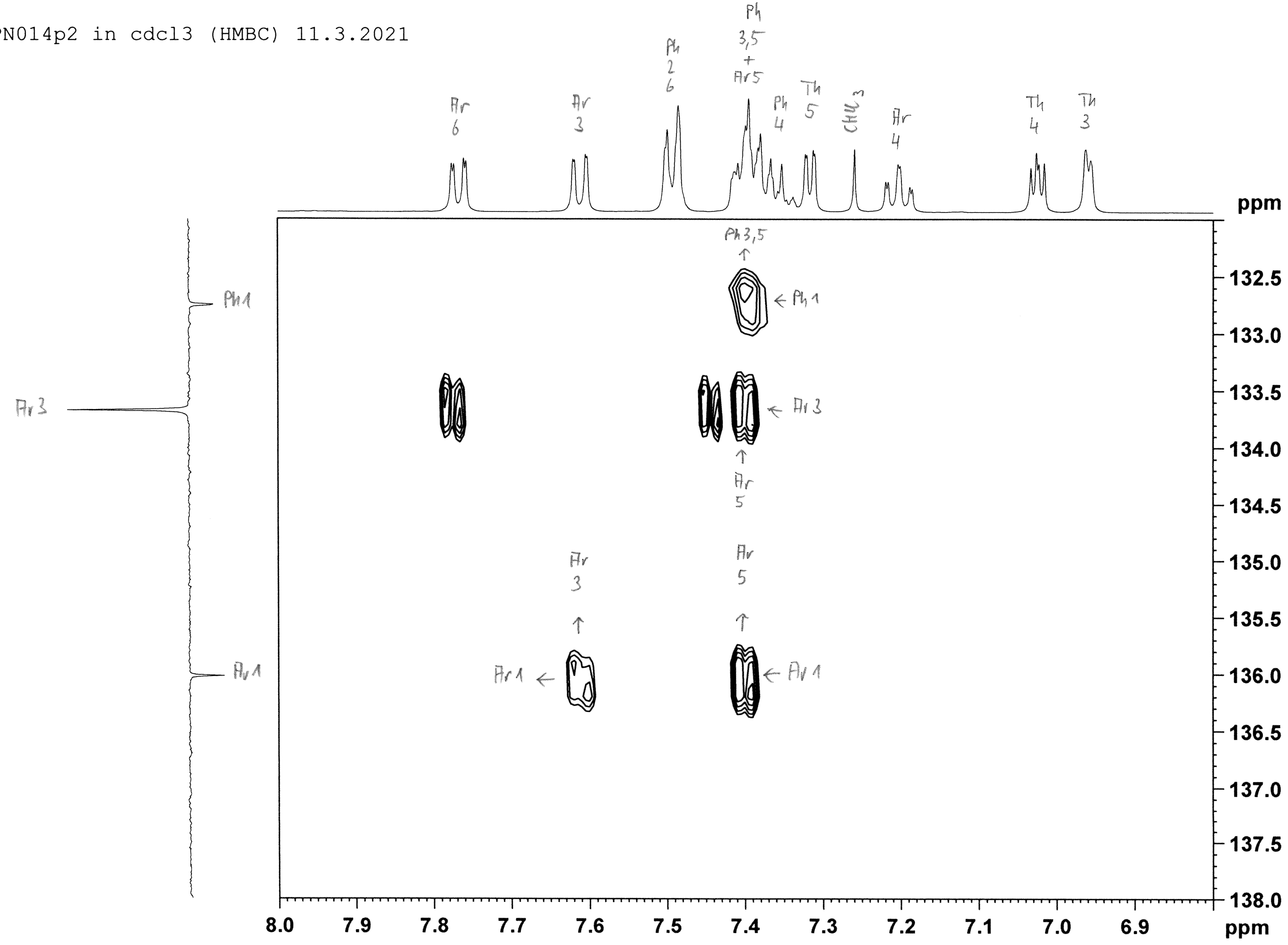

PN014p2 in cdcl3 (HMBC) 11.3.2021

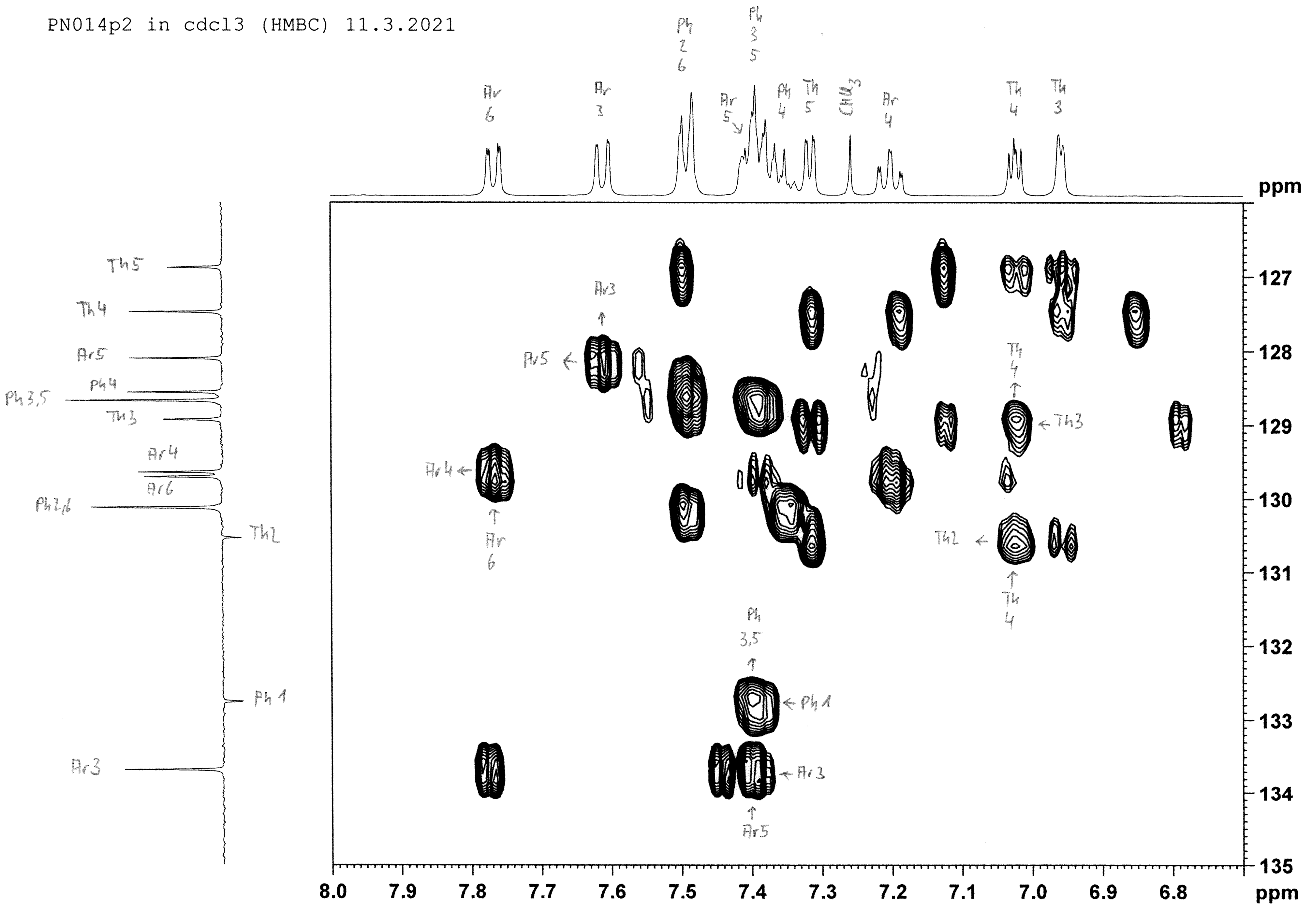

PN014p2 in cdcl3 (HMBC) 11.3.2021

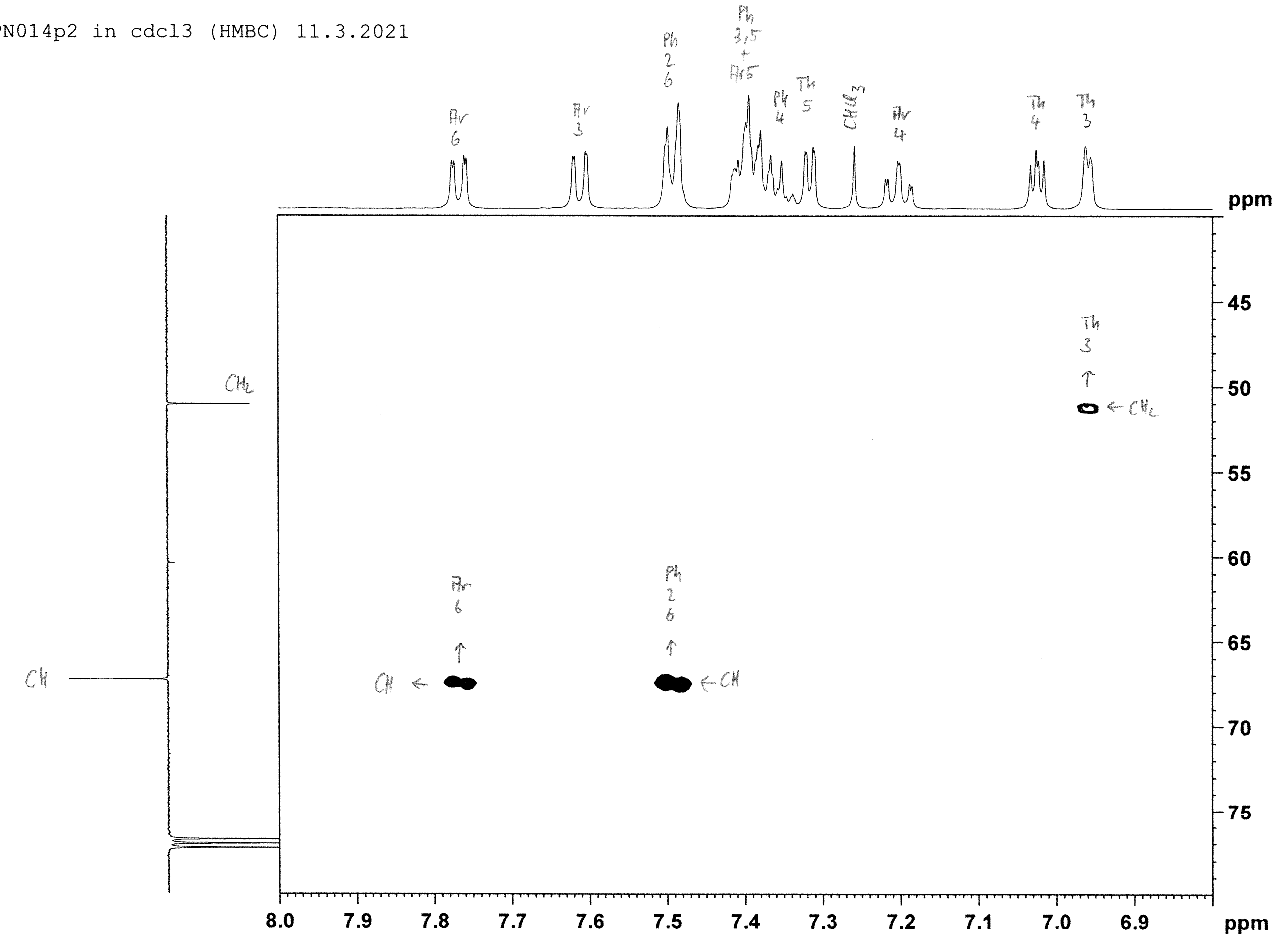

PN014p2 in cdcl3 (HMBC) 11.3.2021

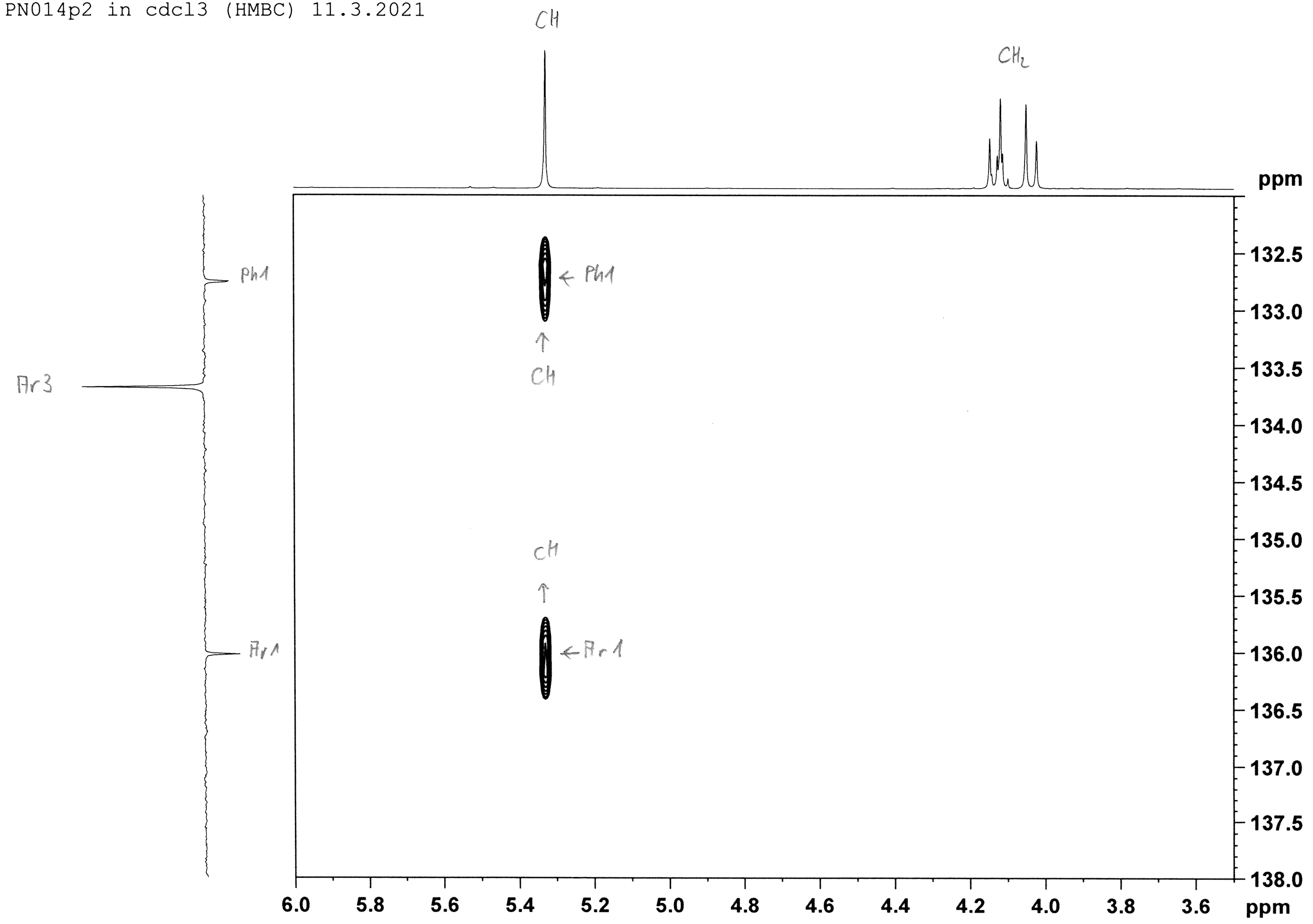

PN014p2 in cdcl3 (HMBC) 11.3.2021

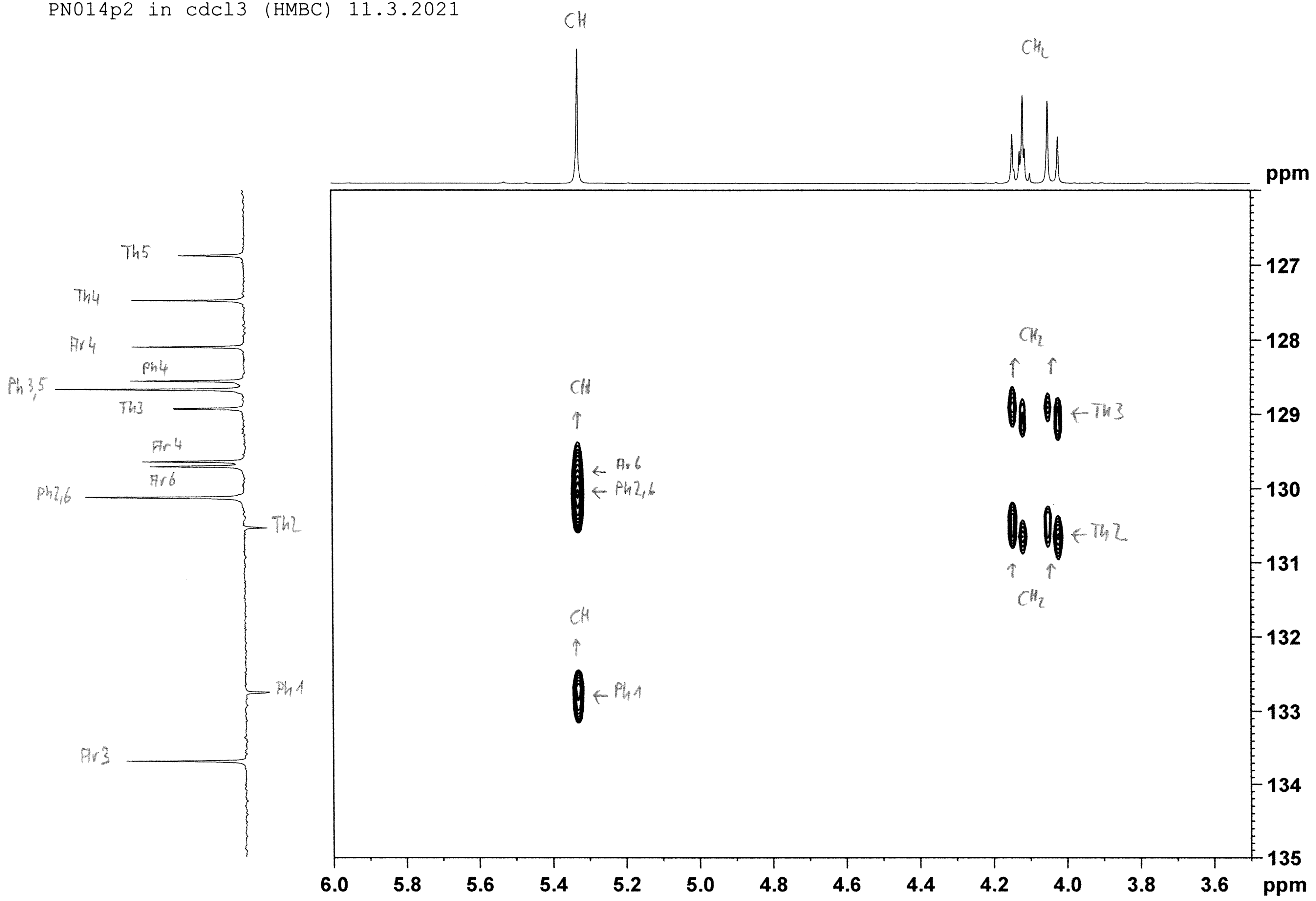

PN014p2 in cdcl3 (HMBC) 11.3.2021

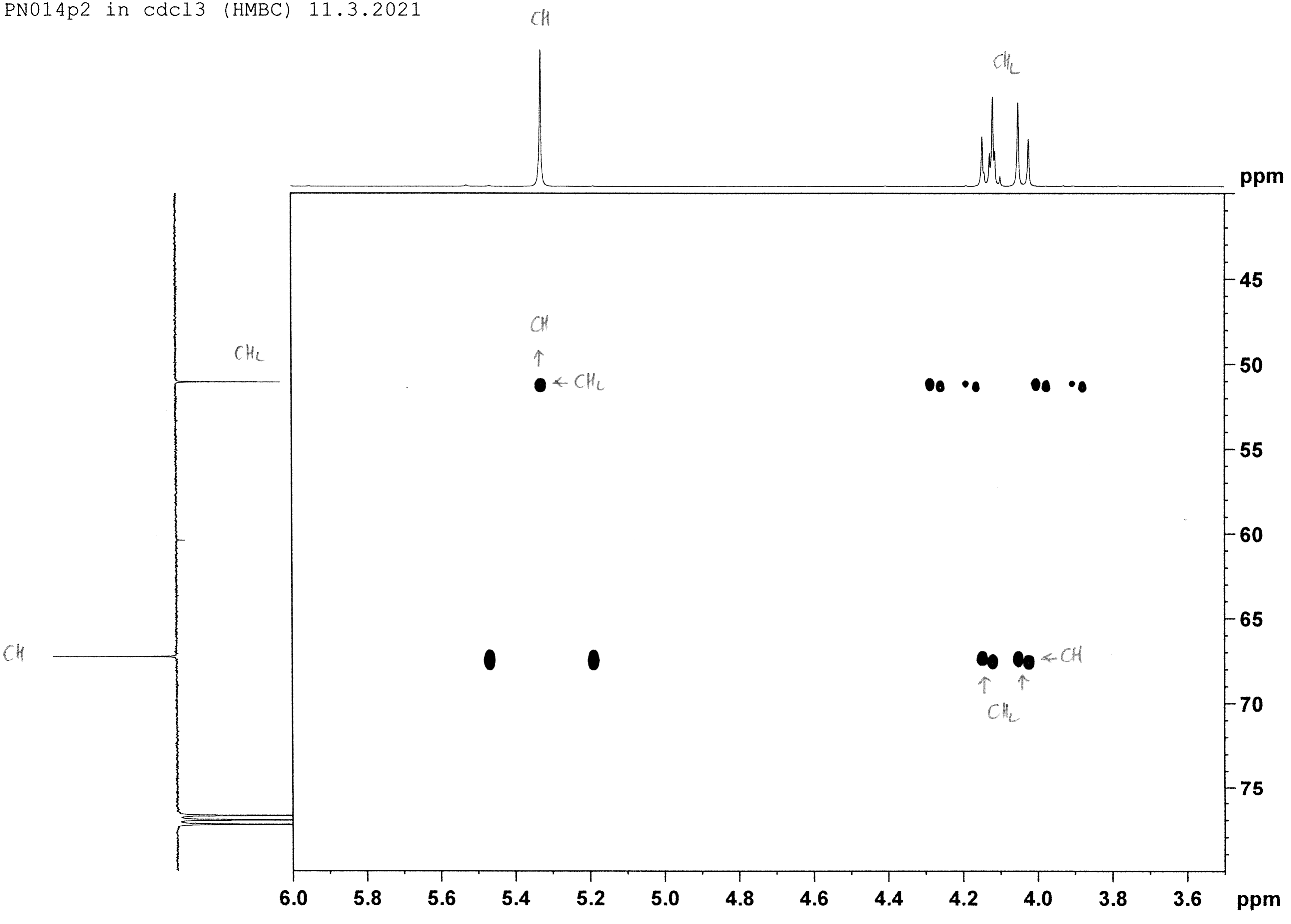

Figure S27c. NMR spectra of compound **7g.**

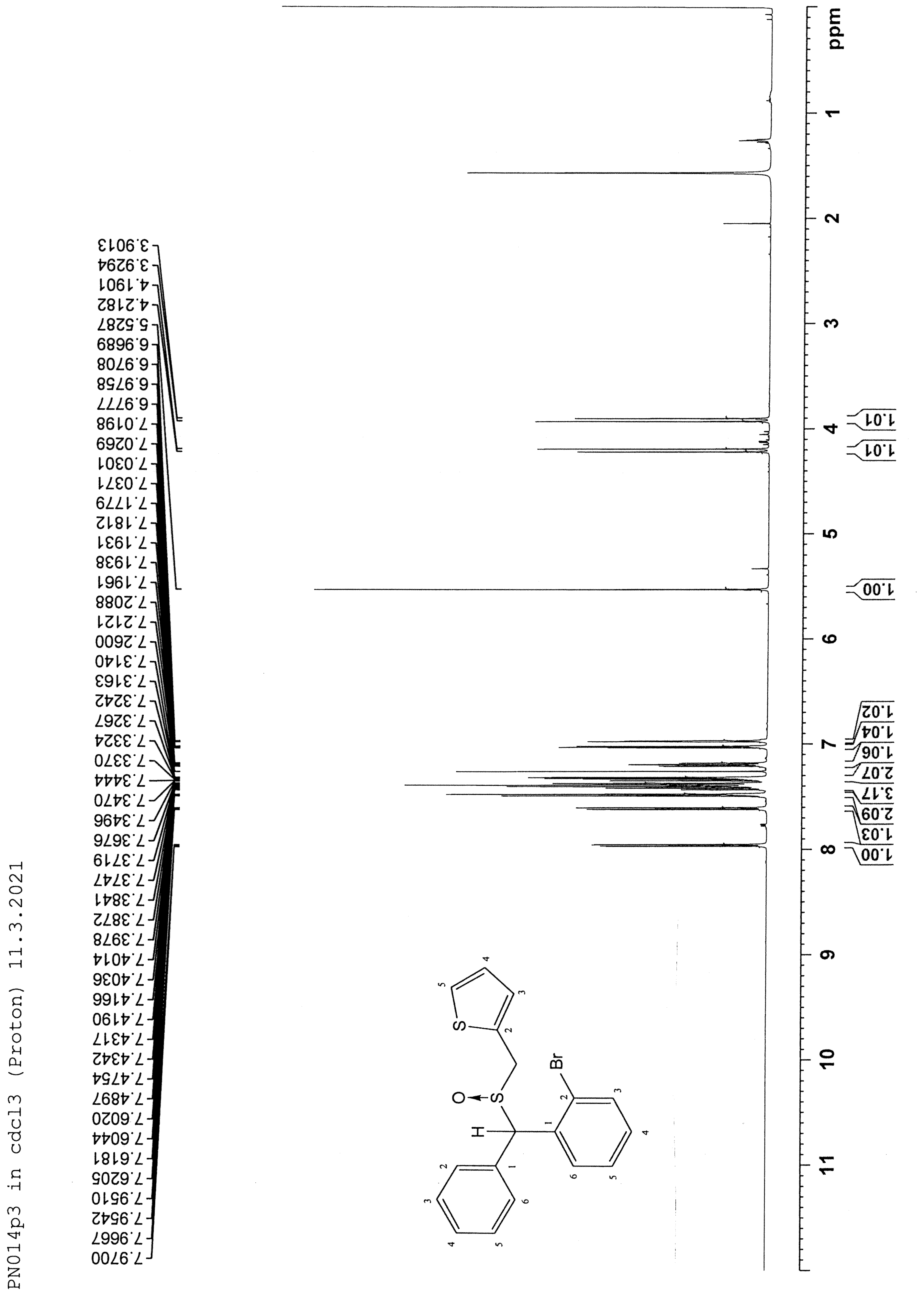

PN014p3 in cdcl3 (Proton) 11.3.2021

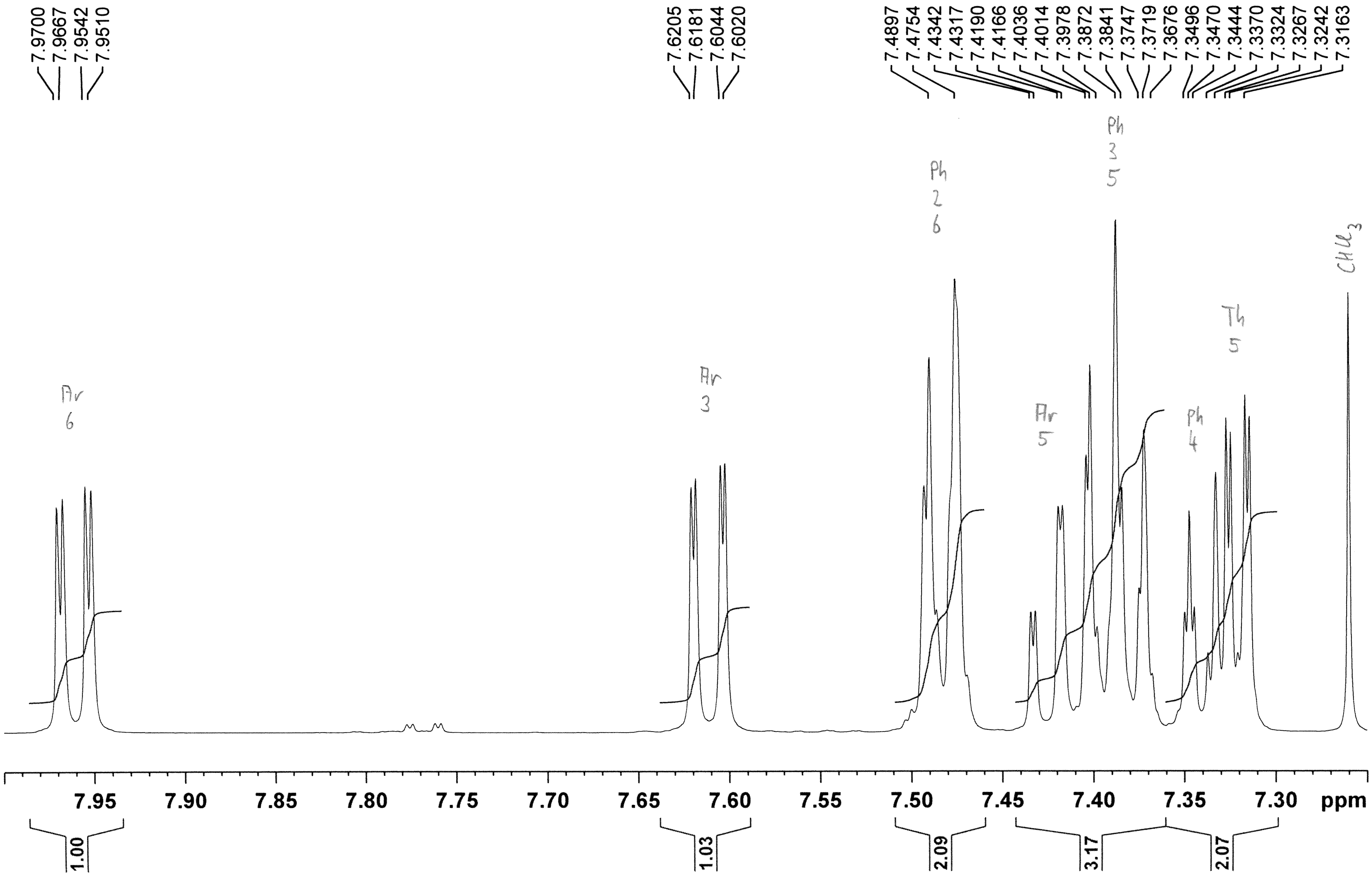

PN014p3 in cdcl3 (Proton) 11.3.2021

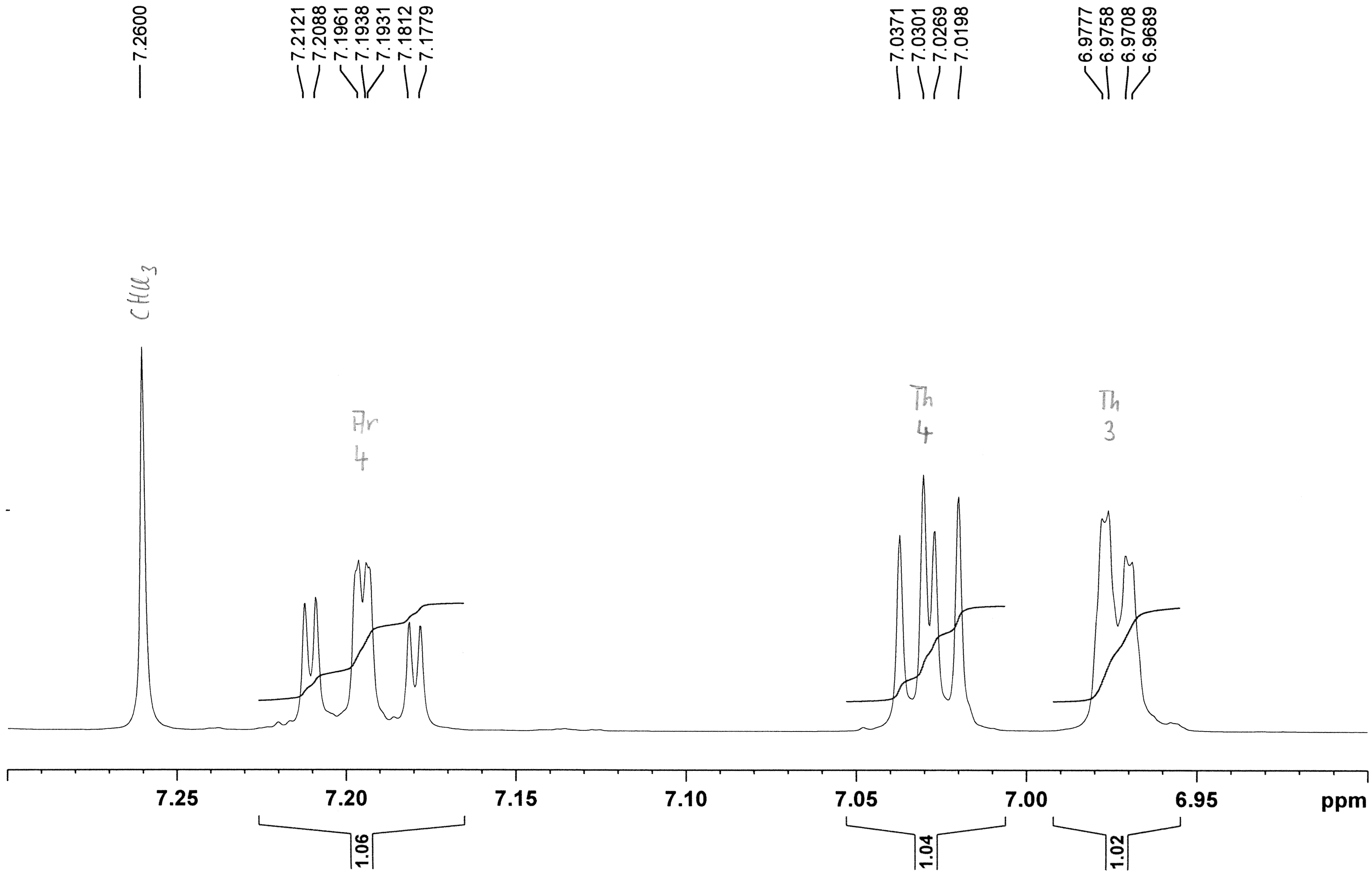

PN014p3 in cdcl3 (Proton) 11.3.2021

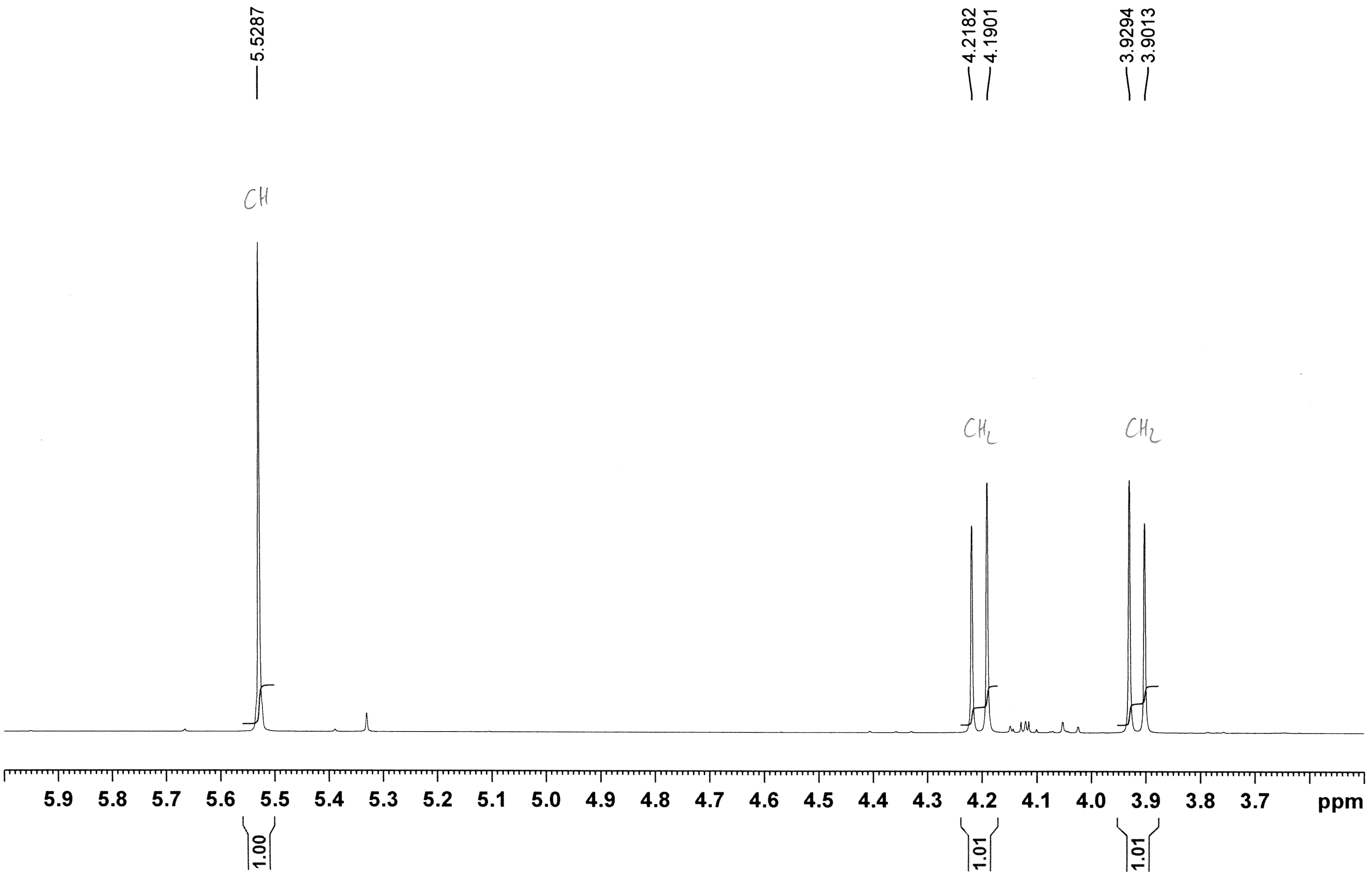

PN014p3 in cdcl3 (APT) 11.3.2021

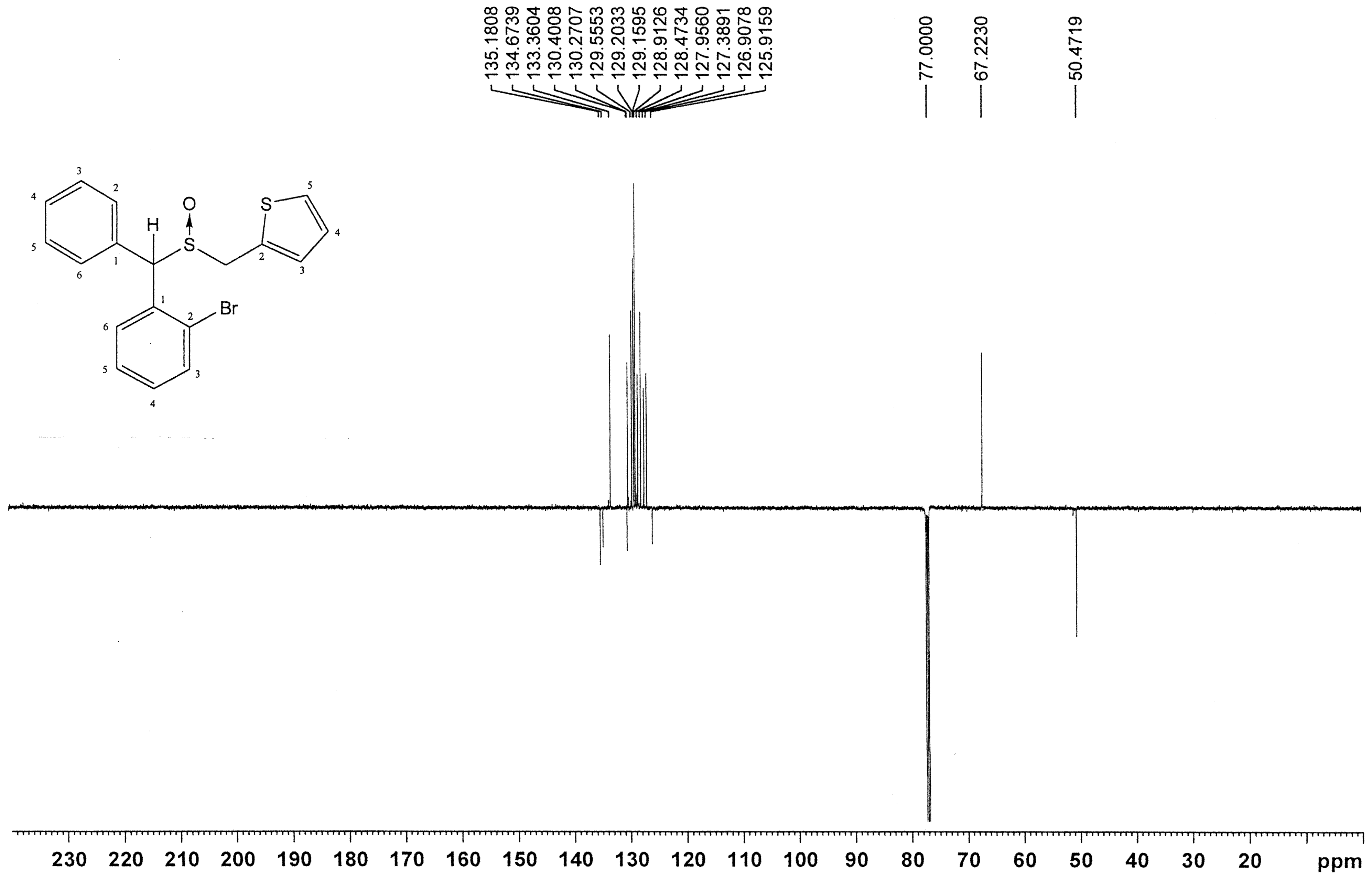

PN014p3 in cdcl3 (APT) 11.3.2021

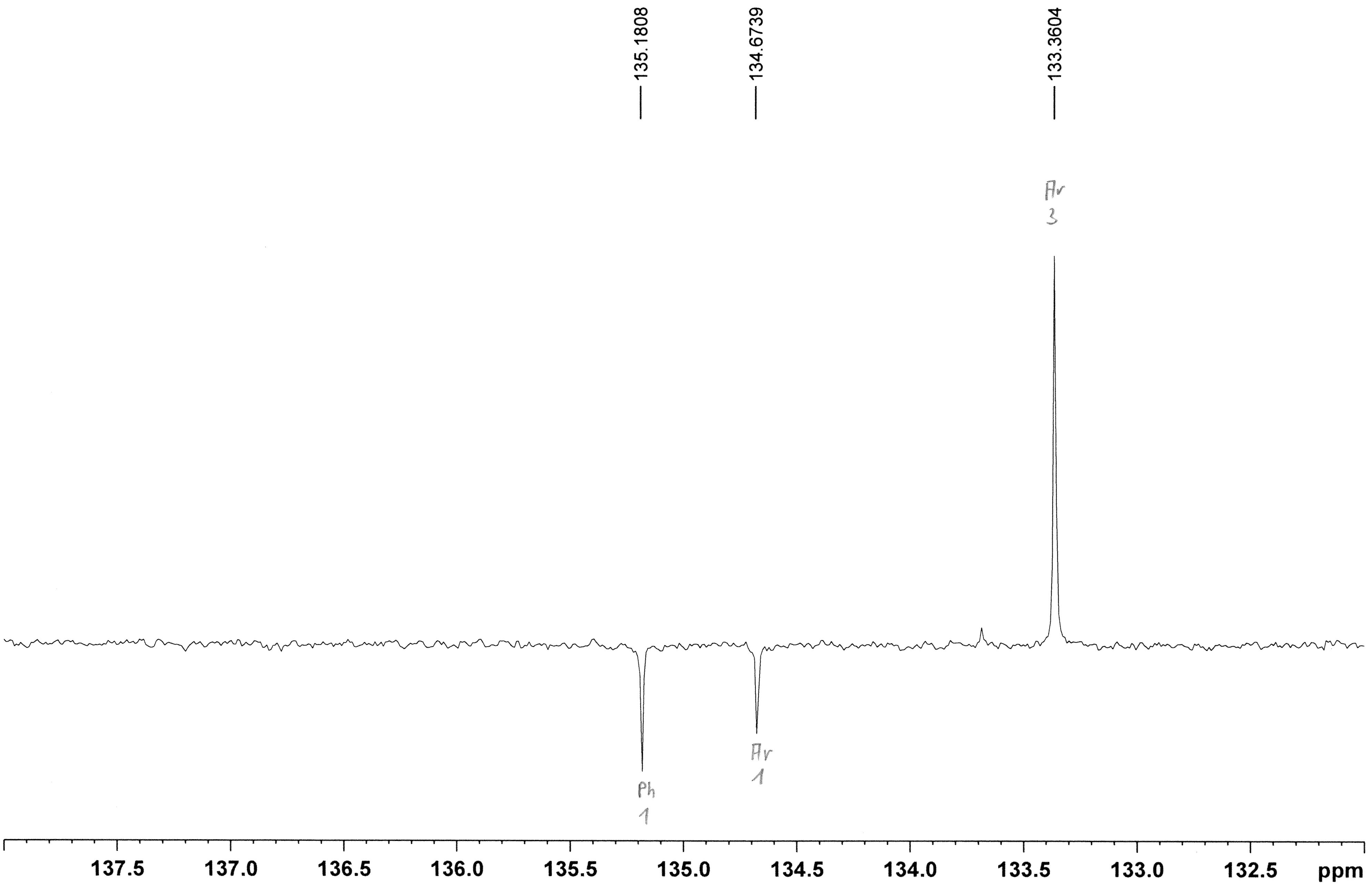

PN014p3 in cdcl3 (APT) 11.3.2021

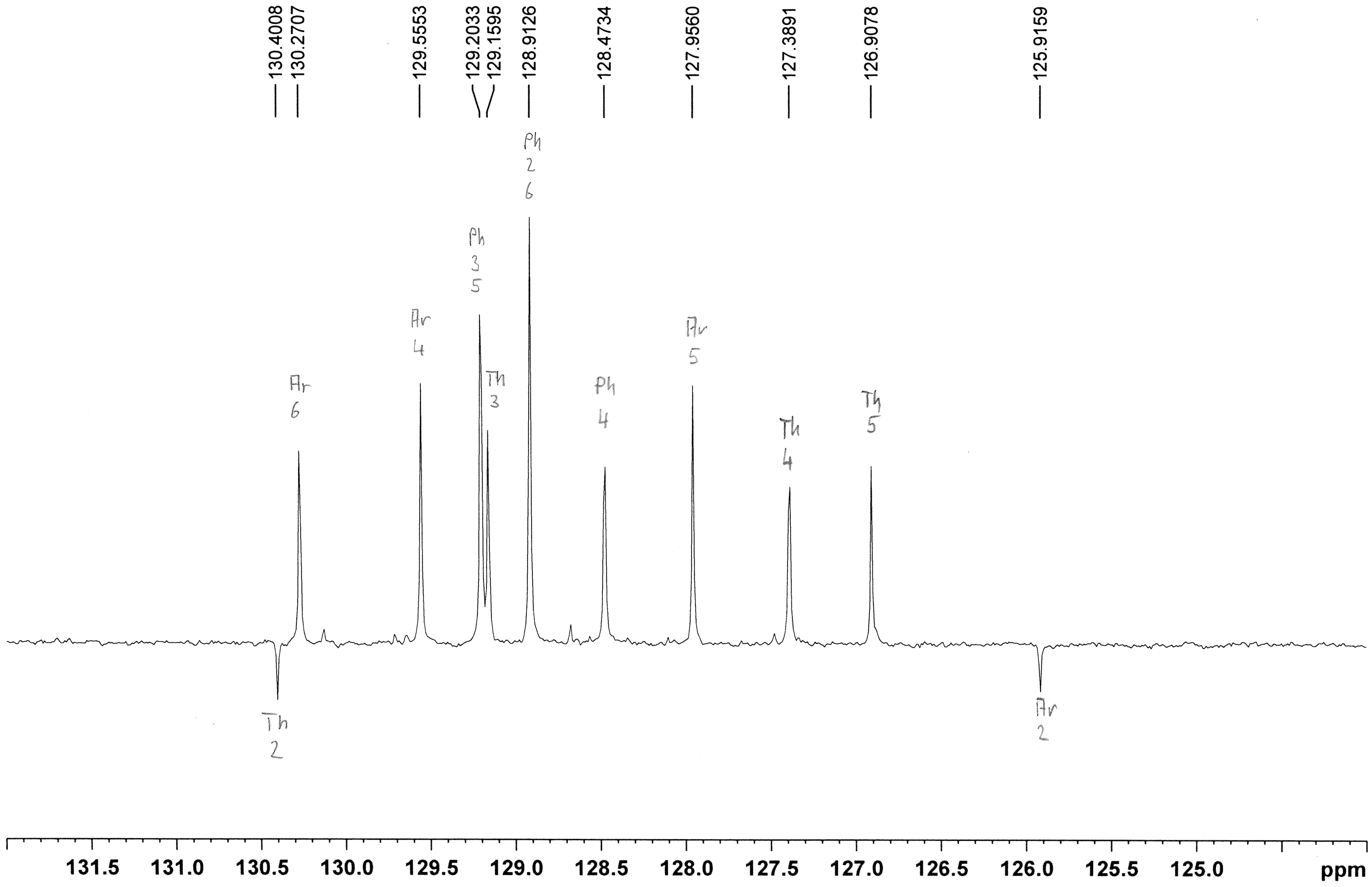

PN014p3 in cdcl3 (APT) 11.3.2021

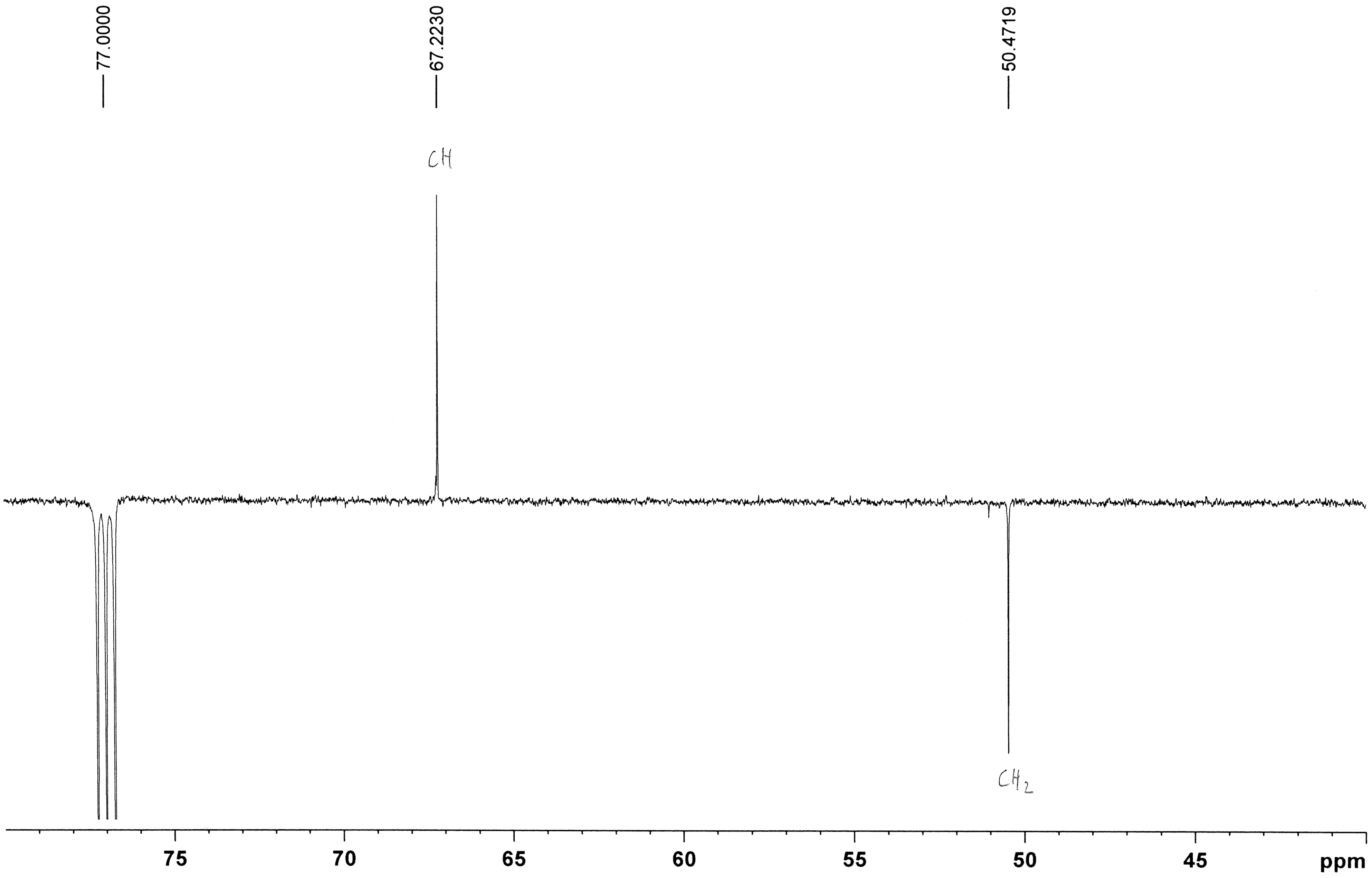

PN014p3 in cdcl3 (COSY) 11.3.2021

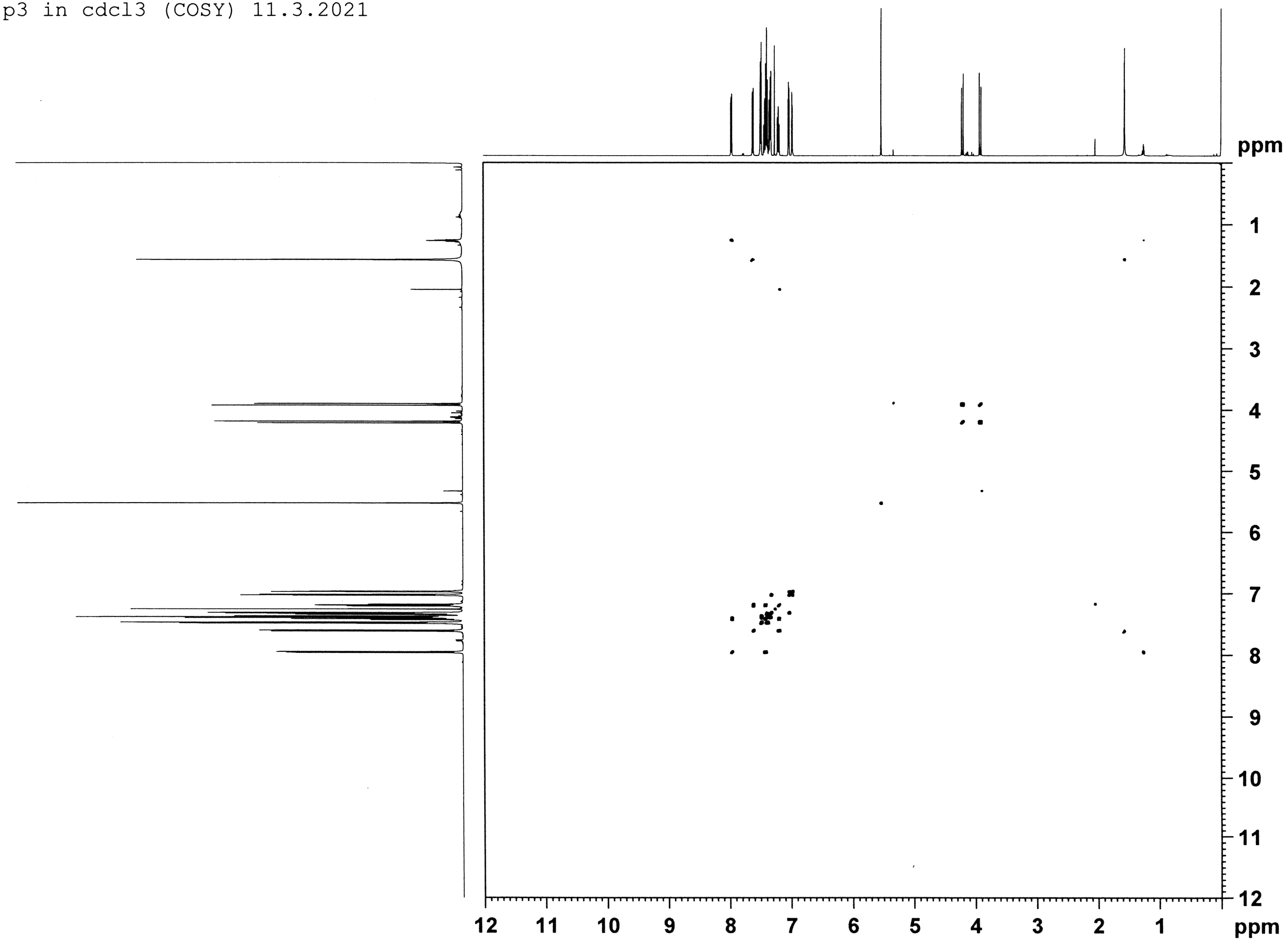

PN014p3 in cdcl3 (COSY) 11.3.2021

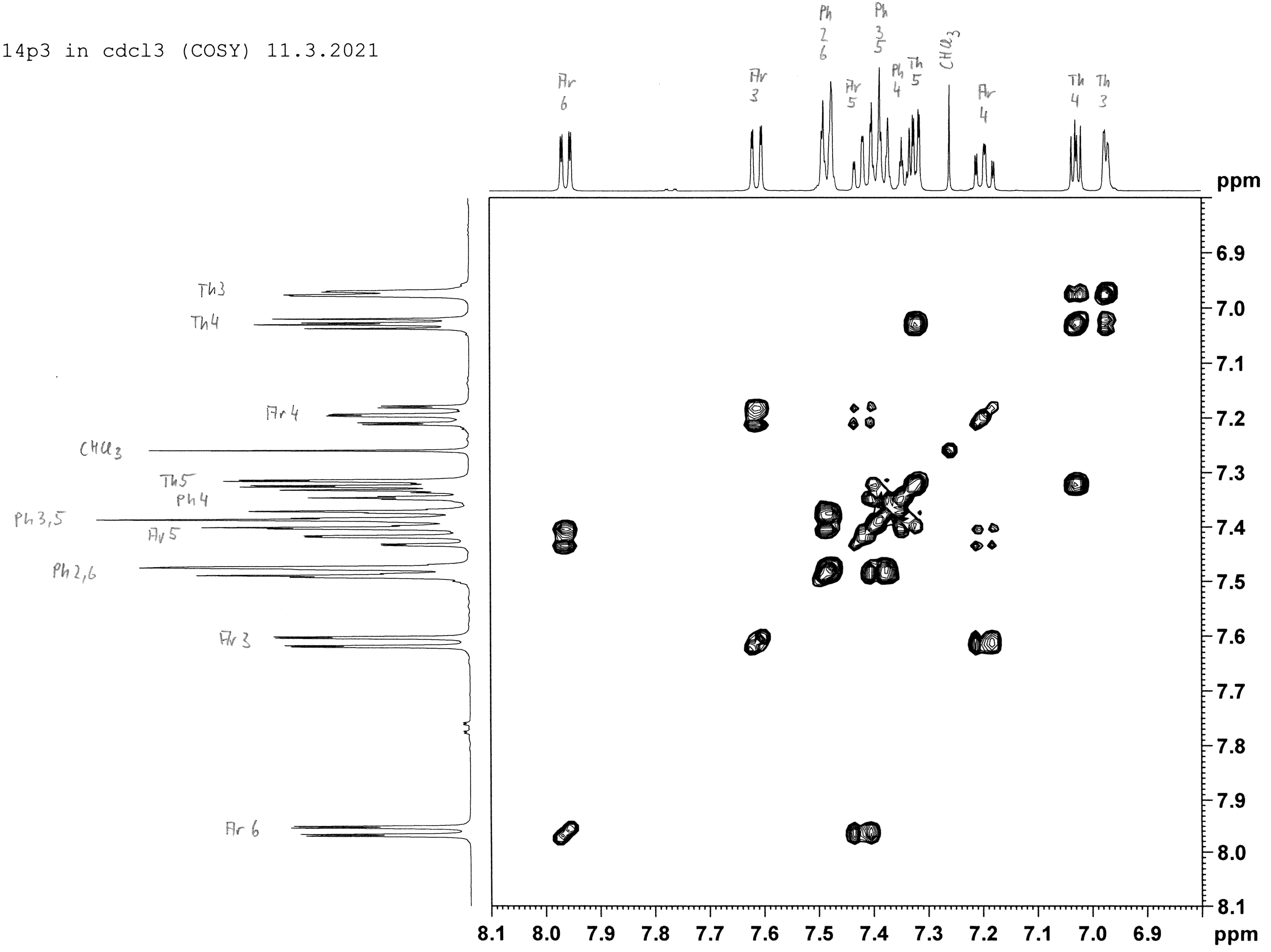

PN014p3 in cdcl3 (COSY) 11.3.2021

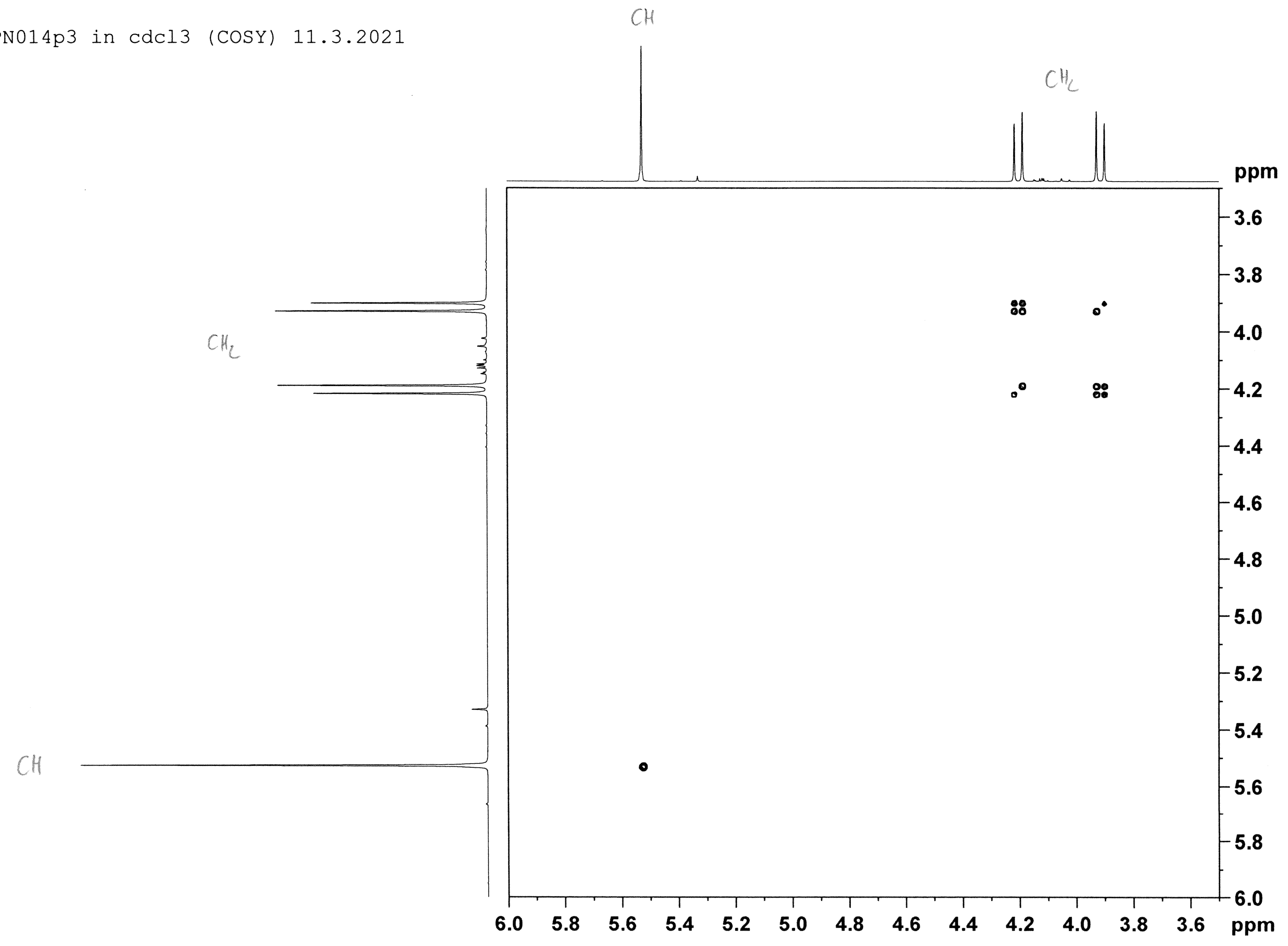

PN014p3 in cdcl3 (HSQC) 11.3.2021

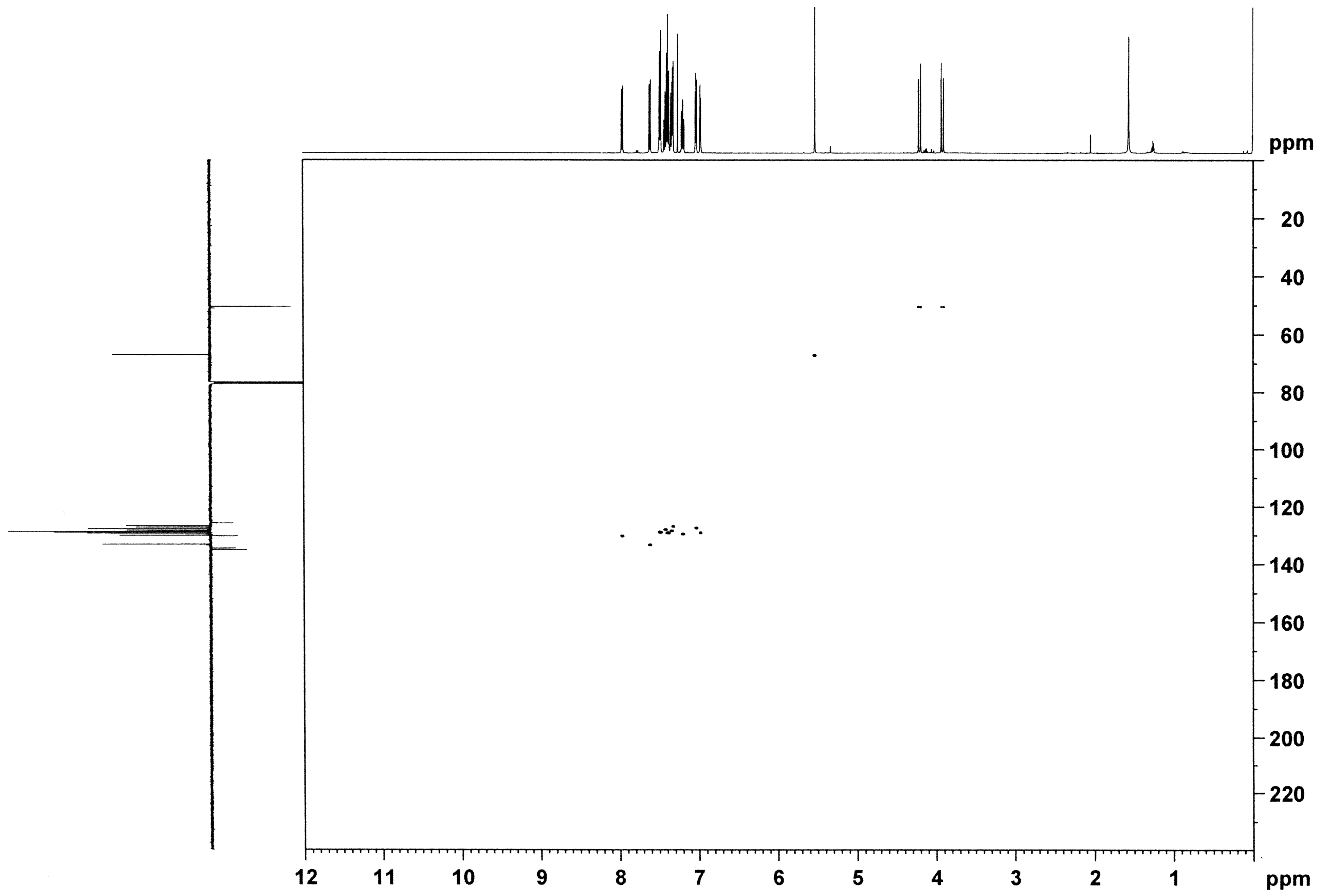

PN014p3 in cdcl3 (HSQC) 11.3.2021

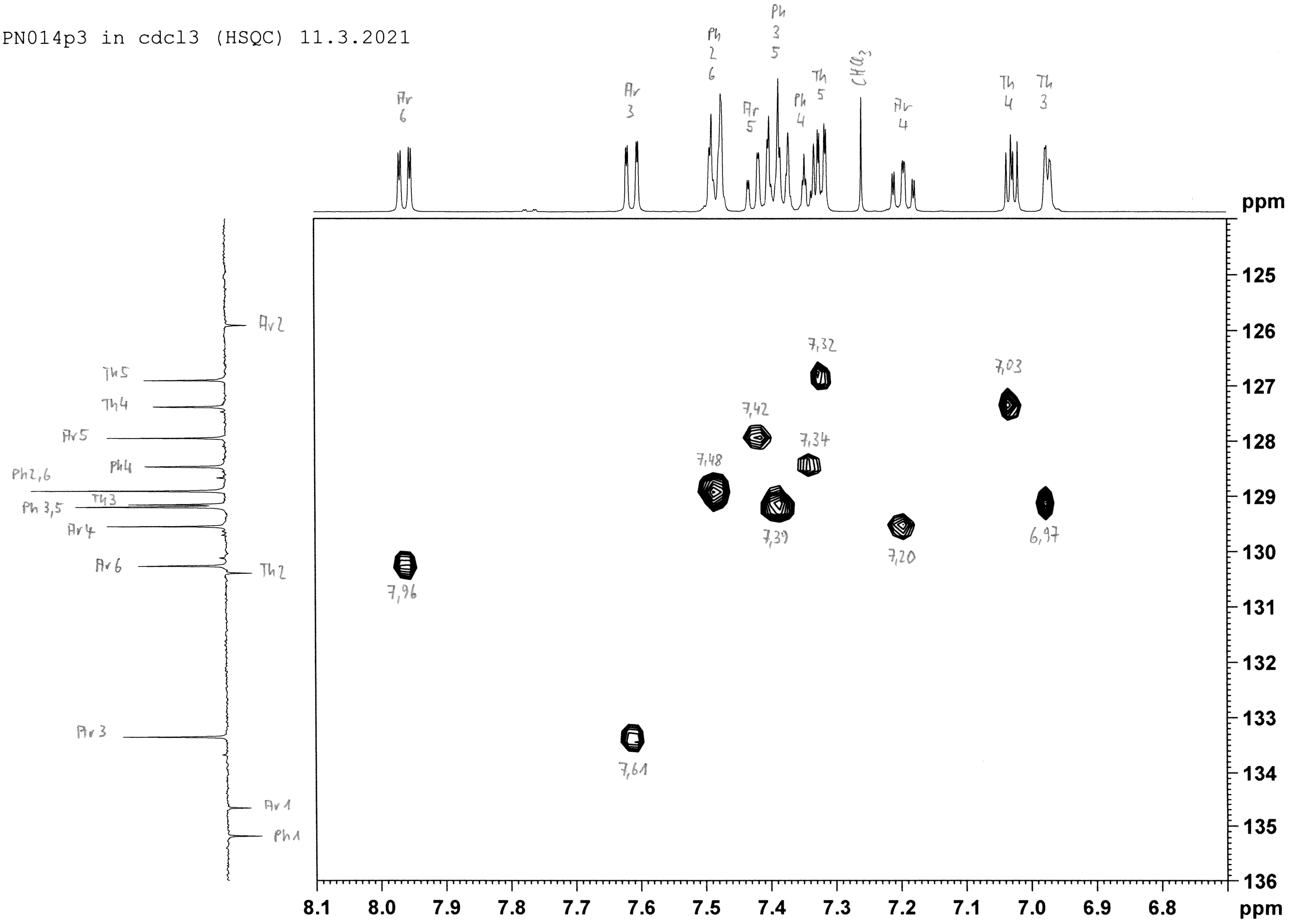

PN014p3 in cdcl3 (HSQC) 11.3.2021

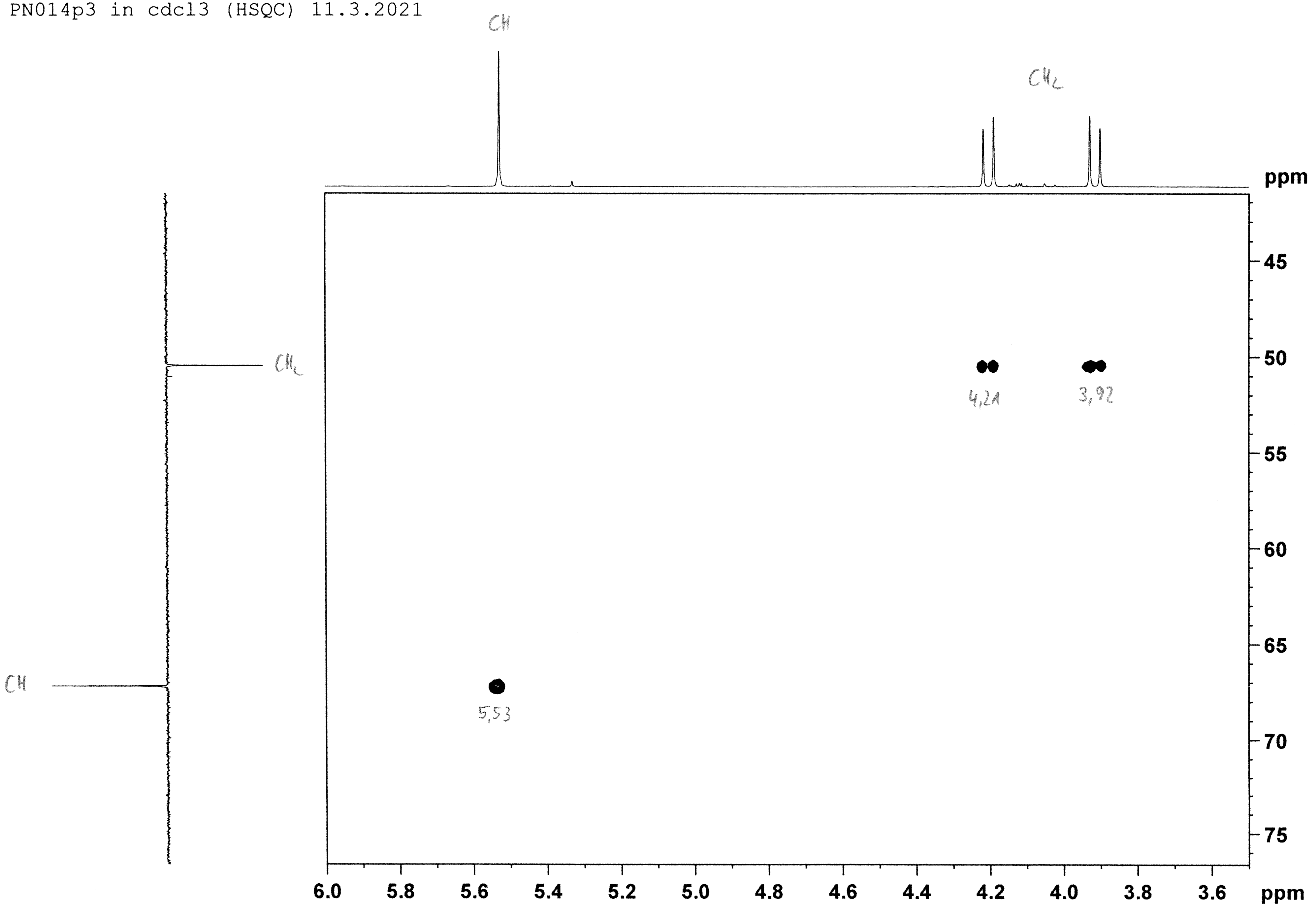

PN014p3 in cdcl3 (HMBC) 11.3.2021

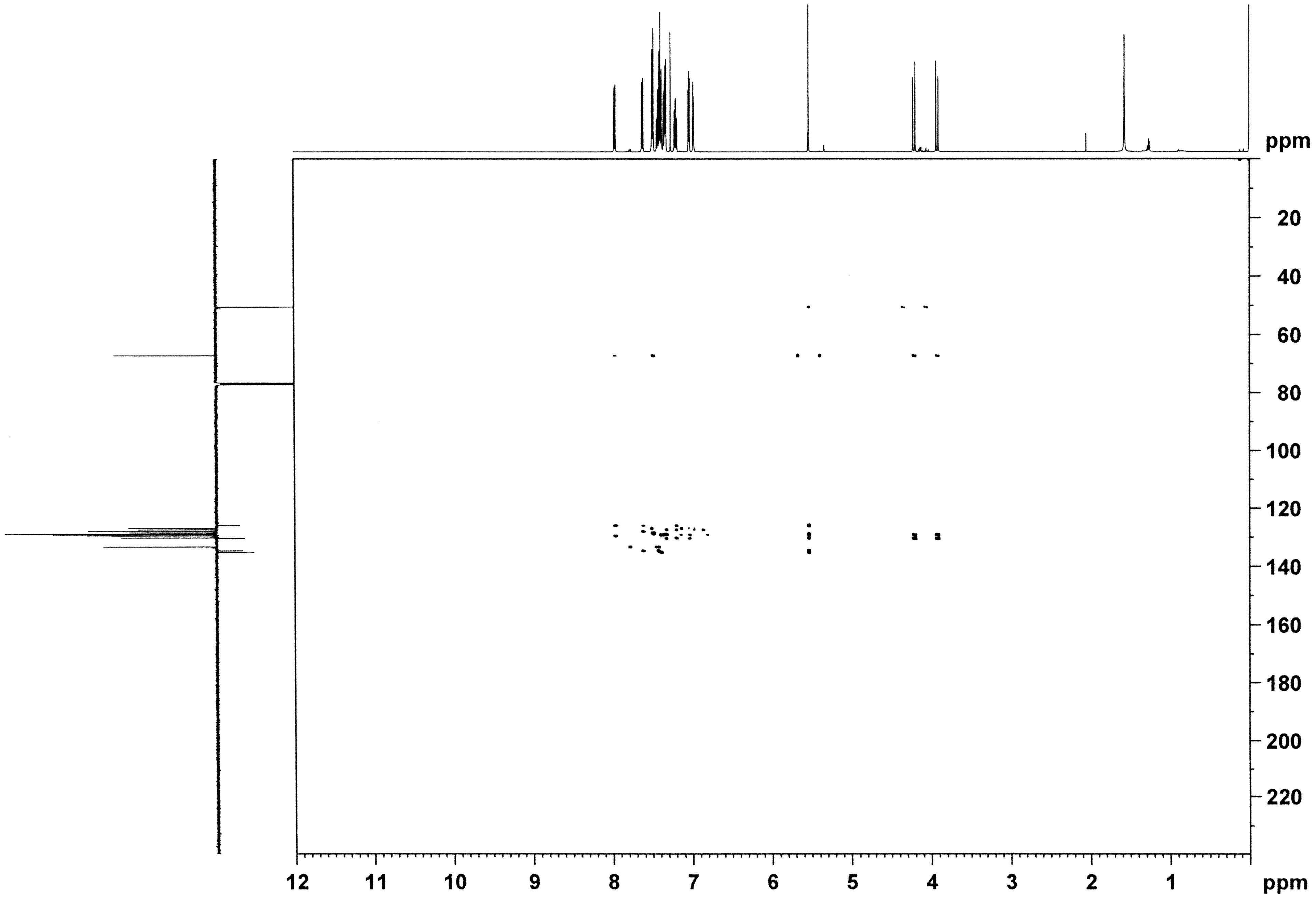

PN014p3 in cdcl3 (HMBC) 11.3.2021

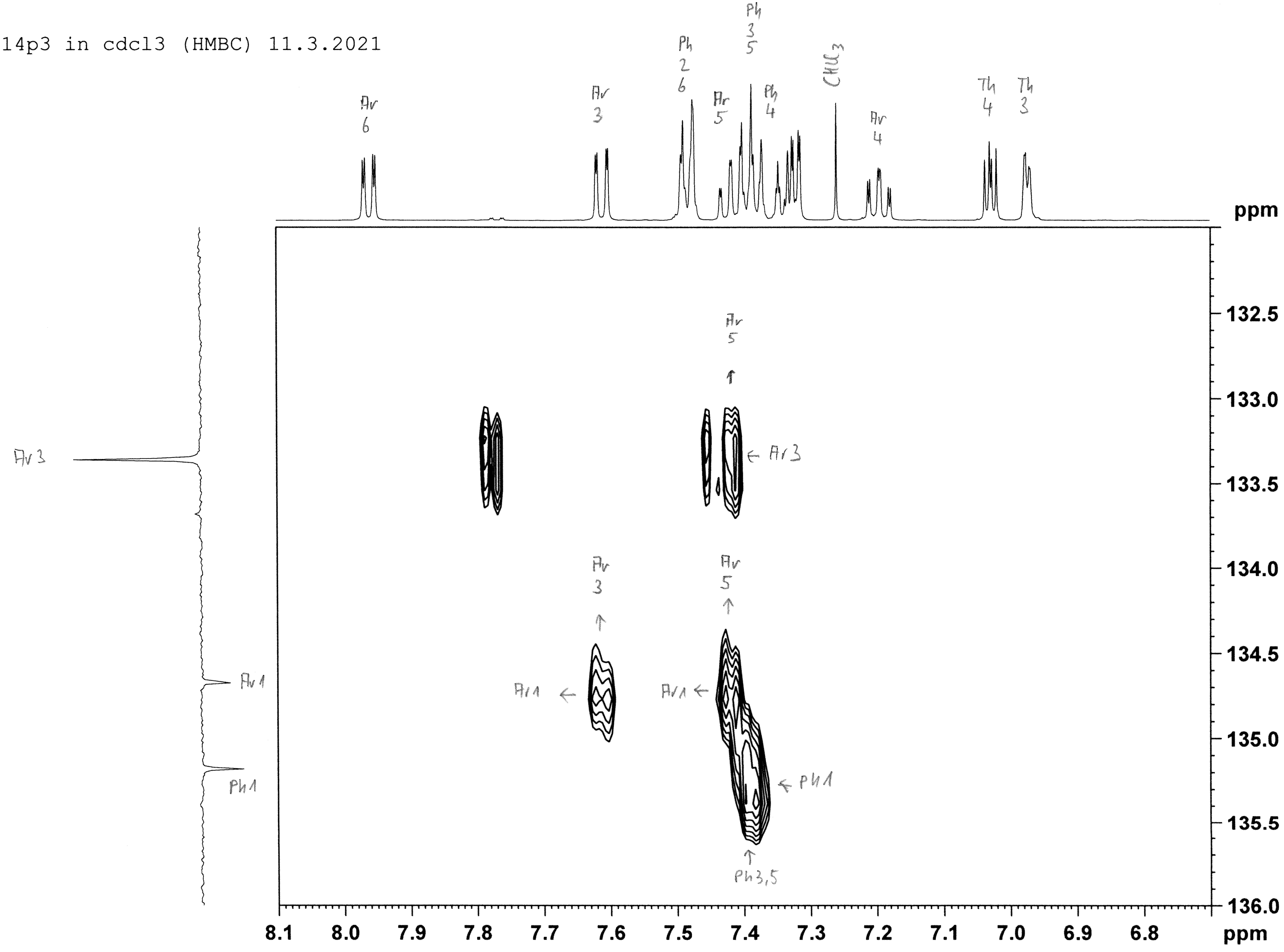

PN014p3 in cdcl3 (HMBC) 11.3.2021

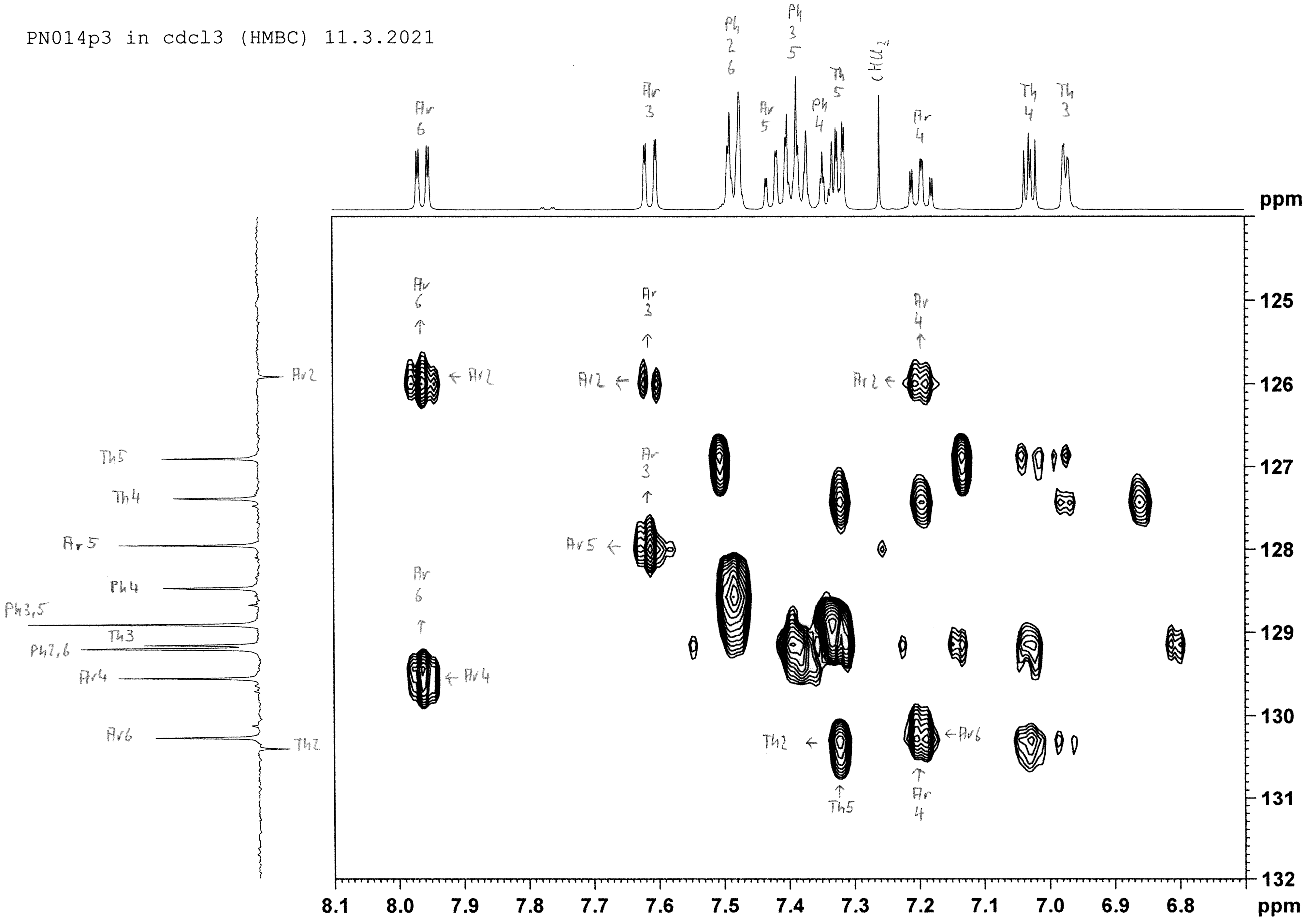

PN014p3 in cdcl3 (HMBC) 11.3.2021

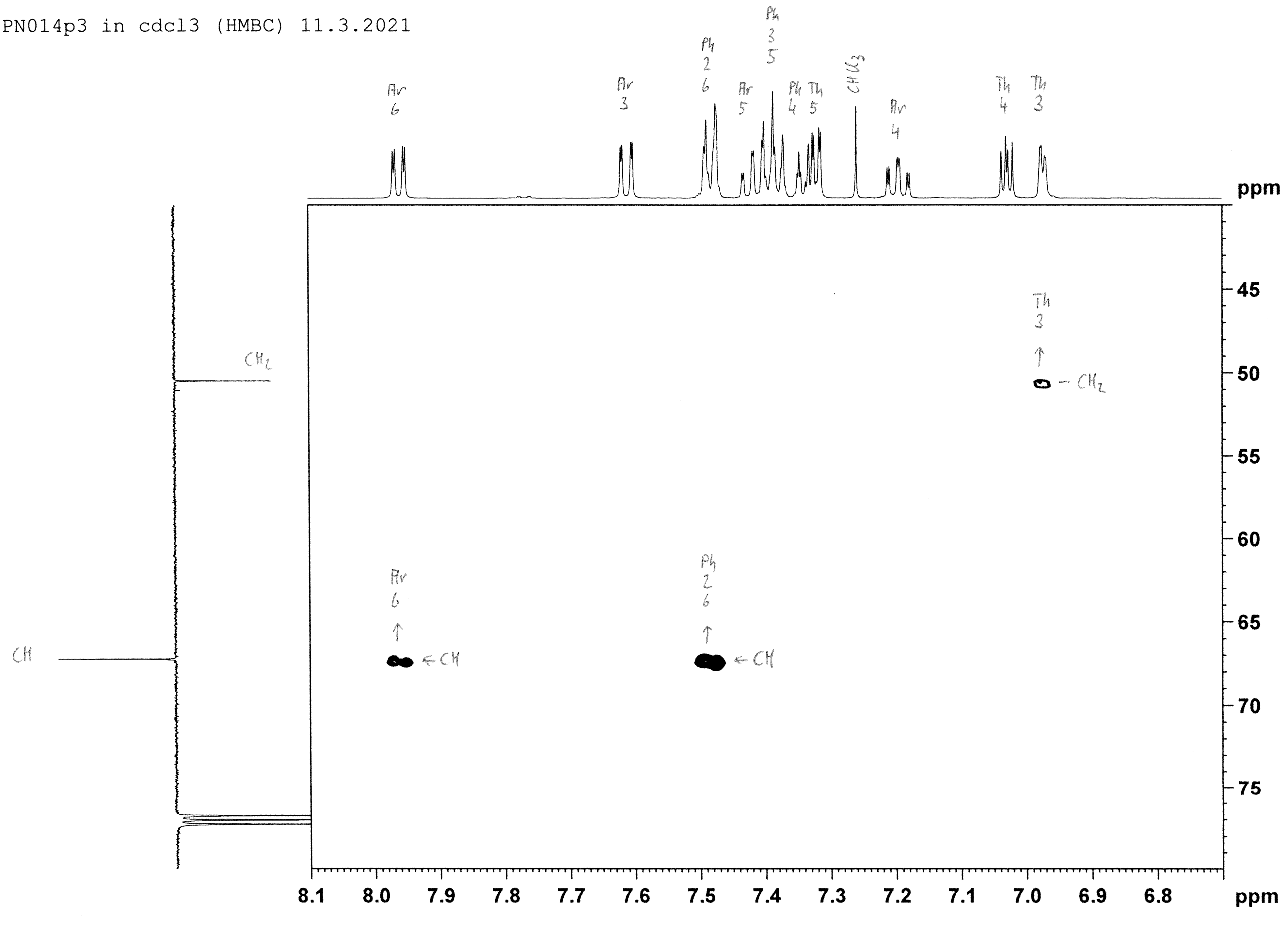

PN014p3 in cdcl3 (HMBC) 11.3.2021

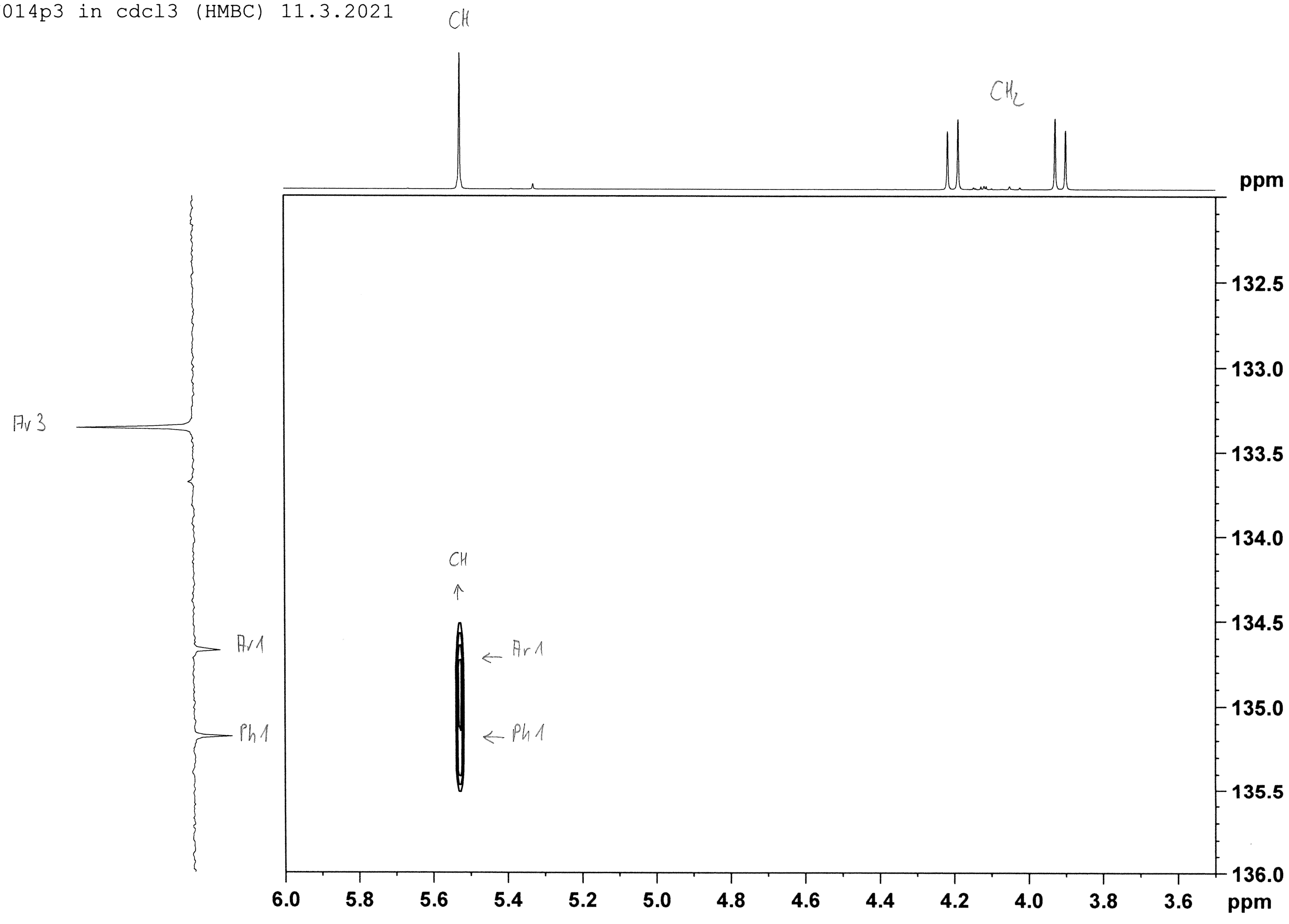

PN014p3 in cdcl3 (HMBC) 11.3.2021

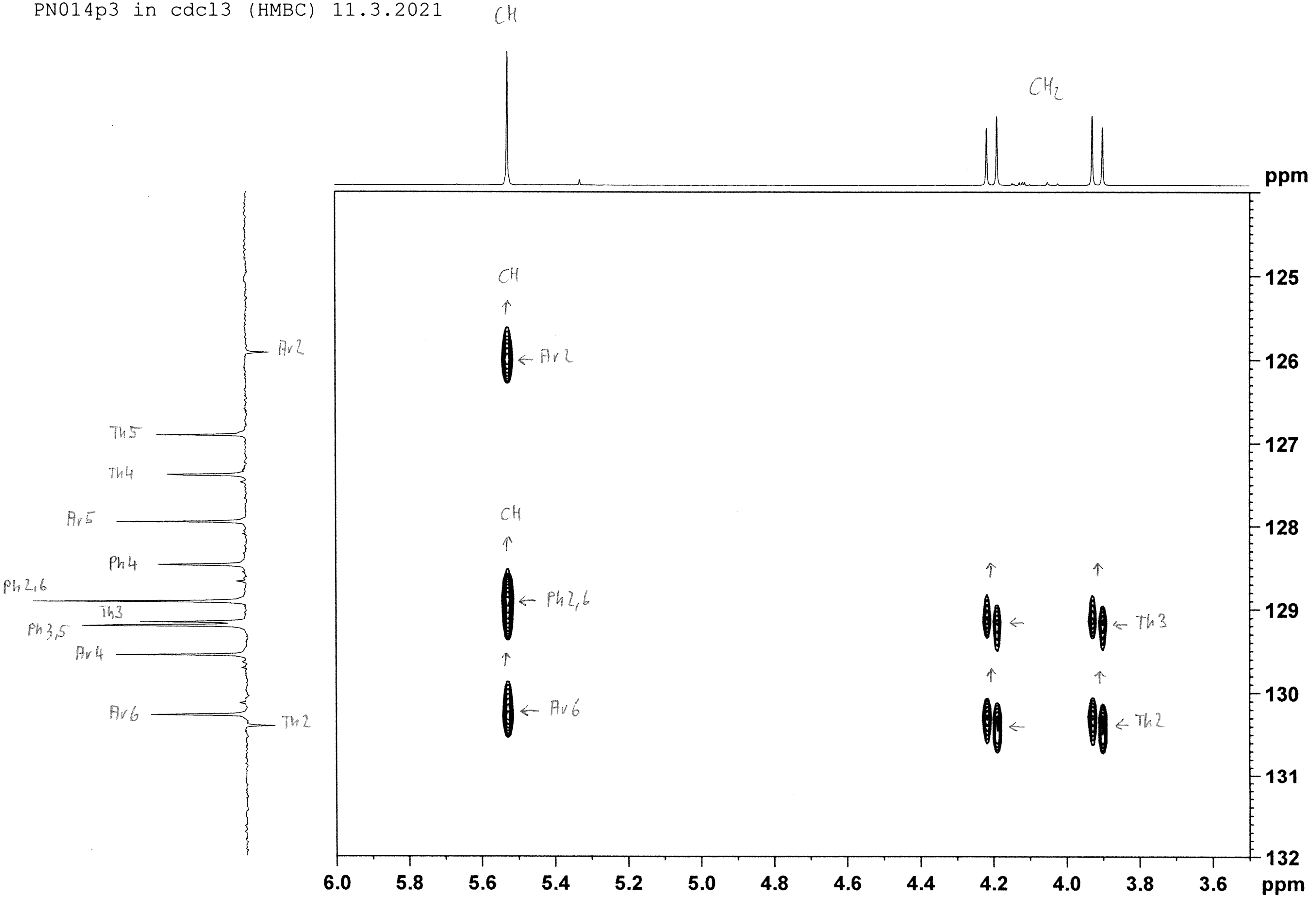

PN014p3 in cdcl3 (HMBC) 11.3.2021

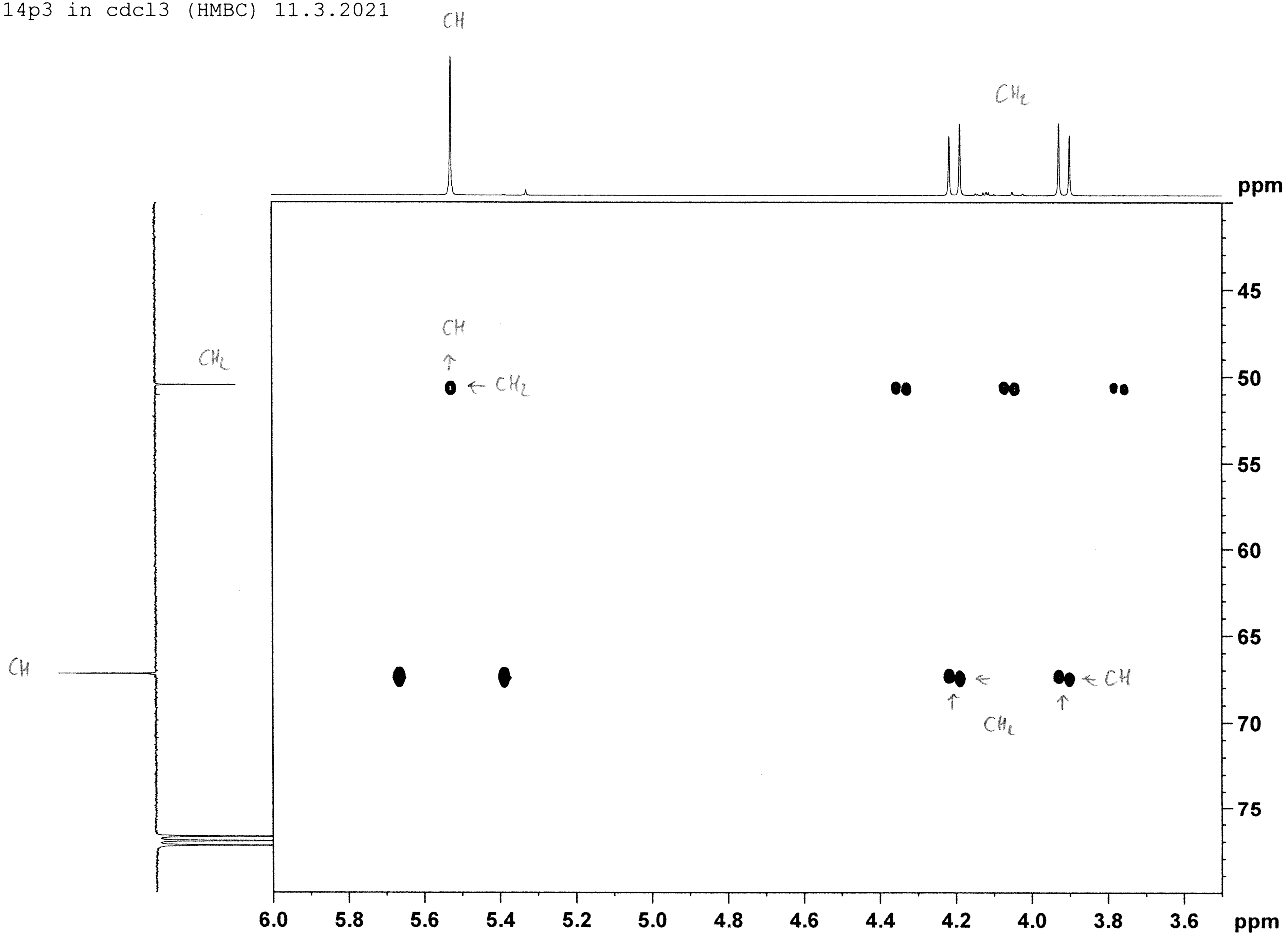

Figure S28c. NMR spectra of compound **8g**.

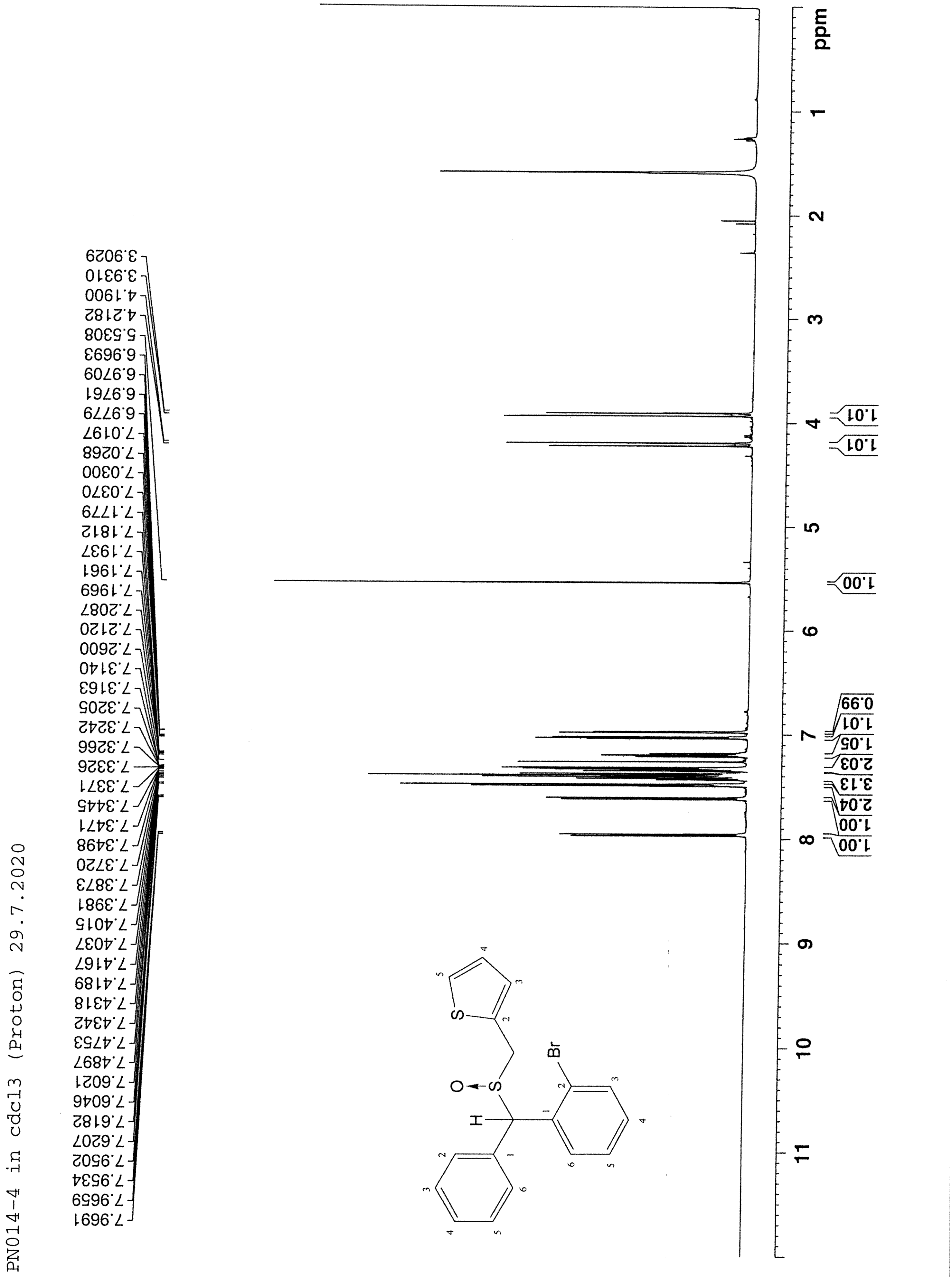

PN014-4 in cdcl3 (Proton) 29.7.2020

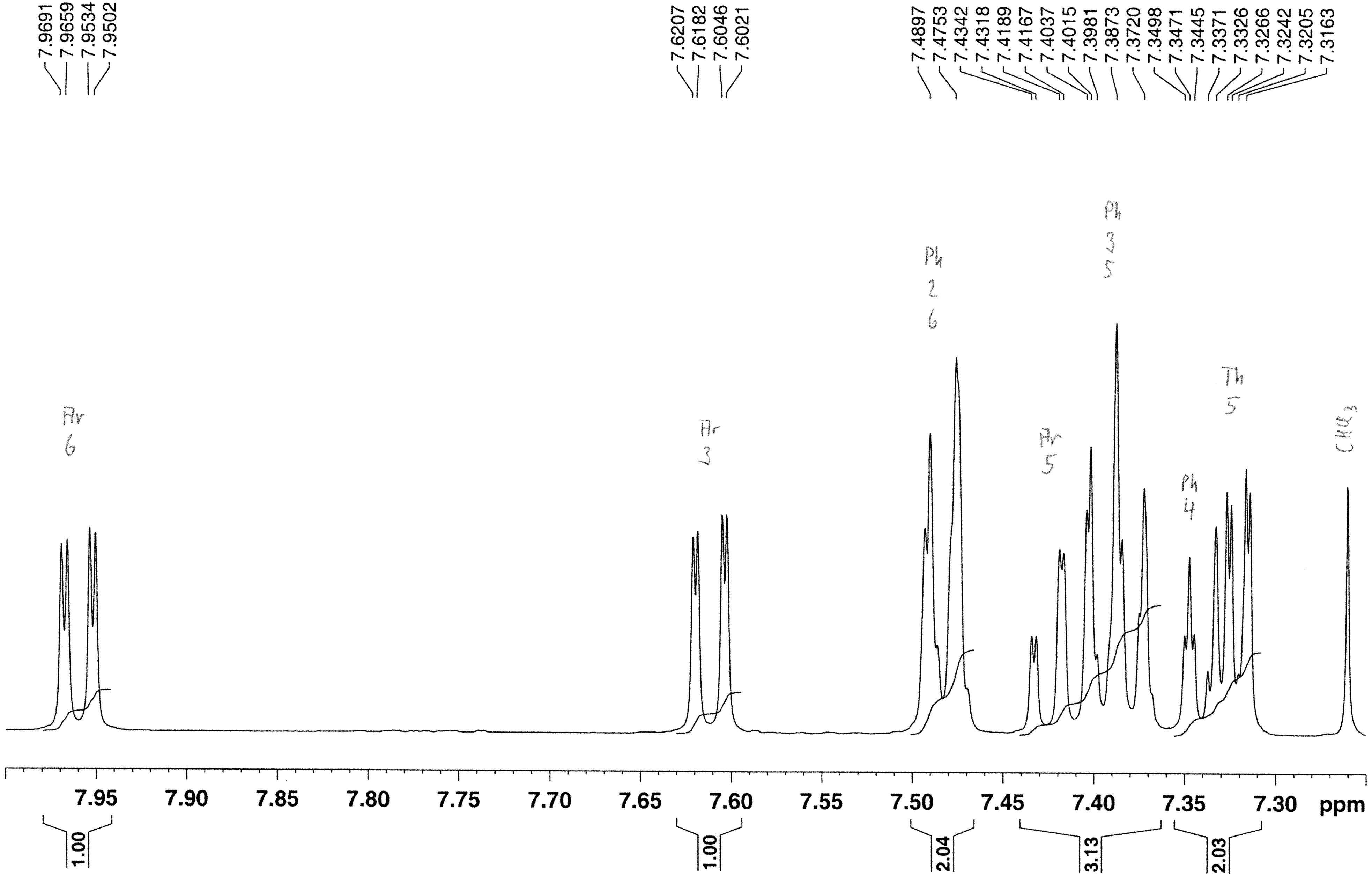

PN014-4 in cdcl3 (Proton) 29.7.2020

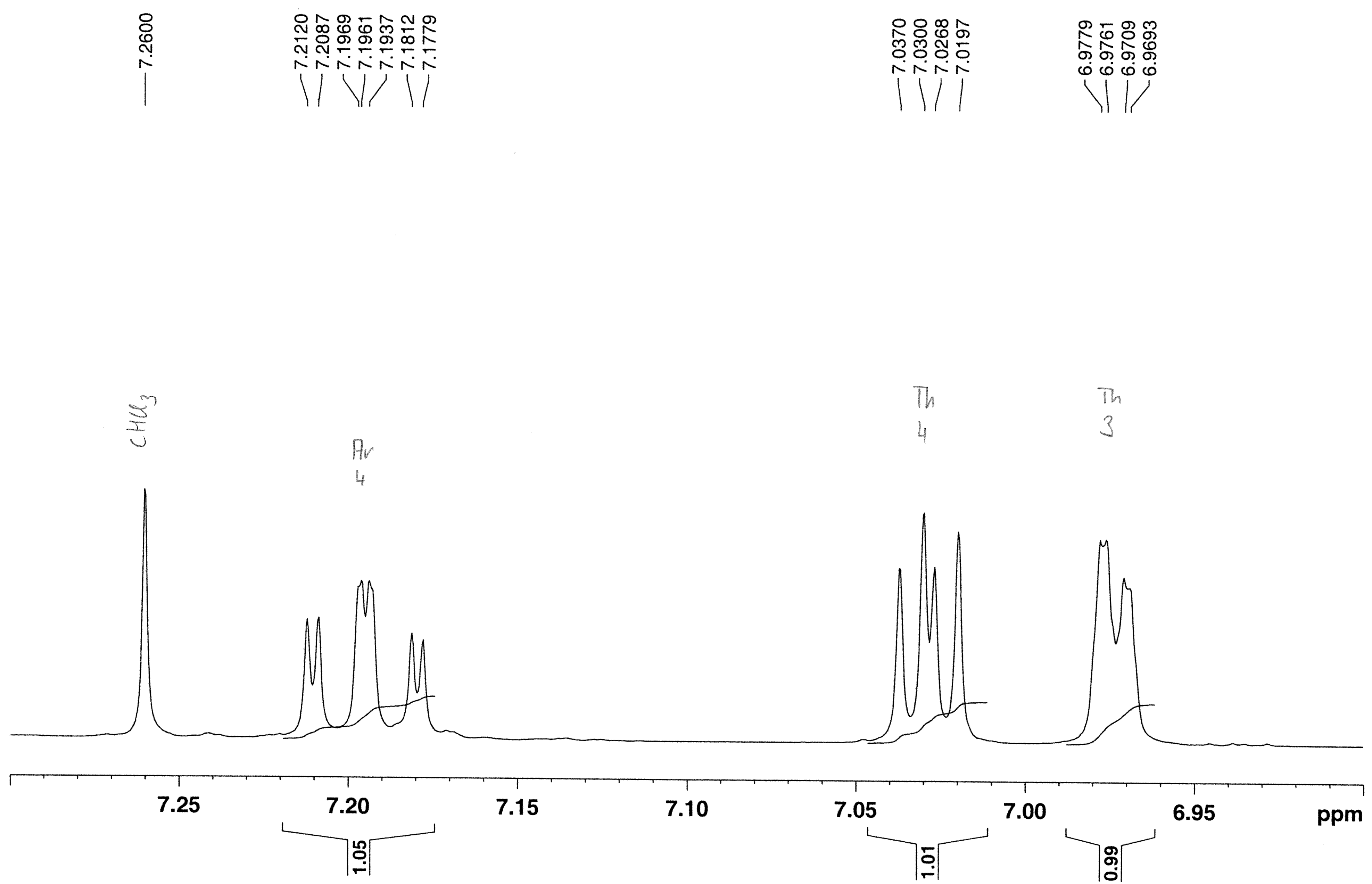

PN014-4 in cdcl3 (Proton) 29.7.2020

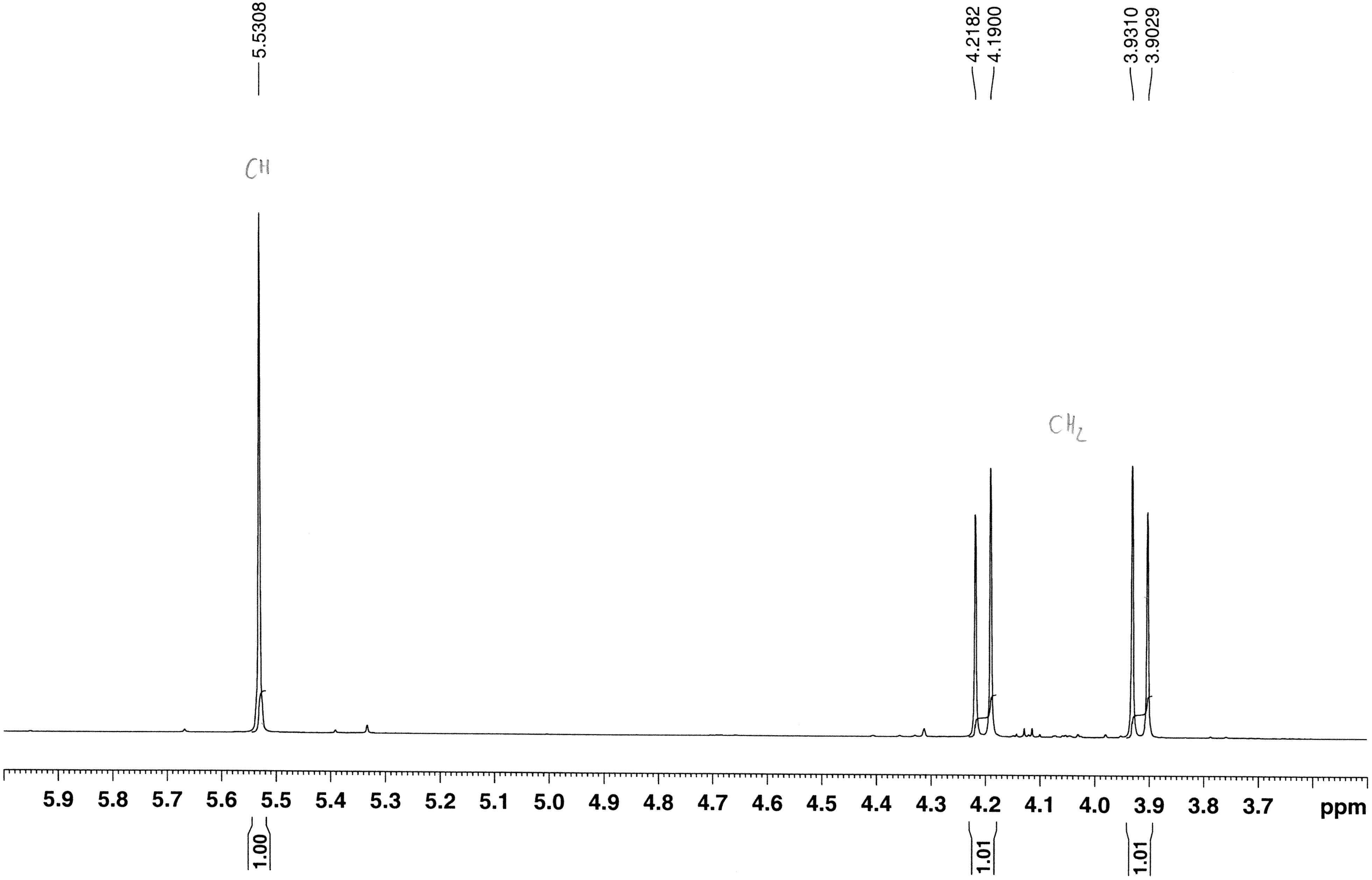

PN014-4 in cdcl3 (APT) 29.7.2020

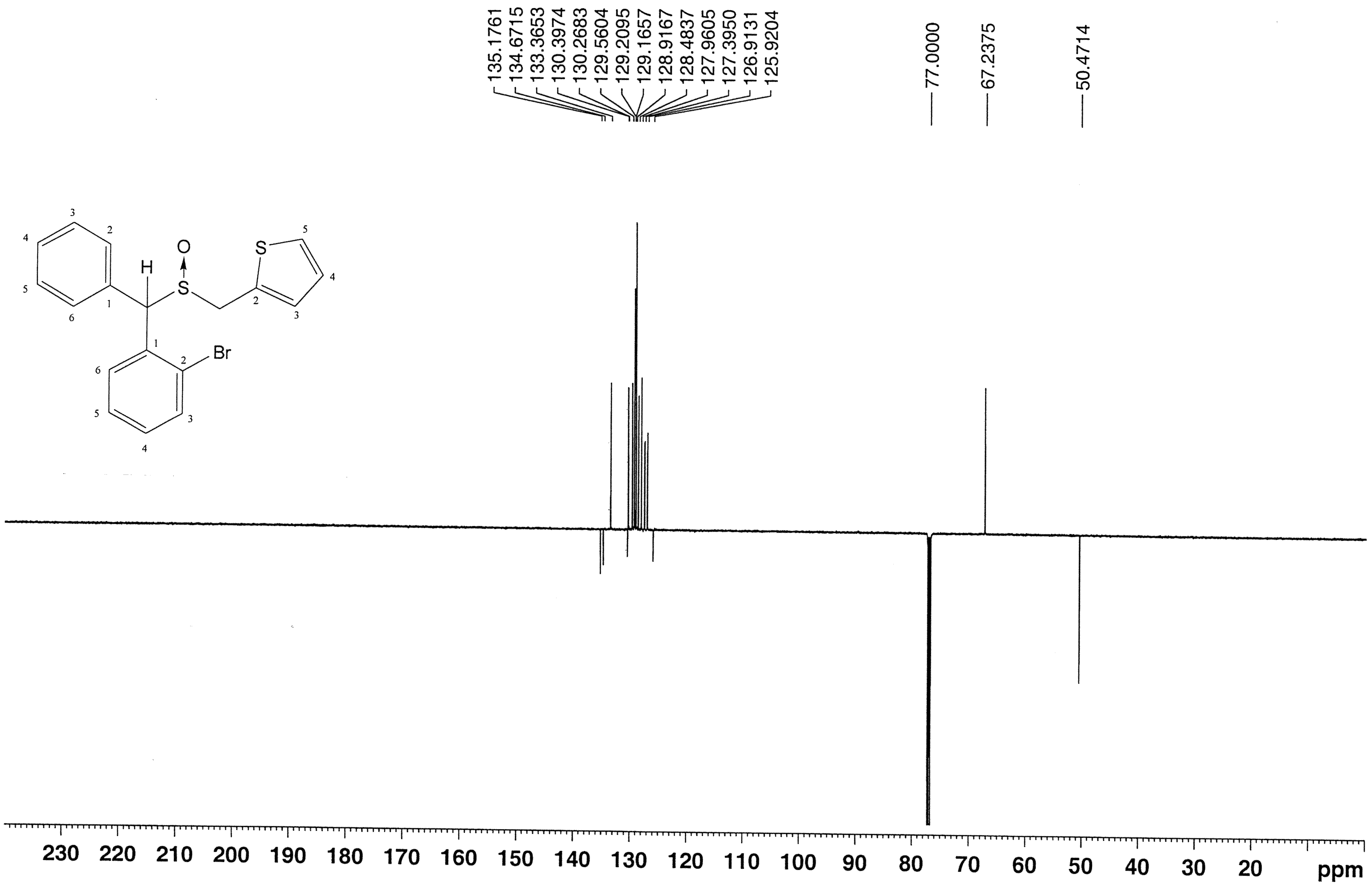

PN014-4 in cdcl3 (APT) 29.7.2020

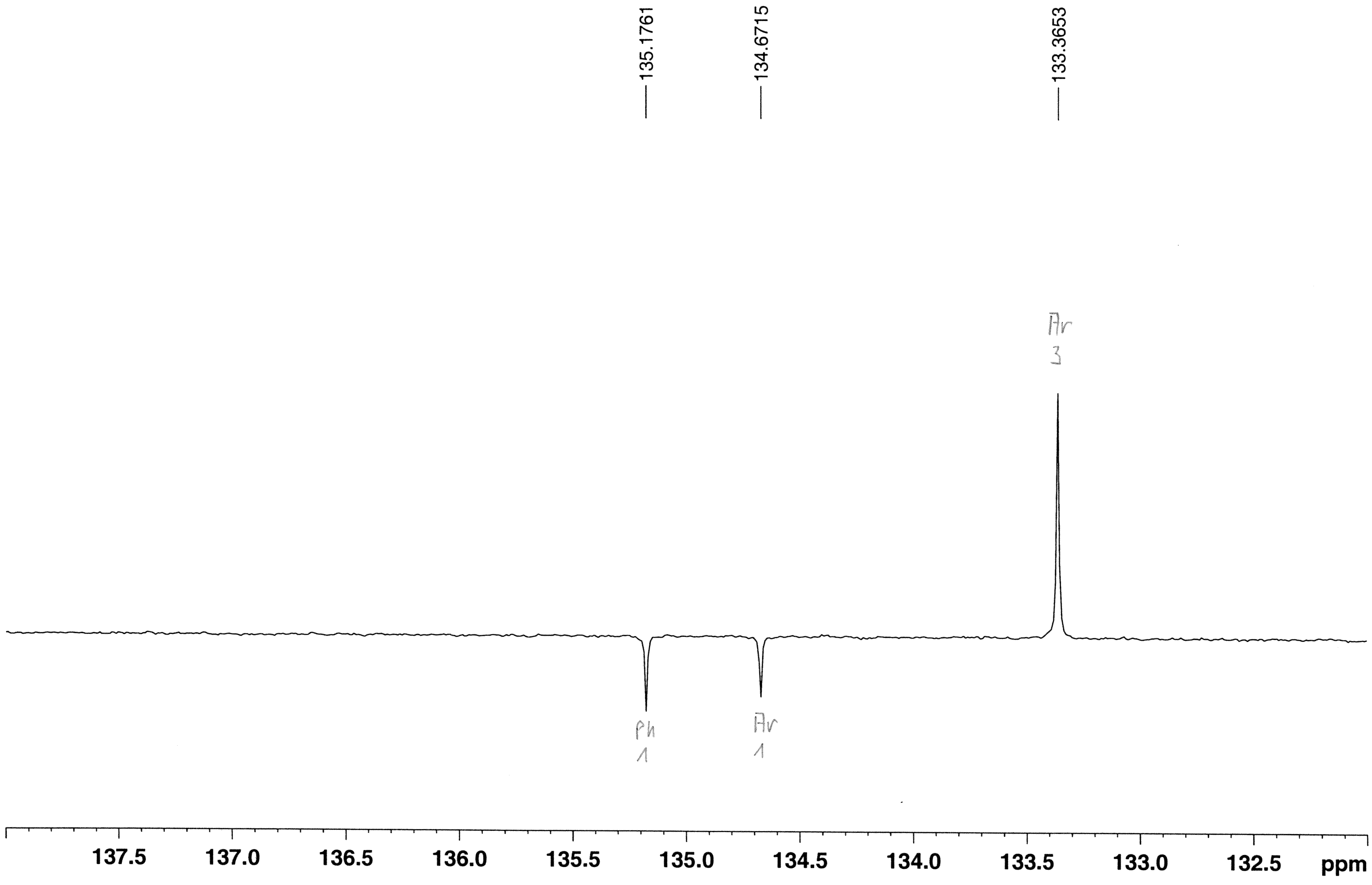

PN014-4 in cdcl3 (APT) 29.7.2020

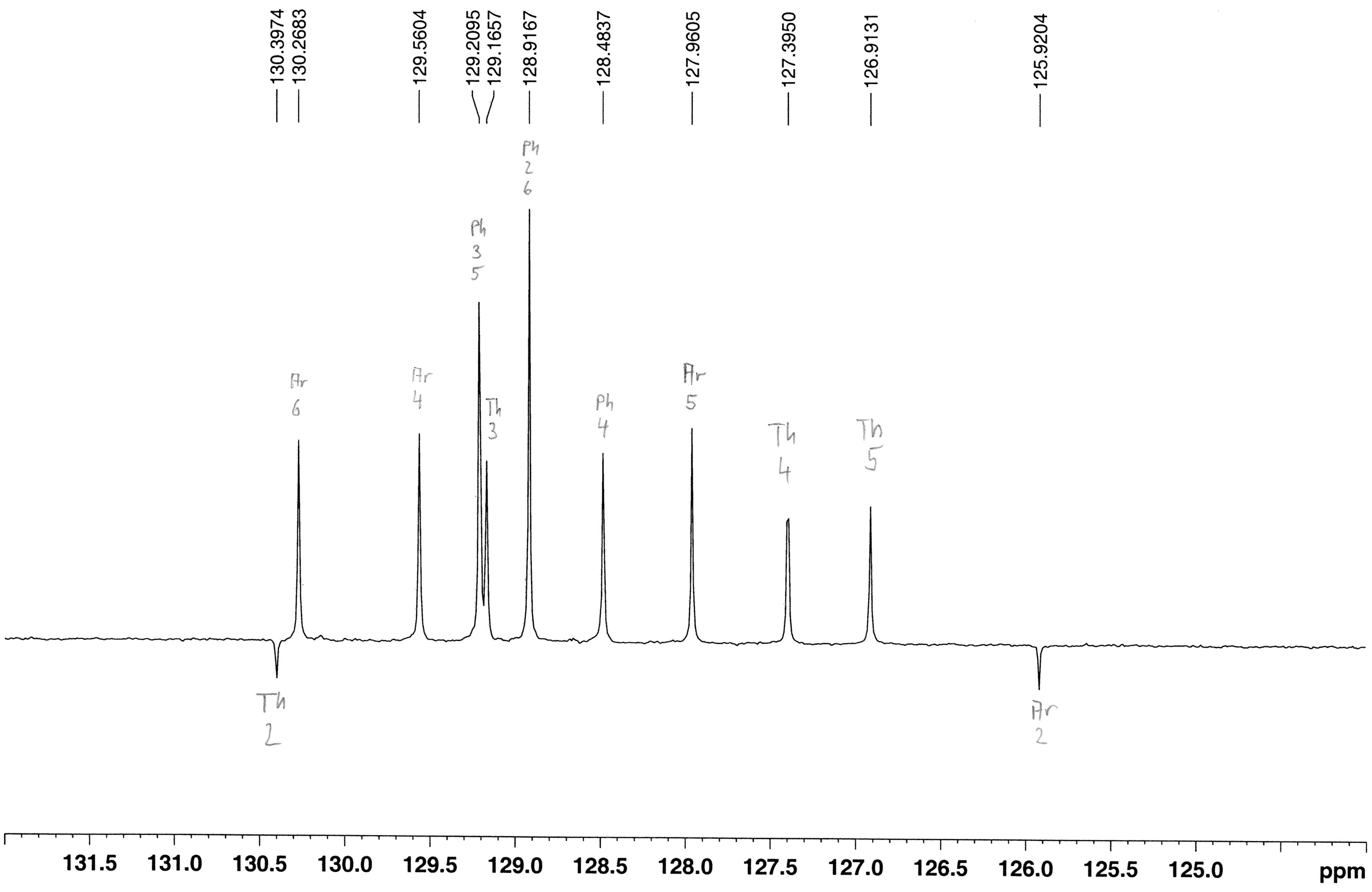

PN014-4 in cdcl3 (APT) 29.7.2020

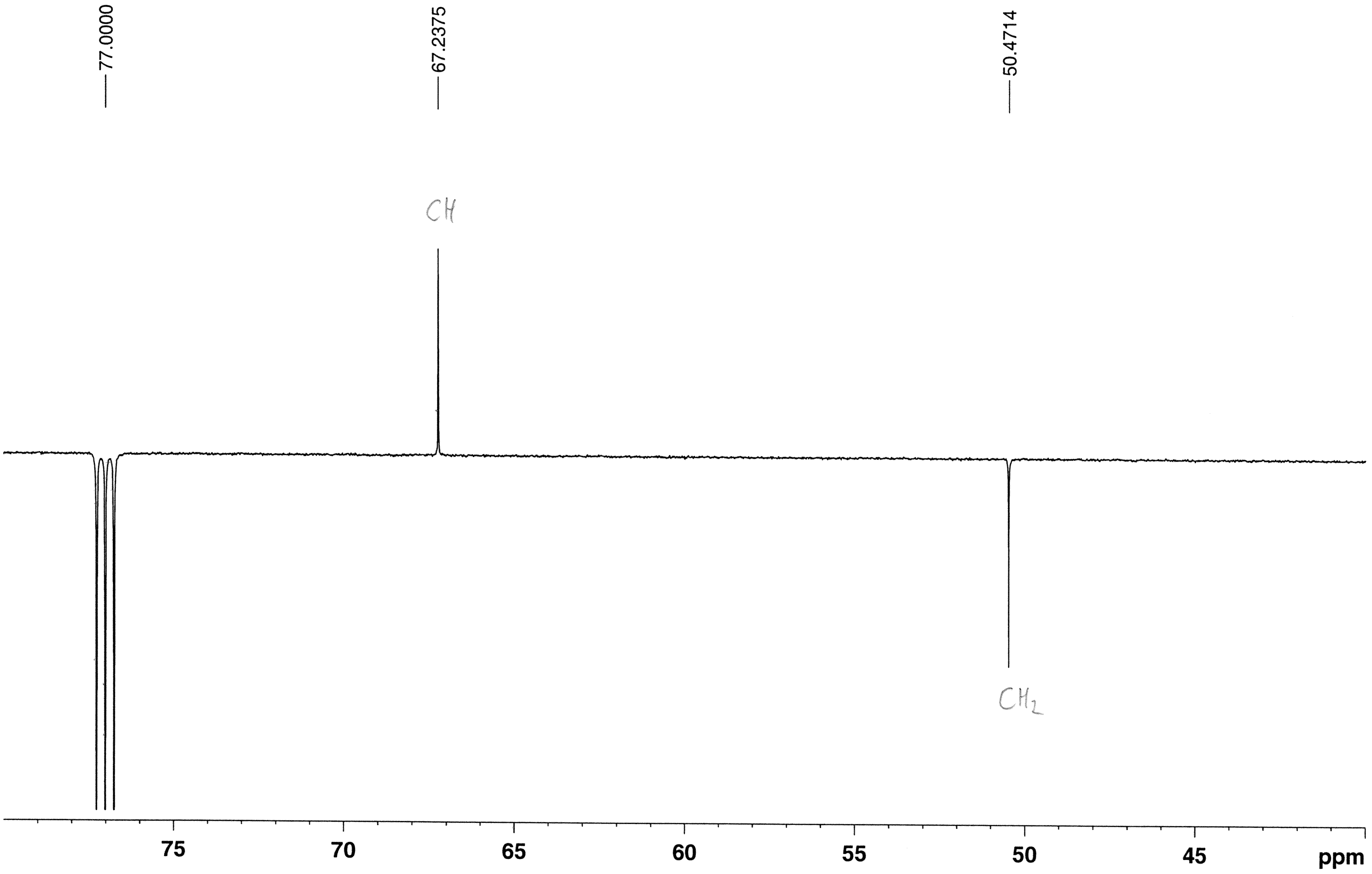

PN014-4 in cdcl3 (COSY) 29.7.2020

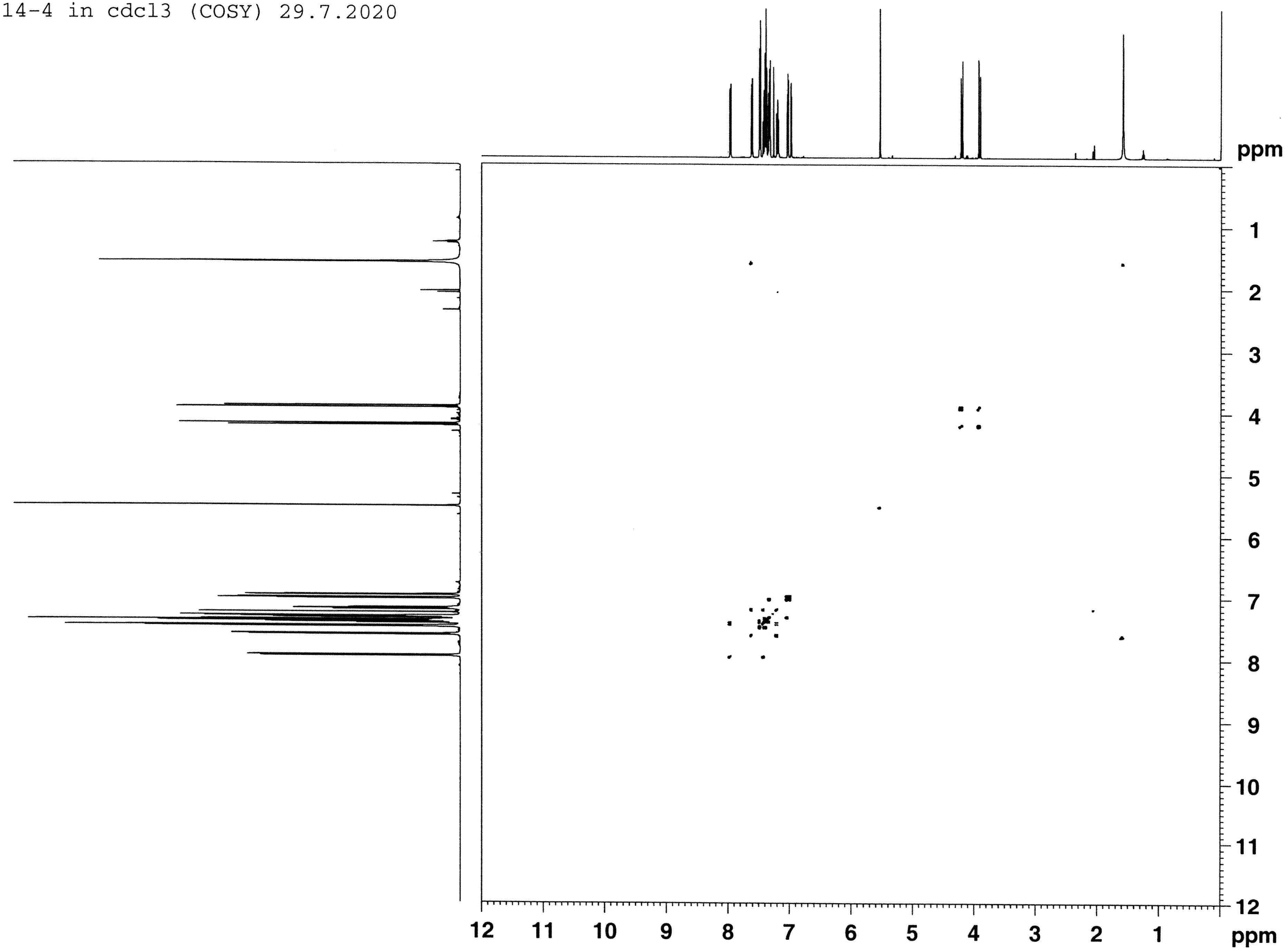

PN014-4 in cdcl3 (COSY) 29.7.2020

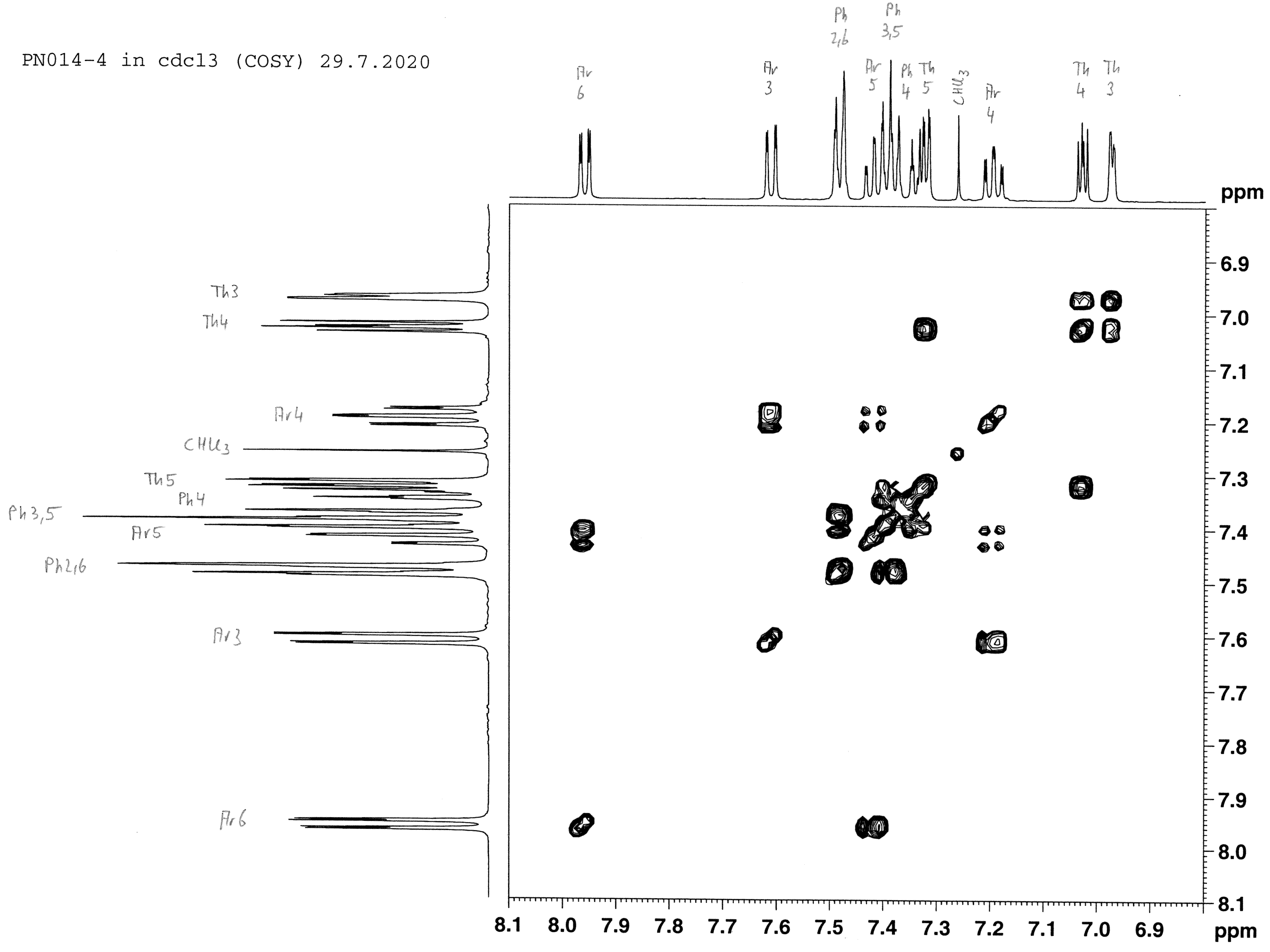

PN014-4 in cdcl3 (COSY) 29.7.2020

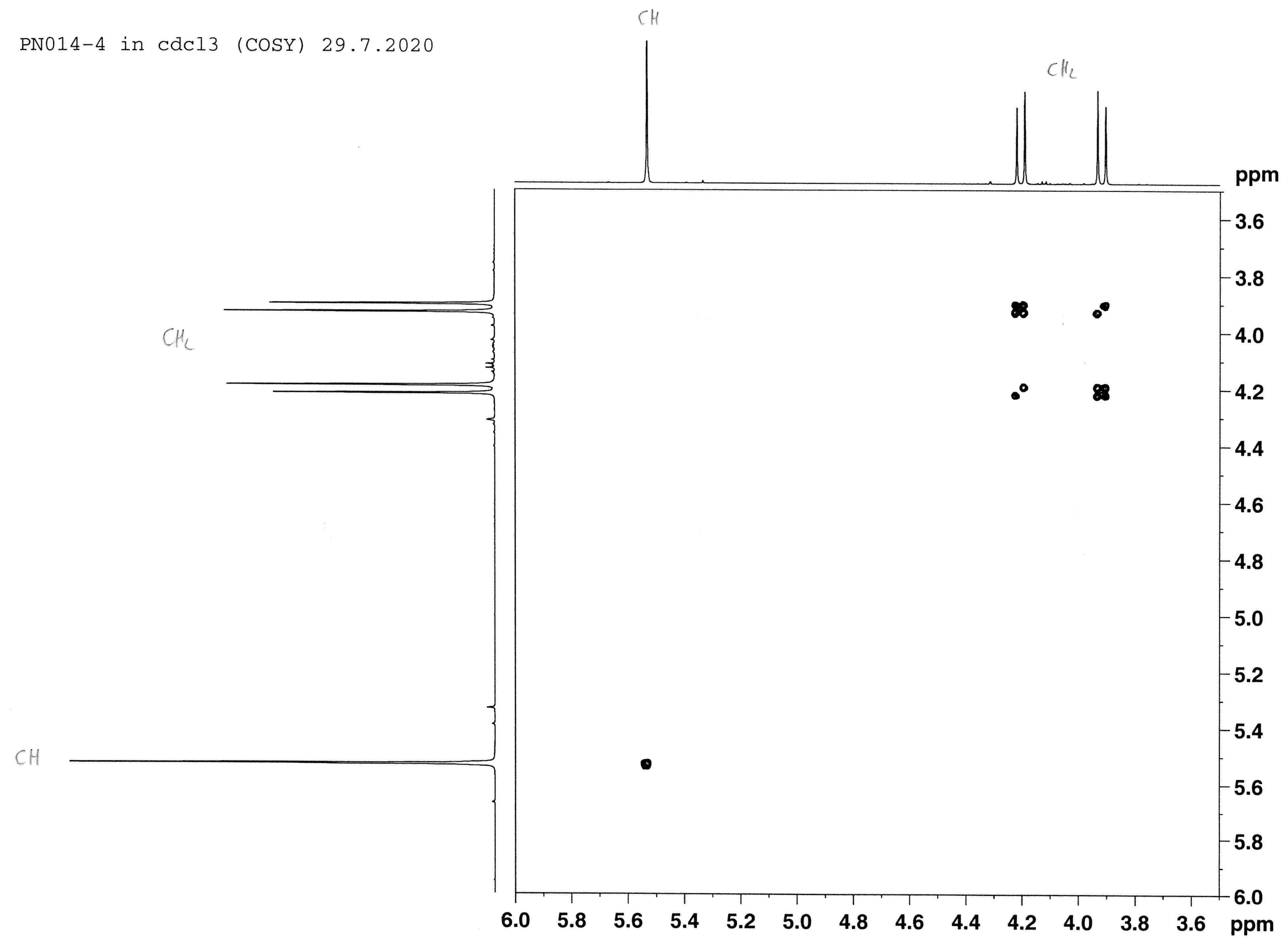

PN014-4 in cdcl3 (HSQC) 29.7.2020

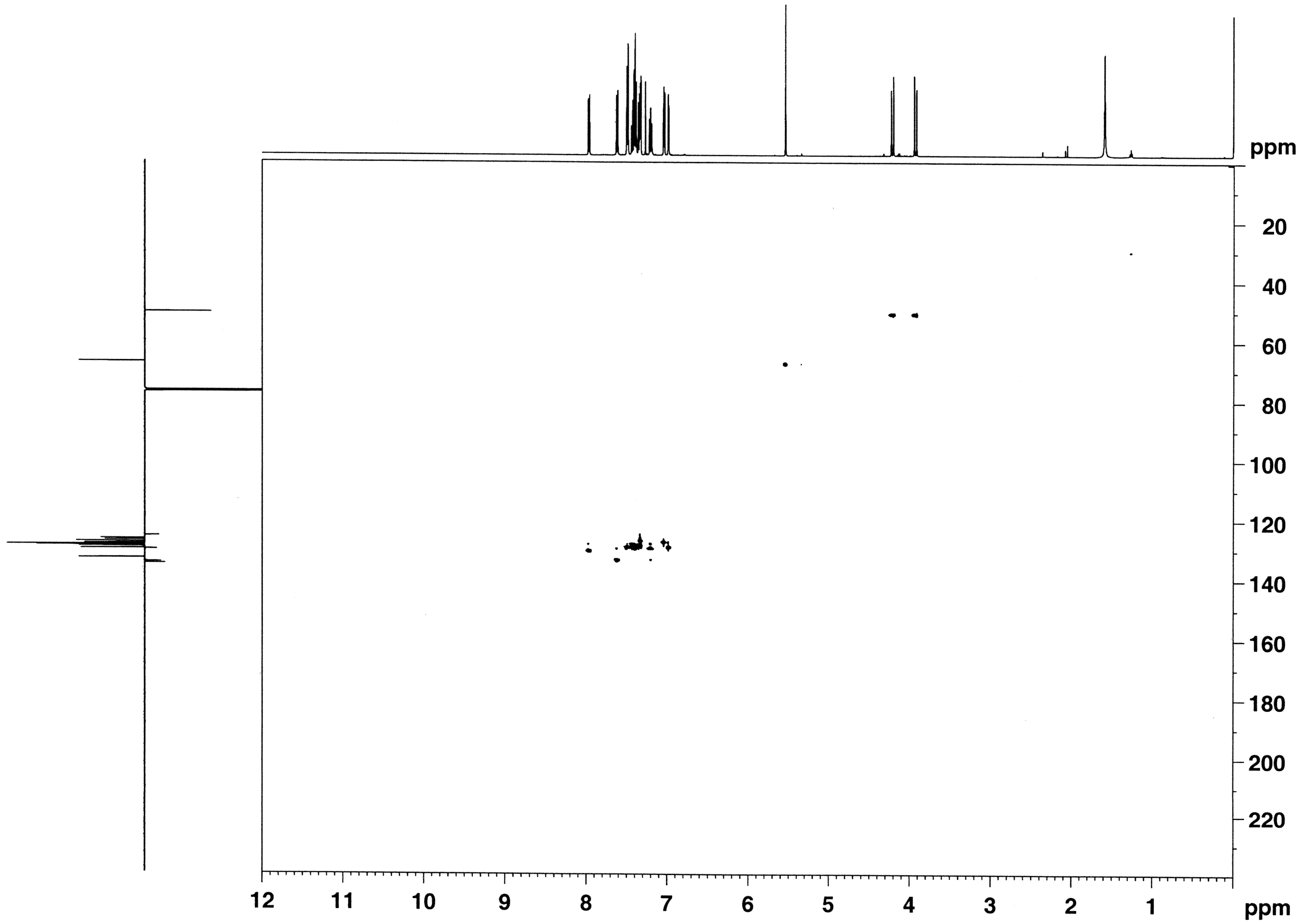

PN014-4 in cdcl3 (HSQC) 29.7.2020

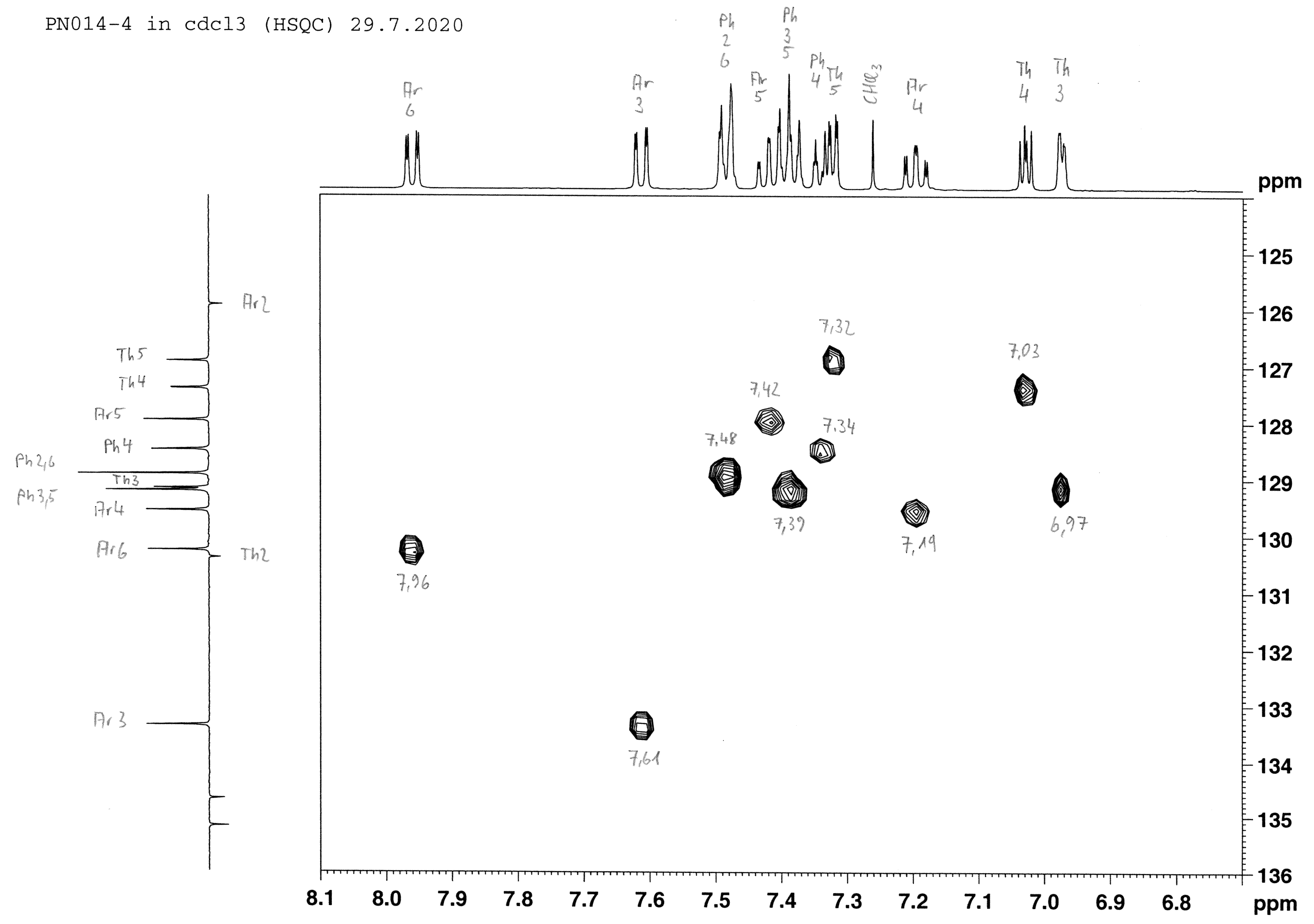

PN014-4 in cdcl3 (HSQC) 29.7.2020

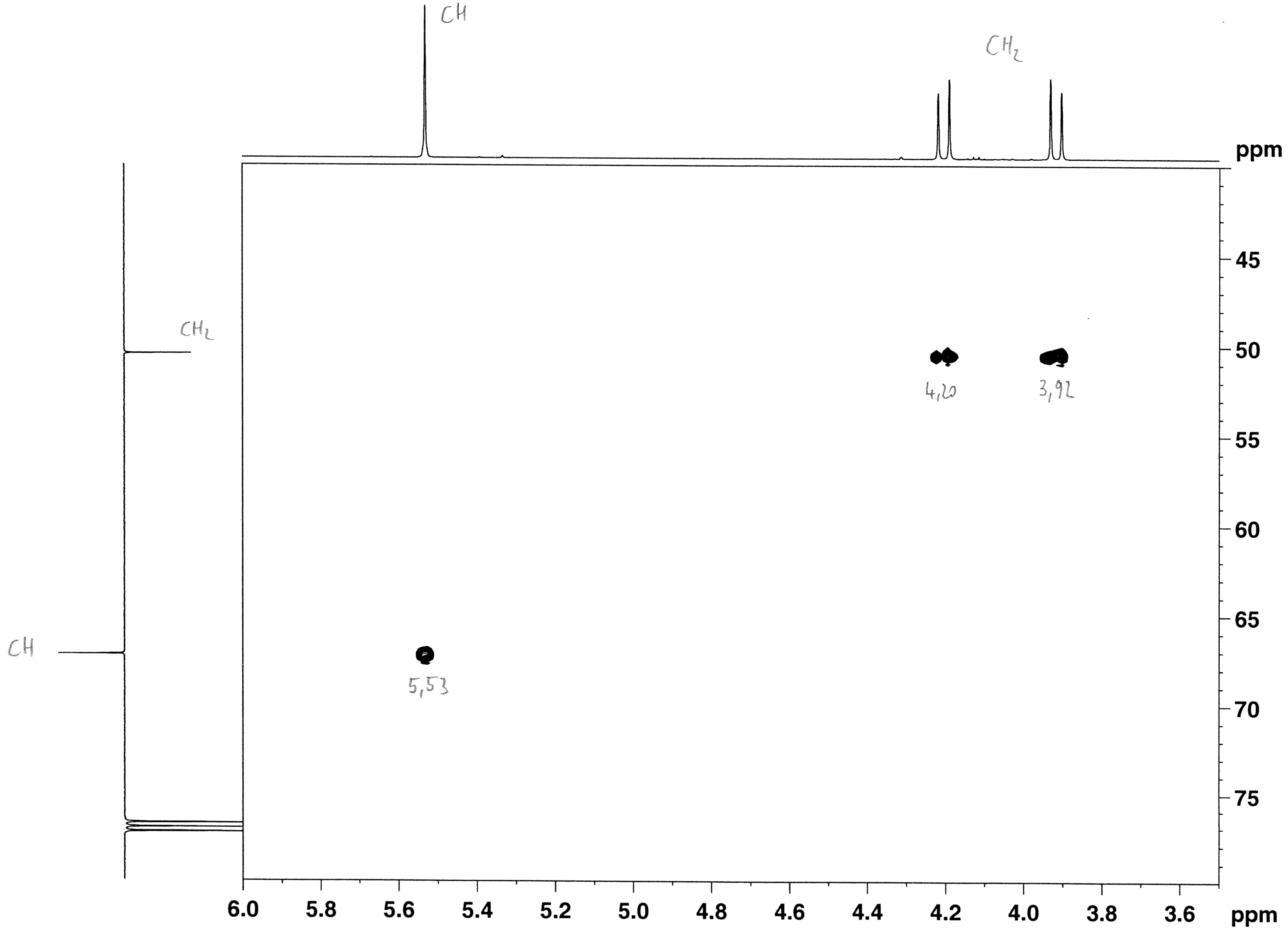

PN014-4 in cdcl3 (HMBC) 29.7.2020

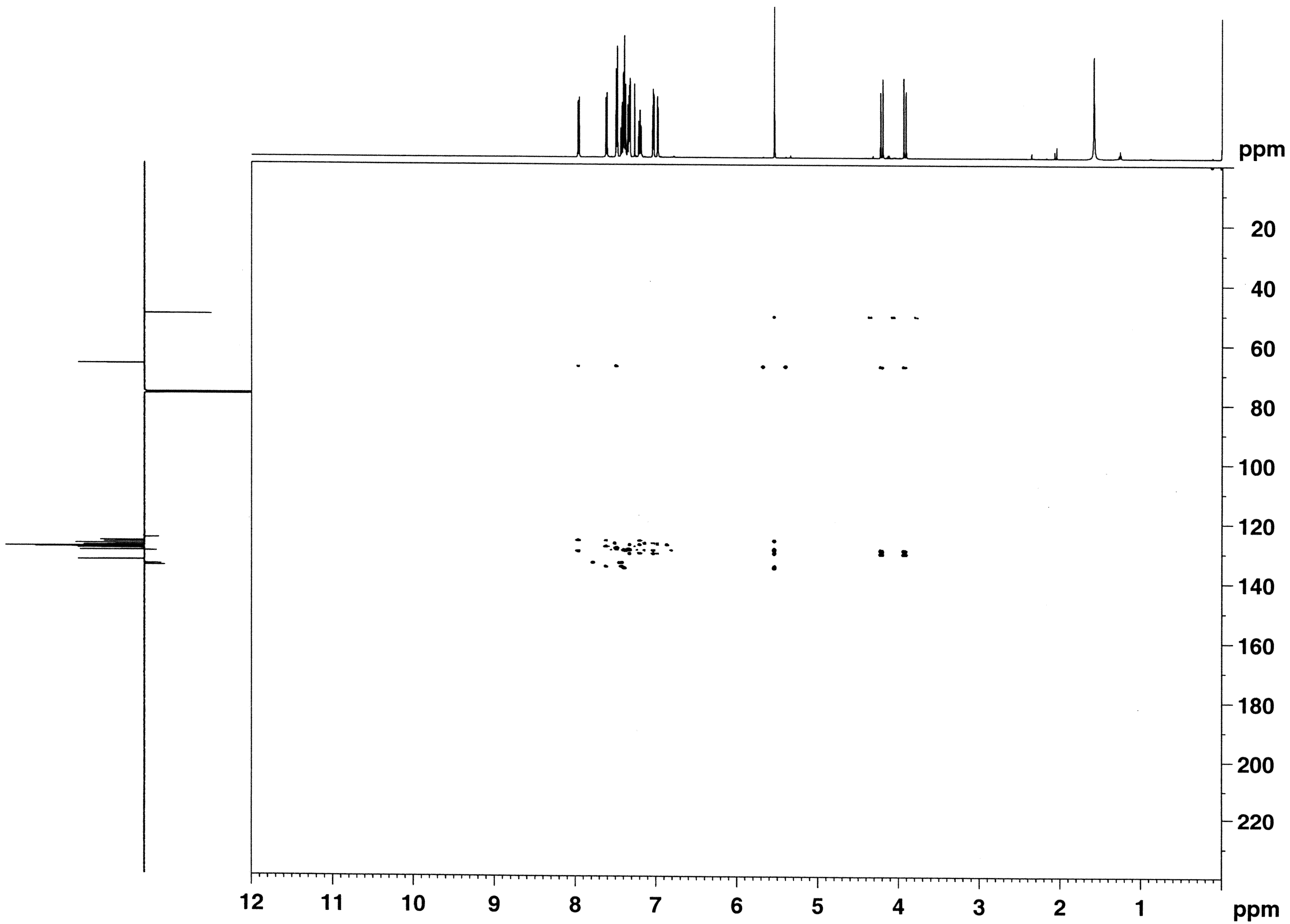

PN014-4 in cdcl3 (HMBC) 29.7.2020

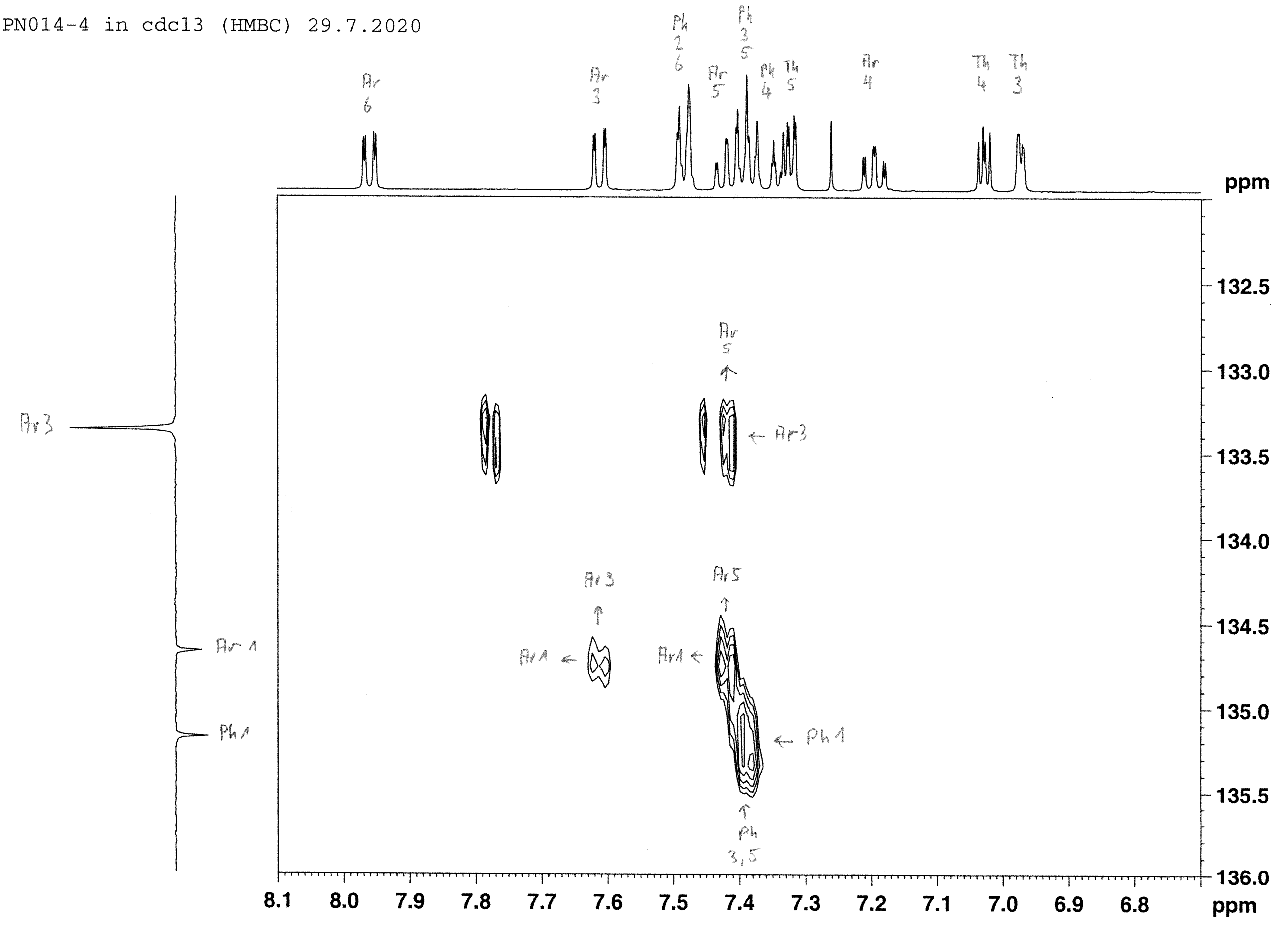

PN014-4 in cdcl3 (HMBC) 29.7.2020

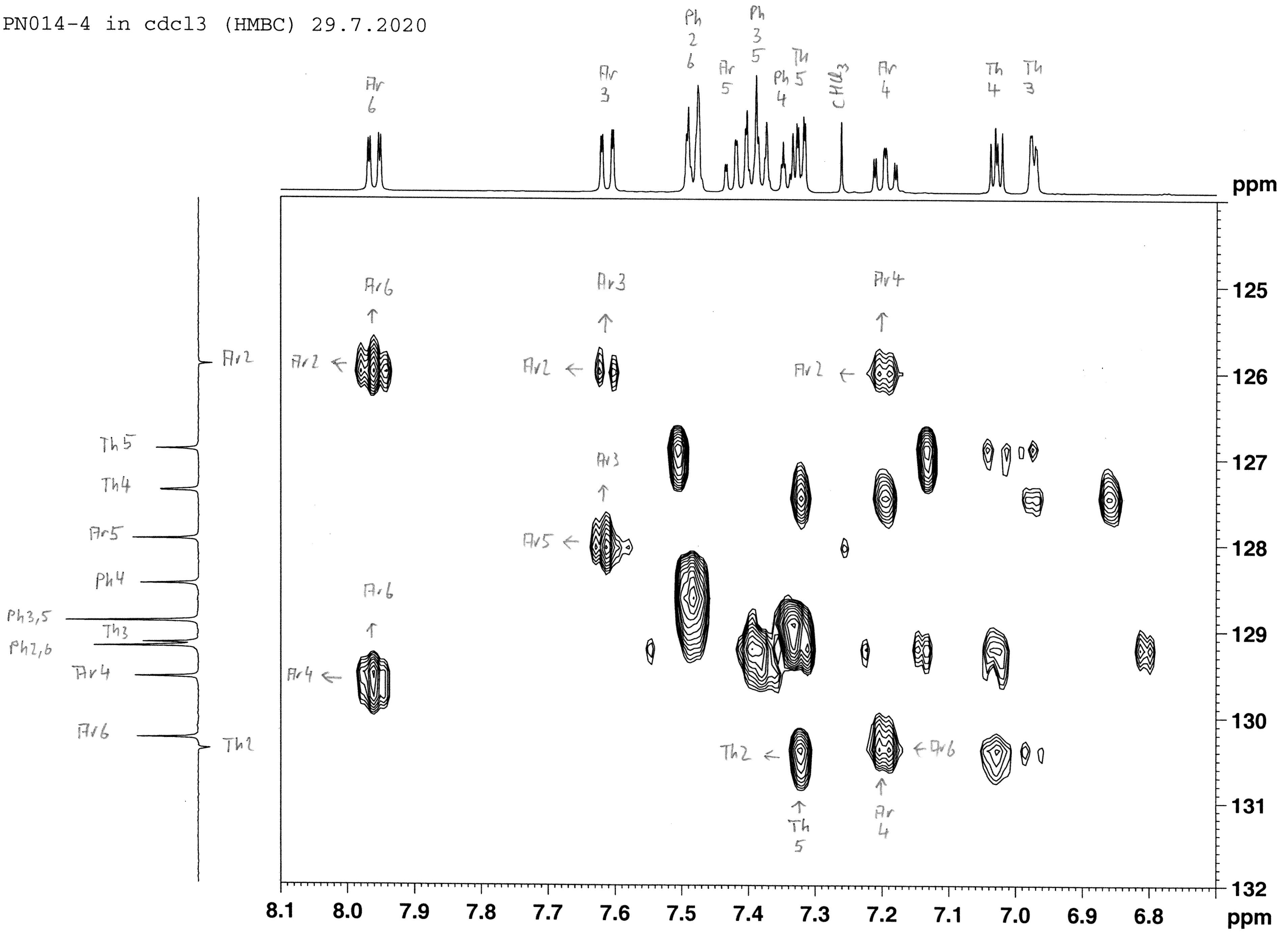

PN014-4 in cdcl3 (HMBC) 29.7.2020

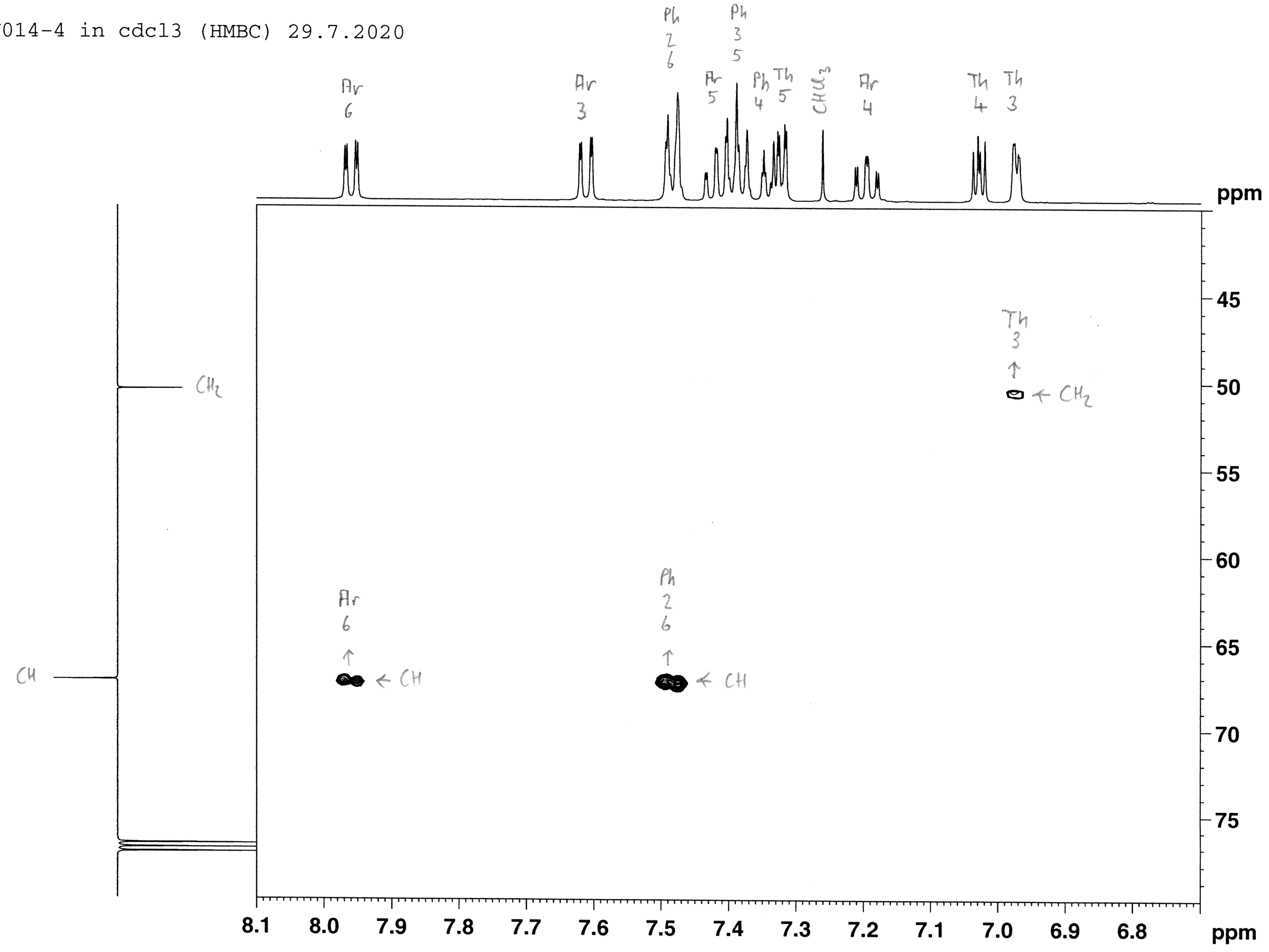

PN014-4 in cdcl3 (HMBC) 29.7.2020

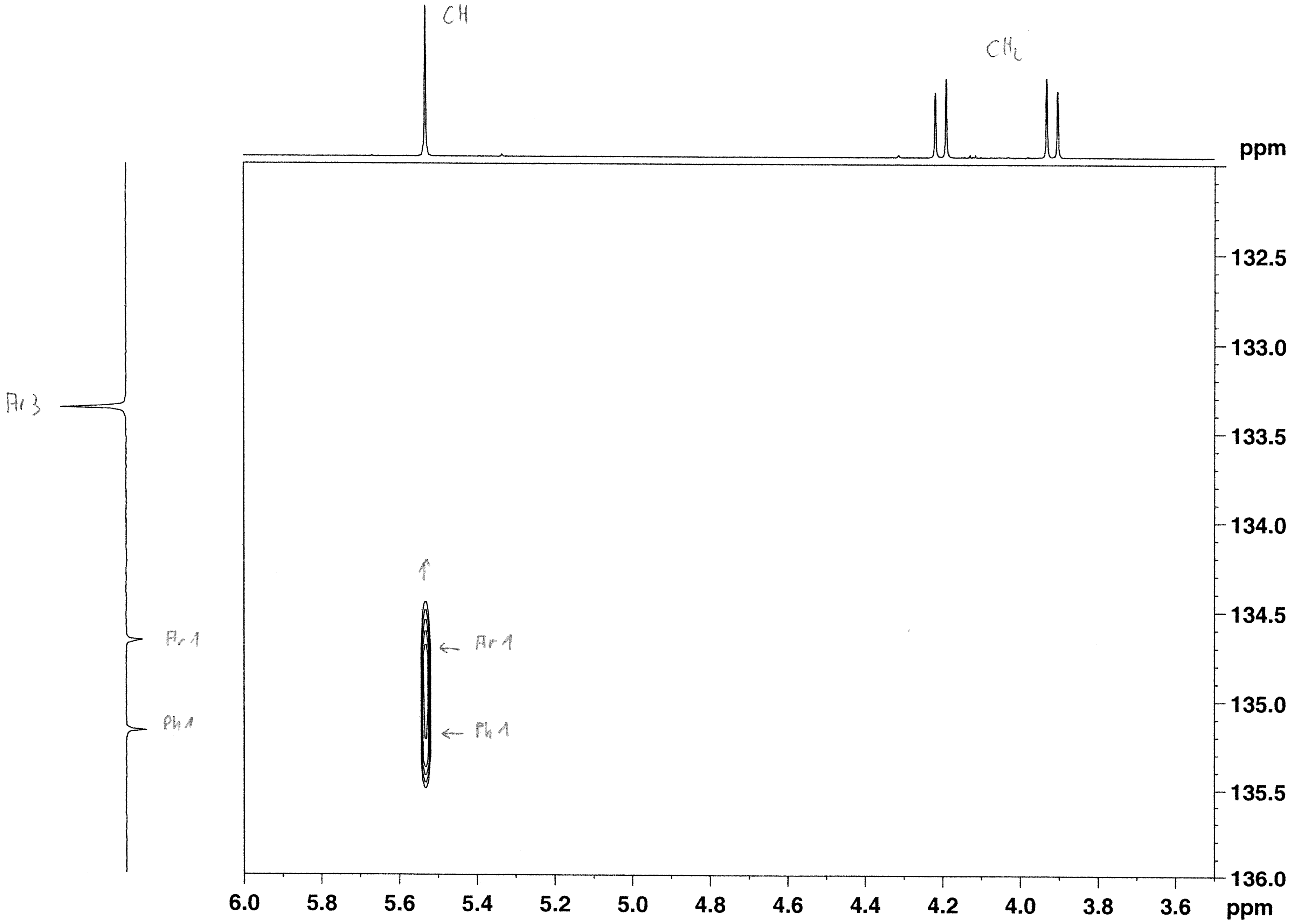

PN014-4 in cdcl3 (HMBC) 29.7.2020

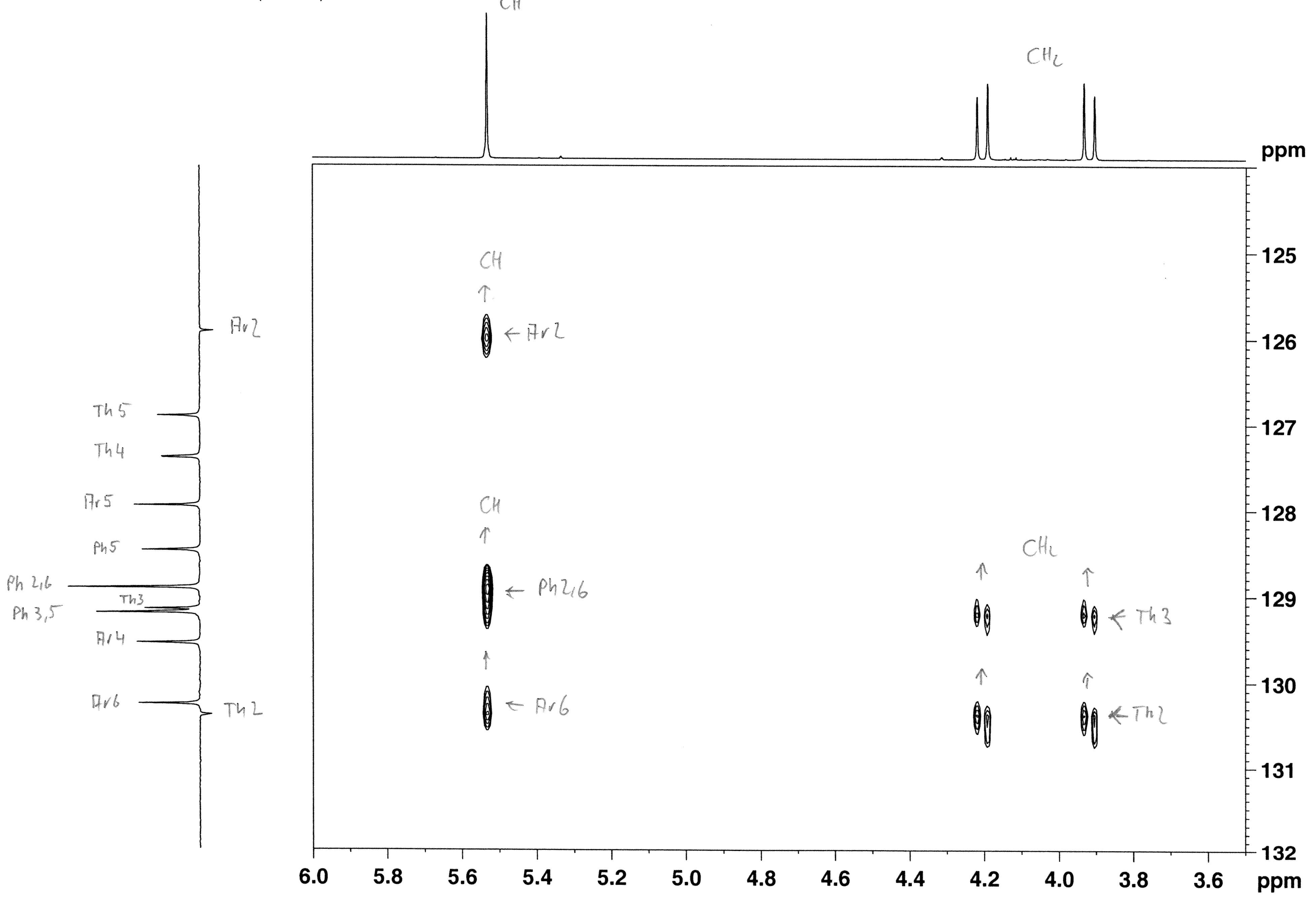

PN014-4 in cdcl3 (HMBC) 29.7.2020

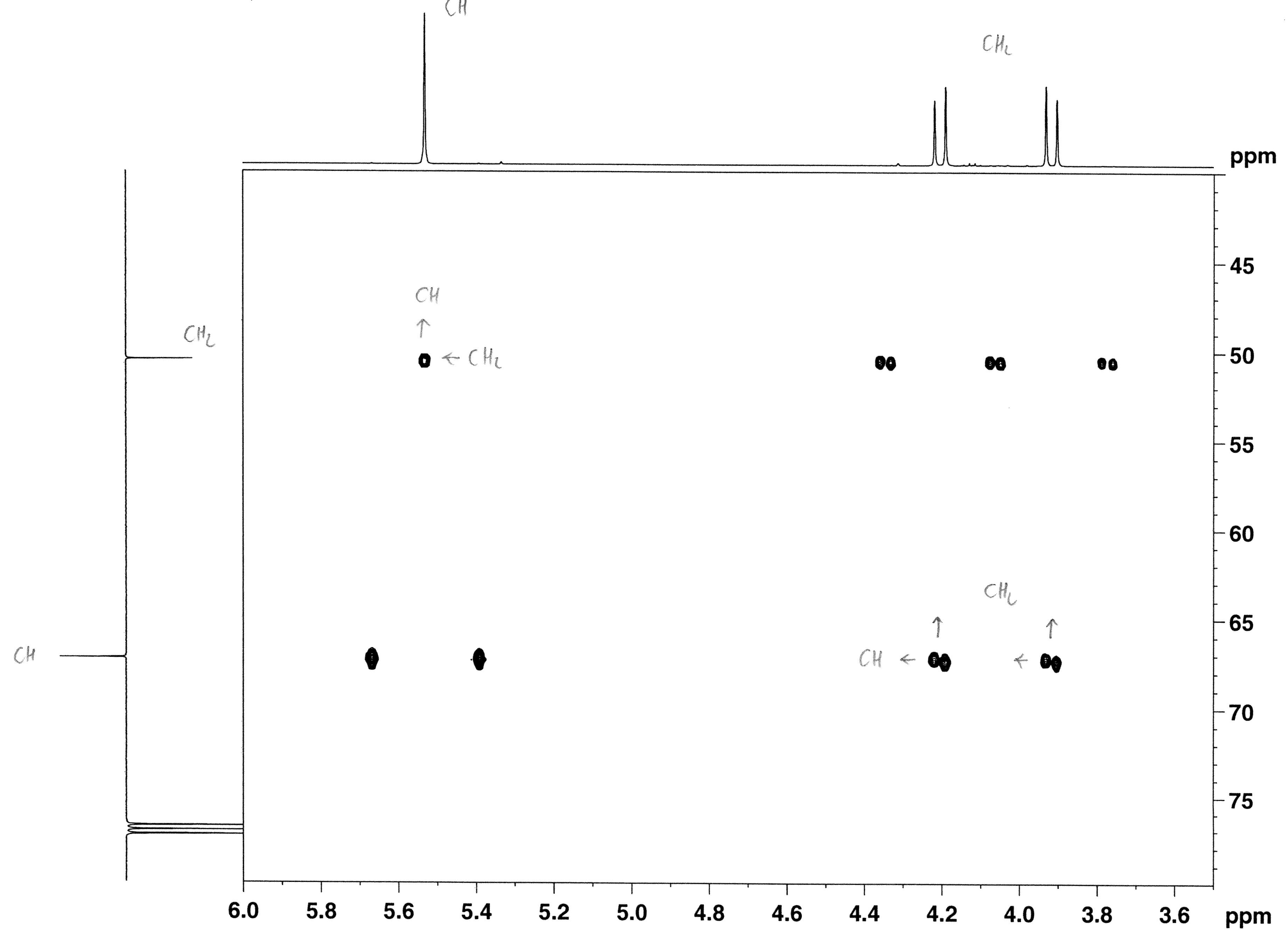

Figure S29c. NMR spectra of compound **5h.**

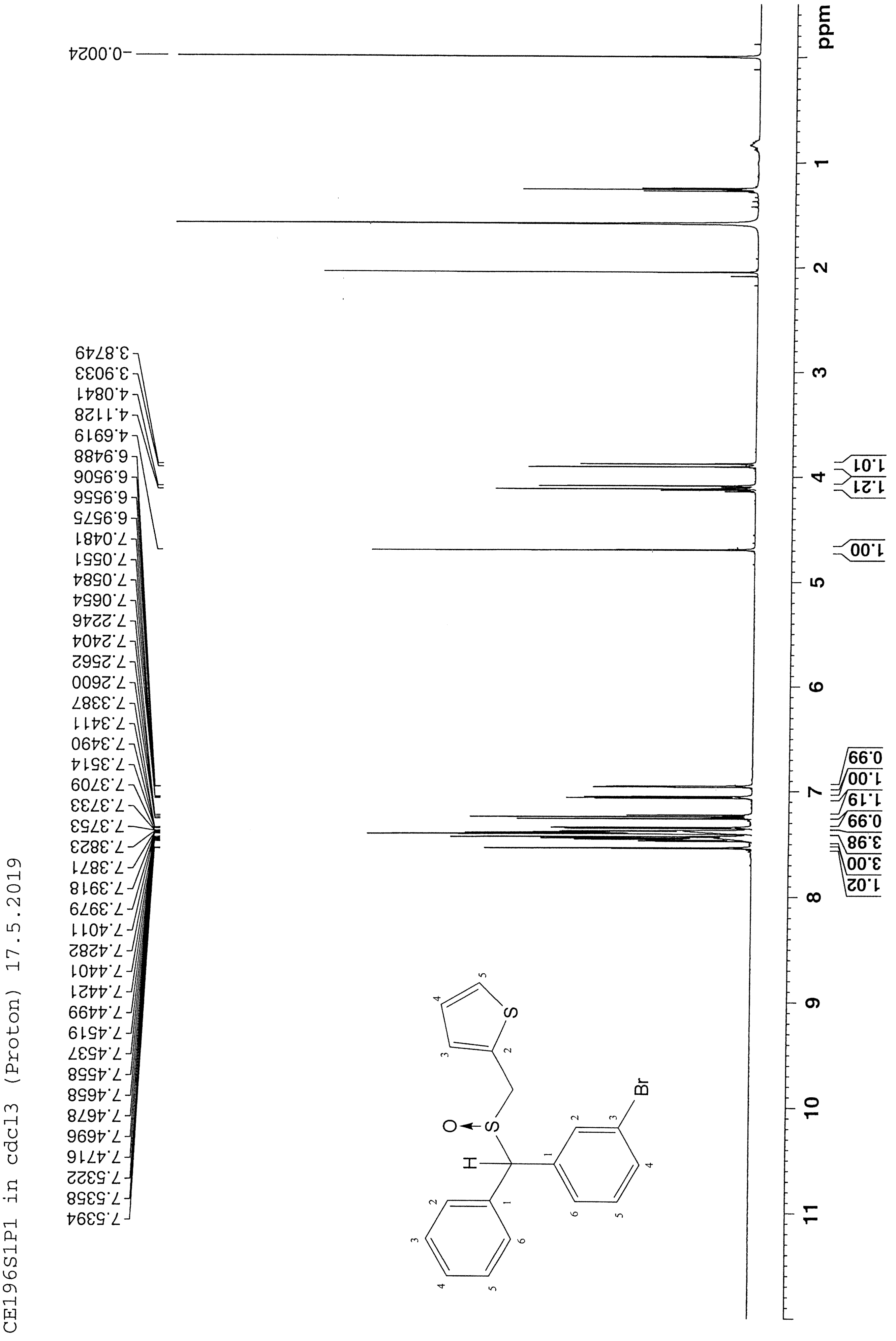

CE196S1P1 in cdcl3 (Proton) 17.5.2019

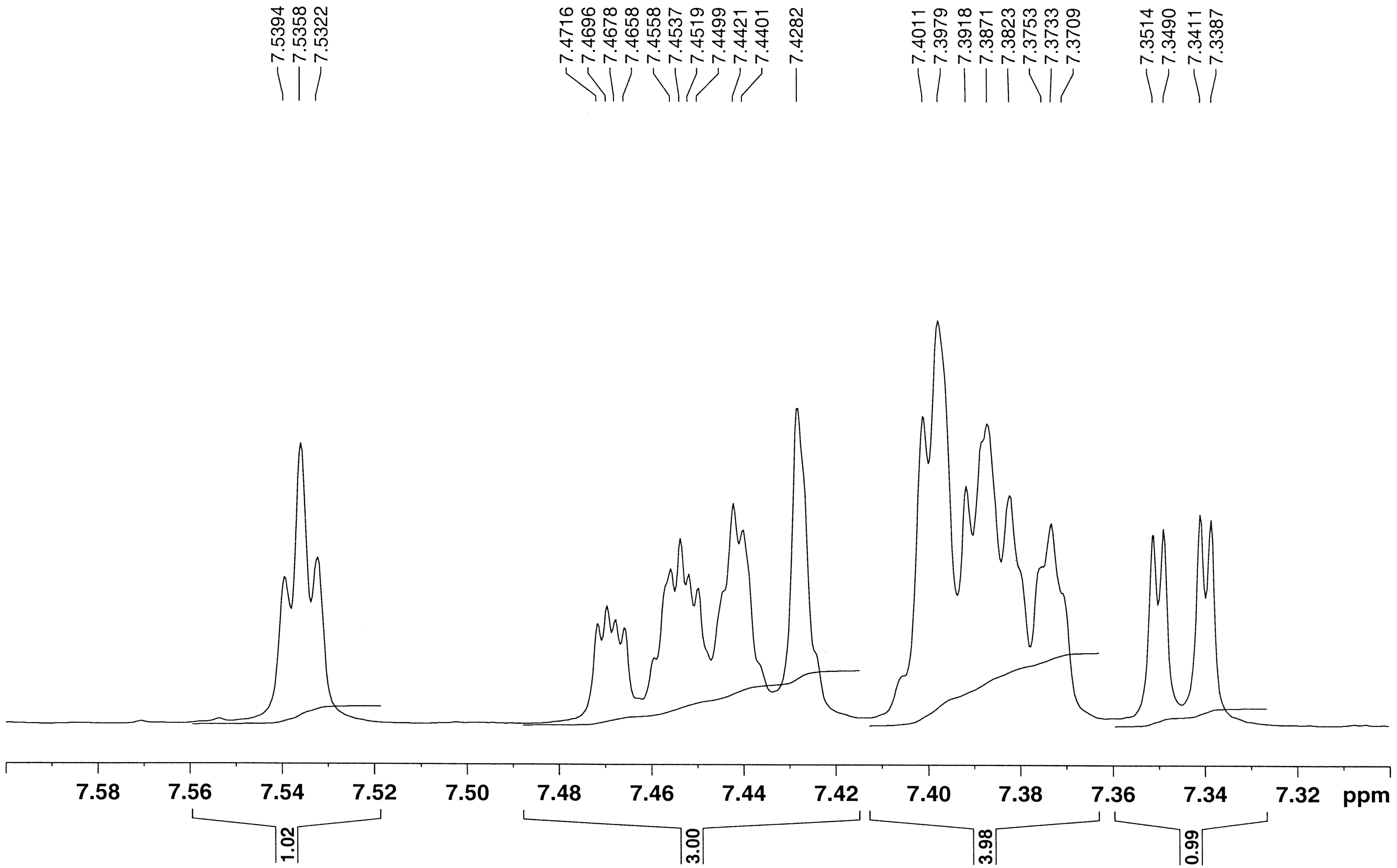

CE196S1P1 in cdcl3 (Proton) 17.5.2019

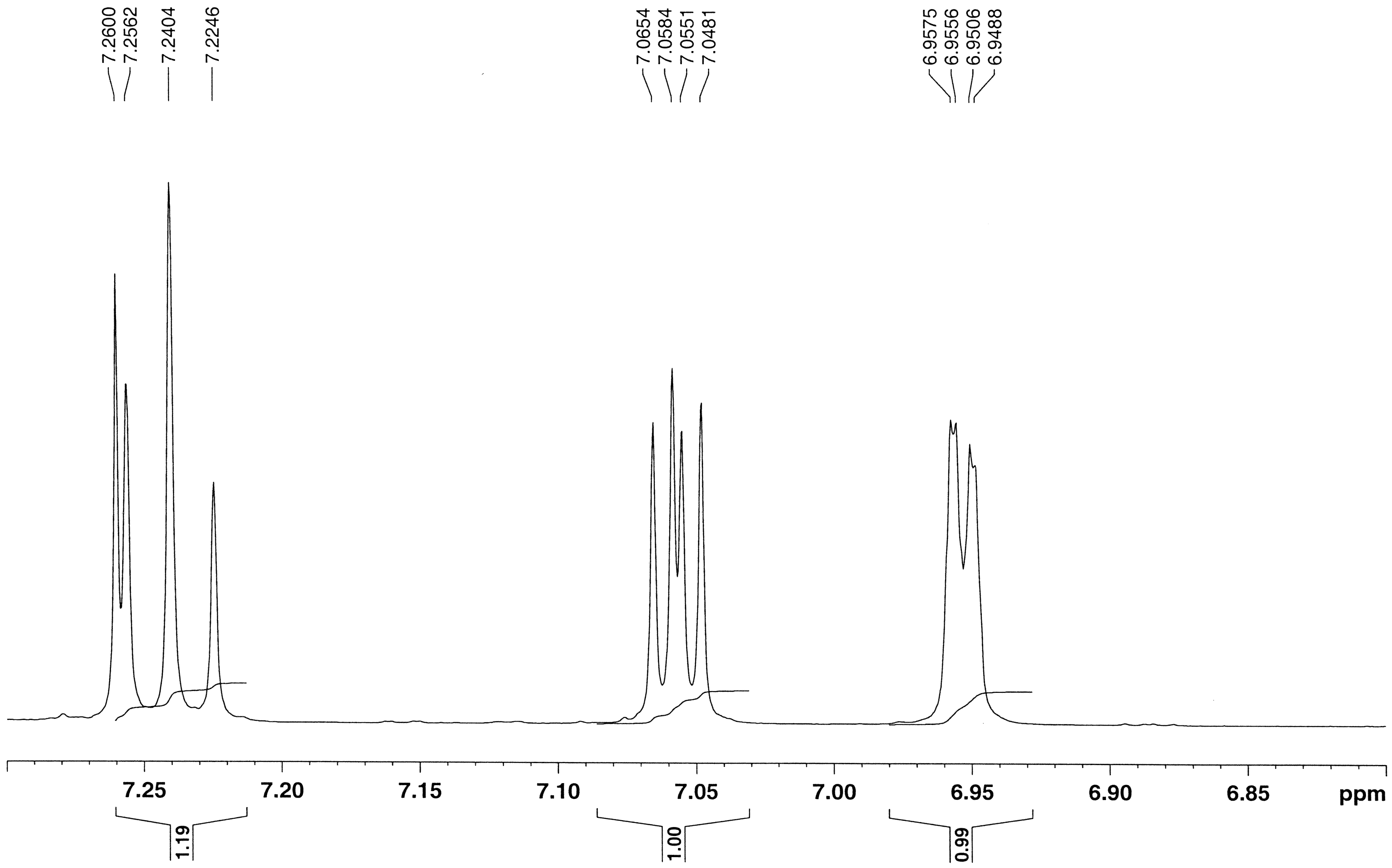

CE196S1P1 in cdcl3 (Proton) 17.5.2019

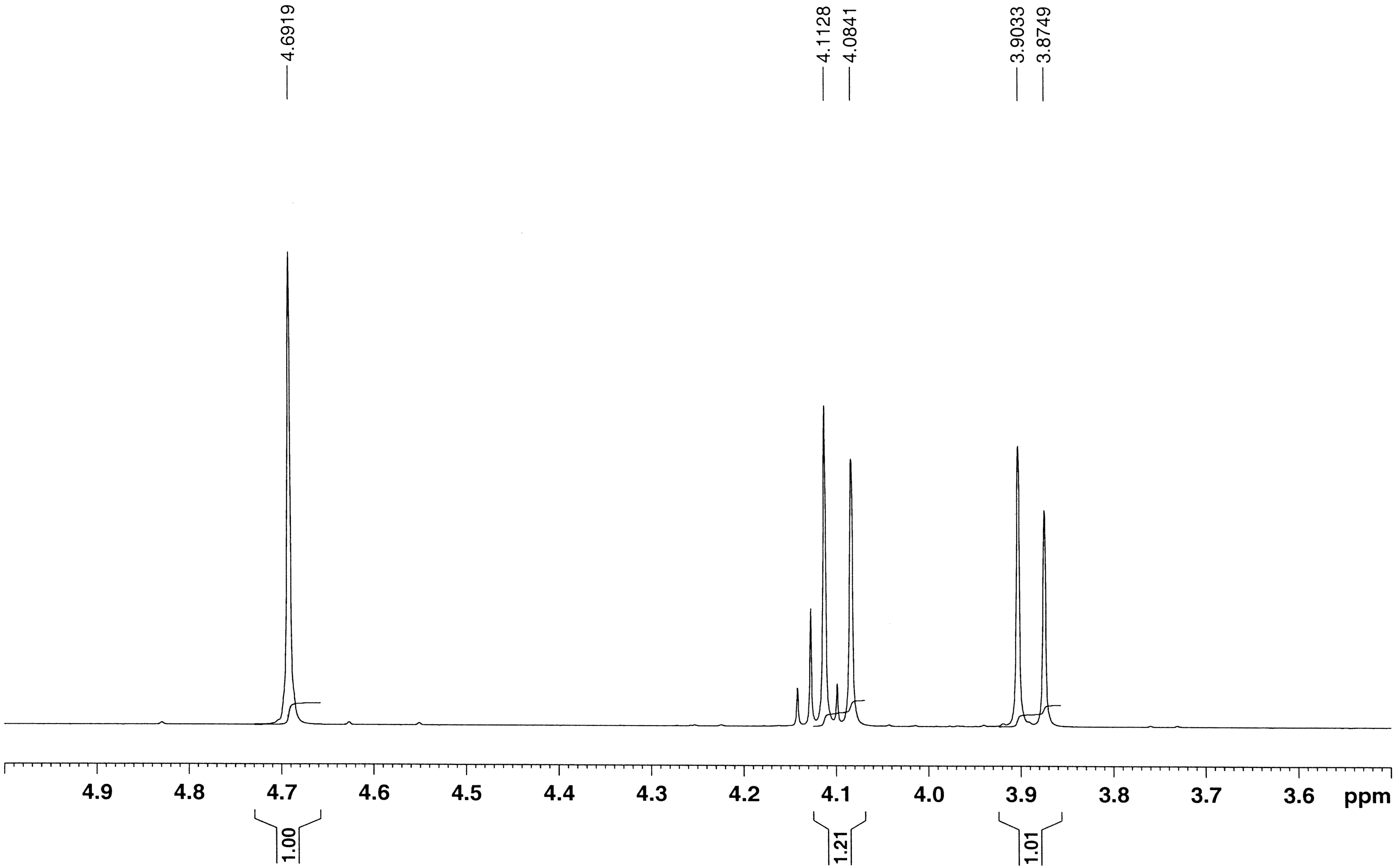

CE196S1P1 in cdcl3 (APT) 17.5.2019

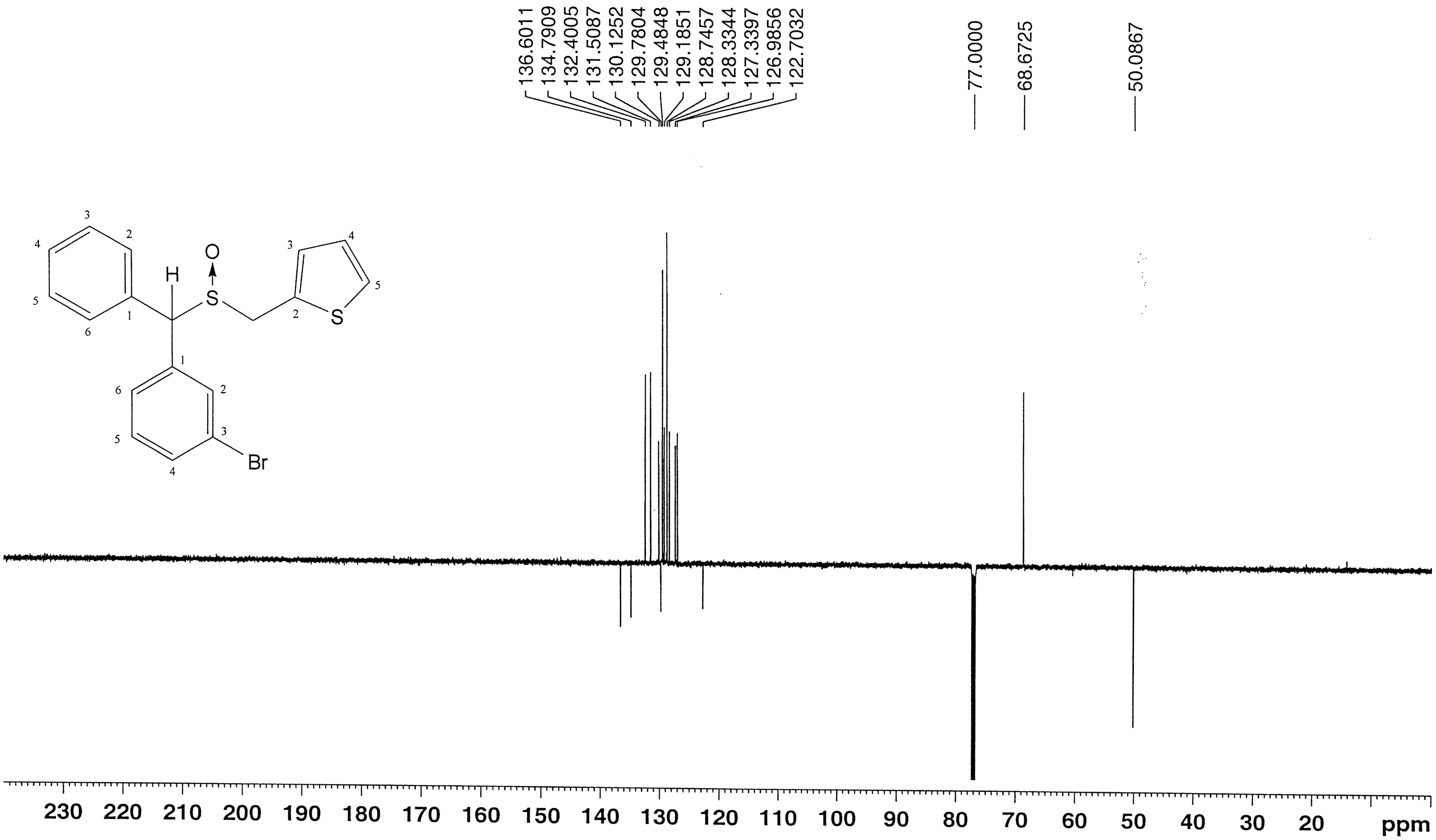

CE196S1P1 in cdcl3 (APT) 17.5.2019

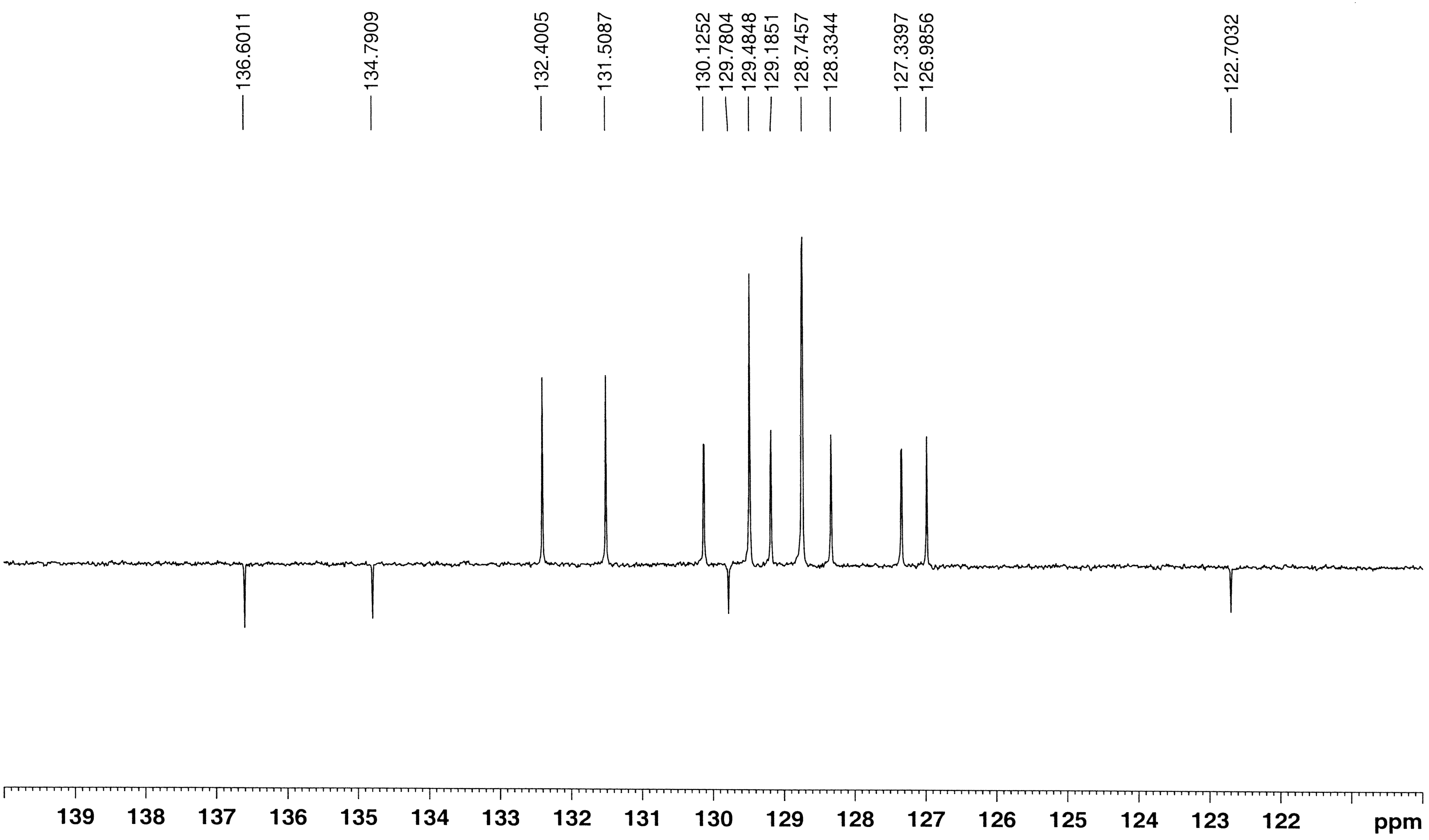

CE196S1P1 in cdcl3 (APT) 17.5.2019

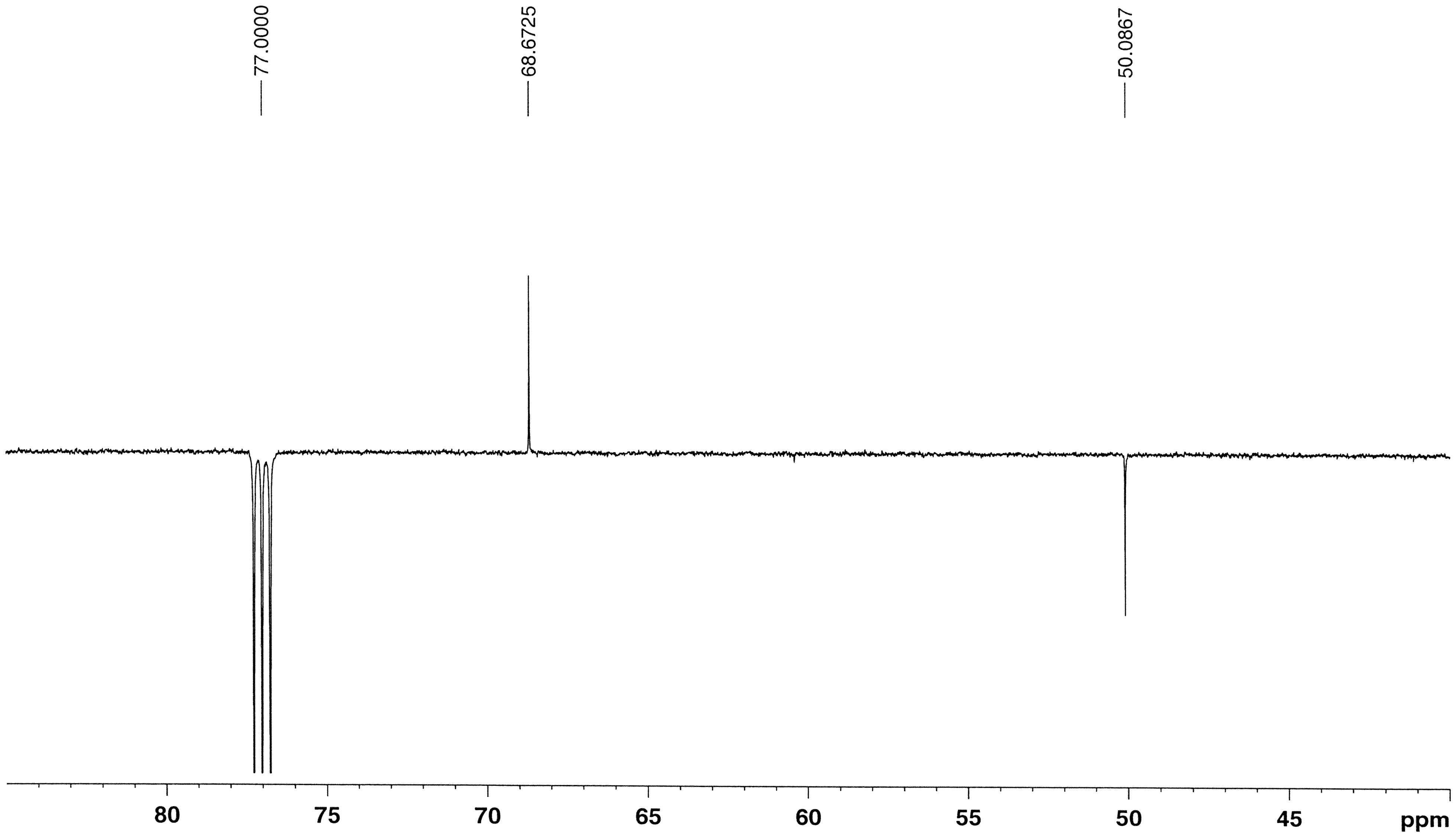

Figure S30c. NMR spectra of compound **6h**.

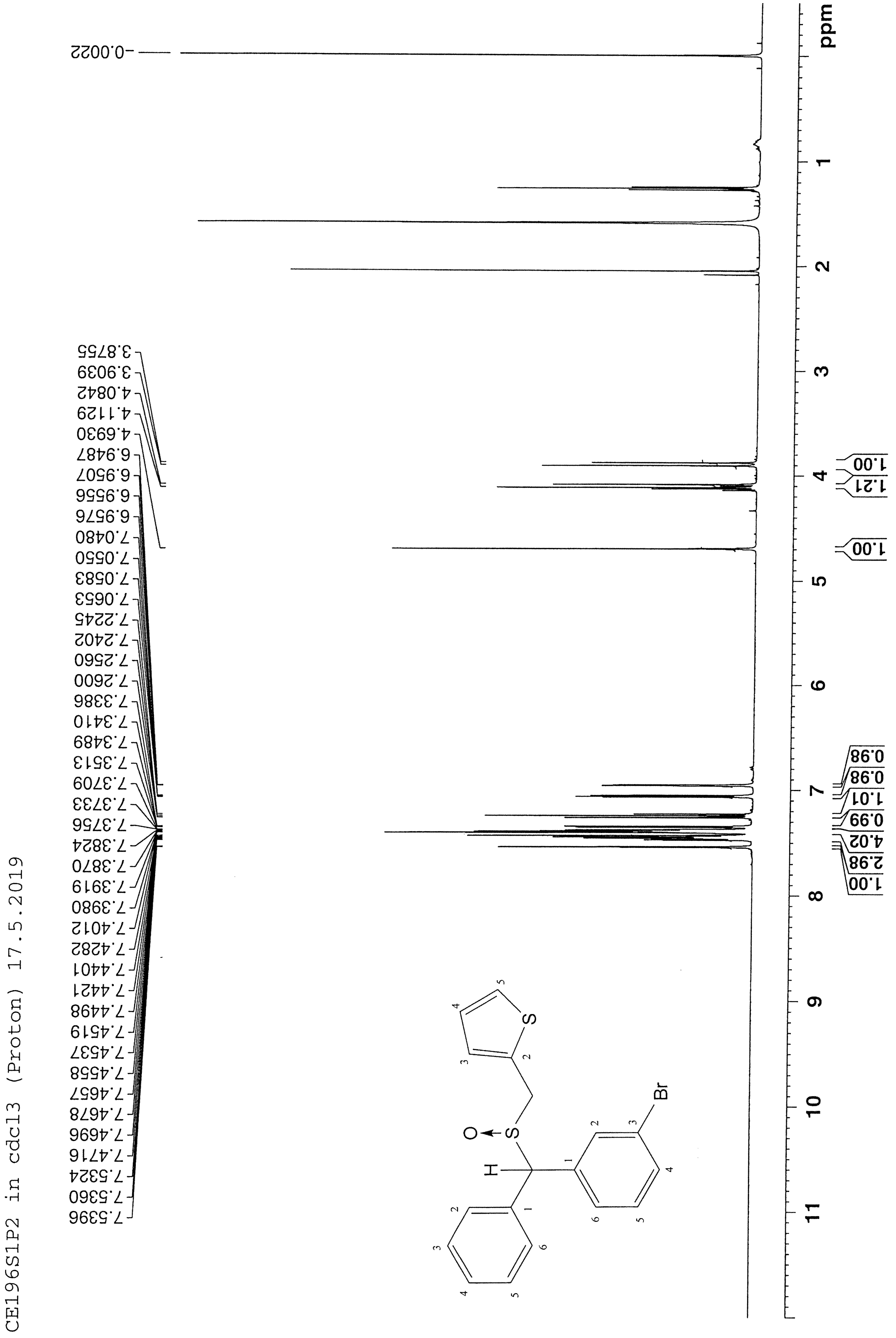

CE196S1P2 in cdcl3 (Proton) 17.5.2019

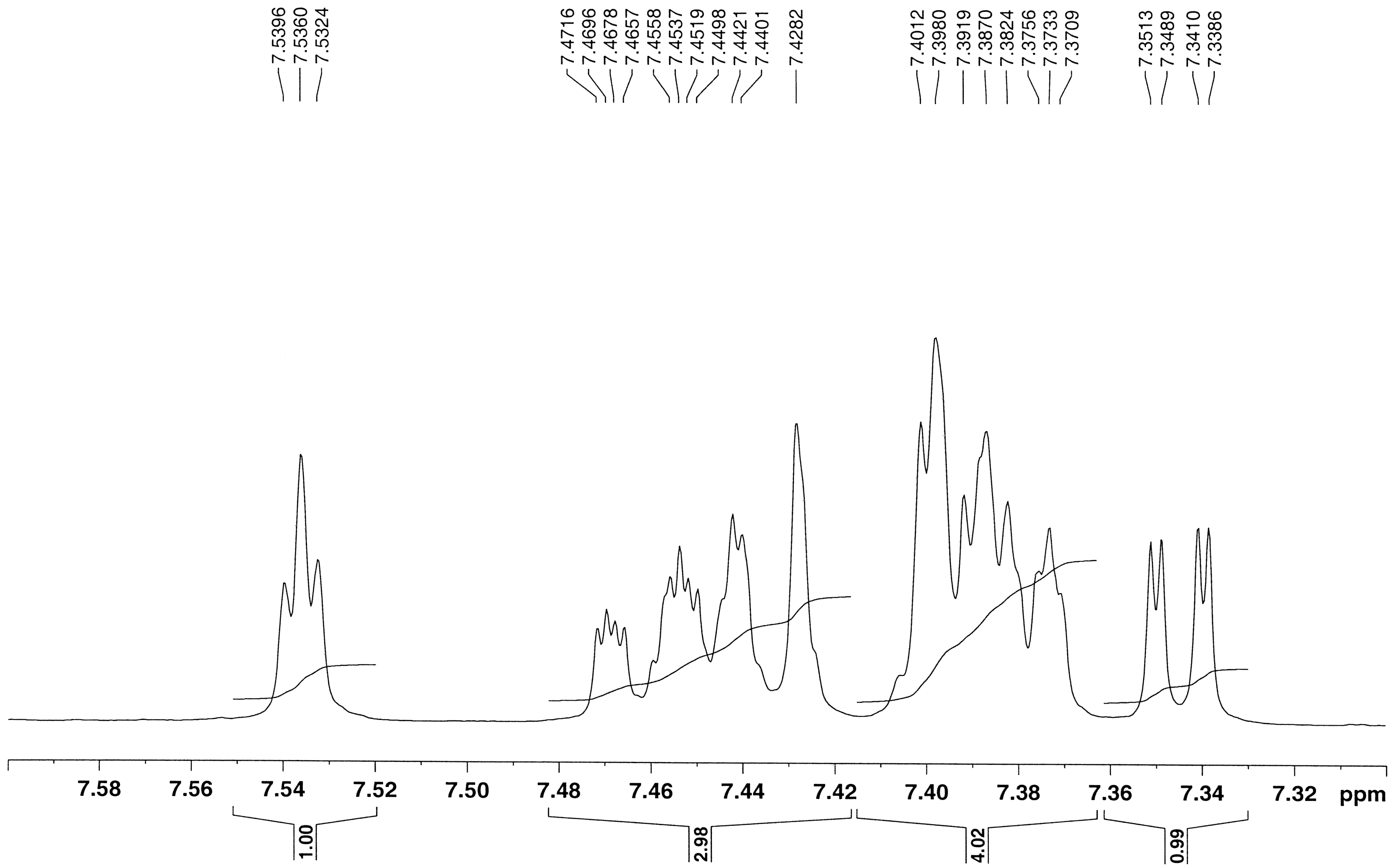

CE196S1P2 in cdcl3 (Proton) 17.5.2019

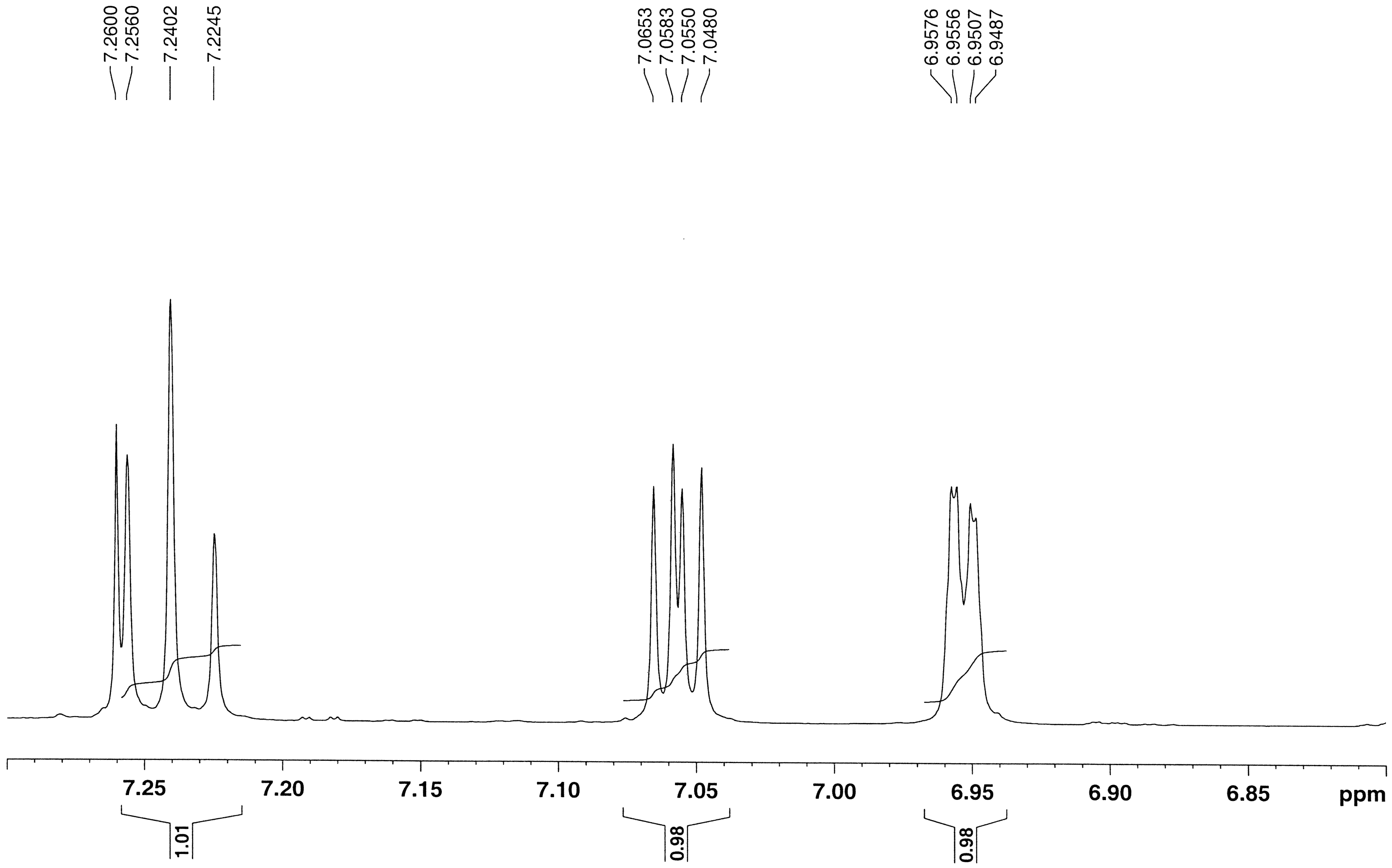

CE196S1P2 in cdcl3 (Proton) 17.5.2019

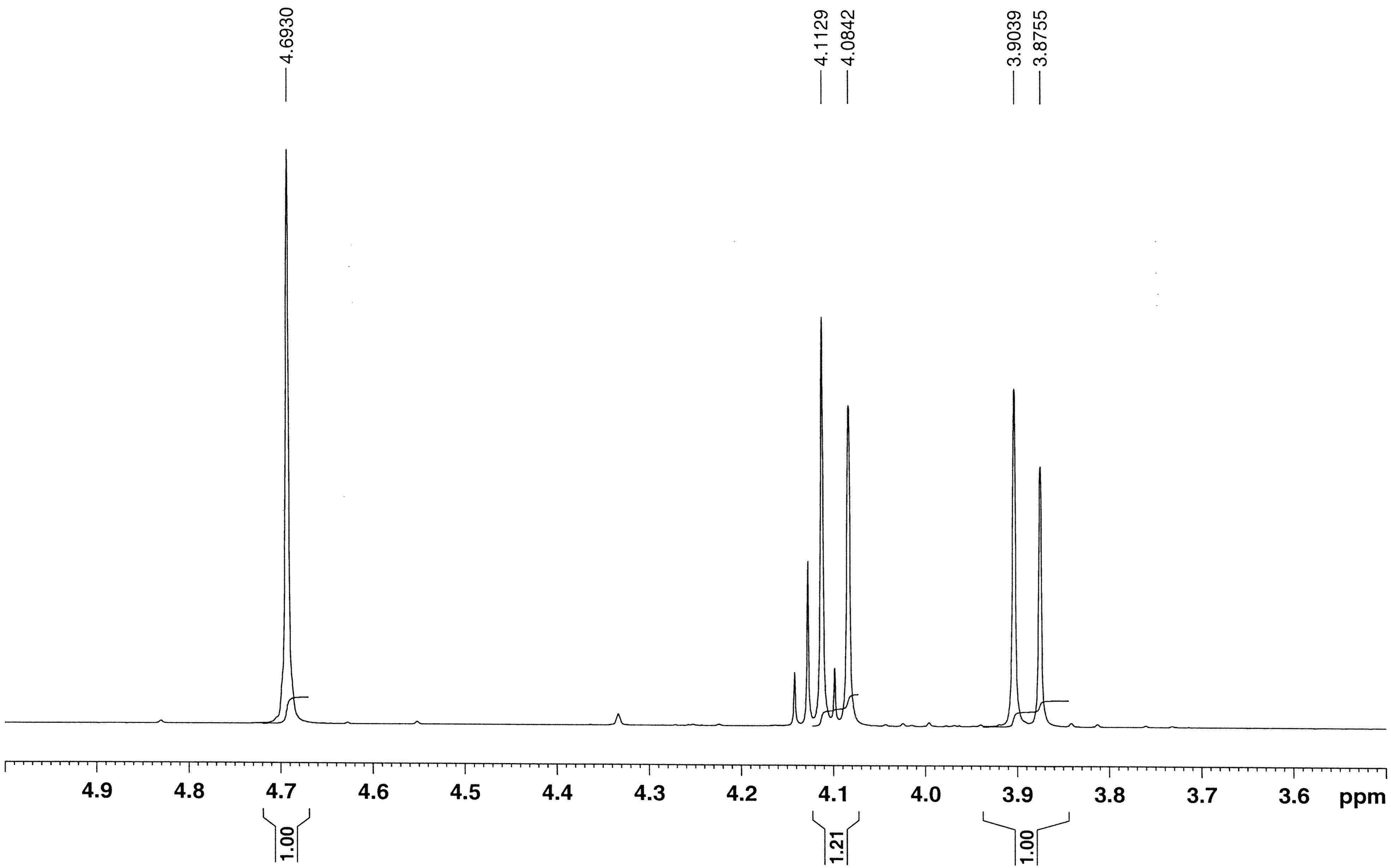

CE196S1P2 in cdcl3 (APT) 17.5.2019

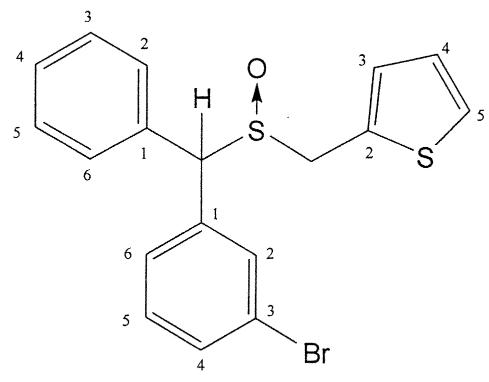

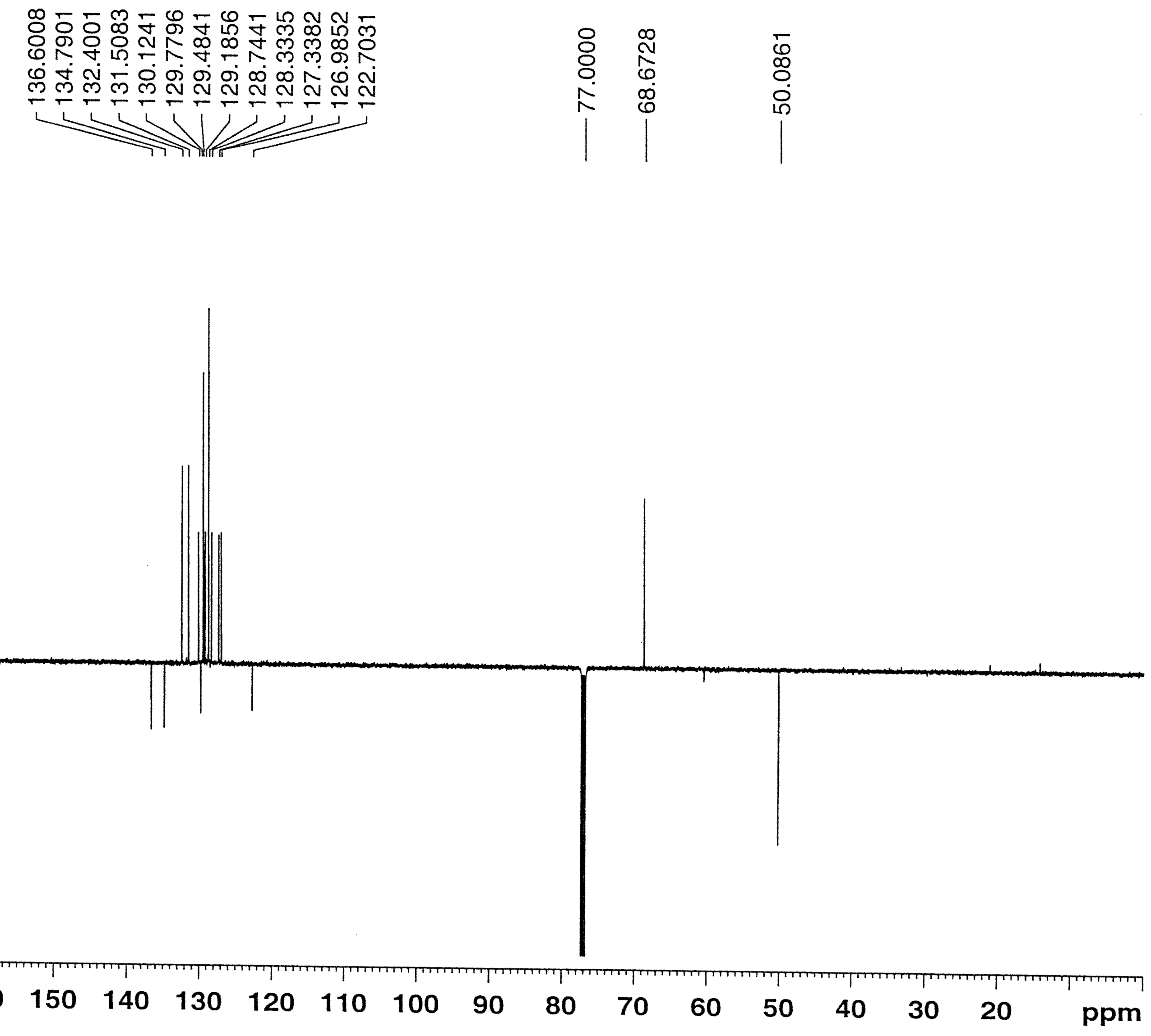

CE196S1P2 in cdcl3 (APT) 17.5.2019

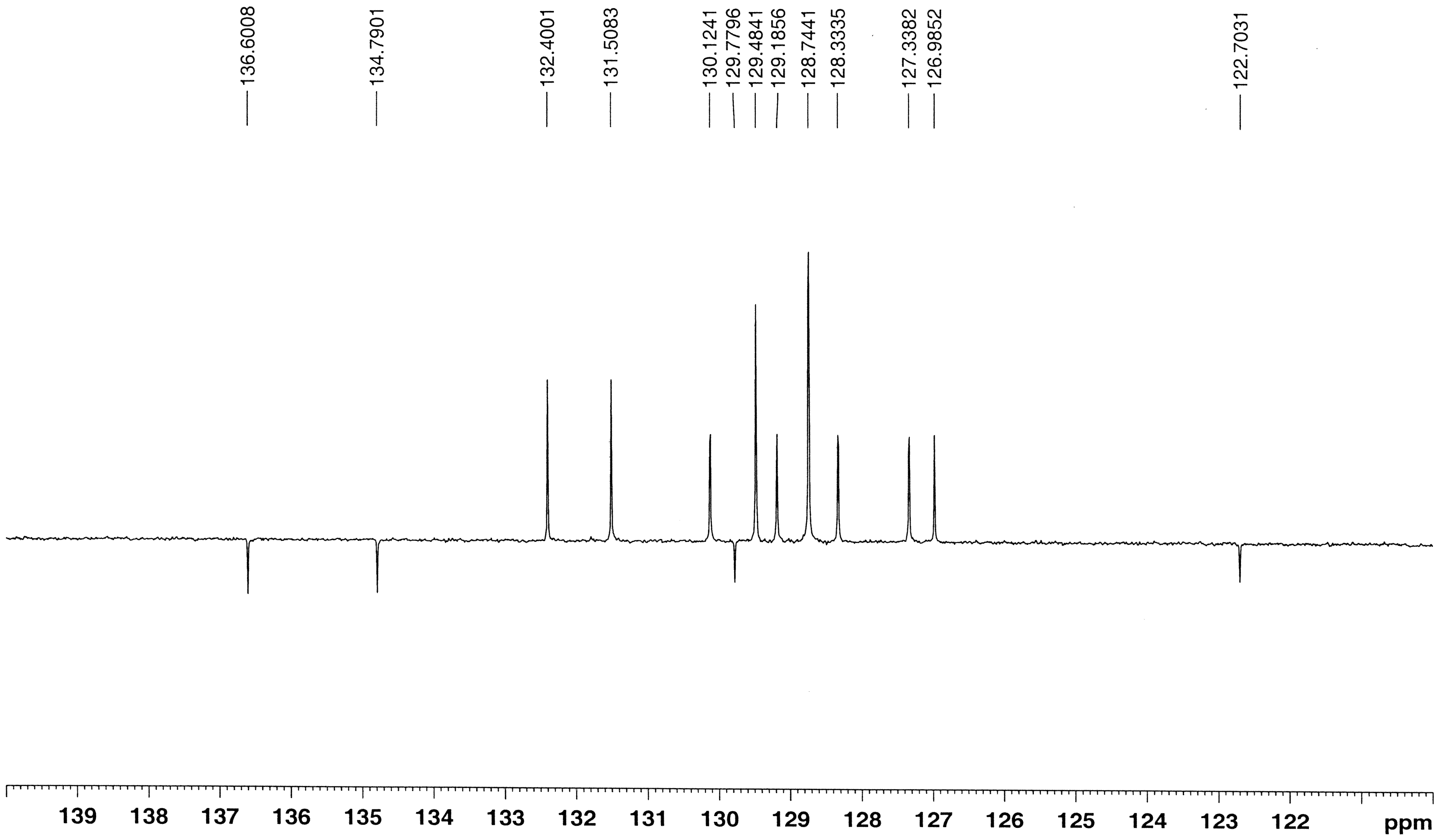

CE196S1P2 in cdcl3 (APT) 17.5.2019

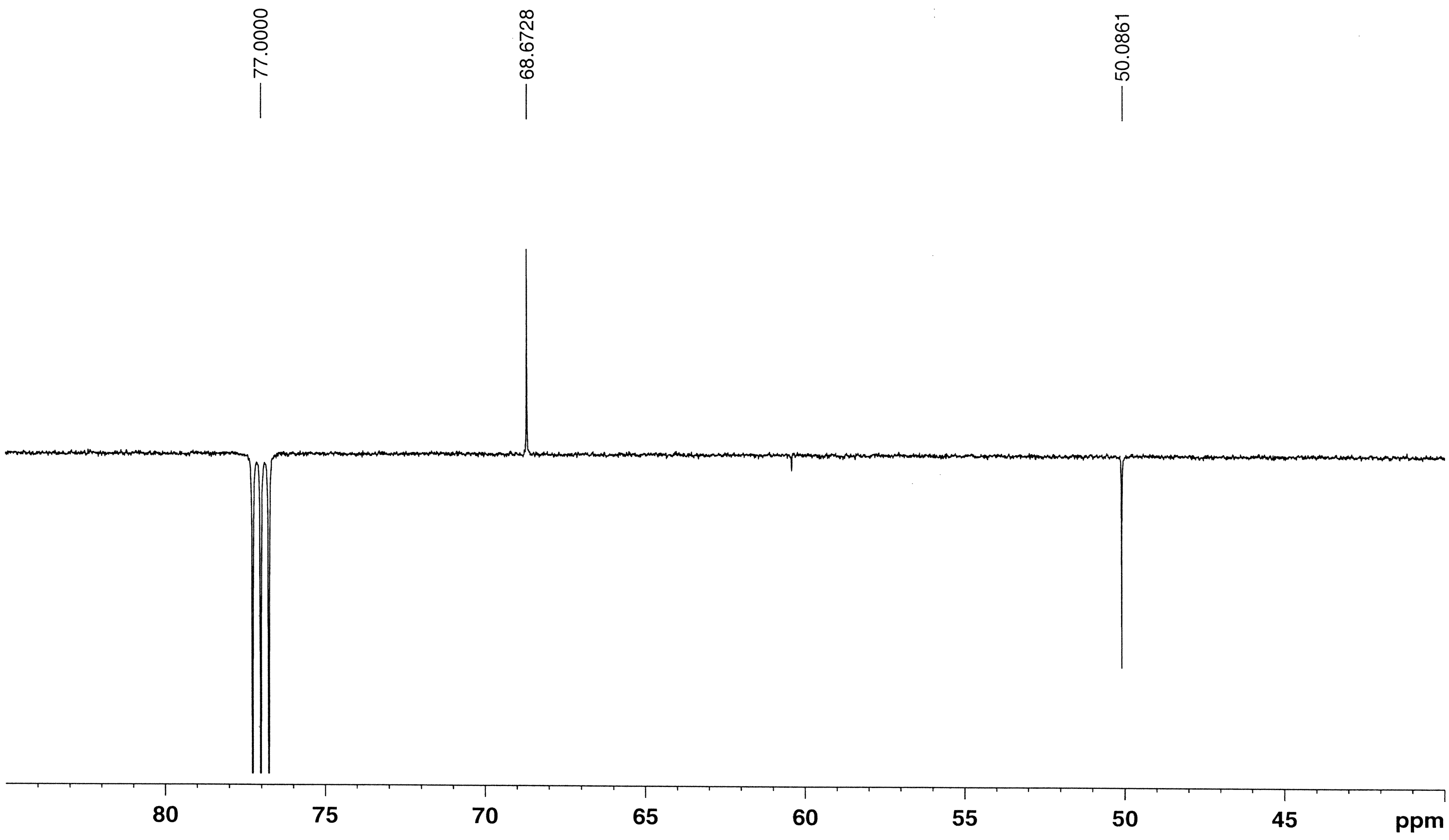

Figure S31c. NMR spectra of compound **7h.**

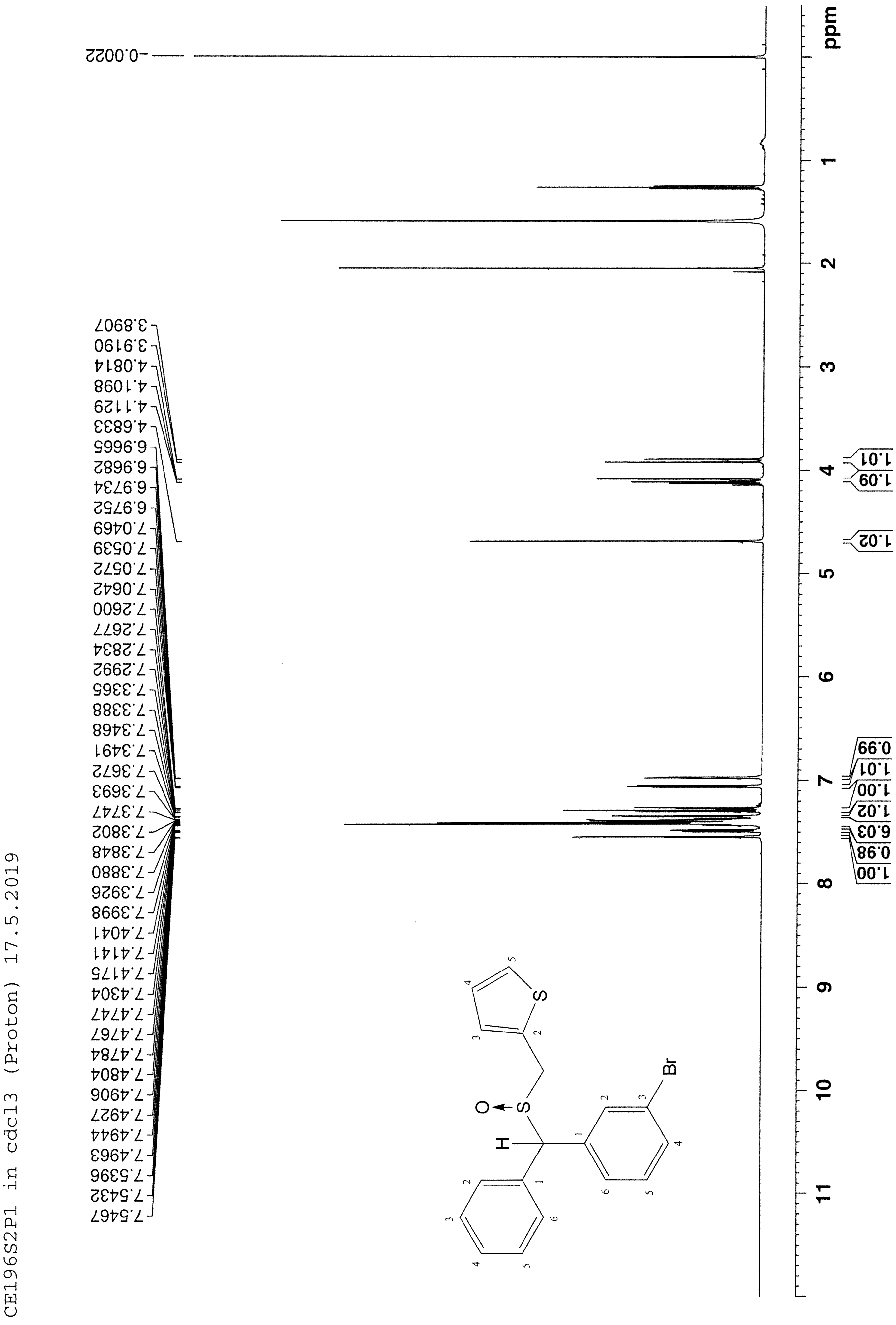

CE196S2P1 in cdcl3 (Proton) 17.5.2019

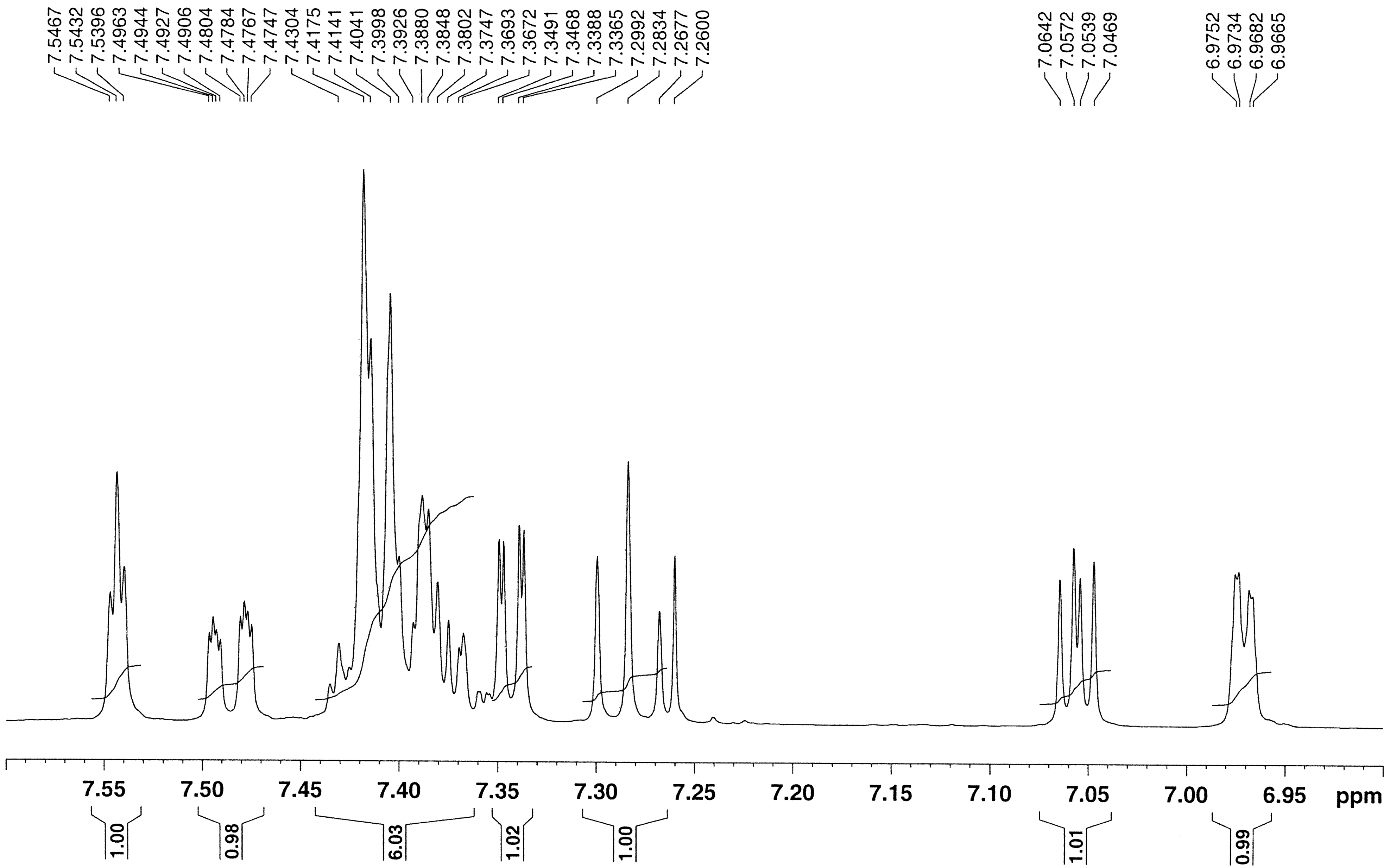

CE196S2P1 in cdcl3 (Proton) 17.5.2019

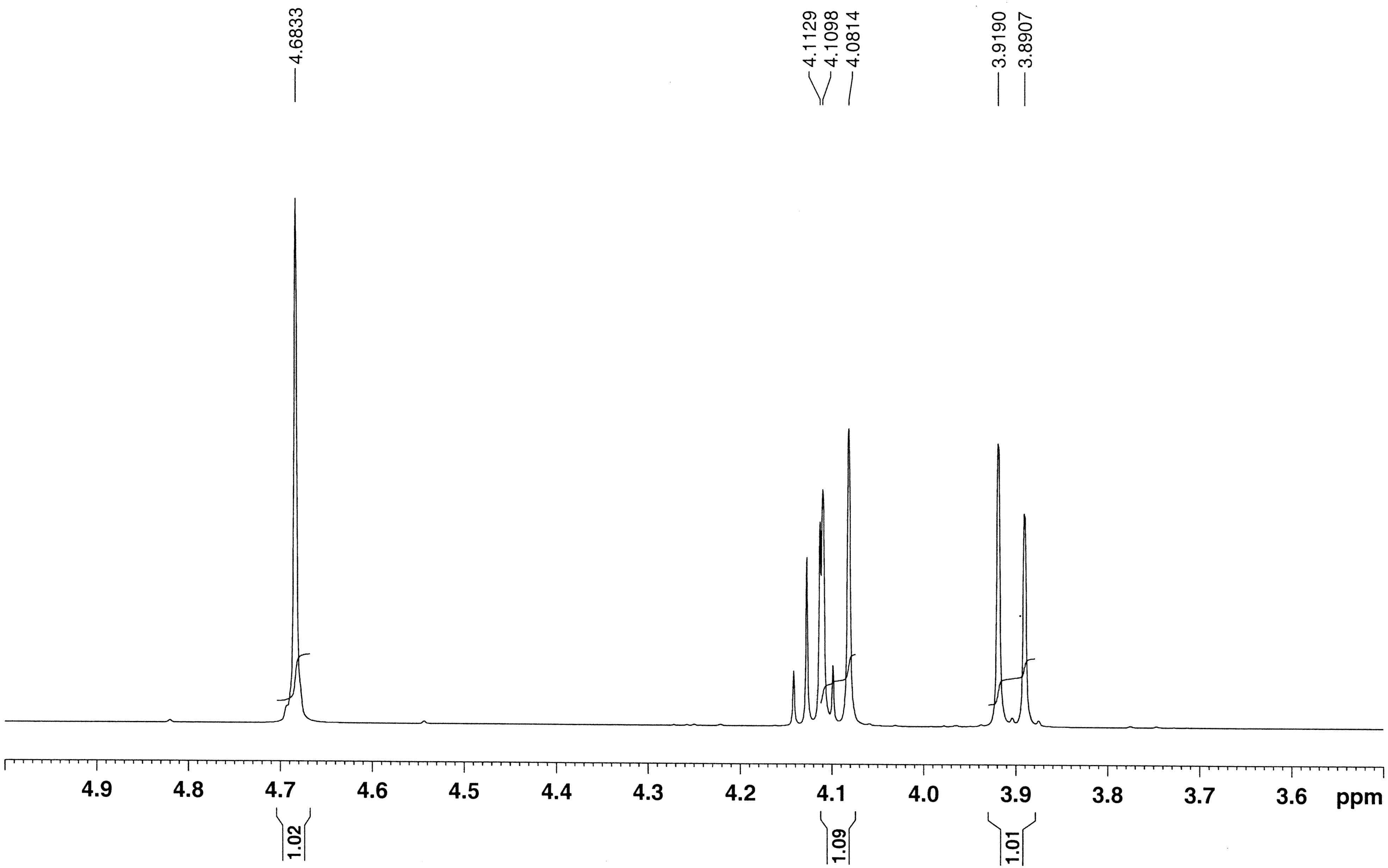

CE196S2P1 in cdcl3 (APT) 17.5.2019

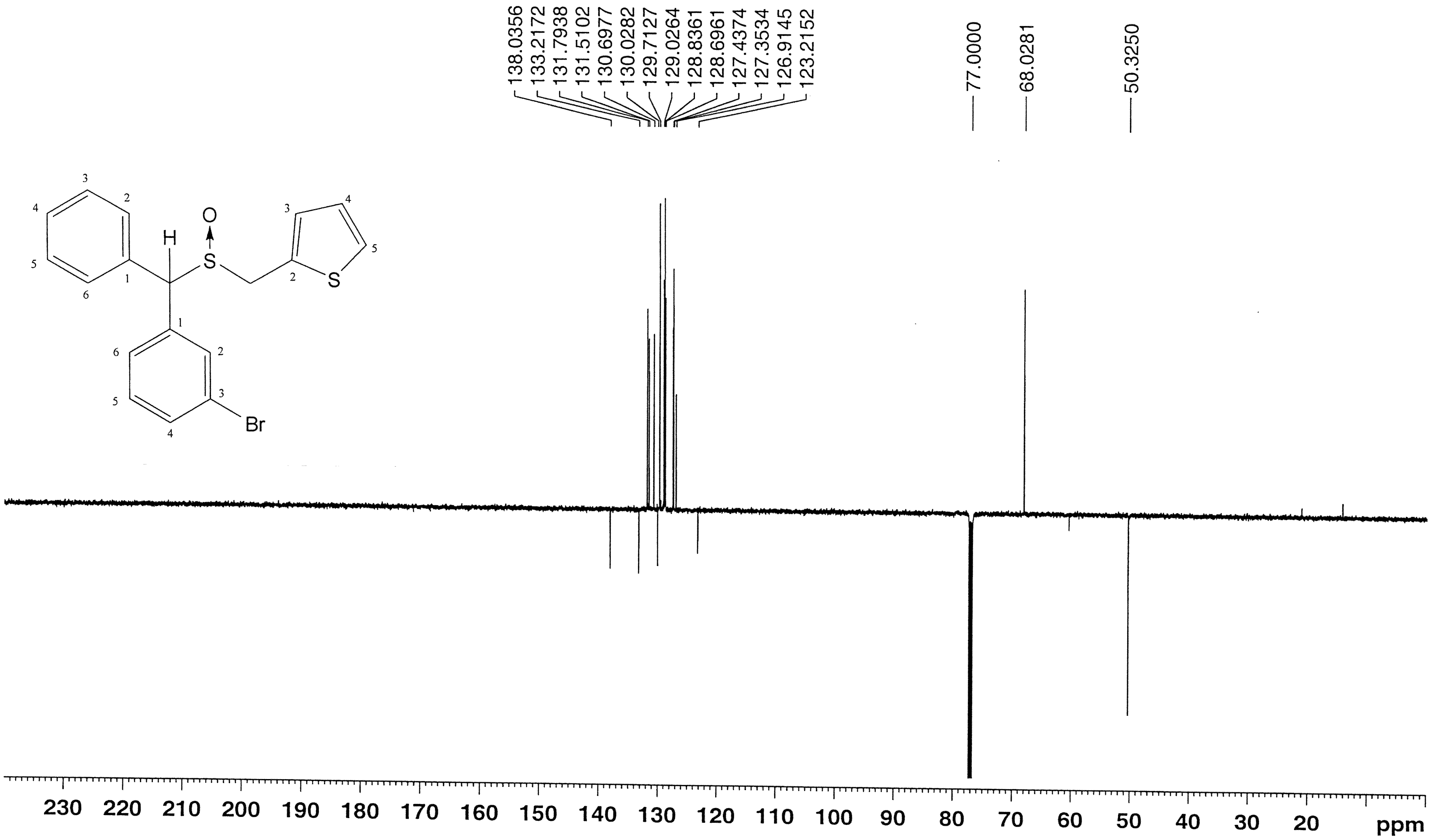

CE196S2P1 in cdcl3 (APT) 17.5.2019

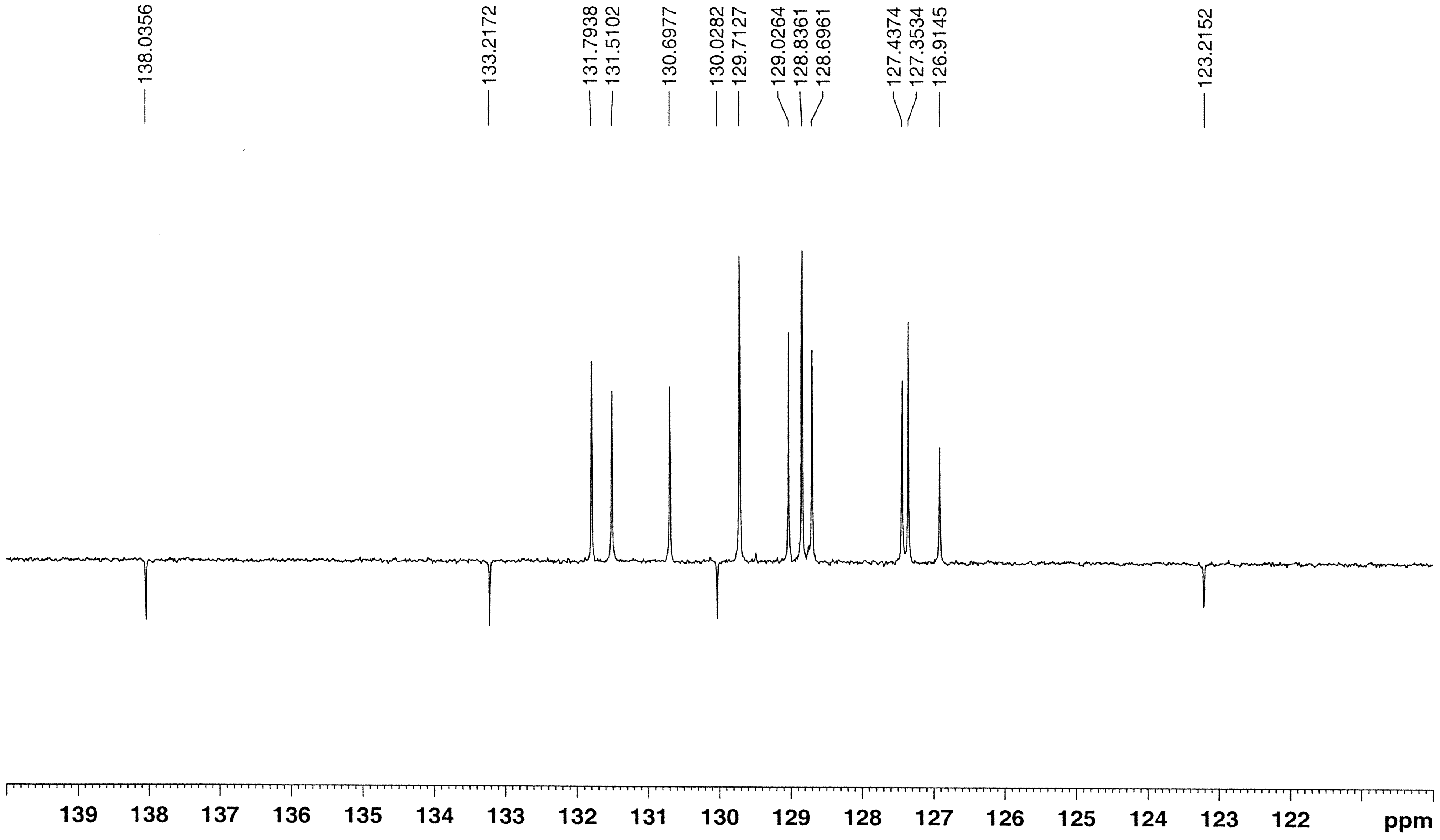

CE196S2P1 in cdcl3 (APT) 17.5.2019

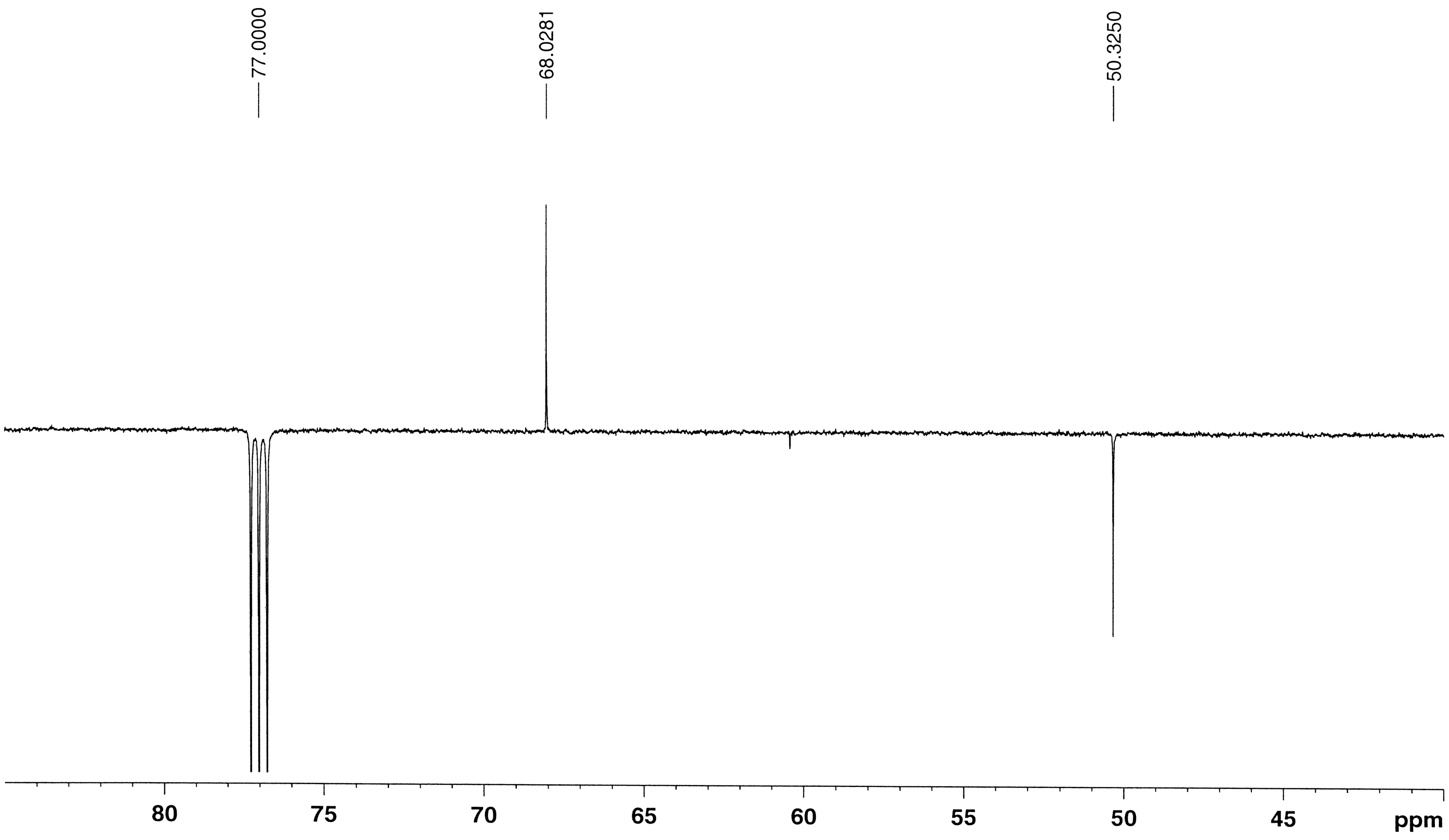

Figure S32c. NMR spectra of compound **8h**.

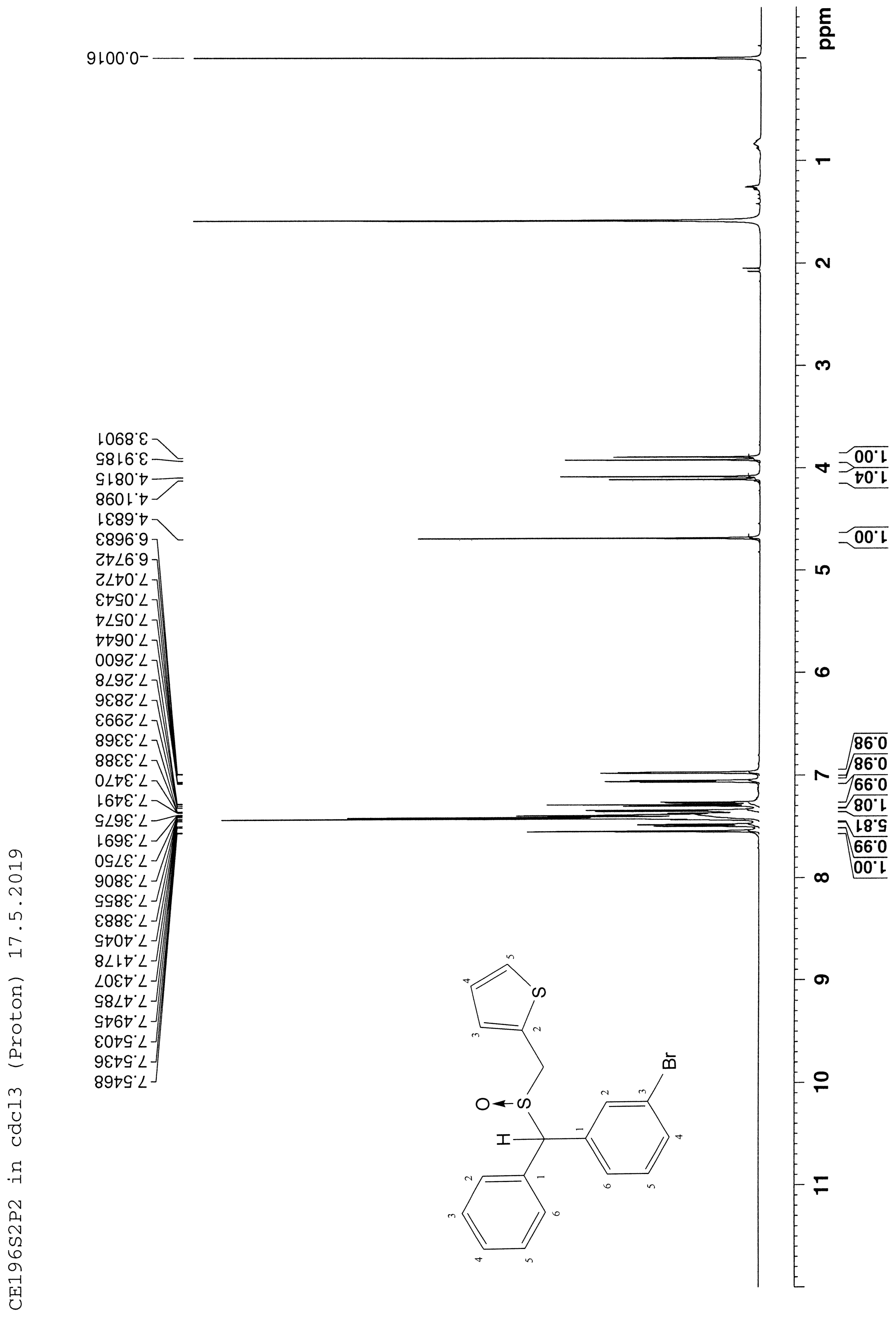

CE196S2P2 in cdcl3 (Proton) 17.5.2019

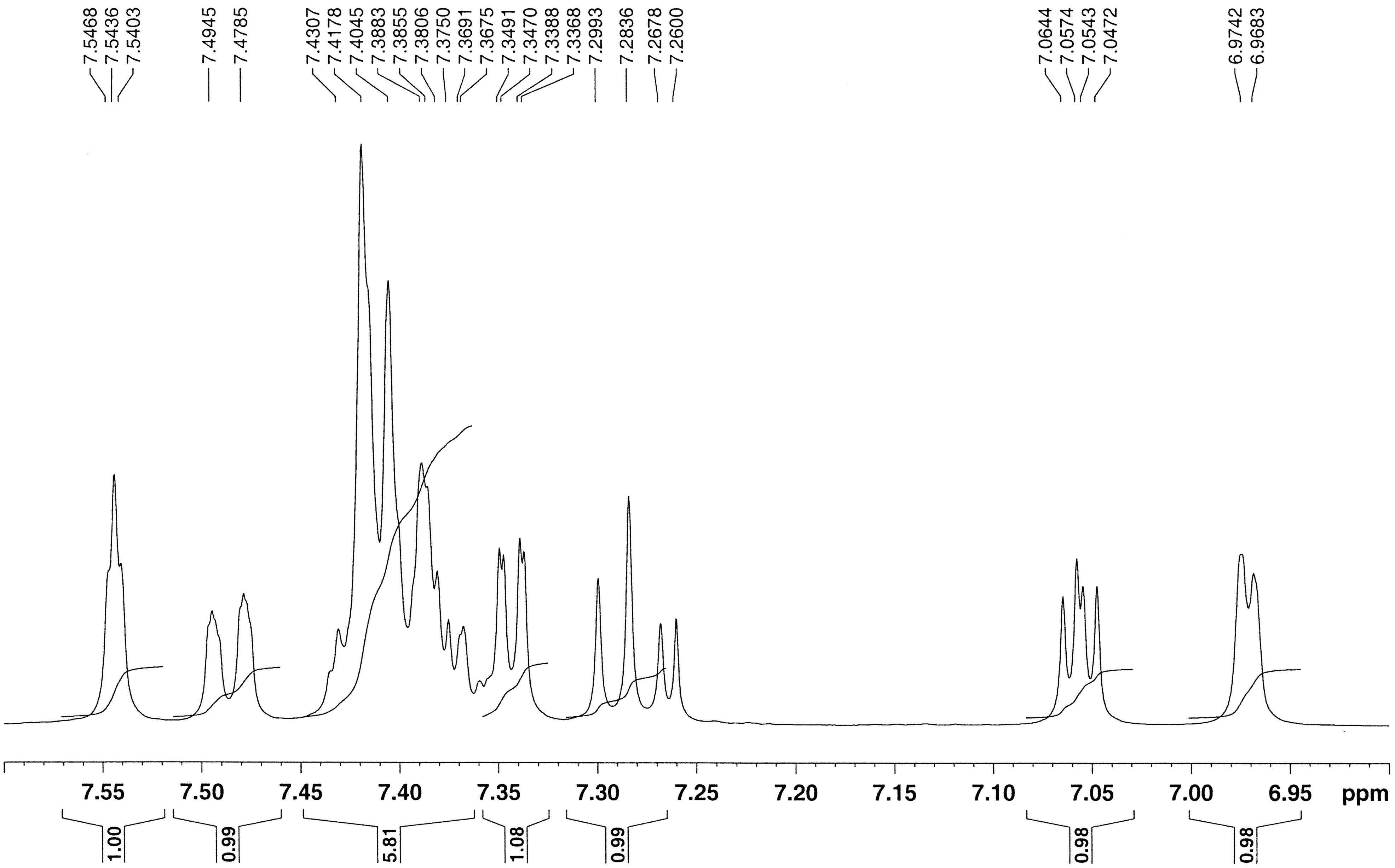

CE196S2P2 in cdcl3 (Proton) 17.5.2019

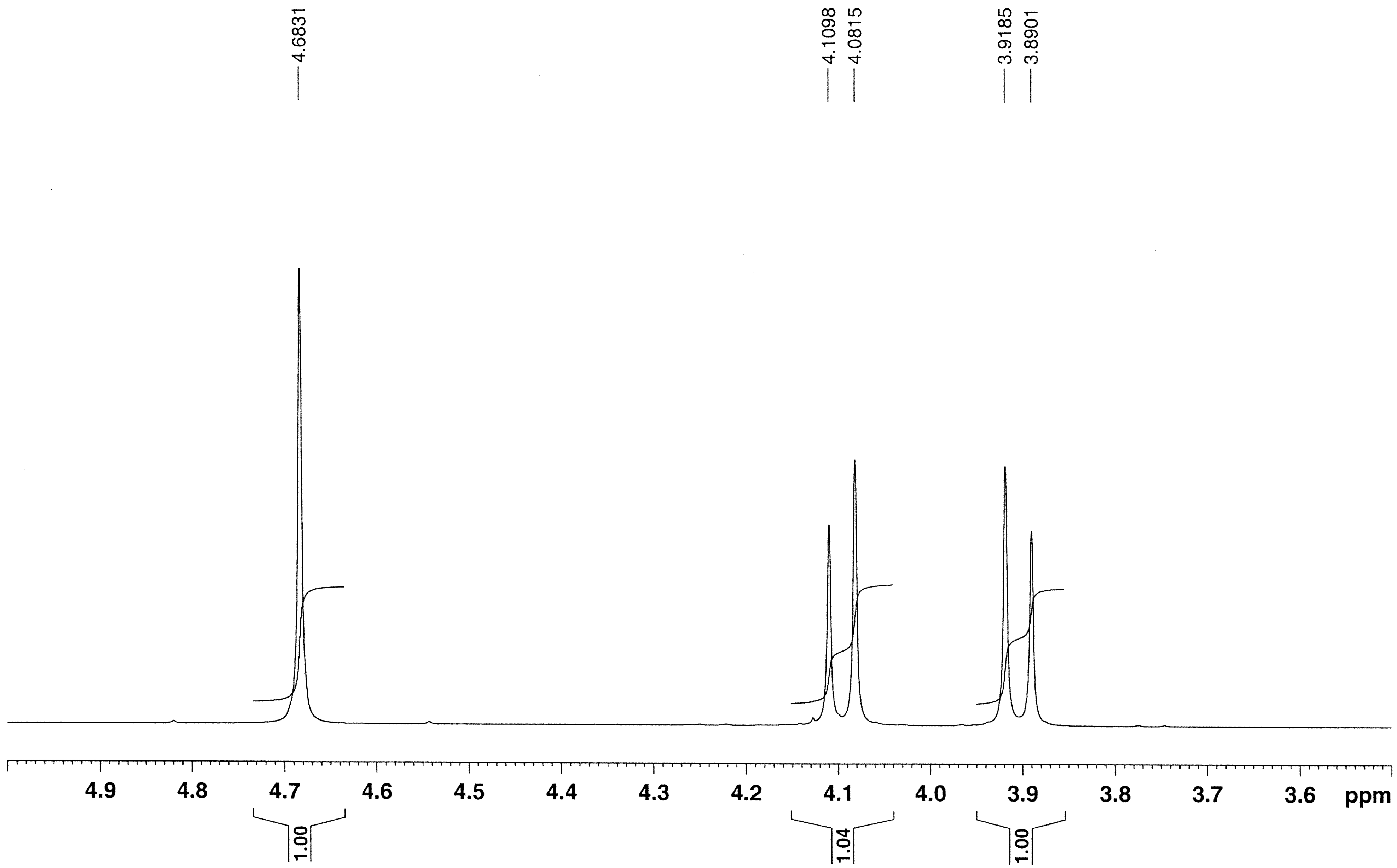

CE196S2P2 in cdcl3 (APT) 17.5.2019

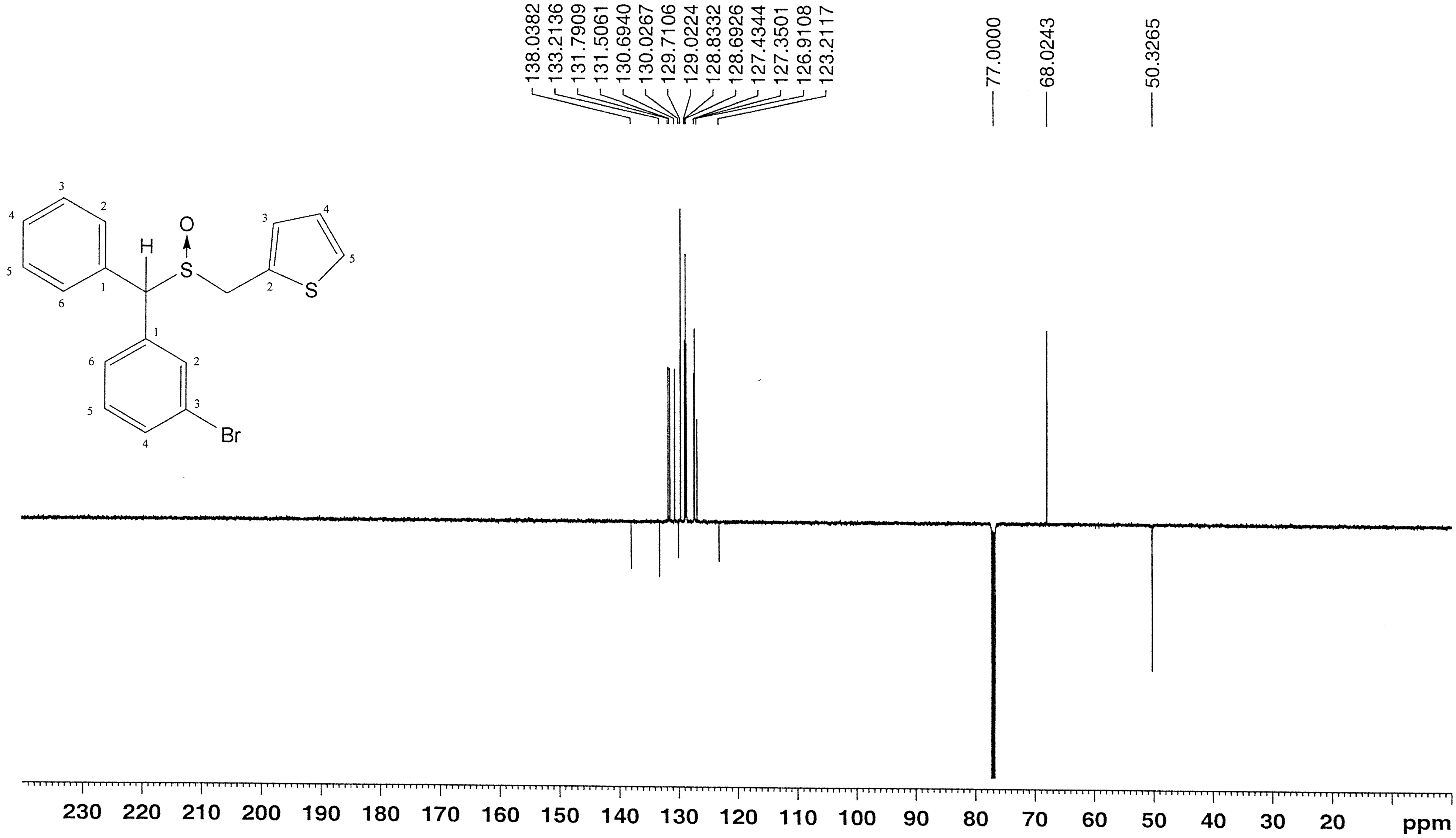

CE196S2P2 in cdcl3 (APT) 17.5.2019

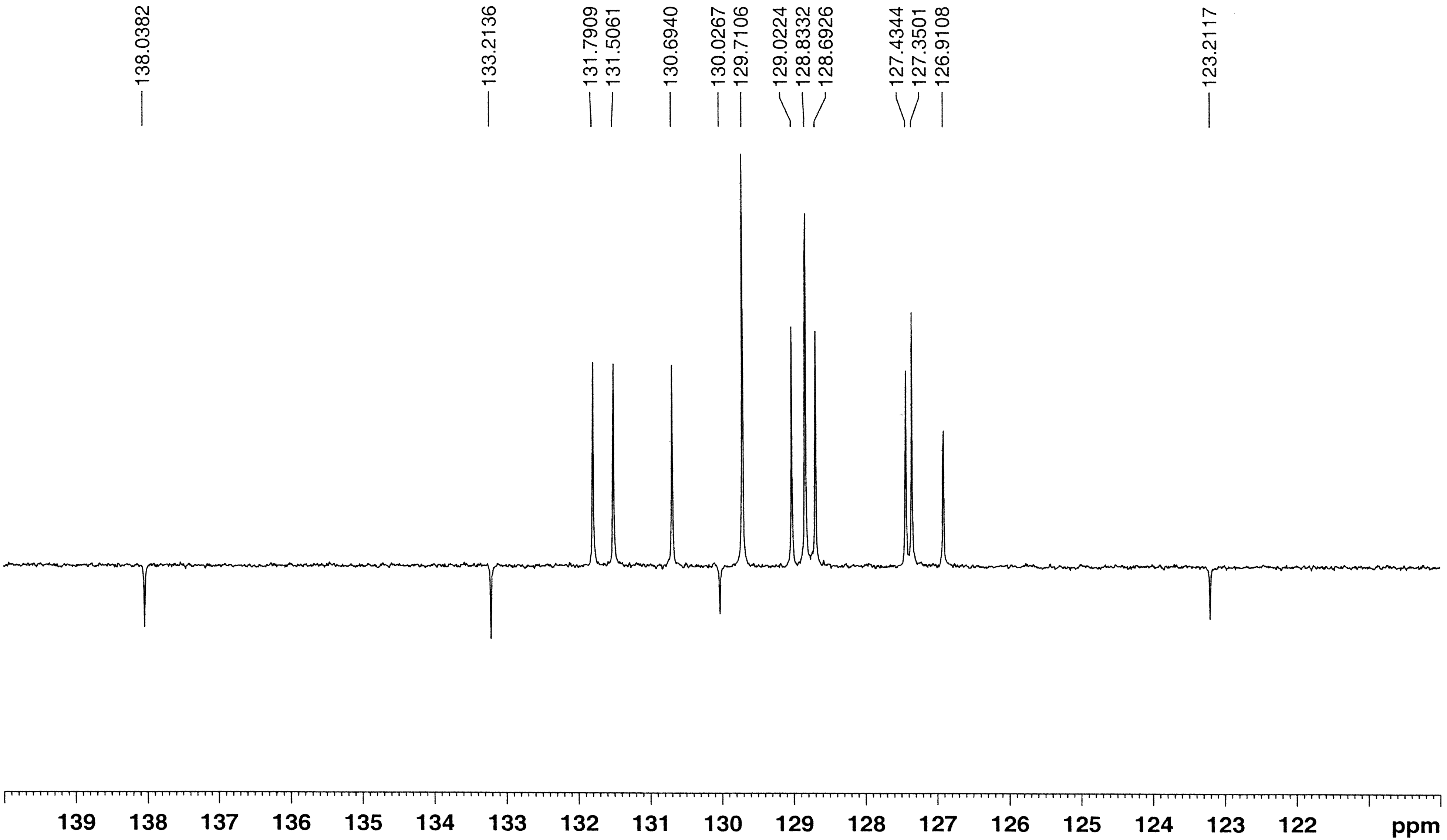

CE196S2P2 in cdcl3 (APT) 17.5.2019

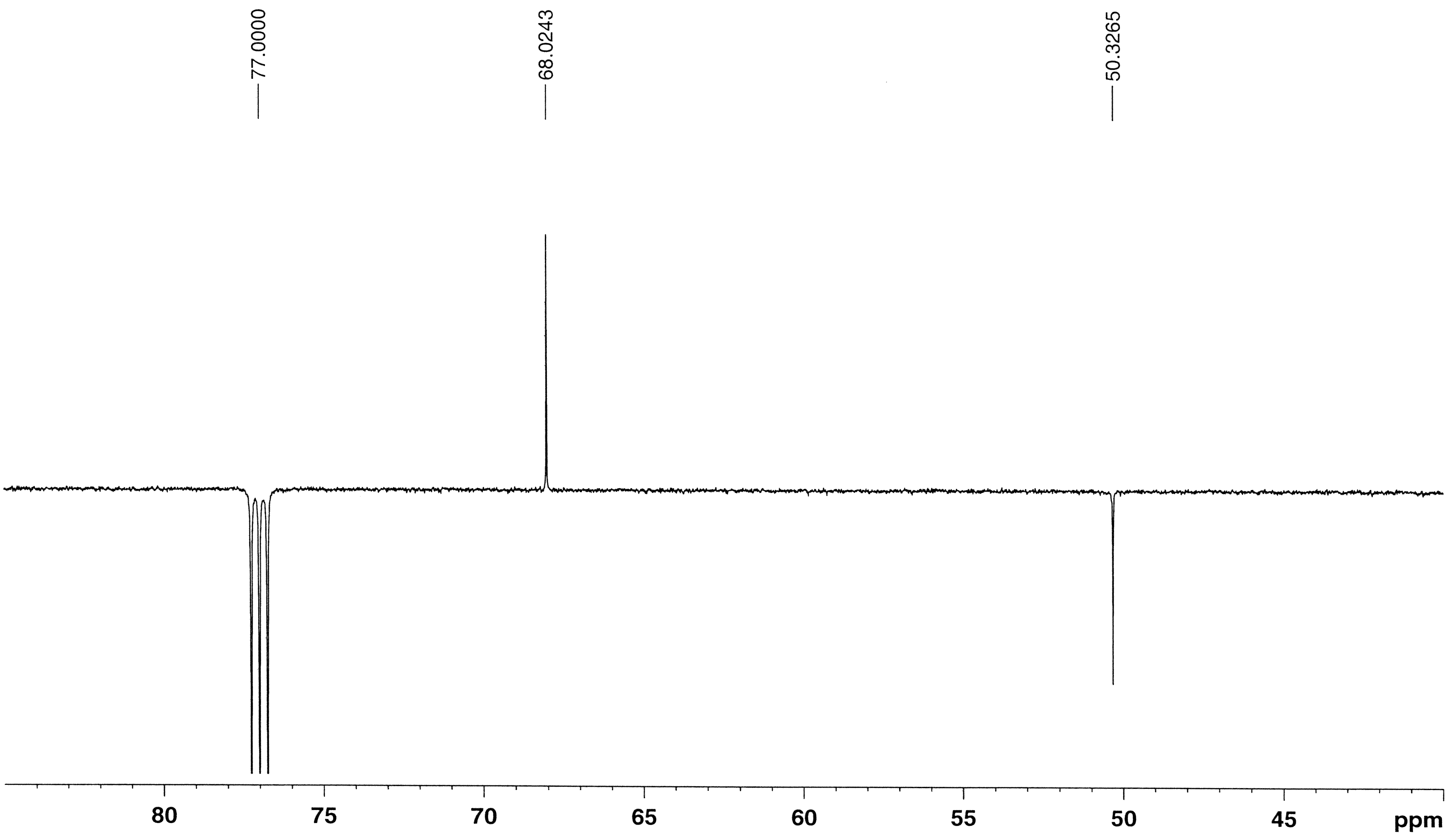

Figure S33c. NMR spectra of compound **5i.**

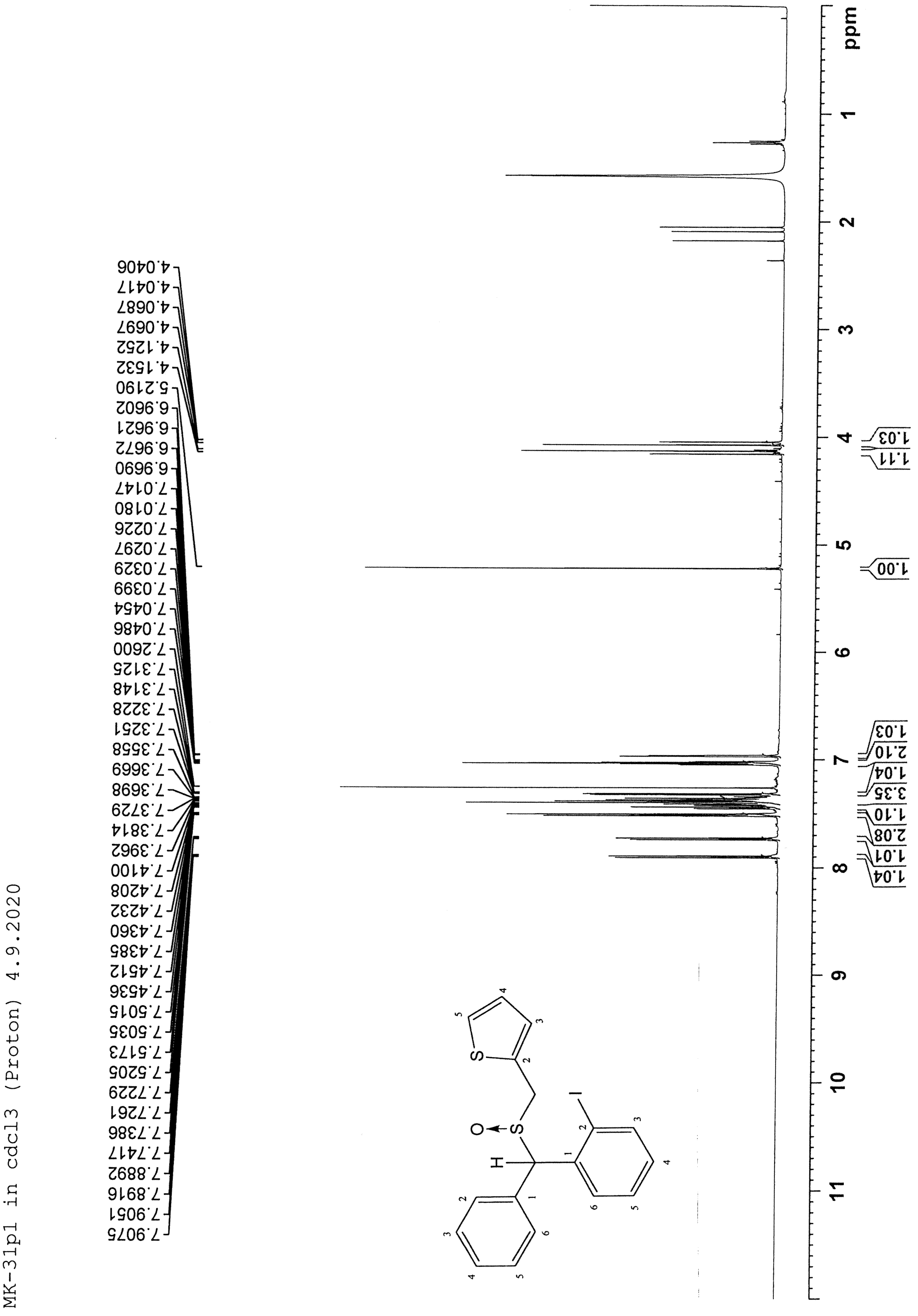

MK-31p1 in cdcl3 (Proton) 4.9.2020

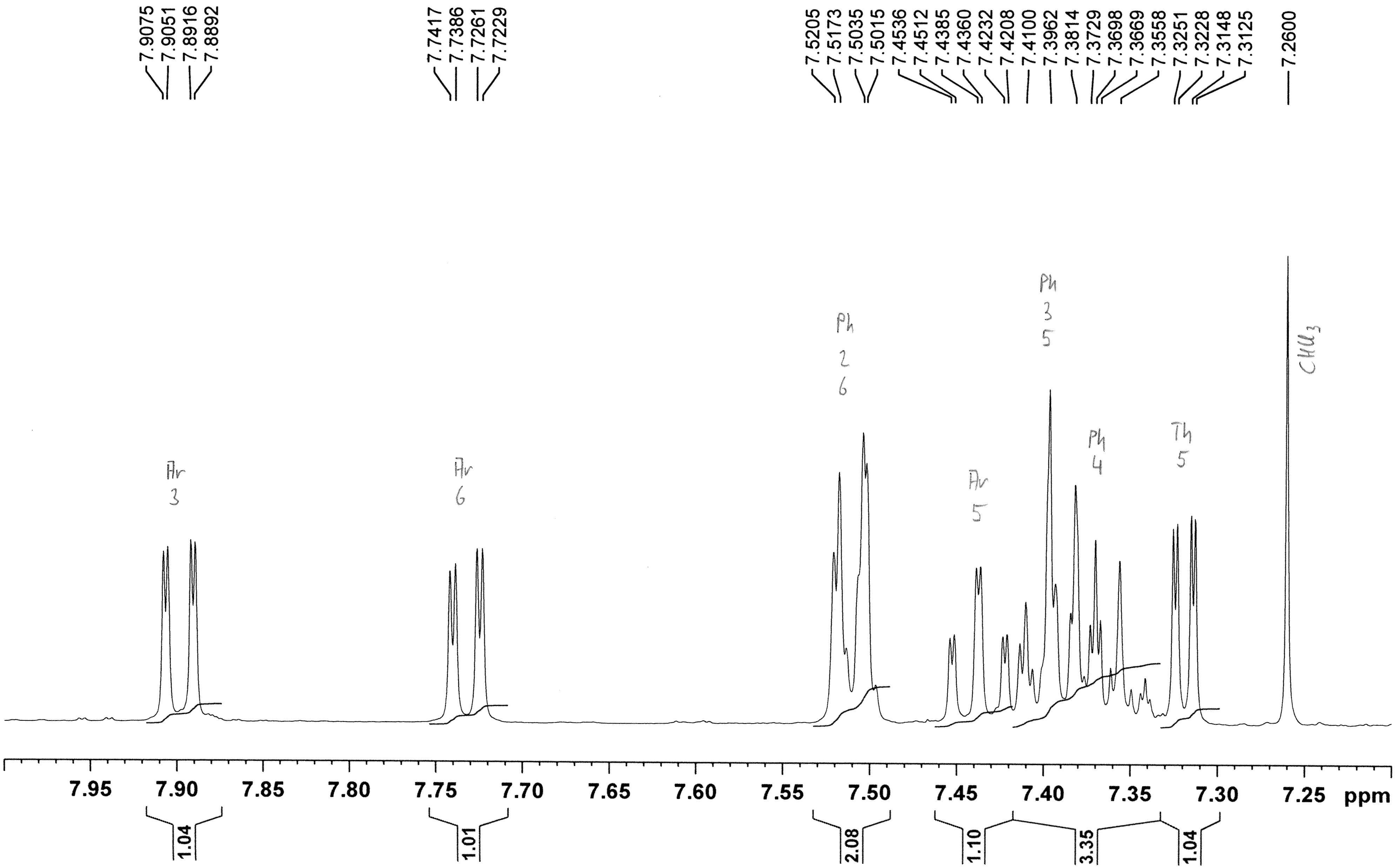

MK-31p1 in cdcl3 (Proton) 4.9.2020

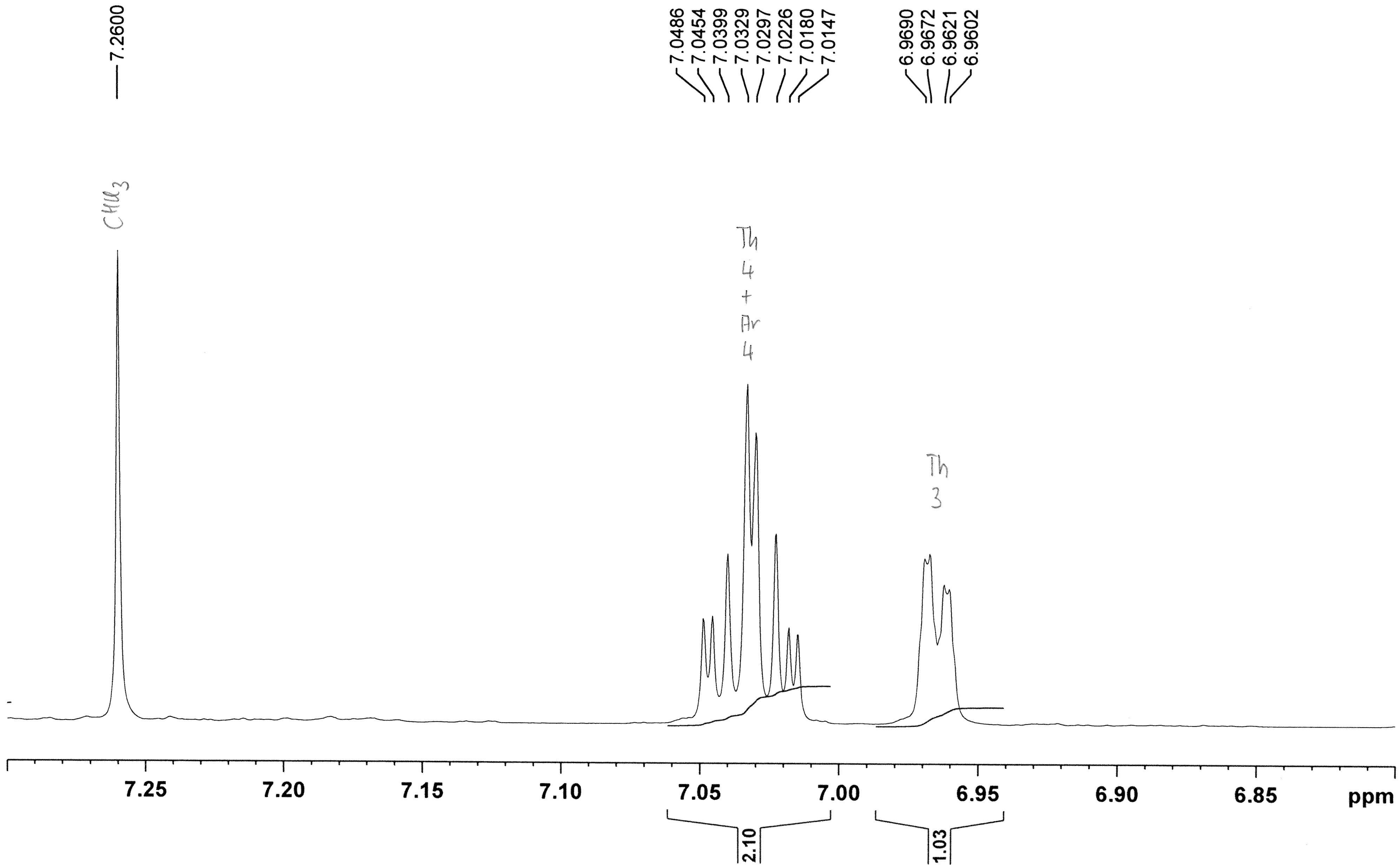

MK-31p1 in cdcl3 (Proton) 4.9.2020

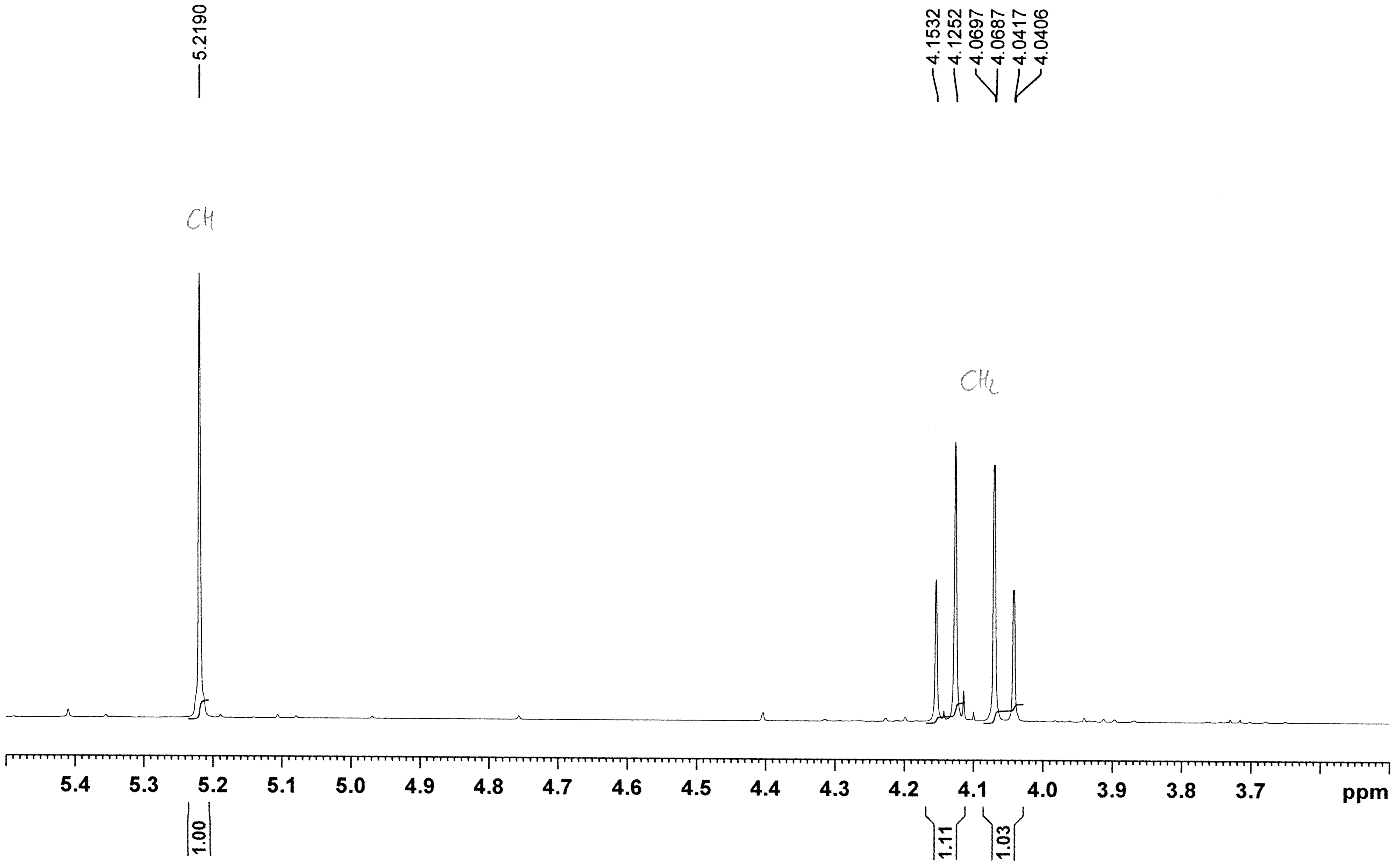

MK-31p1 in cdcl3 (APT) 4.9.2020

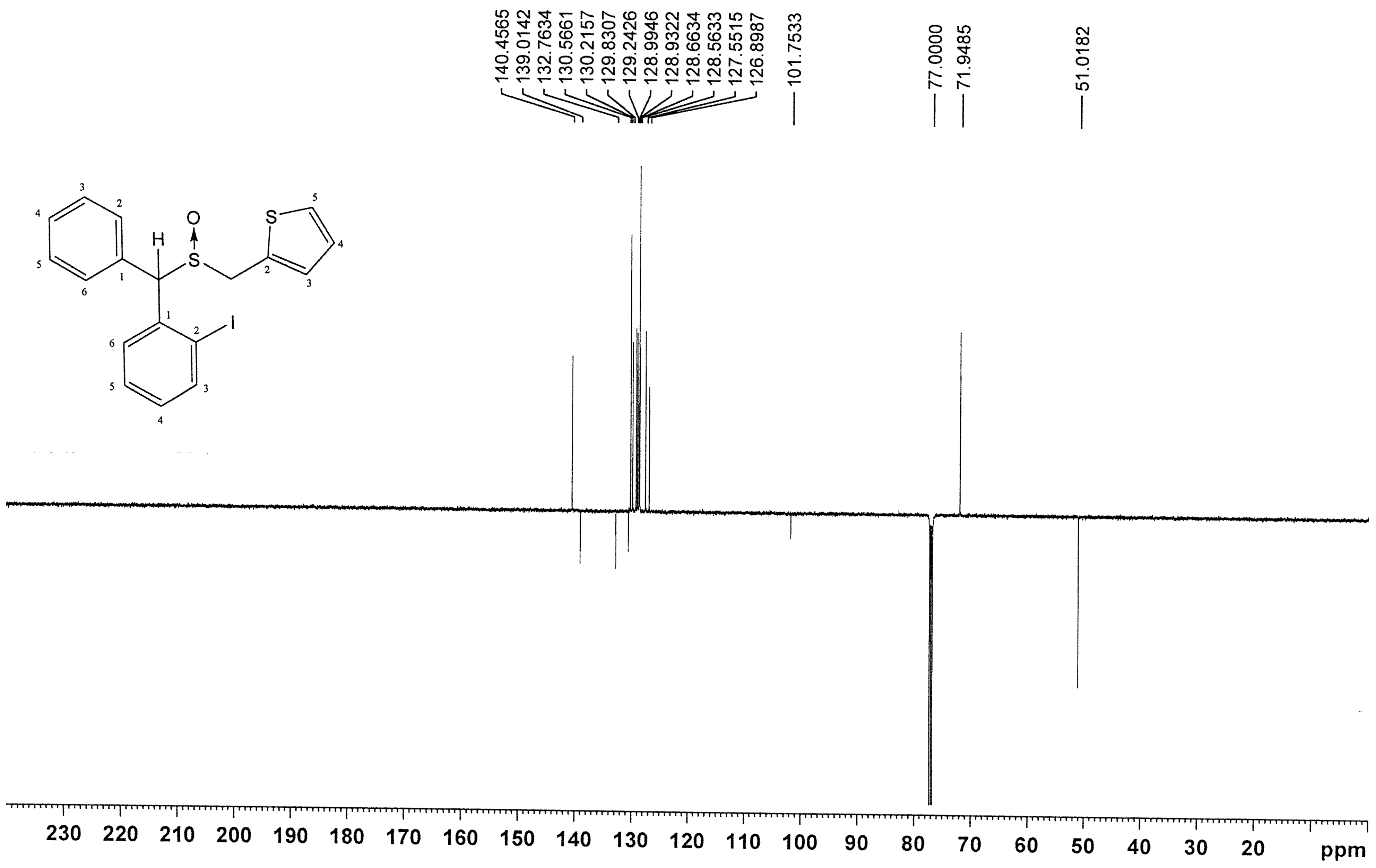

MK-31p1 in cdcl3 (APT) 4.9.2020

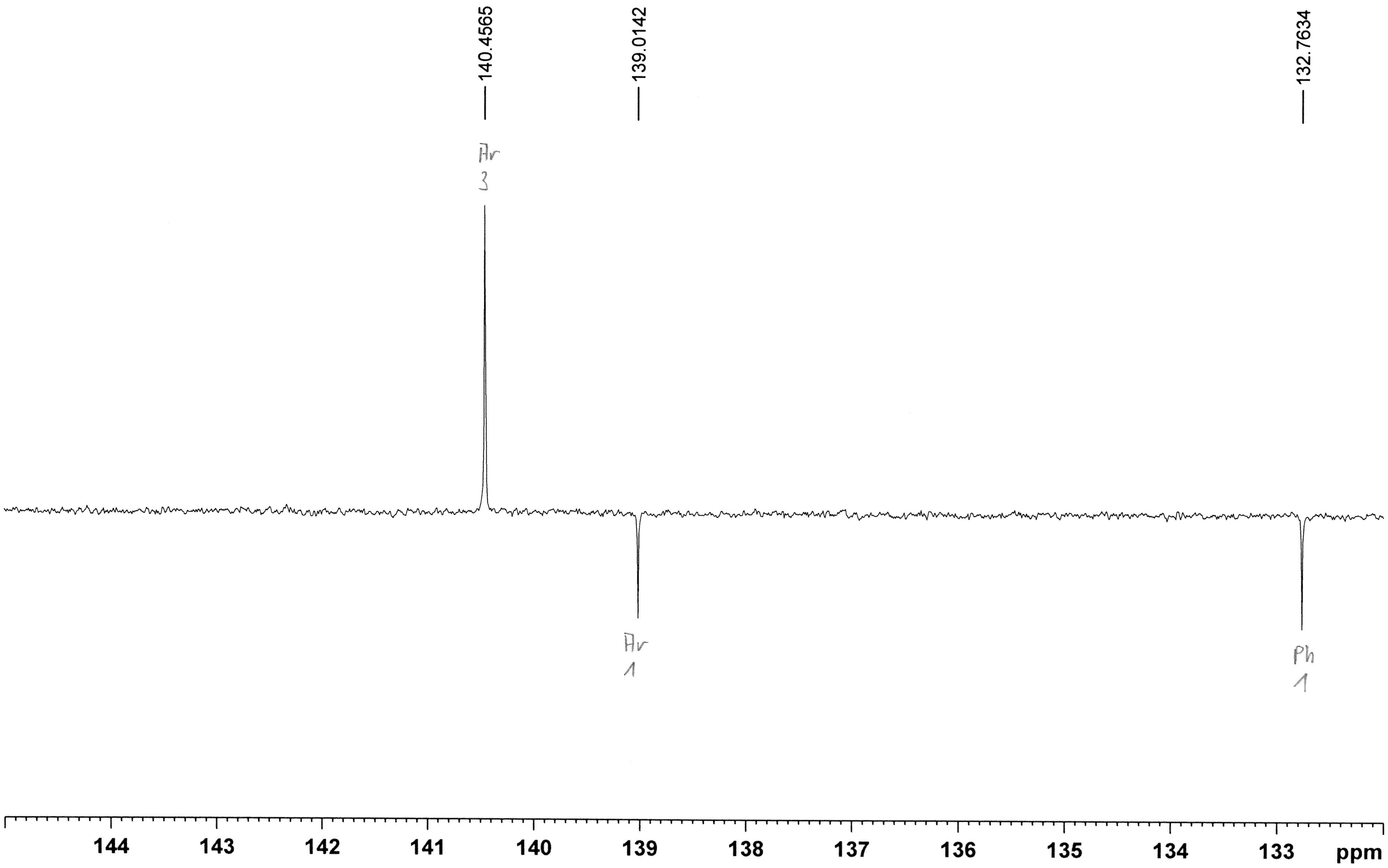

MK-31p1 in cdcl3 (APT) 4.9.2020

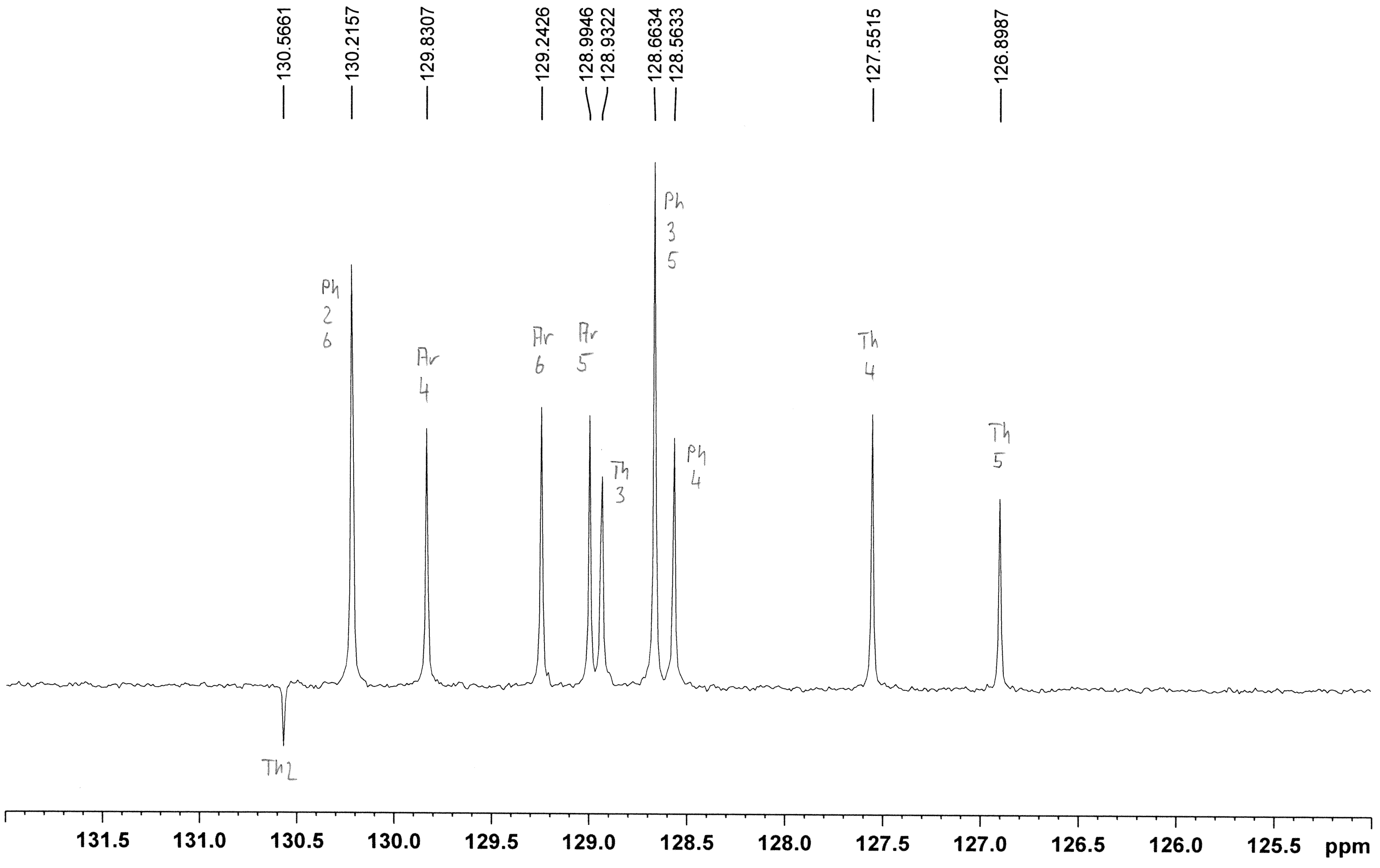

MK-31p1 in cdcl3 (APT) 4.9.2020

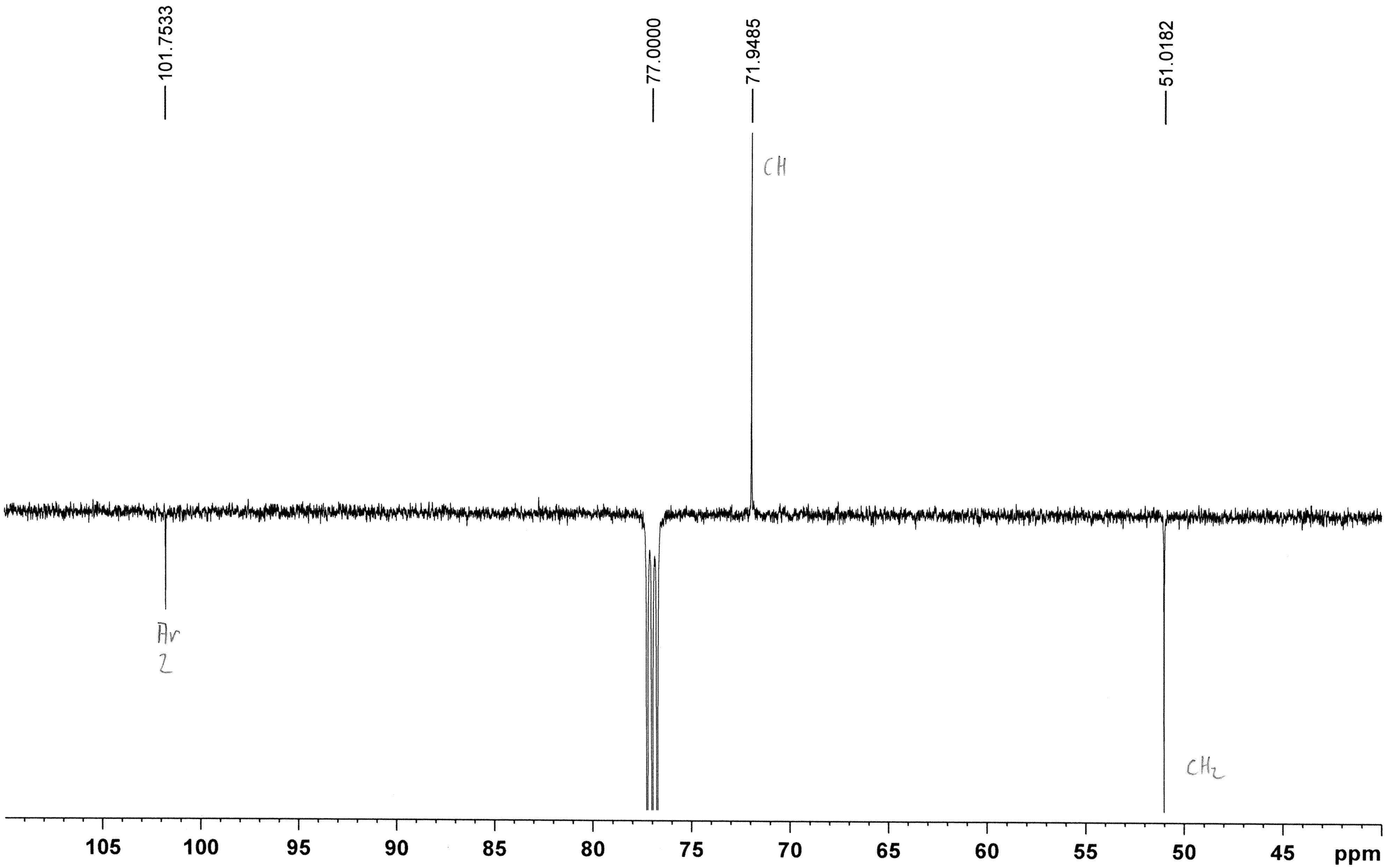

Figure S34c. NMR spectra of compound **6i**.

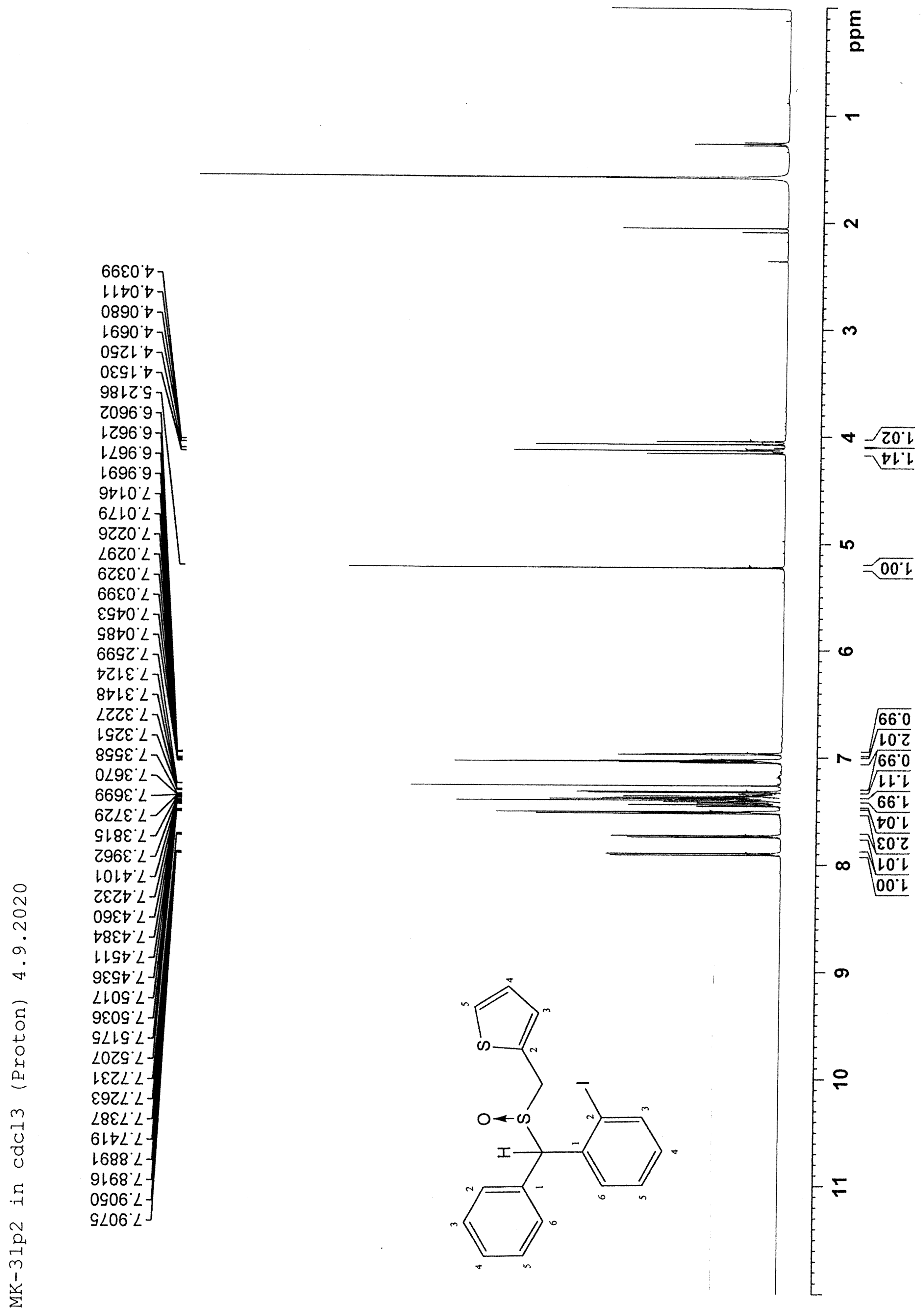

MK-31p2 in cdcl3 (Proton) 4.9.2020

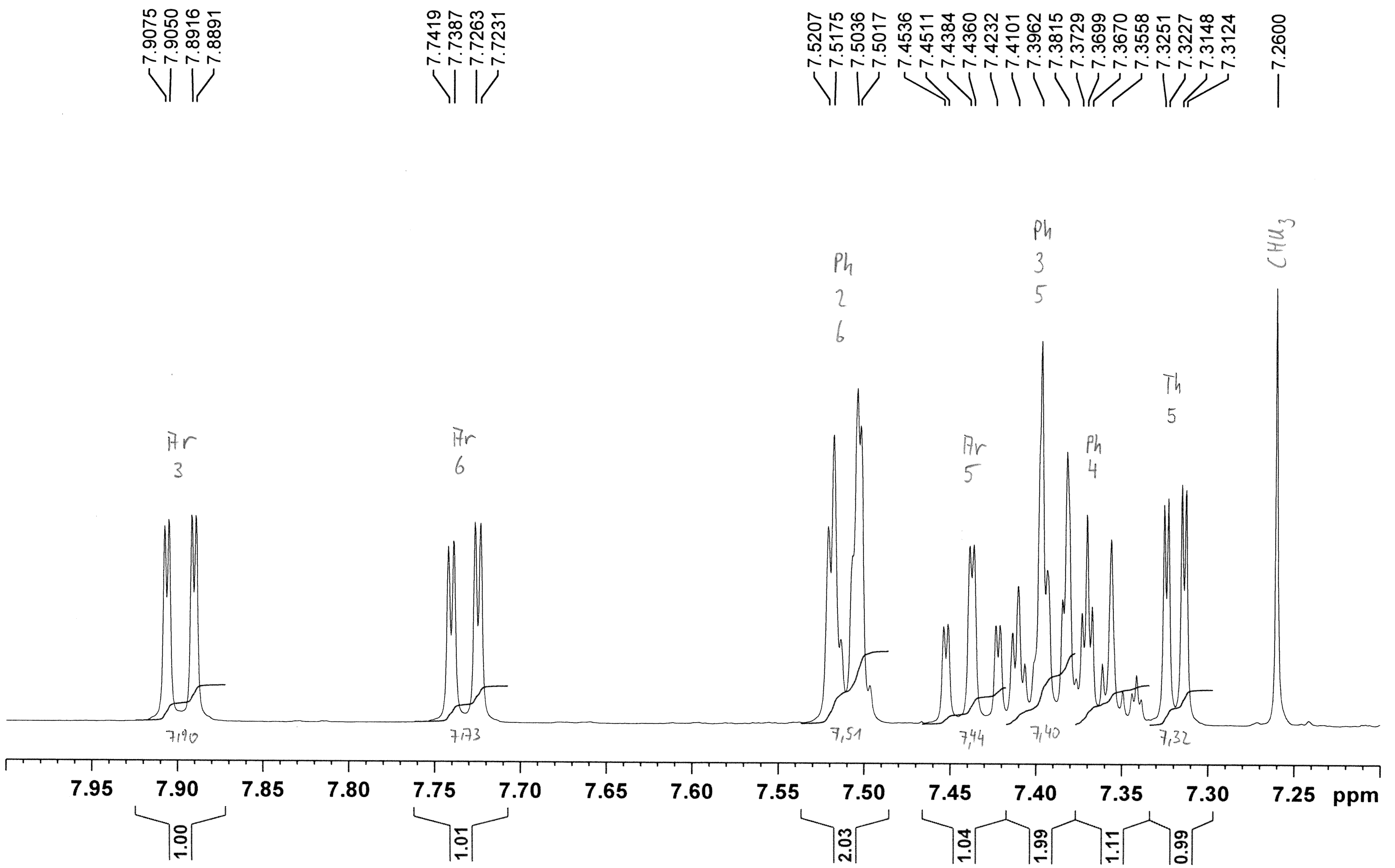

MK-31p2 in cdcl3 (Proton) 4.9.2020

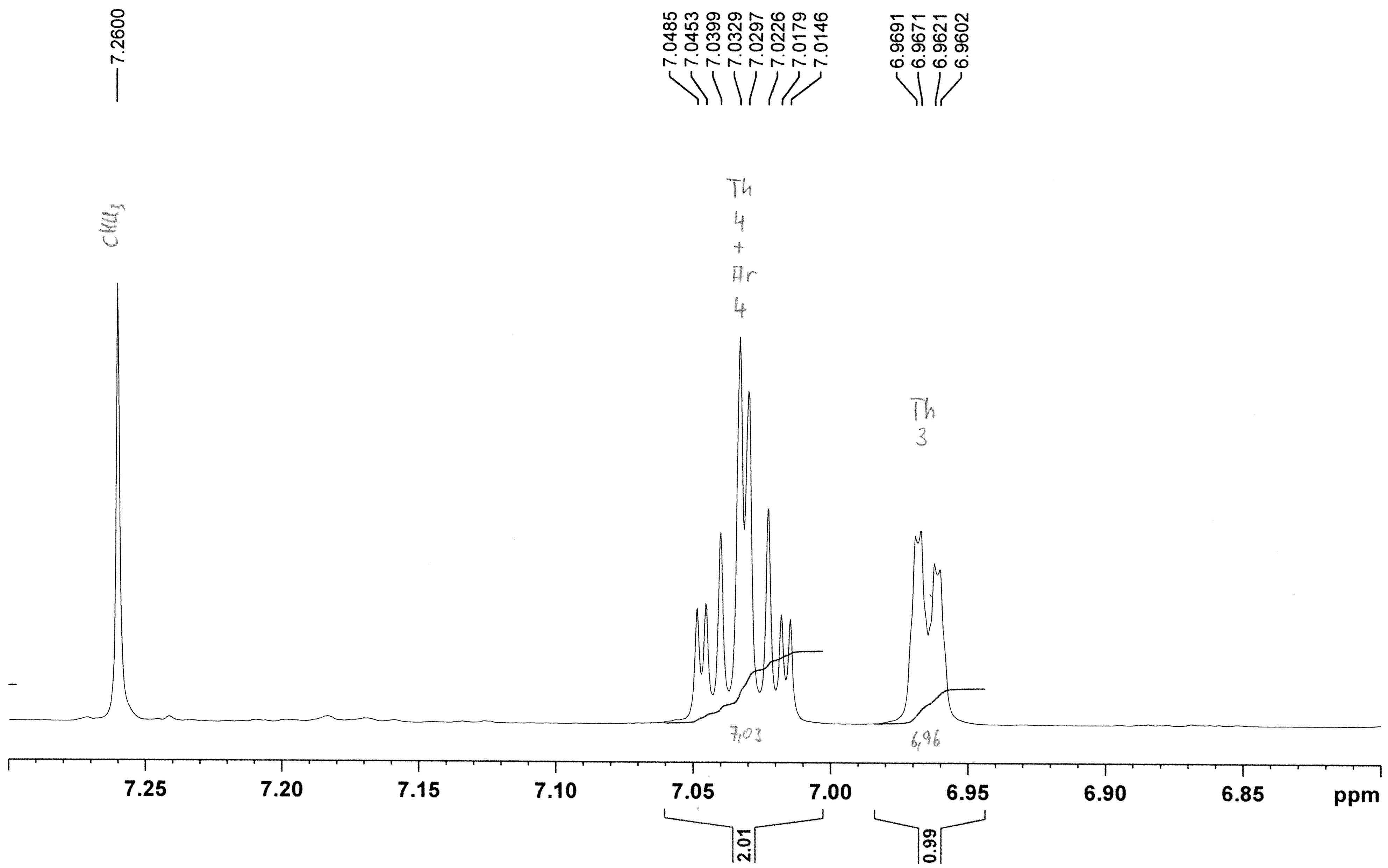

MK-31p2 in cdcl3 (Proton) 4.9.2020

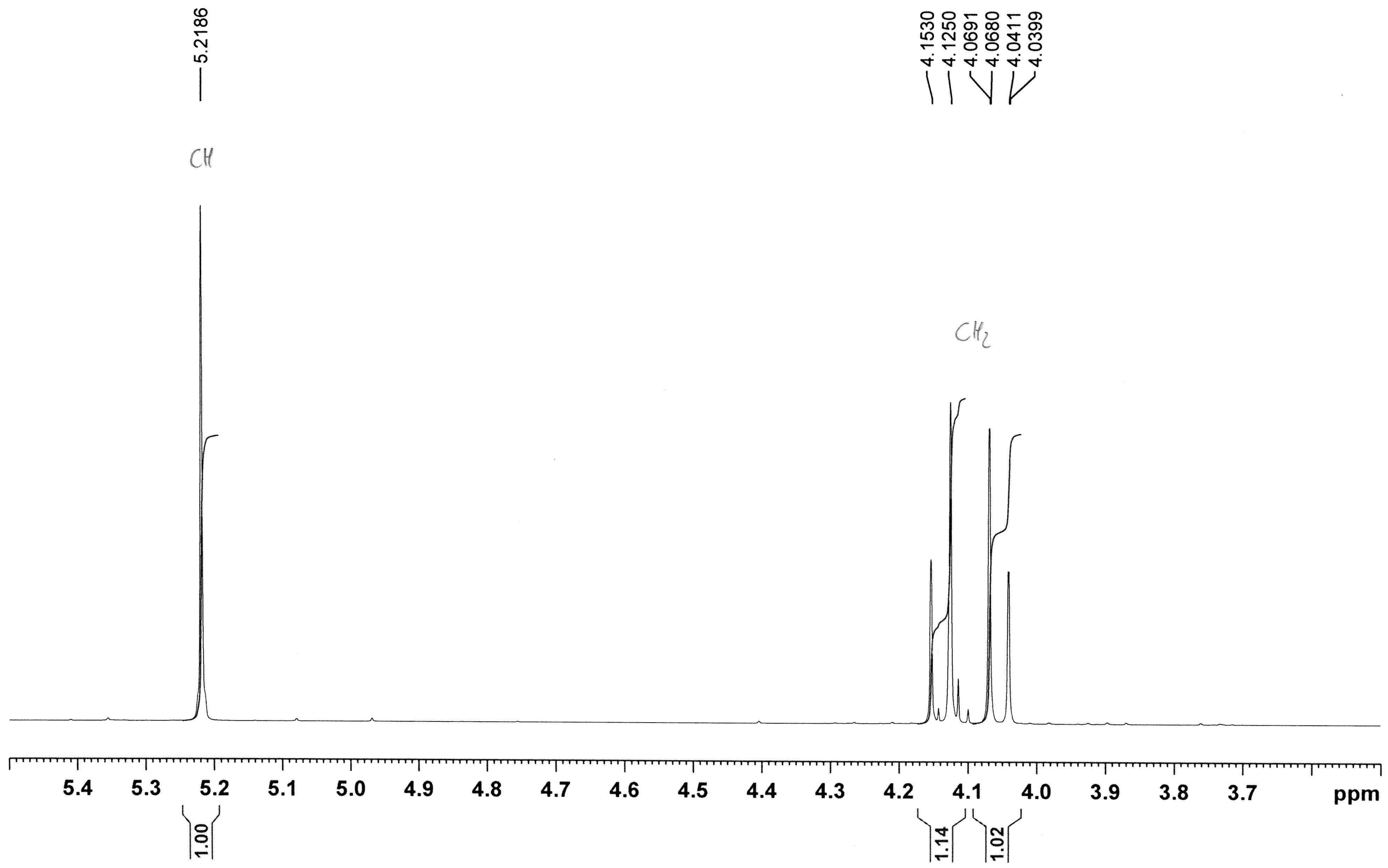

MK-31p2 in cdcl3 (APT) 4.9.2020

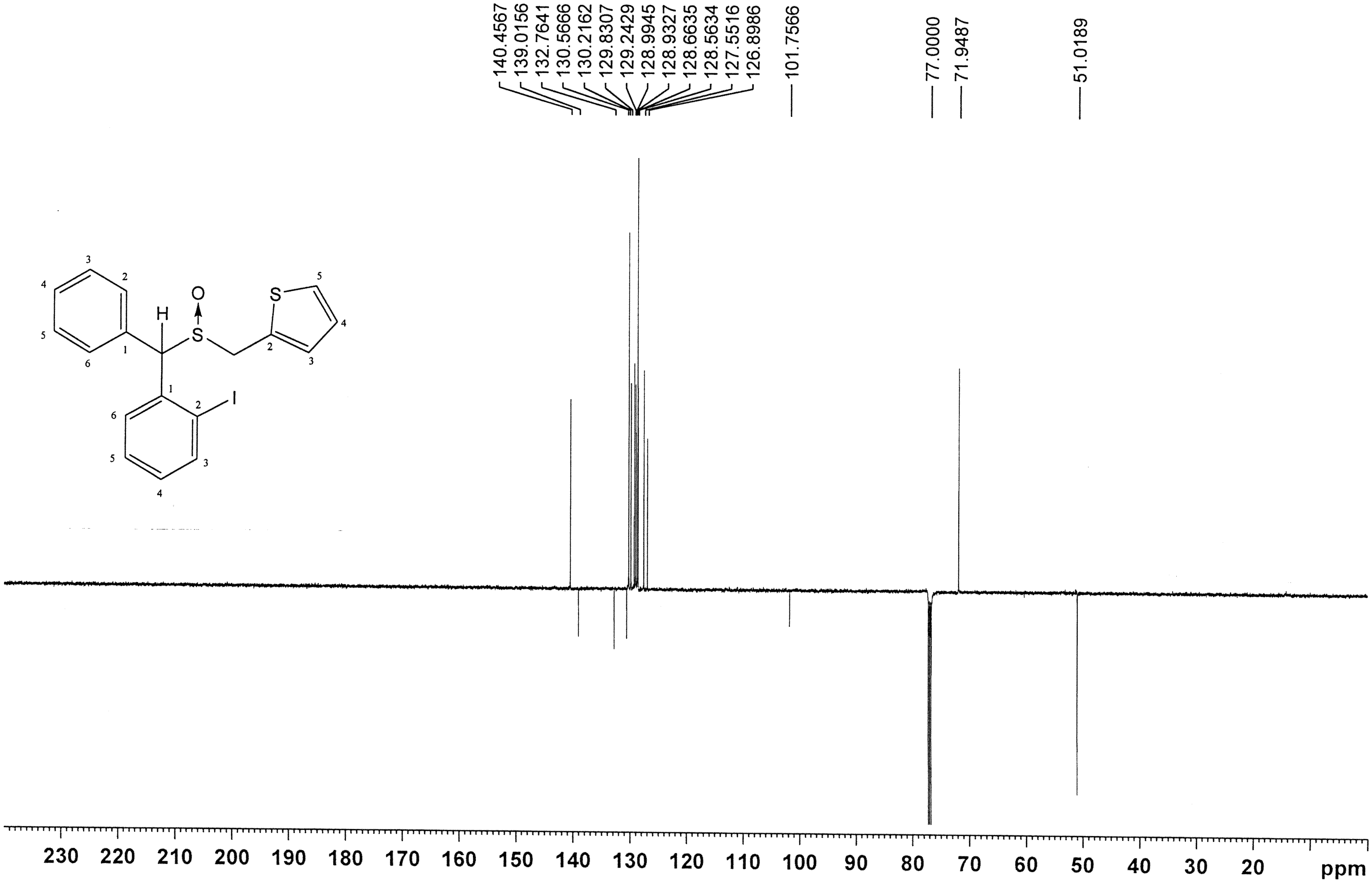

MK-31p2 in cdcl3 (APT) 4.9.2020

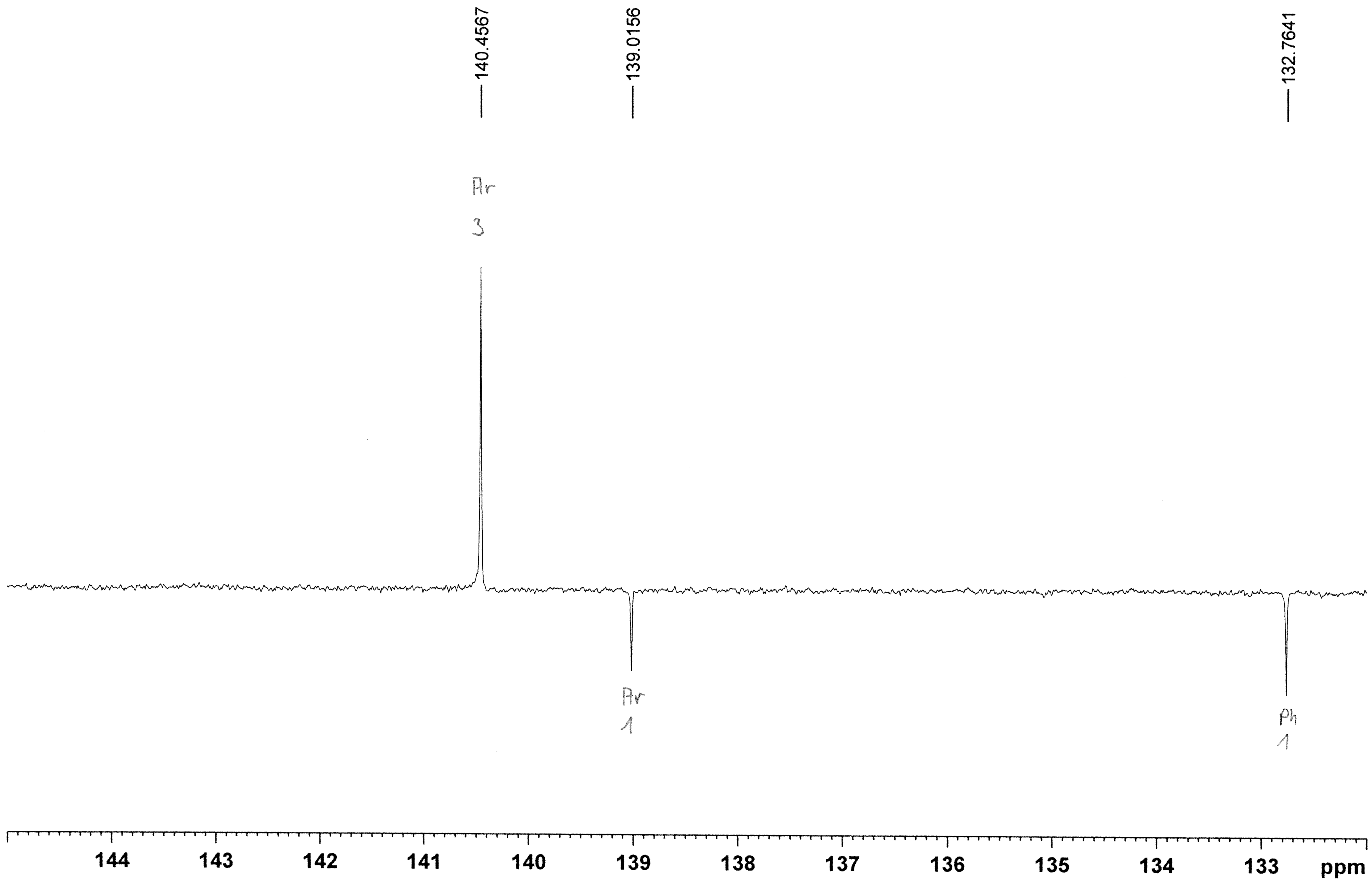

MK-31p2 in cdcl3 (APT) 4.9.2020

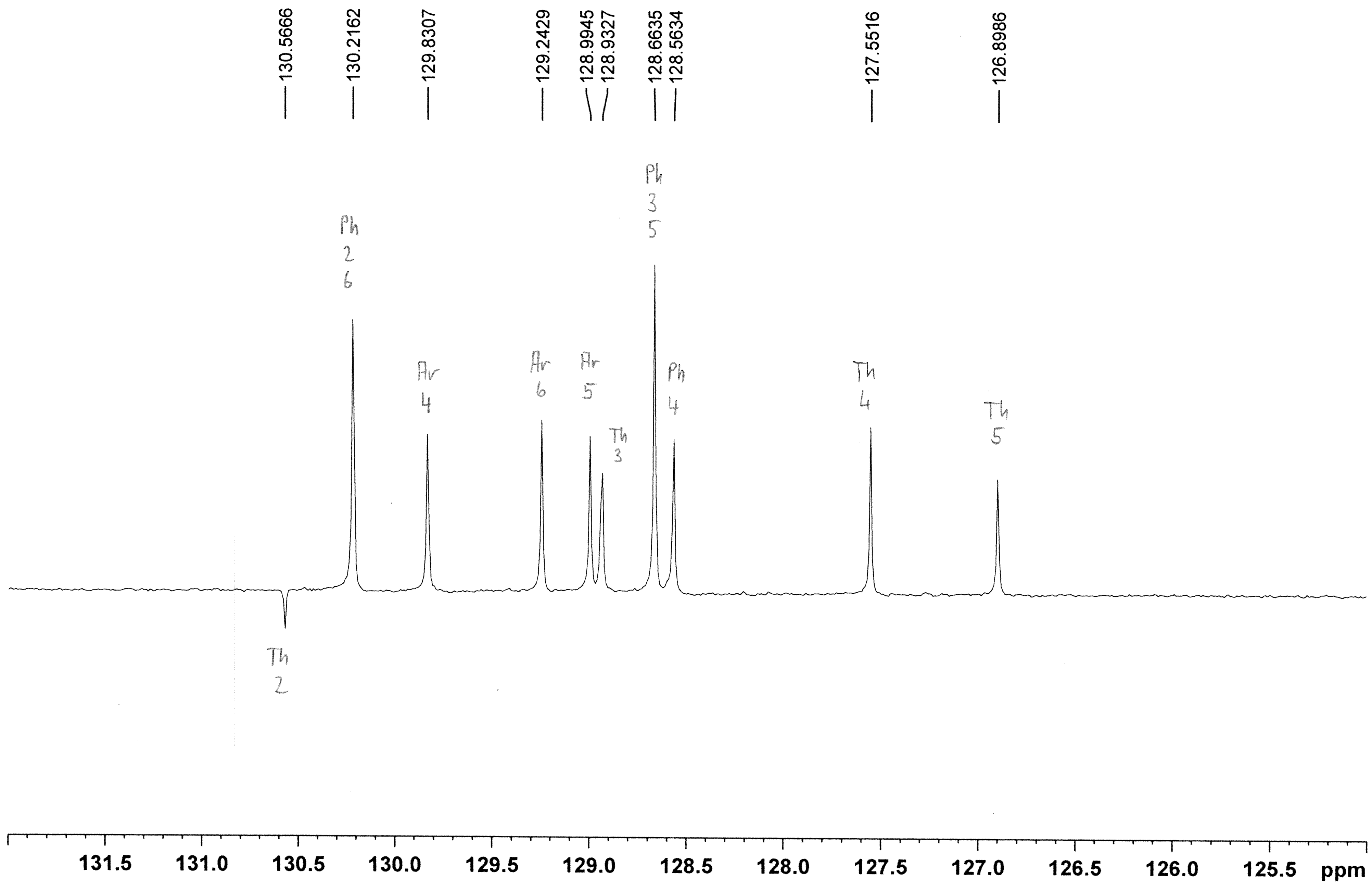

MK-31p2 in cdcl3 (APT) 4.9.2020

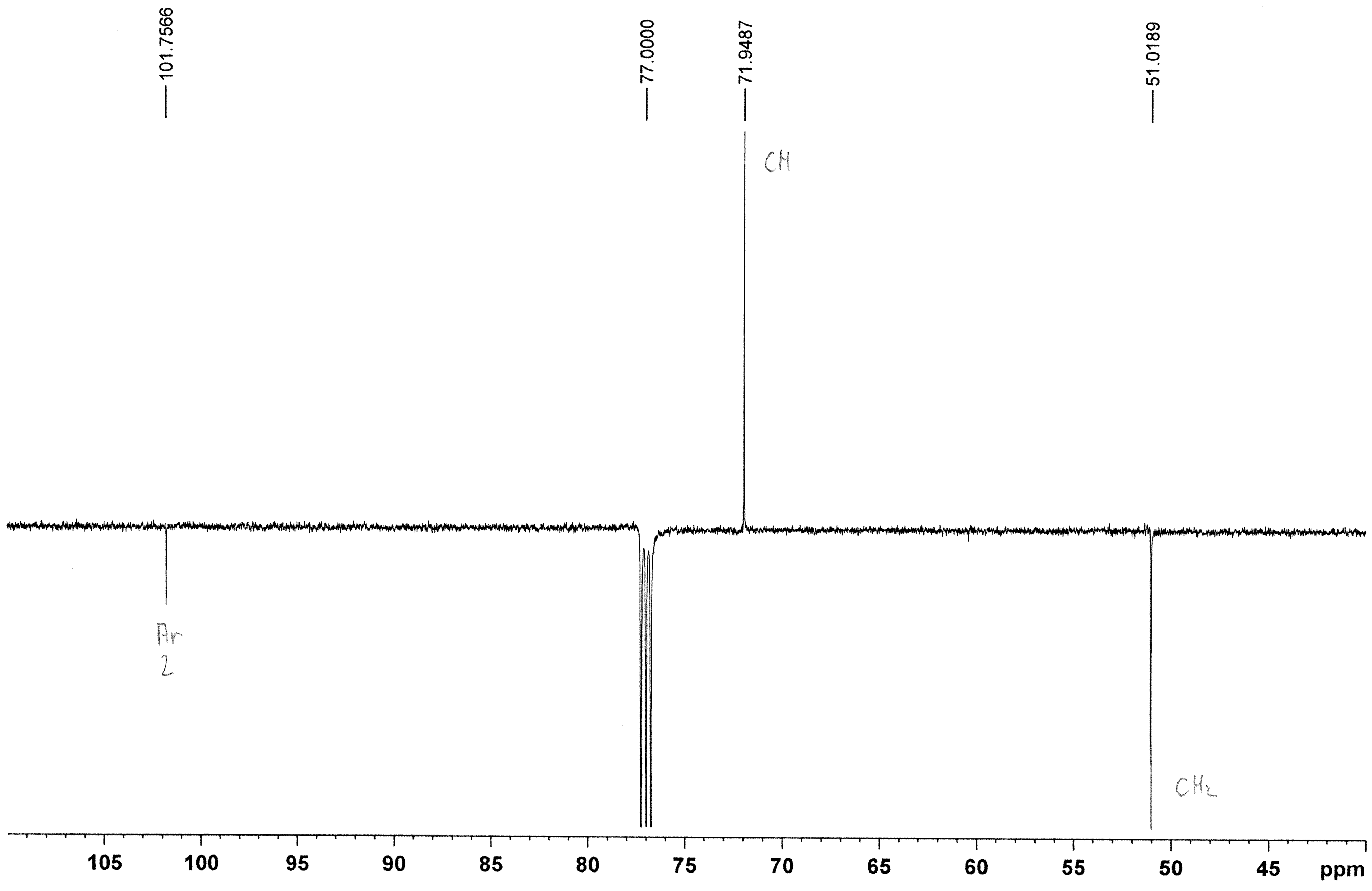

MK-31p2 in cdcl3 (COSY) 4.9.2020

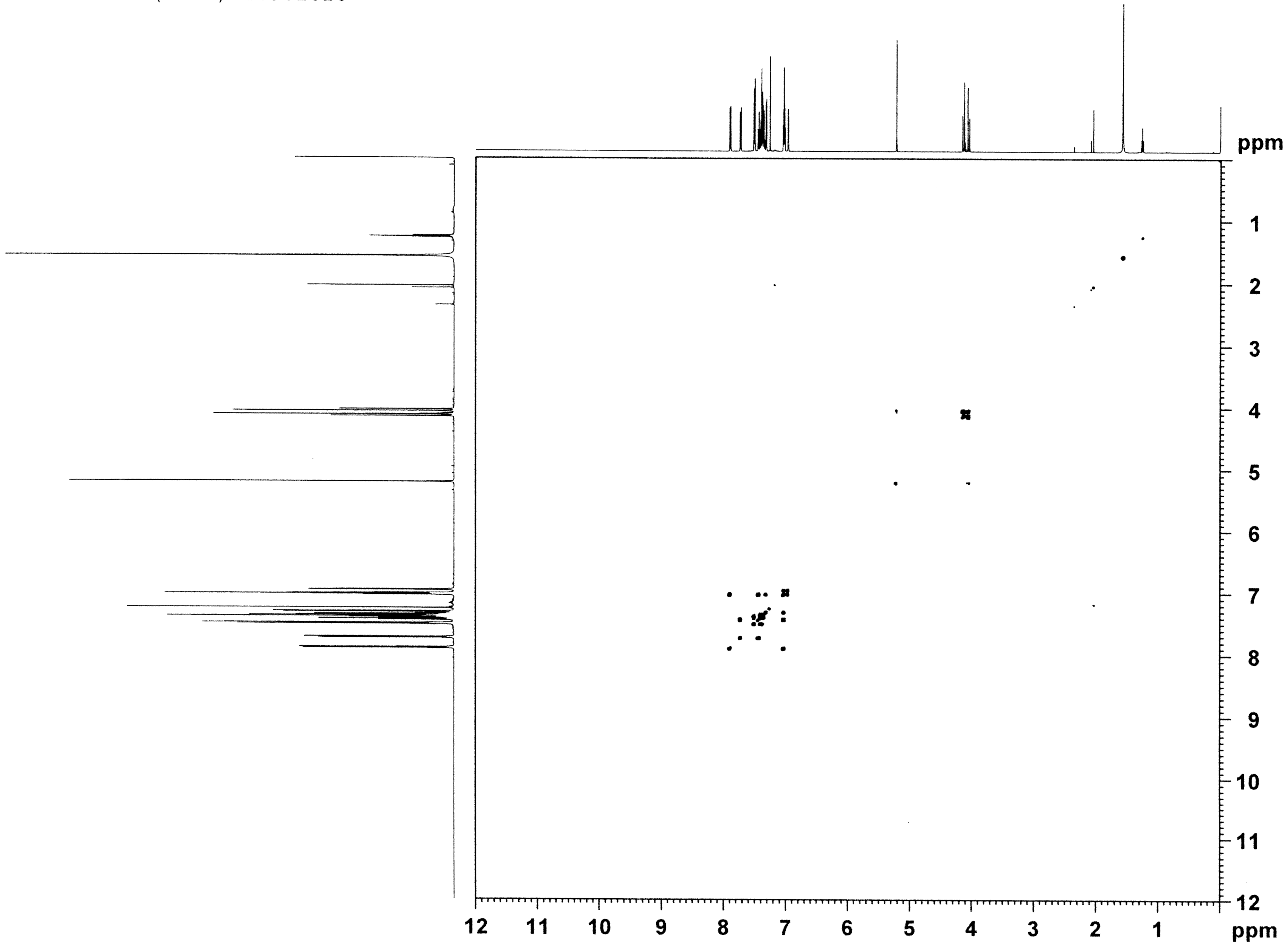

MK-31p2 in cdcl3 (COSY) 4.9.2020

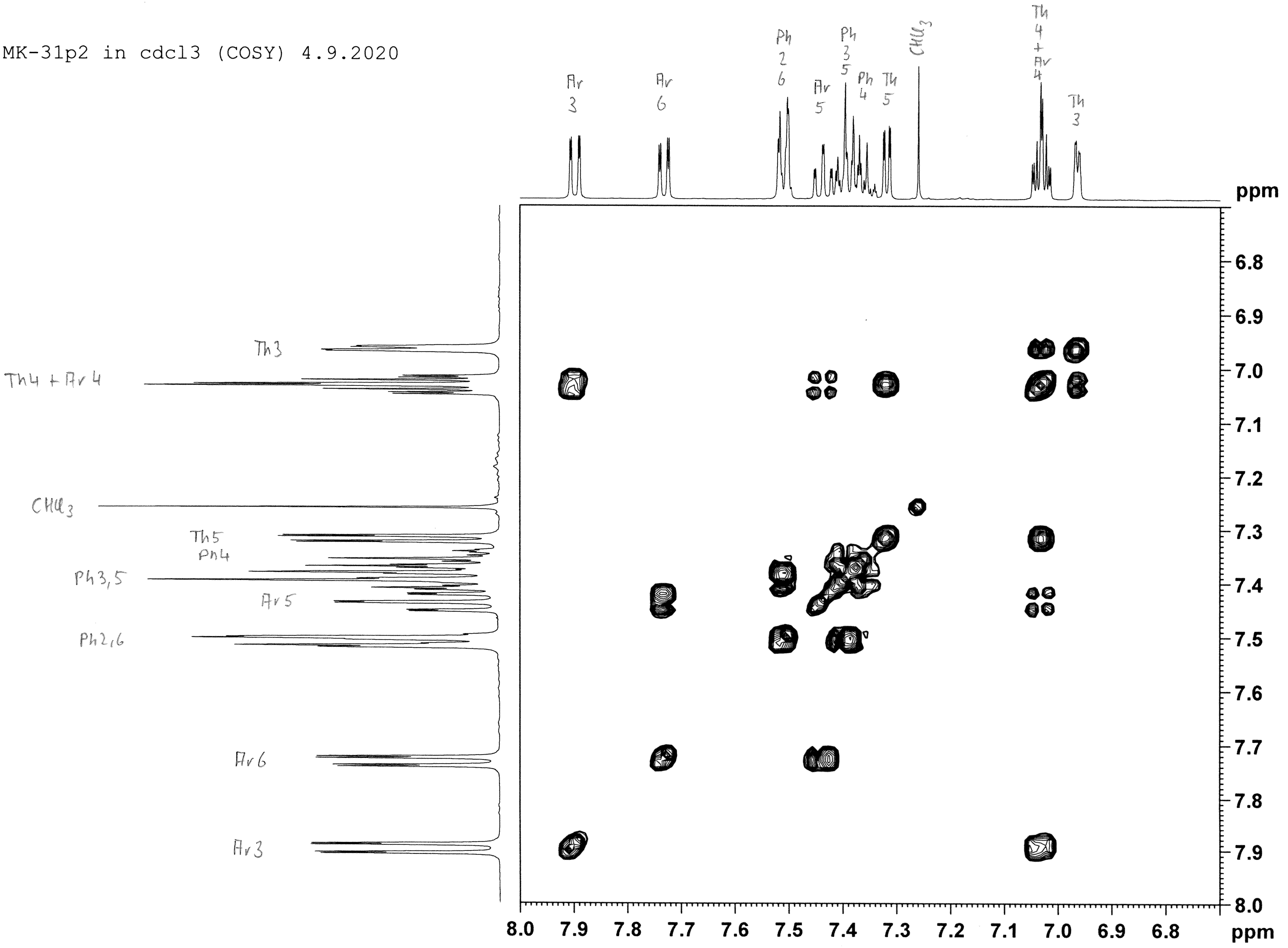

MK-31p2 in cdcl3 (COSY) 4.9.2020

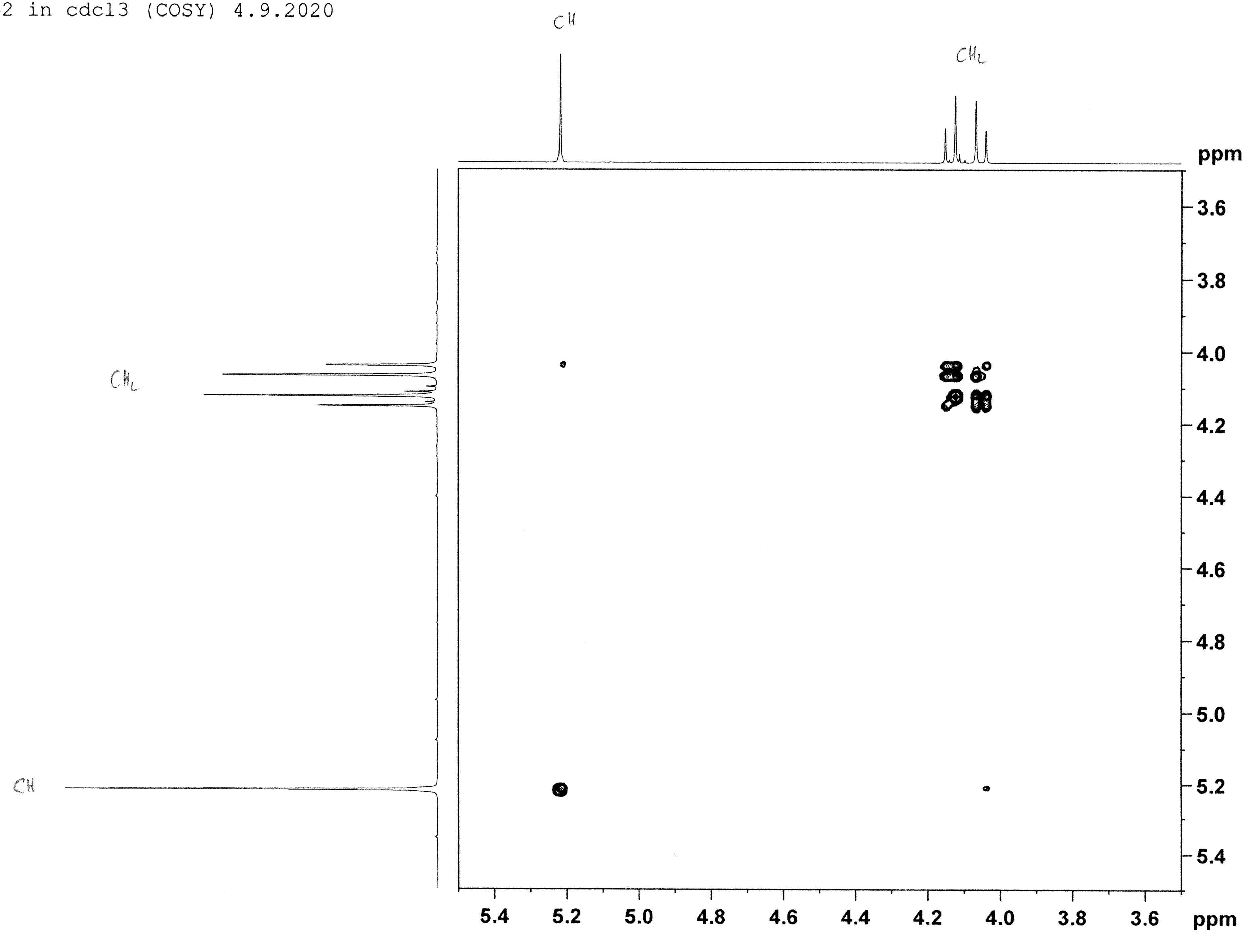

MK-31p2 in cdcl3 (HSQC) 4.9.2020

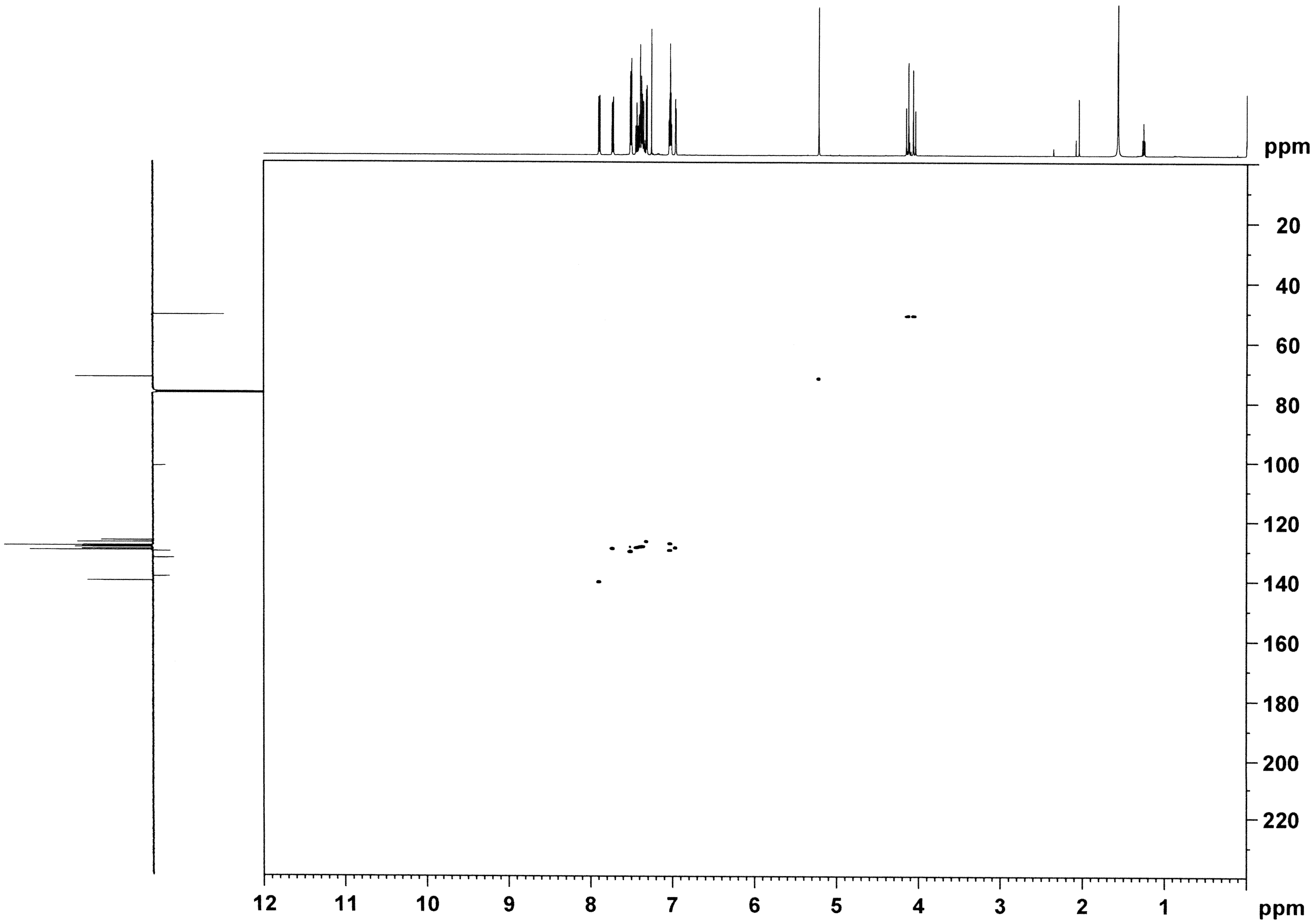

MK-31p2 in cdcl3 (HSQC) 4.9.2020

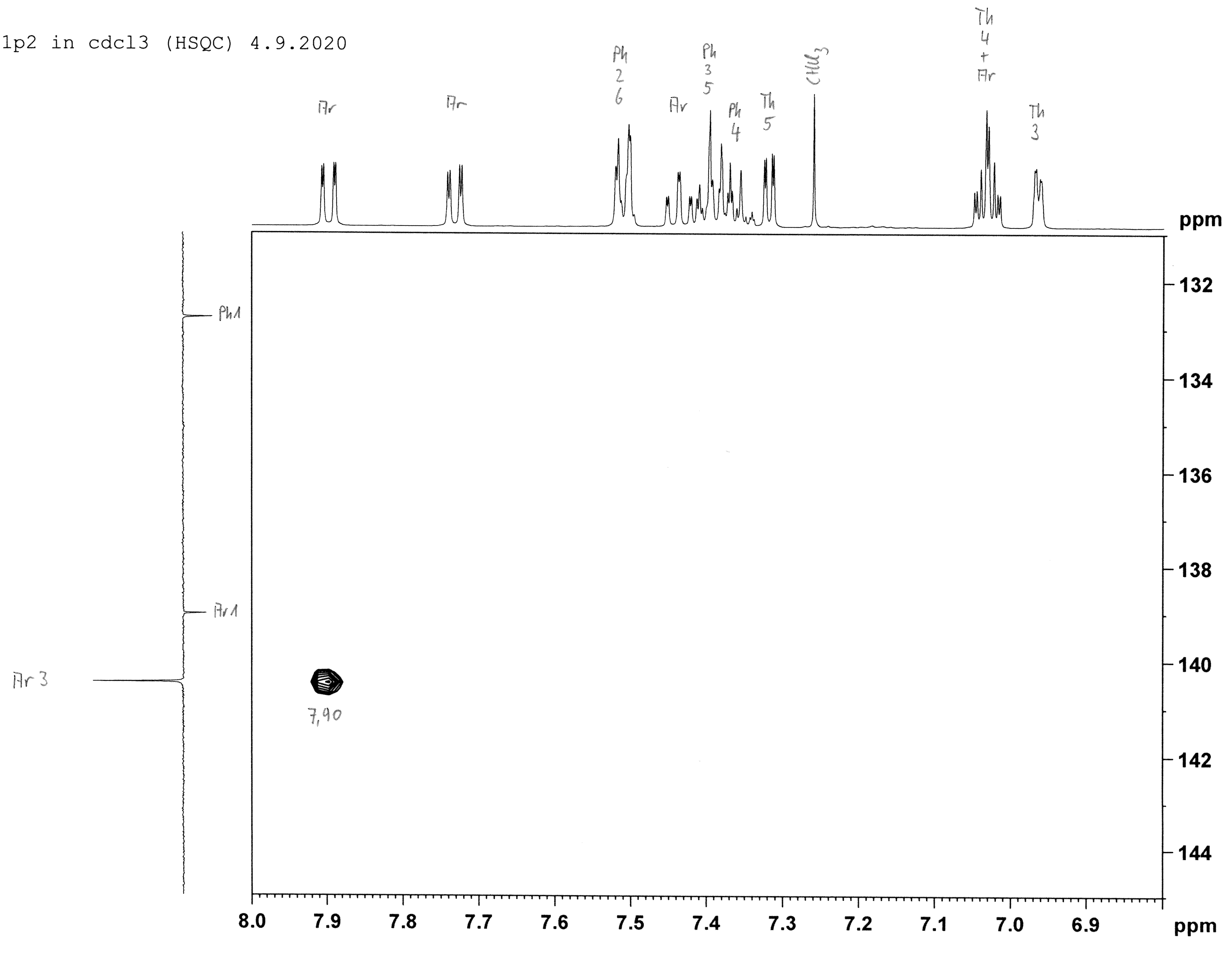

MK-31p2 in cdcl3 (HSQC) 4.9.2020

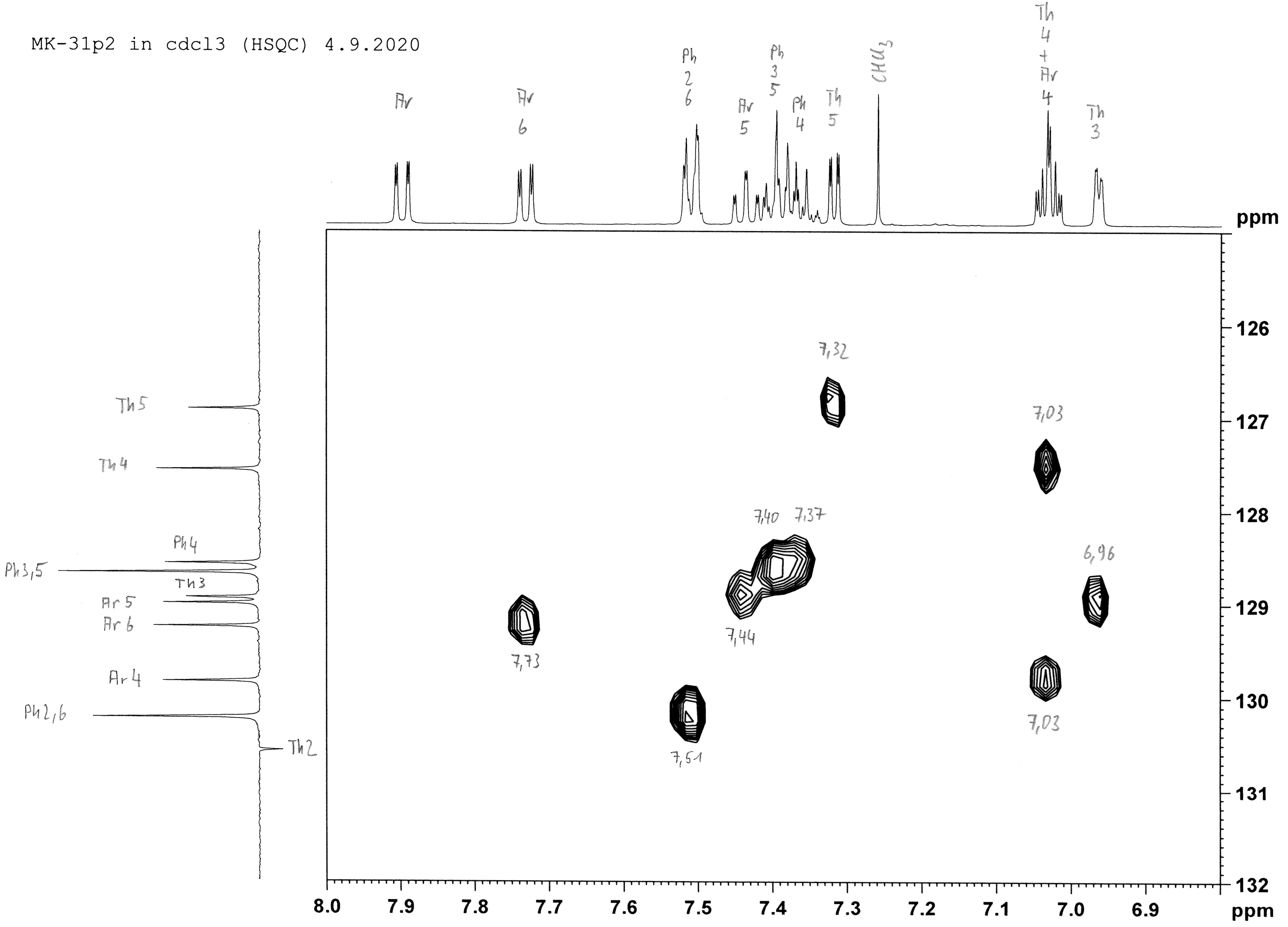

MK-31p2 in cdcl3 (HSQC) 4.9.2020

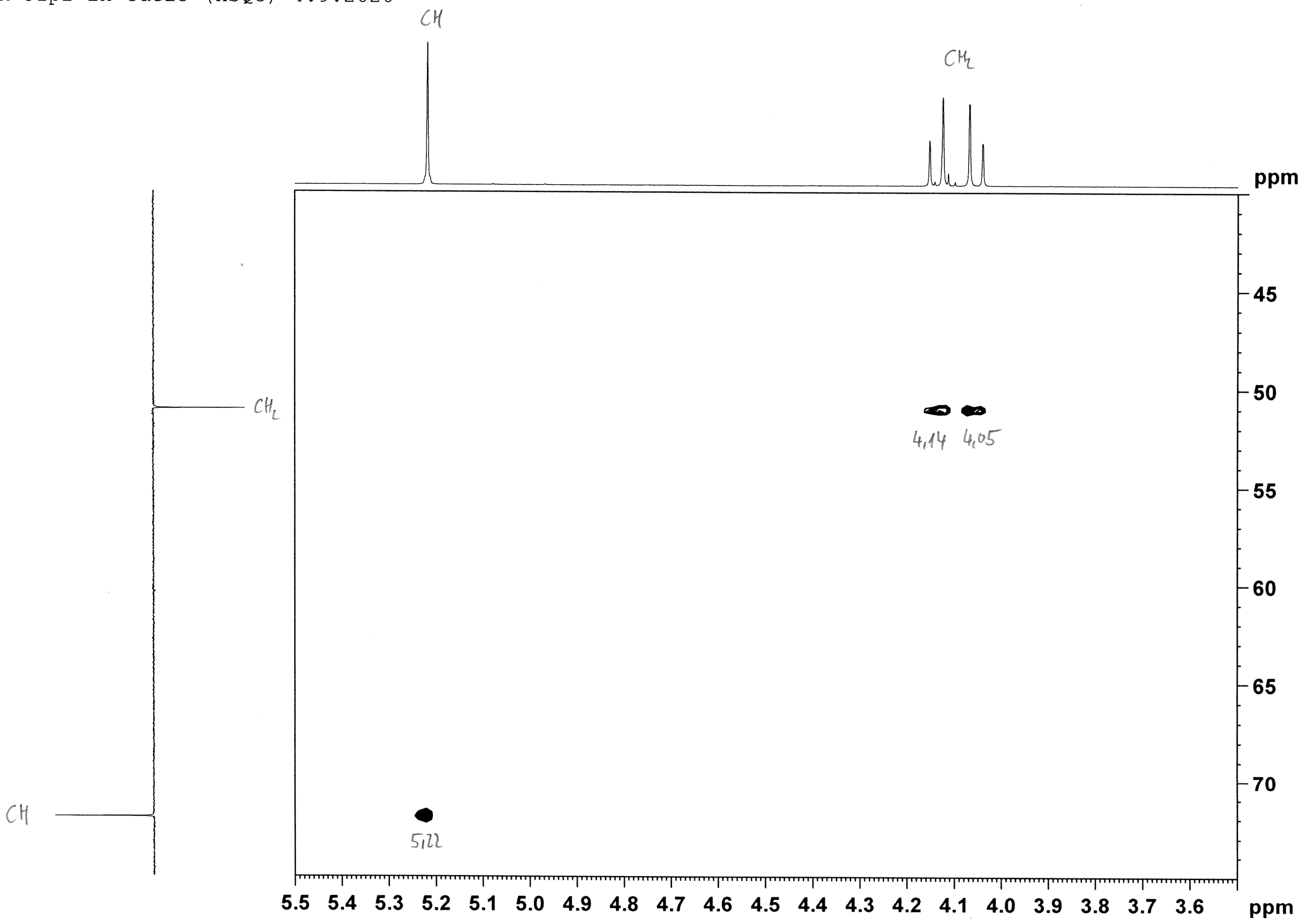

MK-31p2 in cdcl3 (HMBC) 4.9.2020

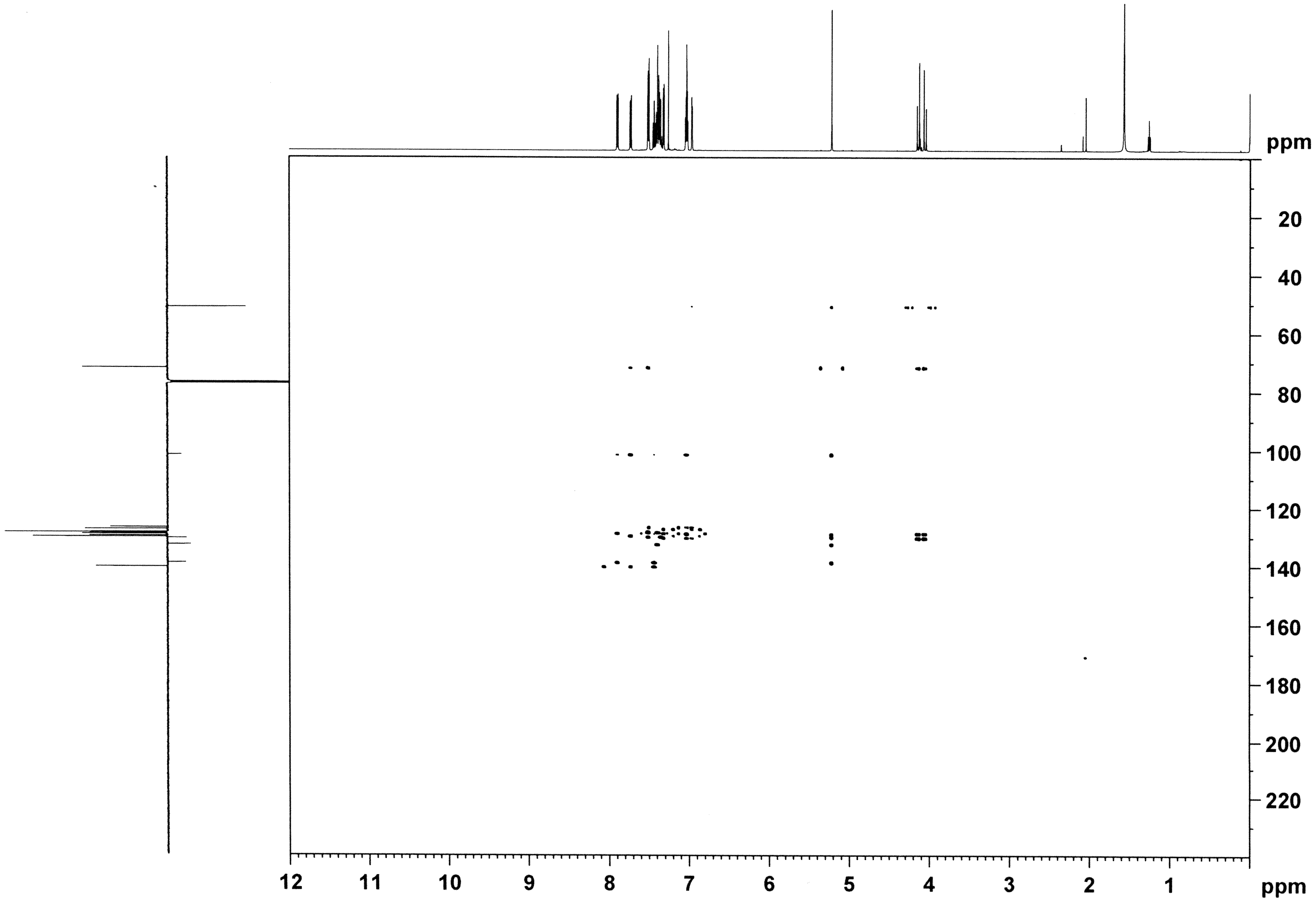

MK-31p2 in cdcl3 (HMBC) 4.9.2020

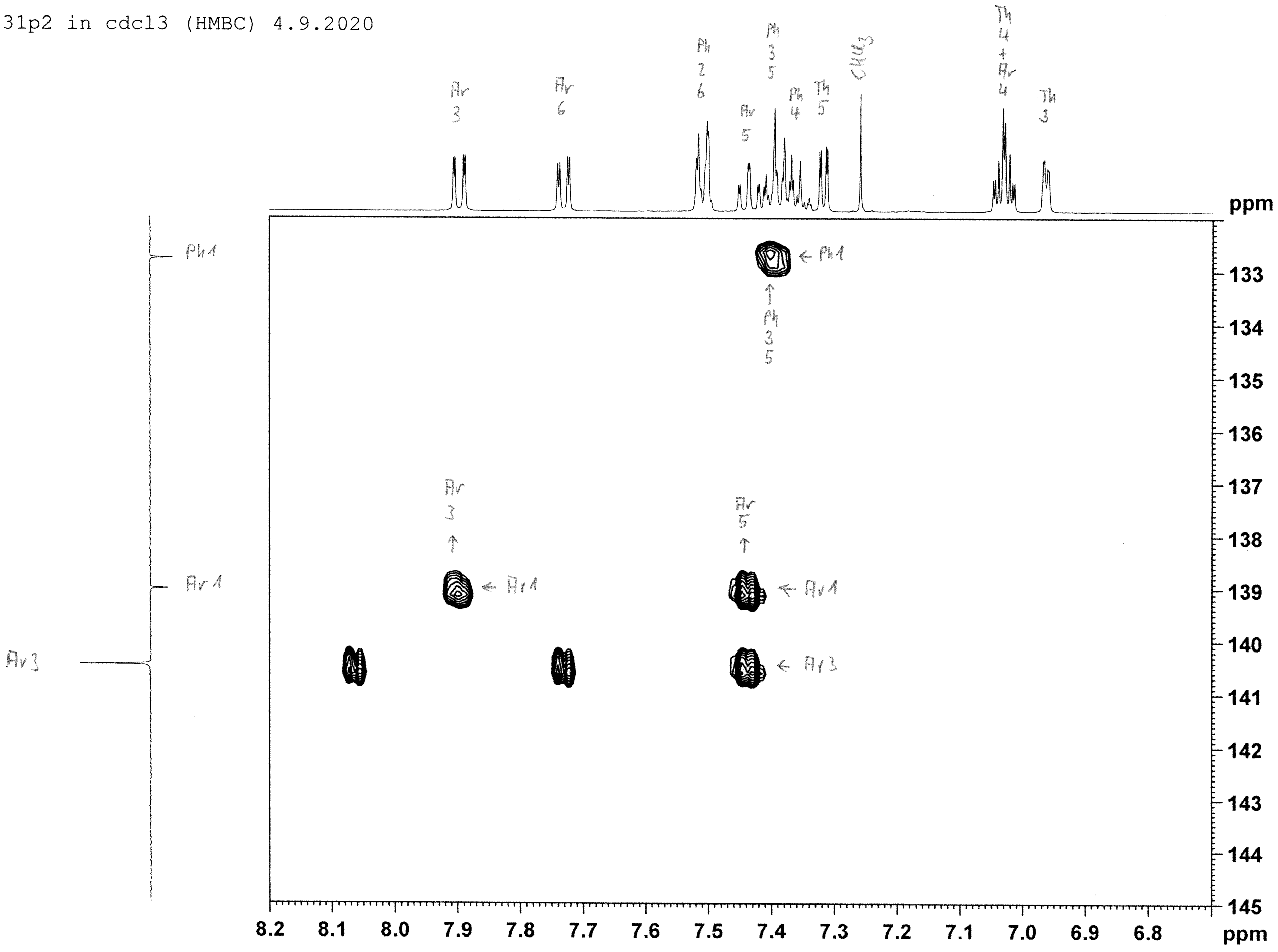

MK-31p2 in cdcl3 (HMBC) 4.9.2020

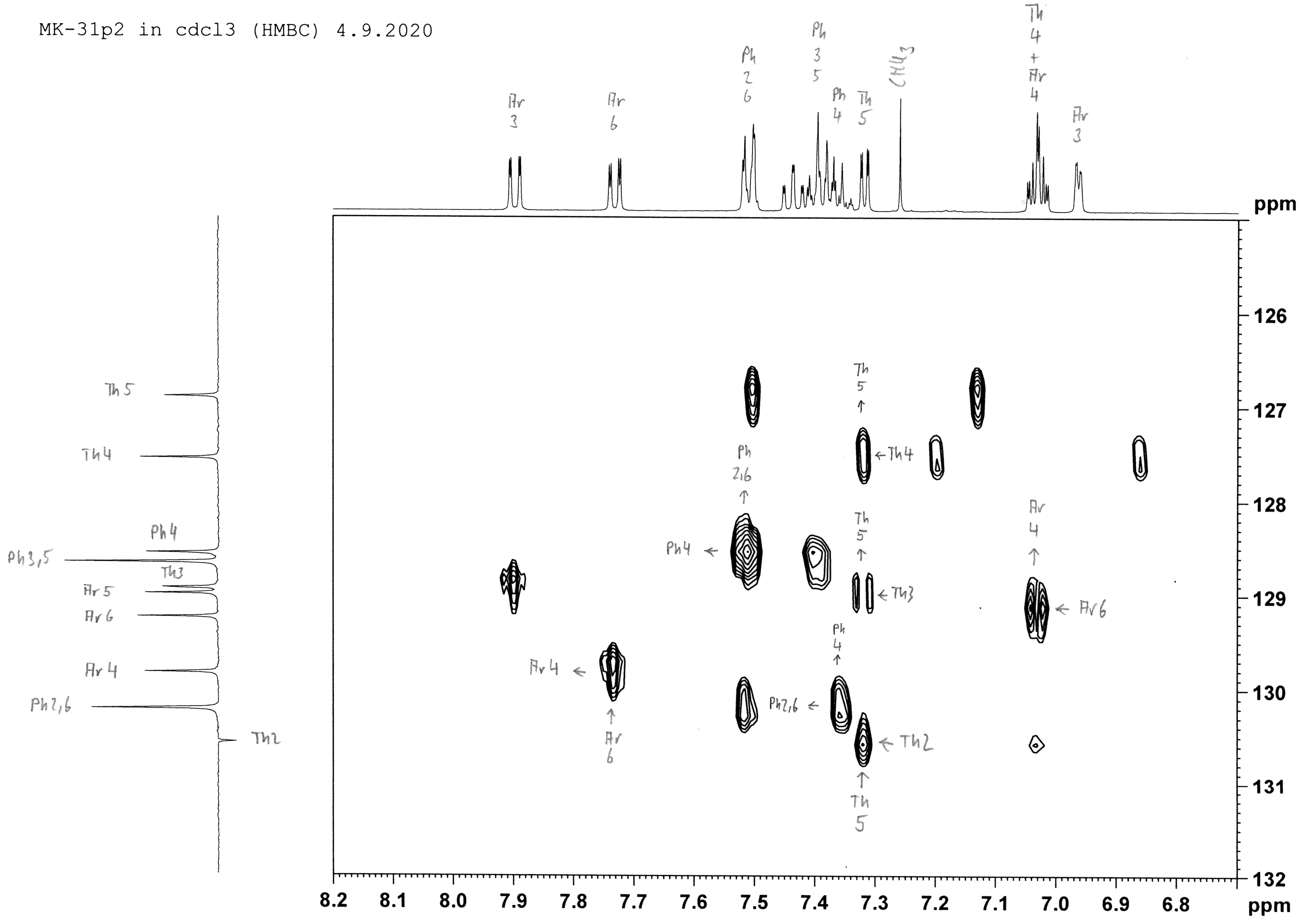

MK-31p2 in cdcl3 (HMBC) 4.9.2020

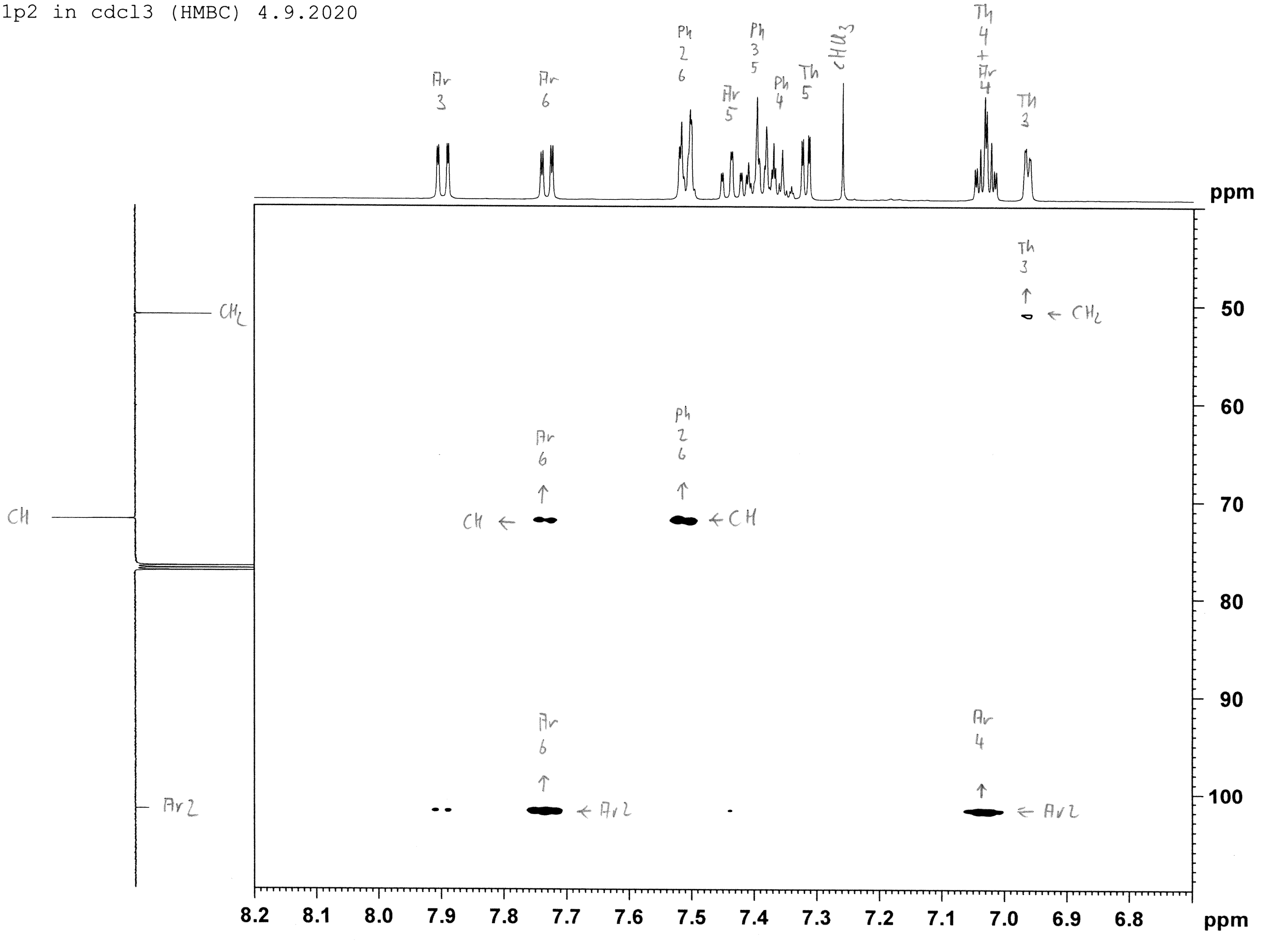

MK-31p2 in cdcl3 (HMBC) 4.9.2020

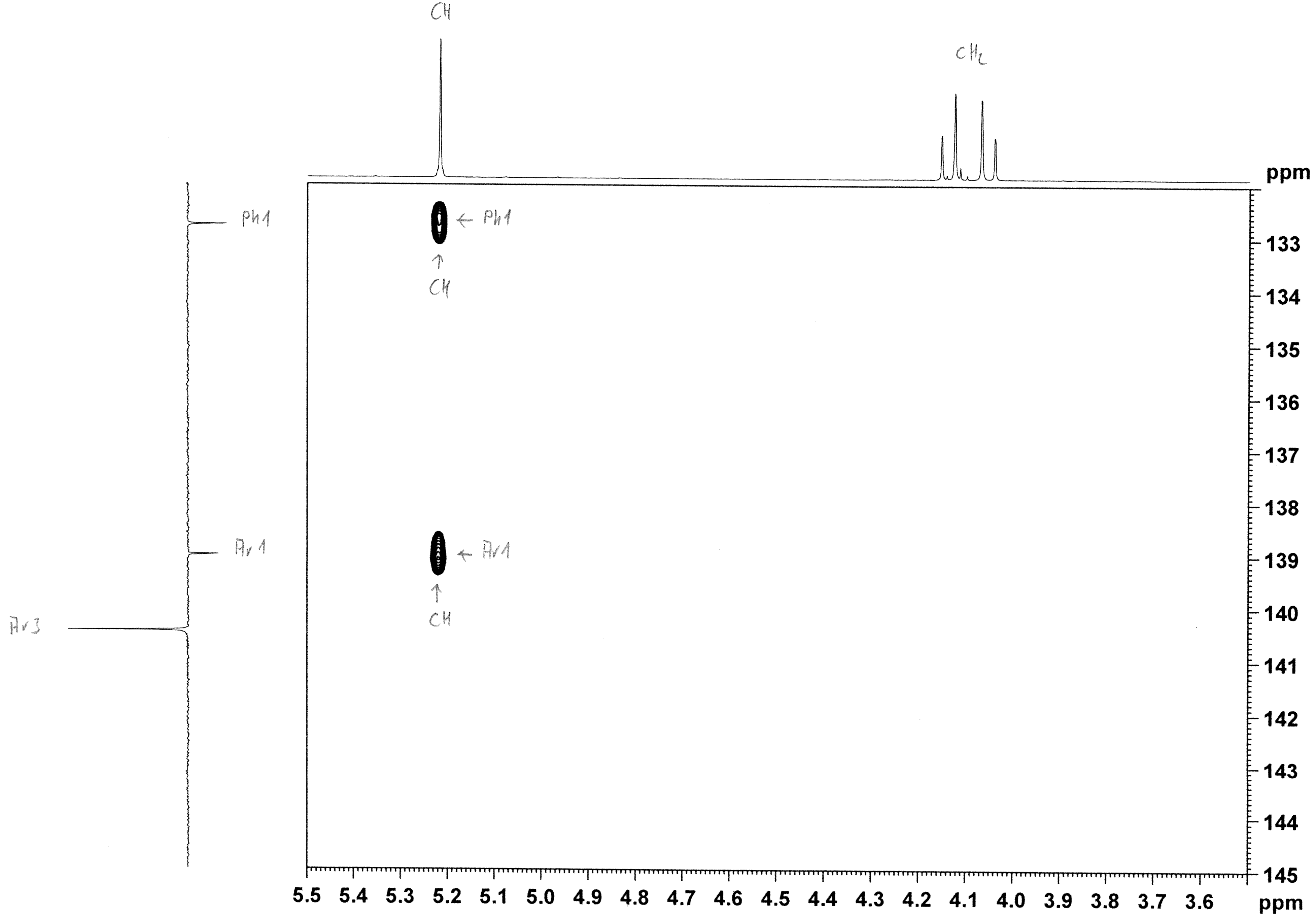

MK-31p2 in cdcl3 (HMBC) 4.9.2020

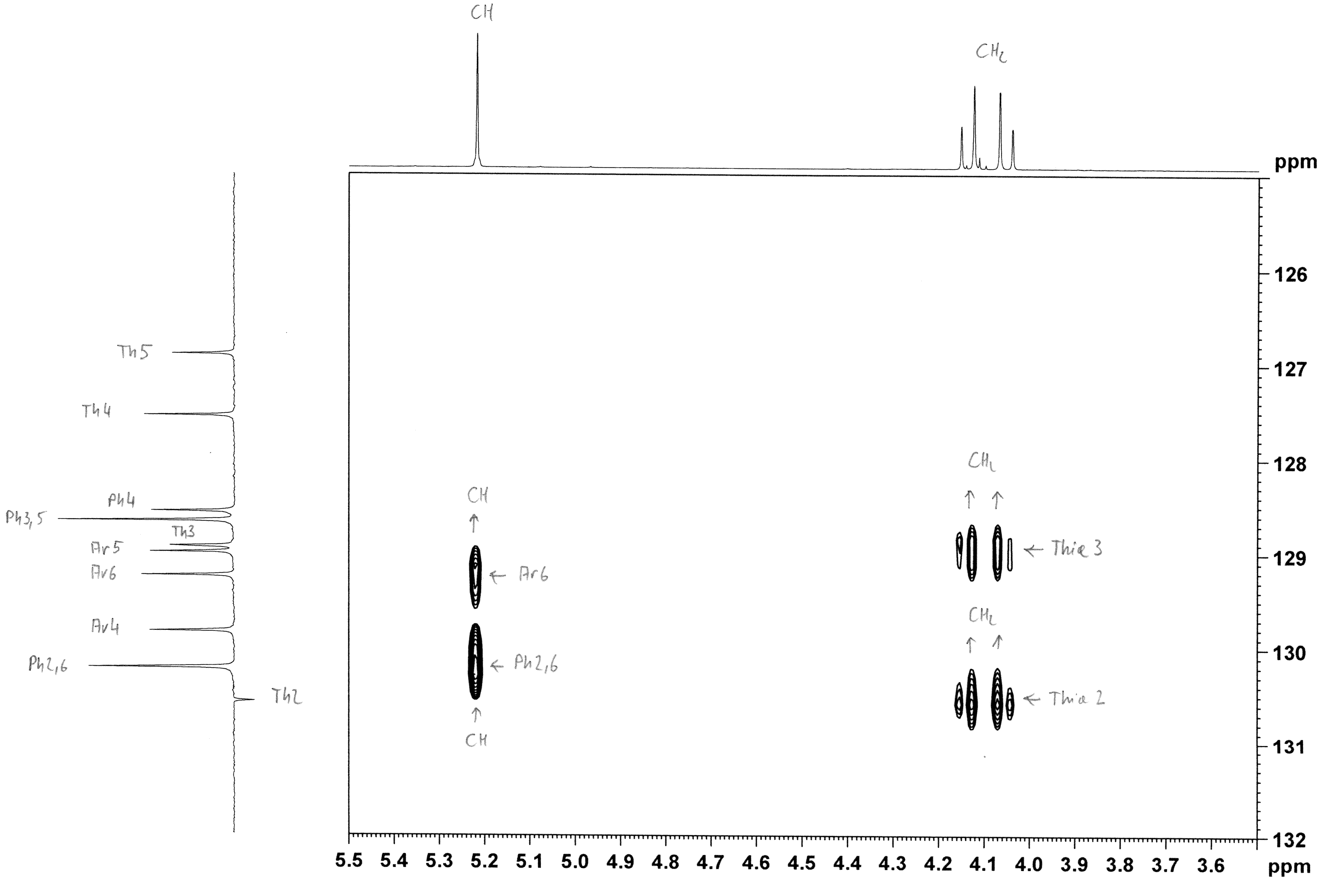

MK-31p2 in cdcl3 (HMBC) 4.9.2020

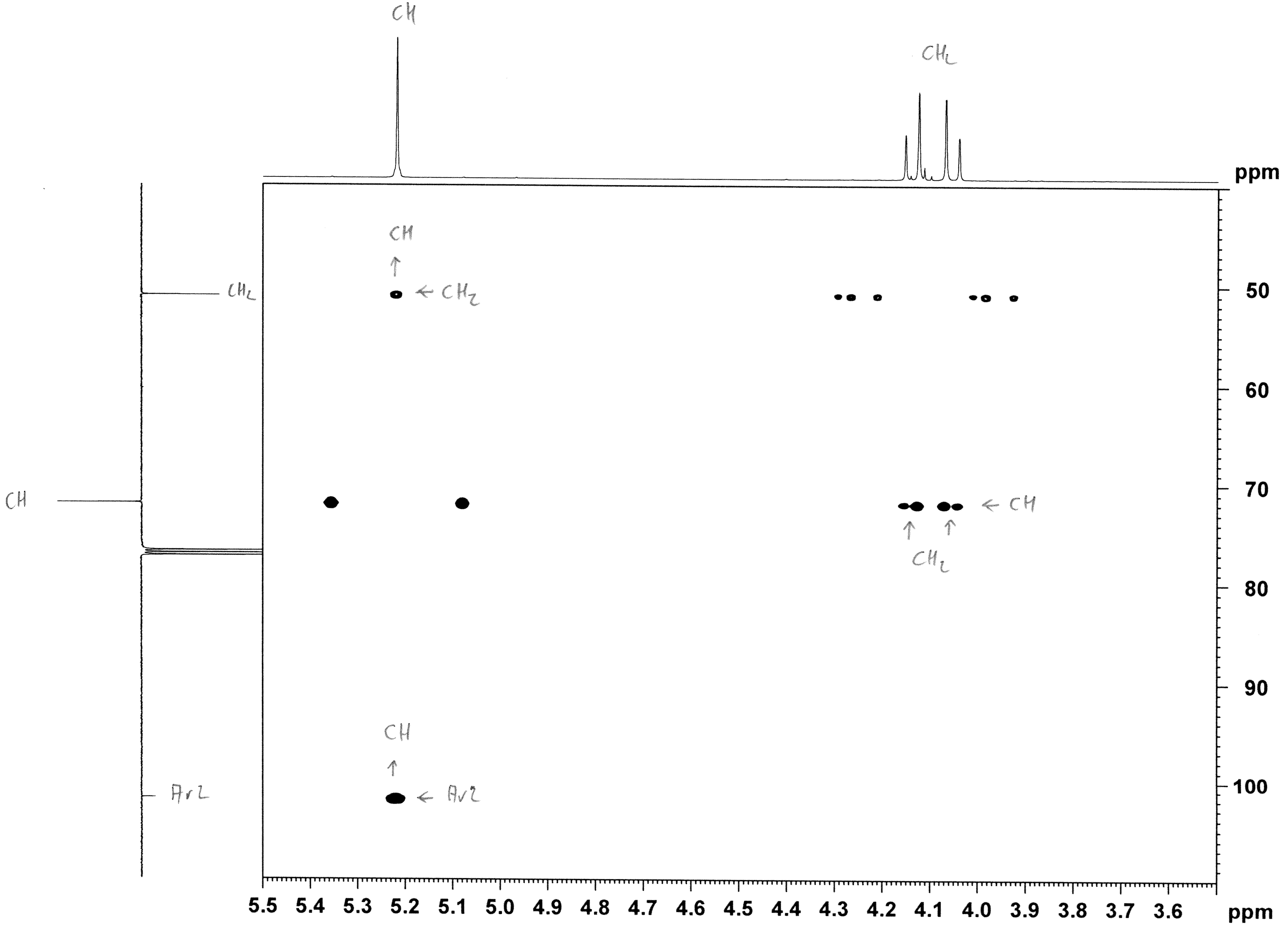

Figure S35c. NMR spectra of compound **7i.**

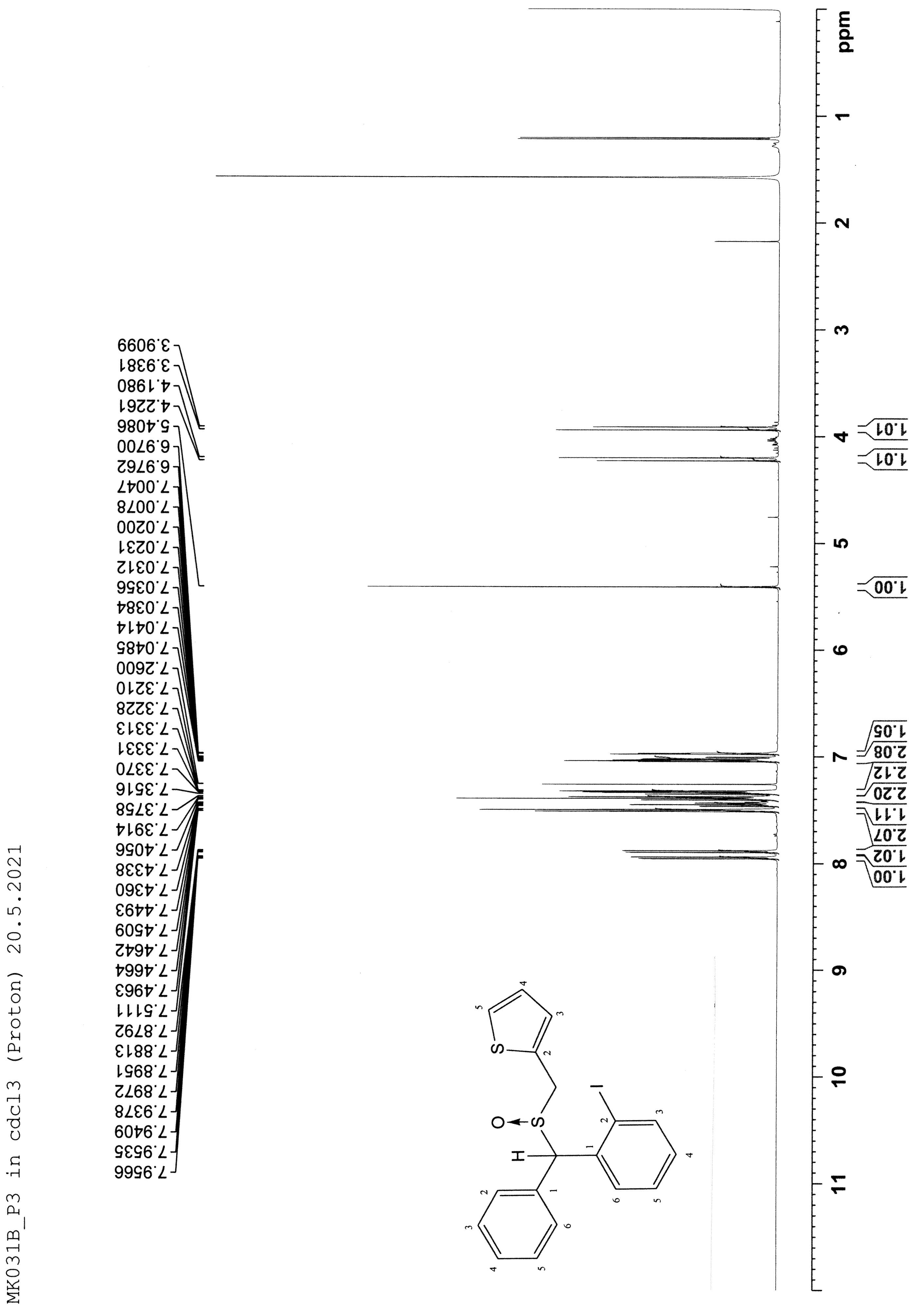

MK031B_P3 in cdcl3 (Proton) 20.5.2021

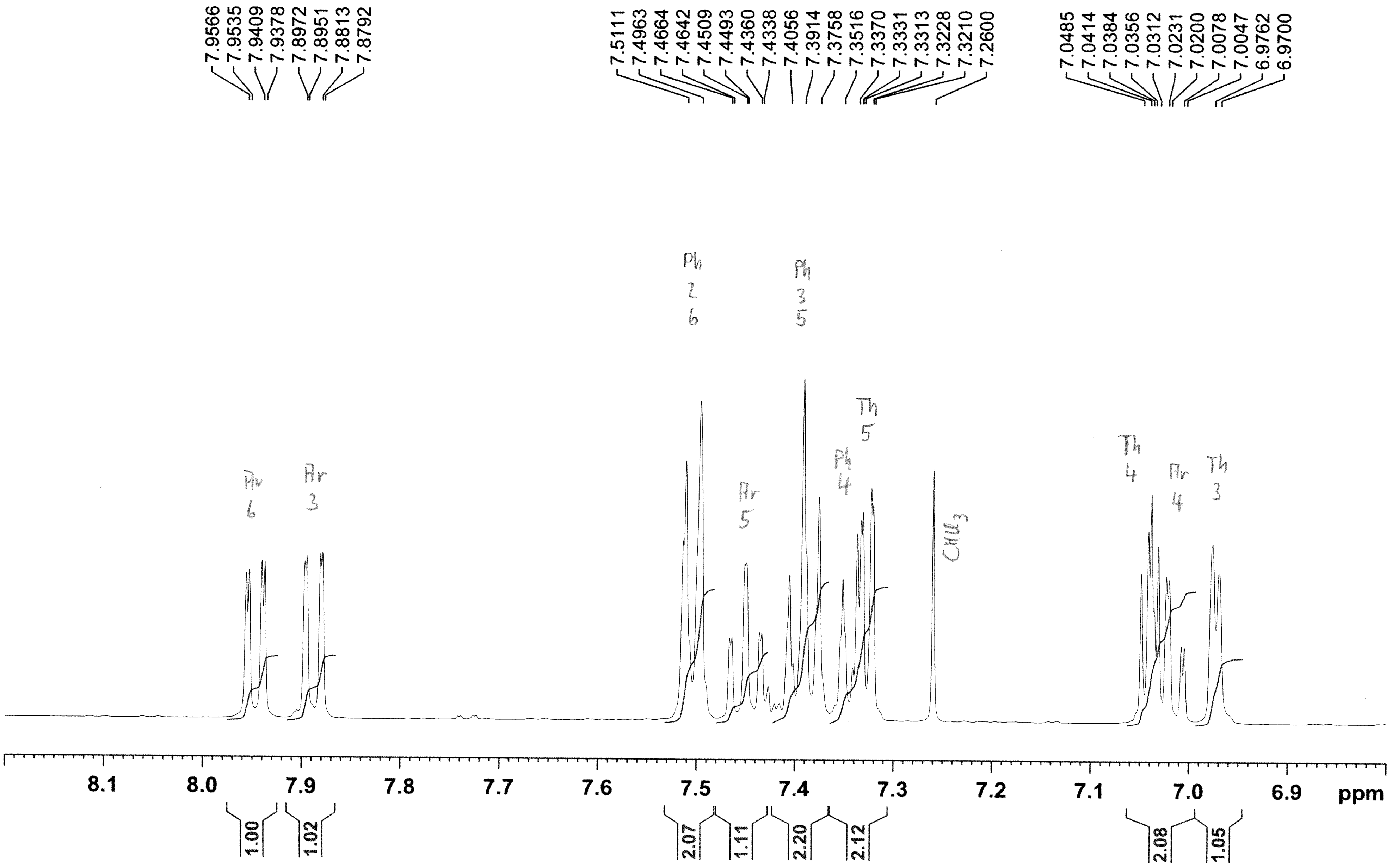

MK031B_P3 in cdcl3 (Proton) 20.5.2021

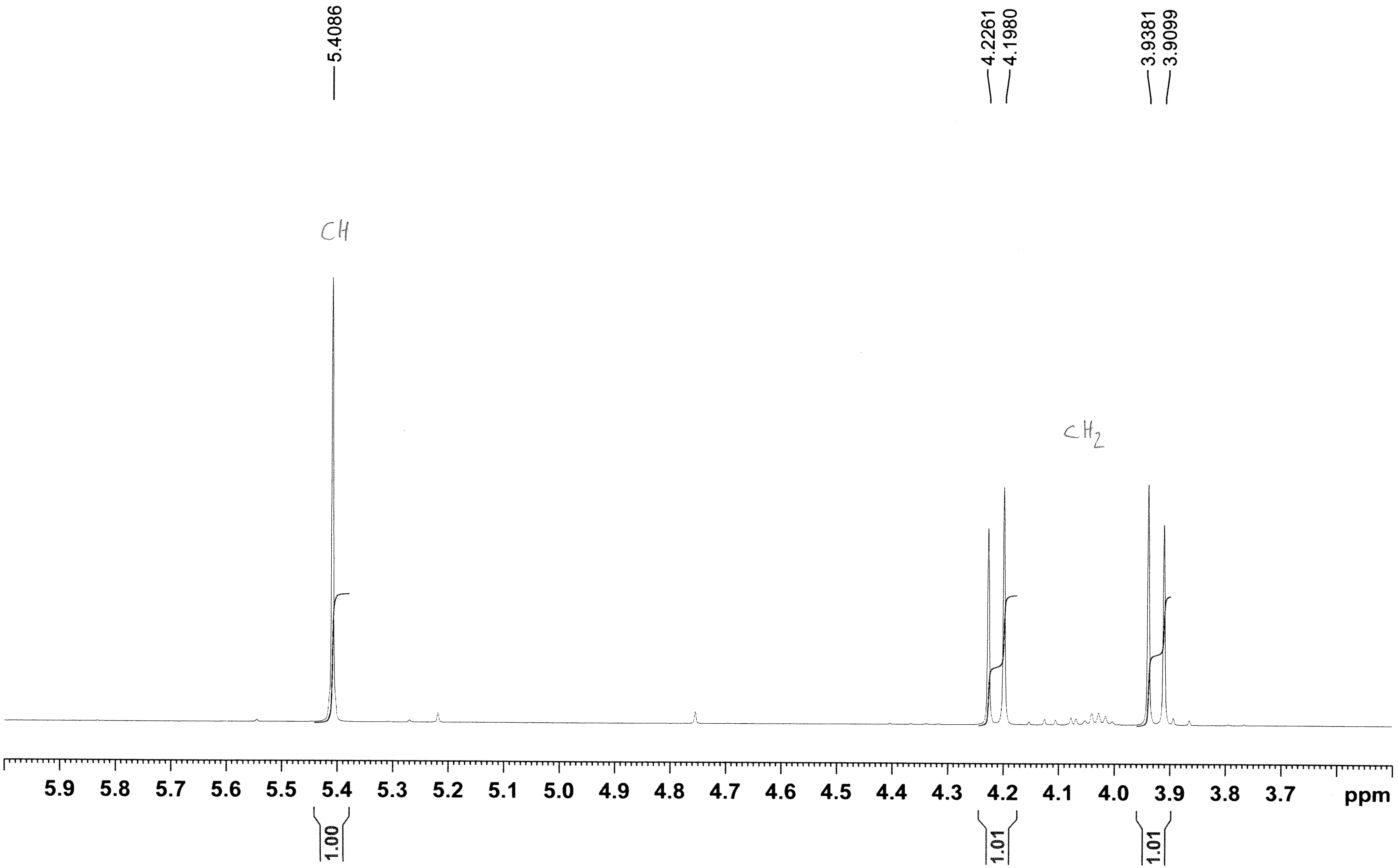

MK031B_P3 in cdcl3 (APT) 20.5.2021

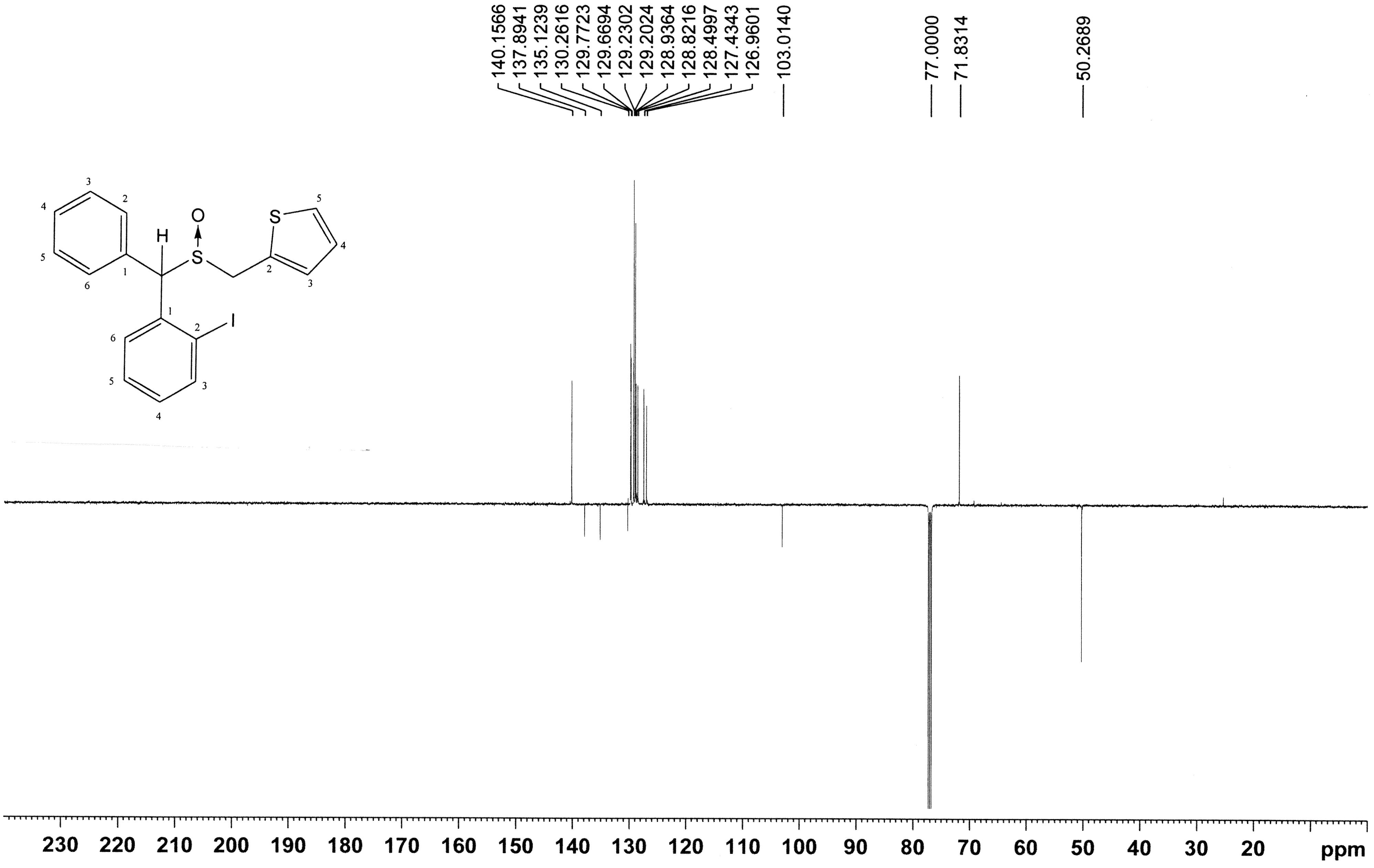

MK031B_P3 in cdcl3 (APT) 20.5.2021

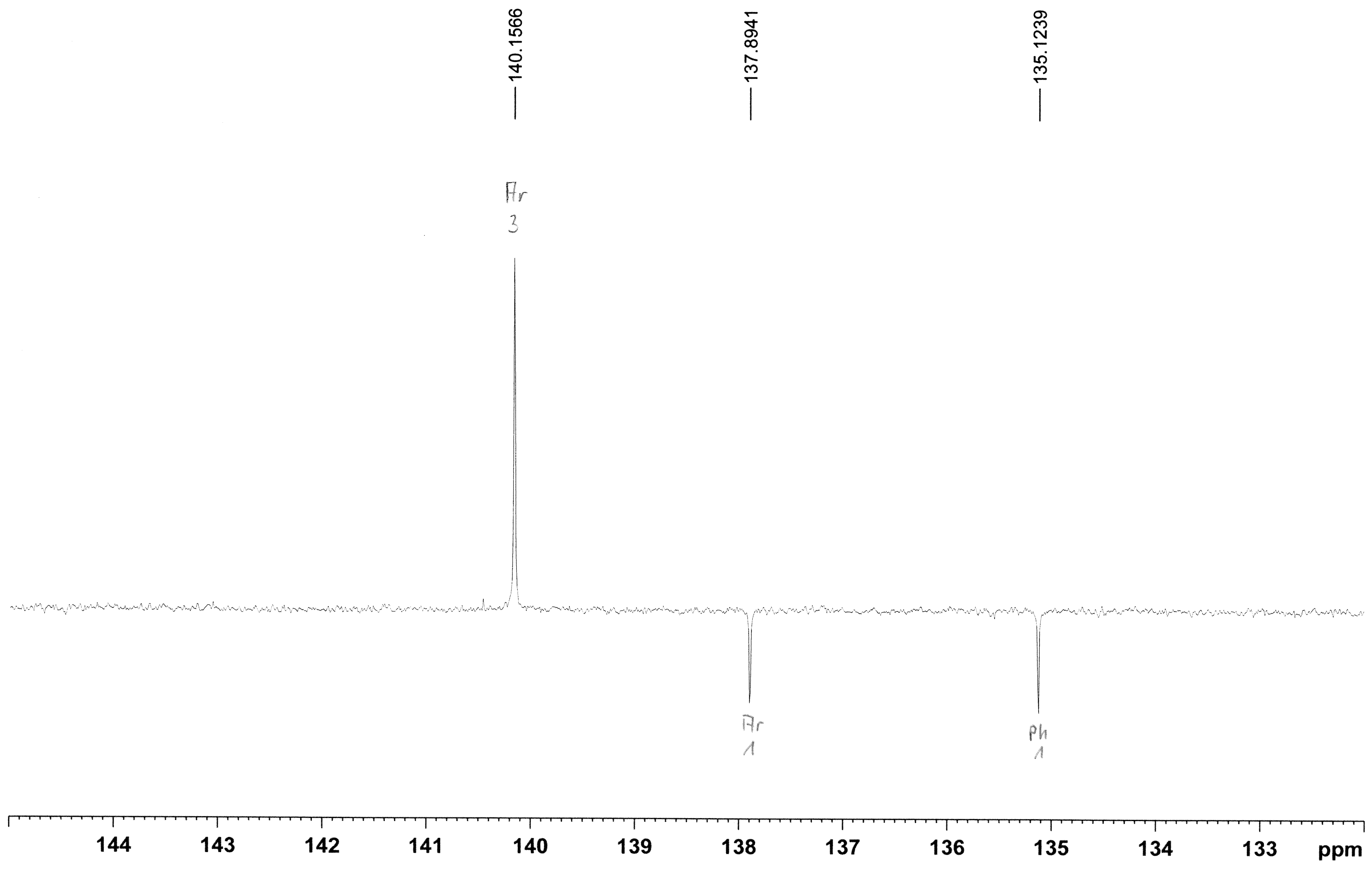

MK031B_P3 in cdcl3 (APT) 20.5.2021

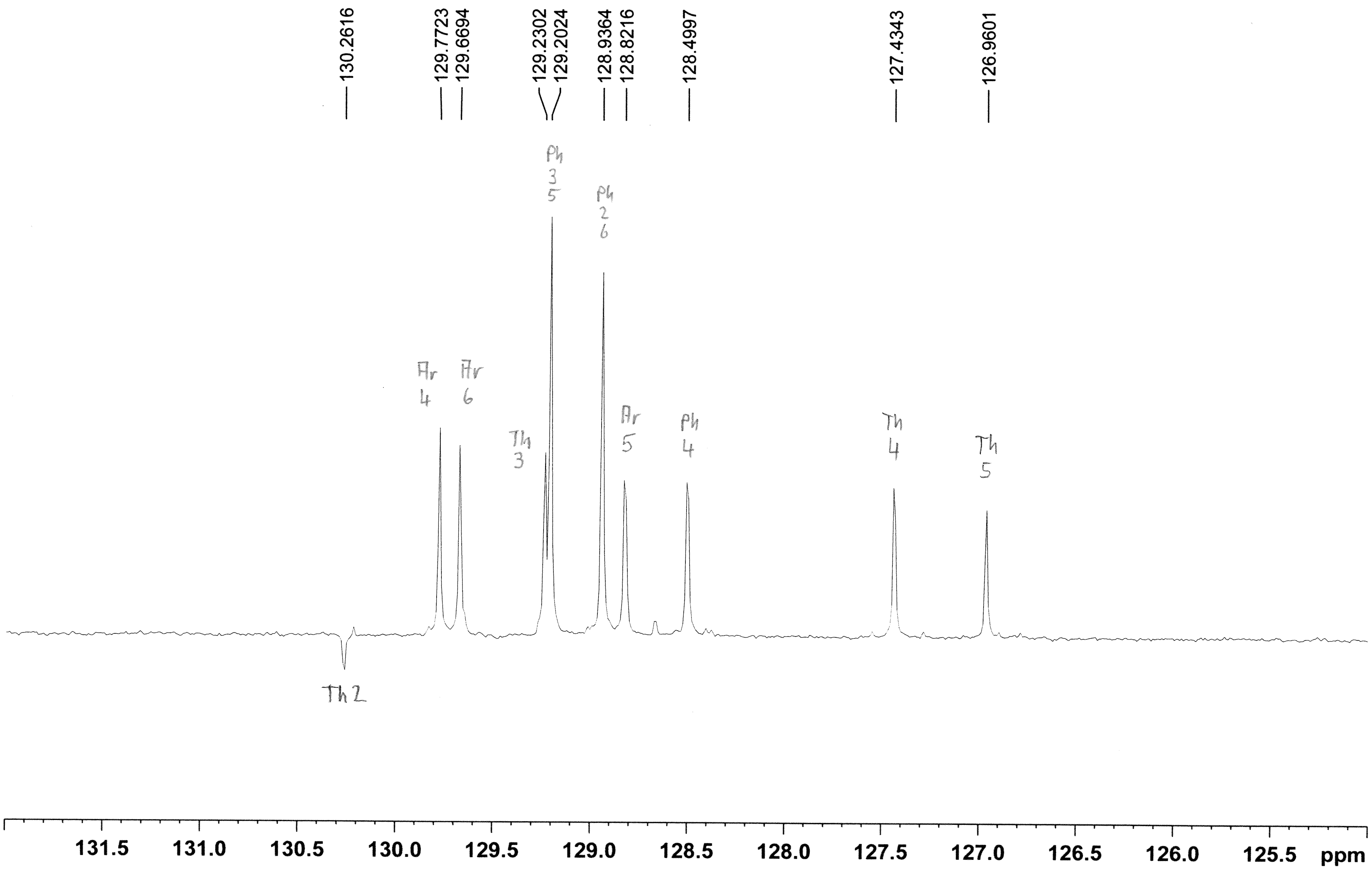

MK031B_P3 in cdcl3 (APT) 20.5.2021

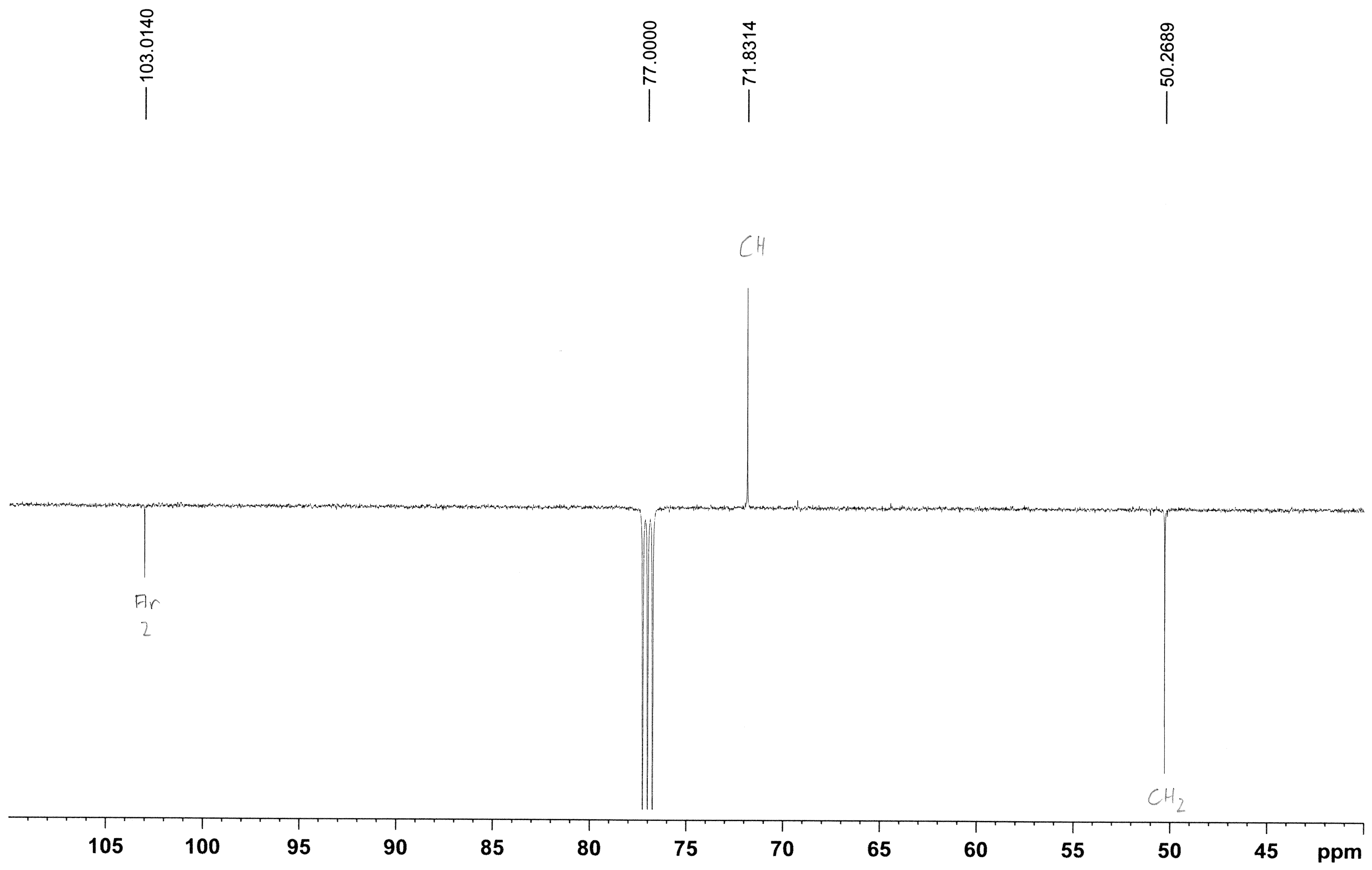

MK031B_P3 in cdcl3 (COSY) 20.5.2021

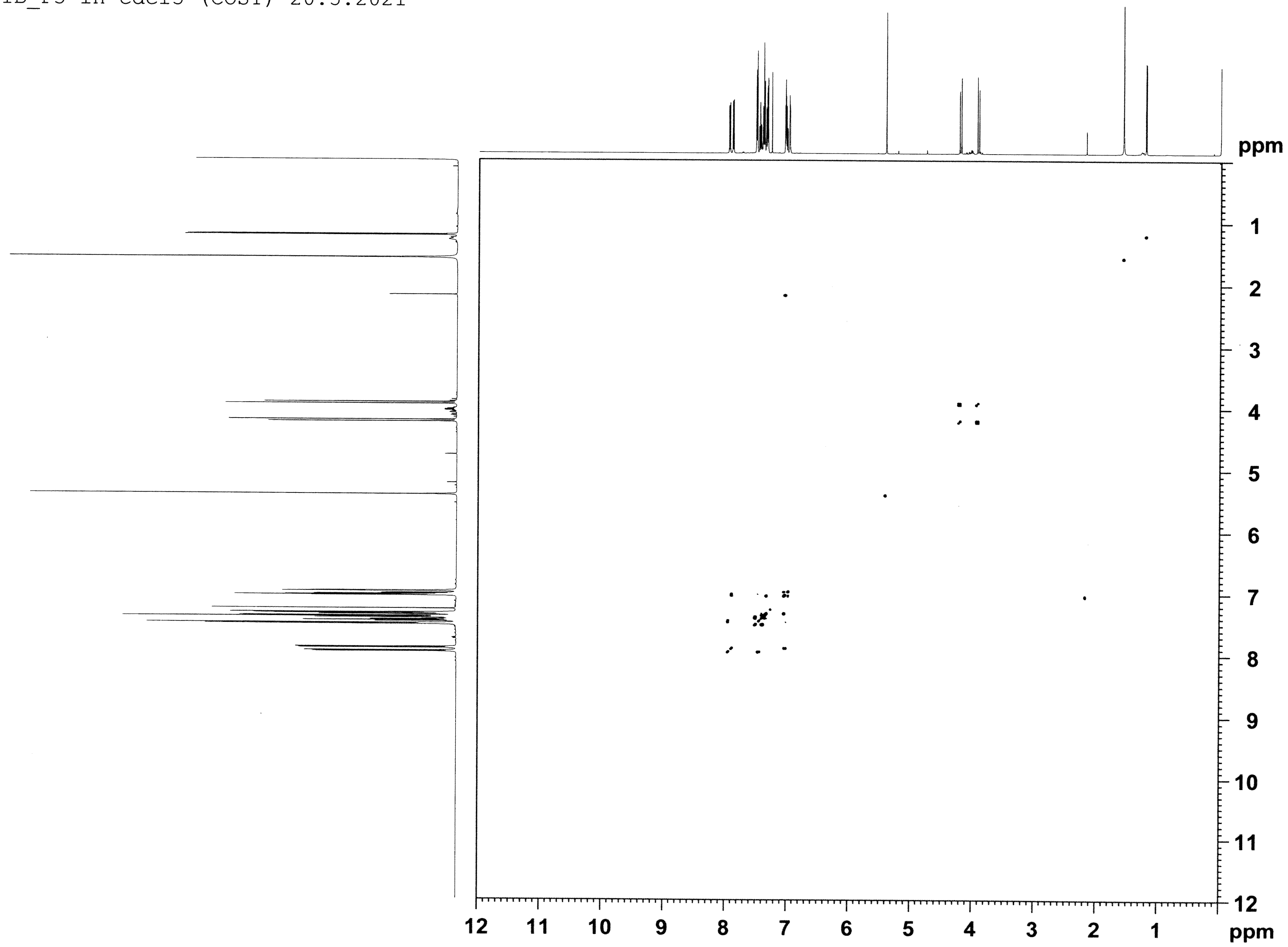

MK031B_P3 in cdcl3 (COSY) 20.5.2021

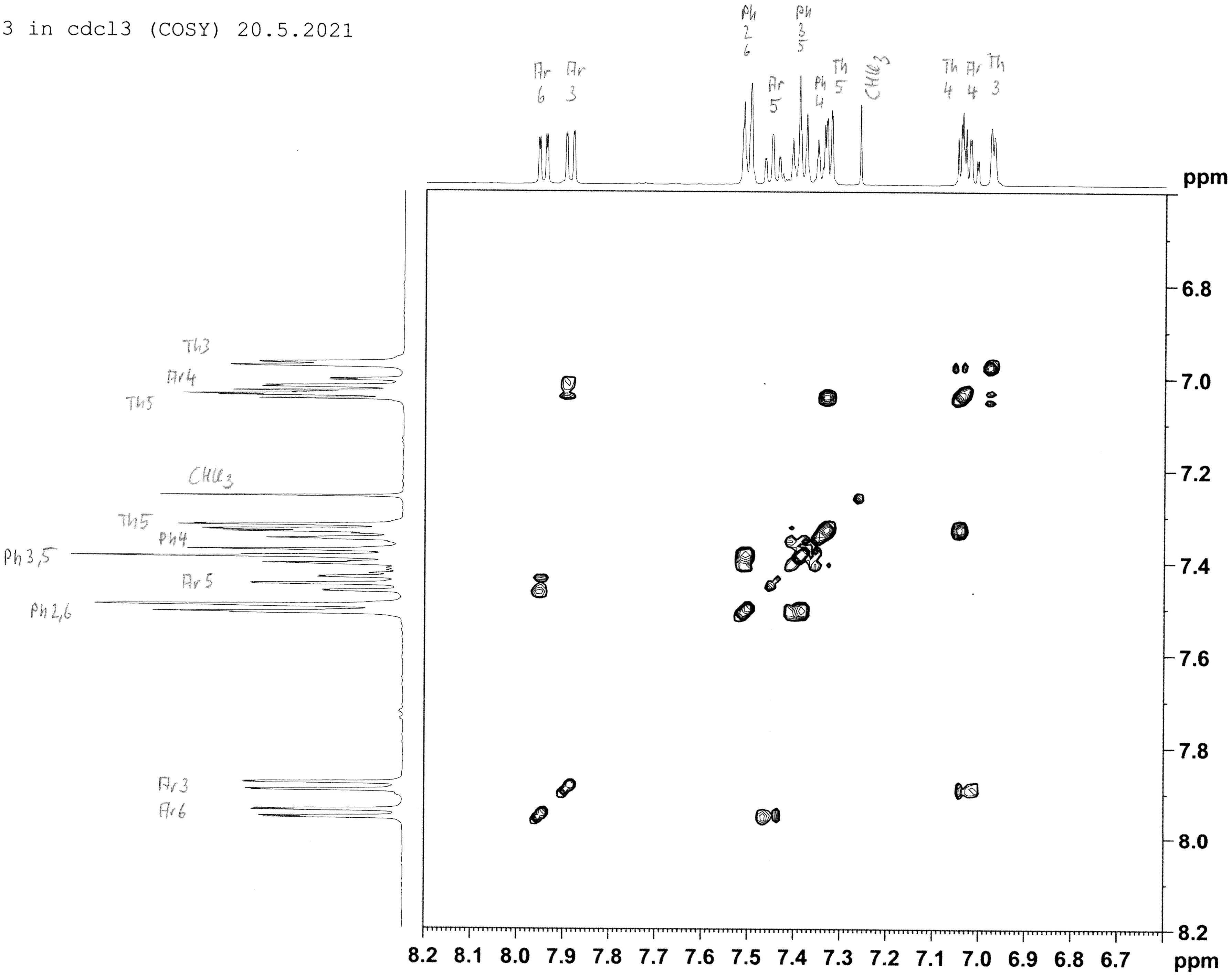

MK031B_P3 in cdcl3 (COSY) 20.5.2021

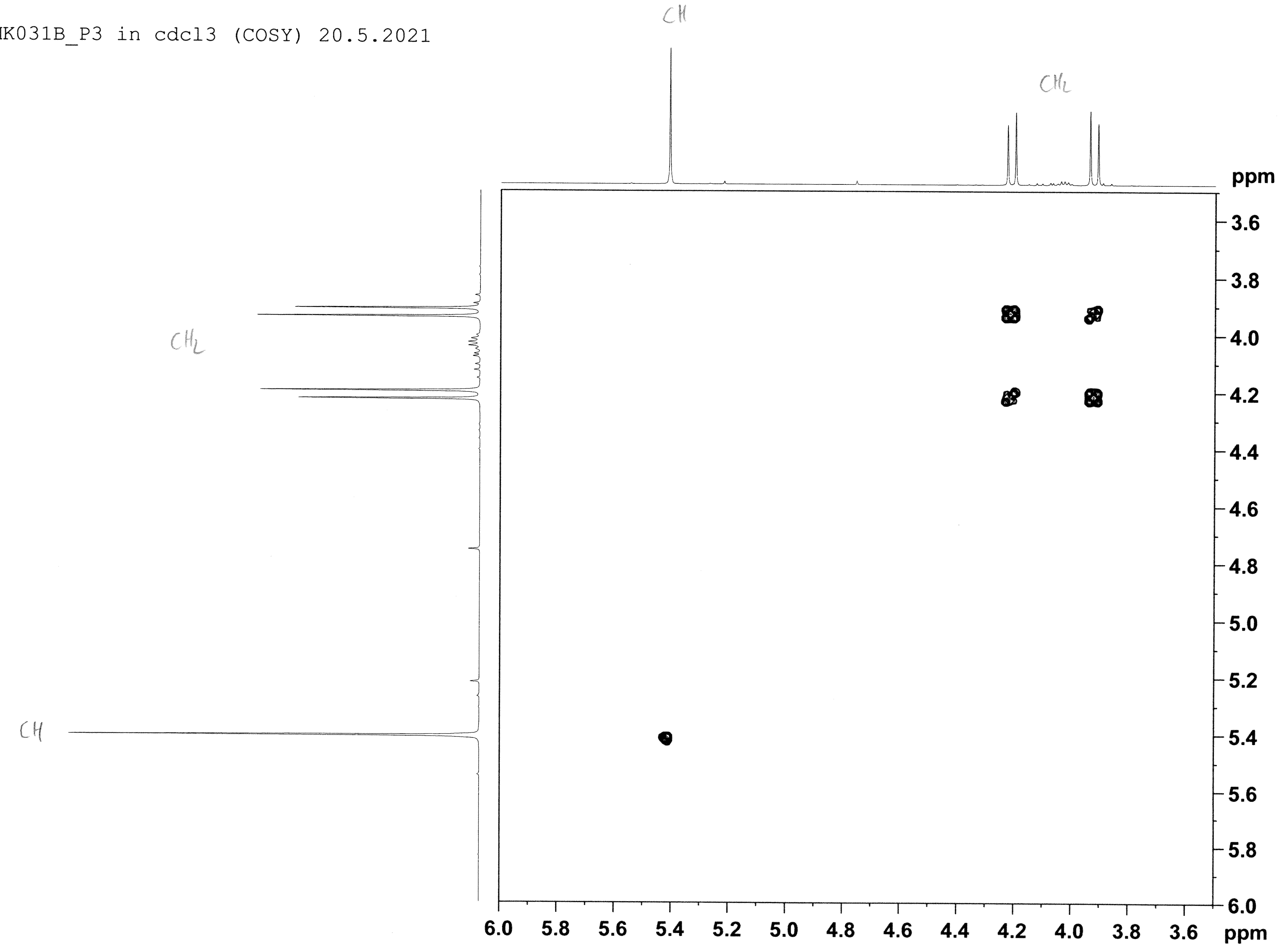

MK031B_P3 in cdcl3 (HSQC) 20.5.2021

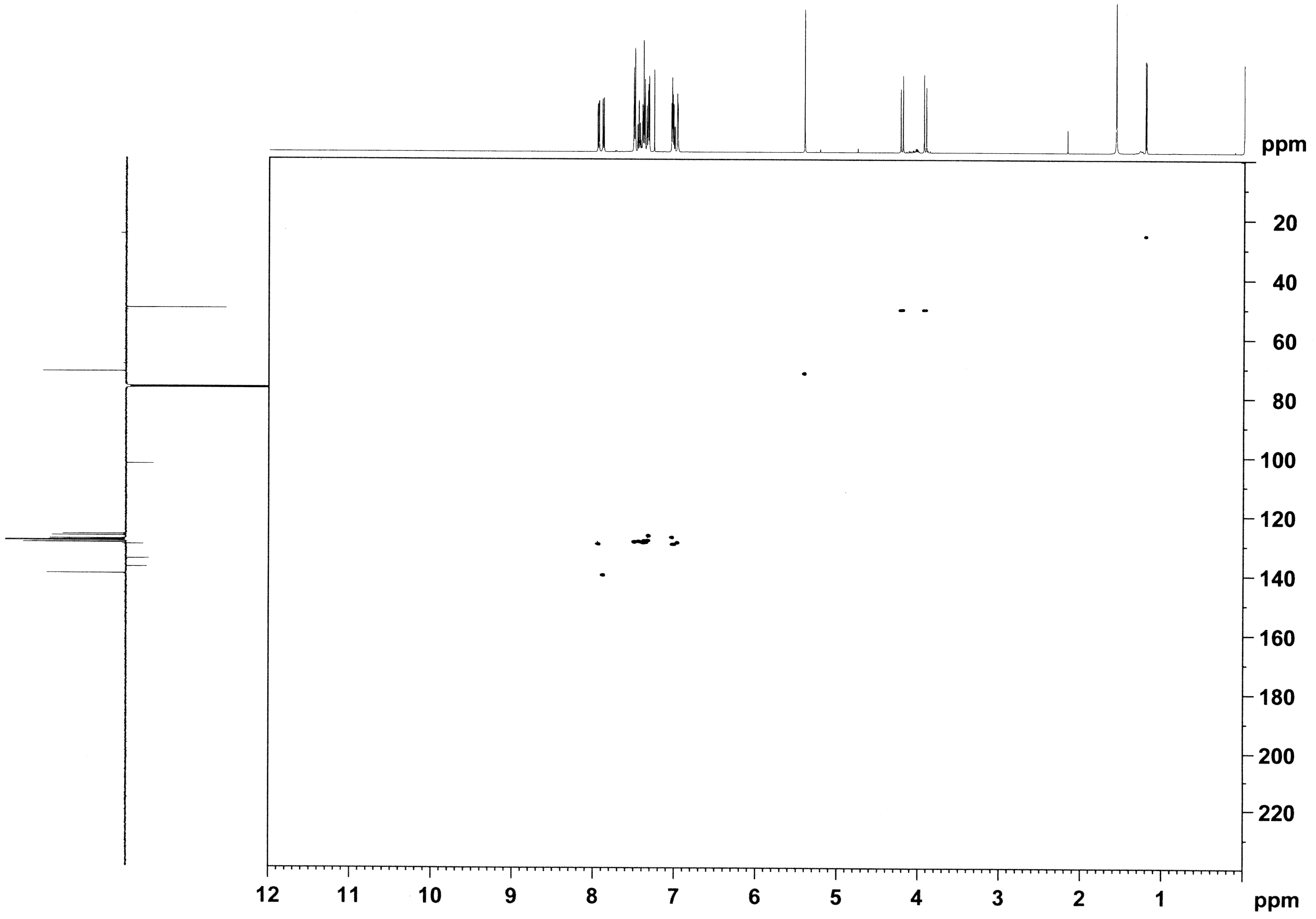

MK031B_P3 in cdcl3 (HSQC) 20.5.2021

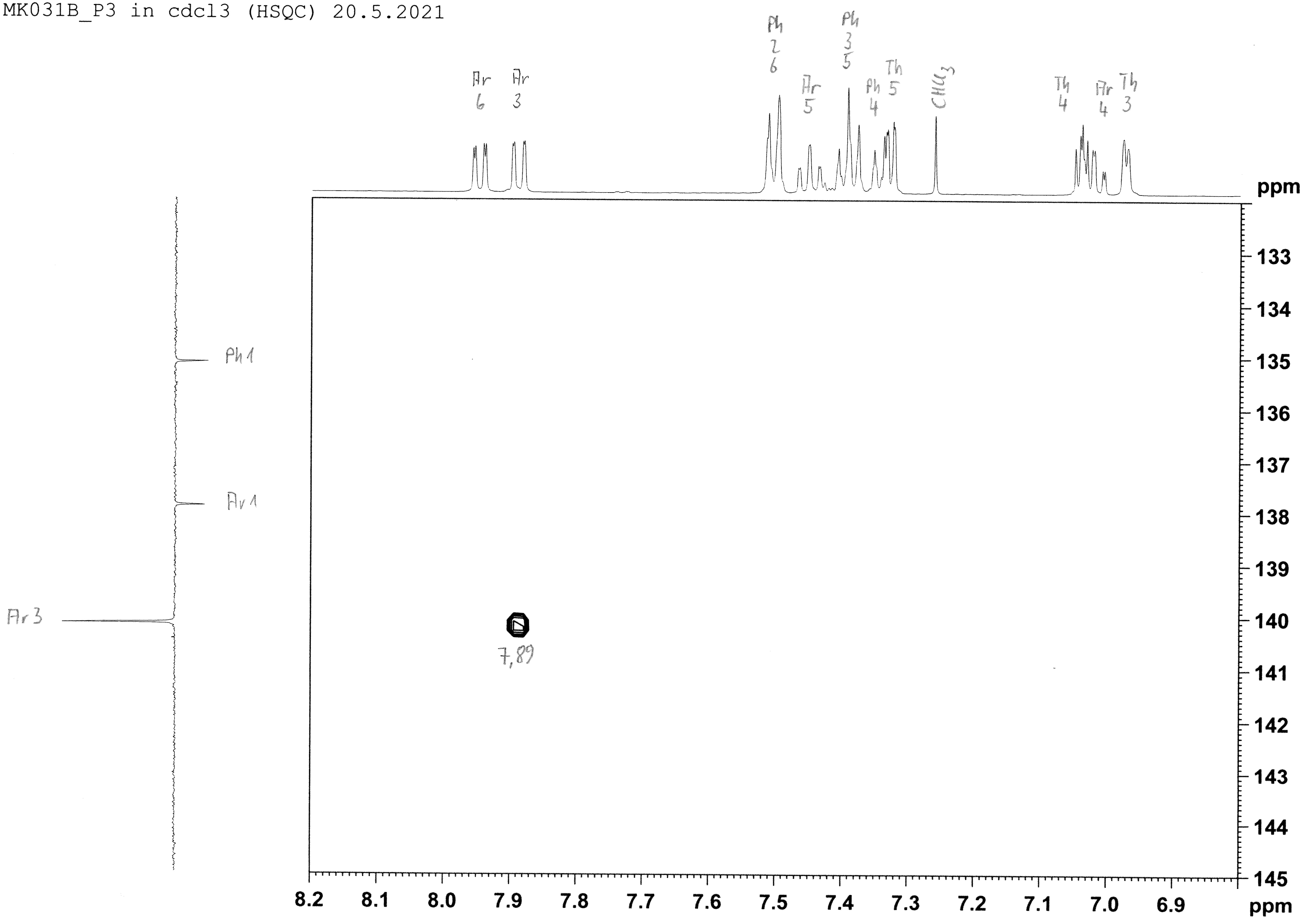

MK031B_P3 in cdcl3 (HSQC) 20.5.2021

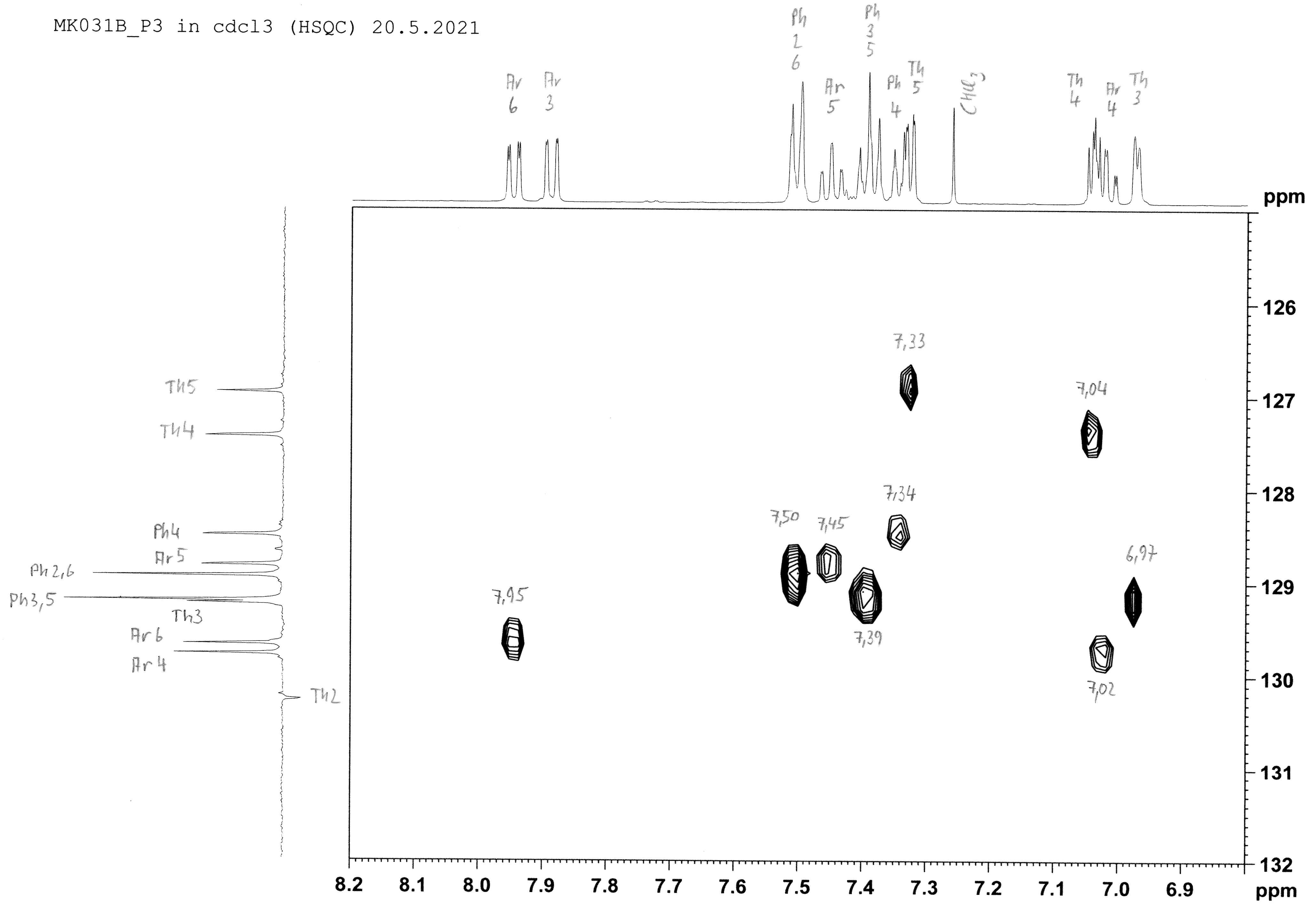

MK031B_P3 in cdcl3 (HSQC) 20.5.2021

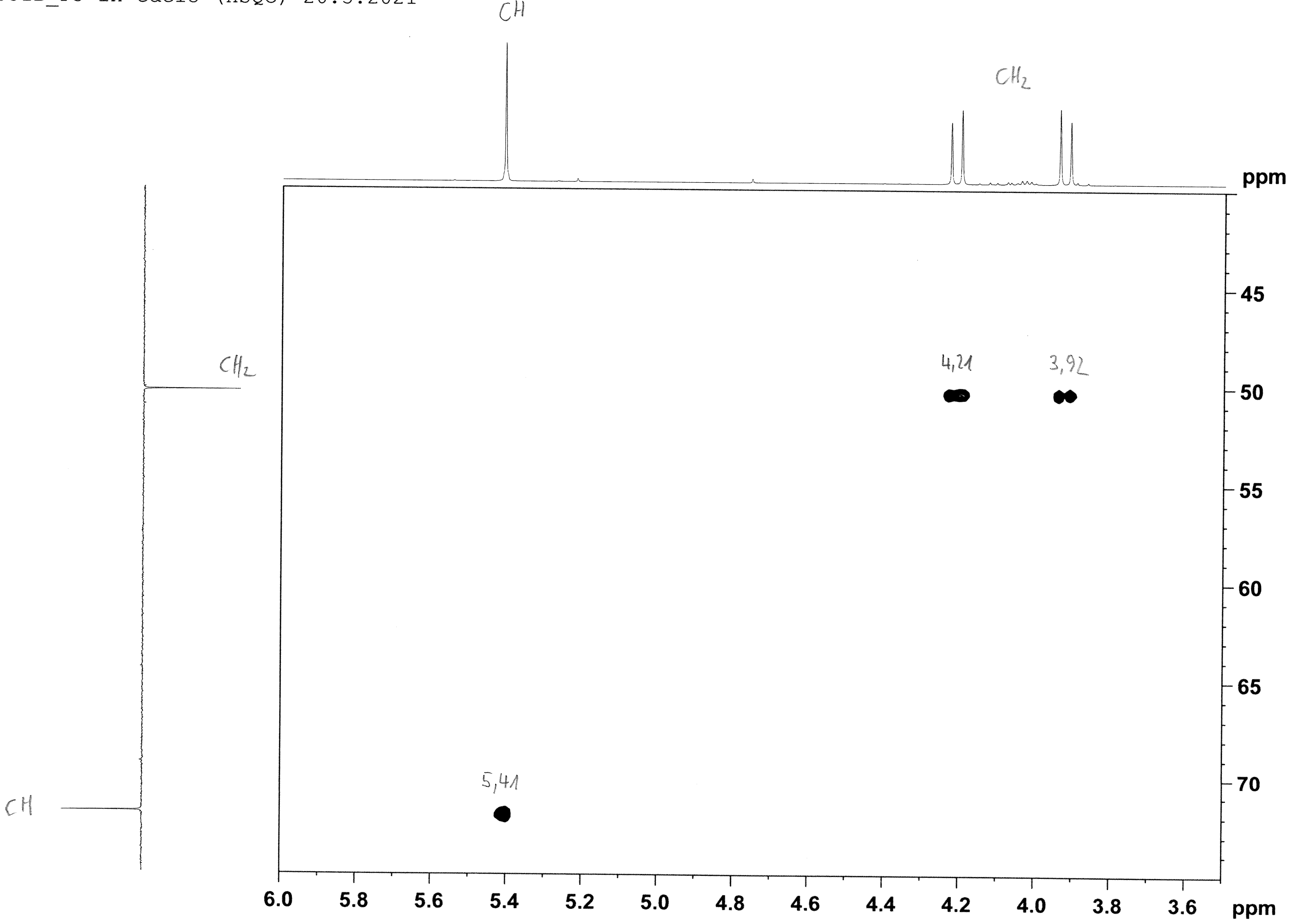

MK031B_P3 in cdcl3 (HMBC) 20.5.2021

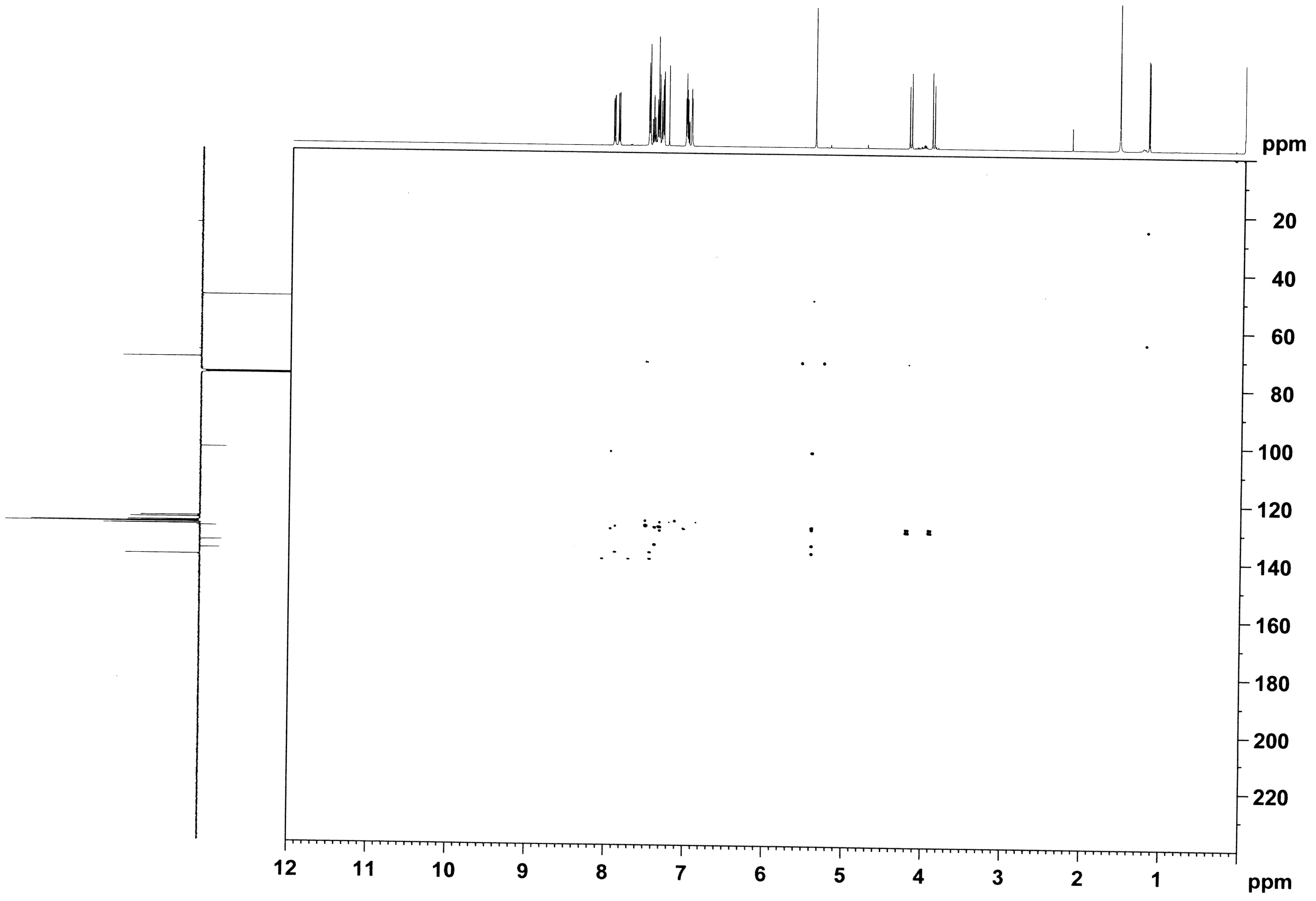

MK031B_P3 in cdcl3 (HMBC) 20.5.2021

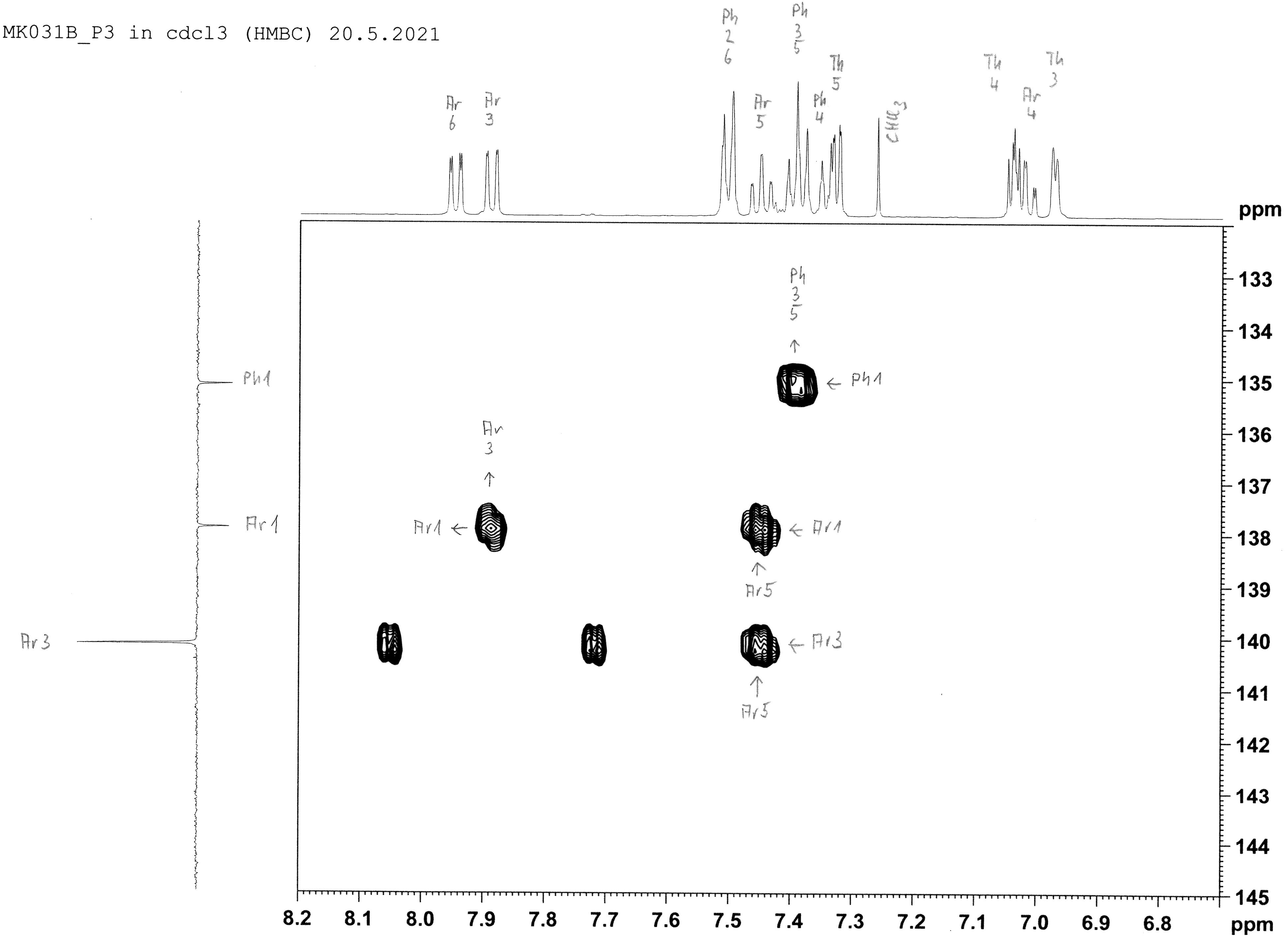

MK031B_P3 in cdcl3 (HMBC) 20.5.2021

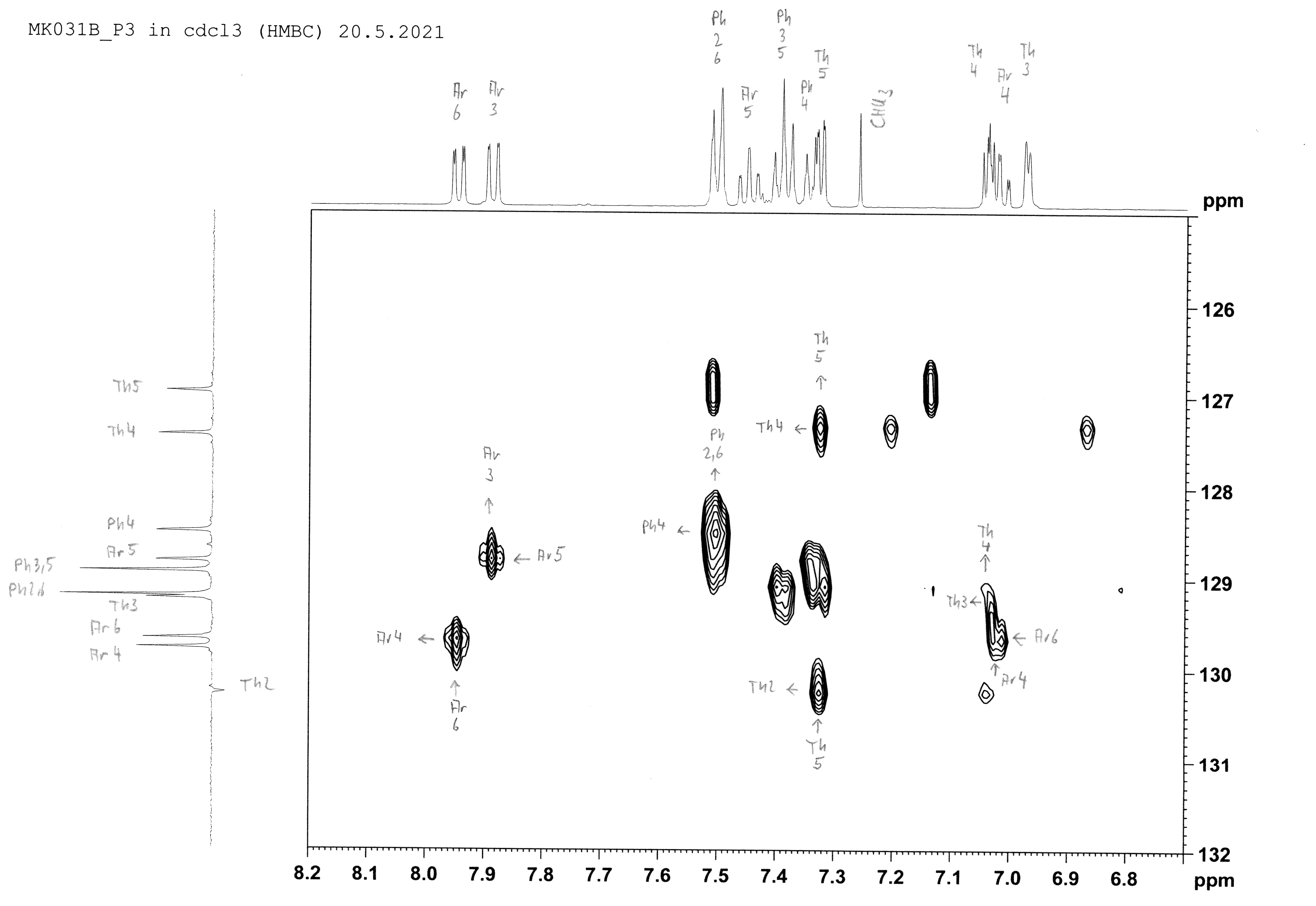

MK031B_P3 in cdcl3 (HMBC) 20.5.2021

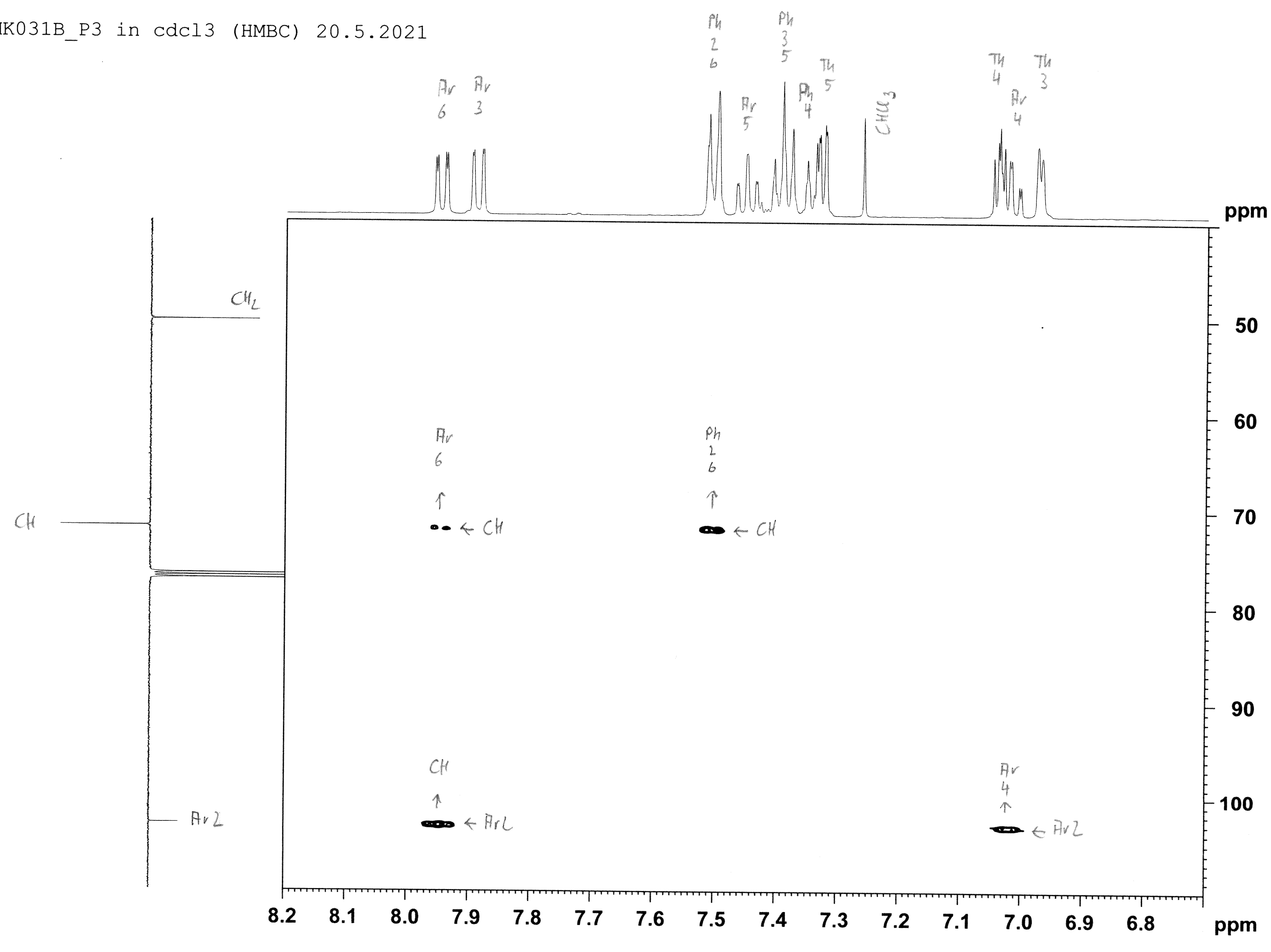

MK031B_P3 in cdcl3 (HMBC) 20.5.2021

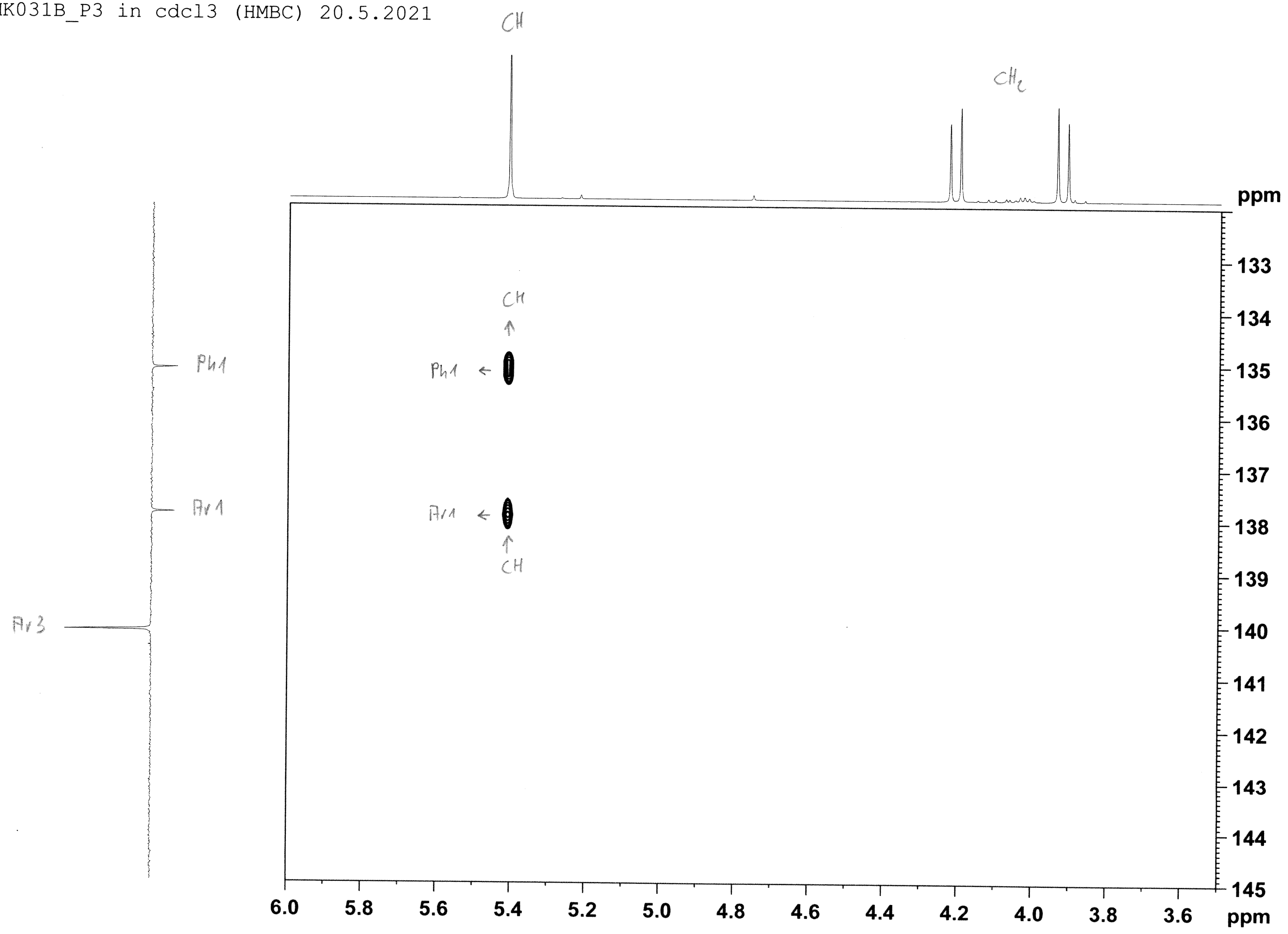

MK031B_P3 in cdcl3 (HMBC) 20.5.2021

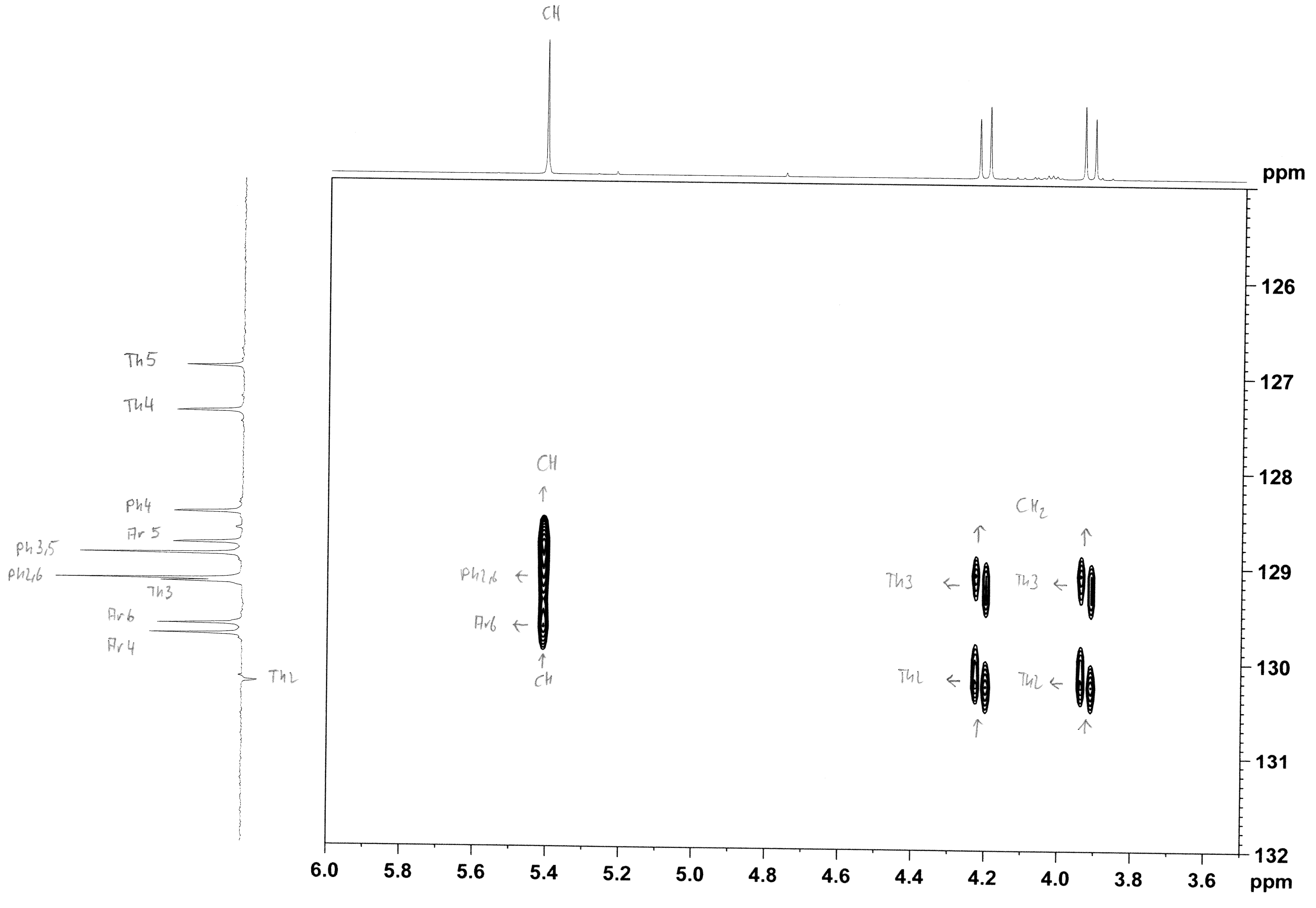

MK031B_P3 in cdcl3 (HMBC) 20.5.2021

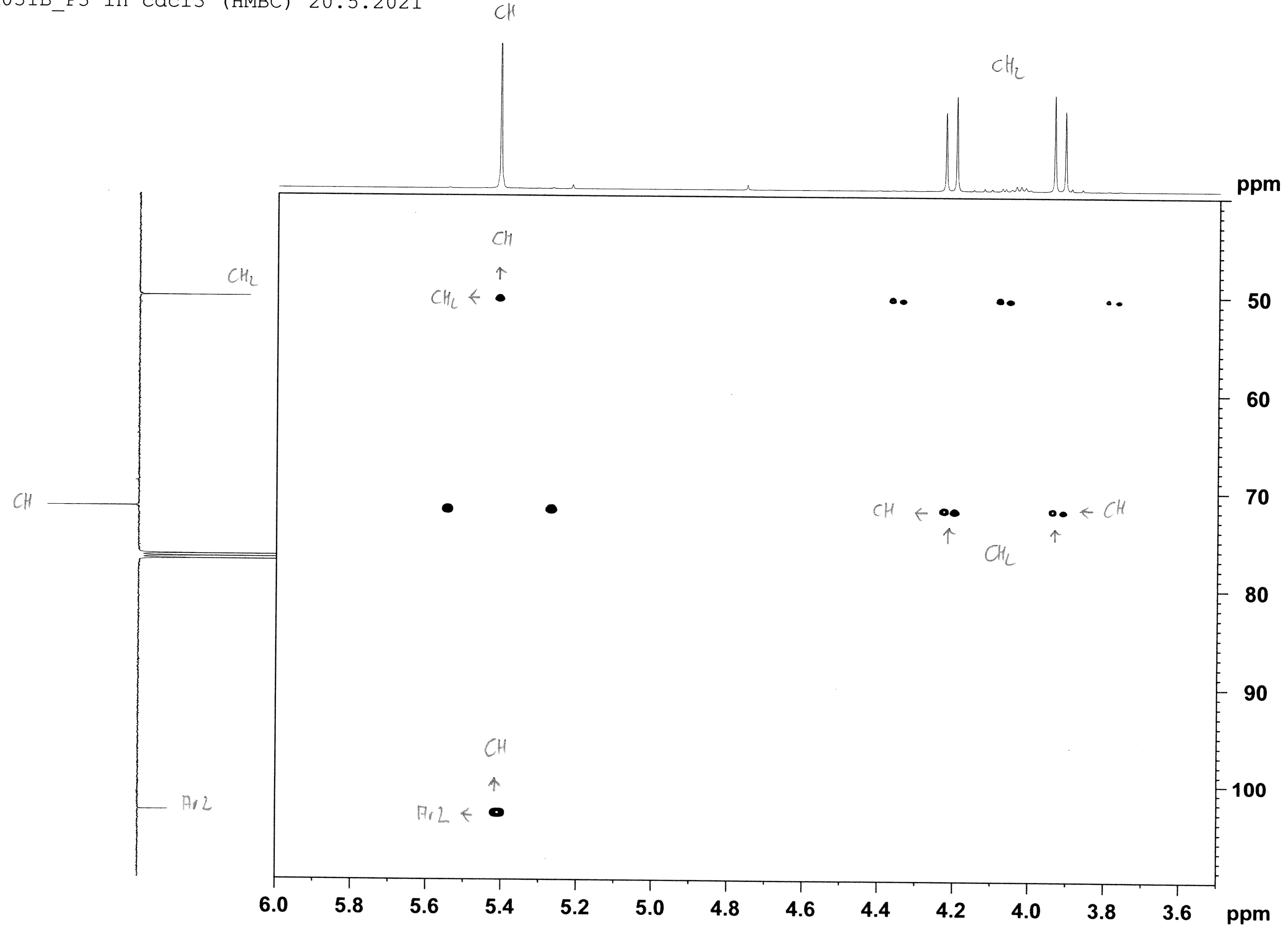

Figure S36c. NMR spectra of compound **8i**.

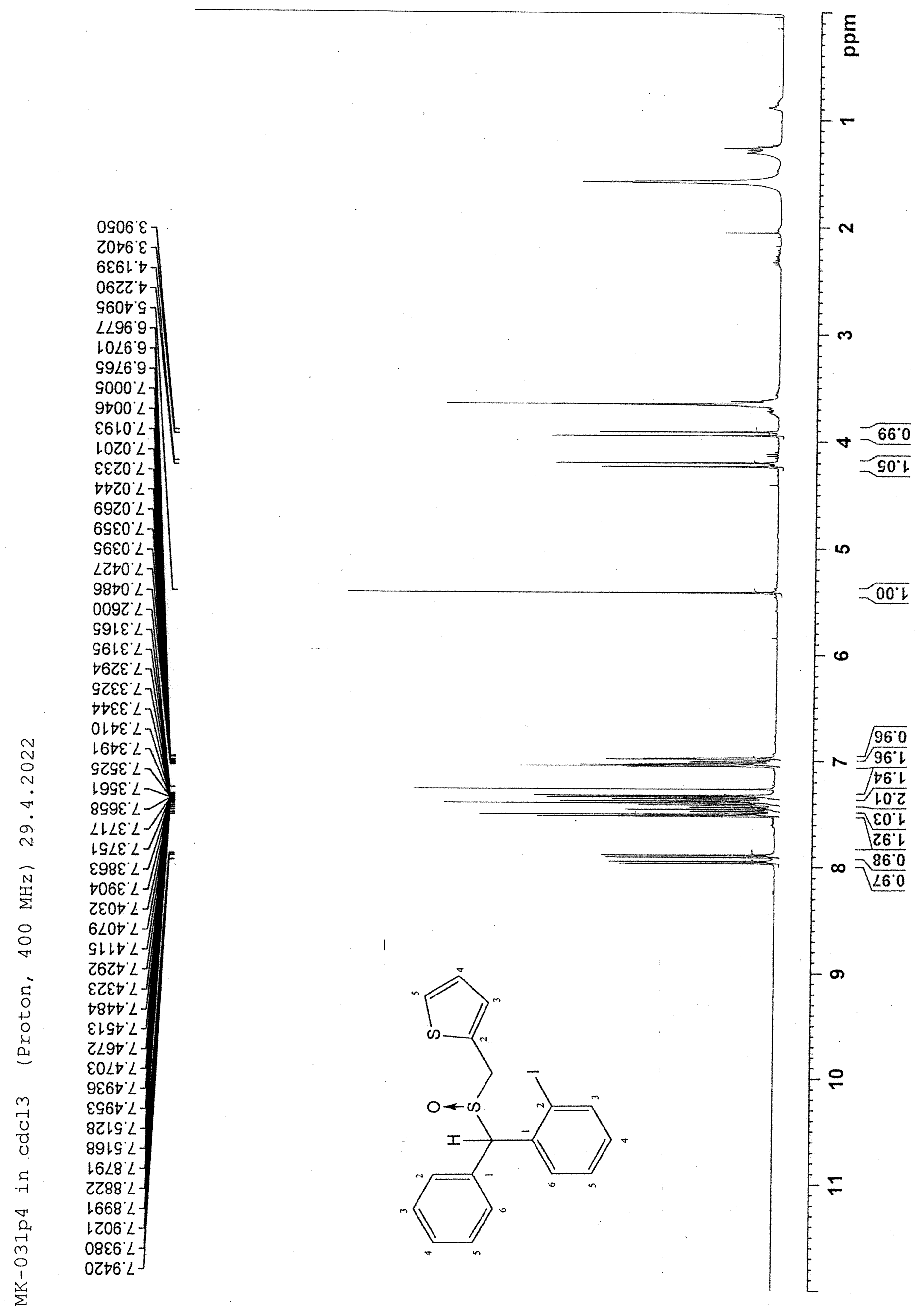

MK-031p4 in cdcl3 (Proton, 400 MHz) 29.4.2022

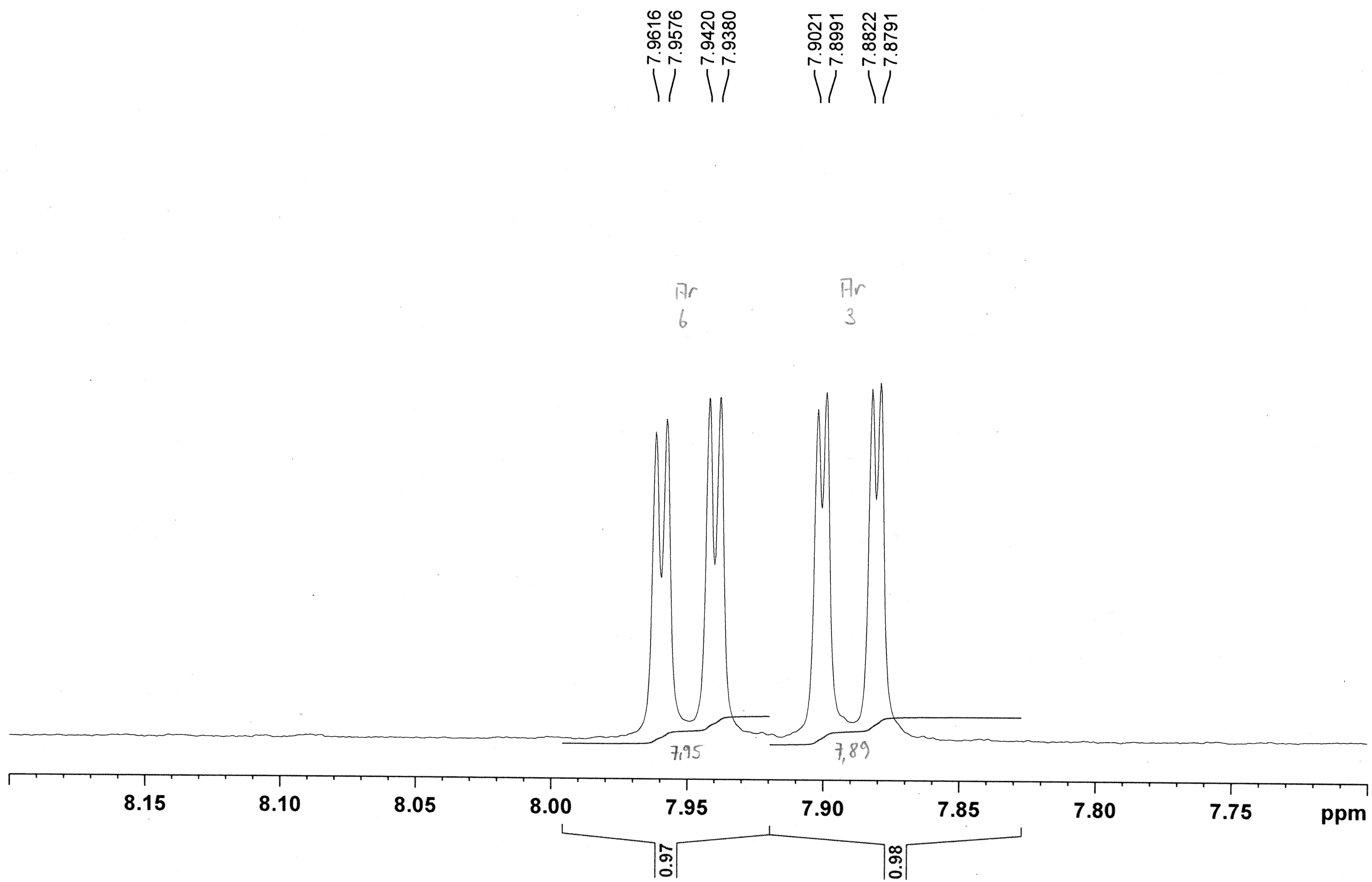

MK-031p4 in cdcl3 (Proton, 400 MHz) 29.4.2022

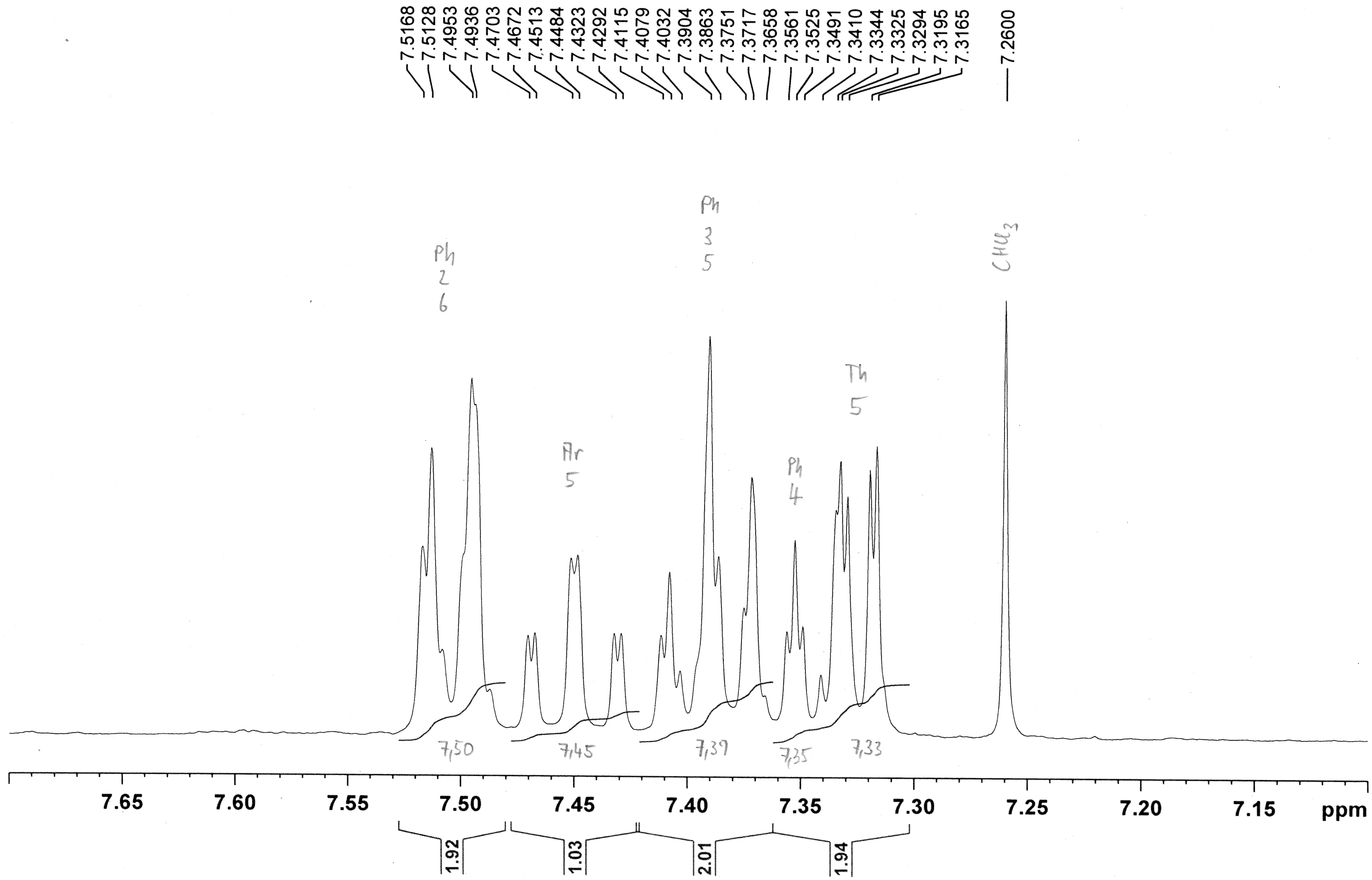

MK-031p4 in cdcl3 (Proton, 400 MHz) 29.4.2022

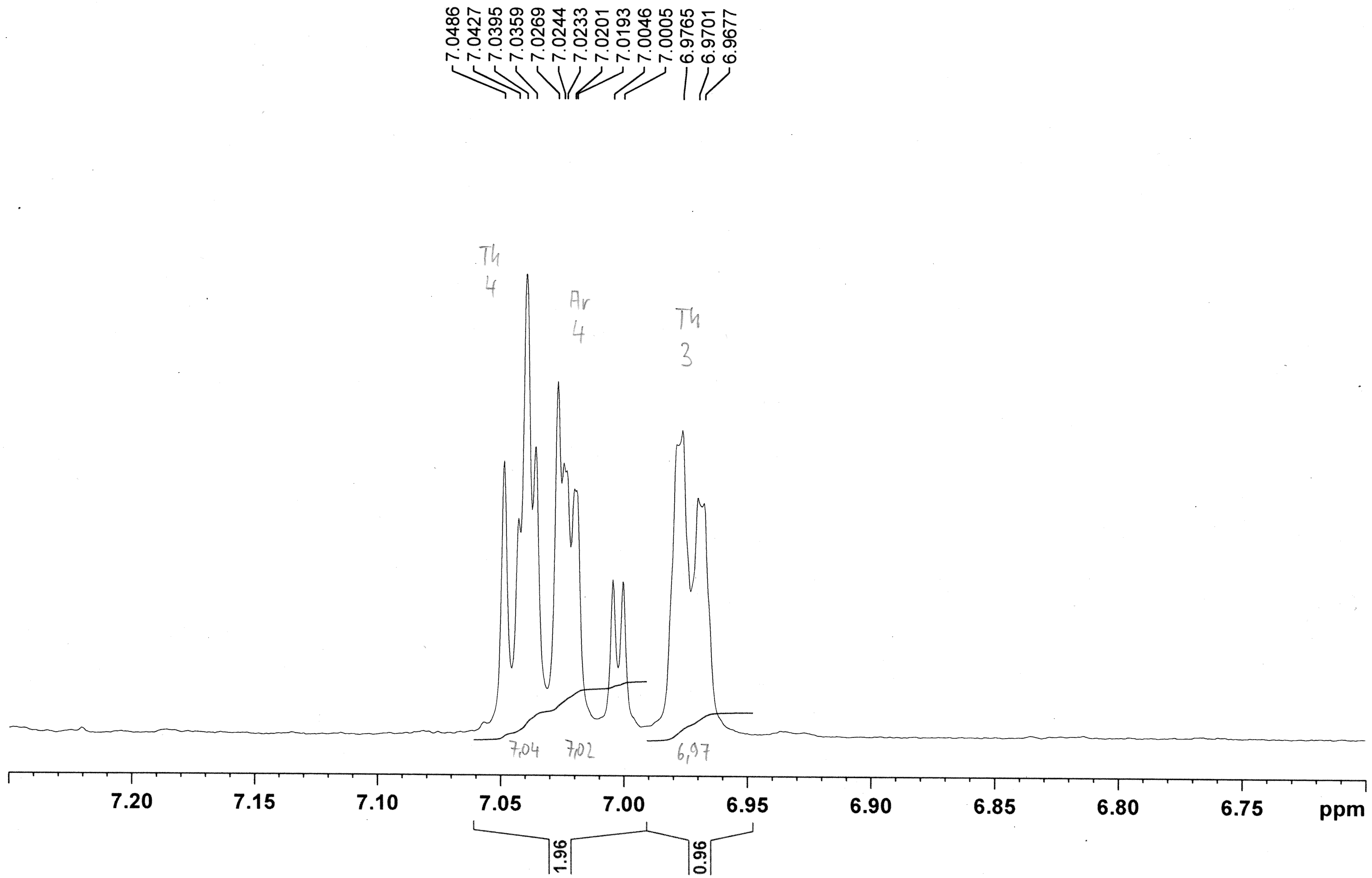

MK-031p4 in cdcl3 (Proton, 400 MHz) 29.4.2022

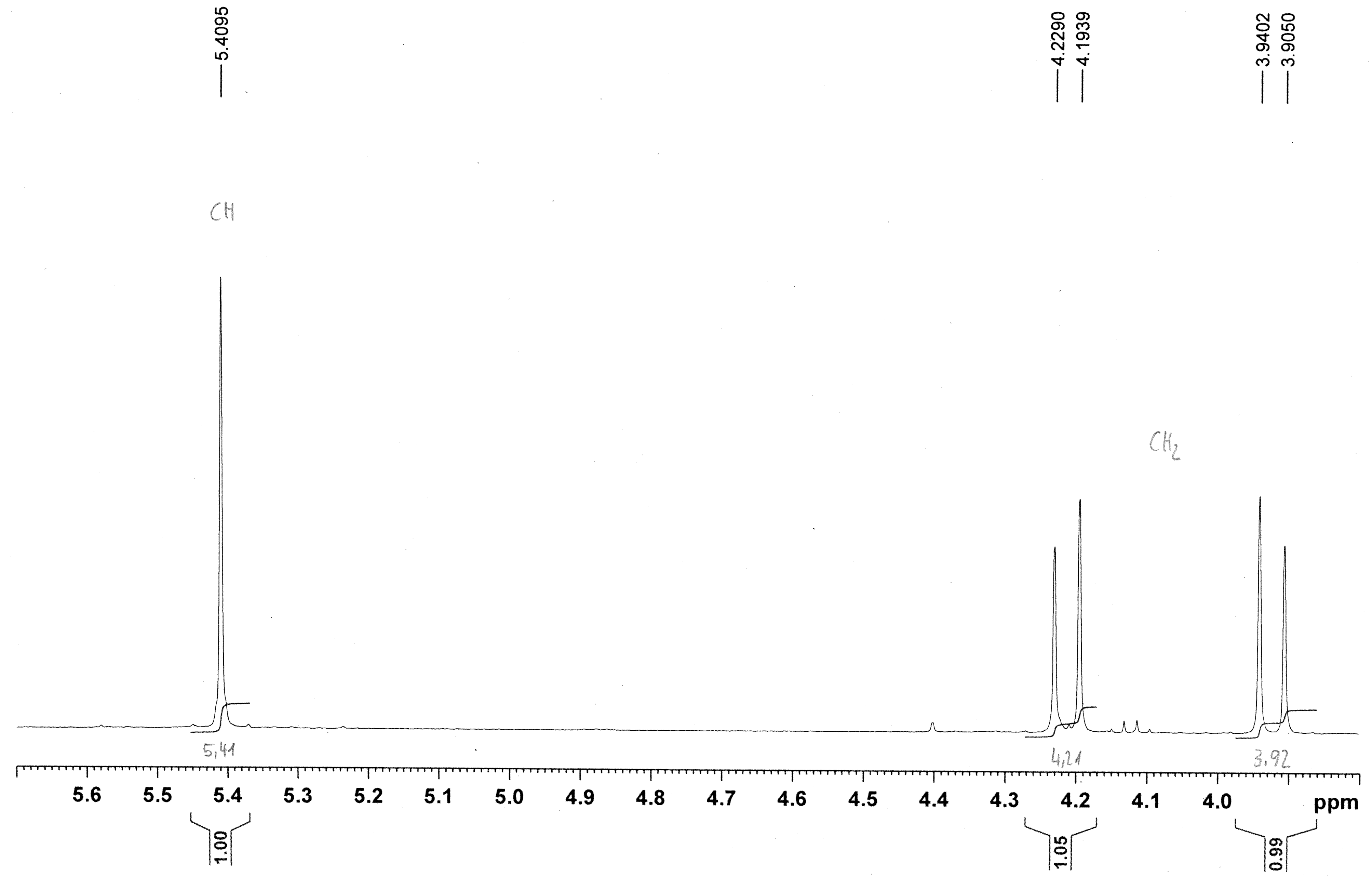

MK-031p4 in cdcl3 (APT, 100 MHz) 29.4.2022

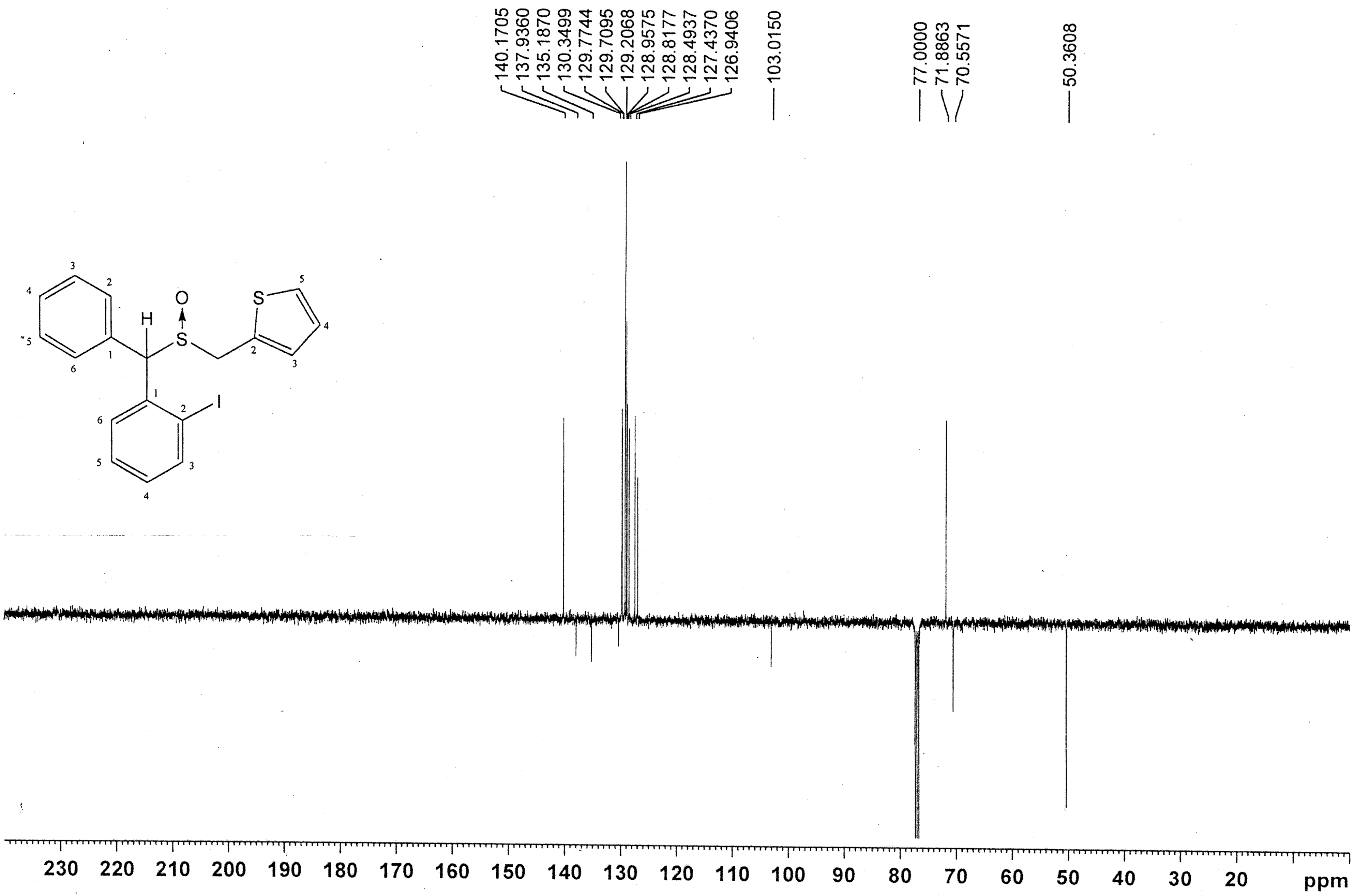

MK-031p4 in cdcl3 (APT, 100 MHz) 29.4.2022

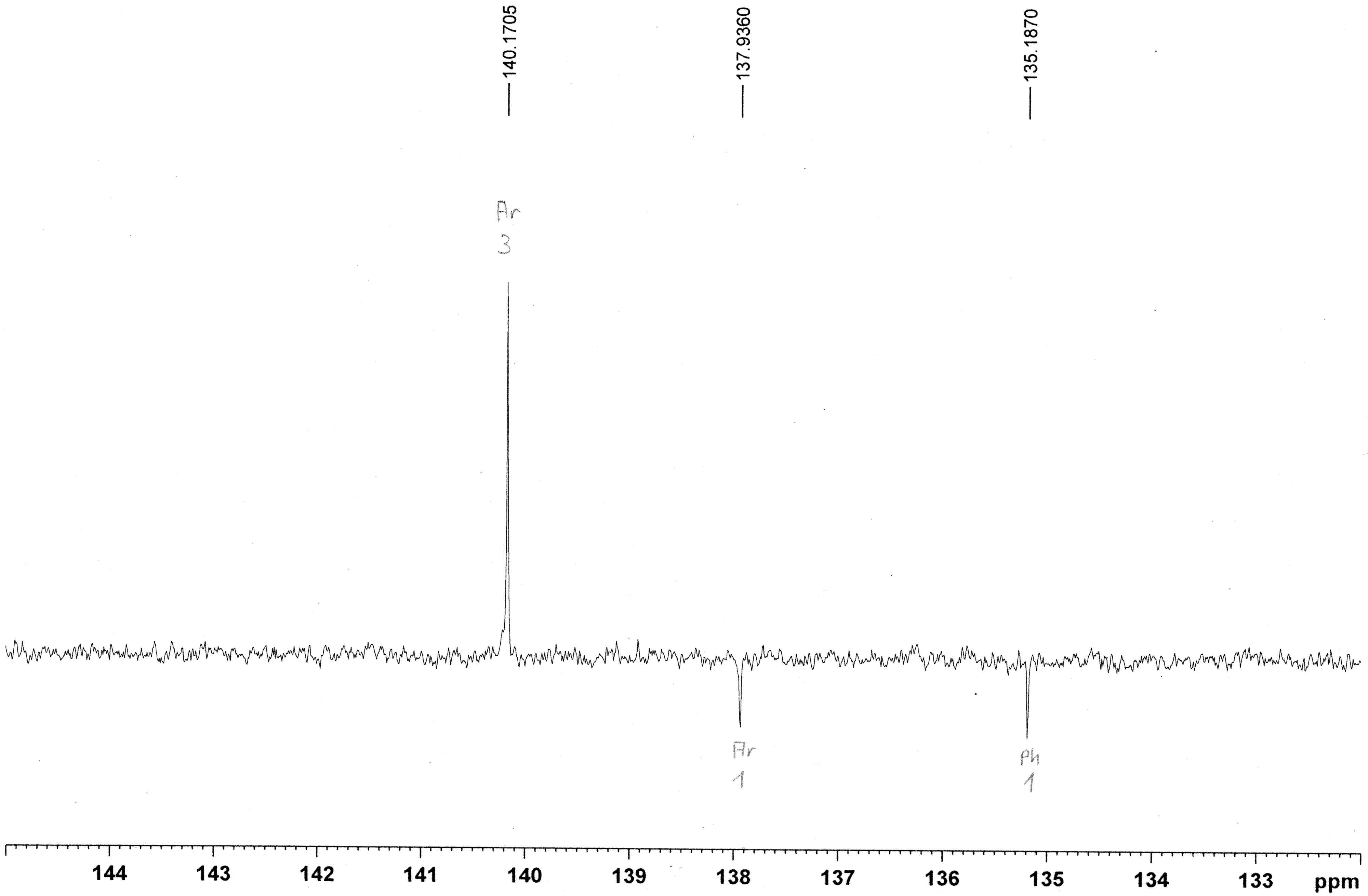

MK-031p4 in cdcl3 (APT, 100 MHz) 29.4.2022

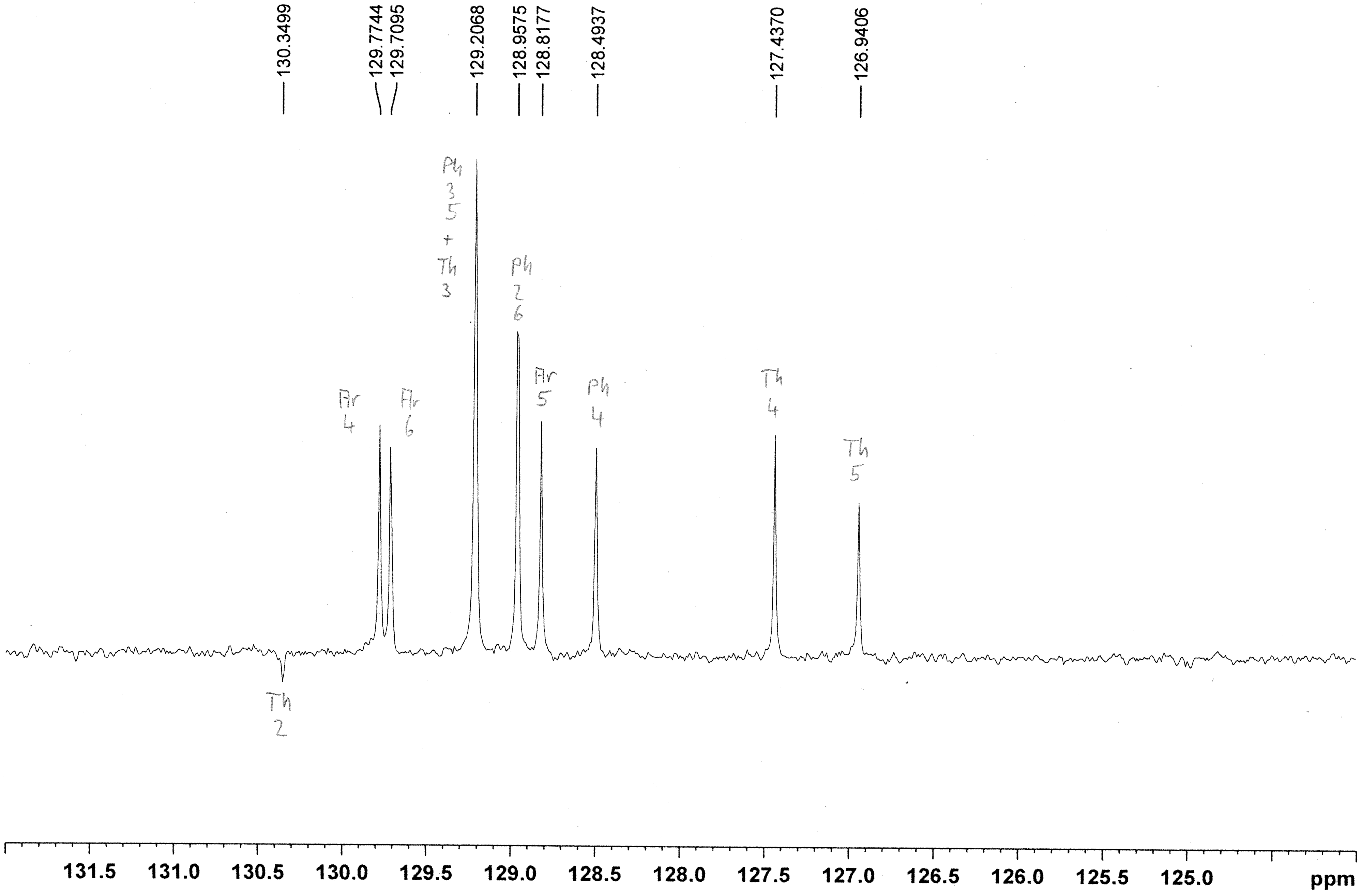

MK-031p4 in cdcl3 (APT, 100 MHz) 29.4.2022

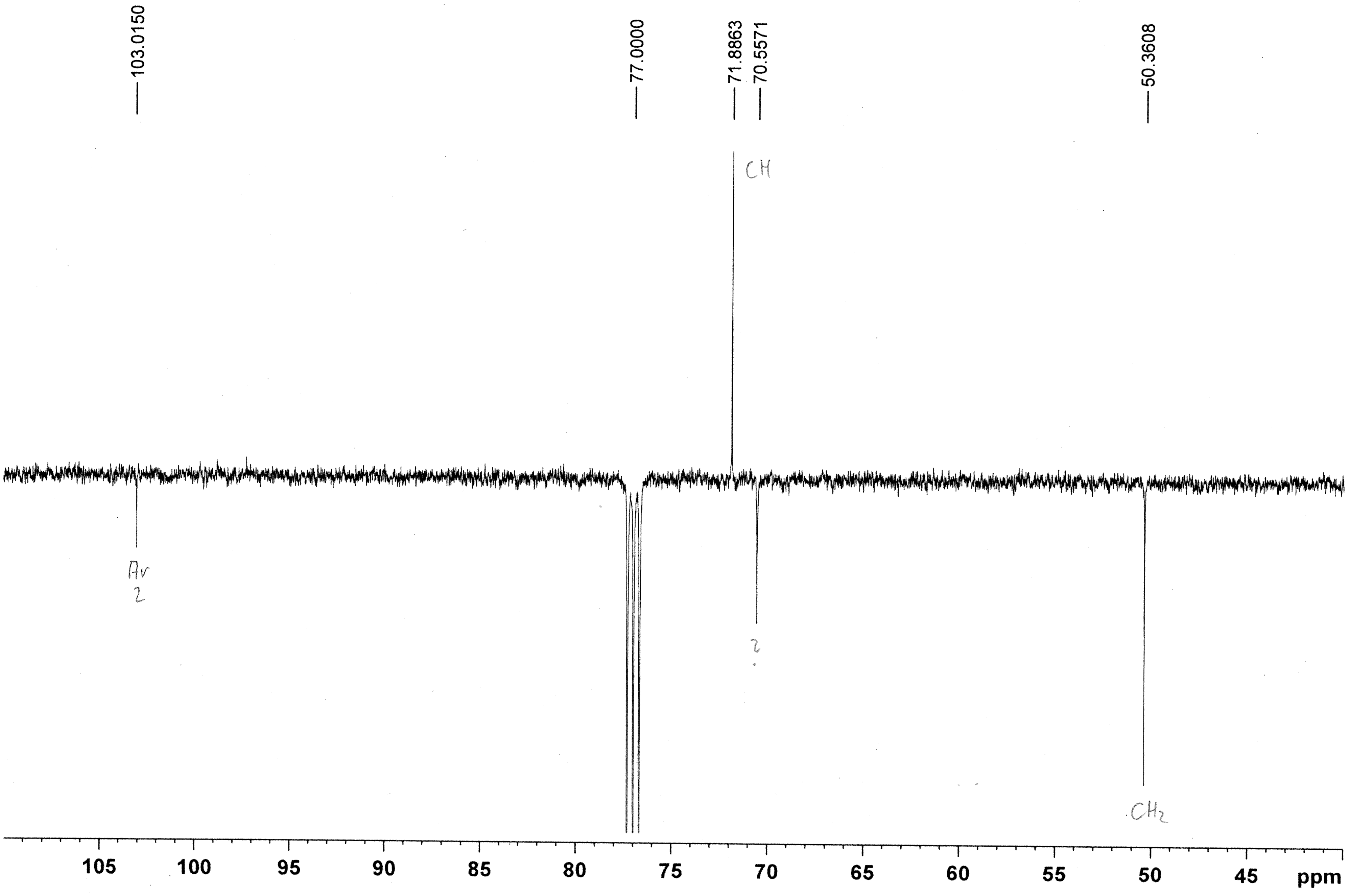

MK-031p4 in cdcl3 (COSY, 400 MHz) 29.4.2022

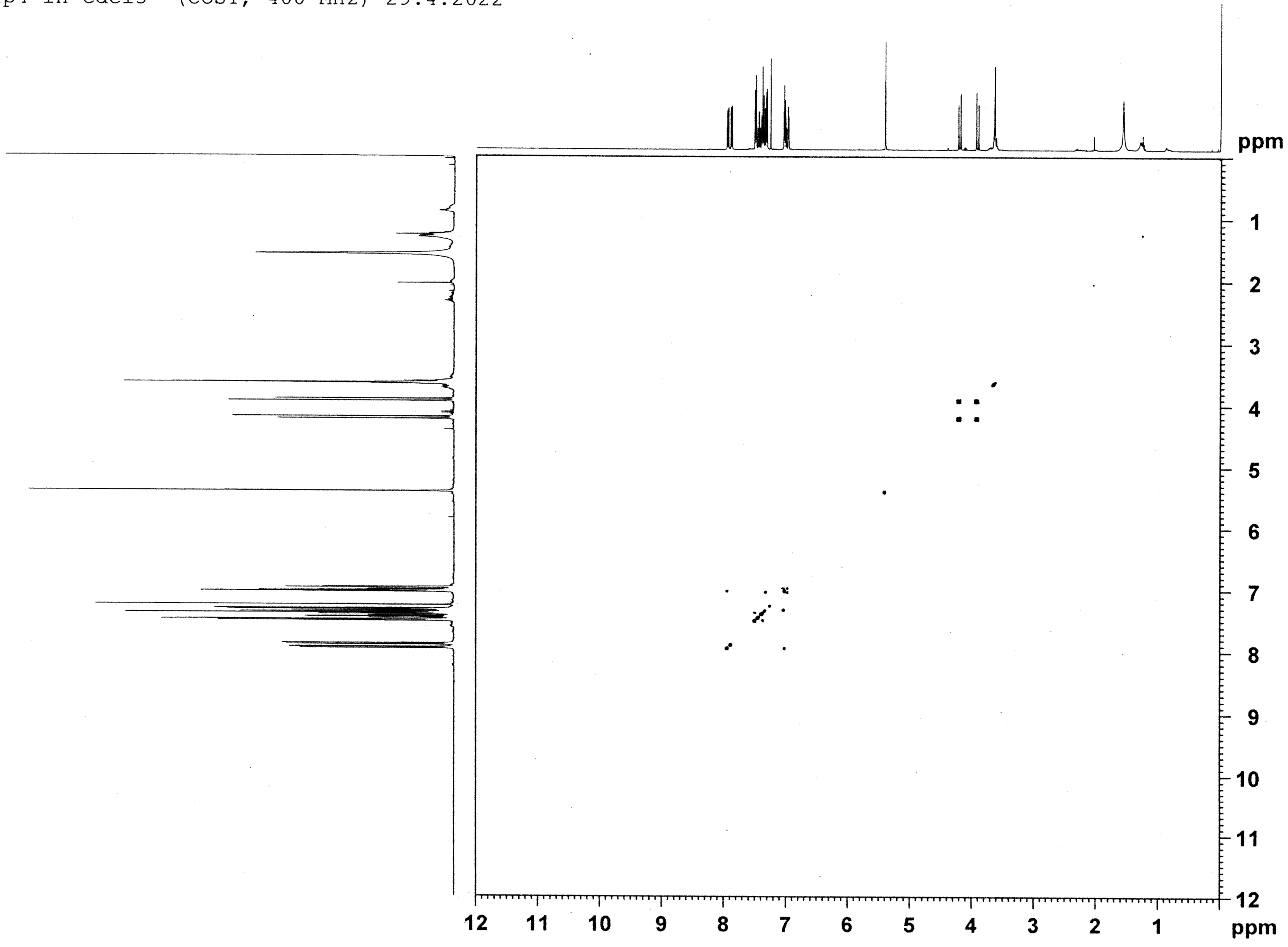

MK-031p4 in cdcl3 (COSY, 400 MHz) 29.4.2022

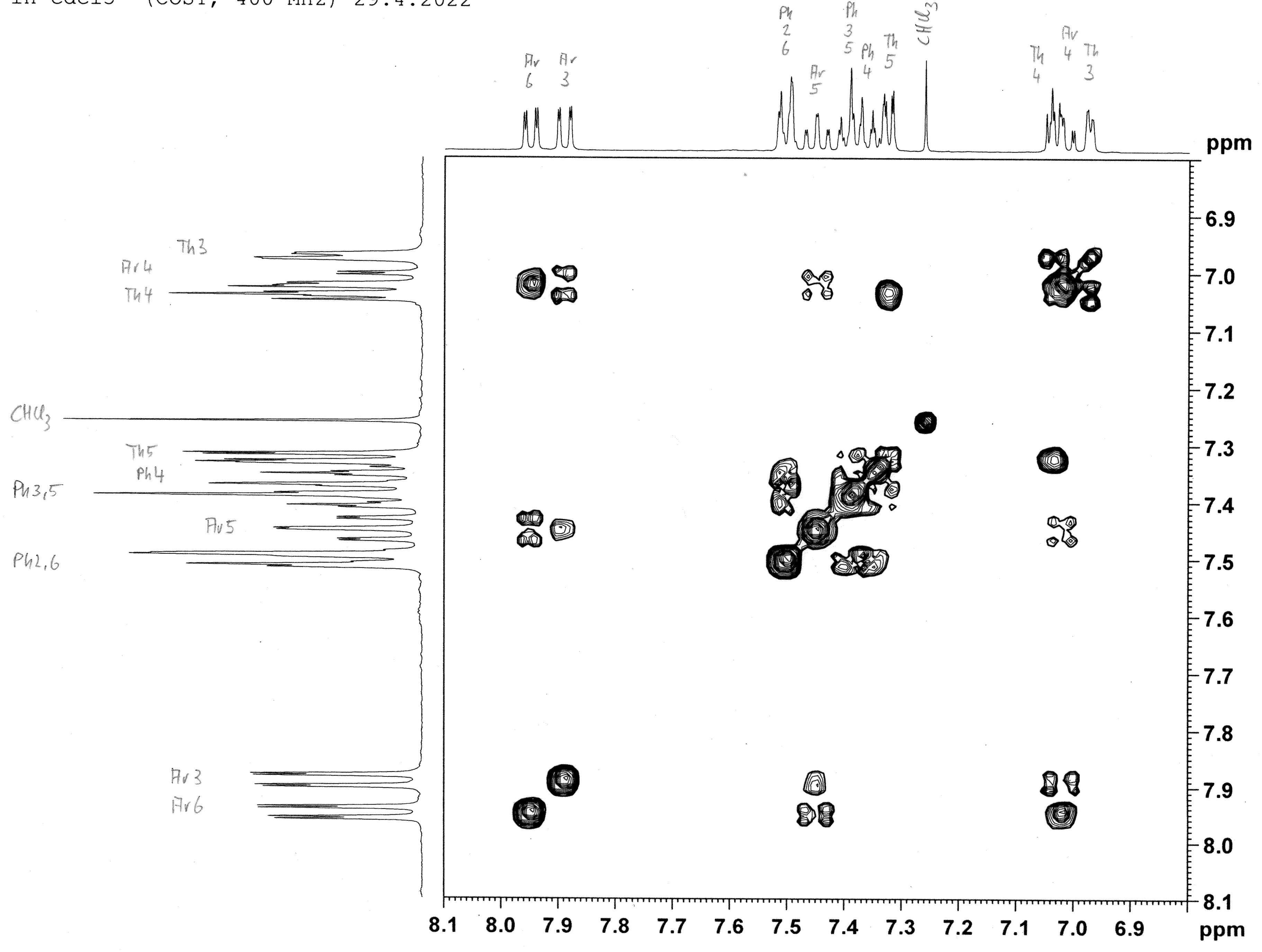

MK-031p4 in cdcl3 (COSY, 400 MHz) 29.4.2022

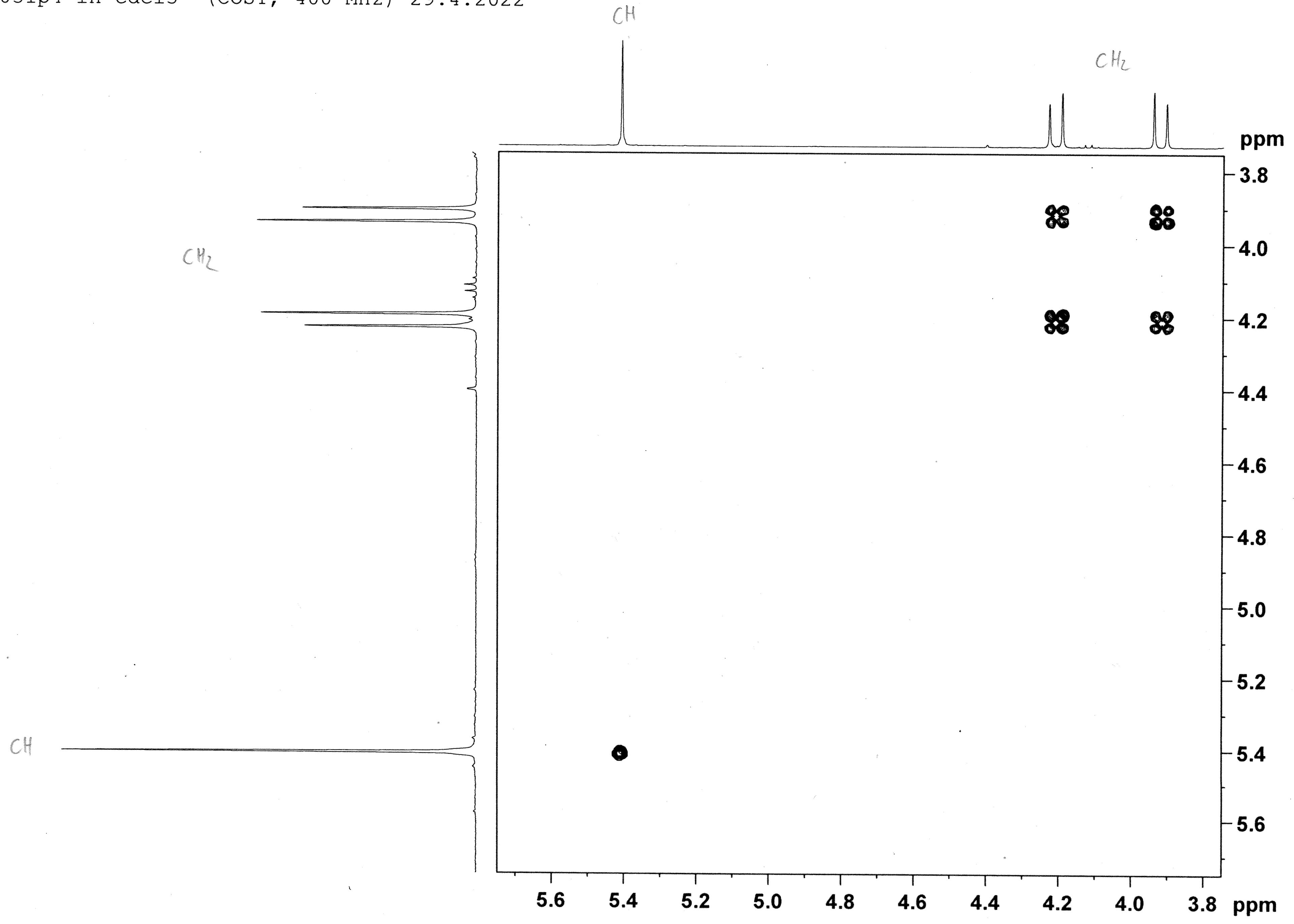

MK-031p4 in cdcl3 (HSQC, 400 MHz) 29.4.2022

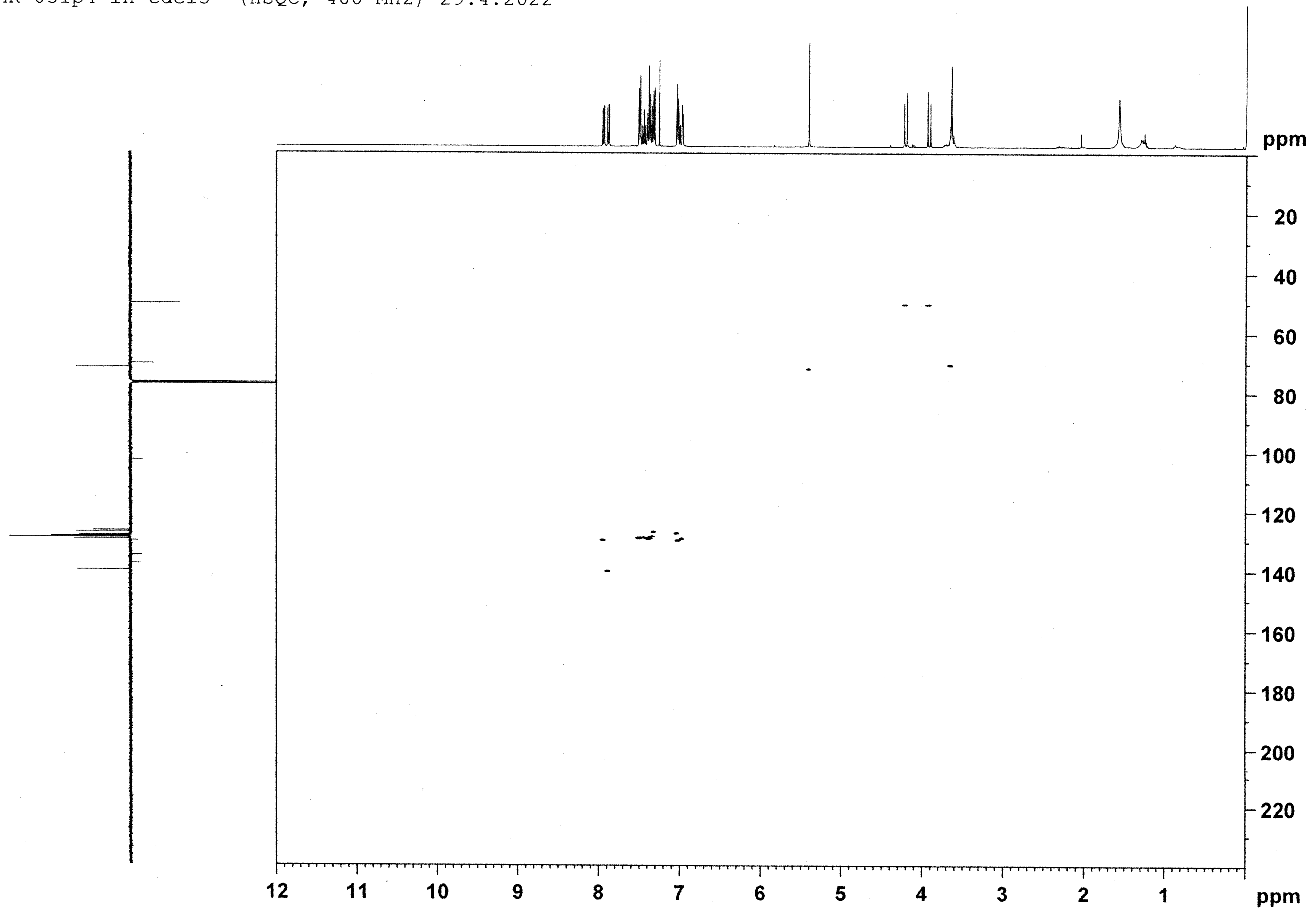

MK-031p4 in cdcl3 (HSQC, 400 MHz) 29.4.2022

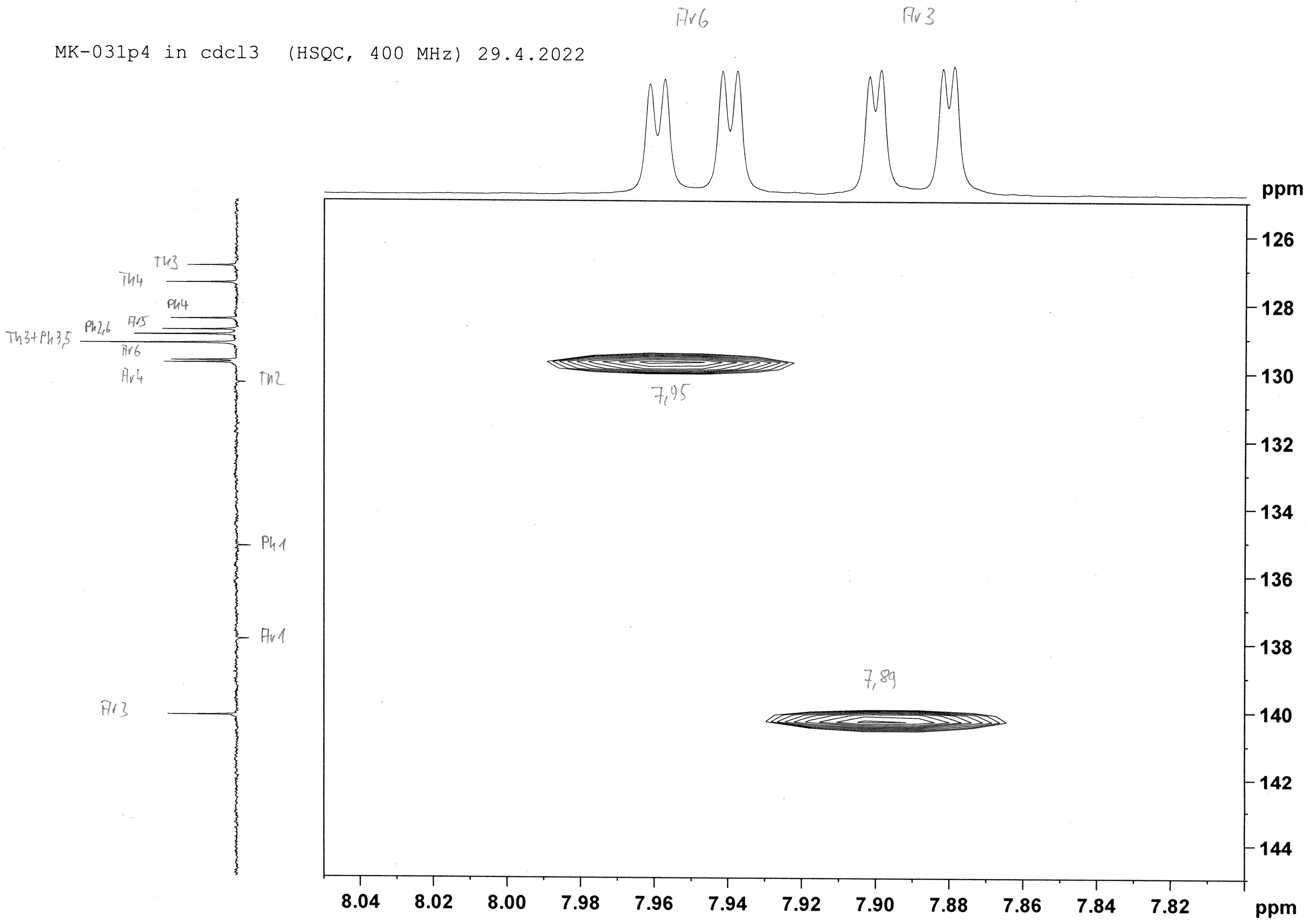

MK-031p4 in cdcl3 (HSQC, 400 MHz) 29.4.2022

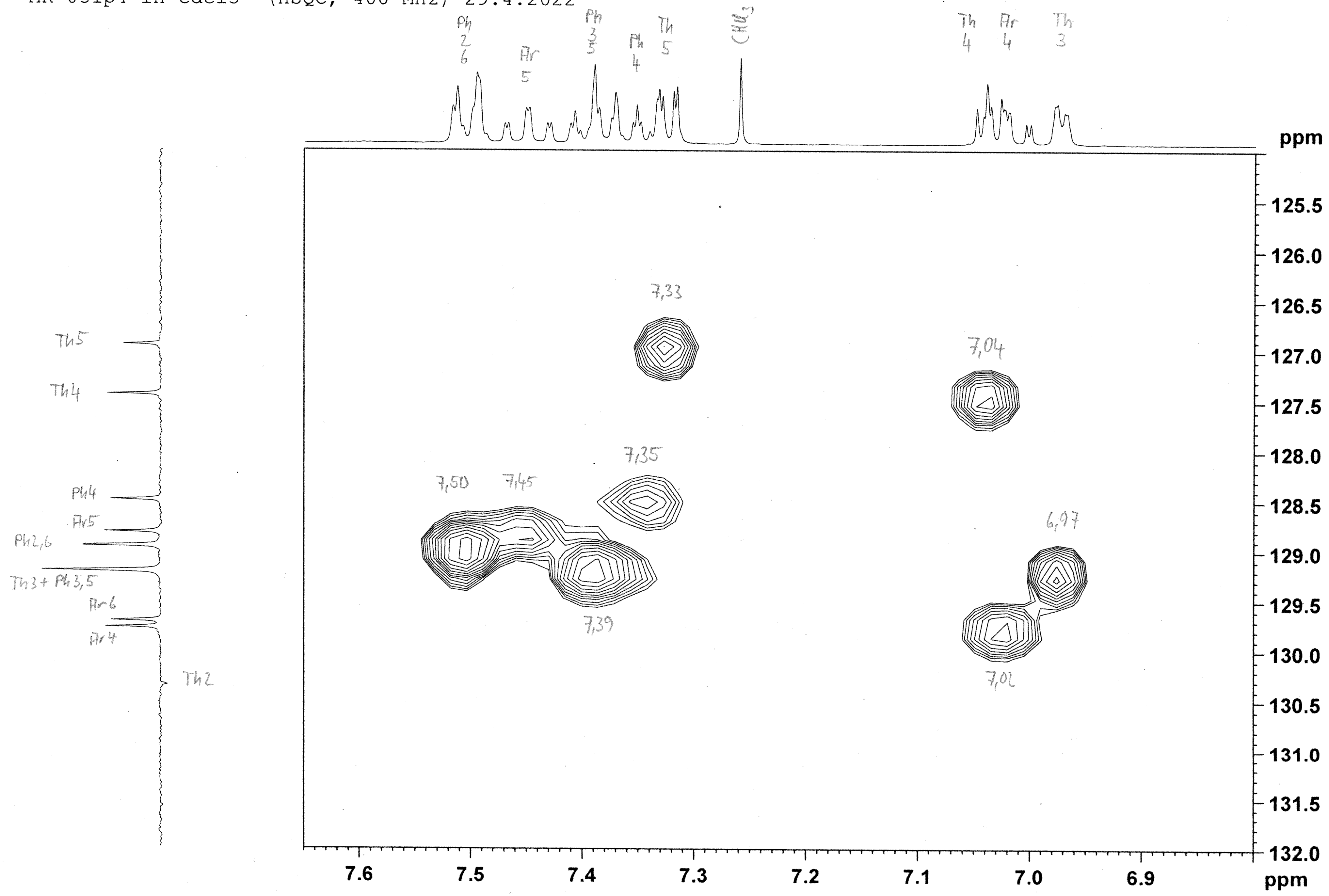

MK-031p4 in cdcl3 (HSQC, 400 MHz) 29.4.2022

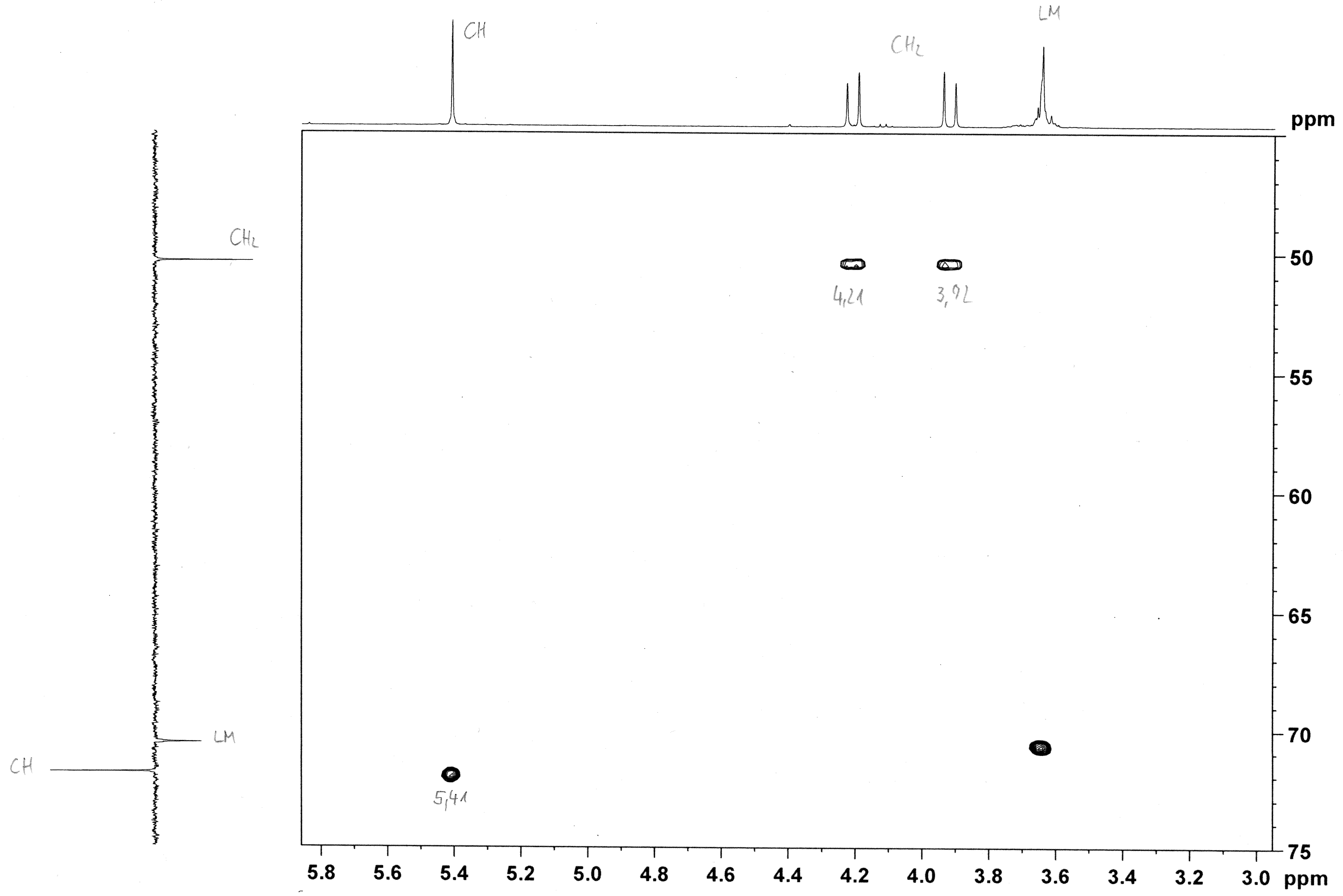

MK-031p4 in cdcl3 (HMBC, 400 MHz) 29.4.2022

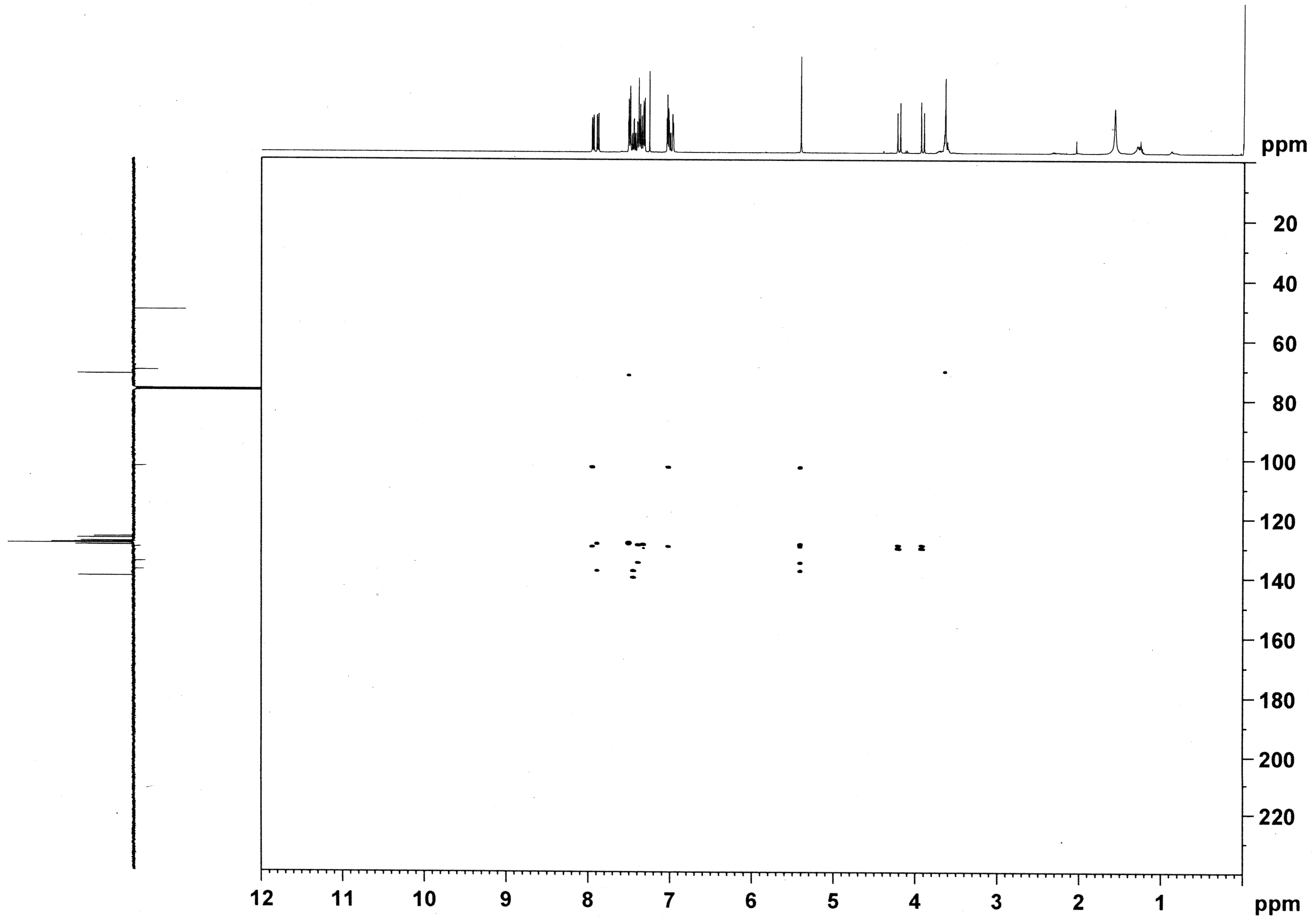

MK-031p4 in cdcl3 (HMBC, 400 MHz) 29.4.2022

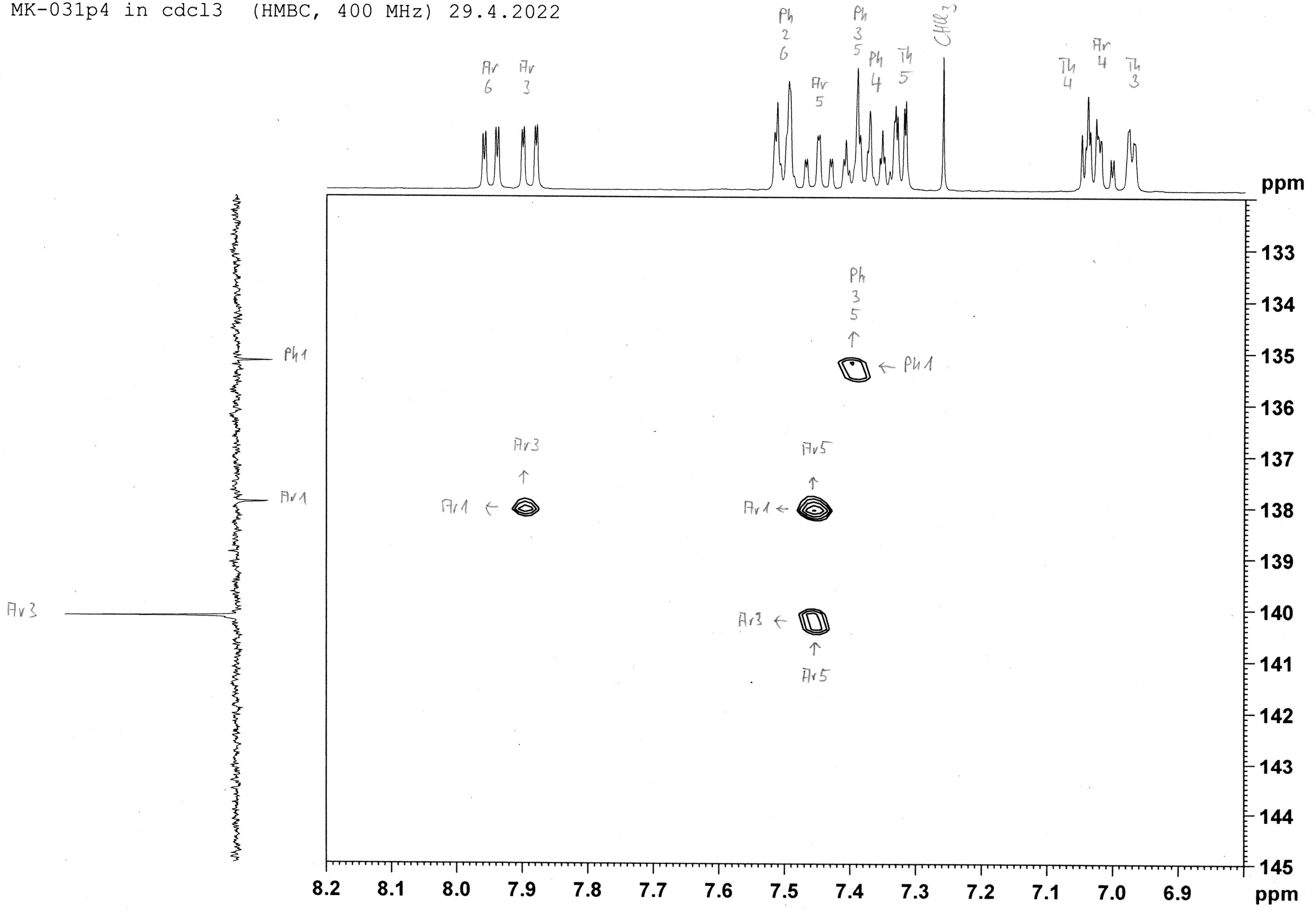

MK-031p4 in cdcl3 (HMBC, 400 MHz) 29.4.2022

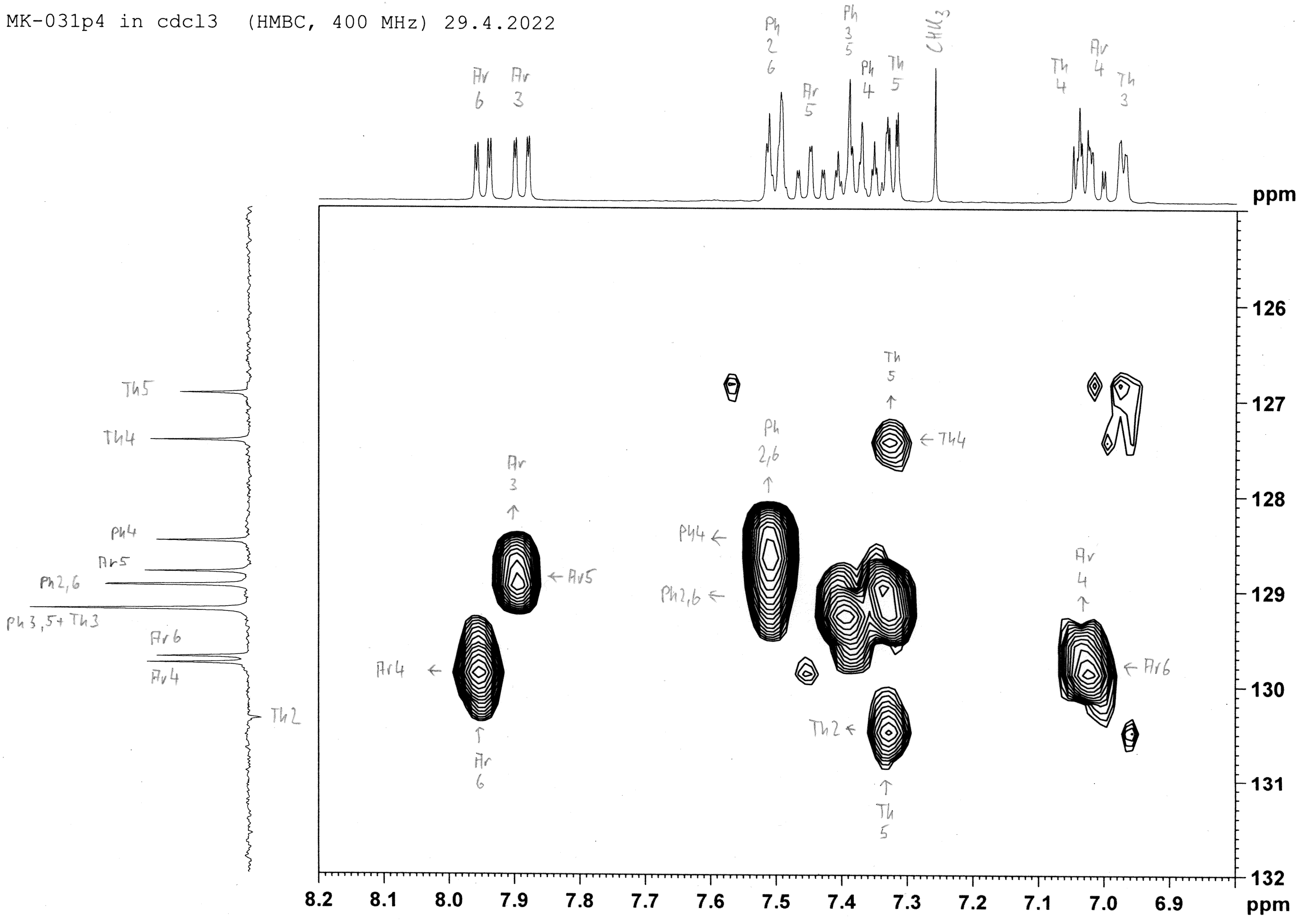

MK-031p4 in cdcl3 (HMBC, 400 MHz) 29.4.2022

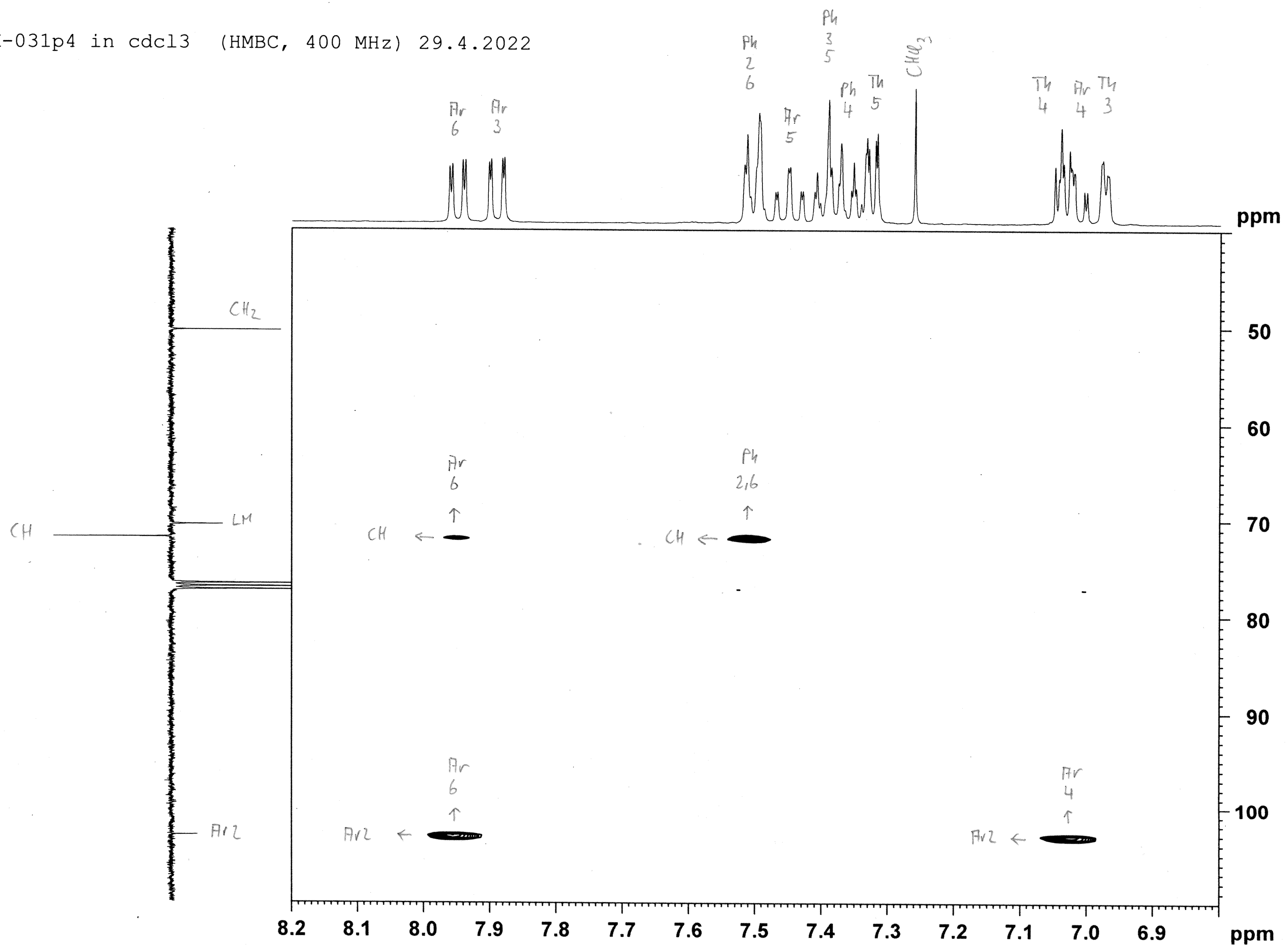

MK-031p4 in cdcl3 (HMBC, 400 MHz) 29.4.2022

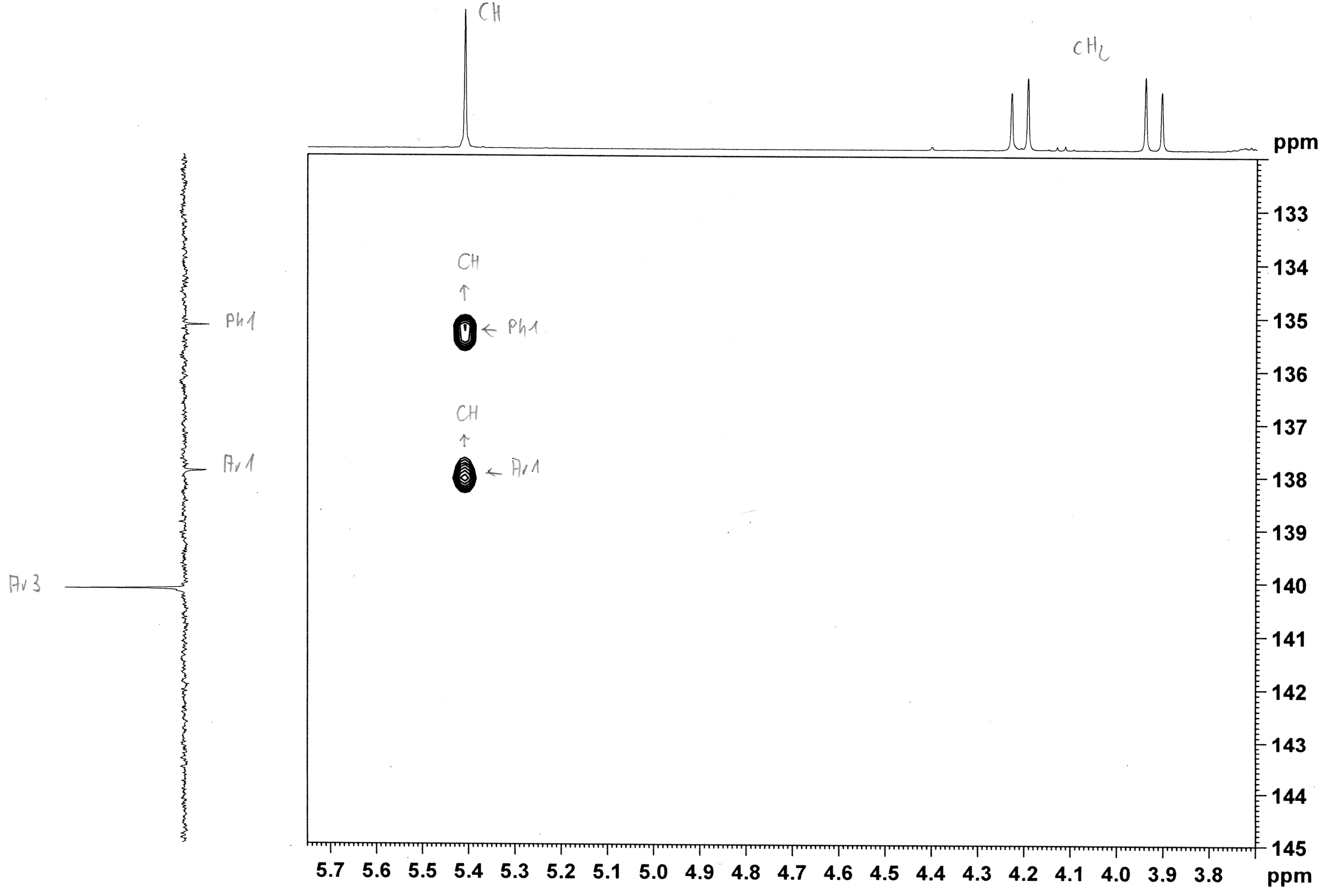

MK-031p4 in cdcl3 (HMBC, 400 MHz) 29.4.2022

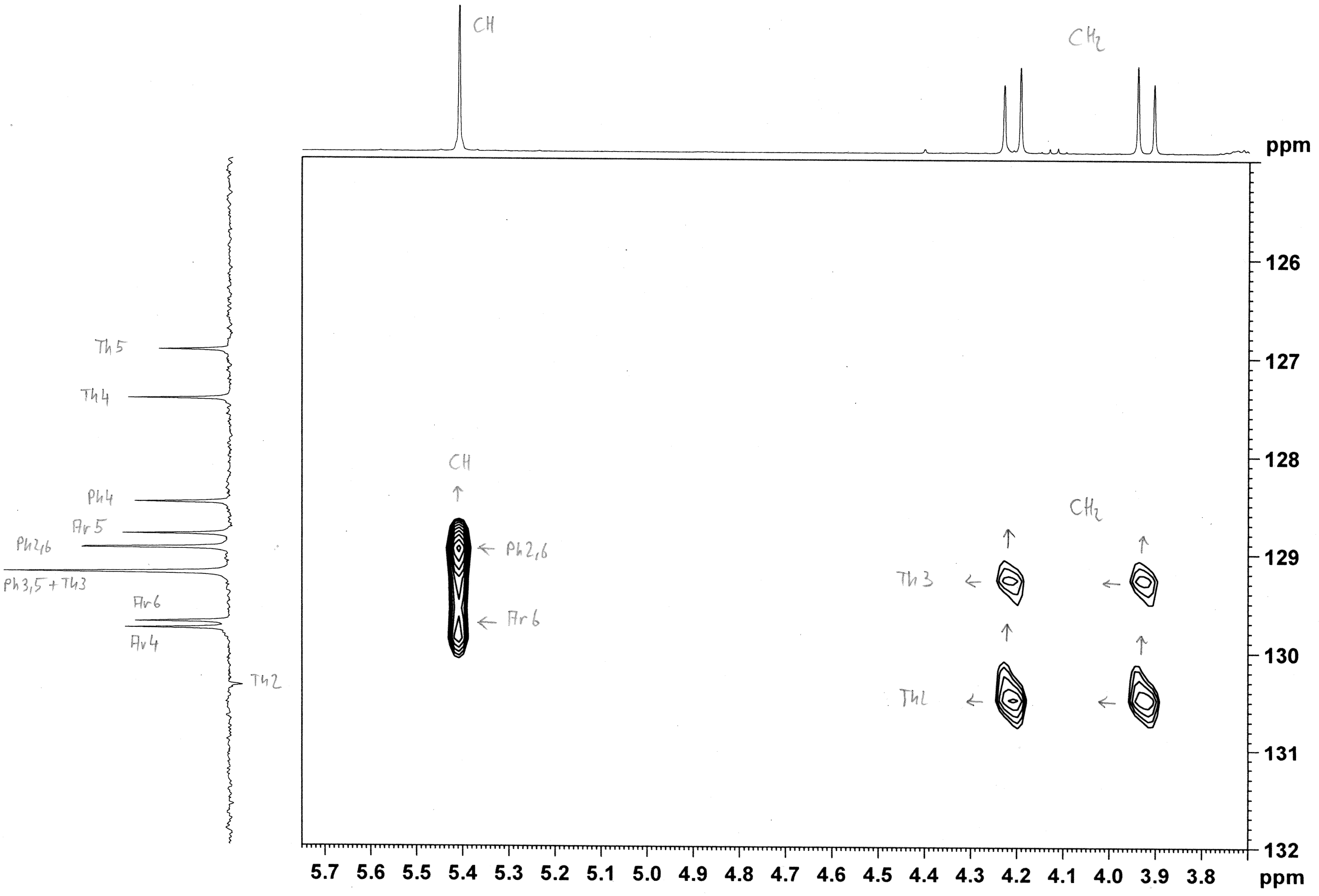

MK-031p4 in cdcl3 (HMBC, 400 MHz) 29.4.2022

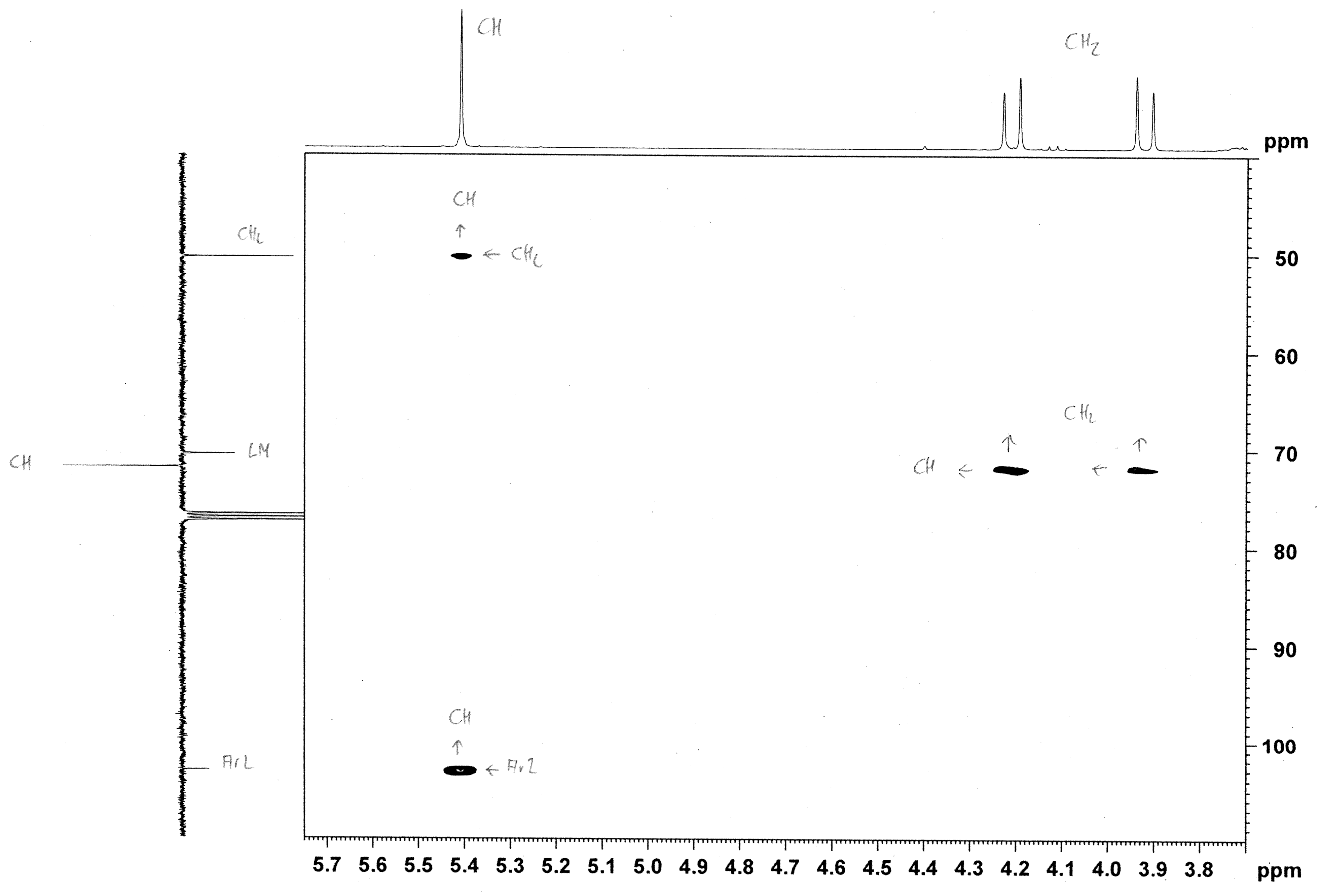

Figure S37c. NMR spectra of compound **5j.**

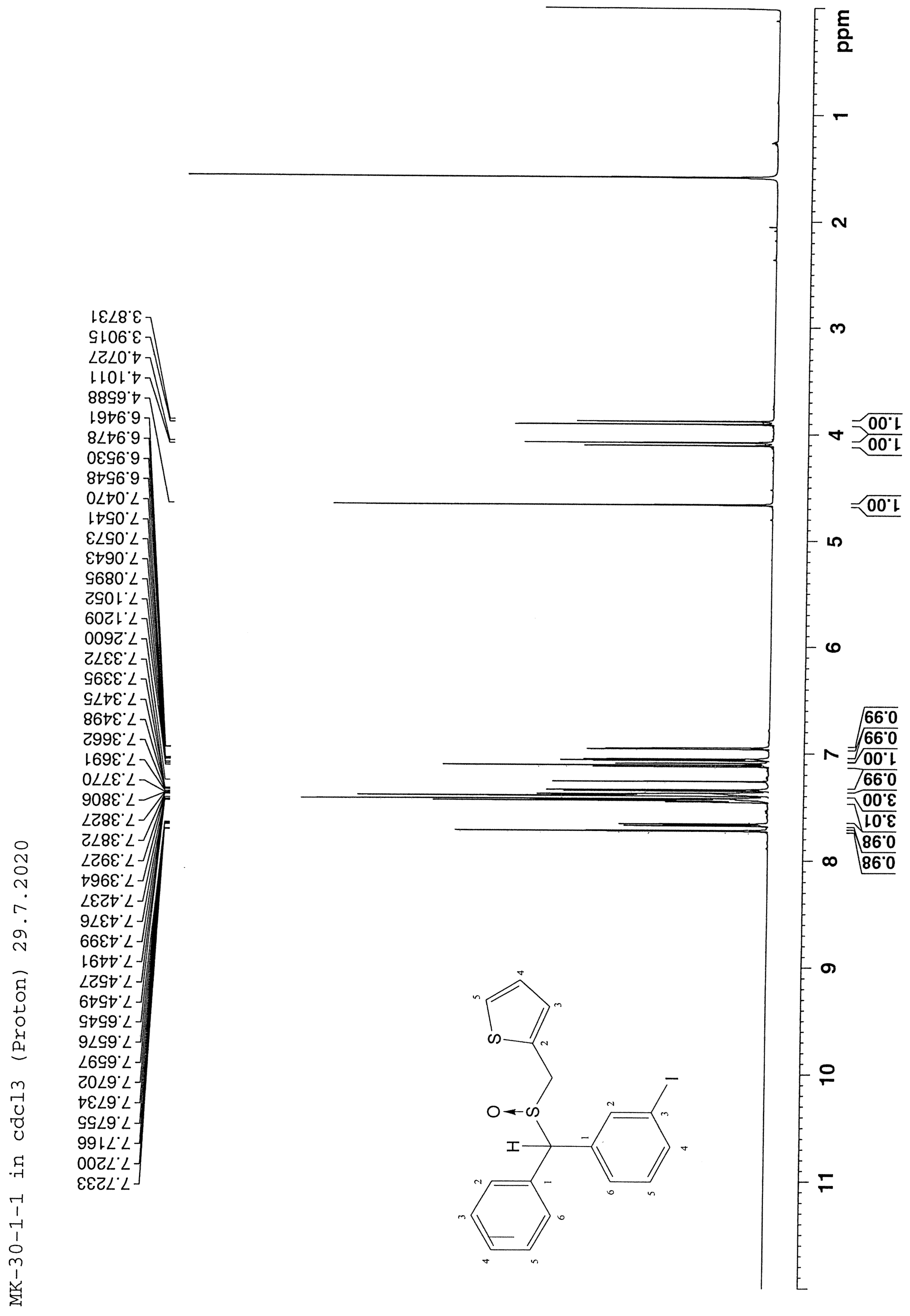

MK-30-1-1 in cdcl3 (Proton) 29.7.2020

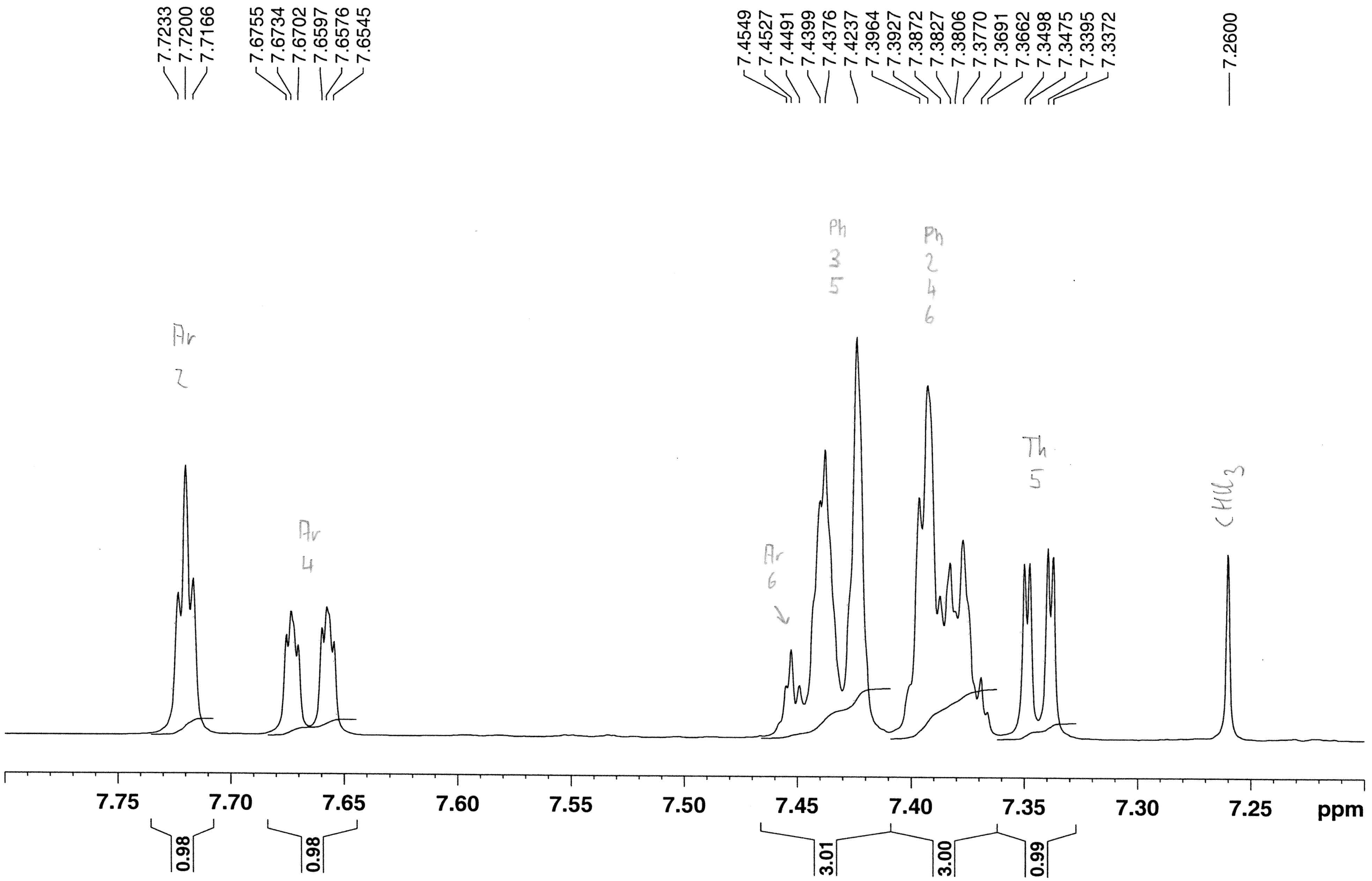

MK-30-1-1 in cdcl3 (Proton) 29.7.2020

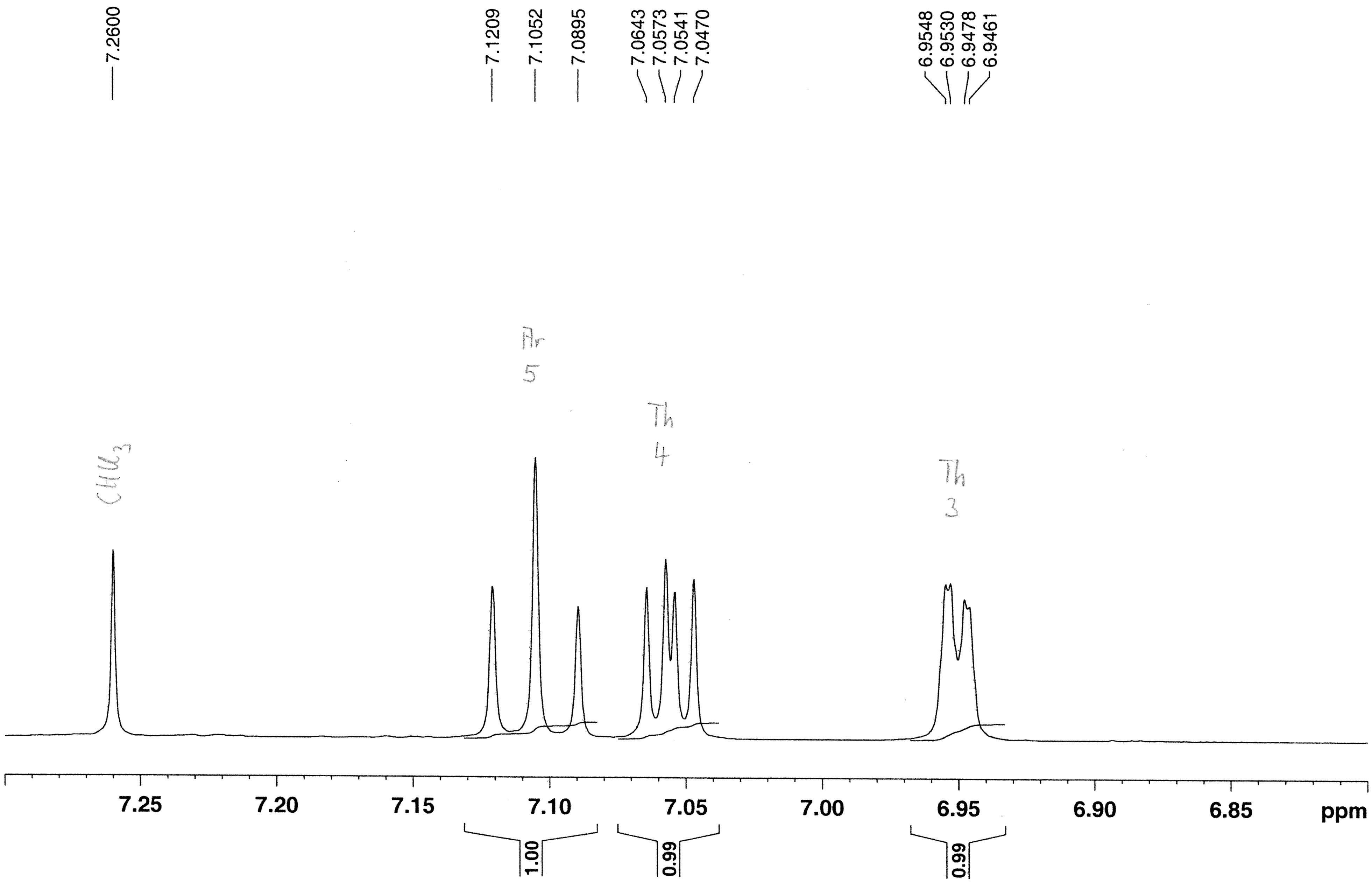

MK-30-1-1 in cdcl3 (Proton) 29.7.2020

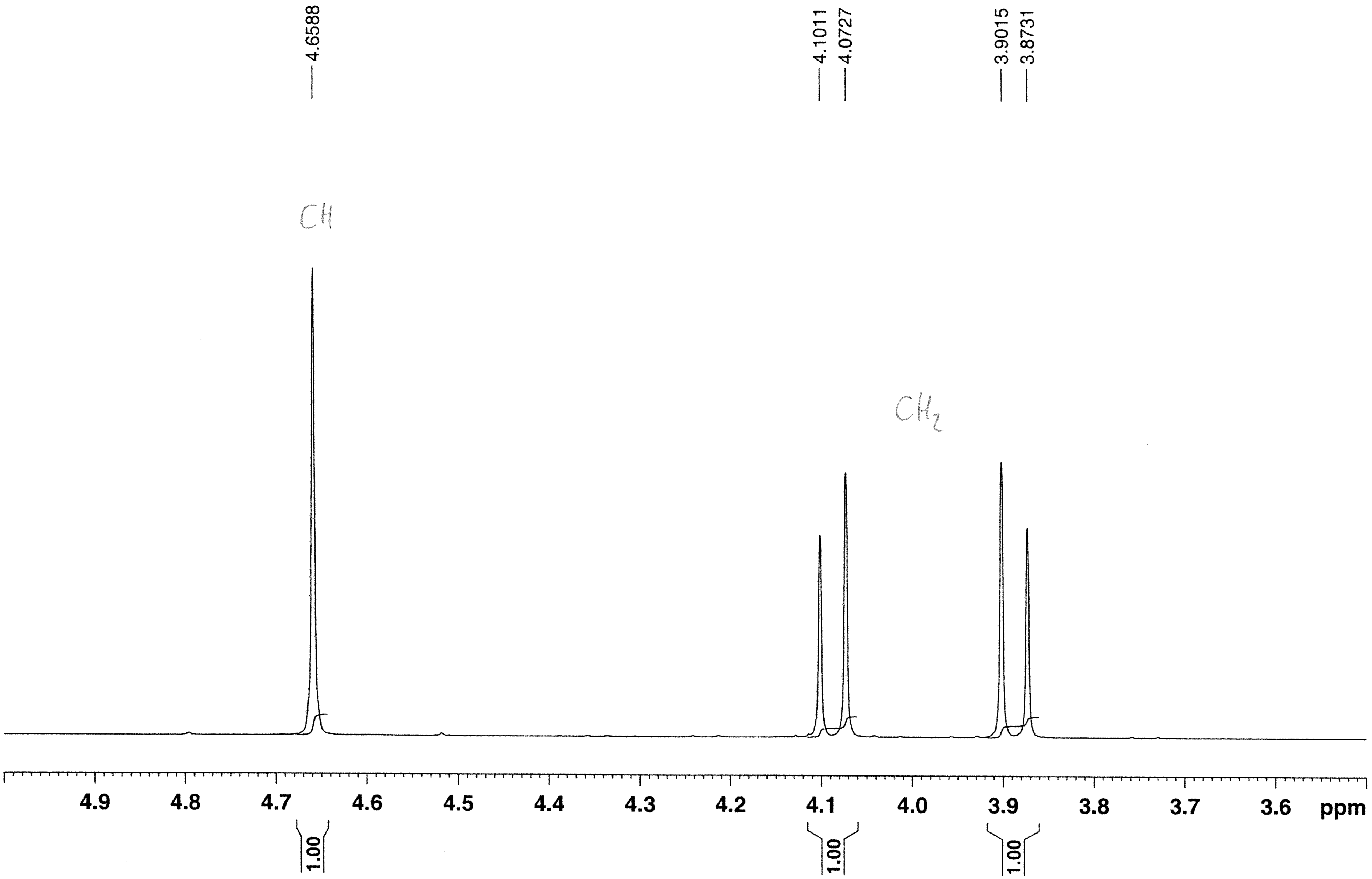

MK-30-1-1 in cdcl3 (APT) 29.7.2020

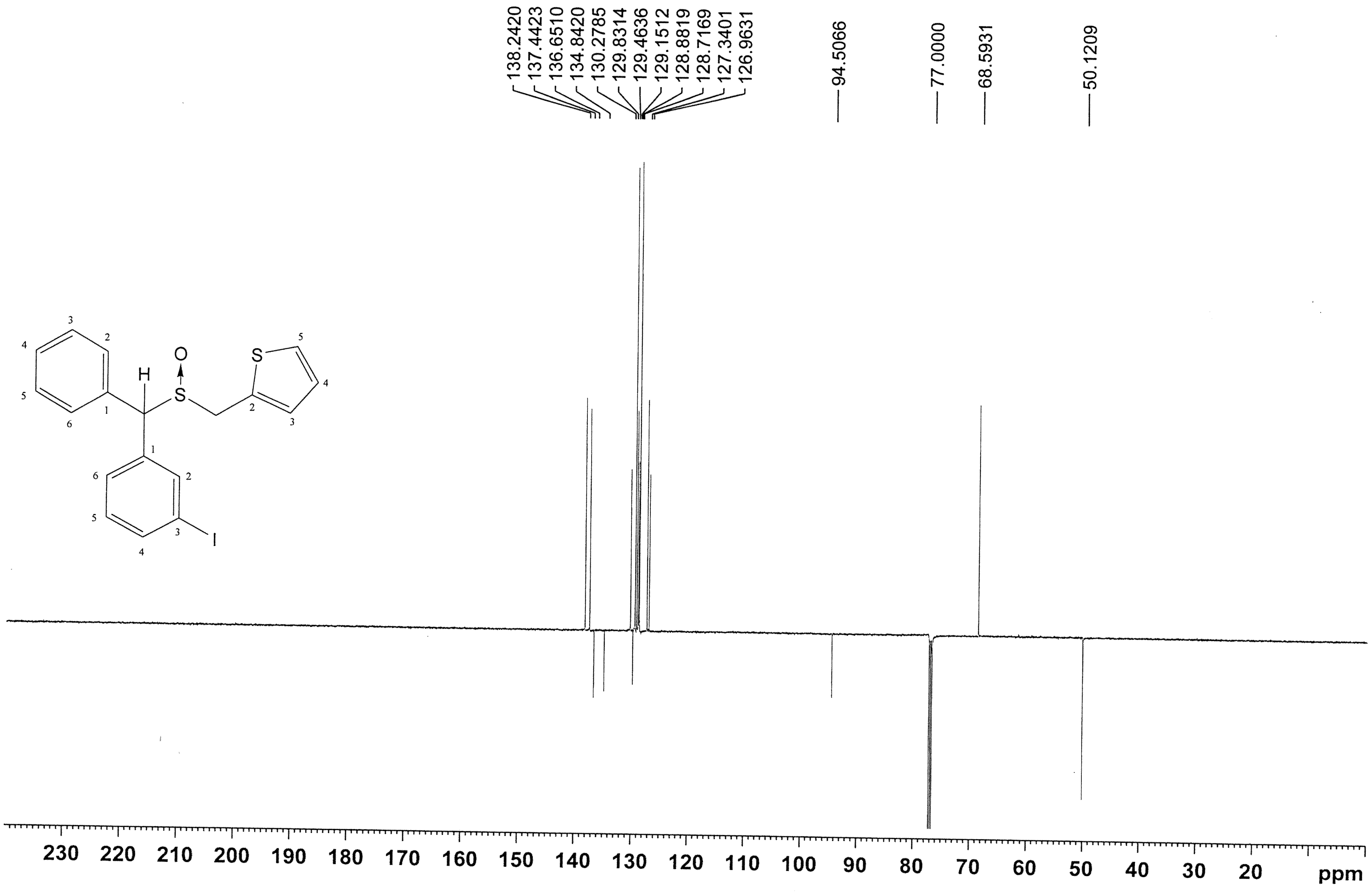

MK-30-1-1 in cdcl3 (APT) 29.7.2020

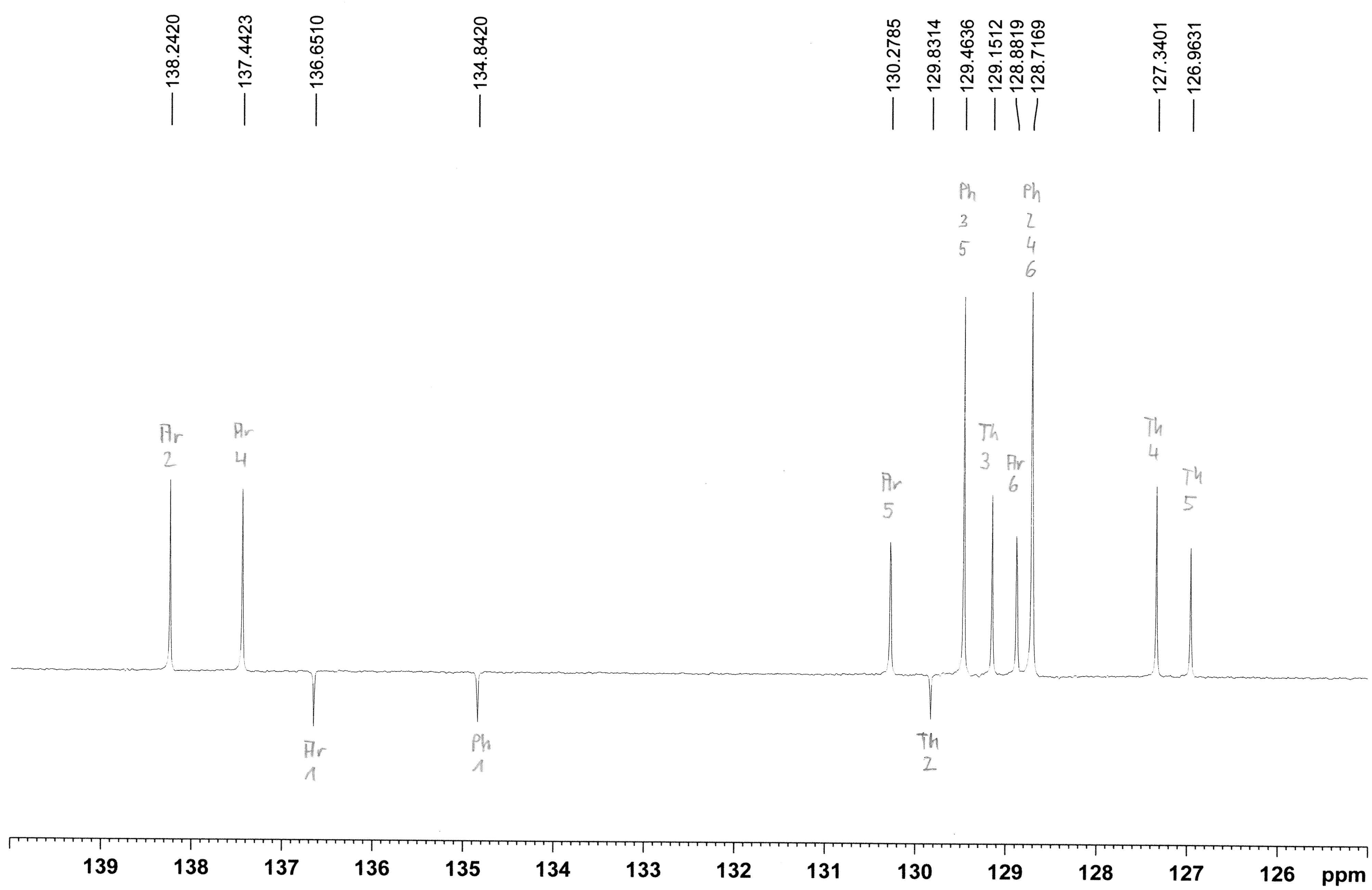

MK-30-1-1 in cdcl3 (APT) 29.7.2020

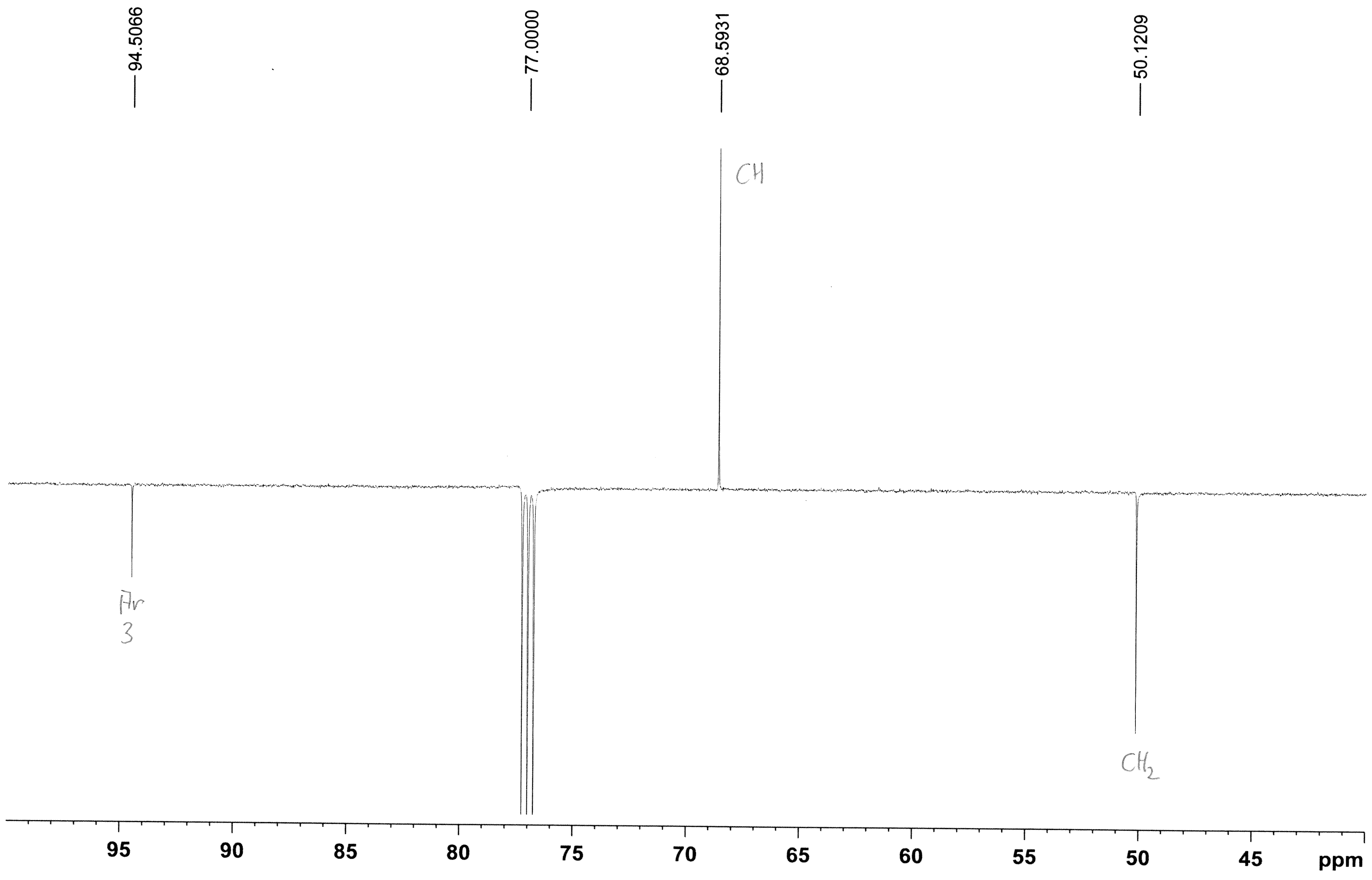

MK-30-1-1 in cdcl3 (COSY) 29.7.2020

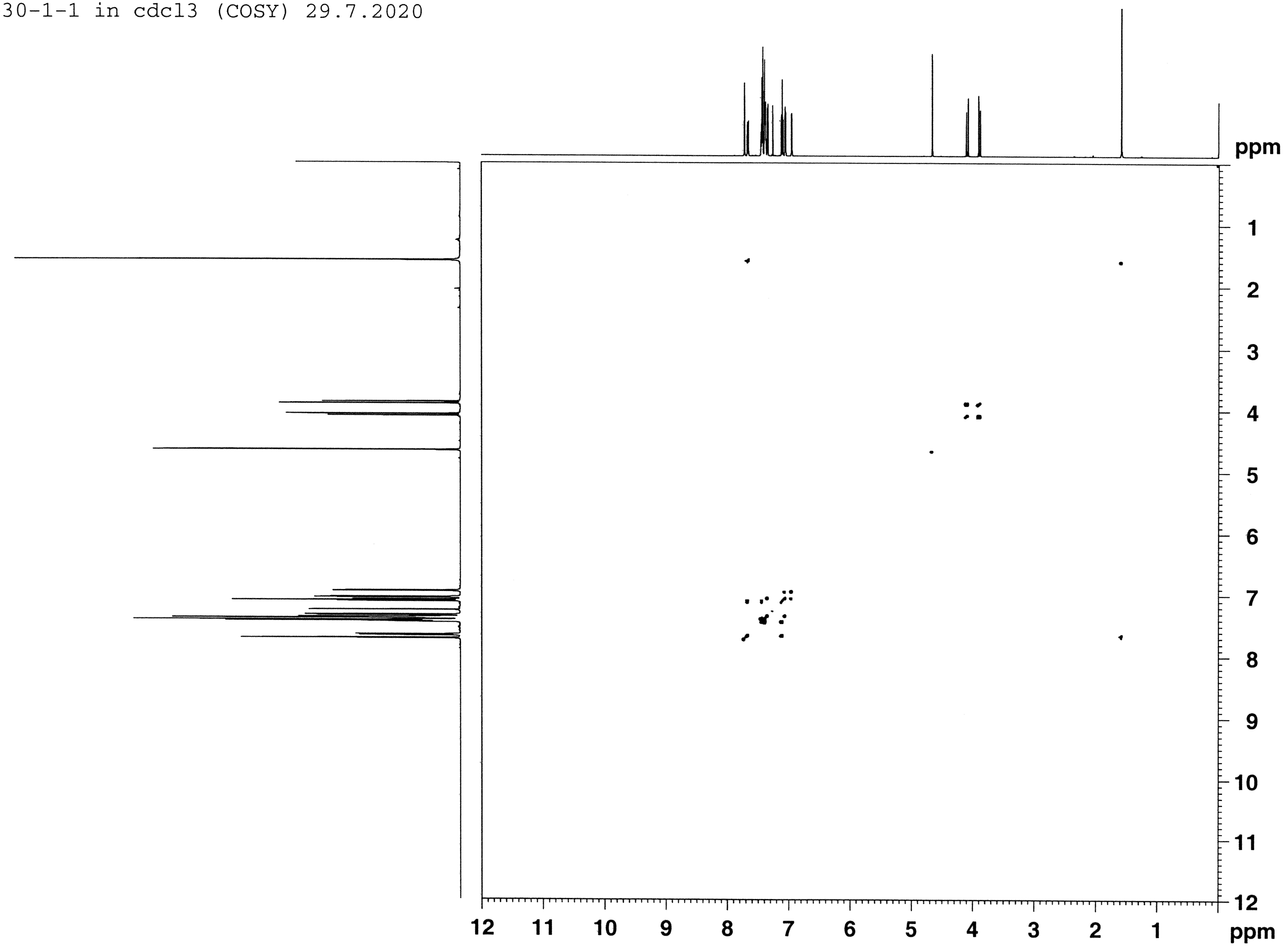

MK-30-1-1 in cdcl3 (COSY) 29.7.2020

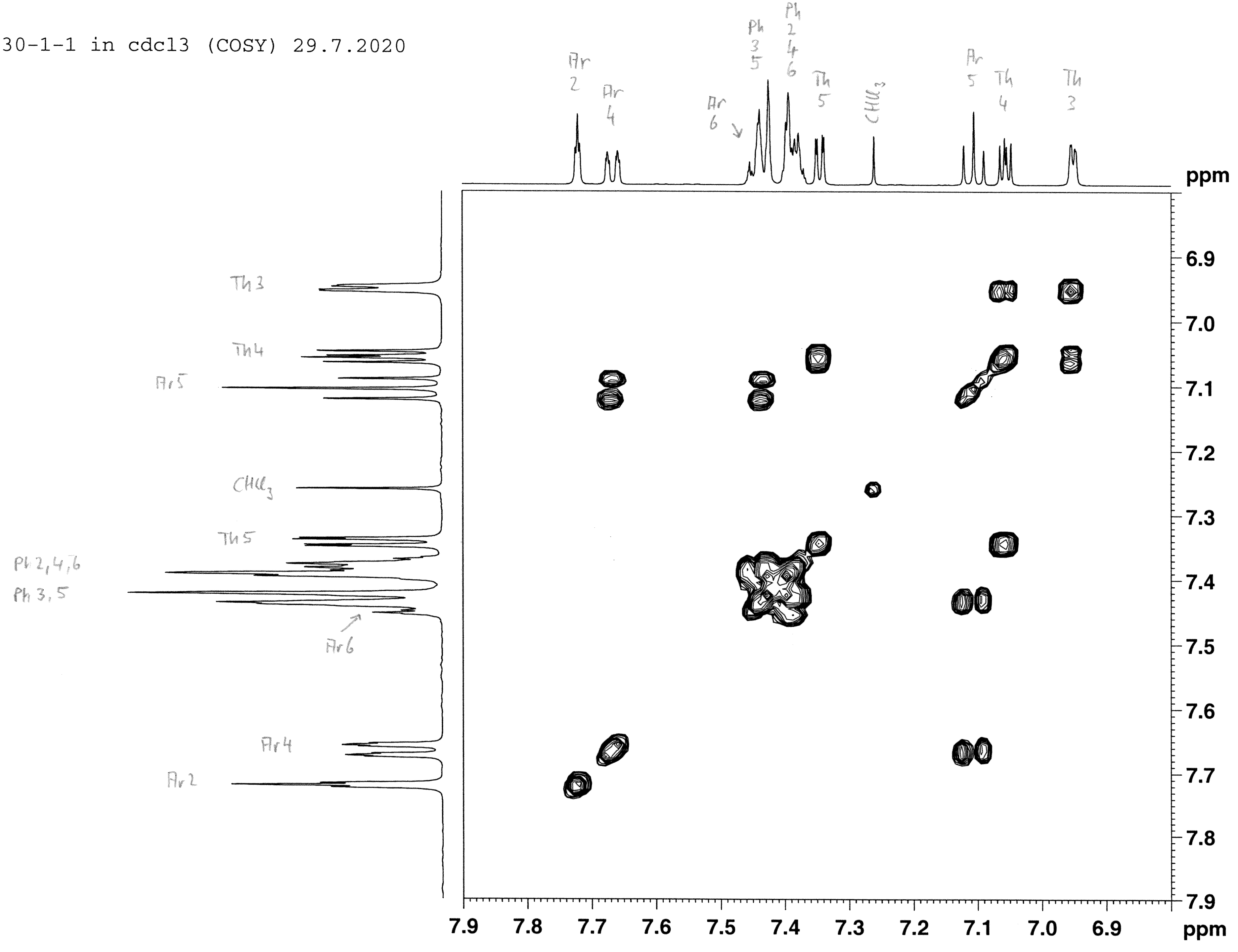

MK-30-1-1 in cdcl3 (COSY) 29.7.2020

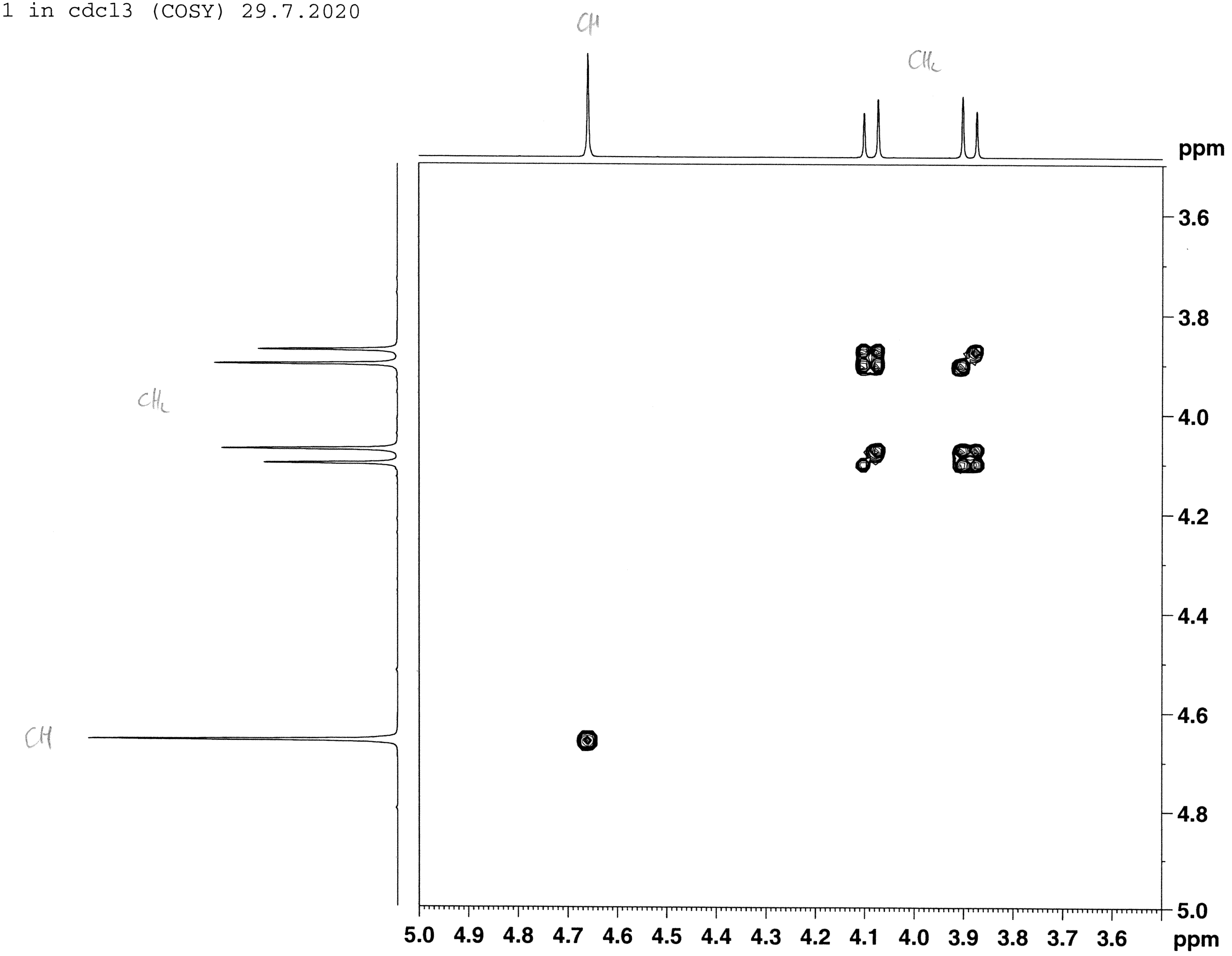

MK-30-1-1 in cdcl3 (HSQC) 29.7.2020

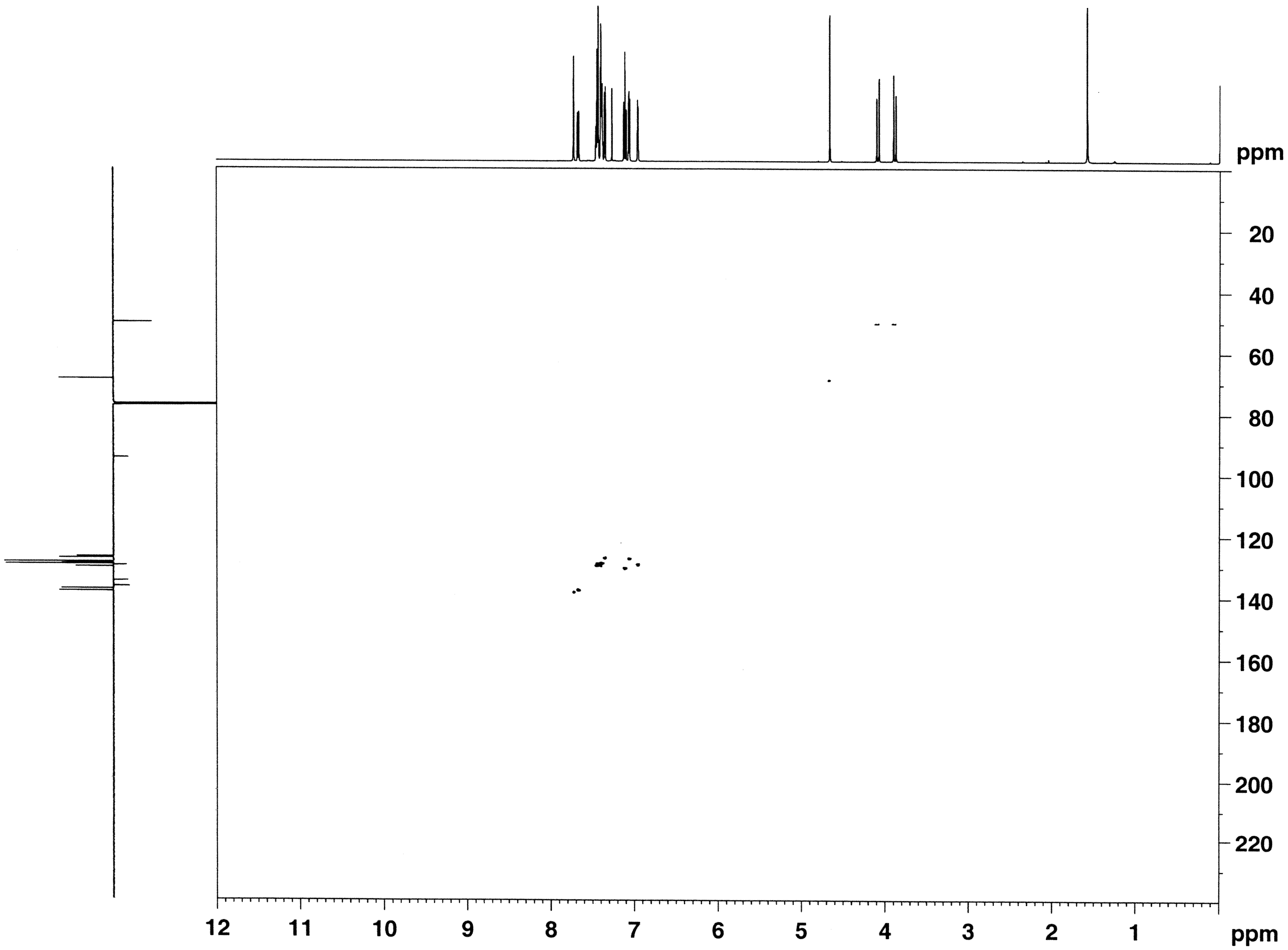

MK-30-1-1 in cdcl3 (HSQC) 29.7.2020

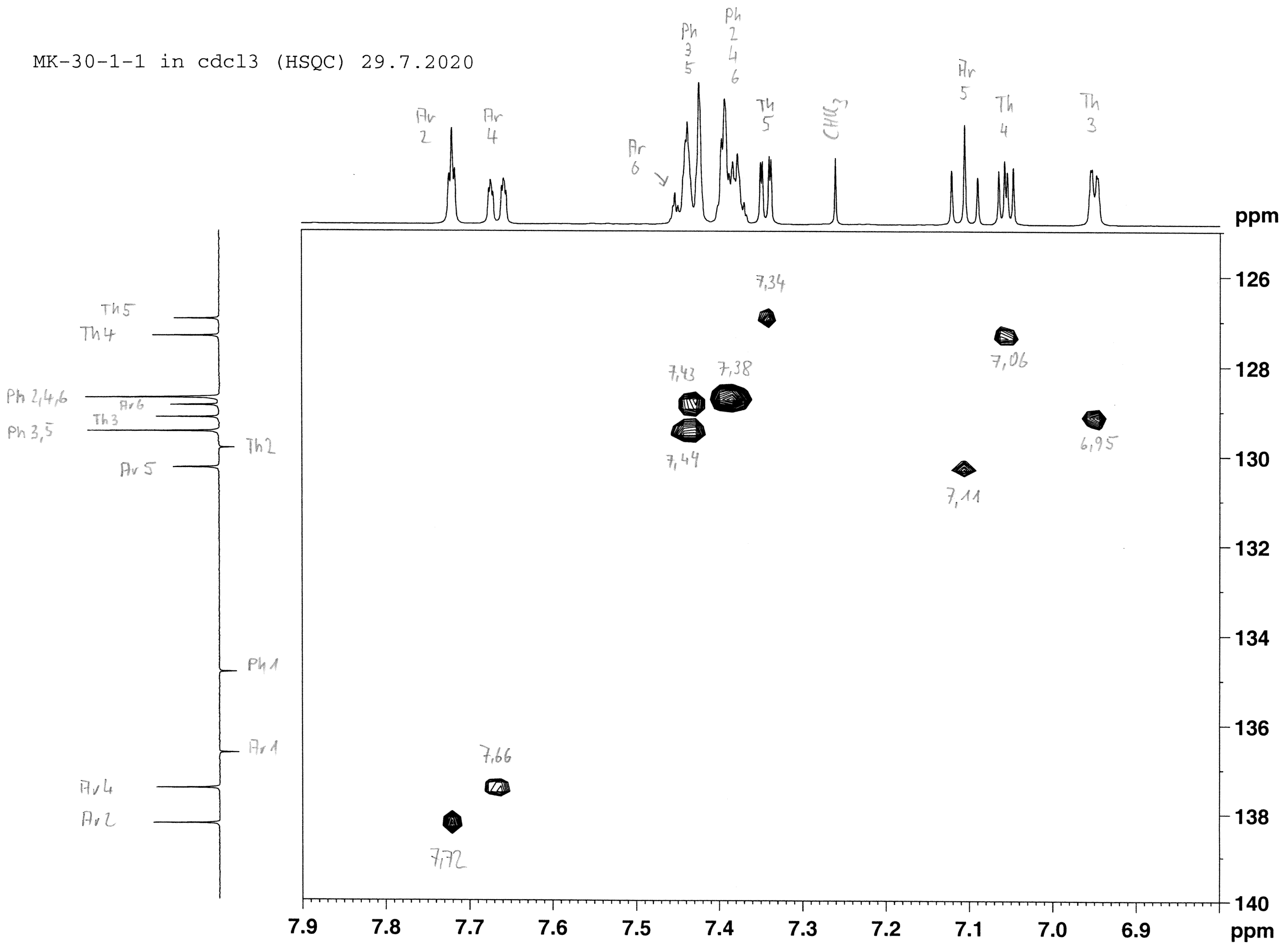

MK-30-1-1 in cdcl3 (HSQC) 29.7.2020

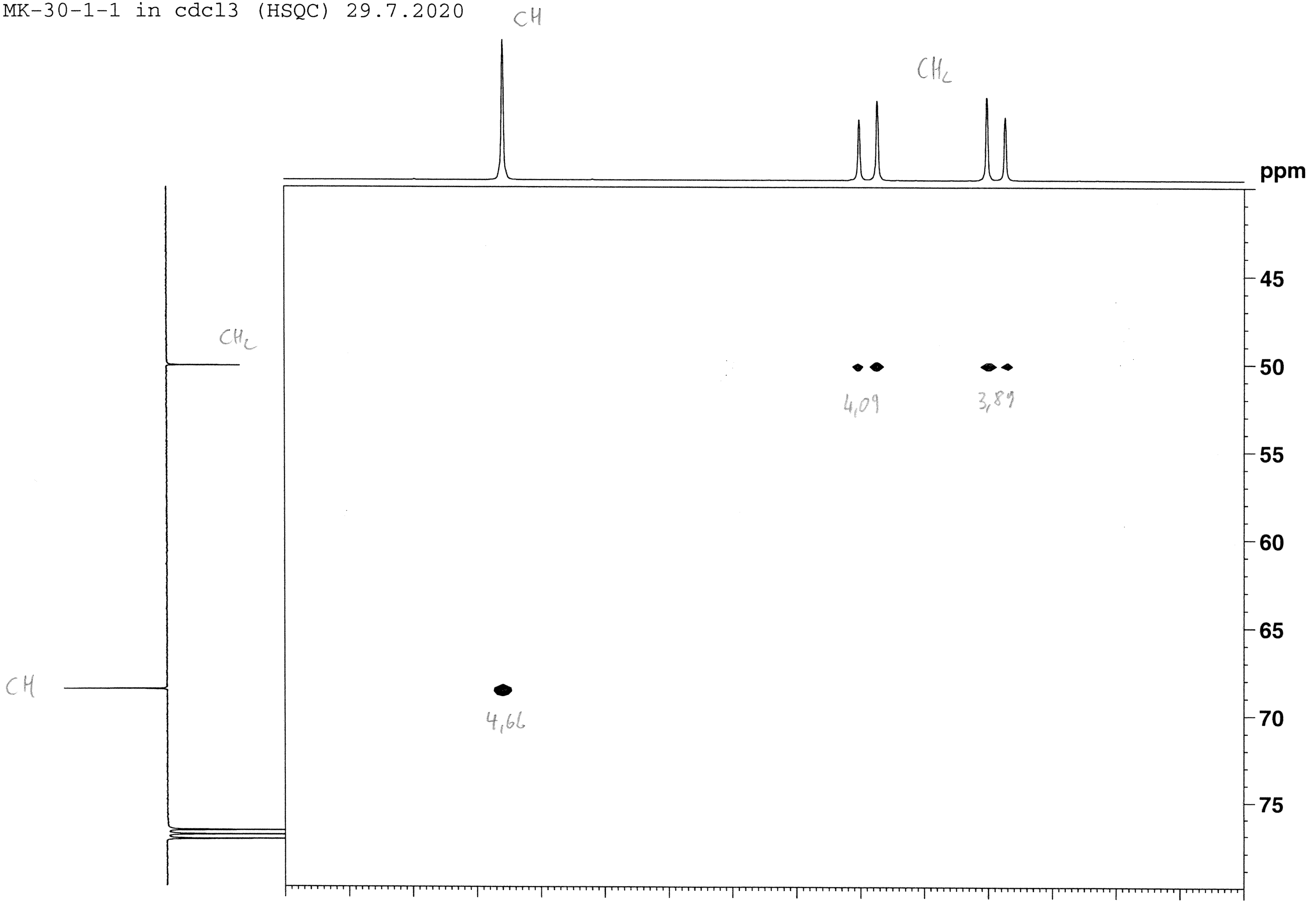

MK-30-1-1 in cdcl3 (HMBC) 29.7.2020

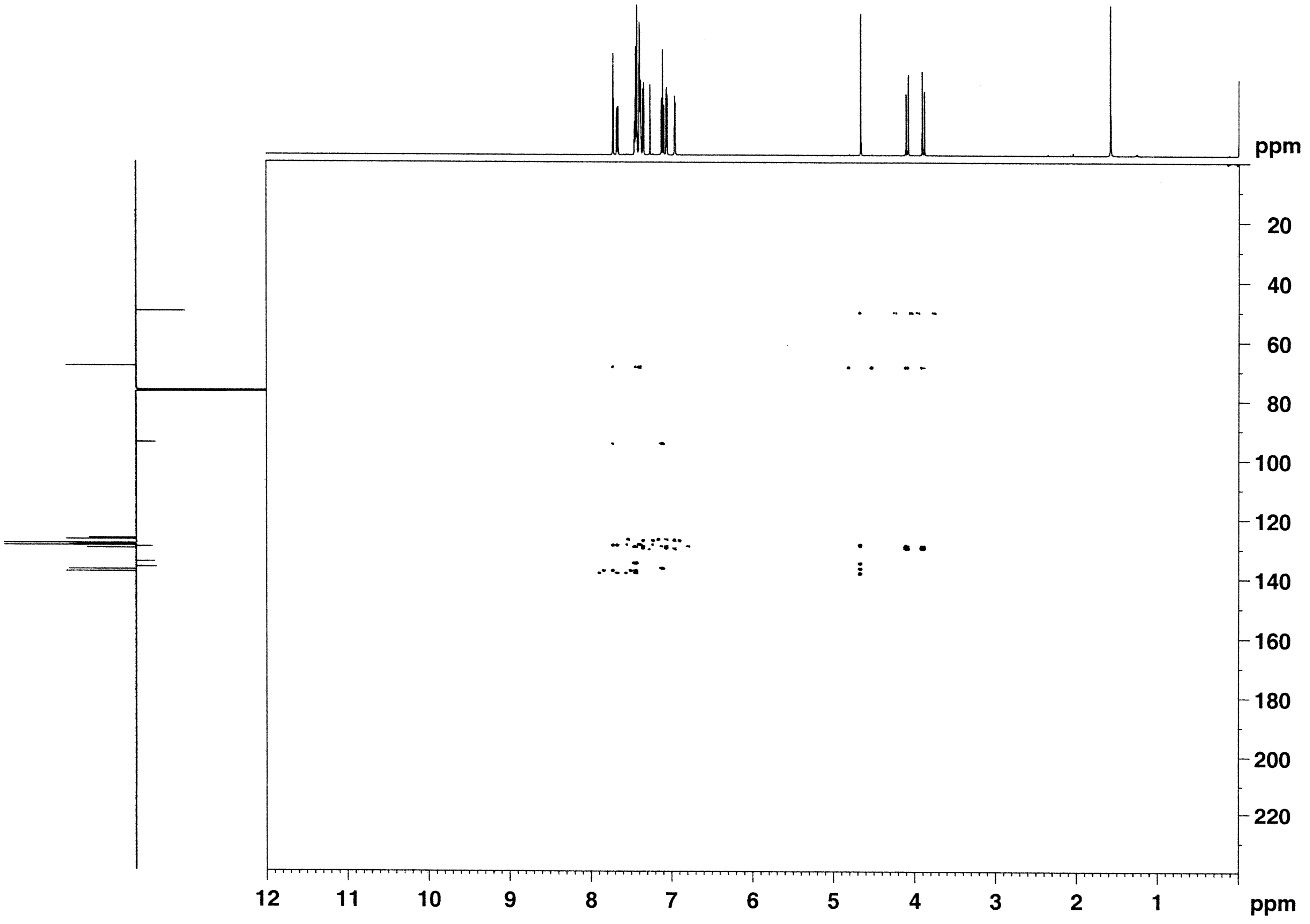

MK-30-1-1 in cdcl3 (HMBC) 29.7.2020

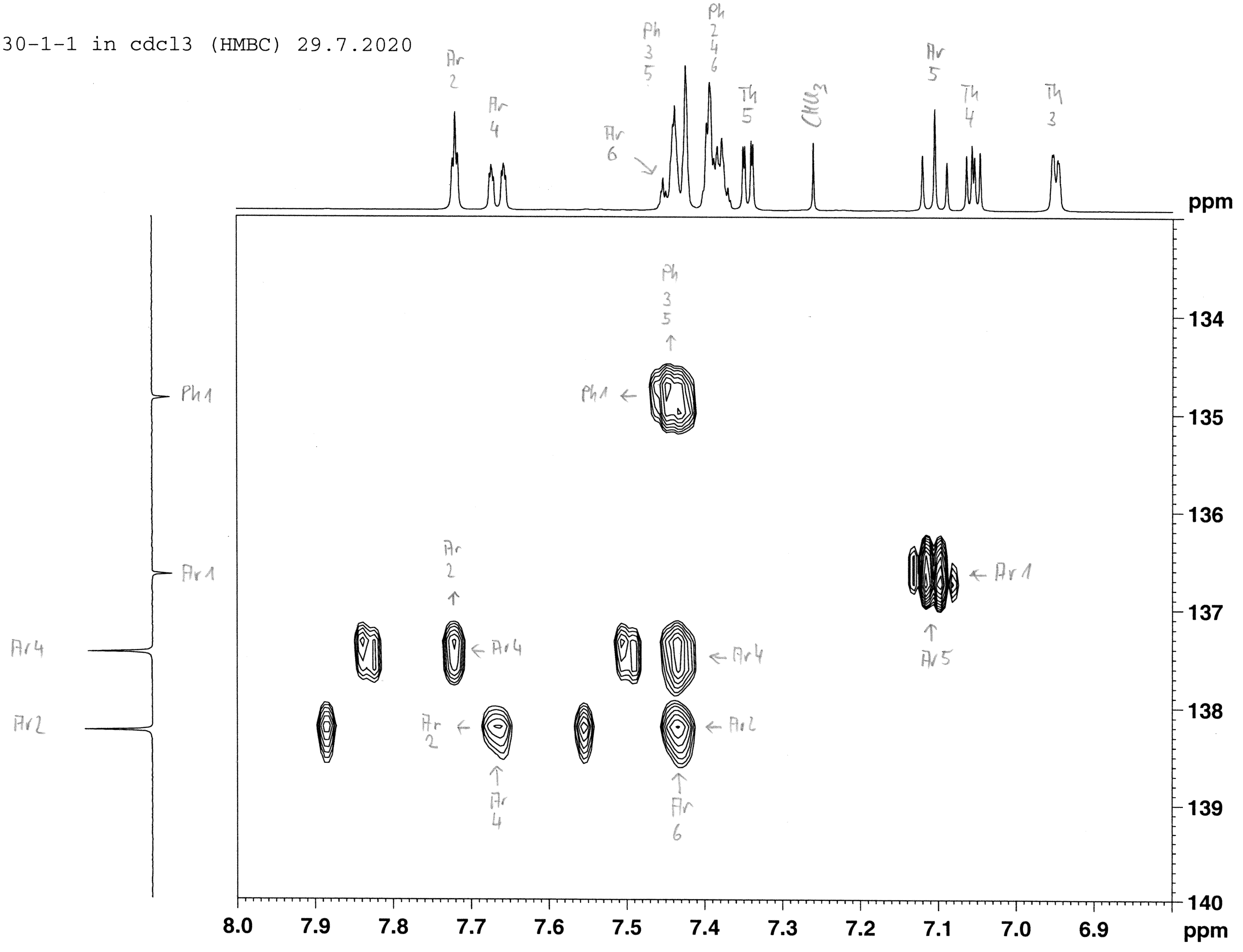

MK-30-1-1 in cdcl3 (HMBC) 29.7.2020

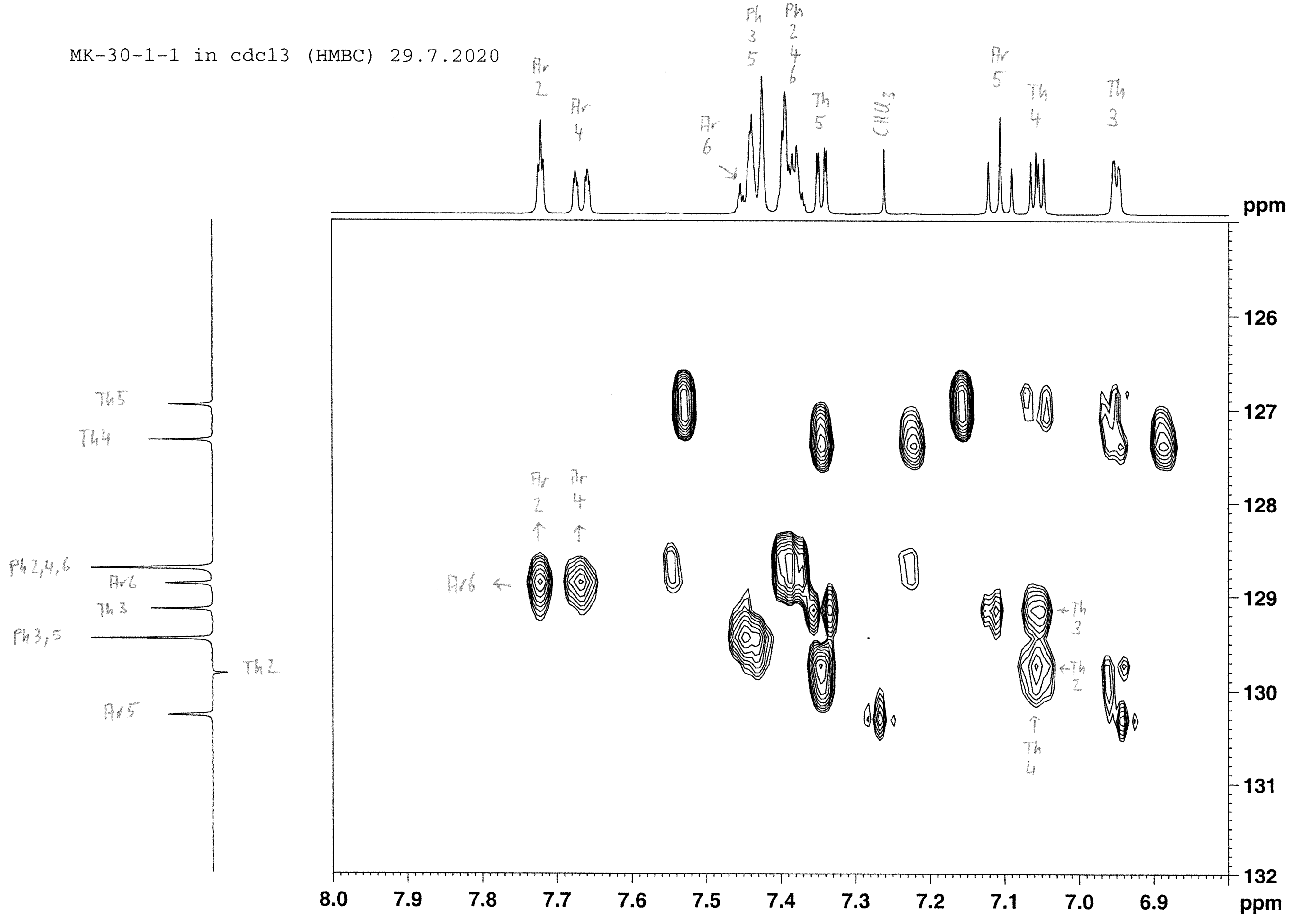

MK-30-1-1 in cdcl3 (HMBC) 29.7.2020

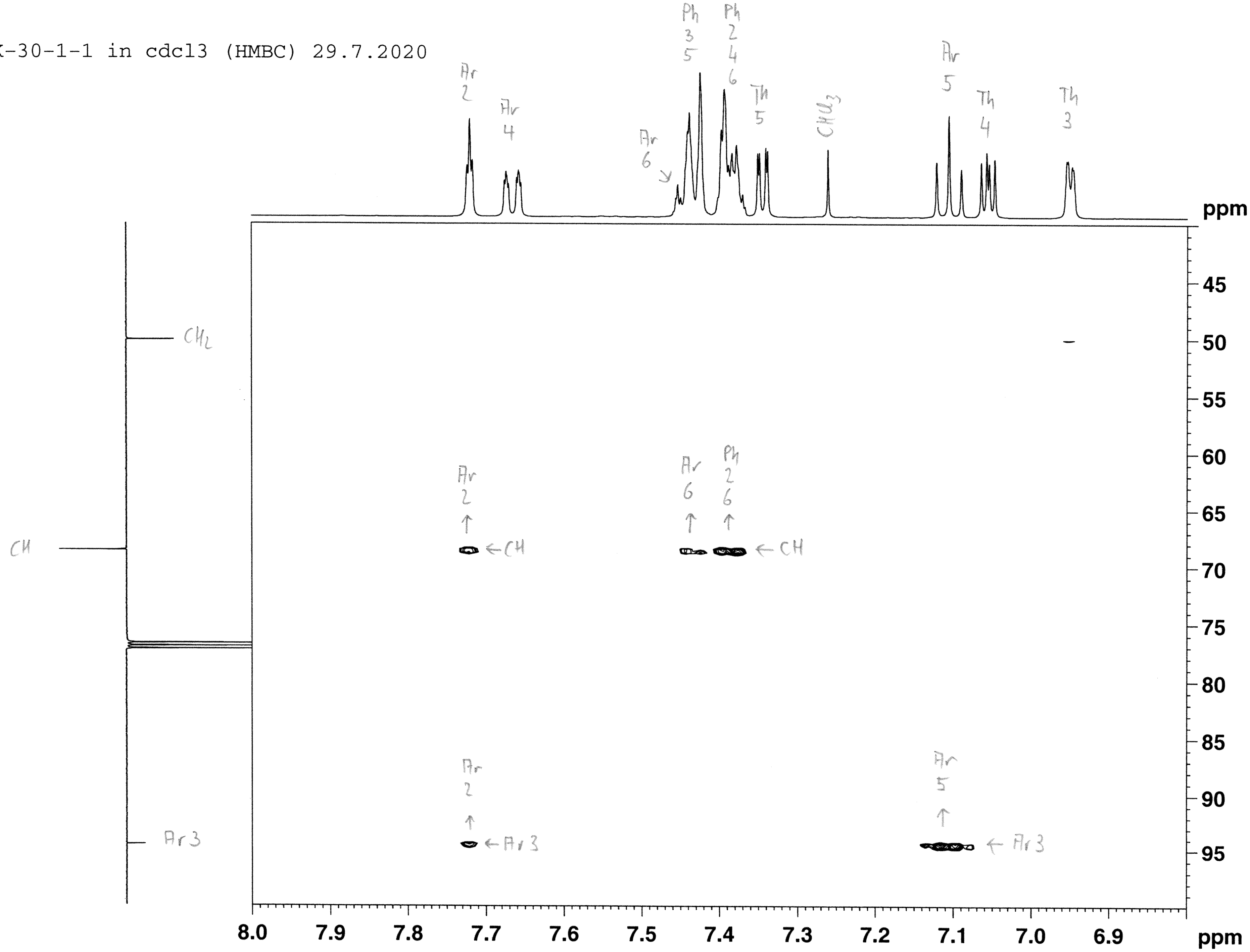

MK-30-1-1 in cdcl3 (HMBC) 29.7.2020

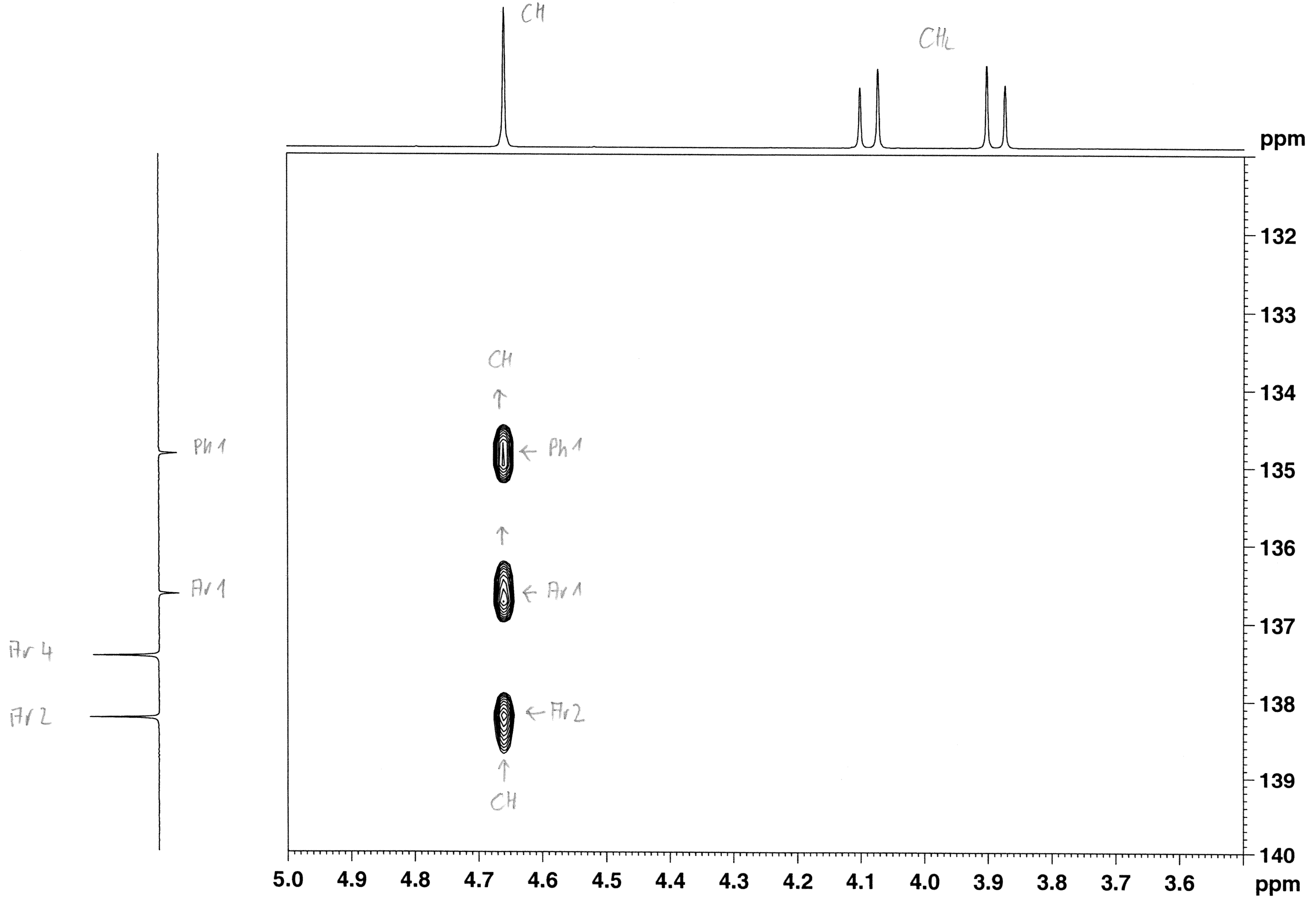

MK-30-1-1 in cdcl3 (HMBC) 29.7.2020

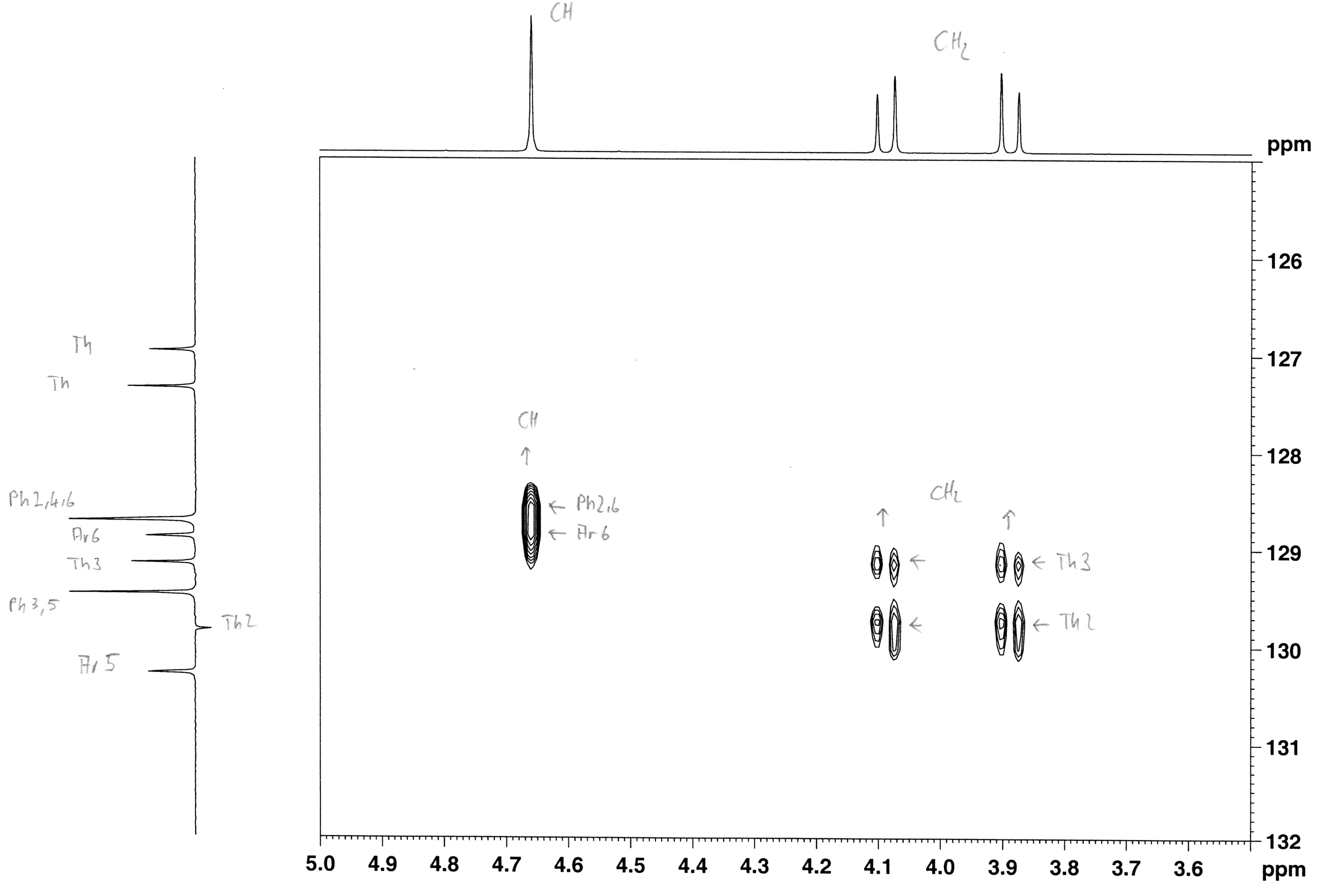

MK-30-1-1 in cdcl3 (HMBC) 29.7.2020

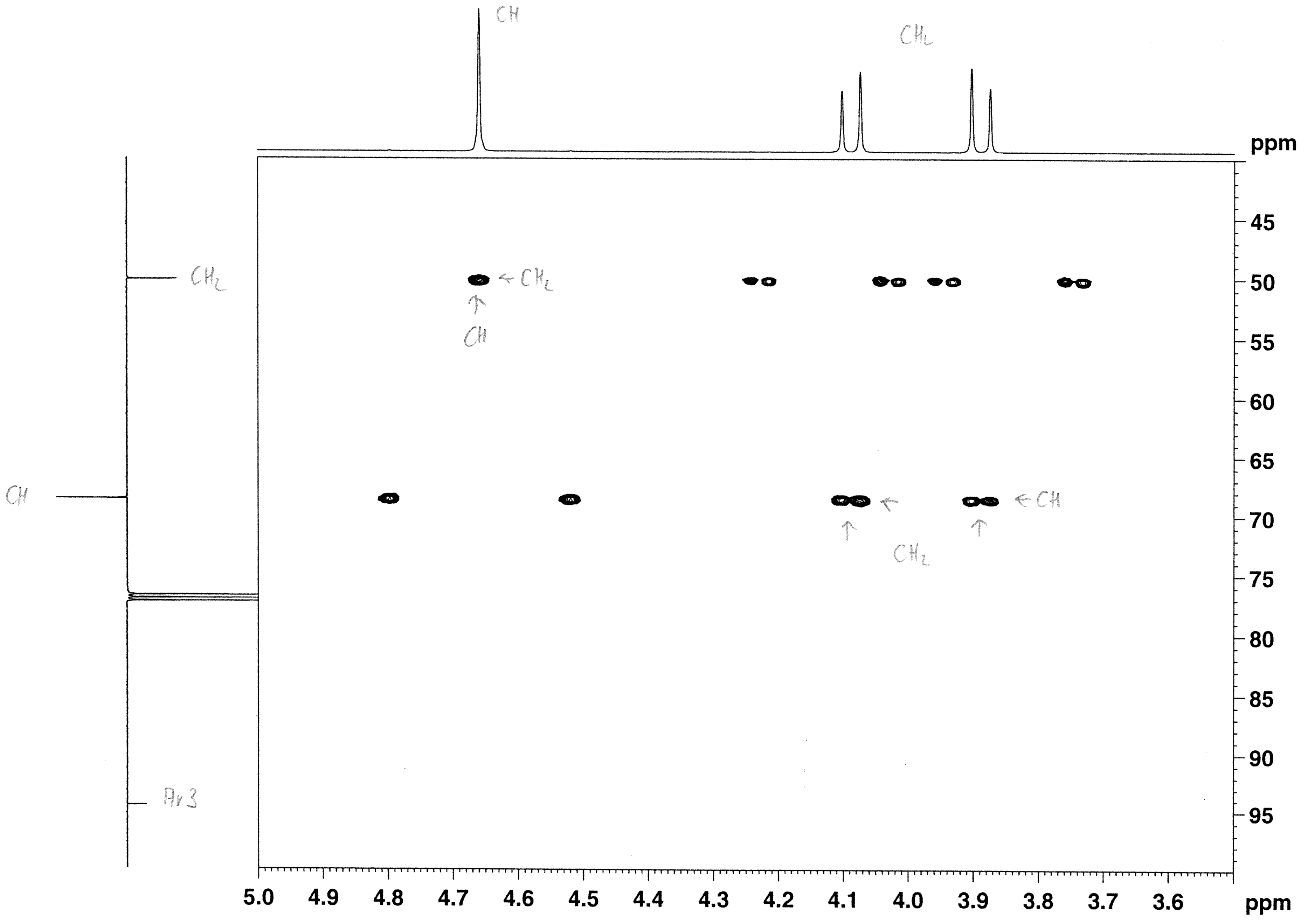

Figure S38c. NMR spectra of compound **6j**.

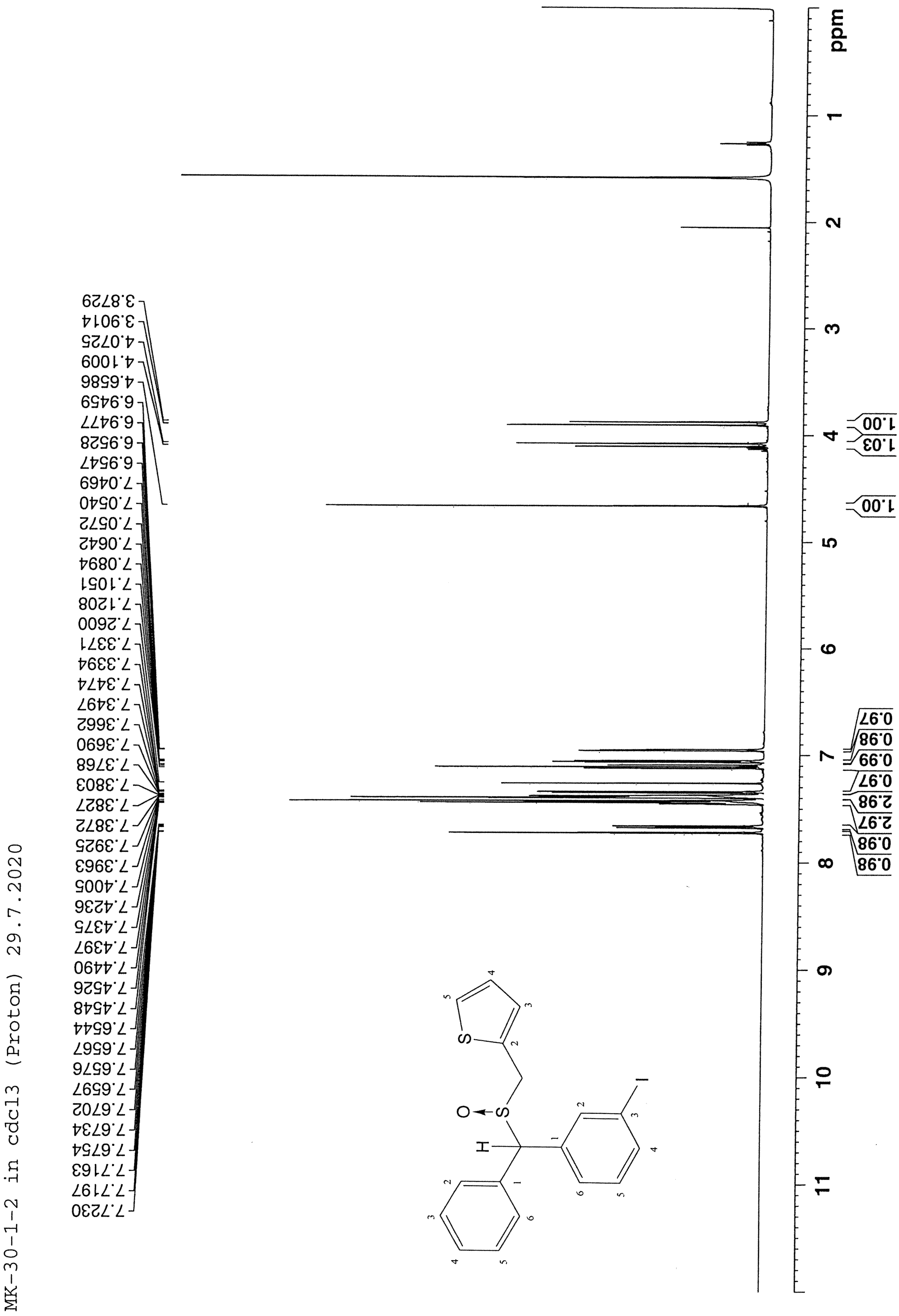

MK-30-1-2 in cdcl3 (Proton) 29.7.2020

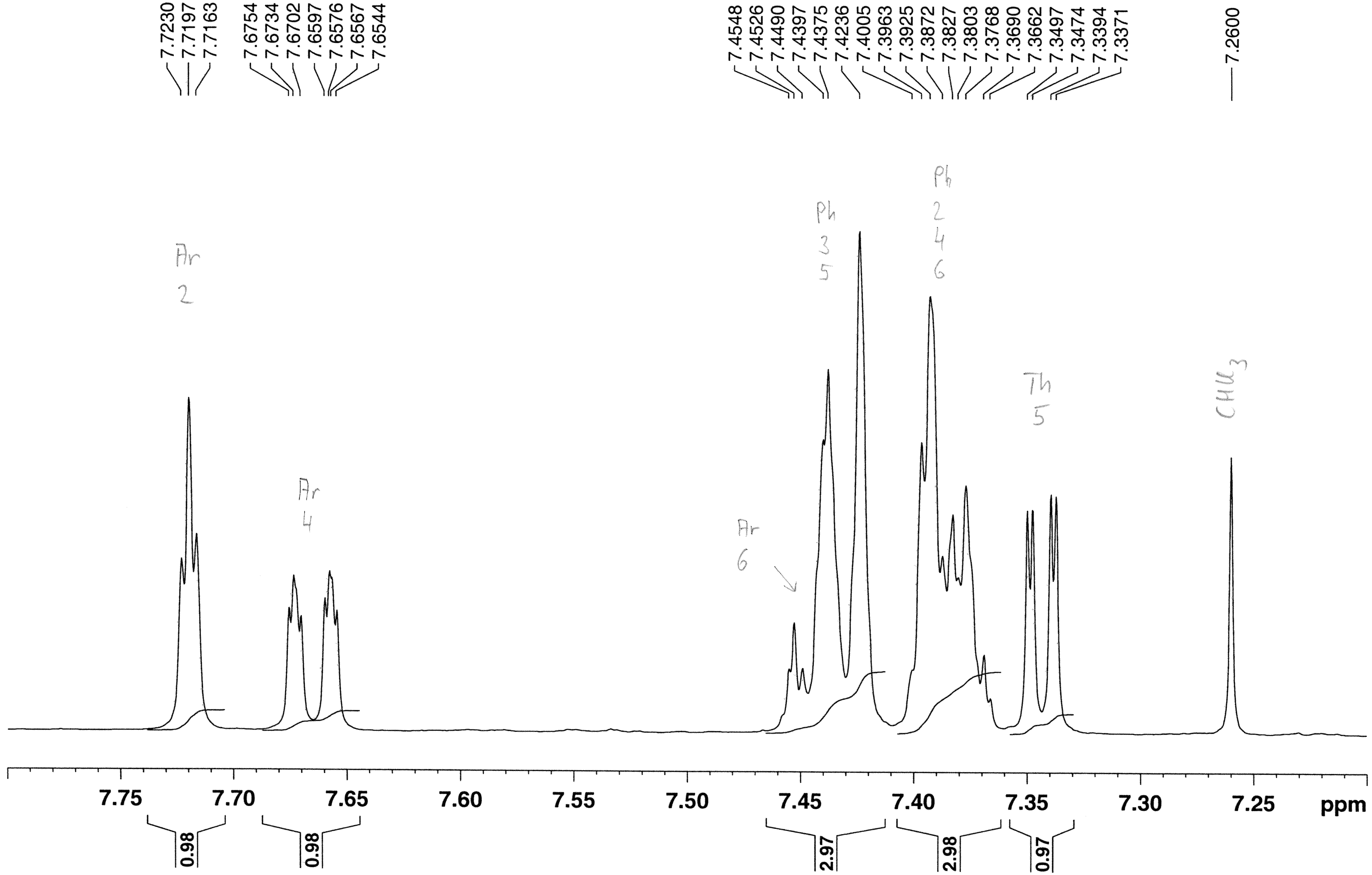

MK-30-1-2 in cdcl3 (Proton) 29.7.2020

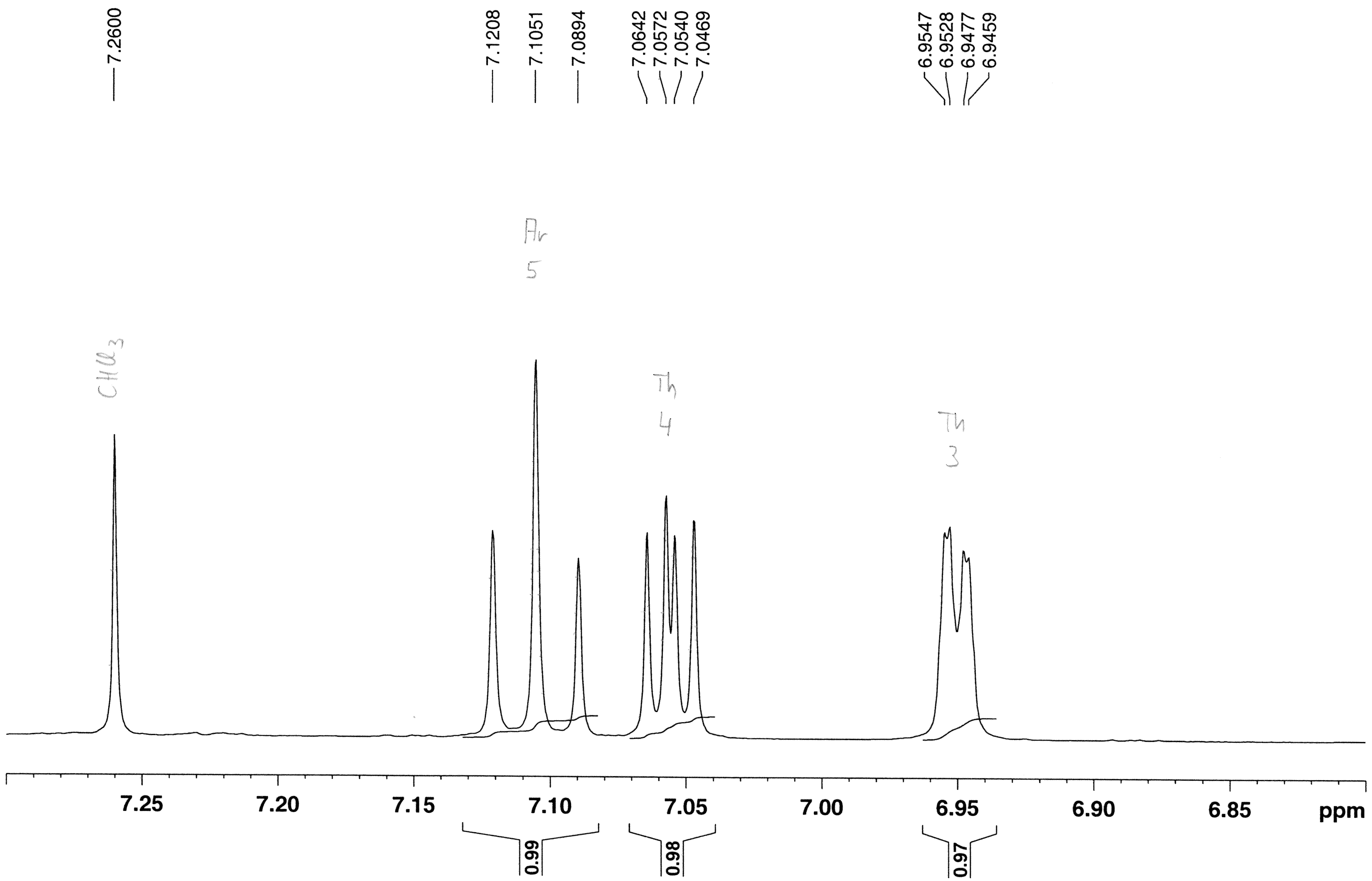

MK-30-1-2 in cdcl3 (Proton) 29.7.2020

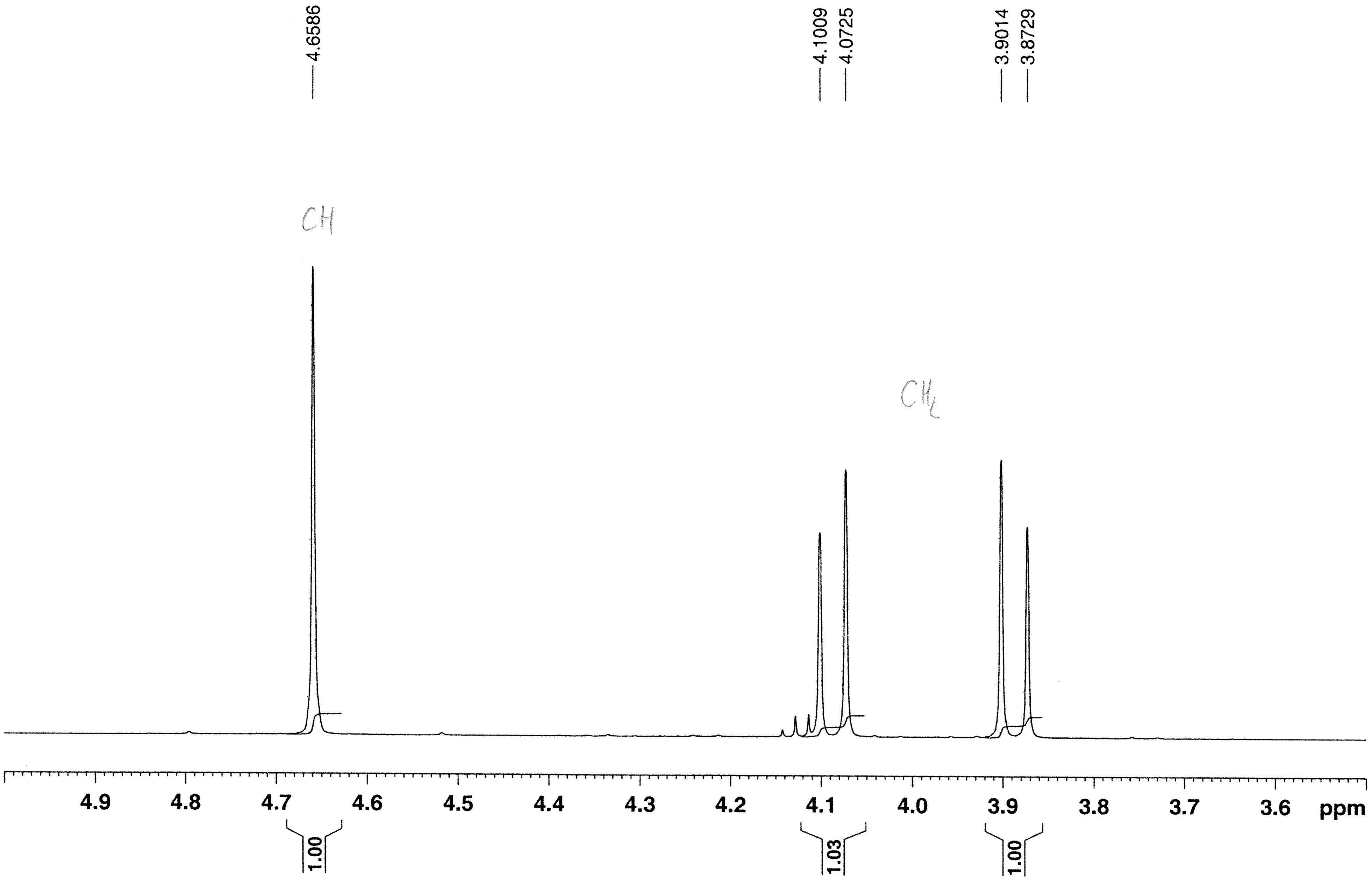

MK-30-1-2 in cdcl3 (APT) 29.7.2020

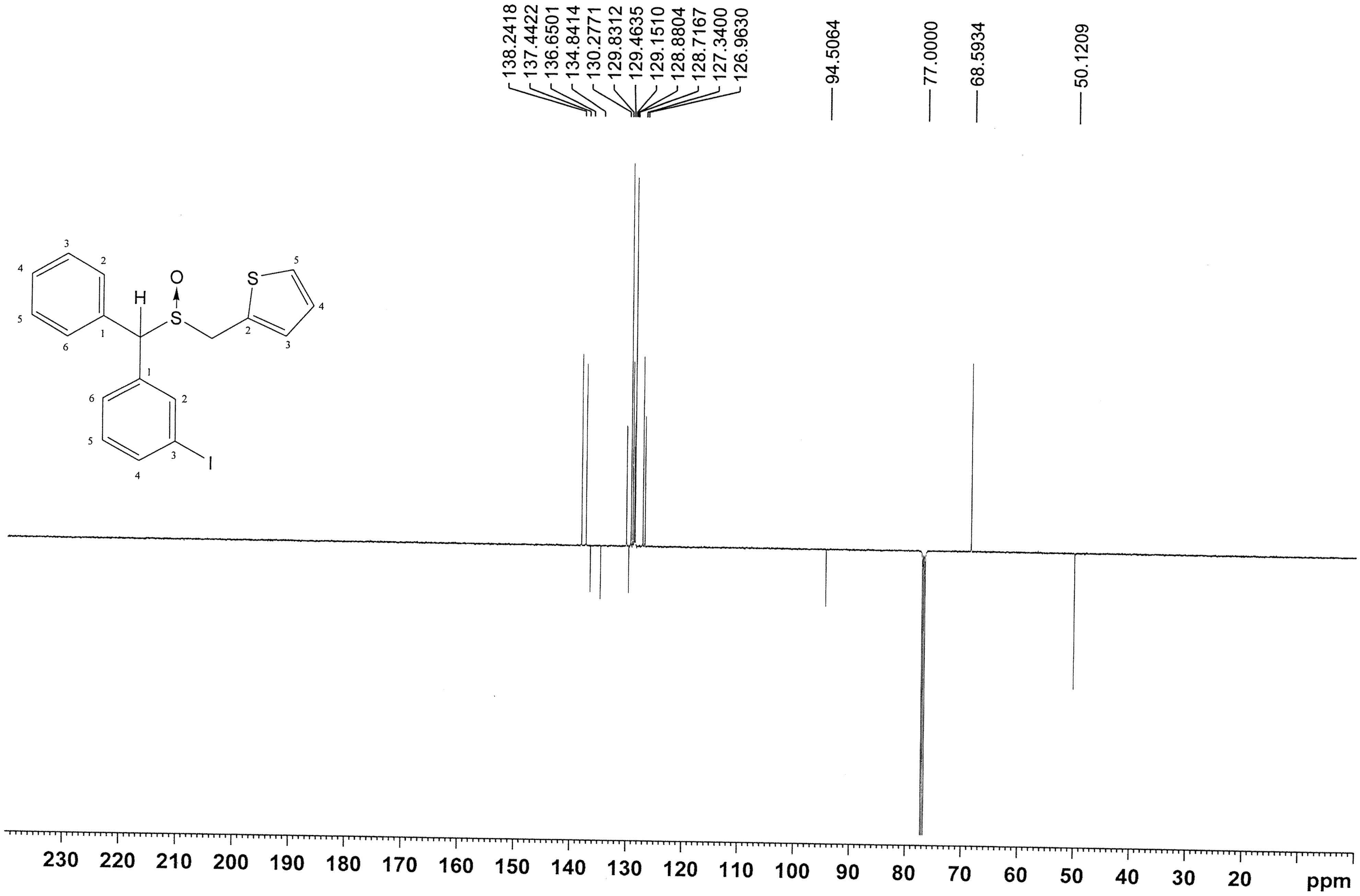

MK-30-1-2 in cdcl3 (APT) 29.7.2020

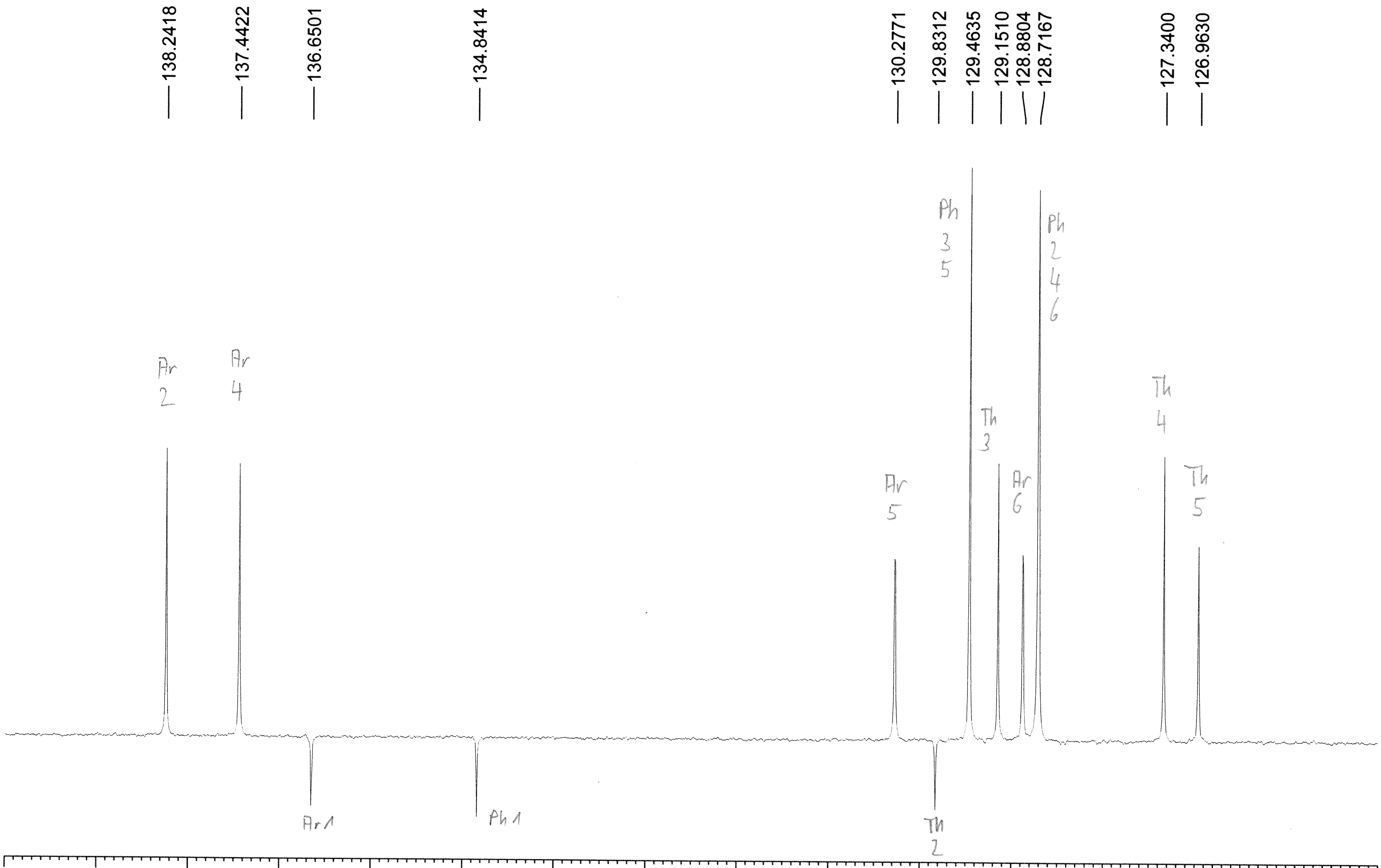

MK-30-1-2 in cdcl3 (APT) 29.7.2020

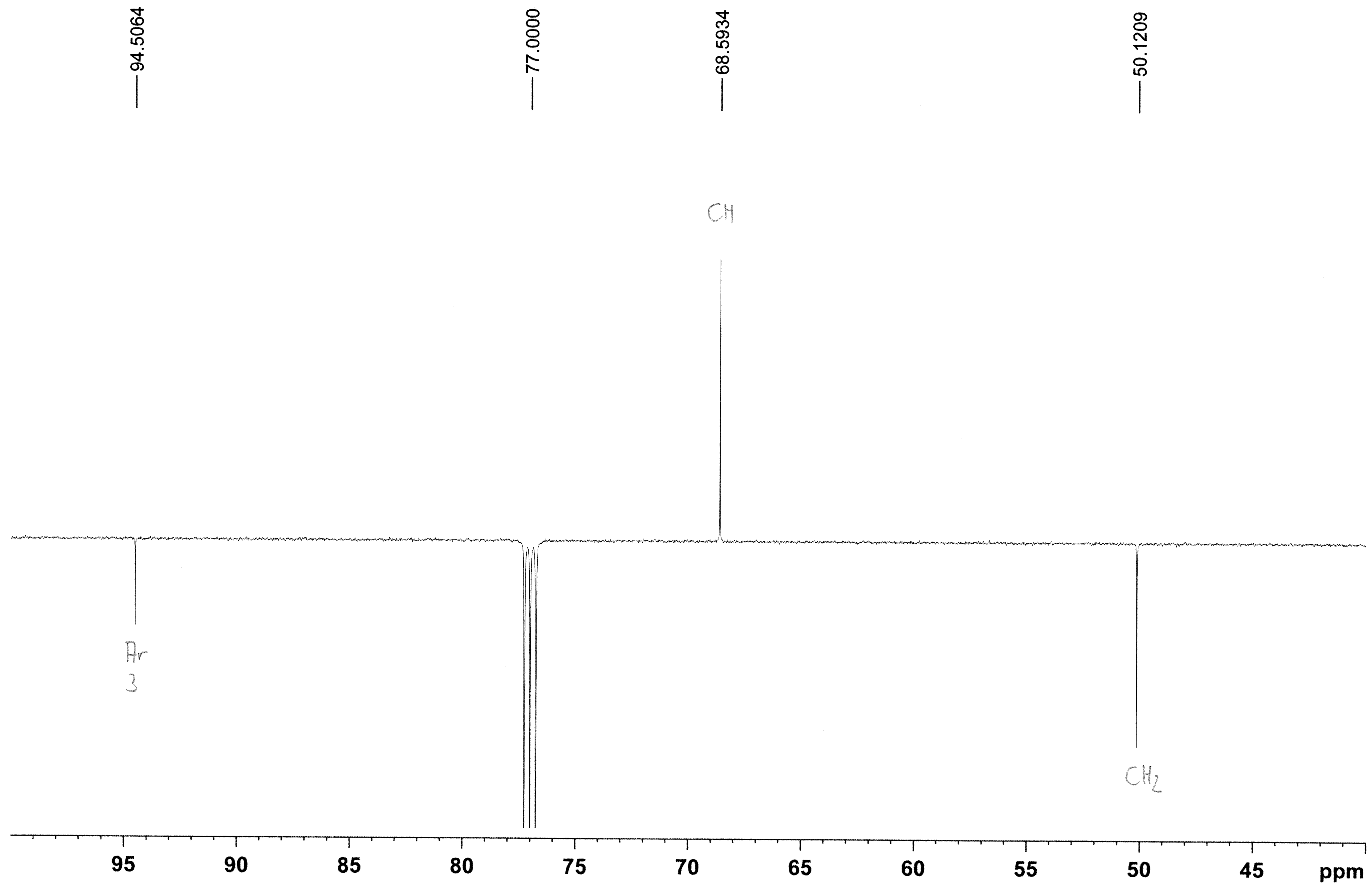

Figure S39c. NMR spectra of compound **7j.**

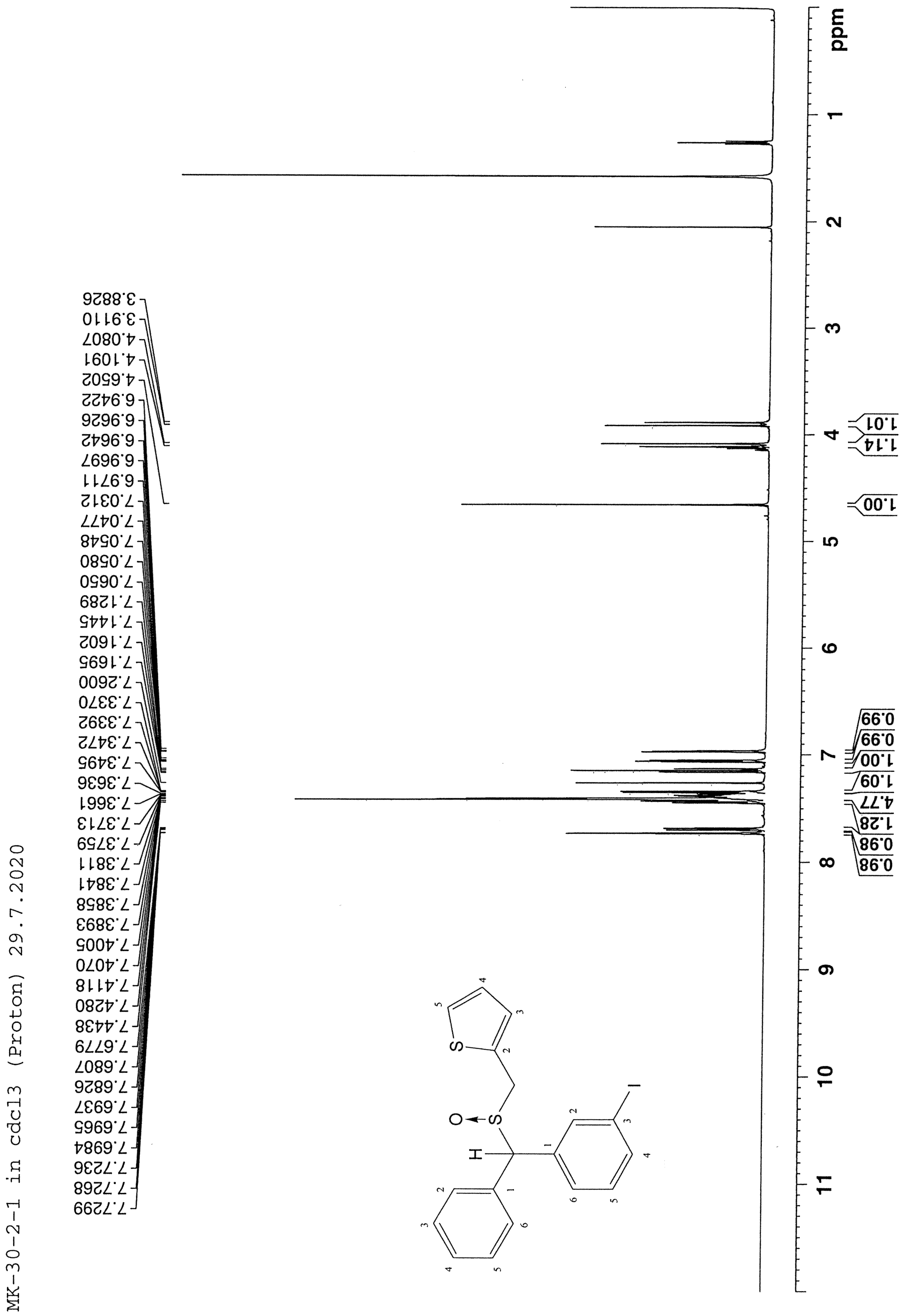

MK-30-2-1 in cdcl3 (Proton) 29.7.2020

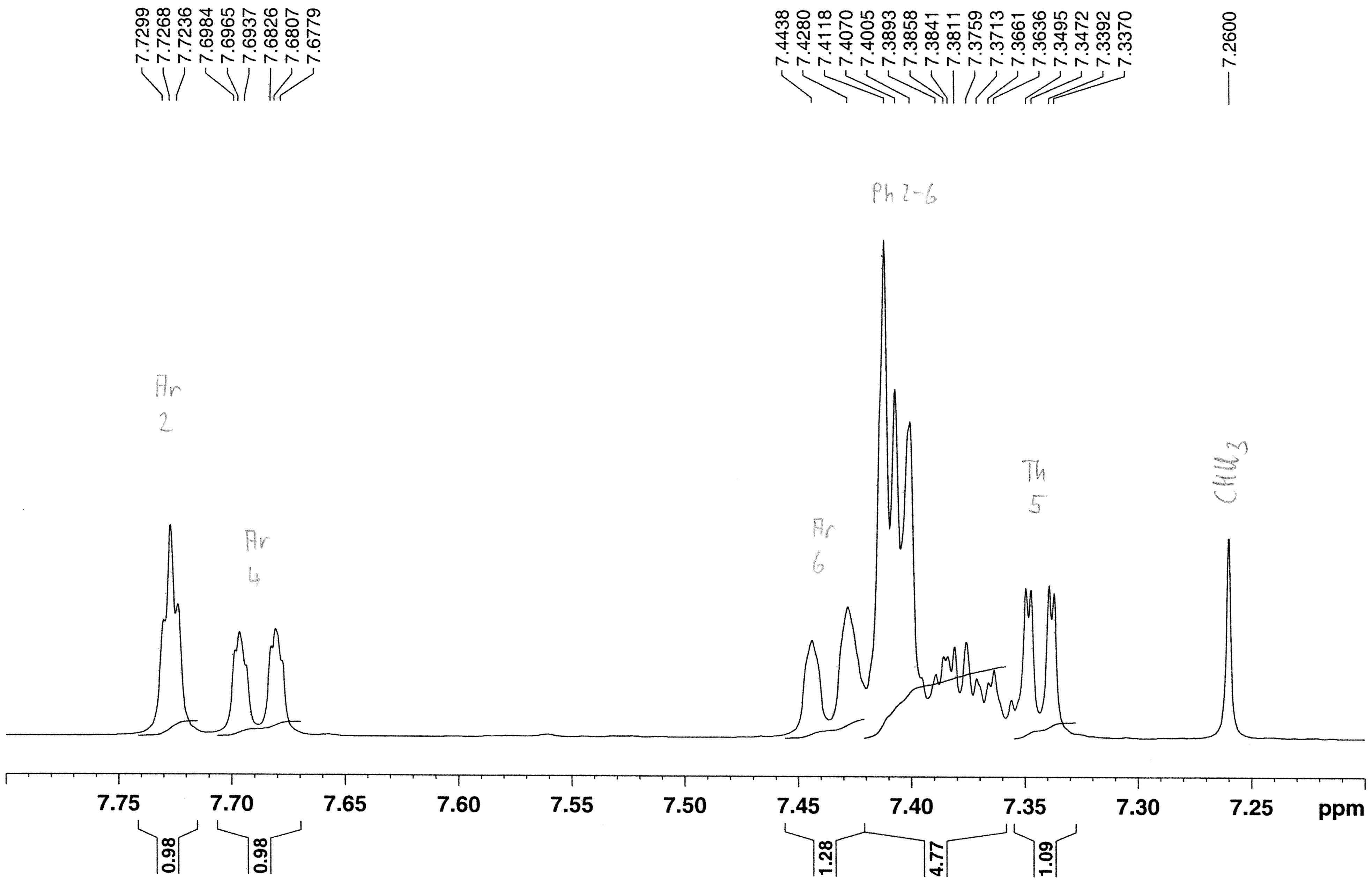

MK-30-2-1 in cdcl3 (Proton) 29.7.2020

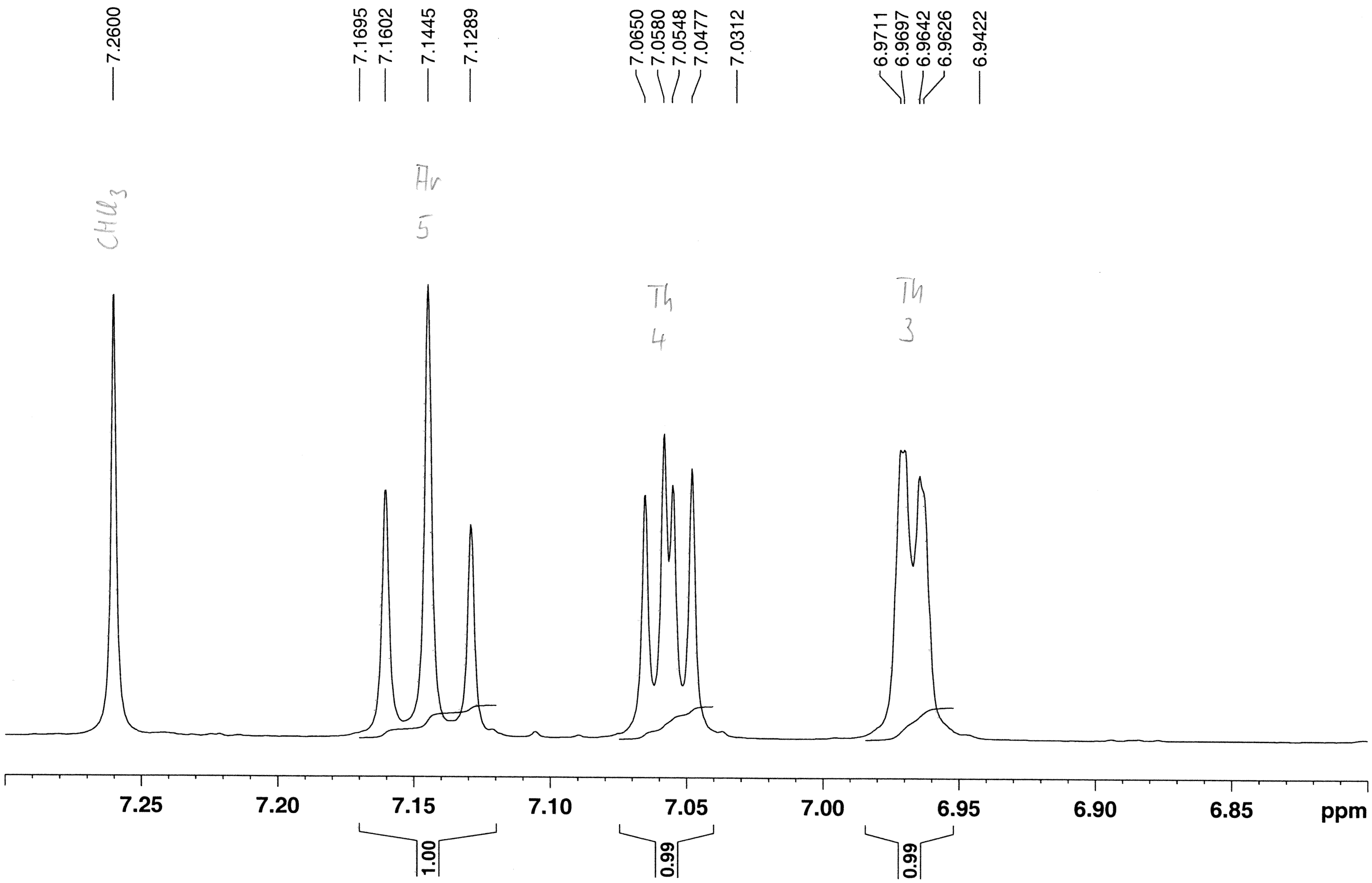

MK-30-2-1 in cdcl3 (Proton) 29.7.2020

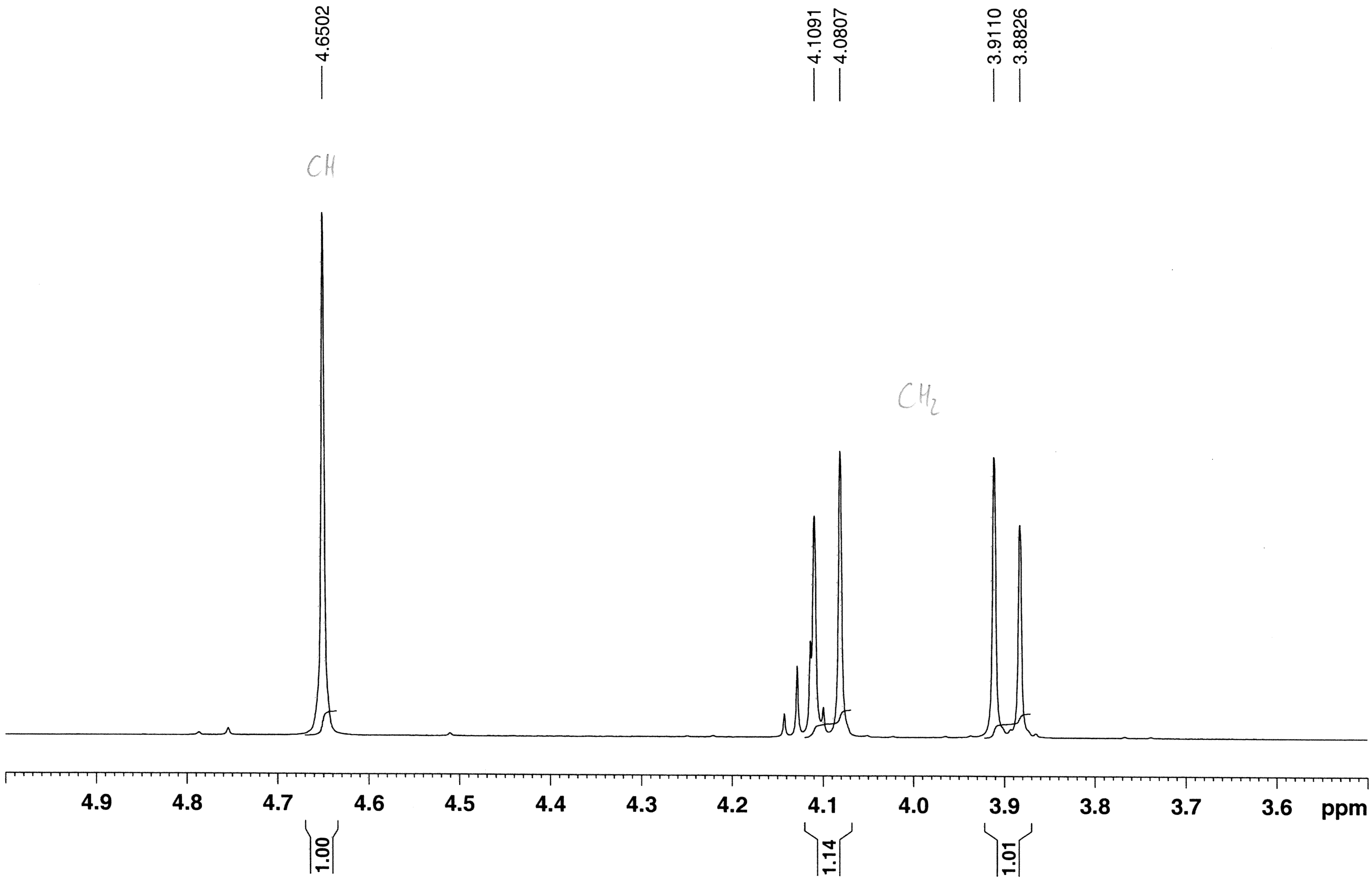

MK-30-2-1 in cdcl3 (APT) 29.7.2020

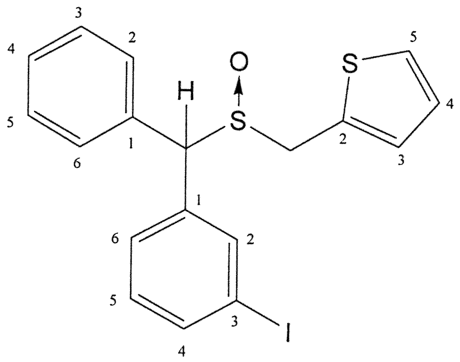

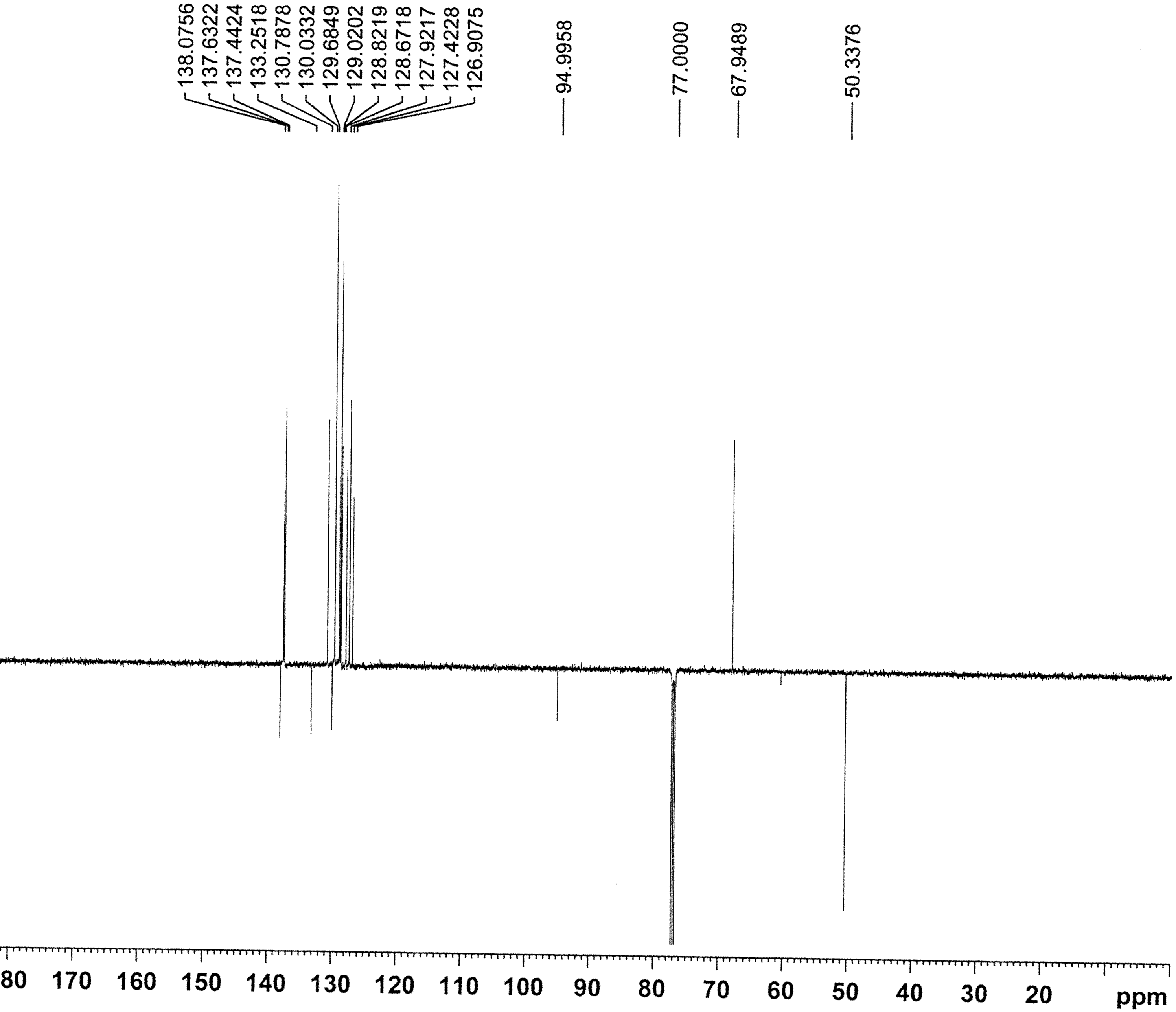

MK-30-2-1 in cdcl3 (APT) 29.7.2020

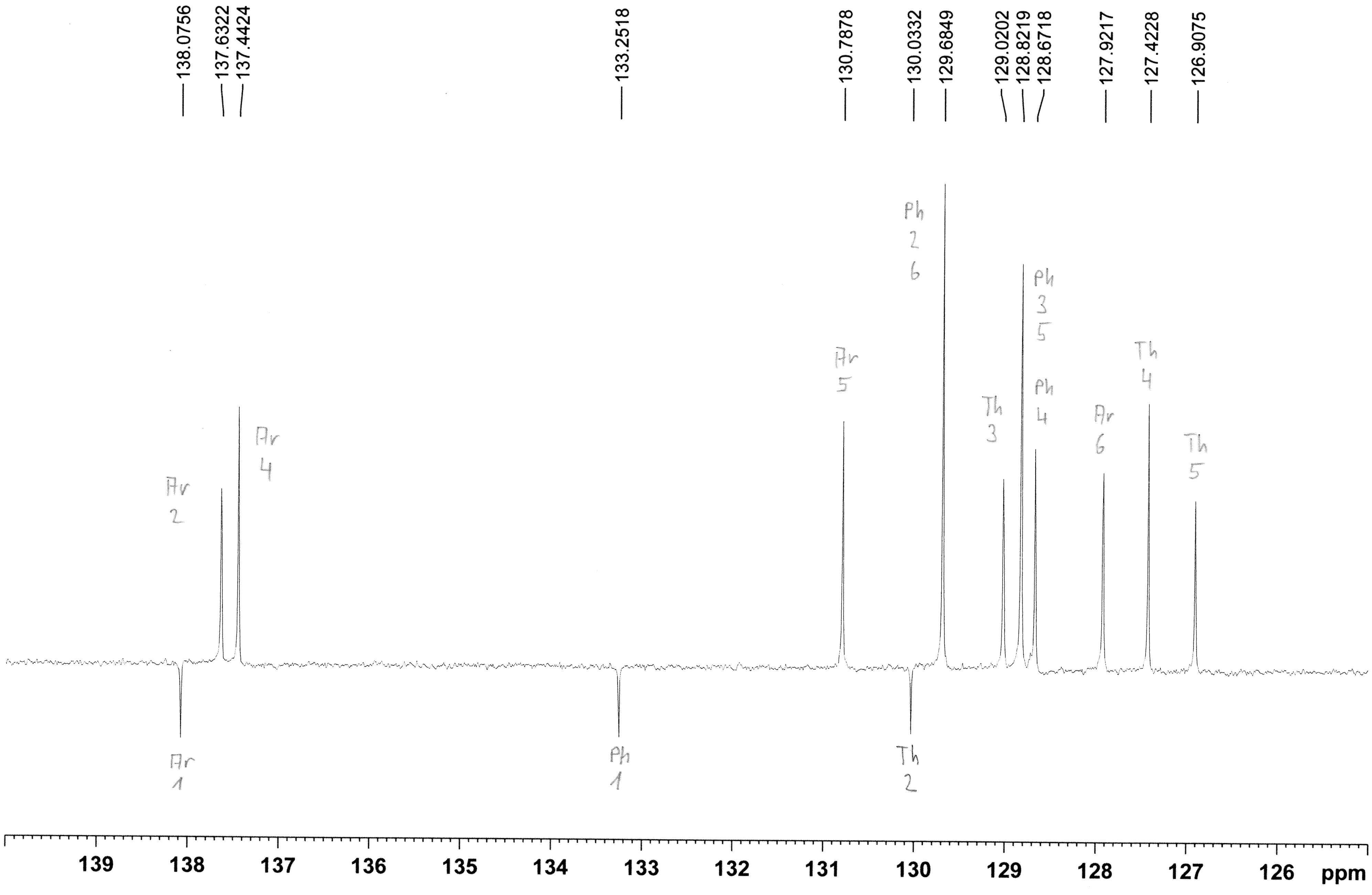

MK-30-2-1 in cdcl3 (APT) 29.7.2020

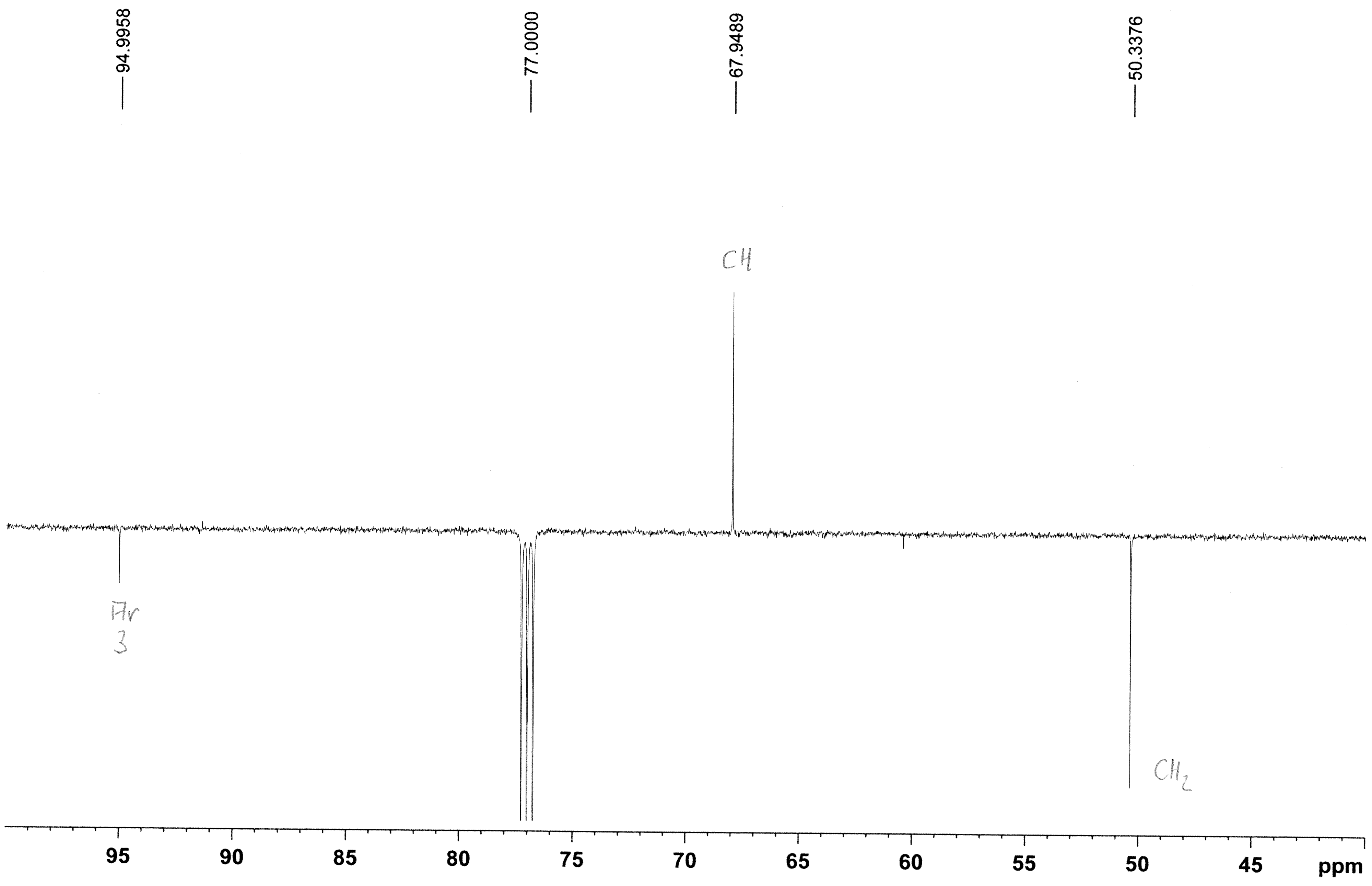

MK-30-2-1 in cdcl3 (COSY) 29.7.2020

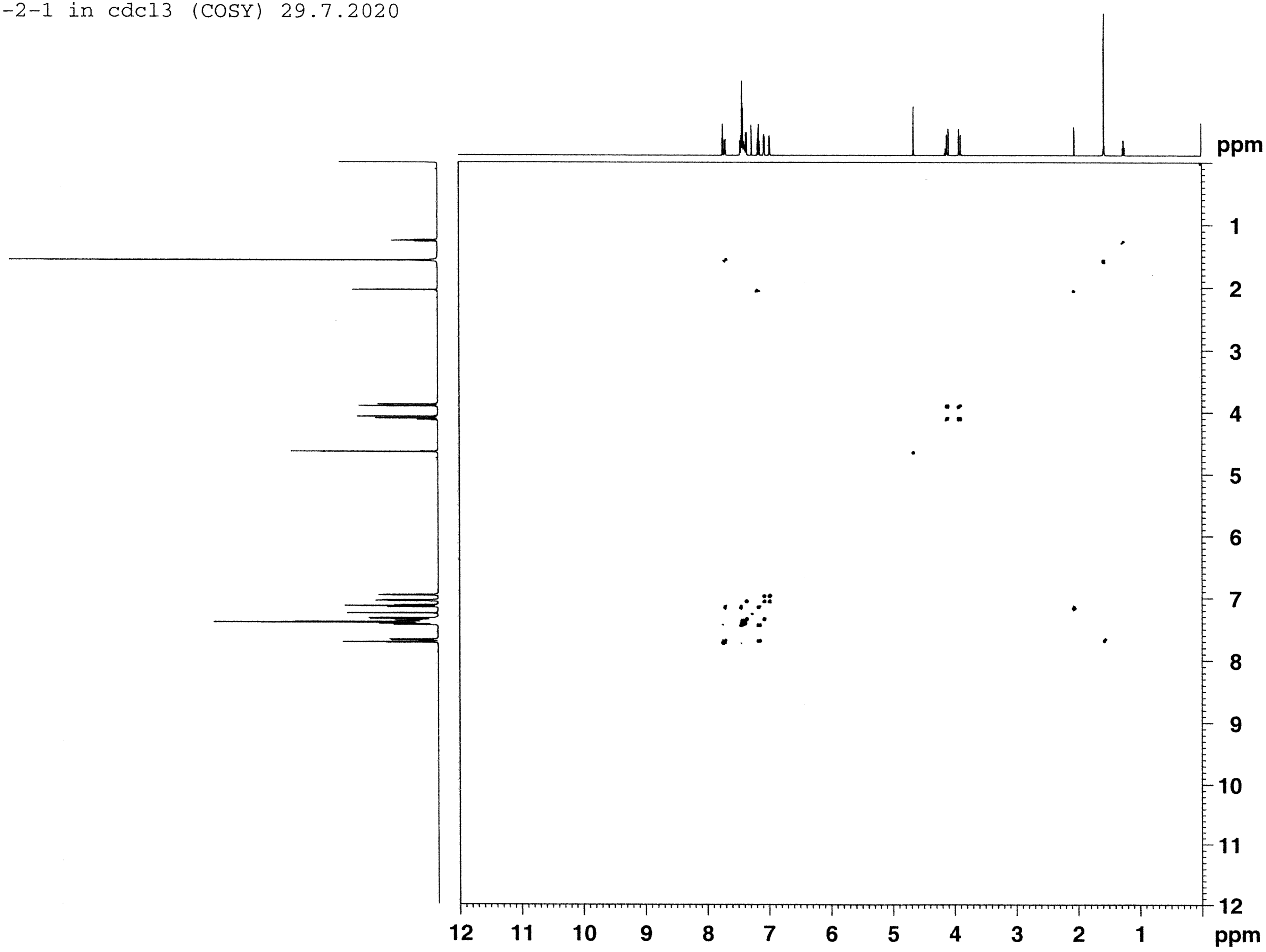

MK-30-2-1 in cdcl3 (COSY) 29.7.2020

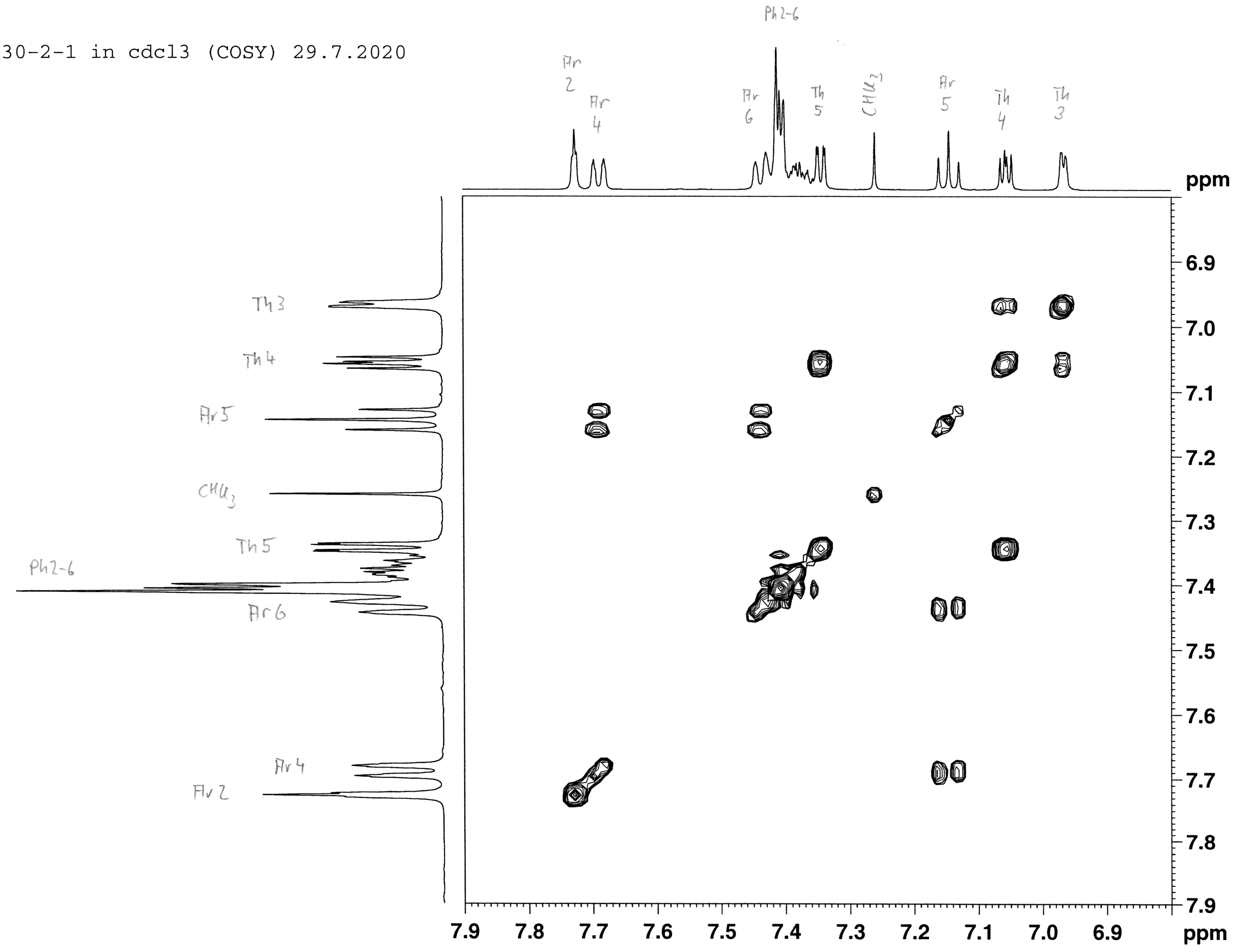

MK-30-2-1 in cdcl3 (COSY) 29.7.2020

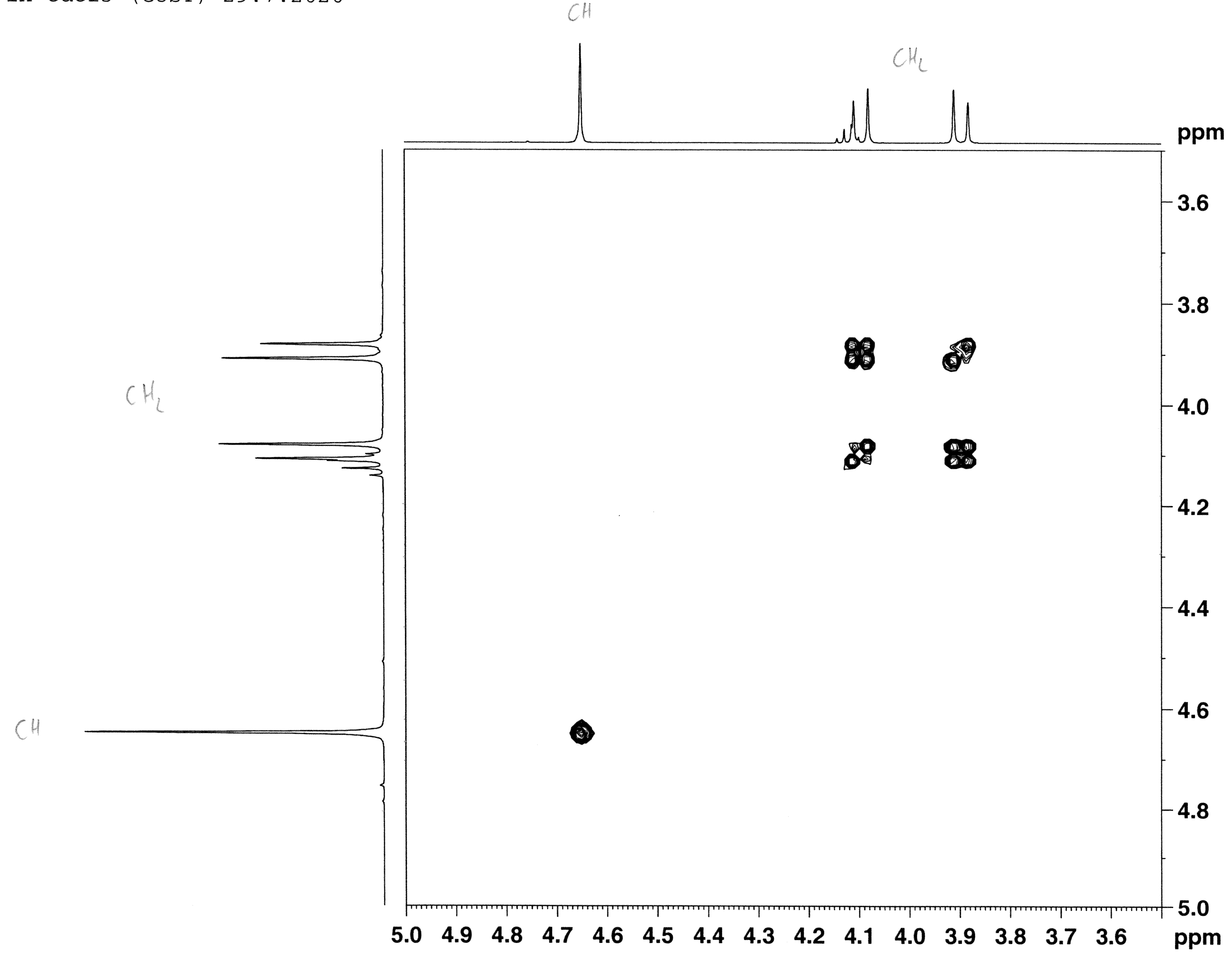

MK-30-2-1 in cdcl3 (HSQC) 29.7.2020

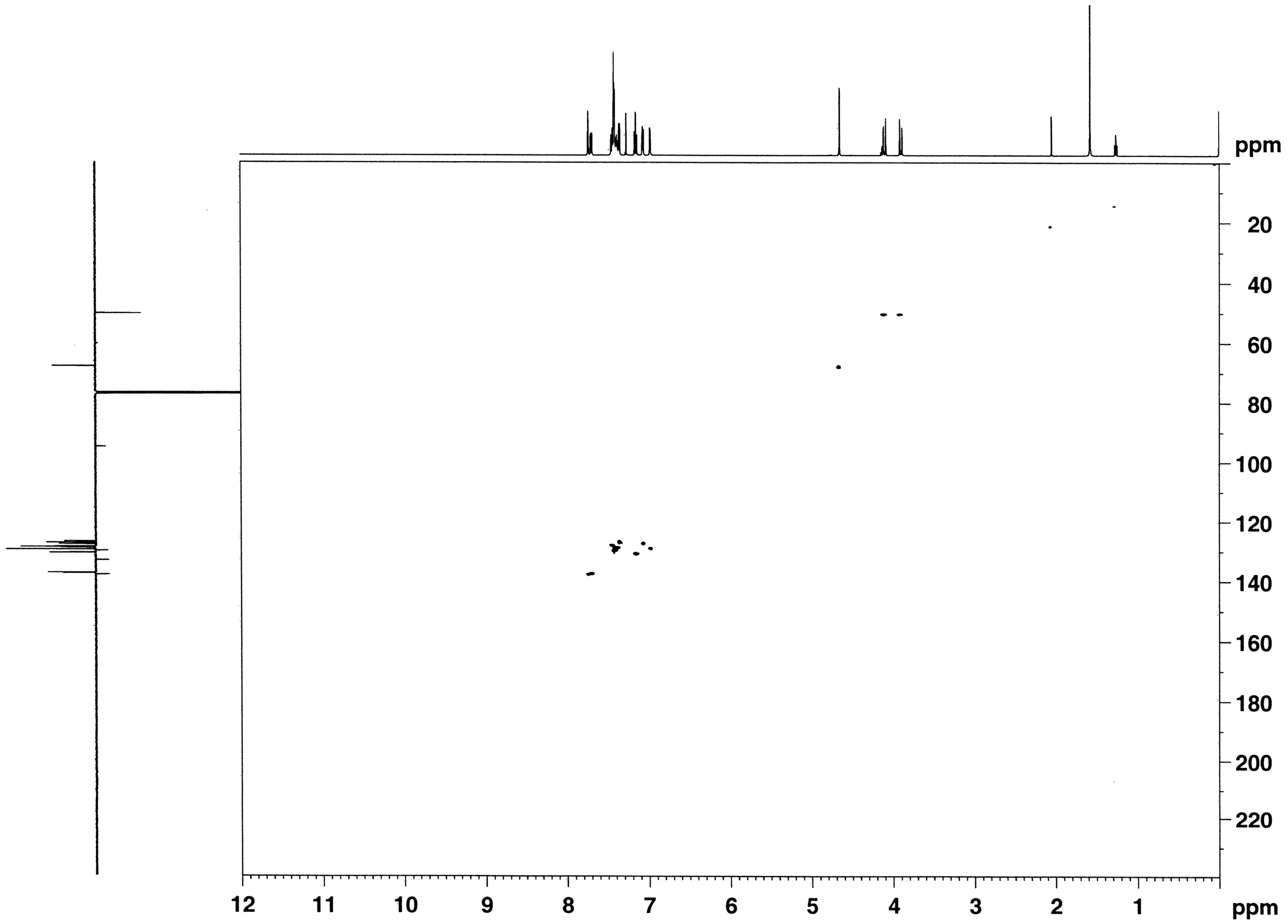

MK-30-2-1 in cdcl3 (HSQC) 29.7.2020

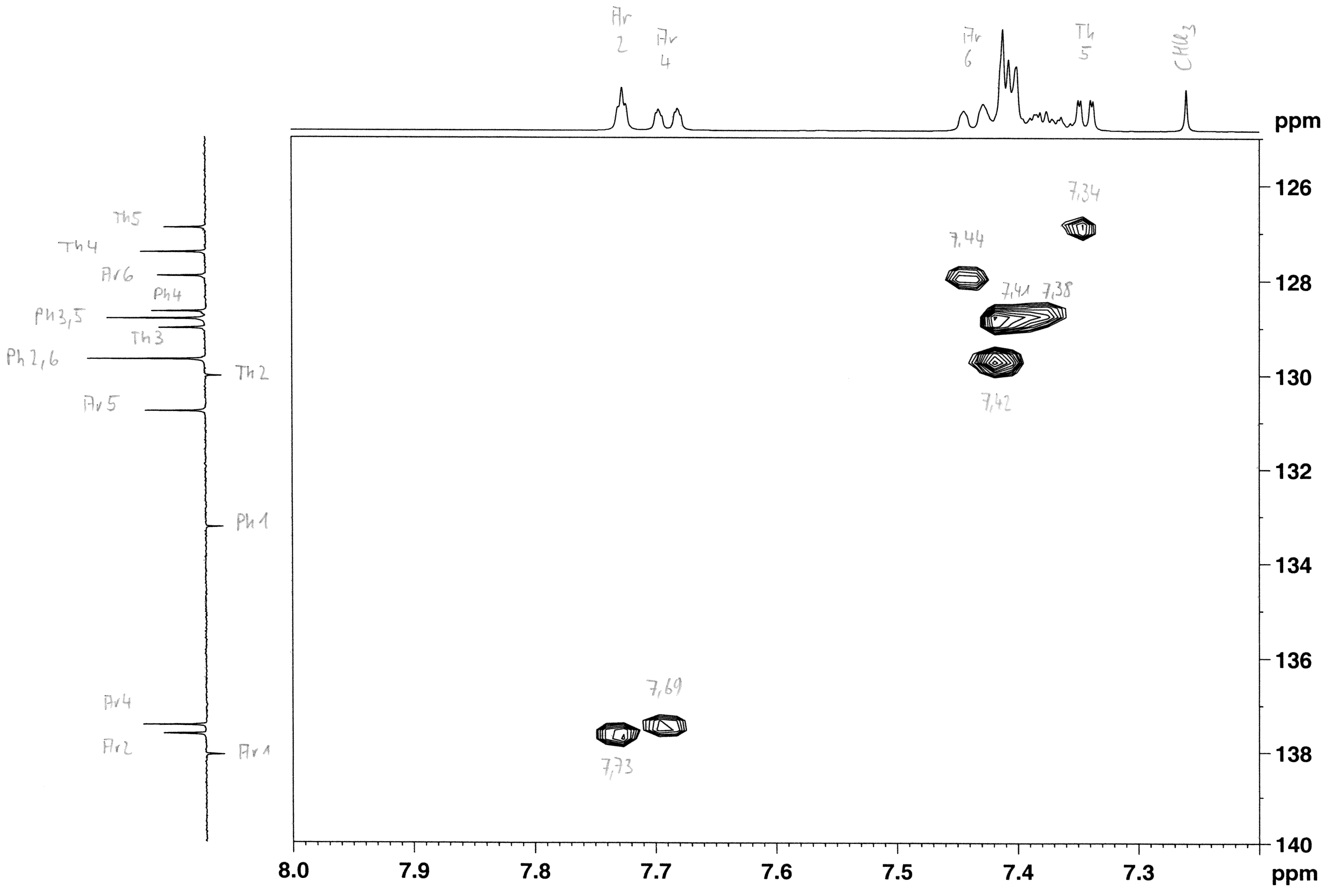

MK-30-2-1 in cdcl3 (HSQC) 29.7.2020

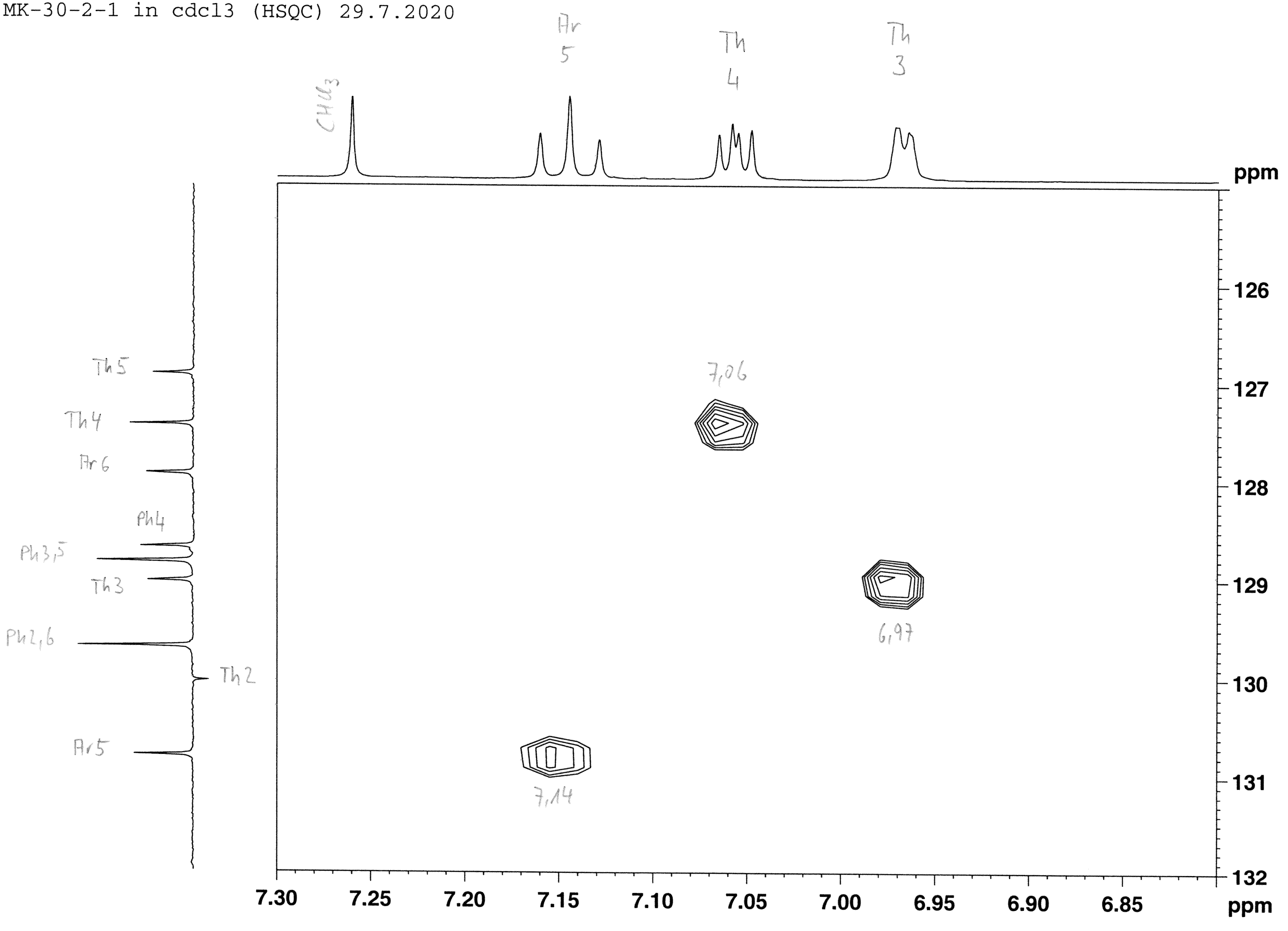

MK-30-2-1 in cdcl3 (HSQC) 29.7.2020

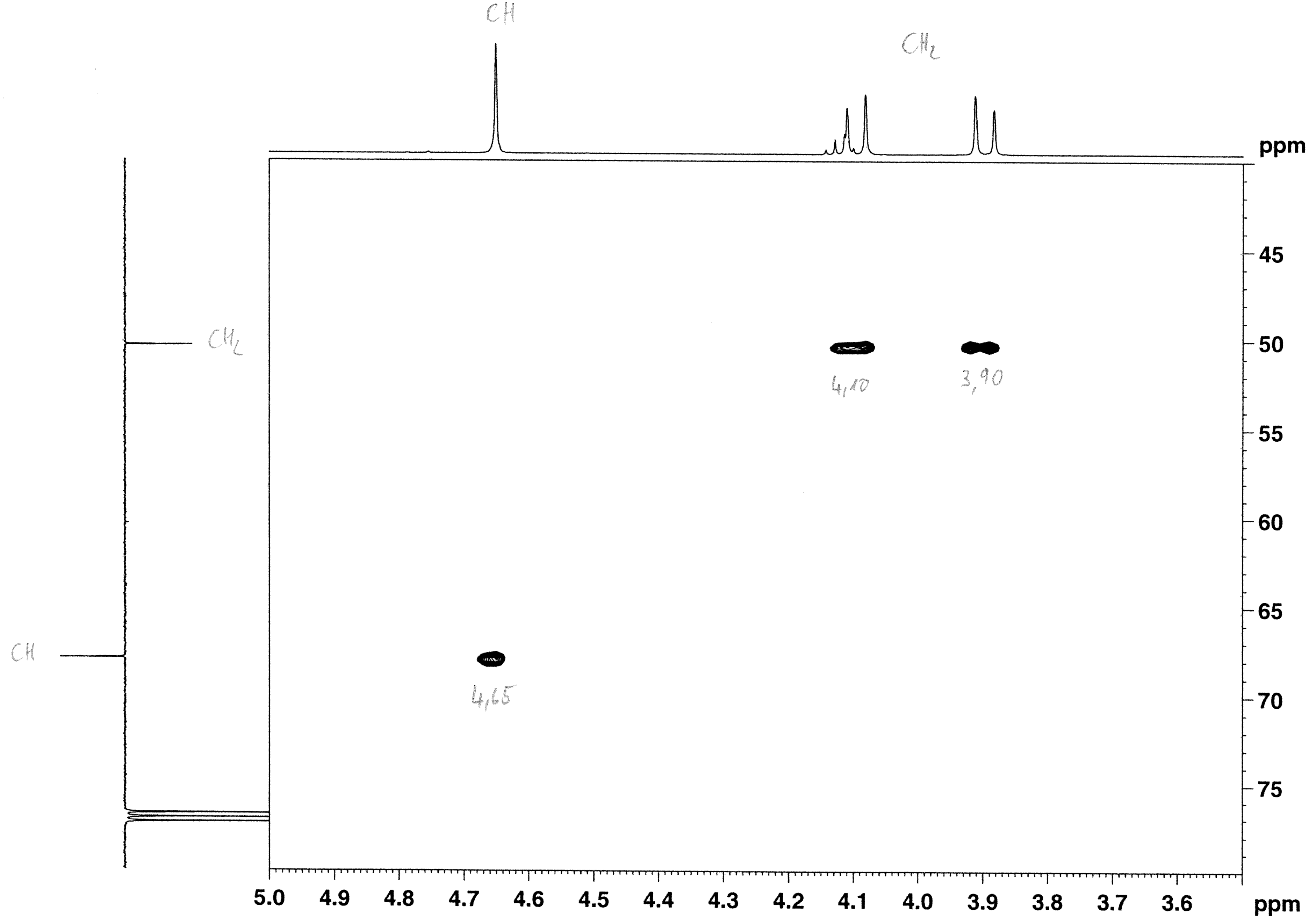

MK-30-2-1 in cdcl3 (HMBC) 29.7.2020

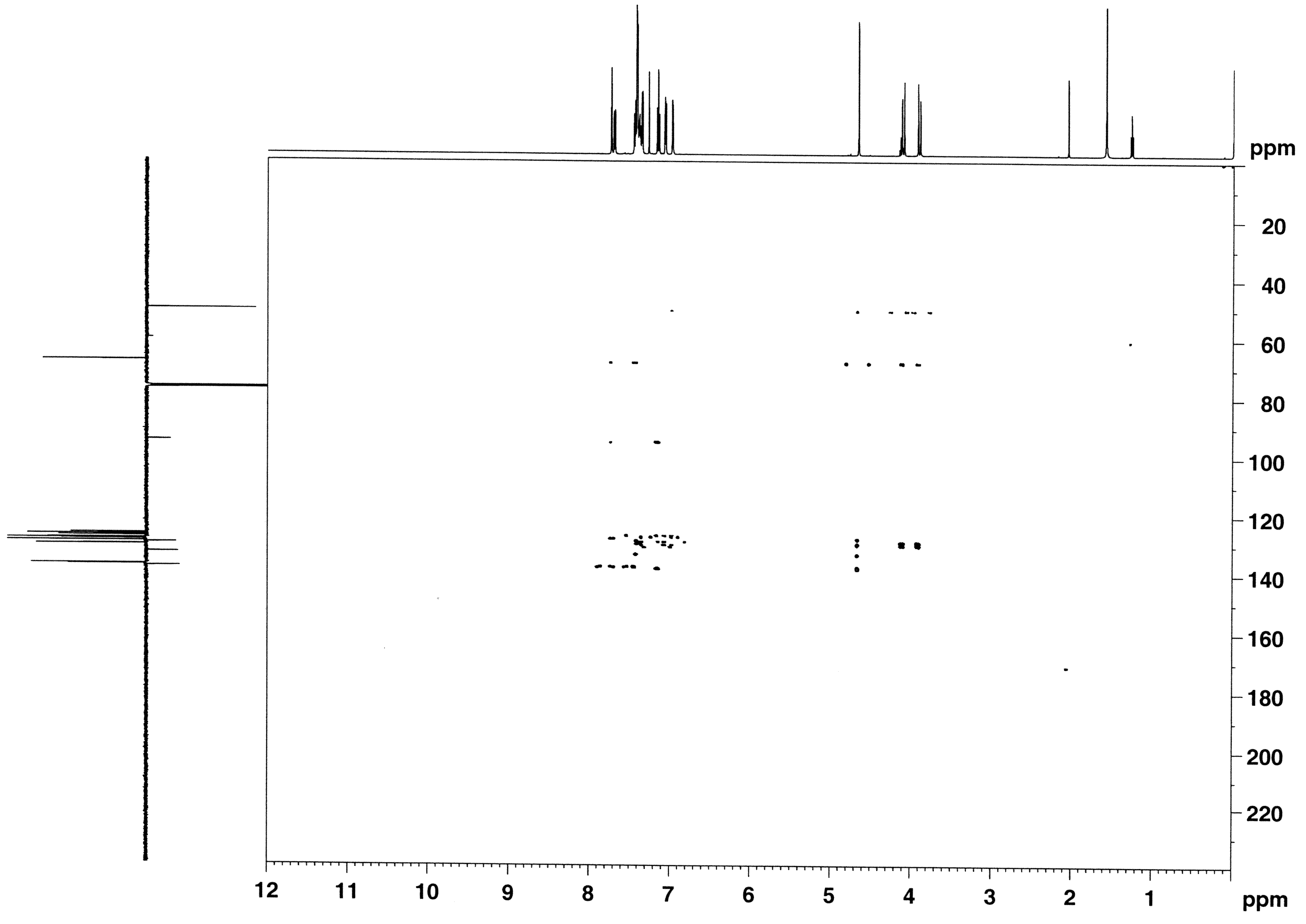

MK-30-2-1 in cdcl3 (HMBC) 29.7.2020

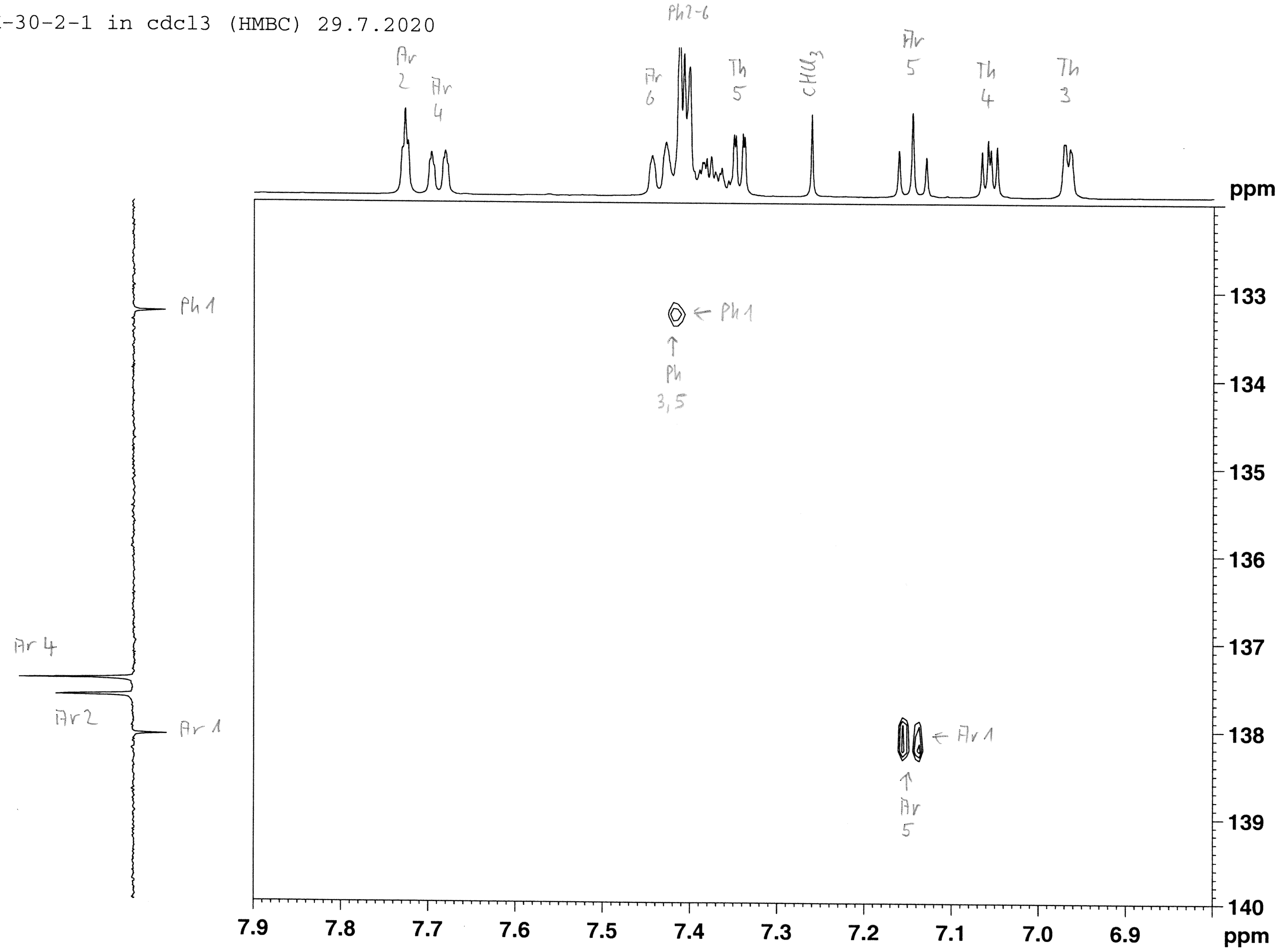

MK-30-2-1 in cdcl3 (HMBC) 29.7.2020

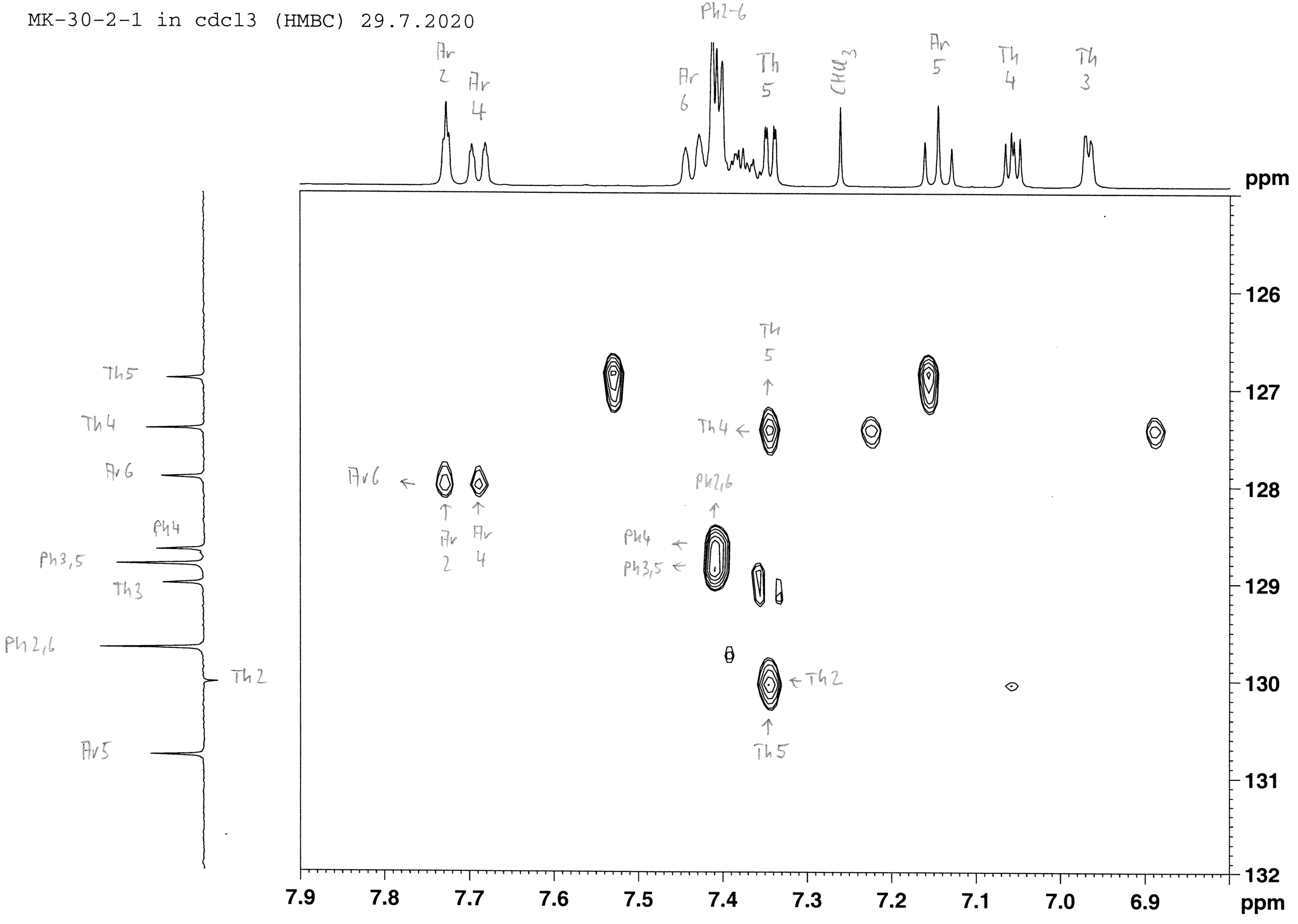

MK-30-2-1 in cdcl3 (HMBC) 29.7.2020

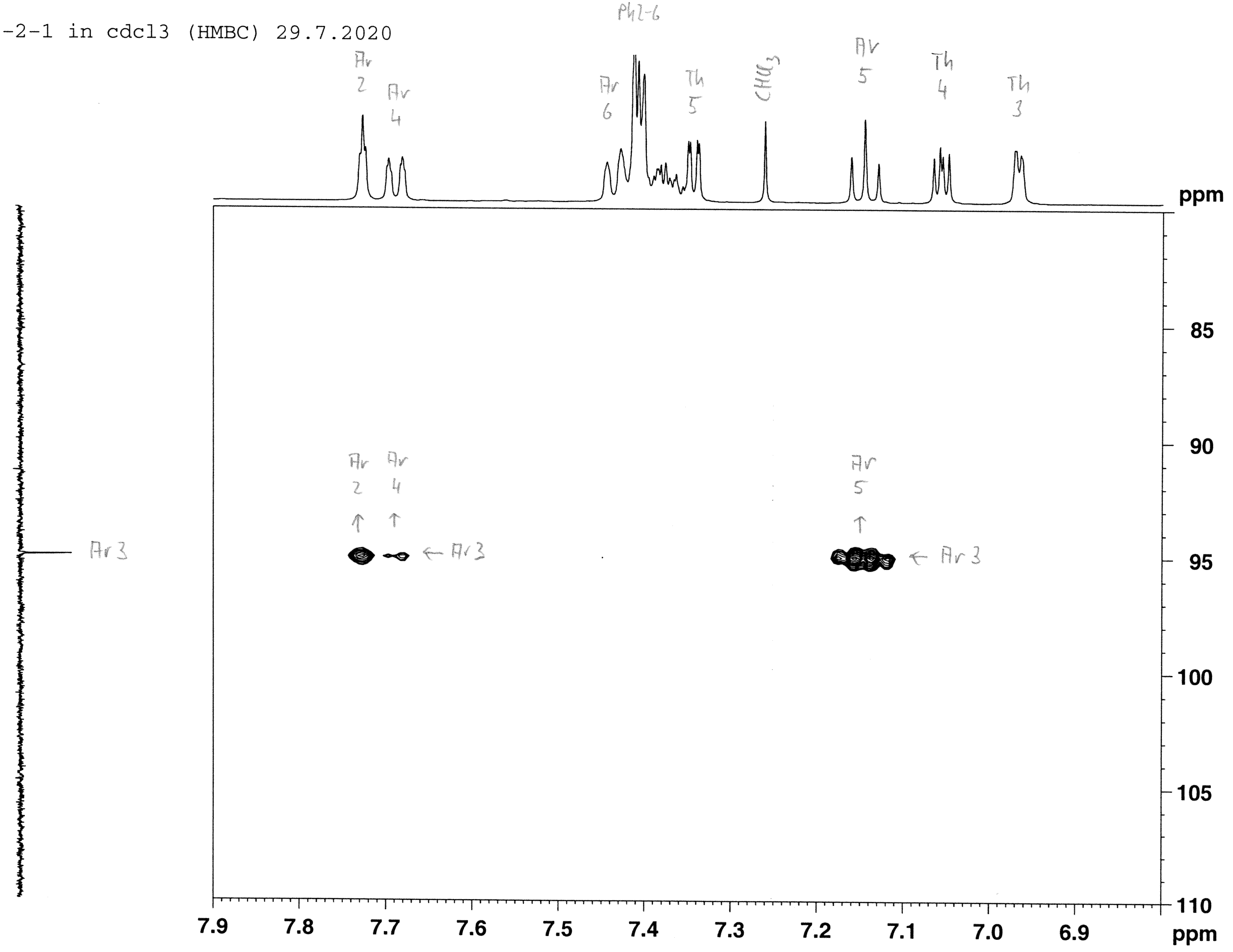

MK-30-2-1 in cdcl3 (HMBC) 29.7.2020

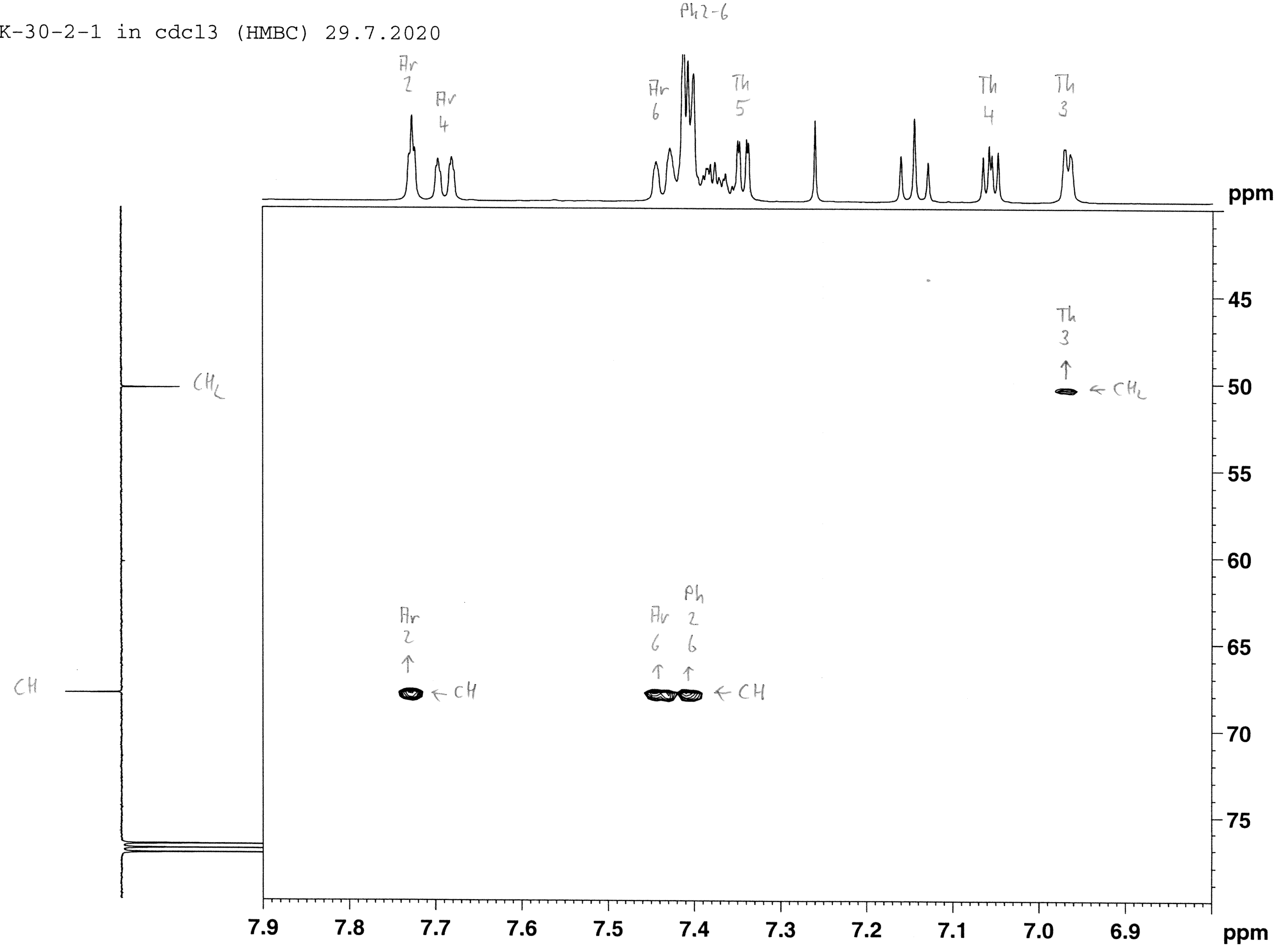

MK-30-2-1 in cdcl3 (HMBC) 29.7.2020

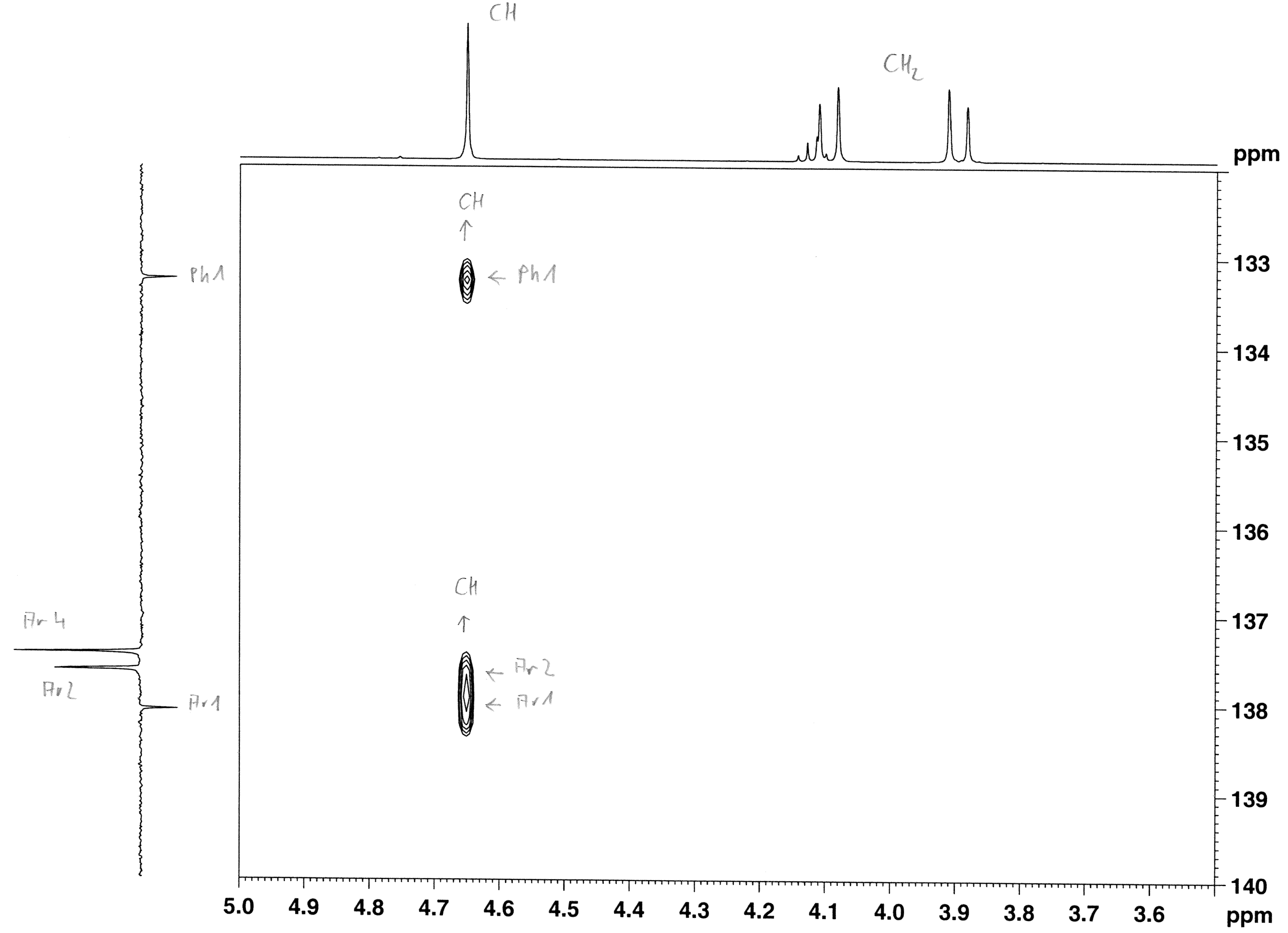

MK-30-2-1 in cdcl3 (HMBC) 29.7.2020

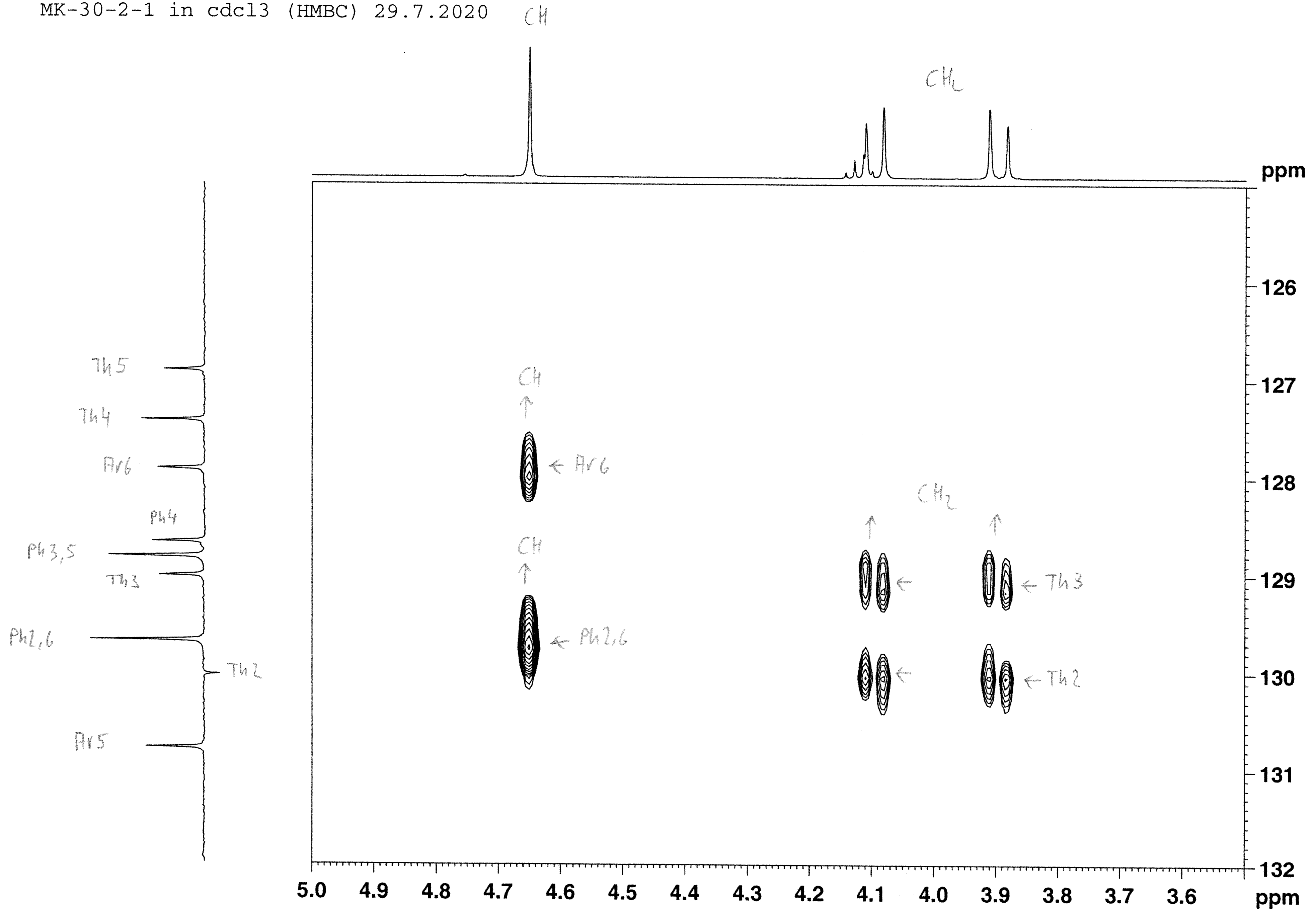

MK-30-2-1 in cdcl3 (HMBC) 29.7.2020

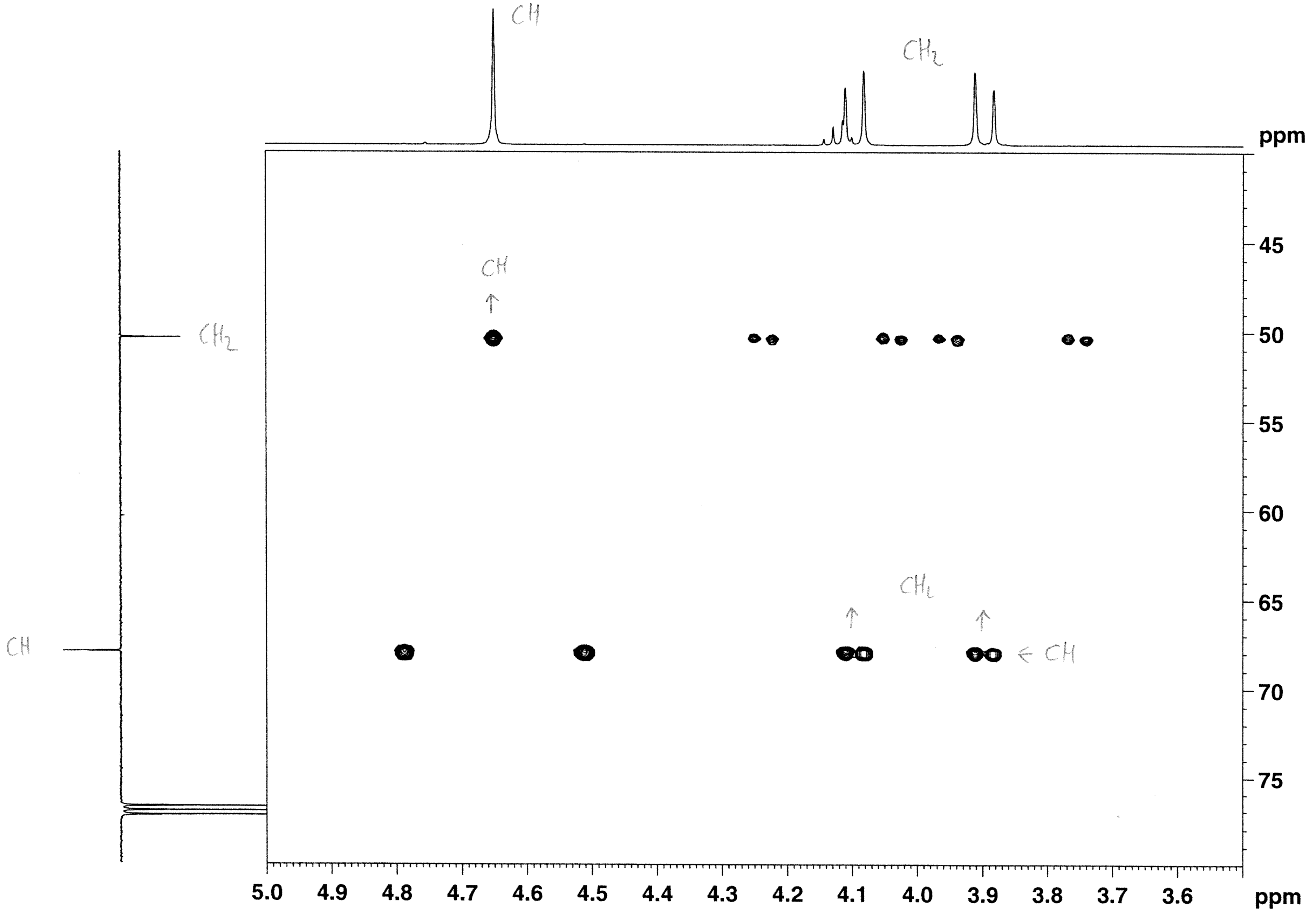

Figure S40c. NMR spectra of compound **8j**.

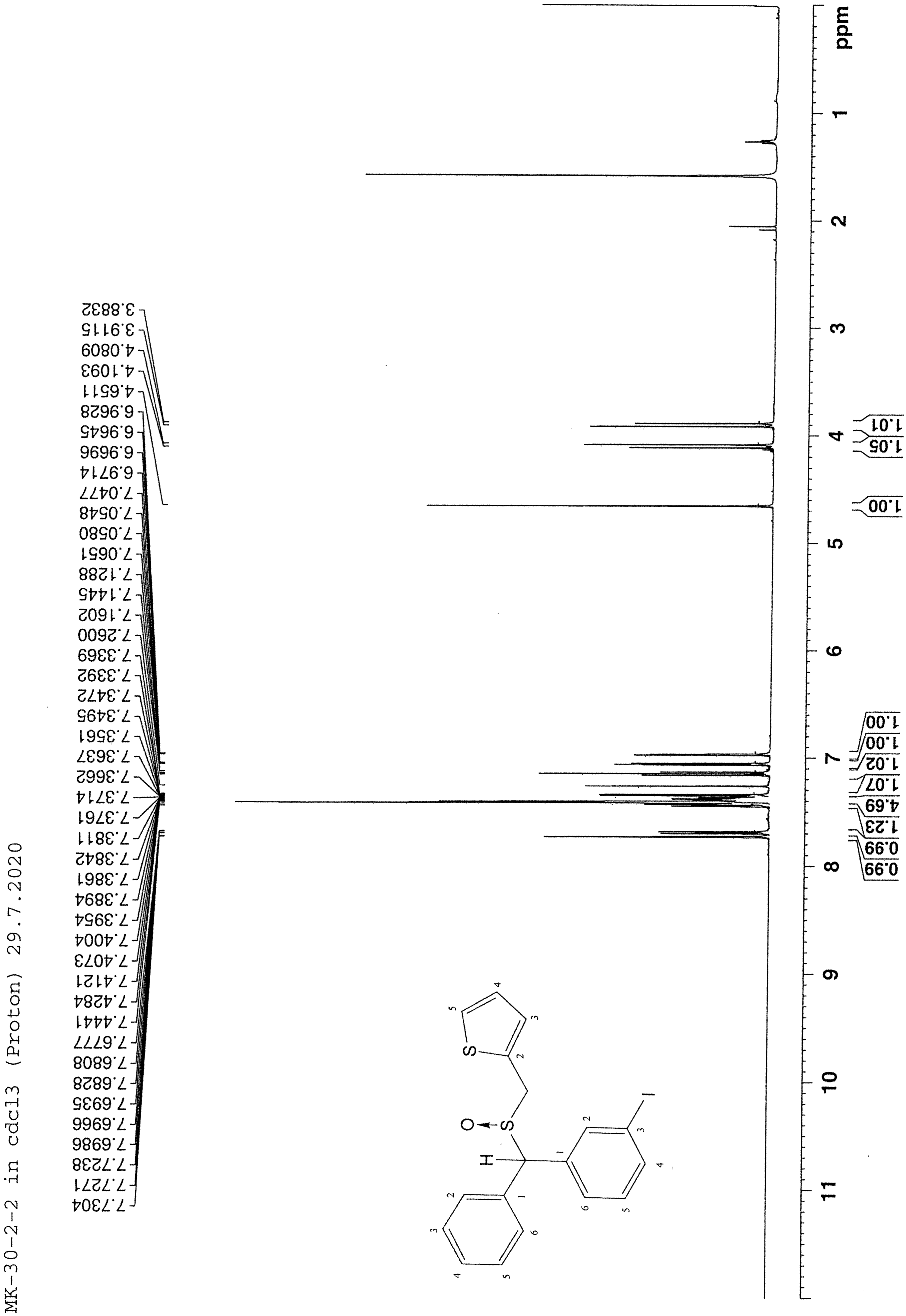

MK-30-2-2 in cdcl3 (Proton) 29.7.2020

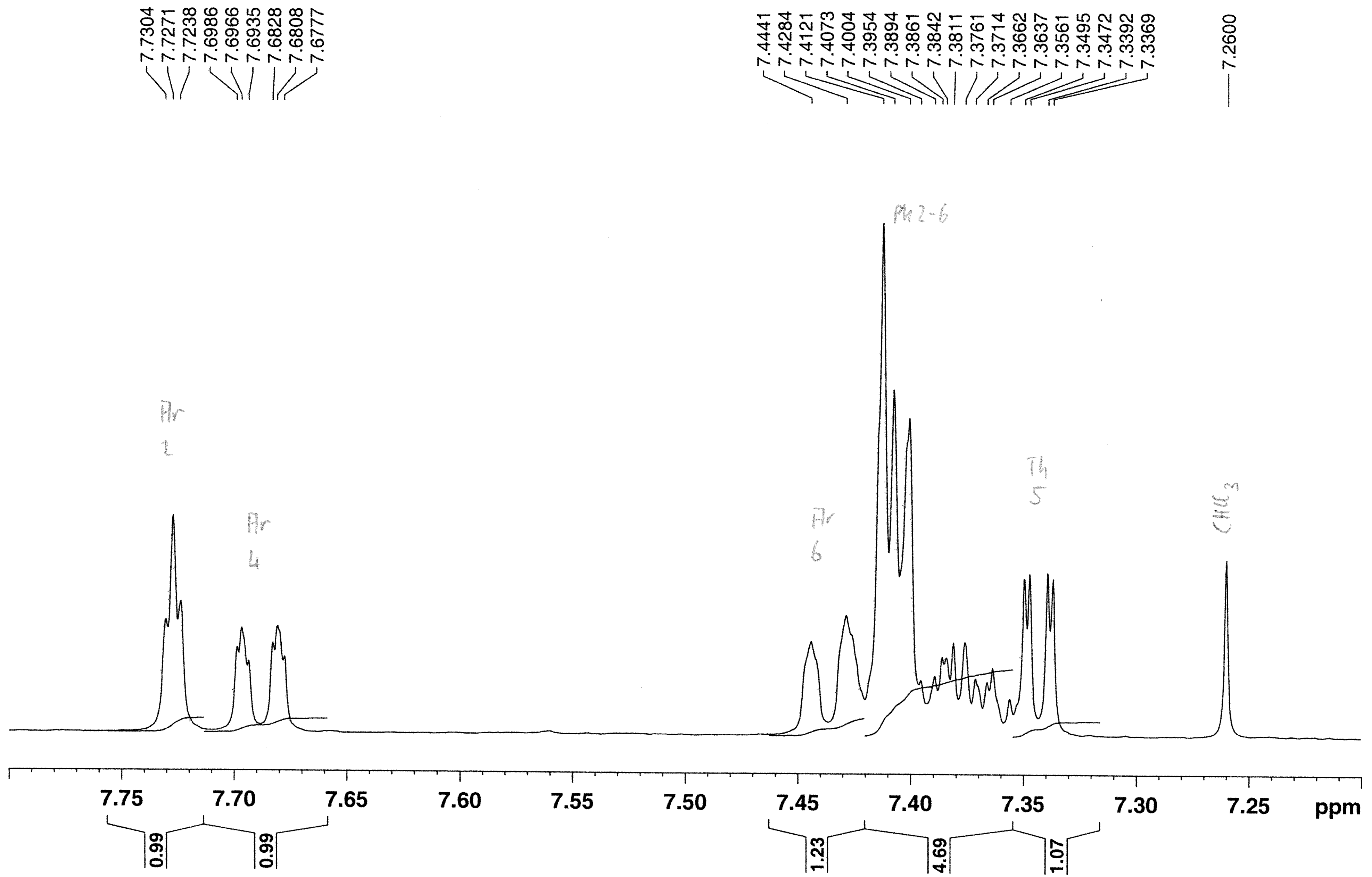

MK-30-2-2 in cdcl3 (Proton) 29.7.2020

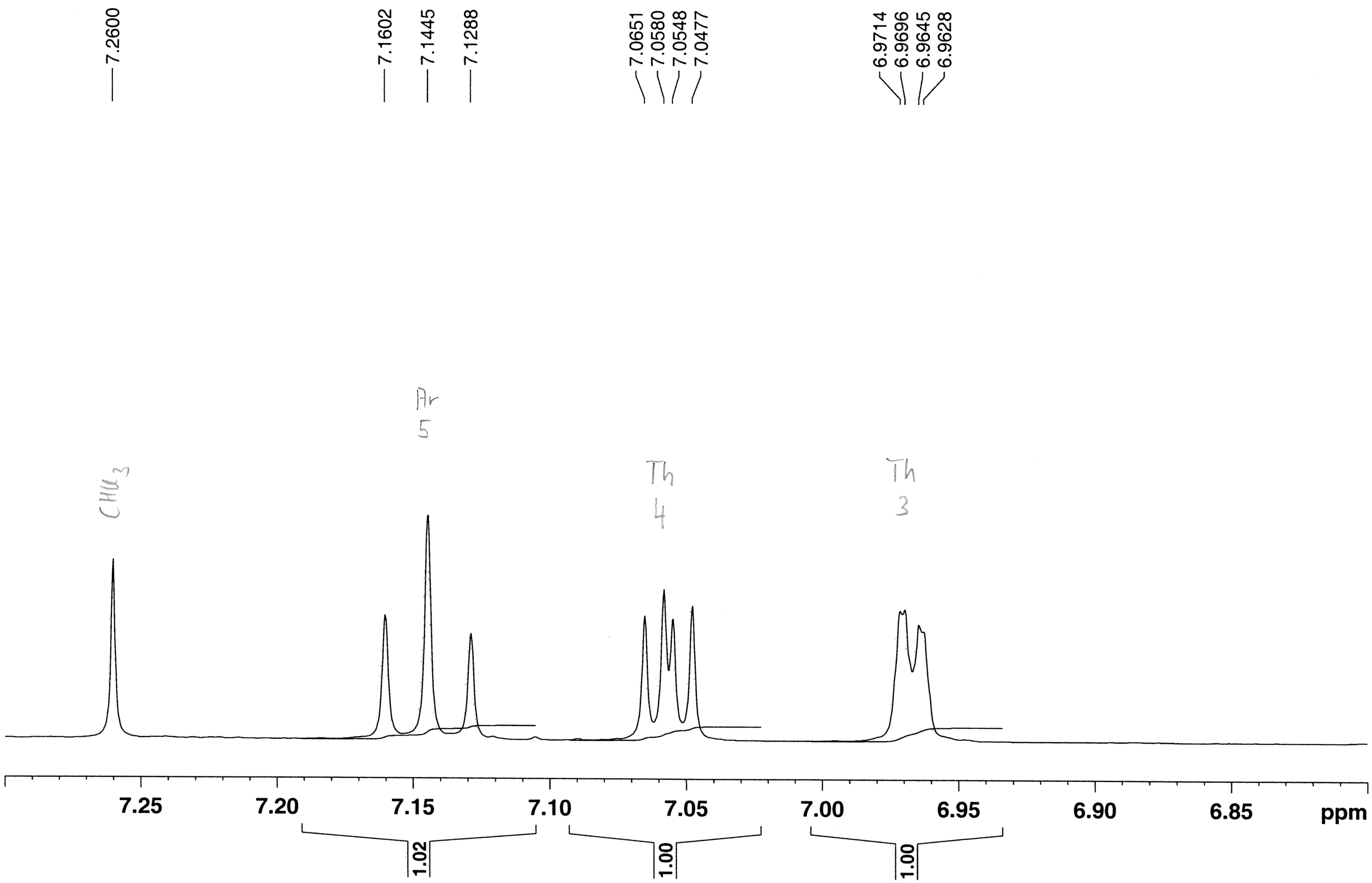

MK-30-2-2 in cdcl3 (Proton) 29.7.2020

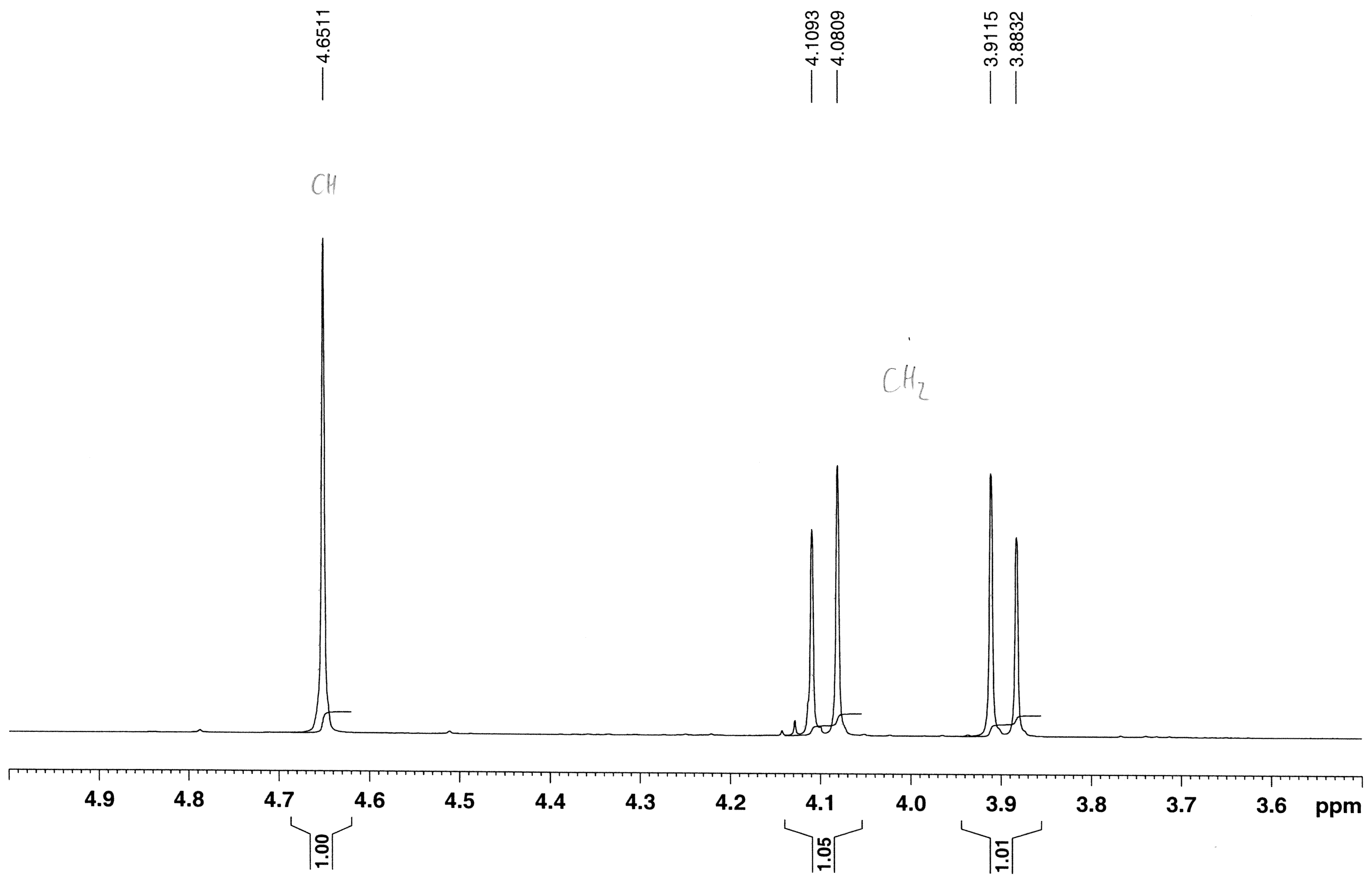

MK-30-2-2 in cdcl3 (APT) 29.7.2020

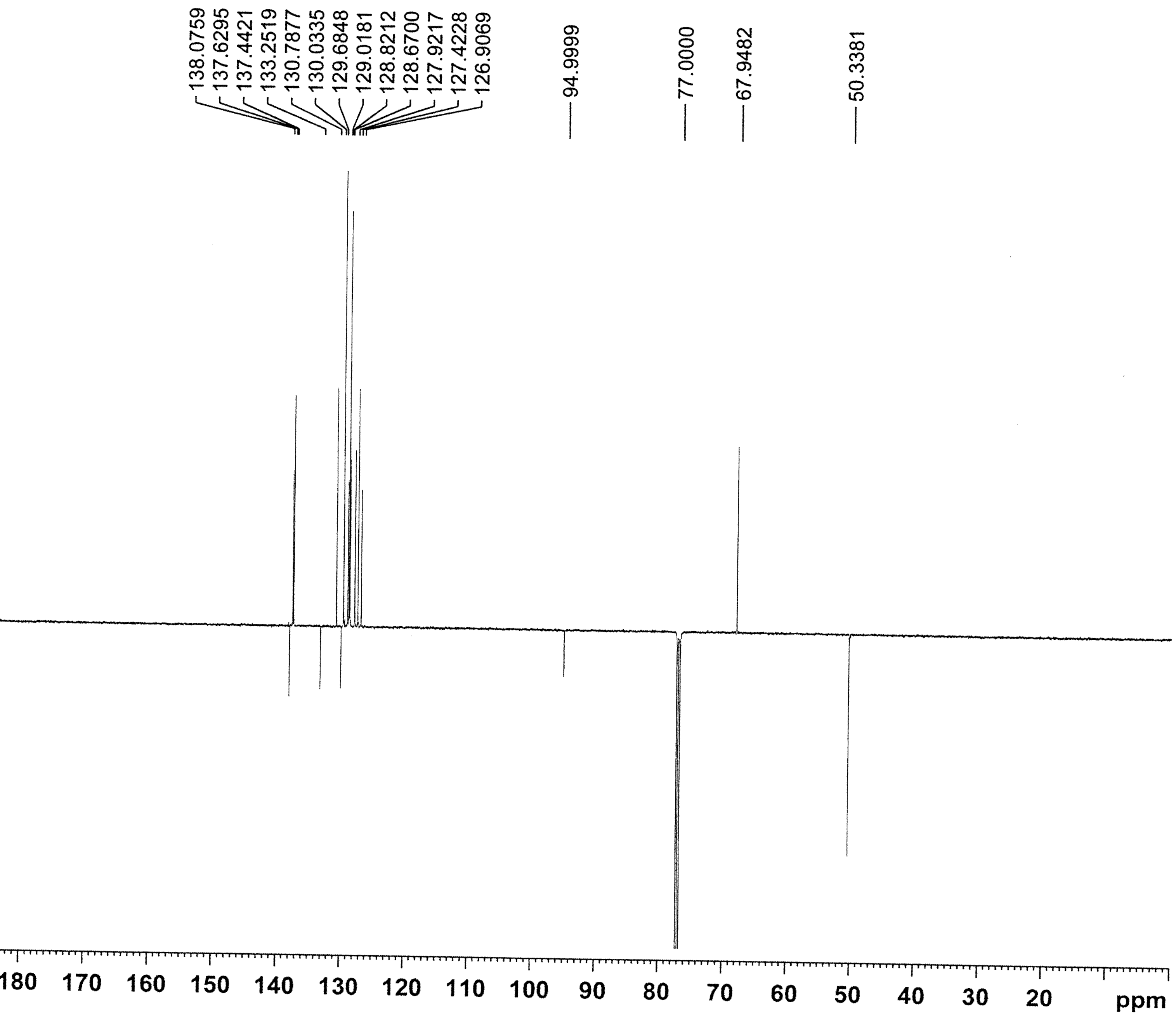

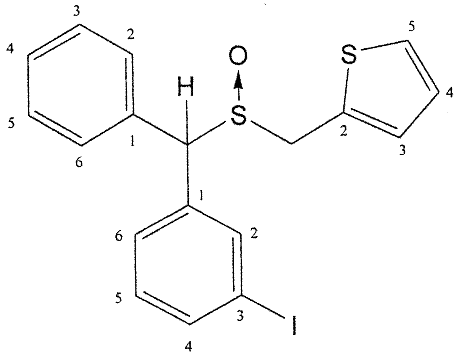

MK-30-2-2 in cdcl3 (APT) 29.7.2020

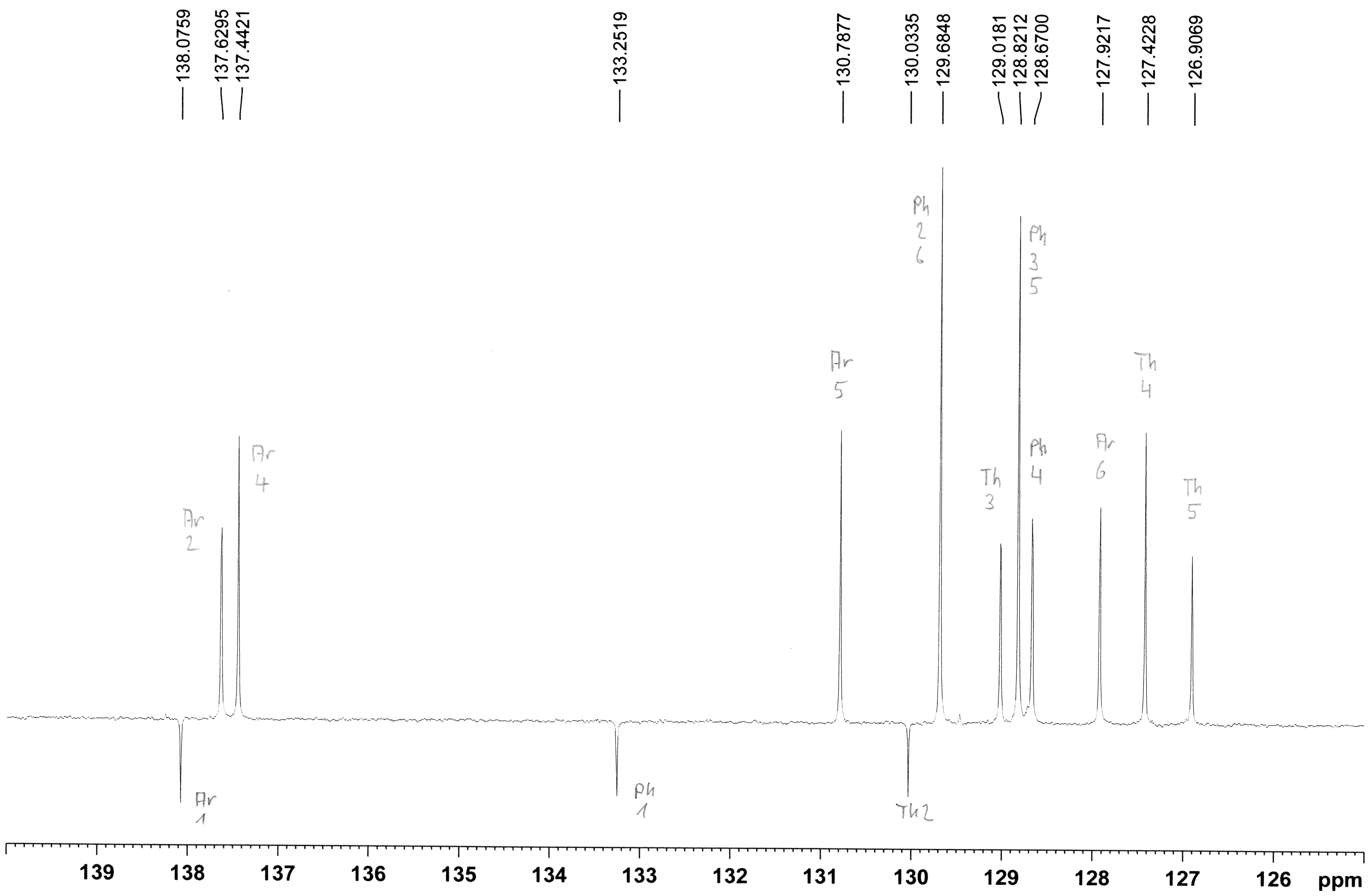

MK-30-2-2 in cdcl3 (APT) 29.7.2020

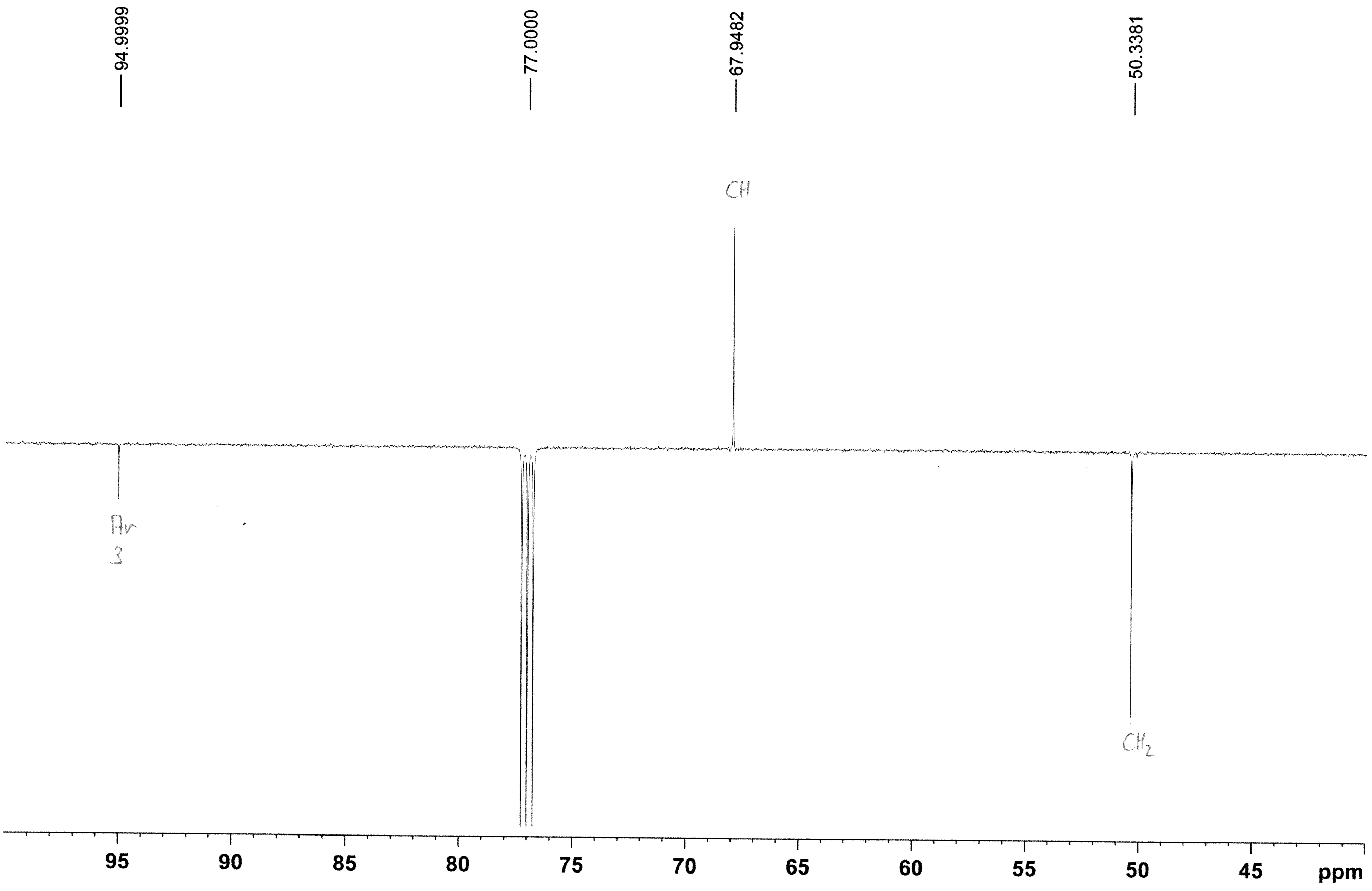

Figure S41c. NMR spectra of compound **5k.**

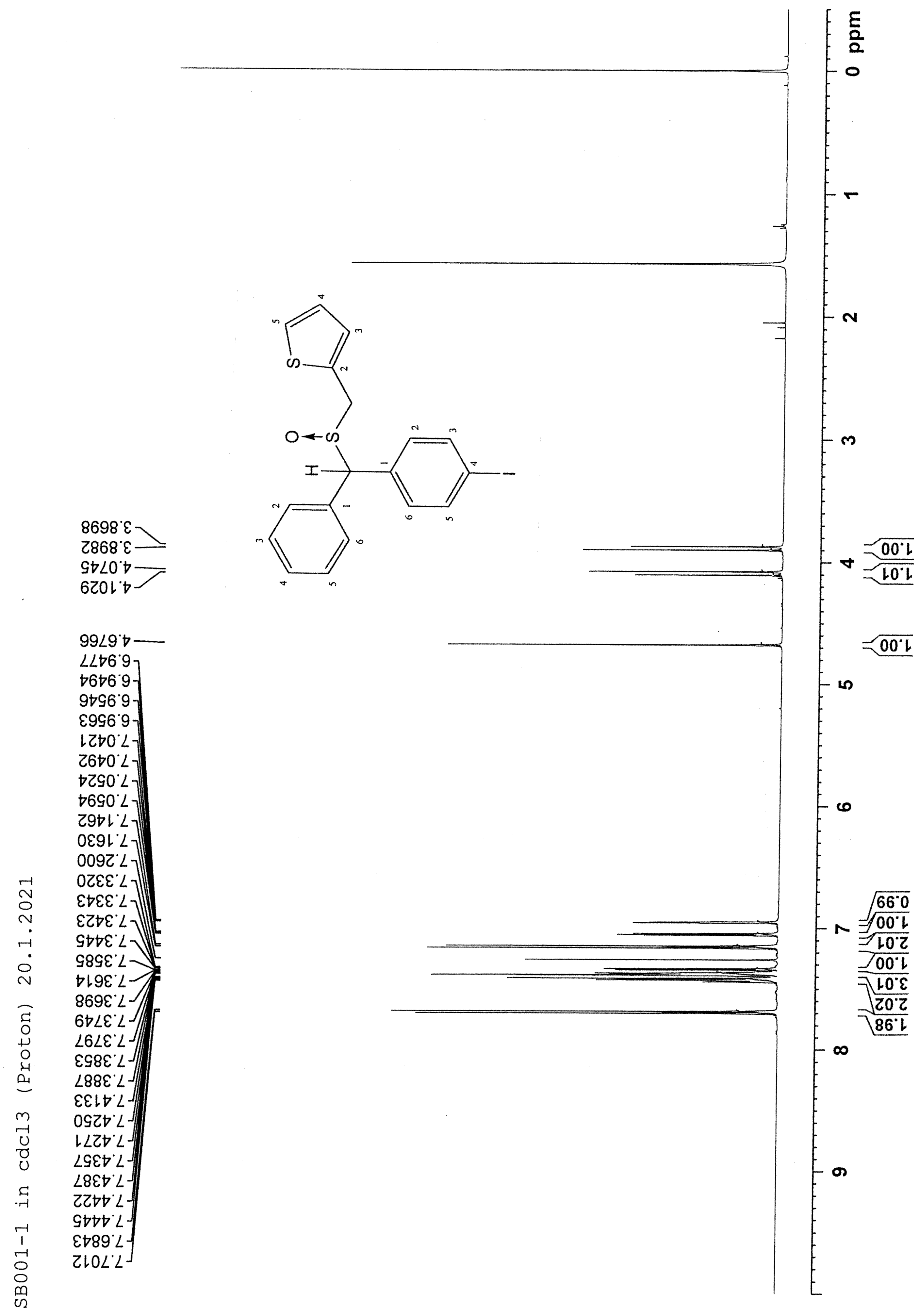

SB001-1 in cdcl3 (Proton) 20.1.2021

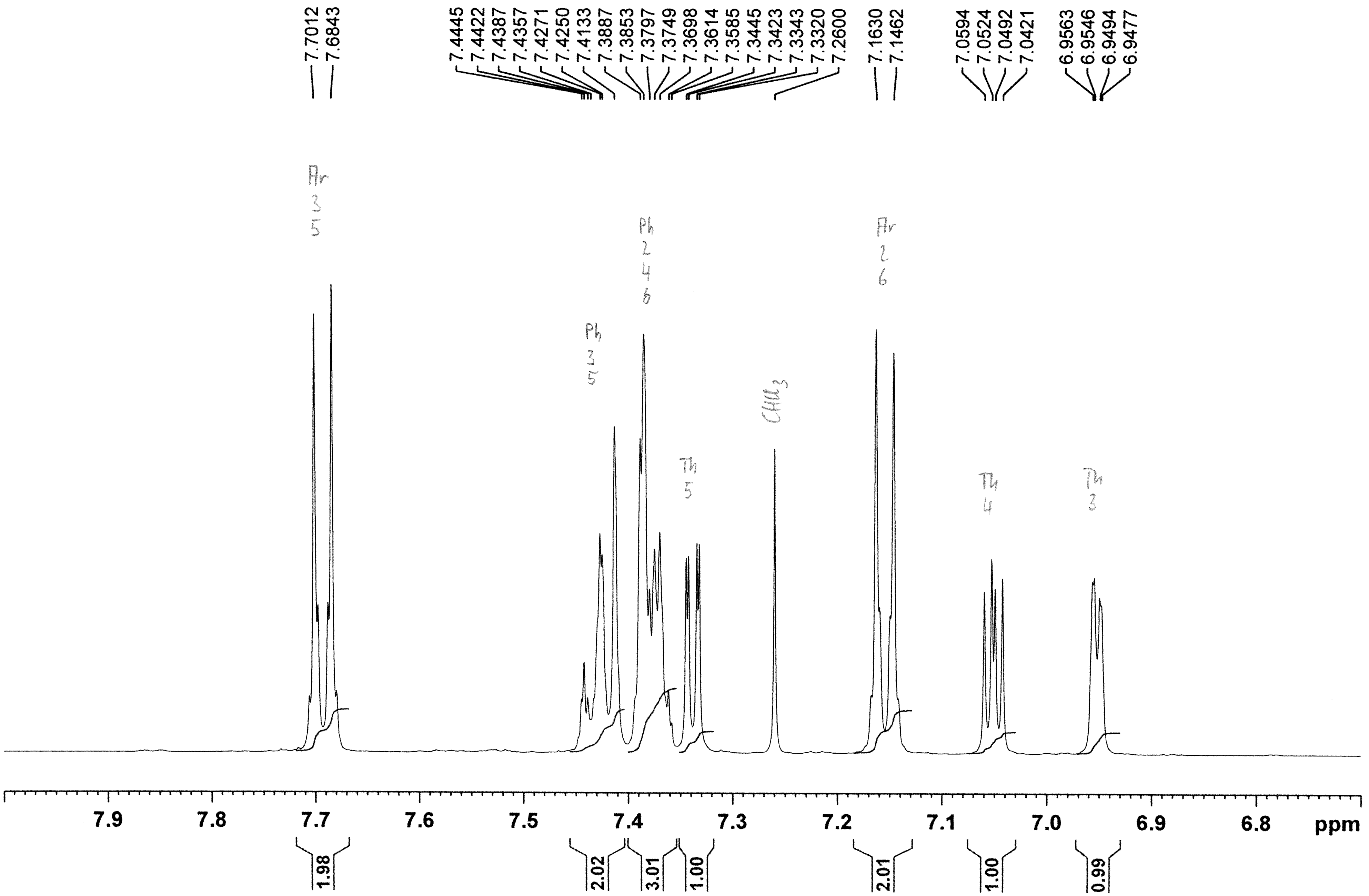

SB001-1 in cdcl3 (Proton) 20.1.2021

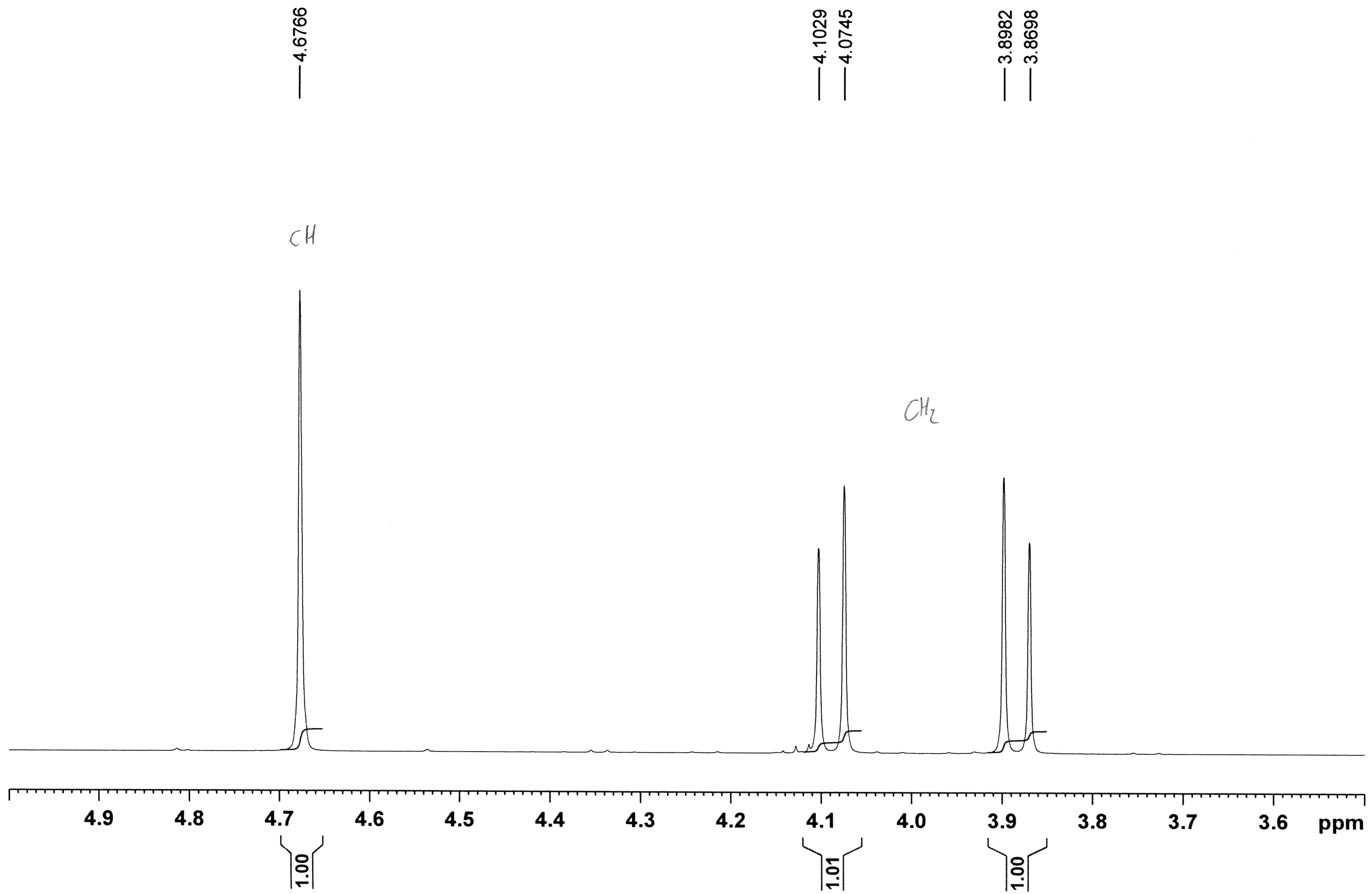

SB001-1 in cdcl3 (APT) 20.1.2021

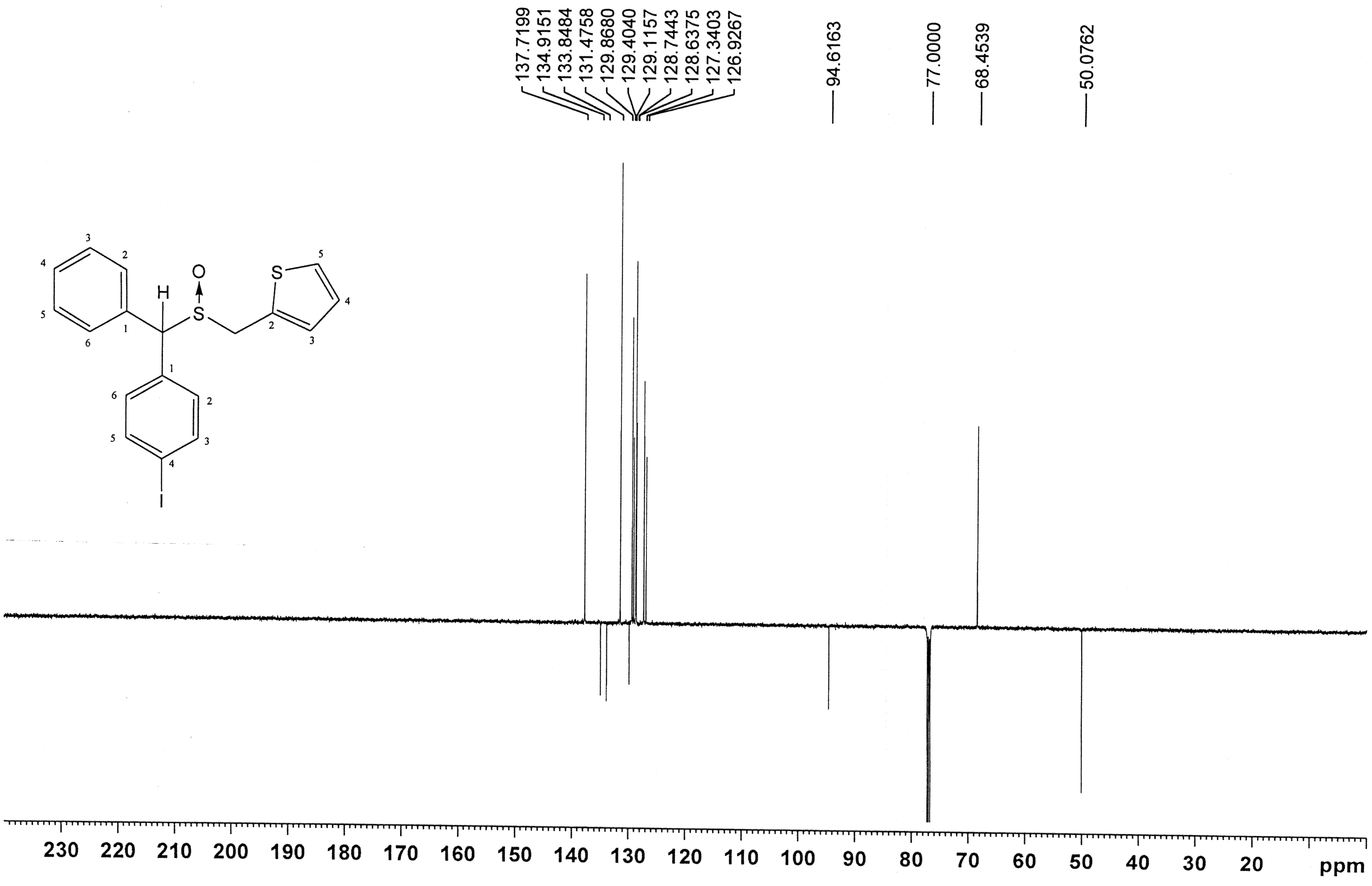

SB001-1 in cdcl3 (APT) 20.1.2021

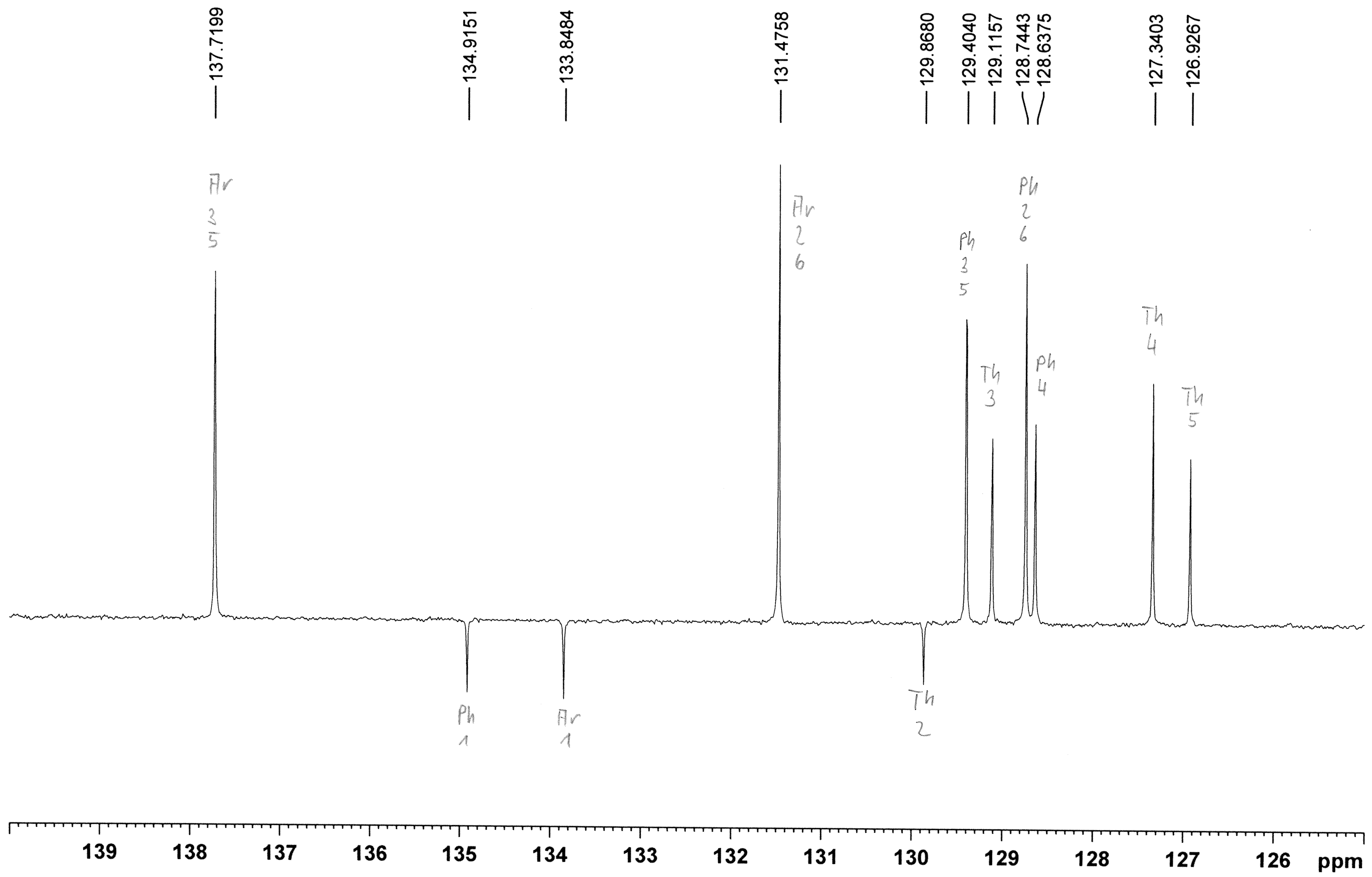

SB001-1 in cdcl3 (APT) 20.1.2021

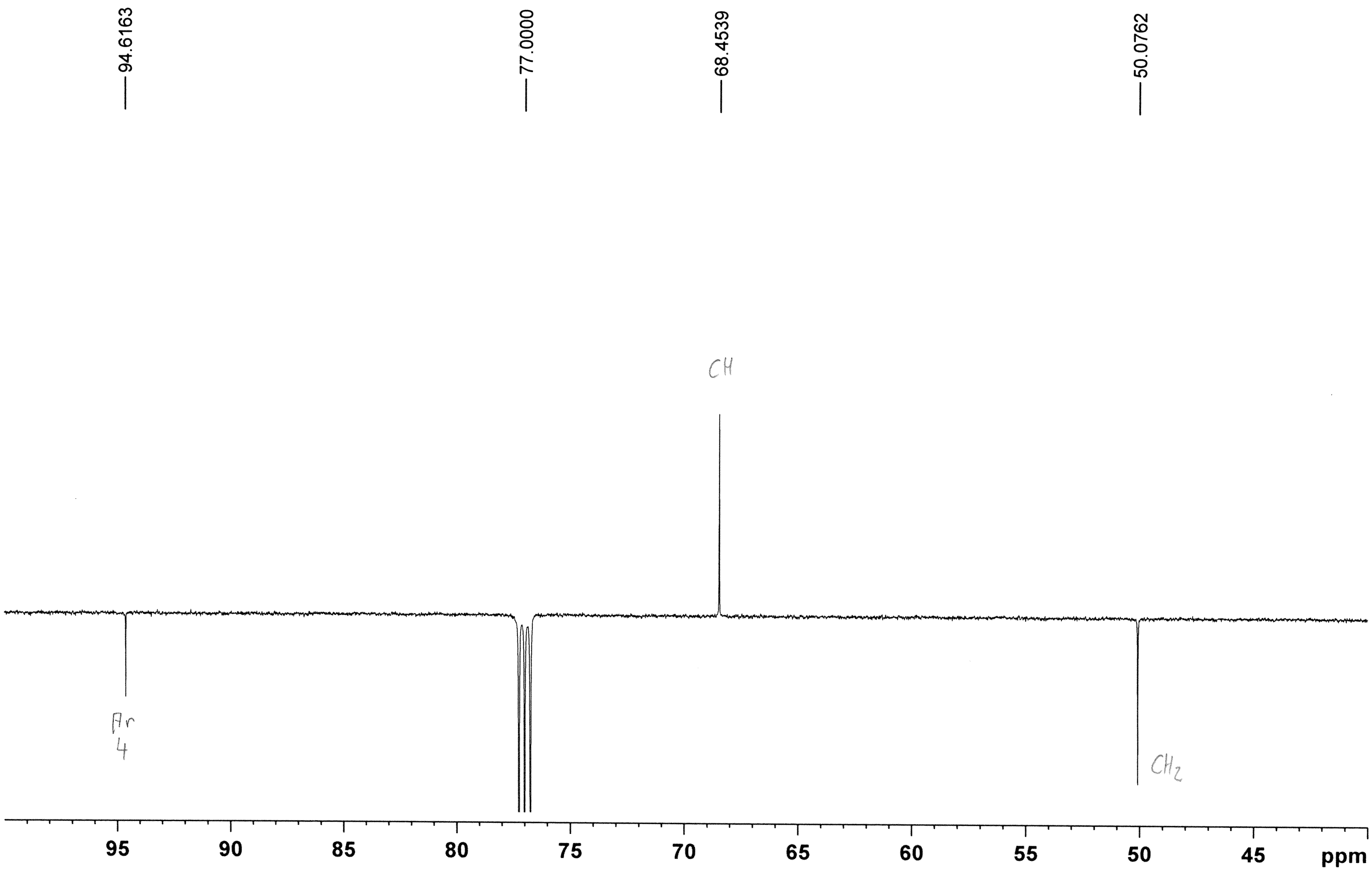

SB001-1 in cdcl3 (COSY) 20.1.2021

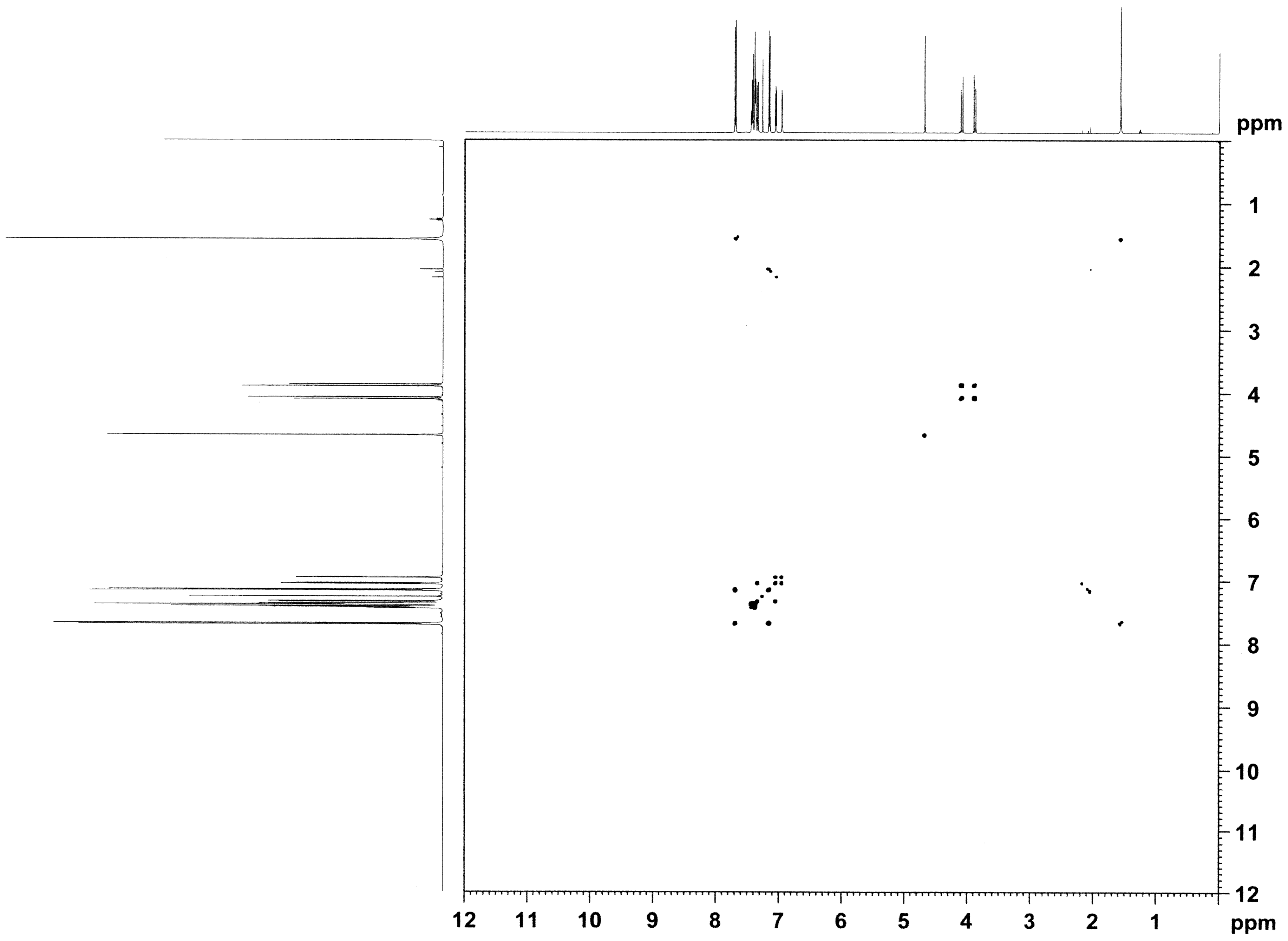

SB001-1 in cdcl3 (COSY) 20.1.2021

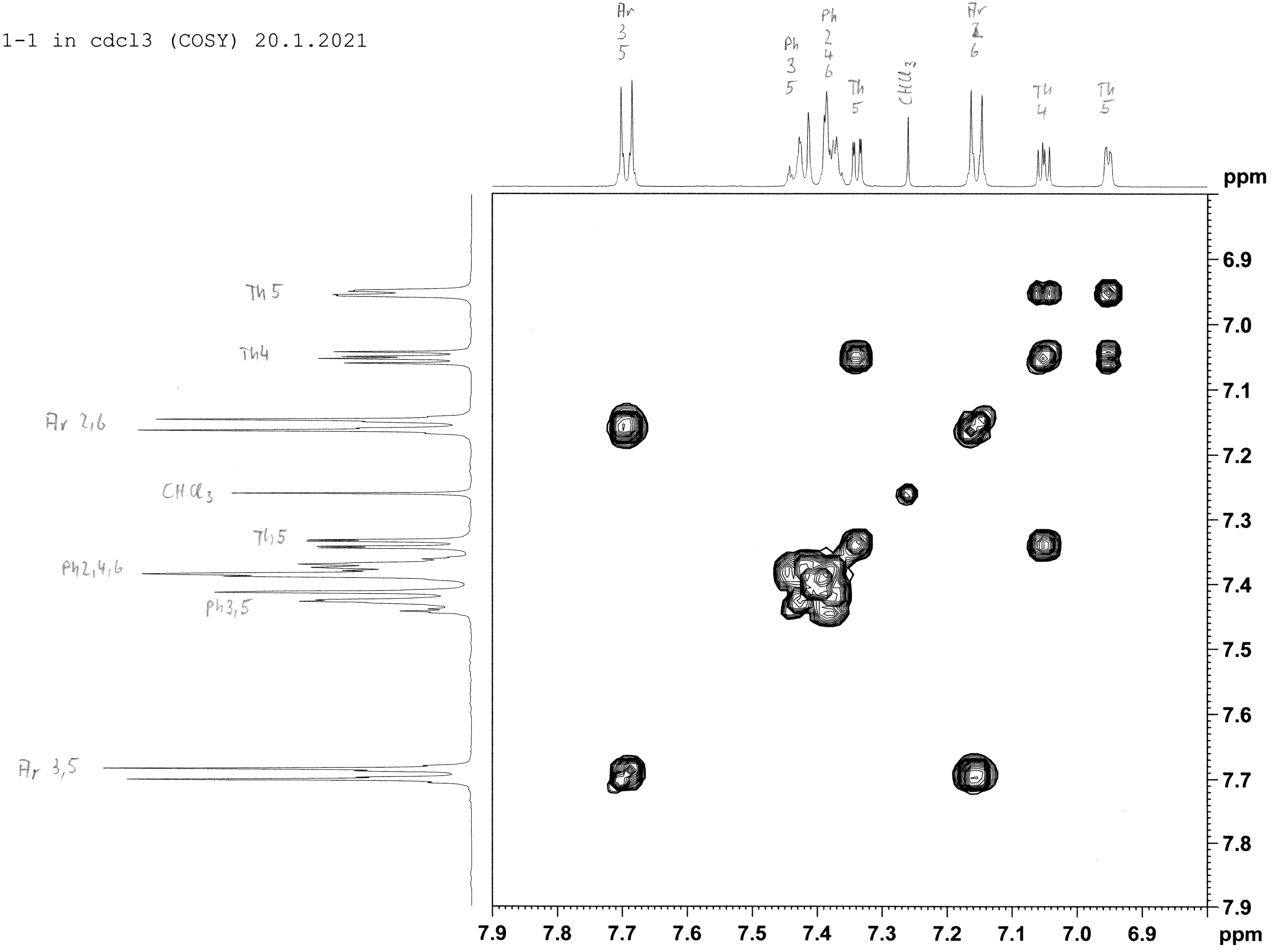

SB001-1 in cdcl3 (COSY) 20.1.2021

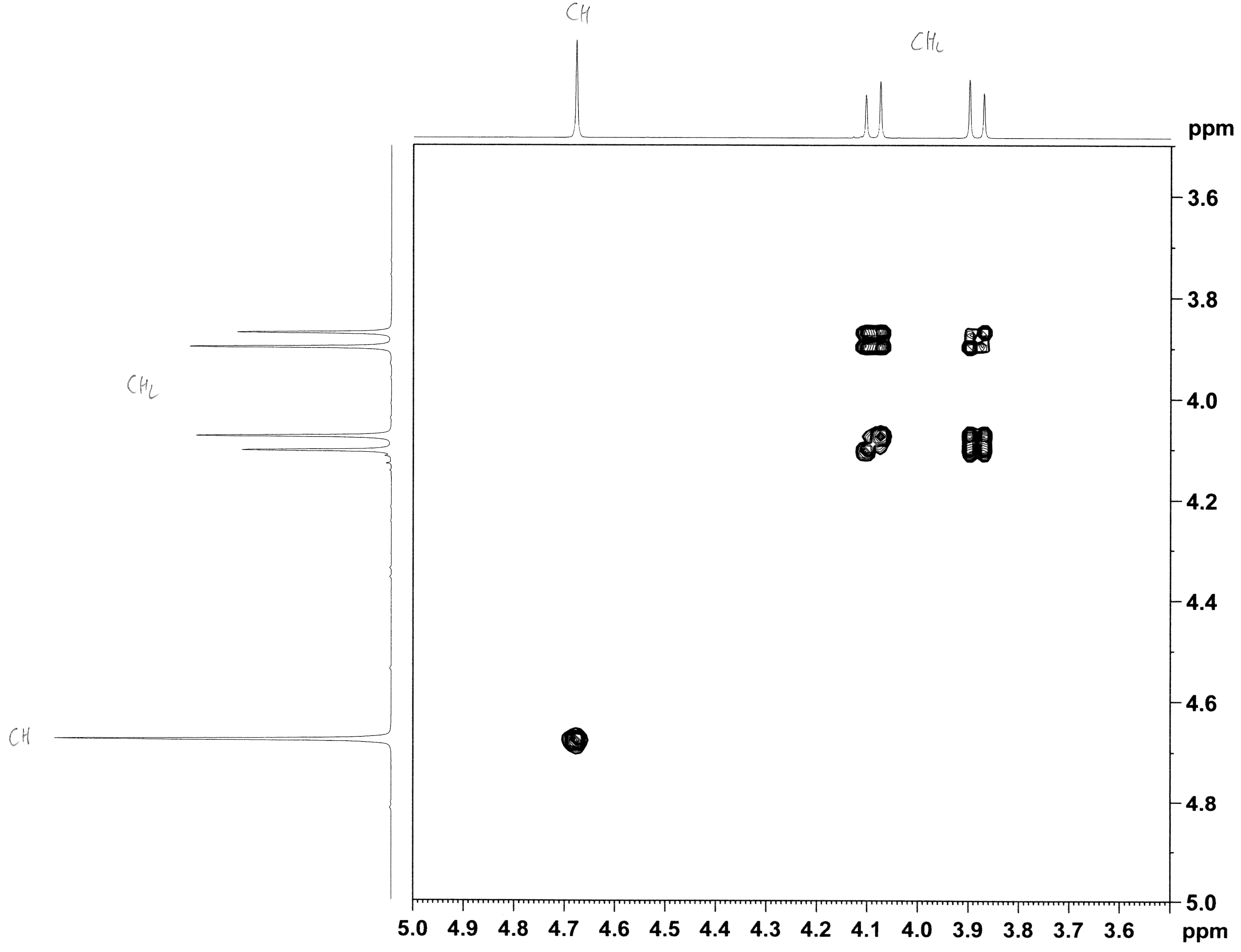

SB001-1 in cdcl3 (HSQC) 20.1.2021

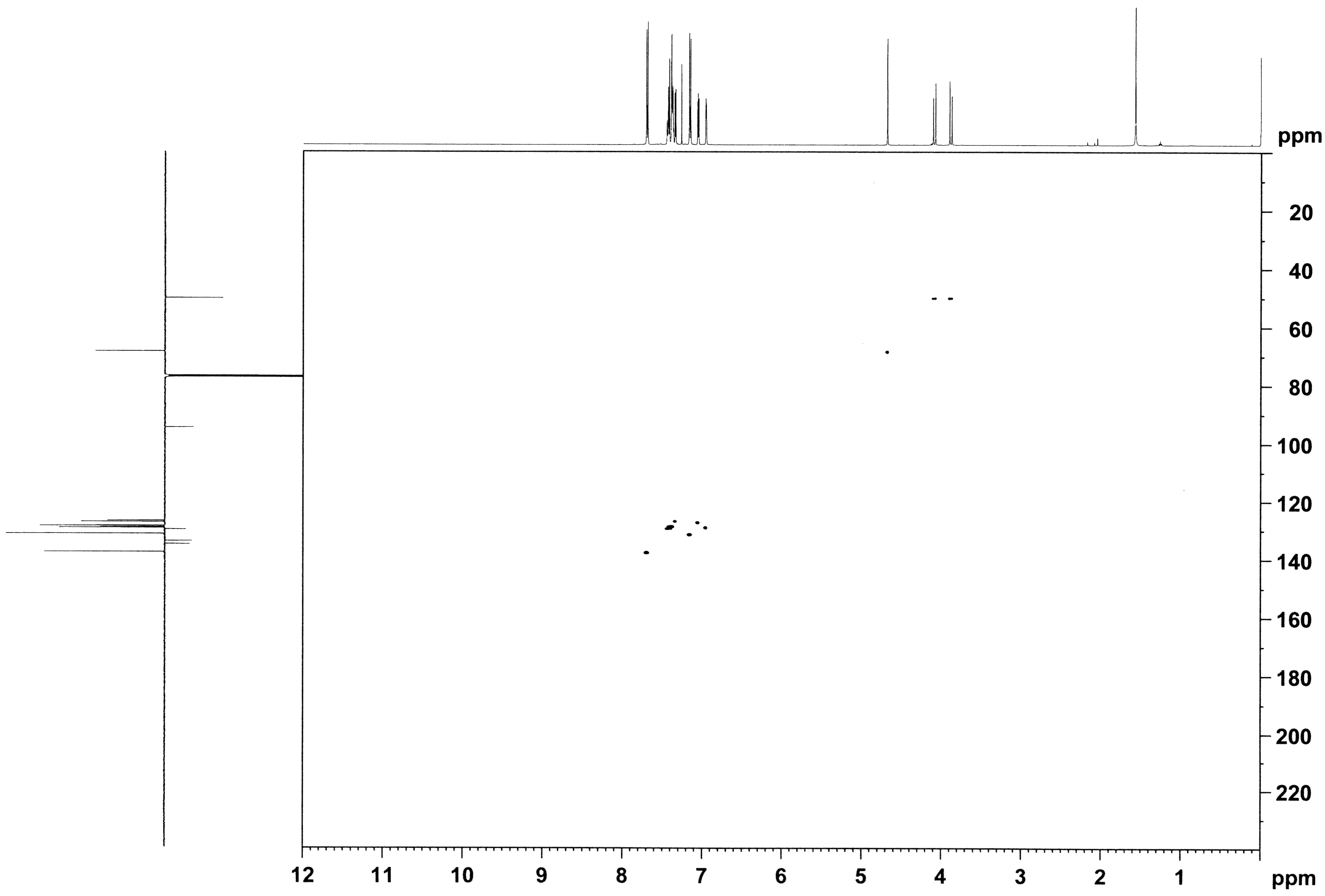

SB001-1 in cdcl3 (HSQC) 20.1.2021

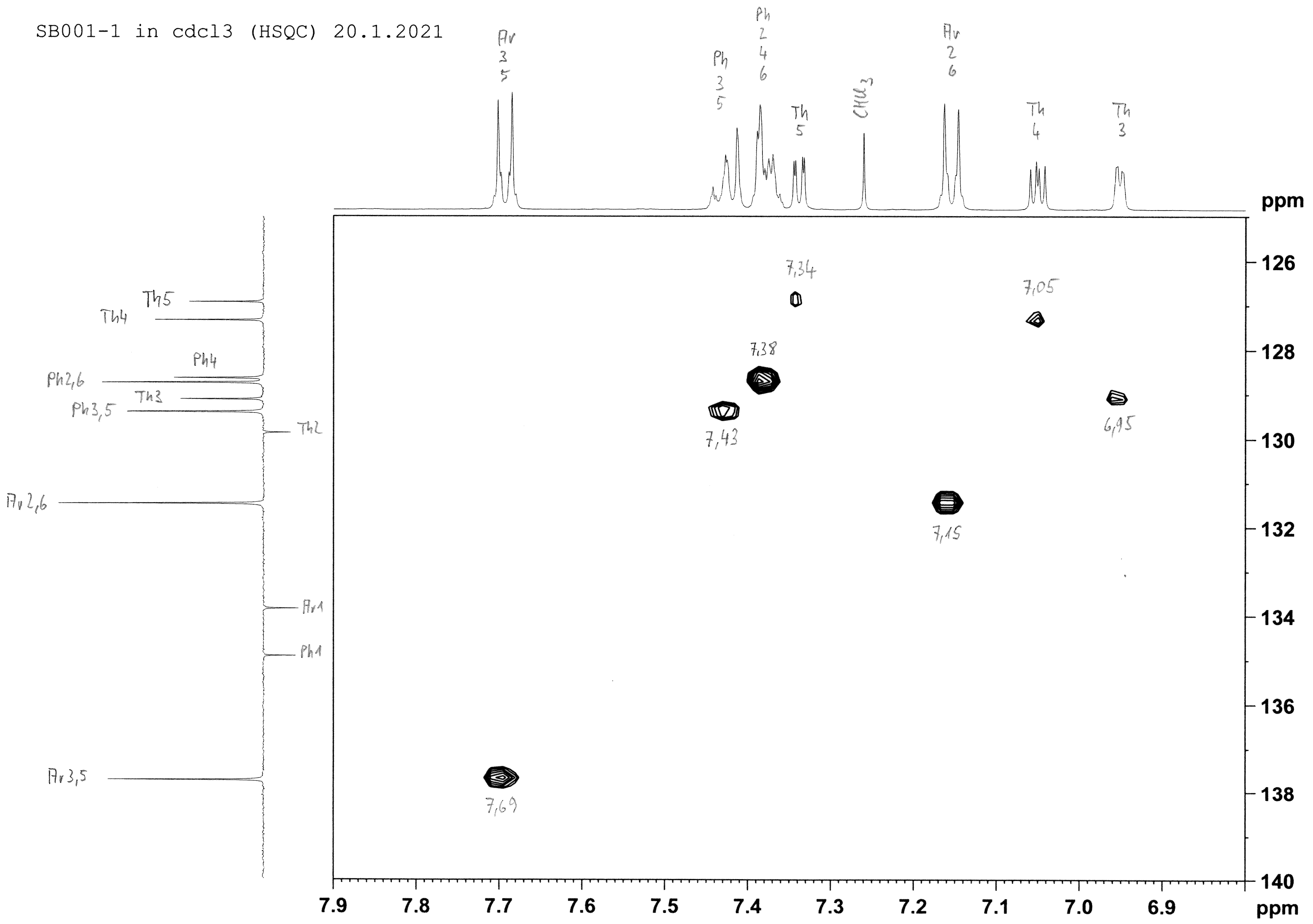

SB001-1 in cdcl3 (HSQC) 20.1.2021

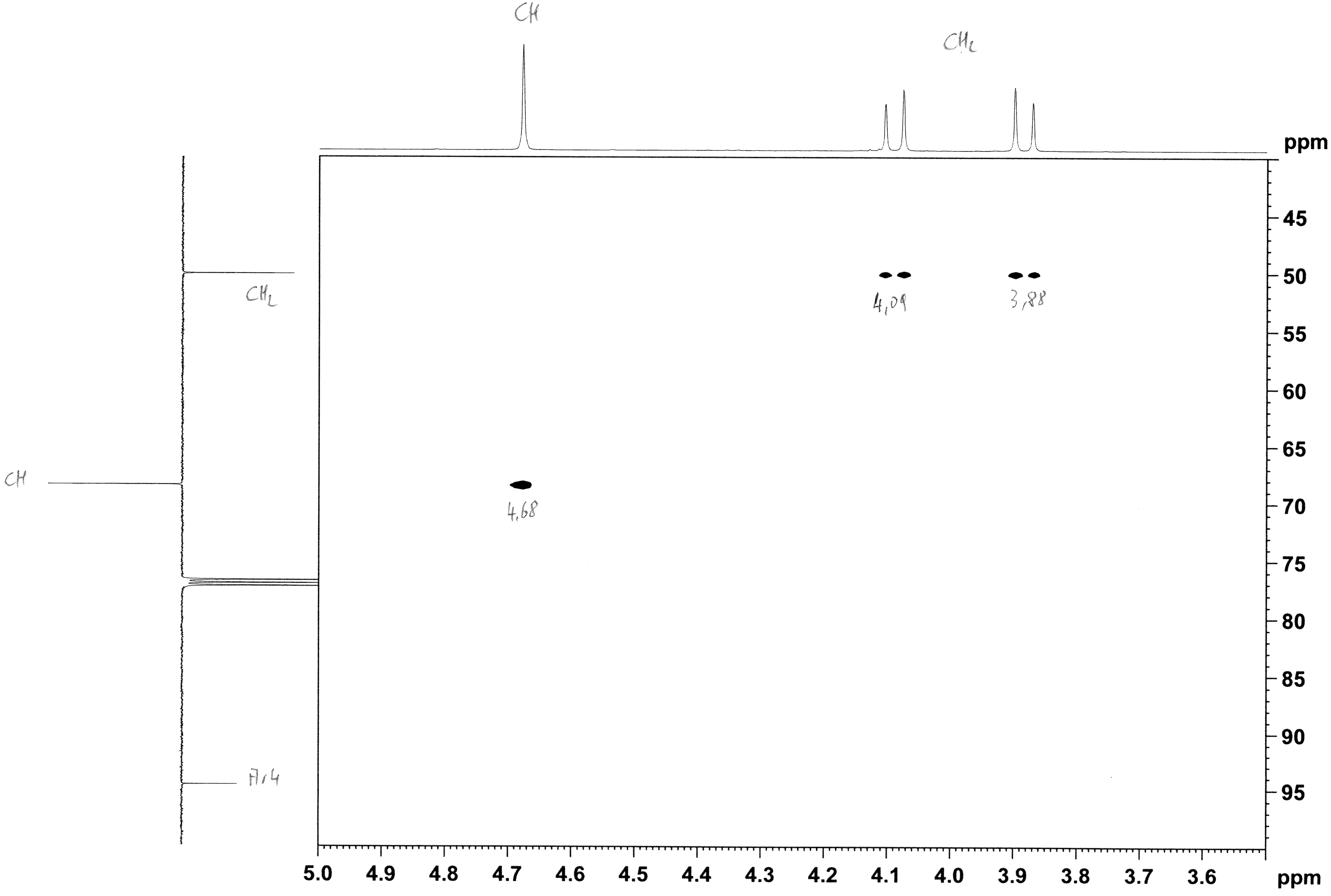

SB001-1 in cdcl3 (HMBC) 20.1.2021

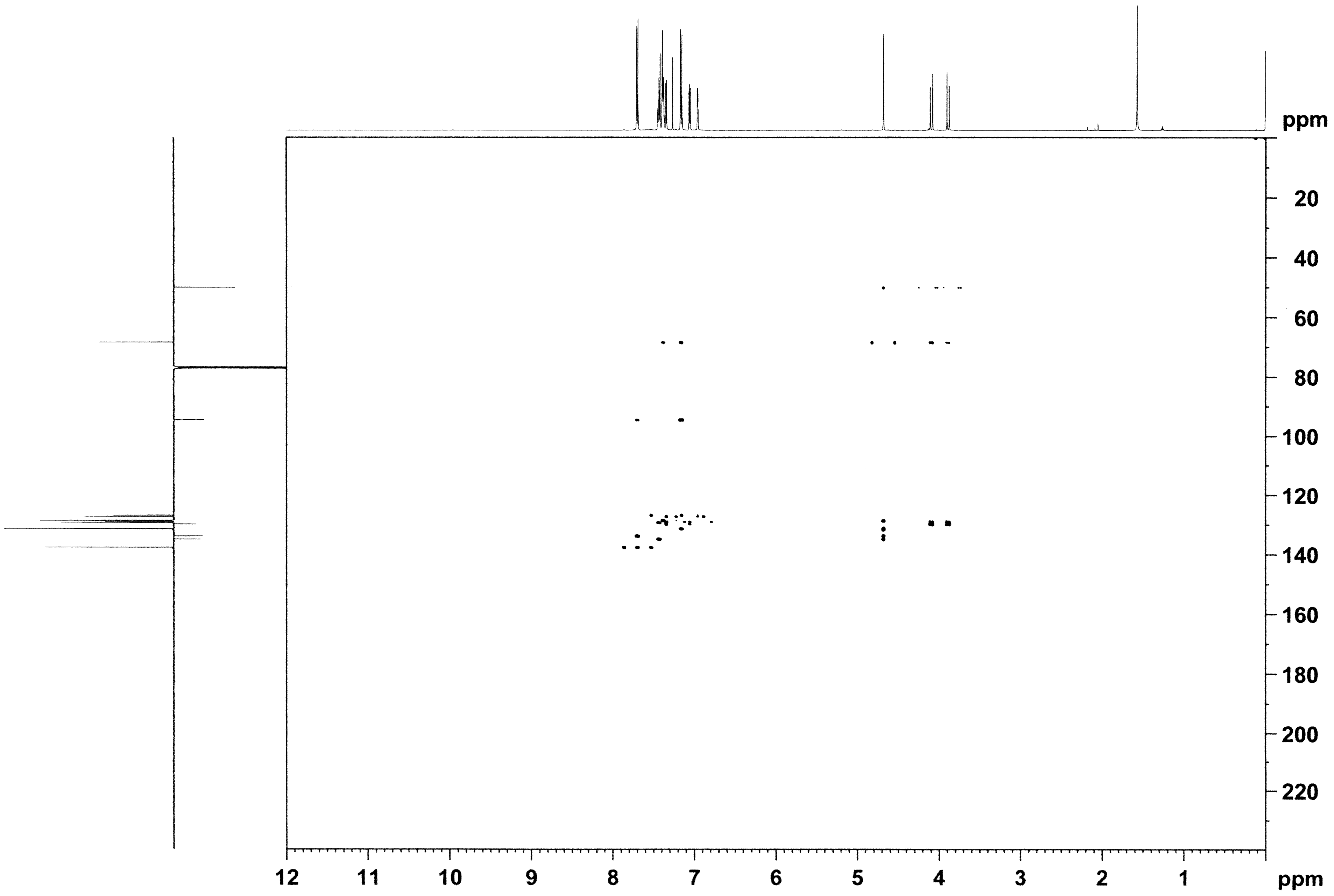

SB001-1 in cdcl3 (HMBC) 20.1.2021

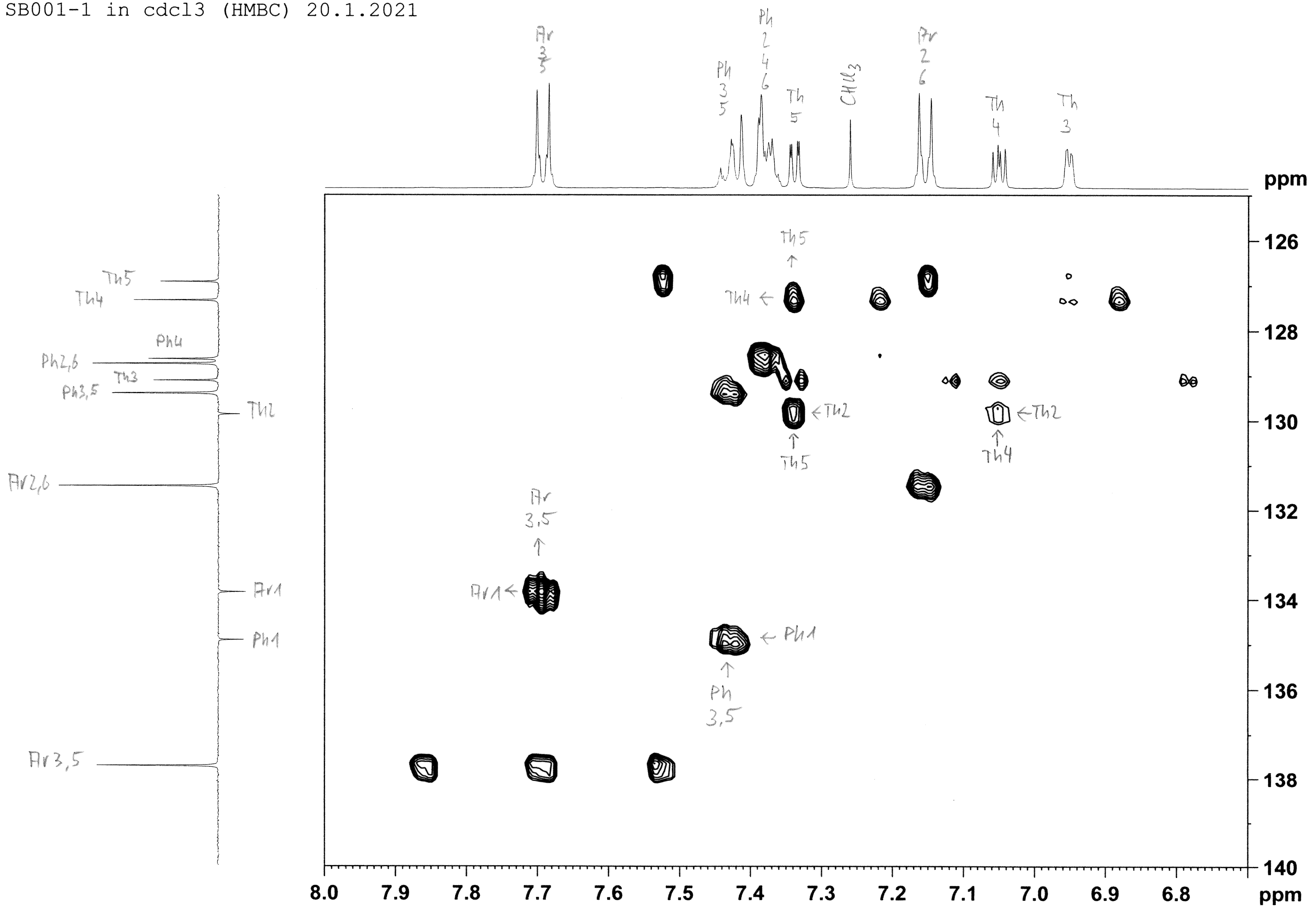

SB001-1 in cdcl3 (HMBC) 20.1.2021

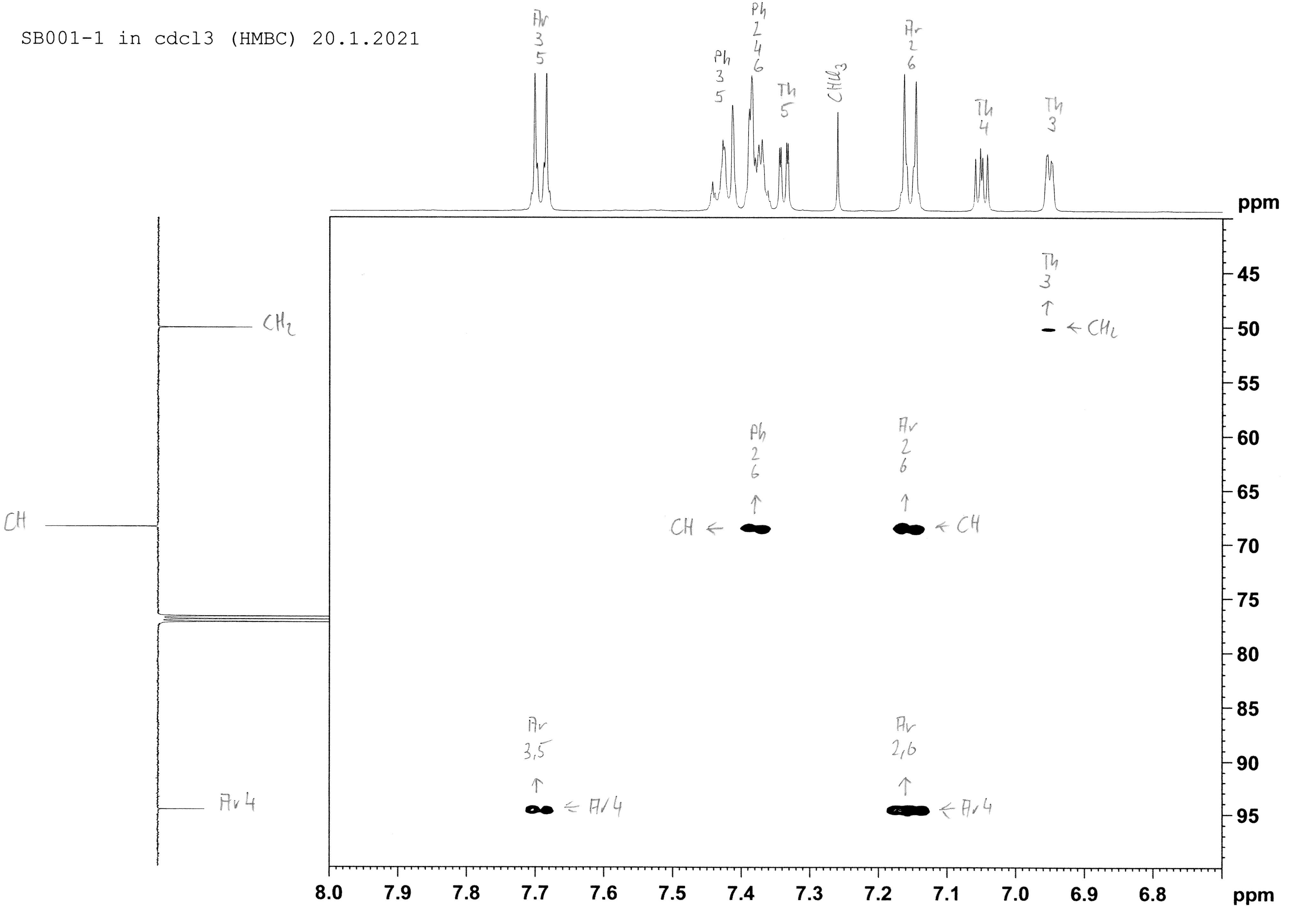

SB001-1 in cdcl3 (HMBC) 20.1.2021

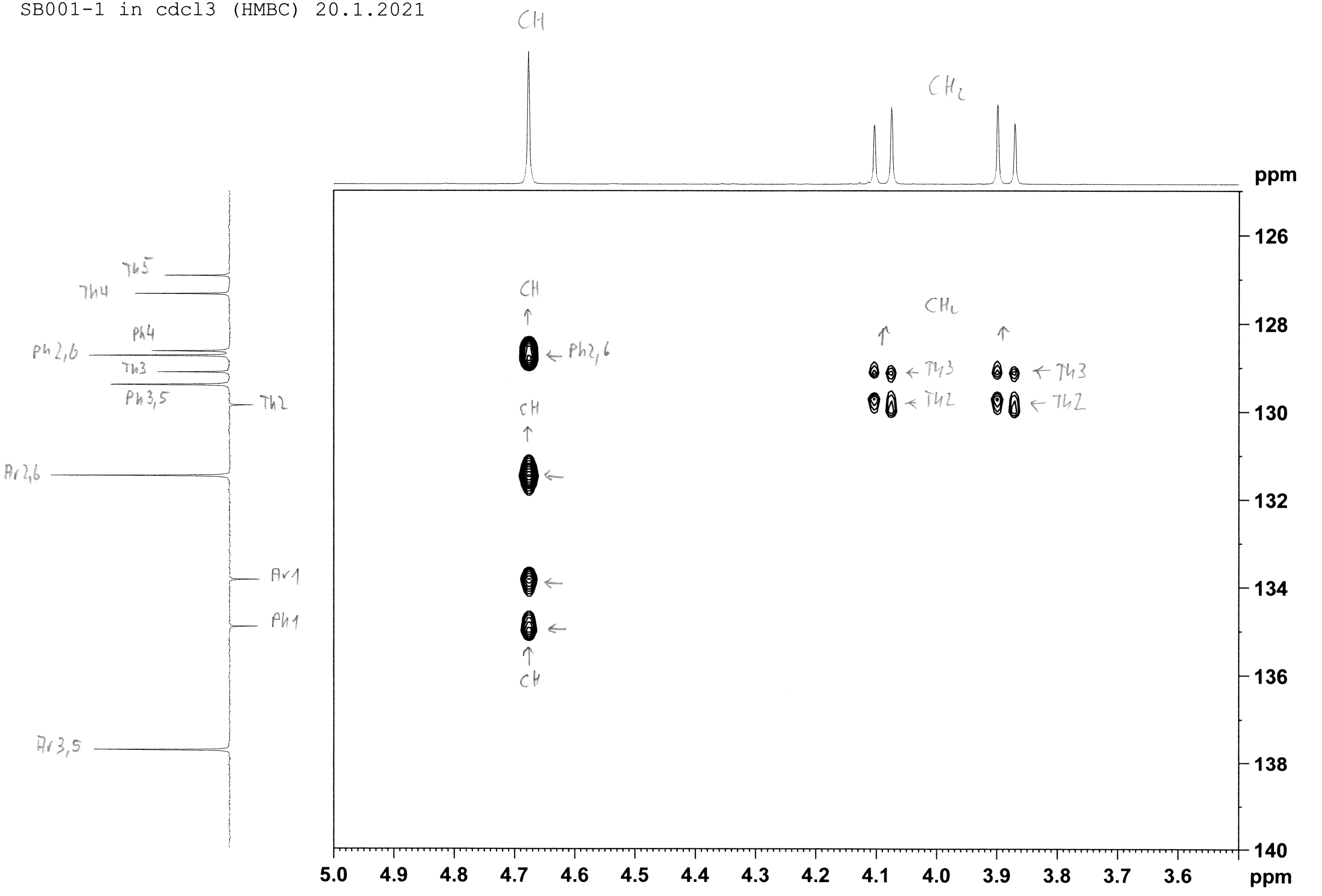

SB001-1 in cdcl3 (HMBC) 20.1.2021

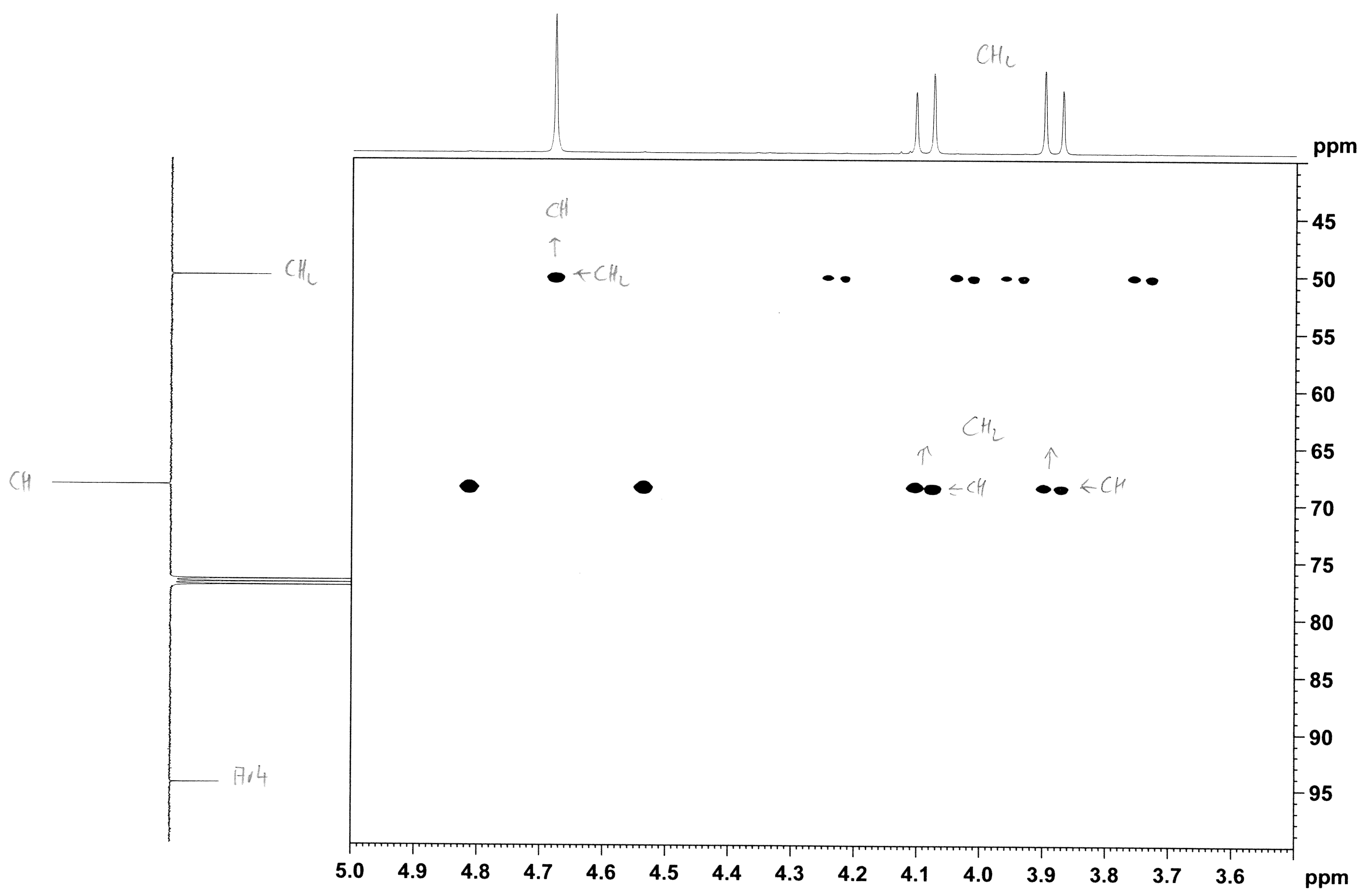

Figure S42c. NMR spectra of compound **6k**.

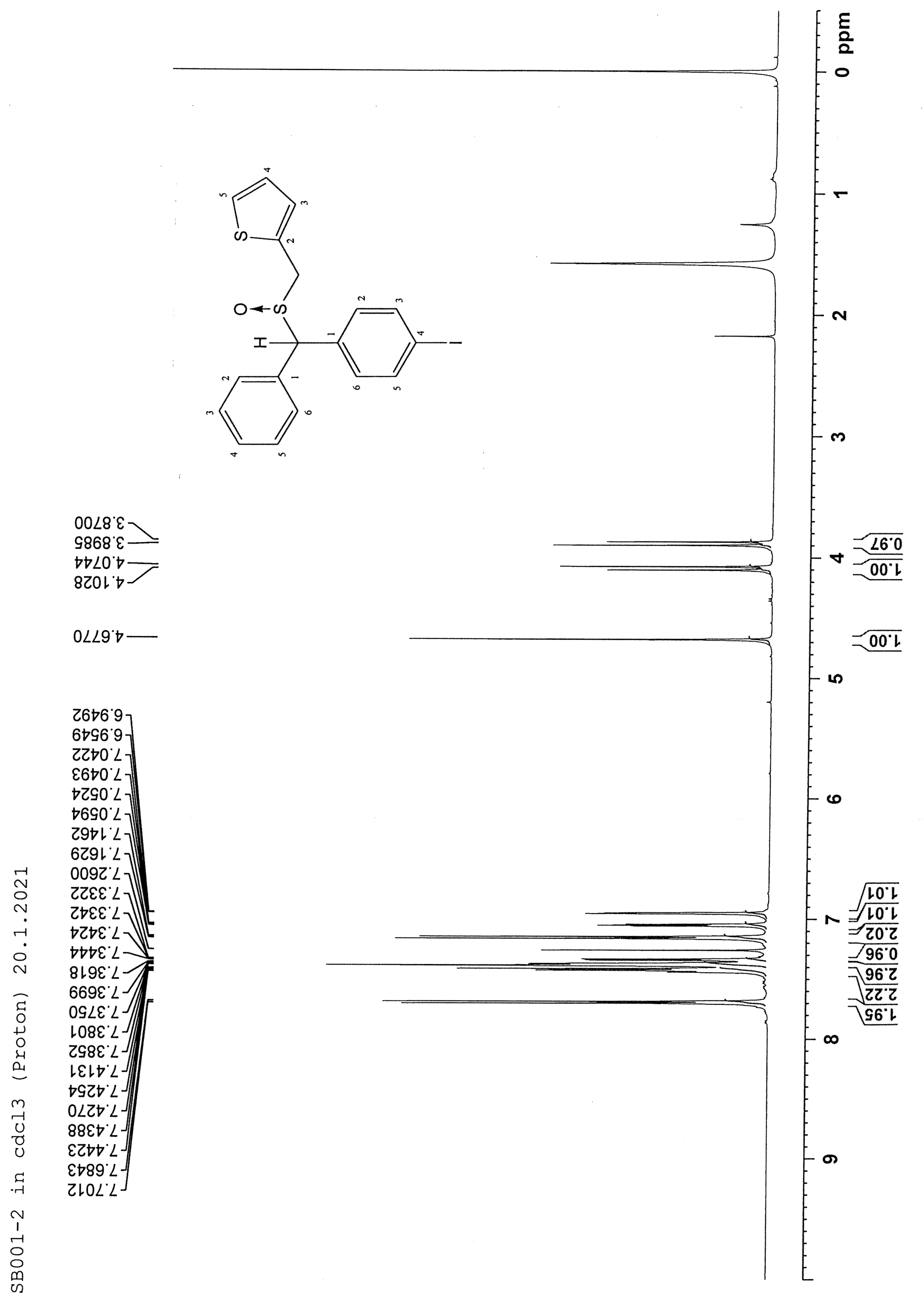

SB001-2 in cdcl3 (Proton) 20.1.2021

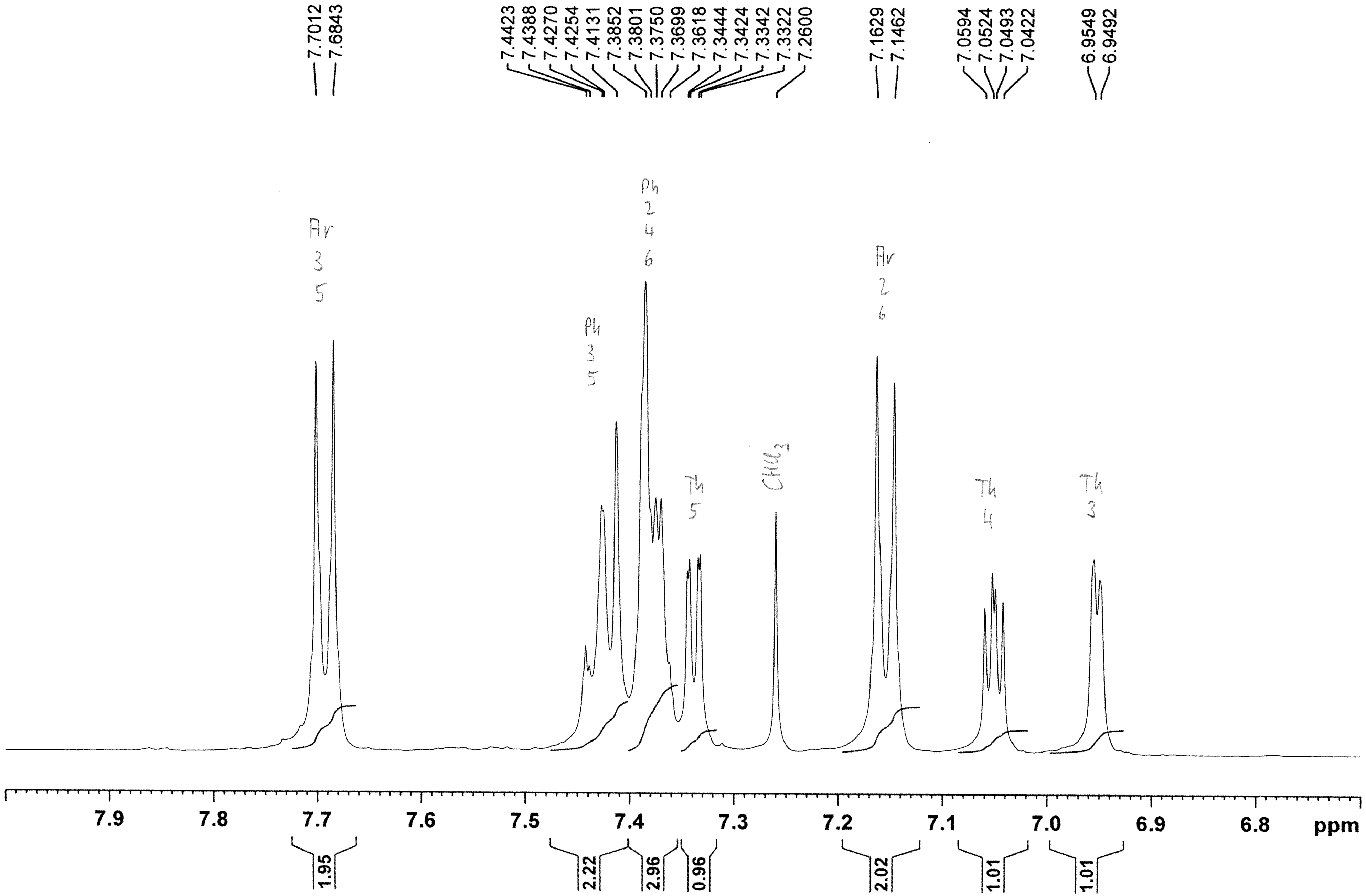

SB001-2 in cdcl3 (Proton) 20.1.2021

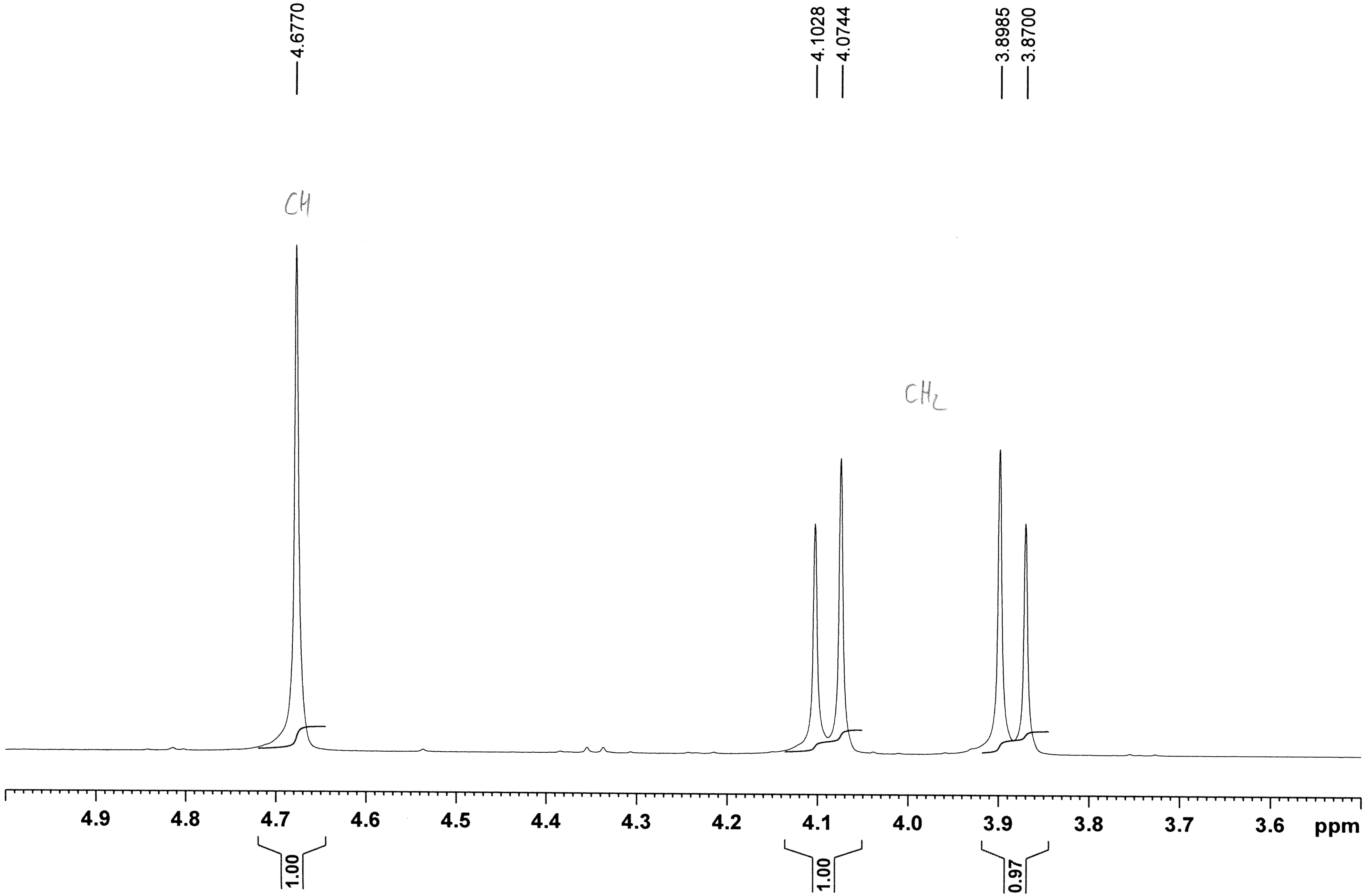

SB001-2 in cdcl3 (APT) 20.1.2021

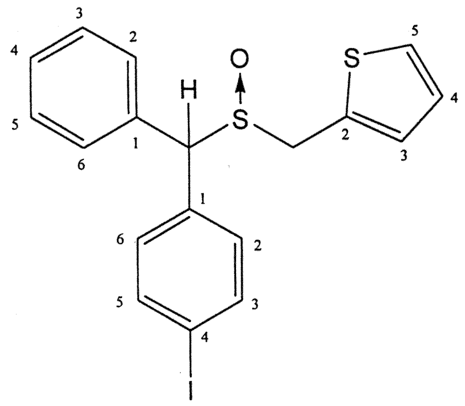

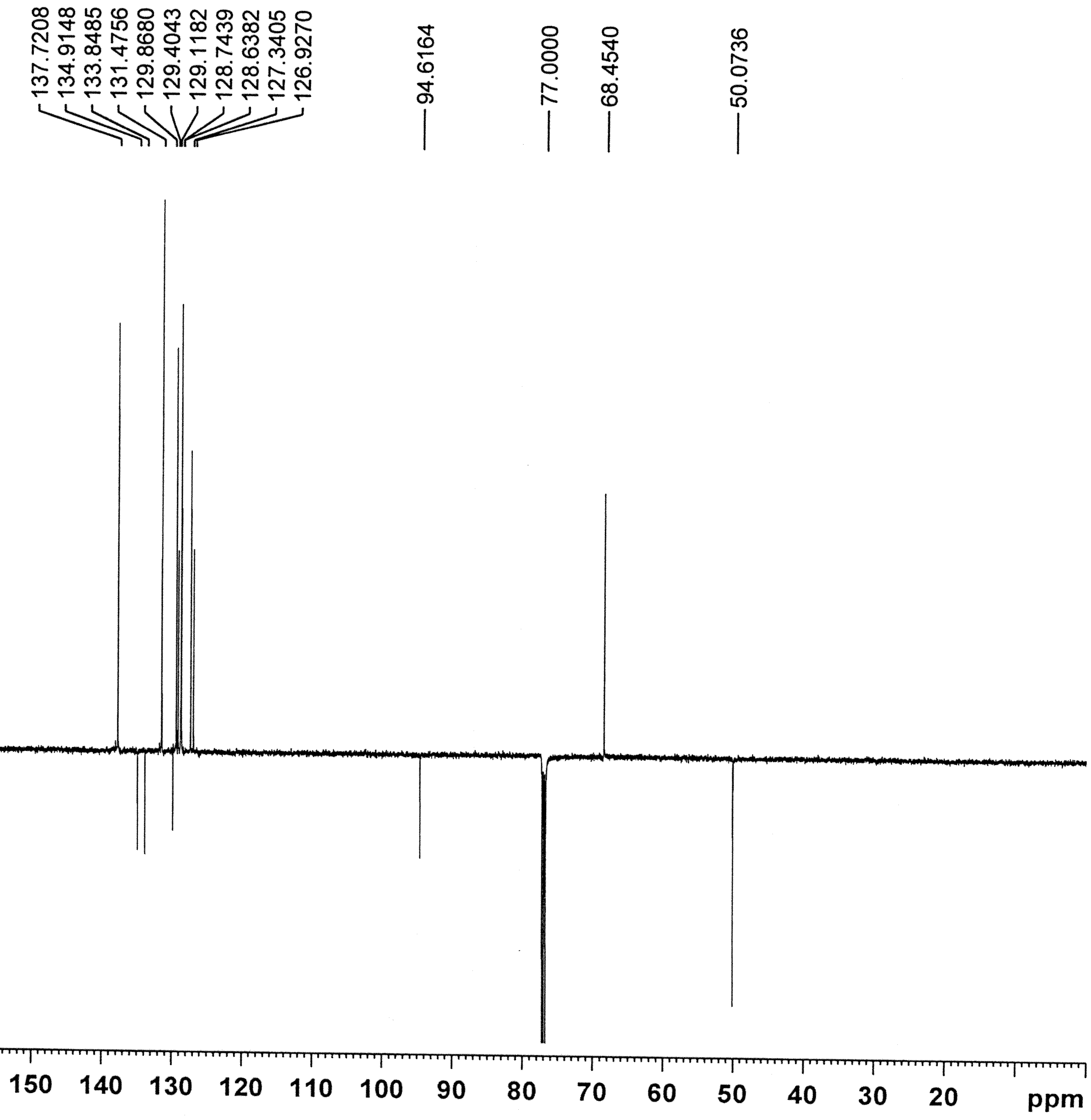

SB001-2 in cdcl3 (APT) 20.1.2021

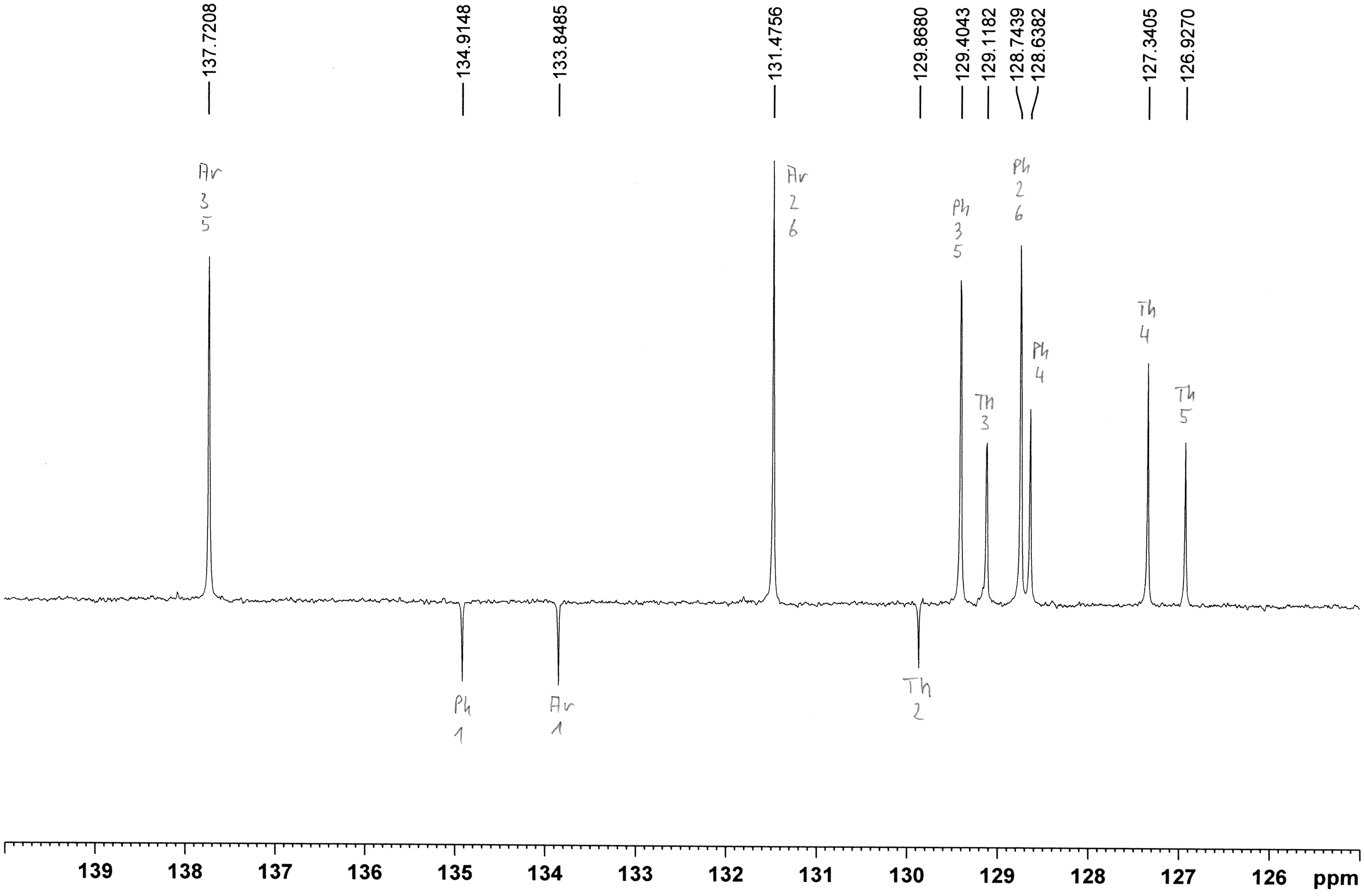

SB001-2 in cdcl3 (APT) 20.1.2021

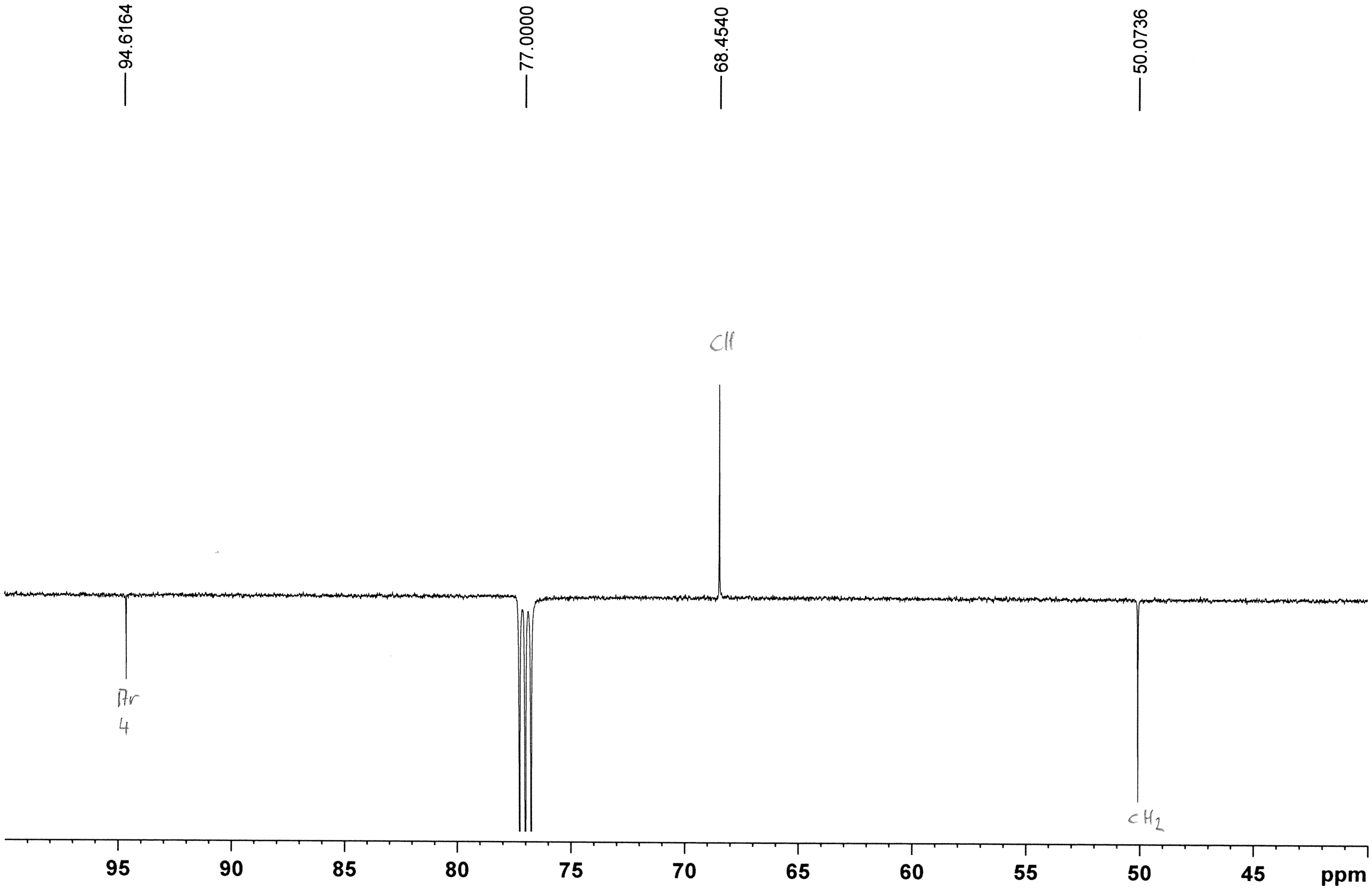

Figure S43c. NMR spectra of compound **7k.**

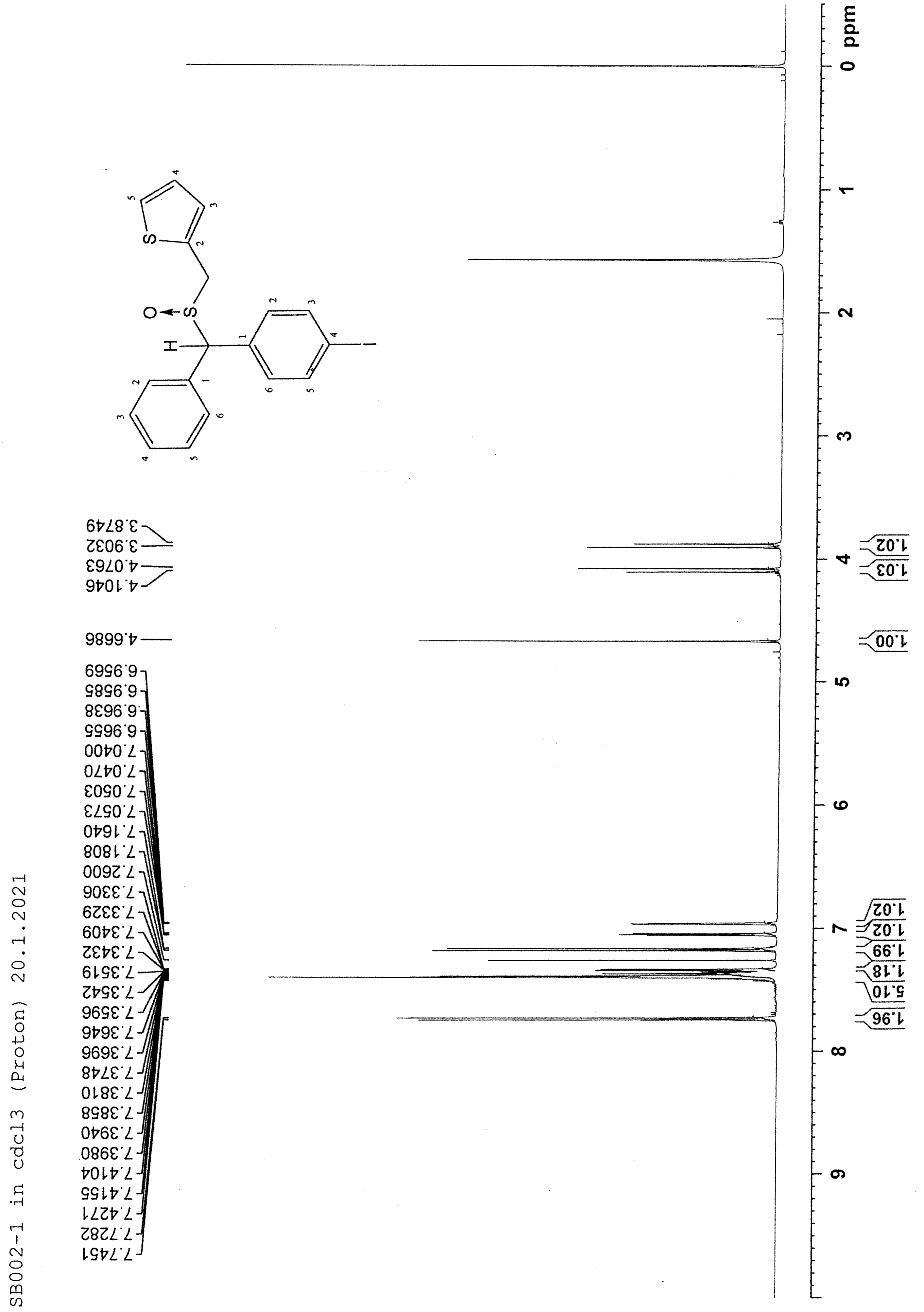

SB002-1 in cdcl3 (Proton) 20.1.2021

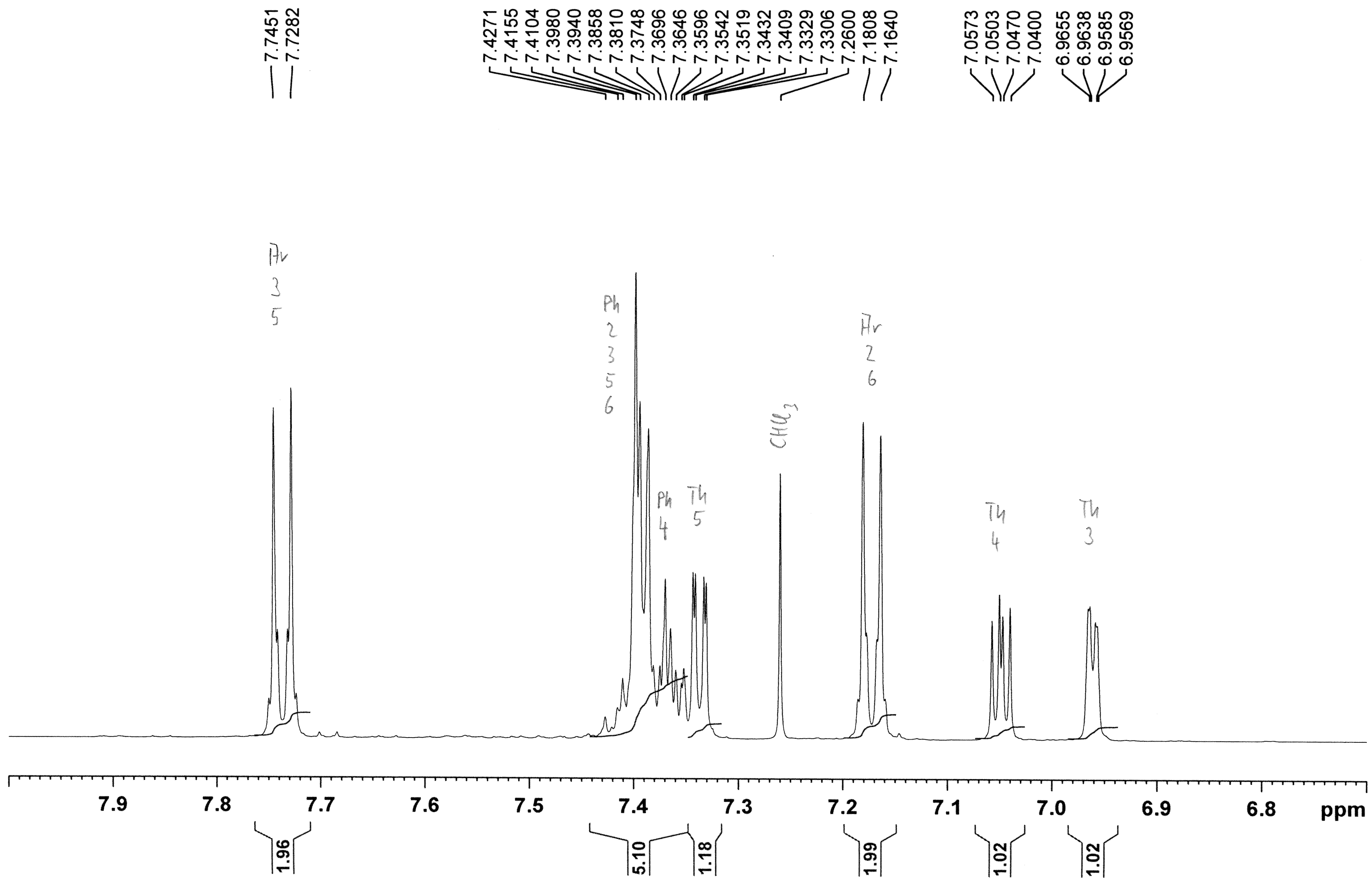

SB002-1 in cdcl3 (Proton) 20.1.2021

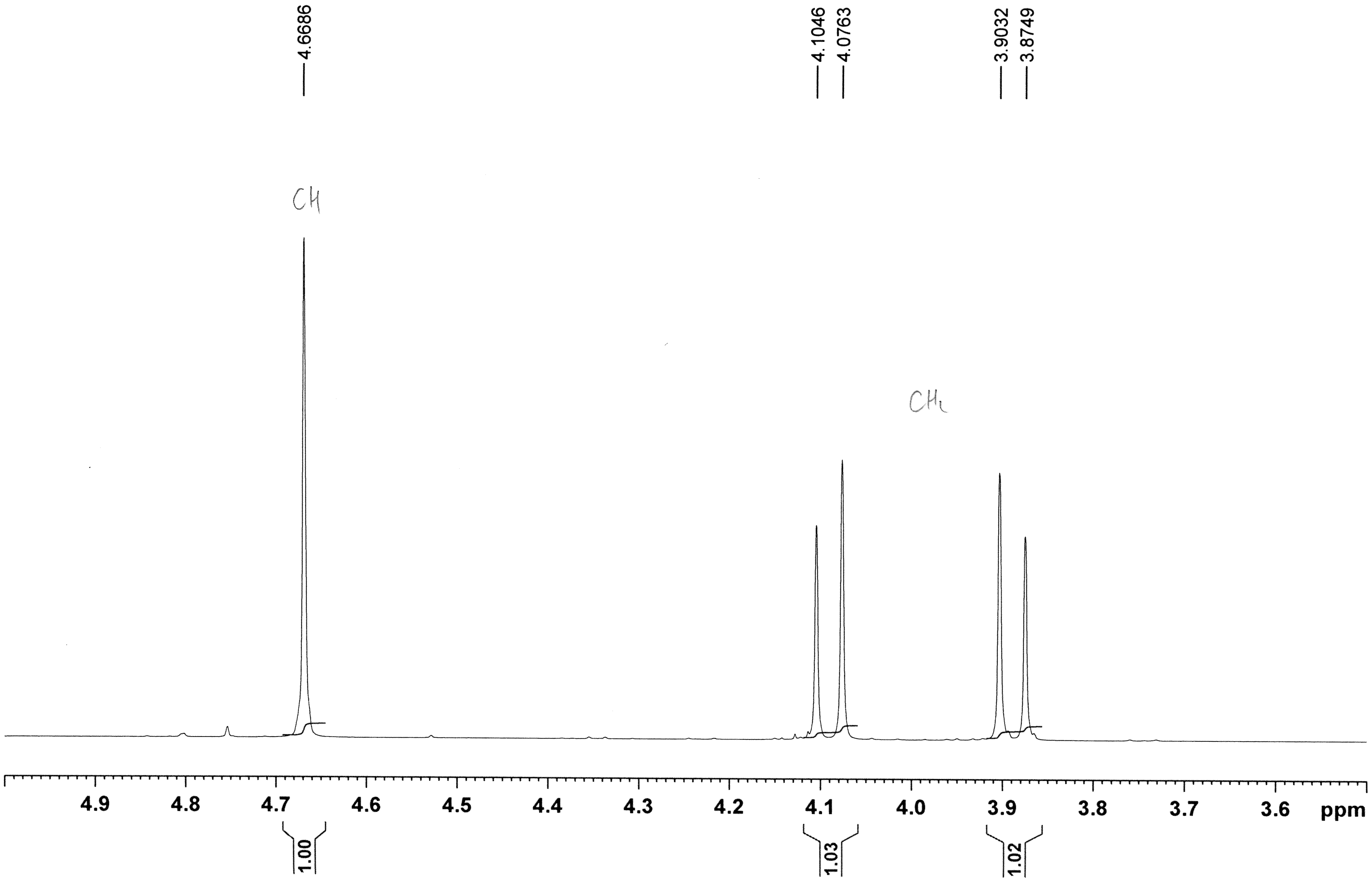

SB002-1 in cdcl3 (APT) 20.1.2021

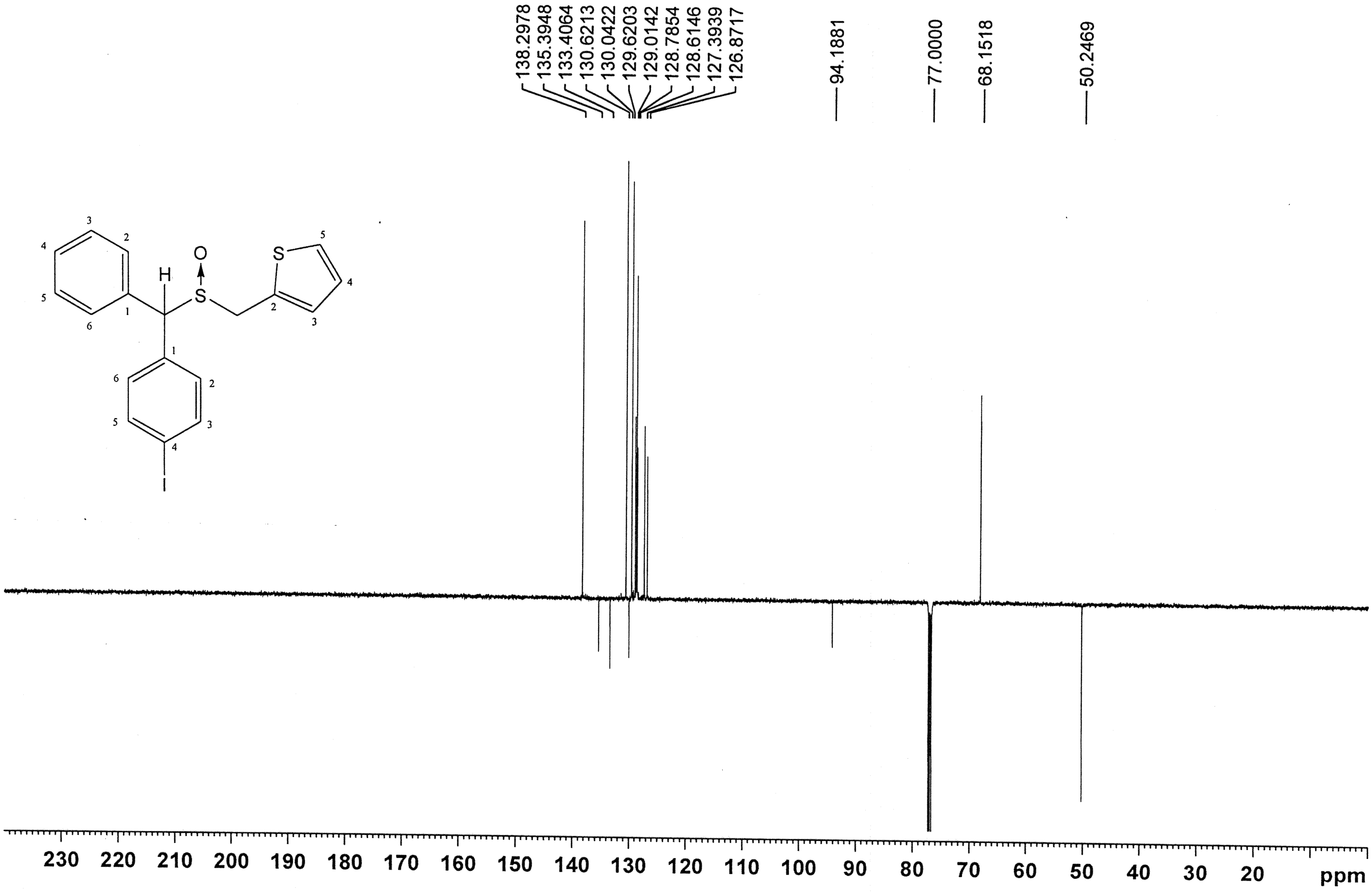

SB002-1 in cdcl3 (APT) 20.1.2021

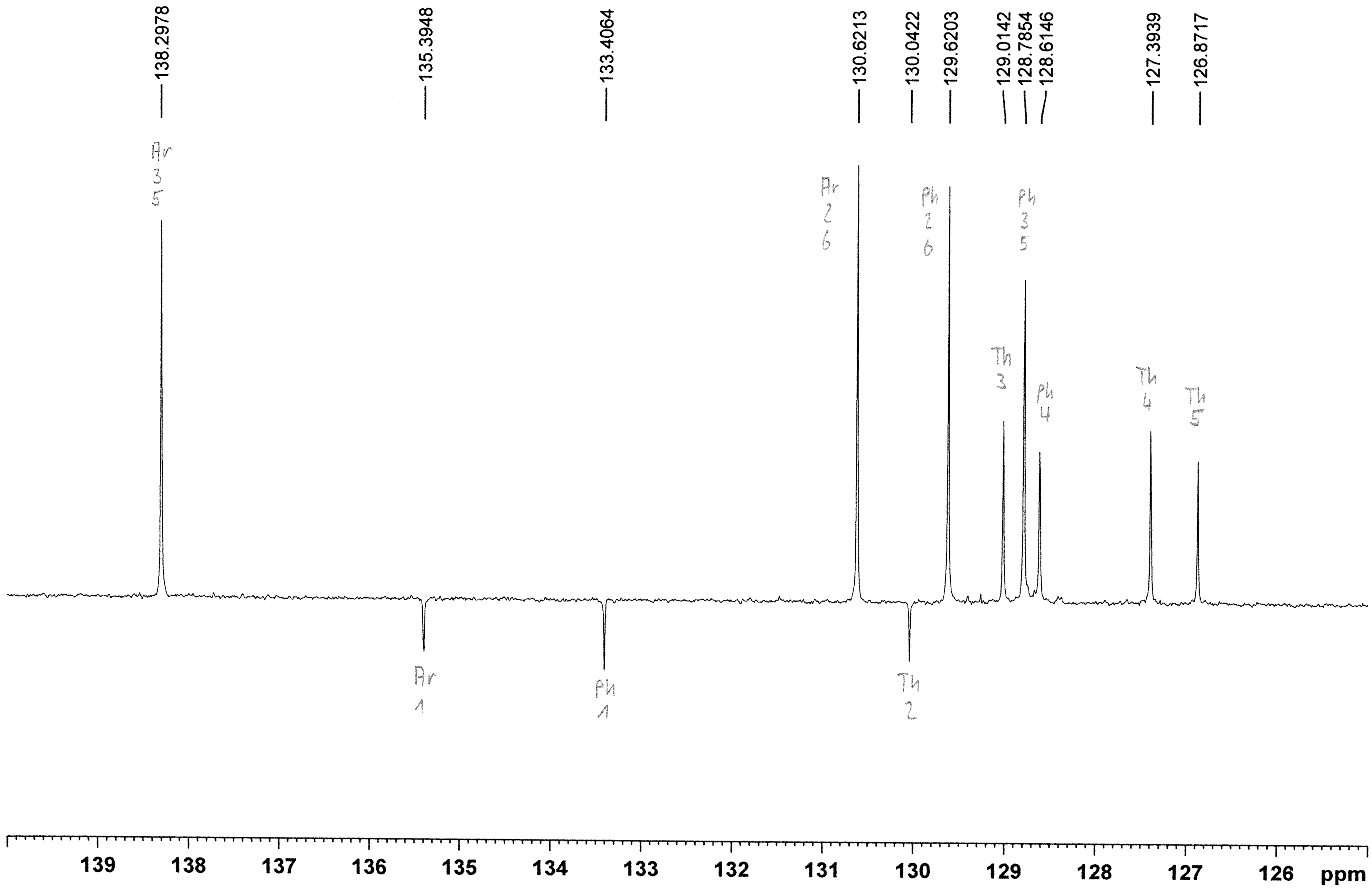

SB002-1 in cdcl3 (APT) 20.1.2021

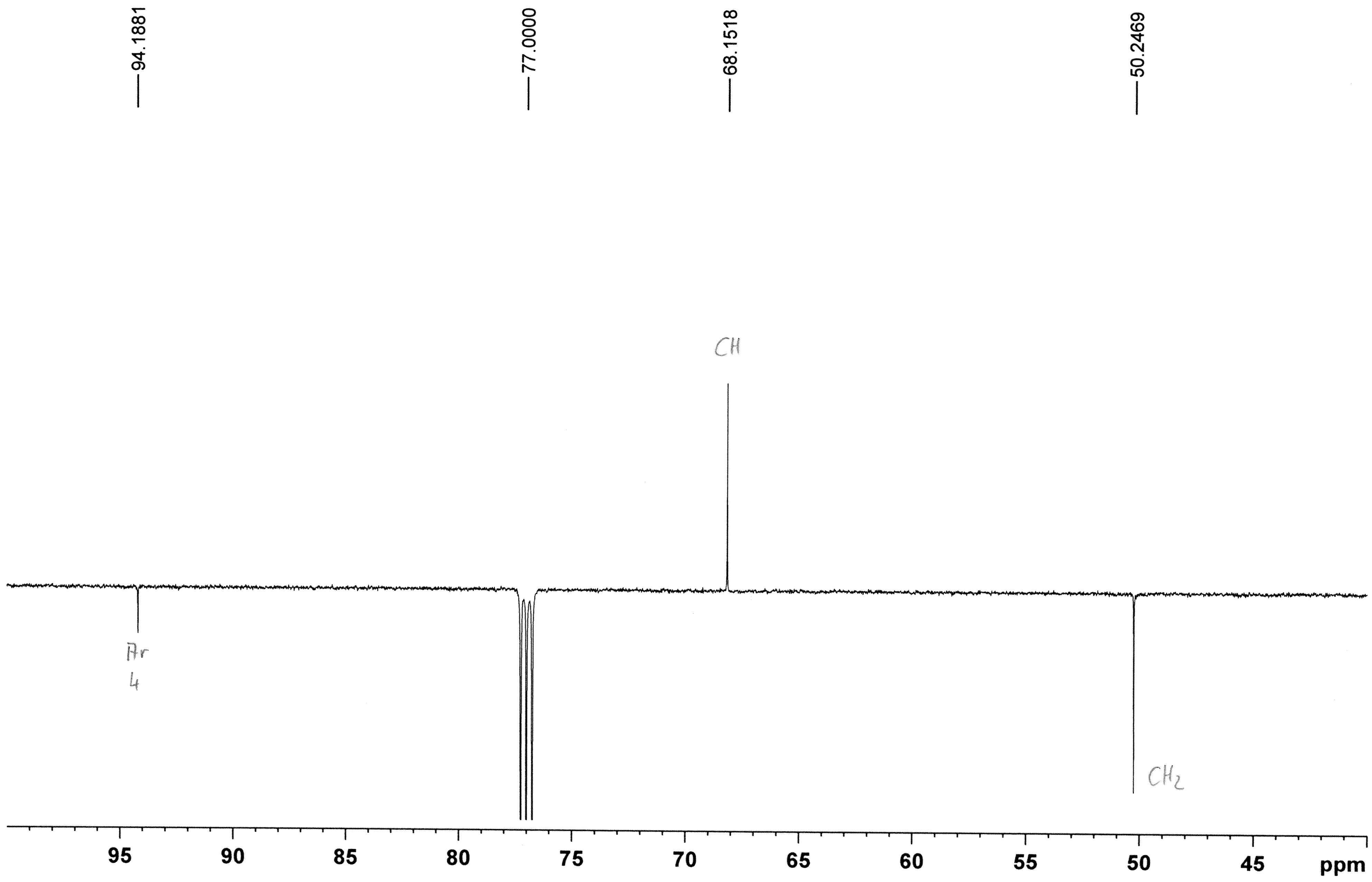

SB002-1 in cdcl3 (COSY) 20.1.2021

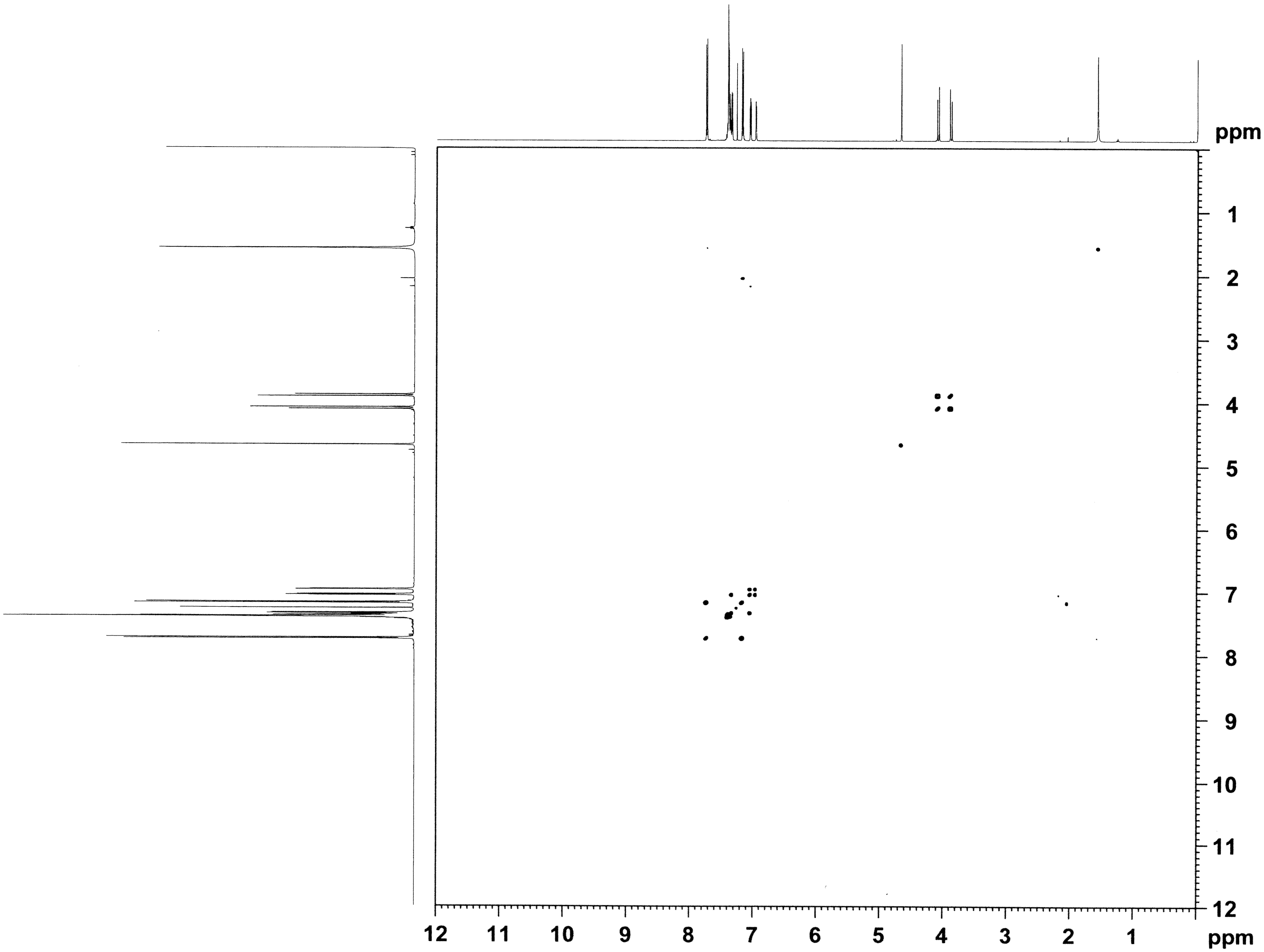

SB002-1 in cdcl3 (COSY) 20.1.2021

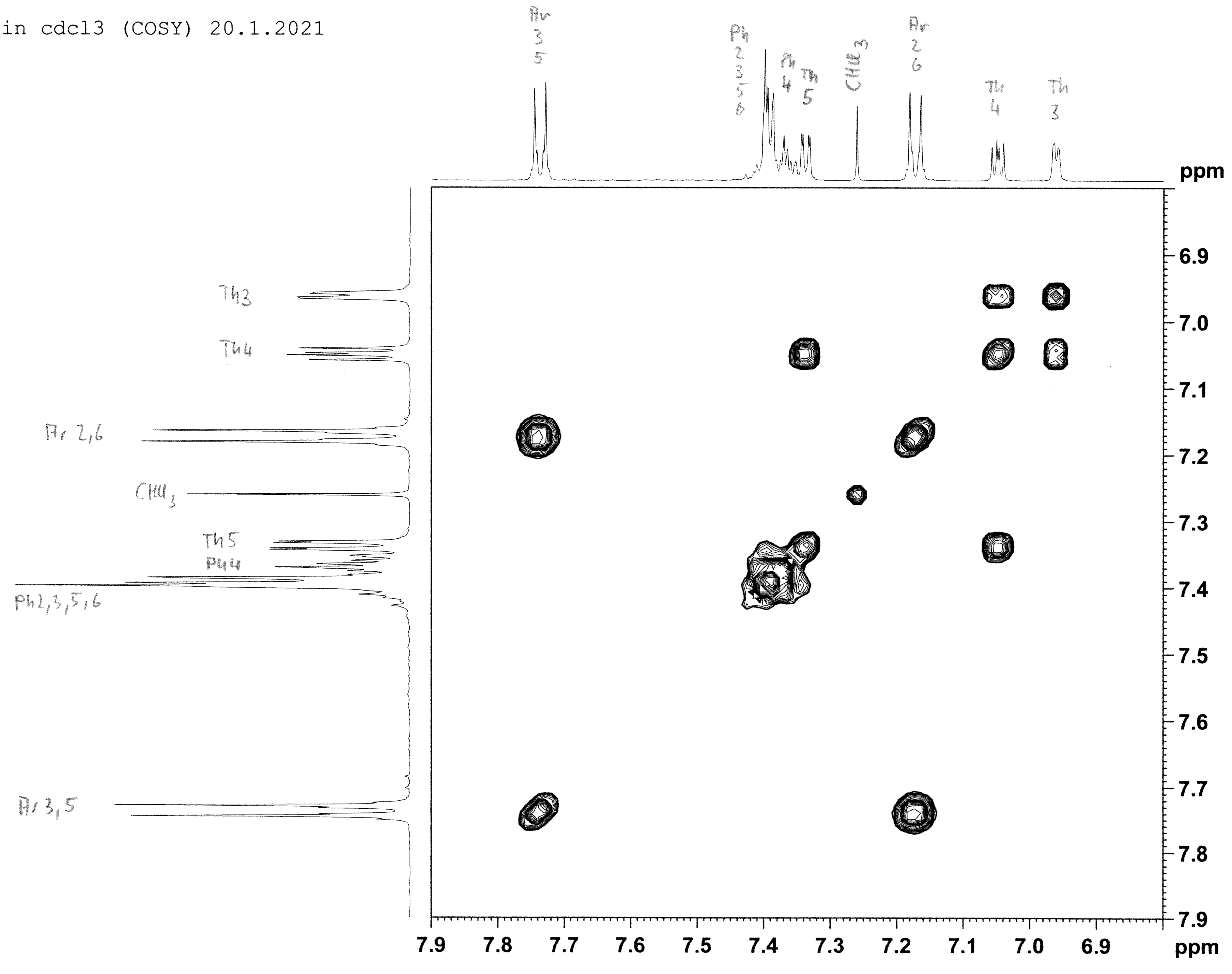

SB002-1 in cdcl3 (COSY) 20.1.2021

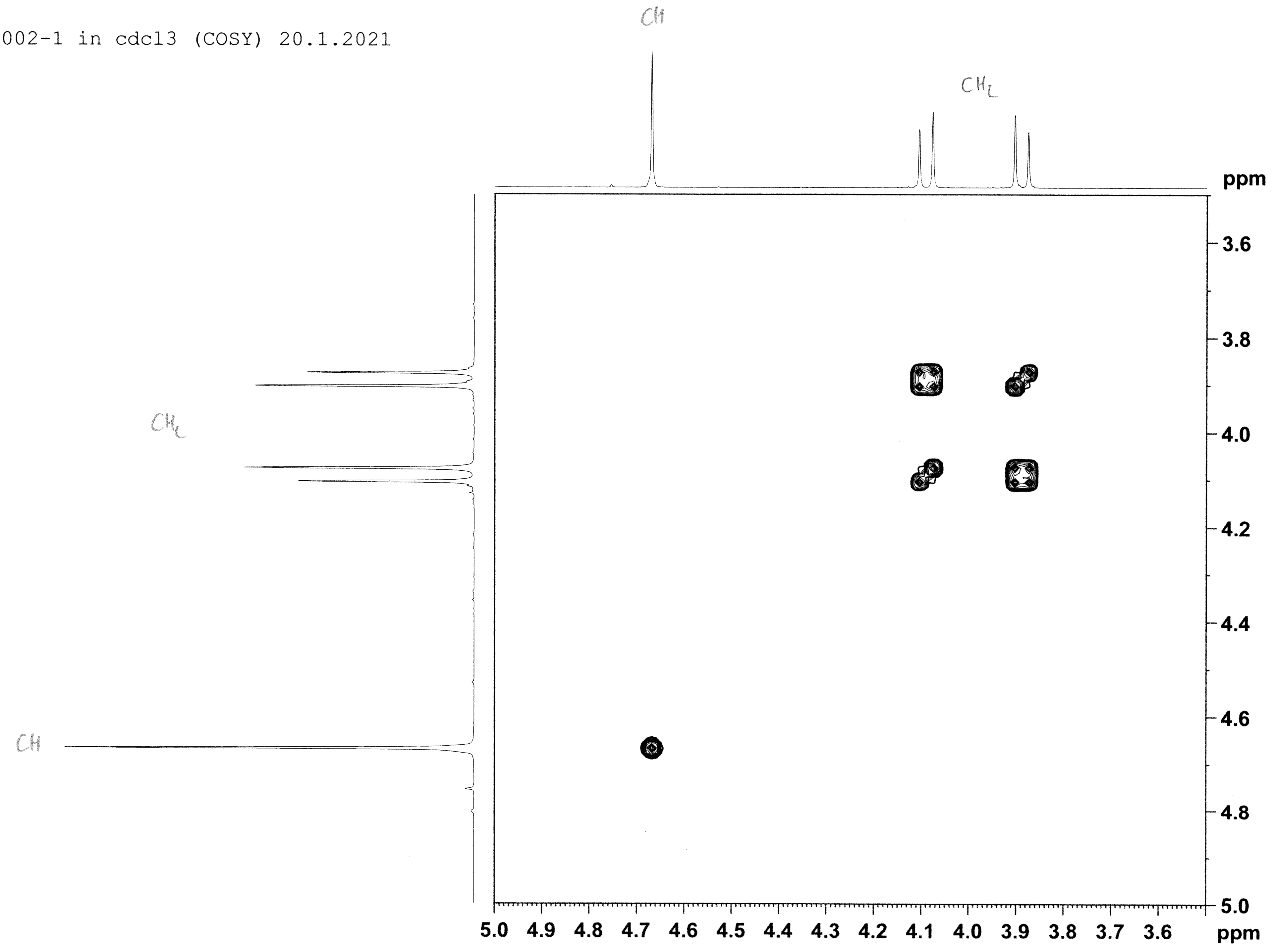

SB002-1 in cdcl3 (HSQC) 20.1.2021

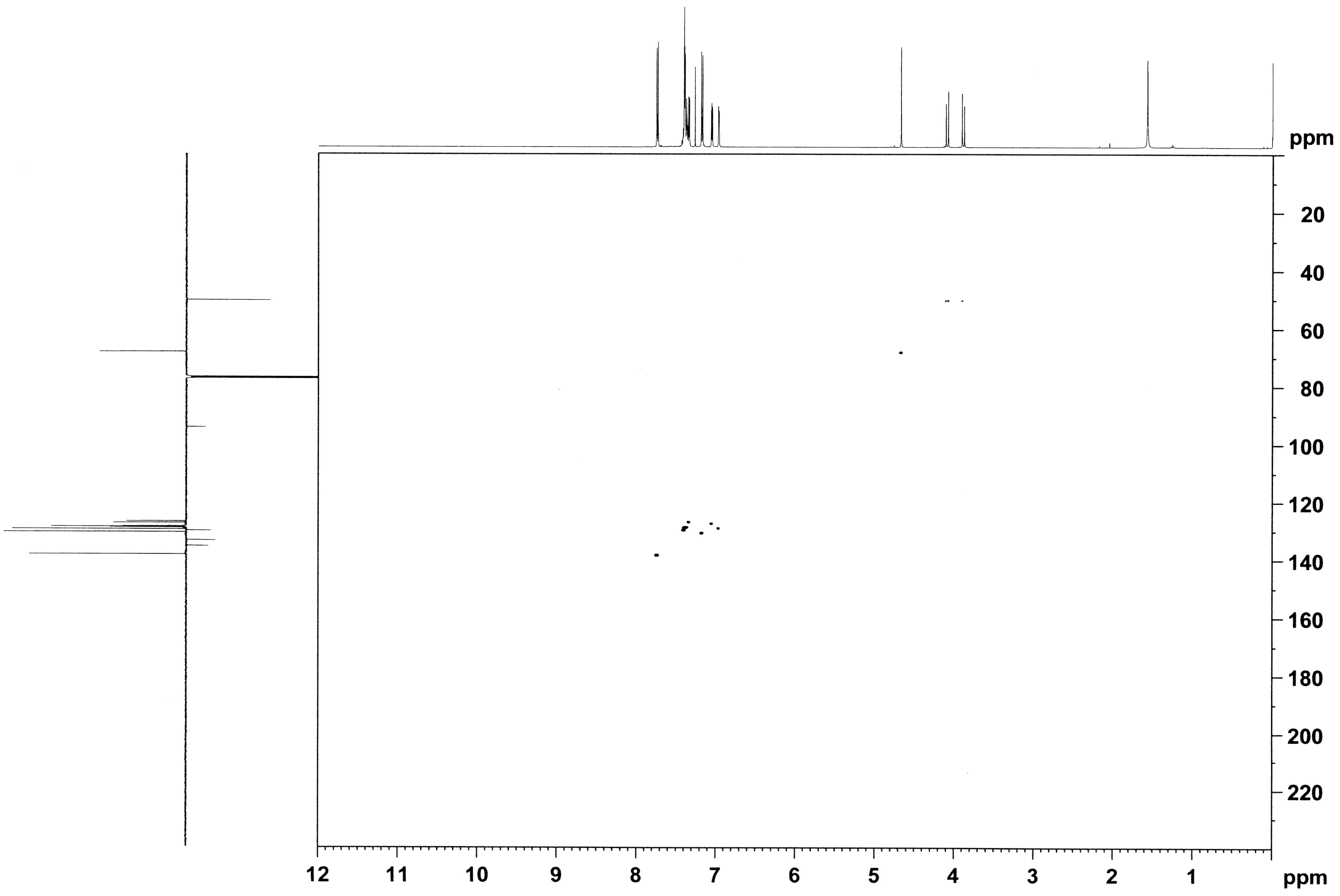

SB002-1 in cdcl3 (HSQC) 20.1.2021

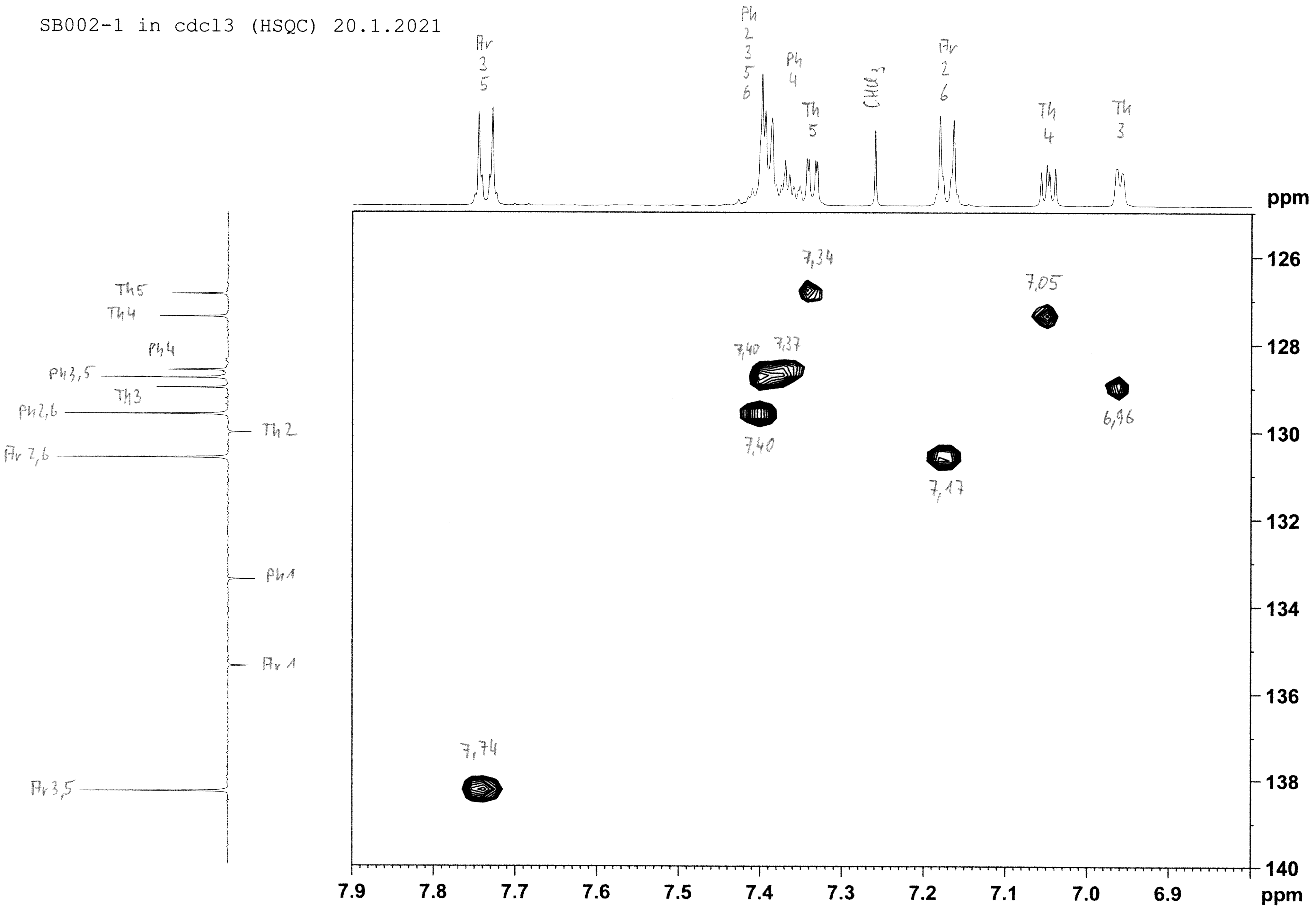

SB002-1 in cdcl3 (HSQC) 20.1.2021

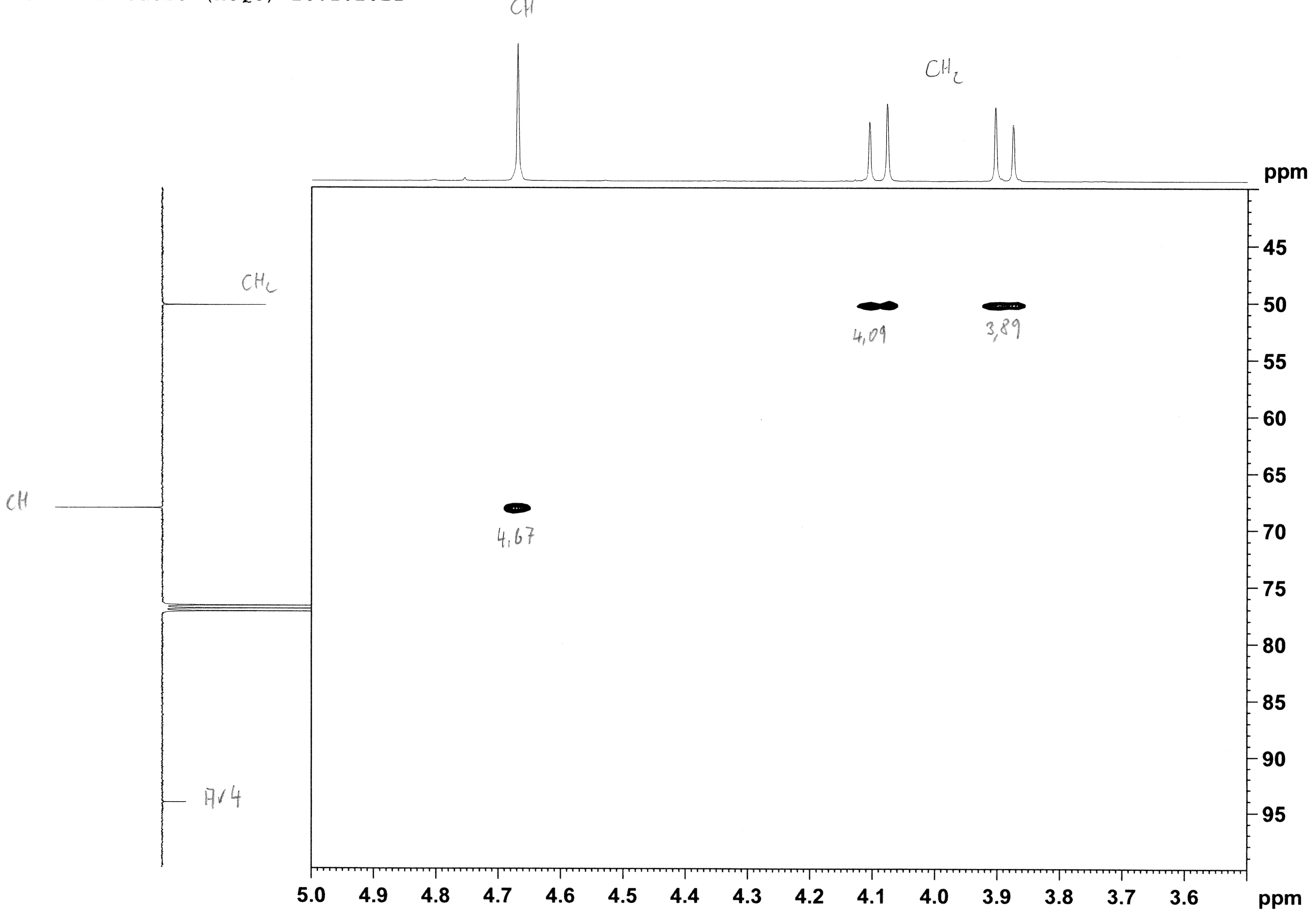

SB002-1 in cdcl3 (HMBC) 20.1.2021

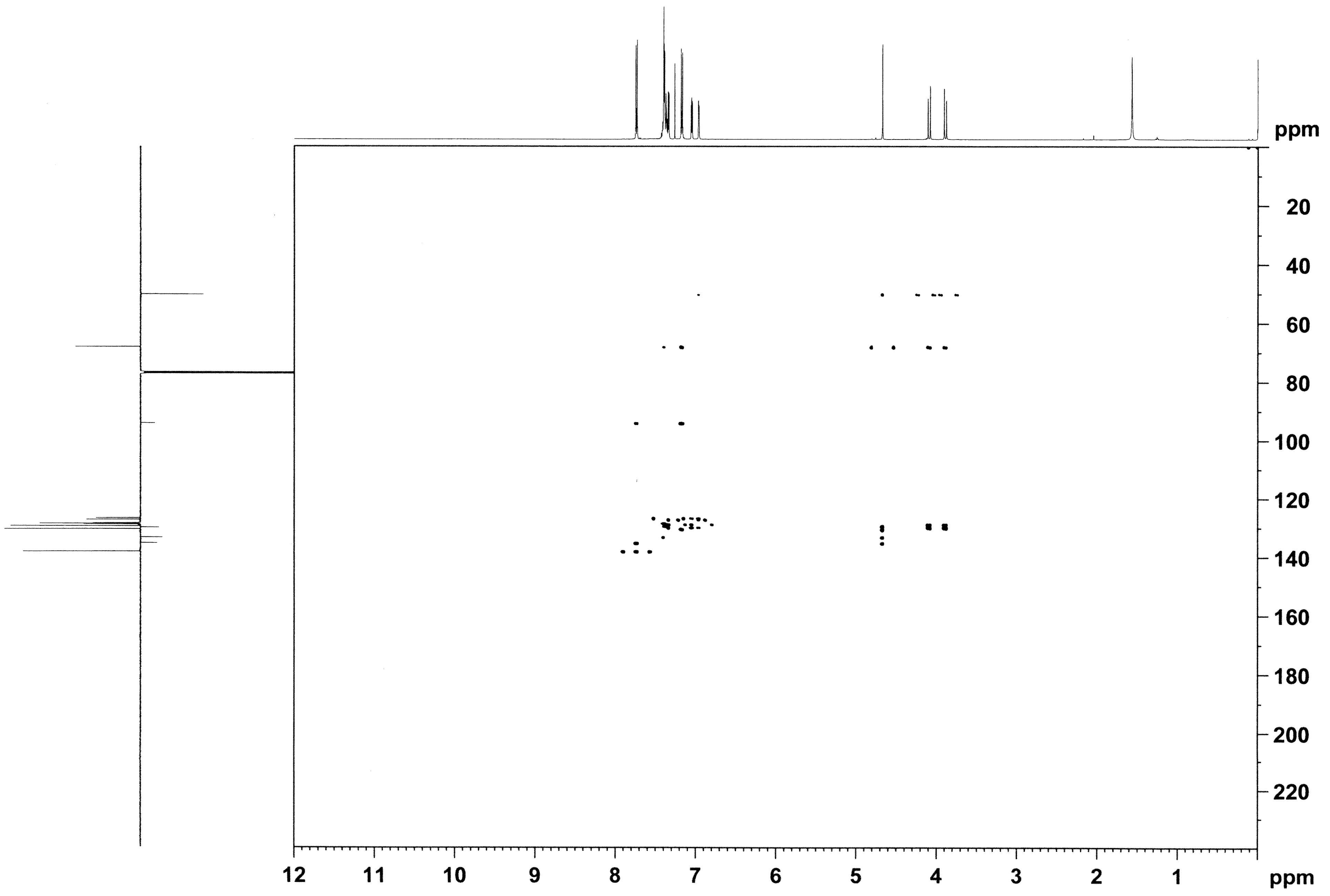

SB002-1 in cdcl3 (HMBC) 20.1.2021

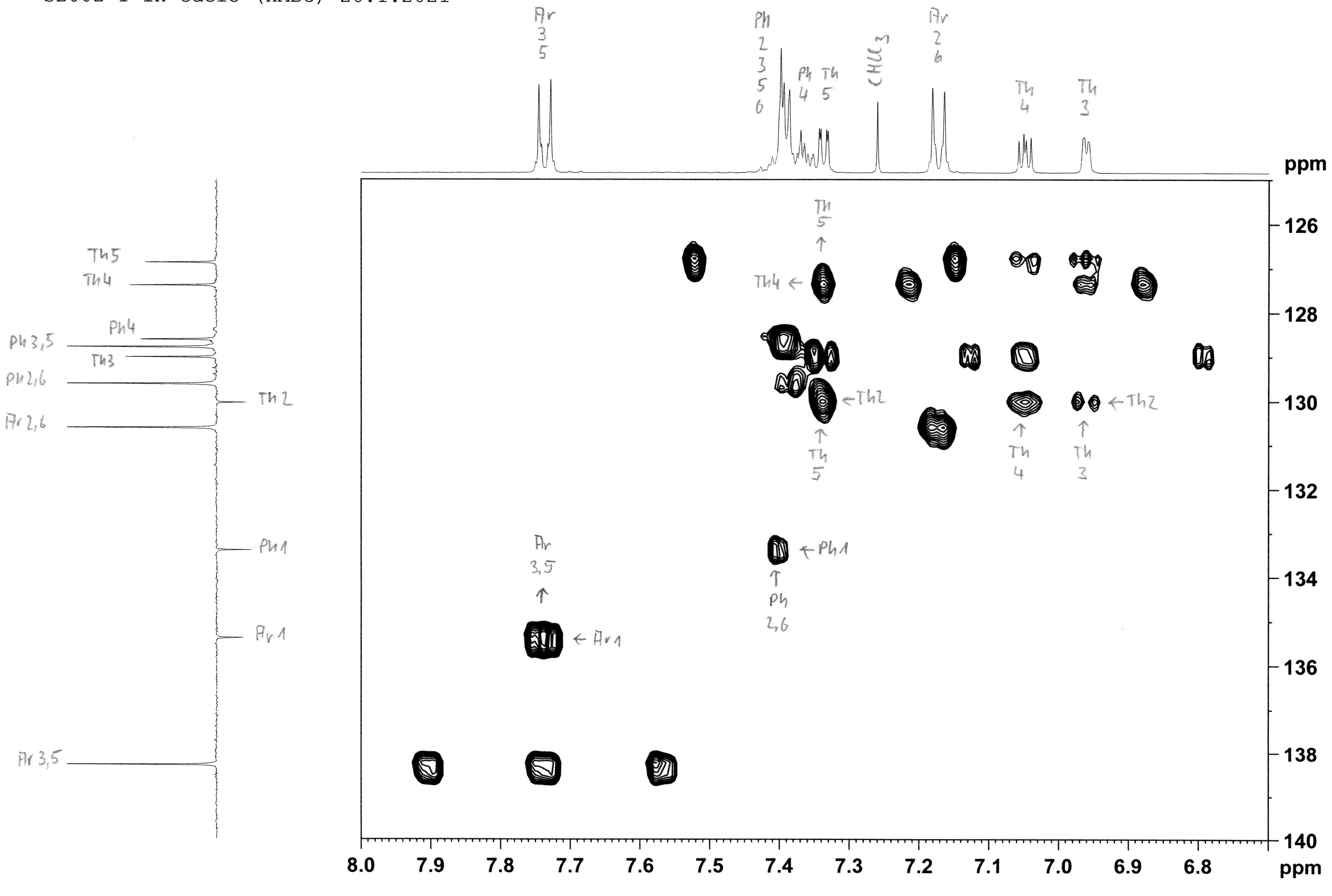

SB002-1 in cdcl3 (HMBC) 20.1.2021

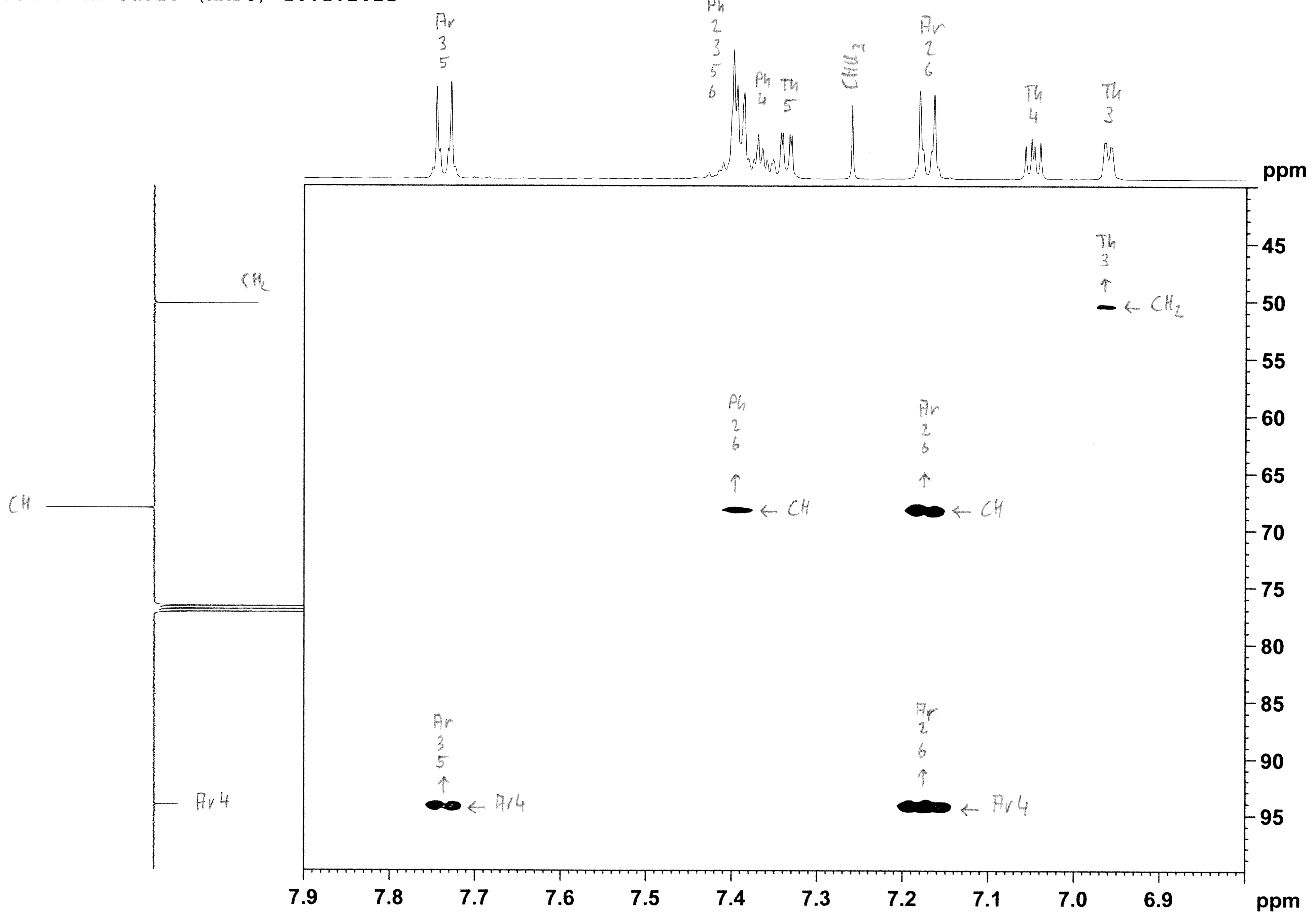

SB002-1 in cdcl3 (HMBC) 20.1.2021

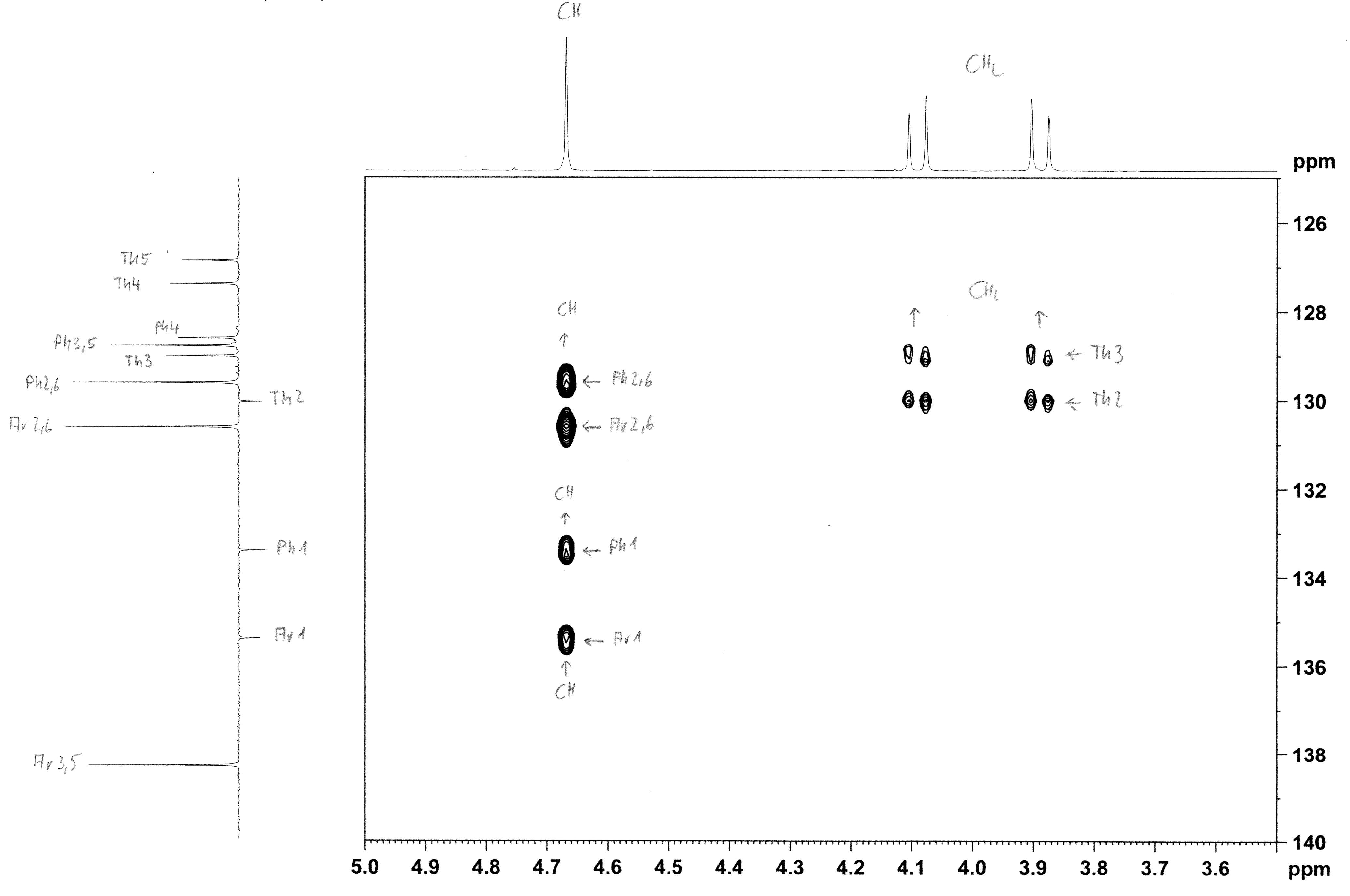

SB002-1 in cdcl3 (HMBC) 20.1.2021

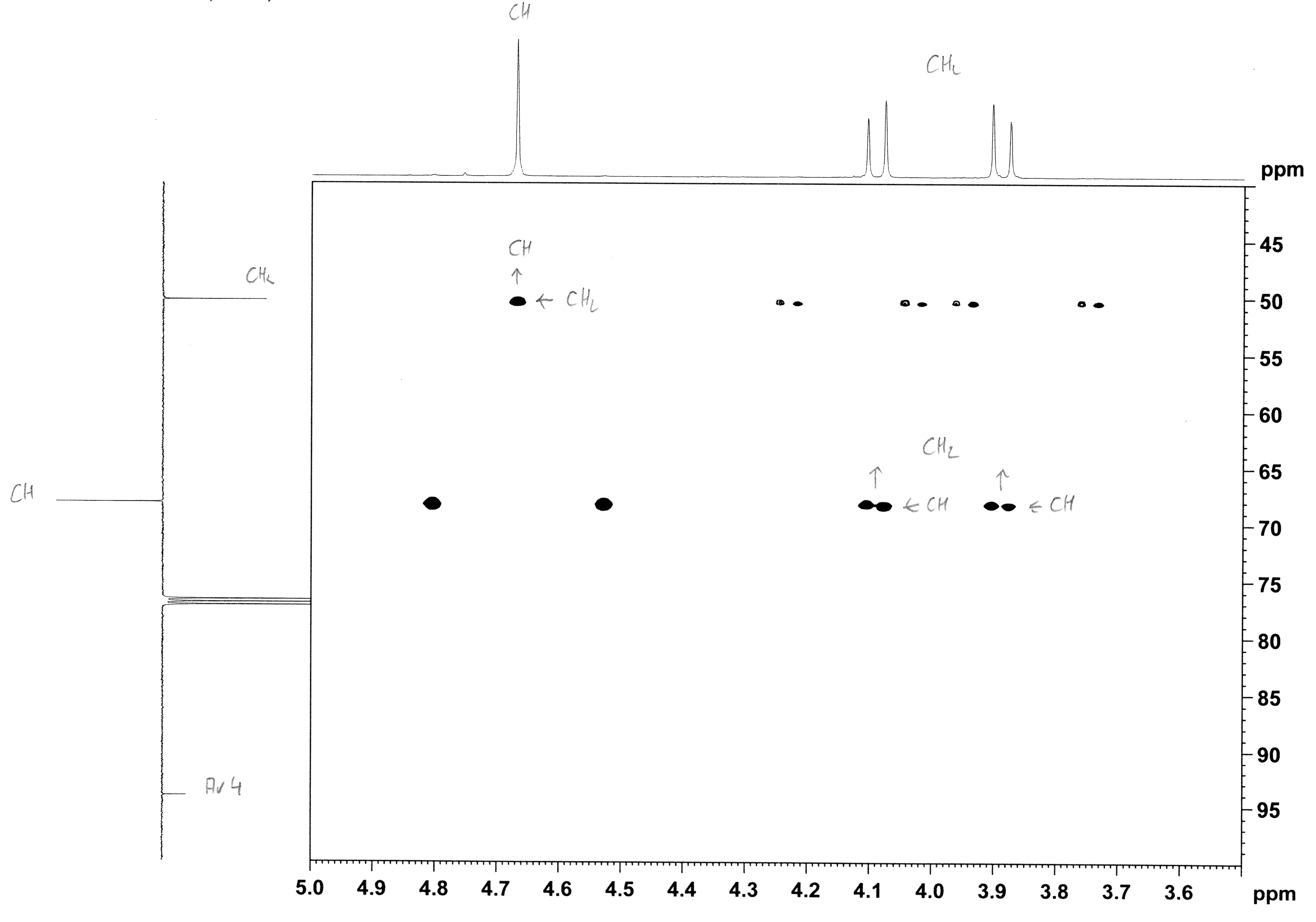

Figure S44c. NMR spectra of compound **8k**.

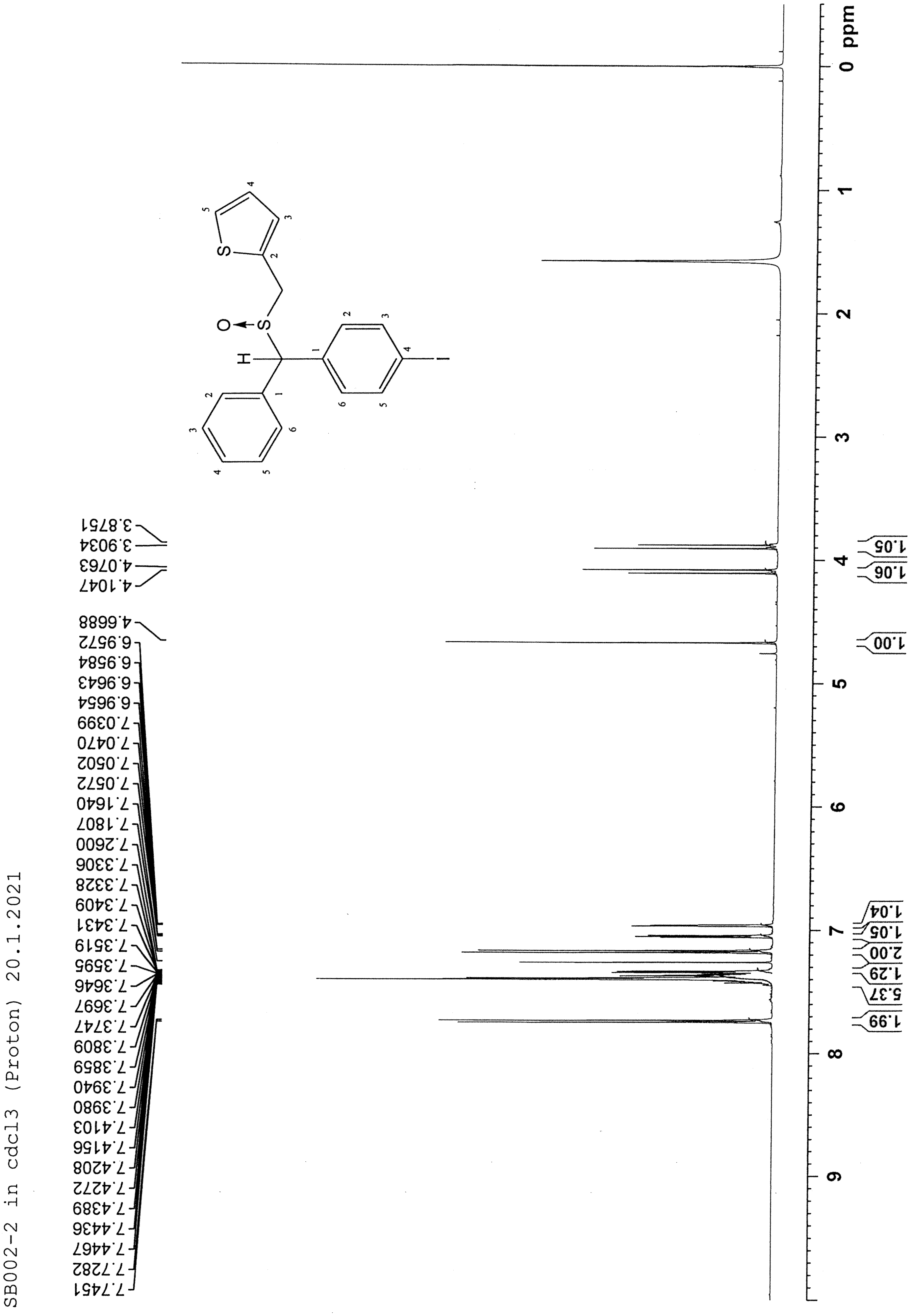

SB002-2 in cdcl3 (Proton) 20.1.2021

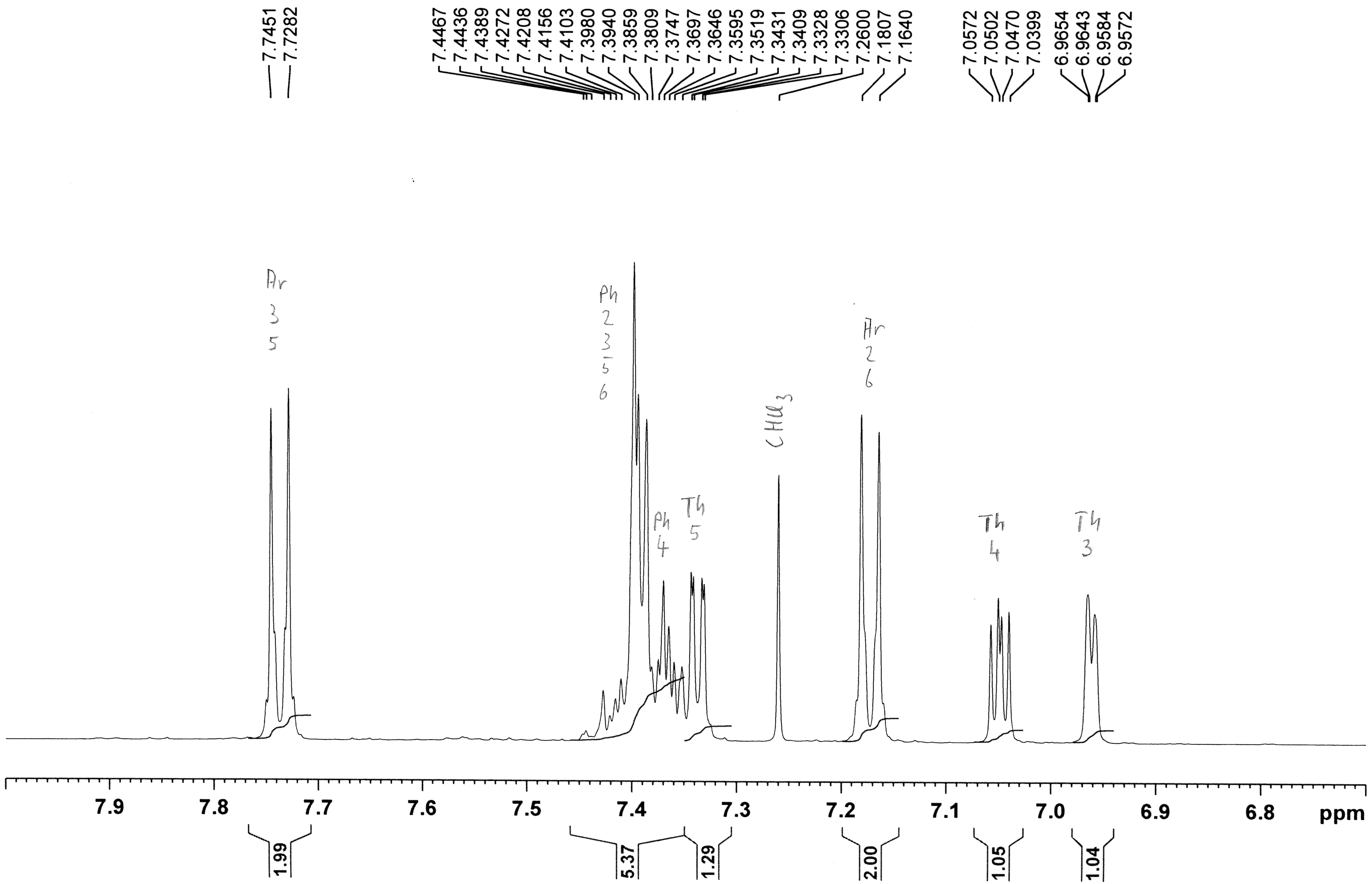

SB002-2 in cdcl3 (Proton) 20.1.2021

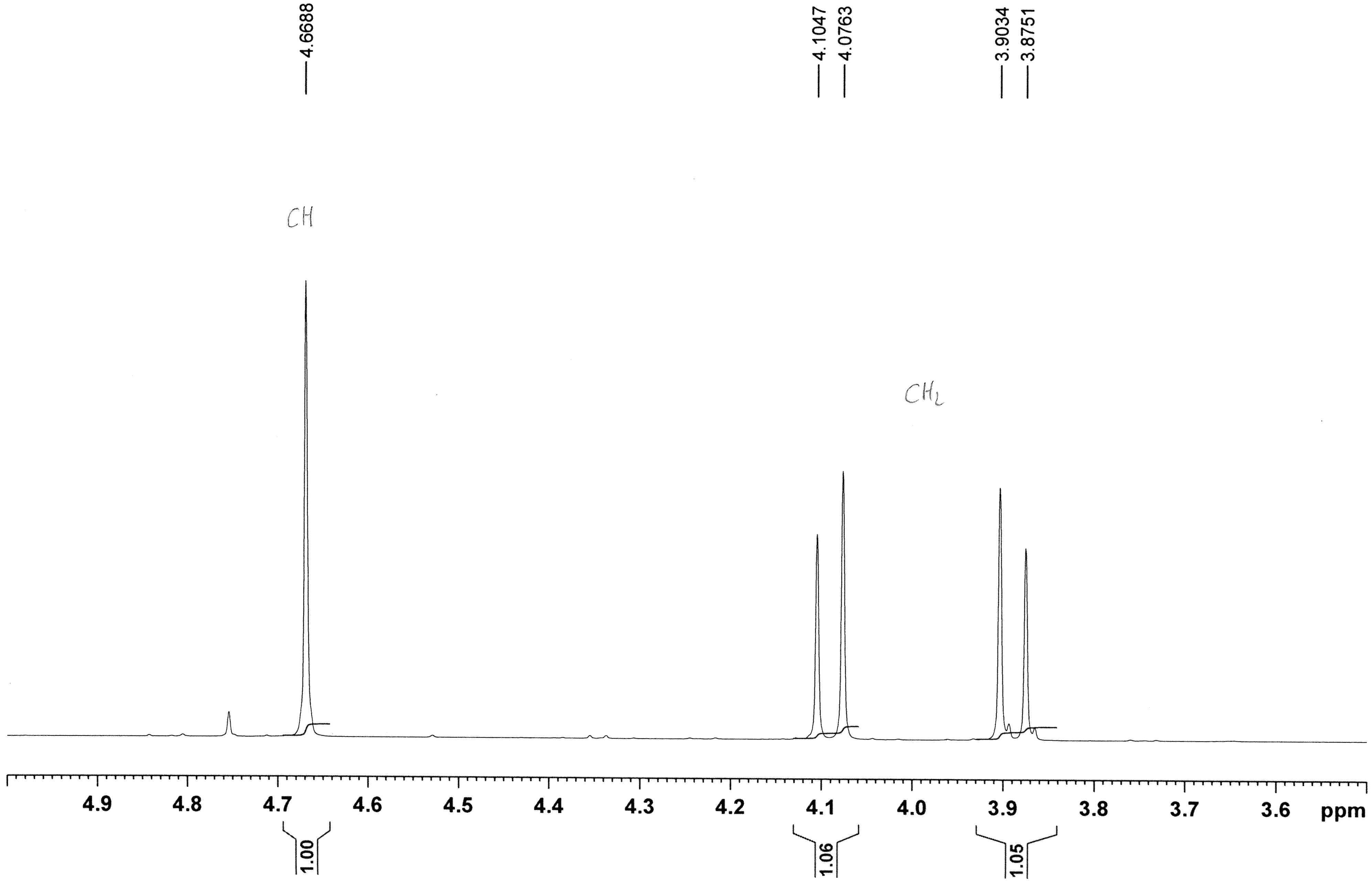

SB002-2 in cdcl3 (APT) 20.1.2021

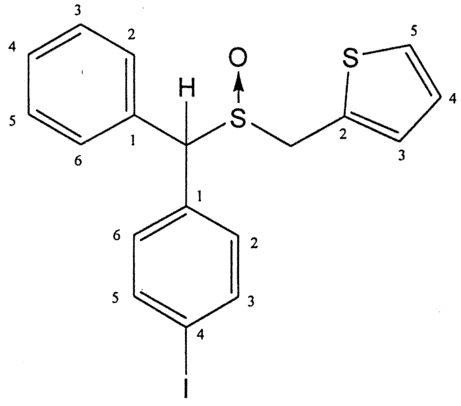

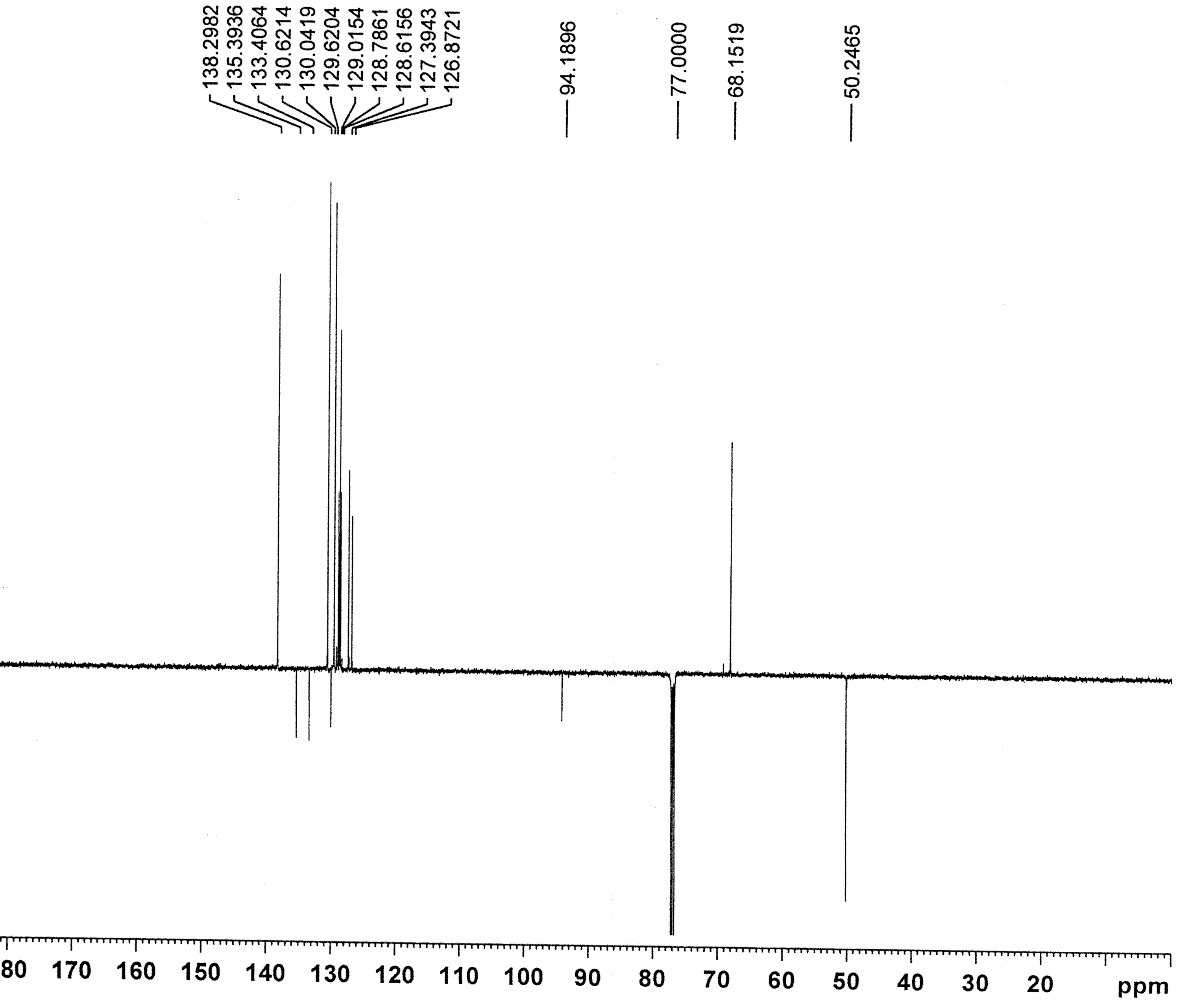

SB002-2 in cdcl3 (APT) 20.1.2021

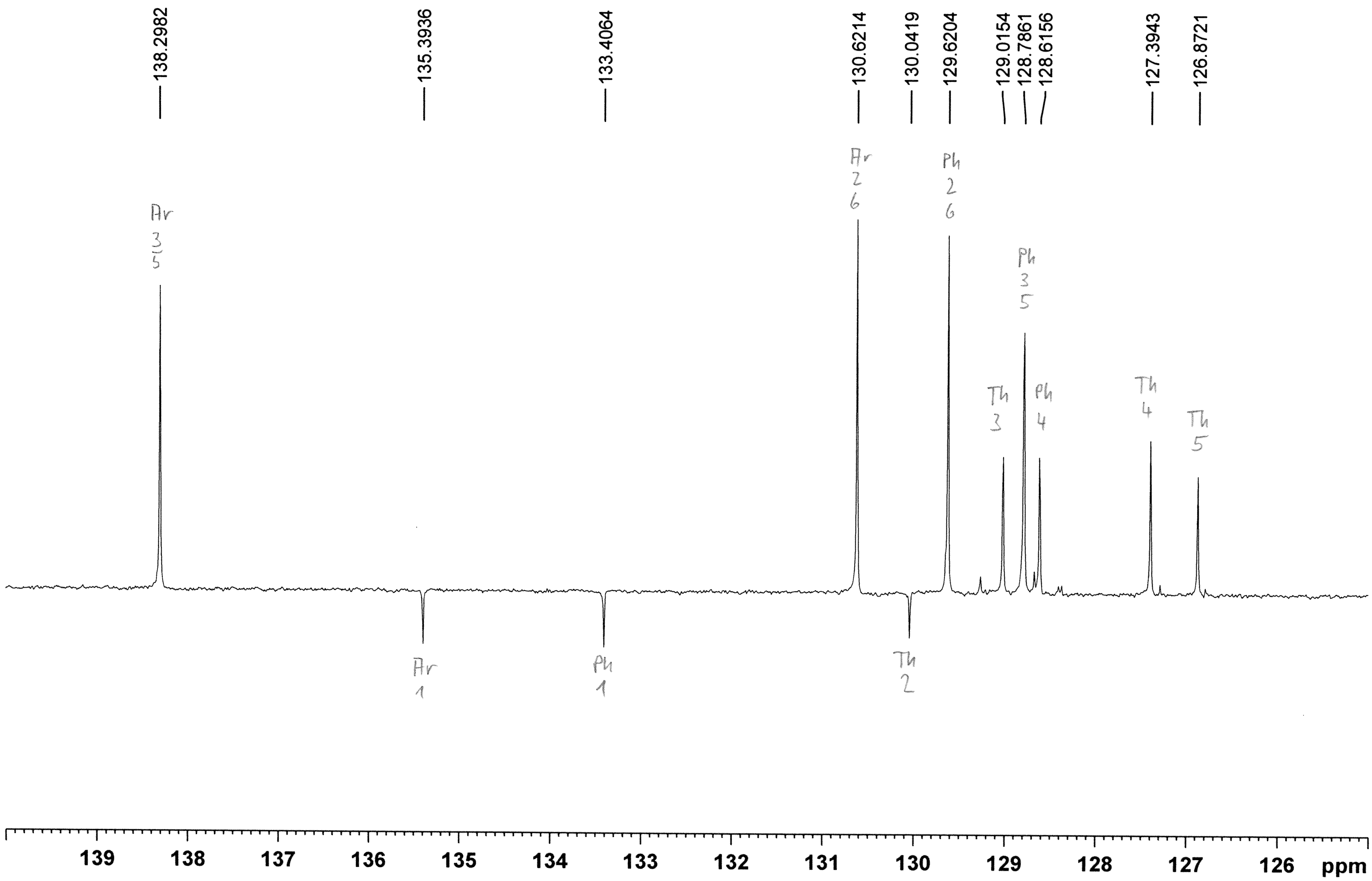

SB002-2 in cdcl3 (APT) 20.1.2021

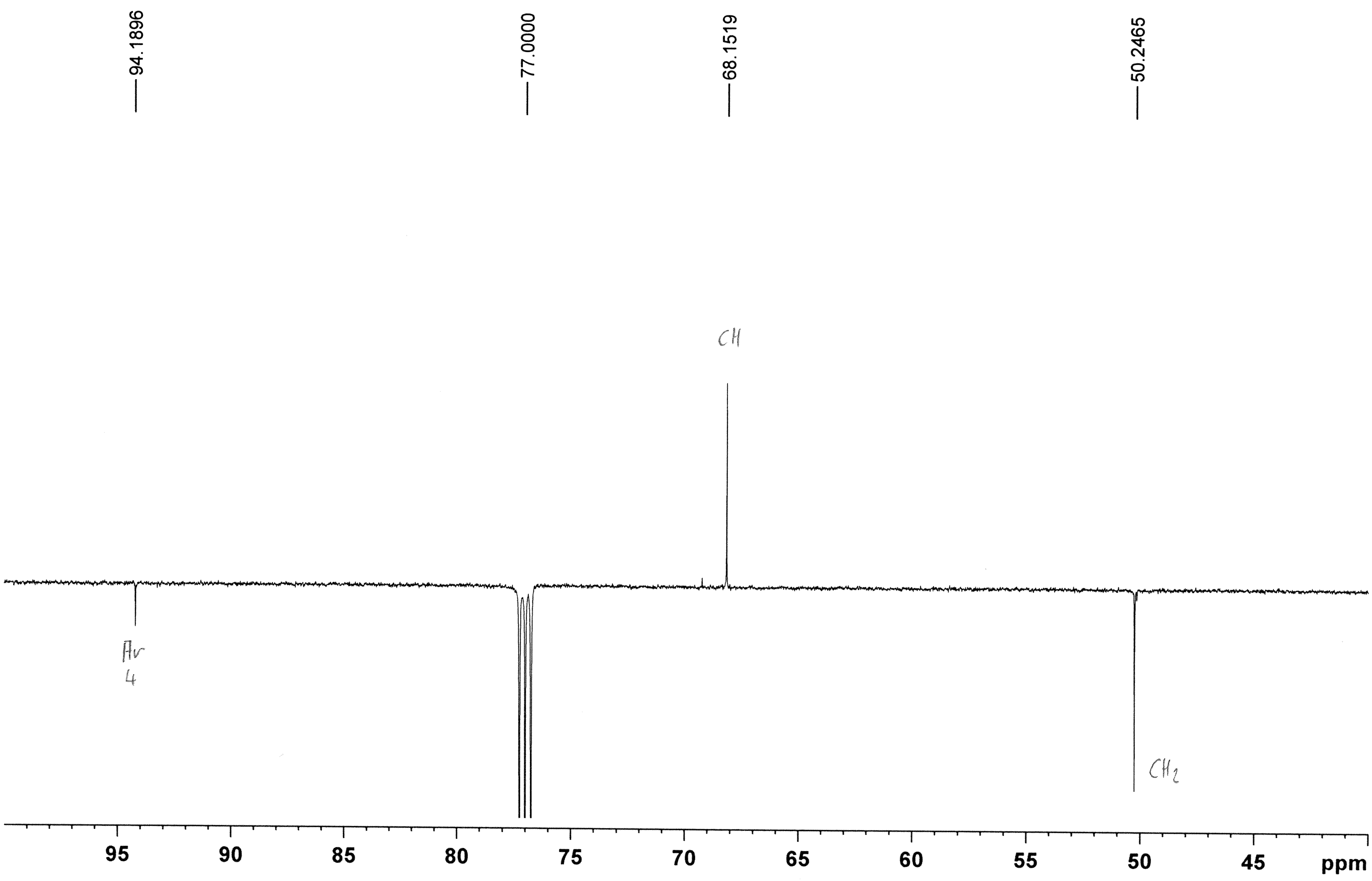

Figure S45c. NMR spectra of compound **5l.**

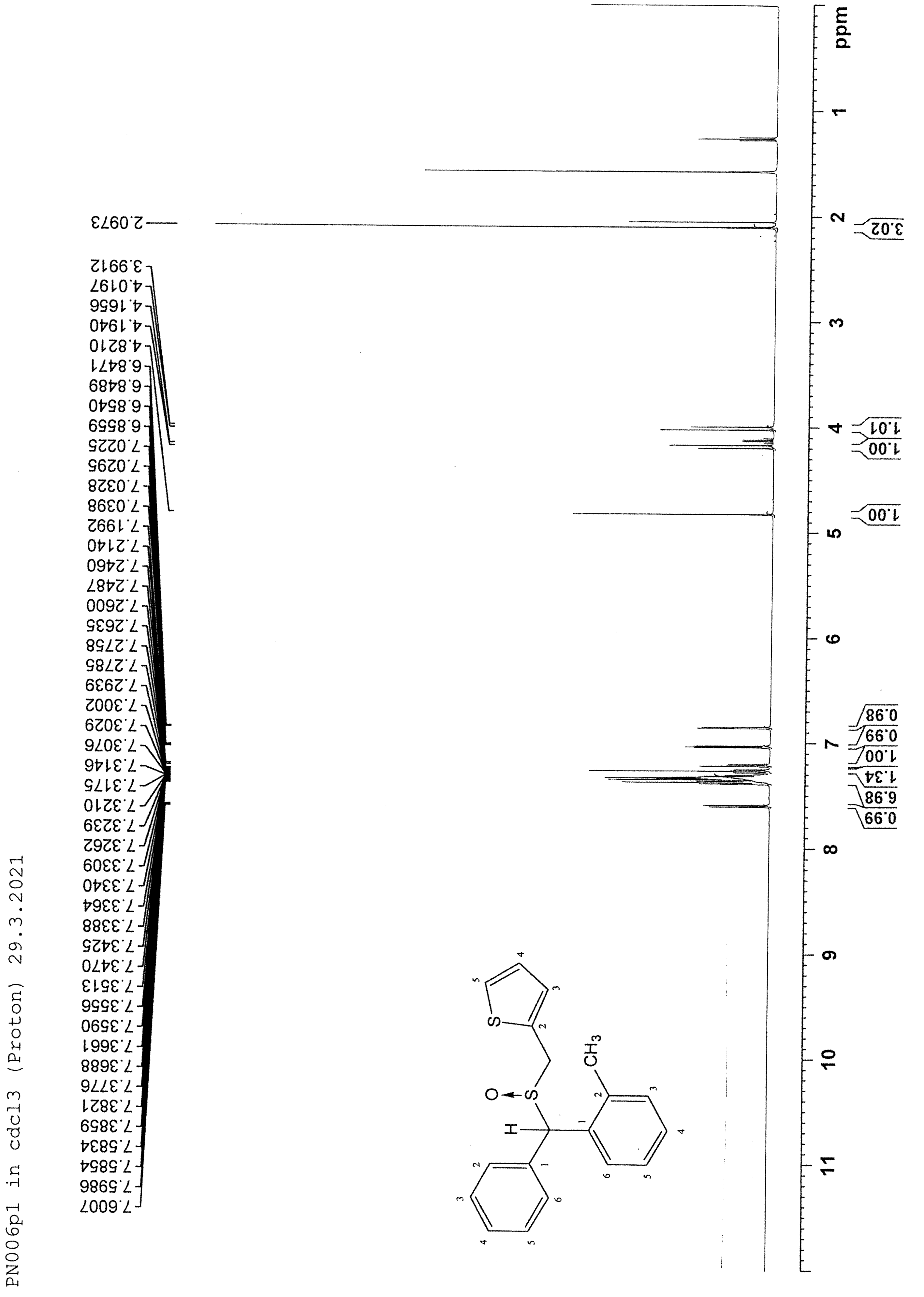

PN006p1 in cdcl3 (Proton) 29.3.2021

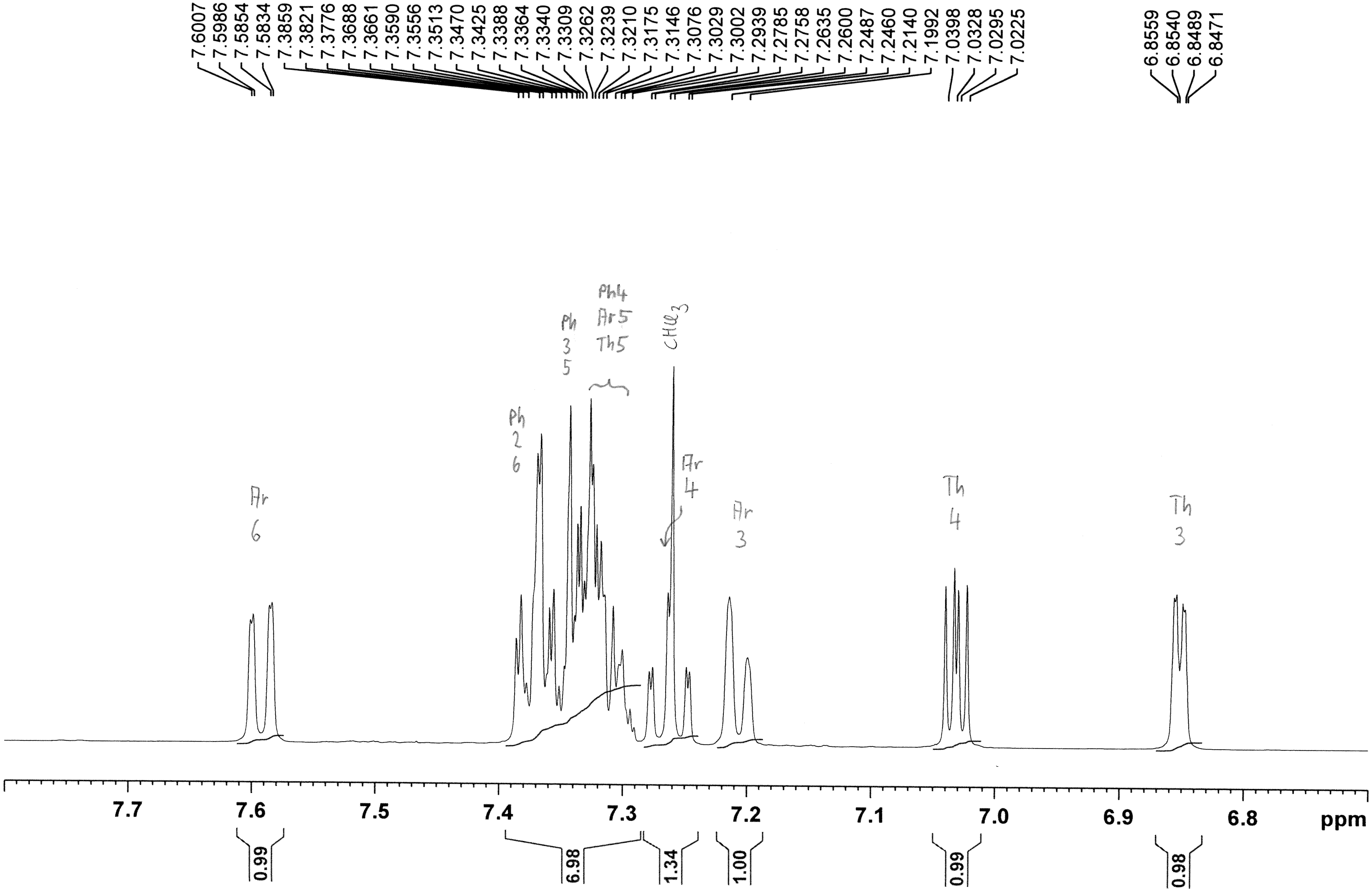

PN006p1 in cdcl3 (Proton) 29.3.2021

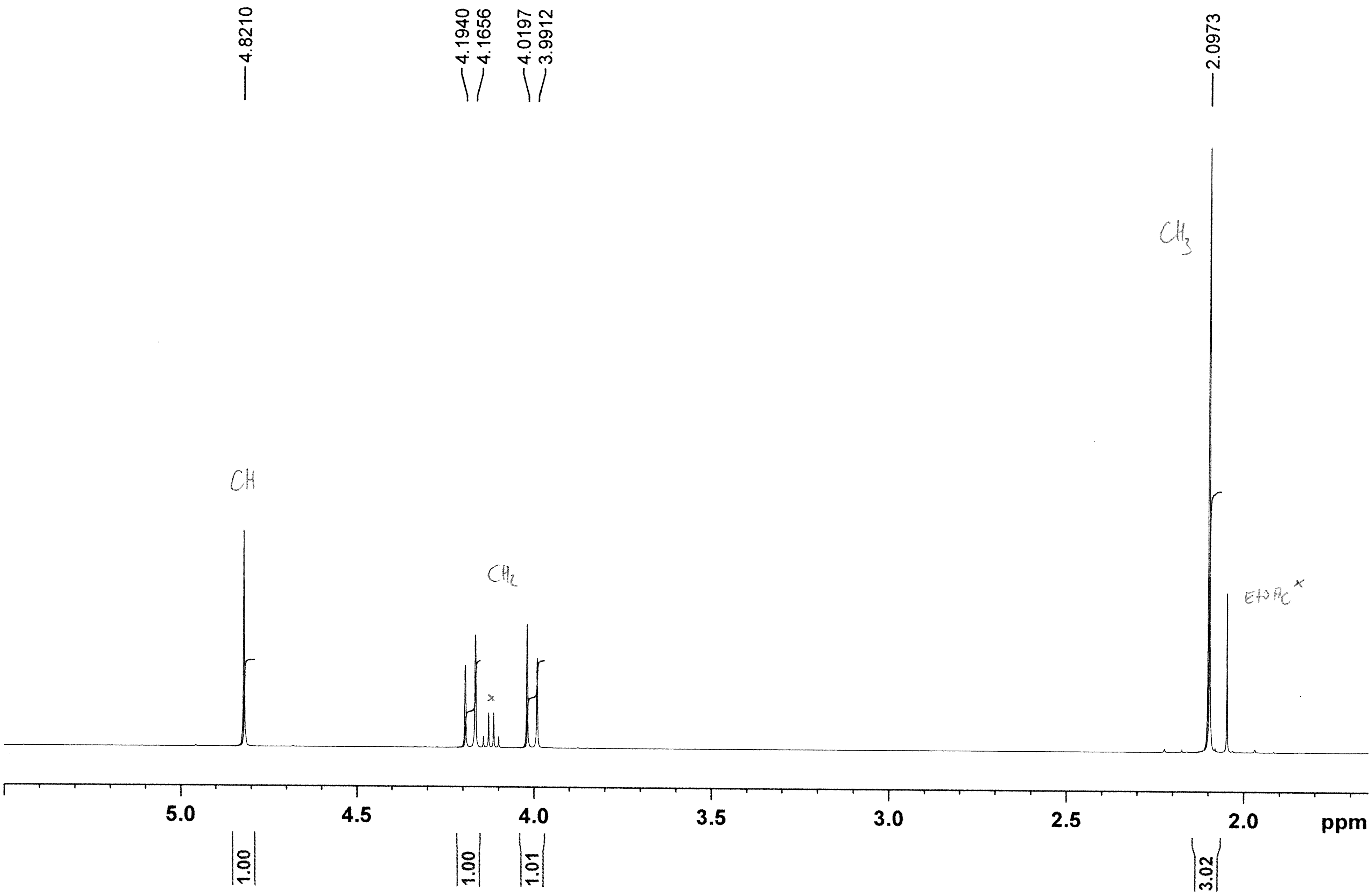

PN006p1 in cdcl3 (APT) 29.3.2021

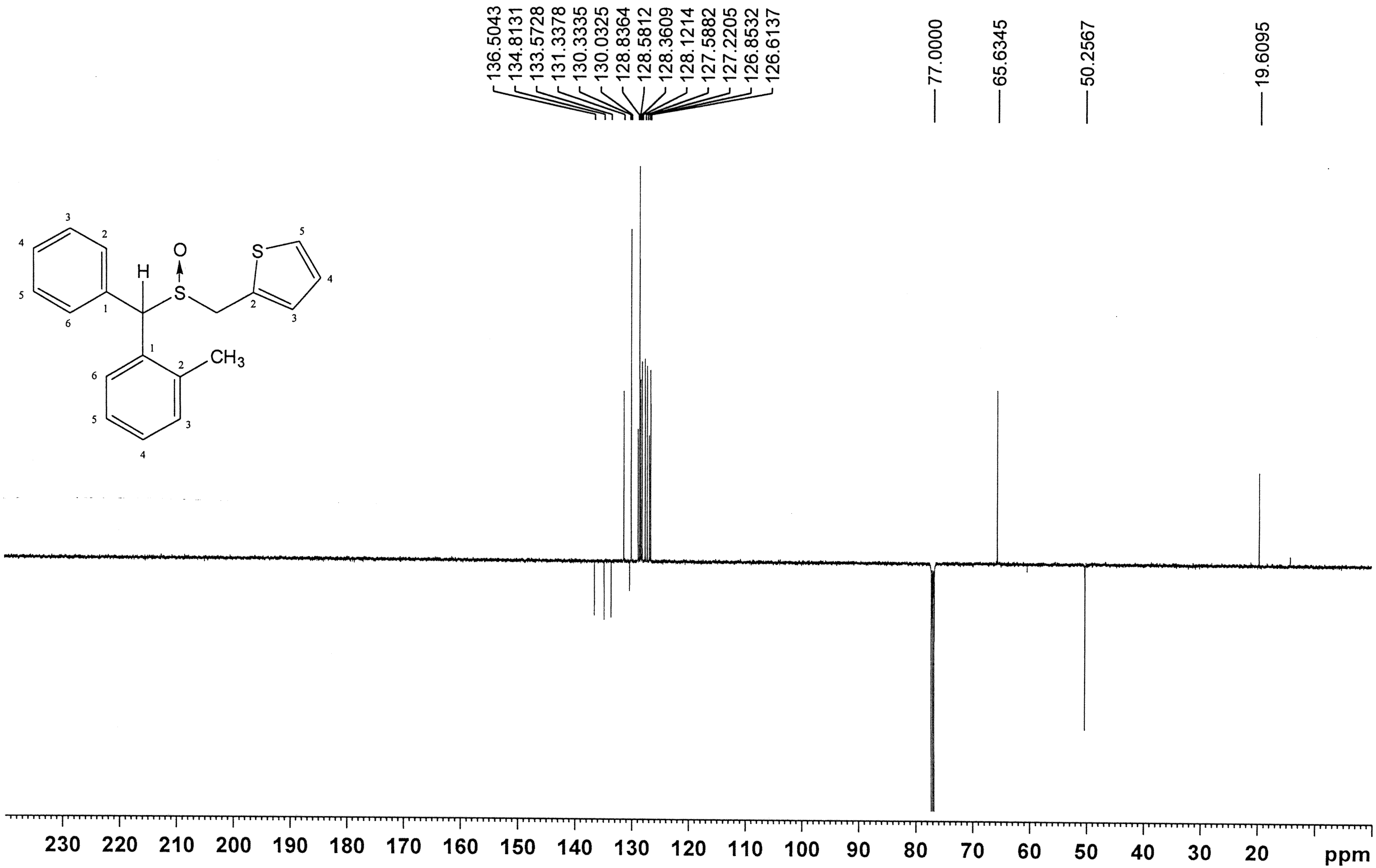

PN006p1 in cdcl3 (APT) 29.3.2021

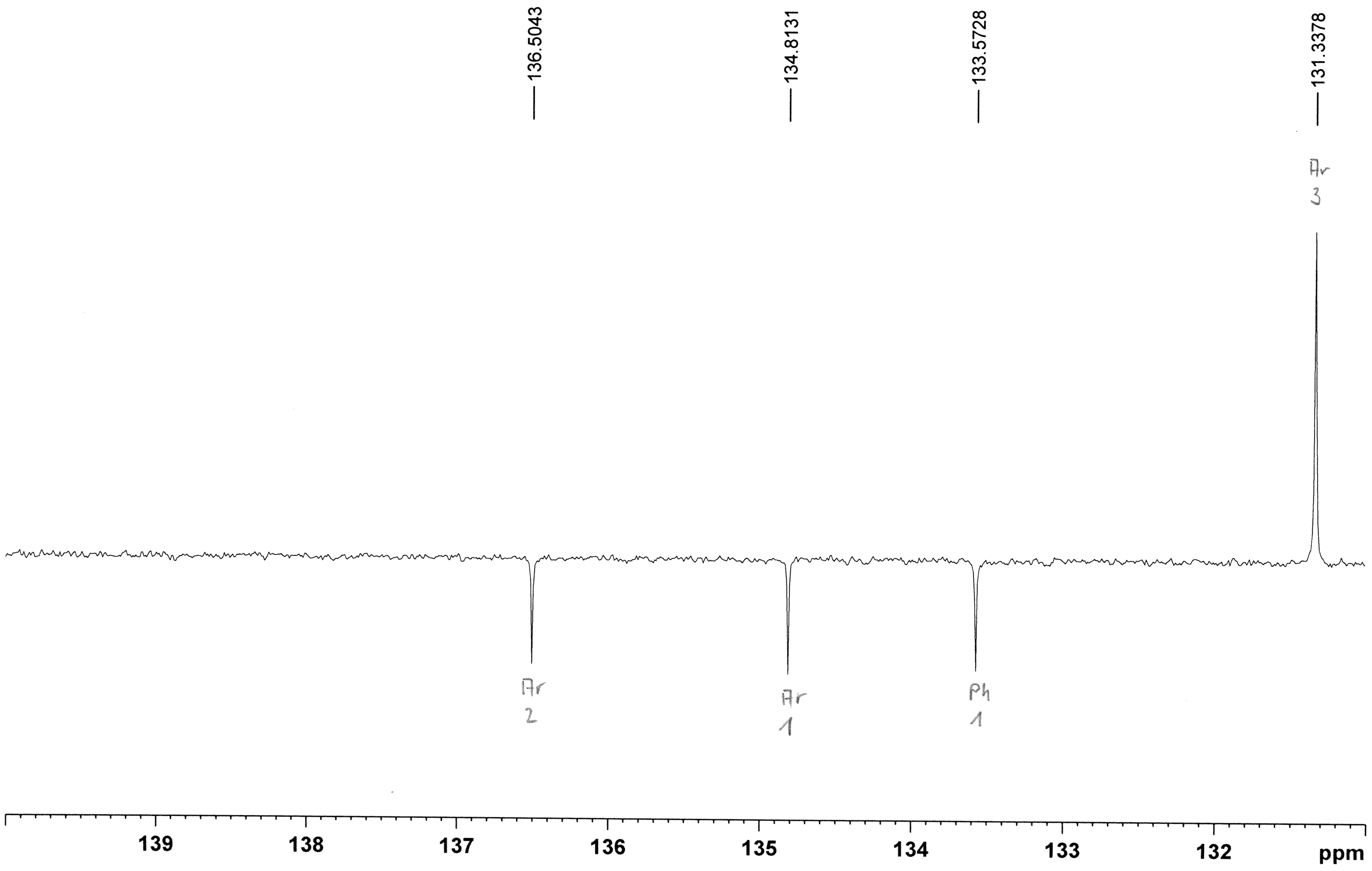

PN006p1 in cdcl3 (APT) 29.3.2021

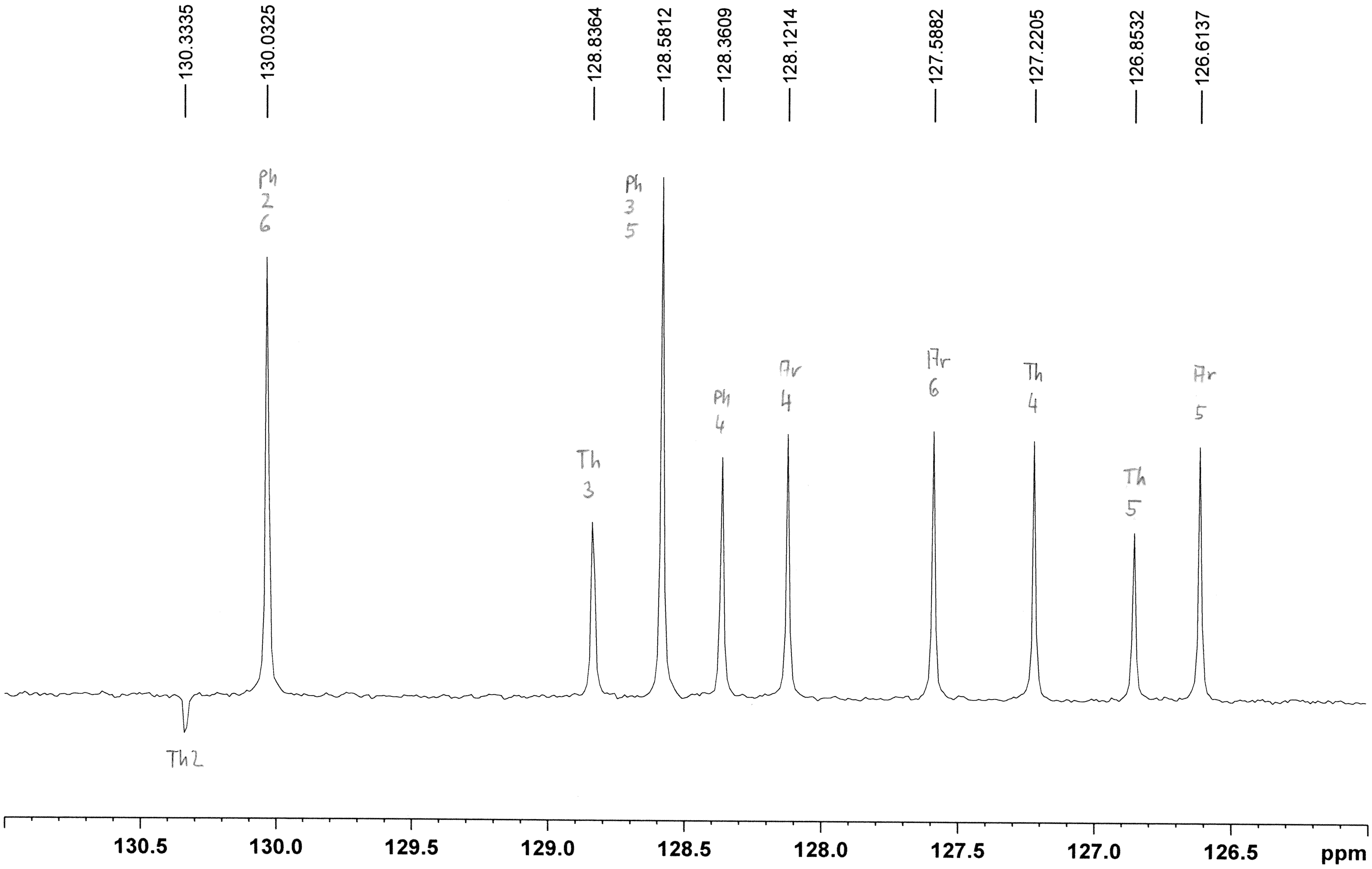

PN006p1 in cdcl3 (APT) 29.3.2021

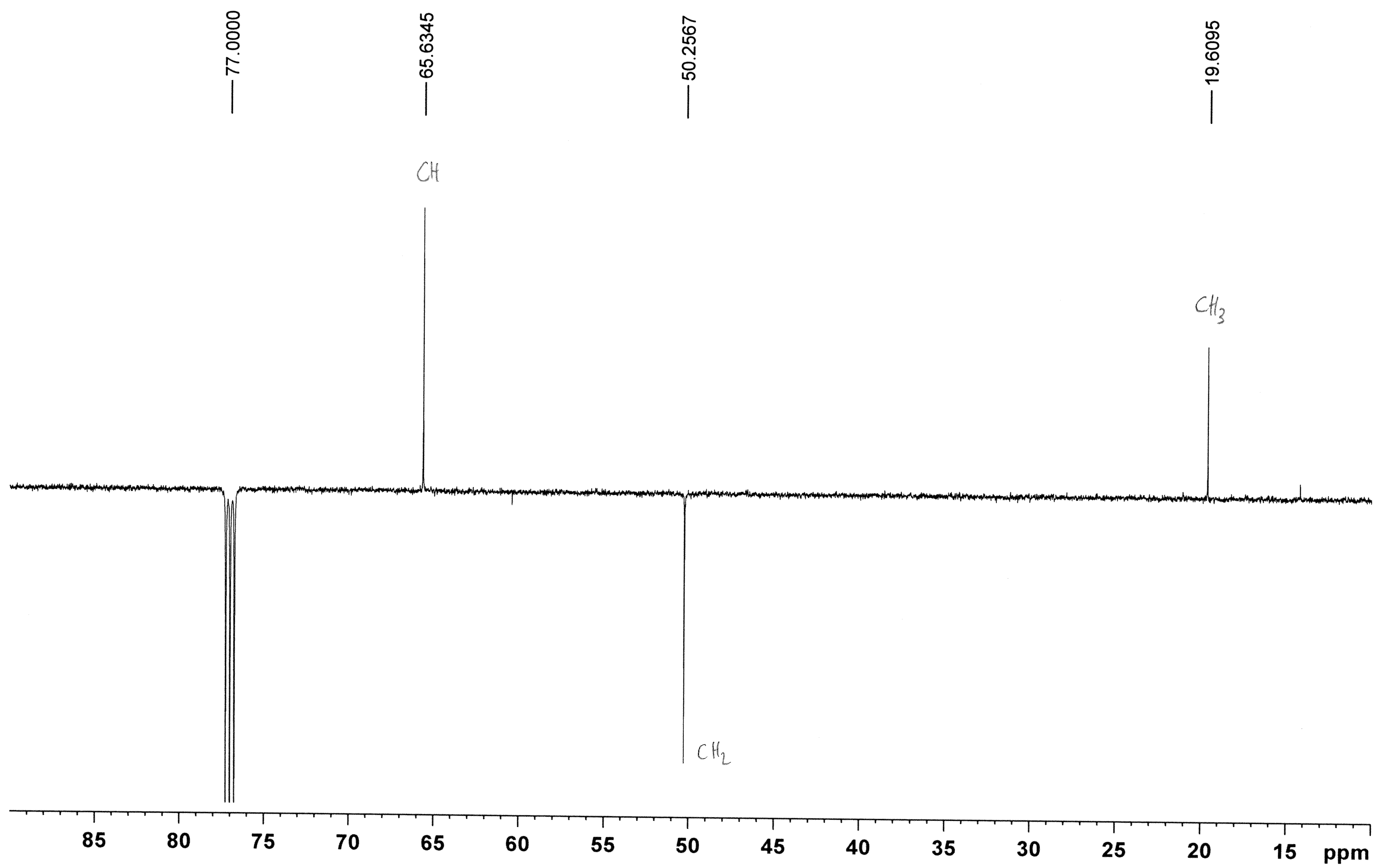

PN006p1 in cdcl3 (COSY) 29.3.2021

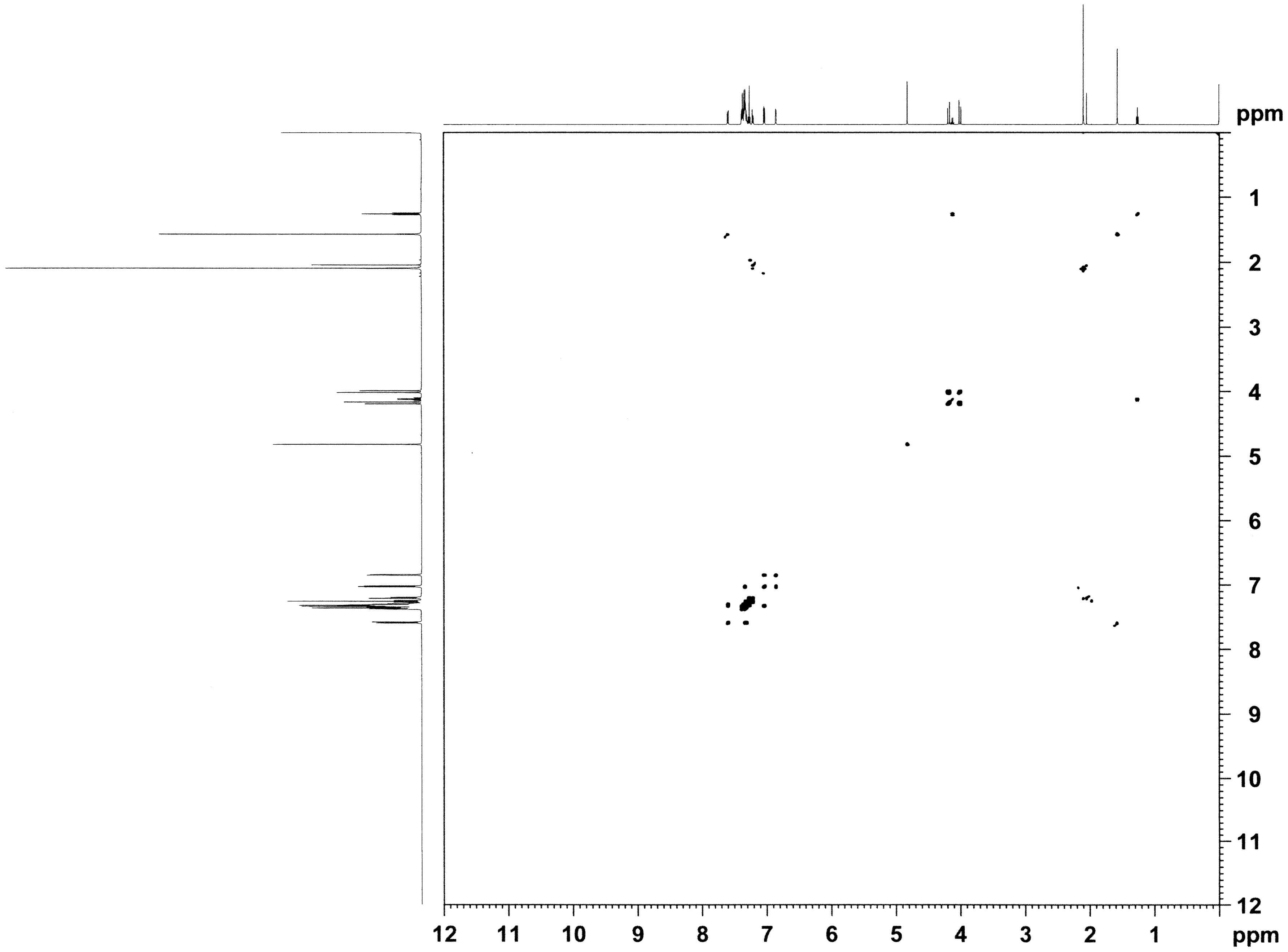

PN006p1 in cdcl3 (COSY) 29.3.2021

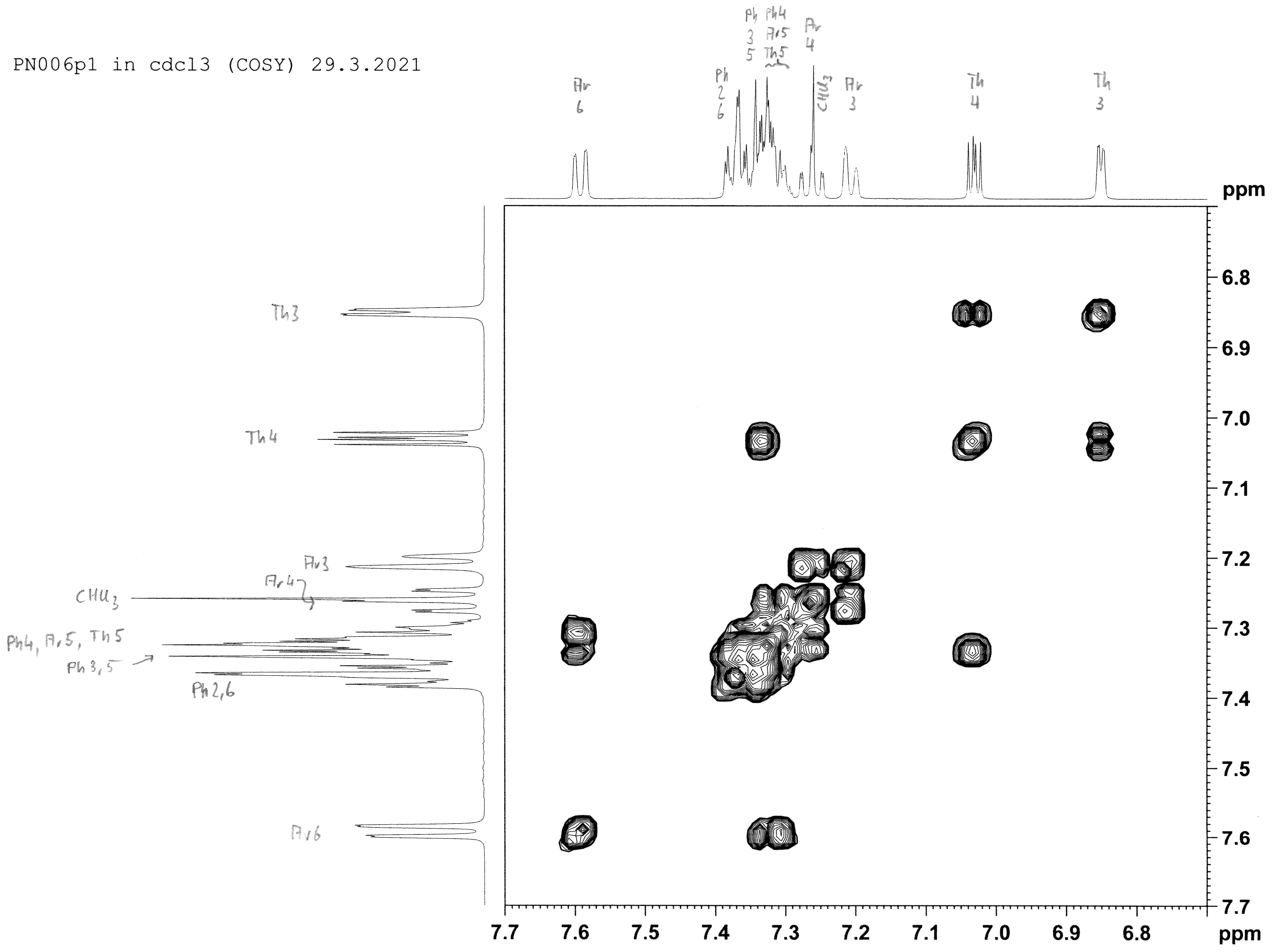

PN006p1 in cdcl3 (COSY) 29.3.2021

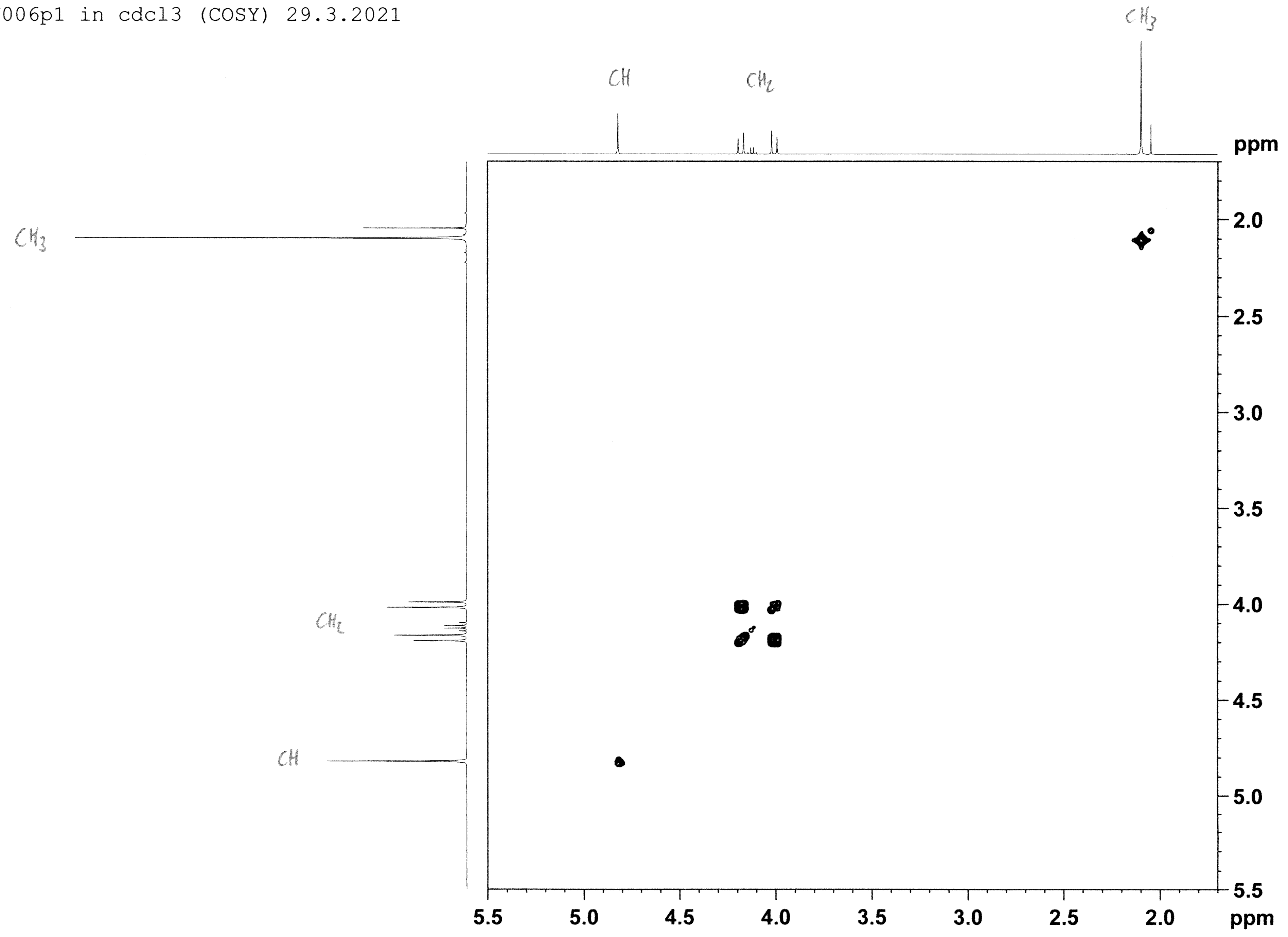

PN006p1 in cdcl3 (HSQC) 29.3.2021

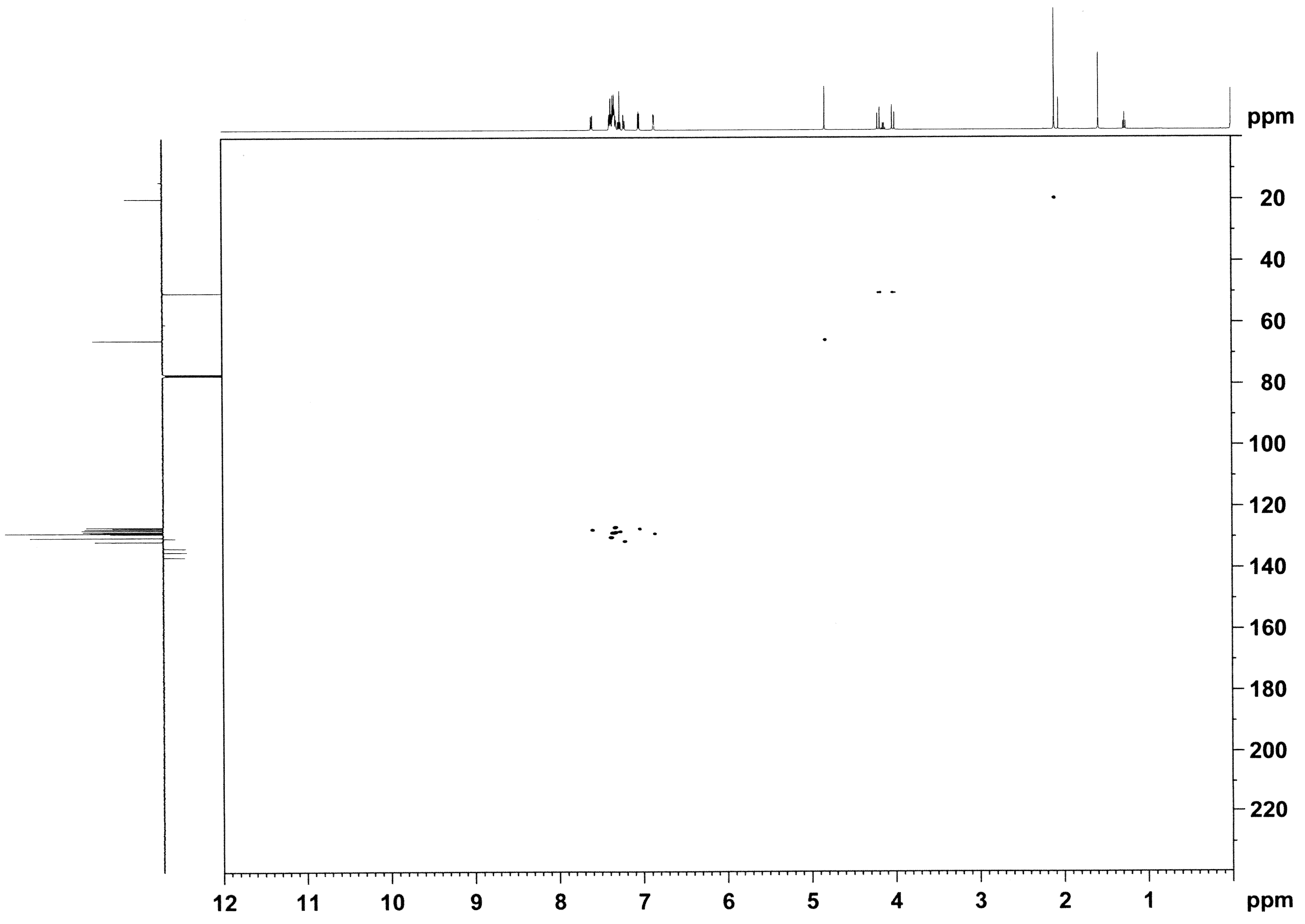

PN006p1 in cdcl3 (HSQC) 29.3.2021

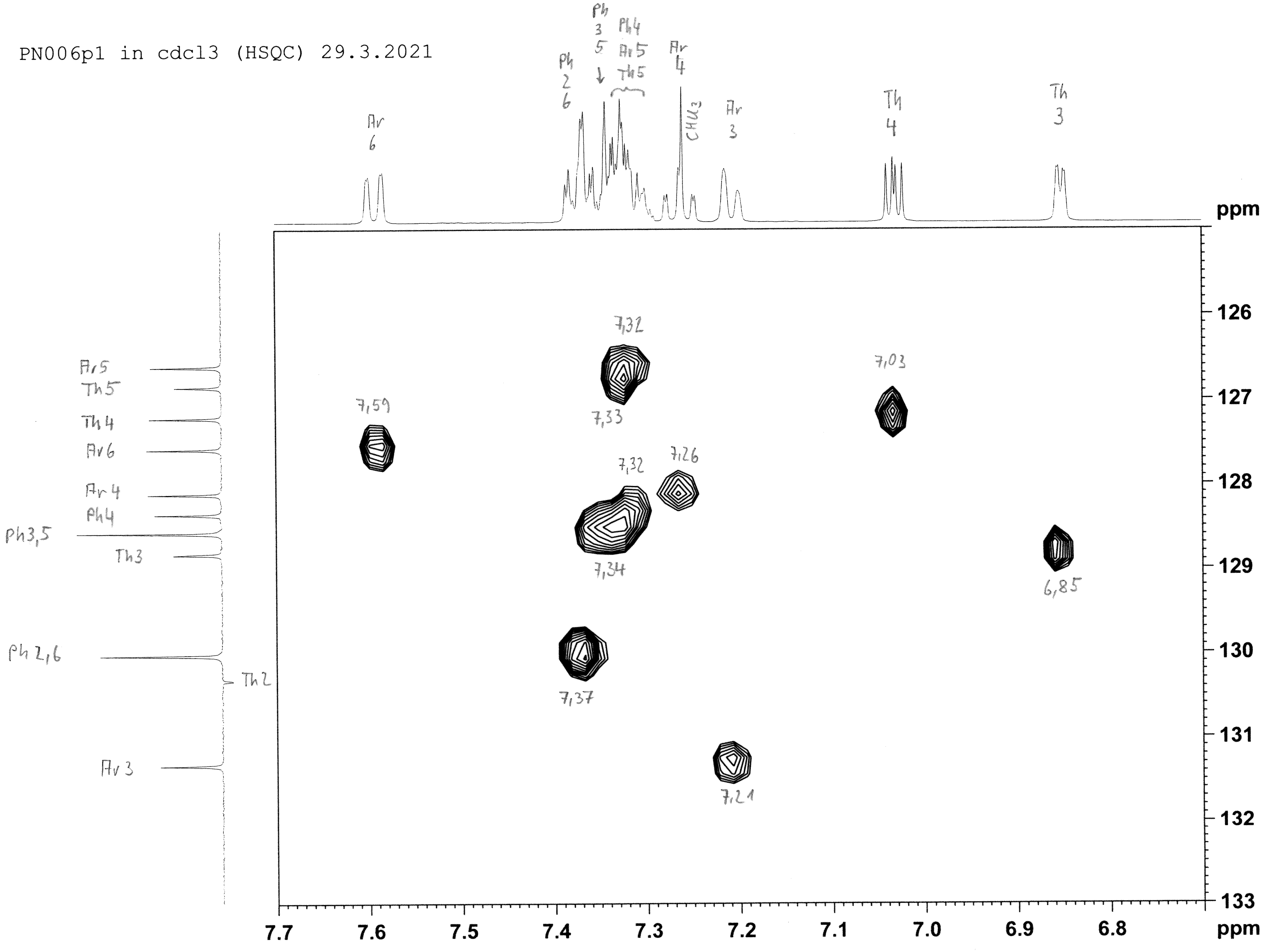

PN006p1 in cdcl3 (HSQC) 29.3.2021

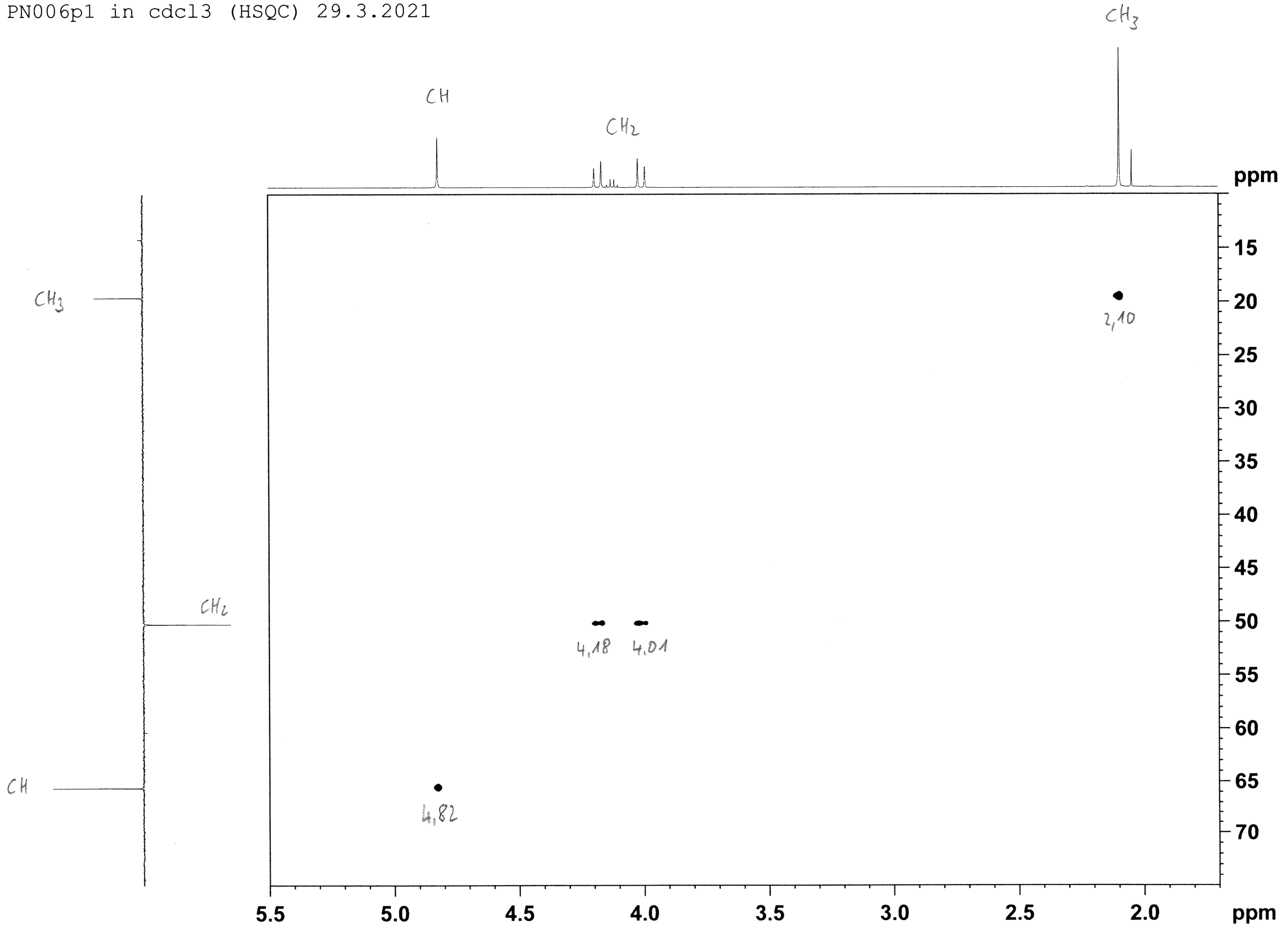

PN006p1 in cdcl3 (HMBC) 29.3.2021

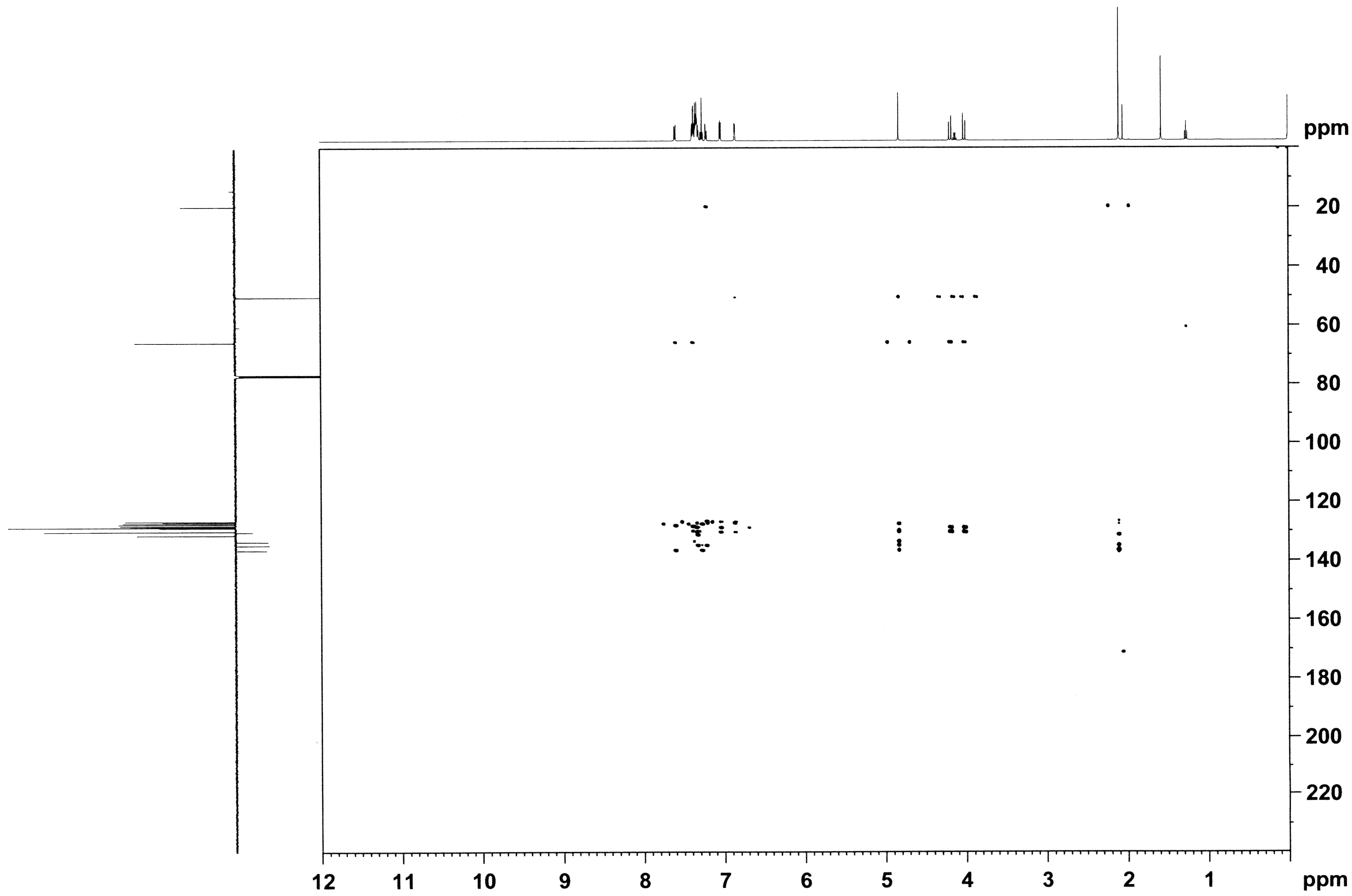

PN006p1 in cdcl3 (HMBC) 29.3.2021

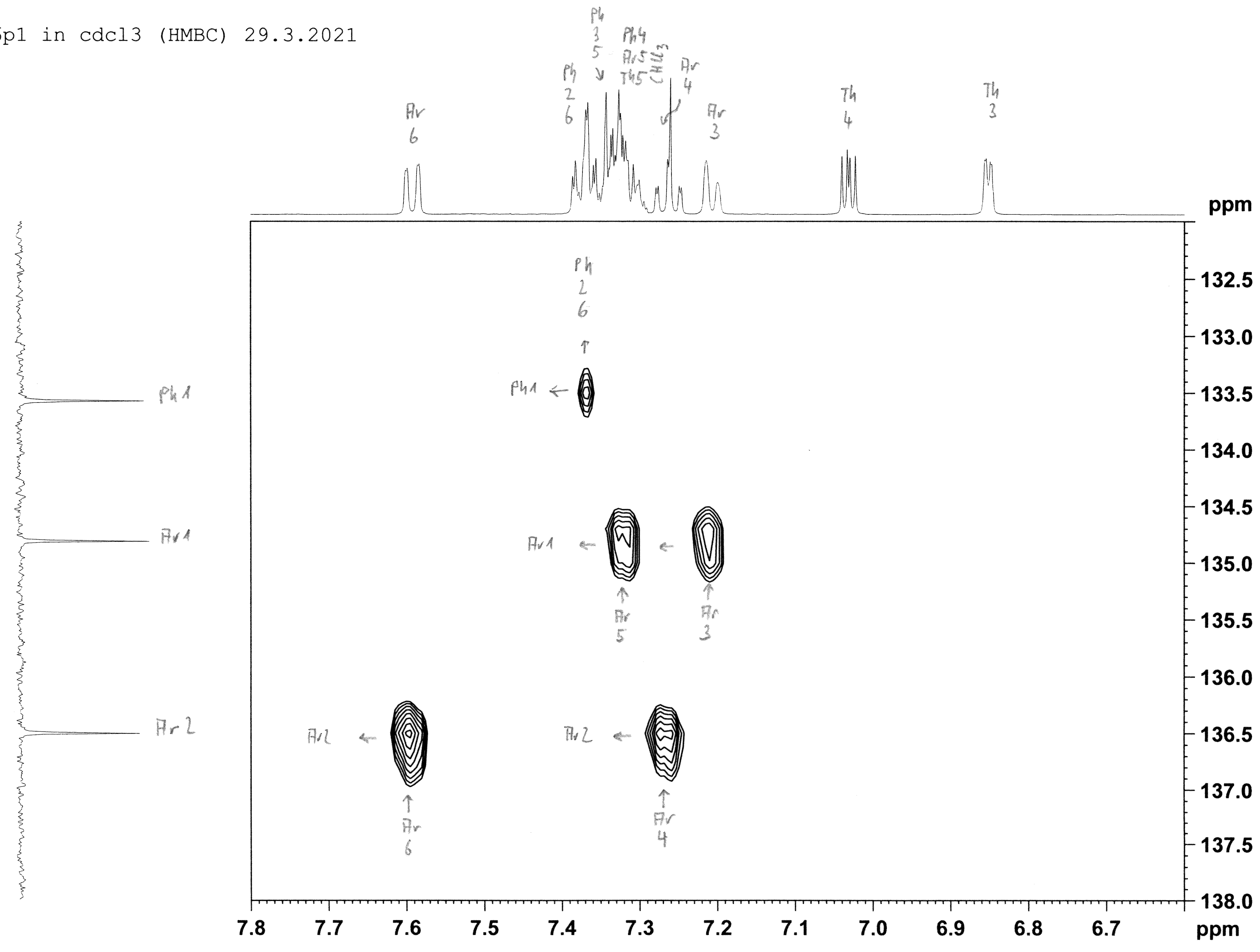

PN006p1 in cdcl3 (HMBC) 29.3.2021

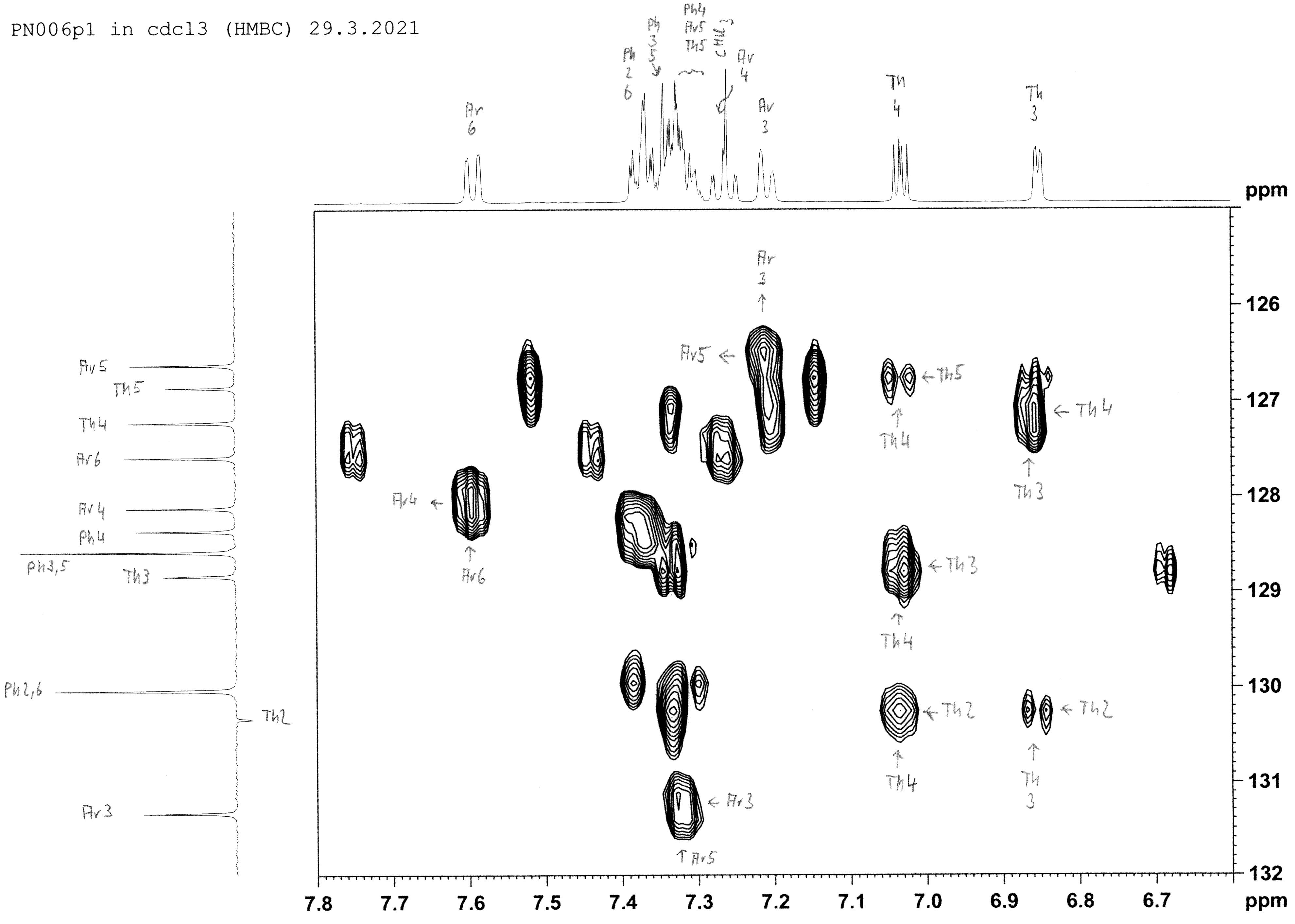

PN006p1 in cdcl3 (HMBC) 29.3.2021

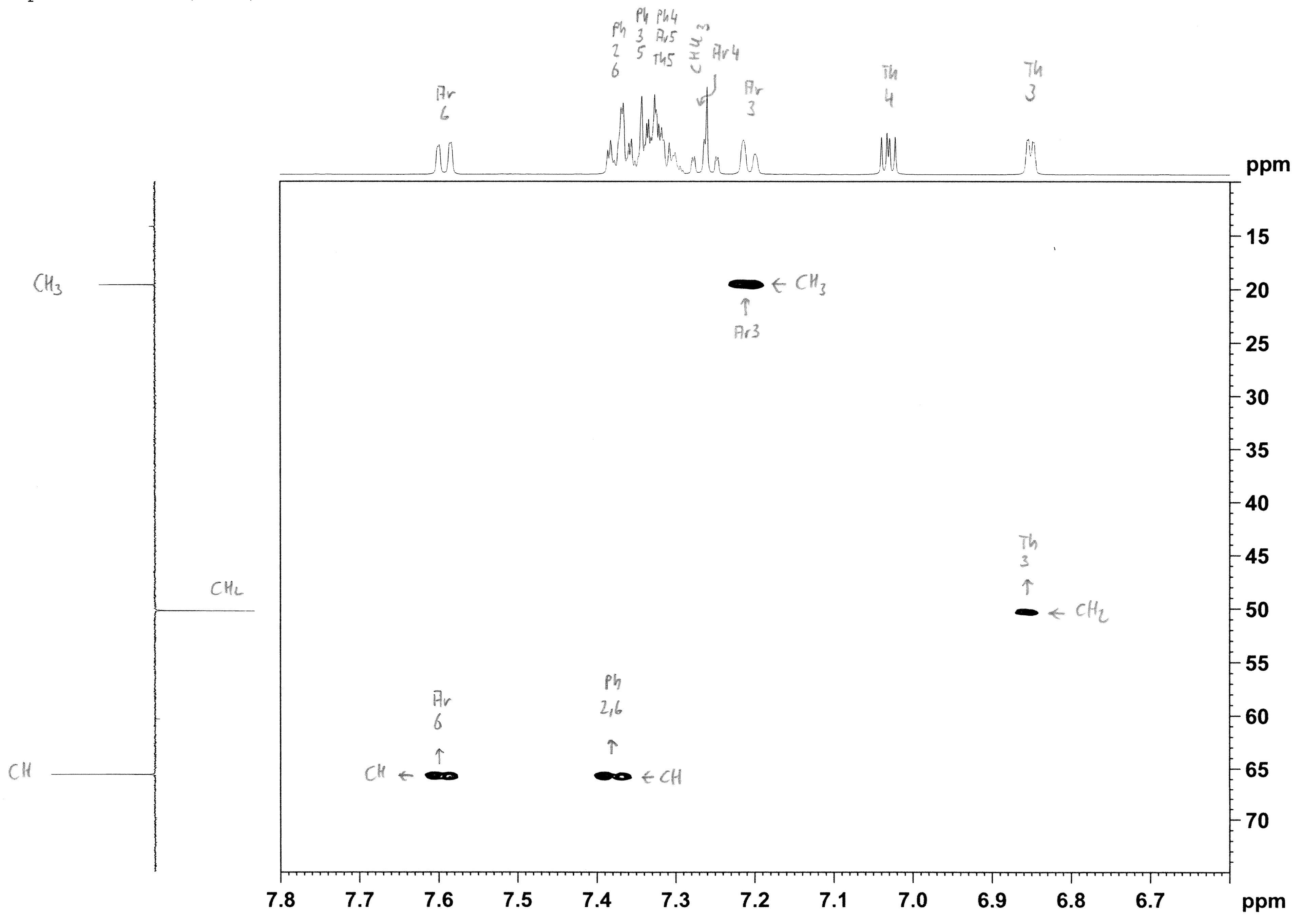

PN006p1 in cdcl3 (HMBC) 29.3.2021

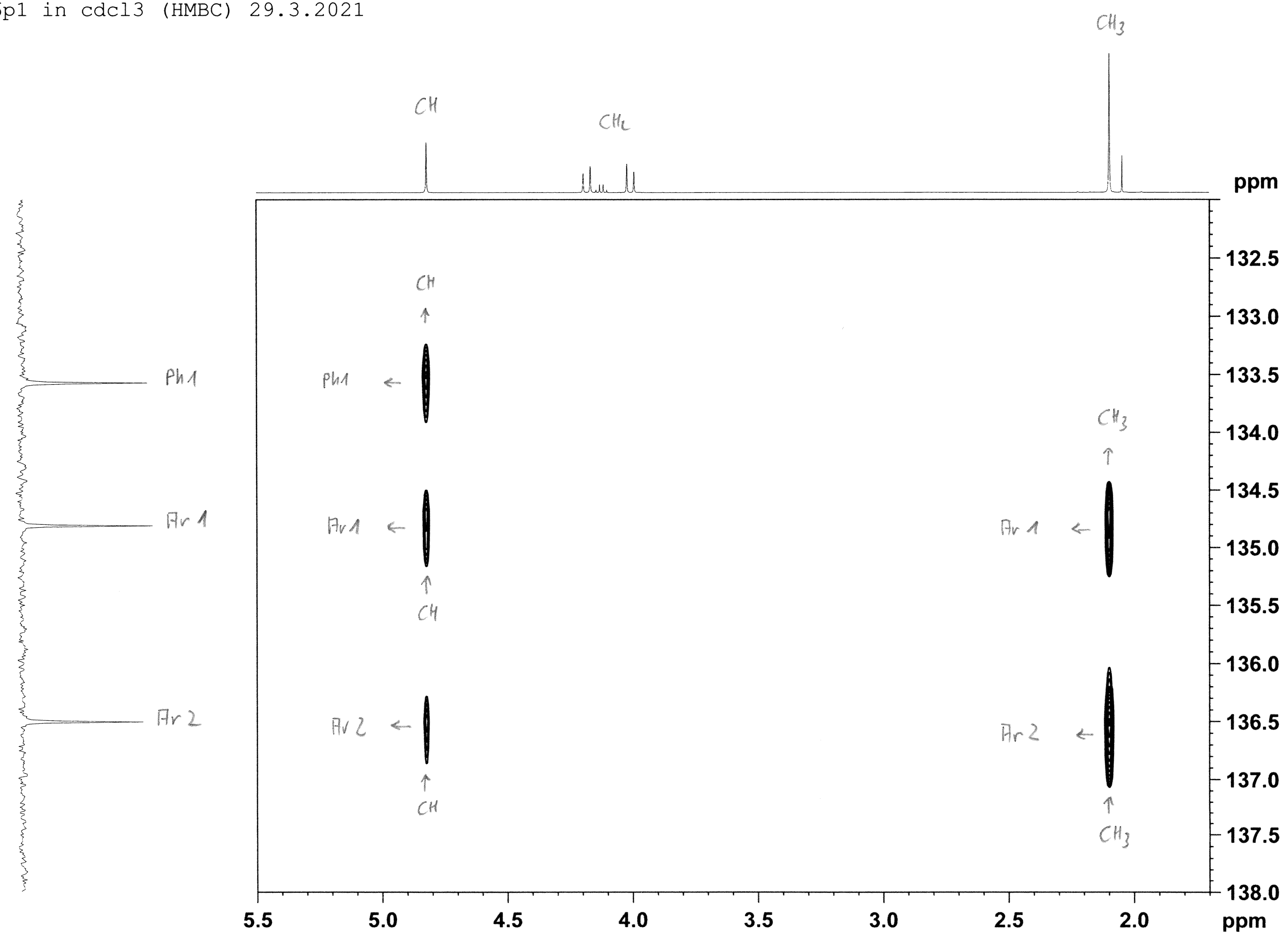

PN006p1 in cdcl3 (HMBC) 29.3.2021

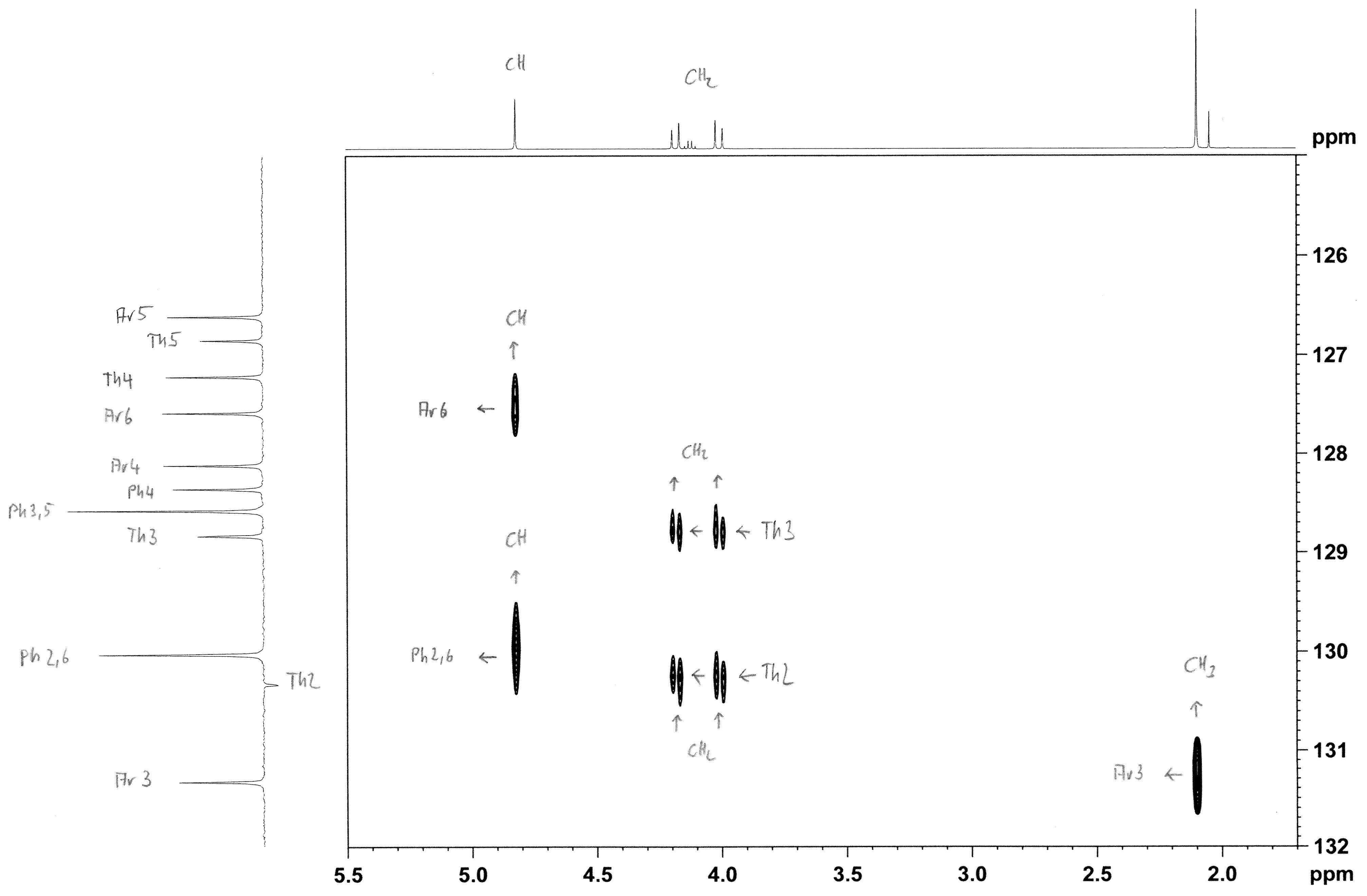

PN006p1 in cdcl3 (HMBC) 29.3.2021

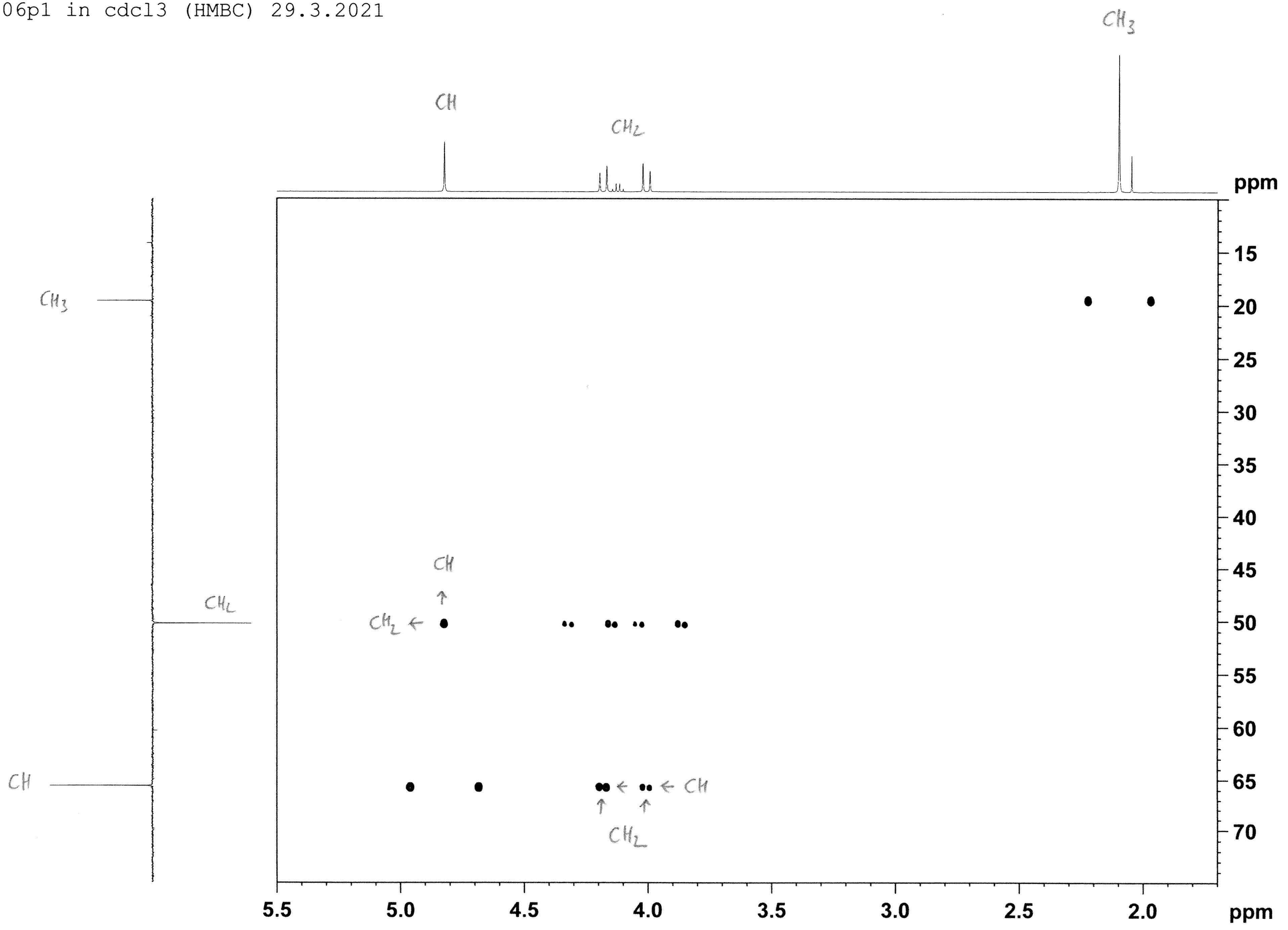

Figure S46c. NMR spectra of compound **6l**.

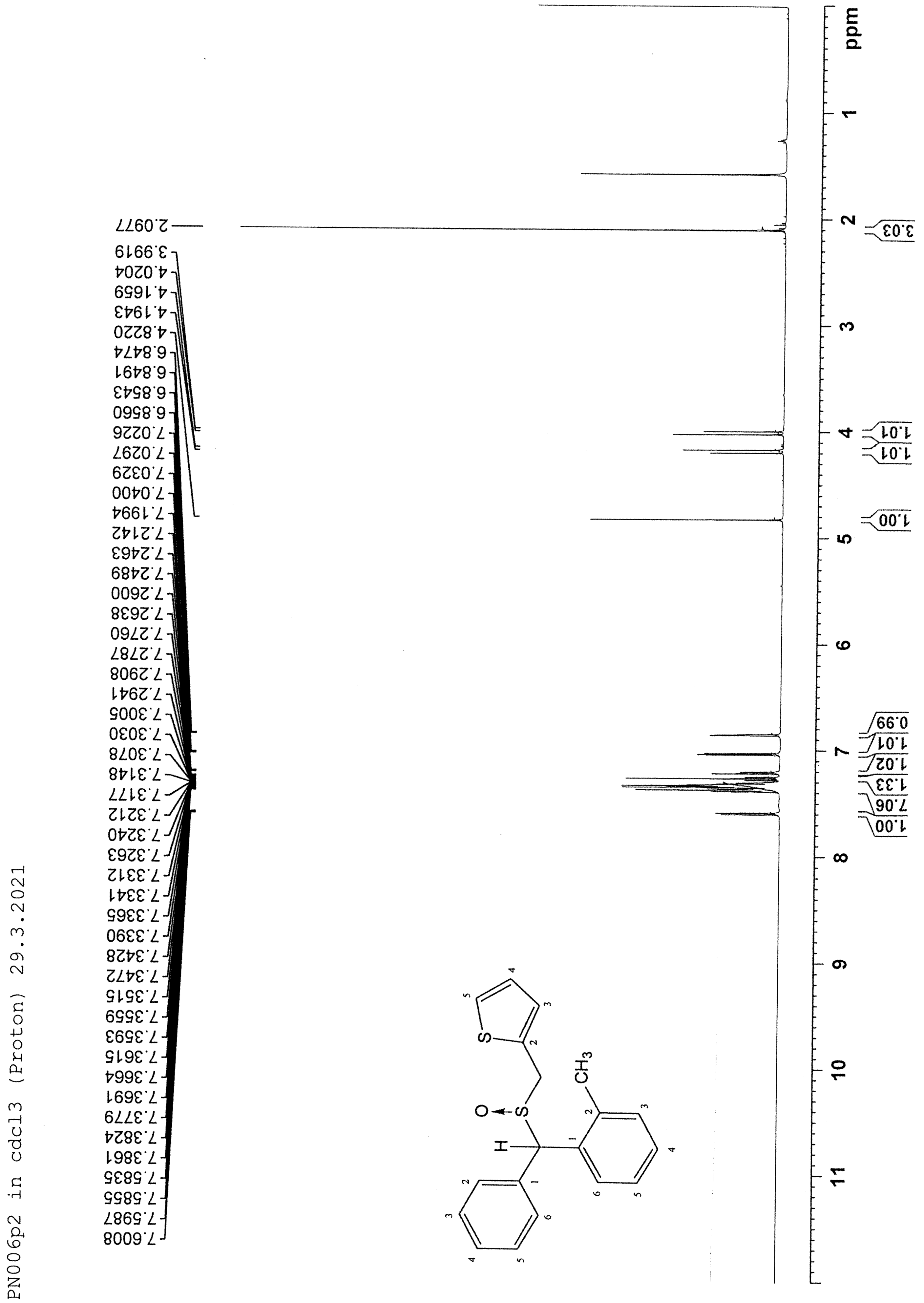

PN006p2 in cdcl3 (Proton) 29.3.2021

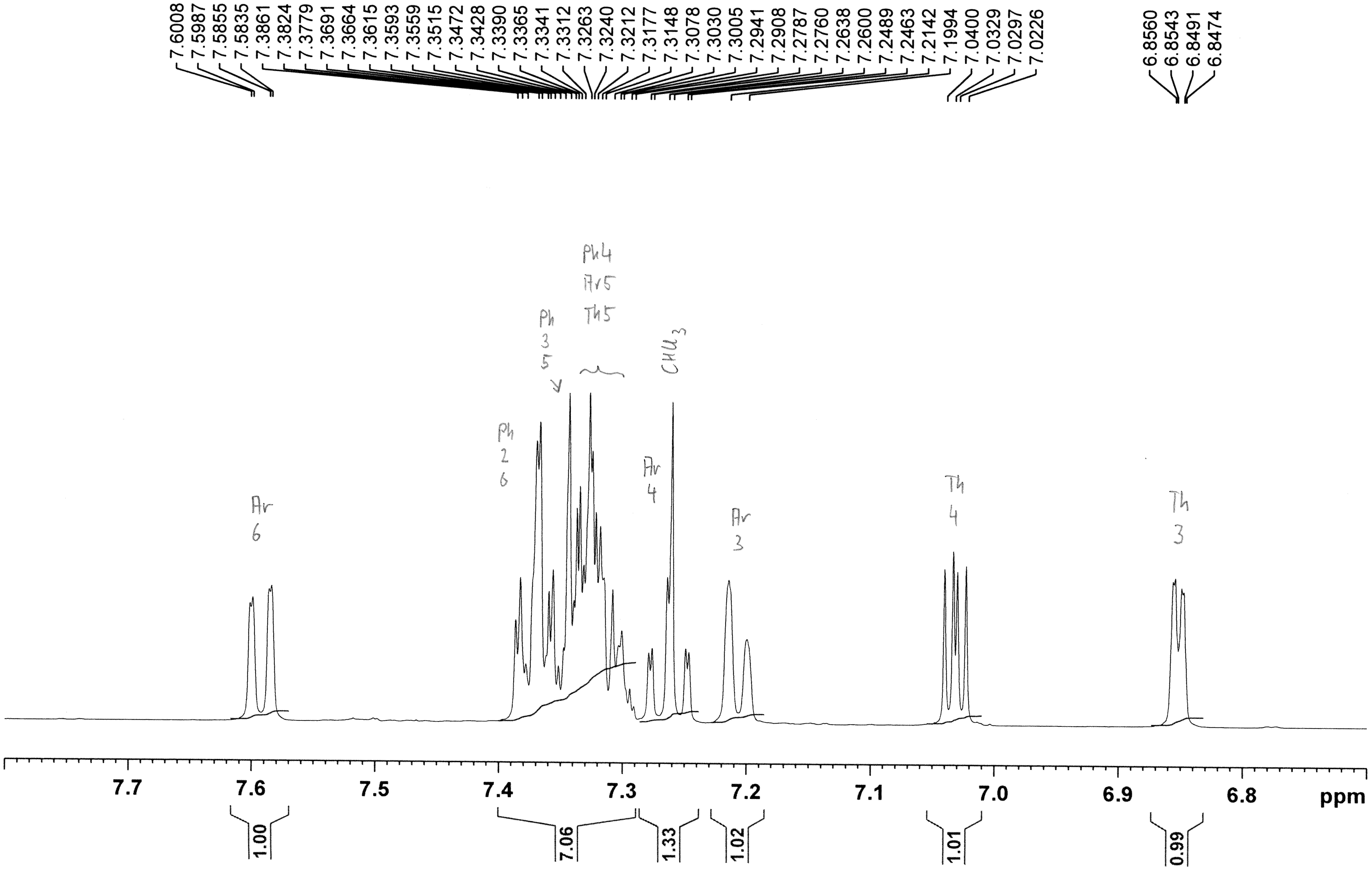

PN006p2 in cdcl3 (Proton) 29.3.2021

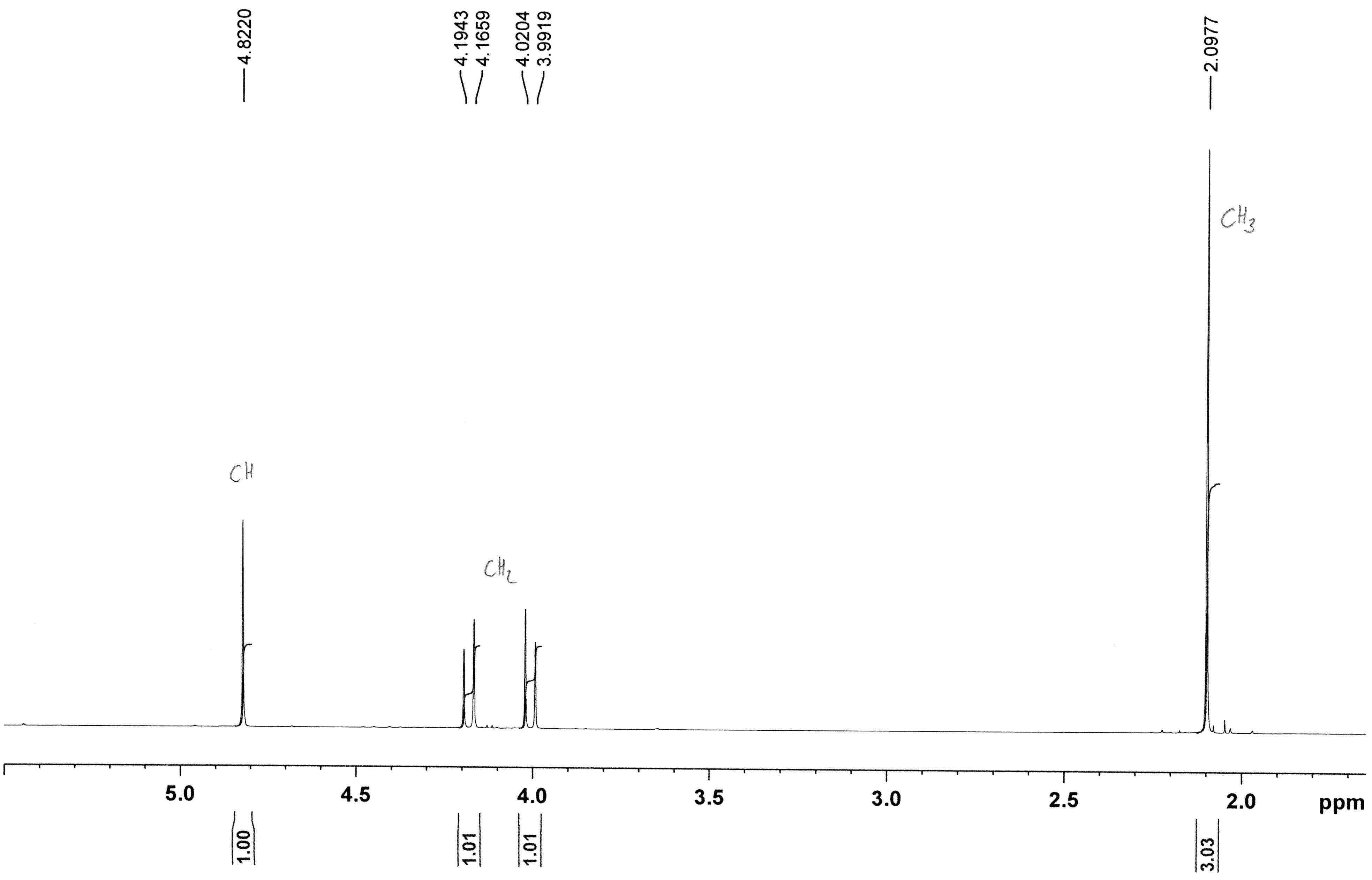

PN006p2 in cdcl3 (APT) 29.3.2021

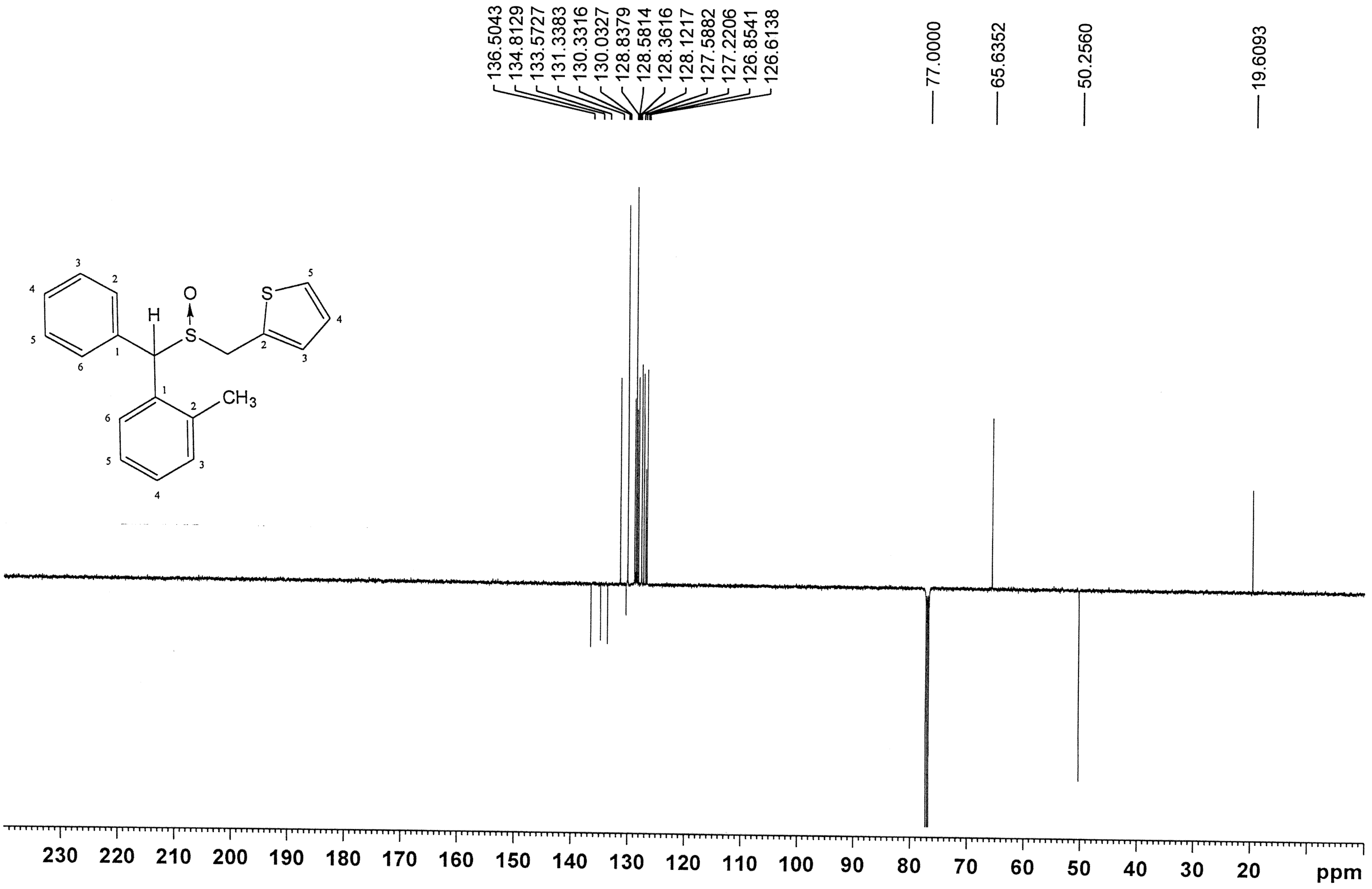

PN006p2 in cdcl3 (APT) 29.3.2021

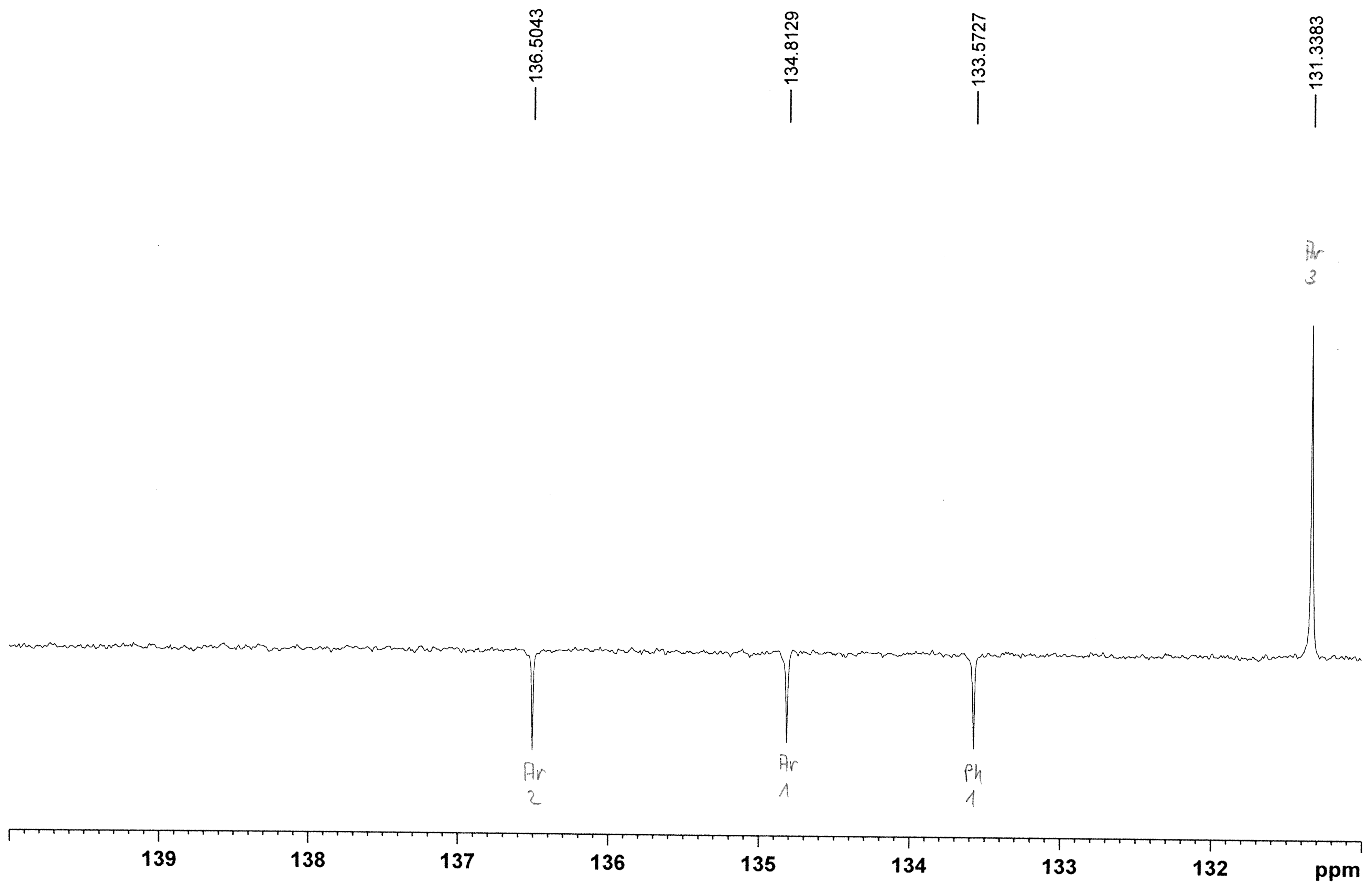

PN006p2 in cdcl3 (APT) 29.3.2021

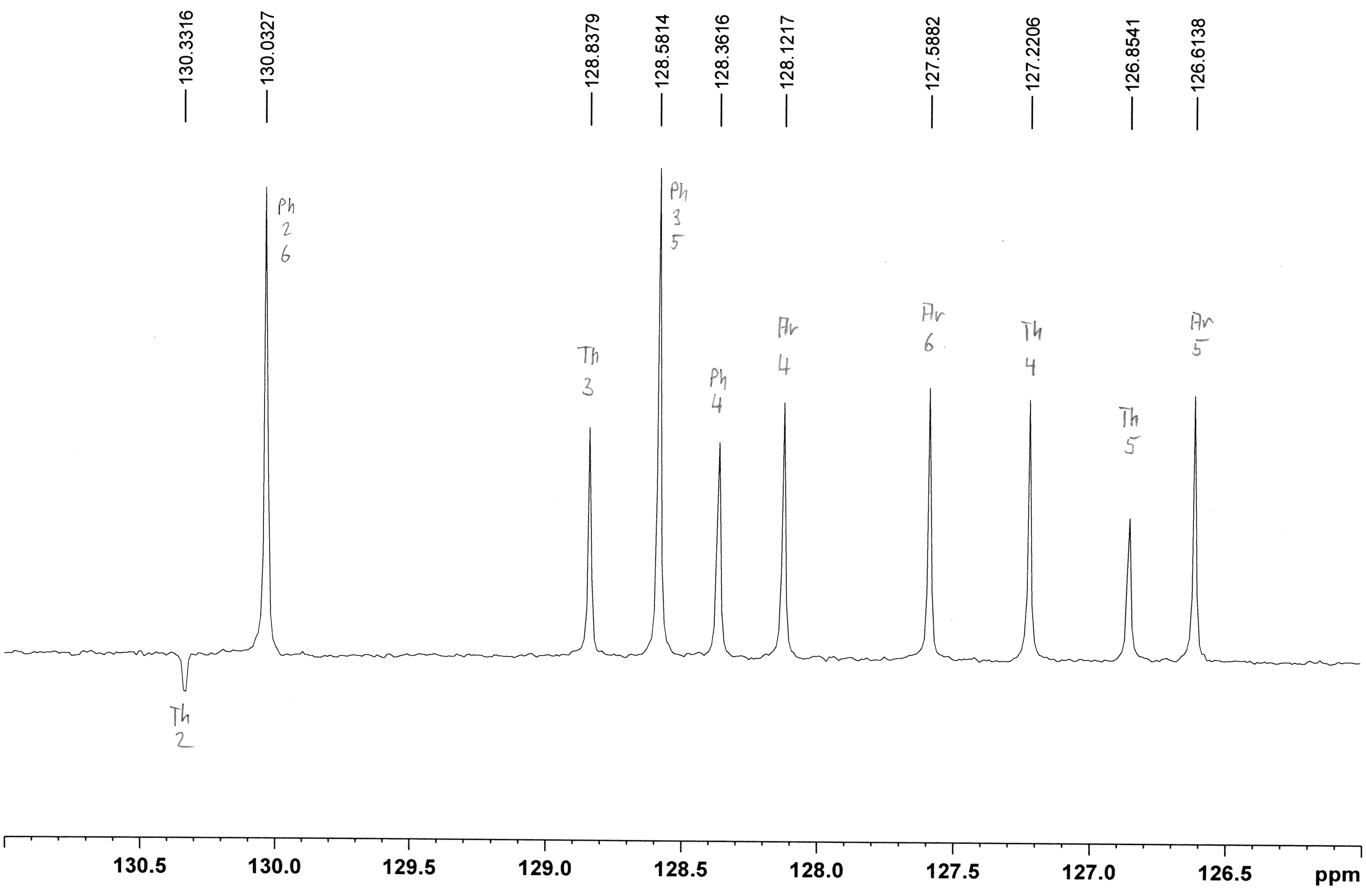

PN006p2 in cdcl3 (APT) 29.3.2021

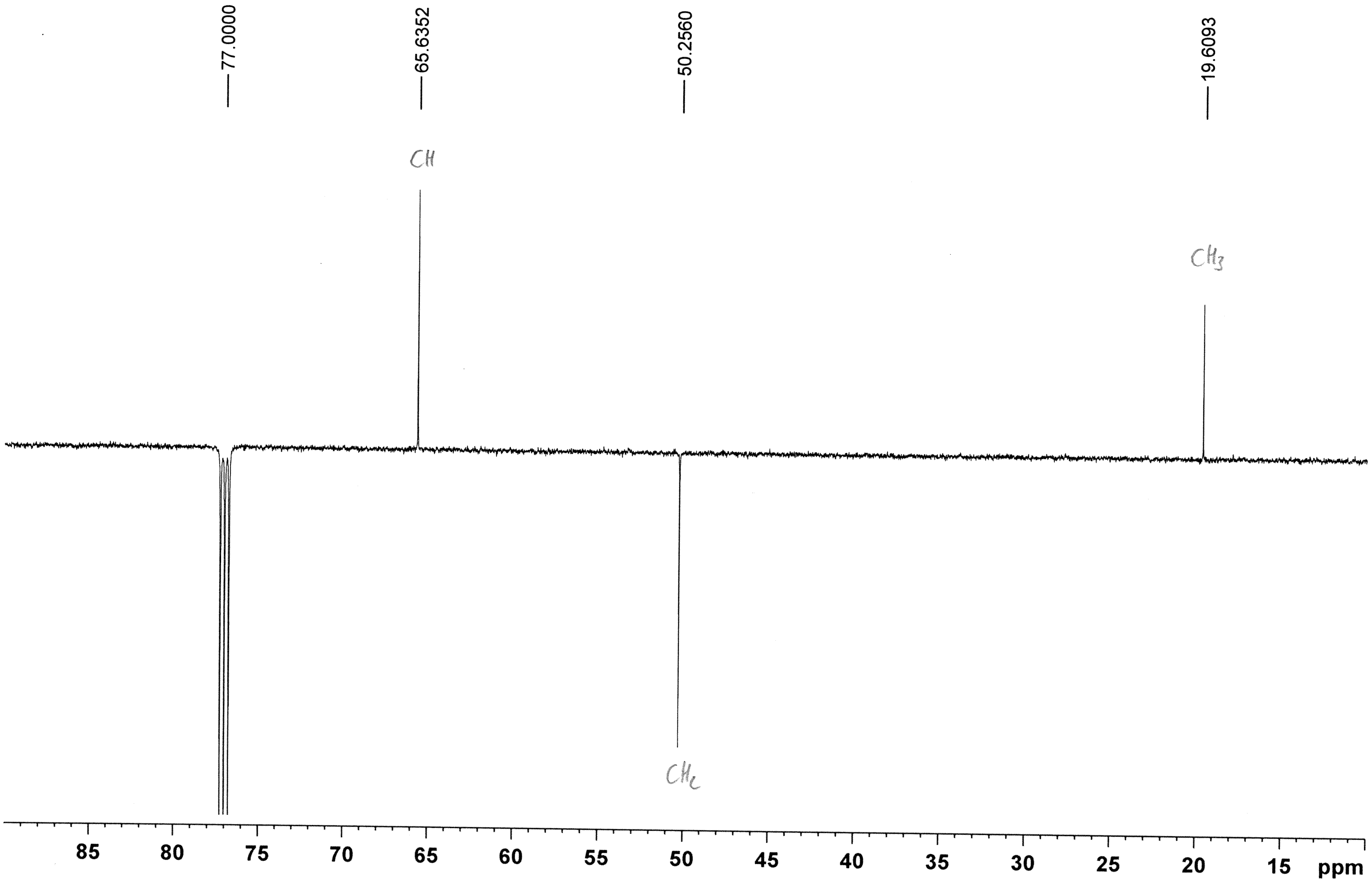

PN006p2 in cdcl3 (COSY) 29.3.2021

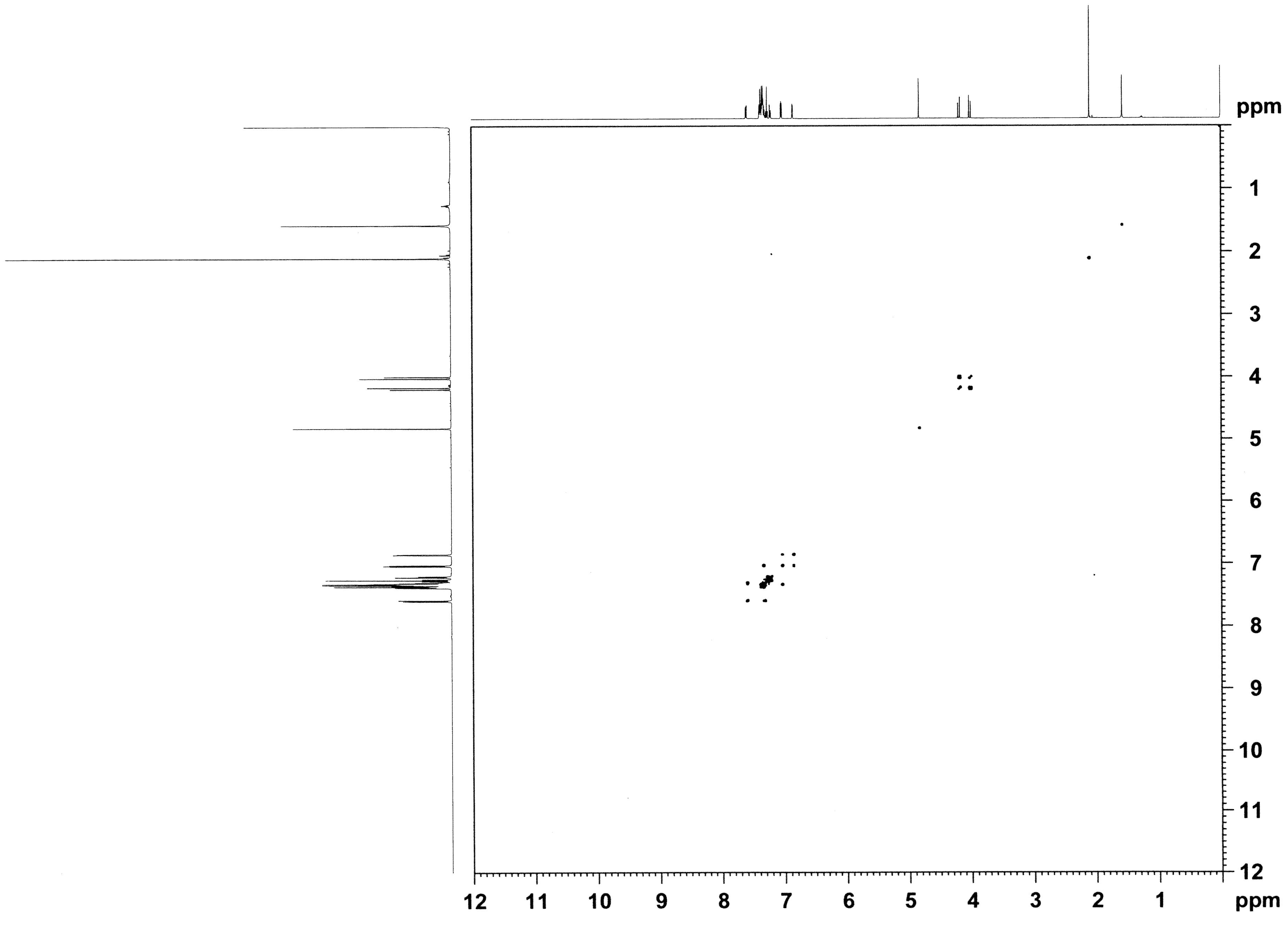

PN006p2 in cdcl3 (COSY) 29.3.2021

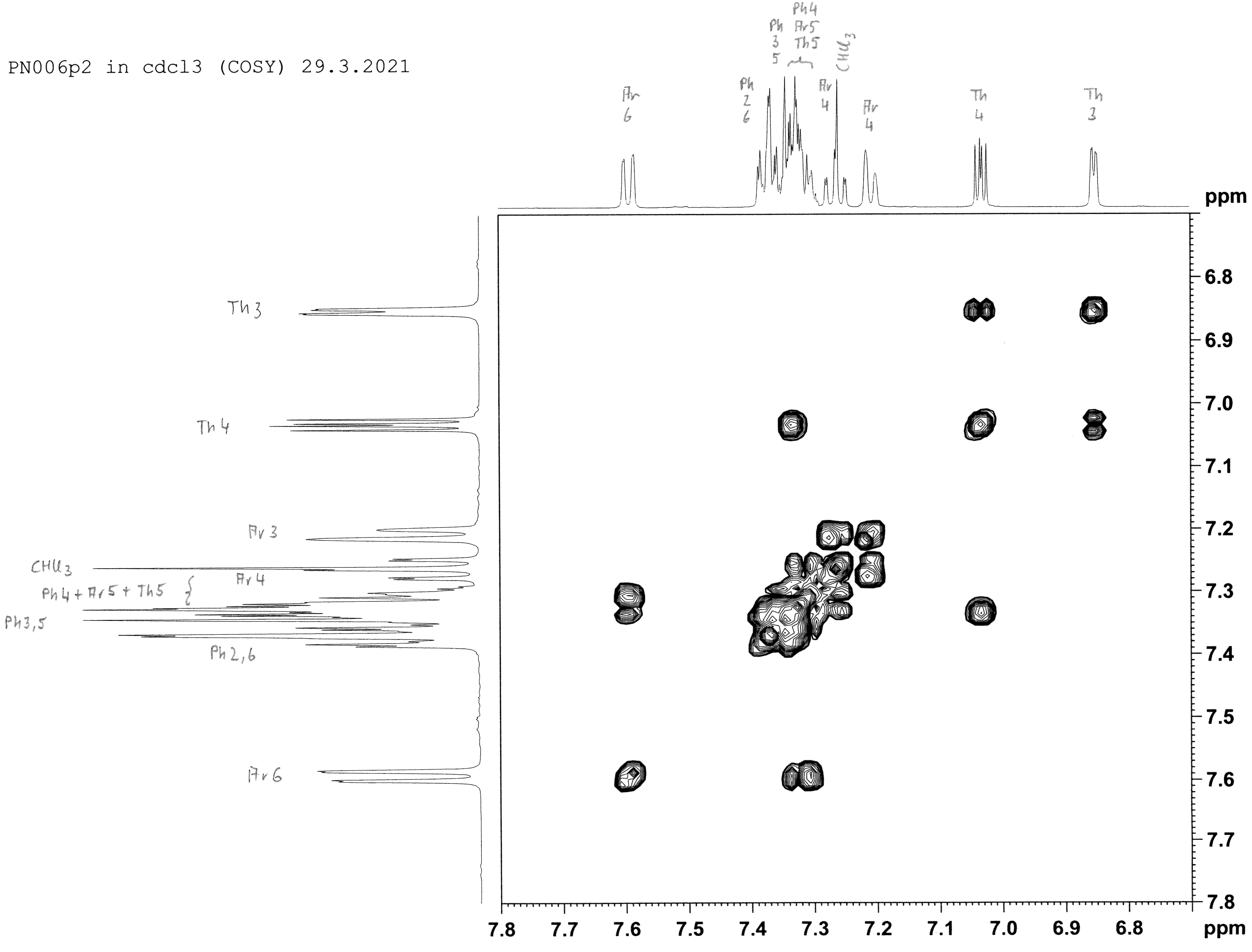

PN006p2 in cdcl3 (COSY) 29.3.2021

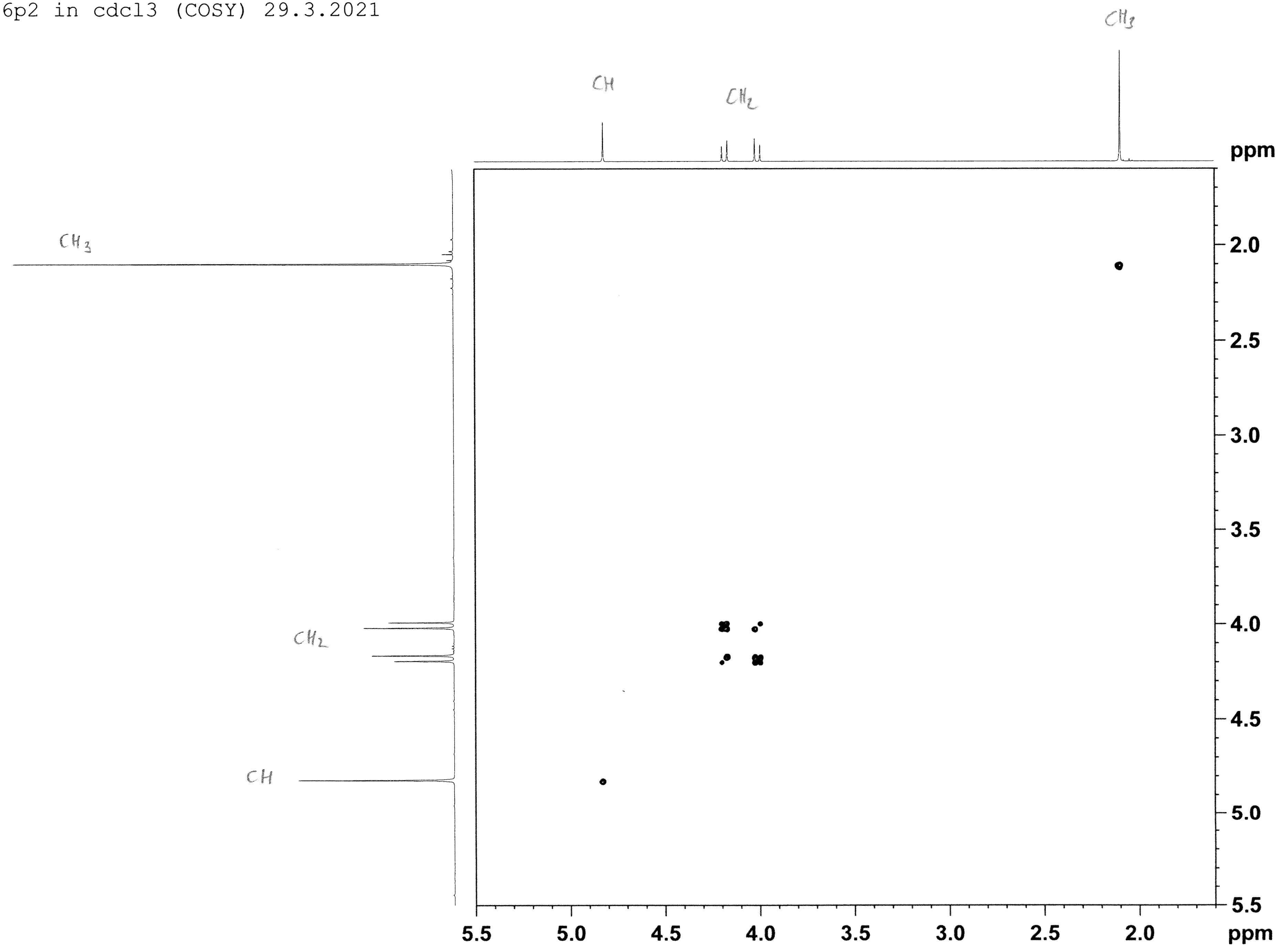

PN006p1 in cdcl3 (HSQC) 29.3.2021

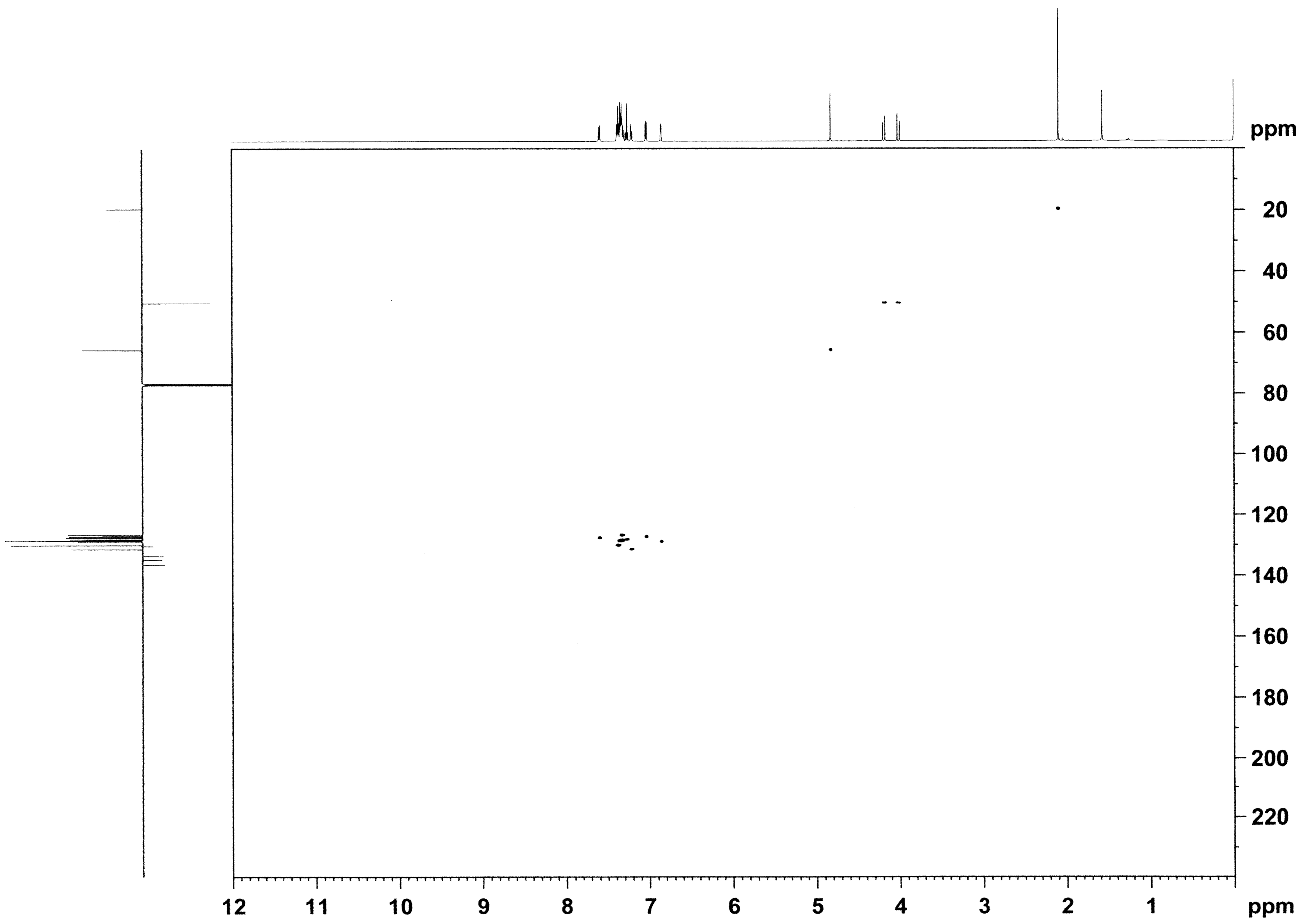

PN006p1 in cdcl3 (HSQC) 29.3.2021

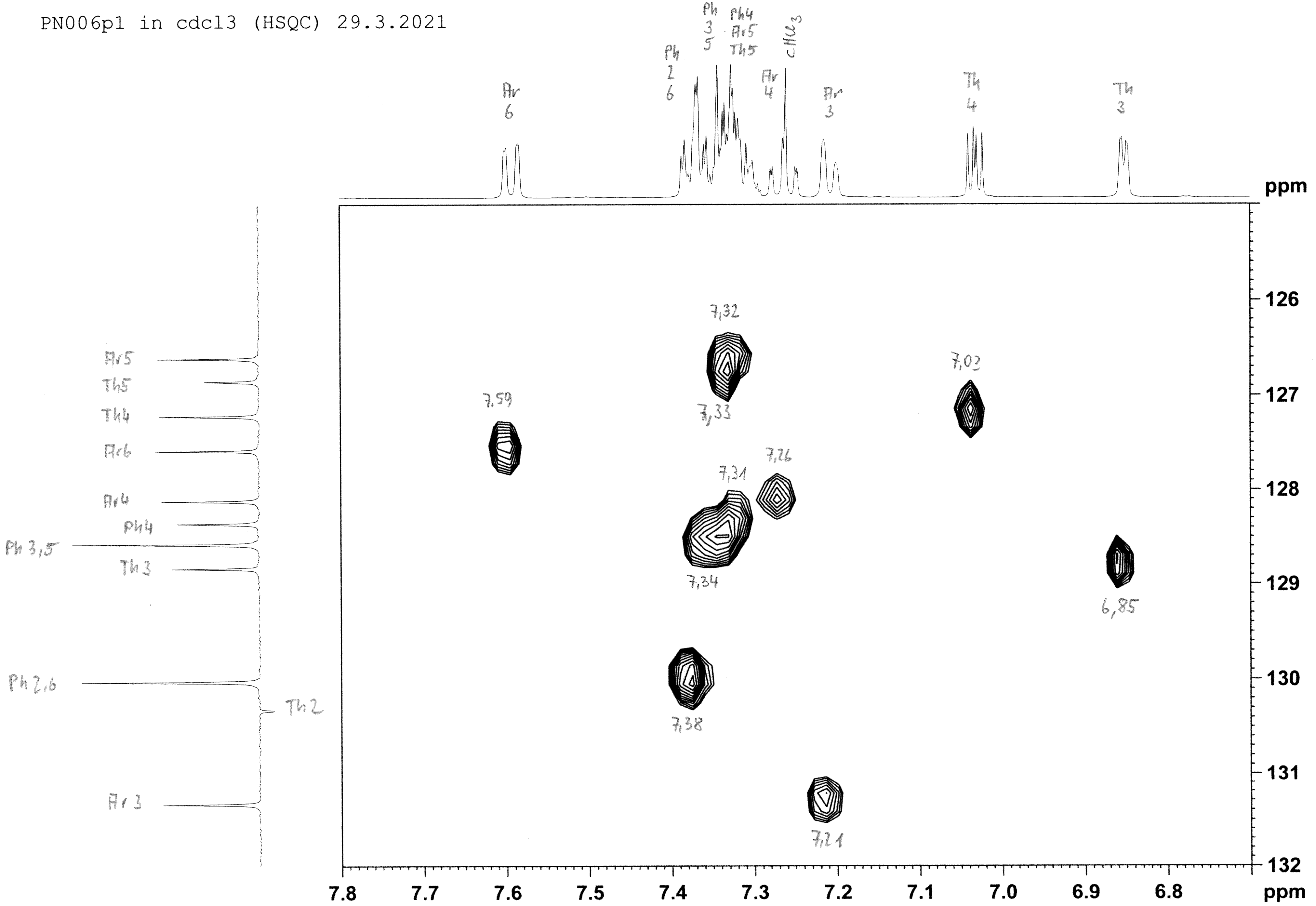

PN006p1 in cdcl3 (HSQC) 29.3.2021

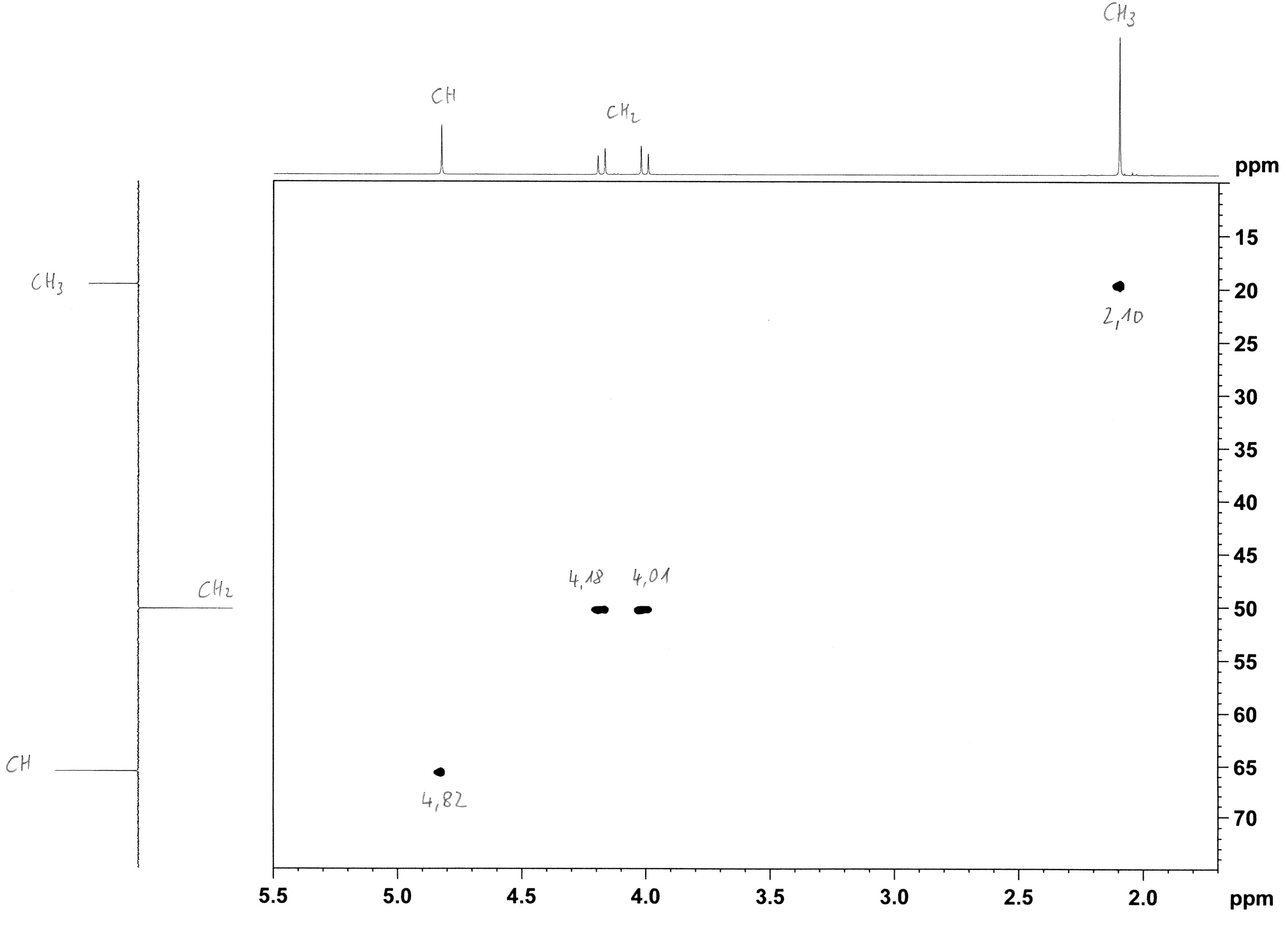

PN006p2 in cdcl3 (HMBC) 29.3.2021

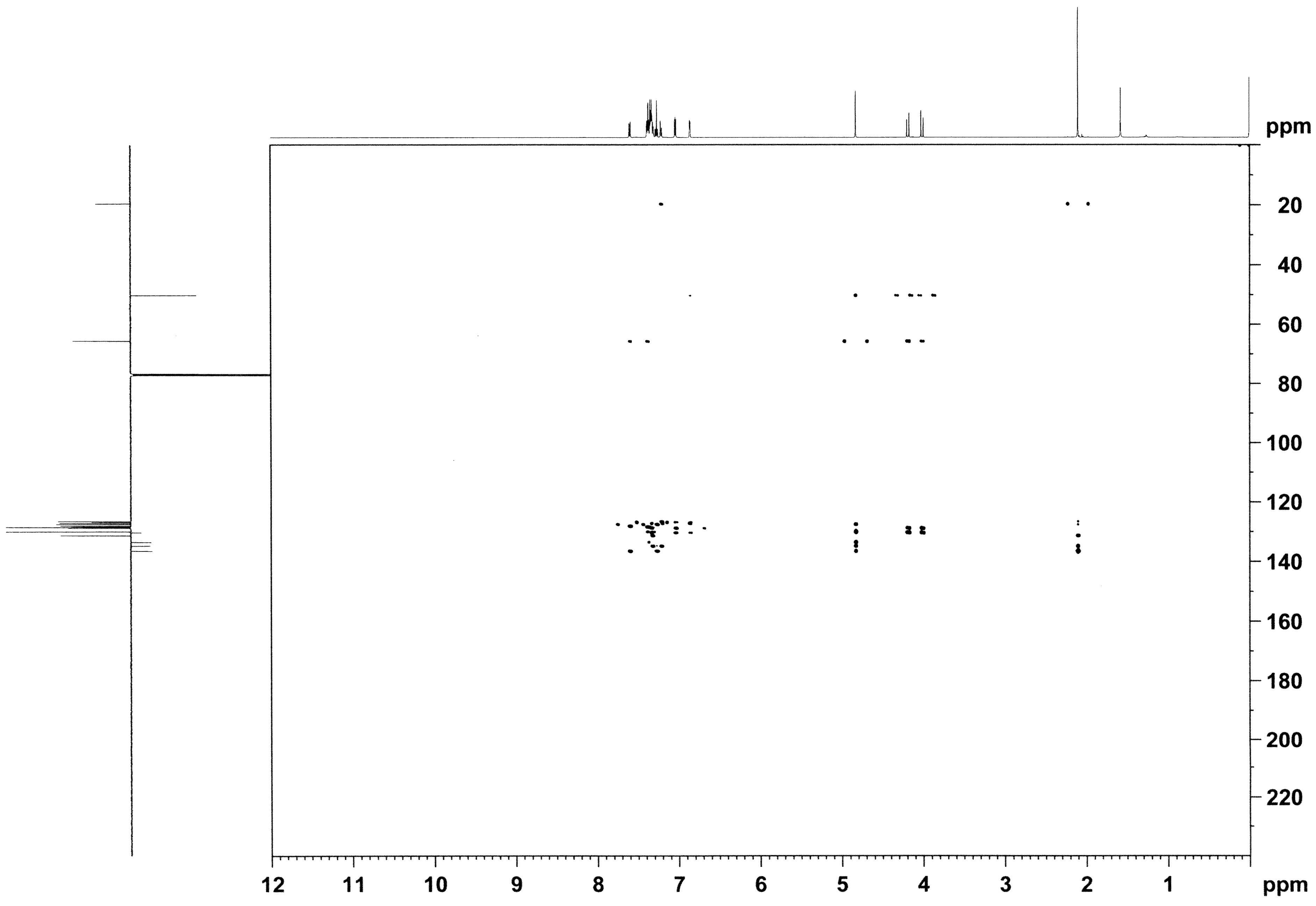

PN006p2 in cdcl3 (HMBC) 29.3.2021

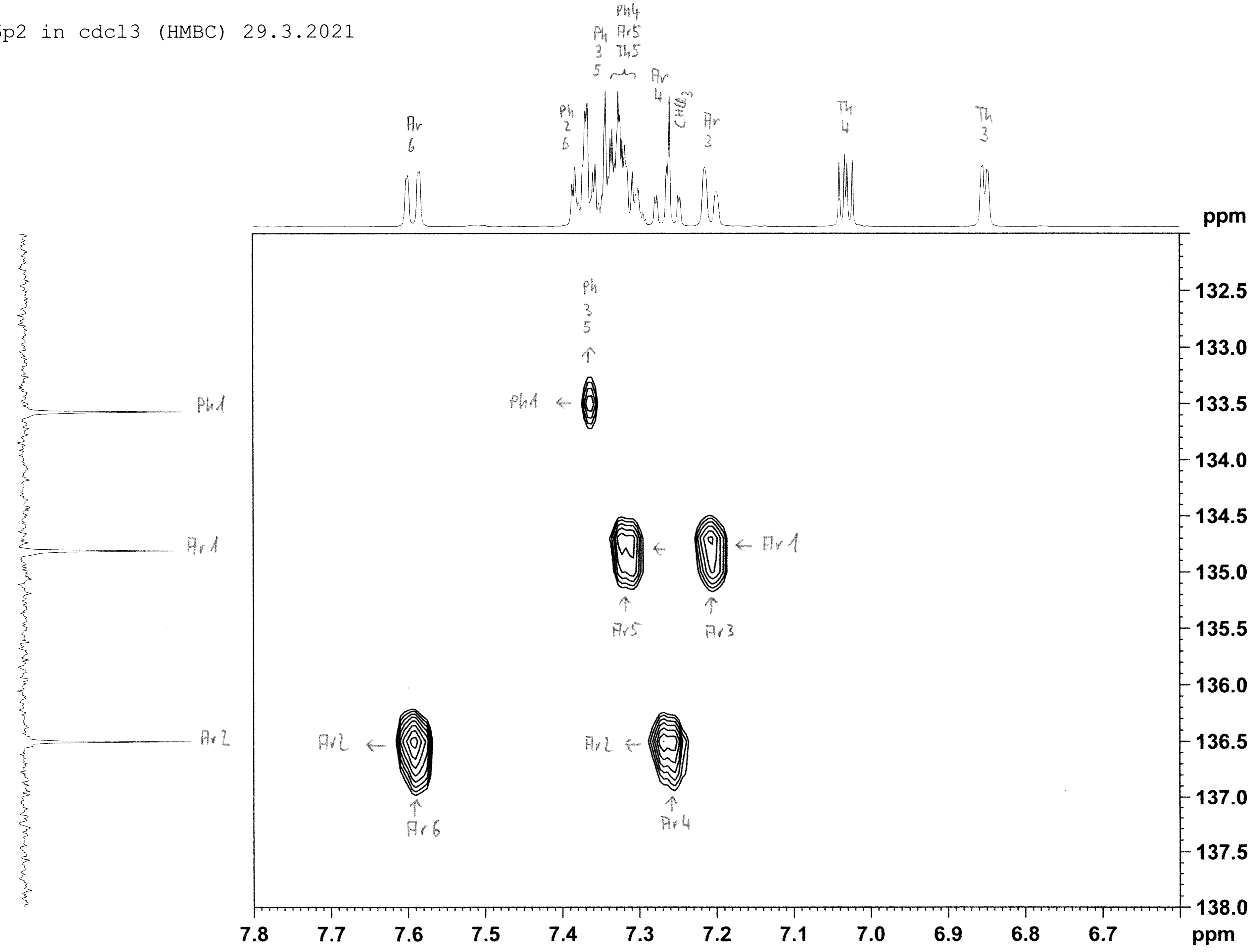

PN006p2 in cdcl3 (HMBC) 29.3.2021

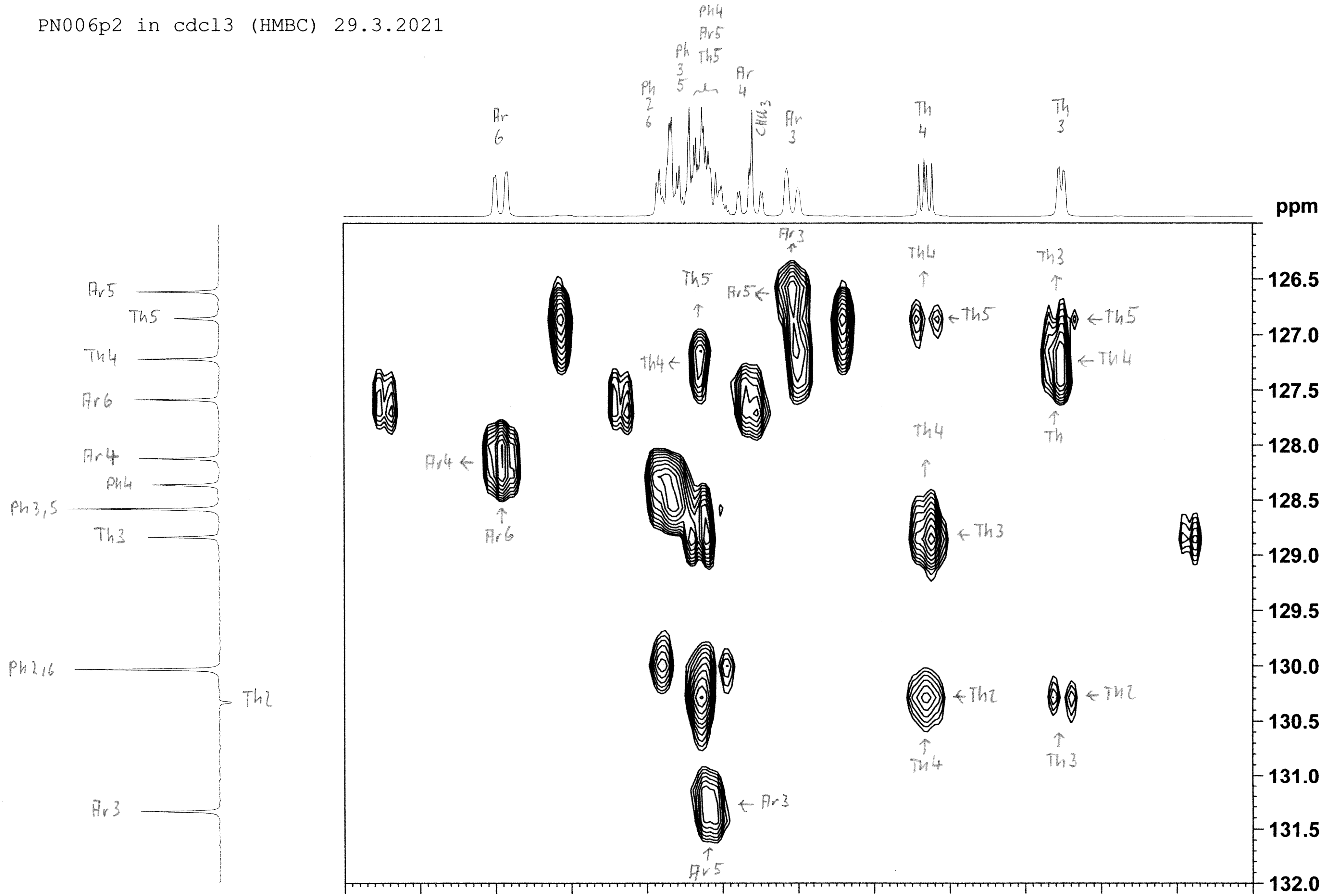

PN006p2 in cdcl3 (HMBC) 29.3.2021

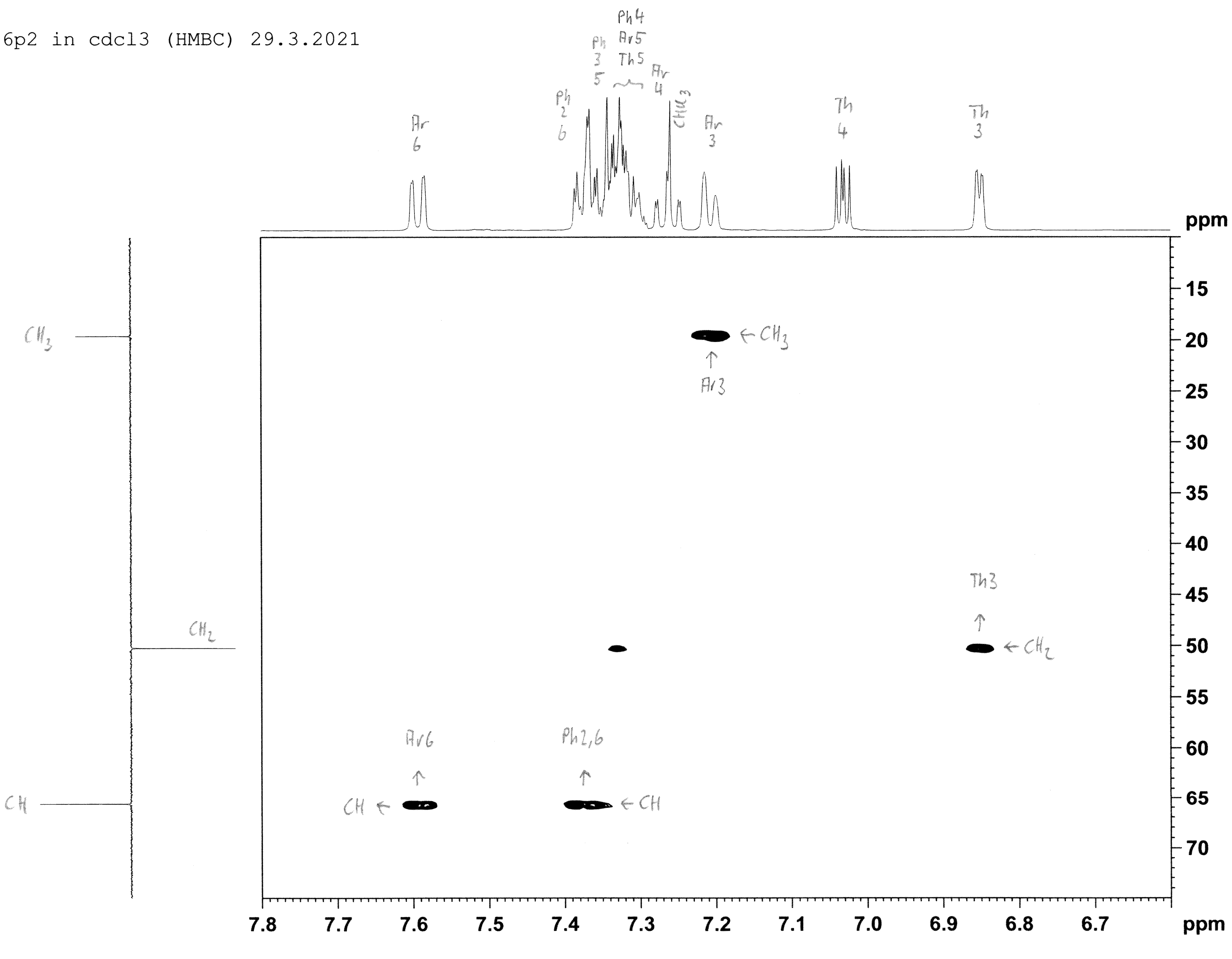

PN006p2 in cdcl3 (HMBC) 29.3.2021

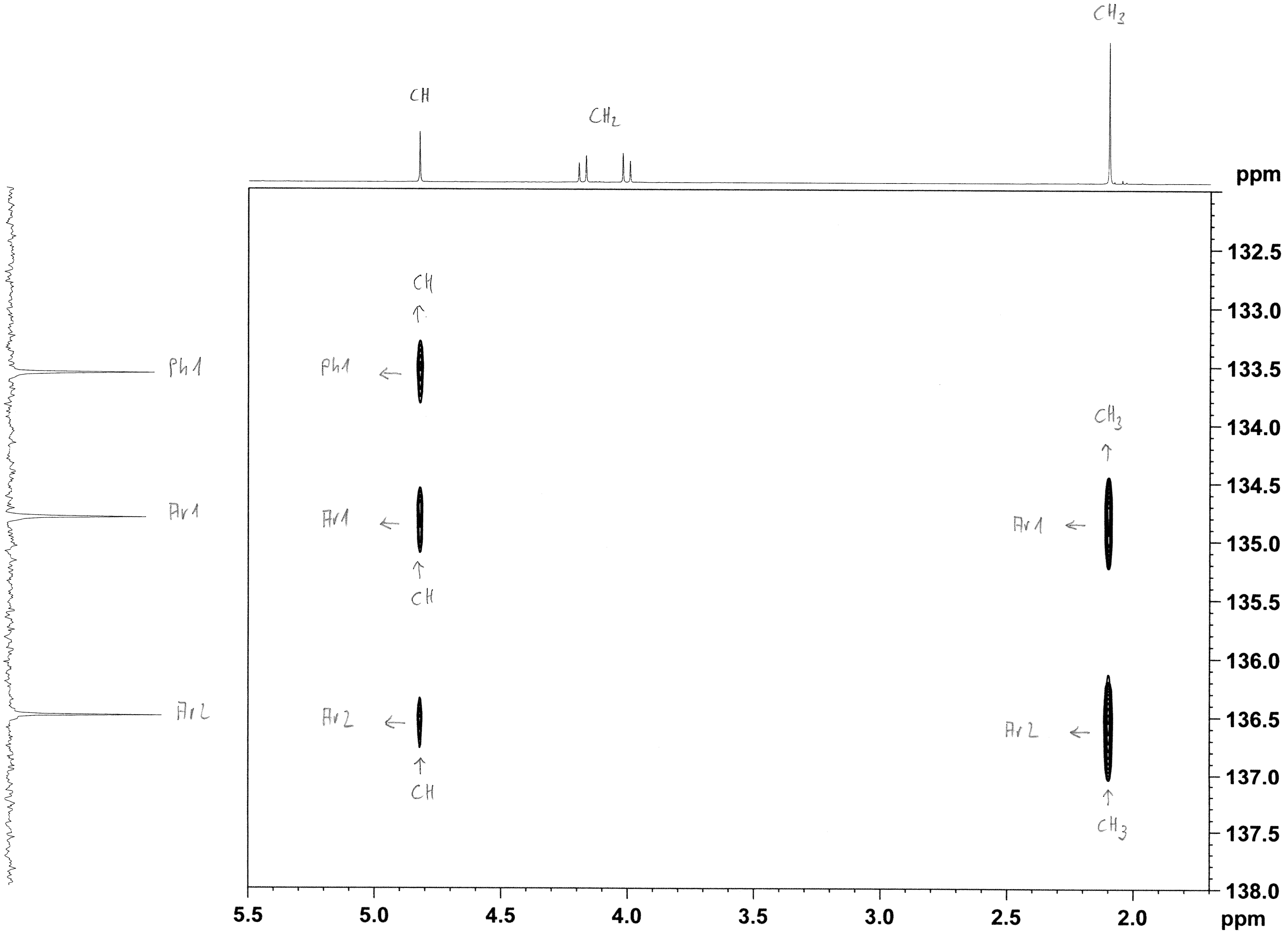

PN006p2 in cdcl3 (HMBC) 29.3.2021

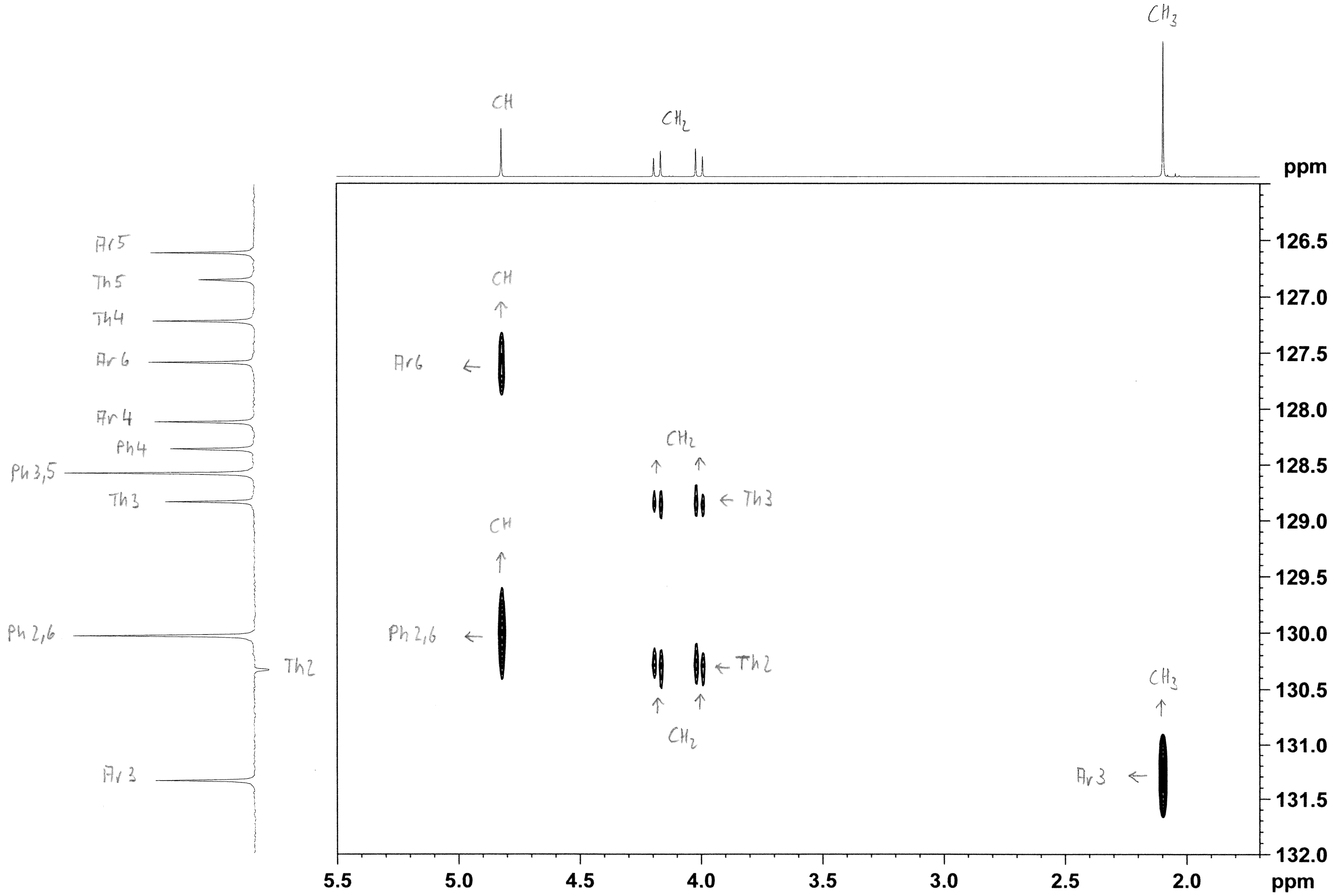

PN006p2 in cdcl3 (HMBC) 29.3.2021

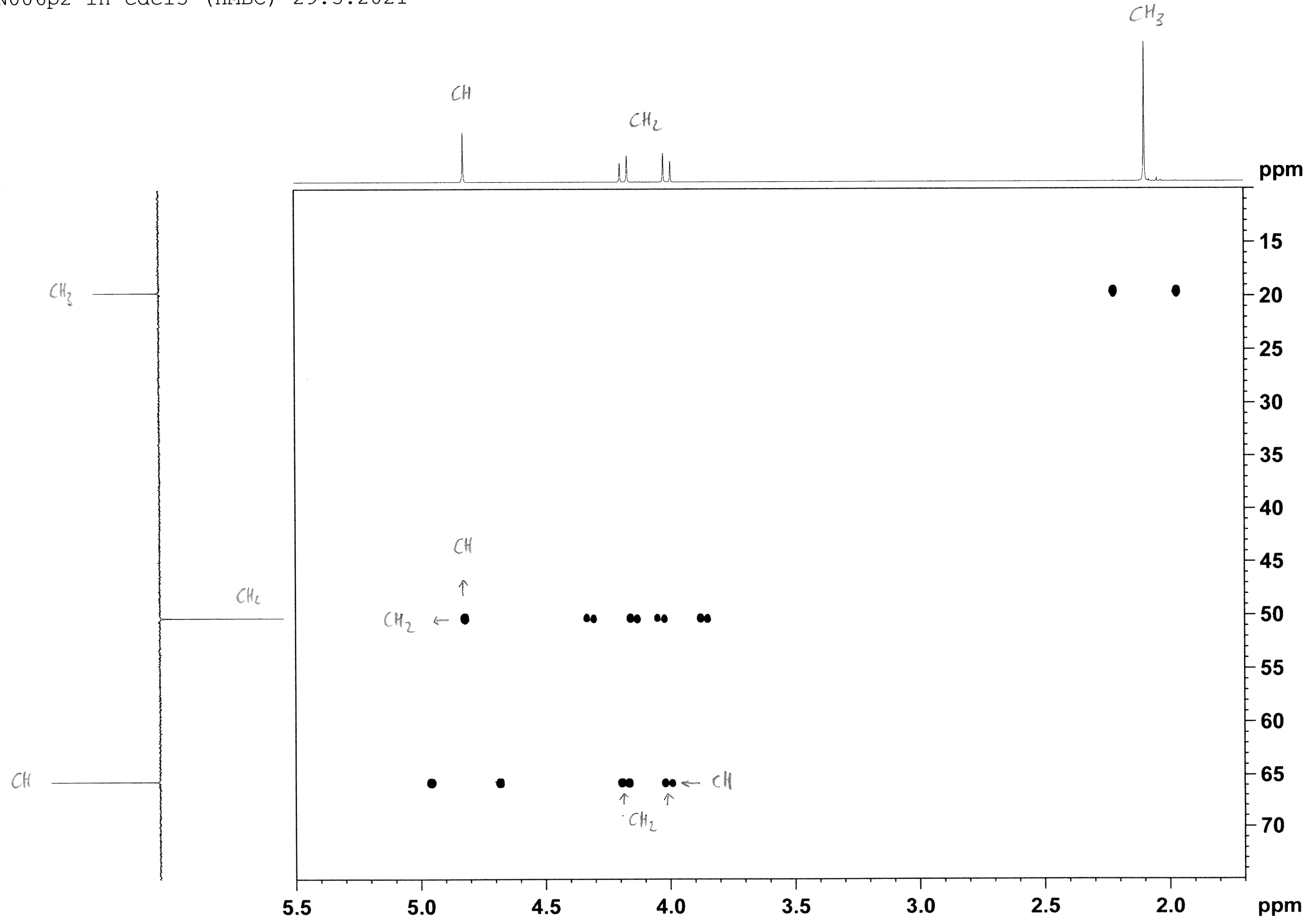

Figure S47c. NMR spectra of compound **7l**.

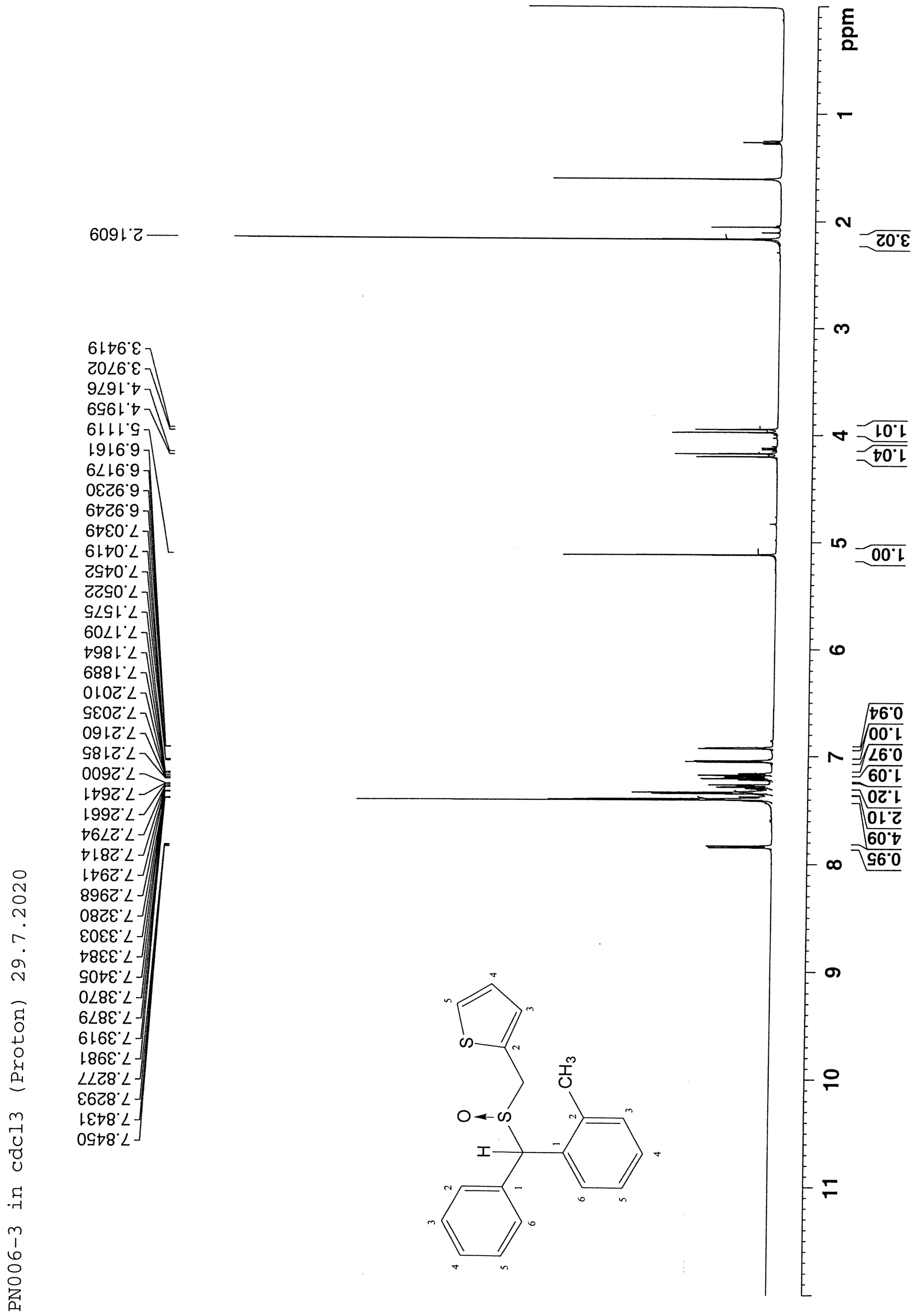

PN006-3 in cdcl3 (Proton) 29.7.2020

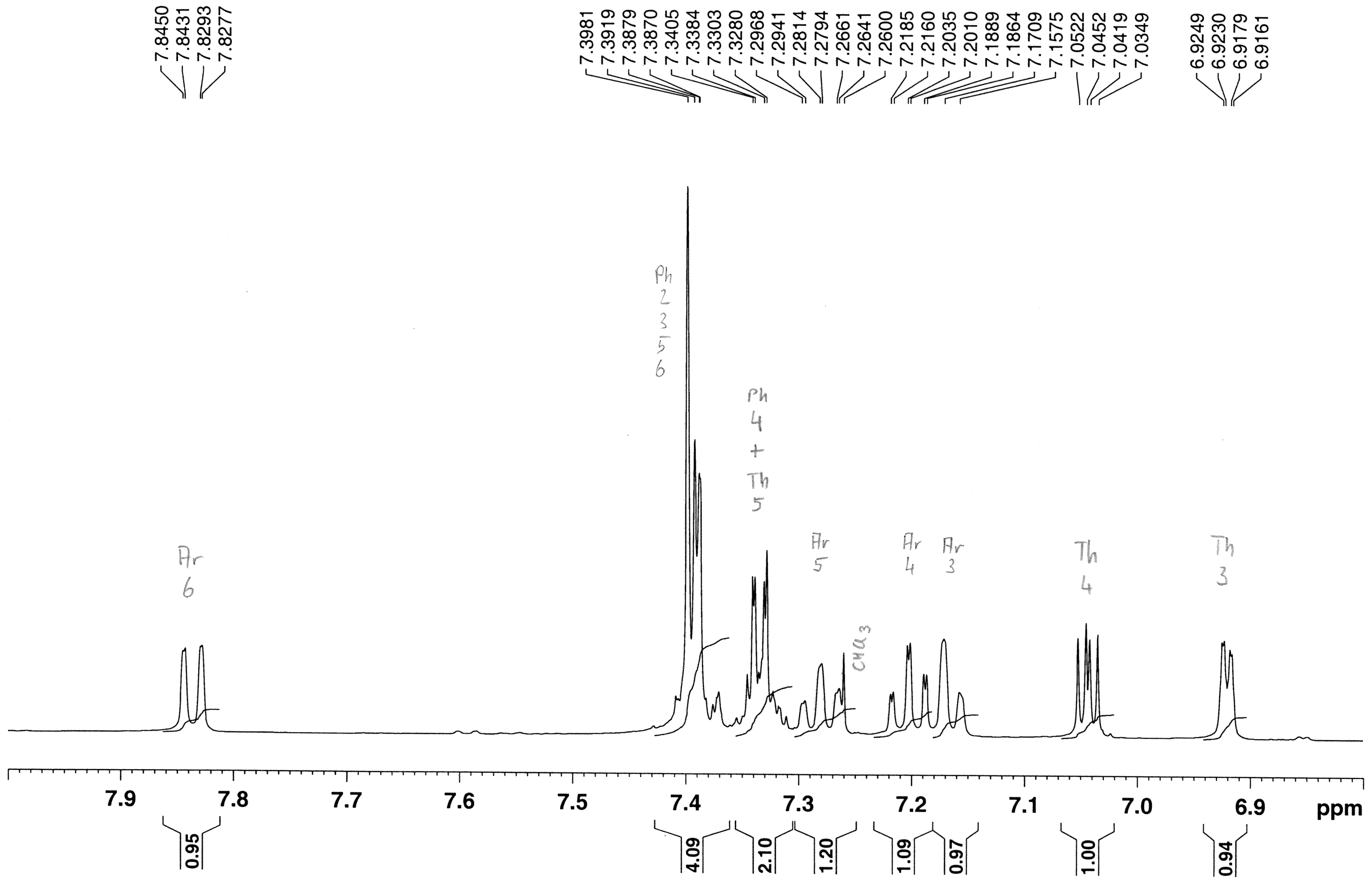

PN006-3 in cdcl3 (Proton) 29.7.2020

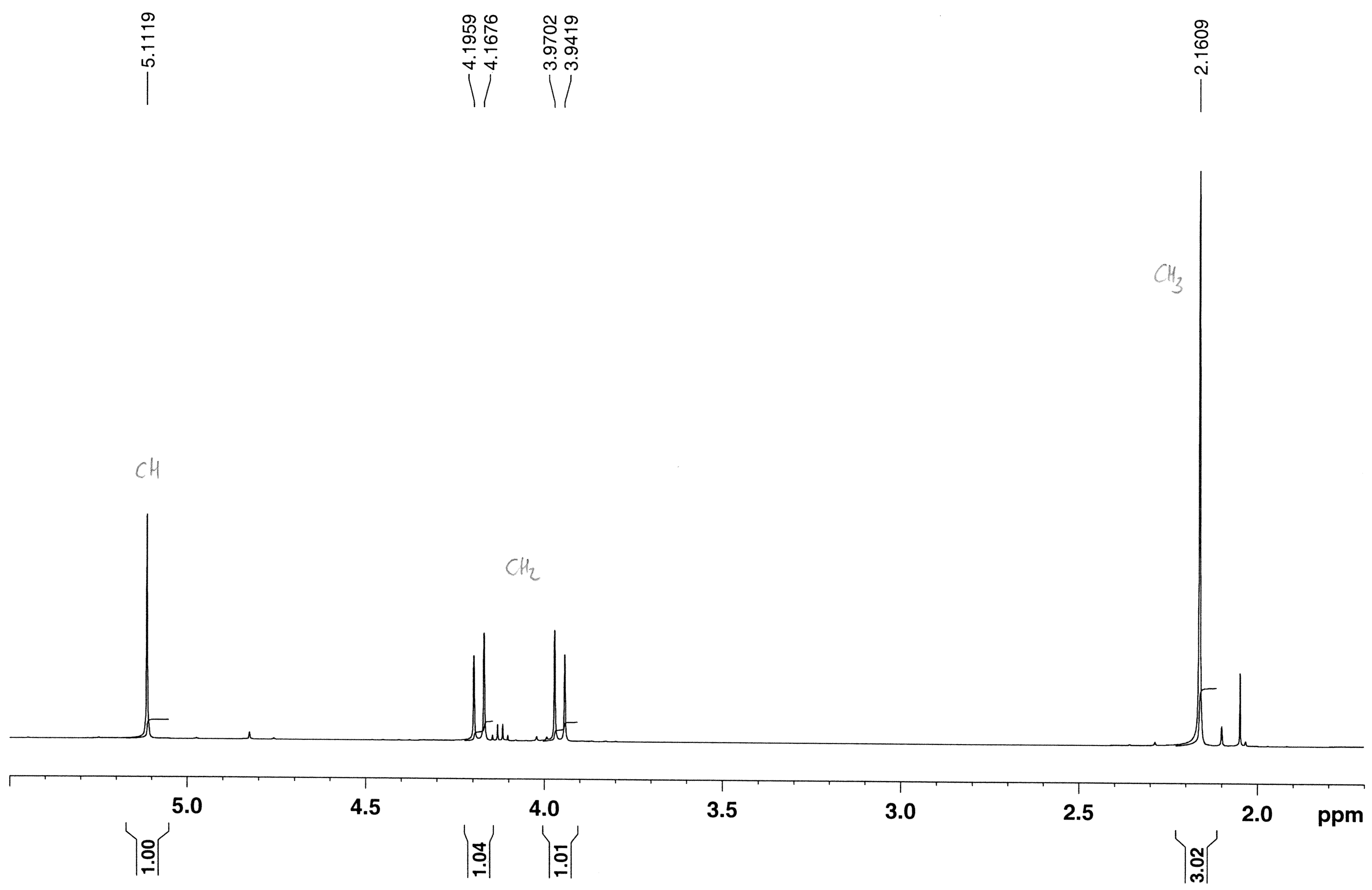

PN006-3 in cdcl3 (APT) 29.7.2020

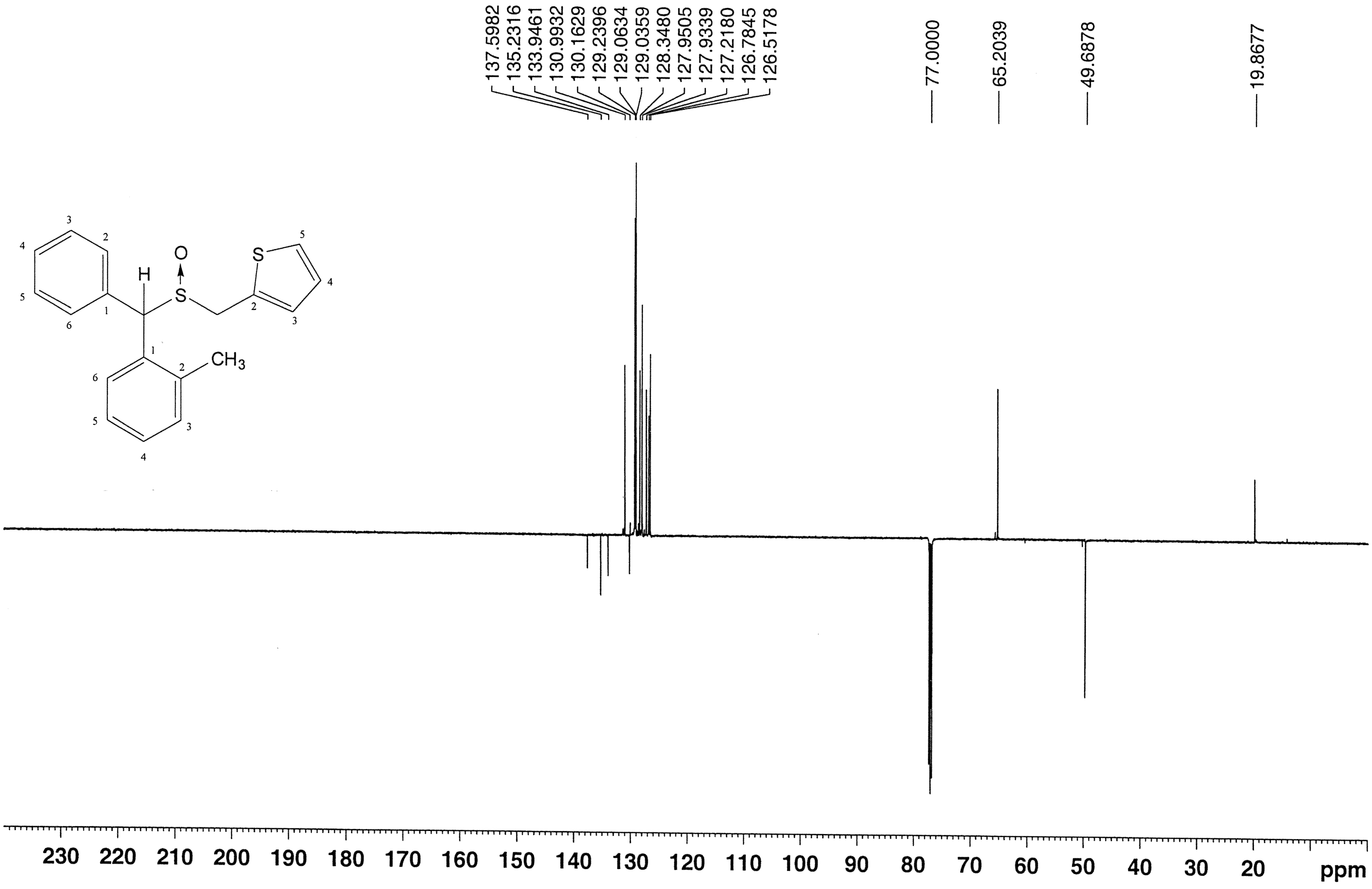

PN006-3 in cdcl3 (APT) 29.7.2020

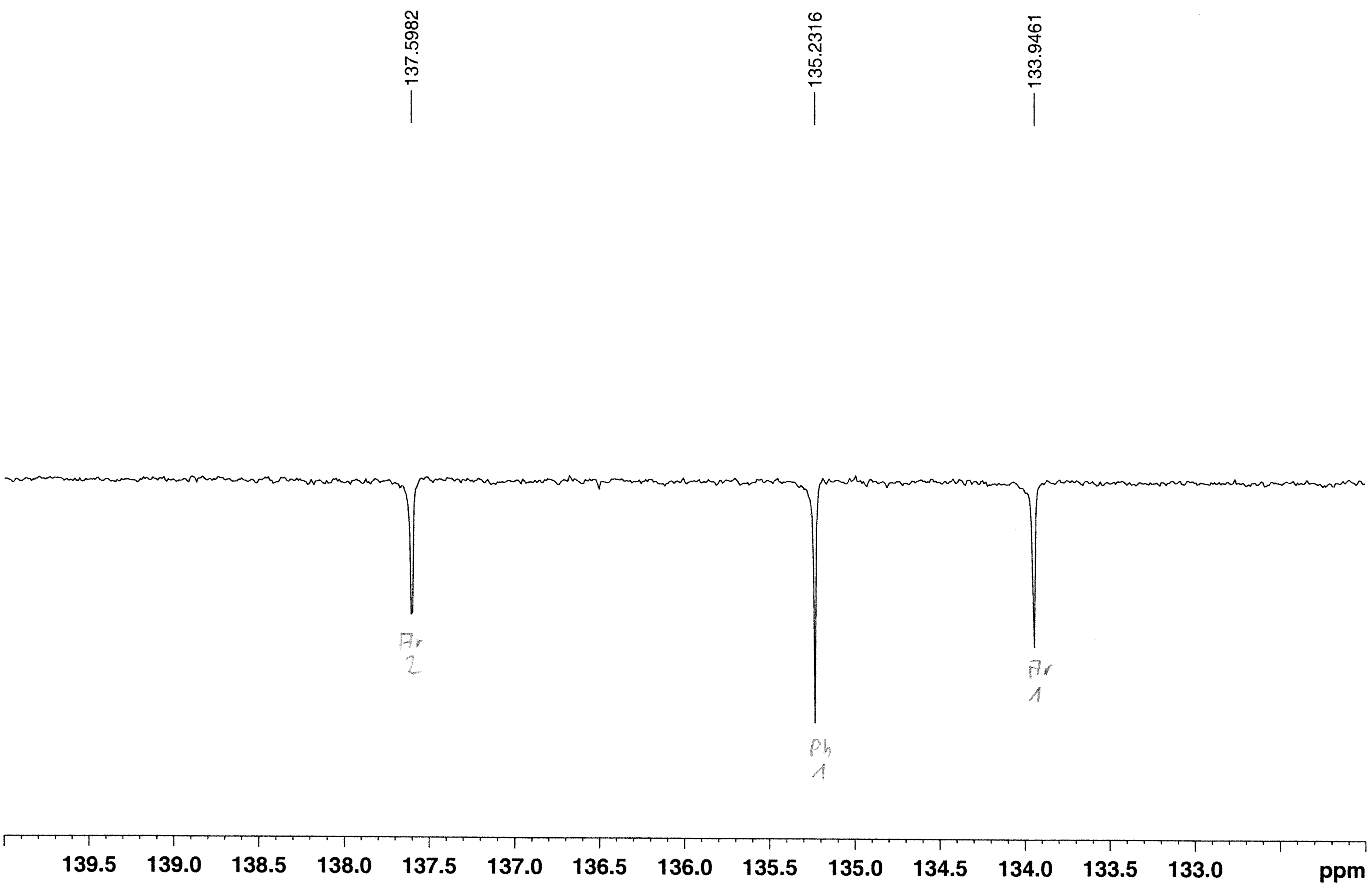

PN006-3 in cdcl3 (APT) 29.7.2020

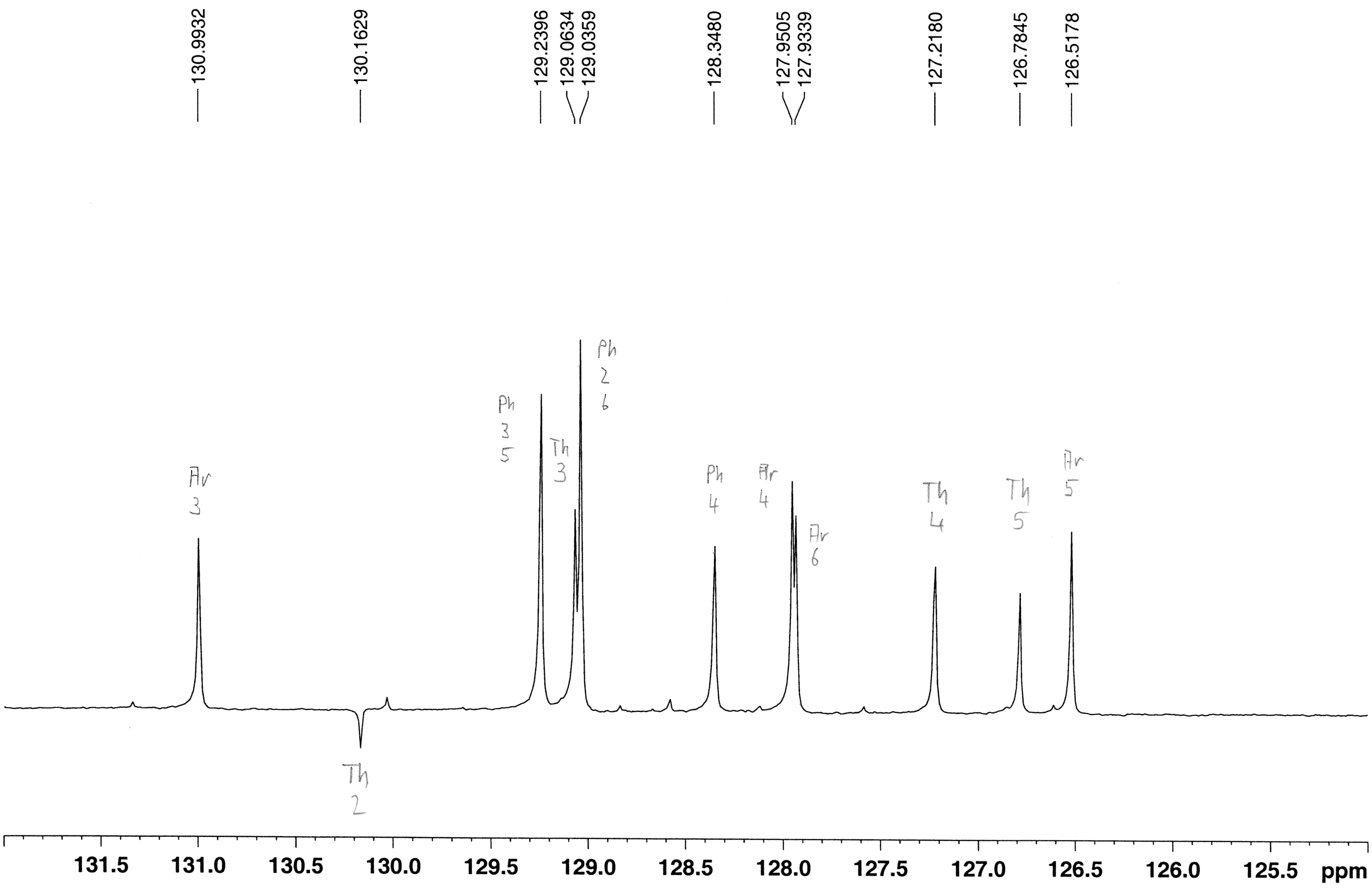

PN006-3 in cdcl3 (APT) 29.7.2020

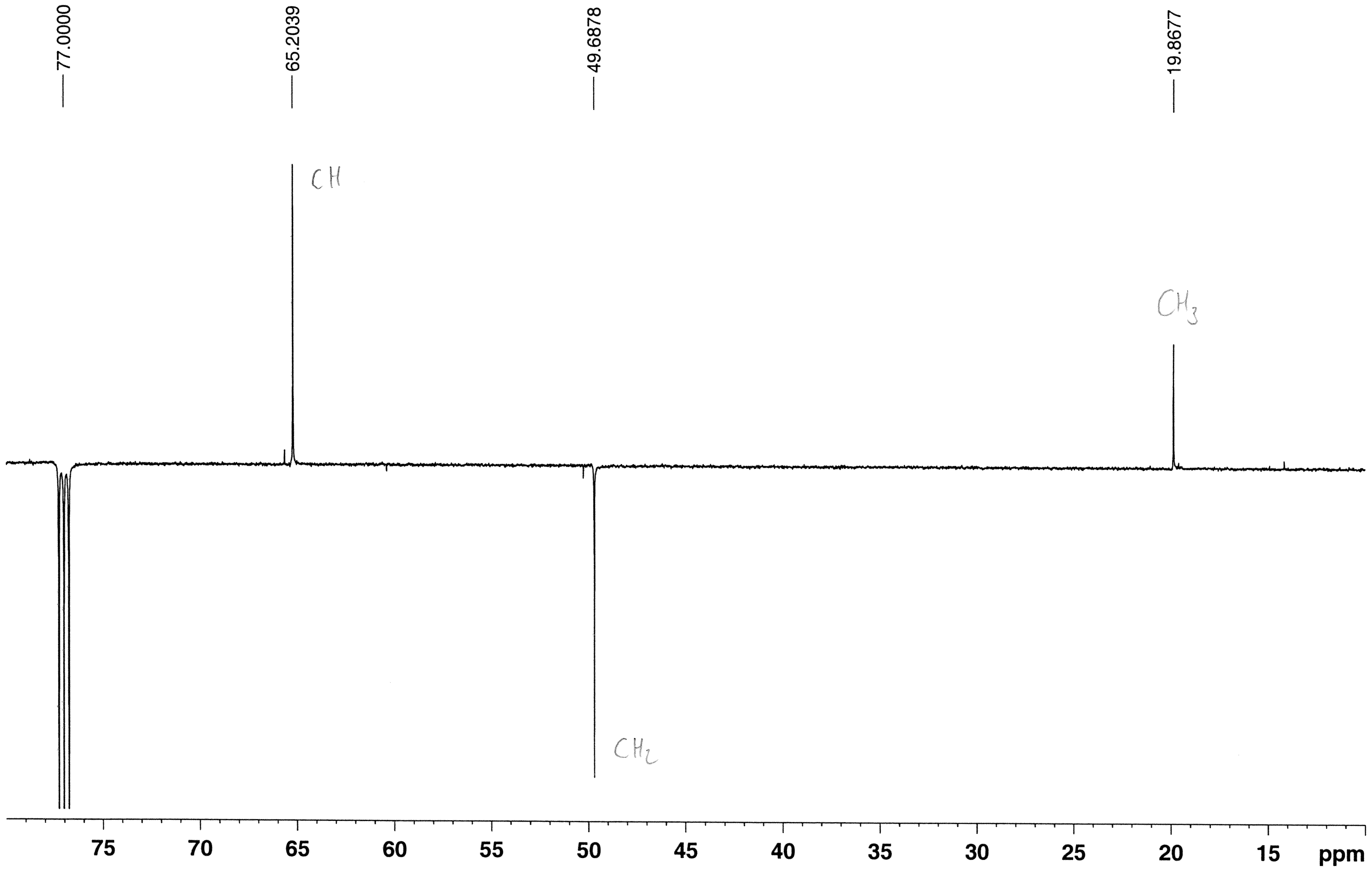

PN006-3 in cdcl3 (COSY) 29.7.2020

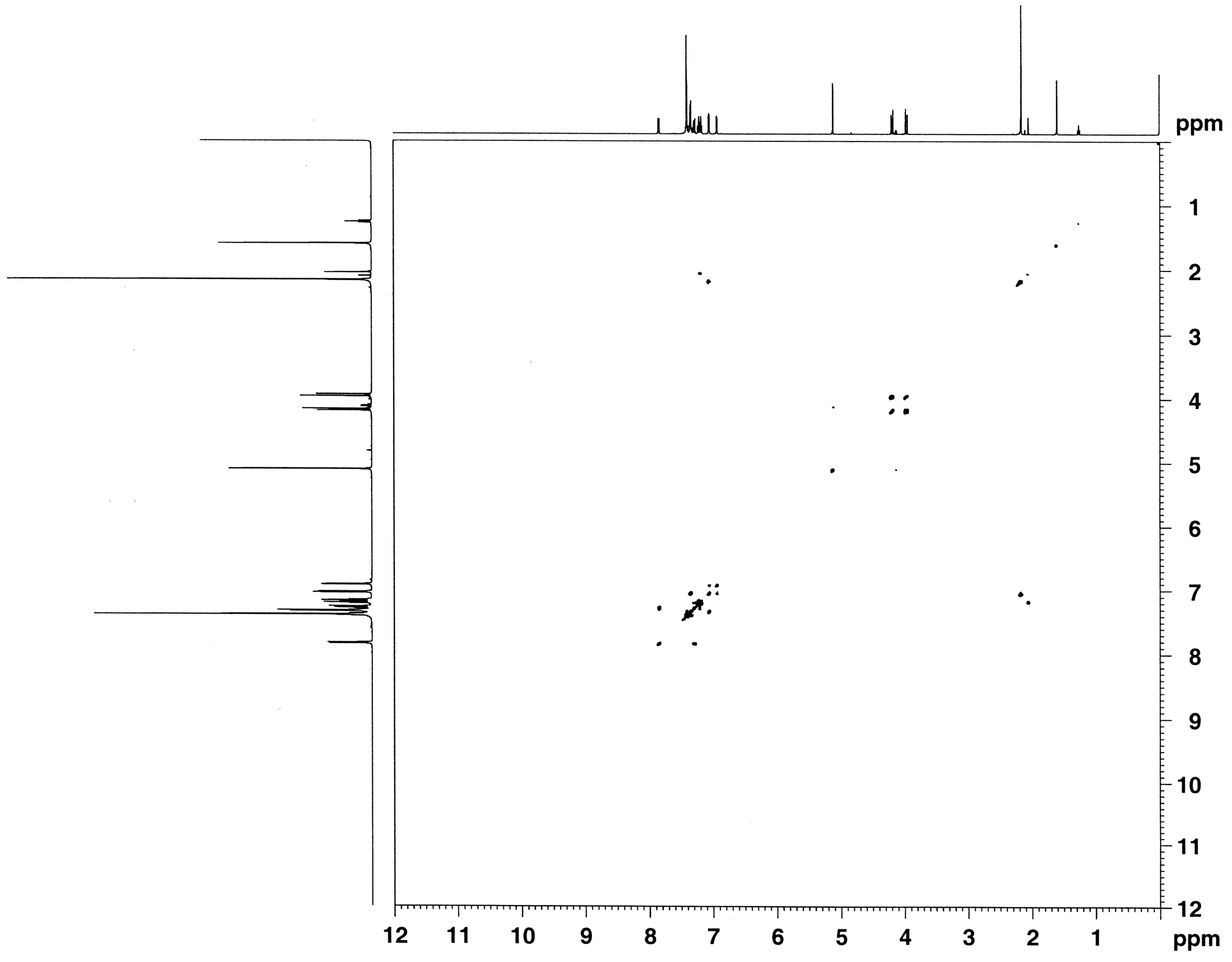

PN006-3 in cdcl3 (COSY) 29.7.2020

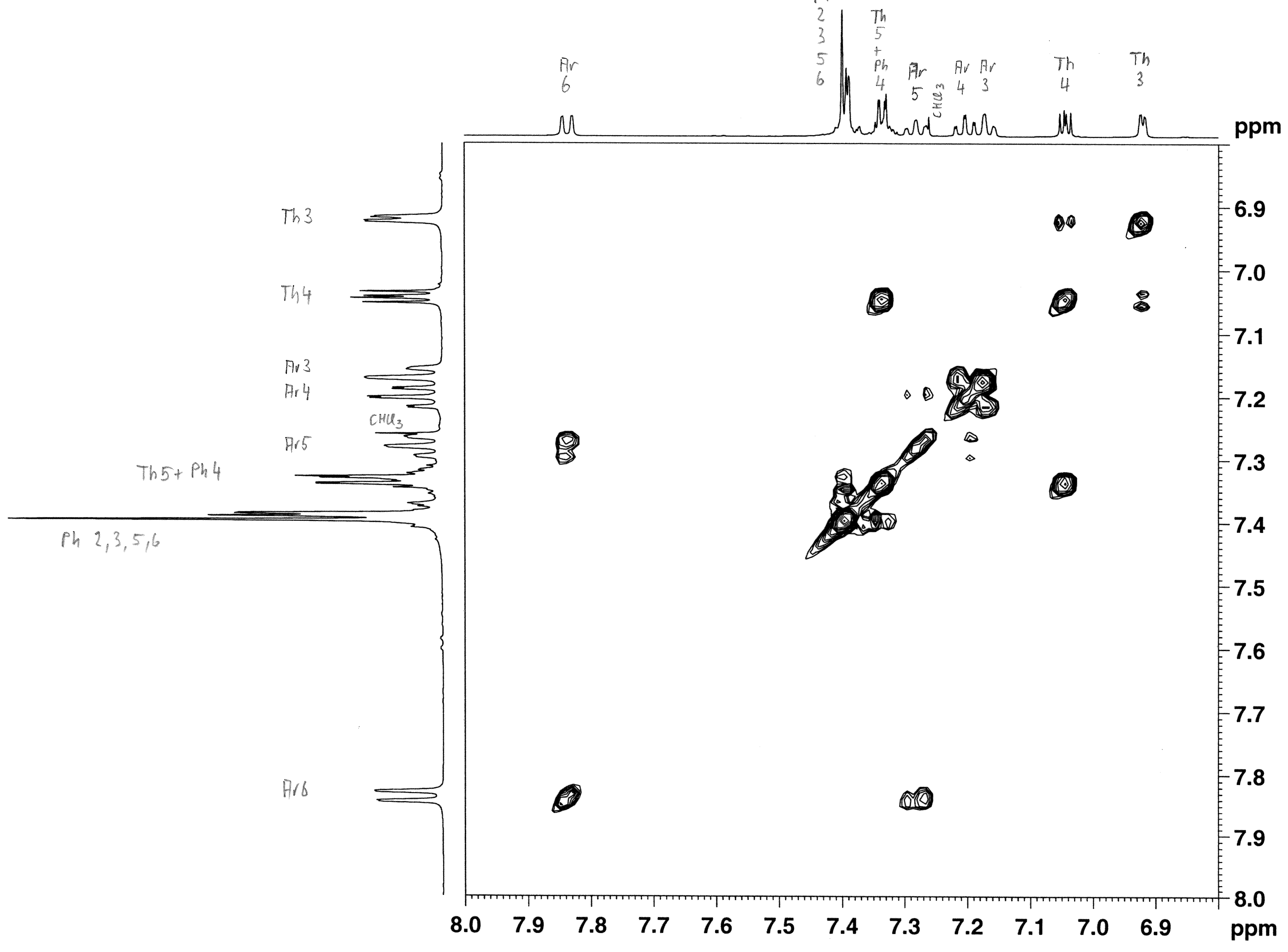

PN006-3 in cdcl3 (COSY) 29.7.2020

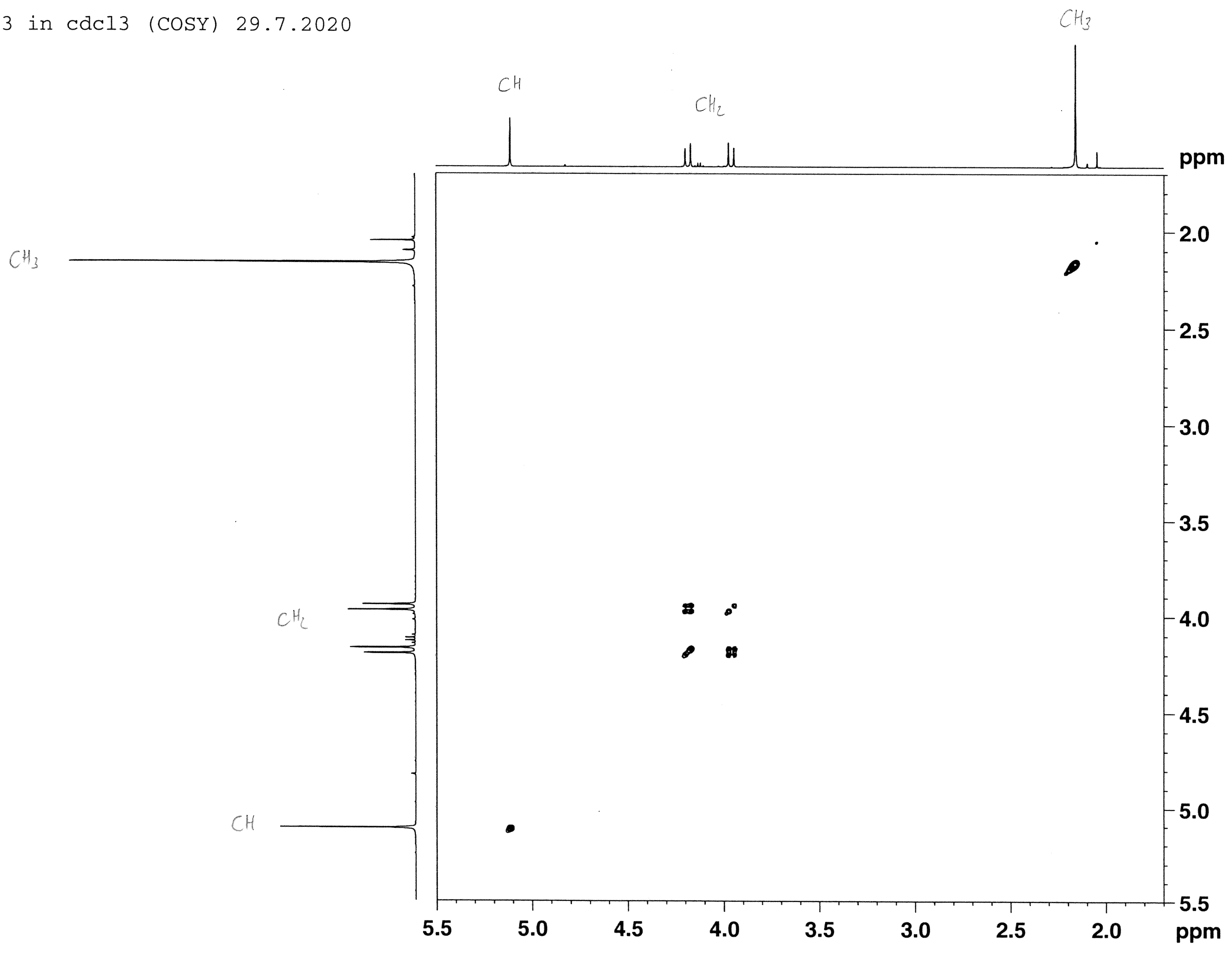

PN006-3 in cdcl3 (HSQC) 29.7.2020

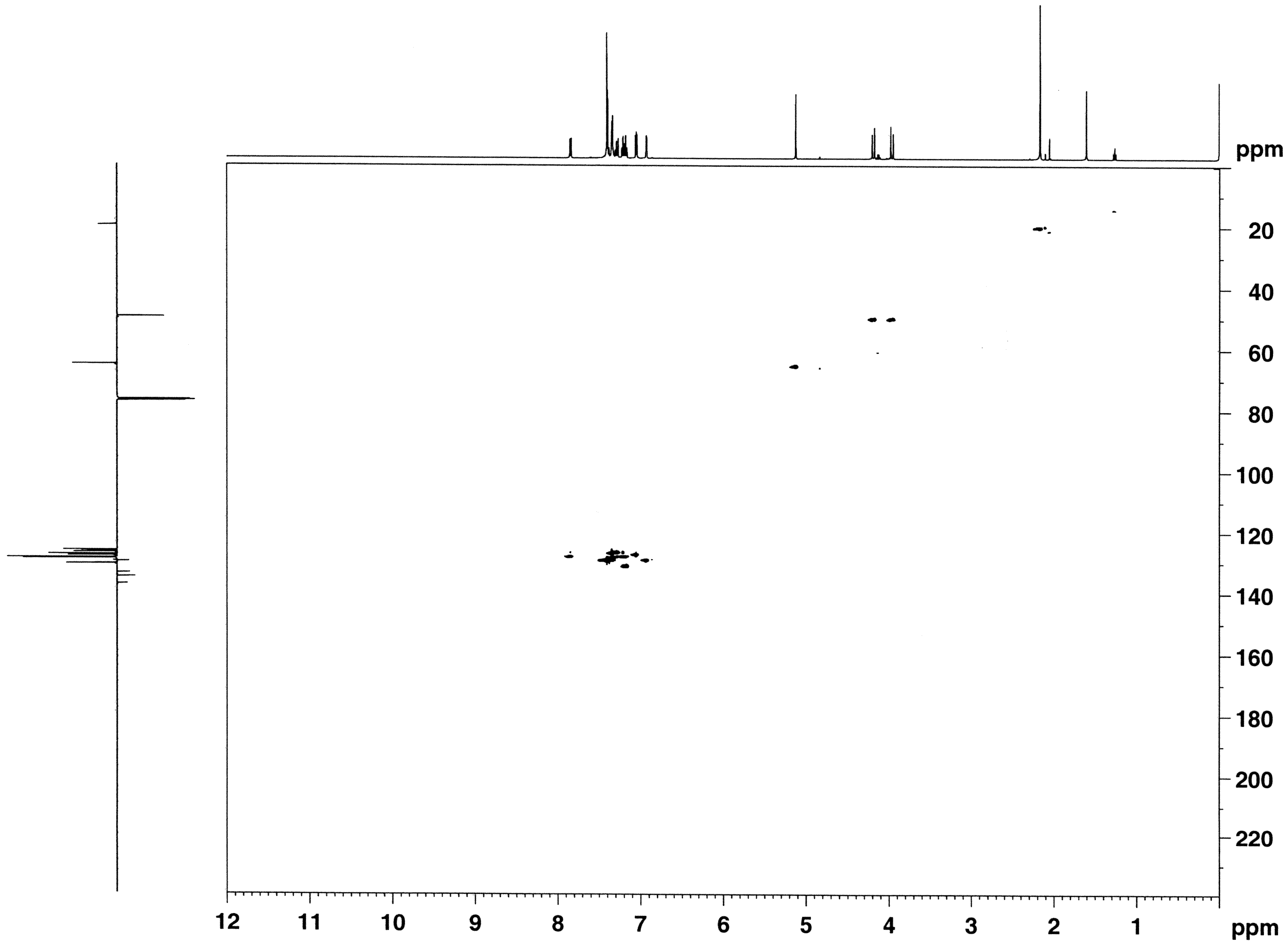

PN006-3 in cdcl3 (HSQC) 29.7.2020

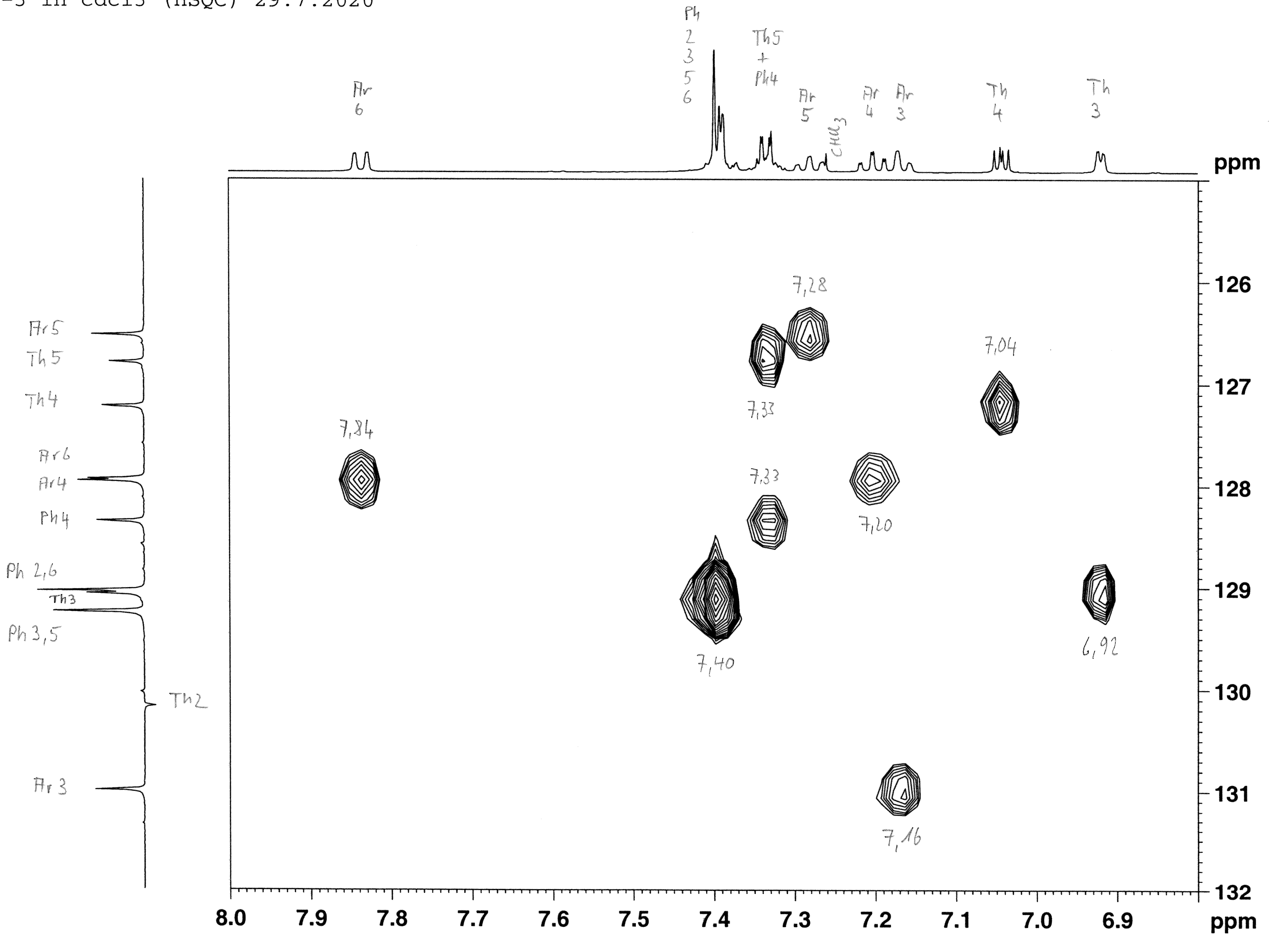

PN006-3 in cdcl3 (HSQC) 29.7.2020

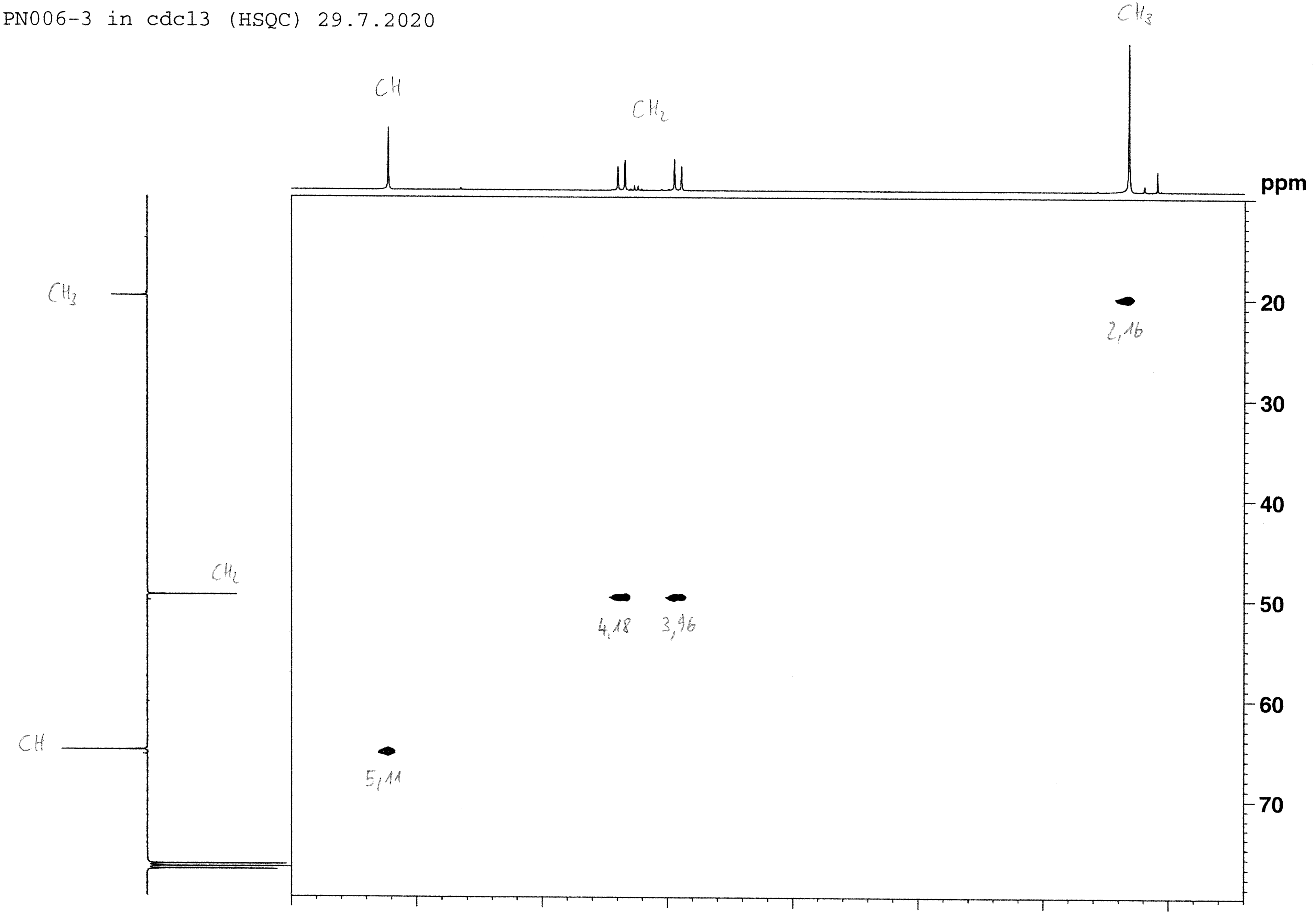

PN006-3 in cdcl3 (HMBC) 29.7.2020

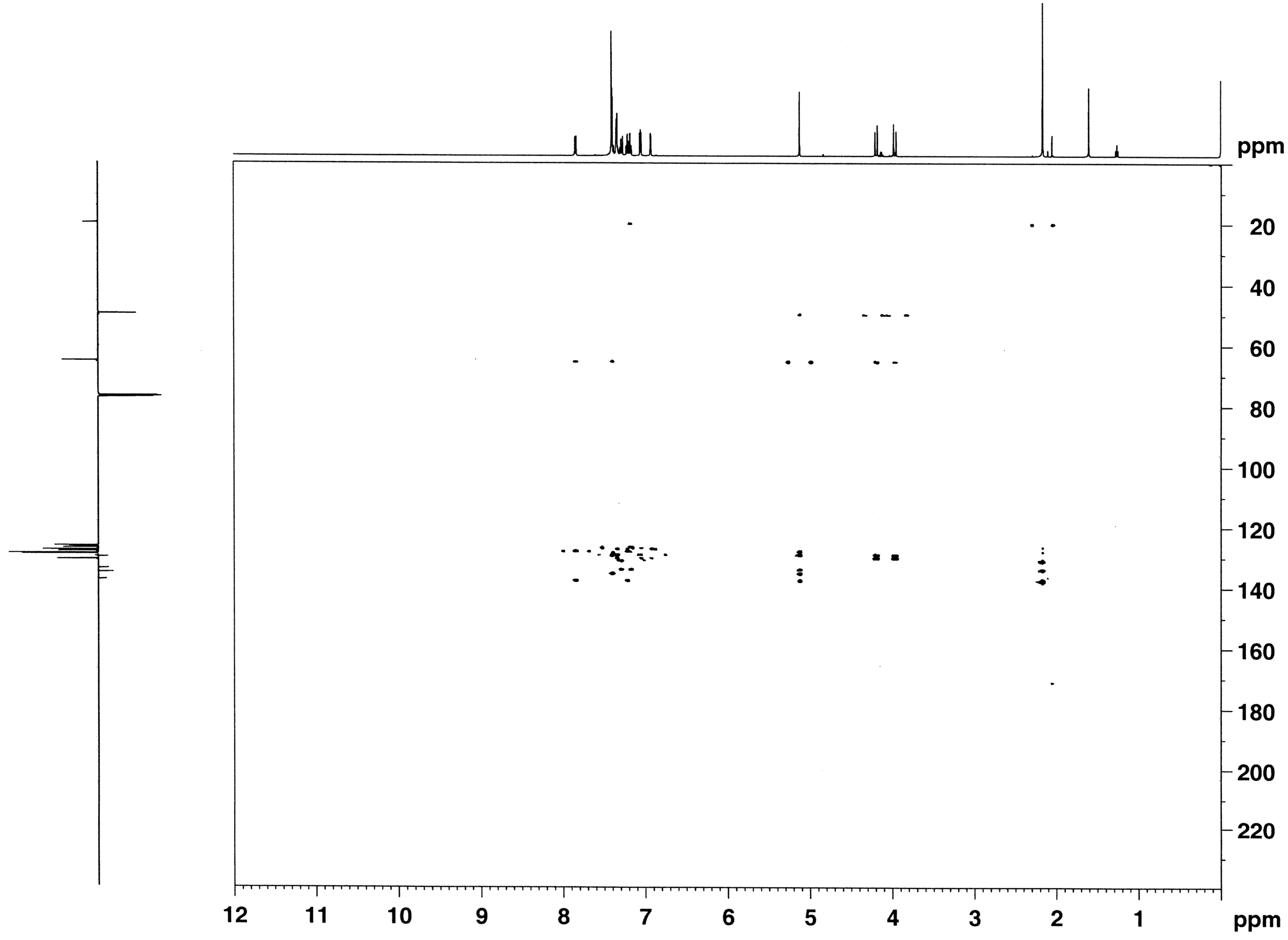

PN006-3 in cdcl3 (HMBC) 29.7.2020

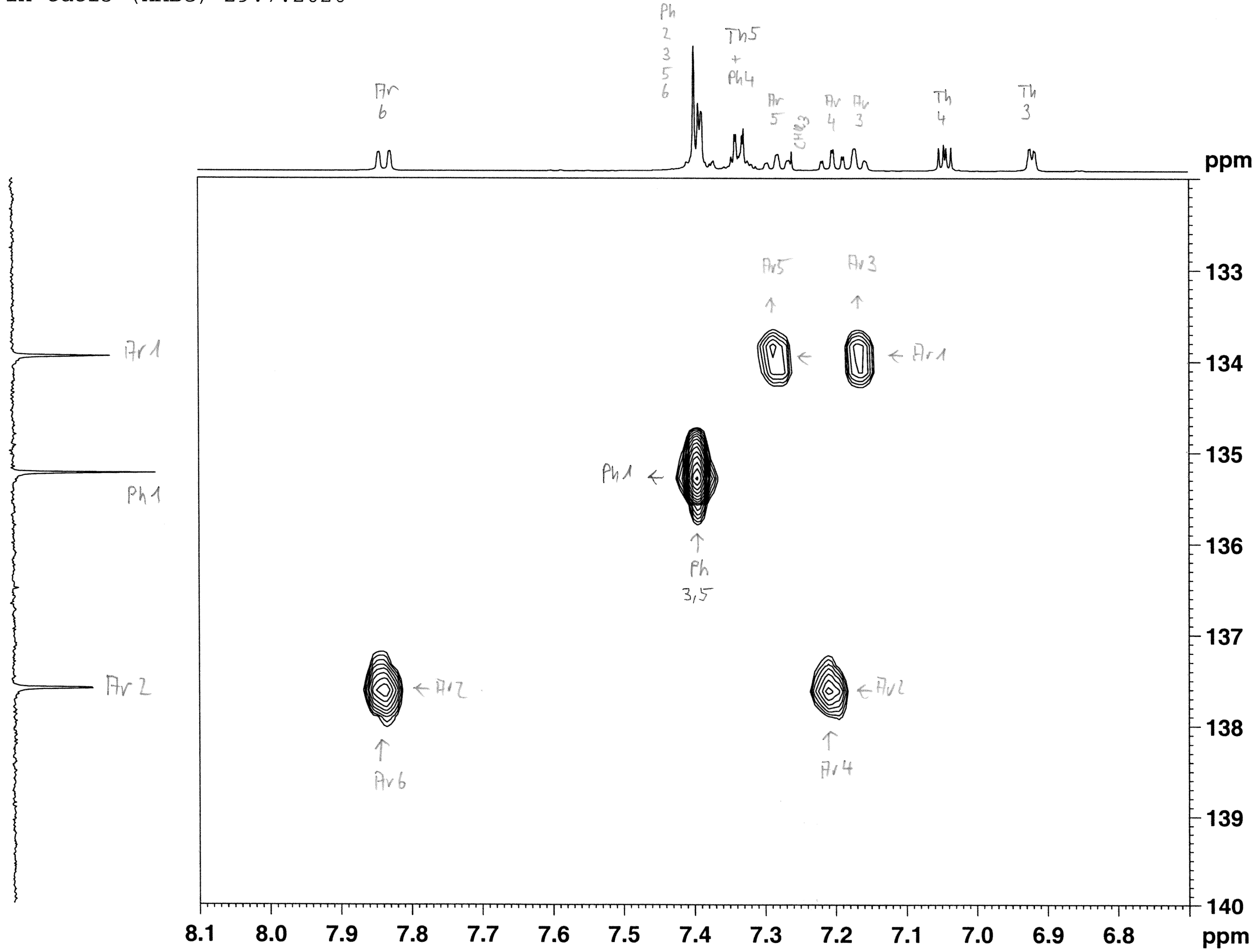

PN006-3 in cdcl3 (HMBC) 29.7.2020

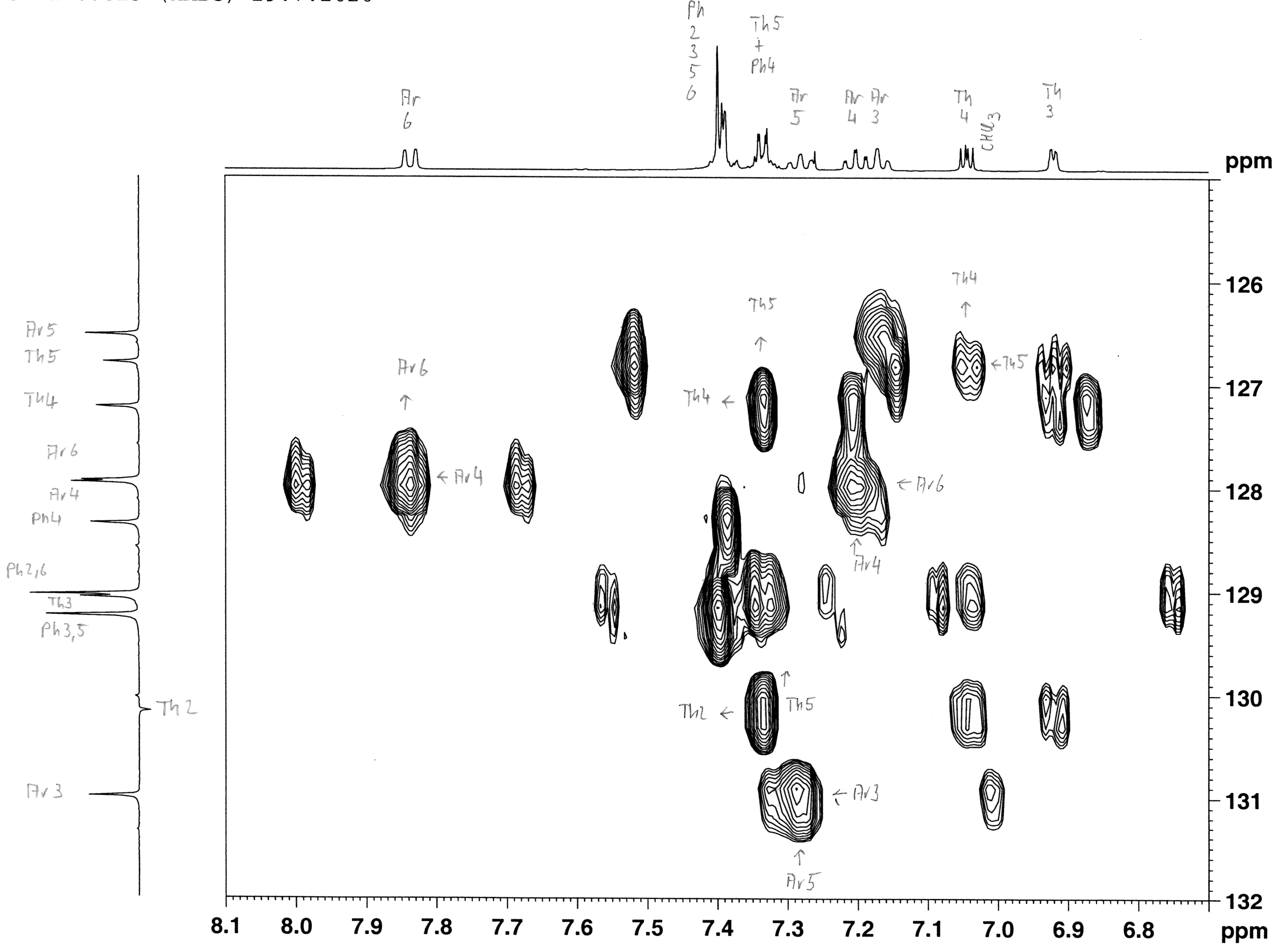

PN006-3 in cdcl3 (HMBC) 29.7.2020

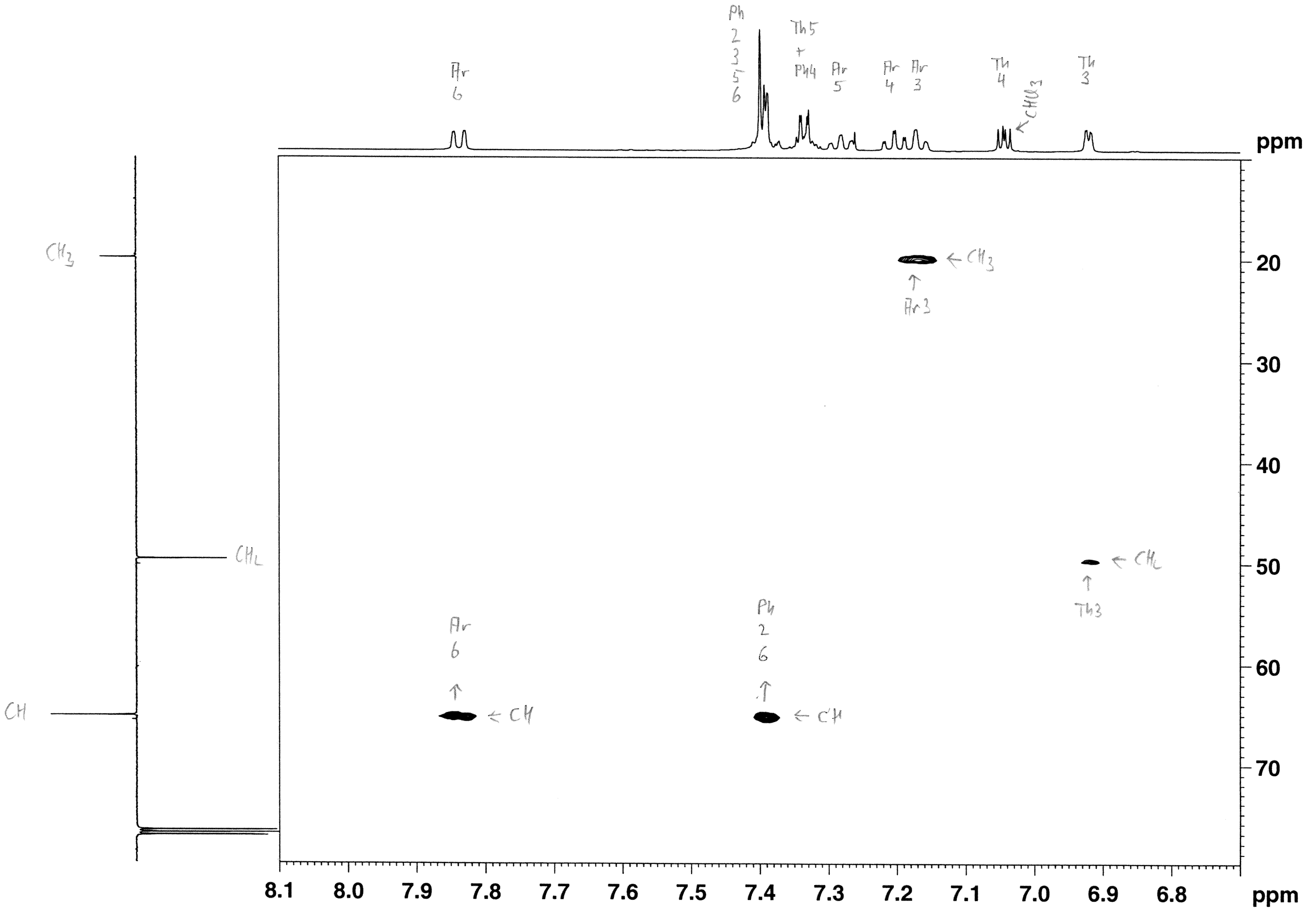

PN006-3 in cdcl3 (HMBC) 29.7.2020

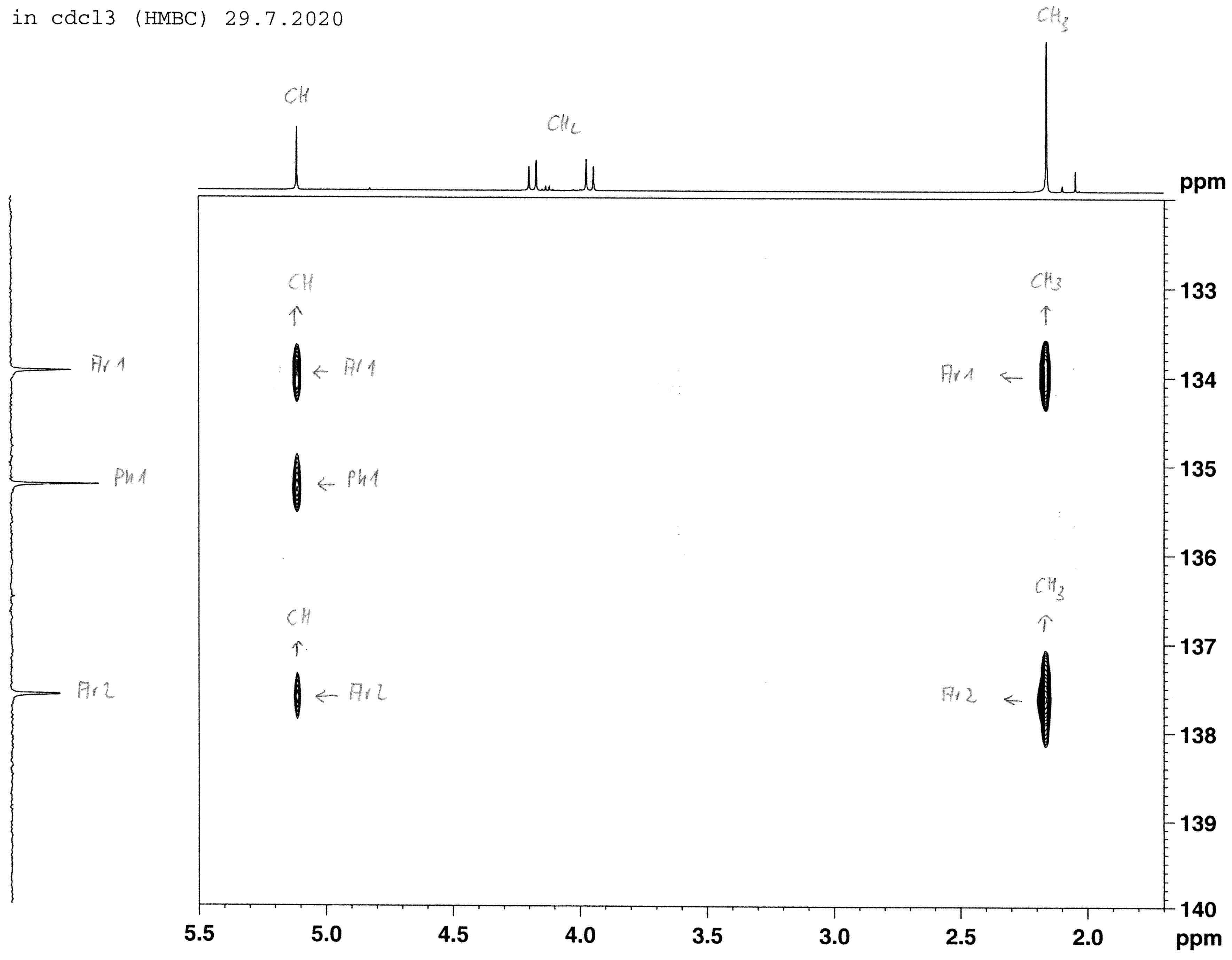

PN006-3 in cdcl3 (HMBC) 29.7.2020

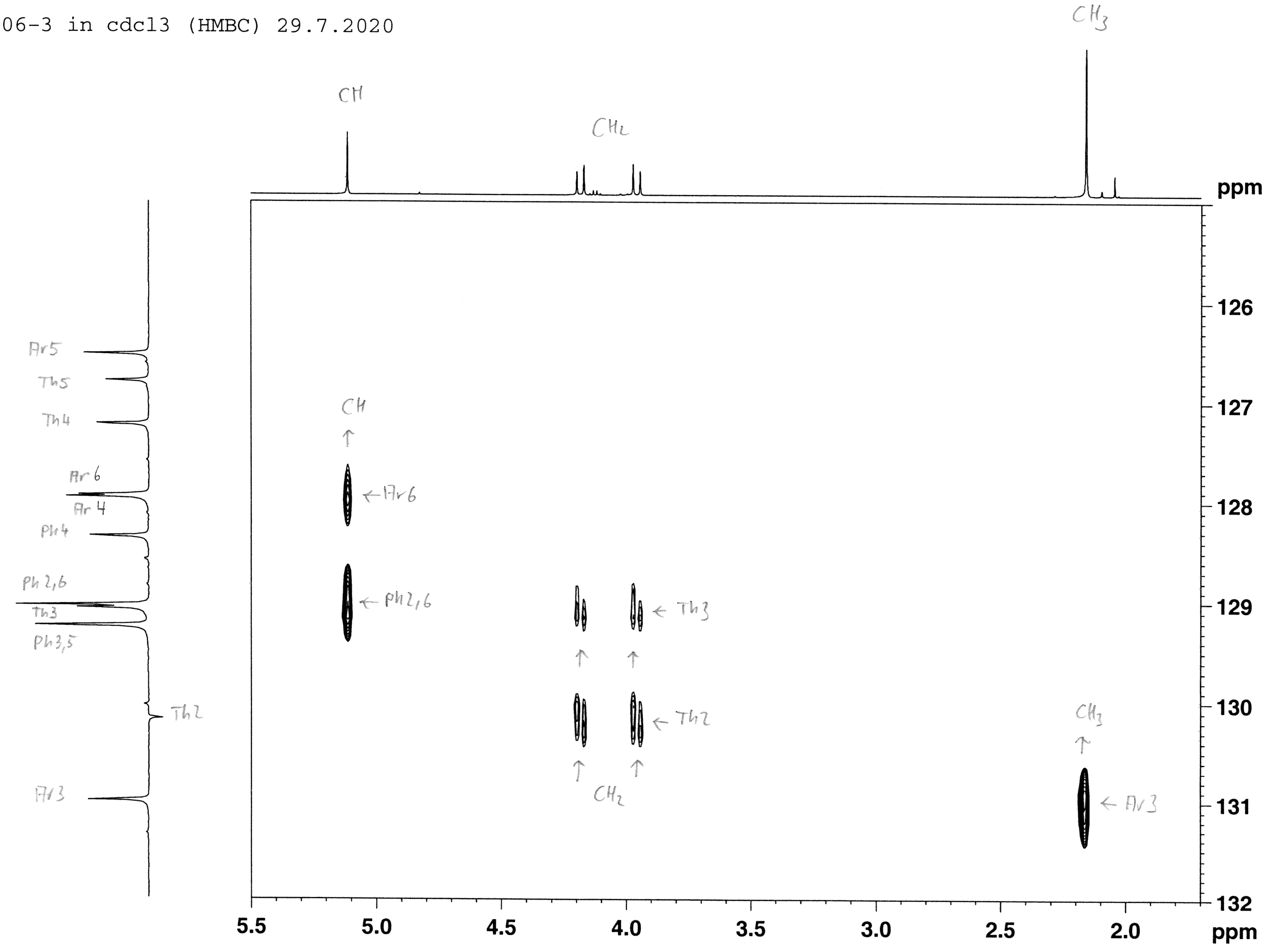

PN006-3 in cdcl3 (HMBC) 29.7.2020

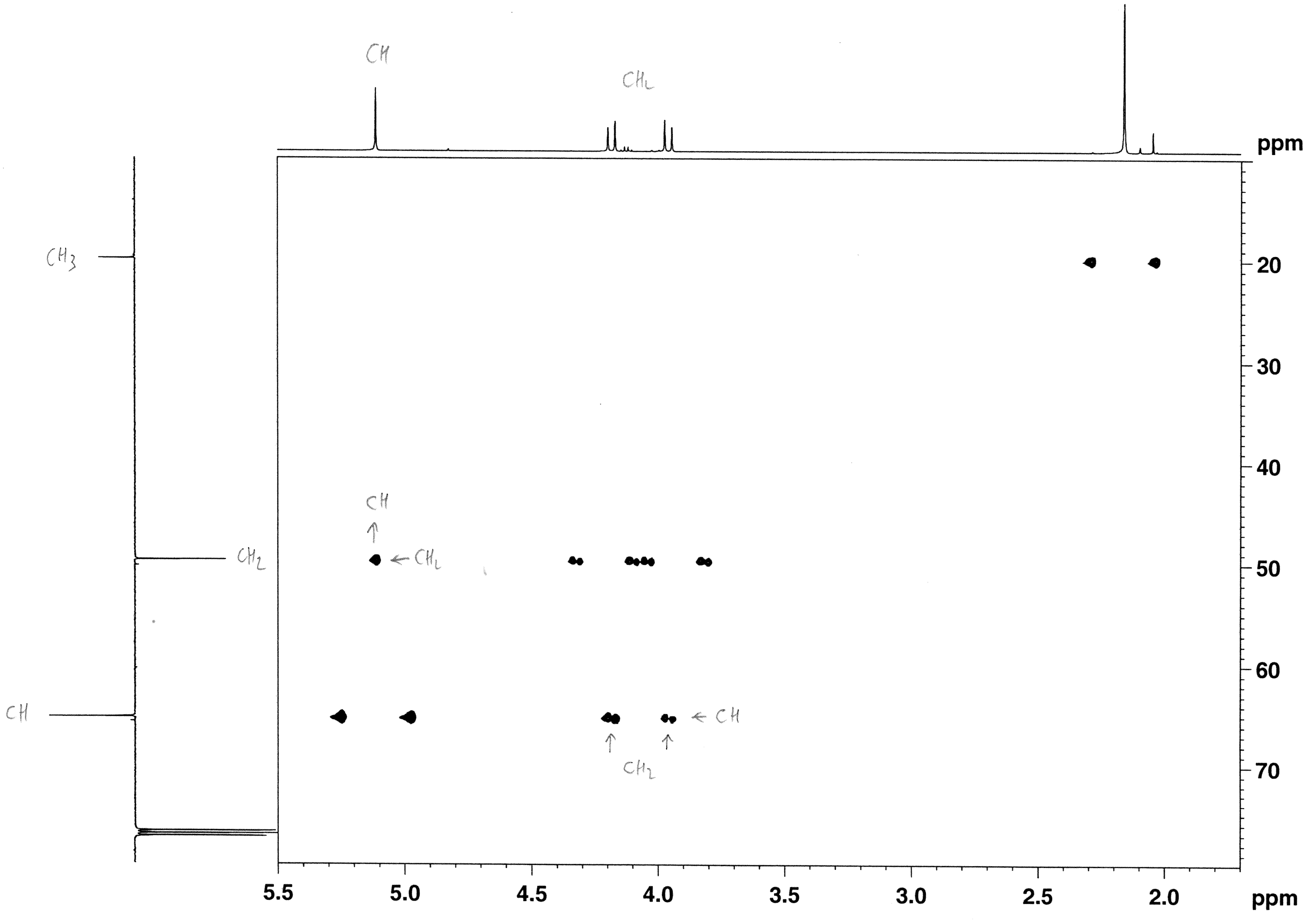

Figure S48c. NMR spectra of compound **8l**.

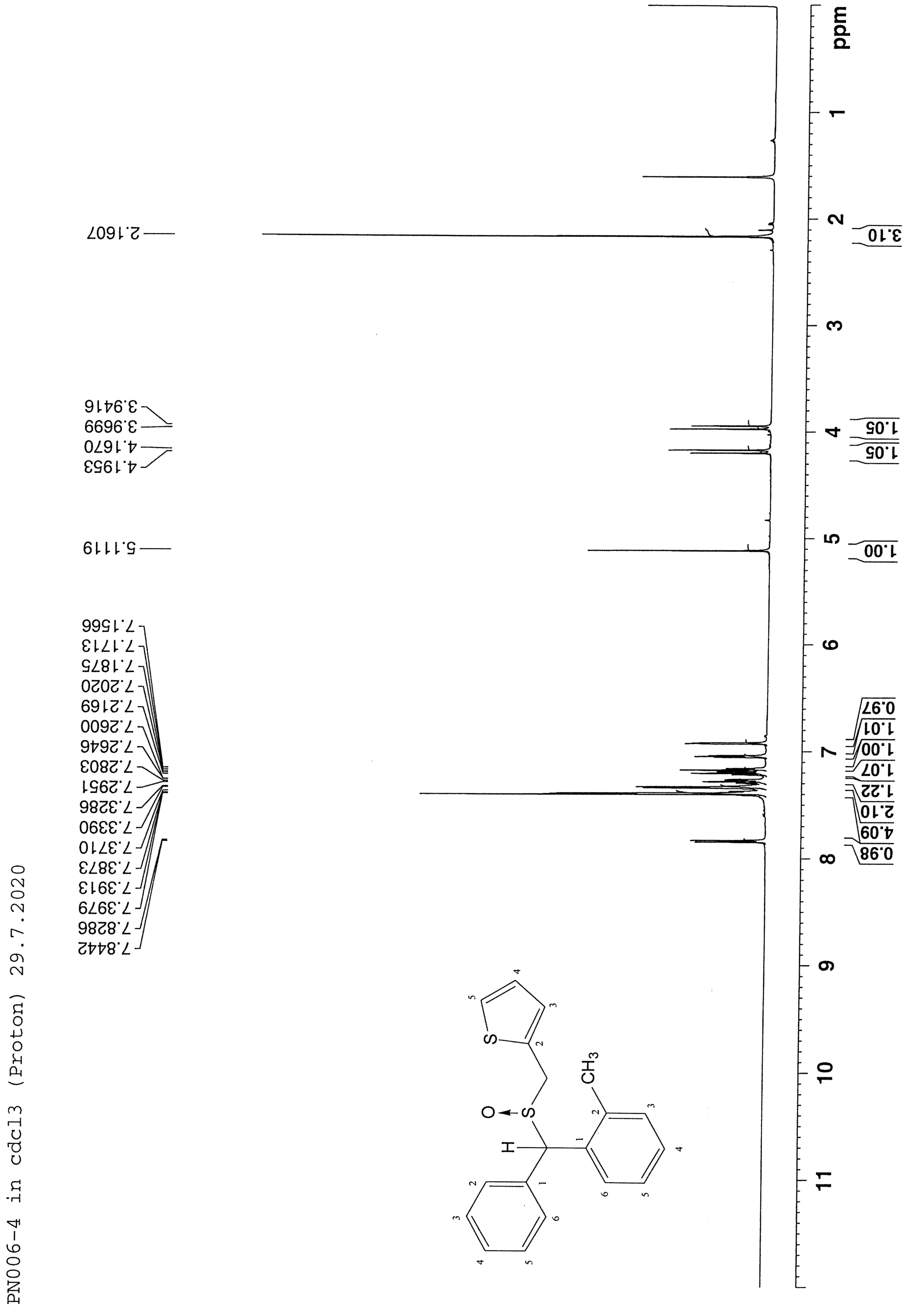

PN006-4 in cdcl3 (Proton) 29.7.2020

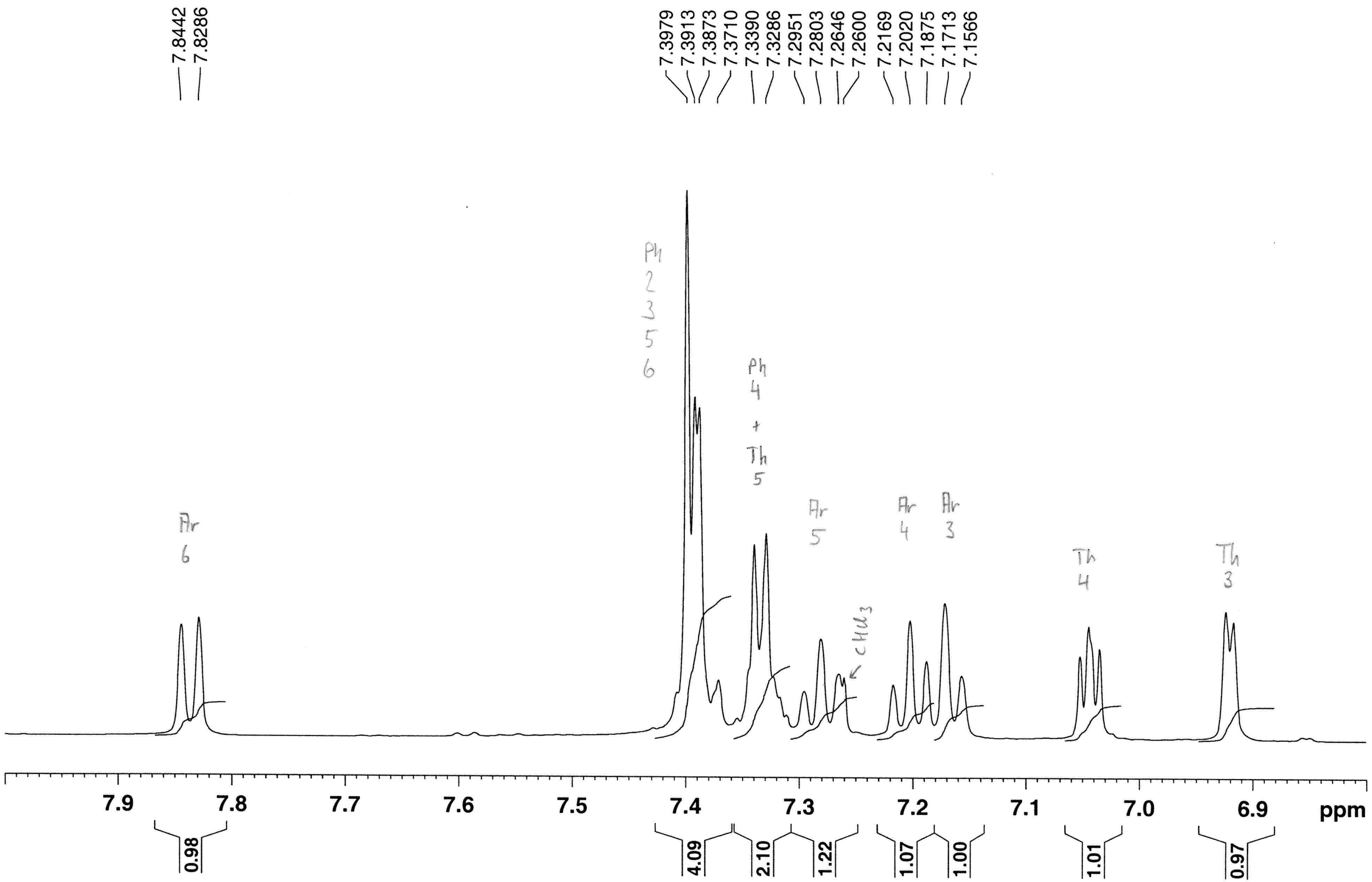

PN006-4 in cdcl3 (Proton) 29.7.2020

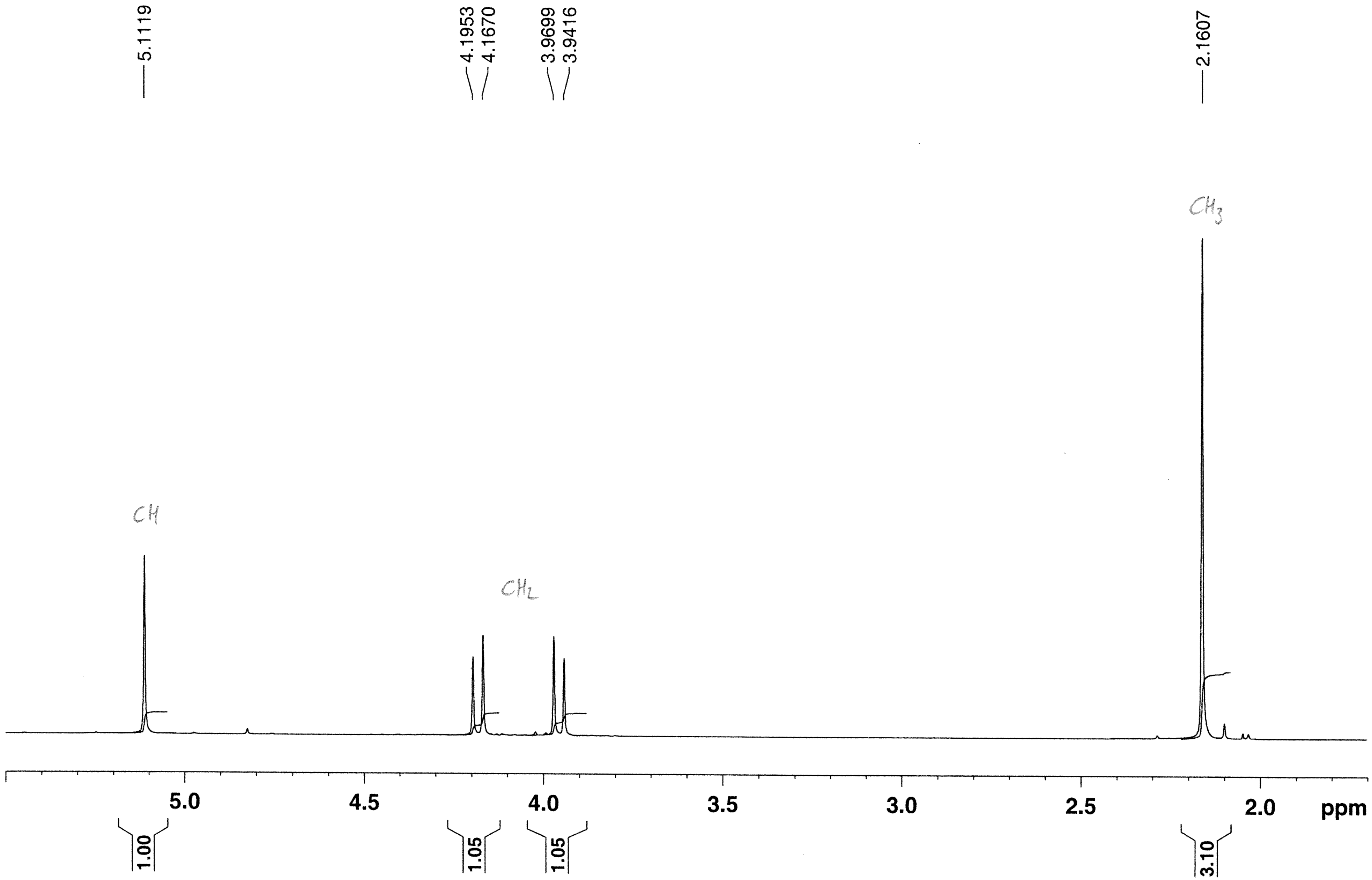

PN006-4 in cdcl3 (APT) 29.7.2020

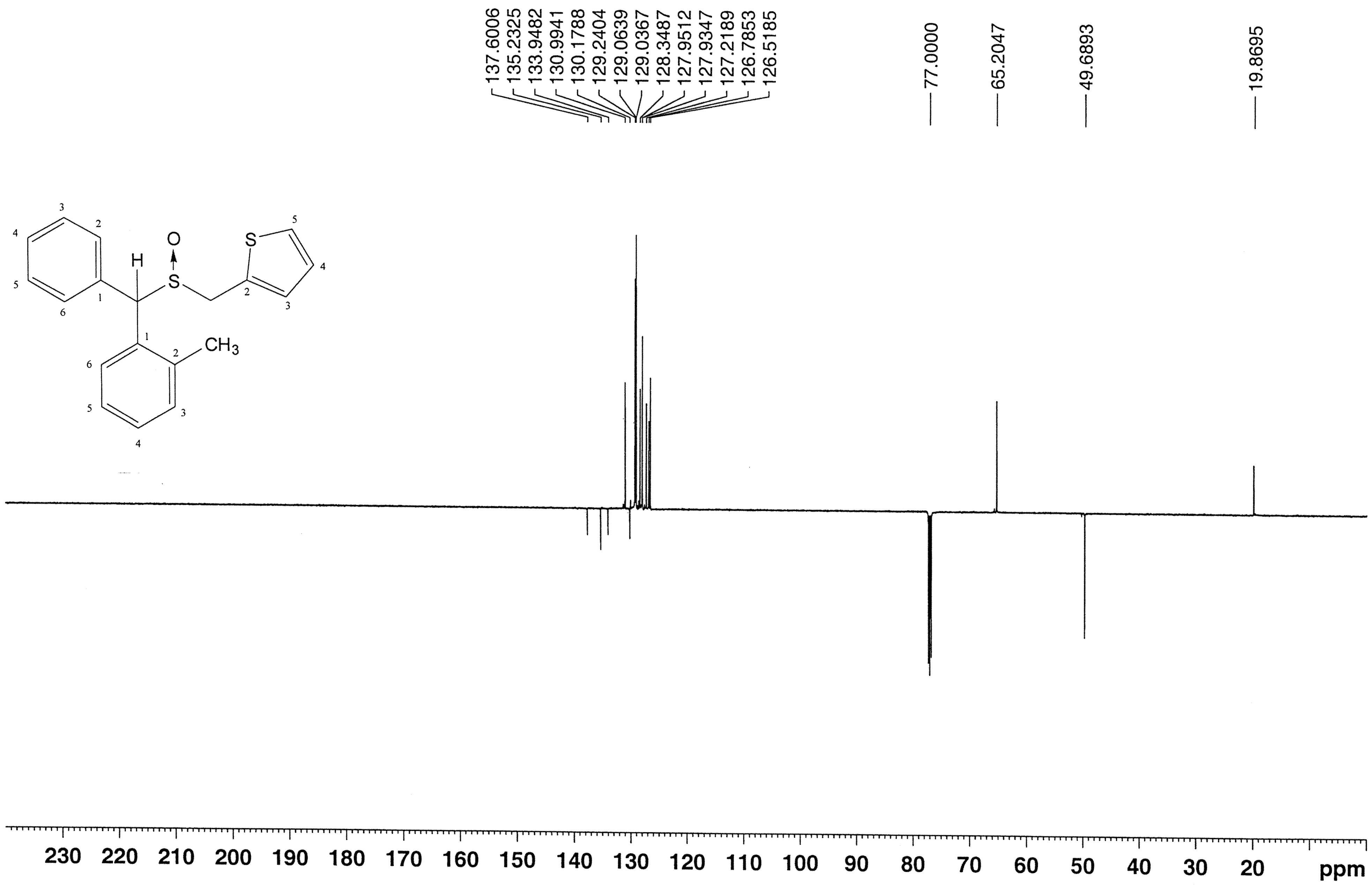

PN006-4 in cdcl3 (APT) 29.7.2020

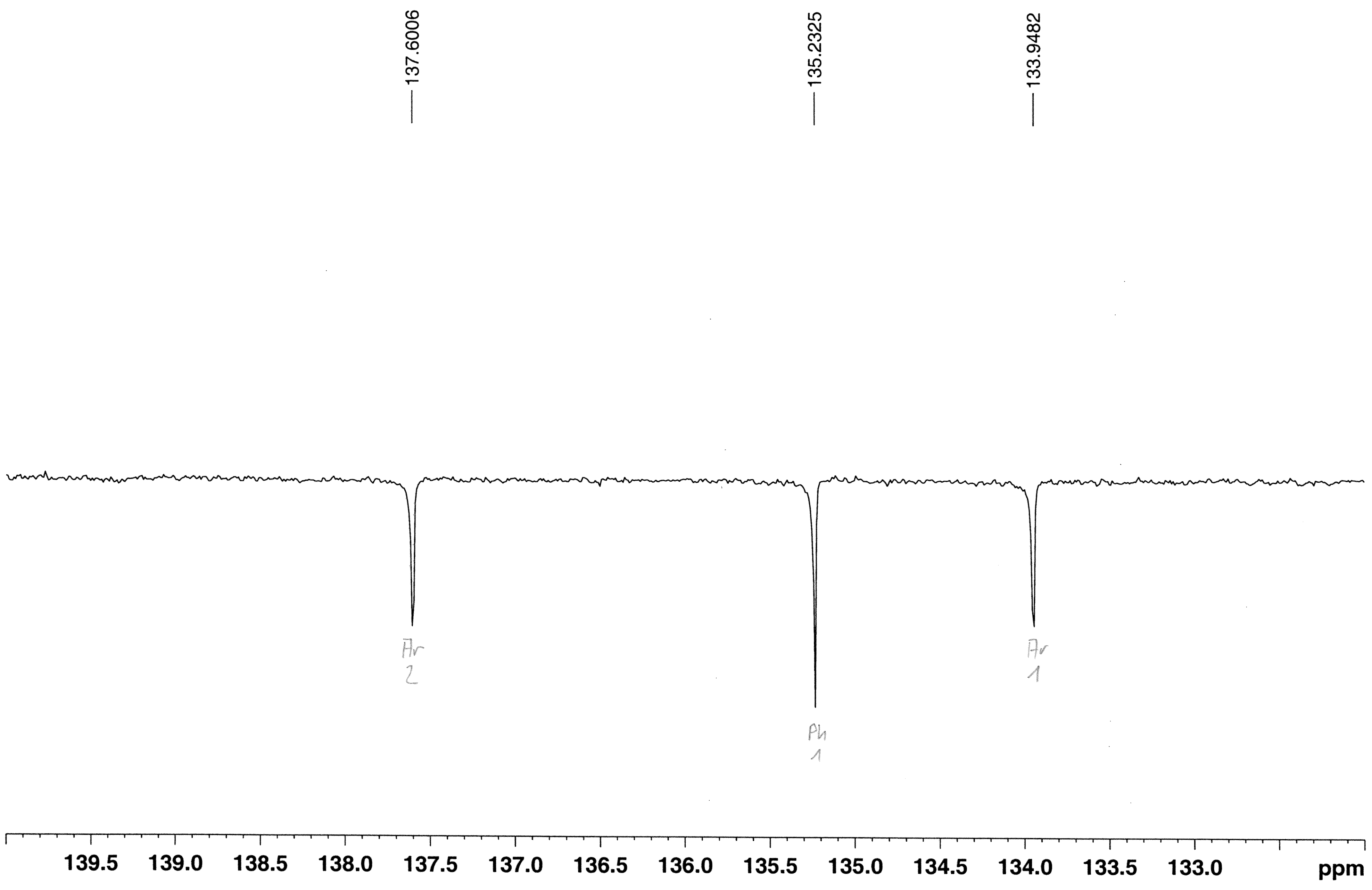

PN006-4 in cdcl3 (APT) 29.7.2020

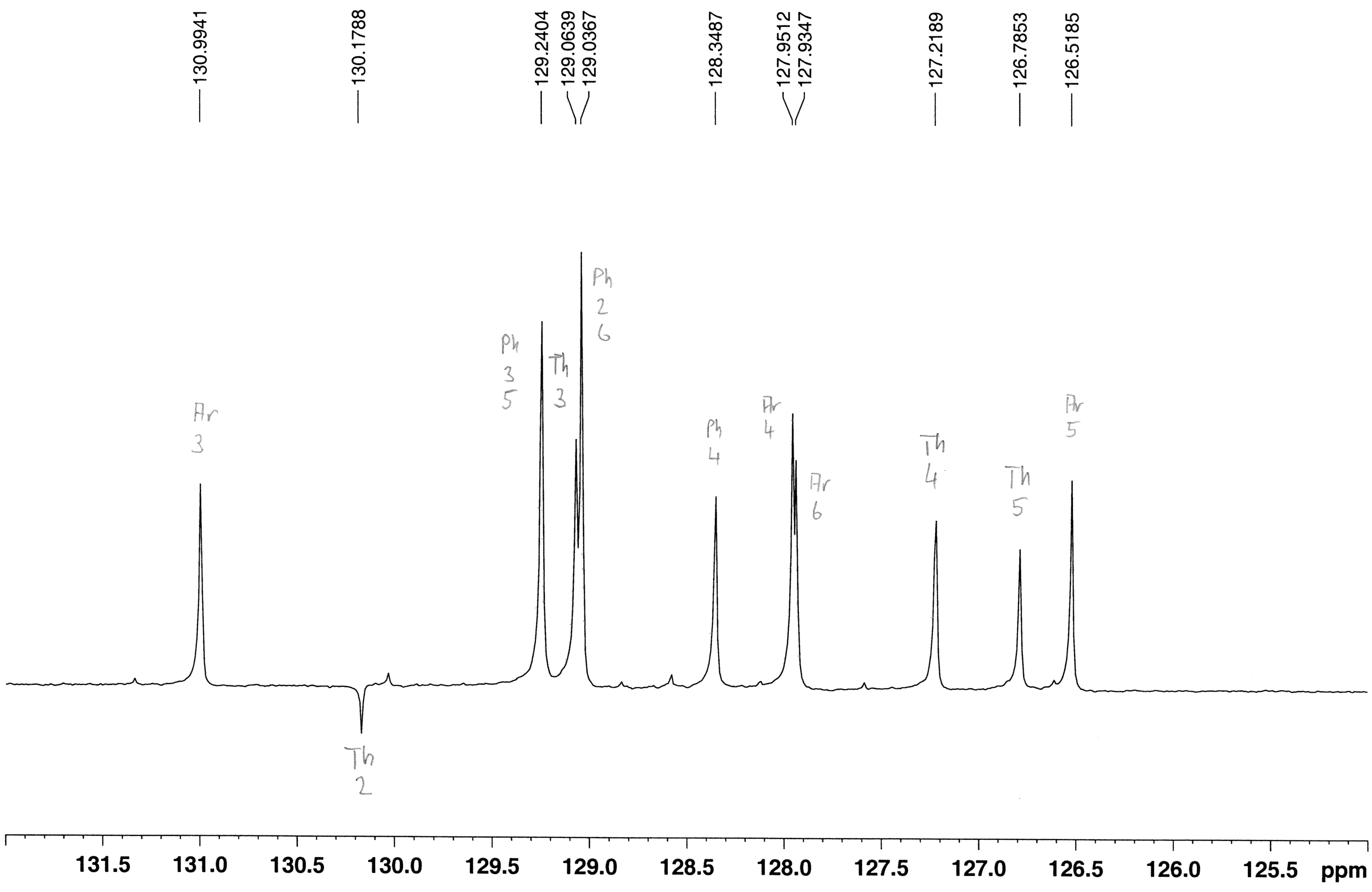

PN006-4 in cdcl3 (APT) 29.7.2020

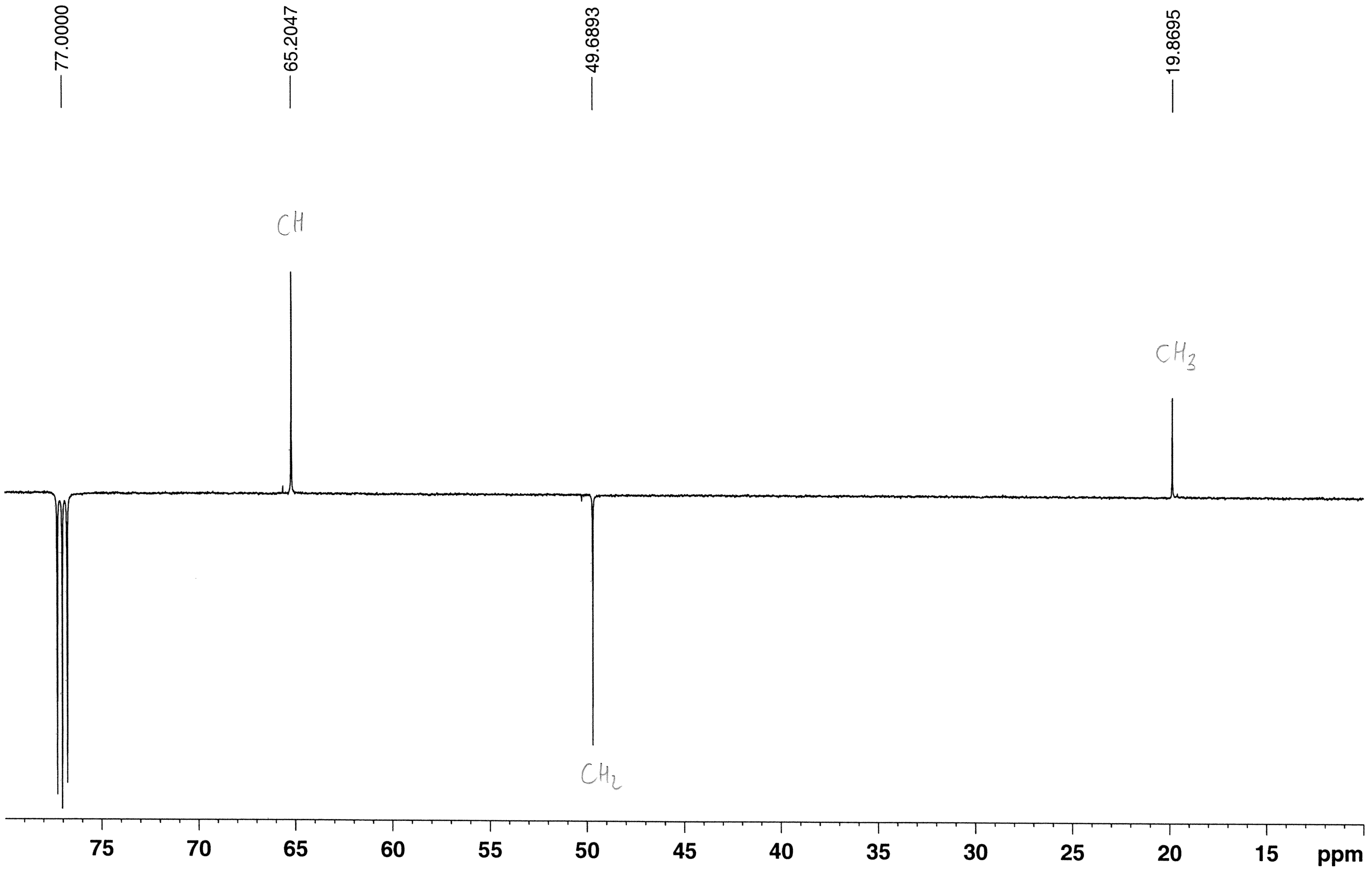

Figure S49c. NMR spectra of compound **5m.**

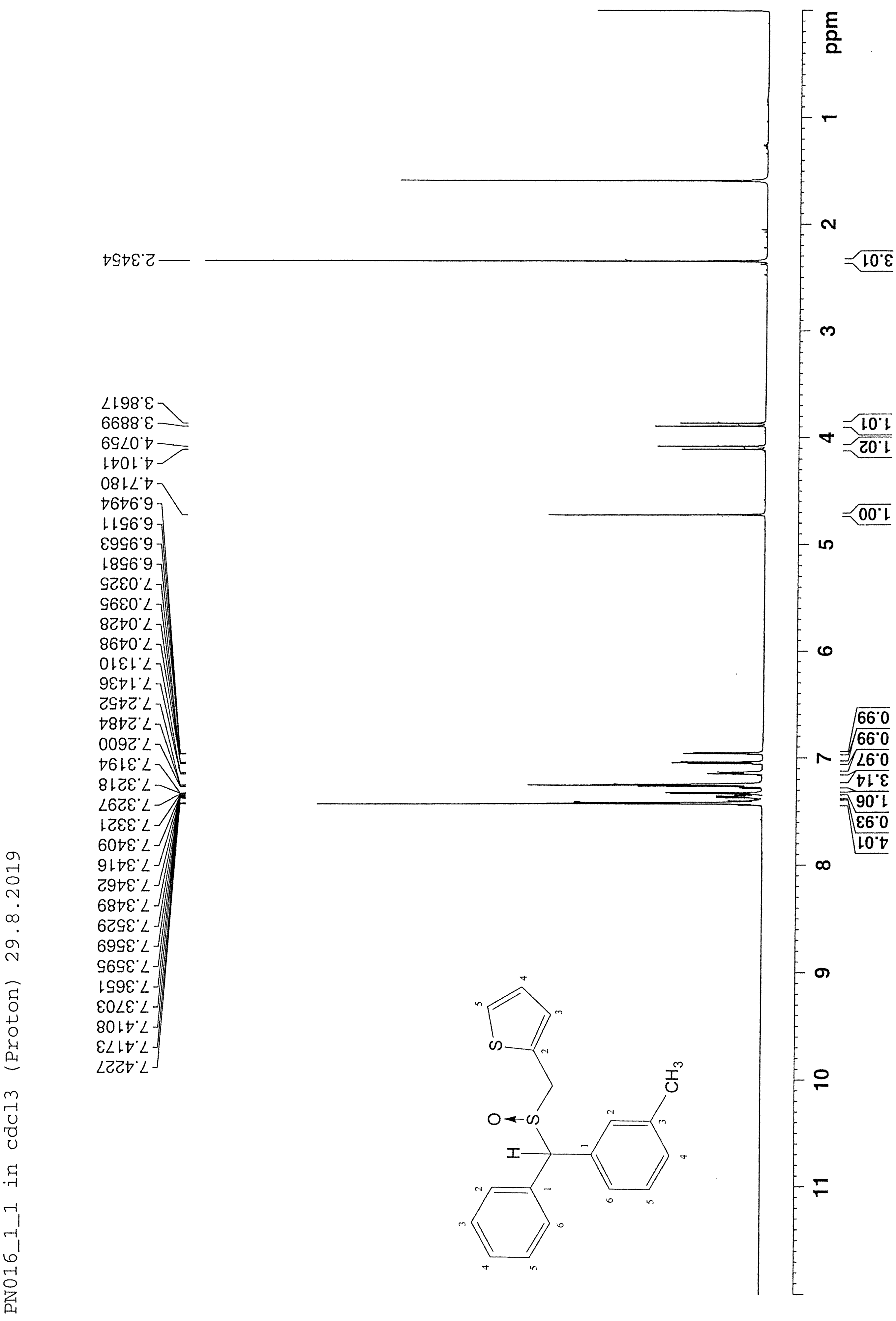

PN016_1_1 in cdcl3 (Proton) 29.8.2019

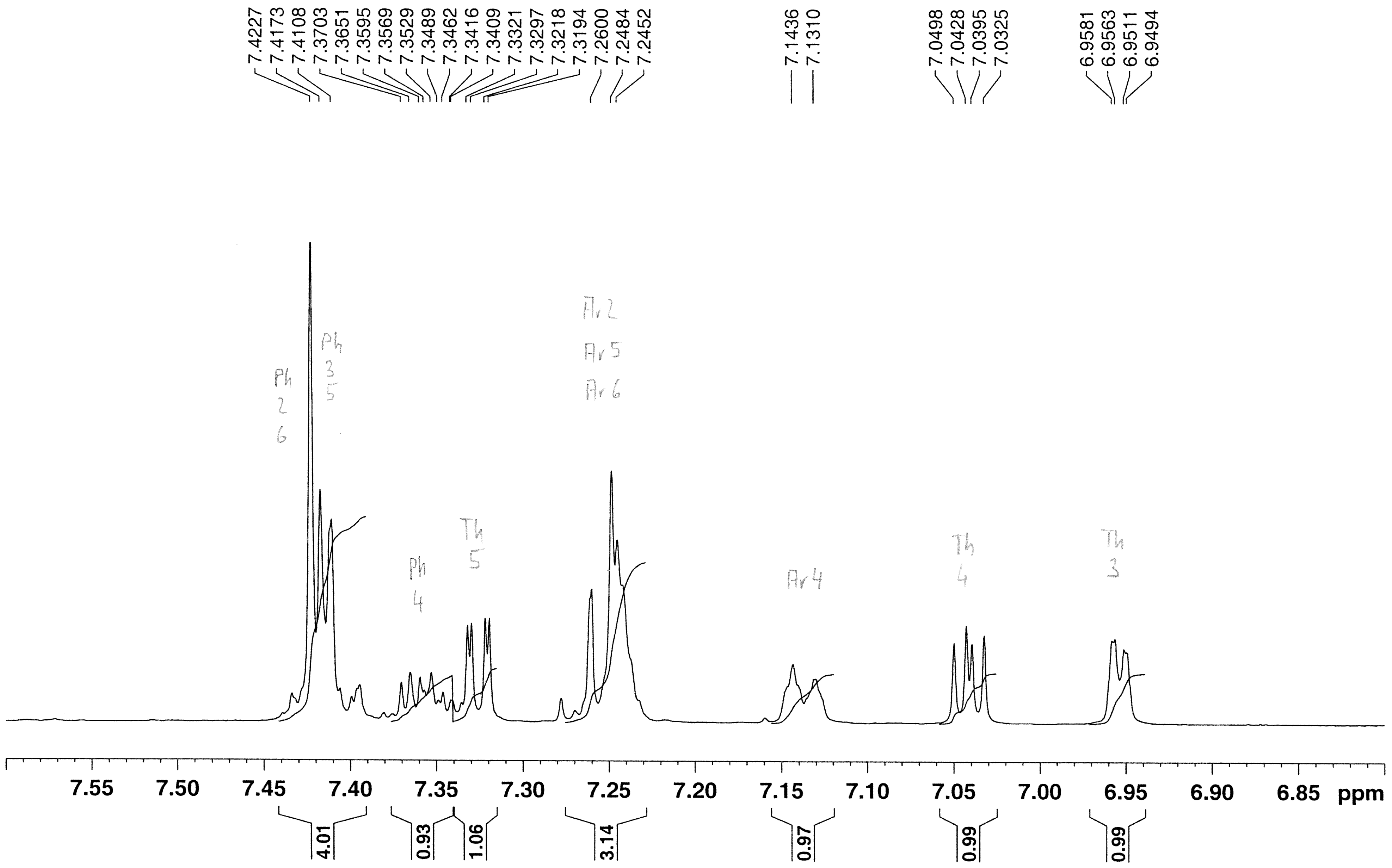

PN016_1_1 in cdcl3 (Proton) 29.8.2019

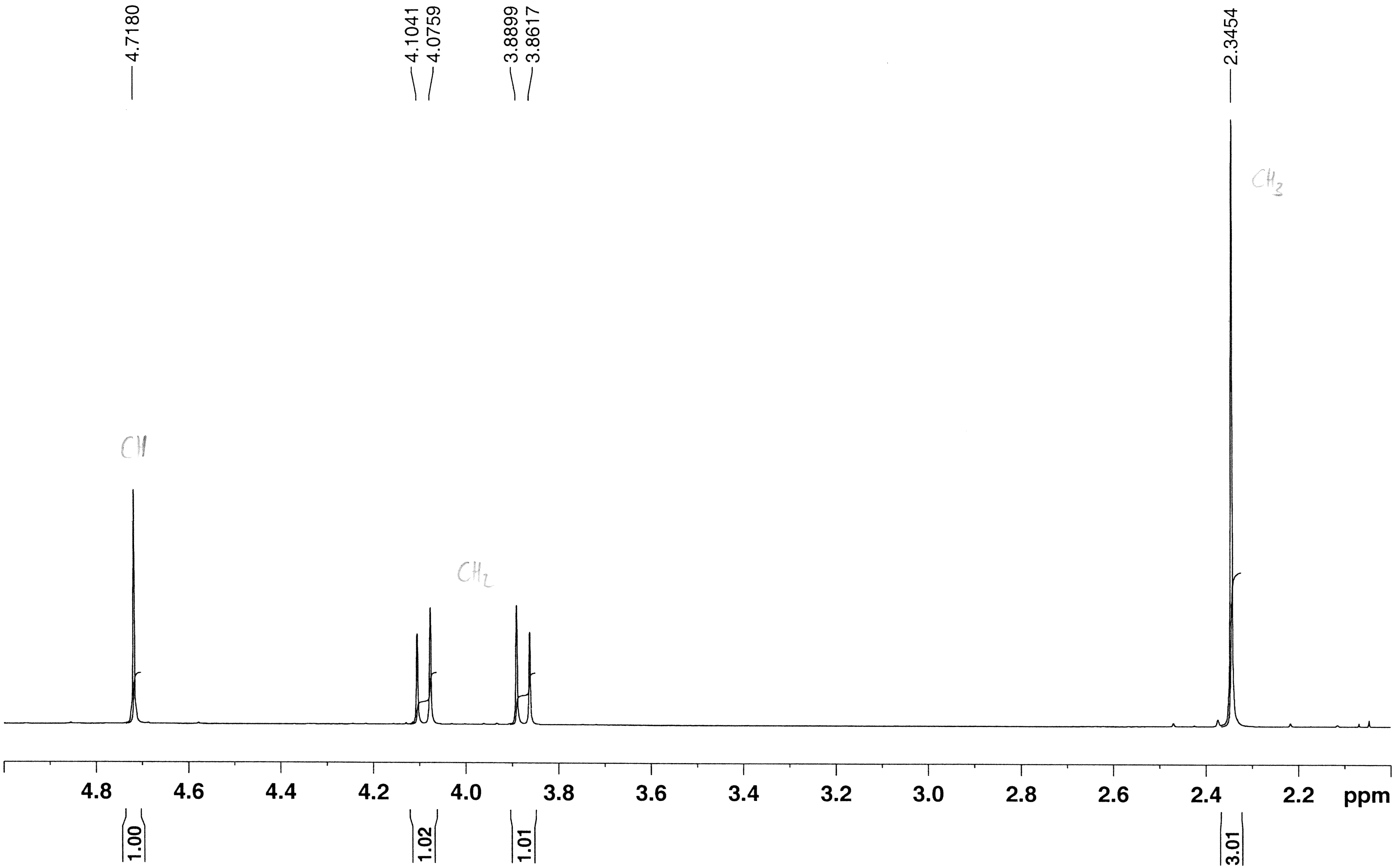

PN016_1_1 in cdcl3 (APT) 29.8.2019

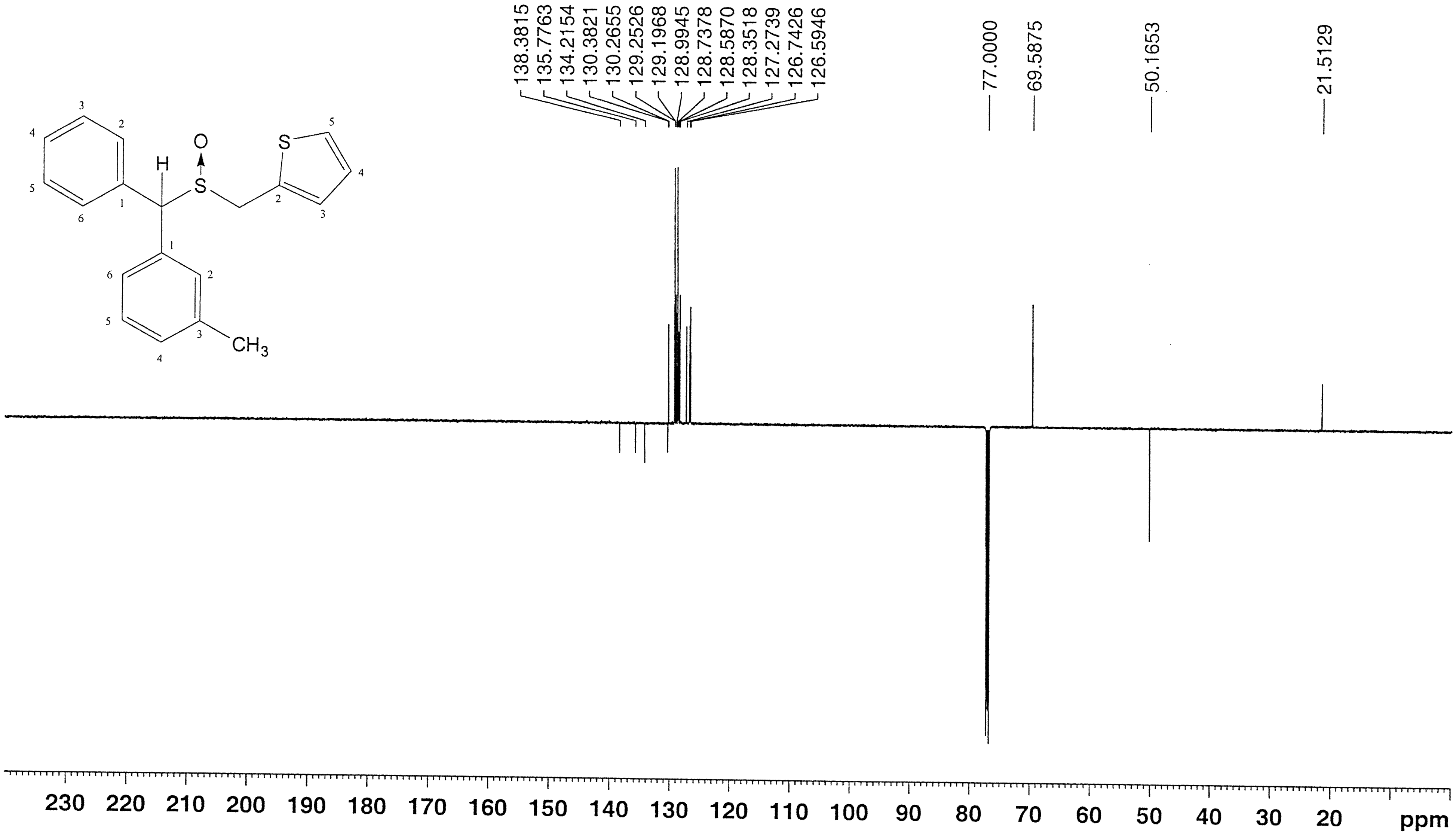

PN016_1_1 in cdcl3 (APT) 29.8.2019

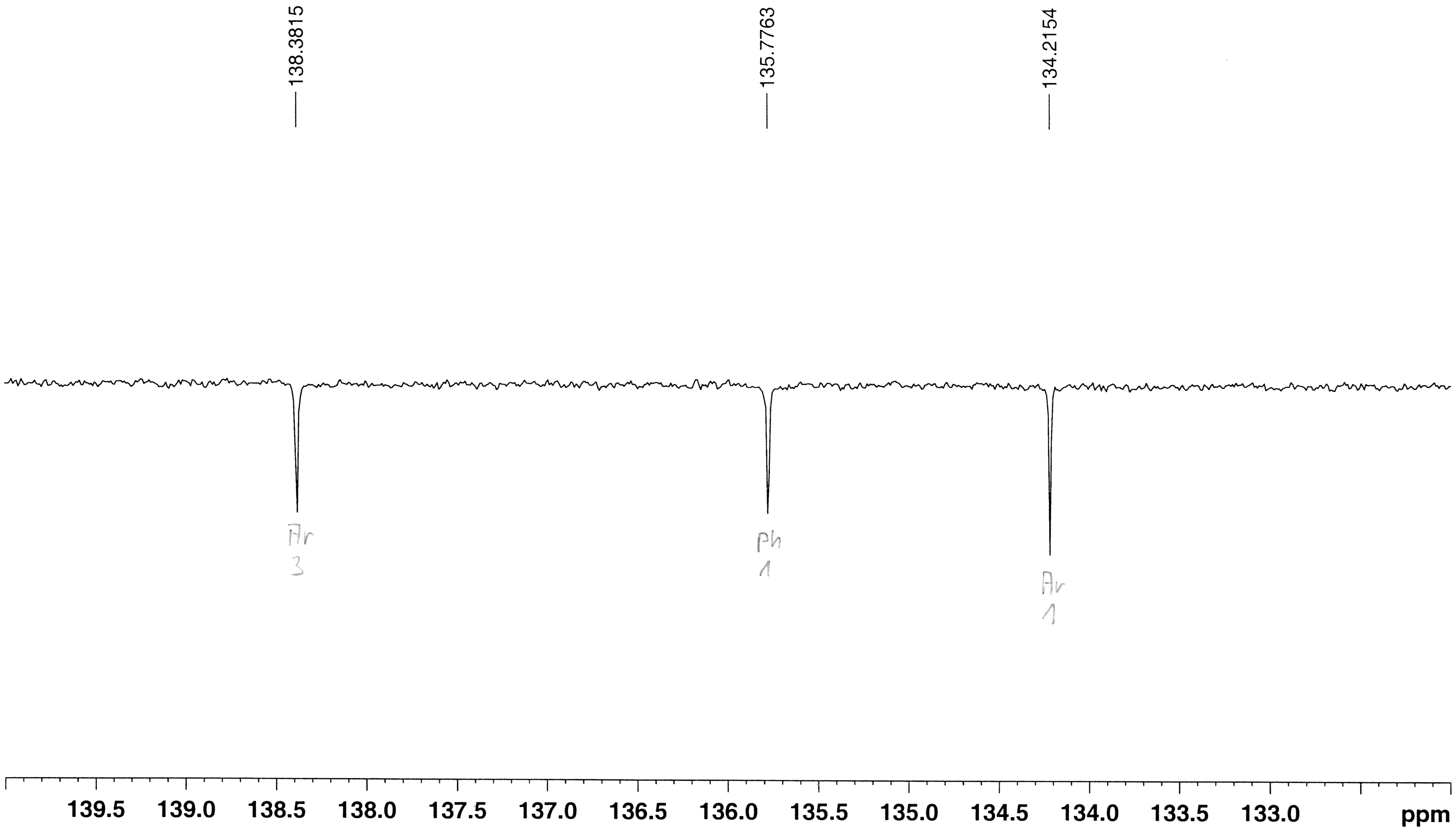

PN016_1_1 in cdcl3 (APT) 29.8.2019

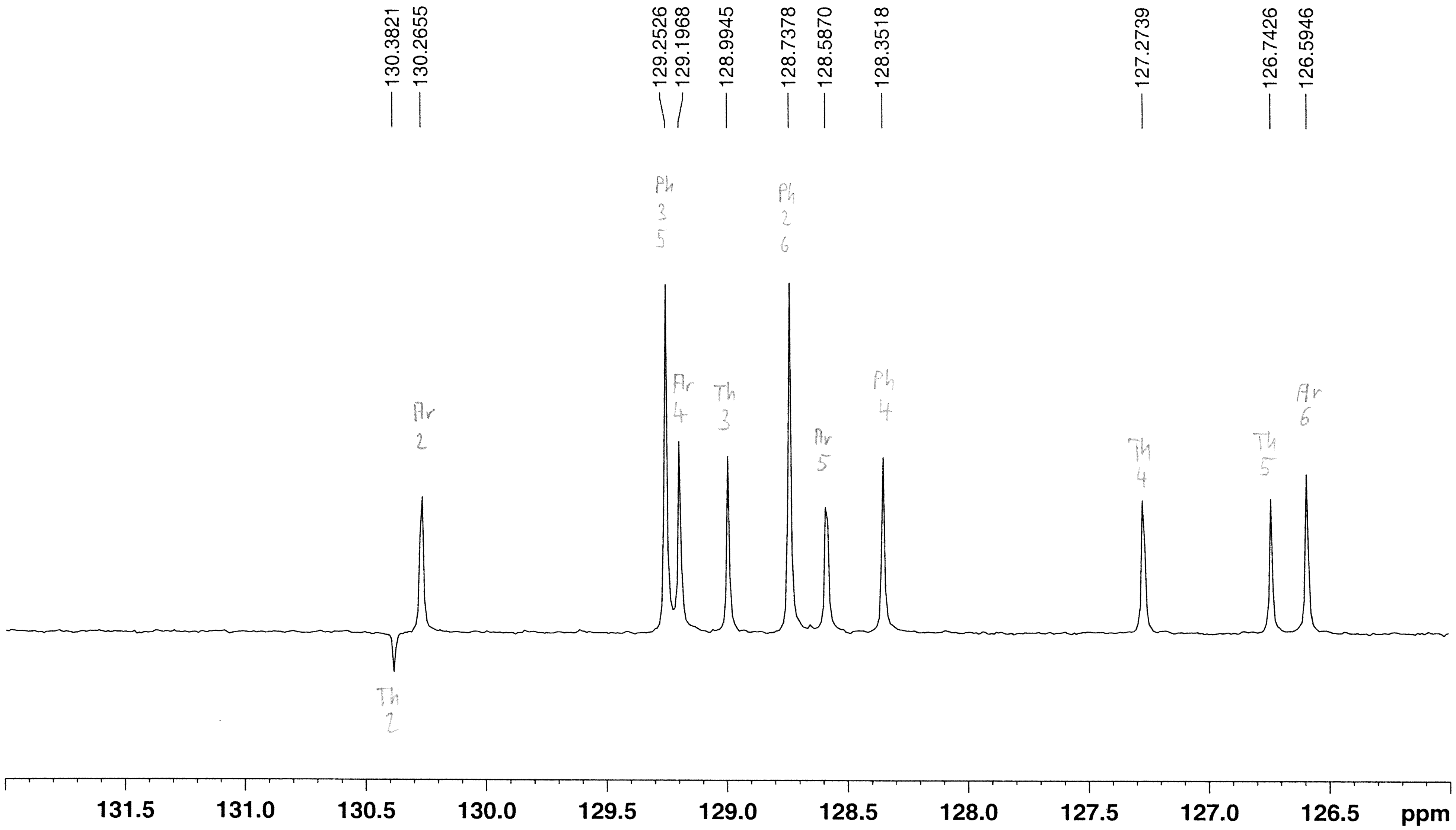

PN016_1_1 in cdcl3 (APT) 29.8.2019

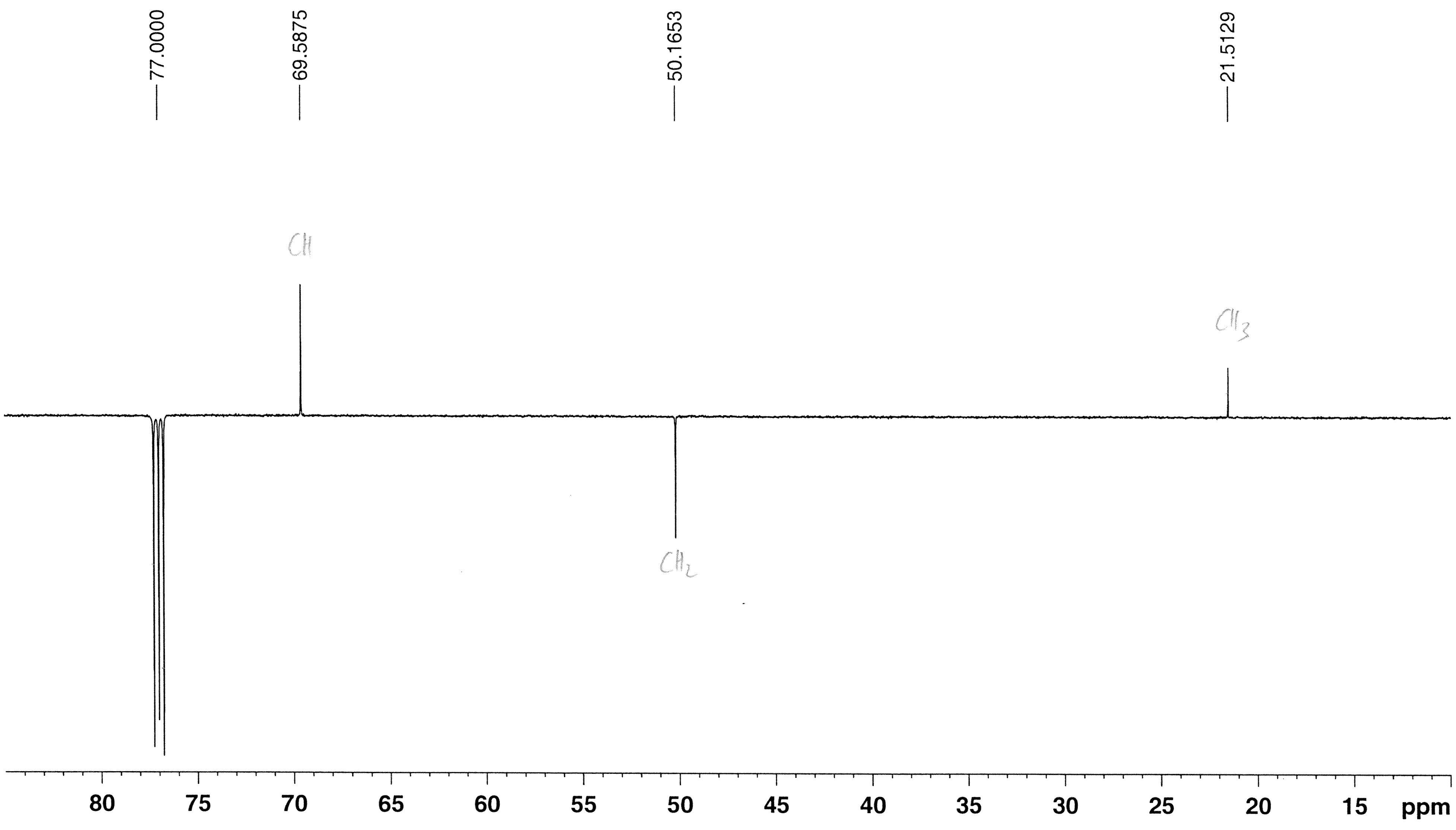

PN016_1_1 in cdcl3 (COSY) 29.8.2019

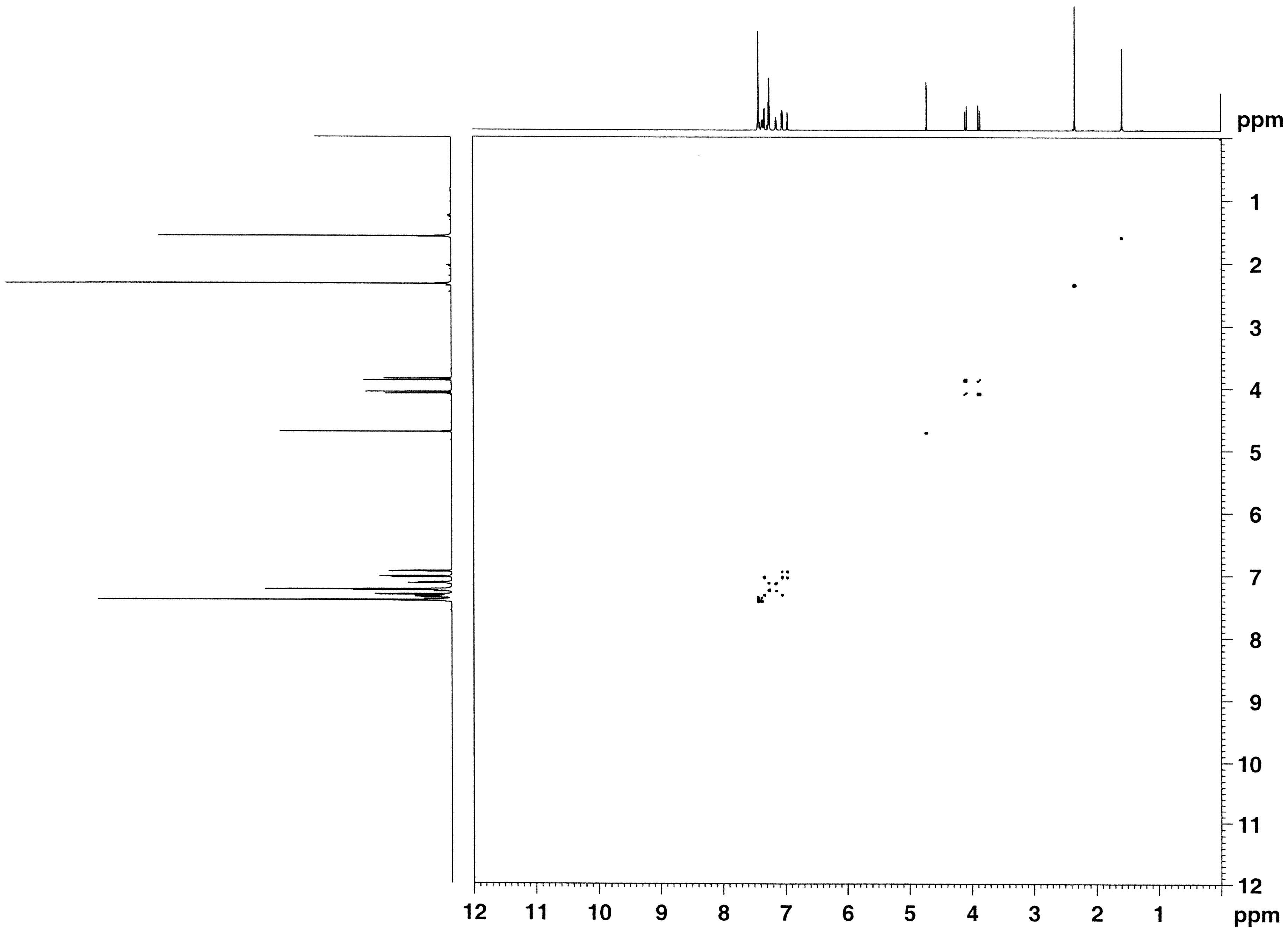

PN016_1_1 in cdcl3 (COSY) 29.8.2019

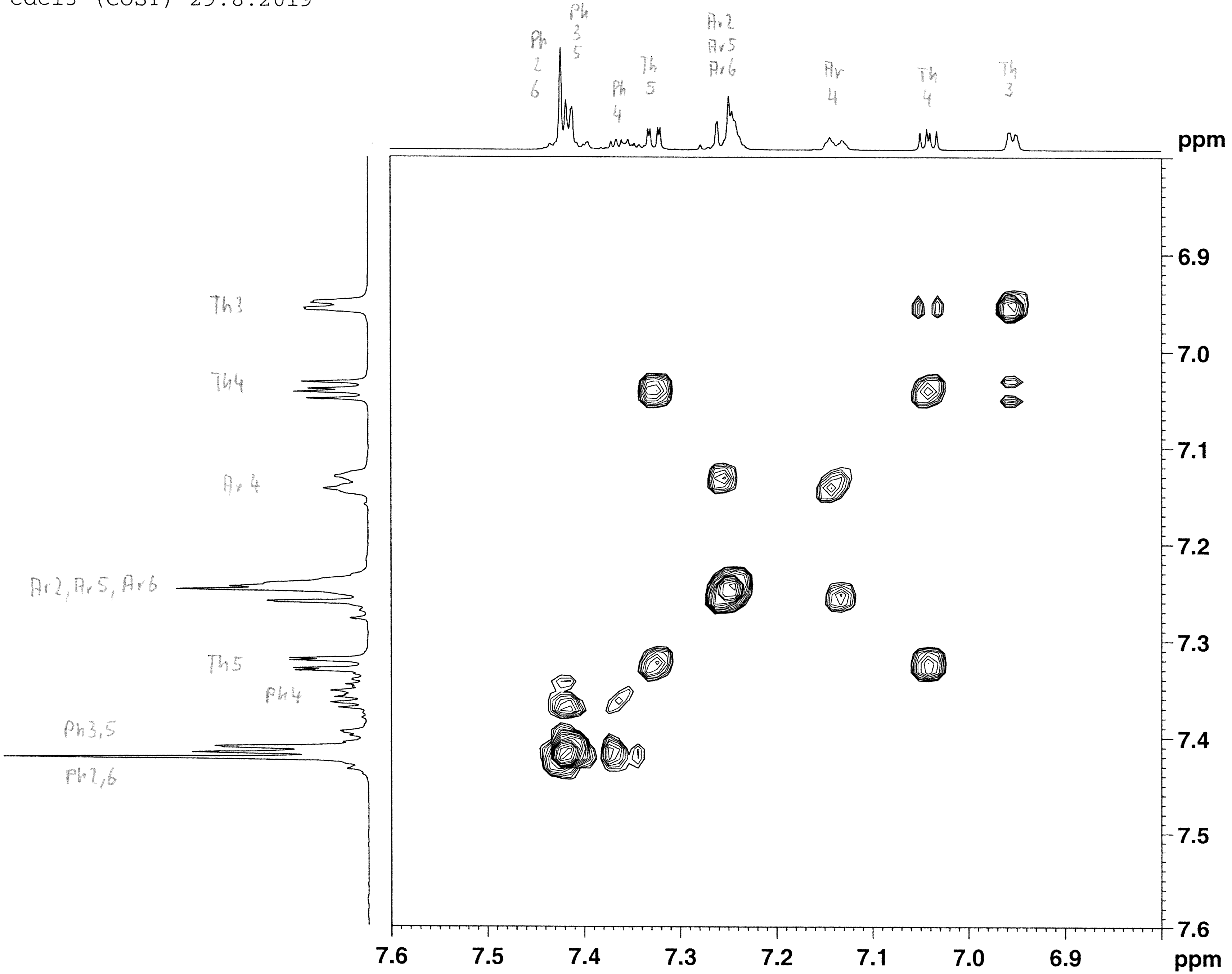

PN016_1_1 in cdcl3 (COSY) 29.8.2019

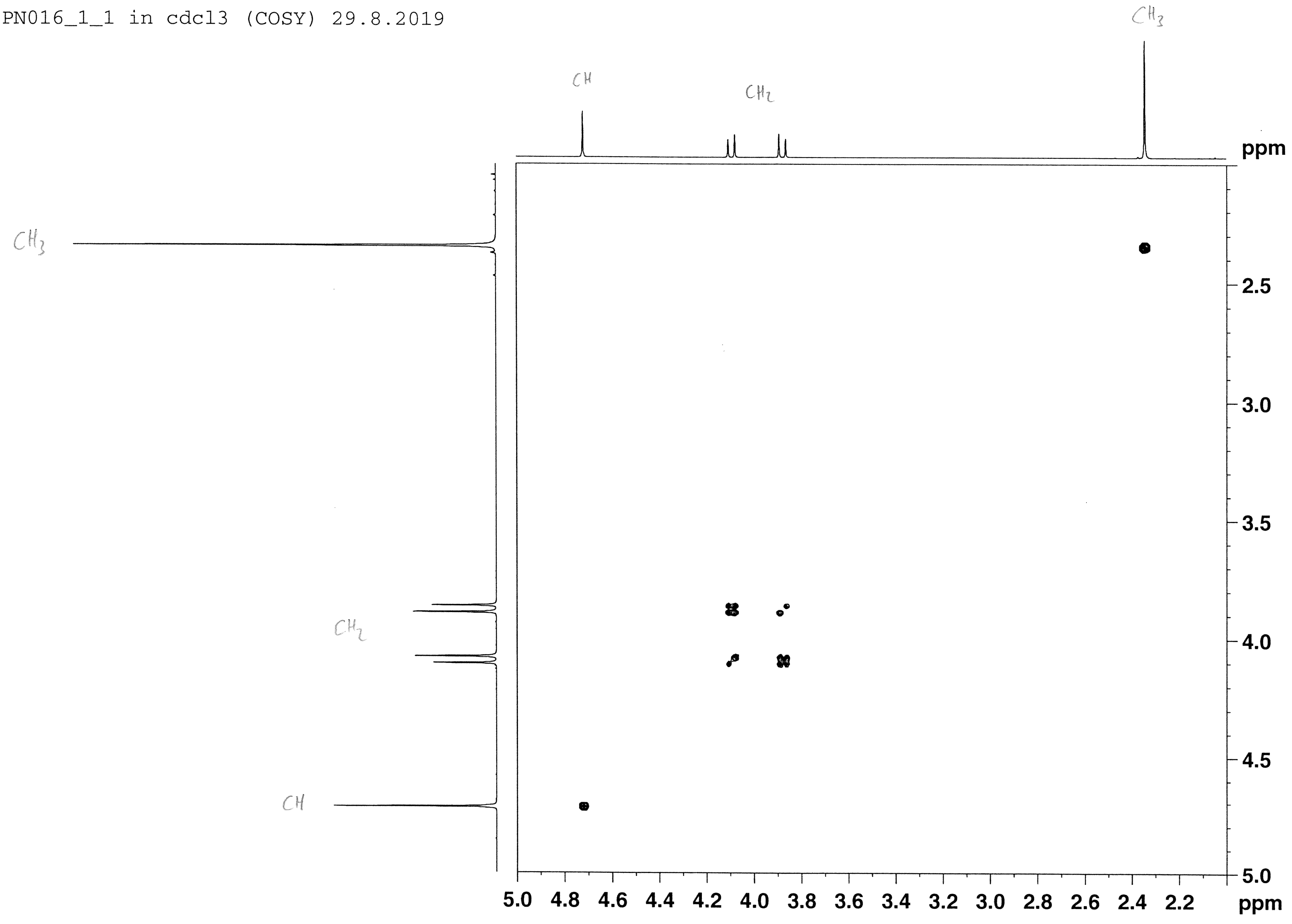

PN016_1_1 in cdcl3 (HSQC) 29.8.2019

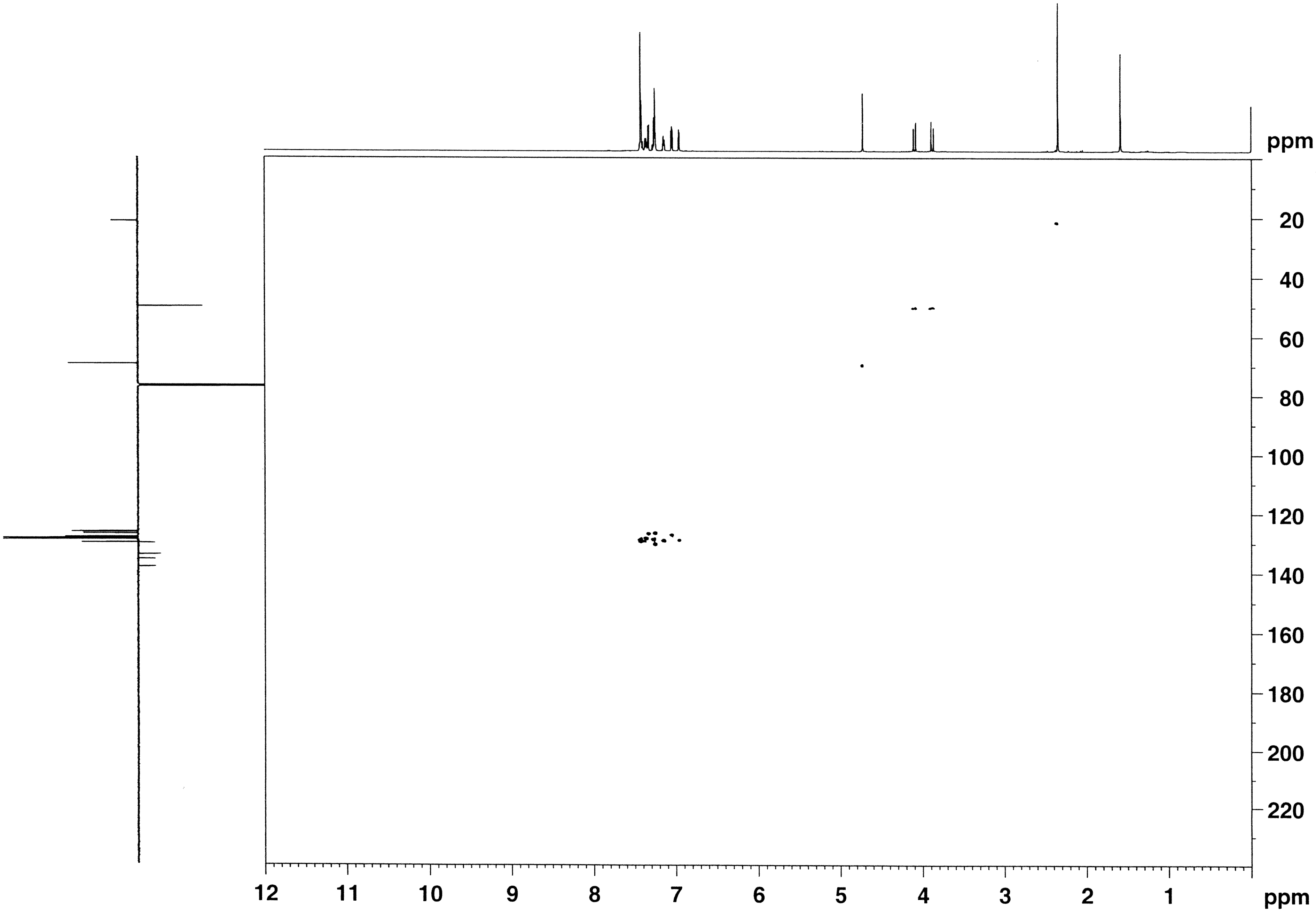

PN016_1_1 in cdcl3 (HSQC) 29.8.2019

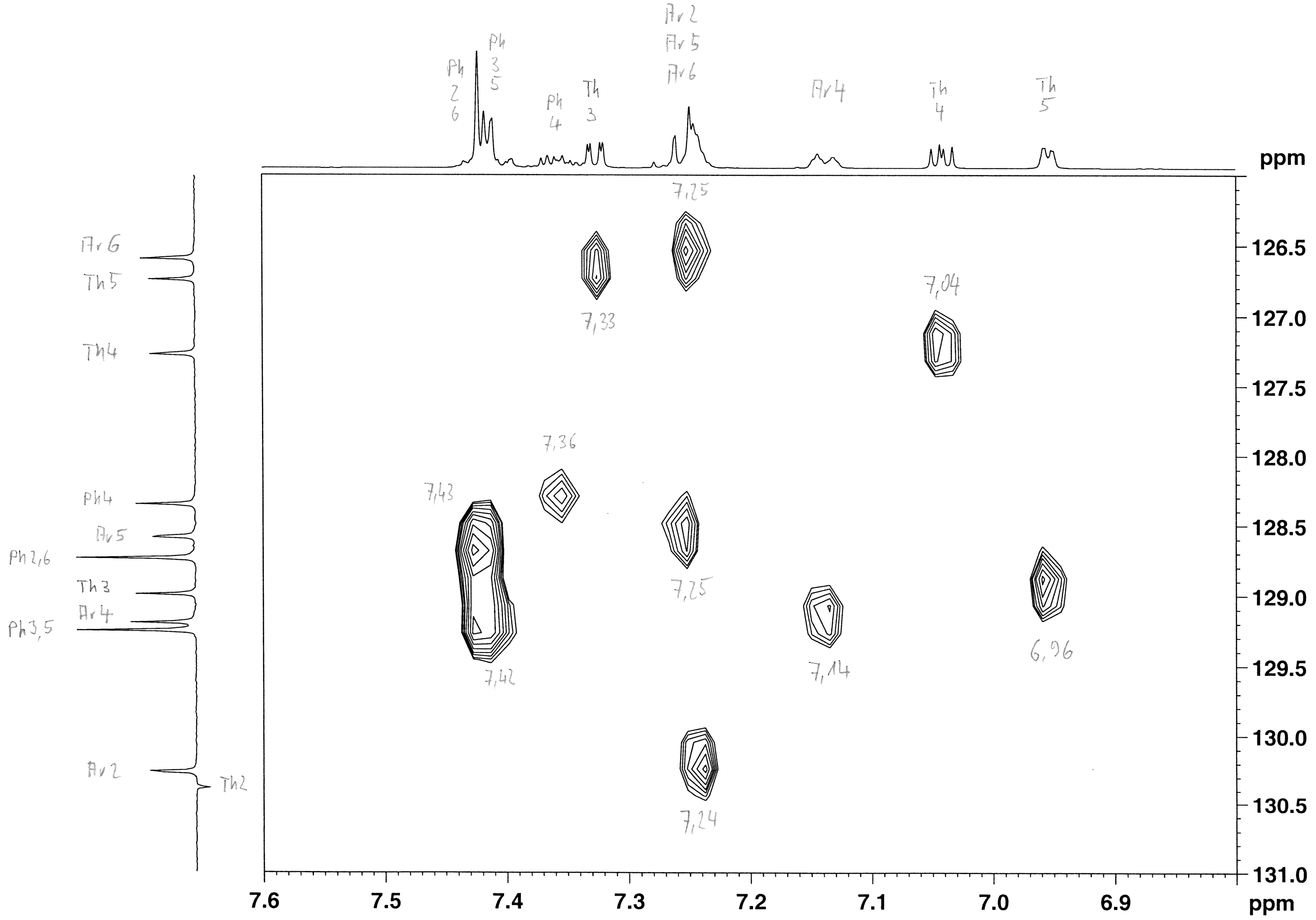

PN016_1_1 in cdcl3 (HSQC) 29.8.2019

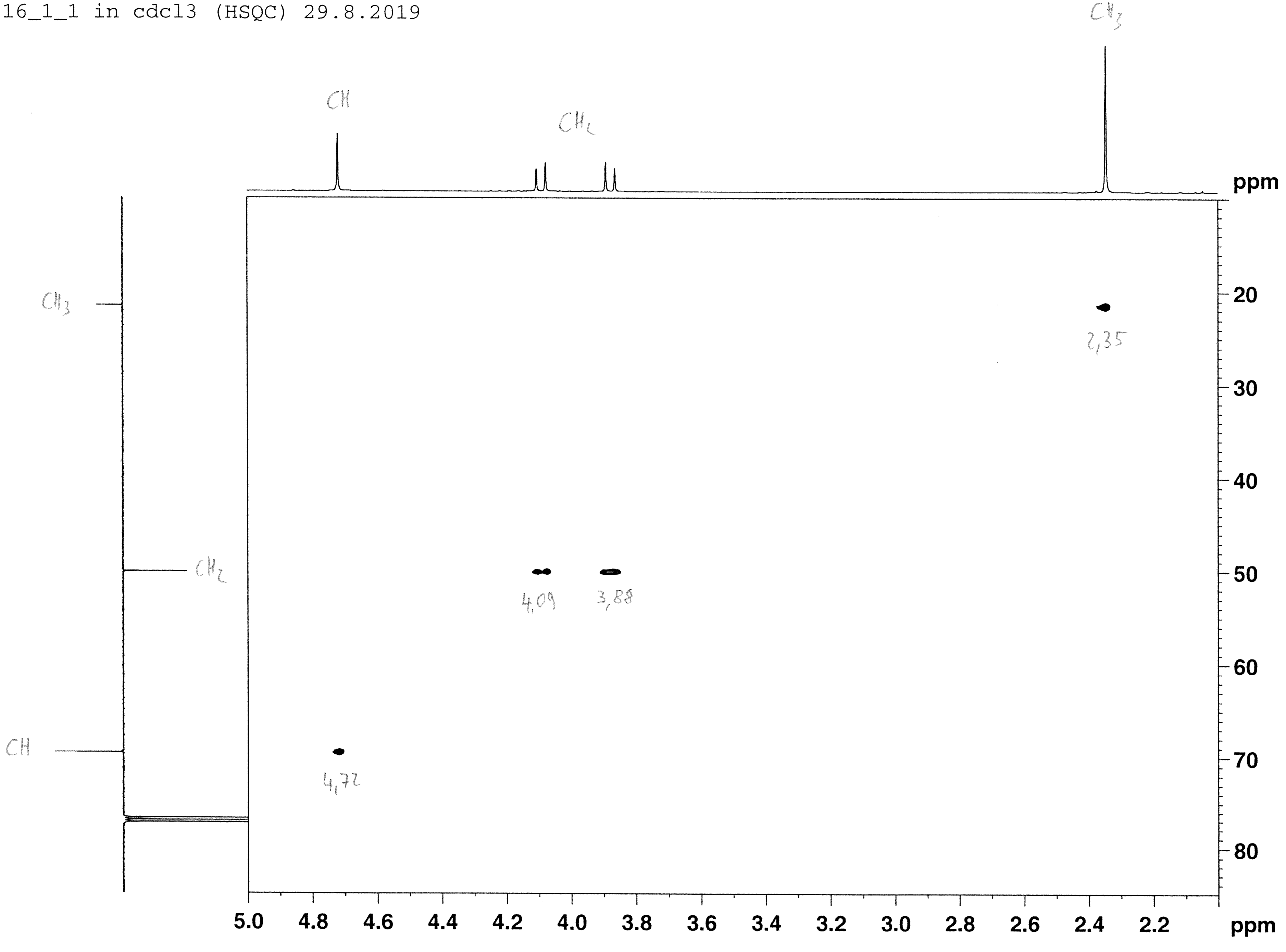

PN016_1_1 in cdcl3 (HMBC) 29.8.2019

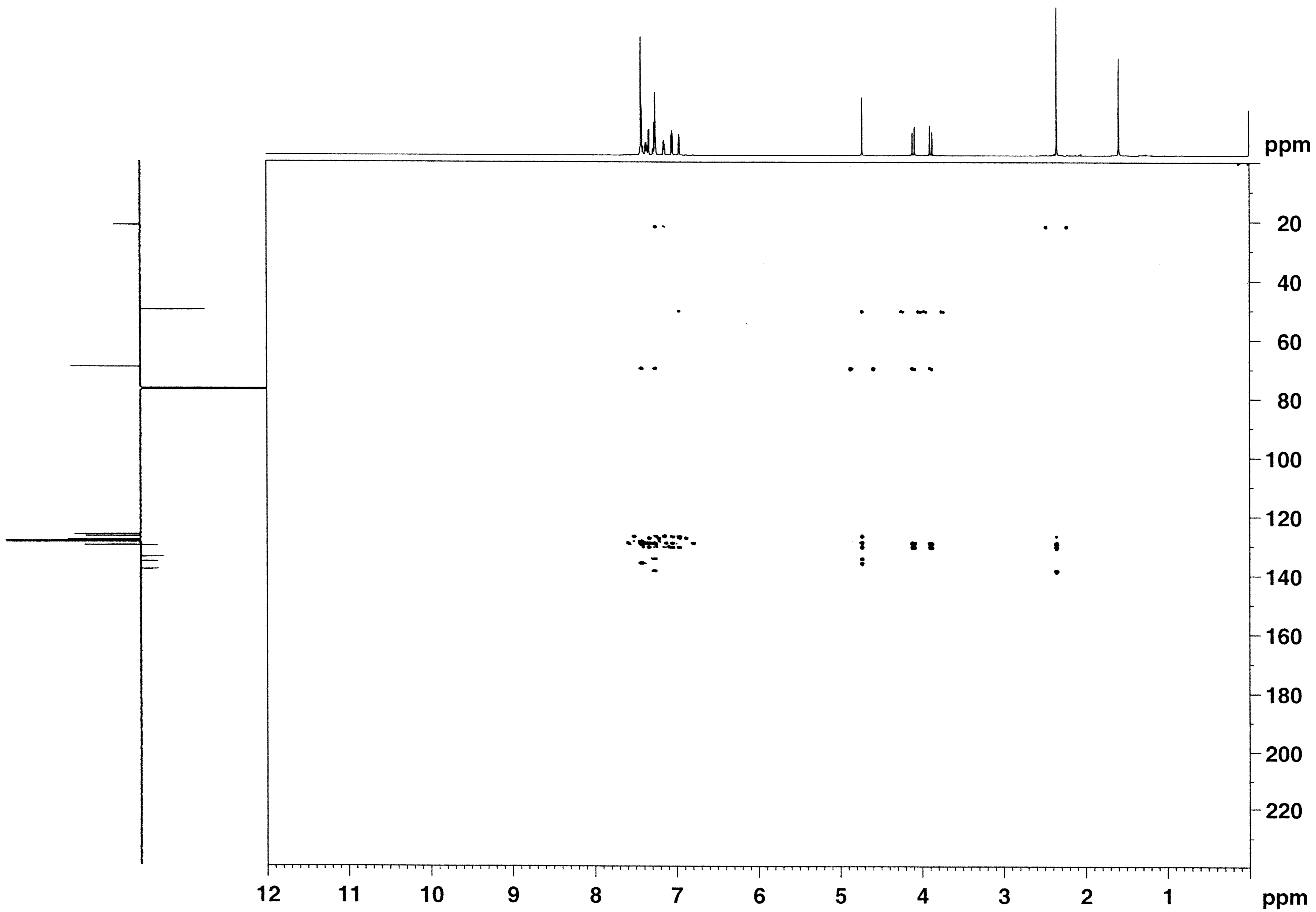

PN016_1_1 in cdcl3 (HMBC) 29.8.2019

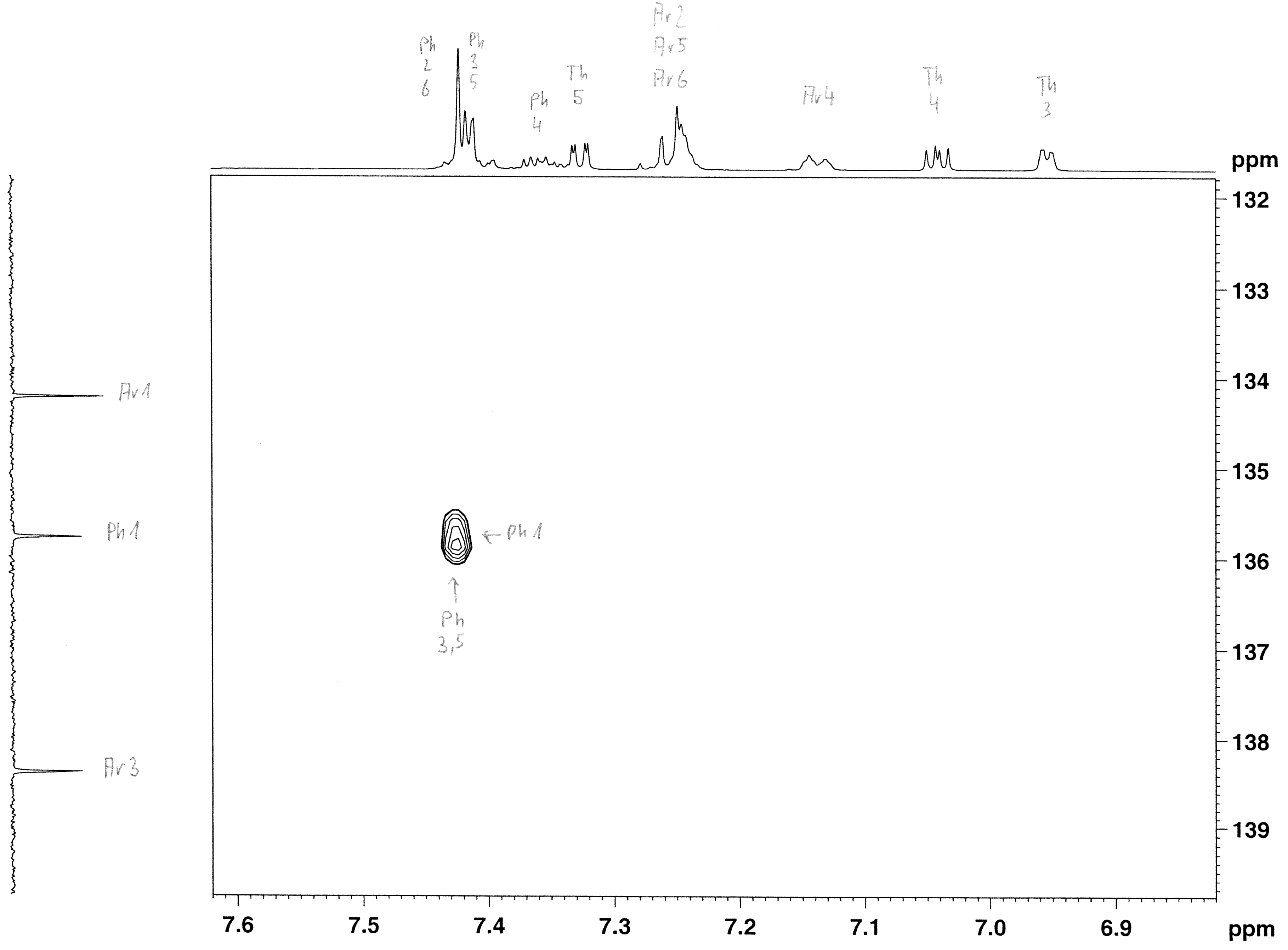

PN016_1_1 in cdcl3 (HMBC) 29.8.2019

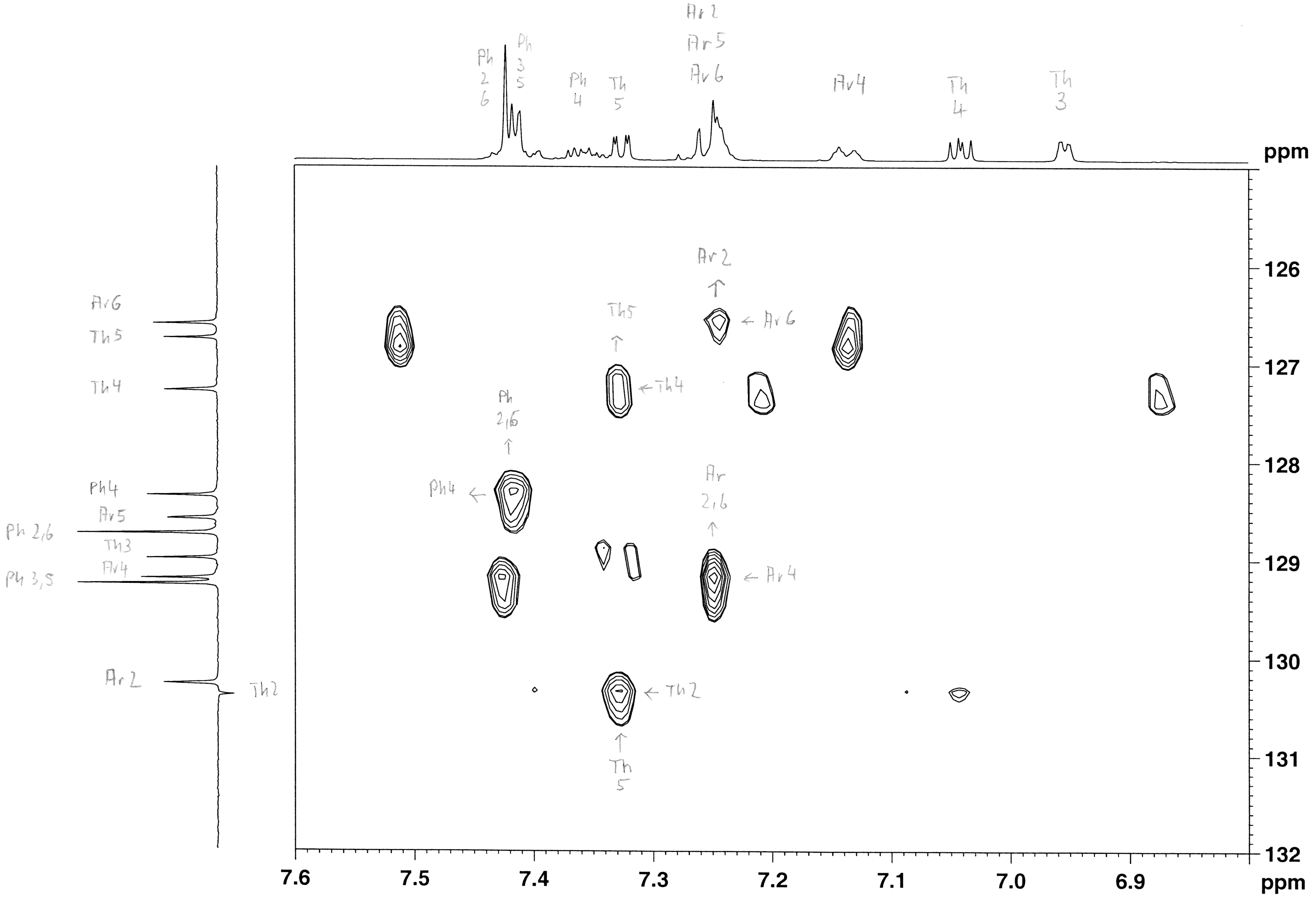

PN016_1_1 in cdcl3 (HMBC) 29.8.2019

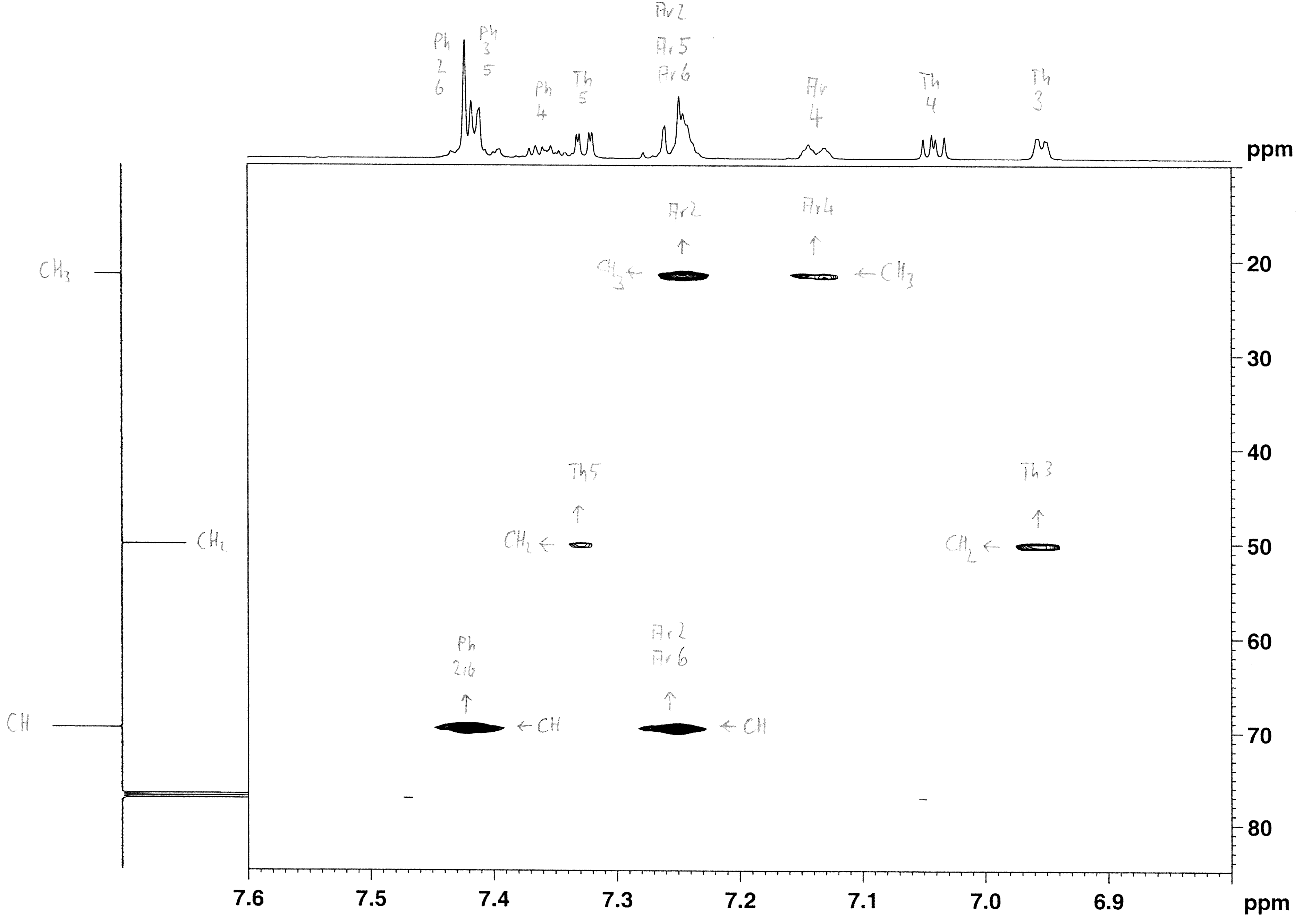

PN016_1_1 in cdcl3 (HMBC) 29.8.2019

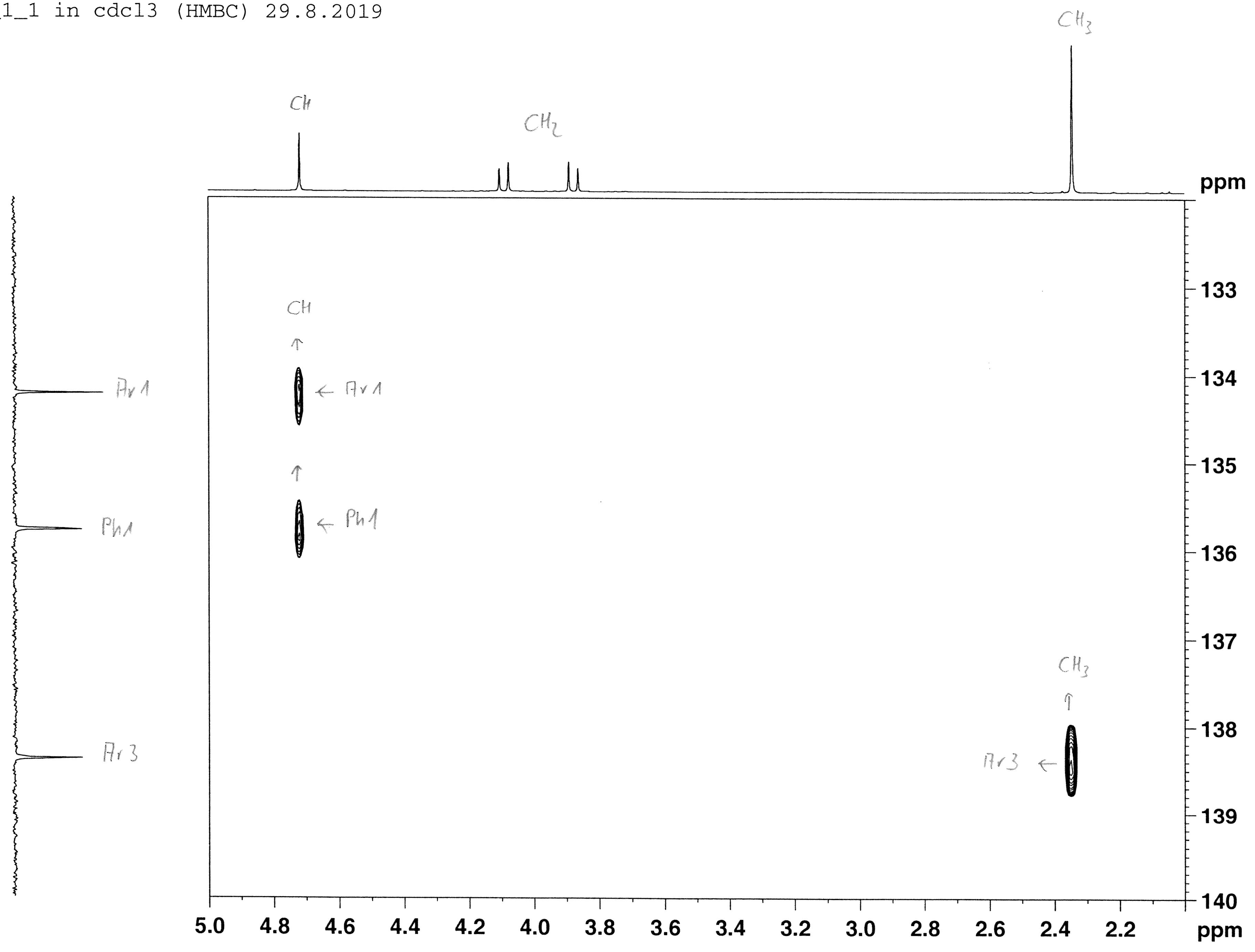

PN016_1_1 in cdcl3 (HMBC) 29.8.2019

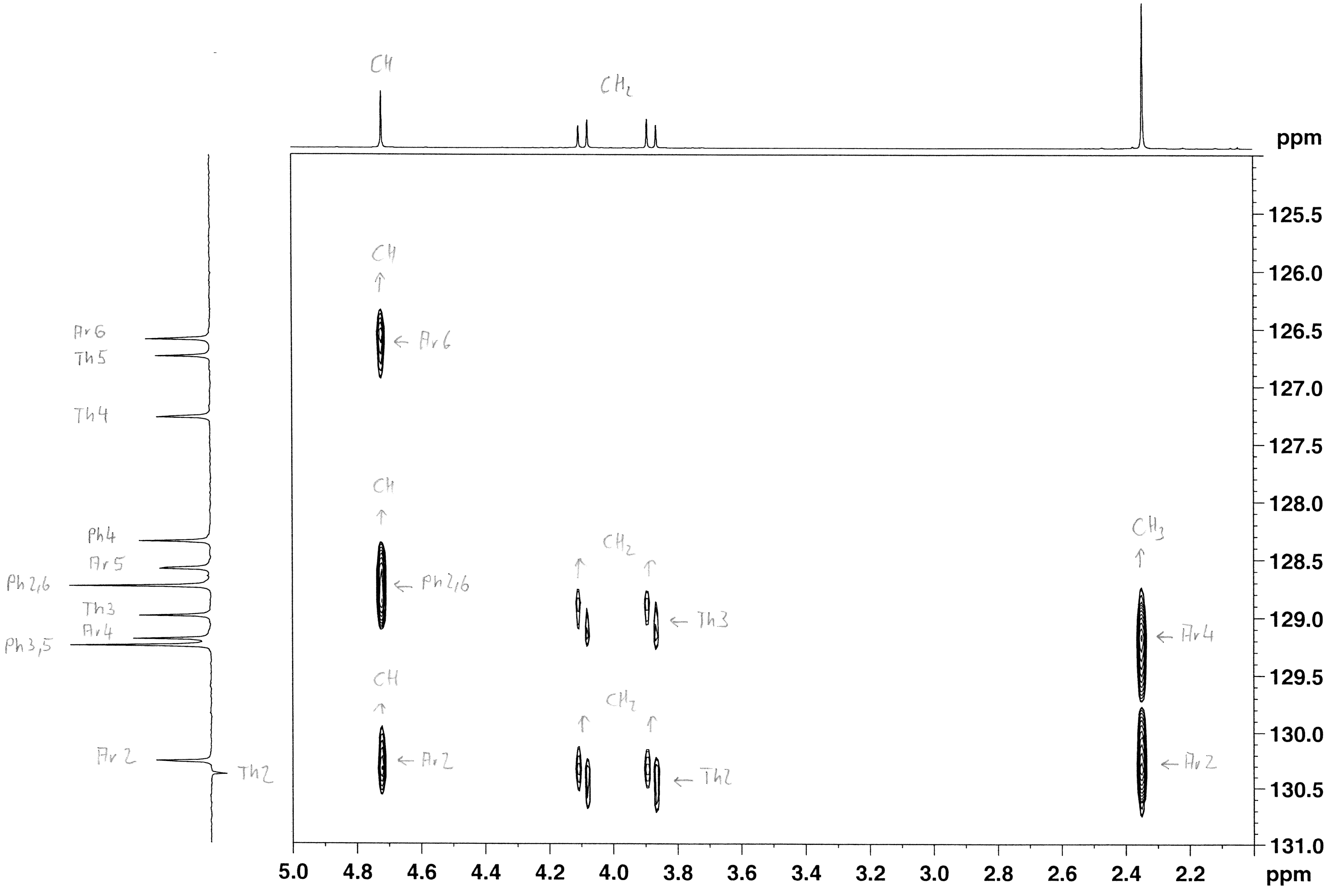

PN016_1_1 in cdcl3 (HMBC) 29.8.2019

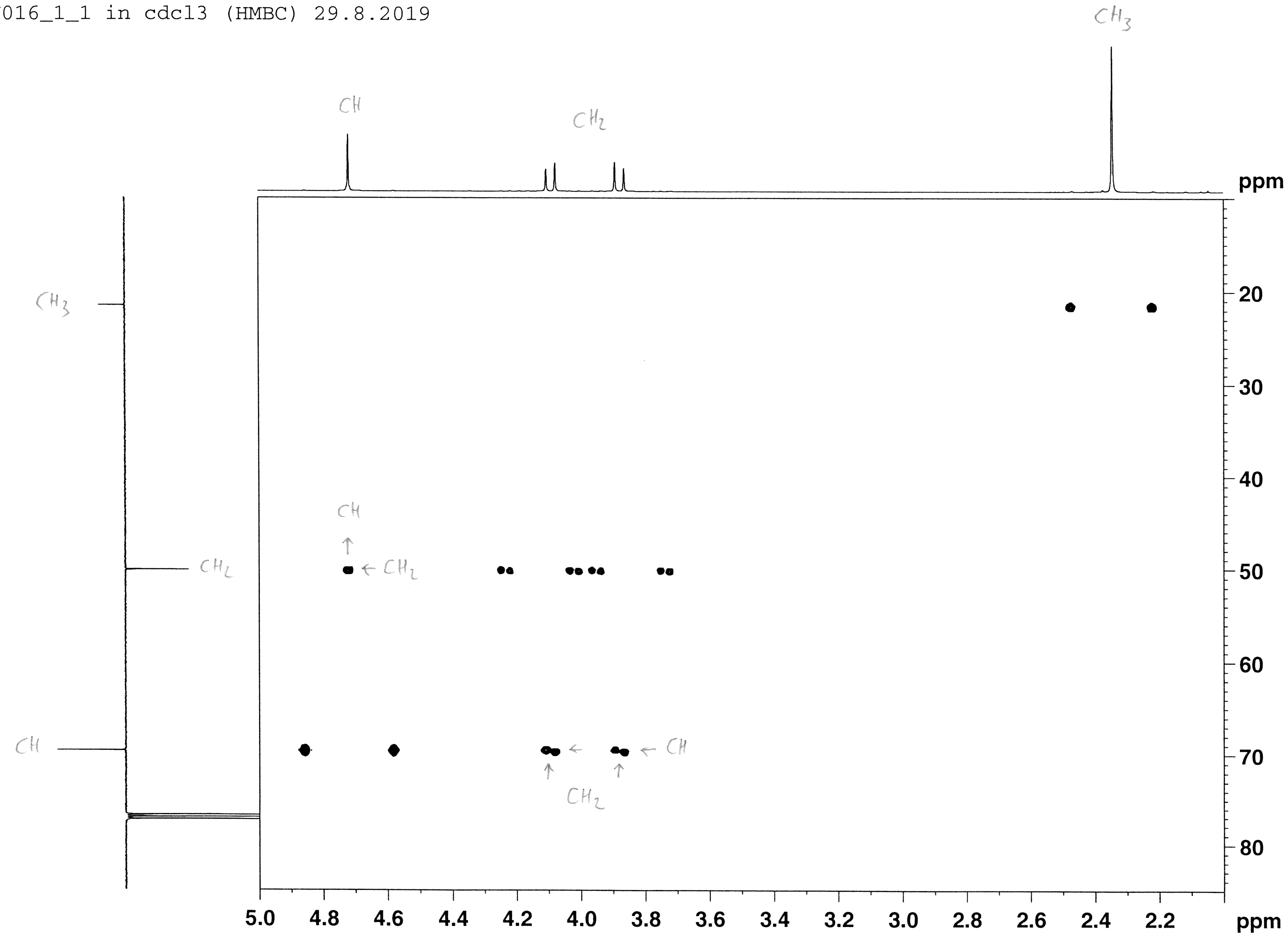

Figure S50c. NMR spectra of compound 6m.

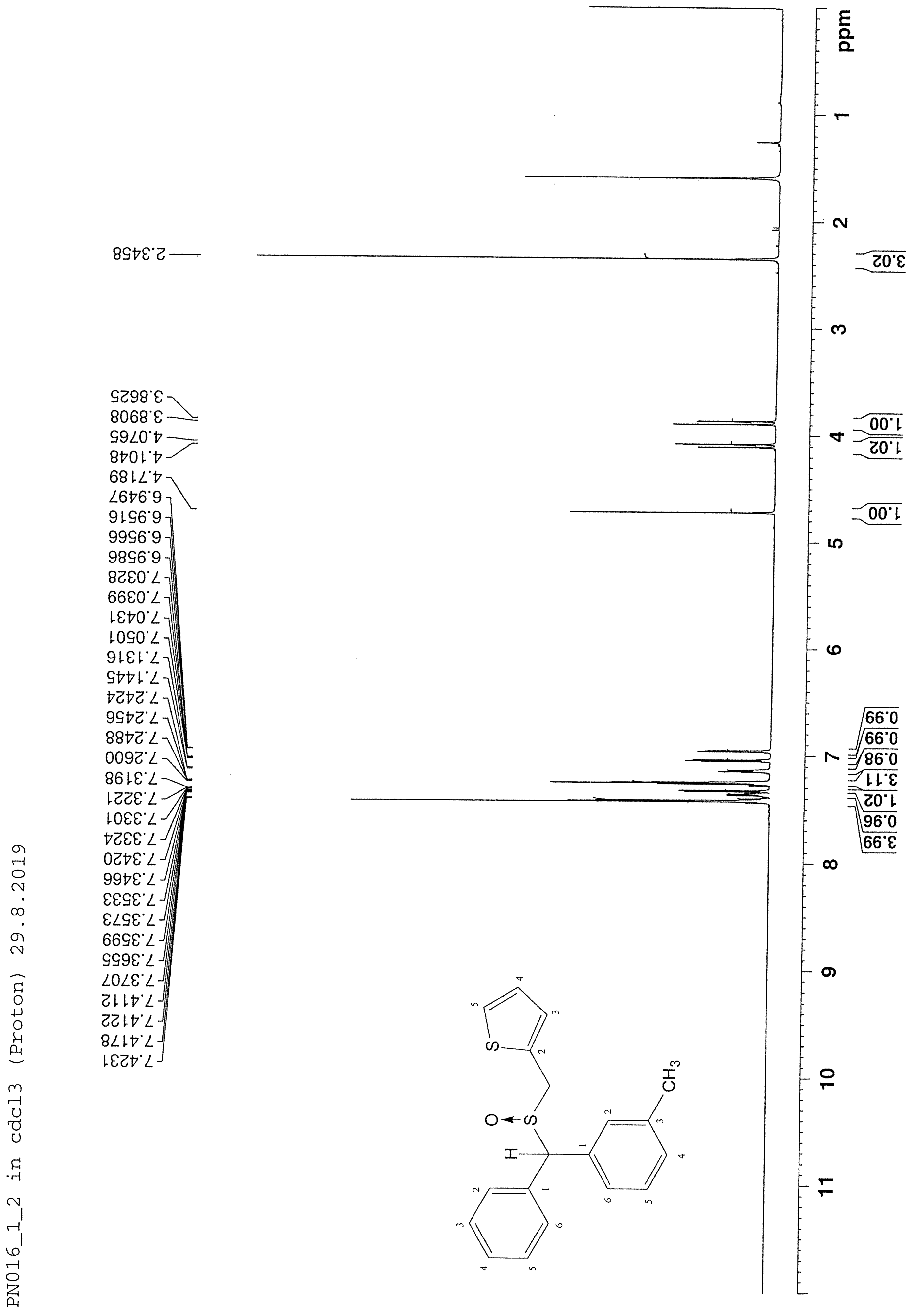

PN016_1_2 in cdcl3 (Proton) 29.8.2019

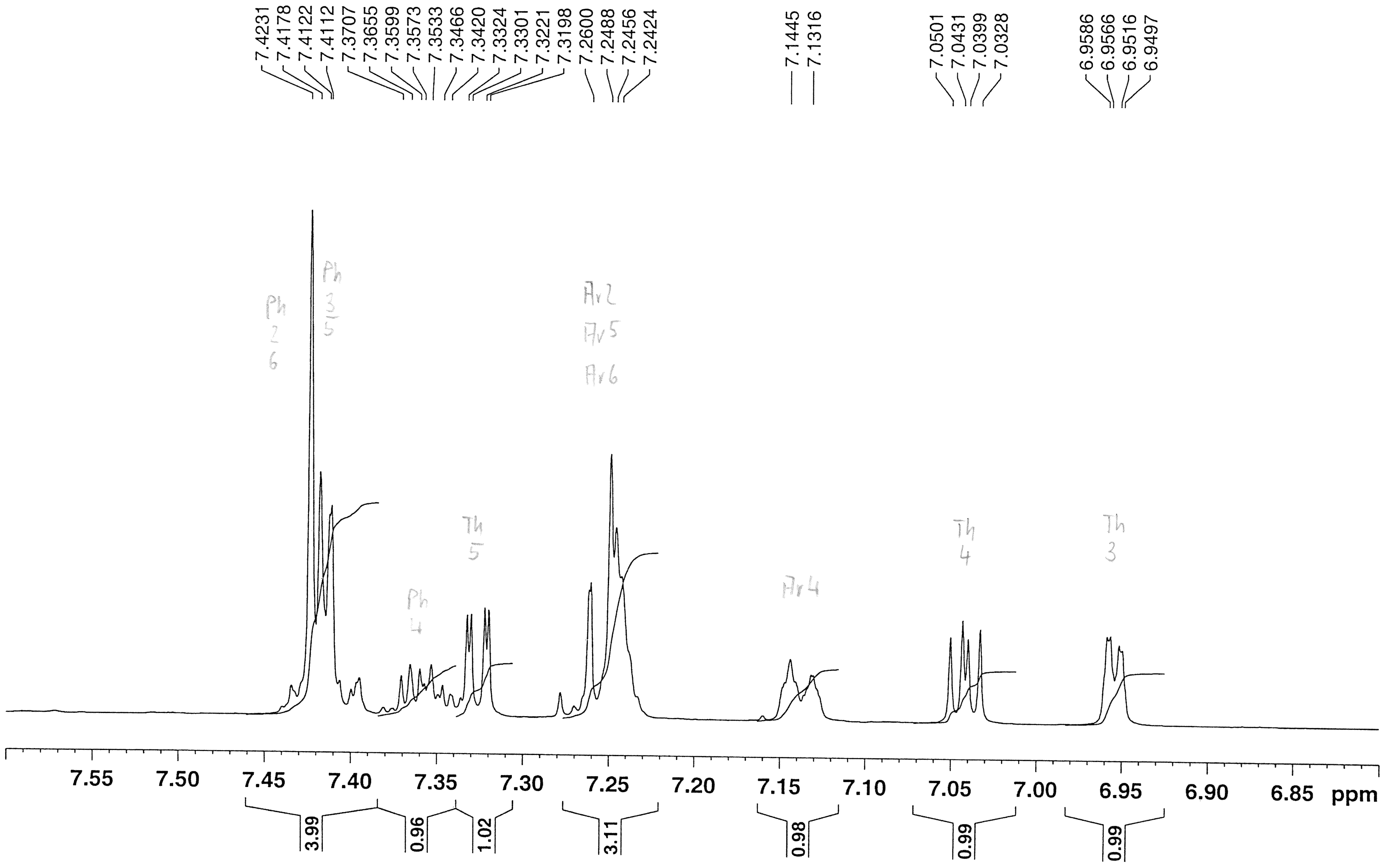

PN016_1_2 in cdcl3 (Proton) 29.8.2019

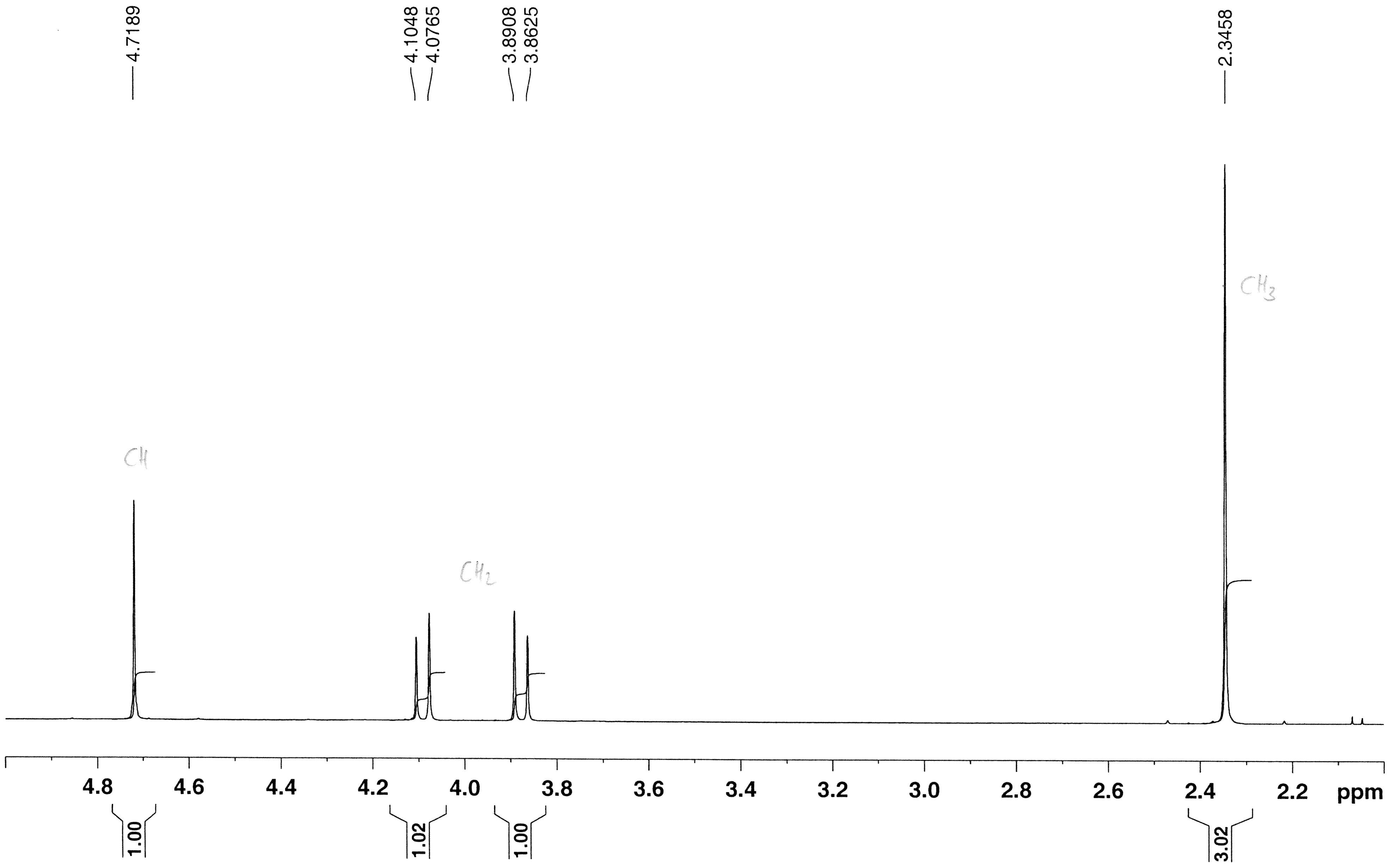

PN016_1_2 in cdcl3 (APT) 29.8.2019

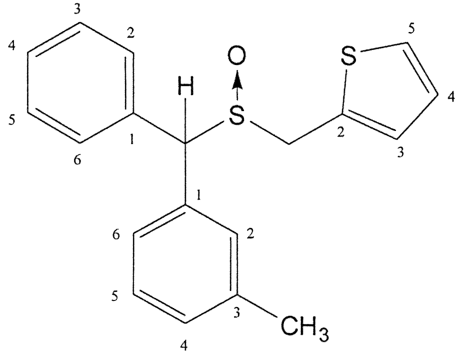

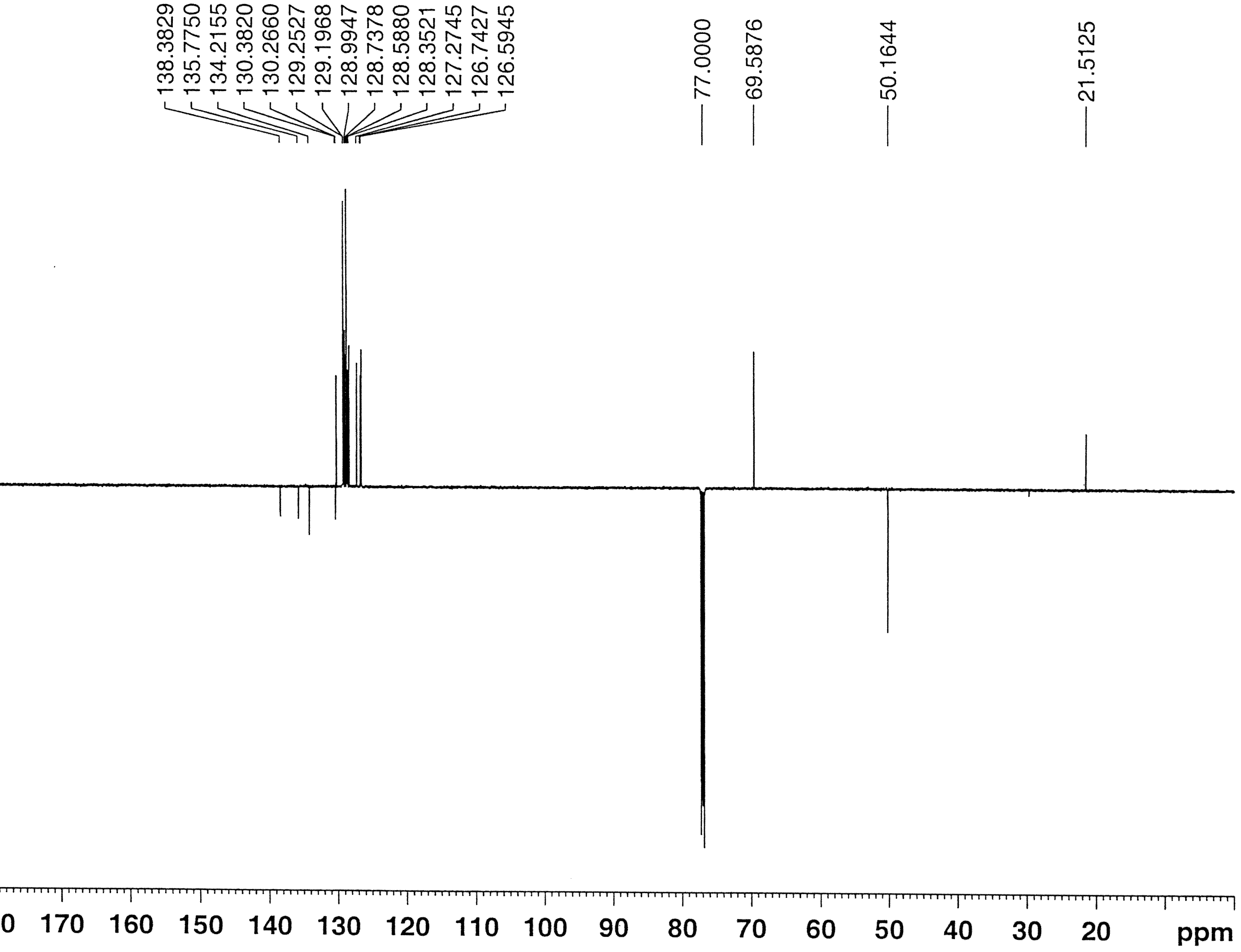

PN016_1_2 in cdcl3 (APT) 29.8.2019

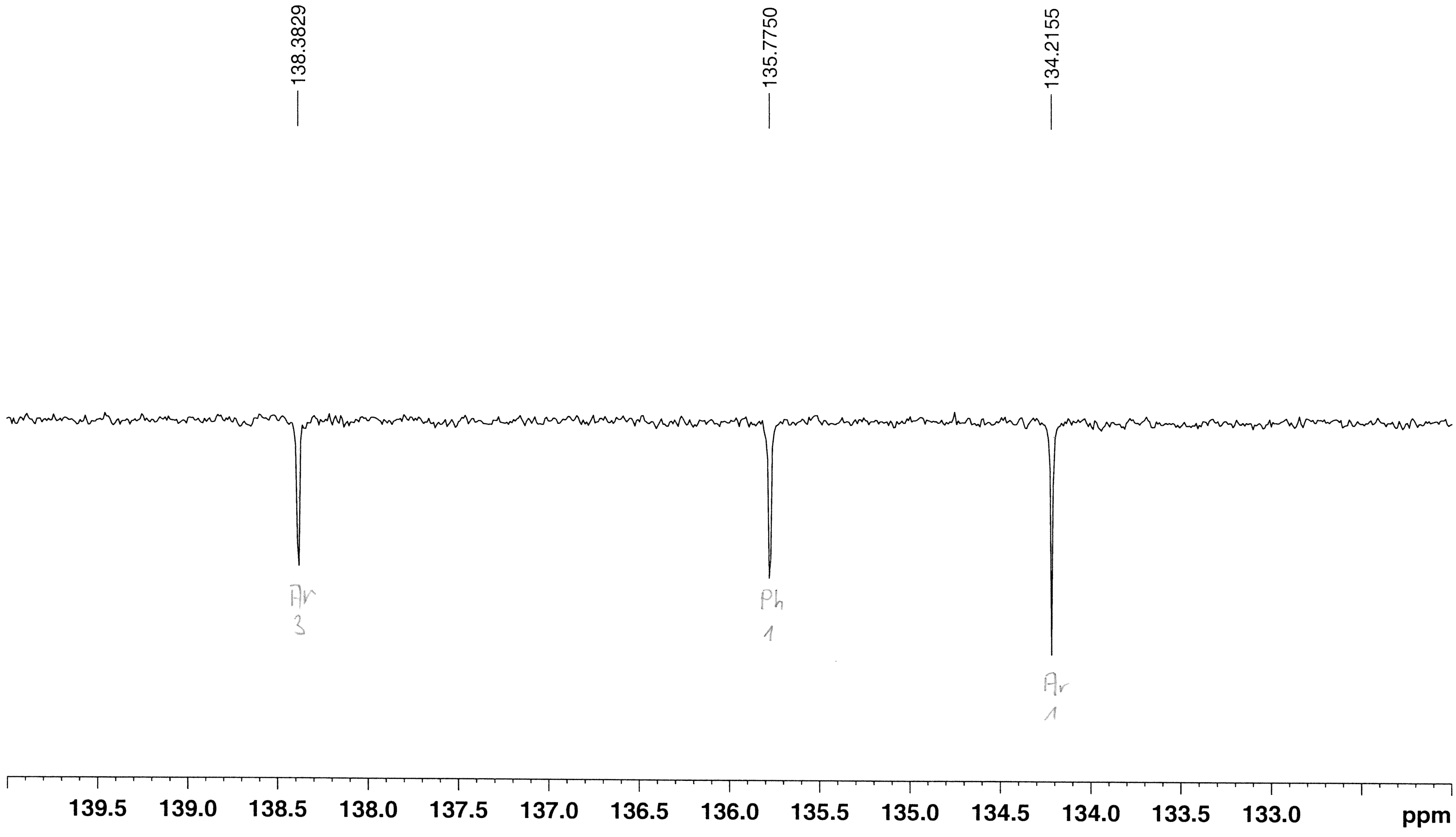

PN016_1_2 in cdcl3 (APT) 29.8.2019

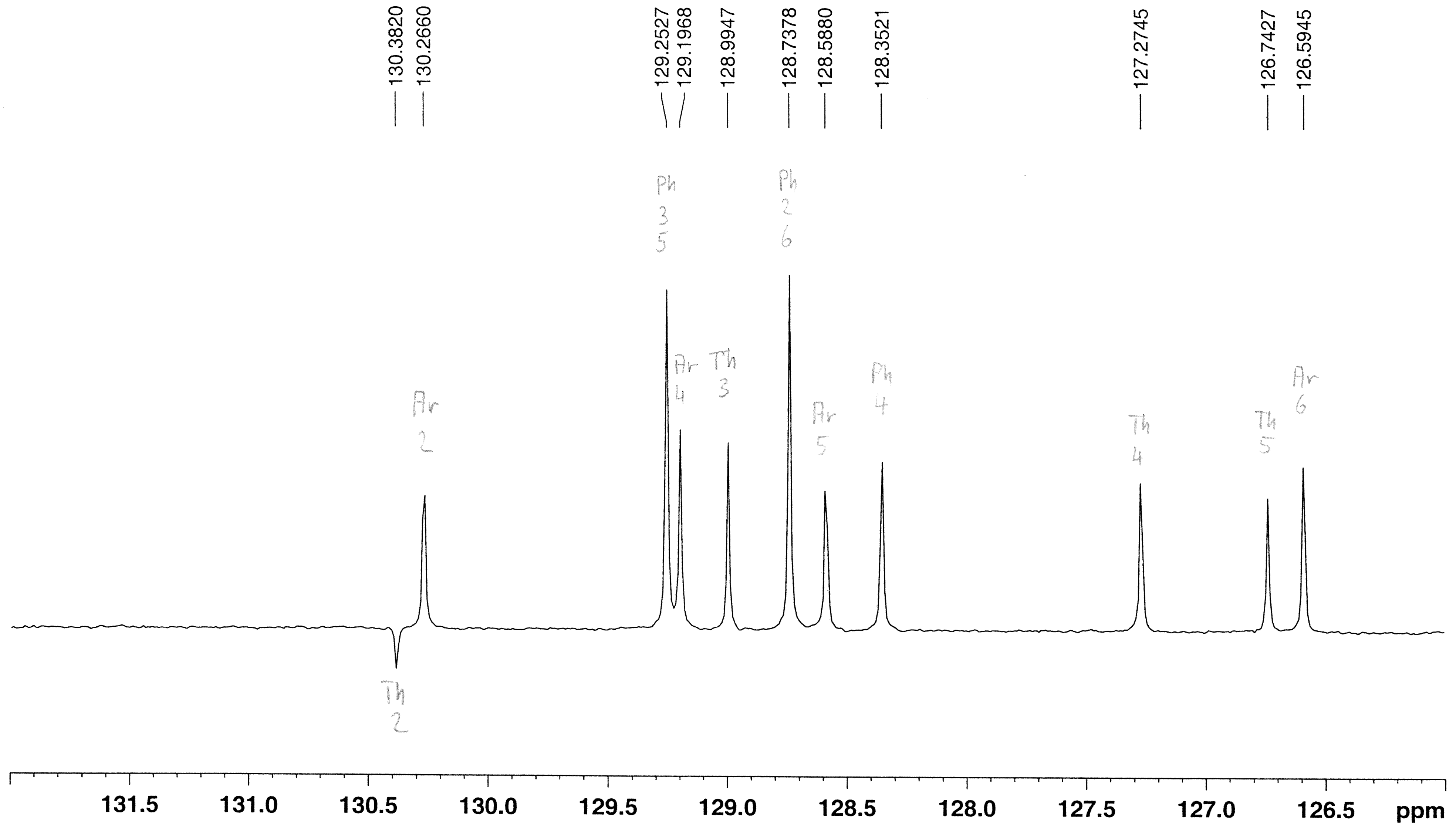

PN016_1_2 in cdcl3 (APT) 29.8.2019

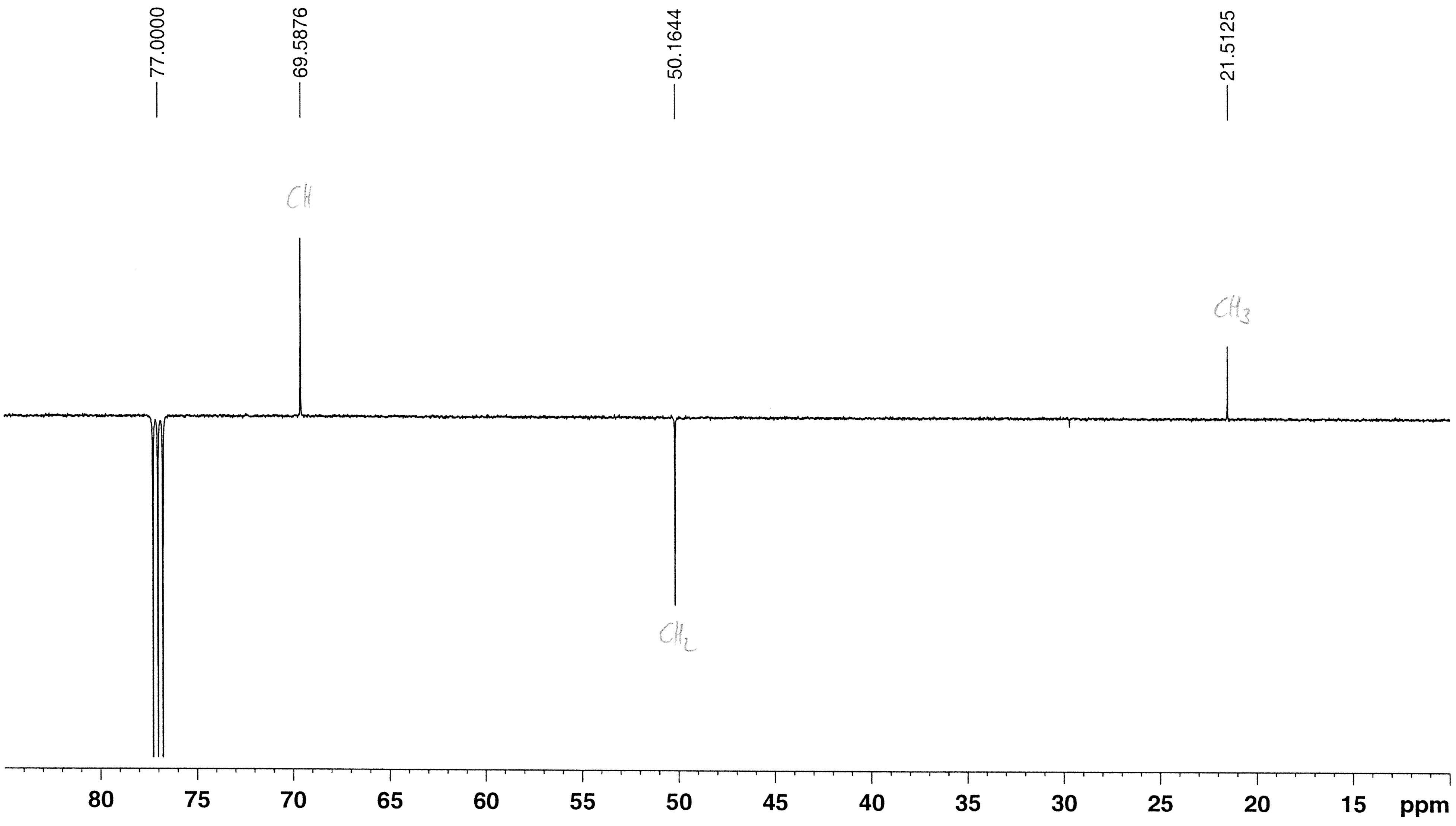

Figure S51c. NMR spectra of compound **7m.**

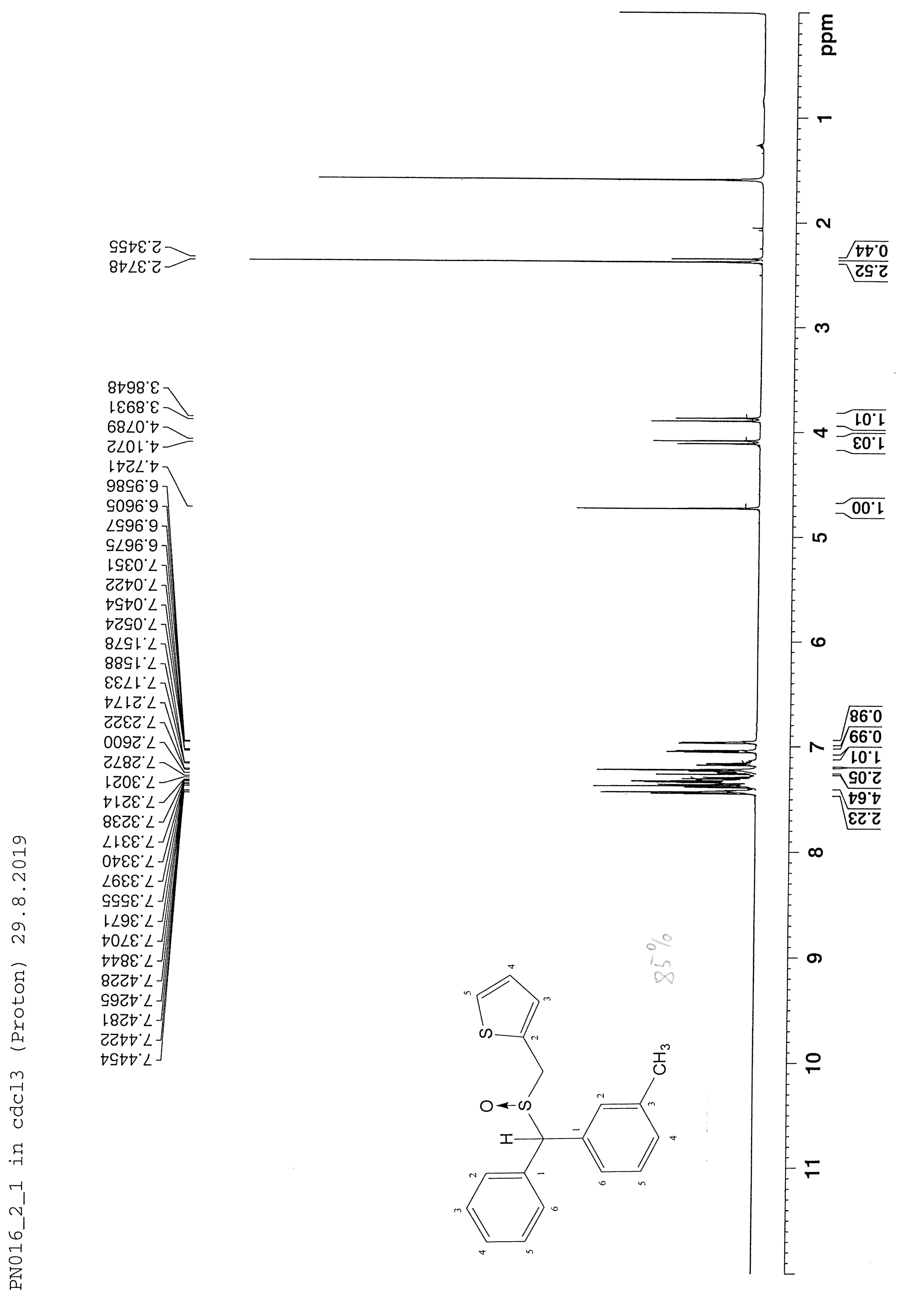

PN016_2_1 in cdcl3 (Proton) 29.8.2019

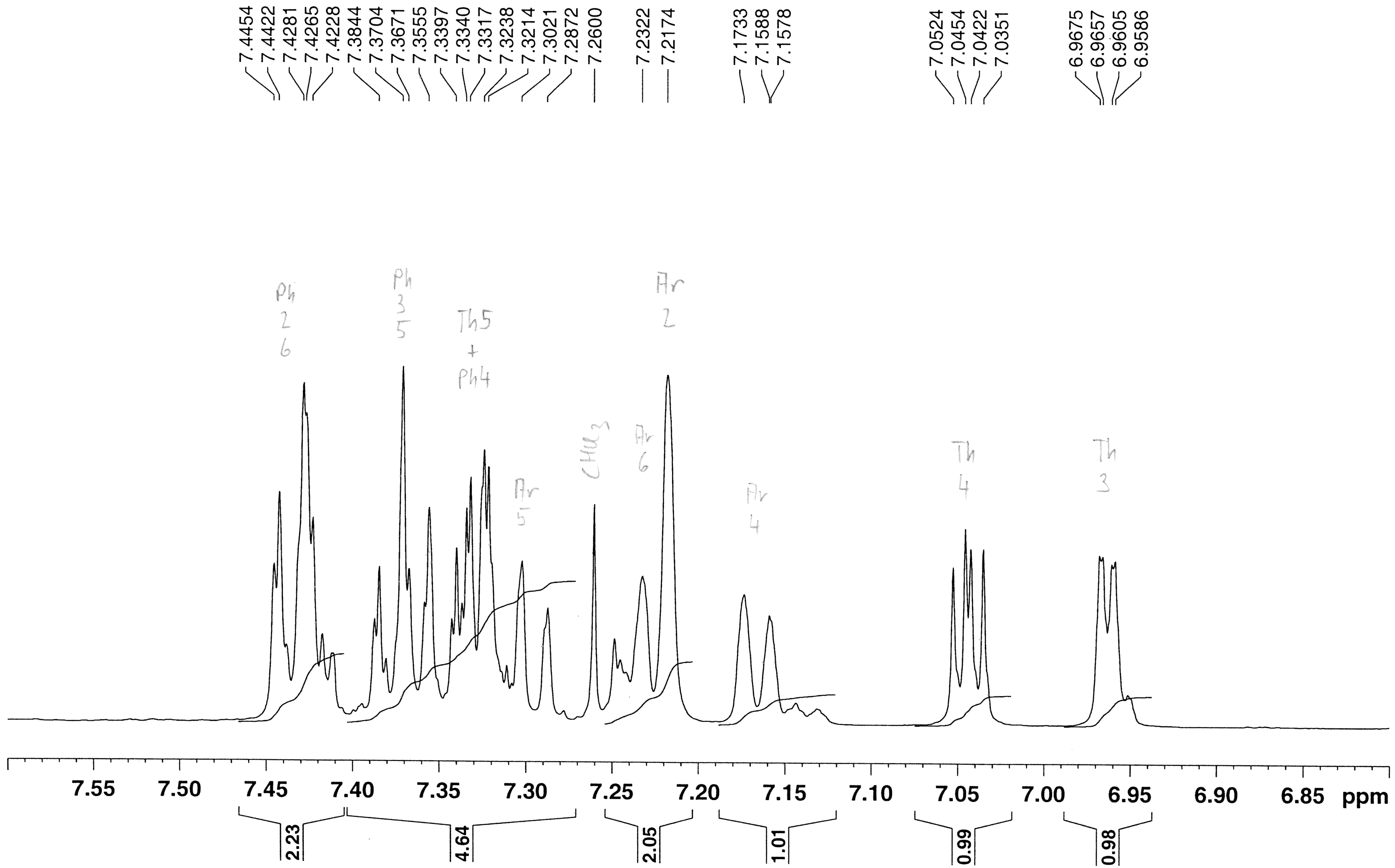

PN016_2_1 in cdcl3 (Proton) 29.8.2019

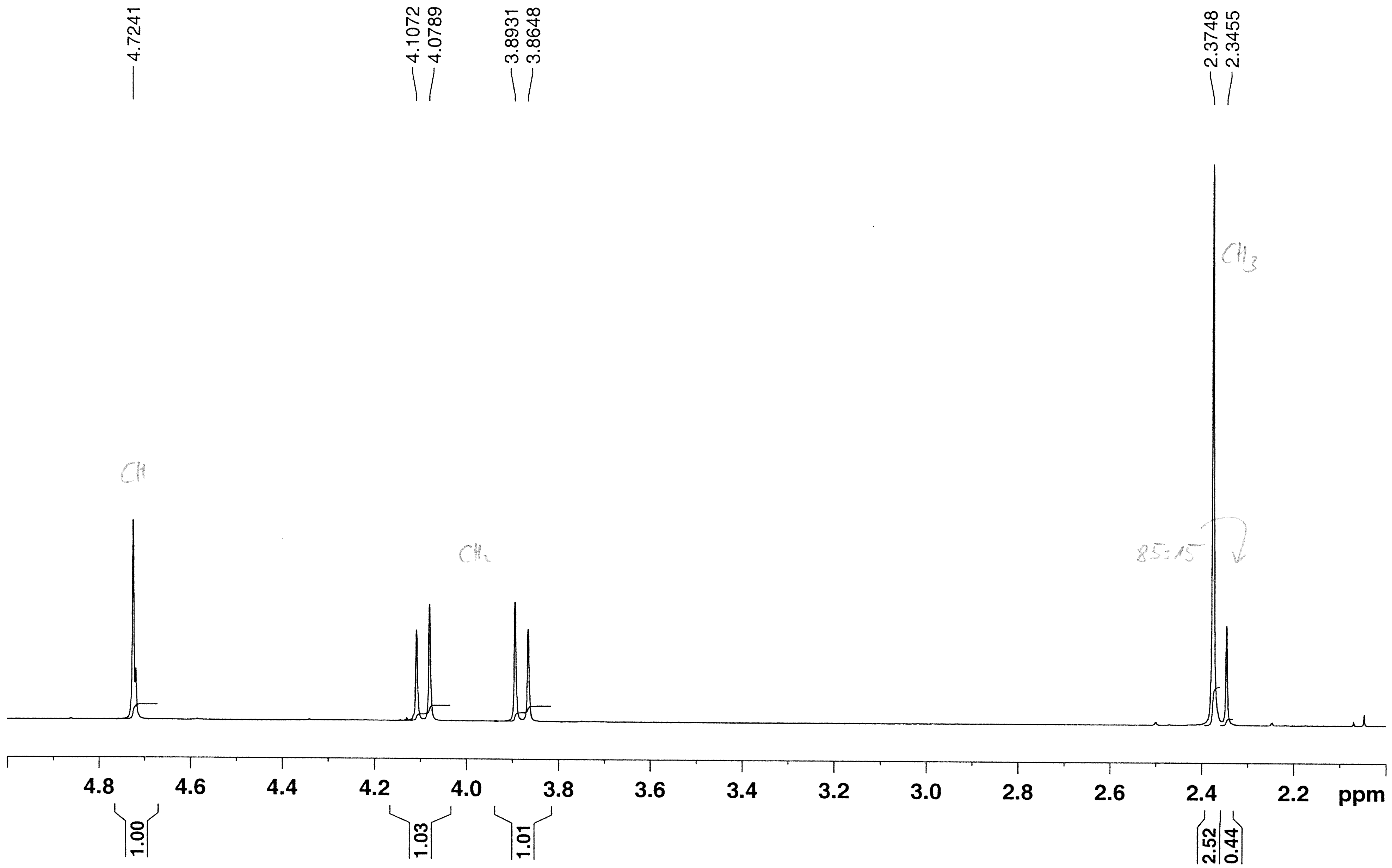

PN016_2_1 in cdcl3 (APT) 29.8.2019

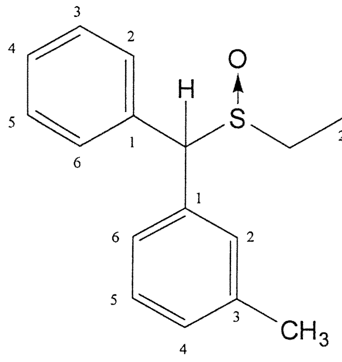

85%

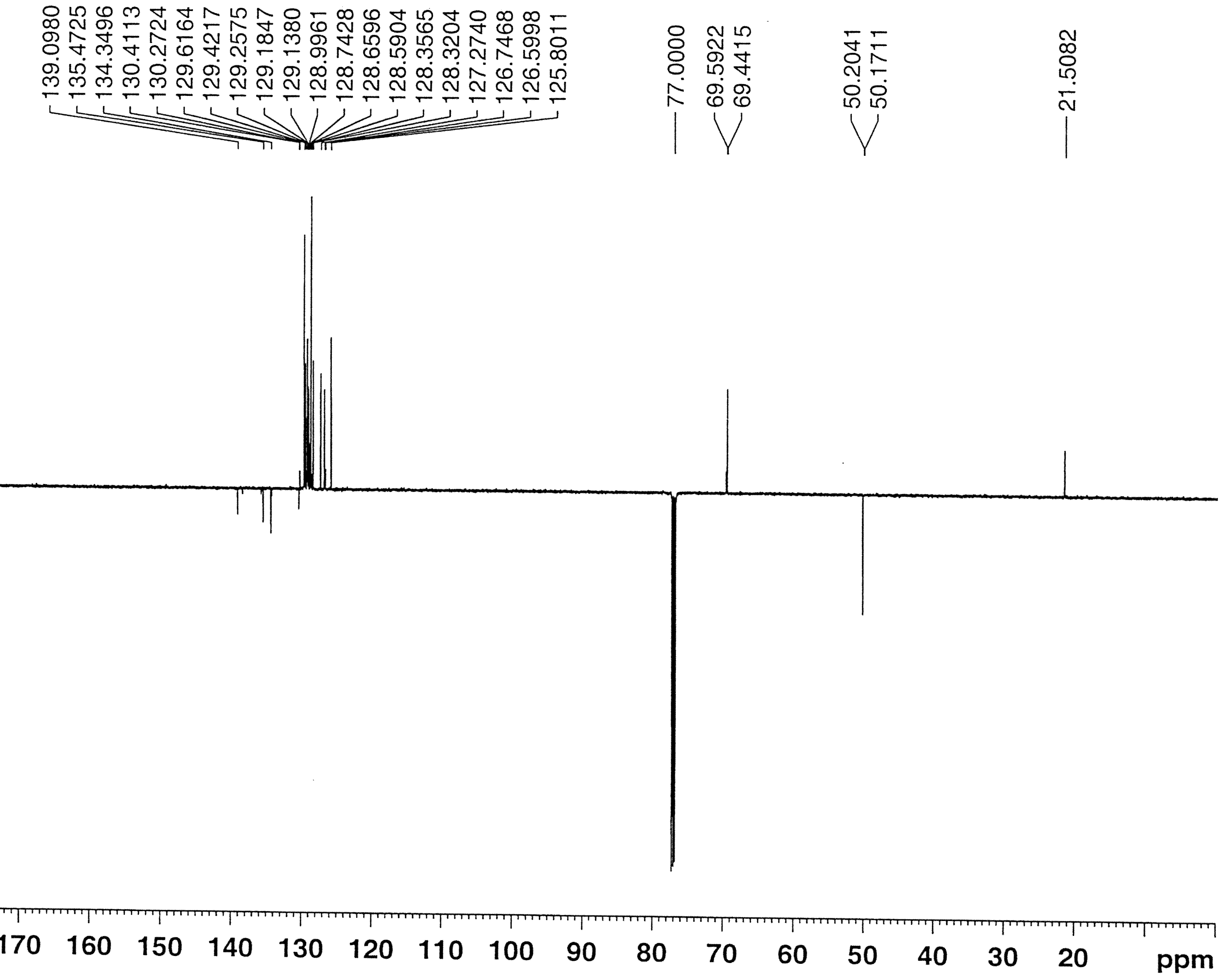

PN016_2_1 in cdcl3 (APT) 29.8.2019

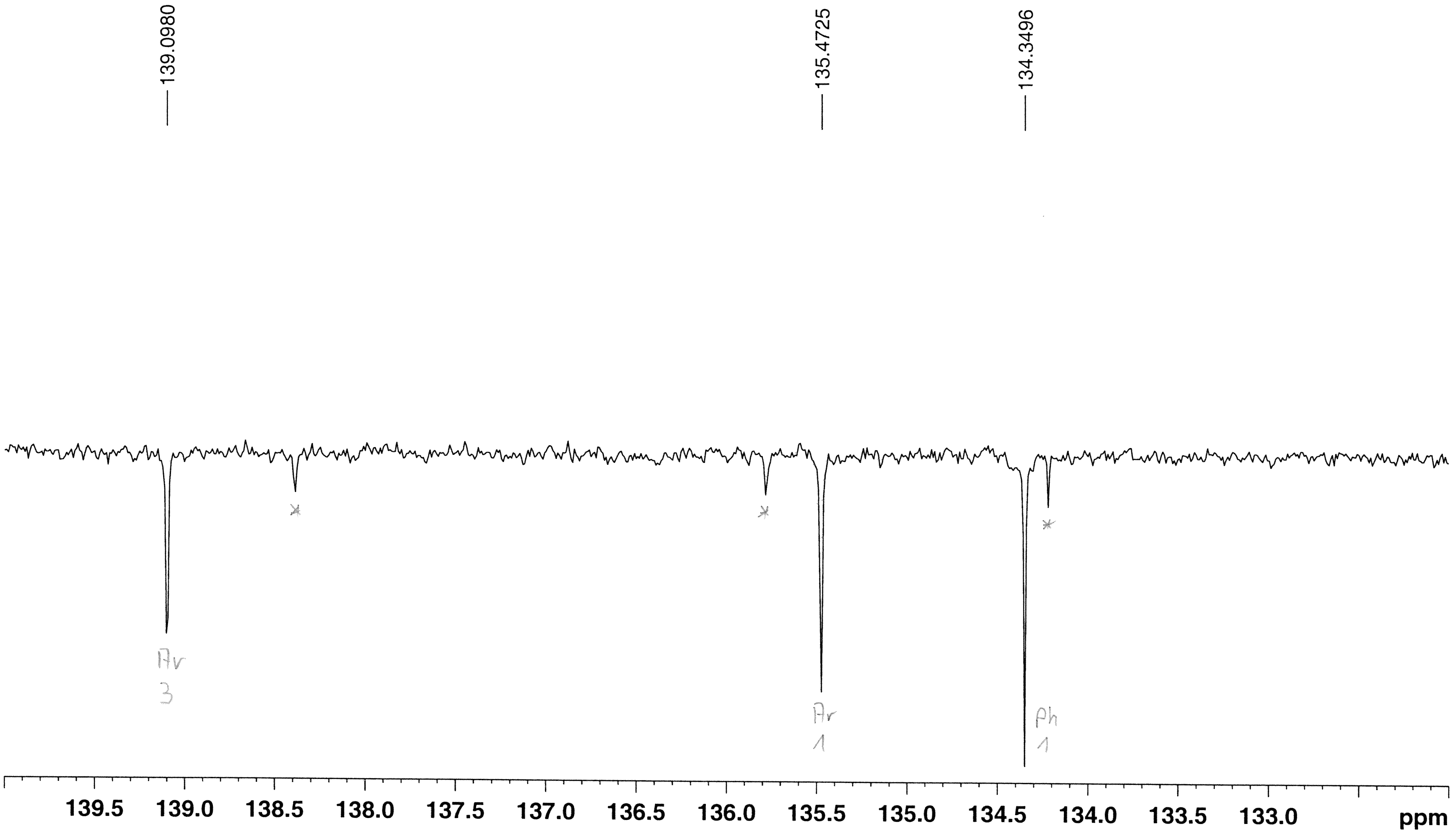

PN016_2_1 in cdcl3 (APT) 29.8.2019

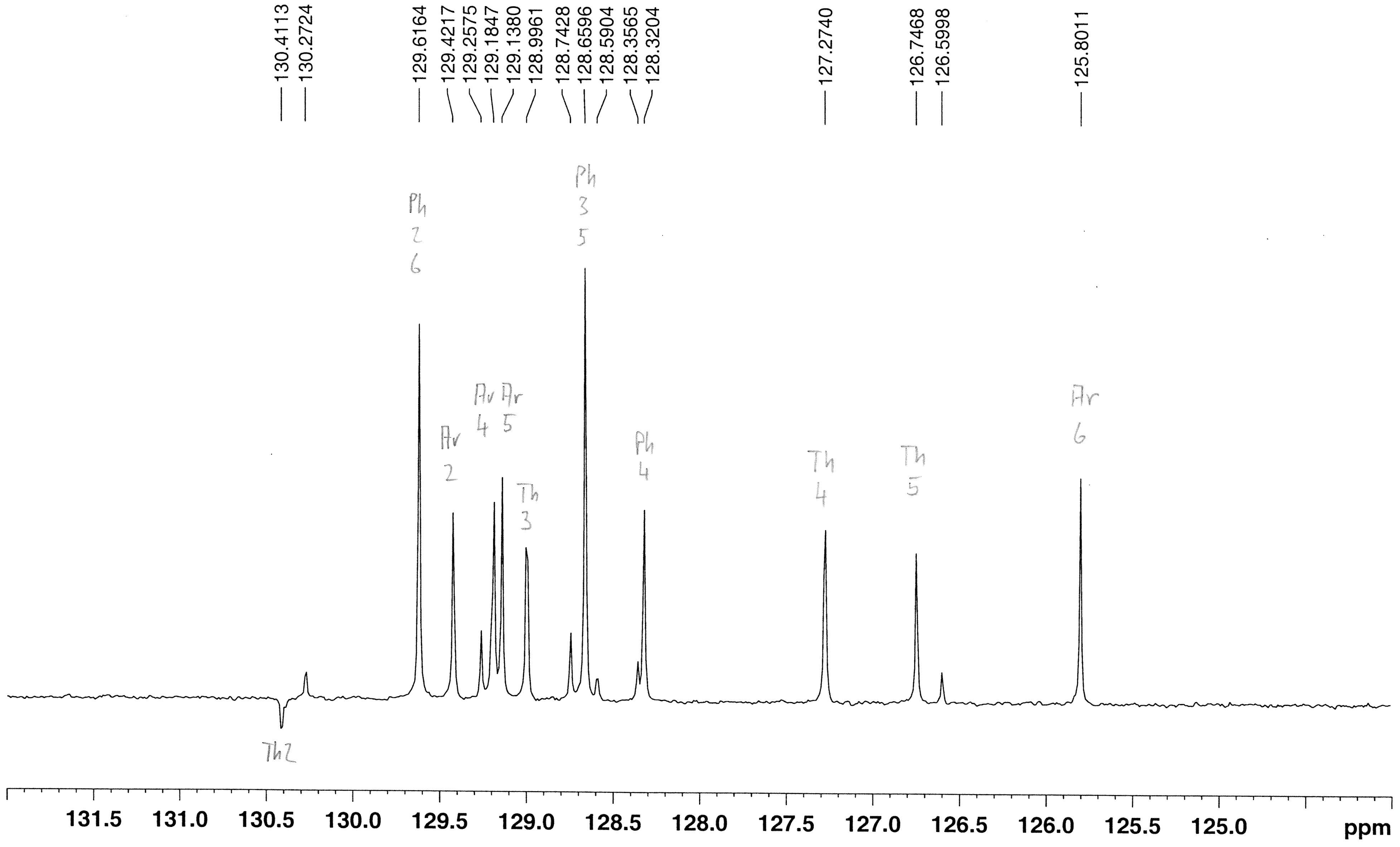

PN016_2_1 in cdcl3 (APT) 29.8.2019

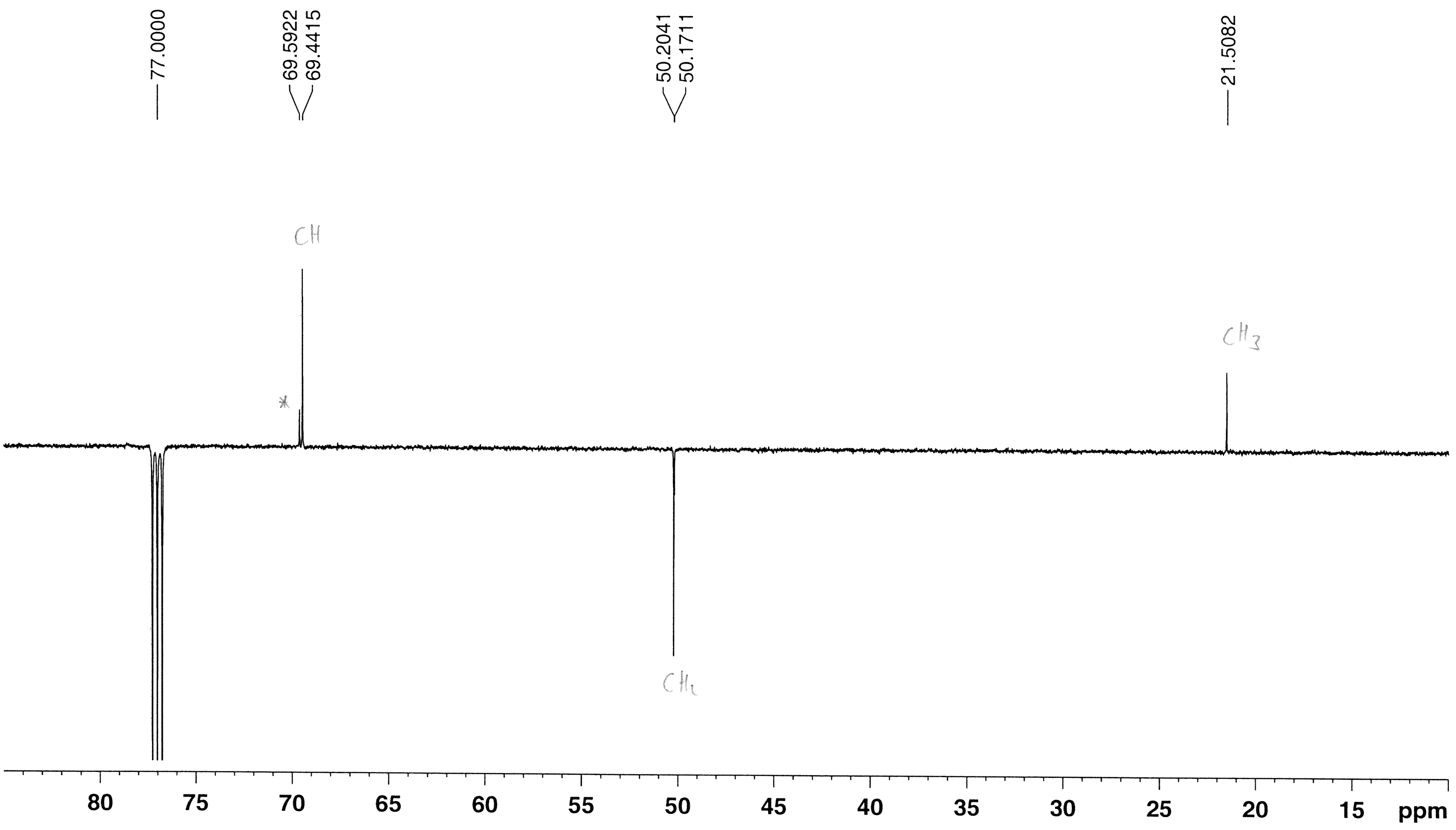

Figure S52c. NMR spectra of compound **8m**.

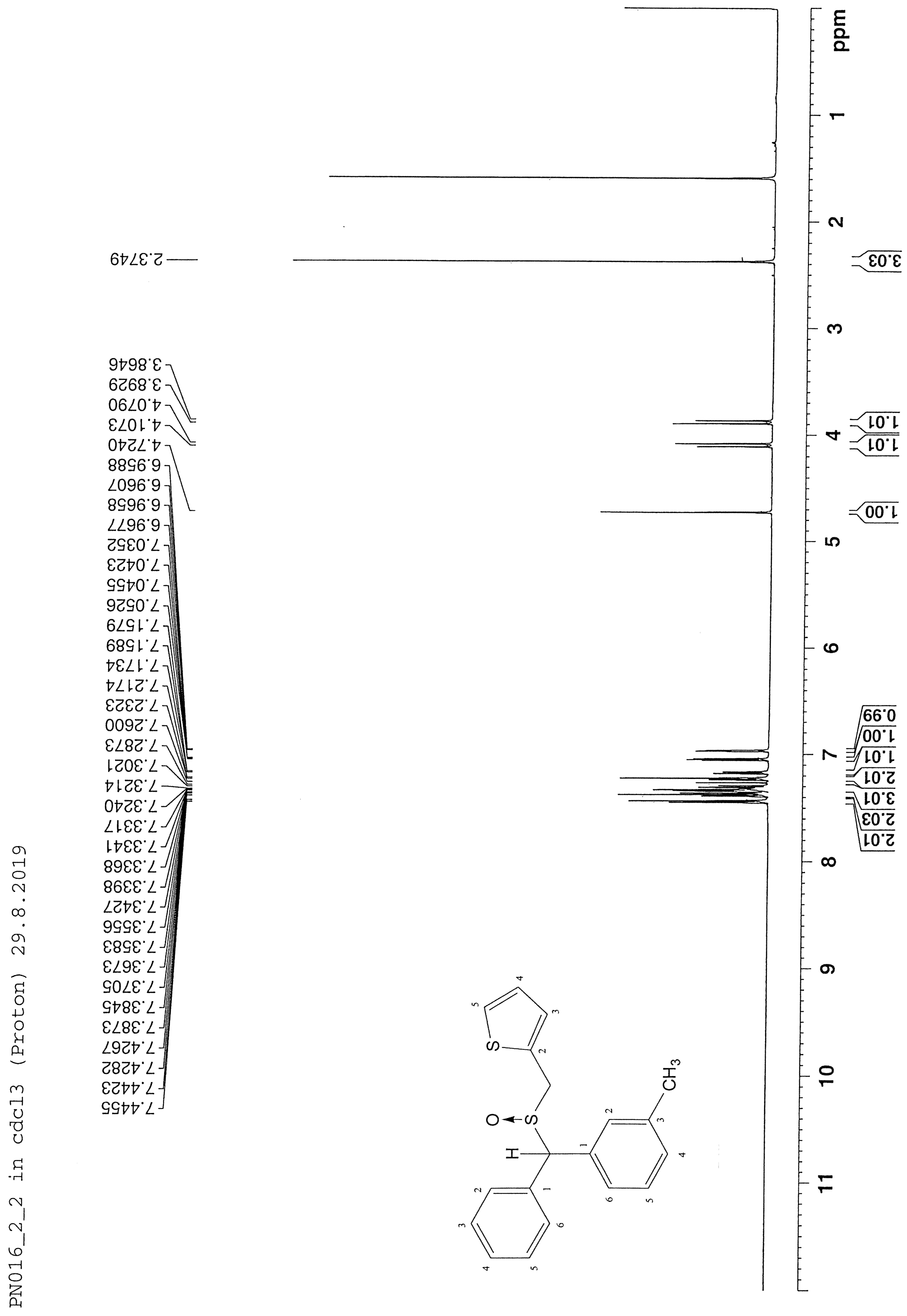

PN016_2_2 in cdcl3 (Proton) 29.8.2019

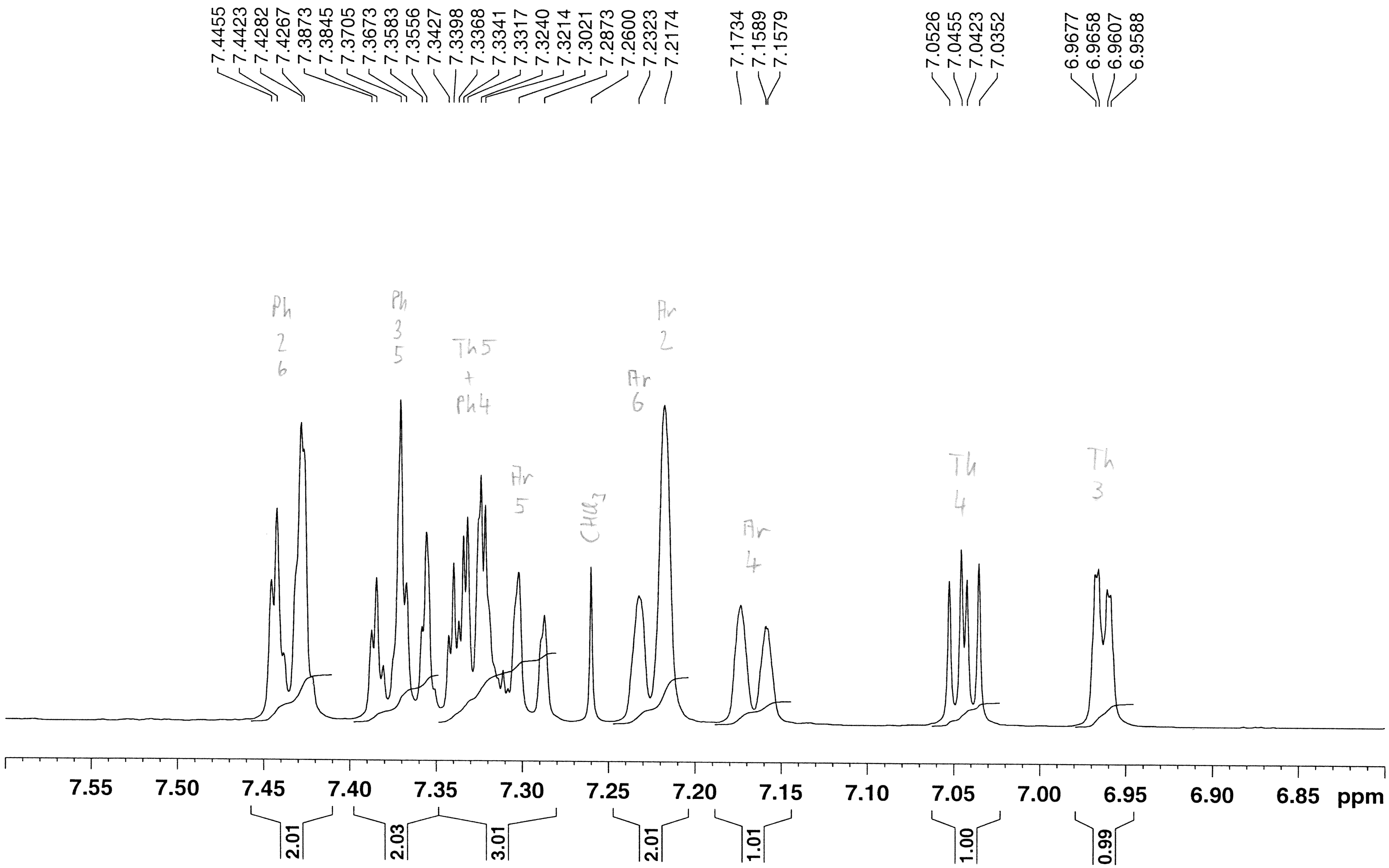

PN016_2_2 in cdcl3 (Proton) 29.8.2019

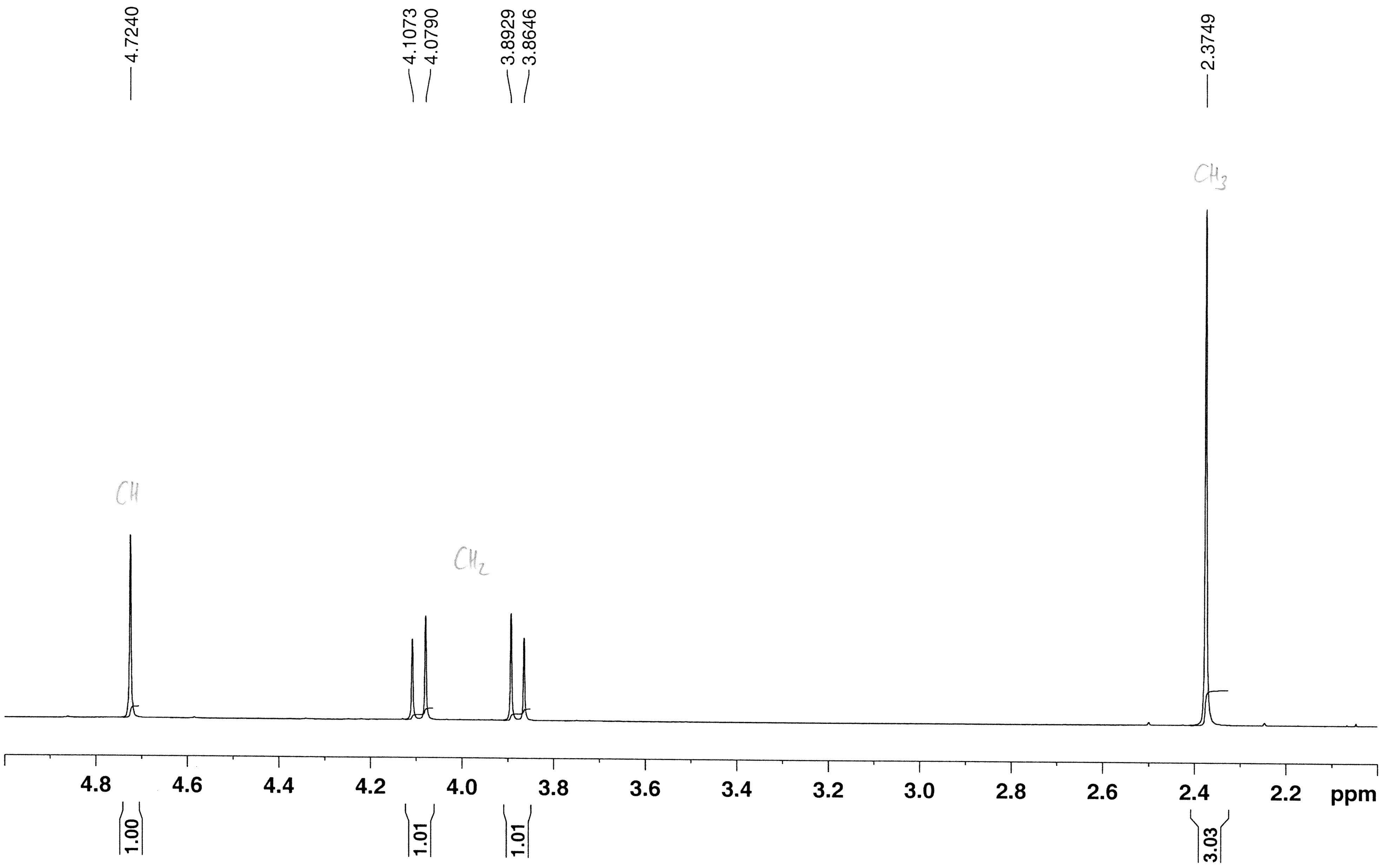

PN016_2_2 in cdcl3 (APT) 29.8.2019

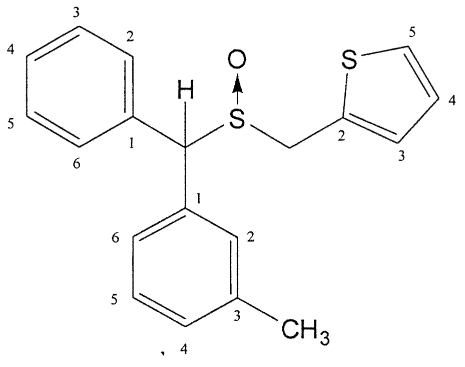

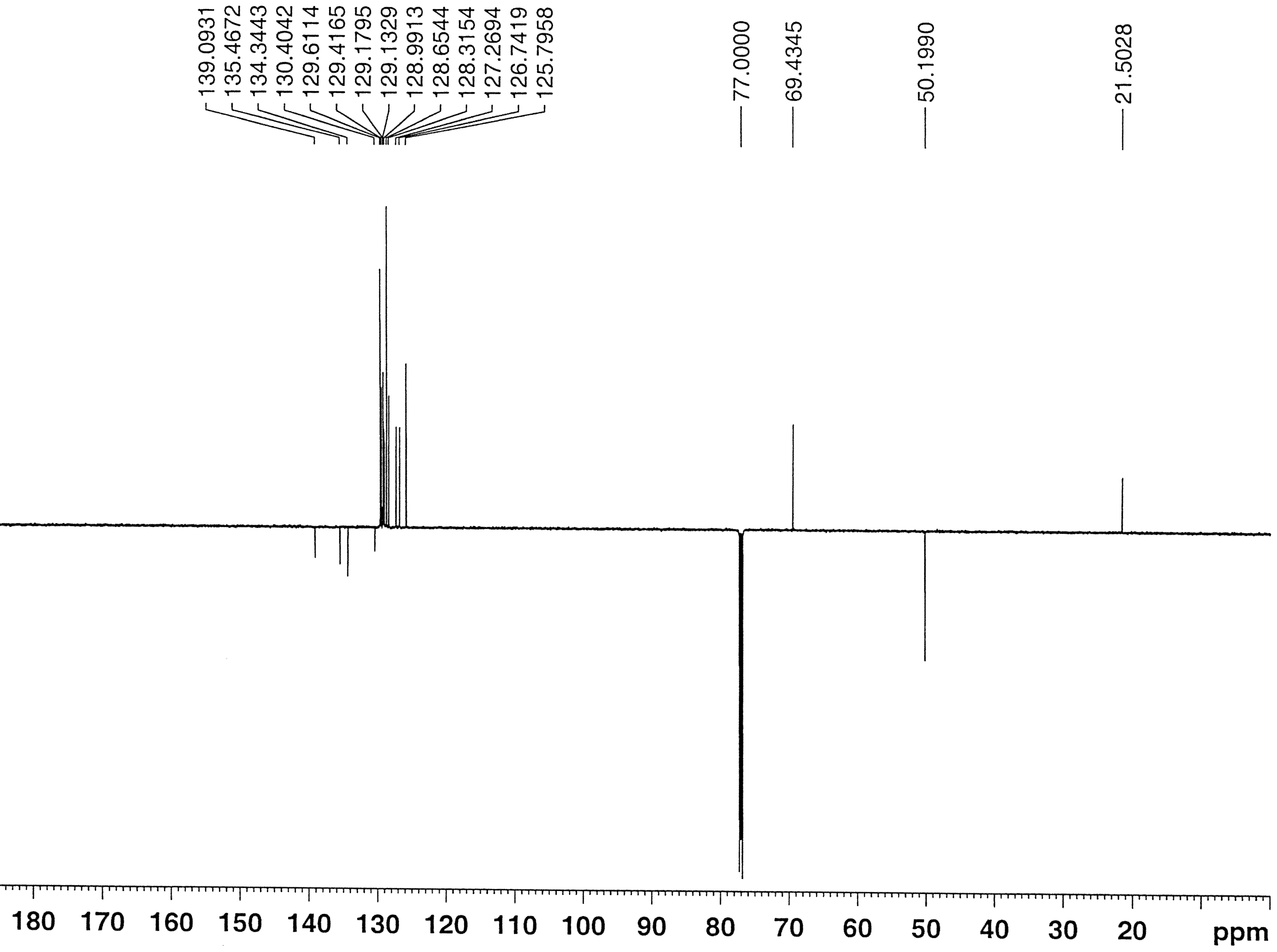

PN016_2_2 in cdcl3 (APT) 29.8.2019

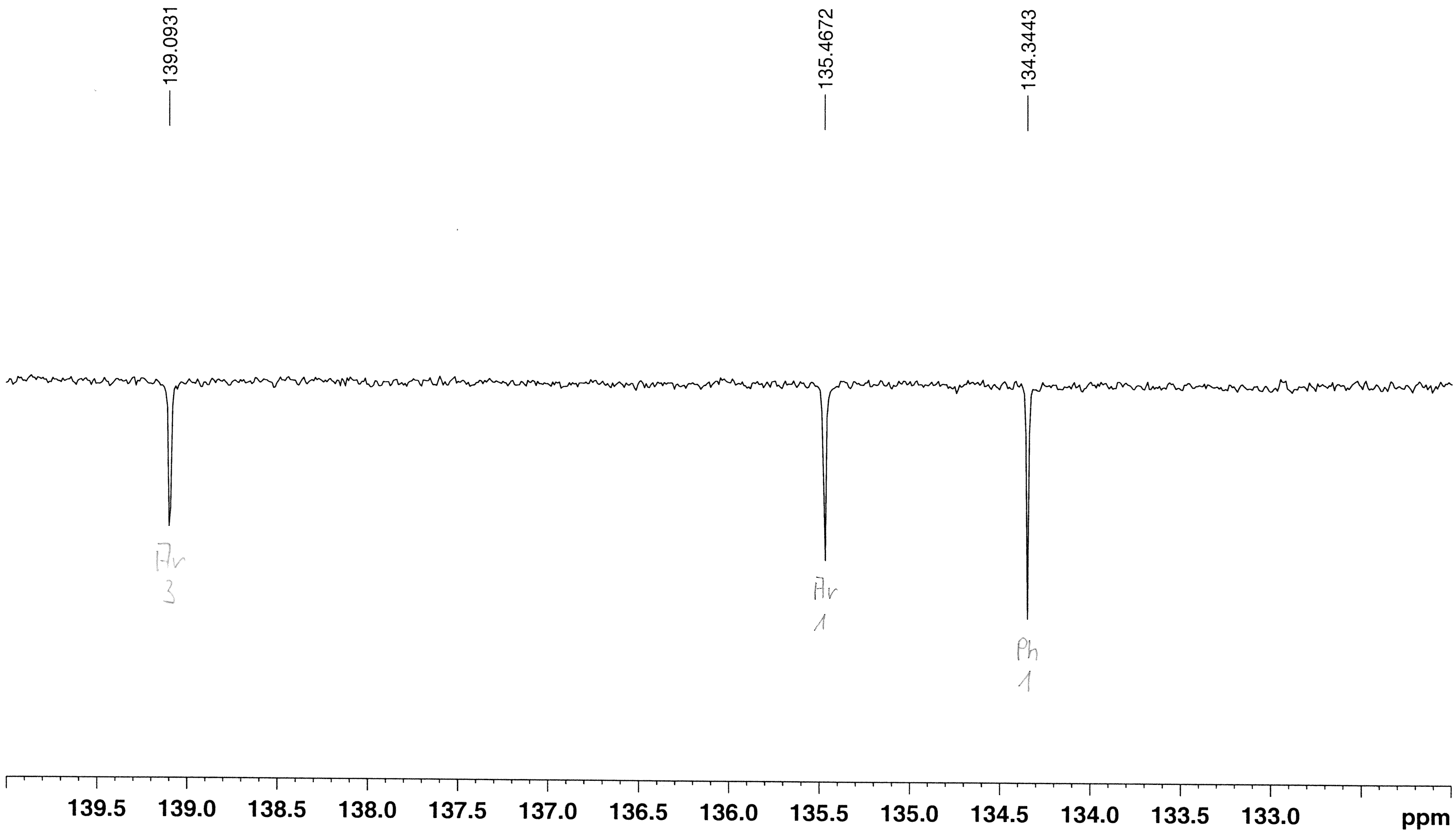

PN016_2_2 in cdcl3 (APT) 29.8.2019

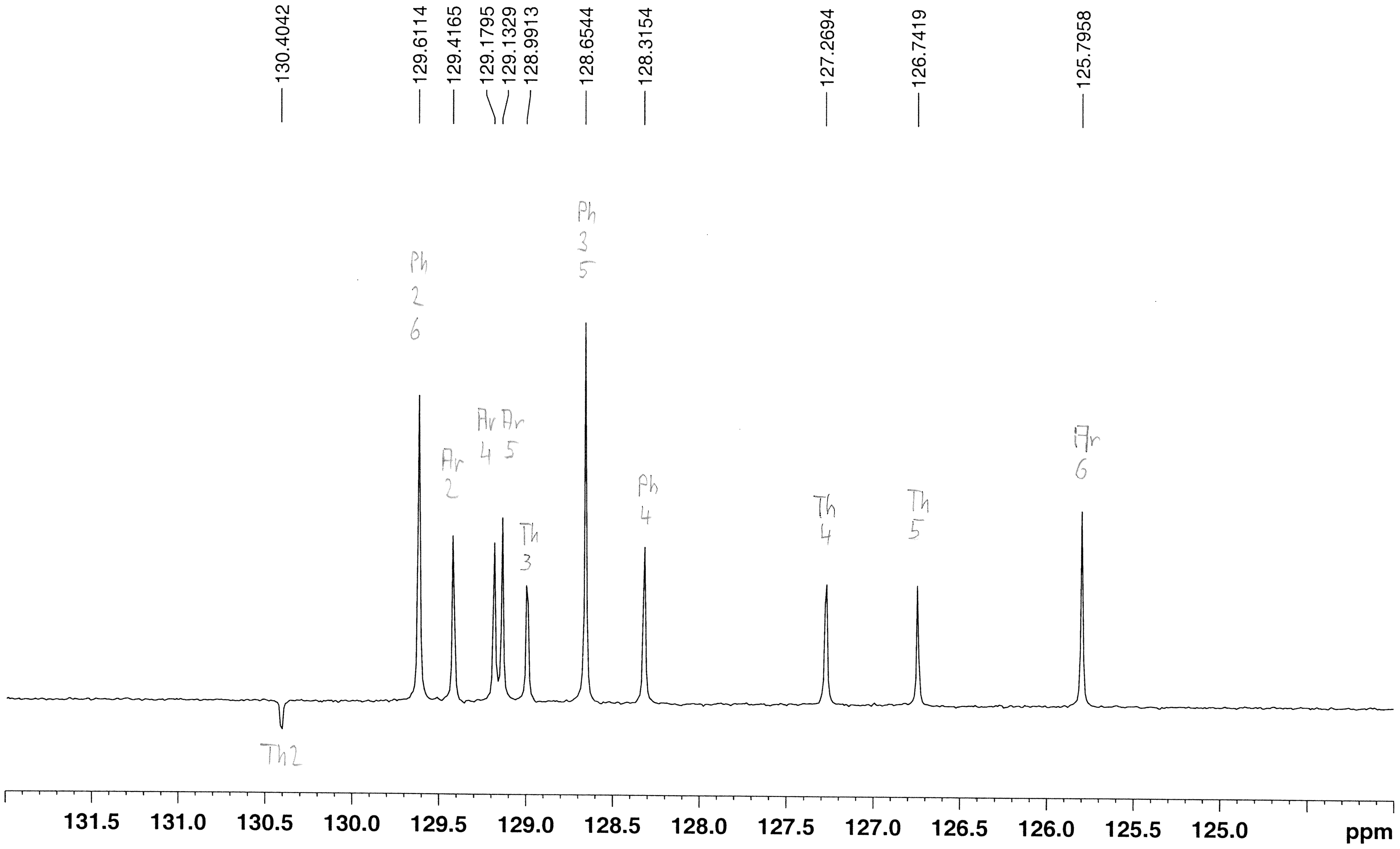

PN016_2_2 in cdcl3 (APT) 29.8.2019

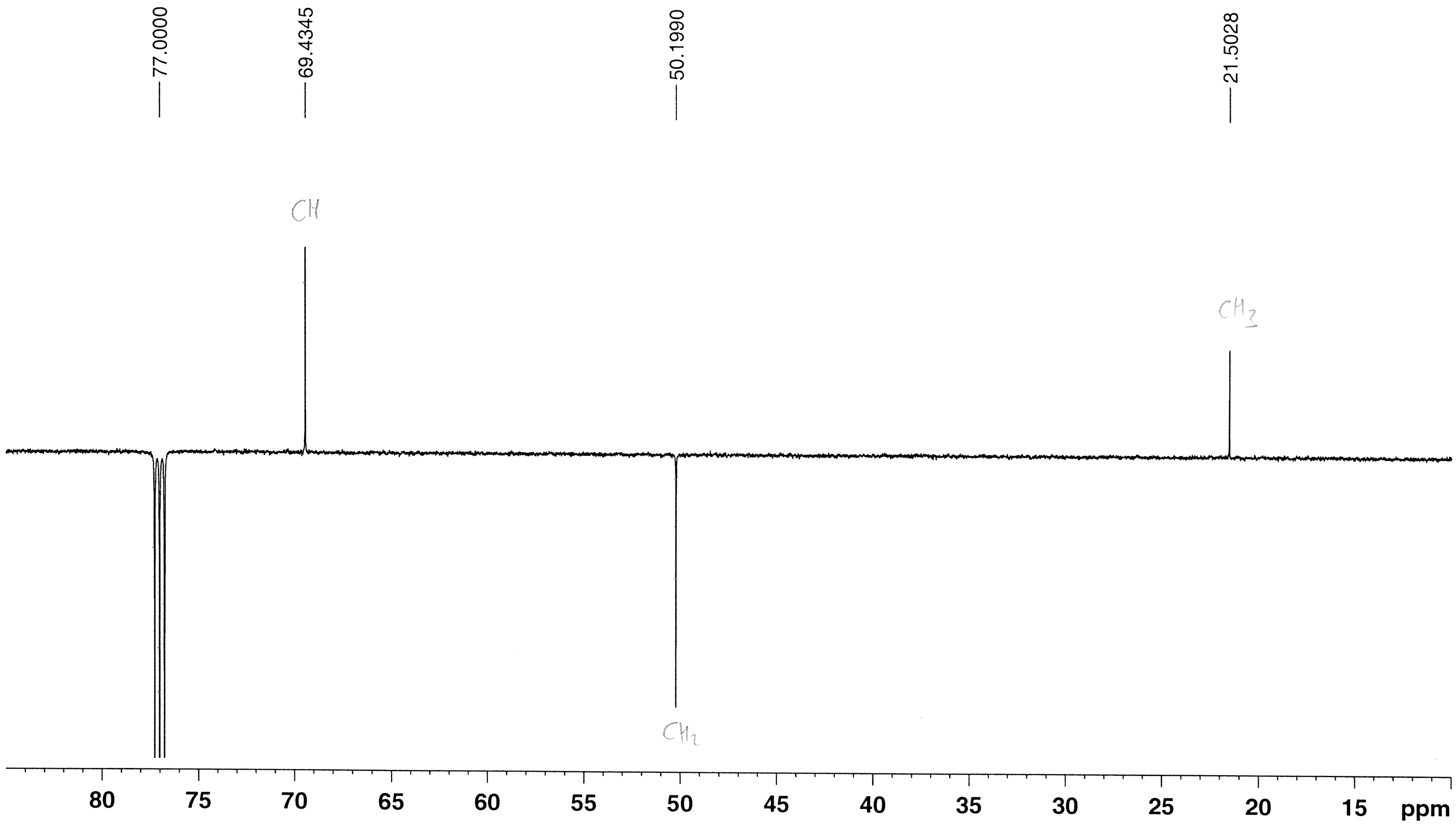

PN016_2_2 in cdcl3 (COSY) 29.8.2019

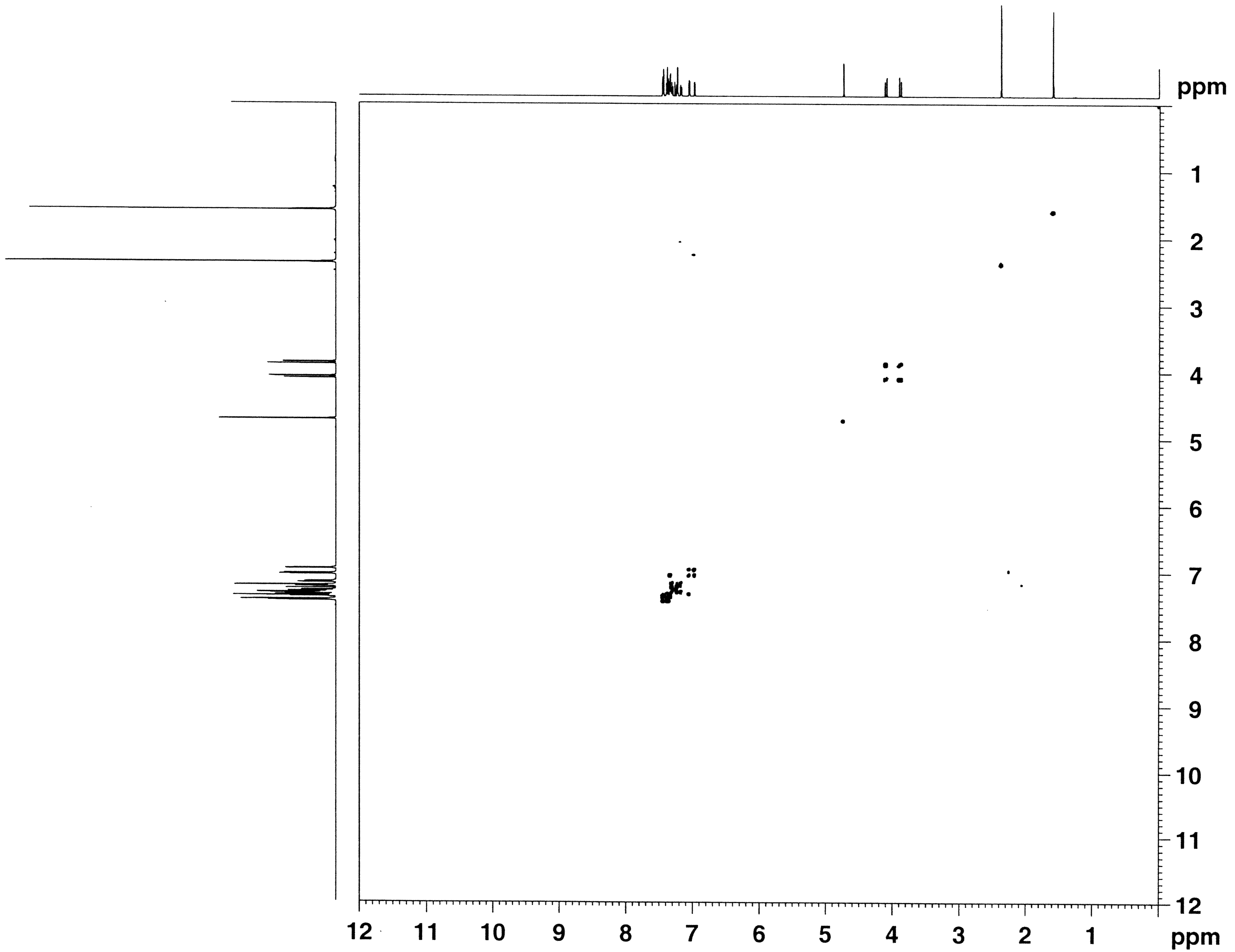

PN016_2_2 in cdcl3 (COSY) 29.8.2019

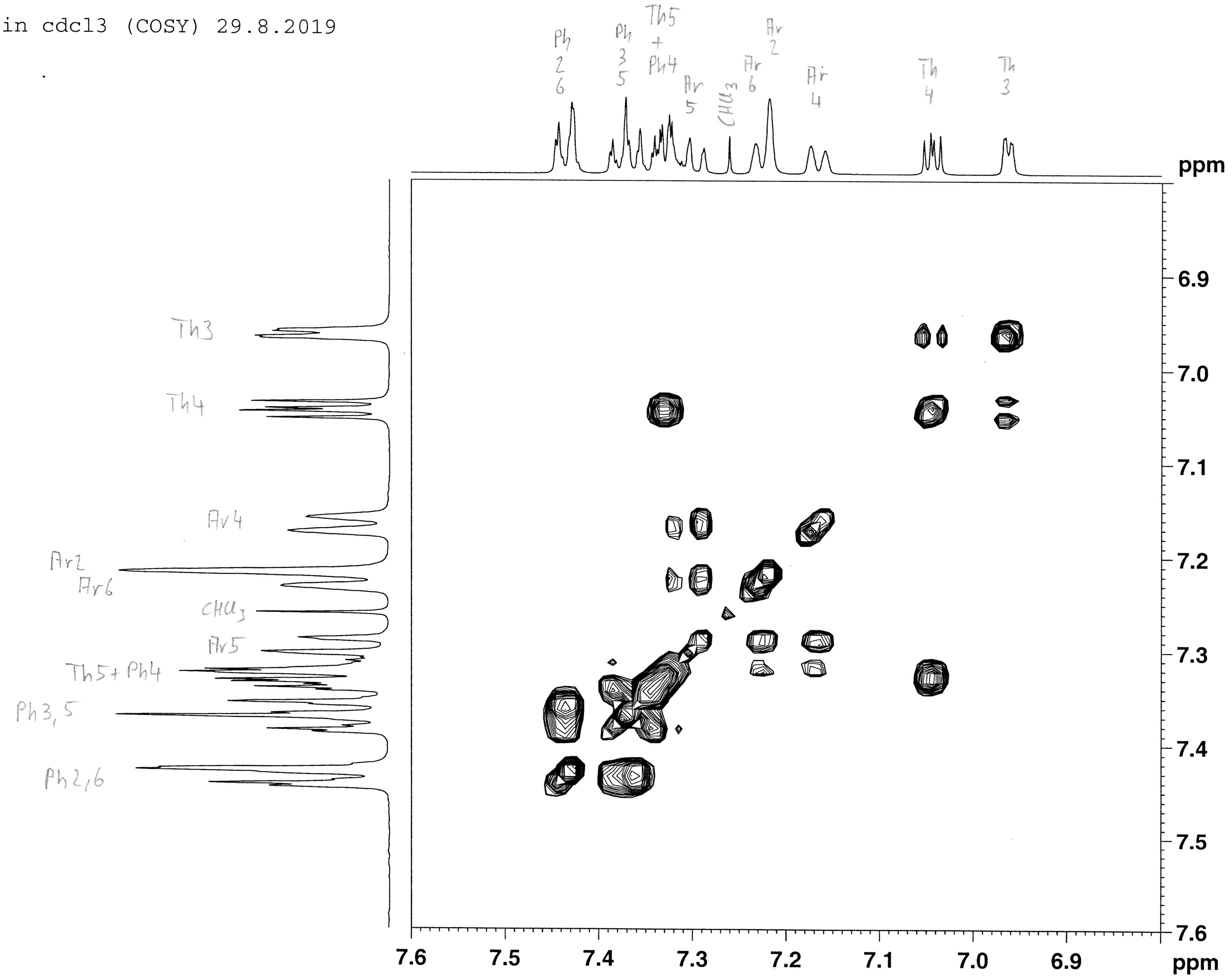

PN016_2_2 in cdcl3 (COSY) 29.8.2019

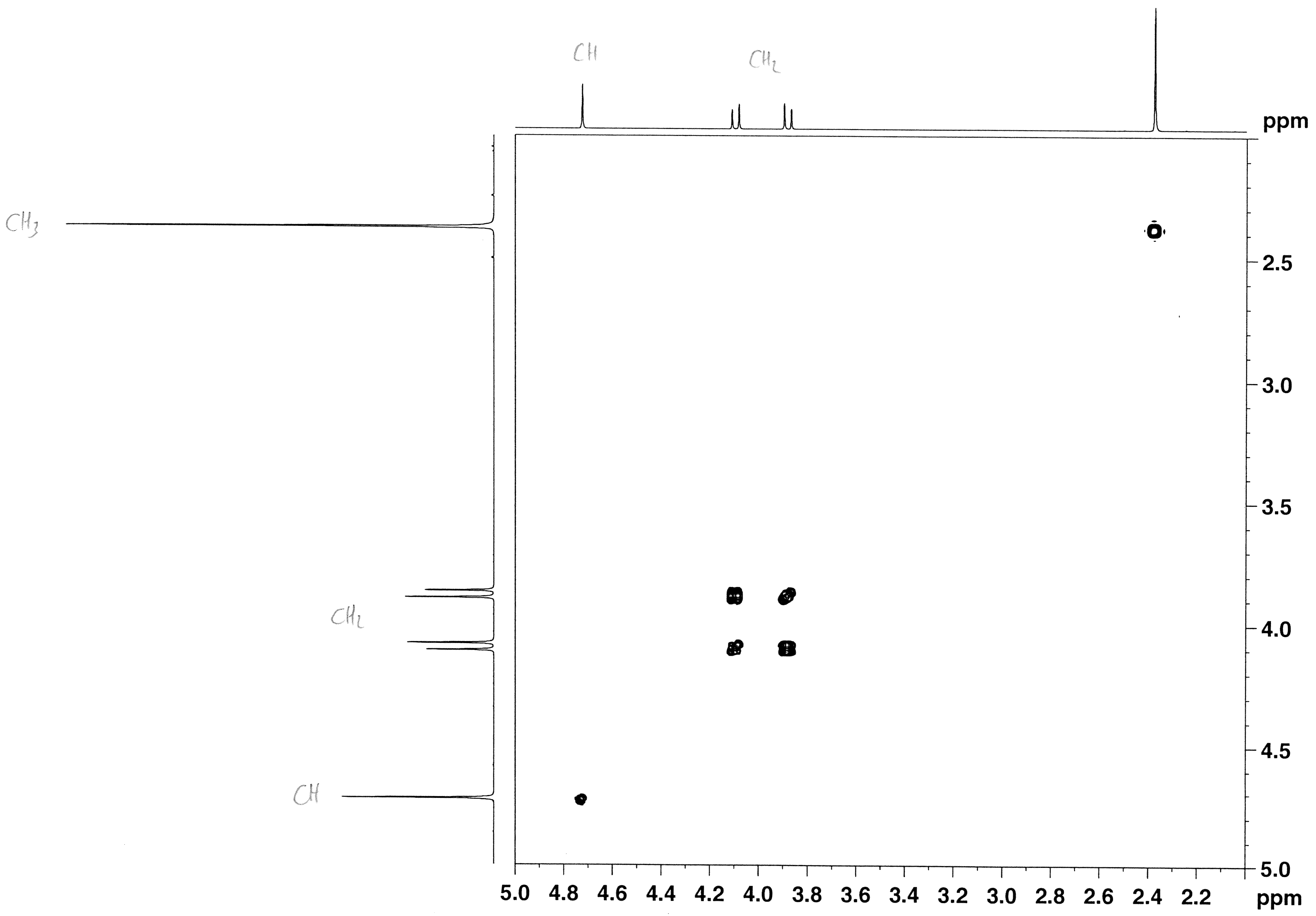

PN016_2_2 in cdcl3 (HSQC) 29.8.2019

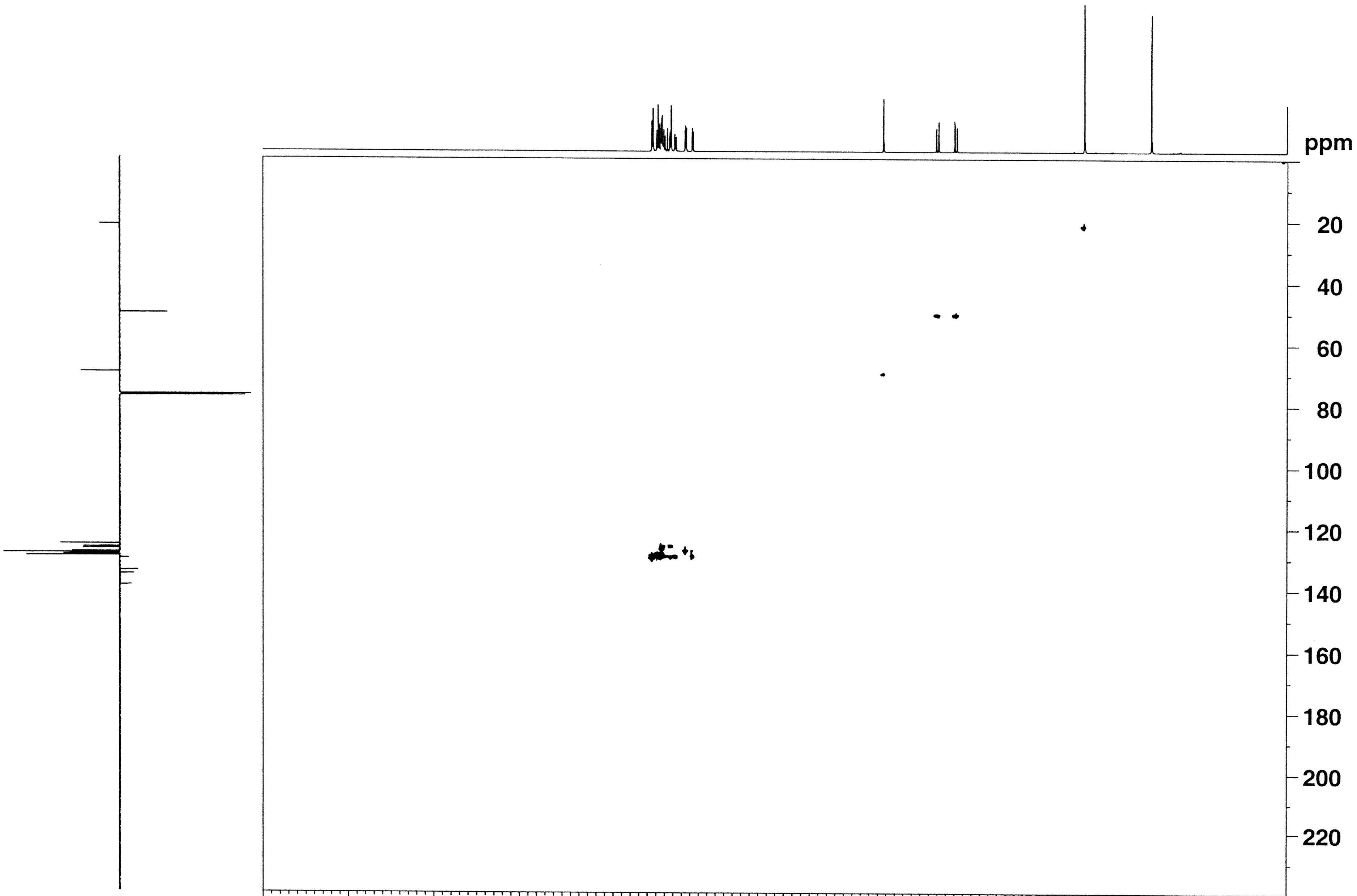

PN016_2_2 in cdcl3 (HSQC) 29.8.2019

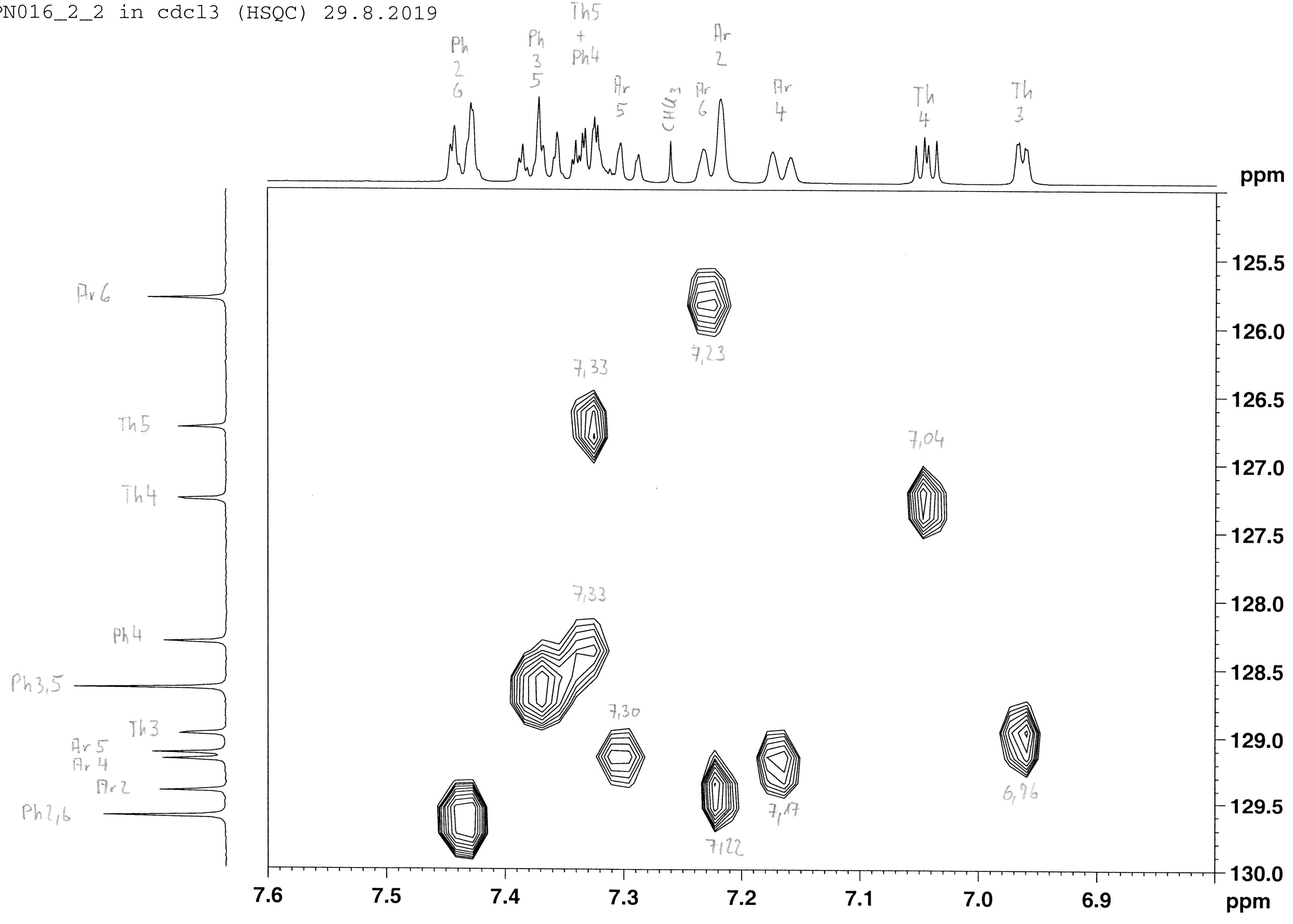

PN016_2_2 in cdcl3 (HSQC) 29.8.2019

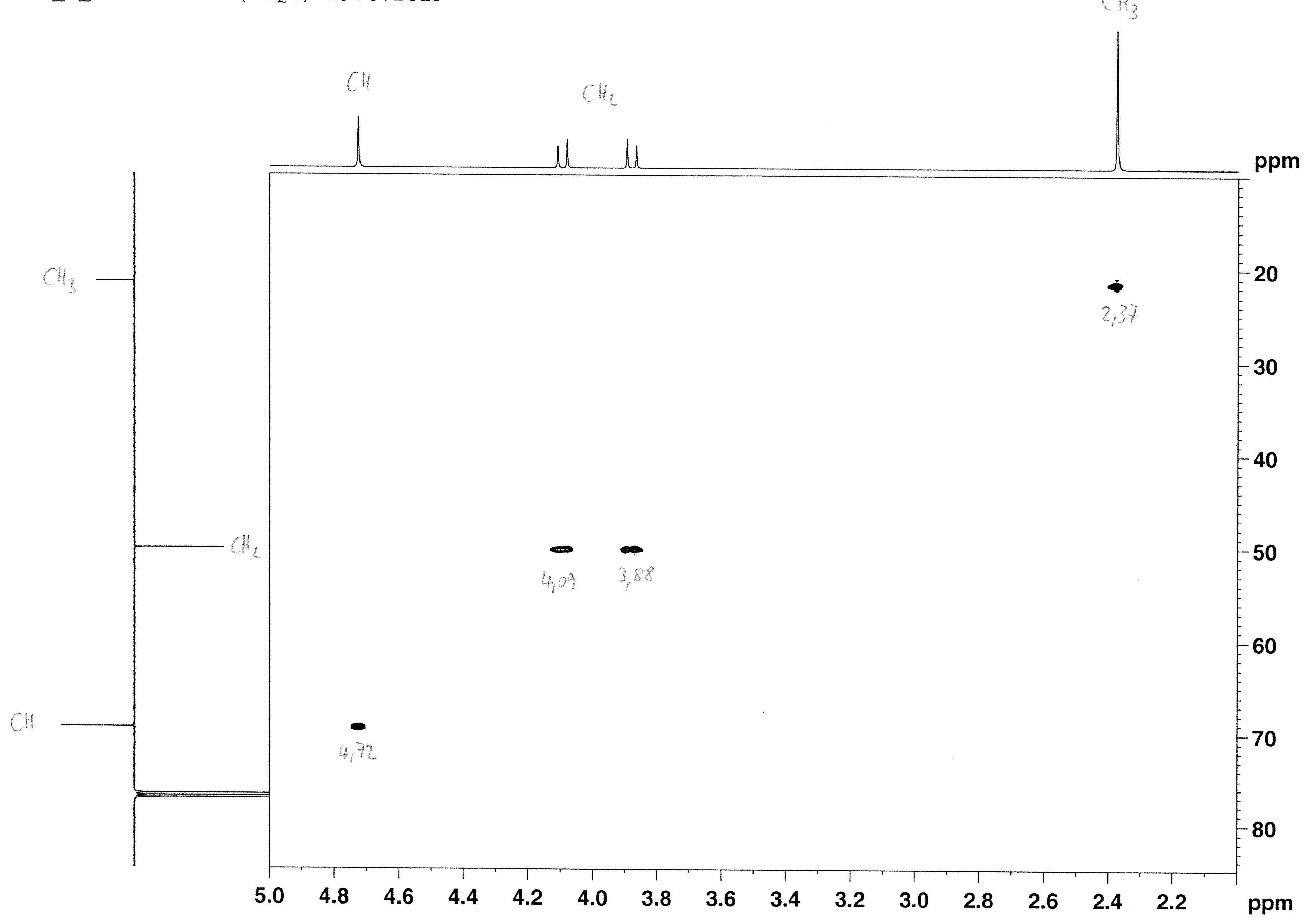

PN016_2_2 in cdcl3 (HMBC) 29.8.2019

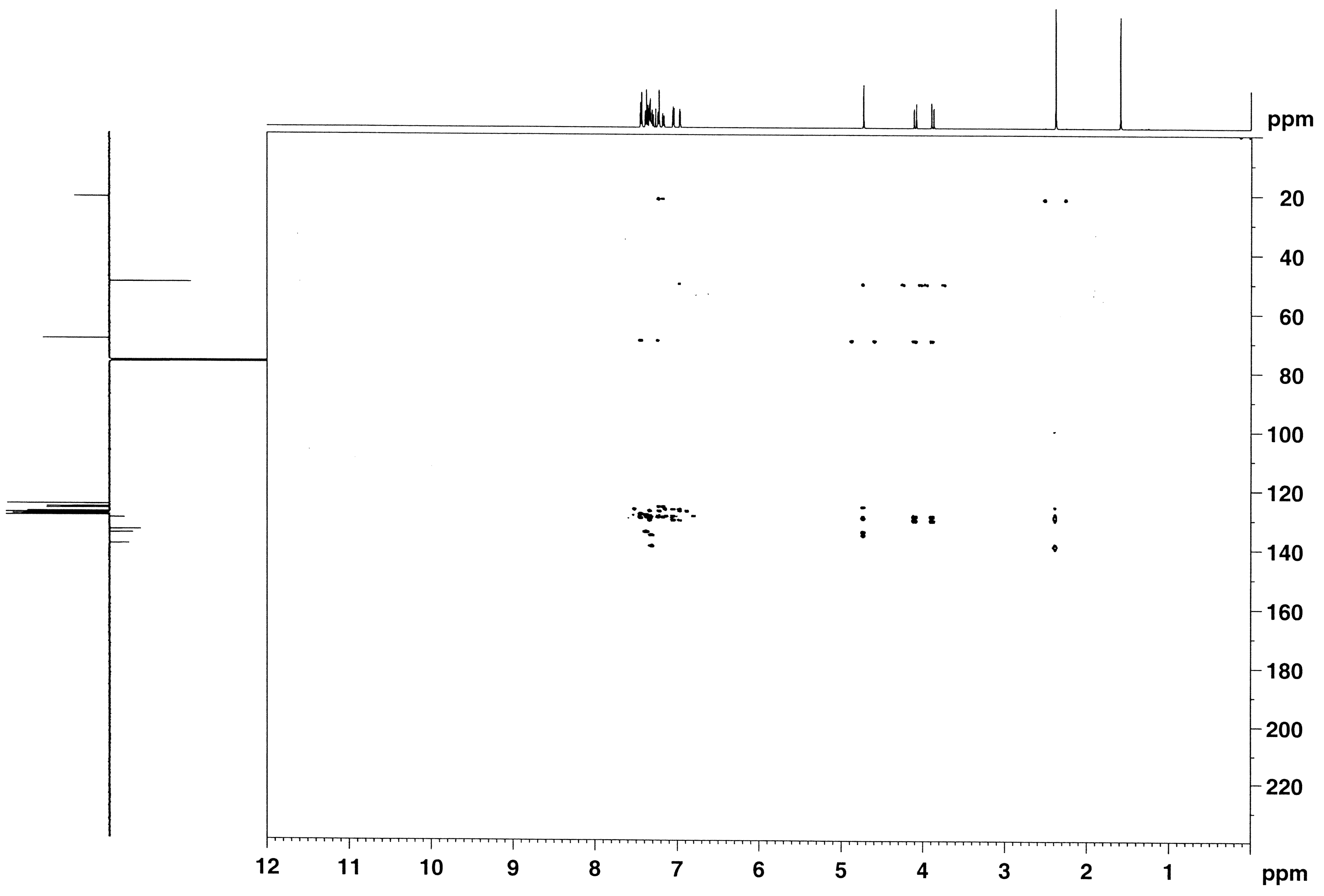

PN016_2_2 in cdcl3 (HMBC) 29.8.2019

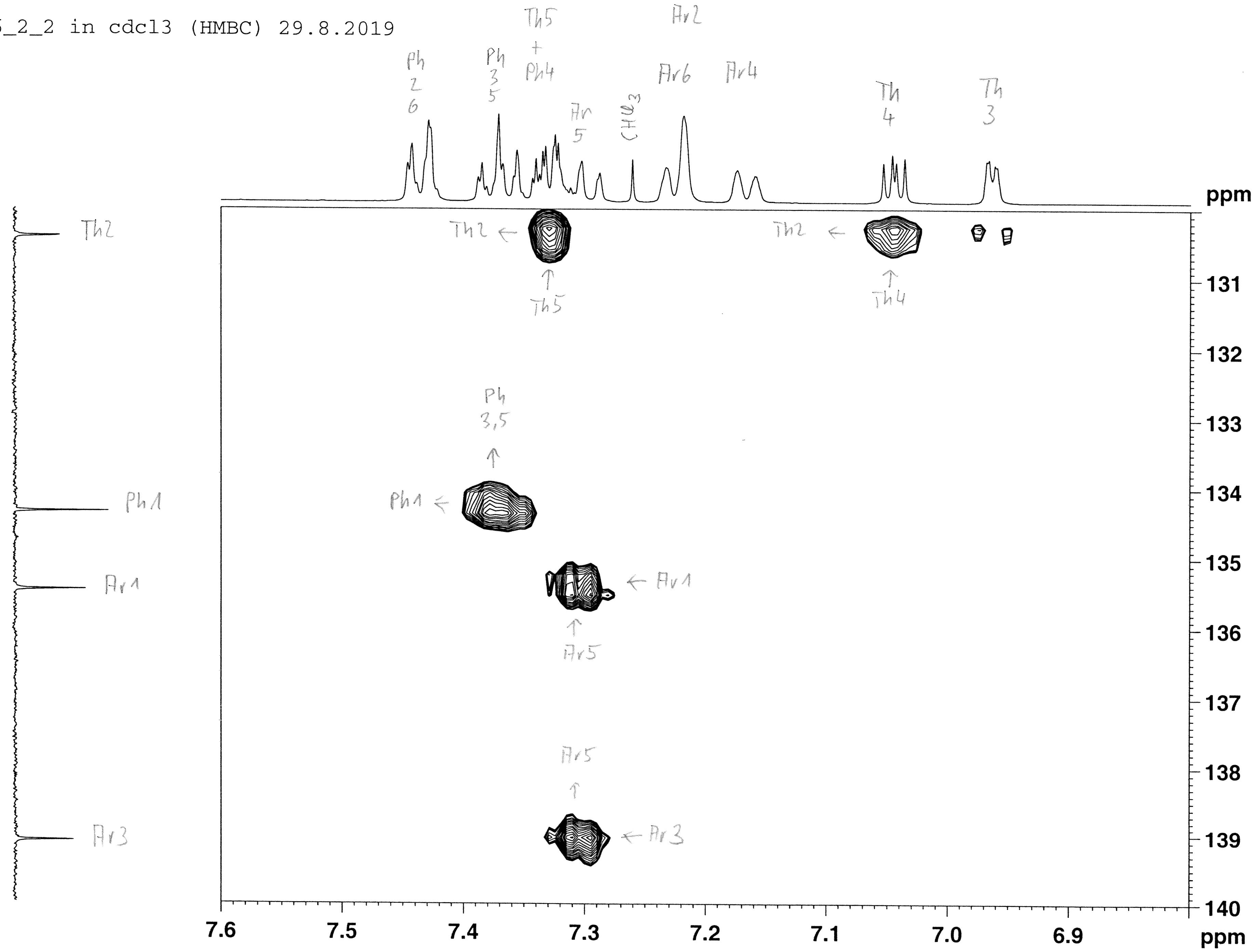

PN016_2_2 in cdcl3 (HMBC) 29.8.2019

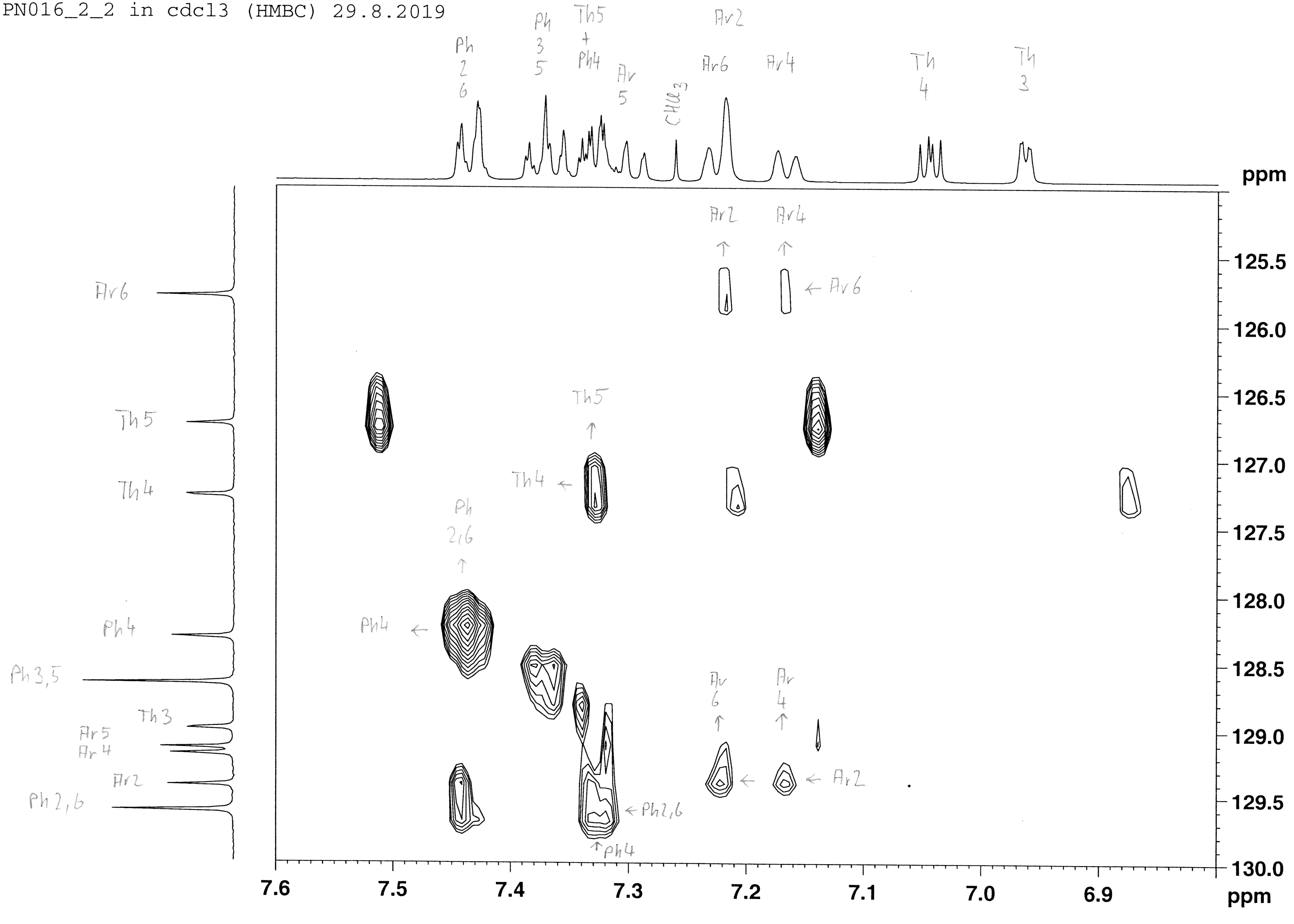

PN016_2_2 in cdcl3 (HMBC) 29.8.2019

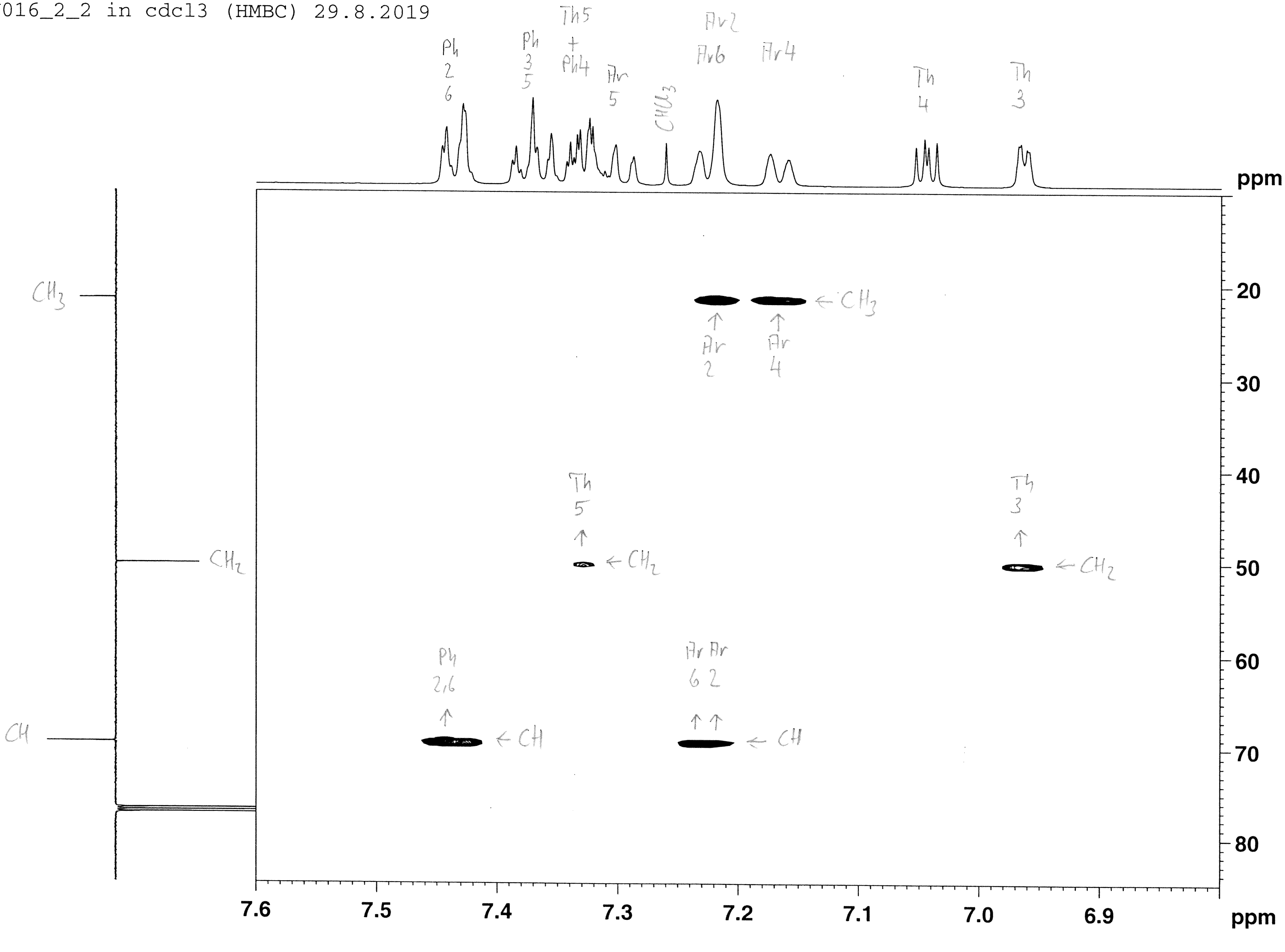

PN016_2_2 in cdcl3 (HMBC) 29.8.2019

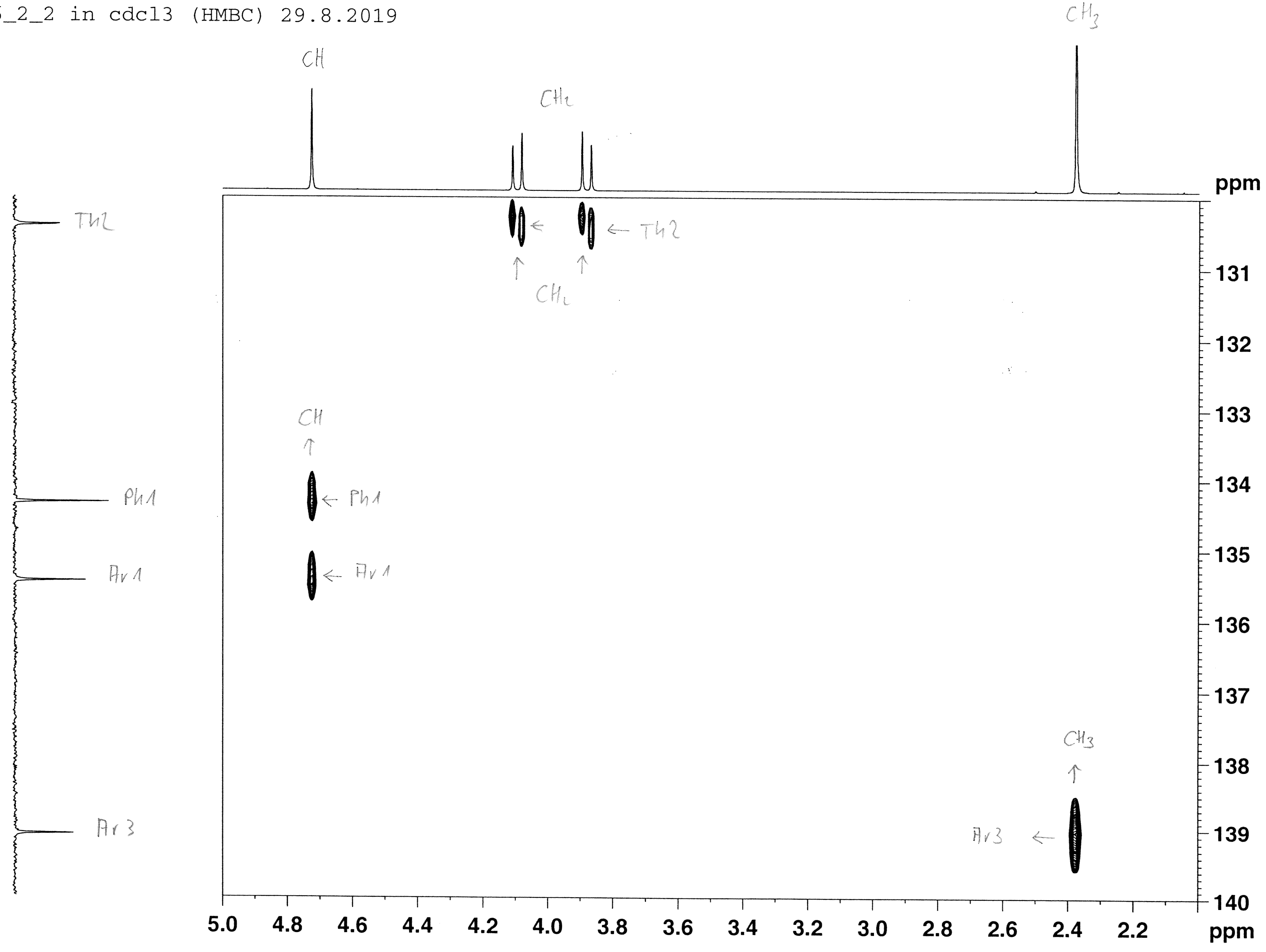

PN016_2_2 in cdcl3 (HMBC) 29.8.2019

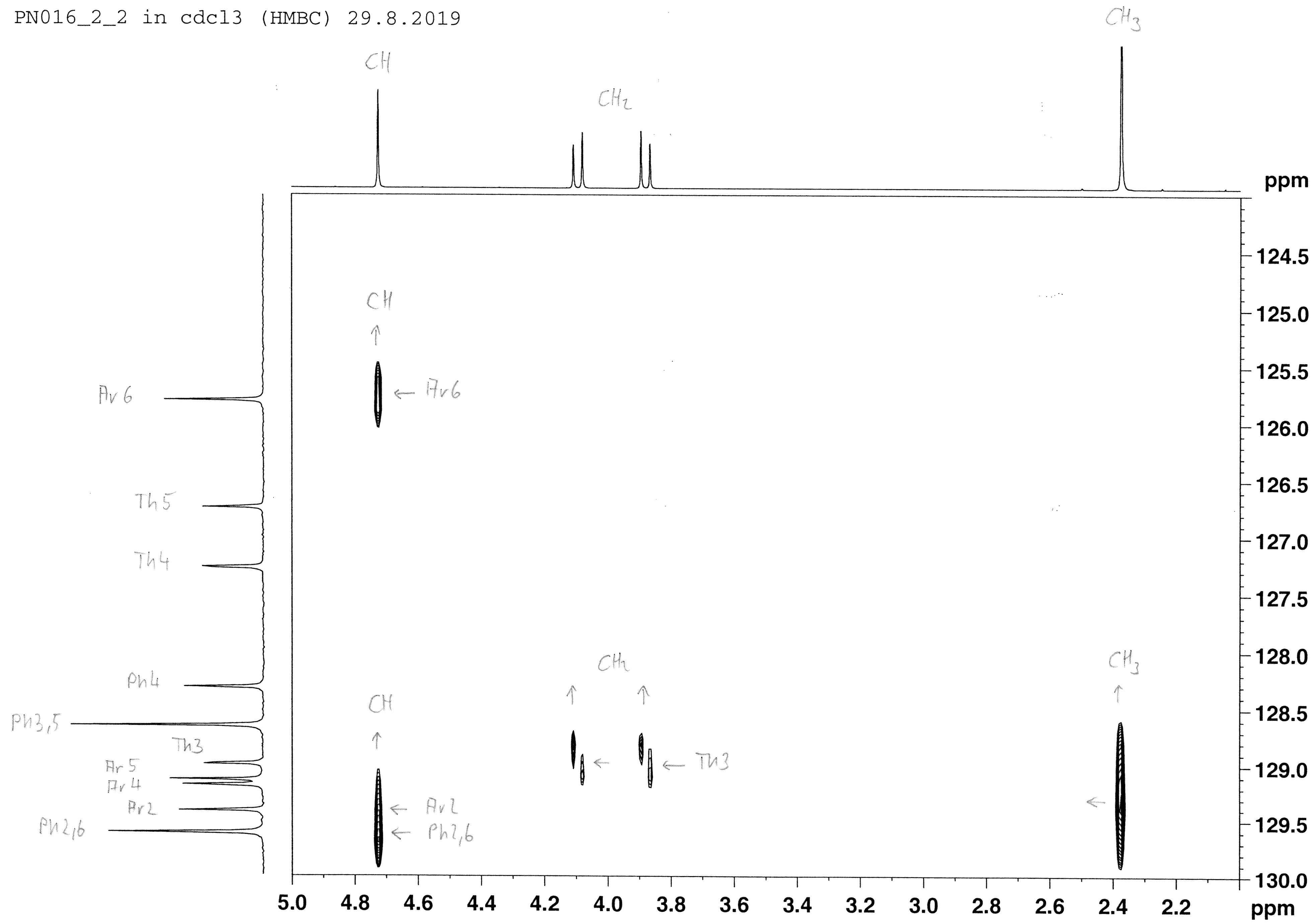

PN016_2_2 in cdcl3 (HMBC) 29.8.2019

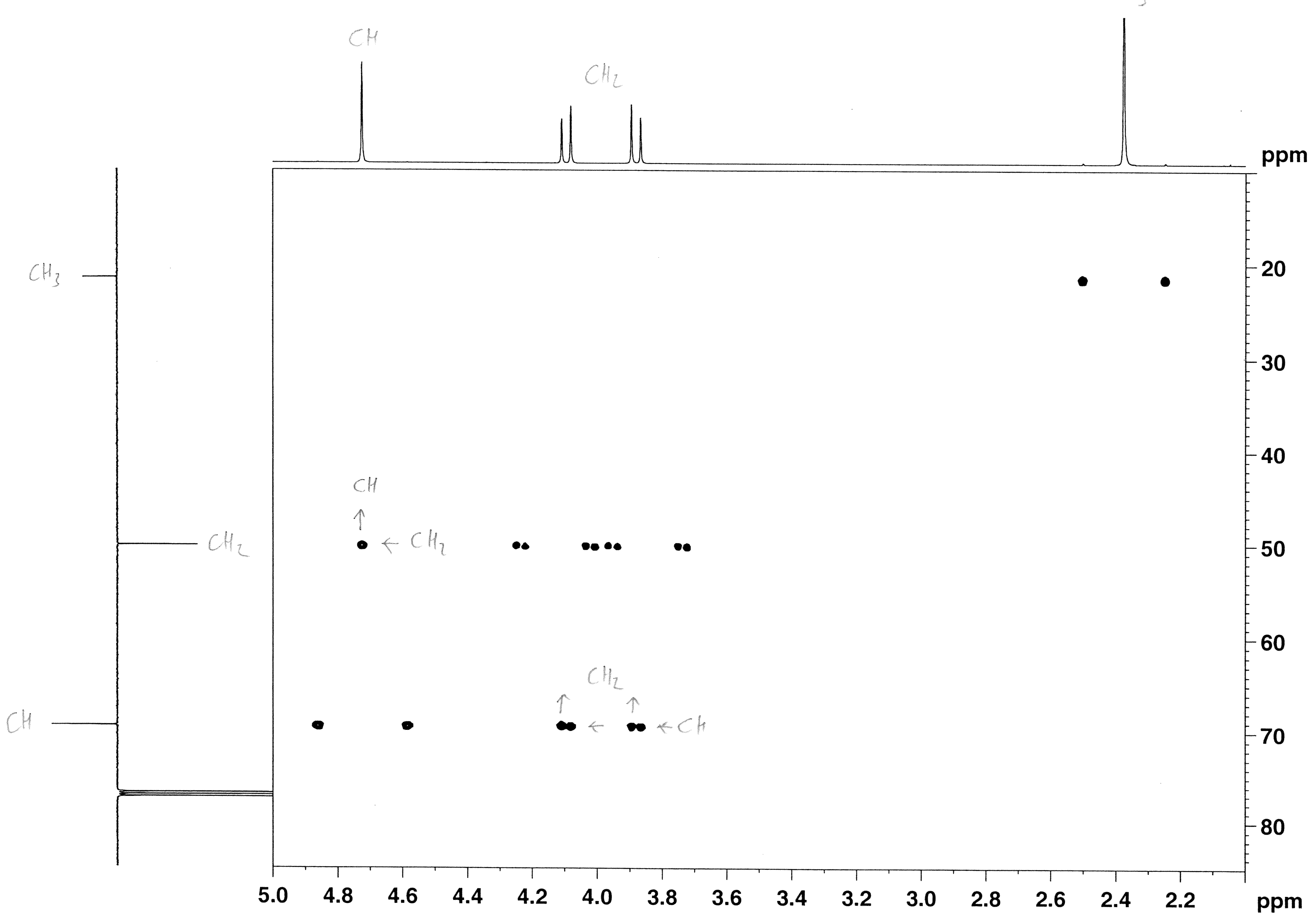

Figure S53c. NMR spectra of compound **5n.**

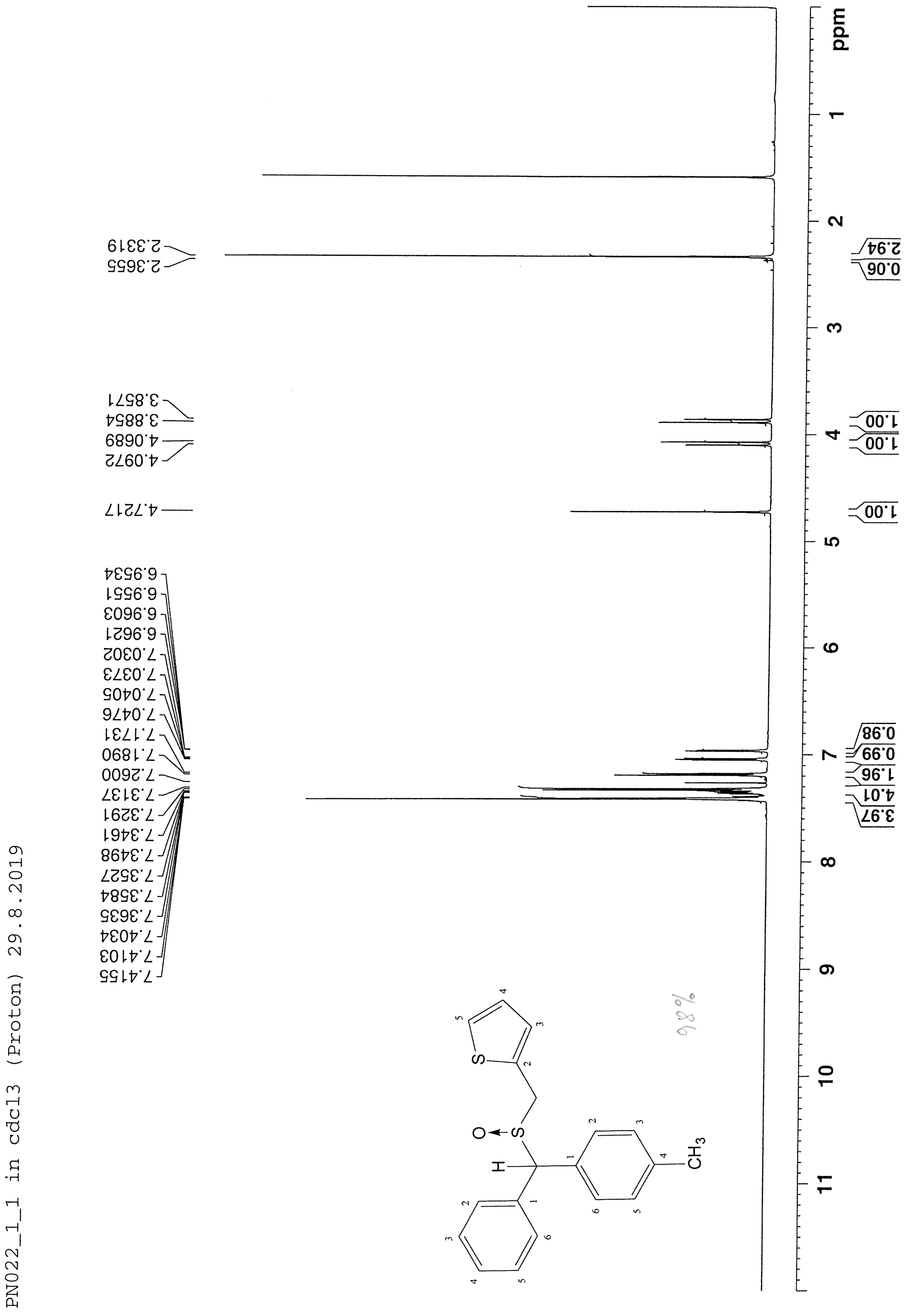

PN022_1_1 in cdcl3 (Proton) 29.8.2019

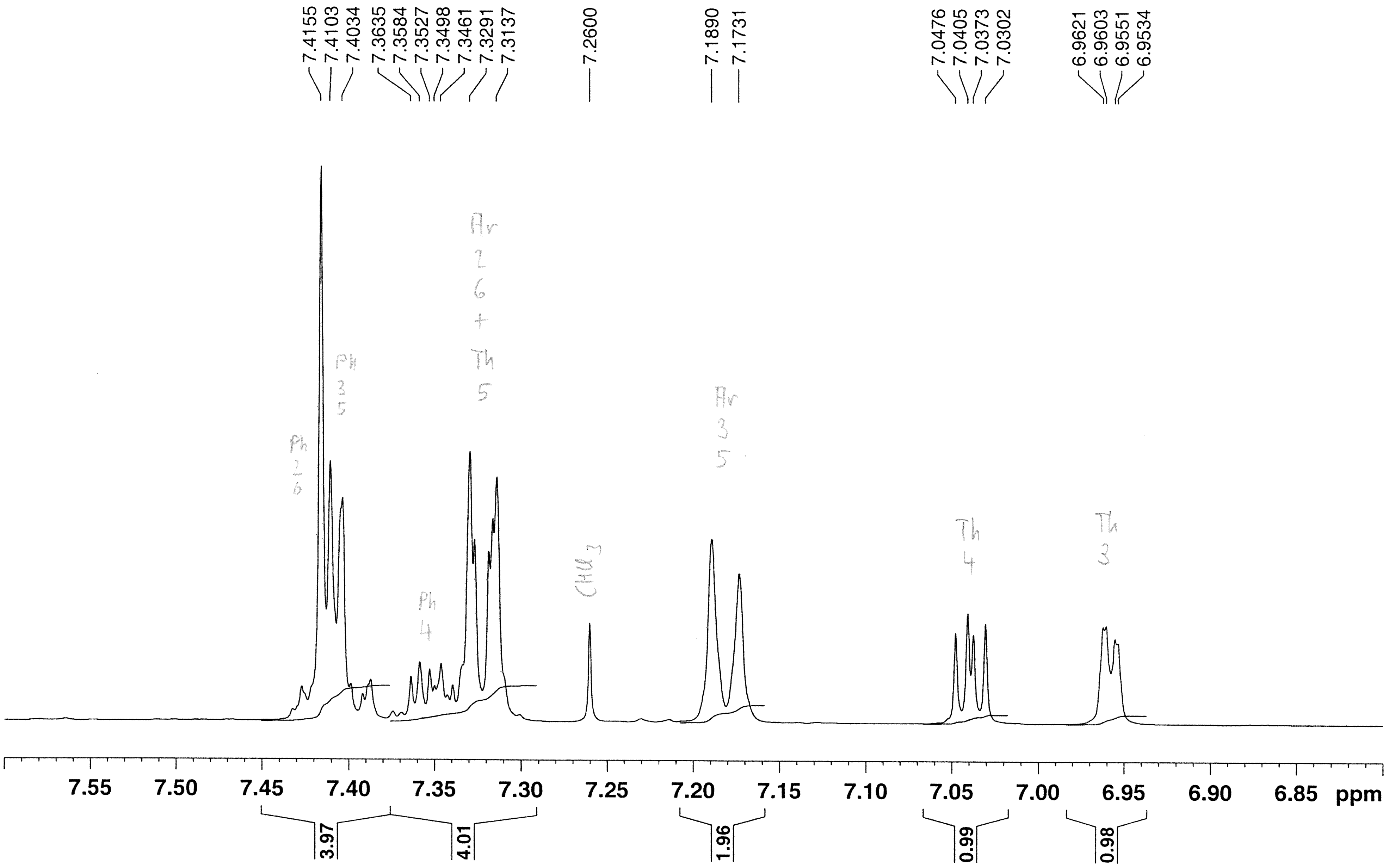

PN022_1_1 in cdcl3 (Proton) 29.8.2019

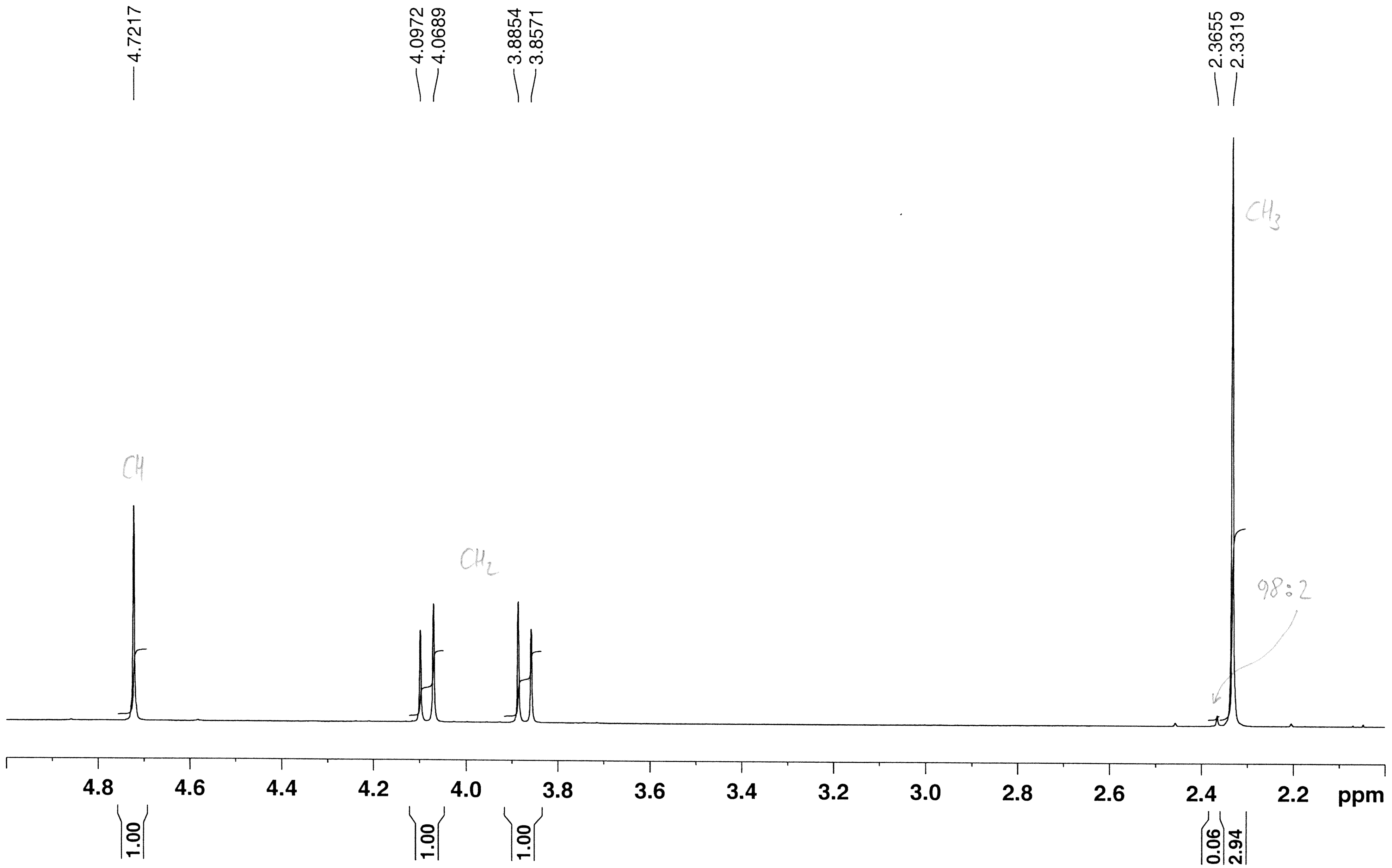

PN022_1_1 in cdcl3 (APT) 29.8.2019

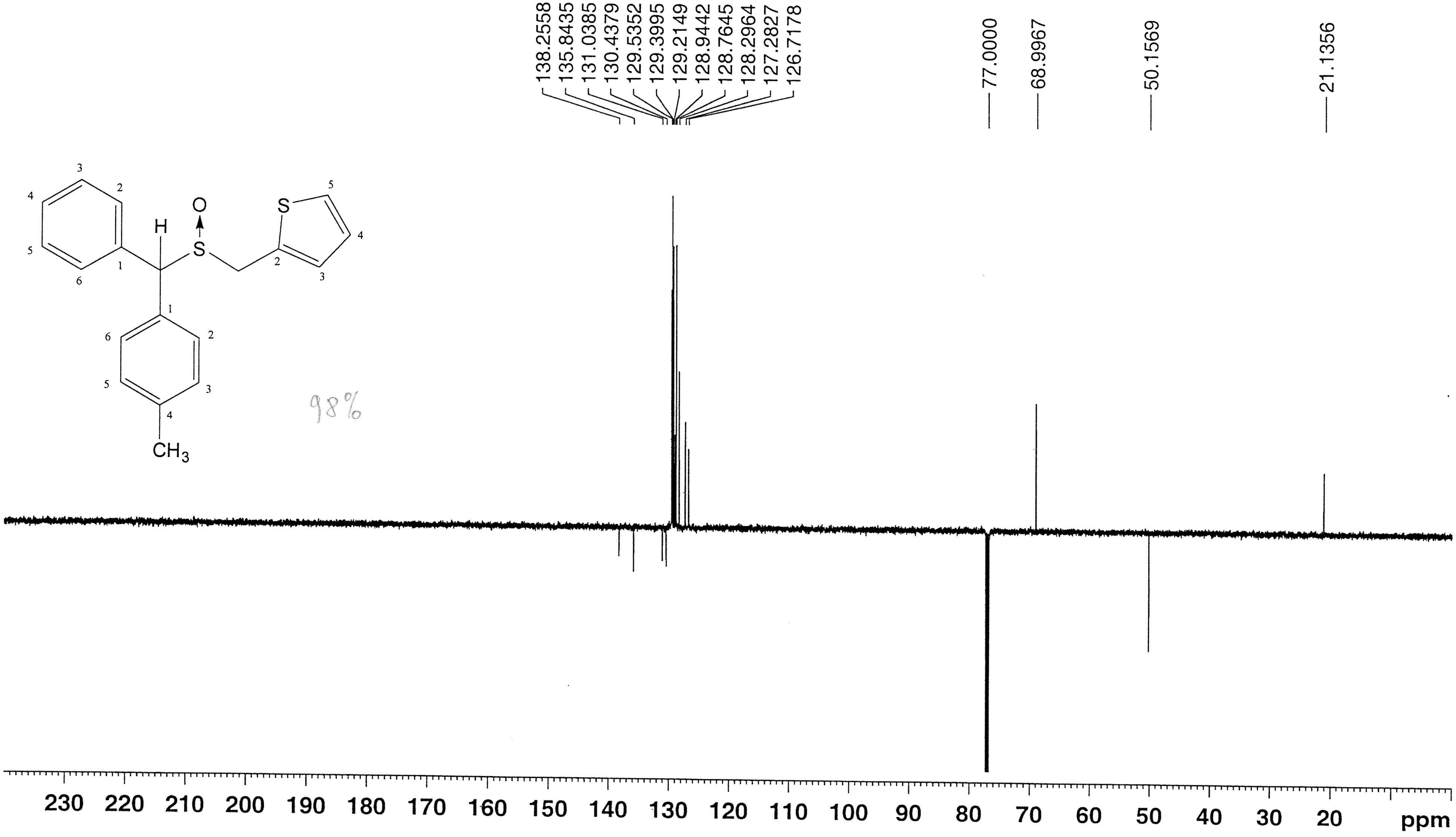

PN022_1_1 in cdcl3 (APT) 29.8.2019

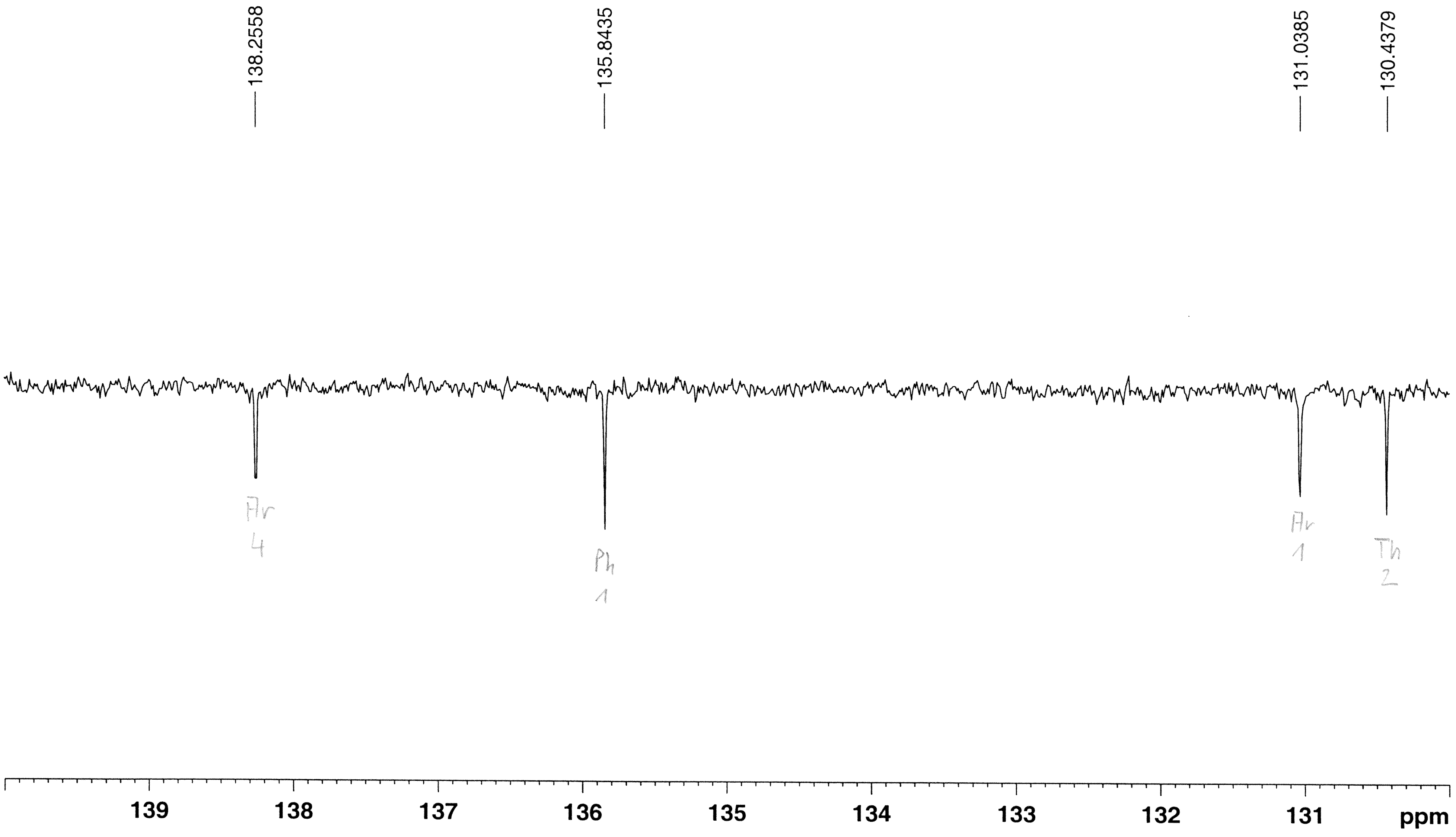

PN022_1_1 in cdcl3 (APT) 29.8.2019

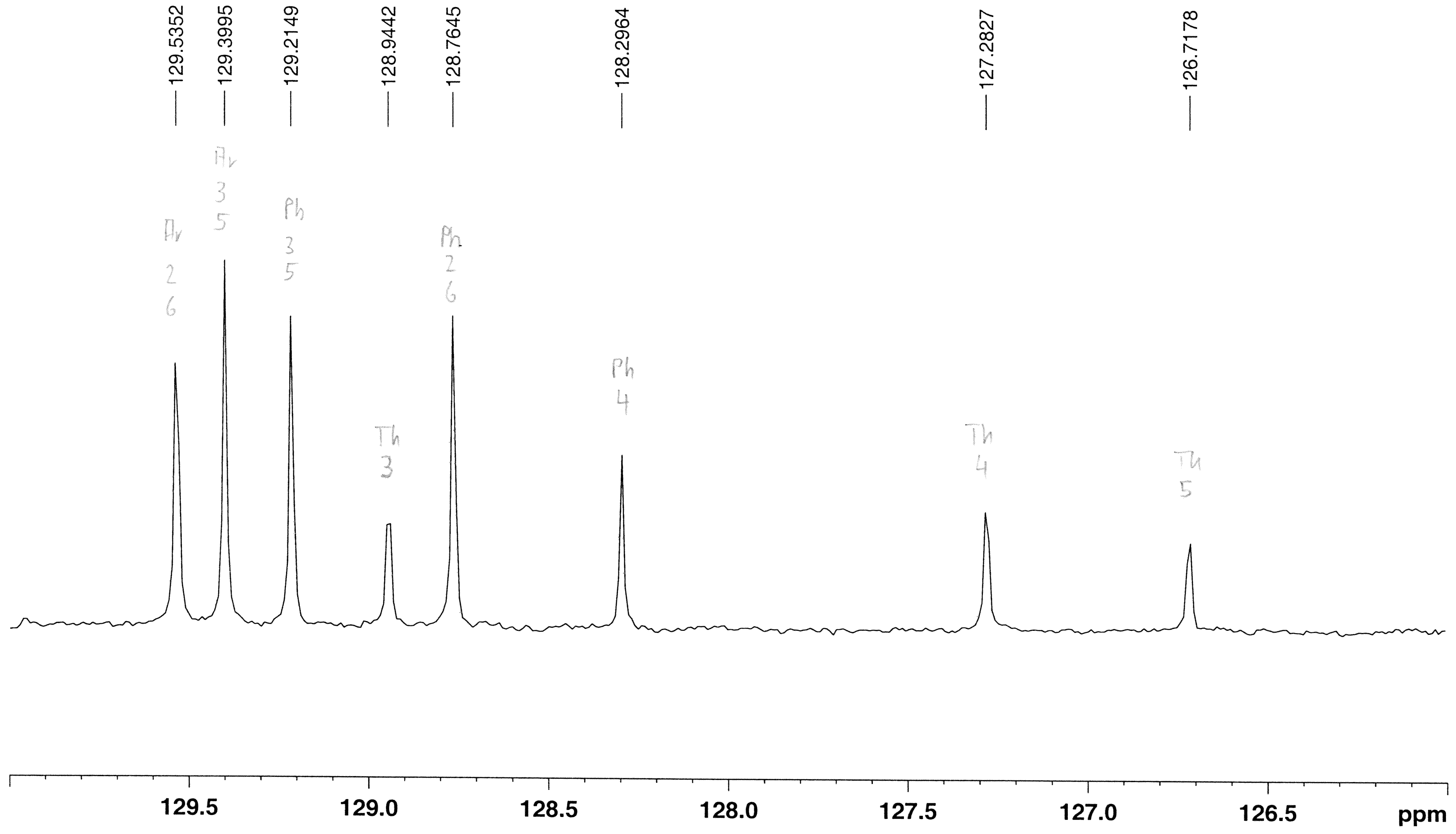

PN022_1_1 in cdcl3 (APT) 29.8.2019

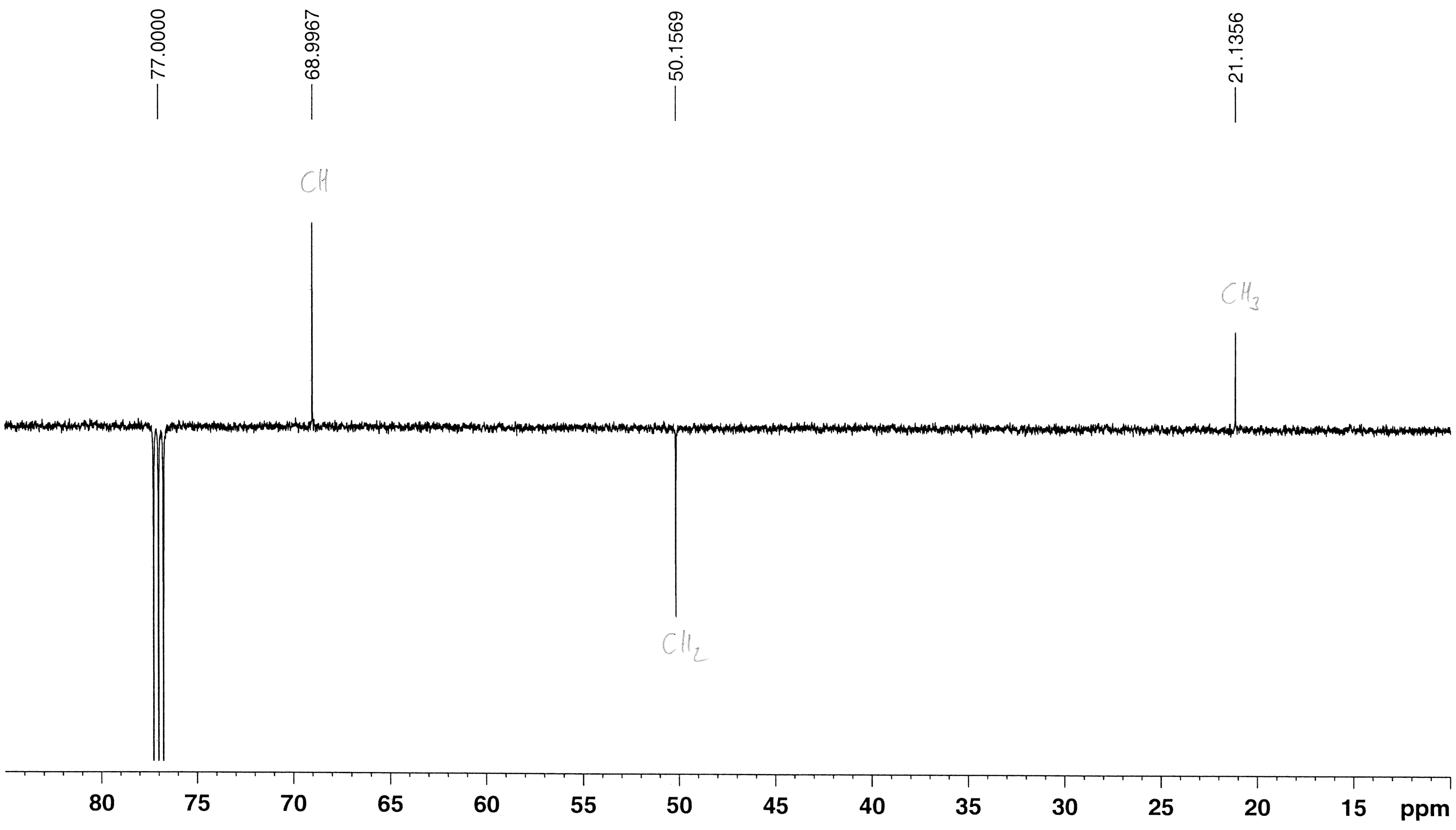

PN022_1_1 in cdcl3 (COSY) 29.8.2019

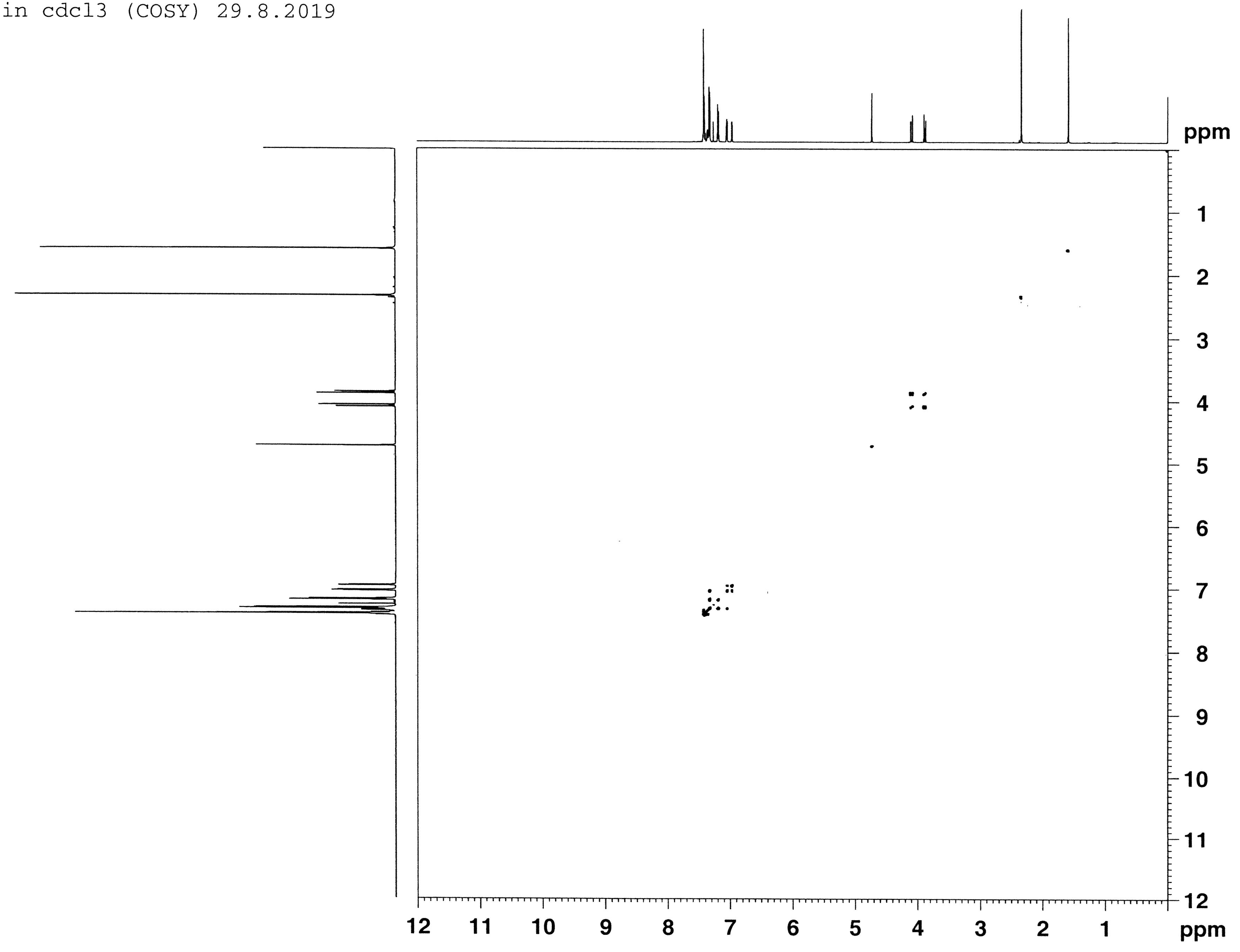

PN022_1_1 in cdcl3 (COSY) 29.8.2019

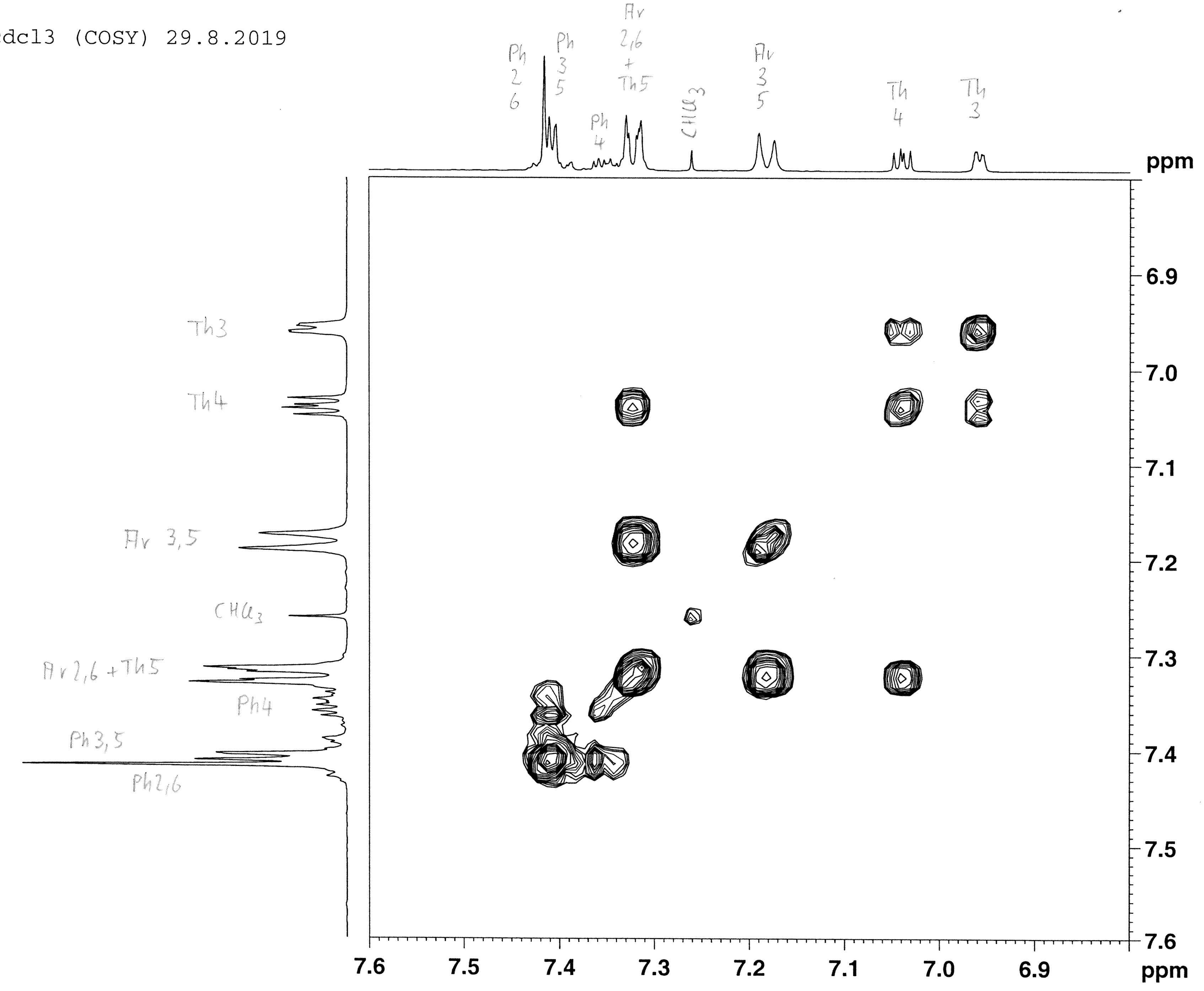

PN022_1_1 in cdcl3 (COSY) 29.8.2019

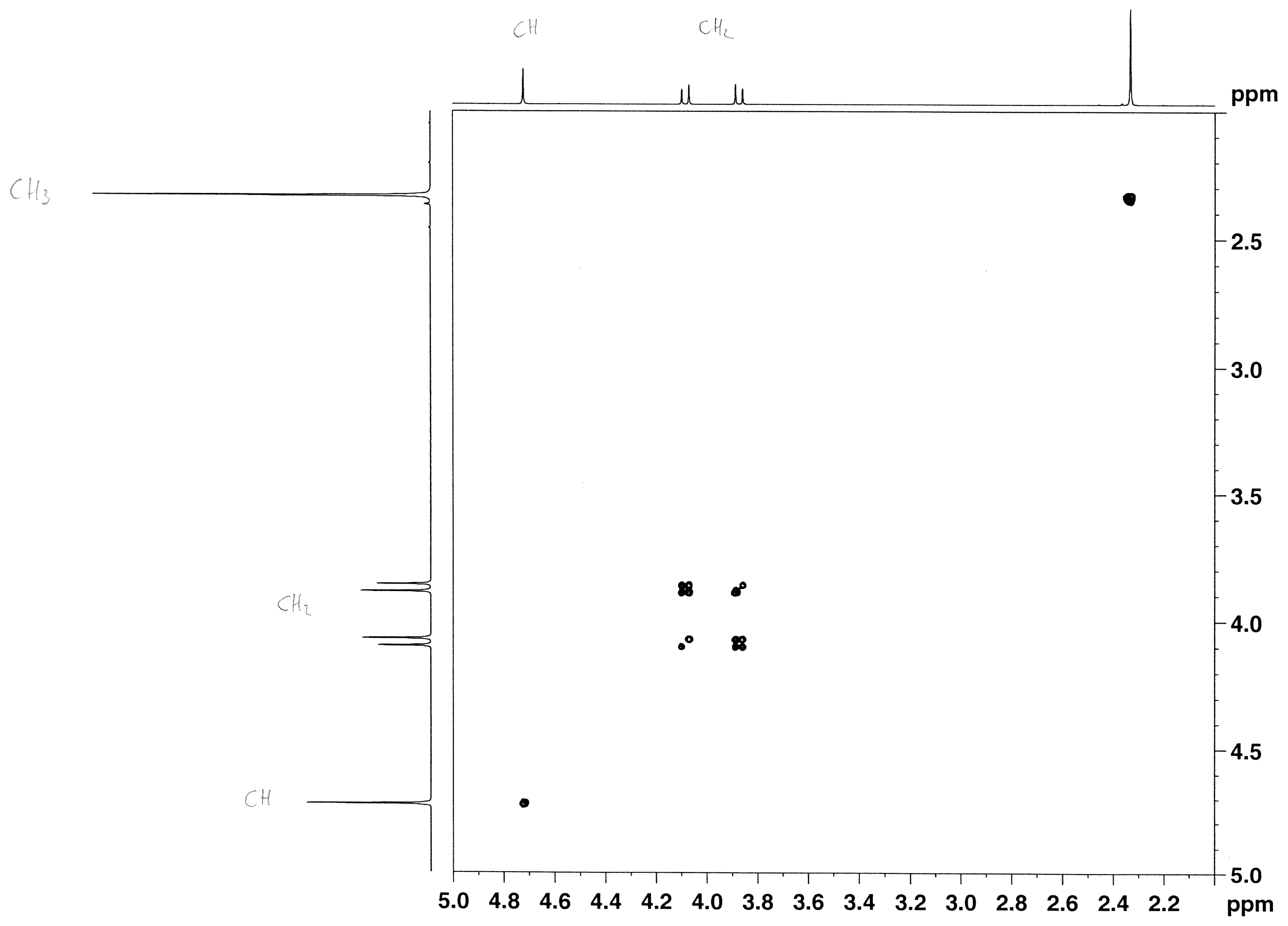

PN022_1_1 in cdcl3 (HSQC) 29.8.2019

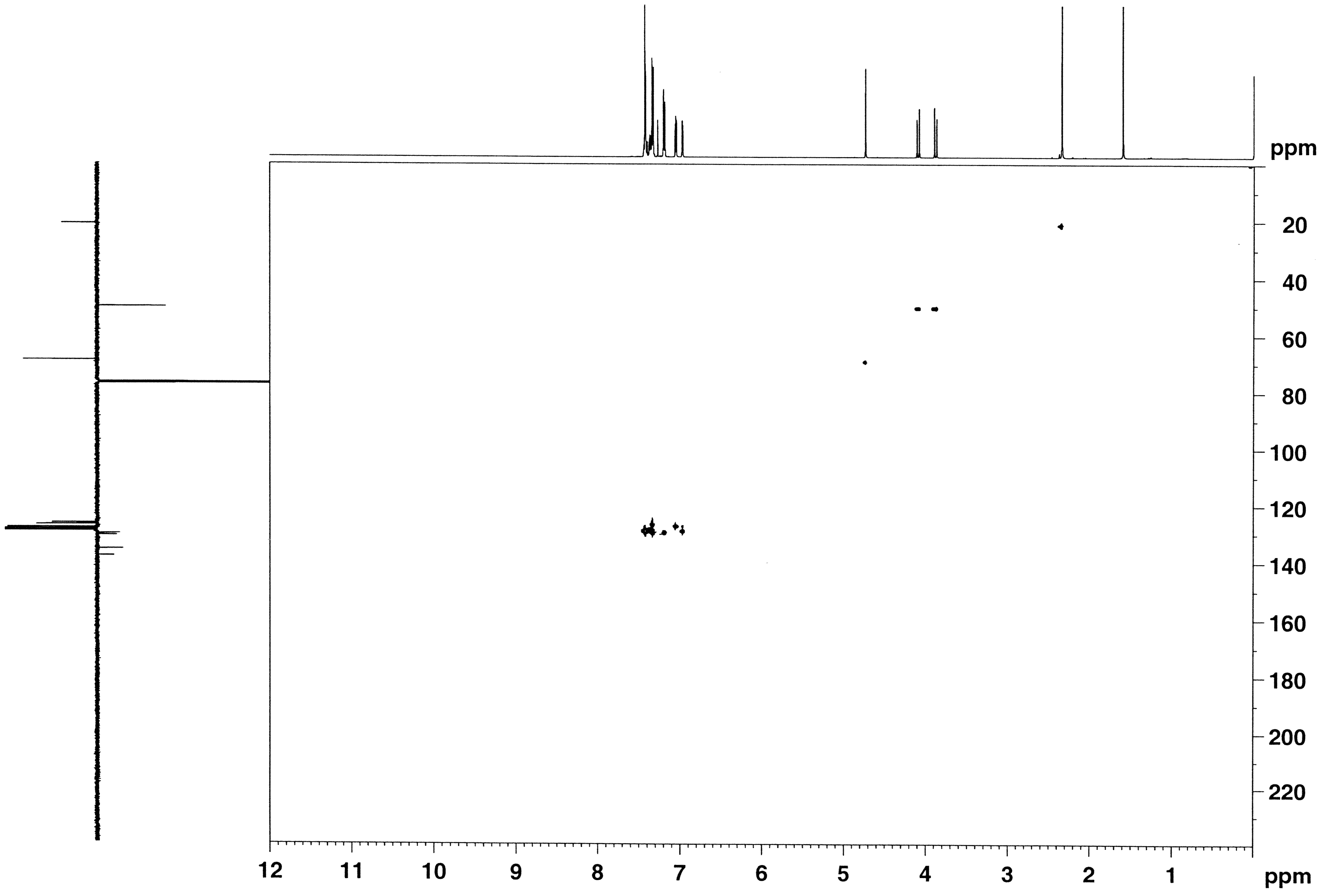

PN022_1_1 in cdcl3 (HSQC) 29.8.2019

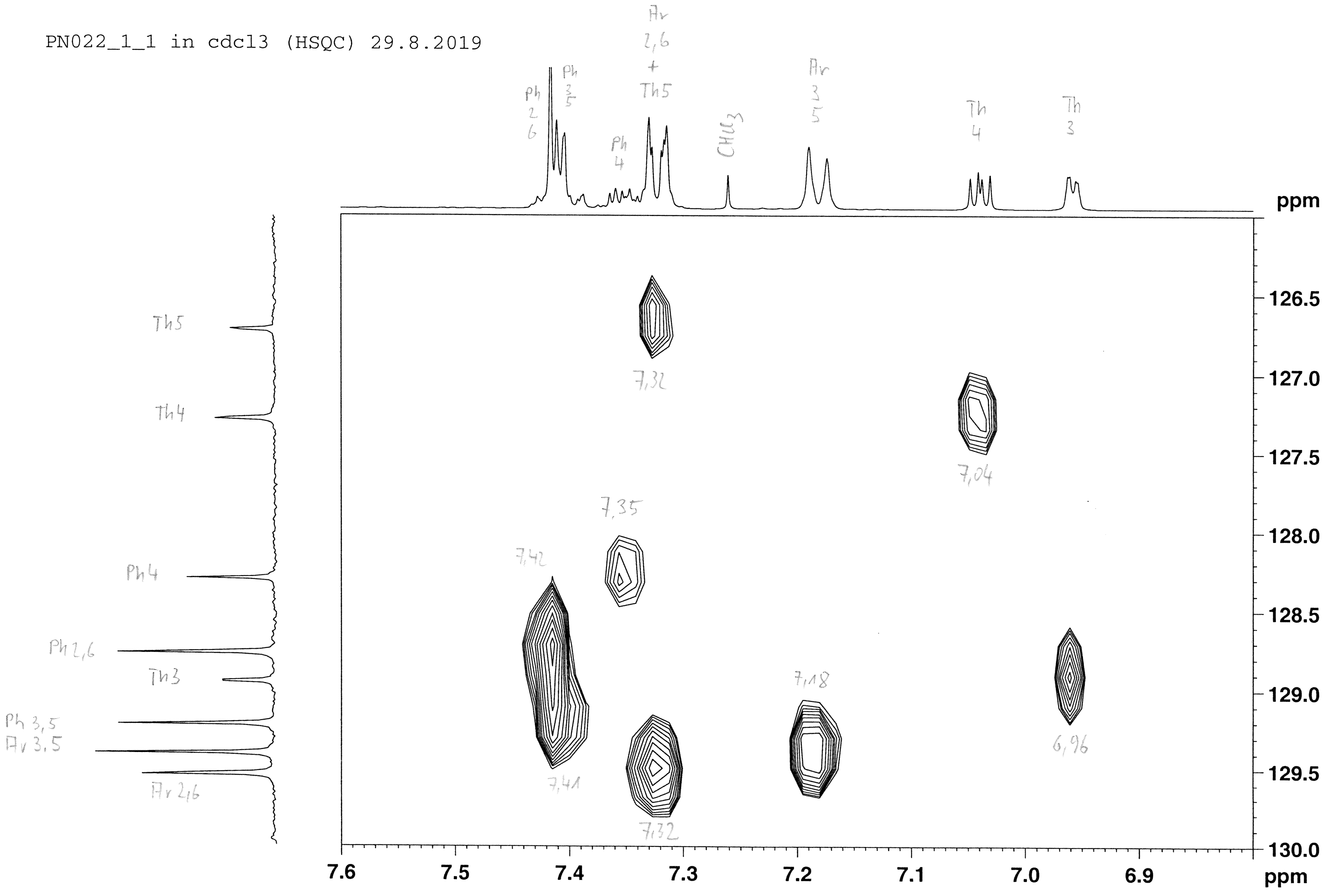

PN022_1_1 in cdcl3 (HSQC) 29.8.2019

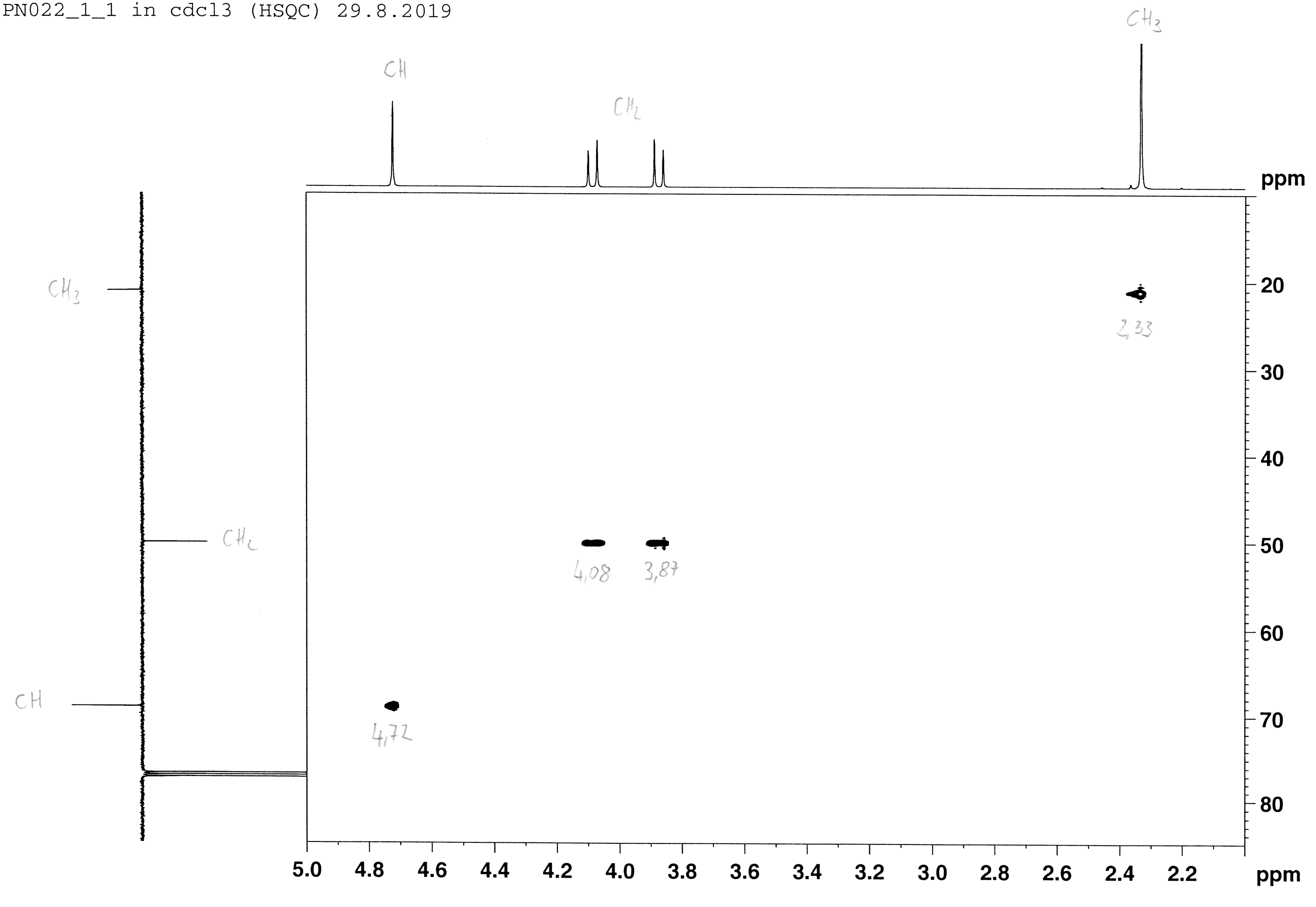

PN022_1_1 in cdcl3 (HMBC) 29.8.2019

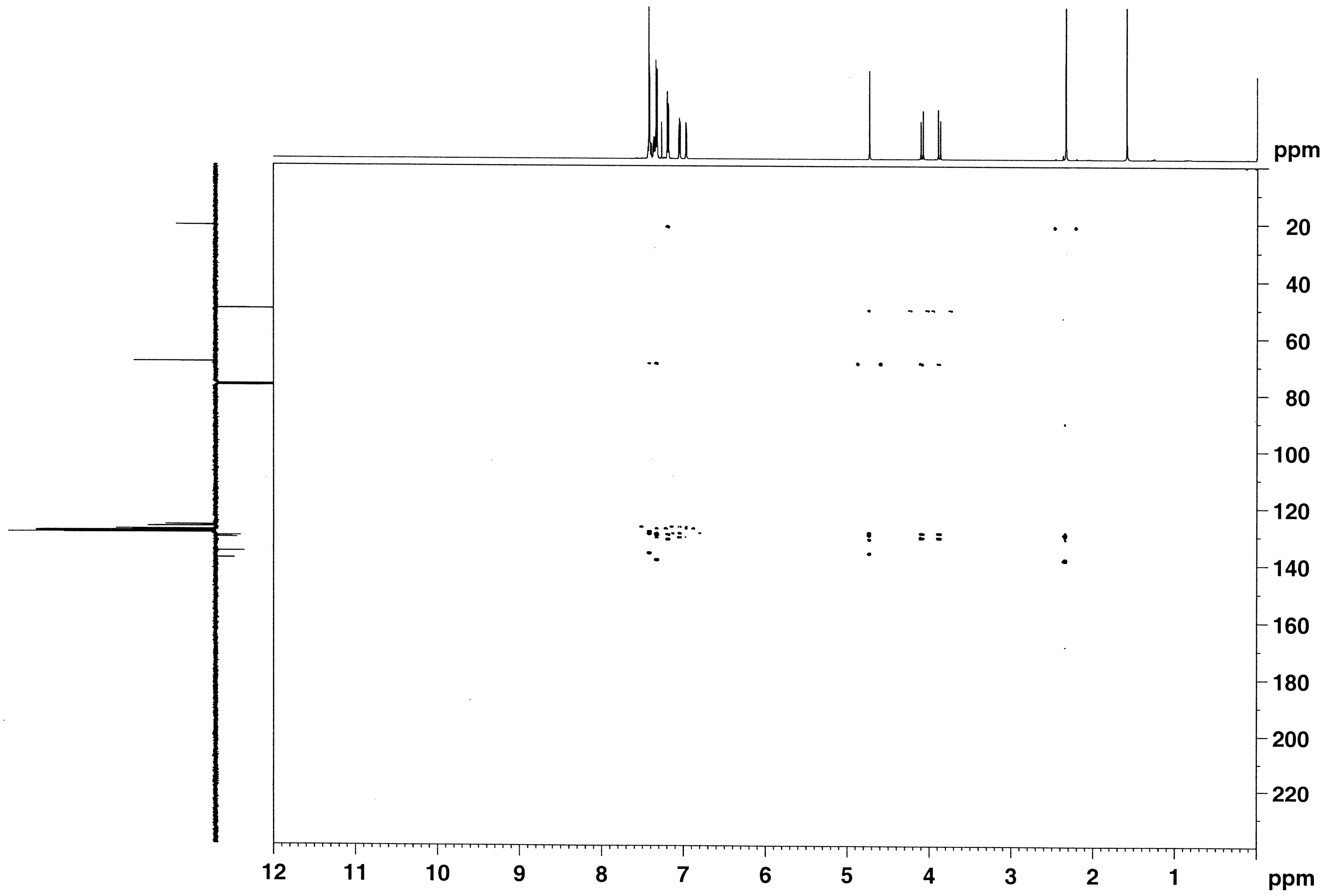

PN022_1_1 in cdcl3 (HMBC) 29.8.2019

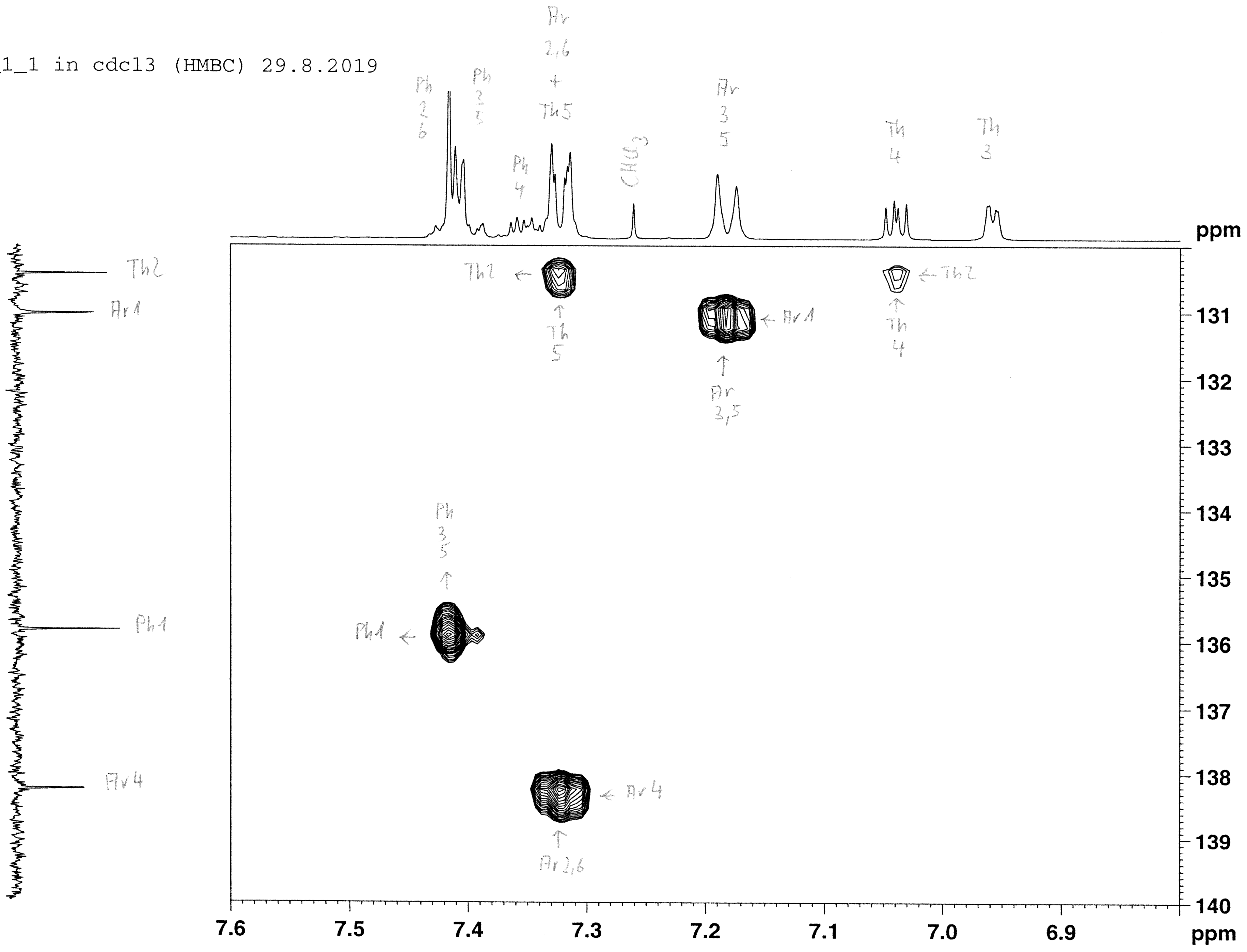

PN022_1_1 in cdcl3 (HMBC) 29.8.2019

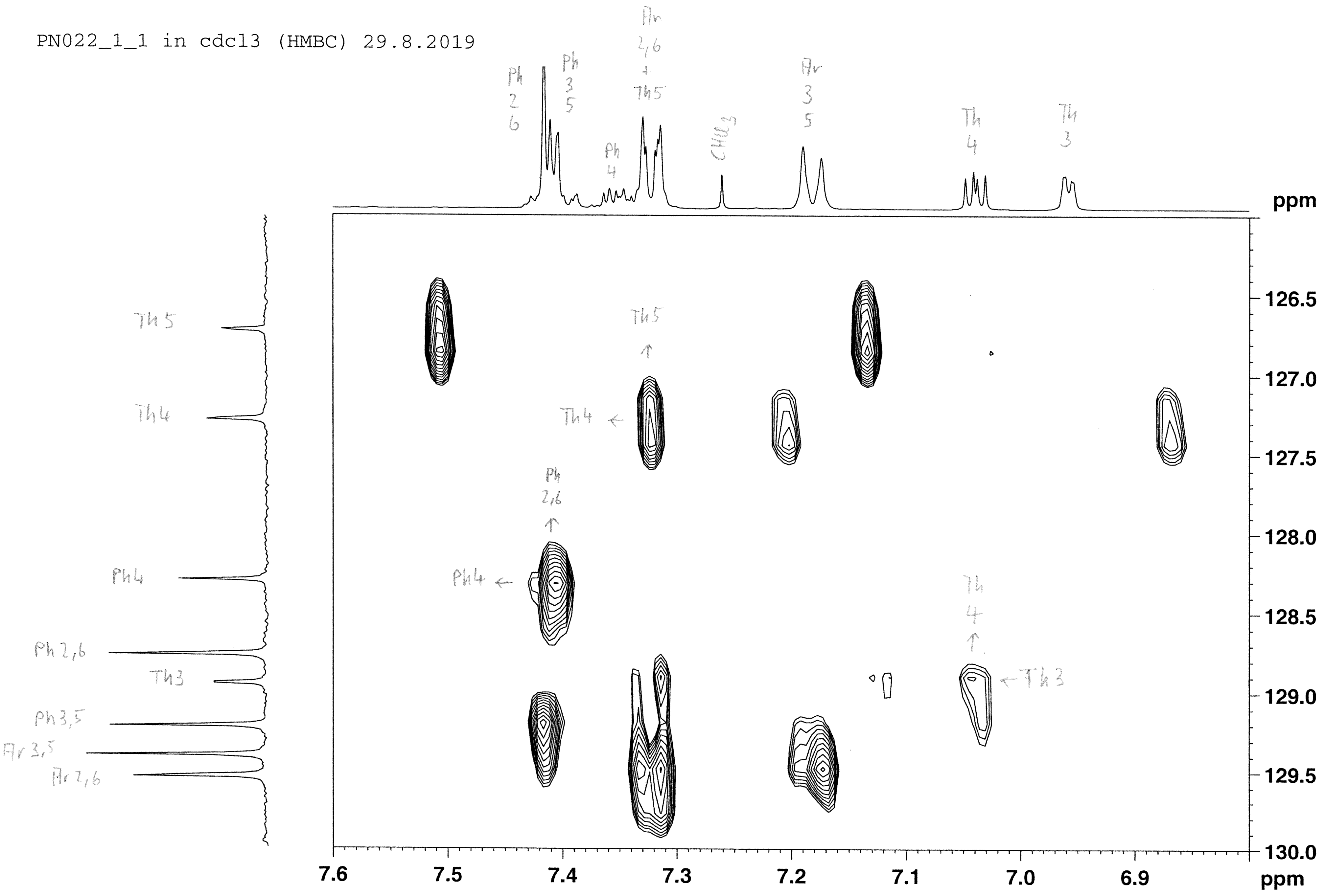

PN022_1_1 in cdcl3 (HMBC) 29.8.2019

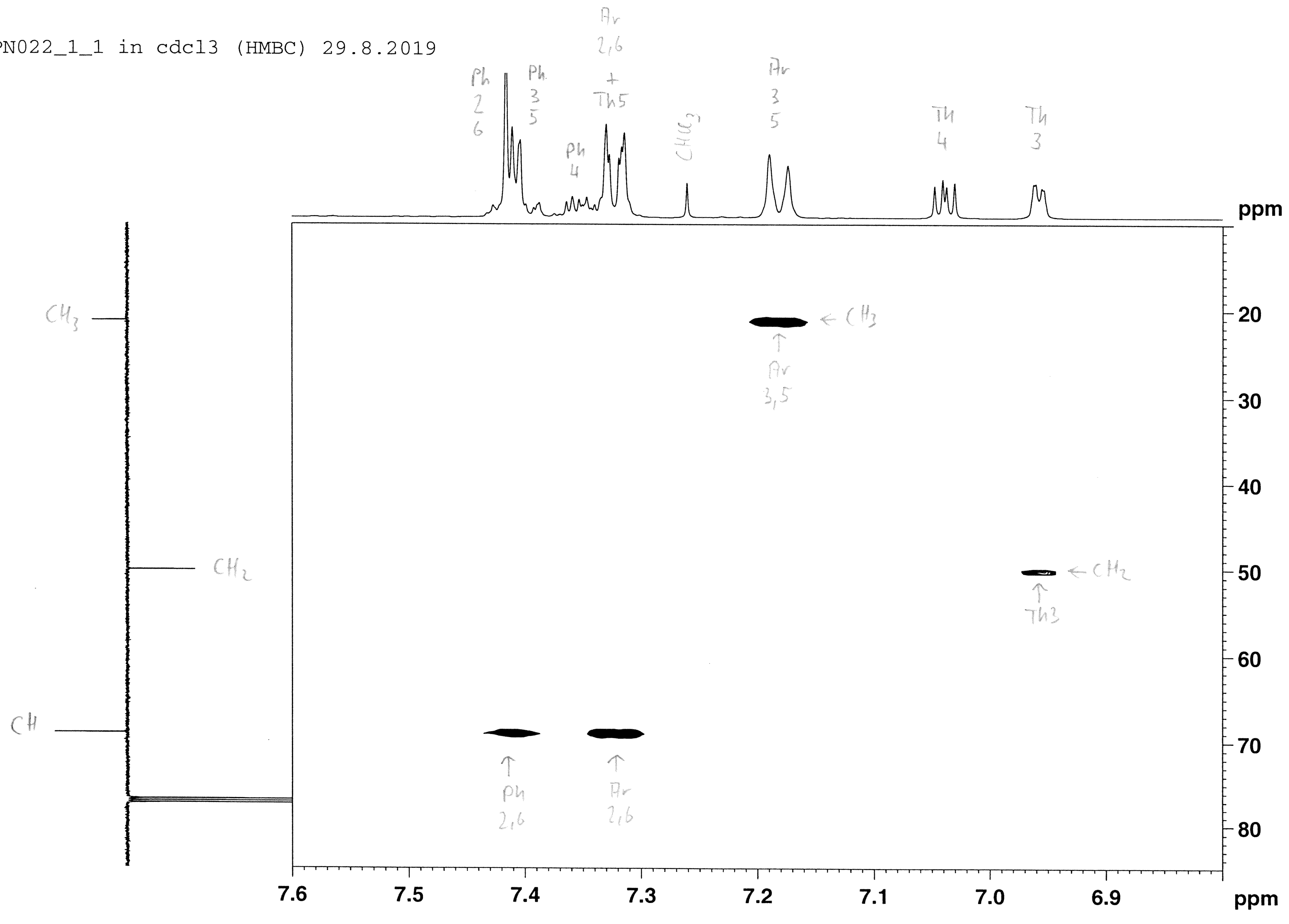

PN022_1_1 in cdcl3 (HMBC) 29.8.2019

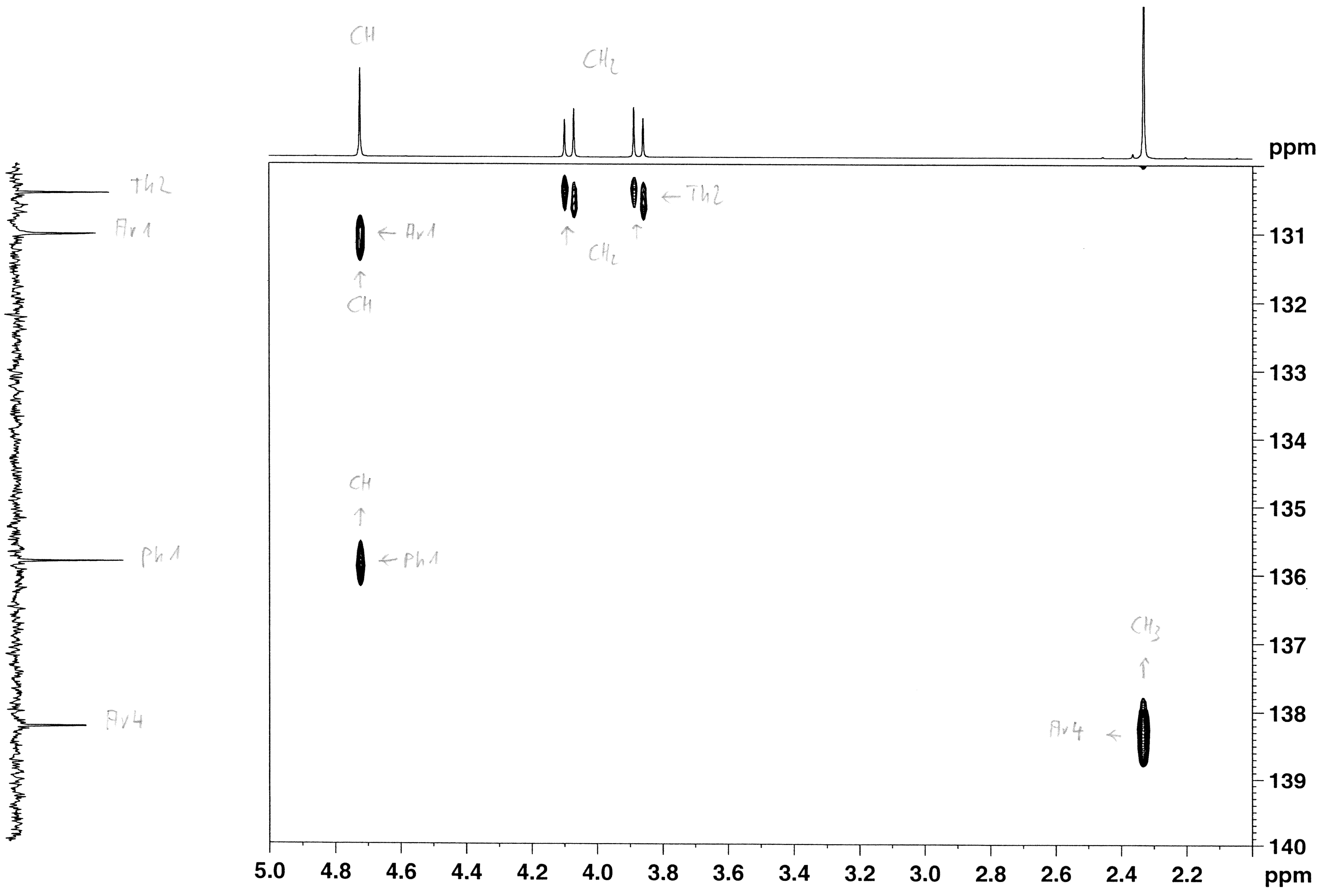

PN022_1_1 in cdcl3 (HMBC) 29.8.2019

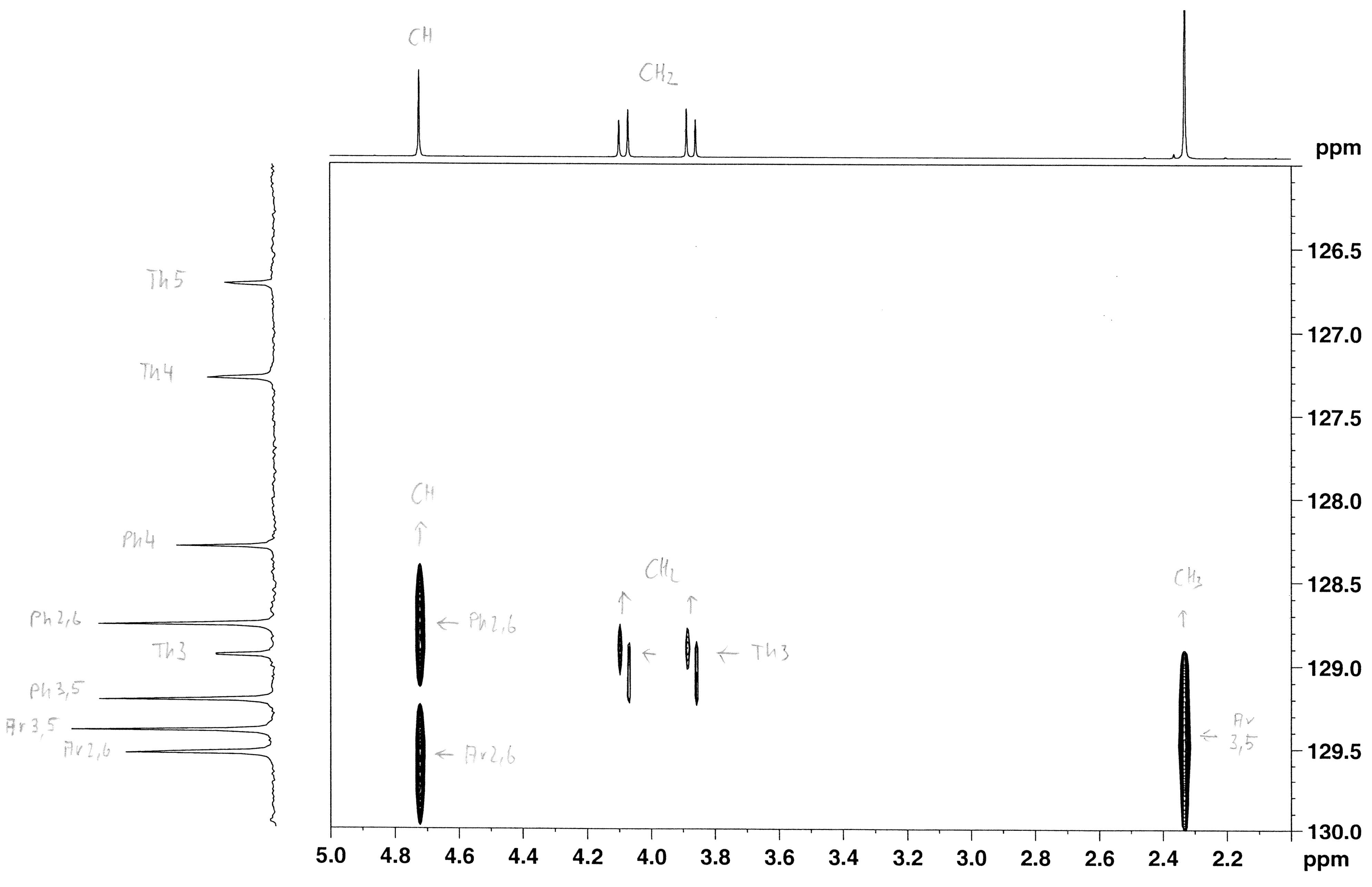

PN022_1_1 in cdcl3 (HMBC) 29.8.2019

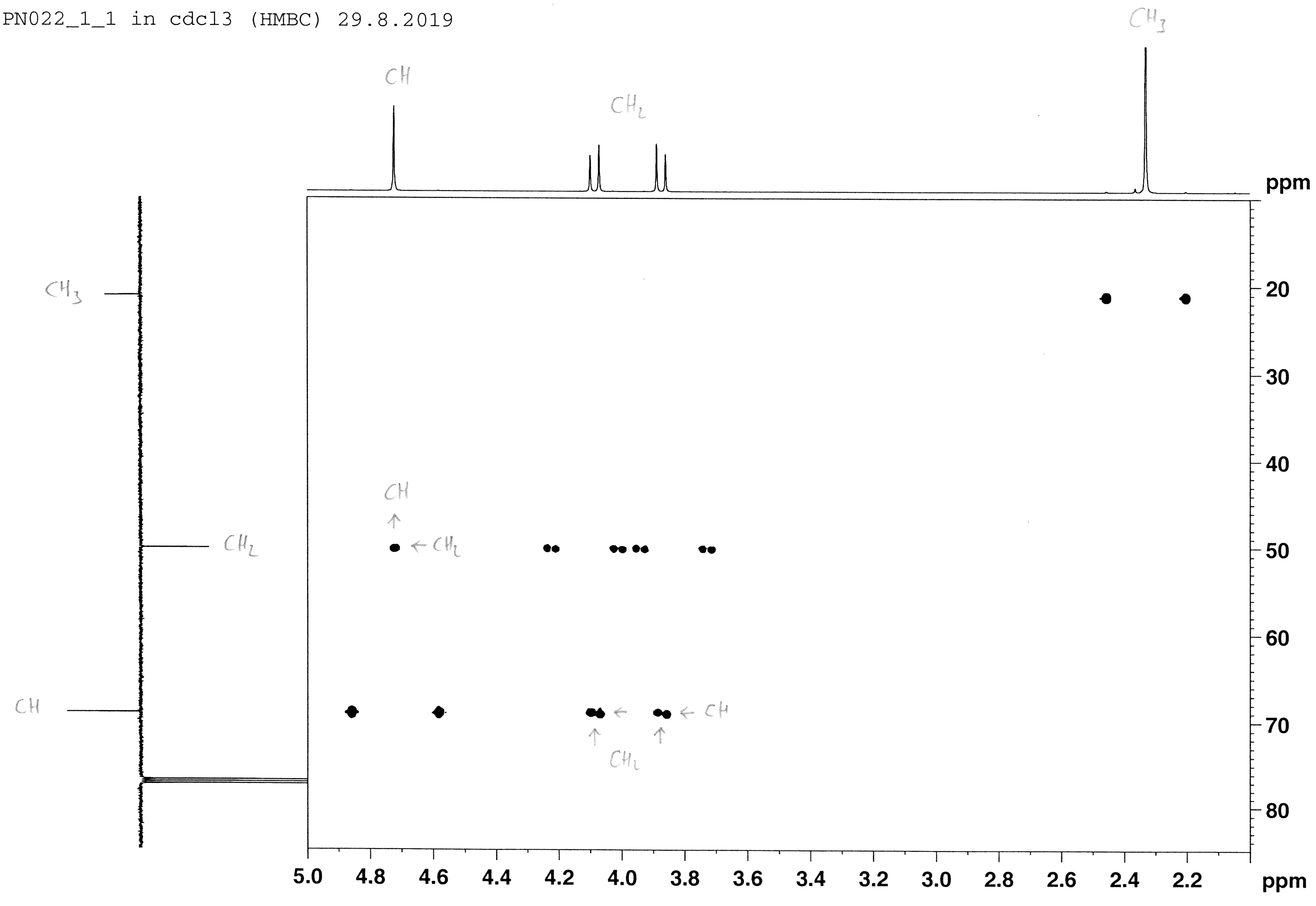

Figure S54c. NMR spectra of compound **6n**.

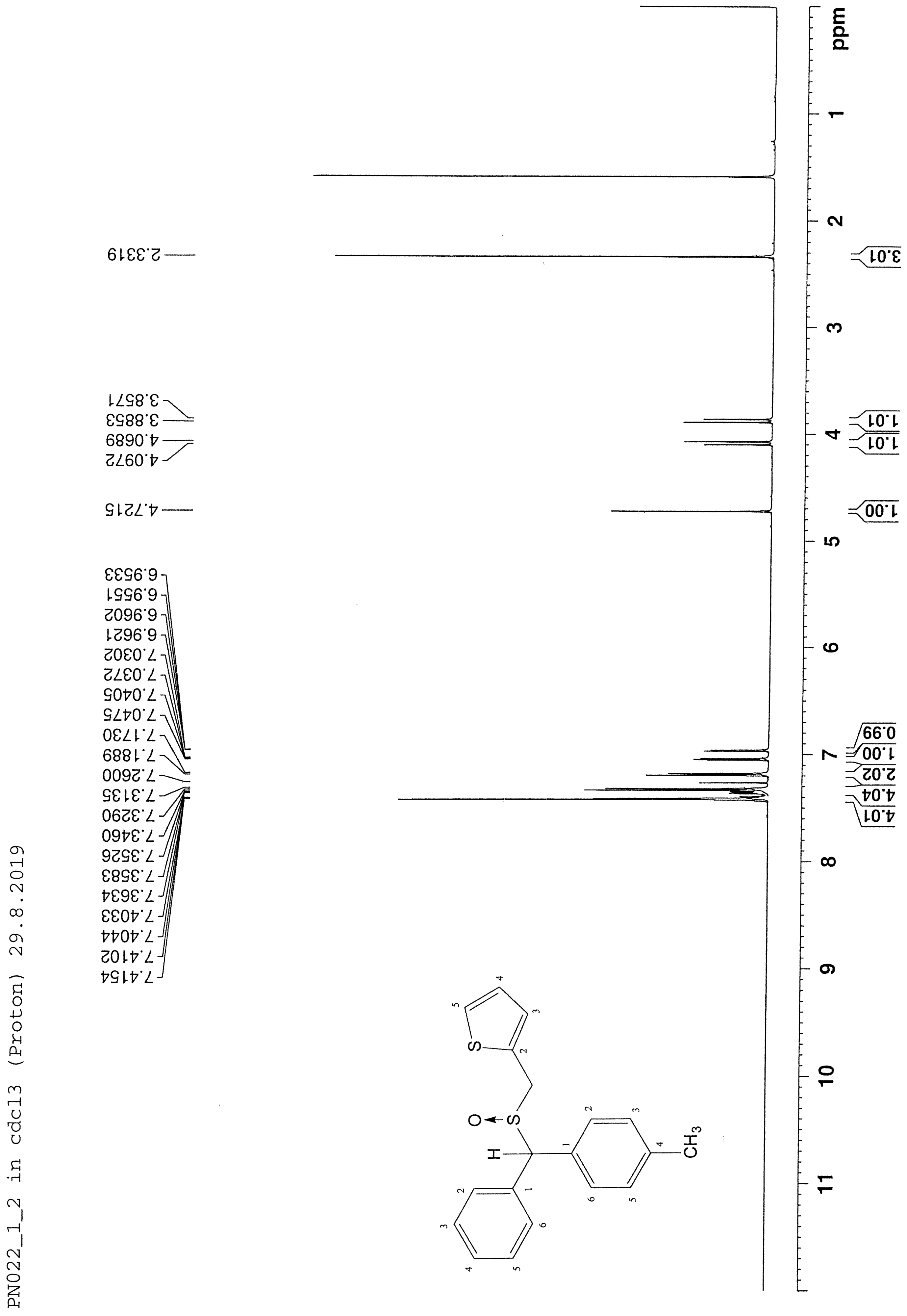

PN022_1_2 in cdcl3 (Proton) 29.8.2019

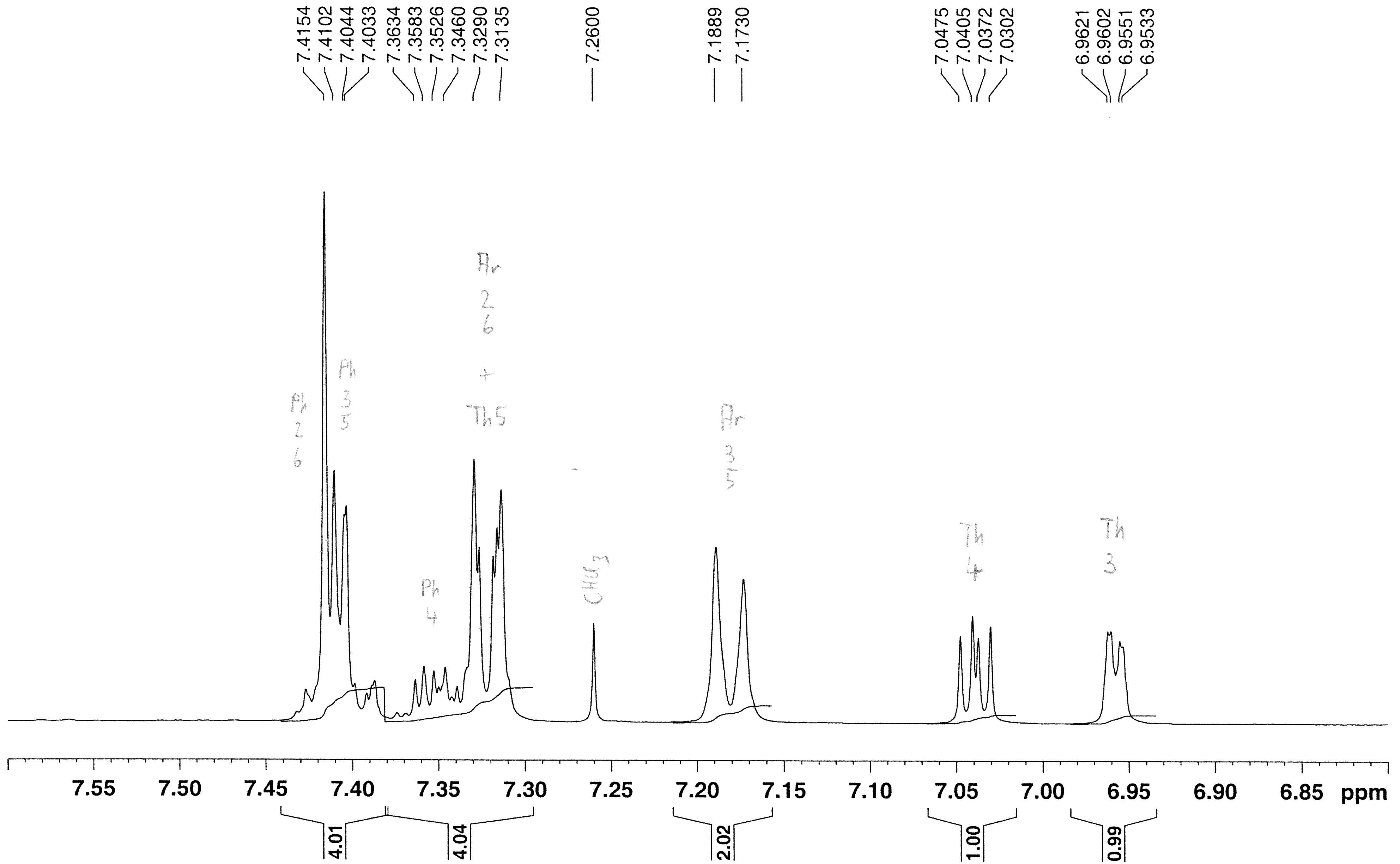

PN022_1_2 in cdcl3 (Proton) 29.8.2019

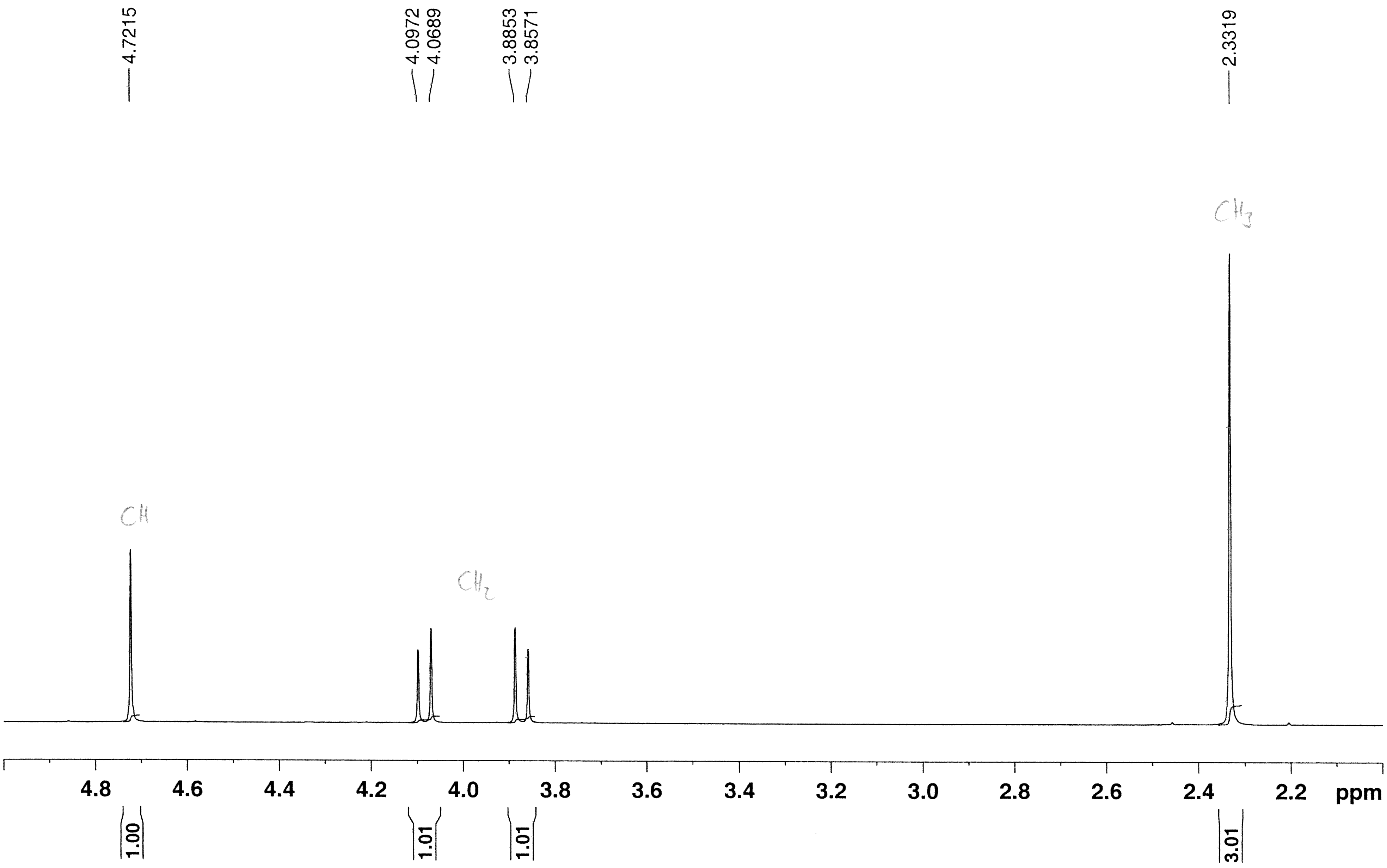

PN022_1_2 in cdcl3 (APT) 29.8.2019

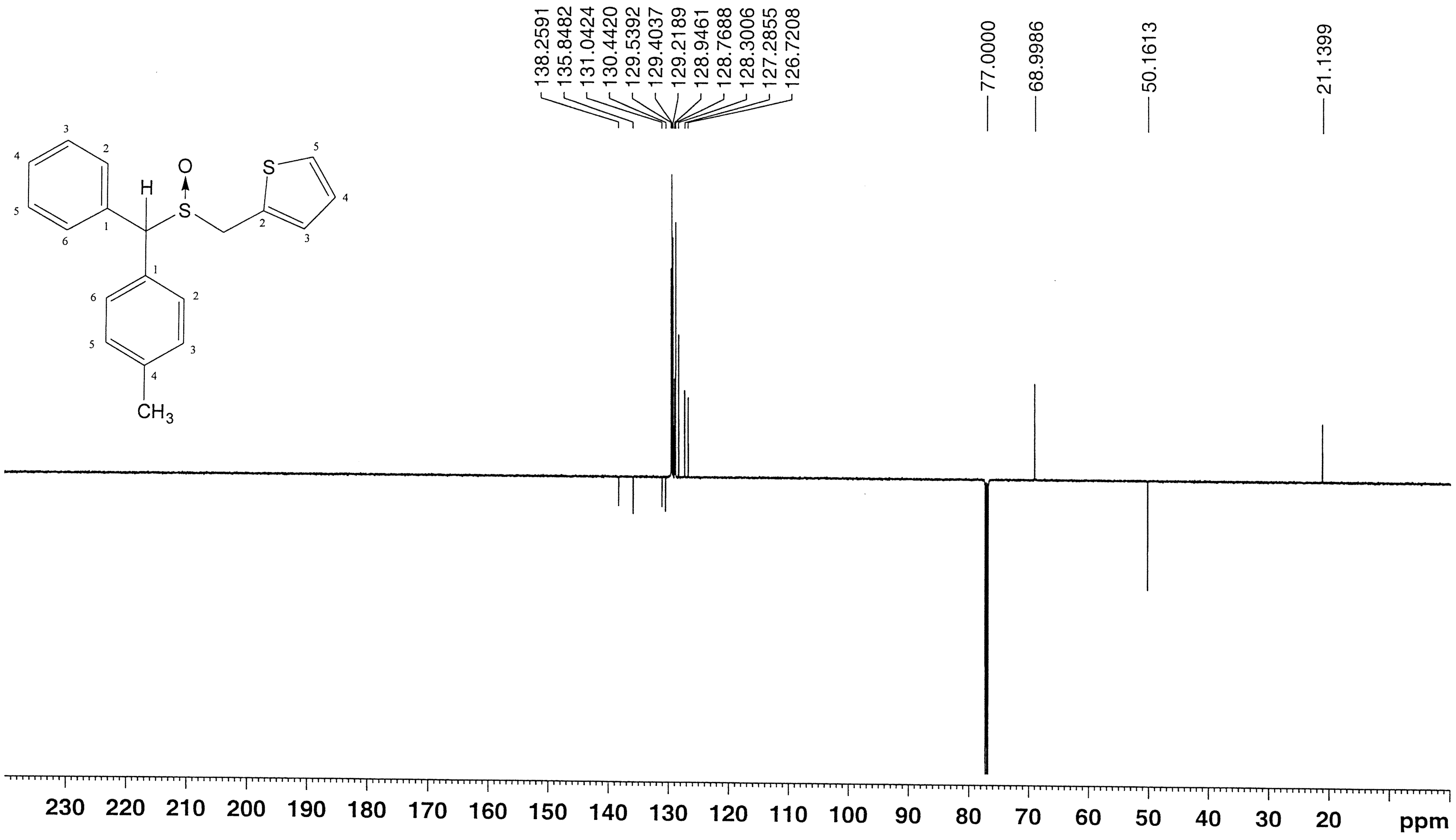

PN022_1_2 in cdcl3 (APT) 29.8.2019

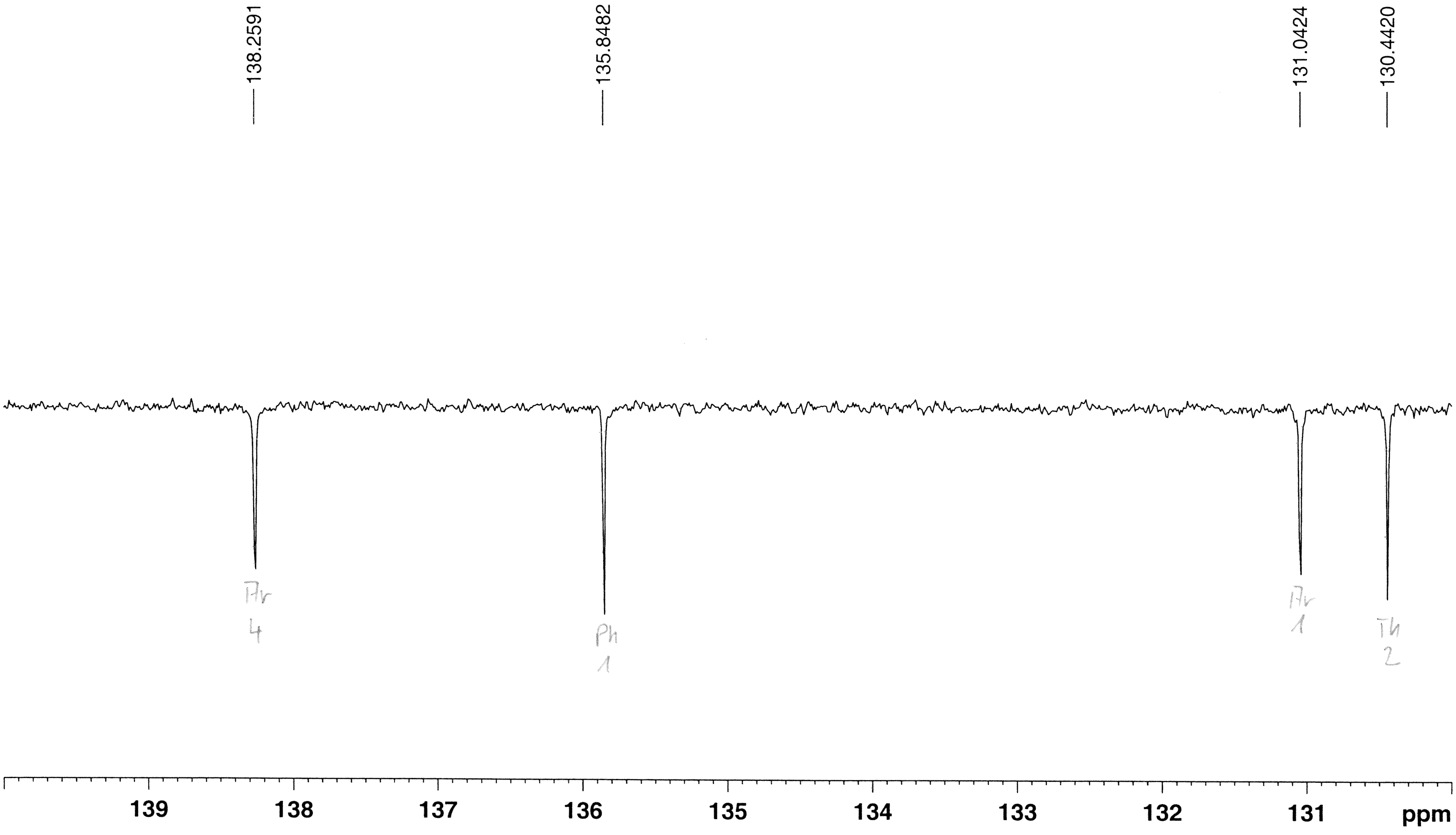

PN022_1_2 in cdcl3 (APT) 29.8.2019

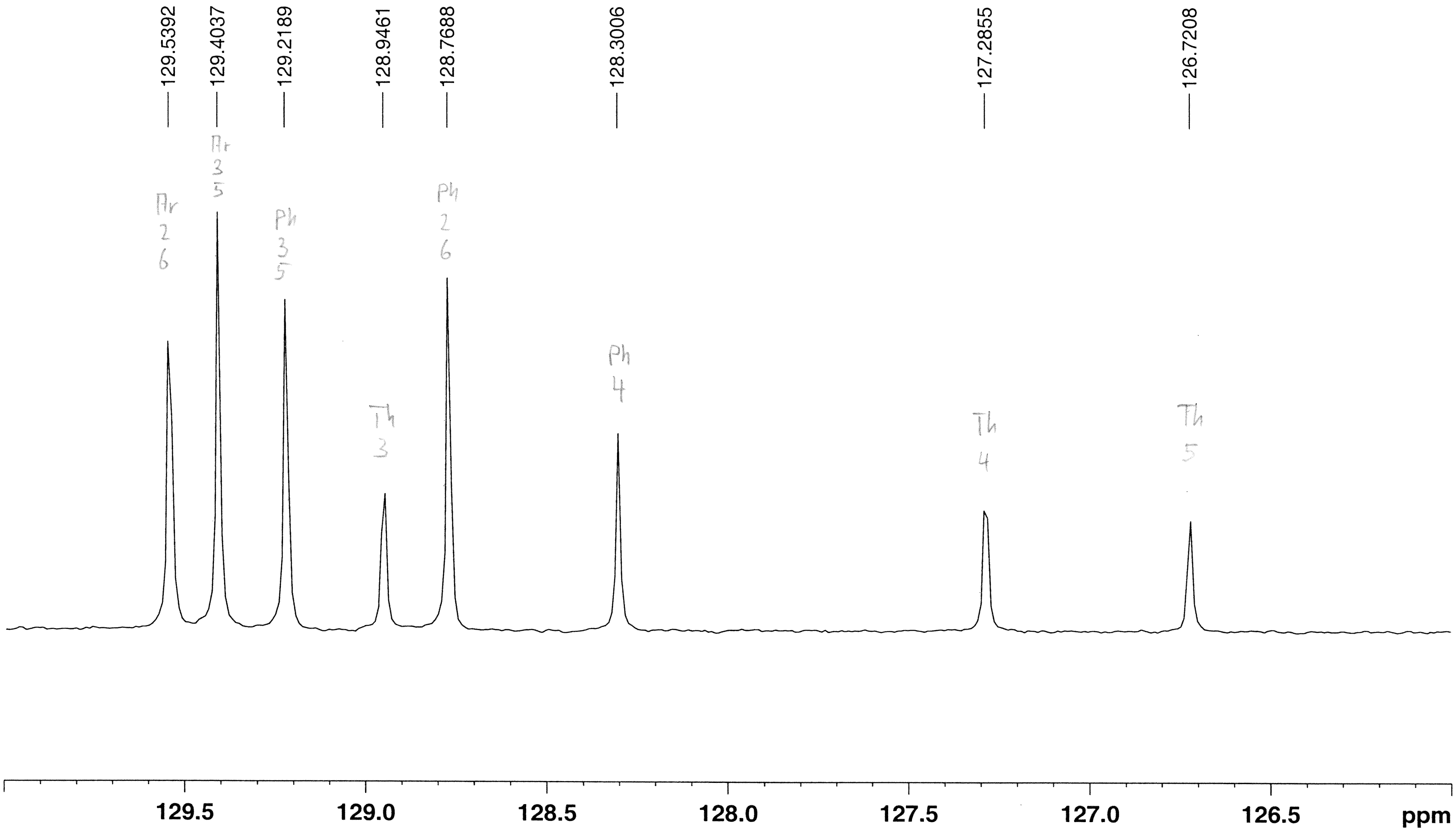

PN022_1_2 in cdcl3 (APT) 29.8.2019

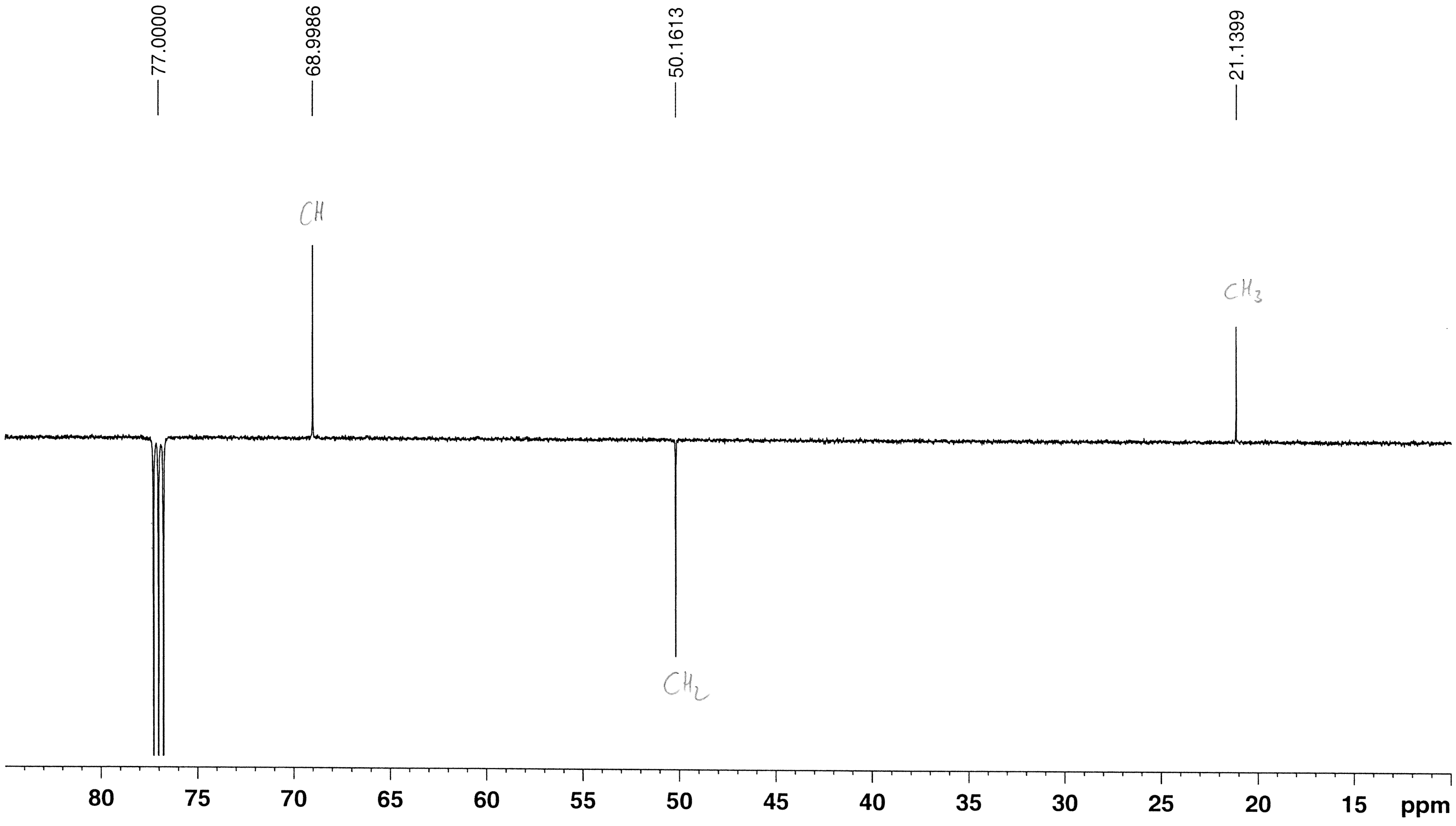

Figure S55c. NMR spectra of compound **7n.**

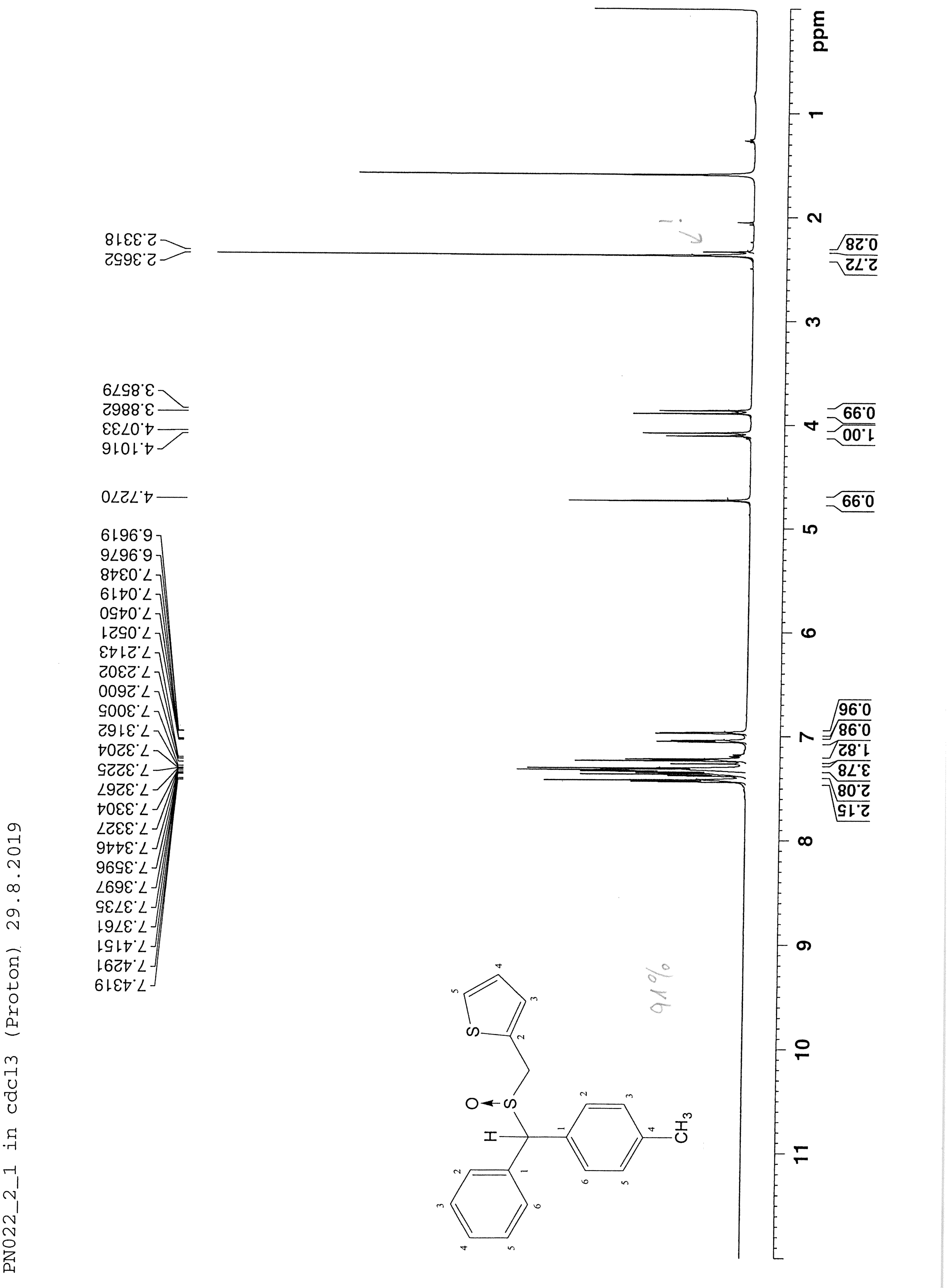

PN022_2_1 in cdcl3 (Proton) 29.8.2019

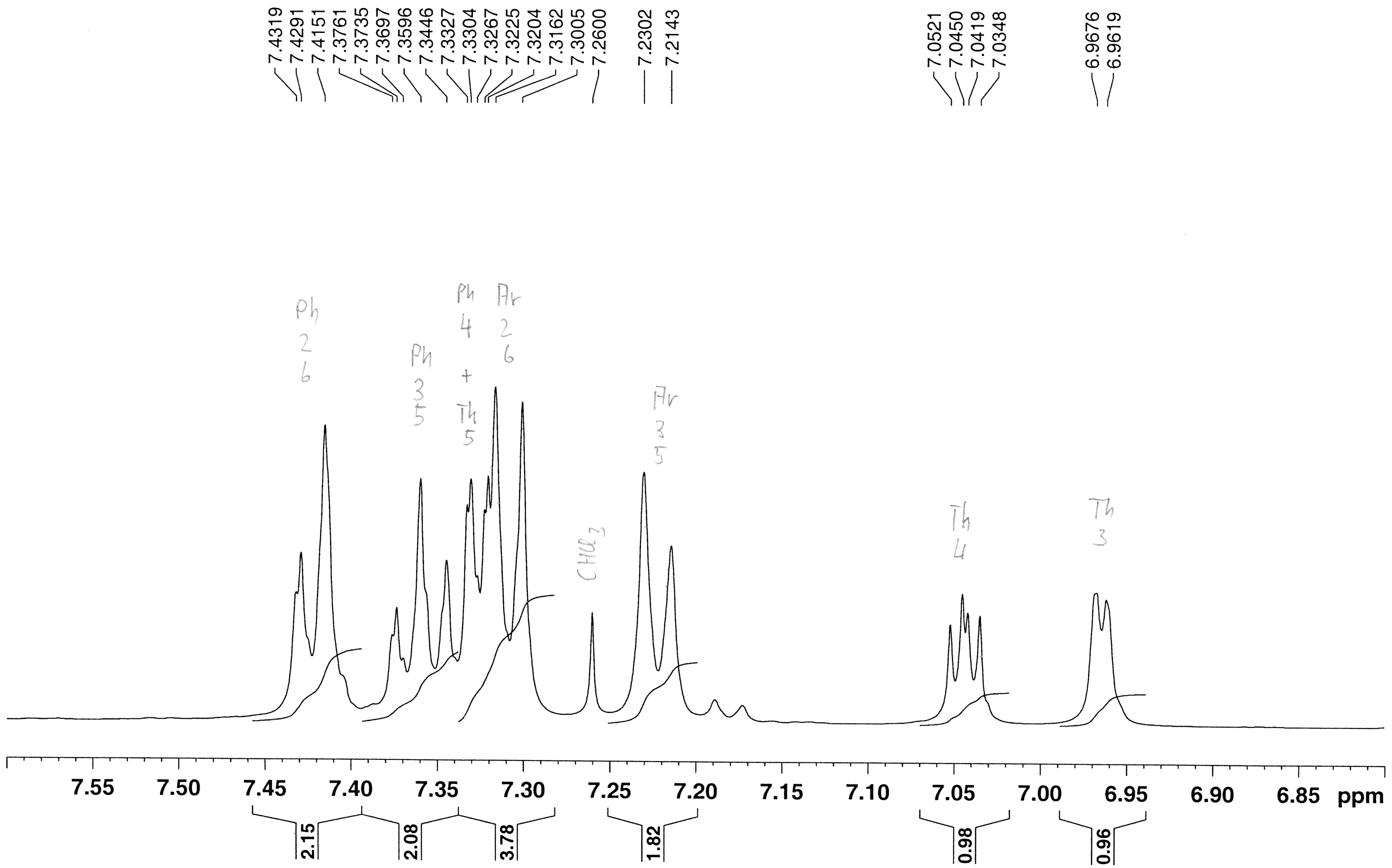

PN022_2_1 in cdcl3 (Proton) 29.8.2019

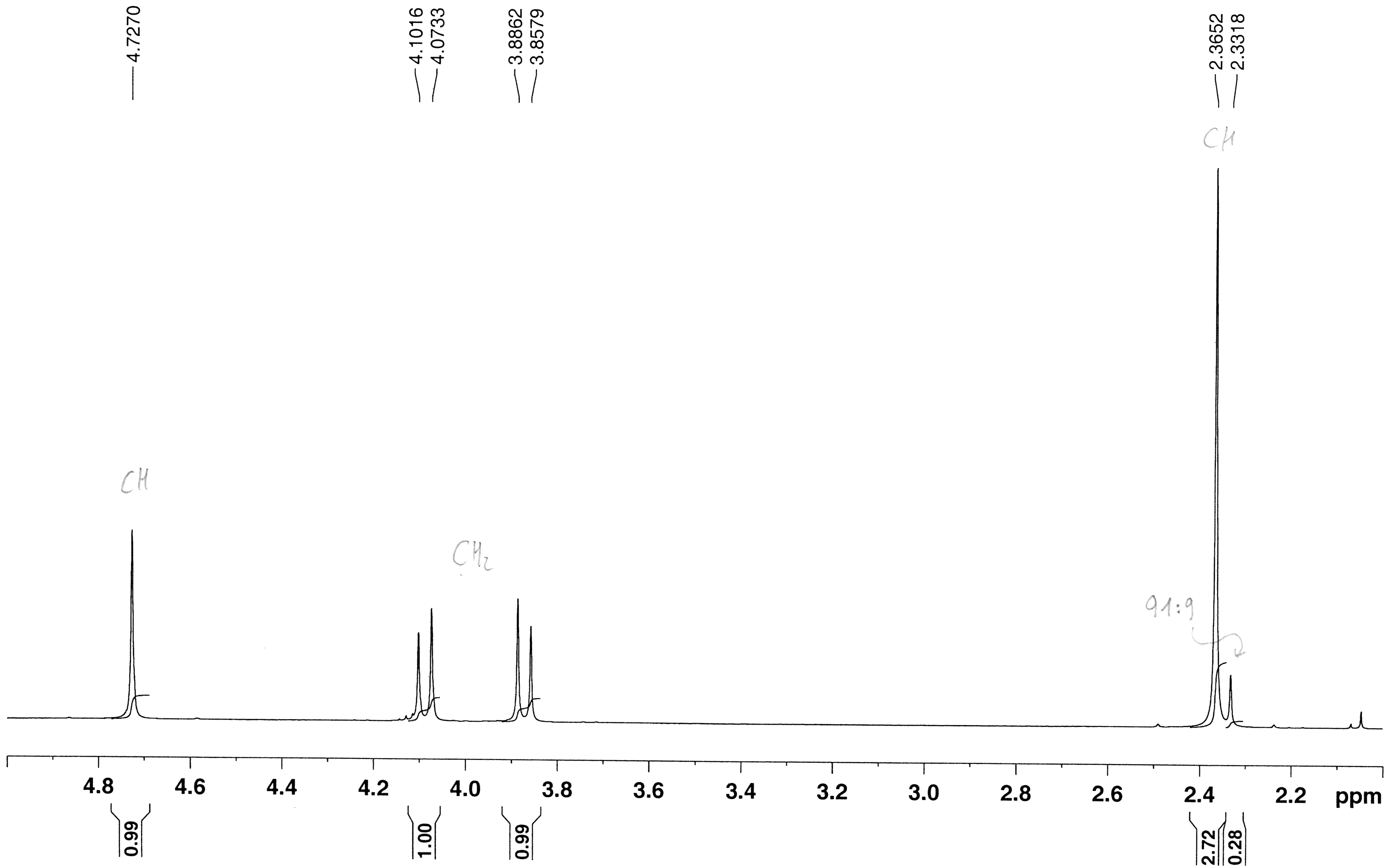

PN022_2_1 in cdcl3 (APT) 29.8.2019

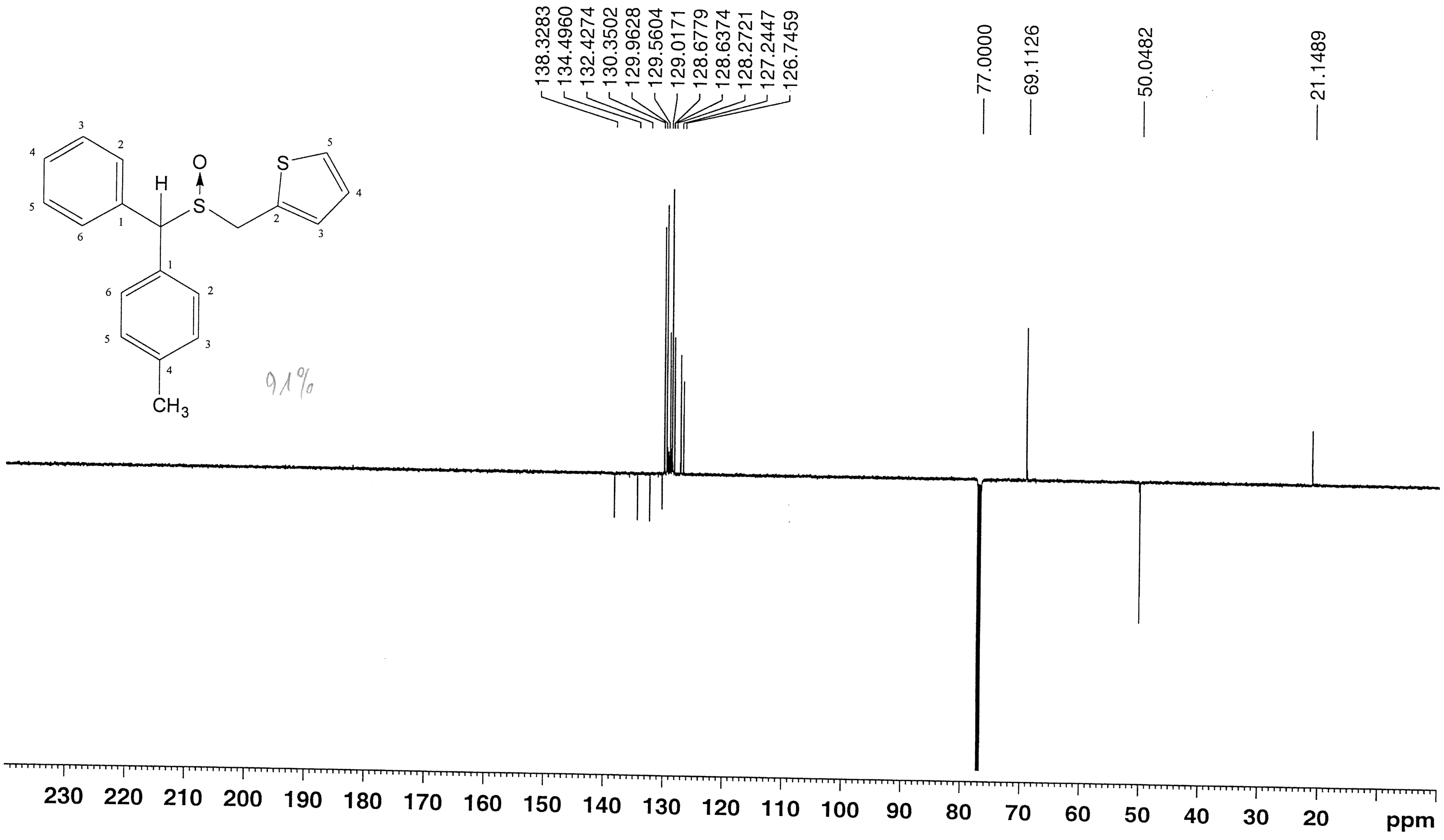

PN022_2_1 in cdcl3 (APT) 29.8.2019

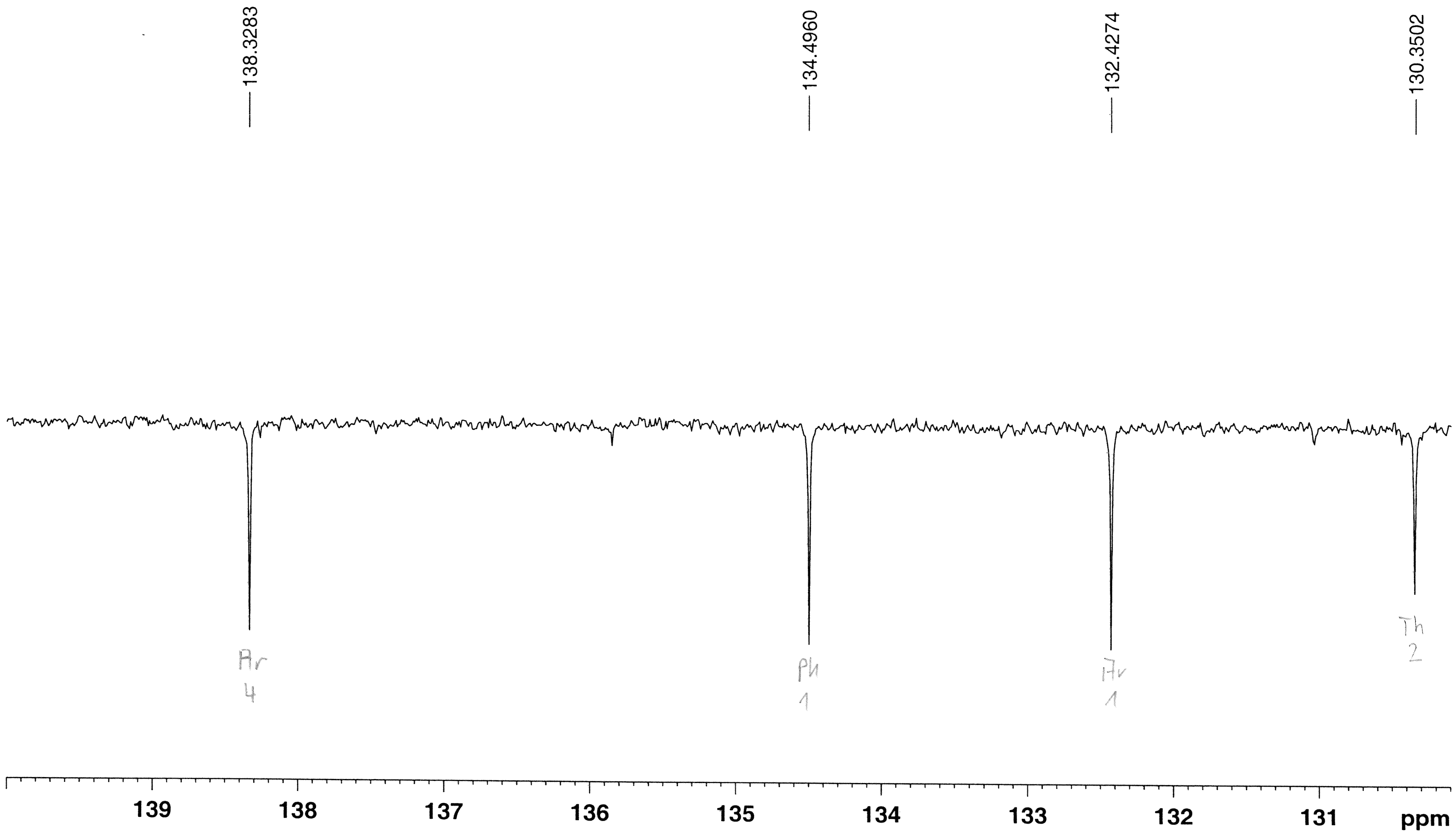

PN022_2_1 in cdcl3 (APT) 29.8.2019

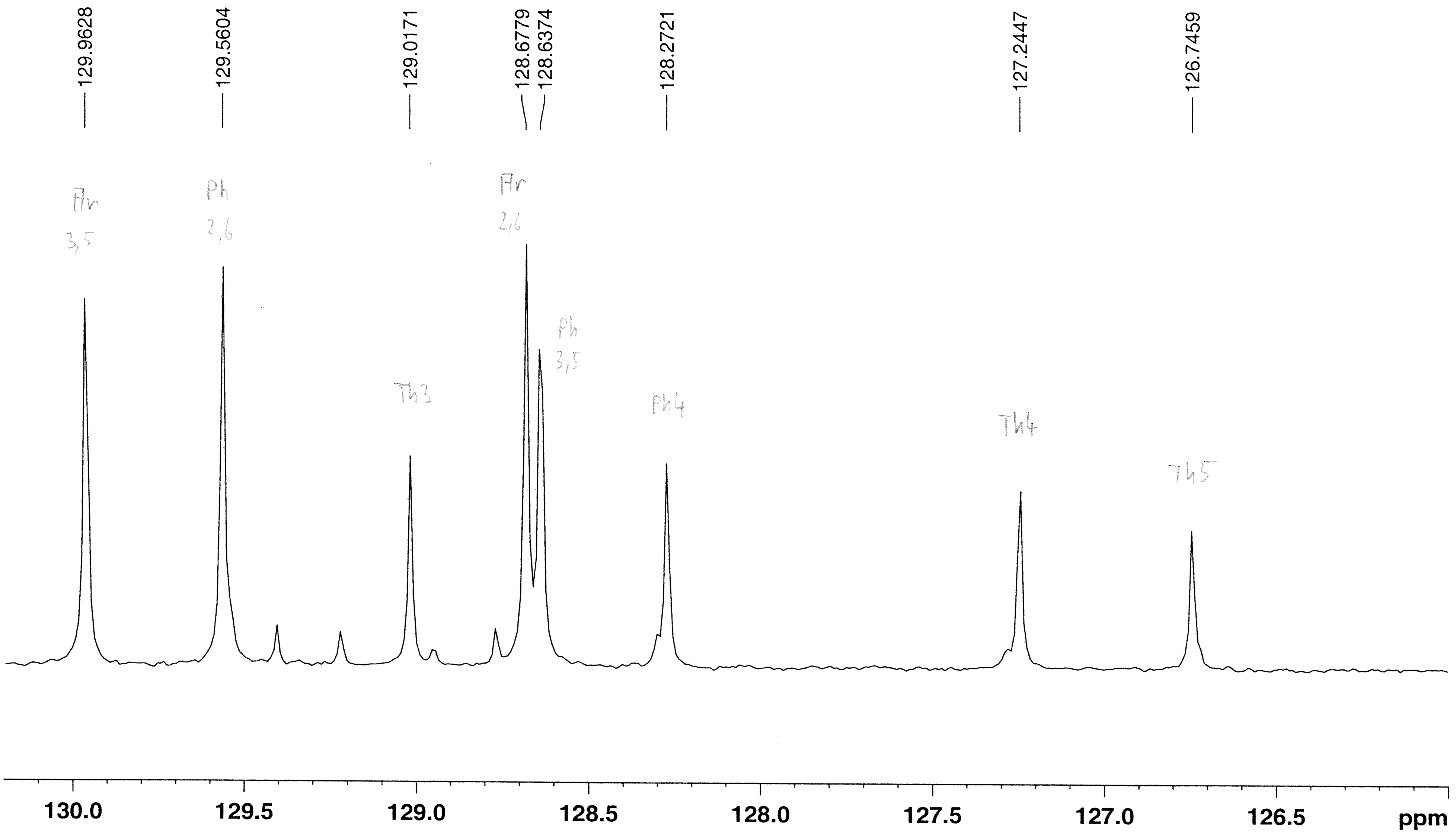

PN022_2_1 in cdcl3 (APT) 29.8.2019

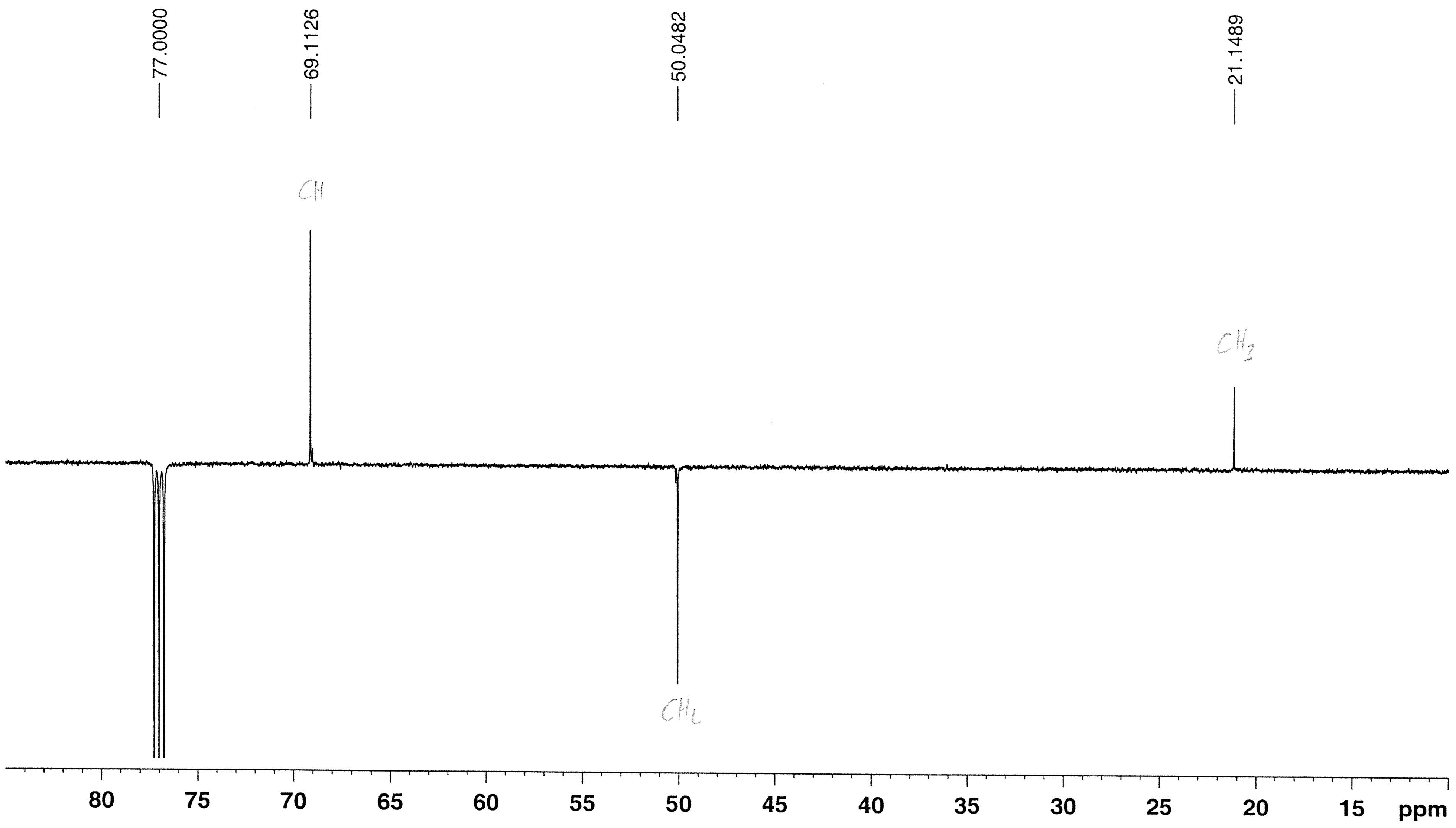

PN022_2_1 in cdcl3 (COSY) 29.8.2019

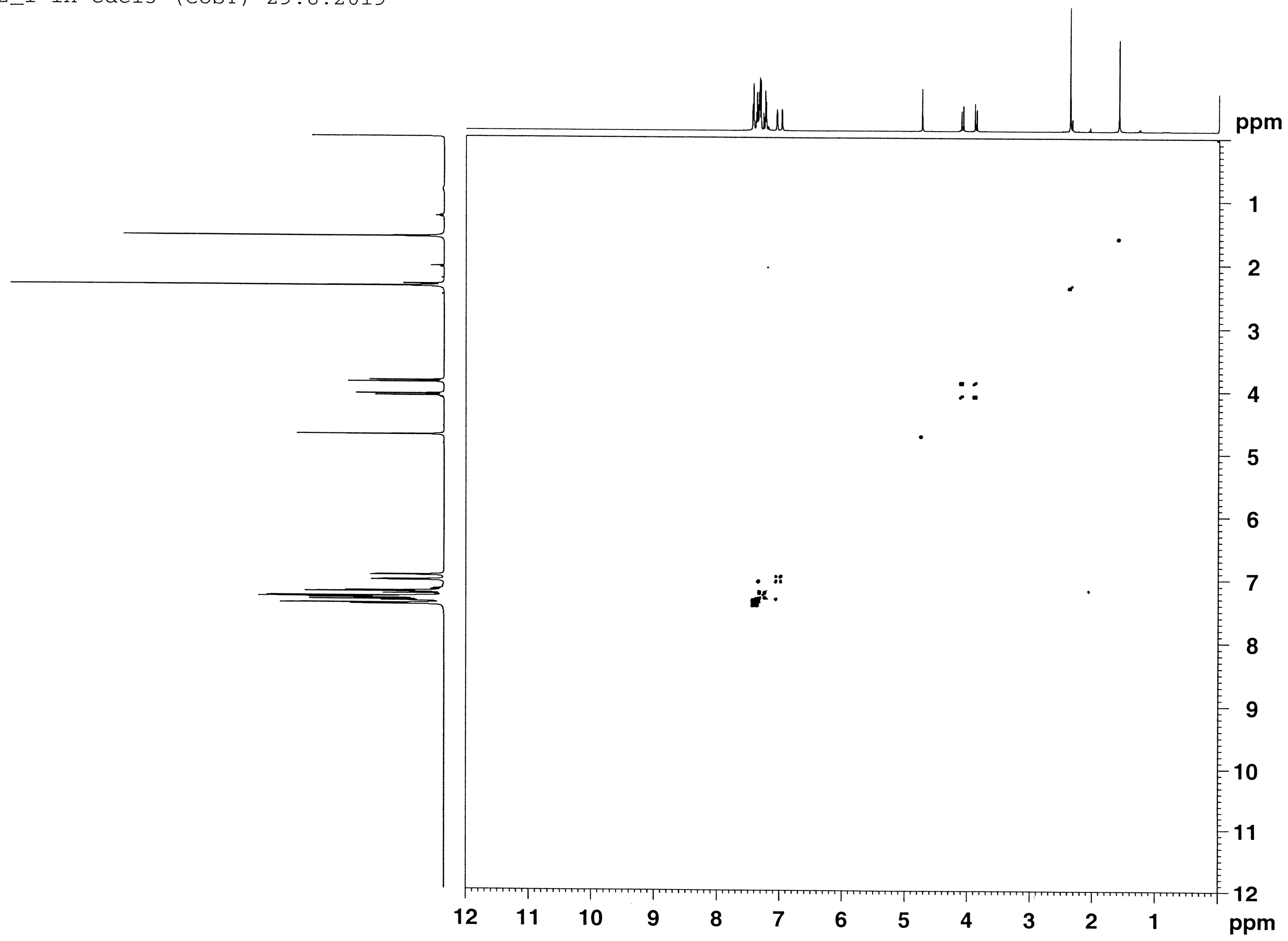

PN022_2_1 in cdcl3 (COSY) 29.8.2019

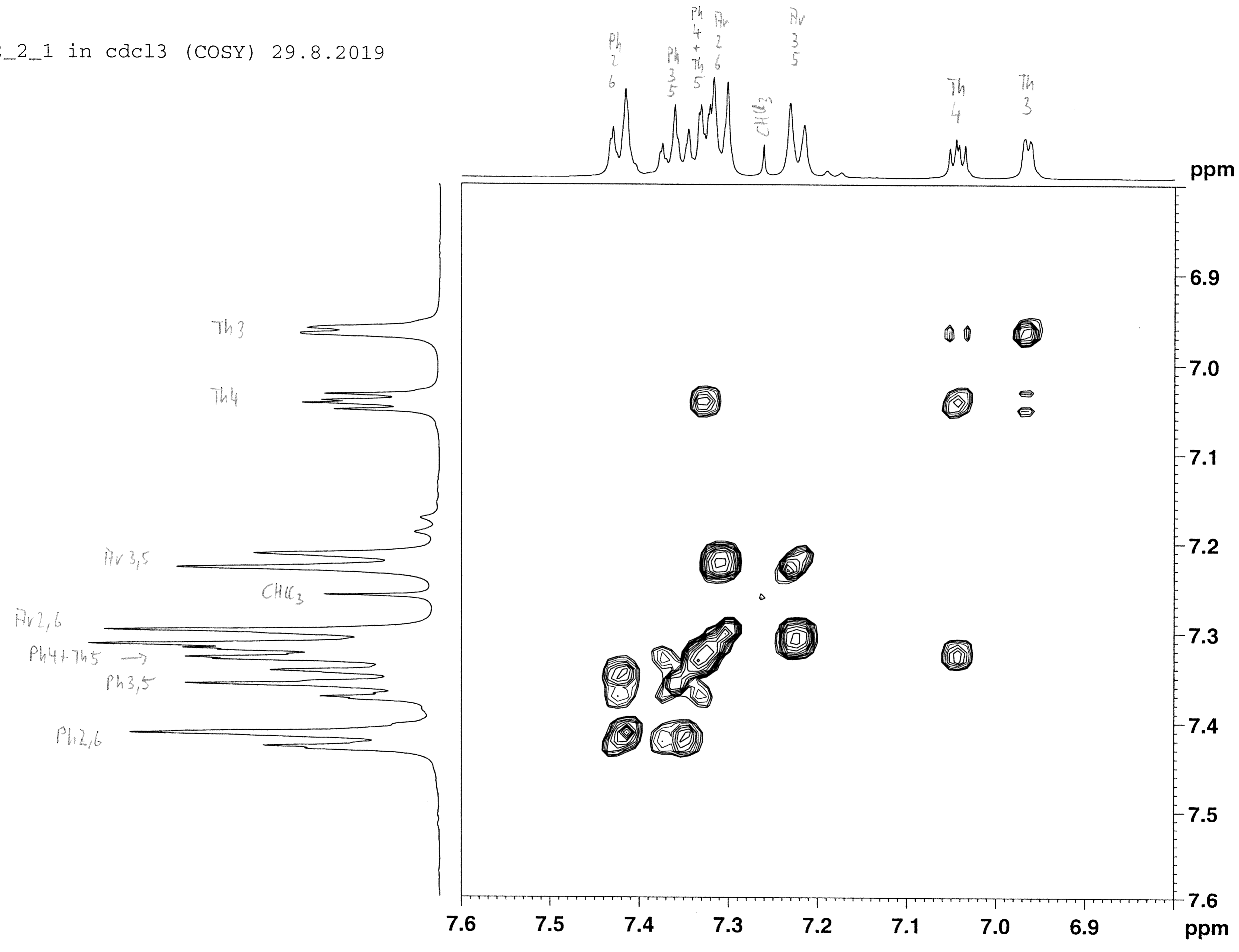

PN022_2_1 in cdcl3 (COSY) 29.8.2019

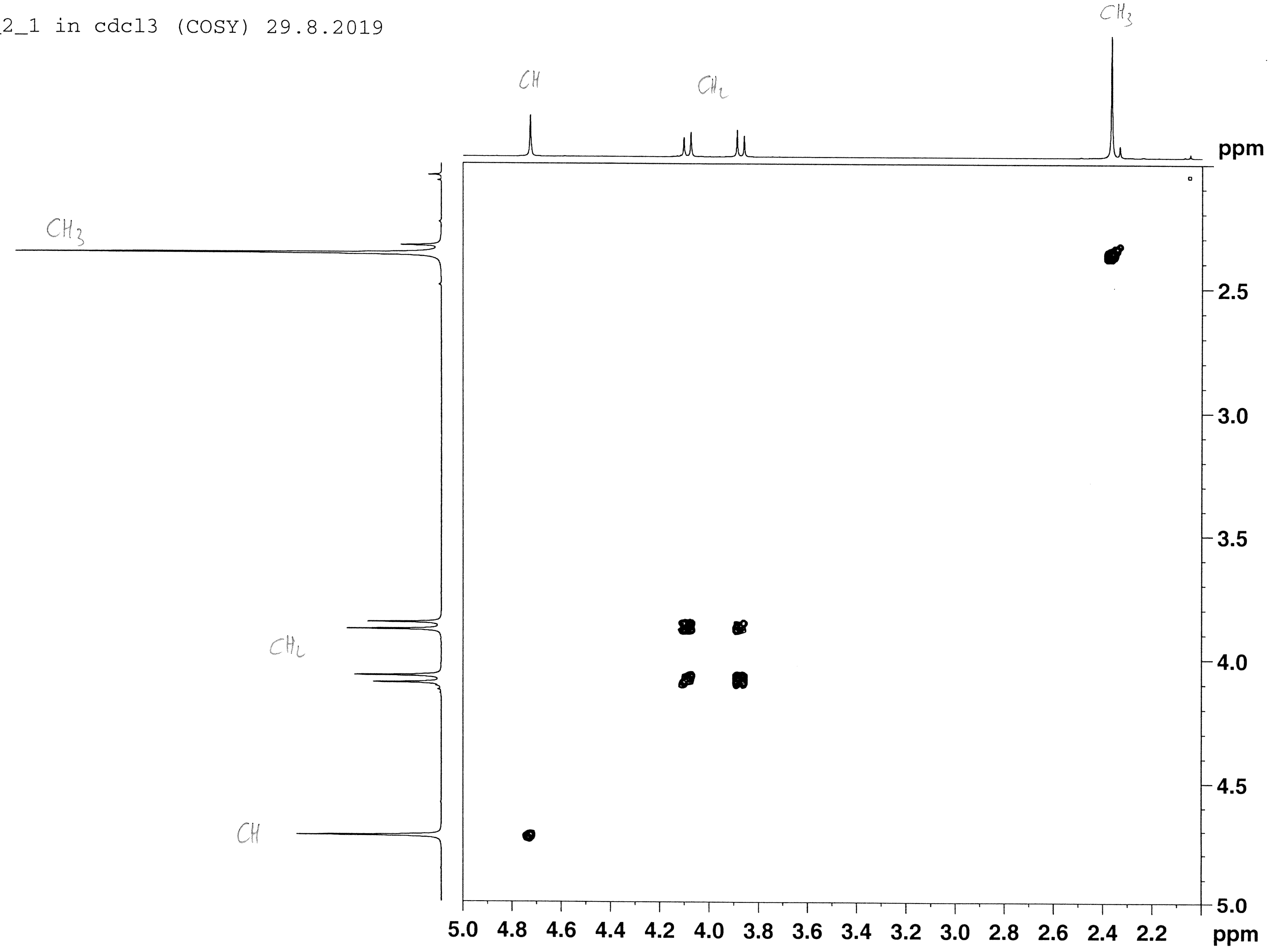

PN022_2_1 in cdcl3 (HSQC) 29.8.2019

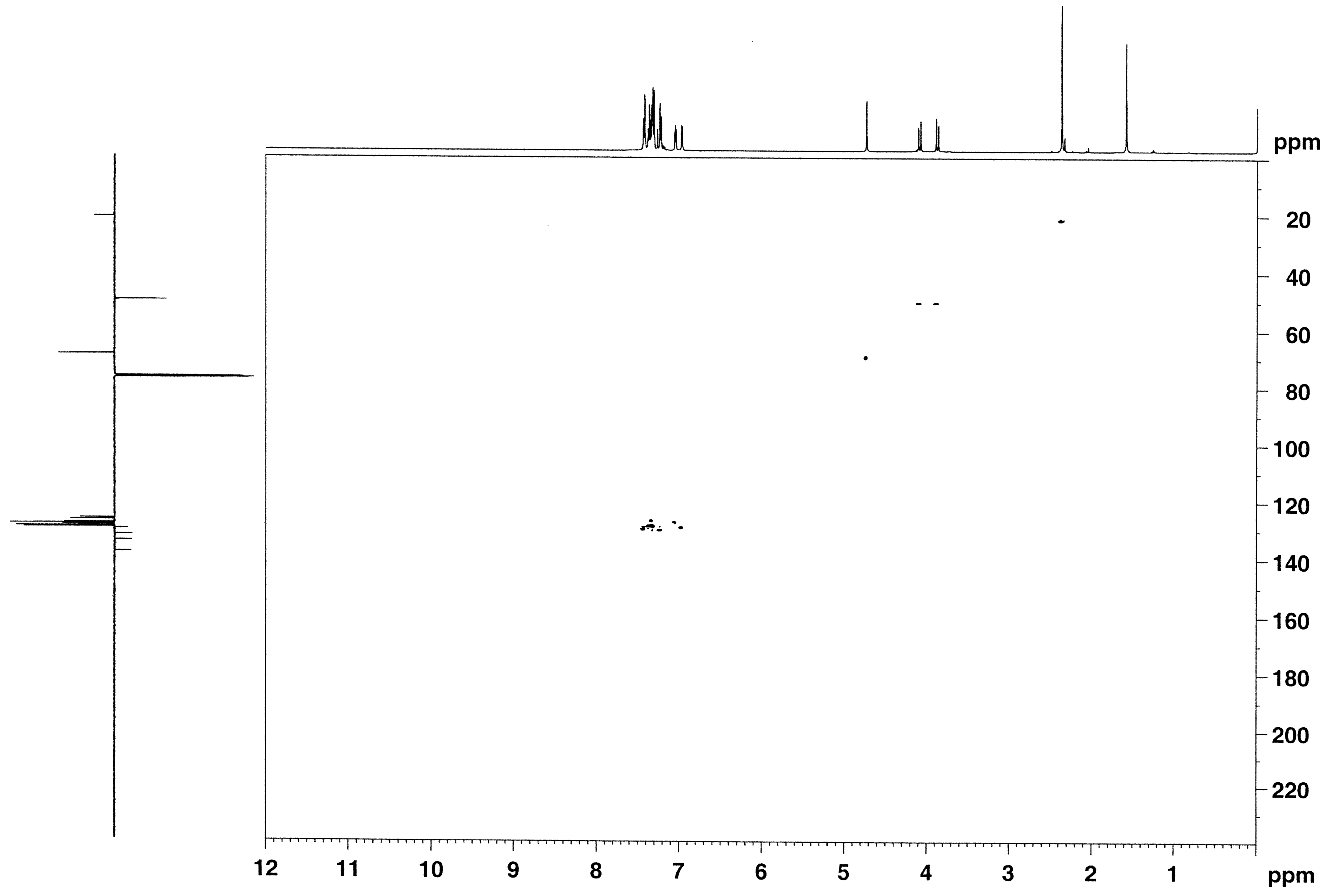

PN022_2_1 in cdcl3 (HSQC) 29.8.2019

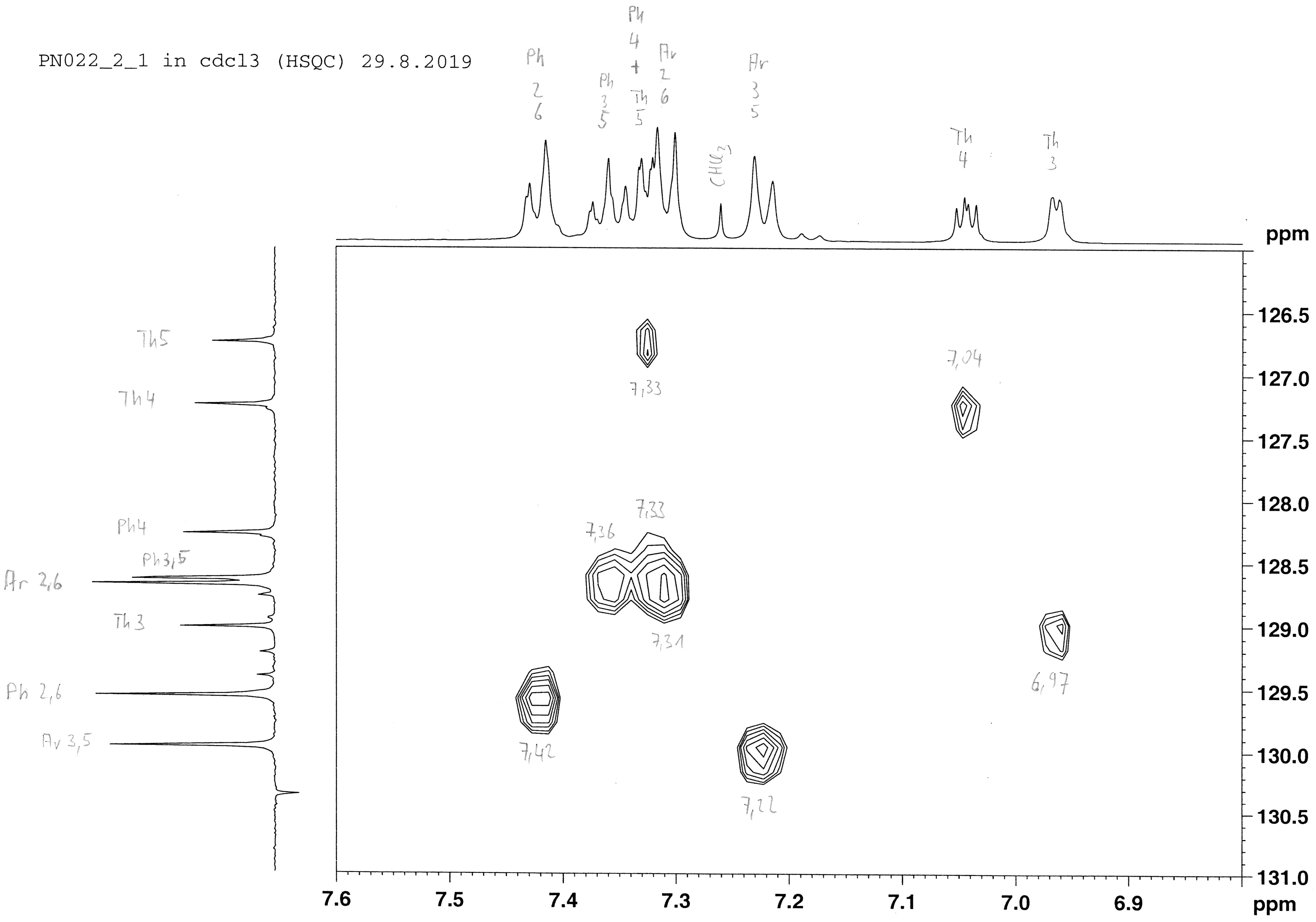

PN022_2_1 in cdcl3 (HSQC) 29.8.2019

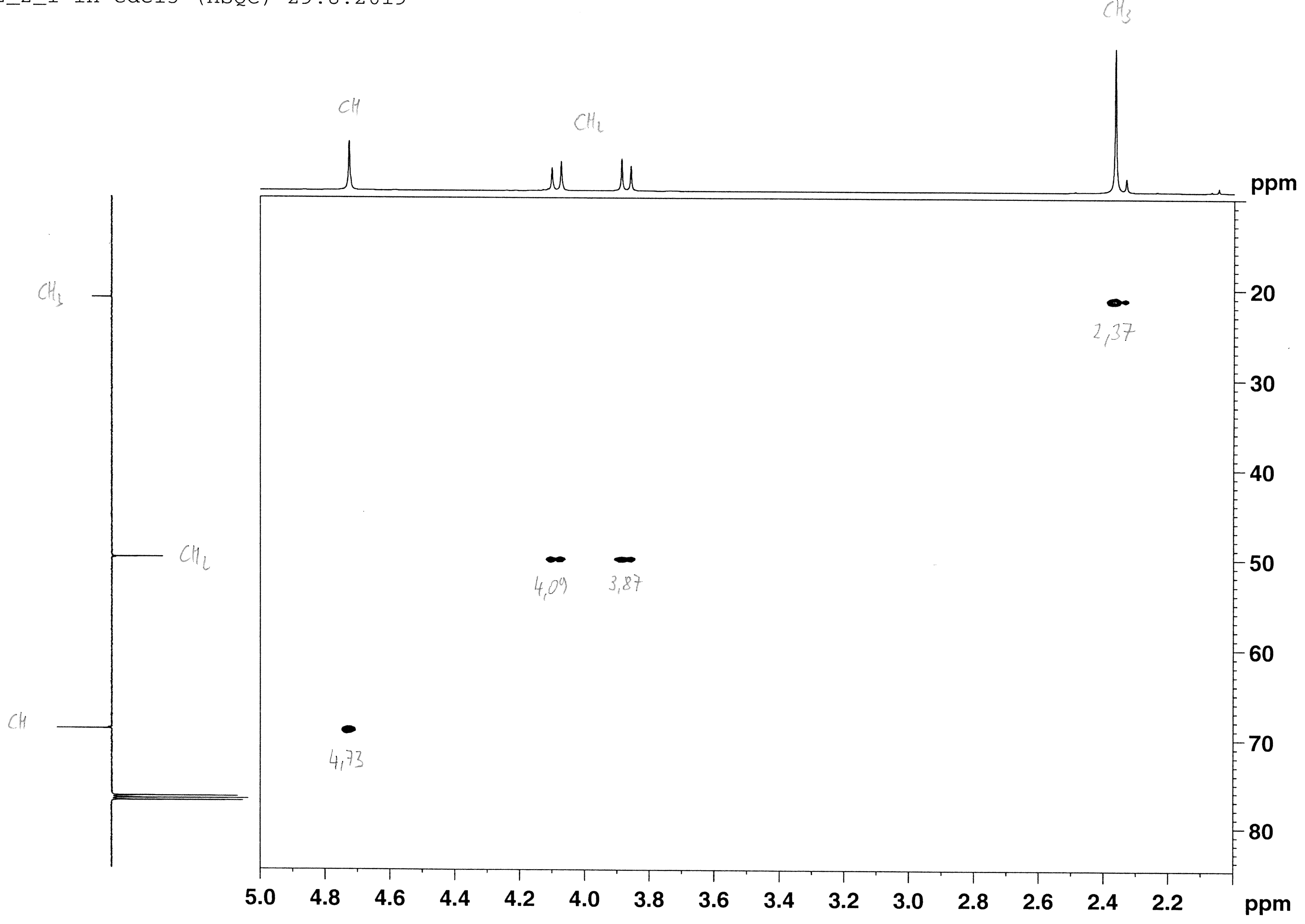

PN022_2_1 in cdcl3 (HMBC) 29.8.2019

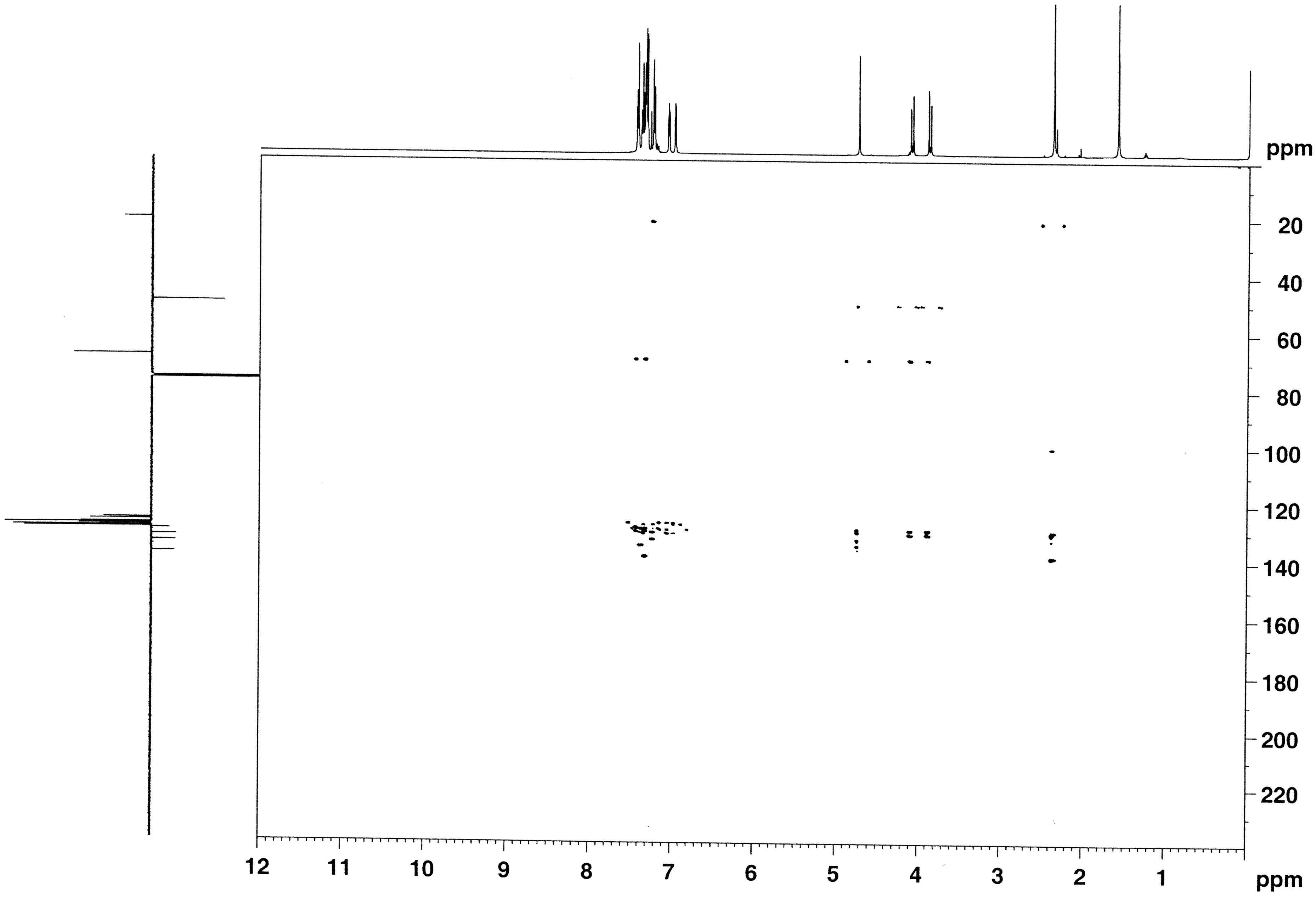

PN022_2_1 in cdcl3 (HMBC) 29.8.2019

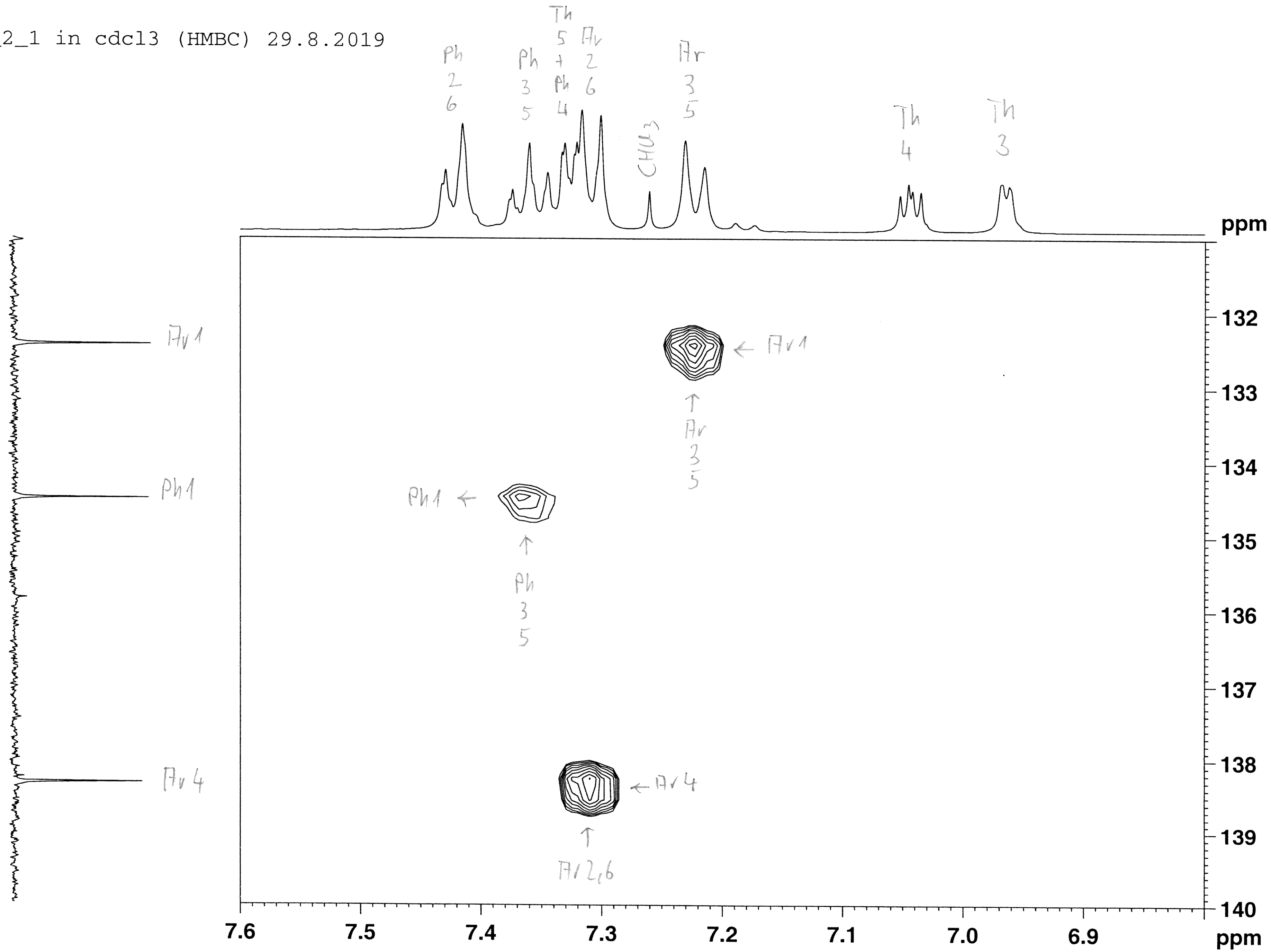

PN022_2_1 in cdcl3 (HMBC) 29.8.2019

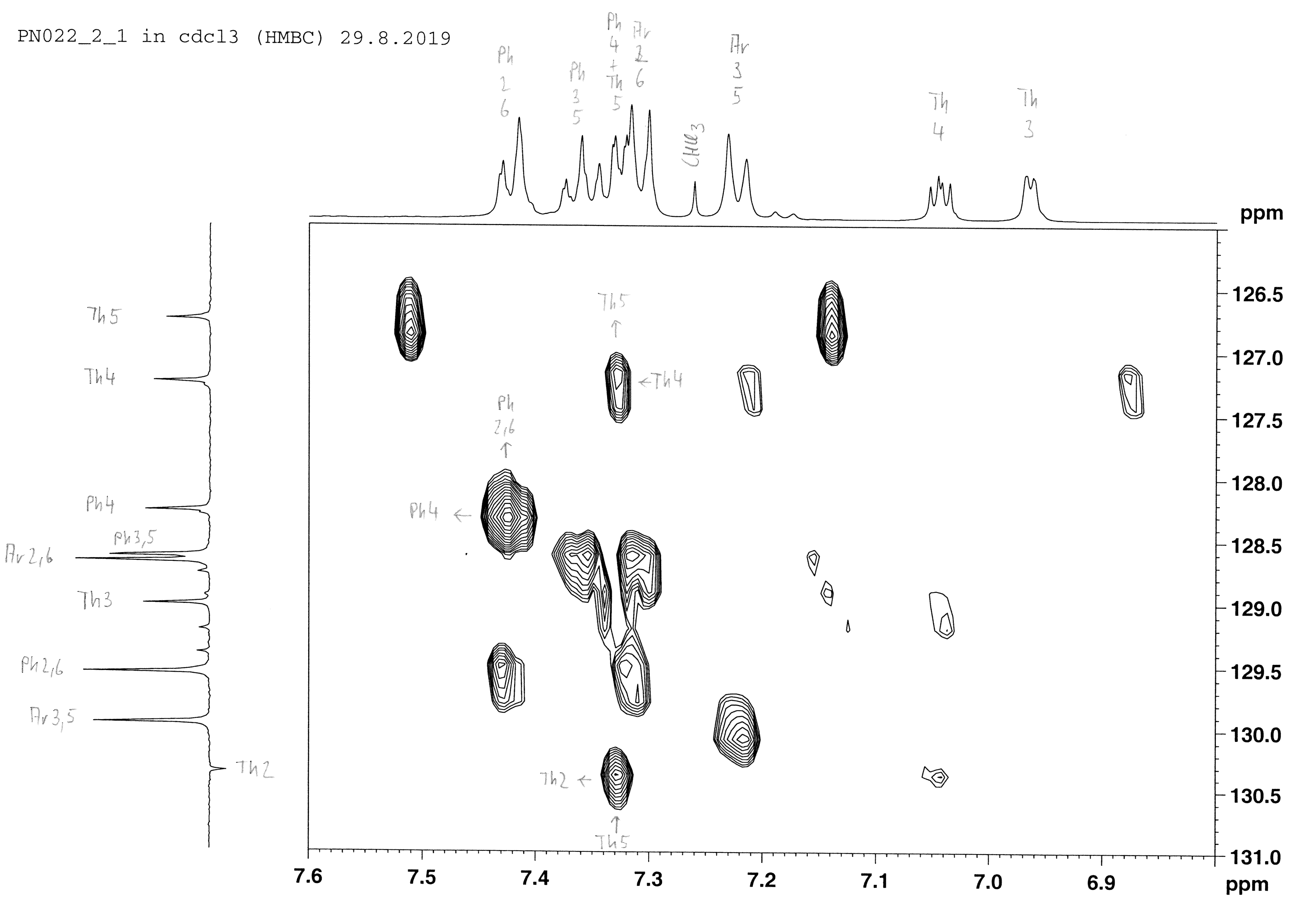

PN022_2_1 in cdcl3 (HMBC) 29.8.2019

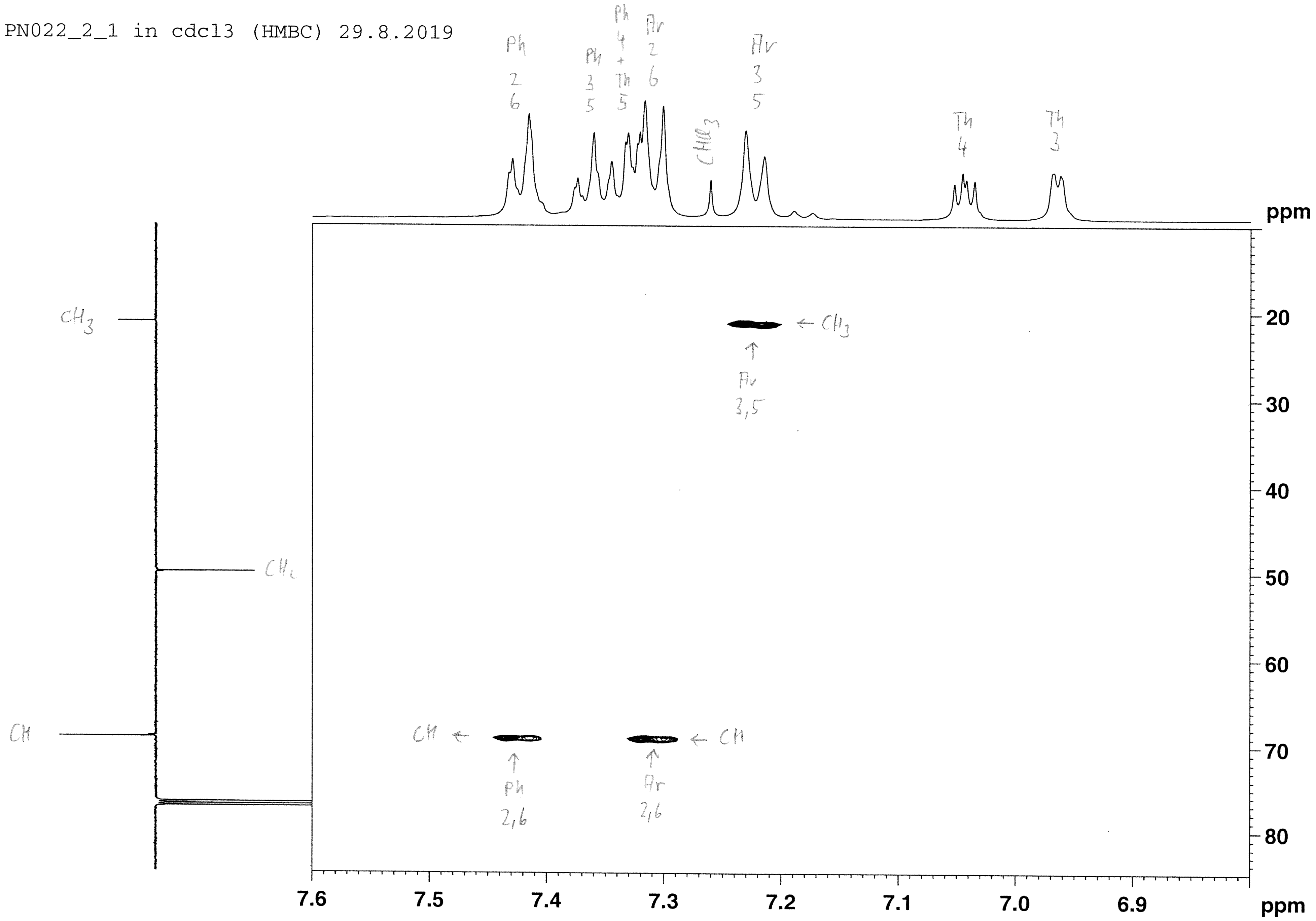

PN022_2_1 in cdcl3 (HMBC) 29.8.2019

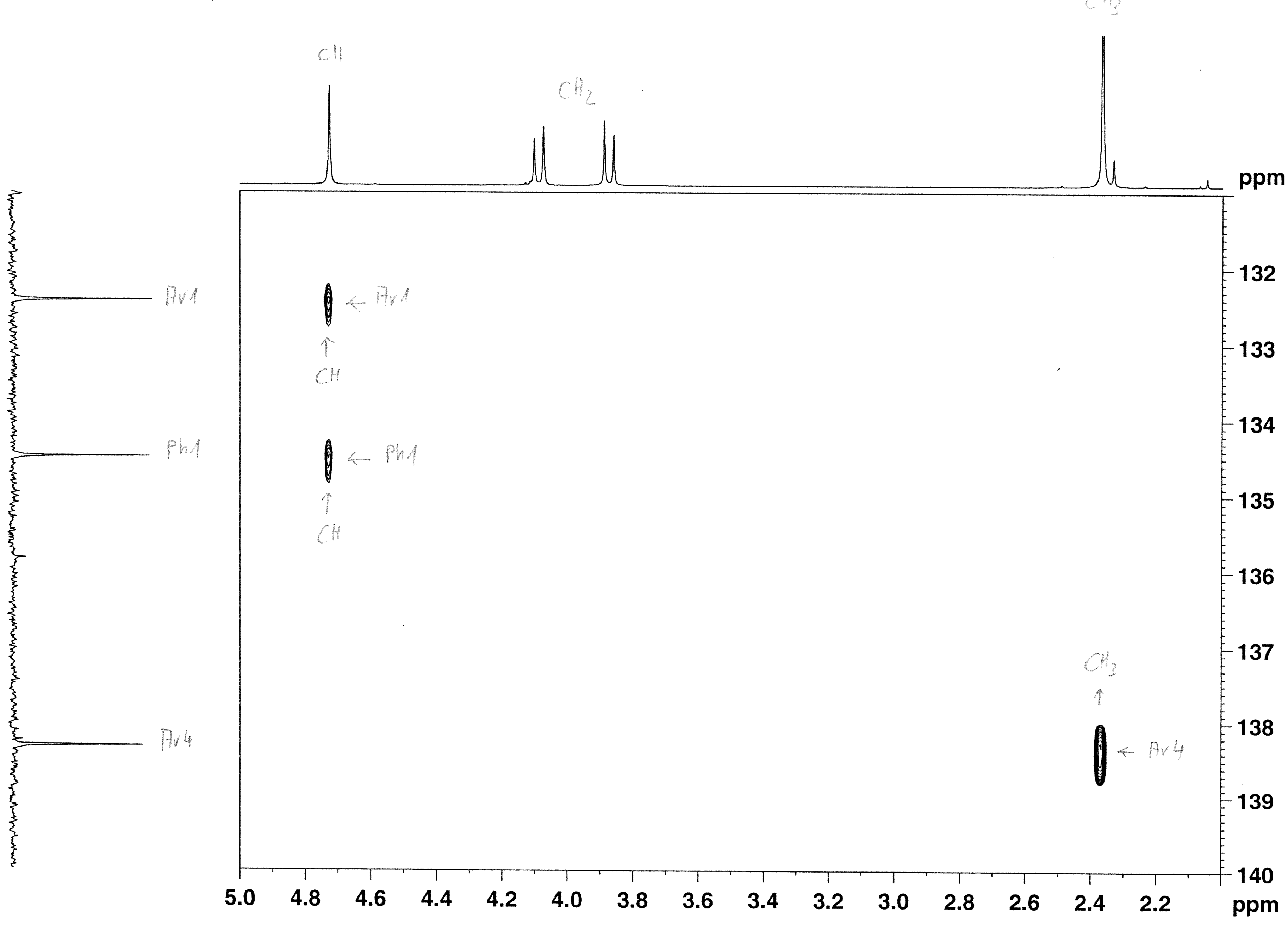

PN022_2_1 in cdcl3 (HMBC) 29.8.2019

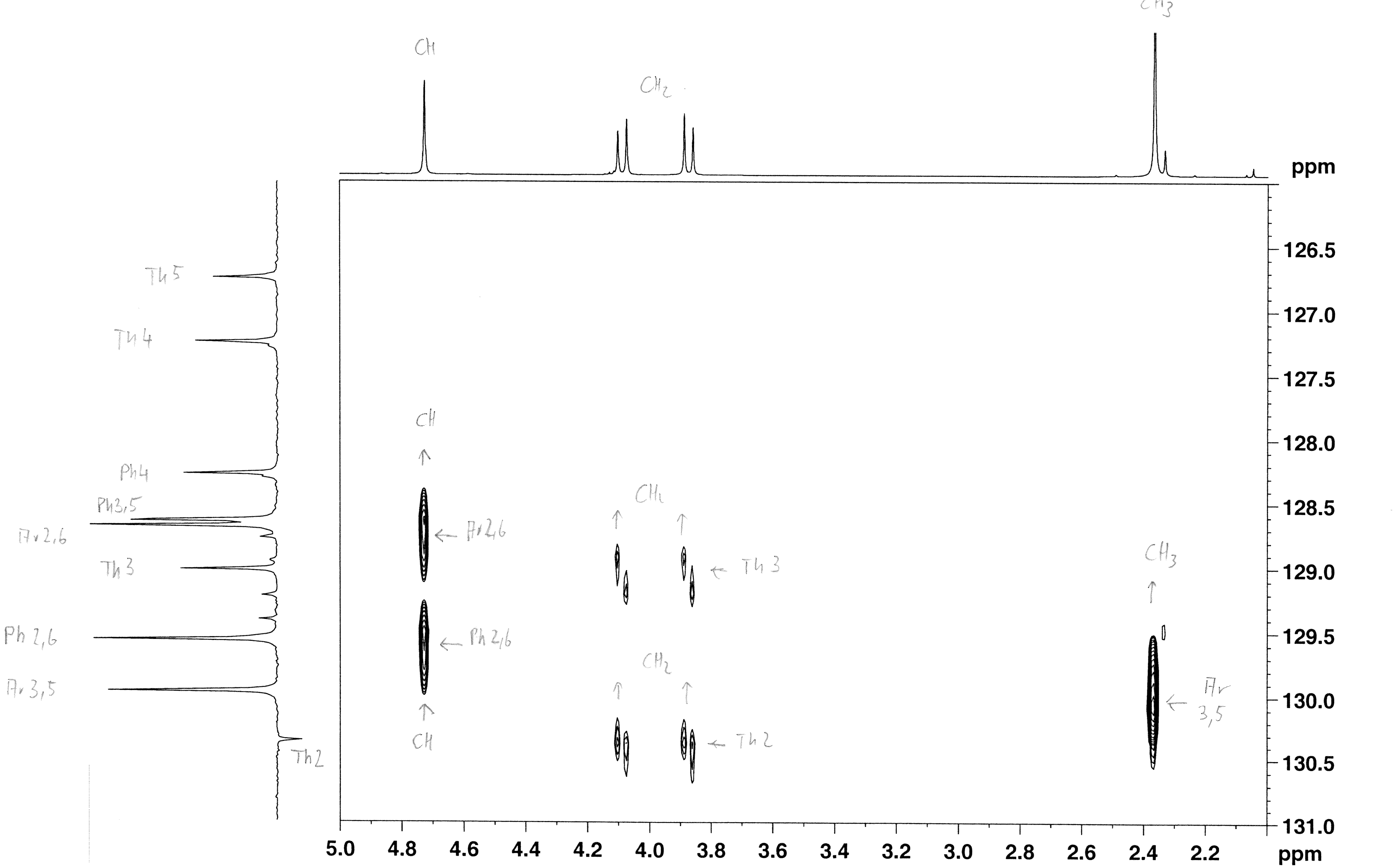

PN022_2_1 in cdcl3 (HMBC) 29.8.2019

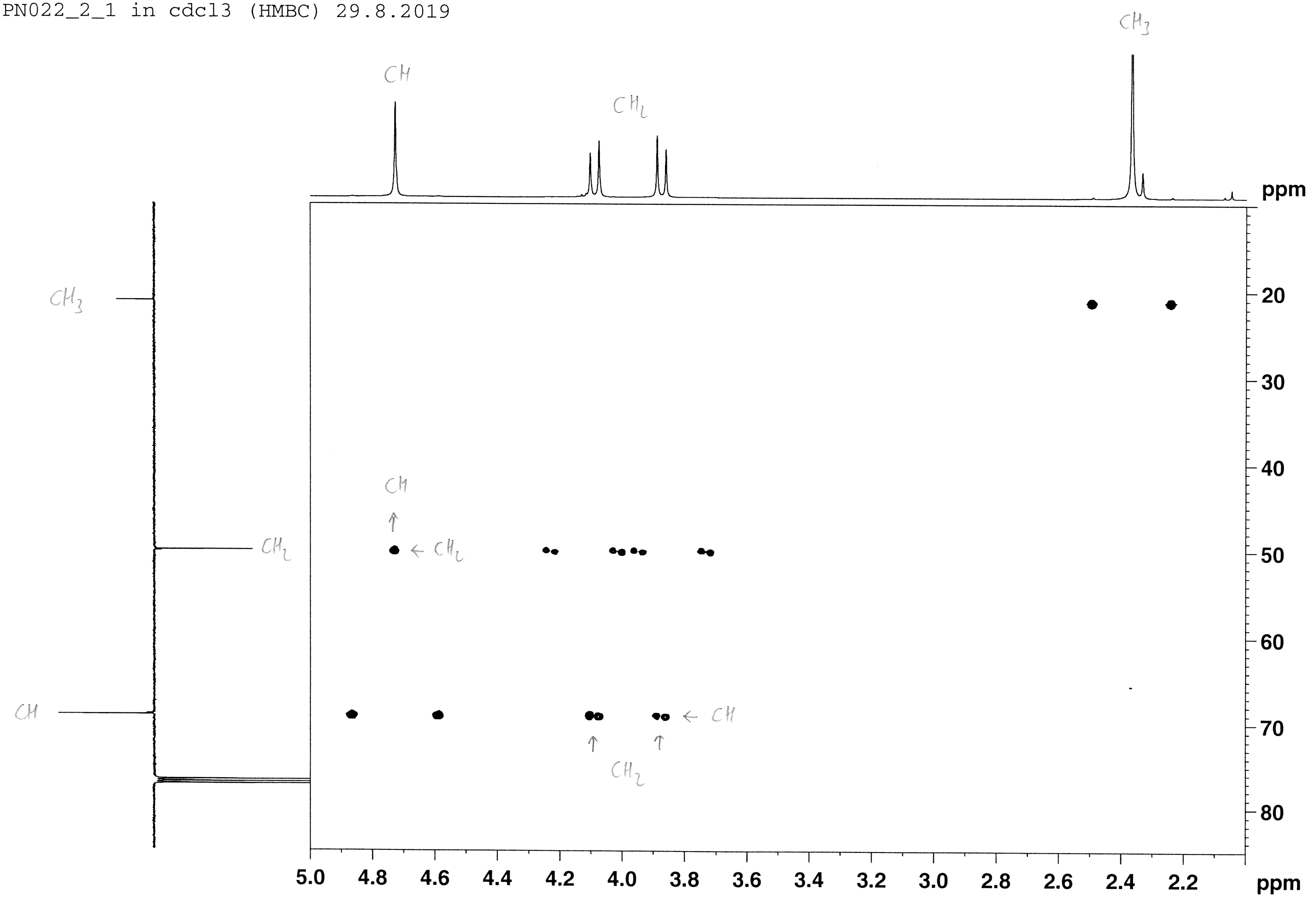

Figure S56c. NMR spectra of compound **8n**.

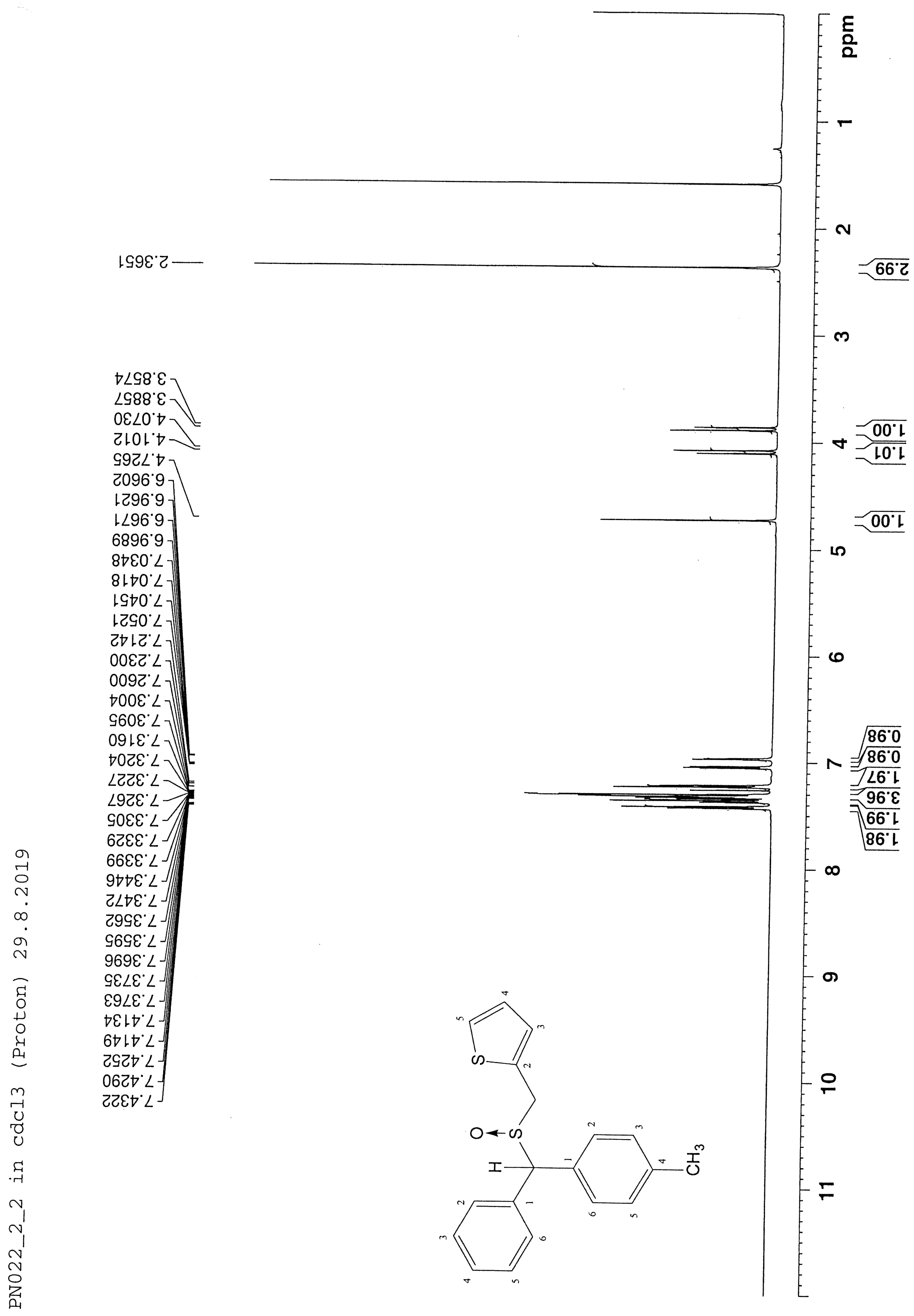

PN022_2_2 in cdcl3 (Proton) 29.8.2019

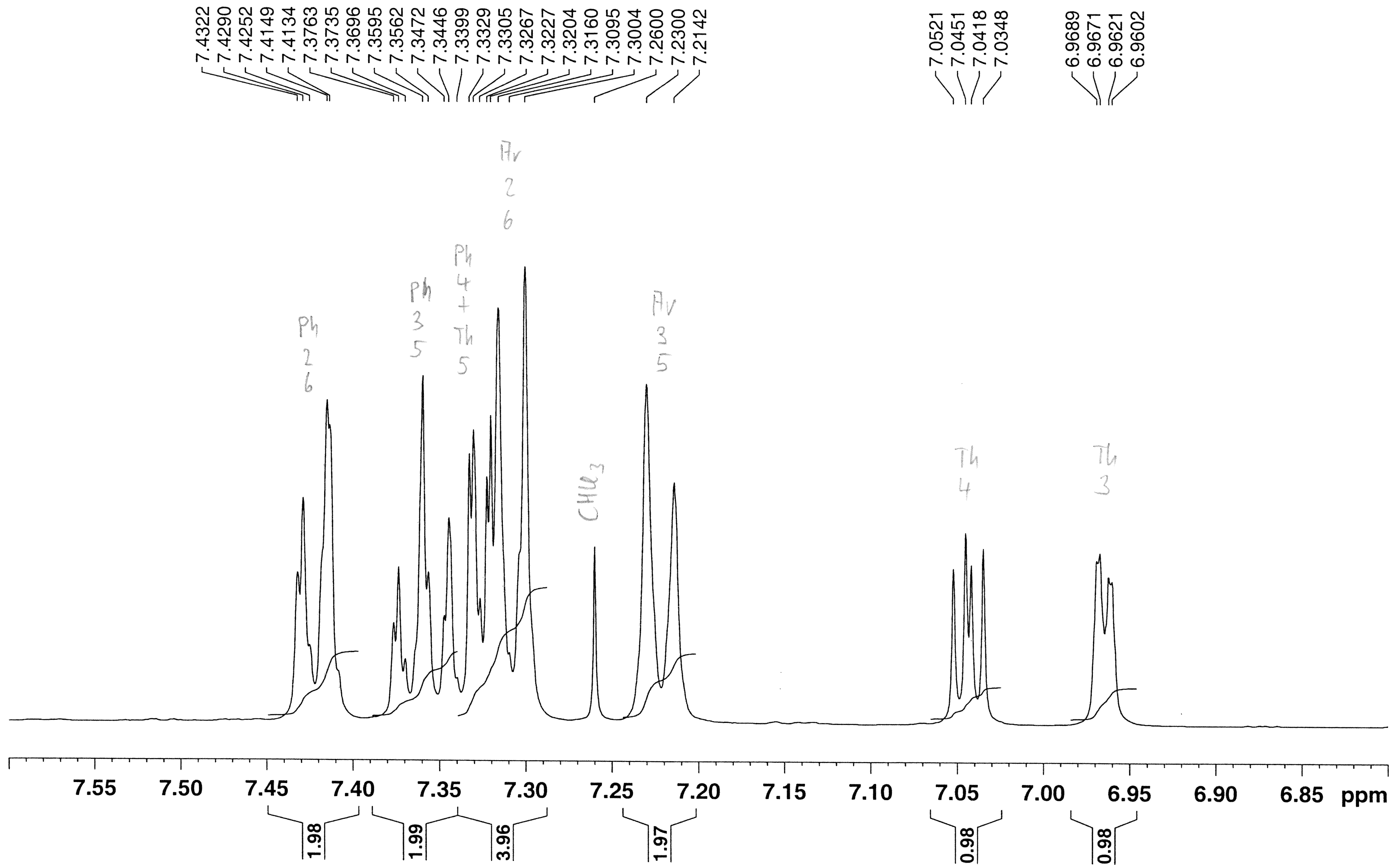

PN022_2_2 in cdcl3 (Proton) 29.8.2019

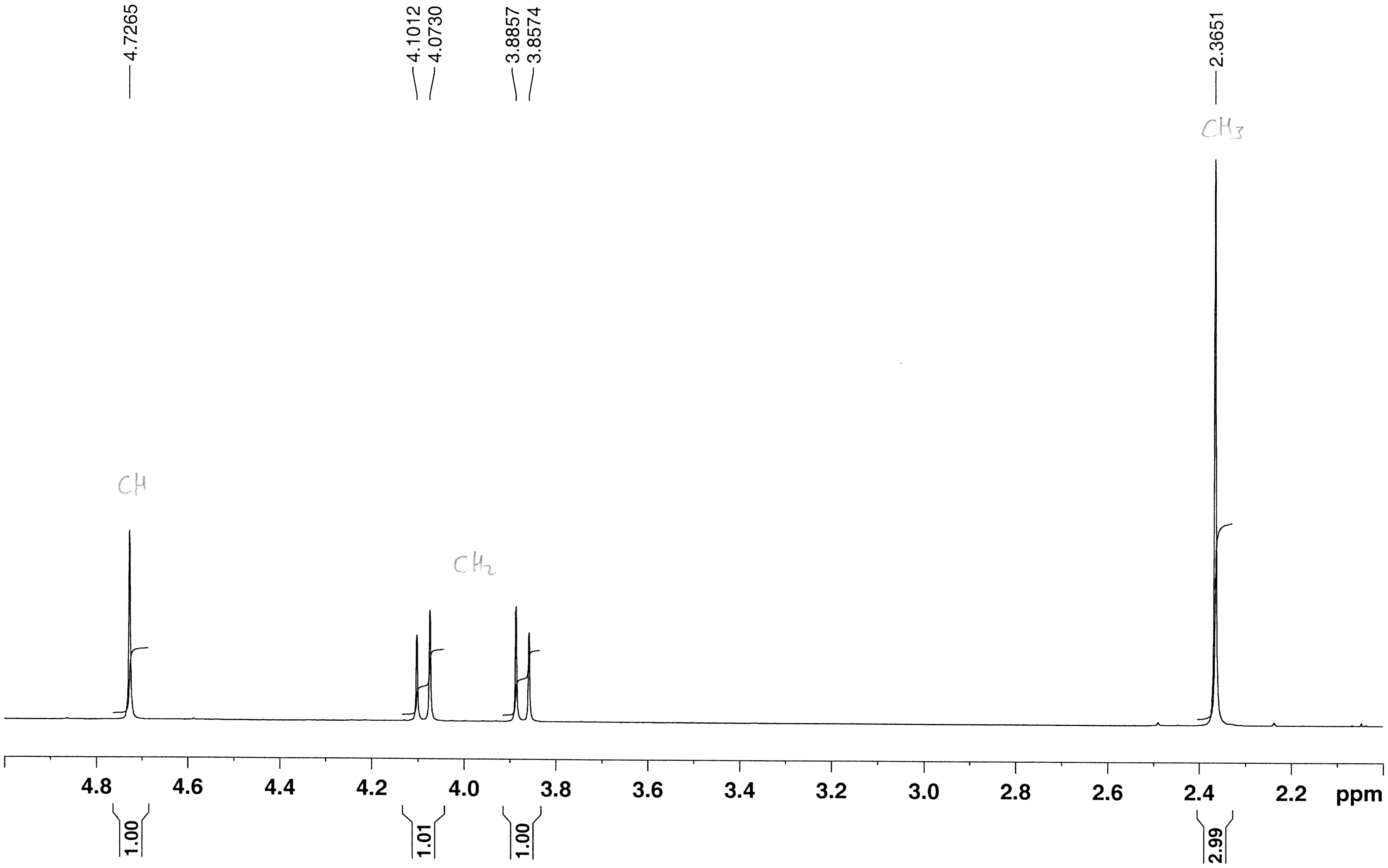

PN022_2_2 in cdcl3 (APT) 29.8.2019

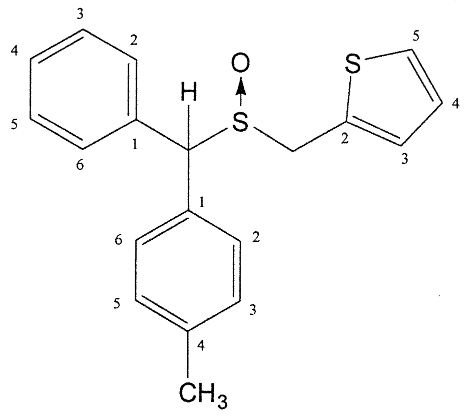

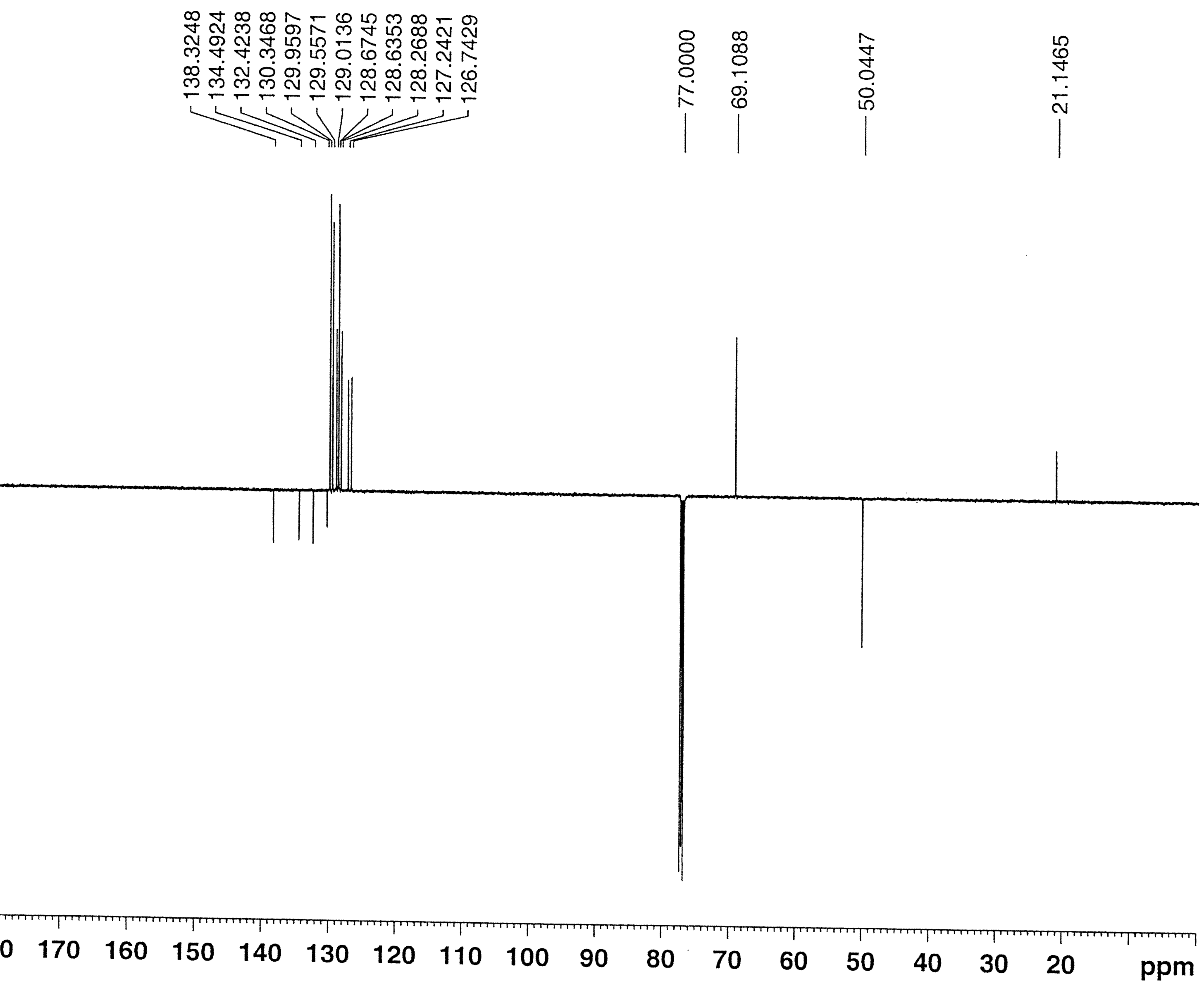

PN022_2_2 in cdcl3 (APT) 29.8.2019

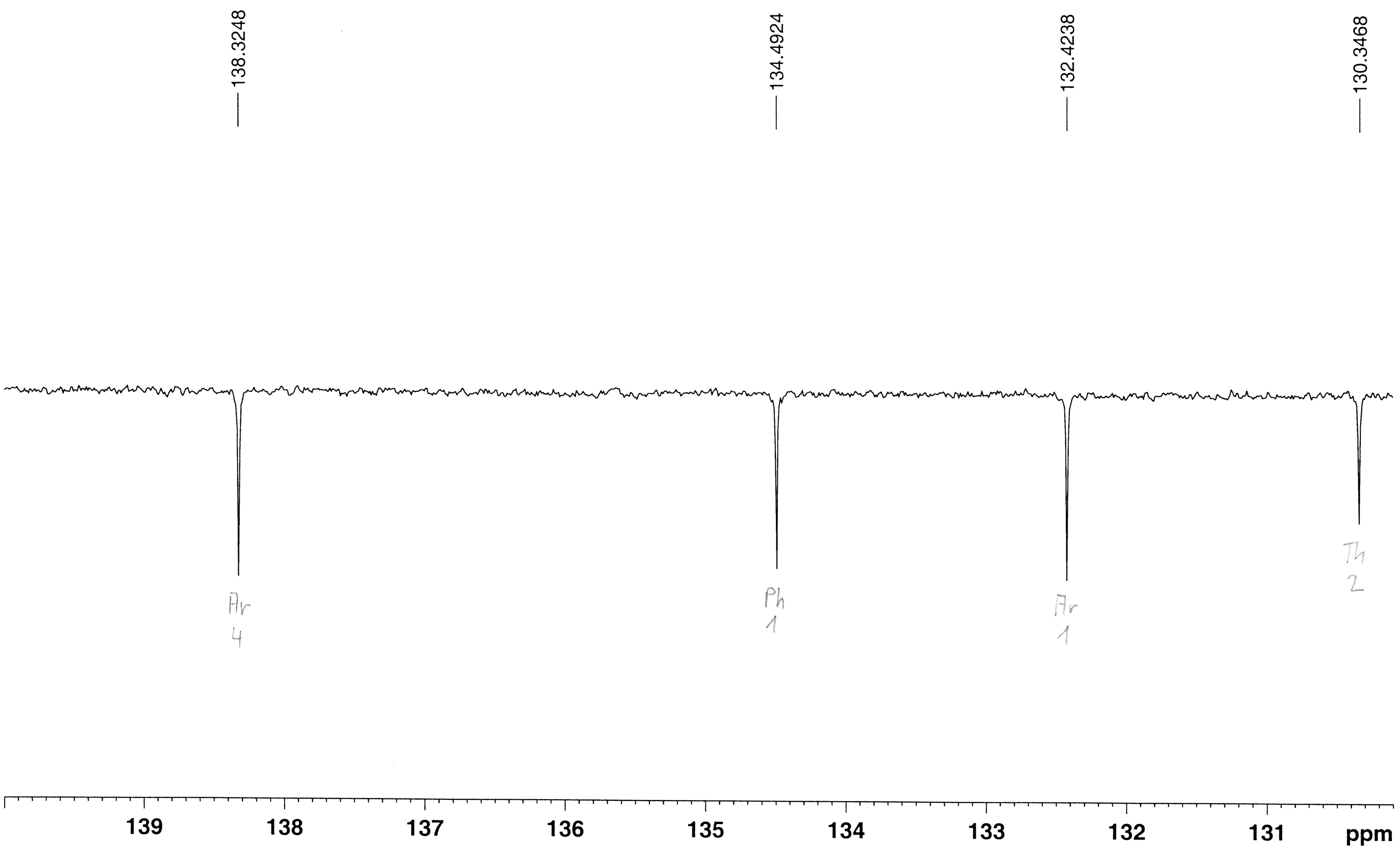

PN022_2_2 in cdcl3 (APT) 29.8.2019

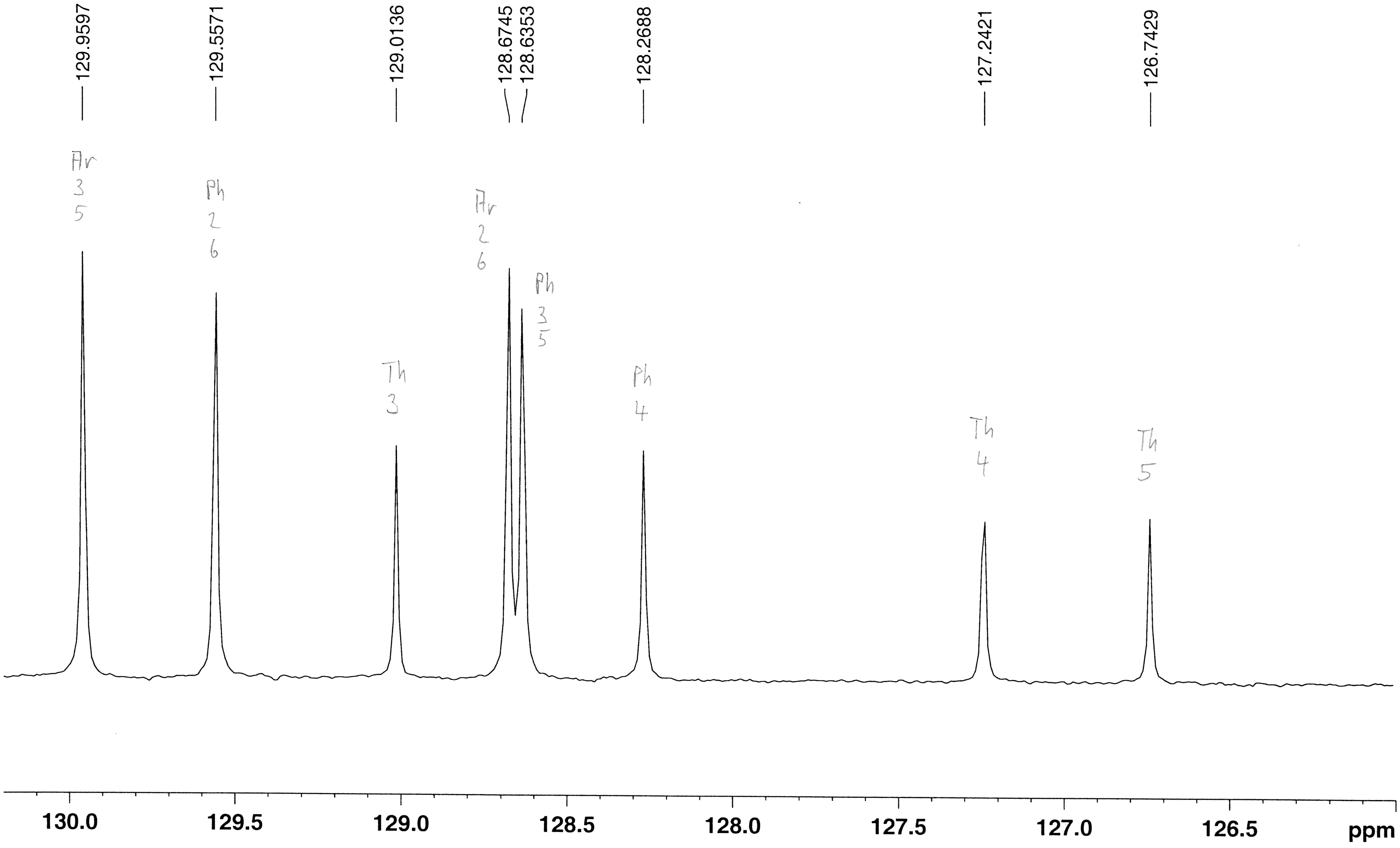

PN022_2_2 in cdcl3 (APT) 29.8.2019

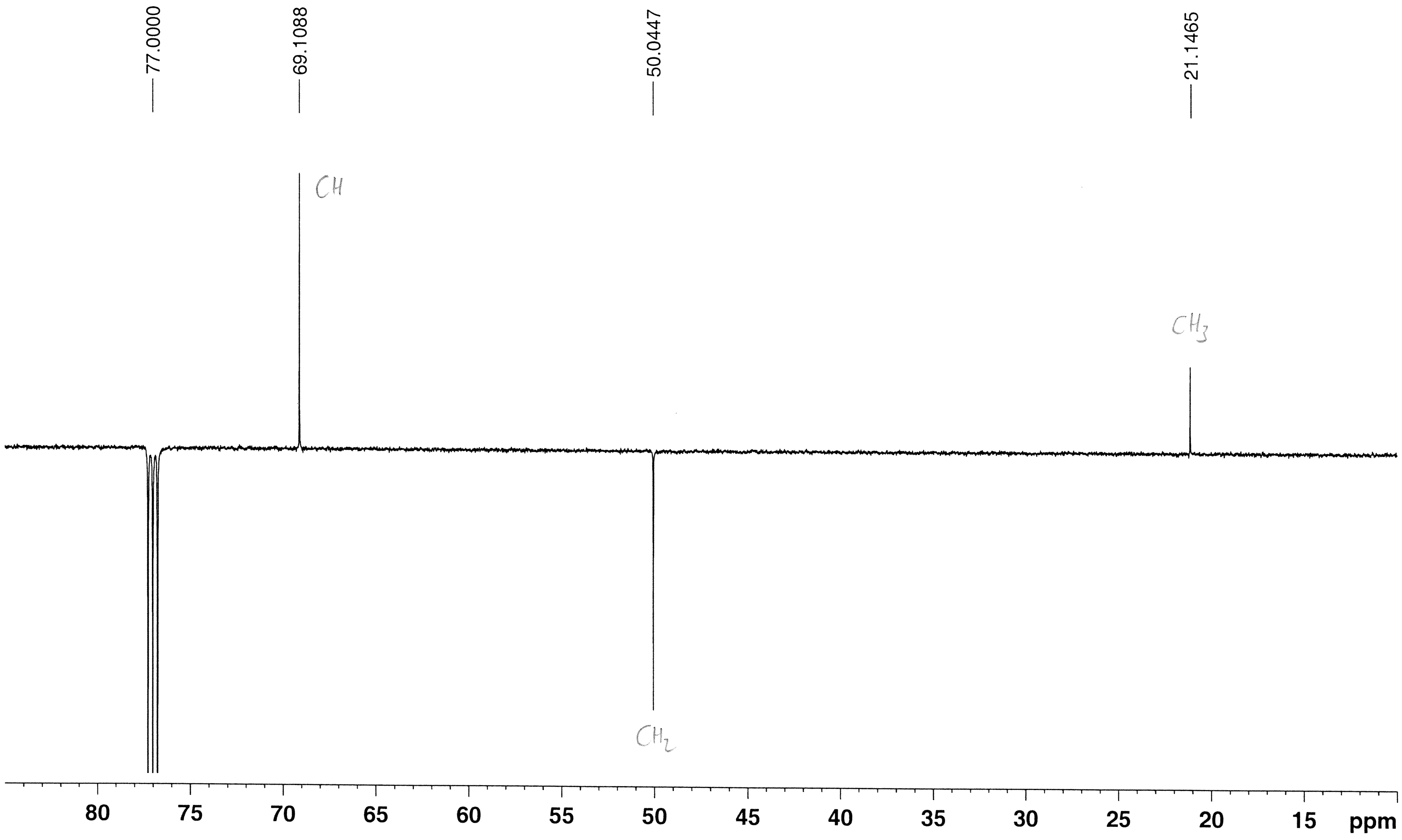

PN022_2_2 in cdcl3 (COSY) 29.8.2019

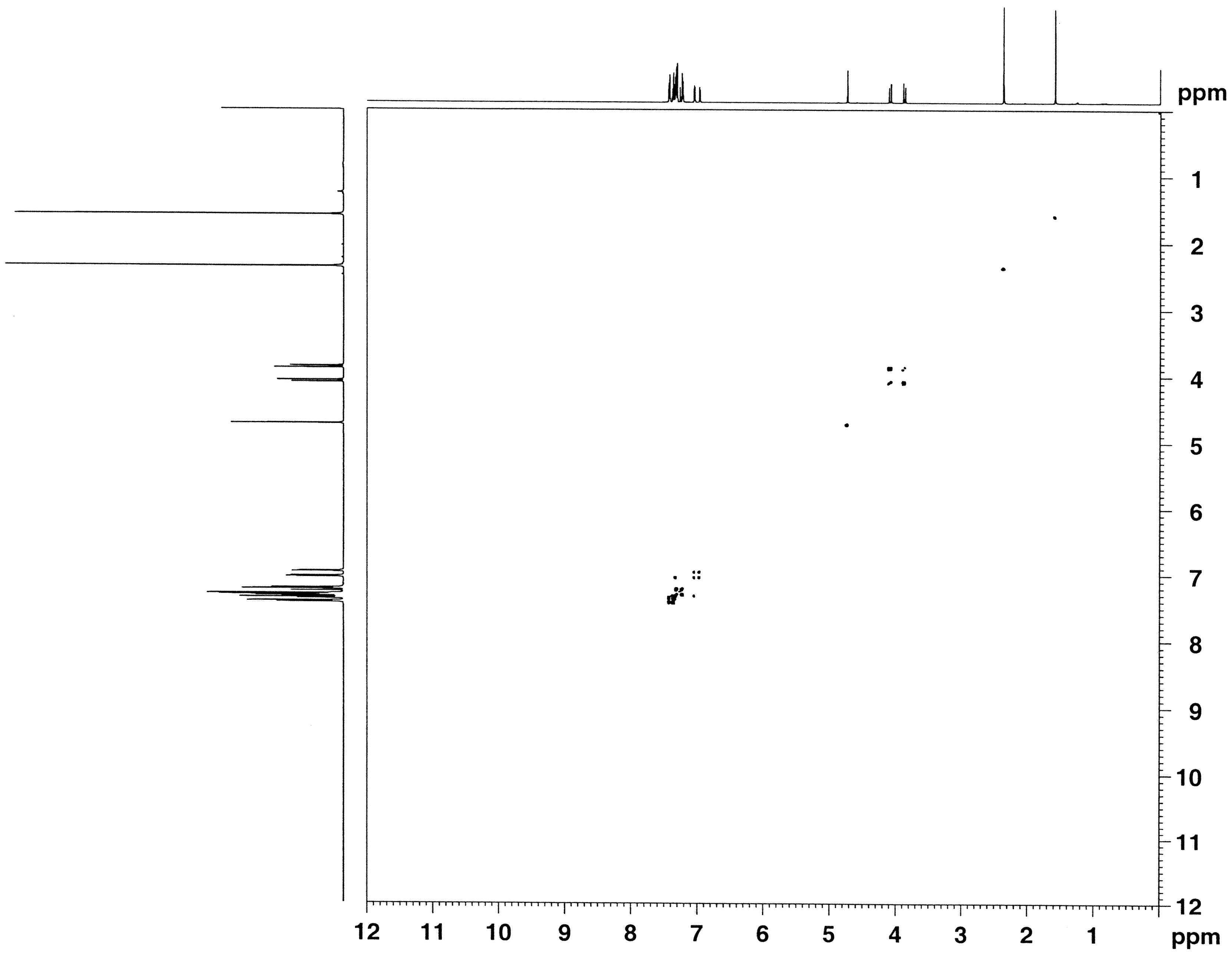

PN022_2_2 in cdcl3 (COSY) 29.8.2019

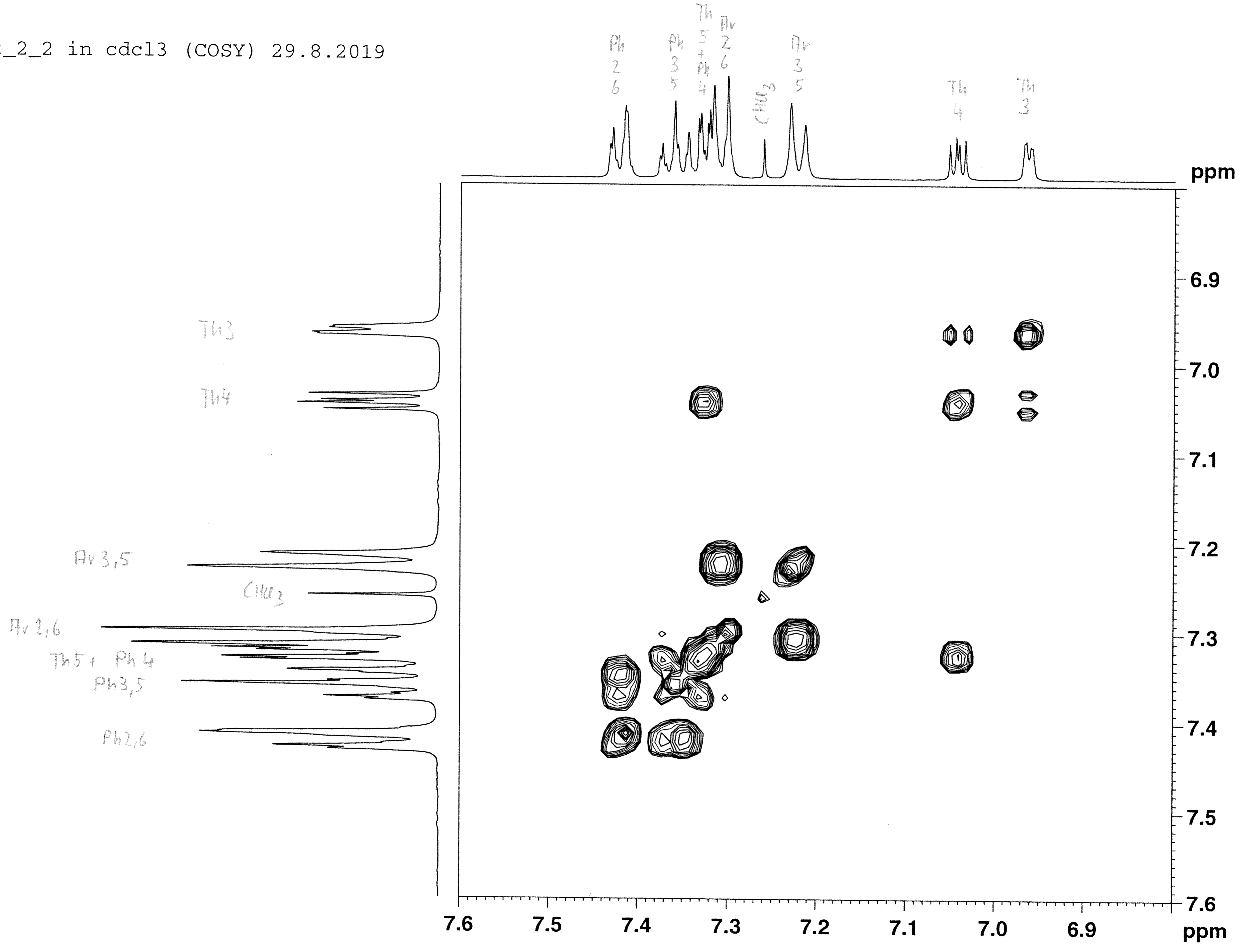

PN022_2_2 in cdcl3 (COSY) 29.8.2019

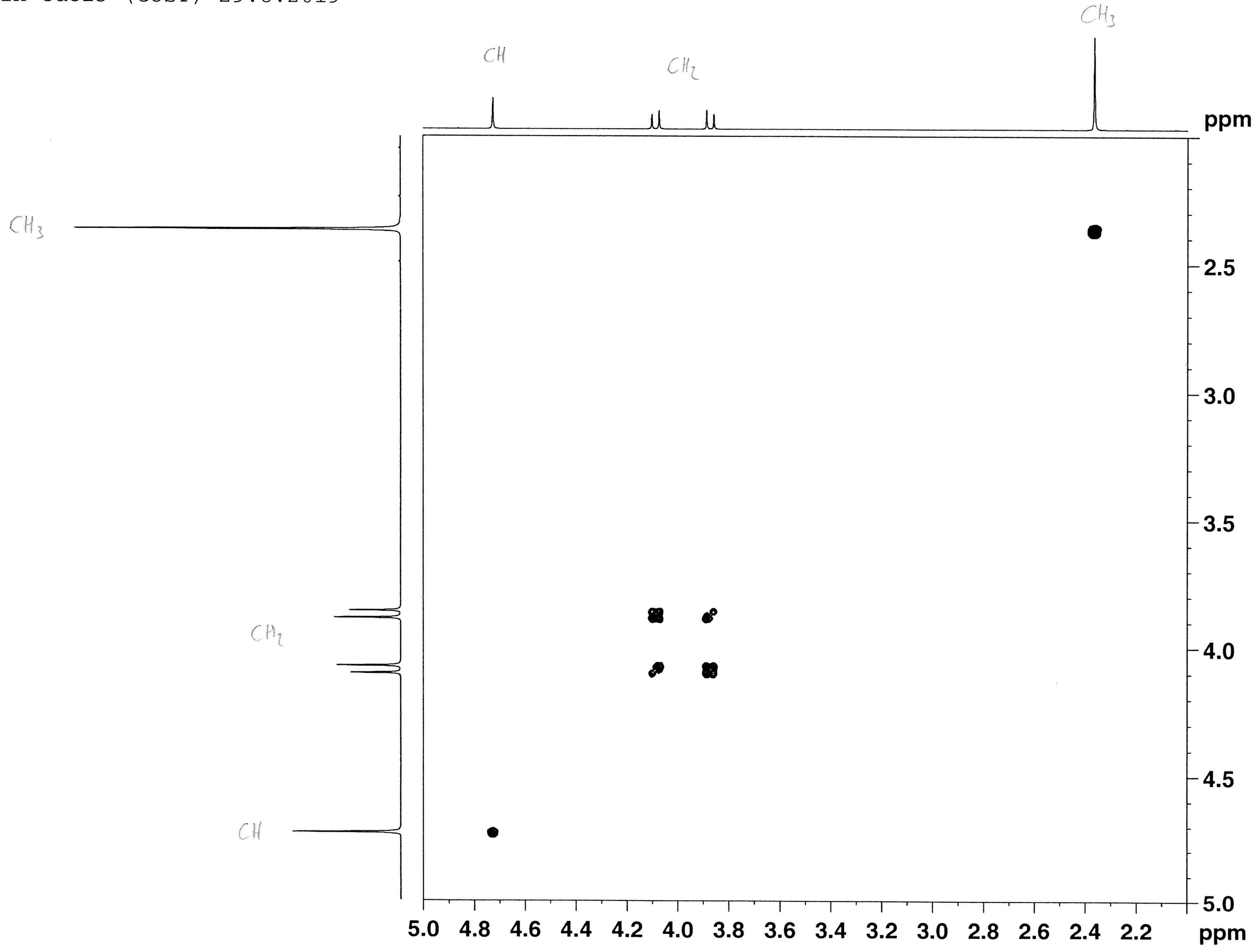

PN022_2_2 in cdcl3 (HSQC) 29.8.2019

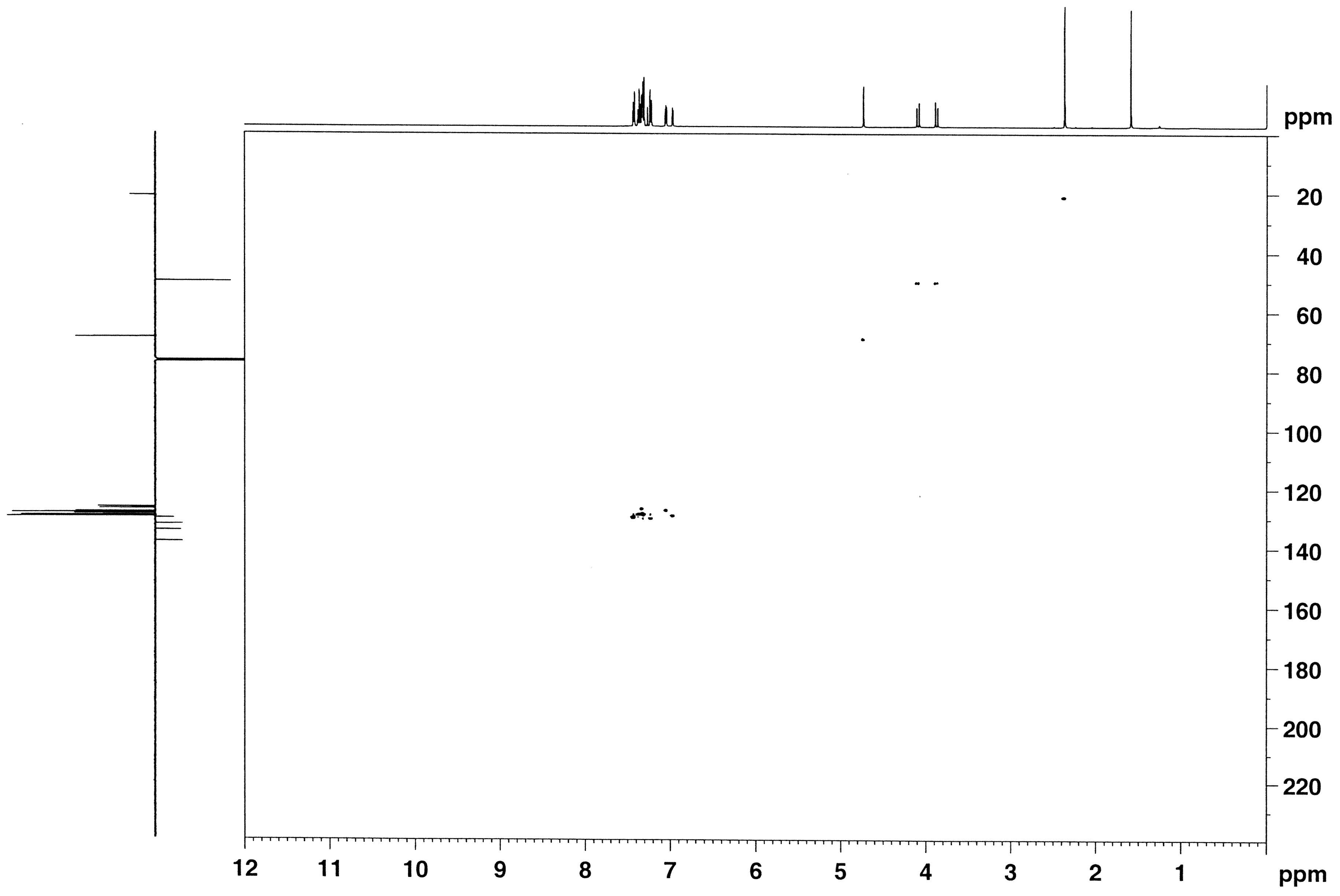

PN022_2_2 in cdcl3 (HSQC) 29.8.2019

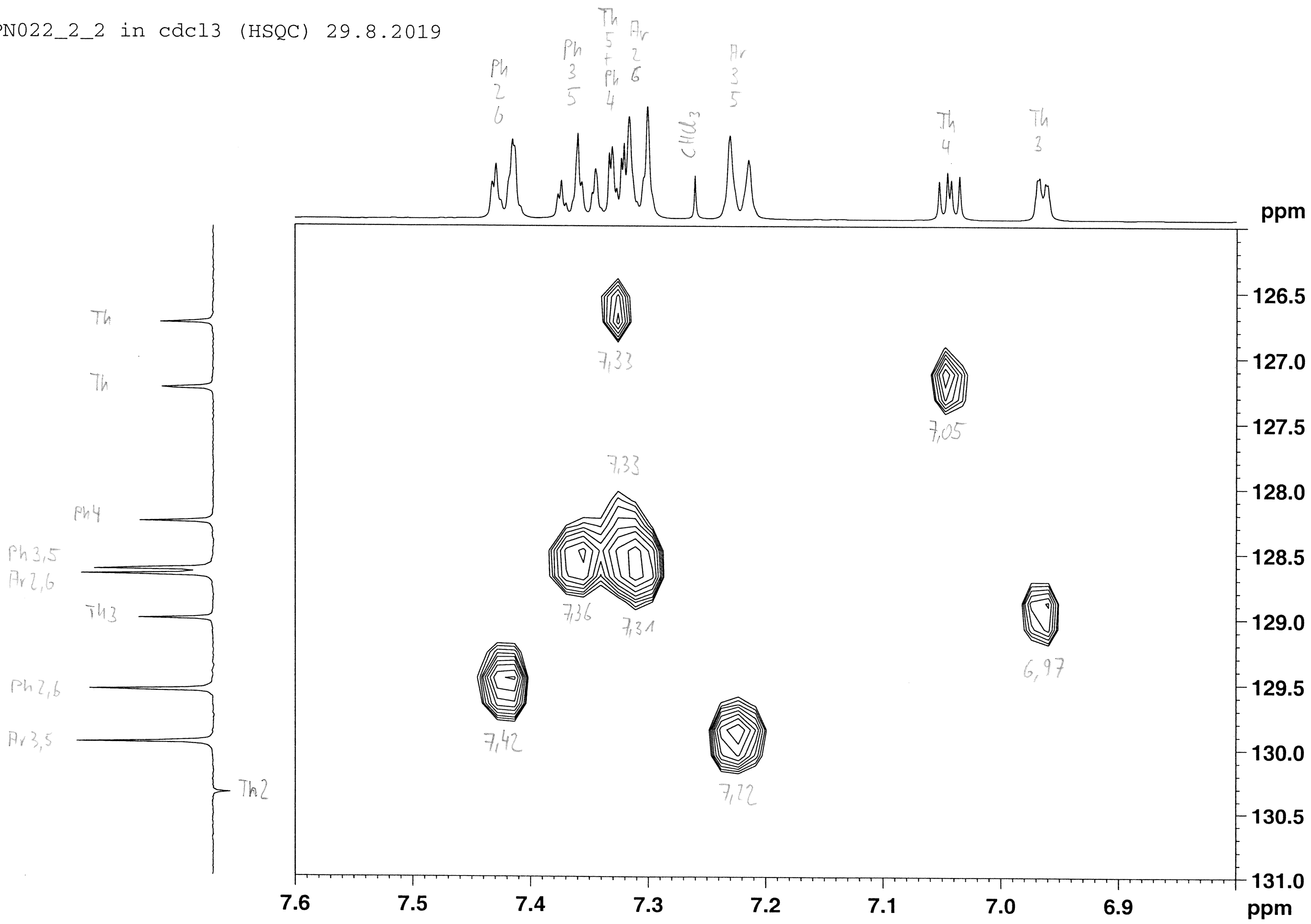

PN022_2_2 in cdcl3 (HSQC) 29.8.2019

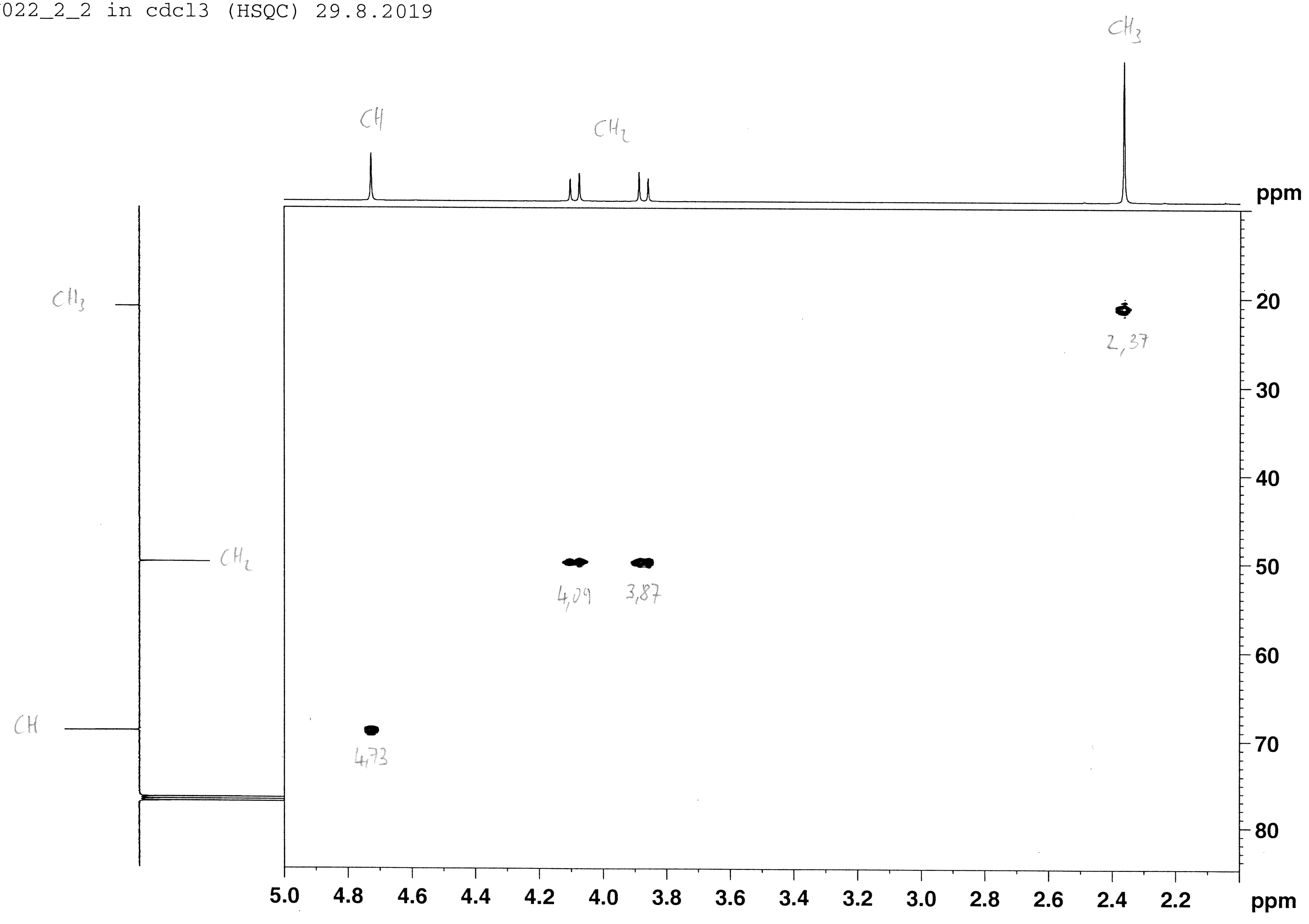

PN022_2_2 in cdcl3 (HMBC) 29.8.2019

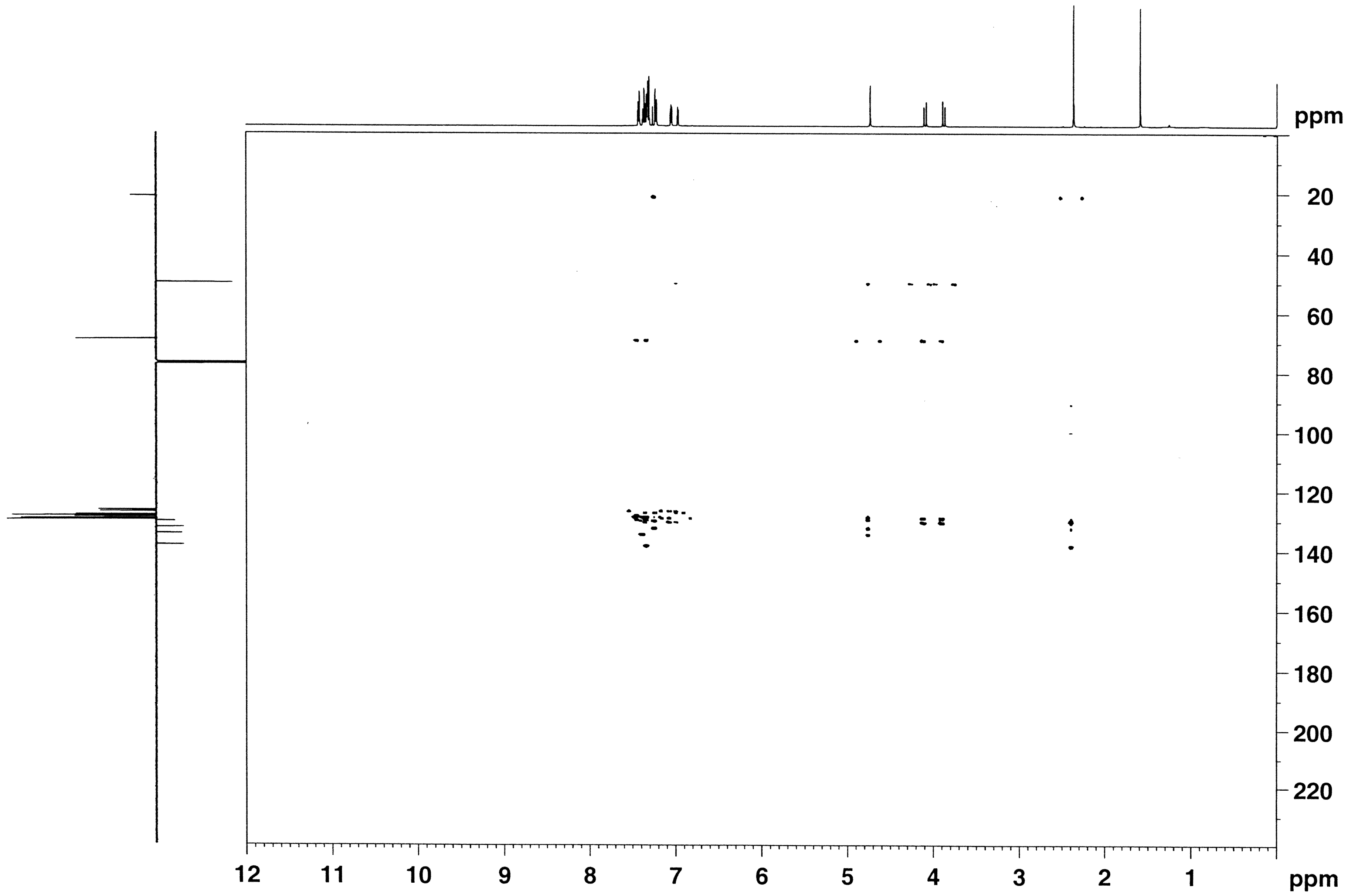

PN022_2_2 in cdcl3 (HMBC) 29.8.2019

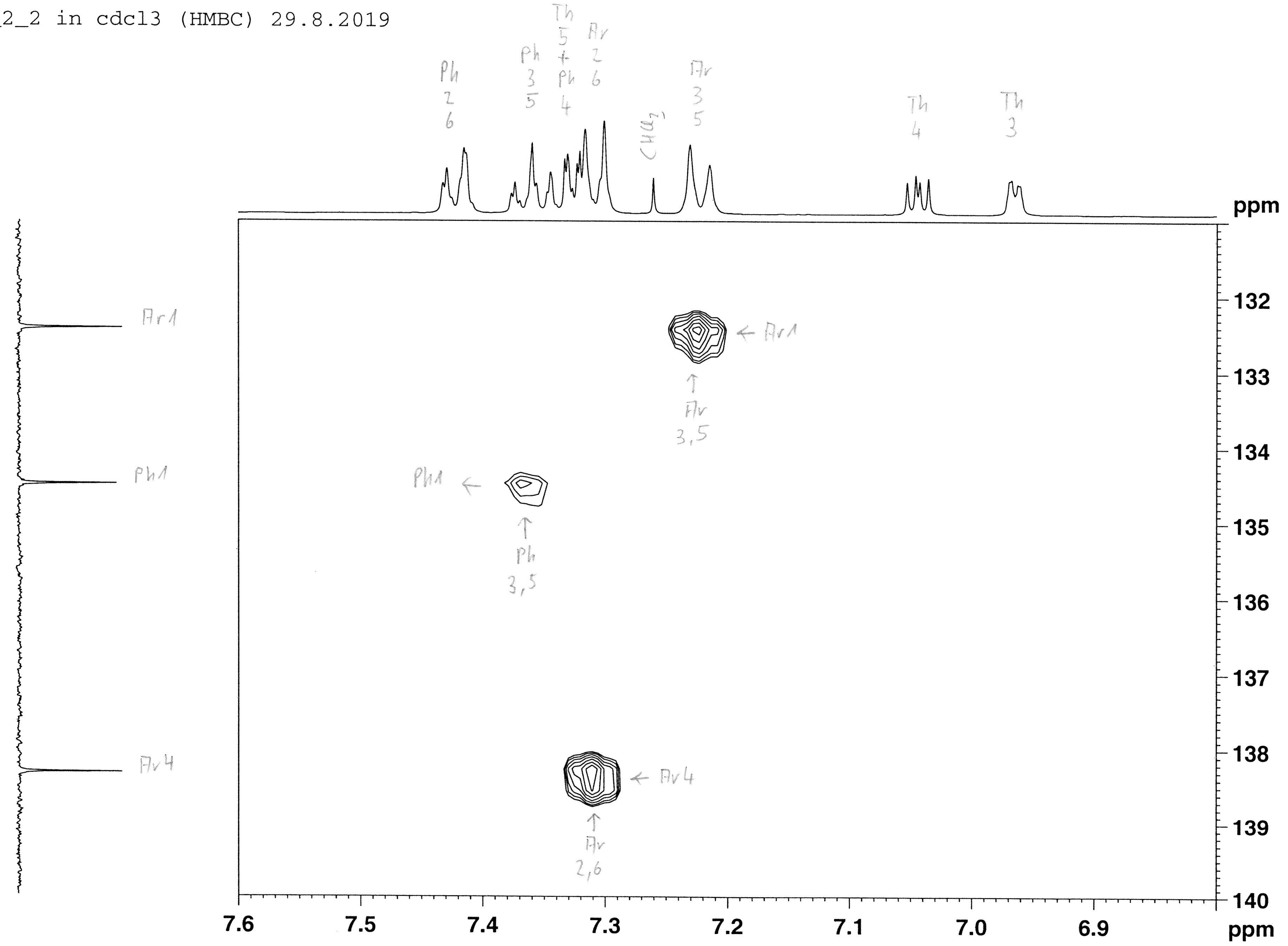

PN022_2_2 in cdcl3 (HMBC) 29.8.2019

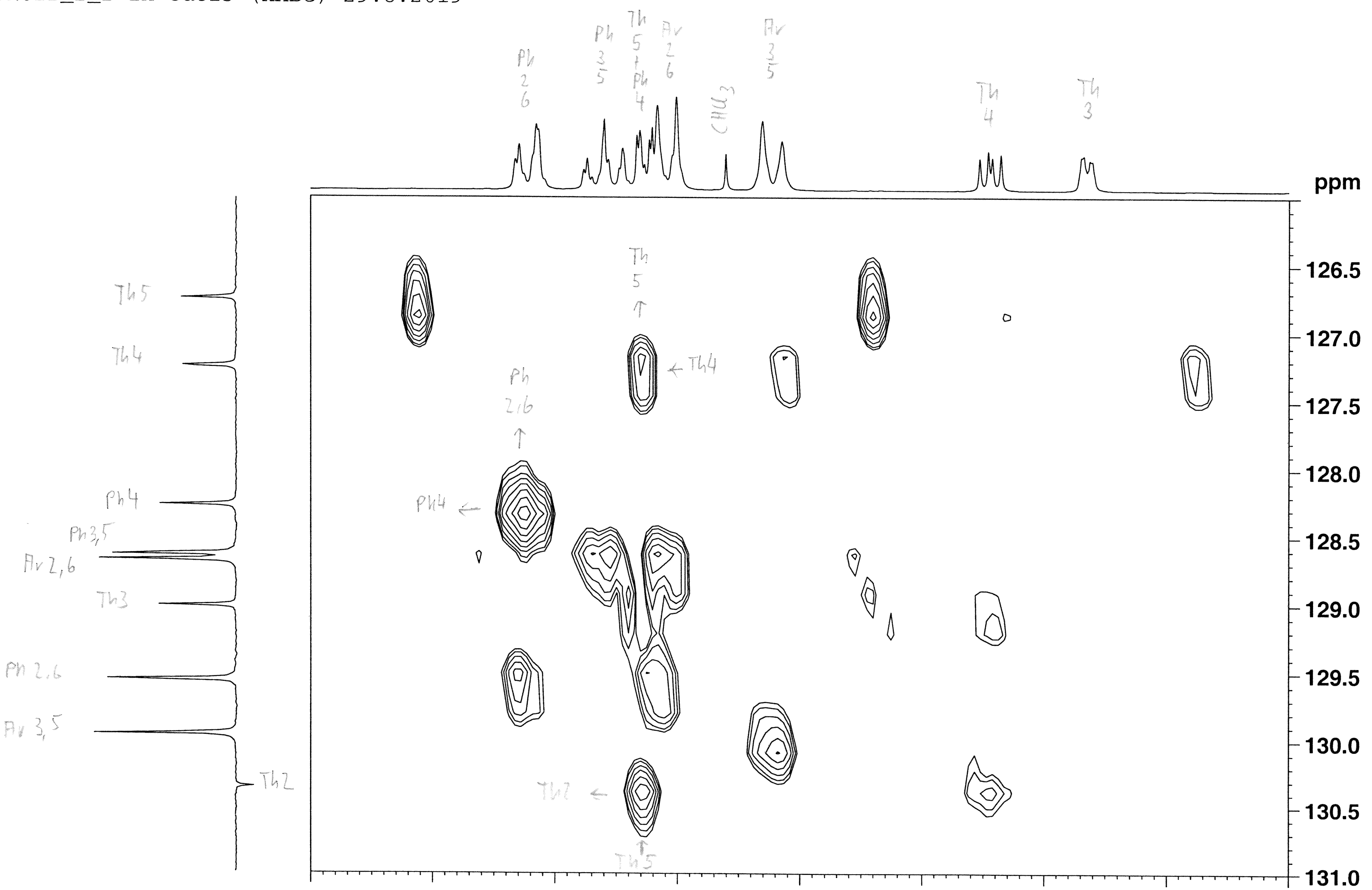

PN022_2_2 in cdcl3 (HMBC) 29.8.2019

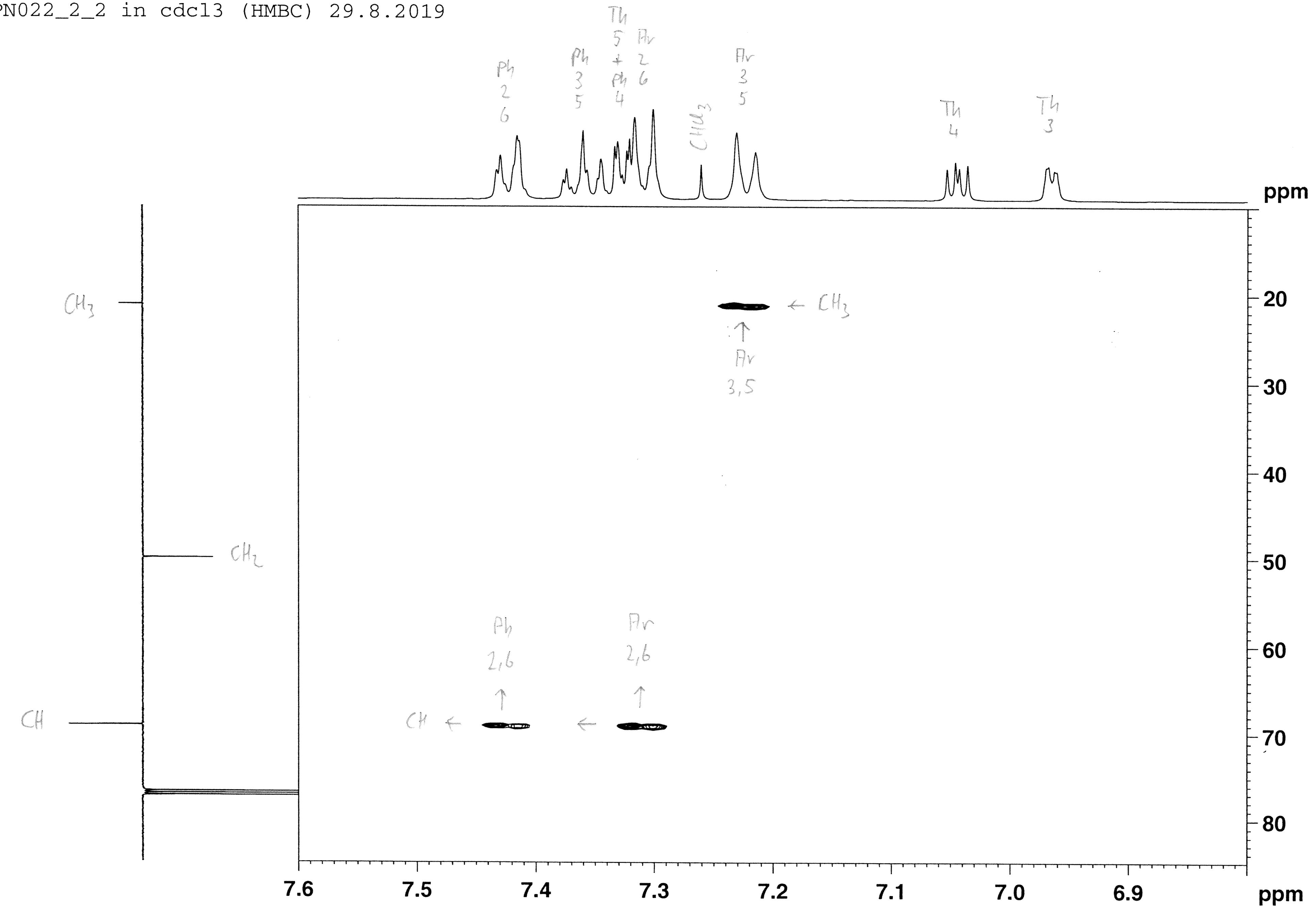

PN022_2_2 in cdcl3 (HMBC) 29.8.2019

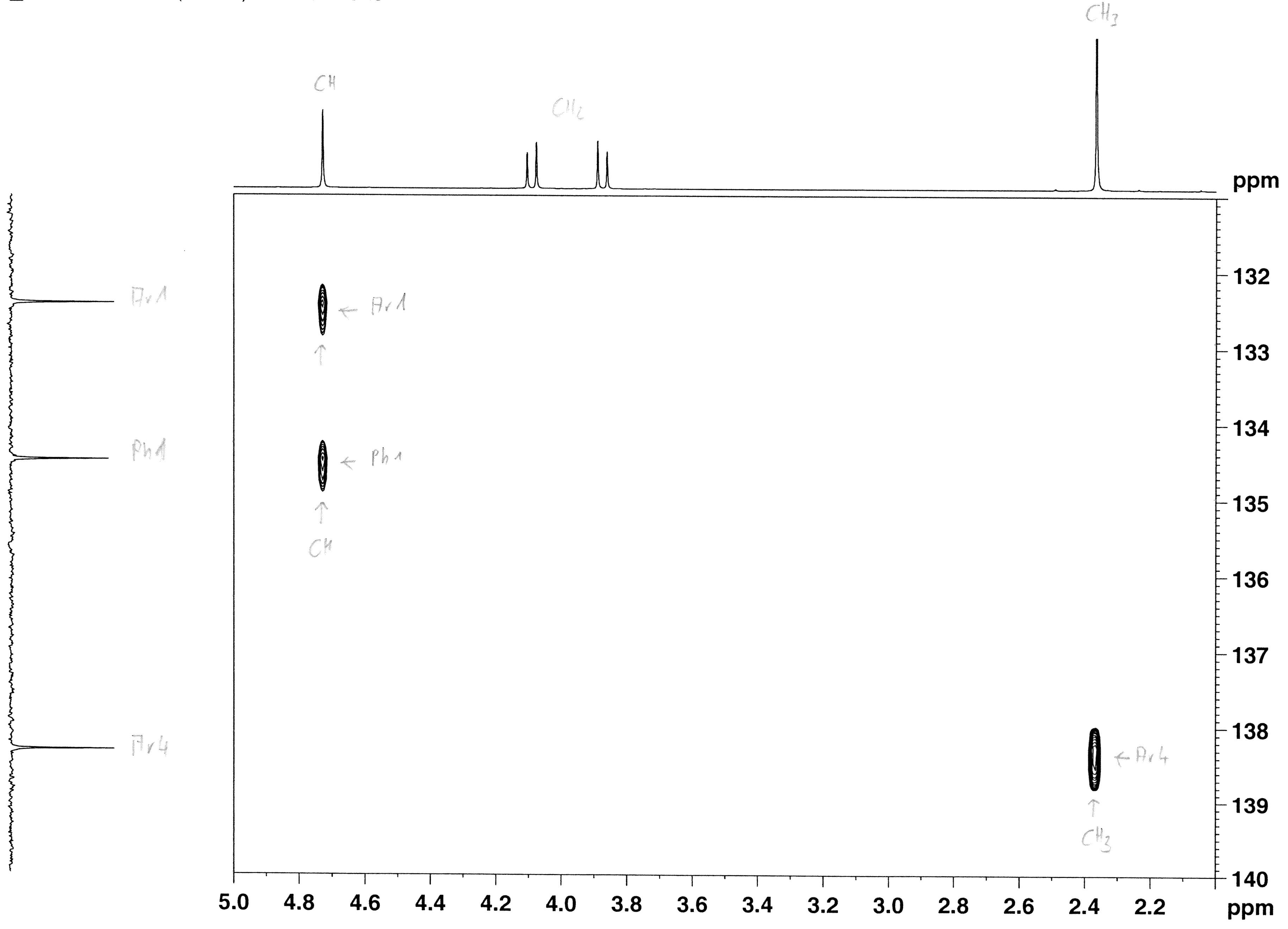

PN022_2_2 in cdcl3 (HMBC) 29.8.2019

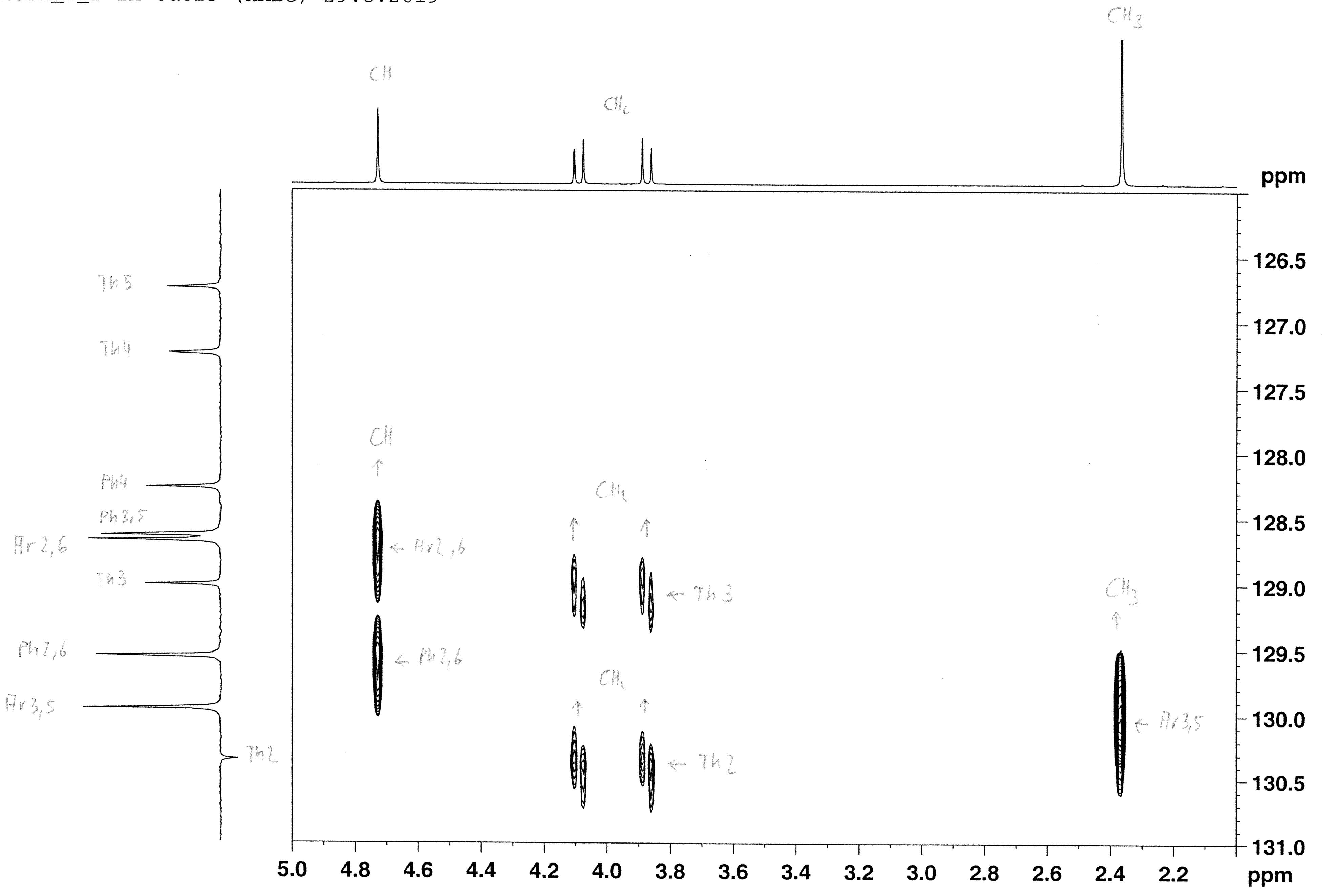

PN022_2_2 in cdcl3 (HMBC) 29.8.2019

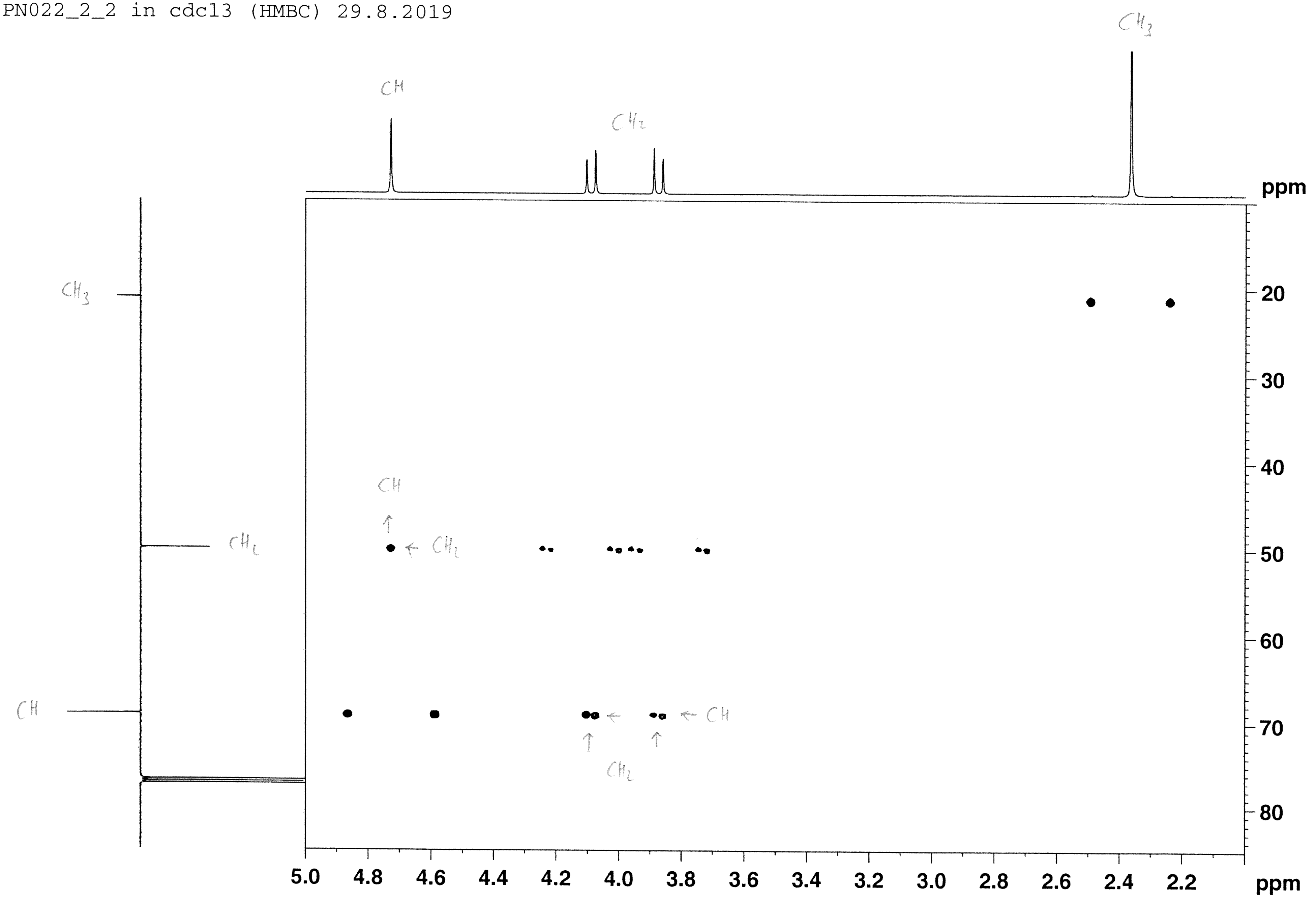

Figure S57c. NMR spectra of compound **5o.**

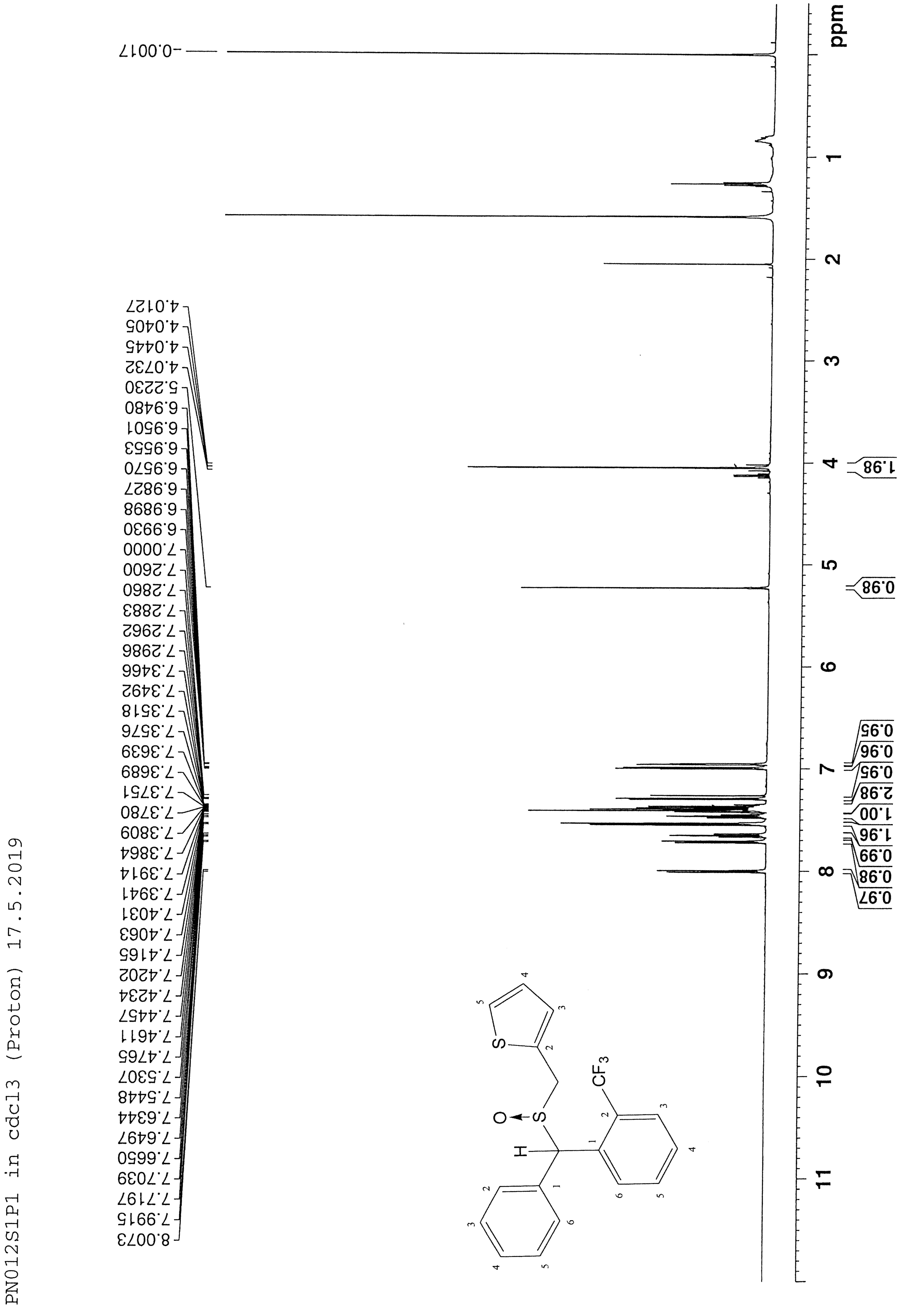

PN012S1P1 in cdcl3 (Proton) 17.5.2019

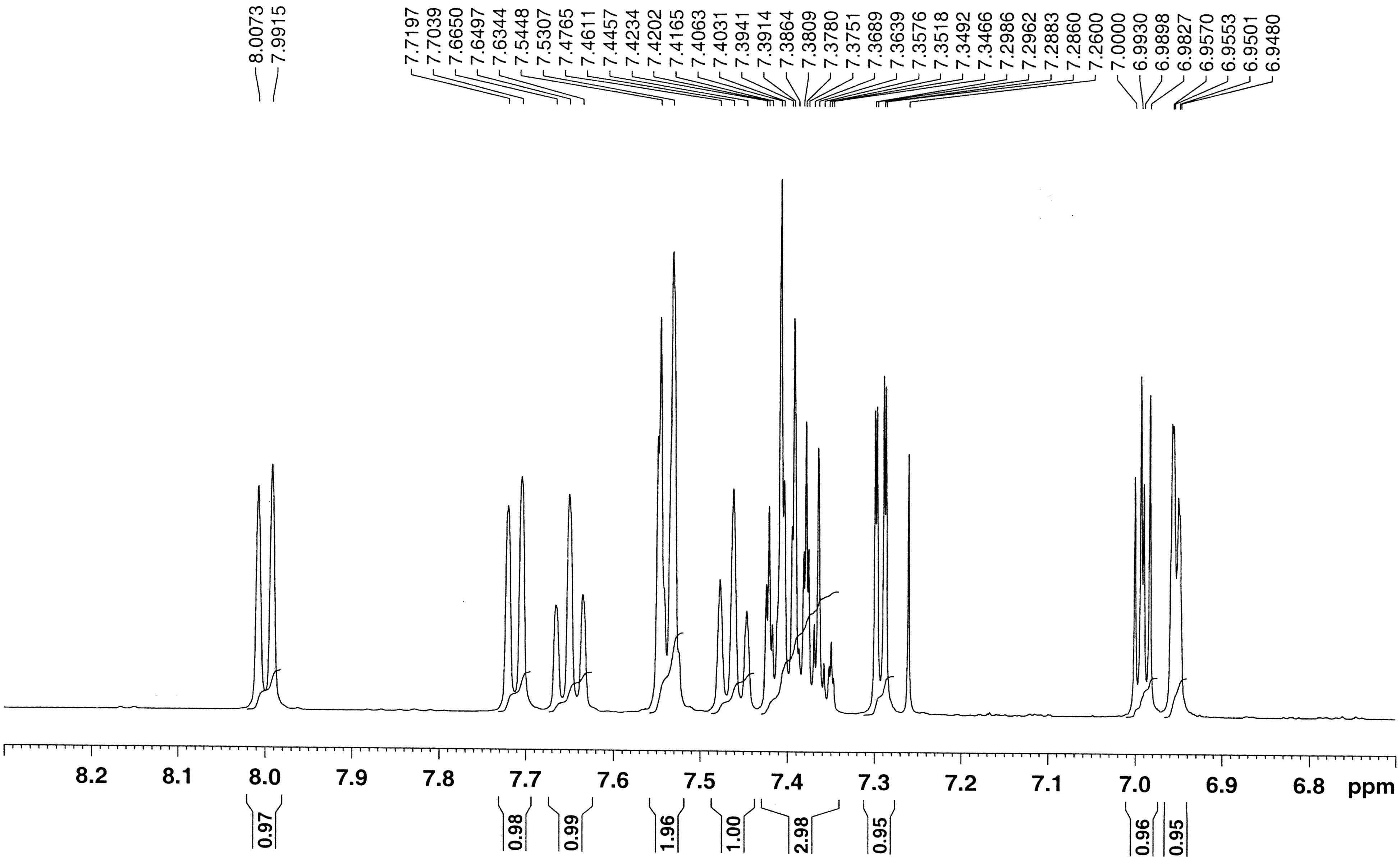

PN012S1P1 in cdcl3 (Proton) 17.5.2019

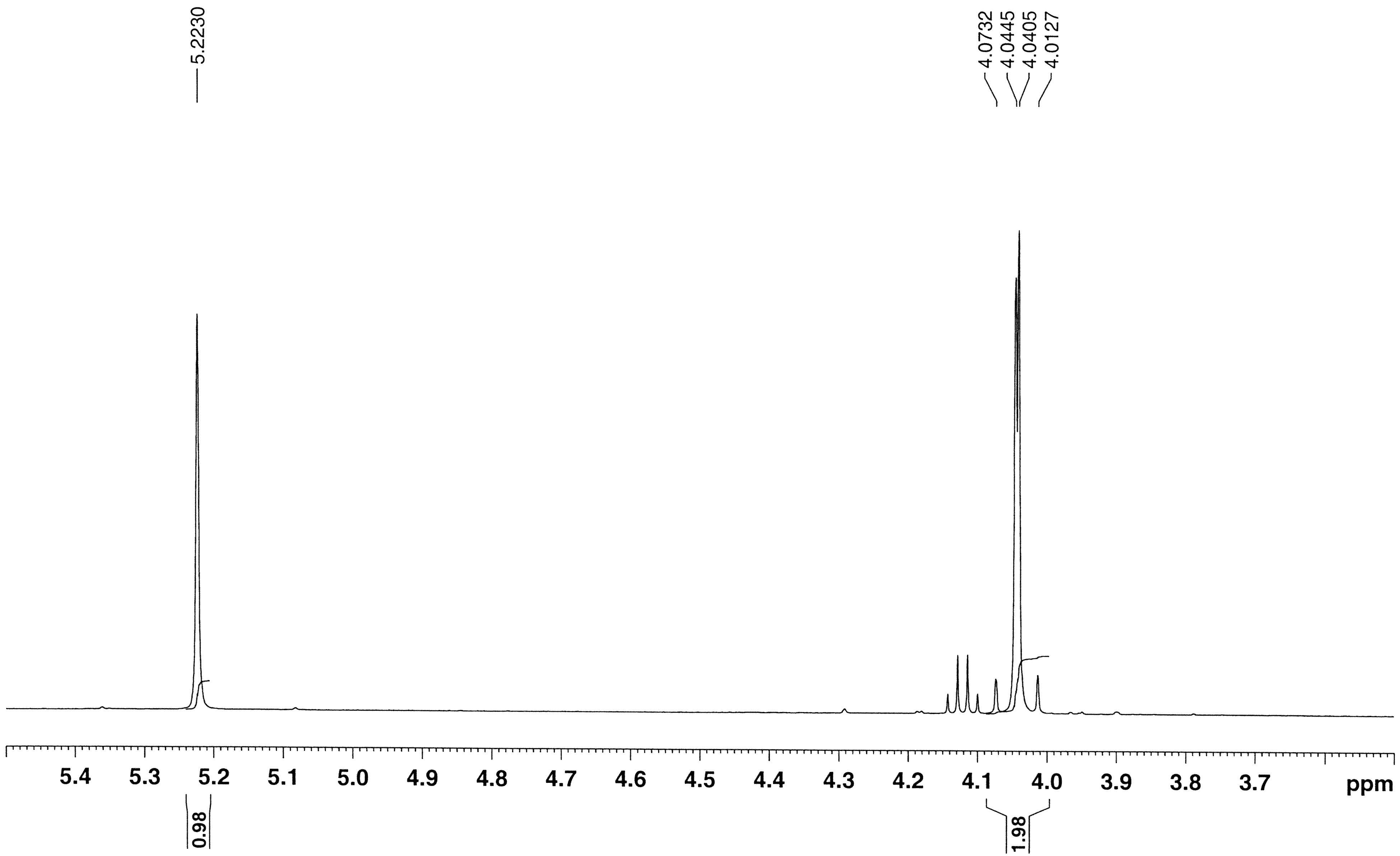

PN012S1P1 in cdcl3 (APT) 17.5.2019

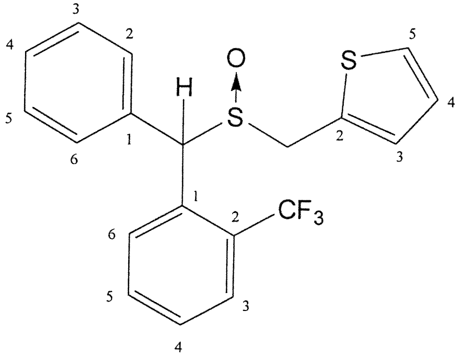

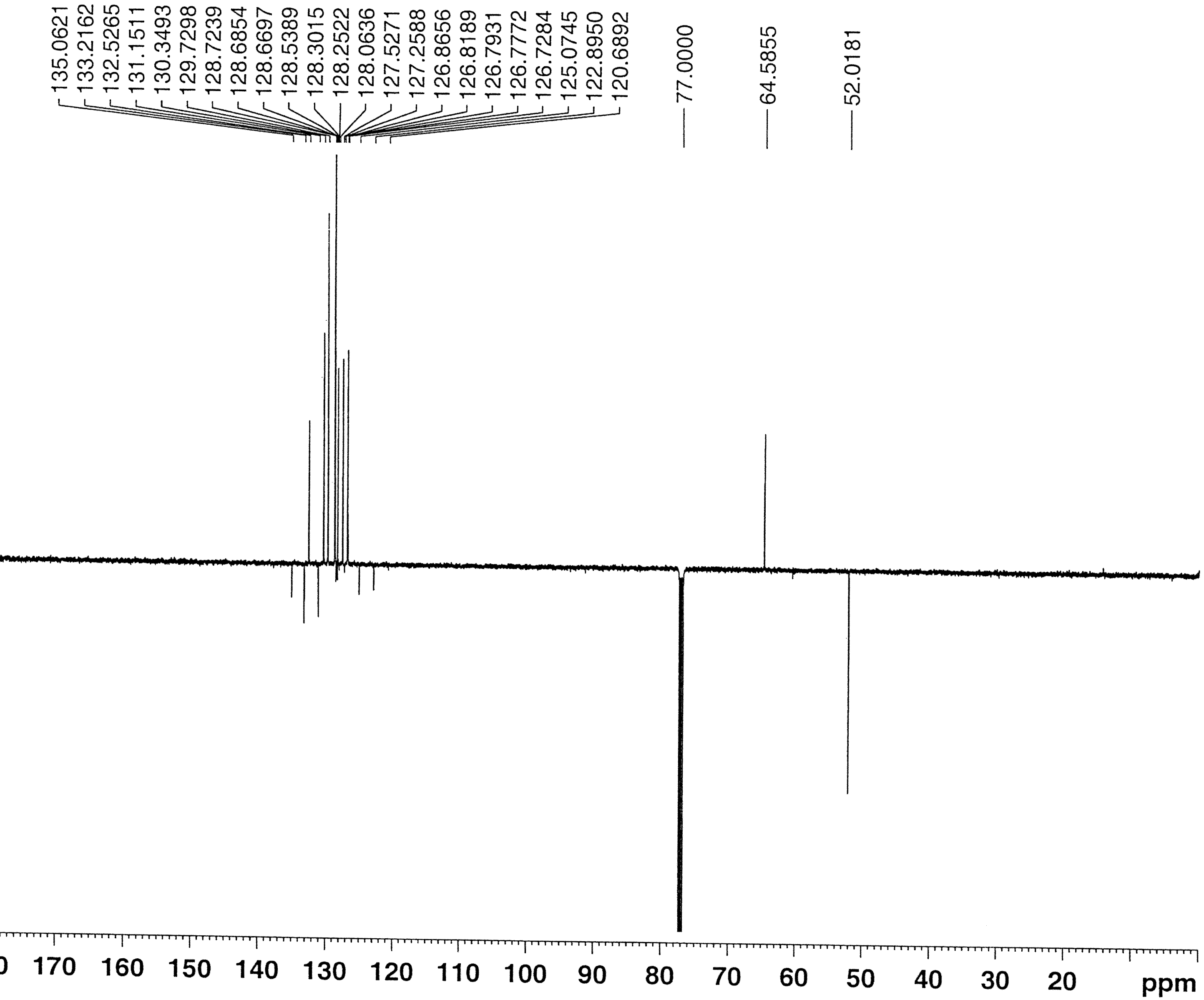

PN012S1P1 in cdcl3 (APT) 17.5.2019

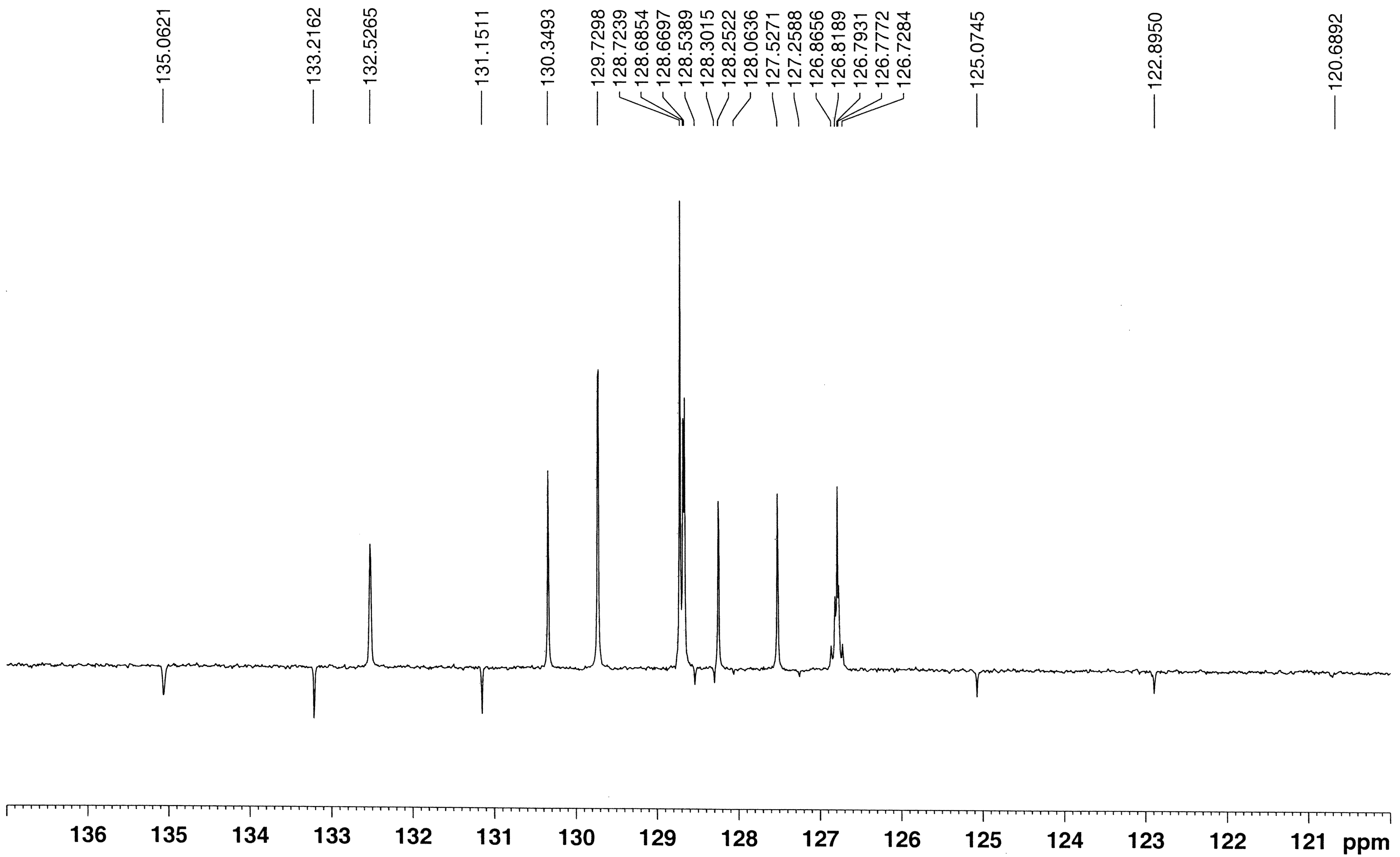

PN012S1P1 in cdcl3 (APT) 17.5.2019

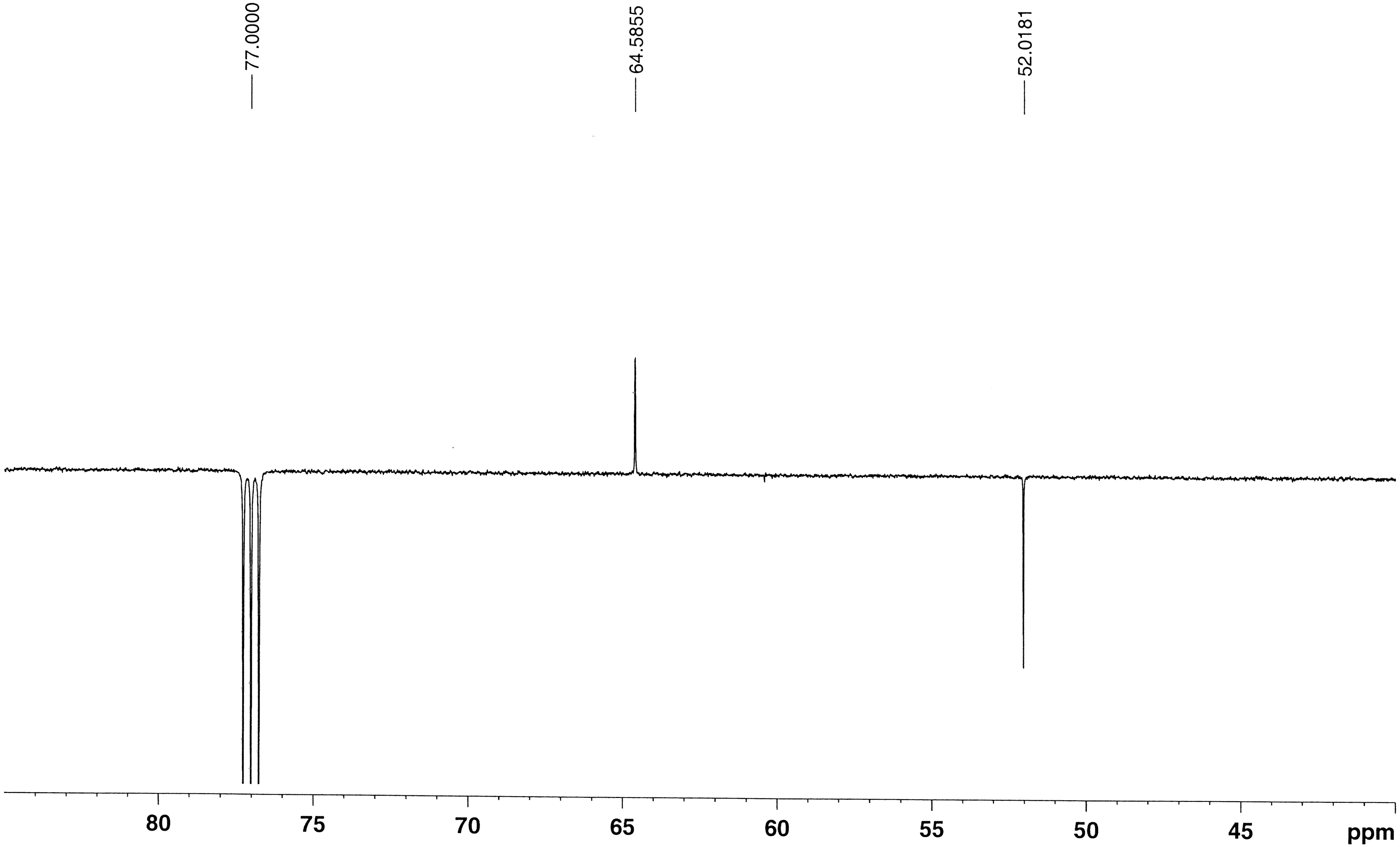

Figure S58c. NMR spectra of compound **6o**.

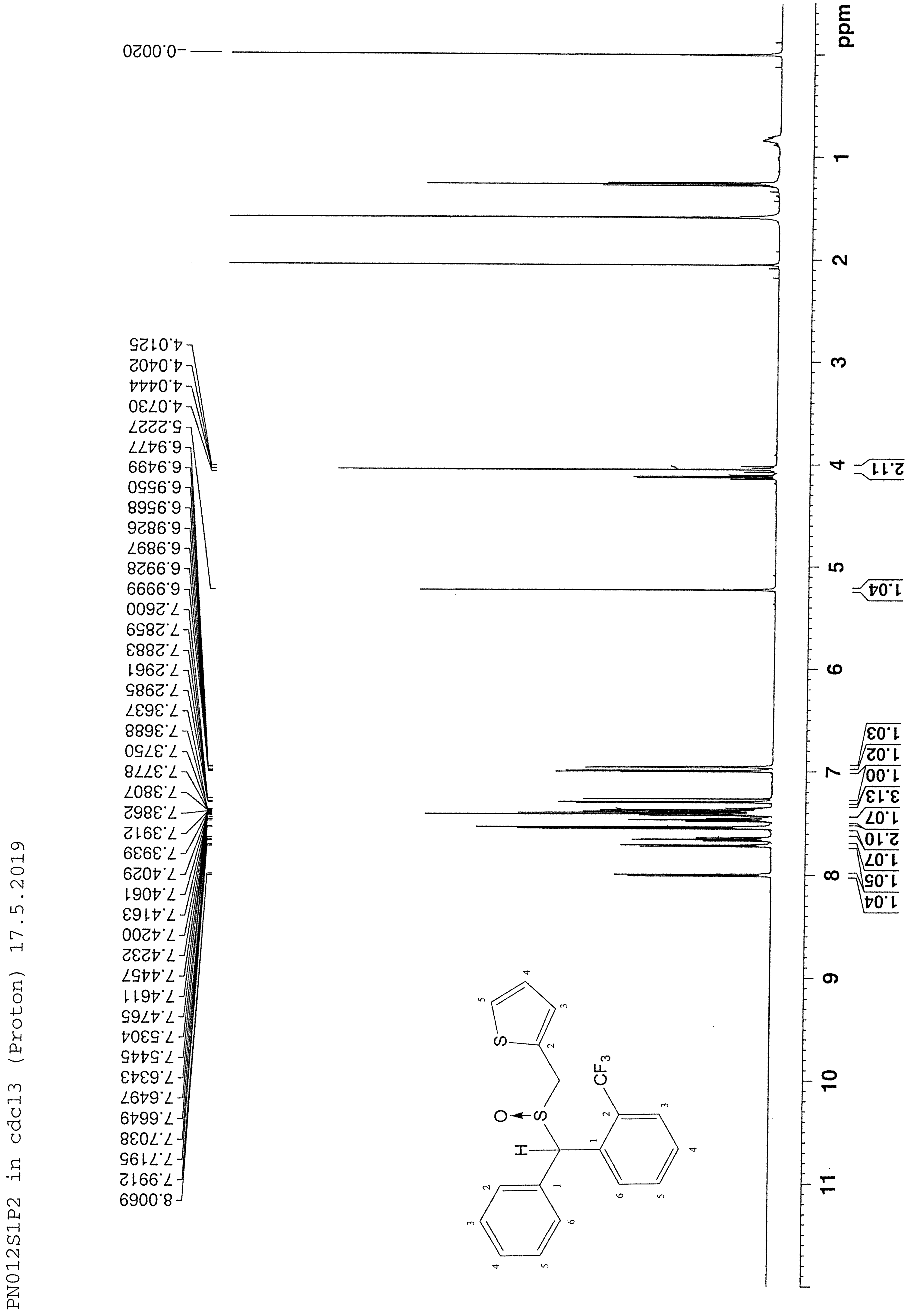

PN012S1P2 in cdcl3 (Proton) 17.5.2019

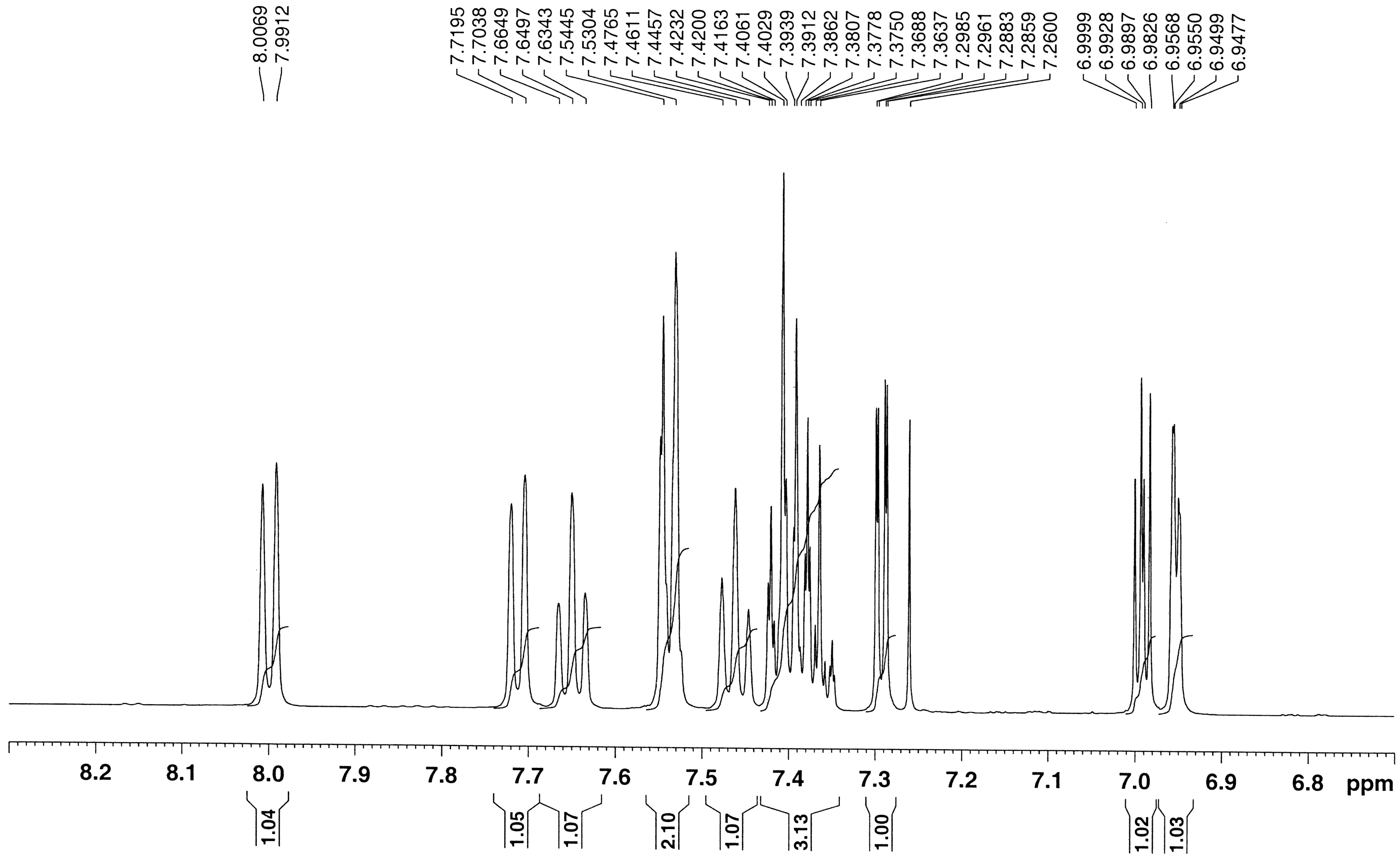

PN012S1P2 in cdcl3 (Proton) 17.5.2019

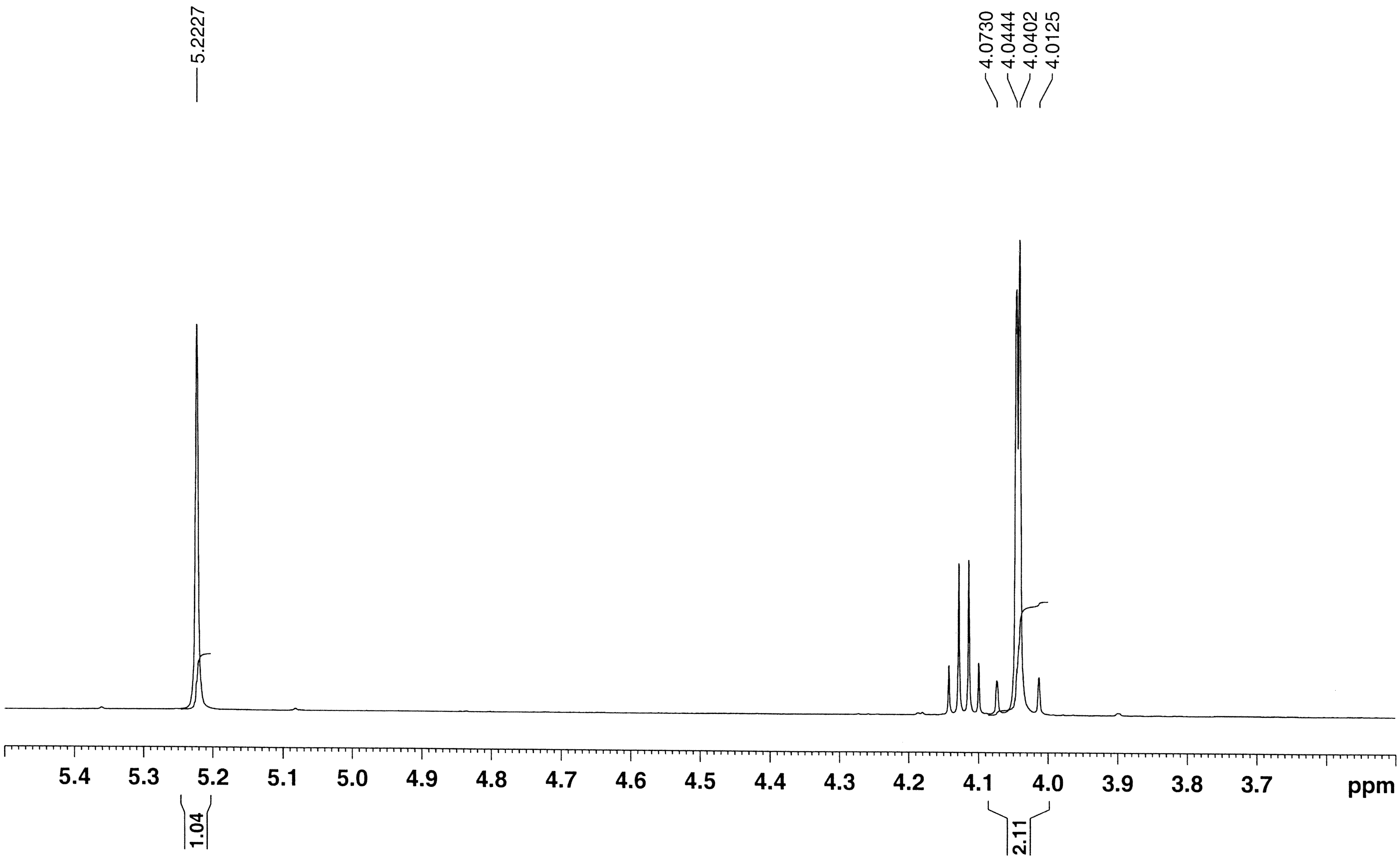

PN012S1P2 in cdcl3 (APT) 17.5.2019

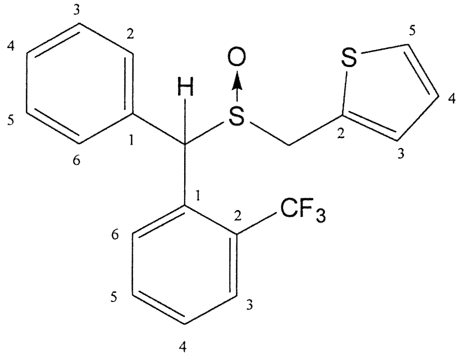

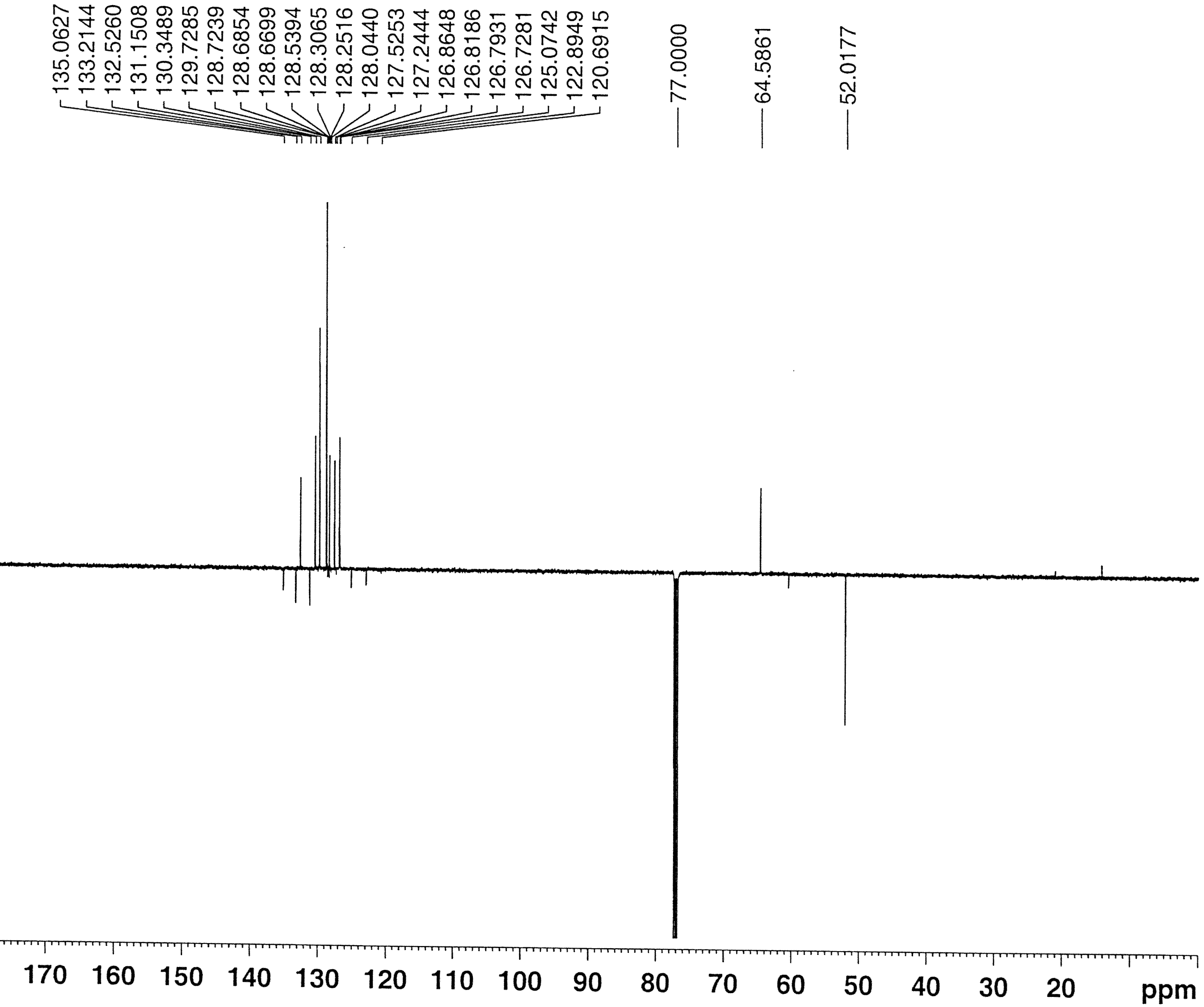

PN012S1P2 in cdcl3 (APT) 17.5.2019

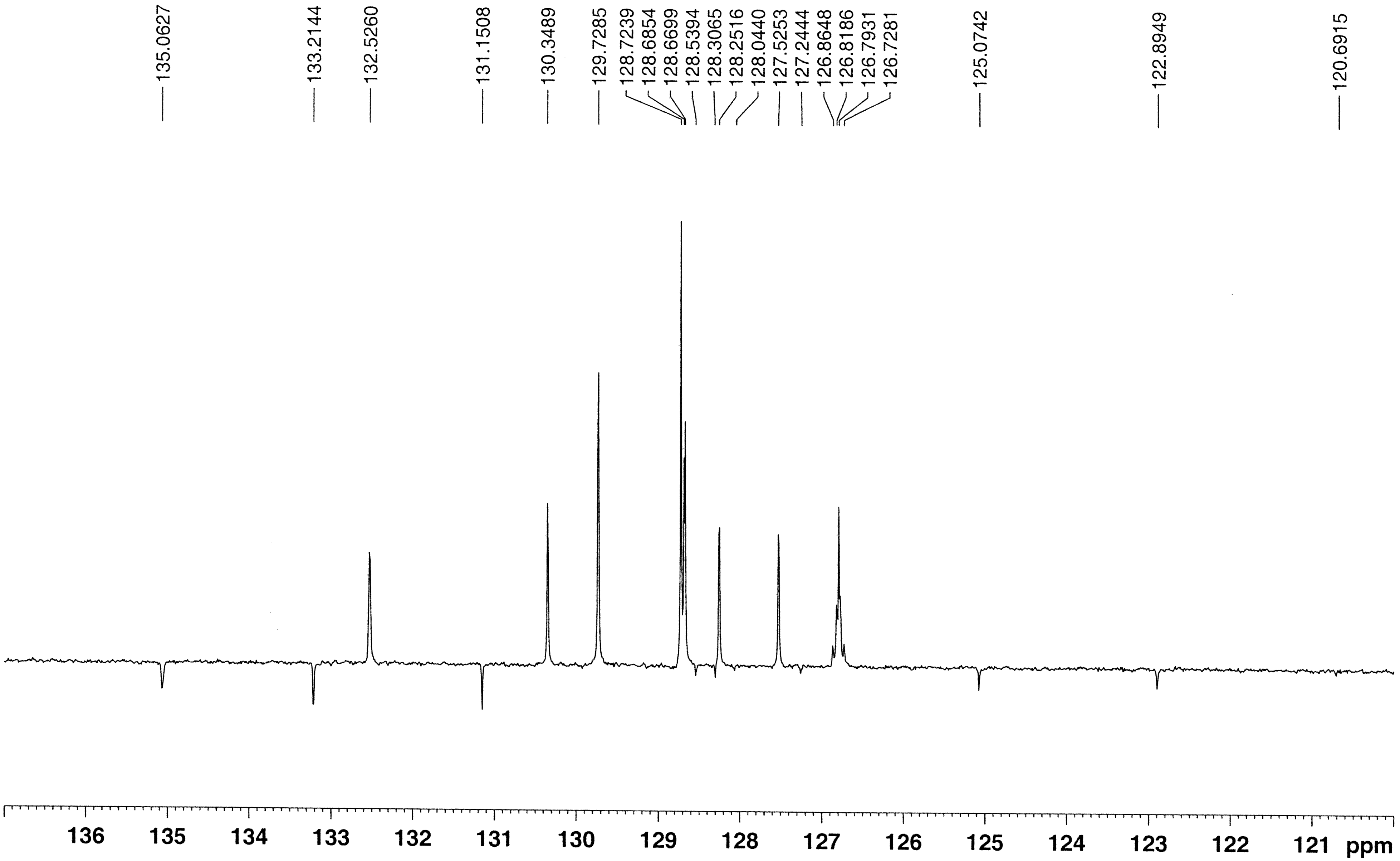

PN012S1P2 in cdcl3 (APT) 17.5.2019

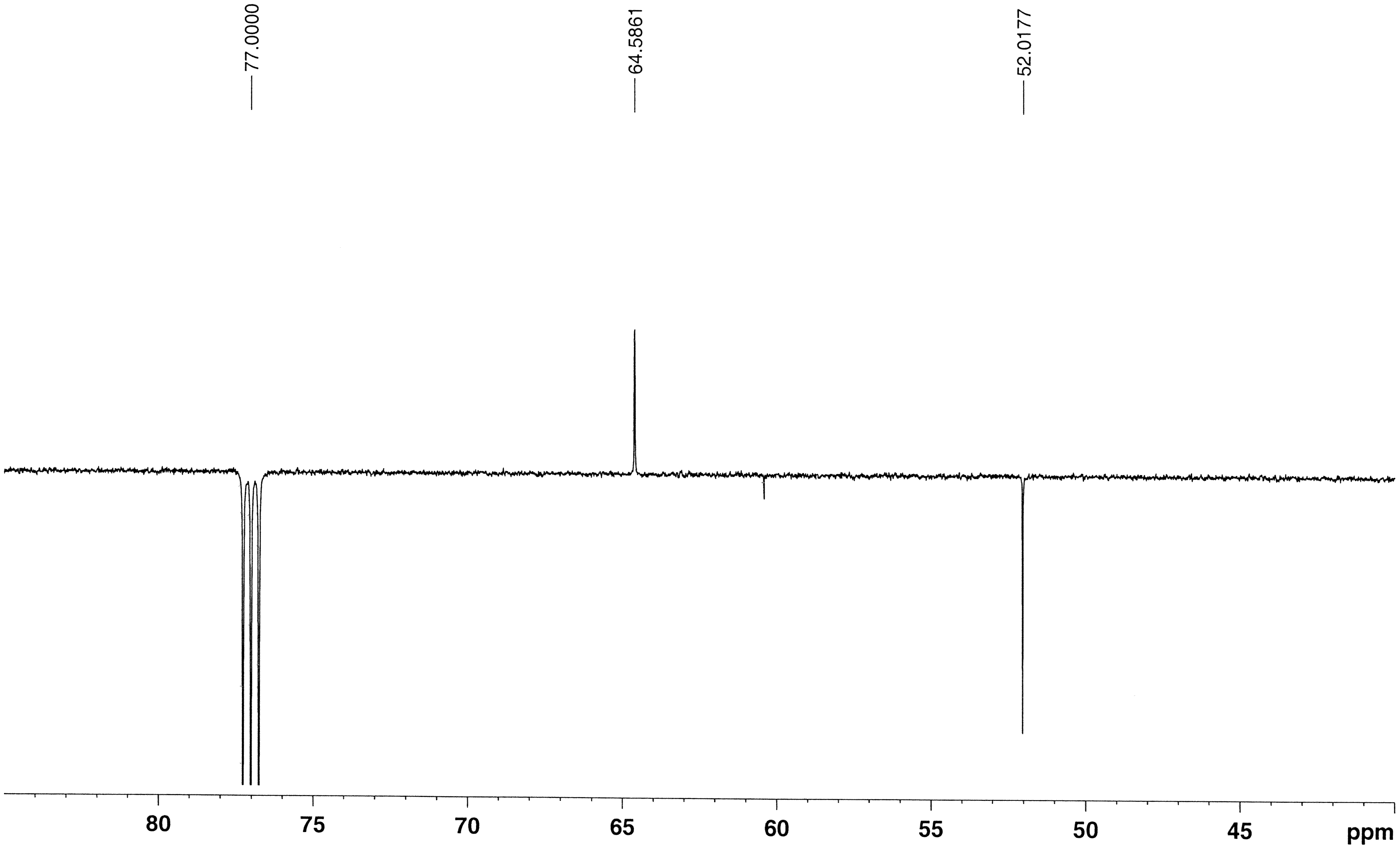

Figure S59c. NMR spectra of compound **7o.**

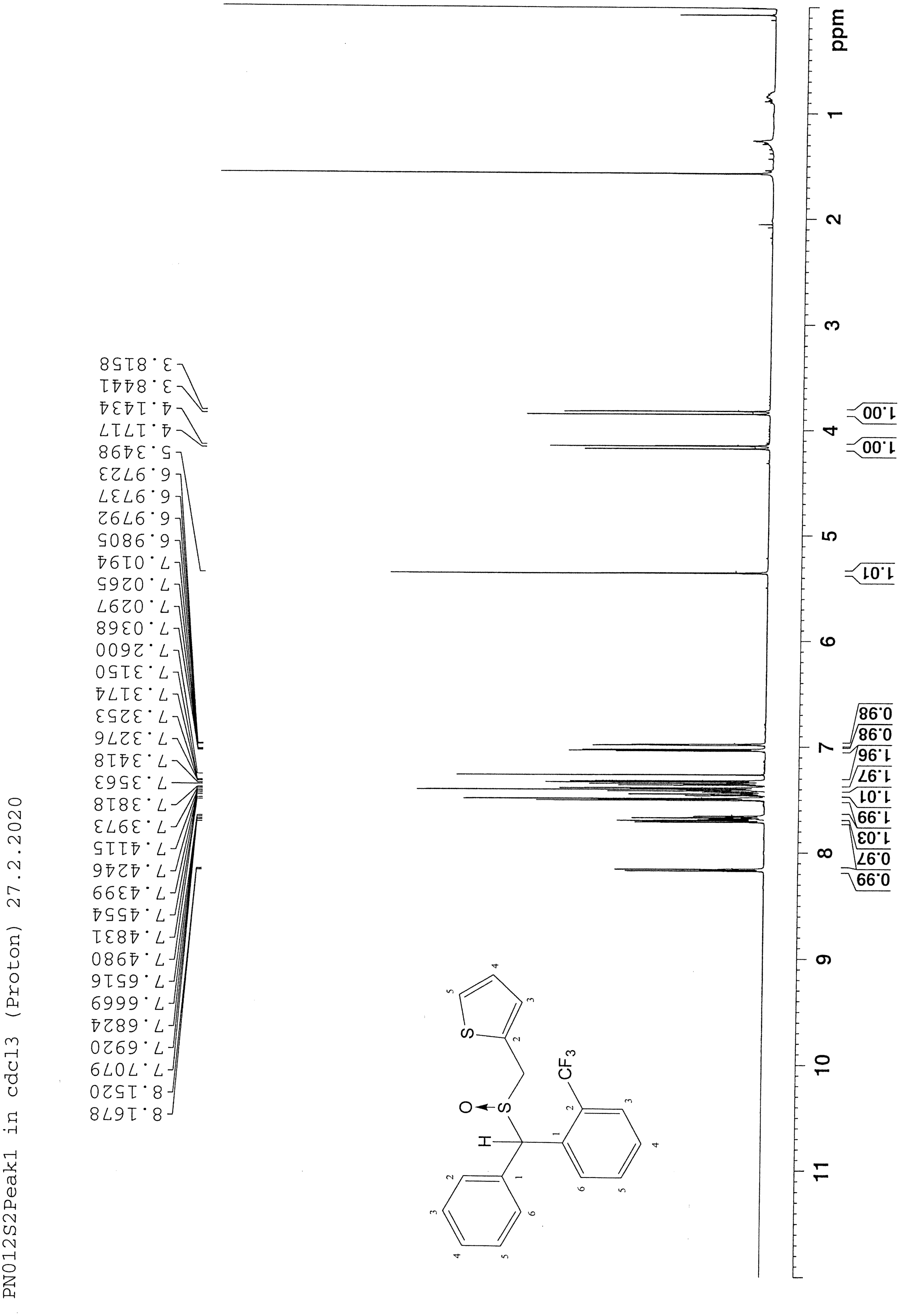

PN012S2Peak1 in cdcl3 (Proton) 27.2.2020

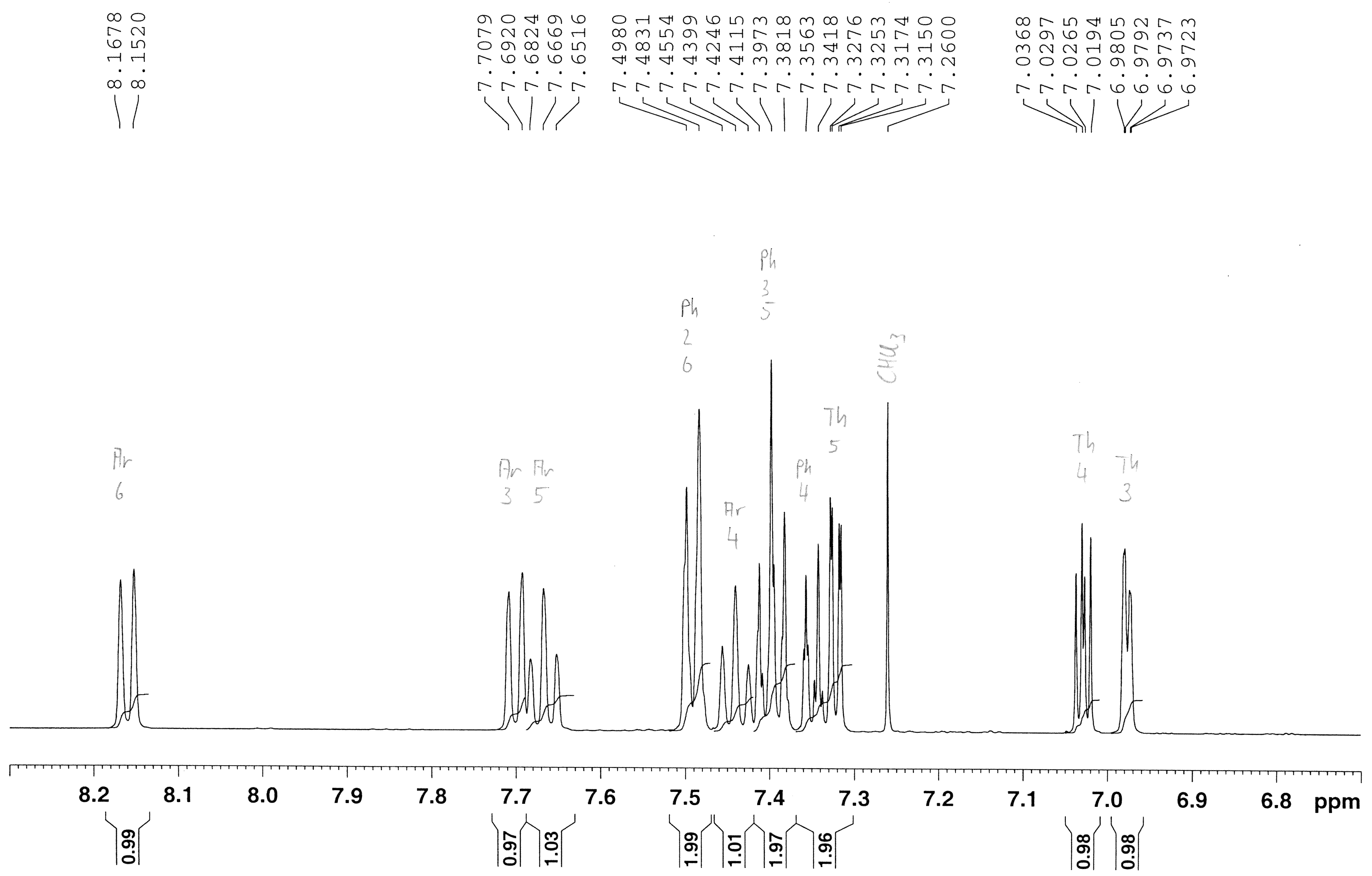

PN012S2Peak1 in cdcl3 (Proton) 27.2.2020

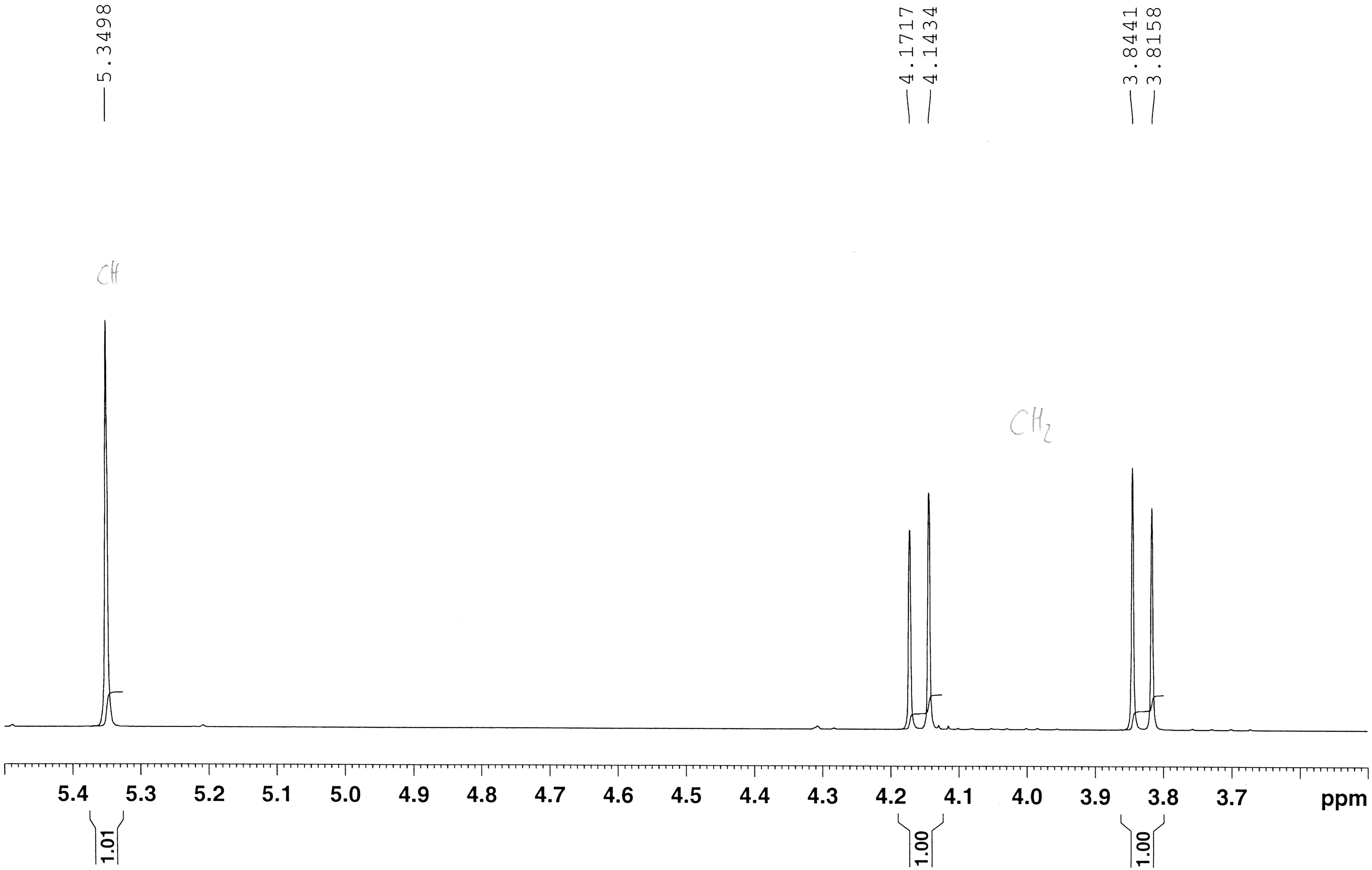

PN012S2Peak1 in cdcl3 (APT) 27.2.2020

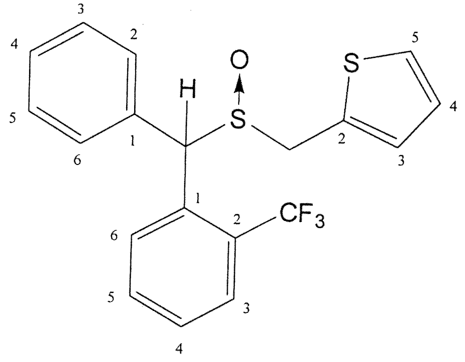

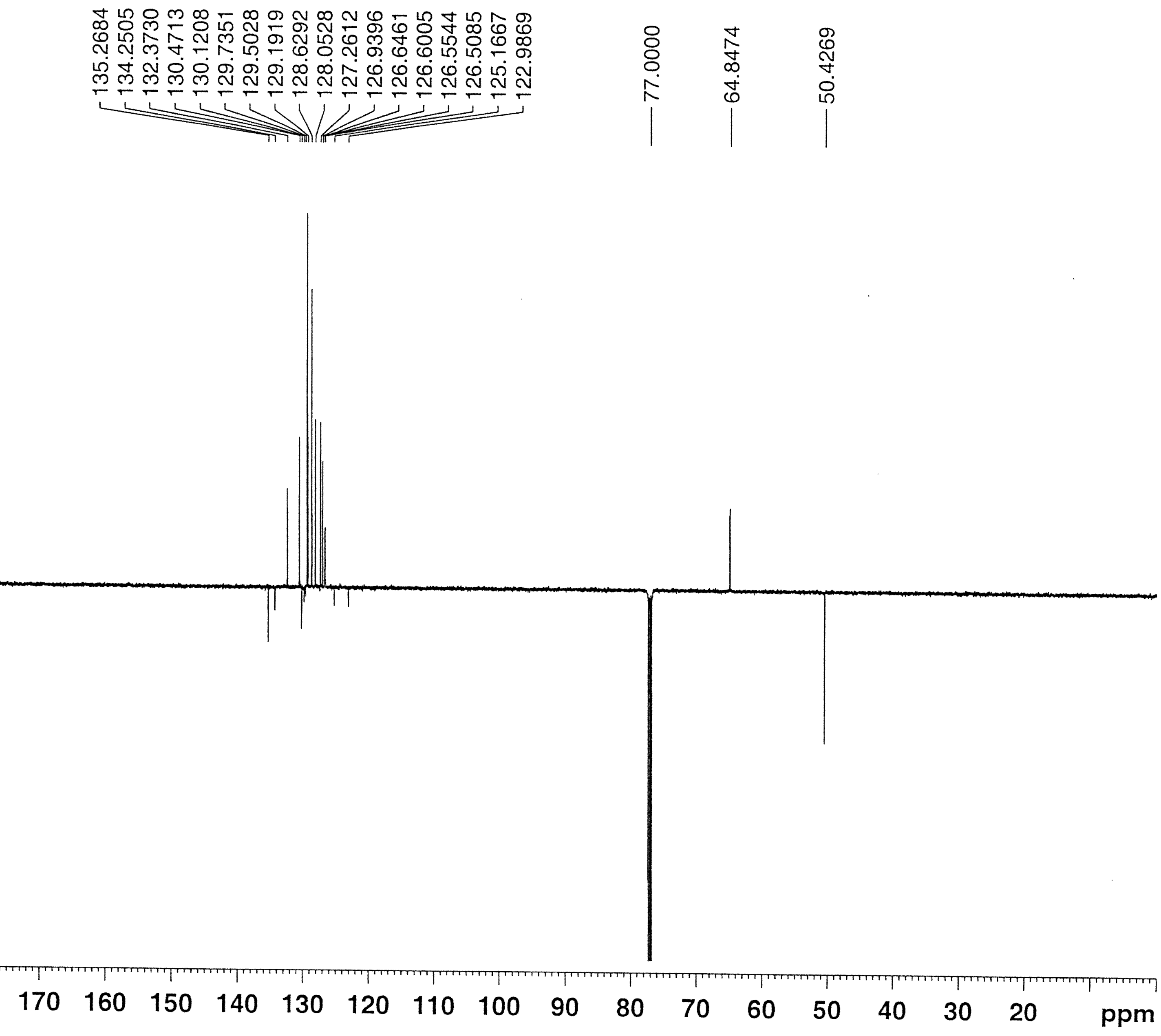

PN012S2Peak1 in cdcl3 (APT) 27.2.2020

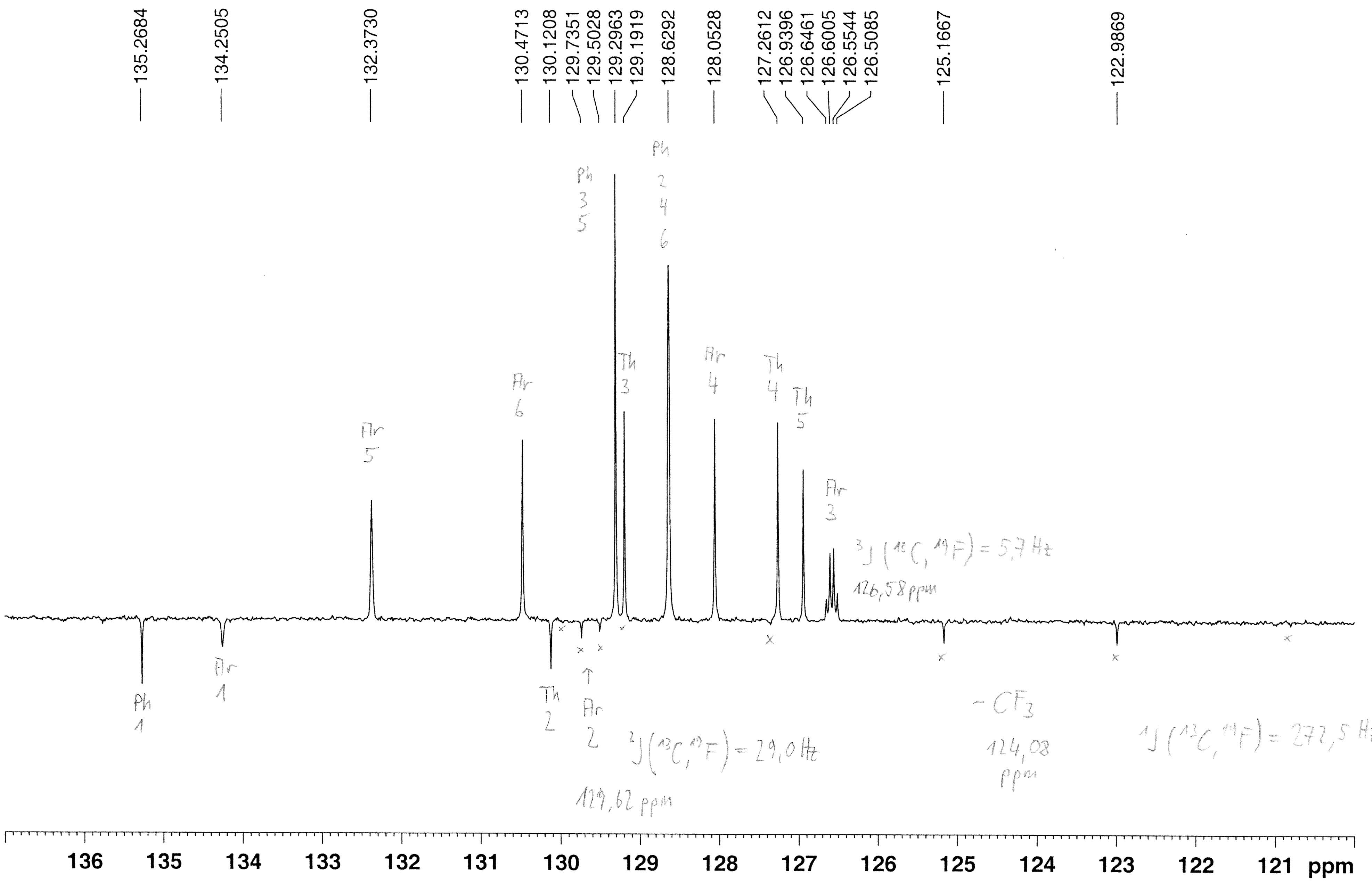

PN012S2Peak1 in cdcl3 (APT) 27.2.2020

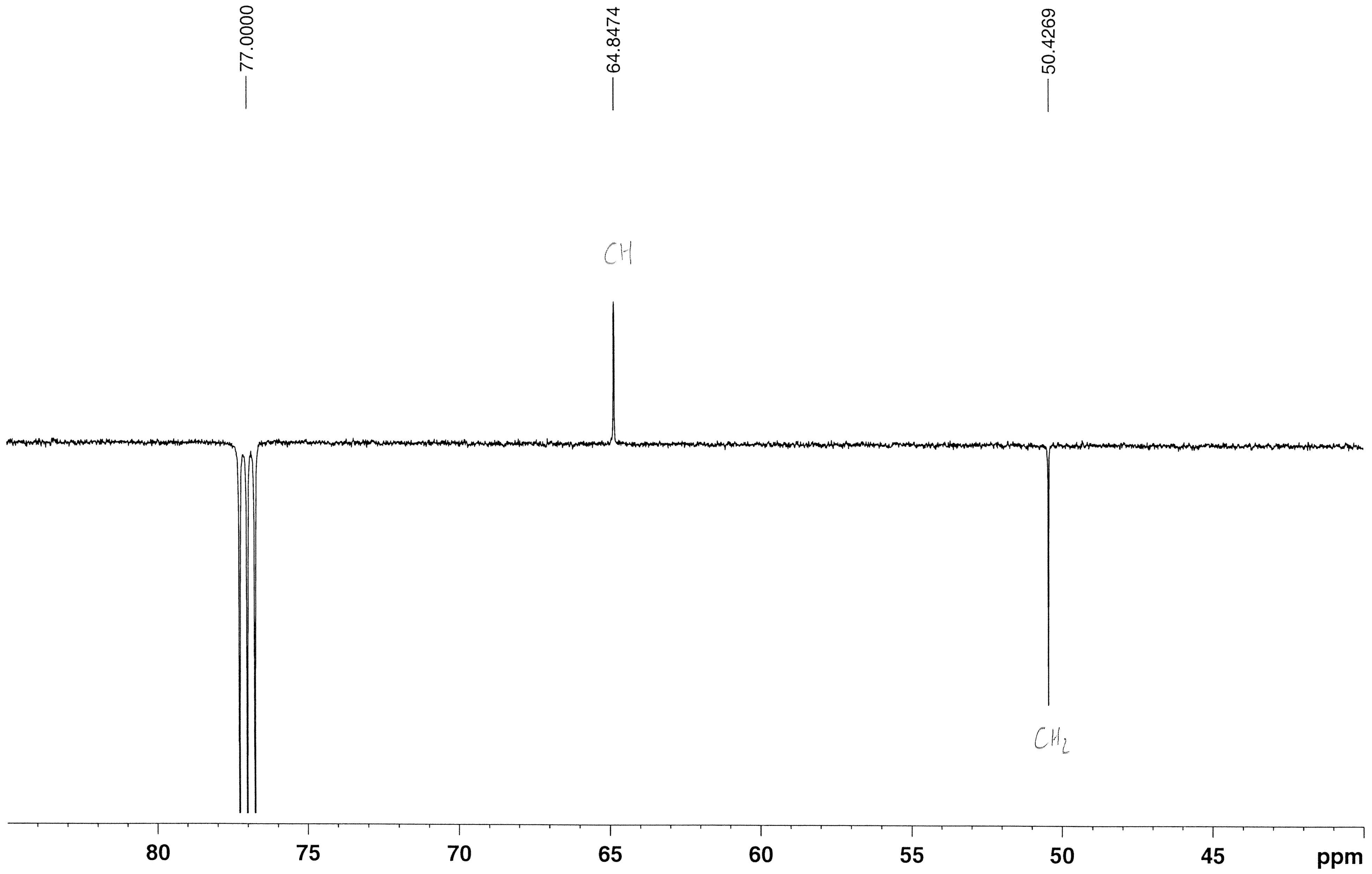

PN012S2Peak1 in cdcl3 (COSY) 27.2.2020

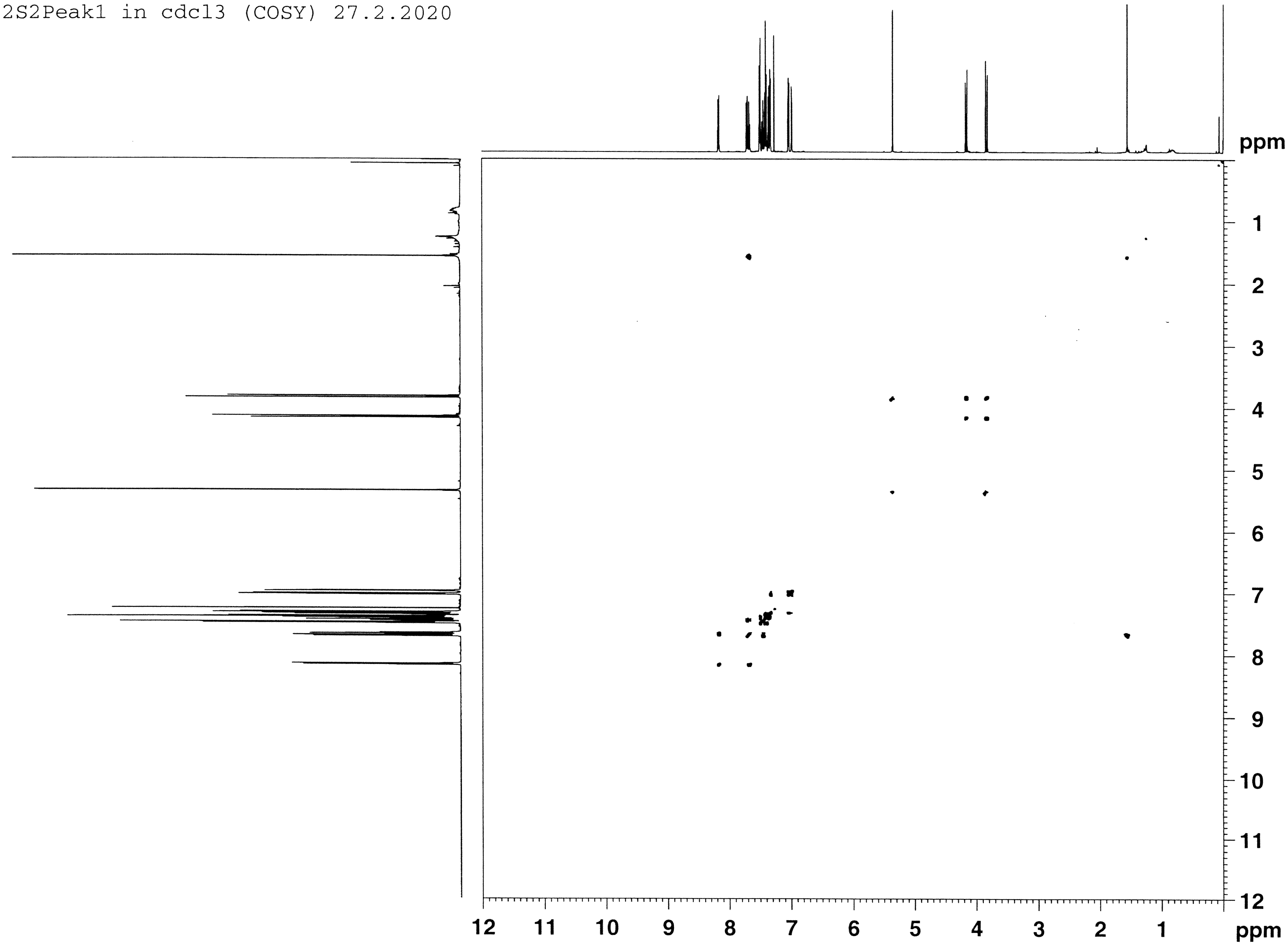

PN012S2Peak1 in cdcl3 (COSY) 27.2.2020

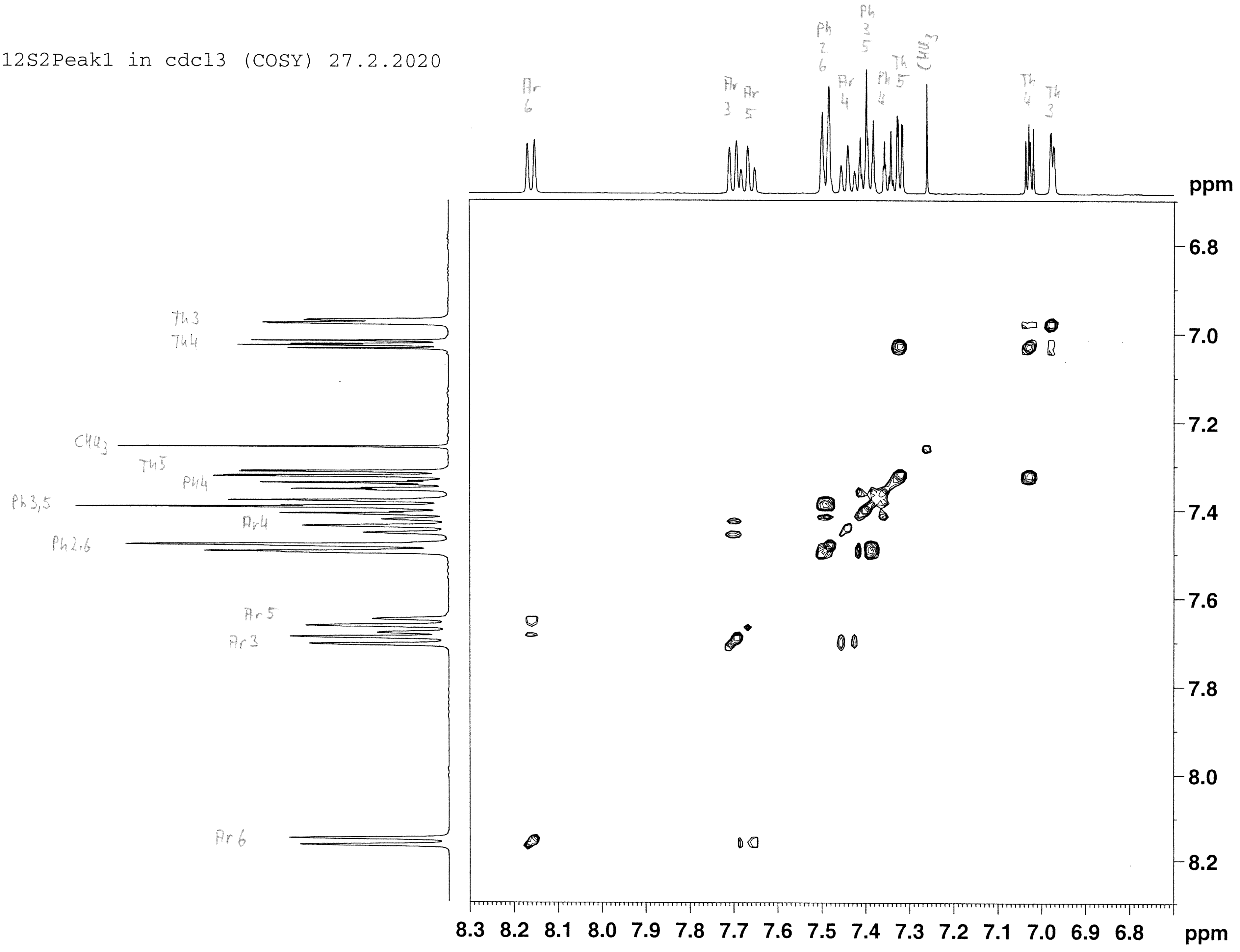

PN012S2Peak1 in cdcl3 (COSY) 27.2.2020

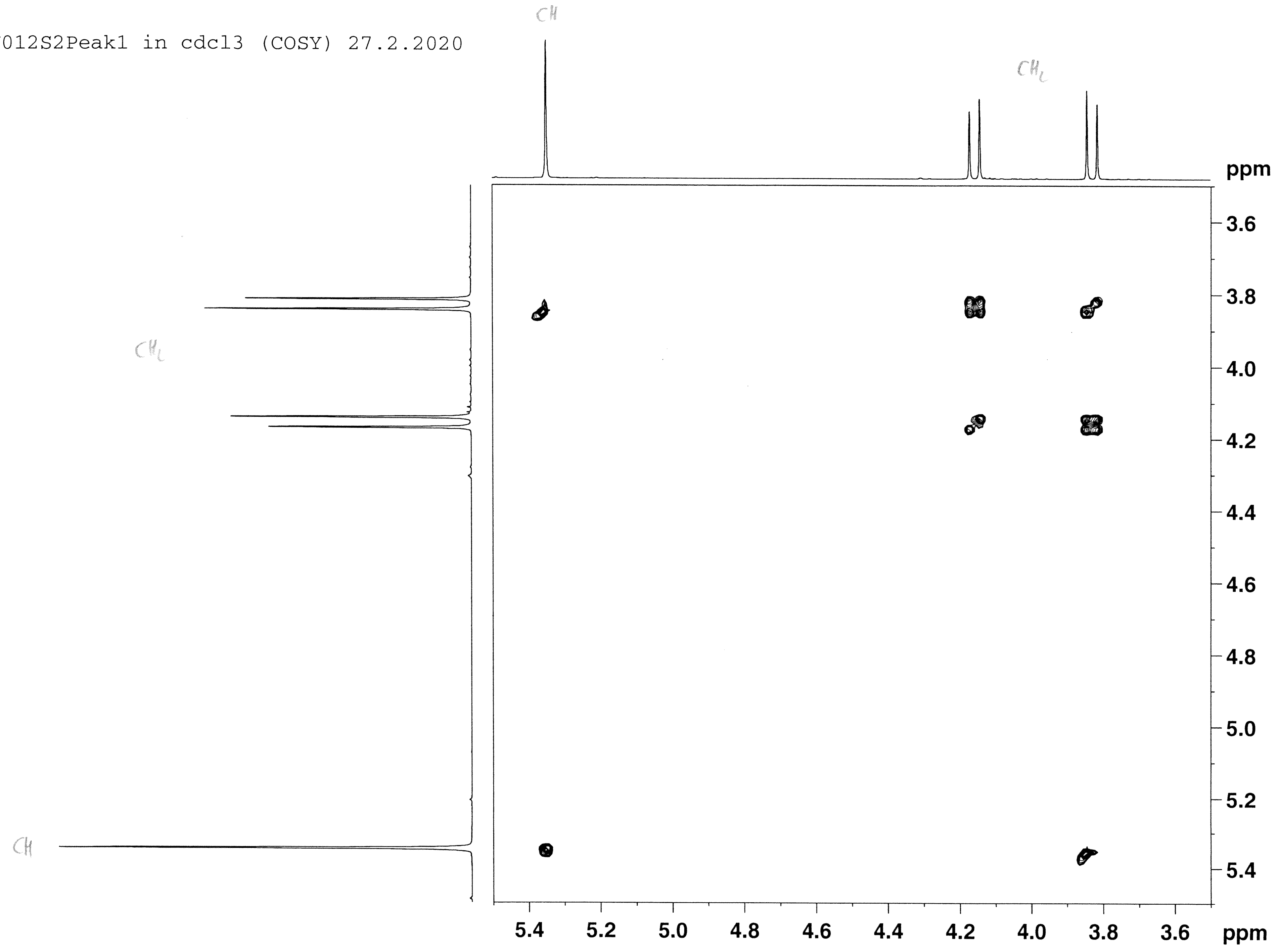

PN012S2Peak1 in cdcl3 (HSQC) 27.2.2020

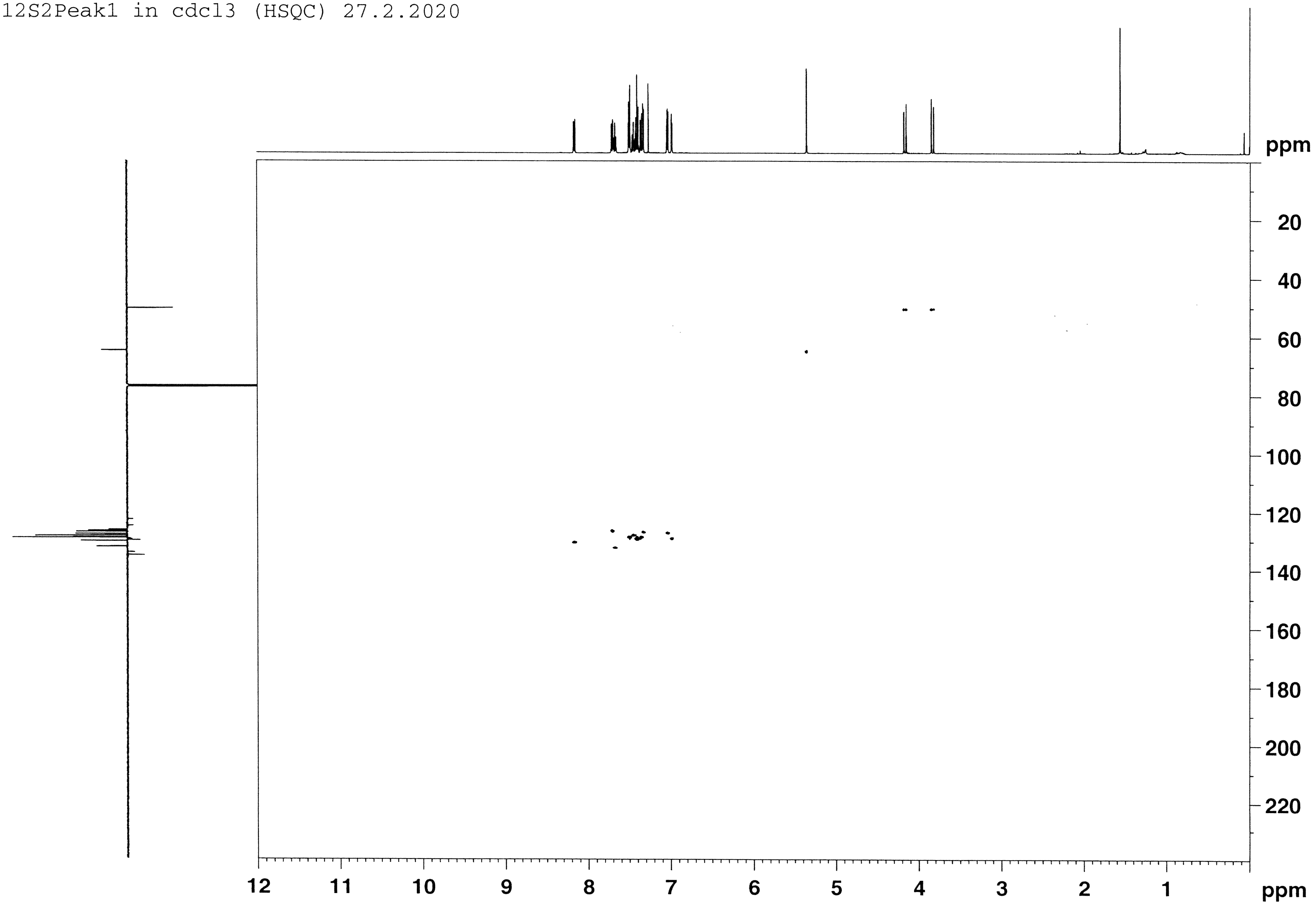

PN012S2Peak1 in cdcl3 (HSQC) 27.2.2020

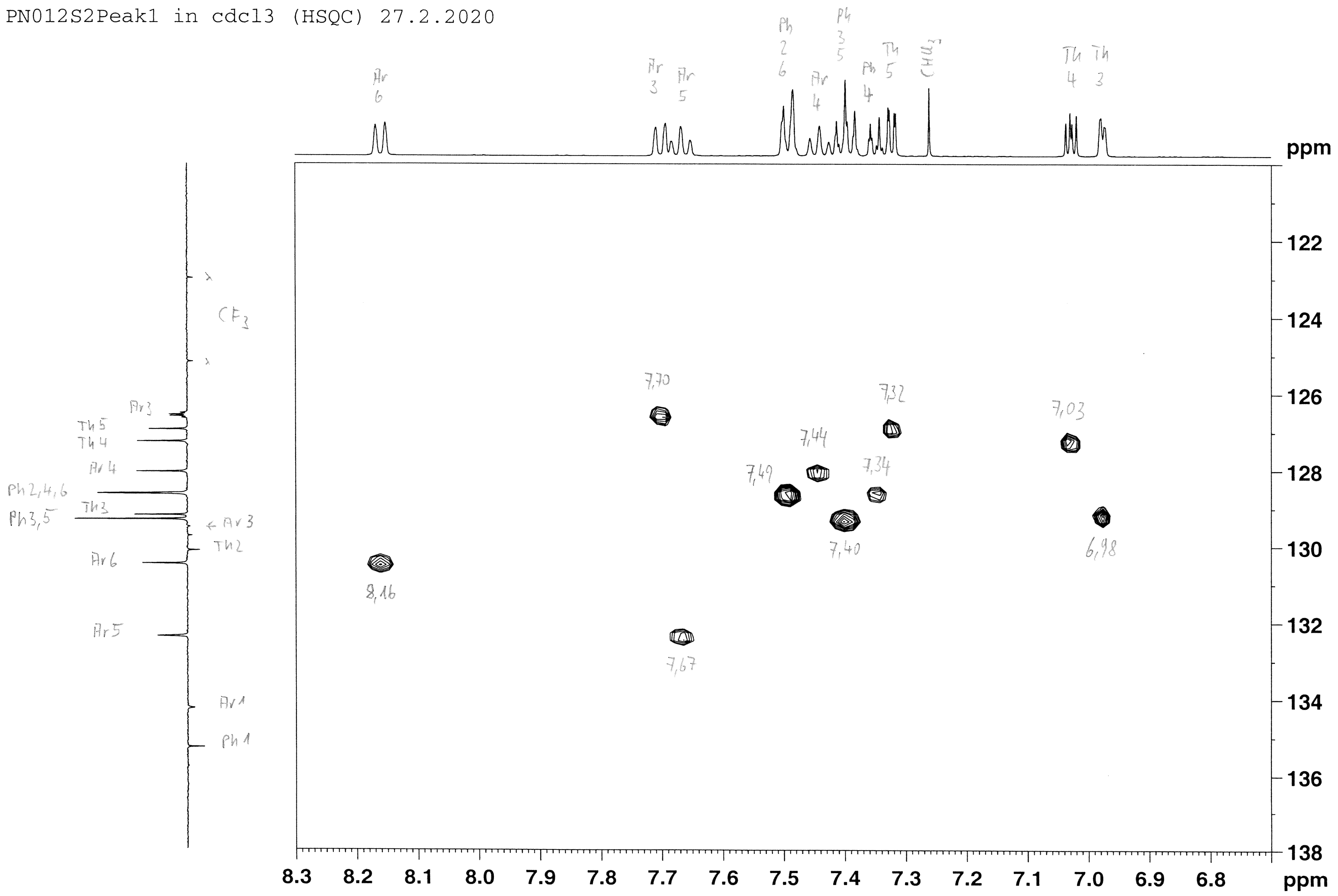

PN012S2Peak1 in cdcl3 (HSQC) 27.2.2020

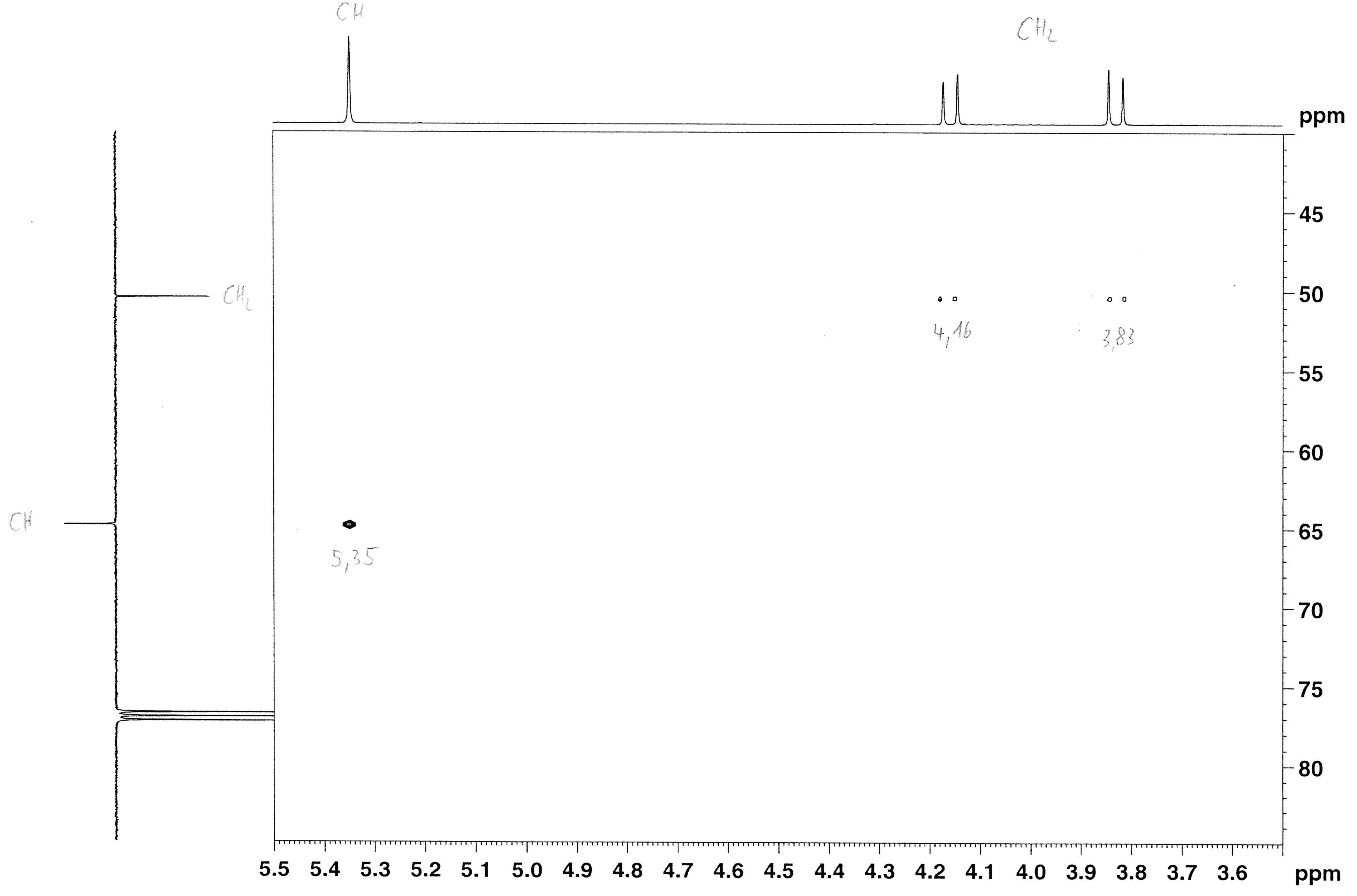

PN012S2Peak1 in cdcl3 (HMBC) 27.2.2020

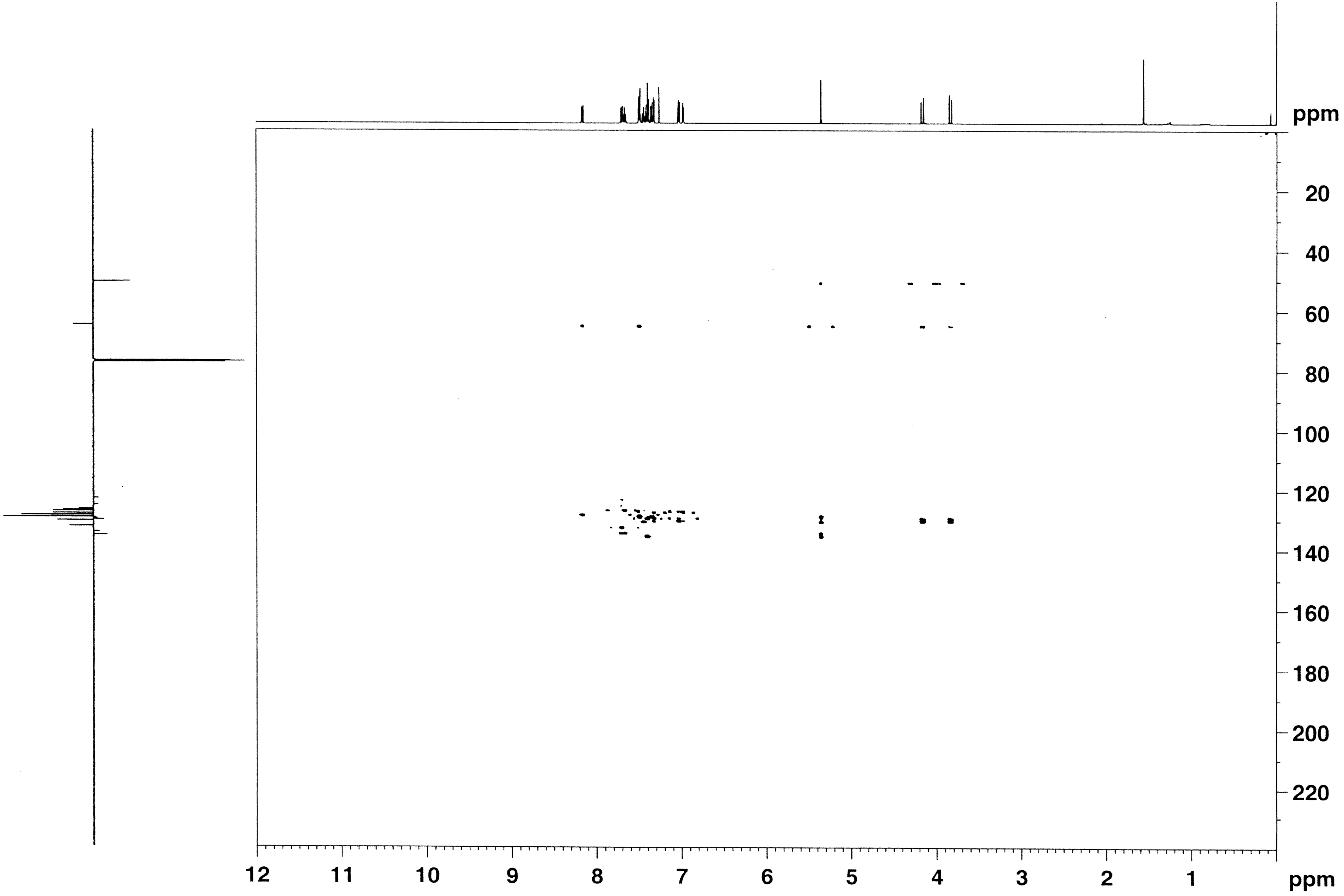

PN012S2Peak1 in cdcl3 (HMBC) 27.2.2020

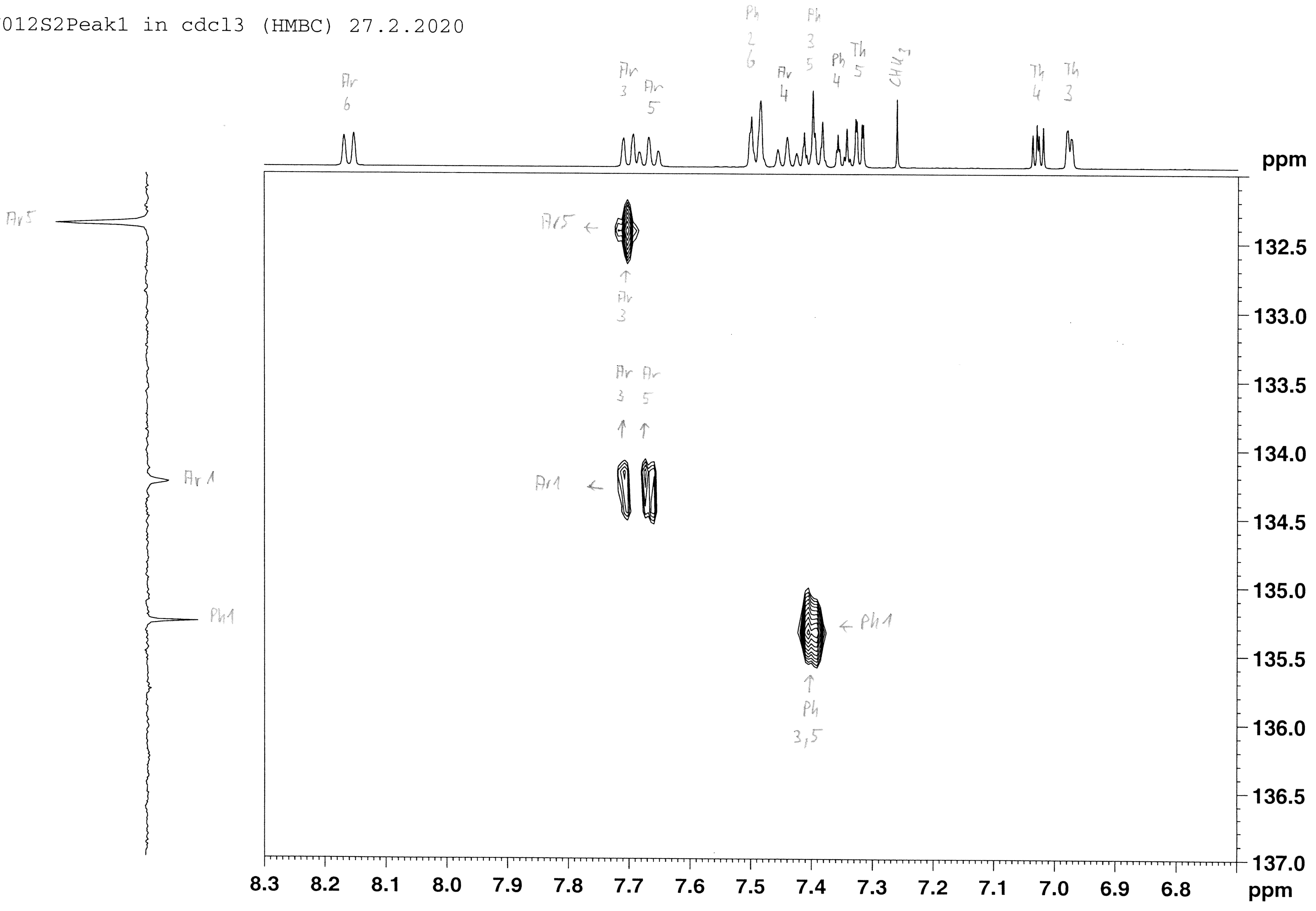

PN012S2Peak1 in cdcl3 (HMBC) 27.2.2020

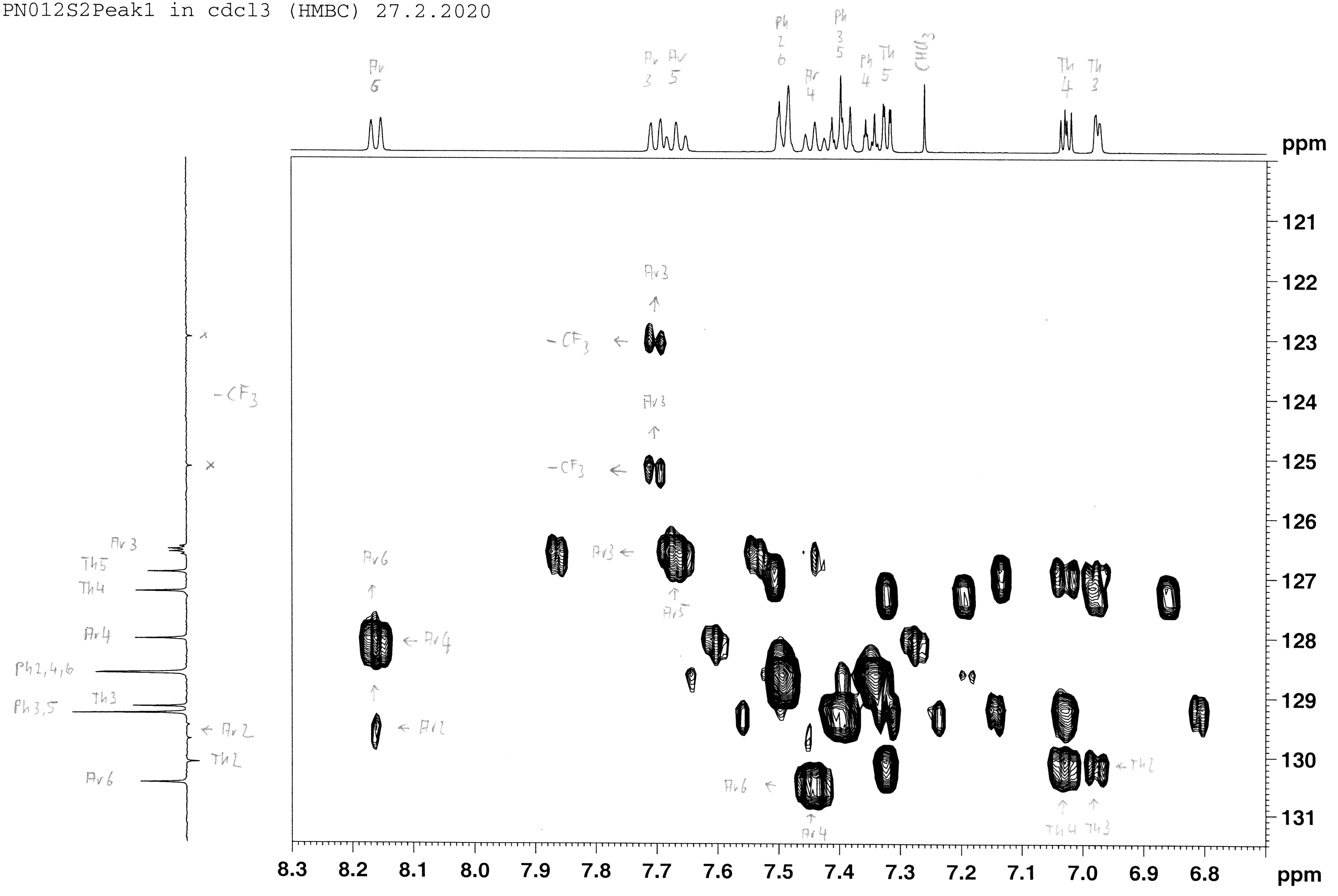

PN012S2Peak1 in cdcl3 (HMBC) 27.2.2020

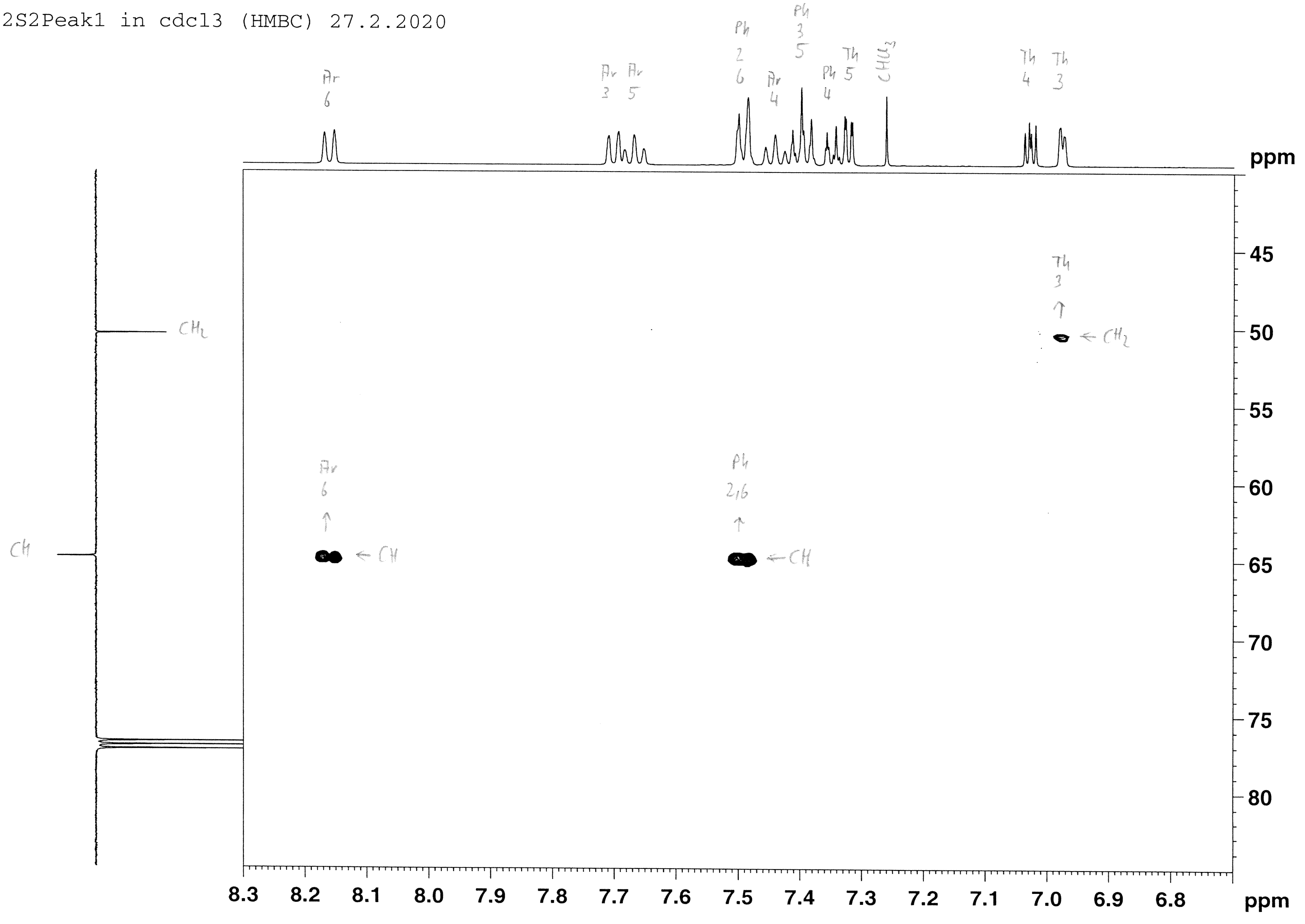

PN012S2Peak1 in cdcl3 (HMBC) 27.2.2020

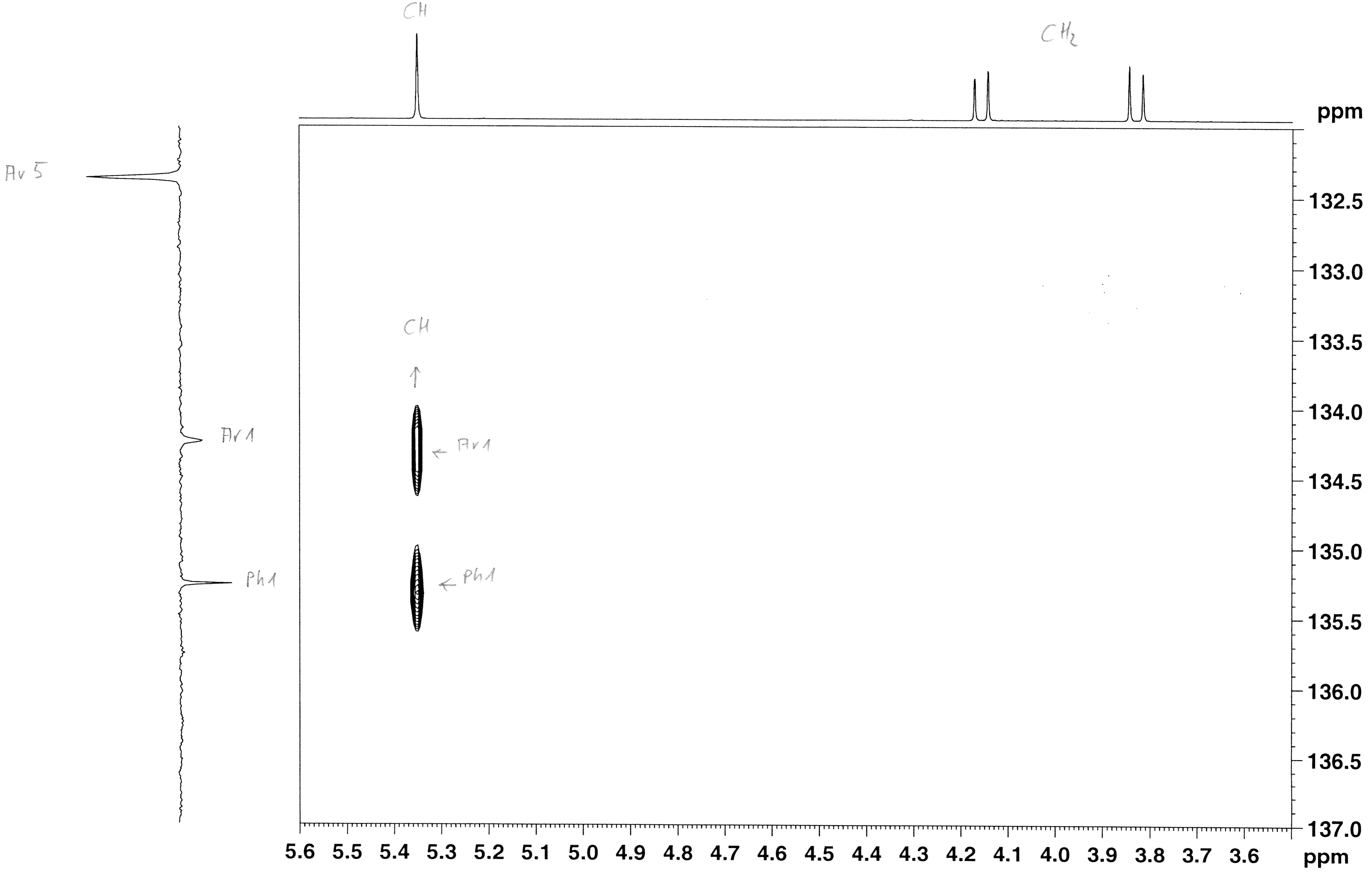

PN012S2Peak1 in cdcl3 (HMBC) 27.2.2020

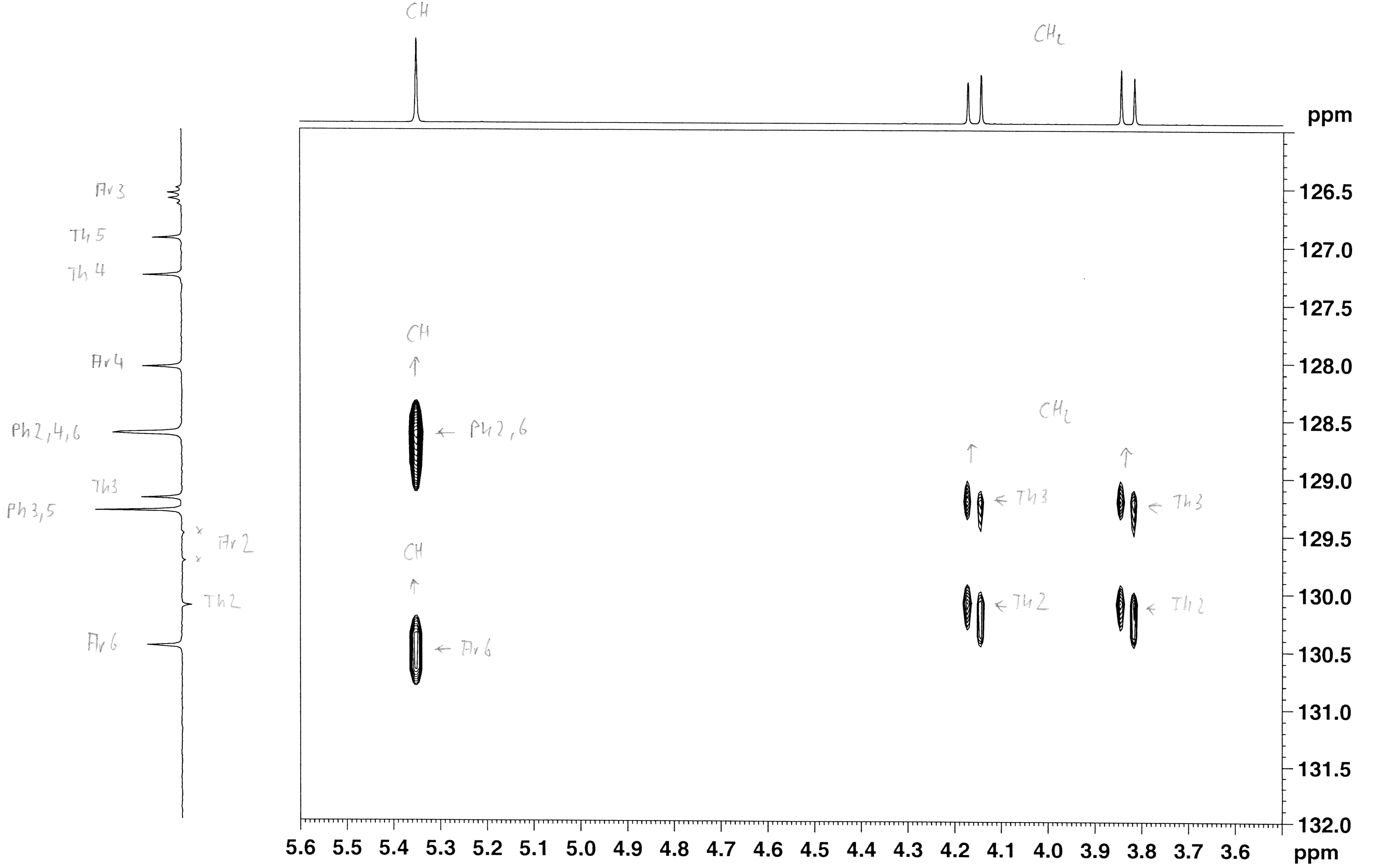

PN012S2Peak1 in cdcl3 (HMBC) 27.2.2020

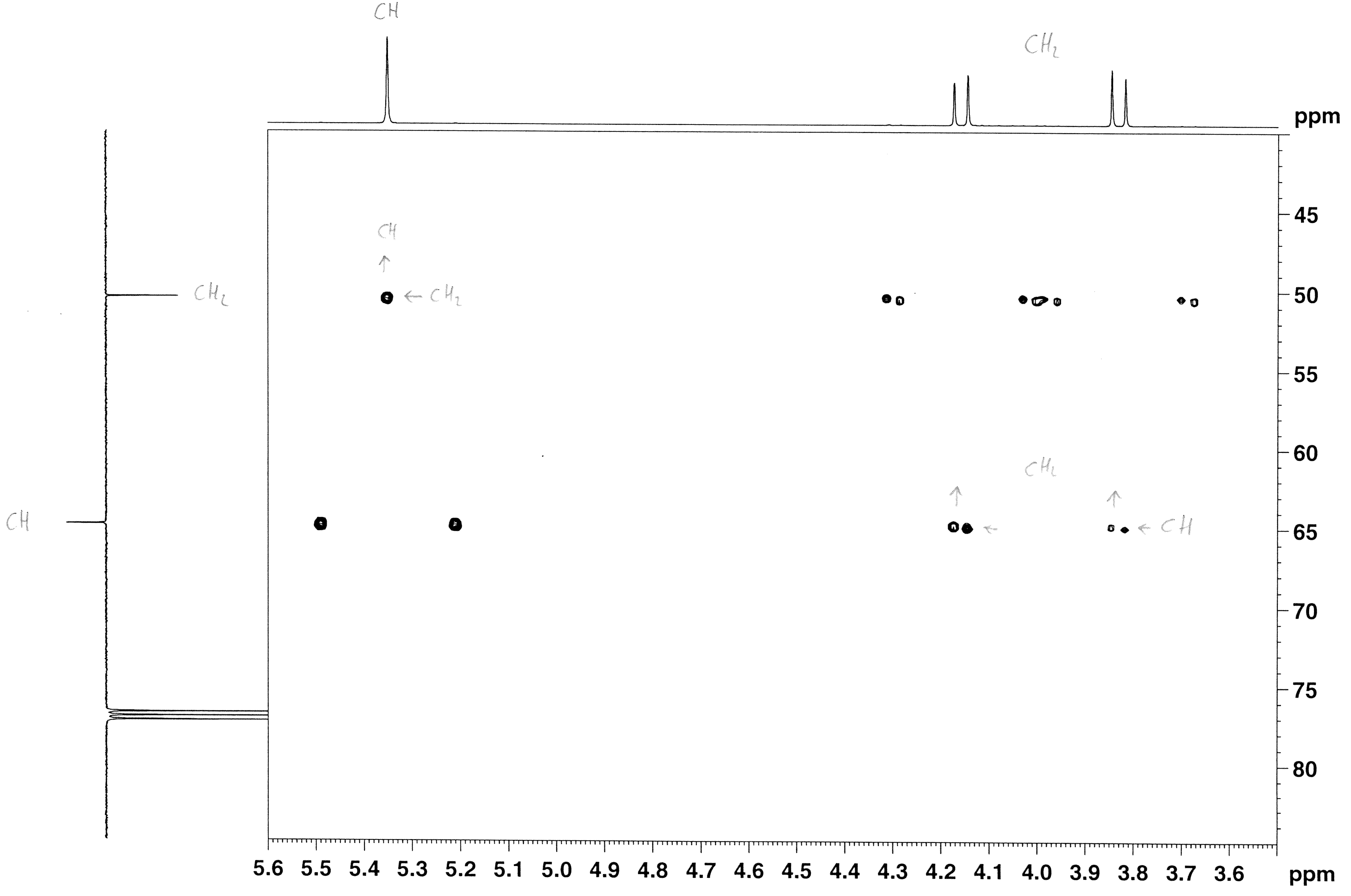

Figure S60c. NMR spectra of compound **8o.**

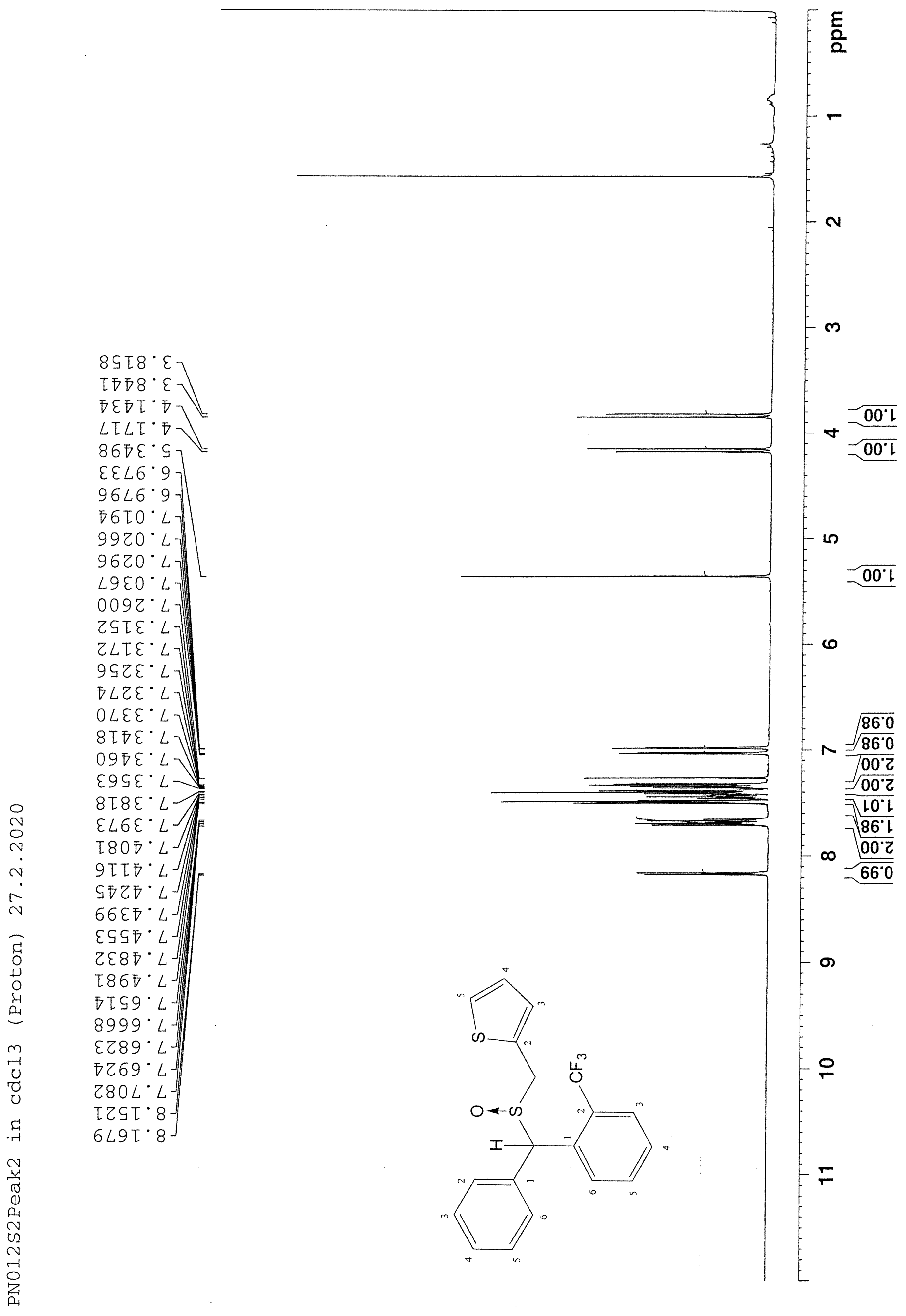

PN012S2Peak2 in cdcl3 (Proton) 27.2.2020

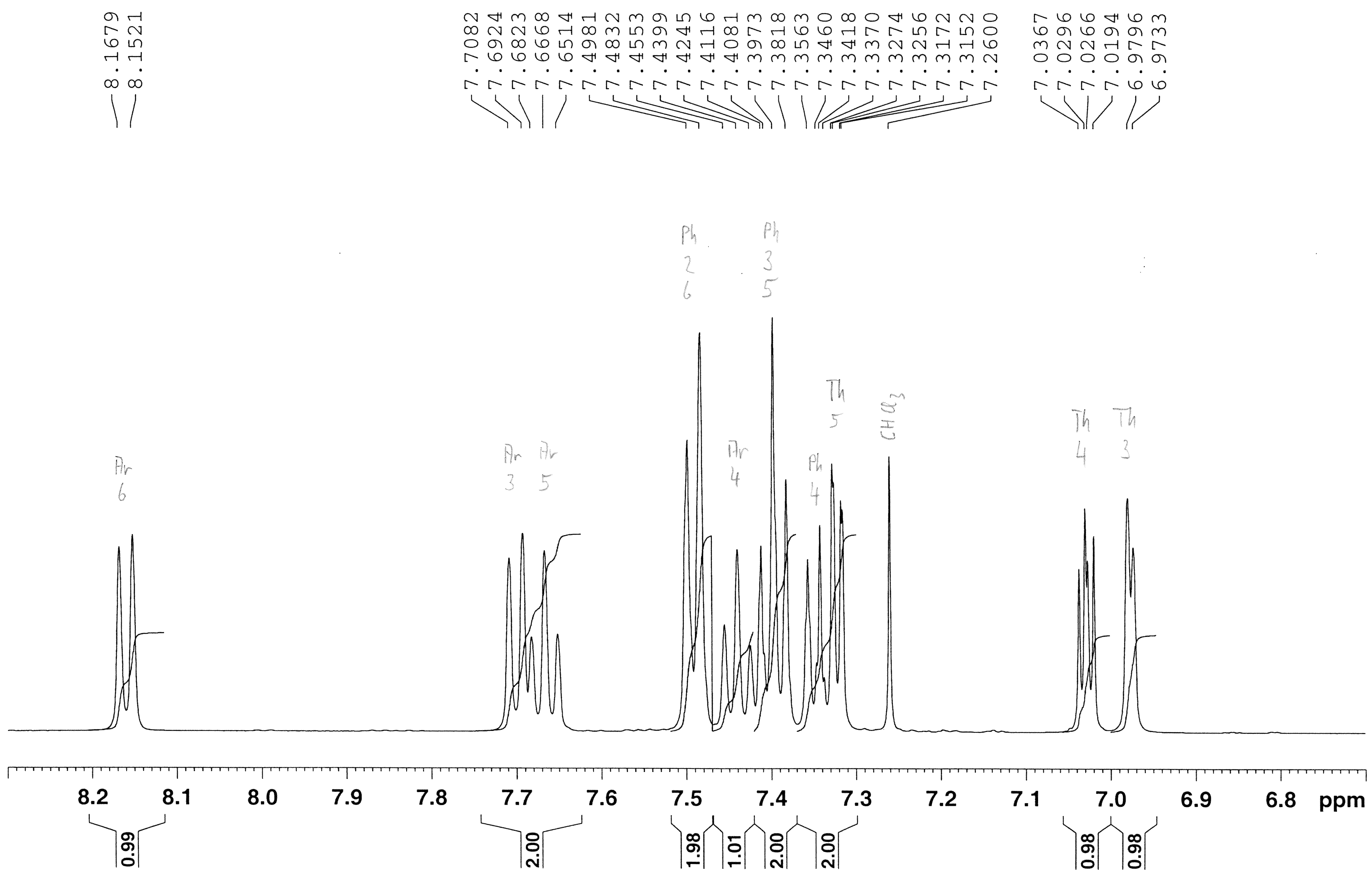

PN012S2Peak2 in cdcl3 (Proton) 27.2.2020

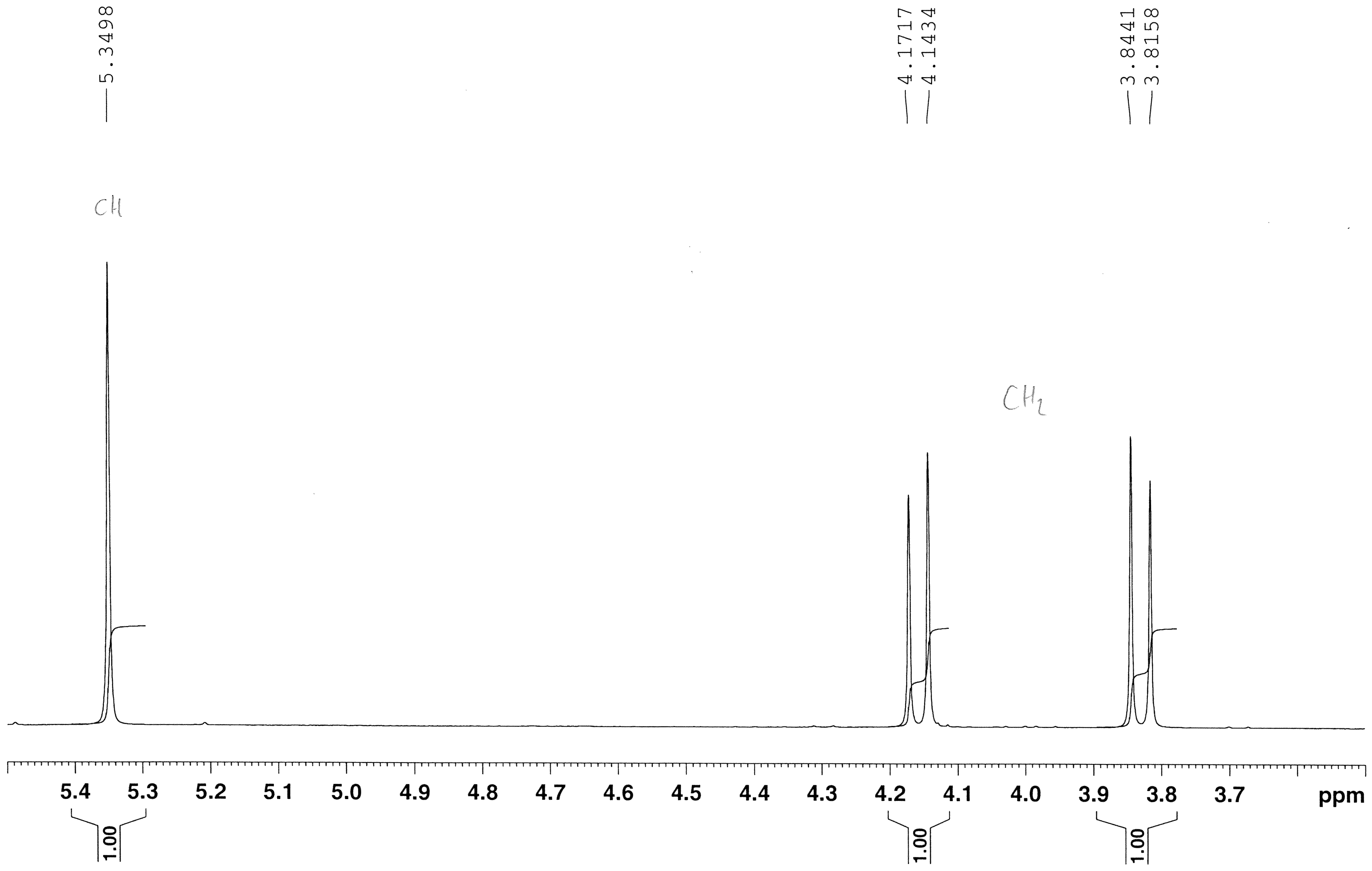

PN012S2Peak2 in cdcl3 (APT) 27.2.2020

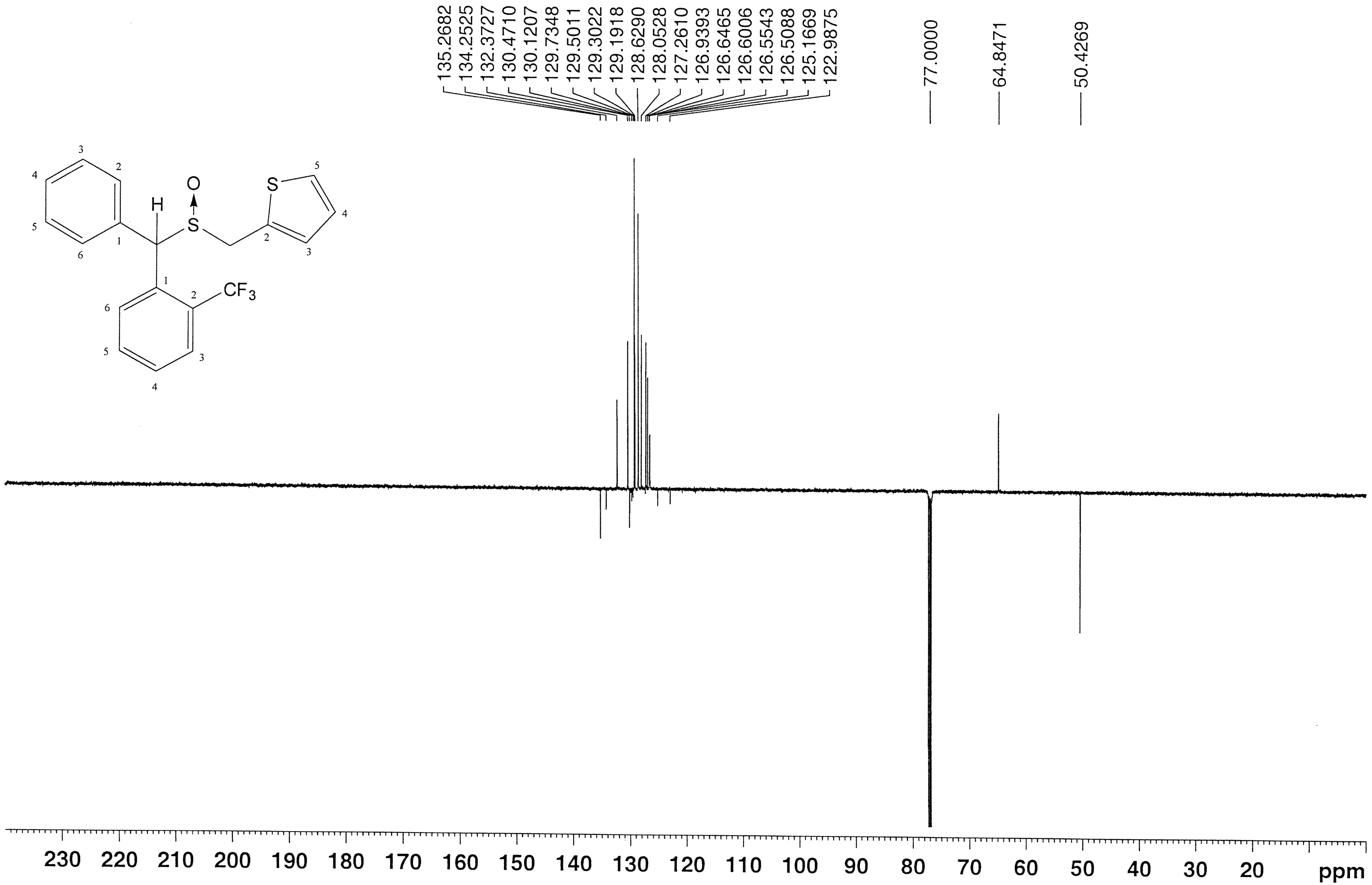

PN012S2Peak2 in cdcl3 (APT) 27.2.2020

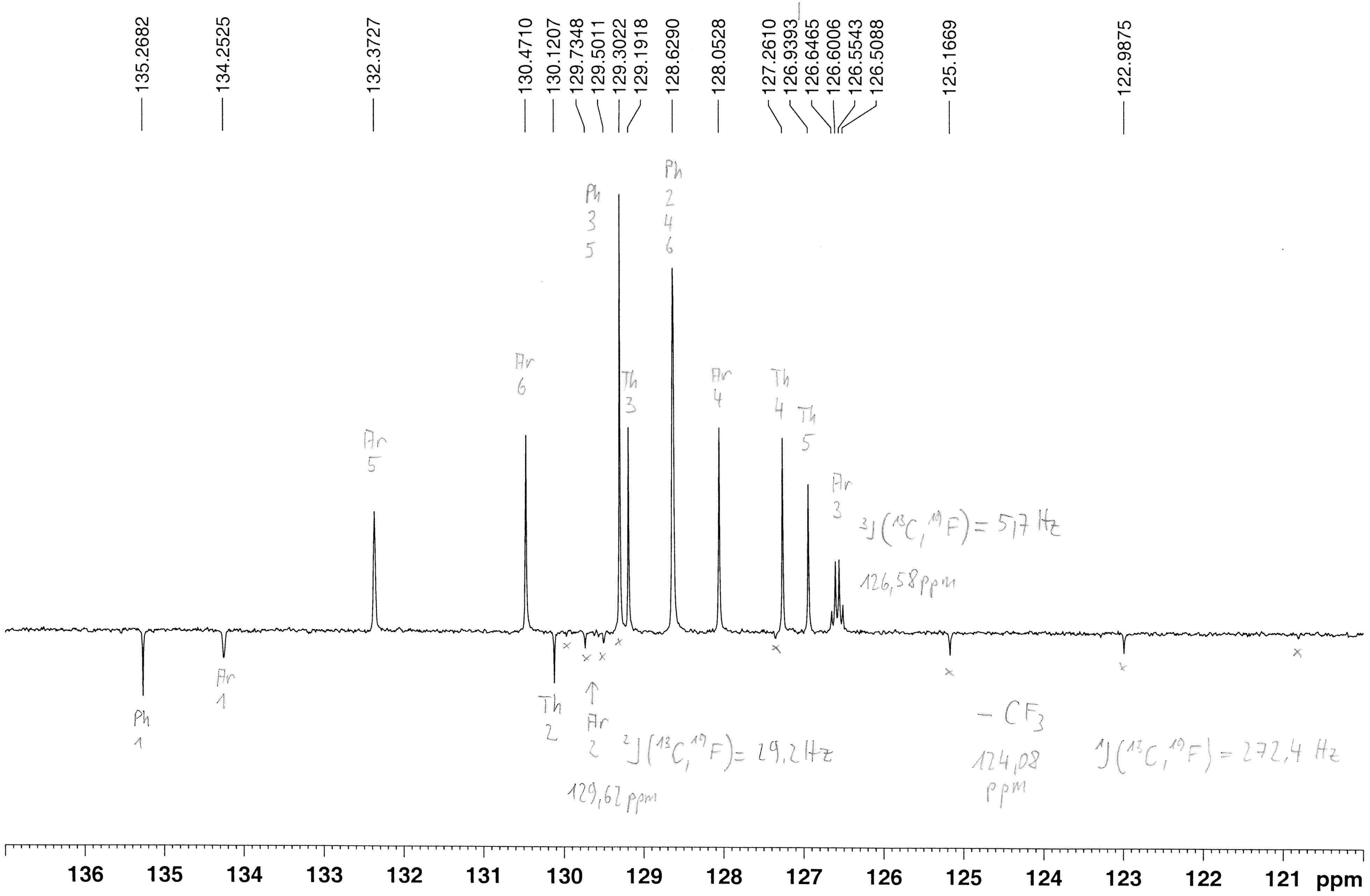

PN012S2Peak2 in cdcl3 (APT) 27.2.2020

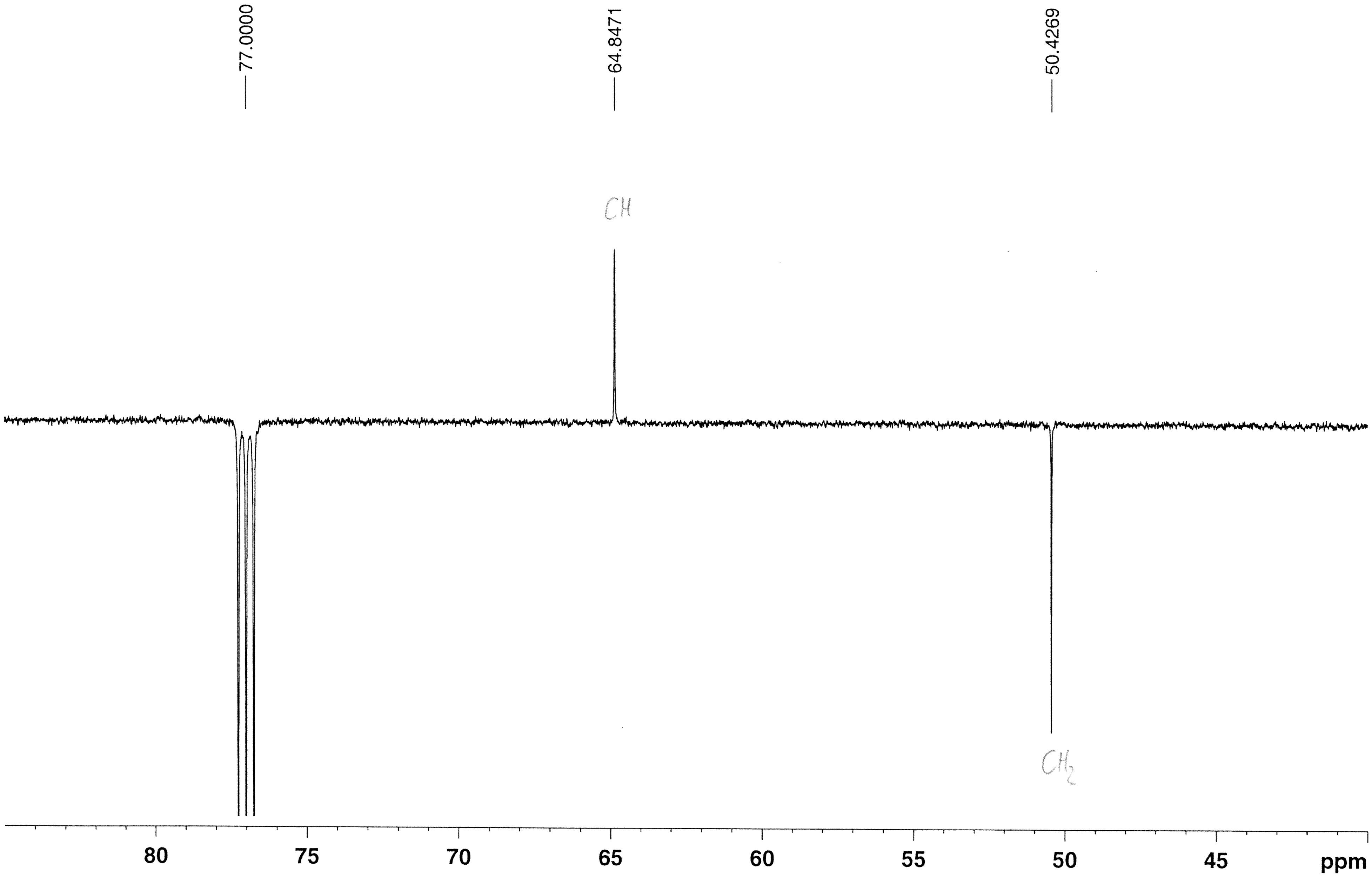

PN012S2Peak2 in cdcl3 (COSY) 27.2.2020

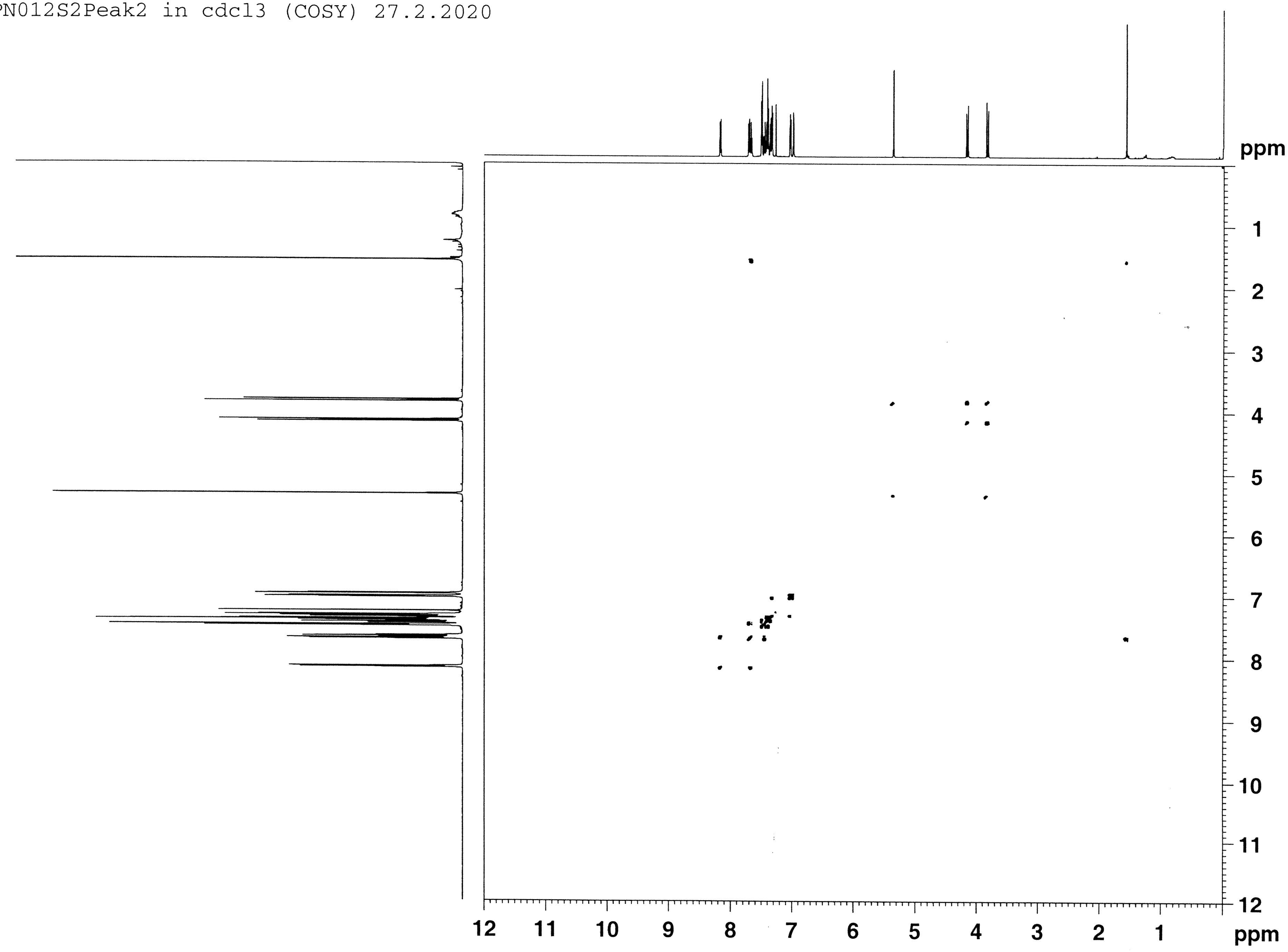

PN012S2Peak2 in cdcl3 (COSY) 27.2.2020

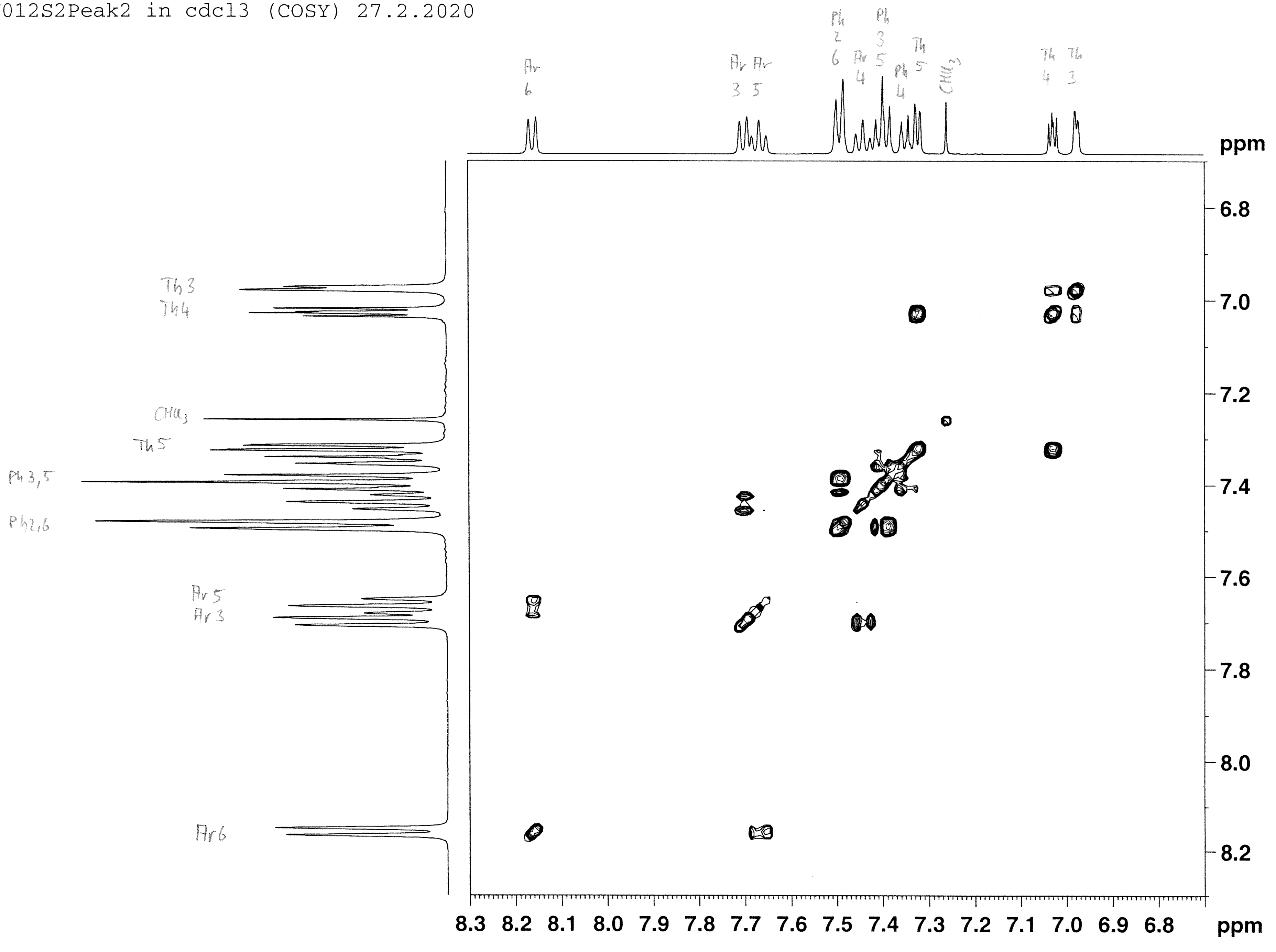

PN012S2Peak2 in cdcl3 (COSY) 27.2.2020

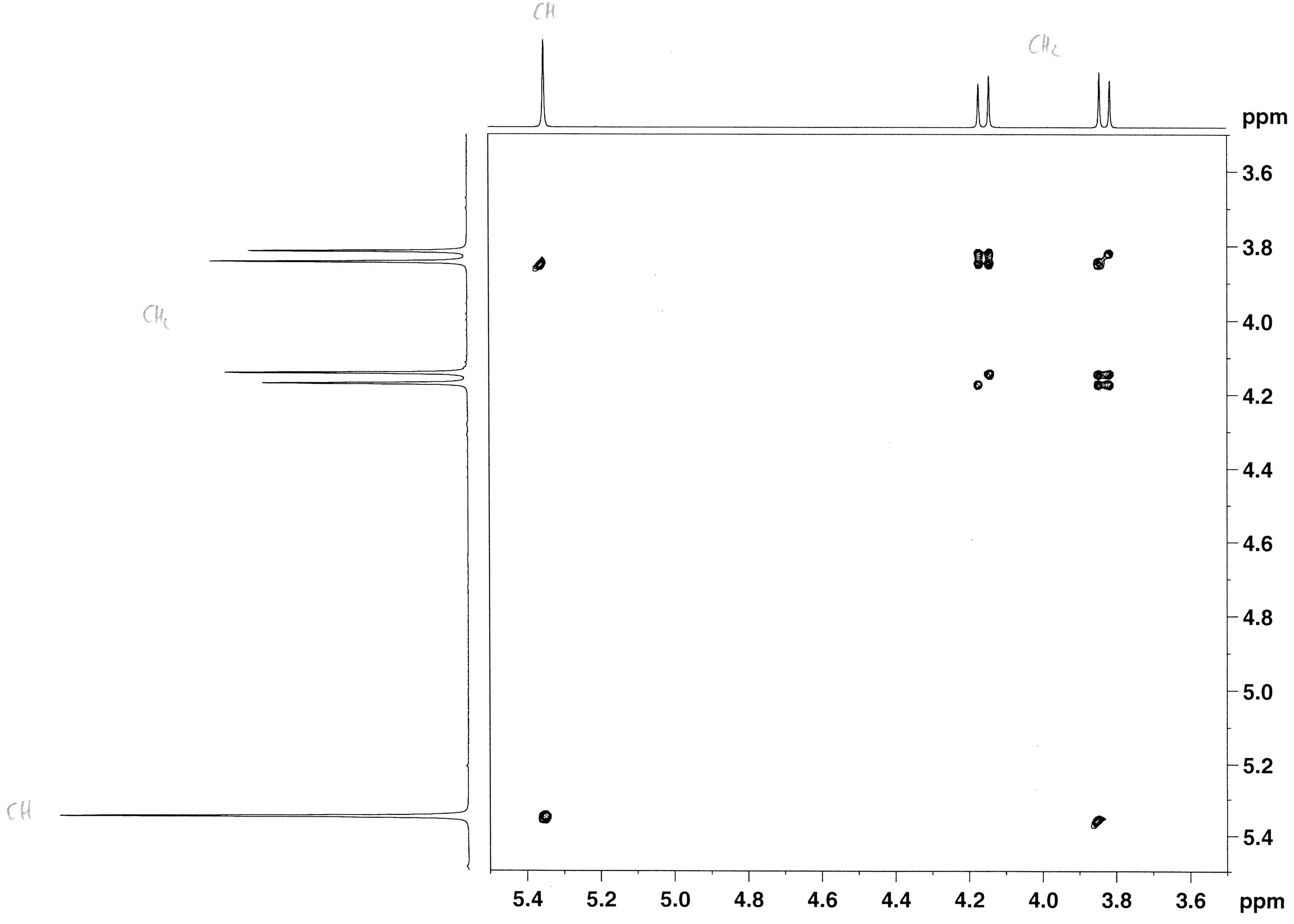

PN012S2Peak2 in cdcl3 (HSQC) 27.2.2020

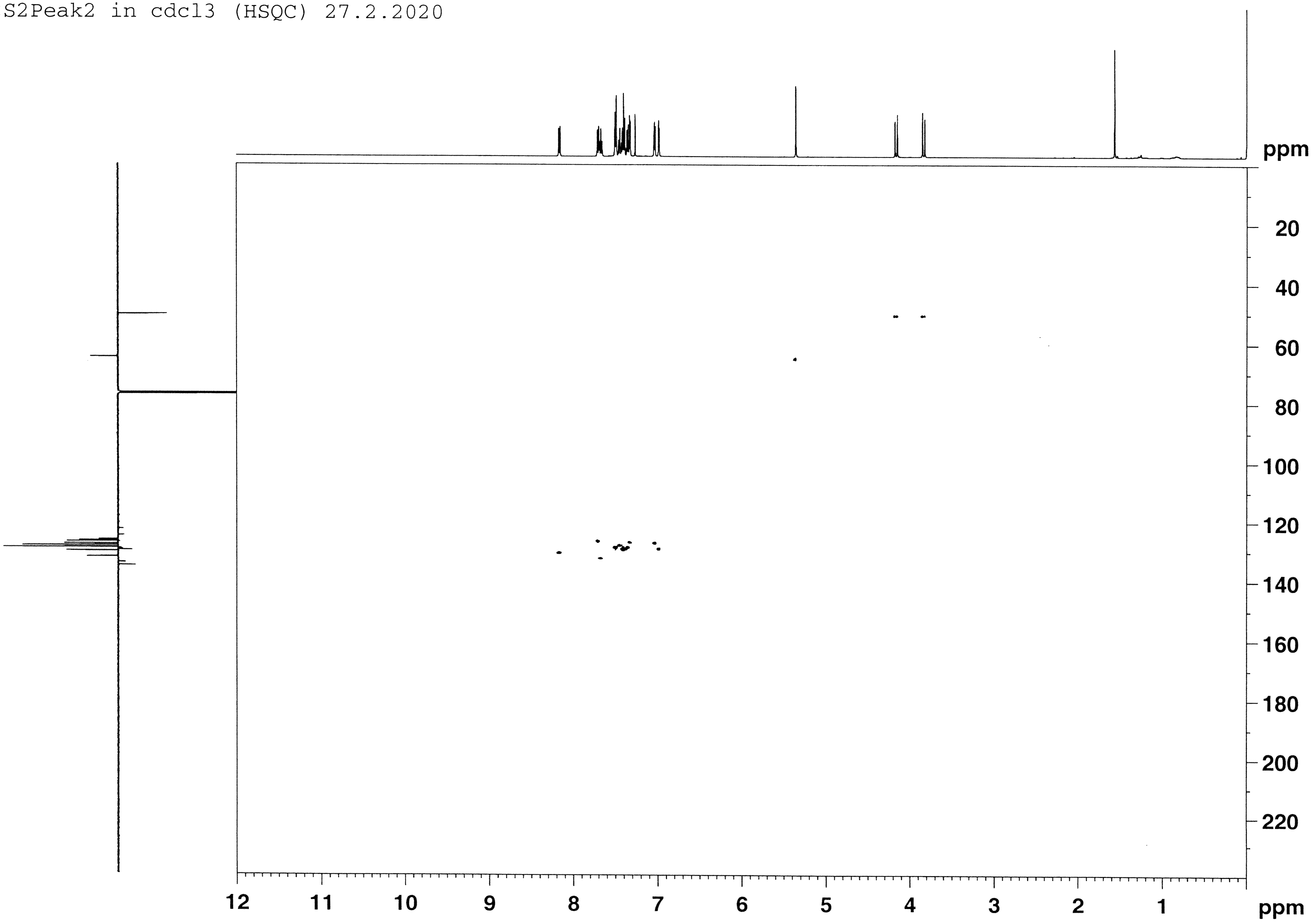

PN012S2Peak2 in cdcl3 (HSQC) 27.2.2020

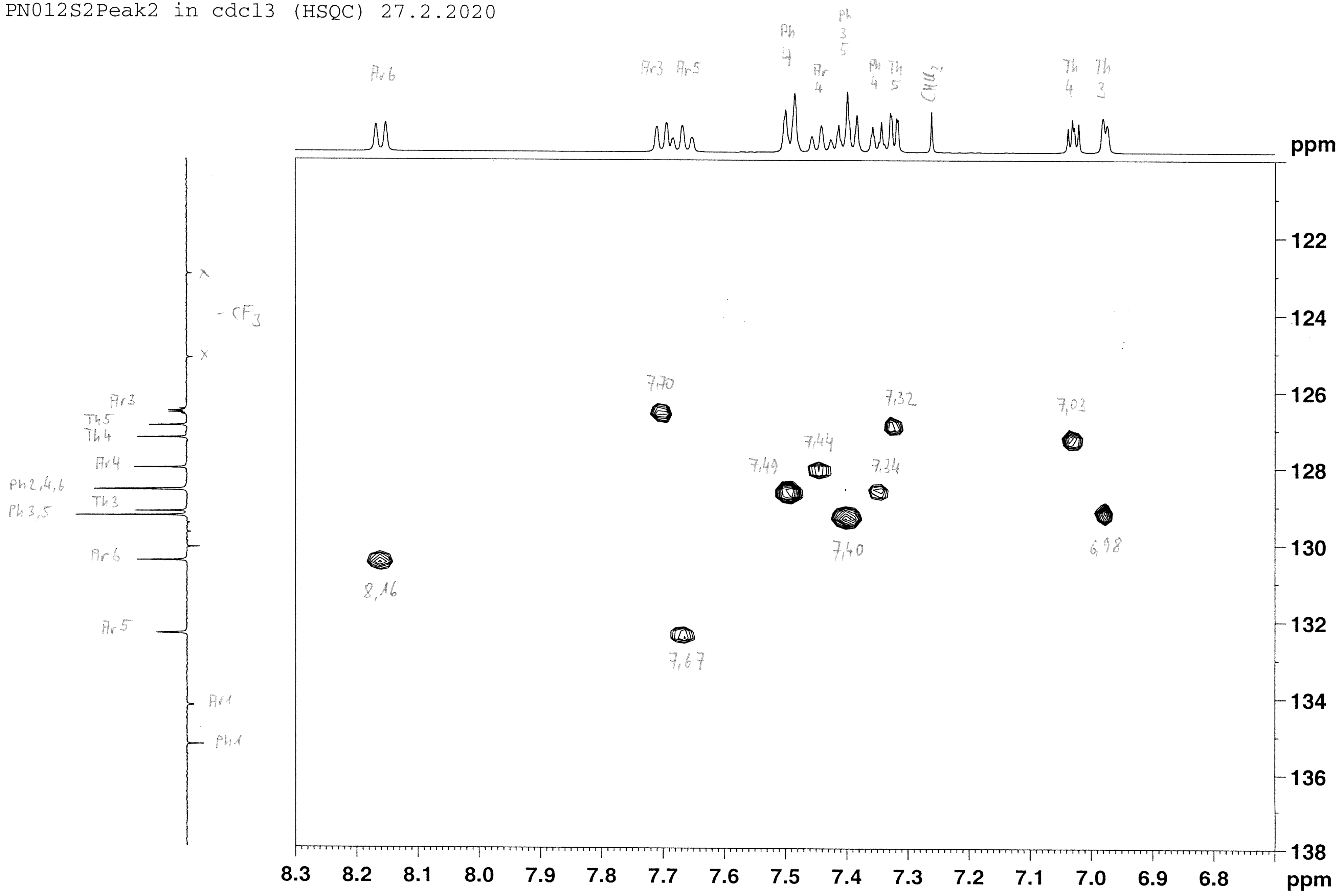

PN012S2Peak2 in cdcl3 (HSQC) 27.2.2020

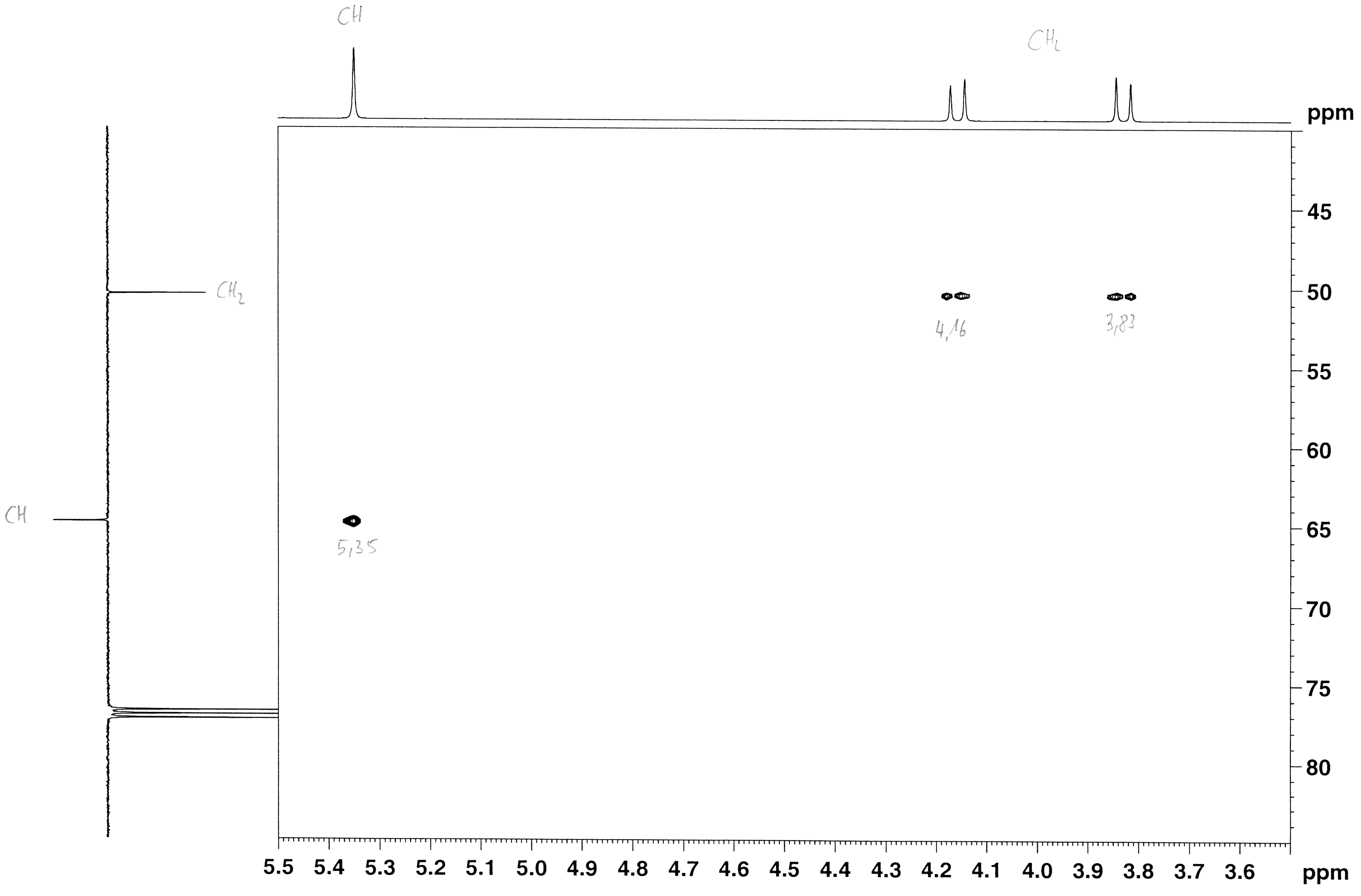

PN012S2Peak2 in cdcl3 (HMBC) 27.2.2020

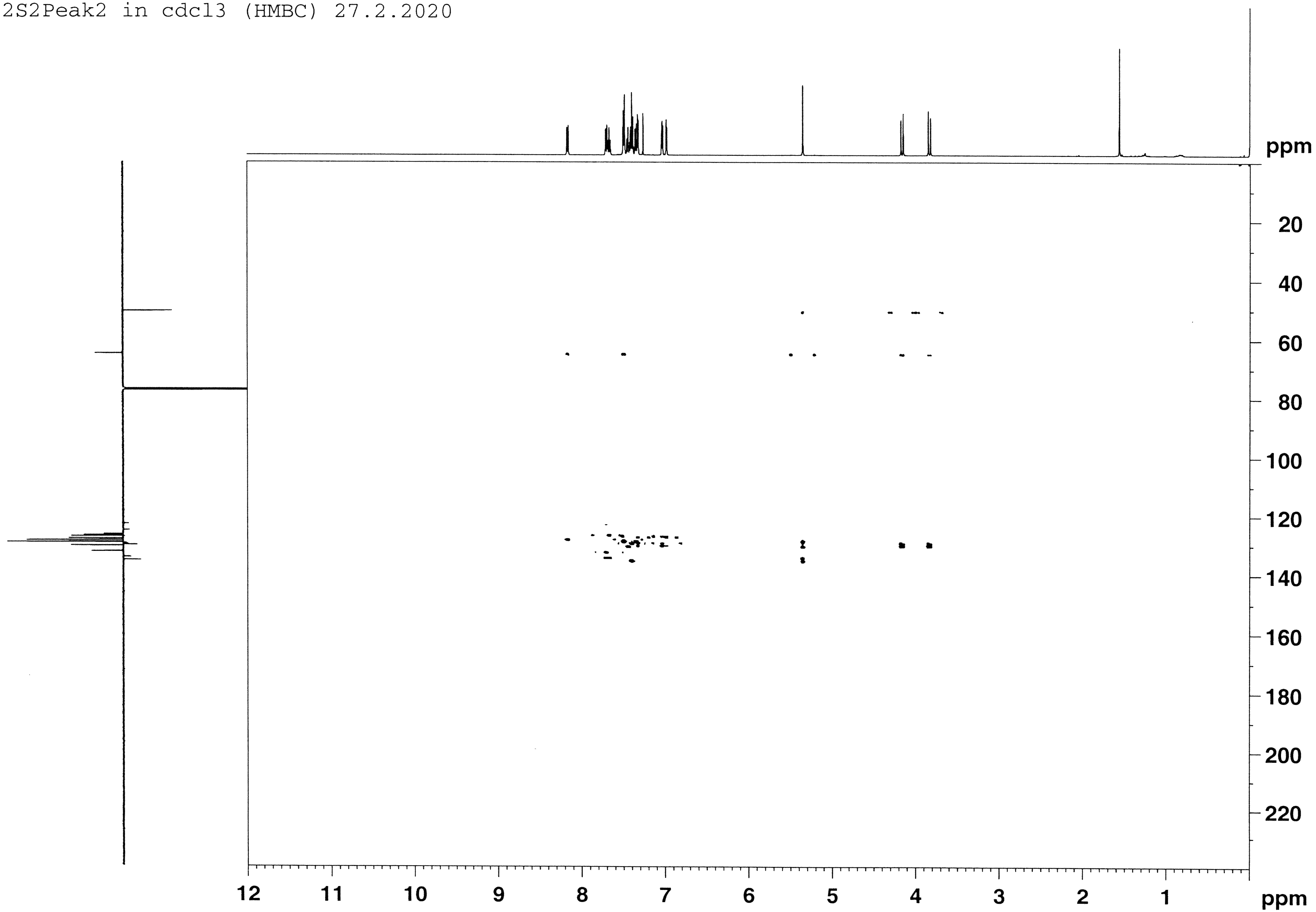

PN012S2Peak2 in cdcl3 (HMBC) 27.2.2020

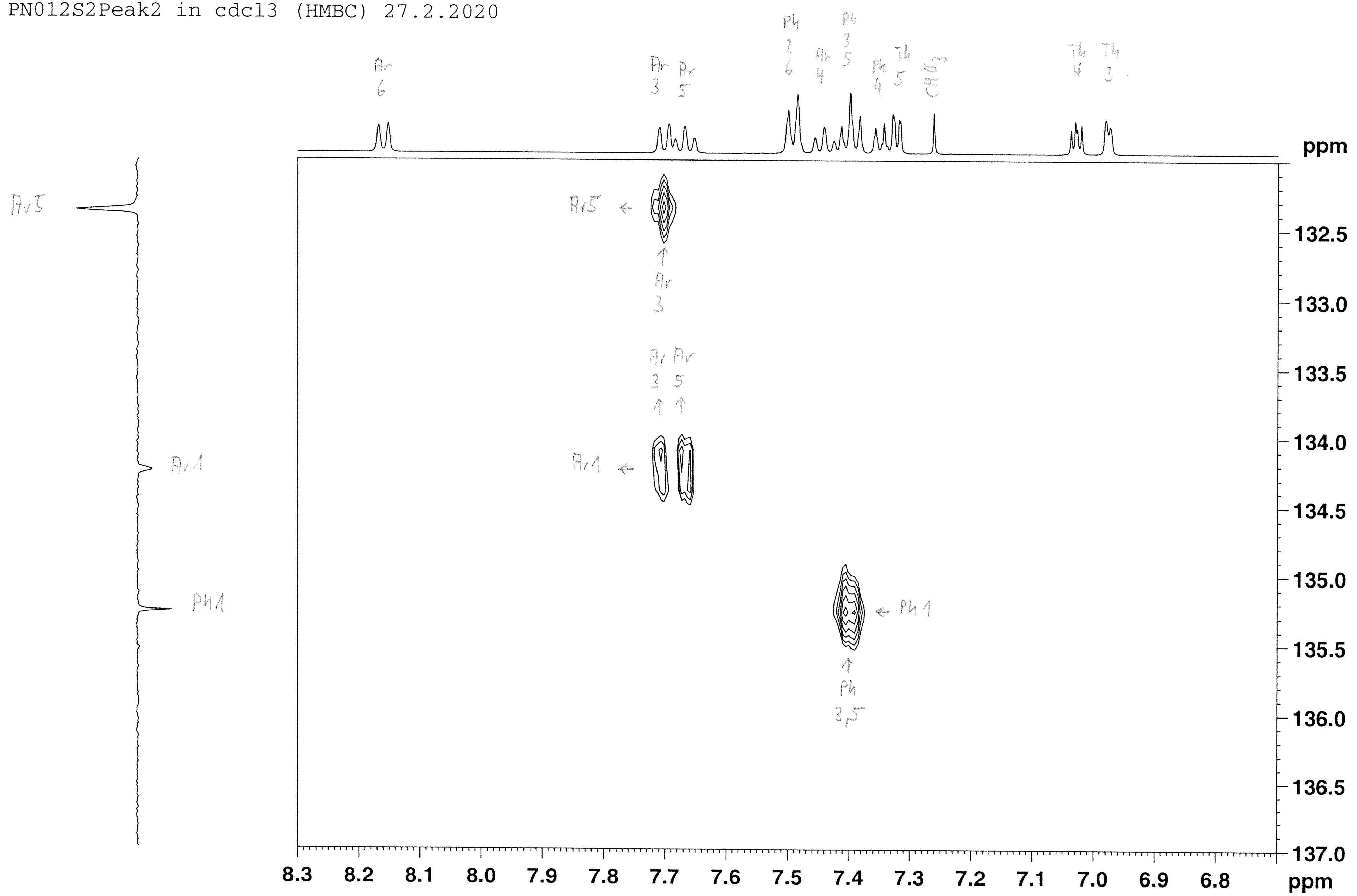

PN012S2Peak2 in cdcl3 (HMBC) 27.2.2020

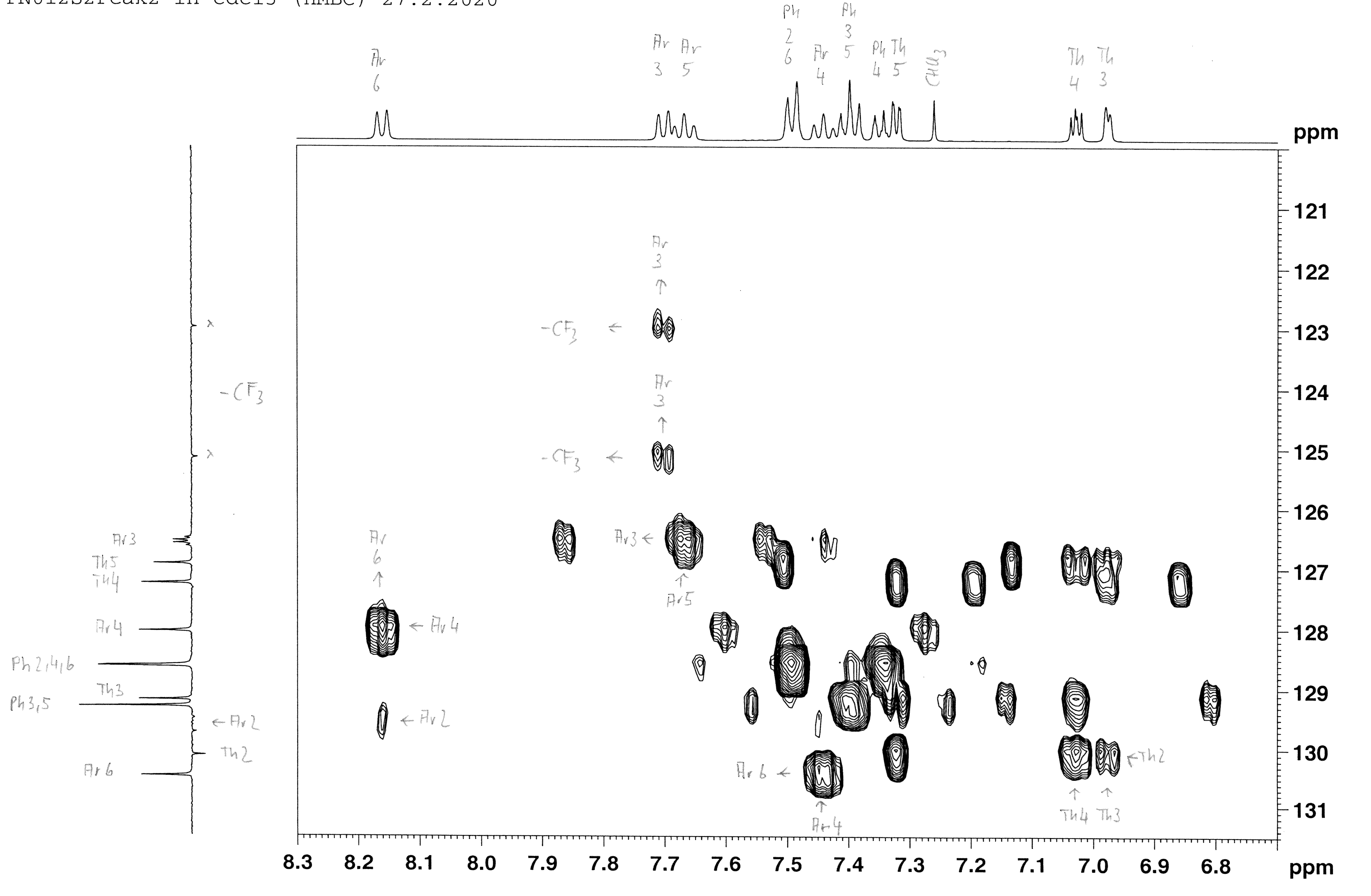

PN012S2Peak2 in cdcl3 (HMBC) 27.2.2020

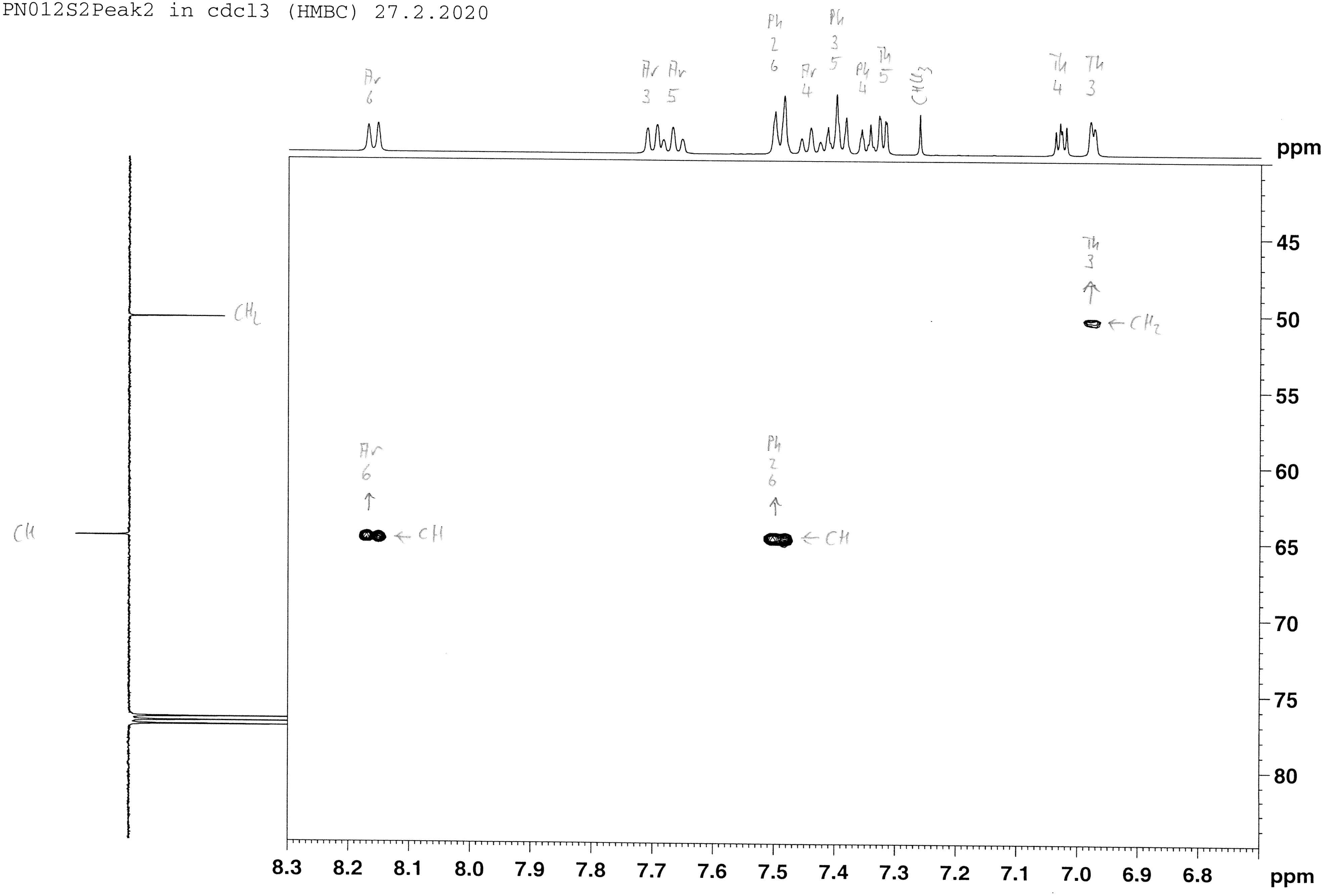

PN012S2Peak2 in cdcl3 (HMBC) 27.2.2020

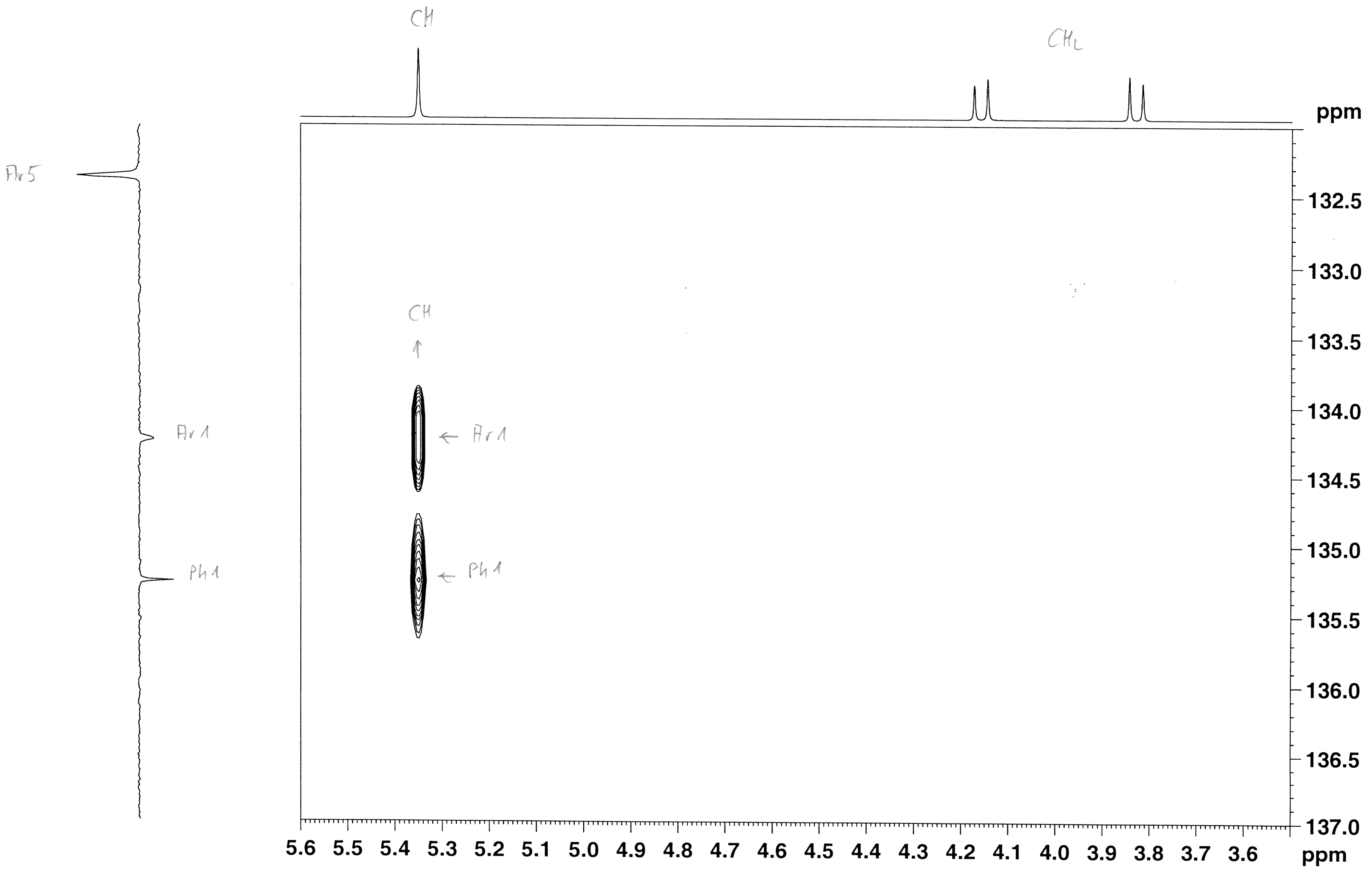

PN012S2Peak2 in cdcl3 (HMBC) 27.2.2020

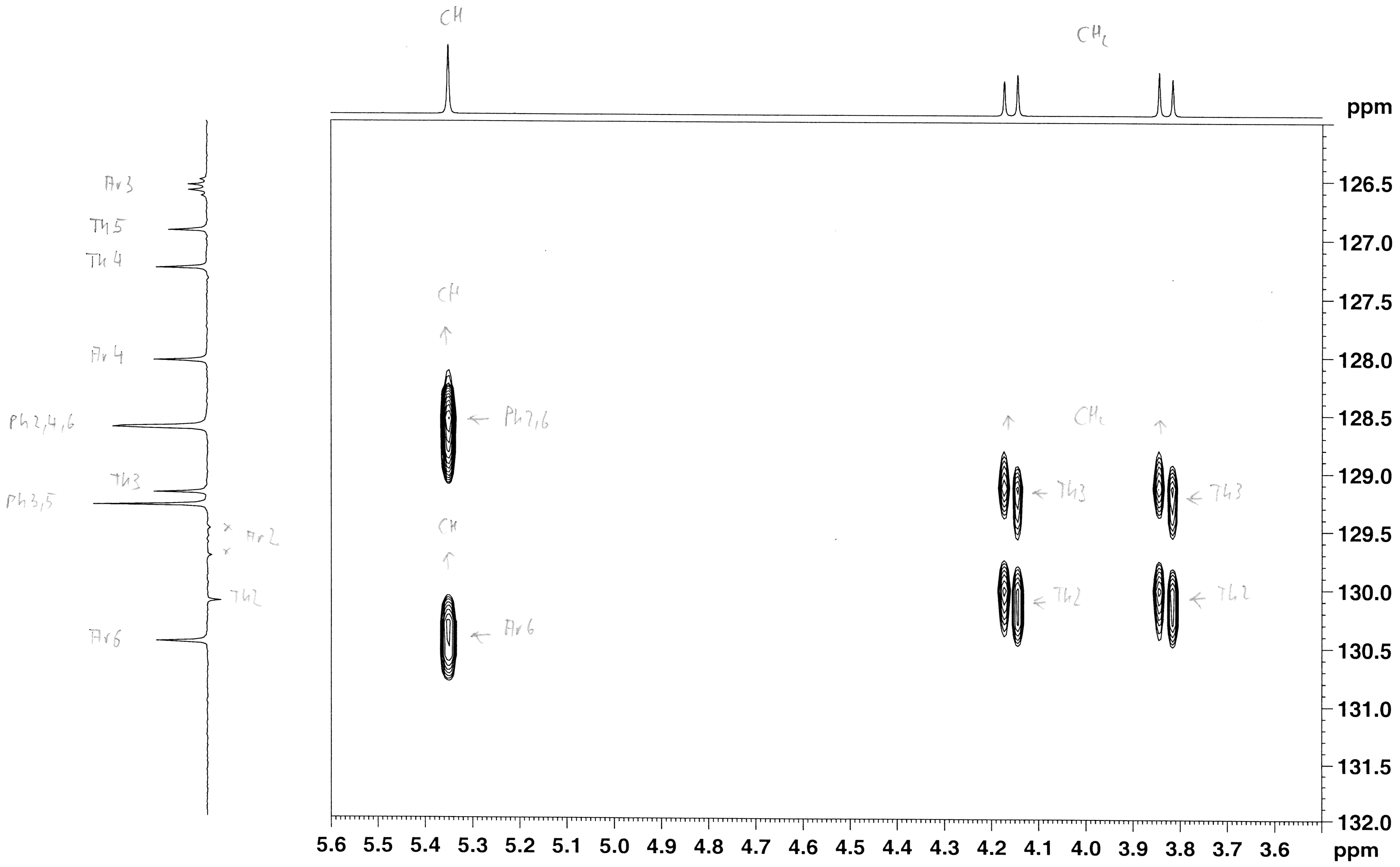

PN012S2Peak2 in cdcl3 (HMBC) 27.2.2020

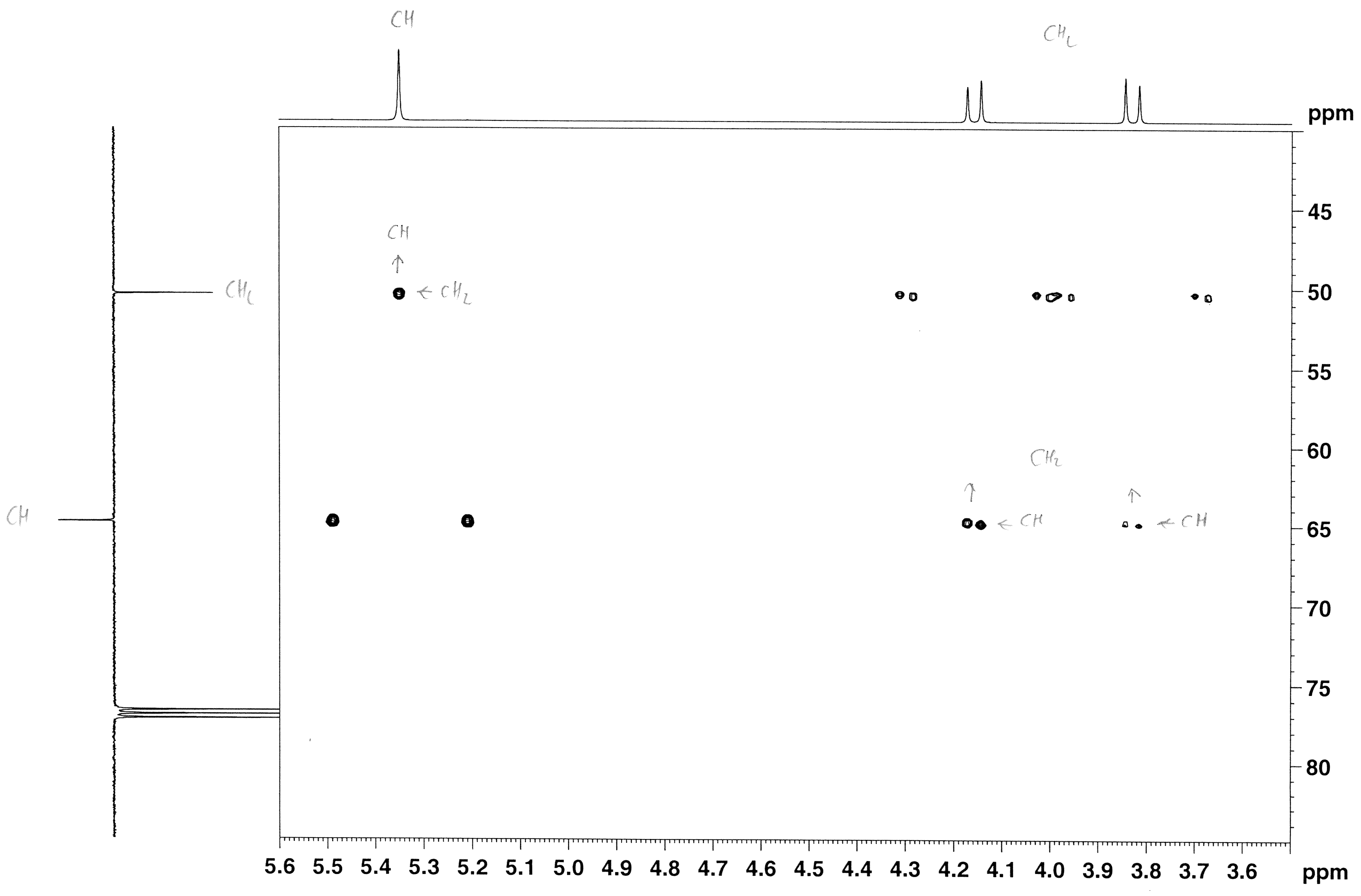

Figure S61c. NMR spectra of compound **5p.**

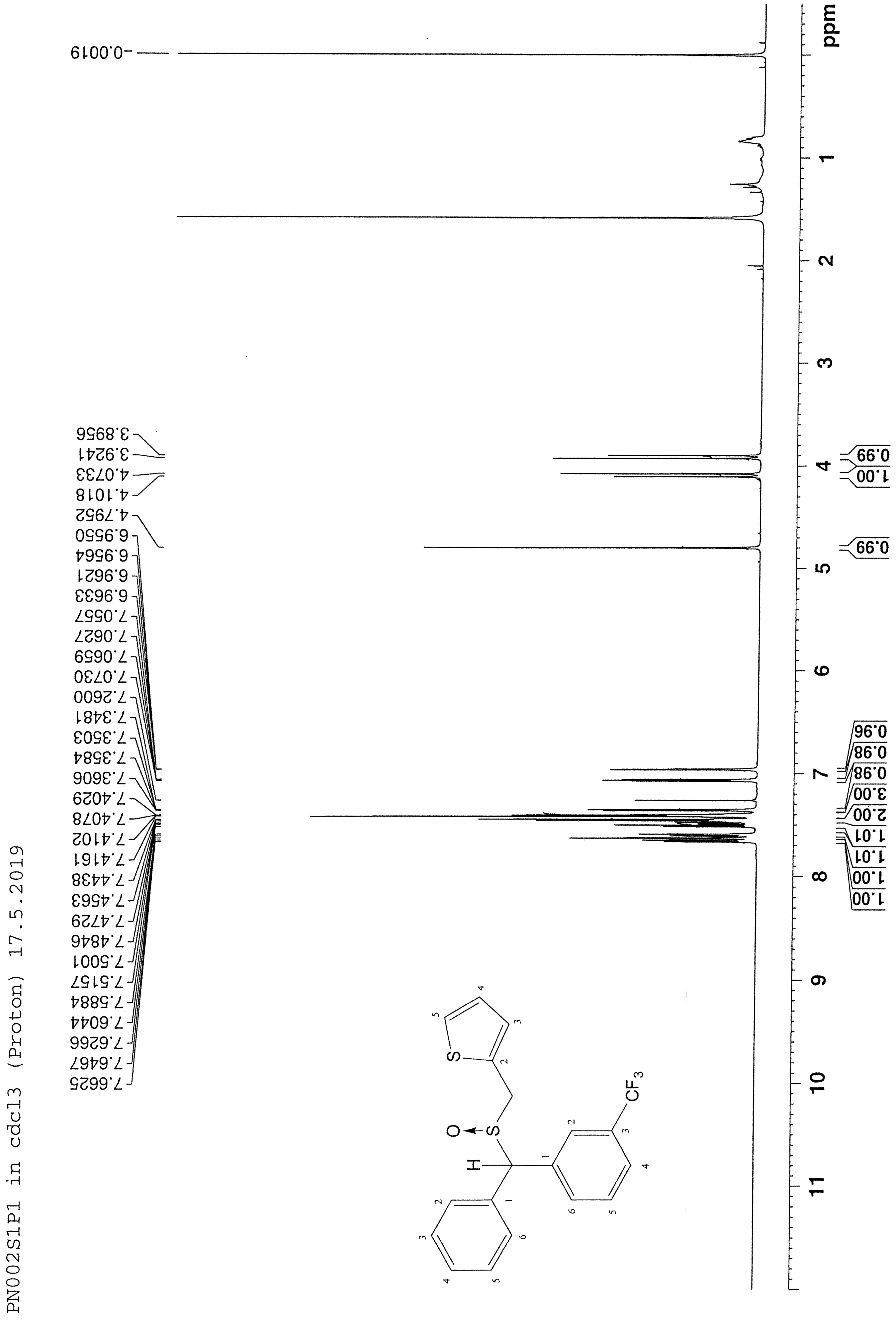

PN002S1P1 in cdcl3 (Proton) 17.5.2019

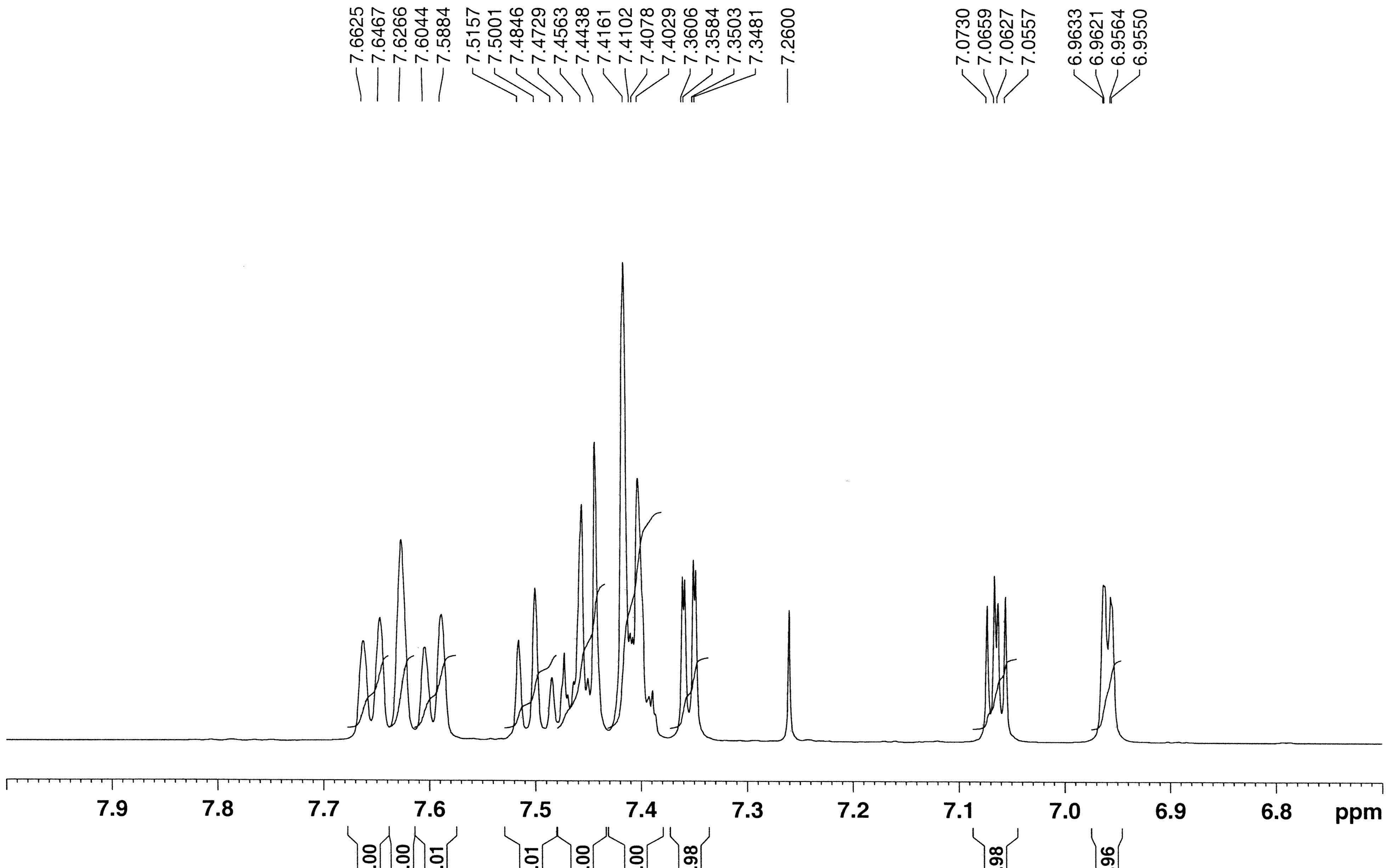

PN002S1P1 in cdcl3 (Proton) 17.5.2019

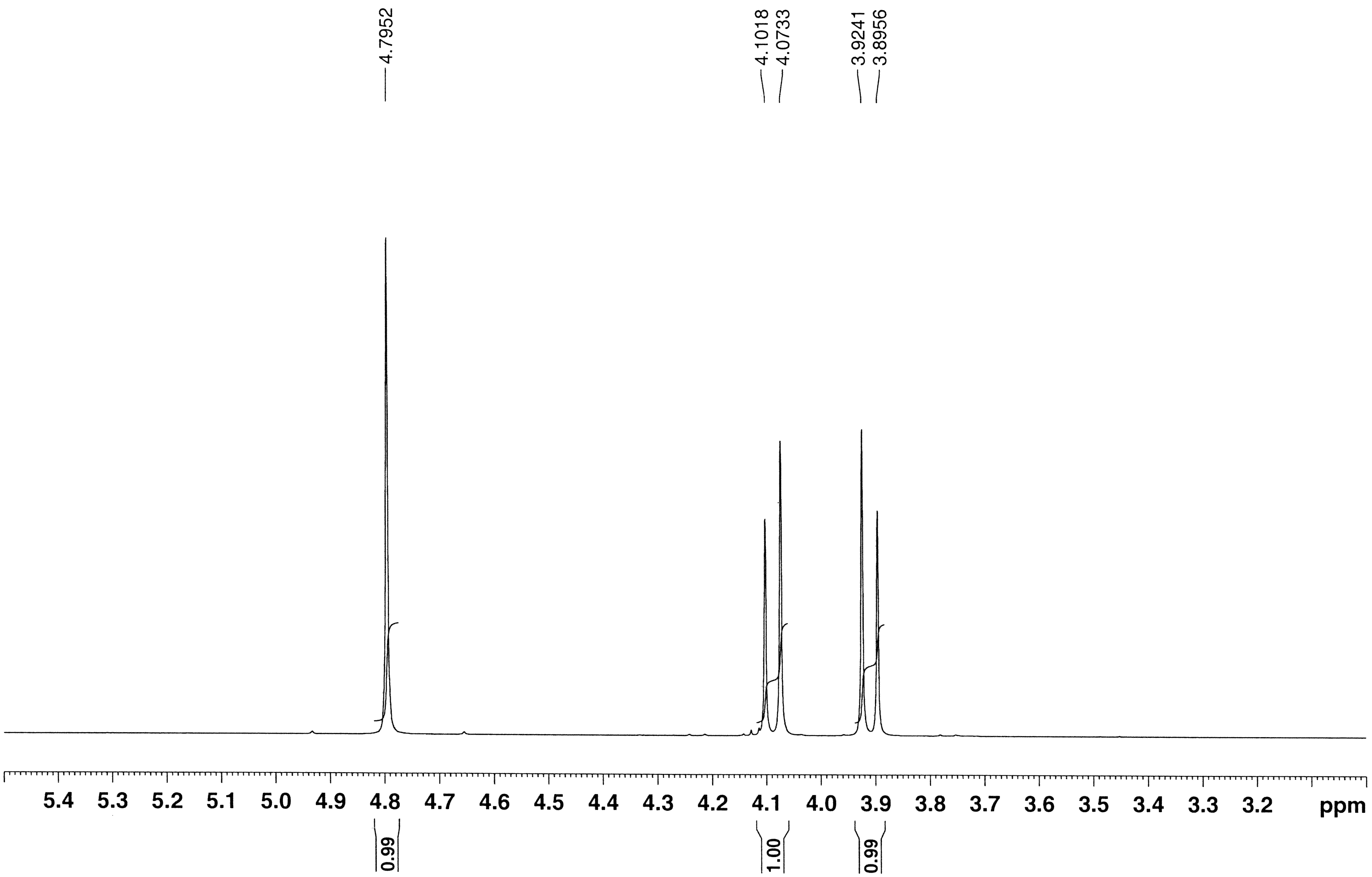

PN002S1P1 in cdcl3 (APT) 17.5.2019

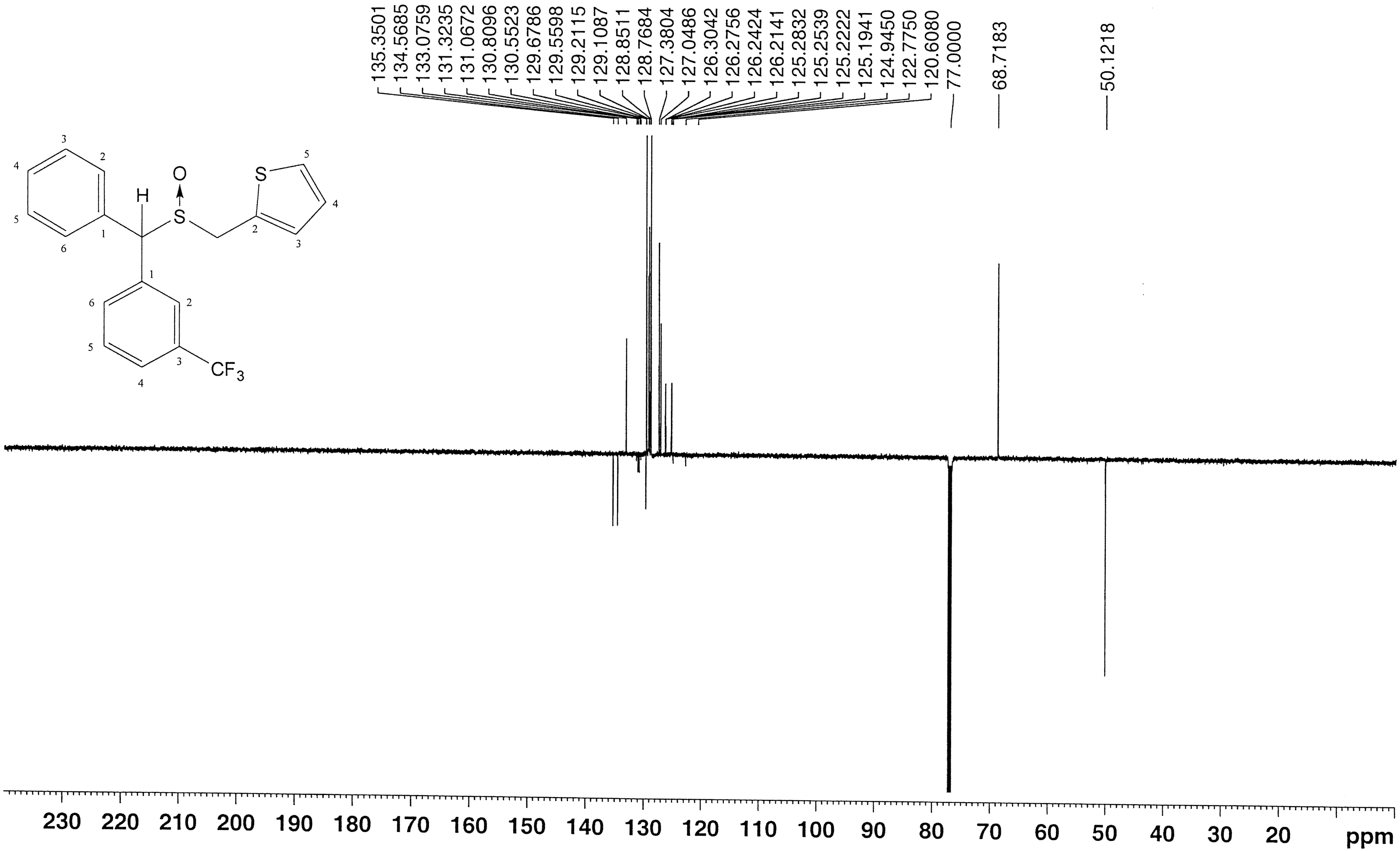

PN002S1P1 in cdcl3 (APT) 17.5.2019

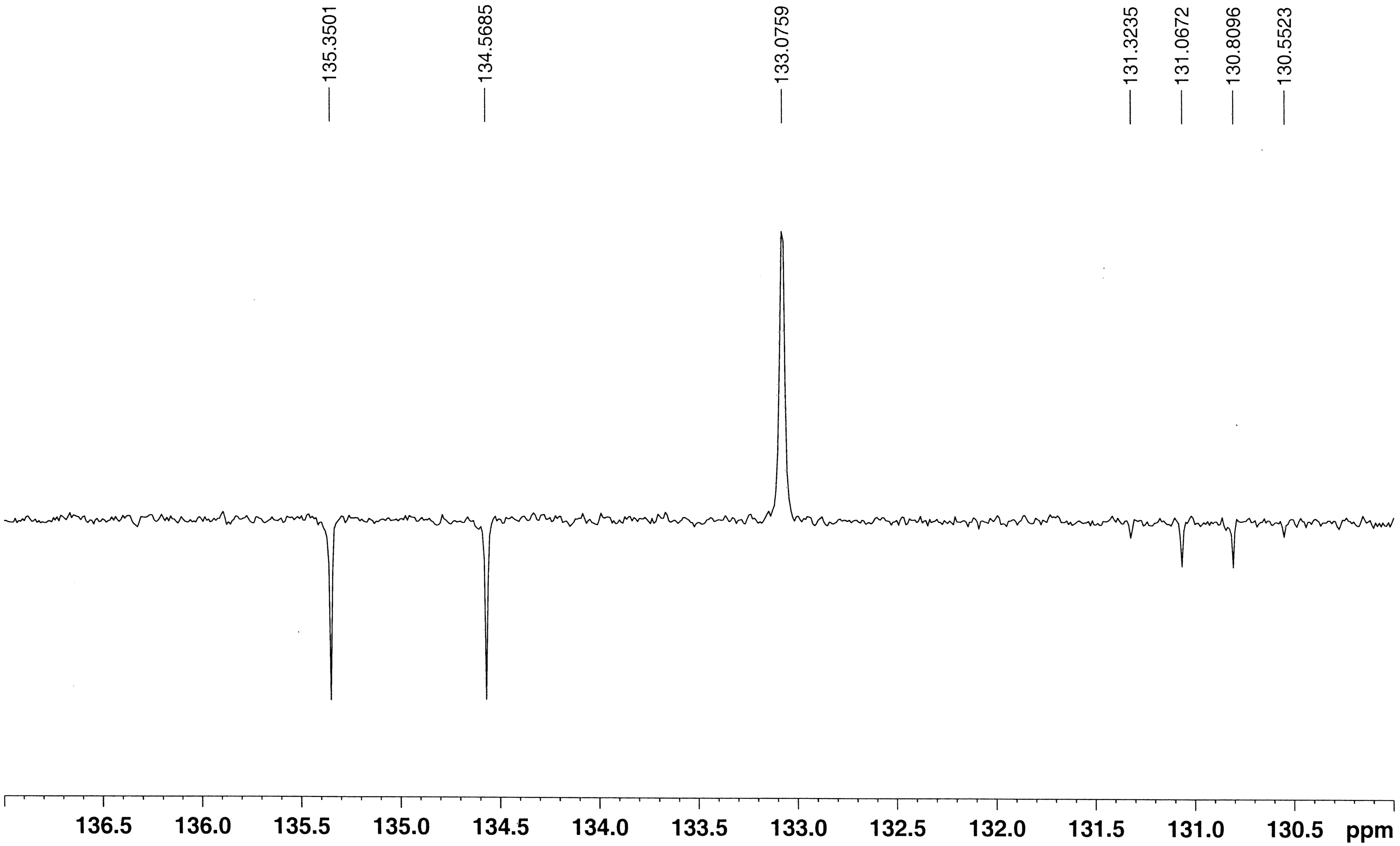

PN002S1P1 in cdcl3 (APT) 17.5.2019

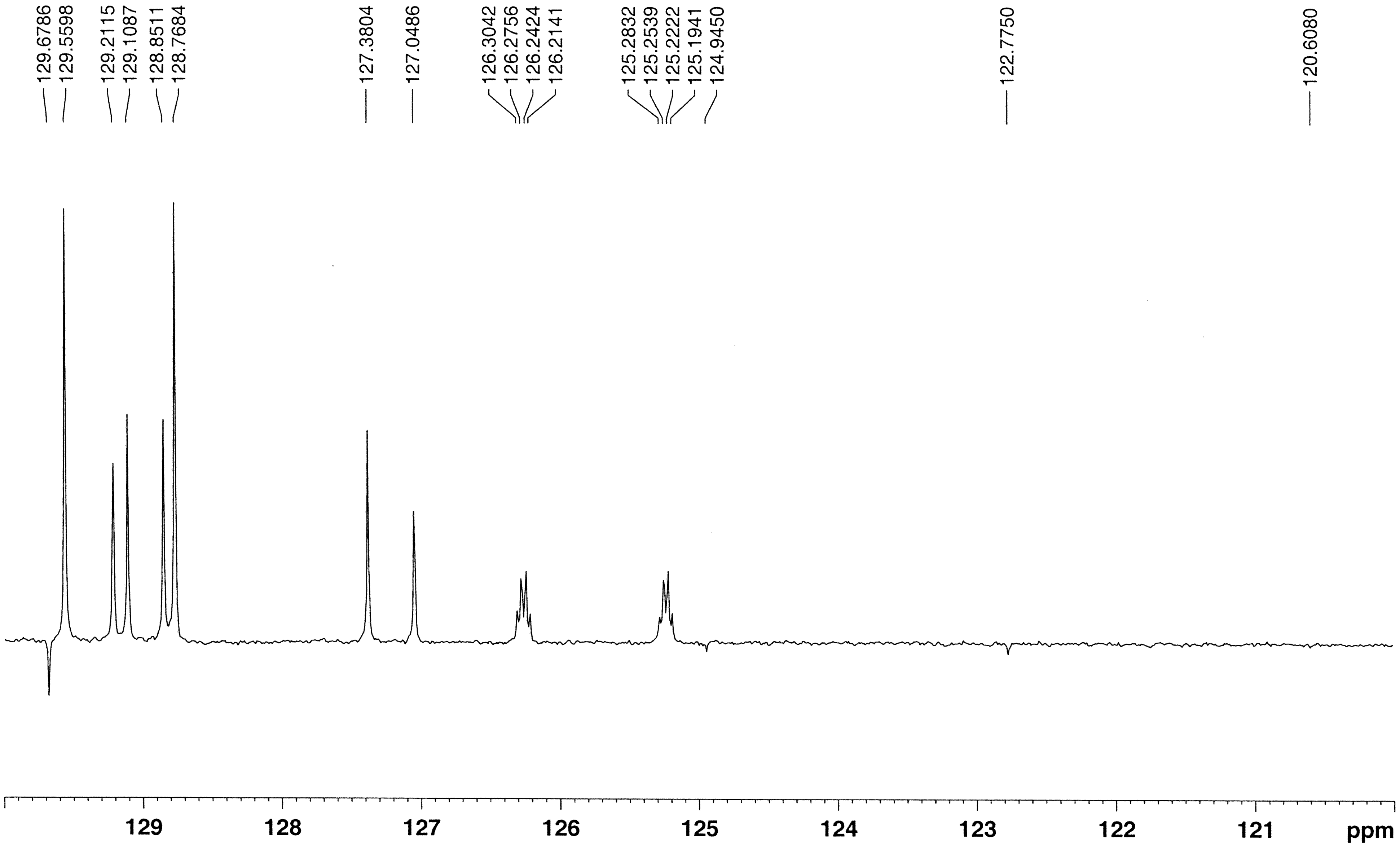

PN002S1P1 in cdcl3 (APT) 17.5.2019

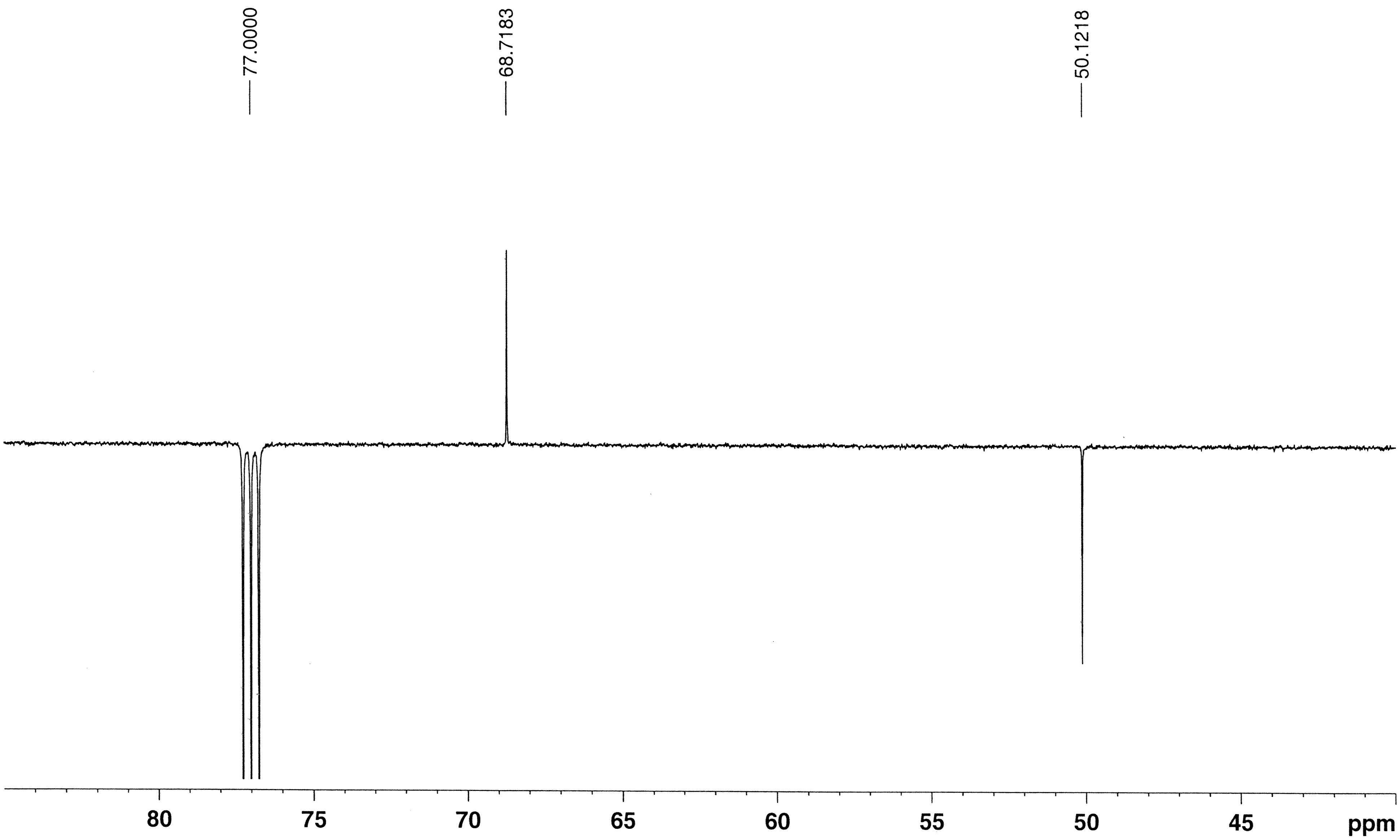

Figure S62c. NMR spectra of compound **6p**.

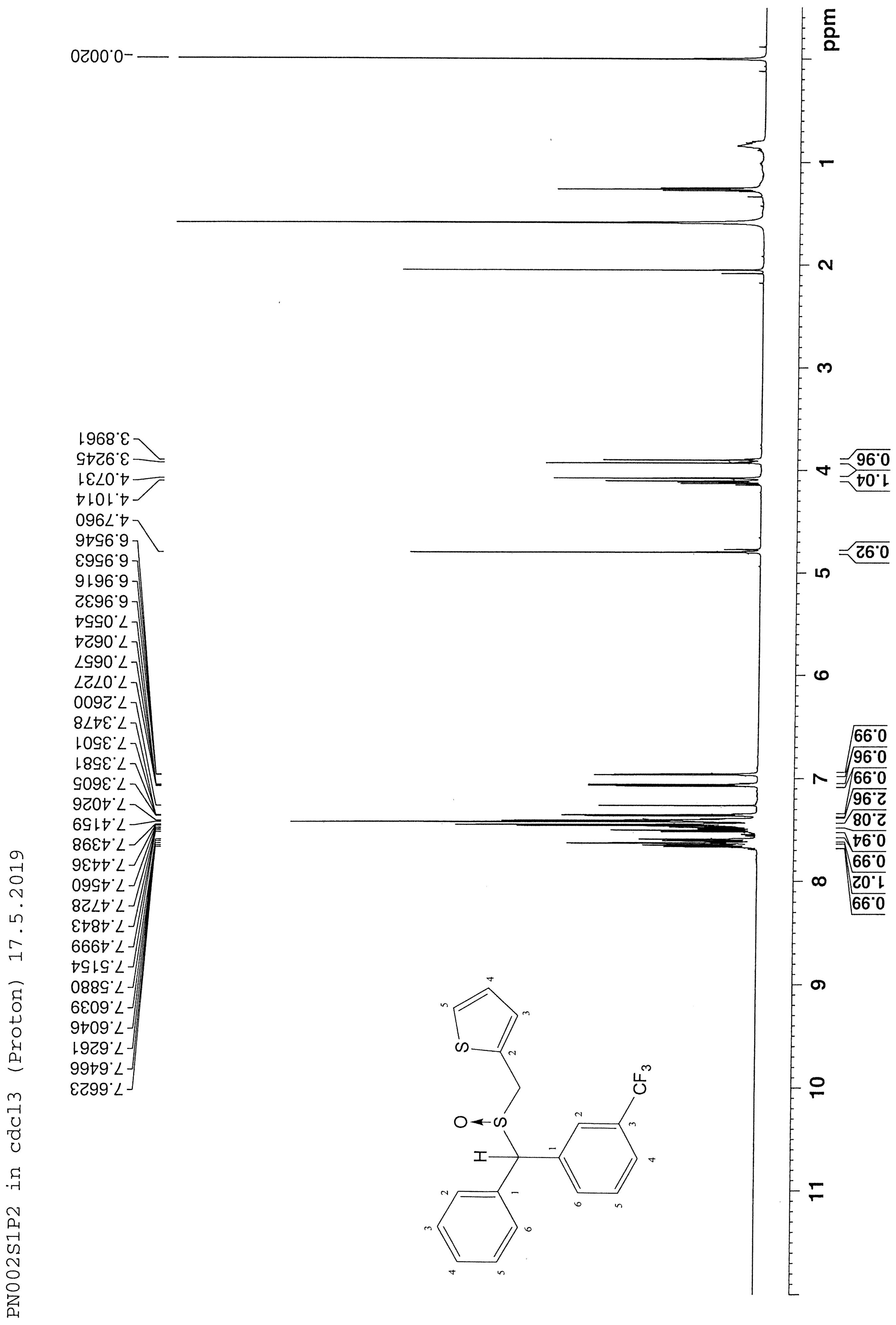

PN002S1P2 in cdcl3 (Proton) 17.5.2019

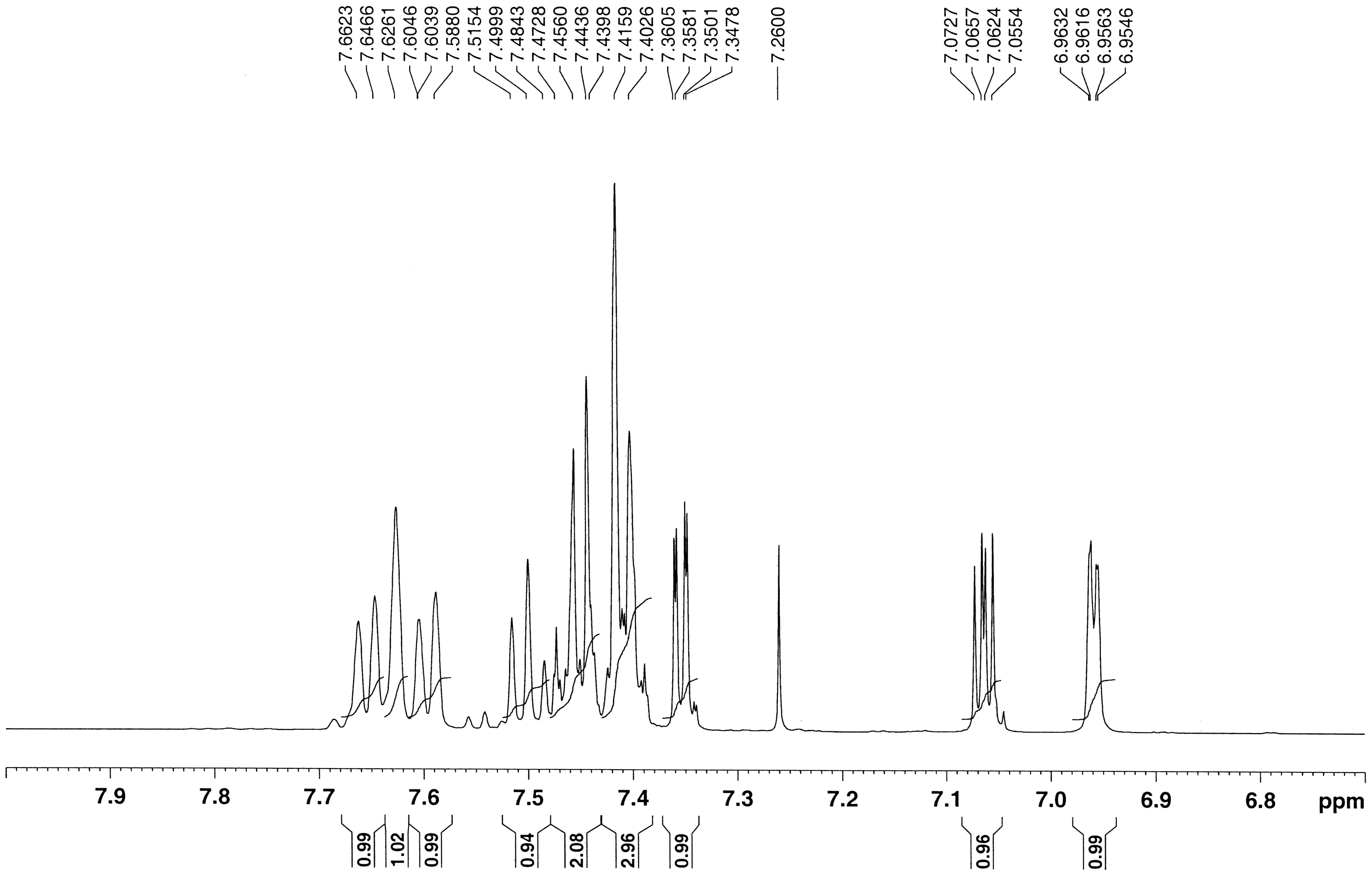

PN002S1P2 in cdcl3 (Proton) 17.5.2019

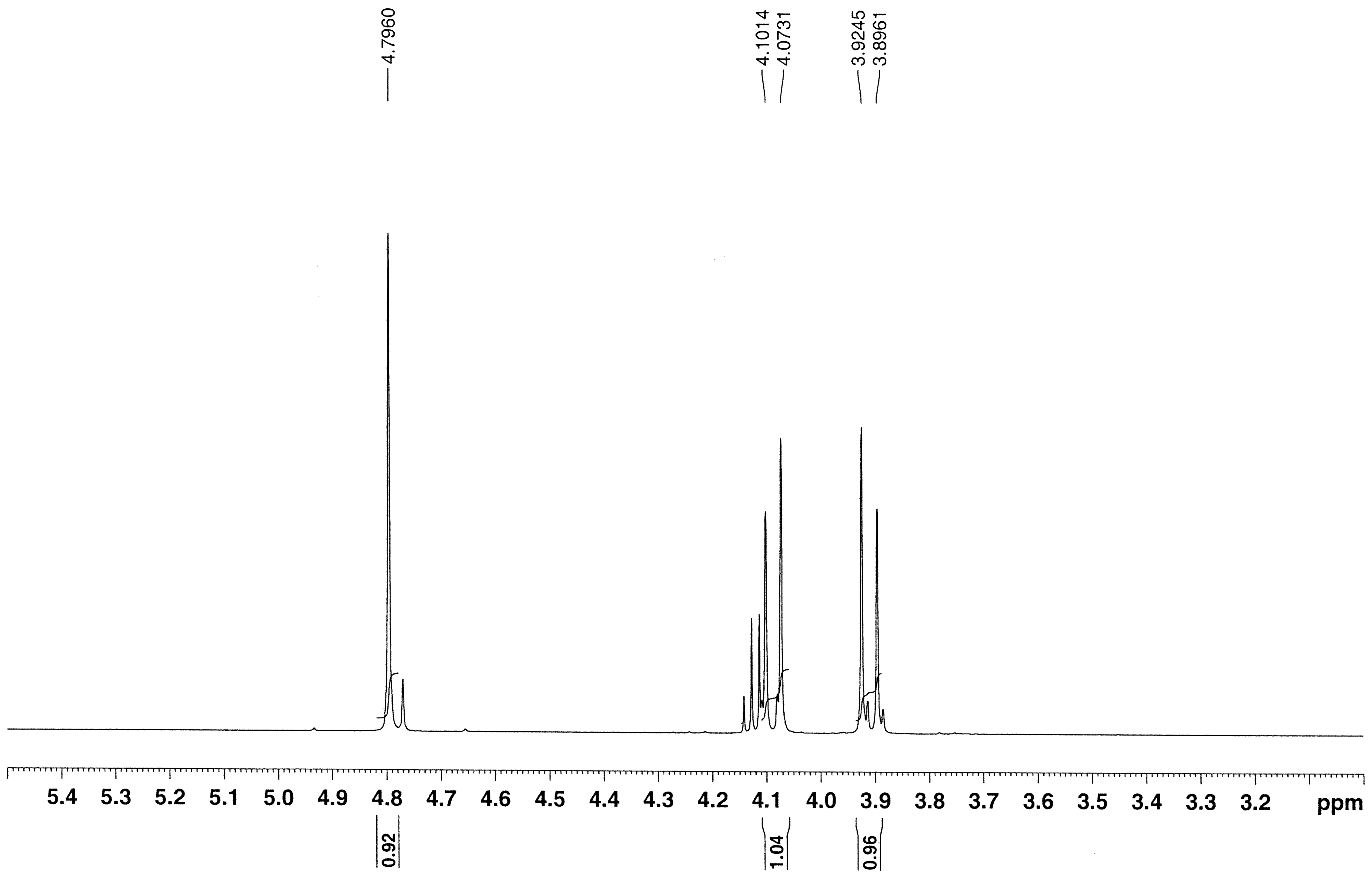

PN002S1P2 in cdcl3 (APT) 17.5.2019

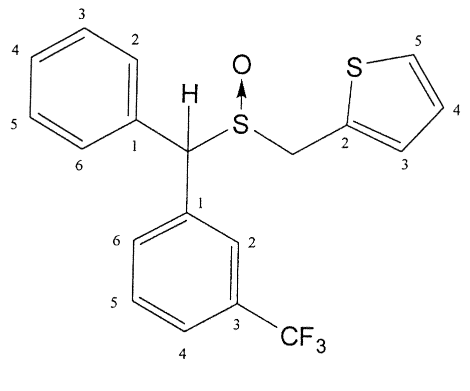

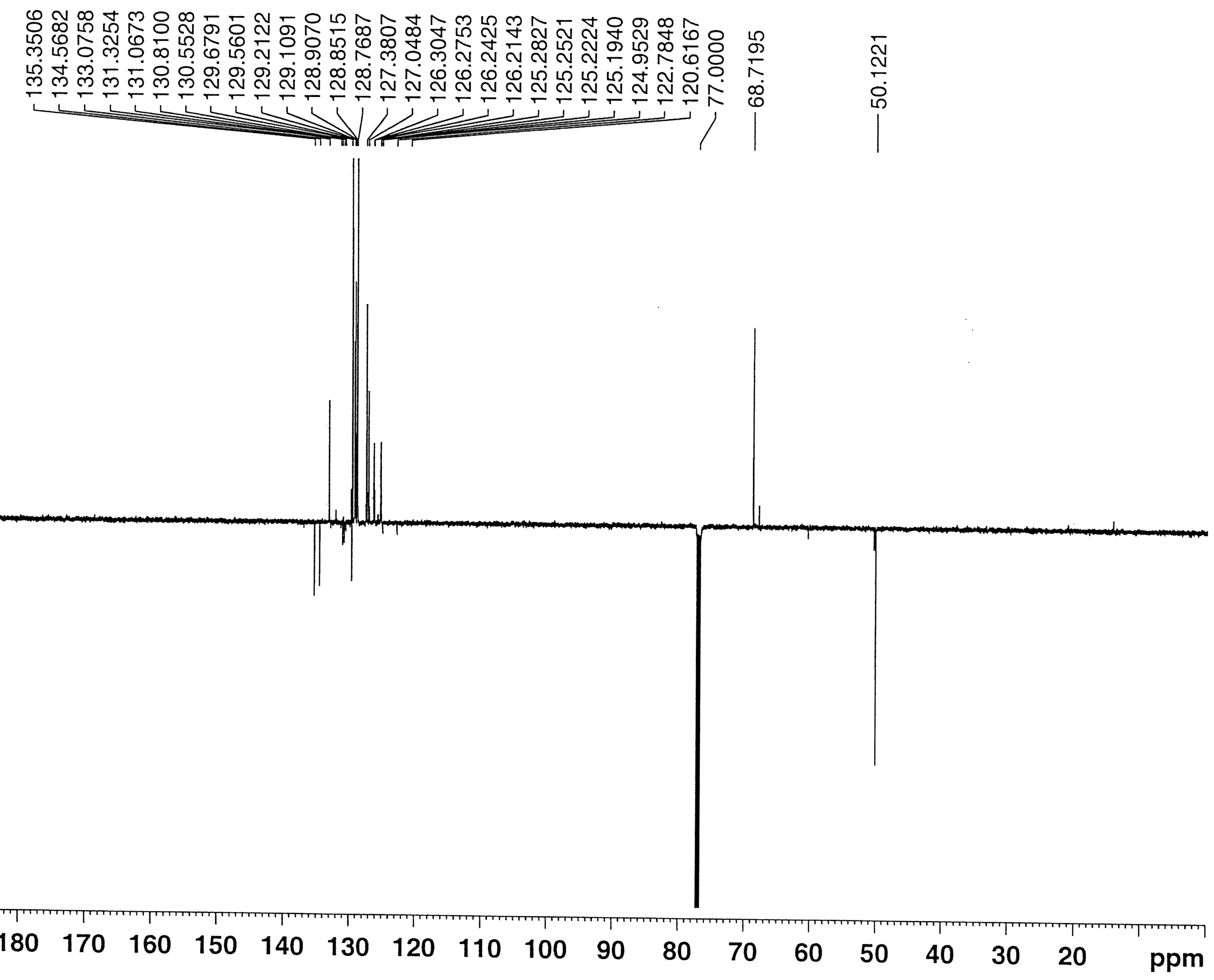

PN002S1P2 in cdcl3 (APT) 17.5.2019

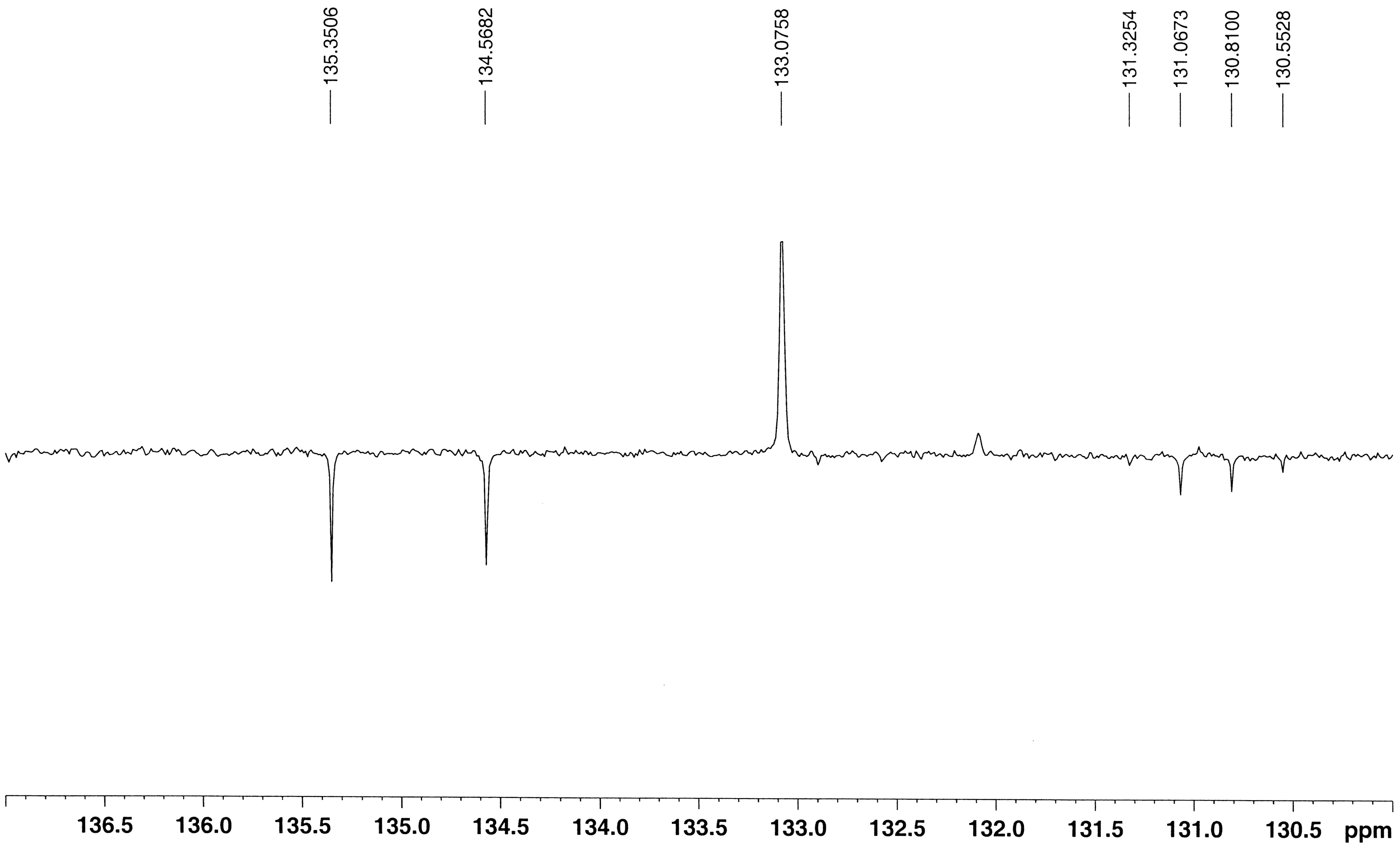

PN002S1P2 in cdcl3 (APT) 17.5.2019

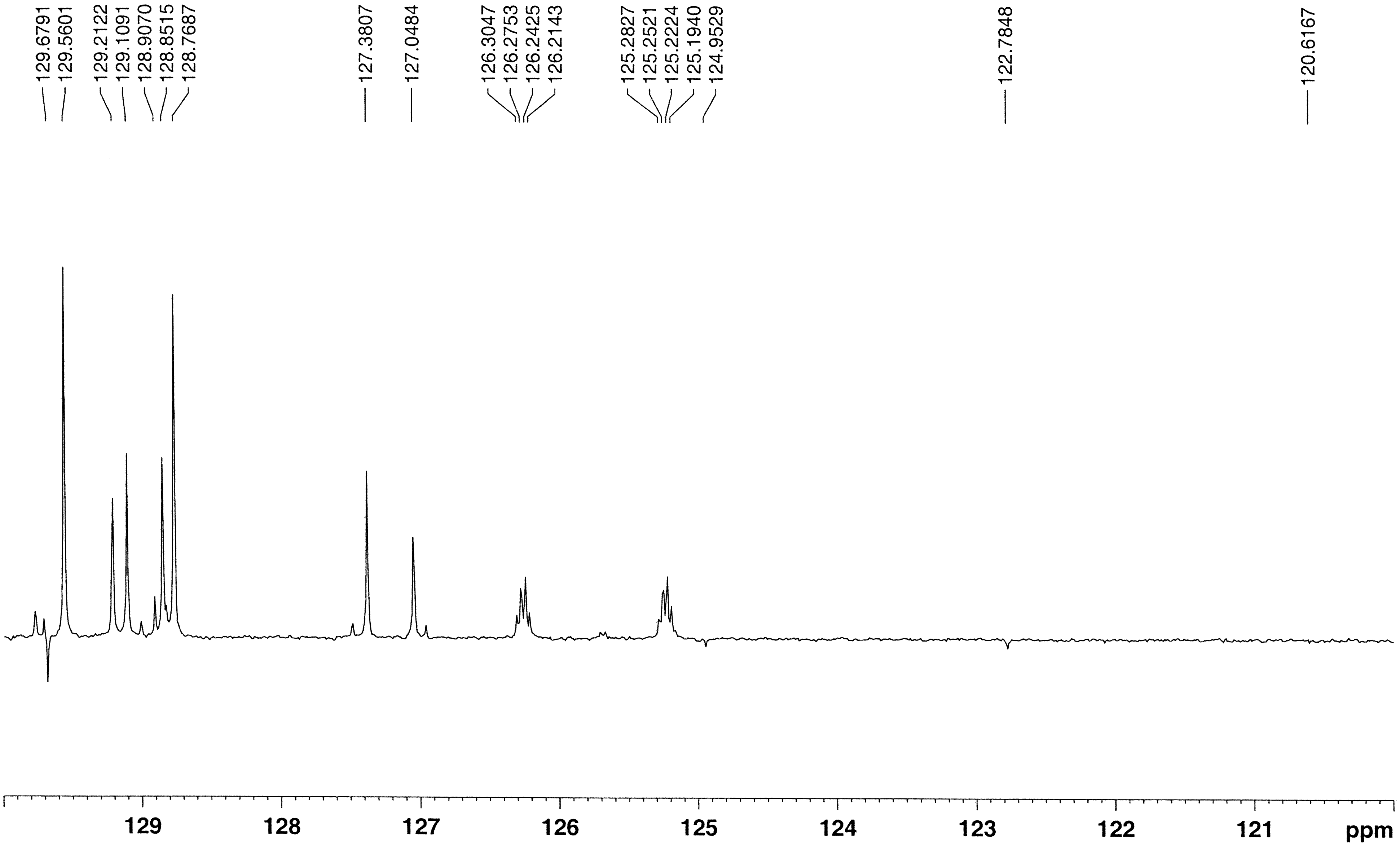

PN002S1P2 in cdcl3 (APT) 17.5.2019

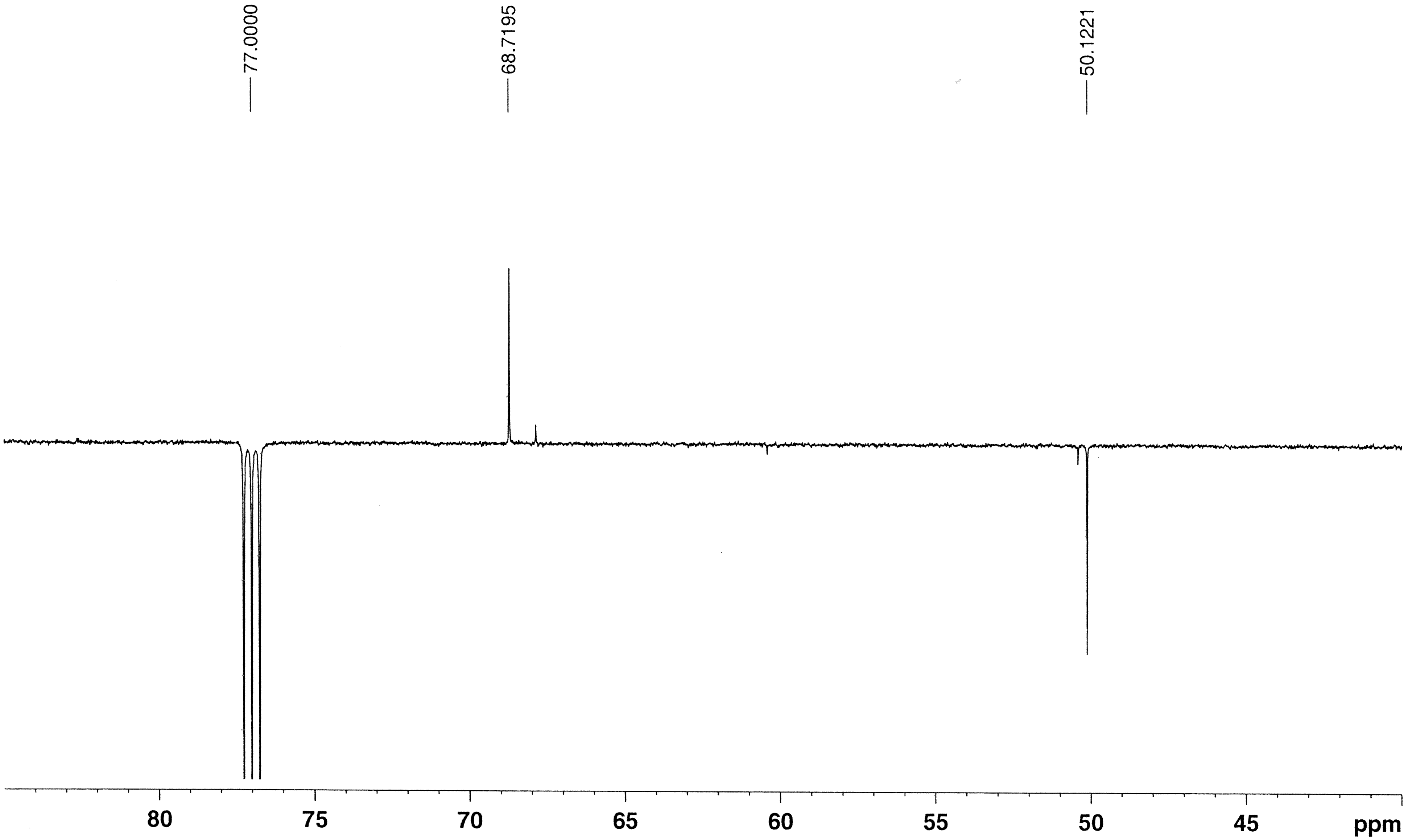

Figure S63c. NMR spectra of compound **7p.**

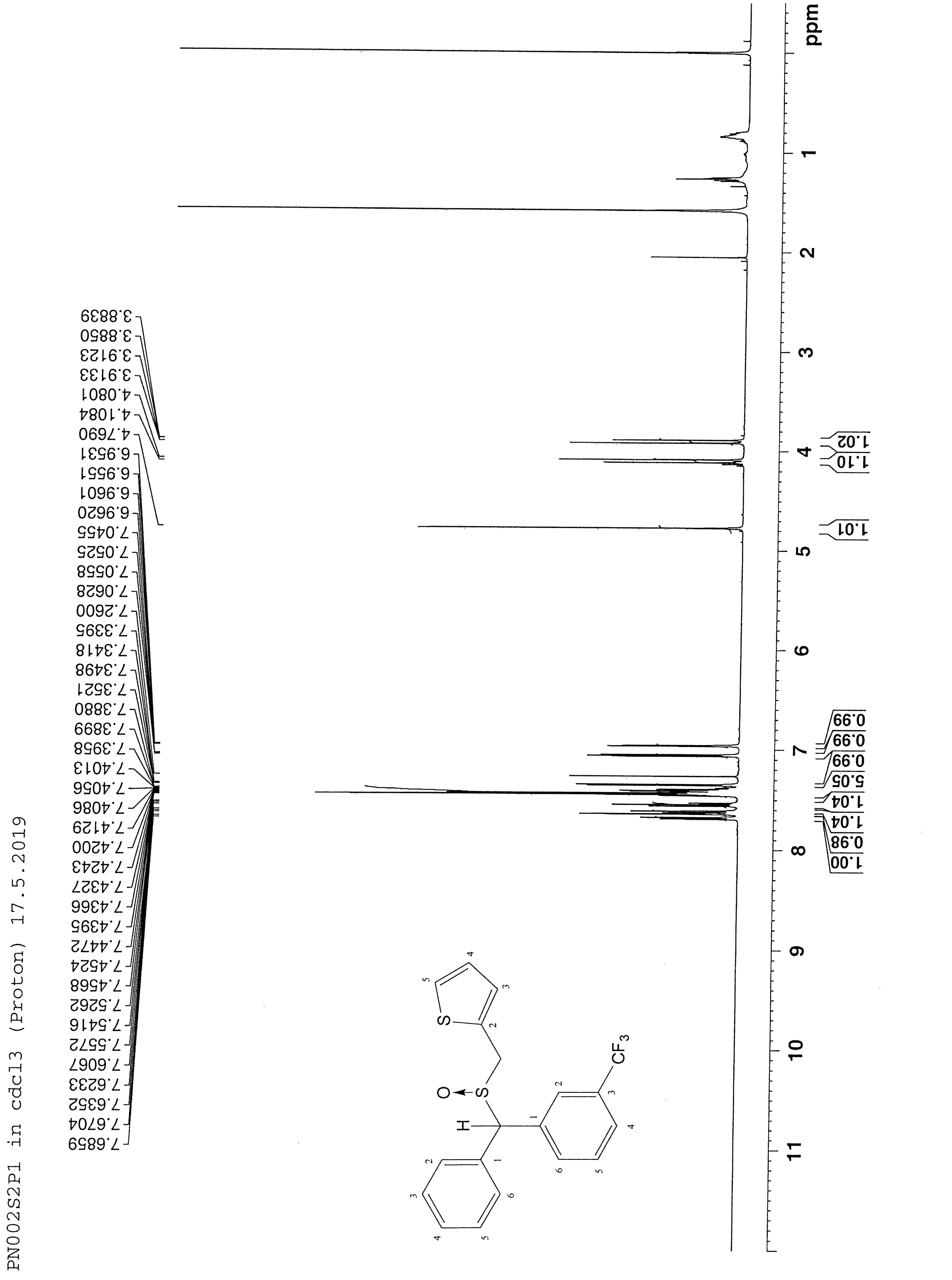

PN002S2P1 in cdcl3 (Proton) 17.5.2019

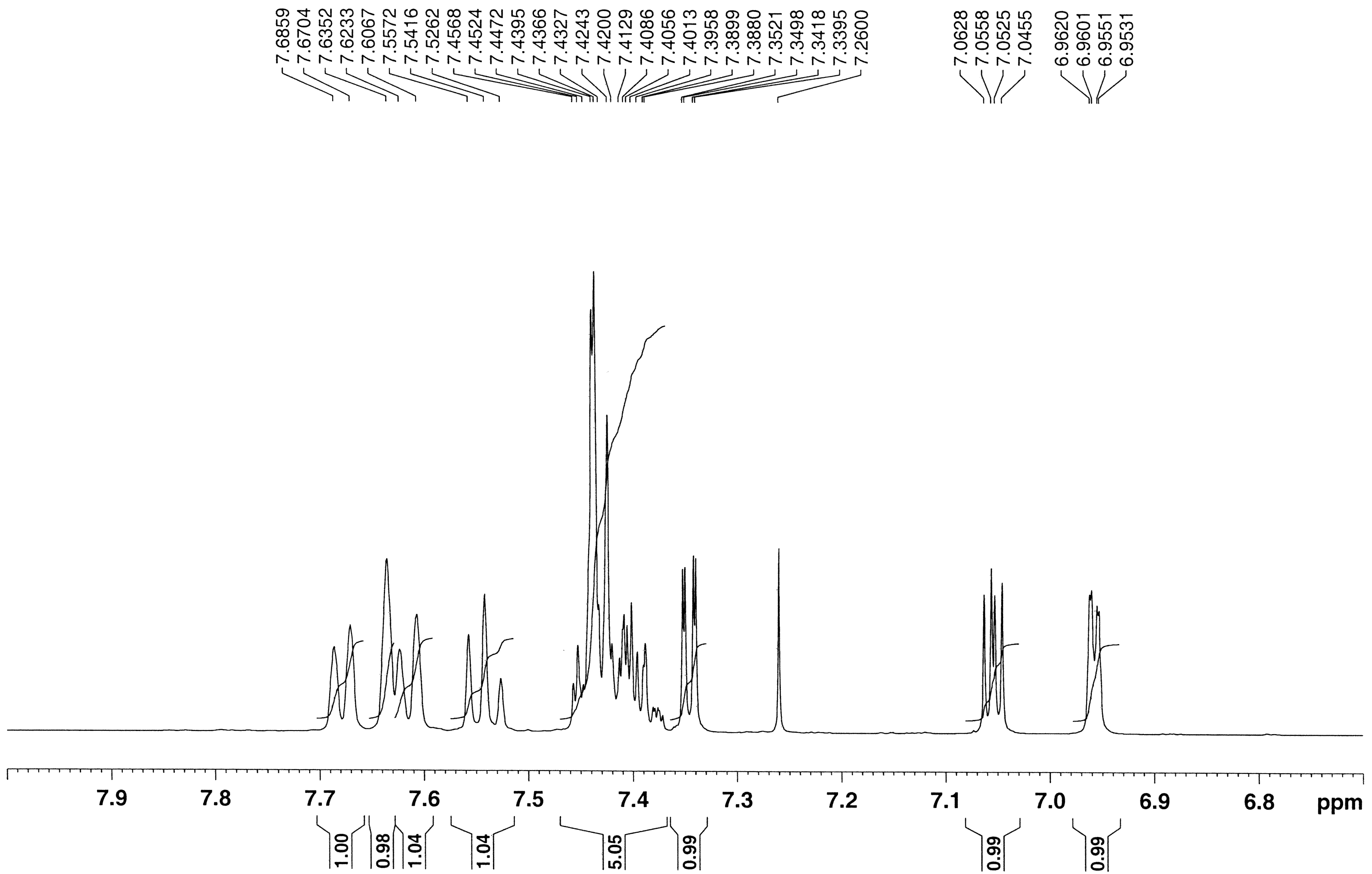

PN002S2P1 in cdcl3 (Proton) 17.5.2019

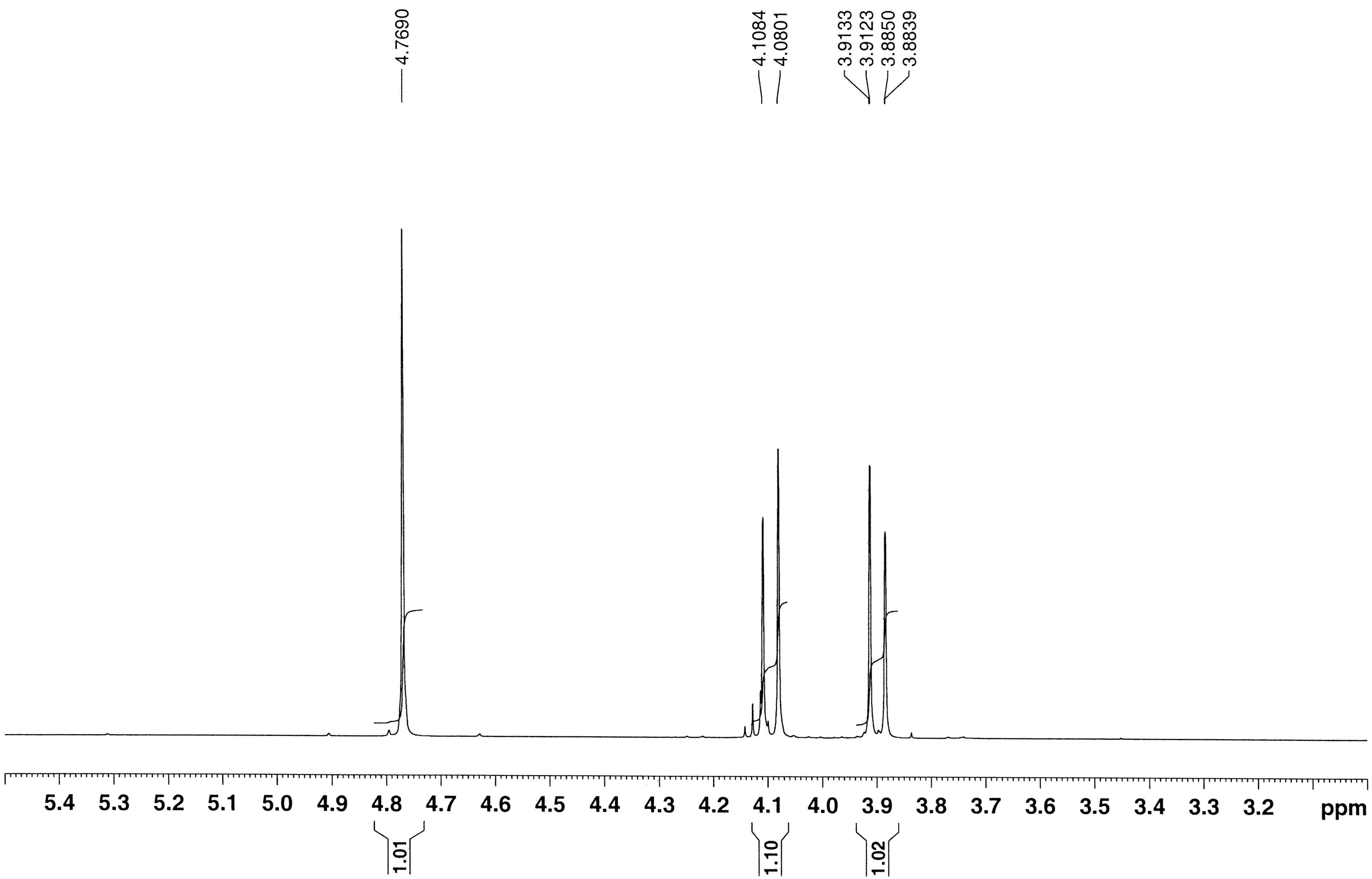

PN002S2P1 in cdcl3 (APT) 17.5.2019

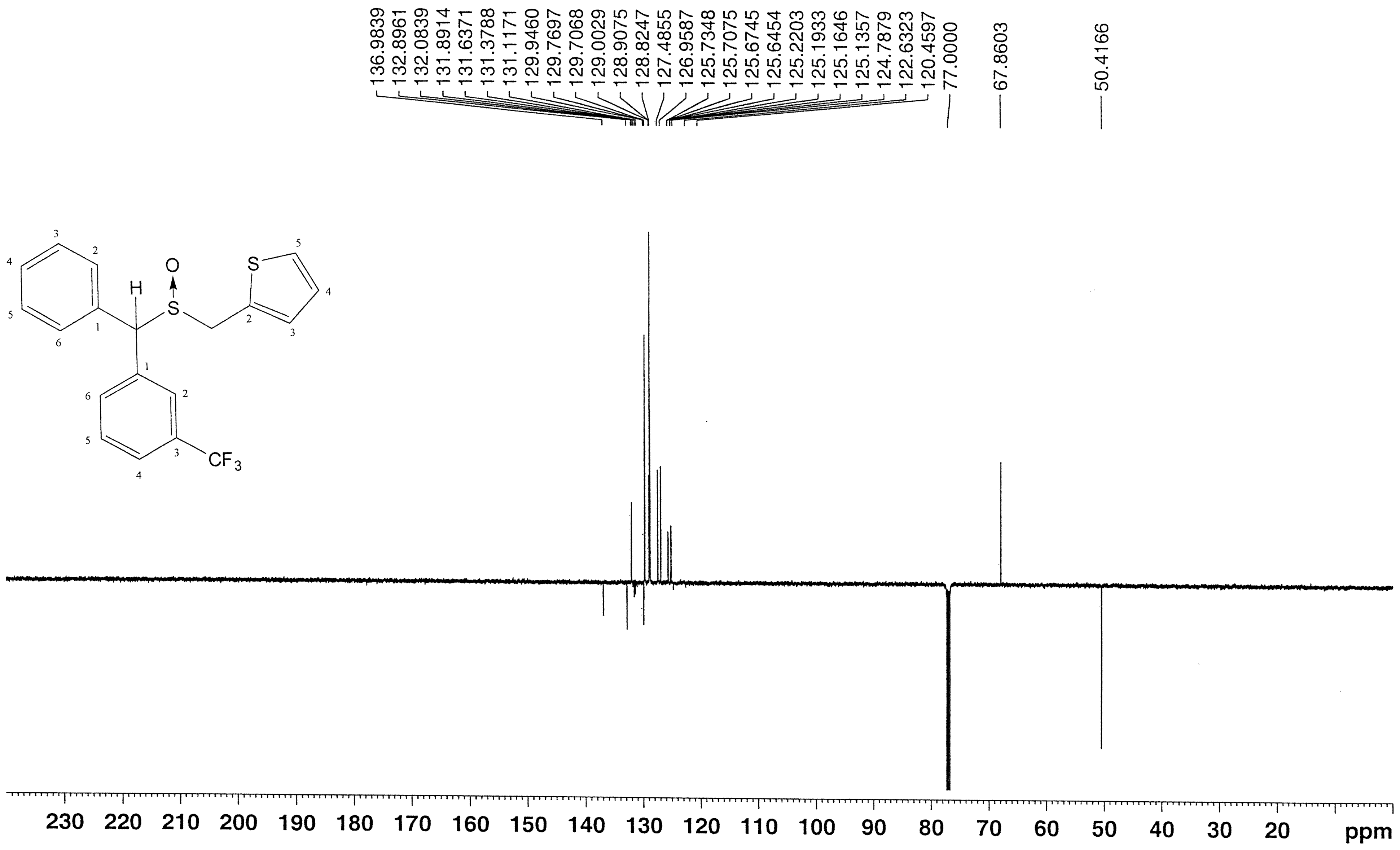

PN002S2P1 in cdcl3 (APT) 17.5.2019

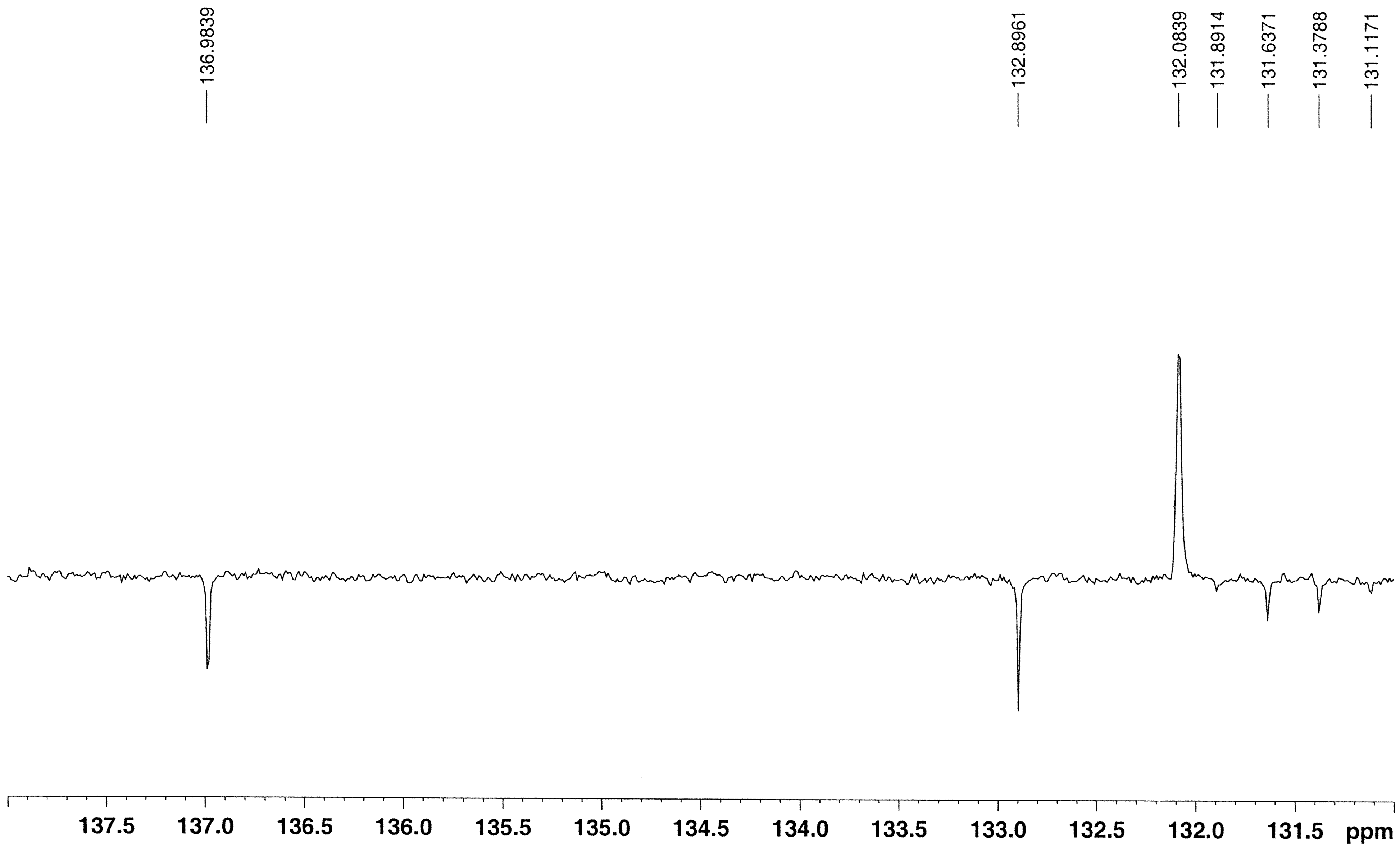

PN002S2P1 in cdcl3 (APT) 17.5.2019

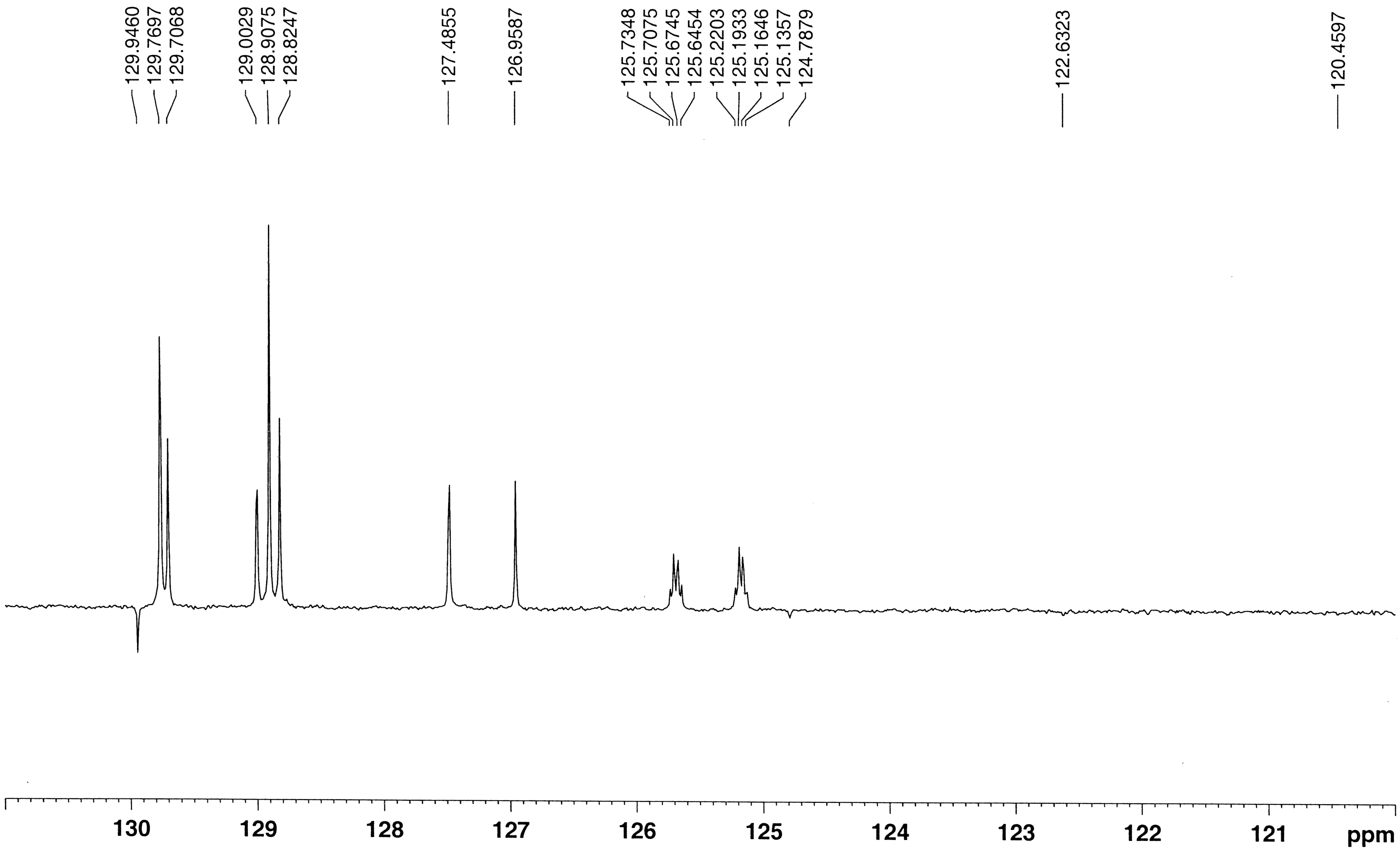

PN002S2P1 in cdcl3 (APT) 17.5.2019

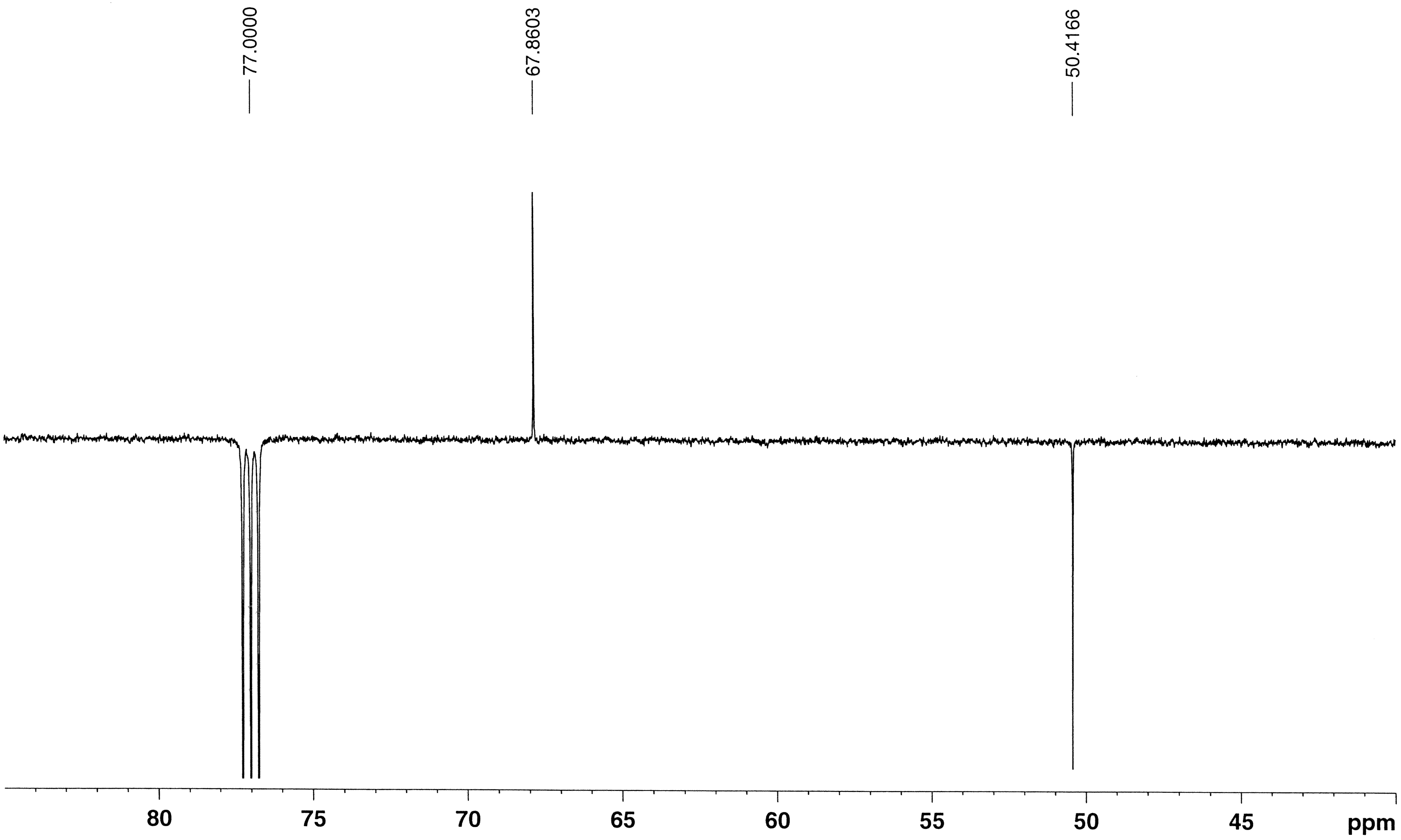

Figure S64c. NMR spectra of compound **8p**.

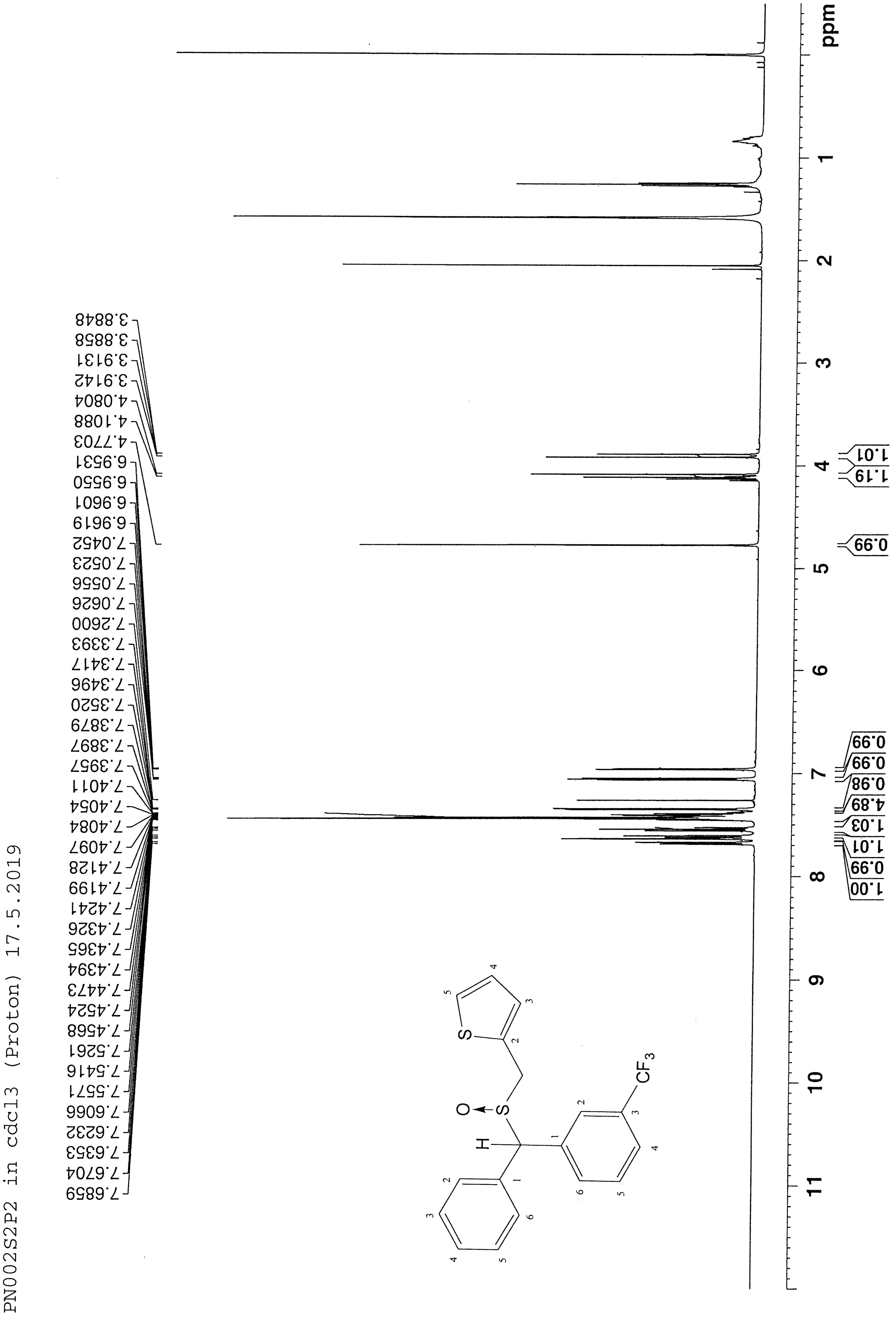

PN002S2P2 in cdcl3 (Proton) 17.5.2019

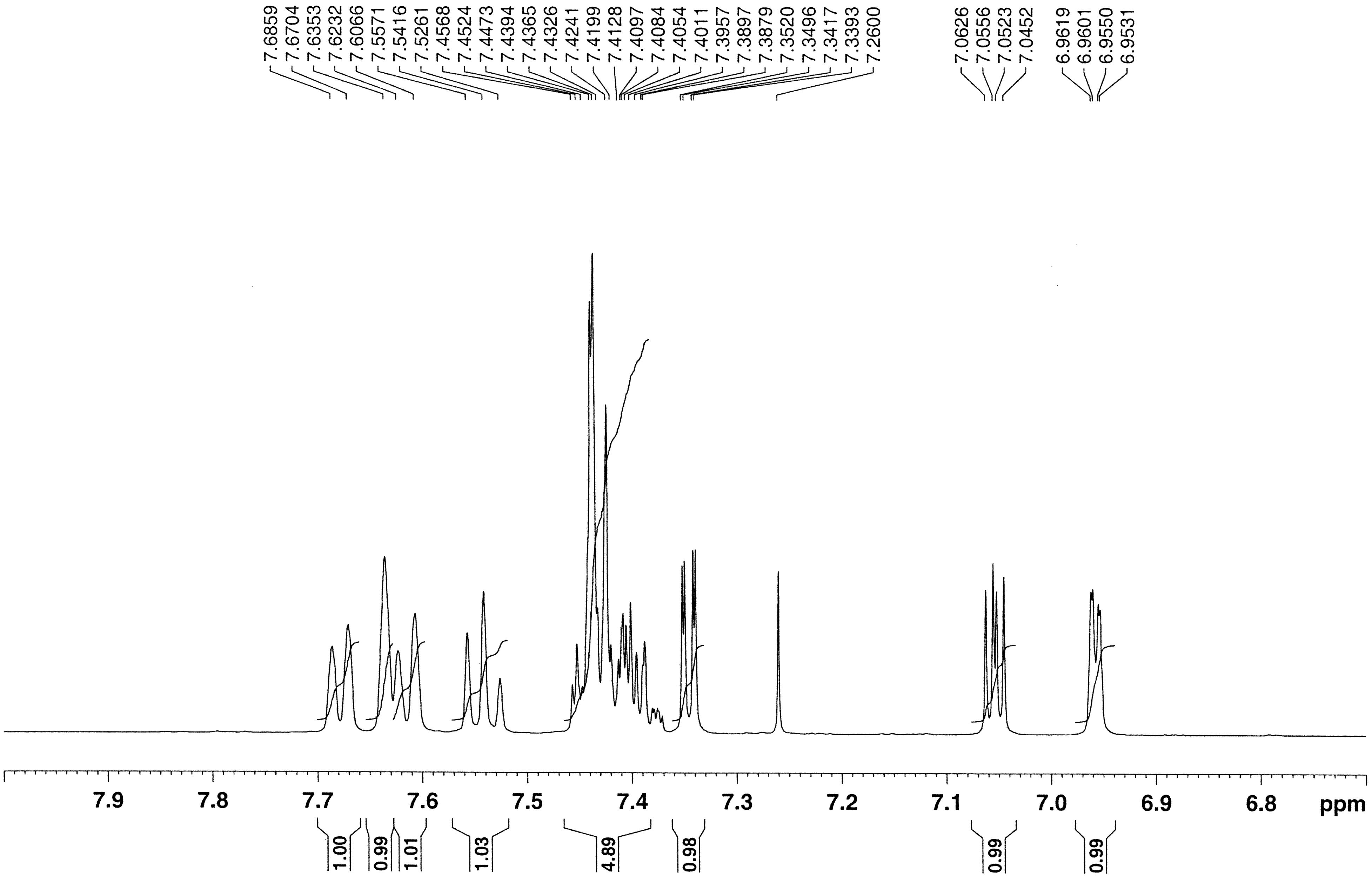

PN002S2P2 in cdcl3 (Proton) 17.5.2019

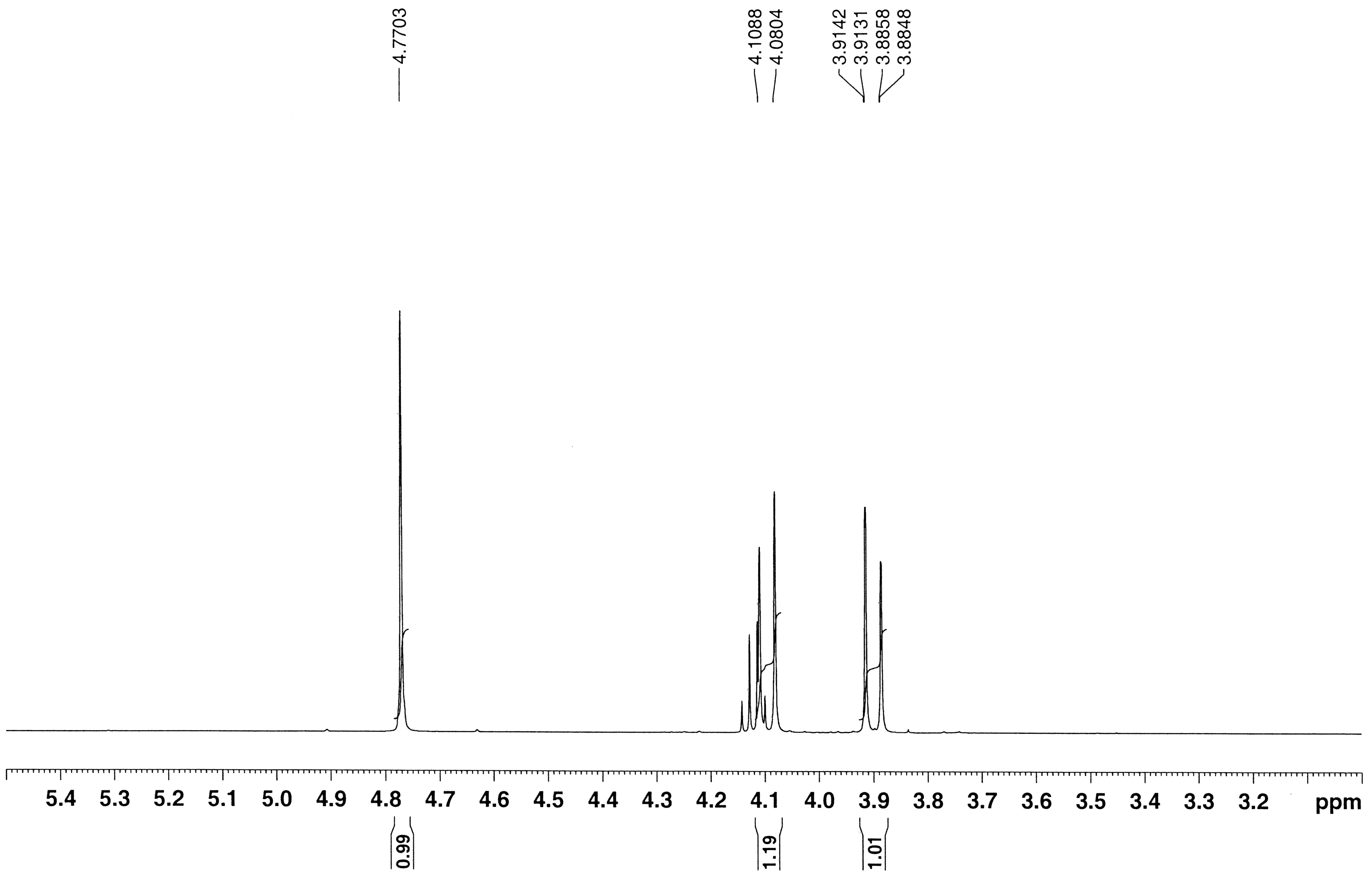

PN002S2P2 in cdcl3 (APT) 17.5.2019

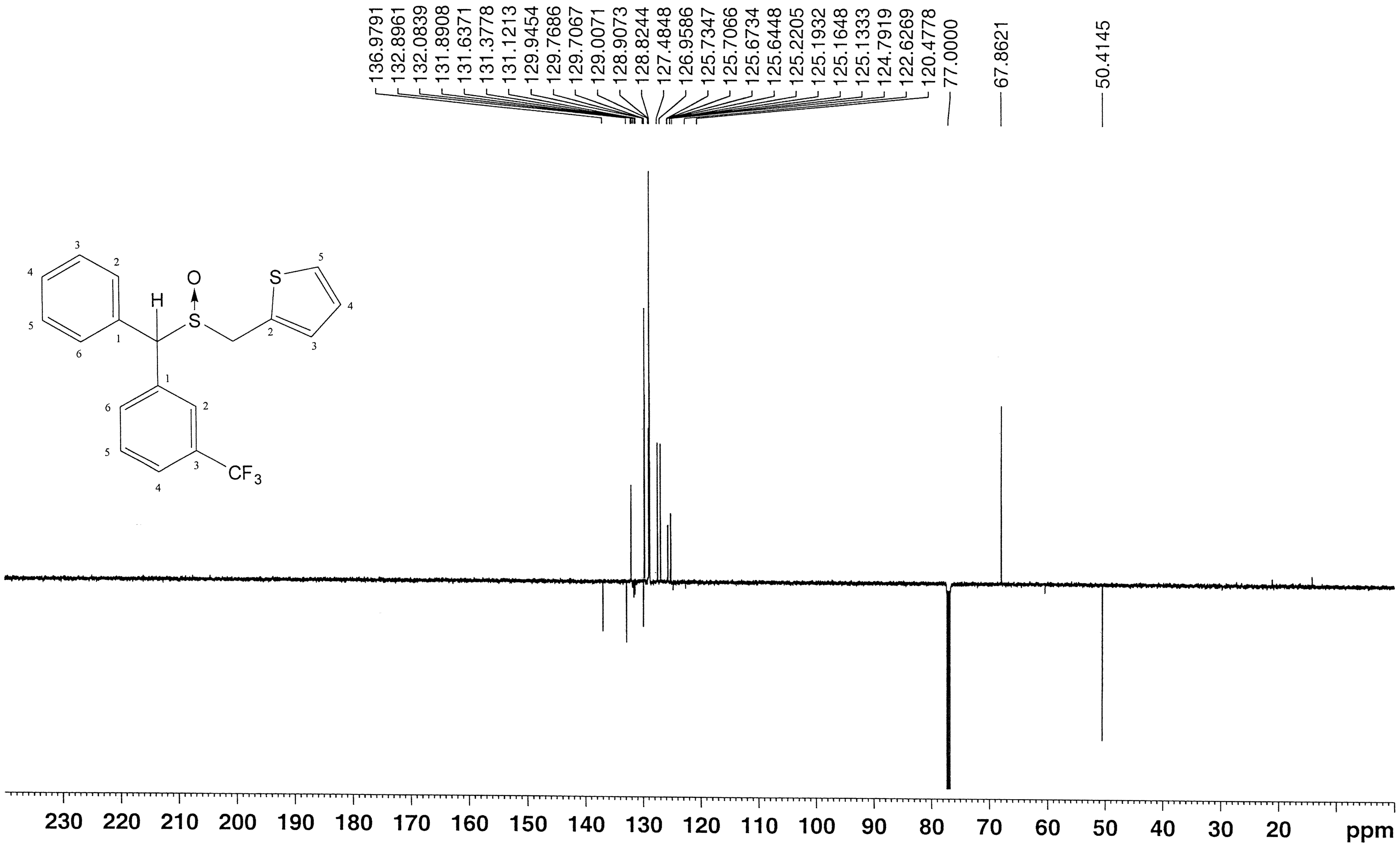

PN002S2P2 in cdcl3 (APT) 17.5.2019

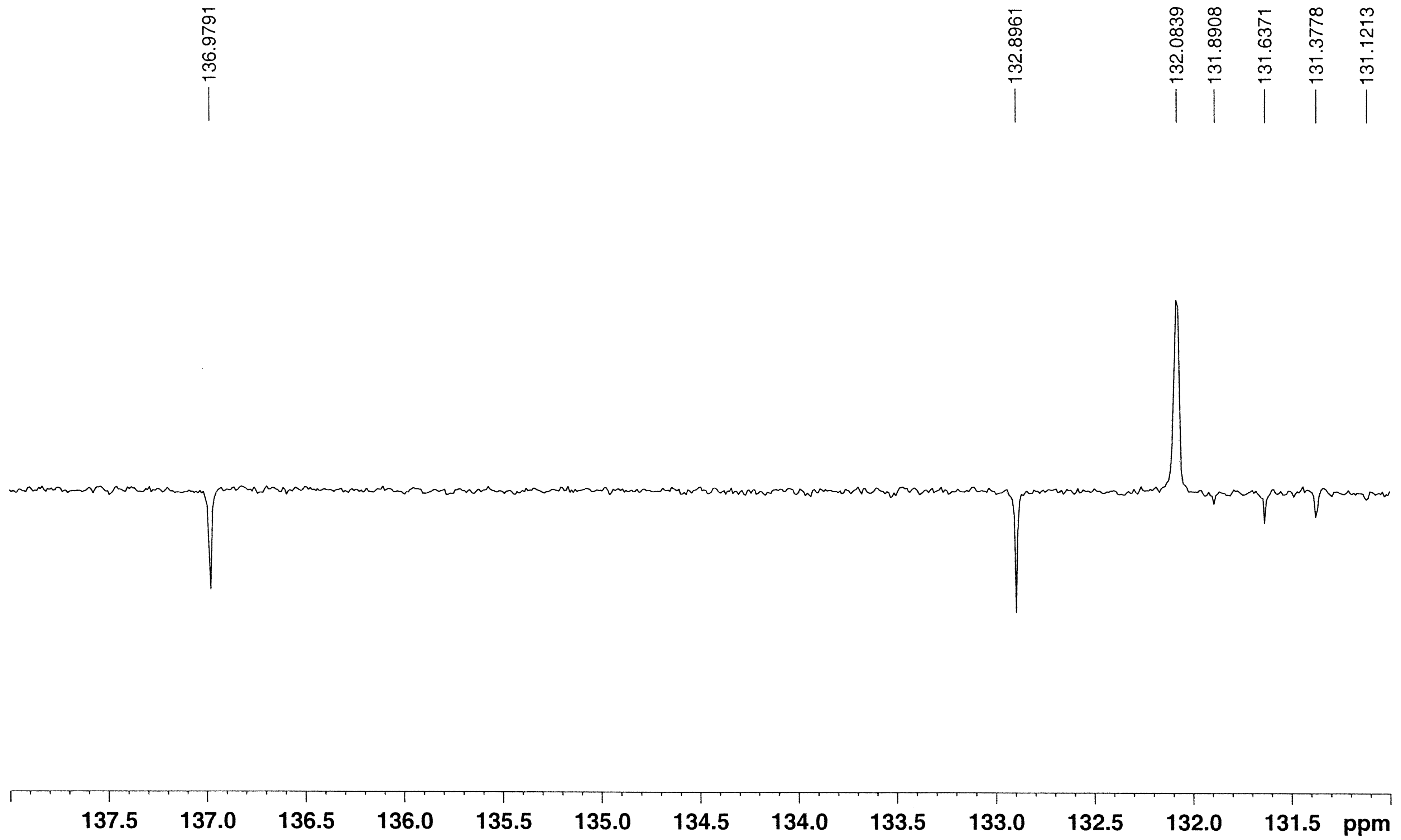

PN002S2P2 in cdcl3 (APT) 17.5.2019

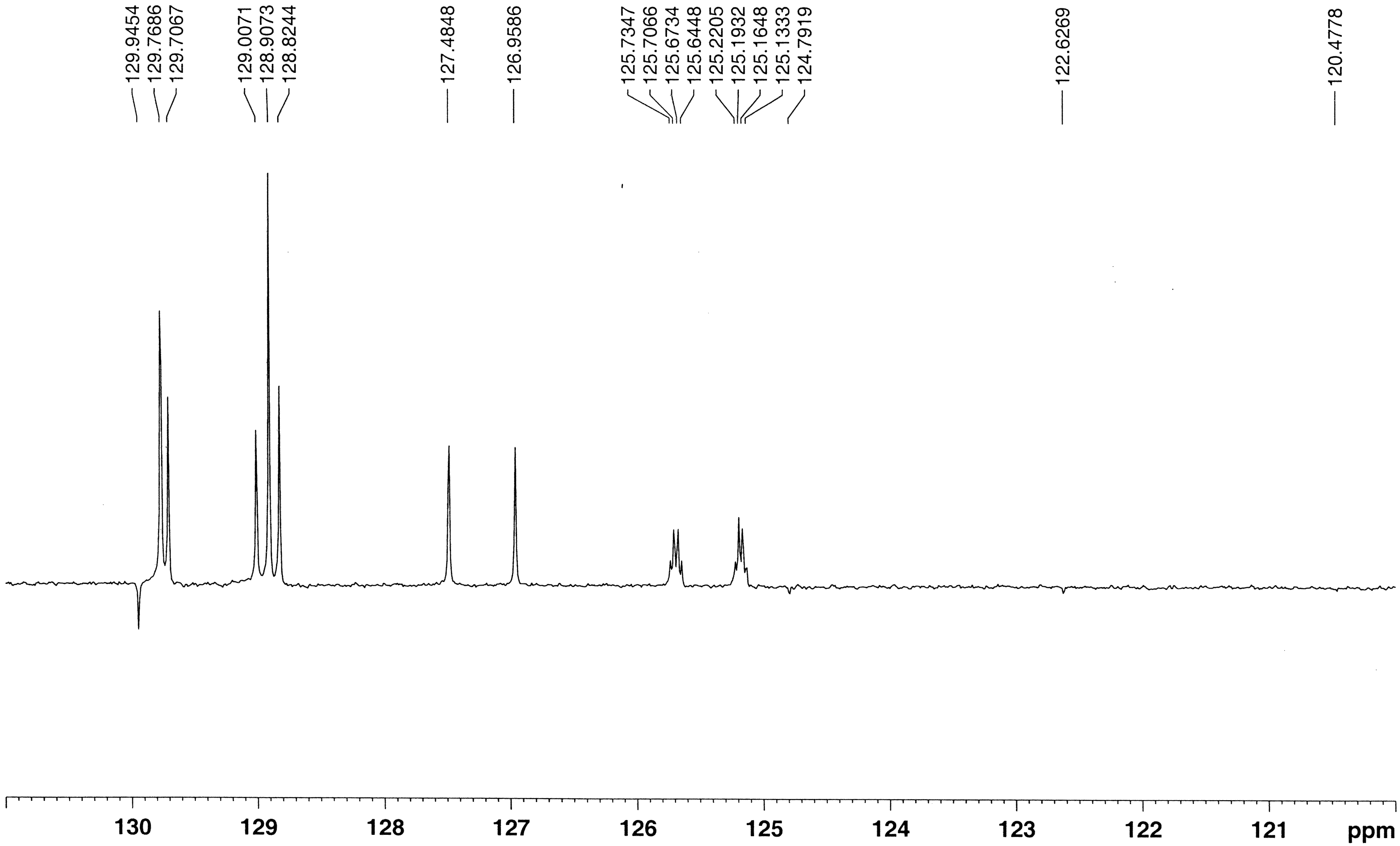

PN002S2P2 in cdcl3 (APT) 17.5.2019

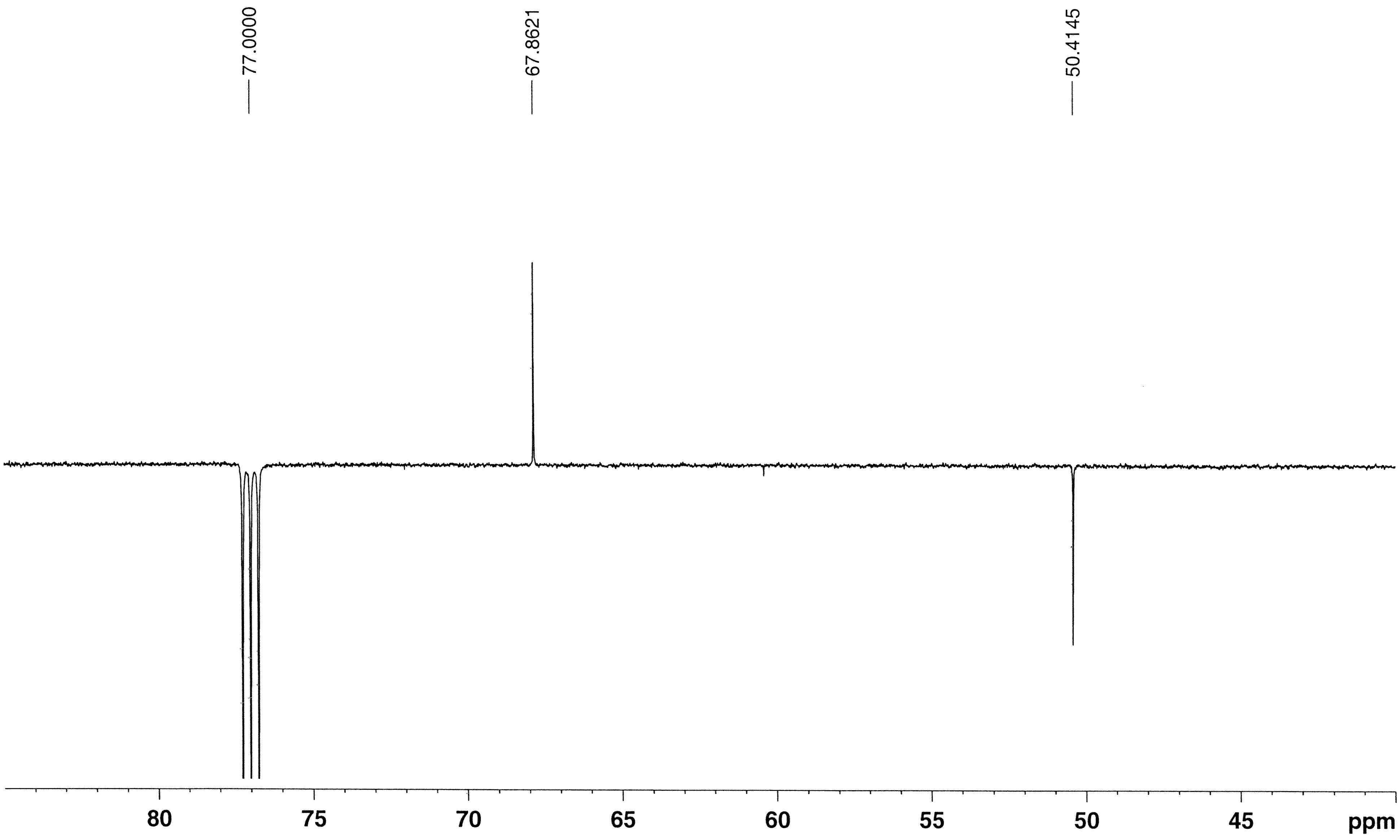

Figure S65c. NMR spectra of compound **5q.**

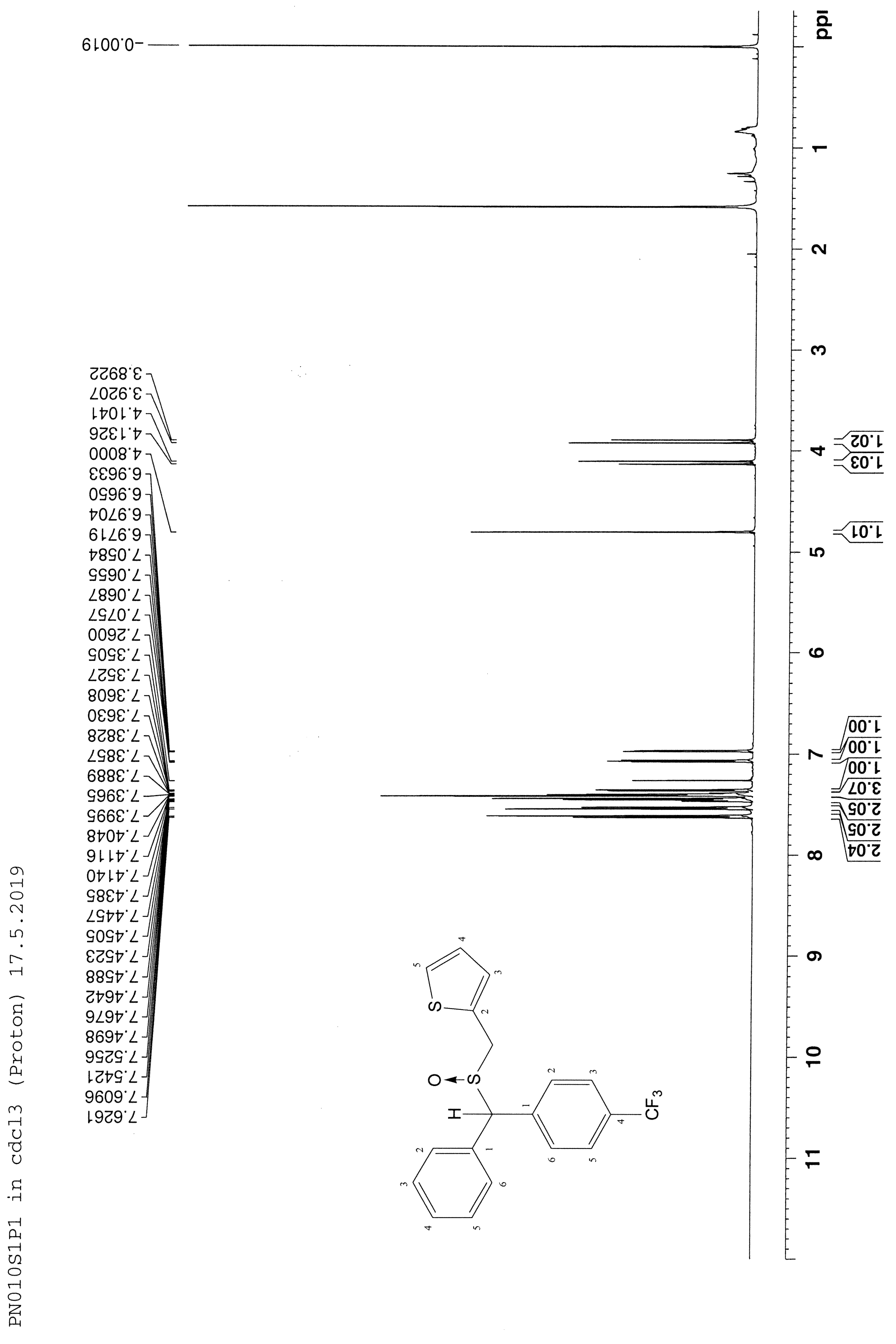

PN010S1P1 in cdcl3 (Proton) 17.5.2019

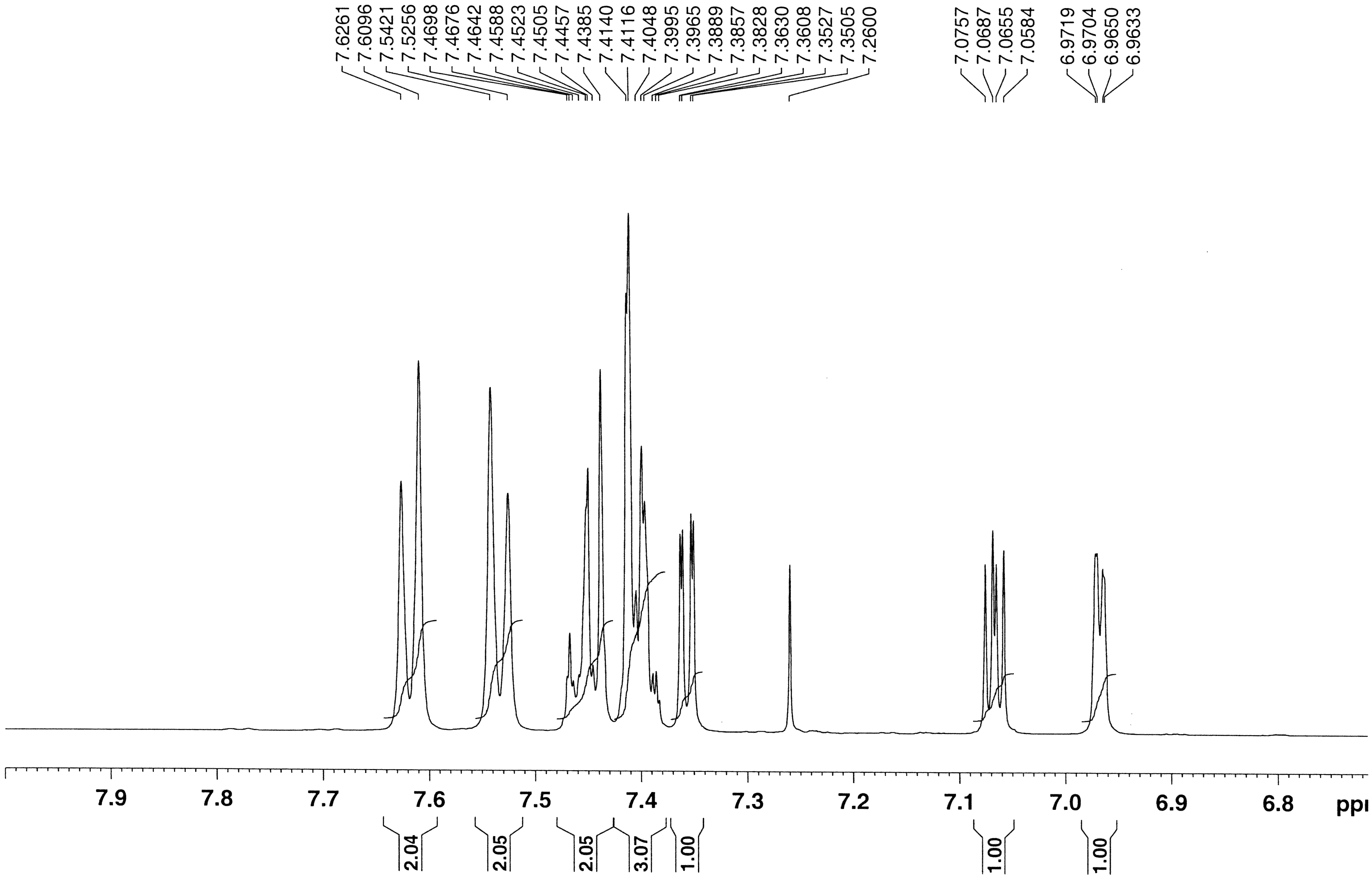

PN010S1P1 in cdcl3 (Proton) 17.5.2019

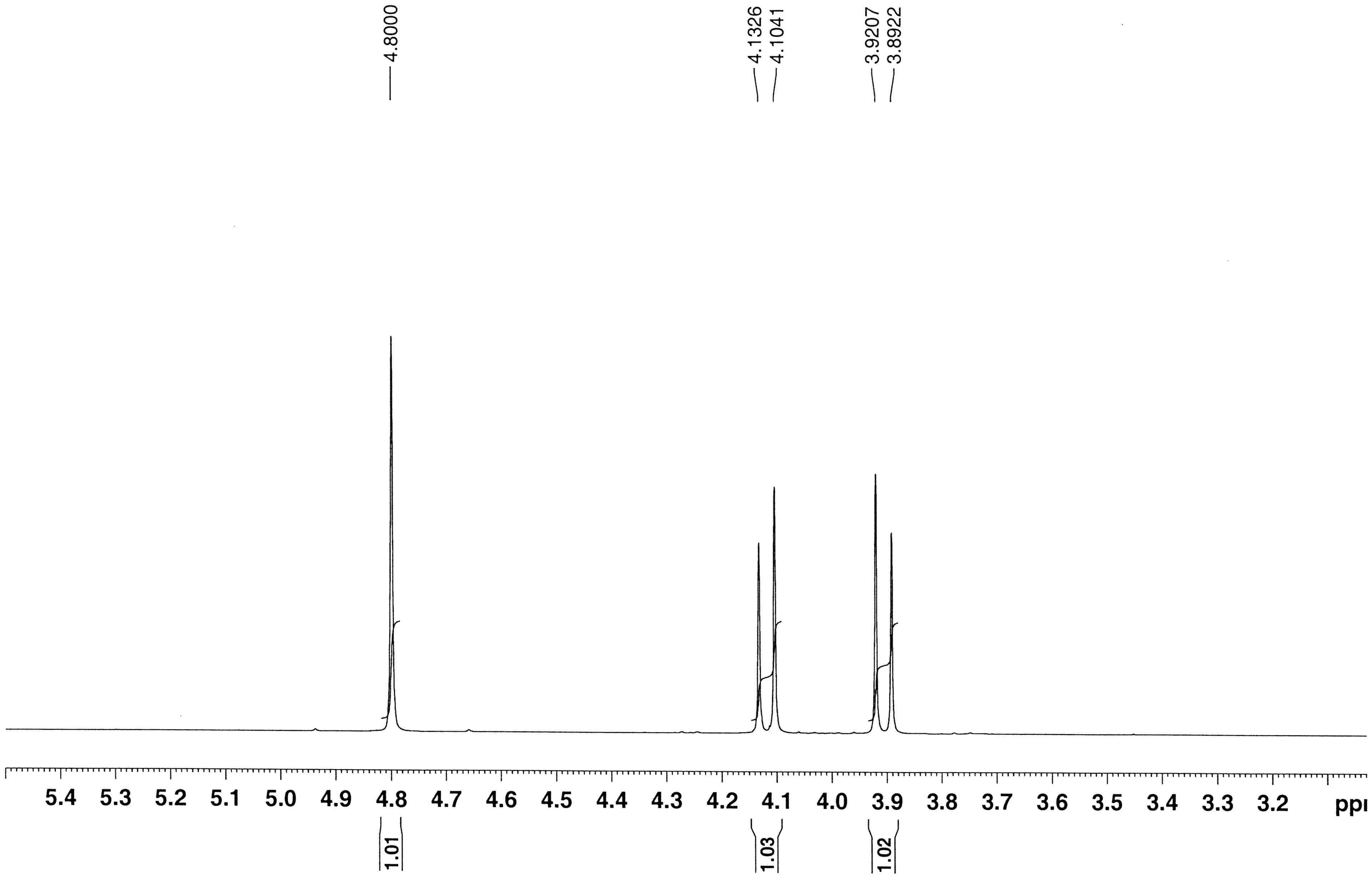

PN010S1P1 in cdcl3 (APT) 17.5.2019

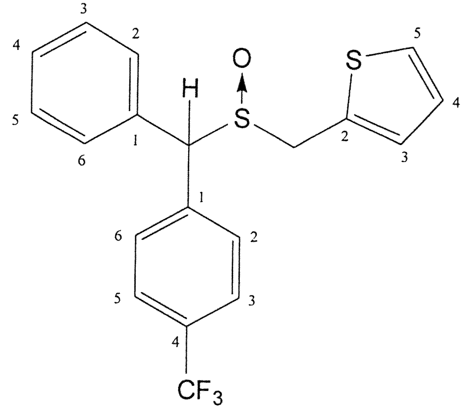

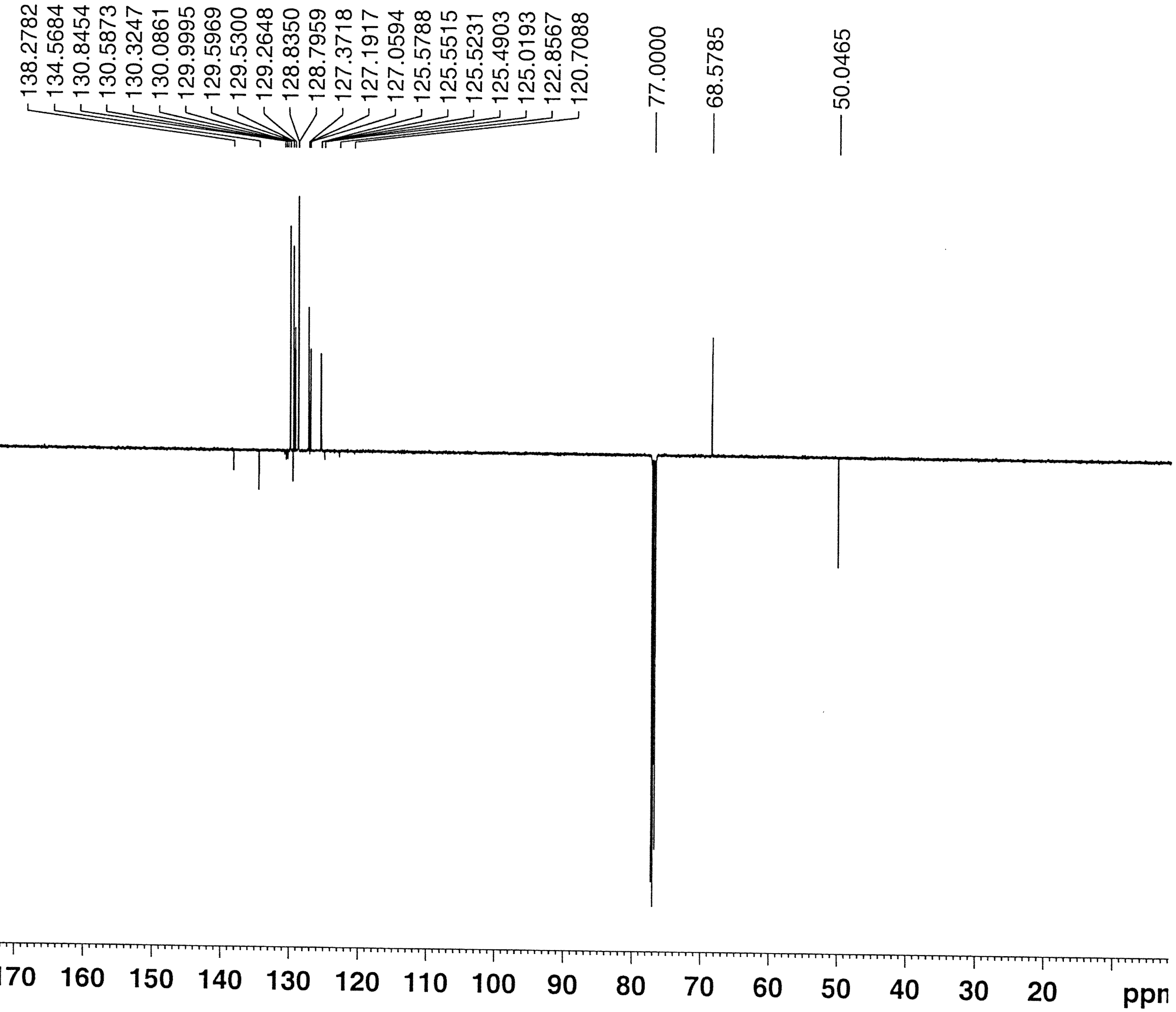

PN010S1P1 in cdcl3 (APT) 17.5.2019

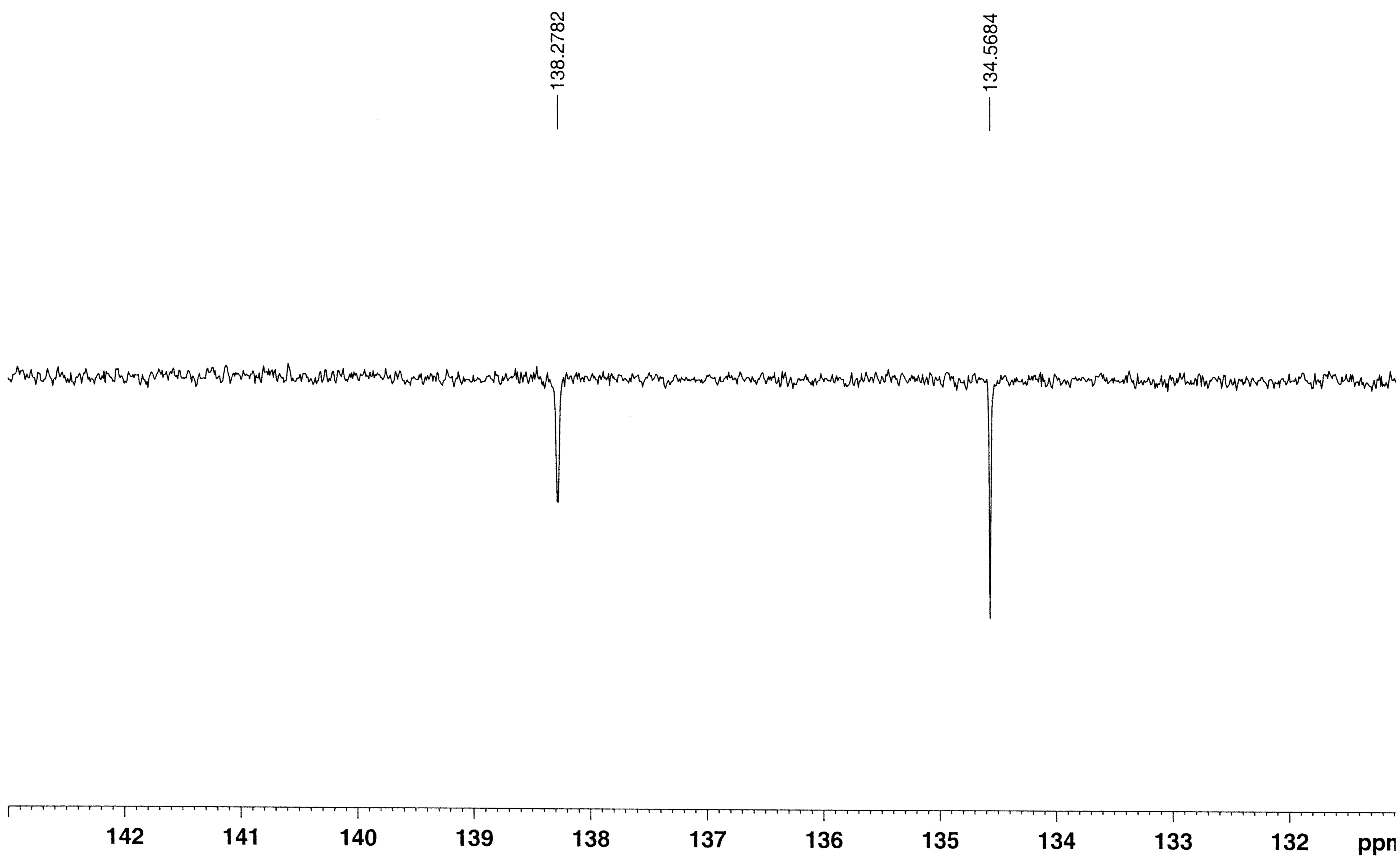

PN010S1P1 in cdcl3 (APT) 17.5.2019

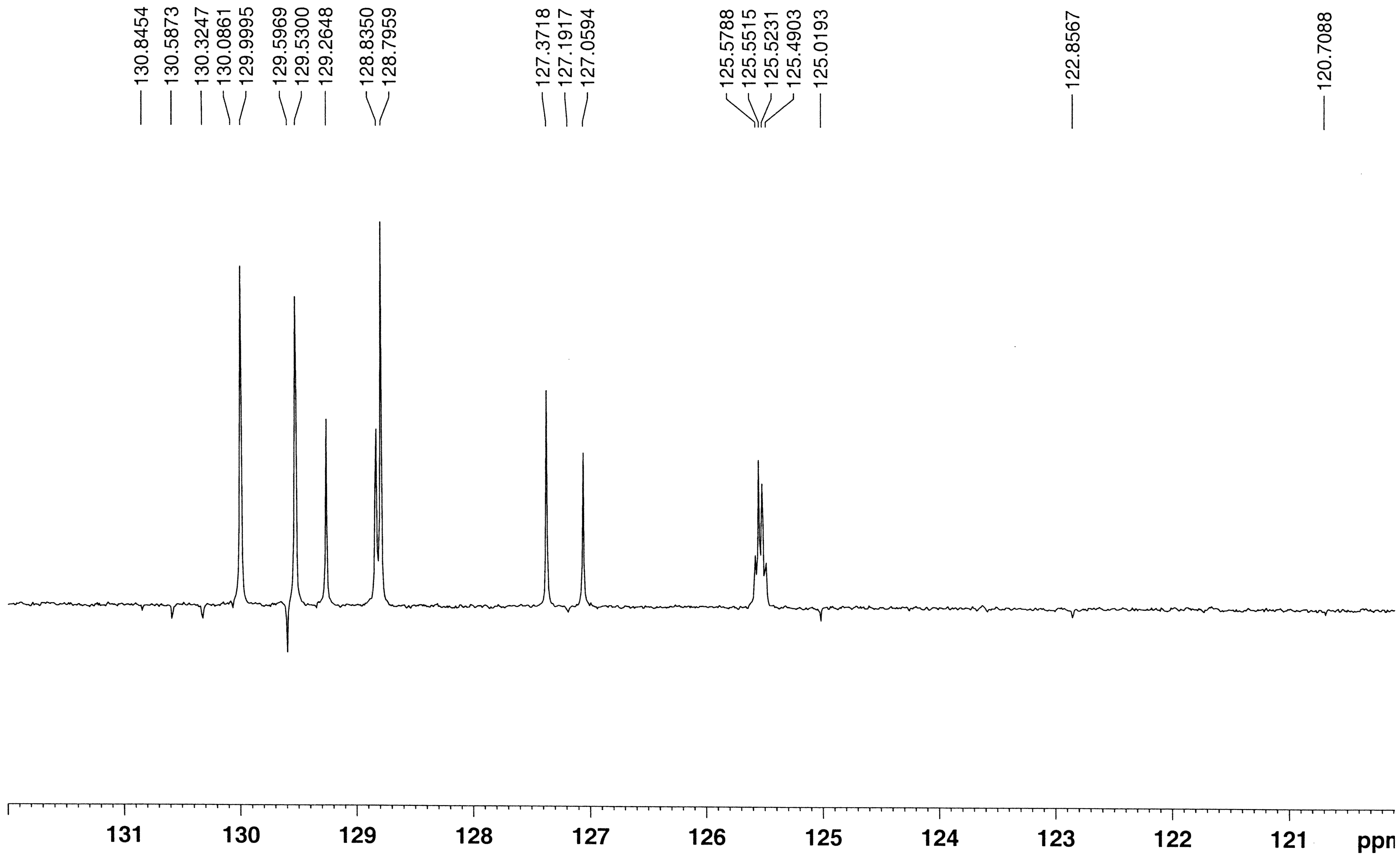

PN010S1P1 in cdcl3 (APT) 17.5.2019

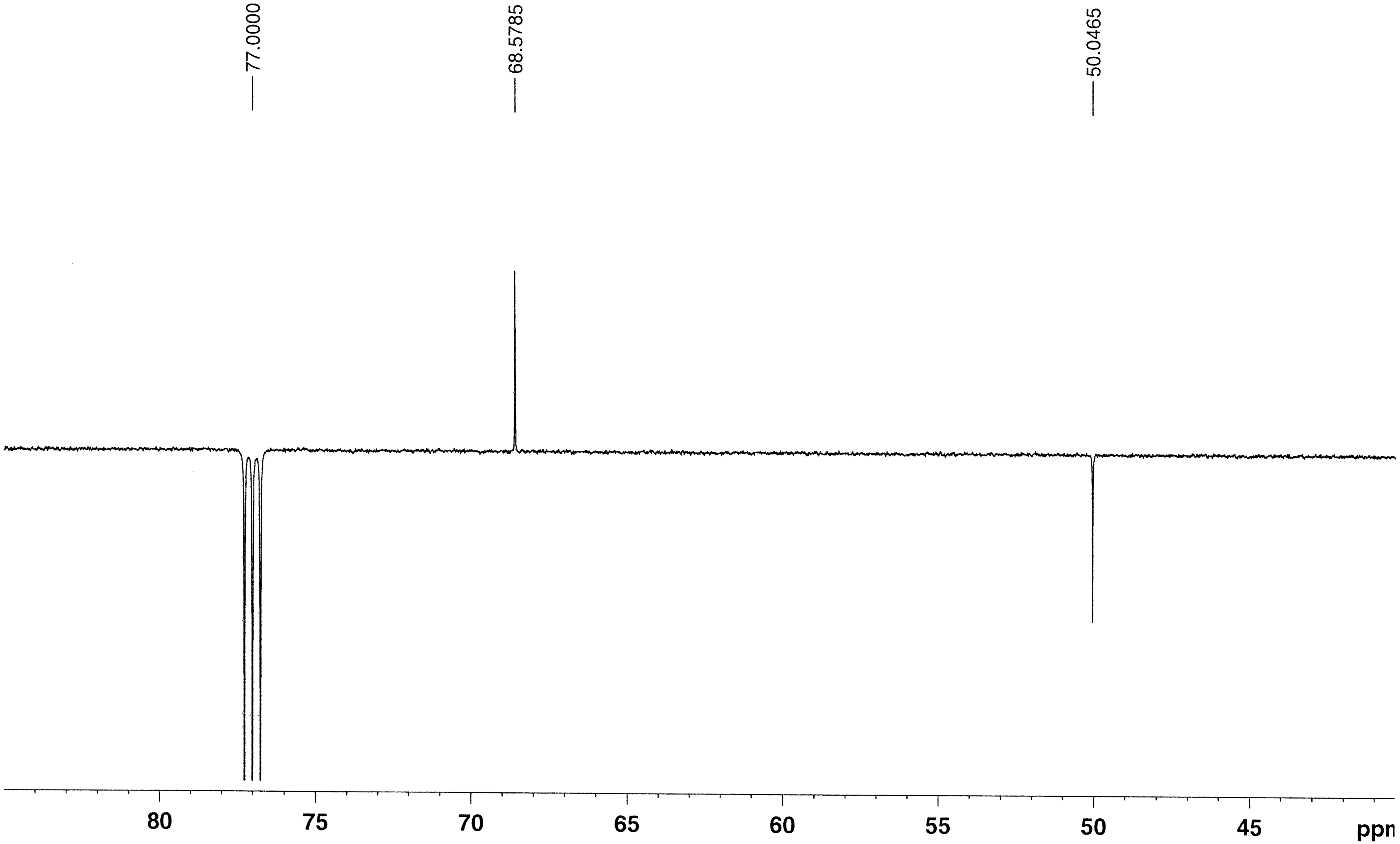

Figure S66c. NMR spectra of compound **6q**.

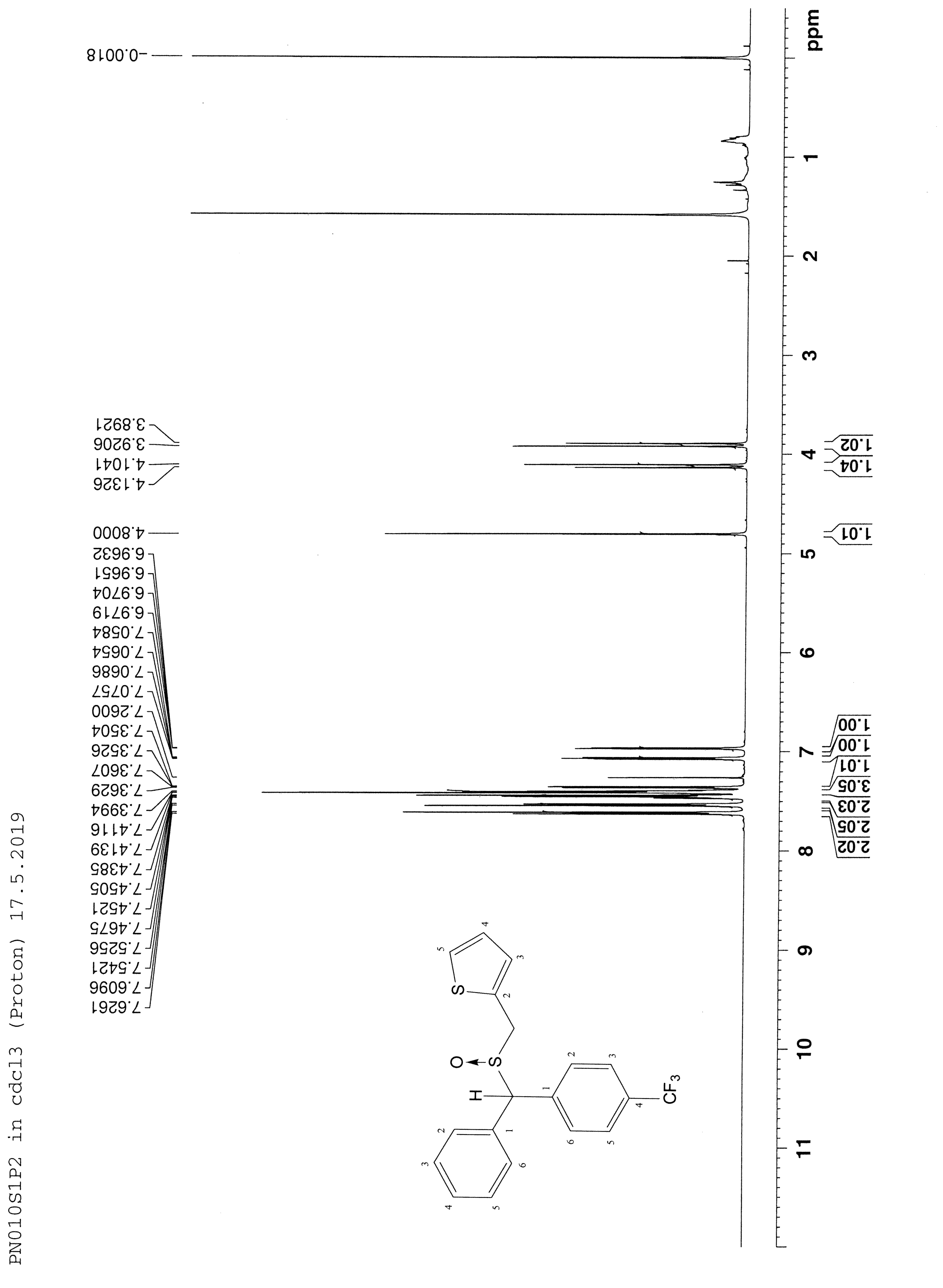

PN010S1P2 in cdcl3 (Proton) 17.5.2019

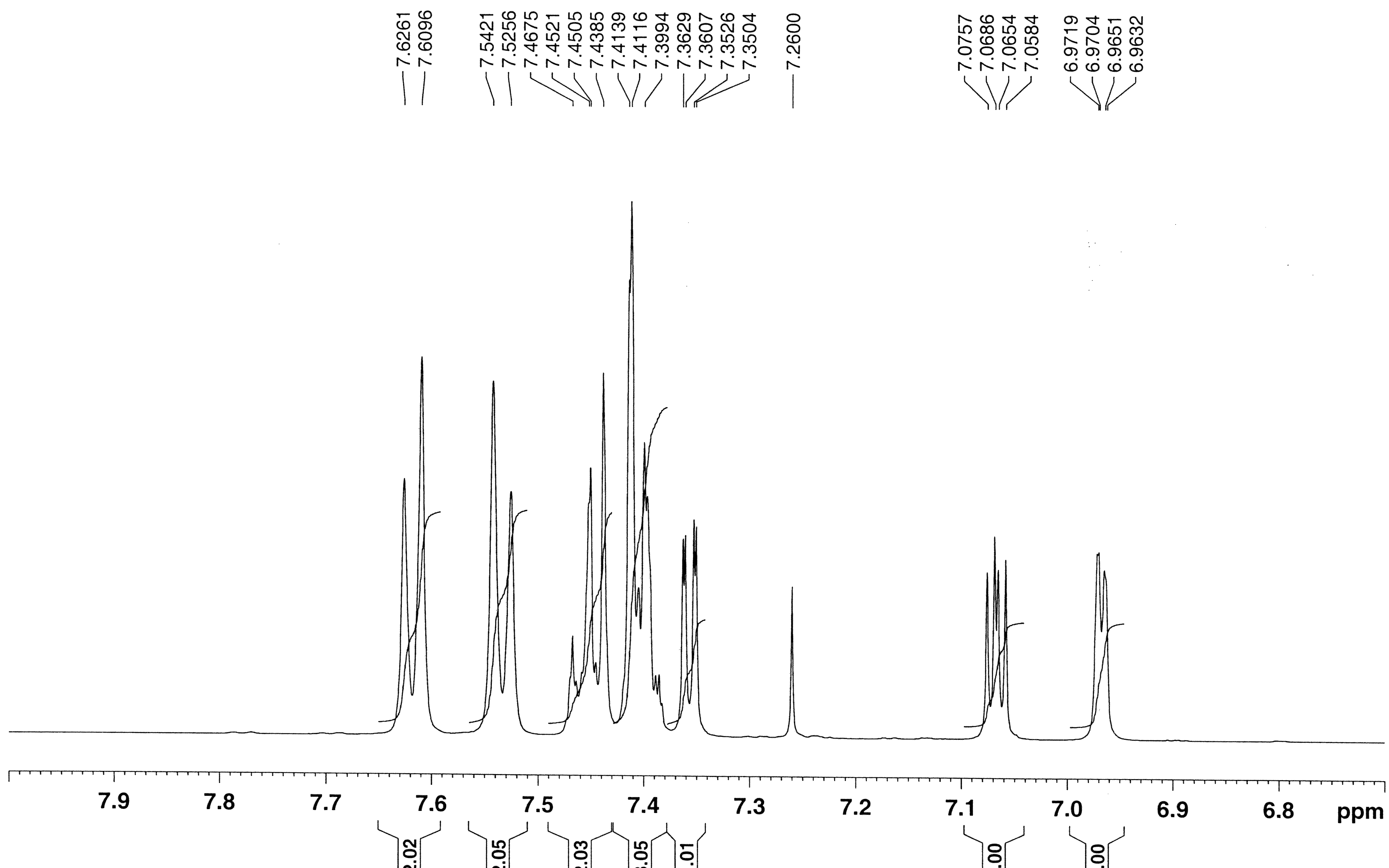

PN010S1P2 in cdcl3 (Proton) 17.5.2019

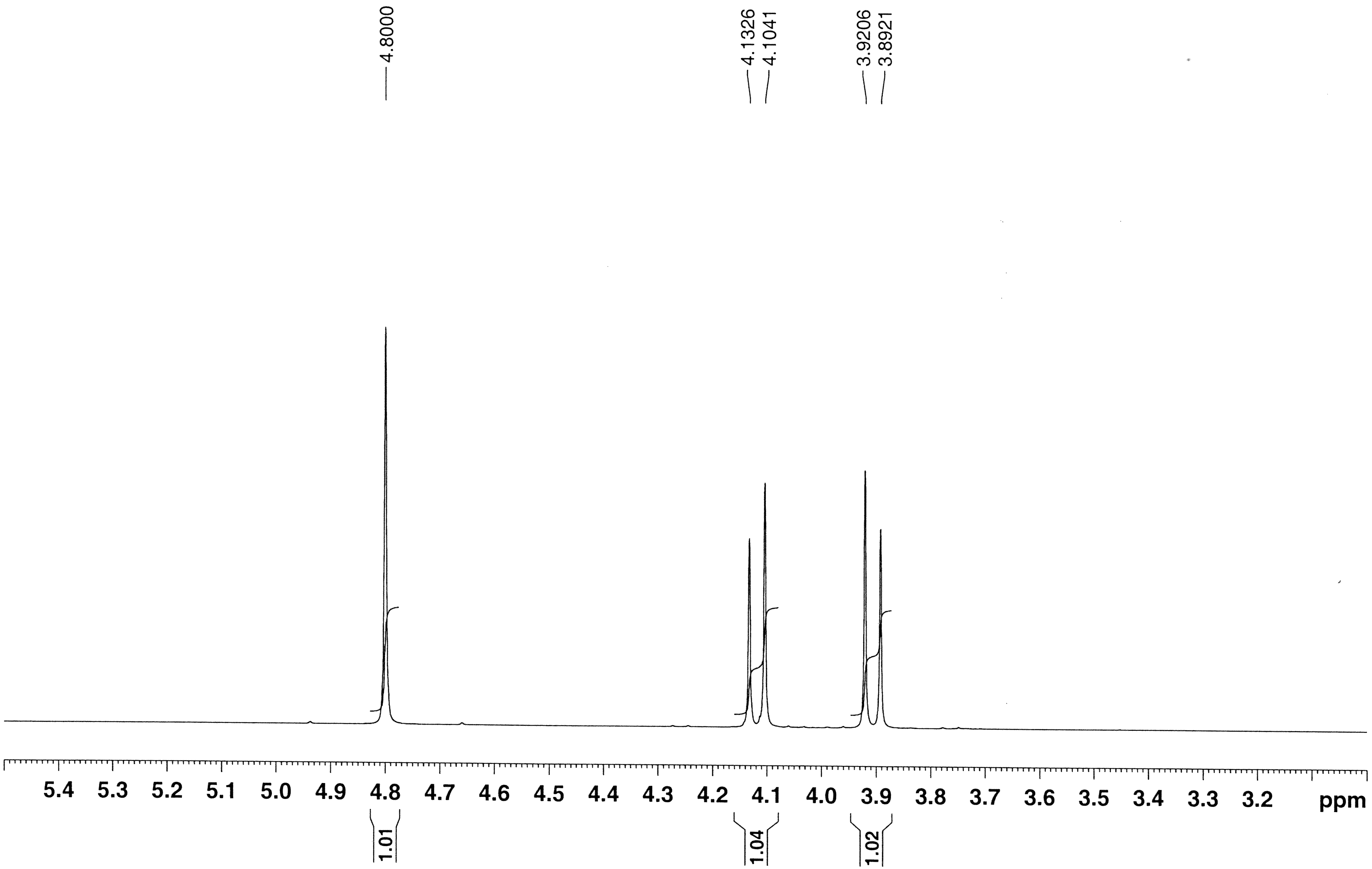

PN010S1P2 in cdcl3 (APT) 17.5.2019

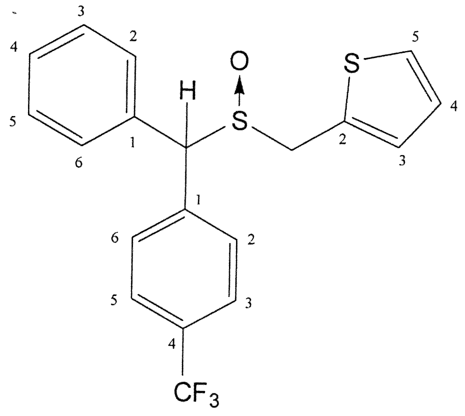

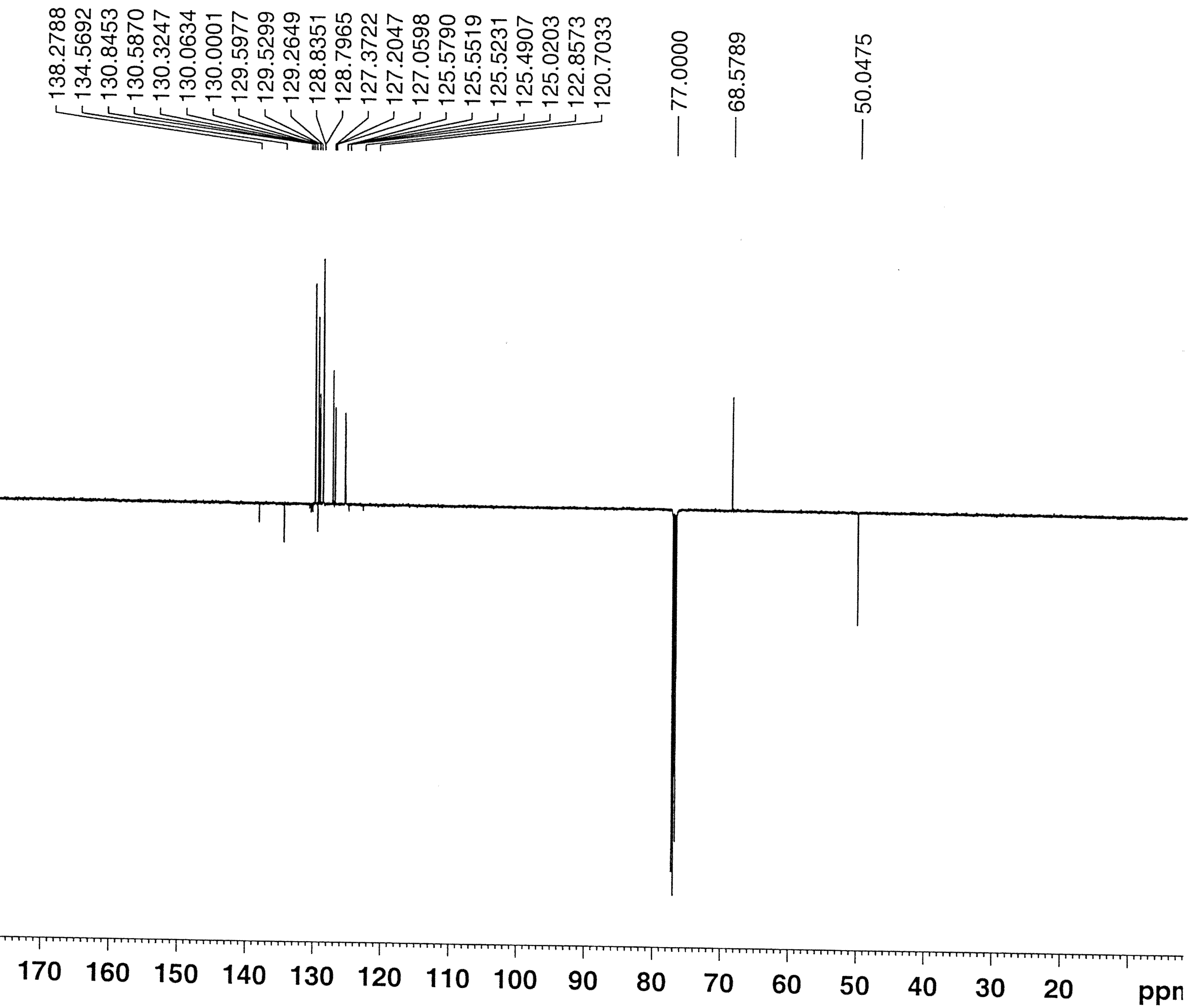

PN010S1P2 in cdcl3 (APT) 17.5.2019

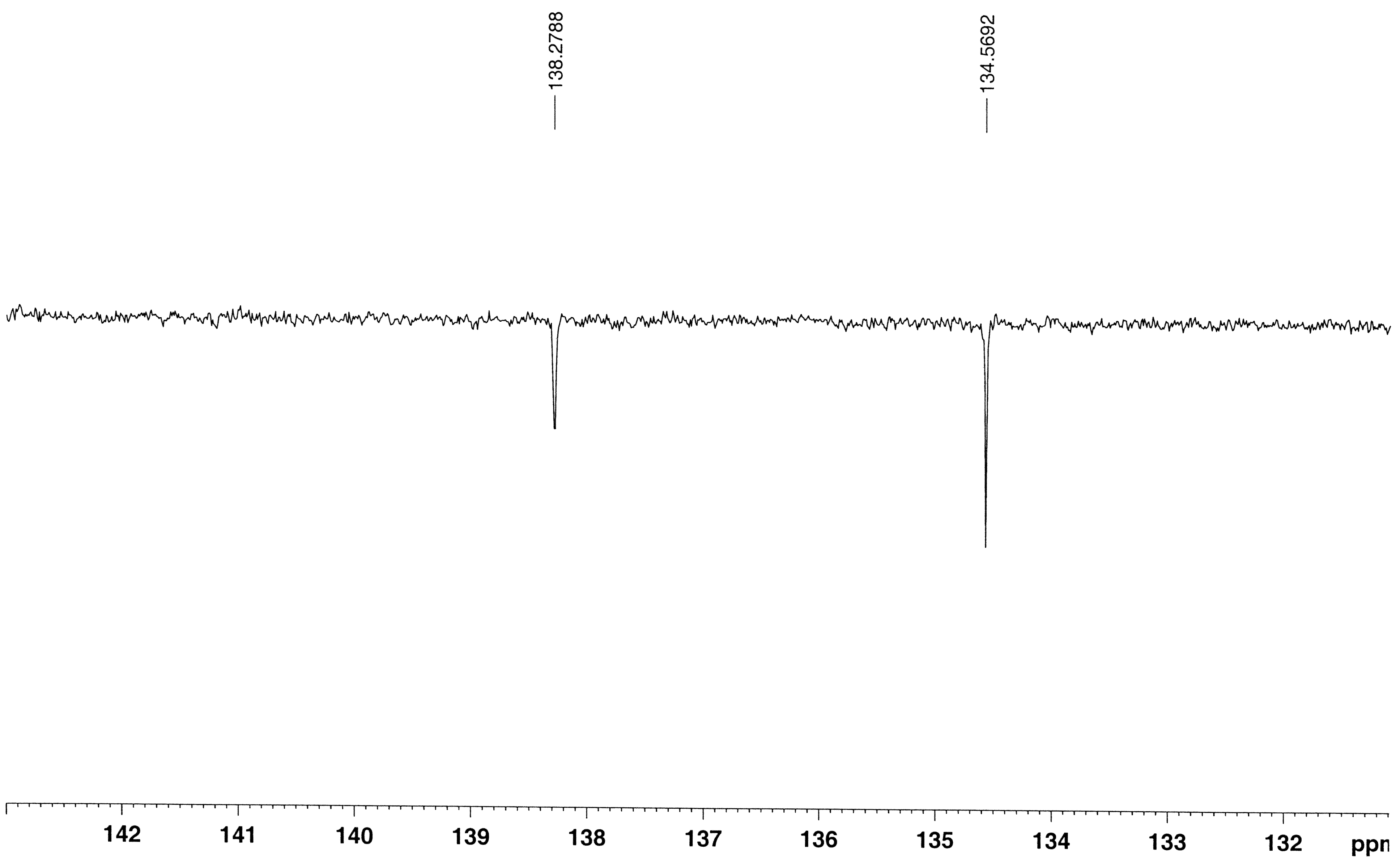

PN010S1P2 in cdcl3 (APT) 17.5.2019

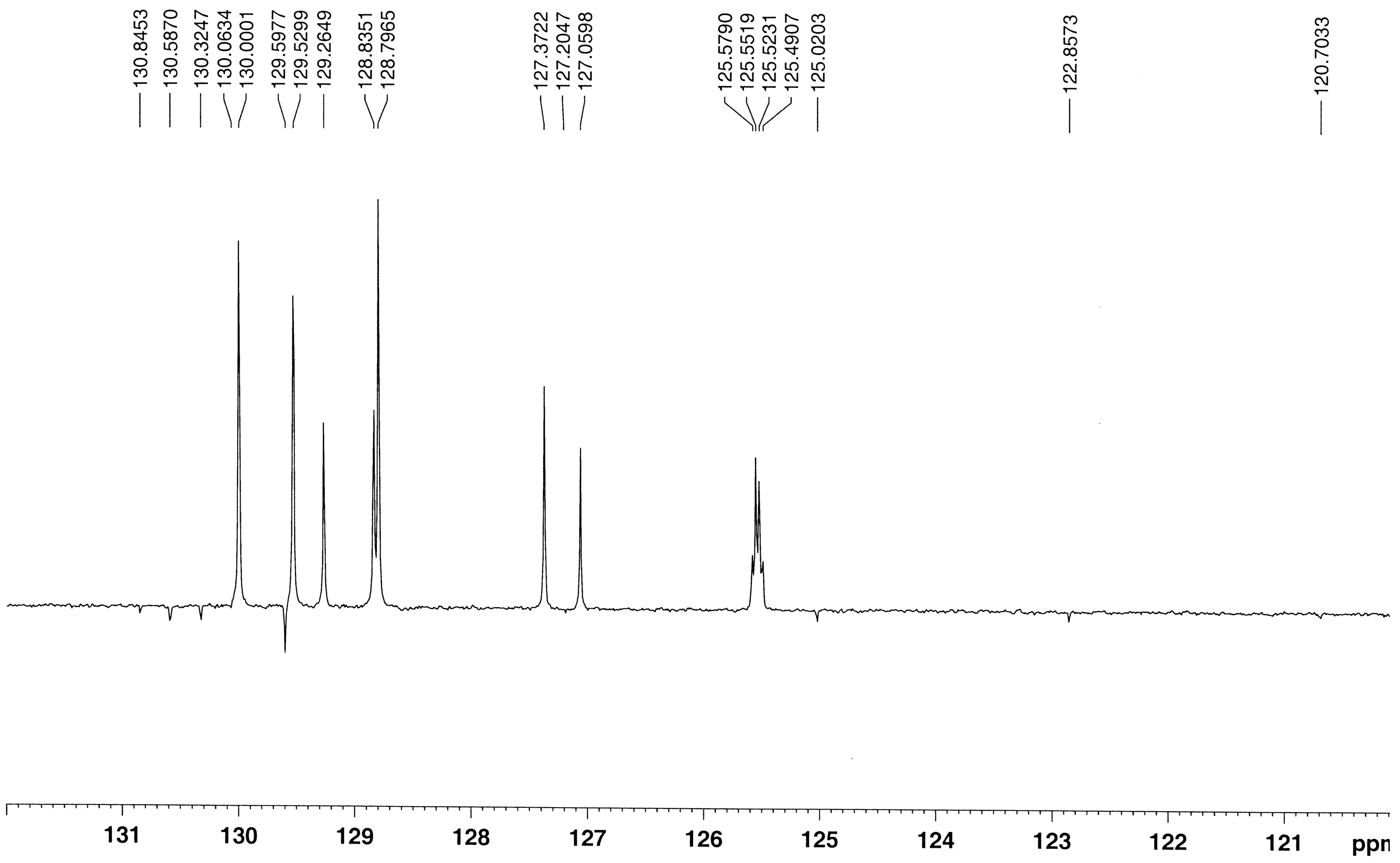

PN010S1P2 in cdcl3 (APT) 17.5.2019

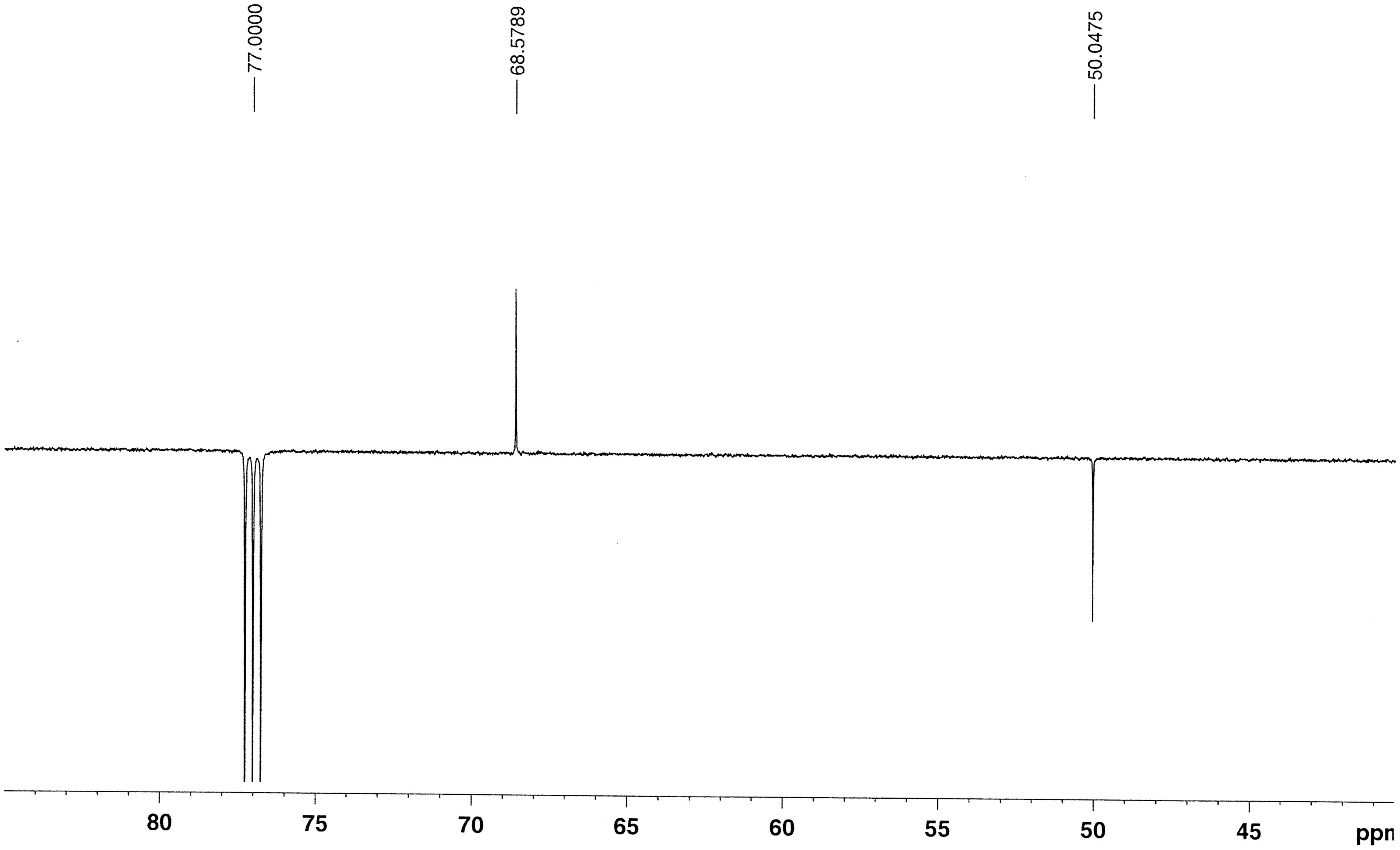

Figure S67c. NMR spectra of compound **7q.**

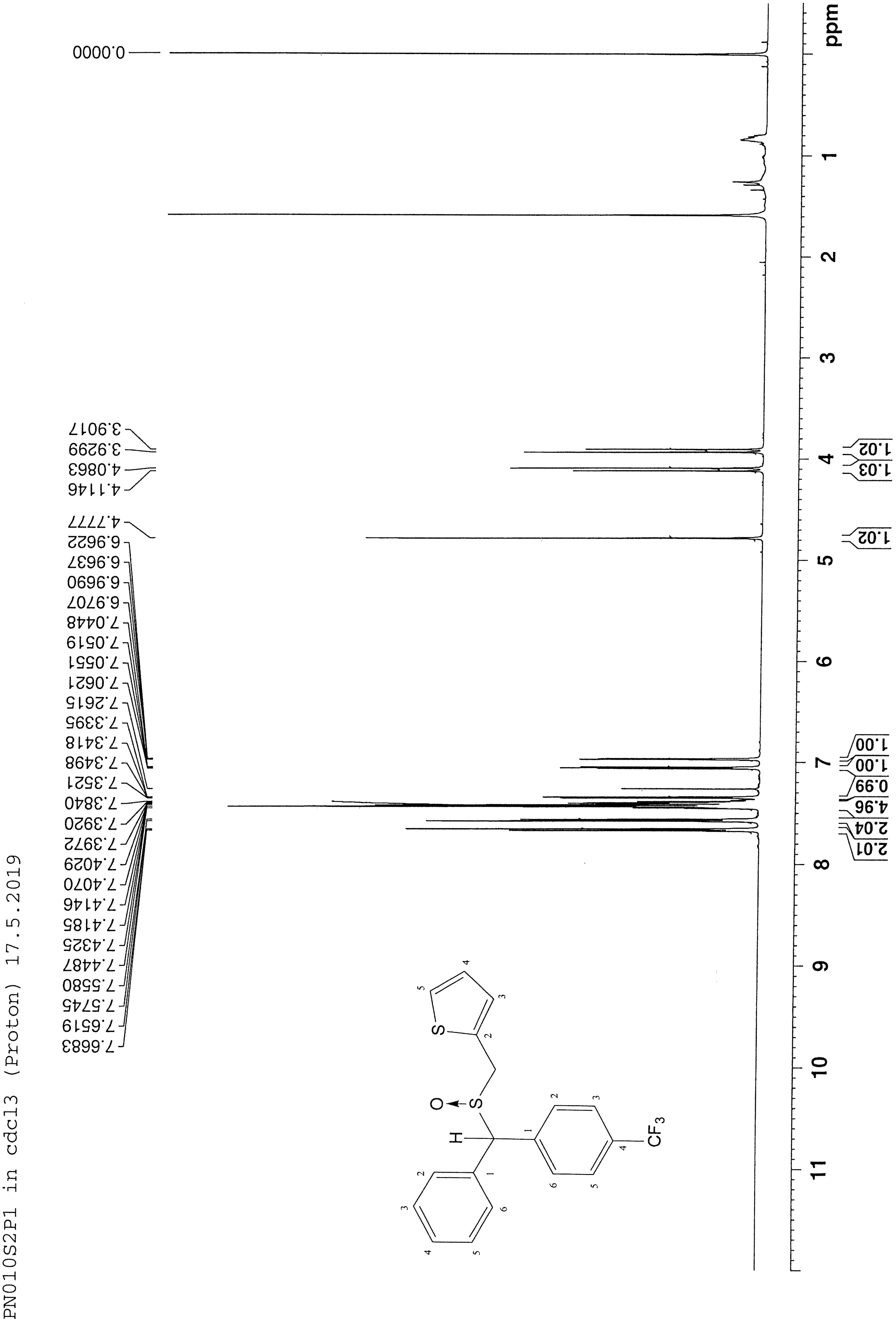

PN010S2P1 in cdcl3 (Proton) 17.5.2019

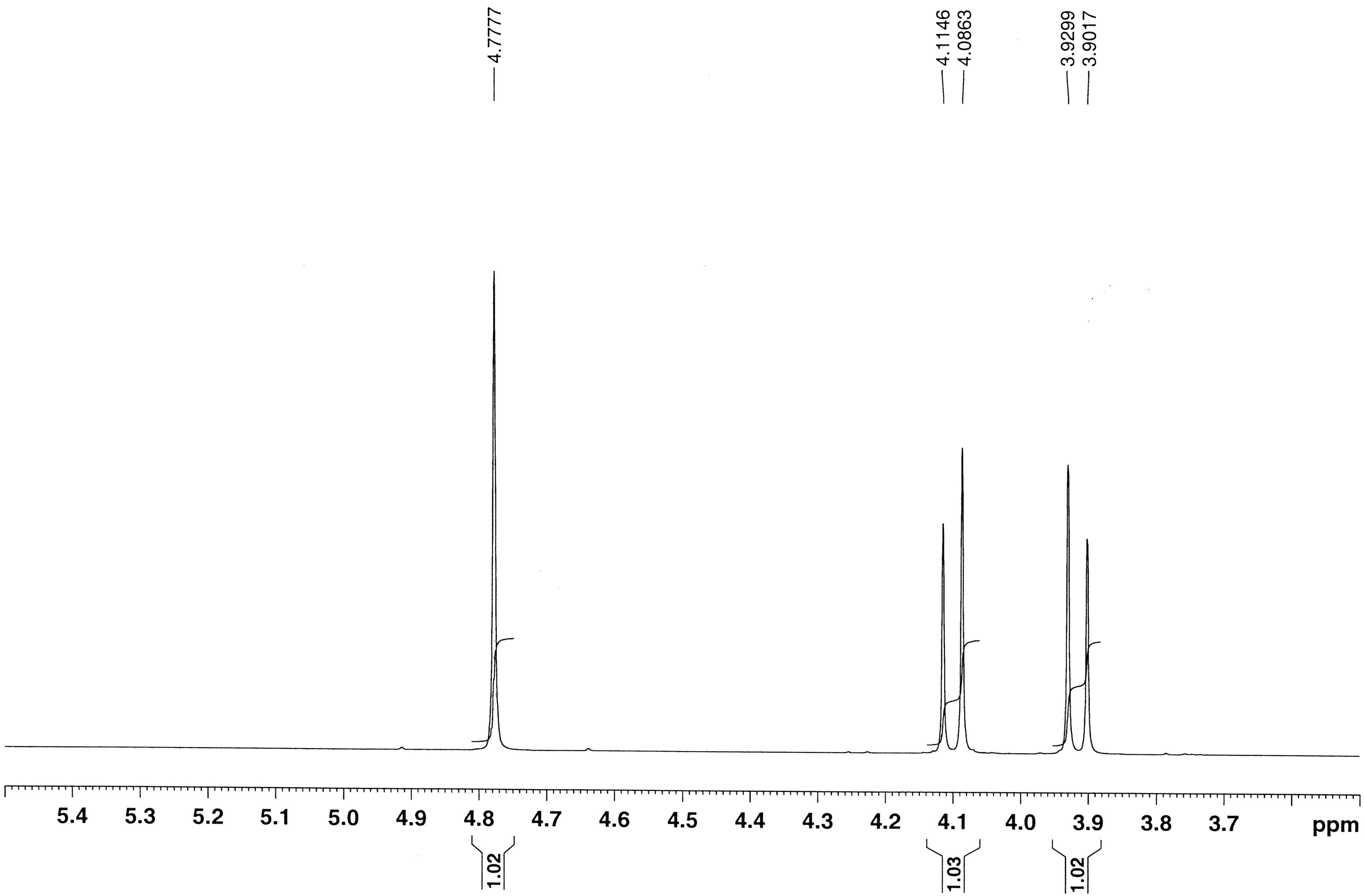

PN010S2P1 in cdcl3 (Proton) 17.5.2019

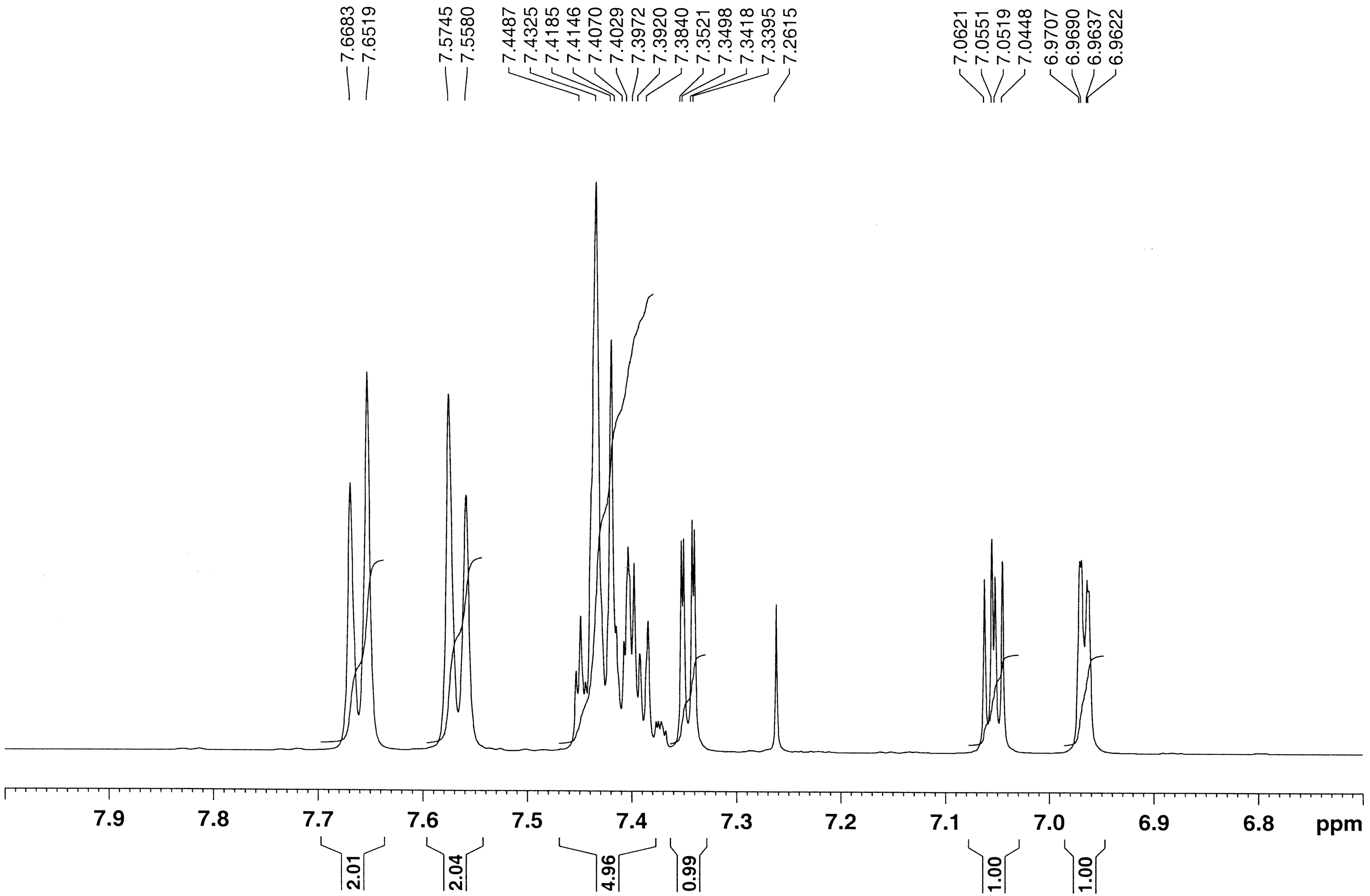

PN010S2P1 in cdcl3 (APT) 17.5.2019

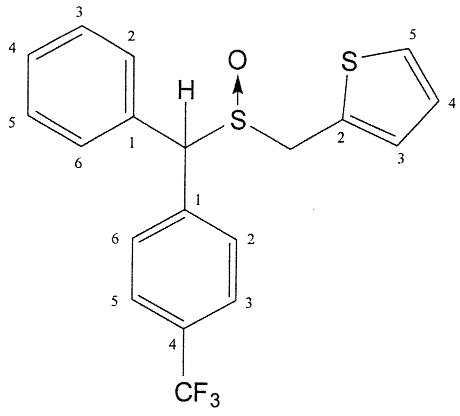

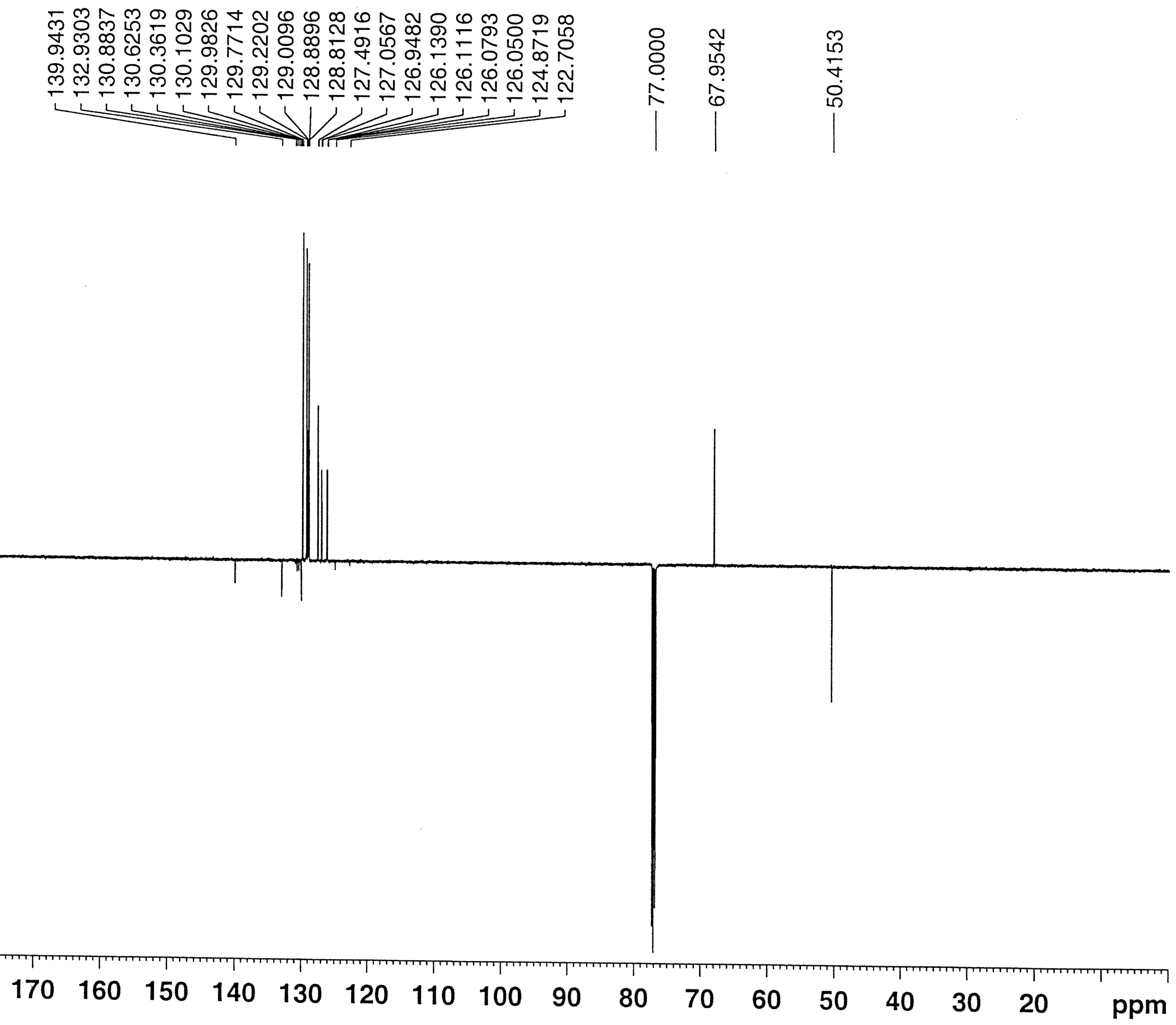

PN010S2P1 in cdcl3 (APT) 17.5.2019

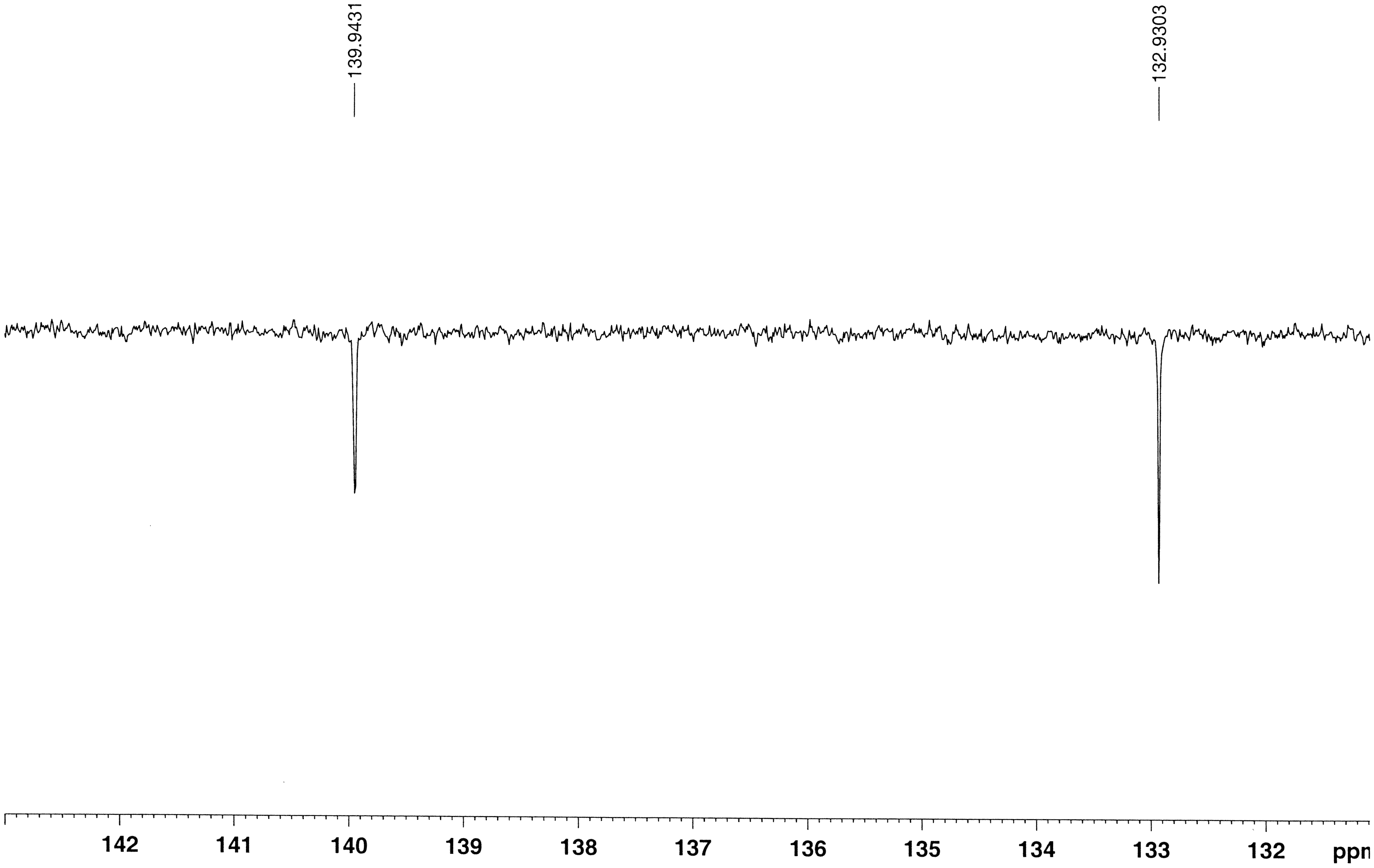

PN010S2P1 in cdcl3 (APT) 17.5.2019

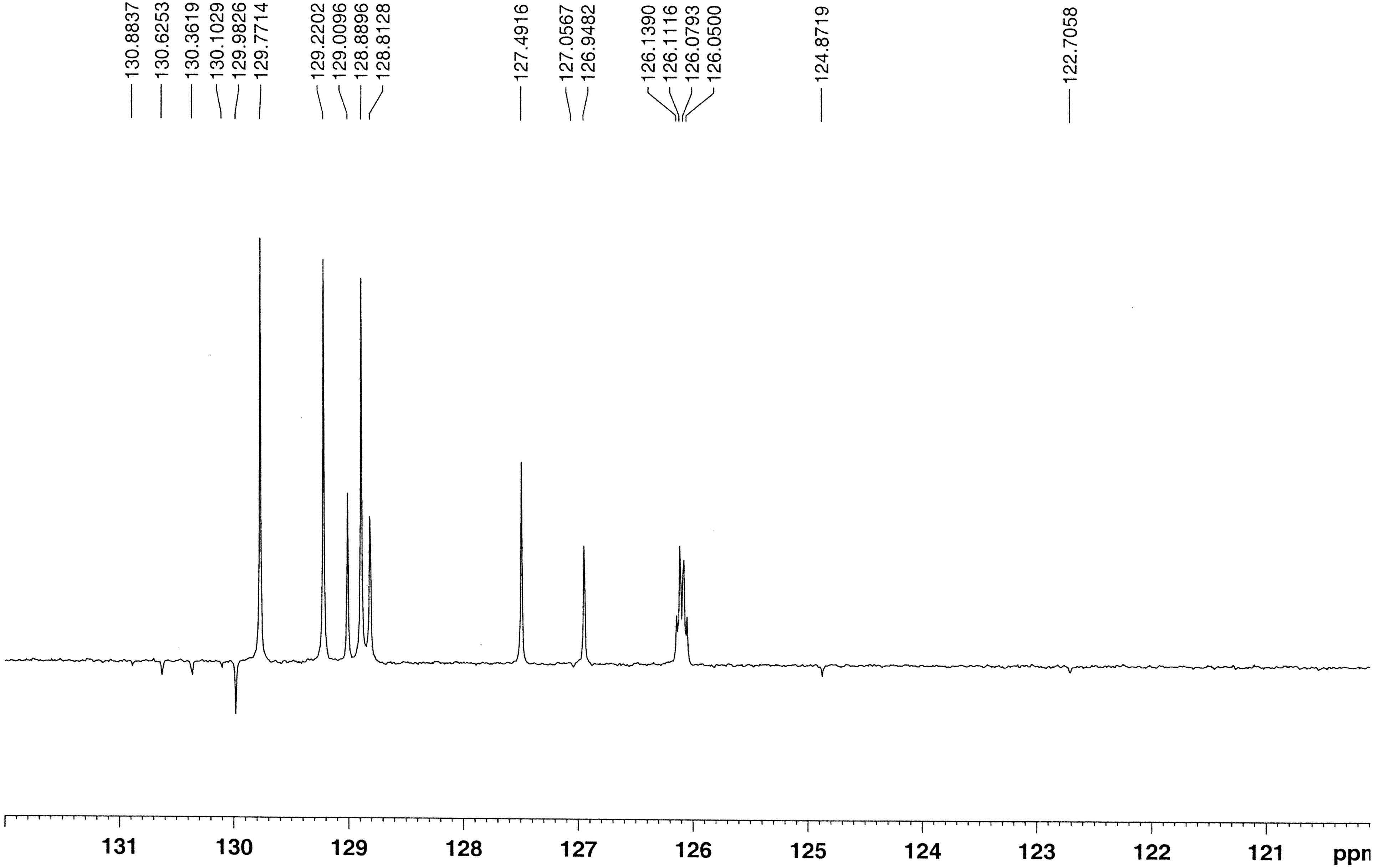

PN010S2P1 in cdcl3 (APT) 17.5.2019

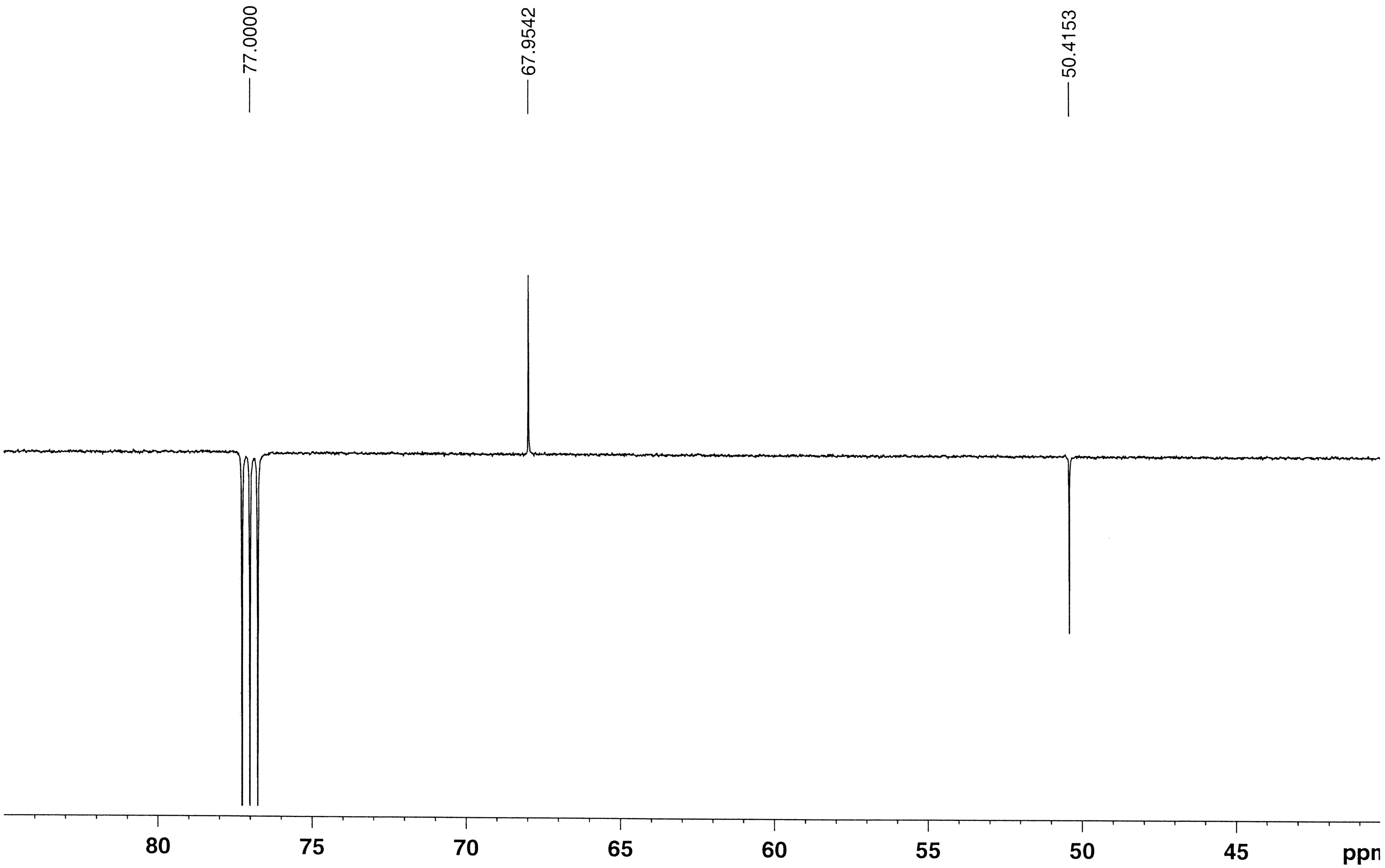

Figure S68c. NMR spectra of compound **8q**.

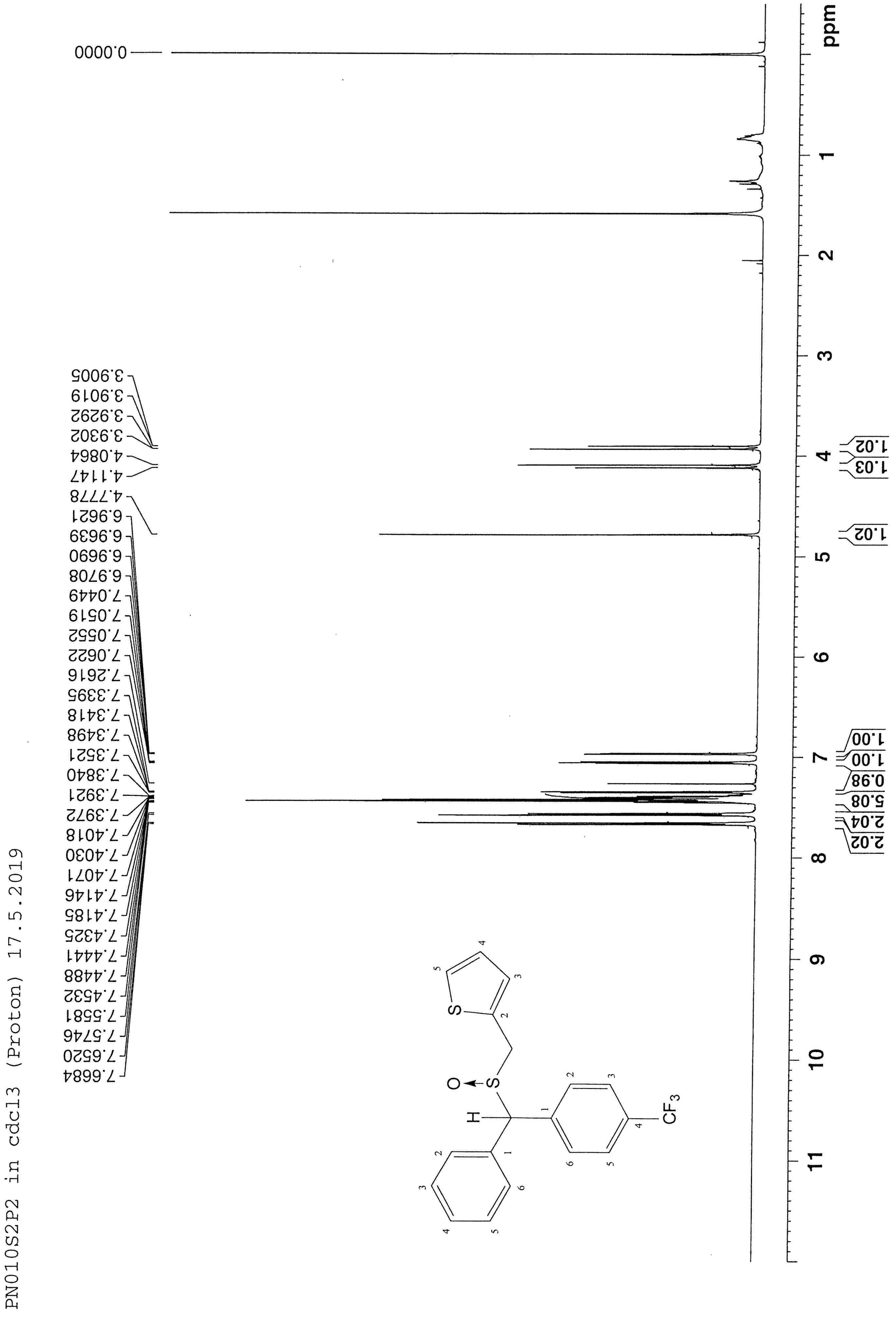

PN010S2P2 in cdcl3 (Proton) 17.5.2019

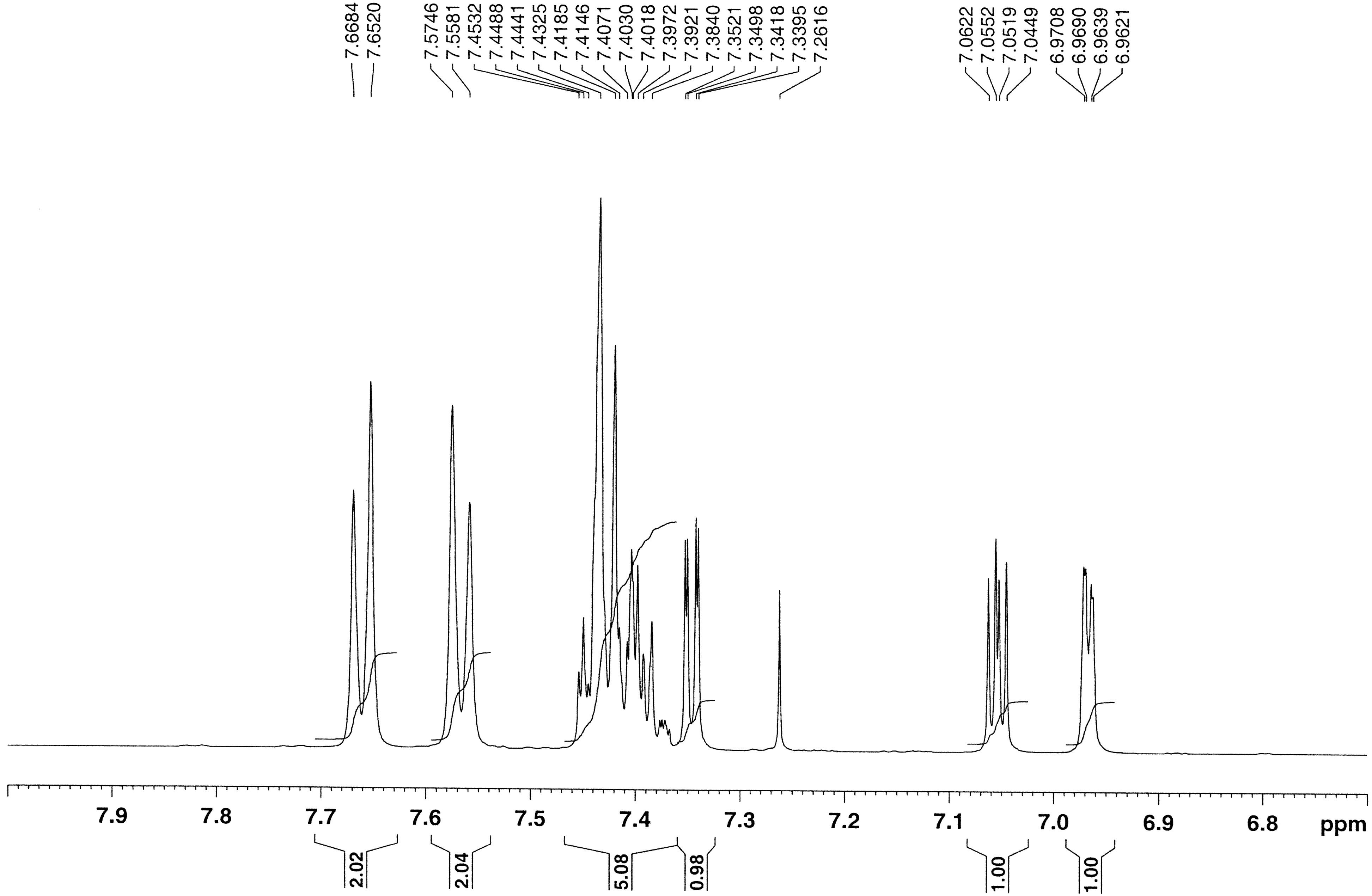

PN010S2P2 in cdcl3 (Proton) 17.5.2019

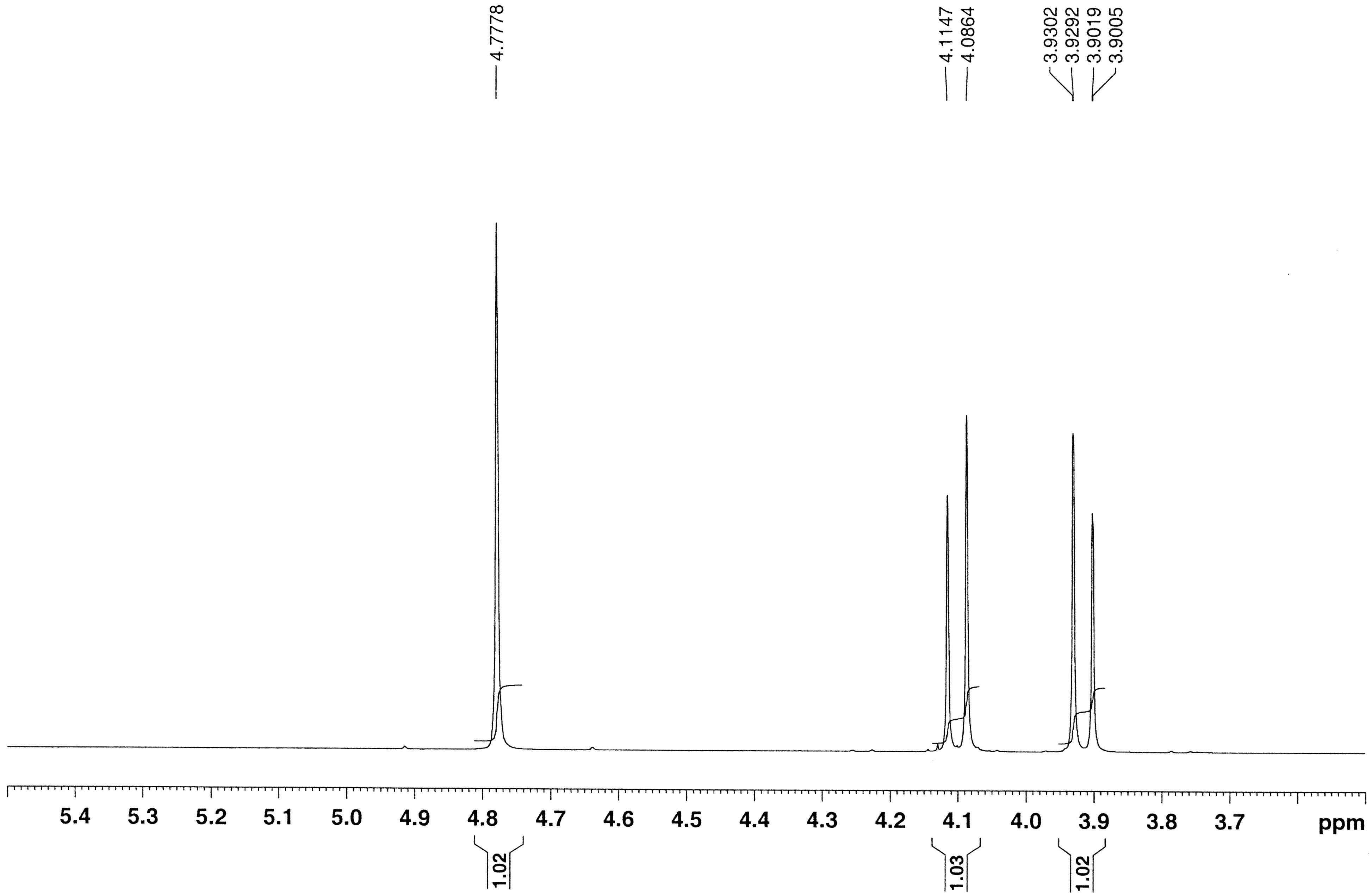

PN010S2P2 in cdcl3 (APT) 17.5.2019

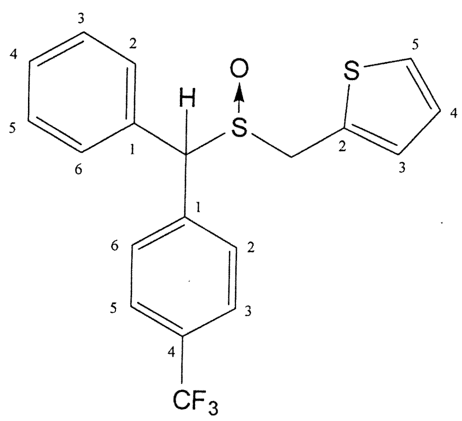

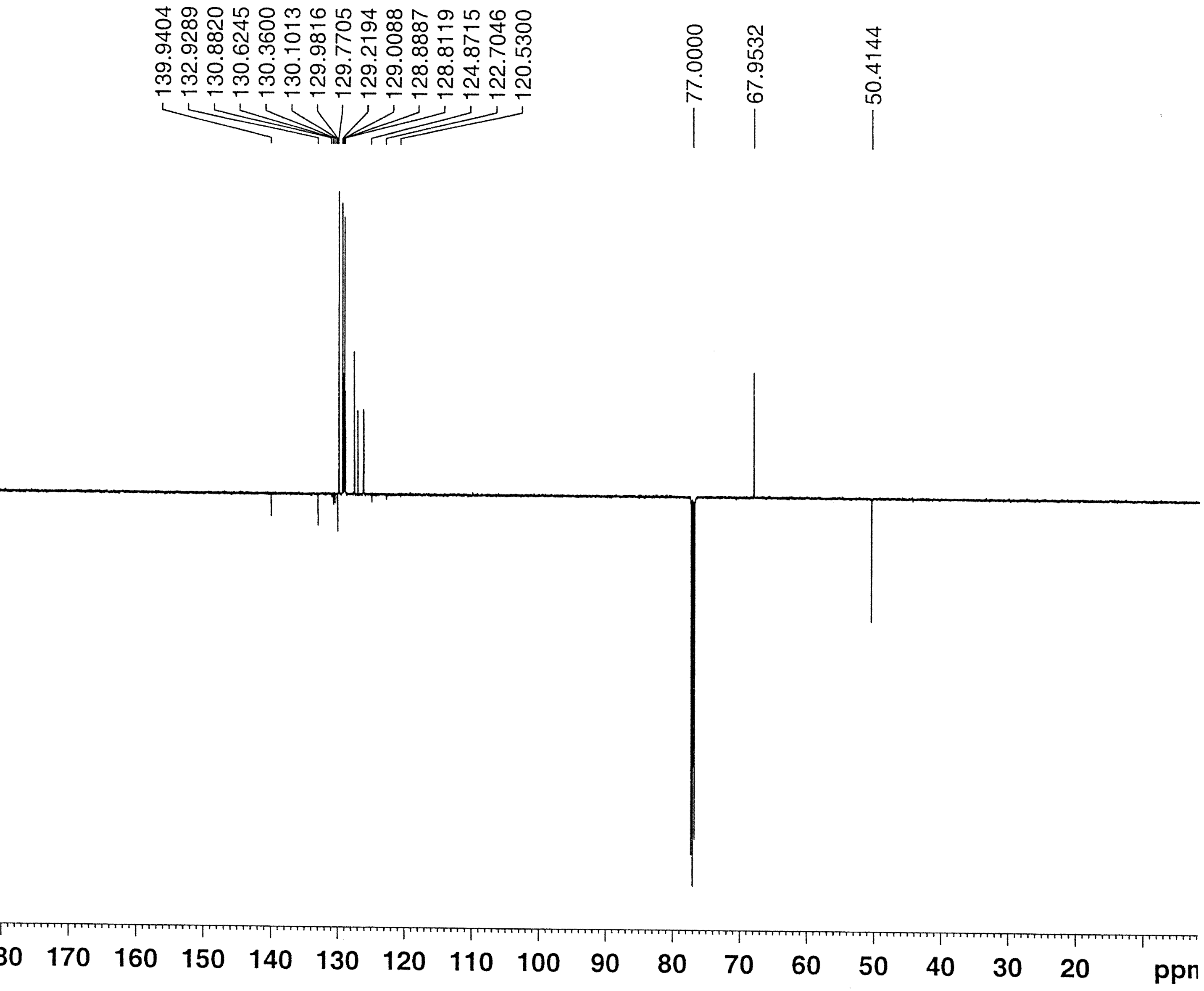

PN010S2P2 in cdcl3 (APT) 17.5.2019

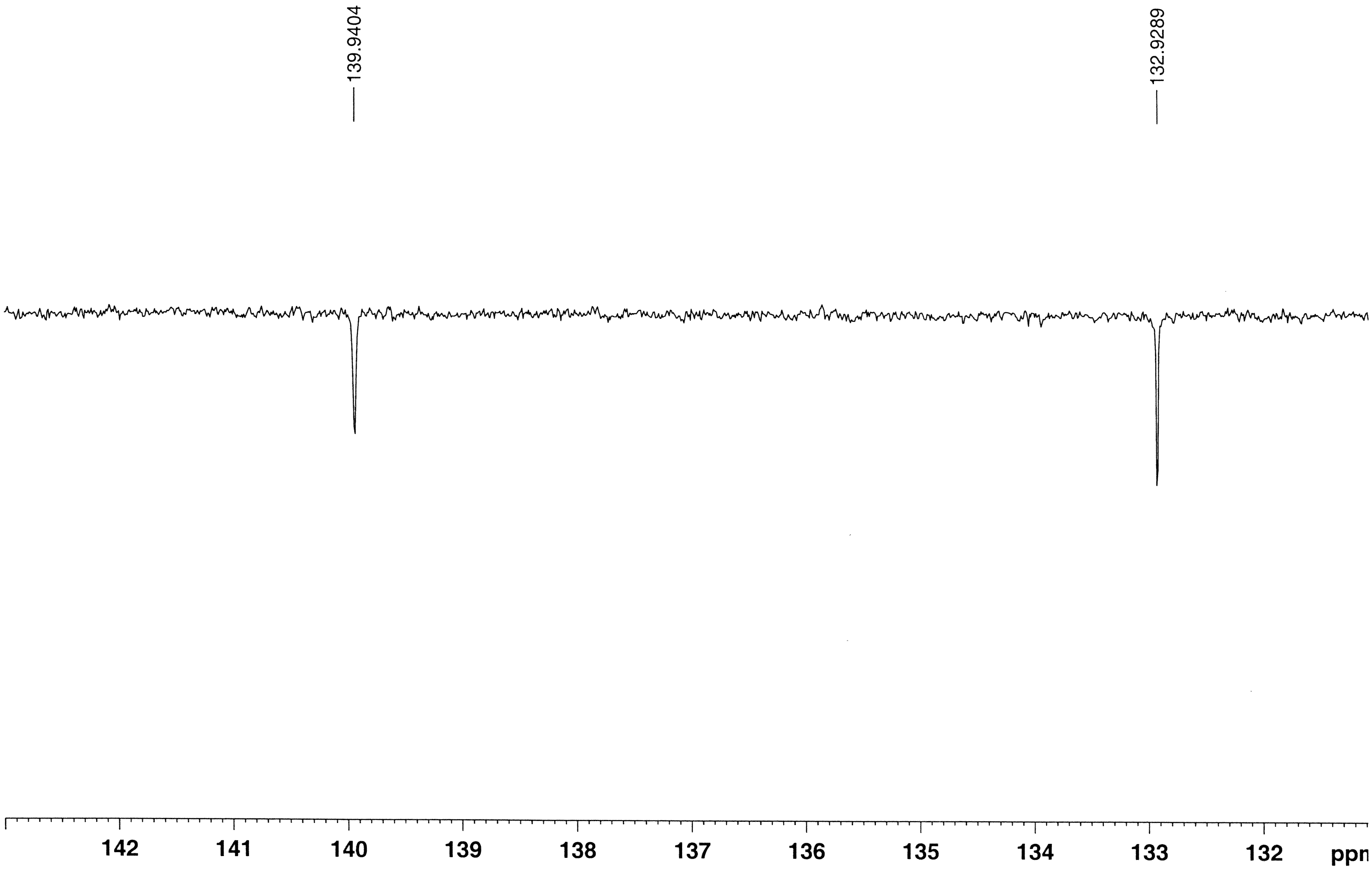

PN010S2P2 in cdcl3 (APT) 17.5.2019

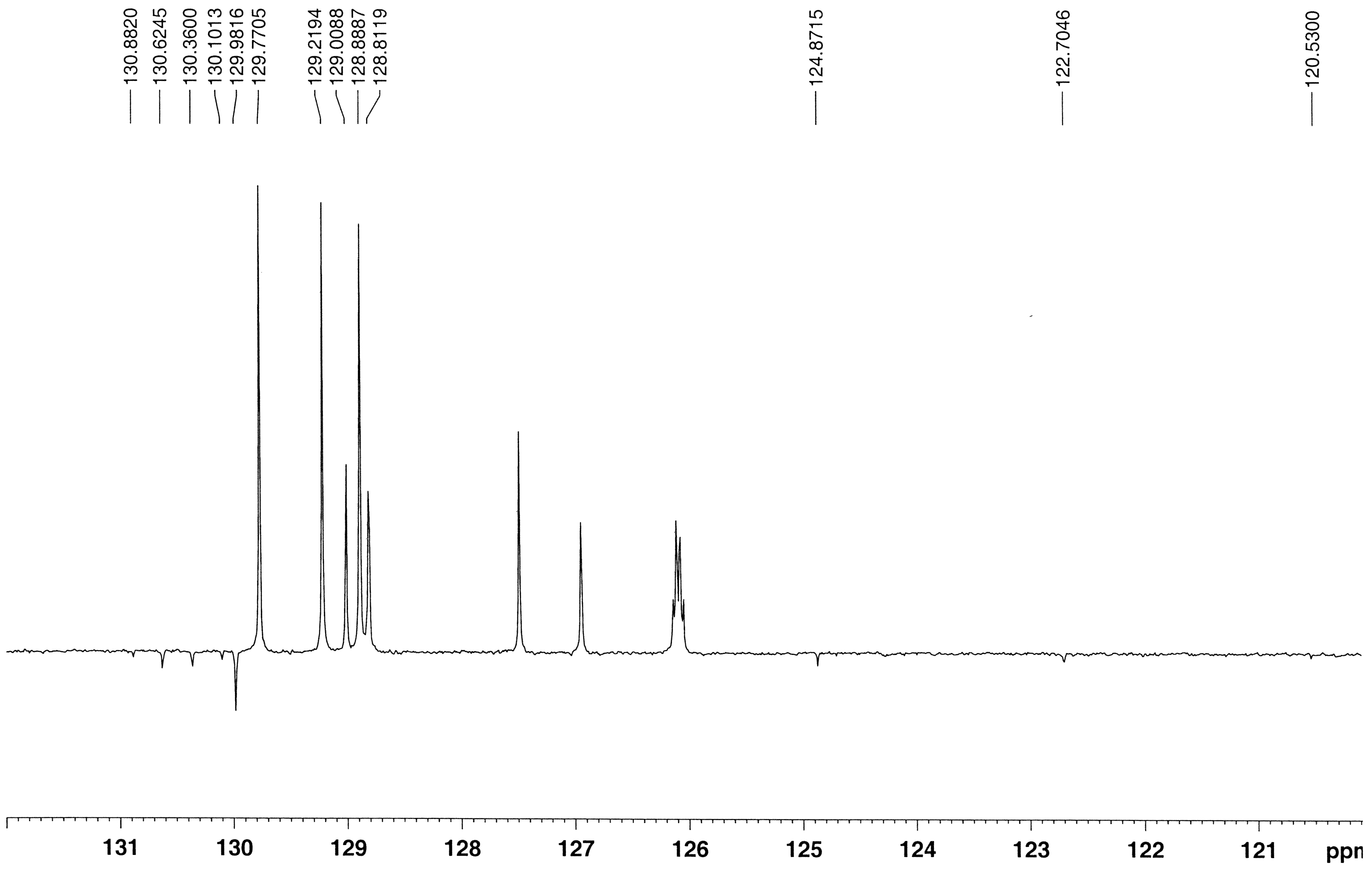

PN010S2P2 in cdcl3 (APT) 17.5.2019

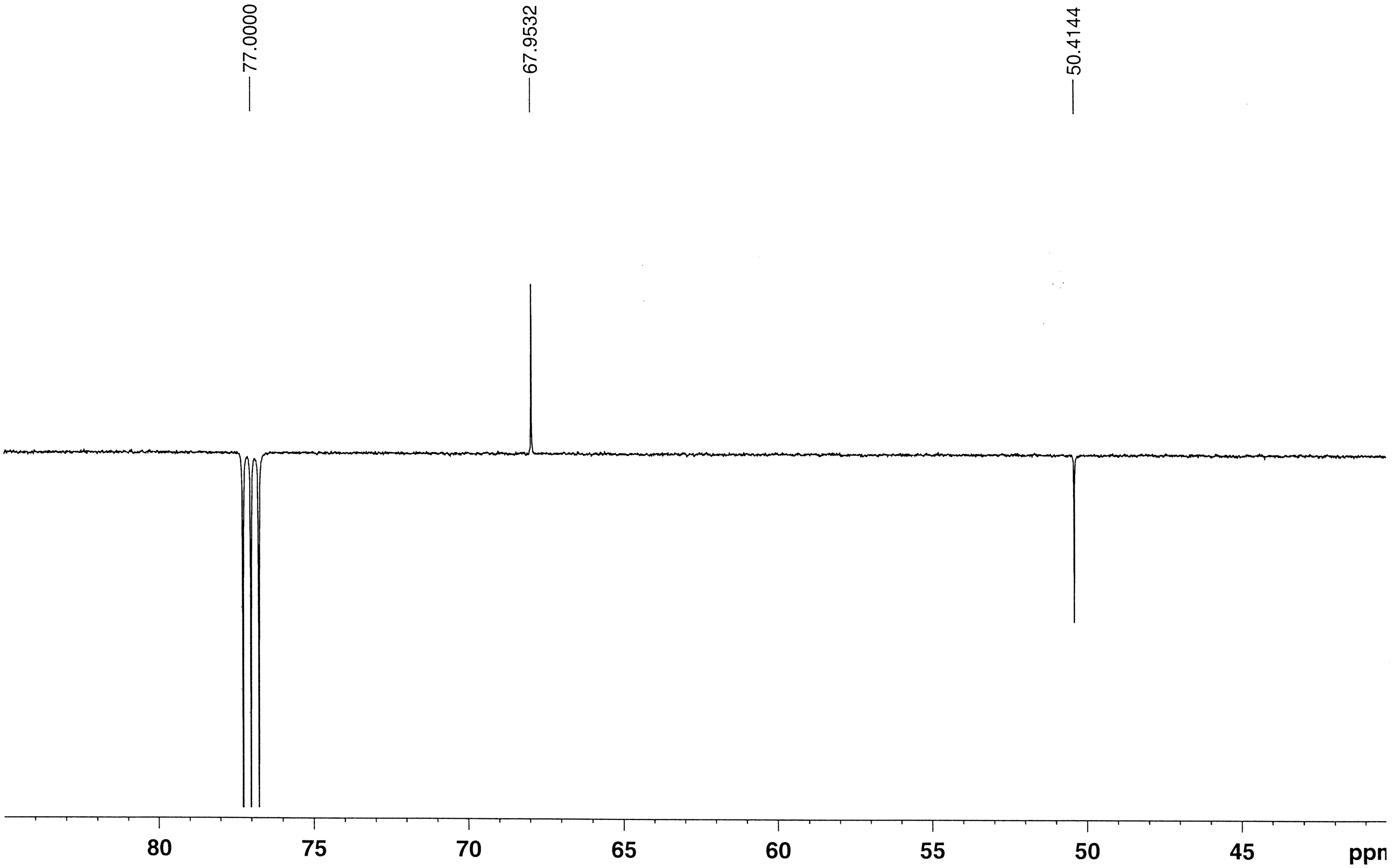

Figure S69c. NMR spectra of compound **5r.**

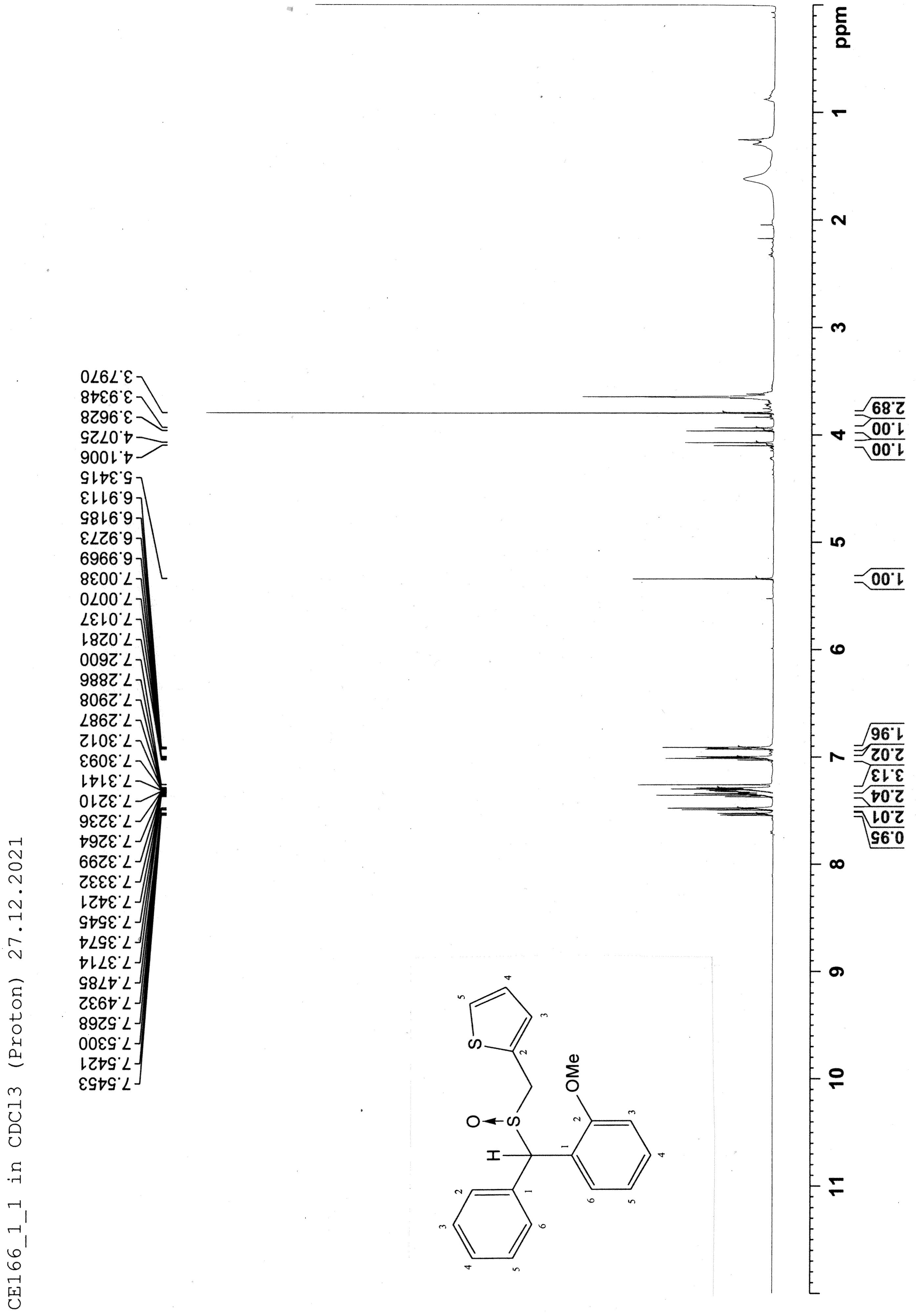

CE166_1_1 in CDCl3 (Proton) 27.12.2021

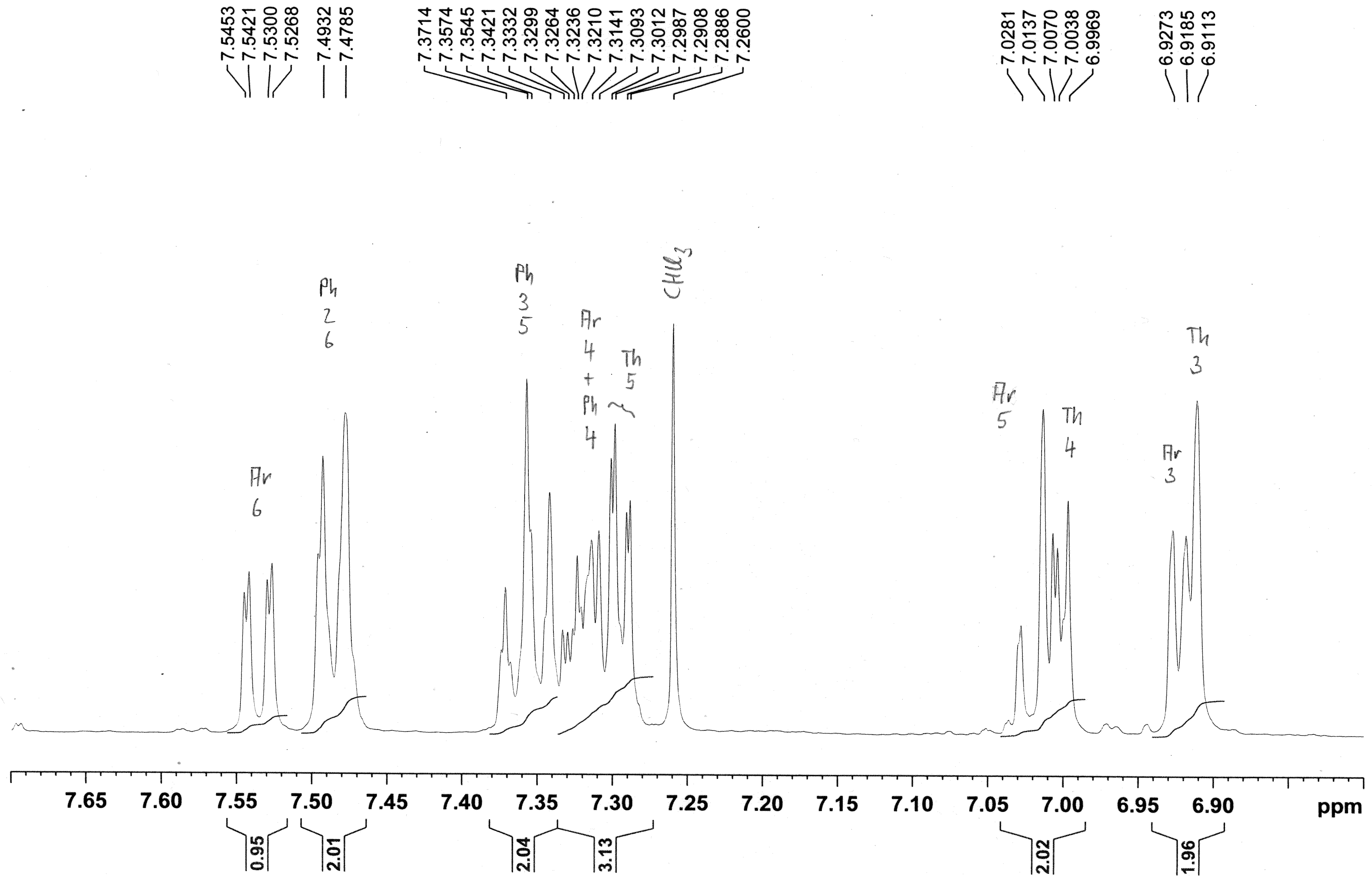

CE166_1_1 in CDCl3 (Proton) 27.12.2021

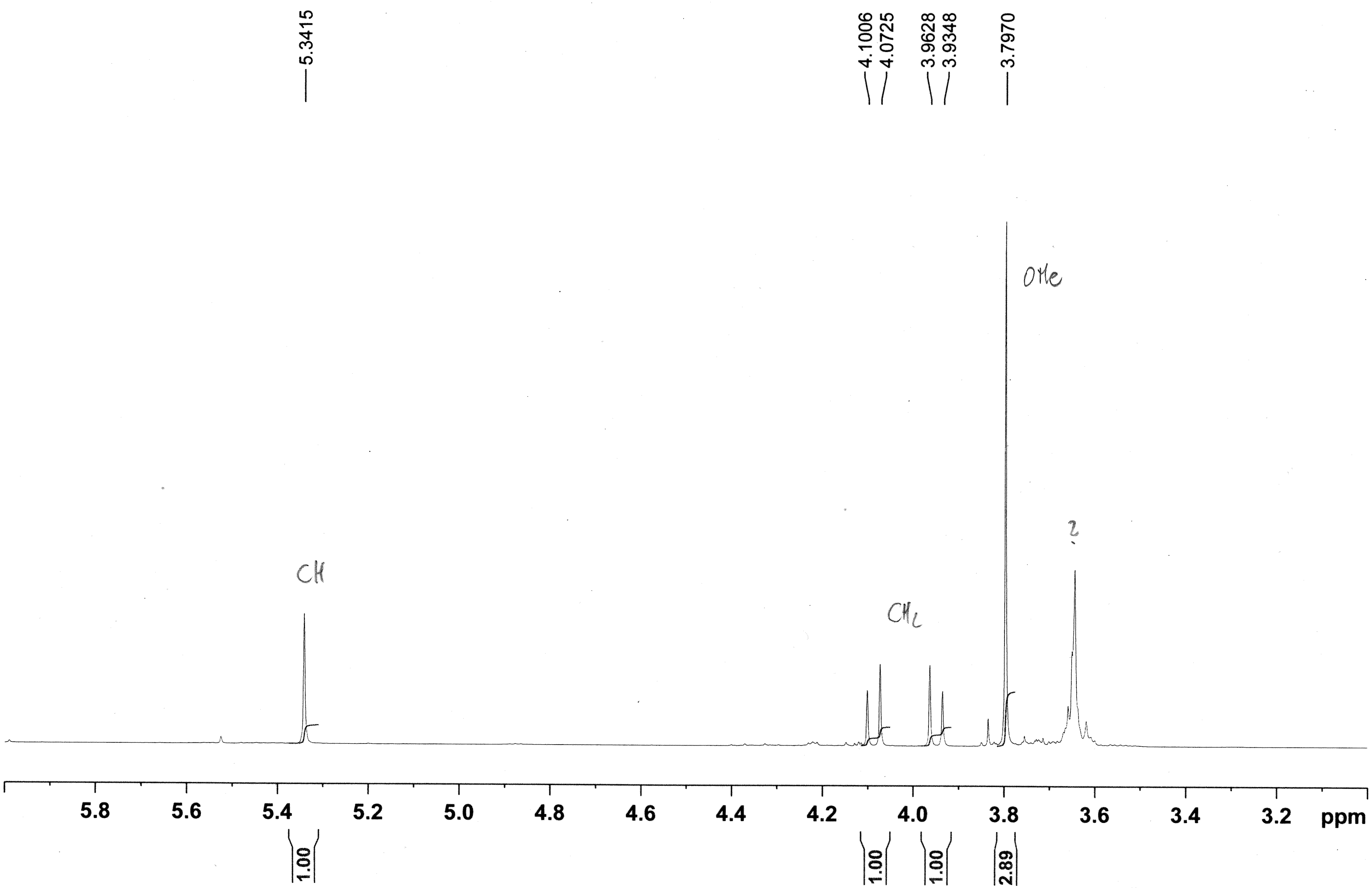

CE166_1_1 in CDCl3 (APT) 27.12.2021

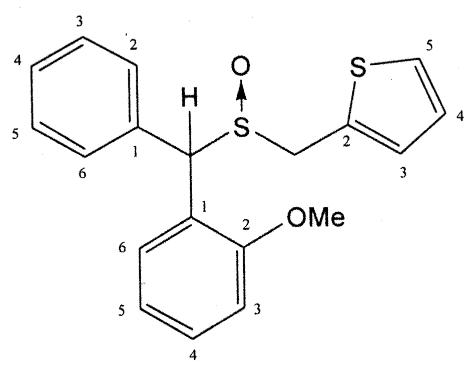

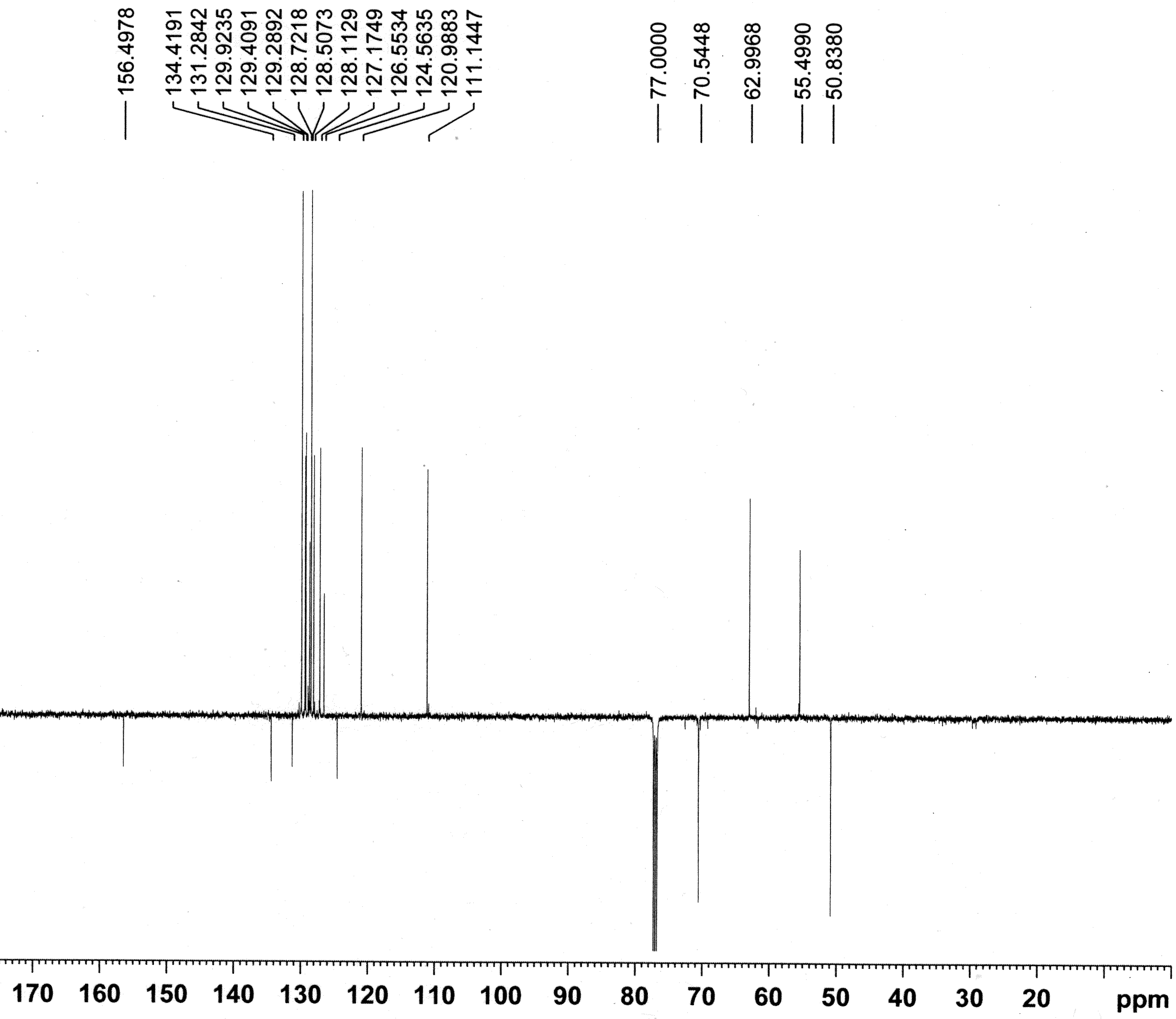

CE166_1_1 in CDCl3 (APT) 27.12.2021

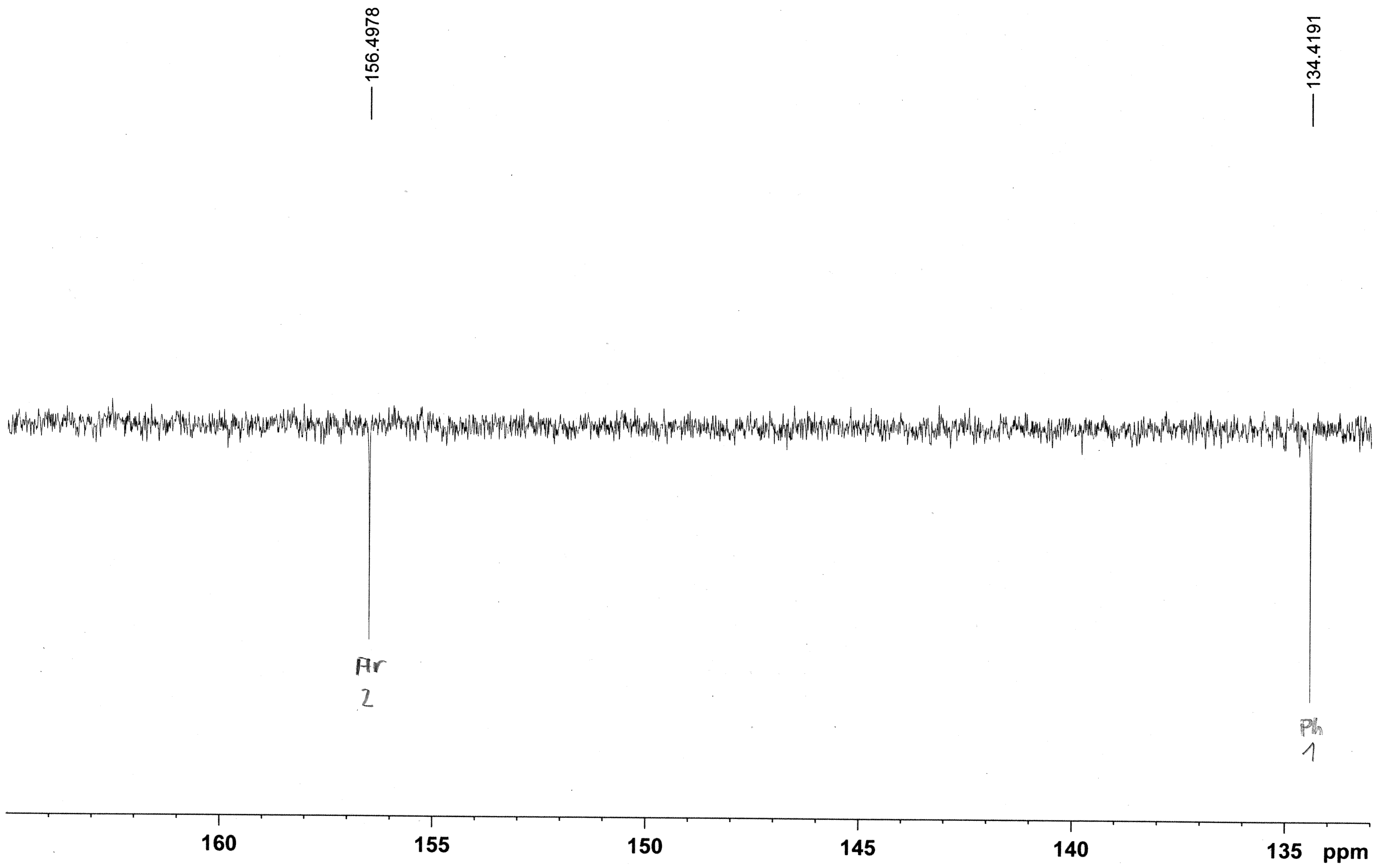

CE166_1_1 in CDCl3 (APT) 27.12.2021

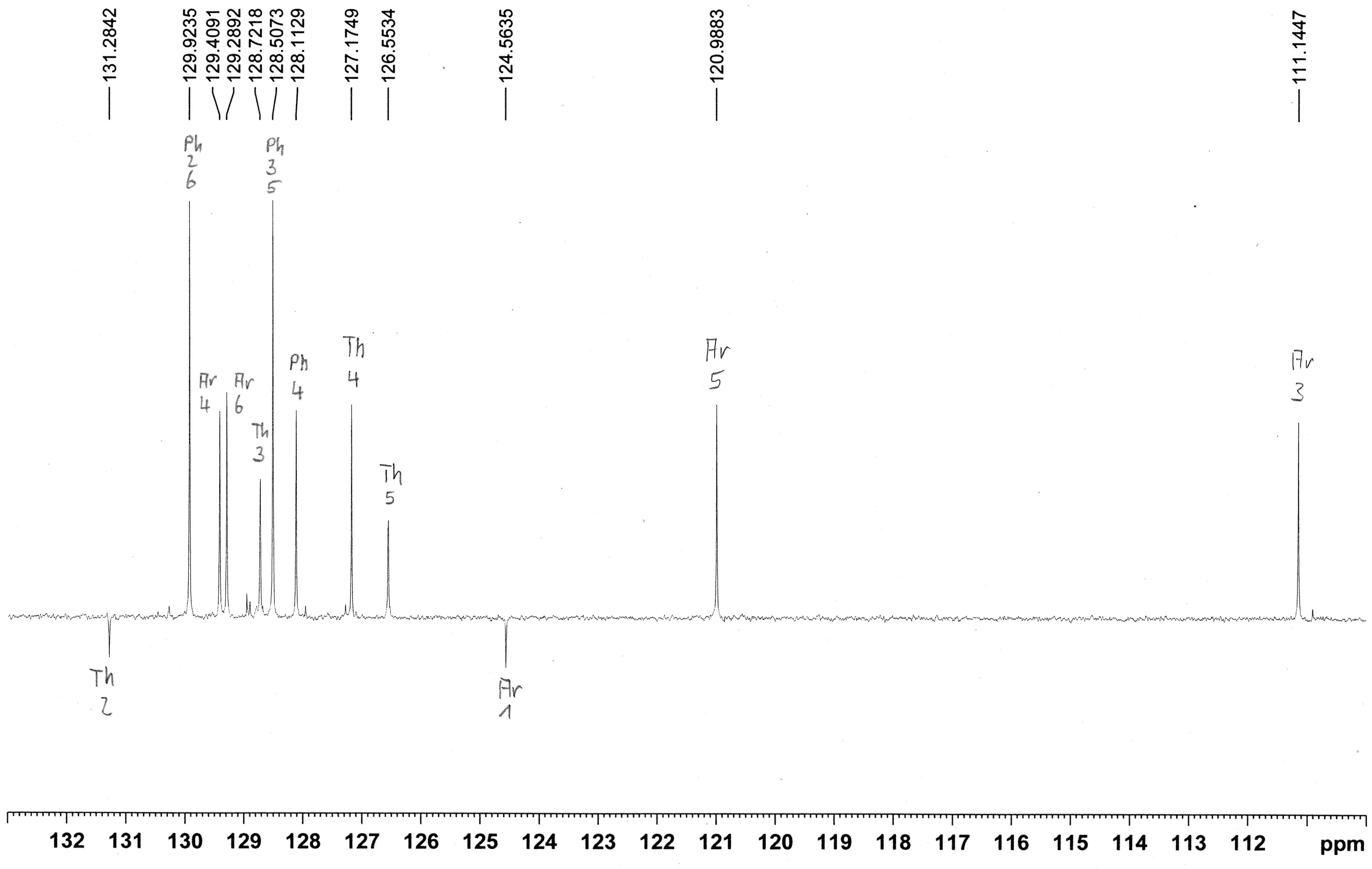

CE166_1_1 in CDCl3 (APT) 27.12.2021

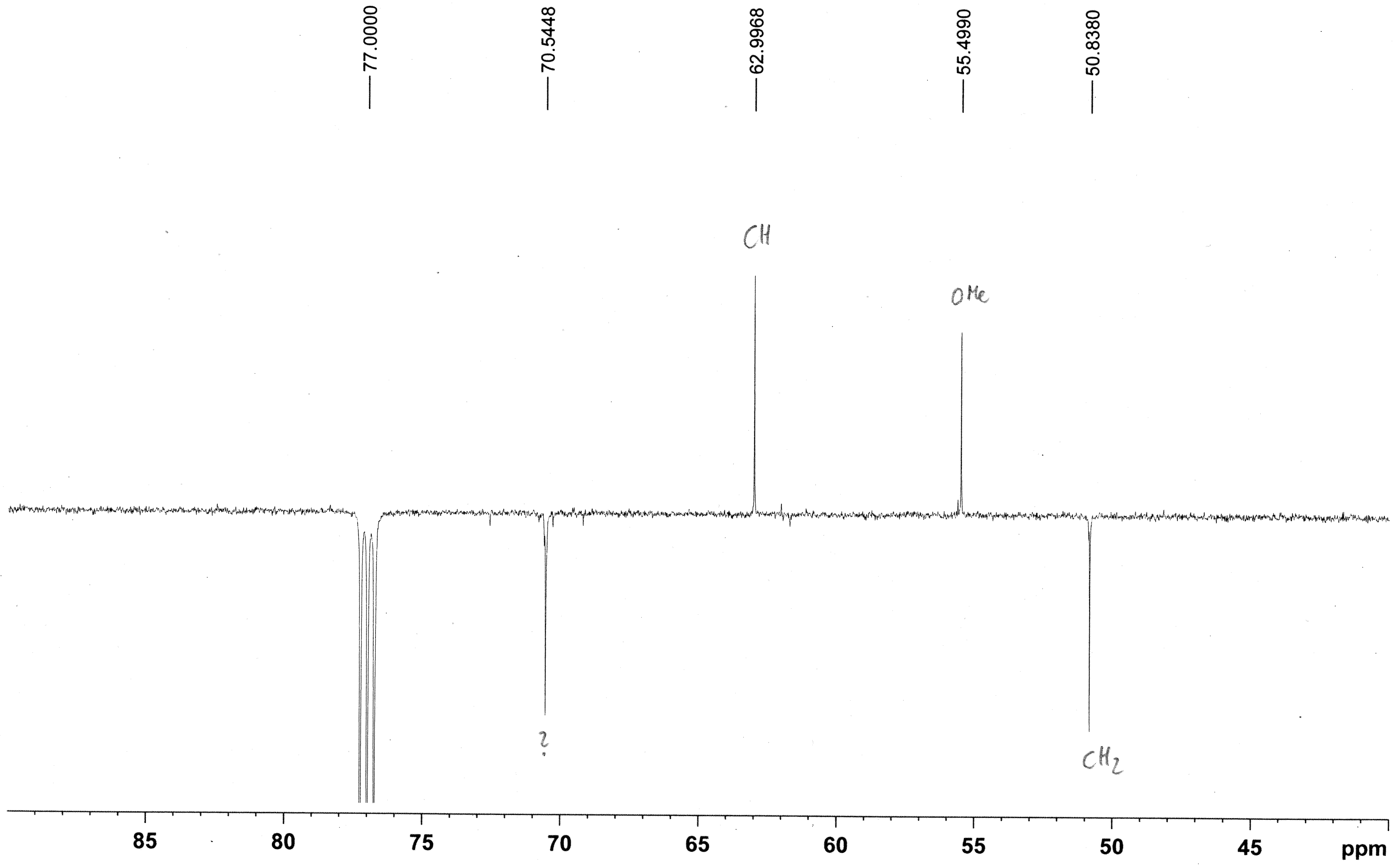

CE166_1_1 in CDCl3 (COSY) 27.12.2021

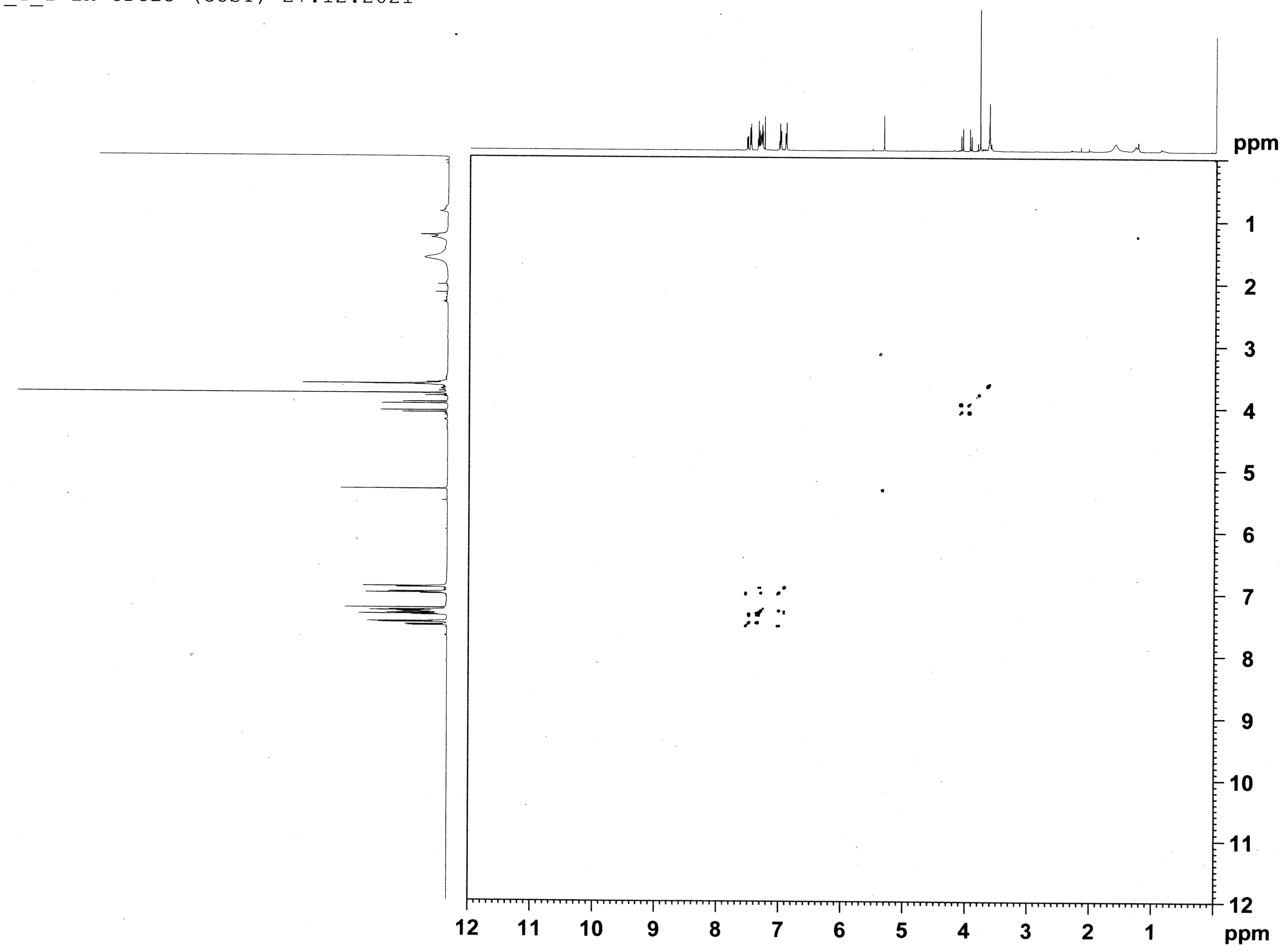

CE166_1_1 in CDCl3 (COSY) 27.12.2021

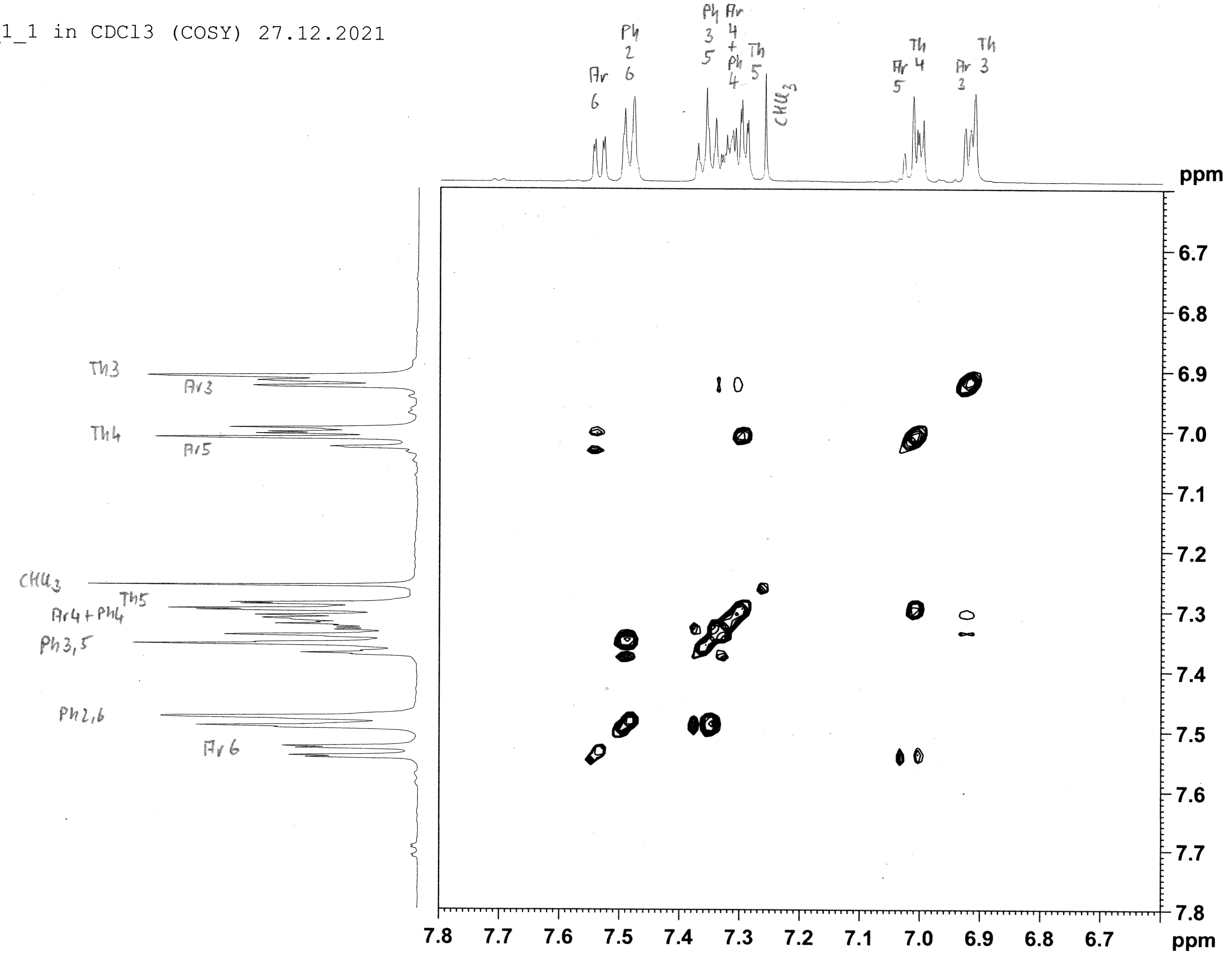

CE166_1_1 in CDCl3 (COSY) 27.12.2021

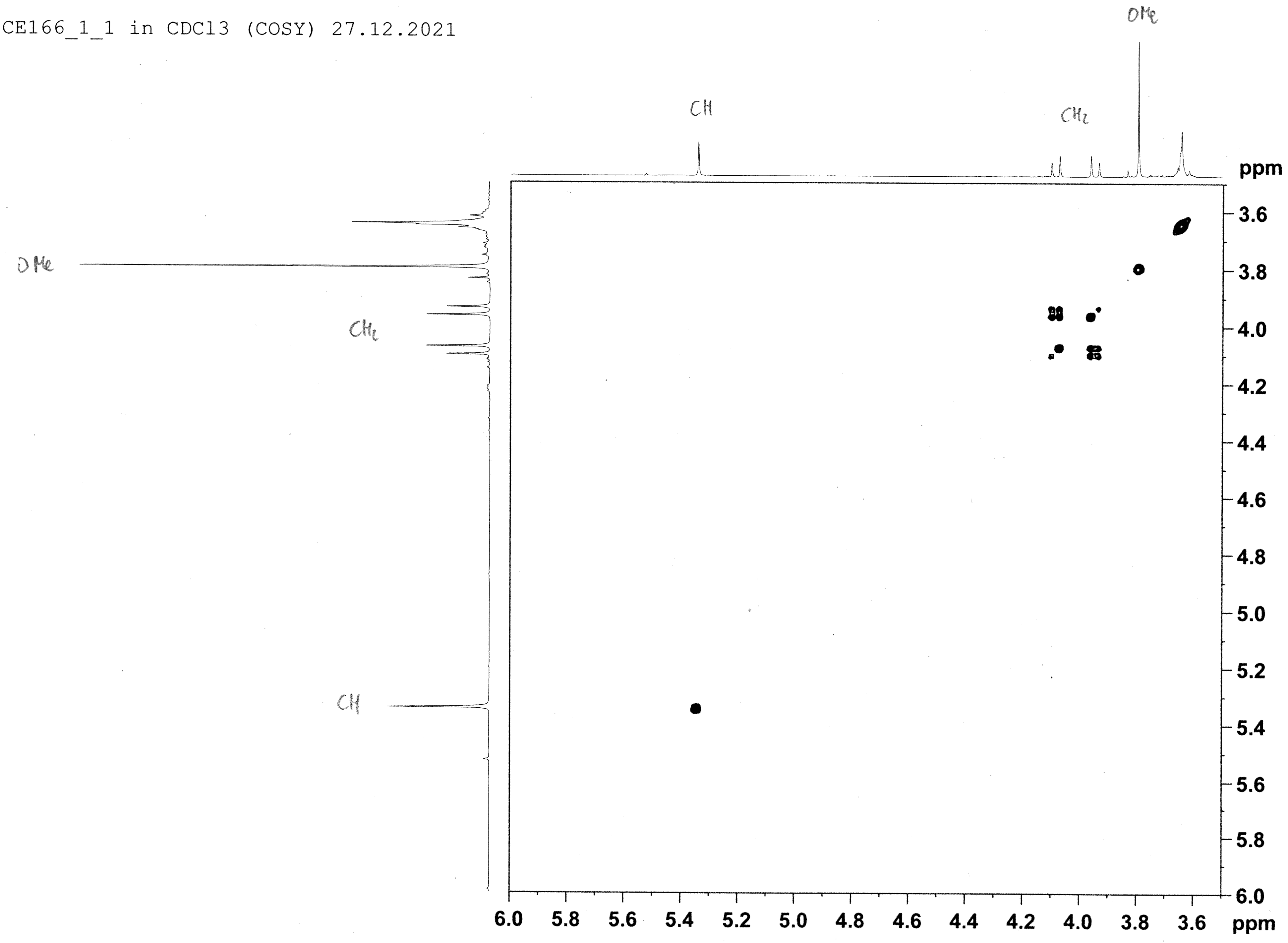

CE166_1_1 in CDCl3 (HSQC) 27.12.2021

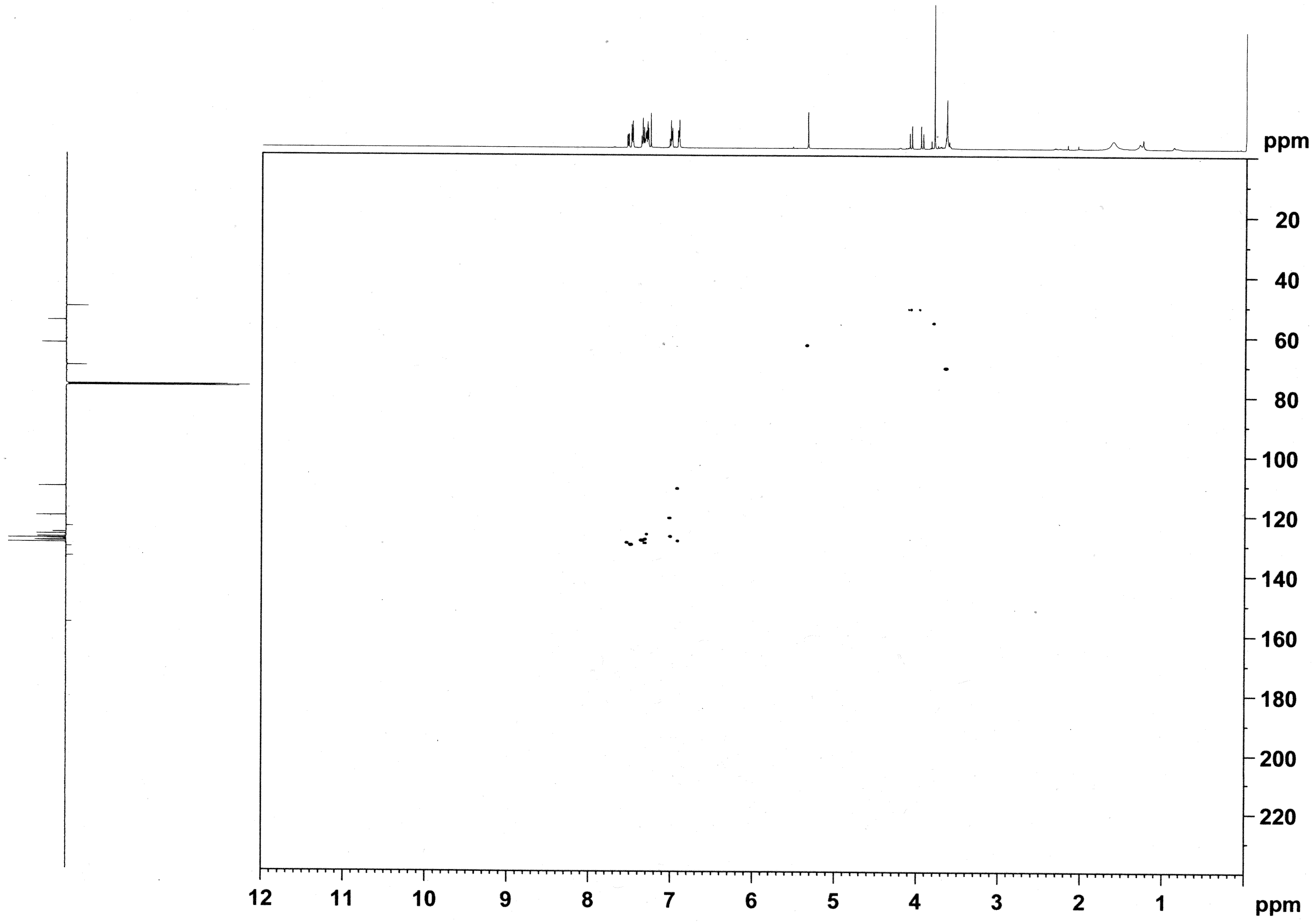

CE166_1_1 in CDCl3 (HSQC) 27.12.2021

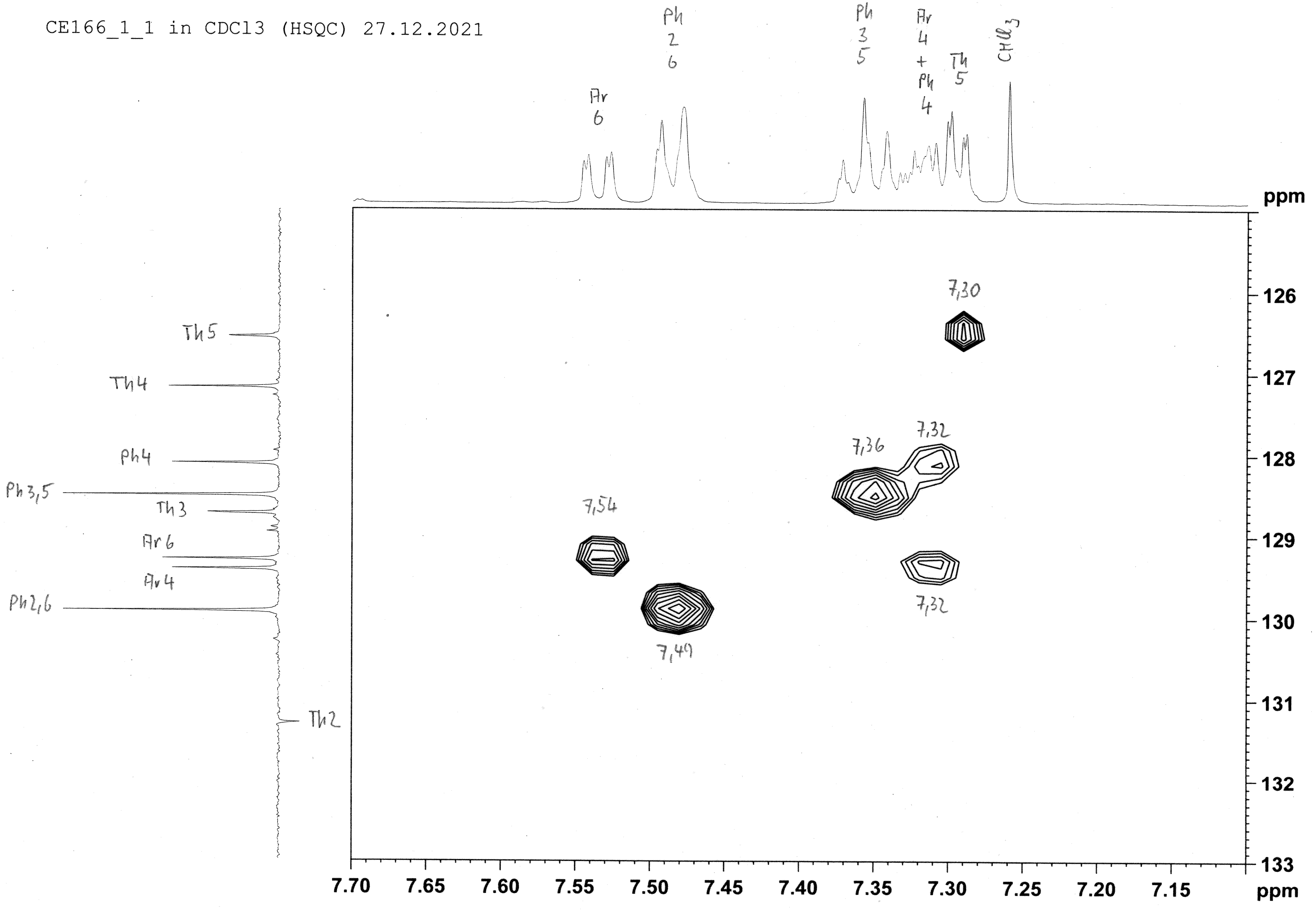

CE166_1_1 in CDCl3 (HSQC) 27.12.2021

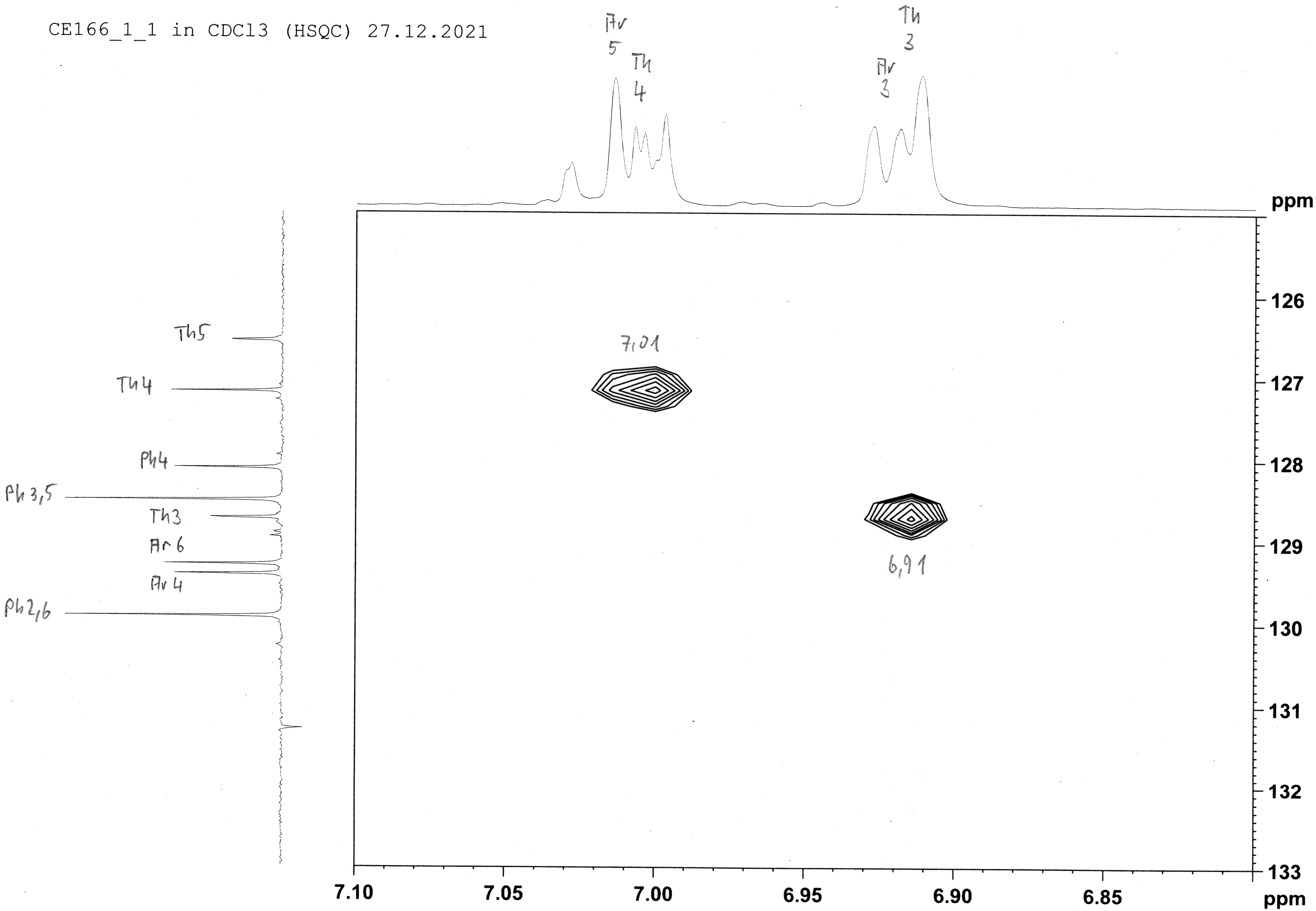

CE166_1_1 in CDCl3 (HSQC) 27.12.2021

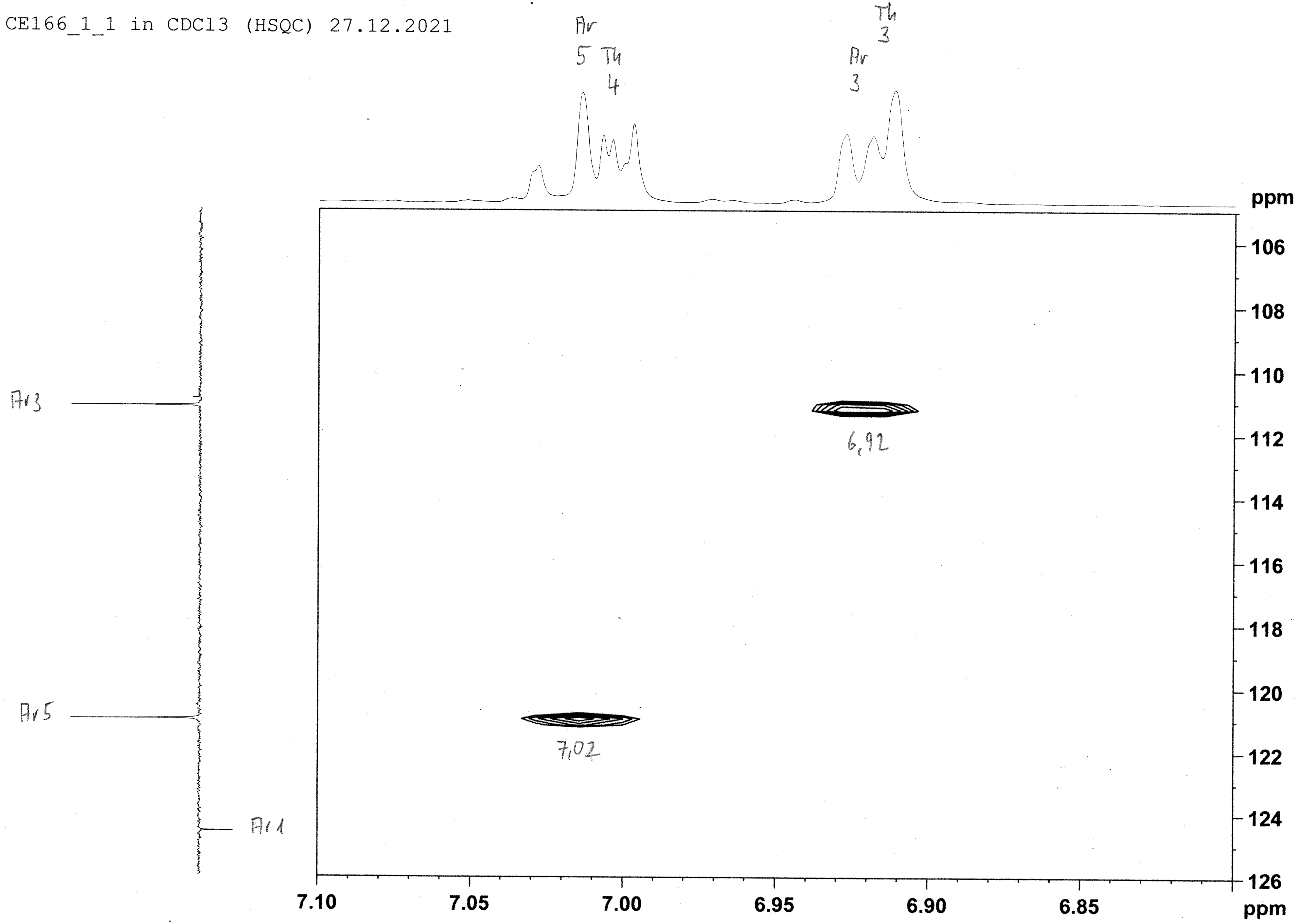

CE166_1_1 in CDCl3 (HSQC) 27.12.2021

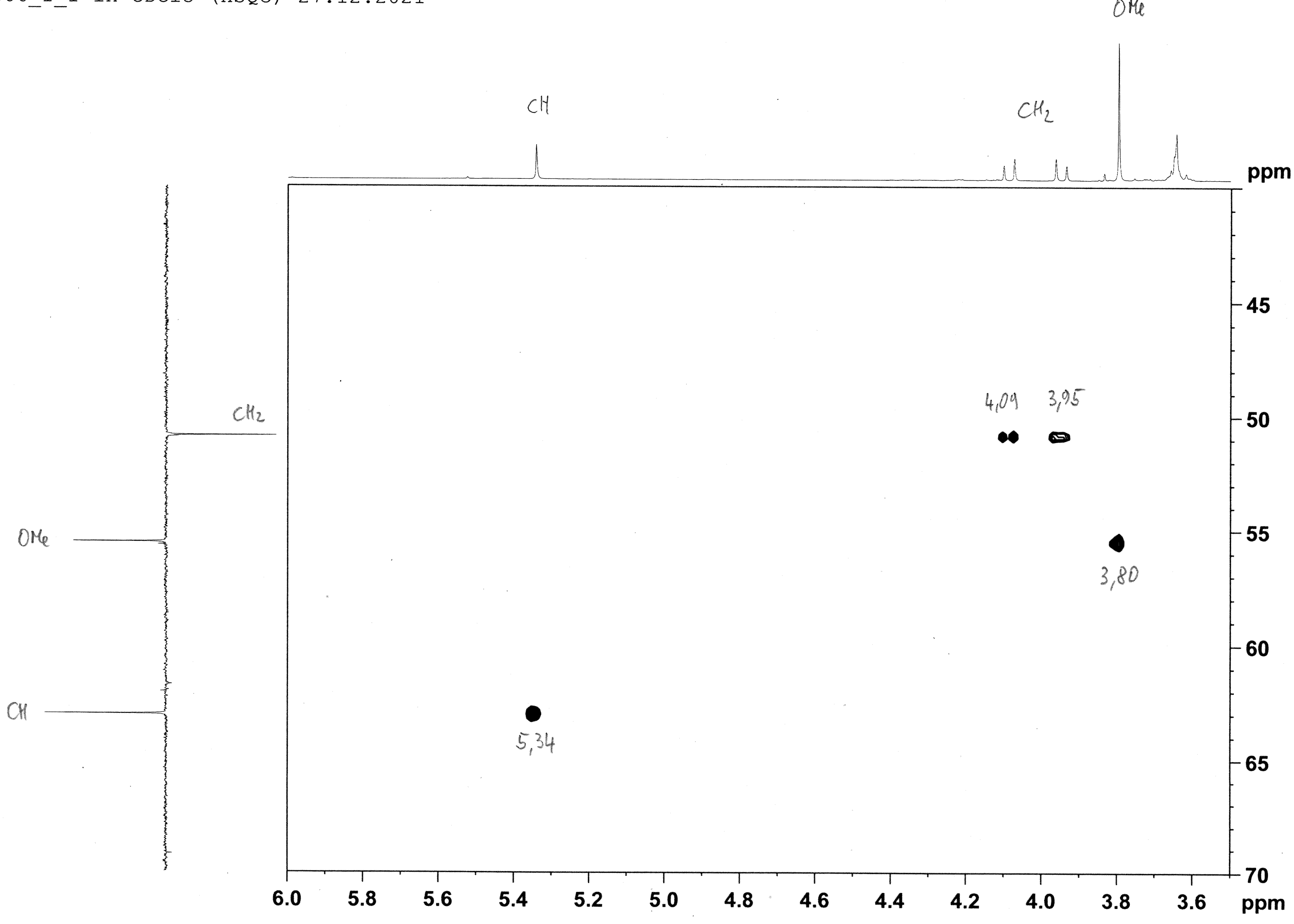

CE166_1_1 in CDCl3 (HMBC) 27.12.2021

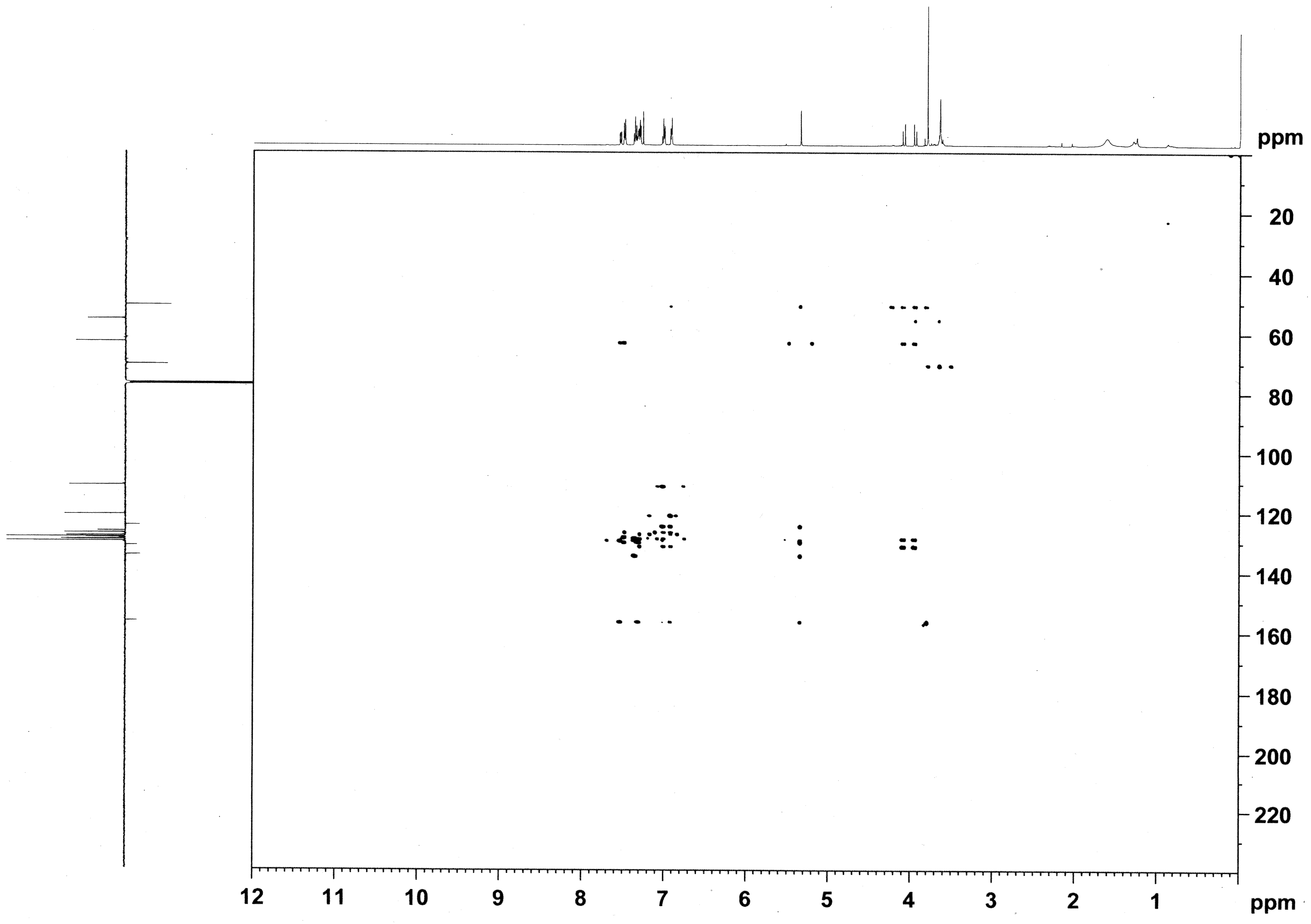

CE166_1_1 in CDCl3 (HMBC) 27.12.2021

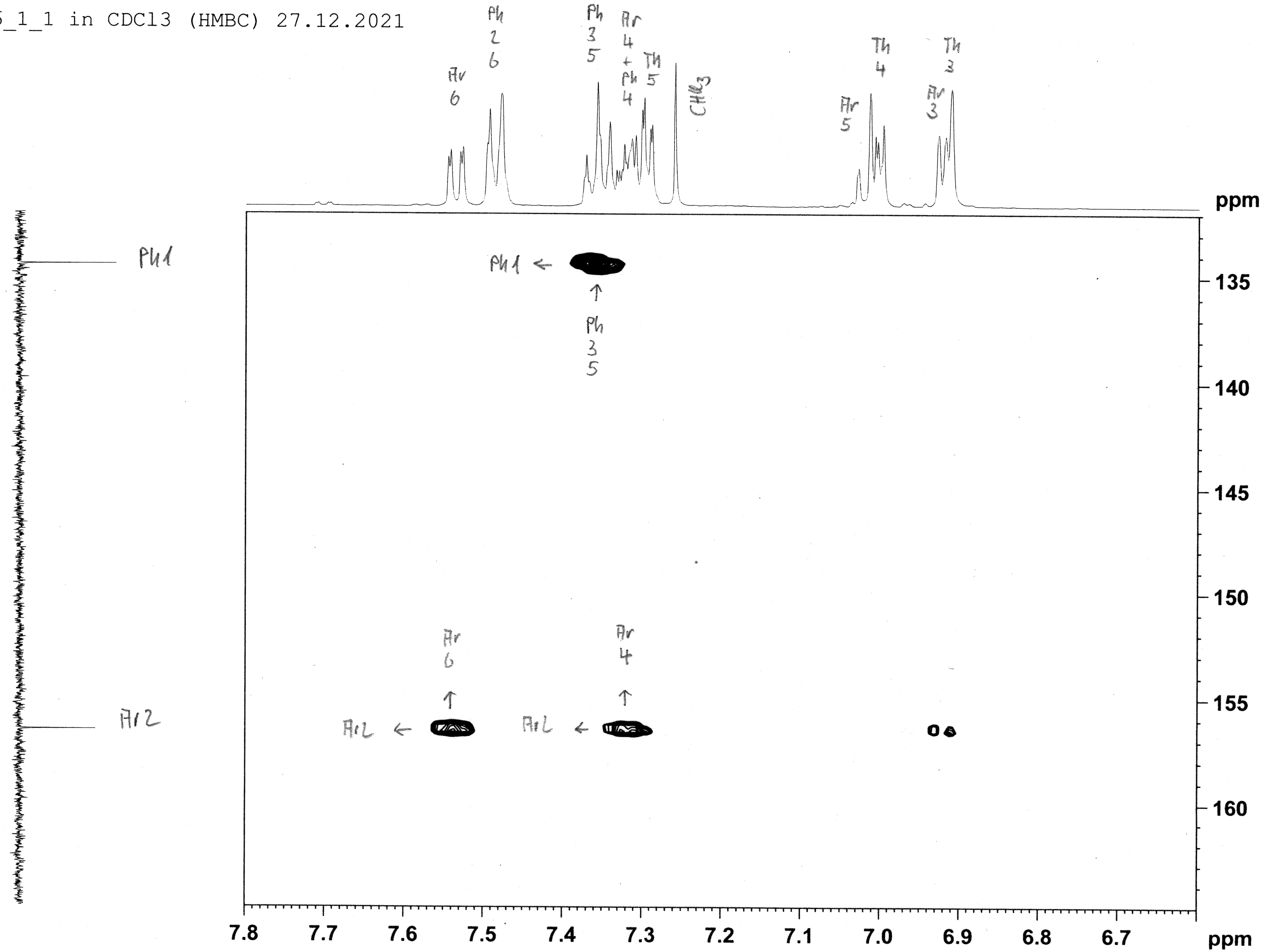

CE166_1_1 in CDCl3 (HMBC) 27.12.2021

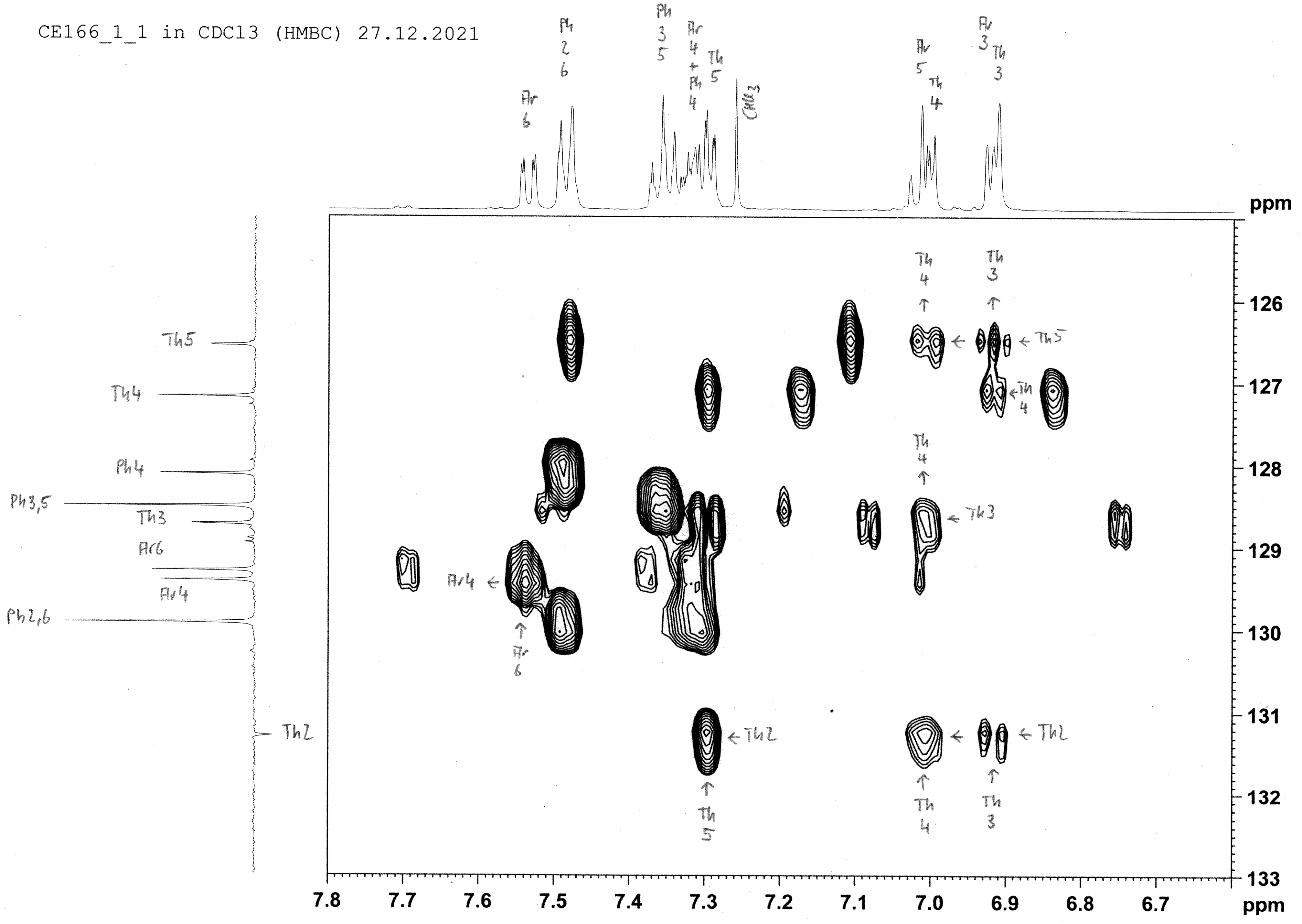

CE166_1_1 in CDCl3 (HMBC) 27.12.2021

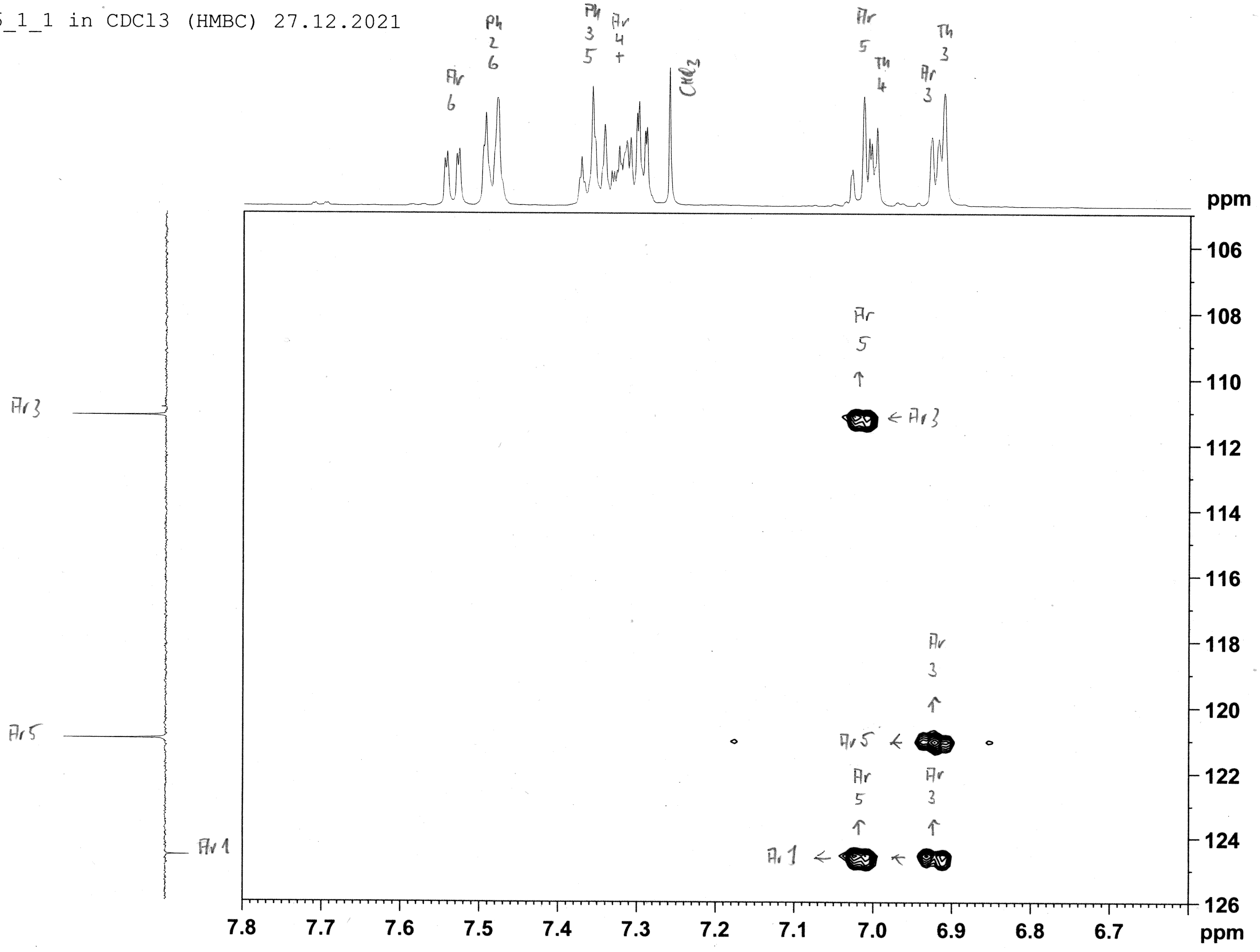

CE166_1_1 in CDCl3 (HMBC) 27.12.2021

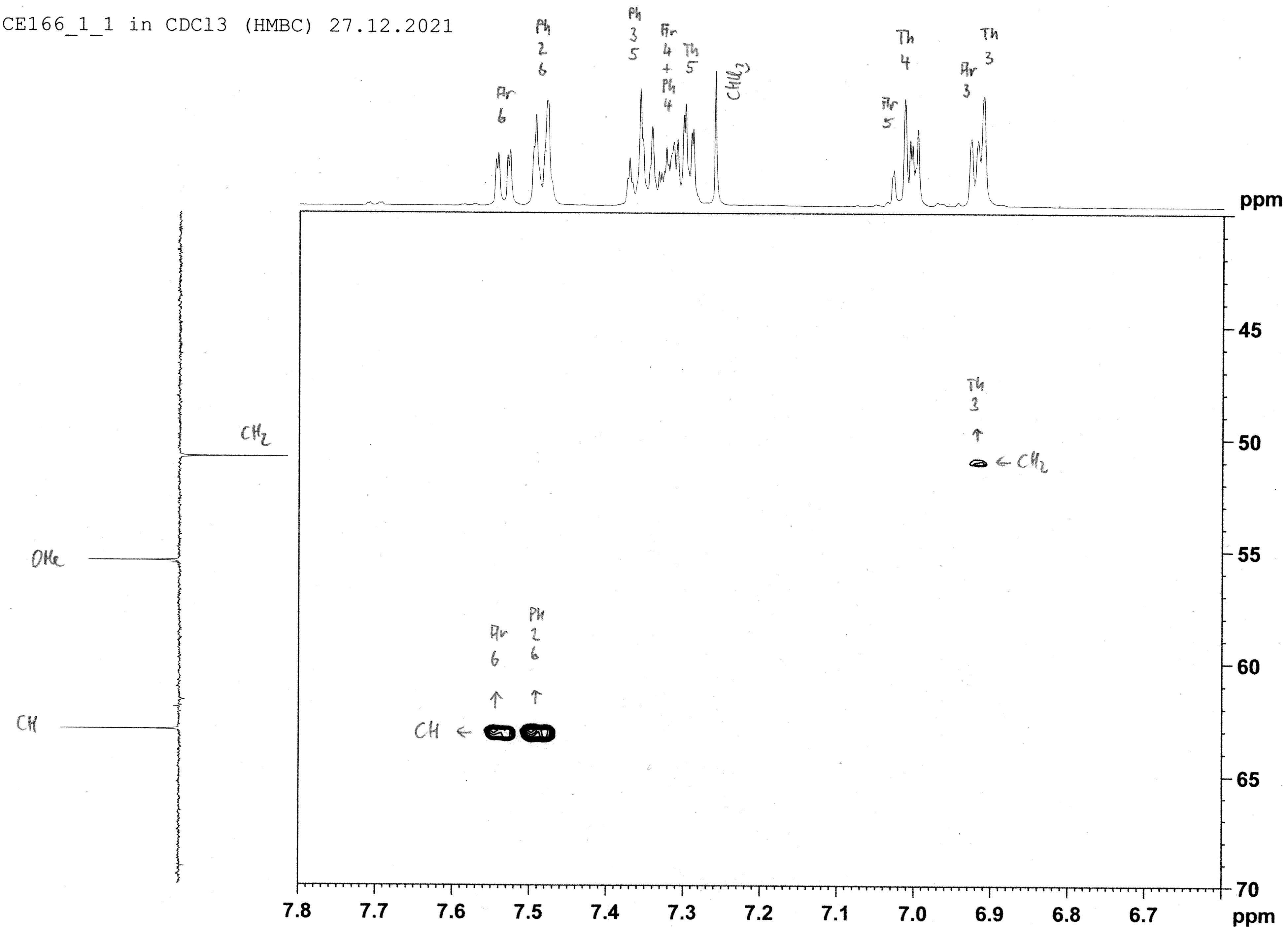

CE166_1_1 in CDCl3 (HMBC) 27.12.2021

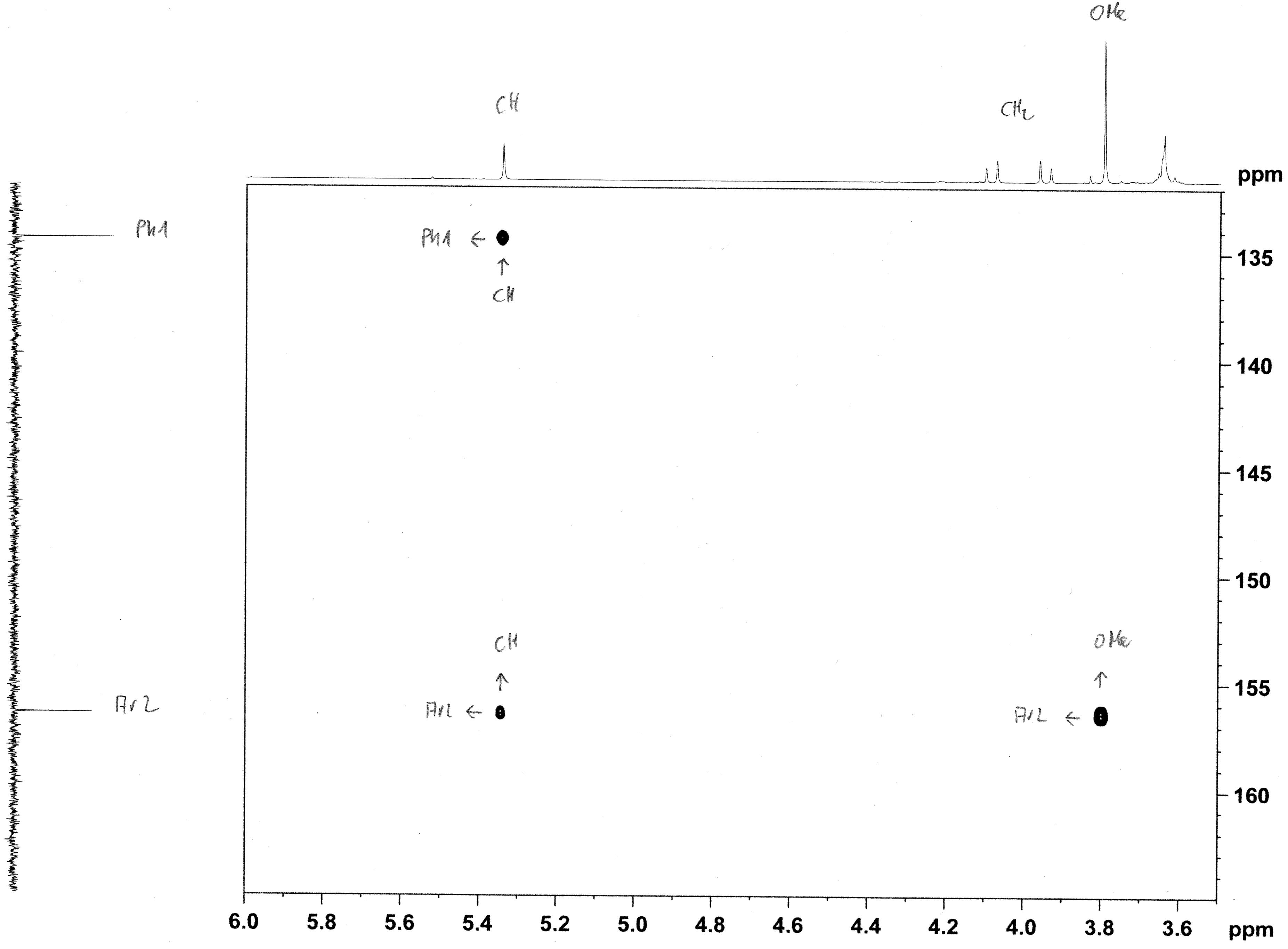

CE166_1_1 in CDCl3 (HMBC) 27.12.2021

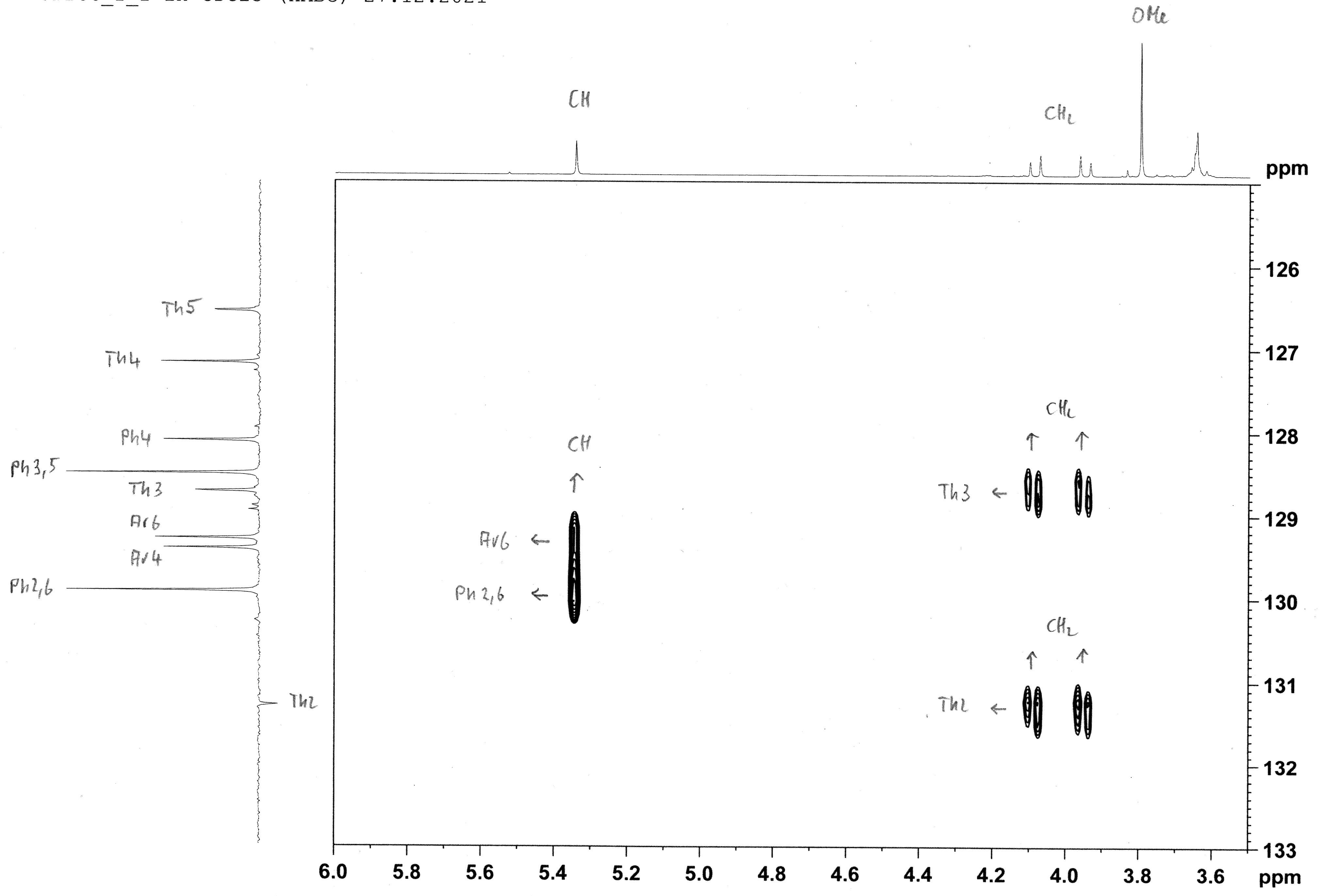

CE166_1_1 in CDCl3 (HMBC) 27.12.2021

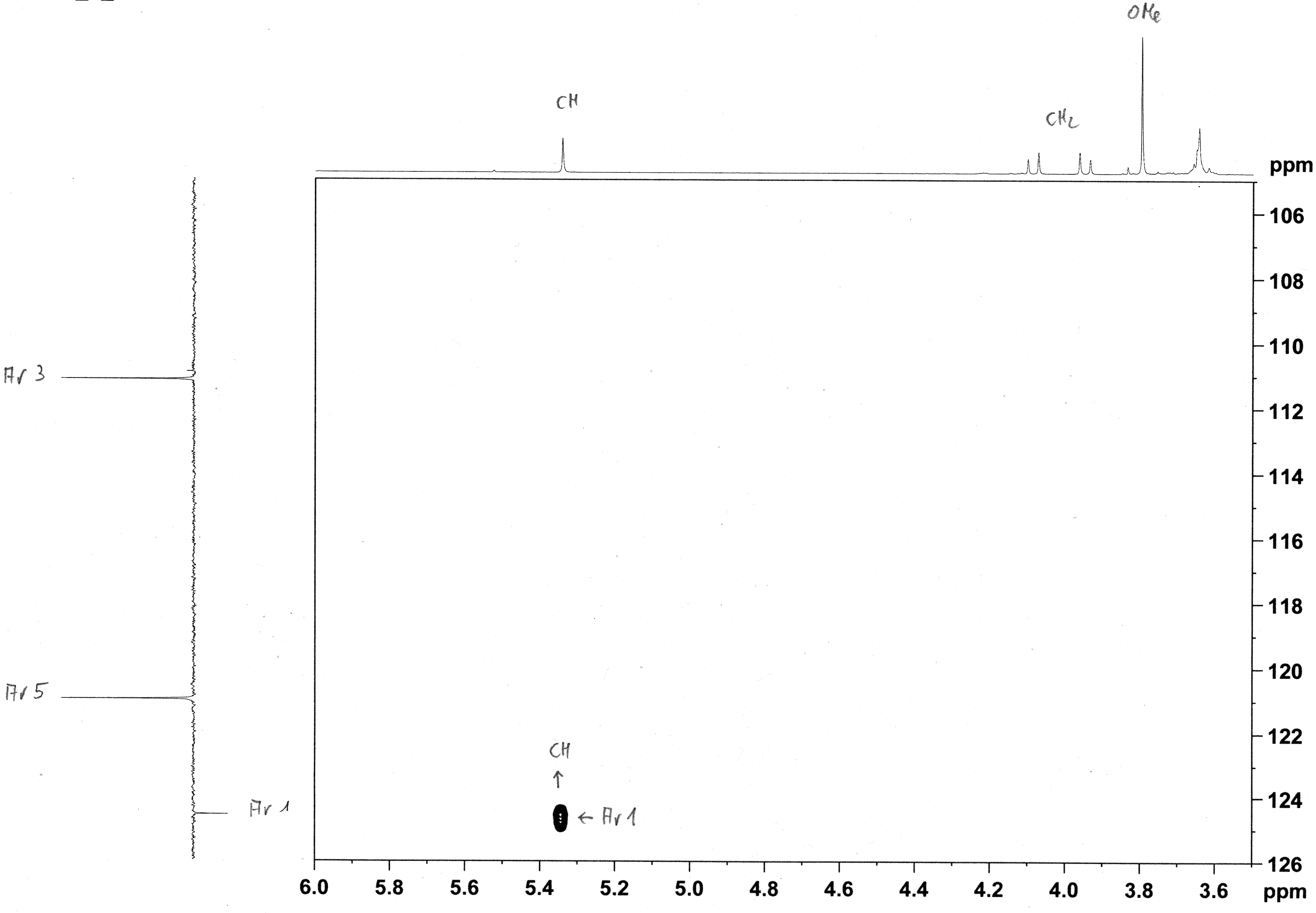

CE166_1_1 in CDCl3 (HMBC) 27.12.2021

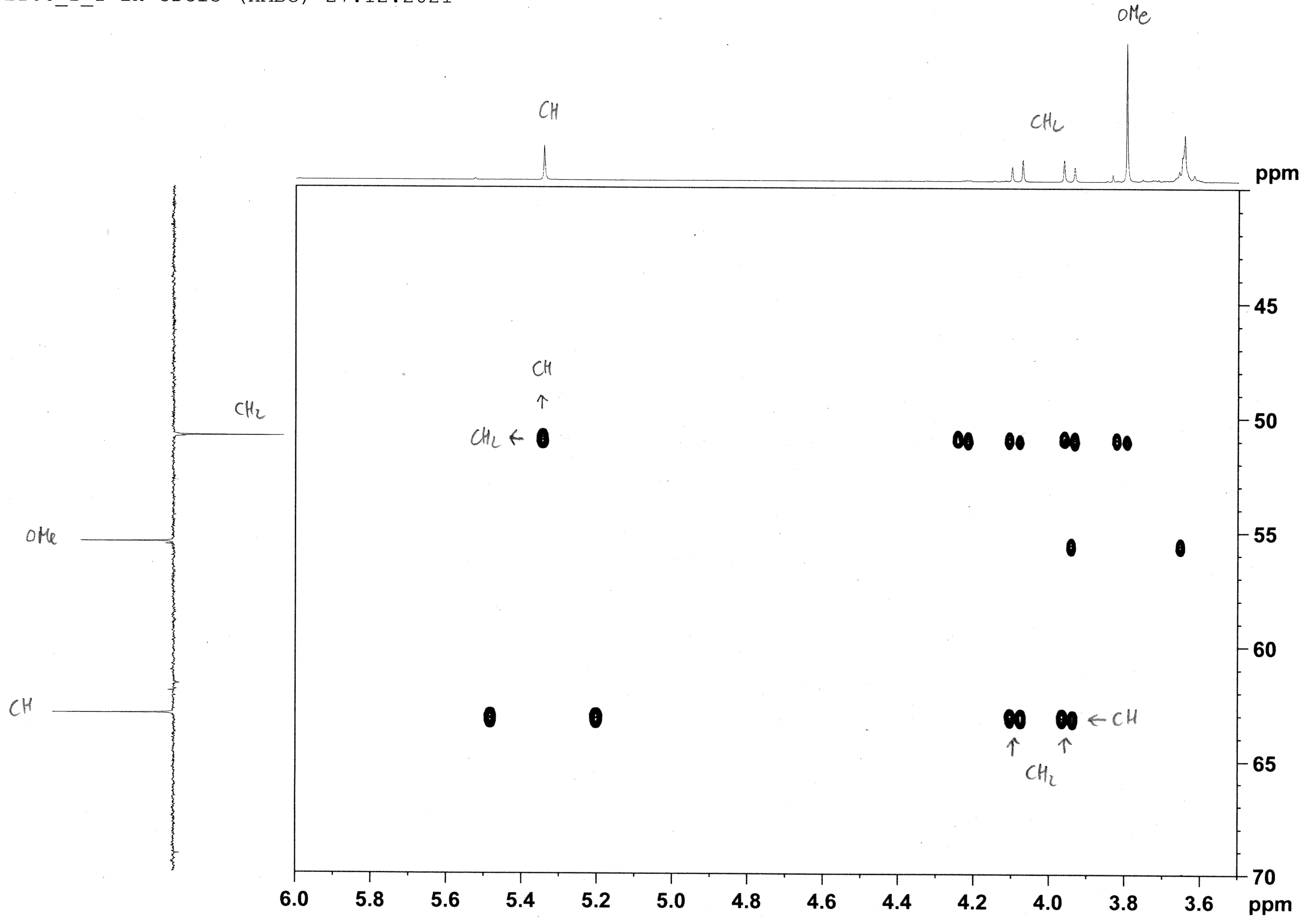

Figure S70c. NMR spectra of compound **6r**.

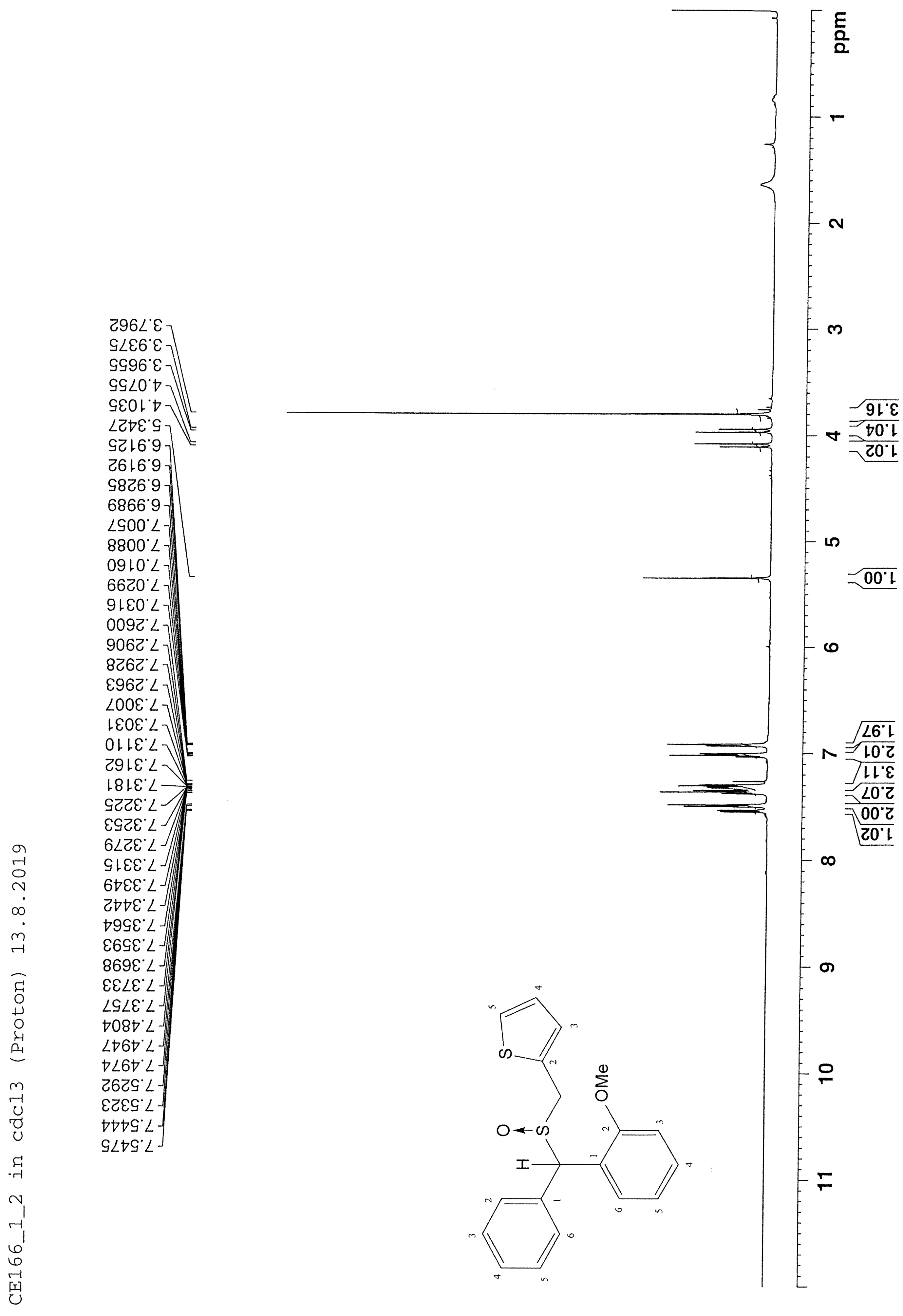

CE166_1_2 in cdcl3 (Proton) 13.8.2019

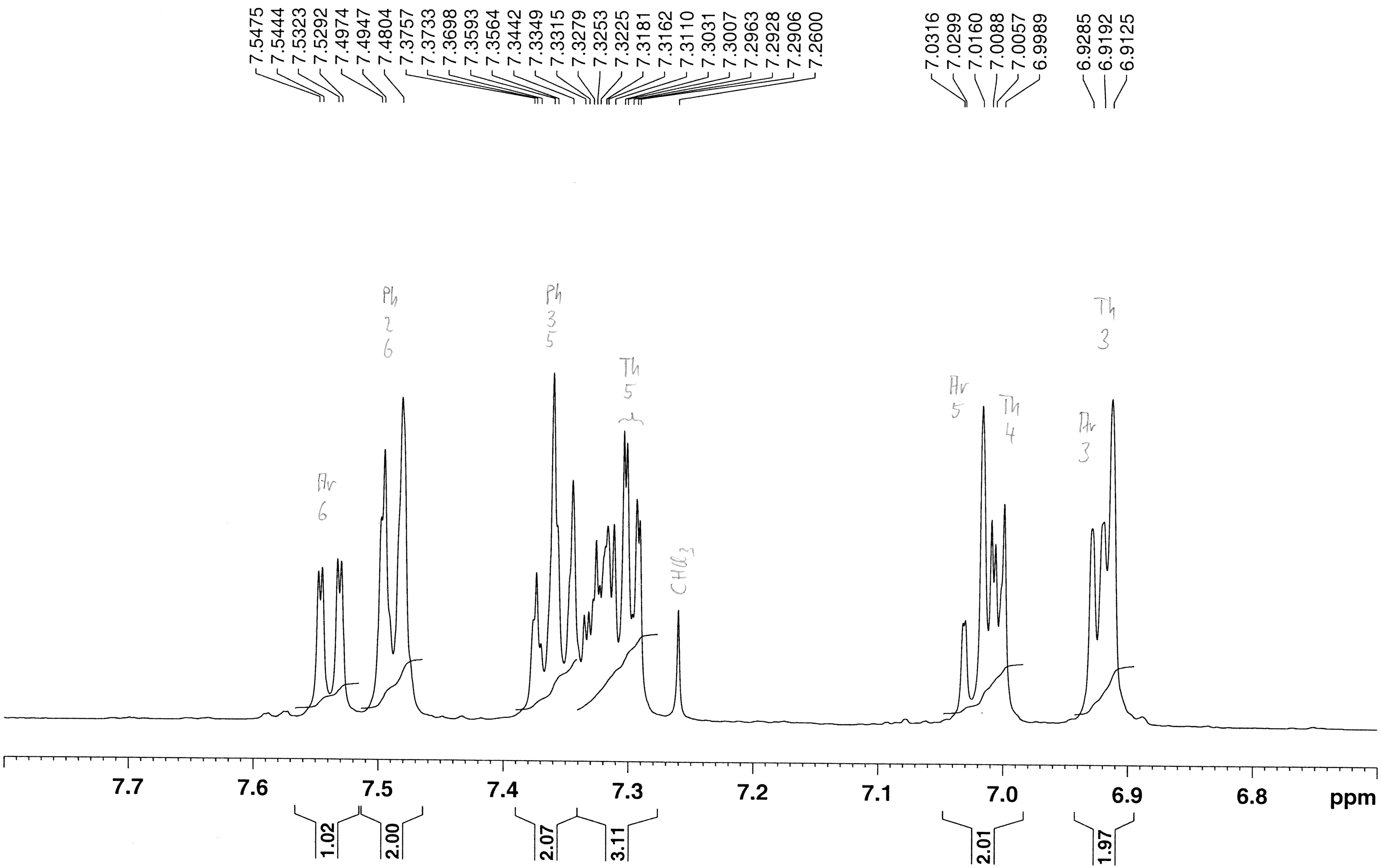

CE166_1_2 in cdcl3 (Proton) 13.8.2019

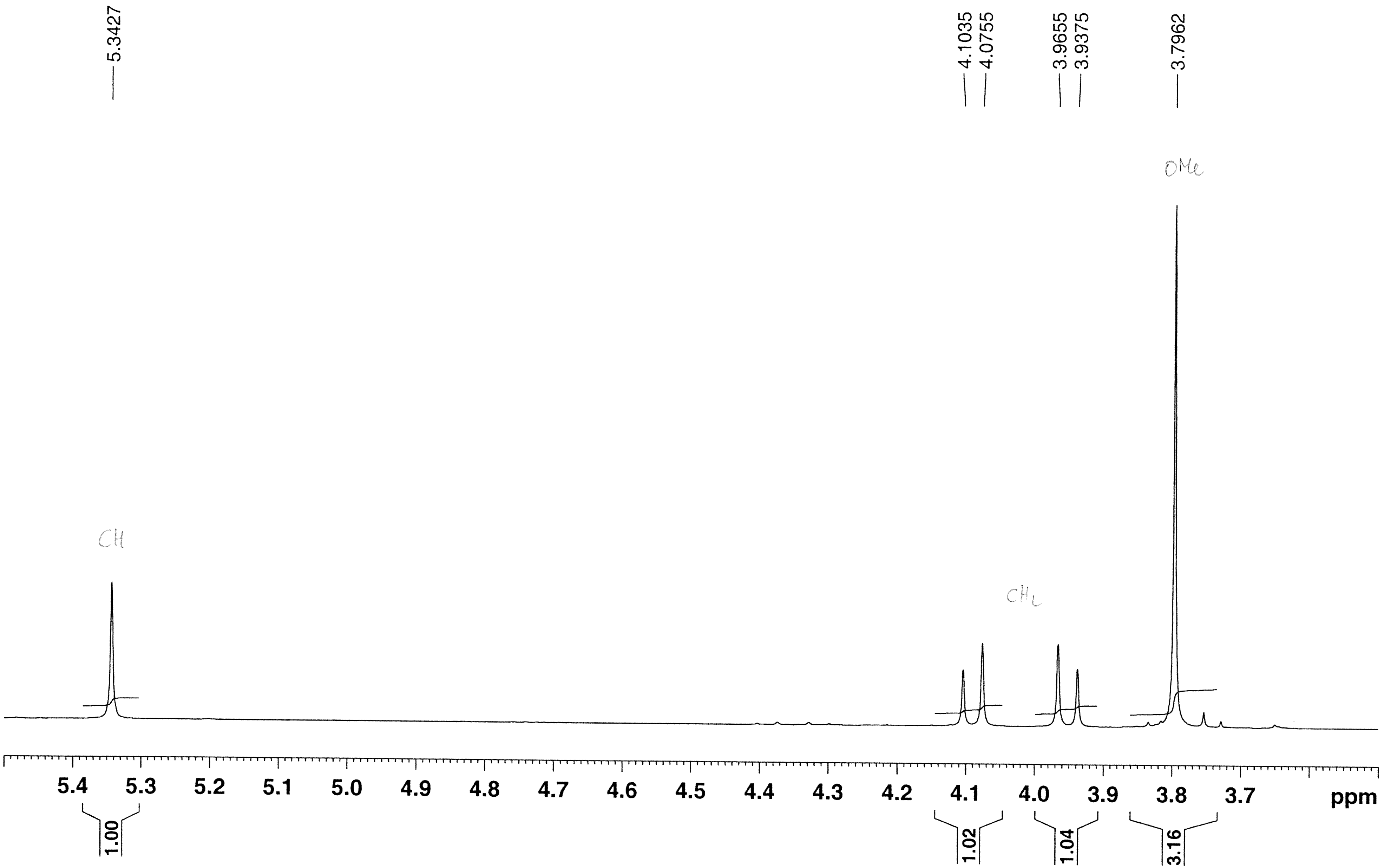

CE166_1_2 in cdcl3 (APT) 13.8.2019

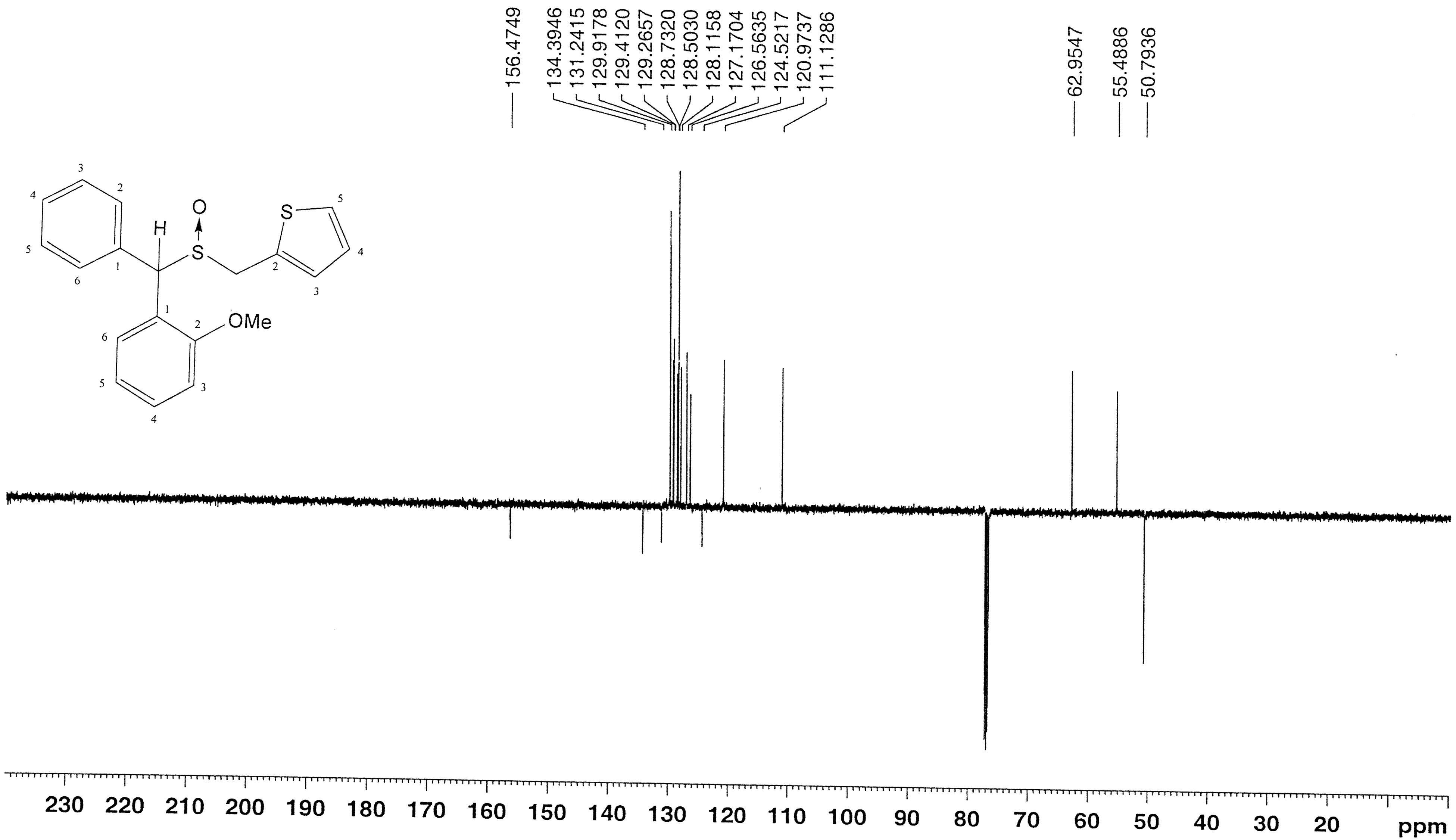

CE166_1_2 in cdcl3 (APT) 13.8.2019

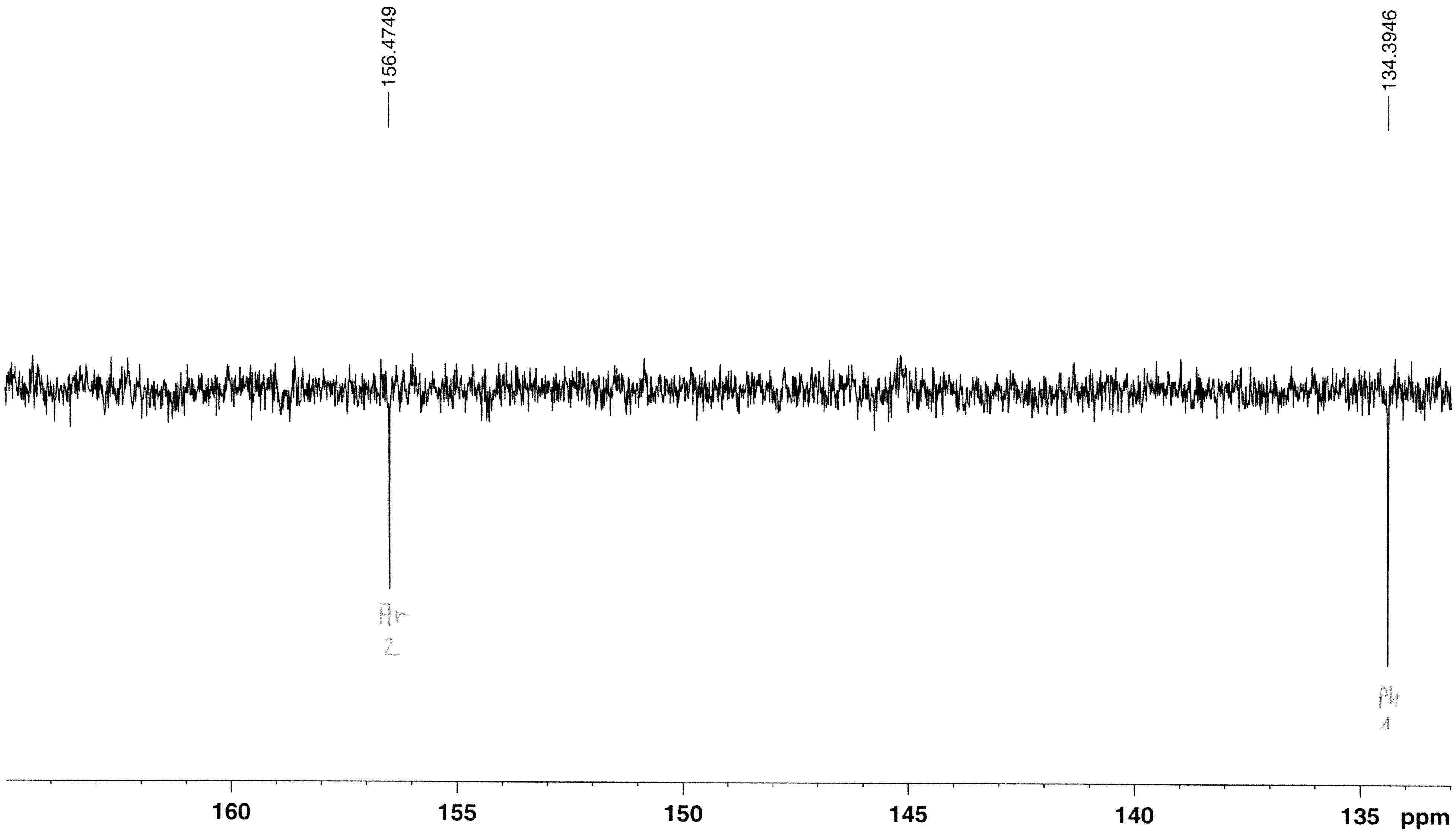

CE166_1_2 in cdcl3 (APT) 13.8.2019

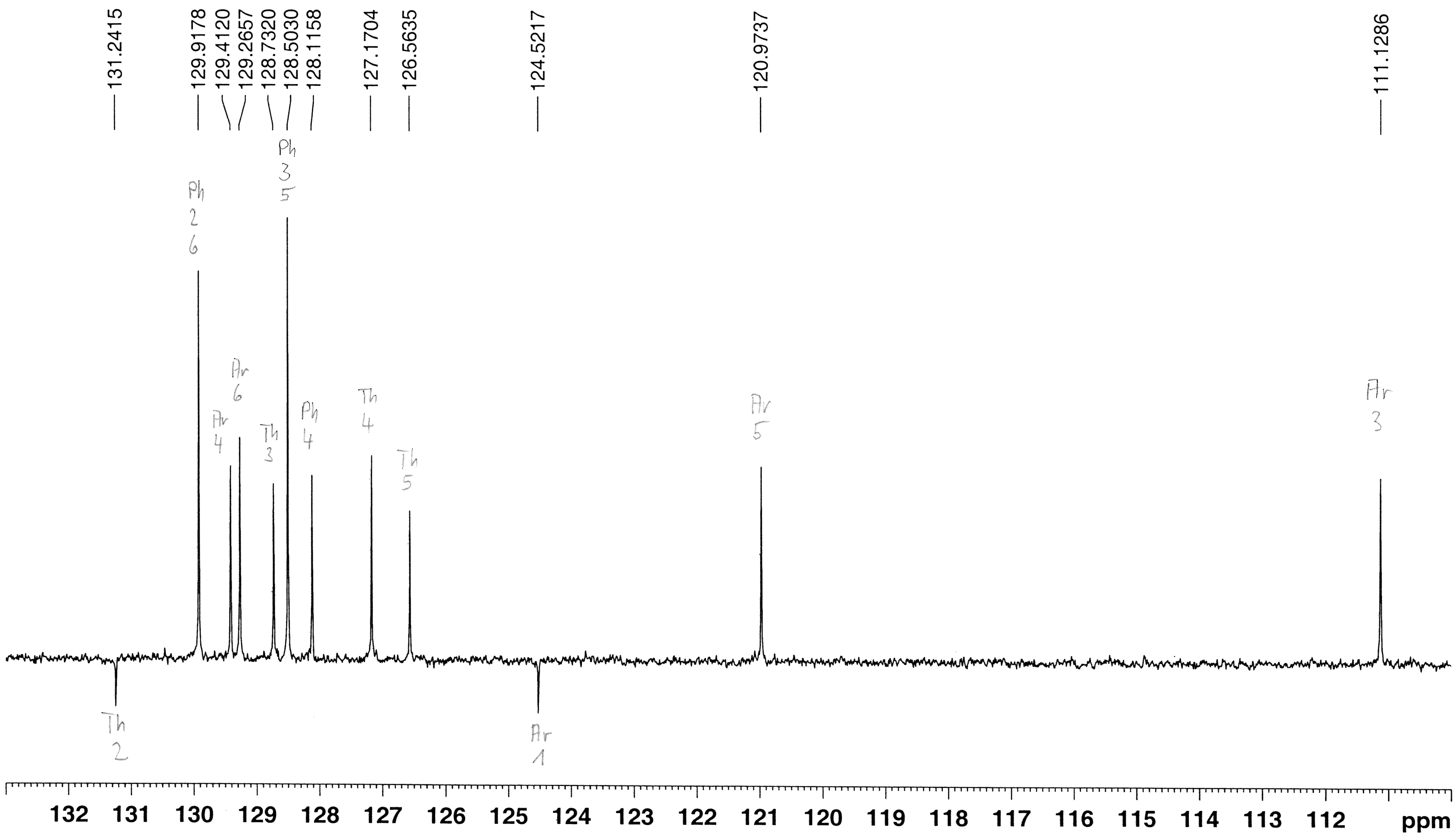

CE166_1_2 in cdcl3 (APT) 13.8.2019

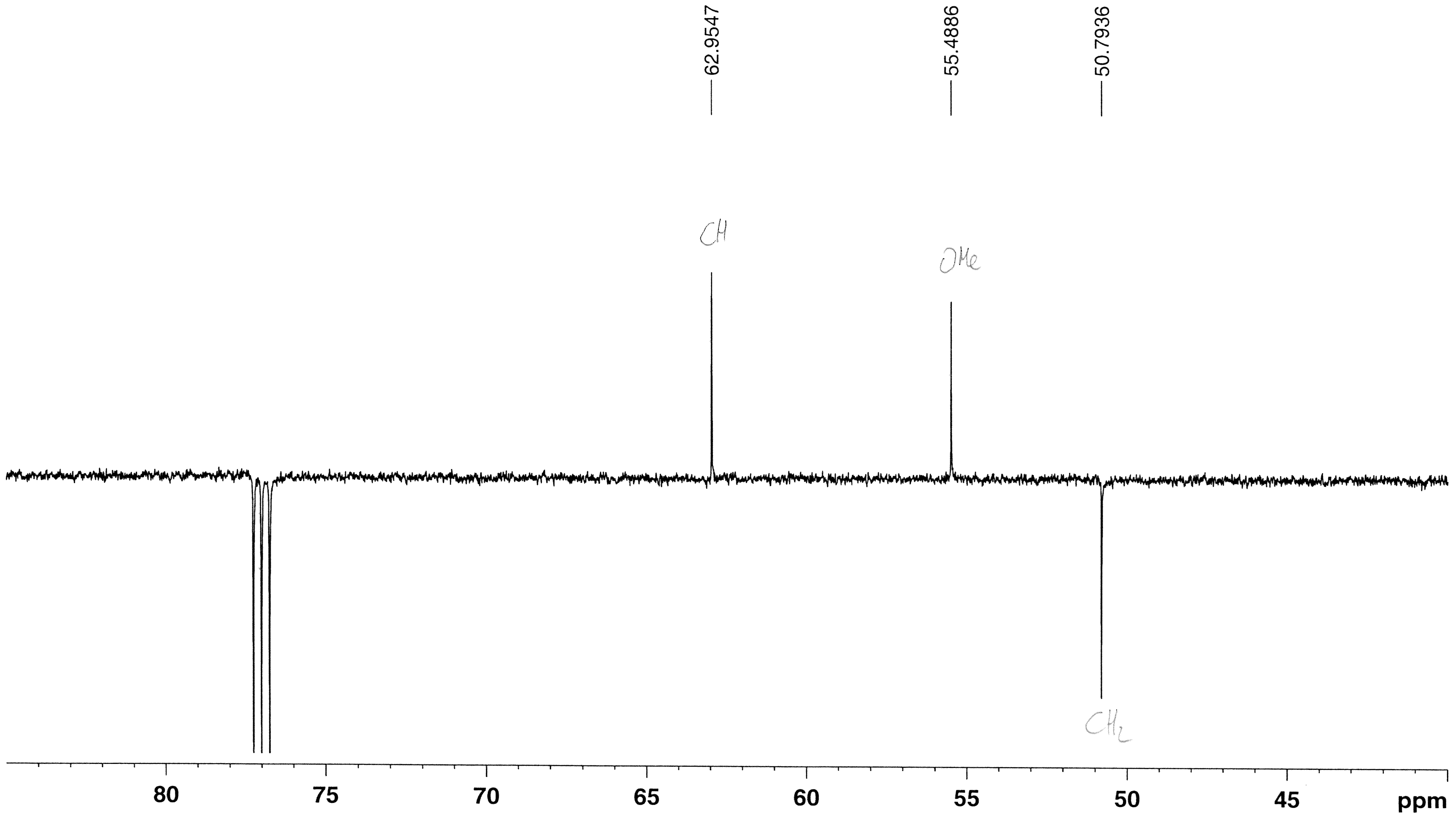

Figure S71c. NMR spectra of compound **7r.**

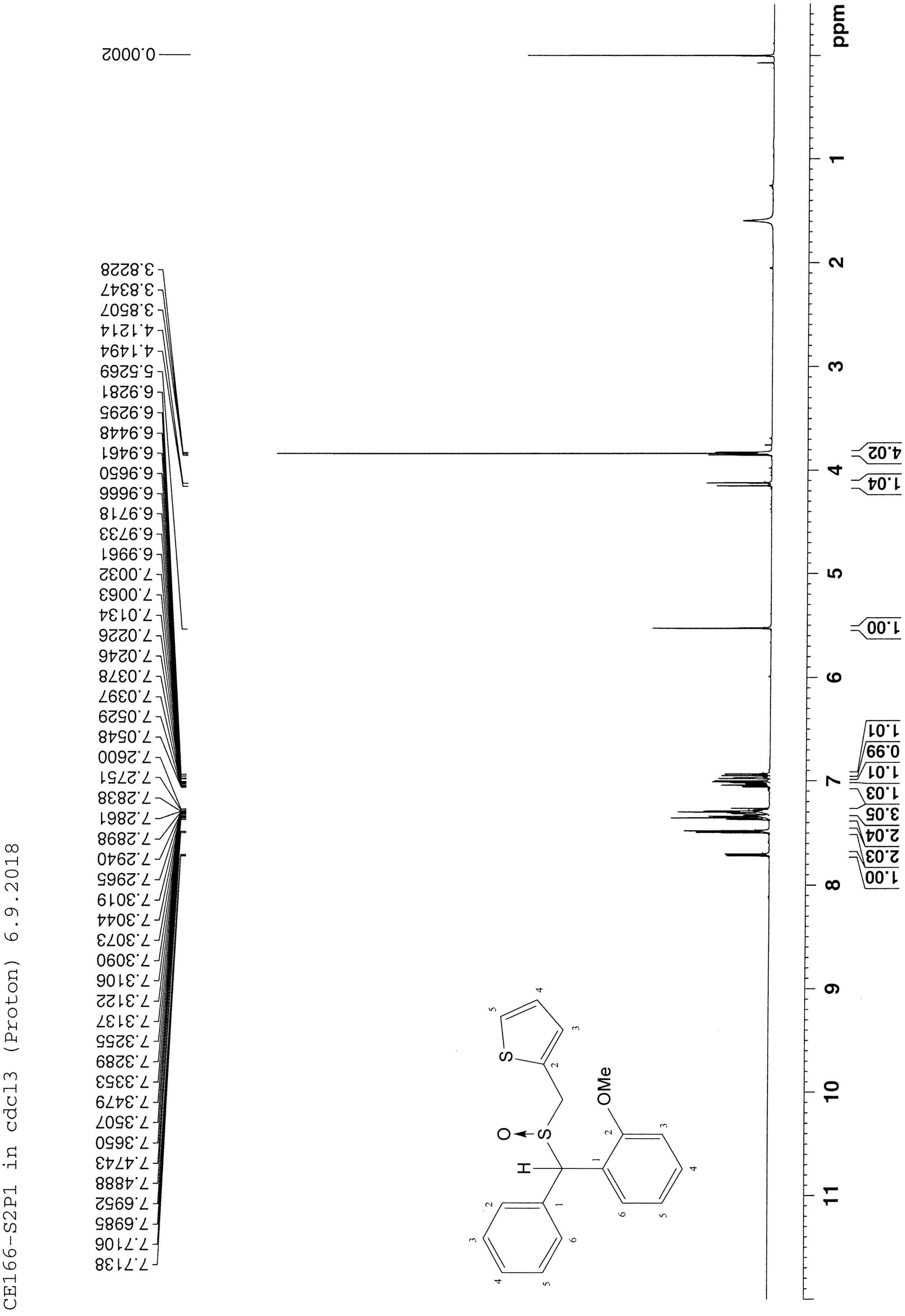

CE166-S2P1 in cdcl3 (Proton) 6.9.2018

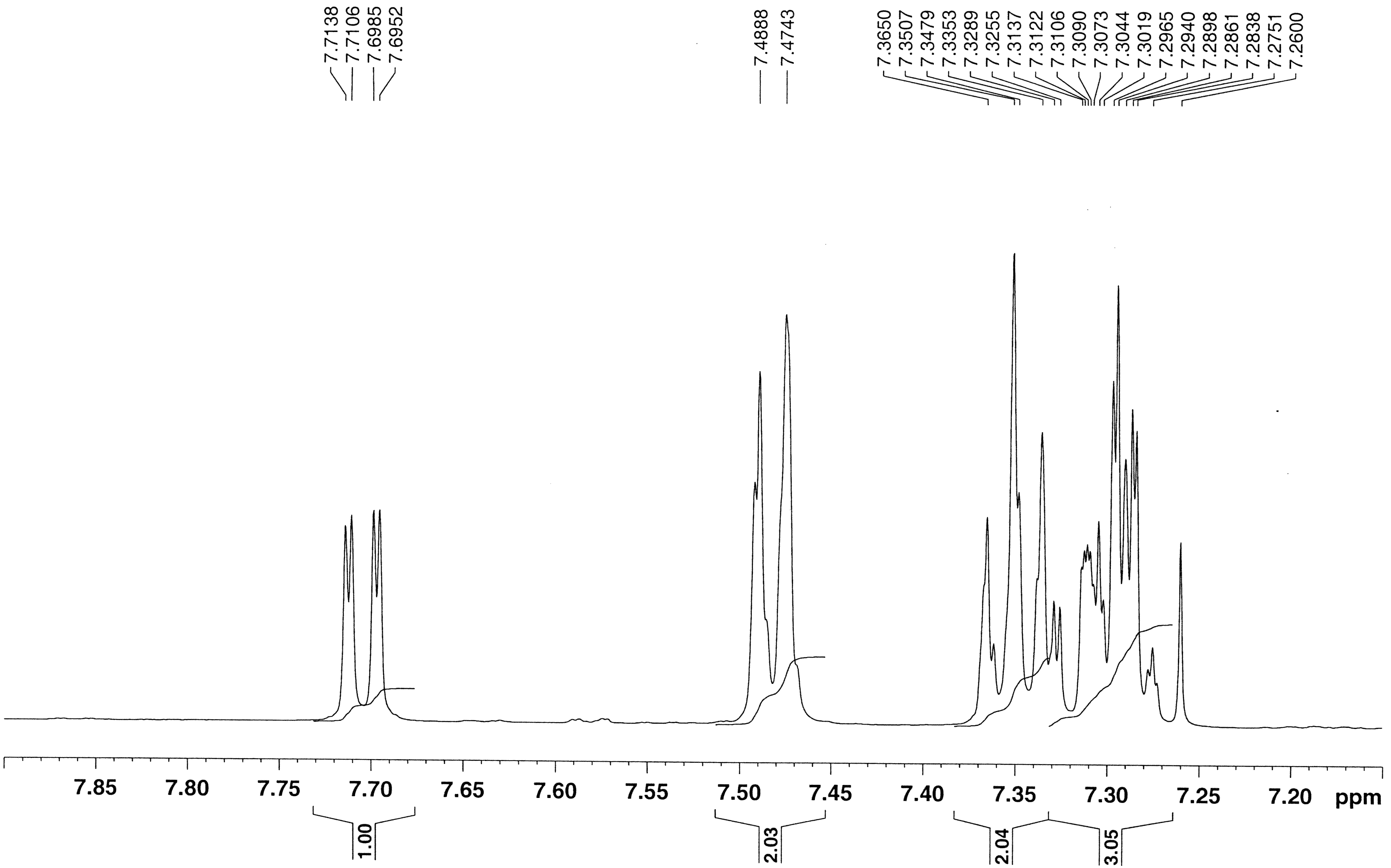

CE166-S2P1 in cdcl3 (Proton) 6.9.2018

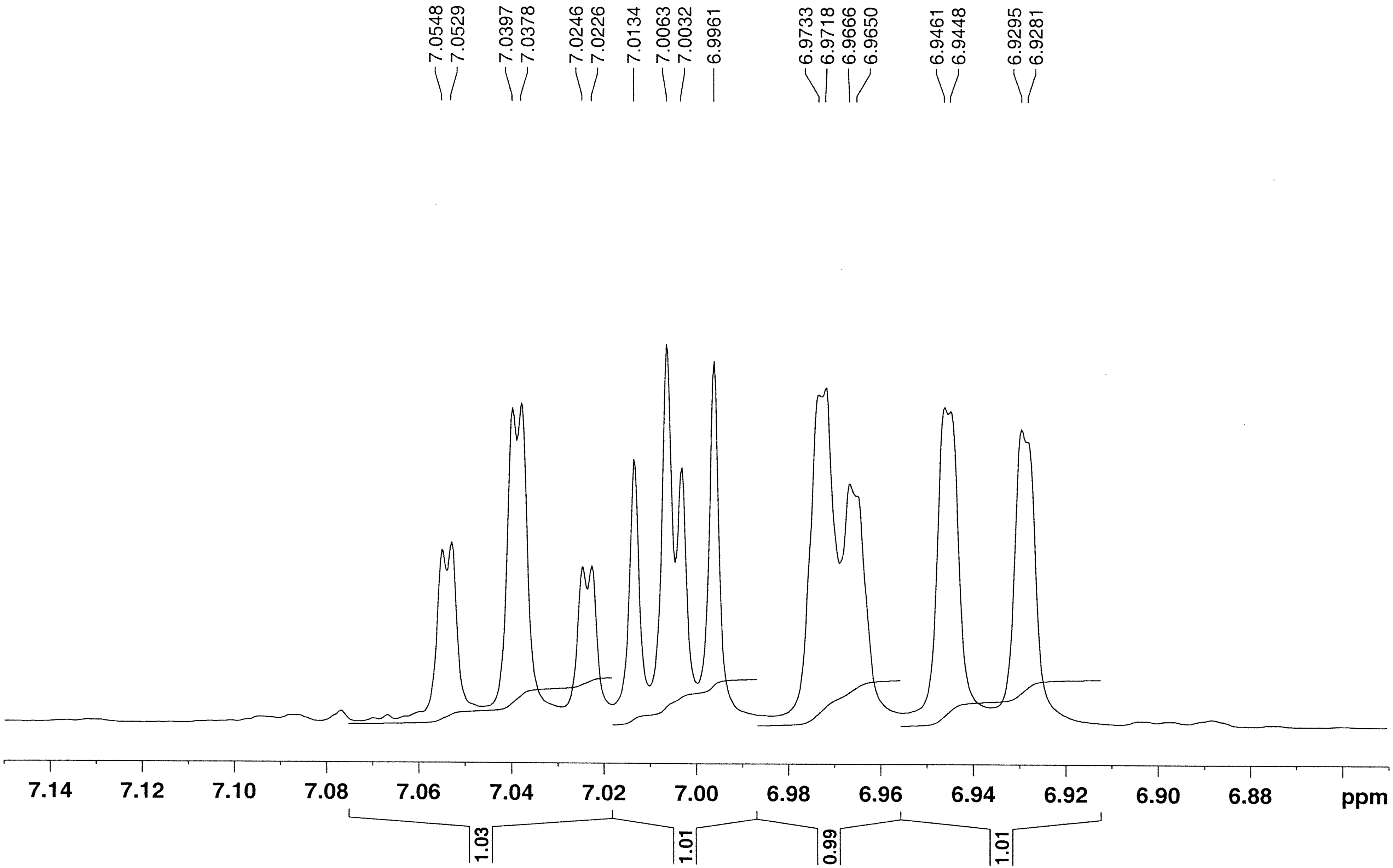

CE166-S2P1 in cdcl3 (Proton) 6.9.2018

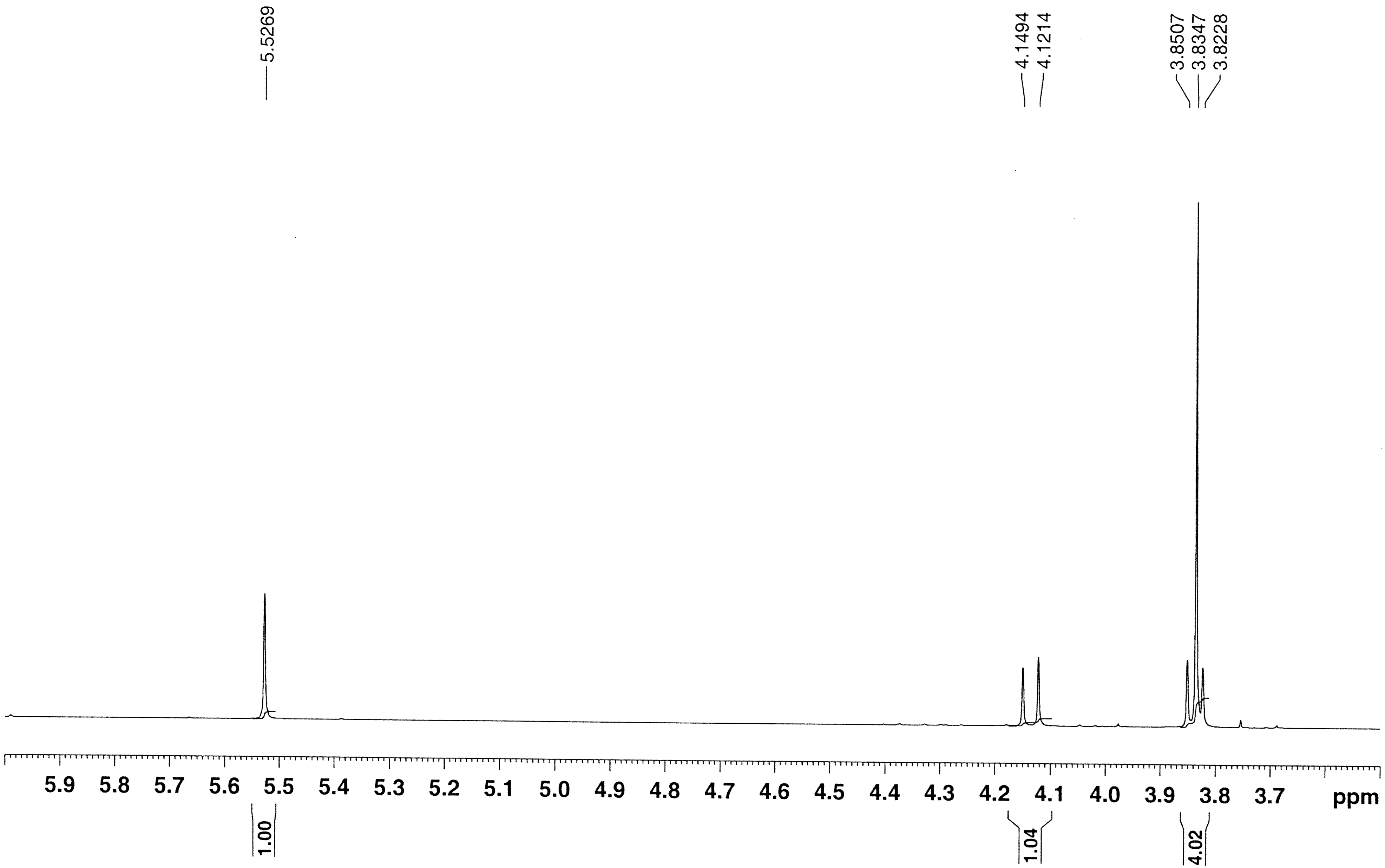

CE166-S2P1 in cdcl3 (APT) 6.9.2018

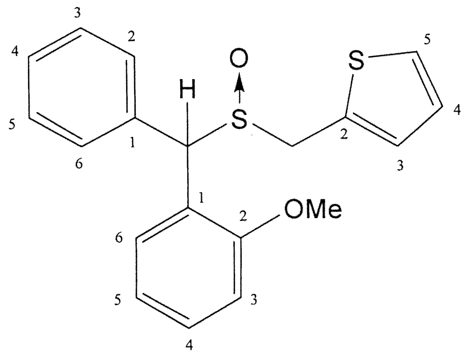

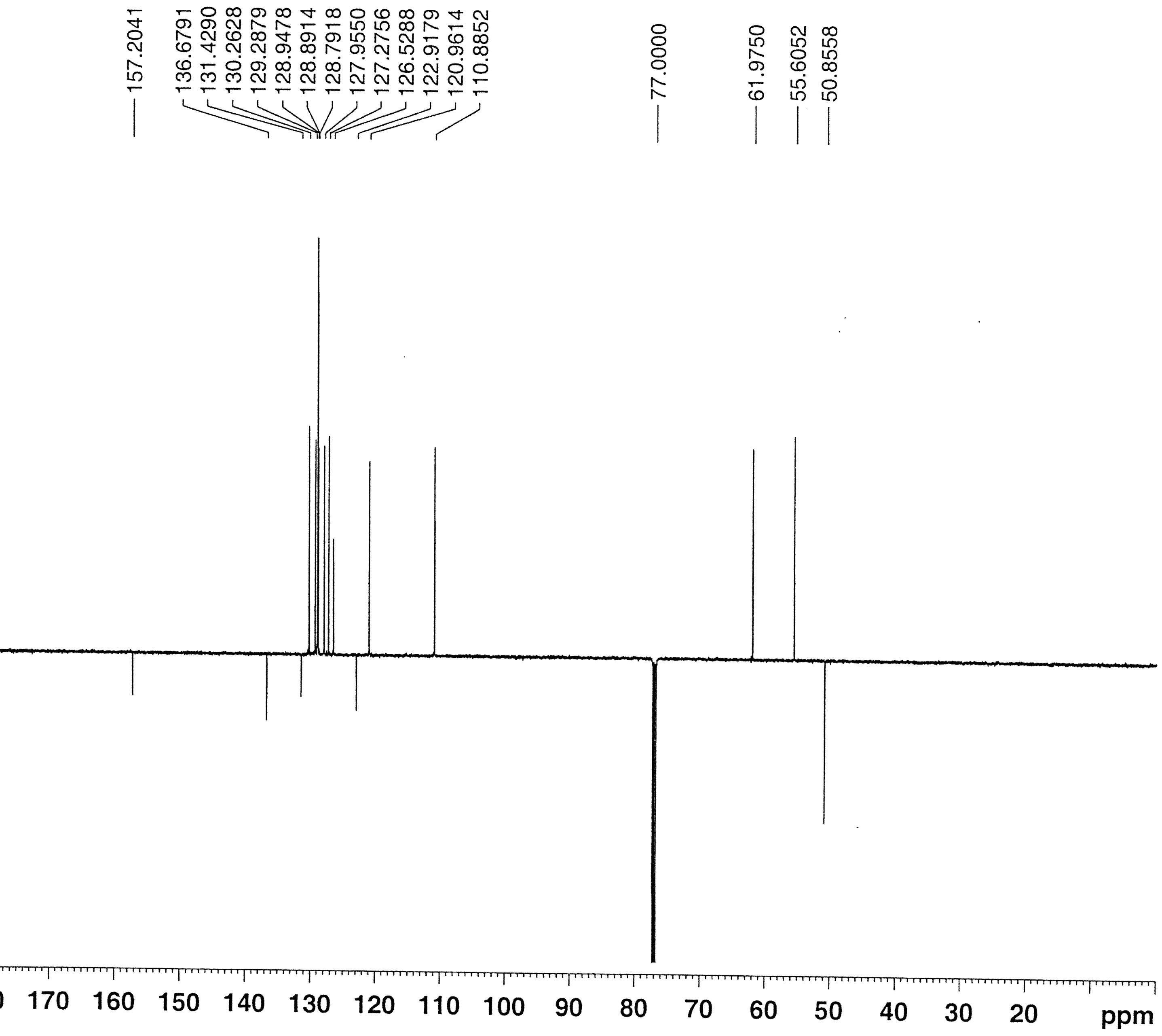

CE166-S2P1 in cdcl3 (APT) 6.9.2018

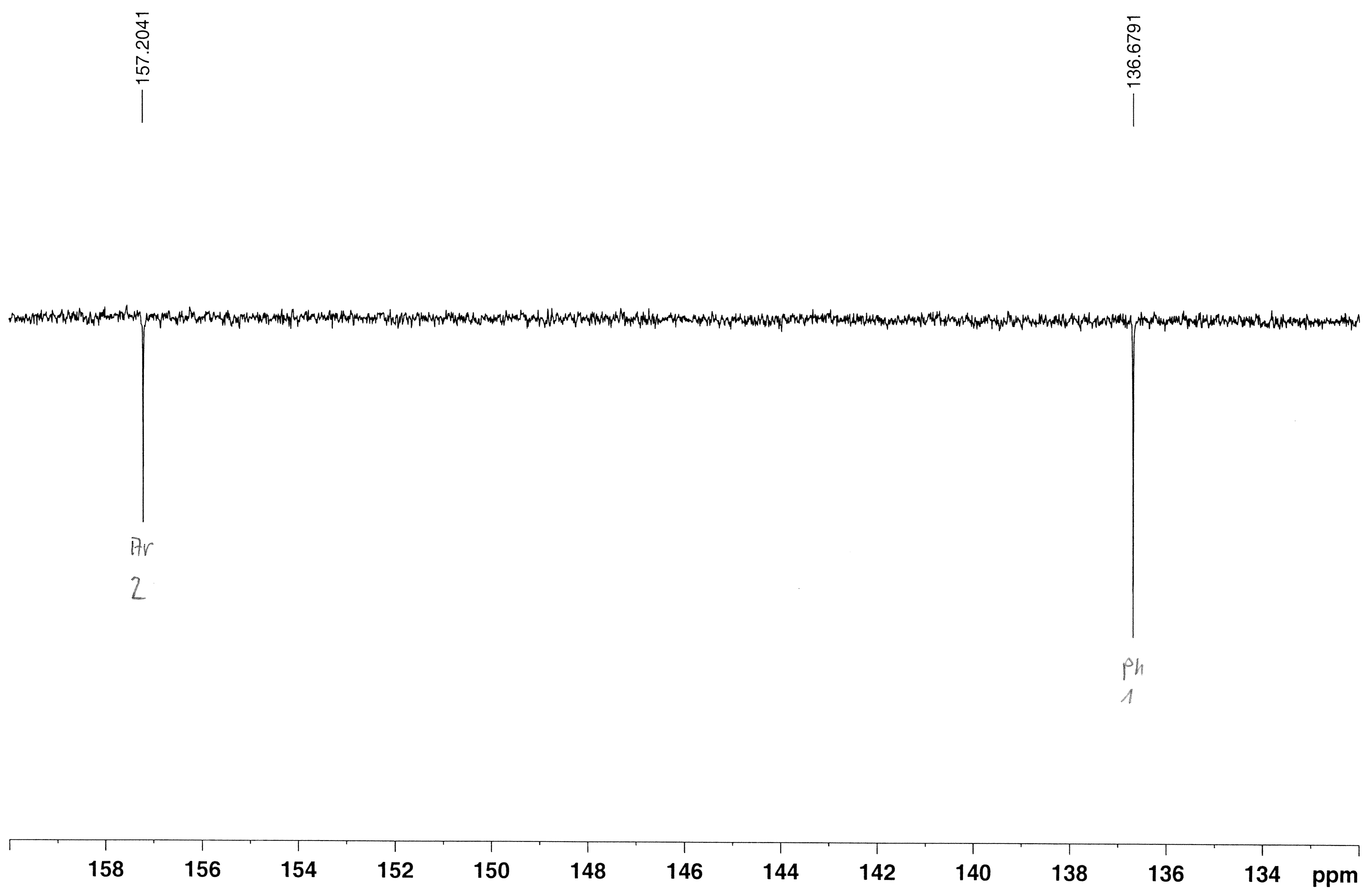

CE166-S2P1 in cdcl3 (APT) 6.9.2018

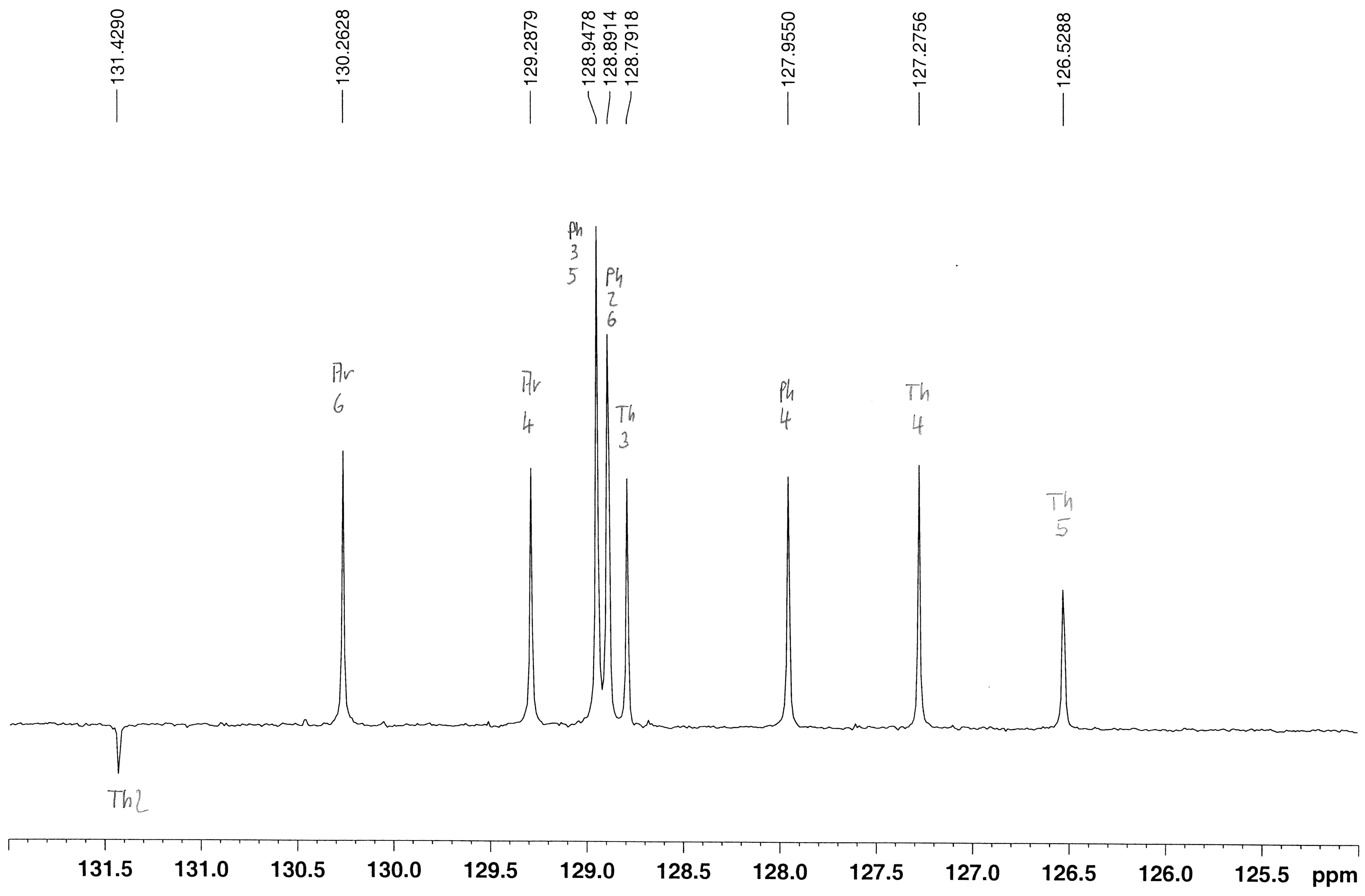

CE166-S2P1 in cdcl3 (APT) 6.9.2018

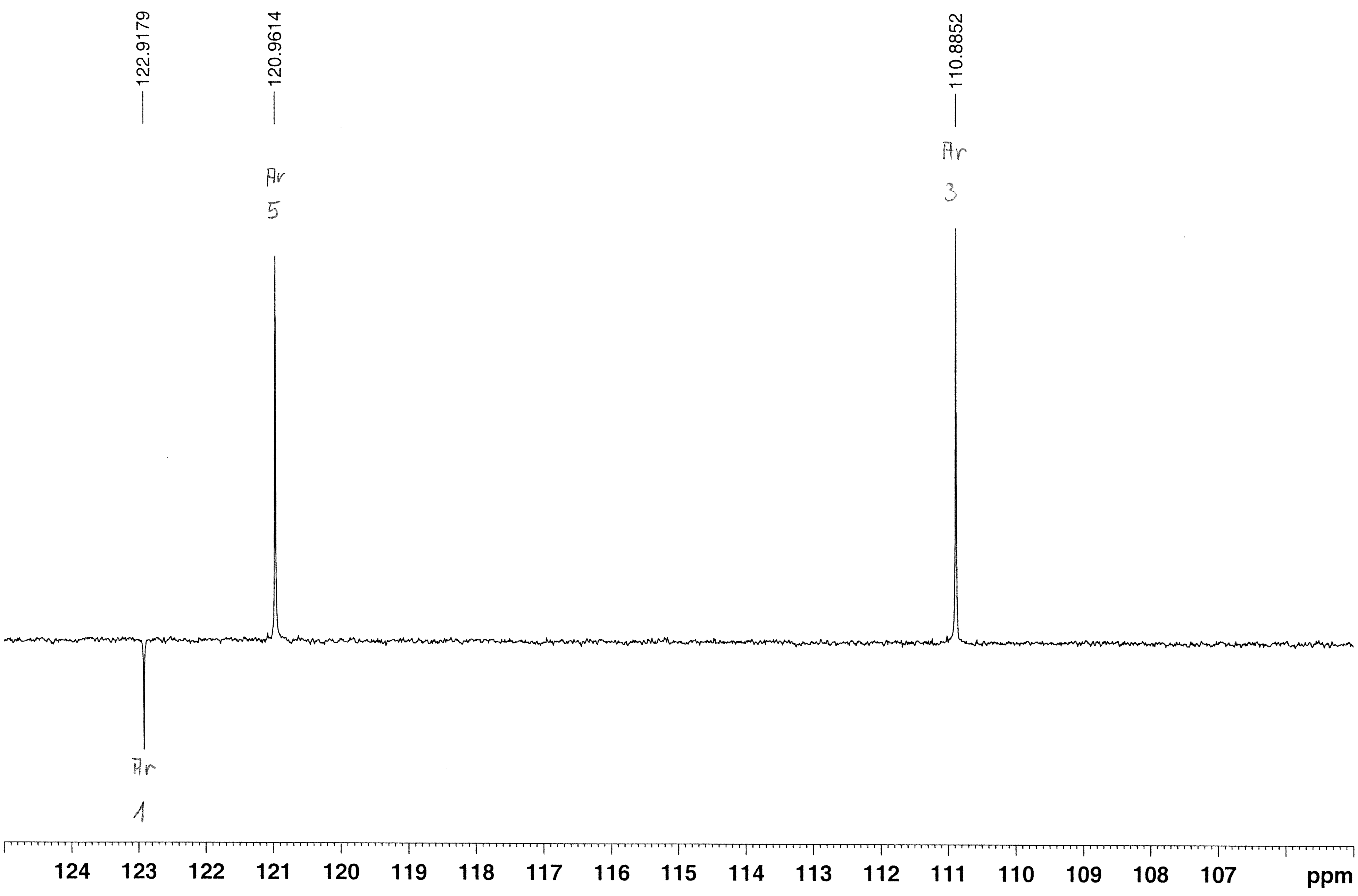

CE166-S2P1 in cdcl3 (APT) 6.9.2018

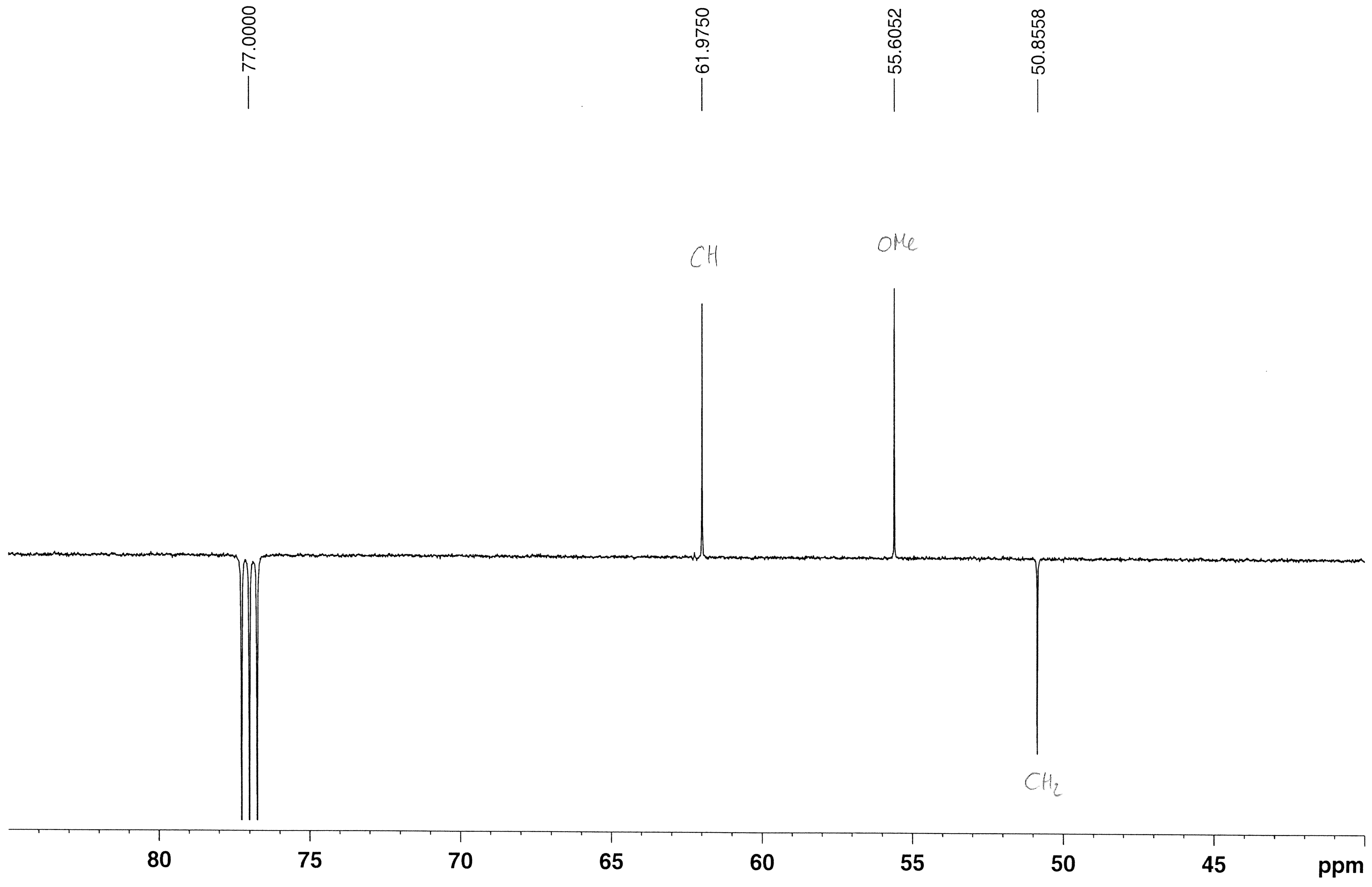

Figure S72c. NMR spectra of compound **8r**.

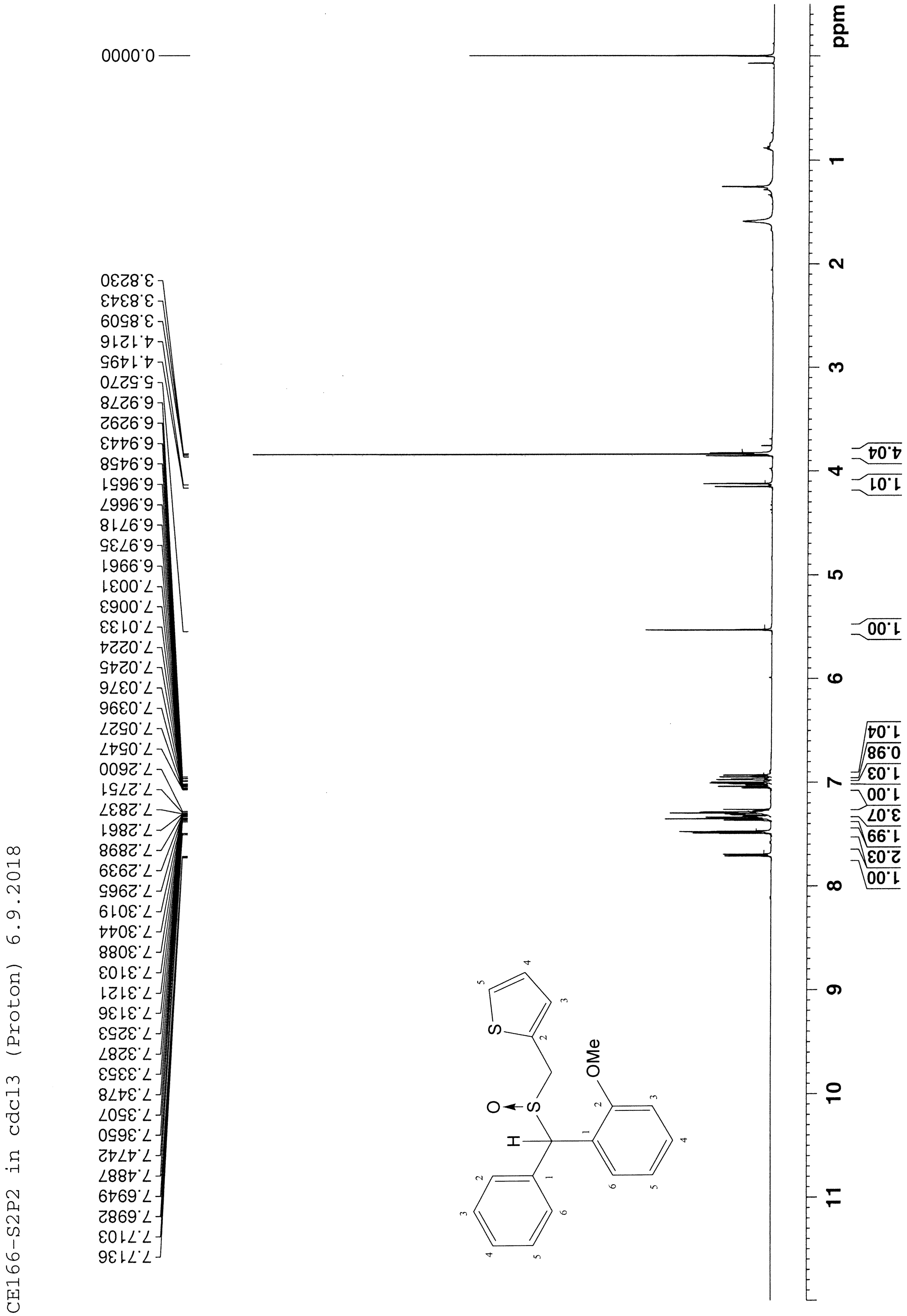

CE166-S2P2 in cdcl3 (Proton) 6.9.2018

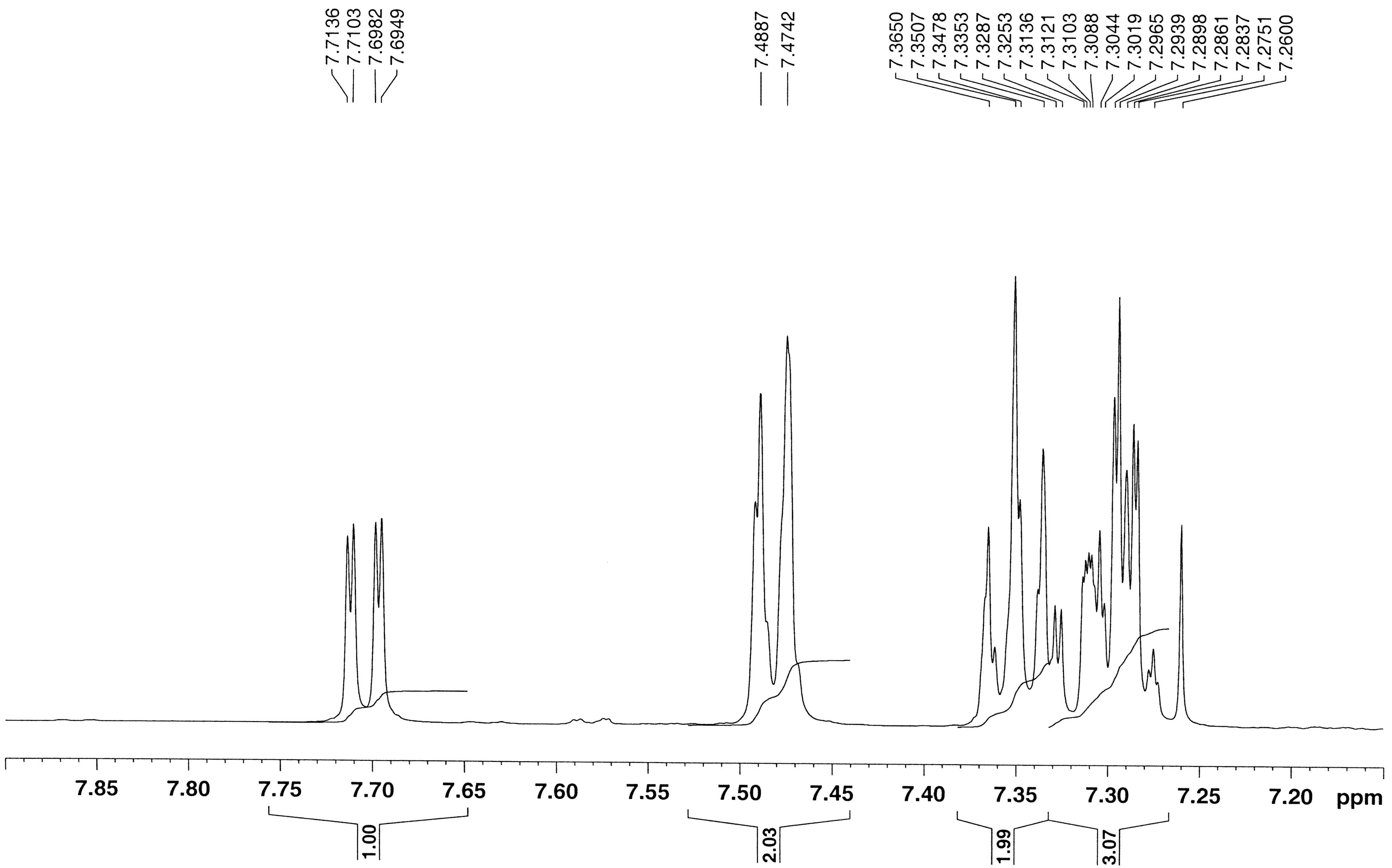

CE166-S2P2 in cdcl3 (Proton) 6.9.2018

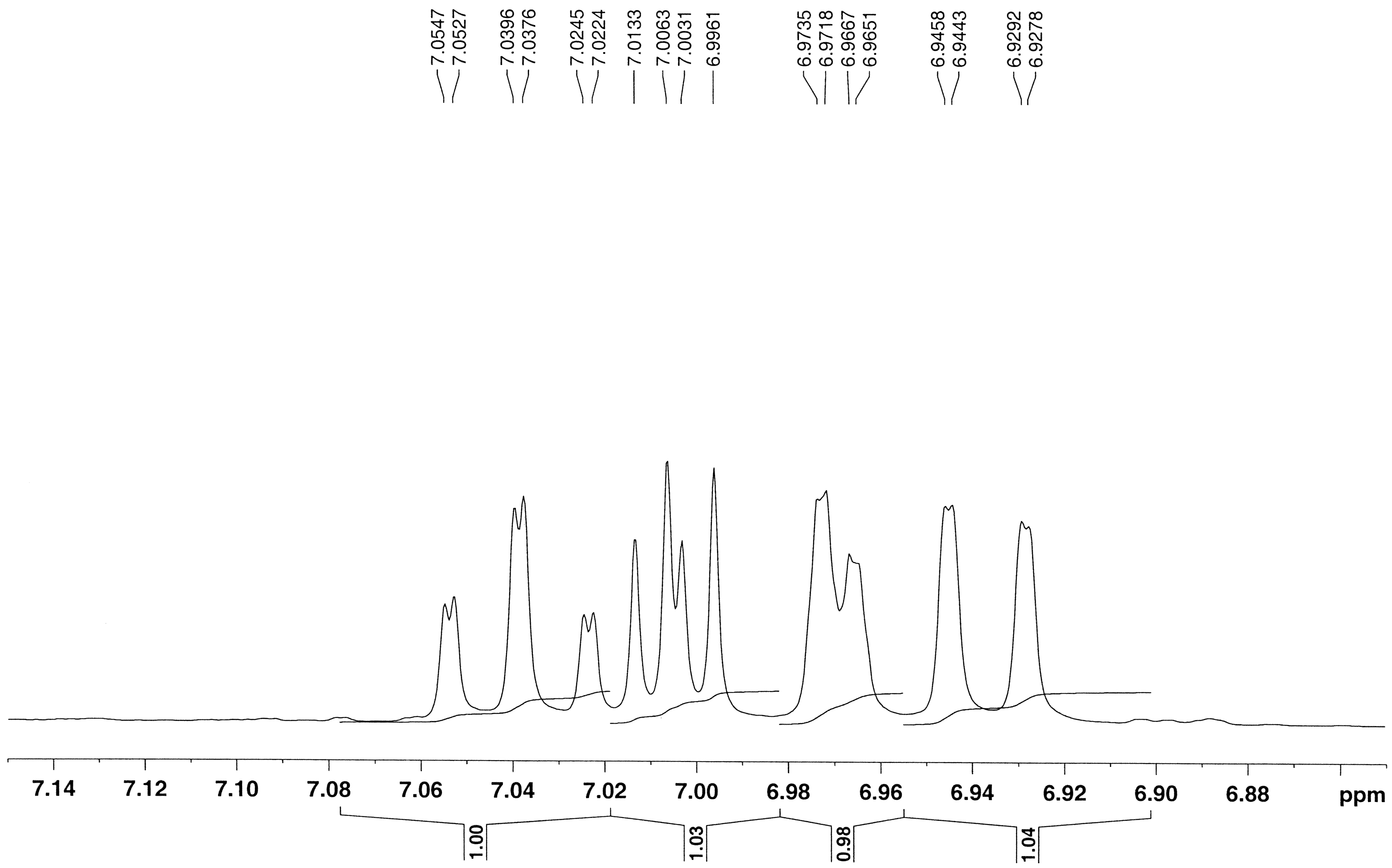

CE166-S2P2 in cdcl3 (Proton) 6.9.2018

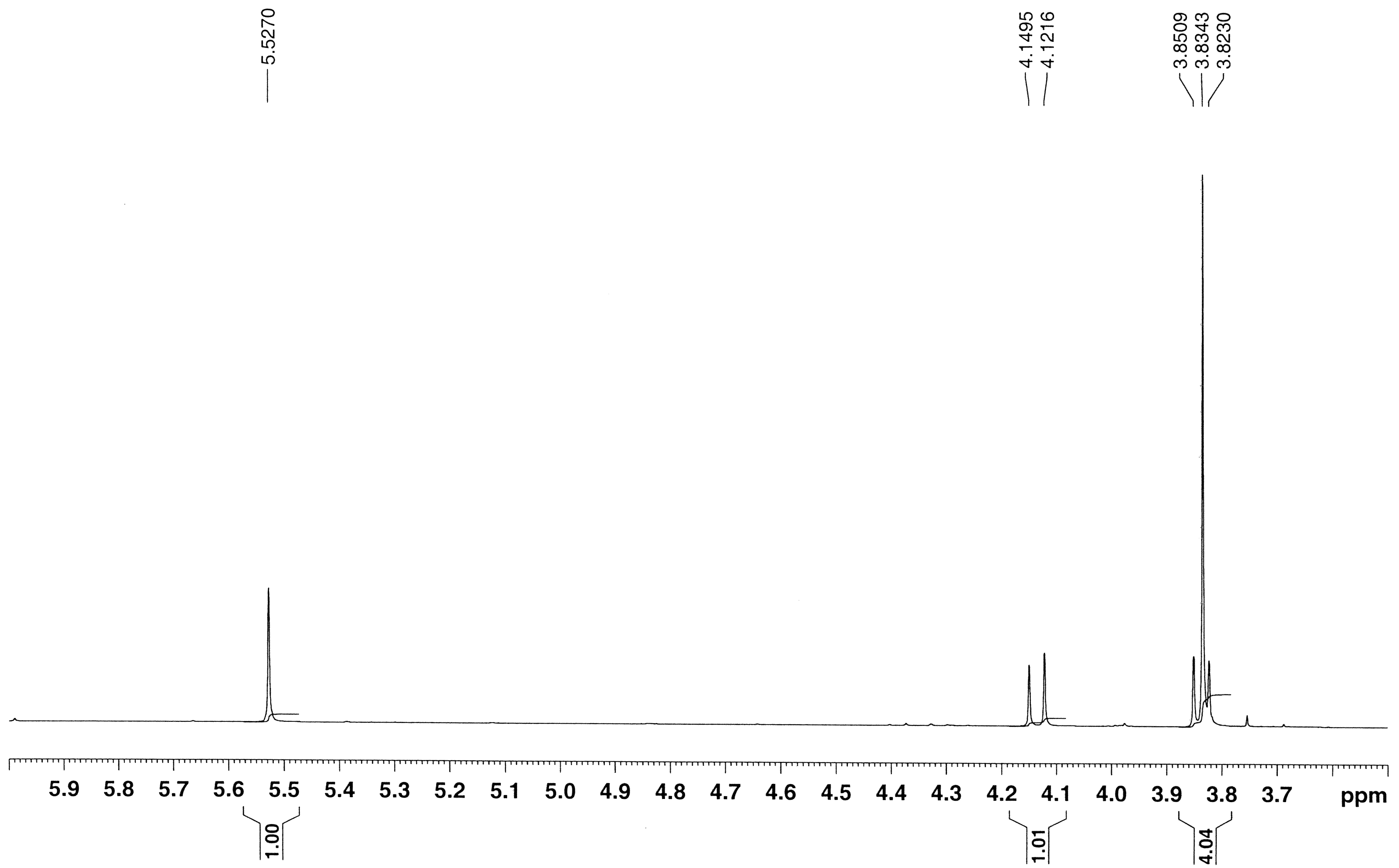

CE166-S2P2 in cdcl3 (APT) 6.9.2018

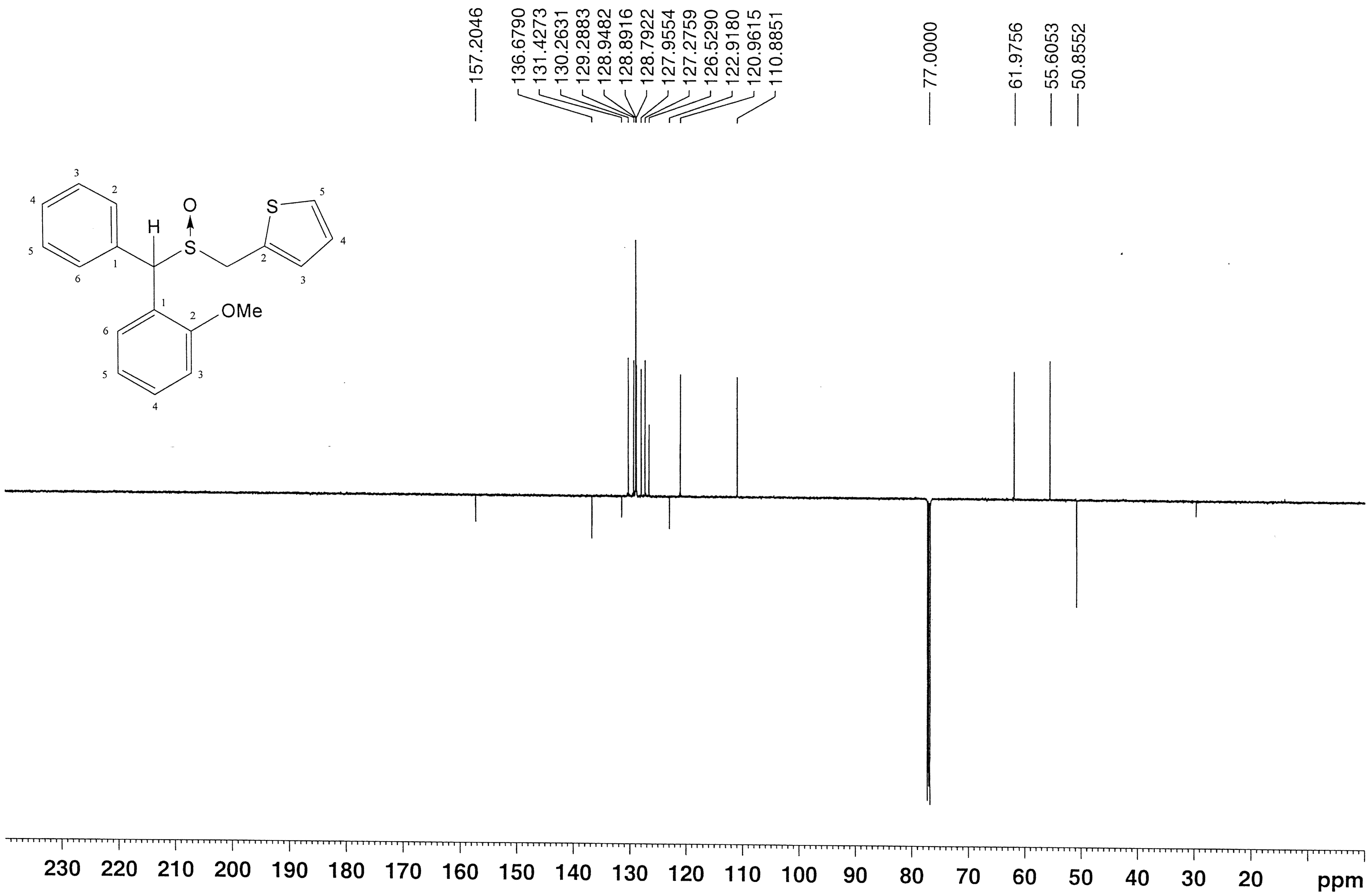

CE166-S2P2 in cdcl3 (APT) 6.9.2018

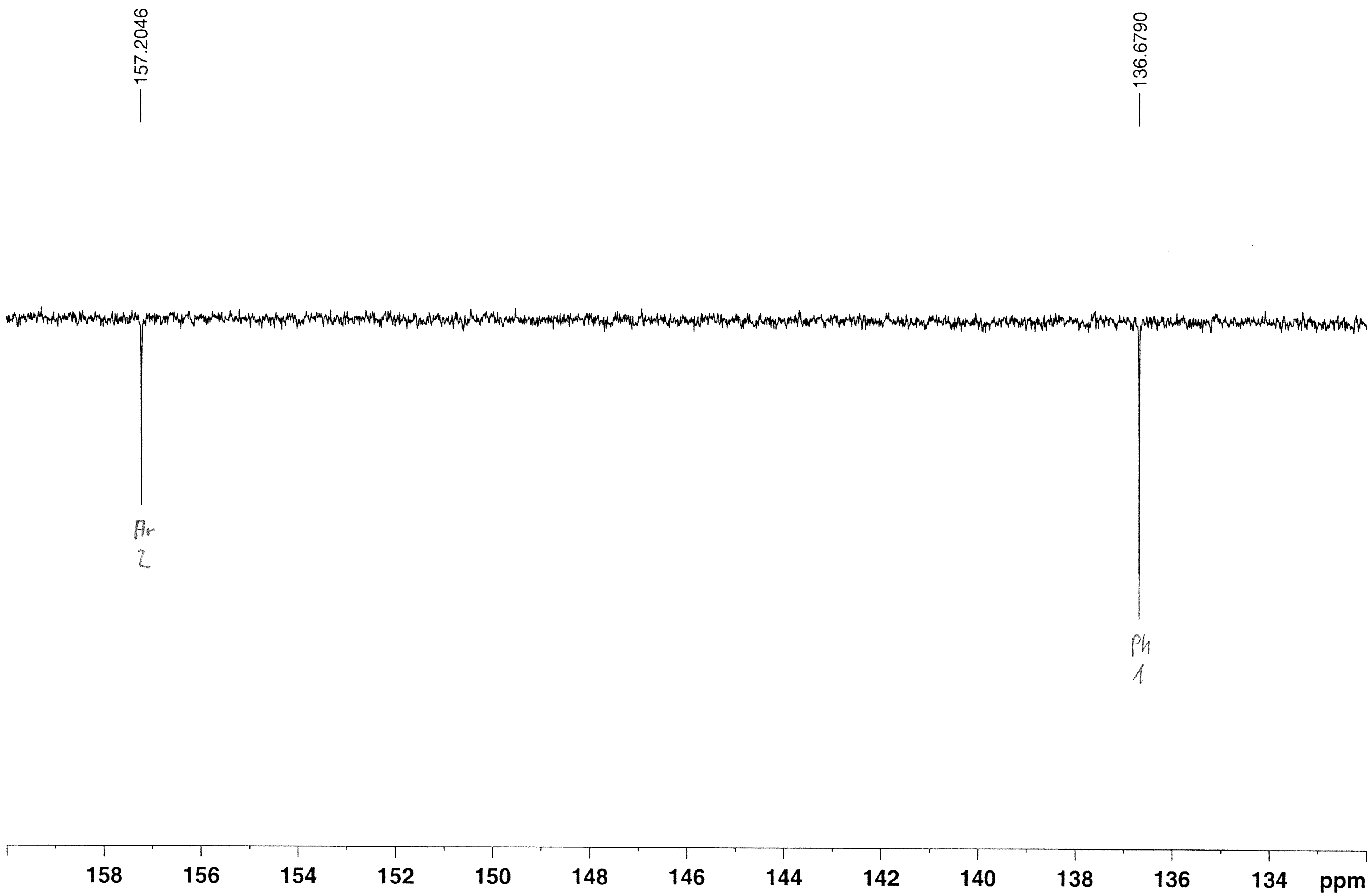

CE166-S2P2 in cdcl3 (APT) 6.9.2018

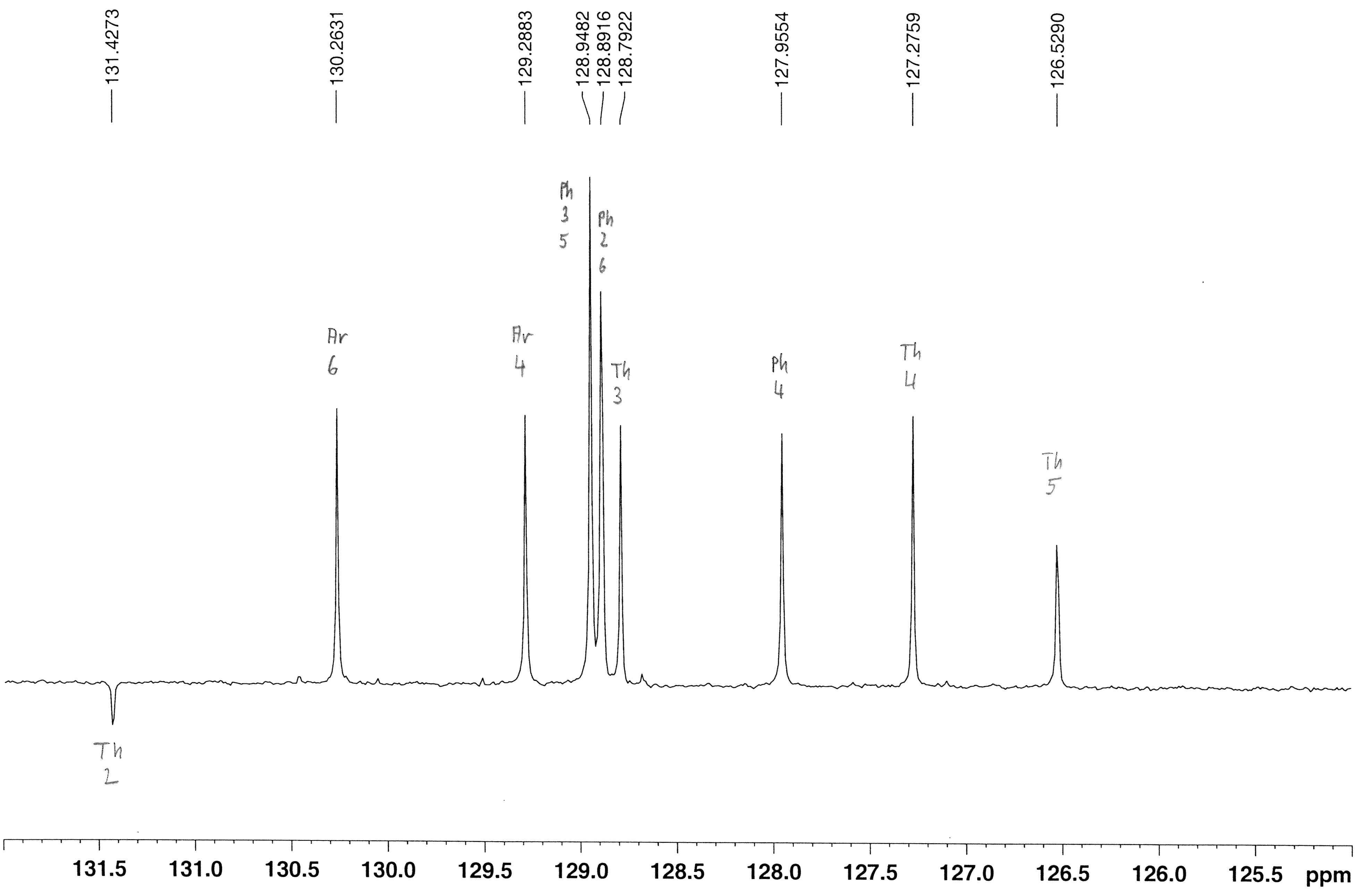

CE166-S2P2 in cdcl3 (APT) 6.9.2018

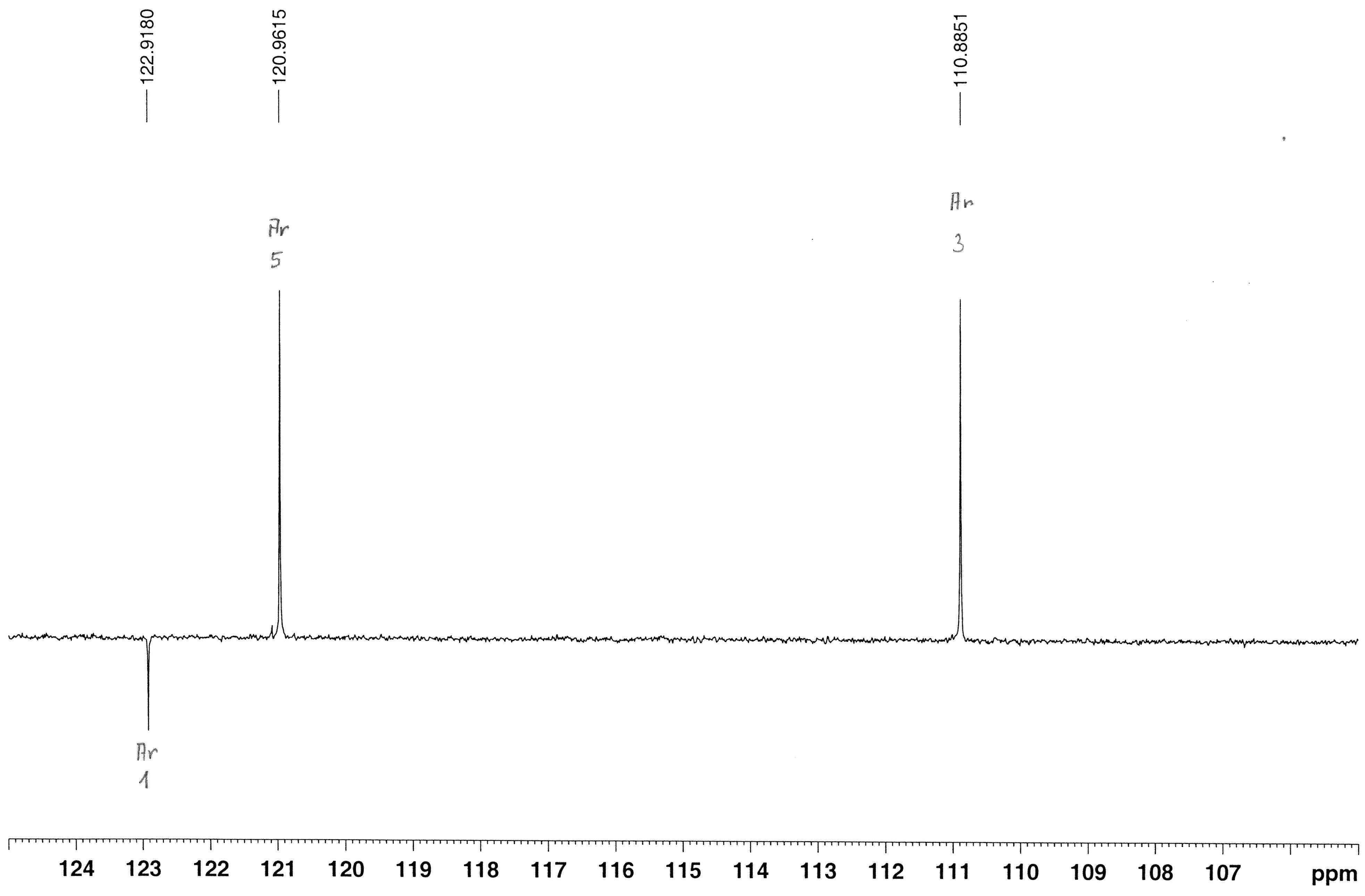

CE166-S2P2 in cdcl3 (APT) 6.9.2018

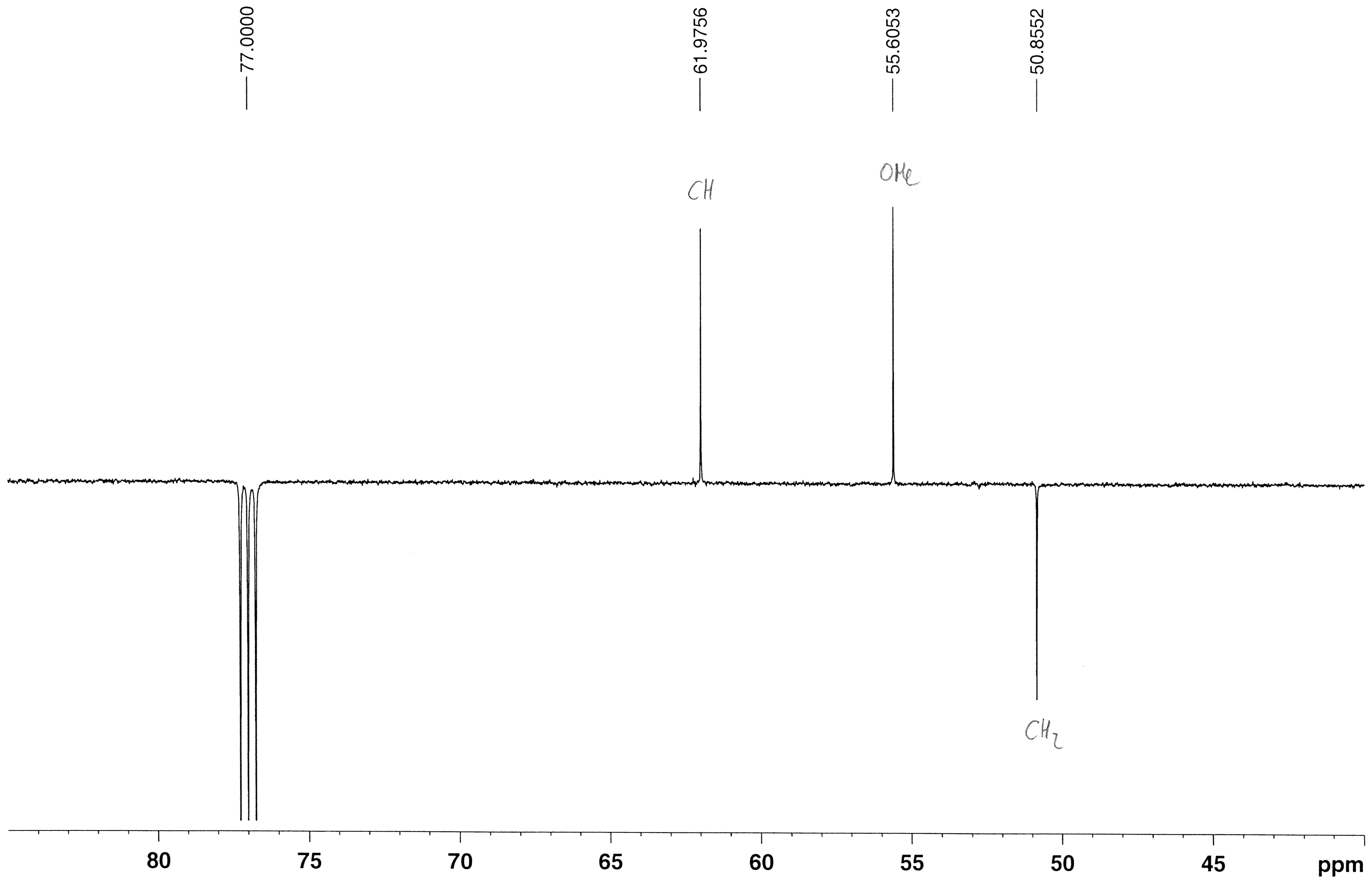

Figure S73c. NMR spectra of compound **5s.**

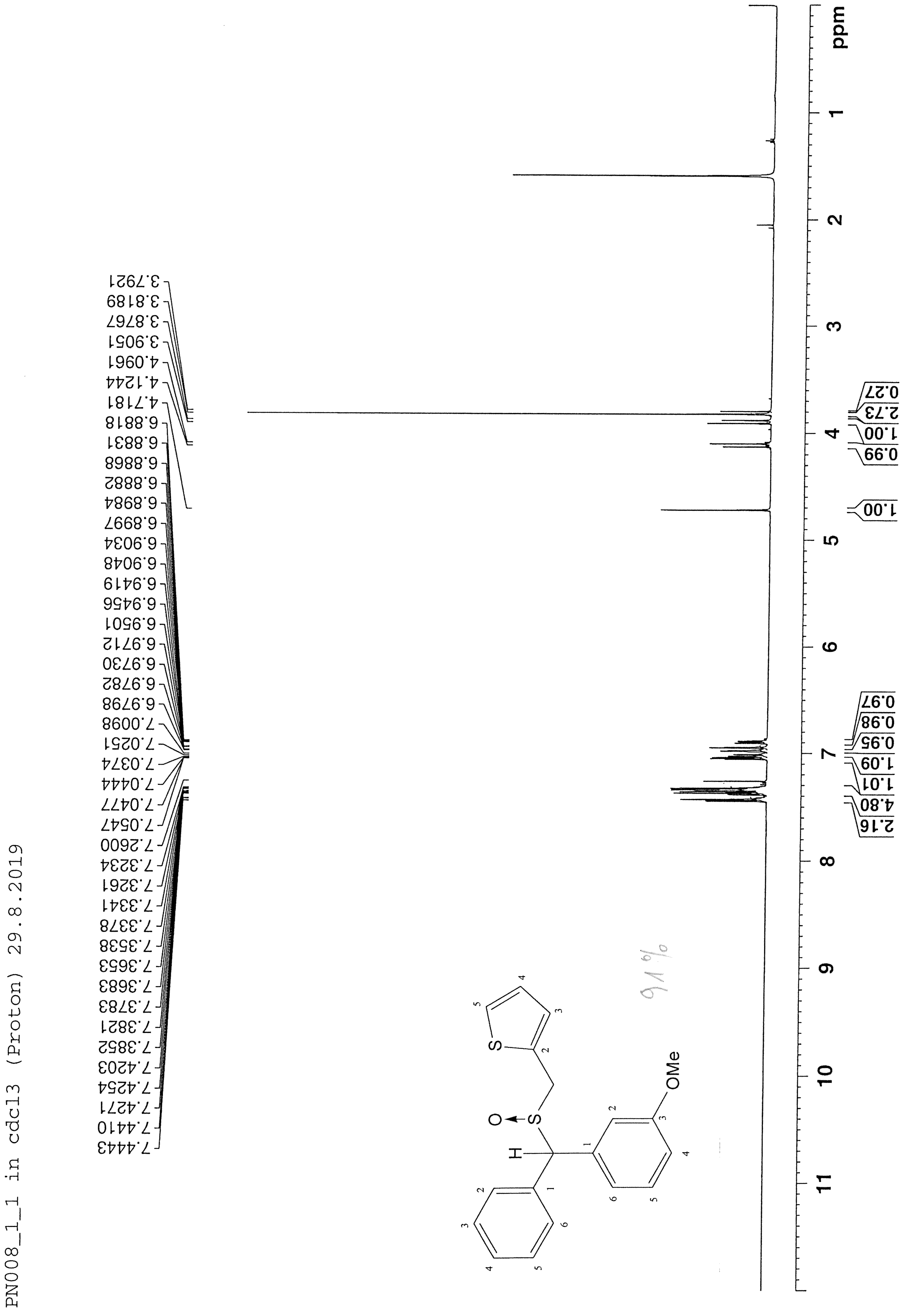

PN008_1_1 in cdcl3 (Proton) 29.8.2019

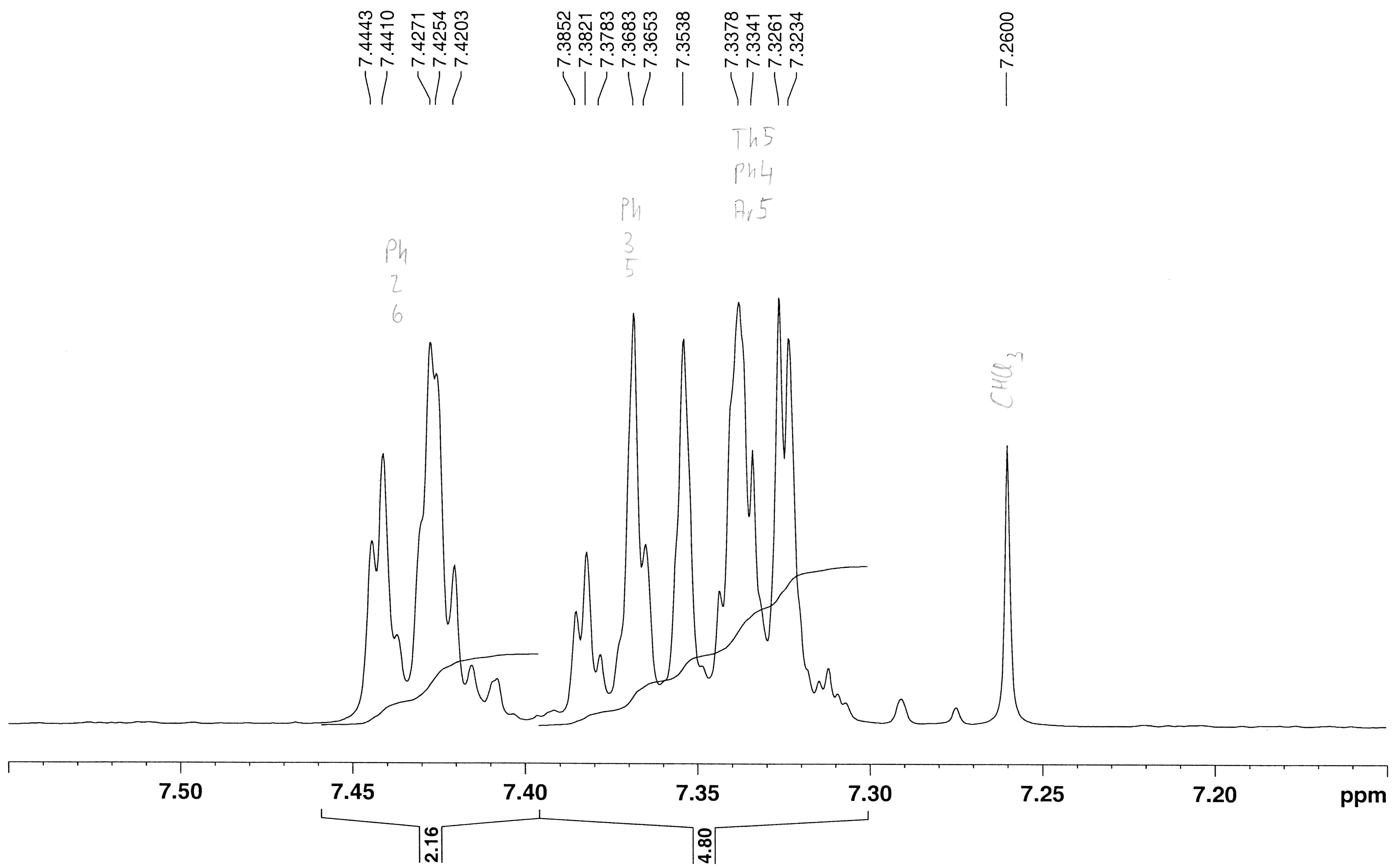

PN008_1_1 in cdcl3 (Proton) 29.8.2019

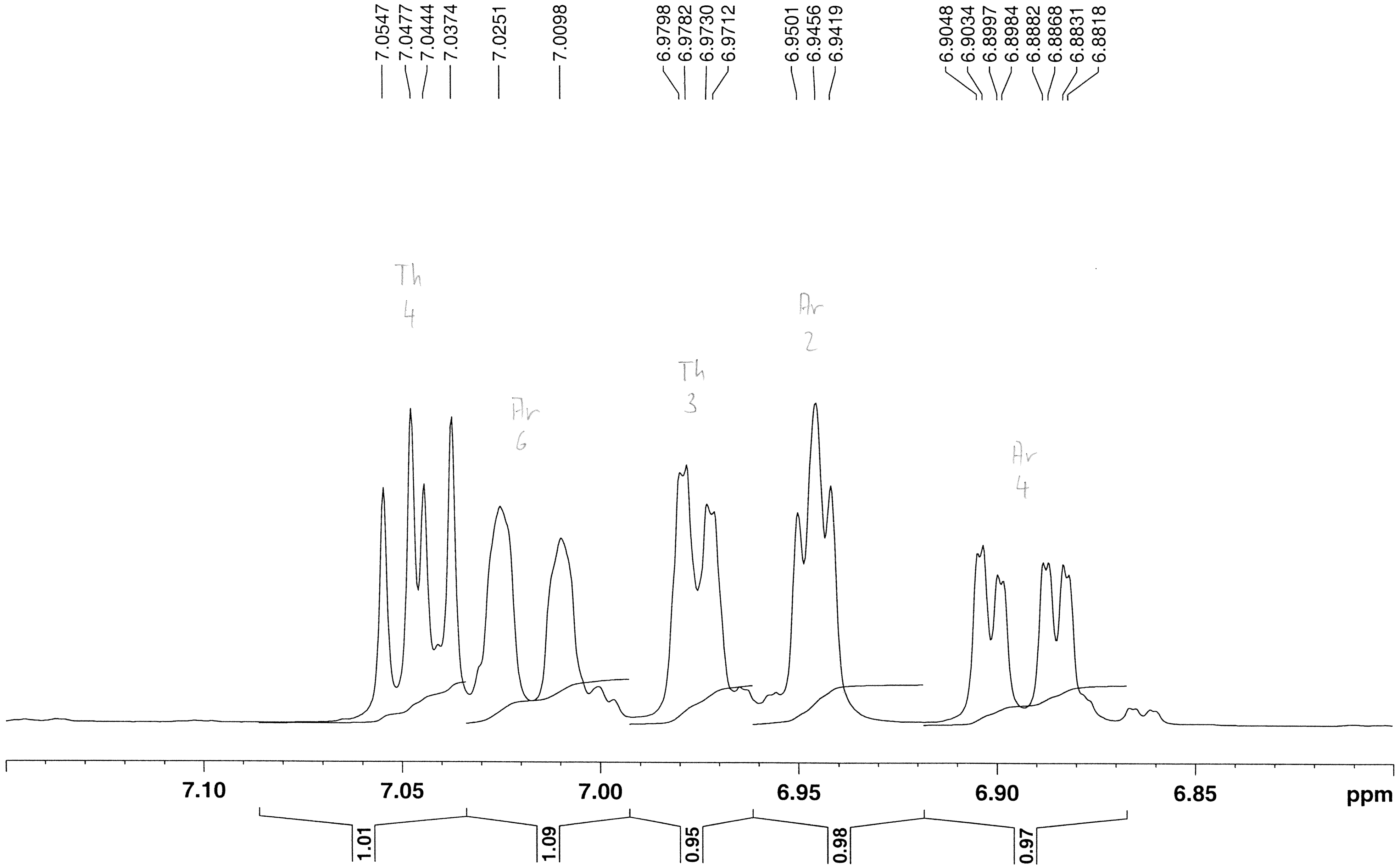

PN008_1_1 in cdcl3 (Proton) 29.8.2019

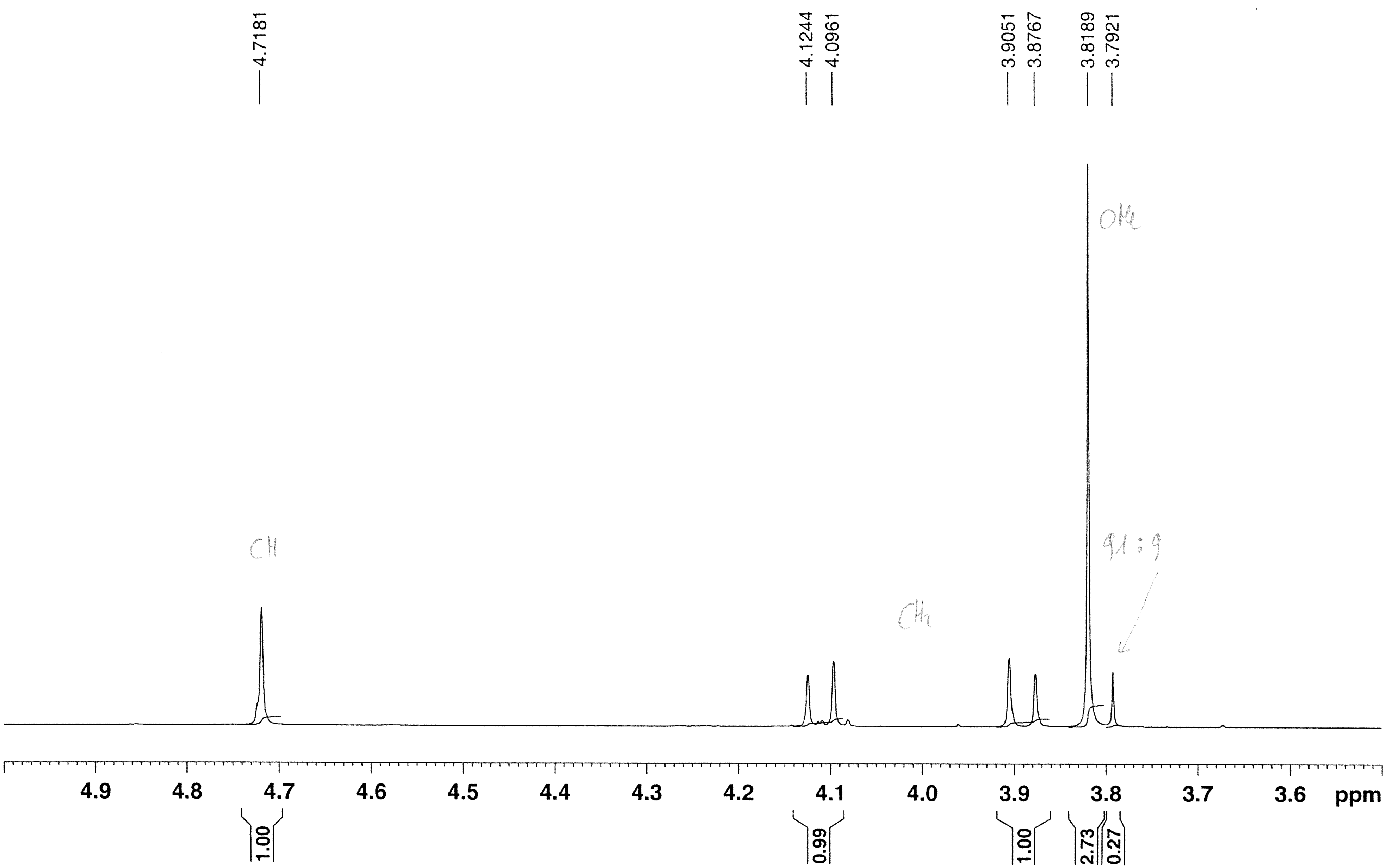

PN008_1_1 in cdcl3 (APT) 29.8.2019

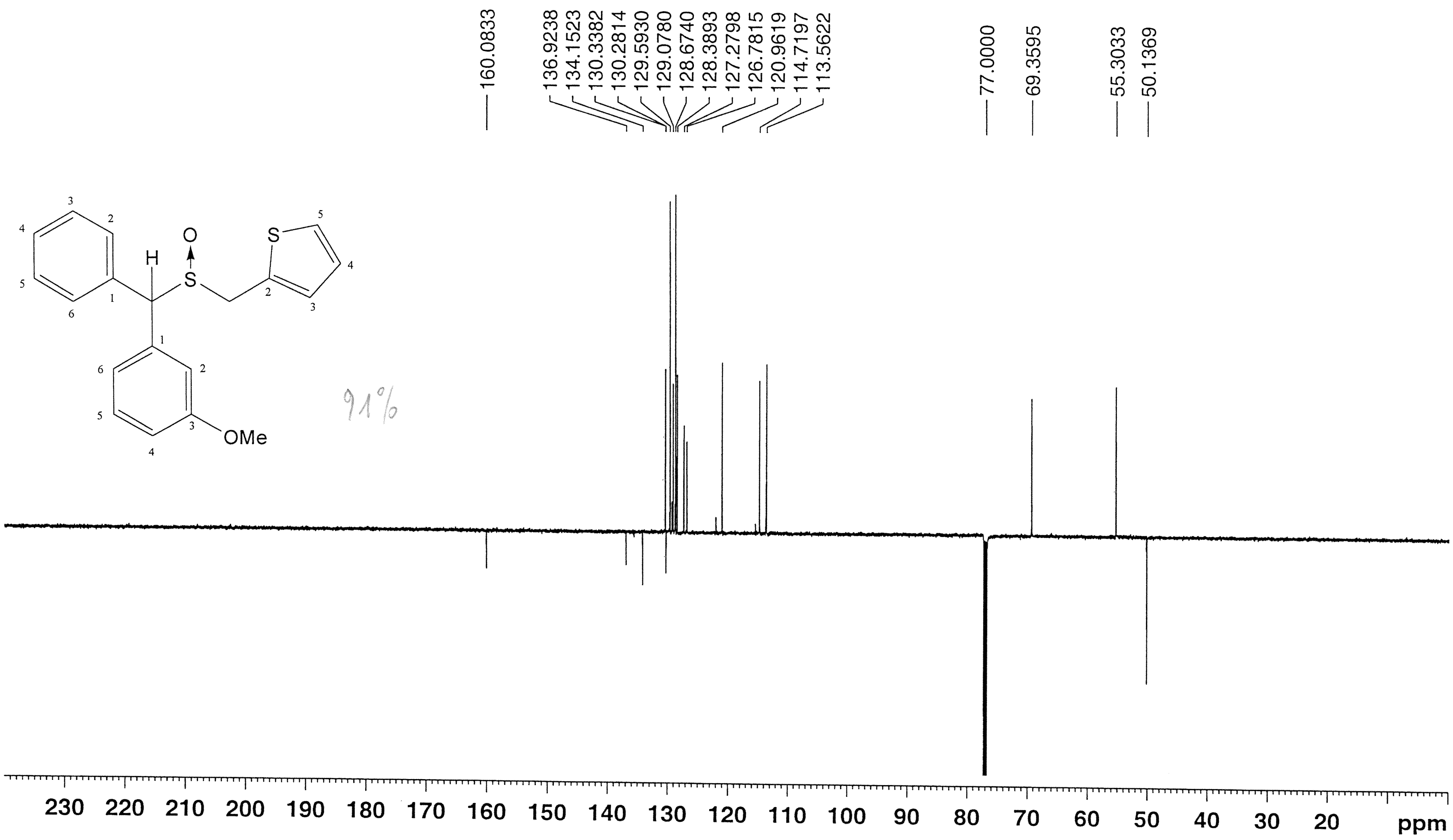

PN008_1_1 in cdcl3 (APT) 29.8.2019

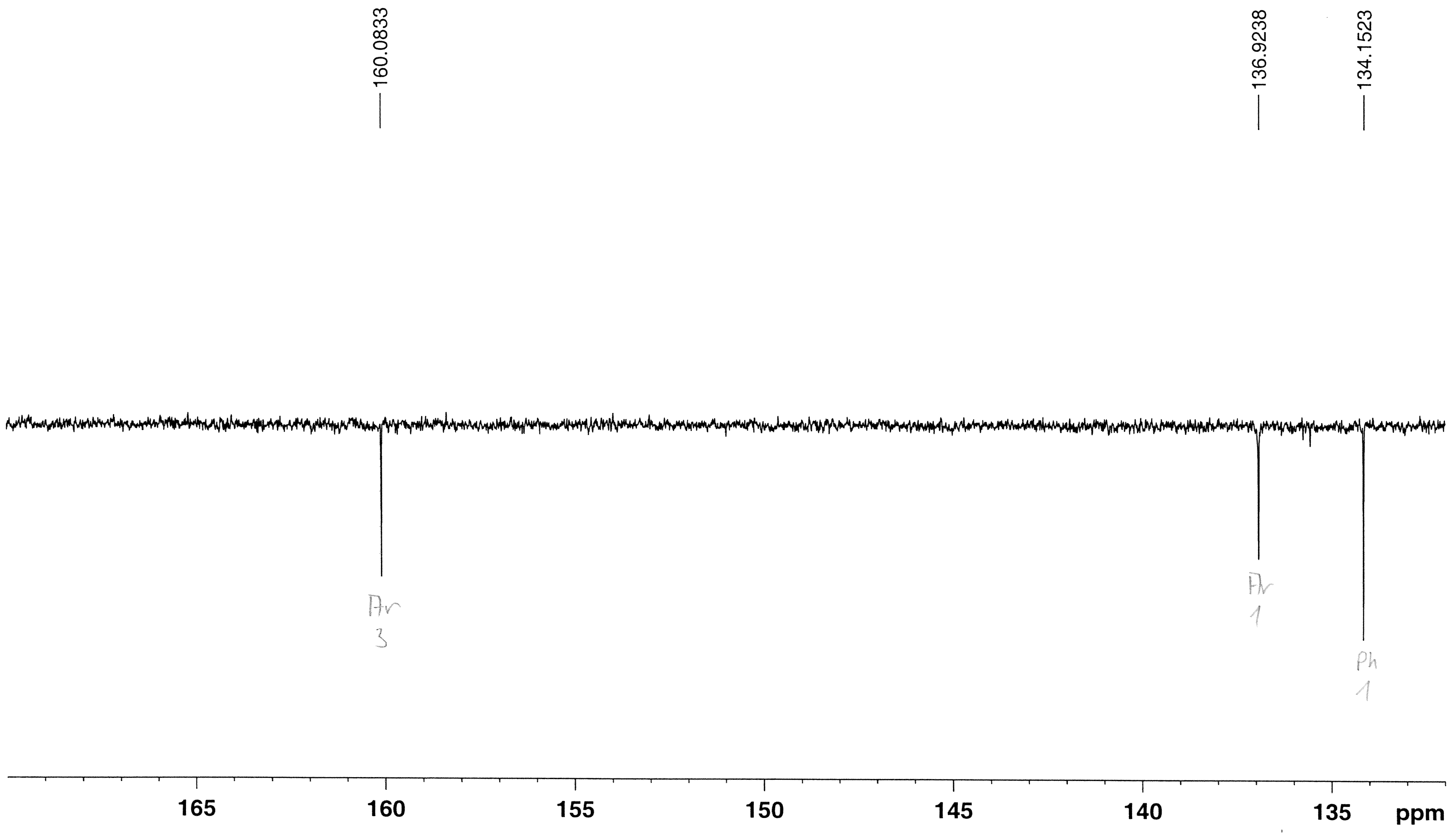

PN008_1_1 in cdcl3 (APT) 29.8.2019

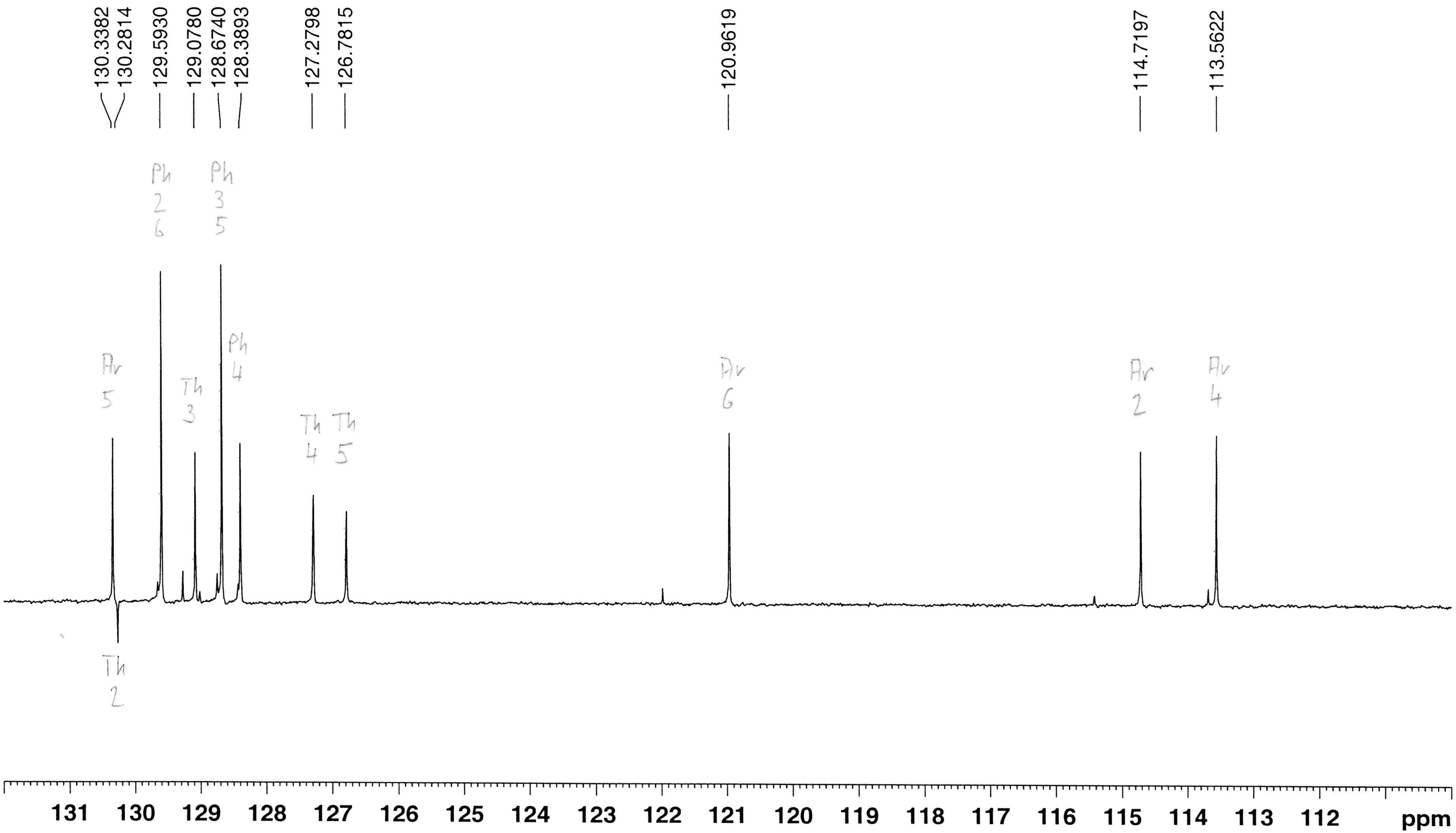

PN008_1_1 in cdcl3 (APT) 29.8.2019

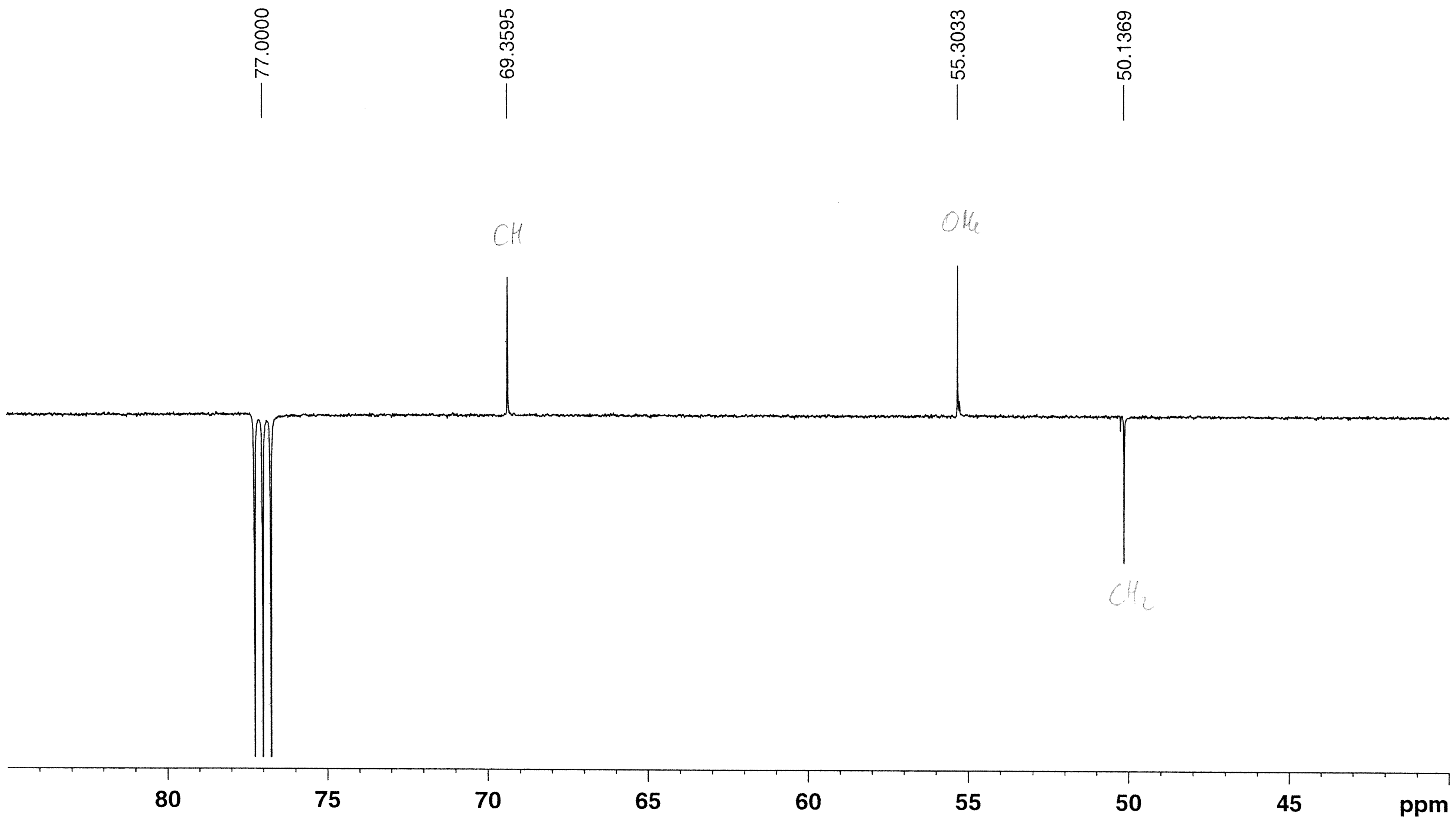

Figure S74c. NMR spectra of compound **6s**.

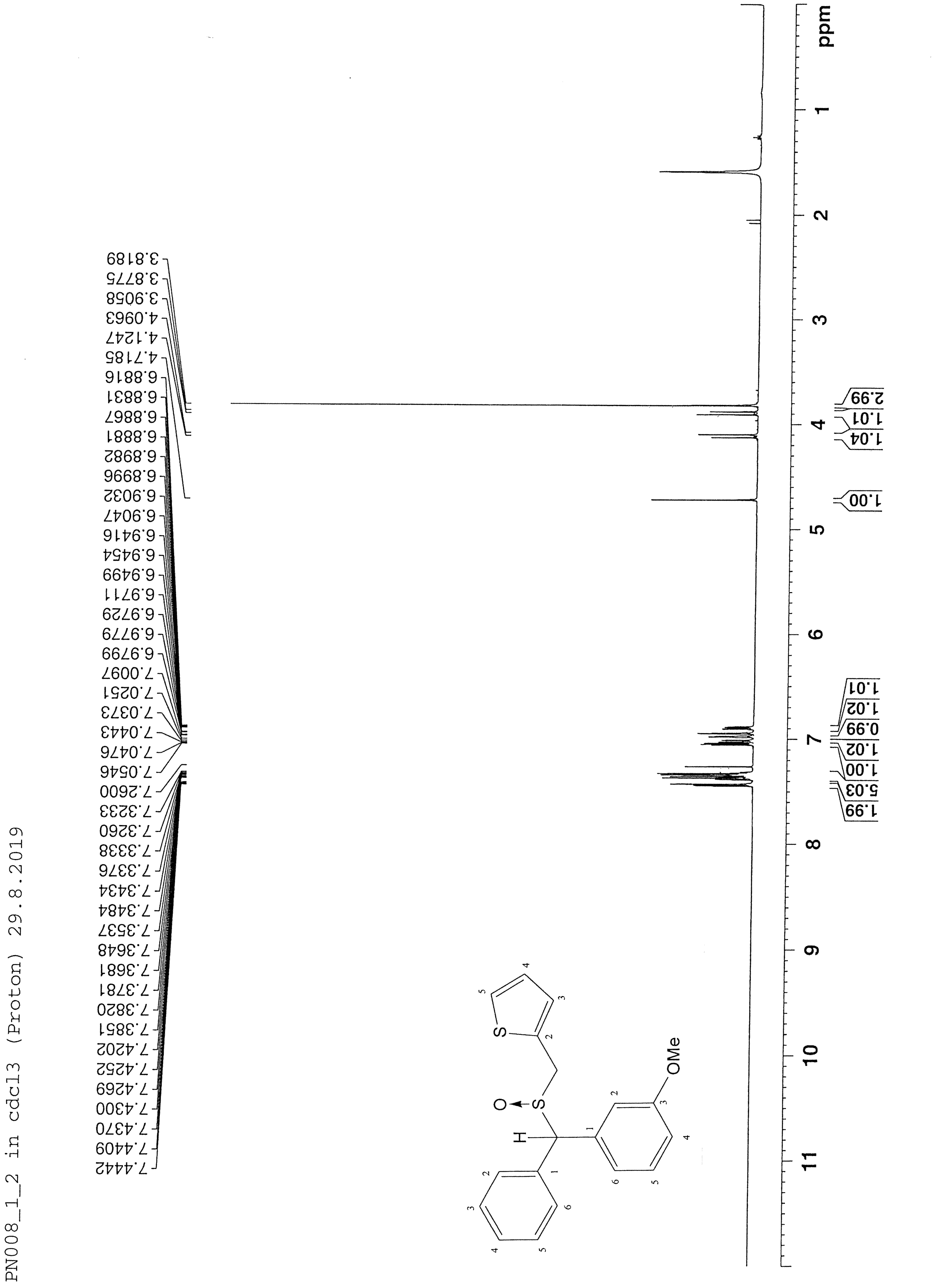

PN008_1_2 in cdcl3 (Proton) 29.8.2019

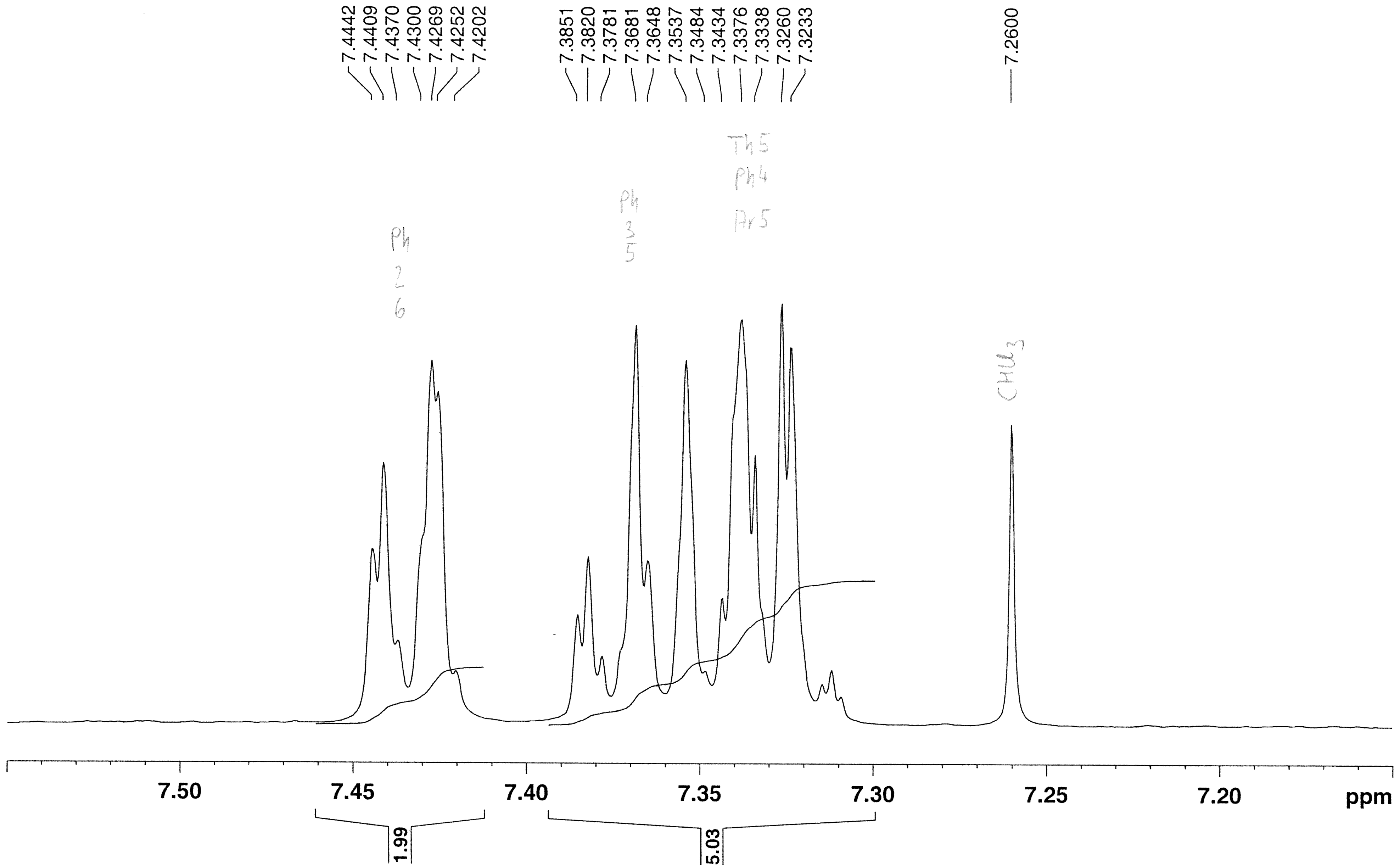

PN008_1_2 in cdcl3 (Proton) 29.8.2019

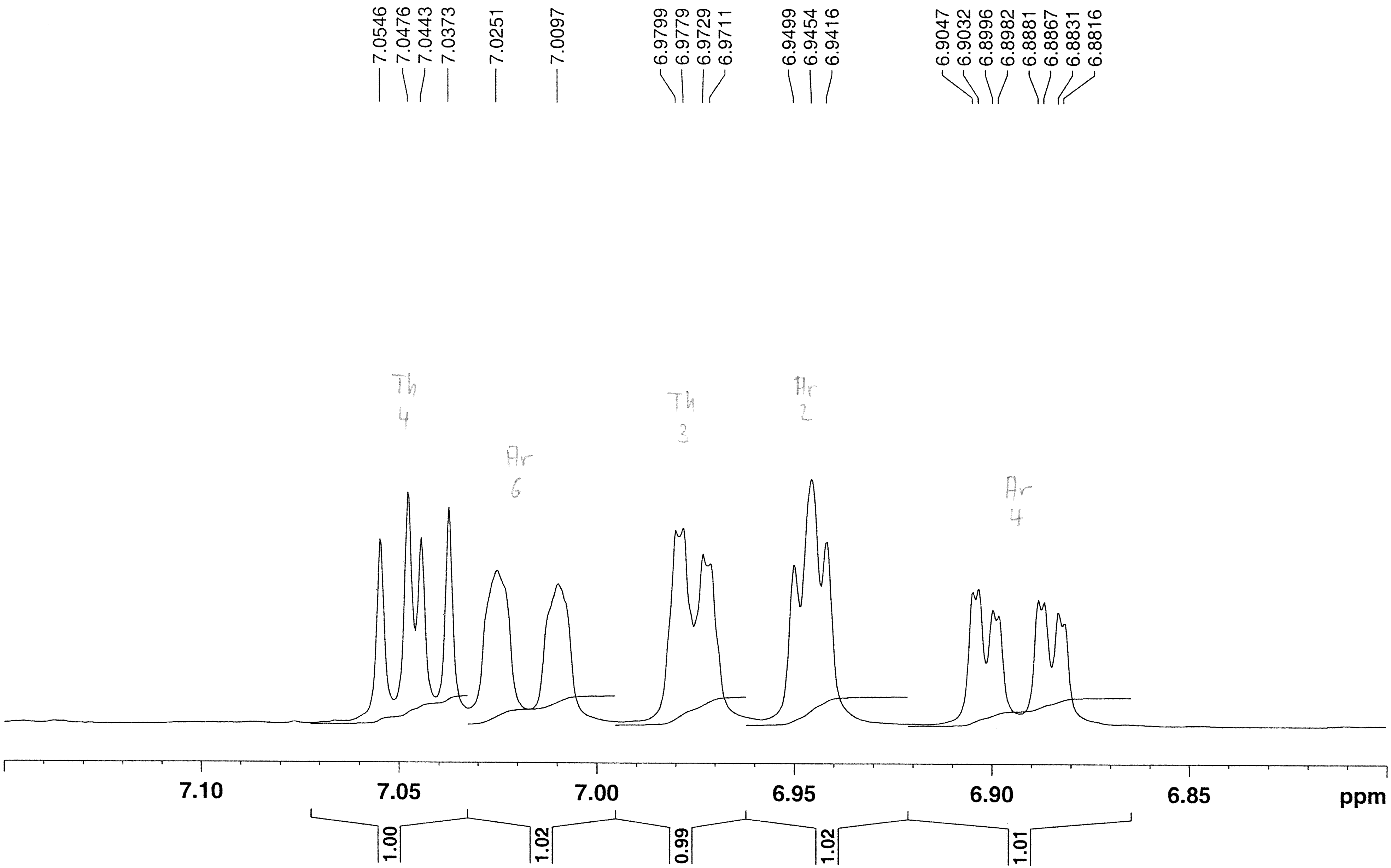

PN008_1_2 in cdcl3 (Proton) 29.8.2019

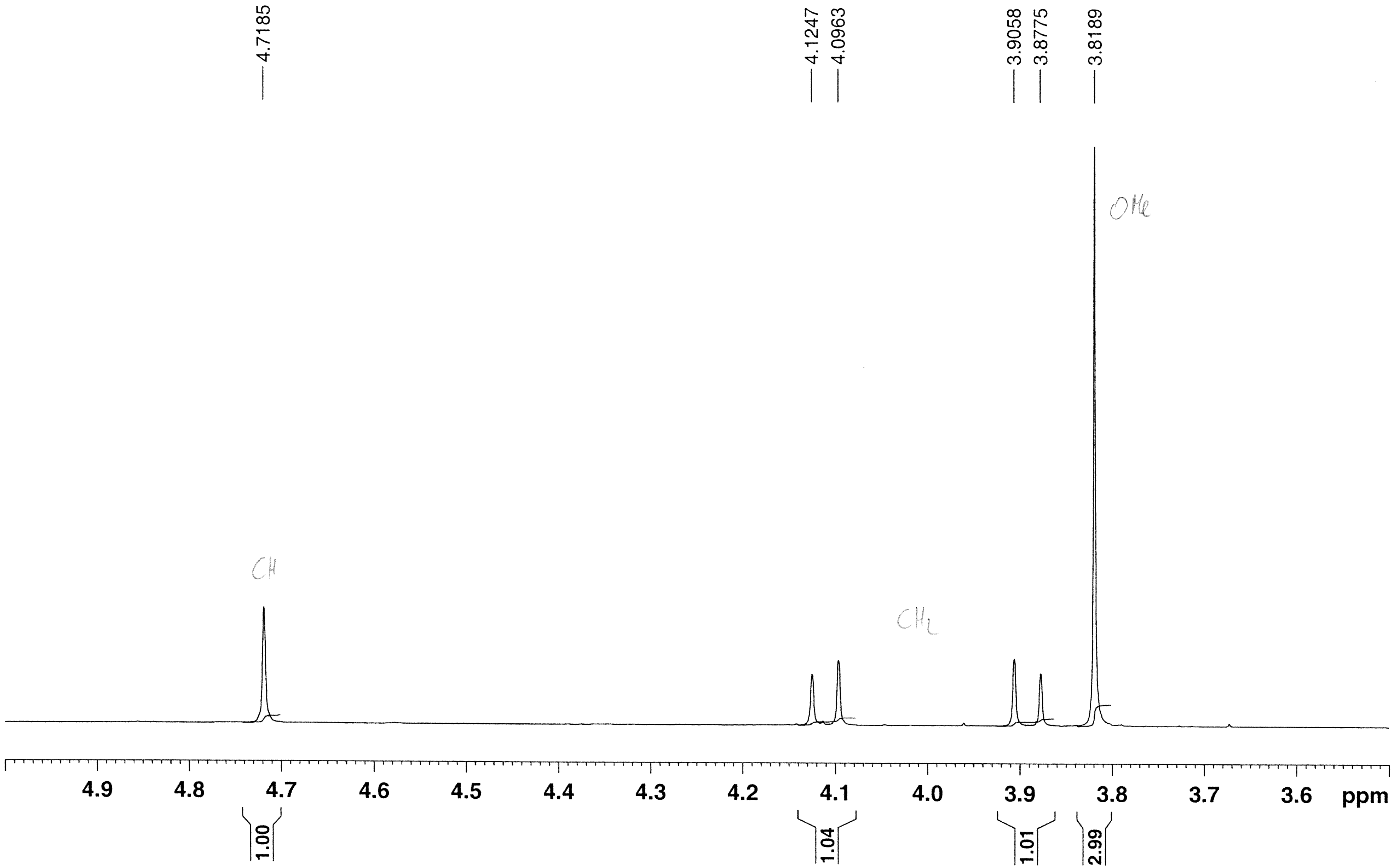

PN008_1_2 in cdcl3 (APT) 29.8.2019

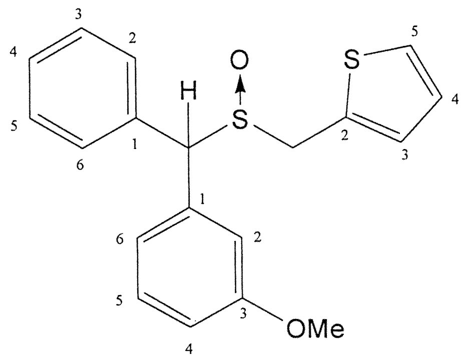

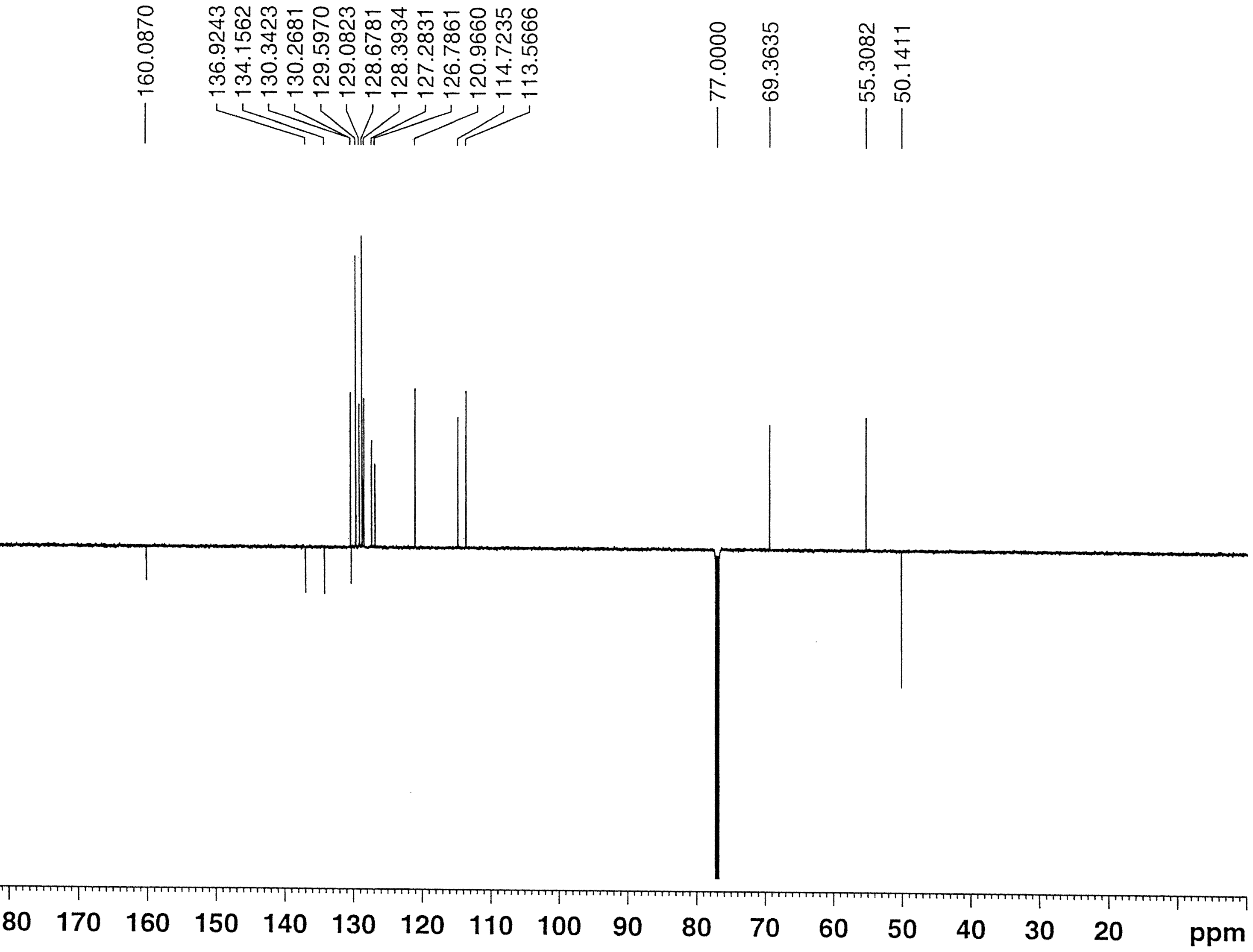

PN008_1_2 in cdcl3 (APT) 29.8.2019

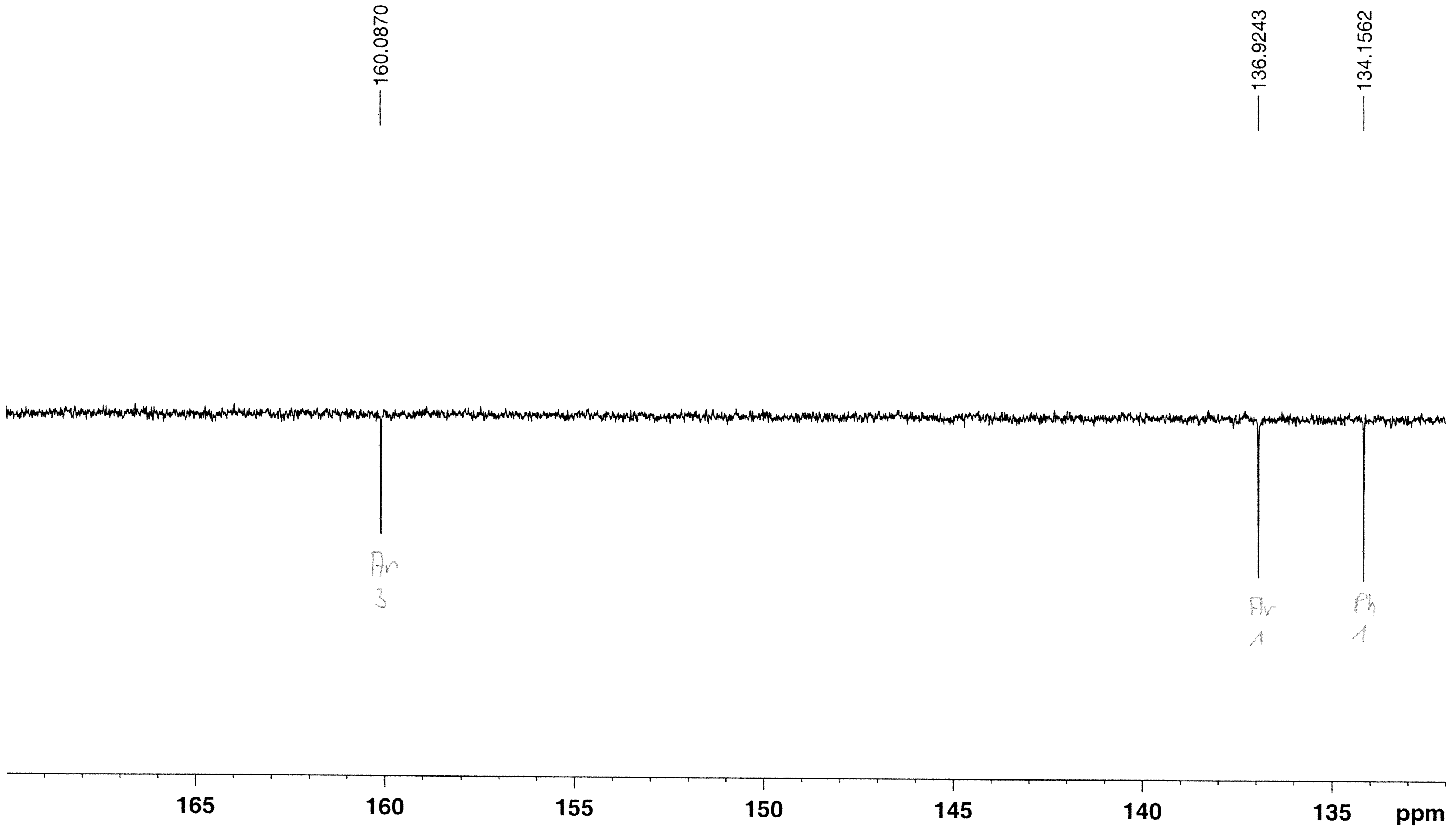

PN008_1_2 in cdcl3 (APT) 29.8.2019

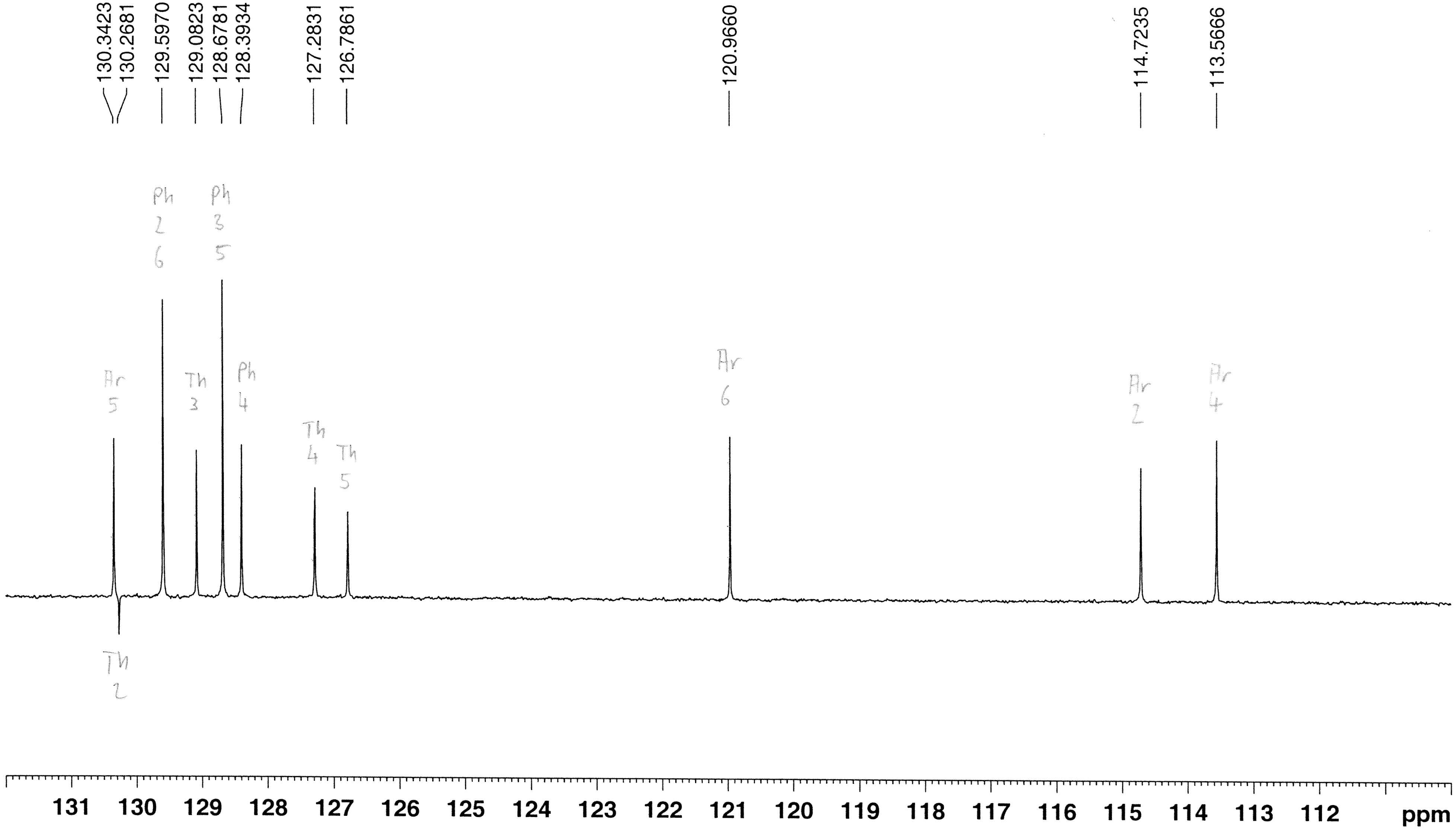

PN008_1_2 in cdcl3 (APT) 29.8.2019

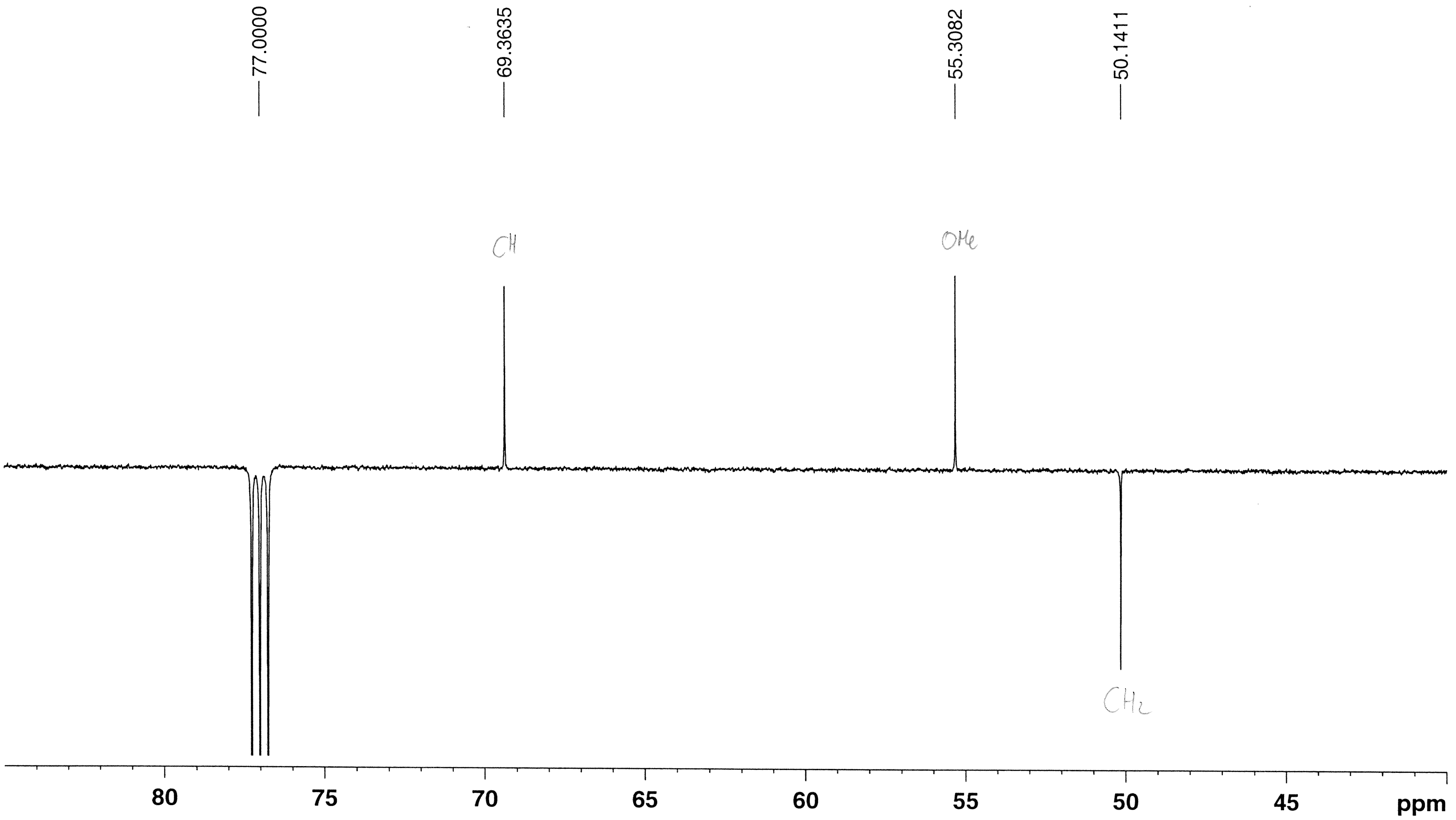

PN008_1_2 in cdcl3 (COSY) 29.8.2019

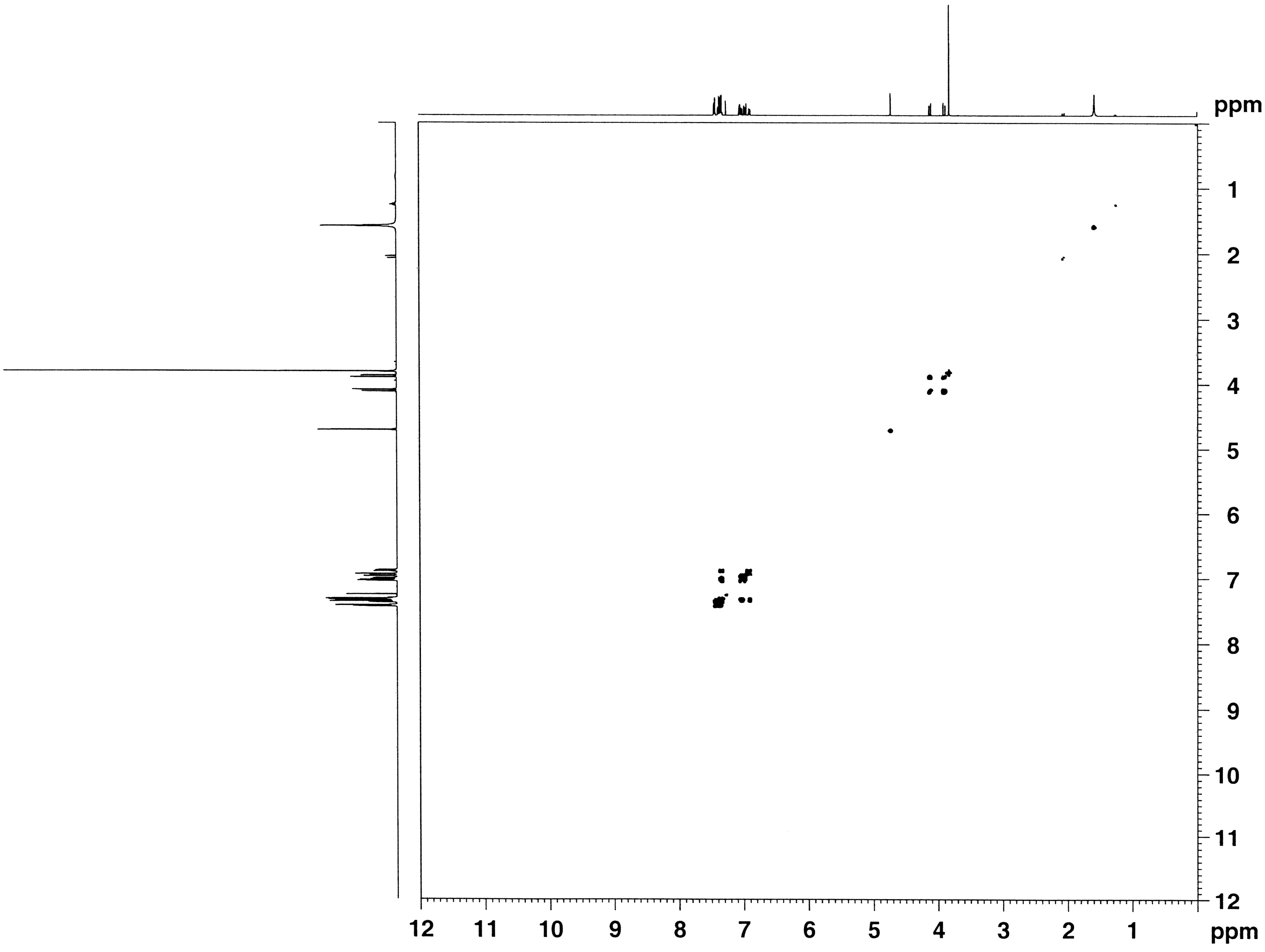

PN008_1_2 in cdcl3 (COSY) 29.8.2019

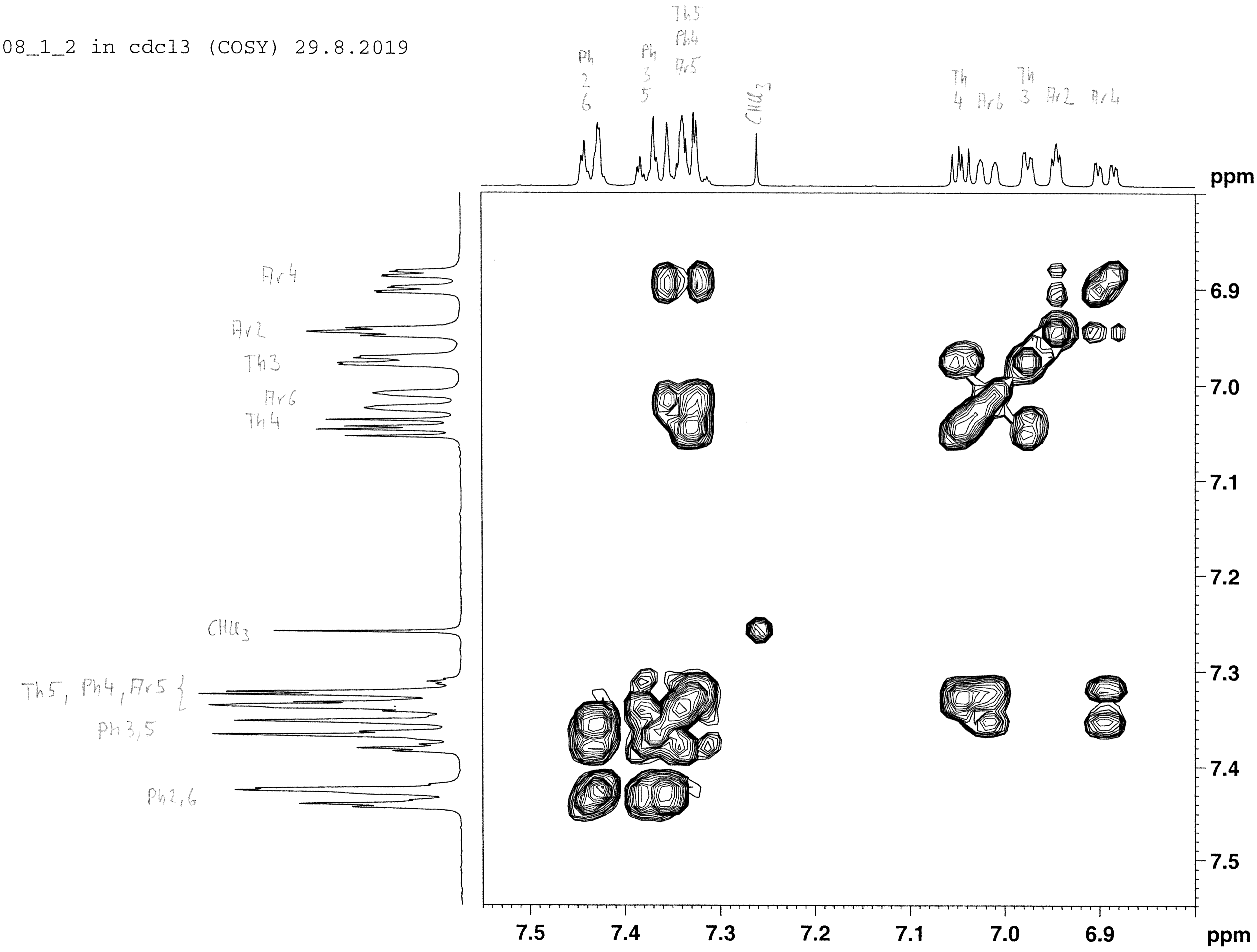

PN008_1_2 in cdcl3 (COSY) 29.8.2019

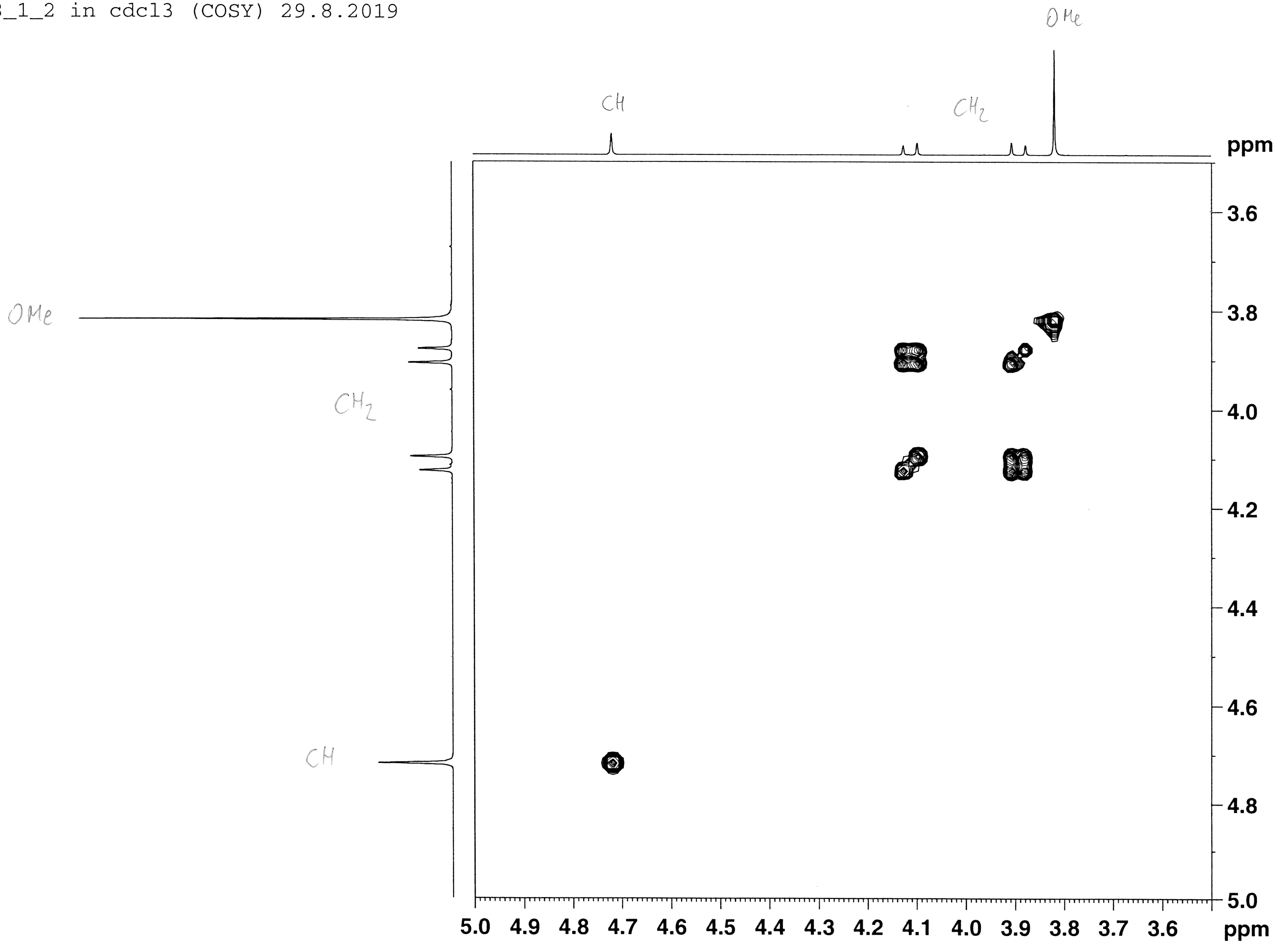

PN008_1_2 in cdcl3 (HSQC) 29.8.2019

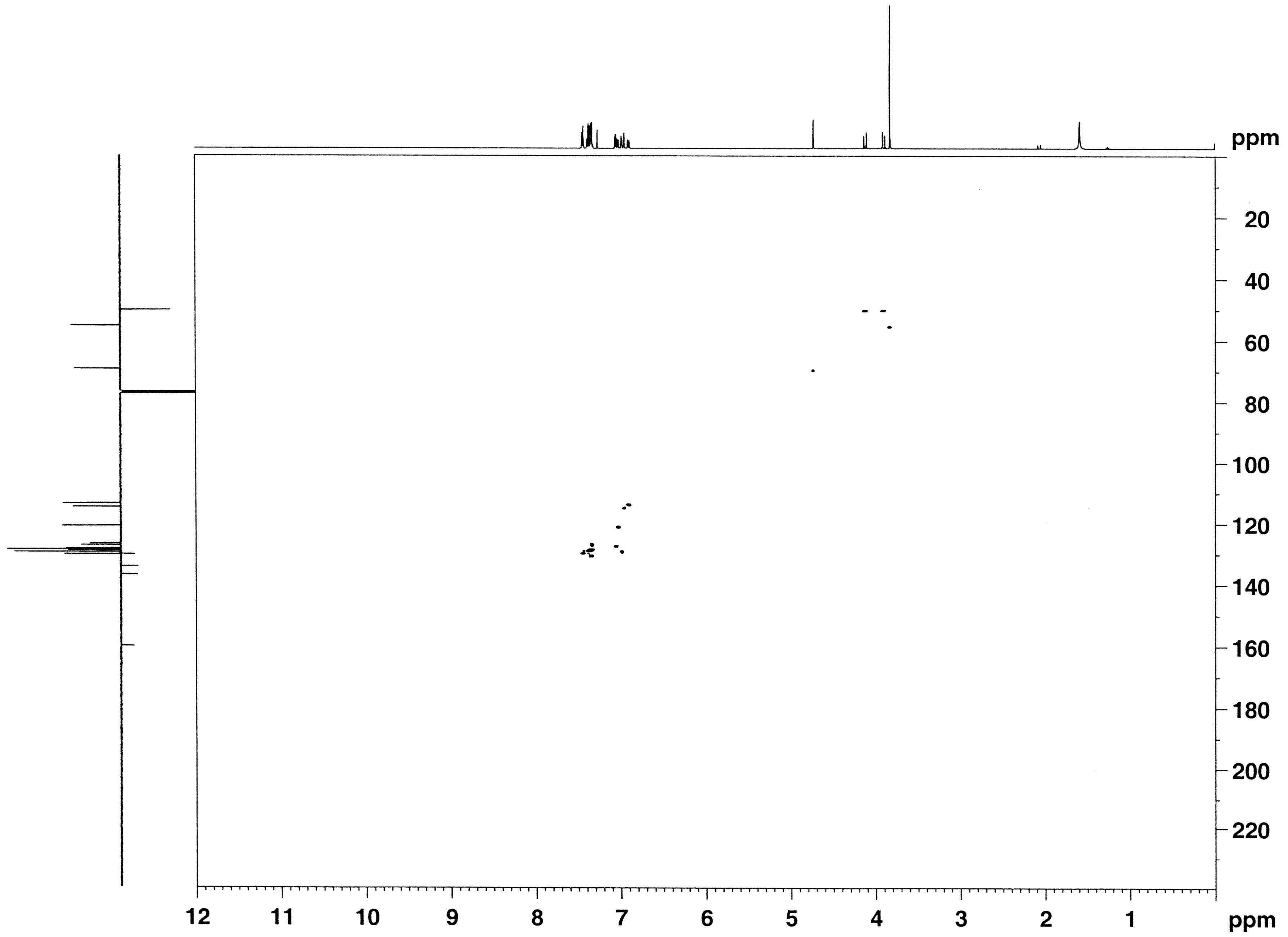

PN008_1_2 in cdcl3 (HSQC) 29.8.2019

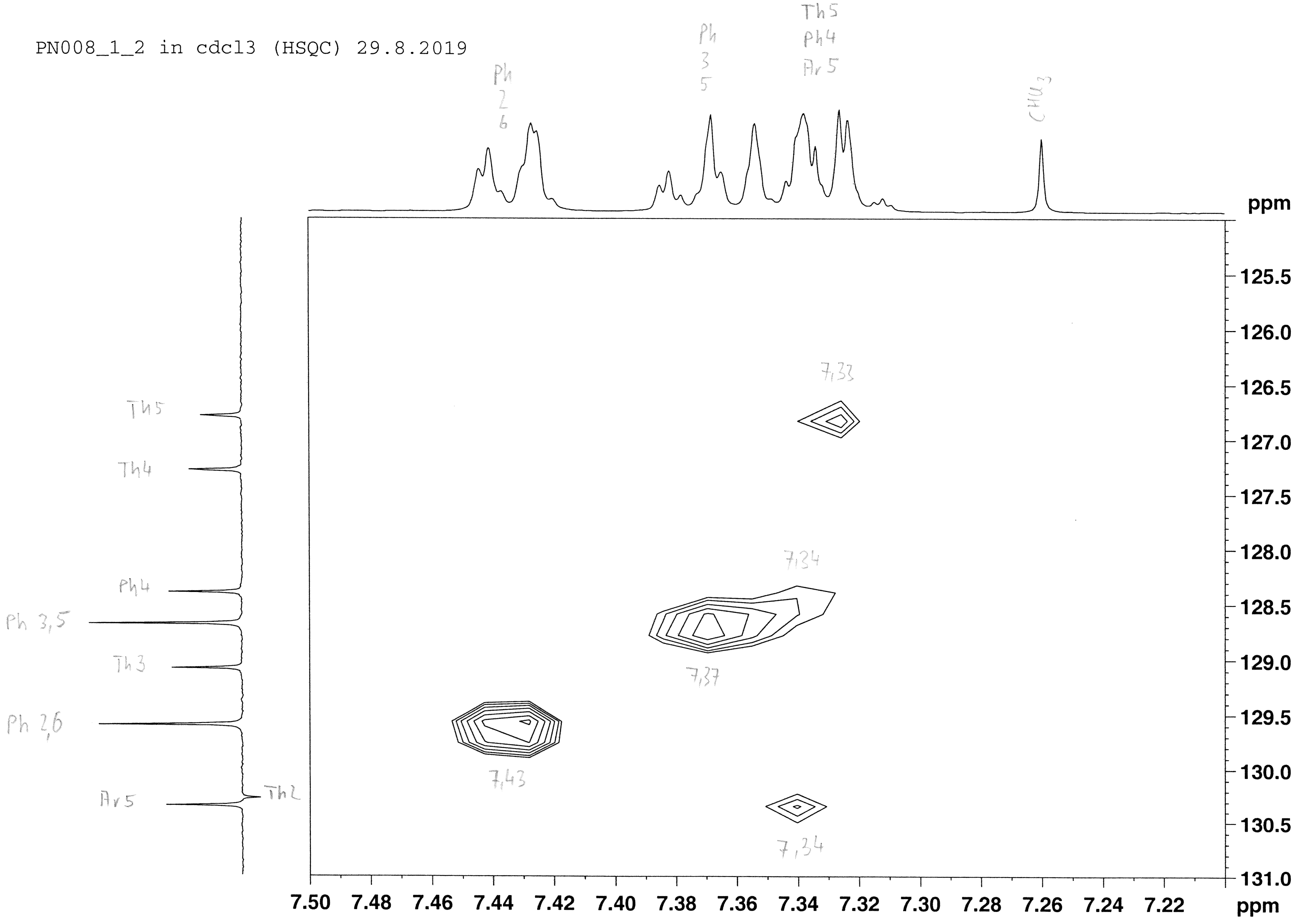

PN008_1_2 in cdcl3 (HSQC) 29.8.2019

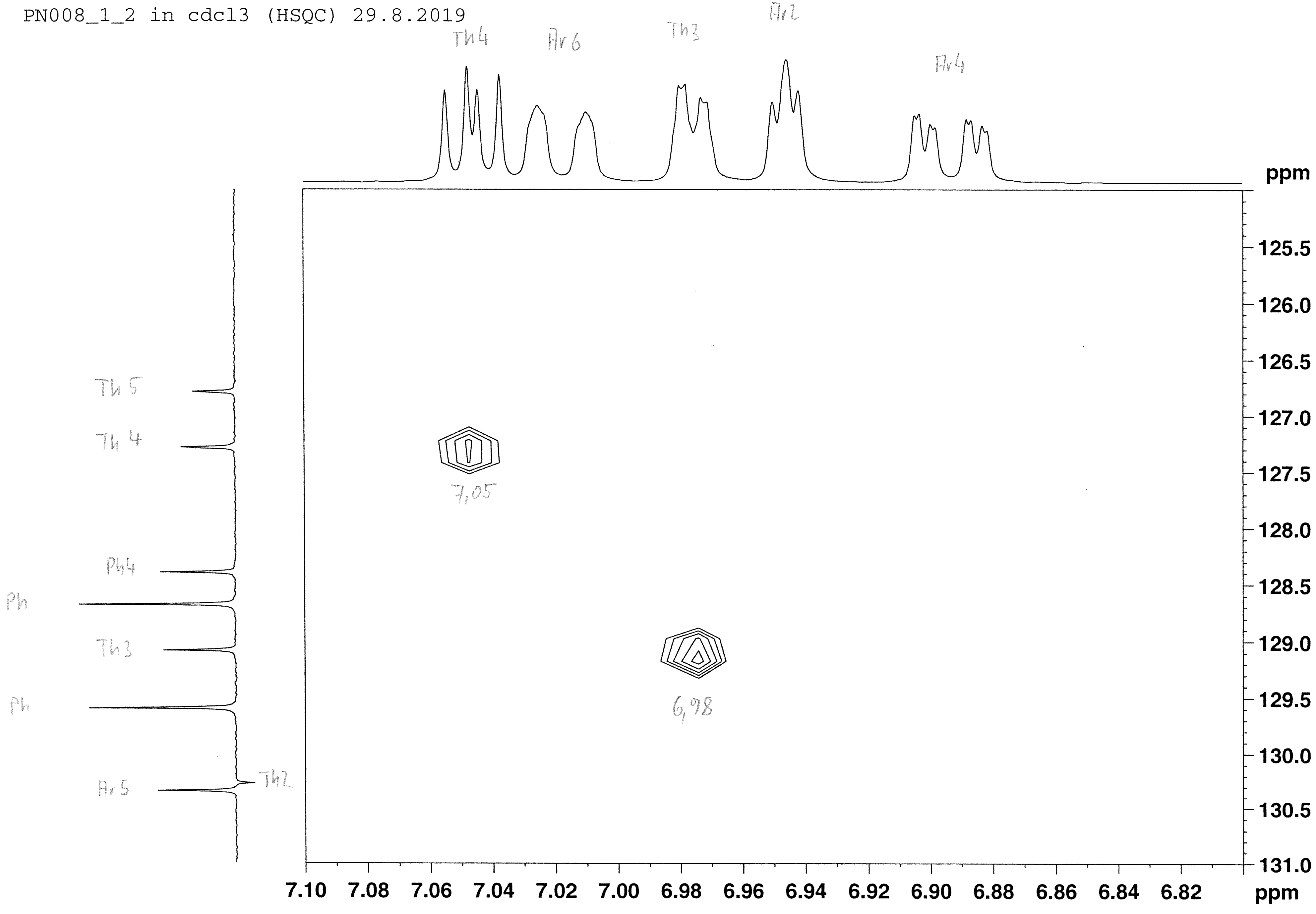

PN008_1_2 in cdcl3 (HSQC) 29.8.2019

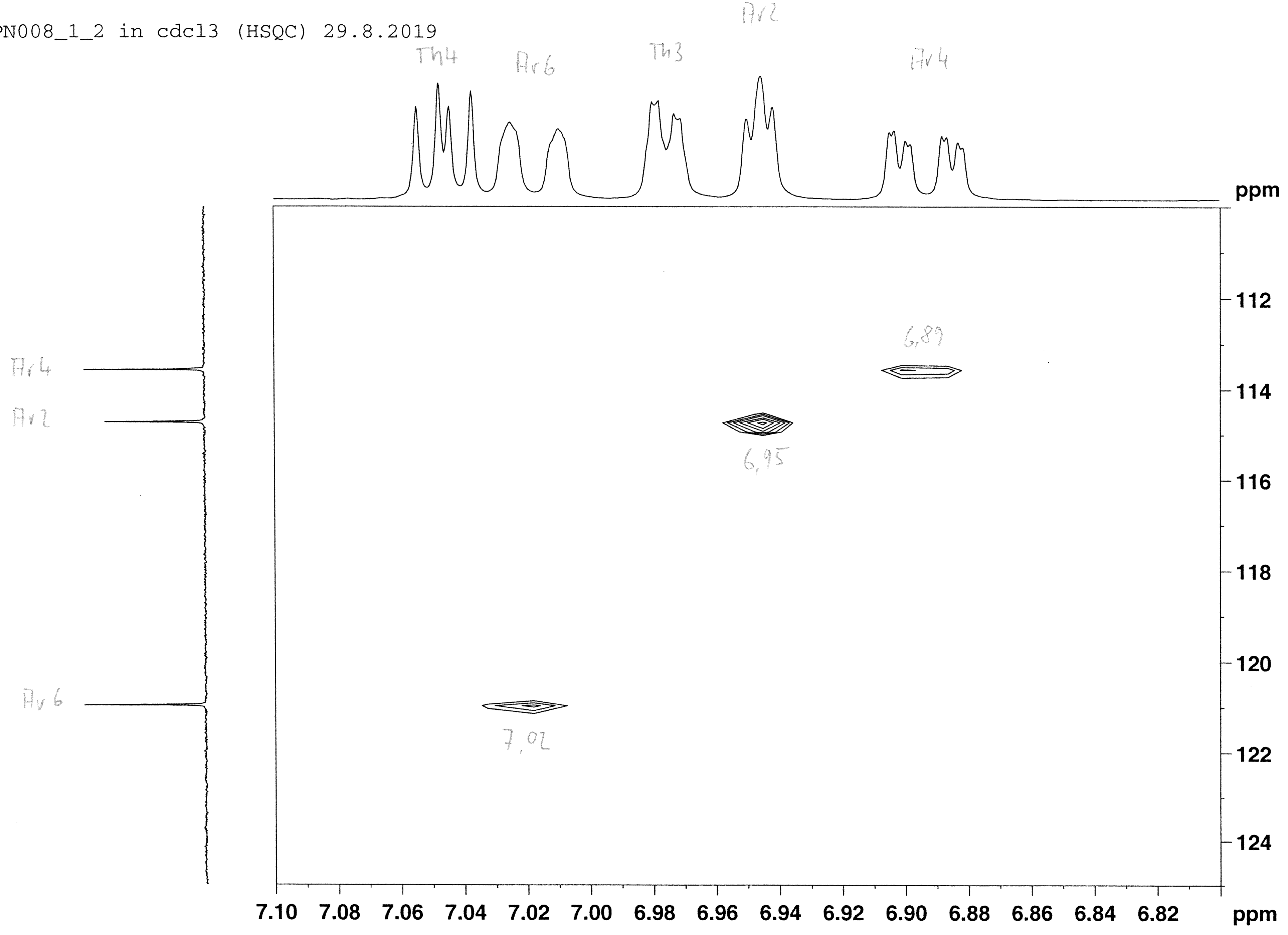

PN008_1_2 in cdcl3 (HSQC) 29.8.2019

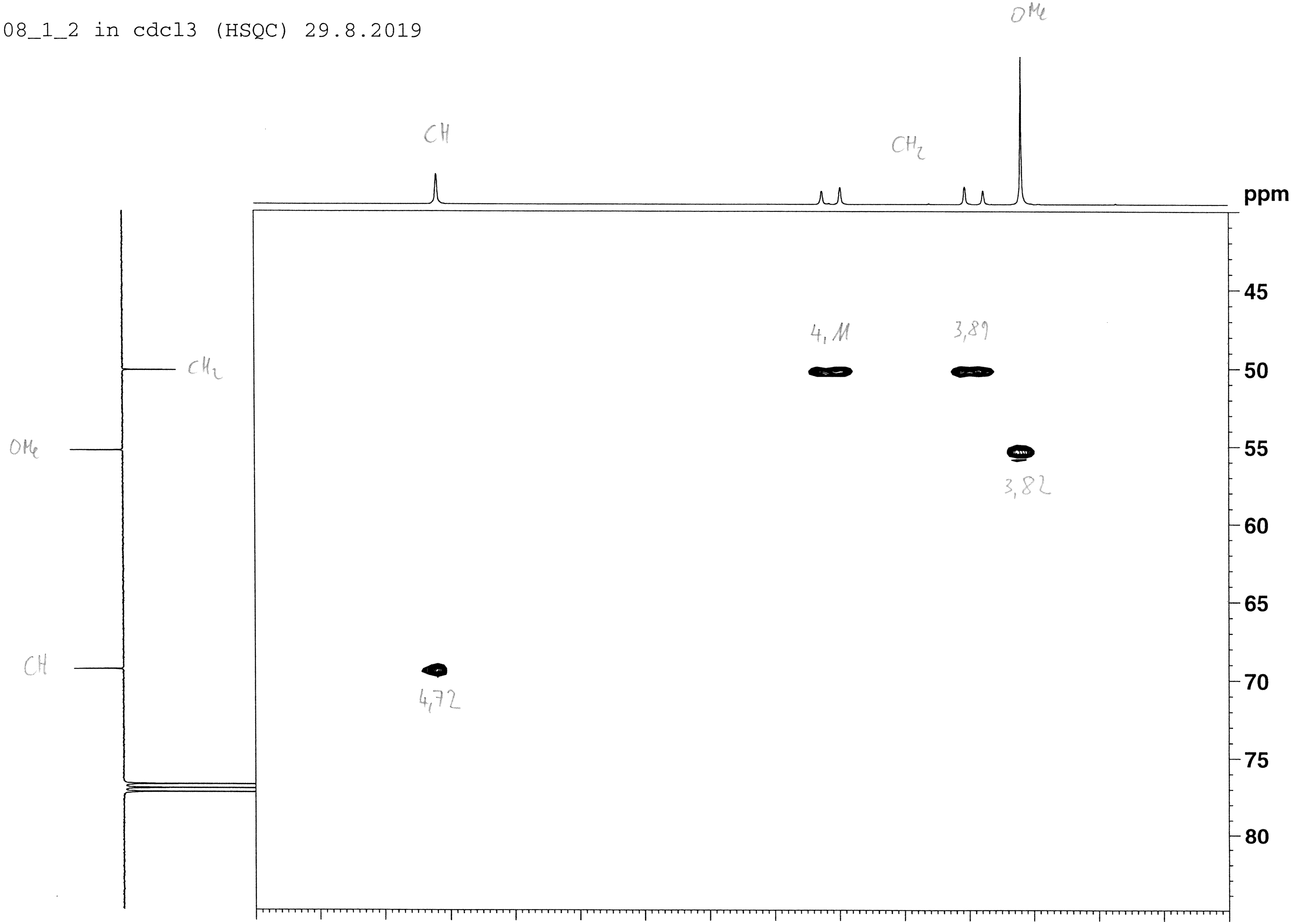

PN008_1_2 in cdcl3 (HMBC) 29.8.2019

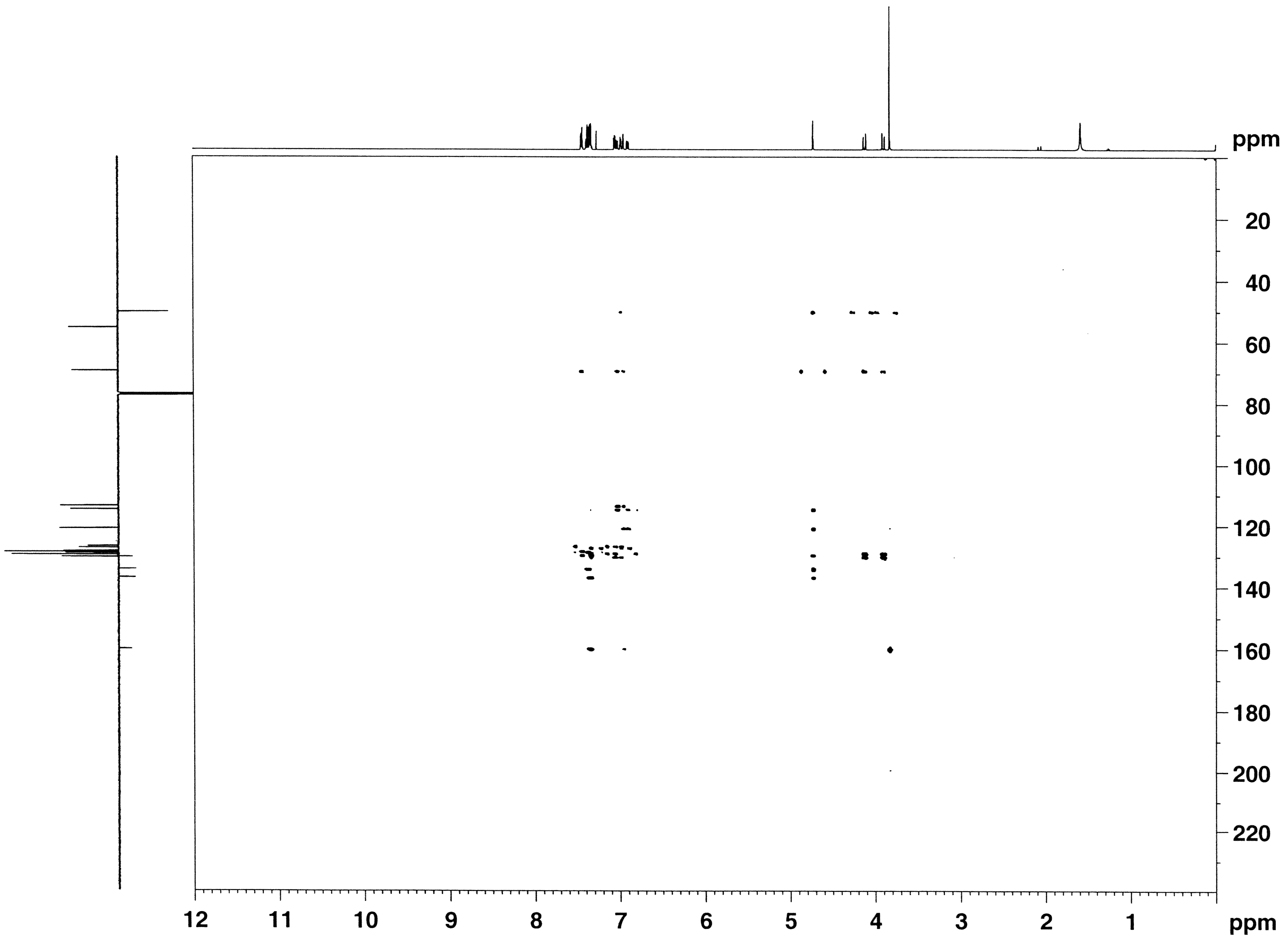

PN008_1_2 in cdcl3 (HMBC) 29.8.2019

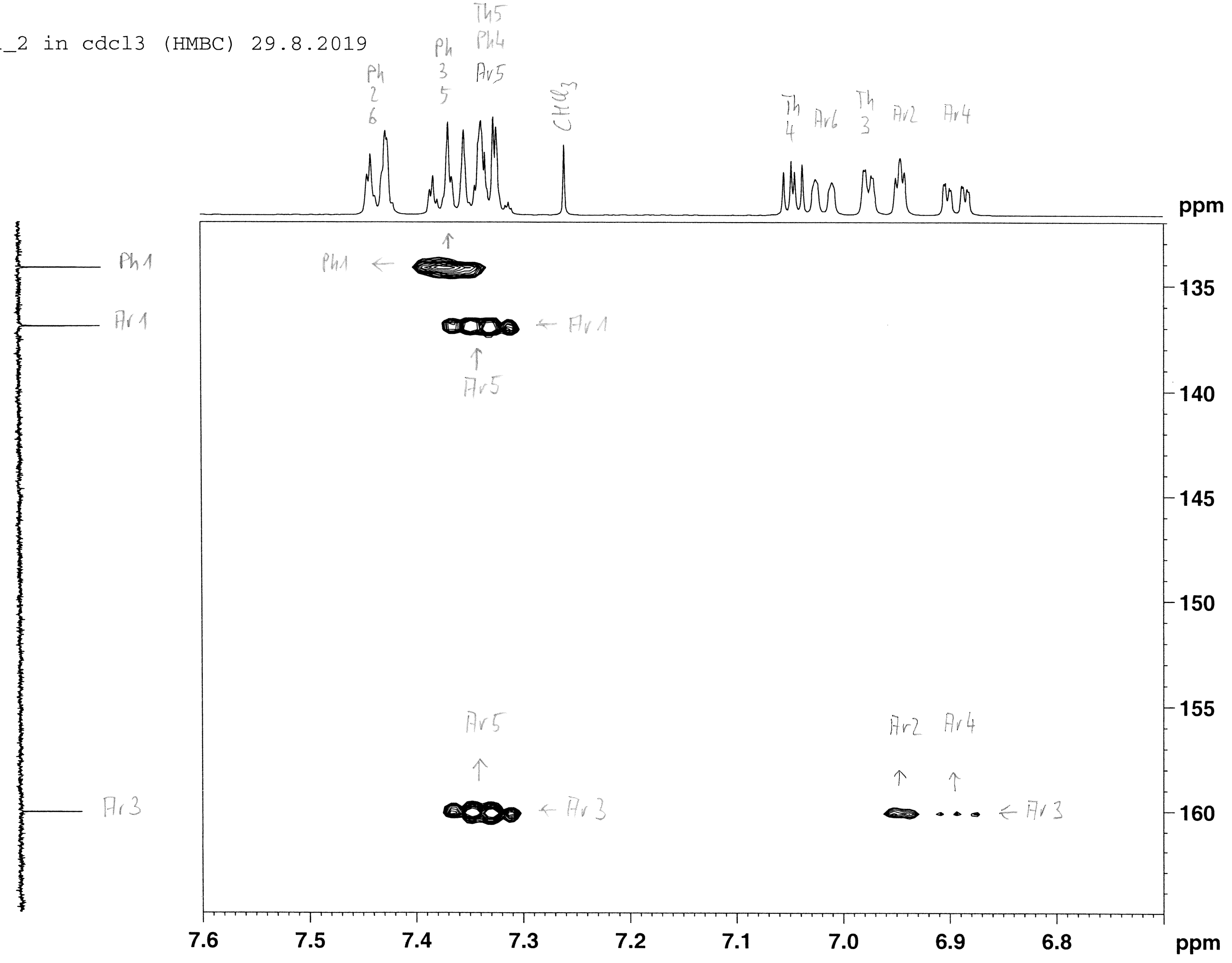

PN008_1_2 in cdcl3 (HMBC) 29.8.2019

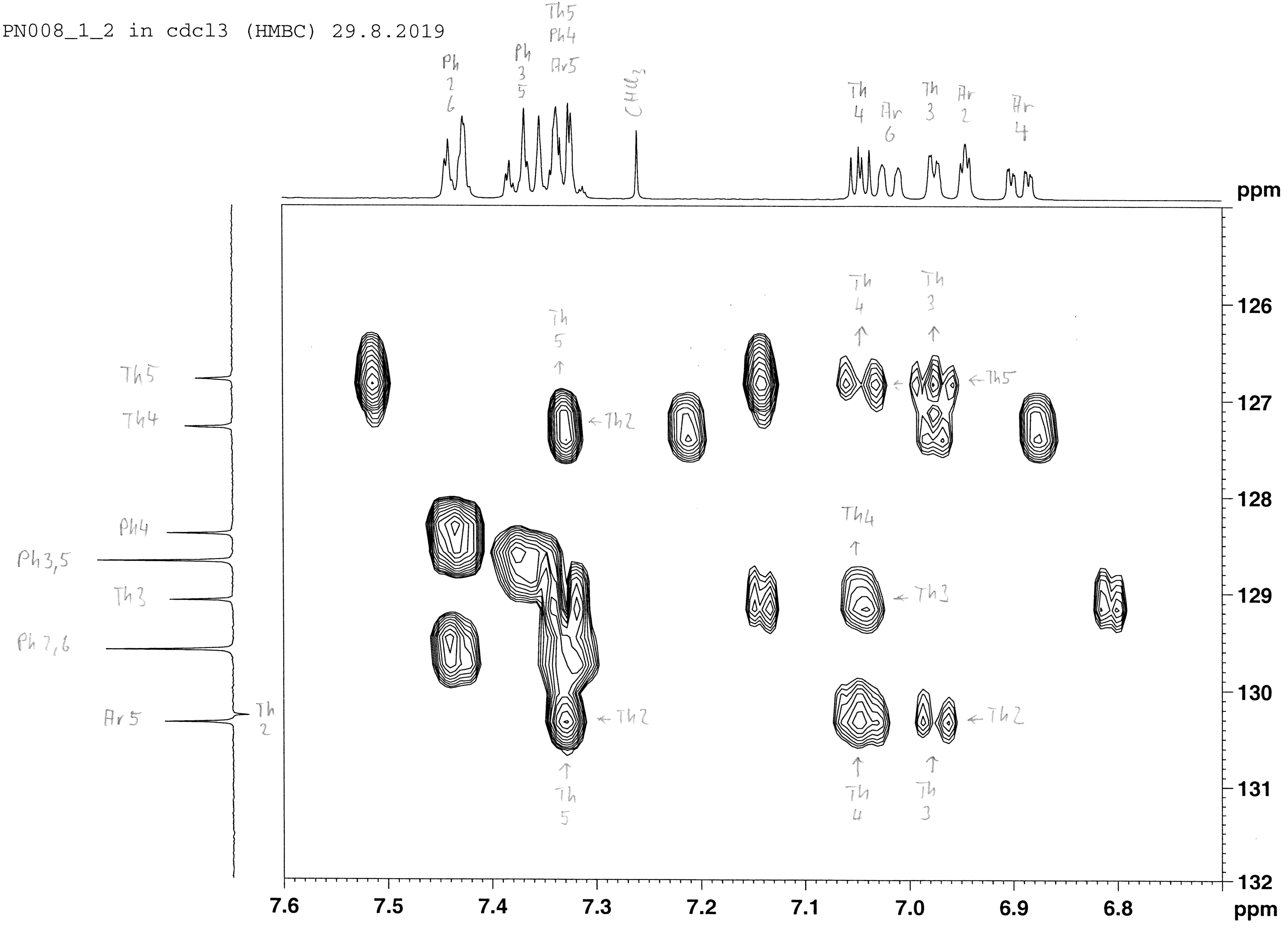

PN008_1_2 in cdcl3 (HMBC) 29.8.2019

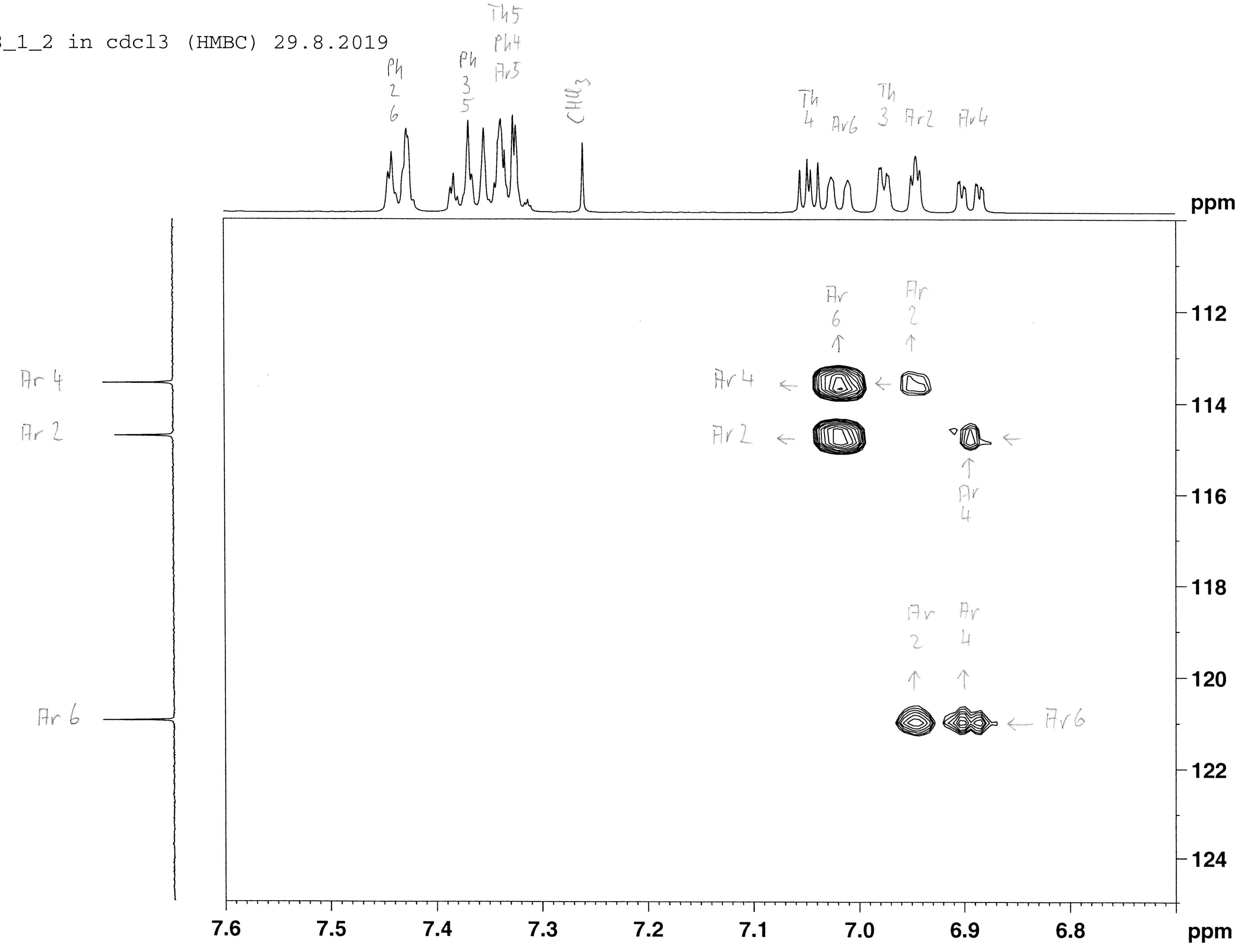

PN008_1_2 in cdcl3 (HMBC) 29.8.2019

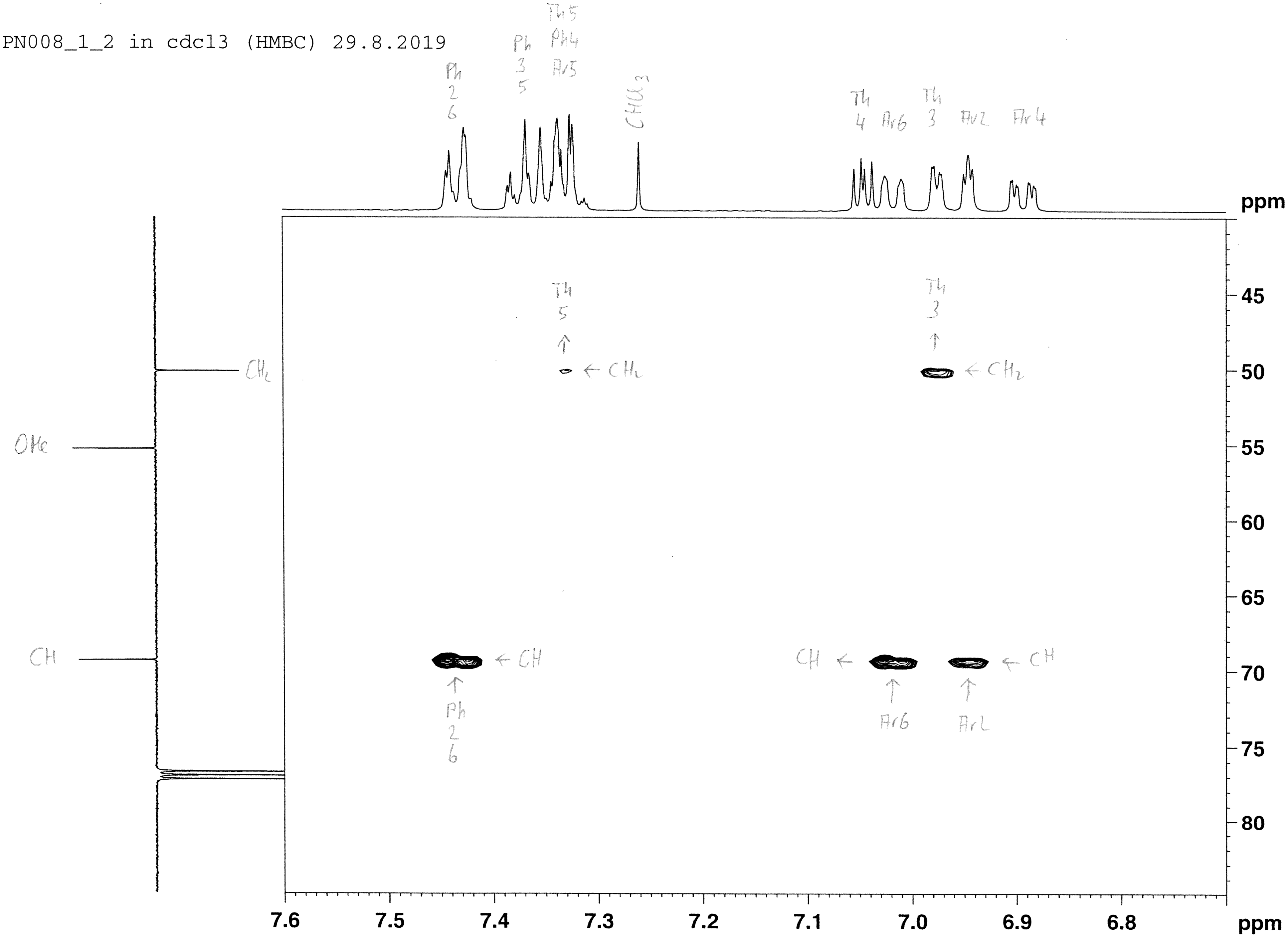

PN008_1_2 in cdcl3 (HMBC) 29.8.2019

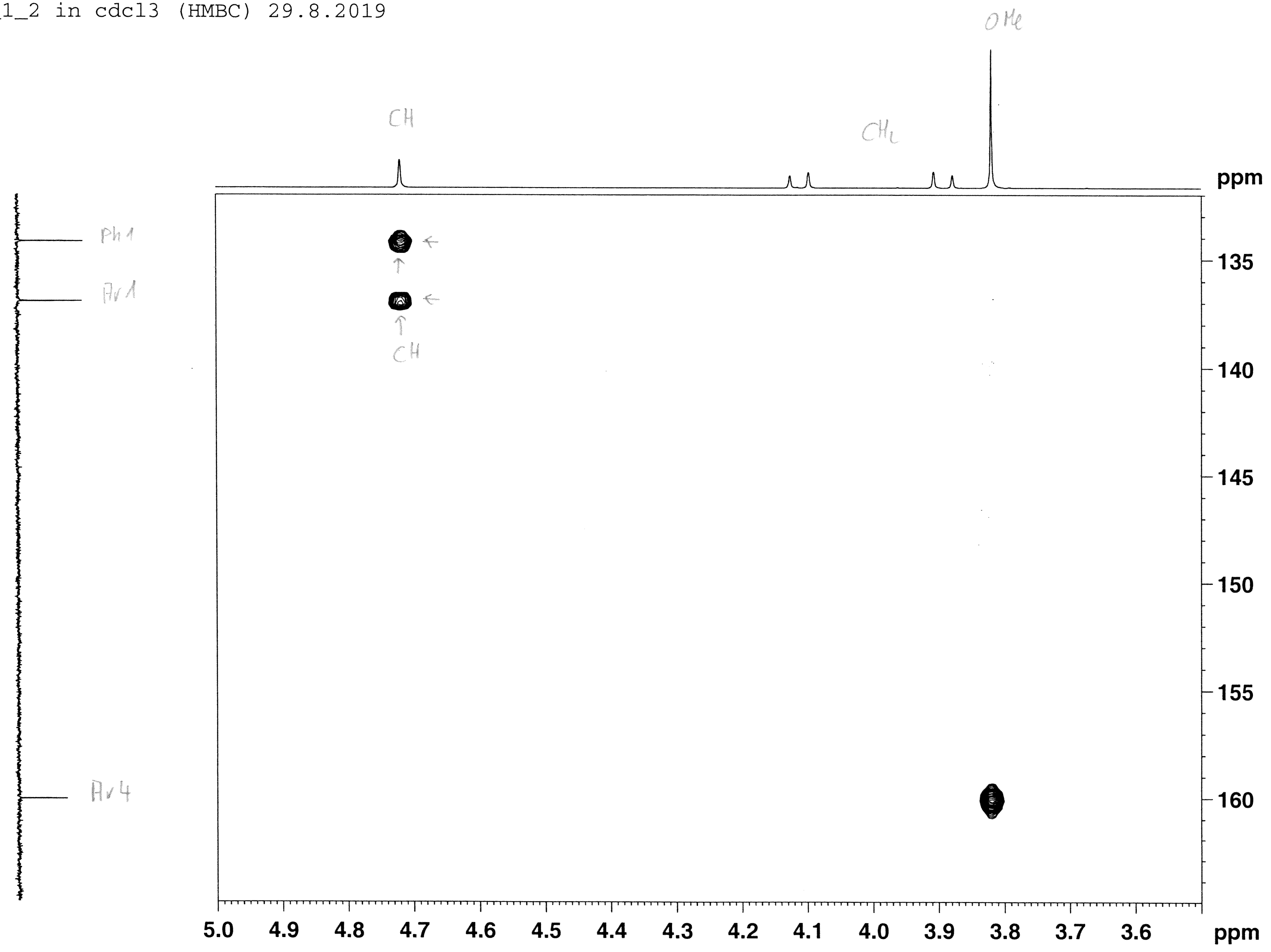

PN008_1_2 in cdcl3 (HMBC) 29.8.2019

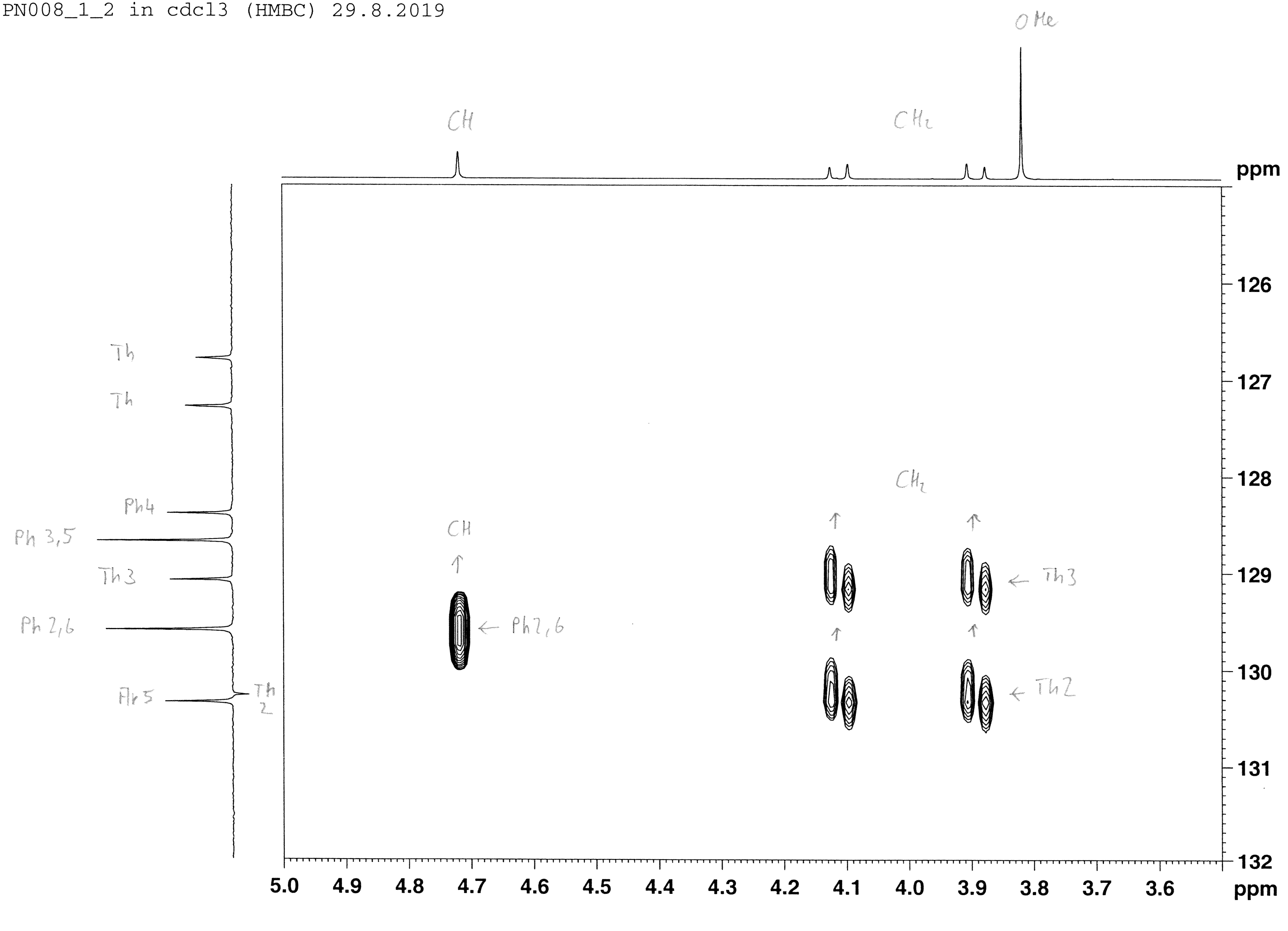

PN008_1_2 in cdcl3 (HMBC) 29.8.2019

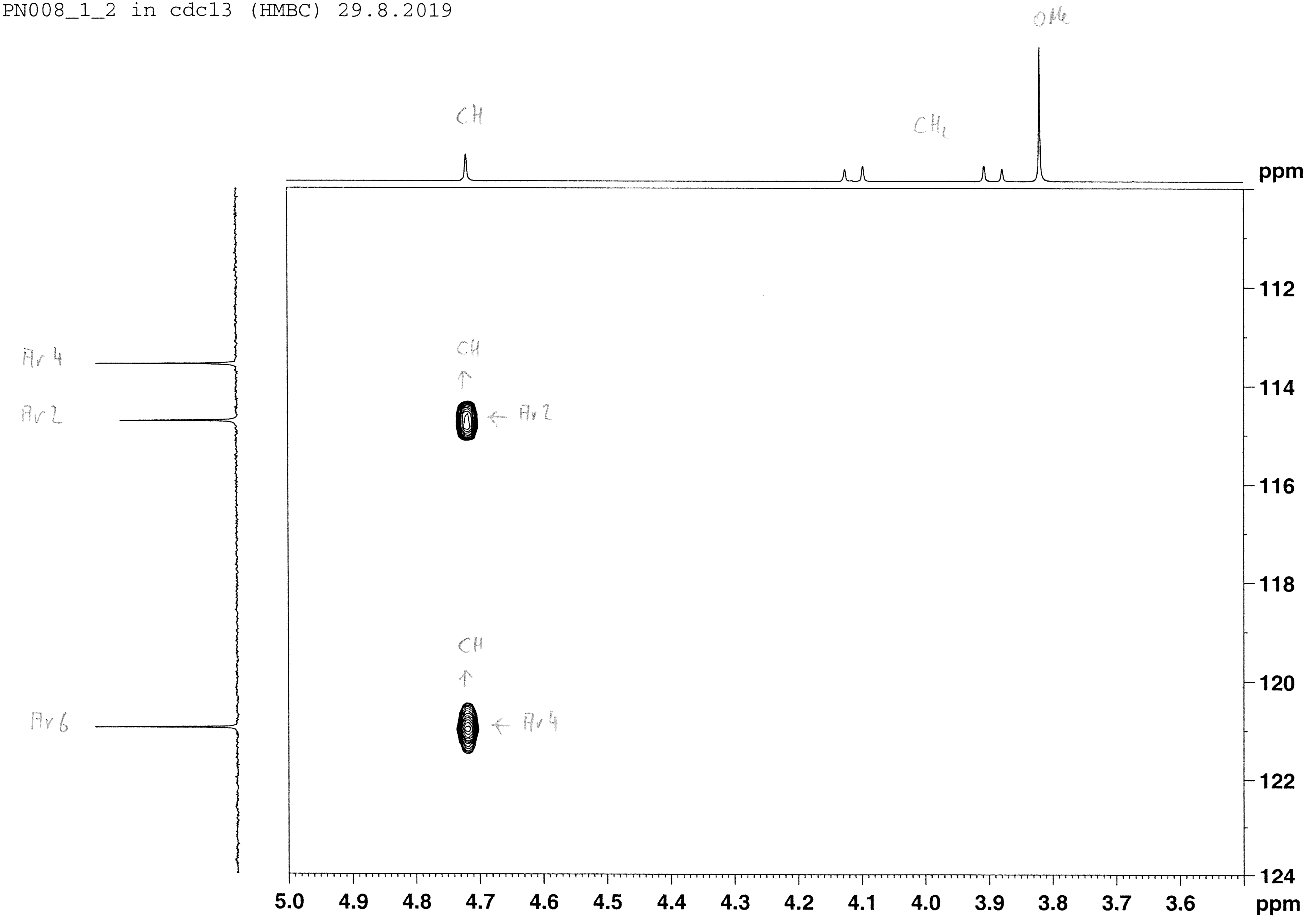

PN008_1_2 in cdcl3 (HMBC) 29.8.2019

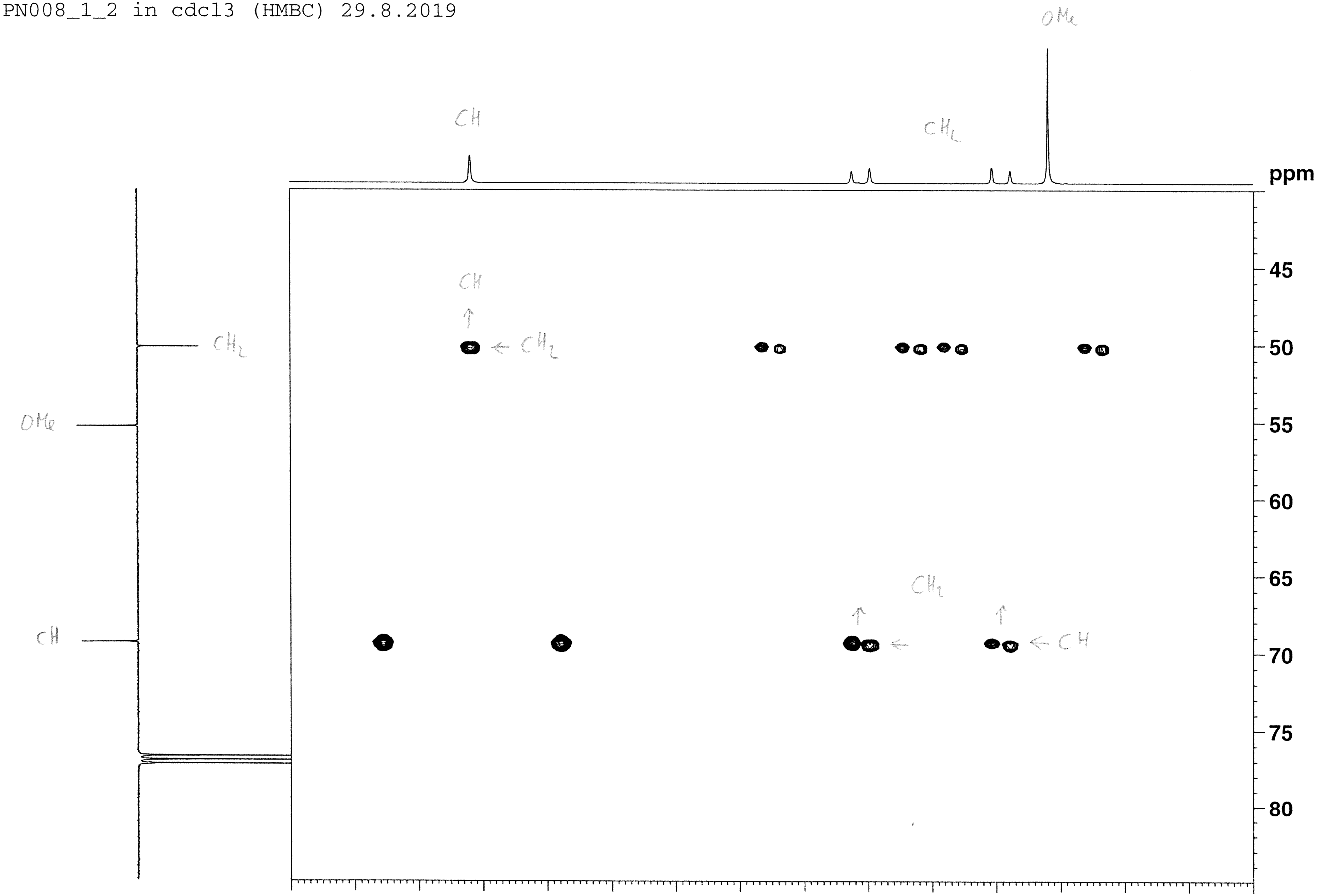

Figure S75c. NMR spectra of compound **7s.**

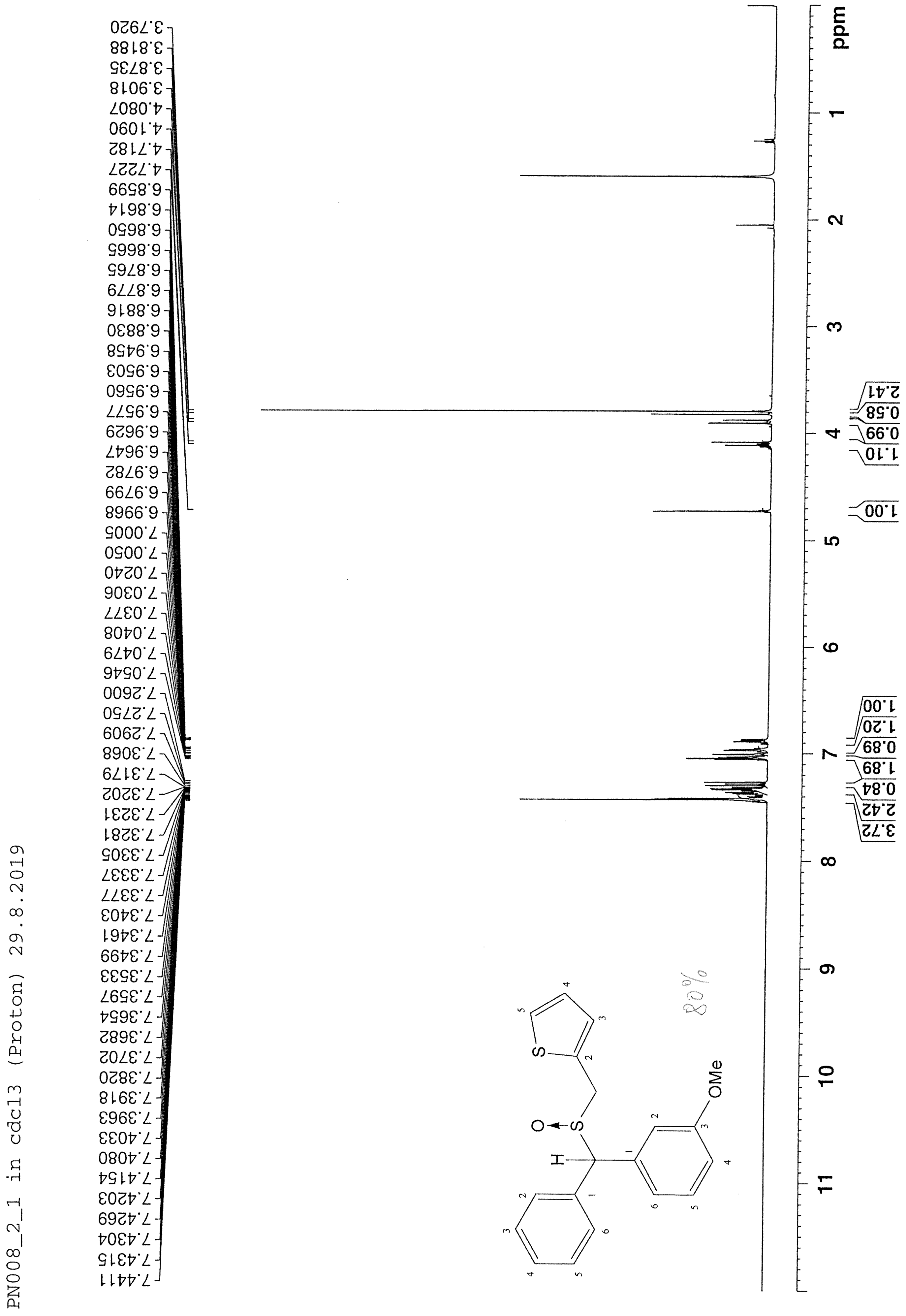

PN008_2_1 in cdcl3 (Proton) 29.8.2019

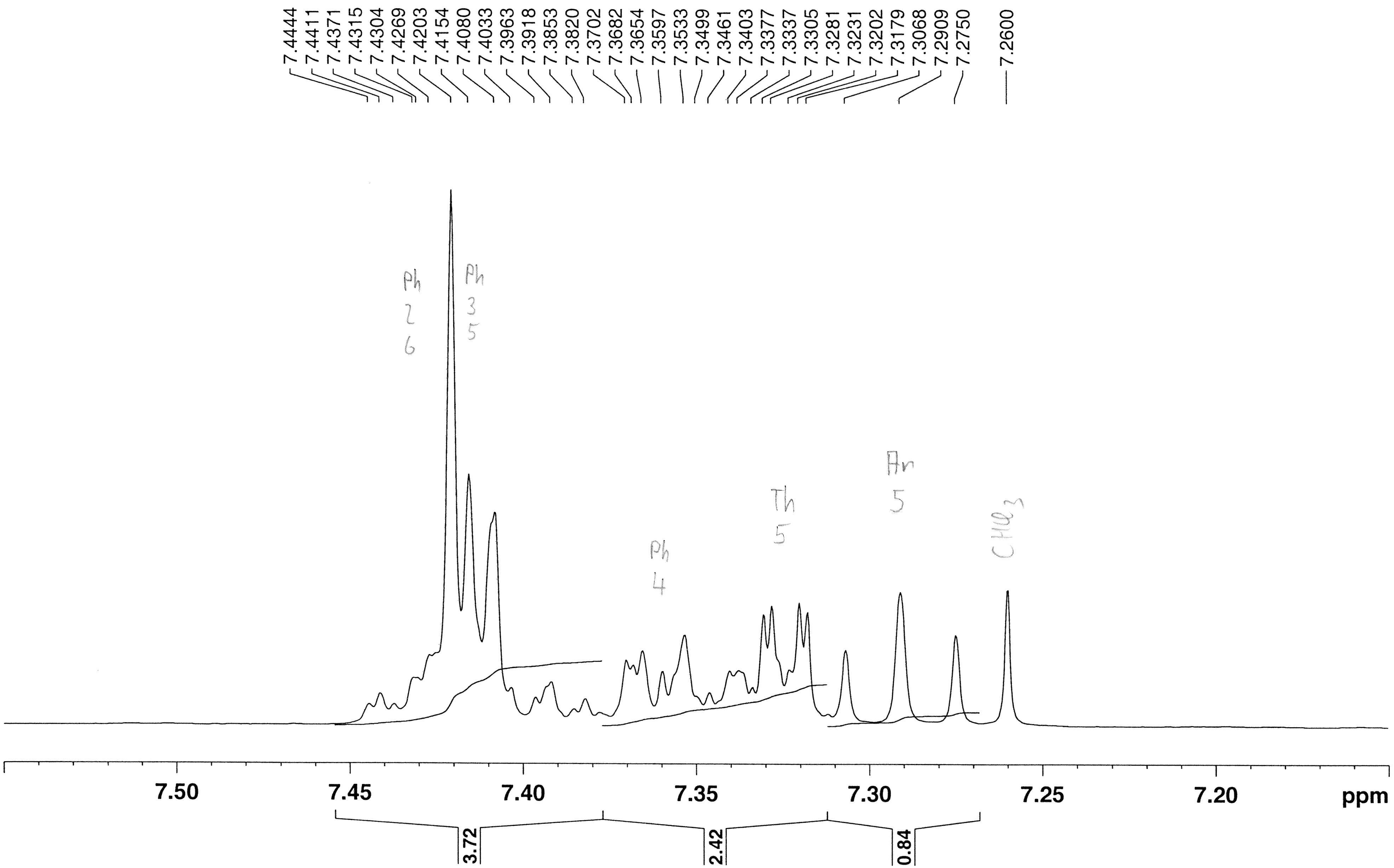

PN008_2_1 in cdcl3 (Proton) 29.8.2019

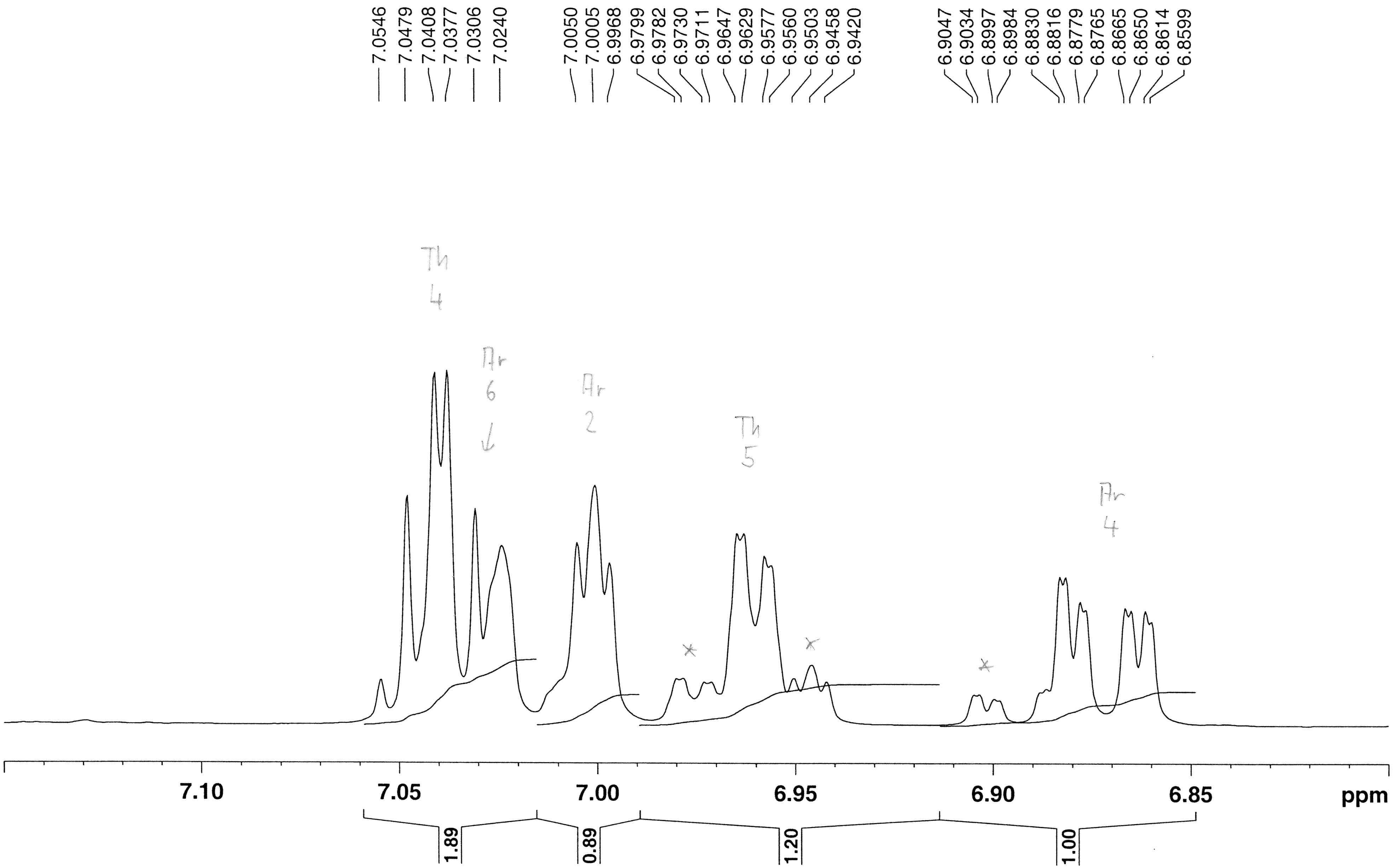

PN008_2_1 in cdcl3 (Proton) 29.8.2019

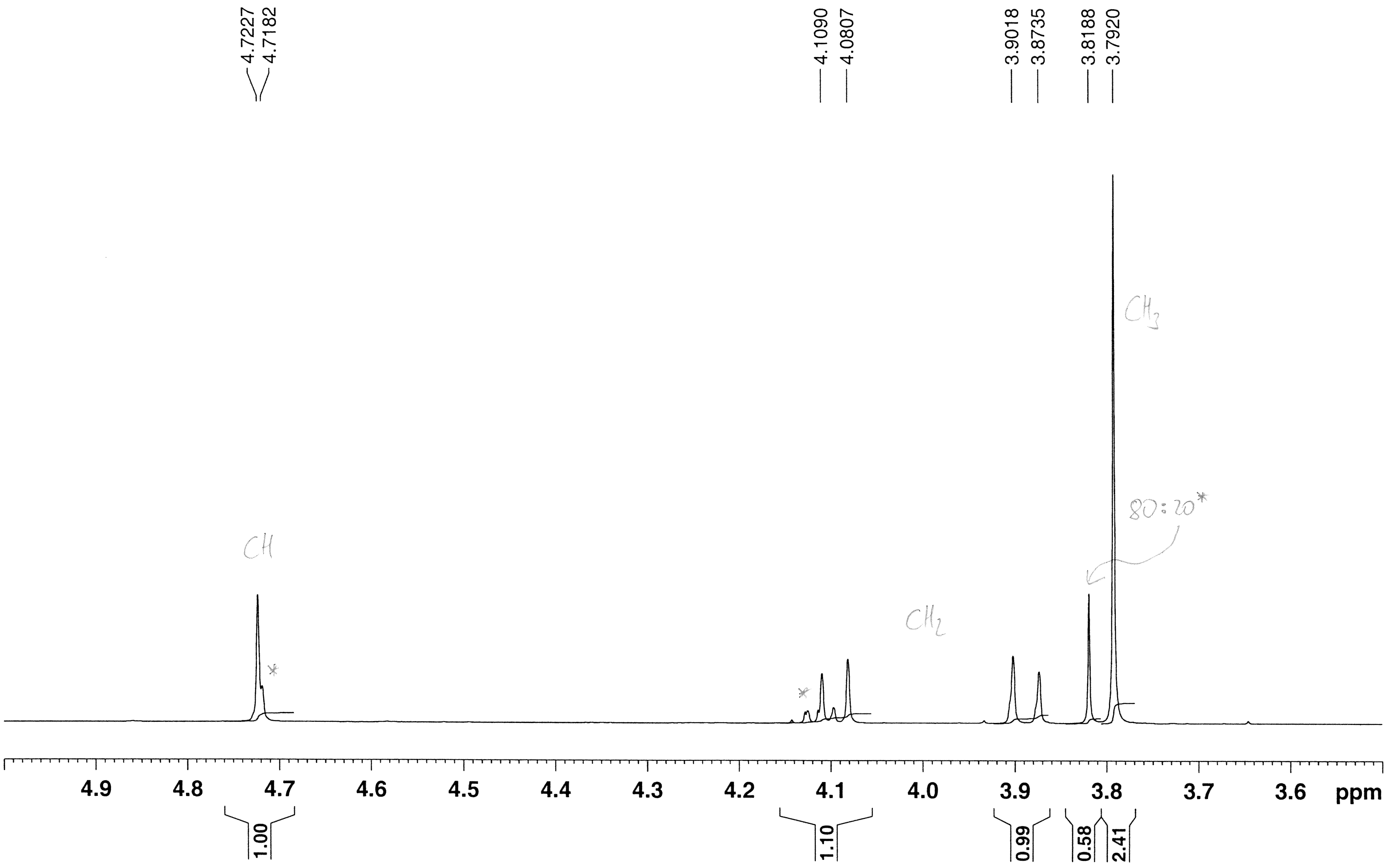

PN008_2_1 in cdcl3 (APT) 29.8.2019

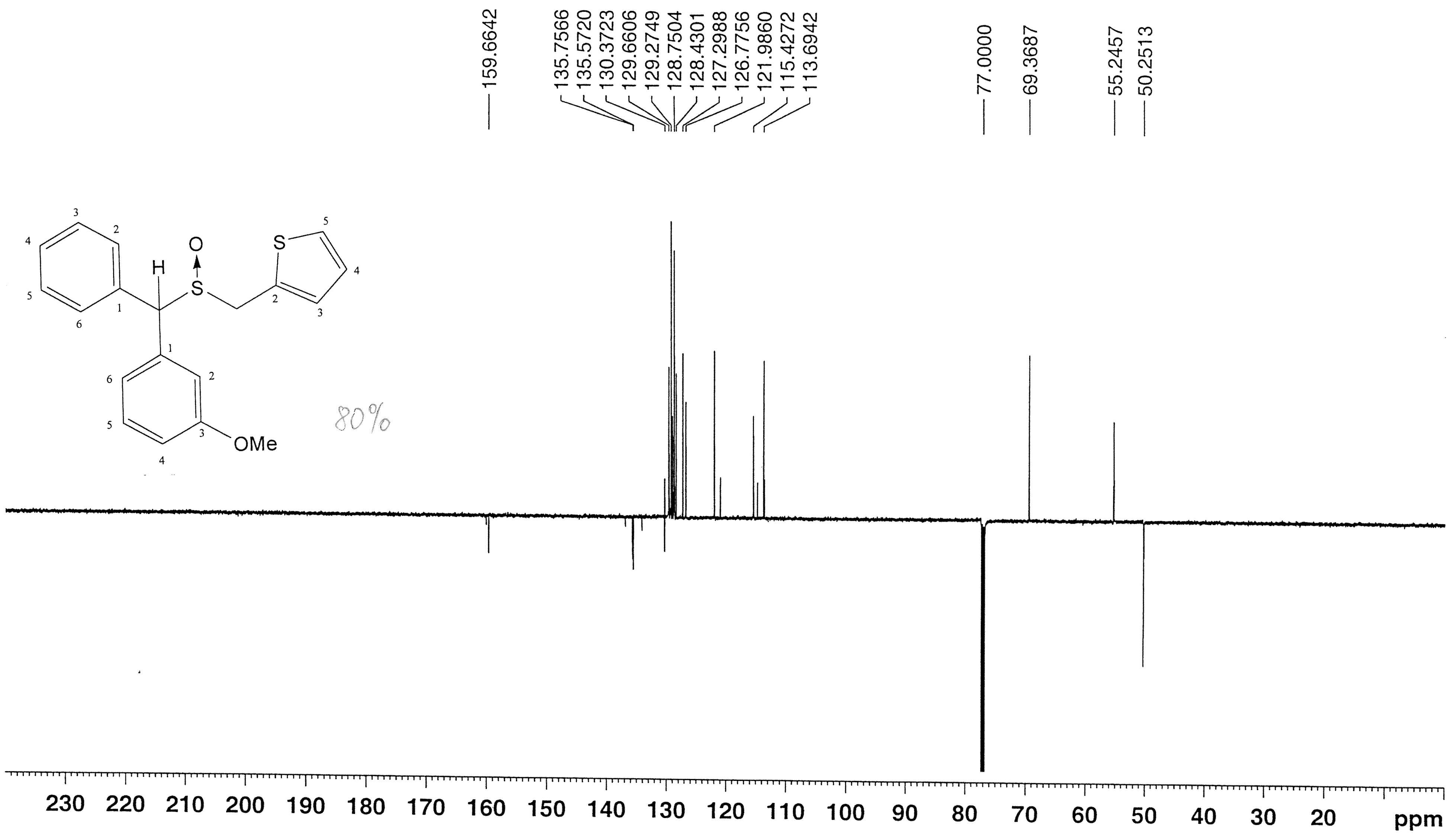

PN008_2_1 in cdcl3 (APT) 29.8.2019

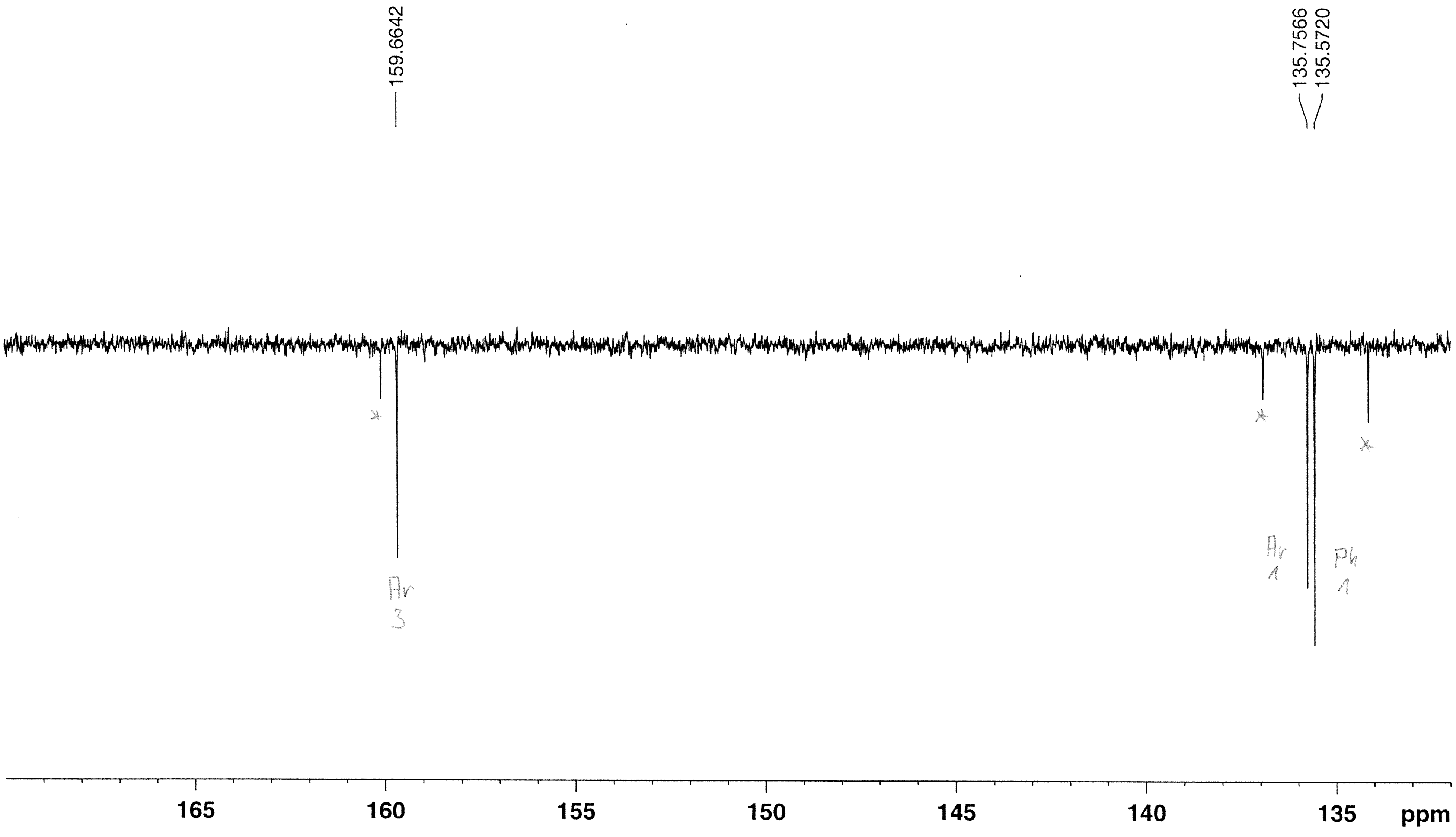

PN008_2_1 in cdcl3 (APT) 29.8.2019

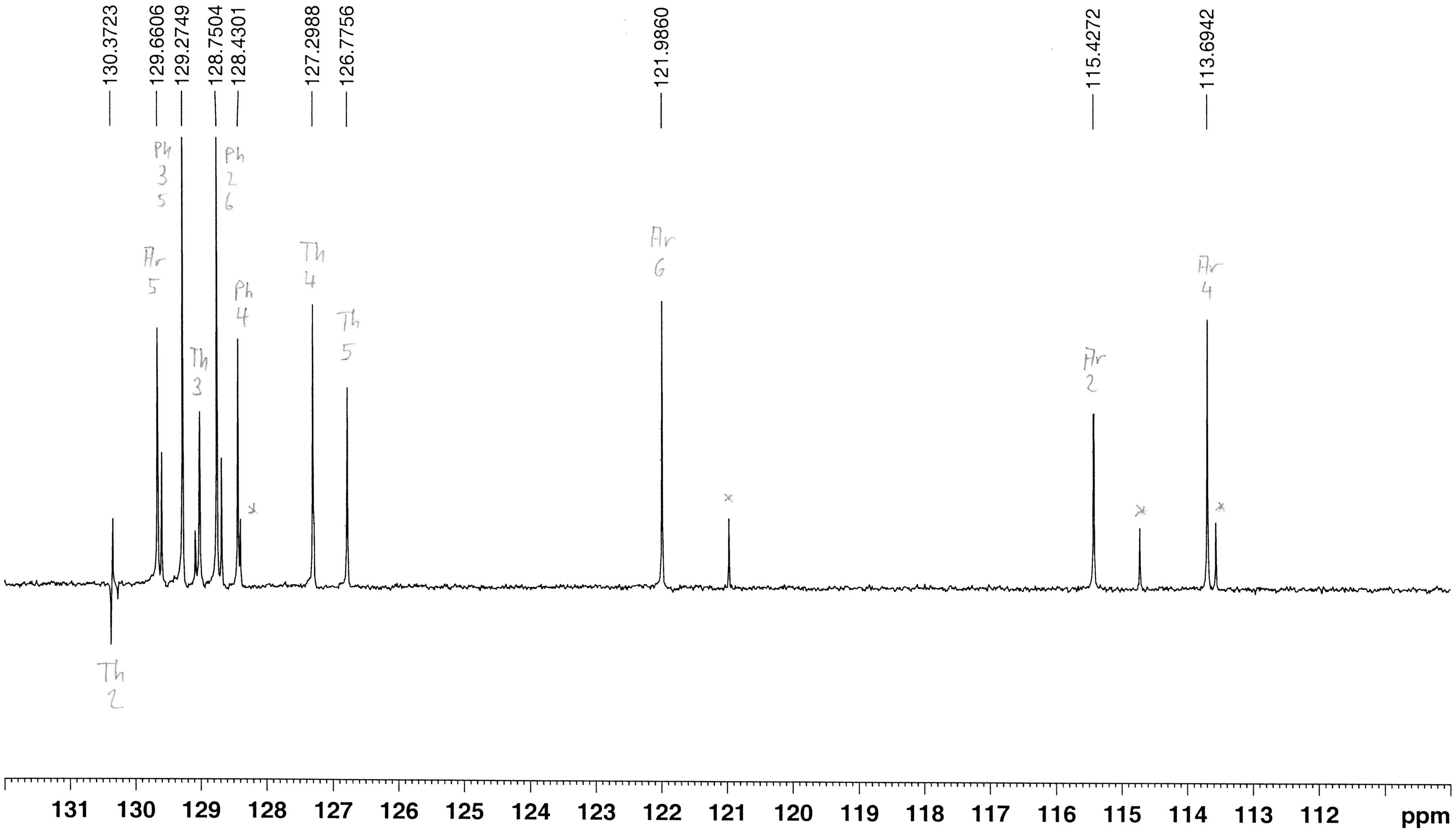

PN008_2_1 in cdcl3 (APT) 29.8.2019

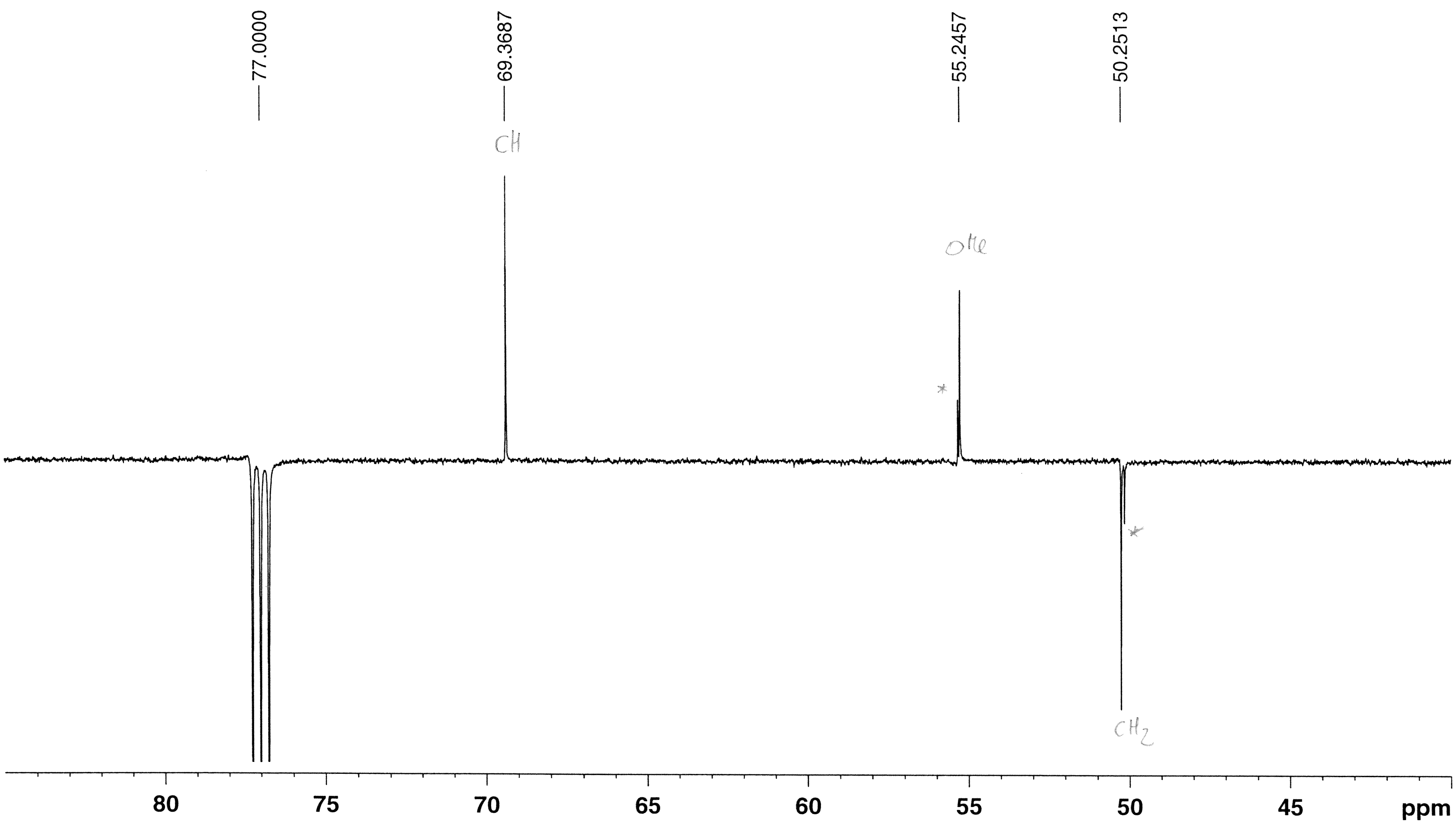

Figure S76c. NMR spectra of compound **8s**.

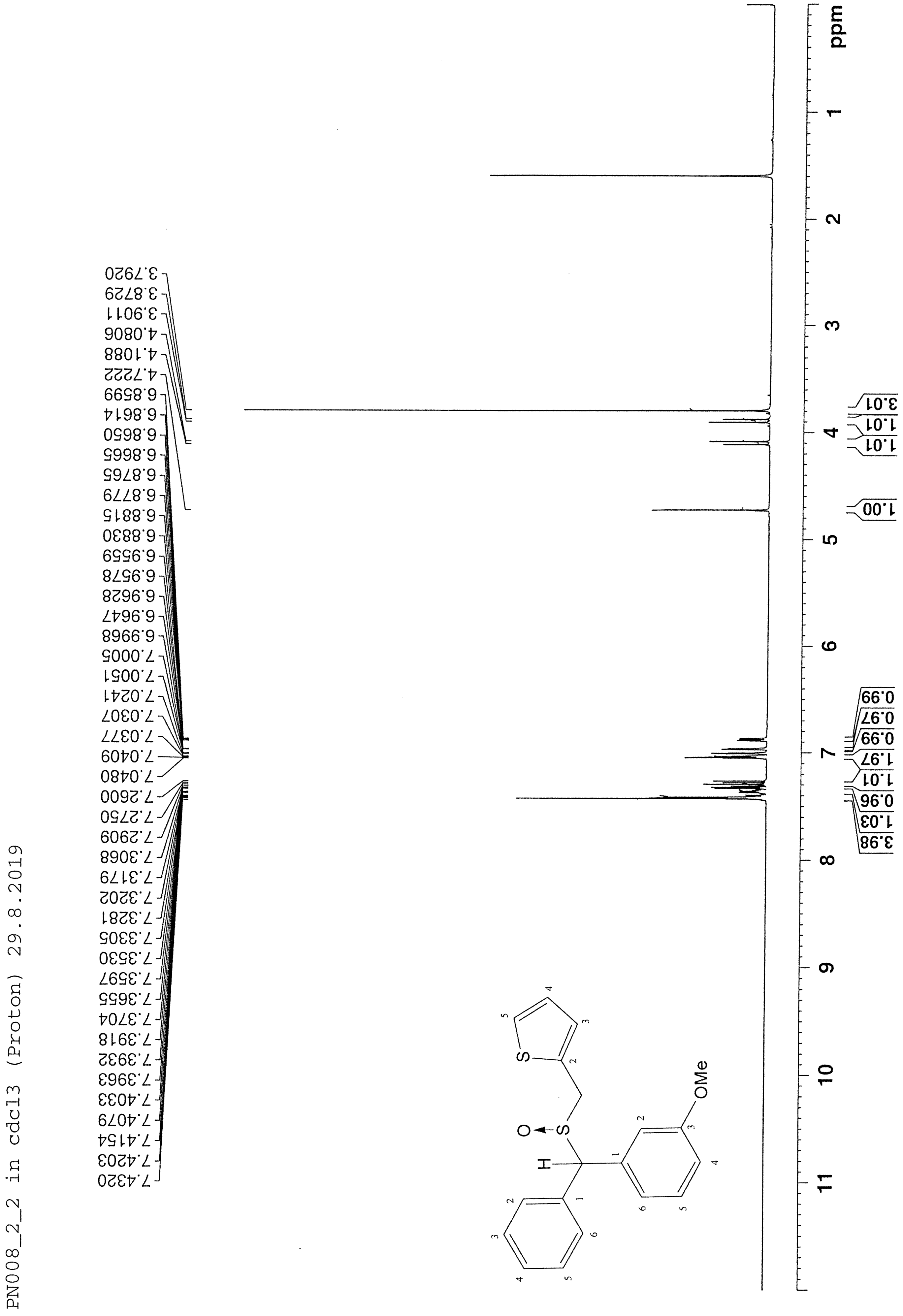

PN008_2_2 in cdcl3 (Proton) 29.8.2019

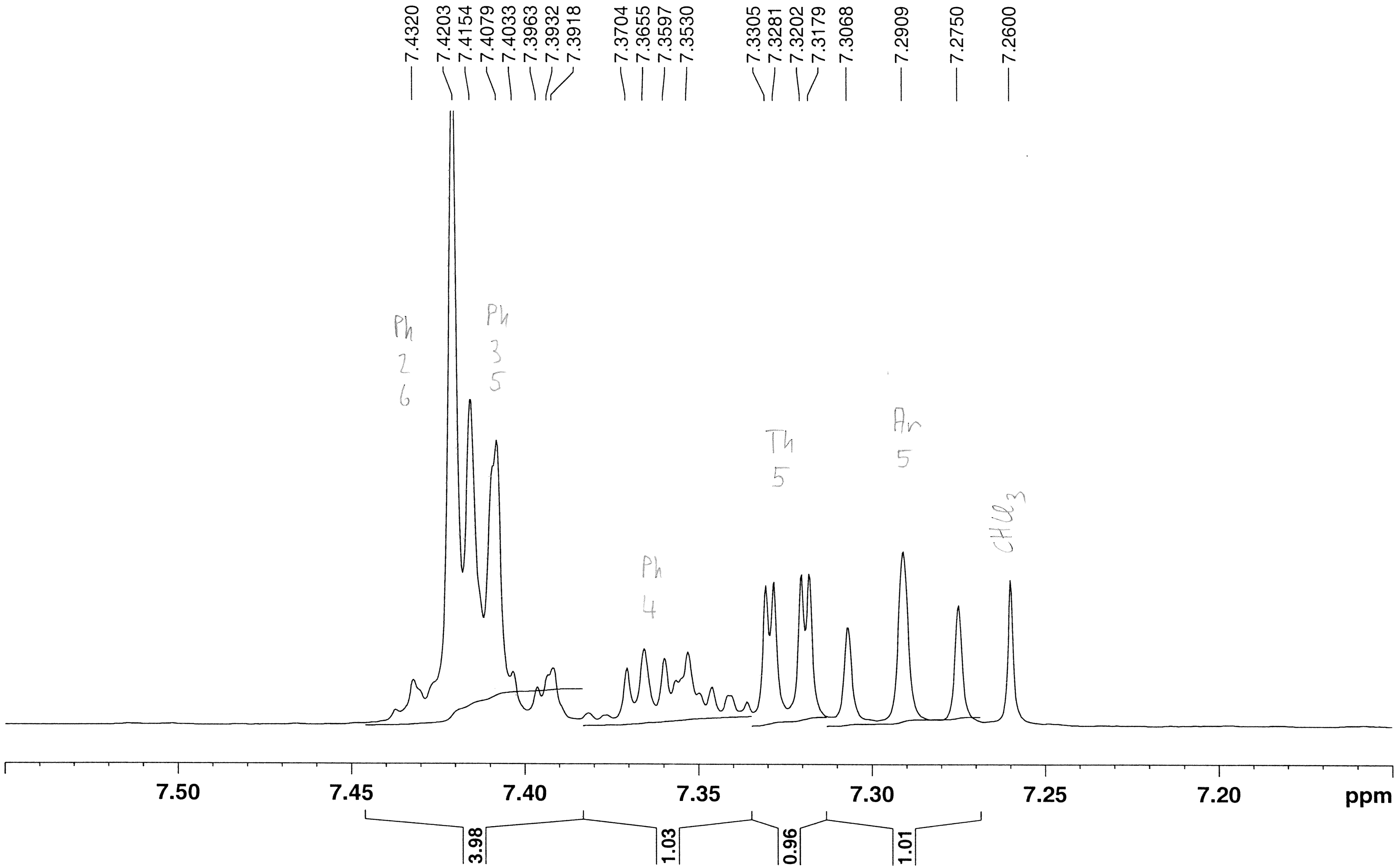

PN008_2_2 in cdcl3 (Proton) 29.8.2019

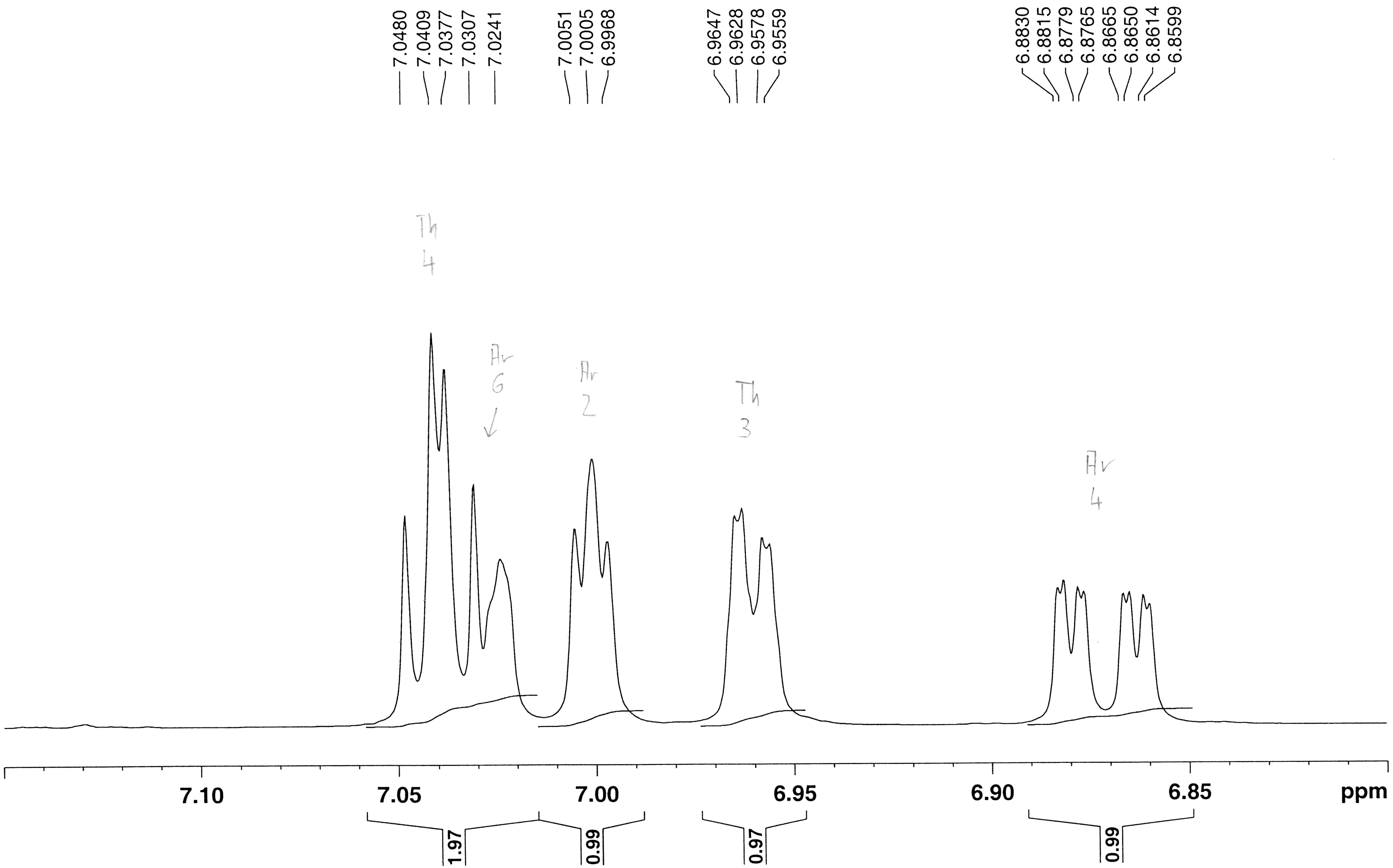

PN008_2_2 in cdcl3 (Proton) 29.8.2019

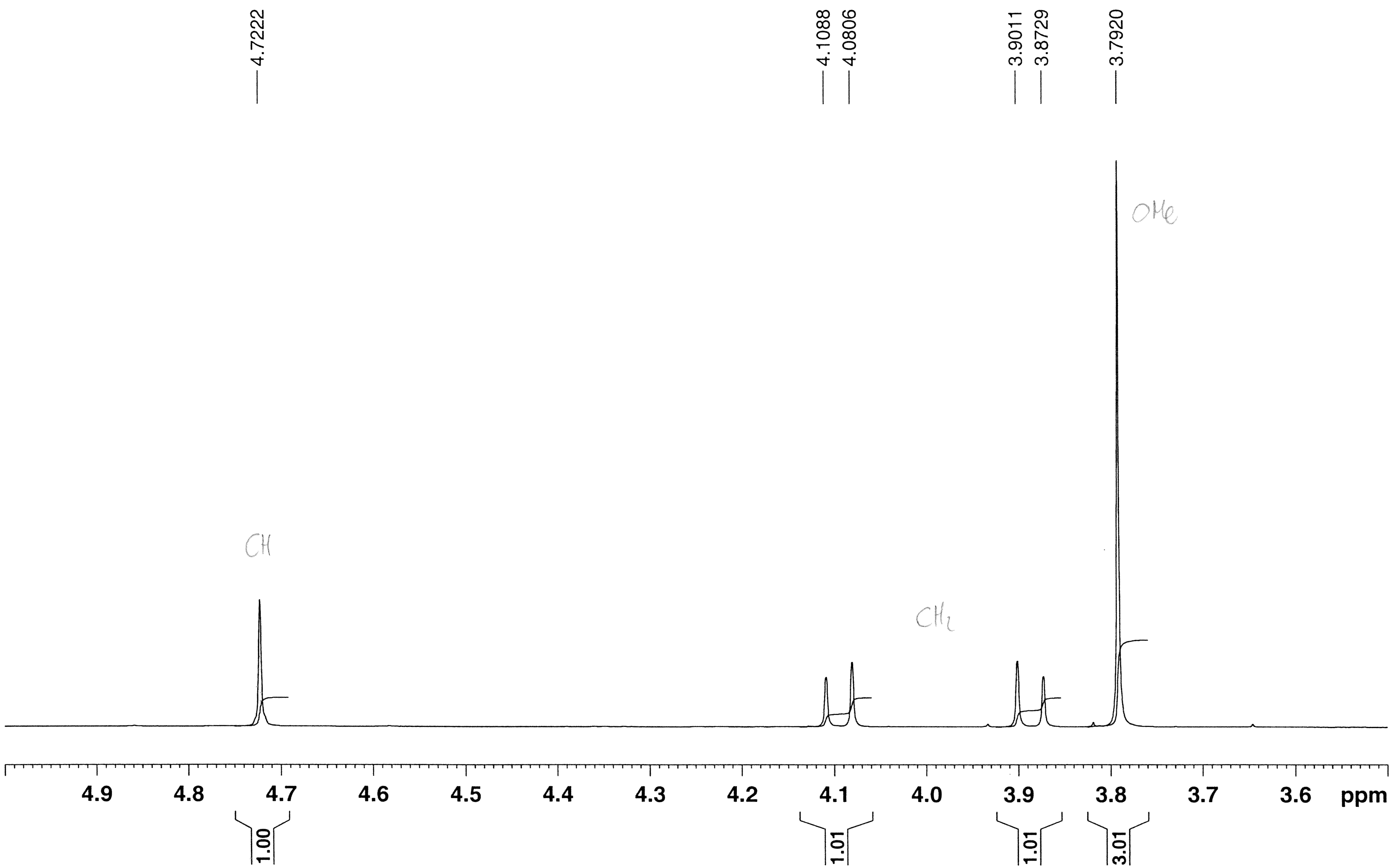

PN008_2_2 in cdcl3 (APT) 29.8.2019

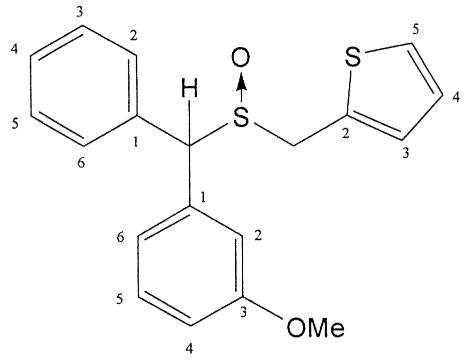

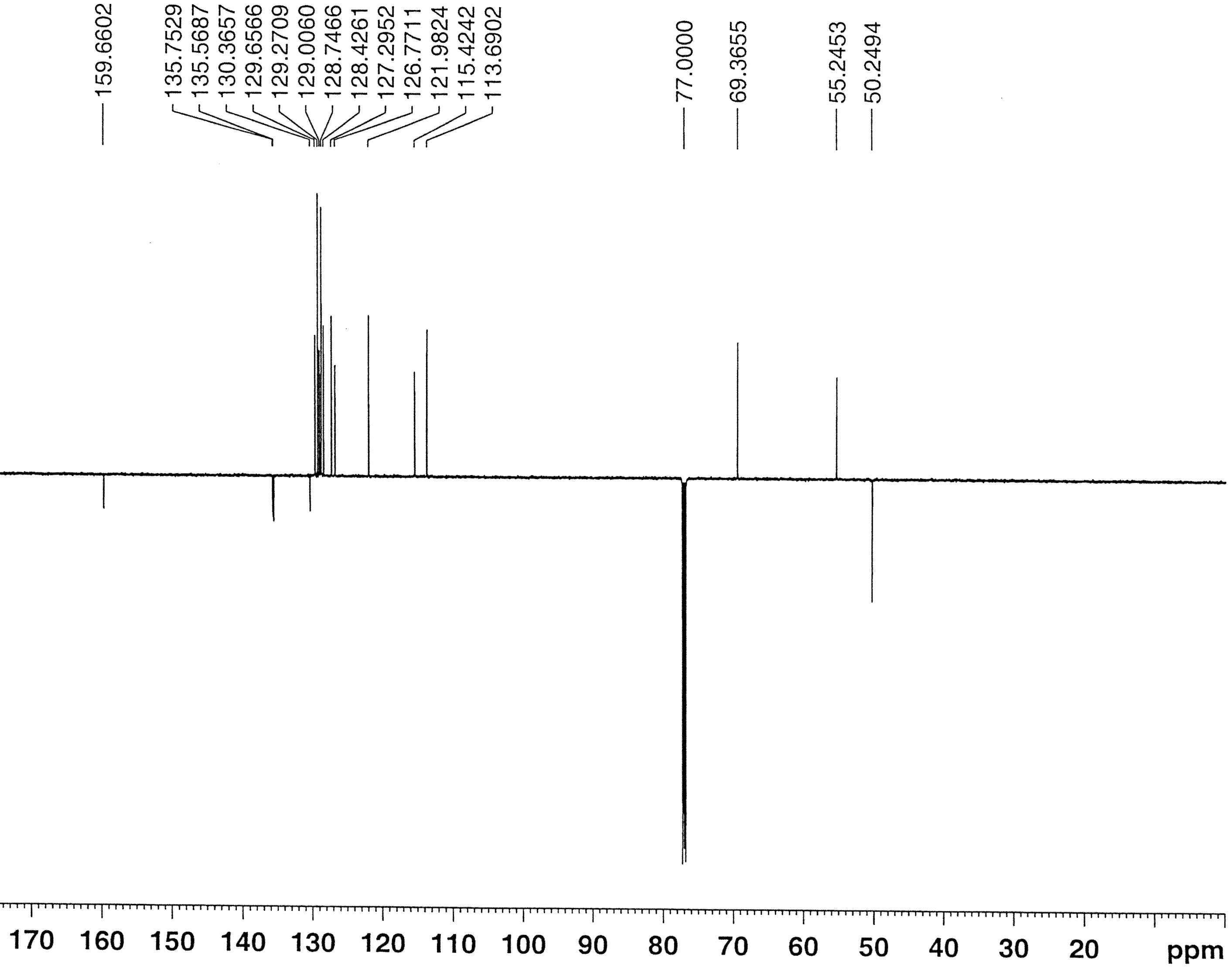

PN008_2_2 in cdcl3 (APT) 29.8.2019

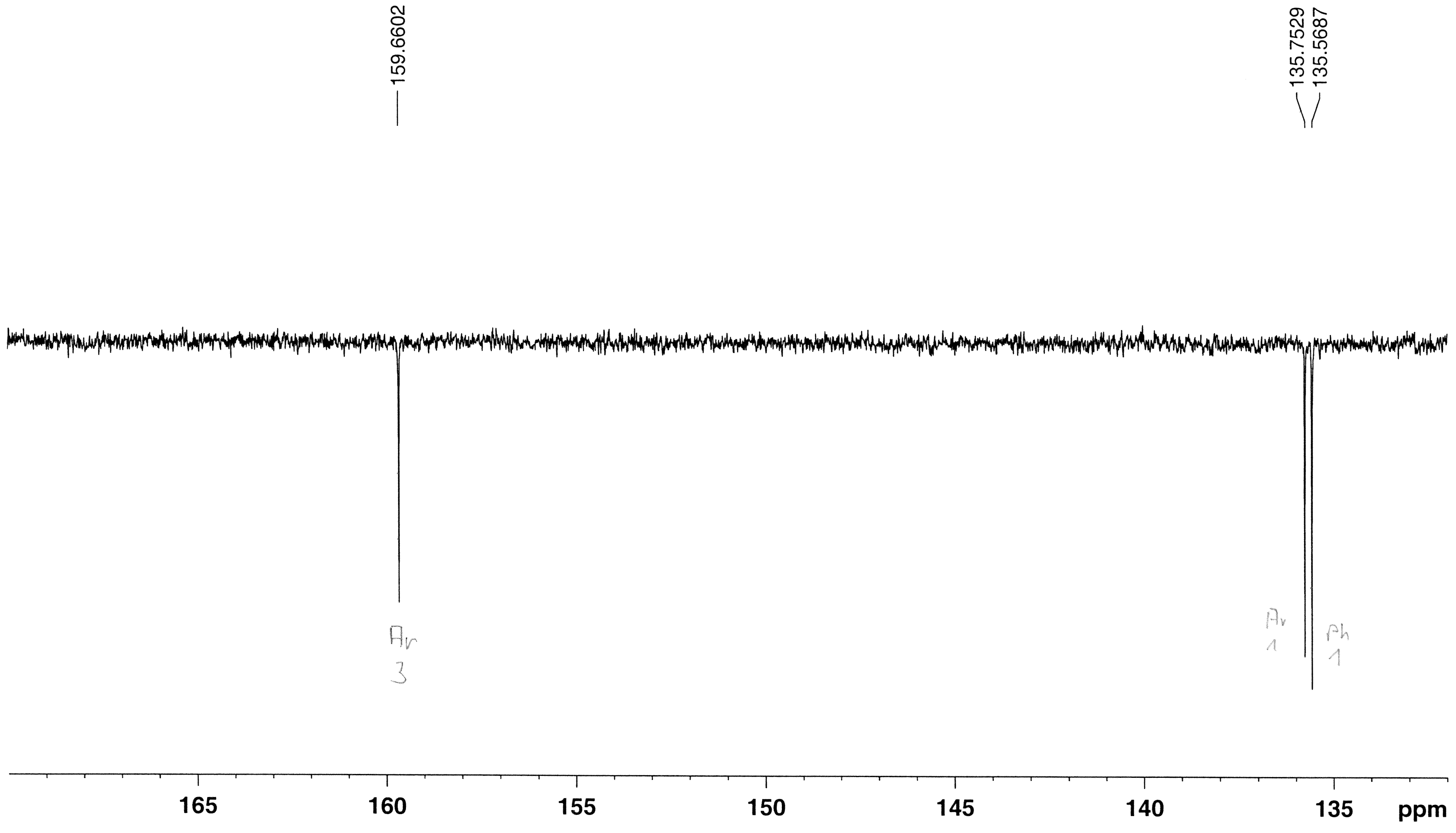

PN008_2_2 in cdcl3 (APT) 29.8.2019

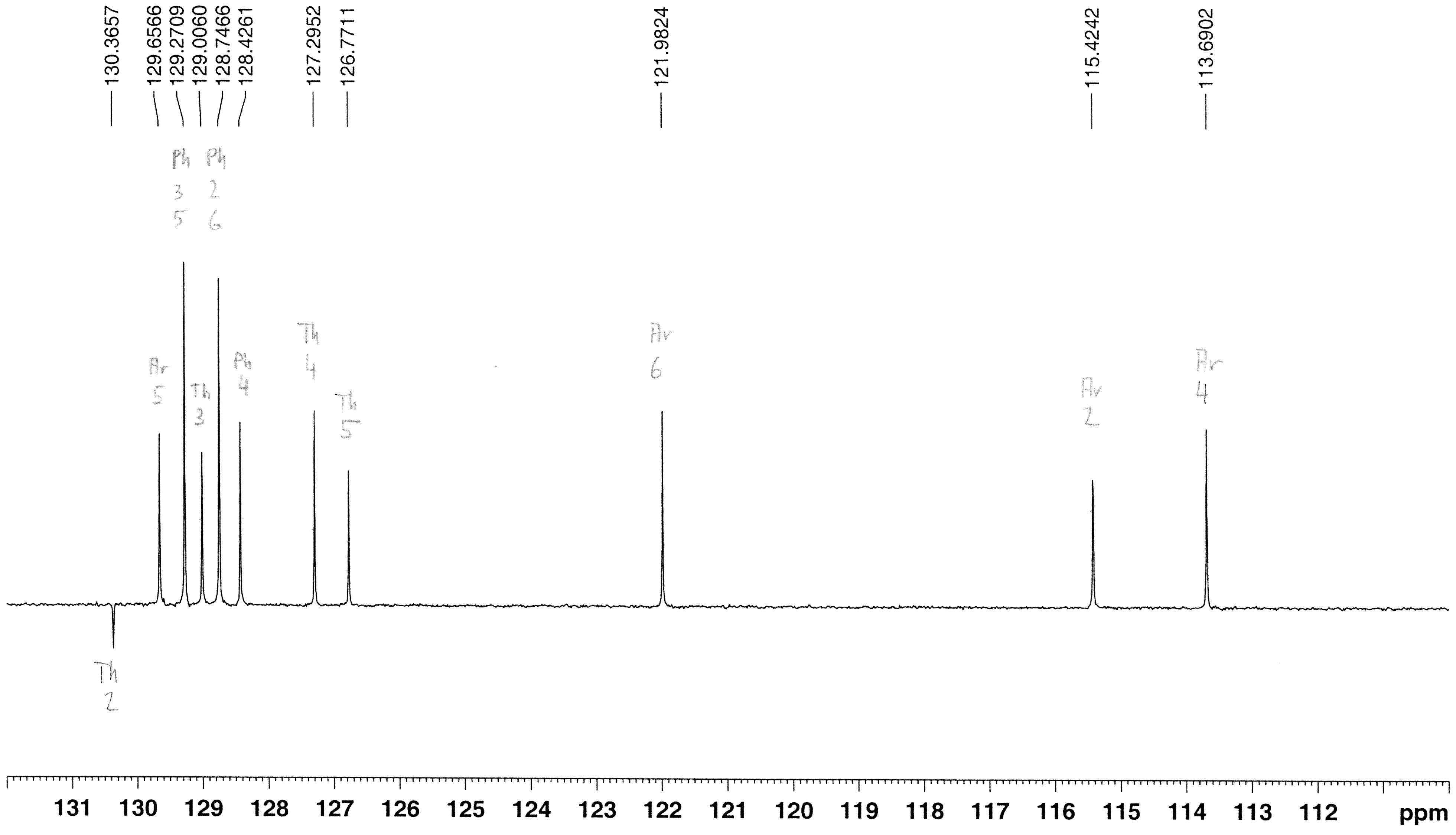

PN008_2_2 in cdcl3 (APT) 29.8.2019

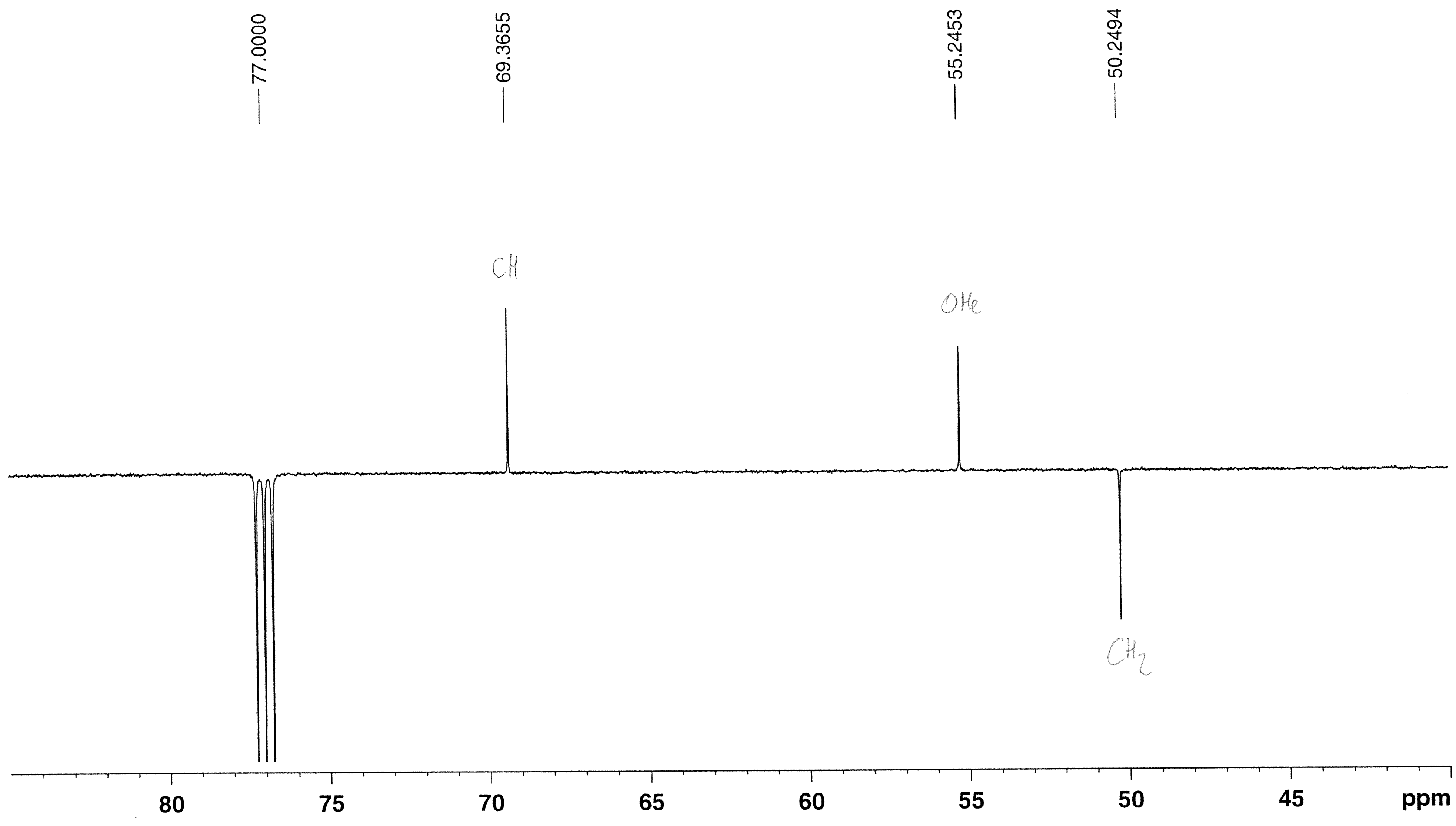

PN008_2_2 in cdcl3 (COSY) 29.8.2019

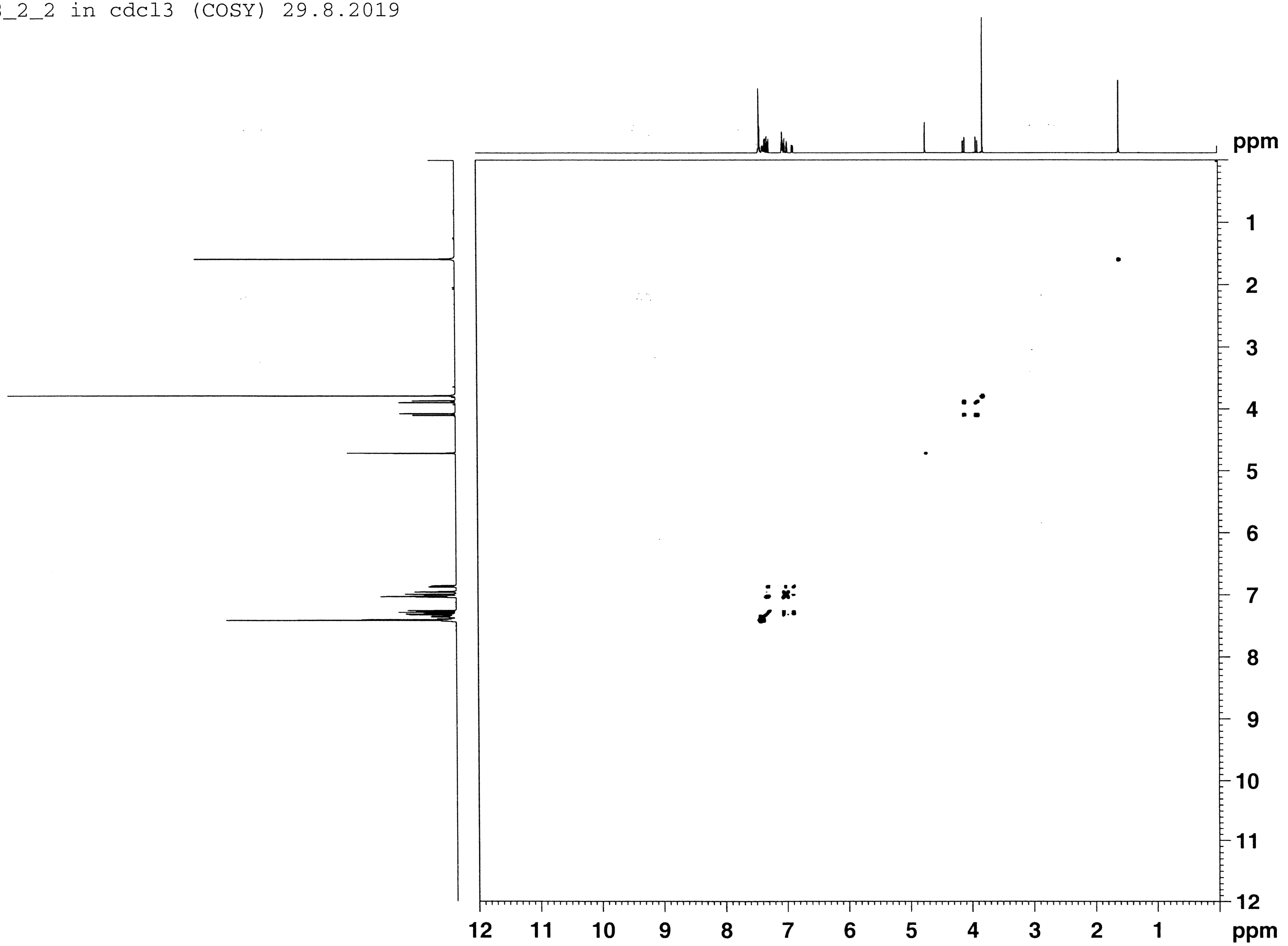

PN008_2_2 in cdcl3 (COSY) 29.8.2019

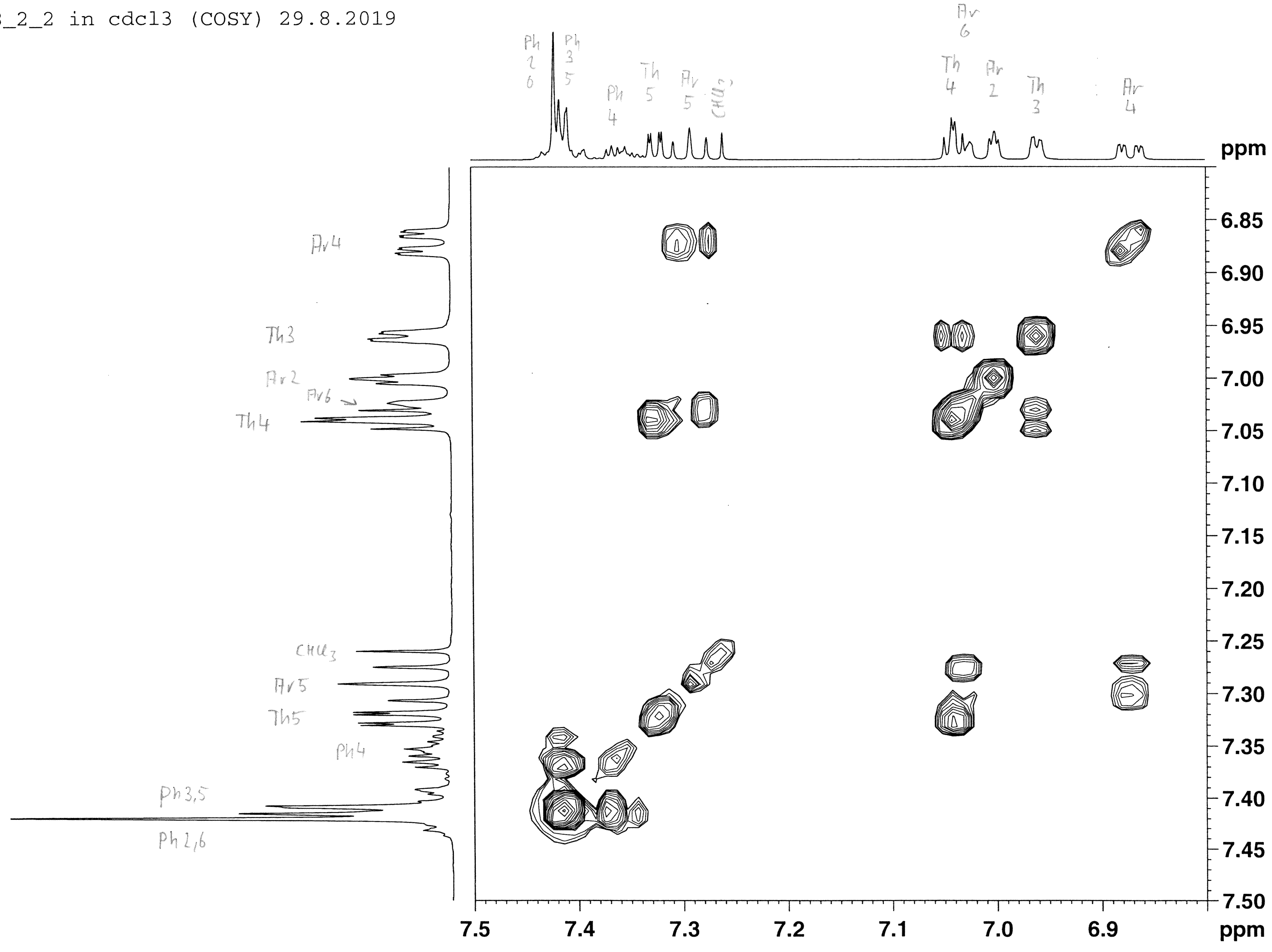

PN008_2_2 in cdcl3 (COSY) 29.8.2019

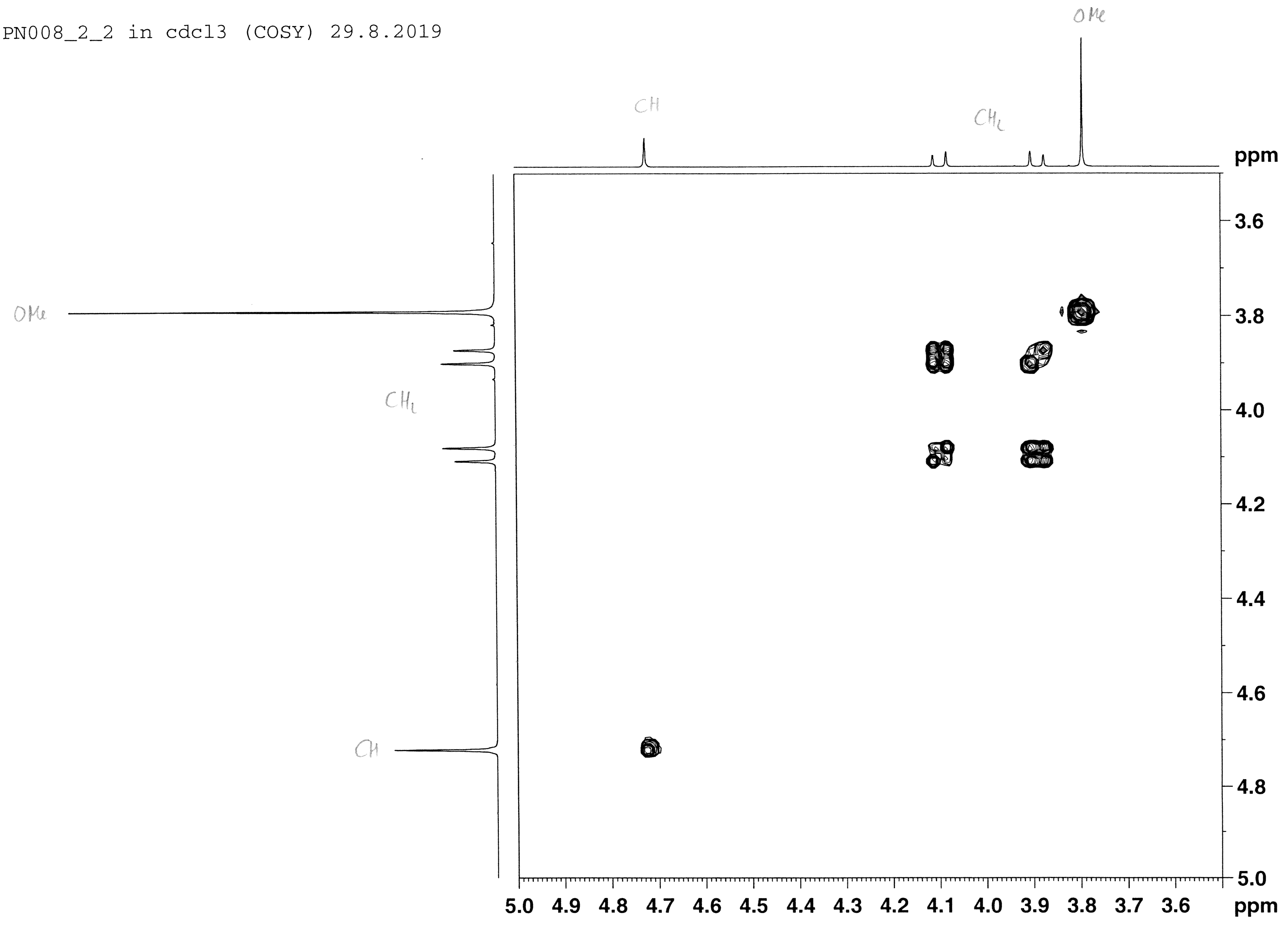

PN008_2_2 in cdcl3 (HSQC) 29.8.2019

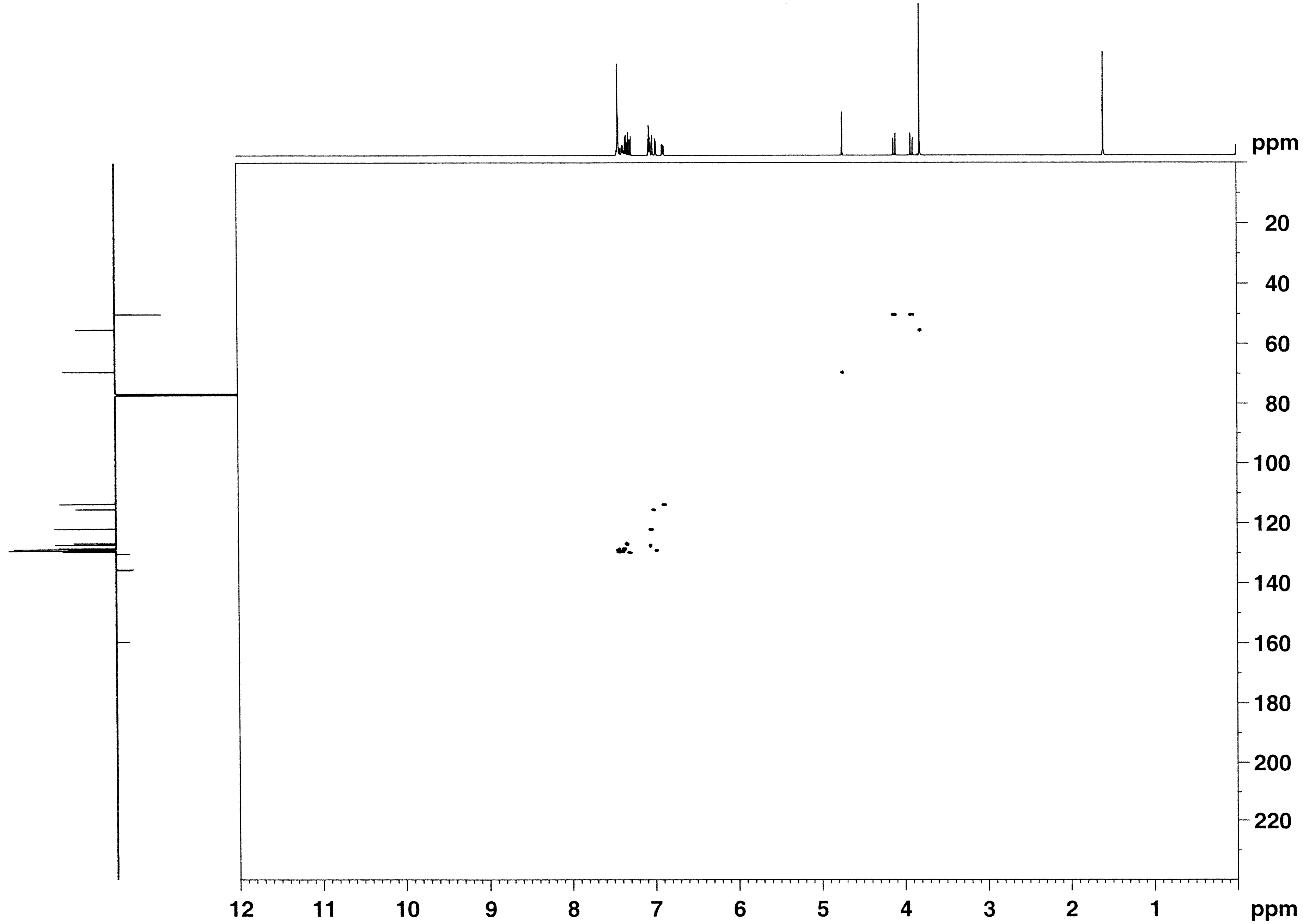

PN008_2_2 in cdcl3 (HSQC) 29.8.2019

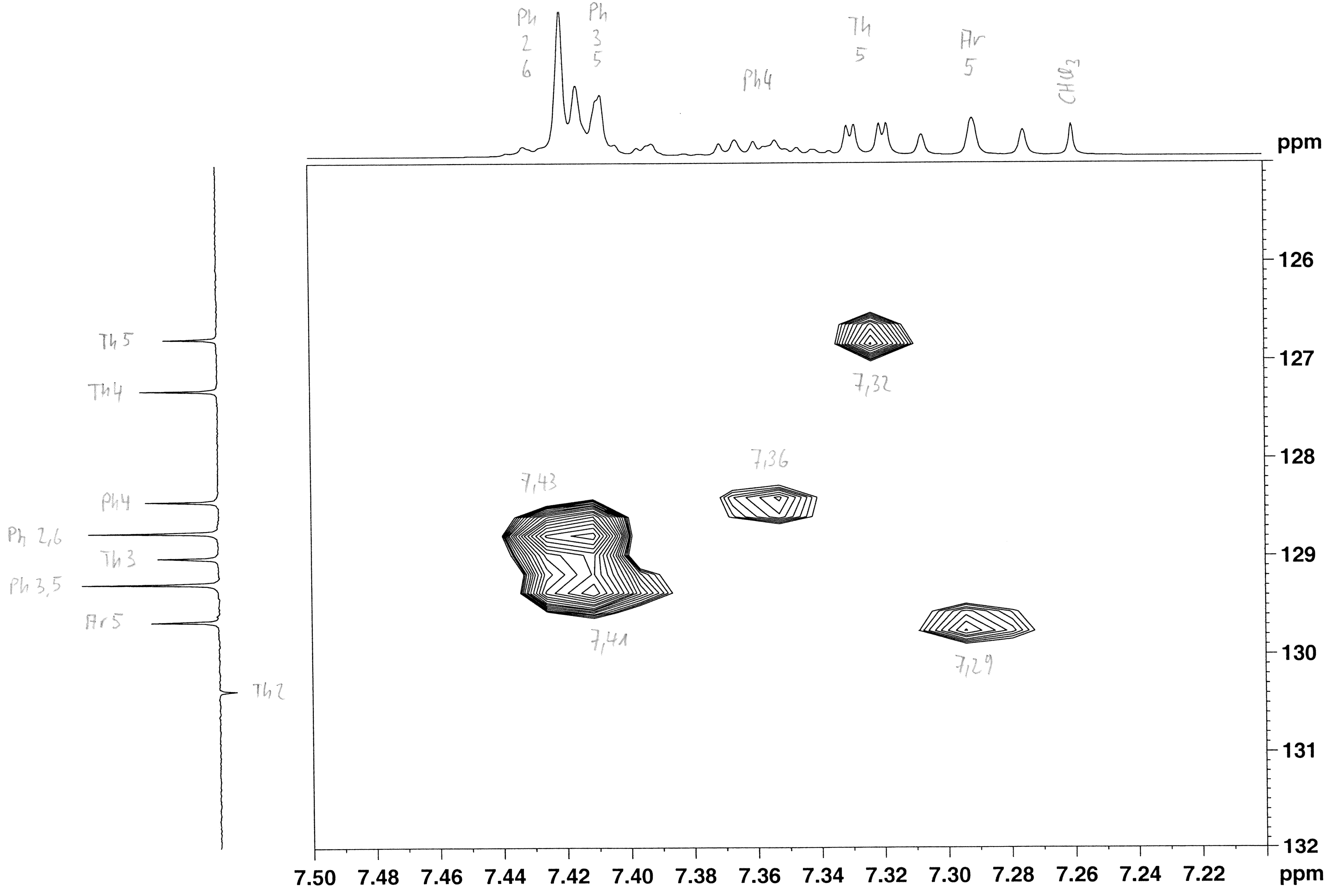

PN008_2_2 in cdcl3 (HSQC) 29.8.2019

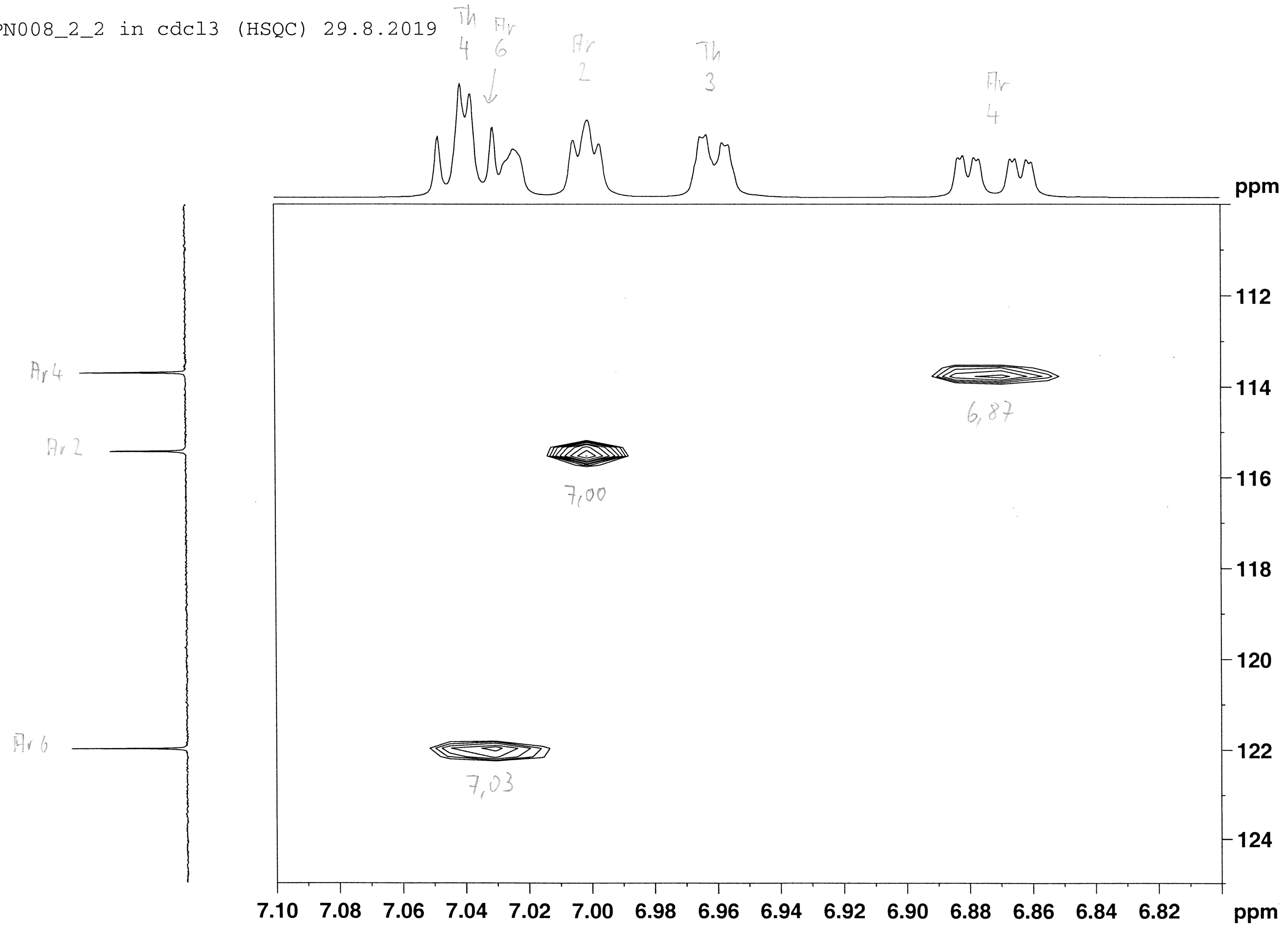

PN008_2_2 in cdcl3 (HSQC) 29.8.2019

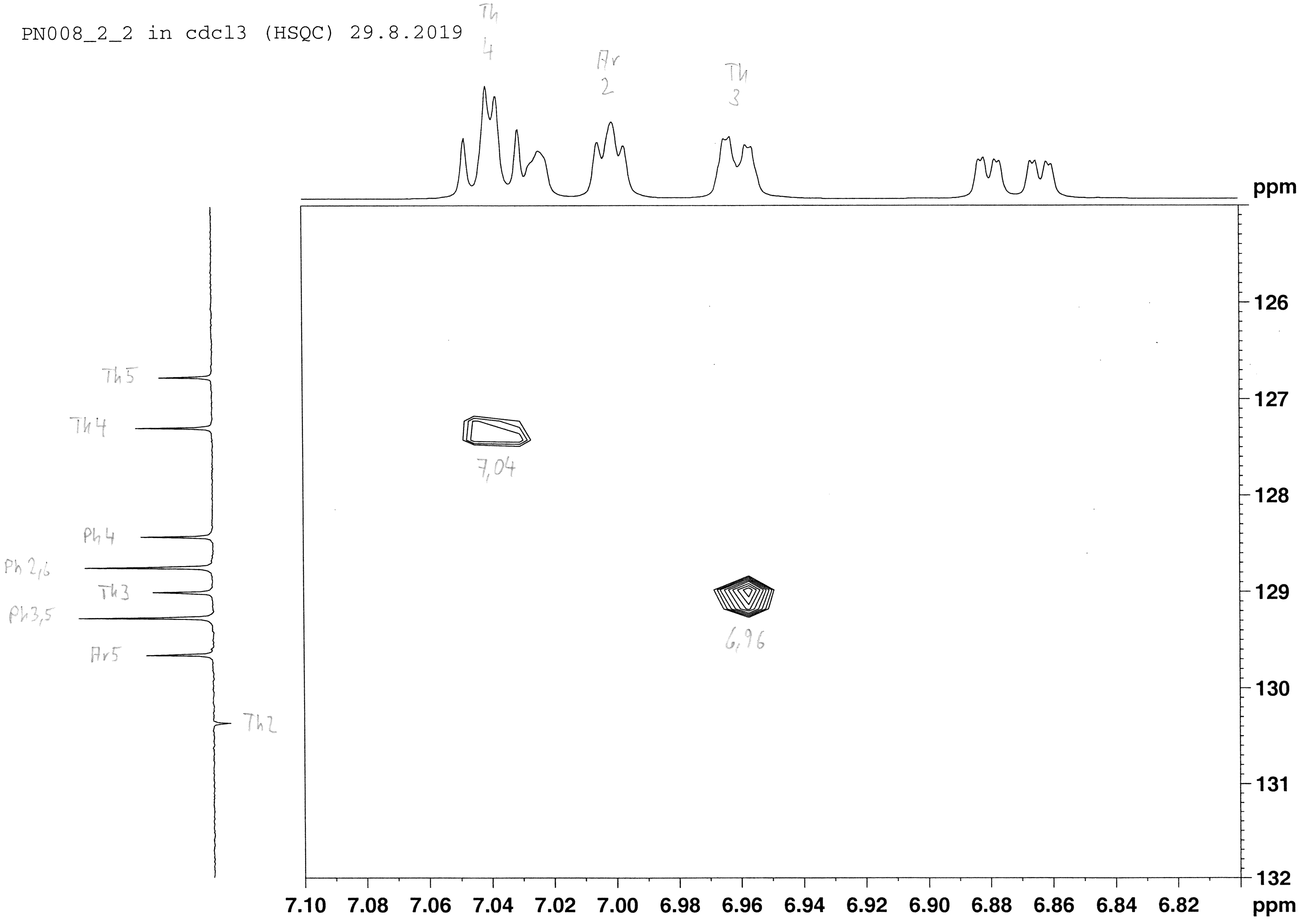

PN008_2_2 in cdcl3 (HSQC) 29.8.2019

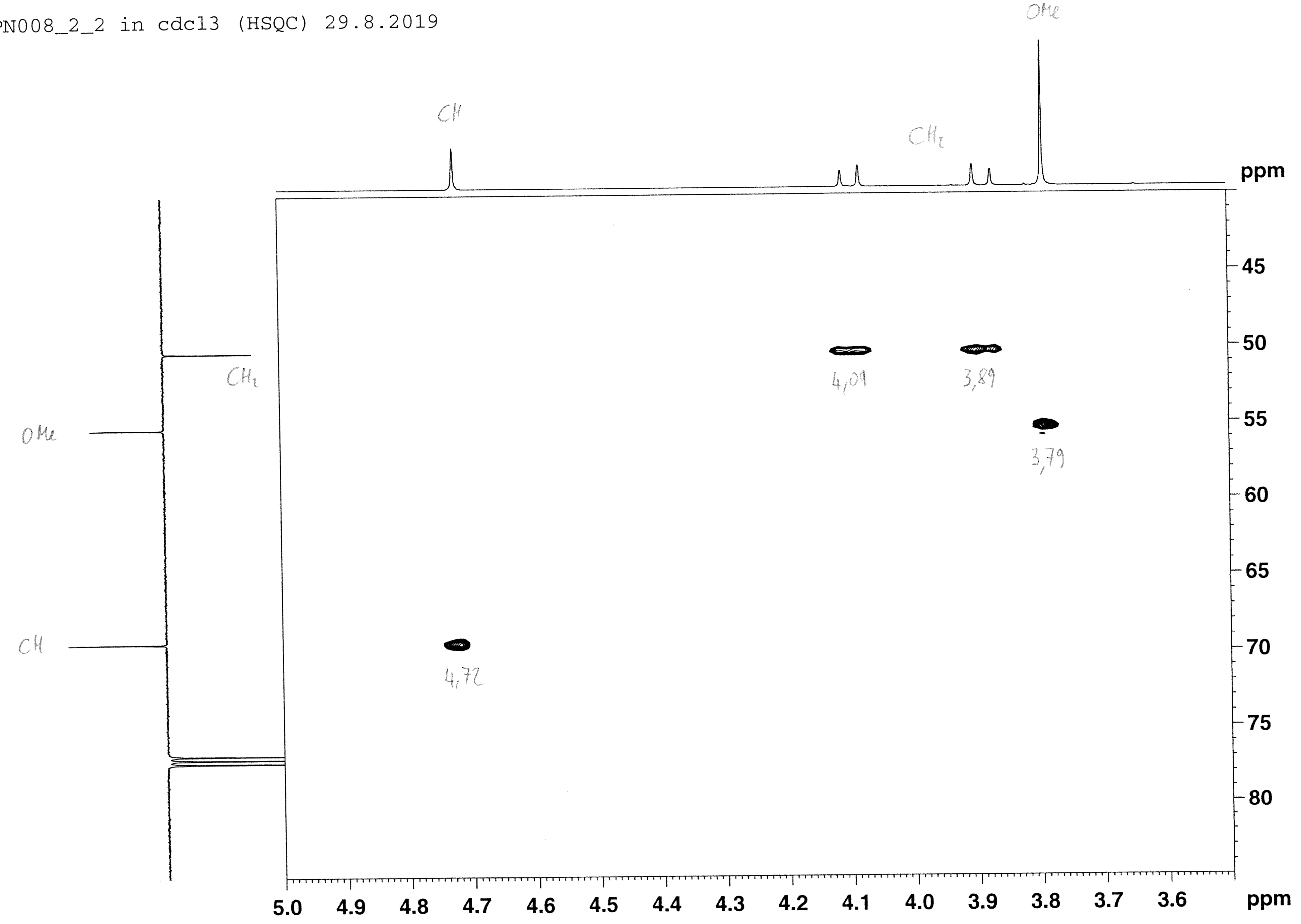

PN008_2_2 in cdcl3 (HMBC) 29.8.2019

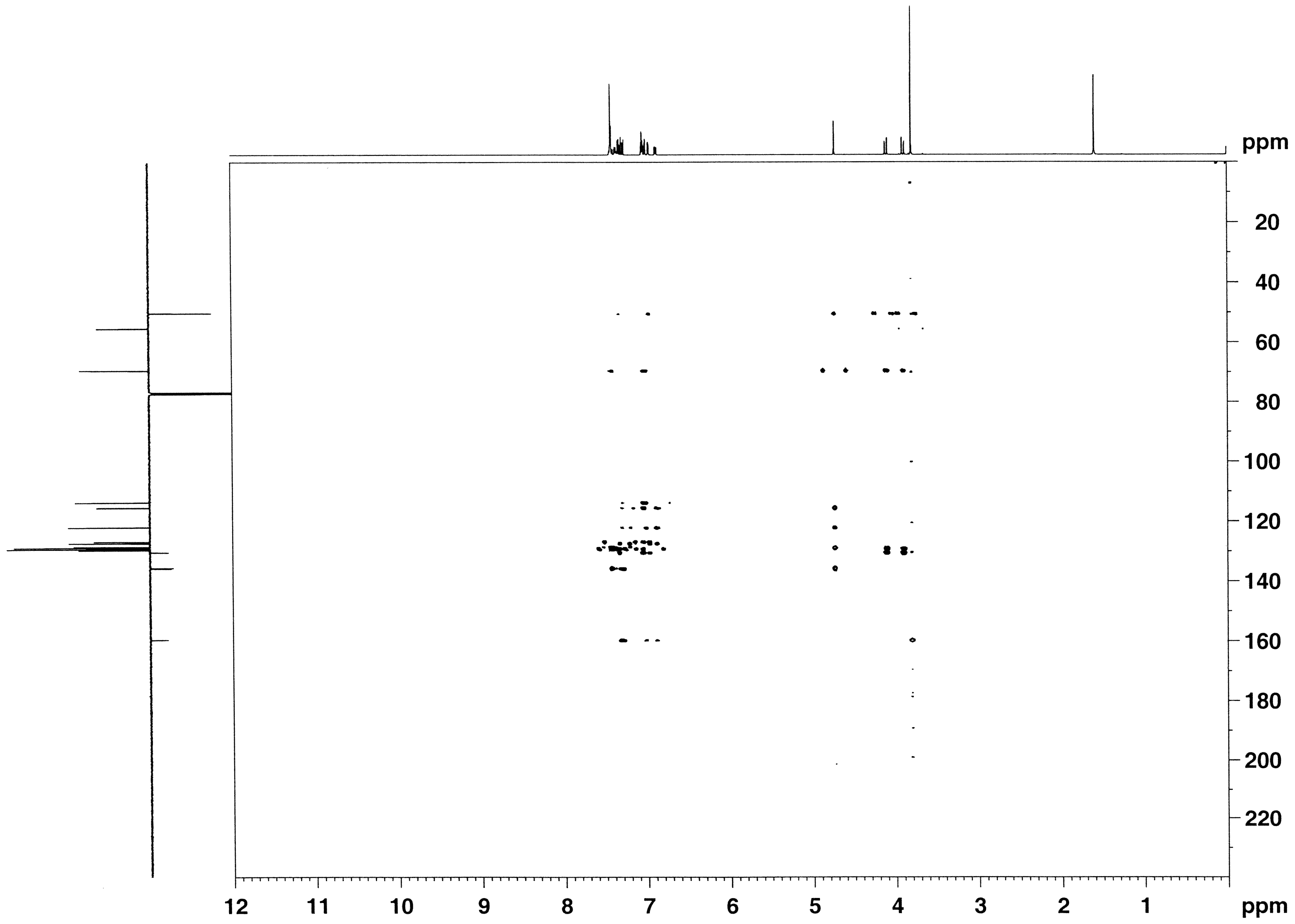

PN008_2_2 in cdcl3 (HMBC) 29.8.2019

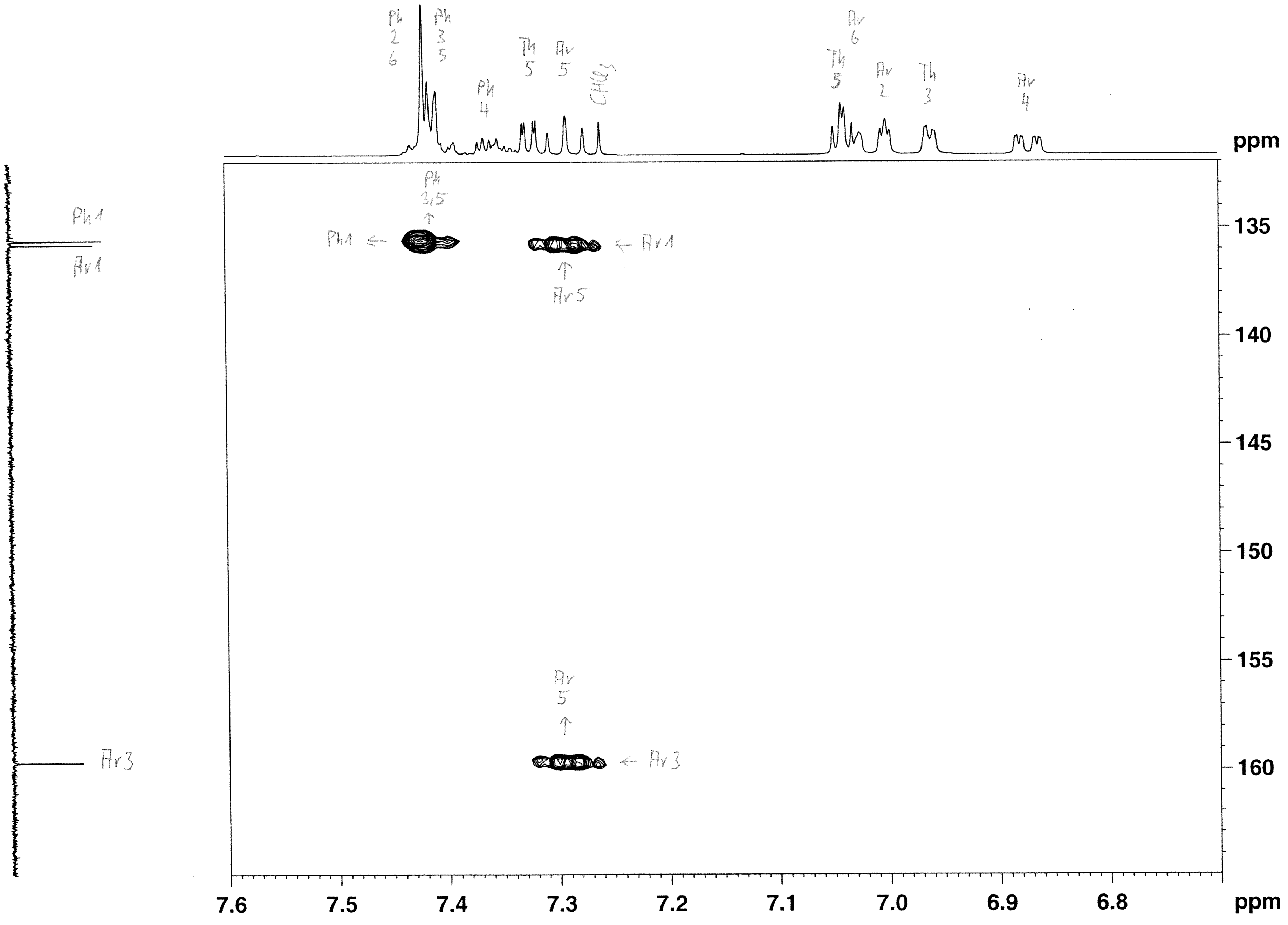

PN008_2_2 in cdcl3 (HMBC) 29.8.2019

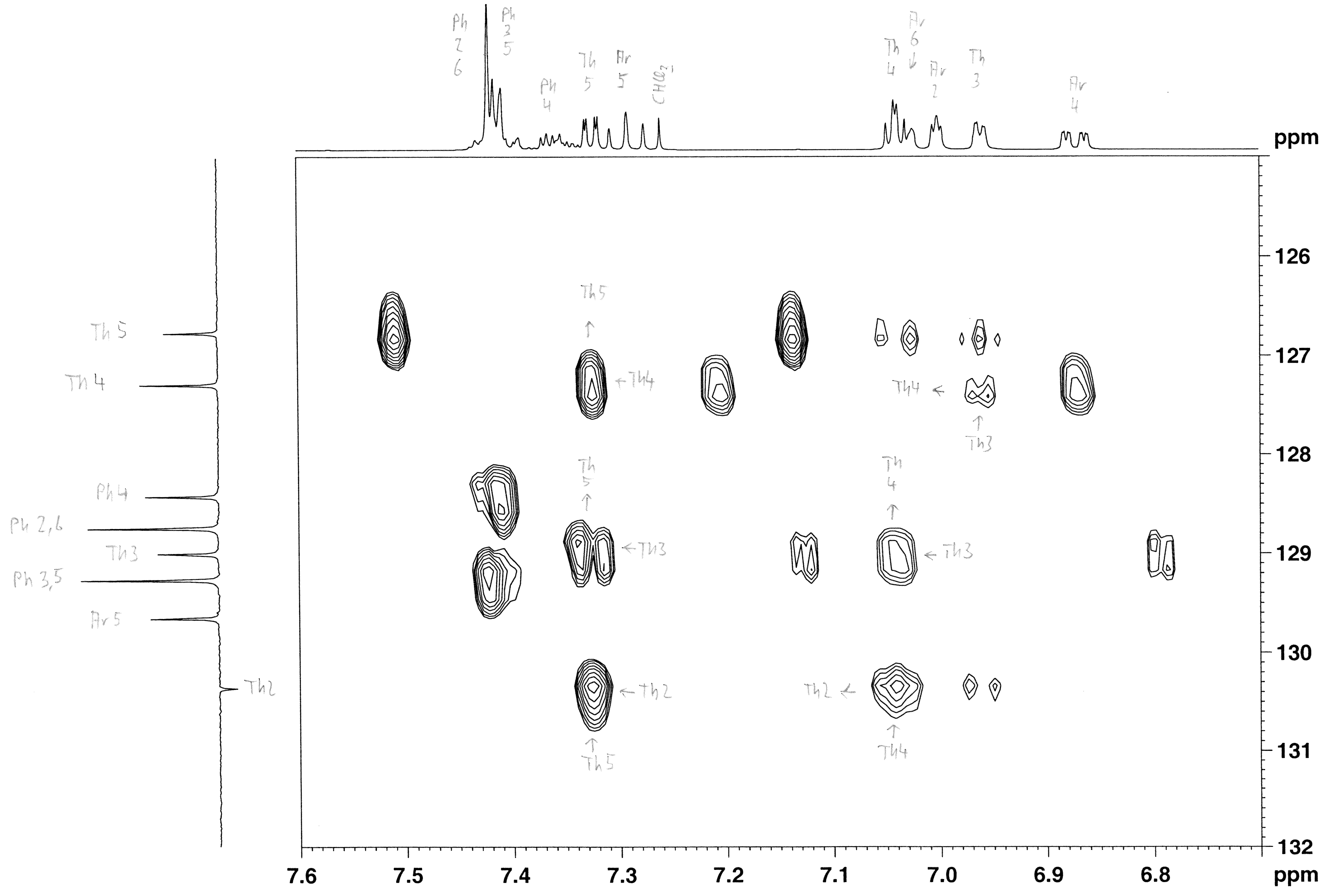

PN008_2_2 in cdcl3 (HMBC) 29.8.2019

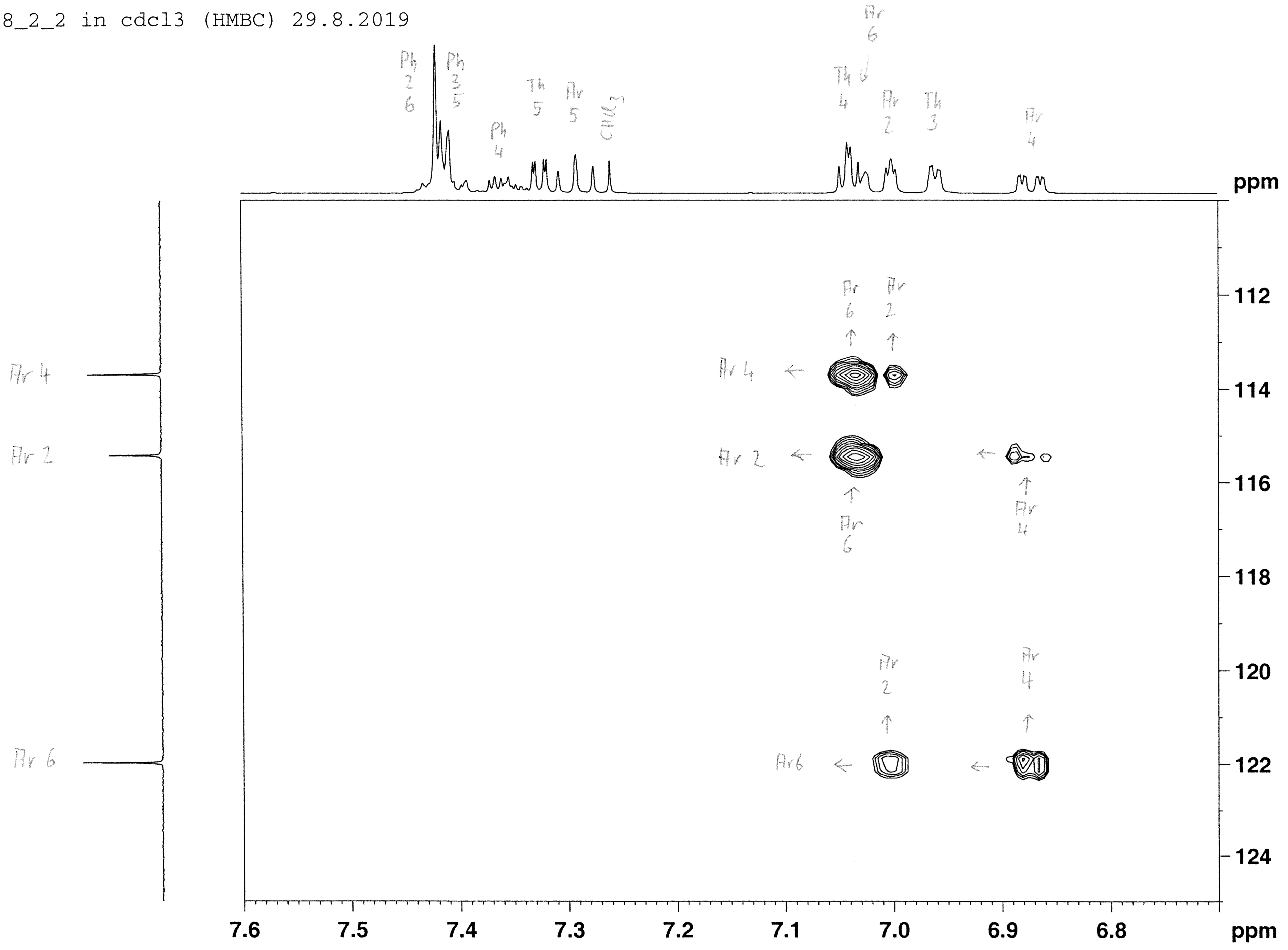

PN008_2_2 in cdcl3 (HMBC) 29.8.2019

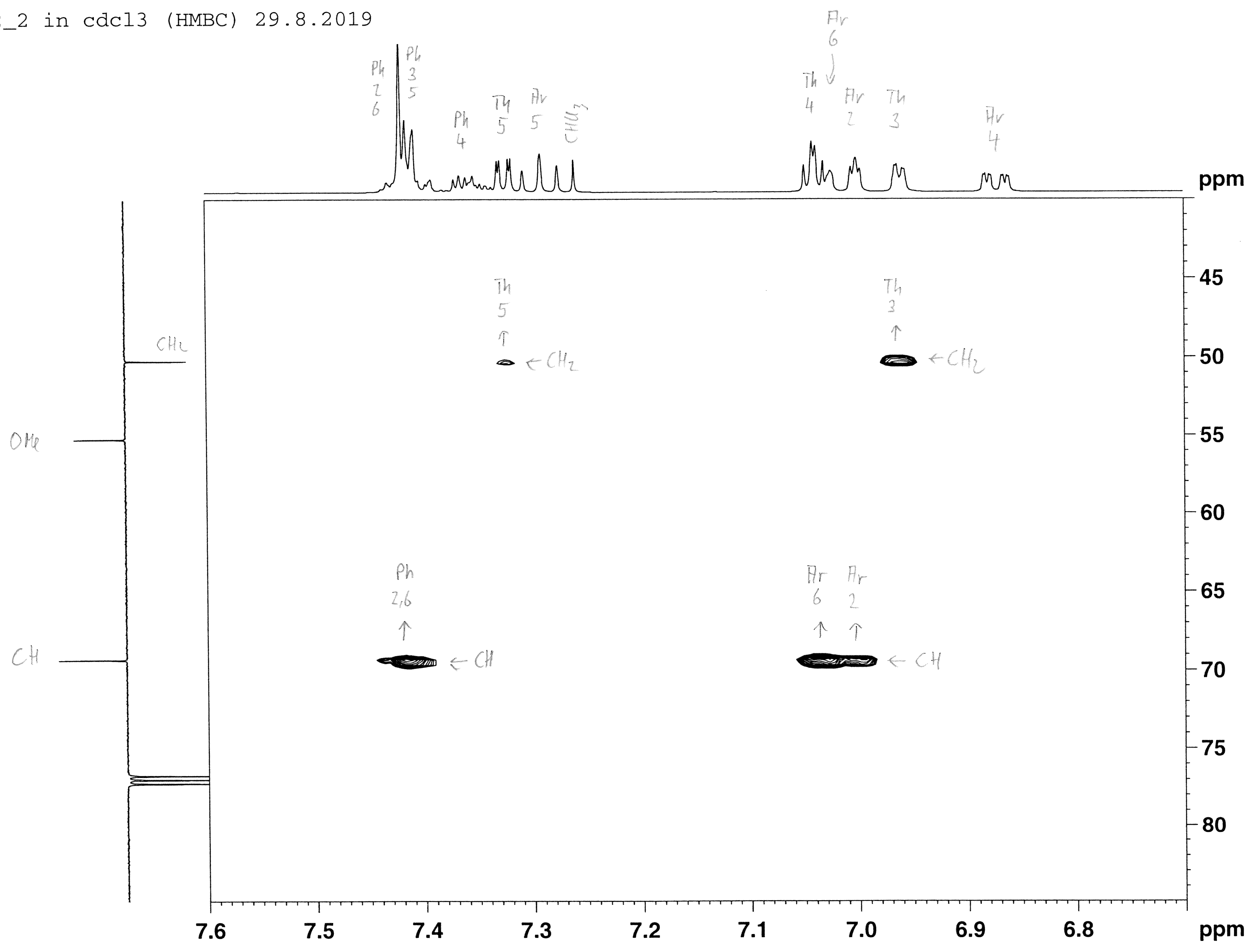

PN008_2_2 in cdcl3 (HMBC) 29.8.2019

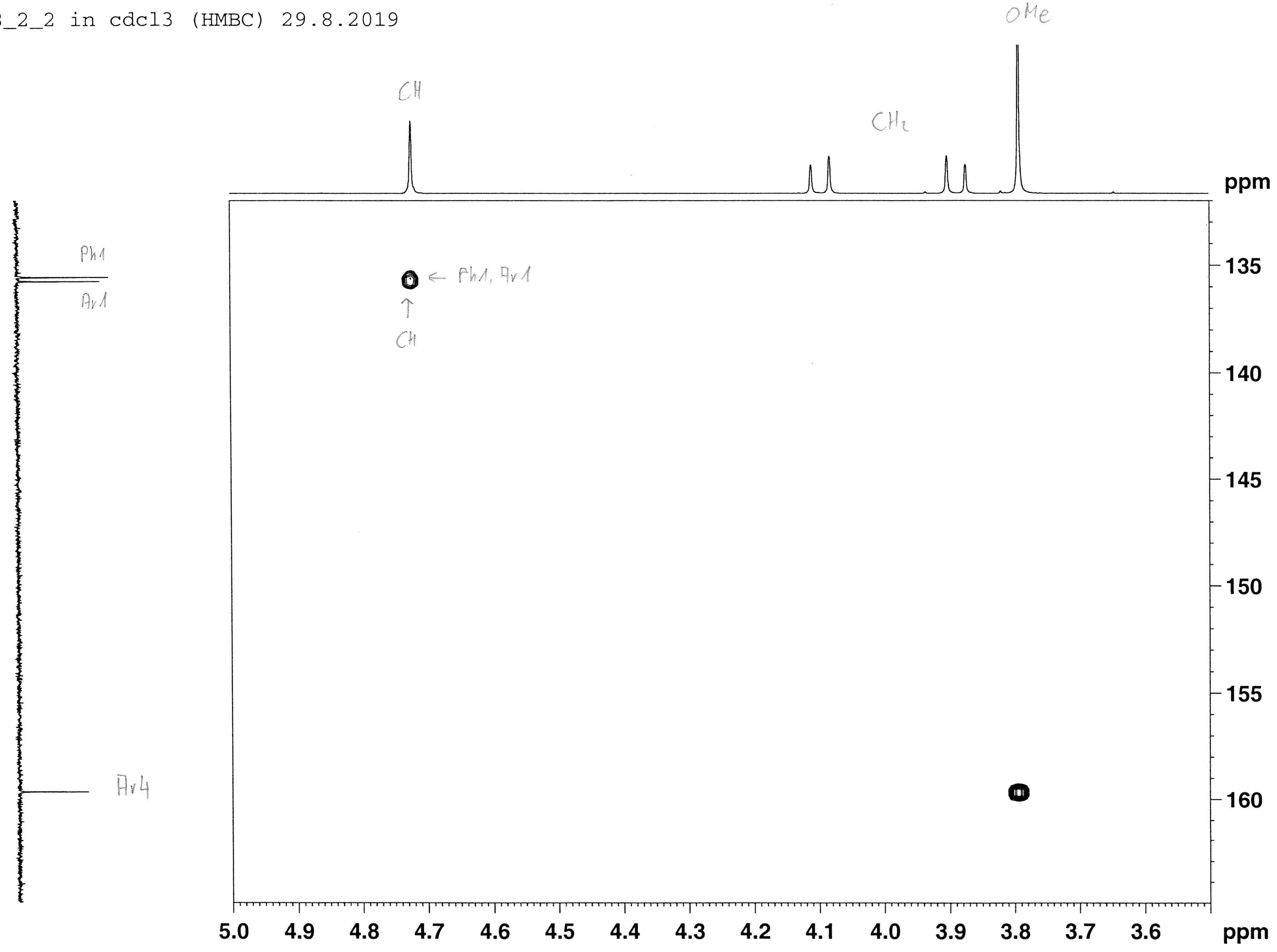

PN008_2_2 in cdcl3 (HMBC) 29.8.2019

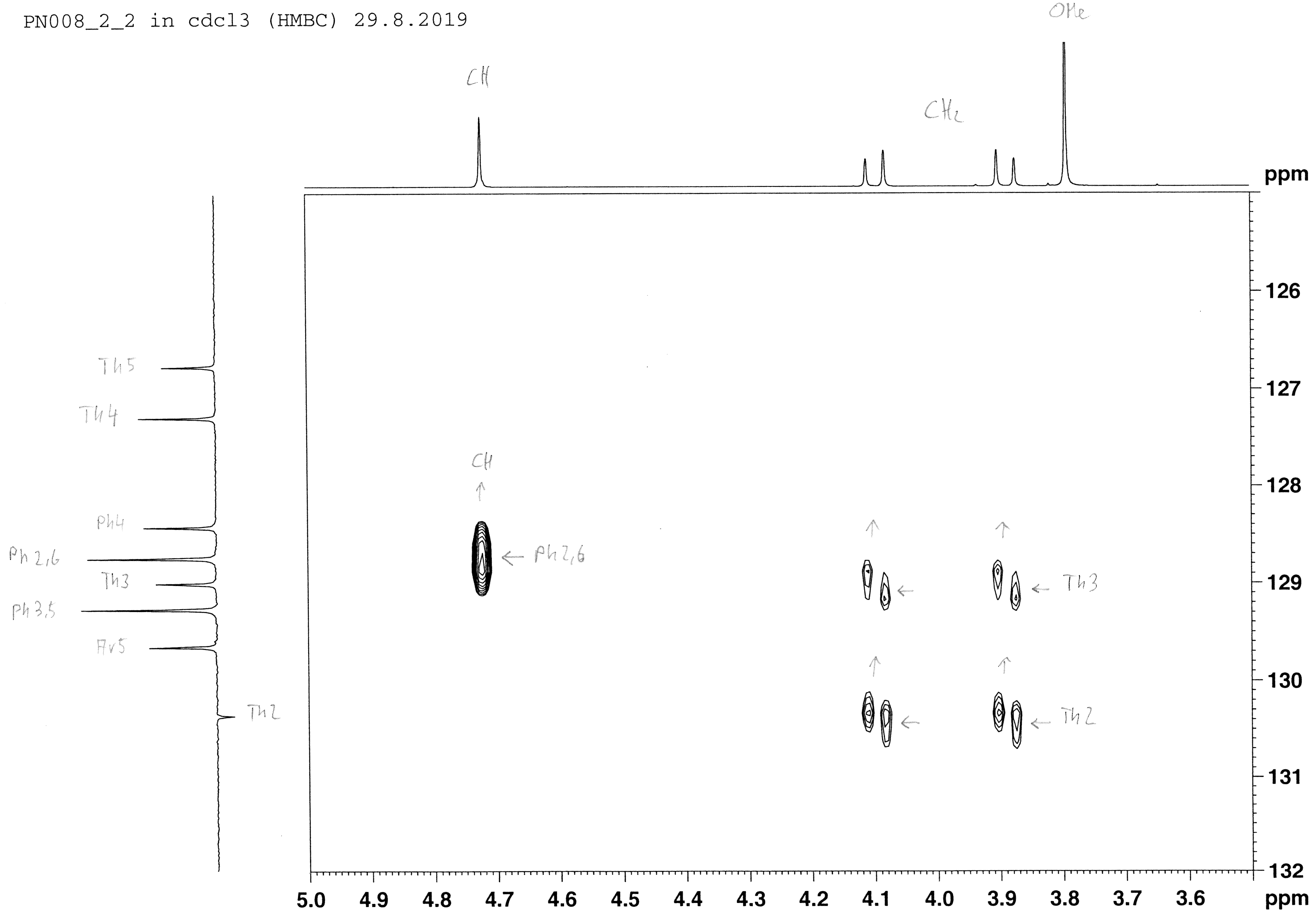

PN008_2_2 in cdcl3 (HMBC) 29.8.2019

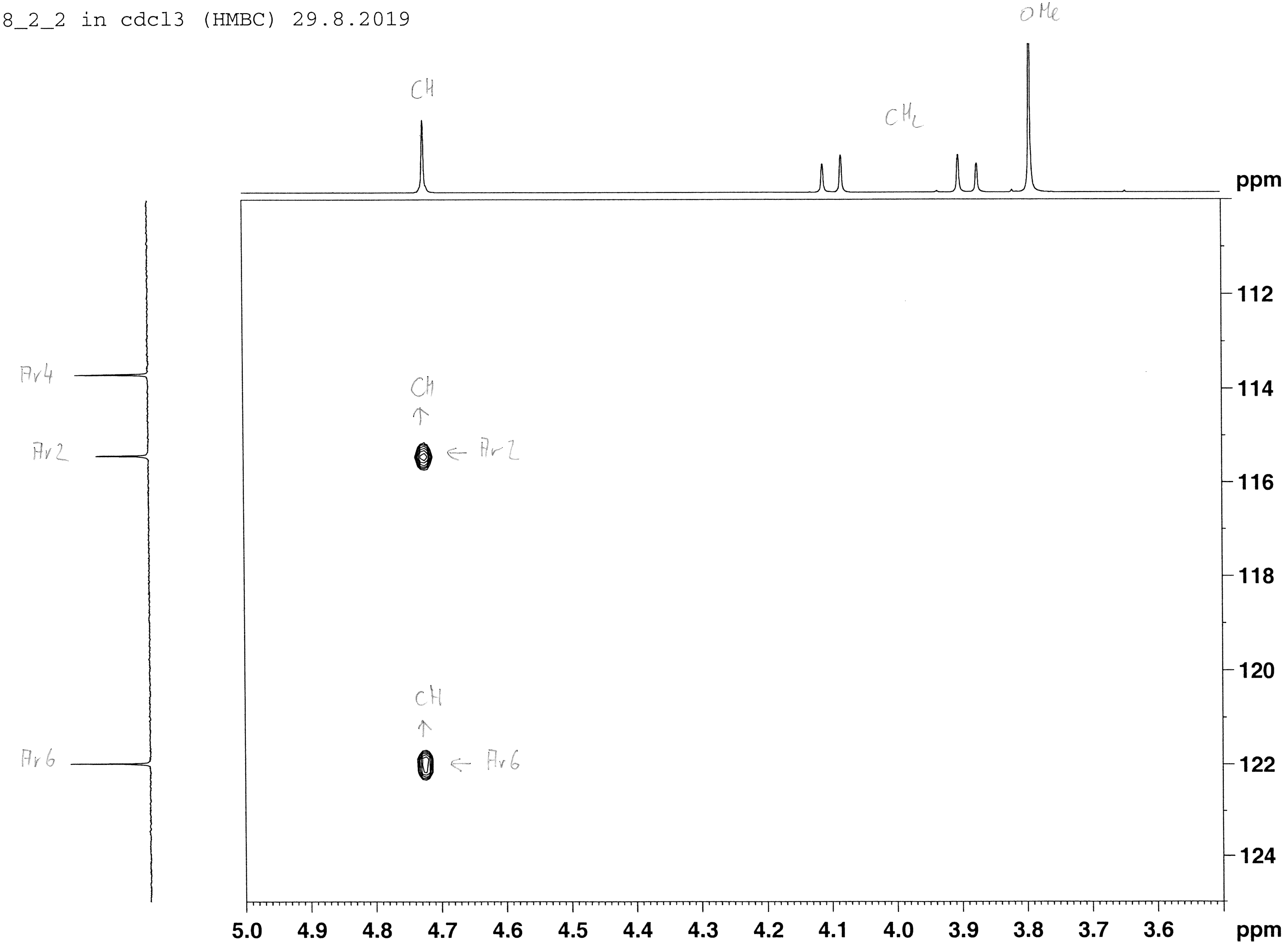

PN008_2_2 in cdcl3 (HMBC) 29.8.2019

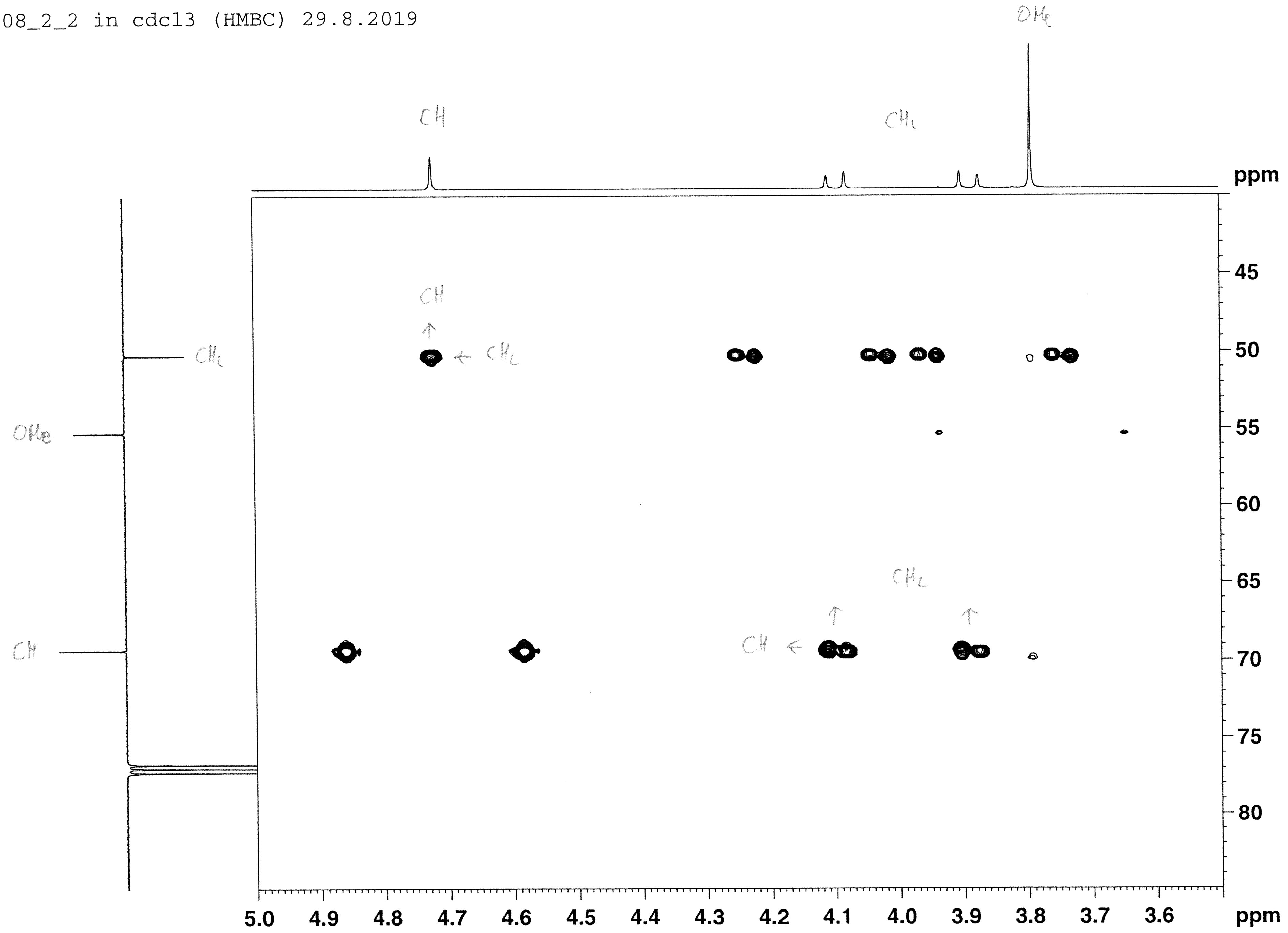

Figure S77c. NMR spectra of compound **5t.**

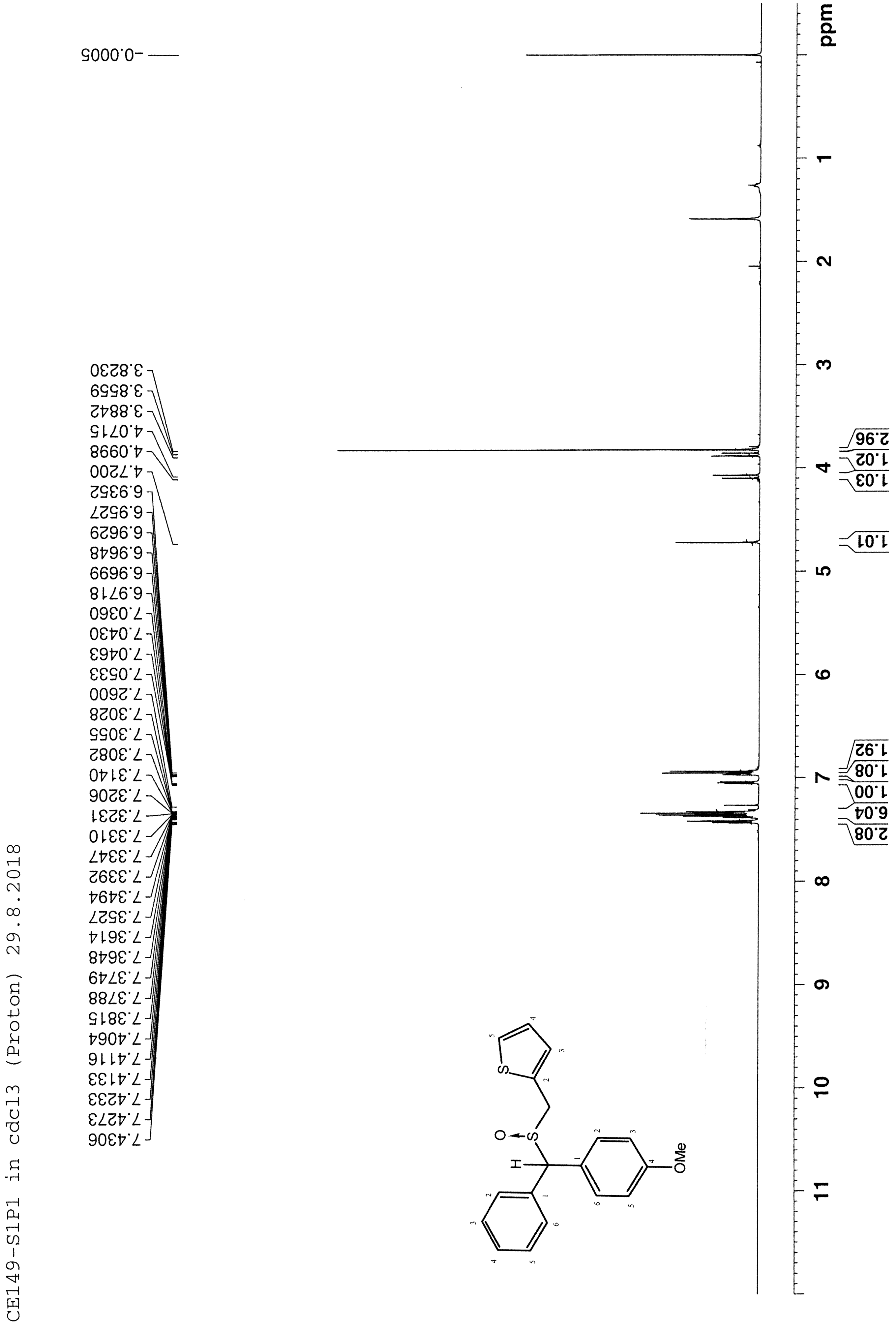

CE149-S1P1 in cdcl3 (Proton) 29.8.2018

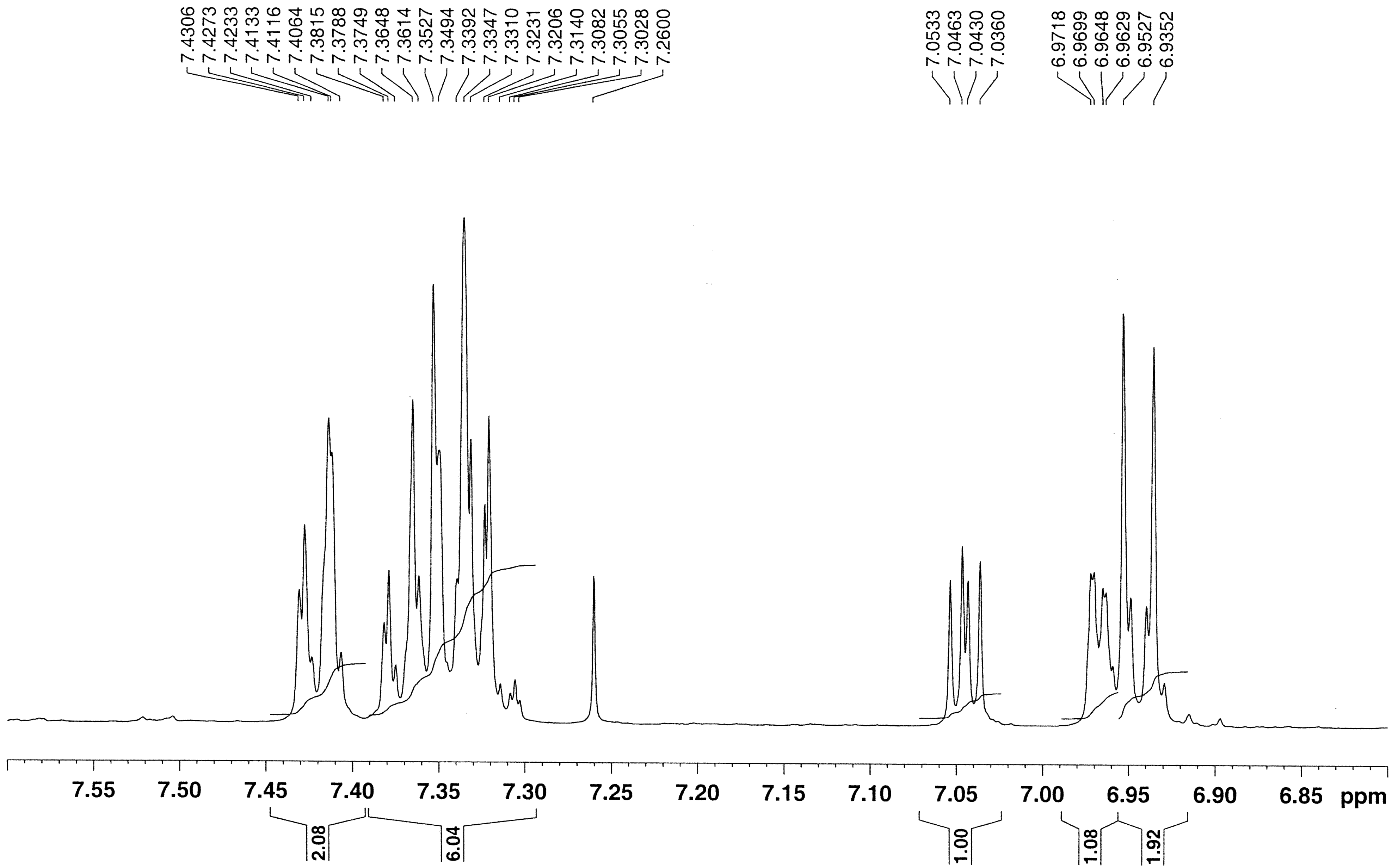

CE149-S1P1 in cdcl3 (Proton) 29.8.2018

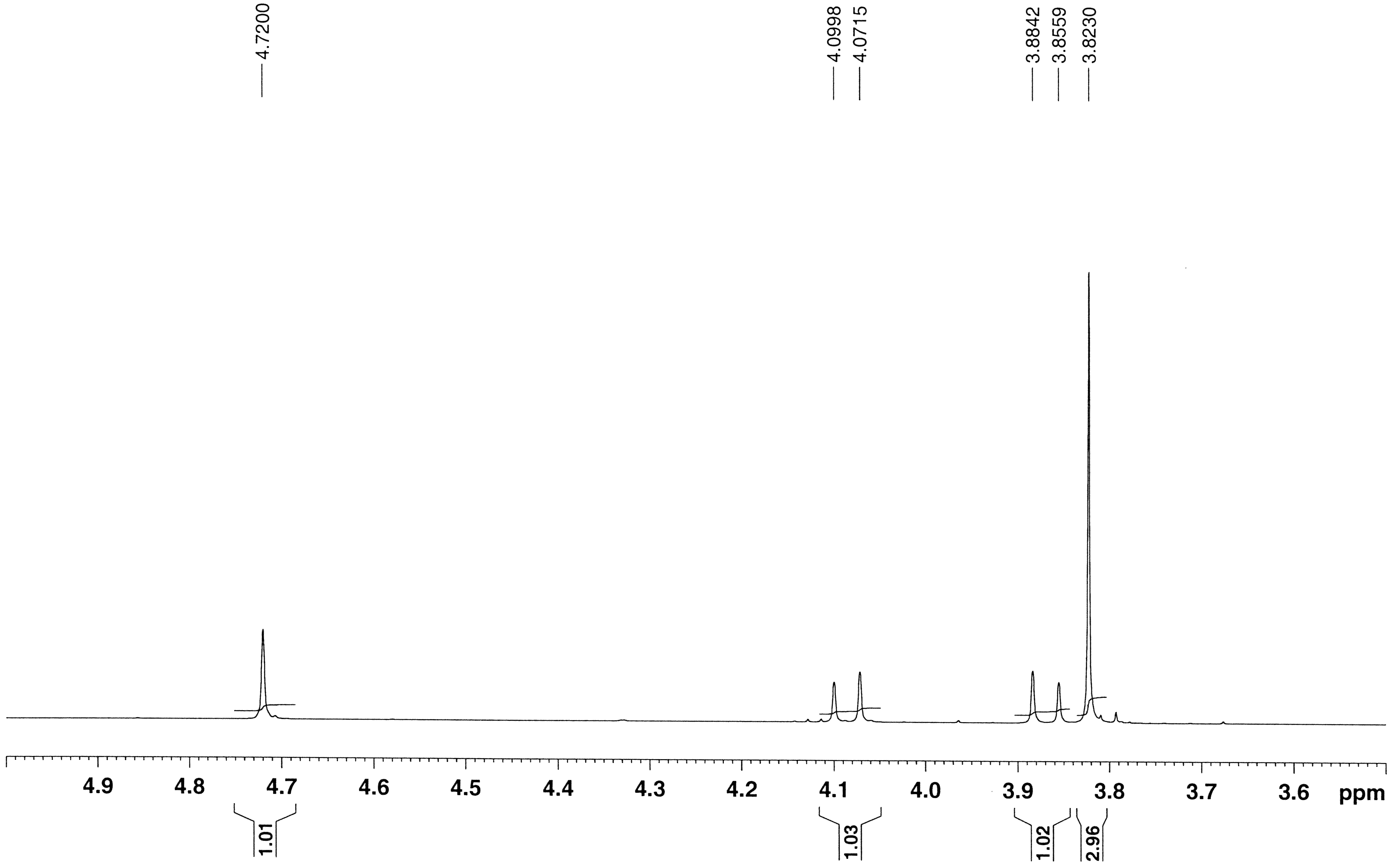

CE149-S1P1 in cdcl3 (APT) 29.8.2018

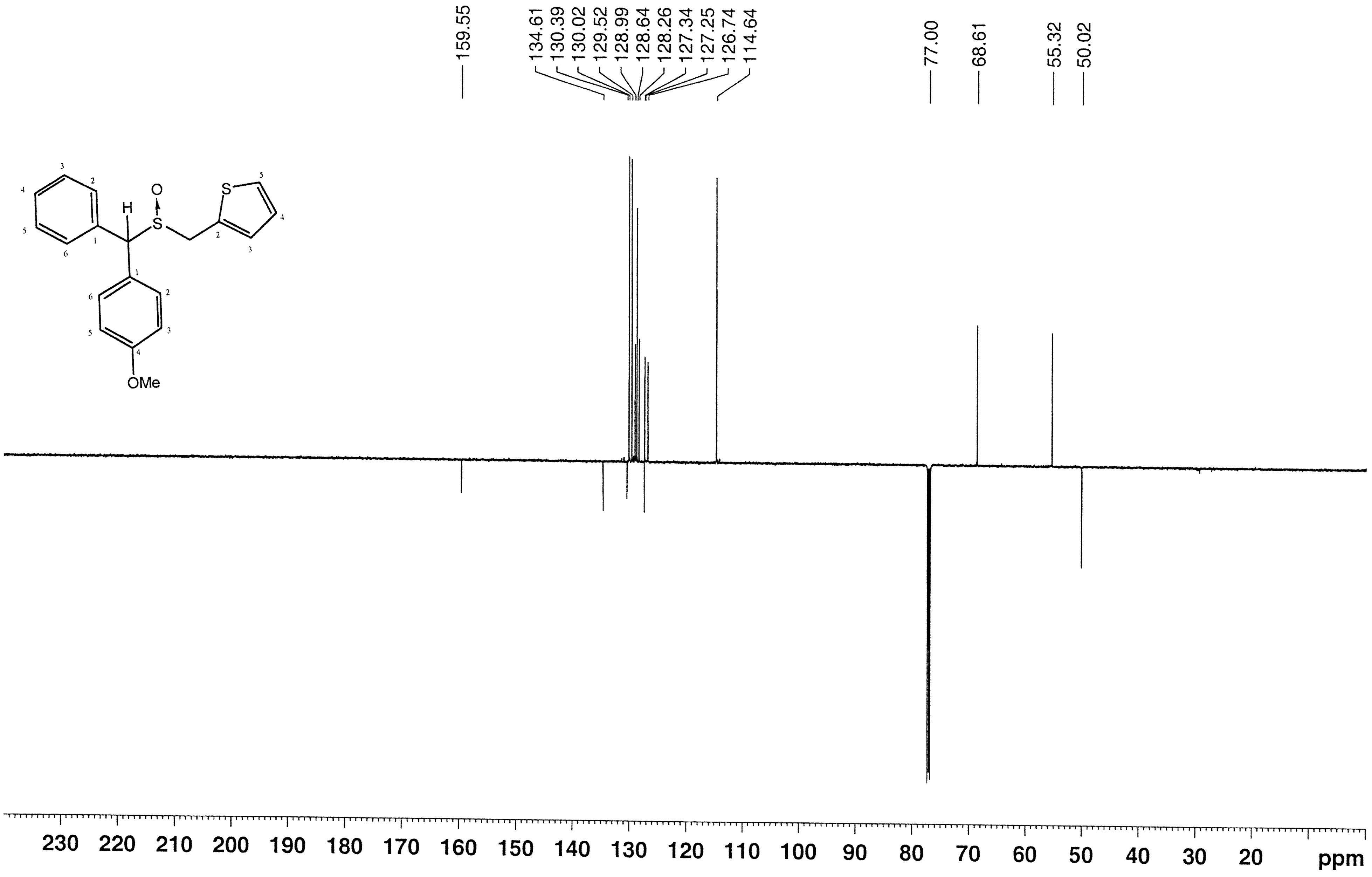

CE149-S1P1 in cdcl3 (APT) 29.8.2018

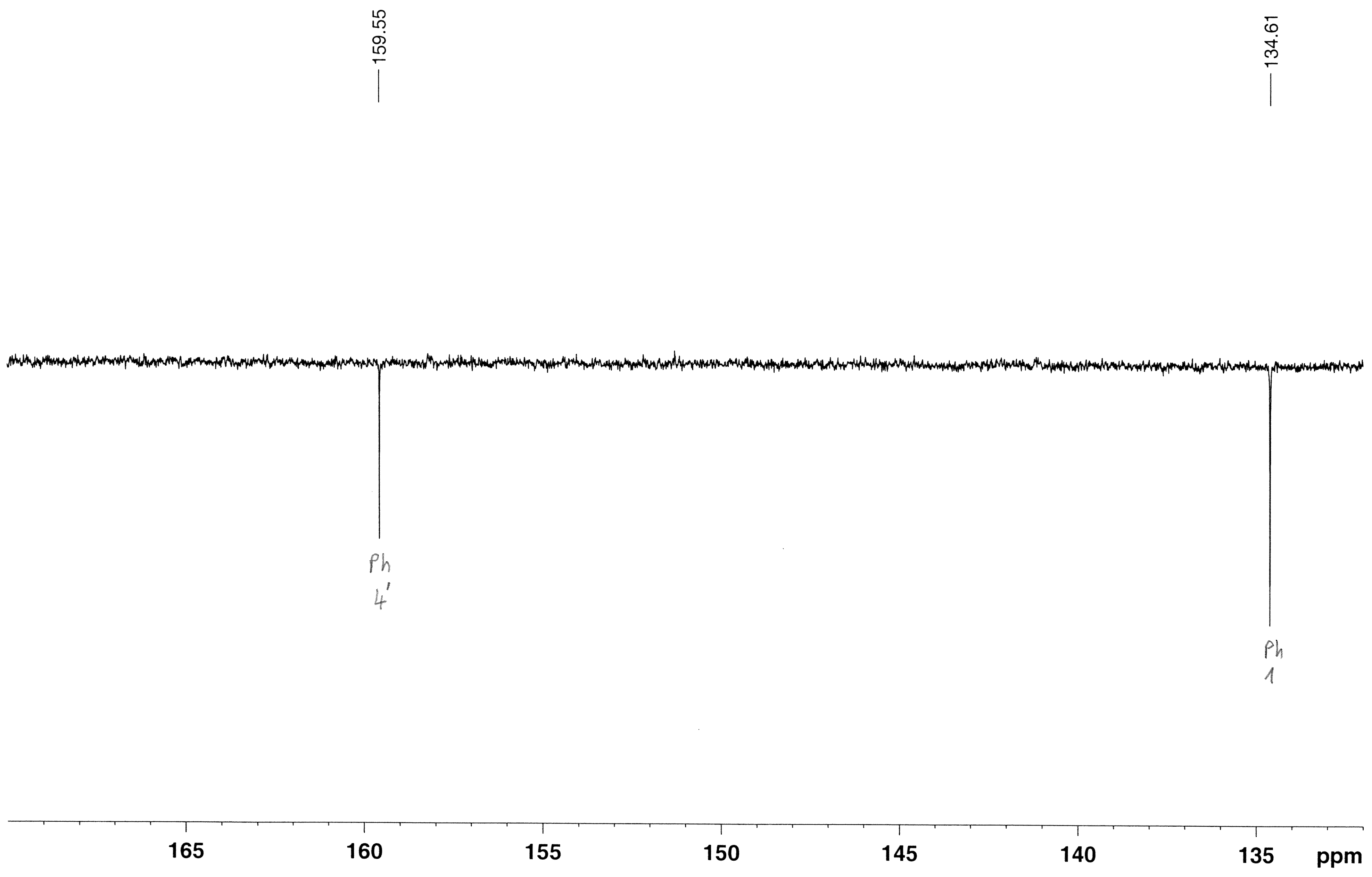

CE149-S1P1 in cdcl3 (APT) 29.8.2018

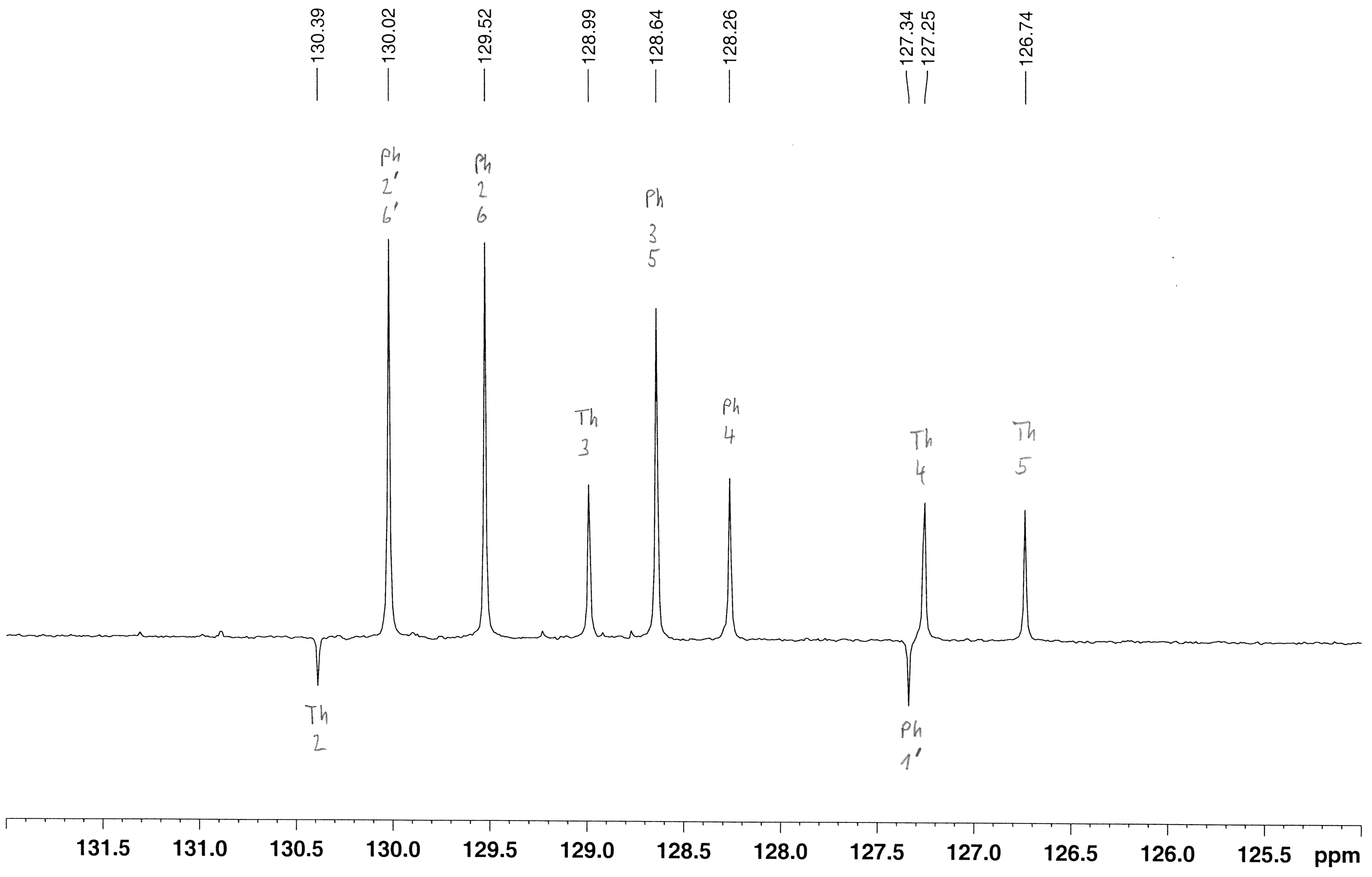

CE149-S1P1 in cdcl3 (APT) 29.8.2018

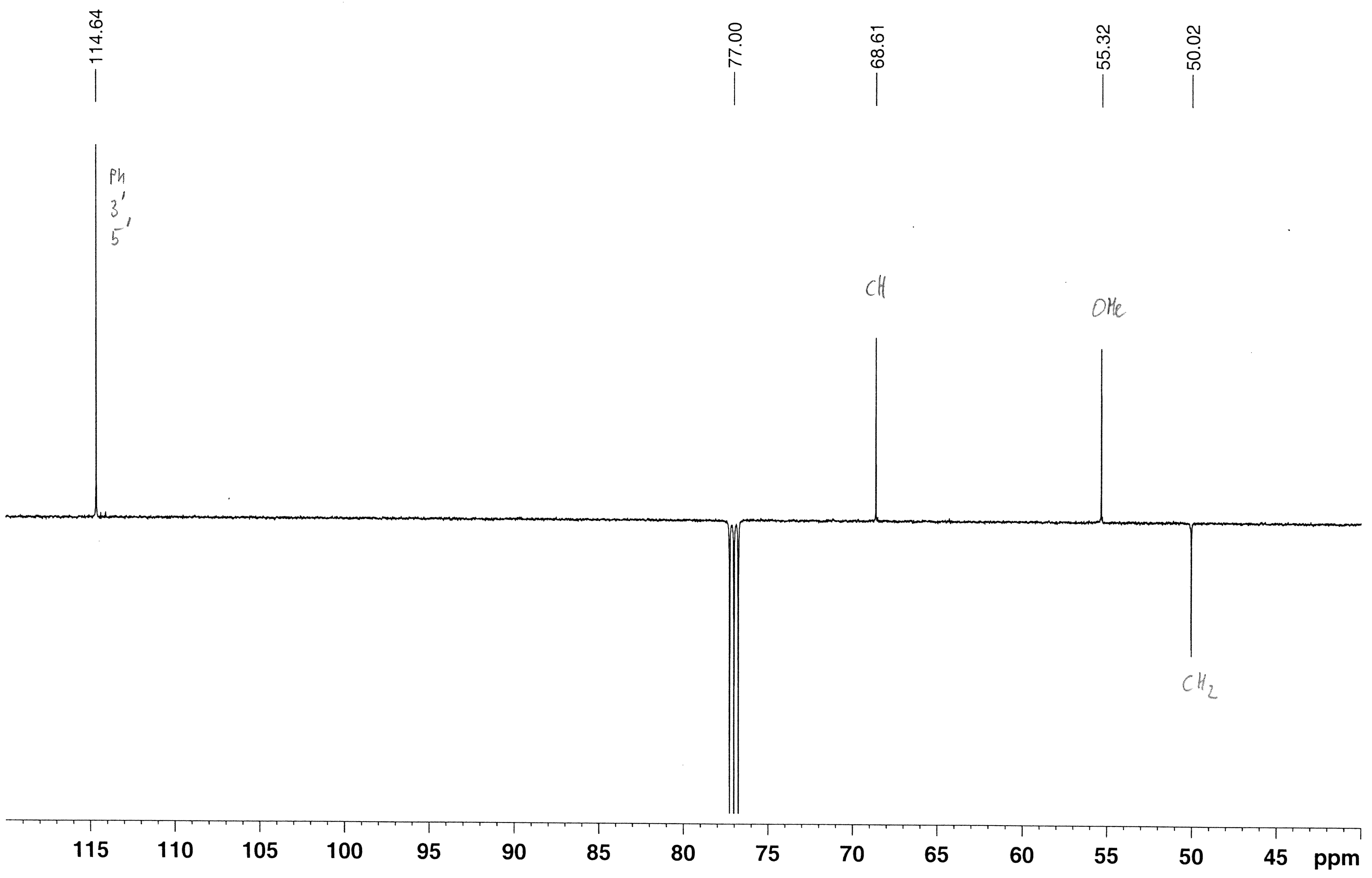

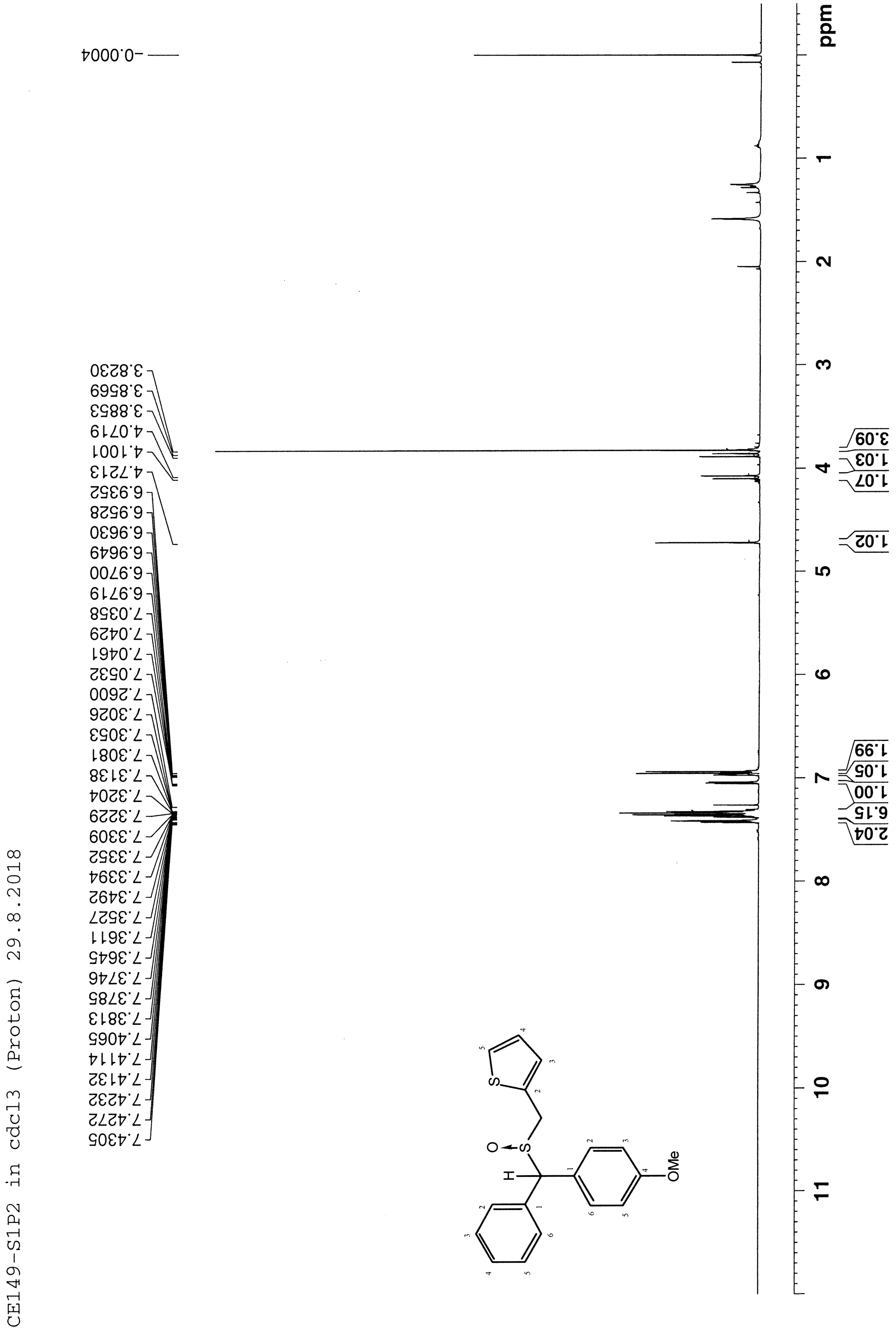

Figure S78c. NMR spectra of compound **6t**.

CE149-S1P2 in cdcl3 (Proton) 29.8.2018

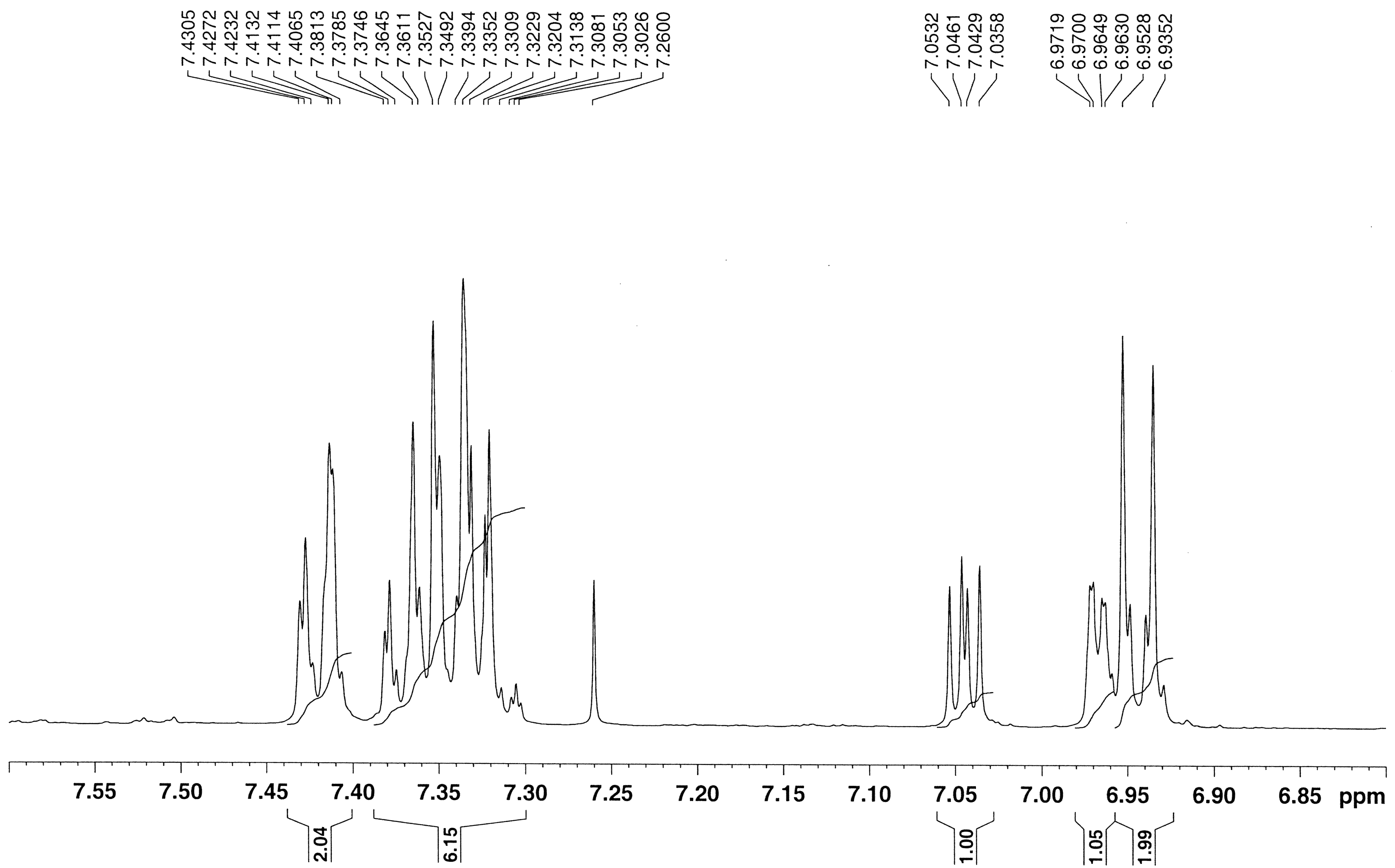

CE149-S1P2 in cdcl3 (Proton) 29.8.2018

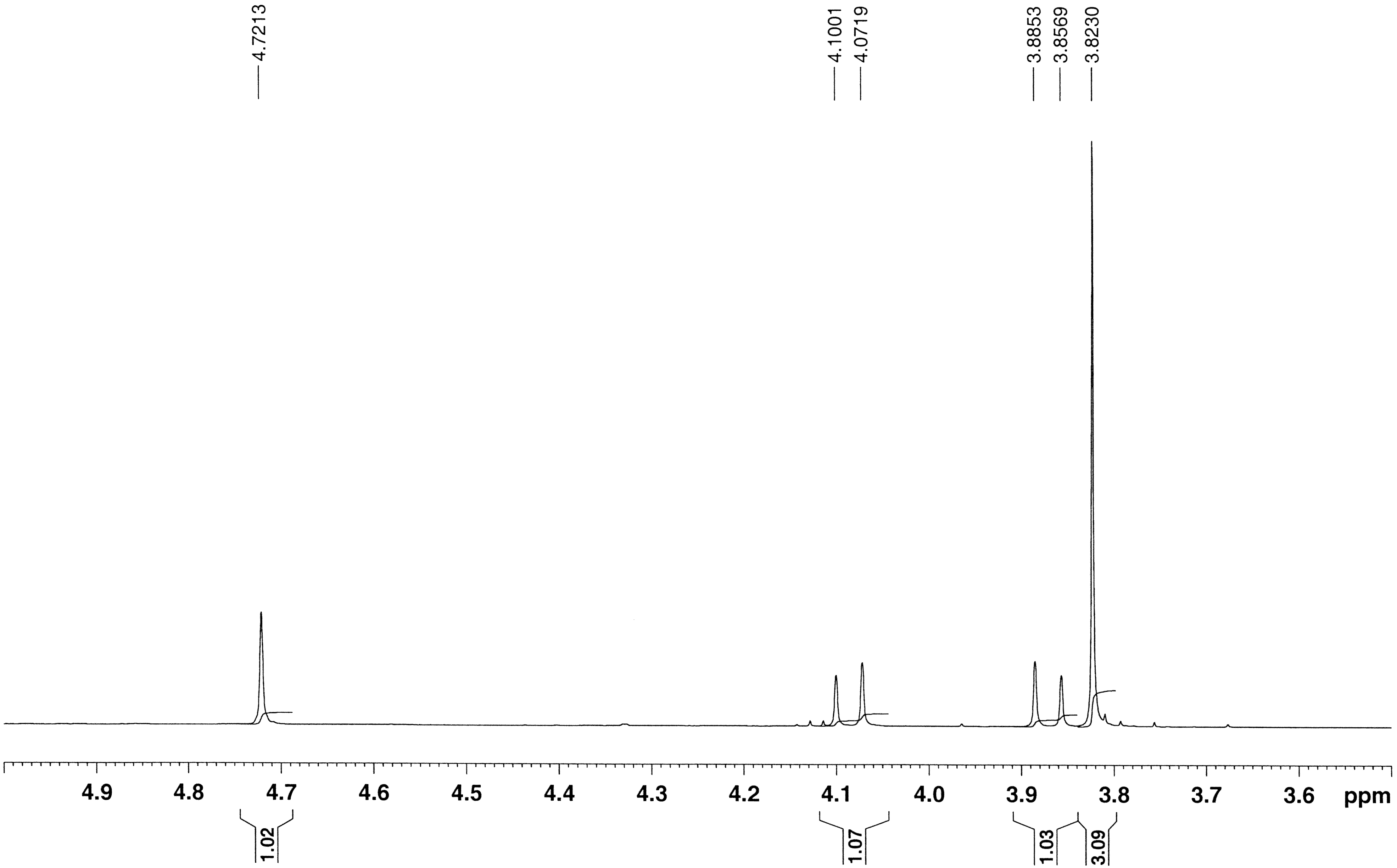

CE149-S1P2 in cdcl3 (APT) 29.8.2018

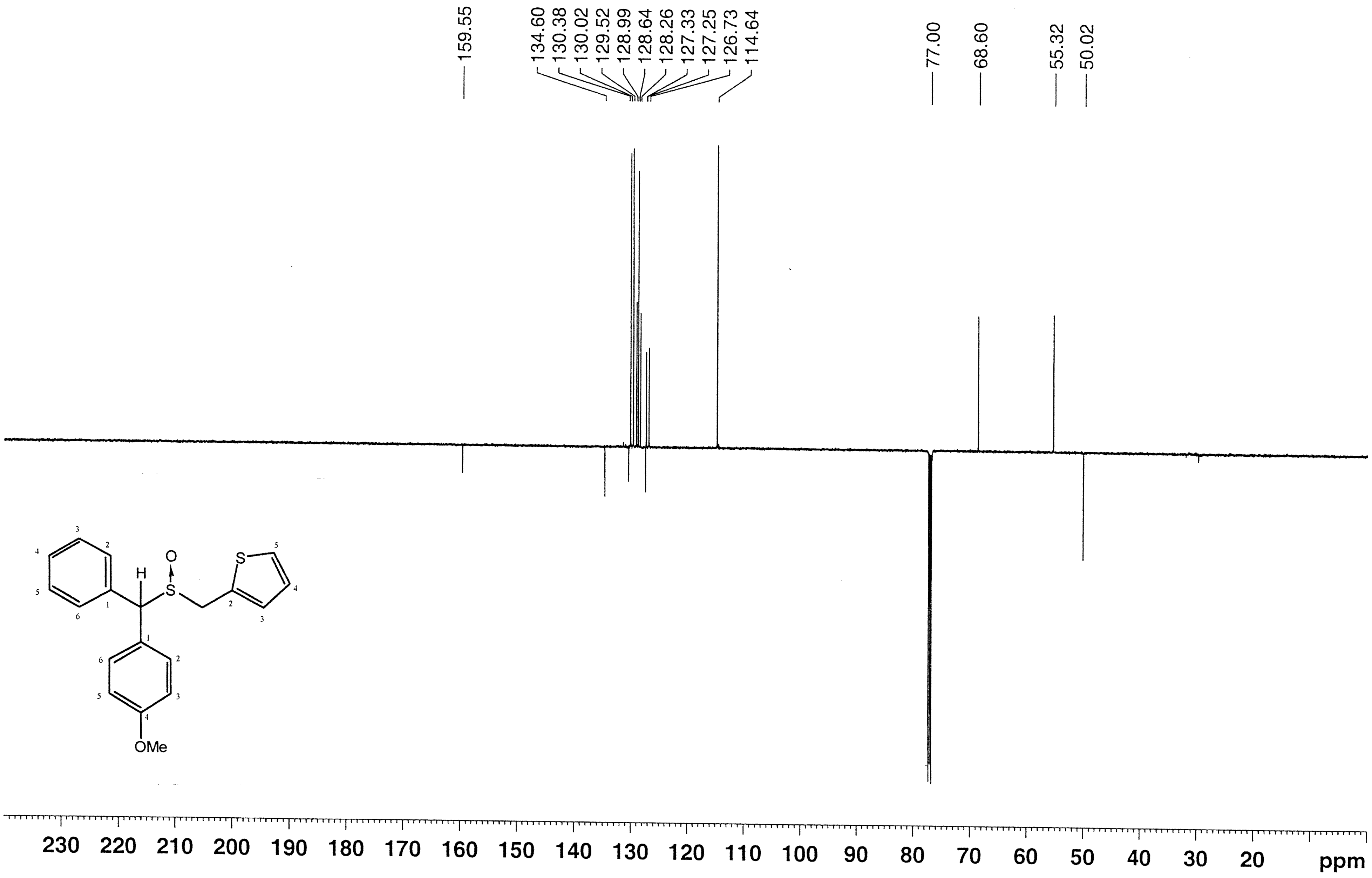

CE149-S1P2 in cdcl3 (APT) 29.8.2018

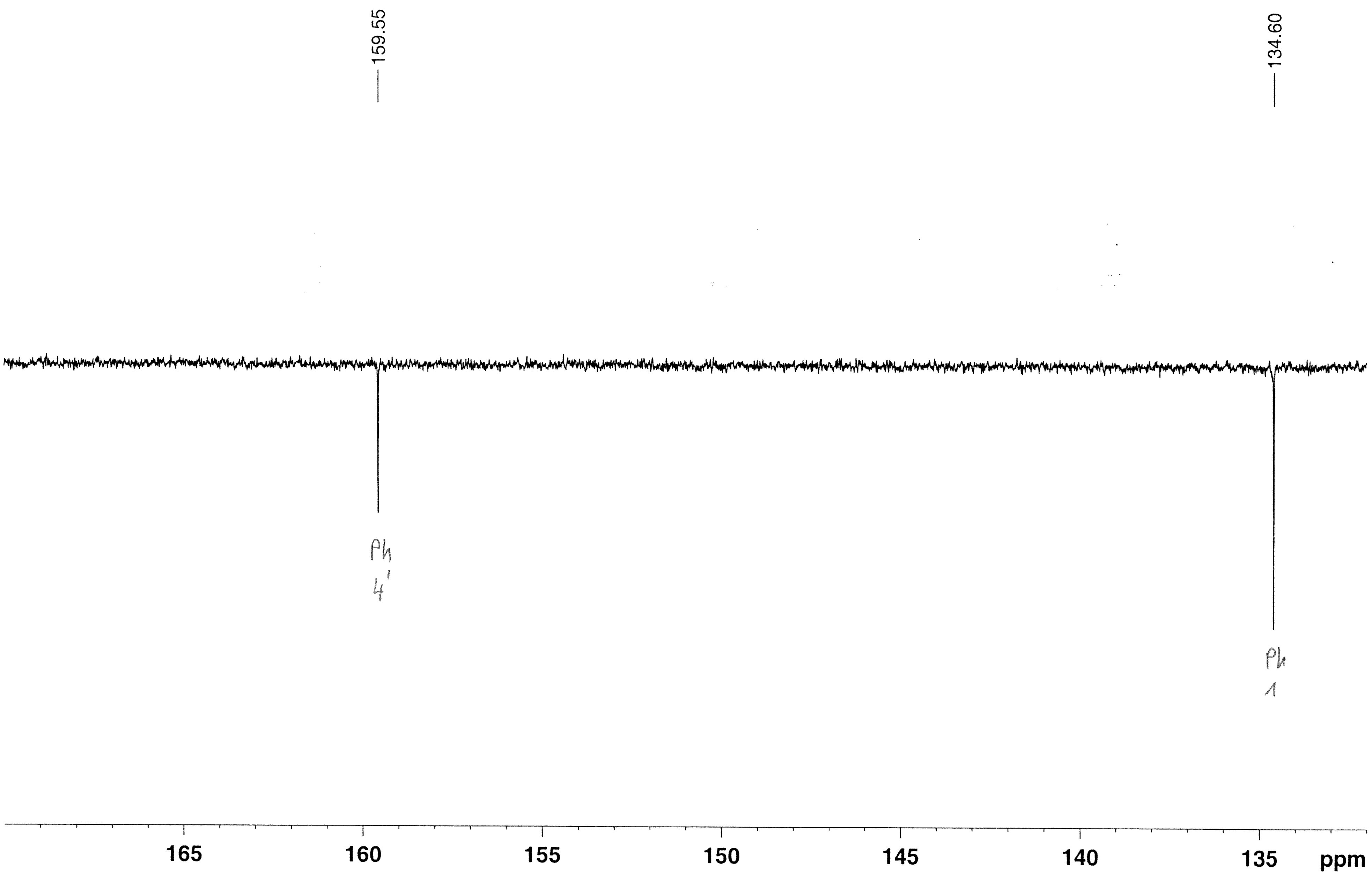

CE149-S1P2 in cdcl3 (APT) 29.8.2018

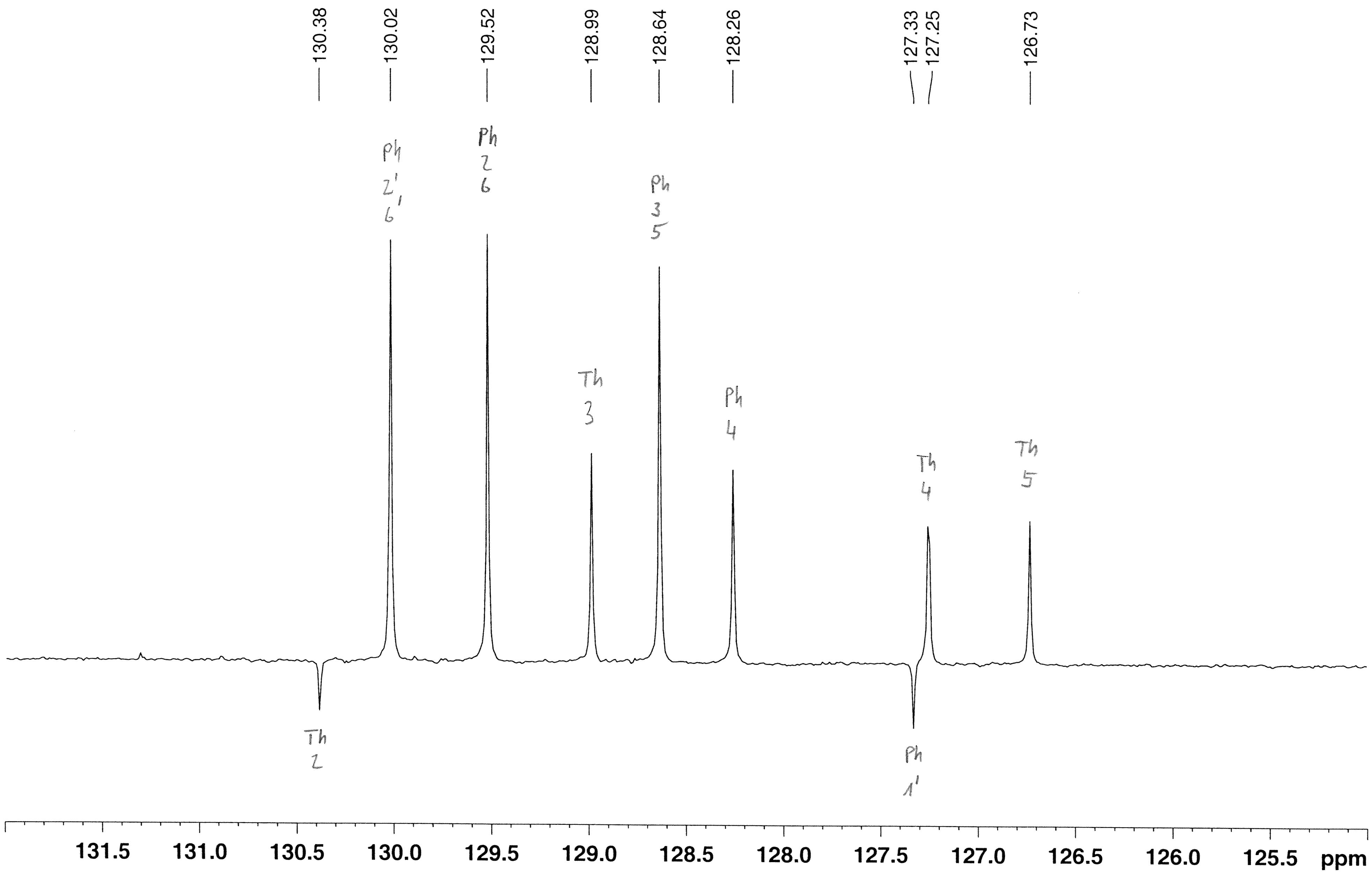

CE149-S1P2 in cdcl3 (APT) 29.8.2018

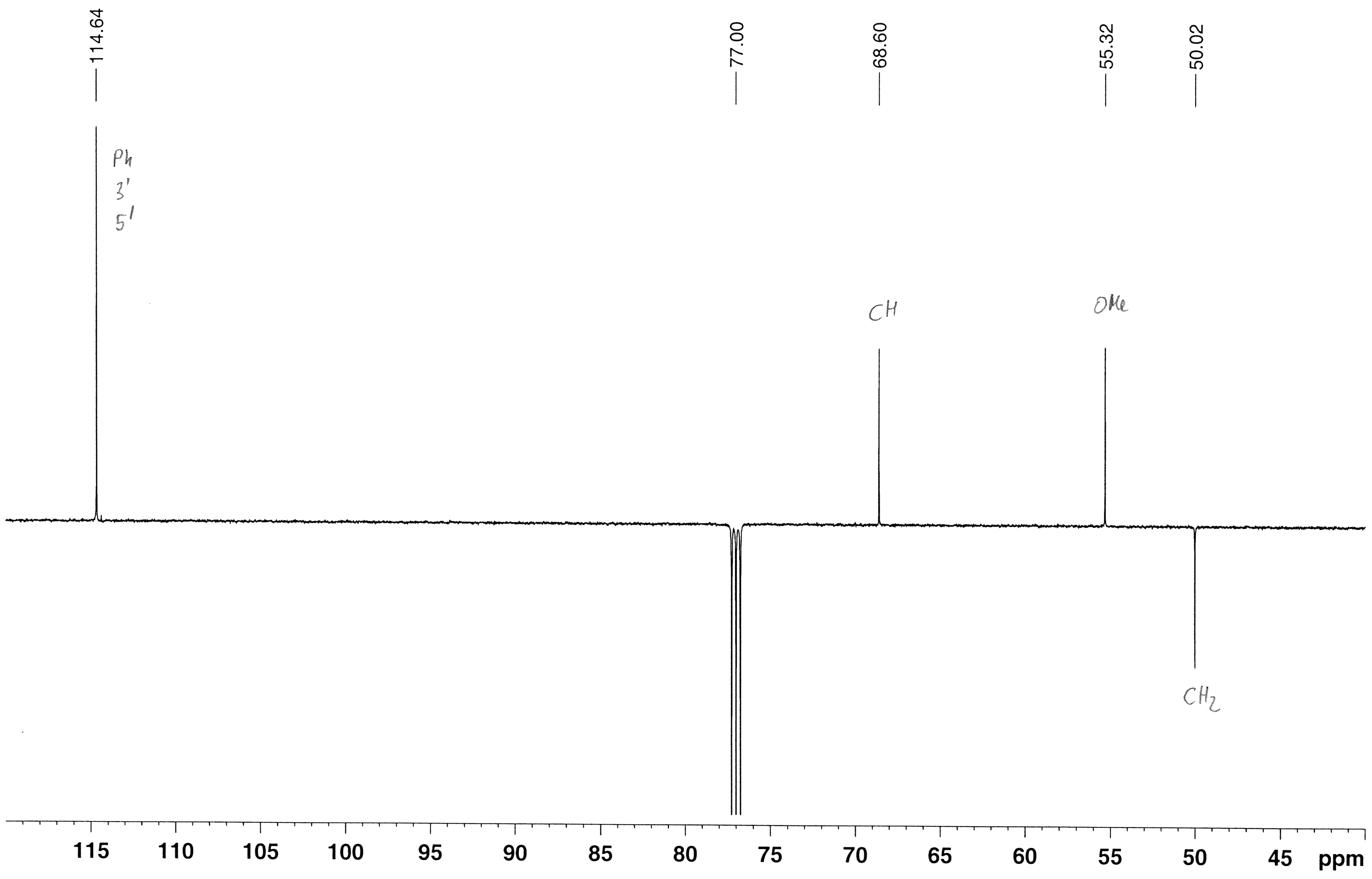

Figure S79c. NMR spectra of compound **7t.**

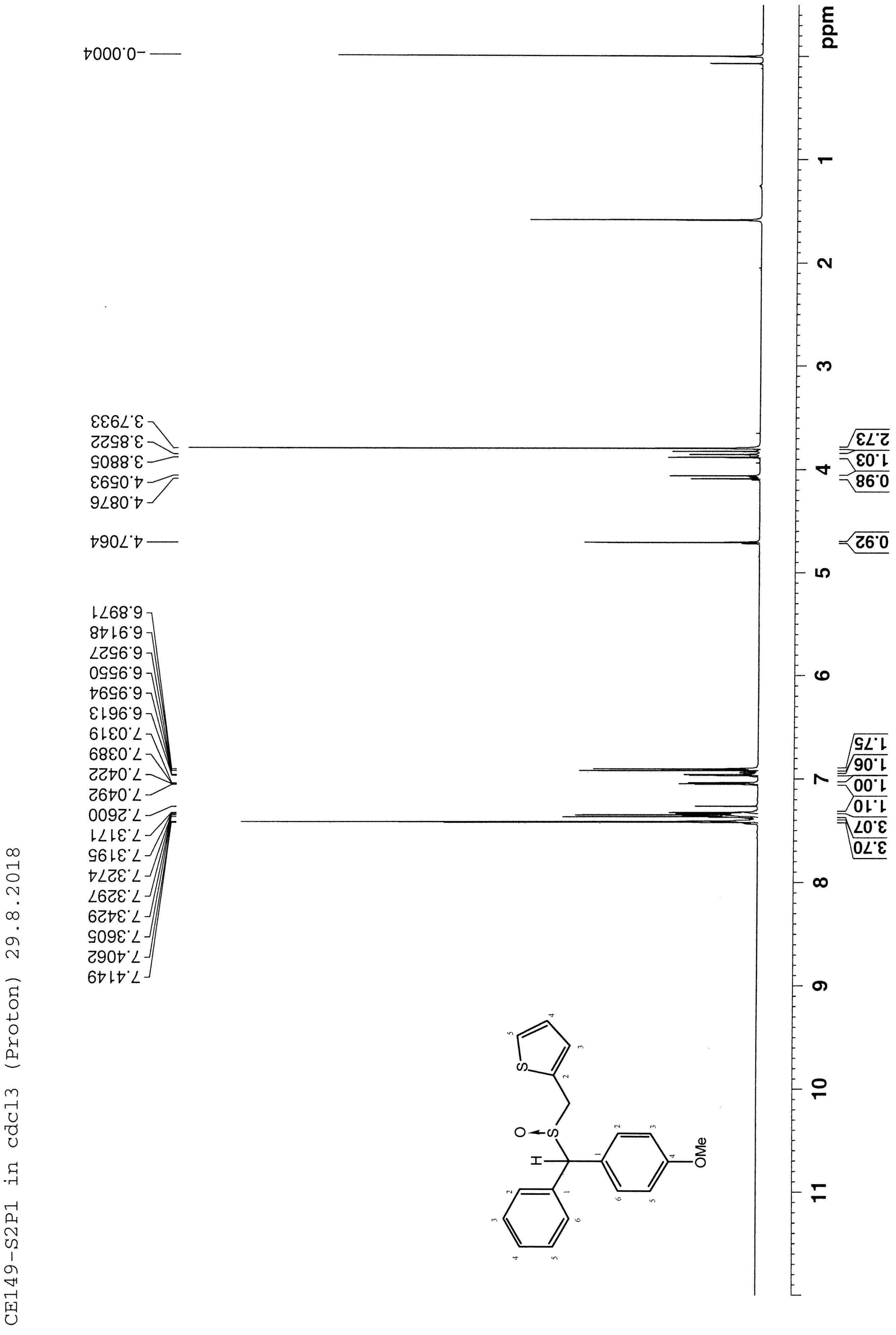

CE149-S2P1 in cdcl3 (Proton) 29.8.2018

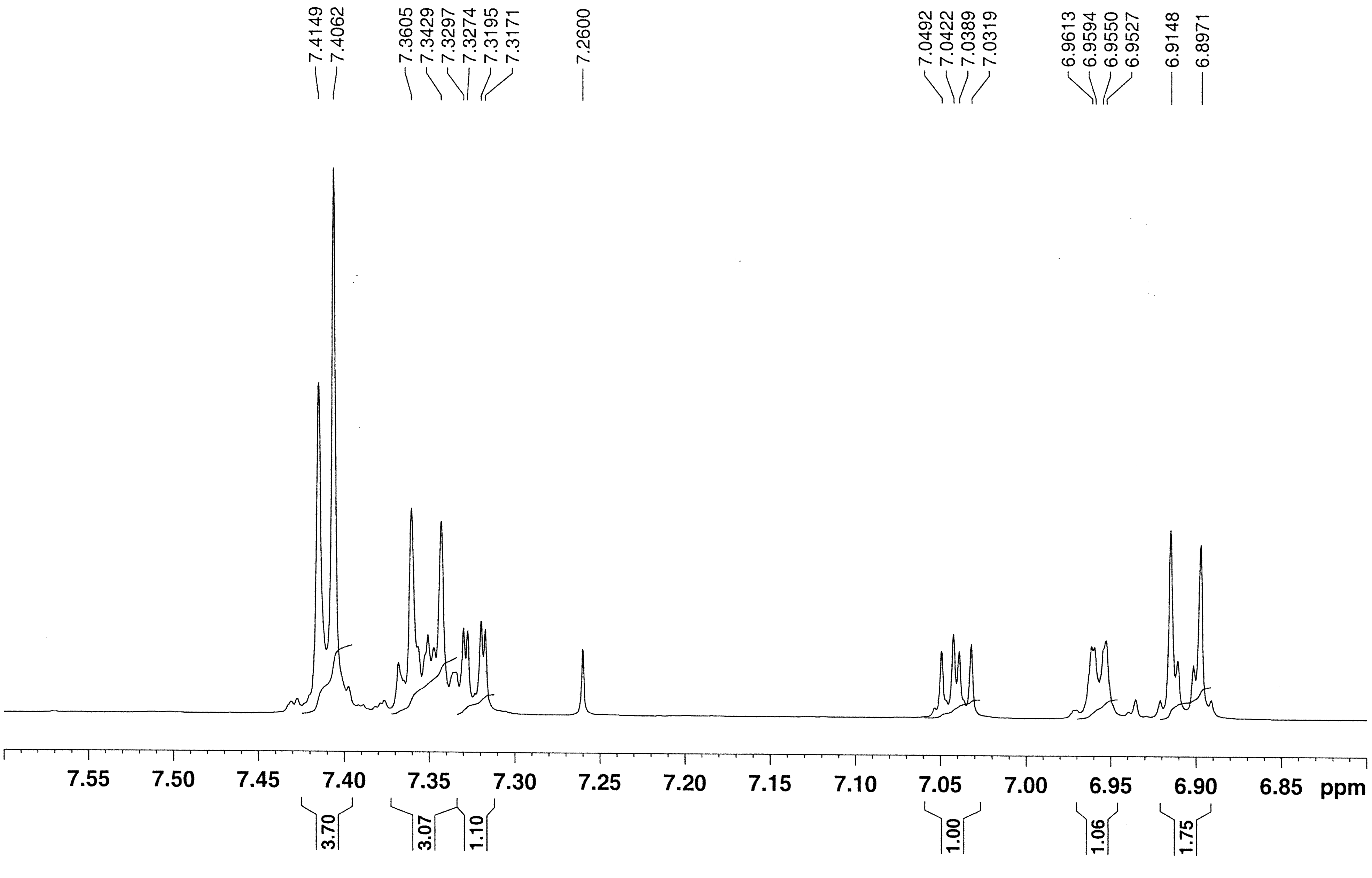

CE149-S2P1 in cdcl3 (Proton) 29.8.2018

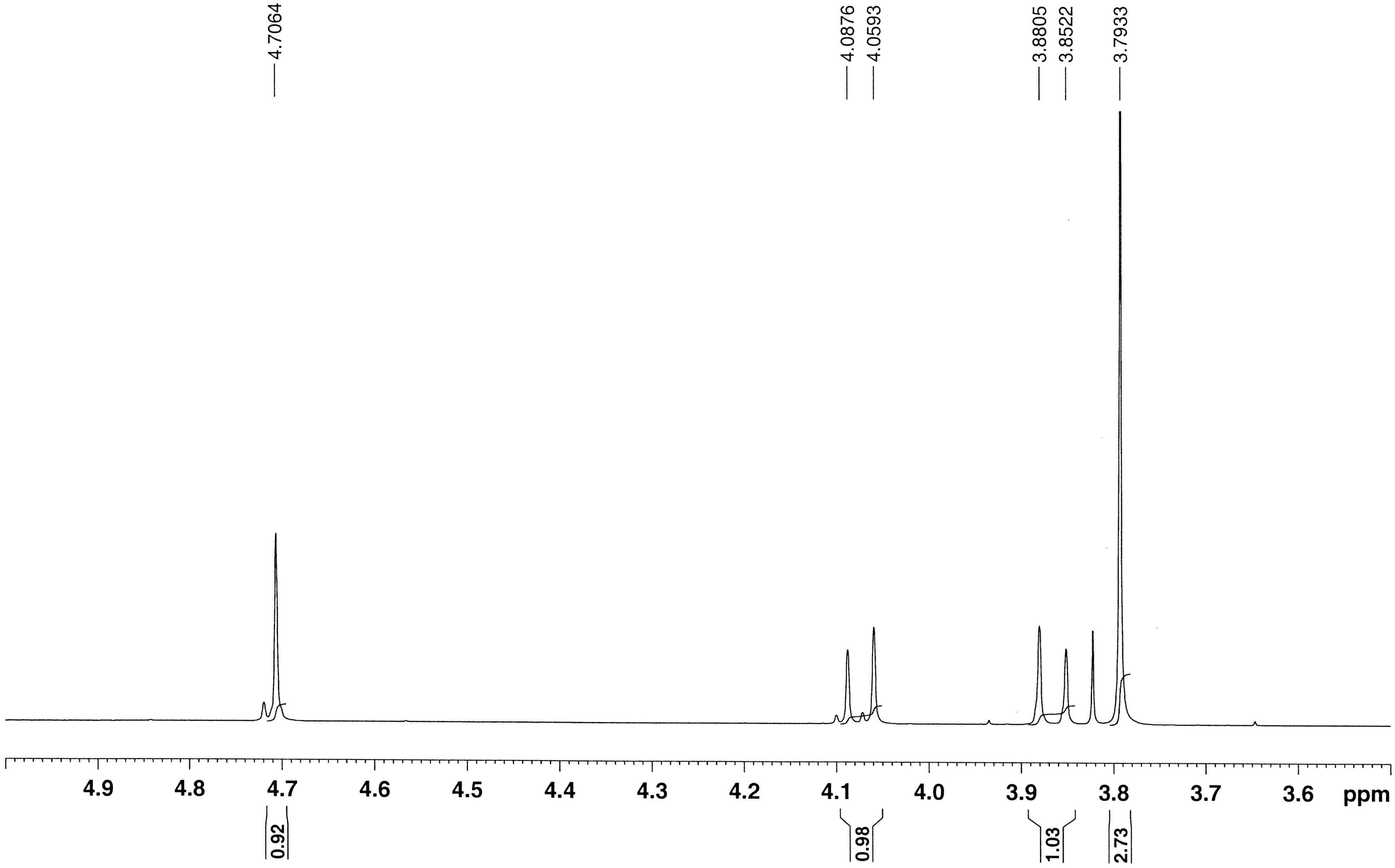

CE149-S2P1 in cdcl3 (APT) 29.8.2018

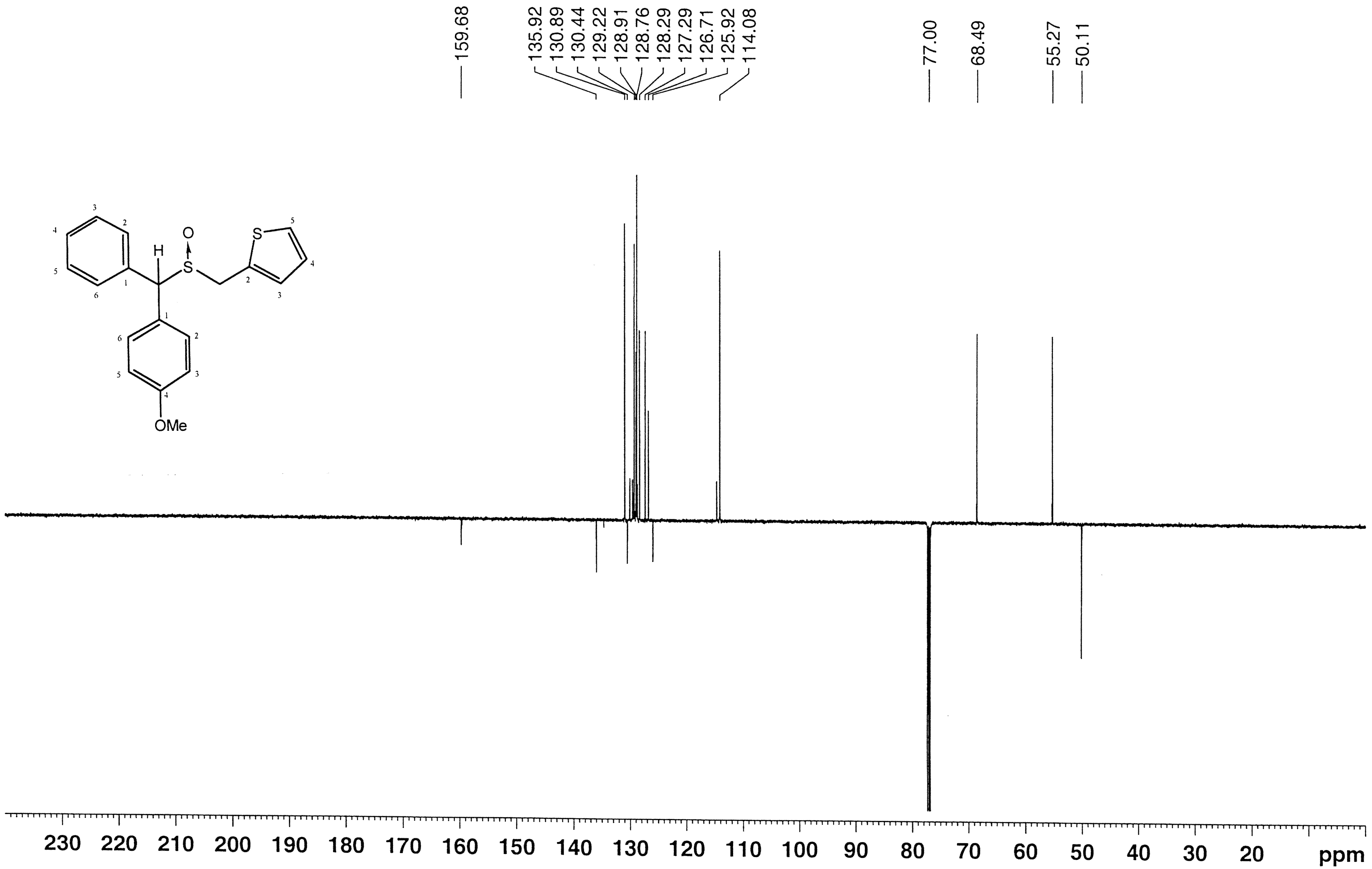

CE149-S2P1 in cdcl3 (APT) 29.8.2018

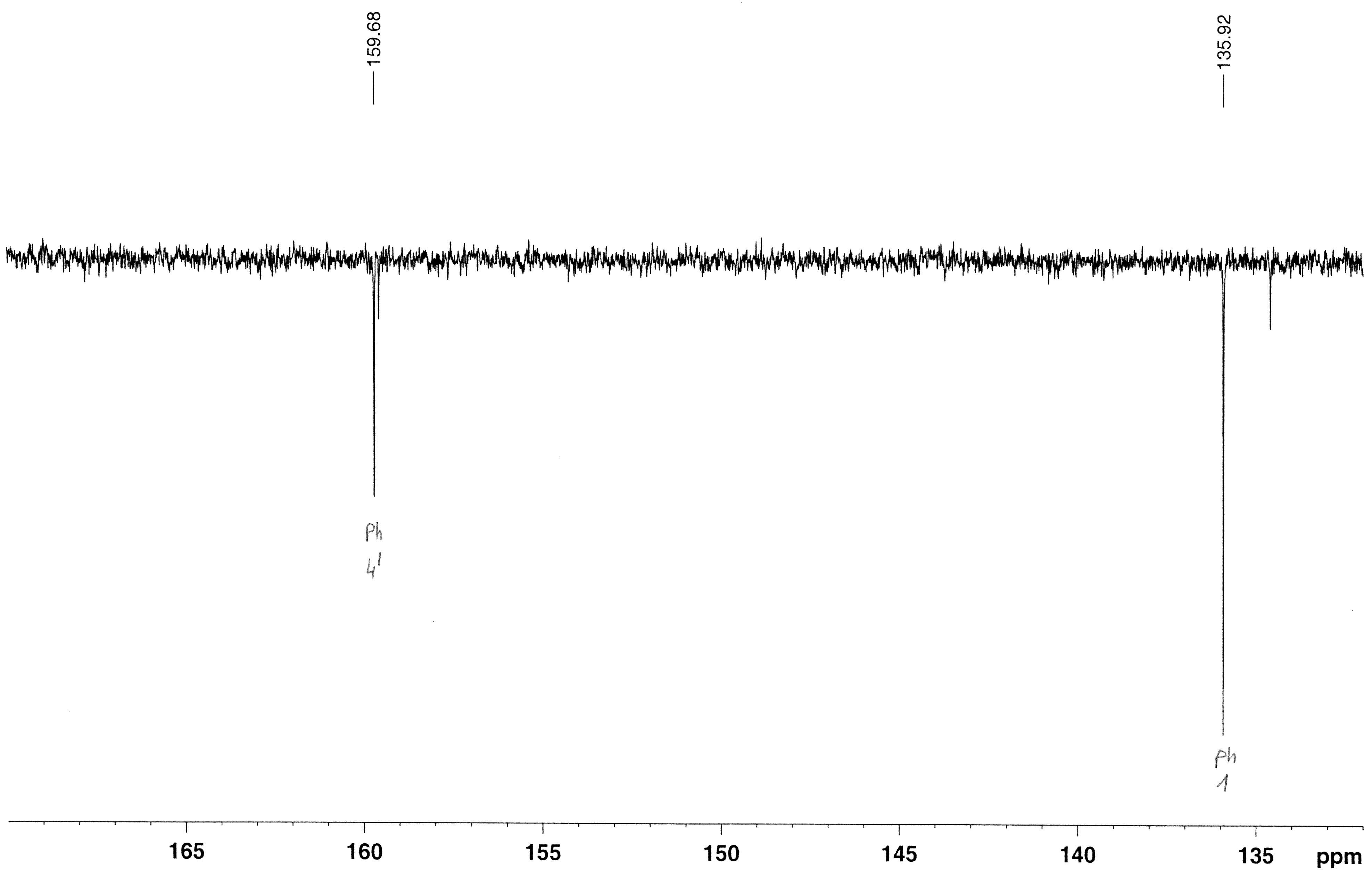

CE149-S2P1 in cdcl3 (APT) 29.8.2018

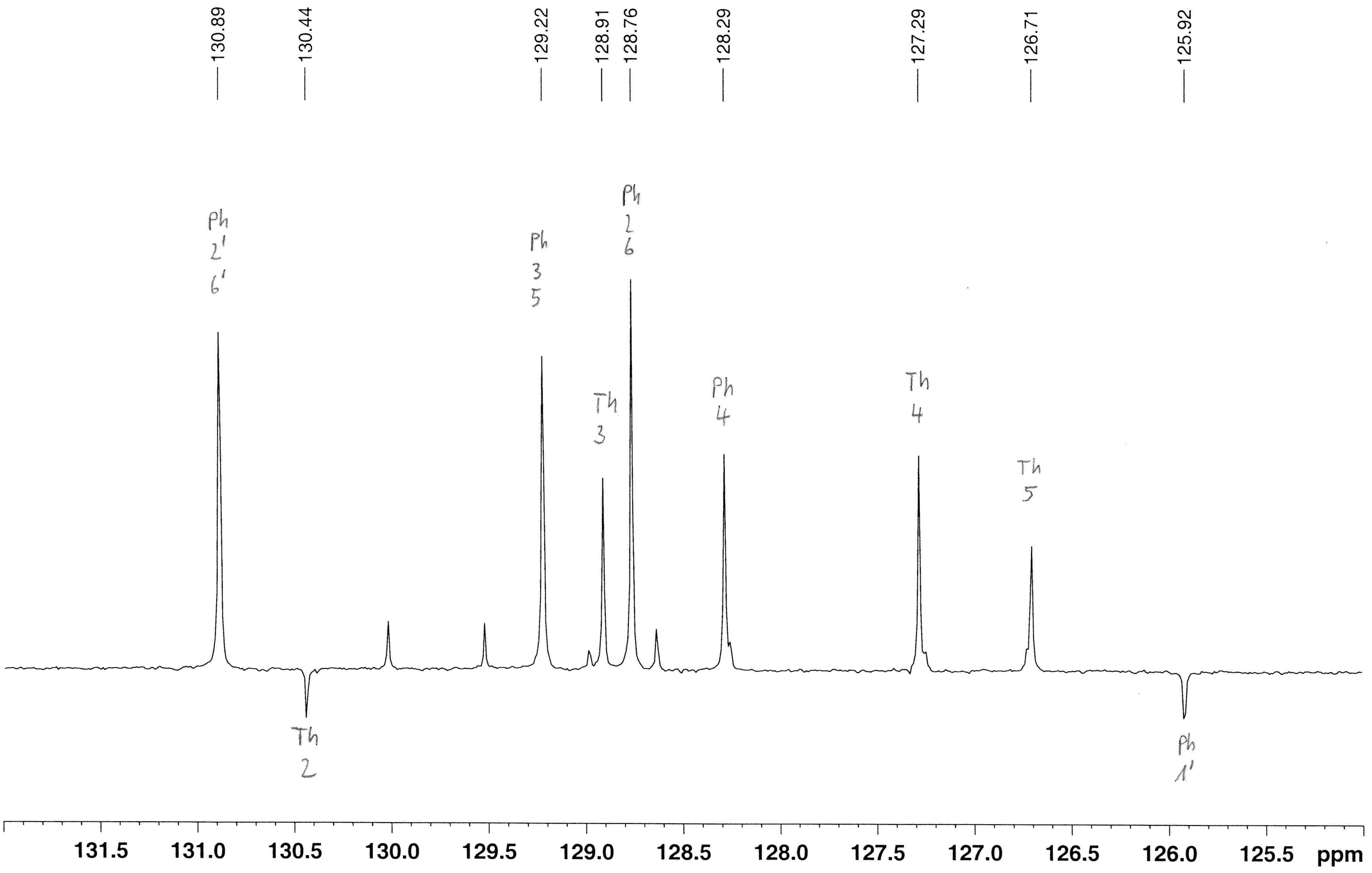

CE149-S2P1 in cdcl3 (APT) 29.8.2018

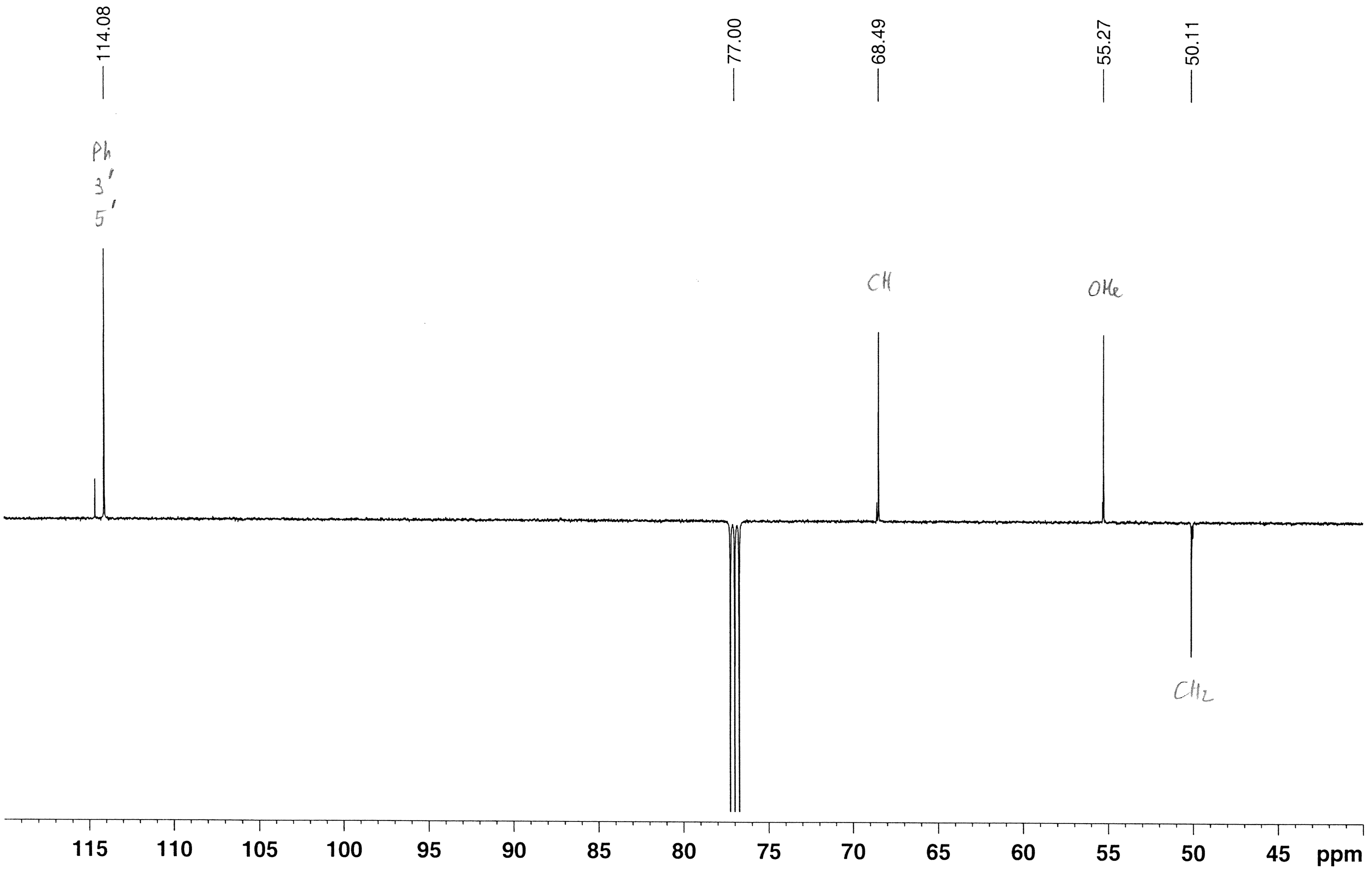

Figure S80c. NMR spectra of compound **8t**.

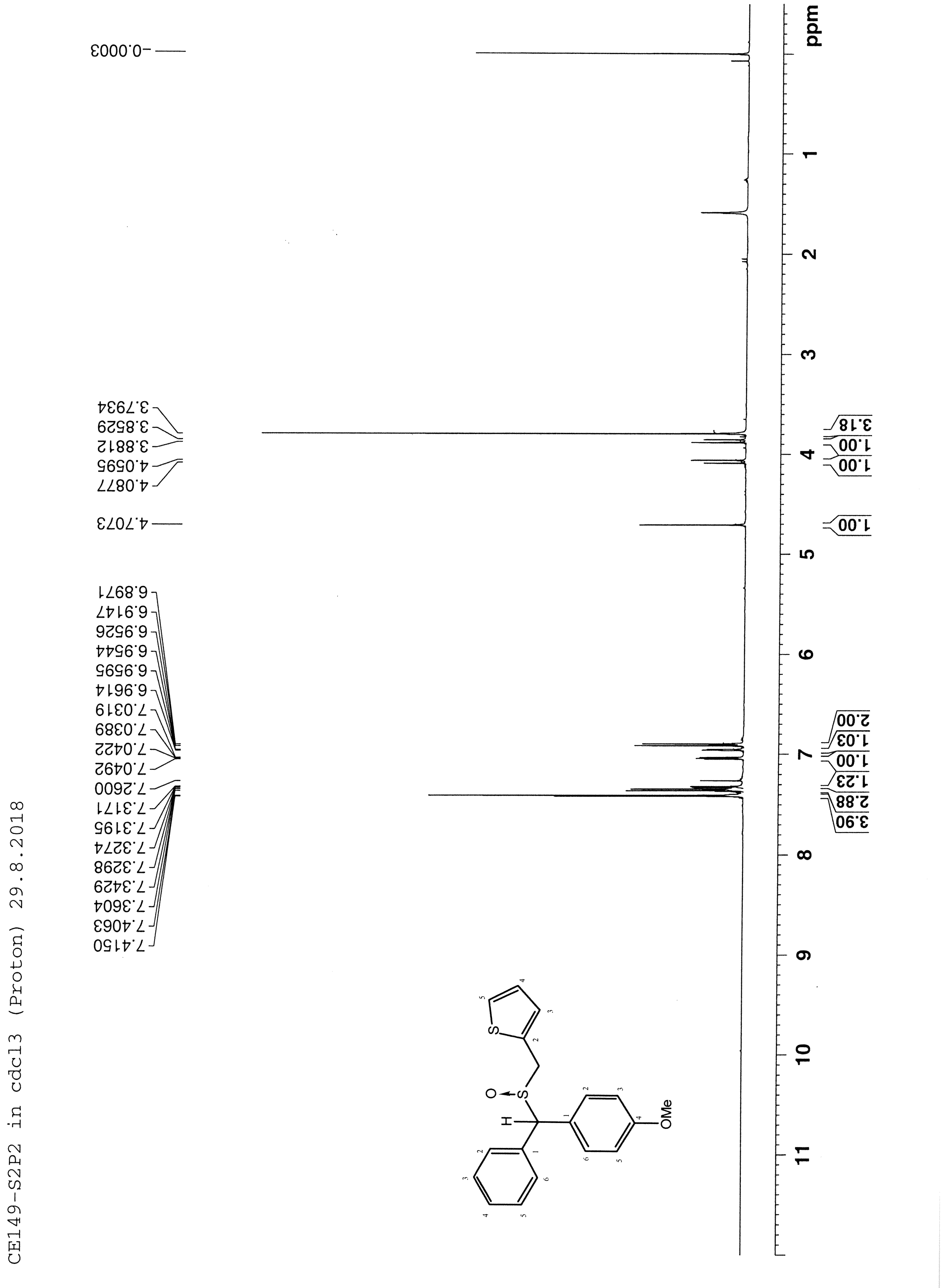

CE149-S2P2 in cdcl3 (Proton) 29.8.2018

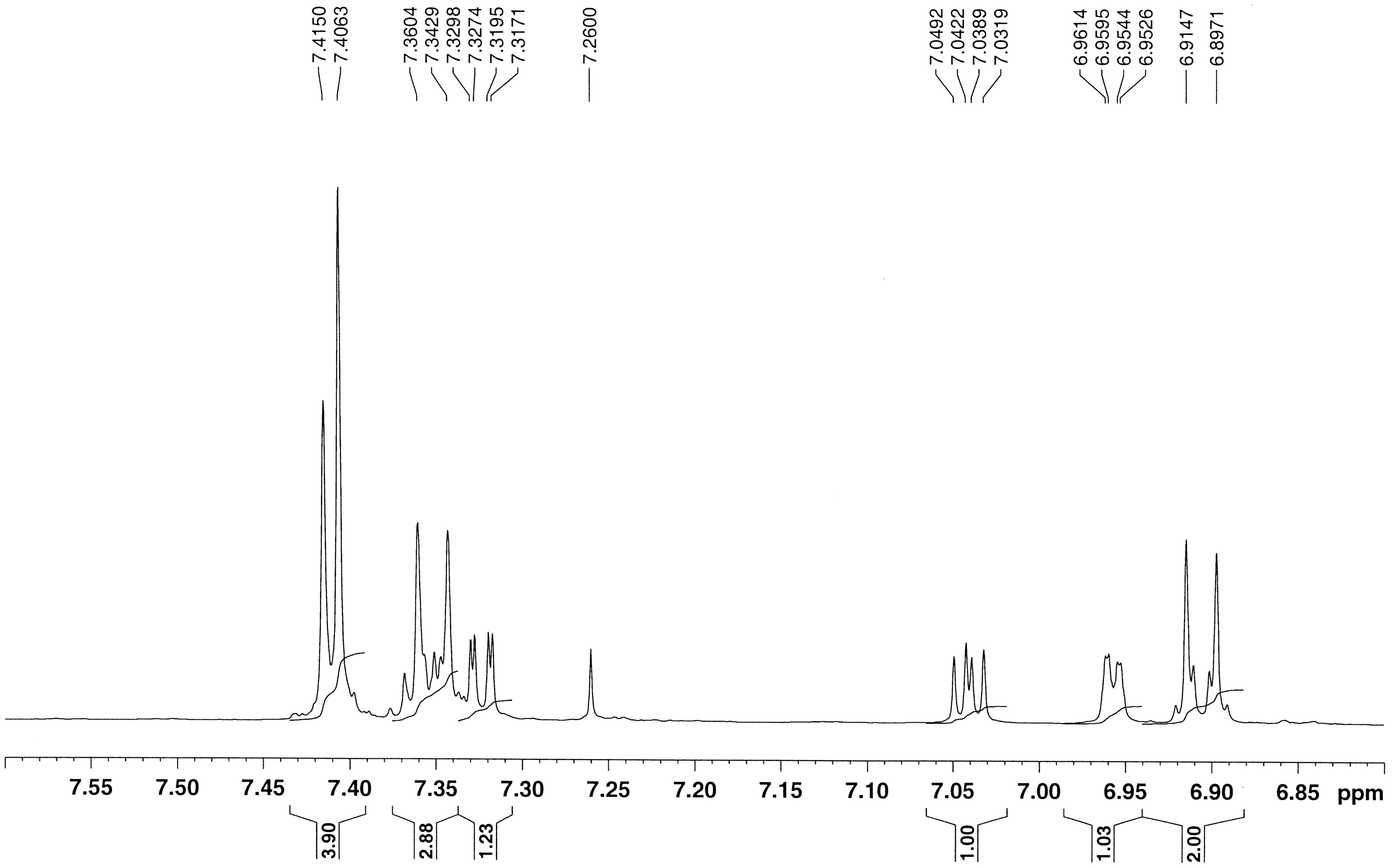

CE149-S2P2 in cdcl3 (Proton) 29.8.2018

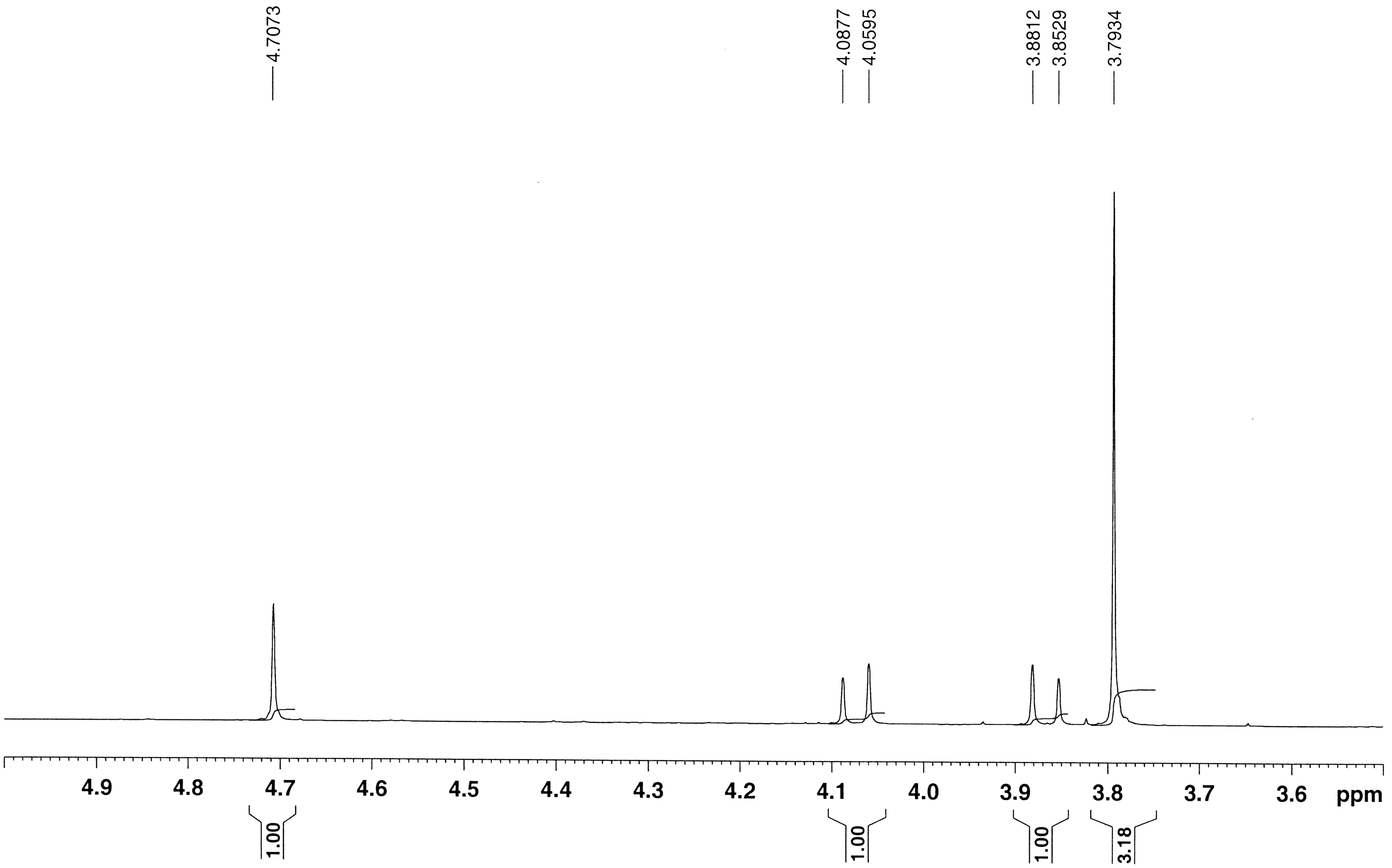

CE149-S2P2 in cdcl3 (APT) 29.8.2018

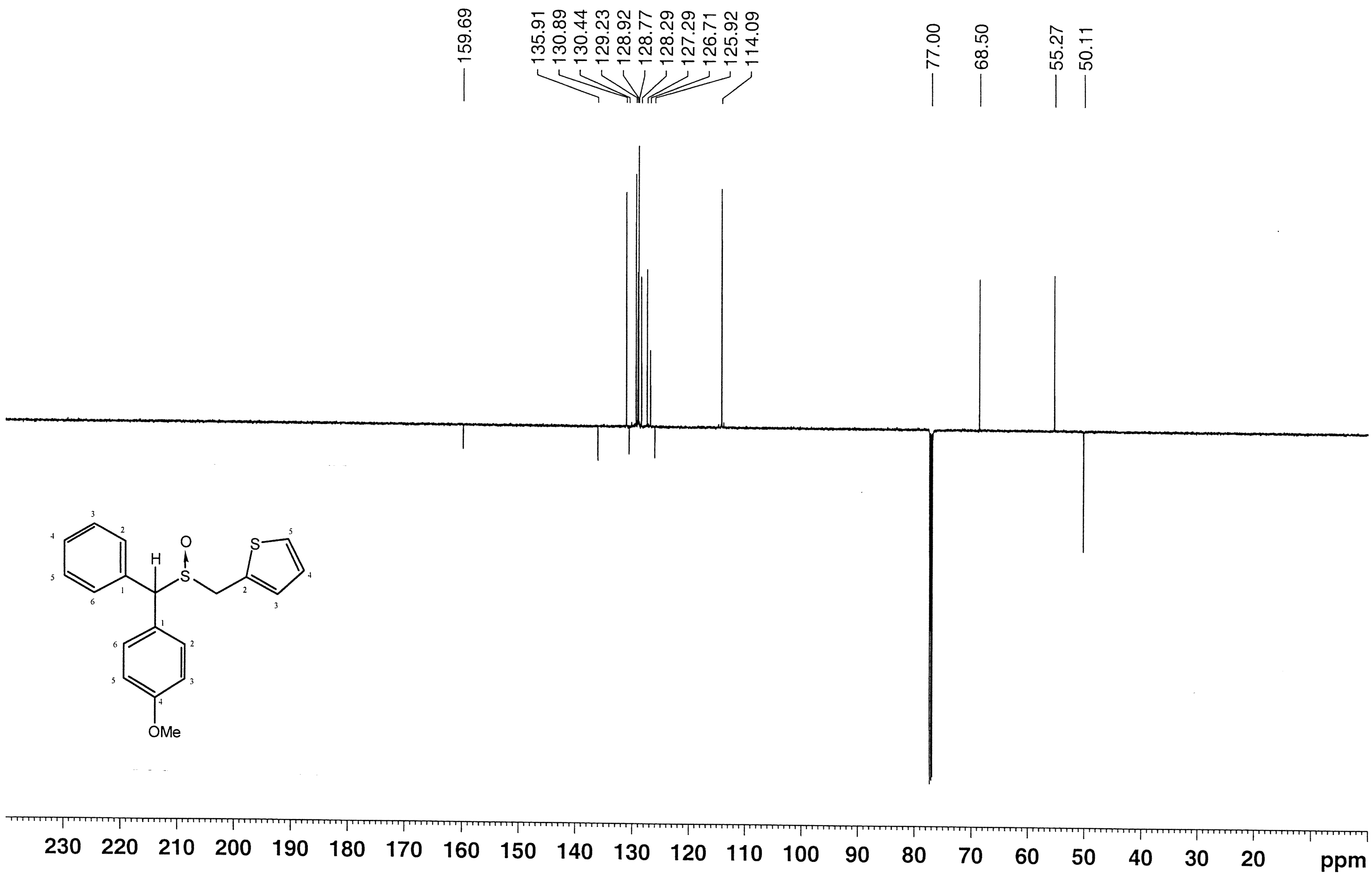

CE149-S2P2 in cdcl3 (APT) 29.8.2018

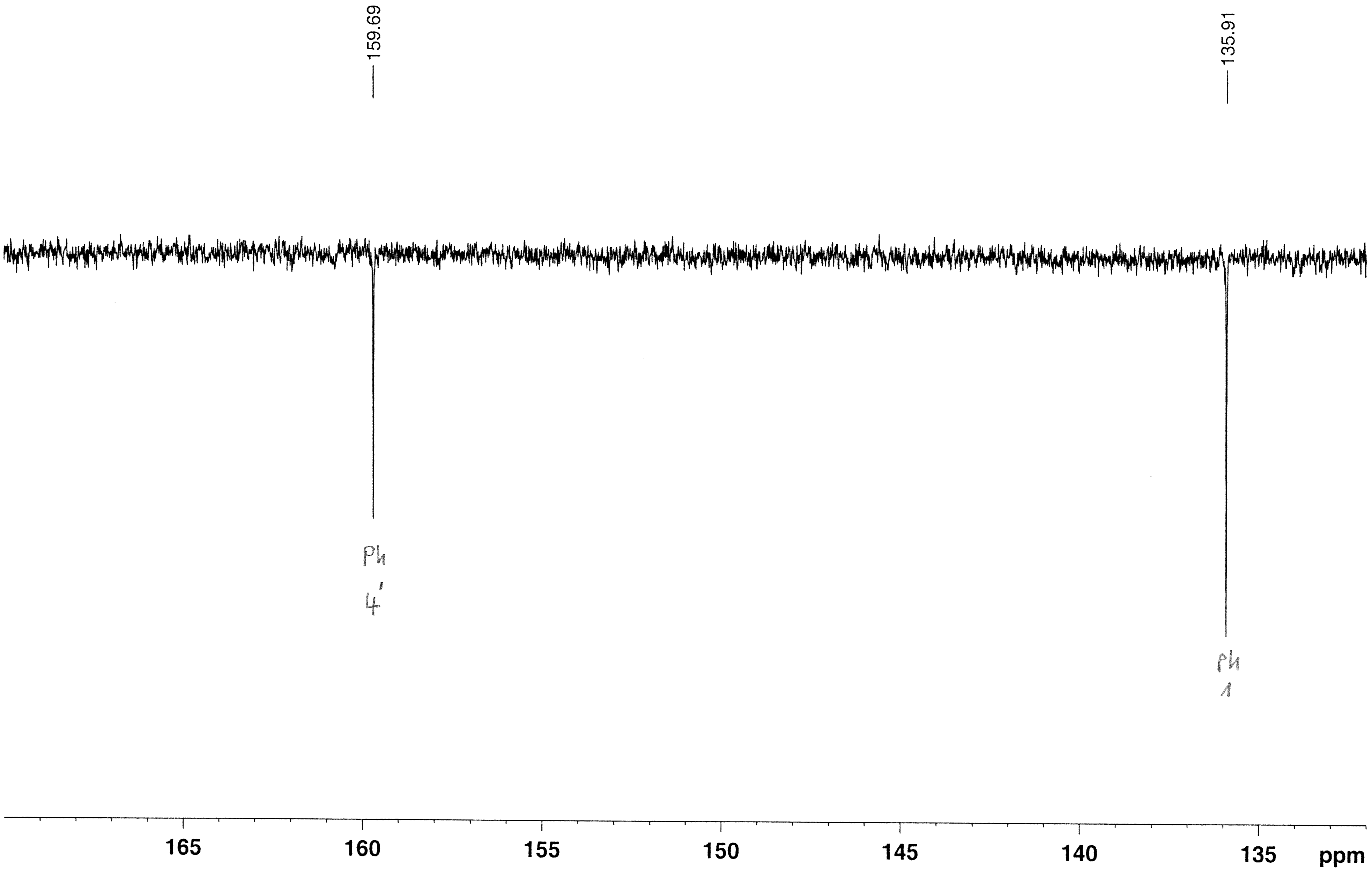

CE149-S2P2 in cdcl3 (APT) 29.8.2018

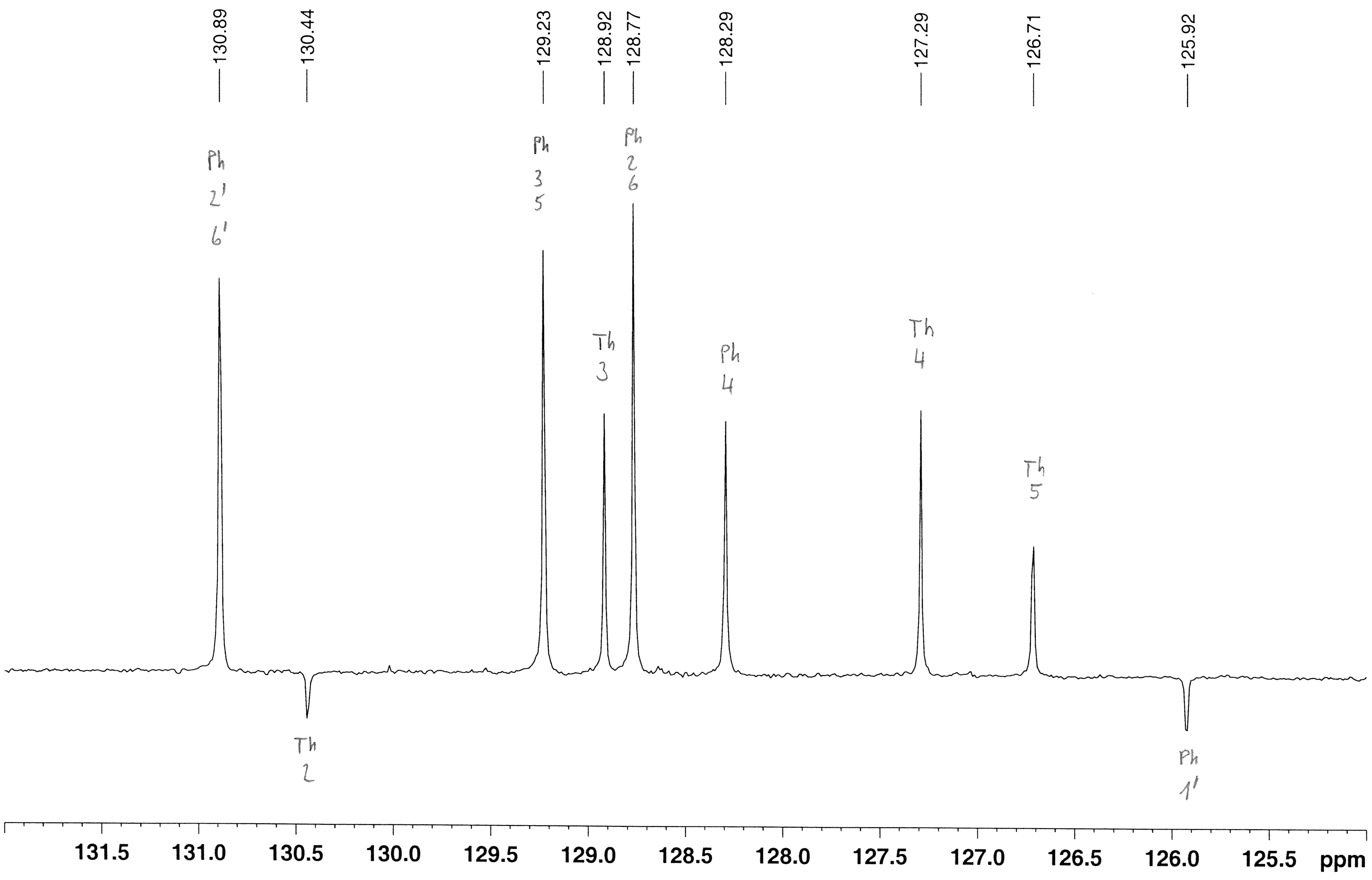

CE149-S2P2 in cdcl3 (APT) 29.8.2018

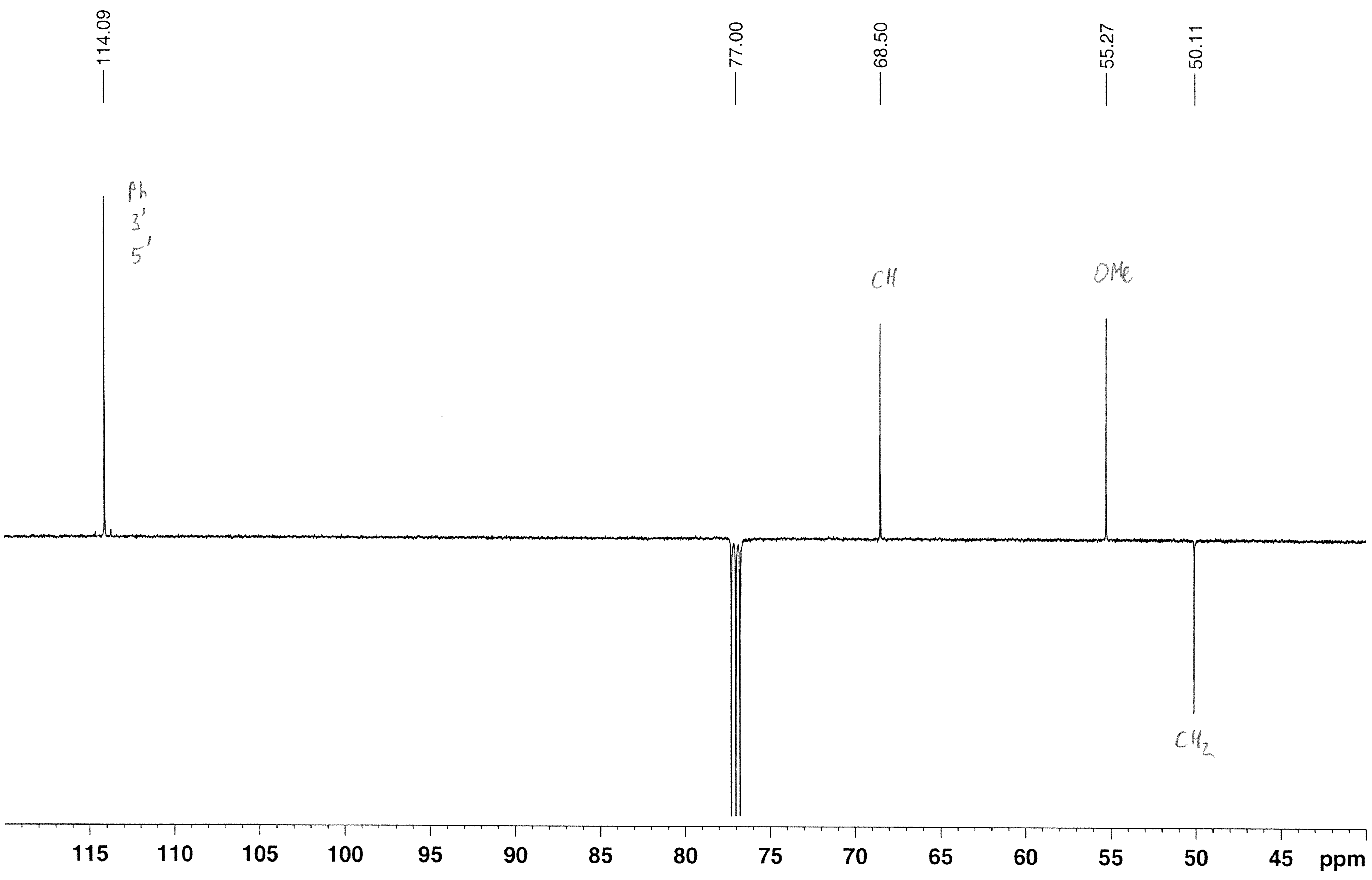

Figure S81c. NMR spectra of compound **5u.**

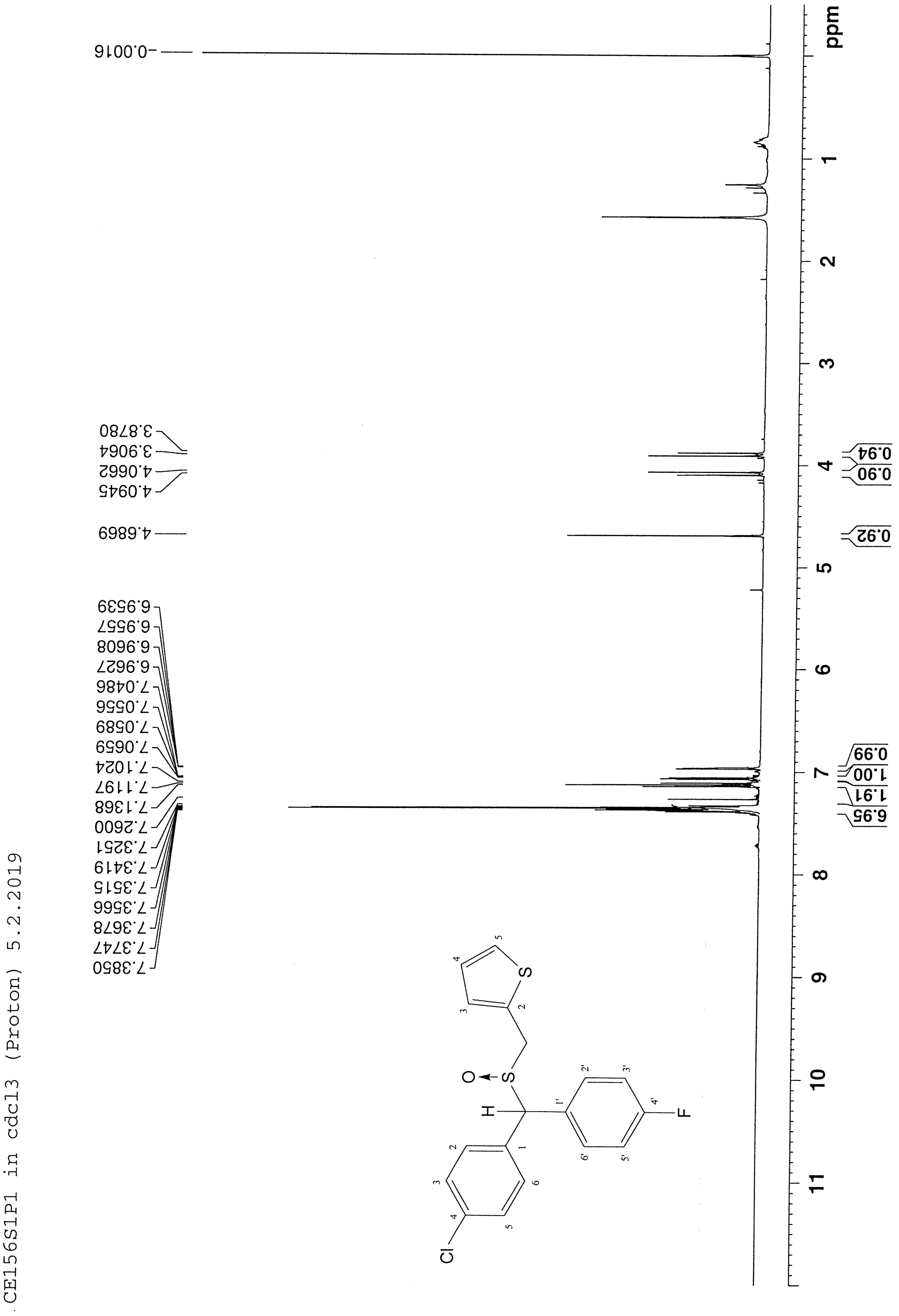

CE156S1P1 in cdcl3 (Proton) 5.2.2019

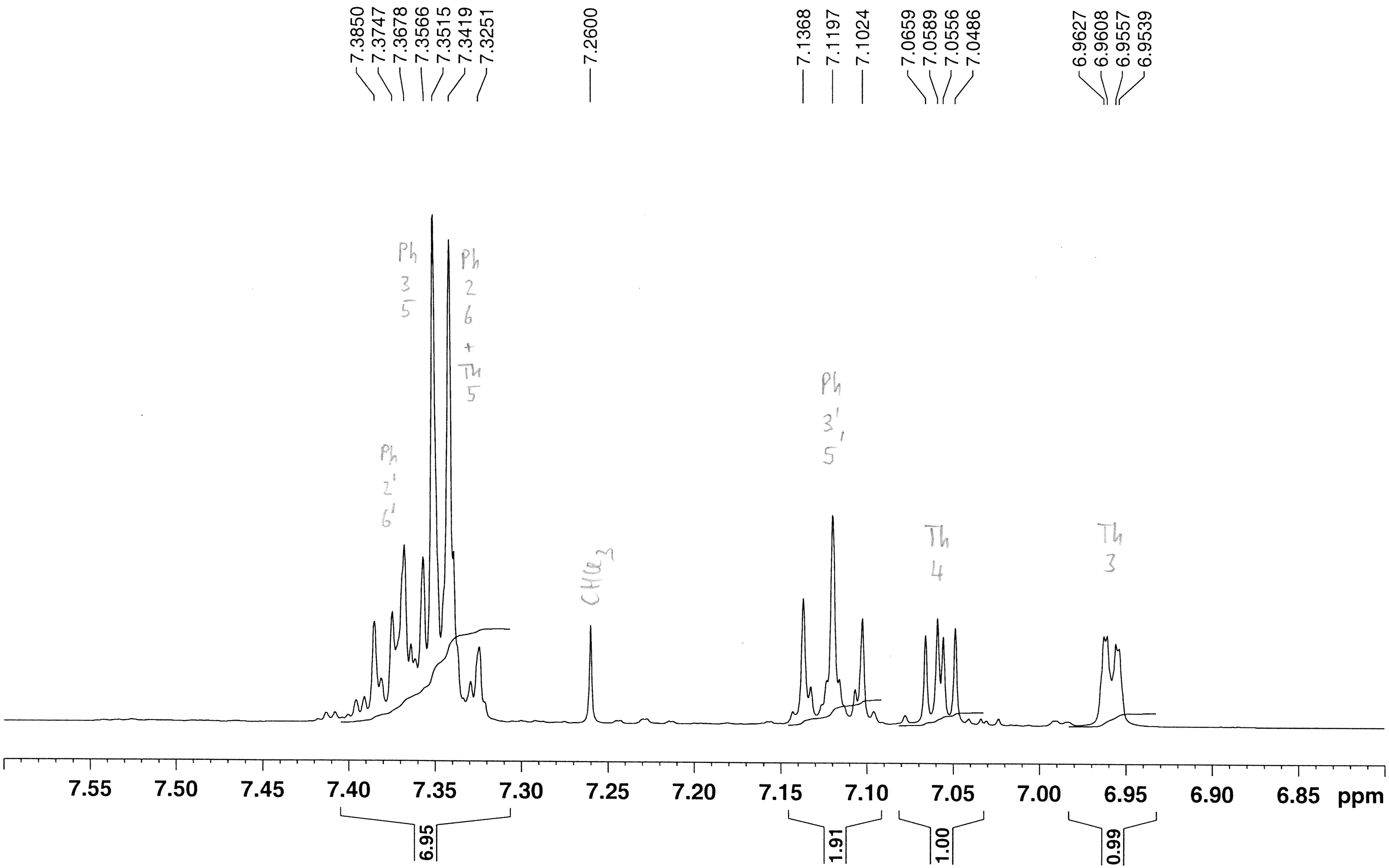

CE156S1P1 in cdcl3 (Proton) 5.2.2019

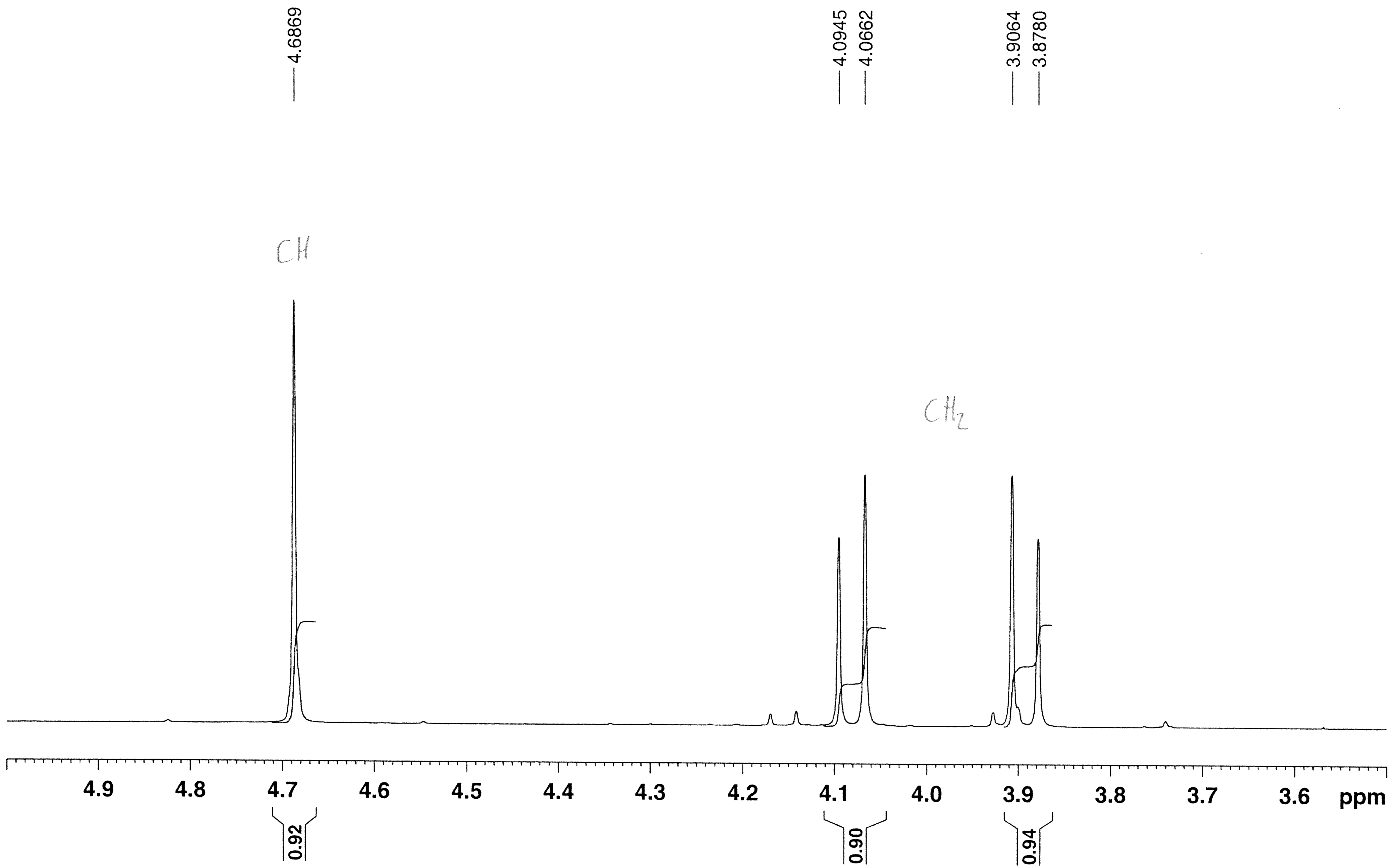

CE156S1P1 in cdcl3 (APT) 5.2.2019

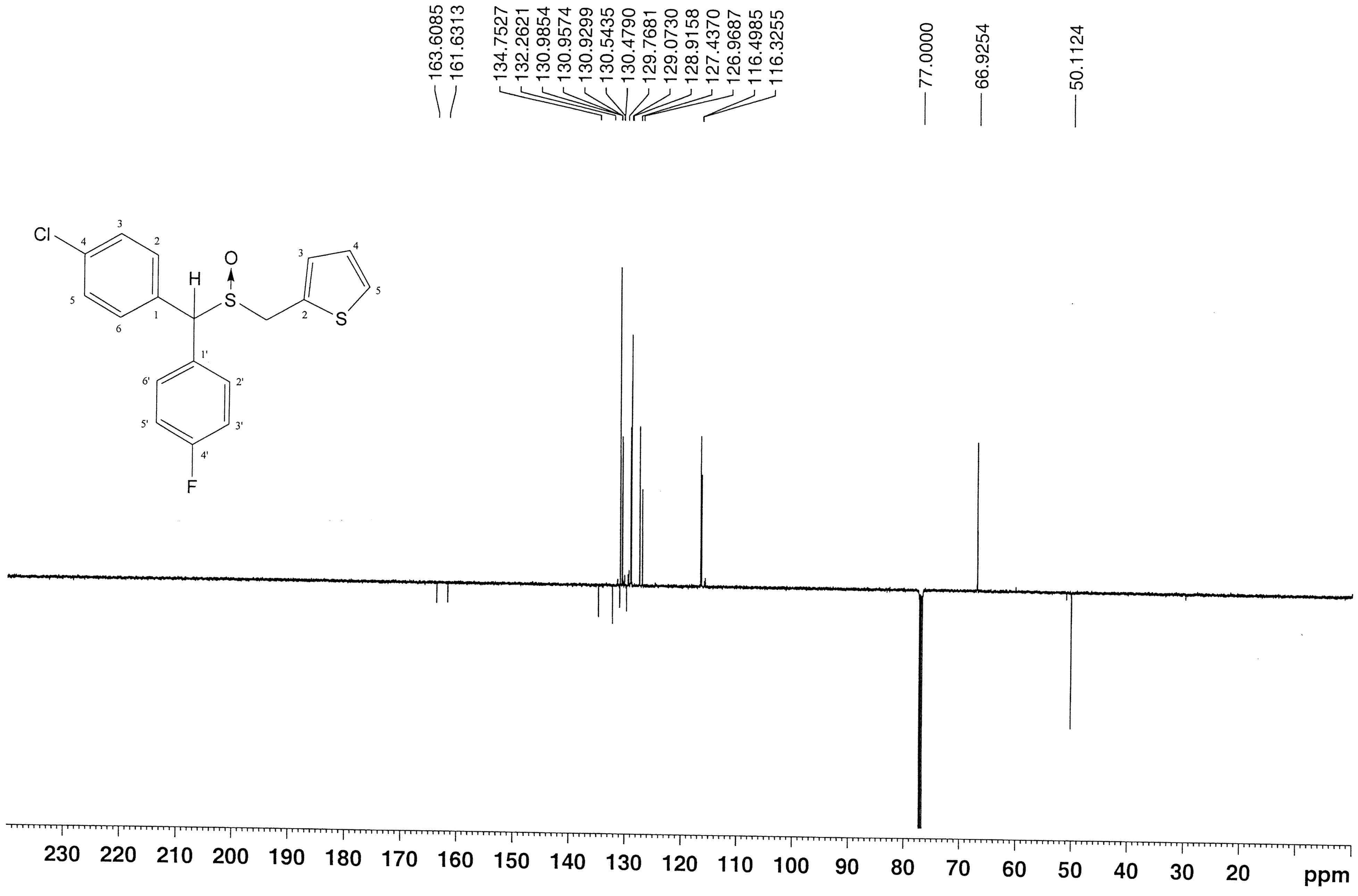

CE156S1P1 in cdcl3 (APT) 5.2.2019

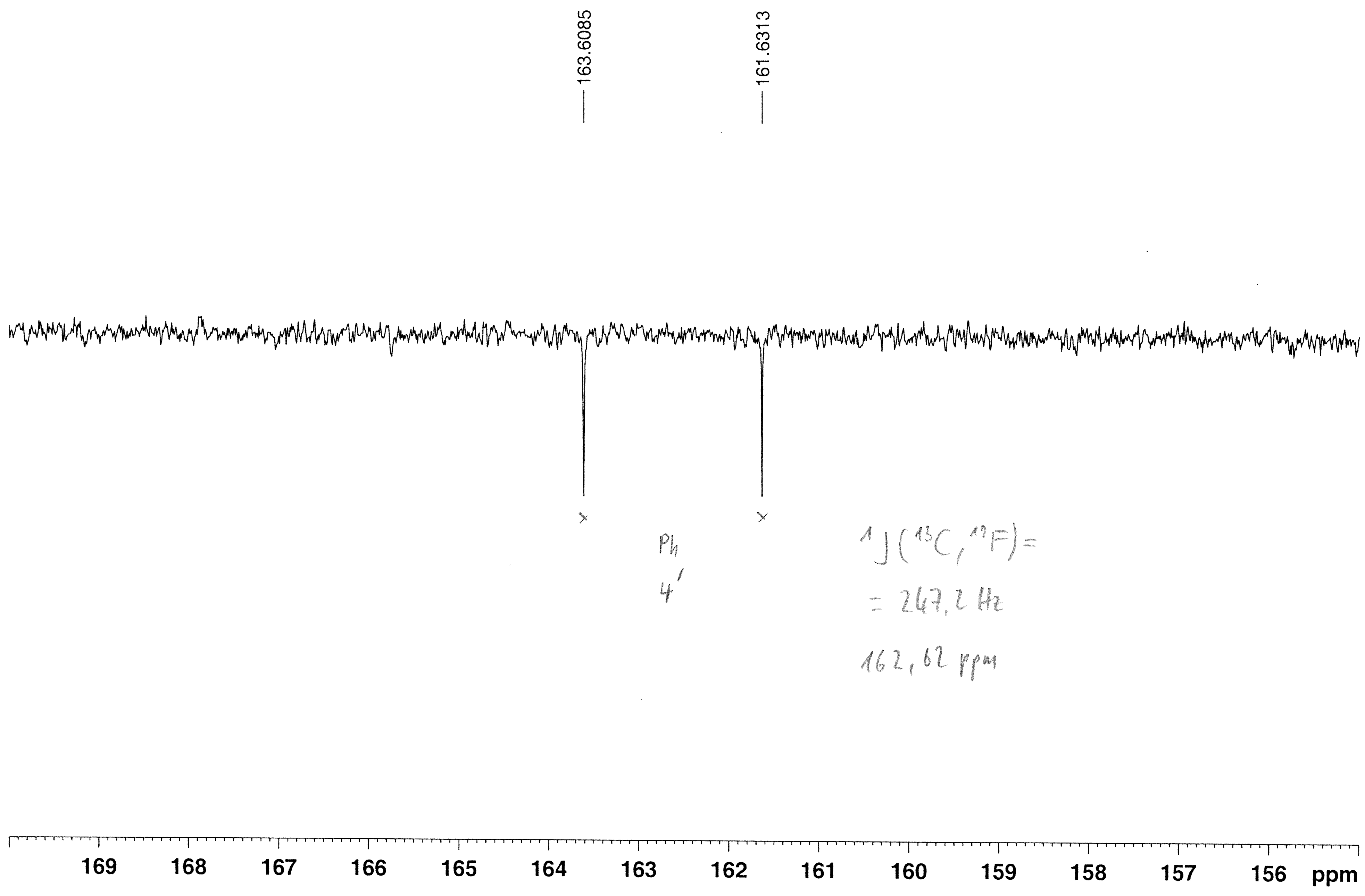

CE156S1P1 in cdcl3 (APT) 5.2.2019

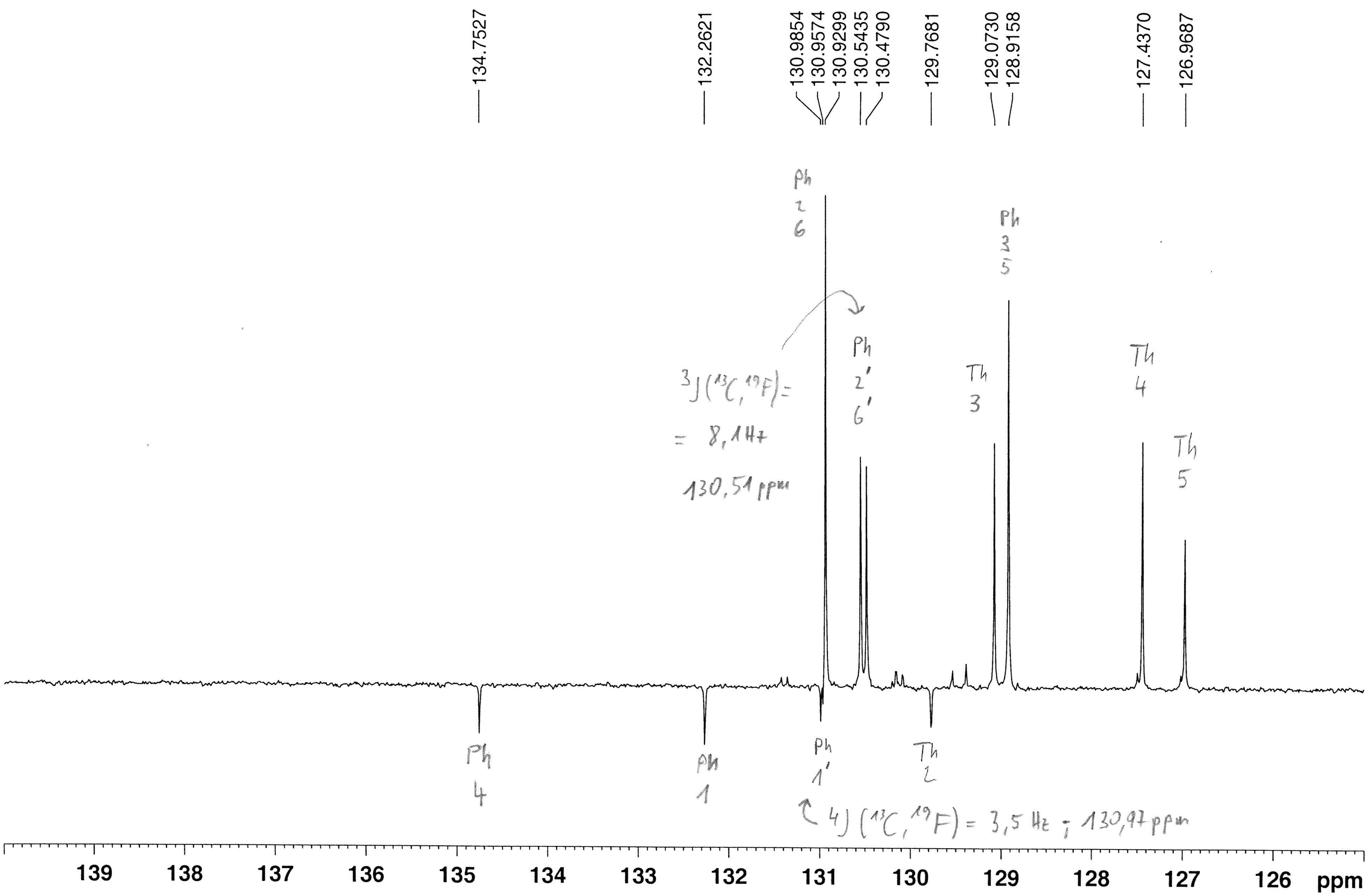

CE156S1P1 in cdcl3 (APT) 5.2.2019

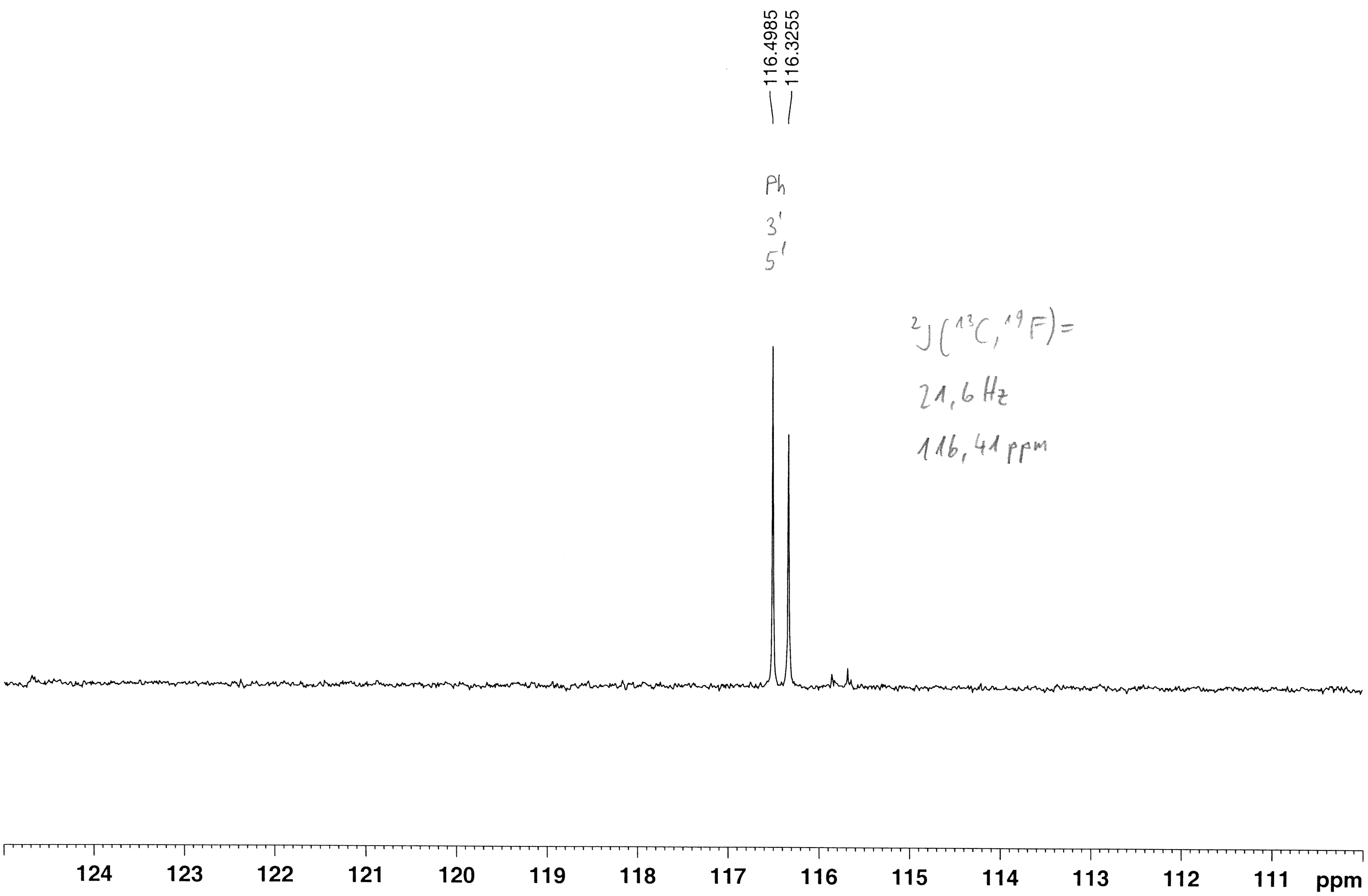

CE156S1P1 in cdcl3 (APT) 5.2.2019

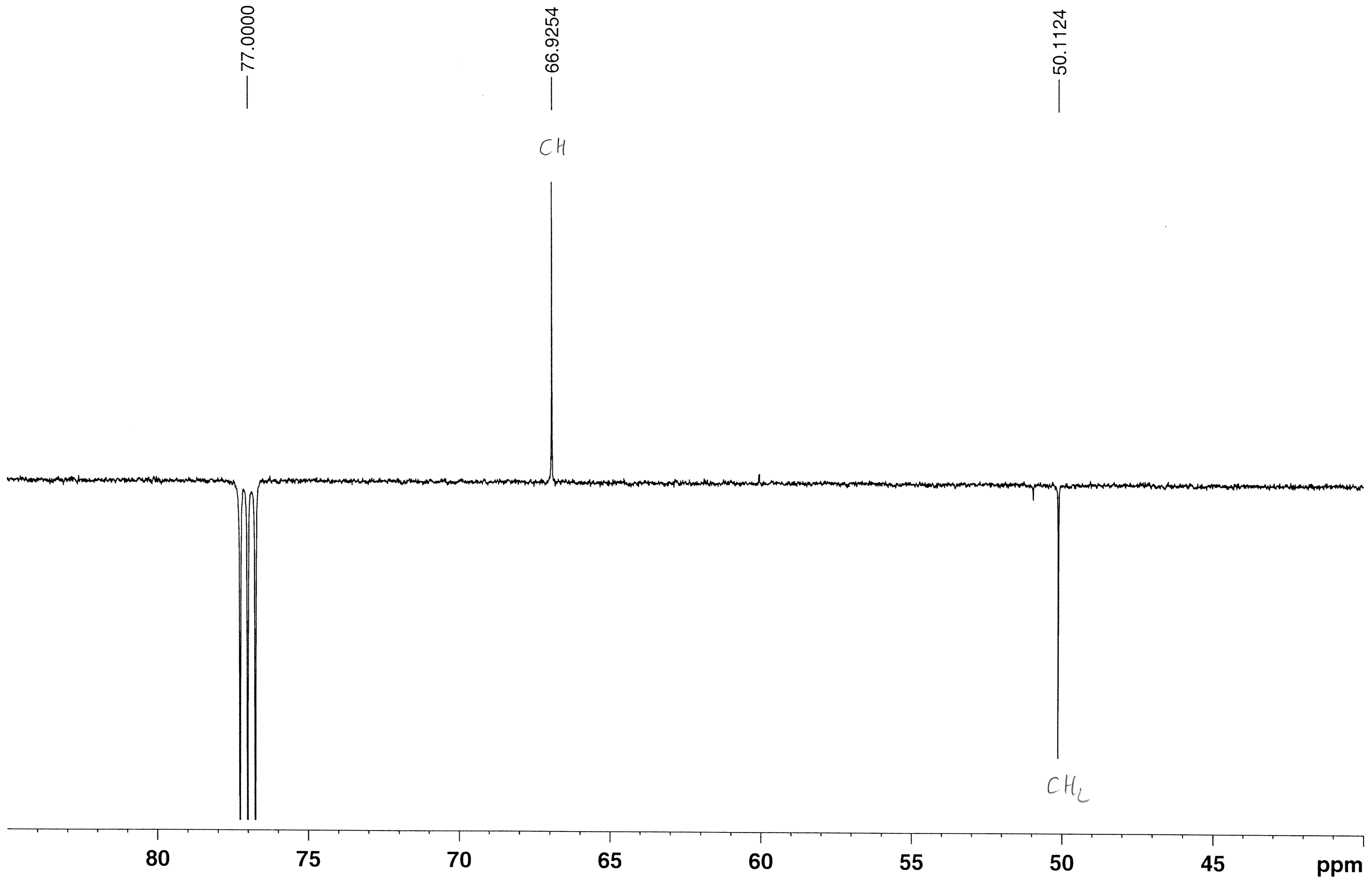

CE156S1P1 in cdcl3 (COSY) 5.2.2019

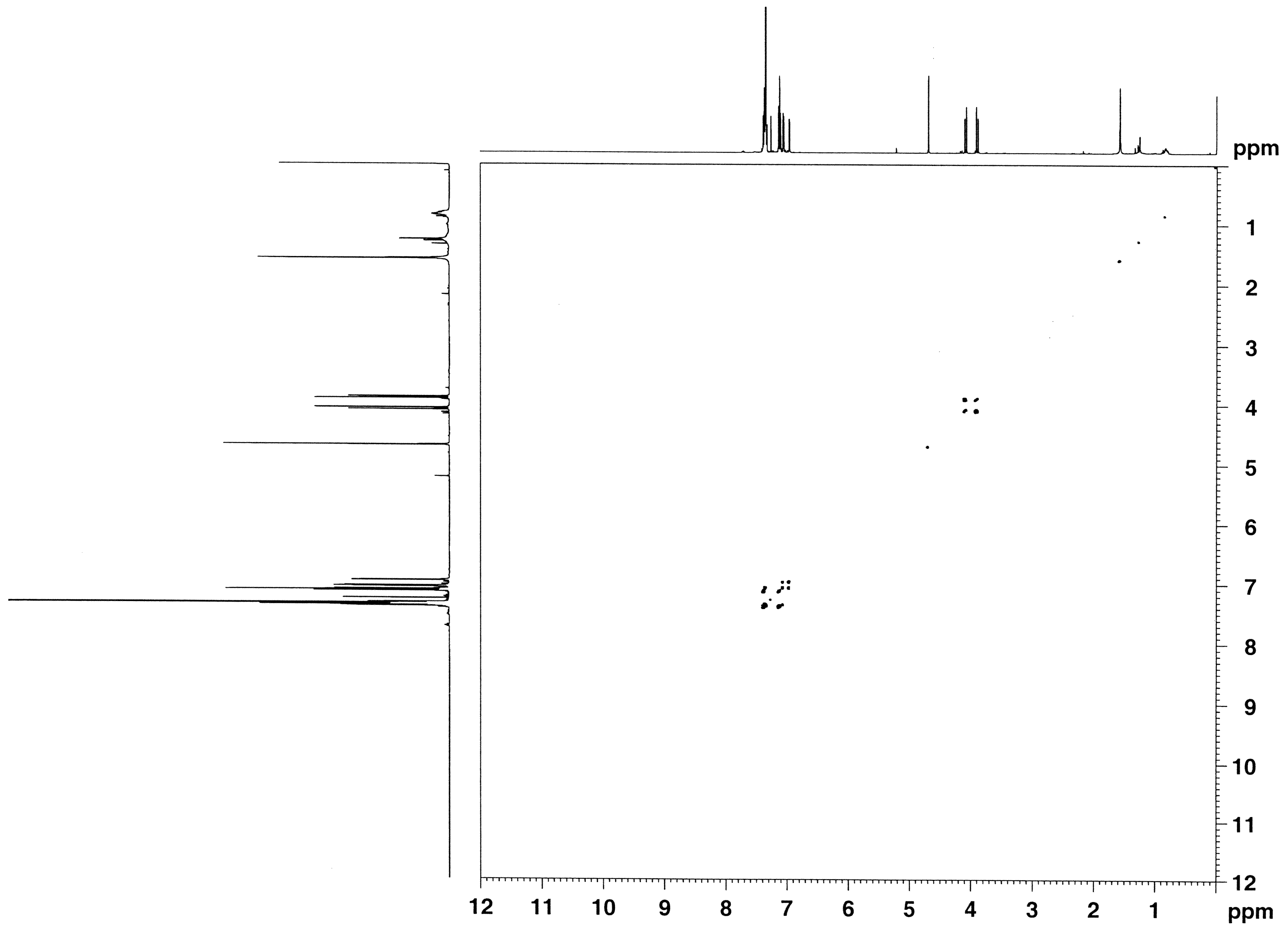

CE156S1P1 in cdcl3 (COSY) 5.2.2019

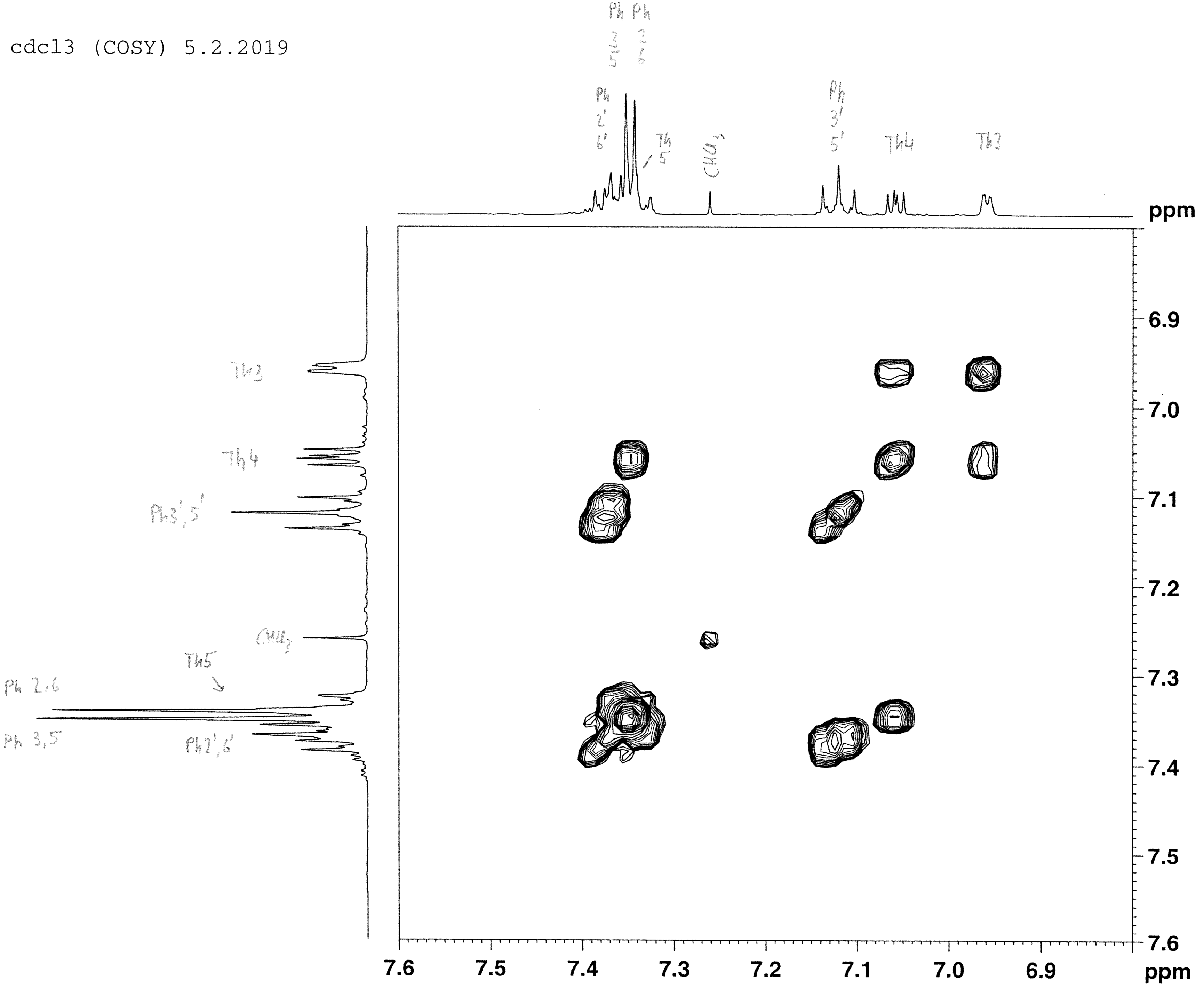

CE156S1P1 in cdcl3 (COSY) 5.2.2019

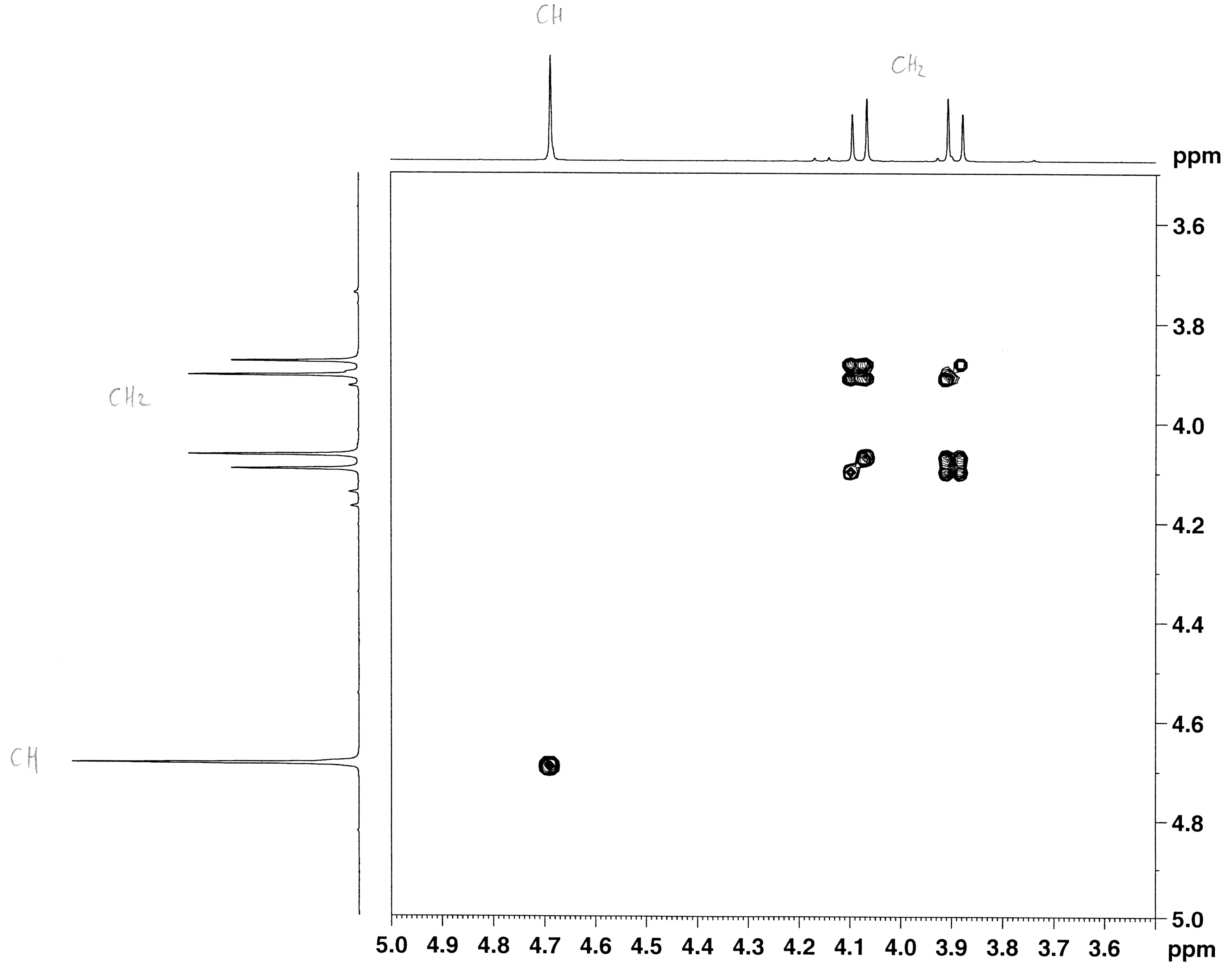

CE156S1P1 in cdcl3 (HSQC) 5.2.2019

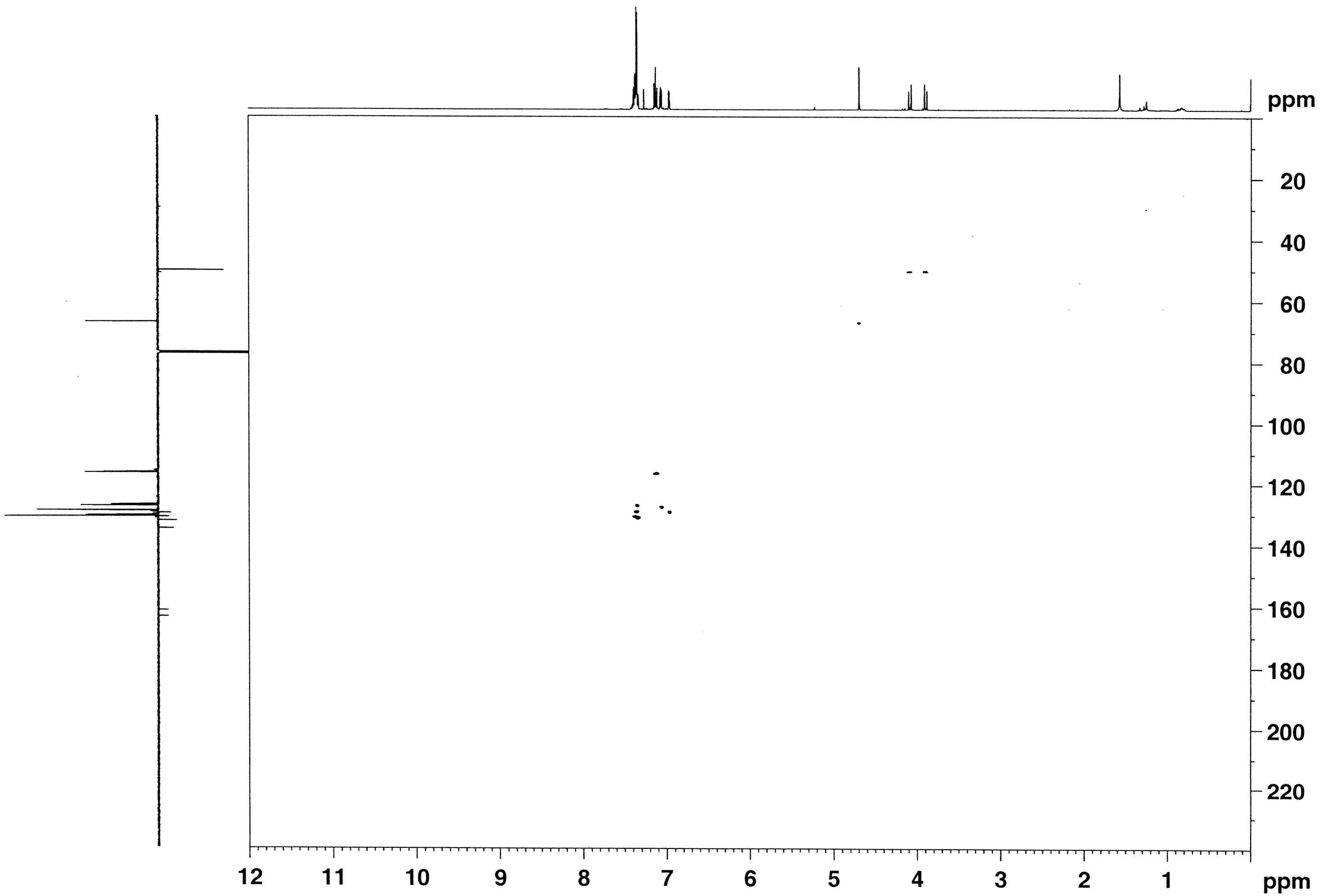

CE156S1P1 in cdcl3 (HSQC) 5.2.2019

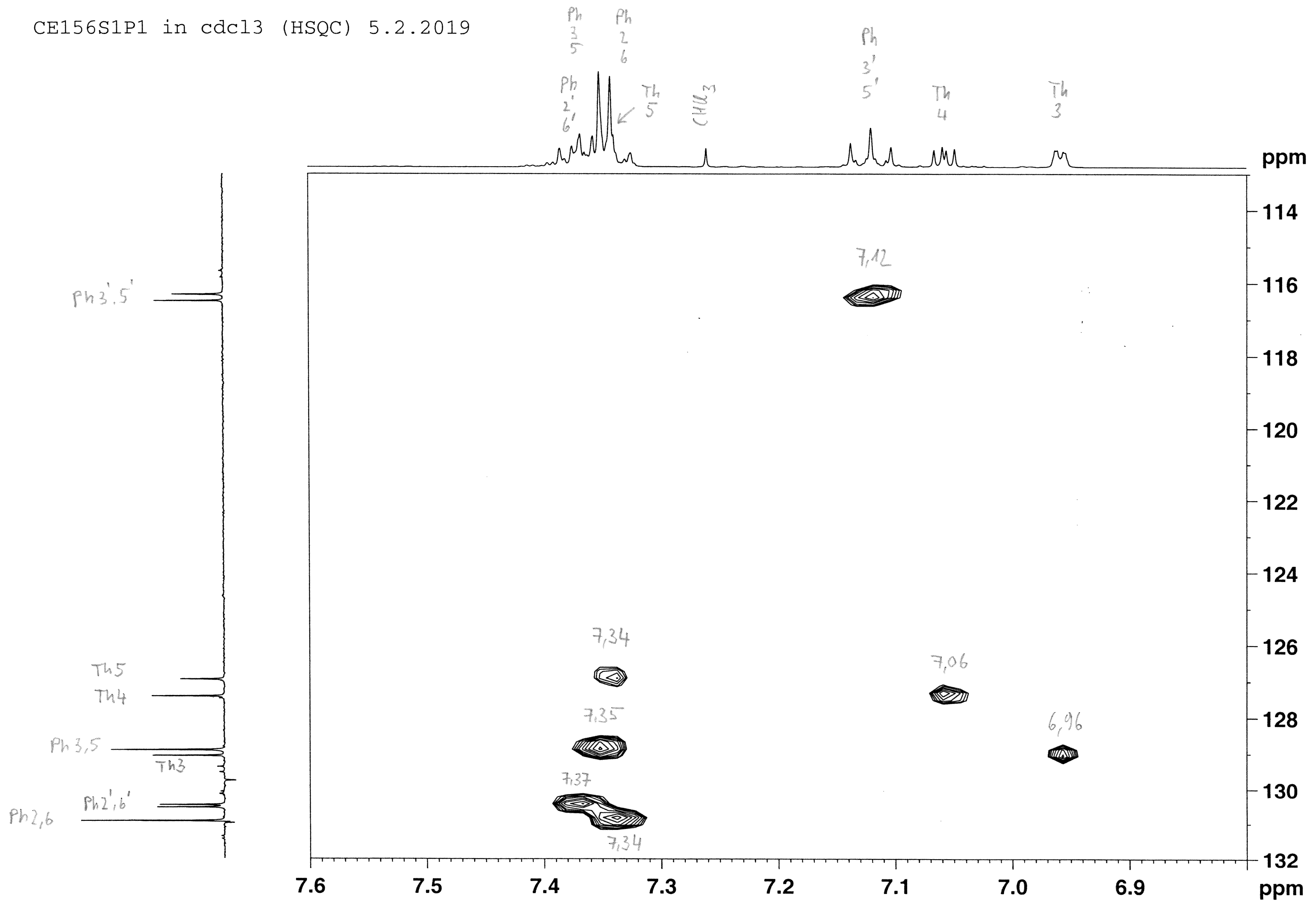

CE156S1P1 in cdcl3 (HSQC) 5.2.2019

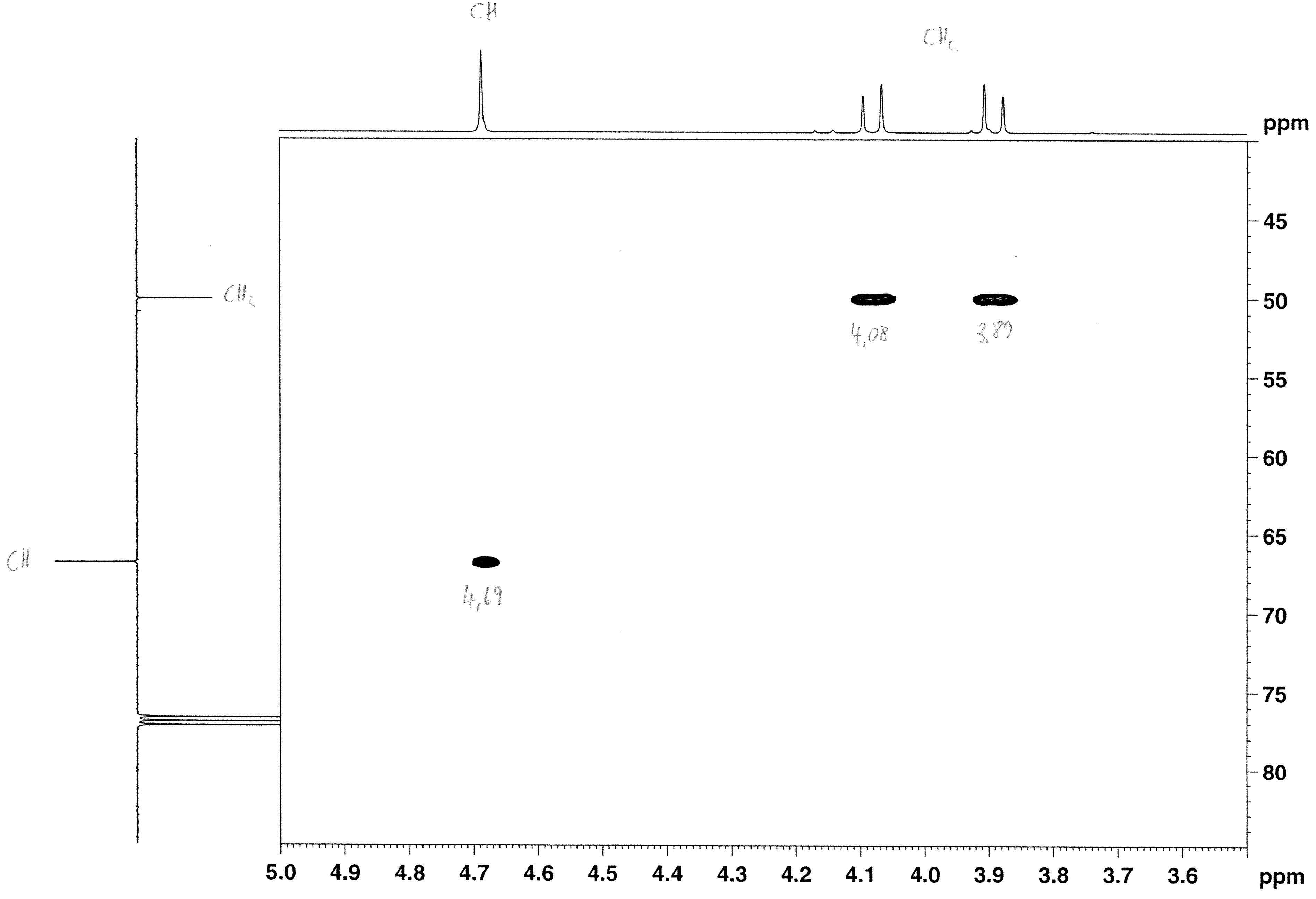

CE156S1P1 in cdcl3 (HMBC) 5.2.2019

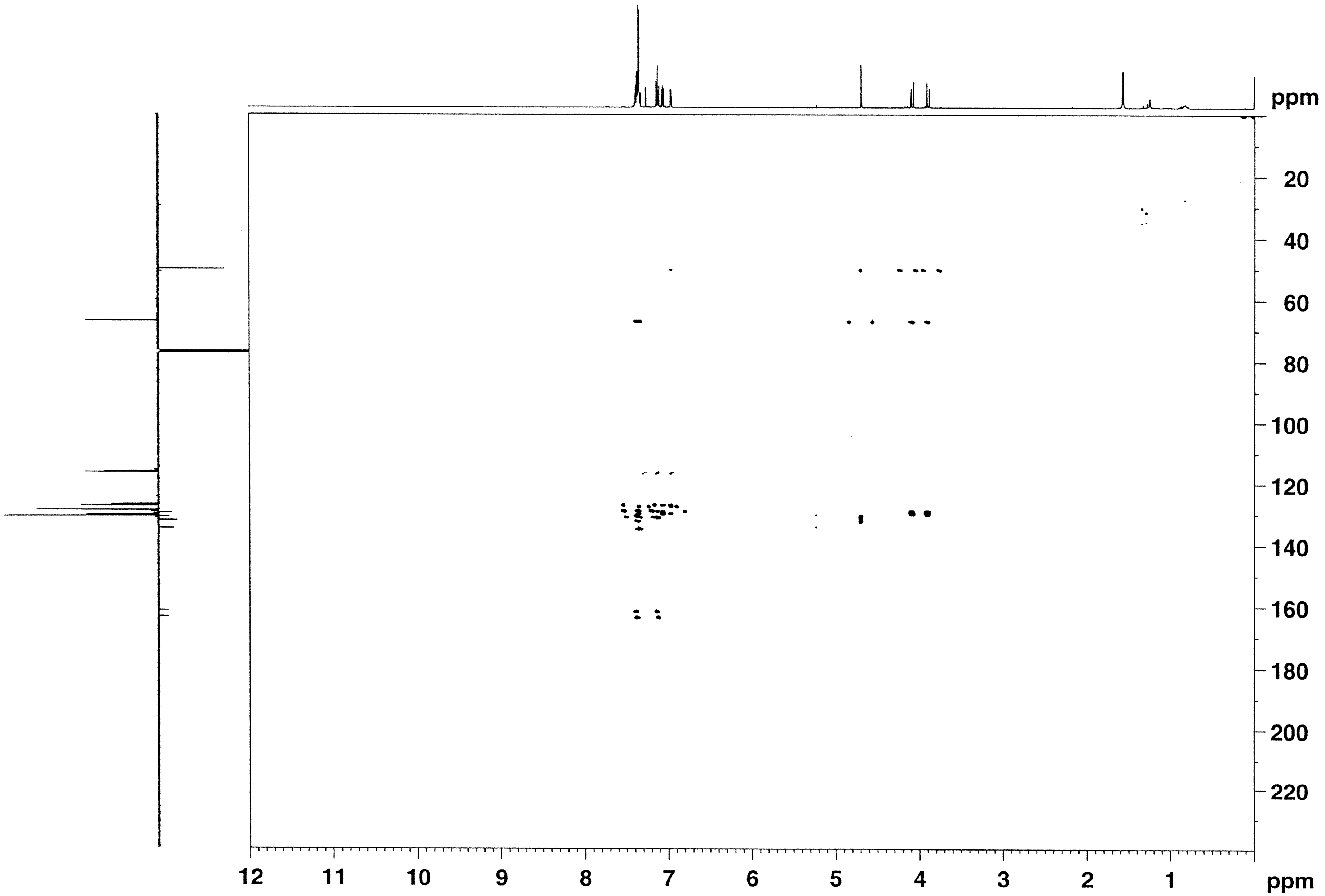

CE156S1P1 in cdcl3 (HMBC) 5.2.2019

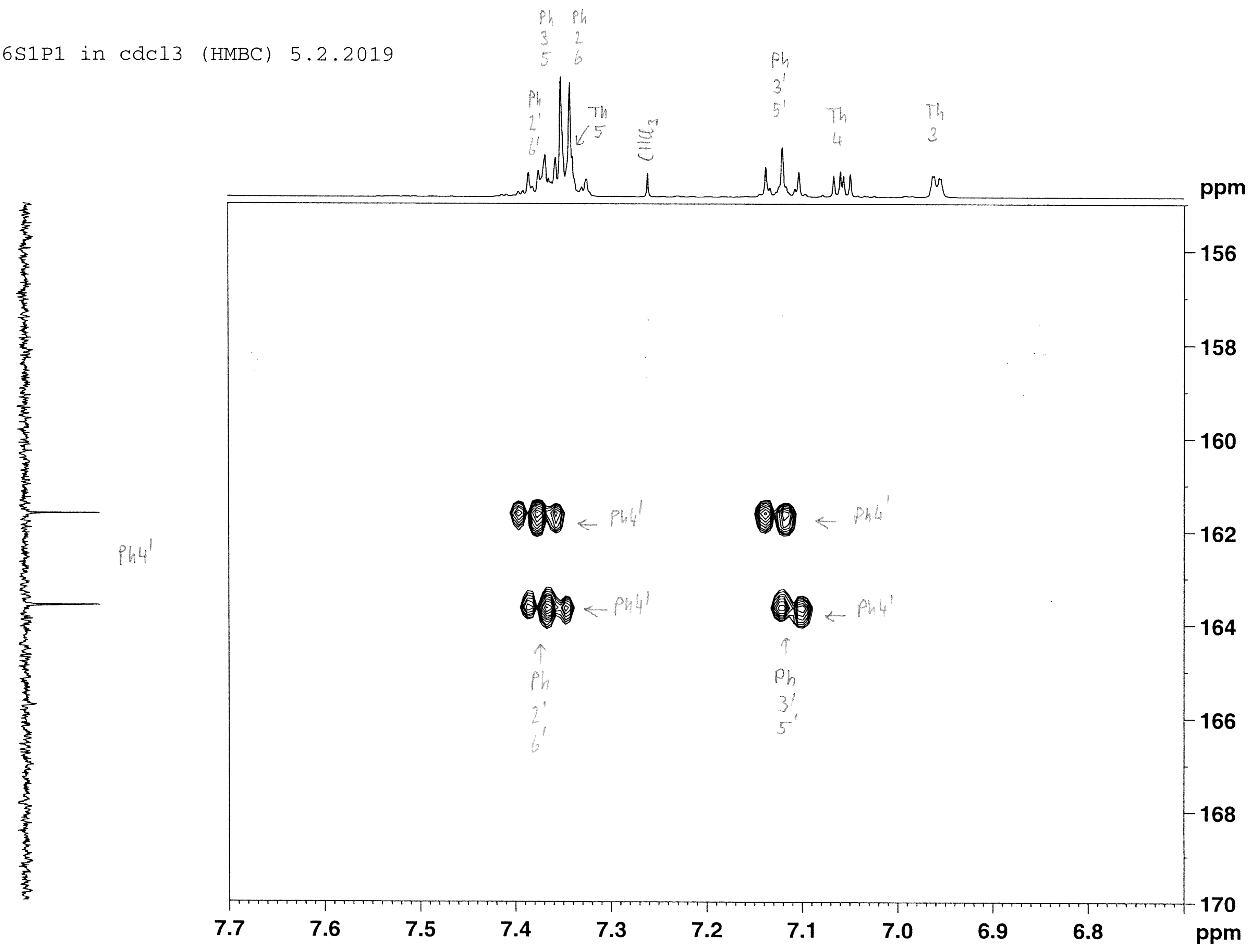

CE156S1P1 in cdcl3 (HMBC) 5.2.2019

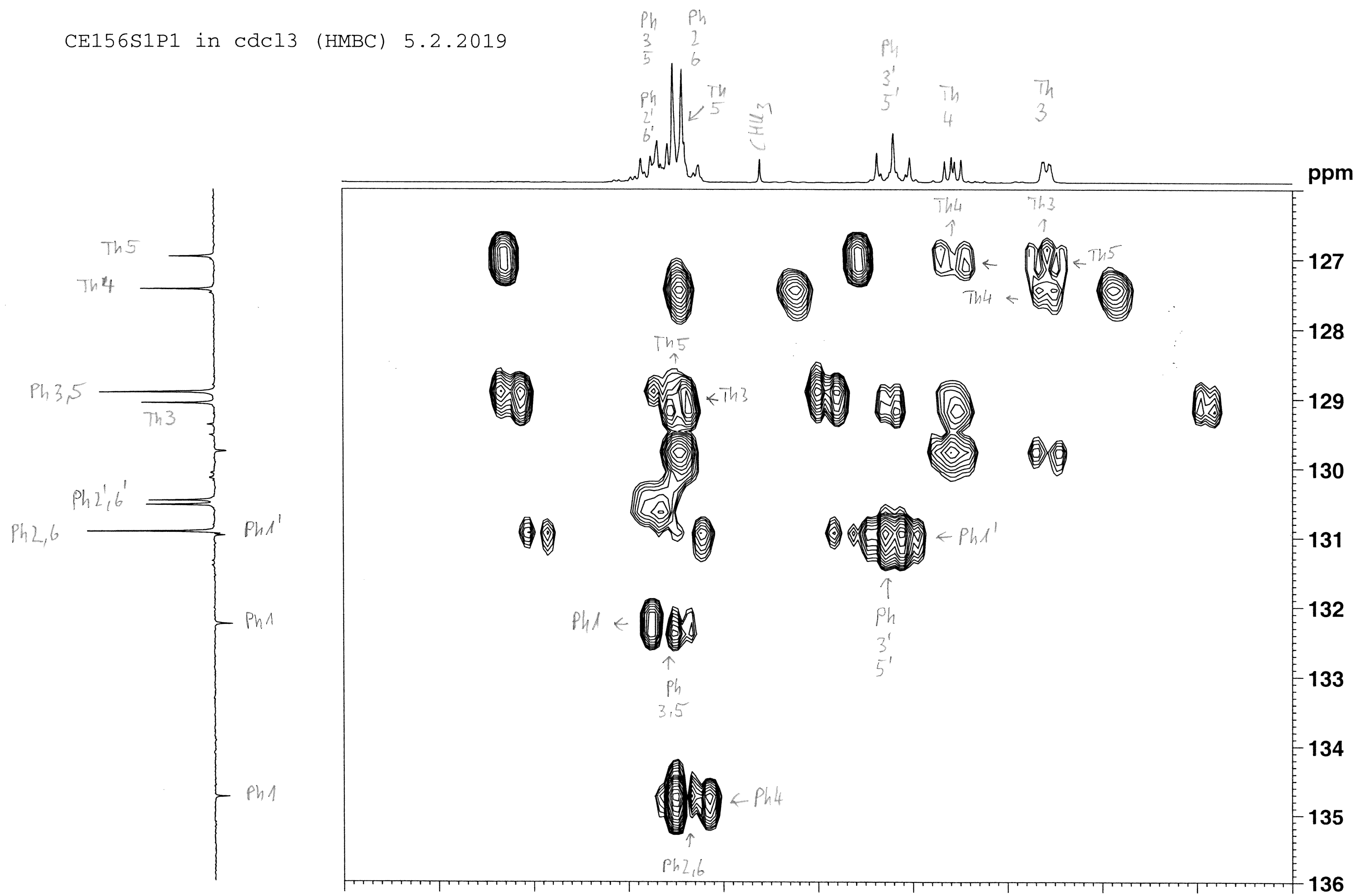

CE156S1P1 in cdcl3 (HMBC) 5.2.2019

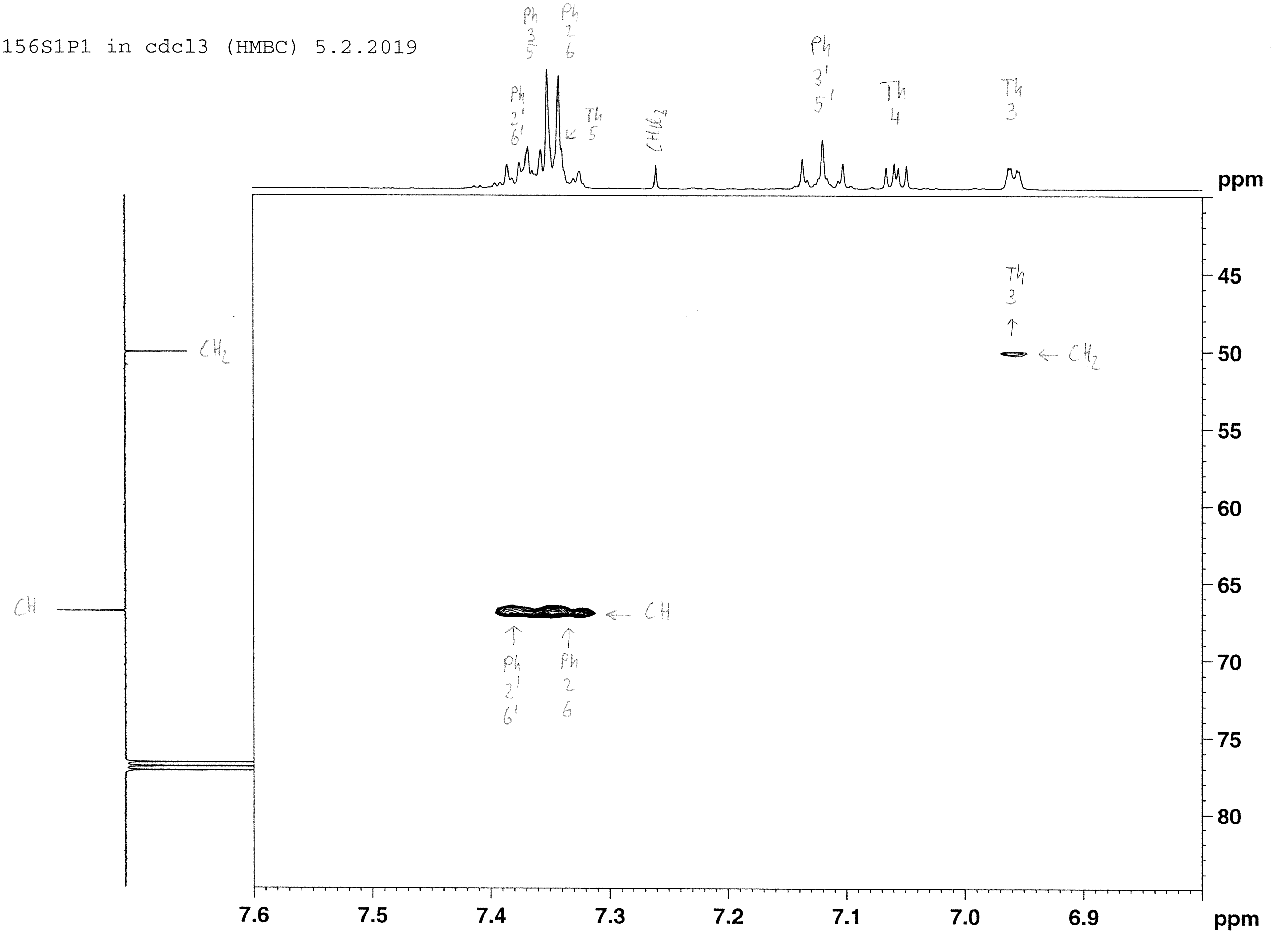

CE156S1P1 in cdcl3 (HMBC) 5.2.2019

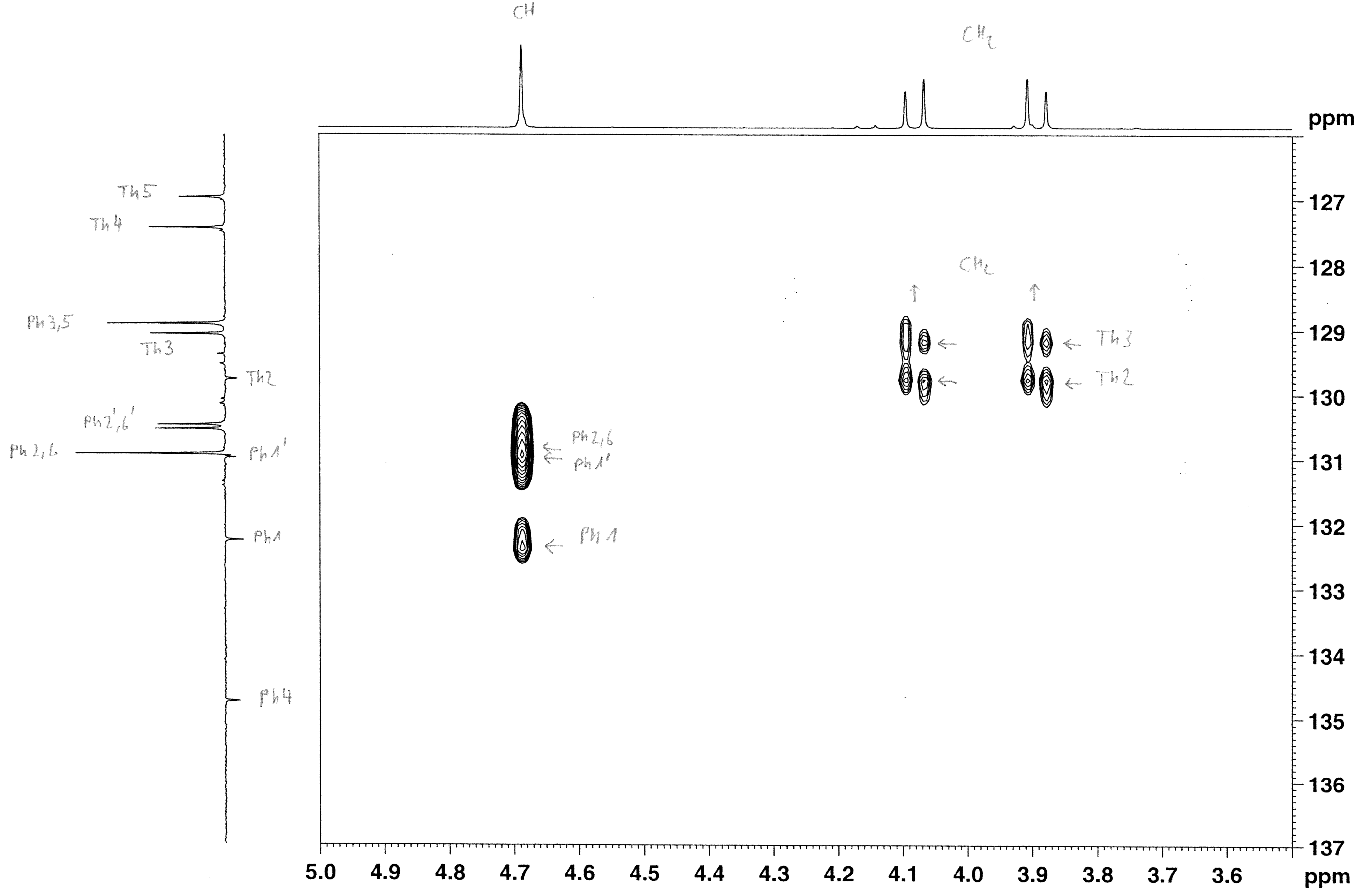

CE156S1P1 in cdcl3 (HMBC) 5.2.2019

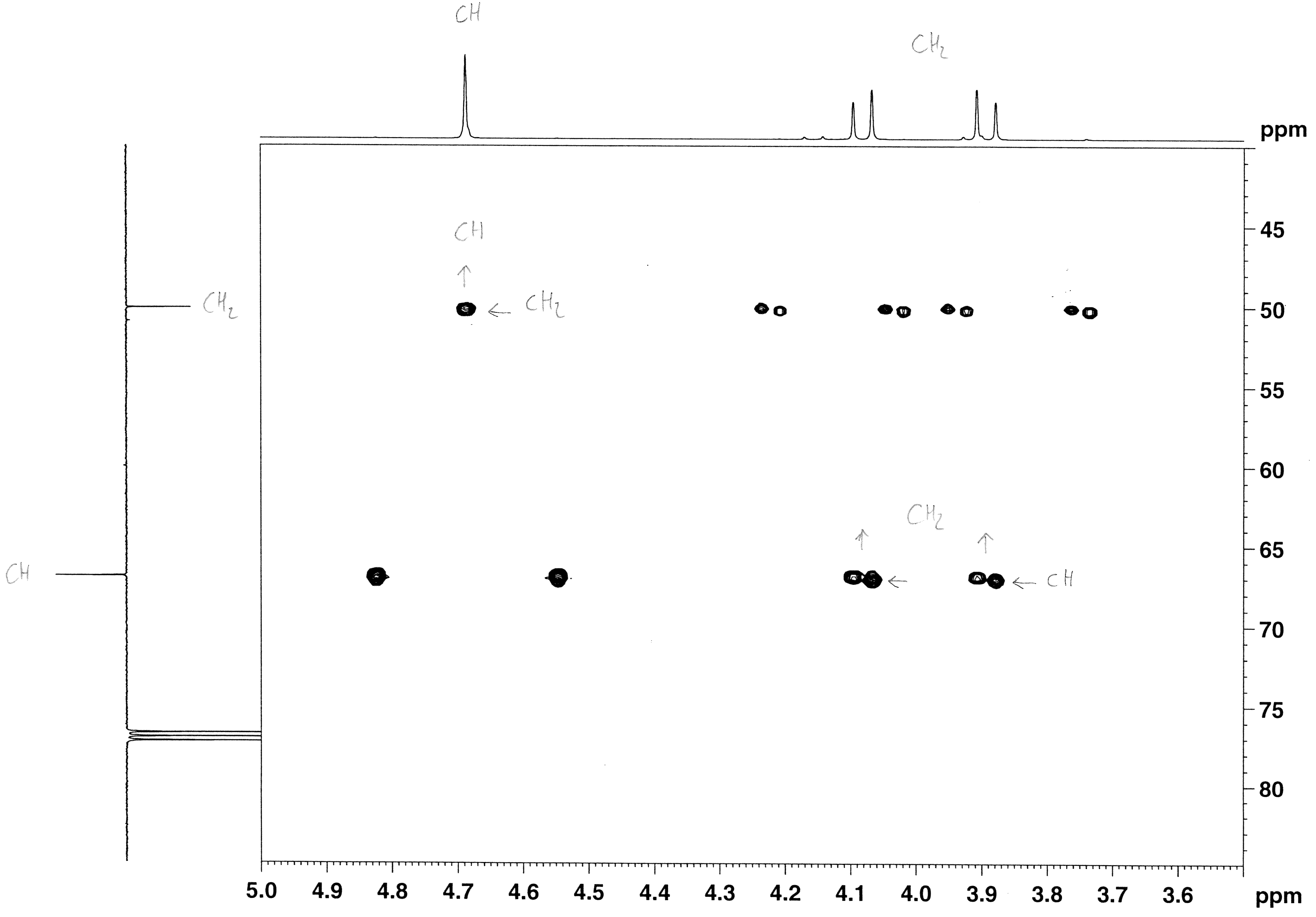

Figure S82c. NMR spectra of compound **6u**.

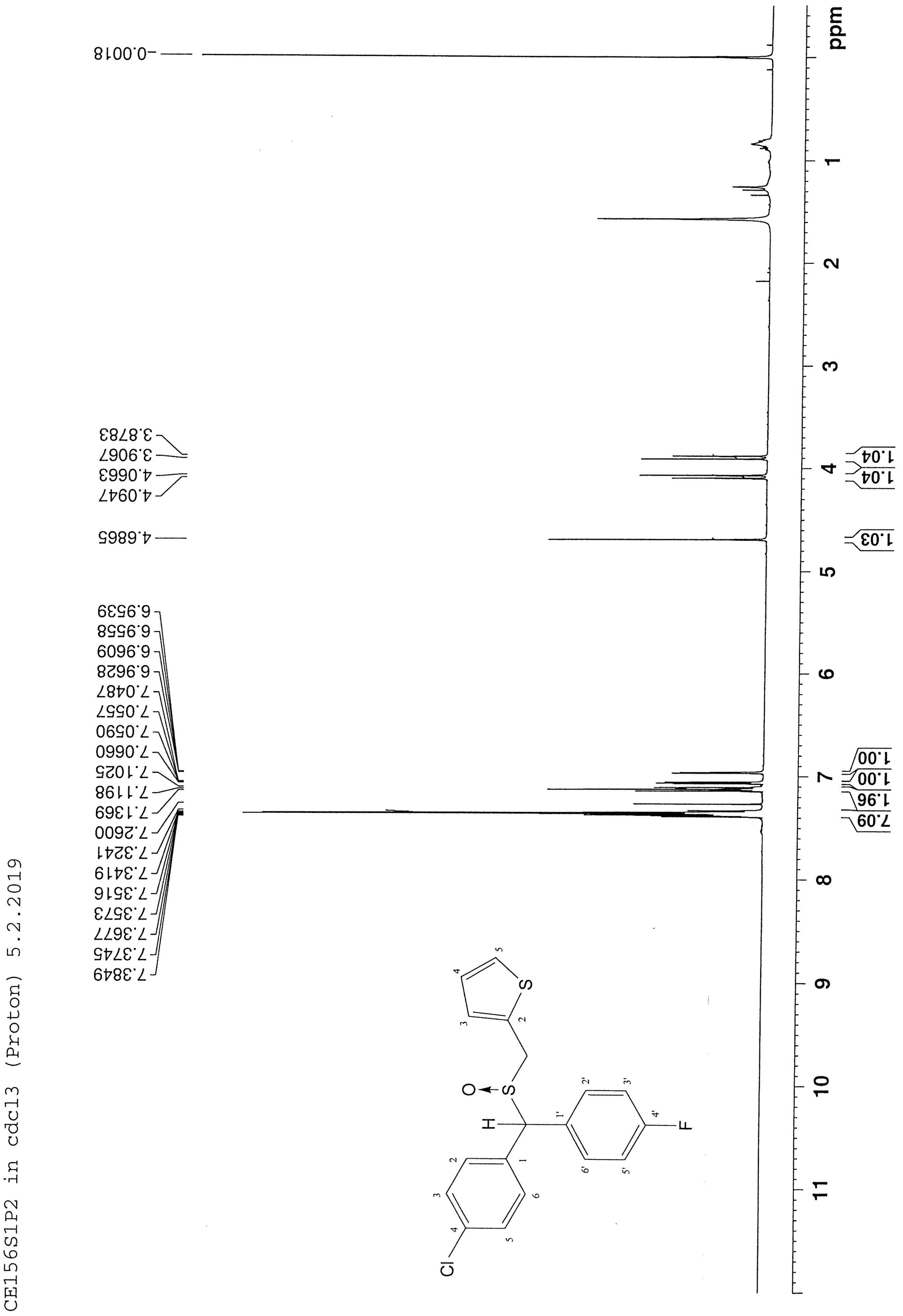

CE156S1P2 in cdcl3 (Proton) 5.2.2019

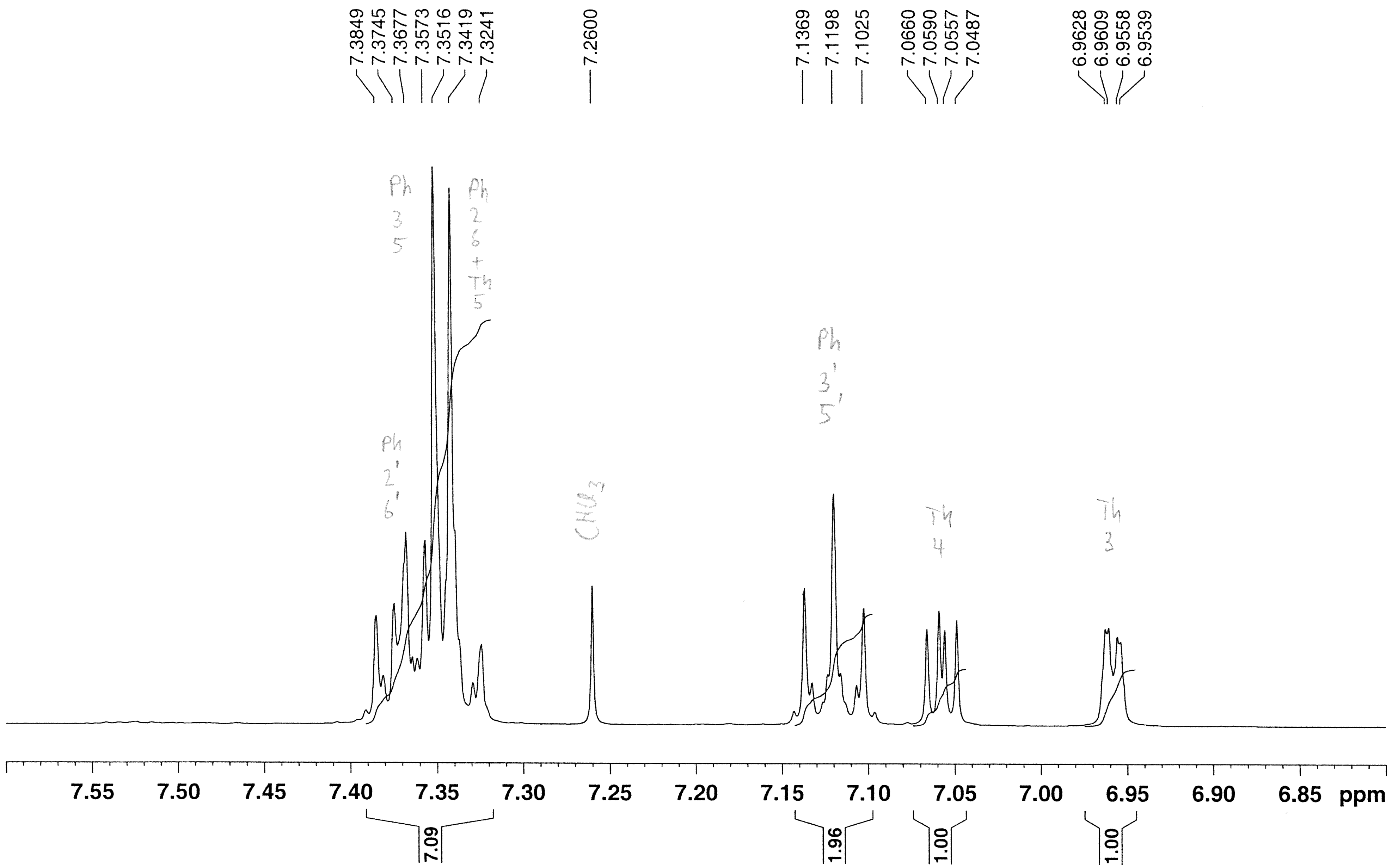

CE156S1P2 in cdcl3 (Proton) 5.2.2019

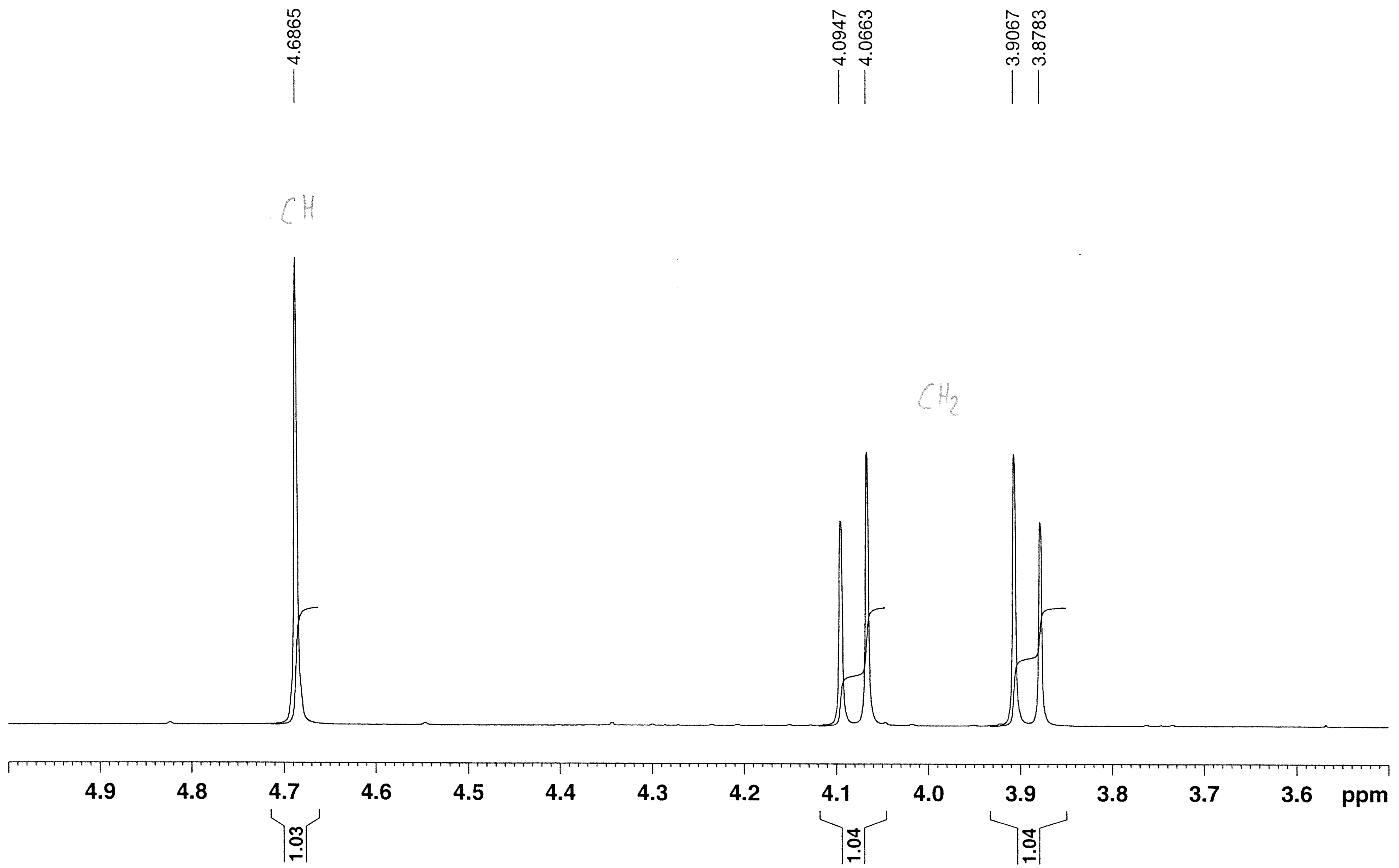

CE156S1P2 in cdcl3 (APT) 5.2.2019

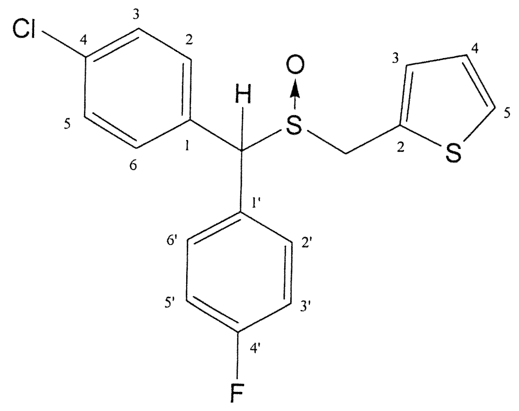

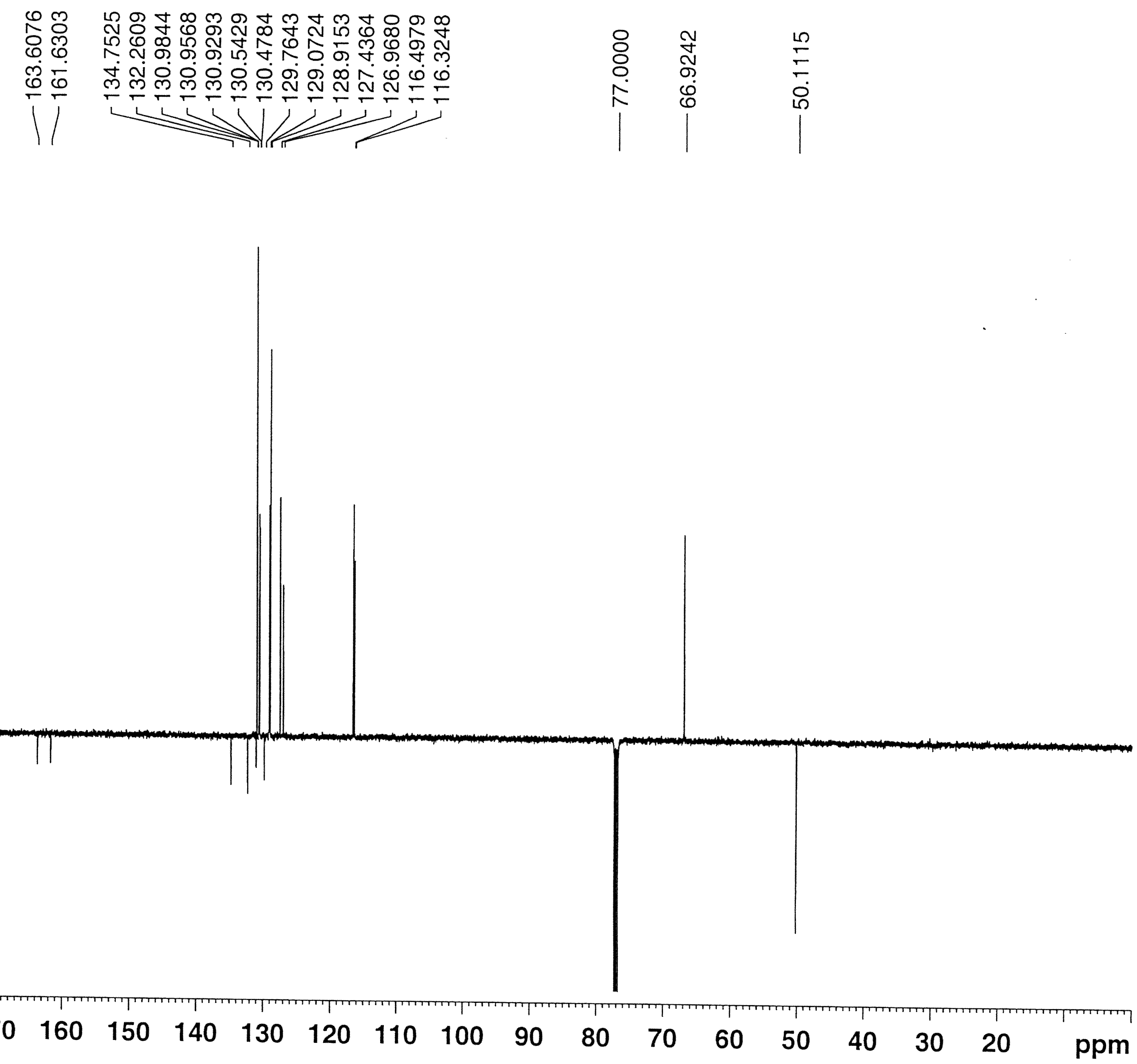

CE156S1P2 in cdcl3 (APT) 5.2.2019

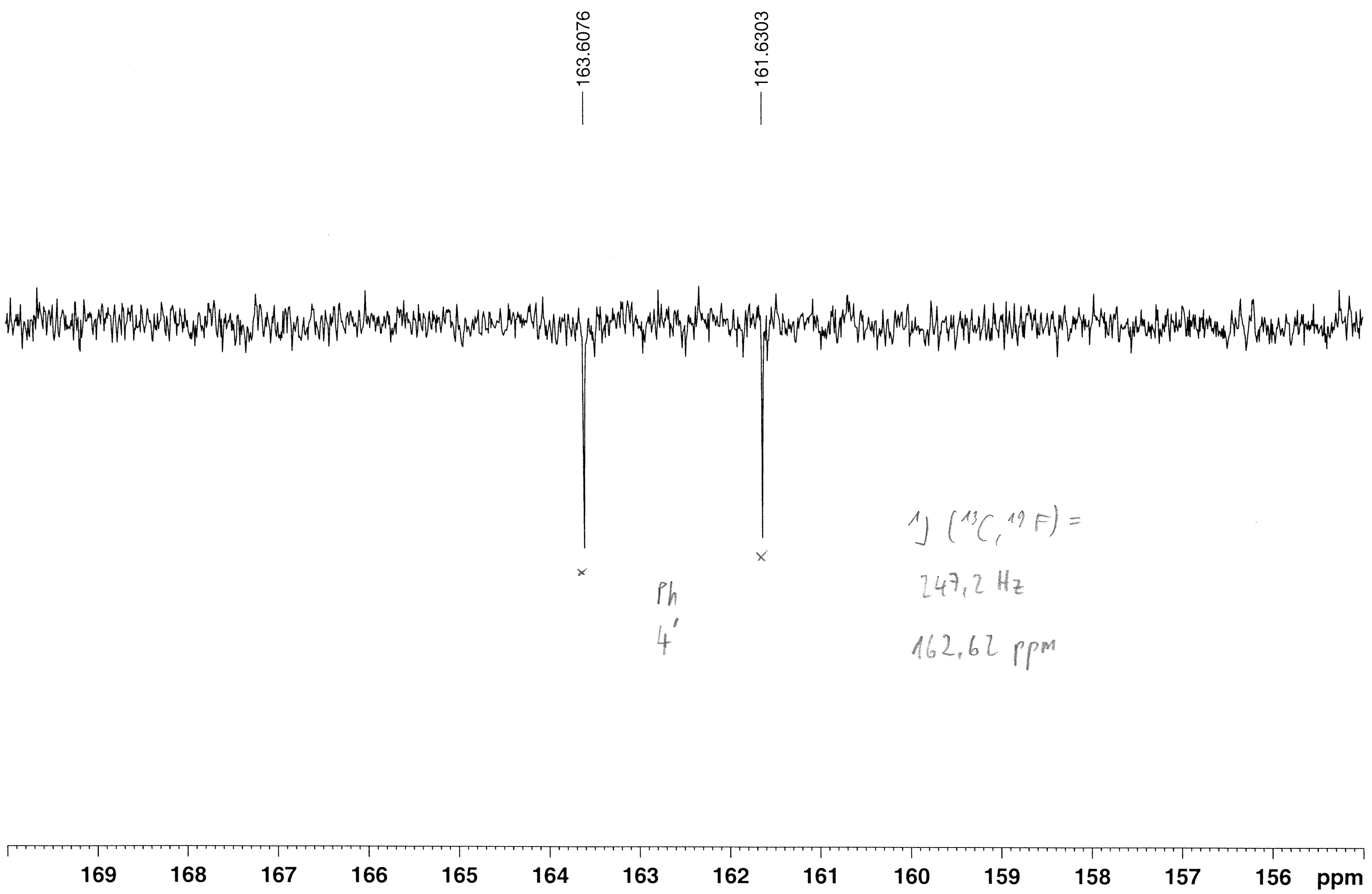

CE156S1P2 in cdcl3 (APT) 5.2.2019

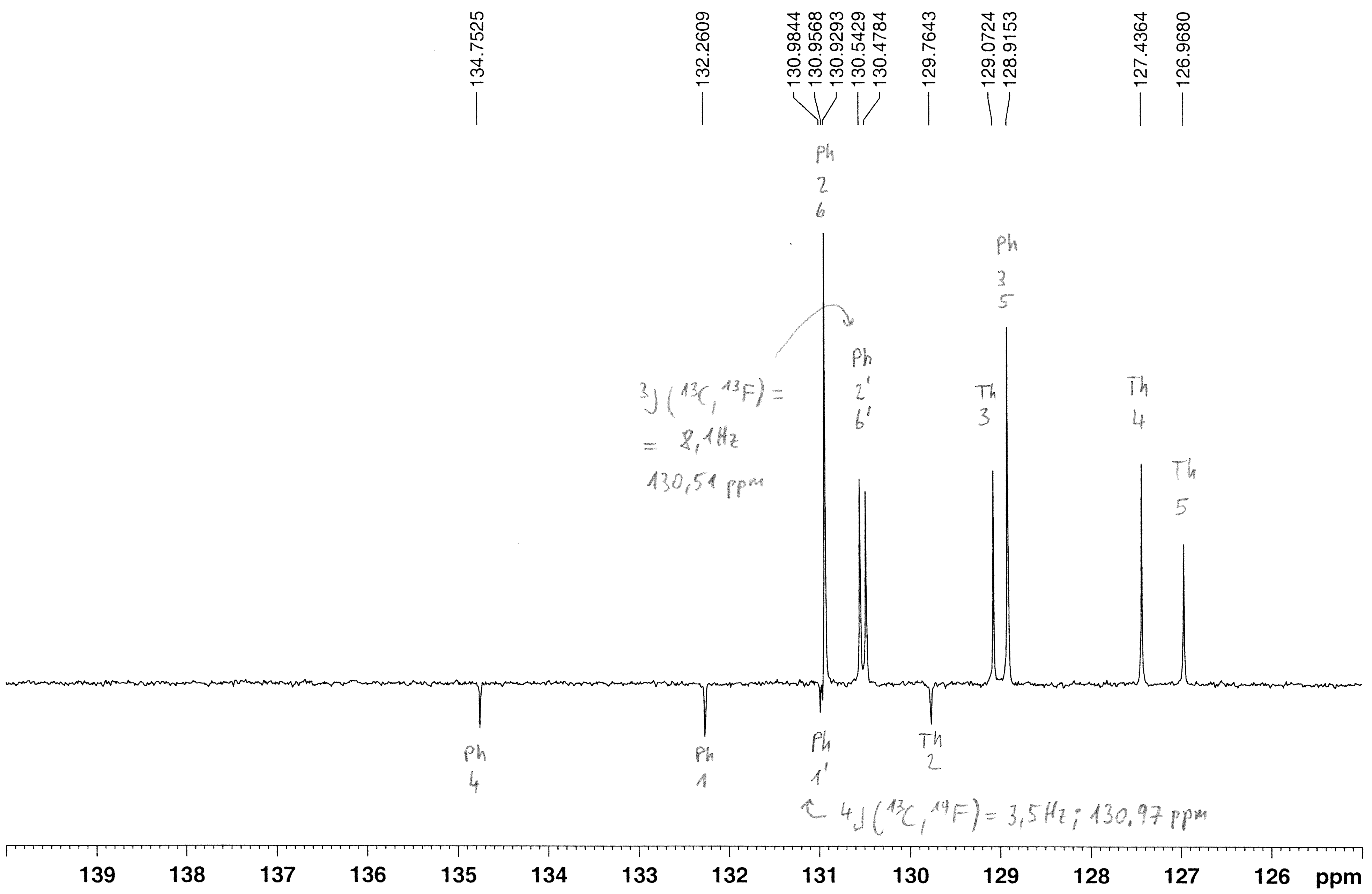

CE156S1P2 in cdcl3 (APT) 5.2.2019

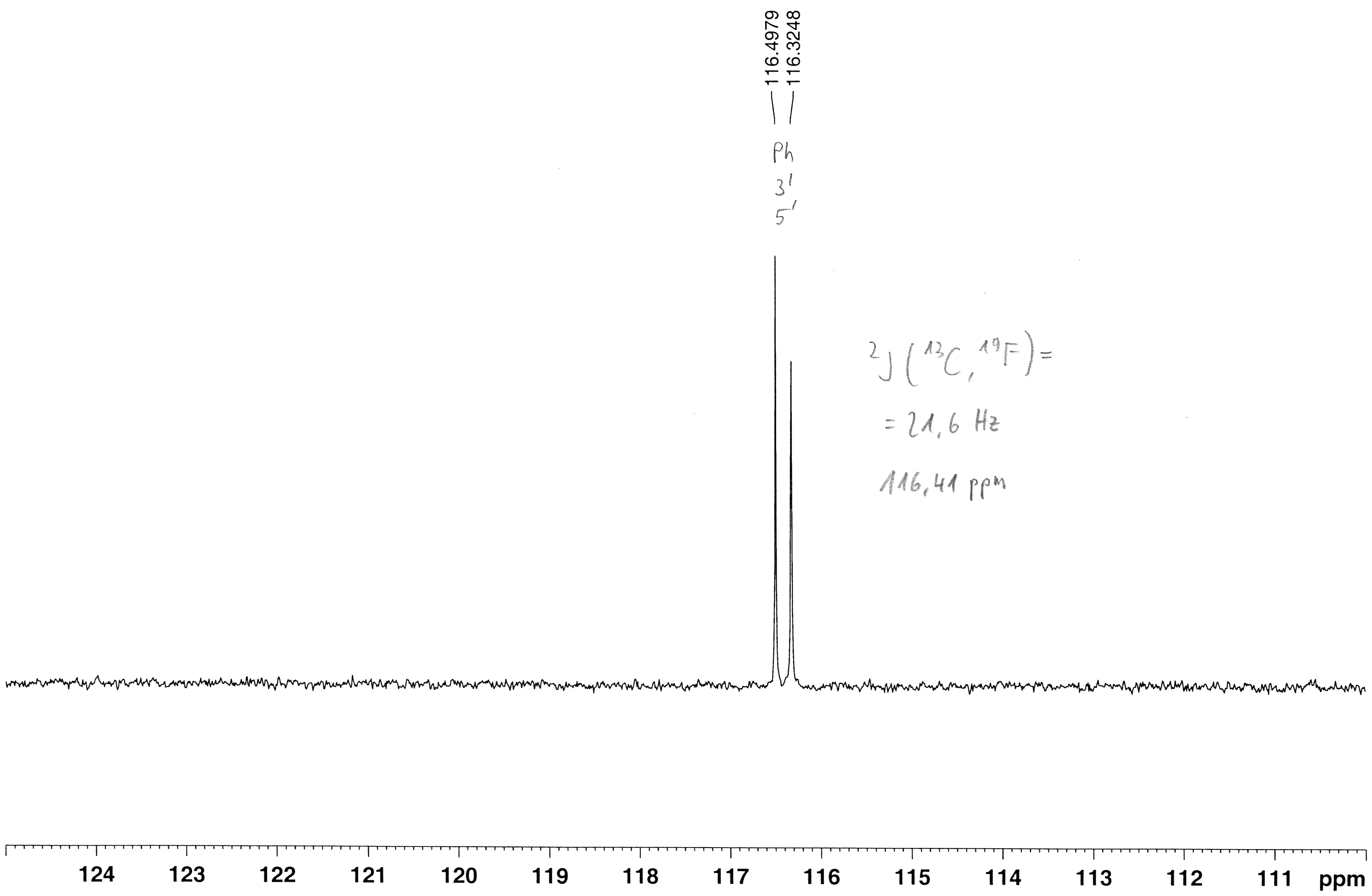

CE156S1P2 in cdcl3 (APT) 5.2.2019

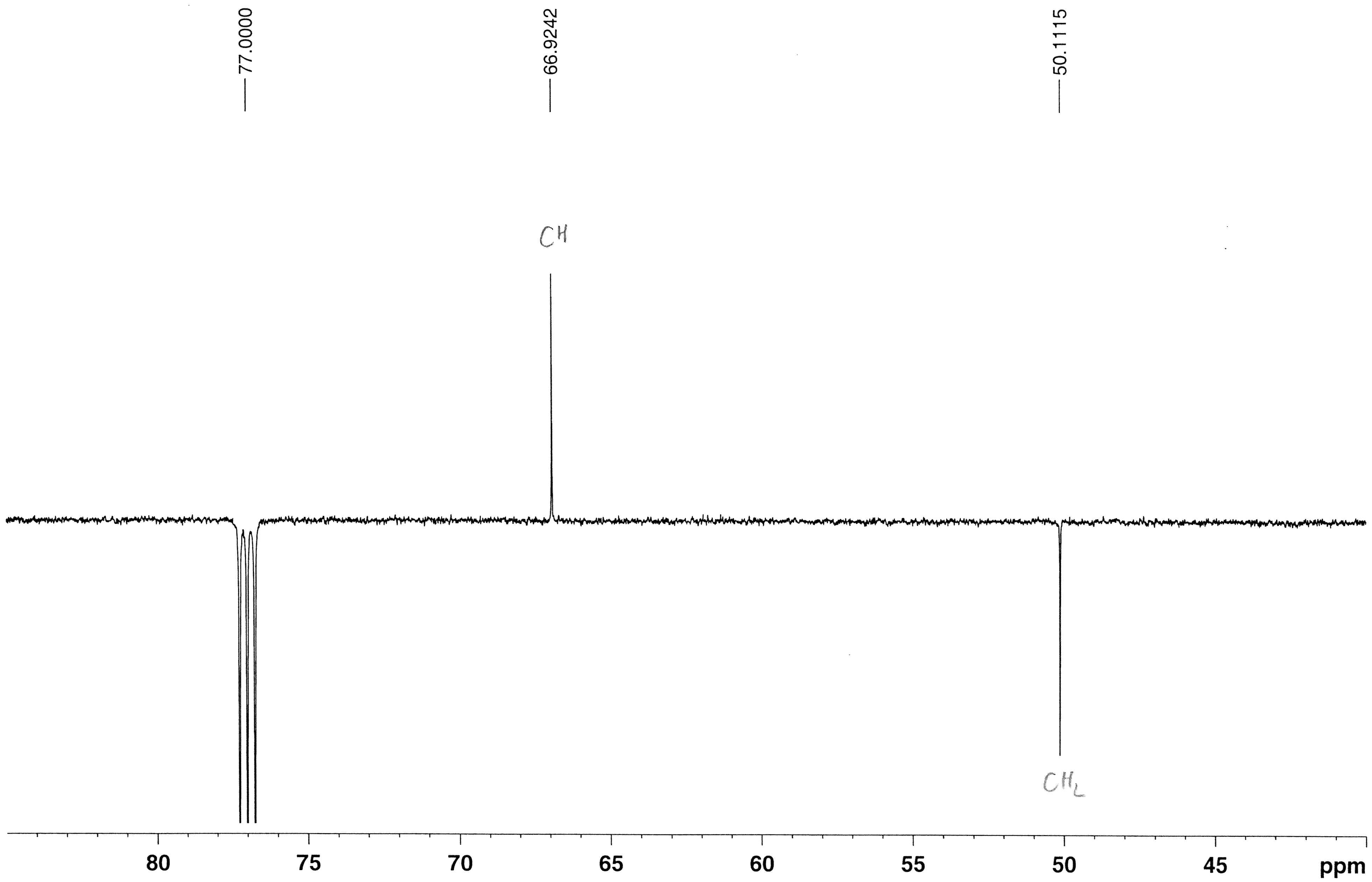

Figure S83c. NMR spectra of compound **7u.**

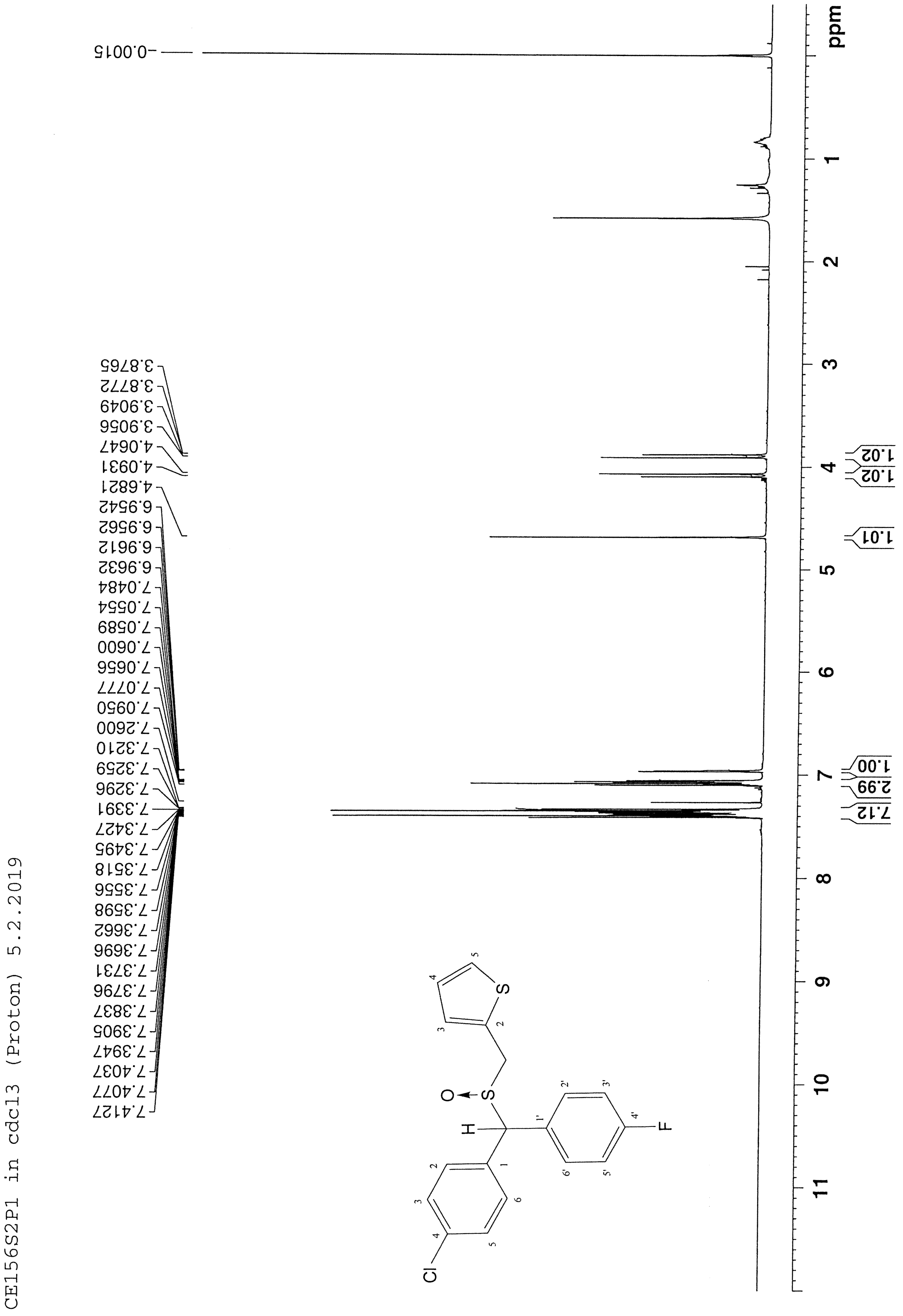

CE156S2P1 in cdcl3 (Proton) 5.2.2019

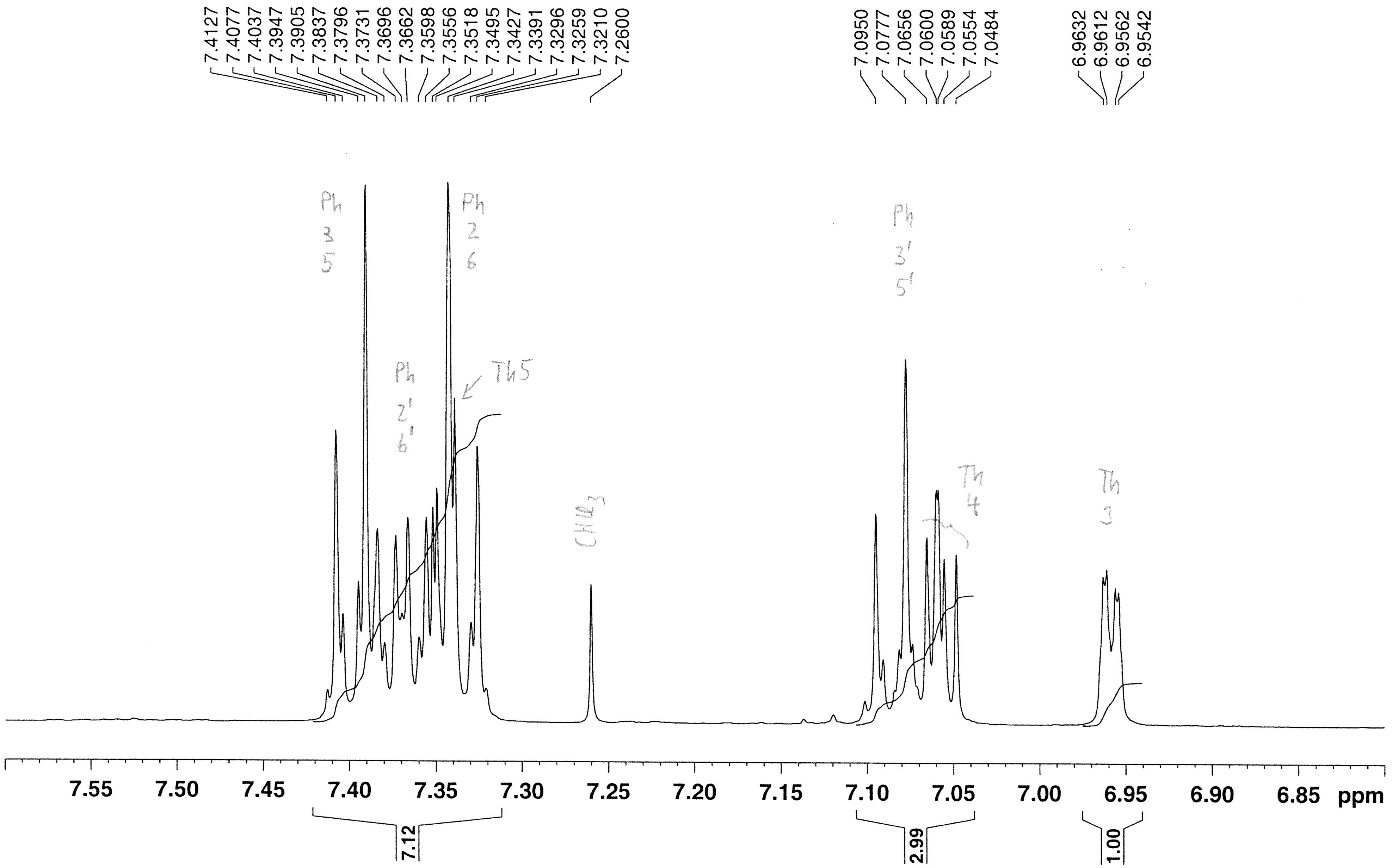

CE156S2P1 in cdcl3 (Proton) 5.2.2019

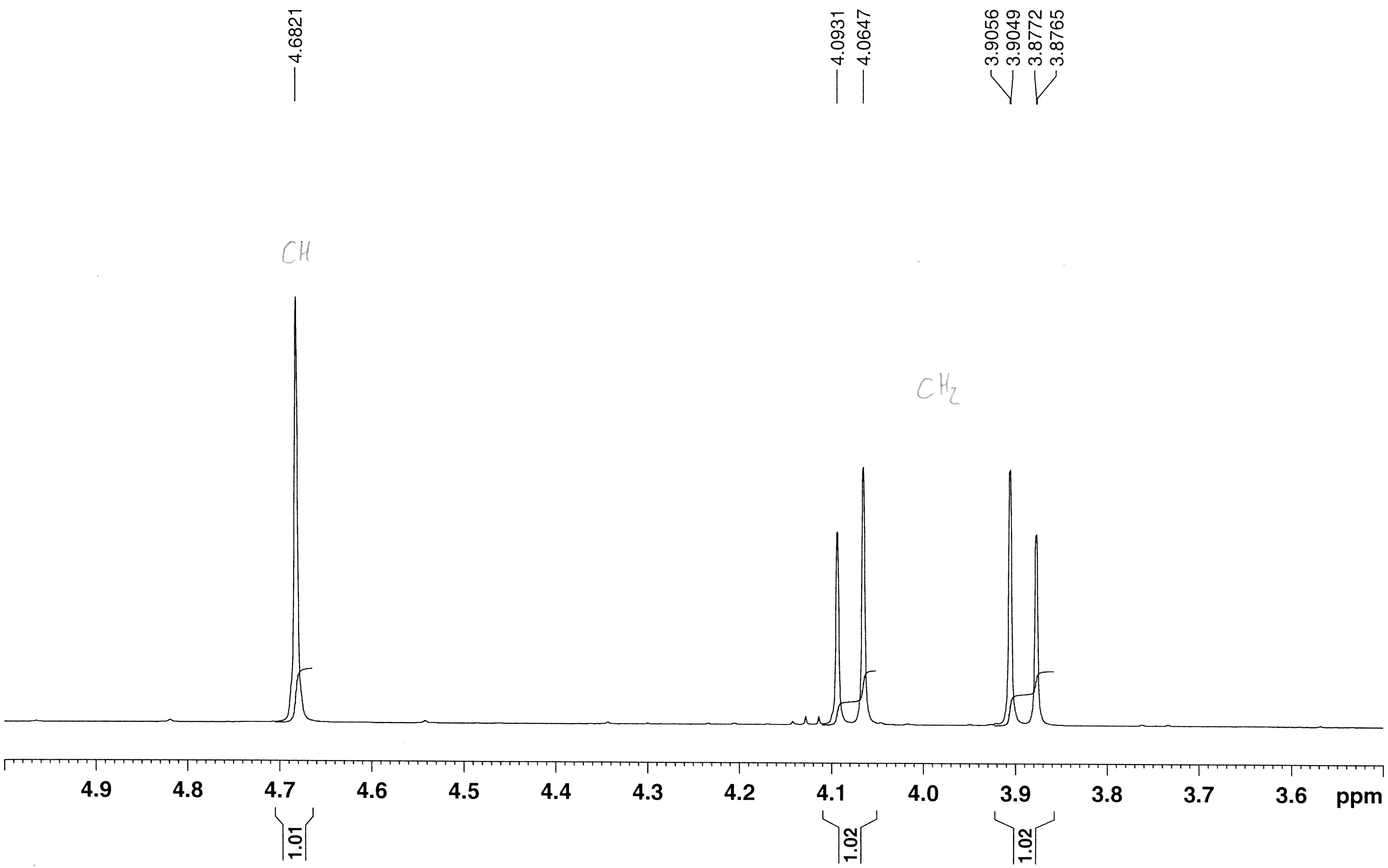

CE156S2P1 in cdcl3 (APT) 5.2.2019

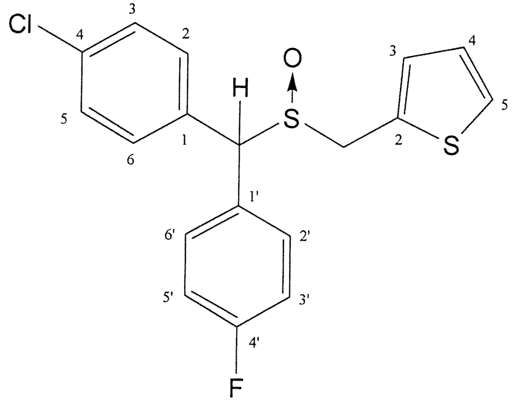

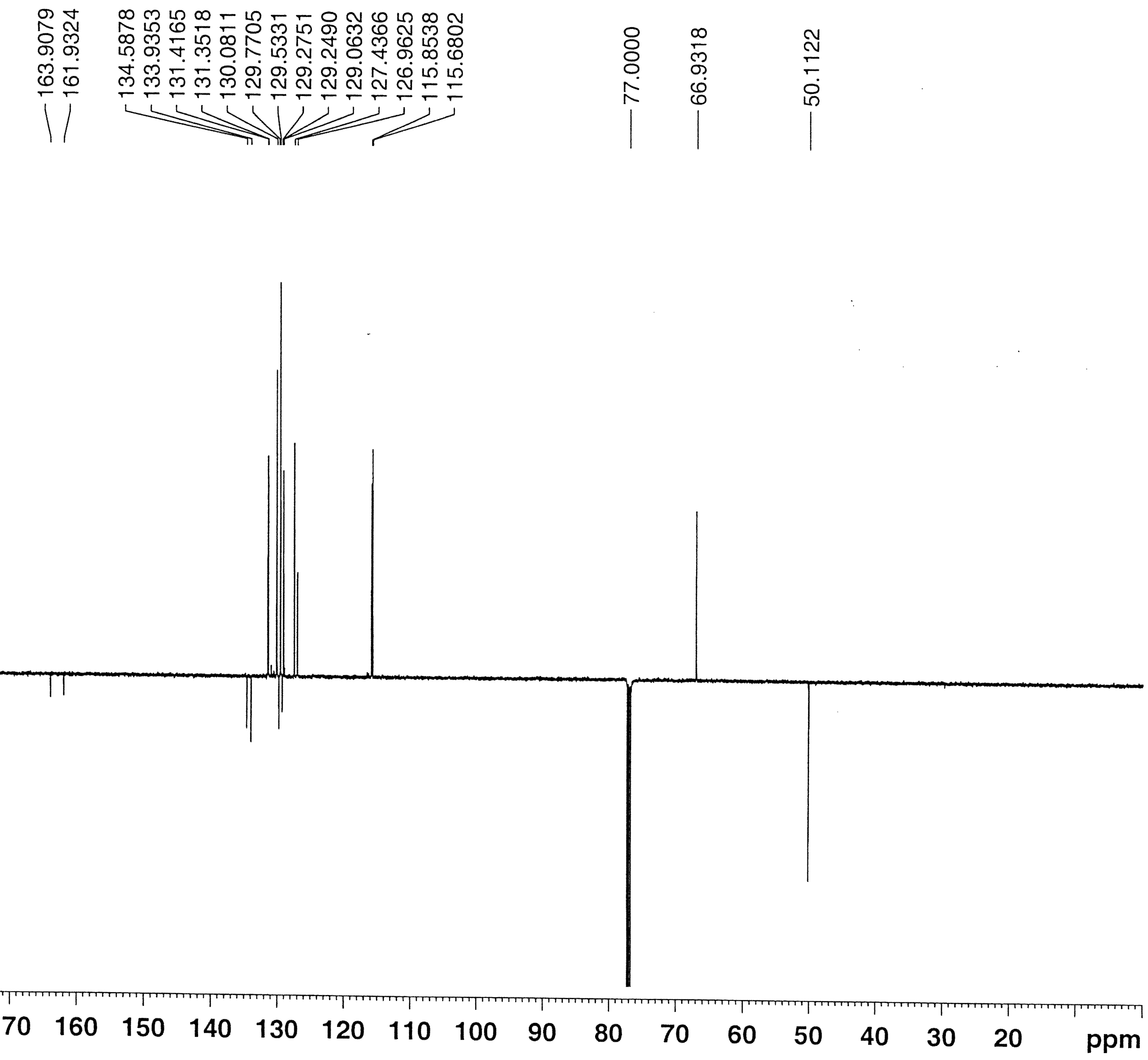

CE156S2P1 in cdcl3 (APT) 5.2.2019

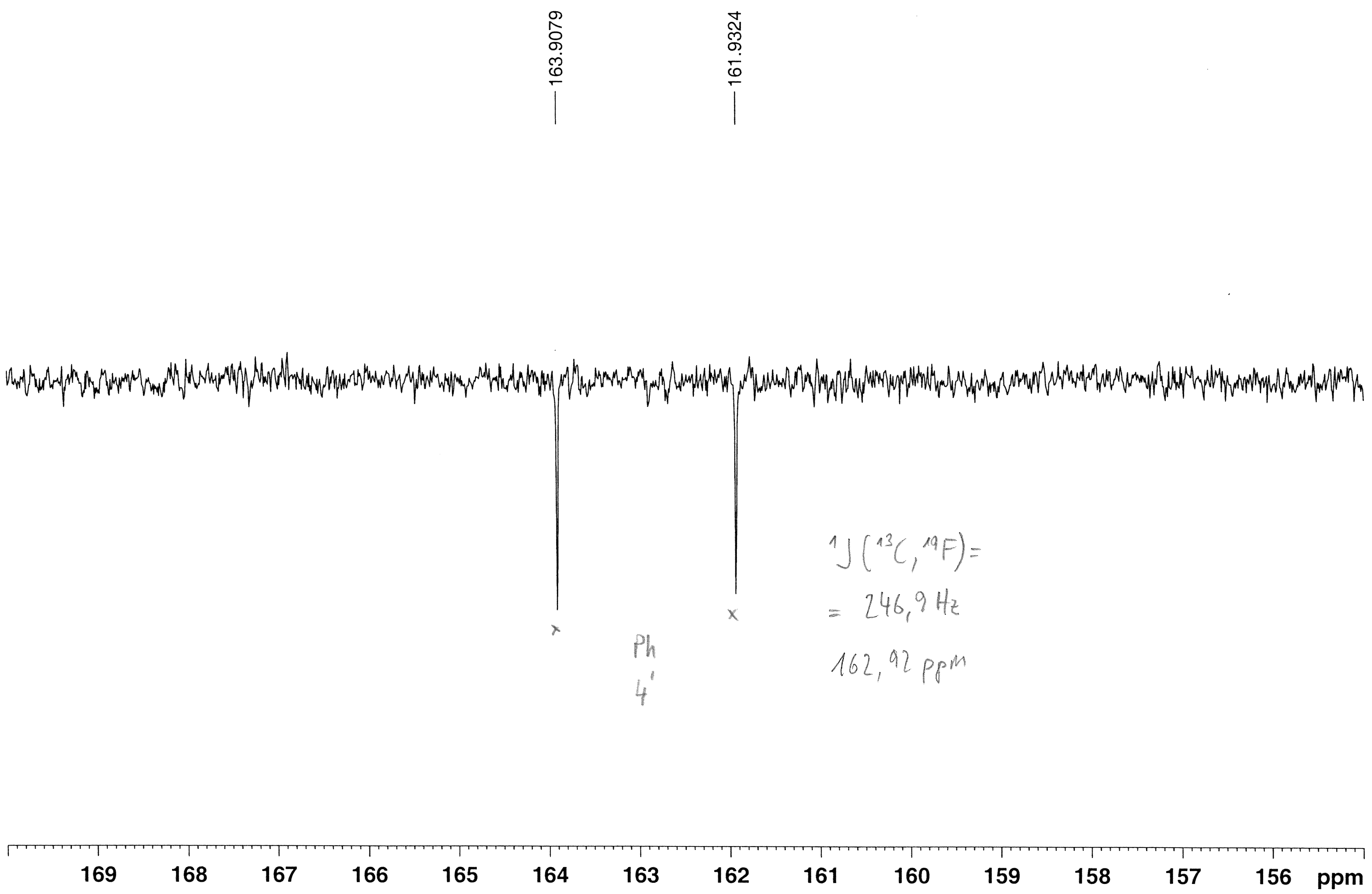

CE156S2P1 in cdcl3 (APT) 5.2.2019

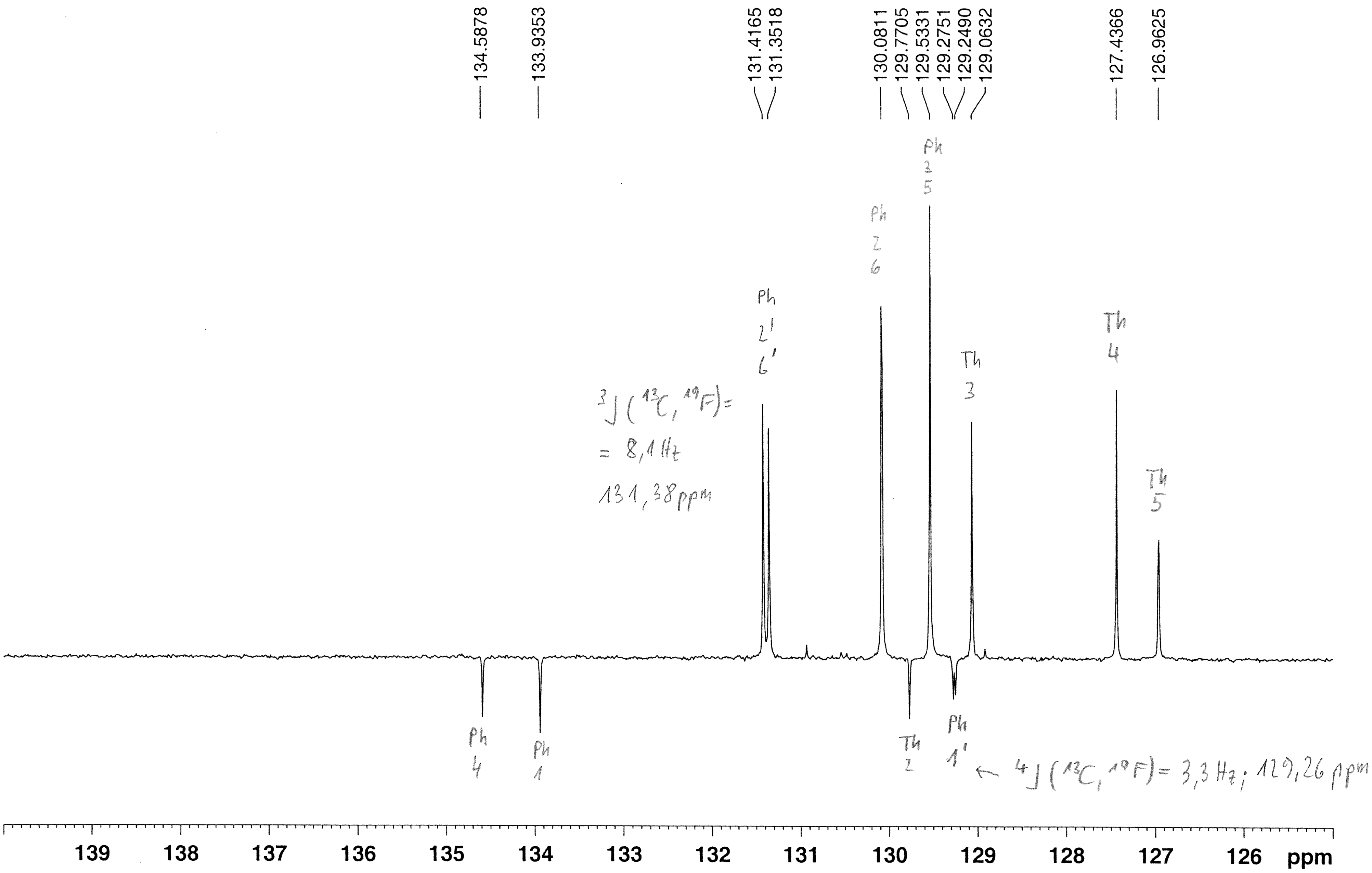

CE156S2P1 in cdcl3 (APT) 5.2.2019

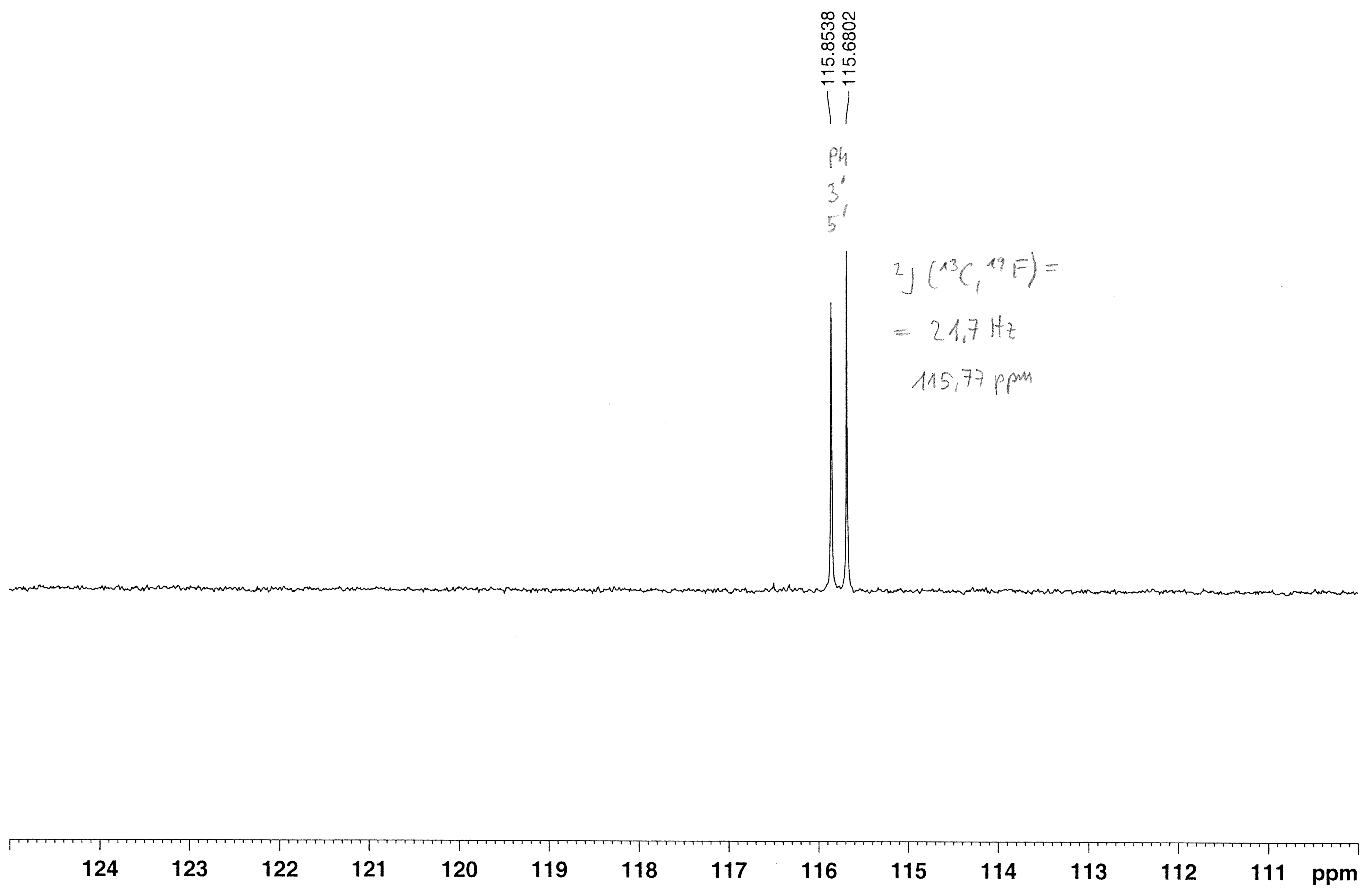

CE156S2P1 in cdcl3 (APT) 5.2.2019

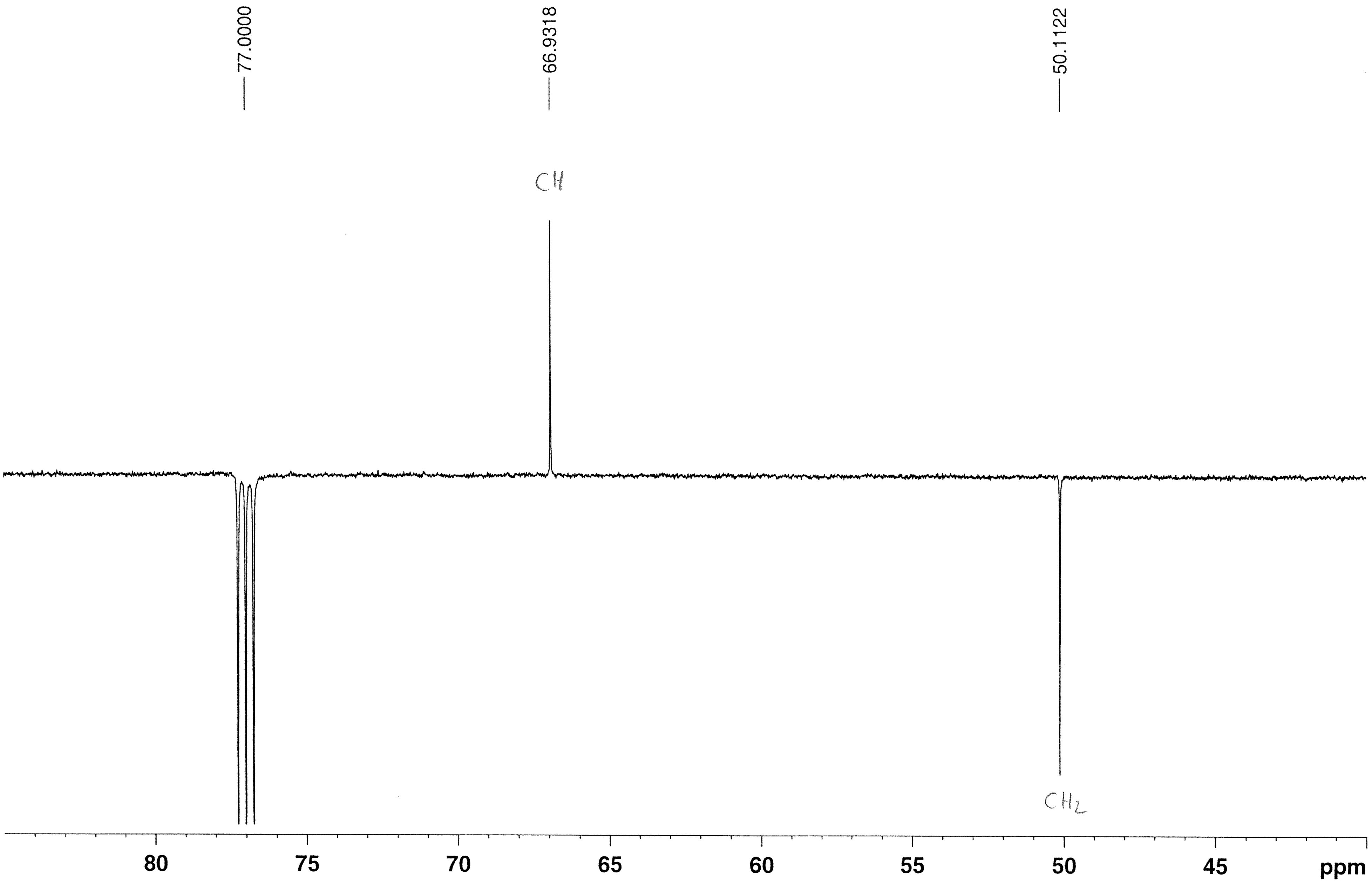

CE156S2P1 in cdcl3 (COSY) 5.2.2019

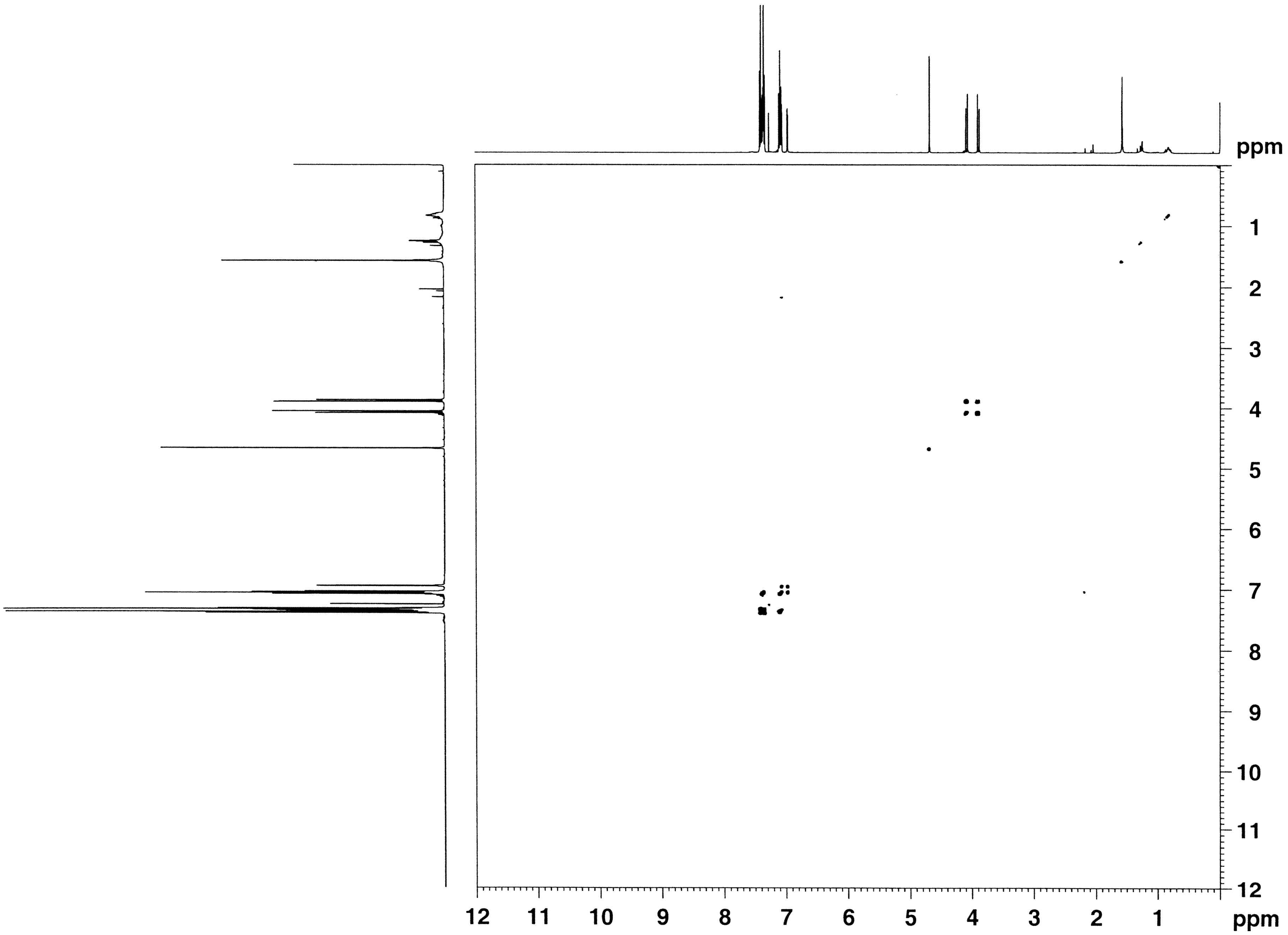

CE156S2P1 in cdcl3 (COSY) 5.2.2019

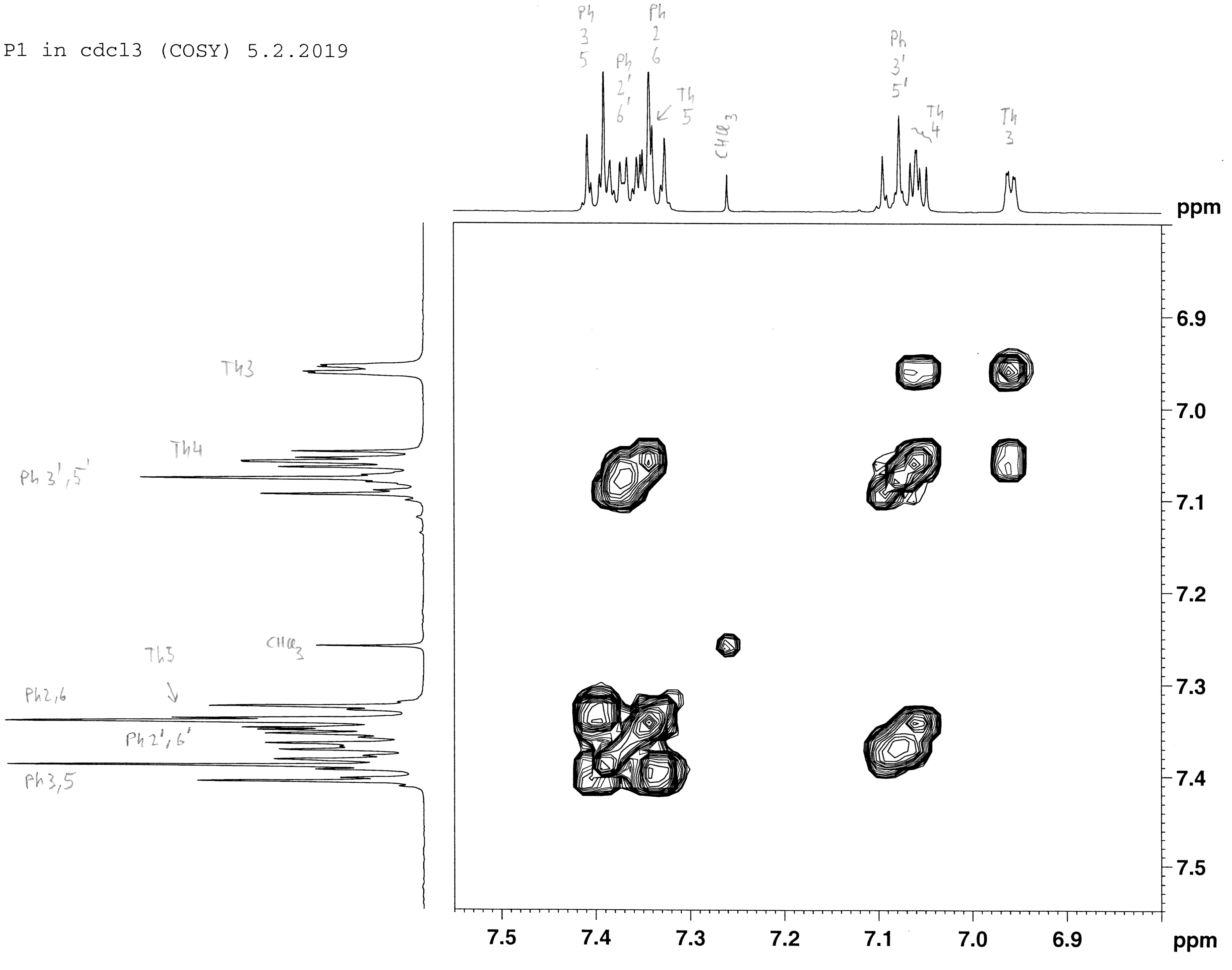

CE156S2P1 in cdcl3 (COSY) 5.2.2019

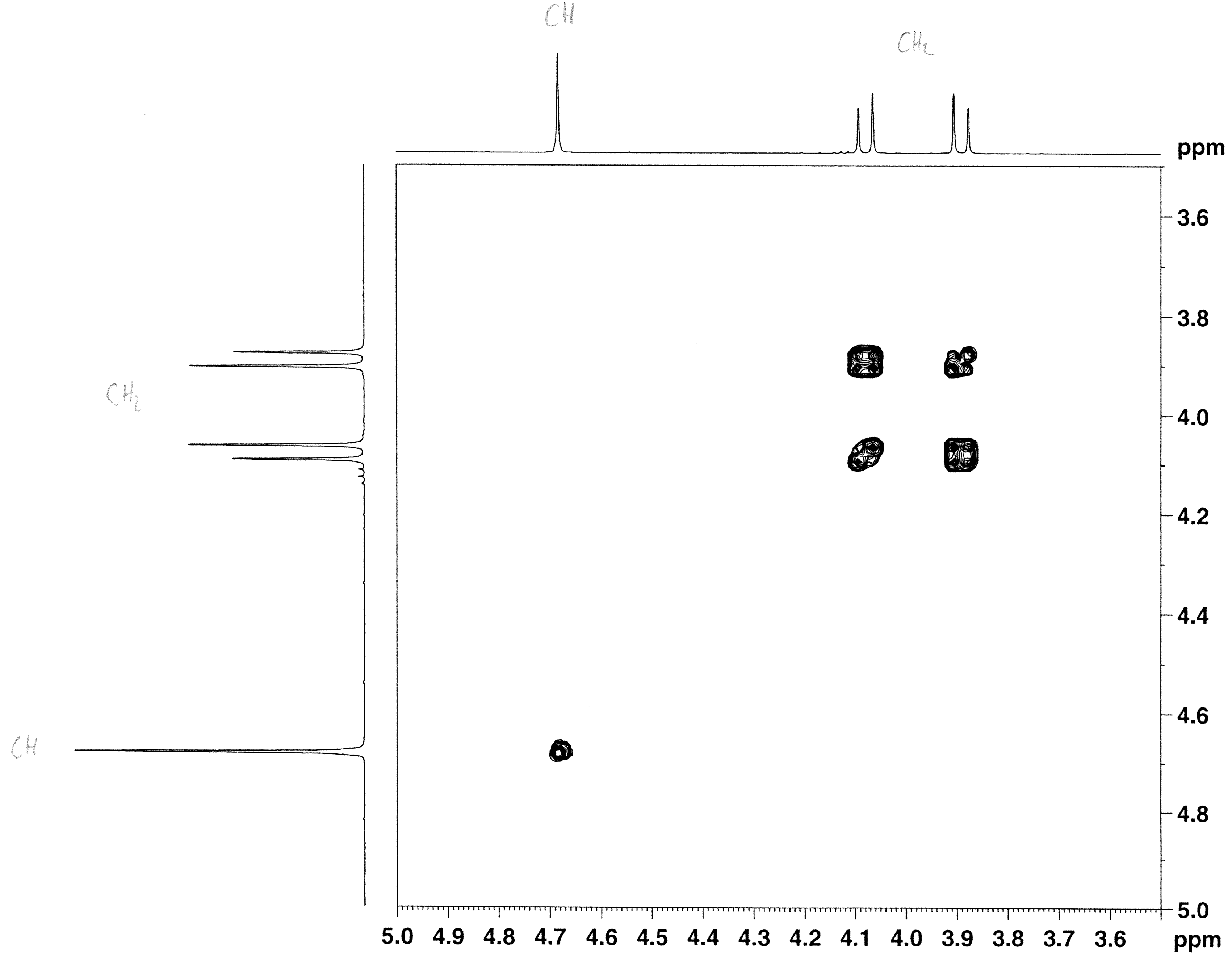

CE156S2P1 in cdcl3 (HSQC) 5.2.2019

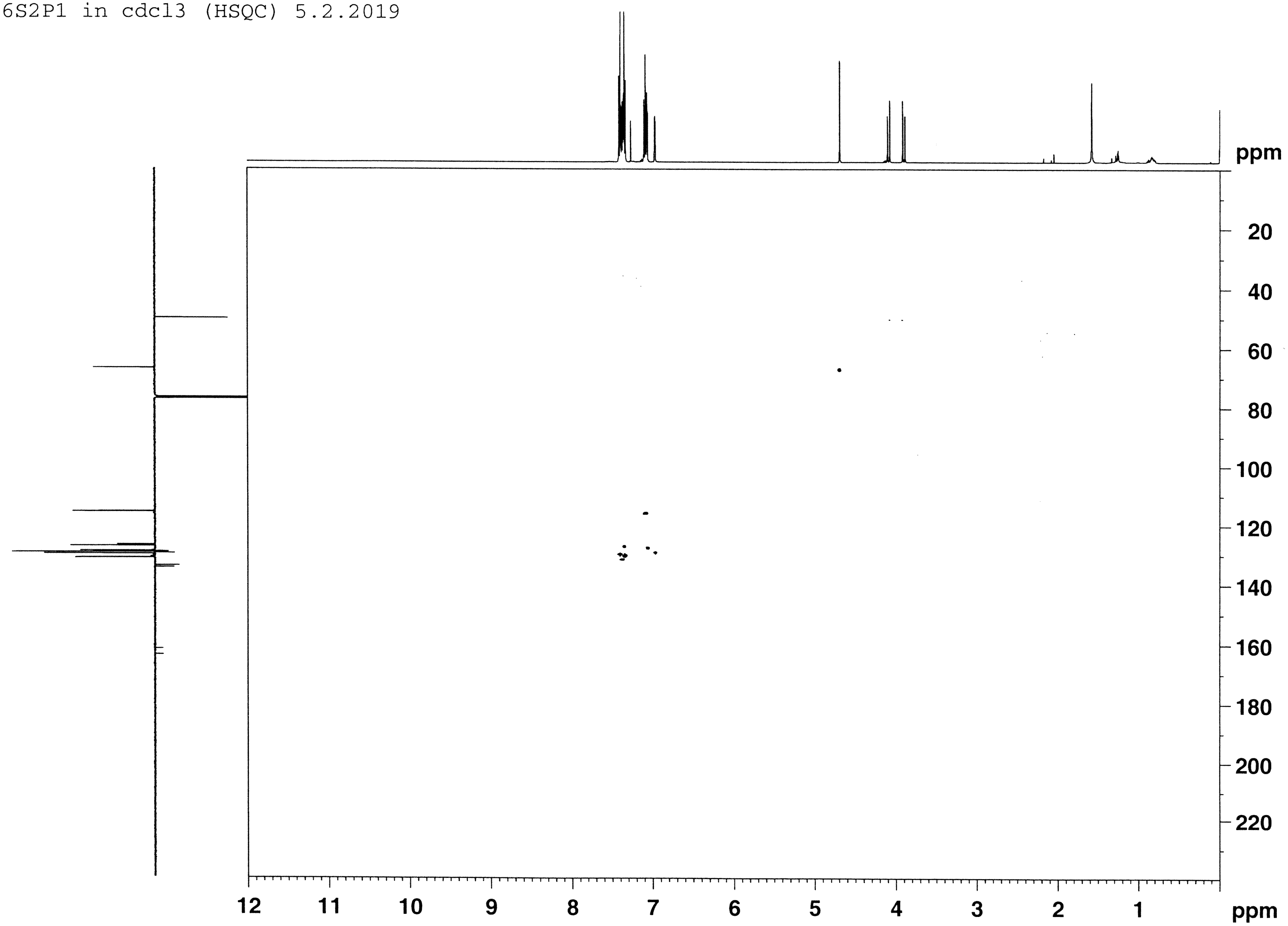

CE156S2P1 in cdcl3 (HSQC) 5.2.2019

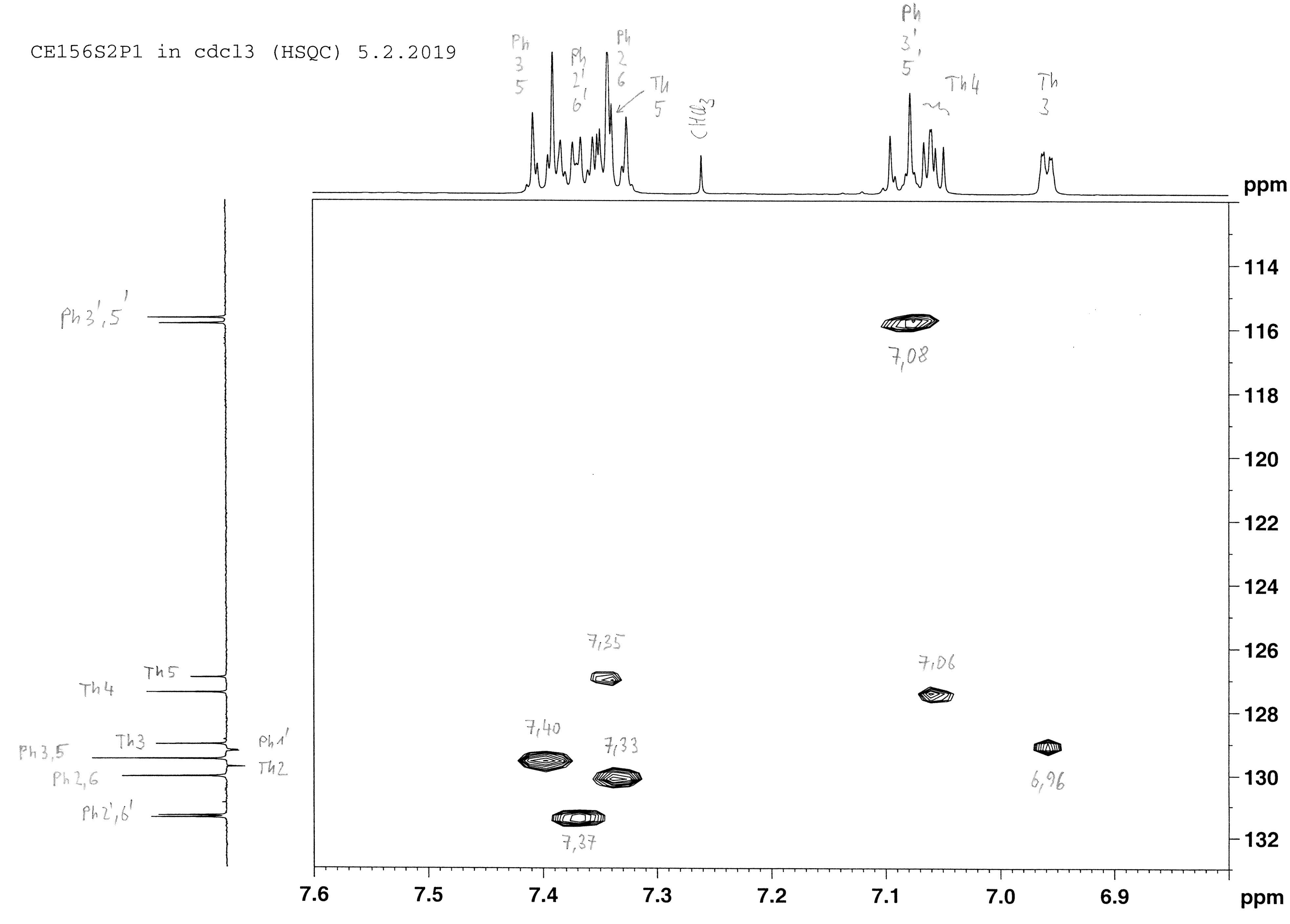

CE156S2P1 in cdcl3 (HSQC) 5.2.2019

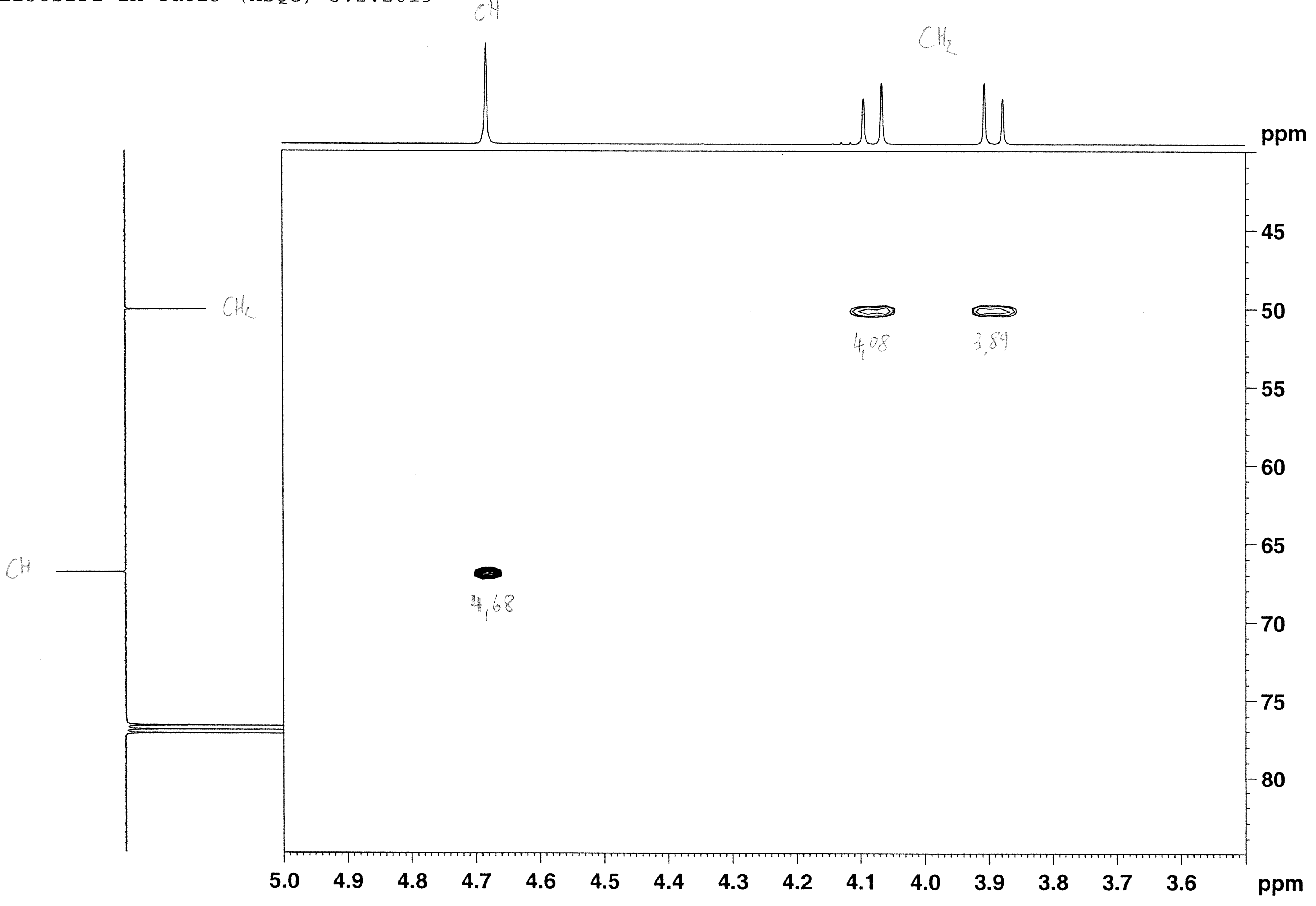

CE156S2P1 in cdcl3 (HMBC) 5.2.2019

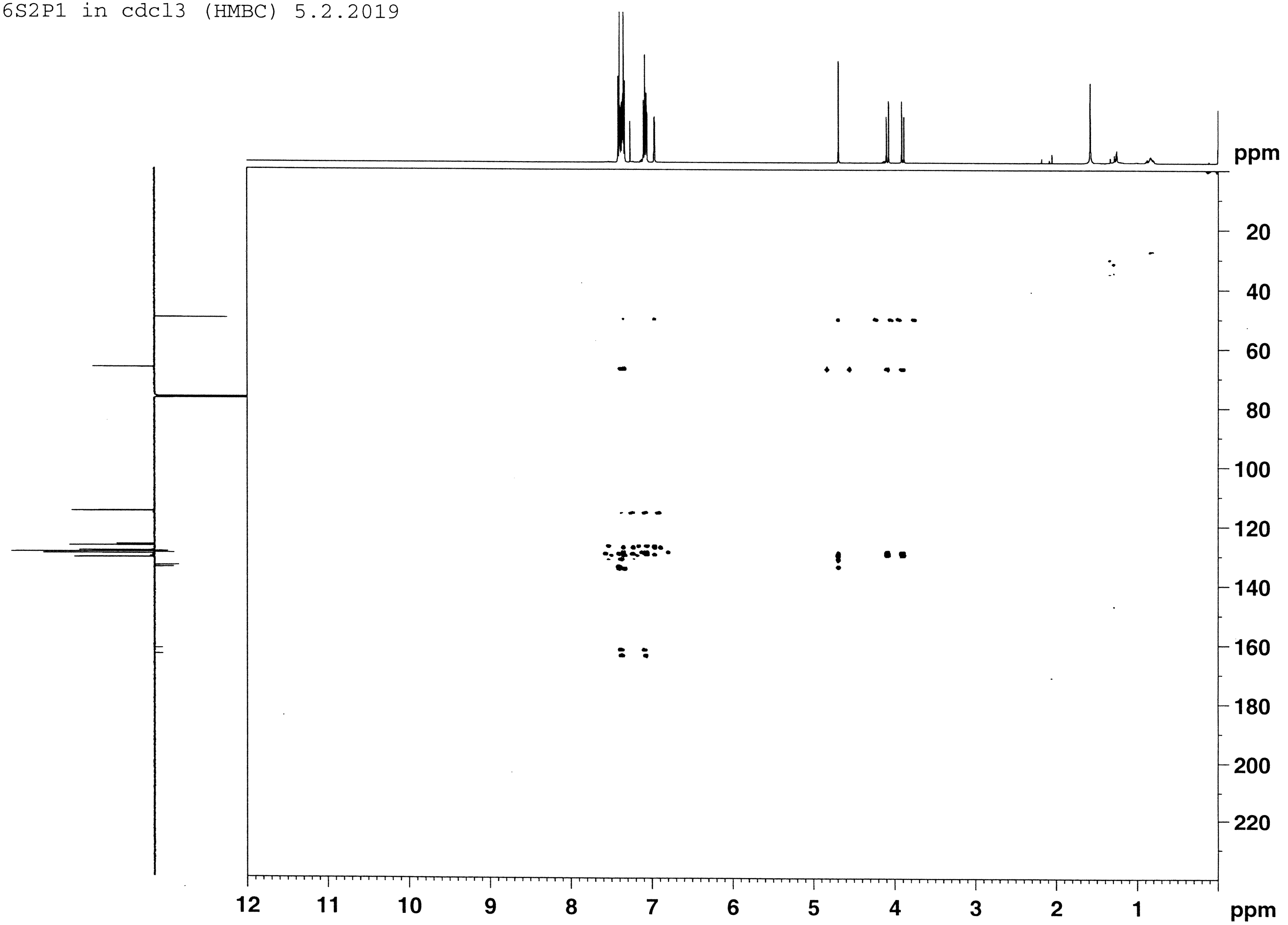

CE156S2P1 in cdcl3 (HMBC) 5.2.2019

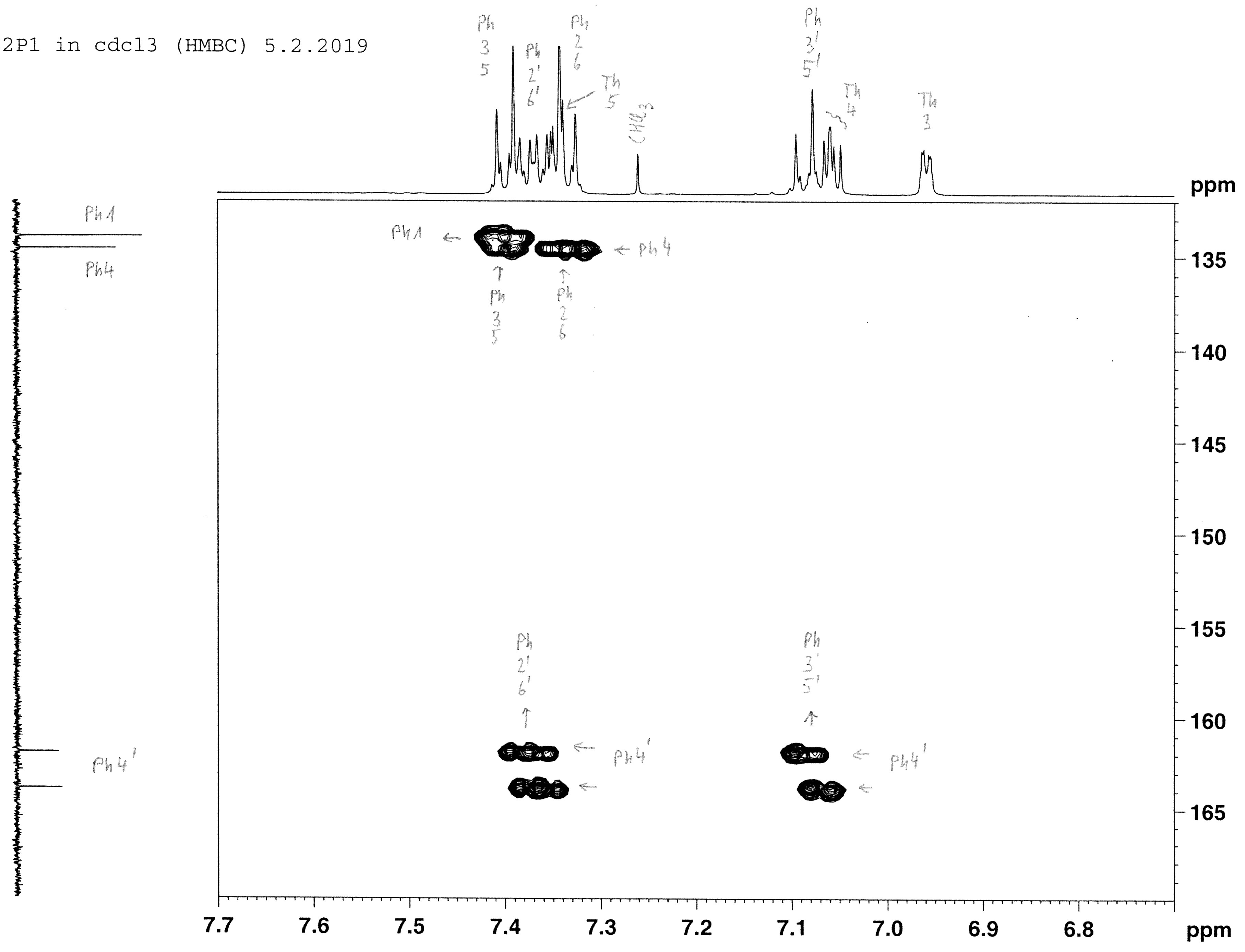

CE156S2P1 in cdcl3 (HMBC) 5.2.2019

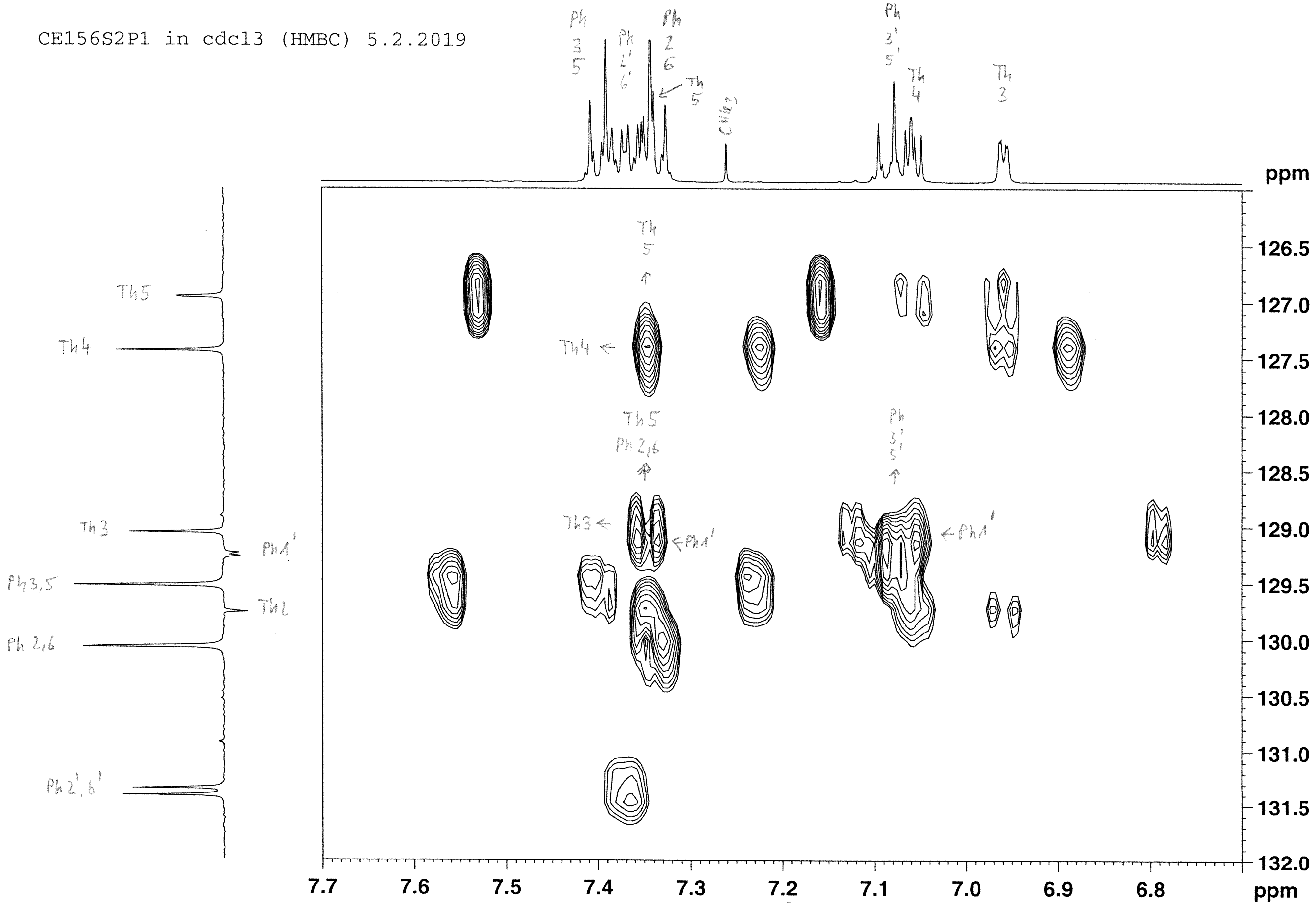

CE156S2P1 in cdcl3 (HMBC) 5.2.2019

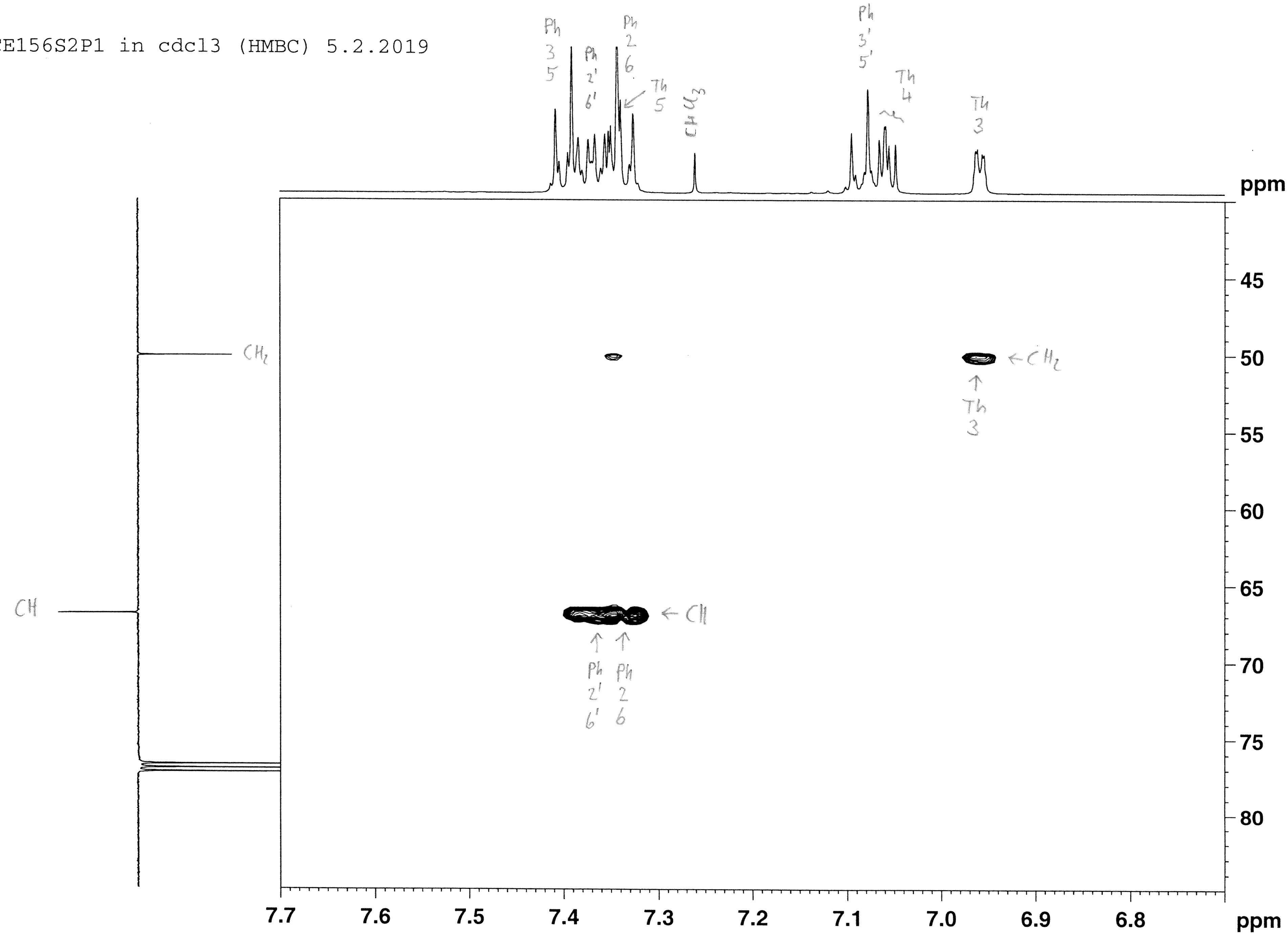

CE156S2P1 in cdcl3 (HMBC) 5.2.2019

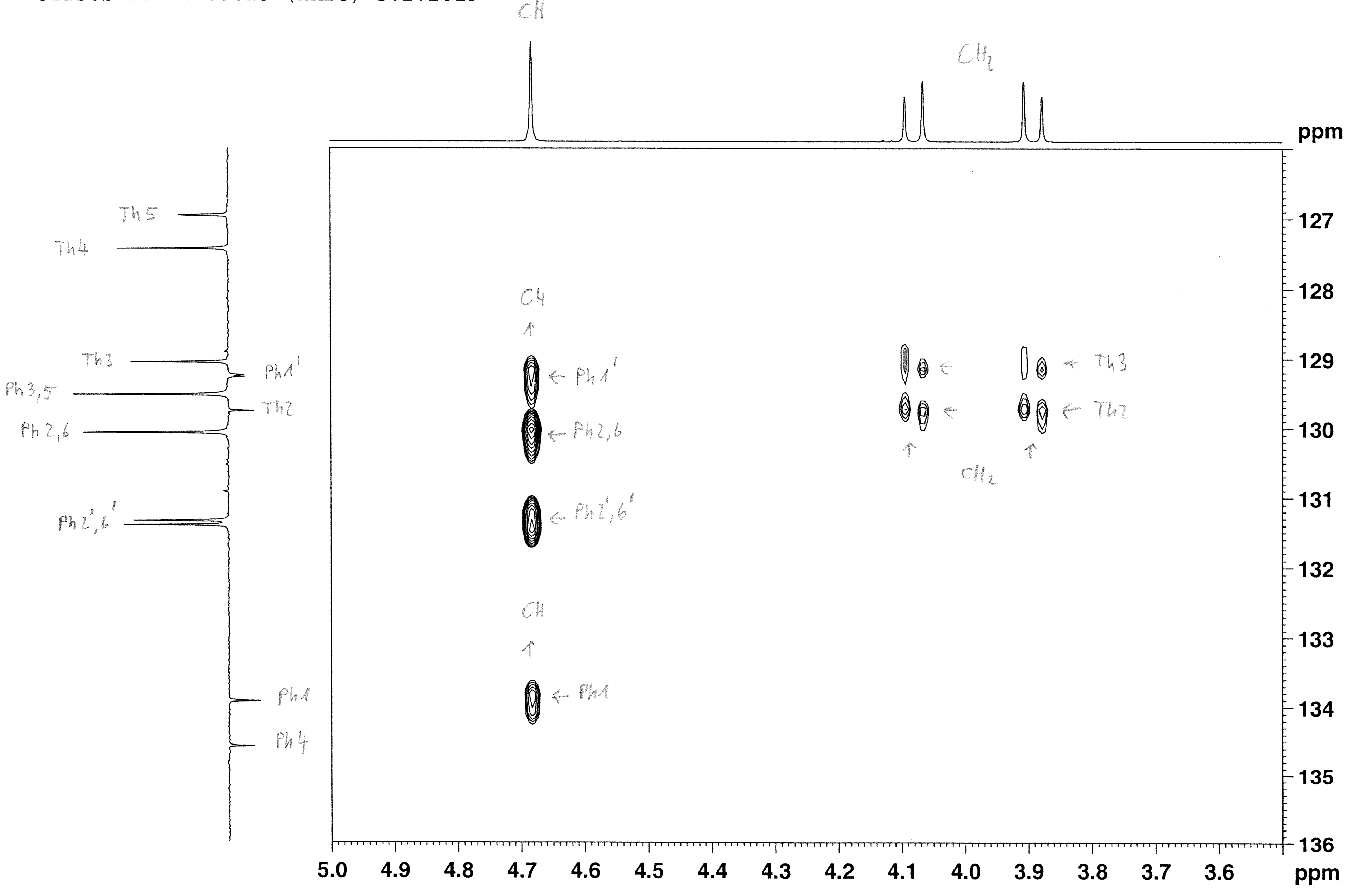

CE156S2P1 in cdcl3 (HMBC) 5.2.2019

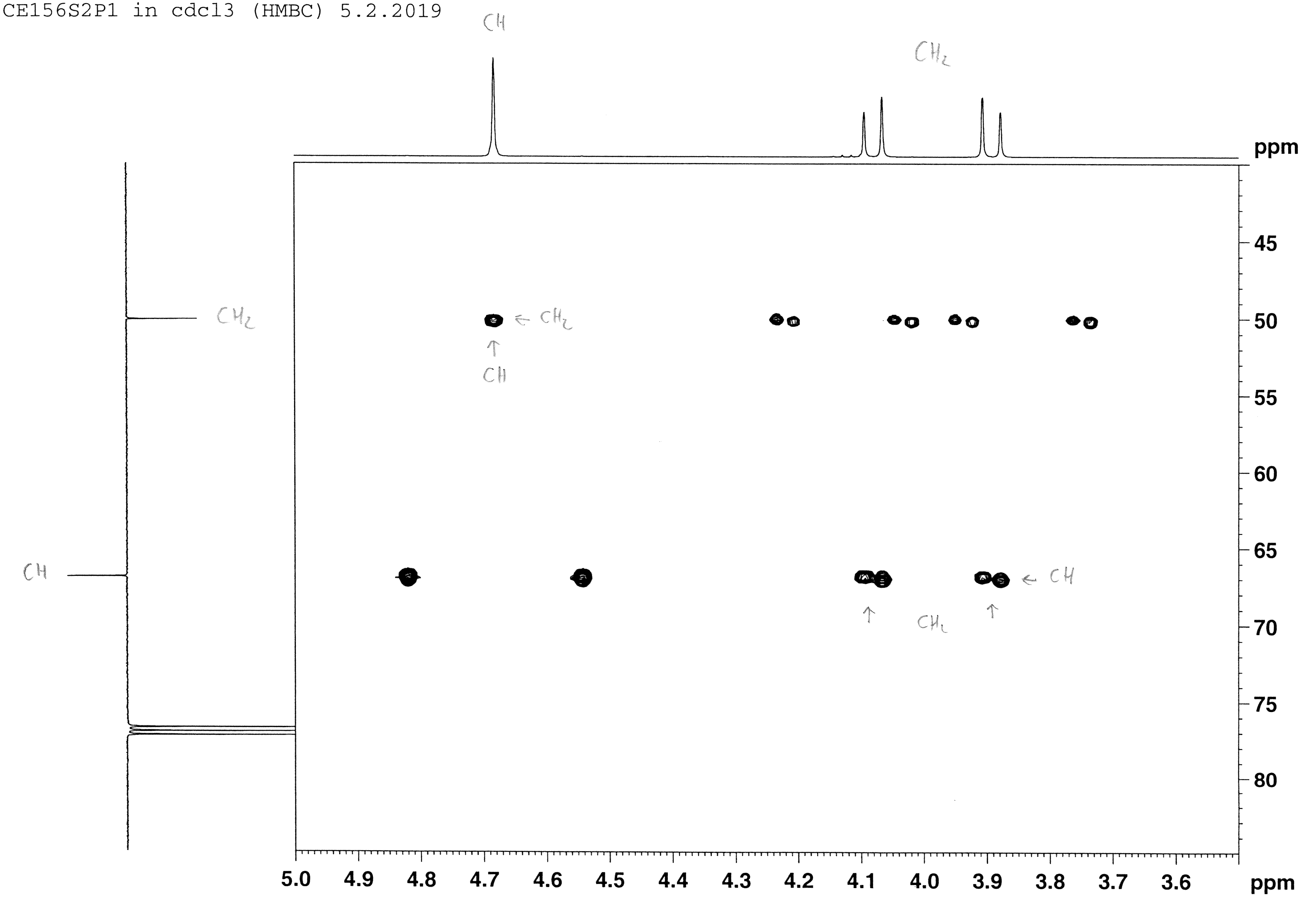

Figure S84c. NMR spectra of compound **8u**.

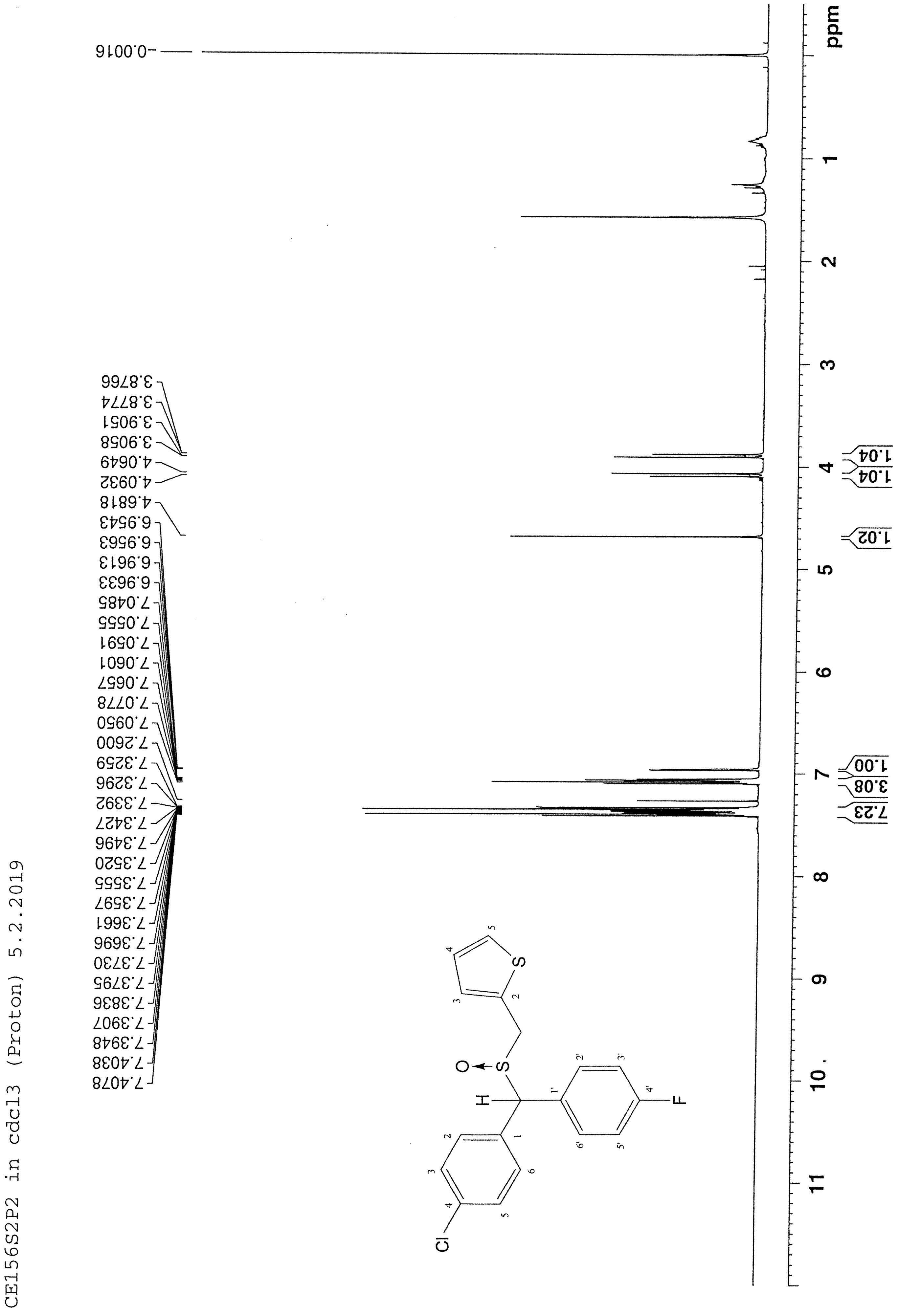

CE156S2P2 in cdcl3 (Proton) 5.2.2019

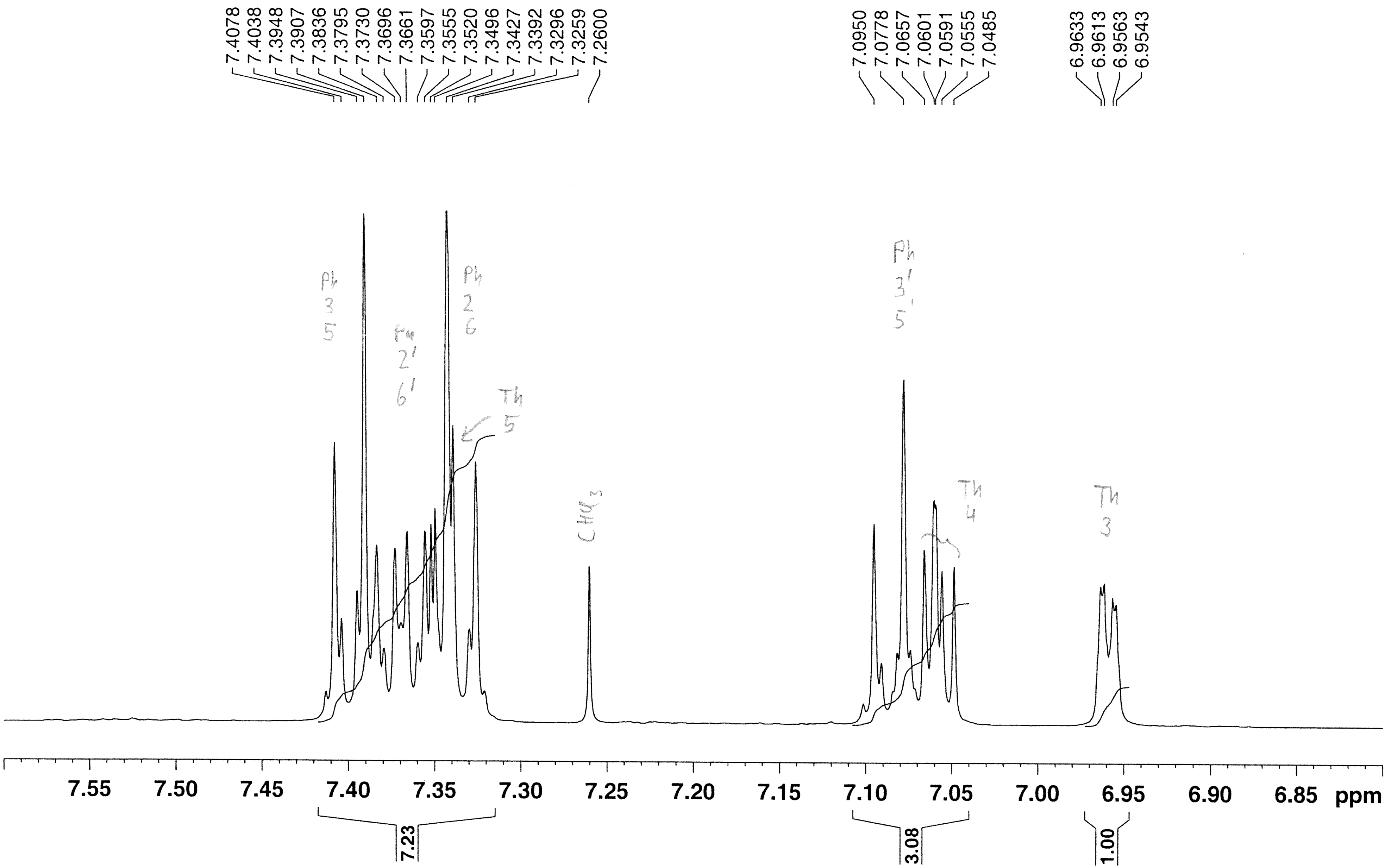

CE156S2P2 in cdcl3 (Proton) 5.2.2019

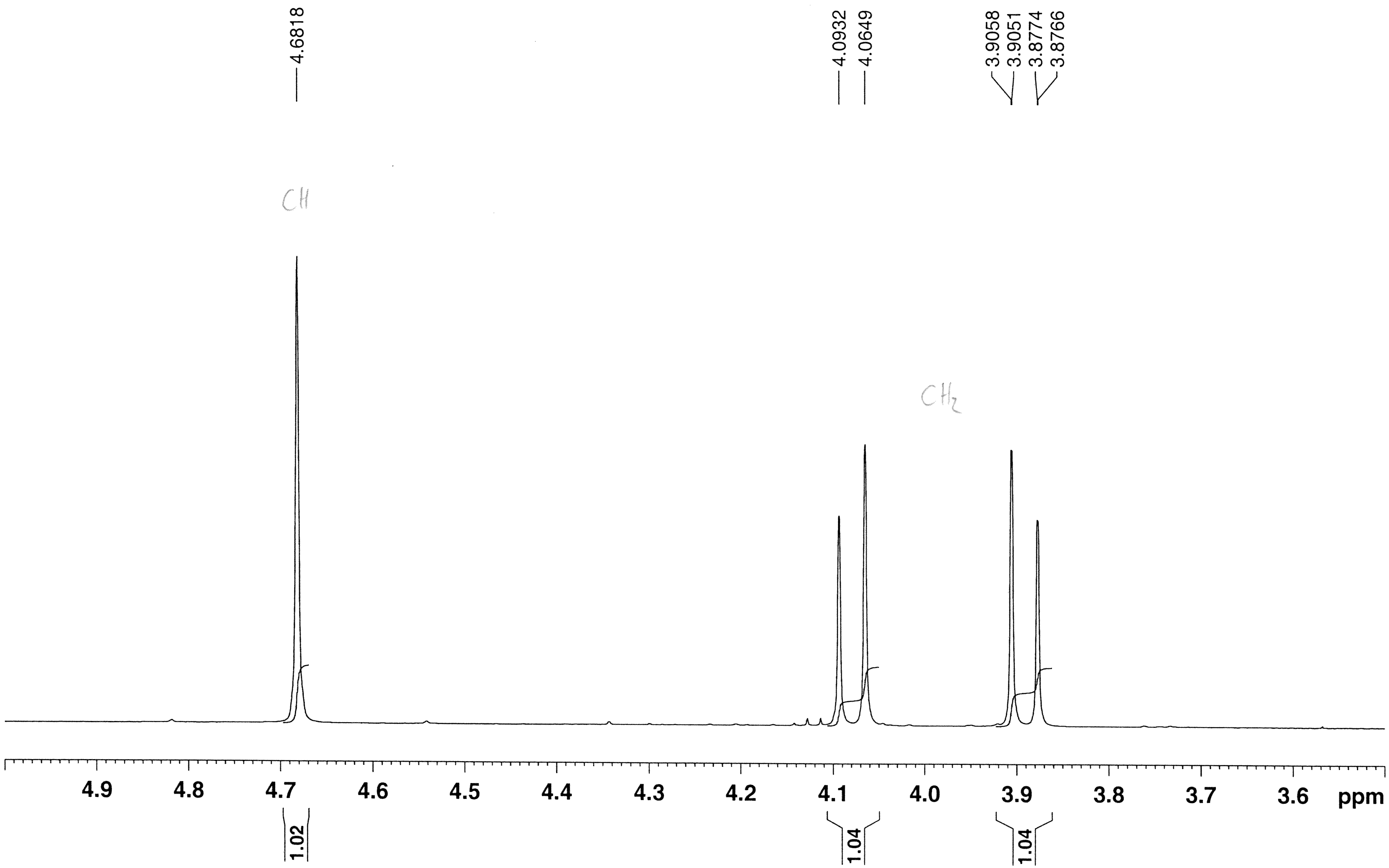

CE156S2P2 in cdcl3 (APT) 5.2.2019

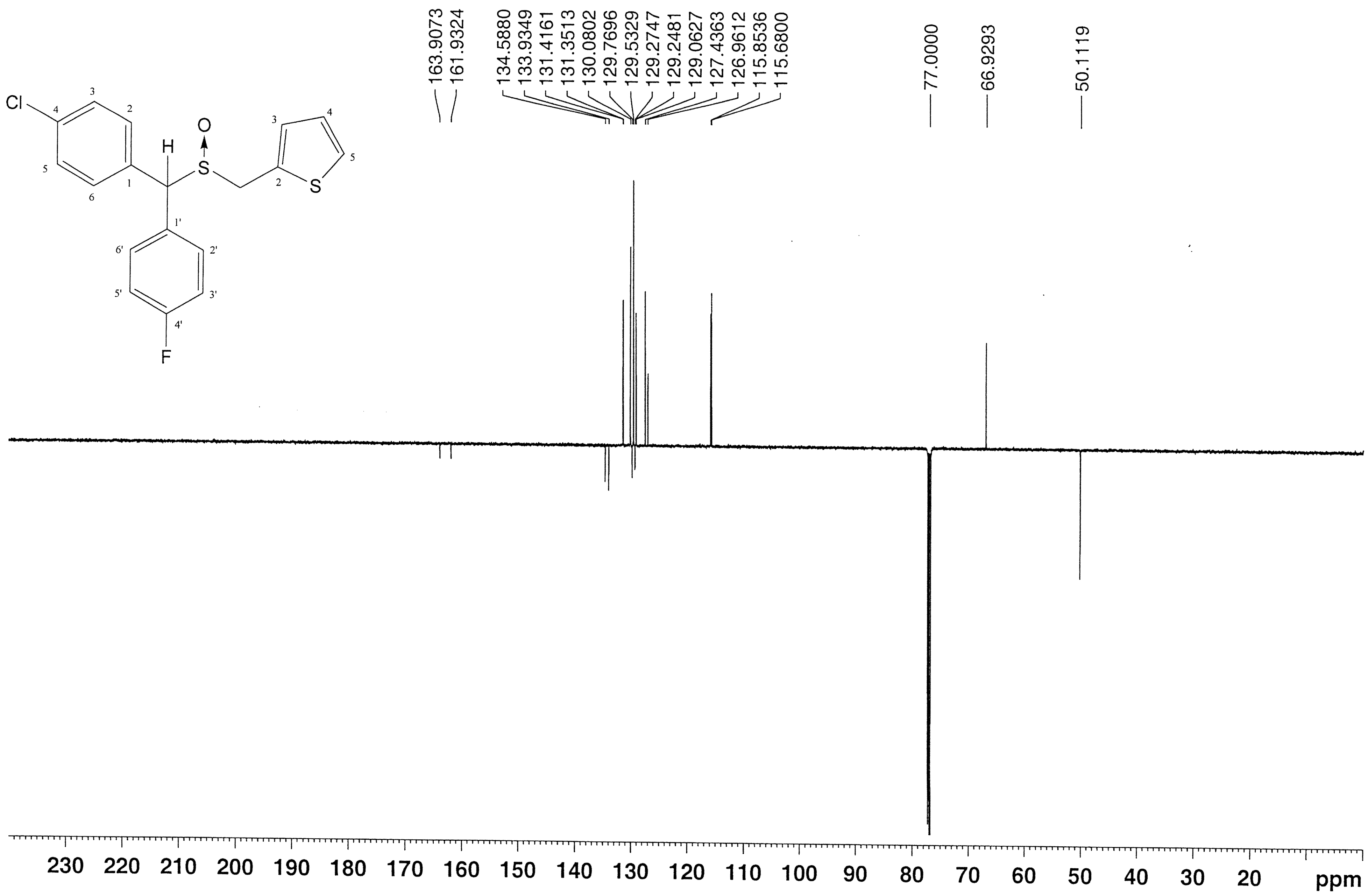

CE156S2P2 in cdcl3 (APT) 5.2.2019

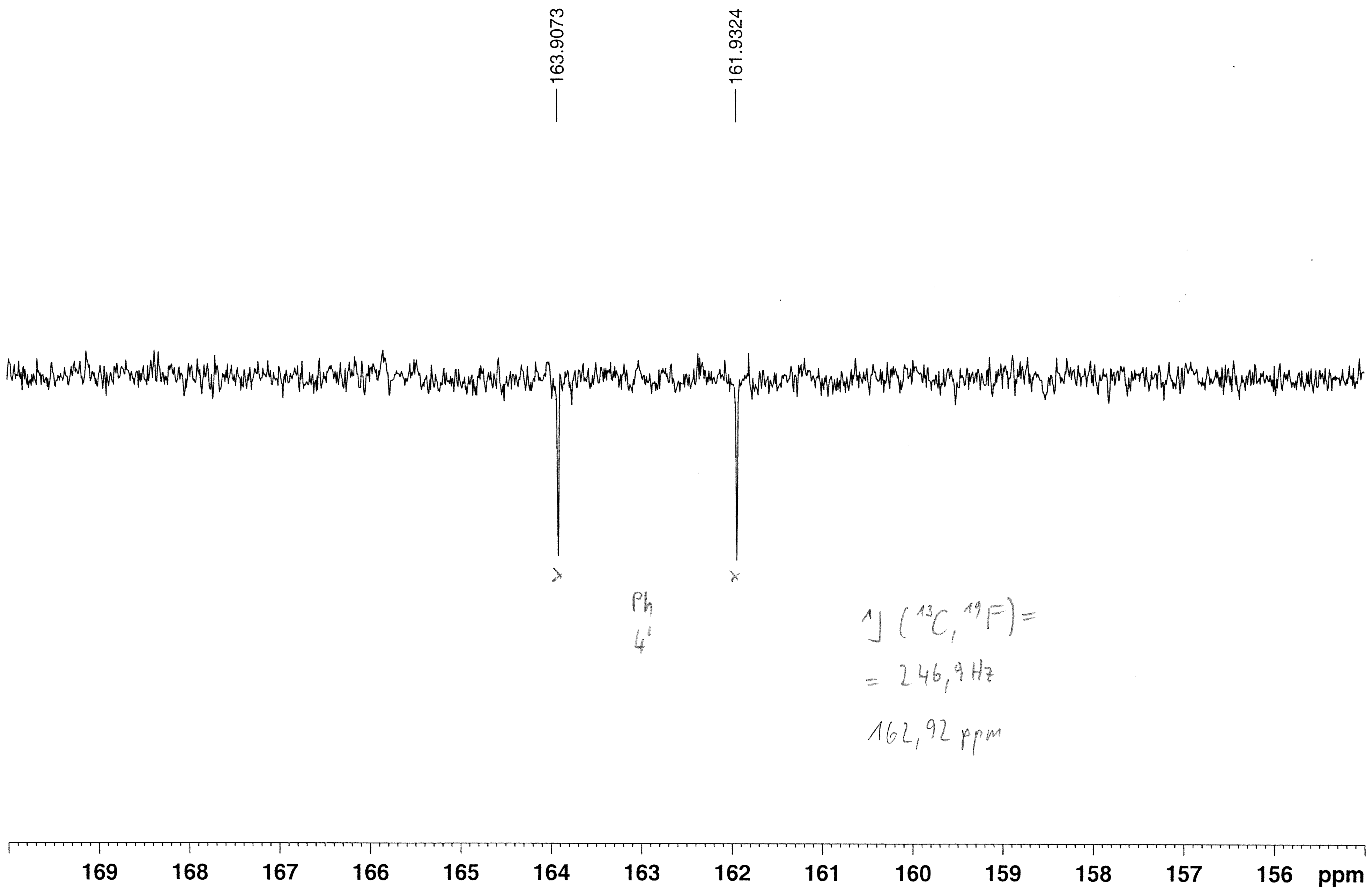

CE156S2P2 in cdcl3 (APT) 5.2.2019

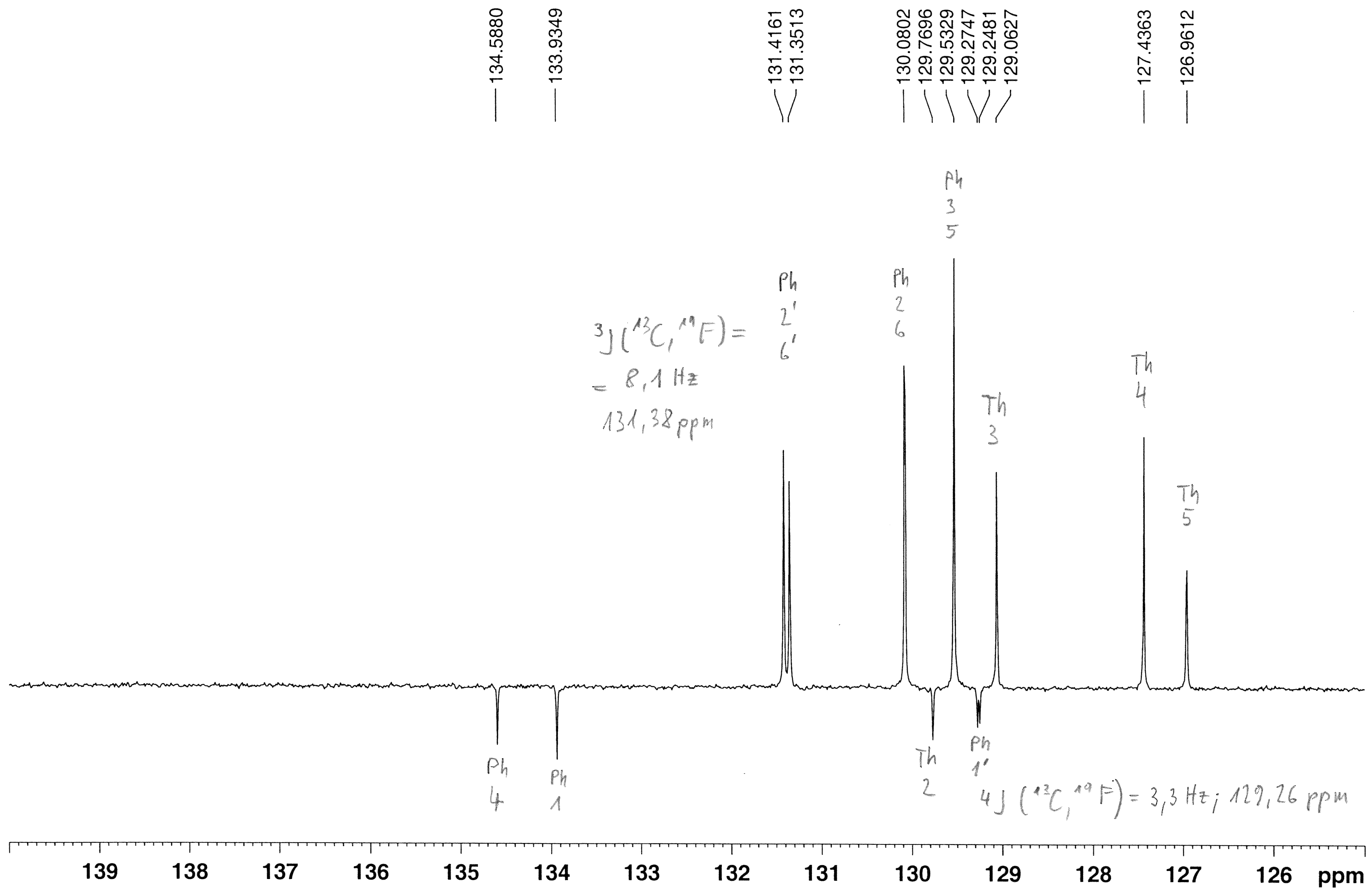

CE156S2P2 in cdcl3 (APT) 5.2.2019

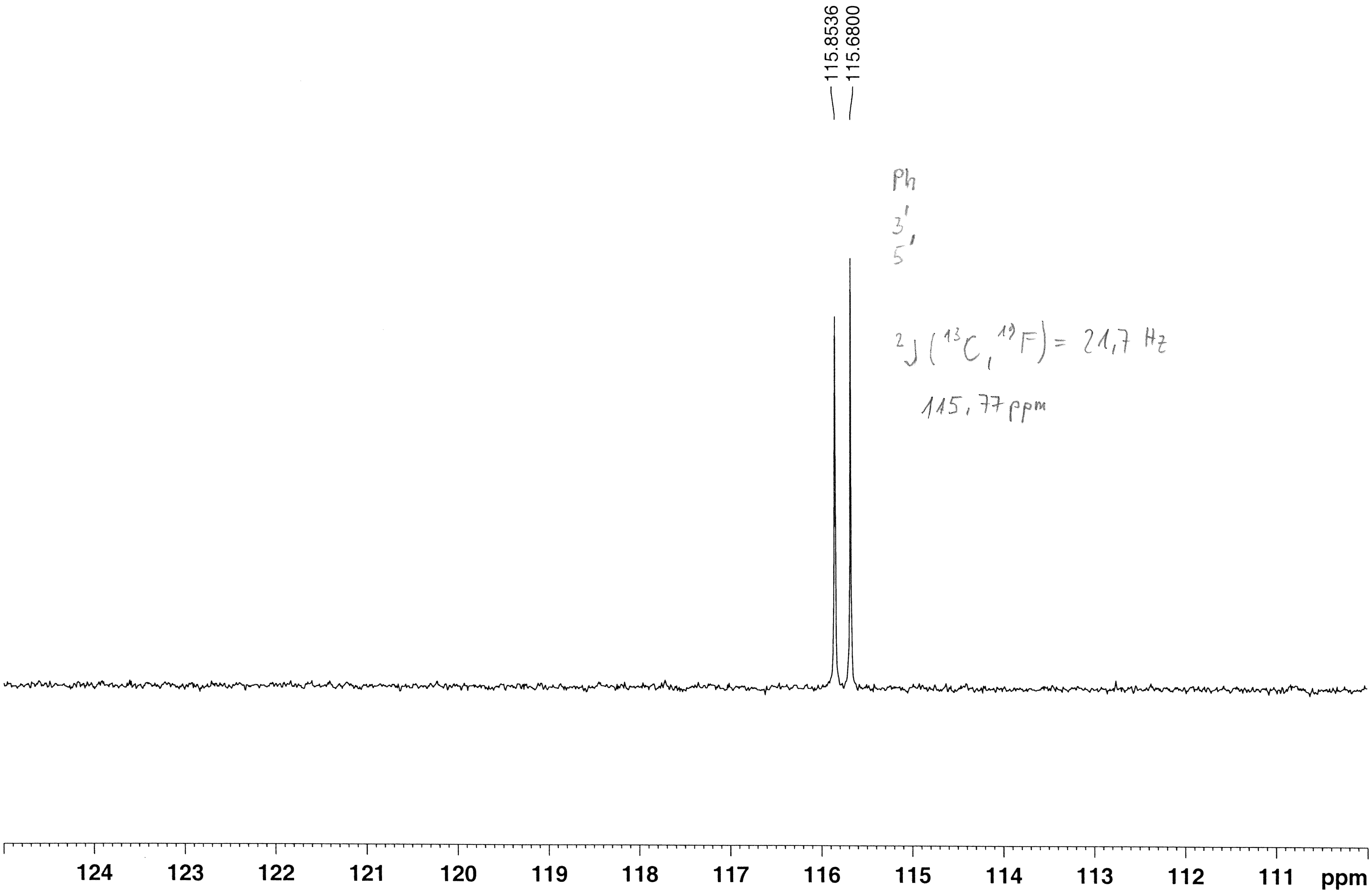

CE156S2P2 in cdcl3 (APT) 5.2.2019

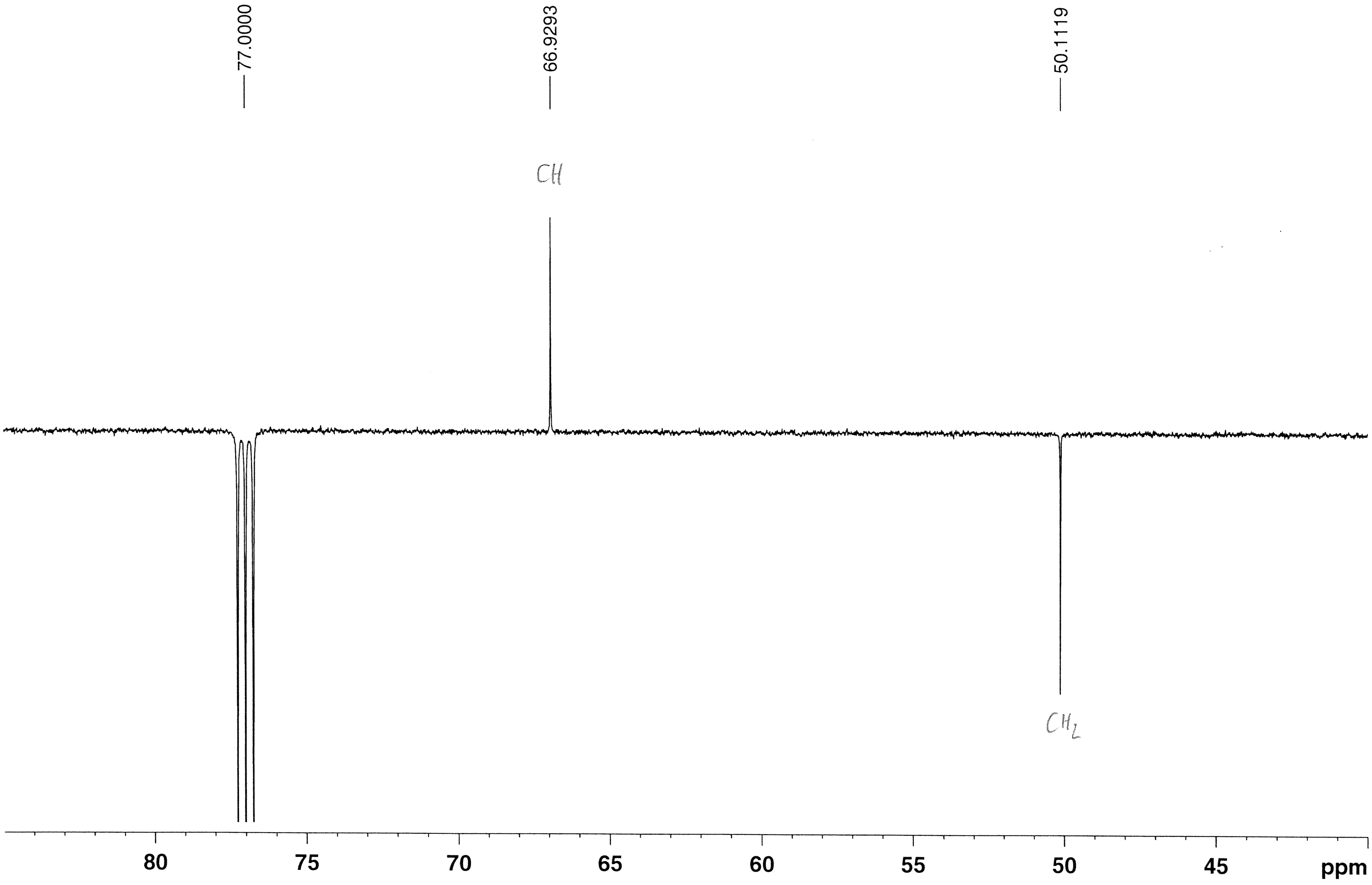

X-ray Analysis

The X-ray intensity data were measured on Bruker D8 Venture and Stoe Stadivari diffractometer equipped with multilayer monochromator, Mo K/α and Cu K/α INCOATEC and AXO micro focus sealed tubes and Oxford cooling systems. The structures were solved by *Direct and Iterative Methods*. Non-hydrogen atoms were refined with *anisotropic displacement parameters*. Hydrogen atoms were inserted at calculated positions and refined with riding model. The following software was used: *Bruker SAINT software package*[i] using a narrow-frame algorithm for frame integration, *SADABS*[ii] for absorption correction, X-Area Integrate Software *package*[iii], *X-Area LANA*[iv] for scaling, *OLEX2*[v] for structure solution, refinement, molecular diagrams and graphical user-interface, *Shelxle*[vi] for refinement and graphical user-interface *SHELXS-2015*[vii] for structure solution, *SHELXL-2015*[viii] for refinement, *Platon*[ix] for symmetry check. Experimental data and CCDC-Codes Experimental data (Available online: http://www.ccdc.cam.ac.uk/conts/retrieving.html) can be found in **Supp. Table 1**. Crystal data, data collection parameters, and structure refinement details are given in **Supporting Tables** below.

Supp. Table 1. Experimental parameter and CCDC-Code.

Sample – Compound ID	Machine	Source	Temp.	Detector Distance	Time/ Frame	#Frames	Frame width	CCDC
			[K]	[mm]	[s]		[°]	
**1**	Stoe Stadivari	Mo	100	40	2.0	4152	0.5	2221111
**3**	Bruker D8	Mo	100	40	1	1000	0.36	2221112
**4**	Stoe Stadivari	Mo	100	40	0.5	6025	0.5	2221113
**13**	Stoe Stadivari	Mo	100	40	2	4898	0.4	2221114
**16**	Stoe Stadivari	Mo	100	40	0.2	1244	0.36	2221115
**17**	Stoe Stadivari	Mo	100	40	8	3013	0.5	2221116
**19**	Stoe Stadivari	Mo	100	40	10	1467	0.36	2221117

**20**	Stoe Stadivari	Mo	100	40	40	5432	0.36	2221118
**21**	Bruker D8	Mo	100	50	10	4065	0,360	2221119
**22**	Stoe Stadivari	Mo	100	40	10	5184	0.36	2221120
**23**	Bruker D8	Mo	100	37	40	720	0.5	2221121
**24**	Bruker D8	Mo	100	37	12	430	0.5	2221122
**41**	Bruker D8	Mo	100	40	1	6021	0.360	2221136
**42**	Bruker D8	Mo	100	40	1	1000	0.36	2221137
**43**	Stoe Stadivari	Mo	100	40	1	5000	0.4	2221138
**44**	Bruker D8	Mo	100	40	20	967	0.36	2221139
**50**	Stoe Stadivari	Mo	100	50	40	6073	0.36	2221127
**51**	Stoe Stadivari	Mo	100	40	2	1812	0.5	2221128
**52**	Stoe Stadivari	Cu	100	40	2	4868	0.5	2221129
**55**	Bruker D8	Mo	100	37	60	1348	1	2221134
**56**	Bruker D8	Mo	100	40	20	2090	0.5	2221135
**70**	Stoe Stadivari	Mo	100	40	0.2	3823	0.4	2221123
**71**	Stoe Stadivari	Mo	100	40	60	3775	0.36	2221125
**72**	Bruker D8	Mo	100	40	4	2855	0.36	2221126
**77**	Stoe Stadivari	Mo	100	40	1	4238	0.4	2221130
**79**	Bruker D8	Mo	100	40	5	14497	0.36	2221131
**80**	Bruker D8	Mo	100	40	1	5431	0.36	2221132

Compound 1

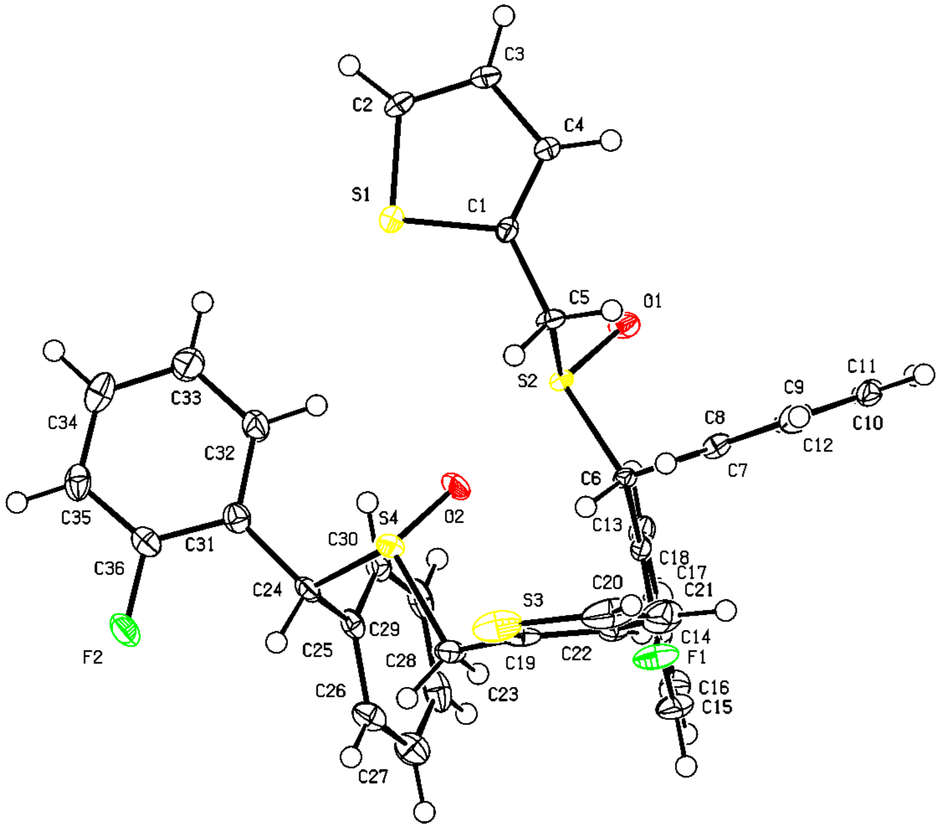

Asymmetric Unit of drawn with 50% displacement ellipsoid. The bond precision for C-C single bonds is 0.0031 Å. Classical Flack parameter = 0.08(4) and 0.10(2) by Parsons' method[1] for S2/S4 (S) C6/C24 (R).

Compound 3

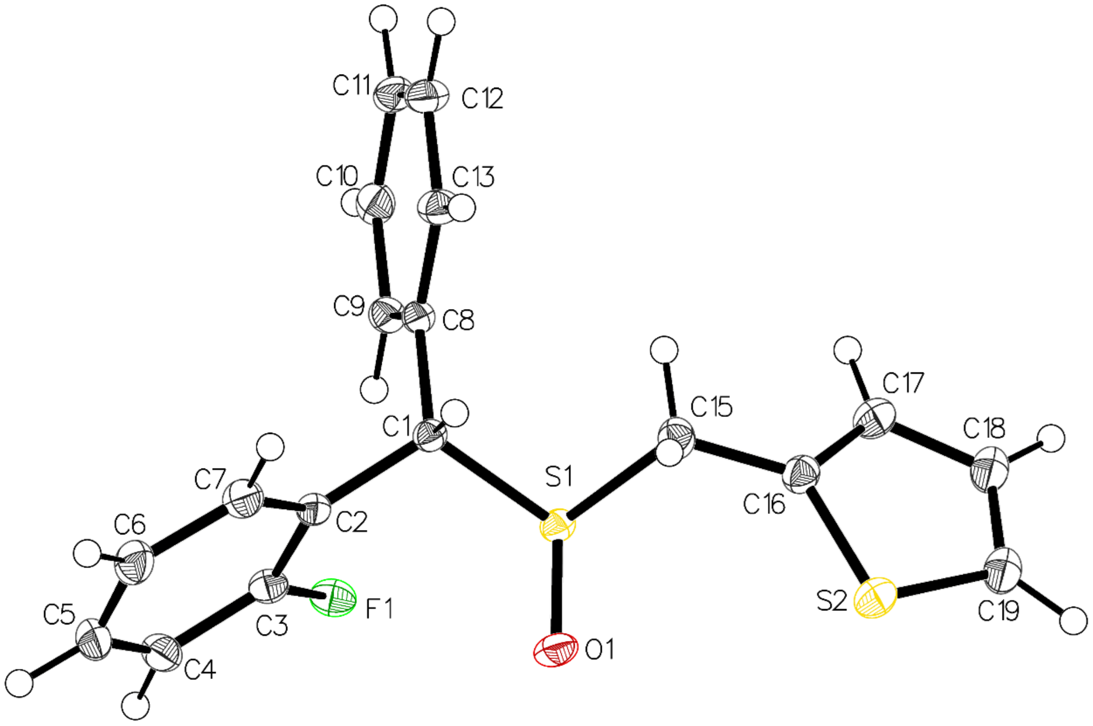

Asymmetric Unit of drawn with 50% displacement ellipsoid. Disorder omitted for clarity. The bond precision for C-C single bonds is 0.0029 Å. Chirality proofed by Hooft (-0.01(2)) and Flack (-0.01(6)) for S1 (R) and C1(S).

Identification code	**Compound 3**
Empirical formula	$C_{18}H_{15}FOS_2$
Formula weight	330.42
Temperature/K	100.00
Crystal system	monoclinic
Space group	$P2_1$
a/Å	9.6927(2)
b/Å	8.6471(2)
c/Å	10.4384(2)

α/°	90
β/°	116.1290(13)
γ/°	90
Volume/Å^3	785.47(3)
Z	2
ρ_{calc}g/cm^3	1.397
μ/mm^{-1}	0.347
F(000)	344.0
Crystal size/mm^3	0.4 × 0.3 × 0.2
Radiation	MoKα (λ = 0.71073)
2Θ range for data collection/°	4.346 to 60.202
Index ranges	-11 ≤ h ≤ 13, -12 ≤ k ≤ 12, -14 ≤ l ≤ 13
Reflections collected	10892
Independent reflections	4345 [R_{int} = 0.0287, R_{sigma} = 0.0370]
Data/restraints/parameters	4345/10/200
Goodness-of-fit on F^2	1.061
Final R indexes [I>=2σ (I)]	R_1 = 0.0277, wR_2 = 0.0658
Final R indexes [all data]	R_1 = 0.0315, wR_2 = 0.0675
Largest diff. peak/hole / e Å^{-3}	0.33/-0.24
Flack parameter	0.00(2)

Compound 4

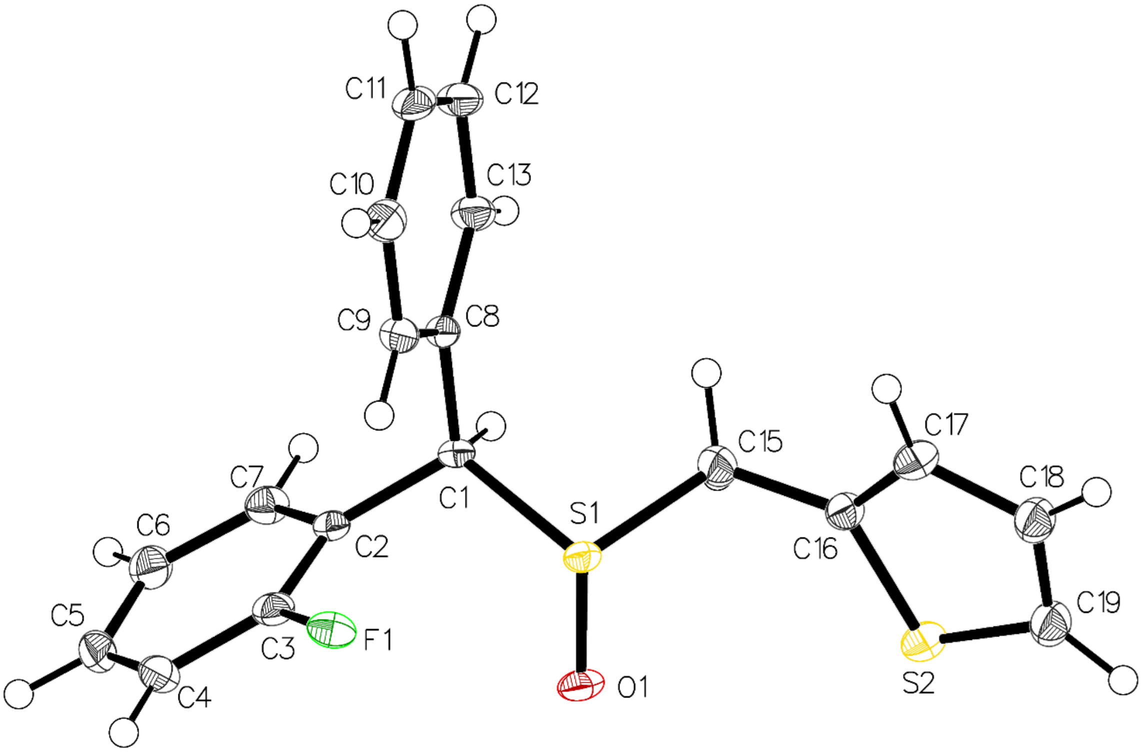

Asymmetric Unit of drawn with 50% displacement ellipsoid. Disorder omitted for clarity. The bond precision for C-C single bonds is 0.0036 Å. Chirality proofed by Hooft (0.044(18)) and Flack (0.02(7)) for S1 (S) and C1(R).

Identification code	**Compound 4**
Empirical formula	$C_{18}H_{15}FOS_2$
Formula weight	330.42
Temperature/K	100
Crystal system	monoclinic
Space group	$P2_1$
a/Å	9.6931(4)
b/Å	8.6156(5)
c/Å	10.4514(5)

α/°	90
β/°	116.357(3)
γ/°	90
Volume/Å^3	782.08(7)
Z	2
ρ_{calc}g/cm^3	1.403
μ/mm^{-1}	0.349
F(000)	344.0
Crystal size/mm^3	0.56 × 0.34 × 0.23
Radiation	Mo Kα (λ = 0.71073)
2Θ range for data collection/°	4.35 to 60.062
Index ranges	-8 ≤ h ≤ 13, -11 ≤ k ≤ 12, -14 ≤ l ≤ 11
Reflections collected	36648
Independent reflections	4463 [R_{int} = 0.0370, R_{sigma} = 0.0333]
Data/restraints/parameters	4463/10/200
Goodness-of-fit on F^2	1.075
Final R indexes [I>=2σ (I)]	R_1 = 0.0315, wR_2 = 0.0797
Final R indexes [all data]	R_1 = 0.0361, wR_2 = 0.0804
Largest diff. peak/hole / e Å^{-3}	0.31/-0.51
Flack parameter	0.07(6)

Compound 13

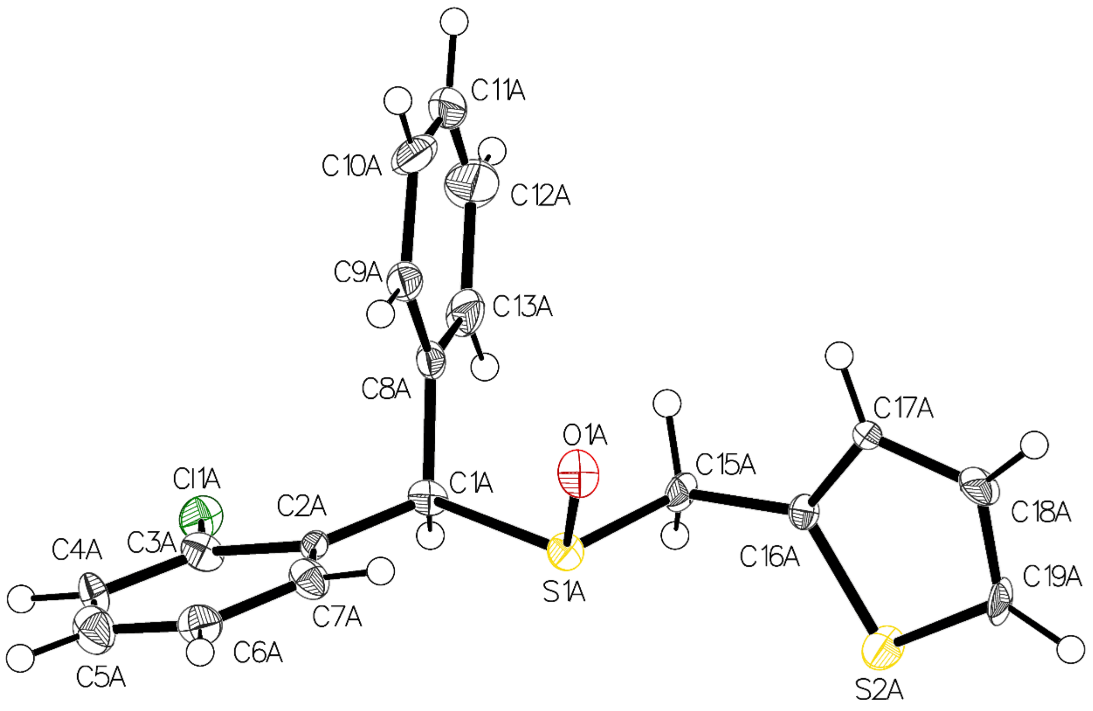

Asymmetric Unit of drawn with 50% displacement ellipsoid. Disorder and second independent molecule omitted for clarity. The bond precision for C-C single bonds is 0.0123 Å. Chirality proofed by Hooft (-0.34(5)) and Flack (-0.19(10)) for S1 (R) and C1(R). Two B-Alerts could not be avoided.

PLAT026_ALERT_3_B Ratio Observed / Unique Reflections (too) Low. 35% Check

PLAT340_ALERT_3_B Low Bond Precision on C-C Bonds .... 0.0123 Ang. To many samples to measure makes sometimes a low coverage necessary.

Identification code	**Compound 13**
Empirical formula	$C_{18}H_{15}ClOS_2$
Formula weight	346.87
Temperature/K	100
Crystal system	triclinic
Space group	P1

a/Å	9.6760(17)
b/Å	9.9117(17)
c/Å	10.6083(19)
α/°	104.107(13)
β/°	115.015(13)
γ/°	99.349(14)
Volume/Å^3	852.4(3)
Z	2
ρ_{calc}g/cm^3	1.351
μ/mm^{-1}	0.467
F(000)	360.0
Crystal size/mm^3	0.4 × 0.193 × 0.08
Radiation	Mo Kα (λ = 0.71073)
2Θ range for data collection/°	4.522 to 60.068
Index ranges	-13 ≤ h ≤ 13, -13 ≤ k ≤ 13, -14 ≤ l ≤ 14
Reflections collected	20622
Independent reflections	9000 [R_{int} = 0.0770, R_{sigma} = 0.4354]
Data/restraints/parameters	9000/669/380
Goodness-of-fit on F^2	0.746
Final R indexes [I>=2σ (I)]	R_1 = 0.0622, wR_2 = 0.1197
Final R indexes [all data]	R_1 = 0.1444, wR_2 = 0.1287
Largest diff. peak/hole / e Å^{-3}	0.55/-0.61
Flack parameter	-0.19(10)

Compound 14

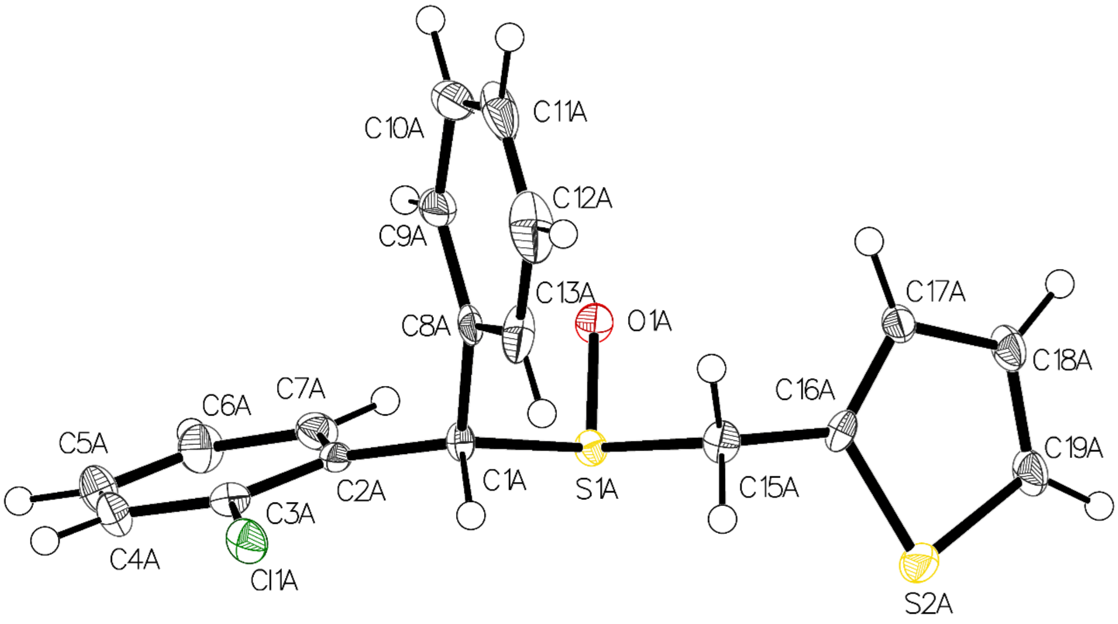

Asymmetric Unit of drawn with 50% displacement ellipsoid. Disorder and second independent molecule omitted for clarity. The bond precision for C-C single bonds is 0.0064 Å. Chirality proofed by Hooft (0.17(4)) and Flack (0.07(6)) for S1 (S) and C1(S). One B-Alert could not avoided.

PLAT915_ALERT_3_B No Flack x Check Done: Low Friedel Pair Coverage 28 %
To many samples to measure makes sometimes a low coverage necessary.

Identification code	**Compound 14**
Empirical formula	$C_{18}H_{15}ClOS_2$
Formula weight	346.87
Temperature/K	150
Crystal system	triclinic
Space group	P1
a/Å	9.6812(8)
b/Å	9.9373(8)

c/Å	10.6047(9)
α/°	104.192(7)
β/°	115.150(6)
γ/°	99.262(7)
Volume/Å^3	853.48(13)
Z	2
ρ_{calc}g/cm^3	1.350
μ/mm^{-1}	0.467
F(000)	360.0
Crystal size/mm^3	0.09 × 0.062 × 0.04
Radiation	Mo Kα (λ = 0.71073)
2Θ range for data collection/°	4.436 to 60.066
Index ranges	-13 ≤ h ≤ 13, -13 ≤ k ≤ 9, -14 ≤ l ≤ 14
Reflections collected	14303
Independent reflections	6293 [R_{int} = 0.0533, R_{sigma} = 0.0775]
Data/restraints/parameters	6293/739/404
Goodness-of-fit on F^2	0.914
Final R indexes [I>=2σ (I)]	R_1 = 0.0388, wR_2 = 0.0816
Final R indexes [all data]	R_1 = 0.0540, wR_2 = 0.0843
Largest diff. peak/hole / e Å^{-3}	0.31/-0.29
Flack parameter	0.07(6)

Compound 16

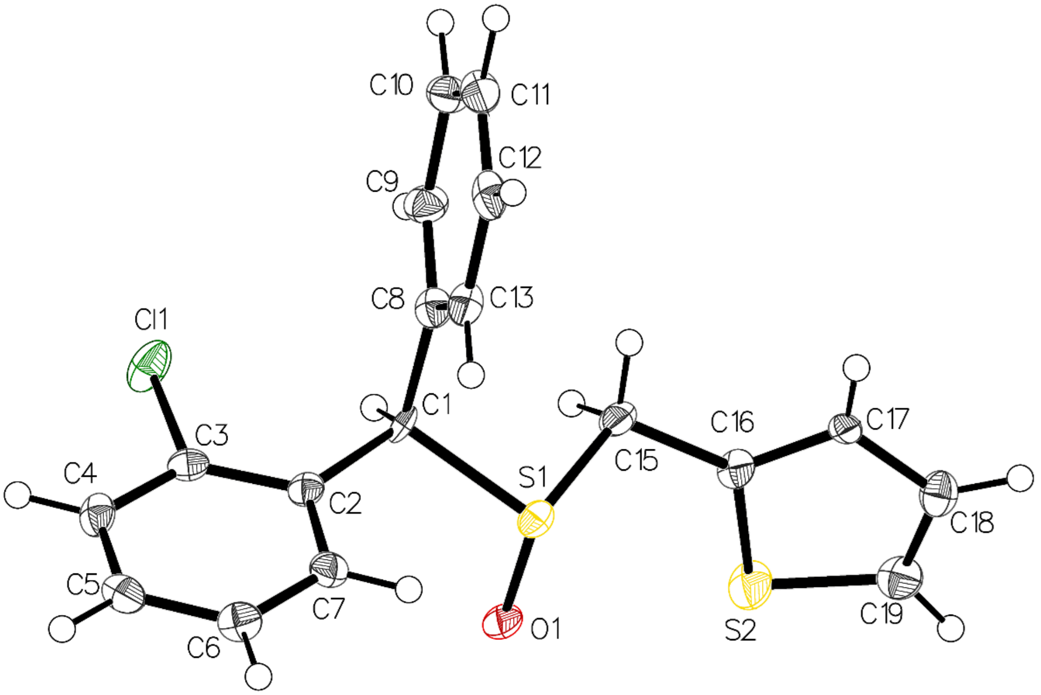

Asymmetric Unit of drawn with 50% displacement ellipsoid. The bond precision for C-C single bonds is 0.0073 Å. Chirality proofed by Hooft (0.34(4)) and Flack (-0.04(11)) for S1 (S) and C1(R).

Identification code	**Compound 16**
Empirical formula	$C_{18}H_{15}ClOS_2$
Formula weight	346.87
Temperature/K	100
Crystal system	monoclinic
Space group	$P2_1$
a/Å	8.5421(11)
b/Å	10.2144(8)
c/Å	9.8299(11)
α/°	90
β/°	107.883(9)

γ/°	90
Volume/Å^3	816.24(16)
Z	2
ρ_{calc}g/cm^3	1.411
μ/mm^{-1}	0.488
F(000)	360.0
Crystal size/mm^3	0.6 × 0.4 × 0.2
Radiation	Mo Kα (λ = 0.71073)
2Θ range for data collection/°	4.354 to 63.158
Index ranges	-10 ≤ h ≤ 12, -14 ≤ k ≤ 14, -14 ≤ l ≤ 14
Reflections collected	10256
Independent reflections	4842 [R_{int} = 0.0443, R_{sigma} = 0.1641]
Data/restraints/parameters	4842/1/199
Goodness-of-fit on F^2	0.904
Final R indexes [I>=2σ (I)]	R_1 = 0.0435, wR_2 = 0.0906
Final R indexes [all data]	R_1 = 0.1108, wR_2 = 0.1189
Largest diff. peak/hole / e Å^{-3}	0.69/-1.00
Flack parameter	0.02(10)

Compound 17

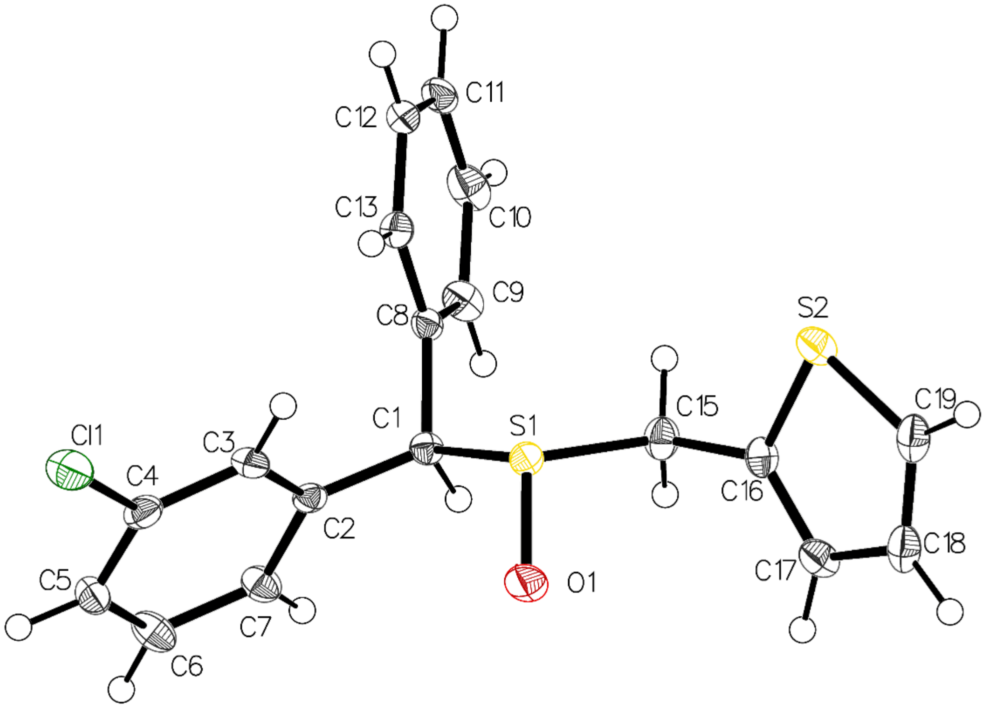

Asymmetric Unit of drawn with 50% displacement ellipsoid. Disorder omitted for clarity. The bond precision for C-C single bonds is 0.0030 Å. Chirality proofed by Hooft (-0.134(18)) and Flack (-0.12(6)) for S1 (S) and C1(R).

Identification code	**Compound 17**
Empirical formula	$C_{18}H_{15}ClOS_2$
Formula weight	346.87
Temperature/K	100
Crystal system	orthorhombic
Space group	$P2_12_12_1$
a/Å	8.2111(3)
b/Å	12.2681(3)
c/Å	16.6181(4)

α/°	90
β/°	90
γ/°	90
Volume/Å^3	1674.02(8)
Z	4
ρ_{calc}g/cm^3	1.376
μ/mm^{-1}	0.476
F(000)	720.0
Crystal size/mm^3	0.16 × 0.117 × 0.09
Radiation	Mo Kα (λ = 0.71073)
2Θ range for data collection/°	4.126 to 62.482
Index ranges	-11 ≤ h ≤ 11, -17 ≤ k ≤ 17, -24 ≤ l ≤ 24
Reflections collected	39509
Independent reflections	5333 [R_{int} = 0.0416, R_{sigma} = 0.0456]
Data/restraints/parameters	5333/9/200
Goodness-of-fit on F^2	0.960
Final R indexes [I>=2σ (I)]	R_1 = 0.0315, wR_2 = 0.0677
Final R indexes [all data]	R_1 = 0.0445, wR_2 = 0.0709
Largest diff. peak/hole / e Å^{-3}	0.42/-0.48
Flack parameter	-0.13(4)

Compound 19

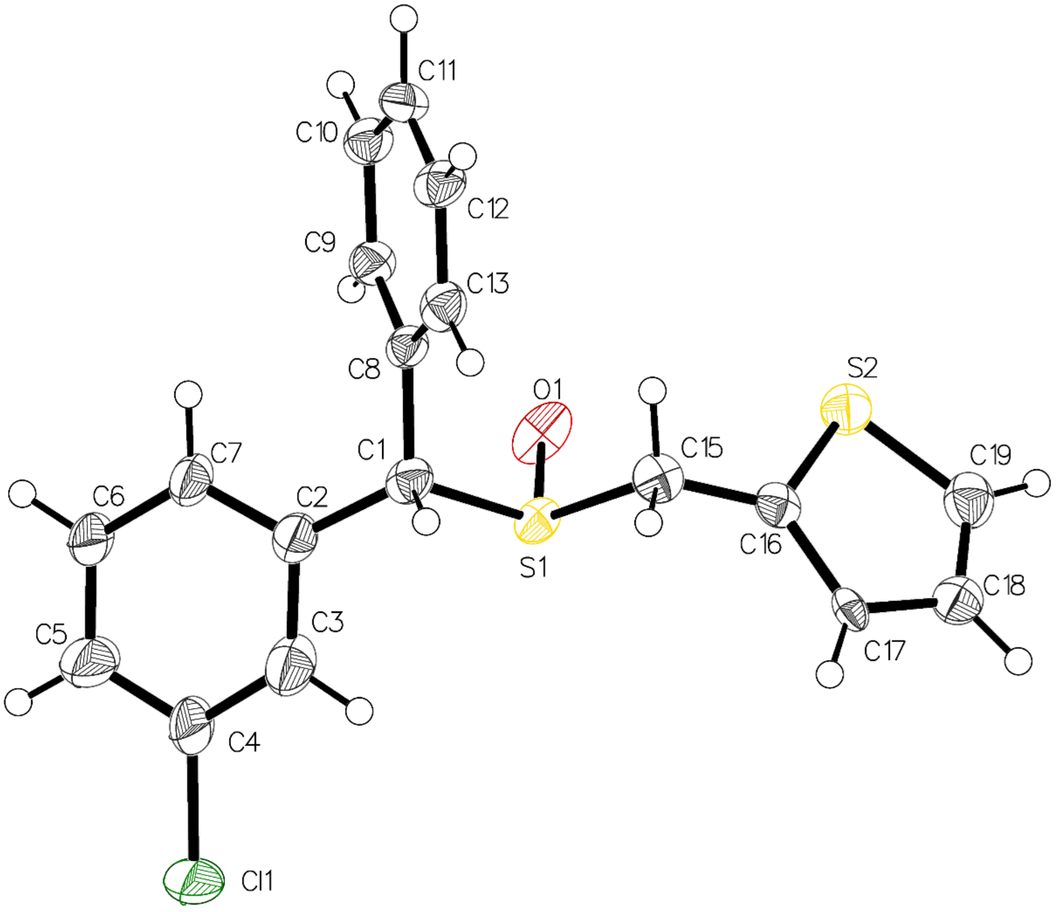

Asymmetric Unit of drawn with 50% displacement ellipsoid. Disorder omitted for clarity. The bond precision for C-C single bonds is 0.0101 Å. Chirality proofed by Hooft (-0.07(6)) and Flack (0.2(2)) for S1 (S) and C1(S). One B-Alert could not be avoided.

PLAT340_ALERT_3_B Low Bond Precision on C-C Bonds ....0.01008 Ang. Related to the quality of the crystal and the disorder.

Identification code	**Compound 19**
Empirical formula	$C_{18}H_{15}ClOS_2$
Formula weight	346.87

Temperature/K	100
Crystal system	orthorhombic
Space group	$P2_12_12_1$
a/Å	5.4961(6)
b/Å	15.672(2)
c/Å	18.777(2)
α/°	90
β/°	90
γ/°	90
Volume/Å^3	1617.4(4)
Z	4
ρ_{calc}g/cm^3	1.425
μ/mm^{-1}	0.492
F(000)	720.0
Crystal size/mm^3	0.18 × 0.083 × 0.03
Radiation	Mo Kα (λ = 0.71073)
2Θ range for data collection/°	3.384 to 63.83
Index ranges	-7 ≤ h ≤ 7, -22 ≤ k ≤ 22, -26 ≤ l ≤ 26
Reflections collected	13674
Independent reflections	5151 [R_{int} = 0.0711, R_{sigma} = 0.1880]
Data/restraints/parameters	5151/78/249
Goodness-of-fit on F^2	1.005
Final R indexes [I>=2σ (I)]	R_1 = 0.0821, wR_2 = 0.1981
Final R indexes [all data]	R_1 = 0.1955, wR_2 = 0.2562
Largest diff. peak/hole / e Å^{-3}	1.10/-1.14
Flack parameter	0.04(12)

Compound 20

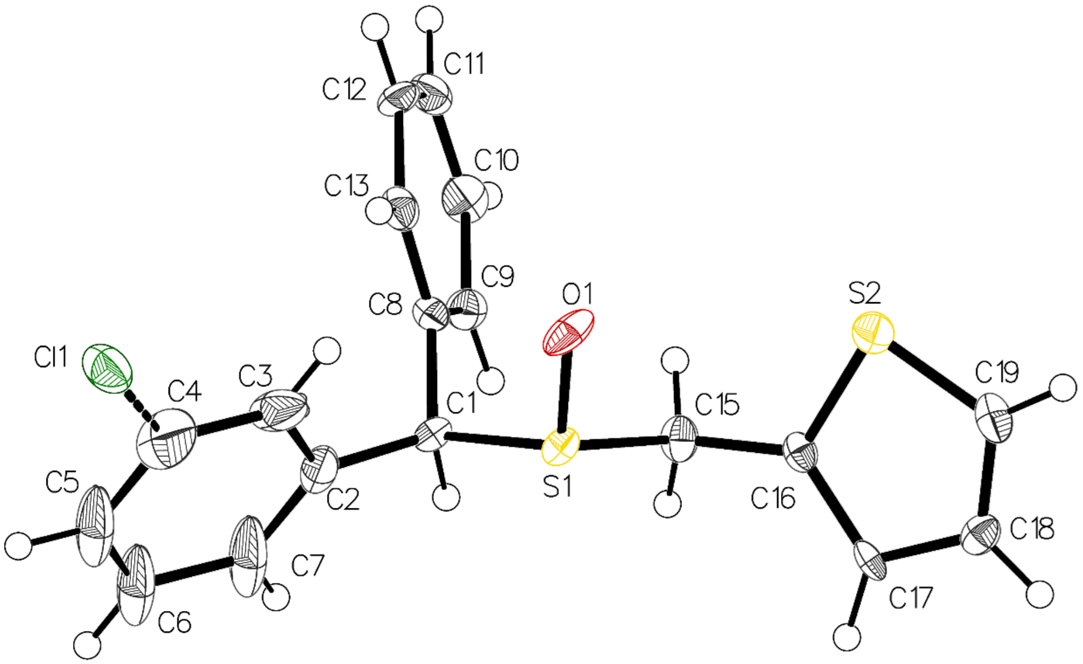

Asymmetric Unit of drawn with 50% displacement ellipsoid. Disorder omitted for clarity. The bond precision for C-C single bonds is 0.0079 Å. Chirality proofed by Flack (-0.1(2)) for S1 (R) and C1(R).

Identification code	**Compound 20**
Empirical formula	$C_{18}H_{15}ClOS_2$
Formula weight	346.87
Temperature/K	100
Crystal system	orthorhombic
Space group	$P2_12_12_1$
a/Å	5.5093(5)
b/Å	15.6870(16)
c/Å	18.8298(17)
α/°	90

β/°	90
γ/°	90
Volume/Å^3	1627.4(3)
Z	4
ρ_{calc}g/cm^3	1.416
μ/mm^{-1}	0.489
F(000)	720.0
Crystal size/mm^3	0.25 × 0.123 × 0.05
Radiation	Mo Kα (λ = 0.71073)
2Θ range for data collection/°	5.046 to 51.968
Index ranges	-6 ≤ h ≤ 6, -19 ≤ k ≤ 19, -23 ≤ l ≤ 23
Reflections collected	43436
Independent reflections	3194 [R_{int} = 0.0530, R_{sigma} = 0.0169]
Data/restraints/parameters	3194/51/217
Goodness-of-fit on F^2	1.099
Final R indexes [I>=2σ (I)]	R_1 = 0.0565, wR_2 = 0.1328
Final R indexes [all data]	R_1 = 0.0605, wR_2 = 0.1389
Largest diff. peak/hole / e Å^{-3}	0.84/-0.91
Flack parameter	0.04(3)

Compound 21

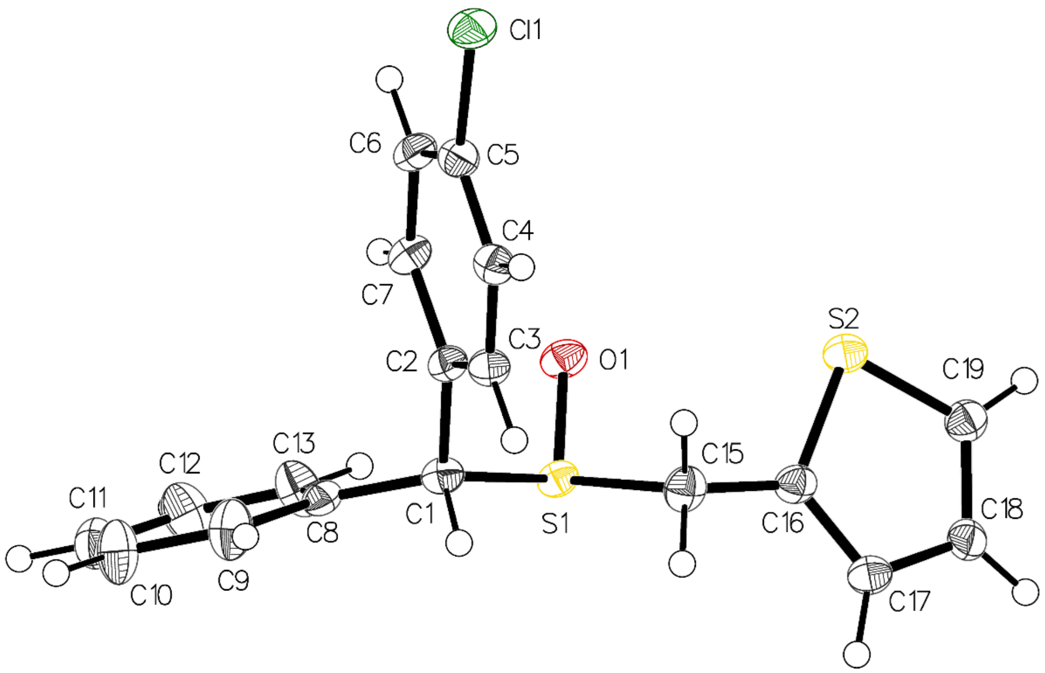

Asymmetric Unit of drawn with 50% displacement ellipsoid. Disorder omitted for clarity. The bond precision for C-C single bonds is 0.0054 Å. Chirality proofed by Hooft (0.02(2)) and Flack (-0.01(11)) for S1 (S) and C1(R).

Identification code	**Compound 21**
Empirical formula	$C_{18}H_{15}OS_2Cl$
Formula weight	346.87
Temperature/K	100.00
Crystal system	orthorhombic
Space group	$P2_12_12_1$
a/Å	5.620(2)
b/Å	16.119(13)
c/Å	18.546(7)
α/°	90

β/°	90
γ/°	90
Volume/Å^3	1679.9(16)
Z	4
ρ_{calc}g/cm^3	1.371
μ/mm^{-1}	0.474
F(000)	720.0
Crystal size/mm^3	0.26 × 0.2 × 0.04
Radiation	MoKα (λ = 0.71073)
2Θ range for data collection/°	4.392 to 60.268
Index ranges	-7 ≤ h ≤ 7, -22 ≤ k ≤ 12, -26 ≤ l ≤ 26
Reflections collected	33251
Independent reflections	4854 [R_{int} = 0.0523, R_{sigma} = 0.0322]
Data/restraints/parameters	4854/9/206
Goodness-of-fit on F^2	1.081
Final R indexes [I>=2σ (I)]	R_1 = 0.0476, wR_2 = 0.1053
Final R indexes [all data]	R_1 = 0.0570, wR_2 = 0.1133
Largest diff. peak/hole / e Å^{-3}	0.60/-0.55
Flack parameter	0.00(4)

Compound 22

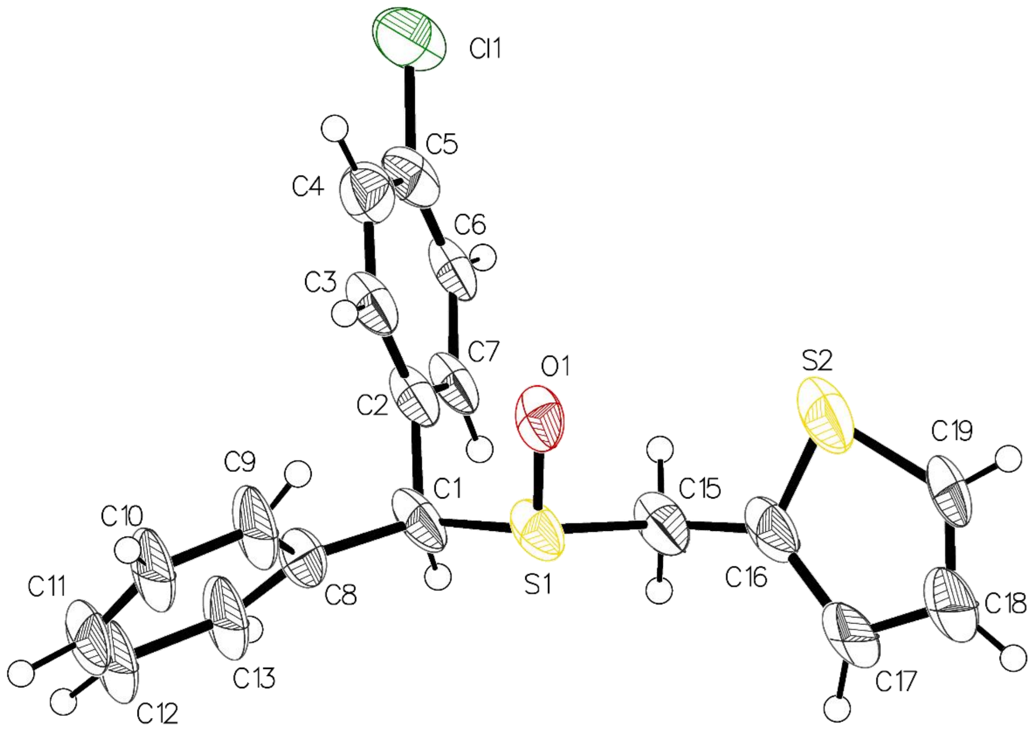

Asymmetric Unit of drawn with 50% displacement ellipsoid. Disorder omitted for clarity. The bond precision for C-C single bonds is 0.0196 Å. Chirality proofed by Hooft (-0.01(3)) and Flack (0.0(4)) for S1 (R) and C1(S). Two B-Alerts could not be avoided. PLAT084_ALERT_3_B High wR2 Value (i.e., > 0.25) .... 0.36 Report

PLAT340_ALERT_3_B Low Bond Precision on C-C Bonds ....0.01958 Ang. Related to the quality of the crystal and the disorder.

Identification code	**Compound 22**
Empirical formula	$C_{18}H_{15}ClOS_2$
Formula weight	346.87
Temperature/K	100
Crystal system	orthorhombic
Space group	$P2_12_12_1$

a/Å	5.4932(3)
b/Å	16.5224(19)
c/Å	18.3715(12)
α/°	90
β/°	90
γ/°	90
Volume/Å^3	1667.4(2)
Z	4
ρ_{calc}g/cm^3	1.382
μ/mm^{-1}	0.478
F(000)	720.0
Crystal size/mm^3	0.25 × 0.123 × 0.05
Radiation	Mo Kα (λ = 0.71073)
2Θ range for data collection/°	4.434 to 51.36
Index ranges	-6 ≤ h ≤ 6, -20 ≤ k ≤ 20, -22 ≤ l ≤ 22
Reflections collected	30961
Independent reflections	3158 [R_{int} = 0.0956, R_{sigma} = 0.0883]
Data/restraints/parameters	3158/422/182
Goodness-of-fit on F^2	1.323
Final R indexes [I>=2σ (I)]	R_1 = 0.1285, wR_2 = 0.3359
Final R indexes [all data]	R_1 = 0.1650, wR_2 = 0.3590
Largest diff. peak/hole / e Å^{-3}	0.95/-0.78
Flack parameter	-0.01(9)

Compound 23

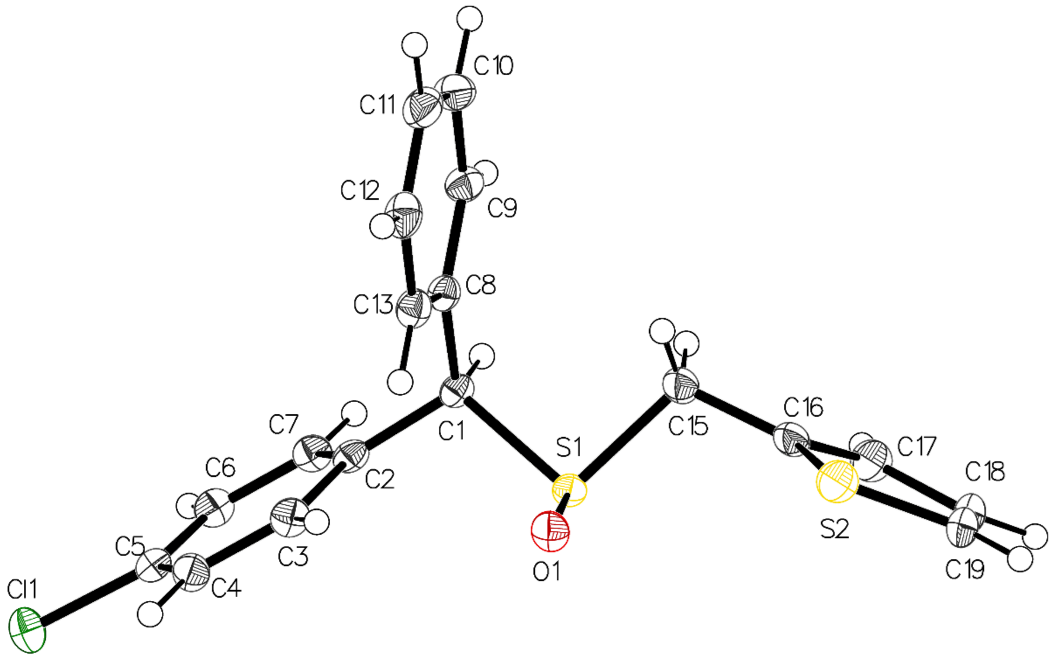

Asymmetric Unit of drawn with 50% displacement ellipsoid. Disorder omitted for clarity. The bond precision for C-C single bonds is 0.0044 Å. Chirality proofed by Hooft (-0.08(5)) and Flack (-0.06(9)) for S1 (S) and C1(S).

Identification code	**Compound 23**
Empirical formula	$C_{18}H_{15}ClOS_2$
Formula weight	346.87
Temperature/K	100.0
Crystal system	orthorhombic
Space group	$P2_12_12_1$
a/Å	5.8552(2)
b/Å	15.5405(7)
c/Å	17.5545(6)
α/°	90

β/°	90
γ/°	90
Volume/Å^3	1597.33(11)
Z	4
ρ_{calc}g/cm^3	1.442
μ/mm^{-1}	0.499
F(000)	720.0
Crystal size/mm^3	0.1 × 0.04 × 0.02
Radiation	MoKα (λ = 0.71073)
2Θ range for data collection/°	4.64 to 60.064
Index ranges	-8 ≤ h ≤ 7, -21 ≤ k ≤ 21, -24 ≤ l ≤ 23
Reflections collected	20655
Independent reflections	4662 [R_{int} = 0.1095, R_{sigma} = 0.0807]
Data/restraints/parameters	4662/12/200
Goodness-of-fit on F^2	1.050
Final R indexes [I>=2σ (I)]	R_1 = 0.0484, wR_2 = 0.0933
Final R indexes [all data]	R_1 = 0.0614, wR_2 = 0.0976
Largest diff. peak/hole / e Å^{-3}	0.38/-0.43
Flack parameter	-0.05(5)

Compound 24

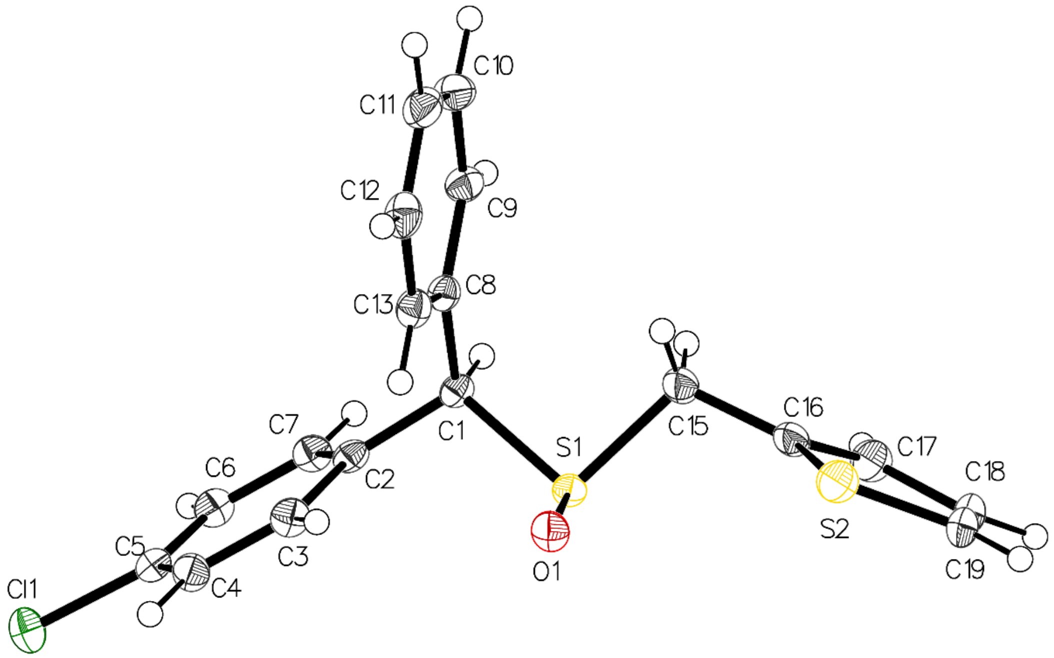

Asymmetric Unit of drawn with 50% displacement ellipsoid. Disorder omitted for clarity. The bond precision for C-C single bonds is 0.0051 Å. Chirality proofed by Hooft (-0.04(5)) and Flack (-0.04(11)) for S1 (R) and C1(R).

Identification code	**Compound 24**
Empirical formula	$C_{18}H_{15}ClOS_2$
Formula weight	346.87
Temperature/K	100.0
Crystal system	orthorhombic
Space group	$P2_12_12_1$
a/Å	5.8511(4)
b/Å	15.5574(9)
c/Å	17.5412(9)
α/°	90

β/°	90
γ/°	90
Volume/Å^3	1596.74(16)
Z	4
ρ_{calc}g/cm^3	1.443
μ/mm^{-1}	0.499
F(000)	720.0
Crystal size/mm^3	0.15 × 0.15 × 0.03
Radiation	MoKα (λ = 0.71073)
2Θ range for data collection/°	4.644 to 60.112
Index ranges	-8 ≤ h ≤ 8, -21 ≤ k ≤ 21, -23 ≤ l ≤ 24
Reflections collected	11148
Independent reflections	4588 [R_{int} = 0.0600, R_{sigma} = 0.0735]
Data/restraints/parameters	4588/12/200
Goodness-of-fit on F^2	1.043
Final R indexes [I>=2σ (I)]	R_1 = 0.0494, wR_2 = 0.0887
Final R indexes [all data]	R_1 = 0.0721, wR_2 = 0.1004
Largest diff. peak/hole / e Å^{-3}	0.36/-0.53
Flack parameter	-0.03(5)

Compound 41

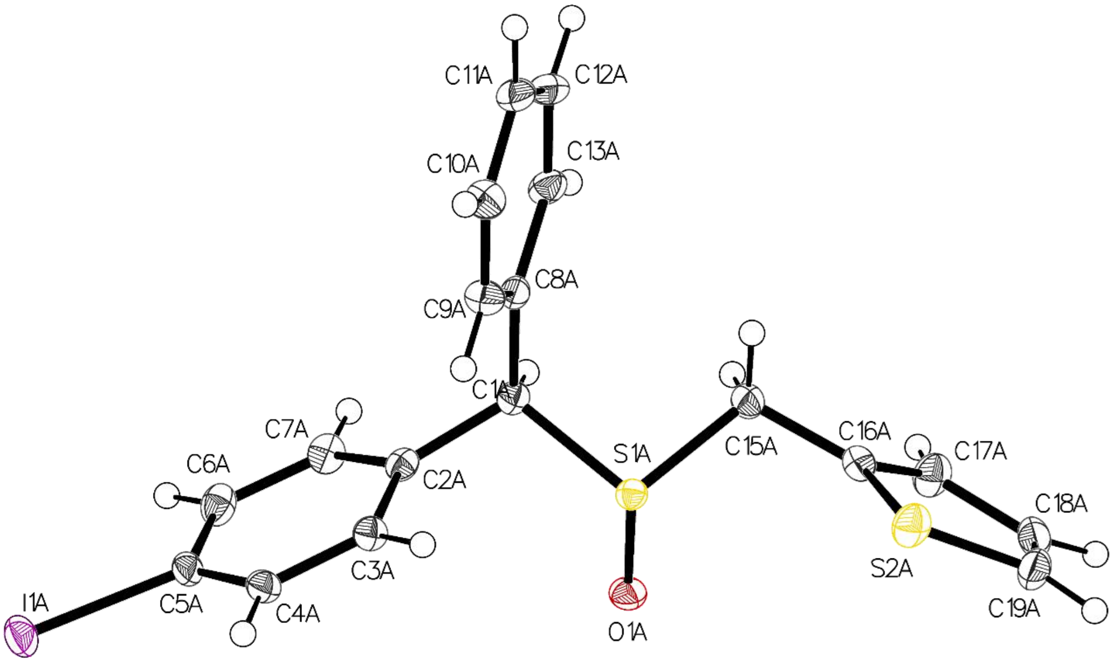

Asymmetric Unit of drawn with 50% displacement ellipsoid. Disorder and second independent molecule omitted for clarity. The bond precision for C-C single bonds is 0.0030 Å. Chirality proofed by Hooft (-0.025(3)) and Flack (-0.022(15)) for S1 (S) and C1(R).

Identification code	**Compound 41**
Empirical formula	$C_{18}H_{15}IOS_2$
Formula weight	438.32
Temperature/K	100.00
Crystal system	monoclinic
Space group	$P2_1$
a/Å	11.0758(2)
b/Å	8.02350(10)
c/Å	19.2404(3)
α/°	90

β/°	94.7416(6)
γ/°	90
Volume/$Å^3$	1703.98(5)
Z	4
$\rho_{calc}g/cm^3$	1.709
μ/mm^{-1}	2.123
F(000)	864.0
Crystal size/mm^3	0.23 × 0.2 × 0.05
Radiation	MoKα (λ = 0.71073)
2Θ range for data collection/°	4.102 to 66.456
Index ranges	-17 ≤ h ≤ 17, -12 ≤ k ≤ 12, -29 ≤ l ≤ 29
Reflections collected	162428
Independent reflections	13065 [R_{int} = 0.0454, R_{sigma} = 0.0218]
Data/restraints/parameters	13065/21/405
Goodness-of-fit on F^2	1.030
Final R indexes [I>=2σ (I)]	R_1 = 0.0212, wR_2 = 0.0420
Final R indexes [all data]	R_1 = 0.0254, wR_2 = 0.0433
Largest diff. peak/hole / e $Å^{-3}$	0.80/-0.56
Flack parameter	-0.029(4)

Compound 42

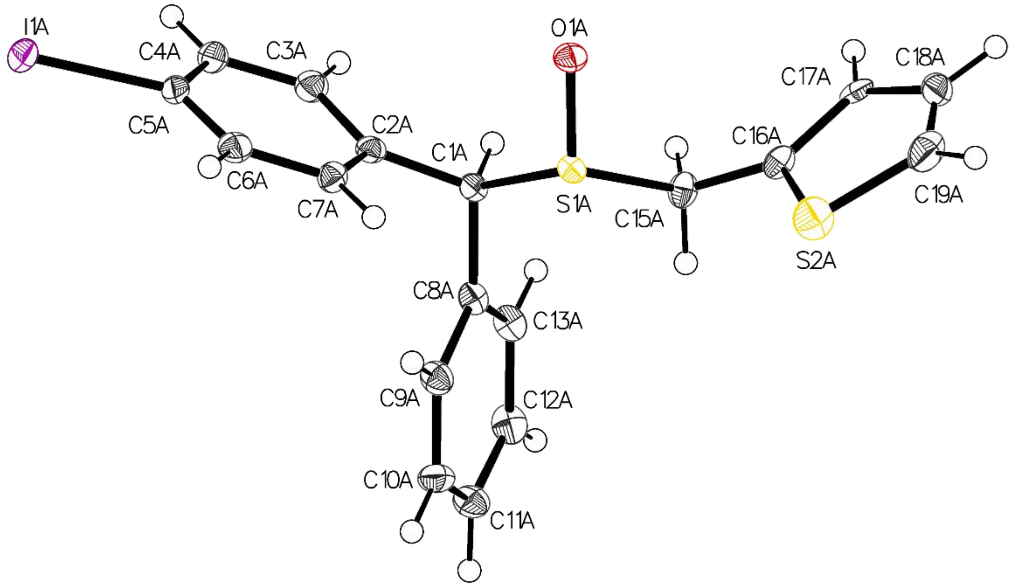

Asymmetric Unit of drawn with 50% displacement ellipsoid. Disorder and second independent molecule omitted for clarity. The bond precision for C-C single bonds is 0.0058 Å. Chirality proofed by Hooft (-0.017(8)) and Flack (-0.020(15)) for S1 (R) and C1(S).

Identification code	**Compound 42**
Empirical formula	$C_{18}H_{15}IOS_2$
Formula weight	438.32
Temperature/K	100.0
Crystal system	monoclinic
Space group	$P2_1$
a/Å	11.0749(2)
b/Å	8.02340(10)
c/Å	19.2284(3)
α/°	90

β/°	94.7476(7)
γ/°	90
Volume/Å^3	1702.74(5)
Z	4
ρ_{calc}g/cm^3	1.710
μ/mm^{-1}	2.124
F(000)	864.0
Crystal size/mm^3	0.2 × 0.2 × 0.15
Radiation	MoKα (λ = 0.71073)
2Θ range for data collection/°	3.69 to 66.43
Index ranges	-16 ≤ h ≤ 16, -11 ≤ k ≤ 12, -29 ≤ l ≤ 26
Reflections collected	20539
Independent reflections	10980 [R_{int} = 0.0310, R_{sigma} = 0.0522]
Data/restraints/parameters	10980/10/398
Goodness-of-fit on F^2	1.021
Final R indexes [I>=2σ (I)]	R_1 = 0.0342, wR_2 = 0.0679
Final R indexes [all data]	R_1 = 0.0449, wR_2 = 0.0714
Largest diff. peak/hole / e Å^{-3}	0.73/-0.66
Flack parameter	-0.017(10)

Compound 43

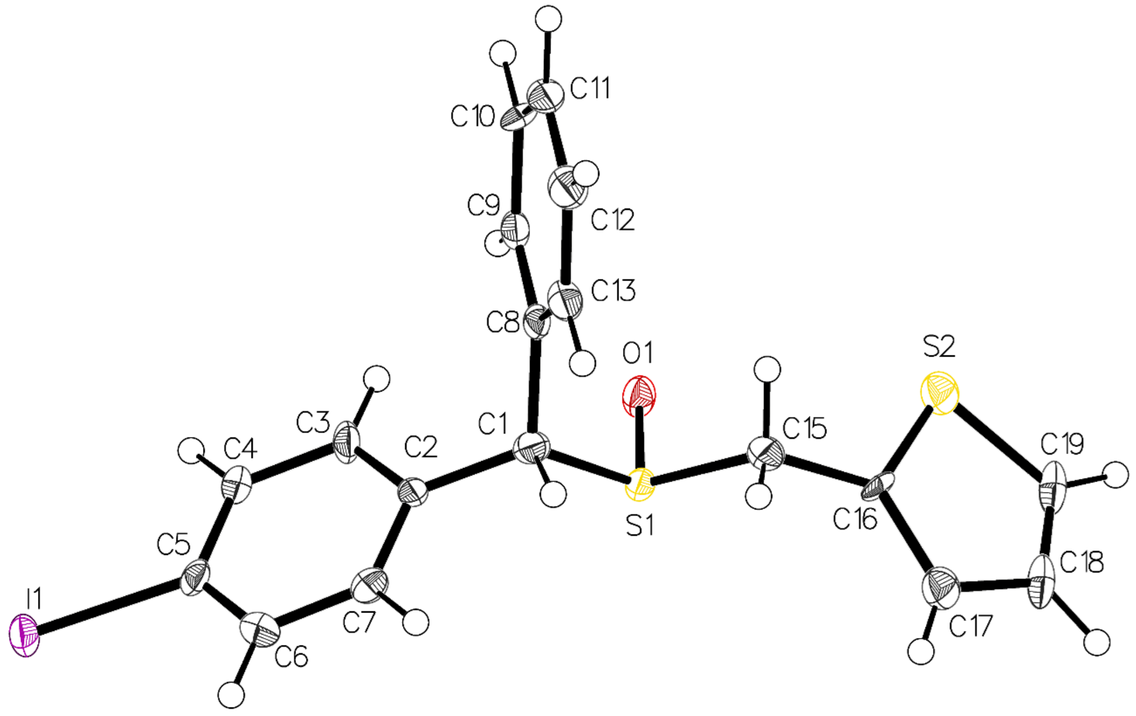

Asymmetric Unit of drawn with 50% displacement ellipsoid. Disorder omitted for clarity. The bond precision for C-C single bonds is 0.0119 Å. Chirality proofed by Hooft (-0.062(16)) and Flack (-0.05(4)) for S1 (S) and C1(S).

Identification code	**Compound 43**
Empirical formula	$C_{18}H_{15}IOS_2$
Formula weight	438.32
Temperature/K	100
Crystal system	orthorhombic
Space group	$P2_12_12_1$
a/Å	5.7190(6)
b/Å	15.8128(17)
c/Å	18.1268(15)

α/°	90
β/°	90
γ/°	90
Volume/Å^3	1639.3(3)
Z	4
ρ_{calc}g/cm^3	1.776
μ/mm^{-1}	2.207
F(000)	864.0
Crystal size/mm^3	0.3 × 0.123 × 0.02
Radiation	Mo Kα (λ = 0.71073)
2Θ range for data collection/°	3.418 to 63.842
Index ranges	-7 ≤ h ≤ 8, -23 ≤ k ≤ 16, -26 ≤ l ≤ 26
Reflections collected	33448
Independent reflections	5476 [R_{int} = 0.1114, R_{sigma} = 0.1391]
Data/restraints/parameters	5476/25/204
Goodness-of-fit on F^2	0.926
Final R indexes [I>=2σ (I)]	R_1 = 0.0568, wR_2 = 0.1276
Final R indexes [all data]	R_1 = 0.0996, wR_2 = 0.1429
Largest diff. peak/hole / e Å^{-3}	1.34/-1.47
Flack parameter	-0.07(4)

Compound 44

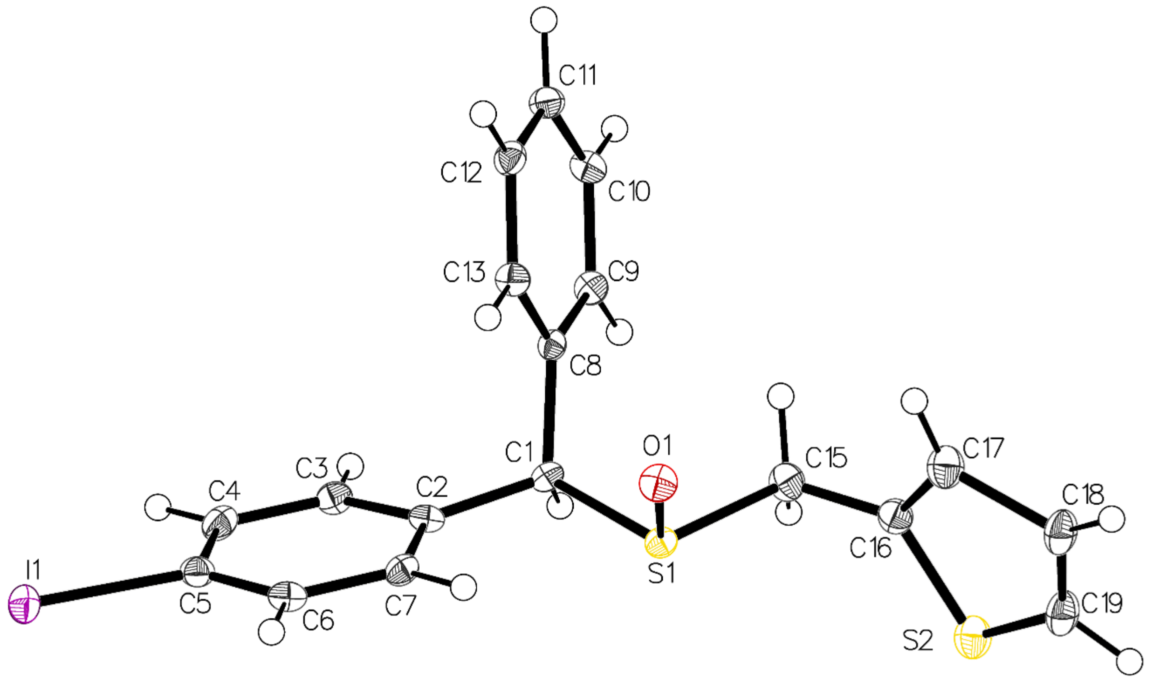

Asymmetric Unit of drawn with 50% displacement ellipsoid. Disorder omitted for clarity. The bond precision for C-C single bonds is 0.0056 Å. Chirality proofed by Hooft (-0.033(12)) and Flack (-0.04(2)) for S1 (R) and C1(R).

Identification code	**Compound 44**
Empirical formula	$C_{18}H_{15}I_{0.97}OS_2$
Formula weight	434.83
Temperature/K	100.0
Crystal system	orthorhombic
Space group	$P2_12_12_1$
a/Å	5.65060(10)
b/Å	11.6257(3)
c/Å	24.9701(7)

α/°	90
β/°	90
γ/°	90
Volume/$Å^3$	1640.34(7)
Z	4
$ρ_{calc}$g/cm^3	1.761
μ/mm^{-1}	2.153
F(000)	858.0
Crystal size/mm^3	0.2 × 0.03 × 0.025
Radiation	MoKα (λ = 0.71073)
2Θ range for data collection/°	3.864 to 61.24
Index ranges	-8 ≤ h ≤ 8, -16 ≤ k ≤ 16, -35 ≤ l ≤ 28
Reflections collected	17037
Independent reflections	5011 [R_{int} = 0.0470, R_{sigma} = 0.0508]
Data/restraints/parameters	5011/9/201
Goodness-of-fit on F^2	1.079
Final R indexes [I>=2σ (I)]	R_1 = 0.0342, wR_2 = 0.0502
Final R indexes [all data]	R_1 = 0.0444, wR_2 = 0.0537
Largest diff. peak/hole / e $Å^{-3}$	0.51/-0.56
Flack parameter	-0.031(13)

Compound 50

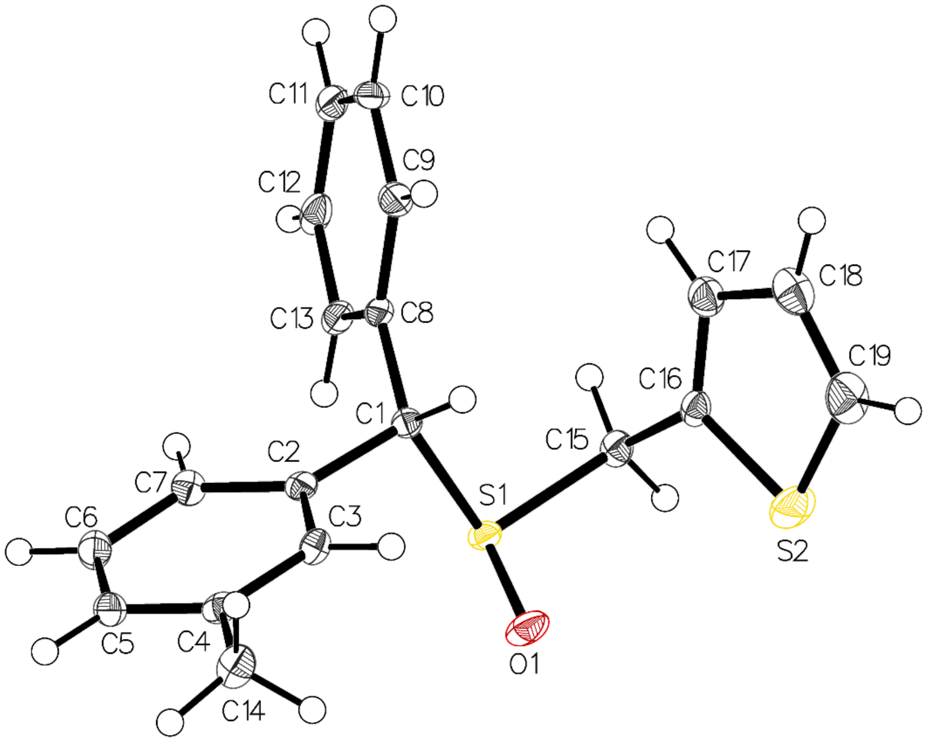

Asymmetric Unit of drawn with 50% displacement ellipsoid. The bond precision for C-C single bonds is 0.0029 Å. Chirality proofed by Hooft (-0.075(14)) and Flack (-0.07(6)) for S1 (R) and C1(S).

Identification code	**Compound 50**
Empirical formula	$C_{19}H_{18}OS_2$
Formula weight	326.45
Temperature/K	100
Crystal system	orthorhombic
Space group	$P2_12_12_1$
a/Å	7.2947(2)
b/Å	13.3002(5)
c/Å	16.7792(6)

α/°	90
β/°	90
γ/°	90
Volume/Å^3	1627.93(10)
Z	4
ρ_{calc}g/cm^3	1.332
μ/mm^{-1}	0.326
F(000)	688.0
Crystal size/mm^3	0.26 × 0.18 × 0.1
Radiation	Mo Kα (λ = 0.71073)
2Θ range for data collection/°	3.908 to 63.316
Index ranges	-10 ≤ h ≤ 10, -19 ≤ k ≤ 19, -24 ≤ l ≤ 24
Reflections collected	68488
Independent reflections	5369 [R_{int} = 0.0547, R_{sigma} = 0.0427]
Data/restraints/parameters	5369/0/200
Goodness-of-fit on F^2	1.067
Final R indexes [I>=2σ (I)]	R_1 = 0.0331, wR_2 = 0.0792
Final R indexes [all data]	R_1 = 0.0499, wR_2 = 0.0854
Largest diff. peak/hole / e Å^{-3}	0.38/-0.59
Flack parameter	-0.07(3)

Compound 51

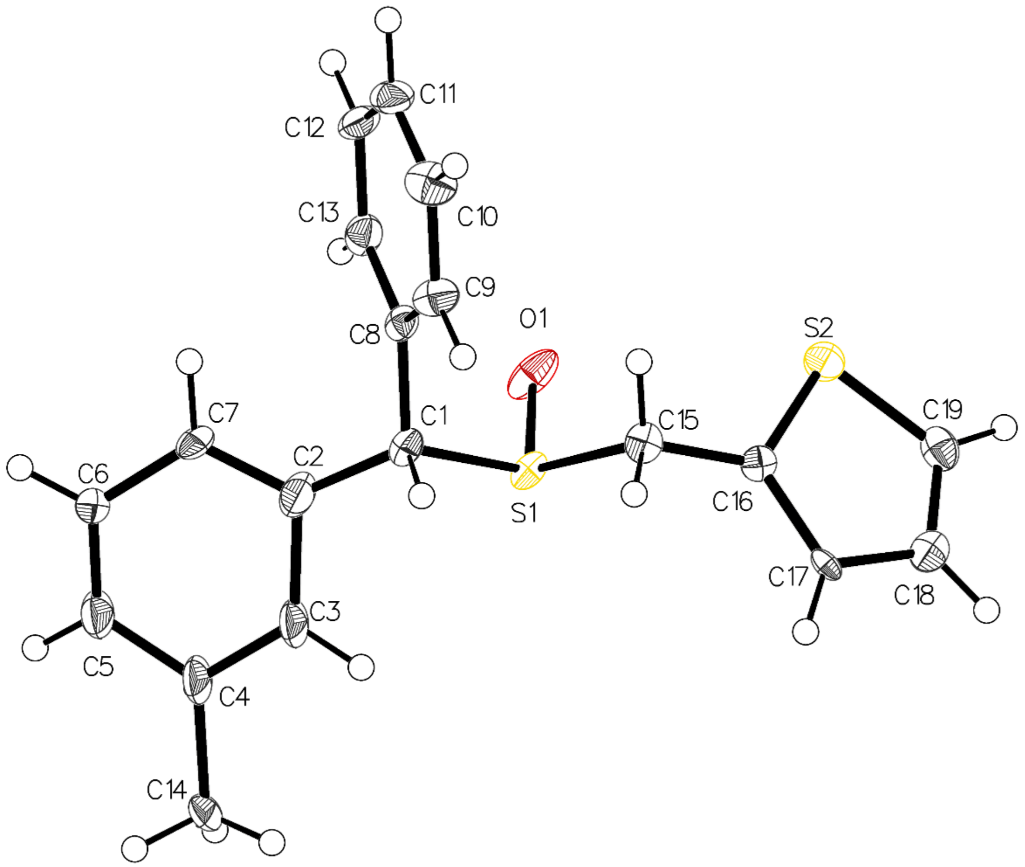

Asymmetric Unit of drawn with 50% displacement ellipsoid. Disorder omitted for clarity. The bond precision for C-C single bonds is 0.0054 Å. Chirality proofed by Hooft (-0.04(4)) and Flack (0.00(14)) for S1 (S) and C1(S). One B-Alert could not be avoided. PLAT220_ALERT_2_B NonSolvent Resd 1 C Ueq(max)/Ueq(min) Range 6.4 Ratio Related to the quality of the crystal and the disorder.

Identification code	**Compound 51**
Empirical formula	$C_{19}H_{18}OS_2$
Formula weight	326.45
Temperature/K	100
Crystal system	orthorhombic

Space group	$P2_12_12_1$
a/Å	5.4801(2)
b/Å	15.7742(5)
c/Å	18.9934(7)
α/°	90
β/°	90
γ/°	90
Volume/Å^3	1641.87(10)
Z	4
ρ_{calc}g/cm^3	1.321
μ/mm^{-1}	0.323
F(000)	688.0
Crystal size/mm^3	0.14 × 0.053 × 0.01
Radiation	Mo Kα (λ = 0.71073)
2Θ range for data collection/°	3.356 to 63.256
Index ranges	-7 ≤ h ≤ 8, -23 ≤ k ≤ 13, -27 ≤ l ≤ 27
Reflections collected	39129
Independent reflections	5308 [R_{int} = 0.0882, R_{sigma} = 0.1329]
Data/restraints/parameters	5308/100/254
Goodness-of-fit on F^2	0.958
Final R indexes [I>=2σ (I)]	R_1 = 0.0534, wR_2 = 0.1098
Final R indexes [all data]	R_1 = 0.1183, wR_2 = 0.1278
Largest diff. peak/hole / e Å^{-3}	0.49/-0.52
Flack parameter	-0.03(7)

Compound 52

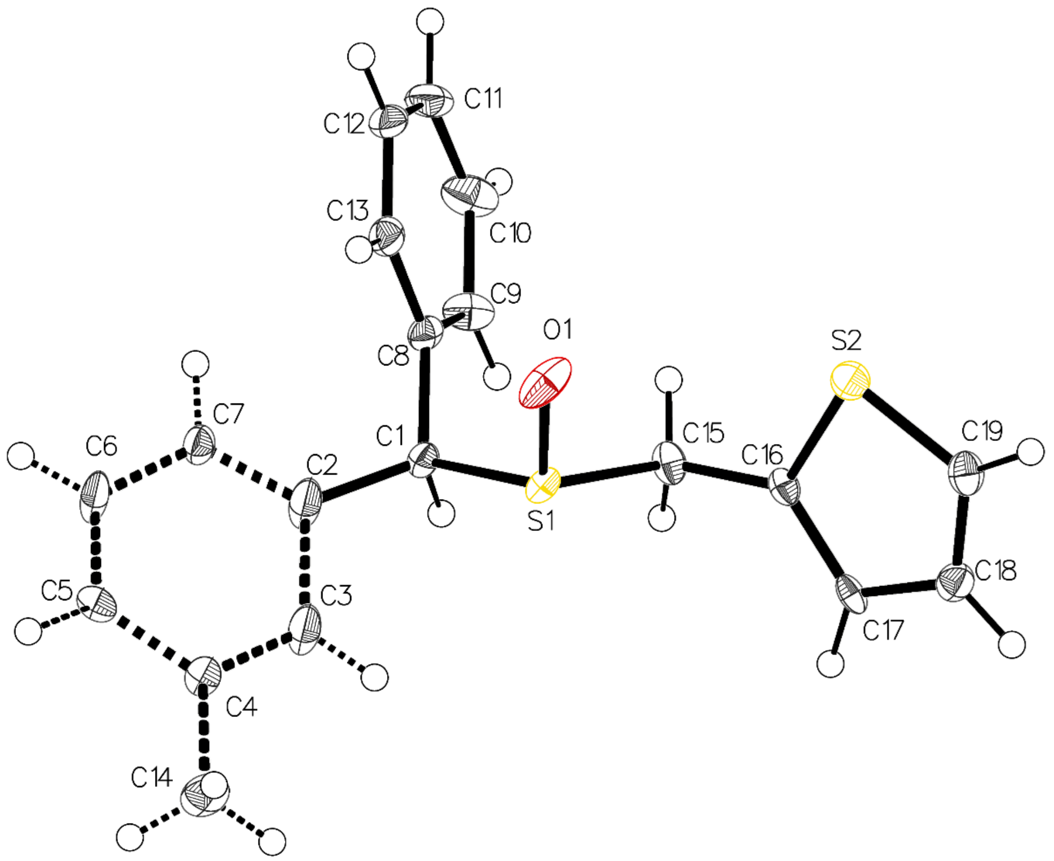

Asymmetric Unit of drawn with 50% displacement ellipsoid. Disorder omitted for clarity. The bond precision for C-C single bonds is 0.0036 Å. Chirality proofed by Hooft (0.12(2)) and Flack (0.1(3)) for S1 (R) and C1(R). Dotted lines because the disorder is splitted in to four parts and negative part numbers because of symmetry reasons.

Identification code	**Compound 52**
Empirical formula	$C_{19}H_{18}OS_2$
Formula weight	326.45
Temperature/K	100.00
Crystal system	orthorhombic
Space group	$P2_12_12_1$

a/Å	5.50580(10)
b/Å	15.7238(3)
c/Å	18.9562(4)
α/°	90
β/°	90
γ/°	90
Volume/Å^3	1641.08(6)
Z	4
ρ_{calc}g/cm^3	1.321
μ/mm^{-1}	0.323
F(000)	688.0
Crystal size/mm^3	0.1 × 0.1 × 0.08
Radiation	MoKα (λ = 0.71073)
2Θ range for data collection/°	4.298 to 60.232
Index ranges	-7 ≤ h ≤ 7, -18 ≤ k ≤ 22, -26 ≤ l ≤ 26
Reflections collected	51946
Independent reflections	4824 [R_{int} = 0.0465, R_{sigma} = 0.0228]
Data/restraints/parameters	4824/28/308
Goodness-of-fit on F^2	1.132
Final R indexes [I>=2σ (I)]	R_1 = 0.0364, wR_2 = 0.0880
Final R indexes [all data]	R_1 = 0.0386, wR_2 = 0.0893
Largest diff. peak/hole / e Å^{-3}	0.40/-0.35
Flack parameter	0.01(2)

Compound 55

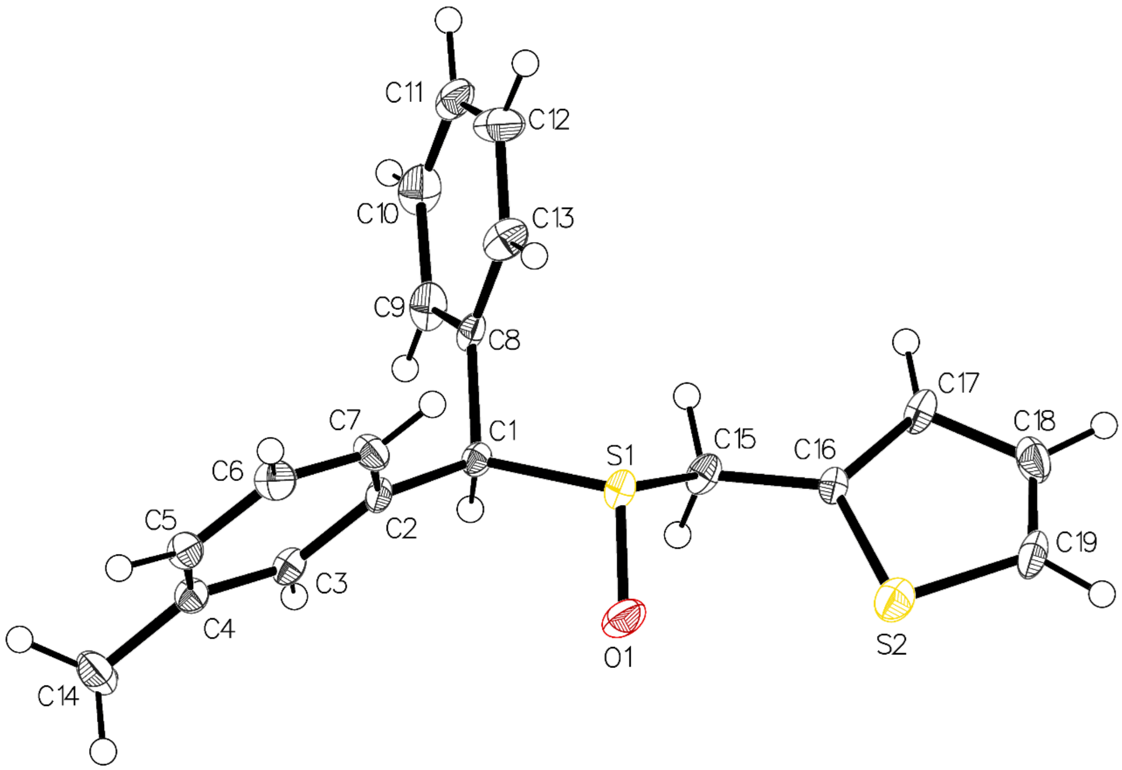

Asymmetric Unit of drawn with 50% displacement ellipsoid. The bond precision for C-C single bonds is 0.0057 Å. Chirality proofed by Hooft (-0.31(4)) and Flack (-0.04(10)) for S1 (S) and C1(R).

Identification code	**Compound 55**
Empirical formula	$C_{19}H_{18}OS_2$
Formula weight	326.45
Temperature/K	100
Crystal system	triclinic
Space group	P1
a/Å	6.4484(6)
b/Å	7.8771(8)
c/Å	9.0594(9)

α/°	109.287(8)
β/°	103.263(8)
γ/°	95.749(8)
Volume/Å^3	414.93(7)
Z	1
ρ_{calc}g/cm^3	1.306
μ/mm^{-1}	0.320
F(000)	172.0
Crystal size/mm^3	0.35 × 0.35 × 0.35
Radiation	Mo Kα (λ = 0.71073)
2Θ range for data collection/°	5.582 to 62.722
Index ranges	-9 ≤ h ≤ 9, -11 ≤ k ≤ 11, -13 ≤ l ≤ 12
Reflections collected	9582
Independent reflections	4636 [R_{int} = 0.0329, R_{sigma} = 0.1052]
Data/restraints/parameters	4636/3/200
Goodness-of-fit on F^2	0.969
Final R indexes [I>=2σ (I)]	R_1 = 0.0453, wR_2 = 0.0968
Final R indexes [all data]	R_1 = 0.0572, wR_2 = 0.0985
Largest diff. peak/hole / e Å^{-3}	0.43/-0.42
Flack parameter	-0.03(10)

Compound 56

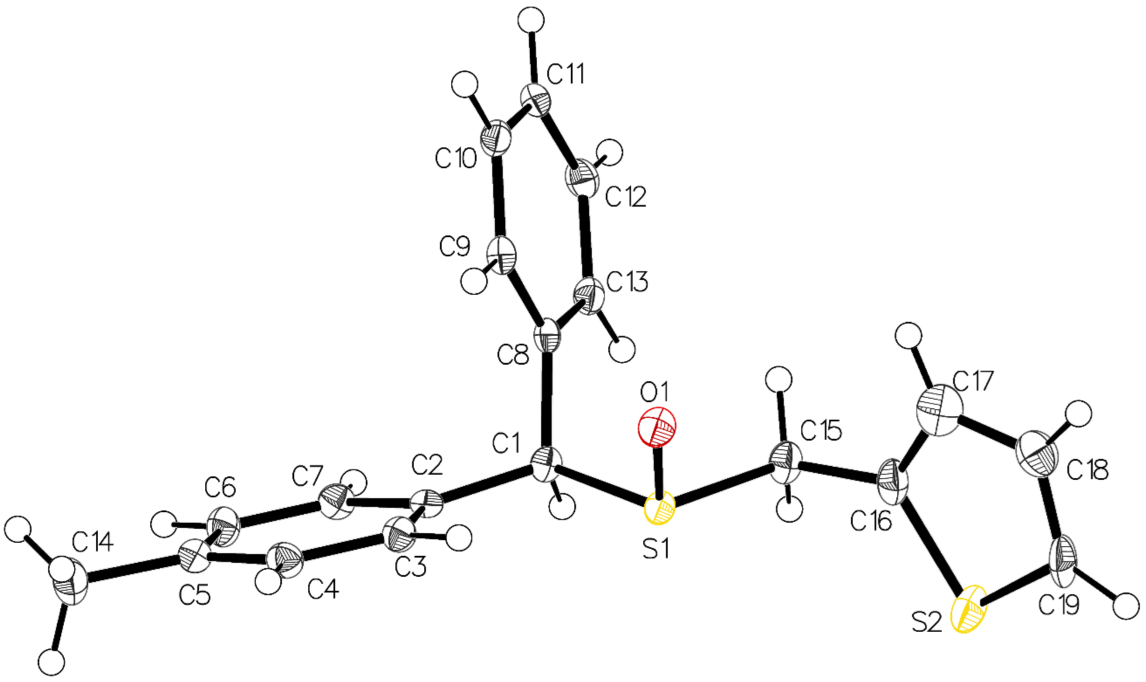

Asymmetric Unit of drawn with 50% displacement ellipsoid. Disorder omitted for clarity. The bond precision for C-C single bonds is 0.0030 Å. Chirality proofed by Hooft (-0.028(6)) and Flack (-0.004(19)) for S1 (R) and C1(R).

Identification code	**Compound 56**
Empirical formula	$C_{19}H_{18}OS_2$
Formula weight	326.45
Temperature/K	100
Crystal system	monoclinic
Space group	$P2_1$
a/Å	11.0717(5)
b/Å	17.9821(5)
c/Å	16.7379(5)
α/°	90

β/°	91.243(3)
γ/°	90
Volume/Å^3	3331.6(2)
Z	8
ρ_{calc}g/cm^3	1.302
μ/mm^{-1}	0.318
F(000)	1376.0
Crystal size/mm^3	0.32 × 0.147 × 0.02
Radiation	Mo Kα (λ = 0.71073)
2Θ range for data collection/°	2.434 to 51.362
Index ranges	-13 ≤ h ≤ 13, -21 ≤ k ≤ 21, -20 ≤ l ≤ 20
Reflections collected	60594
Independent reflections	12564 [R_{int} = 0.0677, R_{sigma} = 0.0789]
Data/restraints/parameters	12564/1/798
Goodness-of-fit on F^2	1.491
Final R indexes [I>=2σ (I)]	R_1 = 0.1248, wR_2 = 0.3441
Final R indexes [all data]	R_1 = 0.1760, wR_2 = 0.3885
Largest diff. peak/hole / e Å^{-3}	1.58/-0.83
Flack parameter	0.14(5)

Compound 70

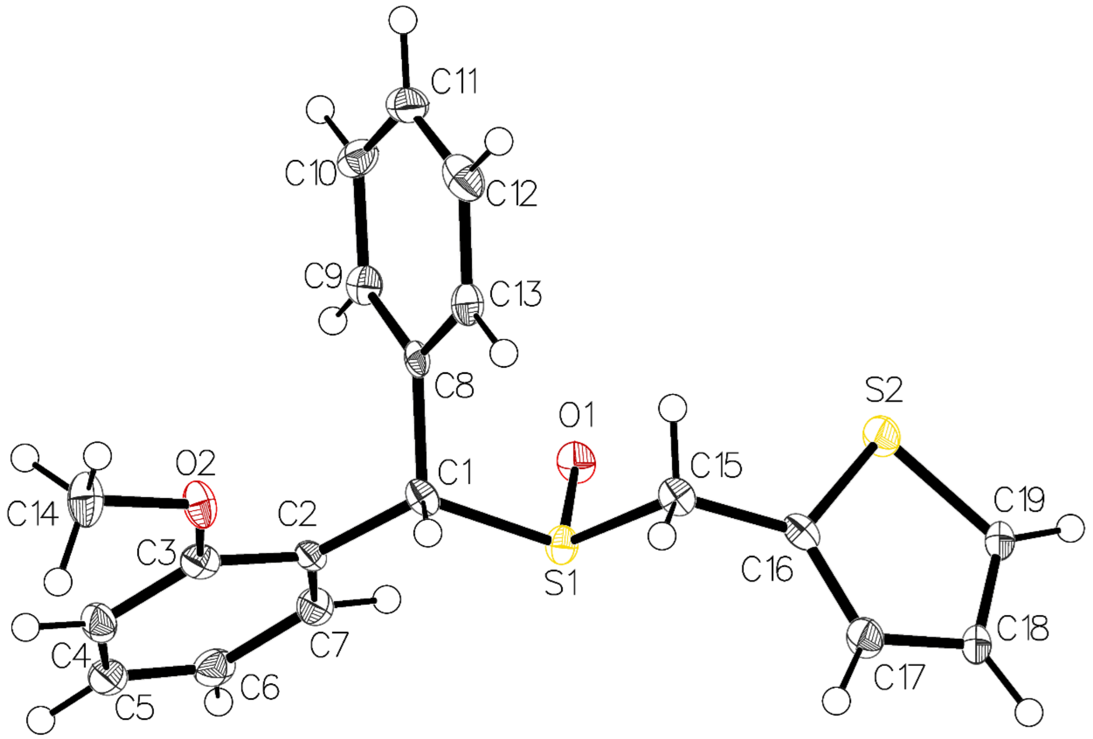

Asymmetric Unit of drawn with 50% displacement ellipsoid. Disorder omitted for clarity. The bond precision for C-C single bonds is 0.0052 Å. Chirality proofed by Hooft (0.003(5)) and Flack (-0.07(10)) for S1 (S) and C1(S).

Identification code	**Compound 70**
Empirical formula	$C_{19}H_{18}O_2S_2$
Formula weight	342.45
Temperature/K	100
Crystal system	orthorhombic
Space group	$P2_12_12_1$
a/Å	5.2845(5)
b/Å	11.8566(14)

c/Å	26.620(2)
α/°	90
β/°	90
γ/°	90
Volume/$Å^3$	1667.9(3)
Z	4
ρ_{calc}g/cm^3	1.364
μ/mm^{-1}	0.326
F(000)	720.0
Crystal size/mm^3	0.4 × 0.2 × 0.1
Radiation	Mo Kα (λ = 0.71073)
2Θ range for data collection/°	3.76 to 63.678
Index ranges	-7 ≤ h ≤ 7, -17 ≤ k ≤ 17, -37 ≤ l ≤ 38
Reflections collected	40306
Independent reflections	5430 [R_{int} = 0.1243, R_{sigma} = 0.2388]
Data/restraints/parameters	5430/8/210
Goodness-of-fit on F^2	0.855
Final R indexes [I>=2σ (I)]	R_1 = 0.0497, wR_2 = 0.0908
Final R indexes [all data]	R_1 = 0.1220, wR_2 = 0.1009
Largest diff. peak/hole / e $Å^{-3}$	0.60/-0.57
Flack parameter	0.06(7)

Compound 71

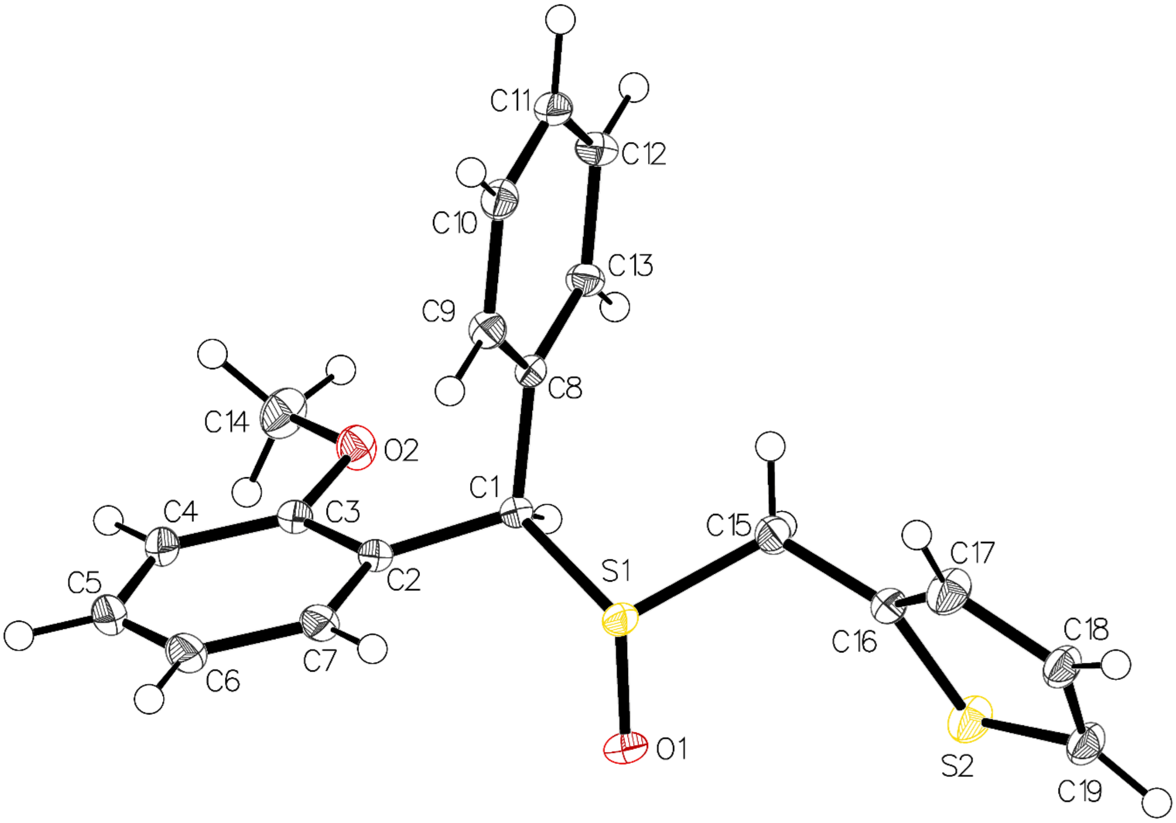

Asymmetric Unit of drawn with 50% displacement ellipsoid. Disorder omitted for clarity. The bond precision for C-C single bonds is 0.0017 Å. Chirality proofed by Hooft (0.0017(6)) and Flack (-0.01(4)) for S1 (S) and C1(R).

Identification code	**Compound 71**
Empirical formula	$C_{19}H_{18}O_2S_2$
Formula weight	342.45
Temperature/K	100.0
Crystal system	triclinic
Space group	P1
a/Å	6.0892(4)

b/Å	8.5758(5)
c/Å	8.8168(5)
α/°	68.282(2)
β/°	76.777(2)
γ/°	84.821(2)
Volume/$Å^3$	416.38(4)
Z	1
ρ_{calc}g/cm^3	1.366
μ/mm^{-1}	0.326
F(000)	180.0
Crystal size/mm^3	0.25 × 0.22 × 0.15
Radiation	MoKα (λ = 0.71073)
2Θ range for data collection/°	5.088 to 66.502
Index ranges	-9 ≤ h ≤ 9, -13 ≤ k ≤ 13, -13 ≤ l ≤ 13
Reflections collected	94774
Independent reflections	6347 [R_{int} = 0.0278, R_{sigma} = 0.0113]
Data/restraints/parameters	6347/12/210
Goodness-of-fit on F^2	1.062
Final R indexes [I>=2σ (I)]	R_1 = 0.0213, wR_2 = 0.0599
Final R indexes [all data]	R_1 = 0.0214, wR_2 = 0.0600
Largest diff. peak/hole / e $Å^{-3}$	0.34/-0.23
Flack parameter	0.018(6)

Compound 72

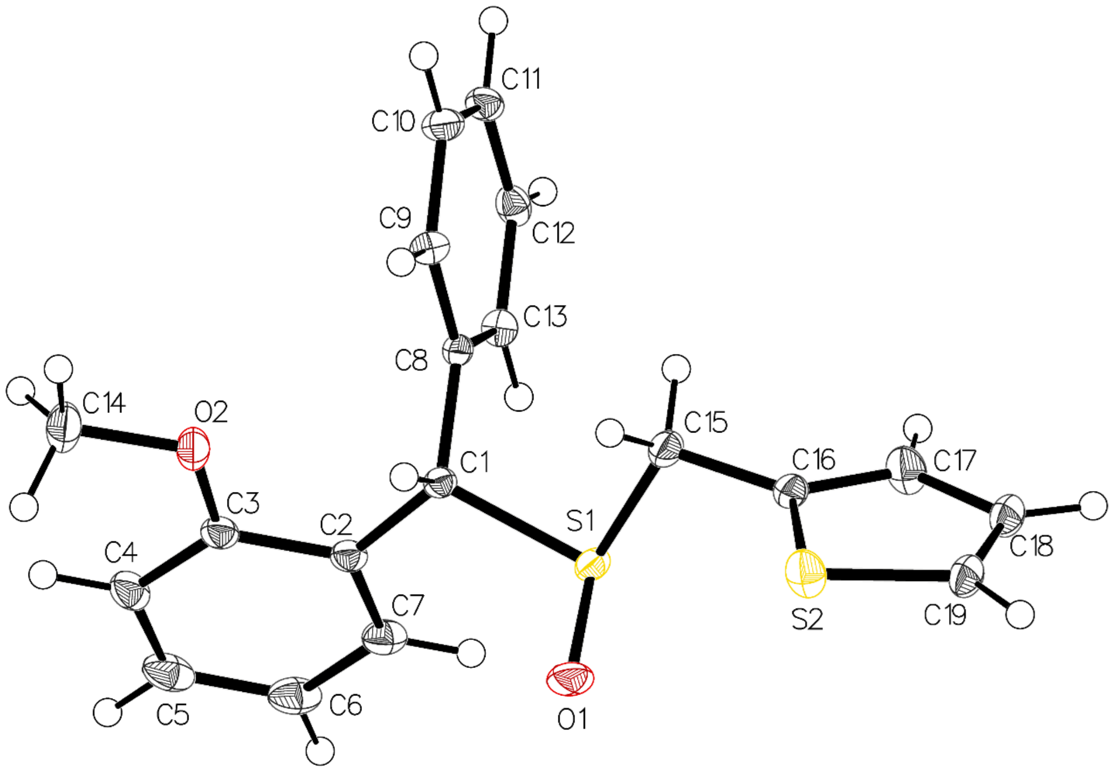

Asymmetric Unit of drawn with 50% displacement ellipsoid. Disorder omitted for clarity. The bond precision for C-C single bonds is 0.0023 Å. Chirality proofed by Hooft (0.001(14)) and Flack (-0.00(5)) for S1 (R) and C1(S).

Identification code	**Compound 72**
Empirical formula	$C_{19}H_{18}O_2S_2$
Formula weight	342.45
Temperature/K	100.0
Crystal system	triclinic
Space group	P1
a/Å	6.0884(2)
b/Å	8.5758(3)

c/Å	8.8177(3)
α/°	68.2567(12)
β/°	76.8362(13)
γ/°	84.7772(16)
Volume/$Å^3$	416.39(2)
Z	1
ρ_{calc}g/cm^3	1.366
μ/mm^{-1}	0.326
F(000)	180.0
Crystal size/mm^3	0.2 × 0.18 × 0.15
Radiation	MoKα (λ = 0.71073)
2Θ range for data collection/°	5.086 to 66.43
Index ranges	-9 ≤ h ≤ 9, -13 ≤ k ≤ 13, -13 ≤ l ≤ 13
Reflections collected	30265
Independent reflections	6391 [R_{int} = 0.0327, R_{sigma} = 0.0285]
Data/restraints/parameters	6391/12/210
Goodness-of-fit on F^2	1.036
Final R indexes [I>=2σ (I)]	R_1 = 0.0286, wR_2 = 0.0697
Final R indexes [all data]	R_1 = 0.0316, wR_2 = 0.0713
Largest diff. peak/hole / e $Å^{-3}$	0.29/-0.24
Flack parameter	0.006(17)

Compound 77

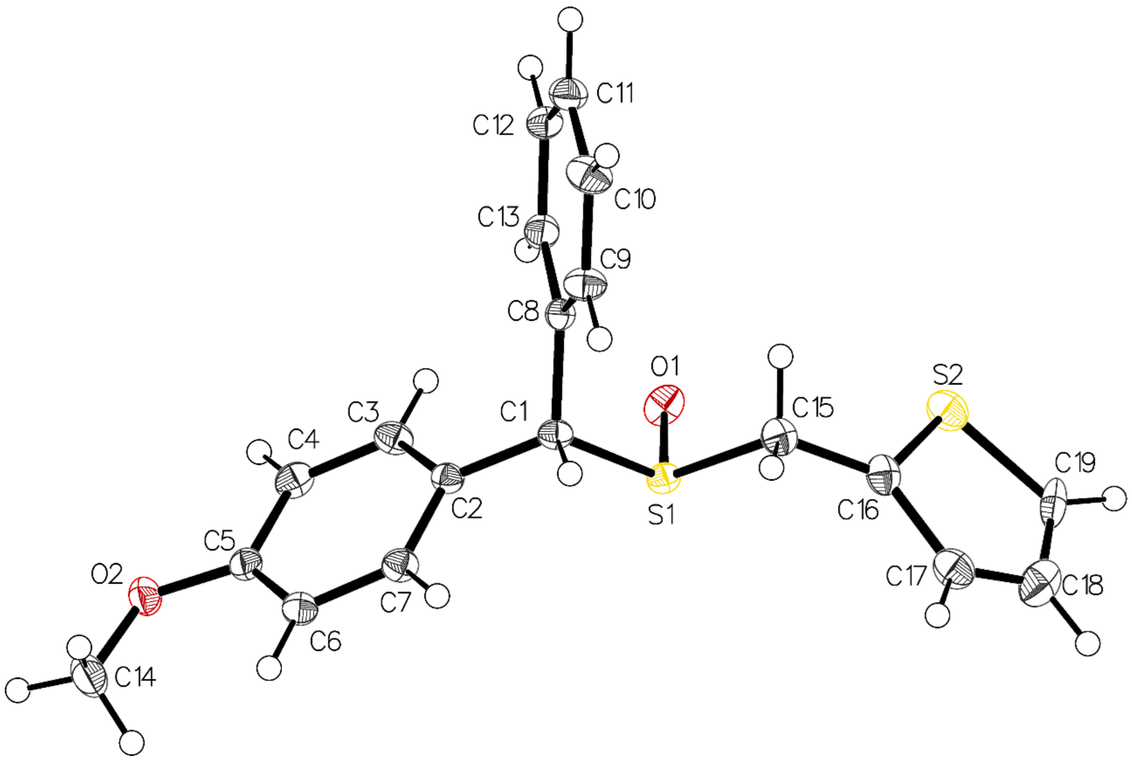

Asymmetric Unit of drawn with 50% displacement ellipsoid. Disorder omitted for clarity. The bond precision for C-C single bonds is 0.0039 Å. Chirality proofed by Hooft (0.11(4)) and Flack (-0.09(10)) for S1 (S) and C1(S).

Identification code	**Compound 77**
Empirical formula	$C_{19}H_{18}O_2S_2$
Formula weight	342.45
Temperature/K	100.00
Crystal system	orthorhombic
Space group	$P2_12_12_1$
a/Å	5.71790(10)
b/Å	15.7296(4)
c/Å	18.1883(4)

α/°	90
β/°	90
γ/°	90
Volume/Å^3	1635.86(6)
Z	4
ρ_{calc}g/cm^3	1.390
μ/mm^{-1}	0.332
F(000)	720.0
Crystal size/mm^3	0.22 × 0.05 × 0.02
Radiation	MoKα (λ = 0.71073)
2Θ range for data collection/°	4.478 to 60.212
Index ranges	-8 ≤ h ≤ 8, -19 ≤ k ≤ 22, -25 ≤ l ≤ 25
Reflections collected	21282
Independent reflections	4771 [R_{int} = 0.0614, R_{sigma} = 0.0512]
Data/restraints/parameters	4771/9/228
Goodness-of-fit on F^2	1.029
Final R indexes [I>=2σ (I)]	R_1 = 0.0405, wR_2 = 0.0807
Final R indexes [all data]	R_1 = 0.0566, wR_2 = 0.0891
Largest diff. peak/hole / e Å^{-3}	0.27/-0.36
Flack parameter	-0.11(4)

Compound 79

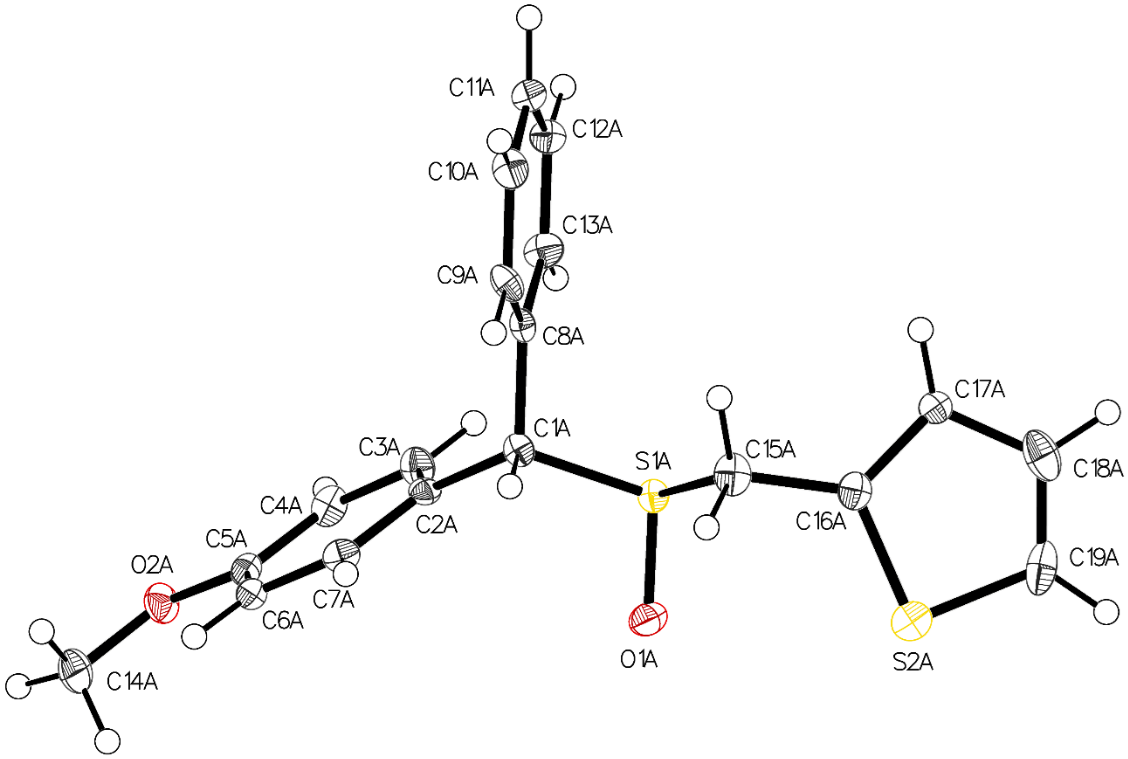

Asymmetric Unit of drawn with 50% displacement ellipsoid. Disorder and second independent molecule omitted for clarity. The bond precision for C-C single bonds is 0.0117 Å. Chirality proofed by Hooft (0.05(6)) and Flack (-0.03(14)) for S1 (S) and C1(R).

Identification code	**Compound 79**
Empirical formula	$C_{19}H_{18}O_2S_2$
Formula weight	342.45
Temperature/K	100.0
Crystal system	triclinic
Space group	P1
a/Å	5.5462(11)
b/Å	9.337(2)
c/Å	16.917(4)

α/°	98.813(7)
β/°	96.293(7)
γ/°	105.117(7)
Volume/Å^3	825.4(3)
Z	2
ρ_{calc}g/cm^3	1.378
μ/mm^{-1}	0.329
F(000)	360.0
Crystal size/mm^3	0.05 × 0.03 × 0.02
Radiation	MoKα (λ = 0.71073)
2Θ range for data collection/°	4.602 to 50.698
Index ranges	-6 ≤ h ≤ 6, -11 ≤ k ≤ 11, -20 ≤ l ≤ 20
Reflections collected	30893
Independent reflections	6039 [R_{int} = 0.1332, R_{sigma} = 0.0982]
Data/restraints/parameters	6039/16/424
Goodness-of-fit on F^2	1.007
Final R indexes [I>=2σ (I)]	R_1 = 0.0594, wR_2 = 0.1334
Final R indexes [all data]	R_1 = 0.0879, wR_2 = 0.1507
Largest diff. peak/hole / e Å^{-3}	0.26/-0.51
Flack parameter	0.05(7)

Compound 80

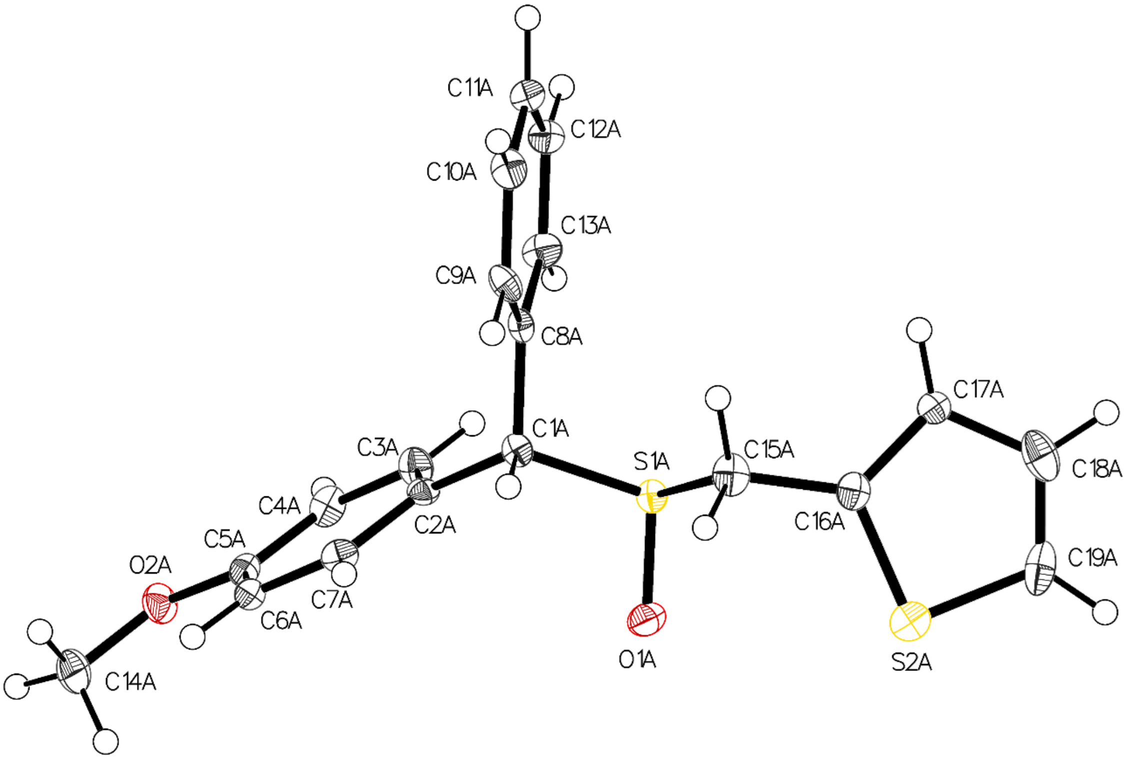

Asymmetric Unit of drawn with 50% displacement ellipsoid. Disorder and second independent molecule omitted for clarity. The bond precision for C-C single bonds is 0.0072 Å. Chirality proofed by Hooft (0.06(5)) and Flack (-0.07(9)) for S1 (R) and C1(S).

Identification code	**Compound 80**
Empirical formula	$C_{19}H_{18}O_2S_2$
Formula weight	342.45
Temperature/K	100.0
Crystal system	triclinic
Space group	P1
a/Å	5.5529(3)

b/Å	9.3431(6)
c/Å	16.9463(11)
α/°	98.786(3)
β/°	96.278(3)
γ/°	105.206(3)
Volume/$Å^3$	828.10(9)
Z	2
ρ_{calc}g/cm^3	1.373
μ/mm^{-1}	0.328
F(000)	360.0
Crystal size/mm^3	0.15 × 0.1 × 0.05
Radiation	MoKα (λ = 0.71073)
2Θ range for data collection/°	4.6 to 52.018
Index ranges	-6 ≤ h ≤ 6, -11 ≤ k ≤ 11, -20 ≤ l ≤ 20
Reflections collected	17255
Independent reflections	6474 [R_{int} = 0.0554, R_{sigma} = 0.0754]
Data/restraints/parameters	6474/18/418
Goodness-of-fit on F^2	1.028
Final R indexes [I>=2σ (I)]	R_1 = 0.0469, wR_2 = 0.0863
Final R indexes [all data]	R_1 = 0.0563, wR_2 = 0.0926
Largest diff. peak/hole / e $Å^{-3}$	0.27/-0.29
Flack parameter	0.03(5)

[i] Bruker SAINT v8.38B & v8.37A Copyright © 2005-2019 Bruker AXS

[ii] Sheldrick, G. M. (1996). *SADABS.* University of Göttingen, Germany.

[iii] X-Area Integrate 2.5.3.0 (STOE, 2021)

[iv] X-Area LANA 2.7.5.0 (STOE, 2022)

[v] Dolomanov, O.V., Bourhis, L.J., Gildea, R.J, Howard, J.A.K. & Puschmann, H., OLEX2, (2009), J. Appl. Cryst. 42, 339-341

[vi] C. B. Huebschle, G. M. Sheldrick and B. Dittrich, ShelXle: a Qt graphical user interface for SHELXL, J. Appl. Cryst., 44, (2011) 1281-1284

[vii] Sheldrick, G. M. (2015). *SHELXS v 2016/4* University of Göttingen, Germany.

[viii] Sheldrick, G. M. (2015). *SHELXL v 2016/4* University of Göttingen, Germany.

[ix] A. L. Spek, Acta Cryst. 2009, D65, 148-155.

References

(1) Parsons, S.; Flack, H. D.; Wagner, T. Use of Intensity Quotients and Differences in Absolute Structure Refinement. *Acta Crystallogr. Sect. B Struct. Sci. Cryst. Eng. Mater.* **2013**, *69* (3), 249–259. https://doi.org/10.1107/S2052519213010014.

5a

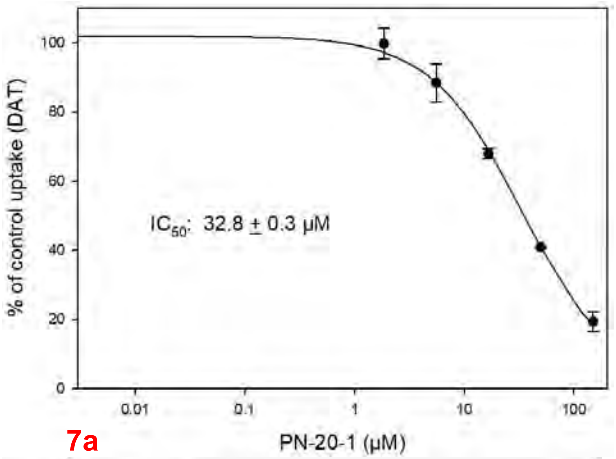

6a

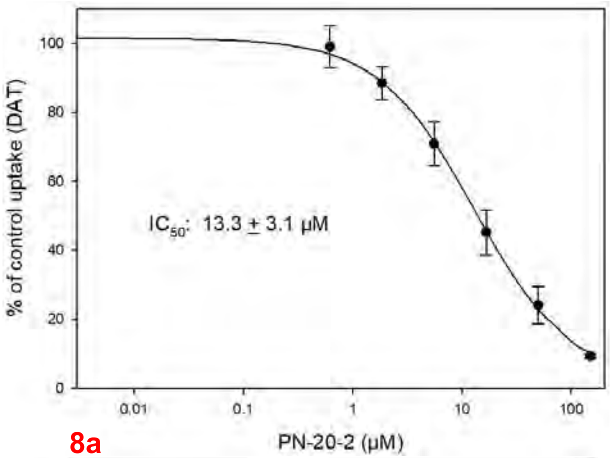

7a

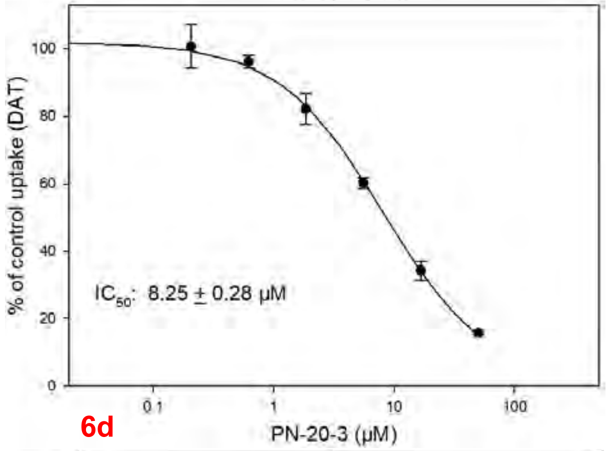

8a

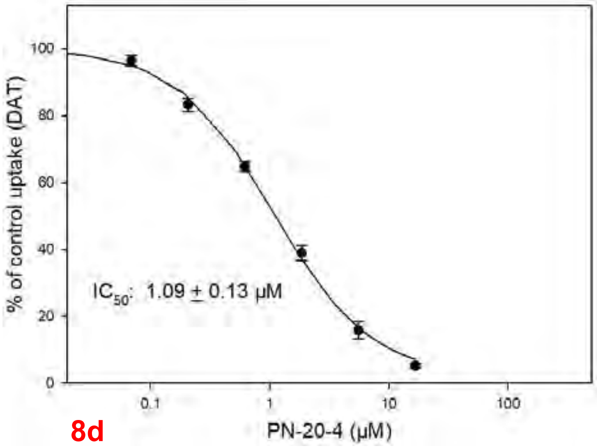

6d

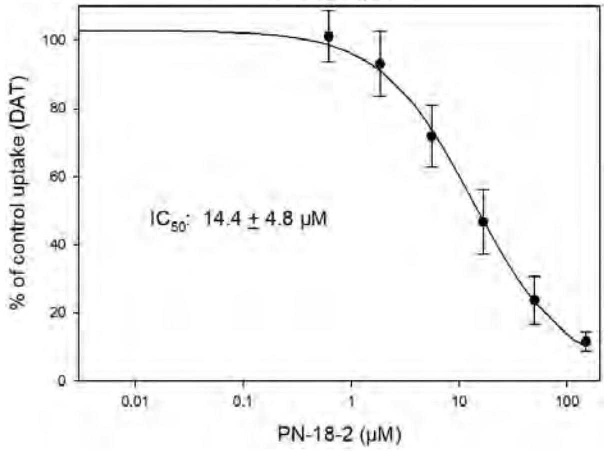

8d

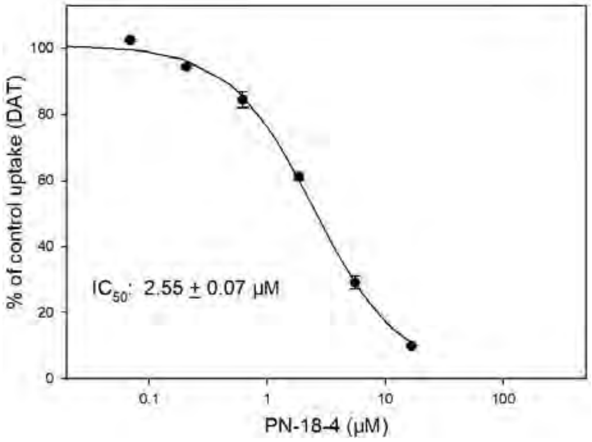

6g

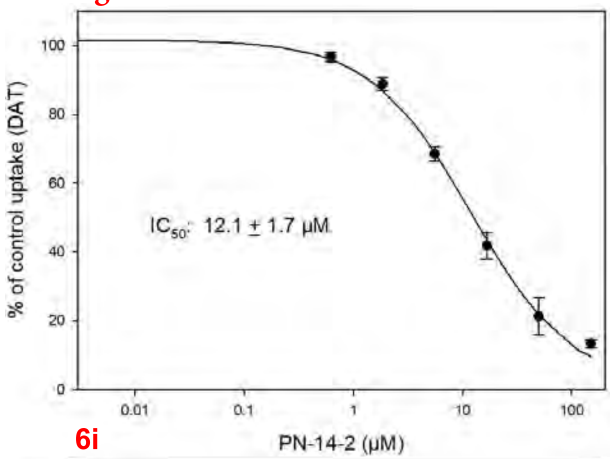

8g

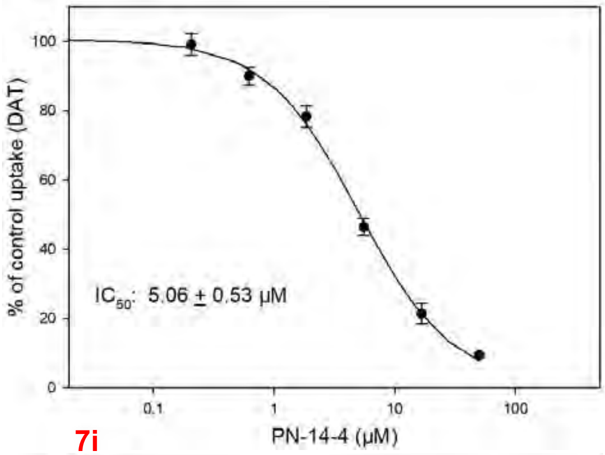

6i

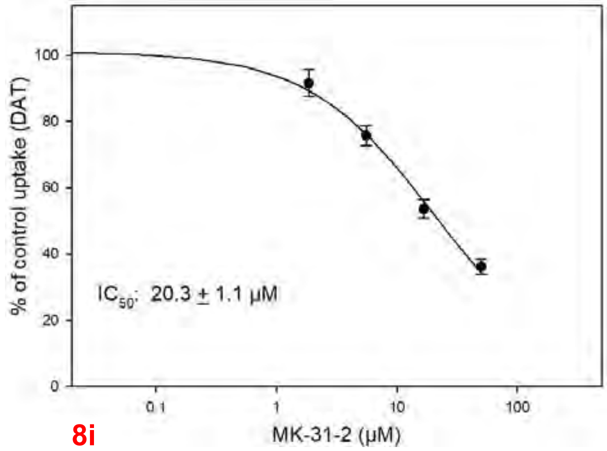

7i

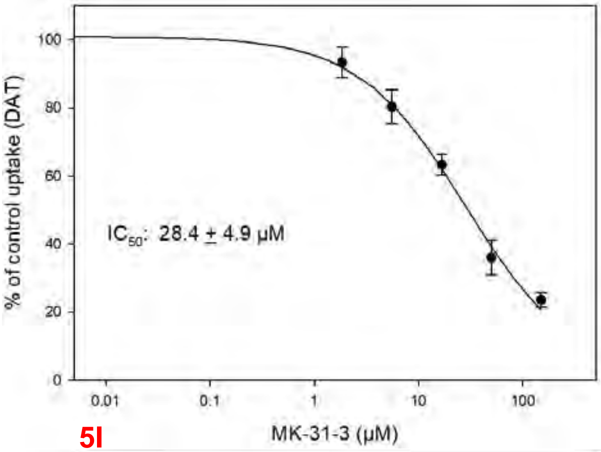

8i

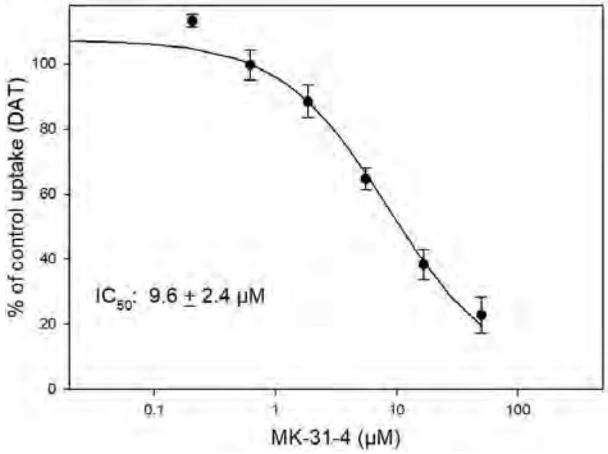

5l

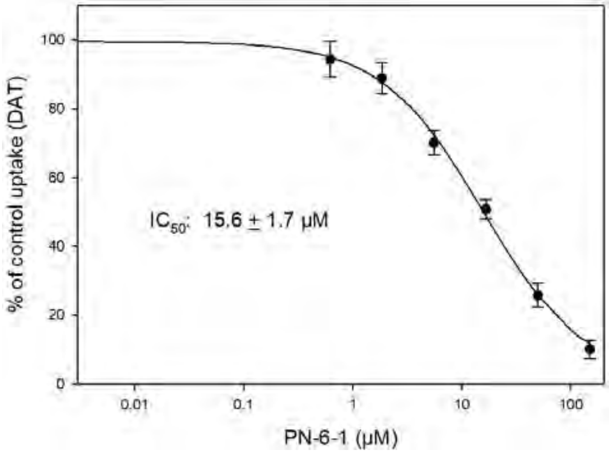

7l

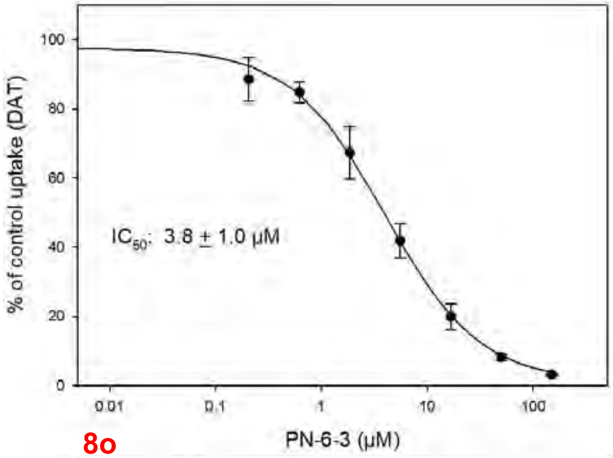

8l

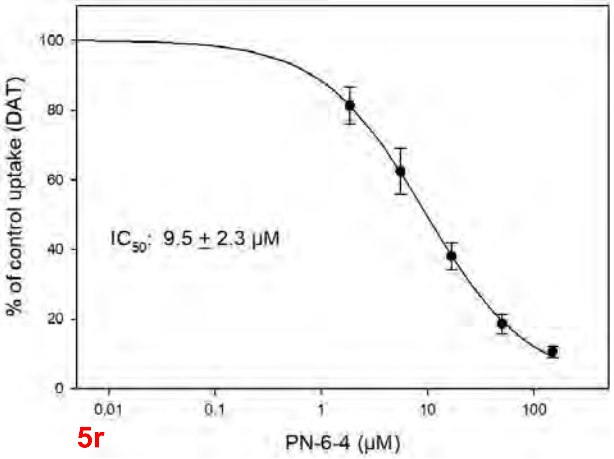

8o

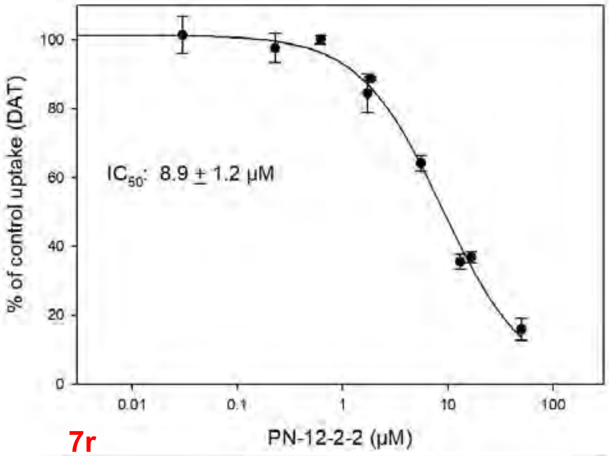

5r

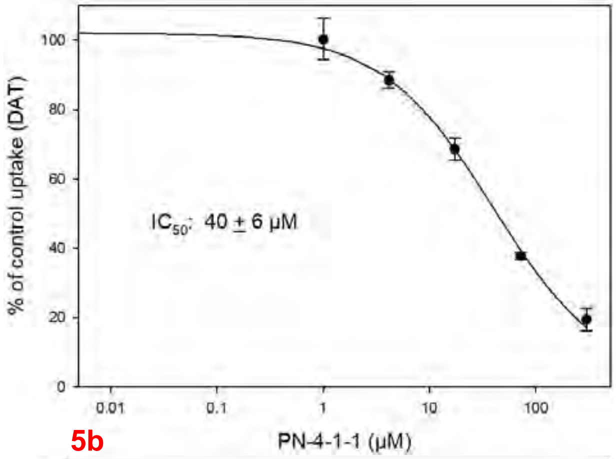

7r

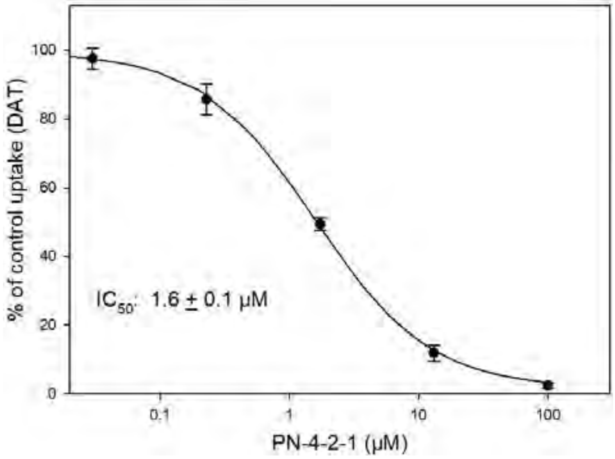

5b

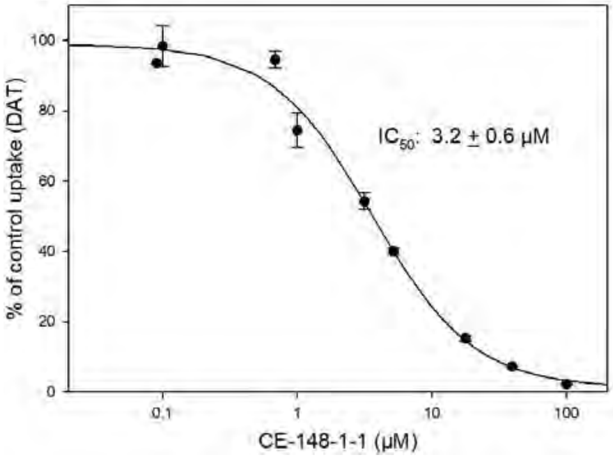

6b

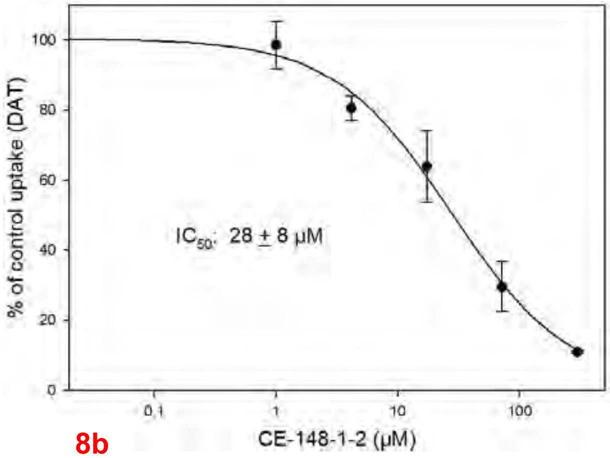

7b

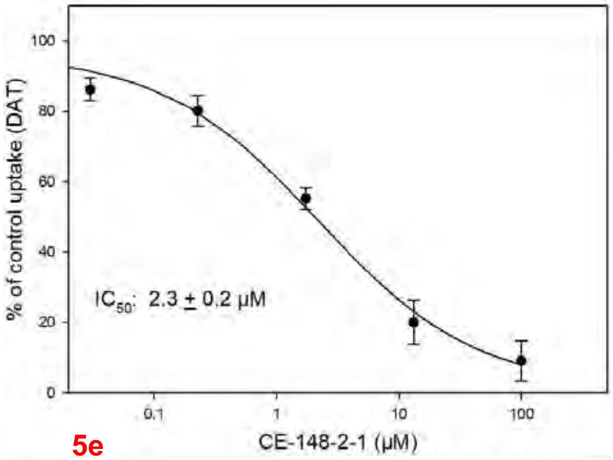

8b

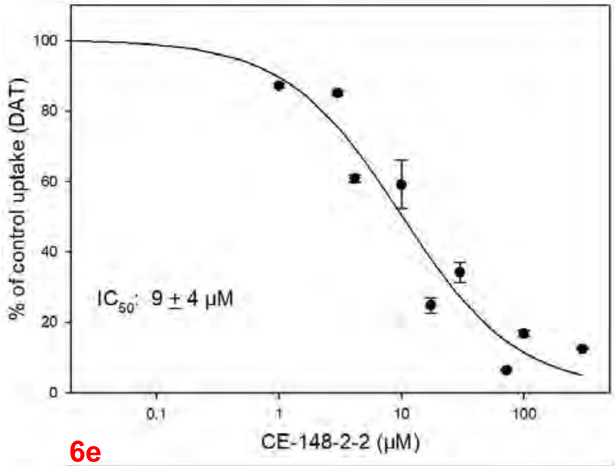

5e

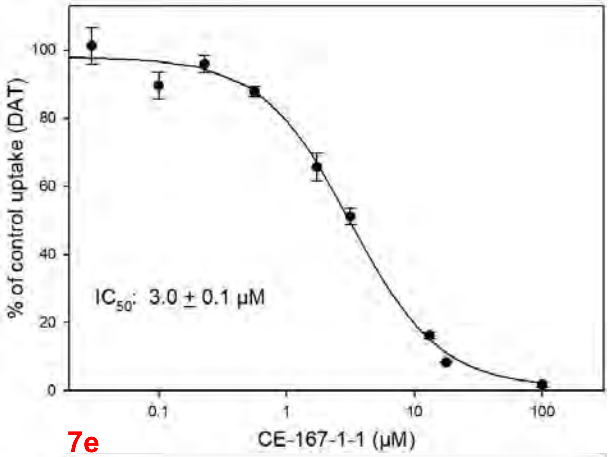

6e

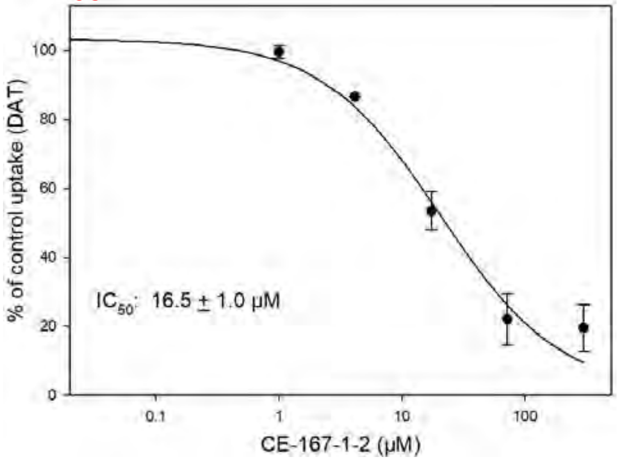

7e

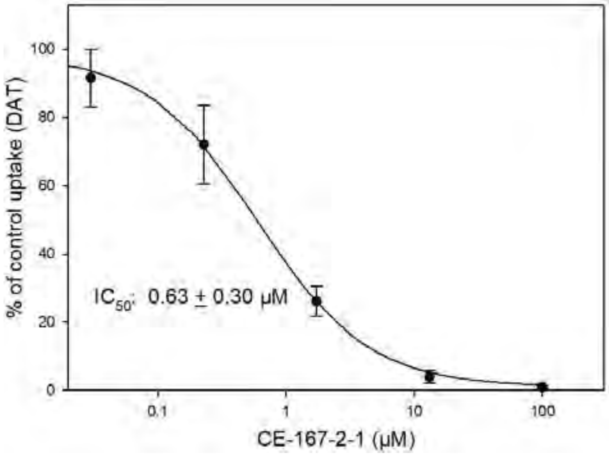

8e

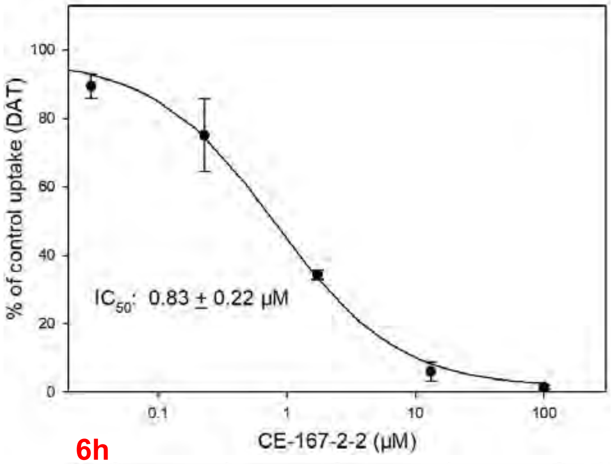

5h

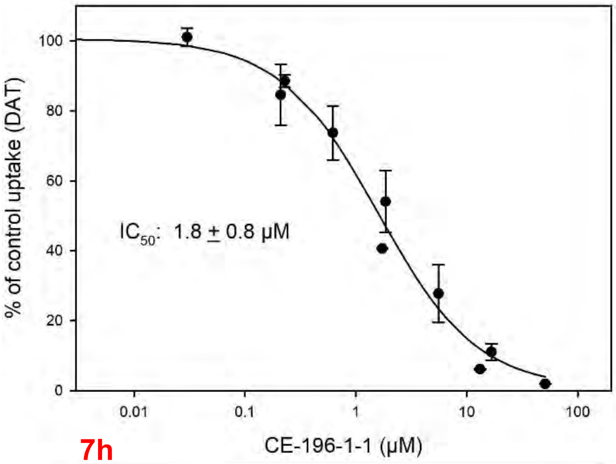

6h

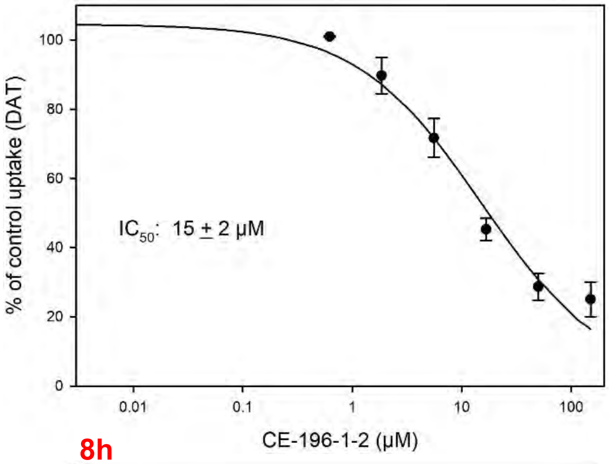

7h

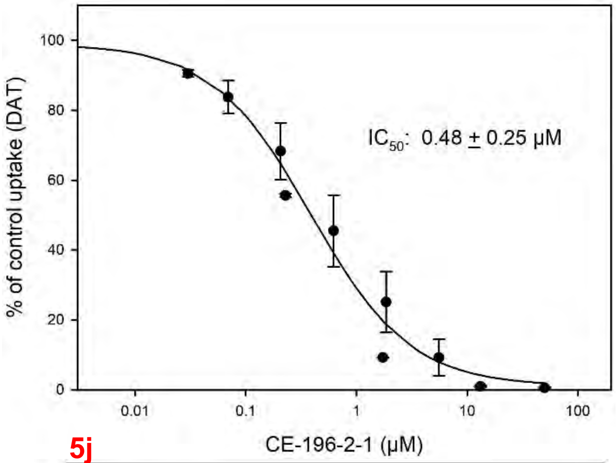

8h

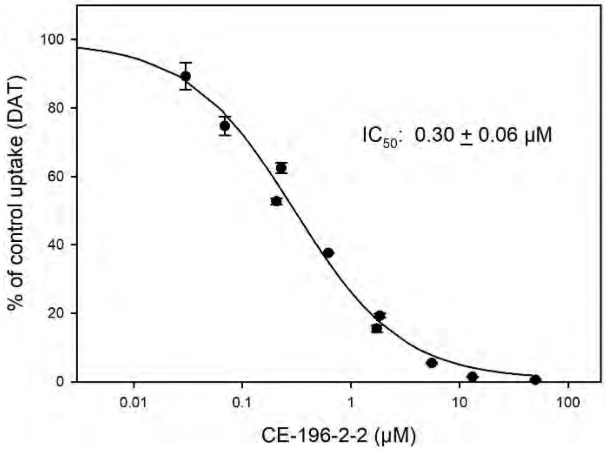

5j

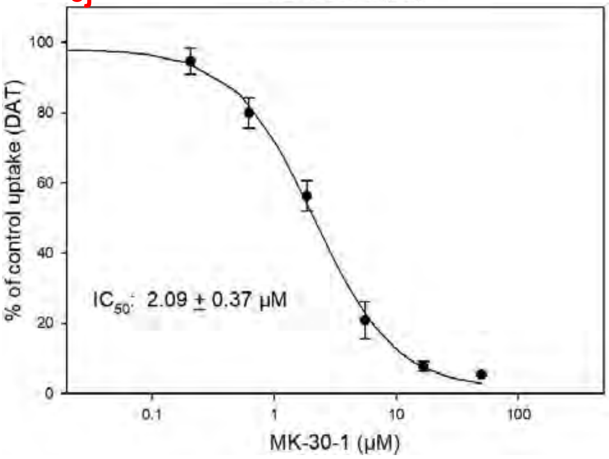

6j

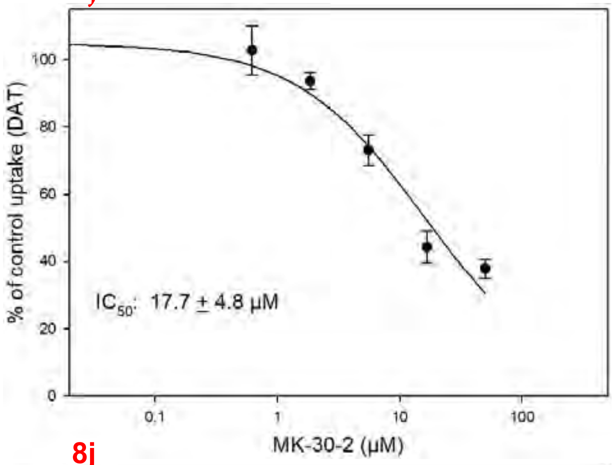

7j

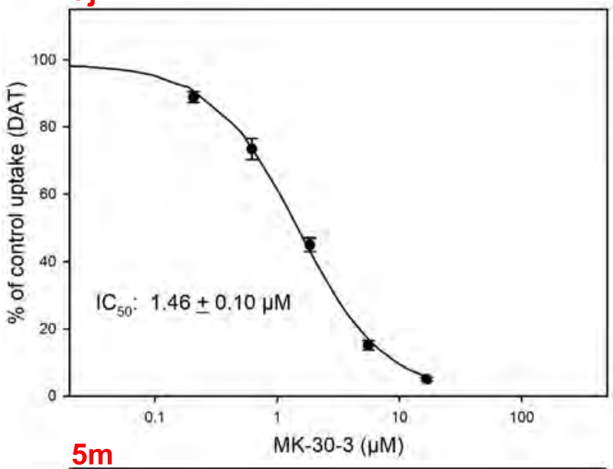

8j

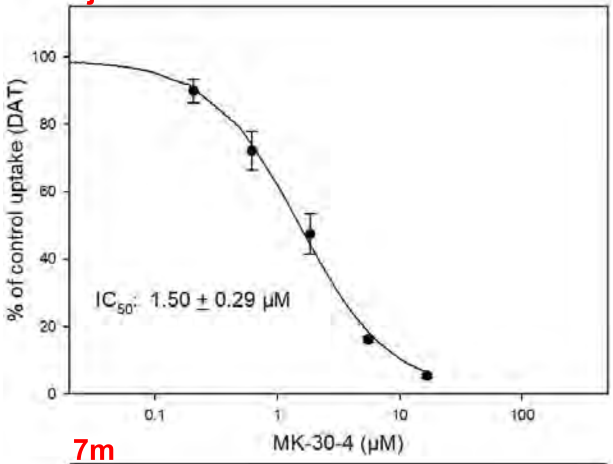

5m

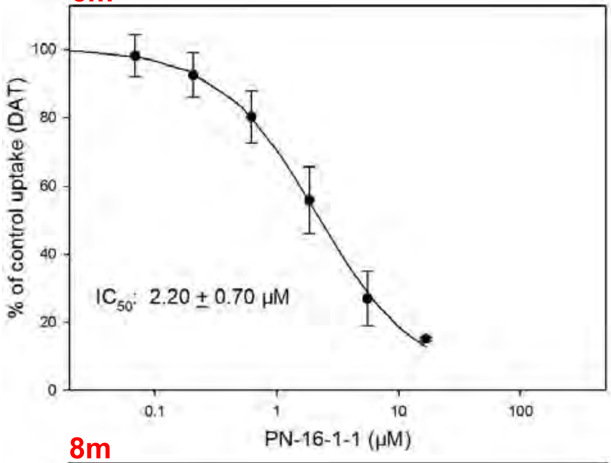

7m

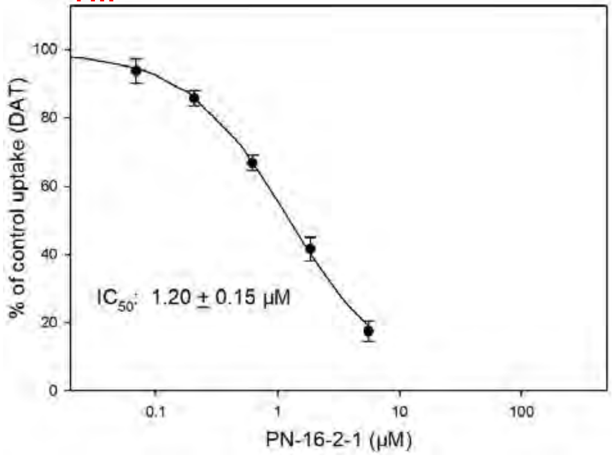

8m

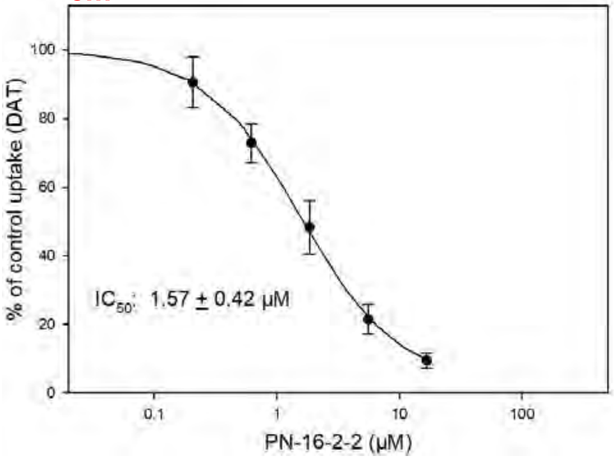

5p

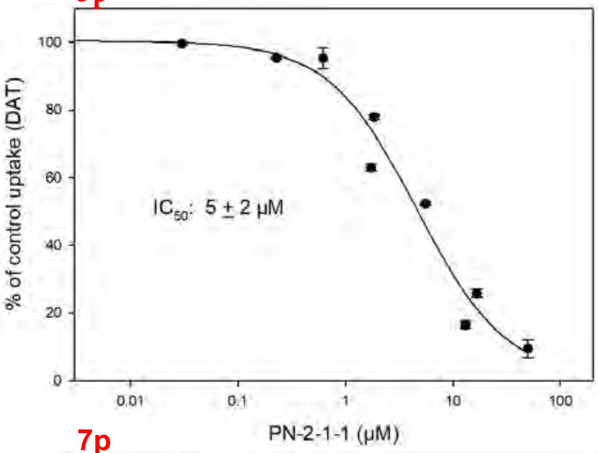

6p

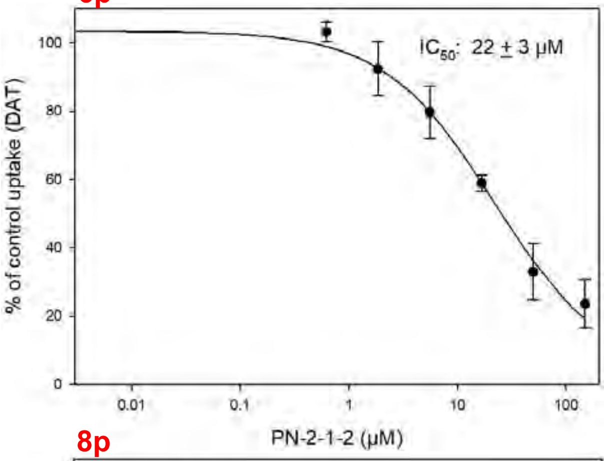

7p

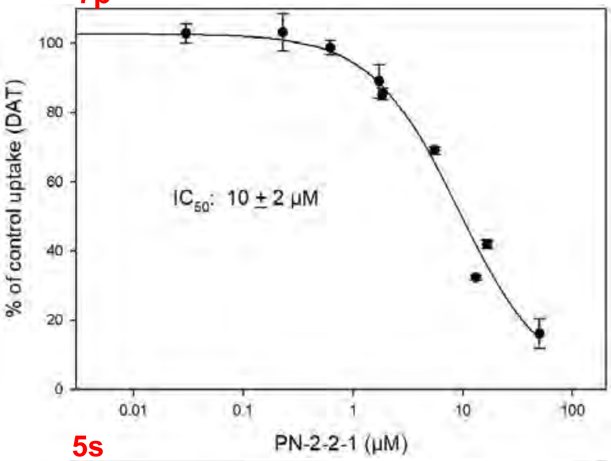

8p

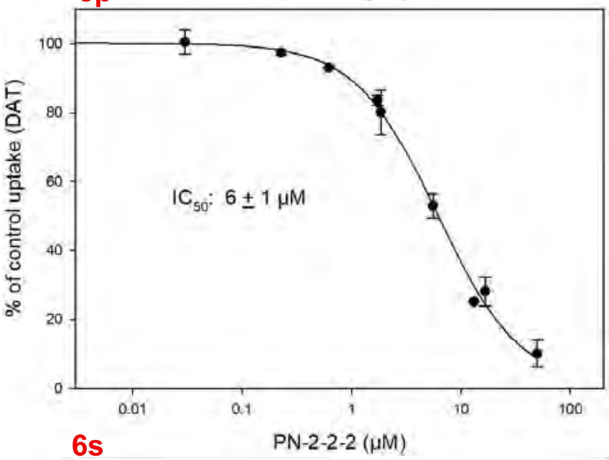

5s

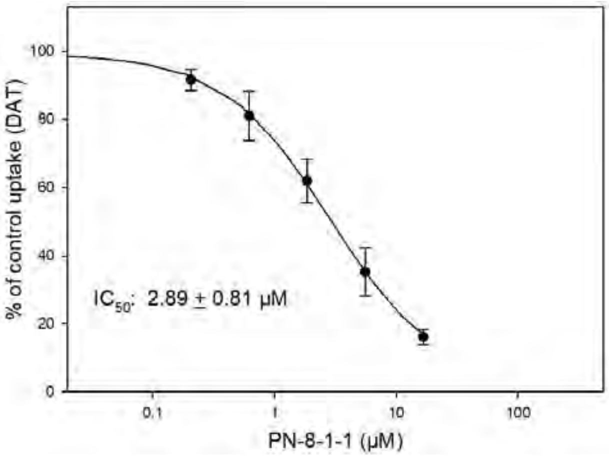

6s

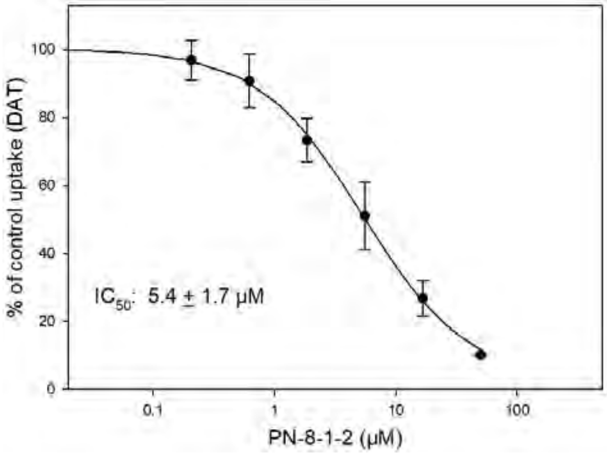

7s

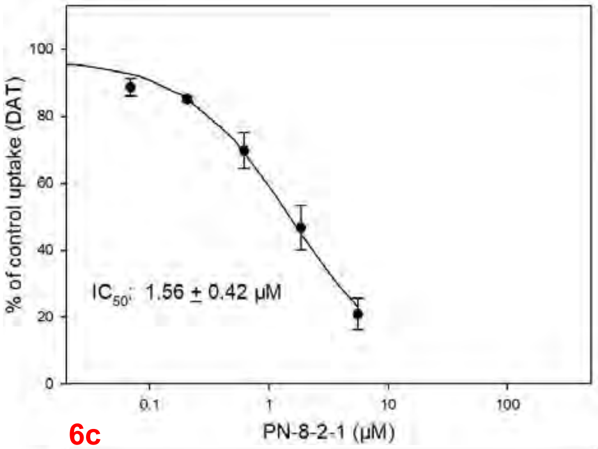

5c

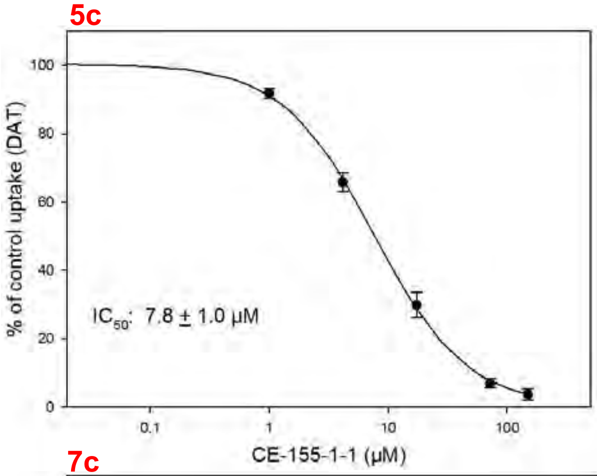

6c

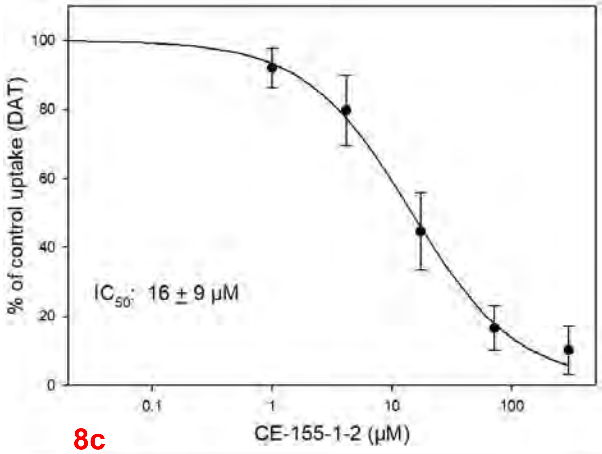

7c

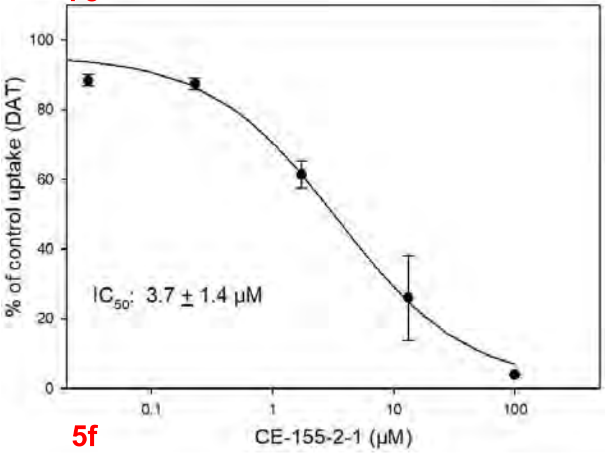

8c

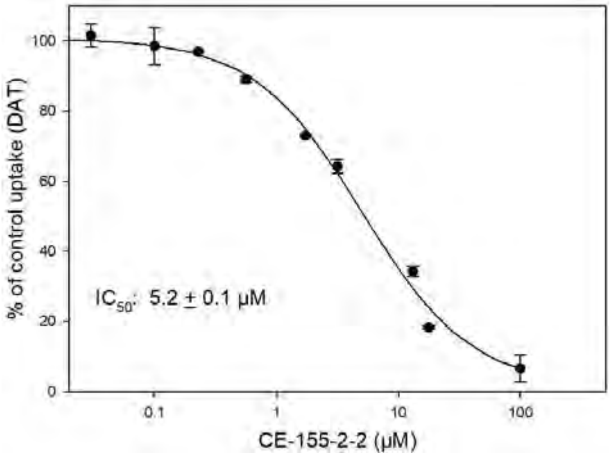

5f

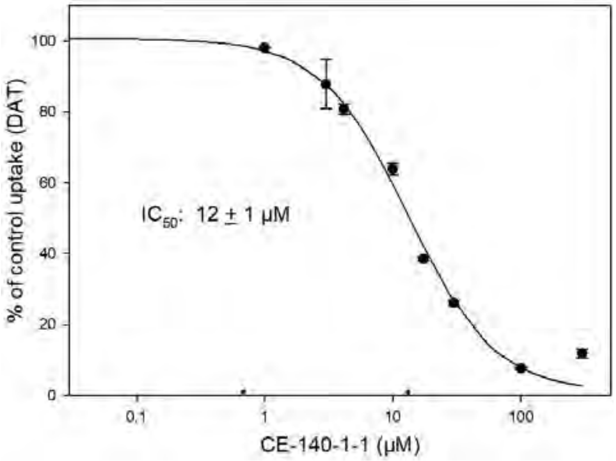

6f

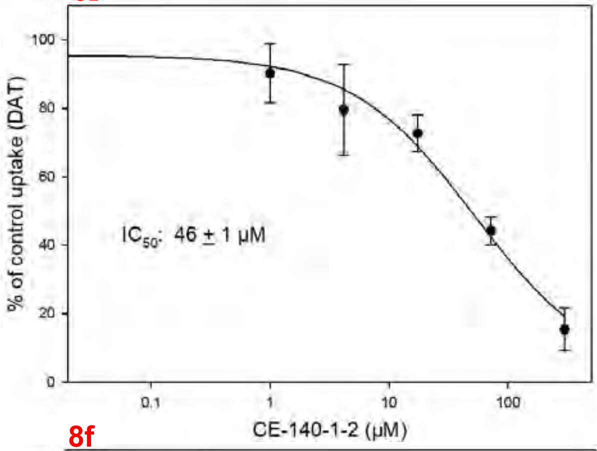

7f

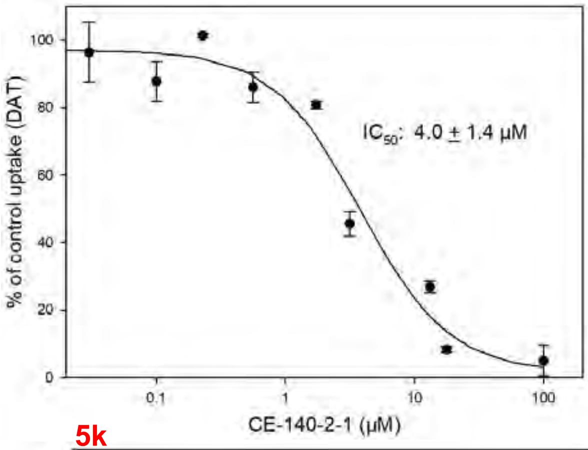

8f

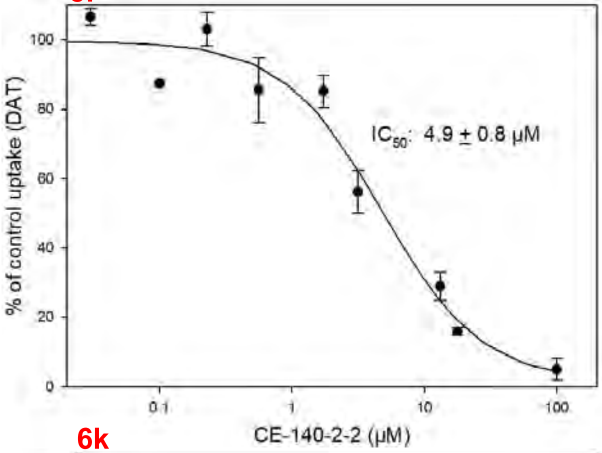

5k

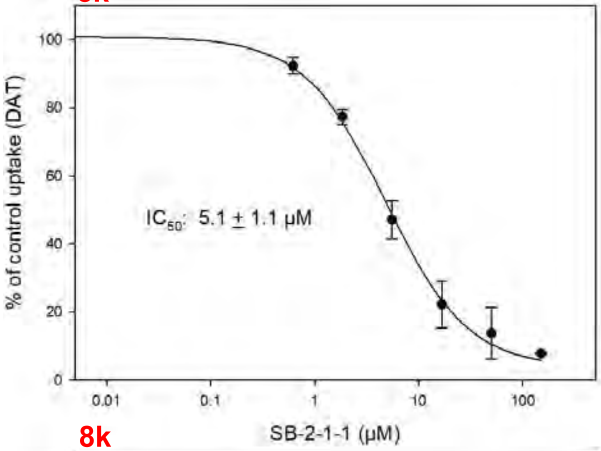

6k

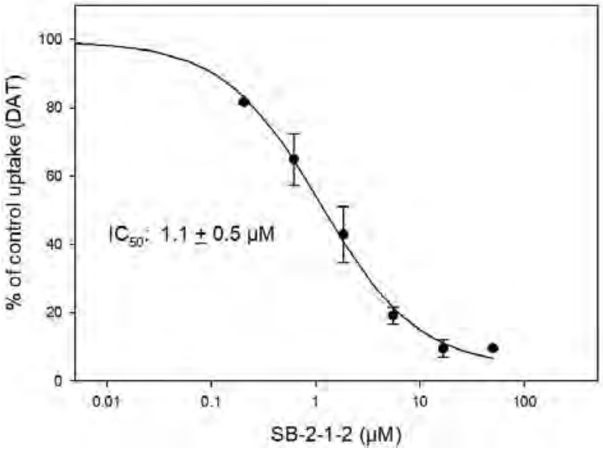

8k

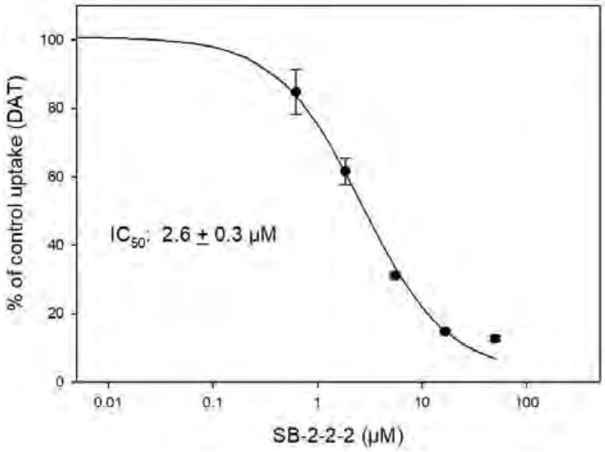

5n

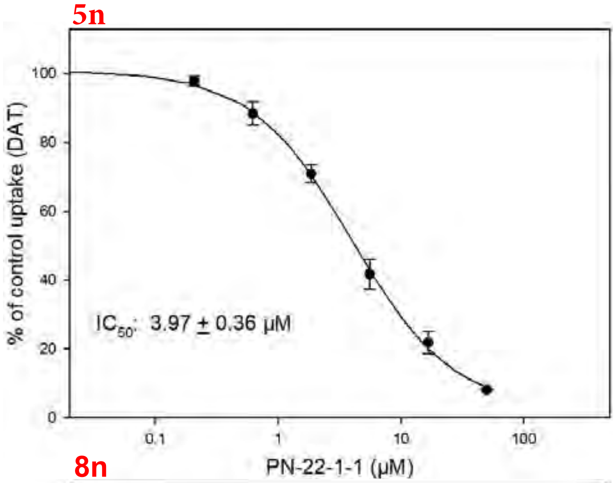

7n

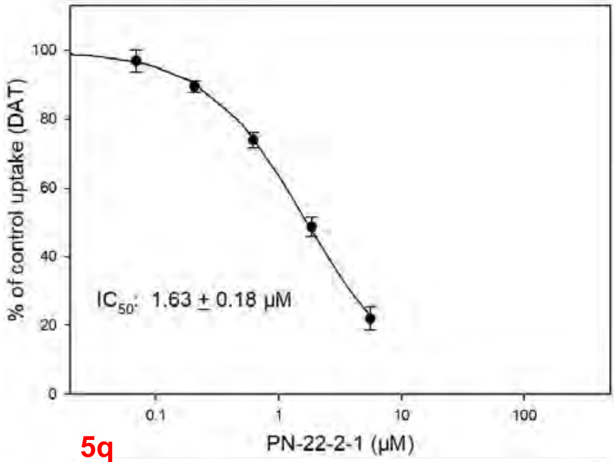

8n

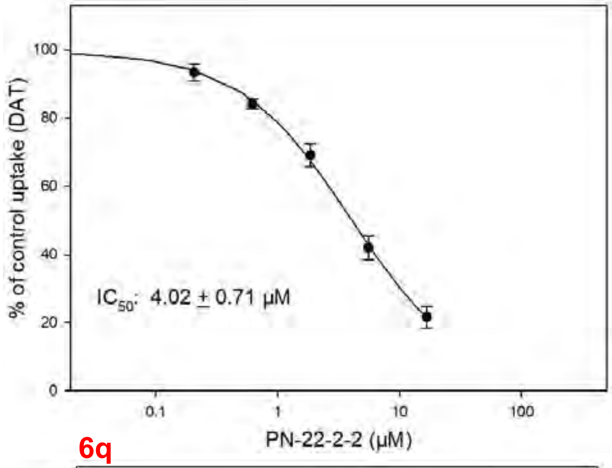

5q

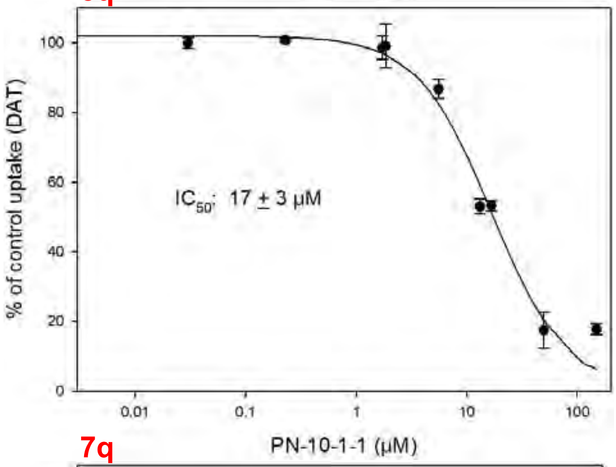

6q

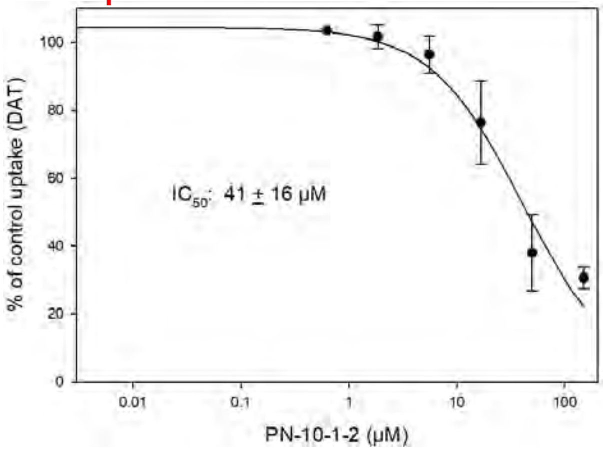

7q

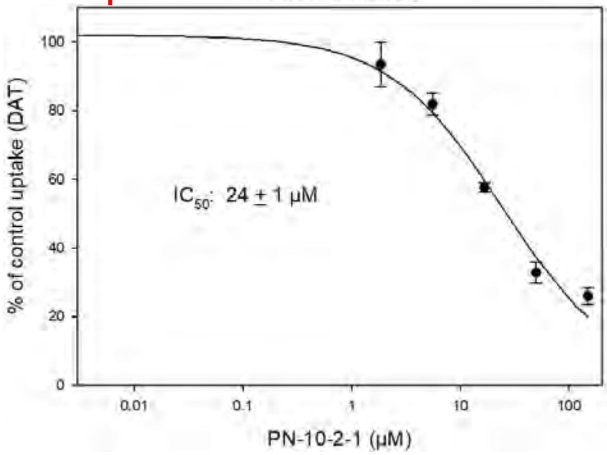

8q

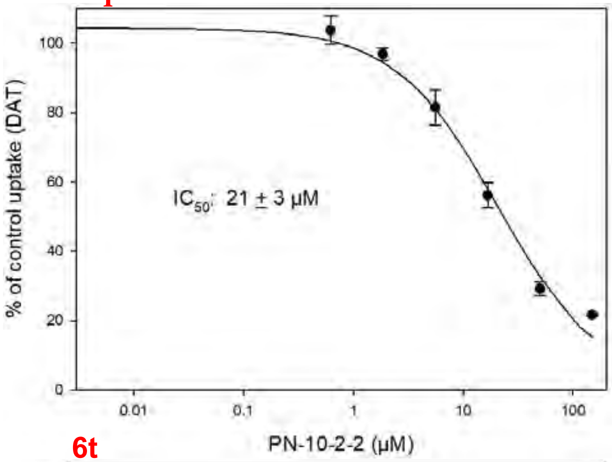

5t

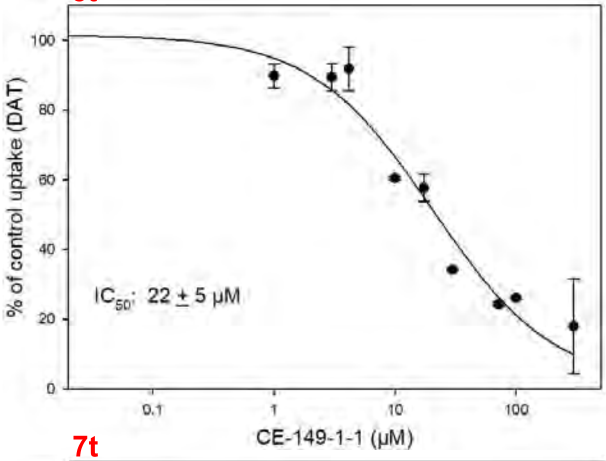

6t

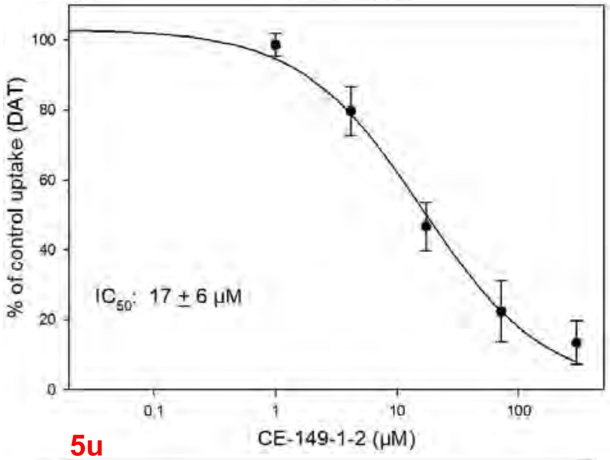

7t

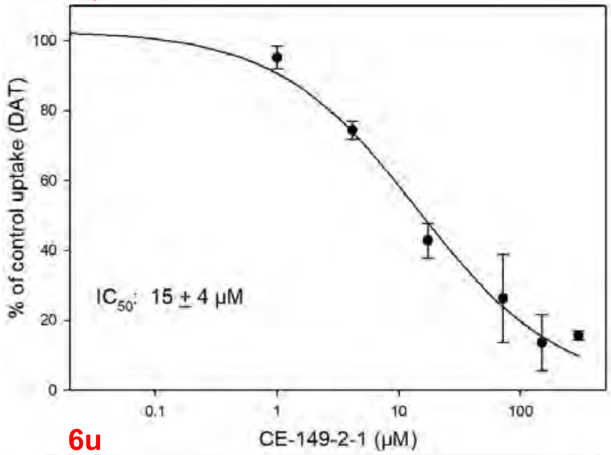

5u

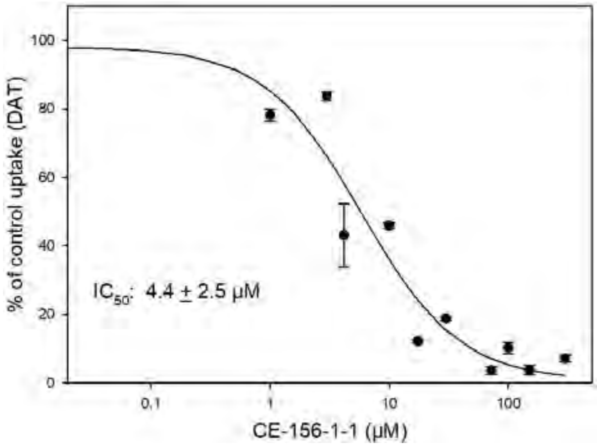

6u

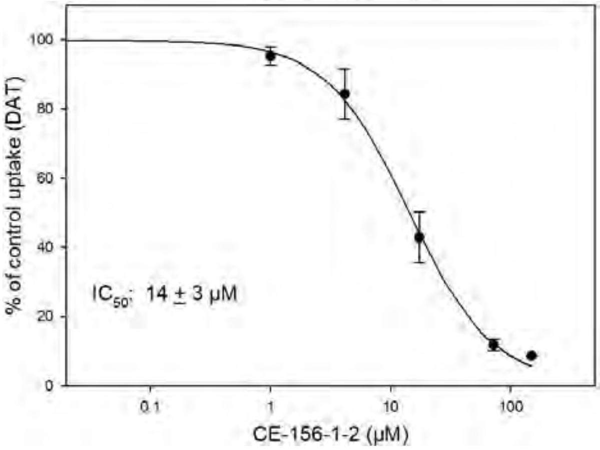

7u

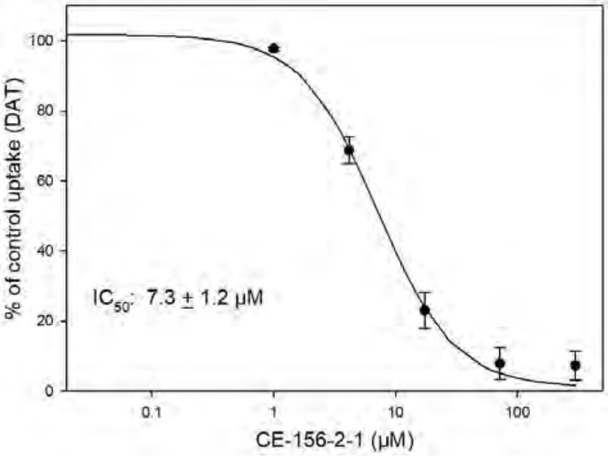

8u

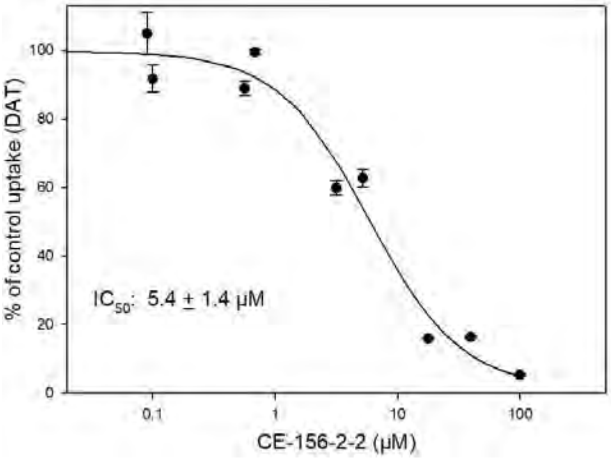

5o

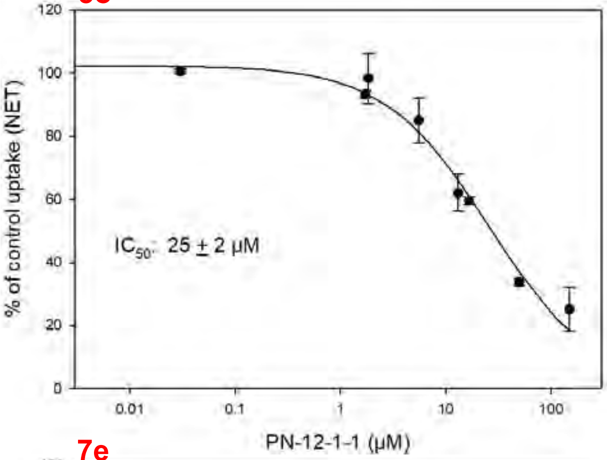

7h

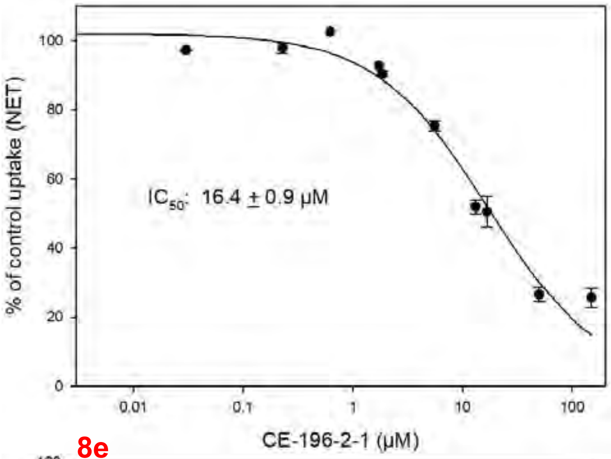

7e

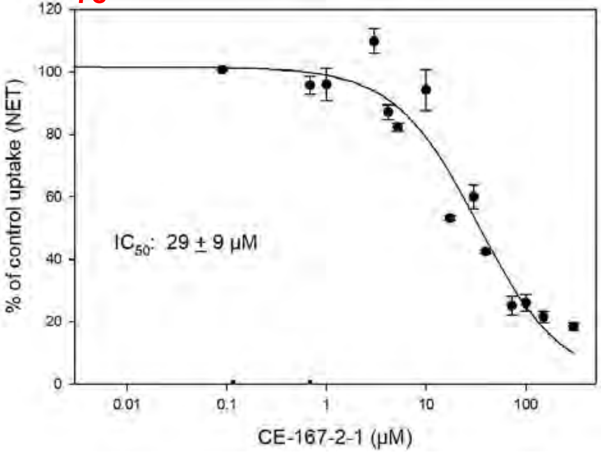

8e

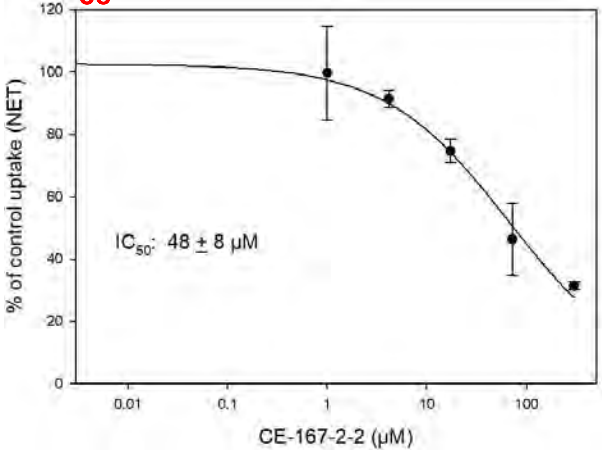

8e

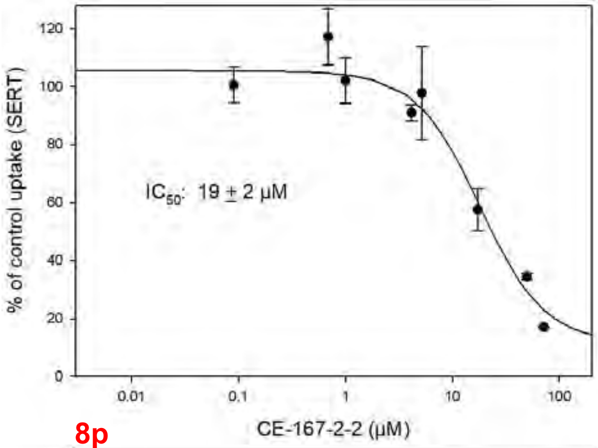

8h

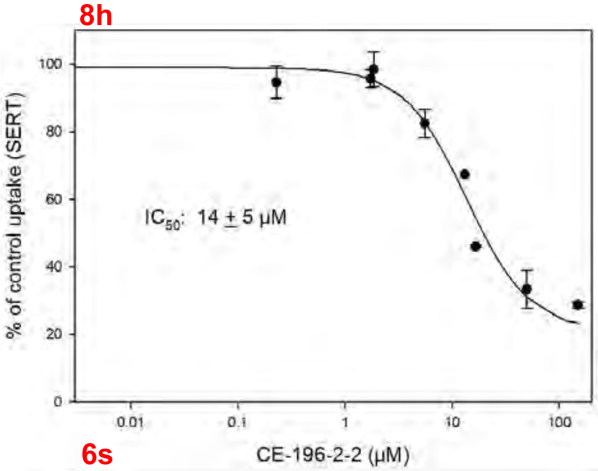

8p

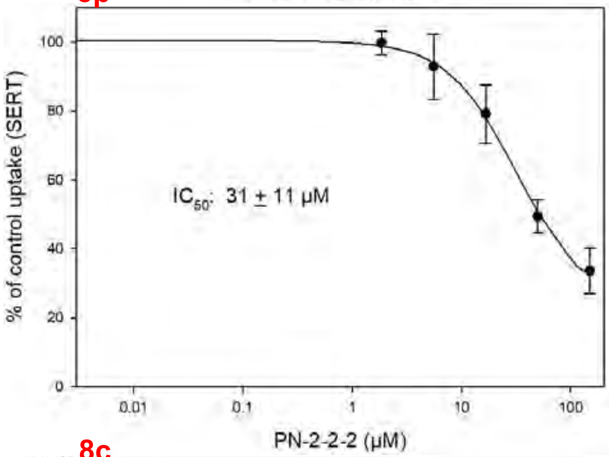

6s

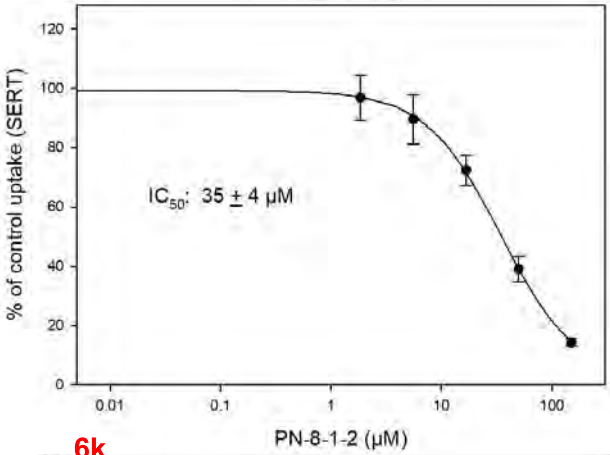

8c

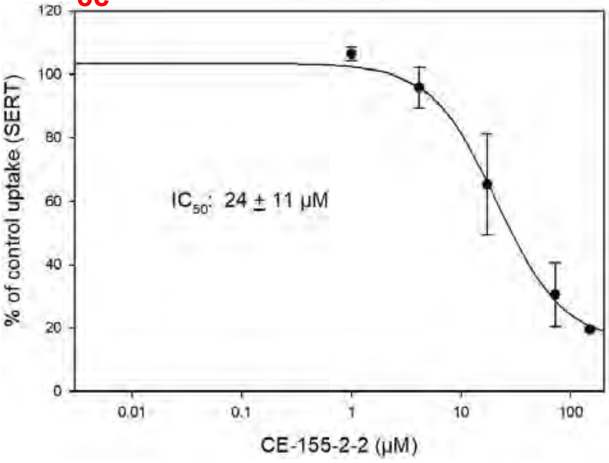

6k

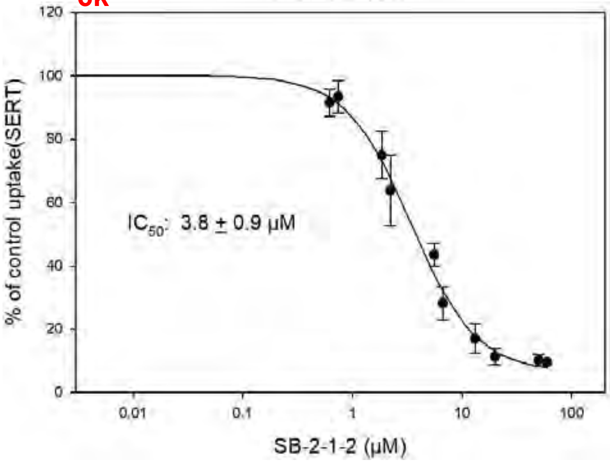

8n

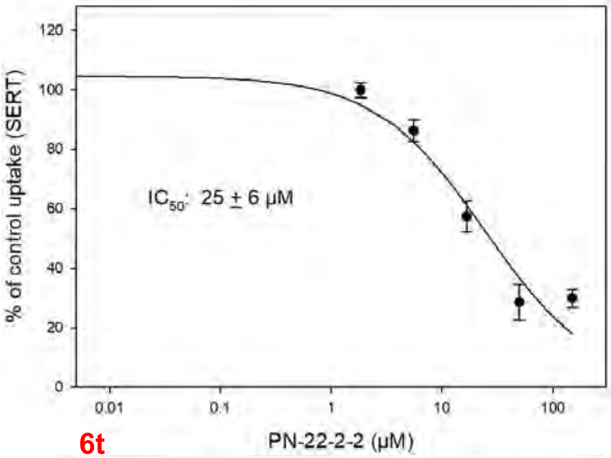

% of control uptake (SERT)
IC50: 25 ± 6 µM
PN-22-2-2 (µM)

8q

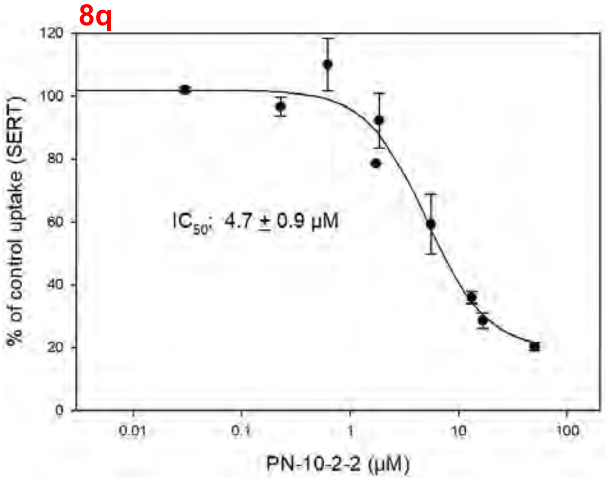

% of control uptake (SERT)
IC50: 4.7 ± 0.9 µM
PN-10-2-2 (µM)

6t

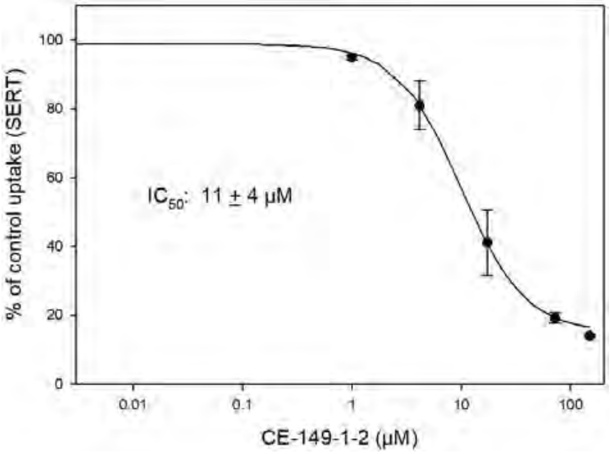

% of control uptake (SERT)
IC50: 11 ± 4 µM
CE-149-1-2 (µM)

FINAL REPORT

eurofins DISCOVERY

In Vitro Pharmacology: Transporters Binding Assays

Study of CE-196-2-1 and CE-196-2-2

STUDY ID: FR095-0020234

STUDY NUMBER
100053935

August 19, 2020

CONFIDENTIAL

1. STUDY REFERENCES

**Study title**	***In Vitro* Pharmacology: Transporters Binding Assays**<br>**Study of CE-196-2-1 and CE-196-2-2**	
**Study number**	**100053935**	**FINAL REPORT**<br>**August 19, 2020**
**Study ID**	**FR095-0020234**	
**Experimental period**	August 17, 2020 - August 18, 2020	

2. PERSONS INVOLVED IN THE STUDY

**Technical contact**	**Eurofins Cerep**<br>Le Bois l'Evêque<br>B.P. 30001<br>86 600 Celle l'Evescault<br>France	**Carsten BROCK,Ph.D.**<br>Study Director<br>CarstenBrock@eurofins.com
**Study sponsor**	**University of Vienna**<br>Dept of Pharm Chemistry<br>1090 Vienna<br>AUSTRIA	**Dr. Predrag KALABA**

3. APPROVAL

Head of laboratory statement

This study was conducted according to the procedures described in this report.

Eurofins Cerep
Le Bois l'Evêque
B.P. 30001
86 600 Celle l'Evescault
France

Sophie SEIGNEURIN,Mrs
Operations Director
SophieSeigneurin@eurofins.com

Signature

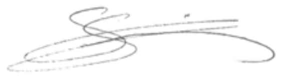

Quality assurance statement

This study was inspected by Eurofins Cerep Quality Control Unit, the results and methods presented in this report accurately reflect the methods used and the data collected for this study.

Eurofins Cerep
Le Bois l'Evêque
B.P. 30001
86 600 Celle l'Evescault
France

Eric BOUCHET
Quality Site Group Leader
EricBouchet@eurofins.com

Signature

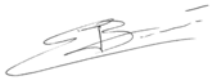

4. TABLE OF CONTENTS

1. STUDY REFERENCES 2

2. PERSONS INVOLVED IN THE STUDY 2

3. APPROVAL 3

4. TABLE OF CONTENTS 4

5. SUMMARY 5

5.1. Study Design 5

5.2. Measurements 5

5.3. Results 5

5.3.1. Compound CE-196-2-1 5

5.3.2. Compound CE-196-2-2 5

6. COMPOUNDS 6

6.1. Test Compounds 6

6.2. Reference Compounds 6

7. RESULTS 7

7.1. *In Vitro* Pharmacology: Binding Assays 7

7.1.1. IC_{50} Determination: Test Compound Results 7

7.1.2. Reference Compound Results 10

8. RESULTS INTERPRETATION GUIDE 11

9. MATERIALS AND METHODS 12

9.1. Experimental Conditions 12

9.1.1. *In Vitro* Pharmacology: Binding Assays 12

9.2. Analysis and expression of results 13

9.2.1. *In Vitro* Pharmacology: Binding Assays 13

10. BIBLIOGRAPHY 14

5. SUMMARY

The purpose of this study was to test CE-196-2-1 and CE-196-2-2 in Binding assays.

5.1. Study Design

CE-196-2-1 and CE-196-2-2 were tested at several concentrations for IC_{50} or EC_{50} determination .

5.2. Measurements

Compound binding was calculated as a % inhibition of the binding of a radioactively labeled ligand specific for each target.

5.3. Results

Results showing an inhibition or stimulation higher than 50% are considered to represent significant effects of the test compounds.

Such effects were observed here and are listed in the following tables.

Only the calculable IC_{50} and EC_{50} are reported below.

5.3.1. Compound CE-196-2-1

Assay	IC_{50}	K_i	K_B	EC_{50}	nH
dopamine transporter*(h)* (antagonist radioligand)	2.5E-08 M	1.3E-08 M			1

5.3.2. Compound CE-196-2-2

Assay	IC_{50}	K_i	K_B	EC_{50}	nH
dopamine transporter*(h)* (antagonist radioligand)	6.5E-08 M	3.5E-08 M			0.8

6. COMPOUNDS

6.1. Test Compounds

Manufacturer: **University of Vienna**

Client Compound ID	Compound ID	Reference Number	Batch Number	FW	MW	Purity	Received Form	Stock solution	Flag
CE-196-2-1	100053935-1	-	-	389.97	-	100.0	Powder	1.E-02 M DMSO	-
CE-196-2-2	100053935-2	-	-	389.97	-	100.0	Powder	1.E-02 M DMSO	-

FW: Formula Weight - MW: Molecular Weight

6.2. Reference Compounds

In each experiment and if applicable, the respective reference compound was tested concurrently with CE-196-2-1 and CE-196-2-2, and the data were compared with historical values determined at Eurofins. The experiment was accepted in accordance with Eurofins validation Standard Operating Procedure.

7. RESULTS

7.1. *In Vitro* Pharmacology: Binding Assays

7.1.1. IC_{50} Determination: Test Compound Results

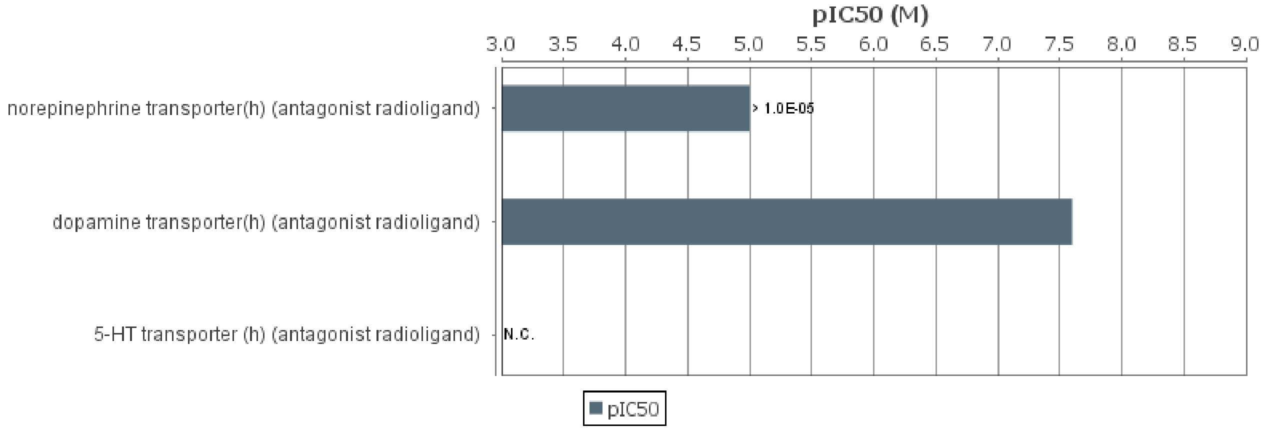

Figure 1. Histogram for CE-196-2-1

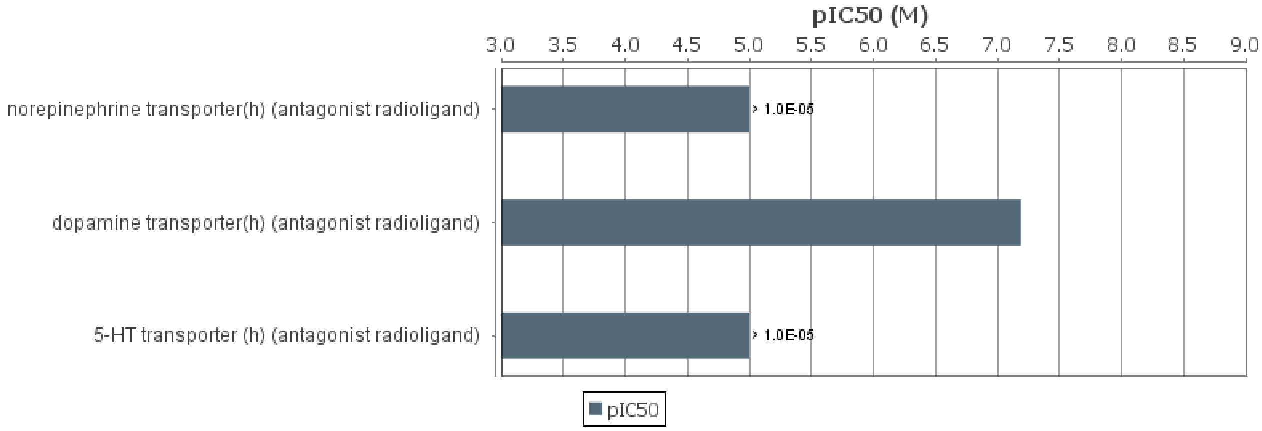

Figure 2. Histogram for CE-196-2-2

Compound I.D.	Client Compound I.D.	IC_{50}(M)	K_i(M)	nH	Test Concentration	% Inhibition of Control Specific Binding 1st	2nd	Mean	Flags 1st	2nd
**norepinephrine transporter*(h)* (antagonist radioligand)**										
100053935-1	CE-196-2-1	> 1.0E-05	n/a	n/a	1.0E-12 M	-22.6	-32.7	-27.6		
					1.0E-11 M	-26.2	-28.4	-27.3		
					1.0E-10 M	-6.6	-25.8	-16.2		
					1.0E-09 M	-18.1	-24.8	-21.5		
					1.0E-08 M	-9.0	-25.6	-17.3		
					1.0E-07 M	-9.8	-19.8	-14.8		
					1.0E-06 M	-16.7	-15.7	-16.2		
					1.0E-05 M	10.5	16.1	13.3		

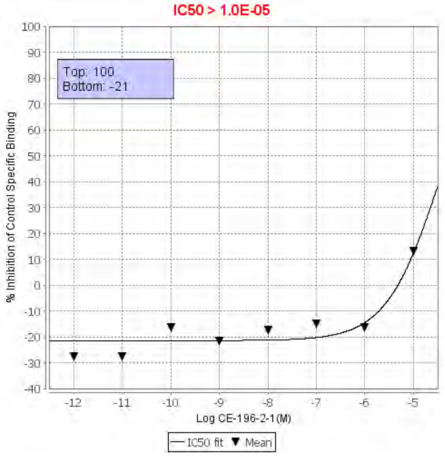

Figure 3. CE-196-2-1 on norepinephrine transporter*(h)* (antagonist radioligand)

Compound I.D.	Client Compound I.D.	IC_{50}(M)	K_i(M)	nH	Test Concentration	1st	2nd	Mean	Flags 1st	2nd
100053935-2	CE-196-2-2	> 1.0E-05	n/a	n/a	1.0E-12 M	-21.0	-21.3	-21.2		
					1.0E-11 M	-22.2	-23.7	-23.0		
					1.0E-10 M	-14.9	-17.9	-16.4		
					1.0E-09 M	-26.2	-14.3	-20.2		
					1.0E-08 M	-18.9	-19.8	-19.4		
					1.0E-07 M	-18.7	-14.5	-16.6		
					1.0E-06 M	-22.7	-16.0	-19.4		
					1.0E-05 M	5.7	18.7	12.2		

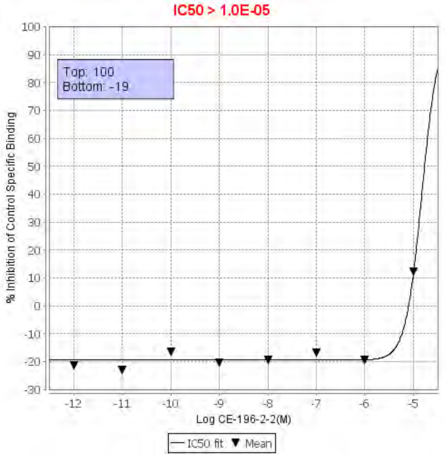

Figure 4. CE-196-2-2 on norepinephrine transporter*(h)* (antagonist radioligand)

Compound I.D.	Client Compound I.D.	IC_{50}(M)	K_i(M)	nH	Test Concen-tration	% Inhibition of Control Specific Binding 1st	2nd	Mean	Flags 1st	2nd
**dopamine transporter*(h)* (antagonist radioligand)**										
100053935-1	CE-196-2-1	**2.5E-08 M**	1.3E-08 M	1.0	1.0E-12 M	-6.7	-12.2	-9.4		
					1.0E-11 M	-3.7	-13.4	-8.5		
					1.0E-10 M	0.8	-10.1	-4.6		
					1.0E-09 M	-2.2	-8.0	-5.1		
					1.0E-08 M	24.6	20.4	22.5		
					1.0E-07 M	76.8	77.5	77.1		
					1.0E-06 M	93.4	95.3	94.4		
					1.0E-05 M	99.2	98.9	99.1		

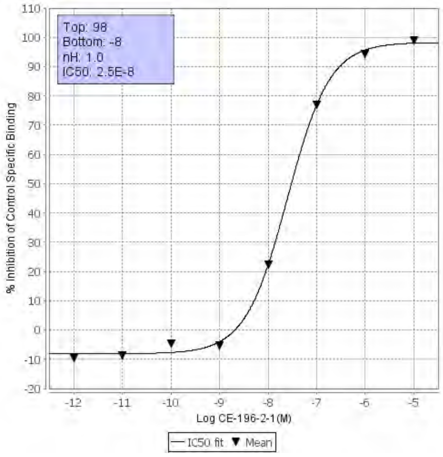

Figure 5. CE-196-2-1 on dopamine transporter*(h)* (antagonist radioligand)

100053935-2	CE-196-2-2	**6.5E-08 M**	3.5E-08 M	0.8	1.0E-12 M	-17.6	-4.9	-11.2		
					1.0E-11 M	-8.9	-6.3	-7.6		
					1.0E-10 M	-5.7	-11.1	-8.4		
					1.0E-09 M	-11.8	-4.3	-8.1		
					1.0E-08 M	13.0	13.7	13.3		
					1.0E-07 M	54.0	53.4	53.7		
					1.0E-06 M	89.3	91.0	90.1		
					1.0E-05 M	98.3	99.1	98.7		

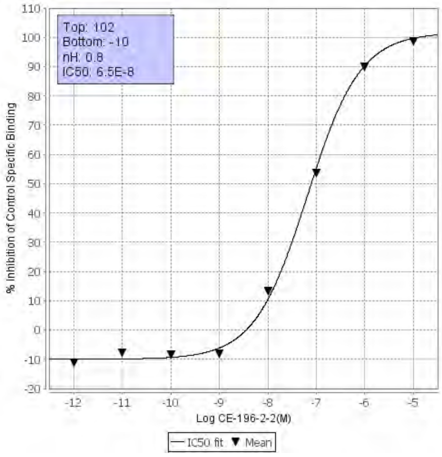

Figure 6. CE-196-2-2 on dopamine transporter*(h)* (antagonist radioligand)

Compound I.D.	Client Compound I.D.	IC_{50}(M)	K_i(M)	nH	Test Concen-tration	% Inhibition of Control Specific Binding 1st	2nd	Mean	Flags 1st	2nd
**5-HT transporter *(h)* (antagonist radioligand)**										
100053935-1	CE-196-2-1	N.C.	n/a	n/a	1.0E-12 M	-1.1	-4.2	-2.7		
					1.0E-11 M	1.2	3.1	2.2		
					1.0E-10 M	-1.9	-11.4	-6.6		
					1.0E-09 M	-2.5	0.8	-0.9		
					1.0E-08 M	3.0	-4.4	-0.7		
					1.0E-07 M	0.2	-1.7	-0.7		
					1.0E-06 M	-9.8	-8.7	-9.3		
					1.0E-05 M	-13.9	-11.4	-12.6		

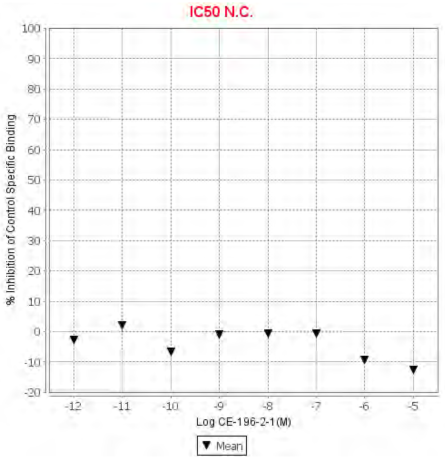

Figure 7. CE-196-2-1 on 5-HT transporter *(h)* (antagonist radioligand)

Compound I.D.	Client Compound I.D.	IC_{50}(M)	K_i(M)	nH	Test Concen-tration	1st	2nd	Mean	Flags 1st	2nd
100053935-2	CE-196-2-2	> 1.0E-05	n/a	n/a	1.0E-12 M	-9.0	-1.9	-5.4		
					1.0E-11 M	-4.8	-7.2	-6.0		
					1.0E-10 M	-3.7	-5.0	-4.3		
					1.0E-09 M	1.2	-4.1	-1.4		
					1.0E-08 M	0.2	-0.6	-0.2		
					1.0E-07 M	-2.8	1.4	-0.7		
					1.0E-06 M	0.1	-6.3	-3.1		
					1.0E-05 M	24.2	28.0	26.1		

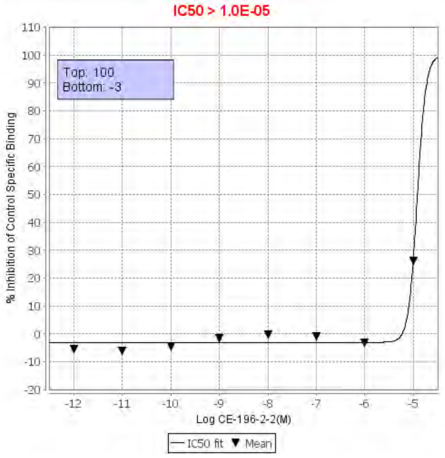

Figure 8. CE-196-2-2 on 5-HT transporter *(h)* (antagonist radioligand)

> *Conc.:* IC_{50} value above the highest test concentration. Concentration-response curve shows less than 50 % effect at the highest validated testing concentration
N.C.: IC_{50} value not calculable. Concentration-response curve shows less than 25% effect at the highest validated testing concentration.

7.1.2. Reference Compound Results

Compound I.D.	IC_{50}(M)	K_i(M)	nH
**norepinephrine transporter*(h)* (antagonist radioligand)**			
protriptyline	5.2E-09 M	3.9E-09 M	1.6
**dopamine transporter*(h)* (antagonist radioligand)**			
BTCP	1.5E-08 M	8.1E-09 M	1.2
**5-HT transporter *(h)* (antagonist radioligand)**			
imipramine	2.5E-09 M	1.2E-09 M	1.0

8. RESULTS INTERPRETATION GUIDE

In Vitro Pharmacology

Results showing an inhibition (or stimulation for assays run in basal conditions) higher than 50% are considered to represent significant effects of the test compounds. 50% is the most common cut-off value for further investigation (determination of IC_{50} or EC_{50} values from concentration-response curves) that we would recommend.
Results showing an inhibition (or stimulation) between 25% and 50% are indicative of weak to moderate effects (in most assays, they should be confirmed by further testing as they are within a range where more inter-experimental variability can occur).
Results showing an inhibition (or stimulation) lower than 25% are not considered significant and mostly attributable to variability of the signal around the control level.
Low to moderate negative values have no real meaning and are attributable to variability of the signal around the control level. High negative values (≥ 50%) that are sometimes obtained with high concentrations of test compounds are generally attributable to non-specific effects of the test compounds in the assays. On rare occasion they could suggest an allosteric effect of the test compound.

9. MATERIALS AND METHODS

9.1. Experimental Conditions

Minor variations to the experimental protocol described below may have occurred during the testing, they have no impact on the quality of the results obtained.

9.1.1. *In Vitro* Pharmacology: Binding Assays

Assay	Source	Ligand	Conc.	Kd	Non Specific	Incubation	Detection Method	Bibl.
**Transporters**								
**norepinephrine transporter *(h)* (antagonist radioligand)**	human recombinant (CHO cells)	[^{3}H]nisoxetine	1 nM	2.9 nM	desipramine (1 µM)	120 min 4°C	Scintillation counting	180
**dopamine transporter *(h)* (antagonist radioligand)**	human recombinant (CHO cells)	[^{3}H]BTCP	4 nM	4.5 nM	BTCP (10 µM)	120 min 4°C	Scintillation counting	190
**5-HT transporter *(h)* (antagonist radioligand)**	human recombinant (CHO cells)	[^{3}H]imipramine	2 nM	1.7 nM	imipramine (10 µM)	60 min RT	Scintillation counting	566

9.2. Analysis and expression of results

9.2.1. *In Vitro* Pharmacology: Binding Assays

The results are expressed as a percent of control specific binding

$$\frac{\text{measured specific binding}}{\text{control specific binding}} * 100$$

and as a percent inhibition of control specific binding

$$100 - \left(\frac{\text{measured specific binding}}{\text{control specific binding}} * 100\right)$$

obtained in the presence of the test compounds.

The IC_{50} values (concentration causing a half-maximal inhibition of control specific binding) and Hill coefficients (nH) were determined by non-linear regression analysis of the competition curves generated with mean replicate values using Hill equation curve fitting

$$Y = D + \left[\frac{A - D}{1 + (C/C_{50})^{nH}}\right]$$

where Y = specific binding, A = left asymptote of the curve, D = right asymptote of the curve, C = compound concentration, C_{50} = IC_{50}, and nH = slope factor. This analysis was performed using software developed at Cerep (Hill software) and validated by comparison with data generated by the commercial software SigmaPlot® 4.0 for Windows® (© 1997 by SPSS Inc.).
The inhibition constants (K_i) were calculated using the Cheng Prusoff equation

$$K_i = \frac{IC_{50}}{(1 + L/K_D)}$$

where L = concentration of radioligand in the assay, and K_D = affinity of the radioligand for the receptor. A scatchard plot is used to determine the K_D.

10. BIBLIOGRAPHY

180. Pacholczyk, T. et al. (1991), *Nature,* 350*: 350-354.*
190. Pristupa, Z.B. et al. (1994), *Mol. Pharmacol.,* 45*: 125-135.*
566. Tatsumi, M. et al. (1999), *Eur. J. Pharmacol.,* 368*: 277-283.*

University of Vienna

CYP2052 –R1

Final Report
October 15, 2019

Neurite Outgrowth

Cryopreserved Rat Cortical Neurons

cyprotex
AN EVOTEC COMPANY

Authentication Statement

I, the undersigned, hereby declare that the work described in this report was performed according to the study protocol and/or standard procedures, and to the best of my knowledge, this report provides a correct record of the results obtained.

	November 8, 2019
Study Manager	Date

Neurite Outgrowth Conclusion

Compounds CE-158-1-1, CE-196-2-1, CE-144-2-1, CE-196-2-2 and S-CE-123 were evaluated in the neurite outgrowth assay using cryopreserved rat cortical neurons (QBM Cell Science). CE-158-1-1 and CE-144-2-1 had nominal effects on valid neuron count, mean neurite average length and neurite total length per neuron. CE-196-2-1 and CE-196-2-2 caused significant decreases in the valid neuron count, mean neurite average length and neurite total length per neuron at the top concentration (100 µM). Compound S-CE-123 did not affect valid neuron count but appeared to cause a nominal increase in the neurite outgrowth endpoints. The negative and positive controls performed as expected.

Materials and Methods

Protocol

Cryopreserved rat cortical neurons (QBM Cell Science) were plated in laminin-coated 384-well plates 1 hour prior to treatment at a density of 10,000 cells per well. After treatment with test articles and control compounds, the neurons were maintained in a humidified environment at 37°C with 5% CO_2 for 72 hrs. At the end of the treatment period, cells were fixed, permeabilized and stained for evaluation of neurite outgrowth and cell health using the high content imaging platform, ArrayScan VTi, with an optimized neuronal profiling bioapplication.

Table 1. Test Compounds

Test Articles	Molecular Weight	Concentrations (µM)
CE-158-1-1	390.97	100, 50, 25, 12.5, 6.25, 3.13, 1.56, 0.78, 0.39, 0.2
CE-196-2-1	389.97	100, 50, 25, 12.5, 6.25, 3.13, 1.56, 0.78, 0.39, 0.2
CE-144-2-1	390.97	100, 50, 25, 12.5, 6.25, 3.13, 1.56, 0.78, 0.39, 0.2
CE-196-2-2	389.97	100, 50, 25, 12.5, 6.25, 3.13, 1.56, 0.78, 0.39, 0.2
S-CE-123	313.06	100, 50, 25, 12.5, 6.25, 3.13, 1.56, 0.78, 0.39, 0.2

Cell Health Parameters

Valid cell count, mean neurite average length and neurite total length per neuron are reported.

Analysis

This assay assesses cell health and neurite outgrowth utilizing a neuronal profiling bioapplication applied to images obtained on an ArrayScan VTi. Valid neuron count, mean neurite average length and neurite total length per neuron are determined and reported. Raw data are normalized to the vehicle control and reported as a normalized % response. Dose response graphs are generated with GraphPad Prism using a Sigmoidal Dose-response (variable slope) algorithm.

Results

Control compounds

Positive control, nocodazole, had an IC50 of 2.22 µM for valid neuron count, an IC50 of 0.097 µM for mean neurite average length and an IC50 of 0.0997 µM for neurite total length per neuron. Negative control, chlorpromazine, had an IC50 of 7.99 µM for valid neuron count, an IC50 of 9.022 µM for mean neurite average length and an IC50 of 13.8 µM for neurite total length per neuron. Nocodazole was positive for substantially effecting neurite outgrowth while nominally effecting cell viability. Chlorpromazine's effect on neurite outgrowth was not independent of its effect on cell viability (Table 2).

Test Compounds

Test compounds, CE-158-1-1 and CE-144-2-1 had nominal effects (> 100 µM) on all three endpoints. Compound S-CE-123 did not have significant measurable effects on cell viability but slight effects (increase) on neurite outgrowth. CE-196-2-1 had an IC50 of 92.16 µM for valid neuron count, and IC50 of 65.82 µM for mean neurite average length, and IC50 of 59.76 µM for neurite total length per neuron. CE-196-2-2 had an IC50 of 94.6 µM for valid neuron count, and IC50 of 75.22 µM for mean neurite average length, and IC50 of 72.15 µM for neurite total length per neuron The effects on neurite outgrowth for CE-196-2-1 and CE-196-2-2 were not independent of their effect on cell viability (Table 2).

Table 2. Results for Valid Neuron Count, Mean Neurite Average Length and Neurite Total Length per Neuron.

Compound	Top Conc. Tested (µM)	Valid Neuron Count IC50 (µM)	Mean Neurite Avg Length IC50 (µM)	Neurite Total Length per Neuron IC50 (µM)	Comments
Nocodazole	1	2.22	0.097	0.0997	Positive control
Chlorpromazine	100	7.99	9.022	13.8	Negative control
CE-158-1-1	100	>100	>100	>100	Test compound
CE-196-2-1	100	92.16	65.82	59.76	Test compound
CE-144-2-1	100	>100	>100	>100	Test compound
CE-196-2-2	100	94.6	75.22	72.15	Test compound
S-CE-123	100	NR	NR	NR	Test compound

NR=No Response

Results
Test Compounds

Neurite Outgrowth

Client compound CE-158-1-1

Data Summary

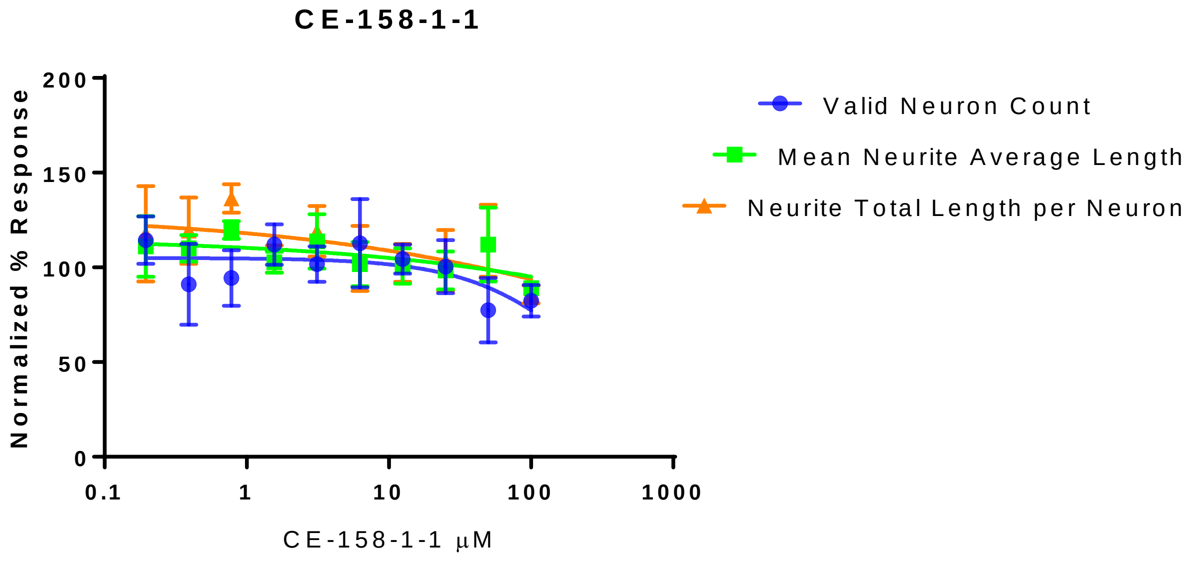

CE-158-1-1 (µM)	Valid Neuron Count			Mean Neurite Avg Length			Neurite Total Length per Neuron		
100	92	77	78	90	91	86	82	91	84
50	61	76	95	132	111	93	132	116	94
25	99	87	115	88	108	99	89	121	101
12.5	102	98	113	90	108	104	91	110	106
6.25	130	86	122	97	115	93	99	124	91
3.13	105	109	91	103	108	130	108	115	134
1.56	121	100	115	109	99	100	109	96	108
0.78	111	89	83	125	118	116	138	128	143
0.39	98	67	108	110	117	103	121	136	101
0.2	124	119	100	96	109	128	95	113	145

Client compound CE-196-2-1

Data Summary

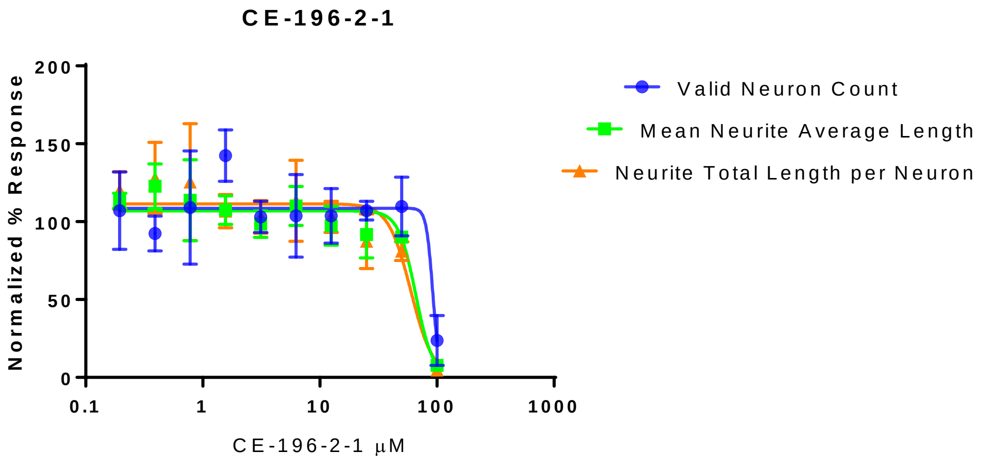

	Valid Neuron Count	Mean Neurite Average Length	Neurite Total Length per Neuron
EC50	~ 92.16	65.82	59.76

CE-196-2-1 (µM)	Valid Neuron Count			Mean Neurite Avg Length			Neurite Total Length per Neuron		
100	42	12	17	7	8	8	4	5	5
50	126	89	114	92	89	89	87	75	81
25	103	114	104	107	91	77	105	87	70
12.5	124	93	94	85	110	97	92	112	105
6.25	78	102	131	122	111	97	139	114	87
3.13	112	92	105	108	95	92	113	104	92
1.56	127	160	140	110	97	115	116	95	109
0.78	151	91	85	89	111	141	86	129	161
0.39	95	80	102	131	131	106	137	145	102
0.2	129	112	80	111	110	119	112	114	134

Client compound CE-144-2-1

Data Summary

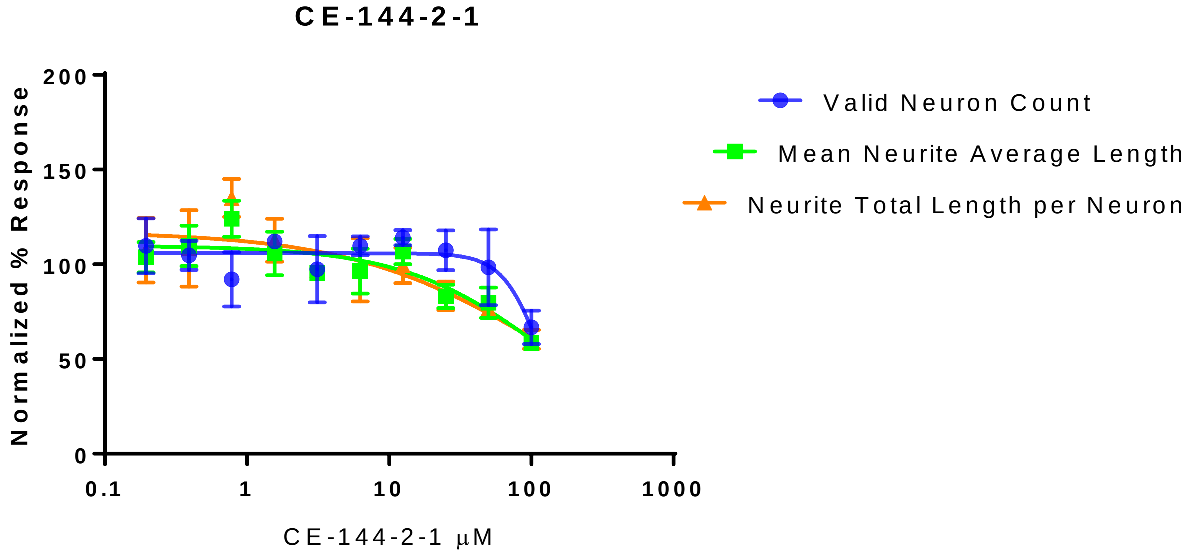

CE-144-2-1 (µM)	Valid Neuron Count			Mean Neurite Avg Length			Neurite Total Length per Neuron		
100	62	77	61	55	61	59	56	66	59
50	99	78	118	72	79	88	76	80	74
25	96	117	109	90	78	81	90	75	85
12.5	110	118	114	109	99	112	96	92	110
6.25	111	104	114	88	110	91	84	116	91
3.13	77	108	107	97	96	93	98	99	94
1.56	113	109	114	94	117	106	100	116	122
0.78	108	80	88	117	135	120	135	145	125
0.39	111	96	107	103	122	104	102	131	92
0.2	94	123	112	105	95	111	124	90	108

Client compound CE-196-2-2

Data Summary

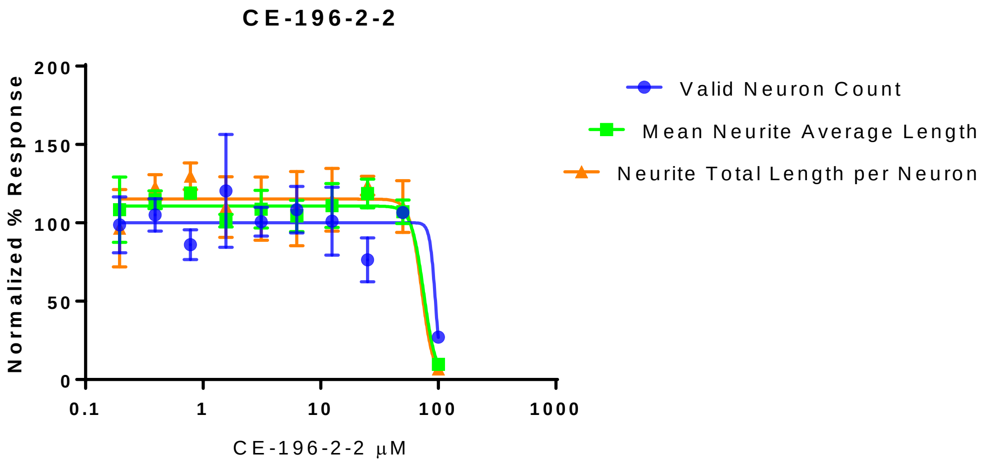

	Valid Neuron Count	Mean Neurite Average Length	Neurite Total Length per Neuron
EC50	~ 94.6	75.22	72.15

CE-196-2-2 (µM)	Valid Neuron Count			Mean Neurite Avg Length			Neurite Total Length per Neuron		
100	26	27	28	9	10	10	7	7	6
50	105	108	169*	108	114	99	115	124	92
25	91	63	75	108	124	124	117	125	129
12.5	115	112	76	105	101	127	112	96	136
6.25	112	121	92	98	99	116	91	100	136
3.13	105	107	90	118	95	113	117	86	124
1.56	136	146	79	100	98	106	103	95	132
0.78	85	96	77	121	119	117	136	120	133
0.39	98	100	117	121	113	110	132	119	117
0.2	78	109	109	132	92	101	166*	79	114

*outlier removed from graph

Client compound S-CE-123

Data Summary

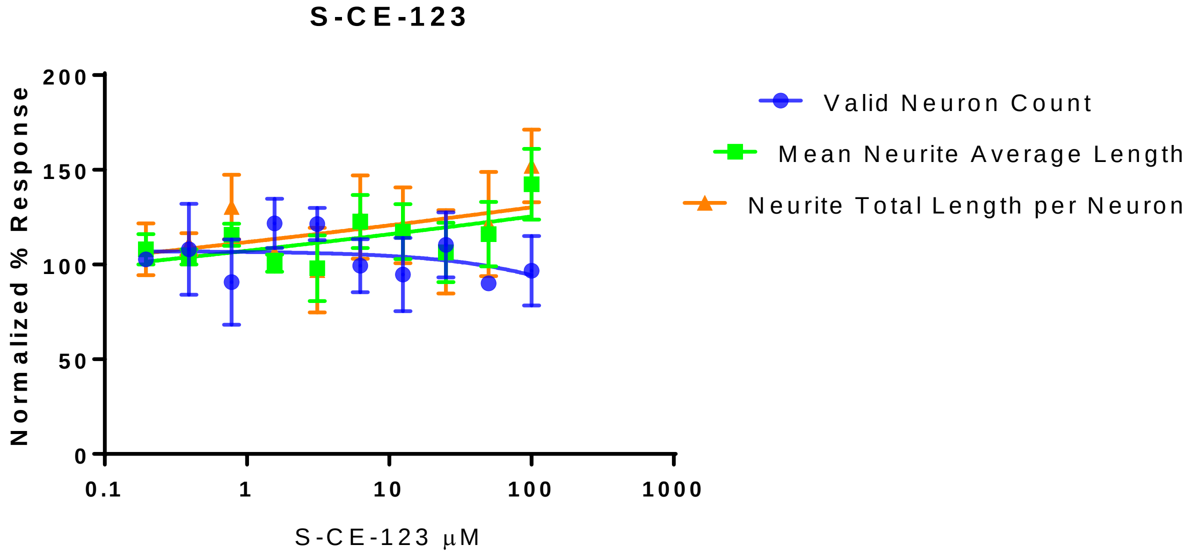

S-CE-123 (µM)	Valid Neuron Count			Mean Neurite Avg Length			Neurite Total Length per Neuron		
100	117	81	92	122	146	159	130	160	166
50	153*	90	90	100	114	134	93	123	148
25	95	129	107	124	94	101	130	86	104
12.5	117	84	83	102	131	119	98	136	128
6.25	85	113	100	139	115	114	147	125	103
3.13	115	131	118	78	107	109	72	104	115
1.56	126	132	107	105	96	101	106	103	109
0.78	83	116	73	118	109	120	133	112	146
0.39	100	135	89	104	100	108	115	104	113
0.2	101	107	100	108	100	116	93	111	120

*outlier removed from graph

Results
Control Compounds

Neurite Outgrowth

Nocodazole

Data Summary

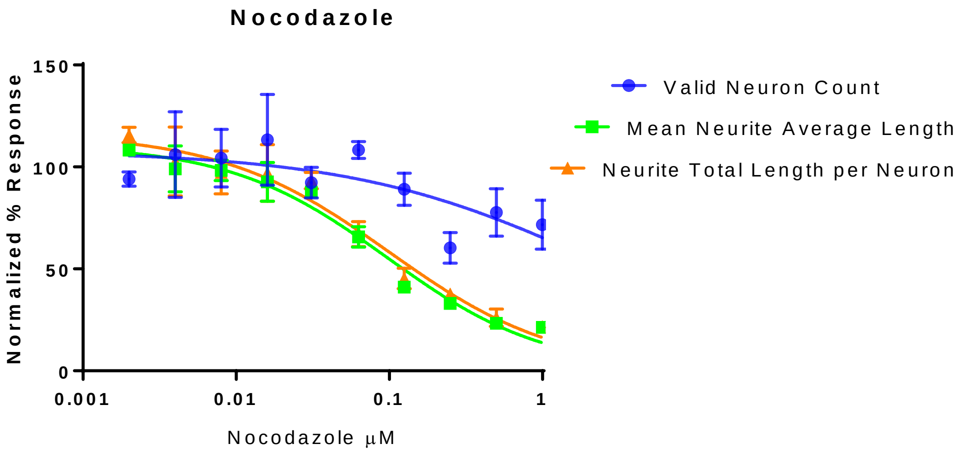

	Valid Neuron Count	Mean Neurite Average Length	Neurite Total Length per Neuron
EC50	2.22	0.097	0.0997

Nocodazole (µM)	Valid Neuron Count			Mean Neurite Avg Length			Neurite Total Length per Neuron		
1	71	60	84	19	23	22	21	22	22
0.5	80	88	65	23	23	24	28	21	29
0.25	69	56	56	31	34	34	38	38	37
0.125	80	95	92	42	42	39	46	50	40
0.063	107	113	105	65	61	71	69	60	72
0.031	101	87	89	89	85	89	89	97	94
0.016	122	130	88	93	83	102	90	88	113
0.008	88	112	113	103	93	99	108	87	97
0.004	128	104	86	93	92	112	84	107	117
0.002	91	98	93	106	108	111	114	113	120

Chlorpromazine

Data Summary

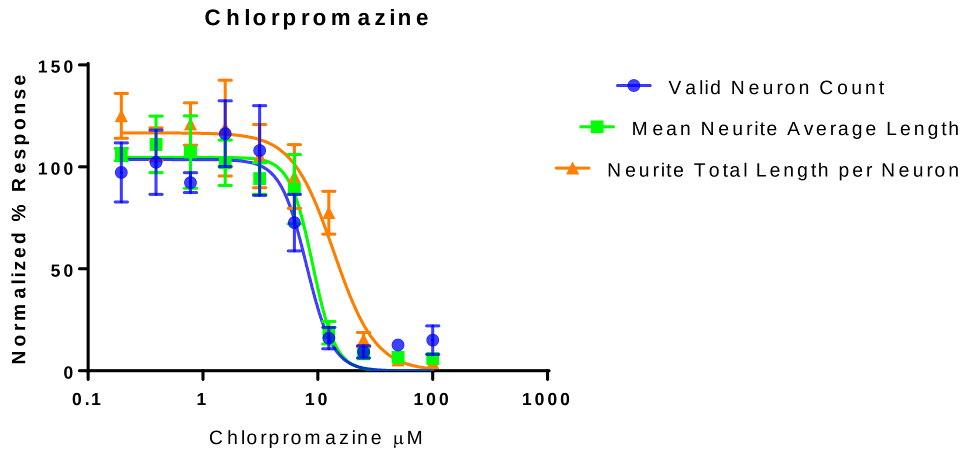

	Valid Neuron Count	Mean Neurite Average Length	Neurite Total Length per Neuron
EC50	7.986	9.022	13.8

Chlorpromazine (μM)	Valid Neuron Count			Mean Neurite Avg Length			Neurite Total Length per Neuron		
100	22	8	15	5	5	8	4	4	4
50	16	11	11	7	6	7	4	6	6
25	12	10	6	6	11	8	15	19	12
12.5	20	18	10	15	25	16	70	85	127*
6.25	88	69	61	73	87	107	83	90	113
3.13	131	106	87	88	92	103	105	90	121
1.56	99	131	119	92	100	114	93	125	139
0.78	89	98	90	88	111	123	114	116	133
0.39	116	106	85	102	104	127	102	113	118
0.2	112	97	83	106	103	109	114	125	136

Images

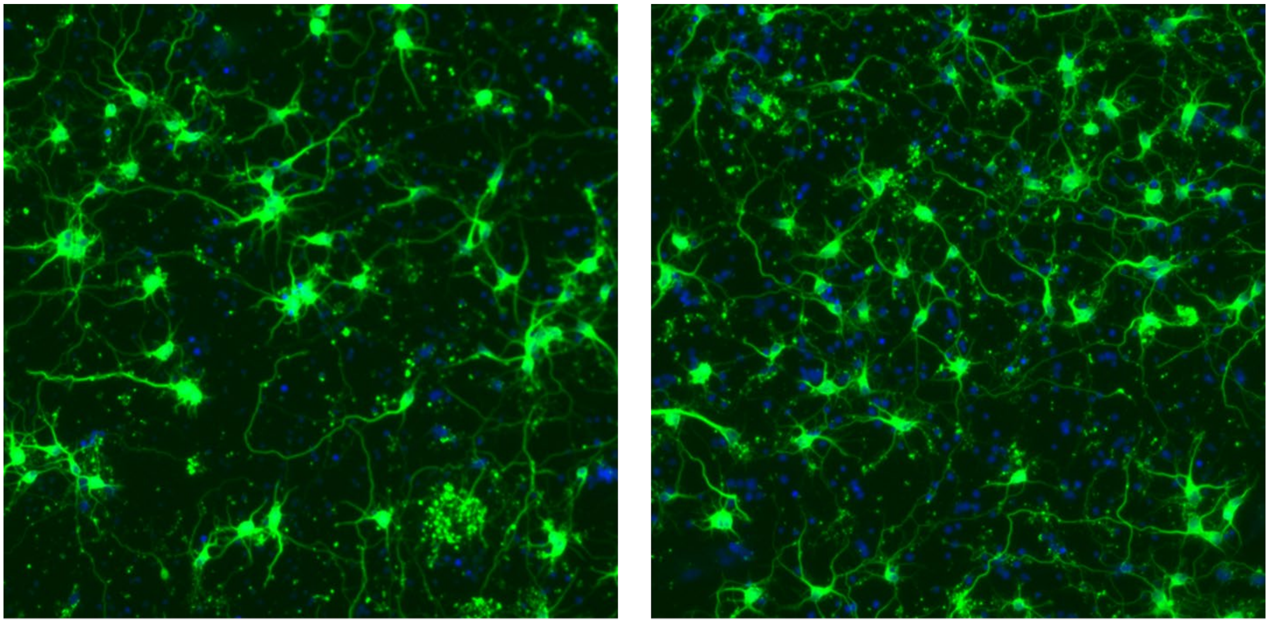

DMSO 0.5%

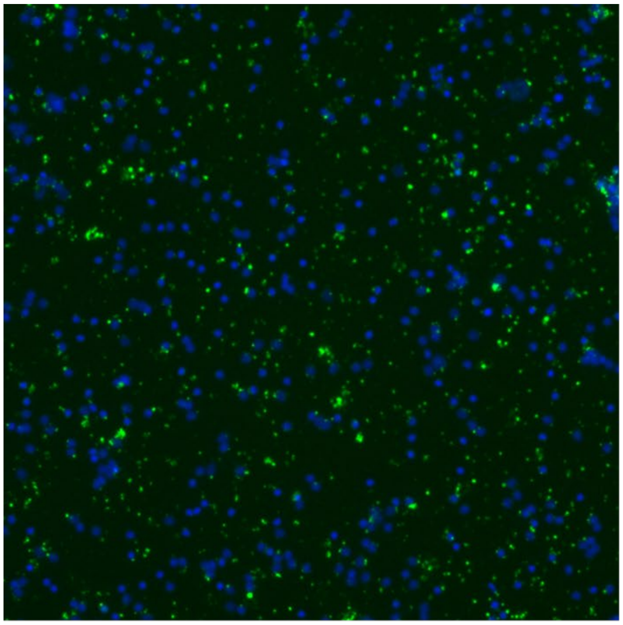

Chlorpromazine 100 µM

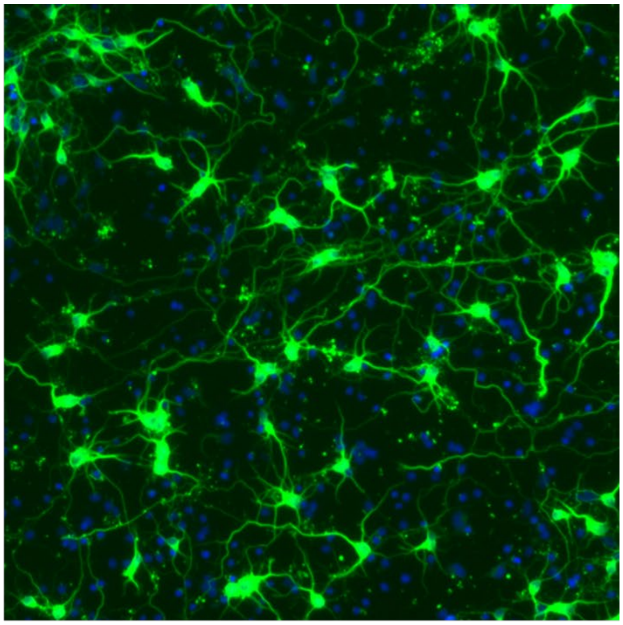

Chlorpromazine 0.2 µM

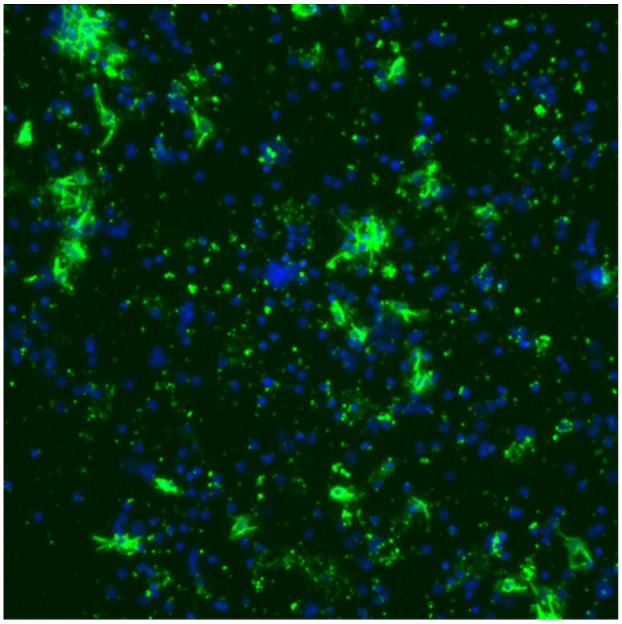

Nocodazole 1 µM

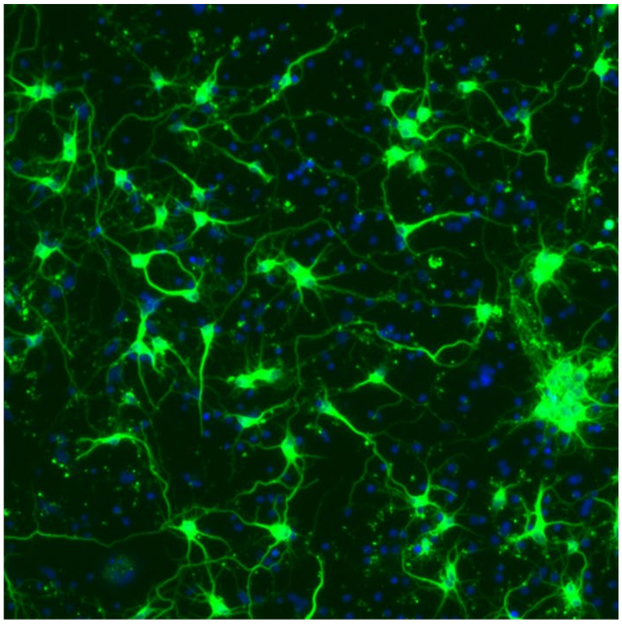

Nocodazole 0.002 µM

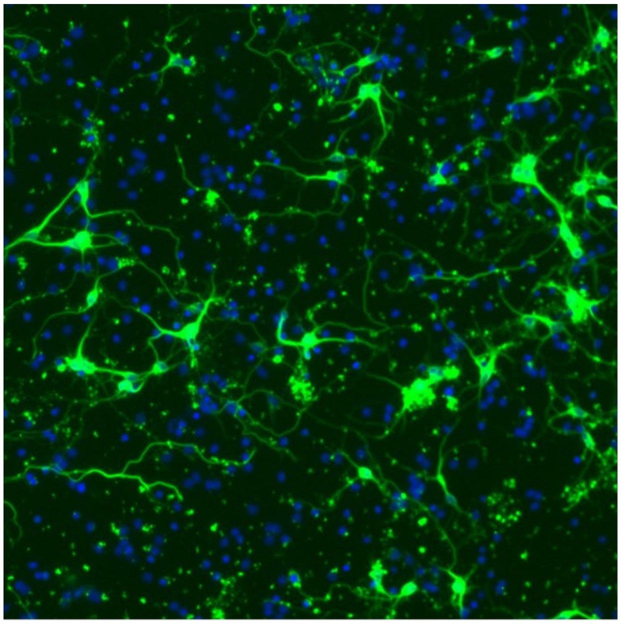

CE-158-1-1 100 µM

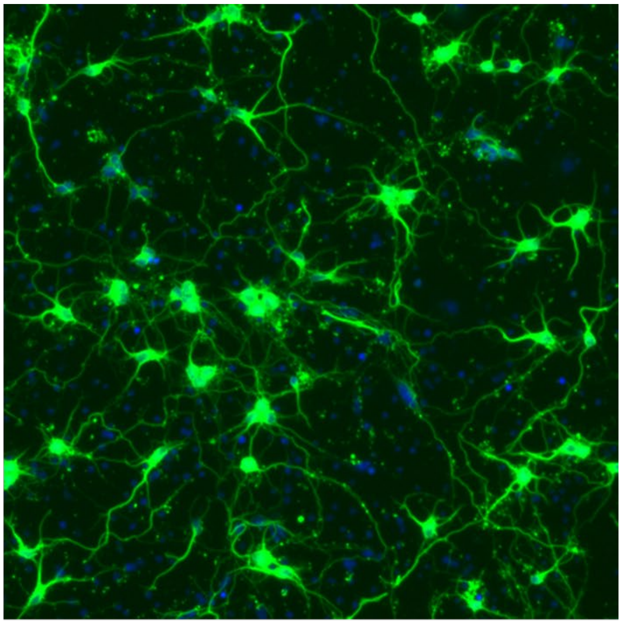

CE-158-1-1 0.2 µM

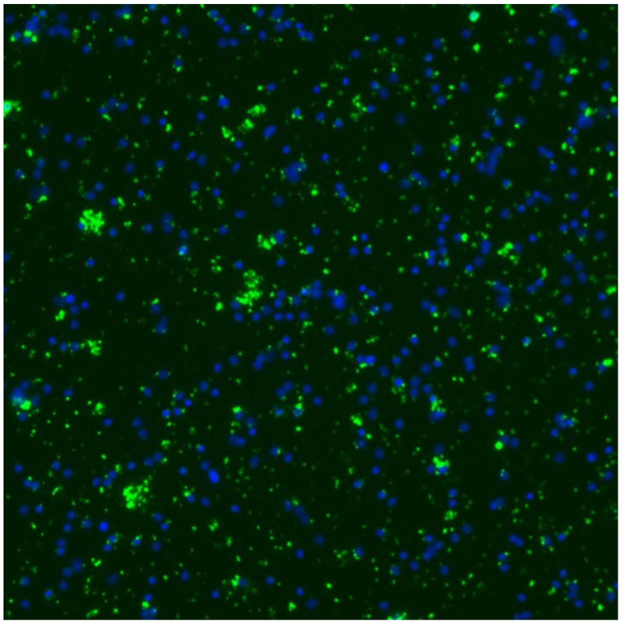

CE-196-2-1 100 µM

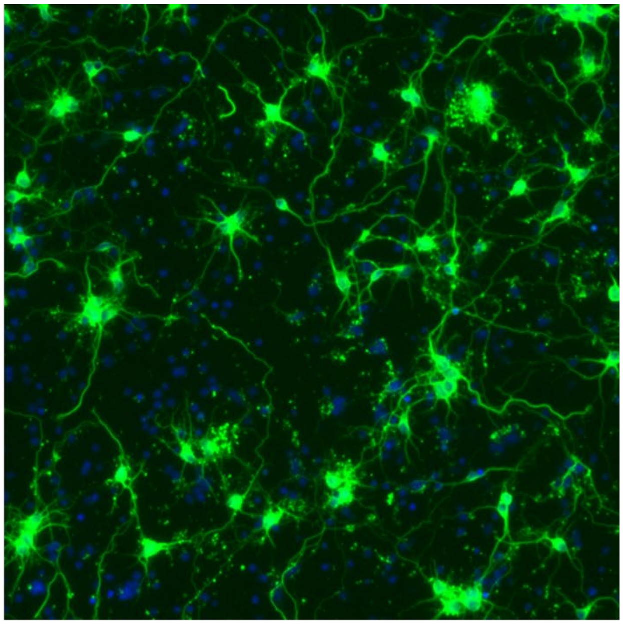

CE-196-2-1 0.2 µM

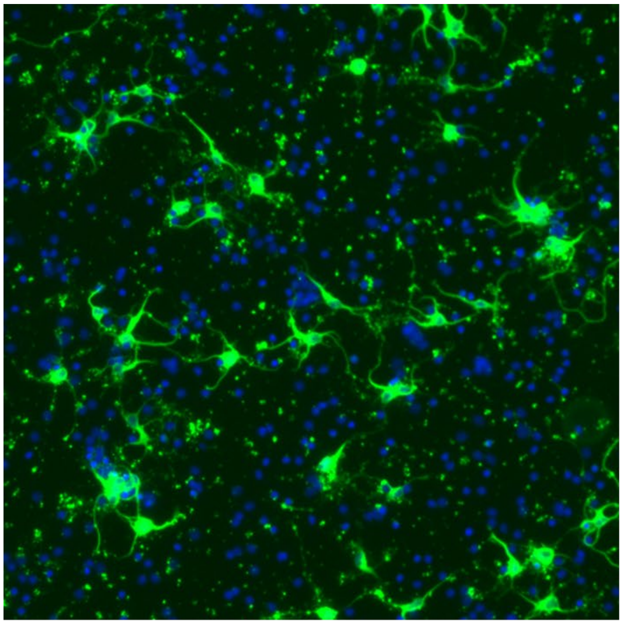

CE-144-2-1 100 µM

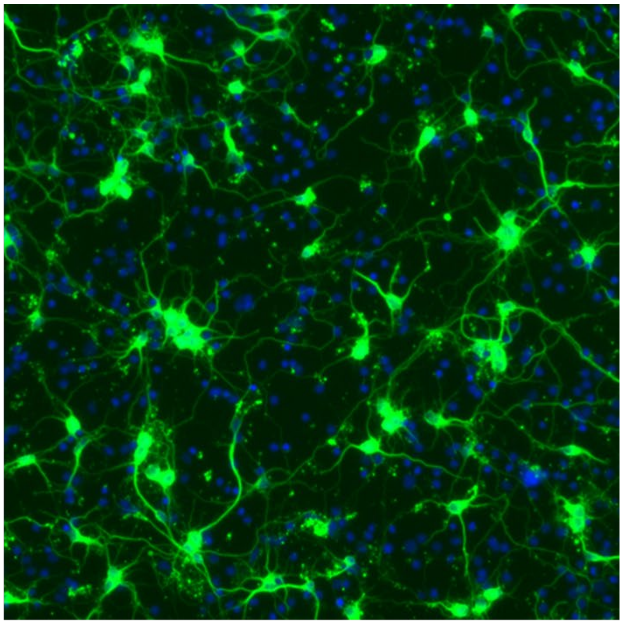

CE-144-2-1 0.2 µM

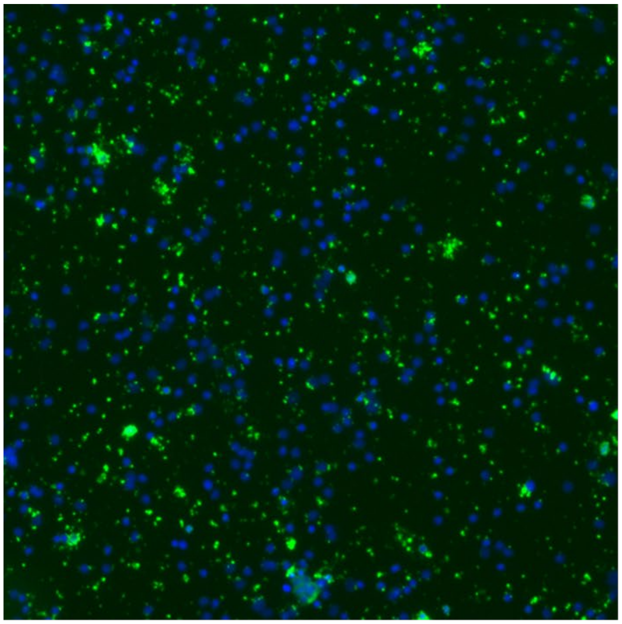

CE-196-2-2 100 μM

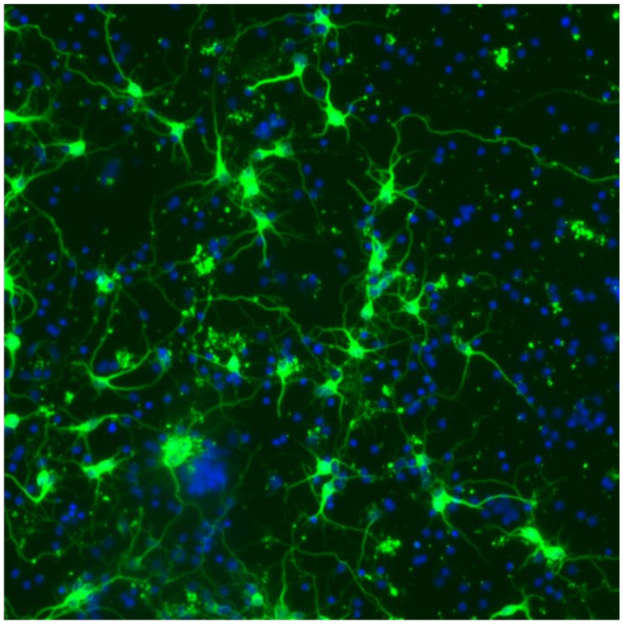

CE-196-2-2 0.2 μM

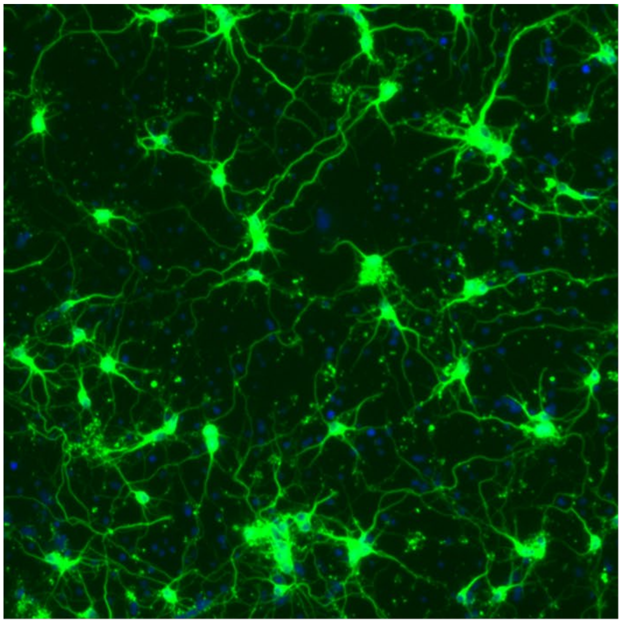

g-CE-123 100 μM

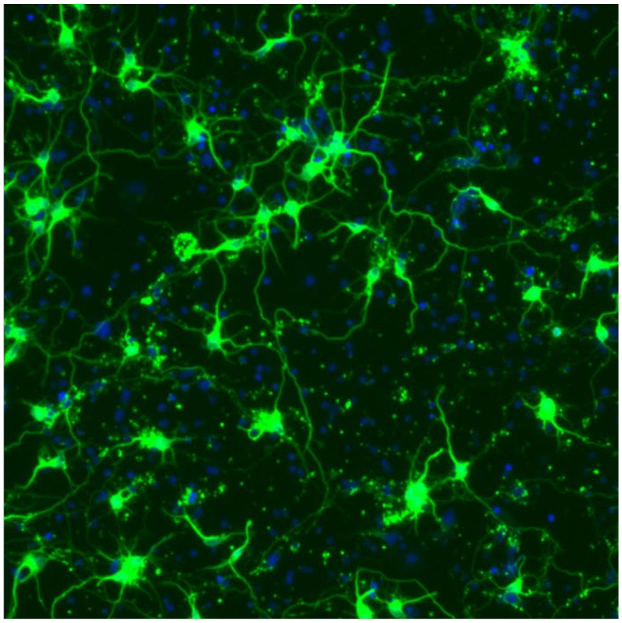

g-CE-123 0.2 μM

DRAFT REPORT

Pharmacokinetics study of CE-196-2-1 in rats after single intraperitoneal administration of CE-196-2-1 at 10 mg/kg.

DMPK_2020_494

FOR

UNIVERSITY OF VIENNA

AUTHOR(S):	Patrick Vinclair
CONTRIBUTORS:	Anne Larvor, Didier Boureme, Eric Erdociain, Jeanne Jaen, Anaïs Giry and Aude Gendras
DATE:	May 27th , 2020

Authentication Statement

This study was performed at the Evotec facilities
195 route d'Espagne
31036 TOULOUSE CEDEX

I, the undersigned, hereby declare that the findings provide a true and accurate record of the results obtained.

20 July 2020

Eric Erdociain
Study Director
eric.erdociain @evotec.com

20 July 2020

Aude Gendras
In-life Study leader
aude.gendras@evotec.com

20 July 2020

Jennifer Dana
Bioanalytical leader
jennifer.dana@evotec.com

TABLE OF CONTENTS

1.0 Study SUMMARY 4
2.0 STATEMENT OF COMPLIANCE 4
3.0 GENERAL INFORMATION 5
3.1 Study Dates 5
3.2 Evotec Contributing Scientists 5
3.3 Objectives 5
4.0 MATERIALS AND METHODS 6
4.1 Test articles 6
4.2 Test items Formulation 6
4.3 Test System 6
4.6 Dose Administration 8
4.7 Blood collection 8
4.8 Bioanalysis, PK interpretation and modelisation 9
4.8.1 Bioanalytical Methods 9
4.8.2 Pharmacokinetic analysis 11
4.9 Descriptive analysis 11
4.10 Study Plan adherence and deviation 11
5.0 Results 12
5.1 Administered dose 12
5.2 Clinical Observations and mortality 12
5.3 Sampling time 12
Brain were harvested at the exact time 12
5.4 Brain sampling 12
5.5 Brain concentrations measurement 13
5.6 Brain penetrance parameters 14
6.0 ConclusionS 14
7.0 Appendix 15
7.1 Individual body weights 15
7.2 Individual administered doses 16
7.3 Actual blood sampling times 17
7.4 Individual brain weights 18
7.5 Individual brain concentrations of CE-196-2-1 PO – 10 mg/kg 19

1.0 STUDY SUMMARY

The aim of this study was to assess the pharmacokinetics in brain of CE-196-2-1 in rats after single intraperitoneal administration at 10 mg/kg, for the compound.

Seven groups of three male Sprague Dawley rats were dosed with CE-196-2-1 at 10 mg/kg by intraperitoneal route. The sampling times were 0.083, 0.25, 0.5, 1, 3, 7 and 10 hours post-administration. A group of three rats was sacrificed and brains collected at each sampling time.

After an intra-cardiac infusion of NaCl 0.9% solution (50 mL) over 1 to 2 min, brains were collected as expected for bioanalyses assay.

For CE-196-2-1, the nominal IP dose formulation concentration was 1 mg/mL. The measured IP dose formulation was 107% of nominal concentration for CE-196-2-1, and was a clear yellow solution with some particles. Nominal dose is used in PK parameter determinations.

No adverse reactions were observed following oral administration of CE-196-2-1 throughout the study.

The main PK outcomes are summarized as follows:

The dose formulation analysis achieved 107 % of the nominal concentration. The outcomes was within our acceptance criteria. Nominal doses are used in PK parameter determinations.
Following a single IP administration of CE-196-2-1 at 10 mg/kg, a mean Cmax of 478 ng/g was observed in brain at a median Tmax of 1h with a long half-life of 3.0 h.

2.0 STATEMENT OF COMPLIANCE

The animals included in the study were housed at the Evotec France SAS animal facility. This facility was accredited by the French Ministry of Agriculture and by the Association for Assessment and Accreditation of Laboratory Animal Care International (AAALAC).
This study is compliant with the corresponding project APAFIS#04.4932.02. This project was reviewed by Evotec France Ethical Committee (identified below as CEPAL: CE 029) and authorized by the French Ministry of Education, Advanced Studies and Research.
Furthermore, the procedure related to this study was reviewed by the SBEA (internal Animal Welfare Body).

3.0 GENERAL INFORMATION

3.1 Study Dates

Acclimation start:	May 14th, 2020
In-life phase start:	May 19th, 2020
Bioanalytical phase start:	May 20th and 25th, 2020
Pharmacokinetic reporting:	May 27th, 2020

Responsible Personnel

Study Director:	Eric Erdociain
In life Study Leader:	Aude Gendras
Bioanalytical Leader:	Jennifer Dana
Study Sponsor:	Predrag Kalaba – University of Vienna

3.2 Evotec Contributing Scientists

Bioanalytical scientist:	Camille Lahille
In life study scientists:	Anaïs Giry, Emmanuelle Cortiade, Fanny Placide, Perrine Comptour and Aude Gendras

3.3 Objectives

The aim of this study was to assess the pharmacokinetics in brain of CE-196-2-1 after single intraperitoneal administration of CE-196-2-1 at 10 mg/kg in rat.

4.0 MATERIALS AND METHODS

4.1 Test articles

Test Item	Batch number	Molecular weight (g/mol)
CE-196-2-1	NC	390.98

NC : no communicated

The test items were kept at room temperature until formulation preparation.

4.2 Test items Formulation

For the compound, the formulation for intraperitoneal administration was prepared in Kolliphor® EL 30% at a nominal concentration of 1 mg/mL. A volume of 98.45 mL (98.45 g) of Kolliphor/water (30/70 – v/v) was added to 99.4 mg of CE-196-2-1.

The formulation preparation was:

- put under magnetic stirring for 1h45 at 37°C
- sonicated for 15 minutes
- put under magnetic stirring for 15 min at 37°C
- sonicated for 15 minutes
- put under magnetic stirring for 4h15 at 37°C

The solution was prepared extemporaneously and left under stirring until the end of administrations. The obtained formulation was a clear yellow solution with particles.

For the compounds, based on the 1 mg/mL, the IPdose formulation was 107% of the nominal concentration for CE-196-2-1.

4.3 Test System

Compound : CE-196-2-1
Animals: 21 male Sprague Dawley (Rj Han:SD) rats
Weight: 290g to 327g at the day of treatment (see 7.1, Individual body weight)
Breeder: Janvier Labs, Le Genest-Saint-Isle, 53940 Saint Berthevin Cedex, France.
Pre-treatment period: after their arrival, the animals were acclimated for a period of 5 days before the beginning of the dosing period.
Identification: the rats were individually identified using a permanent marker on tails.

Group	Sampling Time	Rat Id
Group 1 -10 mg/kg	Destructive<br>0.083h (brain)	1,2,3
Group 2 -10 mg/kg	Destructive<br>0.25h (brain)	4,5,6
Group 3 -10 mg/kg	Destructive<br>0.5h (brain)	7,8,9
Group 4 -10 mg/kg	Destructive<br>1h (brain)	10,11,12
Group 5 -10 mg/kg	Destructive<br>3h (brain)	13,14,15
Group 6 -10 mg/kg	Destructive<br>7h (brain)	16,17,18
Group 7 -10 mg/kg	Destructive<br>10h (brain)	19,20,21

a. Environmental Conditions

Upon their arrival at Evotec France, rats were group-housed in dedicated home cages (1291H; size 820 cm^{2,} Tecniplast®).

The animal room conditions were set as follows:

- temperature: 22 ± 2°C,
- relative humidity: 50 ± 10%,
- light/dark cycle: 12 h/12 h,
- air change rate: 12 to 15 cycles/hour of filtered, 100% fresh air

The corresponding instrumentation and equipment are checked and calibrated at regular intervals. The temperature and relative humidity are recorded continuously using a Building Information Management System (recording devices equipped with alarm systems).
All animals had free access to filtered tap water. Bacterial and chemical analyses of water are performed regularly by external laboratories. These analyses include the detection of possible contaminants (pesticides and heavy metals).
All rats were fed with a maintenance chow (AO4C, Safe, 89290 Augy) during the acclimation and the study.

4.4 Study design

Group	Number of animals/ Group	Test Article	Dose Level (mg/kg)	Route	Dose Volume (mL/kg)	Sampling time number
Group 1	3	CE-196-2-1	10	IP	10	0.083
Group 2						0.25
Group 3						0.5
Group 4						1
Group 5						3
Group 6						7
Group 7						10

4.5 Randomization

On the day of dosing, rats have been randomized to obtain groups of three rats with homogeneous weights between groups for the study.

4.6 Dose Administration

For intraperitoneal route, the dose formulation was administered by injection on animal's lower right quadrant of the abdomen, using needle fitted on a plastic syringe. Needles were changed between each animal.

The volume of dose formulation administered to each animal was adjusted according to the body weight. A constant dosage volume of 10 mL/kg was used.

During the dosing, minor deviations were observed without any impact on the outcomes of the study:

- Rat # 10 : small dose formulation drop observed at the injection site after removing the needle
- Rat # 17: injection in two times
- Rat # 19 : blood drop at the injection site

The exact dose administered to each animal was measured by weighting the dosing system before and after administration.

The dose formulations were kept at room temperature and protected from light throughout the dosing procedure.

4.7 Blood collection

a. Destructive design:

At each scheduled sampling time, rats were anesthetized and exsanguinated, then an intra-cardiac infusion of NaCl 0.9% solution (50 mL) was performed over 1 to 2 min. After, brains

were collected and weighed and placed in IKA tubes. Brain samples were stored at -20°C until bioanalysis

b. Clinical observation

All animals were observed for mortality and general condition at least twice daily (once in the morning and once in the afternoon) during the week and once daily during the weekend.

4.8 Bioanalysis, PK interpretation and modelisation

4.8.1 Bioanalytical Methods

a. Preparation of brain samples

For each Brain, 4 volumes of water were added to ensure an efficient grinding (diluted 5-fold post-grinding). Samples were then ground using IKA tubes.

b. Treatment of samples

Brain homogenates (20 µL) were mixed with 120µL of the precipitant solution (ACN + Tolbutamide as ISTD). Half of the supernatant (70 µL) was then diluted 1/1 into water and 5µL were injected.

CE-196-2-1 was quantified by LC-MS/MS following standard conditions (see below) or equivalent.

c. Instrument configuration

Test article	CE-196-2-1
**Pump**	Agilent 1100 series
**Auto-sampler**	HTC PAL – CTC analytics
**Column Oven**	Agilent 1100 series
**Mass Spectrometer**	THERMO Quantum EMR Ultra
**Software**	Xcalibur 2.1.0 SP1

d. Chromatography

Test article	CE-196-2-1
**Column**	Hypersil Gold C18 1.9µm 50x2.1mm
**Flow**	700 µl/mn
**Oven temperature**	40°C
**Mobile phase**	Phase A: HPLC water with 0.1% formic acid<br>Phase B: Acetonitrile with 0.1% formic acid

**Gradient mode**	Time (min): 0.00, 0.10, 0.98, 1.48, 1.50, 1.70<br>% Phase A: 98, 98, 5, 5, 98, 98<br>% Phase B: 2, 2, 95, 95, 2, 2
**Injection volume**	5 µL

e. Mass spectrometry

**Test article**	**CE-196-2-1**
**Ionization mode**	ESI positive mode
**Source Temperature**	380°C
**Skimmer**	Tolbutamide : -<br>CE-196-2-1 : 8
**Collision Energy Voltage**	CE=11 V for CE-196-2-1<br>CE=30 V for Tolbutamide
**Multiple Reaction Monitoring**	CE-196-2-1 m/z 391→ 245.1<br>Tolbutamide m/z 271.4→ 91.2
**Retention Time**	1.44 mn for CE-196-2-1<br>1.27 mn for Tolbutamide

f. Preparation of quality control and calibration samples

Quality Control (QC) samples and Calibration Samples were prepared daily by spiking Sprague-Dawley plasma or Brain homogenates with working solutions prepared from independent weighing. The calibration curve were calculated from calibration levels at 2.5, 5, 10, 25, 50, 100, 200, 500, 1000, 2000 and 5000ng/mL with at least six non-zero concentrations that back-calculate to ± 20% of their nominal value. The concentrations for Quality Controls were 12.5, 50, 450, 750, 1500 and 4000 ng/mL (At least 66% of all QC samples must be within ± 20% of their nominal value and at least 50% of QC samples at a given concentration must be within +/- 20% of their nominal value). For CE-196_2_1, regressions used were quadratic for both plasma and brain, not forced through the origin, and weighted by 1/x (with R2 >0.98).

g. Sensitivity of the method

The LLOQ was expressed as ng/g for Brain (one gram of Brain being ground with 4 volumes of water).

CE-196-2-1, LLOQ were 5 ng/ml for plasma and 25 ng/g for Brain.

4.8.2 Pharmacokinetic analysis

The pharmacokinetic analysis was performed using Phoenix 64 (WinNonLin) with NCA model 200.

4.9 Descriptive analysis

Results are expressed in mean ± standard deviation (sd).

Descriptive analysis were performed using Excel software.

When 1 out of 3 values is BLQ, it is set to 1/2 LLOQ for mean and SD calculations

4.10 Study Plan adherence and deviation

A minor deviation was observed during the dosing without any impact on the outcomes of the study, some particles were observed in the formulation at administration.

5.0 RESULTS

5.1 Administered dose

For both compounds, the administered dose is targeted at 10 mg/kg.

The administered dose are presented in 7.2 Individual administered dose.

For CE-196-2-1, all the administered individual doses were within the range 100-109% of the targeted dose.

5.2 Clinical Observations and mortality

No morbidity or mortality were observed after IP administration. No adverse events or clinical signs were reported in the study book.

5.3 Sampling time

Brain were harvested at the exact time.
Individual times of collect brain are presented in Appendix 7.3.

5.4 Brain sampling

Individual weights of collected brain are presented in Appendix 7.4.

5.5 Brain concentrations measurement

a. Brain CE-196-2-1 concentration after single intraperintoneal dosing at 10 mg/kg

Individual brain concentrations are presented in Appendix 7.5 and mean brain concentration levels are reported in the table 1 below.

Table 1: Mean Brain concentration measurements of CE-196-2-1 (ng/g)

Time (H)	Brain Concentration (mean in ng/g ± SD)
0.08	139±24.4
0.25	314 (n=2)
0.5	429±140
1	478 (n=2)
3	147±105
7	80.4±26.8
10	28.6±15.5

As shown in Figure 1, following the single intraperitoneal dosing, mean brain concentration levels of CE-196-2-1 reached a C_{max} of 478 ng/g at 1h post-dosing, then concentration in brain decreased slowly up to reach 28.6±15.5 ng/g at 10h post-dosing.

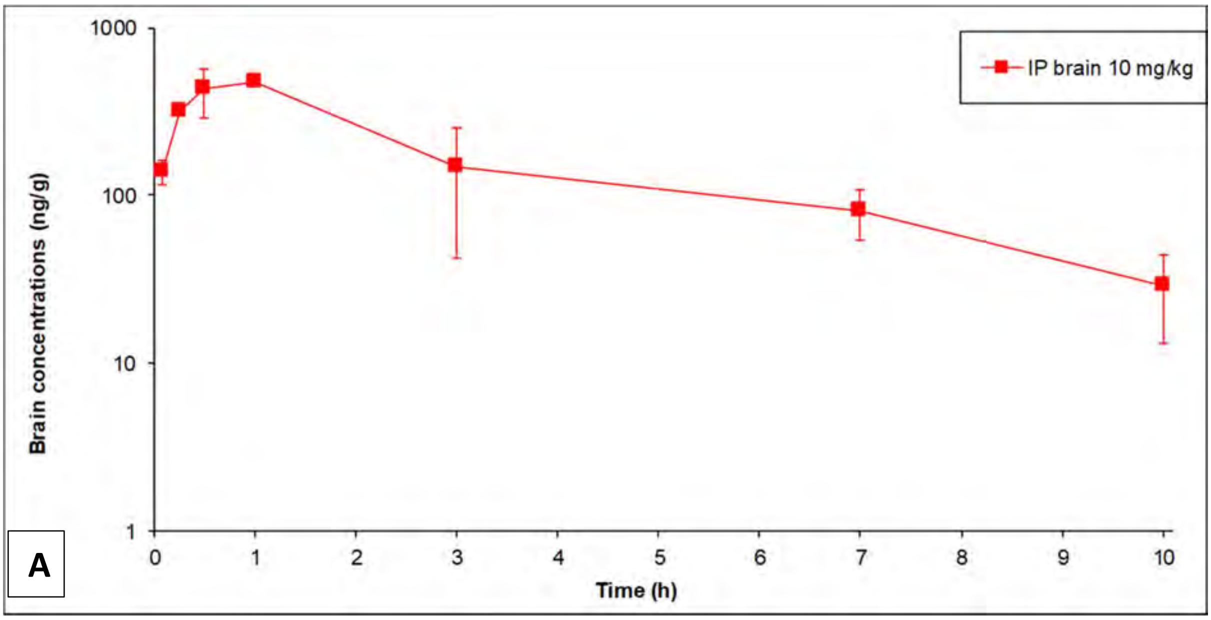

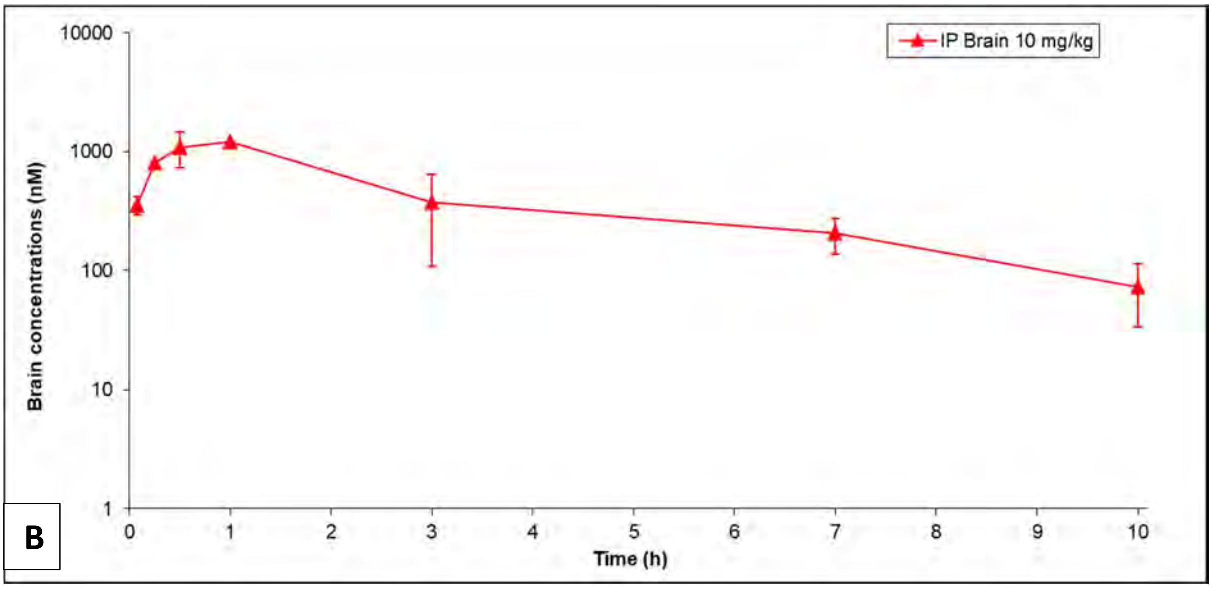

Figure 1: Graphical representation of mean total brain concentrations of CE-196-2-1 in ng/g (**A**) and nM (**B**) following IP administration to Male Sprague Dawley Rat at 10 mg/kg (each point represent the mean of 3 animals ± SD, except for 0.25 and 1 h time points with only 2 animals w/o SD calculation)

5.6 Brain penetrance parameters

Brain penetrance parameters are reported in the table 2.

Table 2: Pharmacokinetics parameters after intraperitoneal administration of CE-196-2-1 in rats:

**Matrix**	C_{max} (ng/g)	t_{max} (h)	AUC (0-last) (ng./g)	AUC (0-inf) (ng.h/g)	t_{last} (h)	$t_{1/2z}$ (h)
Brain	478	1	1518	1643	10	3.0

6.0 CONCLUSIONS

For CE-196-2-1, all rats were administered at the expected dose level of 10 mg/kg. All brain were collected after infusion at the exact time and were transferred for bioanalysis.

Following a single administration of CE-196-2-1 at 10 mg/kg by IP route, a high inter-individual variability was observed probably due to the non-homogeneous administered solution. The maximum concentration C_{max} of 478 ng/g was observed at T_{max} of 1h. A long half-life of 3.0 h was observed in the brain.

7.0 APPENDIX

7.1 Individual body weights

Treatment group	Animal id	Bodyweight (g)	Mean bodyweight per group (g)
Group 1 CE-196-2-1	1	292	305
	2	297	
	3	327	
Group 2 CE-196-2-1	4	294	307
	5	309	
	6	317	
Group 3 CE-196-2-1	7	295	302
	8	301	
	9	311	
Group 4 CE-196-2-1	10	290	306
	11	308	
	12	321	
Group 5 CE-196-2-1	13	294	307
	14	307	
	15	321	
Group 6 CE-196-2-1	16	291	307
	17	306	
	18	323	
Group 7 CE-196-2-1	19	290	305
	20	310	
	21	314	

7.2 Individual administered doses

Treatment group	Animal id	Bodyweight (g)	Weight of administered formulation (g)	Dose (mg/kg)
Group 1 CE-196-2-1	1	292	2.99	10.3
	2	297	3.01	10.2
	3	327	3.50	10.8
Group 2 CE-196-2-1	4	294	3.02	10.4
	5	309	3.15	10.3
	6	317	3.30	10.5
Group 3 CE-196-2-1	7	295	3.12	10.6
	8	301	3.16	10.6
	9	311	3.33	10.8
Group 4 CE-196-2-1	10	290	3.03	10.6
	11	308	3.12	10.2
	12	320	3.24	10.2
Group 5 CE-196-2-1	13	294	3.05	10.5
	14	307	3.22	10.6
	15	320	3.18	10.0
Group 6 CE-196-2-1	16	291	3.02	10.5
	17	306	3.16	10.4
	18	323	3.50	10.9
Group 7 CE-196-2-1	19	290	3.06	10.5
	20	310	3.17	10.2
	21	314	3.20	10.2

7.3 Actual blood sampling times

Animal ID	Theoretical sampling time (hh:mm)						
	00:05	00:15	00:30	01:00	03:00	07:00	10:00
1	00:05						
2	00:05						
3	00:05						
4		00:15					
5		00:15					
6		00:15					
7			00:30				
8			00:30				
9			00:30				
10				**01:03**			
11				01:00			
12				01:00			
13					03:00		
14					03:00		
15					03:00		
16						07:00	
17						07:00	
18						07:00	
19							10:00
20							10:00
21							10:00

7.4 Individual brain weights

Animal id	Brain weight (g)
**1**	1.786
**2**	1.774
**3**	2.018
**4**	1.901
**5**	1.885
**6**	1.983
**7**	1.895
**8**	1.926
**9**	2.028
**10**	1.995
**11**	1.890
**12**	1.852

Animal id	Brain weight (g)
**13**	2.073
**14**	1.989
**15**	2.075
**16**	1.957
**17**	1.903
**18**	2.072
**19**	1.891
**20**	2.020
**21**	2.009

7.5 Individual brain concentrations of CE-196-2-1 PO – 10 mg/kg

Time (hours)	Rat Id	Brain concentrations	
		(ng/g)	(nM)
**0.083**	**1**	152	388
	**2**	154	394
	**3**	111	283
**0.25**	**4**	313	800
	**5**	BLQ (*)	
	**6**	314	804
**0.5**	**7**	513	1312
	**8**	267	683
	**9**	506	1294
**1**	**10**	550	1408
	**11**	405	1035
	**12**	BLQ (*)	
**3**	**13**	250	640
	**14**	40.3	103
	**15**	152	388
**7**	**16**	66.2	169
	**17**	111	285
	**18**	63.8	163
**10**	**19**	43.3	111
	**20**	30.0	76.8
	**21**	BLQ	

BLQ: Below the limit of quantification
BLQ (*): not used in the descriptive statistics as outlier compared to the 2 other rats

Title:

Absolute Configuration Determination Report

**GENERAL INFORMATION**	
**Customer**	University of Vienna
**Sales Order Number**	
**Sample code (Our ref.)**	**CE196-S1P1-S1P2**
**Sample description (Your ref.)**	**CE196-S1P1-S1P2**
**VCD-spectrometer**	ChiralIR w/ Dual*PEM*
**Report prepared by**	Jordan Nafie
**Report validated and signed by**	Rina K Dukor
**Date**	May 27, 2020
**RESULTS**	
Absolute Configuration of CE196-S1P1 is (CR,SS)<br>Absolute Configuration of CE196-S1P2 is (CS,SR)	Confidence Level: **88%**
**MEASUREMENT PARAMETERS**	
**Concentration**	15mg / 100uL
**Solvent**	$CDCl_3$
**Resolution**	4 cm^{-1}
**PEM setting**	1400 cm^{-1}
**Number of scans/Measurement time**	12 hours per enantiomer
**Sample cell**	BaF_2
**Path length**	100 μm
**CALCULATION DETAILS**	
**Gaussian version**	Gaussian 09
**Total low-energy conformers used for Boltzmann sum**	26
**Methodology and basis set for DFT calculations**	B3LYP / 6311G3df2pd w/cpcm ($CDCl_3$)
**Enantiomer used for calculation**	R,S = (CR,SS)
**Total calculated conformers**	28
**Number of low-energy conformations shown in report**	1

COMMENTS

The confidence level is a measure of the degree of congruence between a calculated and measured spectrum. If identical spectra are being compared the confidence level is 100%. The confidence level (CL) is not the likelihood that the assignment is correct. Rather it's a measure of quality or degree of agreement between calculated and measured spectra. With a CL of 88% for this molecule, the visual agreement between measured and calculated spectra is very good despite some minor frequency shifts. Since the objective of this experiment was to distinguish diastereomers, the experimental spectra were compared against each calculated diastereomer. Using a direct comparison of VCD spectra, as well as an enhancement of subtracted VCD spectra, the absolute and relative configurations were determined with high confidence.

Title:

Absolute Configuration Determination Report

Structure of CE196-S1P1:

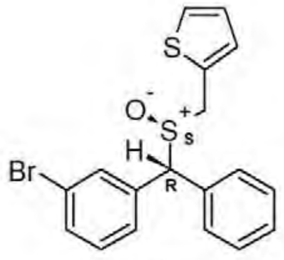

CE-196 S1P1

Structure of CE196-S1P2:

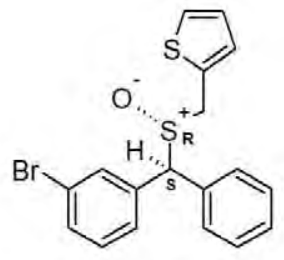

CE-196 S1P2

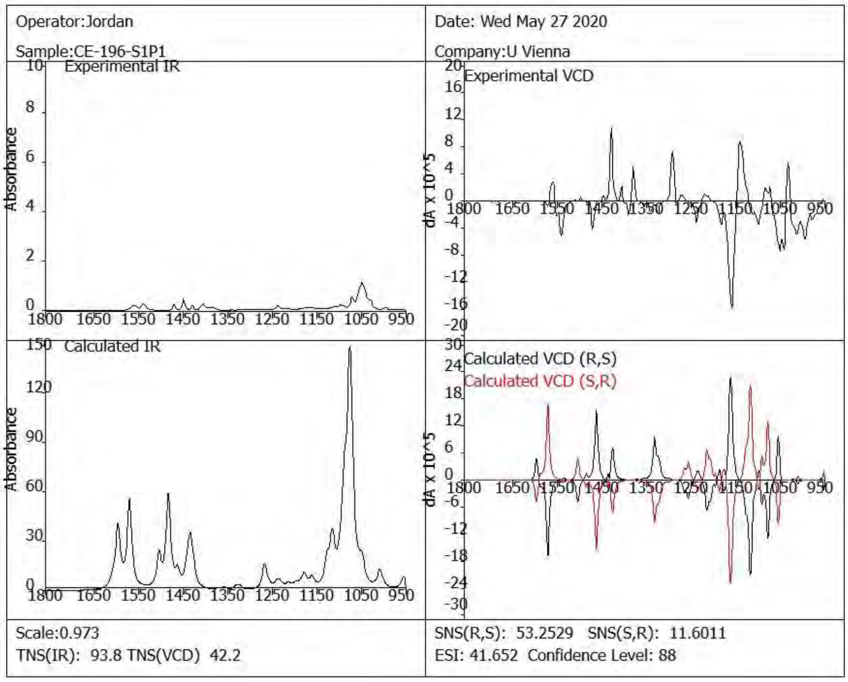

CompareVOA Results.
Please note: In this plot the frequency scaling factor is not applied.

Table 1. Numerical comparison describing the similarity in the range of 950 - 1800 cm^{-1} between the calculated IR and VCD spectra for the **(R,S) = (CR,SS)** enantiomer at the B3LYP/6311G3df2pd (w/cpcm – chloroform) level and the observed IR and VCD spectra for **CE196-S1P1**.

Cal. (950-1800cm^{-1})	Numerical comparison	Observed
		**CE196-S1P1**
**(R,S)**	scaling factor	0.973
	IR similarity (%)	93.8
	$^{a}\sum$ (%)	53.2529
	$^{b}\Delta$ (%)	41.652
	Confidence Level (%)	88

$^{a}\sum$: single VCD similarity, gives the similarity between the calculated and observed VCD spectra.
$^{b}\Delta$: enatiomeric similarity index, gives the difference between the values of $\sum$ for both enantiomers of a given diastereoisomer.

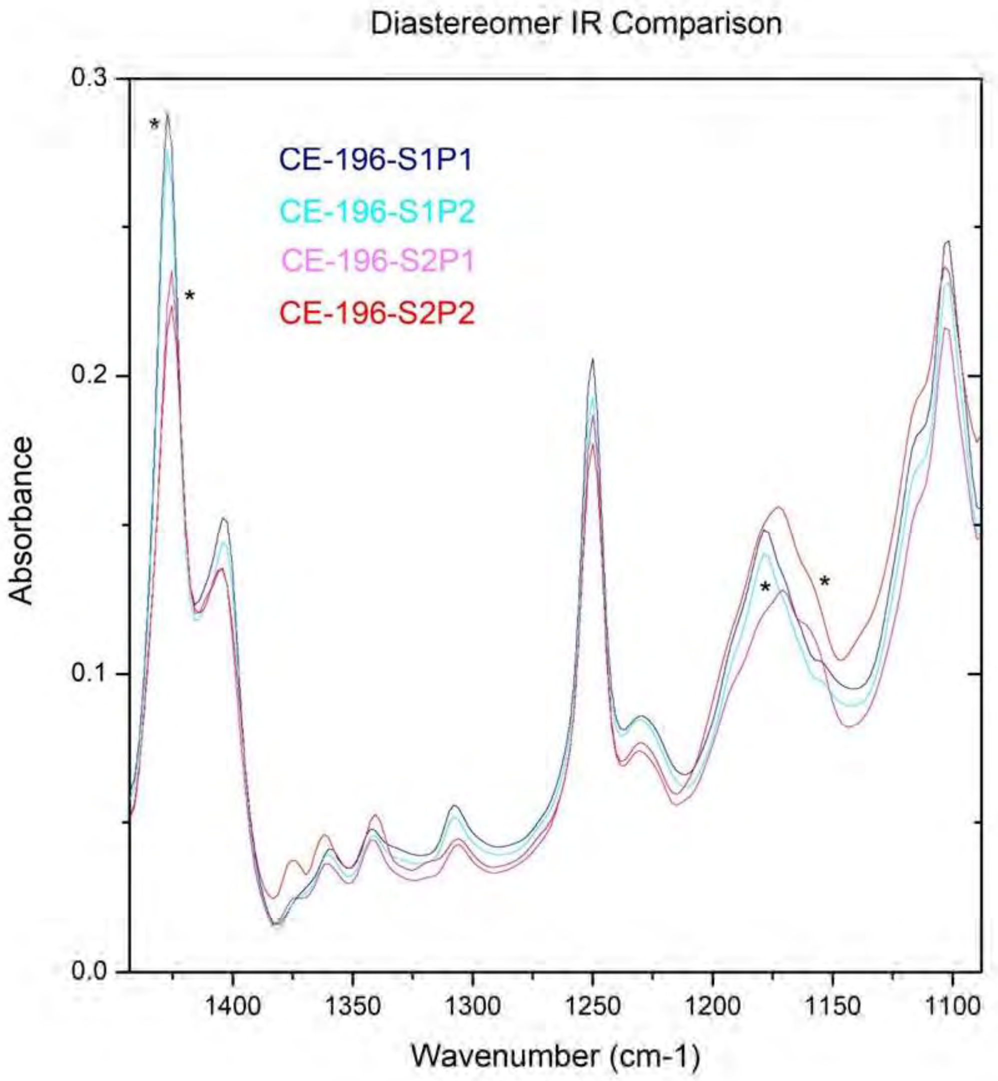

As a double check, the enantiomer pairs were determined to be S1P1 / S1P2 as one pair and S2P1 / S2P2 as the other pair – as confirmed by the small differences in the IR spectra shown above.

Title:

Absolute Configuration Determination Report

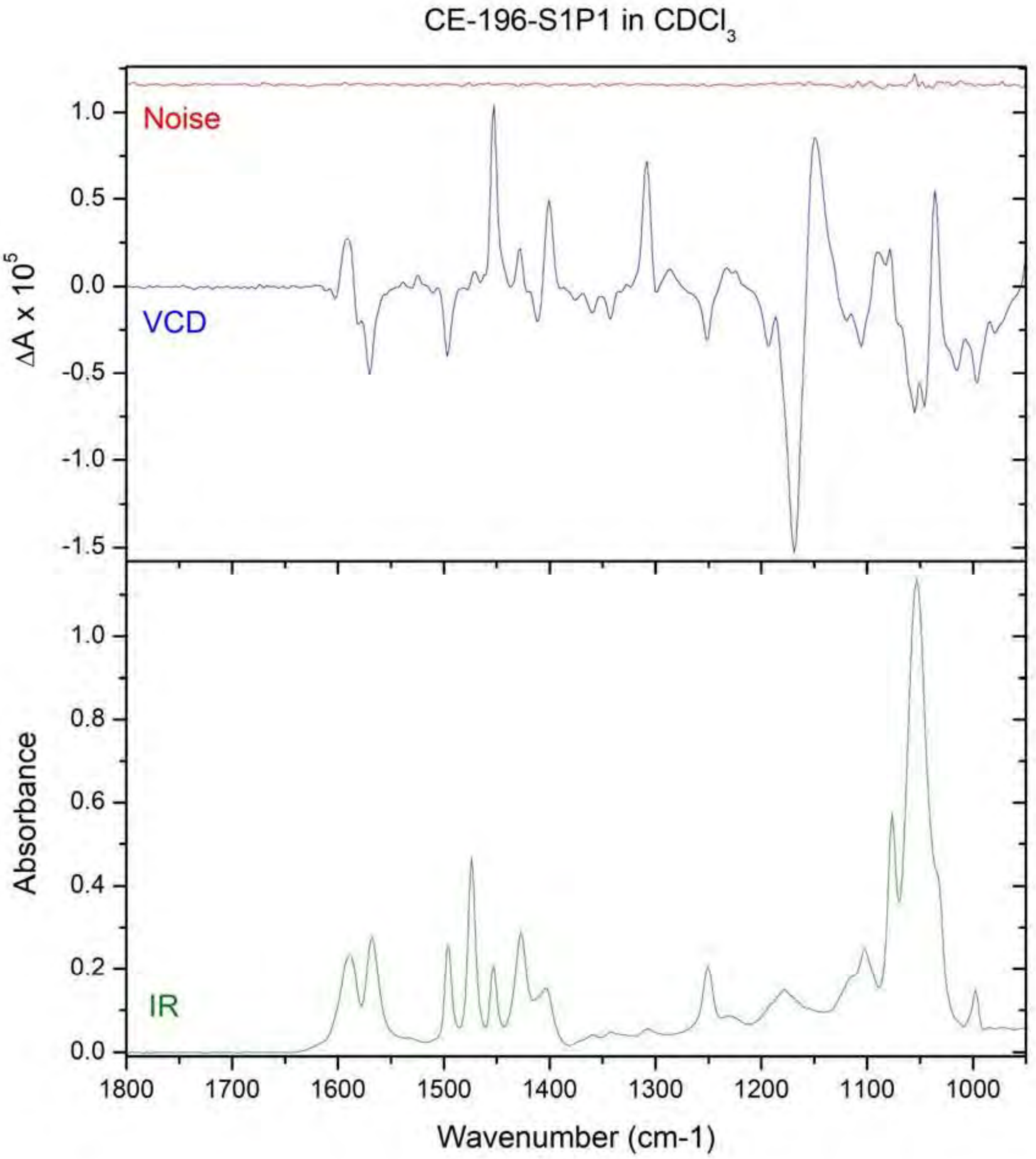

IR (lower frame) and VCD (upper frame) spectra of **CE196-S1P1** in $CDCl_3$; 100um path-length cell with BaF_2 windows; 12 h collection for each enantiomer; instrument optimized at 1400 cm^{-1}. Solvent subtracted IR and enantiomer subtracted VCD spectra are shown. Uppermost trace is the VCD noise spectrum.

Title:

Absolute Configuration Determination Report

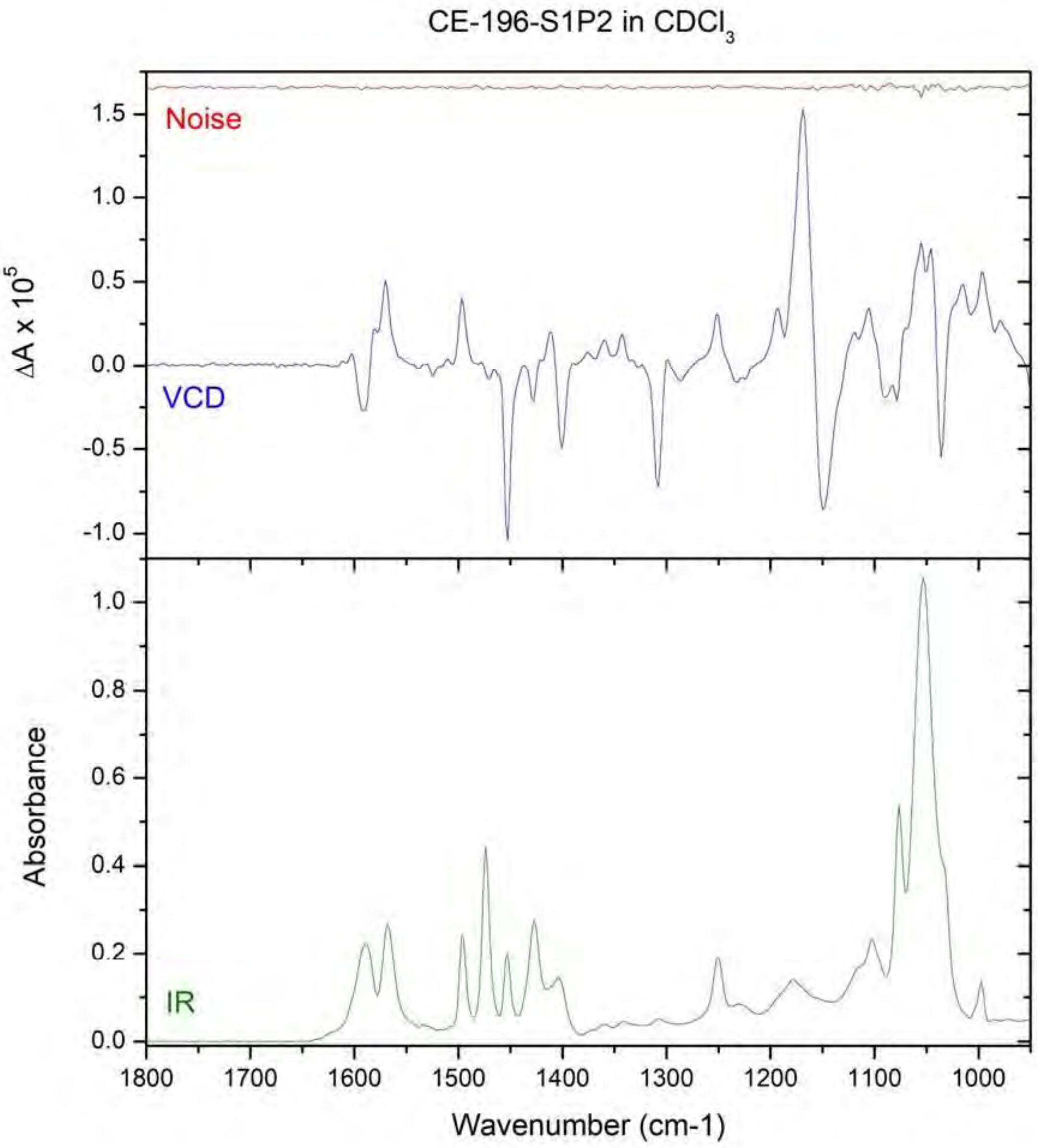

IR (lower frame) and VCD (upper frame) spectra of **CE196-S1P2** in $CDCl_3$; 100um path-length cell with BaF_2 windows; 12 h collection for each enantiomer; instrument optimized at 1400 cm^{-1}. Solvent subtracted IR and enantiomer subtracted VCD spectra are shown. Uppermost trace is the VCD noise spectrum.

Title:

Absolute Configuration Determination Report

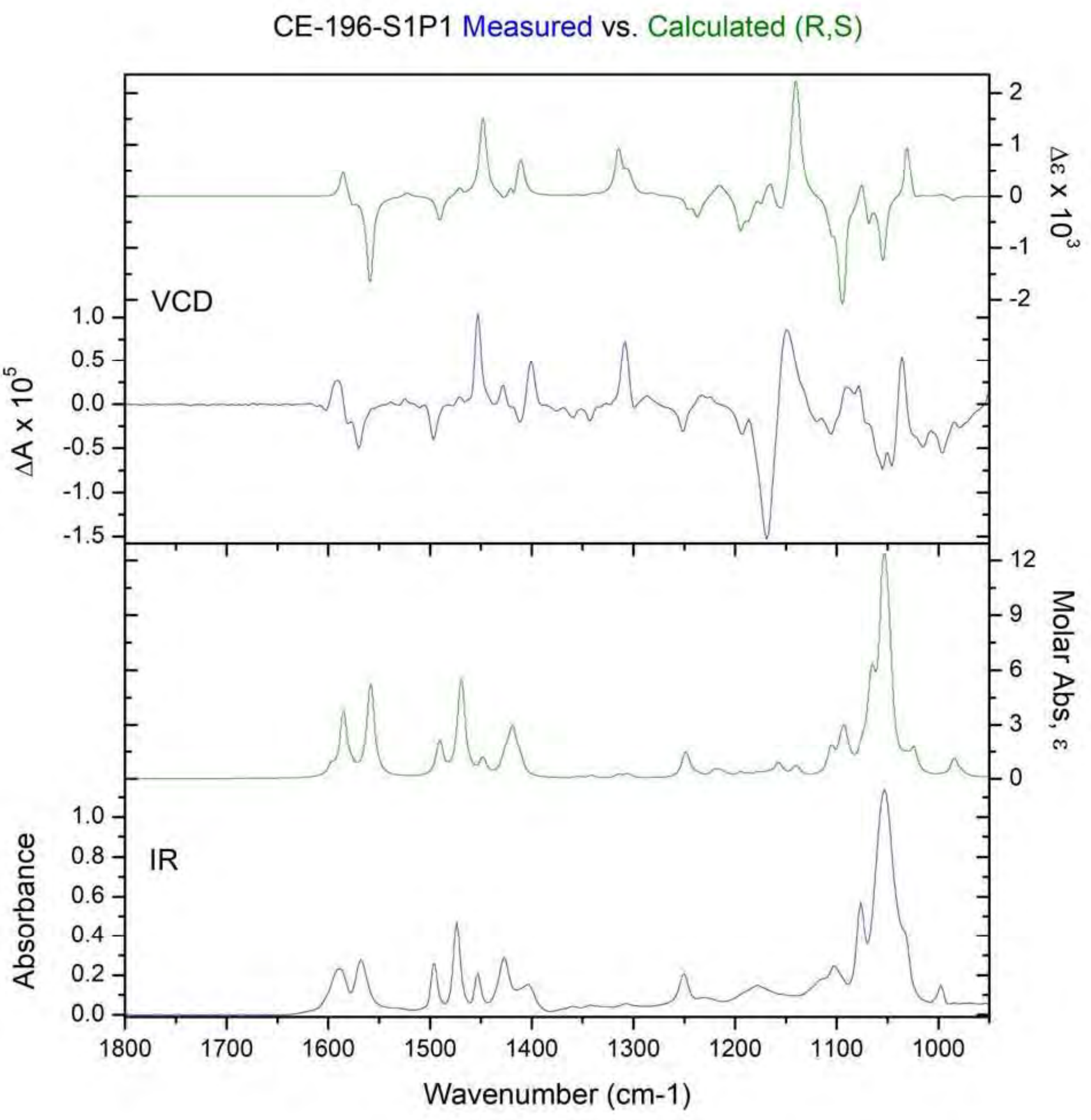

IR (lower frame) and VCD (upper frame) spectra observed for **CE196-S1P1** (left axes) compared with Boltzmann-averaged spectra of the calculated conformations for the **(R,S) = (CR,SS)** configuration, (right axes).

17546 Bee Line Hwy | Jupiter, Florida 33458, USA
ph. 561 625.0133 | fax. 561 625.0717 | www.btools.com

Title:

Absolute Configuration Determination Report

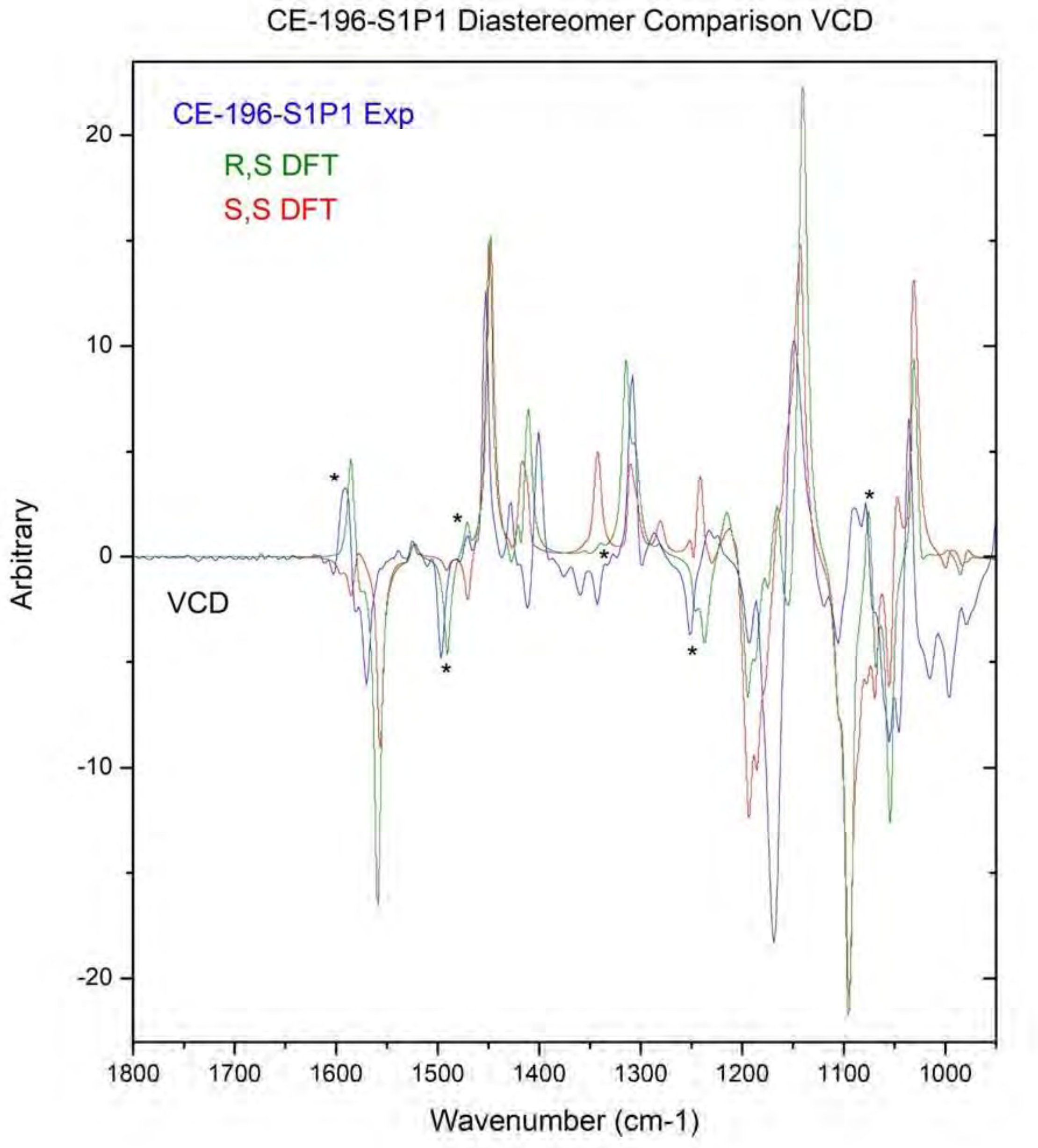

Overlay of experimental VCD for CE196-S1P1 with DFT spectra for each diastereomer. Asterisks indicate areas of agreement with the R,S (CR,SS) diastereomer but not with the S,S(CS,SS) diastereomer. The “difficult” stereochemistry to determine was the chiral center at Carbon – as seen above there was a very small difference for the specified diastereomers.

Title:

Absolute Configuration Determination Report

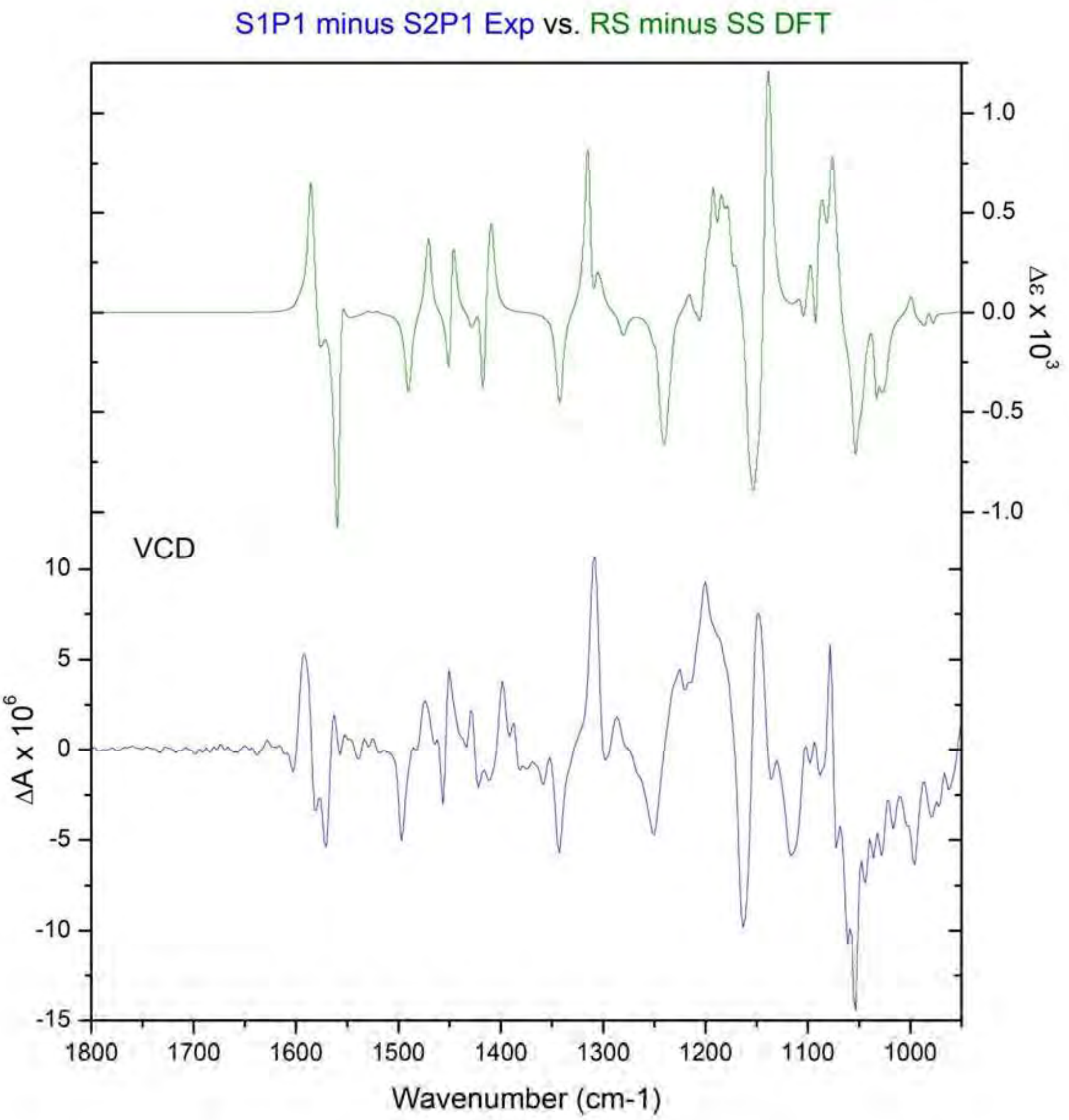

Subtracted VCD spectra
CE196-S1P1 minus CE196-S2P1 Exp
(R,S) DFT minus (S,S) DFT (CR,SS minus CS,SS)
This is an enhancement technique in which the VCD component coming from the chiral center both have in common is removed - in this case the Sulfur S, leaving (mostly) the influence of the differing center – the Carbon R center is what is shown here. This strongly suggests that the diastereomers have been correctly identified.

17546 Bee Line Hwy | Jupiter, Florida 33458, USA
ph. 561 625.0133 | fax. 561 625.0717 | www.btools.com

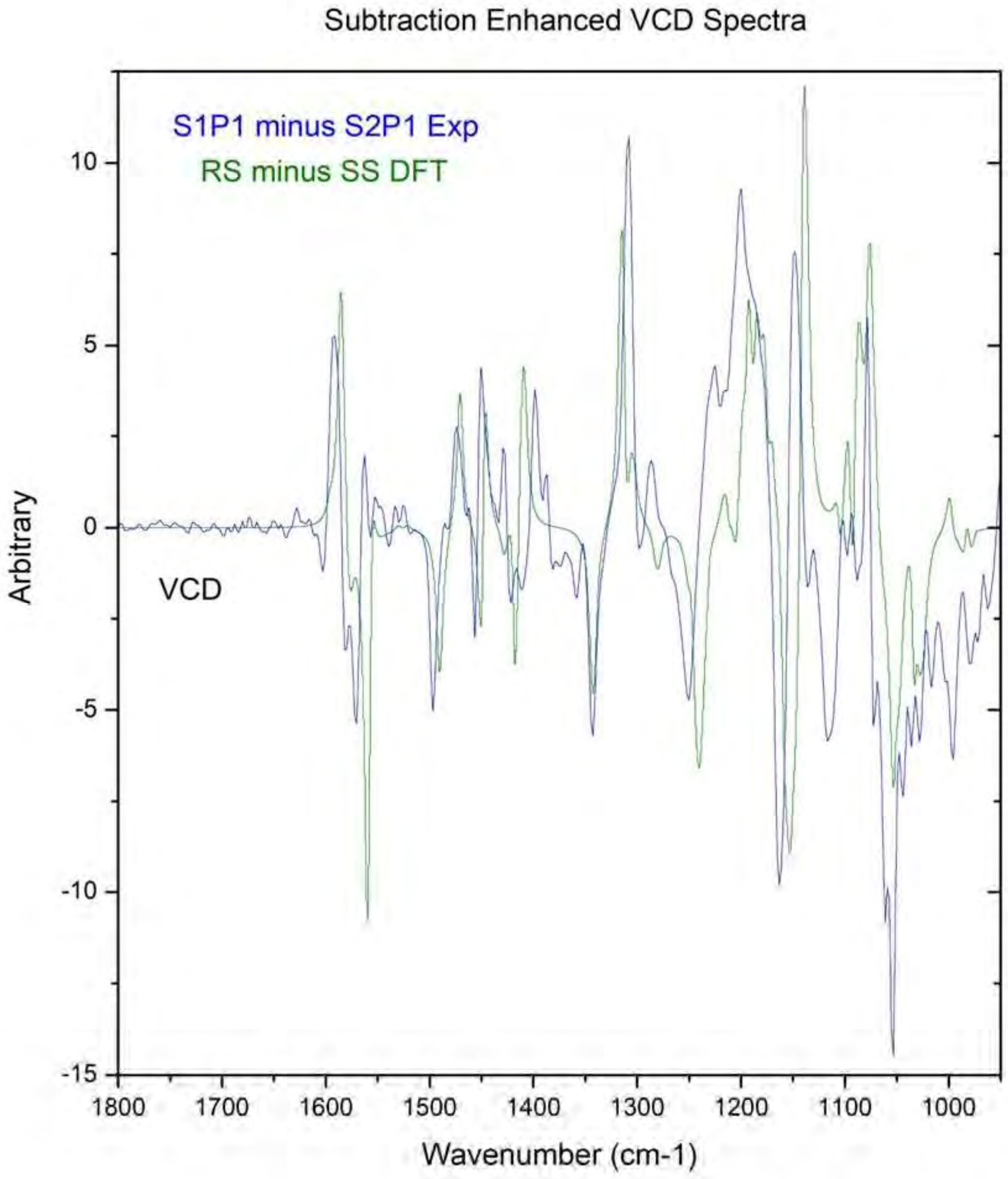

This is the same subtracted data shown above, in an overlay instead of offset – the similarity gives very high confidence in the diastereomer assignment.

Absolute Configuration Determination Report

Lowest energy conformer (2 different views) - (R,S) = (CR,SS) Configuration:

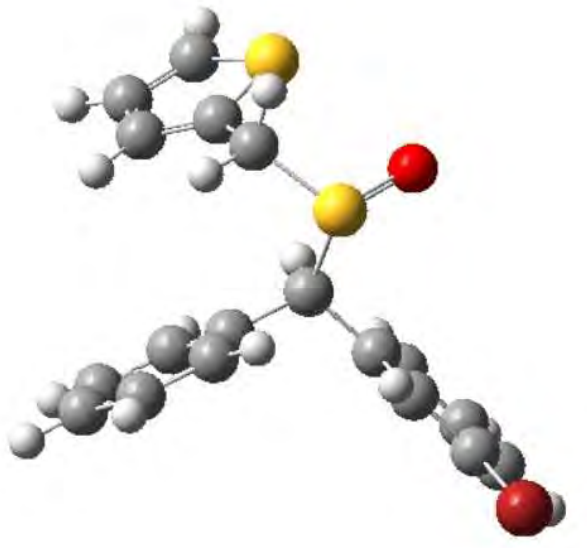

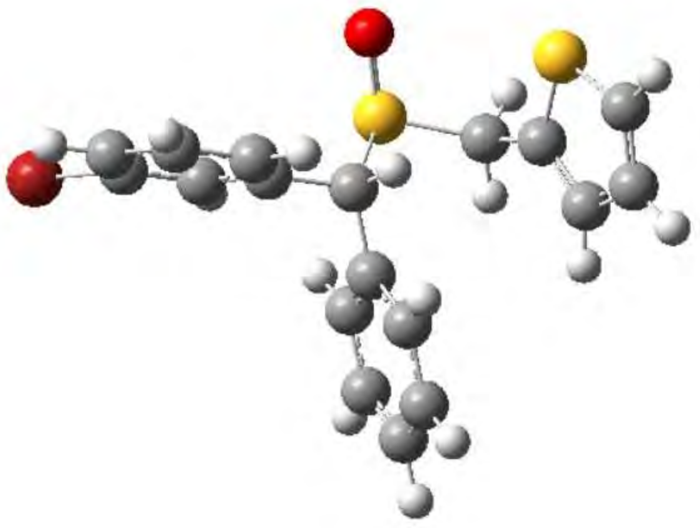

Title:

Absolute Configuration Determination Report

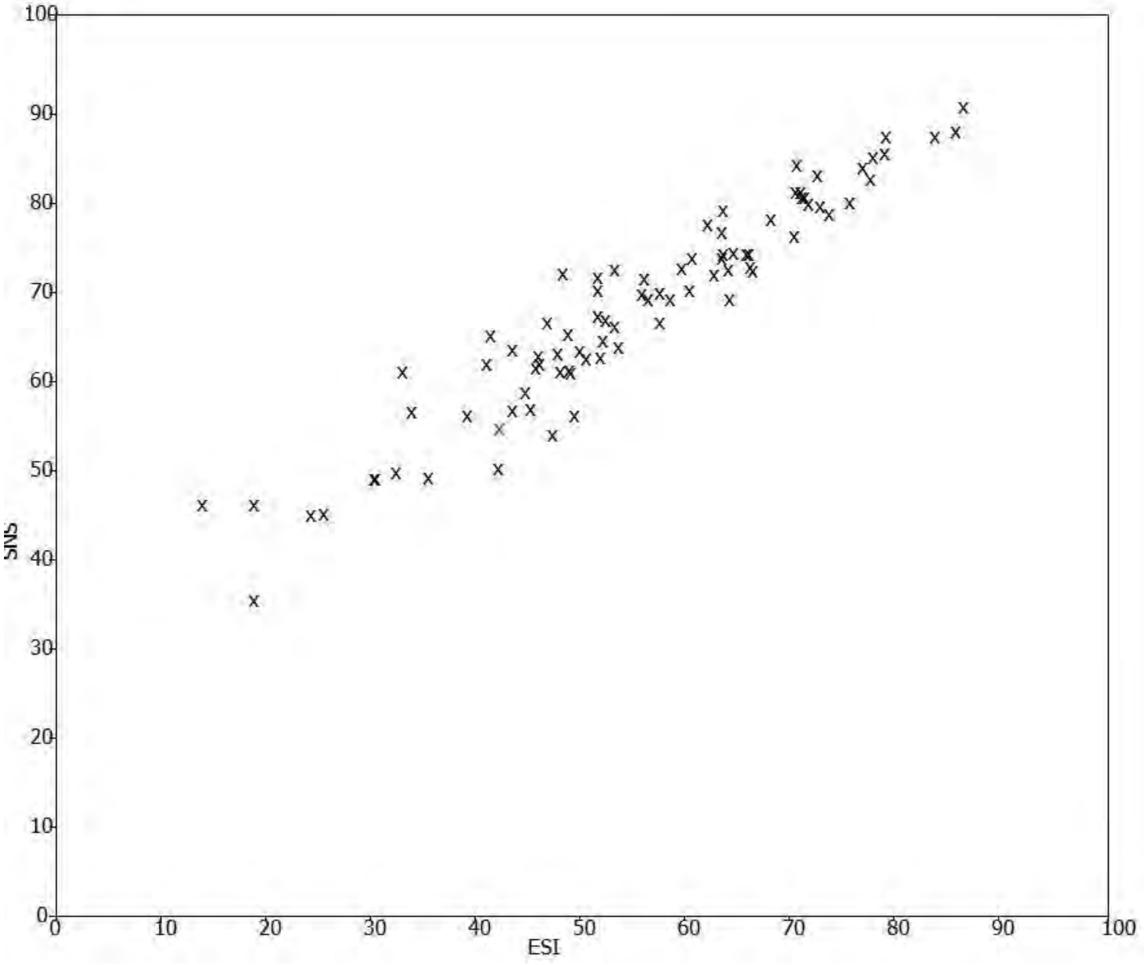

Plot of ESI (similarity of correct enantiomer minus incorrect enantiomer to calculated) vs SNS (overall similarity of correct enantiomer to calculated) for a library of correct assignments verified independently by X-Ray other method (Black X marks). Red X is **CE196-S1P1**.

17546 Bee Line Hwy | Jupiter, Florida 33458, USA
ph. 561 625.0133 | fax. 561 625.0717 | www.btools.com

Title:

Absolute Configuration Determination Report

GENERAL INFORMATION	
**Customer**	University of Vienna
**Sales Order Number**	
**Sample code (Our ref.)**	**CE196-S2P1-S2P2**
**Sample description (Your ref.)**	**CE196-S2P1-S2P2**
**VCD-spectrometer**	ChiralIR w/ Dual*PEM*
**Report prepared by**	Jordan Nafie
**Report validated and signed by**	Rina K Dukor
**Date**	May 27, 2020
**RESULTS**	
Absolute Configuration of CE196-S2P1 is (CS,SS)<br>Absolute Configuration of CE196-S2P2 is (CR,SR)	Confidence Level: **85%**
**MEASUREMENT PARAMETERS**	
**Concentration**	15mg / 100uL
**Solvent**	$CDCl_3$
**Resolution**	4 cm^{-1}
**PEM setting**	1400 cm^{-1}
**Number of scans/Measurement time**	12 hours per enantiomer
**Sample cell**	BaF_2
**Path length**	100 μm
**CALCULATION DETAILS**	
**Gaussian version**	Gaussian 09
**Total low-energy conformers used for Boltzmann sum**	26
**Methodology and basis set for DFT calculations**	B3LYP / 6311G3df2pd w/cpcm ($CDCl_3$)
**Enantiomer used for calculation**	R,R = (CR,SR)
**Total calculated conformers**	27
**Number of low-energy conformations shown in report**	1

COMMENTS

The confidence level is a measure of the degree of congruence between a calculated and measured spectrum. If identical spectra are being compared the confidence level is 100%. The confidence level (CL) is not the likelihood that the assignment is correct. Rather it's a measure of quality or degree of agreement between calculated and measured spectra. With a CL of 85% for this molecule, the visual agreement between measured and calculated spectra is very good despite some minor frequency shifts. Since the objective of this experiment was to distinguish diastereomers, the experimental spectra were compared against each calculated diastereomer. Using a direct comparison of VCD spectra, as well as an enhancement of subtracted VCD spectra, the absolute and relative configurations were determined with high confidence.

Title:

Absolute Configuration Determination Report

Structure of CE196-S2P2:

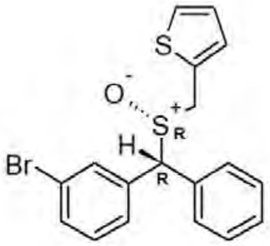

CE-196 S2P2

Structure of CE196-S2P1:

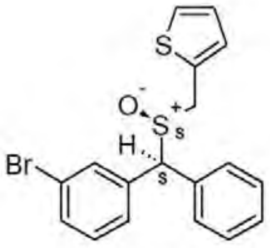

CE-196 S2P1

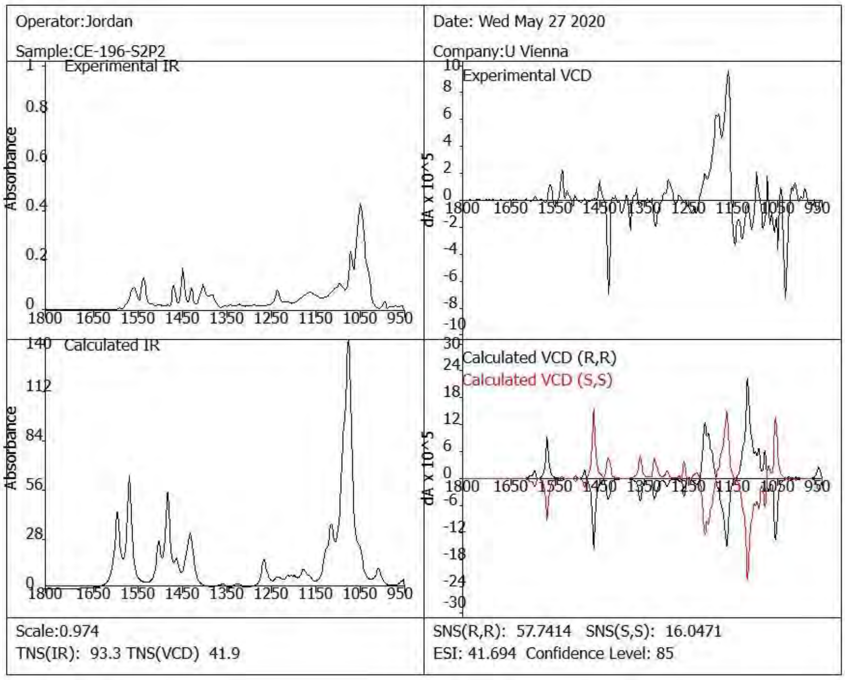

CompareVOA Results.
Please note: In this plot the frequency scaling factor is not applied.

Table 1. Numerical comparison describing the similarity in the range of 950 - 1800 cm^{-1} between the calculated IR and VCD spectra for the **(R,R) = (CR,SR)** enantiomer at the B3LYP/6311G3df2pd (w/cpcm – chloroform) level and the observed IR and VCD spectra for **CE196-S2P2**.

Cal. (950-1800cm^{-1})	Numerical comparison	Observed **CE196-S2P2**
**(R,R)**	scaling factor	0.974
	IR similarity (%)	93.3
	$^{a}\sum$ (%)	57.7414
	$^{b}\Delta$ (%)	41.694
	Confidence Level (%)	85

$^{a}\sum$: single VCD similarity, gives the similarity between the calculated and observed VCD spectra.
$^{b}\Delta$: enatiomeric similarity index, gives the difference between the values of ∑ for both enantiomers of a given diastereoisomer.

Absolute Configuration Determination Report

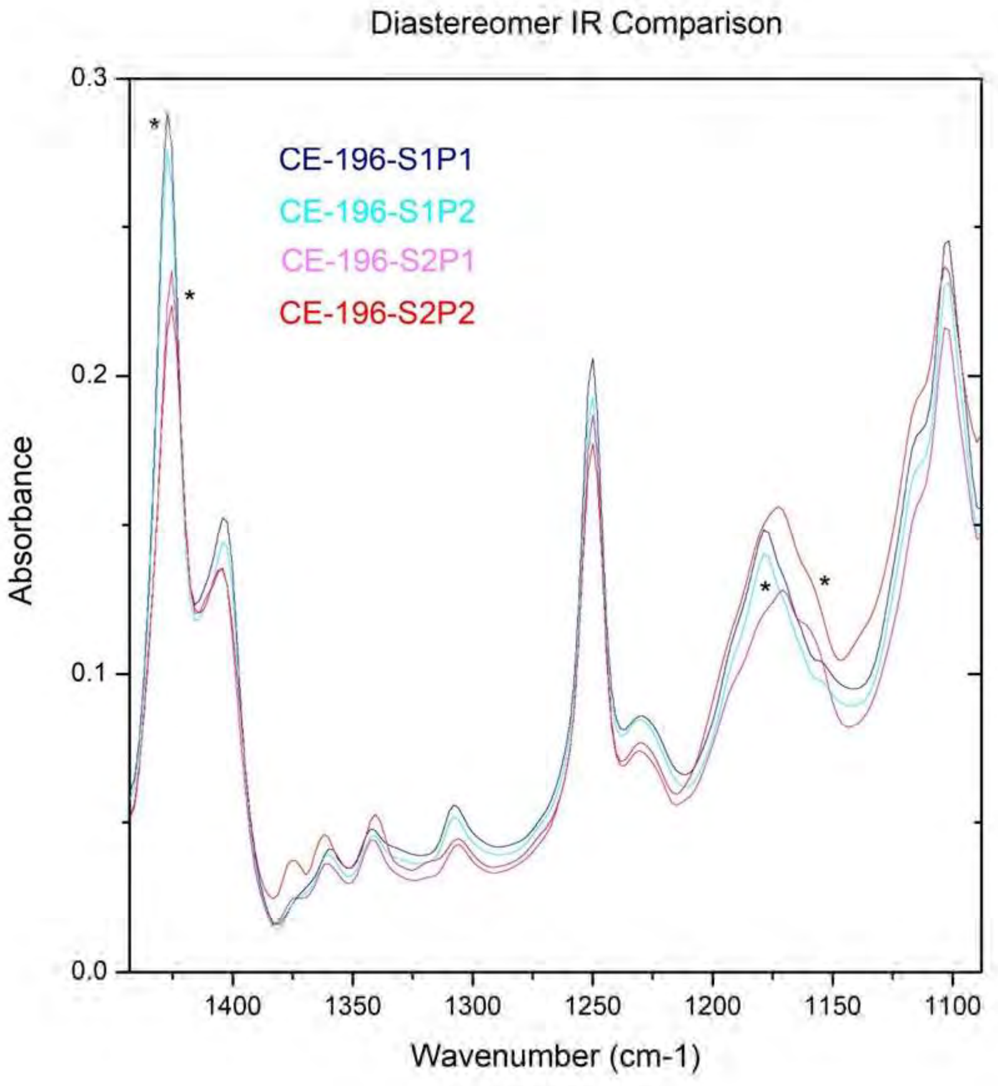

As a double check, the enantiomer pairs were determined to be S1P1 / S1P2 as one pair and S2P1 / S2P2 as the other pair – as confirmed by the small differences in the IR spectra shown above.

Title:

Absolute Configuration Determination Report

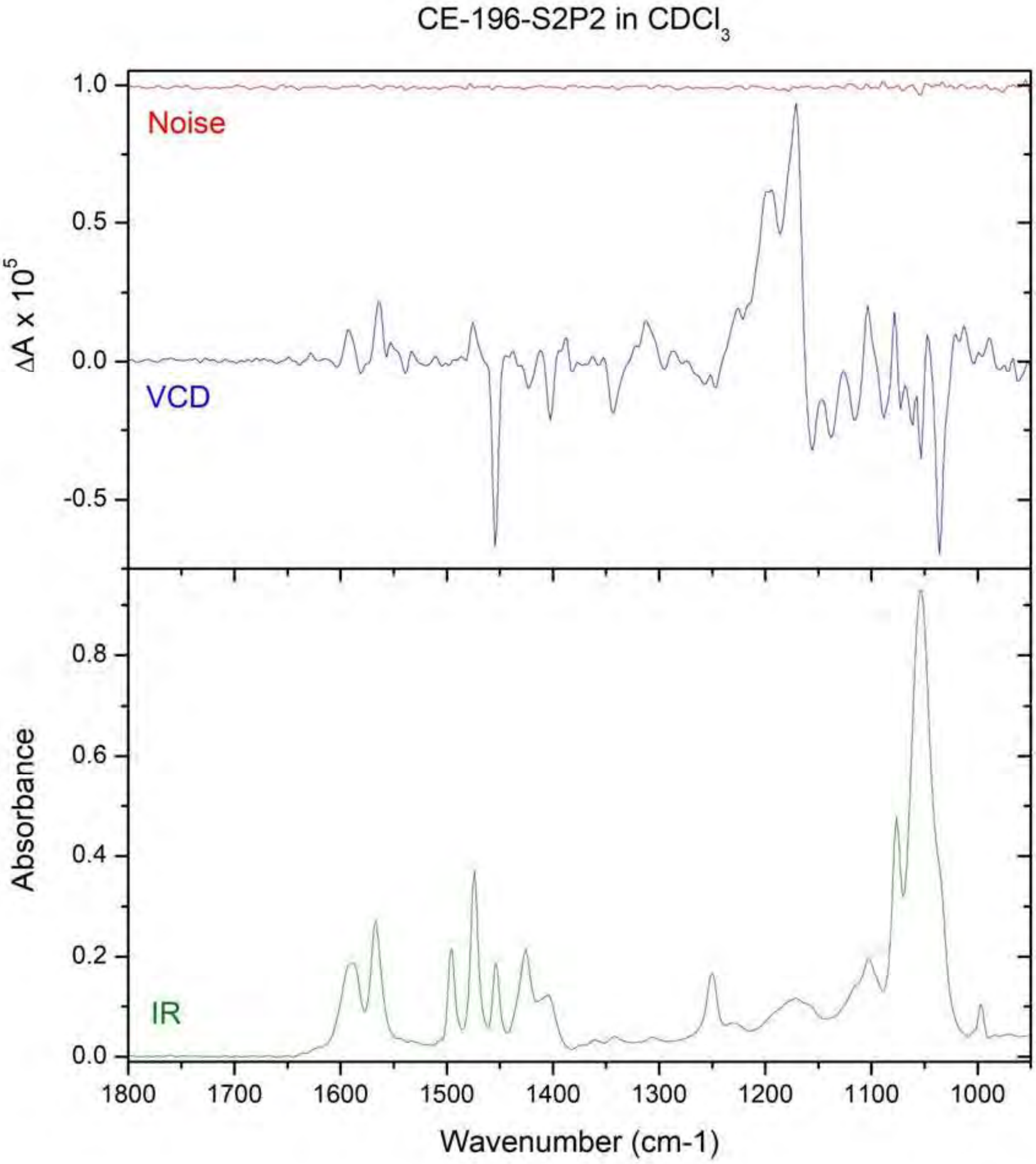

IR (lower frame) and VCD (upper frame) spectra of **CE196-S2P2** in $CDCl_3$; 100um path-length cell with BaF_2 windows; 12 h collection for each enantiomer; instrument optimized at 1400 cm^{-1}. Solvent subtracted IR and enantiomer subtracted VCD spectra are shown. Uppermost trace is the VCD noise spectrum.

Title:

Absolute Configuration Determination Report

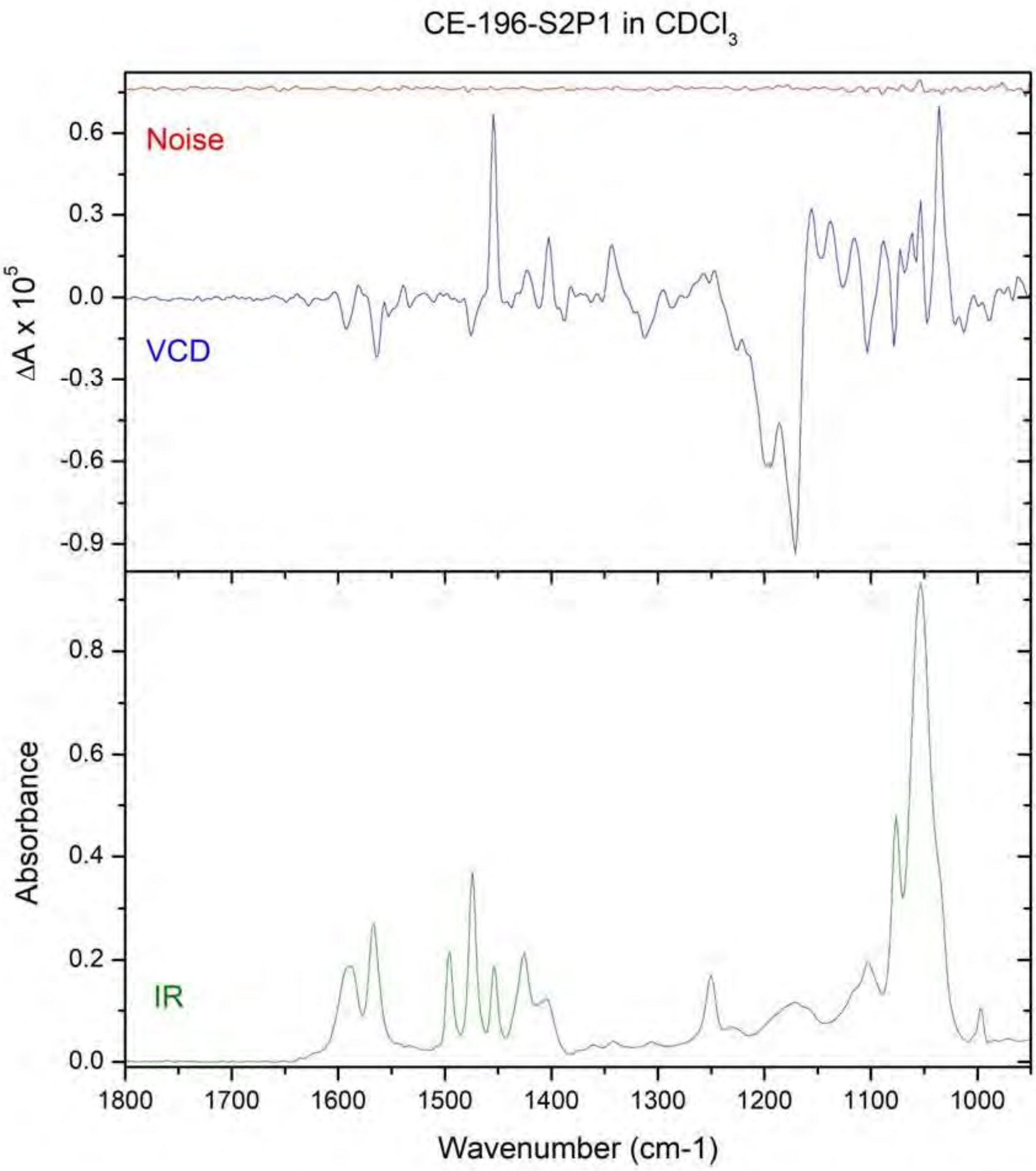

IR (lower frame) and VCD (upper frame) spectra of **CE196-S2P1** in $CDCl_3$; 100um path-length cell with BaF_2 windows; 12 h collection for each enantiomer; instrument optimized at 1400 cm^{-1}. Solvent subtracted IR and enantiomer subtracted VCD spectra are shown. Uppermost trace is the VCD noise spectrum.

'Tools for Discoveries of Life'

Title:

Absolute Configuration Determination Report

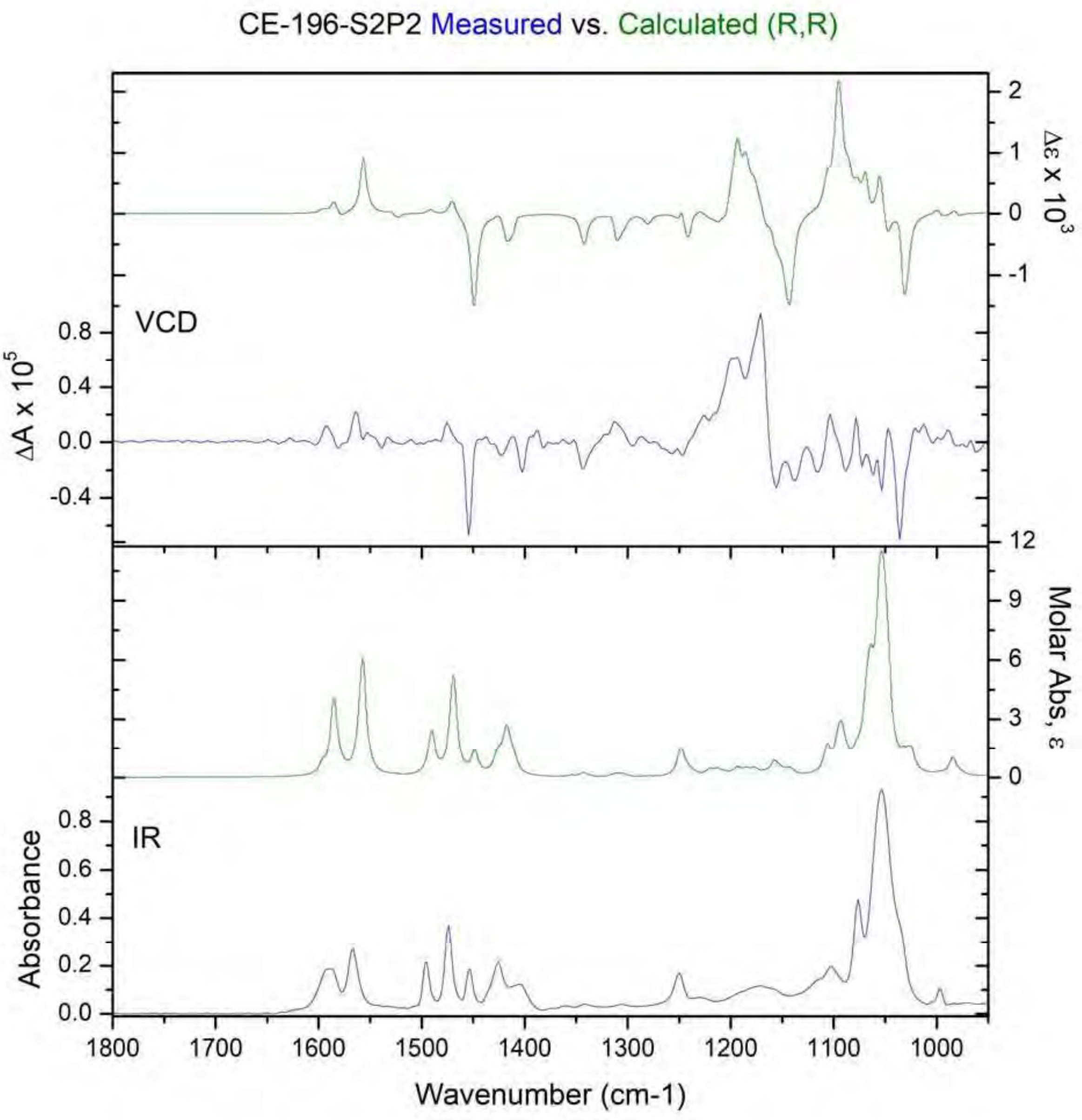

IR (lower frame) and VCD (upper frame) spectra observed for **CE196-S2P2** (left axes) compared with Boltzmann-averaged spectra of the calculated conformations for the **(R,R) = (CR,SR)** configuration, (right axes).

17546 Bee Line Hwy | Jupiter, Florida 33458, USA
ph. 561 625.0133 | fax. 561 625.0717 | www.btools.com

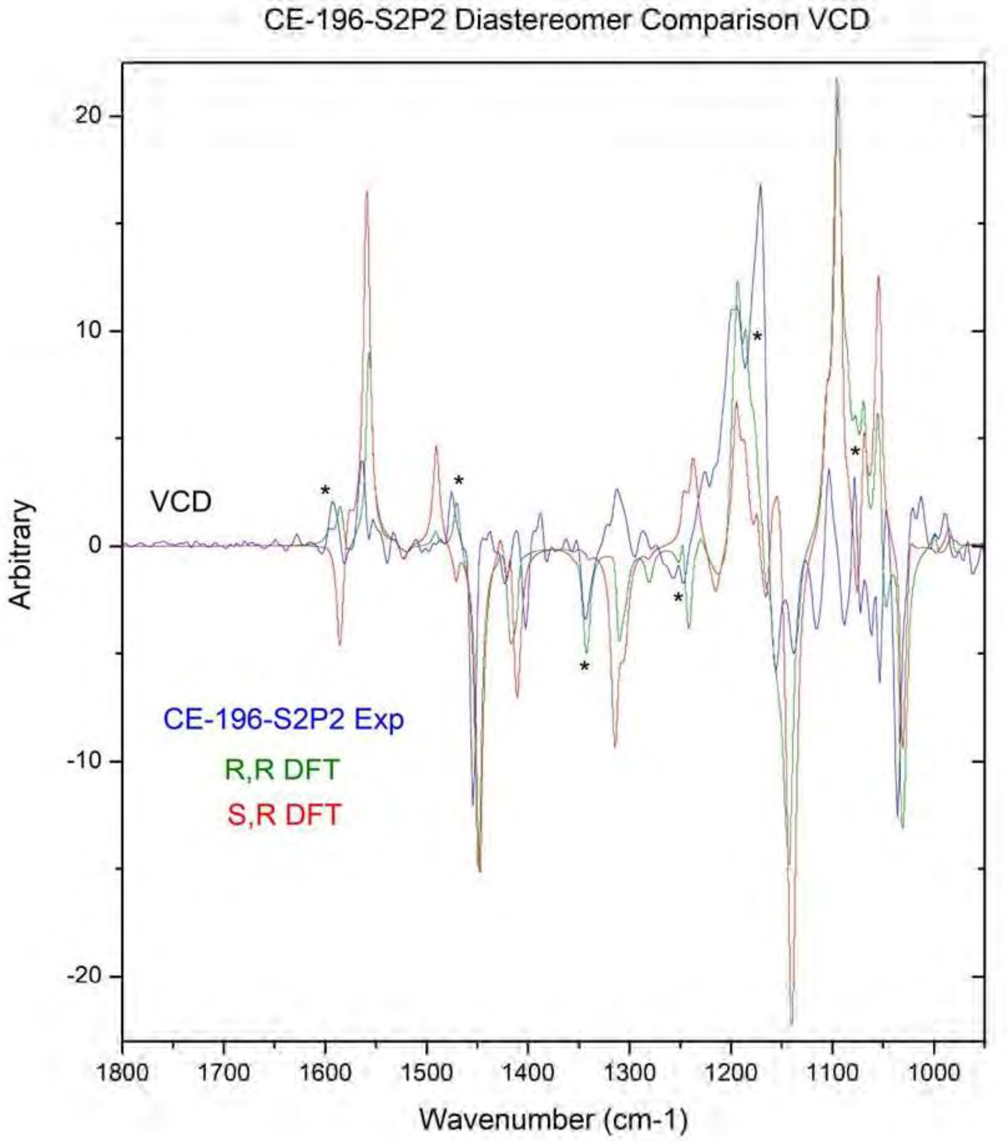

Overlay of experimental VCD for CE196-S2P2 with DFT spectra for each diastereomer. Asterisks indicate areas of agreement with the R,R (CR,SR) diastereomer but not with the S,R (CS,SR) diastereomer. The “difficult” stereochemistry to determine was the chiral center at Carbon – as seen above there was a very small difference for the specified diastereomers.

Title:
Absolute Configuration Determination Report

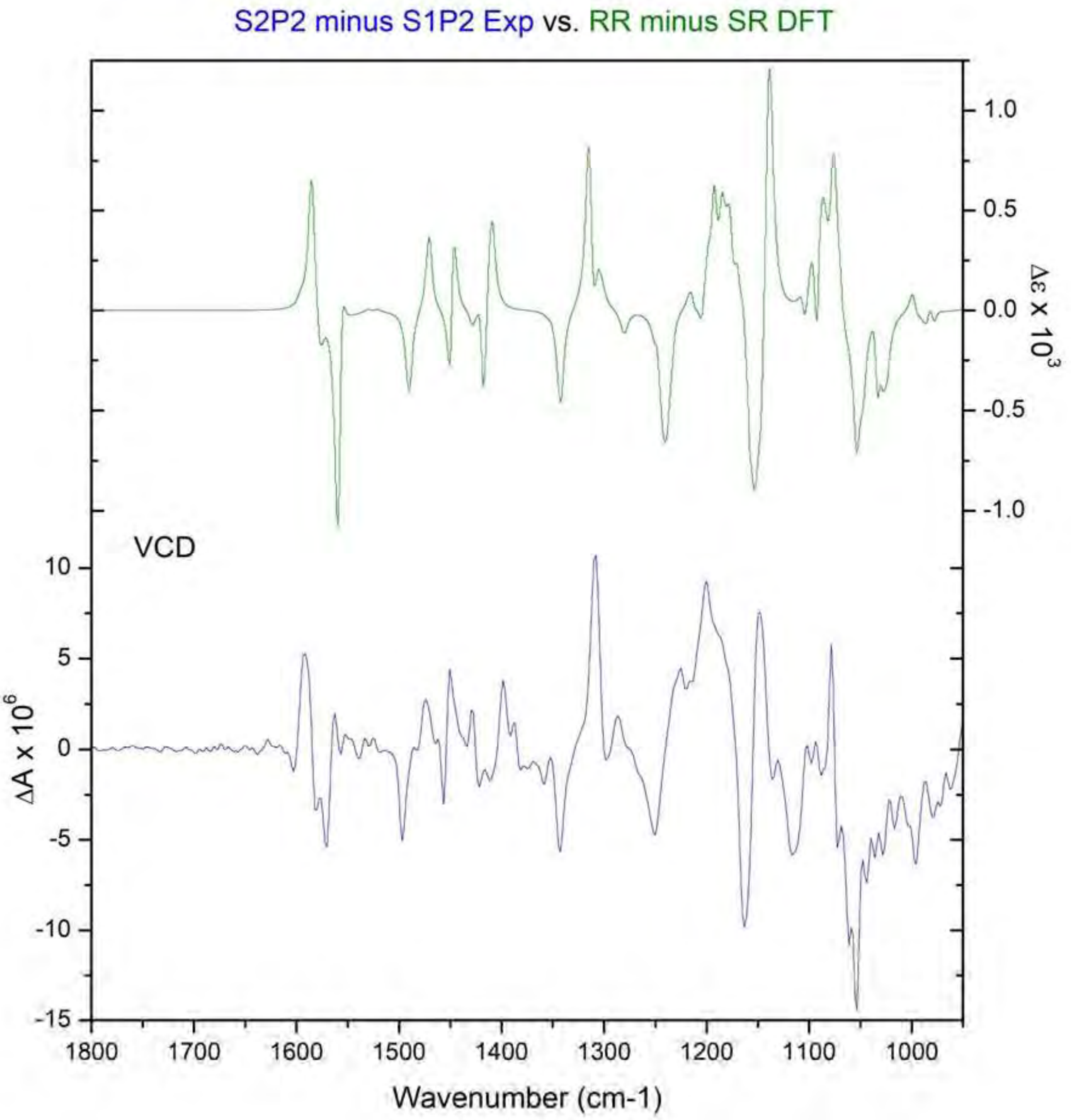

Subtracted VCD spectra
CE196-S2P2 minus CE196-S1P2 Exp
(R,R) DFT minus (S,R) DFT (CR,SR minus CS,SR)
This is an enhancement technique in which the VCD component coming from the chiral center both have in common is removed - in this case the Sulfur S, leaving (mostly) the influence of the differing center – the Carbon R center is what is shown here. This strongly suggests that the diastereomers have been correctly identified.

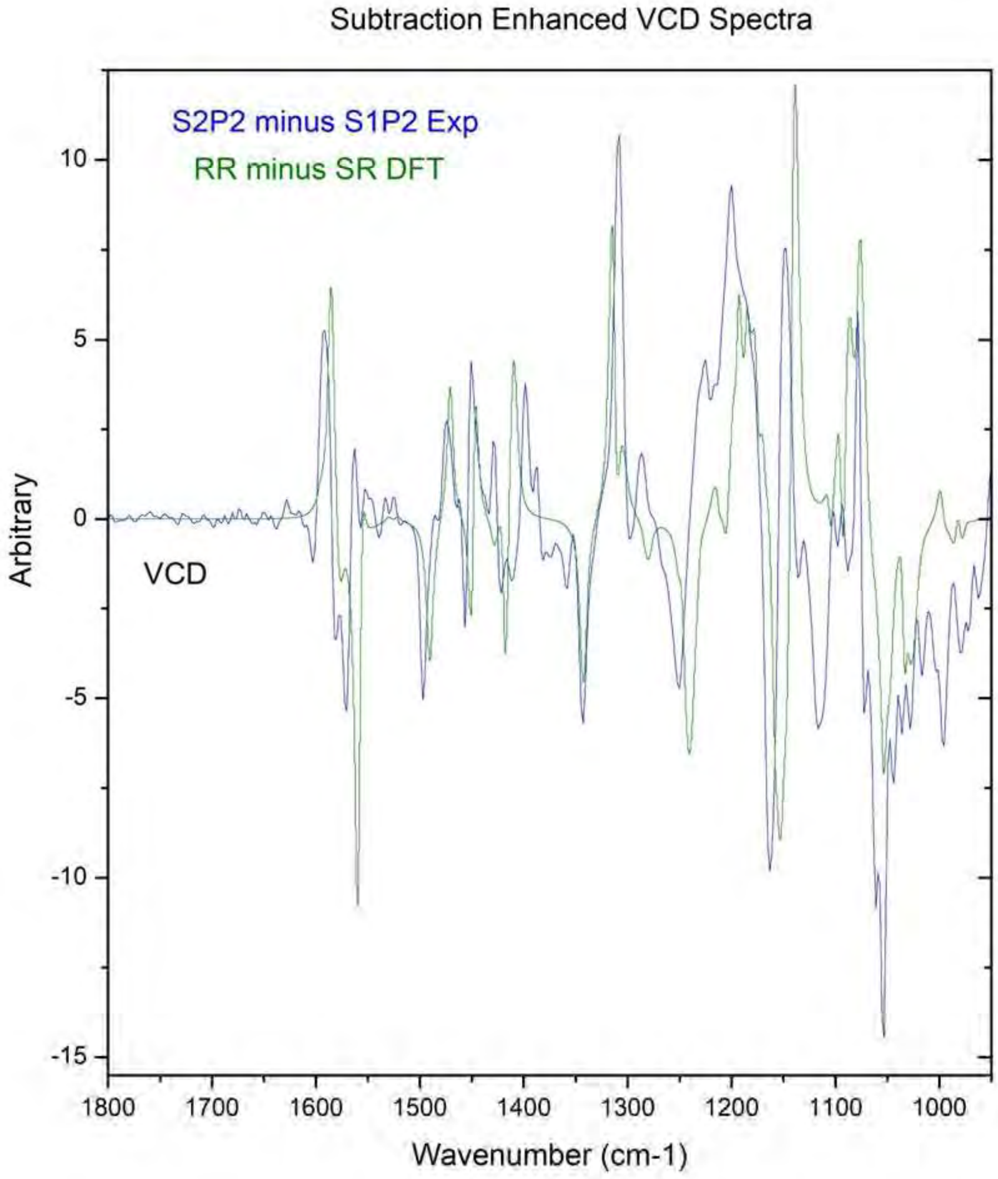

This is the same subtracted data shown above, in an overlay instead of offset – the similarity gives very high confidence in the diastereomer assignment.

Title:

Absolute Configuration Determination Report

Lowest energy conformer (2 different views) - (R,R) = (CR,SR) Configuration:

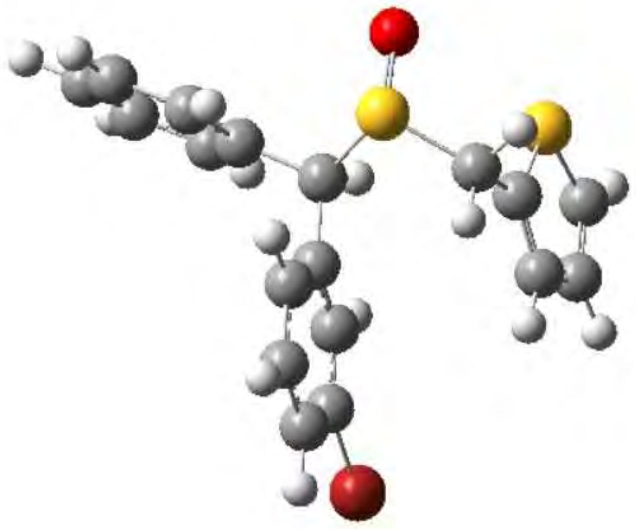

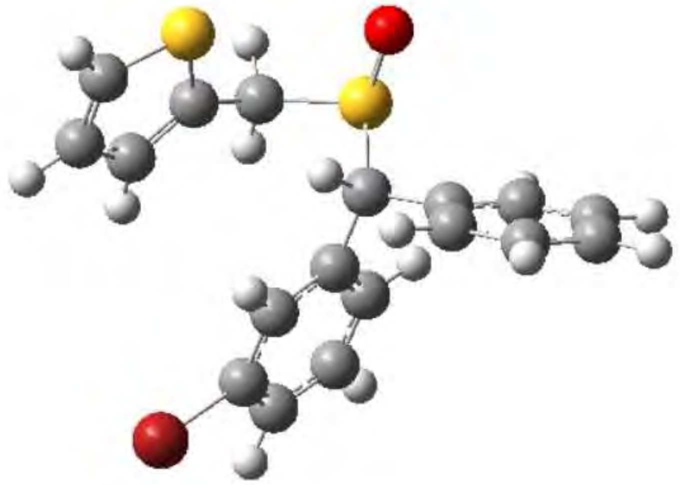

Absolute Configuration Determination Report

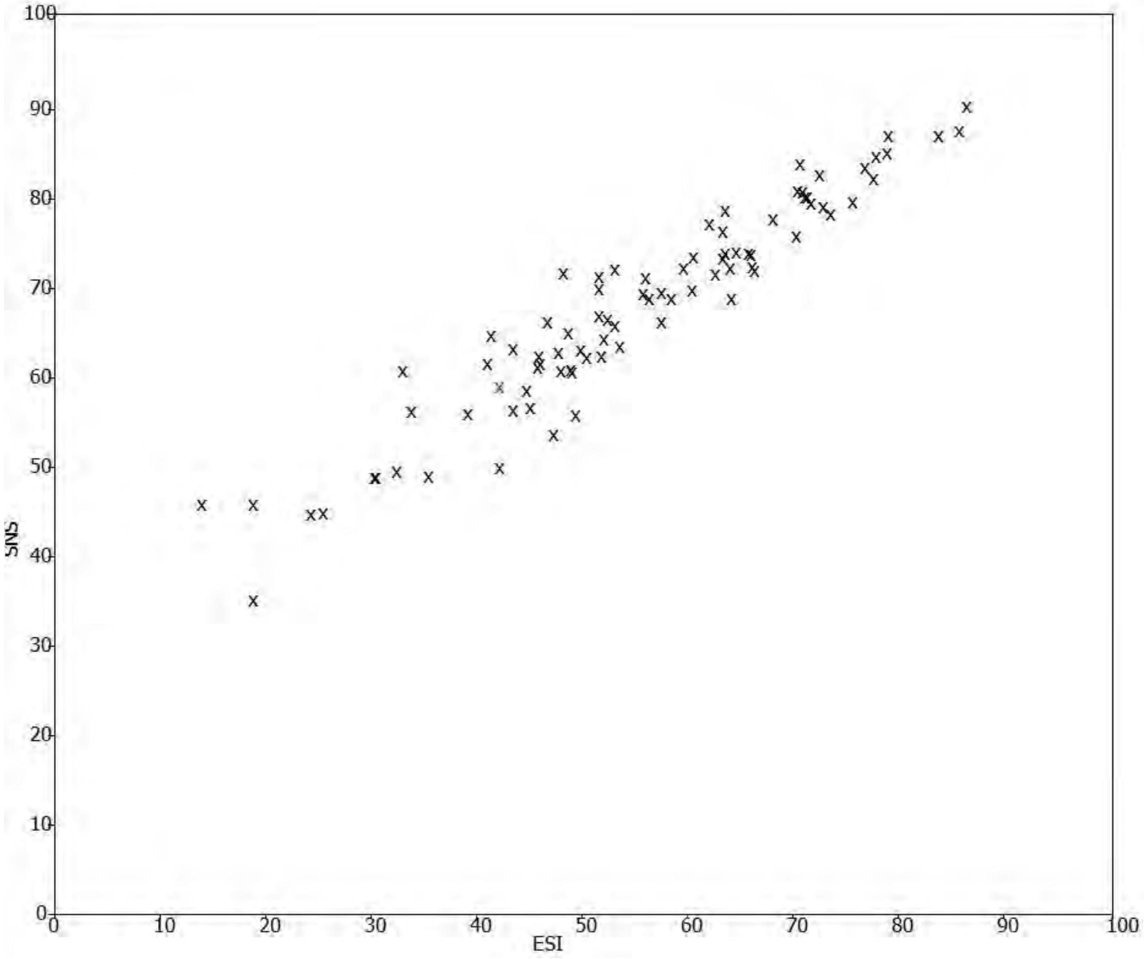

Plot of ESI (similarity of correct enantiomer minus incorrect enantiomer to calculated) vs SNS (overall similarity of correct enantiomer to calculated) for a library of correct assignments verified independently by X-Ray other method (Black X marks). Red X is **CE196-S2P2**.

17546 Bee Line Hwy | Jupiter, Florida 33458, USA
ph. 561 625.0133 | fax. 561 625.0717 | www.btools.com
